# Global, regional, and national comparative risk assessment of 84 behavioural, environmental and occupational, and metabolic risks or clusters of risks for 195 countries and territories, 1990–2017: a systematic analysis for the Global Burden of Disease Study 2017

**DOI:** 10.1016/S0140-6736(18)32225-6

**Published:** 2018-11-10

**Authors:** GBD 2017 Risk Factor Collaborators, Jeffrey D Stanaway, Ashkan Afshin, Emmanuela Gakidou, Stephen S Lim, Degu Abate, Kalkidan Hassen Abate, Cristiana Abbafati, Nooshin Abbasi, Hedayat Abbastabar, Foad Abd-Allah, Jemal Abdela, Ahmed Abdelalim, Ibrahim Abdollahpour, Rizwan Suliankatchi Abdulkader, Molla Abebe, Zegeye Abebe, Semaw F Abera, Olifan Zewdie Abil, Haftom Niguse Abraha, Aklilu Roba Abrham, Laith Jamal Abu-Raddad, Niveen ME Abu-Rmeileh, Manfred Mario Kokou Accrombessi, Dilaram Acharya, Pawan Acharya, Abdu A Adamu, Akilew Awoke Adane, Oladimeji M Adebayo, Rufus Adesoji Adedoyin, Victor Adekanmbi, Zanfina Ademi, Olatunji O Adetokunboh, Mina G Adib, Amha Admasie, Jose C Adsuar, Kossivi Agbelenko Afanvi, Mohsen Afarideh, Gina Agarwal, Anju Aggarwal, Sargis Aghasi Aghayan, Anurag Agrawal, Sutapa Agrawal, Alireza Ahmadi, Mehdi Ahmadi, Hamid Ahmadieh, Muktar Beshir Ahmed, Amani Nidhal Aichour, Ibtihel Aichour, Miloud Taki Eddine Aichour, Mohammad Esmaeil Akbari, Tomi Akinyemiju, Nadia Akseer, Ziyad Al-Aly, Ayman Al-Eyadhy, Hesham M Al-Mekhlafi, Fares Alahdab, Khurshid Alam, Samiah Alam, Tahiya Alam, Alaa Alashi, Seyed Moayed Alavian, Kefyalew Addis Alene, Komal Ali, Syed Mustafa Ali, Mehran Alijanzadeh, Reza Alizadeh-Navaei, Syed Mohamed Aljunid, Ala'a Alkerwi, François Alla, Ubai Alsharif, Khalid Altirkawi, Nelson Alvis-Guzman, Azmeraw T Amare, Walid Ammar, Nahla Hamed Anber, Jason A Anderson, Catalina Liliana Andrei, Sofia Androudi, Megbaru Debalkie Animut, Mina Anjomshoa, Mustafa Geleto Ansha, Josep M Antó, Carl Abelardo T Antonio, Palwasha Anwari, Lambert Tetteh Appiah, Seth Christopher Yaw Appiah, Jalal Arabloo, Olatunde Aremu, Johan Ärnlöv, Al Artaman, Krishna K Aryal, Hamid Asayesh, Zerihun Ataro, Marcel Ausloos, Euripide F G A Avokpaho, Ashish Awasthi, Beatriz Paulina Ayala Quintanilla, Rakesh Ayer, Tambe B Ayuk, Peter S Azzopardi, Arefeh Babazadeh, Hamid Badali, Alaa Badawi, Kalpana Balakrishnan, Ayele Geleto Bali, Kylie Ball, Shoshana H Ballew, Maciej Banach, Joseph Adel Mattar Banoub, Aleksandra Barac, Suzanne Lyn Barker-Collo, Till Winfried Bärnighausen, Lope H Barrero, Sanjay Basu, Bernhard T Baune, Shahrzad Bazargan-Hejazi, Neeraj Bedi, Ettore Beghi, Masoud Behzadifar, Meysam Behzadifar, Yannick Béjot, Bayu Begashaw Bekele, Eyasu Tamru Bekru, Ezra Belay, Yihalem Abebe Belay, Michelle L Bell, Aminu K Bello, Derrick A Bennett, Isabela M Bensenor, Gilles Bergeron, Adugnaw Berhane, Eduardo Bernabe, Robert S Bernstein, Mircea Beuran, Tina Beyranvand, Neeraj Bhala, Ashish Bhalla, Suraj Bhattarai, Zulfiqar A Bhutta, Belete Biadgo, Ali Bijani, Boris Bikbov, Ver Bilano, Nigus Bililign, Muhammad Shahdaat Bin Sayeed, Donal Bisanzio, Tuhin Biswas, Tone Bjørge, Brigette F Blacker, Archie Bleyer, Rohan Borschmann, Ibrahim R Bou-Orm, Soufiane Boufous, Rupert Bourne, Oliver J Brady, Michael Brauer, Alexandra Brazinova, Nicholas J K Breitborde, Hermann Brenner, Andrey Nikolaevich Briko, Gabrielle Britton, Traolach Brugha, Rachelle Buchbinder, Richard T Burnett, Reinhard Busse, Zahid A Butt, Leah E Cahill, Lucero Cahuana-Hurtado, Ismael R Campos-Nonato, Rosario Cárdenas, Giulia Carreras, Juan J Carrero, Félix Carvalho, Carlos A Castañeda-Orjuela, Jacqueline Castillo Rivas, Franz Castro, Ferrán Catalá-López, Kate Causey, Kelly M Cercy, Ester Cerin, Yazan Chaiah, Hsing-Yi Chang, Jung-Chen Chang, Kai-Lan Chang, Fiona J Charlson, Aparajita Chattopadhyay, Vijay Kumar Chattu, Miao Li Chee, Ching-Yu Cheng, Adrienne Chew, Peggy Pei-Chia Chiang, Odgerel Chimed-Ochir, Ken Lee Chin, Abdulaal Chitheer, Jee-Young J Choi, Rajiv Chowdhury, Hanne Christensen, Devasahayam J Christopher, Sheng-Chia Chung, Flavia M Cicuttini, Massimo Cirillo, Aaron J Cohen, Daniel Collado-Mateo, Cyrus Cooper, Owen R Cooper, Josef Coresh, Leslie Cornaby, Paolo Angelo Cortesi, Monica Cortinovis, Megan Costa, Ewerton Cousin, Michael H Criqui, Elizabeth A Cromwell, David K Cundiff, Alemneh Kabeta Daba, Berihun Assefa Dachew, Abel Fekadu Dadi, Albertino Antonio Moura Damasceno, Lalit Dandona, Rakhi Dandona, Sarah C Darby, Paul I Dargan, Ahmad Daryani, Rajat Das Gupta, José Das Neves, Tamirat Tesfaye Dasa, Aditya Prasad Dash, Dragos Virgil Davitoiu, Kairat Davletov, Vanessa De la Cruz-Góngora, Fernando Pio De La Hoz, Diego De Leo, Jan-Walter De Neve, Louisa Degenhardt, Selina Deiparine, Robert P Dellavalle, Gebre Teklemariam Demoz, Edgar Denova-Gutiérrez, Kebede Deribe, Nikolaos Dervenis, Aniruddha Deshpande, Don C Des Jarlais, Getenet Ayalew Dessie, Gabrielle Aline Deveber, Subhojit Dey, Samath Dhamminda Dharmaratne, Meghnath Dhimal, Mesfin Tadese Dinberu, Eric L Ding, Helen Derara Diro, Shirin Djalalinia, Huyen Phuc Do, Klara Dokova, David Teye Doku, Kerrie E Doyle, Tim R Driscoll, Manisha Dubey, Eleonora Dubljanin, Eyasu Ejeta Duken, Bruce B Duncan, Andre R Duraes, Natalie Ebert, Hedyeh Ebrahimi, Soheil Ebrahimpour, David Edvardsson, Andem Effiong, Anne Elise Eggen, Charbel El Bcheraoui, Ziad El-Khatib, Iqbal Rf Elyazar, Ahmadali Enayati, Aman Yesuf Endries, Benjamin Er, Holly E Erskine, Sharareh Eskandarieh, Alireza Esteghamati, Kara Estep, Hamed Fakhim, Mahbobeh Faramarzi, Mohammad Fareed, Talha A Farid, Carla Sofia E sá Farinha, Andrea Farioli, Andre Faro, Maryam S Farvid, Mohammad Hosein Farzaei, Batool Fatima, Kairsten A Fay, Ali Akbar Fazaeli, Valery L Feigin, Andrea B Feigl, Seyed-Mohammad Fereshtehnejad, Eduarda Fernandes, Joao C Fernandes, Giannina Ferrara, Alize J Ferrari, Manuela L Ferreira, Irina Filip, Jonas David Finger, Florian Fischer, Nataliya A Foigt, Kyle J Foreman, Takeshi Fukumoto, Nancy Fullman, Thomas Fürst, João M Furtado, Neal D Futran, Seana Gall, Silvano Gallus, Amiran Gamkrelidze, Morsaleh Ganji, Alberto L Garcia-Basteiro, William M Gardner, Abadi Kahsu Gebre, Amanuel Tesfay Gebremedhin, Teklu Gebrehiwo Gebremichael, Tilayie Feto Gelano, Johanna M Geleijnse, Yilma Chisha Dea Geramo, Peter W Gething, Kebede Embaye Gezae, Reza Ghadimi, Keyghobad Ghadiri, Khalil Ghasemi Falavarjani, Maryam Ghasemi-Kasman, Mamata Ghimire, Rakesh Ghosh, Aloke Gopal Ghoshal, Simona Giampaoli, Paramjit Singh Gill, Tiffany K Gill, Richard F Gillum, Ibrahim Abdelmageed Ginawi, Giorgia Giussani, Elena V Gnedovskaya, William W Godwin, Srinivas Goli, Hector Gómez-Dantés, Philimon N Gona, Sameer Vali Gopalani, Alessandra C Goulart, Ayman Grada, Morgan E Grams, Giuseppe Grosso, Harish Chander Gugnani, Yuming Guo, Rahul Gupta, Rajeev Gupta, Tanush Gupta, Reyna Alma Gutiérrez, Daniela S Gutiérrez-Torres, Juanita A Haagsma, Tesfa Dejenie Habtewold, Vladimir Hachinski, Nima Hafezi-Nejad, Tekleberhan B Hagos, Tewodros Tesfa Hailegiyorgis, Gessessew Bugssa Hailu, Arvin Haj-Mirzaian, Arya Haj-Mirzaian, Randah R Hamadeh, Samer Hamidi, Alexis J Handal, Graeme J Hankey, Yuantao Hao, Hilda L Harb, Sivadasanpillai Harikrishnan, Josep Maria Haro, Hadi Hassankhani, Hamid Yimam Hassen, Rasmus Havmoeller, Caitlin N Hawley, Simon I Hay, Akbar Hedayatizadeh-Omran, Behzad Heibati, Behnam Heidari, Mohsen Heidari, Delia Hendrie, Andualem Henok, Ileana Heredia-Pi, Claudiu Herteliu, Fatemeh Heydarpour, Sousan Heydarpour, Desalegn T Hibstu, Tarig B Higazi, Esayas Haregot Hilawe, Hans W Hoek, Howard J Hoffman, Michael K Hole, Enayatollah Homaie Rad, Praveen Hoogar, H Dean Hosgood, Seyed Mostafa Hosseini, Mehdi Hosseinzadeh, Mihaela Hostiuc, Sorin Hostiuc, Damian G Hoy, Mohamed Hsairi, Thomas Hsiao, Guoqing Hu, Howard Hu, John J Huang, Mamusha Aman Hussen, Chantal K Huynh, Kim Moesgaard Iburg, Nayu Ikeda, Olayinka Stephen Ilesanmi, Usman Iqbal, Seyed Sina Naghibi Irvani, Caleb Mackay Salpeter Irvine, Sheikh Mohammed Shariful Islam, Farhad Islami, Maria D Jackson, Kathryn H Jacobsen, Leila Jahangiry, Nader Jahanmehr, Sudhir Kumar Jain, Mihajlo Jakovljevic, Spencer L James, Simerjot K Jassal, Achala Upendra Jayatilleke, Panniyammakal Jeemon, Ravi Prakash Jha, Vivekanand Jha, John S Ji, Jost B Jonas, Jitendra Jonnagaddala, Zahra Jorjoran Shushtari, Ankur Joshi, Jacek Jerzy Jozwiak, Mikk Jürisson, Zubair Kabir, Amaha Kahsay, Rizwan Kalani, Tanuj Kanchan, Surya Kant, Chittaranjan Kar, Manoochehr Karami, Behzad Karami Matin, André Karch, Corine Karema, Narges Karimi, Seyed M Karimi, Amir Kasaeian, Dessalegn H Kassa, Getachew Mullu Kassa, Tesfaye Dessale Kassa, Nicholas J Kassebaum, Srinivasa Vittal Katikireddi, Anil Kaul, Norito Kawakami, Zhila Kazemi, Ali Kazemi Karyani, Adane Teshome Kefale, Peter Njenga Keiyoro, Grant Rodgers Kemp, Andre Pascal Kengne, Andre Keren, Chandrasekharan Nair Kesavachandran, Yousef Saleh Khader, Behzad Khafaei, Morteza Abdullatif Khafaie, Alireza Khajavi, Nauman Khalid, Ibrahim A Khalil, Gulfaraz Khan, Muhammad Shahzeb Khan, Muhammad Ali Khan, Young-Ho Khang, Mona M Khater, Mohammad Khazaei, Habibolah Khazaie, Abdullah T Khoja, Ardeshir Khosravi, Mohammad Hossein Khosravi, Aliasghar A Kiadaliri, Daniel N Kiirithio, Cho-Il Kim, Daniel Kim, Young-Eun Kim, Yun Jin Kim, Ruth W Kimokoti, Yohannes Kinfu, Adnan Kisa, Katarzyna Kissimova-Skarbek, Mika Kivimäki, Luke D Knibbs, Ann Kristin Skrindo Knudsen, Sonali Kochhar, Yoshihiro Kokubo, Tufa Kolola, Jacek A Kopec, Soewarta Kosen, Parvaiz A Koul, Ai Koyanagi, Michael A Kravchenko, Kewal Krishan, Kristopher J Krohn, Hans Kromhout, Barthelemy Kuate Defo, Burcu Kucuk Bicer, G Anil Kumar, Manasi Kumar, Igor Kuzin, Hmwe Hmwe Kyu, Carl Lachat, Deepesh P Lad, Sheetal D Lad, Alessandra Lafranconi, Ratilal Lalloo, Tea Lallukka, Faris Hasan Lami, Justin J Lang, Van C Lansingh, Samantha Leigh Larson, Arman Latifi, Jeffrey V Lazarus, Paul H Lee, James Leigh, Mostafa Leili, Cheru Tesema Leshargie, Janni Leung, Miriam Levi, Sonia Lewycka, Shanshan Li, Yichong Li, Juan Liang, Xiaofeng Liang, Yu Liao, Misgan Legesse Liben, Lee-Ling Lim, Shai Linn, Shiwei Liu, Rakesh Lodha, Giancarlo Logroscino, Alan D Lopez, Stefan Lorkowski, Paulo A Lotufo, Rafael Lozano, Tim C D Lucas, Raimundas Lunevicius, Stefan Ma, Erlyn Rachelle King Macarayan, Ísis Eloah Machado, Fabiana Madotto, Hue Thi Mai, Marek Majdan, Reza Majdzadeh, Azeem Majeed, Reza Malekzadeh, Deborah Carvalho Malta, Abdullah A Mamun, Ana-Laura Manda, Helena Manguerra, Mohammad Ali Mansournia, Lorenzo Giovanni Mantovani, Joemer C Maravilla, Wagner Marcenes, Ashley Marks, Randall V Martin, Sheila C O Martins, Francisco Rogerlândio Martins-Melo, Winfried März, Melvin B Marzan, Benjamin Ballard Massenburg, Manu Raj Mathur, Prashant Mathur, Kunihiro Matsushita, Pallab K Maulik, Mohsen Mazidi, Colm McAlinden, John J McGrath, Martin McKee, Ravi Mehrotra, Kala M Mehta, Varshil Mehta, Toni Meier, Fantahun Ayenew Mekonnen, Yohannes A Melaku, Addisu Melese, Mulugeta Melku, Peter T N Memiah, Ziad A Memish, Walter Mendoza, Desalegn Tadese Mengistu, George A Mensah, Gert B M Mensink, Seid Tiku Mereta, Atte Meretoja, Tuomo J Meretoja, Tomislav Mestrovic, Haftay Berhane Mezgebe, Bartosz Miazgowski, Tomasz Miazgowski, Anoushka I Millear, Ted R Miller, Molly Katherine Miller-Petrie, G K Mini, Mojde Mirarefin, Andreea Mirica, Erkin M Mirrakhimov, Awoke Temesgen Misganaw, Habtamu Mitiku, Babak Moazen, Bahram Mohajer, Karzan Abdulmuhsin Mohammad, Moslem Mohammadi, Noushin Mohammadifard, Mousa Mohammadnia-Afrouzi, Shafiu Mohammed, Farnam Mohebi, Ali H Mokdad, Mariam Molokhia, Fatemeh Momeniha, Lorenzo Monasta, Yoshan Moodley, Ghobad Moradi, Maziar Moradi-Lakeh, Mehdi Moradinazar, Paula Moraga, Lidia Morawska, Joana Morgado-Da-Costa, Shane Douglas Morrison, Marilita M Moschos, Simin Mouodi, Seyyed Meysam Mousavi, Dariush Mozaffarian, Kalayu Brhane Mruts, Achenef Asmamaw Muche, Kindie Fentahun Muchie, Ulrich Otto Mueller, Oumer Sada Muhammed, Satinath Mukhopadhyay, Kate Muller, Kamarul Imran Musa, Ghulam Mustafa, Ashraf F Nabhan, Mohsen Naghavi, Aliya Naheed, Azin Nahvijou, Gurudatta Naik, Nitish Naik, Farid Najafi, Vinay Nangia, Jobert Richie Nansseu, Bruno Ramos Nascimento, Bruce Neal, Nahid Neamati, Ionut Negoi, Ruxandra Irina Negoi, Subas Neupane, Charles Richard James Newton, Josephine W Ngunjiri, Anh Quynh Nguyen, Grant Nguyen, Ha Thu Nguyen, Huong Lan Thi Nguyen, Huong Thanh Nguyen, Minh Nguyen, Nam Ba Nguyen, Emma Nichols, Jing Nie, Dina Nur Anggraini Ningrum, Yirga Legesse Nirayo, Nobuo Nishi, Molly R Nixon, Marzieh Nojomi, Shuhei Nomura, Ole F Norheim, Mehdi Noroozi, Bo Norrving, Jean Jacques Noubiap, Hamid Reza Nouri, Malihe Nourollahpour Shiadeh, Mohammad Reza Nowroozi, Elaine O Nsoesie, Peter S Nyasulu, Carla M Obermeyer, Christopher M Odell, Richard Ofori-Asenso, Felix Akpojene Ogbo, In-Hwan Oh, Olanrewaju Oladimeji, Andrew T Olagunju, Tinuke O Olagunju, Pedro R Olivares, Helen Elizabeth Olsen, Bolajoko Olubukunola Olusanya, Jacob Olusegun Olusanya, Kanyin L Ong, Sok King Ong, Eyal Oren, Heather M Orpana, Alberto Ortiz, Erika Ota, Stanislav S Otstavnov, Simon Øverland, Mayowa Ojo Owolabi, Mahesh P A, Rosana Pacella, Abhijit P Pakhare, Amir H Pakpour, Adrian Pana, Songhomitra Panda-Jonas, Eun-Kee Park, Charles D H Parry, Hadi Parsian, Shanti Patel, Sanghamitra Pati, Snehal T Patil, Ajay Patle, George C Patton, Deepak Paudel, Katherine R Paulson, Wayra Citlali Paz Ballesteros, Neil Pearce, Alexandre Pereira, David M Pereira, Norberto Perico, Konrad Pesudovs, Max Petzold, Hai Quang Pham, Michael R Phillips, Julian David Pillay, Michael A Piradov, Meghdad Pirsaheb, Tobias Pischon, Farhad Pishgar, Oleguer Plana-Ripoll, Dietrich Plass, Suzanne Polinder, Kevan R Polkinghorne, Maarten J Postma, Richie Poulton, Akram Pourshams, Hossein Poustchi, Dorairaj Prabhakaran, Swayam Prakash, Narayan Prasad, Caroline A Purcell, Manorama B Purwar, Mostafa Qorbani, Amir Radfar, Anwar Rafay, Alireza Rafiei, Fakher Rahim, Zohreh Rahimi, Afarin Rahimi-Movaghar, Vafa Rahimi-Movaghar, Mahfuzar Rahman, Mohammad Hifz ur Rahman, Muhammad Aziz Rahman, Rajesh Kumar Rai, Fatemeh Rajati, Sasa Rajsic, Sree Bhushan Raju, Usha Ram, Chhabi Lal Ranabhat, Prabhat Ranjan, Goura Kishor Rath, David Laith Rawaf, Salman Rawaf, K Srinath Reddy, Colin D Rehm, Jürgen Rehm, Robert C Reiner, Marissa B Reitsma, Giuseppe Remuzzi, Andre M N Renzaho, Serge Resnikoff, Luz Myriam Reynales-Shigematsu, Satar Rezaei, Antonio Luiz P Ribeiro, Juan A Rivera, Kedir Teji Roba, Sonia Rodríguez-Ramírez, Leonardo Roever, Yesenia Román, Luca Ronfani, Gholamreza Roshandel, Ali Rostami, Gregory A Roth, Dietrich Rothenbacher, Ambuj Roy, Enrico Rubagotti, Lesley Rushton, Charumathi Sabanayagam, Perminder S Sachdev, Basema Saddik, Ehsan Sadeghi, Sahar Saeedi Moghaddam, Hosein Safari, Yahya Safari, Roya Safari-Faramani, Mahdi Safdarian, Sare Safi, Saeid Safiri, Rajesh Sagar, Amirhossein Sahebkar, Mohammad Ali Sahraian, Haniye Sadat Sajadi, Nasir Salam, Payman Salamati, Zikria Saleem, Yahya Salimi, Hamideh Salimzadeh, Joshua A Salomon, Devashri Digvijay Salvi, Inbal Salz, Abdallah M Samy, Juan Sanabria, Maria Dolores Sanchez-Niño, Tania G Sánchez-Pimienta, Taren Sanders, Yingying Sang, Damian Francesco Santomauro, Itamar S Santos, João Vasco Santos, Milena M Santric Milicevic, Bruno Piassi Sao Jose, Mayank Sardana, Abdur Razzaque Sarker, Rodrigo Sarmiento-Suárez, Nizal Sarrafzadegan, Benn Sartorius, Shahabeddin Sarvi, Brijesh Sathian, Maheswar Satpathy, Arundhati R Sawant, Monika Sawhney, Mete Saylan, Mehdi Sayyah, Elke Schaeffner, Maria Inês Schmidt, Ione J C Schneider, Ben Schöttker, Aletta Elisabeth Schutte, David C Schwebel, Falk Schwendicke, James G Scott, Soraya Seedat, Mario Sekerija, Sadaf G Sepanlou, Marc L Serre, Edson Serván-Mori, Seyedmojtaba Seyedmousavi, Hosein Shabaninejad, Gavin Shaddick, Azadeh Shafieesabet, Mehdi Shahbazi, Amira A Shaheen, Masood Ali Shaikh, Teresa Shamah Levy, Mehran Shams-Beyranvand, Mohammadbagher Shamsi, Heidar Sharafi, Kiomars Sharafi, Mehdi Sharif, Mahdi Sharif-Alhoseini, Hamid Sharifi, Jayendra Sharma, Meenakshi Sharma, Rajesh Sharma, Jun She, Aziz Sheikh, Peilin Shi, Kenji Shibuya, Mekonnen Sisay Shiferaw, Mika Shigematsu, Min-Jeong Shin, Rahman Shiri, Reza Shirkoohi, Ivy Shiue, Farhad Shokraneh, Haitham Shoman, Mark G Shrime, Matthew S Shupler, Si Si, Soraya Siabani, Abla Mehio Sibai, Tariq J Siddiqi, Inga Dora Sigfusdottir, Rannveig Sigurvinsdottir, Diego Augusto Santos Silva, João Pedro Silva, Dayane Gabriele Alves Silveira, Jasvinder A Singh, Narinder Pal Singh, Virendra Singh, Dhirendra Narain Sinha, Eirini Skiadaresi, Vegard Skirbekk, David L Smith, Mari Smith, Badr Hasan Sobaih, Soheila Sobhani, Ranjani Somayaji, Moslem Soofi, Reed J D Sorensen, Joan B Soriano, Ireneous N Soyiri, Angela Spinelli, Luciano A Sposato, Chandrashekhar T Sreeramareddy, Vinay Srinivasan, Vladimir I Starodubov, Nadine Steckling, Dan J Stein, Murray B Stein, Goran Stevanovic, Leo Stockfelt, Mark A Stokes, Lela Sturua, Michelle L Subart, Agus Sudaryanto, Mu'awiyyah Babale Sufiyan, Gerhard Sulo, Bruno F Sunguya, Patrick John Sur, Bryan L Sykes, Cassandra E I Szoeke, Rafael Tabarés-Seisdedos, Takahiro Tabuchi, Santosh Kumar Tadakamadla, Ken Takahashi, Nikhil Tandon, Segen Gebremeskel Tassew, Mohammad Tavakkoli, Nuno Taveira, Arash Tehrani-Banihashemi, Tigist Gashaw Tekalign, Shishay Wahdey Tekelemedhin, Merhawi Gebremedhin Tekle, Habtamu Temesgen, Mohamad-Hani Temsah, Omar Temsah, Abdullah Sulieman Terkawi, Belay Tessema, Mebrahtu Teweldemedhin, Kavumpurathu Raman Thankappan, Andrew Theis, Sathish Thirunavukkarasu, Hannah J Thomas, Matthew Lloyd Thomas, Nihal Thomas, George D Thurston, Binyam Tilahun, Taavi Tillmann, Quyen G To, Myriam Tobollik, Marcello Tonelli, Roman Topor-Madry, Anna E Torre, Miguel Tortajada-Girbés, Mathilde Touvier, Marcos Roberto Tovani-Palone, Jeffrey A Towbin, Bach Xuan Tran, Khanh Bao Tran, Thomas Clement Truelsen, Nu Thi Truong, Afewerki Gebremeskel Tsadik, Lorainne Tudor Car, E Murat Tuzcu, Hayley D Tymeson, Stefanos Tyrovolas, Kingsley N Ukwaja, Irfan Ullah, Rachel L Updike, Muhammad Shariq Usman, Olalekan A Uthman, Muthiah Vaduganathan, Afsane Vaezi, Pascual R Valdez, Aaron Van Donkelaar, Elena Varavikova, Santosh Varughese, Tommi Juhani Vasankari, Vidhya Venkateswaran, Narayanaswamy Venketasubramanian, Santos Villafaina, Francesco S Violante, Sergey Konstantinovitch Vladimirov, Vasily Vlassov, Stein Emil Vollset, Theo Vos, Kia Vosoughi, Giang Thu Vu, Isidora S Vujcic, Fasil Shiferaw Wagnew, Yasir Waheed, Stephen G Waller, Judd L Walson, Yafeng Wang, Yanping Wang, Yuan-Pang Wang, Elisabete Weiderpass, Robert G Weintraub, Fitsum Weldegebreal, Andrea Werdecker, Adhena Ayaliew Werkneh, J Jason West, Ronny Westerman, Harvey A Whiteford, Justyna Widecka, Tissa Wijeratne, Andrea Sylvia Winkler, Alison B Wiyeh, Charles Shey Wiysonge, Charles D A Wolfe, Tien Yin Wong, Shouling Wu, Denis Xavier, Gelin Xu, Simon Yadgir, Ali Yadollahpour, Seyed Hossein Yahyazadeh Jabbari, Tomohide Yamada, Lijing L Yan, Yuichiro Yano, Mehdi Yaseri, Yasin Jemal Yasin, Alex Yeshaneh, Ebrahim M Yimer, Paul Yip, Engida Yisma, Naohiro Yonemoto, Seok-Jun Yoon, Marcel Yotebieng, Mustafa Z Younis, Mahmoud Yousefifard, Chuanhua Yu, Zoubida Zaidi, Sojib Bin Zaman, Mohammad Zamani, Luis Zavala-Arciniega, Anthony Lin Zhang, Hao Zhang, Kai Zhang, Maigeng Zhou, Stephanie R M Zimsen, Sanjay Zodpey, Christopher J L Murray

## Abstract

**Background:**

The Global Burden of Diseases, Injuries, and Risk Factors Study (GBD) 2017 comparative risk assessment (CRA) is a comprehensive approach to risk factor quantification that offers a useful tool for synthesising evidence on risks and risk–outcome associations. With each annual GBD study, we update the GBD CRA to incorporate improved methods, new risks and risk–outcome pairs, and new data on risk exposure levels and risk–outcome associations.

**Methods:**

We used the CRA framework developed for previous iterations of GBD to estimate levels and trends in exposure, attributable deaths, and attributable disability-adjusted life-years (DALYs), by age group, sex, year, and location for 84 behavioural, environmental and occupational, and metabolic risks or groups of risks from 1990 to 2017. This study included 476 risk–outcome pairs that met the GBD study criteria for convincing or probable evidence of causation. We extracted relative risk and exposure estimates from 46 749 randomised controlled trials, cohort studies, household surveys, census data, satellite data, and other sources. We used statistical models to pool data, adjust for bias, and incorporate covariates. Using the counterfactual scenario of theoretical minimum risk exposure level (TMREL), we estimated the portion of deaths and DALYs that could be attributed to a given risk. We explored the relationship between development and risk exposure by modelling the relationship between the Socio-demographic Index (SDI) and risk-weighted exposure prevalence and estimated expected levels of exposure and risk-attributable burden by SDI. Finally, we explored temporal changes in risk-attributable DALYs by decomposing those changes into six main component drivers of change as follows: (1) population growth; (2) changes in population age structures; (3) changes in exposure to environmental and occupational risks; (4) changes in exposure to behavioural risks; (5) changes in exposure to metabolic risks; and (6) changes due to all other factors, approximated as the risk-deleted death and DALY rates, where the risk-deleted rate is the rate that would be observed had we reduced the exposure levels to the TMREL for all risk factors included in GBD 2017.

**Findings:**

In 2017, 34·1 million (95% uncertainty interval [UI] 33·3–35·0) deaths and 1·21 billion (1·14–1·28) DALYs were attributable to GBD risk factors. Globally, 61·0% (59·6–62·4) of deaths and 48·3% (46·3–50·2) of DALYs were attributed to the GBD 2017 risk factors. When ranked by risk-attributable DALYs, high systolic blood pressure (SBP) was the leading risk factor, accounting for 10·4 million (9·39–11·5) deaths and 218 million (198–237) DALYs, followed by smoking (7·10 million [6·83–7·37] deaths and 182 million [173–193] DALYs), high fasting plasma glucose (6·53 million [5·23–8·23] deaths and 171 million [144–201] DALYs), high body-mass index (BMI; 4·72 million [2·99–6·70] deaths and 148 million [98·6–202] DALYs), and short gestation for birthweight (1·43 million [1·36–1·51] deaths and 139 million [131–147] DALYs). In total, risk-attributable DALYs declined by 4·9% (3·3–6·5) between 2007 and 2017. In the absence of demographic changes (ie, population growth and ageing), changes in risk exposure and risk-deleted DALYs would have led to a 23·5% decline in DALYs during that period. Conversely, in the absence of changes in risk exposure and risk-deleted DALYs, demographic changes would have led to an 18·6% increase in DALYs during that period. The ratios of observed risk exposure levels to exposure levels expected based on SDI (O/E ratios) increased globally for unsafe drinking water and household air pollution between 1990 and 2017. This result suggests that development is occurring more rapidly than are changes in the underlying risk structure in a population. Conversely, nearly universal declines in O/E ratios for smoking and alcohol use indicate that, for a given SDI, exposure to these risks is declining. In 2017, the leading Level 4 risk factor for age-standardised DALY rates was high SBP in four super-regions: central Europe, eastern Europe, and central Asia; north Africa and Middle East; south Asia; and southeast Asia, east Asia, and Oceania. The leading risk factor in the high-income super-region was smoking, in Latin America and Caribbean was high BMI, and in sub-Saharan Africa was unsafe sex. O/E ratios for unsafe sex in sub-Saharan Africa were notably high, and those for alcohol use in north Africa and the Middle East were notably low.

**Interpretation:**

By quantifying levels and trends in exposures to risk factors and the resulting disease burden, this assessment offers insight into where past policy and programme efforts might have been successful and highlights current priorities for public health action. Decreases in behavioural, environmental, and occupational risks have largely offset the effects of population growth and ageing, in relation to trends in absolute burden. Conversely, the combination of increasing metabolic risks and population ageing will probably continue to drive the increasing trends in non-communicable diseases at the global level, which presents both a public health challenge and opportunity. We see considerable spatiotemporal heterogeneity in levels of risk exposure and risk-attributable burden. Although levels of development underlie some of this heterogeneity, O/E ratios show risks for which countries are overperforming or underperforming relative to their level of development. As such, these ratios provide a benchmarking tool to help to focus local decision making. Our findings reinforce the importance of both risk exposure monitoring and epidemiological research to assess causal connections between risks and health outcomes, and they highlight the usefulness of the GBD study in synthesising data to draw comprehensive and robust conclusions that help to inform good policy and strategic health planning.

**Funding:**

Bill & Melinda Gates Foundation.

## Introduction

The environmental, behavioural, and metabolic risks that drive injury and disease are the mechanisms by which public health efforts can most efficiently and effectively prevent health loss. Effecting population health improvements, therefore, requires understanding of not only the injuries and diseases that drive health burdens, but also the risks that drive injury and disease. Through a constantly evolving collection of cohort studies, randomised trials, and case-control studies, decades of epidemiological research have worked to quantify the nature and magnitude of associations between risk exposures and outcomes in studied populations. Moving beyond individual studies of individual populations, this raw evidence can be synthesised to draw the comprehensive and robust conclusions that are necessary to inform good public health policy. The Global Burden of Diseases, Injuries, and Risk Factors Study (GBD) comparative risk assessment (CRA) is a comprehensive and comparable approach to risk factor quantification that offers a useful tool for synthesising evidence on risks and risk–outcome associations. With each annual GBD, we update the GBD CRA to incorporate new data on risk–outcome pairs, risk exposure levels, and risk–outcome associations.

**Research in context**

**Evidence before the study**

Population-level estimates of individual risks have been produced periodically by both WHO and UNICEF, whereas independent scientific publications provide risk estimates that are limited in the number of risks assessed and population size evaluated. Since 2010, the Global Burden of Diseases, Injuries, and Risk Factors Study (GBD) has produced comprehensive assessments of risk factor burden by age, sex, cause, and location. The previous iteration of this study, GBD 2016, assessed 84 behavioural, environmental and occupational, and metabolic risks between 1990 and 2016, with major updates in the assessment of second-hand smoke, alcohol use, and diet. The GBD study remains the only peer-reviewed, comprehensive, and annual assessment of risk factor burden by age, sex, cause, location, and year that complies with the Guidelines for Accurate and Transparent Health Estimates Reporting.

**Added value of this study**

GBD 2017 expands the scope of GBD 2016 with the estimation of one new risk factor—bullying victimisation—and 80 new risk–outcome pairs, with a total of 476 risk–outcome pairs. GBD 2017 incorporates 46 749 sources. We have expanded our estimation locations with the addition of subnational locations for Ethiopia, Iran, Norway, and Russia, and estimates for Māori and non-Māori populations in New Zealand. We implemented broad improvements to methods to better estimate risk factor exposures and relative risks. Notably, we have moved from total cholesterol to low-density lipoprotein cholesterol, implemented continuous measures of exposure for smoking, and updated the ambient particulate matter pollution model with new ground measurement data from almost 4000 sites. We expanded upon our decomposition analyses to investigate the drivers of risk-attributable burden and the changes in burden by country, and to decompose risk-attributable changes between broad categories of risks, thus providing deeper insight into changing patterns of risk-attributable burden and their underlying causes. We broadened our analyses of geographical and temporal trends in risk exposure and burden by estimating expected risk-weighted prevalence of exposures based on Socio-demographic Index. We explored the observed relationship between development status and risk exposure across all locations and years, and for the first time we described spatiotemporal patterns in the ratio of observed-to-expected levels of risk exposure.

**Implications of all the available evidence**

Decomposing trends by their underlying drivers reveals improvements in risk-deleted burden (ie, burden not attributed to risks in the GBD analysis), and broadly, improvements in exposure to environmental and behavioural risks. Conversely, increasing exposure to metabolic risks is driving increases in burden, indicating a crucial need for risk mitigation policies in this area. By quantifying the relationship between development and risk exposure, we highlight which risks appear sensitive to development and, of those, which are likely to improve or worsen with development. This analysis highlights areas where countries are either overperforming or underperforming relative to their economic peers and provides insight into areas where risk-modification strategies might be the best targets to improve health.

Previous GBD studies have assessed the relationship between development, as measured by the Socio-demographic Index (SDI), and both the magnitude and composition of disease burden.^1^, ^2^, ^3^, ^4^ The results of those analyses highlighted the dramatic declines in communicable, maternal, neonatal, and nutritional diseases (CMNNDs) that have generally occurred with increases in socioeconomic development as well as the subsequent increases in life expectancy and absolute burden of non-communicable diseases (NCDs)—a pattern referred to as the epidemiological transition. Previous GBD analyses also estimated the expected burden for each cause in every location and year, based on that location's SDI. The comparison of observed burden to the burden expected based on SDI offered insight into the relative performance of countries at similar levels of development. Here, we extend those methods to analyse epidemiological transition with regards to risk exposure and risk-attributable burden. This analysis allows the identification of risks that are positively associated with development, negatively associated with development, or independent of development status. By estimating the levels of risk exposure and risk-attributable burden on the basis of SDI, and comparing these expectations to observed levels, it is possible to identify locations that either underperform or overperform compared with similarly developed countries.

The GBD 2017 CRA includes 84 risk factors and 476 associated risk–outcome pairs. We expanded the scope of GBD 2016 with the inclusion of 80 new outcomes for existing risks and one new risk factor: bullying victimisation. The study provides estimates of exposure and attributable deaths and disability-adjusted life-years (DALYs) for 195 countries and territories for 1990 through 2017, including new subnational estimates for Ethiopia, Iran, New Zealand, Norway, and Russia. We explored changes in risk-attributable DALYs by decomposing those changes into six main component drivers of change, explored the relationship between risk exposure and SDI, and estimated the ratio of observed-to-expected levels of exposure and risk-attributable burden by SDI. As with previous iterations of GBD, the GBD 2017 CRA results presented here supersede all previously published GBD CRA estimates.

**Key messages**

• The Global Burden of Diseases, Injuries, and Risk Factors Study (GBD) 2017 expands on GBD 2016 with the estimation of one new risk factor—bullying victimisation—and 80 new risk–outcome pairs, making a total of 476 risk–outcome pairs. The study further investigates the drivers of changes in risk-attributable burden and explores the relationship between development and risk exposure.
• In 2017, 34·1 million (95% uncertainty interval [UI] 33·3–35·0) deaths and 1·21 billion (1·14–1·28) disability-adjusted life-years (DALYs) were attributable to risk factors included in GBD 2017. All included risks combined contributed to 61·0% (59·6–62·4) of deaths and 48·3% (46·3–50·2) of DALYs worldwide.
• The five leading risks in 2017 were high systolic blood pressure, smoking, high fasting plasma glucose, high body-mass index, and short gestation for birthweight.
• DALY-based ranks for all metabolic risks increased between 1990 and 2017 for both males and females. Consequently, four of the five leading risks were behavioural risks in 1990, whereas three of the five leading risks were metabolic risks in 2017.
• Between 2007 and 2017, the absolute number of risk-attributable DALYs declined by 3·44% (95% UI 2·47–4·40). During that period, exposures to behavioural, environmental, and occupational risks declined (improved), but these gains were somewhat offset by increases in exposure to metabolic risks, population growth, and population ageing.
• Socioeconomic development was strongly associated with exposure levels for many risks. Among the leading risks, unsafe water, household air pollution, and child wasting show pronounced decreasing trends with development. Conversely, smoking, alcohol use, drug use, and high low-density lipoprotein cholesterol all show a pronounced increasing trend with development.

## Methods

### Overview

The CRA conceptual framework was developed by Murray and Lopez,^5^ who established a causal web of hierarchically organised risks that contribute to health outcomes and facilitate the quantification of risks at any level in the framework. In GBD 2017, as in previous GBDs, we assessed a set of behavioural, environmental or occupational, and metabolic risks that were organised into five hierarchical levels (appendix 1 section 5). At Level 0, GBD 2017 reports estimates for all risk factors combined. Nested within Level 0, Level 1 includes three risk categories: environmental and occupational, metabolic, and behavioural risk factors. This hierarchical structure continues, with each subsequent level including more detailed risks factors that are nested within the broader category above it. There are 19 risks at Level 2, 39 risks at Level 3, and 22 risks at Level 4, for a total of 84 risks or risk groups, where all risks (Level 0) is included as a risk group. Although we have added bullying as a new risk factor, the total number of risk factors remains unchanged from GBD 2016 because of the merging of two risk factors: we previously estimated second-hand smoke and occupational exposure to second-hand smoke as two separate risks but have incorporated the two exposures into one second-hand smoke Level 3 risk for GBD 2017. Each risk factor is associated with an outcome or outcomes, and each combination of risk and outcome included in the GBD is referred to as a risk–outcome pair. Risk–outcome pairs were included on the basis of evidence rules (appendix 1 section 5). To date, we have not quantified the contribution of distal social, cultural, and economic risk factors; however, our analysis of the relationship between risk exposures and sociodemographic development, measured with SDI, offers insights into the relationship between economic context and risk factors.

This analysis largely follows the CRA methods used in GBD 2016.^2^ Given the scope of the analysis, we offer a high-level overview of the study methods and analytical logic, detailing areas of notable change and innovation since GBD 2016 and include risk-specific details in appendix 1 (section 4). This study complies with the Guidelines for Accurate and Transparent Health Estimates Reporting statement^6^ (appendix 1 section 5).

### Geographical units of analysis and years for estimation

For GBD 2017, we have estimated risk factor exposure and attributable burden by age, sex, cause, and location from 1990 to 2017. GBD locations are arranged in a nested hierarchy: 195 countries and territories are within 21 regions and these 21 regions are within seven super-regions. Each year, GBD includes subnational analyses for a few new countries and continues to provide subnational estimates for countries that were added in previous cycles. Subnational estimation in GBD 2017 includes five new countries (Ethiopia, Iran, New Zealand, Norway, Russia) and countries previously estimated at subnational levels (GBD 2013: China, Mexico, and the UK [regional level]; GBD 2015: Brazil, India, Japan, Kenya, South Africa, Sweden, and the USA; GBD 2016: Indonesia and the UK [local government authority level]). All analyses are at the first level of administrative organisation within each country except for New Zealand (by Māori ethnicity), Sweden (by Stockholm and non-Stockholm), and the UK (by local government authorities). All subnational estimates for these countries were incorporated into model development and evaluation as part of GBD 2017. To meet data use requirements, in this publication we present all subnational estimates excluding those pending publication (Brazil, India, Japan, Kenya, Mexico, Sweden, the UK, and the USA; appendix 2). Subnational estimates for countries with populations larger than 200 million (measured using the most recent year of published estimates) that have not yet been published elsewhere are presented wherever estimates are illustrated with maps but are not included in data tables.

### Attributable burden estimation

Four components were used for the calculations to estimate the attributable burden for a given risk–outcome pair: (1) the estimate of the burden metric being assessed for the cause (ie, number of deaths, years of life lost [YLLs], years lived with disability [YLDs], or DALYs); (2) the exposure levels for the risk factor; (3) the counterfactual level of risk factor exposure or theoretical minimum risk exposure level (TMREL); and (4) the relative risk of the outcome relative to the TMREL. For a given risk–outcome pair, we estimated attributable DALYs as total DALYs for the outcome multiplied by the population attributable fraction (PAF) for the risk–outcome pair for each age, sex, location, and year. The same logic applies to estimating attributable deaths, YLLs, and YLDs. The PAF is the proportion by which the outcome would be reduced in a given population and in a given year if the exposure to a risk factor in the past were reduced to the counterfactual level of the TMREL. The PAF for each individual risk–outcome pair is estimated independently and incorporates all burden for the outcome that is attributable to the risk, whether directly or indirectly. For example, the burden of ischaemic heart disease attributable to high body-mass index (BMI) includes the burden resulting from the direct effect of BMI on ischaemic heart disease risk, as well as the burden through the effects of BMI on ischaemic heart disease that are mediated through other risks (eg, high systolic blood pressure [SBP] and high low-density lipoprotein [LDL] cholesterol). When aggregating PAFs across multiple risks we used a mediation adjustment to compute the excess attenuated risk for each of 205 mediation-risk-cause sets (appendix 1 section 5).

### Estimation process

Information about the data sources, estimation methods, computational tools, and statistical analyses used to derive our estimates are provided in appendix 1 (sections 1–4). The analytical steps for estimating the burden attributable to single or clusters of risk–outcome pairs are summarised in the appendix 1 (section 2). Table 1 provides definitions of exposure for each risk factor and the TMREL used. Although the approach taken is largely similar to GBD 2016, we have implemented improvements to methods and incorporated new data sources. Appendix 1 (section 4) details each analytical step by risk. Citation information for the data sources used for relative risks is provided in an online source tool.

**Table 1:** GBD 2017 risk factor hierarchy and accompanying exposure definitions, theoretical minimum risk exposure level, and data representativeness index for each risk factor, pre-2007, 2007–17, and total (across all years)

			**Risk factors**	**Exposure definition**	**Theoretical minimum risk exposure level**	**Data representativeness index**		
						Before 2007	2007–17	Total
**0**	**All**			**..**	**..**	**100·0%**	**100·0%**	**100·0%**
**1**	**Environmental and occupational risks**			**..**	**..**	**100·0%**	**100·0%**	**100·0%**
**2**		**Unsafe water, sanitation, and handwashing**		**..**	**..**	**80·3%**	**63·7%**	**82·4%**
3		Unsafe water source		Proportion of individuals with access to different water sources (unimproved, improved except piped, or piped water supply) and reported use of household water treatment methods (boiling or filtering, chlorinating or solar filtering, or no treatment)	All individuals have access to water from a piped water supply that is also boiled or filtered before drinking	78·2%	61·1%	79·8%
3		Unsafe sanitation		Proportion of individuals with access to different sanitation facilities (unimproved, improved except sewer, or sewer connection)	All individuals have access to toilets with sewer connection	75·7%	54·9%	78·8%
3		No access to handwashing facility		Proportion of individuals with access to handwashing facility with soap, water, and wash station	All individuals have access to handwashing facility with soap, water, and wash station	13·5%	34·7%	39·4%
**2**	**Air pollution**			**..**	**..**	**100·0%**	**100·0%**	**100·0%**
3	Particulate matter pollution			..	..	82·9%	88·6%	96·4%
4		Ambient particulate matter pollution		Annual average daily exposure to outdoor air concentrations of particulate matter with an aerodynamic diameter of ≤2·5 μm (PM_2·5_), measured in μg/m^3^	Joint theoretical minimum risk exposure level for both household and ambient particulate matter pollution is a uniform distribution between 2·4 and 5·9 μg/m^3^, with burden attributed proportionally between household and particulate matter pollution on the basis of source of PM_2·5_ exposure in excess of theoretical minimum risk exposure level	17·1%	57·0%	58·0%
4		Household air pollution from solid fuels		Individual exposure to PM_2·5_ due to use of solid cooking fuel	See ambient particulate matter pollution	82·9%	63·4%	85·5%
3	Ambient ozone pollution			Seasonal (6-month period with highest ozone) 8-h daily maximum ozone concentrations, measured in ppb	Uniform distribution between 29·1 and 35·7 ppb	100·0%	100·0%	100·0%
**2**	**Other environmental risks**			**..**	**..**	**47·2%**	**30·1%**	**48·7%**
3	Residential radon			Average daily exposure to indoor air radon levels measured in becquerels (radon disintegrations per second) per cubic metre (Bq/m^3^)	10 Bq/m^3^, corresponding to the outdoor concentration of radon	36·8%	8·8%	36·8%
3	Lead exposure			Blood lead levels in μg/dL of blood, bone lead levels in μg/g of bone	2 μg/dL, corresponding to lead levels in pre-industrial humans as natural sources of lead prevent the feasibility of zero exposure	35·8%	26·9%	40·9%
**2**		**Occupational risks**		**..**	**..**	**100·0%**	**100·0%**	**100·0%**
3		Occupational carcinogens		..	..	100·0%	100·0%	100·0%
4			Occupational exposure to asbestos	Proportion of the population with cumulative lifetime exposure to occupational asbestos	No occupational exposure to asbestos	100·0%	100·0%	100·0%
4			Occupational exposure to arsenic	Proportion of the population ever exposed to arsenic at work or through their occupation	No occupational exposure to arsenic	100·0%	100·0%	100·0%
4			Occupational exposure to benzene	Proportion of the population ever exposed to benzene at work or through their occupation	No occupational exposure to benzene	100·0%	100·0%	100·0%
4			Occupational exposure to beryllium	Proportion of the population ever exposed to beryllium at work or through their occupation	No occupational exposure to beryllium	100·0%	100·0%	100·0%
4			Occupational exposure to cadmium	Proportion of the population ever exposed to cadmium at work or through their occupation	No occupational exposure to cadmium	100·0%	100·0%	100·0%
4			Occupational exposure to chromium	Proportion of the population ever exposed to chromium at work or through their occupation	No occupational exposure to chromium	100·0%	100·0%	100·0%
4			Occupational exposure to diesel engine exhaust	Proportion of the population ever exposed to diesel engine exhaust at work or through their occupation	No occupational exposure to diesel engine exhaust	100·0%	100·0%	100·0%
4			Occupational exposure to formaldehyde	Proportion of the population ever exposed to formaldehyde at work or through their occupation	No occupational exposure to formaldehyde	100·0%	100·0%	100·0%
4			Occupational exposure to nickel	Proportion of the population ever exposed to nickel at work or through their occupation	No occupational exposure to nickel	100·0%	100·0%	100·0%
4			Occupational exposure to polycyclic aromatic hydrocarbons	Proportion of the population ever exposed to polycyclic aromatic hydrocarbons at work or through their occupation	No occupational exposure to polycyclic aromatic hydrocarbons	100·0%	100·0%	100·0%
4			Occupational exposure to silica	Proportion of the population ever exposed to silica at work or through their occupation	No occupational exposure to silica	100·0%	100·0%	100·0%
4			Occupational exposure to sulphuric acid	Proportion of the population ever exposed to sulphuric acid at work or through their occupation	No occupational exposure to sulphuric acid	100·0%	100·0%	100·0%
4			Occupational exposure to trichloroethylene	Proportion of the population ever exposed to trichloroethylene at work or through their occupation	No occupational exposure to trichloroethylene	100·0%	100·0%	100·0%
3		Occupational asthmagens		Proportion of the population currently exposed to asthmagens at work or through their occupation	Background asthmagen exposures	88·1%	82·9%	91·2%
3		Occupational particulate matter, gases, and fumes		Proportion of the population ever exposed to particulates, gases, or fumes at work or through their occupation	No occupational exposure to particulates, gases, or fumes	86·5%	81·9%	89·6%
3		Occupational noise		Proportion of the population ever exposed to noise greater than 85 decibels at work or through their occupation	Background noise exposure	86·5%	81·0%	89·6%
3		Occupational injuries		Proportion of the population at risk to injuries related to work or through their occupation	The rate of injury deaths per 100 000 person-years is zero	88·1%	82·9%	92·2%
3		Occupational ergonomic factors		Proportion of the population who are exposed to ergonomic risk factors for low back pain at work or through their occupation	All individuals have the ergonomic factors of clerical and related workers	84·5%	81·9%	89·6%
**1**	**Behavioural risks**			**..**	**..**	**100·0%**	**100·0%**	**100·0%**
**2**		**Child and maternal malnutrition**		**..**	**..**	**98·5%**	**97·4%**	**98·5%**
3		Suboptimal breastfeeding		..	..	75·1%	60·6%	83·4%
4			Non-exclusive breastfeeding	Proportion of children younger than 6 months who receive predominant, partial, or no breastfeeding	All children are exclusively breastfed for first 6 months of life	75·1%	60·6%	83·4%
4			Discontinued breastfeeding	Proportion of children aged 6–23 months who do not receive any breast milk	All children continue to receive breast milk until 2 years of age	75·1%	60·6%	83·4%
3		Child growth failure		..	..	76·2%	65·3%	77·2%
4			Child underweight	Proportion of children ≥3 SDs, 2–3 SDs, and 1–2 SDs lower than the WHO 2006 standard weight-for-age curve	All children are <1 SD below the WHO 2006 standard weight-for-age curve	75·1%	63·7%	76·7%
4			Child wasting	Proportion of children ≥3 SDs, 2–3 SDs, and 1–2 SDs lower than the WHO 2006 standard weight-for-length curve	All children are <1 SD below the WHO 2006 standard weight-for-height curve	75·1%	65·3%	77·2%
4			Child stunting	Proportion of children ≥3 SDs, 2–3 SDs, and 1–2 SDs lower than the WHO 2006 standard height-for-age curve	All children are <1 SD below the WHO 2006 standard height-for-age curve	75·1%	64·8%	77·2%
3		Low birthweight and short gestation		..	..	75·7%	78·2%	86·0%
4			Low birthweight for gestation	Proportion of births occurring in 2-week gestational age categories from [0–24) weeks to [40–42) weeks, for each 500-g birthweight category starting from [0–500) g to [4000–4500) g*	500-g birthweight category with lowest risk within each gestational age category	75·7%	78·2%	86·0%
4			Short gestation for birthweight	Proportion of births occurring in 500-g birthweight categories from [0–500) g to [4000–4500) g, for each 2-week gestational age category starting from [0–24) weeks to [40–42) weeks*	2-week gestational age category with lowest risk within each birthweight category	75·7%	78·2%	86·0%
3		Iron deficiency		Peripheral blood haemoglobin concentration in g/L for all iron-responsive causes	Counterfactual haemoglobin concentration in the absence of iron deficiency in g/L for all iron-responsive causes	75·1%	78·2%	86·0%
3		Vitamin A deficiency		Proportion of children aged 0–5 years with serum retinol concentration <0·7 μmol/L	No childhood vitamin A deficiency	63·7%	43·5%	64·8%
3		Zinc deficiency		Proportion of the population with inadequate zinc intake versus loss	No inadequate zinc intake	92·2%	92·2%	92·2%
**2**		**Tobacco**		**..**	**..**	**99·0%**	**99·0%**	**100·0%**
3		Smoking		Prevalence of current use of any smoked tobacco product and prevalence of former use of any smoked tobacco product; among current smokers, cigarette equivalents smoked per smoker per day and cumulative pack-years of exposure; among former smokers, number of years since quitting	All individuals are lifelong non-smokers	98·5%	98·5%	99·5%
3		Chewing tobacco		Current use of any chewing tobacco product	All individuals are lifelong non-users of chewing tobacco products	33·2%	70·5%	73·6%
3		Second-hand smoke		Average daily exposure to air particulate matter from second-hand smoke with an aerodynamic diameter smaller than 2·5 μg, measured in μg/m^3^, among non-smokers	No second-hand smoke exposure	80·3%	73·1%	88·1%
**2**		**Alcohol use**		**Average daily alcohol consumption of pure alcohol (measured in g per day) in current drinkers who had consumed alcohol during the past 12 months**	**Estimated distribution 0–10 g per day**	**52·3%**	**33·2%**	**59·6%**
**2**		**Drug use**		**Proportion of the population dependent upon opioids, cannabis, cocaine, or amphetamines; proportion of the population who have ever injected drugs**	**No drug use**	**17·6%**	**30·1%**	**39·4%**
**2**		**Dietary risks**		**..**	**..**	**100·0%**	**100·0%**	**100·0%**
3		Diet low in fruits		Average daily consumption of fruits (fresh, frozen, cooked, canned, or dried, excluding fruit juices and salted or pickled fruits)	Consumption of fruit 200–300 g per day	68·9%	38·3%	78·8%
3		Diet low in vegetables		Average daily consumption of vegetables (fresh, frozen, cooked, canned, or dried, excluding legumes and salted or pickled vegetables, juices, nuts and seeds, and starchy vegetables such as potatoes or corn)	Consumption of vegetables 290–430 g per day	100·0%	100·0%	100·0%
3		Diet low in legumes		Average daily consumption of legumes (fresh, frozen, cooked, canned, or dried legumes)	Consumption of legumes 50–70 g per day	100·0%	100·0%	100·0%
3		Diet low in whole grains		Average daily consumption of whole grains (bran, germ, and endosperm in their natural proportion) from breakfast cereals, bread, rice, pasta, biscuits, muffins, tortillas, pancakes, and other sources	Consumption of whole grains 100–150 g per day	58·6%	28·0%	68·9%
3		Diet low in nuts and seeds		Average daily consumption of nut and seed foods	Consumption of nuts and seeds 16–25 g per day	100·0%	100·0%	100·0%
3		Diet low in milk		Average daily consumption of milk, including non-fat, low-fat, and full-fat milk, excluding soy milk and other plant derivatives	Consumption of milk 350–520 g per day	100·0%	100·0%	100·0%
3		Diet high in red meat		Average daily consumption of red meat (beef, pork, lamb, and goat but excluding poultry, fish, eggs, and all processed meats)	Consumption of red meat 18–27 g per day	100·0%	100·0%	100·0%
3		Diet high in processed meat		Average daily consumption of meat preserved by smoking, curing, salting, or addition of chemical preservatives	Consumption of processed meat 0–4 g per day	100·0%	100·0%	100·0%
3		Diet high in sugar-sweetened beverages		Average daily consumption of beverages with ≥50 kcal per 226·8 g serving, including carbonated beverages, sodas, energy drinks, fruit drinks, but excluding 100% fruit and vegetable juices	Consumption of sugar-sweetened beverages 0–5 g per day	13·0%	16·1%	26·9%
3		Diet low in fibre		Average daily intake of fibre from all sources including fruits, vegetables, grains, legumes, and pulses	Consumption of fibre 19–28 g per day	100·0%	100·0%	100·0%
3		Diet low in calcium		Average daily intake of calcium from all sources, including milk, yogurt, and cheese	Consumption of calcium 1·0–1·5 g per day	100·0%	100·0%	100·0%
3		Diet low in seafood omega 3 fatty acids		Average daily intake of eicosapentaenoic acid and docosahexaenoic acid	Consumption of seafood omega 3 fatty acids 200–300 mg per day	100·0%	100·0%	100·0%
3		Diet low in polyunsaturated fatty acids		Average daily intake of omega 6 fatty acids from all sources, mainly liquid vegetable oils, including soybean oil, corn oil, and safflower oil	Consumption of polyunsaturated fatty acids as 9–13% of total daily energy	61·1%	31·1%	67·9%
3		Diet high in trans fatty acids		Average daily intake of trans fat from all sources, mainly partially hydrogenated vegetable oils and ruminant products	Consumption of trans fatty acids as 0–1% of total daily energy	35·8%	36·8%	36·8%
3		Diet high in sodium		24-h urinary sodium measured in g per day	24-h urinary sodium 1–5 g per day	13·5%	17·6%	21·8%
**2**		**Intimate partner violence**		**Proportion of the population who have ever experienced one or more acts of physical or sexual violence by a present or former intimate partner since age 15 years**	**No intimate partner violence**	**65·8%**	**70·5%**	**84·5%**
**2**		**Childhood maltreatment**		**..**	**..**	**44·6%**	**62·2%**	**70·5%**
3		Childhood sexual abuse		Proportion of the population ever having had the experience of intercourse or other contact abuse (ie, fondling and other sexual touching) when aged 15 years or younger, and the perpetrator or partner was more than 5 years older than the victim	No childhood sexual abuse	31·1%	20·7%	38·9%
3		Bullying victimisation		Proportion of population attending school who have been exposed to bullying victimisation within the past year	No bullying victimisation	26·4%	52·3%	58·6%
**2**		**Unsafe sex**		**Proportion of the population with exposure to sexual encounters that convey the risk of disease**	**No exposure to disease-causing pathogen through sex**	**18·7%**	**49·2%**	**50·3%**
**2**		**Low physical activity**		**Average weekly physical activity at work, home, transport-related and recreational measured by MET min per week**	**All adults experience 3000–4500 MET min per week**	**51·3%**	**32·1%**	**67·4%**
**1**	**Metabolic risks**			**..**	**..**	**100·0%**	**100·0%**	**100·0%**
**2**		**High fasting plasma glucose**		**Serum fasting plasma glucose measured in mmol/L**	**4·8–5·4 mmol/L**	**50·3%**	**50·3%**	**67·9%**
**2**		**High low-density lipoprotein cholesterol**		**Serum low-density lipoprotein, measured in mmol/L**	**0·7–1·3 mmol/L**	**49·7%**	**48·2%**	**71·5%**
**2**		**High systolic blood pressure**		**Systolic blood pressure, measured in mm Hg**	**110–115 mm Hg**	**61·1%**	**64·8%**	**81·4%**
**2**		**High body-mass index**		**Body-mass index, measured in kg/m^2^**	**20–25 kg/m^2^**	**100·0%**	**100·0%**	**100·0%**
**2**		**Low bone mineral density**		**Standardised mean bone mineral density values measured by dual x-ray absorptiometry at the femoral neck in g/cm^2^**	**99th percentile of NHANES 1988–2014 by age and sex**	**23·8%**	**10·4%**	**25·9%**
**2**		**Impaired kidney function**		**Proportion of the population with ACR >30 mg/g or GFR <60 mL/min/1·73 m^2^, excluding end-stage renal disease**	**GFR >60 mL/min/1·73 m^2^ and ACR <30 mg/g**	**16·1%**	**28·5%**	**31·1%**

The data representativeness index is calculated as the percentage of locations for which we have data in a given time period. ACR=albumin-to-creatine ratio. GBD=Global Burden of Diseases, Injuries, and Risk Factors Study. GFR=glomerular filtration rate. MET=metabolic equivalent. NHANES=National Health and Nutrition Examination Survey. PM_2·5_=particulate matter with an aerodynamic diameter smaller than 2·5 μm, measured in μm/m^3^. ppb=parts per billion.

* In numbered intervals, square brackets indicate included endpoints and round brackets indicate excluded endpoints.

We report all point estimates with 95% uncertainty intervals (UIs). To ensure that UIs capture uncertainty from all relevant sources (uncertainty in exposures, relative risks, TMRELs, and burden estimates) we propagate uncertainty through the estimation chain using posterior simulation using 1000 draws, from which we derive the lower and upper bounds of the UI based on the 2·5th and 97·5th percentiles. Where reported, estimates of percentage change were computed on the basis of the point estimates for the timepoints being compared.

### Summary exposure values

For each risk, we produced a summary measure of exposure, called the summary exposure value (SEV). The metric is a risk-weighted prevalence of an exposure, and it offers an easily comparable single-number summary of exposure to each risk. SEVs range from 0% to 100%, where 0% reflects no risk exposure in a population and 100% indicates that an entire population is exposed to the maximum possible level for that risk. We show estimates of SEVs for each risk factor (table 2; appendix 2) and provide details on how SEVs are computed for categorical and continuous risks in the appendix 1 (section 2).

**Table 2:** Global age-standardised summary exposure values for all risk factors, 1990, 2007, and 2017, with mean percentage change for 1990–2007, 2007–17, and 1990–2017

	**Risk**			**Both sexes**	**Males**						**Females**					
				Percentage change, 1990–2017	1990	2007	2017	Percentage change, 1990–2007	Percentage change, 2007–17	Percentage change, 1990–2017	1990	2007	2017	Percentage change, 1990–2007	Percentage change, 2007–17	Percentage change, 1990–2017
**1**	**Environmental and occupational risks**															
**2**		**Unsafe water, sanitation, and handwashing**														
3		Unsafe water source		−21·24% (−25·26 to −17·26)*	43·22 (41·16 to 44·81)	36·07 (33·72 to 37·87)	33·57 (30·81 to 35·60)	−16·53% (−19·34 to −13·99)*	−6·93% (−9·73 to −3·91)*	−22·32% (−26·29 to −18·32)*	42·46 (40·42 to 44·03)	35·94 (33·62 to 37·70)	33·87 (31·09 to 35·88)	−15·35% (−18·19 to −12·85)*	−5·76% (−8·53 to −2·77)*	−20·23% (−24·28 to −16·19)*
3		Unsafe sanitation		−47·80% (−52·10 to −43·25)*	58·10 (53·42 to 64·54)	40·88 (36·53 to 46·98)	29·88 (25·66 to 35·37)	−29·64% (−33·19 to −26·07)*	−26·92% (−31·82 to −21·86)*	−48·58% (−52·90 to −44·00)*	57·19 (52·49 to 63·65)	40·82 (36·45 to 46·95)	30·28 (26·02 to 35·86)	−28·62% (−32·16 to −25·19)*	−25·81% (−30·56 to −20·97)*	−47·04% (−51·30 to −42·56)*
3		No access to handwashing facility		−12·72% (−16·05 to −9·07)*	37·81 (36·77 to 38·82)	34·52 (33·41 to 35·60)	32·51 (31·36 to 33·63)	−8·72% (−11·64 to −5·64)*	−5·82% (−8·35 to −3·11)*	−14·04% (−17·38 to −10·35)*	37·33 (36·35 to 38·30)	34·58 (33·52 to 35·62)	33·05 (31·93 to 34·14)	−7·37% (−10·17 to −4·35)*	−4·43% (−6·89 to −1·78)*	−11·47% (−14·82 to −7·86)*
**2**		**Air pollution**														
3		Particulate matter pollution														
4			Ambient particulate matter pollution	41·21% (32·15 to 51·99)*	30·08 (23·45 to 38·60)	39·91 (31·90 to 50·67)	41·90 (33·87 to 52·75)	32·71% (25·60 to 40·41)*	4·97% (−0·59 to 10·91)	39·30% (29·87 to 50·14)*	26·83 (21·00 to 34·70)	36·13 (28·60 to 46·32)	38·48 (30·87 to 49·05)	34·66% (27·56 to 42·79)*	6·50% (0·68 to 13·17)*	43·41% (33·87 to 55·06)*
4			Household air pollution from solid fuels	−45·83% (−49·75 to −41·46)*	45·57 (39·19 to 54·22)	31·57 (26·36 to 39·05)	23·90 (19·50 to 30·22)	−30·73% (−34·45 to −26·65)*	−24·30% (−28·40 to −19·95)*	−47·56% (−51·58 to −42·78)*	46·00 (39·75 to 54·60)	33·13 (27·85 to 40·66)	25·67 (21·04 to 32·02)	−27·98% (−31·53 to −24·27)*	−22·50% (−26·61 to −18·30)*	−44·19% (−48·30 to −39·43)*
3		Ambient ozone pollution		3·02% (2·30 to 4·82)*	41·72 (18·06 to 51·11)	41·88 (18·13 to 51·37)	42·89 (18·89 to 52·32)	0·38% (0·16 to 0·61)*	2·42% (1·77 to 4·14)*	2·81% (2·22 to 4·43)*	41·25 (17·72 to 50·99)	41·50 (17·88 to 51·27)	42·58 (18·65 to 52·26)	0·62% (0·41 to 0·91)*	2·60% (1·88 to 4·36)*	3·24% (2·40 to 5·22)*
**2**		**Other environmental risks**														
3		Residential radon		−0·23% (−5·56 to 5·43)	23·73 (14·82 to 33·91)	23·64 (14·79 to 33·99)	23·68 (14·72 to 34·19)	−0·40% (−3·44 to 2·75)	0·18% (−1·97 to 2·32)	−0·22% (−5·27 to 5·15)	23·72 (14·85 to 33·65)	23·64 (14·80 to 33·92)	23·66 (14·75 to 34·15)	−0·33% (−3·57 to 3·21)	0·10% (−2·29 to 2·38)	−0·23 (−5·72 to 5·66)
3		Lead exposure		−27·66% (−33·58 to −22·08)*	15·65 (11·46 to 19·44)	13·94 (9·97 to 17·65)	11·14 (7·54 to 14·68)	−10·93% (−13·35 to −8·81)*	−20·12% (−24·35 to −16·16)*	−28·85% (−34·39 to −23·60)*	9·56 (5·94 to 13·25)	8·79 (5·34 to 12·41)	7·15 (4·10 to 10·63)	−8·11% (−10·42 to −6·13)*	−18·67% (−23·43 to −14·24)*	−25·26 (−31·19 to −19·60)*
**2**		**Occupational risks**														
3		Occupational carcinogens														
4			Occupational exposure to asbestos	−13·76% (−26·71 to 2·19)	2·67 (2·33 to 3·05)	2·47 (2·38 to 2·60)	2·36 (2·28 to 2·45)	−7·69% (−15·84 to 3·65)	−4·24% (−8·89 to −0·25)*	−11·61% (−22·98 to 2·54)	0·98 (0·76 to 1·25)	0·79 (0·73 to 0·88)	0·74 (0·70 to 0·79)	−18·62% (−30·99 to −2·32)*	−6·58% (−13·55 to −0·85)*	−23·97% (−40·00 to −3·78)*
4			Occupational exposure to arsenic	5·12% (−0·36 to 25·19)	0·32 (0·09 to 0·61)	0·33 (0·10 to 0·60)	0·34 (0·10 to 0·61)	2·14% (−1·61 to 13·84)	2·96% (0·27 to 8·66)*	5·16% (−0·22 to 22·82)	0·29 (0·08 to 0·57)	0·30 (0·09 to 0·57)	0·31 (0·10 to 0·57)	3·10% (−1·78 to 18·37)	2·08% (−1·89 to 9·22)	5·25% (−1·93 to 27·75)
4			Occupational exposure to benzene	26·04% (18·96 to 41·92)*	0·54 (0·26 to 1·11)	0·60 (0·31 to 1·20)	0·65 (0·35 to 1·27)	11·09% (7·19 to 19·40)*	7·85% (5·67 to 11·95)*	19·81% (13·79 to 33·14)*	0·51 (0·22 to 1·10)	0·60 (0·28 to 1·26)	0·68 (0·34 to 1·37)	18·18% (13·55 to 28·19)*	12·38% (8·91 to 18·81)*	32·80% (24·14 to 51·85)*
4			Occupational exposure to beryllium	16·89% (14·86 to 18·95)*	0·06 (0·06 to 0·06)	0·07 (0·07 to 0·07)	0·07 (0·07 to 0·07)	8·52% (6·79 to 10·34)*	4·84% (3·52 to 6·14)*	13·77% (11·32 to 16·13)*	0·05 (0·05 to 0·06)	0·06 (0·06 to 0·06)	0·07 (0·06 to 0·07)	13·36% (10·83 to 16·02)*	6·40% (4·63 to 8·17)*	20·61% (17·00 to 23·99)*
4			Occupational exposure to cadmium	19·99% (16·30 to 23·69)*	0·13 (0·12 to 0·13)	0·14 (0·14 to 0·14)	0·15 (0·14 to 0·15)	10·87% (7·77 to 14·21)*	6·98% (4·25 to 9·51)*	18·60% (14·62 to 23·00)*	0·11 (0·11 to 0·11)	0·13 (0·12 to 0·13)	0·14 (0·13 to 0·14)	13·74% (8·91 to 18·80)*	6·97% (2·93 to 11·70)*	21·67% (15·26 to 28·32)*
4			Occupational exposure to chromium	27·30% (23·48 to 31·27)*	0·27 (0·26 to 0·27)	0·31 (0·30 to 0·32)	0·34 (0·33 to 0·35)	14·76% (11·61 to 18·16)*	9·57% (6·88 to 12·06)*	25·75% (21·56 to 30·31)*	0·23 (0·23 to 0·24)	0·28 (0·26 to 0·29)	0·30 (0·29 to 0·32)	17·53% (12·32 to 22·84)*	9·97% (5·72 to 14·97)*	29·25% (22·17 to 36·37)*
4			Occupational exposure to diesel engine exhaust	35·56% (32·42 to 38·55)*	1·51 (1·48 to 1·54)	1·83 (1·80 to 1·87)	2·07 (2·03 to 2·11)	21·61% (18·81 to 24·46)*	13·01% (10·71 to 15·21)*	37·44% (33·50 to 41·49)*	0·94 (0·92 to 0·97)	1·12 (1·09 to 1·15)	1·25 (1·22 to 1·29)	19·06% (15·56 to 22·55)*	11·94% (8·84 to 15·03)*	33·27% (28·17 to 38·41)*
4			Occupational exposure to formaldehyde	21·55% (17·49 to 25·59)*	0·59 (0·57 to 0·60)	0·66 (0·63 to 0·68)	0·71 (0·68 to 0·74)	12·38% (8·98 to 16·13)*	8·08% (5·06 to 10·93)*	21·45% (16·98 to 26·18)*	0·49 (0·47 to 0·51)	0·55 (0·53 to 0·58)	0·60 (0·57 to 0·63)	12·83% (7·23 to 18·52)*	7·96% (3·51 to 12·96)*	21·81% (14·97 to 28·83)*
4			Occupational exposure to nickel	1·54% (−5·26 to 19·70)	0·35 (0·08 to 1·10)	0·35 (0·09 to 1·08)	0·36 (0·10 to 1·08)	0·75% (−3·92 to 11·90)	2·38% (−0·40 to 8·20)	3·15% (−3·54 to 20·18)	0·28 (0·06 to 0·90)	0·28 (0·07 to 0·85)	0·28 (0·07 to 0·84)	−0·34% (−5·50 to 14·23)	0·17% (−3·46 to 8·49)	−0·17% (−7·70 to 20·71)
4			Occupational exposure to polycyclic aromatic hydrocarbons	27·66% (23·85 to 31·43)*	0·55 (0·54 to 0·56)	0·64 (0·62 to 0·65)	0·70 (0·68 to 0·72)	14·82% (11·95 to 18·02)*	9·71% (7·23 to 12·04)*	25·97% (21·96 to 30·35)*	0·48 (0·47 to 0·49)	0·56 (0·54 to 0·59)	0·62 (0·59 to 0·65)	17·49% (12·50 to 22·50)*	10·47% (6·20 to 15·11)*	29·79% (23·18 to 36·31)*
4			Occupational exposure to silica	2·21% (−2·51 to 12·86)	3·71 (1·52 to 9·28)	3·86 (1·70 to 9·29)	4·05 (1·85 to 9·56)	4·01% (0·32 to 11·54)*	4·82% (2·21 to 8·77)*	9·02% (3·50 to 21·16)*	2·50 (1·00 to 6·31)	2·36 (1·01 to 5·64)	2·32 (1·01 to 5·48)	−5·37% (−9·14 to 2·24)	−2·09% (−5·24 to 2·77)	−7·35% (−12·39 to 2·21)
4			Occupational exposure to sulphuric acid	6·40% (0·16 to 15·05)*	0·65 (0·39 to 1·34)	0·68 (0·42 to 1·36)	0·69 (0·44 to 1·35)	4·15% (0·11 to 10·25)*	2·25% (−0·37 to 5·61)	6·50% (0·25 to 15·14)*	0·58 (0·34 to 1·20)	0·61 (0·38 to 1·23)	0·61 (0·39 to 1·22)	5·26% (0·24 to 12·06)*	1·15% (−2·49 to 6·06)	6·47% (−0·62 to 16·38)
4			Occupational exposure to trichloroethylene	30·29% (27·26 to 33·55)*	0·16 (0·15 to 0·16)	0·18 (0·18 to 0·19)	0·20 (0·20 to 0·21)	16·76% (14·28 to 19·65)*	10·21% (8·22 to 12·23)*	28·67% (25·25 to 32·40)*	0·13 (0·13 to 0·13)	0·16 (0·15 to 0·16)	0·17 (0·17 to 0·18)	19·59% (15·55 to 23·76)*	10·72% (7·41 to 14·55)*	32·42% (26·78 to 38·05)*
3		Occupational asthmagens		−4·99% (−9·49 to −0·40)*	16·13 (13·44 to 19·43)	15·59 (13·03 to 18·78)	15·39 (12·87 to 18·38)	−3·33% (−7·27 to 0·67)	−1·27% (−4·61 to 1·74)	−4·55% (−9·66 to 0·49)	8·50 (6·78 to 10·63)	8·26 (6·70 to 10·20)	8·04 (6·59 to 9·74)	−2·78% (−9·20 to 3·95)	−2·68% (−7·59 to 2·70)	−5·38% (−12·99 to 2·95)
3		Occupational particulate matter, gases, and fumes		1·85% (−0·08 to 3·94)	8·45 (6·44 to 11·42)	8·50 (6·55 to 11·39)	8·48 (6·57 to 11·31)	0·55% (−0·73 to 2·07)	−0·24% (−1·20 to 0·80)	0·31% (−1·86 to 2·66)	5·00 (3·80 to 6·73)	5·22 (4·01 to 6·96)	5·20 (4·02 to 6·95)	4·35% (2·46 to 6·58)*	−0·28% (−1·60 to 1·19)	4·06% (1·28 to 7·34)*
3		Occupational noise		6·30% (5·01 to 7·56)*	8·60 (8·24 to 9·12)	8·80 (8·44 to 9·31)	8·91 (8·55 to 9·42)	2·27% (1·35 to 3·32)*	1·28% (0·69 to 1·87)*	3·58% (2·16 to 5·19)*	5·21 (4·96 to 5·53)	5·58 (5·34 to 5·92)	5·74 (5·50 to 6·10)	7·15% (5·65 to 8·90)*	2·91% (1·94 to 3·84)*	10·28% (7·99 to 12·79)*
3		Occupational injuries†		..	..	..	..	..	..	..	..	..	..	..	..	..
3		Occupational ergonomic factors		−14·08% (−18·25 to −9·86)*	17·09 (15·92 to 18·44)	15·91 (14·73 to 17·38)	14·71 (13·50 to 16·15)	−6·90% (−11·17 to −2·80)*	−7·54% (−10·64 to −4·41)*	−13·92% (−18·71 to −9·08)*	11·25 (10·41 to 12·22)	10·54 (9·70 to 11·50)	9·65 (8·75 to 10·74)	−6·28% (−12·78 to 0·55)	−8·42% (−13·25 to −3·44)*	−14·17% (−21·15 to −6·81)*
**1**	**Behavioural risks**															
**2**		**Child and maternal malnutrition**														
3		Suboptimal breastfeeding														
4			Non-exclusive breastfeeding	−10·89% (−13·64 to −7·53)*	0·44 (0·30 to 0·62)	0·40 (0·28 to 0·57)	0·39 (0·27 to 0·53)	−7·10% (−8·99 to −5·22)*	−4·20% (−6·88 to −1·09)*	−11·01% (−13·78 to −7·61)*	0·43 (0·30 to 0·62)	0·41 (0·28 to 0·57)	0·39 (0·27 to 0·54)	−6·81% (−8·62 to −4·99)*	−4·25% (−6·87 to −1·20)*	−10·77% (−13·48 to −7·43)*
4			Discontinued breastfeeding	−1·40% (−3·55 to 0·89)	1·16 (1·13 to 1·18)	1·08 (1·07 to 1·09)	1·14 (1·13 to 1·16)	−6·72% (−8·55 to −4·80)*	5·92% (4·43 to 7·42)*	−1·20% (−3·41 to 1·12)	1·16 (1·14 to 1·18)	1·07 (1·06 to 1·09)	1·14 (1·12 to 1·15)	−7·27% (−9·03 to −5·45)*	6·11% (4·67 to 7·53)*	−1·61% (−3·71 to 0·62)
3		Child growth failure														
4			Child underweight	−44·35% (−48·14 to −41·23)*	1·65 (1·48 to 1·79)	1·22 (1·06 to 1·36)	0·94 (0·80 to 1·06)	−26·04% (−29·95 to −22·70)*	−23·14% (−25·36 to −21·61)*	−43·15% (−47·32 to −39·79)*	1·60 (1·42 to 1·76)	1·14 (0·99 to 1·29)	0·87 (0·73 to 0·99)	−28·37% (−32·92 to −24·87)*	−24·16% (−26·33 to −22·44)*	−45·67% (−50·14 to −42·24)*
4			Child wasting	−22·51% (−25·73 to −19·67)*	0·57 (0·48 to 0·65)	0·50 (0·42 to 0·58)	0·45 (0·37 to 0·52)	−11·87% (−16·36 to −8·34)*	−11·30% (−13·24 to −9·44)*	−21·82% (−26·23 to −18·21)*	0·52 (0·44 to 0·59)	0·46 (0·37 to 0·53)	0·40 (0·33 to 0·46)	−11·72% (−16·12 to −8·29)*	−13·18% (−15·04 to −11·42)*	−23·36% (−27·37 to −19·99)*
4			Child stunting	−36·16% (−41·11 to −32·42)*	2·75 (1·89 to 3·06)	2·35 (1·64 to 2·60)	1·81 (1·27 to 2·05)	−14·29% (−18·93 to −9·98)*	−22·97% (−26·72 to −20·02)*	−33·97% (−39·71 to −29·38)*	2·70 (1·89 to 3·01)	2·24 (1·55 to 2·50)	1·66 (1·17 to 1·91)	−16·94% (−22·01 to −12·76)*	−26·01% (−30·13 to −22·76)*	−38·54% (−44·24 to −33·98)*
3		Low birthweight and short gestation														
4			Short gestation for birthweight	25·26% (21·38 to 29·51)*	0·00 (0·00 to 0·01)	0·01 (0·00 to 0·01)	0·01 (0·00 to 0·01)	18·60% (14·85 to 22·55)*	7·38% (3·85 to 10·34)*	27·35% (21·83 to 32·59)*	0·00 (0·00 to 0·00)	0·00 (0·00 to 0·01)	0·00 (0·00 to 0·01)	16·51% (13·09 to 19·73)*	5·10% (2·43 to 7·72)*	22·45% (17·29 to 27·17)*
4			Low birthweight for gestation	5·92% (1·12 to 11·21)*	0·01 (0·00 to 0·01)	0·01 (0·00 to 0·01)	0·01 (0·00 to 0·01)	2·67% (−2·39 to 8·23)	3·46% (0·62 to 7·00)*	6·22% (−0·73 to 14·61)	0·01 (0·00 to 0·01)	0·01 (0·00 to 0·01)	0·01 (0·00 to 0·01)	2·28% (−1·86 to 7·30)	3·25% (0·71 to 5·60)*	5·60% (−0·11 to 11·72)
3		Iron deficiency†		−23·44% (−27·96 to −19·44)*	..	..	..	..	..	..	12·69 (10·50 to 15·30)	10·78 (8·67 to 13·27)	9·70 (7·68 to 12·17)	−15·03% (−18·63 to −11·98)*	−10·04% (−12·97 to −7·26)*	−23·56% (−28·07 to −19·59)*
3		Vitamin A deficiency		−14·93% (−17·56 to −12·48)*	2·56 (2·21 to 2·95)	2·41 (2·08 to 2·79)	2·17 (1·85 to 2·54)	−5·88% (−7·27 to −4·57)*	−10·08% (−12·43 to −7·74)*	−15·37% (−18·17 to −12·89)*	2·49 (2·15 to 2·88)	2·36 (2·03 to 2·73)	2·13 (1·82 to 2·50)	−5·35% (−6·77 to −4·01)*	−9·63% (−12·19 to −7·27)*	−14·46% (−17·36 to −11·81)*
3		Zinc deficiency		−30·29% (−35·62 to −24·00)*	0·92 (0·28 to 1·77)	0·76 (0·24 to 1·45)	0·64 (0·19 to 1·26)	−17·99% (−23·55 to −8·44)*	−14·85% (−22·51 to −8·70)*	−30·17% (−36·78 to −21·56)*	0·93 (0·27 to 1·76)	0·76 (0·24 to 1·45)	0·64 (0·20 to 1·29)	−18·32% (−23·86 to −9·54)*	−14·81% (−22·91 to −8·51)*	−30·42% (−37·34 to −22·61)*
**2**		**Tobacco**														
3		Smoking		−27·01% (−29·04 to −25·04)*	11·28 (9·97 to 12·66)	9·86 (8·74 to 11·12)	8·70 (7·72 to 9·79)	−12·55% (−15·17 to −9·96)*	−11·77% (−13·43 to −10·01)*	−22·84% (−25·22 to −20·48)*	3·03 (2·69 to 3·42)	2·14 (1·87 to 2·45)	1·76 (1·52 to 2·02)	−29·41% (−32·09 to −26·70)*	−17·69% (−19·36 to −16·15)*	−41·90% (−44·51 to −39·26)*
3		Chewing tobacco		2·29% (−7·00 to 12·86)	3·87 (3·50 to 4·28)	3·84 (3·62 to 4·07)	3·75 (3·49 to 3·99)	−0·70% (−10·25 to 10·88)	−2·38% (−8·94 to 5·11)	−3·07% (−13·75 to 9·32)	2·26 (1·99 to 2·56)	2·51 (2·29 to 2·76)	2·51 (2·26 to 2·78)	10·97% (−3·08 to 26·36)	0·01% (−9·14 to 10·78)	10·99% (−5·66 to 29·80)
3		Second-hand smoke		−21·43% (−23·56 to −19·30)*	37·72 (36·74 to 38·70)	31·63 (30·95 to 32·28)	30·28 (29·49 to 31·03)	−16·14% (−18·81 to −13·41)*	−4·29% (−6·12 to −2·50)*	−19·73% (−22·82 to −16·64)*	55·74 (54·84 to 56·65)	46·24 (45·64 to 46·83)	43·06 (42·34 to 43·73)	−17·04% (−18·84 to −15·22)*	−6·87% (−7·86 to −5·78)*	−22·74% (−24·68 to −20·68)*
**2**		**Alcohol use**		**5·06% (−3·78 to 16·70)**	**14·70 (10·85 to 19·08)**	**15·60 (11·66 to 19·90)**	**16·23 (12·08 to 20·62)**	6·16% (1·44 to 12·04)*	**4·04% (−3·29 to 12·12)**	10·45% (0·63 to 22·45)*	**5·20 (3·28 to 8·08)**	**4·77 (3·02 to 7·43)**	**4·65 (2·96 to 7·16)**	−8·27% (−11·62 to −4·80)*	**−2·55% (−7·82 to 3·98)**	−10·62% (−17·02 to −2·18)*
**2**		**Drug use**		**6·12% (−1·31 to 12·81)**	**0·80 (0·66 to 0·98)**	**0·81 (0·68 to 0·97)**	**0·86 (0·72 to 1·04)**	**1·62% (−2·63 to 5·43)**	6·46% (2·56 to 10·64)*	8·19% (0·33 to 15·62)*	**0·41 (0·34 to 0·52)**	**0·40 (0·34 to 0·50)**	**0·42 (0·35 to 0·52)**	**−2·11% (−6·24 to 1·44)**	4·62% (1·89 to 7·45)*	**2·42% (−3·92 to 8·46)**
**2**		**Dietary risks**														
3		Diet low in fruits		−16·58% (−20·93 to −13·20)*	41·15 (35·95 to 43·87)	37·76 (32·37 to 40·96)	34·72 (29·05 to 38·30)	−8·23% (−10·79 to −6·35)*	−8·07% (−10·70 to −6·07)*	−15·64% (−19·96 to −12·32)*	38·99 (33·82 to 42·00)	35·35 (29·94 to 38·89)	32·14 (26·64 to 36·06)	−9·33% (−12·16 to −7·13)*	−9·08% (−12·00 to −6·77)*	−17·57% (−22·33 to −13·68)*
3		Diet low in vegetables		−25·60% (−31·95 to −20·57)*	35·04 (31·55 to 38·18)	29·42 (25·21 to 33·30)	25·63 (21·20 to 29·72)	−16·03% (−21·02 to −12·12)*	−12·88% (−17·07 to −9·52)*	−26·84% (−33·90 to −21·22)*	36·41 (33·12 to 39·35)	31·17 (26·96 to 34·90)	27·51 (23·05 to 31·60)	−14·40% (−18·99 to −10·80)*	−11·75% (−15·63 to −8·63)*	−24·46% (−30·99 to −19·15)*
3		Diet low in legumes		−6·18% (−9·08 to −2·69)*	21·06 (17·40 to 24·33)	21·13 (18·06 to 23·93)	19·88 (16·89 to 22·61)	0·34% (−3·84 to 5·80)	−5·93% (−8·59 to −3·70)*	−5·61% (−10·07 to 0·10)	24·79 (21·35 to 27·99)	24·52 (21·44 to 27·34)	23·17 (20·20 to 25·82)	−1·09% (−4·01 to 2·82)	−5·51% (−7·37 to −3·55)*	−6·54% (−9·76 to −2·67)*
3		Diet low in whole grains		−1·99% (−2·77 to −1·31)*	39·23 (36·80 to 41·29)	39·21 (36·79 to 41·28)	38·46 (35·92 to 40·64)	−0·04% (−0·73 to 0·65)	−1·93% (−2·80 to −1·24)*	−1·97% (−2·99 to −1·09)*	40·16 (37·90 to 42·09)	40·10 (37·80 to 42·05)	39·36 (36·95 to 41·44)	−0·14% (−0·76 to 0·52)	−1·84% (−2·63 to −1·16)*	−1·99% (−2·91 to −1·16)*
3		Diet low in nuts and seeds		−8·05% (−9·80 to −6·60)*	50·92 (50·10 to 51·59)	48·71 (47·51 to 49·73)	46·66 (45·03 to 48·05)	−4·34% (−5·29 to −3·53)*	−4·19% (−5·31 to −3·27)*	−8·35% (−10·24 to −6·76)*	51·05 (50·27 to 51·71)	49·00 (47·85 to 49·97)	47·09 (45·46 to 48·40)	−4·02% (−4·92 to −3·27)*	−3·91% (−5·02 to −3·08)*	−7·77% (−9·62 to −6·30)*
3		Diet low in milk		−0·17% (−0·45 to 0·10)	45·53 (43·68 to 47·09)	45·56 (43·72 to 47·13)	45·35 (43·45 to 46·95)	0·07% (−0·23 to 0·32)	−0·47% (−0·77 to −0·21)*	−0·41% (−0·81 to −0·05)*	45·57 (43·74 to 47·13)	45·69 (43·88 to 47·21)	45·57 (43·77 to 47·11)	0·26% (0·01 to 0·53)*	−0·24% (−0·52 to −0·00)*	0·01% (−0·36 to 0·35)
3		Diet high in red meat		24·36% (12·39 to 37·31)*	11·92 (10·57 to 13·60)	13·20 (10·85 to 15·28)	15·11 (12·67 to 17·16)	10·73% (−1·26 to 23·05)	14·51% (4·18 to 25·74)*	26·80% (13·02 to 42·06)*	5·69 (4·55 to 6·96)	5·50 (3·77 to 7·28)	6·81 (4·68 to 8·84)	−3·36% (−18·75 to 13·13)	23·71% (7·41 to 42·85)*	19·55% (−0·62 to 43·50)
3		Diet high in processed meat		−9·26% (−17·94 to −1·01)*	5·14 (4·22 to 6·43)	5·04 (4·16 to 6·49)	4·70 (3·59 to 6·30)	−1·86% (−7·74 to 3·41)	−6·86% (−14·14 to −1·12)*	−8·60% (−18·72 to 0·86)	3·67 (2·72 to 4·89)	3·56 (2·72 to 4·88)	3·31 (2·37 to 4·78)	−3·07% (−9·76 to 3·22)	−7·01% (−15·25 to −0·53)*	−9·87% (−20·20 to −0·10)*
3		Diet high in sugar-sweetened beverages		17·14% (8·34 to 28·02)*	5·40 (2·78 to 6·11)	5·78 (2·98 to 6·46)	6·58 (3·37 to 7·53)	7·07% (−0·77 to 16·86)	13·84% (7·17 to 22·04)*	21·88% (10·56 to 36·93)*	4·26 (2·19 to 4·93)	4·32 (2·24 to 4·90)	4·74 (2·43 to 5·52)	1·48% (−8·10 to 12·66)	9·71% (2·50 to 18·05)*	11·34% (−1·05 to 27·07)
3		Diet low in fibre		−8·91% (−11·91 to −6·62)*	33·29 (29·03 to 36·82)	31·63 (27·12 to 35·34)	29·89 (25·25 to 33·84)	−4·98% (−7·48 to −3·12)*	−5·49% (−8·09 to −3·59)*	−10·20% (−14·35 to −7·24)*	37·43 (33·84 to 40·34)	36·00 (32·26 to 39·08)	34·51 (30·41 to 37·81)	−3·80% (−5·65 to −2·43)*	−4·15% (−6·08 to −2·76)*	−7·79% (−10·94 to −5·47)*
3		Diet low in calcium		−8·74% (−11·17 to −6·88)*	40·27 (37·48 to 42·55)	38·34 (35·16 to 40·93)	36·24 (32·61 to 39·15)	−4·81% (−6·29 to −3·71)*	−5·47% (−7·29 to −4·14)*	−10·01% (−13·01 to −7·83)*	41·68 (39·09 to 43·76)	40·22 (37·39 to 42·57)	38·52 (35·36 to 41·16)	−3·52% (−4·57 to −2·67)*	−4·21% (−5·56 to −3·23)*	−7·58% (−9·83 to −5·91)*
3		Diet low in seafood omega 3 fatty acids		−6·77% (−8·52 to −5·29)*	44·02 (42·36 to 45·64)	42·10 (40·09 to 44·08)	40·58 (38·22 to 42·83)	−4·37% (−5·47 to −3·41)*	−3·62% (−4·82 to −2·64)*	−7·83% (−9·93 to −6·06)*	44·84 (43·28 to 46·33)	43·42 (41·57 to 45·17)	42·25 (40·08 to 44·25)	−3·17% (−4·03 to −2·44)*	−2·70% (−3·66 to −1·93)*	−5·78% (−7·42 to −4·39)*
3		Diet low in polyunsaturated fatty acids		−12·92% (−16·36 to −10·15)*	39·12 (36·71 to 41·43)	35·30 (32·30 to 38·06)	33·81 (30·64 to 36·77)	−9·75% (−12·76 to −7·20)*	−4·23% (−5·79 to −2·96)*	−13·57% (−17·22 to −10·52)*	39·10 (36·62 to 41·39)	35·61 (32·53 to 38·35)	34·29 (31·08 to 37·22)	−8·93% (−11·82 to −6·58)*	−3·72% (−5·16 to −2·57)*	−12·31% (−15·97 to −9·43)*
3		Diet high in trans fatty acids		−46·57% (−66·28 to −32·85)*	4·26 (2·41 to 6·95)	2·85 (1·23 to 5·40)	2·26 (0·77 to 4·67)	−33·08% (−49·65 to −22·64)*	−20·61% (−37·67 to −12·32)*	−46·87% (−68·26 to −32·36)*	5·73 (3·62 to 8·61)	3·91 (1·96 to 6·68)	3·08 (1·28 to 5·71)	−31·76% (−45·68 to −22·25)*	−21·33% (−35·58 to −13·42)*	−46·32% (−64·75 to −33·34)*
3		Diet high in sodium		−8·75% (−16·90 to −3·84)*	17·26 (12·52 to 22·33)	16·84 (11·76 to 21·97)	16·56 (11·68 to 21·75)	−2·42% (−10·60 to 3·97)	−1·66% (−6·07 to 2·60)	−4·04% (−13·12 to 3·03)	16·88 (12·31 to 21·94)	15·62 (10·79 to 20·87)	14·62 (9·86 to 19·92)	−7·47% (−16·41 to −2·36)*	−6·43% (−11·89 to −2·61)*	−13·42% (−23·48 to −6·76)*
**2**		**Intimate partner violence**		−4·50% (−6·66 to −2·42)*	..	..	..	..	..	..	**6·80 (5·72 to 7·88)**	**6·47 (5·60 to 7·37)**	**6·46 (5·49 to 7·42)**	−4·82% (−8·10 to −1·03)*	**−0·19% (−2·16 to 1·54)**	−4·99% (−7·15 to −2·88)*
**2**		**Childhood maltreatment**														
3		Childhood sexual abuse		5·52% (4·00 to 7·07)*	6·19 (5·08 to 7·61)	6·42 (5·33 to 7·78)	6·81 (5·63 to 8·33)	3·60% (1·22 to 6·01)*	6·16% (4·19 to 8·06)*	9·98% (8·09 to 11·92)*	6·79 (5·53 to 8·23)	6·53 (5·38 to 7·84)	6·87 (5·59 to 8·33)	−3·86% (−5·76 to −1·92)*	5·25% (2·75 to 7·94)*	1·18% (−1·11 to 3·42)
3		Bullying victimisation		32·17% (25·93 to 41·72)*	5·92 (2·59 to 11·93)	7·11 (3·19 to 14·32)	7·56 (3·40 to 15·32)	20·06% (14·26 to 27·46)*	6·40% (4·67 to 8·45)*	27·74% (21·09 to 37·84)*	4·35 (1·87 to 8·68)	5·48 (2·42 to 10·72)	6·01 (2·66 to 11·89)	25·93% (19·70 to 34·55)*	9·74% (7·97 to 11·82)*	38·20% (30·75 to 48·42)*
**2**		**Unsafe sex**†		..	..	..	..	..	..	..	..	..	..	..	..	..
**2**		**Low physical activity**		**0·12% (−0·20 to 0·38)**	**0·40 (0·18 to 0·75)**	**0·40 (0·18 to 0·75)**	**0·40 (0·18 to 0·76)**	**0·28% (−0·00 to 0·55)**	0·67% (0·35 to 1·01)*	0·96% (0·56 to 1·36)*	**0·37 (0·16 to 0·70)**	**0·36 (0·16 to 0·69)**	**0·36 (0·16 to 0·69)**	−1·29% (−2·01 to −0·52)*	0·43% (0·20 to 0·69)*	−0·87% (−1·54 to −0·18)*
**1**	**Metabolic risks**															
**2**		**High fasting plasma glucose**		37·71% (29·15 to 48·42)*	1·93 (1·46 to 2·50)	2·44 (1·83 to 3·19)	2·72 (2·08 to 3·50)	26·67% (20·02 to 33·69)*	11·44% (4·82 to 19·39)*	41·16% (31·63 to 52·98)*	1·76 (1·34 to 2·29)	2·19 (1·63 to 2·89)	2·35 (1·79 to 3·07)	24·51% (17·30 to 31·74)*	7·26% (−0·08 to 15·53)	33·55% (23·51 to 45·92)*
**2**		**High low-density lipoprotein cholesterol**		−6·82% (−7·88 to −5·82)*	**11·22 (9·83 to 12·77)**	**10·32 (8·93 to 11·89)**	**10·23 (8·86 to 11·81)**	−7·98% (−9·26 to −6·78)*	−0·86% (−1·28 to −0·46)*	−8·77% (−10·10 to −7·52)*	**11·72 (10·40 to 13·25)**	**11·05 (9·71 to 12·58)**	**11·18 (9·83 to 12·72)**	−5·73% (−6·78 to −4·78)*	1·15% (0·78 to 1·55)*	−4·65% (−5·64 to −3·71)*
**2**		**High systolic blood pressure**		**−1·37% (−3·03 to 0·26)**	**7·12 (6·46 to 7·78)**	**7·21 (6·53 to 7·89)**	**7·39 (6·67 to 8·12)**	**1·26% (−0·66 to 3·14)**	2·54% (0·95 to 4·22)*	3·83% (1·65 to 6·05)*	**7·18 (6·64 to 7·75)**	**6·81 (6·25 to 7·39)**	**6·77 (6·19 to 7·33)**	−5·16% (−7·15 to −3·11)*	**−0·56% (−2·45 to 1·33)**	−5·68% (−8·11 to −3·31)*
**2**		**High body-mass index**		70·39% (57·13 to 84·52)*	**5·70 (4·04 to 8·07)**	**8·15 (6·18 to 11·15)**	**9·95 (7·76 to 13·28)**	43·04% (34·24 to 53·89)*	22·09% (18·44 to 26·34)*	74·63% (59·63 to 92·99)*	**6·50 (4·75 to 8·85)**	**9·11 (7·01 to 11·99)**	**10·88 (8·54 to 14·17)**	40·22% (32·65 to 49·58)*	19·41% (15·97 to 23·19)*	67·43% (54·96 to 82·04)*
**2**		**Low bone mineral density**		−2·17% (−3·46 to −0·97)*	**5·31 (4·53 to 6·08)**	**5·15 (4·42 to 5·90)**	**5·10 (4·34 to 5·87)**	−2·94% (−4·06 to −1·79)*	**−1·02% (−2·37 to 0·25)**	−3·93% (−5·37 to −2·55)*	**6·90 (6·05 to 7·85)**	**6·86 (6·04 to 7·79)**	**6·86 (5·94 to 7·87)**	**−0·63% (−1·75 to 0·58)**	**0·02% (−1·65 to 1·75)**	**−0·61% (−2·19 to 0·93)**
**2**		**Impaired kidney function**		**1·48% (−1·94 to 4·14)**	**2·46 (1·13 to 6·12)**	**2·49 (1·14 to 6·17)**	**2·49 (1·14 to 6·16)**	**0·95% (−1·26 to 3·10)**	**0·31% (−1·42 to 2·46)**	**1·25% (−1·90 to 3·81)**	**2·93 (1·40 to 6·81)**	**2·94 (1·39 to 6·86)**	**2·99 (1·42 to 6·96)**	**0·25% (−2·44 to 2·56)**	**1·75% (−0·01 to 4·02)**	**2·00% (−1·47 to 4·69)**

Data in parentheses are 95% uncertainty intervals.

* Statistically significant increase or decrease (p<0·05).

† Estimation methods for these risks precludes the estimation of summary exposure values.

### Updates to spatiotemporal Gaussian process regression

Spatiotemporal Gaussian process regression has been used in previous versions of GBD to estimate exposure for many risks, typically those with rich age-sex-specific data. It synthesises noisy data by borrowing strength across space, time, and age to best estimate the underlying trends for a given risk. With sufficient data, spatiotemporal Gaussian process regression is a fast and flexible modelling strategy for fitting non-linear temporal trends. Although methods were detailed for previous iterations of GBD,^2^ we have implemented several improvements for GBD 2017. First, we have added a space-time interaction weight, which flexibly adjusts the spatial weight of datapoints as an inverse function of data density over time. Second, we refined our method for calculating model uncertainty to ensure that modelling CIs aligned better with observed data variance and were more resilient to parameter changes. Finally, we improved raking, a post-processing step that ensures internal consistency between nested locations (subnationals) and their parents. Specifically, we implemented an option to rake in logit space, ensuring that raked estimates of prevalence data are naturally constrained between 0 and 1. More details are given in appendix 1 (section 2).

### Drivers of trends in DALYs

We decomposed temporal changes in DALYs into six main component drivers of change: (1) population growth; (2) changes in population age structures; (3) changes in exposure to environmental and occupational risks; (4) changes in exposure to behavioural risks, (5) changes in exposure to metabolic risks; and (6) changes due to all other factors, approximated as the risk-deleted death and DALY rates. The risk-deleted rate is the death or DALY rate that would be observed had we removed all risk factors included in GBD 2017. In other words, the risk-deleted rate is the rate that would be observed had we reduced exposure levels to the TMREL for all risk factors included in GBD 2017. Changes in risk-deleted rates might reflect changes in risks or risk–outcome pairs that are not included in our analysis, or changes in other factors like improved treatments. We used methods developed by Das Gupta^7^ and adapted in GBD 2016 to ensure that decomposition results are linear aggregates over time or risk. We did a decomposition analysis for the 10-year period of 2007–17, for individual risks and the all-risk aggregate, accounting for risk mediation at the Level 4 risk and cause level. The contribution of changes in exposure to the individual risks was scaled to the all-risk effect. The contribution of risk exposures at higher cause and risk aggregates (eg, all-cause attributable to Level 1 GBD risks), or for all ages and both sexes combined, were calculated as the linear aggregate of the effect of individual risks for each cause, age, and sex.

### Epidemiological transition

SDI is a composite indicator of development status that was originally constructed for GBD 2015, and is derived from components that correlate strongly with health outcomes. It is the geometric mean for indices of the total fertility rate among women younger than 25 years, mean education for those aged 15 years or older, and lag-distributed income per capita. The resulting metric ranges from 0 to 1, with higher values corresponding to higher levels of development. SDI estimation methods and estimates are detailed in appendix 1 (section 2). We examined the relationship between SDI and SEV to understand the relationship between development status and risk factor exposure levels. For each risk factor, we fit a separate generalised additive model with a Loess smoother on SDI for each combination of age group and sex. Inputs to this model were age-sex-specific SEVs for all Level 4 risks in the GBD risk hierarchy, for all national GBD locations and years between 1990 and 2017. Using an analogous modelling framework, we estimated the expected age and sex structure by SDI and used these expected age and sex proportions to calculate age and sex aggregates of expected exposure. For each risk–outcome pair, we used the expected SEVs to calculate expected PAFs. Because the SEVs for a given risk are not cause specific, the expected PAF estimates were then corrected using cause-specific correction factors that were derived by calibrating expected PAFs against empirical PAFs. To estimate expected risk-attributable burden, we drew from the CRA methods, first calculating the joint adjusted expected PAF for all risks for a cause using mediation factors (appendix 1 section 2). We then drew from the methods for observed risk-attributable burden calculation, using expected YLLs, deaths, and YLDs (appendix 1 section 2) to generate expected burden for a given SDI.

### New risks and risks with substantial changes in the estimation methods compared with GBD 2016

Bullying victimisation is a new risk factor for GBD 2017. We estimate two outcomes for bullying in the GBD analysis: anxiety disorders and major depressive disorder. Bullying is commonly conceptualised as the intentional and repeated harm of a less powerful individual by peers and defined in the GBD as bullying victimisation of children and adolescents attending school by peers. This does not mean that bullying occurs exclusively at school and includes bullying that might occur to and from school as well as cyberbullying. We developed inclusion criteria that were robust while adaptable to the heterogeneity in largely non-health literature. Prevalence data were sourced from multicountry survey series including the Global School-based Student Health Survey and the Health Behavior in School-aged Children survey, as well as peer-reviewed studies, and were available for 153 GBD locations, covering all seven GBD super-regions. To reflect the exposure data and the definition of bullying victimisation in GBD, we adjusted prevalence estimates for the proportion of young people attending school using data published by the UN Educational, Scientific, and Culture Organization. Because the effect of bullying on depressive and anxiety disorders has been reported to wane over time and because prevalence estimates were from surveys of young people reporting current bullying victimisation rather than estimates of past exposure at the time the outcomes occur (ie, retrospective estimates), we developed a cohort method in which the prevalence of bullying victimisation exposure was tracked for the cohort of interest and relative risks varied with time between exposure to bullying and the point of estimation.

In GBD 2017, the modelling process for air pollution, including ambient, household, and ozone exposure sources, was substantially improved. We adjusted the risk hierarchy, retaining air pollution as a Level 2 risk, adding particulate matter pollution at Level 3, and moving both household air pollution due to exposure to smoke from solid cooking fuels and ambient particulate matter pollution to Level 4 of the hierarchy. Developed for risk attribution for particulate matter pollution, the integrated exposure response curves combine epidemiological data from ambient, household, second-hand, and active smoking sources to construct a risk curve for the full exposure range. We updated the integrated exposure responses to include studies on ambient air pollution cohorts that were published after we completed our literature review for GBD 2016, systematic reviews of all active smoking cohorts, and a systematic review of second-hand smoke and chronic obstructive pulmonary disease (COPD). We also developed a strategy to map cohort studies of household air pollution to exposure levels of particulate matter less than 2·5 μm in diameter (PM_2·5_) to incorporate them into the curves.

We did a systematic search of the scientific literature of health outcomes resulting from long-term exposure to ambient particulate matter pollution and, consequently, included type 2 diabetes as a new outcome for both ambient and household air pollution. Evidence suggests that exposure to PM_2·5_ might be mechanistically linked to type 2 diabetes through altered lung function, vascular inflammation, and insulin sensitivity.^8^

We estimated ambient PM_2·5_ exposure by combining satellite data with a chemical transport model and land use information. We calibrated satellite measurements to ground measurements using the Data Integration Model for Air Quality (DIMAQ).^9^ We made three notable improvements as follows: (1) we expanded our database of ground measurements from approximately 6000 to 9700 sites; (2) we made updates so the calibration model varies smoothly over space and time in data-dense regions; and (3) we improved uncertainty estimation by sampling from the DIMAQ's poster distribution in each grid cell (appendix 1 section 4).

For previous GBDs, we have calculated relative risks from the integrated exposure response curves to produce PAFs and attributable burden for ambient particulate matter and household air pollution using the same TMREL for both risk factors. However, were a population to reduce one of the component exposures (ie, either household or ambient pollution), the other would remain. To capture this, we used a proportional PAF approach in which the integrated exposure response is used to calculate a relative risk and PAF for exposure to particulate matter from both ambient and household sources, and these are then weighted by the proportion of individuals exposed to each source (appendix 1 section 4).

In GBD 2016, we estimated the burden attributable to low intake of polyunsaturated fatty acids, where low intake was the result of polyunsaturated fatty acids being replaced by saturated fats. Considering that it is equally harmful to replace polyunsaturated fatty acids with either saturated fat or carbohydrates,^10^ we have redefined the risk factor as low polyunsaturated fatty acids intake where these were replaced by either saturated fatty acids or carbohydrates. In this approach, the TMREL for polyunsaturated fatty acids does not account for saturated fat intake.

For estimating consumption of whole grains, we developed an approach to use UN Food and Agriculture Organization (FAO) data, notably increasing our data coverage across countries and through time. First, we separately estimated total grain and refined grain availability, where availability includes domestic production, adjusted for imports, exports, waste, and animal feed. With whole grains and refined grains representing the entirety of all grain available, we calculated the availability of whole grains as the difference between total and refined grains. Finally, we adjusted these estimates using 24-h dietary recall data to represent consumption.

In past cycles of GBD, given the strength of the causal relationship between sugar-sweetened beverage intake and BMI compared with the association between sugar-sweetened beverages and disease endpoints, we estimated the disease burden of high intake of sugar-sweetened beverages through its effect on BMI. This decision was based on the observation that evidence supporting a causal relationship between sugar-sweetened beverages and BMI was stronger than evidence for a direct causal relationship between sugar-sweetened beverages and disease endpoints. In GBD 2017, we reassessed all existing evidence on causal relationships between sugar-sweetened beverages and disease endpoints, and found sufficient evidence for a causal relationship between sugar-sweetened beverages and ischaemic heart disease and type 2 diabetes. Therefore, we have updated our approach and quantified the burden of disease attributable to the direct effect of sugar-sweetened beverages on disease endpoints.

We added four new outcomes for high BMI as follows: type 2 diabetes, liver cancer due to non-alcoholic fatty liver disease, subarachnoid haemorrhage, and intracerebral haemorrhage. We applied the relative risk of diabetes only to type 2 diabetes. Relative risks for the association between high BMI and all liver cancers were used for both liver cancer due to non-alcoholic fatty liver and liver cancer due to other causes. Similarly, relative risks for the association between high BMI and haemorrhagic stroke were used for both subarachnoid haemorrhage and intracerebral haemorrhage.

We added five additional outcomes for high fasting plasma glucose (FPG) as follows: type 1 diabetes, type 2 diabetes, liver cancer due to non-alcoholic fatty liver disease,^11^ subarachnoid haemorrhage, and intracerebral haemorrhage.^12^ Because an increased FPG concentration is the hallmark of diabetes, we assumed the PAFs were 1·0 for FPG and both type 1 diabetes and type 2 diabetes. Relative risks for the association between high FPG and all liver cancers were used for liver cancer due to non-alcoholic fatty liver and liver cancer due to other causes. Similarly, relative risks for the association between high FPG and haemorrhagic stroke were used for both subarachnoid haemorrhage and intracerebral haemorrhage.

We made four important changes related to the estimation of burden attributable to iron deficiency. First, the definitions of the GBD cause “dietary iron deficiency” and the risk factor “iron deficiency” are no longer identical. The GBD cause name was changed from “iron-deficiency anaemia” to “dietary iron deficiency” to clarify the focus on inadequate intake and exclusion of other causes that can manifest as absolute or functional iron deficiency. Second, although the GBD risk factor name remained “iron deficiency”, the exposure estimates were expanded to include all iron deficiency, irrespective of whether or not inadequate dietary intake is the underlying cause (appendix 1 section 4). This change was based on review of the Child Health Epidemiology Research Group (CHERG) Iron Report,^13^ whose component studies revealed no distinction as to the aetiology of iron deficiency. Third, on the basis of the studies included in the CHERG Iron Report,^13^ which only assessed overall maternal mortality as an outcome, we added all subcauses of maternal disorders as outcomes of iron deficiency (the risk), leading to higher estimates of the burden attributable to iron deficiency among women of reproductive age. Fourth, on the basis of the absence of evidence supporting dietary iron deficiency as a primary cause of death, dietary iron deficiency was removed from the GBD 2017 cause of death analysis, resulting in zero mortality burden and lower overall estimates of burden for dietary iron deficiency (the cause). Dietary iron deficiency (the cause) is expressed in terms of prevalence and YLDs, but the exposure to iron deficiency (the risk) remains expressed as the counterfactual haemoglobin concentration that would be present in a given population group in the absence of all causes of anaemia that manifest as iron deficiency.

We made three major improvements to our analysis of low birthweight for gestation and short gestation for birthweight. First, we added individual-level linked birth and death cohort data from nearly 25 million births in Japan and Singapore to strengthen our analysis of the joint mortality risk surface. Second, we drew on the strong correlation between birthweight and gestational age that we identified in our microdata analysis and used birthweight data to inform exposure estimates of short gestation. We also strengthened the link between non-fatal and risk analyses to ensure estimates of preterm birth were fully consistent throughout GBD 2017. The addition of individual-level linked birth and death cohort data resulted in higher estimates for low birthweight prevalence, mostly in data-sparse locations, whereas the consistency changes resulted in higher exposure estimates for both low birthweight and for short gestation, particularly in the late neonatal period. Third, we corrected an error where the risk attributable to low birthweight was mistakenly attributed to short gestation and vice versa in GBD 2016. This correction has no effect on the aggregate risk of low birthweight and short gestation but is the chief driver of differences in each individually.

We have moved from estimating total cholesterol in GBD 2016 to estimating LDL cholesterol for GBD 2017. During the past two decades, substantially more data have been collected on LDL cholesterol than total cholesterol concentrations. The strong statistical relationship between total and LDL cholesterol also allows us to model LDL cholesterol when other cholesterol subfractions, such as high-density lipoprotein, are reported, but LDL cholesterol concentrations are not.^14^ The use of LDL cholesterol improves the policy relevance of our estimates, because LDL cholesterol is the key target of cholesterol-lowering medications and is the most commonly used laboratory biomarker for clinical decision making. We applied this change to the full dataset, including data that were newly extracted for GBD 2017 and data that had been extracted in previous iterations of GBD.

To estimate smoking-attributable burden for GBD 2017, we transitioned from using 5-year lagged daily smoking prevalence (ie, the prevalence of smoking 5 years before the date for which estimates are being produced) and the smoking impact ratio to using continuous measures of exposure that incorporate cumulative effects among daily, occasional, and former smokers for 47 smoking-attributable health outcomes. We continue to use 5-year lagged daily smoking prevalence as the measure of exposure for ten outcomes. We estimated exposure among current smokers for two continuous indicators: cigarettes per smoker per day, and pack-years. We estimated exposure among former smokers using years since cessation. We estimated non-linear dose-response curves using a Bayesian meta-regression model for each of these continuous exposures. For nine outcomes with significant differences in effect size by sex or age, we produced sex-specific or age-specific risk curves (appendix 1 section 5). We included all forms of smoked tobacco in our exposure estimates and, given data limitations, assume that the risk of non-cigarette smoked tobacco products is the same as the risk of cigarettes; given the scarcity of data, we do not include electronic cigarette or vaporiser use in our exposure estimates.

We added two new outcomes for high SBP: subarachnoid haemorrhage and calcific aortic valve disease. For both outcomes, we estimated relative risks on the basis of data from a pooled cohort study of 1·2 million participants.^15^ We know of no large cohort that has reported age-sex-specific relative risks of either subarachnoid haemorrhage or calcific aortic valve disease due to increased SBP, and used proxy causes for each as follows: we estimated the relative risks for subarachnoid haemorrhage on the basis of all stroke and those for calcific aortic valve disease on the basis of other cardiovascular disease. For each cause, we estimated age-sex-specific relative risks associated with a 10 mm Hg increase in SBP using the DisMod meta-regression tool (appendix 1 section 2).

We have improved the exposure-modelling framework for unsafe water and sanitation. We estimate exposure levels for unsafe water and sanitation using ordinal categories. For example, we estimate the prevalence of exposure to three levels of unsafe water: piped, improved, and unimproved drinking water. Previously, the prevalences of piped and improved water were modelled independently, and we derived the prevalence of unimproved water as one minus the sum of piped and improved water. For GBD 2017, we modelled the prevalence of piped water as before, but now explicitly model the prevalence of improved and unimproved water separately as proportions of the unpiped envelope. This approach enables us to use the exposure category for which we have the most data (ie, piped water access) while also ensuring that the three exposure categories sum to one. The modelling process for unsafe sanitation was revised in an analogous way.

### Role of the funding source

The funders of the study had no role in study design, data collection, data analysis, data interpretation, or writing of the report. All authors had full access to all data in the study and had final responsibility for the decision to submit for publication.

## Results

### Global exposure to risks

We observed diverse temporal trends for levels of exposure to different risk factors between 1990 and 2017 (table 2). During that time, SEVs for two risks increased by more than 40%: SEVs for high BMI increased by 70·4% (95% UI 57·1–84·5) and SEVs for ambient particulate matter pollution increased by 41·2% (32·1–52·0). Conversely, SEVs for four risks decreased by more than 40%: SEVs for child underweight decreased by 44·4% (41·2–48·1), household air pollution by 45·8% (41·5–49·8), diet high in trans fatty acids by 46·6% (32·8–66·3), and unsafe sanitation by 47·8% (43·2–52·1).

**Figure 1:**
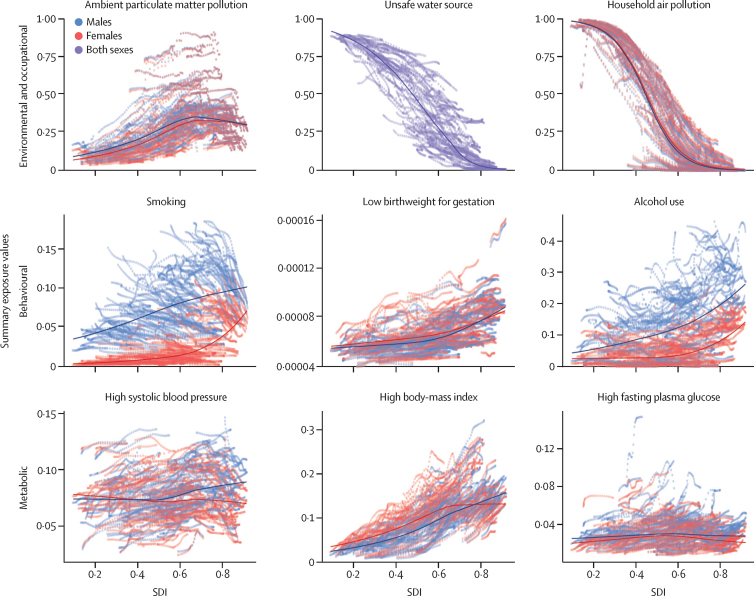
Relationship between age-standardised summary exposure values and SDI for three of the top environmental and occupational, behavioural, and metabolic risk factors by number of attributable DALYs globally The three leading risks for each Level 1 risk group are shown, except alcohol (fourth leading behavioural risk), which was included for variety instead of short gestation for birthweight. Each point corresponds to the age-standardised SEV in a country for males (blue), females (red), or both sexes (purple) for SEVs that are not sex specific. Points depict all country-years, 1990–2017. Lines show the expected SEV by SDI for each sex. Note that the *y*-axis scales differ by risk to correspond to the range of observed SEVs. DALYs=disability-adjusted life-years. SDI=Socio-demographic Index. SEV=summary exposure value.

SDI and SEVs were strongly associated for many risks (figure 1; for all risks, see appendix 2). Unsafe water source, unsafe sanitation, household air pollution, lead exposure, child underweight, child wasting, child stunting, iron deficiency, vitamin A deficiency, low calcium, and intimate partner violence all show a pronounced decreasing trend with increasing SDI. Conversely, discontinued breastfeeding, smoking, alcohol use, drug use, high red meat consumption, high processed meat consumption, high sugar-sweetened beverage consumption, and high LDL cholesterol all show a pronounced increasing trend with increased SDI. Other risks show no clear association with SDI, including ozone, radon, low whole grains, and low fibre.

### Global attributable burden for all risk factors combined

Globally, 61·0% (95% UI 59·6–62·4) of deaths and 48·3% (46·3–50·2) of DALYs were attributed to the risk factors addressed in GBD 2017 (appendix 2). The largest percentage of deaths attributable to risk factors were among NCDs at 64·8% (63·1–66·6), followed by CMNNDs at 61·6% (59·0–64·4), and injuries at 24·4% (22·2–26·5). For DALYs, the largest risk-attributable proportion was among CMNNDs (64·0%, 61·8–66·0), followed by NCDs (45·6%, 42·8–48·4), and injuries (22·1%, 20·2–24·0). Risk-attributable DALYs declined 4·9% (3·3–6·5) and deaths declined 8·3% (6·9–9·5) between 2007 and 2017. The numbers of risk-attributable deaths and DALYs in 2007 and 2017 are provided for each cause in table 3.

**Table 3:** Global all-age attributable deaths and DALYs, 2007–17, and percentage change of deaths and DALYs and age-standardised death rates and DALY rates, 2007–17, for all risk-outcome pairs

	**Risk factors and outcomes**				**2007 deaths (thousands)**	**2017 deaths (thousands)**	**Percentage change in deaths, 2007–17**	**Percentage change in age-standardised death rate, 2007–17**	**2007 DALYs (thousands)**	**2017 DALYs (thousands)**	**Percentage change in DALYs, 2007–17**	**Percentage change in age-standardised DALY rate, 2007–17**
**0**	**All risk factors: all causes**				**31 500 (30 900 to 32 200)**	**34 100 (33 300 to 35 000)**	8·3% (6·9 to 9·5)*	−15·7% (−16·6 to −14·8)*	**1 270 000 (1 210 000 to 1 340 000)**	**1 210 000 (1 140 000 to 1280 000)**	−4·9% (−6·5 to −3·3)*	−19·9% (−21·3 to −18·5)*
**1**	**Environmental and occupational risks: all causes**				**8150 (7590 to 8810)**	**8320 (7690 to 9020)**	**2·2% (−0·3 to 4·7)**	−20·1% (−21·8 to −18·2)*	**345 000 (319 000 to 370 000)**	**308 000 (283 000 to 333 000)**	−10·7% (−13·7 to −7·5)*	−24·9% (−27·4 to −22·3)*
**2**		**Unsafe water, sanitation, and handwashing: all causes**			**1990 (1610 to 2570)**	**1610 (1230 to 2230)**	−18·8% (−26·2 to −10·1)*	−31·6% (−36·9 to −25·0)*	**120 000 (103 000 to 138 000)**	**84 400 (71 800 to 102 000)**	−29·6% (−35·3 to −23·0)*	−36·0% (−41·2 to −30·3)*
3		Unsafe water source: all causes			1500 (938 to 2110)	1230 (736 to 1830)	−18·0% (−26·9 to −8·0)*	−31·2% (−37·2 to −23·3)*	90 100 (58 500 to 113 000)	63 900 (40 800 to 83 100)	−29·1% (−35·4 to −21·5)*	−35·7% (−41·3 to −29·0)*
..			Diarrhoeal diseases		1500 (938 to 2110)	1230 (736 to 1830)	−18·0% (−26·9 to −8·0)*	−31·2% (−37·2 to −23·3)*	90 100 (58 500 to 113 000)	63 900 (40 800 to 83 100)	−29·1% (−35·4 to −21·5)*	−35·7% (−41·3 to −29·0)*
3		Unsafe sanitation: all causes			1110 (856 to 1450)	774 (566 to 1060)	−30·2% (−37·4 to −21·6)*	−41·2% (−46·4 to −34·2)*	66 500 (55 600 to 78 100)	41 500 (34 100 to 49 700)	−37·6% (−43·3 to −30·6)*	−43·2% (−48·4 to −37·1)*
..			Diarrhoeal diseases		1110 (856 to 1450)	774 (566 to 1060)	−30·2% (−37·4 to −21·6)*	−41·2% (−46·4 to −34·2)*	66 500 (55 600 to 78 100)	41 500 (34 100 to 49 700)	−37·6% (−43·3 to −30·6)*	−43·2% (−48·4 to −37·1)*
3		No access to handwashing facility: all causes			892 (528 to 1250)	707 (416 to 1020)	−20·7% (−26·4 to −13·1)*	−32·5% (−36·9 to −26·9)*	55 300 (33 200 to 75 300)	38 400 (22 800 to 52 100)	−30·5% (−35·6 to −24·7)*	−36·4% (−41·0 to −31·1)*
..			Lower respiratory infections		229 (148 to 306)	188 (122 to 249)	−17·9% (−21·9 to −13·6)*	−29·0% (−32·4 to −25·3)*	14 700 (9630 to 19 700)	10 300 (6780 to 13 800)	−29·8% (−34·1 to −25·0)*	−35·0% (−39·0 to −30·4)*
..			Diarrhoeal diseases		663 (312 to 1010)	520 (240 to 839)	−21·7% (−29·6 to −12·0)*	−33·7% (−39·6 to −26·4)*	40 500 (19 500 to 59 500)	28 100 (13 500 to 41 500)	−30·7% (−37·1 to −22·9)*	−36·9% (−42·8 to −29·5)*
**2**		**Air pollution: all causes**			**4630 (4210 to 5040)**	**4900 (4420 to 5390)**	5·8% (3·0 to 8·8)*	−18·7% (−20·7 to −16·7)*	**158 000 (142 000 to 172 000)**	**147 000 (132 000 to 162 000)**	−6·6% (−9·8 to −2·8)*	−23·5% (−26·0 to −20·8)*
3		Particulate matter pollution: all causes			4380 (3960 to 4780)	4580 (4130 to 5030)	4·6% (1·7 to 7·5)*	−19·5% (−21·5 to −17·4)*	154 000 (138 000 to 168 000)	143 000 (129 000 to 156 000)	−7·3% (−10·8 to −3·4)*	−23·9% (−26·6 to −21·1)*
4			Ambient particulate matter pollution: all causes		2420 (2080 to 2760)	2940 (2500 to 3360)	21·6% (16·2 to 26·8)*	−7·8% (−11·7 to −3·8)*	73 600 (63 300 to 83 400)	83 000 (71 400 to 94 300)	12·8% (5·6 to 20·2)*	−9·3% (−15·1 to −3·0)*
..				Lower respiratory infections	420 (337 to 500)	433 (343 to 527)	2·9% (−8·8 to 15·4)	−15·3% (−24·3 to −5·1)*	22 300 (17 700 to 27 200)	18 500 (14 400 to 23 400)	−16·9% (−29·4 to −0·9)*	−24·3% (−35·8 to −9·2)*
..				Tracheal, bronchus, and lung cancer	205 (143 to 270)	265 (183 to 351)	29·3% (22·0 to 36·3)*	−2·0% (−7·6 to 3·4)	4680 (3280 to 6150)	5860 (4050 to 7730)	25·1% (17·8 to 31·7)*	−3·9% (−9·5 to 1·3)
..				Ischaemic heart disease	780 (671 to 901)	977 (839 to 1120)	25·3% (20·6 to 30·2)*	−5·8% (−9·2 to −2·4)*	17 800 (15 300 to 20 600)	21 900 (18 900 to 25 400)	23·1% (18·4 to 28·3)*	−4·2% (−7·8 to −0·5)*
..				Ischaemic stroke	147 (113 to 183)	184 (140 to 228)	24·6% (18·8 to 30·9)*	−7·8% (−11·4 to −3·8)*	3060 (2360 to 3780)	3950 (3040 to 4870)	28·9% (22·7 to 35·4)*	−2·0% (−6·2 to 2·4)
..				Intracerebral haemorrhage	194 (152 to 238)	226 (176 to 280)	17·0% (11·1 to 23·2)*	−11·3% (−15·2 to −6·9)*	4770 (3800 to 5850)	5520 (4340 to 6840)	15·8% (9·5 to 22·2)*	−9·8% (−14·0 to −5·2)*
..				Subarachnoid haemorrhage	29 (22 to 36)	35 (27 to 44)	22·1% (14·8 to 29·8)*	−5·4% (−10·9 to 0·1)	877 (686 to 1100)	1040 (809 to 1310)	18·8% (12·2 to 25·9)*	−5·0% (−10·3 to 0·2)
..				Chronic obstructive pulmonary disease	519 (347 to 679)	633 (416 to 838)	22·1% (13·7 to 29·8)*	−10·5% (−16·5 to −4·8)*	12 800 (8460 to 16 700)	15 700 (10 300 to 20 800)	23·5% (16·1 to 30·7)*	−6·0% (−11·6 to −0·5)*
..				Type 2 diabetes mellitus	122 (82 to 149)	184 (123 to 227)	50·7% (44·8 to 56·0)*	11·1% (6·8 to 15·1)*	7380 (4720 to 9790)	10 500 (6700 to 13 900)	41·9% (35·0 to 48·7)*	10·5% (5·3 to 15·6)*
4			Household air pollution from solid fuels: all causes		1960 (1700 to 2270)	1640 (1400 to 1930)	−16·3% (−20·2 to −11·9)*	−34·2% (−37·3 to −31·0)*	80 100 (68 300 to 92 400)	59 500 (50 800 to 68 900)	−25·8% (−30·2 to −20·9)*	−37·4% (−41·1 to −33·6)*
..				Lower respiratory infections	669 (536 to 797)	459 (367 to 552)	−31·4% (−36·6 to −25·9)*	−40·4% (−44·9 to −35·8)*	43 600 (34 500 to 52 000)	25 900 (20 300 to 31 300)	−40·7% (−46·2 to −35·0)*	−44·9% (−50·1 to −39·6)*
..				Tracheal, bronchus, and lung cancer	94 (69 to 120)	85 (60 to 113)	−10·0% (−16·4 to −3·7)*	−31·5% (−36·4 to −26·7)*	2280 (1660 to 2890)	1990 (1410 to 2640)	−12·7% (−18·7 to −6·9)*	−32·6% (−37·2 to −28·0)*
..				Ischaemic heart disease	435 (371 to 512)	410 (344 to 490)	−5·8% (−10·1 to −1·5)*	−28·2% (−31·4 to −25·0)*	11 100 (9400 to 13 000)	10 200 (8450 to 12 100)	−8·2% (−12·7 to −3·9)*	−27·9% (−31·4 to −24·5)*
..			Ischaemic stroke		88 (69 to 112)	81 (62 to 104)	−8·2% (−13·0 to −3·3)*	−31·3% (−35·0 to −27·8)*	1980 (1540 to 2470)	1830 (1390 to 2330)	−7·2% (−12·4 to −2·6)*	−29·0% (−32·8 to −25·5)*
..			Intracerebral haemorrhage		156 (123 to 191)	132 (102 to 165)	−15·2% (−19·5 to −10·9)*	−35·3% (−38·5 to −32·1)*	4080 (3210 to 5010)	3440 (2660 to 4310)	−15·5% (−19·9 to −11·2)*	−34·0% (−37·2 to −30·9)*
..			Subarachnoid haemorrhage		20 (16 to 27)	18 (14 to 24)	−9·2% (−15·0 to −2·8)*	−29·0% (−33·6 to −24·2)*	664 (506 to 870)	591 (443 to 777)	−11·0% (−16·5 to −5·1)*	−28·4% (−32·7 to −23·9)*
..			Chronic obstructive pulmonary disease		421 (296 to 548)	362 (248 to 482)	−13·9% (−20·5 to −7·4)*	−36·3% (−41·1 to −31·6)*	10 800 (7740 to 13 900)	9370 (6480 to 12 400)	−13·0% (−19·0 to −7·4)*	−33·5% (−38·1 to −29·3)*
..			Type 2 diabetes mellitus		77 (54 to 92)	92 (63 to 113)	19·6% (12·6 to 27·1)*	−10·6% (−15·8 to −5·1)*	4360 (2900 to 5780)	4750 (3110 to 6190)	9·0% (2·6 to 15·5)*	−14·6% (−19·6 to −9·5)*
..			Cataract		..	..	..	..	1320 (696 to 2010)	1440 (732 to 2250)	9·2% (4·6 to 12·7)*	−17·3% (−20·9 to −14·7)*
3		Ambient ozone pollution: all causes			392 (146 to 638)	472 (177 to 768)	20·4% (15·9 to 24·5)*	−11·6% (−14·8 to −8·6)*	6330 (2370 to 10 300)	7370 (2740 to 12 000)	16·4% (12·0 to 20·6)*	−12·2% (−15·6 to −9·1)*
..			Chronic obstructive pulmonary disease		392 (146 to 638)	472 (177 to 768)	20·4% (15·9 to 24·5)*	−11·6% (−14·8 to −8·6)*	6330 (2370 to 10 300)	7370 (2740 to 12 000)	16·4% (12·0 to 20·6)*	−12·2% (−15·6 to −9·1)*
**2**		**Other environmental risks: all causes**			**929 (646 to 1240)**	**1140 (794 to 1530)**	23·0% (19·2 to 26·6)*	−7·9% (−10·4 to −5·3)*	**23 500 (16 500 to 30 600)**	**26 400 (18 400 to 34 800)**	12·3% (8·9 to 15·6)*	−12·0% (−14·4 to −10·1)*
3		Residential radon: all causes			68 (38 to 107)	88 (50 to 139)	28·1% (21·4 to 34·9)*	−2·9% (−6·9 to 1·0)	1570 (859 to 2420)	1930 (1080 to 3020)	23·3% (16·1 to 30·2)*	−5·2% (−9·3 to −1·0)*
..			Tracheal, bronchus, and lung cancer		68 (38 to 107)	88 (50 to 139)	28·1% (21·4 to 34·9)*	−2·9% (−6·9 to 1·0)	1570 (859 to 2420)	1930 (1080 to 3020)	23·3% (16·1 to 30·2)*	−5·2% (−9·3 to −1·0)*
3		Lead exposure: all causes			860 (586 to 1160)	1050 (709 to 1430)	22·6% (18·7 to 26·4)*	−8·3% (−10·8 to −5·7)*	21 900 (15 200 to 28 900)	24 400 (16 700 to 32 800)	11·5% (7·8 to 14·8)*	−12·5% (−15·0 to −10·5)*
..			Rheumatic heart disease		8 (5 to 15)	8 (4 to 14)	−2·9% (−12·8 to 6·5)	−25·0% (−31·6 to −18·8)*	255 (145 to 430)	219 (120 to 368)	−14·2% (−23·1 to −5·5)*	−31·1% (−38·1 to −25·4)*
..			Ischaemic heart disease		391 (256 to 547)	488 (317 to 685)	24·6% (20·7 to 28·4)*	−6·9% (−9·1 to −4·6)*	8350 (5480 to 11 500)	9600 (6210 to 13 300)	15·0% (11·6 to 18·1)*	−11·5% (−13·9 to −9·5)*
..			Ischaemic stroke		110 (69 to 159)	139 (87 to 200)	25·8% (21·1 to 31·5)*	−6·7% (−9·7 to −3·3)*	2380 (1510 to 3340)	3000 (1920 to 4220)	26·2% (21·7 to 30·6)*	−4·2% (−7·4 to −1·0)*
..			Intracerebral haemorrhage		191 (125 to 270)	205 (132 to 292)	7·0% (2·4 to 10·9)*	−19·4% (−22·5 to −16·8)*	4420 (2920 to 6090)	4470 (2850 to 6300)	1·0% (−4·0 to 5·0)	−22·2% (−26·0 to −19·2)*
..			Subarachnoid haemorrhage		23 (14 to 33)	26 (16 to 38)	14·8% (6·1 to 25·2)*	−12·0% (−18·4 to −4·4)*	655 (387 to 964)	693 (409 to 1040)	5·9% (−1·6 to 14·5)	−16·8% (−22·9 to −10·5)*
..			Hypertensive heart disease		66 (25 to 137)	98 (33 to 213)	48·0% (22·7 to 63·0)*	9·2% (−8·9 to 19·8)	1310 (620 to 2560)	1710 (725 to 3480)	31·0% (12·4 to 45·5)*	0·2% (−14·0 to 10·2)
..			Non-rheumatic calcific aortic valve disease		2 (1 to 3)	2 (1 to 4)	30·7% (22·7 to 37·8)*	−5·4% (−10·2 to −1·8)*	28 (16 to 45)	34 (18 to 54)	20·3% (12·8 to 26·9)*	−8·8% (−14·1 to −4·7)*
..			Other cardiomyopathy		5 (3 to 7)	6 (3 to 9)	22·2% (14·6 to 28·8)*	−8·2% (−13·4 to −3·7)*	111 (61 to 176)	126 (68 to 201)	12·8% (4·9 to 19·9)*	−11·8% (−18·1 to −6·7)*
..			Atrial fibrillation and flutter		5 (3 to 8)	8 (5 to 11)	48·1% (43·4 to 53·0)*	5·2% (2·6 to 8·3)*	150 (91 to 218)	193 (118 to 283)	29·0% (25·3 to 32·5)*	−2·9% (−5·2 to −0·8)*
..			Aortic aneurysm		4 (2 to 5)	4 (3 to 6)	19·3% (13·7 to 24·8)*	−10·3% (−14·2 to −6·6)*	75 (44 to 113)	84 (48 to 127)	12·5% (6·8 to 17·9)*	−13·5% (−18·0 to −9·4)*
..			Peripheral vascular disease		1 (0 to 1)	1 (0 to 2)	54·1% (30·4 to 74·7)*	10·5% (−6·0 to 25·2)	19 (9 to 32)	25 (12 to 43)	31·2% (21·0 to 42·9)*	−1·6% (−9·1 to 6·8)
..			Endocarditis		1 (1 to 2)	2 (1 to 3)	30·2% (19·4 to 36·6)*	−1·4% (−8·7 to 2·6)	36 (19 to 59)	42 (22 to 67)	17·1% (5·9 to 24·4)*	−7·4% (−16·0 to −2·3)*
..			Other cardiovascular and circulatory diseases		11 (7 to 16)	13 (8 to 18)	19·1% (12·3 to 24·1)*	−9·7% (−14·6 to −6·3)*	347 (208 to 528)	383 (227 to 580)	10·2% (4·1 to 14·3)*	−13·9% (−19·1 to −10·8)*
..			Idiopathic developmental intellectual disability		..	..	..	..	2670 (1070 to 4880)	2540 (1000 to 4700)	−4·8% (−9·1 to −2·7)*	−13·6% (−17·1 to −11·7)*
**..**			Chronic kidney disease due to type 1 diabetes mellitus		3 (1 to 4)	3 (2 to 5)	11·2% (3·8 to 17·6)*	−12·6% (−18·6 to −8·0)*	90 (48 to 150)	93 (47 to 159)	2·8% (−5·5 to 9·8)	−17·6% (−24·1 to −12·5)*
**..**			Chronic kidney disease due to type 2 diabetes mellitus		13 (8 to 20)	18 (10 to 27)	34·6% (27·8 to 39·0)*	0·4% (−4·5 to 3·2)	303 (179 to 458)	380 (218 to 578)	25·4% (19·4 to 29·6)*	−4·9% (−9·2 to −1·8)*
**..**			Chronic kidney disease due to hypertension		12 (7 to 19)	17 (10 to 26)	38·2% (31·8 to 41·9)*	1·4% (−3·2 to 3·9)	255 (148 to 382)	320 (187 to 485)	25·6% (19·9 to 29·3)*	−4·4% (−8·6 to −1·6)*
**..**			Chronic kidney disease due to glomerulonephritis		6 (3 to 9)	7 (4 to 12)	25·9% (20·3 to 30·2)*	−4·3% (−8·2 to −1·6)*	160 (86 to 260)	177 (93 to 294)	10·7% (5·0 to 15·2)*	−12·6% (−17·2 to −9·1)*
..			Chronic kidney disease due to other and unspecified causes		9 (5 to 13)	11 (6 to 17)	27·7% (22·2 to 32·1)*	−3·3% (−7·3 to −0·6)*	286 (161 to 454)	327 (183 to 526)	14·2% (9·1 to 18·3)*	−11·0% (−15·0 to −8·0)*
**2**		**Occupational risks: all causes**			**1090 (995 to 1190)**	**1160 (1050 to 1280)**	6·5% (3·5 to 9·4)*	−16·6% (−18·7 to −14·7)*	**59 800 (52 300 to 68 100)**	**63 700 (54 900 to 73 200)**	6·7% (4·1 to 9·3)*	−11·6% (−13·7 to −9·4)*
3		Occupational carcinogens: all causes			271 (220 to 322)	334 (271 to 397)	23·3% (19·1 to 27·1)*	−7·4% (−10·5 to −4·5)*	5600 (4560 to 6710)	6750 (5490 to 8120)	20·6% (16·5 to 24·5)*	−8·0% (−11·3 to −5·0)*
4			Occupational exposure to asbestos: all causes		194 (148 to 243)	232 (177 to 289)	19·6% (14·6 to 23·6)*	−10·5% (−14·2 to −7·5)*	3410 (2570 to 4310)	3930 (2980 to 4950)	15·3% (9·9 to 19·8)*	−12·7% (−16·7 to −9·4)*
..				Larynx cancer	3 (2 to 5)	4 (2 to 6)	20·9% (13·4 to 27·0)*	−9·6% (−15·0 to −5·2)*	63 (35 to 95)	74 (41 to 112)	17·1% (9·4 to 23·1)*	−11·6% (−17·3 to −7·0)*
..				Tracheal, bronchus, and lung cancer	161 (115 to 208)	191 (137 to 247)	18·5% (13·5 to 22·6)*	−11·3% (−15·0 to −8·3)*	2740 (1910 to 3590)	3120 (2180 to 4110)	14·0% (8·7 to 18·4)*	−13·8% (−17·8 to −10·6)*
..				Ovarian cancer	5 (3 to 8)	6 (3 to 10)	17·4% (9·6 to 24·2)*	−14·0% (−19·6 to −9·1)*	88 (44 to 138)	100 (49 to 156)	14·3% (6·4 to 21·4)*	−14·3% (−20·1 to −9·2)*
..				Mesothelioma	22 (21 to 23)	27 (27 to 28)	27·7% (20·5 to 33·7)*	−3·6% (−8·8 to 0·7)	464 (434 to 512)	569 (542 to 598)	22·4% (14·5 to 29·4)*	−6·0% (−11·7 to −1·0)*
..				Asbestosis	3 (2 to 3)	3 (2 to 4)	23·3% (15·2 to 33·9)*	−8·3% (−14·1 to −0·4)*	58 (41 to 70)	69 (52 to 81)	19·2% (11·8 to 30·4)*	−8·5% (−14·1 to 0·3)
4			Occupational exposure to arsenic: all causes		7 (1 to 12)	9 (2 to 16)	33·8% (27·3 to 55·2)*	1·9% (−3·0 to 18·6)	189 (43 to 346)	245 (65 to 436)	29·5% (23·2 to 49·0)*	−0·6% (−5·5 to 15·1)
..				Tracheal, bronchus, and lung cancer	7 (1 to 12)	9 (2 to 16)	33·8% (27·3 to 55·2)*	1·9% (−3·0 to 18·6)	189 (43 to 346)	245 (65 to 436)	29·5% (23·2 to 49·0)*	−0·6% (−5·5 to 15·1)
4			Occupational exposure to benzene: all causes		2 (1 to 3)	2 (1 to 3)	18·6% (13·0 to 24·6)*	0·3% (−5·1 to 6·1)	73 (23 to 120)	84 (26 to 137)	15·4% (10·1 to 21·2)*	−0·2% (−5·2 to 5·2)
..				Leukaemia	2 (1 to 3)	2 (1 to 3)	18·6% (13·0 to 24·6)*	0·3% (−5·1 to 6·1)	73 (23 to 120)	84 (26 to 137)	15·4% (10·1 to 21·2)*	−0·2% (−5·2 to 5·2)
..				Acute lymphoid leukaemia	0 (0 to 0)	0 (0 to 1)	29·4% (17·7 to 39·6)*	12·2% (2·1 to 21·3)*	14 (4 to 23)	18 (5 to 29)	26·3% (15·0 to 36·0)*	11·4% (1·4 to 20·1)*
..				Chronic lymphoid leukaemia	0 (0 to 0)	0 (0 to 0)	27·9% (16·8 to 45·2)*	2·0% (−7·4 to 17·1)	3 (1 to 4)	4 (1 to 6)	31·4% (19·4 to 48·4)*	7·3% (−3·4 to 23·2)
..				Acute myeloid leukaemia	0 (0 to 1)	1 (0 to 1)	29·2% (20·2 to 39·2)*	8·6% (0·4 to 17·8)*	20 (7 to 33)	25 (8 to 41)	25·8% (16·6 to 35·6)*	8·2% (−0·3 to 17·2)
..				Chronic myeloid leukaemia	0 (0 to 0)	0 (0 to 0)	7·0% (−1·1 to 15·2)	−9·6% (−16·8 to −2·3)*	6 (2 to 10)	7 (2 to 11)	5·6% (−2·4 to 13·7)	−9·0% (−16·3 to −1·9)*
..				Other leukaemia	1 (0 to 1)	1 (0 to 1)	8·2% (3·0 to 14·7)*	−8·4% (−13·1 to −2·8)*	30 (9 to 49)	31 (10 to 52)	4·1% (−1·0 to 10·4)	−9·9% (−14·6 to −4·3)*
4			Occupational exposure to beryllium: all causes		0 (0 to 0)	0 (0 to 0)	45·4% (38·9 to 52·2)*	11·0% (6·5 to 15·6)*	5 (5 to 6)	8 (6 to 9)	40·1% (33·4 to 47·3)*	8·0% (3·5 to 12·8)*
..				Tracheal, bronchus, and lung cancer	0 (0 to 0)	0 (0 to 0)	45·4% (38·9 to 52·2)*	11·0% (6·5 to 15·6)*	5 (5 to 6)	8 (6 to 9)	40·1% (33·4 to 47·3)*	8·0% (3·5 to 12·8)*
4			Occupational exposure to cadmium: all causes		0 (0 to 1)	1 (1 to 1)	47·7% (40·1 to 56·0)*	12·7% (7·2 to 18·7)*	13 (11 to 15)	18 (15 to 22)	42·4% (34·6 to 50·8)*	9·7% (4·3 to 15·6)*
..				Tracheal, bronchus, and lung cancer	0 (0 to 1)	1 (1 to 1)	47·7% (40·1 to 56·0)*	12·7% (7·2 to 18·7)*	13 (11 to 15)	18 (15 to 22)	42·4% (34·6 to 50·8)*	9·7% (4·3 to 15·6)*
4		Occupational exposure to chromium: all causes			1 (1 to 1)	1 (1 to 2)	49·3% (42·0 to 56·6)*	14·0% (9·0 to 19·3)*	27 (24 to 30)	38 (34 to 43)	44·1% (36·8 to 51·2)*	11·0% (6·0 to 16·3)*
..		Tracheal, bronchus, and lung cancer			1 (1 to 1)	1 (1 to 2)	49·3% (42·0 to 56·6)*	14·0% (9·0 to 19·3)*	27 (24 to 30)	38 (34 to 43)	44·1% (36·8 to 51·2)*	11·0% (6·0 to 16·3)*
4		Occupational exposure to diesel engine exhaust: all causes			12 (11 to 13)	18 (16 to 20)	48·9% (41·6 to 55·4)*	13·5% (8·5 to 18·1)*	344 (304 to 386)	494 (434 to 559)	43·9% (36·8 to 50·5)*	10·8% (5·7 to 15·3)*
..		Tracheal, bronchus, and lung cancer			12 (11 to 13)	18 (16 to 20)	48·9% (41·6 to 55·4)*	13·5% (8·5 to 18·1)*	344 (304 to 386)	494 (434 to 559)	43·9% (36·8 to 50·5)*	10·8% (5·7 to 15·3)*
4		Occupational exposure to formaldehyde: all causes			1 (1 to 1)	1 (1 to 1)	20·6% (13·4 to 28·5)*	1·3% (−3·7 to 7·1)	40 (33 to 48)	46 (38 to 56)	16·1% (9·4 to 23·6)*	−0·3% (−5·4 to 5·4)
..		Nasopharynx cancer			0 (0 to 1)	0 (0 to 1)	23·9% (10·4 to 39·0)*	2·1% (−7·0 to 11·9)	15 (10 to 21)	18 (12 to 25)	18·8% (5·4 to 34·1)*	−0·1% (−9·3 to 10·1)
..		Acute lymphoid leukaemia			0 (0 to 0)	0 (0 to 0)	31·7% (21·7 to 40·9)*	14·4% (6·1 to 22·0)*	5 (4 to 6)	6 (5 to 8)	27·8% (18·3 to 36·9)*	12·9% (4·6 to 20·5)*
..		Chronic lymphoid leukaemia			0 (0 to 0)	0 (0 to 0)	41·3% (32·6 to 51·4)*	14·5% (7·3 to 22·2)*	1 (1 to 1)	1 (1 to 1)	45·0% (34·0 to 57·3)*	20·6% (11·8 to 30·2)*
..				Acute myeloid leukaemia	0 (0 to 0)	0 (0 to 0)	32·0% (24·7 to 38·4)*	12·0% (5·9 to 17·3)*	6 (5 to 7)	8 (6 to 9)	27·8% (20·3 to 34·2)*	10·7% (4·3 to 16·3)*
..				Chronic myeloid leukaemia	0 (0 to 0)	0 (0 to 0)	7·9% (2·7 to 13·8)*	−8·3% (−12·8 to −3·4)*	2 (2 to 2)	2 (2 to 3)	5·6% (0·2 to 11·7)*	−8·7% (−13·6 to −3·5)*
..				Other leukaemia	0 (0 to 0)	0 (0 to 0)	5·9% (−1·3 to 15·0)	−10·0% (−15·6 to −2·6)*	11 (9 to 14)	11 (9 to 14)	1·3% (−5·8 to 10·1)	−12·0% (−17·9 to −4·7)*
4			Occupational exposure to nickel: all causes		7 (1 to 18)	9 (1 to 23)	33·9% (25·4 to 55·2)*	2·0% (−4·5 to 18·6)	184 (26 to 493)	238 (36 to 607)	29·5% (21·2 to 49·1)*	−0·5% (−6·9 to 15·0)
..				Tracheal, bronchus, and lung cancer	7 (1 to 18)	9 (1 to 23)	33·9% (25·4 to 55·2)*	2·0% (−4·5 to 18·6)	184 (26 to 493)	238 (36 to 607)	29·5% (21·2 to 49·1)*	−0·5% (−6·9 to 15·0)
4			Occupational exposure to polycyclic aromatic hydrocarbons: all causes		3 (3 to 4)	5 (4 to 6)	48·9% (41·4 to 56·4)*	13·7% (8·4 to 19·0)*	94 (80 to 107)	134 (114 to 156)	43·7% (36·4 to 51·2)*	10·8% (5·8 to 16·0)*
..				Tracheal, bronchus, and lung cancer	3 (3 to 4)	5 (4 to 6)	48·9% (41·4 to 56·4)*	13·7% (8·4 to 19·0)*	94 (80 to 107)	134 (114 to 156)	43·7% (36·4 to 51·2)*	10·8% (5·8 to 16·0)*
4			Occupational exposure to silica: all causes		47 (27 to 69)	60 (34 to 88)	27·2% (20·5 to 33·7)*	−3·5% (−8·7 to 1·5)	1280 (702 to 1880)	1590 (870 to 2330)	24·6% (18·4 to 31·4)*	−4·2% (−9·0 to 1·3)
..				Tracheal, bronchus, and lung cancer	37 (17 to 58)	49 (22 to 77)	31·2% (24·3 to 41·4)*	0·0% (−5·3 to 7·9)	1050 (470 to 1630)	1330 (595 to 2080)	27·1% (20·2 to 36·5)*	−2·4% (−7·7 to 5·1)
..				Silicosis	10 (9 to 12)	11 (10 to 12)	12·0% (1·2 to 22·8)*	−15·5% (−23·6 to −7·4)*	230 (205 to 266)	261 (233 to 286)	13·2% (1·2 to 24·1)*	−12·3% (−21·6 to −3·9)*
4			Occupational exposure to sulphuric acid: all causes		3 (1 to 6)	4 (2 to 7)	25·5% (19·2 to 32·4)*	−3·8% (−8·7 to 1·5)	101 (43 to 182)	124 (53 to 224)	22·5% (16·1 to 29·5)*	−4·9% (−10·0 to 0·4)
..				Larynx cancer	3 (1 to 6)	4 (2 to 7)	25·5% (19·2 to 32·4)*	−3·8% (−8·7 to 1·5)	101 (43 to 182)	124 (53 to 224)	22·5% (16·1 to 29·5)*	−4·9% (−10·0 to 0·4)
4			Occupational exposure to trichloroethylene: all causes		0 (0 to 0)	0 (0 to 0)	51·9% (46·4 to 59·7)*	16·6% (12·4 to 22·5)*	1 (0 to 2)	2 (0 to 3)	48·8% (43·2 to 56·4)*	15·7% (11·4 to 21·7)*
..				Kidney cancer	0 (0 to 0)	0 (0 to 0)	51·9% (46·4 to 59·7)*	16·6% (12·4 to 22·5)*	1 (0 to 2)	2 (0 to 3)	48·8% (43·2 to 56·4)*	15·7% (11·4 to 21·7)*
3		Occupational asthmagens: all causes			38 (21 to 52)	34 (22 to 47)	−9·4% (−16·1 to 0·5)	−28·8% (−34·5 to −20·6)*	1930 (1460 to 2430)	1910 (1500 to 2410)	−1·2% (−6·9 to 6·9)	−18·6% (−23·9 to −11·3)*
..			Asthma		38 (21 to 52)	34 (22 to 47)	−9·4% (−16·1 to 0·5)	−28·8% (−34·5 to −20·6)*	1930 (1460 to 2430)	1910 (1500 to 2410)	−1·2% (−6·9 to 6·9)	−18·6% (−23·9 to −11·3)*
3		Occupational particulate matter, gases, and fumes: all causes			432 (354 to 510)	488 (397 to 584)	13·0% (9·2 to 17·0)*	−16·2% (−18·7 to −13·5)*	10 500 (9050 to 12 100)	12 100 (10 200 to 13 900)	14·4% (11·1 to 17·8)*	−12·8% (−15·2 to −10·5)*
..			Chronic obstructive pulmonary disease		425 (347 to 503)	481 (391 to 577)	13·1% (9·3 to 17·2)*	−16·1% (−18·7 to −13·4)*	10 400 (8870 to 11 900)	11 900 (10 100 to 13 700)	14·6% (11·2 to 18·0)*	−12·7% (−15·1 to −10·4)*
..			Coal worker pneumoconiosis		3 (3 to 4)	3 (3 to 4)	−2·2% (−12·0 to 11·7)	−26·6% (−33·8 to −16·7)*	81 (65 to 99)	81 (69 to 100)	0·1% (−8·8 to 12·9)	−23·0% (−29·8 to −13·3)*
..			Other pneumoconiosis		3 (3 to 4)	4 (3 to 4)	8·8% (0·0 to 25·4)*	−17·5% (−24·1 to −5·0)*	90 (74 to 110)	97 (82 to 117)	7·6% (0·6 to 20·4)*	−15·9% (−21·4 to −5·5)*
3		Occupational noise: all causes			..	..	..	..	4830 (3300 to 6830)	5980 (4080 to 8430)	23·7% (22·4 to 25·0)*	0·7% (−0·2 to 1·6)
..			Age-related and other hearing loss		..	..	..	..	4830 (3300 to 6830)	5980 (4080 to 8430)	23·7% (22·4 to 25·0)*	0·7% (−0·2 to 1·6)
3		Occupational injuries: all causes			348 (327 to 370)	304 (288 to 323)	−12·8% (−17·3 to −7·8)*	−24·5% (−28·4 to −20·1)*	22 700 (20 800 to 24 800)	21 100 (19 200 to 23 500)	−7·0% (−11·8 to −1·4)*	−18·4% (−22·7 to −13·5)*
..		Pedestrian road injuries			71 (64 to 81)	58 (53 to 66)	−18·1% (−24·3 to −11·2)*	−29·6% (−35·0 to −23·7)*	3840 (3460 to 4350)	3190 (2880 to 3600)	−16·8% (−23·0 to −10·0)*	−27·2% (−32·6 to −21·3)*
..		Cyclist road injuries			10 (9 to 12)	10 (8 to 11)	−8·4% (−17·8 to 2·2)	−21·8% (−29·8 to −12·6)*	709 (600 to 821)	705 (588 to 832)	−0·6% (−9·2 to 8·1)	−13·9% (−21·4 to −6·1)*
..		Motorcyclist road injuries			45 (39 to 50)	39 (34 to 43)	−13·8% (−21·5 to −5·8)*	−23·7% (−30·5 to −16·7)*	2790 (2460 to 3110)	2470 (2190 to 2740)	−11·6% (−18·6 to −4·4)*	−21·4% (−27·4 to −14·8)*
..		Motor vehicle road injuries			68 (62 to 76)	62 (58 to 68)	−9·0% (−13·3 to −3·9)*	−20·0% (−23·9 to −15·5)*	3860 (3540 to 4220)	3540 (3310 to 3860)	−8·1% (−12·4 to −3·4)*	−18·3% (−22·1 to −14·1)*
..		Other road injuries			2 (1 to 2)	2 (1 to 2)	−17·5% (−24·5 to 1·9)	−28·6% (−34·6 to −12·0)*	162 (133 to 196)	170 (136 to 211)	4·9% (−3·9 to 17·0)	−8·7% (−16·3 to 1·4)
..		Other transport injuries			17 (15 to 20)	15 (13 to 18)	−13·5% (−20·0 to −6·8)*	−24·5% (−30·2 to −18·6)*	1300 (1130 to 1500)	1200 (1020 to 1400)	−7·4% (−13·8 to −1·1)*	−18·4% (−24·2 to −12·8)*
..		Falls			37 (32 to 41)	36 (32 to 39)	−3·0% (−10·7 to 6·6)	−19·0% (−25·5 to −11·0)*	3390 (2850 to 4090)	3700 (3030 to 4540)	9·1% (2·6 to 16·4)*	−6·6% (−12·2 to −0·5)*
..		Drowning			26 (25 to 28)	23 (21 to 24)	−13·9% (−19·3 to −8·1)*	−24·5% (−29·3 to −19·4)*	1380 (1290 to 1480)	1170 (1090 to 1260)	−15·3% (−20·6 to −9·6)*	−24·2% (−29·0 to −19·1)*
..		Fire, heat, and hot substances			10 (8 to 11)	8 (7 to 9)	−14·2% (−18·6 to −5·3)*	−26·3% (−30·0 to −18·4)*	898 (753 to 1060)	878 (719 to 1070)	−2·1% (−7·5 to 4·2)	−14·2% (−18·9 to −8·7)*
..		Poisoning by carbon monoxide			5 (4 to 5)	4 (3 to 4)	−14·8% (−23·9 to −4·5)*	−27·0% (−34·9 to −18·3)*	230 (182 to 257)	197 (147 to 223)	−14·3% (−22·8 to −4·4)*	−25·2% (−32·6 to −16·6)*
..		Poisoning by other means			4 (3 to 5)	4 (3 to 4)	−6·3% (−16·3 to 5·4)	−19·4% (−28·0 to −9·4)*	234 (183 to 265)	230 (174 to 266)	−1·6% (−9·8 to 9·1)	−13·6% (−20·8 to −4·3)*
..		Unintentional firearm injuries			4 (3 to 4)	3 (3 to 4)	−10·3% (−16·0 to −4·2)*	−20·9% (−25·7 to −15·5)*	237 (210 to 270)	224 (199 to 256)	−5·3% (−10·7 to 0·6)	−15·7% (−20·5 to −10·5)*
..		Other exposure to mechanical forces			18 (15 to 19)	15 (13 to 16)	−17·5% (−23·3 to −11·0)*	−28·8% (−33·7 to −23·2)*	1420 (1220 to 1660)	1390 (1160 to 1690)	−2·0% (−9·0 to 5·4)	−14·8% (−20·7 to −8·4)*
..		Venomous animal contact			7 (4 to 8)	6 (3 to 7)	−19·2% (−29·8 to −5·9)*	−30·6% (−39·7 to −19·1)*	430 (269 to 524)	368 (245 to 440)	−14·3% (−25·0 to −2·1)*	−24·6% (−34·0 to −14·0)*
..		Non-venomous animal contact			1 (1 to 1)	1 (1 to 1)	−9·7% (−18·5 to 1·3)	−23·1% (−30·6 to −13·5)*	93 (69 to 125)	90 (68 to 121)	−3·4% (−11·0 to 3·5)	−16·0% (−22·6 to −10·0)*
..			Pulmonary aspiration and foreign body in airway		5 (5 to 5)	5 (5 to 6)	3·5% (−0·6 to 7·2)	−12·2% (−15·7 to −9·2)*	254 (241 to 271)	263 (247 to 281)	3·5% (−0·5 to 7·5)	−10·2% (−13·6 to −6·8)*
..			Foreign body in other body part		1 (1 to 1)	1 (1 to 1)	−2·1% (−8·5 to 4·0)	−15·4% (−20·8 to −10·2)*	109 (85 to 135)	120 (92 to 150)	10·9% (5·1 to 16·5)*	−3·4% (−8·4 to 1·5)
..			Other unintentional injuries		18 (17 to 20)	14 (13 to 16)	−23·3% (−29·2 to −16·8)*	−32·9% (−38·0 to −27·1)*	1360 (1200 to 1550)	1200 (1030 to 1430)	−11·9% (−18·4 to −5·0)*	−22·7% (−28·2 to −16·8)*
3		Occupational ergonomic factors: all causes			..	..	..	..	14 200 (10 000 to 19 400)	15 900 (11 200 to 21 800)	12·5% (10·6 to 14·5)*	−5·0% (−6·3 to −3·6)*
..			Low back pain		..	..	..	..	14 200 (10 000 to 19 400)	15 900 (11 200 to 21 800)	12·5% (10·6 to 14·5)*	−5·0% (−6·3 to −3·6)*
**1**		**Behavioural risks: all causes**			**23 200 (22 600 to 23 800)**	**23 800 (23 100 to 24 600)**	2·7% (1·2 to 4·1)*	−18·7% (−19·9 to −17·7)*	**1 020 000 (970 000 to 1 060 000)**	**913 000 (863 000 to 961 000)**	−10·1% (−11·9 to −8·4)*	−23·1% (−24·6 to −21·6)*
**2**		**Child and maternal malnutrition: all causes**			**4650 (4450 to 4880)**	**3190 (3020 to 3360)**	−31·4% (−34·4 to −28·6)*	−34·2% (−37·1 to −31·5)*	**455 000 (430 000 to 482 000)**	**327 000 (303 000 to 352 000)**	−28·2% (−31·2 to −25·3)*	−31·5% (−34·3 to −28·8)*
3		Suboptimal breastfeeding: all causes			270 (200 to 338)	169 (132 to 210)	−37·1% (−42·4 to −30·9)*	−39·4% (−44·4 to −33·4)*	23 800 (17 700 to 29 900)	15 000 (11 700 to 18 600)	−36·8% (−42·0 to −30·6)*	−39·0% (−44·1 to −33·1)*
4			Non-exclusive breastfeeding: all causes		256 (191 to 324)	161 (124 to 201)	−37·1% (−42·4 to −30·8)*	−39·3% (−44·4 to −33·2)*	22 600 (16 800 to 28 500)	14 200 (11 000 to 17 800)	−36·9% (−42·1 to −30·6)*	−39·0% (−44·1 to −33·0)*
..				Lower respiratory infections	97 (52 to 150)	60 (34 to 91)	−38·1% (−43·0 to −30·8)*	−40·2% (−44·9 to −33·1)*	8530 (4530 to 13 100)	5280 (2940 to 7960)	−38·1% (−43·0 to −30·8)*	−40·2% (−44·9 to −33·1)*
..				Diarrhoeal diseases	159 (118 to 199)	101 (77 to 127)	−36·5% (−44·0 to −28·1)*	−38·7% (−45·9 to −30·6)*	14 000 (10 500 to 17 600)	8970 (6850 to 11 300)	−36·1% (−43·5 to −27·8)*	−38·4% (−45·5 to −30·3)*
4			Discontinued breastfeeding: all causes		16 (5 to 28)	10 (4 to 18)	−36·7% (−46·5 to −25·2)*	−39·9% (−49·2 to −28·9)*	1420 (488 to 2550)	931 (322 to 1630)	−34·7% (−44·2 to −23·1)*	−37·9% (−47·0 to −26·9)*
..				Diarrhoeal diseases	16 (5 to 28)	10 (4 to 18)	−36·7% (−46·5 to −25·2)*	−39·9% (−49·2 to −28·9)*	1420 (488 to 2550)	931 (322 to 1630)	−34·7% (−44·2 to −23·1)*	−37·9% (−47·0 to −26·9)*
3		Child growth failure: all causes			1980 (1800 to 2170)	1190 (1060 to 1300)	−40·1% (−43·9 to −36·0)*	−43·6% (−47·2 to −39·8)*	170 000 (155 000 to 186 000)	100 000 (89 800 to 110 000)	−40·9% (−44·7 to −37·0)*	−43·8% (−47·5 to −40·1)*
4			Child underweight: all causes		710 (606 to 853)	417 (365 to 498)	−41·3% (−45·7 to −36·1)*	−46·2% (−50·1 to −41·6)*	58 700 (49 700 to 71 300)	32 400 (28 000 to 39 500)	−44·7% (−48·9 to −40·0)*	−47·9% (−51·8 to −43·5)*
..				Lower respiratory infections	181 (121 to 310)	93 (61 to 169)	−48·8% (−53·5 to −44·1)*	−51·2% (−55·6 to −46·6)*	15 700 (10 500 to 26 900)	8050 (5270 to 14 700)	−48·8% (−53·4 to −44·1)*	−51·1% (−55·6 to −46·6)*
..				Diarrhoeal diseases	139 (113 to 177)	66 (52 to 83)	−53·0% (−59·3 to −45·6)*	−55·3% (−61·3 to −48·2)*	12 300 (10 000 to 15 600)	5860 (4670 to 7430)	−52·4% (−58·7 to −45·2)*	−54·7% (−60·7 to −47·8)*
..				Measles	76 (16 to 199)	27 (5 to 75)	−64·9% (−72·1 to −59·0)*	−66·8% (−73·6 to −61·2)*	6560 (1400 to 17 200)	2300 (437 to 6480)	−65·0% (−72·1 to −59·0)*	−66·8% (−73·6 to −61·2)*
..				Protein-energy malnutrition	313 (288 to 339)	232 (212 to 254)	−26·1% (−31·7 to −18·0)*	−34·6% (−39·4 to −27·5)*	24 100 (21 800 to 26 400)	16 200 (14 500 to 18 000)	−32·6% (−38·3 to −24·7)*	−37·2% (−42·5 to −29·7)*
4			Child wasting: all causes		1770 (1480 to 2020)	1080 (891 to 1220)	−39·1% (−43·1 to −35·0)*	−42·8% (−46·6 to −38·9)*	152 000 (127 000 to 173 000)	91 000 (75 400 to 104 000)	−40·1% (−44·2 to −36·0)*	−43·1% (−47·1 to −39·2)*
..				Lower respiratory infections	700 (481 to 841)	426 (281 to 523)	−39·2% (−44·2 to −34·6)*	−41·9% (−46·7 to −37·5)*	60 900 (41 800 to 73 100)	37 000 (24 500 to 45 500)	−39·1% (−44·1 to −34·5)*	−41·8% (−46·6 to −37·4)*
..				Diarrhoeal diseases	680 (534 to 788)	389 (288 to 452)	−42·8% (−50·5 to −33·8)*	−45·5% (−52·9 to −36·9)*	60 300 (47 200 to 69 700)	35 100 (26 000 to 40 800)	−41·8% (−49·6 to −33·1)*	−44·6% (−52·0 to −36·3)*
..				Measles	76 (11 to 246)	30 (4 to 108)	−60·2% (−68·0 to −53·9)*	−62·3% (−69·6 to −56·4)*	6610 (942 to 21 400)	2630 (322 to 9370)	−60·2% (−68·0 to −53·9)*	−62·2% (−69·6 to −56·4)*
..				Protein-energy malnutrition	313 (288 to 339)	232 (212 to 254)	−26·1% (−31·7 to −18·0)*	−34·6% (−39·4 to −27·5)*	24 100 (21 800 to 26 400)	16 200 (14 500 to 18 000)	−32·6% (−38·3 to −24·7)*	−37·2% (−42·5 to −29·7)*
4			Child stunting: all causes		458 (237 to 754)	221 (103 to 395)	−51·8% (−57·8 to −46·0)*	−54·2% (−59·9 to −48·5)*	40 000 (20 700 to 65 700)	19 400 (9070 to 34 600)	−51·5% (−57·3 to −45·8)*	−53·9% (−59·5 to −48·3)*
..				Lower respiratory infections	229 (30 to 505)	119 (13 to 285)	−48·0% (−58·0 to −42·3)*	−50·4% (−59·9 to −44·9)*	19 900 (2600 to 43 900)	10 300 (1120 to 24 800)	−47·9% (−57·9 to −42·2)*	−50·3% (−59·9 to −44·8)*
..				Diarrhoeal diseases	174 (69 to 303)	81 (30 to 149)	−53·3% (−61·1 to −44·7)*	−55·5% (−63·0 to −47·4)*	15 400 (6100 to 26 700)	7310 (2720 to 13 400)	−52·5% (−60·3 to −44·2)*	−54·8% (−62·3 to −46·9)*
..				Measles	56 (6 to 158)	20 (2 to 61)	−63·5% (−72·0 to −57·8)*	−65·4% (−73·5 to −60·1)*	4810 (513 to 13 700)	1760 (168 to 5310)	−63·5% (−72·0 to −57·8)*	−65·4% (−73·5 to −60·1)*
3		Low birthweight and short gestation: all causes			2490 (2400 to 2570)	1880 (1790 to 1970)	−24·4% (−27·4 to −21·2)*	−26·4% (−29·4 to −23·3)*	229 000 (221 000 to 237 000)	178 000 (169 000 to 187 000)	−22·1% (−25·3 to −18·7)*	−24·7% (−27·6 to −21·5)*
4			Short gestation for birthweight: all causes		1890 (1820 to 1970)	1430 (1360 to 1510)	−24·2% (−27·4 to −21·0)*	−26·3% (−29·4 to −23·1)*	176 000 (170 000 to 184 000)	139 000 (131 000 to 147 000)	−21·3% (−24·4 to −17·6)*	−24·0% (−27·0 to −20·6)*
..				Lower respiratory infections	186 (172 to 200)	129 (117 to 142)	−30·7% (−36·5 to −23·8)*	−32·6% (−38·3 to −25·9)*	16 300 (15 100 to 17 600)	11 300 (10 300 to 12 400)	−30·7% (−36·5 to −23·8)*	−32·6% (−38·3 to −25·9)*
..				Upper respiratory infections	0 (0 to 0)	0 (0 to 0)	−38·4% (−48·3 to −21·7)*	−40·2% (−49·8 to −23·9)*	10 (5 to 13)	6 (4 to 9)	−38·4% (−48·3 to −21·7)*	−40·2% (−49·8 to −23·9)*
..				Otitis media	0 (0 to 0)	0 (0 to 0)	−51·6% (−70·5 to −22·3)*	−53·0% (−71·3 to −24·5)*	3 (1 to 11)	2 (1 to 6)	−51·6% (−70·5 to −22·3)*	−53·0% (−71·3 to −24·5)*
..				Diarrhoeal diseases	48 (43 to 53)	31 (27 to 35)	−35·1% (−43·6 to −25·9)*	−37·0% (−45·2 to −28·0)*	4180 (3790 to 4620)	2710 (2410 to 3040)	−35·1% (−43·6 to −25·9)*	−37·0% (−45·2 to −28·0)*
..				Pneumococcal meningitis	2 (2 to 3)	2 (1 to 2)	−20·4% (−31·4 to −6·9)*	−22·6% (−33·3 to −9·4)*	187 (148 to 224)	149 (118 to 183)	−20·4% (−31·4 to −6·8)*	−22·6% (−33·3 to −9·4)*
..				*H influenzae* type B meningitis	4 (4 to 5)	3 (2 to 3)	−40·9% (−48·8 to −31·0)*	−42·6% (−50·2 to −33·0)*	382 (309 to 462)	225 (176 to 277)	−40·9% (−48·8 to −31·0)*	−42·6% (−50·2 to −33·0)*
..				Meningococcal infection	2 (2 to 3)	2 (1 to 2)	−34·6% (−42·8 to −24·4)*	−36·5% (−44·4 to −26·5)*	211 (175 to 246)	138 (109 to 167)	−34·6% (−42·8 to −24·4)*	−36·4% (−44·4 to −26·5)*
..				Other meningitis	7 (5 to 8)	6 (5 to 7)	−11·8% (−22·6 to 2·5)	−14·2% (−24·8 to −0·3)*	573 (475 to 664)	505 (413 to 592)	−11·8% (−22·6 to 2·5)	−14·2% (−24·8 to −0·3)*
..				Encephalitis	1 (1 to 1)	1 (1 to 1)	−16·0% (−33·2 to −1·7)*	−18·4% (−35·1 to −4·4)*	95 (85 to 107)	80 (67 to 89)	−16·0% (−33·2 to −1·7)*	−18·4% (−35·1 to −4·4)*
..				Neonatal preterm birth	879 (830 to 991)	649 (605 to 721)	−26·2% (−31·3 to −21·5)*	−28·1% (−33·2 to −23·6)*	87 700 (82 100 to 97 300)	70 200 (64 400 to 77 200)	−19·9% (−25·3 to −14·4)*	−23·3% (−28·4 to −18·3)*
..				Neonatal encephalopathy due to birth asphyxia and trauma	351 (314 to 379)	281 (248 to 309)	−20·0% (−26·6 to −12·4)*	−22·1% (−28·5 to −14·7)*	30 800 (27 600 to 33 300)	24 700 (21 800 to 27 200)	−20·0% (−26·6 to −12·4)*	−22·1% (−28·5 to −14·7)*
..				Neonatal sepsis and other neonatal infections	120 (106 to 157)	111 (97 to 144)	−8·1% (−17·1 to 2·0)	−10·6% (−19·3 to −0·8)*	10 600 (9280 to 13 800)	9710 (8540 to 12 700)	−8·1% (−17·1 to 2·0)	−10·6% (−19·3 to −0·8)*
..				Haemolytic disease and other neonatal jaundice	44 (38 to 50)	28 (24 to 32)	−36·3% (−44·7 to −26·2)*	−38·0% (−46·2 to −28·2)*	3840 (3340 to 4380)	2450 (2140 to 2810)	−36·3% (−44·7 to −26·2)*	−38·0% (−46·2 to −28·2)*
..				Other neonatal disorders	242 (210 to 266)	188 (160 to 207)	−22·3% (−28·9 to −13·9)*	−24·4% (−30·8 to −16·1)*	21 300 (18 400 to 23 400)	16 500 (14 000 to 18 200)	−22·3% (−28·9 to −13·9)*	−24·4% (−30·8 to −16·1)*
..				Sudden infant death syndrome	3 (1 to 5)	2 (1 to 4)	−20·9% (−31·2 to −4·0)*	−23·2% (−33·2 to −6·8)*	262 (103 to 467)	208 (98 to 351)	−20·9% (−31·2 to −4·0)*	−23·2% (−33·2 to −6·8)*
4			Low birthweight for gestation: all causes		1480 (1410 to 1570)	1100 (1040 to 1160)	−25·7% (−28·9 to −22·2)*	−27·6% (−30·8 to −24·3)*	140 000 (134 000 to 148 000)	110 000 (103 000 to 117 000)	−21·8% (−25·4 to −17·9)*	−24·7% (−28·0 to −21·0)*
..				Lower respiratory infections	95 (86 to 105)	63 (56 to 71)	−33·9% (−40·6 to −26·4)*	−35·6% (−42·2 to −28·3)*	8350 (7530 to 9270)	5520 (4880 to 6260)	−33·8% (−40·6 to −26·4)*	−35·6% (−42·2 to −28·3)*
..				Upper respiratory infections	0 (0 to 0)	0 (0 to 0)	−45·8% (−54·9 to −30·6)*	−47·3% (−56·1 to −32·5)*	5 (3 to 7)	3 (2 to 4)	−45·8% (−54·9 to −30·6)*	−47·3% (−56·1 to −32·5)*
..				Otitis media	0 (0 to 0)	0 (0 to 0)	−48·9% (−68·4 to −16·0)*	−50·3% (−69·3 to −18·4)*	2 (0 to 7)	1 (0 to 4)	−48·9% (−68·4 to −16·0)*	−50·3% (−69·3 to −18·4)*
..				Diarrhoeal diseases	18 (16 to 21)	11 (10 to 13)	−37·4% (−46·1 to −28·8)*	−39·1% (−47·5 to −30·7)*	1610 (1400 to 1830)	1010 (878 to 1150)	−37·4% (−46·1 to −28·8)*	−39·1% (−47·5 to −30·7)*
..				Pneumococcal meningitis	1 (1 to 1)	1 (1 to 1)	−28·4% (−39·6 to −14·4)*	−30·4% (−41·3 to −16·7)*	100 (76 to 124)	72 (56 to 89)	−28·4% (−39·6 to −14·4)*	−30·4% (−41·3 to −16·7)*
..				*H influenzae* type B meningitis	2 (2 to 3)	1 (1 to 1)	−46·5% (−53·9 to −37·0)*	−48·0% (−55·2 to −38·7)*	177 (134 to 227)	95 (71 to 118)	−46·5% (−53·9 to −37·0)*	−48·0% (−55·2 to −38·7)*
..				Meningococcal infection	1 (1 to 1)	1 (1 to 1)	−37·6% (−46·6 to −26·6)*	−39·2% (−48·0 to −28·6)*	104 (81 to 126)	65 (50 to 80)	−37·6% (−46·6 to −26·6)*	−39·2% (−48·0 to −28·6)*
..				Other meningitis	3 (3 to 4)	3 (2 to 3)	−16·0% (−27·6 to −2·3)*	−18·3% (−29·5 to −5·0)*	286 (229 to 340)	240 (191 to 284)	−16·0% (−27·6 to −2·3)*	−18·3% (−29·5 to −5·0)*
..				Encephalitis	0 (0 to 0)	0 (0 to 0)	−16·7% (−32·2 to −3·4)*	−19·0% (−34·1 to −6·0)*	38 (33 to 44)	31 (27 to 36)	−16·7% (−32·2 to −3·4)*	−19·0% (−34·1 to −6·0)*
..				Neonatal preterm birth	879 (830 to 991)	649 (605 to 721)	−26·2% (−31·3 to −21·5)*	−28·1% (−33·2 to −23·6)*	87 700 (82 100 to 97 300)	70 200 (64 400 to 77 200)	−19·9% (−25·3 to −14·4)*	−23·3% (−28·4 to −18·3)*
..				Neonatal encephalopathy due to birth asphyxia and trauma	249 (221 to 271)	188 (166 to 207)	−24·6% (−30·7 to −17·7)*	−26·6% (−32·5 to −19·8)*	21 900 (19 400 to 23 800)	16 500 (14 600 to 18 200)	−24·6% (−30·7 to −17·7)*	−26·6% (−32·5 to −19·8)*
..				Neonatal sepsis and other neonatal infections	64 (55 to 90)	57 (49 to 80)	−10·8% (−20·3 to 0·0)*	−13·2% (−22·4 to −2·7)*	5620 (4830 to 7910)	5010 (4320 to 6990)	−10·8% (−20·3 to 0·0)*	−13·2% (−22·4 to −2·7)*
..				Haemolytic disease and other neonatal jaundice	21 (18 to 24)	13 (12 to 15)	−35·7% (−44·1 to −26·2)*	−37·4% (−45·6 to −28·2)*	1810 (1570 to 2080)	1160 (1010 to 1330)	−35·7% (−44·1 to −26·2)*	−37·4% (−45·6 to −28·2)*
..				Other neonatal disorders	141 (121 to 156)	109 (92 to 121)	−22·8% (−29·7 to −13·9)*	−24·8% (−31·5 to −16·2)*	12 400 (10 600 to 13 700)	9560 (8060 to 10 600)	−22·8% (−29·7 to −13·9)*	−24·8% (−31·5 to −16·2)*
..				Sudden infant death syndrome	1 (0 to 1)	1 (0 to 1)	−22·7% (−33·6 to −7·6)*	−24·9% (−35·5 to −10·3)*	68 (32 to 127)	52 (27 to 93)	−22·7% (−33·6 to −7·6)*	−24·9% (−35·5 to −10·3)*
3		Iron deficiency: all causes			89 (36 to 133)	60 (24 to 91)	−32·6% (−37·0 to −28·4)*	−38·5% (−42·5 to −34·6)*	37 100 (25 800 to 51 300)	33 700 (23 200 to 47 200)	−9·3% (−13·2 to −5·3)*	−17·6% (−21·1 to −14·1)*
..			Maternal haemorrhage		28 (12 to 44)	12 (5 to 19)	−56·9% (−64·0 to −48·6)*	−60·7% (−67·3 to −53·2)*	1650 (670 to 2570)	702 (281 to 1100)	−57·4% (−64·6 to −48·7)*	−60·9% (−67·6 to −53·1)*
..			Maternal sepsis and other pregnancy related infections		11 (4 to 17)	7 (3 to 11)	−36·7% (−49·0 to −23·8)*	−42·1% (−53·2 to −30·3)*	634 (265 to 1010)	397 (156 to 635)	−37·4% (−49·4 to −24·1)*	−42·3% (−53·3 to −30·0)*
..			Maternal hypertensive disorders		10 (4 to 16)	9 (3 to 14)	−16·0% (−32·6 to 2·1)	−22·6% (−37·9 to −5·8)*	659 (262 to 1050)	550 (212 to 863)	−16·6% (−32·7 to 0·5)	−22·7% (−37·6 to −6·9)*
..			Maternal obstructed labour and uterine rupture		6 (2 to 10)	4 (2 to 7)	−26·8% (−44·8 to −5·1)*	−33·5% (−49·6 to −13·6)*	461 (188 to 761)	343 (139 to 561)	−25·6% (−40·2 to −10·8)*	−32·1% (−45·2 to −18·5)*
..				Maternal abortive outcome	6 (3 to 10)	5 (2 to 9)	−17·0% (−32·9 to 0·5)	−24·7% (−38·9 to −8·9)*	374 (152 to 602)	304 (118 to 484)	−18·7% (−35·0 to −0·3)*	−25·5% (−40·1 to −9·0)*
..				Ectopic pregnancy	4 (2 to 9)	3 (1 to 6)	−21·9% (−51·6 to 20·4)	−28·4% (−55·1 to 10·5)	263 (92 to 541)	203 (75 to 378)	−23·0% (−51·4 to 19·5)	−29·0% (−55·2 to 9·9)
..				Indirect maternal deaths	12 (5 to 20)	10 (4 to 17)	−14·5% (−28·4 to −0·6)*	−21·9% (−34·7 to −9·5)*	714 (287 to 1150)	598 (233 to 956)	−16·2% (−30·5 to −1·9)*	−23·0% (−36·3 to −10·3)*
..				Late maternal deaths	1 (0 to 2)	1 (0 to 2)	−13·3% (−19·6 to −7·3)*	−20·7% (−26·2 to −15·2)*	65 (26 to 104)	56 (21 to 92)	−14·3% (−20·4 to −8·3)*	−21·2% (−26·6 to −15·7)*
..				Maternal deaths aggravated by HIV/AIDS	1 (0 to 1)	0 (0 to 1)	−30·3% (−37·6 to −22·8)*	−37·8% (−44·3 to −31·1)*	38 (15 to 63)	25 (9 to 44)	−32·8% (−39·9 to −25·6)*	−39·7% (−46·2 to −33·2)*
..				Other maternal disorders	9 (4 to 15)	8 (3 to 12)	−18·8% (−35·7 to 2·8)	−25·8% (−41·4 to −6·0)*	577 (227 to 936)	470 (184 to 736)	−18·6% (−36·0 to 2·5)	−25·2% (−40·9 to −5·8)*
..				Dietary iron deficiency	..	..	..	..	31 700 (21 400 to 45 200)	30 000 (20 300 to 43 600)	−5·2% (−8·4 to −1·9)*	−14·3% (−17·2 to −11·4)*
3			Vitamin A deficiency: all causes		437 (339 to 550)	233 (179 to 294)	−46·8% (−51·4 to −42·0)*	−49·2% (−53·7 to −44·6)*	48 500 (38 700 to 59 900)	29 000 (23 000 to 35 600)	−40·2% (−44·7 to −35·8)*	−43·3% (−47·6 to −39·0)*
..				Lower respiratory infections	94 (36 to 161)	53 (20 to 92)	−43·4% (−47·6 to −39·3)*	−45·9% (−49·8 to −41·9)*	8130 (3110 to 14 000)	4600 (1750 to 8000)	−43·4% (−47·6 to −39·3)*	−45·8% (−49·8 to −41·9)*
..				Diarrhoeal diseases	263 (217 to 308)	146 (120 to 173)	−44·5% (−51·0 to −37·1)*	−47·1% (−53·3 to −40·0)*	23 300 (19 300 to 27 300)	13 100 (10 800 to 15 500)	−43·7% (−50·1 to −36·5)*	−46·3% (−52·4 to −39·4)*
..				Measles	81 (27 to 181)	34 (11 to 78)	−58·0% (−63·3 to −53·0)*	−60·2% (−65·2 to −55·4)*	6980 (2310 to 15 700)	2930 (961 to 6750)	−58·0% (−63·2 to −53·0)*	−60·2% (−65·1 to −55·4)*
..				Vitamin A deficiency	..	..	..	..	10 000 (6650 to 14 500)	8310 (5400 to 12 200)	−17·3% (−20·8 to −13·6)*	−22·3% (−25·7 to −18·8)*
3			Zinc deficiency: all causes		60 (3 to 157)	29 (1 to 77)	−52·3% (−61·6 to −41·7)*	−55·2% (−64·0 to −45·3)*	5290 (398 to 13 600)	2580 (235 to 6750)	−51·2% (−60·2 to −36·4)*	−54·2% (−62·6 to −40·2)*
..				Lower respiratory infections	24 (0 to 98)	12 (0 to 52)	−49·3% (−58·1 to 0)	−57·2% (−60·4 to 0)	2100 (3 to 8430)	1070 (2 to 4460)	−49·2% (−57·9 to −21·2)*	−52·2% (−60·4 to −26·0)*
..				Diarrhoeal diseases	36 (0 to 102)	16 (0 to 48)	−54·4% (−63·4 to 0)	−52·3% (−65·6 to 0)	3190 (132 to 8890)	1510 (113 to 4270)	−52·6% (−61·0 to −12·4)*	−55·4% (−63·3 to −17·7)*
**2**			**Tobacco: all causes**		**7280 (7010 to 7560)**	**8100 (7790 to 8420)**	11·3% (9·1 to 13·4)*	−15·9% (−17·6 to −14·4)*	**200 000 (188 000 to 212 000)**	**213 000 (201 000 to 227 000)**	6·8% (4·6 to 9·0)*	−16·8% (−18·5 to −15·2)*
3			Smoking: all causes		6380 (6170 to 6590)	7100 (6830 to 7370)	11·2% (8·8 to 13·5)*	−16·2% (−18·0 to −14·4)*	169 000 (160 000 to 177 000)	182 000 (173 000 to 193 000)	8·2% (6·0 to 10·3)*	−16·4% (−18·1 to −14·7)*
..				Drug-susceptible tuberculosis	200 (163 to 234)	156 (124 to 188)	−22·1% (−29·6 to −14·5)*	−38·7% (−44·6 to −32·8)*	6740 (5450 to 7860)	5210 (4140 to 6290)	−22·7% (−29·7 to −15·3)*	−37·5% (−43·1 to −31·7)*
..				Multidrug-resistant tuberculosis without extensive drug resistance	26 (20 to 34)	20 (11 to 32)	−26·0% (−54·4 to 16·5)	−41·4% (−63·9 to −7·6)*	887 (671 to 1130)	636 (368 to 1030)	−28·3% (−54·8 to 10·7)	−41·7% (−63·2 to −9·8)*
..				Extensively drug-resistant tuberculosis	3 (2 to 4)	3 (2 to 4)	−2·6% (−26·1 to 27·8)	−21·8% (−40·8 to 2·5)	110 (83 to 140)	103 (74 to 138)	−6·5% (−28·6 to 20·6)	−23·1% (−41·4 to −1·0)*
..				Lower respiratory infections	241 (180 to 301)	265 (194 to 338)	9·9% (5·2 to 13·8)*	−17·6% (−21·0 to −14·8)*	5200 (3950 to 6410)	5410 (4020 to 6820)	4·0% (−0·6 to 7·9)	−18·9% (−22·5 to −15·9)*
..				Lip and oral cavity cancer	48 (40 to 56)	59 (48 to 69)	22·2% (15·8 to 27·9)*	−7·2% (−12·0 to −2·8)*	1270 (1030 to 1500)	1500 (1190 to 1780)	17·9% (11·3 to 23·6)*	−9·1% (−14·1 to −4·6)*
..				Nasopharynx cancer	15 (11 to 19)	18 (13 to 23)	19·7% (13·9 to 26·5)*	−8·2% (−12·5 to −3·0)*	427 (303 to 541)	492 (350 to 627)	15·3% (9·3 to 22·3)*	−10·0% (−14·5 to −4·9)*
..				Other pharynx cancer	39 (32 to 45)	51 (41 to 59)	29·9% (18·4 to 39·8)*	−1·1% (−9·9 to 6·5)	1060 (867 to 1220)	1330 (1050 to 1580)	25·7% (14·4 to 35·6)*	−3·2% (−12·0 to 4·5)
..				Oesophageal cancer	151 (137 to 164)	170 (156 to 186)	13·1% (8·4 to 18·0)*	−14·4% (−17·9 to −10·6)*	3520 (3170 to 3850)	3810 (3470 to 4170)	8·3% (3·8 to 12·8)*	−17·1% (−20·5 to −13·6)*
..				Stomach cancer	147 (120 to 174)	155 (126 to 183)	5·1% (0·8 to 10·0)*	−20·6% (−23·7 to −17·0)*	3220 (2570 to 3850)	3270 (2640 to 3890)	1·6% (−2·6 to 6·5)	−22·4% (−25·6 to −18·8)*
..				Colon and rectum cancer	101 (68 to 134)	119 (79 to 160)	18·2% (13·6 to 22·6)*	−11·3% (−14·9 to −7·8)*	2190 (1420 to 2870)	2540 (1620 to 3390)	16·0% (11·2 to 20·2)*	−11·8% (−15·5 to −8·5)*
..				Liver cancer due to hepatitis B	53 (28 to 78)	64 (35 to 92)	20·7% (9·5 to 38·7)*	−7·1% (−15·1 to 5·9)	1520 (742 to 2250)	1760 (933 to 2560)	16·0% (5·2 to 34·1)*	−9·6% (−17·0 to 4·0)
..				Liver cancer due to hepatitis C	33 (19 to 46)	41 (23 to 58)	25·9% (19·2 to 34·1)*	−5·4% (−10·1 to 0·4)	712 (388 to 1020)	880 (491 to 1260)	23·5% (17·0 to 31·7)*	−6·2% (−10·7 to −0·5)*
..				Liver cancer due to alcohol use	20 (11 to 30)	26 (14 to 38)	26·0% (19·6 to 33·8)*	−4·1% (−8·8 to 1·2)	484 (266 to 712)	595 (319 to 888)	23·1% (16·4 to 31·1)*	−5·4% (−9·9 to 0·1)
..				Liver cancer due to non-alcoholic steatohepatitis	8 (4 to 12)	11 (6 to 16)	39·6% (30·5 to 51·3)*	5·3% (−1·3 to 13·7)	179 (96 to 261)	244 (128 to 353)	36·1% (26·8 to 47·9)*	4·1% (−2·4 to 12·7)
..				Liver cancer due to other causes	8 (4 to 11)	10 (5 to 14)	29·8% (18·6 to 45·9)*	−0·7% (−8·6 to 10·5)	205 (104 to 304)	255 (135 to 370)	24·2% (13·2 to 41·5)*	−3·4% (−10·8 to 8·2)
..				Pancreatic cancer	75 (67 to 83)	94 (83 to 105)	24·9% (21·1 to 28·4)*	−6·3% (−9·3 to −3·6)*	1570 (1380 to 1750)	1910 (1670 to 2140)	21·6% (17·7 to 24·9)*	−7·6% (−10·5 to −5·1)*
..				Larynx cancer	67 (59 to 73)	77 (68 to 84)	14·9% (11·4 to 18·0)*	−12·7% (−15·4 to −10·3)*	1730 (1540 to 1900)	1930 (1700 to 2130)	11·3% (7·6 to 14·6)*	−14·5% (−17·4 to −12·0)*
..				Tracheal, bronchus, and lung cancer	965 (940 to 992)	1190 (1150 to 1230)	23·3% (19·9 to 26·4)*	−6·9% (−9·4 to −4·6)*	21 000 (20 400 to 21 700)	25 100 (24 100 to 26 100)	19·3% (15·8 to 22·4)*	−9·1% (−11·7 to −6·8)*
..				Breast cancer	16 (12 to 21)	17 (12 to 22)	5·2% (1·9 to 8·4)*	−20·4% (−23·1 to −17·7)*	447 (317 to 582)	451 (320 to 593)	0·8% (−2·5 to 3·8)	−22·0% (−24·9 to −19·5)*
..				Cervical cancer	27 (14 to 43)	27 (14 to 43)	−0·5% (−6·1 to 4·5)	−23·2% (−27·6 to −19·0)*	807 (443 to 1260)	766 (414 to 1190)	−5·0% (−10·0 to −0·4)*	−24·8% (−28·9 to −20·7)*
..				Prostate cancer	22 (10 to 34)	25 (10 to 40)	15·7% (7·1 to 23·3)*	−13·9% (−20·1 to −8·4)*	414 (188 to 640)	475 (206 to 750)	14·7% (6·6 to 22·6)*	−13·6% (−19·8 to −7·6)*
..				Kidney cancer	21 (15 to 27)	26 (18 to 34)	19·7% (14·8 to 24·6)*	−9·8% (−13·5 to −6·0)*	482 (336 to 623)	554 (378 to 721)	15·0% (10·3 to 19·7)*	−12·3% (−16·1 to −8·6)*
..				Bladder cancer	56 (42 to 67)	66 (50 to 81)	19·5% (16·1 to 23·2)*	−10·8% (−13·4 to −7·9)*	1130 (869 to 1350)	1320 (1010 to 1590)	16·9% (13·4 to 20·2)*	−11·2% (−14·0 to −8·7)*
..				Acute lymphoid leukaemia	5 (3 to 8)	7 (4 to 10)	25·7% (16·2 to 33·3)*	−2·7% (−9·5 to 2·5)	149 (78 to 232)	179 (95 to 279)	20·1% (11·1 to 28·0)*	−4·9% (−11·8 to 0·3)
..				Chronic lymphoid leukaemia	8 (5 to 12)	9 (6 to 13)	11·5% (6·3 to 17·8)*	−17·4% (−21·4 to −12·6)*	168 (103 to 234)	187 (116 to 261)	11·3% (5·0 to 18·2)*	−16·0% (−21·0 to −10·3)*
..				Acute myeloid leukaemia	18 (11 to 25)	21 (13 to 30)	18·8% (13·7 to 23·2)*	−9·6% (−13·7 to −6·0)*	412 (248 to 595)	471 (275 to 704)	14·4% (9·2 to 18·7)*	−11·3% (−16·0 to −7·2)*
..				Chronic myeloid leukaemia	5 (3 to 7)	5 (3 to 7)	−6·1% (−10·0 to −2·2)*	−28·8% (−32·2 to −25·6)*	116 (67 to 174)	104 (58 to 157)	−10·4% (−14·4 to −6·6)*	−30·3% (−34·3 to −27·0)*
..				Other leukaemia	24 (15 to 34)	26 (16 to 38)	9·5% (4·0 to 16·8)*	−16·9% (−20·6 to −11·5)*	565 (327 to 830)	588 (346 to 862)	4·2% (−1·3 to 13·1)	−19·0% (−22·8 to −13·1)*
..				Ischaemic heart disease	1500 (1440 to 1570)	1620 (1540 to 1690)	7·8% (4·6 to 11·1)*	−17·8% (−20·2 to −15·3)*	38 400 (36 800 to 40 200)	40 600 (38 700 to 42 500)	5·6% (2·4 to 9·0)*	−18·0% (−20·4 to −15·3)*
..				Ischaemic stroke	295 (276 to 317)	335 (313 to 361)	13·4% (8·6 to 17·8)*	−14·7% (−18·2 to −11·4)*	7540 (6910 to 8260)	9000 (8170 to 9920)	19·3% (14·7 to 23·8)*	−9·0% (−12·5 to −5·6)*
..				Intracerebral haemorrhage	470 (439 to 500)	481 (450 to 514)	2·3% (−2·2 to 6·9)	−21·9% (−25·4 to −18·5)*	12 400 (11 600 to 13 300)	12 600 (11 800 to 13 600)	1·5% (−2·8 to 5·8)	−21·2% (−24·6 to −17·8)*
..				Subarachnoid haemorrhage	67 (60 to 77)	71 (63 to 81)	6·2% (−1·4 to 14·5)	−17·4% (−23·3 to −11·1)*	2190 (1950 to 2510)	2270 (2020 to 2560)	3·5% (−3·2 to 10·7)	−17·9% (−23·2 to −12·4)*
..				Atrial fibrillation and flutter	10 (6 to 13)	12 (8 to 16)	27·2% (22·5 to 32·3)*	−6·4% (−9·8 to −2·9)*	414 (262 to 591)	502 (315 to 718)	21·2% (17·8 to 24·7)*	−7·5% (−9·9 to −5·2)*
..				Aortic aneurysm	51 (47 to 56)	56 (51 to 62)	10·4% (6·0 to 15·3)*	−16·3% (−19·6 to −12·6)*	1160 (1080 to 1270)	1270 (1160 to 1400)	9·5% (4·9 to 14·7)*	−15·4% (−18·9 to −11·4)*
..				Peripheral vascular disease	13 (8 to 21)	17 (11 to 29)	31·6% (9·2 to 47·5)*	−2·2% (−19·0 to 9·8)	376 (240 to 553)	471 (298 to 716)	25·1% (9·1 to 37·0)*	−5·7% (−17·8 to 3·3)
..				Chronic obstructive pulmonary disease	1130 (1030 to 1230)	1230 (1120 to 1350)	9·5% (6·4 to 13·0)*	−19·1% (−21·3 to −16·7)*	26 100 (23 300 to 28 700)	28 200 (25 100 to 31 100)	7·8% (4·7 to 11·1)*	−18·4% (−20·7 to −16·0)*
..				Asthma	69 (34 to 108)	59 (29 to 90)	−15·5% (−23·9 to −3·8)*	−35·8% (−42·2 to −27·0)*	2340 (1220 to 3350)	2130 (1100 to 3080)	−9·0% (−16·4 to 0·5)	−28·7% (−34·7 to −20·9)*
..				Peptic ulcer disease	51 (44 to 58)	46 (39 to 54)	−10·1% (−15·5 to −3·9)*	−31·5% (−35·6 to −26·7)*	1370 (1180 to 1540)	1200 (1020 to 1400)	−11·9% (−17·0 to −6·8)*	−30·9% (−35·0 to −26·8)*
..				Gallbladder and biliary diseases	5 (4 to 6)	6 (4 to 7)	15·1% (9·7 to 22·7)*	−13·6% (−17·6 to −7·9)*	106 (78 to 136)	116 (84 to 151)	9·6% (4·1 to 16·8)*	−14·9% (−19·0 to −9·4)*
..				Alzheimer's disease and other dementias	249 (158 to 338)	318 (198 to 434)	27·6% (21·8 to 32·5)*	−9·5% (−13·4 to −6·0)*	3800 (2410 to 5110)	4650 (2900 to 6310)	22·4% (17·2 to 26·7)*	−10·0% (−13·6 to −6·6)*
..				Parkinson's disease	−24 (−38 to −12)	−29 (−46 to −14)	20·0% (10·5 to 28·2)*	−10·4% (−17·3 to −4·4)*	−481 (−750 to −239)	−567 (−890 to −281)	17·9% (9·1 to 26·2)*	−10·5% (−17·1 to −4·3)*
..				Multiple sclerosis	2 (2 to 3)	3 (2 to 3)	12·1% (−1·6 to 19·2)	−11·4% (−21·9 to −5·7)*	132 (101 to 165)	143 (107 to 179)	8·2% (0·3 to 14·5)*	−12·0% (−18·3 to −6·8)*
..				Type 2 diabetes mellitus	65 (53 to 78)	83 (67 to 100)	27·3% (22·6 to 32·2)*	−4·6% (−8·1 to −0·9)*	4710 (3450 to 6160)	5680 (4140 to 7530)	20·6% (15·2 to 26·2)*	−5·6% (−9·6 to −1·4)*
..				Cataract	..	..	..	..	534 (362 to 747)	610 (408 to 860)	14·3% (8·7 to 19·5)*	−13·0% (−17·1 to −8·9)*
..				Age-related macular degeneration	..	..	..	..	48 (20 to 79)	56 (24 to 95)	17·0% (9·3 to 23·4)*	−12·7% (−18·4 to −8·0)*
..				Rheumatoid arthritis	3 (1 to 6)	4 (1 to 6)	10·4% (2·4 to 20·2)*	−16·8% (−22·8 to −9·3)*	245 (72 to 443)	285 (82 to 519)	16·4% (10·0 to 21·8)*	−9·0% (−13·9 to −4·8)*
..				Low back pain	..	..	..	..	9350 (6160 to 13 000)	10 100 (6700 to 14 100)	8·1% (5·4 to 10·7)*	−12·6% (−14·7 to −10·9)*
..				Pedestrian road injuries	4 (3 to 5)	4 (3 to 5)	0·9% (−8·3 to 7·2)	−22·2% (−29·4 to −17·2)*	126 (91 to 167)	126 (90 to 169)	−0·4% (−7·7 to 5·0)	−21·1% (−27·0 to −16·9)*
..				Cyclist road injuries	1 (0 to 1)	1 (0 to 1)	13·2% (3·4 to 23·5)*	−12·0% (−19·7 to −4·3)*	25 (17 to 34)	29 (20 to 40)	16·8% (10·0 to 23·7)*	−8·1% (−13·2 to −2·7)*
..				Motorcyclist road injuries	1 (1 to 2)	1 (1 to 2)	6·7% (−7·3 to 16·4)	−14·0% (−25·5 to −6·2)*	71 (48 to 96)	76 (51 to 105)	6·9% (−2·7 to 13·7)	−13·3% (−21·1 to −7·9)*
..				Motor vehicle road injuries	3 (2 to 4)	3 (2 to 3)	−1·9% (−9·8 to 2·7)	−22·5% (−28·8 to −18·8)*	105 (71 to 143)	103 (69 to 140)	−2·0% (−7·4 to 1·7)	−21·2% (−25·7 to −18·3)*
..				Other road injuries	0 (0 to 0)	0 (0 to 0)	−2·7% (−12·4 to 16·6)	−24·9% (−32·5 to −10·6)*	8 (6 to 12)	10 (7 to 15)	26·6% (19·1 to 34·2)*	−1·8% (−7·3 to 4·0)
..				Other transport injuries	1 (1 to 2)	1 (1 to 2)	3·3% (−2·3 to 10·4)	−19·5% (−23·9 to −14·1)*	71 (52 to 94)	77 (55 to 104)	8·6% (4·3 to 13·2)*	−14·5% (−17·7 to −11·1)*
..				Falls	15 (11 to 20)	19 (13 to 25)	23·6% (15·1 to 34·6)*	−10·5% (−16·9 to −2·4)*	508 (365 to 694)	630 (443 to 870)	23·9% (19·2 to 29·2)*	−6·4% (−9·9 to −2·5)*
..				Other exposure to mechanical forces	1 (1 to 1)	1 (1 to 1)	1·6% (−3·8 to 6·7)	−21·3% (−25·2 to −17·5)*	64 (45 to 88)	71 (50 to 102)	12·3% (7·5 to 16·4)*	−11·7% (−15·1 to −8·9)*
..				Non-venomous animal contact	0 (0 to 0)	0 (0 to 0)	−0·4% (−11·7 to 15·6)	−23·6% (−32·3 to −11·3)*	5 (3 to 7)	5 (3 to 8)	5·3% (−0·8 to 12·3)	−18·4% (−23·0 to −12·9)*
..				Assault by other means	0 (0 to 0)	0 (0 to 0)	−8·1% (−15·2 to −0·8)*	−27·5% (−33·1 to −21·8)*	17 (12 to 23)	16 (12 to 22)	−2·0% (−6·9 to 2·5)	−22·2% (−25·9 to −18·7)*
3			Chewing tobacco: all causes		59 (48 to 70)	76 (62 to 91)	29·5% (22·0 to 38·3)*	−1·6% (−7·3 to 5·2)	1490 (1220 to 1790)	1890 (1530 to 2270)	26·6% (19·5 to 35·1)*	−1·9% (−7·5 to 4·8)
**..**				Lip and oral cavity cancer	31 (24 to 38)	44 (35 to 54)	41·3% (31·8 to 51·6)*	8·1% (0·9 to 16·0)*	853 (661 to 1050)	1160 (892 to 1440)	36·0% (26·2 to 46·3)*	6·3% (−1·3 to 14·4)
**..**				Oesophageal cancer	27 (20 to 36)	32 (22 to 41)	16·0% (6·0 to 27·2)*	−12·4% (−20·2 to −4·0)*	641 (455 to 833)	731 (507 to 948)	14·0% (5·2 to 24·4)*	−12·6% (−19·5 to −5·1)*
3			Second-hand smoke: all causes		1110 (888 to 1370)	1220 (984 to 1500)	10·2% (6·9 to 13·5)*	−15·9% (−18·2 to −13·6)*	36 600 (28 600 to 46 000)	36 300 (28 600 to 45 100)	−0·9% (−5·6 to 4·5)	−20·0% (−23·0 to −16·5)*
**..**				Lower respiratory infections	208 (123 to 302)	179 (107 to 261)	−13·6% (−16·3 to −10·5)*	−27·8% (−30·0 to −25·4)*	11 900 (6940 to 17 400)	7960 (4630 to 11 600)	−33·2% (−36·4 to −29·8)*	−39·0% (−42·0 to −35·7)*
**..**				Otitis media	0 (0 to 0)	0 (0 to 0)	−60·5% (−72·8 to −40·3)*	−62·4% (−74·1 to −43·1)*	127 (69 to 209)	119 (63 to 199)	−6·1% (−10·8 to −2·6)*	−11·6% (−15·9 to −8·2)*
**..**				Tracheal, bronchus, and lung cancer	78 (44 to 117)	100 (57 to 149)	28·3% (22·5 to 33·8)*	−2·5% (−6·7 to 1·6)	1830 (1050 to 2750)	2240 (1300 to 3360)	22·7% (17·6 to 28·1)*	−5·5% (−9·4 to −1·4)*
**..**				Breast cancer	12 (3 to 21)	15 (4 to 26)	21·2% (14·3 to 26·9)*	−5·4% (−10·6 to −1·0)*	404 (98 to 688)	478 (118 to 829)	18·5% (11·6 to 24·6)*	−4·9% (−10·4 to −0·2)*
**..**				Ischaemic heart disease	330 (269 to 399)	382 (311 to 463)	16·0% (12·8 to 19·3)*	−12·1% (−14·1 to −10·0)*	7890 (6440 to 9490)	8850 (7180 to 10 700)	12·2% (9·2 to 15·3)*	−12·4% (−14·5 to −10·2)*
**..**				Ischaemic stroke	65 (48 to 85)	74 (55 to 98)	14·6% (9·6 to 20·4)*	−14·6% (−17·4 to −11·3)*	1430 (1070 to 1850)	1670 (1250 to 2190)	17·4% (11·9 to 22·6)*	−10·3% (−13·8 to −6·9)*
**..**				Intracerebral haemorrhage	96 (73 to 124)	102 (76 to 132)	5·9% (1·1 to 10·9)*	−19·4% (−22·4 to −16·1)*	2460 (1840 to 3160)	2550 (1880 to 3290)	3·8% (−0·9 to 8·5)	−18·9% (−21·9 to −15·8)*
**..**				Subarachnoid haemorrhage	14 (11 to 19)	16 (12 to 21)	10·9% (5·5 to 16·9)*	−13·4% (−17·1 to −9·2)*	467 (337 to 616)	500 (360 to 656)	7·1% (2·2 to 12·6)*	−14·0% (−17·2 to −10·3)*
**..**				Chronic obstructive pulmonary disease	244 (126 to 363)	266 (136 to 405)	9·0% (3·5 to 14·6)*	−20·0% (−23·9 to −15·8)*	6230 (3190 to 9260)	6910 (3540 to 10 400)	10·8% (6·3 to 15·2)*	−15·3% (−18·8 to −12·0)*
**..**				Type 2 diabetes mellitus	61 (23 to 92)	86 (33 to 131)	41·1% (37·5 to 45·0)*	5·3% (2·7 to 8·1)*	3890 (1390 to 6340)	5040 (1810 to 8230)	29·3% (25·1 to 33·8)*	1·3% (−2·0 to 4·8)
**2**			**Alcohol use: all causes**		**2560 (2230 to 2910)**	**2840 (2440 to 3250)**	11·0% (3·2 to 19·1)*	−11·7% (−18·1 to −4·8)*	**102 000 (92 400 to 114 000)**	**108 000 (96 200 to 120 000)**	5·5% (0·5 to 10·7)*	−13·1% (−17·2 to −8·7)*
..				Drug-susceptible tuberculosis	338 (247 to 415)	287 (209 to 361)	−15·0% (−22·8 to −7·7)*	−32·0% (−38·1 to −25·9)*	12 600 (9340 to 15 400)	10 500 (7730 to 13 100)	−16·6% (−23·9 to −9·9)*	−30·9% (−37·0 to −25·3)*
..				Multidrug-resistant tuberculosis without extensive drug resistance	45 (32 to 60)	38 (20 to 64)	−16·0% (−50·5 to 31·4)	−32·5% (−60·3 to 5·5)	1650 (1170 to 2170)	1330 (697 to 2260)	−19·2% (−51·4 to 24·6)	−33·0% (−59·7 to 3·5)
..				Extensively drug-resistant tuberculosis	5 (3 to 6)	5 (3 to 7)	6·1% (−20·2 to 43·2)	−13·8% (−35·2 to 16·2)	178 (128 to 225)	178 (124 to 254)	−0·4% (−24·4 to 32·7)	−16·9% (−36·8 to 10·7)
..				Lower respiratory infections	96 (36 to 154)	114 (39 to 186)	19·1% (−0·6 to 31·4)	−8·2% (−20·9 to 2·8)	2630 (1310 to 3830)	2840 (1380 to 4230)	8·0% (−5·2 to 18·0)	−13·2% (−22·7 to −5·2)*
..				Lip and oral cavity cancer	59 (51 to 66)	78 (66 to 89)	32·8% (25·1 to 40·2)*	2·1% (−3·8 to 7·8)	1710 (1490 to 1910)	2200 (1900 to 2490)	28·6% (20·6 to 35·9)*	1·1% (−5·3 to 6·8)
..				Nasopharynx cancer	25 (21 to 29)	32 (26 to 37)	25·7% (17·7 to 34·3)*	−2·2% (−8·4 to 4·3)	790 (662 to 900)	956 (801 to 1100)	21·1% (13·1 to 29·7)*	−3·6% (−9·7 to 3·1)
..				Other pharynx cancer	38 (31 to 44)	52 (42 to 62)	38·0% (25·3 to 49·5)*	6·1% (−3·7 to 14·9)	1110 (912 to 1290)	1490 (1190 to 1770)	34·2% (21·6 to 45·3)*	4·9% (−5·0 to 13·5)
..				Oesophageal cancer	120 (97 to 143)	139 (110 to 166)	15·6% (7·8 to 24·5)*	−12·1% (−18·2 to −5·3)*	2980 (2430 to 3510)	3310 (2650 to 3910)	10·9% (3·9 to 18·7)*	−14·7% (−20·2 to −8·6)*
..				Colon and rectum cancer	103 (83 to 124)	126 (98 to 155)	22·3% (14·0 to 31·0)*	−7·9% (−14·1 to −1·3)*	2410 (1970 to 2870)	2900 (2290 to 3500)	20·5% (12·2 to 29·3)*	−7·2% (−13·6 to −0·4)*
..				Liver cancer due to alcohol use	98 (87 to 112)	129 (115 to 147)	31·7% (26·8 to 37·3)*	0·6% (−3·0 to 4·8)	2400 (2100 to 2790)	3080 (2680 to 3590)	27·9% (22·5 to 34·0)*	−0·5% (−4·4 to 4·0)
..				Larynx cancer	32 (21 to 40)	38 (25 to 48)	17·6% (11·3 to 24·4)*	−10·2% (−15·1 to −4·8)*	886 (594 to 1100)	1010 (683 to 1270)	14·4% (8·5 to 20·8)*	−11·5% (−16·1 to −6·6)*
..				Breast cancer	54 (46 to 62)	59 (49 to 69)	9·7% (4·3 to 14·1)*	−16·3% (−20·4 to −13·0)*	1560 (1320 to 1790)	1670 (1390 to 1940)	7·0% (2·0 to 11·3)*	−15·9% (−19·9 to −12·3)*
..				Ischaemic heart disease	−129 (−342 to 78)	−112 (−364 to 123)	−13·4% (−192·5 to 327·3)	−38·7% (−304·3 to 136·6)	−384 (−4880 to 4100)	343 (−4690 to 5200)	−189·5% (−502·2 to 832·6)	−111·5% (−487·6 to 473·6)
..				Ischaemic stroke	74 (11 to 143)	102 (28 to 181)	38·3% (−1·6 to 154·7)	9·2% (−32·8 to 220·5)	2070 (771 to 3370)	2870 (1210 to 4520)	38·2% (11·8 to 88·8)*	7·2% (−14·3 to 53·9)
..				Intracerebral haemorrhage	399 (292 to 503)	462 (338 to 592)	15·7% (3·0 to 31·2)*	−12·1% (−21·8 to −0·3)*	9910 (7550 to 12 200)	11 200 (8440 to 14 000)	13·3% (2·4 to 27·2)*	−11·9% (−20·5 to −1·0)*
..				Hypertensive heart disease	93 (63 to 120)	134 (84 to 179)	43·9% (21·8 to 61·4)*	6·5% (−9·2 to 19·1)	2000 (1410 to 2570)	2680 (1760 to 3520)	34·3% (15·8 to 49·2)*	3·3% (−10·9 to 14·9)
..				Alcoholic cardiomyopathy	119 (91 to 125)	89 (81 to 96)	−25·3% (−29·5 to −8·3)*	−40·5% (−43·7 to −27·6)*	4230 (3190 to 4450)	2990 (2720 to 3210)	−29·4% (−33·5 to −11·1)*	−42·2% (−45·6 to −27·5)*
..				Atrial fibrillation and flutter	20 (15 to 26)	28 (20 to 36)	40·1% (28·9 to 51·6)*	−1·0% (−8·7 to 7·1)	567 (408 to 738)	740 (536 to 966)	30·4% (21·0 to 41·2)*	−2·3% (−9·6 to 5·6)
..				Cirrhosis and other chronic liver diseases due to alcohol use	284 (260 to 309)	332 (303 to 373)	16·9% (11·2 to 23·7)*	−8·8% (−13·2 to −3·4)*	9000 (8290 to 9790)	10 200 (9310 to 11 300)	13·1% (8·1 to 18·9)*	−9·3% (−13·4 to −4·8)*
..				Pancreatitis	31 (24 to 38)	36 (29 to 45)	17·6% (7·5 to 27·4)*	−6·2% (−14·2 to 1·8)	1150 (929 to 1390)	1300 (1050 to 1590)	13·0% (3·4 to 22·1)*	−6·9% (−14·8 to 0·6)
..				Epilepsy	23 (17 to 29)	25 (19 to 32)	8·4% (2·1 to 16·0)*	−9·6% (−15·0 to −2·9)*	2050 (1460 to 2760)	2240 (1550 to 3100)	9·0% (0·4 to 17·7)*	−6·3% (−13·7 to 1·2)
..				Alcohol use disorders	180 (163 to 184)	185 (167 to 193)	2·7% (−2·2 to 7·7)	−16·5% (−20·4 to −12·4)*	16 600 (13 600 to 20 400)	17 500 (14 100 to 21 600)	5·0% (2·4 to 7·4)*	−10·2% (−12·7 to −8·1)*
..				Type 2 diabetes mellitus	−4 (−24 to 15)	−3 (−28 to 23)	−41·1% (−486·6 to 318·0)	−52·3% (−365·2 to 347·8)	240 (−1140 to 1620)	363 (−1460 to 2260)	51·2% (−291·8 to 254·2)	41·4% (−236·8 to 207·0)
..				Pedestrian road injuries	42 (25 to 59)	40 (23 to 56)	−4·4% (−13·2 to 6·4)	−19·7% (−26·9 to −10·7)*	2000 (1180 to 2820)	1880 (1120 to 2630)	−6·0% (−13·7 to 4·2)	−17·6% (−24·3 to −8·9)*
..				Cyclist road injuries	6 (3 to 8)	6 (3 to 9)	9·5% (−1·0 to 22·8)	−7·6% (−16·2 to 3·4)	365 (221 to 528)	404 (246 to 573)	10·6% (2·5 to 21·6)*	−4·3% (−11·2 to 5·1)
..				Motorcyclist road injuries	23 (14 to 33)	24 (14 to 34)	2·5% (−8·5 to 14·5)	−9·0% (−18·7 to 1·4)	1450 (887 to 2050)	1480 (896 to 2070)	1·9% (−7·1 to 12·7)	−8·6% (−16·5 to 0·8)
..				Motor vehicle road injuries	43 (25 to 60)	40 (24 to 56)	−6·6% (−11·6 to −0·8)*	−18·6% (−23·0 to −13·6)*	2380 (1430 to 3320)	2190 (1340 to 3030)	−8·2% (−12·6 to −2·6)*	−18·0% (−21·9 to −13·0)*
..				Other road injuries	1 (1 to 2)	1 (1 to 1)	−8·8% (−16·0 to 7·3)	−22·4% (−28·6 to −8·8)*	81 (47 to 117)	85 (51 to 124)	5·4% (−2·6 to 16·8)	−8·7% (−15·4 to 1·1)
..				Other transport injuries	8 (5 to 12)	8 (5 to 11)	−3·8% (−10·1 to 4·0)	−17·1% (−22·7 to −10·5)*	565 (338 to 803)	577 (345 to 823)	2·1% (−4·1 to 9·7)	−10·2% (−15·5 to −3·7)*
..				Falls	38 (17 to 62)	48 (22 to 80)	25·9% (16·3 to 39·2)*	−3·7% (−11·1 to 6·8)	2540 (1150 to 4230)	2990 (1370 to 4980)	17·9% (11·8 to 27·0)*	−4·6% (−9·4 to 3·1)
..				Drowning	17 (7 to 28)	16 (7 to 27)	−2·3% (−8·4 to 6·8)	−18·0% (−23·0 to −10·3)*	732 (322 to 1220)	669 (284 to 1100)	−8·6% (−14·2 to 0·2)	−20·5% (−25·2 to −13·5)*
..				Fire, heat, and hot substances	8 (4 to 13)	7 (3 to 12)	−11·6% (−17·9 to −5·0)*	−28·9% (−33·6 to −23·9)*	464 (202 to 766)	440 (192 to 743)	−5·1% (−11·8 to 2·0)	−20·4% (−25·9 to −14·4)*
..				Poisoning by carbon monoxide	4 (2 to 7)	3 (1 to 6)	−21·4% (−31·1 to −11·3)*	−35·1% (−42·9 to −26·8)*	177 (77 to 283)	132 (59 to 215)	−25·3% (−33·4 to −16·5)*	−36·6% (−43·4 to −29·4)*
..				Poisoning by other means	2 (1 to 3)	2 (1 to 4)	12·4% (−2·0 to 31·6)	−7·6% (−19·3 to 7·9)	106 (48 to 176)	116 (53 to 197)	9·5% (0·6 to 23·0)*	−7·1% (−14·4 to 4·1)
..				Unintentional firearm injuries	2 (1 to 3)	2 (1 to 3)	−9·0% (−14·8 to −2·7)*	−22·8% (−28·0 to −17·6)*	111 (50 to 192)	105 (47 to 179)	−5·7% (−11·0 to 0·1)	−18·1% (−23·0 to −13·3)*
..				Venomous animal contact	3 (1 to 5)	3 (1 to 6)	14·0% (−1·1 to 35·6)	−6·8% (−19·3 to 10·9)	140 (56 to 246)	157 (63 to 285)	11·7% (−0·8 to 29·8)	−5·6% (−15·9 to 9·1)
..				Non-venomous animal contact	1 (0 to 1)	1 (0 to 1)	2·6% (−7·3 to 16·5)	−16·9% (−25·0 to −5·5)*	42 (18 to 70)	45 (20 to 77)	6·9% (−0·2 to 16·8)	−11·1% (−16·9 to −3·1)*
..				Environmental heat and cold exposure	7 (3 to 12)	5 (2 to 9)	−23·9% (−32·6 to −15·9)*	−38·3% (−45·0 to −31·8)*	354 (154 to 572)	297 (130 to 498)	−16·1% (−23·2 to −8·3)*	−30·2% (−36·1 to −23·6)*
..				Exposure to forces of nature	1 (0 to 1)	1 (0 to 1)	4·6% (−7·6 to 25·0)	−11·1% (−21·6 to 5·9)	42 (19 to 72)	57 (24 to 100)	34·9% (21·8 to 50·6)*	17·5% (6·5 to 31·1)*
..				Other unintentional injuries	9 (4 to 14)	8 (3 to 13)	−8·2% (−16·0 to 4·6)	−22·1% (−28·5 to −11·6)*	670 (287 to 1120)	683 (297 to 1130)	1·9% (−4·7 to 10·6)	−14·0% (−19·3 to −6·8)*
..				Self-harm by firearm	16 (9 to 23)	17 (9 to 25)	5·7% (−6·8 to 18·1)	−11·8% (−22·0 to −1·5)*	691 (389 to 995)	709 (399 to 1020)	2·6% (−8·9 to 14·5)	−11·3% (−21·3 to −1·0)*
..				Self-harm by other specified means	157 (96 to 214)	161 (99 to 225)	2·8% (−4·7 to 11·0)	−14·3% (−20·6 to −7·2)*	6730 (4120 to 9190)	6700 (4150 to 9310)	−0·4% (−7·7 to 7·8)	−14·5% (−20·9 to −7·7)*
..				Assault by firearm	34 (22 to 46)	36 (23 to 49)	6·6% (−0·6 to 13·3)	−5·2% (−11·7 to 1·1)	1840 (1220 to 2510)	1940 (1270 to 2650)	5·2% (−2·1 to 11·8)	−5·0% (−11·8 to 1·1)
..				Assault by sharp object	20 (13 to 29)	18 (11 to 24)	−14·4% (−20·1 to −8·4)*	−25·6% (−30·7 to −20·3)*	1100 (702 to 1550)	944 (598 to 1310)	−14·4% (−19·5 to −8·9)*	−24·4% (−29·2 to −19·5)*
..				Sexual violence	..	..	..	..	114 (50 to 200)	131 (58 to 228)	15·0% (6·7 to 26·4)*	1·5% (−5·8 to 10·6)
..				Assault by other means	21 (14 to 29)	22 (14 to 31)	4·1% (−3·5 to 11·1)	−11·0% (−17·6 to −4·6)*	1260 (792 to 1770)	1340 (854 to 1880)	6·9% (−0·4 to 12·9)	−7·5% (−14·1 to −2·2)*
**2**			**Drug use: all causes**		**459 (416 to 501)**	**585 (535 to 635)**	27·6% (24·0 to 31·4)*	4·1% (1·2 to 7·0)*	**34 900 (29 800 to 40 400)**	**41 700 (35 300 to 48 200)**	19·2% (16·8 to 21·6)*	3·2% (0·9 to 5·4)*
..				HIV/AIDS and drug-susceptible tuberculosis co-infection	14 (9 to 20)	8 (5 to 12)	−42·6% (−47·6 to −37·9)*	−50·7% (−55·2 to −46·9)*	700 (455 to 993)	412 (276 to 582)	−41·1% (−45·9 to −36·2)*	−48·8% (−53·0 to −44·7)*
..				HIV/AIDS and multidrug-resistant tuberculosis without extensive drug resistance co-infection	2 (1 to 4)	1 (1 to 2)	−44·0% (−58·8 to −22·3)*	−51·8% (−64·5 to −33·4)*	113 (68 to 176)	63 (36 to 103)	−43·9% (−58·6 to −23·6)*	−51·2% (−63·9 to −33·7)*
..				HIV/AIDS and extensively drug-resistant tuberculosis co-infection	0 (0 to 0)	0 (0 to 0)	4·1% (−19·4 to 33·5)	−9·6% (−30·3 to 16·0)	14 (8 to 22)	14 (8 to 23)	3·5% (−20·2 to 33·2)	−9·4% (−30·0 to 16·4)
..				HIV/AIDS resulting in other diseases	71 (58 to 88)	56 (46 to 70)	−20·7% (−26·5 to −11·3)*	−31·5% (−36·4 to −23·4)*	3630 (2920 to 4520)	2880 (2340 to 3630)	−20·8% (−26·0 to −11·4)*	−30·9% (−35·5 to −22·8)*
..				Acute hepatitis B	1 (0 to 1)	1 (0 to 1)	26·1% (13·9 to 41·6)*	0·8% (−8·8 to 13·1)	19 (14 to 25)	23 (17 to 30)	17·8% (6·3 to 31·3)*	−3·0% (−12·2 to 7·9)
..				Acute hepatitis C	0 (0 to 1)	1 (0 to 1)	19·9% (2·2 to 41·8)*	−6·3% (−20·3 to 10·9)	15 (8 to 29)	17 (9 to 33)	13·2% (−2·4 to 33·2)	−7·6% (−19·6 to 7·1)
..				Liver cancer due to hepatitis B	3 (2 to 3)	4 (3 to 5)	44·0% (35·2 to 55·2)*	13·2% (6·4 to 21·4)*	85 (68 to 104)	112 (90 to 138)	32·2% (23·5 to 42·1)*	5·8% (−1·1 to 13·9)
..				Liver cancer due to hepatitis C	84 (72 to 97)	127 (110 to 144)	51·0% (44·1 to 59·6)*	14·6% (9·4 to 21·4)*	2040 (1760 to 2320)	2930 (2560 to 3310)	43·9% (37·8 to 51·0)*	10·9% (6·2 to 16·5)*
..				Cirrhosis and other chronic liver diseases due to hepatitis B	3 (2 to 4)	4 (3 to 4)	25·6% (17·6 to 35·0)*	0·3% (−6·4 to 8·0)	103 (82 to 131)	121 (96 to 152)	17·4% (9·7 to 25·7)*	−3·9% (−10·2 to 3·1)
..				Cirrhosis and other chronic liver diseases due to hepatitis C	137 (115 to 160)	175 (150 to 204)	28·4% (22·3 to 35·0)*	1·7% (−3·1 to 7·2)	4730 (3970 to 5520)	5800 (4920 to 6760)	22·5% (17·0 to 28·5)*	−0·6% (−4·8 to 4·2)
..				Opioid use disorders	62 (58 to 65)	110 (106 to 114)	77·0% (68·8 to 88·5)*	49·4% (42·5 to 59·2)*	16 400 (12 400 to 20 800)	21 500 (16 300 to 27 100)	30·8% (27·5 to 34·4)*	15·3% (12·4 to 18·3)*
..				Cocaine use disorders	5 (5 to 6)	7 (7 to 8)	42·2% (30·1 to 58·3)*	19·6% (9·2 to 33·0)*	833 (620 to 1090)	992 (747 to 1290)	19·0% (15·5 to 24·2)*	4·9% (1·9 to 9·3)*
..				Amphetamine use disorders	4 (3 to 4)	5 (3 to 5)	27·2% (0·8 to 41·0)*	8·6% (−14·0 to 20·6)	1120 (708 to 1670)	1180 (757 to 1740)	5·5% (1·5 to 9·4)*	−2·5% (−6·2 to 0·4)
..				Cannabis use disorders	..	..	..	..	496 (314 to 727)	518 (329 to 766)	4·4% (2·2 to 6·6)*	−3·7% (−5·7 to −1·8)*
..				Other drug use disorders	34 (32 to 37)	45 (43 to 48)	35·2% (22·8 to 46·1)*	11·3% (1·2 to 19·9)*	2560 (2130 to 3090)	3010 (2550 to 3540)	17·5% (10·9 to 24·0)*	3·5% (−2·2 to 8·9)
..				Self-harm by firearm	5 (3 to 7)	6 (3 to 9)	24·2% (16·4 to 31·8)*	8·2% (1·5 to 14·7)*	232 (137 to 378)	277 (165 to 441)	19·3% (11·8 to 27·0)*	6·7% (0·1 to 13·5)*
..				Self-harm by other specified means	35 (19 to 60)	36 (19 to 64)	3·1% (−1·8 to 7·1)	−10·0% (−14·0 to −6·6)*	1820 (1010 to 3100)	1820 (980 to 3180)	0·1% (−4·7 to 4·1)	−10·7% (−14·6 to −7·3)*
**2**			**Dietary risks: all causes**		**9160 (8510 to 9810)**	**10 900 (10 100 to 11 700)**	18·9% (17·0 to 20·7)*	−11·2% (−12·6 to −9·8)*	**219 000 (203 000 to 235 000)**	**255 000 (234 000 to 274 000)**	16·4% (14·6 to 18·1)*	−9·9% (−11·3 to −8·6)*
3			Diet low in fruits: all causes		2220 (1410 to 3180)	2420 (1480 to 3530)	9·0% (4·5 to 12·3)*	−17·4% (−20·6 to −15·1)*	60 200 (38 600 to 84 000)	64 800 (40 600 to 92 000)	7·7% (3·2 to 11·1)*	−15·7% (−19·0 to −13·1)*
..				Lip and oral cavity cancer	10 (0 to 21)	12 (0 to 26)	25·3% (17·0 to 1318·4)*	−3·7% (−10·0 to 1002·4)	269 (0 to 582)	325 (0 to 709)	21·0% (12·2 to 1198·1)*	−4·7% (−11·5 to 879·6)
..				Nasopharynx cancer	4 (0 to 8)	4 (0 to 10)	12·9% (5·1 to 1008·3)*	−12·1% (−18·1 to 739·6)	118 (0 to 259)	128 (0 to 284)	8·6% (1·0 to 946·6)*	−13·5% (−19·3 to 712·2)
..				Other pharynx cancer	6 (0 to 13)	7 (0 to 16)	28·4% (15·0 to 42·0)*	−1·3% (−11·5 to 9·0)	163 (0 to 373)	204 (0 to 458)	24·8% (11·9 to 38·3)*	−2·3% (−12·3 to 8·1)
..				Oesophageal cancer	82 (18 to 146)	83 (18 to 152)	1·7% (−5·1 to 6·4)	−22·9% (−27·9 to −19·4)*	1900 (430 to 3400)	1860 (399 to 3390)	−1·8% (−8·4 to 2·8)	−24·4% (−29·4 to −21·0)*
..				Larynx cancer	7 (0 to 15)	8 (0 to 17)	11·4% (5·2 to 218·8)*	−14·9% (−19·5 to 127·1)	183 (0 to 403)	199 (0 to 436)	8·4% (2·3 to 189·4)*	−16·0% (−20·5 to 104·4)
..				Tracheal, bronchus, and lung cancer	152 (64 to 261)	185 (77 to 320)	21·8% (16·2 to 25·8)*	−7·6% (−11·8 to −4·6)*	3470 (1460 to 5920)	4050 (1680 to 6950)	16·6% (10·9 to 20·6)*	−10·2% (−14·4 to −7·1)*
..				Ischaemic heart disease	822 (280 to 1440)	916 (306 to 1650)	11·4% (7·5 to 14·5)*	−16·1% (−18·8 to −14·0)*	19 500 (6770 to 33 700)	20 800 (7110 to 36 600)	7·0% (2·9 to 10·0)*	−16·2% (−19·1 to −13·9)*
..				Ischaemic stroke	376 (195 to 573)	406 (206 to 631)	8·1% (2·9 to 11·9)*	−18·8% (−22·6 to −16·1)*	9860 (5310 to 14 600)	11 200 (5800 to 16 900)	13·6% (7·8 to 18·1)*	−12·4% (−16·7 to −9·1)*
..				Intracerebral haemorrhage	615 (338 to 933)	624 (331 to 972)	1·4% (−3·8 to 5·2)	−22·3% (−26·2 to −19·5)*	16 700 (9480 to 24 800)	16 800 (9210 to 25 400)	0·4% (−4·6 to 4·0)	−21·2% (−24·8 to −18·5)*
..				Subarachnoid haemorrhage	92 (50 to 138)	99 (53 to 152)	7·5% (1·2 to 13·4)*	−15·8% (−20·4 to −11·2)*	3160 (1770 to 4650)	3330 (1820 to 5020)	5·2% (−0·2 to 10·5)	−15·1% (−19·3 to −11·0)*
..				Type 2 diabetes mellitus	60 (12 to 112)	80 (15 to 151)	32·1% (27·1 to 36·2)*	−0·6% (−4·2 to 2·4)	4880 (928 to 9240)	5900 (1090 to 11 300)	21·0% (15·1 to 26·1)*	−3·5% (−7·9 to 0·4)
3			Diet low in vegetables: all causes		1390 (715 to 2240)	1460 (732 to 2400)	5·3% (−0·8 to 9·7)	−20·7% (−25·2 to −17·7)*	33 600 (17 900 to 53 300)	34 200 (17 700 to 55 700)	1·8% (−4·0 to 6·1)	−20·4% (−24·8 to −17·3)*
..				Ischaemic heart disease	947 (370 to 1660)	1020 (396 to 1830)	8·2% (3·0 to 12·0)*	−19·1% (−23·0 to −16·3)*	21 400 (8520 to 37 400)	22 100 (8730 to 39 400)	3·2% (−2·2 to 7·2)	−19·5% (−23·5 to −16·4)*
..				Ischaemic stroke	150 (36 to 280)	154 (36 to 297)	3·1% (−3·3 to 8·1)	−22·6% (−27·4 to −19·0)*	3920 (970 to 7330)	4170 (1010 to 8090)	6·2% (−0·9 to 12·0)	−17·9% (−23·3 to −13·6)*
..				Intracerebral haemorrhage	252 (71 to 474)	243 (66 to 469)	−3·6% (−10·4 to 1·4)	−26·1% (−31·2 to −22·4)*	6910 (1970 to 12 900)	6550 (1800 to 12 500)	−5·2% (−11·5 to −0·5)*	−25·4% (−30·3 to −21·9)*
..				Subarachnoid haemorrhage	39 (11 to 73)	41 (11 to 78)	3·3% (−3·2 to 9·3)	−18·8% (−23·8 to −14·2)*	1360 (381 to 2530)	1380 (371 to 2580)	1·0% (−4·9 to 6·4)	−18·4% (−22·9 to −14·1)*
3			Diet low in legumes: all causes		481 (192 to 819)	535 (214 to 909)	11·2% (9·2 to 13·0)*	−17·3% (−18·8 to −16·0)*	10 600 (4280 to 18 000)	11 000 (4390 to 18 700)	3·3% (0·9 to 5·5)*	−19·3% (−21·1 to −17·6)*
..				Ischaemic heart disease	481 (192 to 819)	535 (214 to 909)	11·2% (9·2 to 13·0)*	−17·3% (−18·8 to −16·0)*	10 600 (4280 to 18 000)	11 000 (4390 to 18 700)	3·3% (0·9 to 5·5)*	−19·3% (−21·1 to −17·6)*
3			Diet low in whole grains: all causes		2630 (1820 to 3490)	3070 (2110 to 4120)	16·7% (14·6 to 18·5)*	−12·2% (−13·6 to −10·8)*	71 500 (51 300 to 93 800)	82 500 (59 000 to 109 000)	15·5% (13·2 to 17·6)*	−9·7% (−11·4 to −8·1)*
..				Ischaemic heart disease	1510 (890 to 2170)	1780 (1050 to 2570)	18·4% (16·4 to 20·2)*	−11·5% (−12·9 to −10·2)*	34 300 (20 600 to 48 800)	39 100 (23 300 to 55 700)	13·9% (11·9 to 15·9)*	−11·2% (−12·8 to −9·7)*
..				Ischaemic stroke	363 (241 to 501)	418 (276 to 580)	15·1% (12·5 to 17·6)*	−13·6% (−15·5 to −11·8)*	9870 (6680 to 13 500)	12 000 (8050 to 16 400)	21·3% (18·0 to 24·4)*	−6·4% (−9·0 to −4·0)*
..				Intracerebral haemorrhage	550 (368 to 745)	597 (401 to 813)	8·5% (5·5 to 11·2)*	−16·9% (−19·2 to −14·9)*	14 900 (10 100 to 20 100)	16 000 (10 800 to 21 400)	7·0% (4·1 to 9·7)*	−16·0% (−18·3 to −14·0)*
..				Subarachnoid haemorrhage	86 (58 to 118)	98 (66 to 134)	13·6% (8·2 to 19·1)*	−11·1% (−15·3 to −7·0)*	2960 (2010 to 3950)	3280 (2240 to 4410)	11·0% (6·5 to 15·6)*	−10·7% (−14·3 to −7·1)*
..				Type 2 diabetes mellitus	122 (69 to 186)	169 (95 to 259)	38·4% (35·1 to 41·4)*	3·7% (1·4 to 5·9)*	9400 (4980 to 15 100)	12 200 (6400 to 19 400)	29·7% (25·6 to 33·9)*	2·8% (−0·4 to 6·0)
3			Diet low in nuts and seeds: all causes		1740 (1130 to 2410)	2060 (1330 to 2880)	18·7% (16·6 to 20·6)*	−11·2% (−12·7 to −9·9)*	43 300 (28 800 to 58 800)	49 900 (33 100 to 68 000)	15·2% (12·9 to 17·3)*	−10·0% (−11·8 to −8·3)*
..				Ischaemic heart disease	1660 (1070 to 2330)	1960 (1230 to 2750)	17·8% (15·7 to 19·7)*	−11·9% (−13·4 to −10·6)*	37 600 (24 500 to 51 800)	42 600 (27 300 to 58 700)	13·2% (10·9 to 15·2)*	−11·8% (−13·4 to −10·3)*
..				Type 2 diabetes mellitus	74 (36 to 116)	103 (50 to 160)	38·4% (35·4 to 41·3)*	3·9% (1·8 to 6·1)*	5700 (2640 to 9390)	7310 (3410 to 12 100)	28·2% (24·1 to 32·4)*	1·8% (−1·4 to 4·9)
3			Diet low in milk: all causes		96 (34 to 171)	126 (45 to 220)	30·6% (26·0 to 34·8)*	−1·9% (−5·2 to 1·2)	2140 (757 to 3770)	2720 (965 to 4730)	27·0% (21·9 to 31·5)*	−2·0% (−6·0 to 1·4)
..				Colon and rectum cancer	96 (34 to 171)	126 (45 to 220)	30·6% (26·0 to 34·8)*	−1·9% (−5·2 to 1·2)	2140 (757 to 3770)	2720 (965 to 4730)	27·0% (21·9 to 31·5)*	−2·0% (−6·0 to 1·4)
3			Diet high in red meat: all causes		17 (7 to 28)	25 (11 to 40)	47·5% (36·9 to 59·6)*	11·2% (3·0 to 20·4)*	880 (352 to 1520)	1310 (508 to 2250)	49·2% (36·7 to 63·8)*	17·8% (7·9 to 29·5)*
..				Colon and rectum cancer	11 (3 to 20)	16 (4 to 29)	45·7% (34·3 to 58·4)*	9·8% (1·4 to 19·6)*	262 (63 to 472)	378 (89 to 678)	44·3% (32·7 to 57·6)*	11·2% (2·6 to 21·2)*
..				Type 2 diabetes mellitus	6 (1 to 11)	9 (1 to 17)	50·8% (40·3 to 62·8)*	13·6% (5·8 to 23·1)*	618 (77 to 1200)	934 (113 to 1830)	51·2% (37·6 to 66·6)*	20·8% (10·1 to 32·9)*
3			Diet high in processed meat: all causes		125 (24 to 239)	130 (26 to 265)	4·3% (−5·0 to 13·2)	−22·3% (−28·9 to −15·6)*	3360 (1050 to 6120)	3570 (1260 to 6770)	6·1% (−4·4 to 19·4)	−17·9% (−25·9 to −6·9)*
..				Colon and rectum cancer	9 (5 to 15)	11 (5 to 18)	13·8% (6·4 to 20·3)*	−14·9% (−20·4 to −10·1)*	205 (107 to 330)	227 (114 to 373)	10·6% (3·0 to 16·9)*	−15·4% (−21·1 to −10·5)*
..				Ischaemic heart disease	105 (5 to 213)	107 (5 to 230)	2·0% (−9·8 to 10·2)	−24·0% (−32·5 to −18·0)*	2320 (120 to 4650)	2270 (111 to 4860)	−2·2% (−13·8 to 6·3)	−24·5% (−33·3 to −17·8)*
..				Type 2 diabetes mellitus	10 (5 to 17)	12 (6 to 21)	18·6% (6·0 to 29·7)*	−11·3% (−20·6 to −2·9)*	838 (404 to 1430)	1070 (490 to 1900)	28·0% (16·3 to 37·0)*	0·2% (−8·9 to 7·6)
3			Diet high in sugar-sweetened beverages: all causes		105 (13 to 206)	137 (20 to 264)	30·4% (23·2 to 52·7)*	−2·5% (−8·2 to 14·4)	3350 (981 to 5910)	4450 (1490 to 7700)	32·8% (24·7 to 54·4)*	4·7% (−1·8 to 23·1)
..				Ischaemic heart disease	92 (0 to 189)	117 (0 to 242)	27·4% (0 to 34·2)	−4·7% (−10·2 to 1·1)	2270 (0 to 4620)	2810 (0 to 5700)	23·7% (0 to 30·2)	−2·9% (−7·8 to 2·4)
..				Type 2 diabetes mellitus	14 (7 to 20)	21 (11 to 30)	50·5% (39·9 to 62·8)*	12·5% (4·6 to 21·7)*	1080 (570 to 1670)	1650 (852 to 2520)	52·0% (43·2 to 62·5)*	21·1% (14·0 to 29·3)*
3			Diet low in fibre: all causes		737 (433 to 1110)	873 (510 to 1330)	18·6% (15·9 to 20·7)*	−11·2% (−13·2 to −9·6)*	17 700 (10 400 to 26 500)	19 900 (11 600 to 30 400)	12·6% (9·5 to 15·0)*	−11·4% (−13·7 to −9·6)*
..				Colon and rectum cancer	84 (44 to 131)	106 (54 to 167)	26·0% (21·7 to 29·8)*	−5·8% (−8·9 to −3·0)*	1800 (926 to 2800)	2200 (1140 to 3470)	22·2% (17·2 to 26·4)*	−5·8% (−9·5 to −2·7)*
..				Ischaemic heart disease	653 (368 to 1010)	768 (429 to 1210)	17·6% (14·9 to 19·8)*	−11·9% (−13·9 to −10·4)*	15 900 (9030 to 24 400)	17 700 (9940 to 27 700)	11·5% (8·3 to 14·0)*	−12·0% (−14·3 to −10·1)*
3			Diet low in calcium: all causes		145 (91 to 205)	185 (116 to 262)	27·4% (22·5 to 31·2)*	−4·5% (−8·0 to −1·8)*	3150 (1990 to 4420)	3890 (2450 to 5480)	23·7% (18·1 to 27·9)*	−4·7% (−8·9 to −1·5)*
..				Colon and rectum cancer	145 (91 to 205)	185 (116 to 262)	27·4% (22·5 to 31·2)*	−4·5% (−8·0 to −1·8)*	3150 (1990 to 4420)	3890 (2450 to 5480)	23·7% (18·1 to 27·9)*	−4·7% (−8·9 to −1·5)*
3			Diet low in seafood omega 3 fatty acids: all causes		1220 (565 to 2000)	1440 (667 to 2380)	18·4% (16·3 to 20·2)*	−10·8% (−12·4 to −9·5)*	28 200 (13 300 to 45 600)	32 400 (15 200 to 52 700)	14·9% (12·8 to 17·0)*	−10·3% (−12·0 to −8·8)*
..				Ischaemic heart disease	1220 (565 to 2000)	1440 (667 to 2380)	18·4% (16·3 to 20·2)*	−10·8% (−12·4 to −9·5)*	28 200 (13 300 to 45 600)	32 400 (15 200 to 52 700)	14·9% (12·8 to 17·0)*	−10·3% (−12·0 to −8·8)*
3			Diet low in polyunsaturated fatty acids: all causes		676 (288 to 1110)	799 (343 to 1310)	18·3% (15·7 to 20·8)*	−11·3% (−13·1 to −9·5)*	15 700 (6740 to 25 500)	17 900 (7770 to 29 000)	13·7% (11·3 to 16·2)*	−11·0% (−12·8 to −9·1)*
..				Ischaemic heart disease	676 (288 to 1110)	799 (343 to 1310)	18·3% (15·7 to 20·8)*	−11·3% (−13·1 to −9·5)*	15 700 (6740 to 25 500)	17 900 (7770 to 29 000)	13·7% (11·3 to 16·2)*	−11·0% (−12·8 to −9·1)*
3			Diet high in trans fatty acids: all causes		250 (93 to 519)	258 (80 to 577)	3·2% (−14·0 to 10·5)	−23·1% (−36·5 to −17·4)*	6050 (2180 to 12 800)	6160 (1870 to 13 800)	1·9% (−14·3 to 8·6)	−20·3% (−33·2 to −15·0)*
..				Ischaemic heart disease	250 (93 to 519)	258 (80 to 577)	3·2% (−14·0 to 10·5)	−23·1% (−36·5 to −17·4)*	6050 (2180 to 12 800)	6160 (1870 to 13 800)	1·9% (−14·3 to 8·6)	−20·3% (−33·2 to −15·0)*
3			Diet high in sodium: all causes		2520 (1070 to 4370)	3200 (1420 to 5450)	26·6% (21·5 to 38·3)*	−5·4% (−9·4 to 4·4)	57 400 (26 500 to 97 000)	70 400 (33 600 to 118 000)	22·7% (18·0 to 32·2)*	−5·9% (−9·4 to 1·7)
..				Stomach cancer	295 (158 to 460)	327 (175 to 506)	10·9% (3·1 to 19·9)*	−15·8% (−20·4 to −9·2)*	6950 (3880 to 10 500)	7350 (4120 to 11 100)	5·8% (−2·1 to 14·9)	−18·2% (−23·3 to −12·0)*
..				Rheumatic heart disease	19 (7 to 37)	18 (7 to 37)	−0·8% (−10·5 to 6·8)	−23·9% (−30·6 to −18·7)*	583 (240 to 1130)	554 (222 to 1100)	−5·0% (−13·4 to 1·2)	−24·3% (−30·6 to −19·9)*
..				Ischaemic heart disease	963 (336 to 1870)	1250 (472 to 2350)	29·8% (24·0 to 47·4)*	−3·8% (−8·1 to 10·4)	20 000 (7450 to 37 200)	25 000 (10 300 to 45 300)	25·3% (20·2 to 40·6)*	−4·2% (−8·2 to 7·9)
..				Ischaemic stroke	318 (120 to 582)	421 (179 to 744)	32·5% (24·5 to 52·5)*	−2·1% (−8·2 to 13·6)	7270 (3110 to 12 300)	10 300 (4840 to 16 700)	41·2% (31·8 to 61·4)*	6·7% (−0·3 to 22·5)
..				Intracerebral haemorrhage	547 (261 to 912)	629 (305 to 1040)	14·9% (8·8 to 23·1)*	−13·4% (−17·8 to −6·7)*	13 000 (6660 to 20 900)	14 500 (7450 to 23 300)	11·5% (5·8 to 18·6)*	−14·2% (−18·4 to −8·4)*
..				Subarachnoid haemorrhage	66 (28 to 116)	82 (36 to 143)	24·0% (13·7 to 37·3)*	−5·6% (−13·3 to 4·6)	1900 (837 to 3310)	2240 (1020 to 3850)	18·1% (8·5 to 29·0)*	−7·8% (−15·2 to 0·3)
..				Hypertensive heart disease	147 (43 to 330)	236 (67 to 512)	60·1% (26·0 to 91·2)*	17·7% (−7·0 to 41·5)	3000 (1060 to 6130)	4380 (1590 to 8810)	46·3% (20·5 to 71·3)*	11·4% (−8·5 to 30·6)
..				Non-rheumatic calcific aortic valve disease	4 (1 to 10)	6 (1 to 14)	39·4% (29·6 to 48·8)*	0·3% (−5·3 to 7·3)	81 (22 to 172)	107 (29 to 225)	32·1% (24·2 to 40·3)*	−0·4% (−5·5 to 6·2)
..				Other cardiomyopathy	12 (3 to 24)	15 (4 to 32)	29·8% (21·3 to 35·7)*	−3·2% (−8·0 to 1·5)	292 (83 to 611)	366 (100 to 774)	25·6% (16·7 to 31·4)*	−2·2% (−8·8 to 2·5)
..				Atrial fibrillation and flutter	15 (4 to 30)	22 (7 to 45)	51·5% (45·3 to 67·2)*	7·7% (3·2 to 21·1)*	446 (165 to 848)	628 (244 to 1170)	40·9% (35·9 to 53·2)*	5·8% (1·9 to 16·2)*
..				Aortic aneurysm	11 (3 to 22)	14 (5 to 28)	27·8% (21·8 to 40·3)*	−4·4% (−8·7 to 5·6)	225 (75 to 447)	280 (98 to 552)	24·5% (17·8 to 37·2)*	−4·6% (−9·6 to 5·3)
..				Peripheral vascular disease	2 (0 to 5)	3 (1 to 8)	60·6% (34·7 to 93·7)*	15·8% (−2·4 to 42·0)	64 (18 to 139)	91 (26 to 195)	42·4% (29·0 to 57·9)*	6·5% (−3·2 to 18·2)
..				Endocarditis	4 (1 to 9)	6 (2 to 12)	36·0% (25·7 to 43·2)*	2·2% (−4·7 to 8·0)	103 (31 to 212)	132 (40 to 272)	28·3% (18·6 to 35·5)*	0·6% (−6·7 to 5·6)
..				Other cardiovascular and circulatory diseases	23 (7 to 48)	30 (9 to 60)	27·1% (21·5 to 41·6)*	−4·3% (−8·3 to 7·1)	832 (275 to 1680)	1020 (350 to 2040)	23·2% (18·7 to 31·0)*	−4·3% (−7·6 to 1·7)
..				Chronic kidney disease due to type 1 diabetes mellitus	6 (2 to 13)	8 (3 to 16)	19·7% (7·3 to 26·9)*	−5·8% (−14·3 to −1·4)*	231 (88 to 467)	262 (95 to 552)	13·2% (0·6 to 21·0)*	−9·0% (−18·4 to −3·8)*
..				Chronic kidney disease due to type 2 diabetes mellitus	31 (11 to 62)	44 (16 to 89)	42·0% (34·4 to 49·7)*	5·3% (0·1 to 11·4)*	743 (282 to 1460)	1000 (383 to 1990)	35·0% (27·8 to 41·0)*	2·2% (−2·9 to 6·7)
..				Chronic kidney disease due to hypertension	29 (10 to 58)	42 (15 to 84)	46·1% (38·9 to 56·9)*	6·9% (1·7 to 16·3)*	619 (223 to 1230)	847 (318 to 1650)	36·9% (30·2 to 45·0)*	3·8% (−0·8 to 10·4)
..				Chronic kidney disease due to glomerulonephritis	13 (4 to 27)	17 (5 to 36)	34·2% (27·5 to 42·0)*	1·7% (−2·7 to 8·4)	372 (126 to 756)	460 (156 to 946)	23·7% (16·5 to 30·2)*	−2·6% (−7·6 to 1·7)
..				Chronic kidney disease due to other and unspecified causes	19 (6 to 38)	25 (8 to 52)	36·3% (29·0 to 44·6)*	2·7% (−2·3 to 9·0)	664 (240 to 1340)	837 (304 to 1700)	26·1% (19·5 to 32·5)*	−2·1% (−6·6 to 2·3)
**2**			**Intimate partner violence: all causes**		**151 (95 to 217)**	**71 (49 to 97)**	−53·2% (−56·6 to −47·9)*	−59·3% (−62·4 to −54·6)*	**10 300 (7270 to 14 000)**	**6700 (5150 to 8580)**	−35·0% (−41·8 to −25·1)*	−43·0% (−49·0 to −34·3)*
..				HIV/AIDS and drug-susceptible tuberculosis co-infection	27 (13 to 44)	10 (5 to 16)	−63·0% (−66·7 to −58·0)*	−68·3% (−71·7 to −64·1)*	1280 (615 to 2110)	500 (248 to 802)	−61·0% (−65·0 to −55·5)*	−66·1% (−69·6 to −61·2)*
..				HIV/AIDS and multidrug-resistant tuberculosis without extensive drug resistance co-infection	3 (1 to 5)	1 (0 to 2)	−59·6% (−73·0 to −42·6)*	−65·4% (−76·9 to −51·0)*	127 (50 to 230)	53 (23 to 94)	−58·0% (−71·7 to −39·8)*	−63·4% (−75·4 to −47·8)*
..				HIV/AIDS and extensively drug-resistant tuberculosis co-infection	0 (0 to 0)	0 (0 to 0)	−19·2% (−39·7 to 4·2)	−30·7% (−48·4 to −10·4)*	2 (1 to 4)	2 (1 to 3)	−18·7% (−39·5 to 4·3)	−29·3% (−47·3 to −9·1)*
..				HIV/AIDS resulting in other diseases	96 (51 to 148)	37 (20 to 58)	−61·3% (−64·4 to −58·2)*	−66·5% (−69·3 to −63·8)*	4890 (2580 to 7620)	1990 (1050 to 3100)	−59·2% (−62·4 to −56·0)*	−64·3% (−67·2 to −61·5)*
..				Maternal abortive outcome	3 (2 to 4)	3 (2 to 4)	−9·2% (−25·8 to 11·4)	−18·4% (−32·9 to 0·7)	162 (99 to 241)	144 (90 to 212)	−10·7% (−26·8 to 9·4)	−19·1% (−33·6 to −0·1)*
..				Major depressive disorder	..	..	..	..	1760 (1080 to 2660)	2000 (1240 to 3030)	13·7% (10·2 to 17·8)*	−2·9% (−4·9 to −1·0)*
..				Assault by firearm	5 (4 to 6)	5 (4 to 6)	−4·8% (−9·7 to 3·0)	−15·7% (−20·1 to −8·8)*	270 (209 to 328)	251 (203 to 309)	−7·1% (−11·7 to 1·0)	−15·9% (−20·2 to −8·5)*
..				Assault by sharp object	7 (5 to 8)	5 (4 to 6)	−17·2% (−21·1 to −10·6)*	−27·5% (−30·8 to −21·8)*	368 (291 to 421)	302 (244 to 352)	−18·0% (−22·1 to −10·6)*	−26·8% (−30·6 to −20·4)*
..				Sexual violence	..	..	..	..	712 (469 to 1040)	787 (520 to 1150)	10·7% (5·2 to 16·0)*	−0·8% (−5·3 to 3·5)
..				Assault by other means	11 (8 to 13)	10 (8 to 12)	−11·9% (−16·6 to −4·4)*	−22·6% (−26·7 to −16·2)*	733 (576 to 871)	669 (561 to 806)	−8·7% (−13·4 to −0·9)*	−19·0% (−22·9 to −12·2)*
**2**			**Childhood maltreatment: all causes**		**7 (6 to 10)**	**8 (6 to 10)**	**6·6% (−0·6 to 13·9)**	−12·6% (−18·5 to −6·9)*	**4670 (3280 to 6460)**	**5210 (3660 to 7220)**	11·6% (9·9 to 13·5)*	1·7% (0·3 to 3·0)*
3			Childhood sexual abuse: all causes		7 (6 to 10)	8 (6 to 10)	6·6% (−0·6 to 13·9)	−12·6% (−18·5 to −6·9)*	2380 (1670 to 3230)	2700 (1900 to 3690)	13·3% (11·2 to 15·6)*	−2·4% (−4·3 to −0·7)*
..				Major depressive disorder	..	..	..	..	1620 (1080 to 2300)	1860 (1250 to 2640)	14·9% (12·8 to 17·2)*	−1·0% (−2·9 to 0·8)
..				Alcohol use disorders	7 (6 to 10)	8 (6 to 10)	6·6% (−0·6 to 13·9)	−12·6% (−18·5 to −6·9)*	760 (528 to 1050)	836 (583 to 1170)	10·0% (6·2 to 13·6)*	−5·2% (−8·7 to −2·2)*
3			Bullying victimisation: all causes		..	..	..	..	2340 (1380 to 3660)	2570 (1540 to 3970)	9·9% (8·1 to 12·5)*	6·3% (4·5 to 8·8)*
..				Major depressive disorder	..	..	..	..	1110 (624 to 1800)	1210 (689 to 1940)	9·5% (6·9 to 12·6)*	6·0% (3·3 to 9·0)*
..				Anxiety disorders	..	..	..	..	1230 (732 to 1950)	1360 (815 to 2130)	10·2% (8·1 to 12·6)*	6·6% (4·6 to 9·0)*
**2**			**Unsafe sex: all causes**		**1750 (1680 to 1830)**	**1030 (980 to 1080)**	−41·2% (−43·2 to −39·2)*	−49·8% (−51·4 to −48·1)*	**86 100 (81 700 to 90 600)**	**50 200 (47 100 to 53 400)**	−41·7% (−43·8 to −39·4)*	−49·2% (−51·0 to −47·2)*
..				HIV/AIDS and drug-susceptible tuberculosis co-infection	330 (223 to 440)	153 (108 to 202)	−53·6% (−56·9 to −49·1)*	−59·9% (−62·8 to −56·2)*	16 000 (10 700 to 21 400)	7900 (5640 to 10 300)	−50·7% (−54·5 to −45·7)*	−56·6% (−60·0 to −52·2)*
..				HIV/AIDS and multidrug-resistant tuberculosis without extensive drug resistance co-infection	35 (20 to 55)	17 (10 to 26)	−51·2% (−66·3 to −31·0)*	−57·9% (−70·9 to −40·6)*	1690 (952 to 2650)	865 (513 to 1310)	−48·8% (−64·4 to −28·1)*	−54·9% (−68·6 to −36·8)*
..				HIV/AIDS and extensively drug-resistant tuberculosis co-infection	1 (1 to 1)	1 (0 to 1)	−8·7% (−29·5 to 16·7)	−21·3% (−39·4 to 0·4)	43 (26 to 65)	40 (24 to 59)	−8·8% (−28·7 to 16·7)	−20·1% (−37·6 to 2·0)
..				HIV/AIDS resulting in other diseases	1160 (1020 to 1290)	590 (526 to 656)	−49·0% (−51·5 to −46·2)*	−55·7% (−58·0 to −53·3)*	59 800 (52 700 to 67 100)	31 700 (28 300 to 35 400)	−47·1% (−49·9 to −44·2)*	−53·4% (−55·9 to −50·9)*
..				Syphilis	2 (1 to 2)	2 (1 to 2)	2·9% (−11·3 to 18·4)	−17·6% (−28·9 to −5·0)*	124 (101 to 151)	132 (108 to 162)	7·1% (−0·1 to 14·1)	−11·2% (−17·2 to −5·6)*
..				Chlamydial infection	1 (1 to 1)	1 (1 to 1)	2·5% (−4·4 to 11·3)	−15·2% (−21·0 to −8·4)*	329 (205 to 555)	355 (219 to 606)	8·1% (5·1 to 10·6)*	−3·0% (−5·9 to −0·8)*
..				Gonococcal infection	3 (2 to 3)	3 (2 to 3)	3·7% (−3·4 to 12·5)	−15·0% (−20·8 to −8·2)*	290 (206 to 437)	303 (216 to 468)	4·5% (0·1 to 8·6)*	−6·1% (−10·7 to −2·1)*
..				Trichomoniasis	..	..	..	..	209 (84 to 454)	243 (98 to 524)	16·0% (14·2 to 17·7)*	2·2% (1·1 to 3·2)*
..				Genital herpes	..	..	..	..	207 (68 to 492)	247 (80 to 594)	19·8% (18·1 to 21·0)*	1·5% (0·8 to 2·3)*
..				Other sexually transmitted infections	2 (1 to 2)	2 (1 to 2)	0·2% (−6·4 to 8·4)	−15·9% (−21·6 to −9·5)*	385 (257 to 604)	416 (276 to 659)	8·1% (5·9 to 10·1)*	−2·6% (−5·0 to −0·7)*
..				Cervical cancer	219 (204 to 231)	260 (241 to 269)	18·8% (12·9 to 22·8)*	−7·2% (−11·6 to −4·0)*	7000 (6500 to 7340)	8060 (7530 to 8400)	15·2% (9·5 to 19·2)*	−7·0% (−11·6 to −3·8)*
**2**			**Low physical activity: all causes**		**1030 (557 to 1640)**	**1260 (681 to 2010)**	22·0% (20·4 to 23·7)*	−10·8% (−12·1 to −9·5)*	**19 700 (10 500 to 31 300)**	**23 700 (12 500 to 37 300)**	20·1% (18·0 to 22·1)*	−8·9% (−10·7 to −7·2)*
..			Colon and rectum cancer		26 (3 to 61)	33 (4 to 77)	27·3% (23·8 to 31·3)*	−5·2% (−7·9 to −1·8)*	525 (70 to 1230)	652 (89 to 1540)	24·1% (20·1 to 28·5)*	−5·1% (−8·2 to −1·2)*
..			Breast cancer		7 (0 to 17)	9 (0 to 21)	25·9% (20·8 to 30·5)*	−4·4% (−8·1 to −0·5)*	205 (4 to 470)	253 (5 to 575)	23·4% (17·7 to 28·5)*	−3·2% (−7·5 to 1·3)
..			Ischaemic heart disease		730 (346 to 1220)	889 (416 to 1480)	21·8% (20·1 to 23·3)*	−10·9% (−12·1 to −9·6)*	13 200 (6020 to 21 900)	15 400 (7120 to 25 700)	17·0% (15·2 to 18·7)*	−11·1% (−12·5 to −9·7)*
..			Ischaemic stroke		246 (86 to 456)	295 (103 to 547)	20·1% (17·9 to 22·4)*	−13·0% (−14·7 to −11·2)*	4410 (1520 to 8280)	5460 (1870 to 10 200)	23·8% (20·7 to 27·1)*	−7·4% (−9·8 to −4·7)*
..			Type 2 diabetes mellitus		26 (6 to 48)	36 (8 to 69)	42·5% (39·8 to 45·2)*	5·3% (3·3 to 7·2)*	1380 (300 to 2710)	1870 (410 to 3660)	35·8% (32·1 to 40·0)*	5·2% (2·4 to 8·3)*
**1**	**Metabolic risks: all causes**				**14 100 (12 900 to 15 100)**	**17 600 (16 100 to 18 900)**	25·0% (23·3 to 26·6)*	−7·3% (−8·5 to −6·1)*	**346 000 (317 000 to 376 000)**	**423 000 (386 000 to 462 000)**	22·2% (20·7 to 23·9)*	−5·4% (−6·6 to −4·1)*
**2**			**High fasting plasma glucose: all causes**		**5140 (4150 to 6490)**	**6530 (5230 to 8230)**	27·1% (22·5 to 31·7)*	−6·0% (−9·4 to −2·9)*	**136 000 (115 000 to 160 000)**	**171 000 (144 000 to 201 000)**	25·5% (21·8 to 29·5)*	**−3·2% (−6·1 to 0·0)**
..				Drug-susceptible tuberculosis	125 (80 to 176)	118 (73 to 166)	−6·1% (−14·1 to 2·4)	−26·6% (−32·6 to −20·1)*	4060 (2680 to 5580)	3740 (2400 to 5200)	−8·0% (−15·6 to 0·1)	−26·0% (−31·9 to −19·6)*
..				Multidrug-resistant tuberculosis without extensive drug resistance	14 (9 to 22)	14 (7 to 25)	−4·3% (−43·5 to 50·2)	−24·9% (−55·9 to 17·7)	464 (285 to 672)	428 (210 to 757)	−7·8% (−44·8 to 44·5)	−25·7% (−55·7 to 16·6)
..				Extensively drug-resistant tuberculosis	1 (1 to 2)	1 (1 to 2)	21·5% (−12·2 to 68·0)	−3·6% (−30·3 to 33·2)	40 (24 to 58)	46 (26 to 73)	14·3% (−16·3 to 56·9)	−7·0% (−31·9 to 27·2)
..				Colon and rectum cancer	60 (15 to 129)	80 (19 to 172)	32·6% (25·5 to 41·0)*	−1·7% (−7·0 to 4·5)	1130 (269 to 2450)	1470 (352 to 3200)	29·8% (22·4 to 38·2)*	−1·8% (−7·3 to 4·5)
..				Liver cancer due to non-alcoholic steatohepatitis	4 (1 to 8)	5 (1 to 12)	44·0% (35·8 to 52·6)*	8·0% (1·8 to 14·7)*	74 (18 to 163)	105 (25 to 230)	40·8% (33·1 to 49·3)*	7·2% (1·2 to 13·9)*
..				Liver cancer due to other causes	3 (1 to 7)	4 (1 to 9)	26·5% (18·8 to 34·8)*	−3·9% (−9·7 to 2·4)	81 (19 to 174)	99 (24 to 217)	22·4% (15·2 to 30·7)*	−5·3% (−10·8 to 1·1)
..				Pancreatic cancer	27 (6 to 57)	39 (9 to 84)	46·3% (38·6 to 55·0)*	8·8% (3·1 to 15·1)*	502 (115 to 1090)	717 (163 to 1570)	42·9% (35·3 to 51·4)*	8·0% (2·4 to 14·5)*
..				Tracheal, bronchus, and lung cancer	119 (27 to 257)	154 (34 to 336)	29·6% (23·0 to 36·9)*	−2·5% (−7·6 to 2·8)	2420 (544 to 5270)	3050 (674 to 6710)	26·4% (19·8 to 33·6)*	−4·1% (−9·1 to 1·3)
..				Breast cancer	33 (6 to 73)	44 (9 to 99)	35·2% (27·9 to 43·9)*	1·3% (−4·2 to 7·6)	805 (153 to 1800)	1070 (205 to 2430)	33·5% (25·4 to 42·4)*	2·8% (−3·2 to 9·5)
..				Ovarian cancer	10 (2 to 23)	14 (3 to 32)	38·1% (31·3 to 46·3)*	3·1% (−1·9 to 9·3)	222 (43 to 521)	307 (61 to 718)	38·6% (31·6 to 46·9)*	5·6% (0·3 to 11·8)*
..				Bladder cancer	14 (3 to 31)	19 (4 to 42)	34·8% (28·0 to 42·5)*	−0·8% (−5·7 to 5·0)	246 (50 to 538)	320 (65 to 697)	30·1% (23·8 to 37·6)*	−2·2% (−6·9 to 3·4)
..				Ischaemic heart disease	1820 (1070 to 2930)	2270 (1340 to 3570)	24·9% (18·6 to 31·5)*	−8·7% (−12·8 to −4·2)*	32 500 (20 800 to 49 400)	39 800 (25 900 to 60 000)	22·7% (17·6 to 28·7)*	−7·2% (−11·3 to −2·5)*
..				Ischaemic stroke	504 (243 to 1010)	594 (301 to 1210)	17·8% (11·0 to 25·2)*	−13·8% (−18·0 to −9·1)*	9580 (5110 to 16 800)	11 800 (6500 to 20 100)	23·0% (16·2 to 31·1)*	−7·7% (−12·8 to −1·4)*
..				Intracerebral haemorrhage	529 (329 to 813)	591 (368 to 878)	11·7% (5·1 to 18·4)*	−16·3% (−21·3 to −11·4)*	11 300 (7280 to 16 500)	12 700 (8210 to 18 000)	12·9% (6·0 to 19·1)*	−13·9% (−19·4 to −8·6)*
..				Subarachnoid haemorrhage	72 (46 to 107)	88 (57 to 129)	23·0% (16·3 to 30·4)*	−7·0% (−12·3 to −1·3)*	1850 (1190 to 2730)	2280 (1460 to 3230)	23·1% (16·5 to 30·3)*	−4·6% (−9·7 to 0·9)
..				Peripheral vascular disease	12 (7 to 20)	19 (11 to 34)	61·8% (34·4 to 84·1)*	15·9% (−3·4 to 31·8)	278 (188 to 409)	402 (269 to 605)	44·8% (28·0 to 60·2)*	7·9% (−4·7 to 19·3)
..				Alzheimer's disease and other dementias	164 (37 to 353)	249 (58 to 532)	52·0% (44·2 to 60·8)*	4·6% (−0·9 to 11·0)	2060 (462 to 4440)	2940 (681 to 6340)	42·8% (35·7 to 50·9)*	2·5% (−2·6 to 8·4)
..				Type 1 diabetes mellitus	300 (276 to 326)	346 (319 to 371)	15·1% (10·5 to 19·0)*	−11·0% (−14·6 to −7·8)*	9370 (8720 to 10 100)	10 400 (9790 to 11 100)	11·4% (7·9 to 14·5)*	−9·8% (−12·8 to −7·2)*
..				Type 2 diabetes mellitus	716 (686 to 748)	1020 (986 to 1070)	43·0% (40·4 to 45·8)*	5·9% (4·1 to 8·0)*	42 900 (33 400 to 53 700)	57 400 (45 000 to 71 900)	34·0% (30·3 to 38·1)*	5·1% (2·3 to 8·1)*
..				Chronic kidney disease due to type 1 diabetes mellitus	63 (51 to 76)	77 (62 to 95)	23·2% (19·0 to 27·4)*	−1·2% (−4·0 to 1·2)	2440 (2010 to 2950)	2890 (2370 to 3500)	18·2% (14·3 to 22·3)*	−2·6% (−5·1 to −0·3)*
..				Chronic kidney disease due to type 2 diabetes mellitus	248 (219 to 282)	349 (307 to 396)	40·5% (36·4 to 43·6)*	4·2% (1·4 to 6·2)*	6050 (5290 to 6850)	8120 (7120 to 9250)	34·3% (30·9 to 37·2)*	2·3% (−0·2 to 4·2)
..				Chronic kidney disease due to hypertension	143 (100 to 181)	208 (150 to 255)	45·0% (37·7 to 53·6)*	4·8% (−0·2 to 10·9)	2670 (1880 to 3420)	3710 (2690 to 4650)	39·2% (32·0 to 47·4)*	4·4% (−0·8 to 10·4)
..				Chronic kidney disease due to glomerulonephritis	66 (46 to 85)	91 (66 to 116)	38·6% (31·3 to 47·4)*	3·6% (−1·5 to 9·7)	1600 (1100 to 2110)	2140 (1520 to 2730)	33·5% (26·6 to 41·2)*	3·7% (−1·4 to 9·6)
..				Chronic kidney disease due to other and unspecified causes	93 (64 to 121)	130 (93 to 165)	40·4% (33·1 to 48·9)*	4·2% (−0·8 to 10·3)	2800 (1920 to 3740)	3760 (2640 to 4880)	34·3% (27·0 to 42·4)*	2·8% (−2·6 to 9·0)
..				Glaucoma	..	..	..	..	46 (11 to 105)	61 (14 to 141)	32·7% (27·1 to 39·4)*	−1·1% (−5·4 to 3·8)
..				Cataract	..	..	..	..	503 (112 to 1170)	687 (153 to 1580)	36·7% (31·0 to 43·1)*	2·4% (−2·0 to 7·1)
**2**			**High low-density lipoprotein cholesterol: all causes**		**3570 (2780 to 4450)**	**4320 (3330 to 5440)**	20·8% (18·2 to 23·2)*	−10·6% (−11·8 to −9·4)*	**81 000 (67 800 to 95 700)**	**94 900 (78 800 to 112 000)**	17·2% (15·3 to 19·1)*	−9·3% (−10·6 to −7·9)*
..				Ischaemic heart disease	3140 (2430 to 3900)	3790 (2890 to 4730)	20·7% (17·9 to 23·3)*	−10·4% (−11·6 to −9·1)*	70 800 (59 400 to 82 400)	82 200 (68 600 to 96 400)	16·1% (14·3 to 17·8)*	−9·9% (−11·3 to −8·6)*
..				Ischaemic stroke	439 (177 to 893)	532 (211 to 1080)	21·2% (16·4 to 24·7)*	−12·1% (−13·6 to −10·2)*	10 200 (6160 to 16 900)	12 700 (7610 to 21 200)	25·0% (21·6 to 28·1)*	−5·0% (−7·5 to −2·1)*
**2**			**High systolic blood pressure: all causes**		**8500 (7640 to 9340)**	**10 400 (9390 to 11 500)**	22·8% (20·5 to 24·7)*	−9·0% (−10·6 to −7·6)*	**182 000 (164 000 to 198 000)**	**218 000 (198 000 to 237 000)**	20·0% (18·0 to 21·8)*	−8·0% (−9·5 to −6·7)*
..				Rheumatic heart disease	69 (46 to 106)	72 (48 to 113)	4·5% (−1·9 to 11·0)	−20·1% (−24·2 to −16·1)*	2070 (1420 to 3130)	2100 (1450 to 3260)	1·6% (−4·0 to 7·2)	−18·8% (−23·0 to −15·0)*
..				Ischaemic heart disease	4030 (3350 to 4740)	4890 (4030 to 5760)	21·2% (19·0 to 23·0)*	−10·4% (−11·6 to −9·2)*	80 500 (69 200 to 91 400)	94 800 (81 100 to 108 000)	17·7% (15·9 to 19·4)*	−10·0% (−11·4 to −8·7)*
..				Ischaemic stroke	1150 (900 to 1390)	1370 (1060 to 1680)	19·4% (16·5 to 22·7)*	−12·3% (−14·0 to −10·6)*	23 500 (18 700 to 27 700)	29 400 (23 400 to 34 700)	25·1% (21·9 to 28·4)*	−5·7% (−8·1 to −3·2)*
..				Intracerebral haemorrhage	1530 (1230 to 1800)	1730 (1400 to 2050)	13·5% (10·5 to 16·6)*	−14·9% (−17·0 to −12·8)*	34 700 (29 000 to 40 100)	38 900 (32 400 to 44 800)	12·0% (9·2 to 14·8)*	−13·8% (−16·0 to −11·7)*
..				Subarachnoid haemorrhage	215 (175 to 258)	258 (211 to 311)	19·7% (14·4 to 26·0)*	−8·8% (−12·8 to −4·4)*	6120 (5000 to 7240)	7140 (5900 to 8480)	16·7% (12·4 to 22·0)*	−8·4% (−11·8 to −4·3)*
..				Hypertensive heart disease	632 (516 to 676)	926 (681 to 995)	46·6% (26·3 to 59·3)*	7·5% (−7·3 to 16·3)	12 200 (10 100 to 13 200)	16 500 (12 700 to 17 900)	35·6% (20·5 to 46·6)*	3·7% (−7·9 to 11·8)
..				Non-rheumatic calcific aortic valve disease	24 (17 to 34)	33 (23 to 47)	35·5% (28·7 to 41·4)*	−3·2% (−7·4 to 0·0)	408 (317 to 514)	528 (410 to 670)	29·6% (24·4 to 34·3)*	−2·9% (−6·1 to 0·2)
..				Other cardiomyopathy	61 (47 to 76)	80 (62 to 101)	31·6% (27·0 to 35·9)*	−2·3% (−5·8 to 1·1)	1460 (1150 to 1770)	1890 (1490 to 2290)	29·5% (23·8 to 34·0)*	1·1% (−3·3 to 4·8)
..				Atrial fibrillation and flutter	70 (56 to 83)	100 (80 to 121)	43·8% (40·2 to 47·0)*	1·4% (−0·7 to 3·4)	1800 (1450 to 2210)	2410 (1930 to 2950)	33·8% (31·9 to 35·7)*	−0·2% (−1·4 to 1·1)
..				Aortic aneurysm	49 (39 to 59)	59 (47 to 72)	21·3% (17·2 to 25·5)*	−9·6% (−12·5 to −6·6)*	968 (801 to 1140)	1150 (952 to 1360)	18·8% (14·4 to 23·5)*	−9·0% (−12·5 to −5·4)*
..				Peripheral vascular disease	12 (7 to 22)	18 (10 to 33)	49·1% (24·9 to 66·2)*	7·2% (−9·4 to 19·7)	300 (186 to 457)	410 (257 to 632)	36·8% (22·9 to 49·1)*	2·1% (−8·0 to 10·7)
..				Endocarditis	20 (15 to 25)	27 (21 to 34)	36·5% (29·4 to 41·4)*	2·4% (−2·5 to 6·6)	478 (375 to 589)	629 (499 to 762)	31·6% (24·3 to 36·8)*	3·9% (−1·4 to 7·8)
..				Other cardiovascular and circulatory diseases	117 (100 to 137)	143 (122 to 167)	22·8% (18·5 to 26·0)*	−7·6% (−10·7 to −5·3)*	3840 (3170 to 4680)	4680 (3850 to 5660)	21·8% (18·8 to 24·2)*	−5·3% (−7·5 to −3·5)*
..				Chronic kidney disease due to type 1 diabetes mellitus	23 (16 to 33)	30 (20 to 42)	29·1% (24·8 to 33·1)*	1·0% (−2·0 to 3·4)	799 (524 to 1130)	999 (660 to 1410)	25·1% (20·6 to 29·2)*	0·4% (−2·4 to 2·9)
..				Chronic kidney disease due to type 2 diabetes mellitus	119 (85 to 154)	168 (120 to 217)	41·4% (37·3 to 44·6)*	4·4% (1·6 to 6·5)*	2710 (1930 to 3550)	3700 (2640 to 4840)	36·5% (33·1 to 39·4)*	3·0% (0·4 to 5·0)*
..				Chronic kidney disease due to hypertension	246 (216 to 276)	347 (305 to 391)	41·4% (37·4 to 44·2)*	3·2% (0·4 to 5·2)*	5550 (4900 to 6230)	7350 (6450 to 8220)	32·4% (29·0 to 35·0)*	2·1% (−0·3 to 4·1)
..				Chronic kidney disease due to glomerulonephritis	57 (40 to 77)	77 (54 to 103)	34·2% (30·8 to 37·5)*	1·4% (−0·9 to 3·3)	1560 (1060 to 2110)	1990 (1350 to 2690)	27·1% (24·0 to 30·1)*	0·1% (−1·8 to 1·9)
..				Chronic kidney disease due to other and unspecified causes	80 (56 to 103)	109 (76 to 142)	36·7% (32·6 to 40·3)*	2·4% (−0·3 to 4·6)	2670 (1850 to 3560)	3450 (2410 to 4620)	29·0% (25·8 to 32·2)*	−0·1% (−2·4 to 2·0)
**2**			**High body-mass index: all causes**		**3470 (2110 to 5030)**	**4720 (2990 to 6700)**	36·3% (31·9 to 42·5)*	**2·3% (−0·9 to 7·2)**	**108 000 (69 000 to 153 000)**	**148 000 (98 600 to 202 000)**	36·7% (31·5 to 44·5)*	6·8% (2·6 to 13·0)*
..				Oesophageal cancer	61 (20 to 121)	81 (27 to 153)	32·7% (24·0 to 45·1)*	0·3% (−6·4 to 9·8)	1470 (464 to 2860)	1900 (622 to 3530)	29·7% (20·9 to 42·0)*	−0·5% (−7·3 to 9·0)
..				Colon and rectum cancer	54 (29 to 84)	73 (41 to 114)	36·4% (32·2 to 41·7)*	2·4% (−0·8 to 6·4)	1210 (662 to 1880)	1640 (920 to 2500)	35·4% (30·6 to 41·1)*	3·9% (0·3 to 8·2)*
..				Liver cancer due to hepatitis B	26 (9 to 55)	40 (15 to 79)	56·1% (42·2 to 86·2)*	21·5% (10·8 to 44·8)*	794 (264 to 1690)	1210 (429 to 2390)	51·8% (37·3 to 82·8)*	20·7% (9·1 to 45·2)*
..				Liver cancer due to hepatitis C	21 (8 to 40)	31 (12 to 58)	52·0% (44·1 to 64·6)*	14·0% (8·0 to 23·3)*	467 (181 to 895)	705 (286 to 1310)	51·0% (42·5 to 64·4)*	15·2% (8·7 to 25·3)*
..				Liver cancer due to alcohol use	13 (5 to 25)	19 (8 to 37)	50·8% (43·4 to 61·8)*	15·0% (9·2 to 23·2)*	317 (123 to 630)	471 (189 to 905)	48·9% (40·6 to 61·0)*	15·4% (9·2 to 24·6)*
..				Liver cancer due to other causes	5 (2 to 10)	8 (3 to 15)	59·4% (47·8 to 80·2)*	22·7% (13·8 to 38·2)*	142 (52 to 285)	220 (85 to 413)	54·7% (42·4 to 77·2)*	22·0% (12·5 to 39·1)*
..				Gallbladder and biliary tract cancer	20 (10 to 34)	27 (14 to 44)	32·7% (27·8 to 39·0)*	−1·2% (−4·9 to 3·5)	422 (219 to 694)	561 (299 to 897)	33·0% (27·0 to 39·9)*	1·3% (−3·2 to 6·6)
..				Pancreatic cancer	19 (7 to 35)	27 (11 to 51)	47·2% (43·5 to 51·8)*	10·0% (7·1 to 13·6)*	398 (153 to 758)	577 (225 to 1090)	45·2% (41·2 to 49·9)*	10·7% (7·7 to 14·3)*
..				Breast cancer	26 (10 to 48)	40 (16 to 71)	54·8% (39·2 to 88·4)*	11·1% (−0·3 to 30·8)	487 (125 to 983)	817 (267 to 1530)	67·7% (45·5 to 153·1)*	16·2% (1·2 to 52·4)*
..				Uterine cancer	25 (17 to 34)	33 (23 to 44)	30·8% (25·9 to 37·6)*	−1·7% (−5·3 to 3·2)	642 (426 to 869)	842 (584 to 1120)	31·2% (25·5 to 38·8)*	0·7% (−3·5 to 6·3)
..				Ovarian cancer	4 (0 to 10)	6 (0 to 13)	34·5% (22·4 to 39·8)*	1·8% (−5·1 to 5·8)	111 (0 to 251)	149 (0 to 334)	35·1% (22·6 to 41·2)*	4·9% (−4·4 to 9·5)
..				Kidney cancer	20 (12 to 30)	27 (16 to 40)	36·7% (32·4 to 42·0)*	3·0% (−0·3 to 7·0)	464 (275 to 690)	619 (370 to 911)	33·5% (29·0 to 39·3)*	2·6% (−0·8 to 7·1)
..				Thyroid cancer	3 (1 to 5)	4 (2 to 7)	43·9% (37·0 to 51·7)*	9·3% (3·9 to 15·2)*	81 (39 to 136)	116 (57 to 191)	43·1% (35·6 to 51·8)*	12·2% (6·3 to 18·8)*
..				Non-Hodgkin lymphoma	9 (4 to 17)	13 (6 to 24)	40·2% (36·2 to 46·1)*	6·4% (3·1 to 11·2)*	238 (100 to 427)	330 (144 to 591)	38·9% (34·5 to 44·6)*	9·0% (5·6 to 13·6)*
..				Multiple myeloma	5 (2 to 10)	8 (3 to 13)	41·2% (35·8 to 47·8)*	5·7% (1·6 to 10·9)*	119 (52 to 210)	168 (75 to 291)	41·0% (35·4 to 47·9)*	7·7% (3·4 to 13·2)*
..				Acute lymphoid leukaemia	2 (1 to 3)	2 (1 to 4)	44·4% (35·2 to 52·4)*	15·2% (8·0 to 21·4)*	56 (26 to 98)	79 (39 to 135)	40·9% (31·9 to 49·9)*	17·0% (9·6 to 24·2)*
..				Chronic lymphoid leukaemia	2 (1 to 4)	3 (2 to 5)	25·5% (21·2 to 30·2)*	−7·8% (−10·9 to −4·2)*	48 (24 to 79)	60 (31 to 98)	25·9% (21·8 to 30·5)*	−4·9% (−8·0 to −1·4)*
..				Acute myeloid leukaemia	5 (3 to 9)	7 (4 to 12)	36·3% (31·4 to 42·2)*	4·7% (0·9 to 9·2)*	138 (67 to 231)	184 (92 to 303)	33·3% (27·8 to 39·6)*	6·2% (2·0 to 11·1)*
..				Chronic myeloid leukaemia	2 (1 to 3)	2 (1 to 3)	10·2% (6·3 to 15·6)*	−15·9% (−18·8 to −11·7)*	40 (19 to 68)	43 (21 to 72)	8·9% (4·5 to 15·0)*	−13·2% (−16·6 to −8·5)*
..				Other leukaemia	7 (3 to 12)	8 (4 to 14)	26·6% (21·2 to 34·8)*	−3·1% (−7·0 to 2·7)	175 (80 to 311)	215 (103 to 364)	22·9% (16·1 to 32·8)*	−1·8% (−6·9 to 5·6)
..				Ischaemic heart disease	1270 (750 to 1890)	1630 (985 to 2380)	28·0% (24·4 to 32·6)*	−3·7% (−6·1 to 0·0)	30 800 (19 100 to 44 100)	39 300 (25 300 to 55 000)	27·6% (23·6 to 33·4)*	−0·6% (−3·7 to 4·2)
..				Ischaemic stroke	259 (143 to 394)	324 (184 to 493)	25·3% (20·9 to 30·2)*	−5·4% (−8·2 to −1·8)*	7410 (4430 to 10 900)	10 100 (6190 to 14 600)	36·4% (31·2 to 43·2)*	5·3% (1·3 to 10·5)*
..				Intracerebral haemorrhage	487 (279 to 736)	622 (380 to 907)	27·7% (20·8 to 37·3)*	−1·2% (−6·5 to 6·4)	14 900 (8960 to 21 400)	18 900 (12 000 to 26 000)	27·0% (19·9 to 37·1)*	0·5% (−5·3 to 8·6)
..				Subarachnoid haemorrhage	93 (59 to 132)	119 (79 to 164)	27·6% (21·7 to 34·5)*	1·0% (−3·8 to 6·6)	3510 (2300 to 4800)	4420 (3010 to 5900)	26·0% (20·4 to 33·2)*	2·2% (−2·6 to 8·1)
..				Hypertensive heart disease	213 (118 to 332)	327 (176 to 522)	53·8% (36·3 to 65·3)*	13·7% (1·6 to 21·2)*	4690 (2930 to 6620)	6830 (4290 to 9600)	45·7% (32·0 to 56·2)*	12·3% (1·9 to 20·2)*
..				Atrial fibrillation and flutter	39 (22 to 61)	60 (34 to 93)	53·5% (49·2 to 59·2)*	6·6% (3·5 to 11·0)*	907 (487 to 1450)	1310 (712 to 2070)	44·6% (41·3 to 49·1)*	6·8% (4·3 to 10·3)*
..				Asthma	58 (28 to 106)	72 (38 to 126)	23·9% (13·7 to 38·8)*	−5·4% (−13·3 to 6·4)	2800 (1540 to 4510)	3550 (2050 to 5580)	27·0% (19·4 to 36·1)*	3·3% (−3·8 to 11·6)
..				Gallbladder and biliary diseases	24 (15 to 35)	34 (22 to 49)	41·8% (36·7 to 49·6)*	3·6% (−0·2 to 9·4)	453 (282 to 653)	611 (391 to 866)	35·0% (28·5 to 44·2)*	4·0% (−0·9 to 10·9)
..				Alzheimer's disease and other dementias	207 (77 to 395)	319 (121 to 599)	54·1% (49·4 to 61·7)*	5·8% (2·0 to 11·7)*	2640 (1030 to 5050)	3900 (1580 to 7270)	47·6% (43·1 to 54·7)*	5·9% (2·4 to 11·6)*
..				Type 1 diabetes mellitus	271 (186 to 363)	422 (299 to 552)	55·7% (49·7 to 64·0)*	16·6% (12·0 to 23·0)*	20 600 (13 500 to 29 500)	31 100 (21 400 to 43 000)	50·7% (42·7 to 61·5)*	19·3% (13·0 to 27·8)*
..				Chronic kidney disease due to type 2 diabetes mellitus	67 (32 to 112)	109 (54 to 177)	62·3% (52·2 to 75·4)*	20·2% (13·8 to 29·2)*	1800 (857 to 2940)	2840 (1400 to 4450)	58·2% (48·9 to 71·5)*	19·4% (13·3 to 27·9)*
..				Chronic kidney disease due to hypertension	63 (27 to 116)	100 (44 to 180)	59·0% (50·2 to 70·2)*	15·9% (10·3 to 26·0)*	1510 (725 to 2590)	2330 (1180 to 3780)	54·7% (45·4 to 67·0)*	17·4% (11·4 to 26·3)*
..				Chronic kidney disease due to glomerulonephritis	37 (17 to 62)	55 (26 to 90)	47·0% (38·4 to 58·2)*	11·9% (7·5 to 18·0)*	1160 (451 to 2030)	1650 (692 to 2760)	42·0% (33·8 to 54·8)*	12·3% (7·8 to 18·7)*
..				Chronic kidney disease due to other and unspecified causes	48 (22 to 80)	75 (35 to 120)	53·7% (44·5 to 66·6)*	16·0% (10·4 to 24·2)*	1850 (806 to 3110)	2710 (1260 to 4380)	46·9% (38·5 to 59·5)*	14·4% (8·7 to 22·4)*
..				Cataract	..	..	..	..	304 (130 to 581)	456 (207 to 847)	49·9% (43·0 to 61·0)*	13·2% (7·9 to 21·8)*
..				Osteoarthritis	..	..	..	..	1360 (520 to 2920)	2030 (801 to 4370)	49·2% (43·8 to 58·0)*	14·3% (10·2 to 21·0)*
..				Low back pain	..	..	..	..	3250 (1690 to 5610)	4370 (2350 to 7400)	34·4% (30·1 to 40·1)*	9·1% (5·8 to 13·4)*
..				Gout	..	..	..	..	284 (142 to 490)	419 (216 to 706)	47·8% (42·8 to 55·0)*	15·6% (11·8 to 21·2)*
**2**			**Low bone mineral density: all causes**		**245 (226 to 256)**	**327 (308 to 347)**	33·7% (27·2 to 41·0)*	**−2·7% (−7·3 to 2·6)**	**7850 (6660 to 9210)**	**10 300 (8690 to 12 200)**	31·5% (28·1 to 34·8)*	**−0·9% (−3·3 to 1·5)**
..				Pedestrian road injuries	29 (28 to 32)	33 (31 to 36)	13·9% (5·3 to 19·6)*	−14·5% (−21·1 to −10·2)*	780 (704 to 857)	886 (792 to 981)	13·6% (6·5 to 18·8)*	−12·5% (−18·1 to −8·7)*
..				Cyclist road injuries	3 (3 to 4)	4 (4 to 4)	27·6% (17·0 to 36·2)*	−2·4% (−10·7 to 4·1)	166 (135 to 198)	218 (177 to 263)	31·6% (25·8 to 36·5)*	1·4% (−2·9 to 5·0)
..				Motorcyclist road injuries	6 (5 to 7)	8 (7 to 9)	25·9% (7·9 to 36·3)*	−1·5% (−15·6 to 6·7)	319 (269 to 373)	406 (334 to 483)	27·2% (16·4 to 33·6)*	0·3% (−8·2 to 5·1)
..				Motor vehicle road injuries	18 (17 to 20)	22 (19 to 24)	17·1% (8·4 to 21·8)*	−10·8% (−17·6 to −7·3)*	610 (534 to 683)	709 (615 to 800)	16·3% (9·9 to 20·1)*	−9·6% (−14·6 to −6·9)*
..				Other road injuries	1 (1 to 1)	1 (1 to 1)	18·1% (7·6 to 36·0)*	−10·9% (−19·0 to 2·2)	84 (64 to 109)	132 (99 to 175)	57·0% (51·0 to 62·7)*	19·2% (14·8 to 23·3)*
..				Other transport injuries	8 (7 to 9)	9 (8 to 10)	18·6% (13·6 to 27·6)*	−9·4% (−13·3 to −2·5)*	481 (397 to 585)	616 (500 to 762)	28·1% (24·6 to 32·0)*	−1·1% (−3·6 to 1·8)
..				Falls	169 (154 to 176)	238 (223 to 256)	41·1% (32·6 to 52·0)*	0·6% (−5·3 to 8·2)	4810 (4010 to 5760)	6590 (5500 to 7860)	37·0% (32·6 to 42·2)*	1·5% (−1·6 to 5·3)
..				Other exposure to mechanical forces	7 (6 to 8)	9 (7 to 10)	21·4% (15·6 to 27·3)*	−9·0% (−13·2 to −4·4)*	443 (347 to 562)	582 (447 to 752)	31·5% (27·8 to 34·8)*	0·9% (−1·6 to 3·2)
..				Non-venomous animal contact	1 (0 to 1)	1 (1 to 1)	17·9% (4·3 to 37·3)*	−11·3% (−21·4 to 3·2)	50 (36 to 69)	62 (44 to 88)	24·1% (17·5 to 30·2)*	−5·9% (−10·7 to −1·2)*
..				Assault by other means	3 (2 to 3)	3 (3 to 3)	7·2% (−1·0 to 13·8)	−17·8% (−24·0 to −12·7)*	114 (97 to 134)	132 (109 to 158)	16·1% (10·9 to 20·0)*	−10·6% (−14·3 to −7·7)*
**2**			**Impaired kidney function: all causes**		**2040 (1880 to 2210)**	**2590 (2390 to 2800)**	26·6% (23·6 to 29·7)*	−5·8% (−7·9 to −3·7)*	**51 000 (47 300 to 54 900)**	**61 300 (56 900 to 66 100)**	20·3% (17·9 to 22·9)*	−5·4% (−7·3 to −3·5)*
..				Ischaemic heart disease	716 (593 to 844)	882 (726 to 1050)	23·2% (17·7 to 28·8)*	−10·5% (−14·1 to −6·8)*	12 500 (10 700 to 14 400)	14 900 (12 800 to 17 200)	18·9% (14·6 to 23·8)*	−9·7% (−12·9 to −6·1)*
..				Ischaemic stroke	187 (137 to 239)	223 (162 to 288)	19·0% (13·2 to 24·8)*	−12·9% (−15·9 to −9·9)*	3760 (3010 to 4550)	4650 (3750 to 5700)	23·7% (19·0 to 28·9)*	−6·6% (−9·9 to −3·2)*
..				Intracerebral haemorrhage	214 (175 to 257)	243 (199 to 293)	13·7% (9·2 to 18·0)*	−14·3% (−17·2 to −11·5)*	4970 (4090 to 5860)	5530 (4580 to 6550)	11·4% (7·6 to 15·4)*	−13·9% (−16·6 to −11·4)*
..				Peripheral vascular disease	6 (4 to 11)	10 (5 to 18)	52·4% (25·2 to 73·7)*	11·5% (−7·5 to 26·5)	184 (121 to 278)	257 (166 to 398)	39·9% (23·4 to 55·0)*	5·7% (−6·6 to 16·8)
..				Chronic kidney disease due to type 1 diabetes mellitus	63 (51 to 76)	77 (62 to 95)	23·2% (19·0 to 27·4)*	−1·2% (−4·0 to 1·2)	2440 (2010 to 2950)	2890 (2370 to 3500)	18·2% (14·3 to 22·3)*	−2·6% (−5·1 to −0·3)*
..				Chronic kidney disease due to type 2 diabetes mellitus	248 (219 to 282)	349 (307 to 396)	40·5% (36·4 to 43·6)*	4·2% (1·4 to 6·2)*	6050 (5290 to 6850)	8120 (7120 to 9250)	34·3% (30·9 to 37·2)*	2·3% (−0·2 to 4·2)
..				Chronic kidney disease due to hypertension	246 (216 to 276)	347 (305 to 391)	41·4% (37·4 to 44·2)*	3·2% (0·4 to 5·2)*	5550 (4900 to 6230)	7350 (6450 to 8220)	32·4% (29·0 to 35·0)*	2·1% (−0·3 to 4·1)
..				Chronic kidney disease due to glomerulonephritis	151 (132 to 172)	190 (165 to 217)	25·5% (22·1 to 28·8)*	−1·3% (−3·2 to 0·7)	5800 (5180 to 6450)	6600 (5860 to 7420)	13·8% (11·0 to 17.0)*	−4·4% (−6·3 to −2·4)*
..				Chronic kidney disease due to other and unspecified causes	212 (186 to 239)	267 (233 to 304)	25·9% (22·4 to 29·4)*	−1·4% (−3·7 to 0·6)	9580 (8570 to 10 700)	10 900 (9660 to 12 200)	13·3% (10·4 to 16·2)*	−6·0% (−8·0 to −4·1)*
..				Gout	..	..	..	..	135 (91 to 187)	191 (130 to 265)	41·5% (35·7 to 47·7)*	6·3% (2·1 to 10·7)*

Results are for both sexes combined. Data in parentheses are 95% uncertainty intervals.

* Statistically significant increase or decrease. DALYs=disability-adjusted life-years.

In 2017, NCDs had the largest risk-attributable burden, with 26·6 million (95% UI 25·8–27·5) deaths and 706 million (659–756) DALYs attributable to all risk factors combined (appendix 2). The five leading NCD causes of absolute risk-attributable DALYs were ischaemic heart disease, intracerebral haemorrhage, type 2 diabetes, COPD, and ischaemic stroke. In 2017, 95·0% (95% UI 93·4–96·3), and 93·8% (91·3–95·9) of ischaemic heart disease DALYs and deaths were risk attributable, resulting in 162 million (158–166) risk-attributable ischaemic heart disease DALYs, and 8·38 million (8·10–8·65) risk-attributable ischaemic heart disease deaths. After ischaemic heart disease, intracerebral haemorrhage had 57·9 million (55·2–60·6) risk-attributable DALYs (89·8% [87·0–92·3] of all intracerebral haemorrhage DALYs), type 2 diabetes had 57·4 million (45·0–71·9), COPD had 54·9 million (48·1–60·4; 67·3% [60·8–72·8] of all COPD DALYs), and ischaemic stroke had 47·8 million (43·4–52·3; 86·7% [82·7–90·8] of ischaemic stroke DALYs). Because we estimated a PAF of 1·0 for high FPG and type 2 diabetes, 100% of type 2 diabetes DALYs and deaths were attributed to risk factors addressed in GBD 2017.

Among CMNNDs, 6·40 million (6·00–7·00) deaths and 446 million (419–475) DALYs were attributable to risks. The five leading causes of absolute risk-attributable DALYs among CMNNDs were lower respiratory infections, diarrhoeal diseases, neonatal preterm birth complications, neonatal encephalopathy due to birth asphyxia and trauma, and HIV/AIDS resulting in other diseases. In 2017, 80·5% (95% UI 77·4–83·4) and 64·0% (59·9–68·1) of lower respiratory infection DALYs and deaths were risk attributable, resulting in 76·9 million (66·1–92·8) risk-attributable lower respiratory infection DALYs and 1·64 million (1·51–1·77) risk-attributable lower respiratory infection deaths. Diarrhoeal diseases had 76·9 million (66·1–92·8) risk-attributable DALYs (94·9% [89·8–97·7] of all diarrhoeal diseases DALYs), neonatal preterm birth had 70·2 million (64·4–77·2; 100% of all neonatal preterm birth DALYs, since we estimate a PAF of 1·0 for this risk factor), neonatal encephalopathy due to birth asphyxia and trauma had 41·2 million (36·7–45·0; 72·9% [66·6–78·3] of neonatal encephalopathy due to birth asphyxia and trauma DALYs), and HIV/AIDS resulting in other diseases had 32·6 million (29·1–36·3; 77·5% [75·6–79·2] of HIV/AIDS resulting in other diseases DALYs).

Among injuries, 1·09 million (0·99–1·19) deaths and 55·9 million (49·6–62·5) DALYs were attributable to risks in 2017. The five leading injury causes of risk-attributable DALYs were falls, self-harm by other specified means, motor vehicle road injuries, pedestrian road injuries, and motorcyclist road injuries. In 2017, 34·1% (95% UI 30·7–38·2) of DALYs and 43·4% (40·6–46·6) of deaths from falls were risk attributable, resulting in 12·2 million (9·93–15·0) risk-attributable DALYs and 302 000 (275 000–330 000) risk-attributable deaths from falls. Self-harm by other specified means had 8·07 million (5·50–10·7) risk-attributable DALYs (25·7% [17·6–33·9] of all DALYs from self-harm by other specified means), motor vehicle road injuries had 5·95 million (5·19–6·70; 24·2% [21·3–27·0] of all motor vehicle road injury DALYs), pedestrian road injuries had 5·50 million (4·76–6·28; 23·4% [20·8–26·1] of pedestrian road injury DALYs), and motorcyclist road injuries had 4·05 million (3·46–4·64; 29·0% [25·3–32·4] of motorcyclist road injury DALYs).

### Levels and trends in the burden attributable to risk factors

In 2017, 34·1 million (95% UI 33·3–35·0) deaths and 1·21 billion (1·14–1·28) DALYs were attributable to GBD risk factors (table 3). Between 2007 and 2017, the number of all-age risk-attributable YLLs declined from 1·04 billion (1·02–1·07) to 944 million (922–968), and age-standardised YLL rates declined from 16 359 (16 005–16 748) per 100 000 to 12 509 (12 205–12 837) per 100 000. Age-standardised risk-attributable death rates declined from 531 (520–544) per 100 000 in 2007 to 448 (437–460) per 100 000 in 2017. Conversely, during that period, the absolute number of risk-attributable deaths increased from 31·5 million (30·9–32·2) to 34·1 million (33·3–35·0). During the same period, there was no significant trend in risk-attributable non-fatal burden because there was no statistically significant trend in non-fatal burden: age-standardised risk-attributable YLD rates were 3442 (2593–4374) per 100 000 in 2007 and 3357 (2528–4275) per 100 000 in 2017; and the absolute numbers of risk-attributable YLDs were 227 million (170–288) in 2007 and 263 million (198–336) in 2017. The largest percentage declines in the number of risk-attributable DALYs are for measles; the largest percentage increases are for osteoarthritis. Globally in 2017, high SBP was the leading risk factor, accounting for 10·4 million (9·39–11·5) deaths and 218 million (198–237) DALYs, followed by smoking (7·10 million [6·83–7·37] deaths and 182 million [173–193] DALYs), high FPG (6·53 million [5·23–8·23] deaths and 171 million [144–201] DALYs), high BMI (4·72 million [2·99–6·70] deaths and 148 million [98·6–202] DALYs), and short gestation (1·43 million [1·36–1·51] deaths and 139 million [131–147] DALYs; table 3).

Behavioural risk factors accounted for 43·6% (95% UI 41·7–45·5) of all DALYs in 1990, followed by environmental and occupational risk factors at 17·4% (15·9–19·0) and metabolic at 10·3% (9·63–11·1). Between 1990 and 2017, these percentages declined by 16·2% for behavioural risk factors, which accounted for 36·5% (34·7–38·4) of all DALYs in 2017, and 29·3% for environmental and occupational risks, which accounted for 12·3% (11·5–13·3) in 2017. Proportions increased by 63·7% for metabolic risks, which accounted for 16·9% (15·6–18·3) of all DALYs in 2017.

The proportion of all DALYs attributable to each Level 1 risk also varies with SDI (appendix 2). Metabolic risks accounted for increasingly large proportions of DALYs with increasing levels of SDI, up to high-middle SDI: metabolic risks accounted for 8·31% (95% UI 7·69–9·00) of DALYs in low SDI, 13·6% (12·6–14·7) in low-middle SDI, 21·3% (19·6–23·0) in middle SDI, 24·8% (22·7–27·2) in high-middle SDI, and 20·7% (18·4–23·1) in high SDI countries. Conversely, both behavioural and environmental and occupational risks accounted for smaller proportions of DALYs in higher SDI regions. Behavioural risks accounted for 38·8% (37·2–40·4) of DALYs in low SDI, 38·5% (36·8–40·2) in low-middle SDI, 35·2% (33·2–37·2) in middle SDI, 37·4% (34·9–39·7) in high-middle SDI, and 30·2% (28·1–32·4) in high SDI countries. Environmental and occupational risks accounted for 16·9% (15·6–18·3) of DALYs in low SDI, 14·1% (13·0–15·3) in low-middle SDI, 11·1% (10·2–12·0) in middle SDI, 9·61% (8·85–10·4) in high-middle SDI, and 6·56% (5·92–7·23) in high SDI countries (appendix 2).

### Changes in leading risk factors

**Figure 2:**
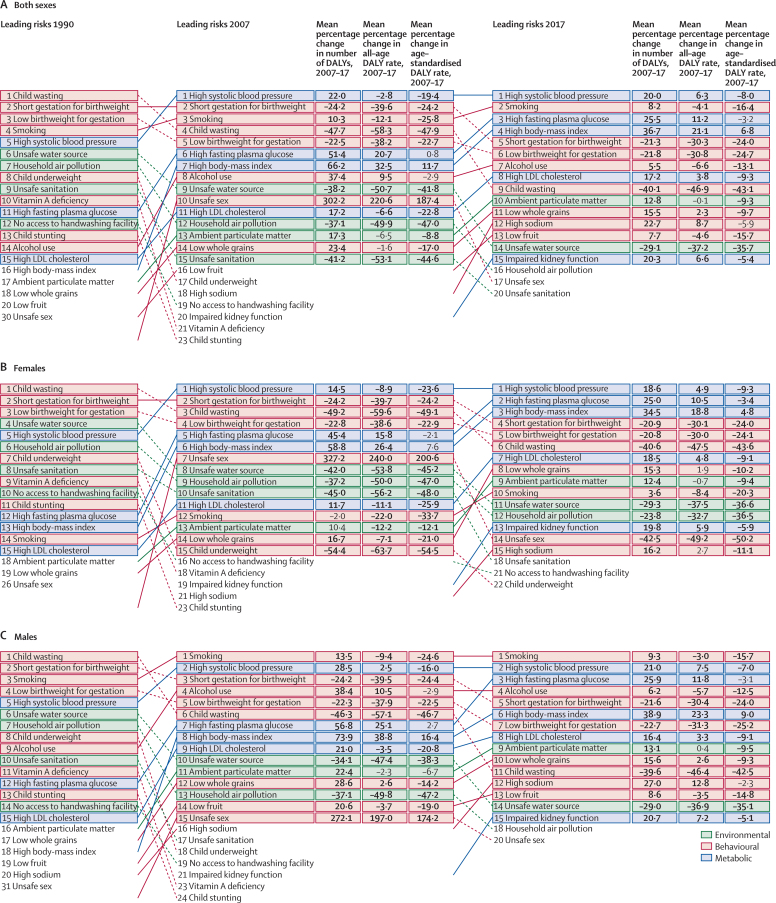
Leading 15 Level 4 risk factors by attributable DALYs at the global level, 1990, 2007, and 2017, for both sexes (A), females (B), and males (C) Risks are connected by lines between time periods; solid lines are increases and dashed lines are decreases. Statistically significant increases or decreases are shown in bold (p<0·05). DALYs=disability-adjusted life-years. LDL=low-density lipoprotein.

Between 1990 and 2017, high SBP was consistently responsible for the largest number of all-cause deaths, followed by smoking then high FPG. The fourth leading risk for mortality in 1990 was child wasting, which was in 21st position in 2017. The fourth leading risk for mortality in 2017 was high BMI, which increased in rank from 1990 when it was ninth. High LDL cholesterol remained the fifth leading cause of risk-attributable deaths in 1990 and 2017. These rankings and trends differ for DALYs. In 1990, the five leading risks for DALYs were child wasting, short gestation, low birthweight, smoking, and high SBP, whereas in 2017, the leading five risks were high SBP, smoking, high FPG, high BMI, and short gestation (figure 2A).

Leading risks differ for males and females. For males, the leading risks in 2017 were (in order of descending rank) smoking, high SBP, high FPG, alcohol use, and short gestation; the leading risks for females were high SBP, high FPG, high BMI, short gestation, and low birthweight. Three of the five leading risks for males were behavioural risks, whereas three of the five leading risks for females were metabolic risks (figure 2B, C).

DALY-based ranks for all metabolic risks increased between 1990 and 2017 for both males and females, whereas changes in ranks were more heterogeneous for environmental and occupational and behavioural risks. In 1990, six of the ten leading risks were behavioural risks, three were environmental, and one was metabolic. Of the ten leading risks in 2017 for both sexes, five were behavioural, four were metabolic, and only one was environmental. Broadly, in terms of their relative importance, metabolic risks rose in rank whereas environmental and occupational risks fell. Within behavioural risks, 13 of the 15 dietary risks increased rank. Appendix 2 shows trends in leading risk factors within each SDI quintile and trends in leading risk factors for deaths and DALYs by location.

### Drivers of changes in risk-attributable burden

**Figure 3:**
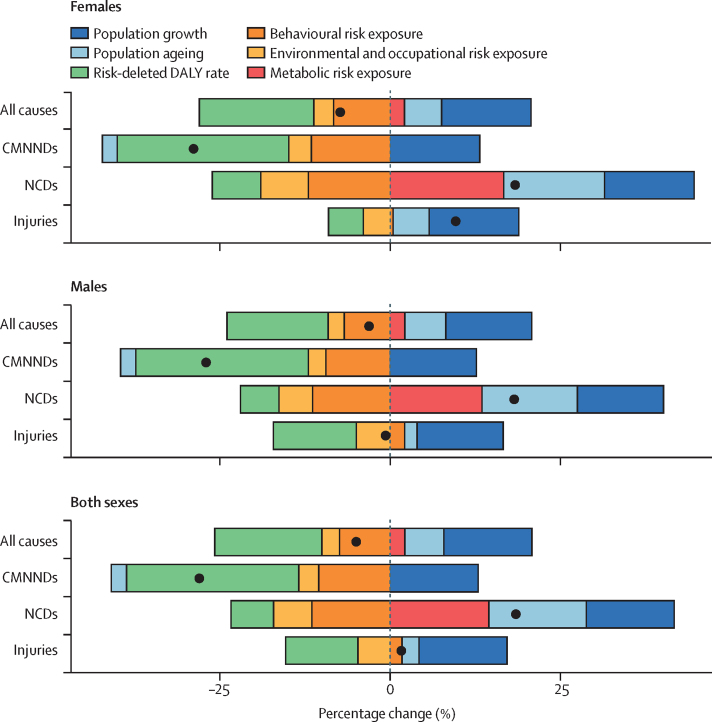
Percentage change in risk-attributable DALYs at the global level in 2007–17, due to population growth, population ageing, changes in exposure to Level 1 risk factors, and changes in risk-deleted DALY rates, for females, males, and both sexes Results are shown for all causes combined, CMNNDs, NCDs, and injuries. The black dot on each bar shows total percentage change. The risk-deleted DALY rate is the expected DALY rate if the exposure level for all risk factors were reduced to the theoretical minimum risk exposure level. Changes in the risk-deleted rate might result from changes in risks and risk–outcome pairs that are not currently included in the Global Burden of Diseases, Injuries, and Risk Factors Study or changes in other factors such as treatment. The change in CMNNDs and injuries due to metabolic risk exposure for both males and females is not zero but is too small to visualise because of the small number of risk–outcome pairs. CMNNDs=communicable, maternal, neonatal, and nutritional diseases. DALYs=disability-adjusted life-years. NCDs=non-communicable diseases.

Changes in the absolute number of DALYs over time are the result of changes in six underlying components: (1) population growth; (2) population ageing; (3) changes in exposure to environmental and occupational risks; (4) changes in exposure to behavioural risks; (5) changes in exposure to metabolic risks; and (6) changes due to all other (ie, risk-deleted or residual) factors. Figure 3 shows the changes in these components for each Level 1 cause, and for all causes combined (for Level 2 causes, see appendix 2). Broadly, in the absence of demographic changes, changes in risk exposure and risk-deleted DALYs would have led to a 23·5% decline in DALYs between 2007 and 2017 in both sexes. Conversely, in the absence of changes in risk exposure and risk-deleted DALYs, demographic changes would have led to an 18·6% increase in DALYs during that period. Comparing drivers of change for males and females, females have generally had greater declines in risk exposure: the aggregate effect of changes in exposure across all risks would have led to a 6·93% decline in DALYs from all causes for males, versus a 9·08% decline for females (figure 3).

Of all individual risk categories, changes in exposure to behavioural risks have driven the largest change in burden: declines in exposure to these risks would have resulted in a 7·41% decline in DALYs from all causes in both sexes combined. For both males and females, changing exposures to behavioural risks drove declines for NCDs and CMNNDs and increases for injuries (figure 3). Changes in exposure to environmental and occupational risks would have resulted a 2·59% decline in DALYs from all causes for both sexes, and it is the only risk category that was a driver of decline for all cause groups and both sexes. Conversely, in the absence of changes from other drivers, changes in metabolic risks would have resulted in a 2·15% increase in all-cause DALYs for both sexes.

Among the three cause groups, CMNNDs are the only Level 1 cause group for which combined effects of improvements in risk exposure and risk-deleted burden have outweighed the effects of demographic changes for both males and females. Declines in risk exposure would have resulted in a 12·0% decline in DALYs from CMNNDs among males and a 14·8% decline in females. For NCDs, increasing exposure to metabolic risks has largely offset health gains from improvements in environmental and behavioural risks. In aggregate, changes in risk exposure would have resulted in a 2·83% decline in DALYs from NCDs in males and a 2·29% decline in females. Among both males and females, changes in exposure to environmental risks, behavioural risks, and all risks combined would have resulted in reduced injury burden.

**Figure 4:**
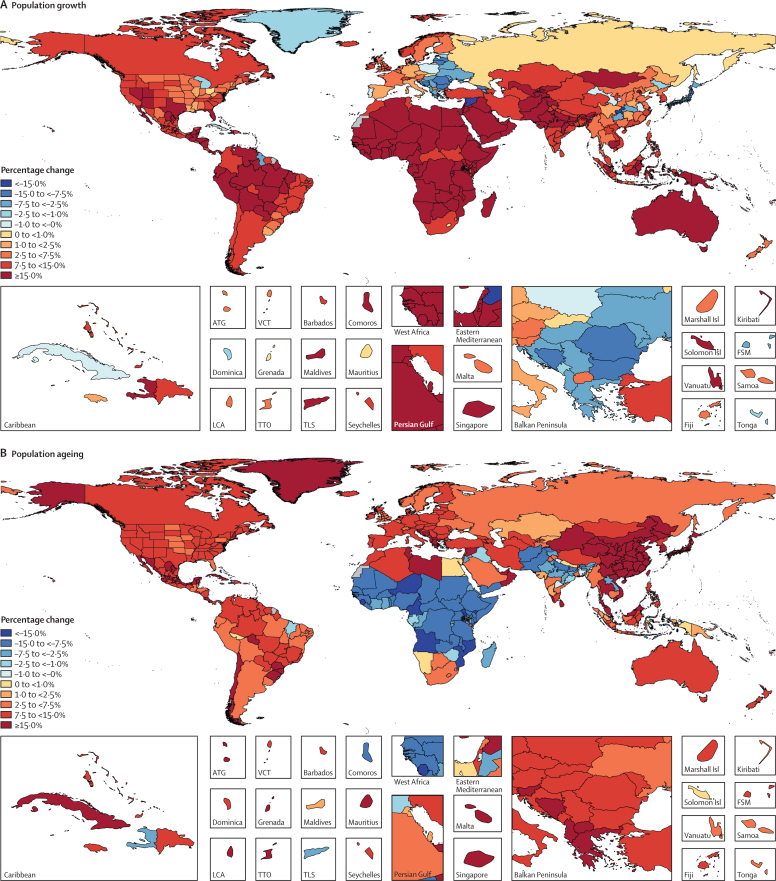

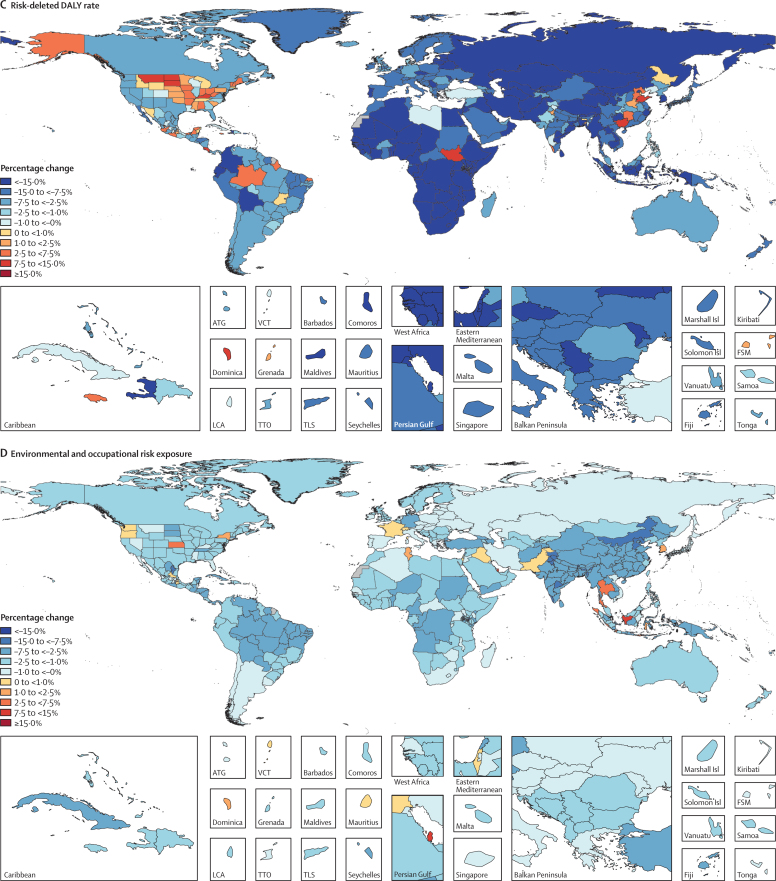

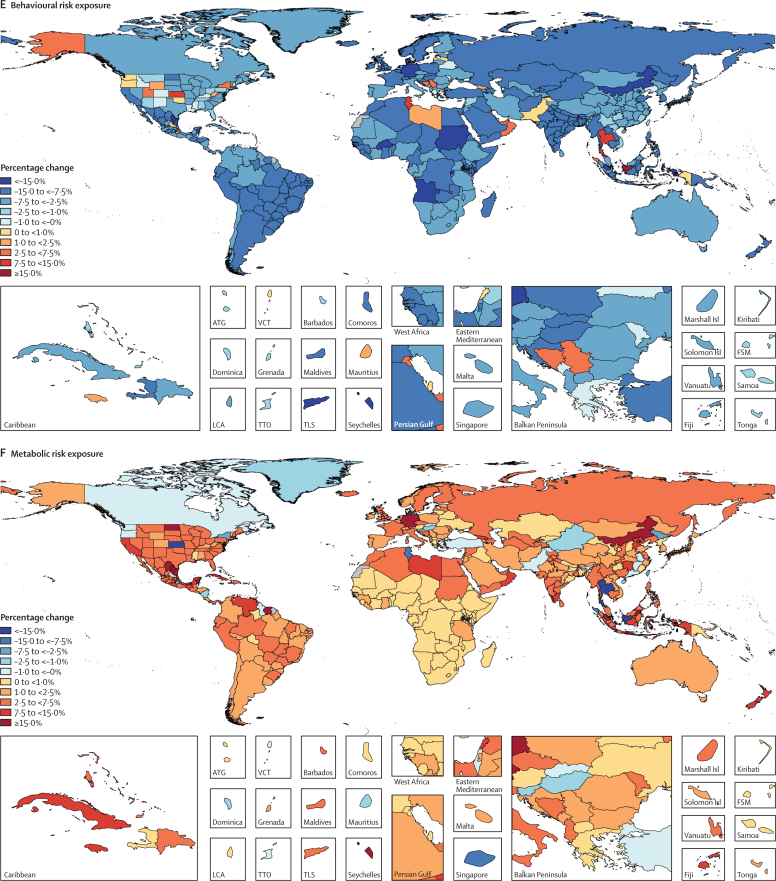
Percentage change in the absolute number of all-cause risk-attributable DALYs for both sexes, by location, 2007–17 Changes due to population growth (A), population ageing (B), changes in risk-deleted DALY rates (C), changes in exposure to environmental and occupational risk factors (D), changes in exposure to behavioural risk factors (E), and changes in exposure to metabolic risk factors (F). The risk-deleted DALY rate is the expected DALY rate if the exposure level for all risk factors were reduced to the theoretical minimum risk exposure level. Changes in the risk-deleted rate might result from changes in risks and risk–outcome pairs not included in the Global Burden of Diseases, Injuries, and Risk Factors Study or changes in other factors such as treatment. ATG=Antigua and Barbuda. DALYs=disability-adjusted life-years. FSM=Federated States of Micronesia. Isl=Islands. LCA=Saint Lucia. TLS=Timor-Leste. TTO=Trinidad and Tobago. VCT=Saint Vincent and the Grenadines.

Drivers of changes in risk-attributable DALYs were spatially heterogeneous (figure 4). Broadly, population growth has driven increases in risk-attributable DALYs across most locations except for Eastern Europe, Cuba, Greenland, Guyana, Japan, and Portugal. Population ageing has driven increases in most locations, with notable exceptions in central, eastern, and western sub-Saharan Africa, and parts of the Middle East and south and southeast Asia. Conversely, all-cause risk-deleted DALY rates have declined across most locations; however, they have increased in a number of subnational locations in Brazil, China, the UK, India, Mexico, and the USA. Broadly, DALYs attributable to metabolic risk exposure have increased in most locations and DALYs attributable to environmental and occupational and behavioural risks have largely declined.

### Observed versus expected summary exposure values

Estimating the expected SEV for a given risk factor at a given level of SDI, a comparison can be made between observed exposure levels in a given place and time and their expected levels based on SDI. An observed to expected ratio (O/E ratio) of 1·0 indicates that observed exposure levels equal our expectation; O/E ratios less than 1·0 indicate that observed exposures are better than expected (ie, at levels associated with lower risk); and O/E ratios greater than 1·0 indicate that observed exposures are worse than expected (ie, at levels associated with greater risk).

**Figure 5:**
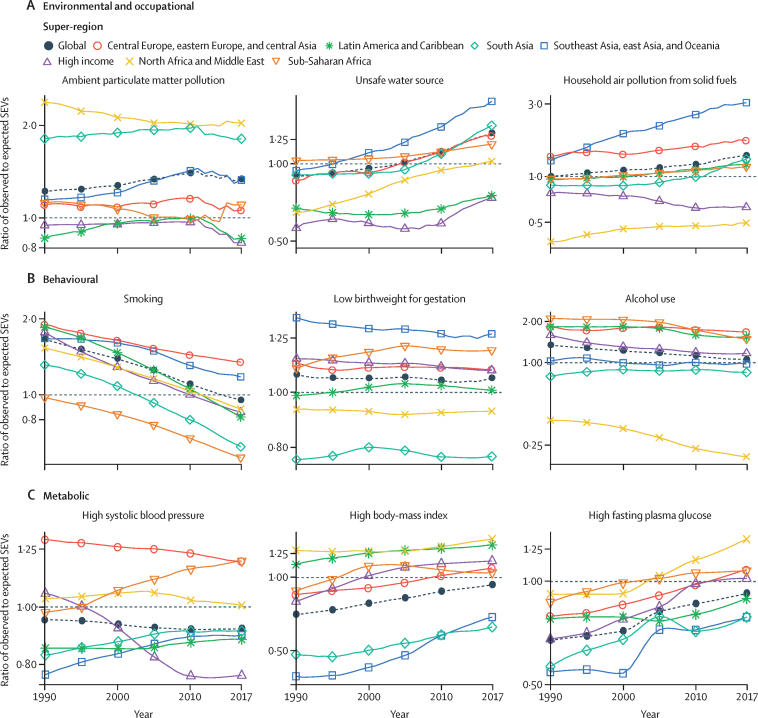
Trends in the ratios of observed SEVs to SEVs expected based on SDI, by super-region, for both sexes, 1990–2017 Trends are for three of the top environmental (A), behavioural (B), and metabolic (C) risk factors by number of attributable DALYs globally. Observed to expected ratios are based on age-standardised SEVs. *y*-axes are on a log scale with the range scaled appropriately for each risk factor. DALYs=disability-adjusted life-years. SDI=Socio-demographic Index. SEV=summary exposure value.

Across leading environmental risks, O/E ratios for ambient particulate matter pollution showed no consistent trends across regions, whereas there were nearly universal increases in O/E ratios for household air pollution and unsafe water (figure 5A). In 2017, O/E ratios for ambient particulate matter pollution were notably high in north Africa and Middle East (2·03) and south Asia (1·81). O/E ratios for household air pollution were notably high in southeast Asia, east Asia, and Oceania (3·05) and central Europe, eastern Europe, and central Asia (1·73). Between 1990 and 2017, O/ E ratios for unsafe water increased across all super-regions, with the largest changes in southeast Asia, east Asia, and Oceania (85·6% increase; ratio of 1·75 in 2017), north Africa and Middle East (57·8% increase; ratio of 1·02 in 2017), and south Asia (55·5% increase; ratio of 1·41 in 2017).

Among the leading behavioural risks between 1990 and 2017, there were near universal declines in O/E ratios for both smoking and alcohol use, and inconsistent trends in O/E ratios for low birthweight (figure 5B). The range of O/E ratios between regions is especially broad for alcohol use. These ratios were consistently high in sub-Saharan Africa (O/E ratios declined from 2·10 to 1·49 between 1990 and 2017), Latin America and the Caribbean (ratios declined from 1·83 to 1·54), and central Europe, eastern Europe, and central Asia (ratios declined from 1·82 to 1·68). O/E ratios for alcohol use were consistently the lowest in north Africa and the Middle East, declining from 0·38 to 0·21 between 1990 and 2017.

Trends in O/E ratios for leading metabolic risks were heterogeneous across super-regions (figure 5C). For high SBP, O/E ratios improved by more than 5% between 1990 and 2017 for two super-regions: 27·1% decrease in the high-income region and 8·05% in the central Europe, eastern Europe, and central Asia region. By contrast, ratios for high SBP increased by more than 5% in three super-regions: 22·0% in sub-Saharan Africa, 16·4% in southeast Asia, east Asia, and Oceania, and 9·67% in south Asia. In 2017, ratios for high SBP were highest in sub-Saharan Africa (1·19) and central Europe, eastern Europe, and central Asia (1·19) and were lowest in the high-income region (0·77). O/E ratios increased for both high FPG and high BMI across all super-regions between 1990 and 2017. The north Africa and Middle East region stands out as having the highest O/E ratios for both high FPG and high BMI across many years.

### Expected attributable burden

**Figure 6:**
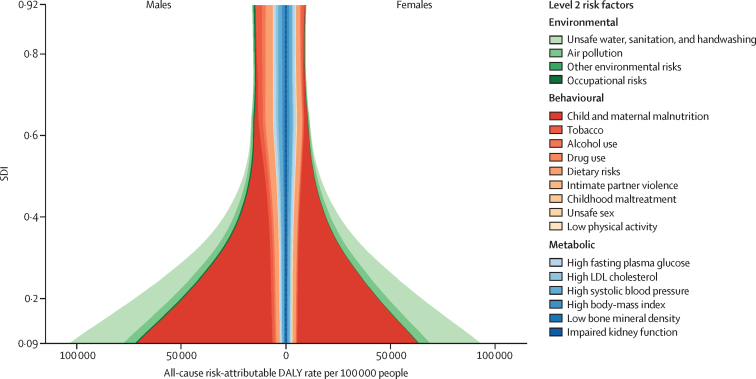
Expected relationship between all-age, all-cause risk-attributable DALY rates and SDI for each GBD Level 2 risk, 1990–2017 Stacked curves show males (left) and females (right) after adjusting for mediation, scaling to account for overlapping risks, and aggregating so that total expected DALY rates reflect the true all-cause total expected DALY rates attributable to all risk factors. The y-axis shows lowest SDI (0·09) to highest SDI (0·92) for all GBD countries and territories, 1990–2017. Coloured regions are the proportion of the total attributable DALY rate corresponding to that risk factor. DALYs=disability-adjusted life-years. GBD=Global Burden of Diseases, Injuries, and Risk Factors Study. LDL=low-density lipoprotein. SDI=Socio-demographic Index.

Broadly, total expected risk-attributable burden declines with increasing SDI (figure 6). At an SDI of 0·1, we expect 101 000 risk-attributable DALYs per 100 000 males and 90 600 per 100 000 females; at an SDI of 0·5, we expect 20 200 per 100 000 males and 16 300 per 100 000 females; and at an SDI of 0·9, we expect 15 900 per 100 000 among males and 9640 per 100 000 among females.

Increasing SDI was associated with dramatic declines in the expected burden attributable to environmental risks in both sexes: the expected burden attributable to environmental risks declines from 41 600 DALYs per 100 000 people (31·6% of total burden) with an SDI of 0·1 to 1870 DALYs per 100 000 people (10·6% of total burden) with an SDI of 0·9. The pattern for behavioural risks was more complex and heterogeneous. Although the expected burden attributable to malnutrition declined with increasing SDI, the expected burden attributable to tobacco use, drug use, alcohol use, and most dietary risks generally increased with increasing SDI. At an SDI value of 0·1, behavioural risks accounted for 65·0% of the total expected risk-attributable burden, at an SDI of 0·75 they accounted for 51·5%, and at an SDI of 0·9 they accounted for 52·9%. The expected burden attributable to metabolic risks increased with increasing SDI up to an SDI of 0·65, then declines with increasing SDI above that level. At an SDI value of 0·1, metabolic risks accounted for 3·44% of the total expected risk-attributable burden, at an SDI of 0·75 they accounted for 37·6%, and at an SDI of 0·9 they accounted for 36·4%.

### Observed versus expected attributable burden

**Figure 7:**
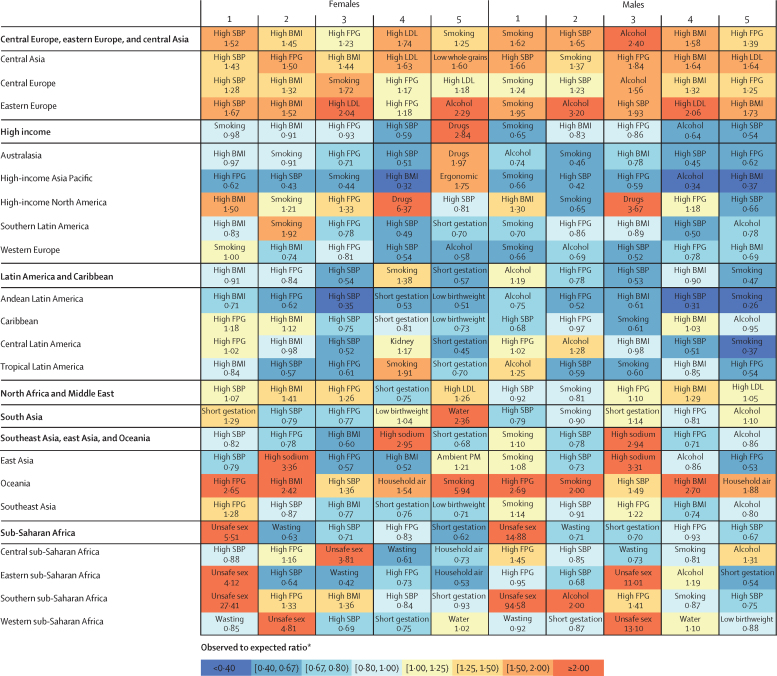
Leading five risk factors for DALYs with the ratio of observed to expected DALYs based on Socio-demographic Index, by super-region and region, and by sex, 2017 Number below each risk factor is its observed to expected ratio. Ratios are based on age-standardised DALY rates. BMI=body-mass index. DALYs=disability-adjusted life-years. Ergonomic=occupational ergonomic factors. FPG=fasting plasma glucose. LDL=low-density lipoprotein. Household air=household air pollution from solid fuels. Kidney=impaired kidney function. Low birthweight=low birthweight for gestation. PM=particulate matter pollution. SBP=systolic blood pressure. Short gestation=short gestation for birthweight. Wasting=child wasting. Water=unsafe water source. *Round brackets indicate excluded endpoints whereas square brackets indicate included endpoints.

In males, the two leading risk factors were smoking and high SBP (in varying order) in four of the seven super-regions (figure 7; appendix 2). The three exceptions were sub-Saharan Africa (unsafe sex was the leading risk with an O/E ratio of 14·9 followed by child wasting with a ratio of 0·71), Latin American and the Caribbean (alcohol use with a ratio of 1·19 then high FPG with a ratio of 0·78), and the high-income super-region (smoking with a ratio of 0·65 then high BMI with a ratio of 0·83). In females, the leading risk factors were either high BMI or high SBP in four of the seven super-regions (figure 7; appendix 2). The three exceptions were sub-Saharan Africa (as for males, unsafe sex was the leading risk among females, with an O/E ratio of 5·51, followed by child wasting with a ratio of 0·63), south Asia (short gestation with a ratio of 0·79, followed by high SBP with a ratio of 0·79), and the high-income super-region (smoking with a ratio of 0·98 followed by high BMI with a ratio of 0·91). High FPG was the second leading risk factor in southeast Asia, east Asia, and Oceania (O/E ratio of 0·78) and Latin America and the Caribbean (O/E ratio of 0·84) for females. Notably, the O/E ratios exceed one for all five leading risk factors for males for the central Europe, eastern Europe, and central Asia regions, and for nearly all countries within those regions.

**Figure 8:**
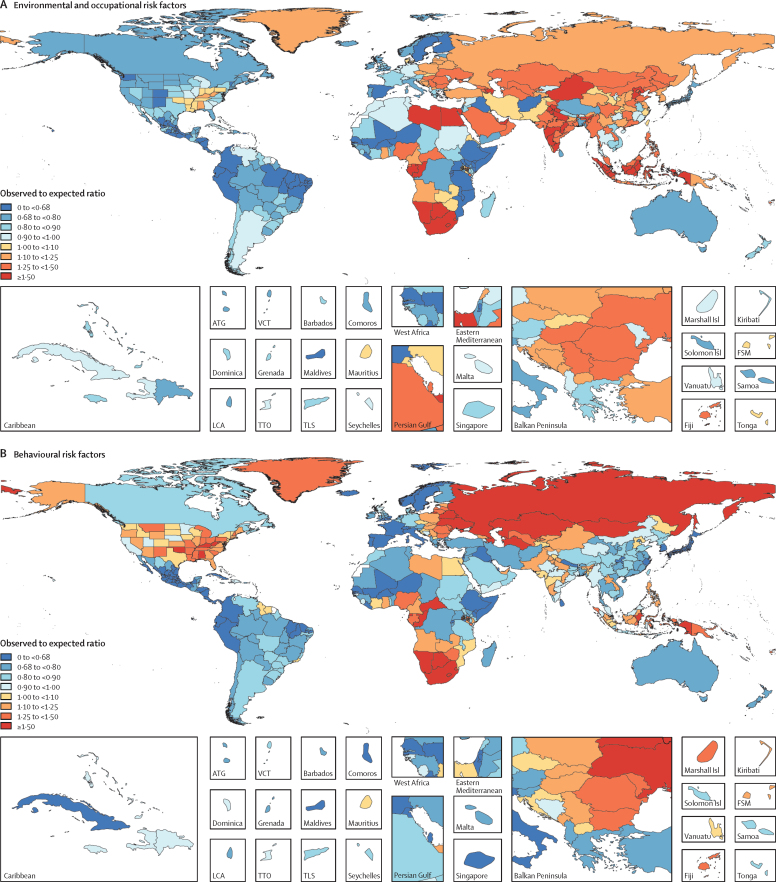

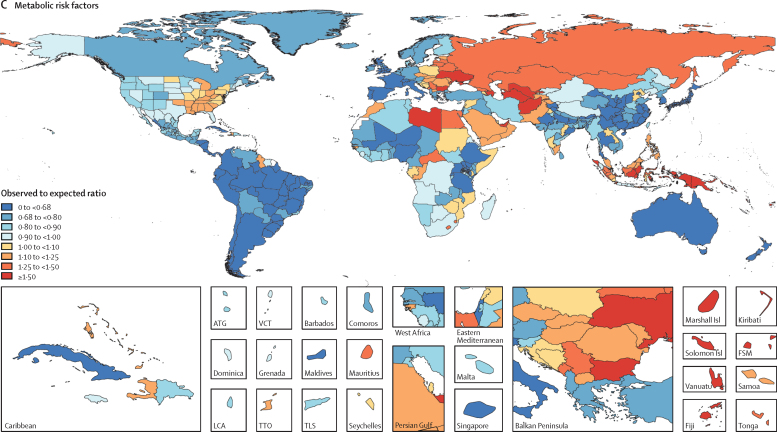
Ratios of observed to expected attributable DALY rates based on Socio-demographic Index for each Level 1 risk for both sexes by location, 2017 Ratios for environmental and occupational risk factors (A), behavioural risk factors (B), and metabolic risk factors (C). Observed to expected ratios are based on age-standardised DALY rates.ATG=Antigua and Barbuda. DALYs=disability-adjusted life-years. FSM=Federated States of Micronesia. Isl=Islands. LCA=Saint Lucia. TLS=Timor-Leste. TTO=Trinidad and Tobago. VCT=Saint Vincent and the Grenadines.

The highest O/E ratios for environmental and occupational risks were in southern sub-Saharan Africa and south Asia, driven largely by air pollution and unsafe water, sanitation, and handwashing in both regions (figure 8). For behavioural risks, high O/E ratios were recorded in southern sub-Saharan Africa, driven largely by higher than expected unsafe sex and intimate partner violence, and eastern Europe, largely due to higher than expected alcohol and drug use. The burden attributable to metabolic risks was higher than expected in Oceania and much of the central Europe, eastern Europe, and central Asia super-region.

### New risks, leading risks, and risks with significant changes in GBD 2017

Globally, bullying victimisation is the leading risk factor for mental disorder DALYs in 2017 (other risks factors are lead exposure, intimate partner violence, and childhood sexual abuse) and the 35th leading risk factor of all 54 risks for all NCDs. However, for major depressive disorder, bullying accounts for fewer DALYs than childhood sexual abuse or intimate partner violence. In 2017, 2·57 million (95% UI 1·54–3·97) DALYs were attributable to bullying victimisation, by peers, of children and adolescents attending school. This was distributed relatively evenly between anxiety disorders (1·36 million DALYs [0·81–2·13]) and major depressive disorder (1·21 million DALYs [0·69–1·94]). Globally, bullying victimisation accounted for 5·01% (3·40–6·83) of all anxiety disorder DALYs and 3·68% (2·37–5·21) of all major depressive disorder DALYs.

In 2017, high SBP was the leading risk factor globally, accounting for 10·4 million (95% UI 9·39–11·5) deaths and 218 million (198–237) DALYs. Overall, 8·74% (7·78–9·68) of total DALYs were attributable to high SBP. Most of the burden attributable to high SBP was due to ischaemic heart disease and stroke, and high SBP accounted for 55·7% (48·1–63·1) of DALYs due to ischaemic heart disease and 57·1% (49·4–63·8) of DALYs due to stroke. Globally, age-standardised DALY rates attributable to high SBP declined between 1990 and 2017 (25·8% decrease [24·3–27·5]). Notable declines occurred in the high-income (54·5% [53·1–56·0] decline), north Africa and Middle East (32·6% [28·7–35·7]), and Latin America and Caribbean (32·4% [30·7–34·1]) super-regions.

In 2017, smoking was the second leading risk for both deaths and DALYs. It was responsible for 7·10 million (95% UI 6·83–7·37) deaths (12·7% [12·2–13·2] of all deaths globally) and accounted for 182 million (173–193) DALYs (7·31% [6·81–7·80] of all DALYs globally). Although smoking-attributable age-standardised mortality rates declined 38·2% (36·4–40·0) between 1990 and 2017, the total number of smoking-attributable deaths has increased by 24·9% (21·3–28·6). In 2017, smoking accounted for a larger proportion of deaths among men in high-middle SDI countries (27·1% [26·3–27·9]) than in low SDI countries (10·2% [9·23–11·0]; appendix 2). Between 1990 and 2017, among men, the percentage of all-cause all-age deaths attributable to smoking in high SDI countries decreased, whereas it increased in all other SDI groups. Levels and trends in smoking prevalence and attributable burden vary by gender. As a risk factor for all-cause all-age deaths, smoking ranks first among men and eighth among women, reflecting both the lower prevalence and lower intensity of smoking among women. In 2017, the four Level 3 causes with the largest number of deaths attributable to smoking were ischaemic heart disease (1·62 million [1·54–1·69] deaths), COPD (1·23 million [1·12–1·35] deaths), tracheal, bronchus, and lung cancer (1·19 million [1·15–1·23] deaths), and stroke (887 000 [833 000–944 000] deaths), and together account for 69·4% of total smoking-attributable deaths (table 3). For lung cancer and COPD, changing from indirect estimation using the smoking impact ratio to direct estimation using measures of cumulative lifetime exposure resulted in large changes in smoking-attributable burden estimates in countries with scarce or low-quality data on lung cancer mortality. At the all-cause level, using measures of continuous and cumulative exposure that take into account risk among former and occasional smokers has resulted in increases in attributable burden in 71·8% of countries compared with previously published estimates.

In 2017, low birthweight and short gestation was the third leading Level 3 risk factor globally for all-age DALYs. Age-standardised DALY rates attributable to this risk declined from 4558 (95% UI 4347–4739) DALYs per 100 000 in 1990 to 2680 (2543–2812) DALYs per 100 000 in 2017. Although exposure to low birthweight and short gestation decreased globally between 1990 and 2017, most of the declines in risk-attributable burden came from declines in mortality from causes attributable to this risk. For low birthweight, the biggest improvements in risk-attributable mortality were in Saudi Arabia, Maldives, and Singapore (appendix 2). Increasing SDI was associated with decreasing exposure and decreasing attributable burden. Exposure and attributable burden were highest in eastern, central, and western sub-Saharan Africa. For short gestation, the biggest improvements were in Saudi Arabia, Maldives, and Turkey. Similar to low birthweight trends, increasing SDI was associated with a decrease in exposure, as well as a decrease in attributable burden. Exposure and attributable burden were highest in south Asia and eastern, central, and western sub-Saharan Africa.

High BMI accounted for 4·72 million (95% UI 2·99–6·70) deaths and 148 million (98·6–202) DALYs globally in 2017 (table 3). It ranked fourth among risk factors for mortality, primarily due to the effect of high BMI on cardiovascular disease. Globally, the SEV for high BMI for both sexes has increased 70·4% (57·1–84·5) since 1990 (table 2), and trends among children suggest that this will continue to rise. The resulting disease burden is large and increasing, with the absolute number of DALYs attributable to high BMI increasing by 36·7% (31·5–44·5) between 2007 and 2017 and by 127% (99·5–172) between 1990 and 2017, and age-standardised DALY rates attributable to high BMI increasing 6·84% (2·60–13·0) between 2007 and 2017 and 19·3% (4·83–43·2) between 1990 and 2017. In 2017, high BMI was the leading cause of premature mortality, as measured by YLLs, in seven countries and territories: Ecuador, Peru, Kuwait, Qatar, Saudi Arabia, United Arab Emirates, and American Samoa. Among risk factors assessed in GBD 2017, BMI prevalence continues to have one of the highest rates of increase over time. Trends in BMI prevalence and burden exhibit marked geographical variation, with the highest levels among wealthier countries.

Globally, high FPG was the third leading risk factor for both mortality and DALYs in 2017, accounting for 6·53 million (95% UI 5·23–8·23) deaths and 171 million (144–201) DALYs. Cardiovascular disease was the leading Level 2 cause of death due to high FPG, accounting for 54·5% of deaths attributed to the risk. Diabetes and kidney diseases was the second ranking Level 2 cause of disability attributable to high FPG and accounted for 51·9% of DALYs attributable to this risk.

In 2017, 5·25% (95% UI 4·49–6·01) of all deaths were attributable to ambient particulate matter pollution, making it the eighth leading risk for deaths, with a total of 2·94 million (2·50–3·36) deaths globally, and 1·05 million (0·88–1·22) deaths in southeast Asia, east Asia, and Oceania. It has increased from 15th leading risk for deaths in 1990, with 1·75 million (1·48–2·03) deaths, with a percentage change of 67·7% (56·6–79·3). It is among the top ten ranked Level 4 risk factors for deaths in 95 of the 195 countries and territories for which we produce estimates. Mortality rates attributable to ambient particulate matter pollution are notably high in Bahrain (11·9% [10·2–13·3] of all deaths were attributable to ambient particulate matter; fourth leading risk factor for deaths), Egypt (11·8% [9·07–14·4]; sixth leading risk factor), and Kuwait (10·6% [8·94–12·3]; sixth leading risk factor). The Level 3 causes with the largest proportion of burden attributable to ambient particulate matter pollution are COPD and lower respiratory infection: ambient particulate matter is responsible for 19·3% (12·6–25·4) of all COPD DALYs, 17·4% (13·7–21·6) of all lower respiratory infection DALYs, and 17·5% (13·2–22·7) of lower respiratory infection DALYs in children younger than 5 years. In GBD 2017, we added type 2 diabetes as an outcome of ambient particulate matter pollution, which contributes 10·5 million (6·70–13·9) or 12·6% of the 83·0 million (71·4–94·3) DALYs attributable to ambient particulate matter pollution in 2017 (table 3).

In 2017, 4·32 million (95% UI 3·33–5·44) deaths and 94·9 million (78·8–112) DALYs were attributable to high LDL cholesterol. Overall, 3·80% (3·14–4·56) of total DALYs were attributable to high LDL cholesterol. The age-standardised DALY rate in males (1480 per 100 000 [1230–1750]) was higher than in females (880 per 100 000 [714–1070]). Globally, between 1990 and 2017, age-standardised DALY rates declined markedly (29·9% [28·1–31·7] decline). Notable reductions in age-standardised DALY rates occurred in both the high-income (61·8% [60·4–63·2] decline) and Latin America and Caribbean super-regions (40·7% [38·9–42·5] decline).

Globally in 2017, dietary risks were the leading Level 2 risk factor for deaths and the second leading Level 2 risk factor for DALYs, accounting for 10·9 million (95% UI 10·1–11·7) deaths and 255 million (234–274) DALYs (table 3). Among dietary factors, high intake of sodium was the leading risk for mortality, accounting for 3·20 million (1·42–5·45) deaths. Low intake of whole grains was the leading risk factor for DALYs, accounting for 82·5 million (59·0–109) DALYs. Diet with high sugar-sweetened beverage consumption continues to show a marked increasing trend in overall attributable burden, with SEVs for this risk increasing 12·1% (7·02–18·2) between 2007 and 2017 and 17·1% (8·34–28·0) since 1990 (table 2).

## Discussion

### General findings

We estimated the burden of disease attributable to 84 metabolic, environmental and occupational, and behavioural risk factors or clusters of risks from 1990 to 2017 in 195 countries and territories, based on 46 749 data sources. In 2017, all included risks combined contributed to 61·0% (95% UI 59·6–62·4) of deaths and 48·3% (46·3–50·2) of DALYs worldwide, compared with 61·4% (59·9–62·7) of deaths and 52·4% (50·4–54·5) of DALYs in 1990. With each iteration of GBD, including GBD 2017, an increasing proportion of burden of disease has been attributed to risk factors. The contributions of risk factors to total deaths in the year 2010 (the most recent year for which we have comparable estimates from the most GBD iterations) were estimated to be 55·7% in GBD 2013, 57·3% in GBD 2015, 60·2% in GBD 2016, and 61·3% in GBD 2017. Similarly, in 2010, the contribution of risk factors to DALYs was 40·4% in GBD 2013, 41·5% in 2015, 46·5% in 2016, and 49·5% in 2017. This increase stems from the growing collection of risk–outcome pairs included in each iteration. With the addition of new risk factors and new risk–outcome pairs, future iterations of the GBD study should explain an increasing portion of the remaining unattributed burden of disease.

The role of changes in risk factors in explaining changes in deaths and DALYs varies considerably across causes and ages. Since 1990, in the 64 individual or Level 4 risks, exposure levels increased significantly for 20 risks, did not change significantly for 14 risks, and decreased significantly for 31 risks. The risks with the highest increases in SEVs globally include high BMI, ambient particulate matter pollution, and high FPG; the risks with the largest decreases in exposure are unsafe sanitation, diet high in trans fatty acids, and household air pollution. These trends are consistent with what are expected with socioeconomic development.

We found considerable heterogeneity across super-regions in the leading risk factors. Some notable patterns are the role of unsafe sexual practices as a driver of the HIV/AIDS epidemic in eastern and southern sub-Saharan Africa and the role of alcohol consumption in eastern Europe and central Asia. There are also marked spatial patterns for other risks such as high BMI in central America, north Africa and the Middle East, and Oceania. Interpretation of spatial patterns needs to take into account the fact that some risks have a strong relationship with socioeconomic development. The numbers of some environmental and behavioural risks, including unsafe water, sanitation, and handwashing, household air pollution, and childhood growth failure, decrease rapidly with increased development. Other risks tend to increase with development, including high BMI, high SBP, red meat consumption, high sugar-sweetened beverage consumption, and drug and alcohol use.

### Risk exposure and socioeconomic development

Risks form clusters with regard to the nature of their association with development. In this regard, we observe four categories of risks as follows: (1) risks positively associated with SDI; (2) risks negatively associated with SDI; (3) risks with clear, but non-monotonic associations with SDI; and (4) risks that show little association with SDI.

The category of risks that are positively associated with SDI includes smoking, high BMI, alcohol use, and high LDL cholesterol. This category includes predominately behavioural and metabolic risks that are associated with NCDs. The category of risks that are negatively associated with SDI includes child wasting, unsafe water source, diet low in fruits, household air pollution, unsafe sanitation, and no access to handwashing facility. This category includes predominately environmental and behavioural risks that are associated with CMNNDs. The category of risks that have non-monotonic associations with SDI includes high FPG and ambient particulate matter pollution, which both follow an inverted U-shaped association with development, and low birthweight, which has a U-shaped association with development. The category of risks showing little association with SDI includes high SBP and diet low in whole grains.

Notably, for some risks, the trends of O/E ratios and SEVs are in opposite directions. For example, O/E ratios for household air pollution and unsafe water increased between 1990 and 2017, indicating that, for a given level of SDI, exposure levels have increased during that period. Conversely, SEVs for those risks declined during the same period. Thus, although exposure to these risks is broadly improving with socioeconomic development and time, improvements in development are occurring more rapidly than are improvements in the underlying risk structure in a population. For other risks, SEVs show temporal trends that run counter to trends expected on the basis of SDI. For example, although SDI was strongly positively associated with smoking, both SEVs and O/E ratios for smoking have declined despite concurrent increases in SDI. The marked declines in O/E ratios for smoking across all super-regions again suggest that the expected increase in smoking has not been fully realised with increasing SDI. In the case of smoking, the declining O/E ratios probably reflect benefits of smoking-reduction policies.^16^ Similarly, alcohol use was strongly positively associated with SDI, and trends in alcohol use SEVs did not change greatly, resulting in reductions in O/E ratios for alcohol use in all super-regions except south Asia.

### Risk exposure and sex

In addition to spatiotemporal variation in risk exposure, there were important differences between males and females within a given location. Females had notably higher SEVs for household air pollution, second-hand smoke, low bone mineral density, and diet low in fibre, legumes, calcium, and vegetables; whereas males had notably higher SEVs for lead exposure, smoking, alcohol use, ambient particulate matter pollution, red meat consumption, and most occupational risks.

For smoking, SEVs were higher for males than for females at all SDI levels, and, for both sexes, SEVs increased with increasing SDI. However, although smoking SEVs for males increase nearly linearly with SDI, smoking SEVs for females remain very low at lower SDI, and then increase rapidly between SDI values of 0·6 and 0·9. This suggests that smoking interventions that target males might be most effective in lower SDI settings, whereas interventions in higher SDI settings should target males and females more equally. Similarly, alcohol use remains low among females across lower and middle SDI levels but increases with SDI at higher SDI levels, suggesting that interventions should target males in low to middle SDI settings, but be targeted more broadly in higher SDI settings.

SEVs for high BMI increased for both sexes between 1990 and 2007, 2007 and 2017, and 1990 and 2017. Although this increase continues for males across the entire range of SDI, the increase for females slows and the curve flattens at an SDI of 0·66. Consequently, although females have higher BMI SEVs than males at low and middle SDI, they have lower SEVs than males at high SDI. Similarly, there was a crossover in sex differences for high SBP. Although high SBP SEVs for females decline slightly with SDI, they increase slightly with SDI for males. Consequently, expected SBP SEVs are lower for males than for females where SDI is 0·36 or below, and they are higher for males than for females where SDI is greater than 0·36.

### Cross-cutting themes

The number of all-cause DALYs declined for both males and females between 2007 and 2017, because the effects of improvements in both risk exposures and risk-deleted DALY rates have outpaced the opposing effects of population growth and ageing. Decreases in risk-deleted DALY rates are probably a function of improvements in treatments and improvements in risk factors that are not captured in the GBD analysis, as well as uncaptured social, cultural, and economic factors. Although changes in environmental and occupational and behavioural risks have driven declines in all-cause DALYs, increasing exposure to metabolic risks has had the opposite effect. This pattern is remarkably consistent across geographies. Consequently, increasing exposure to metabolic risks stands as one of the key drivers of increasing DALYs from NCDs. Although concerns about NCD burden have historically focused on high-income countries, previous iterations of the GBD study, as well as other studies, have clearly shown the large and growing problem of NCDs in low-income and middle-income settings.^4^, ^17^ Similarly, although metabolic risk factors such as high LDL cholesterol, high SBP, and high BMI have historically been viewed mostly as challenges in high-income settings, these key metabolic risk factors are now commonly increased even in low-income settings. Between 1990 and 2017, 47 countries and territories had greater than 100% increases in SEVs for high BMI and, of these, 37 (79%) had an SDI that was less than the median value in 1990. Although these increases have occurred in connection with increasing SDI, increasing O/E ratios show that trends in these metabolic risks are outpacing the expectations based on development.

High LDL cholesterol and high SBP are among the leading risk factors for all-cause risk-attributable burden in 2017. Both are commonly increased even in low-resource settings, and both are increased by obesity. Although both are also easily reduced or treated with inexpensive and cost-effective medications, these treatments remain inaccessible to broad segments of the world's population.^18^ Growing evidence from studies such as the SimCard Trial^19^ in rural India and China suggests that effective methods exist to deliver these kinds of important pharmacotherapy, even in the most resource-limited locations. A 2018 trial of a fixed low-dose triple-combination antihypertensive drug tested in urban Sri Lanka, consisting of amlodipine, telmisartan, and chlorthalidone, showed substantial improvement in control of blood pressure compared with usual care at 6 months, and no difference in adverse events.^20^ Another trial from 2016 showed that lowering LDL cholesterol with a high-potency statin was effective even among individuals only at intermediate risk of cardiovascular events.^21^ Efforts to improve universal health coverage must directly address how these therapies can be delivered even more effectively. Studies have further identified public health interventions that are cost-effective outside of high-income settings. Legislative interventions targeting sodium and trans fat, improvements in food labelling and advertising, and health-focused and diet-focused media campaigns are cost-effective interventions in south Asia, for example.^22^ Fortunately, philanthropic donors are beginning to identify cardiovascular risk as an important target for improving global health.^23^

O/E ratios offer a useful benchmarking tool for national policy makers who are hoping to compare the risk exposure against an empirical standard for countries at a given level of development. Although the burden attributable to a given risk indicates the risks for which exposure reduction can offer the greatest health gains, O/E ratios offer insight into what might be achievable given the resource constraints that exist at a certain level of development. For example, in the southeast Asia, east Asia, and Oceania super-region, high SBP and high FPG are among the leading risks, but both have SEV O/E ratios below 1·0. This suggests that, despite the high risk-attributable burden, the countries in this super-region show better than expected performance in managing exposure to these risks. Conversely, ambient particulate matter pollution and household air pollution are the tenth and 15th leading risks in the super-region, but the corresponding SEV O/E ratios are 1·32 and 3·05, suggesting that the countries in the super-region have greater exposure to these risks than other countries at similar levels of development. The degree of underperformance suggests that gains in managing these risks might be more easily achieved than would be gains from risks in which the super-region is already outperforming its peers.

### Important changes in GBD 2017 compared with GBD 2016

GBD 2017 marks the first time that bullying victimisation by peers of children and adolescents attending school has been included as a risk factor. It is one of only three psychosocial risk factors to be included in GBD, one of only two risk factors for major depressive disorder, and the first-ever risk factor for anxiety disorders in GBD. The substantial proportion of major depressive disorder and anxiety disorder YLDs attributable to bullying victimisation highlights the importance of considering psychosocial risk factors when assessing health outcomes. The small number of risk factors for mental disorders has been an ongoing criticism of GBD, especially considering that mental disorders are a leading cause of disability worldwide.^24^ GBD estimates suggest that the prevalence of mental disorders has changed little in the past few decades despite growing recognition of the associated burden. Psychosocial risk factors can be challenging to assess and quantify, which in turn can make them easy to overlook. However, bullying victimisation has an operationalised definition, can be measured in population surveys, and is consistently associated with risk of mental disorders.^25^, ^26^ Scientifically assessed school-based interventions that address bullying show positive programme effects.^27^ A reduction in the prevalence of bullying victimisation could help reduce the prevalence, and therefore burden, of major depressive disorder and anxiety disorders later in life. Crucially, the inclusion of bullying victimisation as a risk factor in GBD might motivate investment in intervention programmes to reduce bullying. The inclusion of bullying victimisation in GBD also shows the importance of integrating cross-disciplinary research in the identification of the complex aetiology of mental disorders.

Using continuous and cumulative measures of smoking, which capture both former and occasional smokers, has shown that the burden of smoking is higher than previously thought. The implications of the new methods on attributable burden varies across outcomes and locations. For 71·8% of countries, the burden attributable to smoking has increased as a result of the new methods. For the largest causes of burden (ischaemic heart disease, COPD, lung cancer, and stroke) there are differential trends across countries and by sex. By quantifying this dose-response relationship, we are able to highlight the potential health benefits of policies and programmes that reduce smoking. We have also produced a globally complete time series of estimates for age of initiation that shows that the average age of initiation has changed little across time, even in locations with strong and successful tobacco control. Targeting interventions to reduce the initiation rate in the critical age window of 13–23 years will yield substantial health benefits.^28^ Finally, the concept of capturing exposure over an individual's life course can and should be extended to other risk factors, for which the health effects are probably the result of cumulative exposure.

### Comparison of GBD 2017 to other estimates

The GBD study is the most comprehensive population-level CRA across countries and risks. For several risks, including ambient particulate matter pollution, household air pollution,^29^ intimate partner violence,^30^ unsafe water source,^31^, ^32^ breastfeeding,^33^ and lead exposure,^34^ GBD estimates are generally lower than published WHO estimates.^30^, ^31^, ^32^, ^34^, ^35^ These discrepancies can be attributed to different definitions, methods, granularity, and input data. For some findings, annual estimates might disagree, but regional patterns are consistent between WHO and GBD. UNICEF produces estimates for child stunting^36^ that are lower than GBD estimates, with some disagreement in which locations progress has been made. There is more consistency in estimates between UNICEF and GBD for child wasting and child underweight.^36^ GBD estimates for the prevalence of low birthweight and short gestation are slightly lower than the WHO estimates but show similar geographical patterns.^37^ Scientific literature reveals similar results to GBD for impaired kidney function^38^ and low birthweight and short gestation.^39^ Research published on iron-deficiency anaemia^40^ differs from GBD in methods and definitions, resulting in generally higher GBD estimates. GBD estimates were much lower than published research on occupational estimates,^41^, ^42^, ^43^ largely because of different cause–outcome pairs and GBD's application of the CRA approach.

Improvements in our smoking estimation methods have resulted in higher estimates of smoking-attributable burden. Although smoking estimates from previous iterations of the GBD study were slightly lower than those published by WHO,^35^ our GBD 2017 estimates show marked similarities. Among the 142 countries and territories included in the WHO report and estimated in GBD, the correlation coefficient for 2017 smoking prevalence estimates for females was 0·91 and for males was 0·85. Where estimates diverge, these differences can be attributed to differing modelling methods or data sources. For example, the WHO model was fit on 1175 country-year data sources whereas the GBD model was fit on 2870 country-year data sources.

### Future directions

As with previous iterations of GBD, we have continued to use the World Cancer Research Fund criteria of convincing or probable evidence as the threshold for including a risk–outcome pair in GBD 2017.^44^ The subjectivity of some aspects of these definitions might result in differing interpretations of the available evidence and different conclusions about the strength of the evidence supporting the inclusion of a given risk–outcome pair. Moving toward more objective and quantifiable criteria could help reconcile these different interpretations. Quantifiable criteria would also permit simplified evidence scores that could help policy makers who, faced with scarce resources, might choose to prioritise interventions that address risks supported by the strongest evidence base. Objective and quantifiable criteria would facilitate such comparisons, allowing stakeholders to more easily understand the variable strength of evidence supporting the causal connection for each risk–outcome pair. In an effort to summarise the evidence base for each risk–outcome pair in GBD 2017, we present the number of studies that are available, by type of study design. However, we recognise that quantifying the strength of evidence requires moving beyond simple quantification of the number of studies. To that end, we have begun to extract detailed information from all 3638 studies used across risk–outcome pairs. This effort includes details about study design, the study population and sampling, exposure measurement, outcome assessment, potential sources of bias and confounding, and analytic efforts to address bias and confounding. With this information, we can systematically analyse the degree to which different study limitations affect the resulting strength of associations, allowing us to objectively assess study quality. Looking at the studies for a given risk–outcome pair, we can then calculate an objective evidence score based on the number and quality of the supporting studies, and the potential for publication bias. Such a score would facilitate evidence-based decision making in the face of scarce political and financial resources, allowing attention to be focused on risks with both the greatest attributable burden and strongest supporting evidence.

The inclusion of bullying as a new risk in GBD 2017 suggests potential areas for future work. We hope that its inclusion will help in setting a higher standard of measurement in future research on both the occurrence and health effects of bullying. It also provides a starting point for including additional risk factors related to violence against children, such as non-sexual forms of child abuse in addition to childhood sexual abuse, which is the only GBD 2017 risk factor pertaining to child maltreatment.

A growing body of research has shown direct connections between ambient temperature and health outcomes. For example, the Multicountry Observational Study,^45^ the largest study of all-cause mortality risk and ambient temperature to date, estimated that 7·71% (95% CI 7·43–7·91) of deaths were attributable to suboptimal ambient temperature: 7·29% (7·02–7·49) of deaths were attributable to low ambient temperatures and 0·42% (0·39–0·44) were attributable to high temperatures. The growing evidence base, especially in the context of global concerns about the potential health effects of climate change, makes the inclusion of meteorological risk factors a priority for future iterations of GBD. We have already begun work to estimate the cause-specific burden directly attributable to effects of ambient temperature, with plans to include suboptimal ambient temperature as a risk in future estimations of GBD. The inclusion of exposure to suboptimal temperature is intended also to lay the groundwork for exploring other components of weather and climate in future GBD analyses.

### Limitations

A study of this scope has many limitations. The new proportional PAF strategy that we have implemented for particulate matter pollution reflects a broader challenge with disentangling inherently connected risks with non-linear relationships between exposure and disease risk. In the case of particulate matter pollution, risk for a given outcome rises sharply with increased exposure at the low end of the exposure range, and the marginal increase in risk declines as the risk curve flattens at the upper end of the exposure range. Two risks compose particulate matter pollution: ambient particulate matter pollution and household air pollution. If exposure to each of these two sources were sequentially reduced to the TMREL, the reduction in burden per μg/m^3^ reduction in exposure for whichever source is removed first will be less than the reduction for the source that is removed second. This occurs because, for the first source, the exposure reduction occurs where the risk curve is flatter, whereas for the second source, exposure reduction occurs where the risk curve is steeper. No clear solution to this problem that does not raise a new problem exists within the CRA framework. Treating each exposure in isolation, as we did in previous GBD iterations, results in overestimates of attributable burden, because both risks will have their burden estimated from the steepest part of the risk curve. Considering both exposures together and treating each exposure as the first to be removed, in line with the CRA approach, results in underestimates of risk, because both risks will have their burden estimated from the flattest part of the risk curve. We have chosen instead to estimate burden for total particulate matter pollution and divide this total burden proportionately between the two sources. This accurately captures burden but deviates somewhat from the CRA approach. The challenge extends beyond ambient and household particulates. Smoking and second-hand smoke are additional sources of particulate matter exposure that we have not yet incorporated into our proportional burden strategy. This is a limitation of the CRA approach, and other areas probably have similar challenges with non-linear and inherently connected risks that have not yet been identified and corrected.

Bullying is characterised by intention to harm, a power imbalance, and repetition; however, studies vary greatly with regard to how they specifically define these criteria. For example, we were unable to include many studies where the threshold for repetition was either insufficiently specific or not reported. Second, the relative risk data used in the calculation of PAFs were only from high-income countries. The magnitude of the association between bullying victimisation and outcomes might differ between high-income countries and low-income and middle-income countries.

Our updated continuous measures of smoking better capture true risk, but also introduce new challenges. First, because of data limitations, we assume the risk of all smoked tobacco products is equivalent to the risk of cigarettes. Second, smoking histories were reconstructed on the basis of cross-sectional survey data, not longitudinal data. Third, we were not able to effectively adjust for illicit tobacco in our estimates of supply-side consumption. Fourth, we have not incorporated the effects of tobacco-control policies on various exposures. Consequently, if no survey data are collected after implementation of a tobacco-control policy, the impact of that policy will not be reflected in the modelled estimates. Fifth, insufficient evidence is available to estimate risk-reduction curves for individuals with different smoking histories, so the rate of risk reduction has been assumed to be the same for all levels of exposure. Sixth, we recognise that our exposure definitions are summaries of an individual's smoking history, and that more nuanced exposure definitions might perform better for individual-level risk prediction.

### Conclusion

This study provides a comprehensive and comparable assessment of 84 environmental and occupational, behavioural, and metabolic risks across locations and time. By quantifying levels and trends in exposures to risk factors and the resulting disease burden, this assessment offers insights into past programme and policy successes and highlights the current priorities for public health action. Our findings show that improvements in behavioural, environmental, and occupational risks, and decreases in risk-deleted DALY rates have largely outpaced the effects of population growth and ageing, in terms of absolute burden. Sustained efforts in reducing these risks are crucial to maintaining trends in declining burden from injuries and CMNNDs. Conversely, the combination of increasing metabolic risks and population ageing will probably continue to drive the increasing trends in NCDs at the global level, which presents both a public health challenge and opportunity. Working down from the global to national level reveals considerable heterogeneity in levels of risk exposure and risk-attributable burden. Although development underlies some of this heterogeneity, ratios of observed burden to the burden expected based on SDI reveal risks for which countries are overperforming or underperforming relative to their level of socioeconomic development. As such, these ratios provide a benchmarking tool to help focus local decision making. Our findings reinforce the importance of both risk exposure monitoring and epidemiological research to assess causal connections between risks and health outcomes, and they highlight the essential role of GBD in synthesising data to draw the comprehensive and robust conclusions that are necessary to inform evidence-based policy.

Correspondence to: Prof Christopher J L Murray, Institute for Health Metrics and Evalution, Seattle, WA 98121, USA cjlm@uw.edu

## Data sharing
