# Department of Error

**DOI:** 10.1016/S0140-6736(18)33216-1

**Published:** 2019-01-12

*GBD 2017 Risk Factor Collaborators. Global, regional, and national comparative risk assessment of 84 behavioural, environmental and occupational, and metabolic risks or clusters of risks for 195 countries and territories, 1990–2017: a systematic analysis for the Global Burden of Disease Study 2017.* Lancet *2018; **392:** 1923–94—*The funding of this paper has been updated to “Bill & Melinda Gates Foundation and Bloomberg Philanthropies” and the first sentence of the acknowledgments has been changed to “Research reported in this publication was supported by the Bill & Melinda Gates Foundation, Bloomberg Philanthropies, the University of Melbourne, Public Health England, the Norwegian Institute of Public Health, St Jude Children's Research Hospital, the National Institute on Ageing of the National Institutes of Health (NIH; award P30AG047845), and the National Institute of Mental Health of NIH (award R01MH110163)”. These corrections have been made to the online version as of Jan 10, 2019.

