# Department of Error

**DOI:** 10.1016/S0140-6736(19)31429-1

**Published:** 2019-06-22

*GBD 2017 Risk Factor Collaborators. Global, regional, and national comparative risk assessment of 84 behavioural, environmental and occupational, and metabolic risks or clusters of risks for 195 countries and territories, 1990–2017: a systematic analysis for the Global Burden of Disease Study 2017.* Lancet *2018; **392:** 1923–94—*In this Global Health Metrics paper, affiliations have been amended for Yasin Jemal Yasin, Joseph Adel Mattar Banoub, and Tanuj Kanchan; the declaration of interests statement has been amended for Boris Bikbov; and acknowledgments have been added for providers of smoking relative risk data. These corrections have been made to the online version as of June 20, 2019.

