# Supplementary material for: Global, regional, and national comparative risk assessment of 84 behavioural, environmental and occupational, and metabolic risks or clusters of risks for 195 countries and territories, 1990–2017: a systematic analysis for the Global Burden of Disease Study 2017
Source: Lancet. 2018 Nov 10;392(10159):1923–94. doi: 10.1016/S0140-6736(18)32225-6 (PMC6227755; doi:10.1016/S0140-6736(18)32225-6)
Supplement: Supplementary appendix 1 [file mmc1.pdf]

THE LANCET

## Supplementary appendix 1

This appendix formed part of the original submission and has been peer reviewed. We post it as supplied by the authors.

Supplement to: GBD 2017 Risk Factor Collaborators. Global, regional, and national comparative risk assessment of 84 behavioural, environmental and occupational, and metabolic risks or clusters of risks for 195 countries and territories, 1990–2017: a systematic analysis for the Global Burden of Disease Study 2017. *Lancet* 2018; **392:** 1923–45.

# Methods appendix to Global, regional, and national comparative risk assessment of 84 behavioural, environmental and occupational, and metabolic risks or clusters of risks, 1990-2017: a systematic analysis for the Global Burden of Disease Study 2017

This appendix provides further methodological detail, supplemental figures, and more detailed results for risk factors. The appendix is organised into broad sections following the structure of the main paper.

## Preamble

This appendix provides further methodological detail and more detailed results for "Global, regional, and national comparative risk assessment of 84 behavioural, environmental and occupational, and metabolic risks or clusters of risks, 1990-2017: a systematic analysis for the Global Burden of Disease Study 2017." This study complies with the Guidelines for Accurate and Transparent Health Estimates Reporting (GATHER) recommendations. It includes detailed tables and information on data in an effort to maximise transparency in our estimation processes and provide a comprehensive description of analytical steps. We intend this appendix to be a living document, to be updated with each iteration of the Global Burden of Disease Study.

# Contents

Authors contributions.......... 7
Section 1: GBD overview.......... 16
Section 1.1: Locations of the Analysis.......... 16
Section 1.2: Time Period of the Analysis.......... 16
Section 1.3: Statement of GATHER Compliance.......... 16
Section 1.4: GBD risk factor hierarchy.......... 16
Section 1.5: List of abbreviations.......... 16
Section 1.6: GBD results overview.......... 19
Section 1.7: Data input sources overview.......... 19
Section 1.8: Funding Sources.......... 19
Section 2: Risk factor estimation.......... 20
Section 2.1: Overview.......... 20
Section 2.2: Step 1. Effect size estimation.......... 21
Section 2.2.1: Collate relative risk data.......... 21
Section 2.2.2: Determine relative risks.......... 22
Section 2.3: Step 2. Exposure estimation.......... 23
Section 2.3.1: Collate exposure data.......... 23
Section 2.3.2: 2b. Adjust exposure data.......... 24
Section 2.3.3: 2c. Estimate exposure.......... 24
Section 2.4: Step 3. TMREL.......... 33
Section 2.5: Step 4. Estimate population attributable fractions.......... 33
Section 2.6: Step 5. Estimate summary exposure values.......... 34
Section 2.7: Step 6. Mediation.......... 35
Section 2.7.1: Summary.......... 35
Section 2.7.2: Additional detail.......... 35
Section 2.7.3: Calculating burden of multiple risk factors.......... 37
Section 2.7.4: Adjusting for mediation.......... 37
Section 2.7.5: Calculating mediation factor.......... 38
Section 2.7.6: Piecewise aggregation (Pattern 3).......... 40
Section 2.7.7: Uncertainty of aggregated and mediated PAFs.......... 41
Section 2.7.8: Important assumptions in aggregating risk factors and including mediation.... 41
Section 2.8: Step 7. Estimate attributable burden.......... 41
Section 2.9: Decomposition analysis of deaths and DALYs.......... 42
Section 2.10: SDI Analysis.......... 43

Section 2.10.1: Development of SDI…… 43
Section 2.10.2: Development of revised SDI indicator…… 43
Section 2.11: Epidemiological Transition…… 45
Section 2.11.1: Derivation of expected SEVs…… 45
Section 2.11.2: Calculating attributable burden…… 45
Section 2.12: Additional Methods Information…… 46
Section 2.12.1: Risk-specific comparisons to other estimates…… 46
Section 3: References…… 53
Section 4: Risk-specific estimation…… 57
Section 4.1: Unsafe water source…… 58
Section 4.2: Unsafe sanitation…… 62
Section 4.3: Unsafe hygiene…… 65
Section 4.4: Ambient particulate matter pollution…… 67
Section 4.5: Household air pollution…… 77
Section 4.6: Ambient ozone pollution…… 79
Section 4.7: Residential radon…… 81
Section 4.8: Lead exposure…… 83
Section 4.9: Occupational risks…… 87
Section 4.10: Suboptimal breastfeeding…… 92
Section 4.11: Child growth failure…… 95
Section 4.12: Low birth weight and short gestation…… 100
Section 4.13: Iron deficiency…… 107
Section 4.14: Vitamin A deficiency…… 109
Section 4.15: Zinc deficiency…… 115
Section 4.16: Smoking…… 116
Section 4.17: Second-hand smoke…… 121
Section 4.18: Chewing tobacco…… 124
Section 4.19: Alcohol use…… 128
Section 4.20: Drug use…… 137
Section 4.21: Dietary risks…… 144
Section 4.22: Intimate partner violence…… 149
Section 4.23: Childhood sexual assault…… 156
Section 4.24: Bullying…… 161
Section 4.25: Unsafe sex…… 167
Section 4.26: Low physical activity…… 170
Section 4.27: High fasting plasma glucose…… 173

Section 4.28: High LDL cholesterol…… 177
Section 4.29: High systolic blood pressure…… 184
Section 4.30: High body-mass index…… 193
Section 4.31: Bone mineral density…… 200
Section 4.32: Impaired kidney function…… 209
Section 5: Tables and Figures…… 212
**Appendix Figure 1.** Analytical flowchart of the comparative risk assessment for the estimation of population attributable fractions by geography, age, sex, and year for GBD 2017. Ovals represent data inputs, rectangular boxes represent analytical steps, cylinders represent databases, and parallelograms represent intermediate and final results. GBD=Global Burden of Disease. SEVs=Summary exposure values. TMREL=Theoretical minimum-risk exposure level. PAFs=Population attributable fractions. YLLs=years of life lost. YLDs=years lived with disability. DALYs=disability-adjusted life-years…… 213
**Appendix Figure 2.** GBD 2016 DisMod-MR 2.1 analytic cascade…… 214
**Appendix Figure 3.** ST-GPR flowchart…… 215
**Appendix Table 1.** Guidelines for Accurate and Transparent Health Estimates Reporting (GATHER) 18-items checklist with description of compliance and location of information for GBD 2017 risk factors capstone…… 216
**Appendix Table 2.** GBD 2017 risk factor hierarchy with levels, modelling strategies, and the main type of data sources used to estimate exposure levels…… 219
**Appendix Table 3.** Types of Comparative Risk Assessments (CRA) based on the time perspective and the nature of the counterfactual level or distribution of exposure. The shaded box represents the type of CRA currently undertaken in GBD 2017. GBD=Global Burden of Disease…… 221
**Appendix Table 4.** Descriptive cataloguing of the epidemiological evidence used to assess whether each risk-outcome paper meets the causal criteria for inclusion in the Global Burden of Disease Study 2017…… 222
If multiple reports existed from the same study, we counted them as one study. We only assessed the dose-response relationship for continuous risks. To evaluate the magnitude of the effect size for continuous risks, we evaluated the relative risk comparing the 75th percentile with the 25th percentile of the exposure distribution at the global level. RCT=randomised controlled trial. RR=relative risk. *Prospective cohort studies or non-randomised interventions. †Whether or not any biological or mechanistic pathway exists that could potentially explain the relationship of the risk-outcome pair. ‡Whether or not the risk is associated with another outcome from the same category and whether or not any evidence exists that it can cause the current outcome through the same pathway.
**Appendix Table 5.** Epidemiological evidence supporting causality between risk-outcome pairs included in the Global Burden of Disease 2017 study including A. Citations and B. Additional information…… 227
**Appendix Table 6a.** Relative risks used by age and sex for each outcome for all risk factors except for ambient air pollution, alcohol, and smoking…… 259

**Appendix Table 6b.** Relative risks used by age and sex for each outcome for the particulate matter integrated exposure response curve………… 306

**Appendix Table 6c.** Relative risks used by age and sex for each outcome for alcohol use globally………… 310

**Appendix Table 6d.** Relative risks used by age and sex for each outcome for smoking globally 317

**Appendix Table 7.** GBD 2017 location hierarchy with levels………… 333

**Appendix Table 8.** Mediation factors. For IHD, stroke, and diabetes we pooled all available cohorts and estimated relative risks with and without adjustment across all combinations of metabolic risk factors. We then computed the excess attenuated risk for each mediation-risk-cause set………… 347

**Appendix Table 9.** Socio-Demographic Index groupings by location, based on 2017 values……. 352

**Appendix Table 10.** Socio-Demographic Index values for all estimated GBD 2017 locations, 1990–2017………… 366

## Authors' Contributions

### Managing the estimation process

Ashkan Afshin, Tahiya Alam, Brigette Blacker, Michael Brauer, Aaron Cohen, Elizabeth Cromwell, Lalit Dandona, Rakhi Dandona, Louisa Degenhardt, Samath Dharmaratne, Charbel El Bcheraoui, Kara Estep, Valery Feigin, Nancy Fullman, Emmanuela Gakidou, Caitlin Hawley, Simon Hay, Spencer L James, Nicholas J Kassebaum, Ibrahim Khalil, Hmwe Kyu, Stephen Lim, Alan Lopez, Rafael Lozano, Ashley Marks, Awoke Misganaw, Ali Mokdad, Christopher Murray, Mohsen Naghavi, Elaine Nsoesie, Helen Olsen, Robert Reiner, Gregory Roth, David Smith, Mari Smith, Jeffrey Stanaway, Stein Emil Vollset, Theo Vos, and Harvey Whiteford

### Writing the first draft of the manuscript

Jason Anderson, Kate Causey, William Gardner, Sam Larson, Ashley Marks, Molly Nixon, Jeff Stanaway, Anna Torre.

### Providing data or critical feedback on data sources

Aliasghar A Kiadaliri, Kalkidan Hassen Abate, Cristiana Abbafati, Nooshin Abbasi, Ibrahim Abdollahpour, Molla Abebe, Zegeye Abebe, Semaw Ferede Abera, Laith Abu-Raddad, Niveen Abu-Rmeileh, Manfred Accrombessi, Oladimeji Adebayo, Zanfina Ademi, Olatunji Adetokunboh, Mina Adib, José C. Adusar, Mohsen Afarideh , Ashkan Afshin, Sargis Aghayan, Alireza Ahmadi, Mehdi Ahmadi, Muktar Ahmed, Ibtihel Aichour, Mohammad Esmail Akbari, Tomi Akinyemiju, Nadia Akseer, Fares Alahdab, Khurshid Alam, Kefyalew Addis Alene, Syed Aljunid, François Alla, Ubai Alsharif, Dayane Gabriele Alves Silveira, Nelson Alvis-Guzman, Mamusha Aman, Azmeraw T. Amare, Walid Ammar, Catalina Liliana Andrei, Mr. Mustafa Geleto Ansha, Carl Abelardo Antonio, Seth Christopher Y. Appiah, Olatunde Aremu, Johan Ärnlöv, Al Artaman, Krishna Kumar Aryal, Hamid Asayesh, Marcel Ausloos, Ashish Awasthi, Beatriz Paulina Ayala Quintanilla, Rakesh Ayer, Tambe Betrand Ayuk, Peter Azzopardi, Nam Ba Nguyen, Arefeh Babazadeh, Alaa Badawi, Kalpana Balakrishnan, Shoshana Ballew, Maciej Banach, Joseph Banoub, Aleksandra Barac, Till Bärnighausen, Lope Barrero, Sanjay Basu, Bernhard Baune, Neeraj Bedi, Masoud Behzadifar, Meysam Behzadifar, Bayu Begashaw Bekele, Yihalem Abebe Belay, Aminu Bello, Isabela Bensenor, Eduardo Bernabe, Robert Bernstein, Mircea Beuran, Tina Beyranvand, Neeraj Bhala, Suraj Bhattarai, Boris Bikbov, Nigus Bililign, Muhammad Shahdaat Bin Sayeed, Ibrahim Bou-Orm, Rupert Bourne, Michael Brauer, Andrey Briko, Gabrielle Britton, Leah Cahill, Giulia Carreras, Juan J Carrero, Deborah Carvalho Malta, Carlos Castañeda-Orjuela, Jacqueline Castillo Rivas, Franz Castro, Ferrán Catalá-López, Yazan Chaiah, Hsing-Yi Chang, Jung-Chen Chang, Fiona Charlson, Aparajita Chattopadhyay, Miao Li Chee, Ching-Yu Cheng, Peggy Pei-Chia Chiang, Abdulaal Chitheer, Rajiv Chowdhury, Devasahayam Christopher, Sheng-Chia Chung, Massimo Cirillo, Aaron Cohen, Daniel Collado-Mateo, Cyrus Cooper, Josef Coresh, Paolo Angelo Cortesi, Monica Cortinovis, Michael Criqui, Alemneh Kabeta Daba, Albertino Damasceno, Lalit Dandona, Rakhi Dandona, Sarah Darby, Paul Dargan, Ahmad Daryani, José Das Neves, Fernando De La Hoz, Kebede Deribe, Nikolaos Dervenis, Aniruddha Deshpande, Getenet Dessie, Gabrielle Deveber, Samath Dharmaratne, Meghnath Dhimal , Eric L. Ding, Klara Dokova, David Teye Doku, Kerrie Doyle, Manisha Dubey, Eyasu Ejeta Duken, Bruce Duncan, Andre Duraes, Natalie Ebert, Hedyeh Ebrahimi, Soheil Ebrahimpour, Andem Effiong, Anne Elise Eggen, Ziad El-Khatib, Iqbal Elyazar, Benjamin Er, Holly Erskine, Sharareh Eskandarieh, Alireza Esteghamati, Sadaf Esteghamati, Mohammad Fareed, Carla Farinha, Andre Faro, Mohammad Hosen Farzaei, Valery Feigin, Andrea B. Feigl, Seyed-Mohammad Fereshtehnejad, Joao Fernandes, Irina Filip, Jonas D. Finger, Nataliya Foigt,

Takeshi Fukumoto, Thomas Fürst, Neal Futran, Seana Gall, Amiran Gamkrelidze, Tigit Gashaw, Abadi Kahsu Gebre, Amanuel Tesfay Gebremedhin, Merhawi Gebremedhin, Teklu Gebremichael, Tilayie Feto Gelano, Johanna Geleijnse, Ayele Geleto, Mamata Ghimire, Simona Giampaoli, Ibrahim Ginawi, Srinivas Goli, Hector Gomez-Dantes, Philimon Gona, Sameer Gopalani, Ayman Grada, Morgan Grams, Giuseppe Grosso, Yuming Guo, Rahul Gupta, Rajat Das Gupta, Rajeev Gupta, Tanush Gupta, Daniela Sarahí Gutiérrez-Torres, Juanita Haagsma, Nima Hafezi-Nejad, Tekleberhan Beyene Hagos, Gessessew Bugssa Hailu, Arya Haj-Mirzaian, Arvin Ha-Mirzaian, Yuantao Hao, Hilda Harb, Sivadasanpillai Harikrishnan, Josep Maria Haro, Hadi Hassankhani, Hamid Y. Hassen, Rasmus Havmoeller, Behnam Heidari, Mohsen Heidari, Delia Hendrie, Andualem Henok, Ileana B. Heredia-Pi, Claudiu Herteliu, Hans Hoek, Howard J. Hoffman, Michael Hole, Praveen Hoogar, H Dean Hosgood, Mehdi Hosseinzadeh, Mihaela Hostiuc, Sorin Hostiuc, Damian Hoy, Mohamed Hsairi, Guoqing Hu, Kim Moesgaard Iburg, Nayu Ikeda, Usman Iqbal, Farhad Islami, Maria Jackson, Kathryn H. Jacobsen, Mihajlo Jakovljevic, Spencer James, Simerjot Jassal, Achala Jayatilleke, Panniyammakal Jeemon, Vivekanand Jha, Jost B. Jonas, Zahra Jorjoran Shushtari, Jacek Jozwiak, Mikk Jürisson, Zubair Kabir, Amaha Kahsay, Amaha Kahsay, Tanuj Kanchan, André Karch, Corine Karema, Seyed M. Karimi, Amir Kasaeian, Getachew Mullu Kassa, Nicholas Kassebaum, Srinivasa Vittal Katikireddi, Anil Kaul, Norito Kawakami, Peter Keiyoro, Andre Keren, Chandrasekharan Nair Kesavachandran, Yousef Khader, Morteza Abdullatif Khafaie, Ibrahim Khalil, Muhammad Shahzeb Khan, Young-Ho Khang, Mona Khater, Habibolah Khazaie, Abdullah T. Khoja, Ardeshir Khosravi, Mohammad Hossein Khosravi, Daniel Kiirithio, Cho-Il Kim, Daniel Kim, Young-Eun Kim, Yun Jin Kim, Adnan Kisa, Luke Knibbs, Ann Kristin Knudsen, Soewarta Kosen, Parvaiz Koul, Ai Koyanagi, Michael Kravchenko, Kewal Krishan, Hans Kromhout, Barthelemy Kuate Defo, Burcu Kucuk Bicer, G Anil Kumar, Manasi Kumar, Ihor Kuzin, Carl Lachat, Deepesh P. Lad, Sheetal Lad, Alessandra Lafranconi, Huong Lan Nguyen, Justin Lang, Van Lansingh, Samantha Larson, Misgan Legesse, Yirga Legesse, James Leigh, Janni Leung, Shanshan Li, Yichong Li, Juan Liang, Xiaofeng Liang, Yu Liao, Lee-Ling Lim, Shiwei Liu, Alan Lopez, Stefan Lorkowski, Paulo Lotufo, Stefan Ma, Erlyn Rachelle Macarayan, Isis Machado, P A Mahesh, Marek Majdan, Reza Majdzadeh, Azeem Majeed, Reza Malekzadeh, Mohammad Ali Mansournia, Lorenzo Mantovani, Joemer Maravilla, Wagner Marcenes, Randall V. Martin, Francisco Rogerlândio Martins-Melo, Winfried März, Melvin Marzan, Benjamin Massenburg, Manu Mathur, Kunihiro Matsushita, Mohsen Mazidi, Colm Mcalinden, John Mcgrath, Abla Mehio Sibai, Varshil Mehta, Toni Meier, Yohannes Adama Melaku, Addisu Melese, Mulugeta Melku, Peter Memiah, Ziad Memish, Walter Mendoza, Gert B.M. Mensink, Atte Meretoja, Tuomo Meretoja, Tomislav Mestrovic, Haftay Berhane Mezgebe, Tomasz Miazgowski, Ted R Miller, Erkin Mirrakhimov, Babak Moazen, Bahram Mohajer, Karzan Mohammad, Moslem Mohammadi, Noushin Mohammadifard, Shafiu Mohammed, Farnam Mohebi, Ali Mokdad, Lorenzo Monasta, Yoshan Moodley, Ghobad Moradi, Maziar Moradi-Lakeh, Mehdi Moradinazar, Lidia Morawska, Joana Morgado-Da-Costa, Marilita Moschos, Seyyed Meysam Mousavi, Dariush Mozaffarian, Achenef Muche, Ulrich Mueller, Kamarul Imran Musa, Ghulam Mustafa, Ashraf Nabhan, Mohsen Naghavi, Seyed Sina Naghibi Irvani, Azin Nahvijou, Bruno Nascimento, Ionut Negoi, Ruxandra Irina Negoi, Charels Newton, Josephine Ngunjiri, Anh Nguyen, Ha Nguyen, Huong Nguyen, Nobuo Nishi, Marzieh Nojomi, Shuhei Nomura, Mehdi Noroozi , Bo Norrving, Jean Jacques Noubiap, Hamid Reza Nouri, Malihe Nourollahpour, Mohammad Reza Nowroozi, Christopher M Odell, Felix Ogbo, In-Hwan Oh, Olanrewaju Oladimeji, Andrew T. Olagunju, Tinuke O. Olagunju, Bolajoko Olusanya, Jacob Olusanya, Kanyin Ong, Sok King Ong, Eyal Oren, Heather Orpana, Alberto Ortiz, Stanislav Otstavnov, Simon Øverland, Mayowa Owolabi, Rosana Pacella, Adrian Pana, Songhomitra Panda-Jonas, Hadi Parsian, Shanti Patel, Sanghamitra Pati, Snehal Patil, Ajay Patle, Deepak Paudel, Wayra Citlali Paz Ballesteros, Neil Pearce,

Alexandre Pereira, David Pereira, Norberto Perico, Max Petzold, Huyen Phuc Do, Meghdad Pirsaheb, Farhad Pishgar, Dietrich Plass, Suzanne Polinder, Kevan Polkinghorne, Hossein Poustchi, Swayam Prakash, Narayan Prasad, Hai Quang Pham, Amir Radfar, Anwar Rafay, Alireza Rafiei, Fakher Rahim, Zohreh Rahimi, Afarin Rahimi-Movaghar, Vafa Rahimi-Movaghar, Mahfuzar Rahman, Mohammad Hifz Ur Rahman, Rajesh Kumar Rai, Usha Ram, Chhabi Lal Ranabhat, Prabhat Ranjan, Goura K Rath, David Laith Rawaf, Salman Rawaf, Kolli Srinath Reddy, Colin D Rehm, Jürgen Rehm, Giuseppe Remuzzi, Andre Renzaho, Luz Myriam Reynales-Shigematsu, Antonio Luiz Ribeiro, Juan A Rivera, Sonia Rodríguez-Ramírez, Leonardo Roever, Luca Ronfani, Gholamreza Roshandel, Ali Rostami, Ambuj Roy, Enrico Rubagotti, Lesley Rushton, Charumathi Sabanayagam, Basema Saddik, Ehsan Sadeghi, Hosein Safari, Yahya Safari, Saeid Safiri, Mohammad Ali Sahraian, Nasir Salam, Payman Salamati, Yahya Salimi, Hamideh Salimzadeh, Devashri Salvi, Abdallah M. Samy, Juan Sanabria , Tania G Sánchez-Pimienta, Yingying Sang, Milena Santric Milicevic, Bruno Sao Jose, Mayank Sardana, Rodrigo Sarmiento-Suárez, Nizal Sarrafzadegan, Shahabeddin Sarvi, Thirunavukkarasu Sathish, Maheswar Satpathy, Monika Sawhney, Mete Saylan, Mehdi Sayyah, Elke Schaeffner, Maria Inês Schmidt, David C Schwebel, Falk Schwendicke, James Scott, Mario Šekerija, Sadaf Sepanlou, Edson Serván-Mori, Seyedmojtaba Seyedmousavi, Gavin Shaddick, Amira Shaheen, Masood Ali Shaikh, Teresa Shamah-Levy, Mehran Shams-Beyranvand, Kiomars Sharafi, Mehdi Sharif, Hamid Sharifi, Sheikh Mohammed Shariful Islam, Jayendra Sharma, Meenakshi Sharma, Aziz Sheikh, Peilin Shi, Min-Jeong Shin, Ivy Shiue, Farhad Shokraneh, Haitham Shoman, Si Si, Soraya Siabani, Tariq Jamal Siddiqi, Diego Augusto Santos Silva, Jasvinder Singh, Virendra Singh, Dhirendra Narain Sinha, Eirini Skiadaresi, Vegard Skirbekk, Soheila Sobhani, Reed Sorensen, Joan B Soriano, Angela Spinelli, Luciano Sposato, Chandrashekhar T Sreeramareddy, Goran Stevanovic, Leo Stockfelt, Mark Stokes, Lela Sturua, Agus Sudaryanto, Muawiyyah Babale Sufiyan, Rizwan Suliankatchi Abdulkader, Patrick Sur, Bryan L. Sykes, Cassandra Szoeke, Rafael Tabarés-Seisdedos, Mesfin Tadese, Eyasu Tamru , Nuno Taveira, Gebre Teklemariam Demoz, Awoke Temesgen, Mohamad-Hani Temsah, Omar Temsah, Belay Tessema, Mebrahtu Teweldemedhin, Hue Thi Mai, Nu Thi Truong, Matthew L Thomas, Nihal Thomas, Giang Thu Vu, Myriam Tobollik, Marcello Tonelli, Miguel Tortajada-Girbés, Mathilde Touvier, Marcos Roberto Tovani-Palone, Bach Tran, Khanh Bao Tran, Thomas Truelsen, Stefanos Tyrovolas, Kingsley Nnanna Ukwaja, Irfan Ullah, Muhammad Shariq Usman, Olalekan A Uthman, Muthiah Vaduganathan, Afsane Vaezi, Pascual Valdez, Aaron Van Donkelaar, Tommi Vasankari, Vidhya Venkateswaran, Narayanaswamy Venketasubramanian, Santos Villafaina, Sergey Vladimirov, Vasiliy Vlassov, Stein Emil Vollset, Fasil Wagnew, Shishay Wahdey, Yasir Waheed, Yanping Wang, Yuan-Pang Wang, Elisabete Weiderpass, Robert Weintraub, Inbal Weiss Salz, Andrea Werdecker, Ronny Westerman, Harvey Whiteford, Justyna Widecka, Tissa Wijeratne, Charles Shey Wiysonge, Charles Wolfe, Tien Yin Wong, Shouling Wu, Denis Xavier, Gelin Xu, Ali Yadollahpour, Hossein Yahyazadeh, Lijing Yan, Mehdi Yaseri, Yasin Jemal Yasin, Alex Yeshaneh, Ebrahim M. Yimer, Naohiro Yonemoto, Seok-Jun Yoon, Marcel Yotebieng, Mustafa Younis, Chuanhua Yu, Zoubida Zaidi, Sojib Bin Zaman, Mohammad Zamani, Luis Zavala-Arciniega, Anthony Lin Zhang, and Sanjay Zodpey.

## Developing methods or computational machinery

Cristiana Abbafati, Ibrahim Abdollahpour, Ashkan Afshin, Mohammad Esmail Akbari, Mehran Alijanzadeh, Marcel Ausloos, Doanl Bisanzio, Michael Brauer, Kate Causey, Kelly Cercy, Aaron Cohen, Josef Coresh, Ahmad Daryani, Holly Erskine, Kairsten Fay, Kyle Foreman, Morgan Grams, Hamid Y. Hassen, Claudiu Herteliu, Howard Hu, Chantal Huynh, Spencer James, Nicholas Kassebaum, Young-Eun Kim, Adnan Kisa, Van Lansingh, Misgan Legesse, James Leigh, Tim Lucas, Helena Manguerra, Randall V.

Martin, Kunihiro Matsushita, Fantahun Mekonnen, Anoushka Millear, Dariush Mozaffarian, Mohsen Naghavi, Kanyin Ong, Charles Parry, Alireza Rafiei, Chhabi Lal Ranabhat, Jürgen Rehm, Robert Reiner, Marissa Reitsma, Gregory Roth, Lesley Rushton, Abdallah M. Samy, Yingying Sang, Damian Santomauro, Maheswar Satpathy, David C Schwebel, James Scott, Gavin Shaddick, Mehdi Sharif, David Smith, Reed Sorensen, Vinay Srinivasan, Jeff Stanaway, Patrick Sur, Hannah J Thomas, Matthew L Thomas, Hayley Tymeson, Rachel L. Updike, Ronny Westerman, Simon Yadgir, Lijing Yan, and Naohiro Yonemoto.

## Applying analytical methods to produce estimates

Ashkan Afshin, Syed Mustafa Ali, Azmeraw T. Amare, Olatunde Aremu, Marcel Ausloos, Bayu Begashaw Bekele, Doanl Bisanzio, Michael Brauer, Kate Causey, Kelly Cercy, Fiona Charlson, Devasahayam Christopher, Aaron Cohen, Ahmad Daryani, Getenet Dessie, Tim Driscoll, Andem Effiong, Aman Endries, Holly Erskine, Kairsten Fay, Alize Ferrari, William Gardner, Segen Gebremeskel, Rakesh Ghosh, Hamid Y. Hassen, Claudiu Herteliu, Caleb Irvine, Manoochehr Karami, Nicholas Kassebaum, Behzad Khafaie , Yun Jin Kim, Adnan Kisa, Samantha Larson, Misgan Legesse, James Leigh, Tim Lucas, Helena Manguerra, Randall V. Martin, Fantahun Mekonnen, Anoushka Millear, Shafiu Mohammed, Ali Mokdad, Mohsen Naghavi, Grant Nguyen, Emma Nichols, Bolajoko Olusanya, Jacob Olusanya, Kanyin Ong, Katherine Paulson , Swayam Prakash, Caroline Purcell, Chhabi Lal Ranabhat, Marissa Reitsma, Gregory Roth, Enrico Rubagotti, Devashri Salvi, Abdallah M. Samy, Yingying Sang, Damian Santomauro, Shahabeddin Sarvi, Maheswar Satpathy, Seyedmojtaba Seyedmousavi, Gavin Shaddick, Mehdi Sharif, Reed Sorensen, Chandrashekhar T Sreeramareddy, Vinay Srinivasan, Jeff Stanaway, Patrick Sur, Bryan L. Sykes, Andrew Theis, Hannah J Thomas, Matthew L Thomas, Rachel L. Updike, Vidhya Venkateswaran, Inbal Weiss Salz, Ronny Westerman, Tissa Wijeratne, and Simon Yadgi.

## Providing critical feedback on methods or results

Aliasghar A Kiadaliri, Degu Abate, Kalkidan Hassen Abate, Cristiana Abbafati, Nooshin Abbasi, Hedayat Abbastabar, Jemal Abdela, Ibrahim Abdollahpour, Molla Abebe, Zegeye Abebe, Semaw Ferede Abera, Haftom Abraha, Niveen Abu-Rmeileh, Pawan Acharya, Abdu Adamu, Akilew Awoke Adane, Oladimeji Adebayo, Victor Adekanmbi, Olatunji Adetokunboh, Mina Adib, José C. Adusar, Kossivi Afanvi, Mohsen Afarideh , Ashkan Afshin, Gina Agarwal, Anju Aggarwal, Sargis Aghayan, Anurag Agrawal, Alireza Ahmadi, Mehdi Ahmadi, Hamid Ahmadieh, Muktar Ahmed, Amani Nidhal Aichour, Ibtihel Aichour, Miloud Taki Eddine Aichour, Mohammad Esmail Akbari, Tomi Akinyemiju, Nadia Akseer, Fares Alahdab, Ziyad Al-Aly, Khurshid Alam, Tahiya Alam, Seyed Moayed Alavian, Kefyalew Addis Alene, Ayman Al-Eyadhy, Syed Mustafa Ali, Mehran Alijanzadeh, Reza Alizadeh-Navaei, Syed Aljunid, Ala'a Alkerwi, Hesham Al-Mekhlafi, Ubai Alsharif, Khalid Altirkawi, Dayane Gabriele Alves Silveira, Nelson Alvis-Guzman, Mamusha Aman, Azmeraw T. Amare, Walid Ammar, Mirica Andreea, Catalina Liliana Andrei, Sofia Androudi, Mina Anjomshoa, Mr. Mustafa Geleto Ansha, Josep M Antó, Carl Abelardo Antonio, Palwasha Anwari, Lambert Appiah, Olatunde Aremu, Johan Ärnlöv, Al Artaman, Krishna Kumar Aryal, Hamid Asayesh, Zerihun Ataro, Marcel Ausloos, Euripide Avokpaho, Ashish Awasthi, Beatriz Paulina Ayala Quintanilla, Tambe Betrand Ayuk, Peter Azzopardi, Nam Ba Nguyen, Arefeh Babazadeh, Hamid Badali, Alaa Badawi, Kylie Ball, Shoshana Ballew, Joseph Banoub, Aleksandra Barac, Suzanne Barker-Collo, Till Bärnighausen, Lope Barrero, Sanjay Basu, Bernhard Baune, Shahrzad Bazargan-Hejazi, Neeraj Bedi, Ettore Beghi, Masoud Behzadifar, Meysam Behzadifar, Yannick Béjot, Bayu Begashaw Bekele, Ezrt Belay, Yihalem Abebe Belay, Michelle Bell, Aminu Bello, Derrick Bennett, Isabela Bensenor, Gilles Bergeron, Adugnaw Berhane, Eduardo Bernabe, Robert Bernstein, Mircea Beuran, Tina Beyranvand, Neeraj Bhala, Ashish Bhalla, Suraj Bhattarai, Belete Biadgo, Ali Bijani, Boris Bikbov, Ver Bilano, Nigus Bililign, Muhammad

Shahdaat Bin Sayeed, Doanl Bisanzio, Tuhin Biswas, Tone Bjørge, Archie Bleyer, Rohan Borschmann, Soufiane Boufous, Rupert Bourne, Oliver Brady, Michael Brauer, Alexandra Brazinova, Nicholas Breitborde, Hermann Brenner, Andrey Briko, Gabrielle Britton, Rachelle Buchbinder, Reinhard Busse, Zahid Butt, Leah Cahill, Lucero Cahuana-Hurtado, Ismael Campos-Nonato, Rosario Cárdenas, Juan J Carrero, Felix Carvalho, Deborah Carvalho Malta, Carlos Castañeda-Orjuela, Jacqueline Castillo Rivas, Franz Castro, Ferrán Catalá-López, Ester Cerin, Yazan Chaiah, Jung-Chen Chang, Fiona Charlson, Aparajita Chattopadhyay, Vijay Kumar Chattu, Peggy Pei-Chia Chiang, Odgerel Chimed-Ochir, Ken Chin, Yilma Chisha, Jee-Young Choi, Hanne Christensen, Devasahayam Christopher, Sheng-Chia Chung, Flavia Cicuttini, Massimo Cirillo, Aaron Cohen, Daniel Collado-Mateo, Cyrus Cooper, Josef Coresh, Paolo Angelo Cortesi, Monica Cortinovis, Megan Costa, Ewerton Cousin, Michael Criqui, David Cundiff, Alemneh Kabeta Daba, Berihun Dachew, Abel Dadi, Lalit Dandona, Rakhi Dandona, Sarah Darby, Paul Dargan, Ahmad Daryani, José Das Neves, Tamirat Tesfaye Dasa, Kairat Davletov, Vanessa De La Cruz-Góngora, Fernando De La Hoz, Diego De Leo, Jan-Walter De Neve, Megbaru Debalkie, Robert Dellavalle, Edgar Denova-Gutiérrez, Kebede Deribe, Nikolaos Dervenis, Don Des Jarlais, Aniruddha Deshpande, Getenet Dessie, Subhojit Dey, Samath Dharmaratne, Meghnath Dhimal , Eric L. Ding, Helen Diro, Shirin Djalalinia, Klara Dokova, David Teye Doku, Kerrie Doyle, Tim Driscoll, Manisha Dubey, Eleonora Dubljanin, Eyasu Ejeta Duken, Bruce Duncan, Andre Duraes, Natalie Ebert, Hedyeh Ebrahimi, Soheil Ebrahimpour, David Edvardsson, Andem Effiong, Anne Elise Eggen, Ziad El-Khatib, Iqbal Elyazar, Ahmadali Enayati, Aman Endries, Benjamin Er, Holly Erskine, Sharareh Eskandarieh, Alireza Esteghamati, Sadaf Esteghamati, Hamed Fakhim, Mahbobeh Faramarzi, Mohammad Fareed, Talha Farid, Carla Farinha, Andrea Farioli, Andre Faro, Mohammad Hosen Farzaei, Batool Fatima, Valery Feigin, Andrea B. Feigl, Seyed-Mohammad Fereshtehnejad, Eduarda Fernandes, Joao Fernandes, Manuela Ferreira, Irina Filip, Jonas D. Finger, Florian Fischer, Nataliya Foigt, Takeshi Fukumoto, Nancy Fullman, Thomas Fürst, João M. Furtado, Neal Futran, Silvano Gallus, Amiran Gamkrelidze, Morsaleh Ganji, Alberto L. García-Basteiro, Tigit Gashaw, Abadi Kahsu Gebre, Amanuel Tesfay Gebremedhin, Merhawi Gebremedhin, Afewerki Gebremeskel, Teklu Gebremichael, Tilayie Feto Gelano, Johanna Geleijnse, Ayele Geleto, Kebede Embaye Gezae, Reza Ghadimi, Khalil Ghasemi Falavarjani, Maryam Ghasemi-Kasman, Mamata Ghimire, Rakesh Ghosh, Paramjit Gill, Tiffany Gill, Richard Gillum, Giorgia Giussani, Elena Gnedovskaya, Srinivas Goli, Philimon Gona, Aloke Gopal, Sameer Gopalani, Ayman Grada, Morgan Grams, Giuseppe Grosso, Harish Gugnani, Yuming Guo, Rahul Gupta, Rajat Das Gupta, Rajeev Gupta, Tanush Gupta, Reyna Alma Gutiérrez, Juanita Haagsma, Tesfa Dejenie Habtewold, Vladimir Hachinski, Nima Hafezi-Nejad, Tekleberhan Beyene Hagos, Dessalegn Haile, Gessessew Bugssa Hailu, Arya Haj-Mirzaian, Randah Hamadeh, Samer Hamidi, Arvin Ha-Mirzaian, Alexis Jeannine Handal, Graeme Hankey, Yuantao Hao, Hilda Harb, Sivadasanpillai Harikrishnan, Josep Maria Haro, Hadi Hassankhani, Hamid Y. Hassen, Rasmus Havmoeller, Caitlin Hawley, Akbar Hedayatizadeh-Omran, Behzad Heibati, Behnam Heidari, Mohsen Heidari, Delia Hendrie, Ileana B. Heredia-Pi, Claudiu Herteliu, Fatemeh Heydarpour, Sousan Heydarpour, Desalegn Hibstu, Tarig Higazi, Esayas Haregot Hilawe, Michael Hole, Enayatollah Homaie Rad, Praveen Hoogar, H Dean Hosgood, Mostafa Hosseini, Mihaela Hostiuc, Sorin Hostiuc, Damian Hoy, Guoqing Hu, John Huang, Nayu Ikeda, Olayinka Ilesanmi, Usman Iqbal, Farhad Islami, Kathryn H. Jacobsen, Nader Jahanmehr, Sudhir Kumar Jain, Mihajlo Jakovljevic, Spencer James, Achala Jayatilleke, Panniyammakal Jeemon, Ravi Prakash Jha, Vivekanand Jha, Jost B. Jonas, Jitendra Jonnagaddala, Zahra Jorjoran Shushtari, Ankur Joshi, Jacek Jozwiak, Mikk Jürisson, Amaha Kahsay, Amaha Kahsay, Rizwan Kalani, Tanuj Kanchan, Chittaranjan Kar, Manoochehr Karami, Behazad Karami Matin, André Karch, Seyed M. Karimi, Amir Kasaeian, Getachew Mullu Kassa, Tesfaye Kassa, Nicholas Kassebaum, Srinivasa Vittal Katikireddi, Zhila Kazemi, Ali Kazemi

Karyani, Adane Kefale, Peter Keiyoro, Andre P Kengne, Chandrasekharan Nair Kesavachandran, Yousef Khader, Morteza Abdullatif Khafaie, Nauman Khalid, Ibrahim Khalil, Gulfaraz Khan, Muhammad Ali Khan, Muhammad Shahzeb Khan, Young-Ho Khang, Mona Khater, Abdullah T. Khoja, Ardeshir Khosravi, Mohammad Hossein Khosravi, Daniel Kiirithio, Daniel Kim, Yun Jin Kim, Ruth Kimokoti, Adnan Kisa, Katarzyna Kissimova-Skarbek, Mika Kivimaki, Luke Knibbs, Ann Kristin Knudsen, Sonali Kochhar, Yoshihiro Kokubo, Tufa Kolola, Jacek Kopec, Parvaiz Koul, Ai Koyanagi, Kewal Krishan, Barthelemy Kuate Defo, G Anil Kumar, Manasi Kumar, Hmwe Kyu, Deepesh P. Lad, Sheetal Lad, Alessandra Lafranconi, Ratilal Lalloo, Tea Lallukka, Faris Lami, Huong Lan Nguyen, Justin Lang, Van Lansingh, Arman Latifi, Jeffrey Lazarus, Paul Lee, Misgan Legesse, Yirga Legesse, James Leigh, Cheru T Leshargie, Janni Leung, Miriam Levi, Sonia Lewycka, Shanshan Li, Yu Liao, Lee-Ling Lim, Shai Linn, Shiwei Liu, Rakesh Lodha, Alan Lopez, Stefan Lorkowski, Paulo Lotufo, Raimundas Lunevicius, Kala M. Mehta, Stefan Ma, Erlyn Rachelle Macarayan, Isis Machado, F Madotto, P A Mahesh, Marek Majdan, Reza Majdzadeh, Azeem Majeed, Reza Malekzadeh, Abdullah Mamun, Mohammad Ali Mansournia, Lorenzo Mantovani, Joemer Maravilla, Wagner Marcenes, Sheila Martins, Francisco Rogerlândio Martins-Melo, Melvin Marzan, Benjamin Massenburg, Manu Mathur, Prashant Mathur, Kunihiro Matsushita, Pallab K Maulik, Mohsen Mazidi, Colm Mcalinden, John Mcgrath, Martin Mckee, Ravi Mehrotra, Varshil Mehta, Toni Meier, Fantahun Mekonnen, Yohannes Adama Melaku, Addisu Melese, Mulugeta Melku, Peter Memiah, Ziad Memish, Walter Mendoza, Desalegn Tadese Mengistu, George Mensah, Gert B.M. Mensink, Seid Tiku Mereta, Atte Meretoja, Tuomo Meretoja, Tomislav Mestrovic, Haftay Berhane Mezgebe, Bartosz Miazgowski, Tomasz Miazgowski, Ted R Miller, Molly Miller-Petrie, Gk Mini, Mojde Mirarefin, Erkin Mirrakhimov, Habtamu Mitiku, Babak Moazen, Moslem Mohammadi, Shafiu Mohammed, Farnam Mohebi, Ali Mokdad, Mariam Molokhia, Fatemeh Momeniha, Lorenzo Monasta, Ghobad Moradi, Maziar Moradi-Lakeh, Mehdi Moradinazar, Paula Moraga, Lidia Morawska, Joana Morgado-Da-Costa, Shane Morrison, Marilita Moschos, Seyyed Meysam Mousavi, Dariush Mozaffarian, Kalayu Brhane Mruts, Achenef Muche, Kindie Fentahun Muchie, Ulrich Mueller, Satinath Mukhopadhyay, Kamarul Imran Musa, Ghulam Mustafa, Ashraf Nabhan, Mohsen Naghavi, Seyed Sina Naghibi Irvani, Aliya Naheed, Azin Nahvijou, Nitish Naik, Farid Najafi, Vinay Nangia, Jobert Richie Nansseu, Bruno Nascimento, Ionut Negoi, Ruxandra Irina Negoi, Subas Neupane, Charels Newton, Josephine Ngunjiri, Emma Nichols, Jing Nie, Nobuo Nishi, Ole F Norheim, Mehdi Noroozi , Bo Norrving, Jean Jacques Noubiap, Hamid Reza Nouri, Malihe Nourollahpour, Mohammad Reza Nowroozi, Dina Nur Anggraini Ningrum, Peter Nyasulu, Richard Ofori-Asenso, Felix Ogbo, In-Hwan Oh, Olanrewaju Oladimeji, Andrew T. Olagunju, Tinuke O. Olagunju, Pedro Olivares, Helen Elizabeth Olsen, Bolajoko Olusanya, Jacob Olusanya, Kanyin Ong, Eyal Oren, Heather Orpana, Alberto Ortiz, Erika Ota, Stanislav Otstavnov, Simon Øverland, Mayowa Owolabi, Rosana Pacella, Abhijit Pakhare, Amir Pakpour, Adrian Pana, Songhomitra Panda-Jonas, Eun-Kee Park, Hadi Parsian, Shanti Patel, Sanghamitra Pati, Snehal Patil, George Patton, Deepak Paudel, Katherine Paulson , Neil Pearce, Alexandre Pereira, David Pereira, Norberto Perico, Huyen Phuc Do, Julian Pillay, Michael Piradov, Meghdad Pirsaheb, Tobias Pischon, Farhad Pishgar, Oleguer Plana-Ripoll, Dietrich Plass, Suzanne Polinder, Kevan Polkinghorne, Maarten Postma, Richie Poulton, Akram Pourshams, Hossein Poustchi, Dorairaj Prabhakaran, Swayam Prakash, Manorama Purwar, Mostafa Qorbani, Hai Quang Pham, Amir Radfar, Anwar Rafay, Alireza Rafiei, Fakher Rahim, Afarin Rahimi-Movaghar, Vafa Rahimi-Movaghar, Mahfuzar Rahman, Muhammad Aziz Rahman, Rajesh Kumar Rai, Fatemeh Rajati, Sasa Rajsic, Usha Ram, Chhabi Lal Ranabhat, Prabhat Ranjan, Goura K Rath, David Laith Rawaf, Salman Rawaf, Kolli Srinath Reddy, Colin D Rehm, Marissa Reitsma, Giuseppe Remuzzi, Andre Renzaho, Serge Resnikoff, Satar Rezaei, Juan A Rivera, Aklilu Abrham Roba, Kedir Teji Roba, Sonia Rodríguez-Ramírez, Leonardo

Roever, Luca Ronfani, Gholamreza Roshandel, Ali Rostami, Gregory Roth, Dietrich Rothenbacher, Ambuj Roy, Enrico Rubagotti, Lesley Rushton, Perminder Sachdev, Basema Saddik, Hosein Safari, Yahya Safari, Roya Safari-Faramani, Mahdi Safdarian, Sare Safi, Saeid Safiri, Rajesh Sagar, Amirhossein Sahebkar, Mohammad Ali Sahraian, Haniye Sadat Sajadi, Nasir Salam, Yahya Salimi, Hamideh Salimzadeh, Joshua A Salomon, Abdallah M. Samy, Juan Sanabria , Maria Dolores Sanchez-Niño, Tania G Sánchez-Pimienta, Taren Sanders, Yingying Sang, Itamar Santos, João Vasco Santos, Milena Santric Milicevic, Bruno Sao Jose, Mayank Sardana, Abdur Razzaque Sarker, Rodrigo Sarmiento-Suárez, Benn Sartorius, Brijesh Sathian, Thirunavukkarasu Sathish, Maheswar Satpathy, Monika Sawhney, Mete Saylan, Mehdi Sayyah, Elke Schaeffner, Maria Inês Schmidt, Ione Schneider, Ben Schöttker, Aletta Schutte, David C Schwebel, Falk Schwendicke, James Scott, Sadaf Sepanlou, Edson Serván-Mori, Seyedmojtaba Seyedmousavi, Hosein Shabaninejad, Azadeh Shafieesabet, Amira Shaheen, Masood Ali Shaikh, Mehran Shams-Beyranvand, Mohammadbagher Shamsi, Heidar Sharafi, Kiomars Sharafi, Mehdi Sharif, Mahdi Sharif-Alhoseini, Hamid Sharifi, Sheikh Mohammed Shariful Islam, Rajesh Sharma, Jun She, Aziz Sheikh, Mika Shigematsu, Min-Jeong Shin, Rahman Shiri, Ivy Shiue, Haitham Shoman, Mark Shrime, Si Si, Soraya Siabani, Tariq Jamal Siddiqi, Inga Dora Sigfusdottir, Rannveig Sigurvinsdottir, Diego Augusto Santos Silva, João Pedro Silva, Jasvinder Singh, Virendra Singh, Dhirendra Narain Sinha, Mekonnen Sisay, Eirini Skiadaresi, Badr Sobaih, Soheila Sobhani, Ranjani Somayaji, Moslem Soofi, Reed Sorensen, Joan B Soriano, Ireneous Soyiri, Luciano Sposato, Chandrashekhar T Sreeramareddy, Nadine Steckling-Muschack, Dan J Stein, Murray Stein, Goran Stevanovic, Leo Stockfelt, Mark Stokes, Lela Sturua, Muawiyyah Babale Sufiyan, Rizwan Suliankatchi Abdulkader, Gerhard Sulo, Bruno Sunguya, Patrick Sur, Bryan L. Sykes, Cassandra Szoeke, Rafael Tabarés-Seisdedos, Takahiro Tabuchi, Santosh Tadakamadla, Mesfin Tadese, Ken Takahashi, Eyasu Tamru , Nikhil Tandon, Nuno Taveira, Arash Tehrani-Banihashemi, Gebre Teklemariam Demoz, Awoke Temesgen, Habtamu Temesgen, Mohamad-Hani Temsah, Omar Temsah, Abdullah Terkawi, Tewodros Tesfa, Belay Tessema, Mebrahtu Teweldemedhin, Kavumpurathu Thankappan, Hue Thi Mai, Nu Thi Truong, Nihal Thomas, Giang Thu Vu, George Thurston, Binyam Tilahun, Taavi Tillmann, Quyen G To, Myriam Tobollik, Marcello Tonelli, Roman Topor-Madry, Miguel Tortajada-Girbés, Mathilde Touvier, Marcos Roberto Tovani-Palone, Jeffrey Towbin, Bach Tran, Khanh Bao Tran, Thomas Truelsen, Lorainne Tudor Car, E Murat Tuzcu, Stefanos Tyrovolas, Kingsley Nnanna Ukwaja, Irfan Ullah, Muhammad Shariq Usman, Olalekan A Uthman, Muthiah Vaduganathan, Afsane Vaezi, Pascual Valdez, Santosh Varughese, Tommi Vasankari, Narayanaswamy Venketasubramanian, Santos Villafaina, Francesco S Violante, Sergey Vladimirov, Vasiliy Vlassov, Stein Emil Vollset, Kia Vosoughi, Isidora Vujcic, Fasil Wagnew, Shishay Wahdey, Yasir Waheed, Stephen Waller, Judd Walson, Yafeng Wang, Yuan-Pang Wang, Elisabete Weiderpass, Robert Weintraub, Inbal Weiss Salz, Fitsum Weldegebreal, Andrea Werdecker, Adhena A. Werkneh, Ronny Westerman, Tissa Wijeratne, Andrea Winkler, Alison B. Wiyeh, Charles Shey Wiysonge, Charles Wolfe, Denis Xavier, Gelin Xu, Ali Yadollahpour, Tomohide Yamada, Lijing Yan, Yuichiro Yano, Mehdi Yaseri, Yasin Jemal Yasin, Alex Yeshaneh, Ebrahim M. Yimer, Engida Yisma, Naohiro Yonemoto, Seok-Jun Yoon, Marcel Yotebieng, Mustafa Younis, Mahmoud Yousefifard, Chuanhua Yu, Zoubida Zaidi, Sojib Bin Zaman, Mohammad Zamani, Olifan Zewdie, Anthony Lin Zhang, Hao Zhang, and Kai Zhang.

## Drafting the work or revising is critically for important intellectual content

Cristiana Abbafati, Nooshin Abbasi, Olatunji Adetokunboh, Mohsen Afarideh , Sutapa Agrawal, Muktar Ahmed, Mohammad Esmail Akbari, Tomi Akinyemiju, Fares Alahdab, Khurshid Alam, Syed Aljunid, Mamusha Aman, Jason Anderson, Josep M Antó, Marcel Ausloos, Peter Azzopardi, Nam Ba Nguyen, Hamid Badali, Shahrzad Bazargan-Hejazi, Yihalem Abebe Belay, Neeraj Bhala, Oliver Brady, Michael

Brauer, Alessandra C Goulart, Franz Castro, Kate Causey, Devasahayam Christopher, Aaron Cohen, Alemneh Kabeta Daba, Ahmad Daryani, Dragos Davitoiu, Jan-Walter De Neve, Selina Deiparine, Getenet Dessie, David Teye Doku, David Edvardsson, Holly Erskine, Sadaf Esteghamati, Andre Faro, Mohammad Hosen Farzaei, Seyed-Mohammad Fereshtehnejad, Joao Fernandes, Nataliya Foigt, Takeshi Fukumoto, Morsaleh Ganji, Segen Gebremeskel, Kebede Embaye Gezae, Maryam Ghasemi-Kasman, Mamata Ghimire, Rakesh Ghosh, Richard Gillum, Rajat Das Gupta, Alexis Jeannine Handal, Hadi Hassankhani, Rasmus Havmoeller, Behzad Heibati, Behnam Heidari, Claudiu Herteliu, Enayatollah Homaie Rad, Farhad Islami, Ravi Prakash Jha, Mikk Jürisson, Manoochehr Karami, Nicholas Kassebaum, Chandrasekharan Nair Kesavachandran, Morteza Abdullatif Khafaie, Behzad Khafaie , Alireza Khajavi, Gulfaraz Khan, Mona Khater, Yun Jin Kim, Mika Kivimaki, Huong Lan Nguyen, Samantha Larson, James Leigh, Alan Lopez, Azeem Majeed, Mohammad Ali Mansournia, Joemer Maravilla, Ashley Marks, Randall V. Martin, Francisco Rogerlândio Martins-Melo, Melvin Marzan, Benjamin Massenburg, Colm Mcalinden, Fantahun Mekonnen, Tuomo Meretoja, Bahram Mohajer, Karzan Mohammad, Ghobad Moradi, Mehdi Moradinazar, Ghulam Mustafa, Nahid Neamati, Molly Nixon, Olanrewaju Oladimeji, Andrew T. Olagunju, Tinuke O. Olagunju, Bolajoko Olusanya, Jacob Olusanya, Kanyin Ong, Simon Øverland, Konrad Pesudovs, Michael R Phillips, Huyen Phuc Do, Julian Pillay, Richie Poulton, Hai Quang Pham, Alireza Rafiei, Vafa Rahimi-Movaghar, Fatemeh Rajati, Goura K Rath, Marissa Reitsma, Leonardo Roever, Gregory Roth, Saeid Safiri, Juan Sanabria , Damian Santomauro, Milena Santric Milicevic, Bruno Sao Jose, Abdur Razzaque Sarker, Shahabeddin Sarvi, Maheswar Satpathy, James Scott, Seyedmojtaba Seyedmousavi, Mehdi Sharif, Mika Shigematsu, Inga Dora Sigfusdottir, Rannveig Sigurvinsdottir, Diego Augusto Santos Silva, Dhirendra Narain Sinha, Eirini Skiadaresi, Jeff Stanaway, Patrick Sur, Nuno Taveira, Hue Thi Mai, Nu Thi Truong, Hannah J Thomas, Nihal Thomas, Giang Thu Vu, Marcos Roberto Tovani-Palone, Bach Tran, Thomas Truelsen, Olalekan A Uthman, Yuan-Pang Wang, and Robert Weintraub.

### Extracting, cleaning, or cataloging data; designing or coding figures and tables

Hedayat Abbastabar, Olatunji Adetokunboh, Muktar Ahmed, Miloud Taki Eddine Aichour, Khurshid Alam, Komal Ali, Jason Anderson, Doanl Bisanzio, Rupert Bourne, Ferrán Catalá-López, Kate Causey, Leslie Cornaby, Ahmad Daryani, Getenet Dessie, Aman Endries, Benjamin Er, Holly Erskine, Andre Faro, Kairsten Fay, Giannina Ferrara, Alize Ferrari, Takeshi Fukumoto, William Gardner, Segen Gebremeskel, Johanna Geleijnse, Ayele Geleto, Rakesh Ghosh, Hadi Hassankhani, Mehdi Hosseinzadeh, Nayu Ikeda, Caleb Irvine, Manoochehr Karami, André Karch, Tesfaye Kassa, Nicholas Kassebaum, Norito Kawakami, Andre Keren, Mohammad Hossein Khosravi, Yun Jin Kim, Barthelemy Kuate Defo, Samantha Larson, Misgan Legesse, Janni Leung, Stefan Ma, Reza Malekzadeh, Helena Manguerra, Mohammad Ali Mansournia, Ashley Marks, Melvin Marzan, Mohsen Mazidi, Anoushka Millear, Lorenzo Monasta, Maziar Moradi-Lakeh, Dariush Mozaffarian, Bruno Nascimento, Minh Nguyen, Emma Nichols, Nobuo Nishi, Kanyin Ong, Katherine Paulson , Hossein Poustchi, Caroline Purcell, Jürgen Rehm, Aklilu Abrham Roba, Yesenia Roman, Luca Ronfani, Enrico Rubagotti, Lesley Rushton, Hosein Safari, Saeid Safiri, Devashri Salvi, Abdallah M. Samy, Damian Santomauro, Maheswar Satpathy, Monika Sawhney, Sadaf Sepanlou, Seyedmojtaba Seyedmousavi, Mehdi Sharif, Farhad Shokraneh, Soraya Siabani, Patrick Sur, Eyasu Tamru, Mohammad Tavakkoli, Awoke Temesgen, Hannah J Thomas, Anna Torre, Irfan Ullah, Rachel L. Updike, Vidhya Venkateswaran, Tissa Wijeratne, Simon Yadgir, Ali Yadollahpour, Hossein Yahyazadeh, Mahmoud Yousefifard, and Stephanie R M Zimsen.

## Managing the overall research enterprise

Ashkan Afshin, Tahiya Alam, Brigette F Blacker, Michael Brauer, Deborah Carvalho Malta, Aaron Cohen, Elizabeth Cromwell, Lalit Dandona, Rakhi Dandona, Louisa Degenhardt, Samath Dharmaratne, Charbel El Bcheraoui, Kara Estep, Valery Feigin, Kyle Foreman, Nancy Fullman, Thomas Fürst, Emmanuela Gakidou, Caitlin Hawley, Simon Hay, Spencer James, Nicholas Kassebaum, Ibrahim Khalil, Kristopher Krohn, Hmwe Kyu, Xiaofeng Liang, Stephen Lim, Alan Lopez, Rafael Lozano, Ashley Marks, George Mensah, Molly Miller-Petrie, Awoke Misganaw, Ali Mokdad, Kate Muller, Christopher Murray, Mohsen Naghavi, Molly Nixon, Elaine Nsoesie, Helen Olsen, Robert Reiner, Gregory Roth, Joshua A Salomon, Benn Sartorius, David Smith, Mari Smith, Jeffrey Stanaway, Roman Topor-Madry, Stein Emil Vollset, Theo Vos, Andrea Werdecker, and Harvey Whiteford.

## Did not provide contribution information

Dash A P, Dilaram Acharya, Rufus A. Adedoyin, Amha Admasie, Samiah Alam, Nahla Anber, Jalal Arabloo, Traolach Brugha, Maryam Farvid, Ali Abar Fazaeli, John Ji, Narges Karimi, Mohammad Khazaei, Giancarlo Logroscino, Ana Laura Manda, Mousa Mohammadnia-Afrouzi, Simin Mouodi, Sreebhushan Raju, Sahar Saeedi Moghaddam, Zikria Saleem, Soraya Seedat, Mehdi Shahbazi, Reza Shirkoohi, Michelle Subart, and Paul Yip.

# Section 1: GBD overview

## Section 1.1: Locations of the Analysis

The locations included in Global Burden of Diseases, Injuries, and Risk Factors Study 2017 (GBD 2017) have been arranged into a set of hierarchical categories composed of seven super-regions and a further nested set of 21 regions containing 195 countries and territories. The locations for which GBD estimated global, regional, and national risk exposure, relative risk, theoretical minimum-risk exposure level (TMREL), and population attributable fractions (PAFs), have not expanded following GBD 2015. Subnational estimation in GBD 2017 includes Brazil, China, India, Indonesia, Japan, Kenya, Mexico, South Africa, Sweden, the United Kingdom, and the United States, and new subnational assessments at the administrative one level for Ethiopia, Iran, Norway, and Russia and by Maori ethnicity for New Zealand. For this publication, we present subnational estimates in figures only for all subnational countries with the exception of the new assessments, which will be reported in separate publications. Select subnational estimates are also included in supplementary results appendix. Combined, there are a total of 390 locations at the first subnational unit level. Included in subnational Level 1 locations are countries that have been subdivided into the first subnational level, such as states or provinces, for the GBD analysis; subnational Level 2 only applies to India, England, and Russia. For this paper, we present data at the national and territory level.

## Section 1.2: Time Period of the Analysis

A complete set of risk-specific exposure, relative risk, TMREL, and PAFs were computed for the years 1990-2017. All GBD 2017 results and online data visualisations are available at http://vizhub.healthdata.org/gbd-compare with access to results for all GBD metrics.

## Section 1.3: Statement of GATHER Compliance

This study complies with the Guidelines for Accurate and Transparent Health Estimates Reporting (GATHER) recommendations. We have documented the steps involved in our analytical procedures and detailed the data sources used. See Appendix Table 1 for the GATHER checklist.

The GATHER recommendations may be found here: http://gather-statement.org/

## Section 1.4: GBD risk factor hierarchy

In this analysis, we focus on three groups of risk factors: behavioural, environmental and occupational, and metabolic. The GBD 2017 risk factors hierarchy and Levels are summarised in Appendix Table 2.

The GBD risk list continues to evolve to reflect the policy relevance, public health, and medical care importance of major risk factors. The risk factors list expanded following feedback from GBD 2013 and input from GBD 2015 collaborators. Three risks were added to the list for GBD 2017.

## Section 1.5: List of abbreviations

APCSC: Asia-Pacific Cohort Studies Collaboration

BMI: body-mass index

BMD: bone mineral density

CKD: chronic kidney disease

COD: causes of death

CODEm: cause of death ensemble modelling

COPD: chronic obstructive pulmonary disease

CSA: childhood sexual abuse

CSMR: cause-specific mortality rate

CRA: comparative risk assessment

CVD: cardiovascular disease

DALY: disability-adjusted life-year

DHS: Demographic and Health Survey

DRI: data representativeness index

EMR: excess mortality rate

FAO: Food and Agriculture Organization

FPG: fasting plasma glucose

GATHER: Guidelines for Accurate and Transparent Health Estimates Reporting

GBD: Global Burden of Disease

GoF: goodness of fit

ID: Iron deficiency

IDA: Iron deficiency anaemia

IER: integrated exposure response

IHD: ischemic heart disease

ILO: International Labour Organization

IPV: intimate partner violence

JMP: Joint Monitoring Project

LDI: lag distributed income per capita

LMIC: Low and Middle-Income Countries

LRI: lower respiratory infection

MCMC: Markov Chain Monte Carlo simulations

MDG: Millennium Development Goal

MICS: Multiple Indicator Cluster Surveys

MoM: method of moments

NCD: non-communicable disease

OER: observed-to-expected ratio

PAF: population attributable fraction

PDF: probability density function

$PM_{2.5}$: particulate matter <2.5 μm in diameter

PSC: Prospective Cohort Study

RCT: randomised controlled trial

REDCap: Research Electronic Data Capture

RMSE: root mean square error

RR: relative risk

SBP: systolic blood pressure

SD: standard deviation

SDG: sustainable development goal

SDI: Socio-demographic Index

SEER: Surveillance, Epidemiology, and End Results Program

SEV: summary exposure value

SIR: smoking impact ratio

SSB: sugar-sweetened beverages

ST-GPR: spatiotemporal Gaussian process regression

TB: tuberculosis

TMREL: theoretical minimum-risk exposure level

TSNA: tobacco-specific nitrosamines

UI: uncertainty interval

WCRF: World Cancer Research Fund

WHO: World Health Organization

YLD: years lived with disability

YLL: years of life lost

## Section 1.6: GBD results overview

Results from the Global Burden of Disease Study (GBD 2017) are now measured in terabytes. Results are available in an interactive data downloading tool on the Global Health Data exchange (GHDx). Data and underlying code used for this analysis will be made publicly available pending manuscript acceptance.

The current version of the data download tool is available in the GHDx and contains core summary results for the GBD 2017: http://ghdx.healthdata.org/gbd-results-tool. The core summary results include deaths, YLLs, years lived with disability (YLDs), and disability-adjusted life-years (DALYs). The GHDx includes data for causes, risks, cause-risk attribution, aetiologies, and impairments.

In the GBD 2017 version, the GHDx tool also contains measures such as prevalence and incidence as well as rate of change data. Data above a certain size cannot be viewed online but can be downloaded. Depending on the size of the download, users may need to enter an email address; a download location will be sent to them when the files are prepared.

## Section 1.7: Data input sources overview

GBD 2017 incorporated a large number and wide variety of input sources to estimate mortality, causes of death and illness, and risk factors for 195 countries and territories from 1990-2017. These input sources are accessible through an interactive citation tool available in the GHDx.

Users can retrieve citations for a specific GBD component, cause or risk, and location by choosing from the available selection boxes. They can then view and access GHDx records for input sources and export a CSV file that includes the GHDx metadata, citations, and information about where the data were used in GBD. Additional metadata for each input source are available through the citation tool, as required by the GATHER statement.

The citation tool is accessible through the GHDx at http://ghdx.healthdata.org/gbd-2016/data-input-sources.

## Section 1.8: Funding Sources

Research reported in this publication was supported by the Bill & Melinda Gates Foundation, the National Institute on Aging of the National Institutes of Health (award P30AG047845), and the National Institute of Mental Health of the National Institutes of Health (award R01MH110163). The content is solely the responsibility of the authors and does not necessarily represent the official views of the Bill & Melinda Gates Foundation or the National Institutes of Health.

# Section 2: Risk factor estimation

## Section 2.1: Overview

The comparative risk assessment (CRA) conceptual framework was developed by Murray and Lopez,[1] who established a causal web of hierarchically organised risks or causes that contribute to health outcomes, which allows for quantification of risks or causes at any Level in the framework. In GBD 2017, as in previous iterations of the GBD study, we evaluated a set of behavioural, environmental and occupational, and metabolic risks, where risk-outcome pairs were included based on evidence rules (see Section 2.2.1). These risks were organised in four hierarchical Levels, where Level 1 represents the overarching categories (behavioural, environmental and occupational, and metabolic) nested within Level 1 risks; Level 2 contains both single risks and risk clusters (such as child and maternal malnutrition); Level 3 contains the disaggregated single risks from within Level 2 risk clusters (such as low birthweight and short gestation); and Level 4 details risks with the most granular disaggregation, such as for specific occupational carcinogens, the subcomponents of child growth failure (stunting, wasting, underweight), and suboptimal breastfeeding (discontinued and non-exclusive breastfeeding). At each level of risk, we evaluated whether risk combinations were additive, multiplicative, or shared common pathways for intervention. This approach allows the quantification of the proportion of risk-attributable burden shared with another risk or combination of risks and the measurement of potential overlaps between behavioural, environmental and occupational, and metabolic risks. To date in the GBD we have not quantified the contribution of other classes of risk factors illustrated in Appendix Table 3. We do provide some insights into the potential magnitude of distal social, cultural, and economic factors through an analysis of the relationship between risk exposures and development measured using the Socio-demographic Index (SDI) (see Appendix Section 2.9).

Two types of risk assessments are possible within the CRA framework: attributable burden and avoidable burden. Attributable burden is the reduction in current disease burden that would have been possible if past population exposure had shifted to an alternative or counterfactual distribution of risk exposure. Avoidable burden is the potential reduction in future disease burden that could be achieved by changing the current distribution of exposure to a counterfactual distribution of exposure. Murray and Lopez identified four types of counterfactual exposure distributions: (1) theoretical minimum risk; (2) plausible minimum risk; (3) feasible minimum risk; and (4) cost-effective minimum risk.[2] The theoretical minimum risk level (TMREL) is the level of risk exposure that minimises risk at the population level, or the level of risk that captures the maximum attributable burden. Other possible forms of risk quantification include plausible minimum risk – which reflects the distribution of risk that is conceivably possible and would minimise population-level risk if achieved – while feasible minimum risk describes the lowest risk distribution that has been attained within a population, and the cost-effective minimum risk is the lowest risk distribution for a population that can be attained in a cost-effective manner. Because no robust set of forecasts for all components of GBD is available, in this study we focus on quantifying attributable burden using the theoretical minimum risk counterfactual distribution. Appendix Table 3 shows the eight possible types of risk quantification within the CRA framework, with the hatched box representing the type of CRA currently undertaken by the GBD study. As per the definition of avoidable burden, risk reversibility would be incorporated into this type of assessment, as it would involve reducing risk to the counterfactual for the index year, given a history of past risk exposure. Given the focus in this study on attributable burden, risk reversibility is not a criteria used in estimation here.

In general, this analysis follows the CRA methods used since GBD 2015.[3] The methods described here provide a high-level overview of the analytical logic with a focus on areas of notable change from the methods employed in GBD 2015. Here we aim to provide sufficient detail on the methodology and overall structure of the estimation process. This study complies with the GATHER recommendations proposed by the World Health Organization (WHO) and others, which include recommendations on documentation of data sources, estimation methods, and statistical analysis (Appendix Table 1).[4]

## Section 2.2: Step 1. Effect size estimation

### Section 2.2.1: Collate relative risk data

#### *Criteria for inclusion of risk-outcome pairs*

In this study, as in GBD 2016, we have included risk-outcome pairs that we have assessed as meeting the World Cancer Research Fund (WCRF) grades of convincing or probable evidence.[5] In this framework, convincing evidence consists of biologically plausible associations between exposure and disease established from multiple epidemiological studies in different populations. Evidentiary studies must be substantial, include prospective observational studies, and where relevant, randomised controlled trials (RCTs) of sufficient size, duration, and quality, and showing consistent effects. Probable evidence is similarly based on epidemiological studies with consistent associations between exposure and disease, but for which shortcomings in the evidence exist, such as insufficient trials (or prospective observational studies) available.

#### *The World Cancer Research Fund grading system*

##### Convincing evidence

Evidence based on epidemiological studies showing consistent associations between exposure and disease, with little or no evidence to the contrary. The available evidence is based on a substantial number of studies including prospective observational studies and where relevant, randomised controlled trials of sufficient size, duration, and quality showing consistent effects. The association should be biologically plausible.

##### Probable evidence

Evidence based on epidemiological studies showing fairly consistent associations between exposure and disease, but for which there are perceived shortcomings in the available evidence or some evidence to the contrary, which precludes a more definite judgment. Shortcomings in the evidence may be any of the following: insufficient duration of trials (or studies); insufficient trials (or studies) available; inadequate sample sizes; or incomplete follow-up. Laboratory evidence is usually supportive. The association should be biologically plausible.

##### Possible evidence

Evidence based mainly on findings from case-control and cross-sectional studies. Insufficient randomised controlled trials, observational studies, or non-randomised controlled trials are available. Evidence based on non-epidemiological studies, such as clinical and laboratory investigations, is supportive. More trials are needed to support the tentative associations, which should be biologically plausible.

### Insufficient evidence

Evidence based on findings of a few studies which are suggestive, but insufficient to establish an association between exposure and disease. Little or no evidence is available from randomised controlled trials. More well designed research is needed to support the tentative association.

### Causal criteria

As in GBD 2015 and 2016, to be more objective, consistent, and transparent in our evaluation of the causal relationship, we summarized epidemiologic evidence supporting causality for each risk-outcome pair (Appendix Table 4 and Appendix Table 5). For each pair, we collected data on the following domains:

| *Domains* | *Description* |
|---|---|
| RCTs of disease endpoint | Number of independent RCTs evaluating the effect of the risk on the disease endpoint |
| | Percent of independent RCTs showing significant effect in the opposite direction |
| | Percent of independent RCTs showing no effect |
| Prospective observational studies of disease endpoint | Number of independent prospective observational studies evaluating the association of the risk with the disease endpoint |
| | Percent of independent prospective observational studies with significant association in the opposite direction |
| Strength | Lower Limit of relative risk (RR) in observational studies> 1.5 (Yes/No) |
| Dose response | Evidence of the dose-response relationship between the risk and the outcome (Yes/No) |
| Biologic plausibility | Potential biologic mechanism that could explain the effect of the risk on the disease endpoint (Yes/No) |
| Analogy | Evidence on the relationship between the risk factor and a disease endpoint from the same category (Yes/No) |

For risk-outcome pairs with less than five prospective studies, we summarized evidence from case-control studies as well including (a) the number of independent case-control studies evaluating the association of the risk with the disease endpoint and (b) percent of independent case control studies with significant association in the opposite direction.

---

## Section 2.2.2: Determine relative risks

### *Effect size estimation*

The relative risk by level of exposure, or by cause, for mortality or morbidity can be found in published and unpublished primary studies or in secondary studies that summarize relative risks. In Step 1a of the analytical process (Appendix Figure 1), we collated information from randomised controlled trials, cohort, pooled cohort, and case-control studies, and in Step 1b, used these data to determine the relative risk for the risk-outcome pairs included in GBD 2017 (Appendix Table 6). For most risks, data from pooled cohorts, or meta-analyses of cohorts, were used; in the case of the risk of cataracts from household air pollution, cohort data were not available, and instead we used case-control data. We estimated relative risks of mortality and morbidity for 65 risk factors for which we determined attributable burden using relative risk and exposure. We incorporated relative risks from studies that controlled for confounding but not for factors along the causal pathway between exposure and

outcome. For risk-outcome pairs with evidence available for only one of mortality or morbidity, we generally assumed that the estimated relative risks applied equally to both. Given evidence of statistically different relative risks for mortality and morbidity, we incorporated different relative risks for each. We did not find that relative risks were consistently higher or lower for mortality compared with morbidity. Details and citation information for the data sources used for relative risks are provided in searchable form through a new web-tool (http://ghdx.healthdata.org/). Available data sources for determining relative risks varied across risks. Details on how relative risks were calculated for each risk can be found in Appendix Section 4: Risk-specific estimates.

For all outcomes related to unsafe sex, the relative risk and exposure framework was not used to estimate attributable burden. For unsafe sex and HIV, we used a direct attribution approach to address the lack of data on unsafe sexual practices in most populations. The proportion of HIV attributable to unsafe sex was modelled directly using DisMod-MR 2.1 from data on the fraction of cases identified as being through sexual transmission, intravenous drug use, or blood transfusion.

For risks estimated from a continuous exposure distribution where the effect size was reported by categories in pooled or meta-analysis studies, we converted those categories to relative risk per unit increase in exposure. This implies a linear increase in the log of the relative risk and exposure; various studies have suggested this is a reasonable approximation of the dose-response curve for many risks. An example of this is high systolic blood pressure, where data from the Prospective Cohort Study (PSC) and the Asia-Pacific Cohort Studies Collaboration (APCSC) were well-described by a linear increase in the logarithm of the relative risk by a 10-unit increase in high systolic blood pressure. This approximately log-linear relationship suggests that the proportional difference in the age-specific risk of stroke death associated with a given absolute difference in exposure is about the same at all levels of risk. Many meta-analyses convert relative risks to per unit increase for convenience, particularly when studies choose different categories that could not otherwise be compared. The log-linear approximation appears plausible[6] even where there is limited consensus on the appropriate TMREL. Where there were insufficient samples in the primary studies at high levels of exposure to inform the shape of the tail of the distribution, we applied a cap to the maximum relative risk using the midpoint of the last category for which a relative risk was reported.

## Section 2.3: Step 2. Exposure estimation

### Section 2.3.1: Collate exposure data

#### *Systematic reviews*

For GBD 2017, we conducted systematic literature reviews for 23 risks. For other risk factors, only a small fraction of the existing data appears in the published literature and other sources predominate such as survey data and satellite data. Data were systematically screened from household surveys archived in the Global Health Data Exchange (ghdx.healthdata.org), including Demographic and Health Surveys, Multiple Indicator Cluster Surveys, Living Standards Measurement Surveys, and Reproductive Health Surveys. Other national health surveys were identified based on survey series that had yielded usable data for past rounds of GBD, sources suggested to us by in-country collaborators, and surveys identified in major multinational survey data catalogues, such as the International Household Survey Network and the WHO Central Data Catalog, as well as through country Ministry of Health and Central Statistical Office websites. Citations for all data sources used for risk factor estimation in GBD 2017 are

provided in searchable form through a web-tool (http://ghdx.healthdata.org/). A description of the search terms employed for risk-specific systematic reviews are detailed by cause in Appendix Section 4.

Information on systematic reviews were managed using Research Electronic Data Capture (REDCap) electronic data capture tools hosted at the University of Washington.[7] REDCap is a secure, web-based application designed to support data capture for research studies, providing 1) an intuitive interface for validated data entry; 2) audit trails for tracking data manipulation and export procedures; 3) automated export procedures for seamless data downloads to common statistical packages; and 4) procedures for importing data from external sources

#### *Search terms*

Search terms for updates of systematic reviews for GBD 2017 are shown by risk in Appendix Section 4.

#### *Survey data preparation*

For GBD 2017, survey data constitutes a substantial part of the underlying data used in the estimation process. During extraction, we concentrate on demographic variables (such as location, gender, age), survey design variables (such as sampling strategy and sampling weights), and the variables used to define the population estimate (such a prevalence or a proportion) and a measure of uncertainty (standard error, confidence interval or sample size and number of cases).

### Section 2.3.2: 2b. Adjust exposure data

A number of adjustments were applied to extracted exposure sources in order to make the data more consistent and suitable for modelling. Commonly applied adjustments included age-sex splitting, adding study-level covariates, and bias correction. Age-sex splitting was applied to literature data reported by age or sex but not by age and sex assuring that the total number of cases remained as reported. If a source did not report sample size by age or sex, we applied the age-sex distribution of the population for the same location and year to the reported total sample size. We relied on the metaregression component of DisMod-MR 2.1 for most of the bias correction of data for variations in study attributes such as case definitions and measurement method. DisMod-MR 2.1 calculates a single adjustment that is applied regardless of age, sex, or location. If enough data were available to differentiate these adjustments by age, sex, or location, or if detailed survey data were available to make more precise adjustments between different thresholds on a biochemical measure, we applied bias corrections to the data before entry into DisMod-MR 2.1.

### Section 2.3.3: 2c. Estimate exposure

#### *Mean exposure estimation*

In Step 2a of the estimation process, we used systematic literature reviews to identify risk factor exposure studies published or identified since GBD 2016 and combined these with existing data from household and health examination surveys, census, morbidity, or satellite imagery and ground sensor data (used for estimation of $PM_{2.5}$ [particulate matter <2.5 μm in diameter]). Certain risks, such as diet and alcohol consumption, also incorporated administrative record systems. Data sources used in estimating risk factor exposure can be accessed through the data source tool at http://ghdx.healthdata.org/.

Once data were collected and compiled, step 2b of the analytical flowchart describes the adjustments applied, where necessary, to correct for bias. Examples of these adjustments include: use of urban studies for lead; crosswalks between different measurements, methods, and definitions, such as for self-

report of obesity and glycated haemoglobin (HbA1C) for diabetes; and age-sex splitting of data, such as for fasting plasma glucose, cholesterol, and systolic blood pressure that may be reported from broad age-groups.

For the GBD, we developed two modelling approaches, a Bayesian meta-regression model (DisMod-MR 2.1) and a spatiotemporal Gaussian process regression model (ST-GPR), to pool data from different sources, control and adjust for bias in data, and incorporate other types of information such as country-level covariates. DisMod-MR 2.1 and ST-GPR are mixed effect models that borrow information across age, time, and locations to synthesise multiple data sources into unified estimates of levels and trends. A detailed description of the likelihood used for estimation, and a full description of improvements made for DisMod-MR 2.1, are detailed by Vos and colleagues[8] with additional detail in the appendix to that paper. The ST-GPR model has three main hyper-parameters that control for smoothing across time, age, and location. Values for these hyper-parameters were selected based on cross-validation. Cross-validation tests were conducted for different combinations of the hyper-parameters for three types of models: one data-sparse model, one data-moderate model, and one data-dense model. In each test, 20% of the data were held out and the performance of each combination of hyper-parameters evaluated on the held out data. For each hyper-parameter combination, 10 cross-validation tests were conducted. The performance of each model in predicting the withheld 20% of the data was evaluated using a combined measure based on root mean square error (RMSE) and uncertainty interval coverage. A detailed description of the ST-GPR process regression can be found below in Appendix Section 2.3.3.

The main difference between these methods is their power to include unstructured types of data by sex and age group and in their degree of flexibility. Step 2c in Appendix Figure 1 outlines the use of DisMod-MR 2.1 for 10 risk factors where data were available by different age intervals or mixed sex groups; DisMod-MR 2.1 is the preferred tool in these cases because of its ability to integrate over age and adjust for different exposure definitions in the data; however, the use of Bayesian Markov Chain Monte Carlo (MCMC) simulations with large volumes of data renders the analysis computationally intensive and reduces the number of iterations that are possible. If large volumes of standard age-group data are available – as is generally the case for metabolic risks – using ST-GPR becomes the preferred approach.

In some cases, we adapted our methods of modelling exposure to risks where necessary to account for complexities in the risk-outcome relationship or the need for particular handling of data, for example, dietary risks and ambient air pollution (see Appendix Section 4 for more detail). A complete list of risks and the analytical method used is reported in Appendix Table 2. Additional details for adjustments or adaptations to particular risk models are located in Appendix Section 4.

### *DisMod-MR 2.1 Estimation*

#### DisMod-MR 2.1 description

Until GBD 2010, nonfatal estimates in burden of disease assessments were based on a single data source on prevalence, incidence, remission or a mortality risk selected by the researcher as most relevant to a particular location and time. For GBD 2010, we set a more ambitious goal: to evaluate all available information on a disease that passes a minimum quality standard. That required a different analytical tool that would be able to pool disparate information presented in varying age groupings and from data sources using different methods. The DisMod-MR 1.0 tool used in GBD 2010 evaluated and pooled all available data, adjusted data for systematic bias associated with methods that varied from the reference and produced estimates by world regions with uncertainty intervals using Bayesian statistical methods.

For GBD 2013, the improved DisMod-MR 2.0 had increased computational speed allowing computations that were consistent between all disease parameters at the country rather than region Level. The hundred-fold increase in speed of DisMod-MR 2.0 was partly due to a more efficient rewrite of the code in C++ but also by changing to a model specification using log rates rather than a negative binomial model used in DisMod-MR 1.0. In cross-validation tests, the log rates specification worked as well or better than the negative binomial specification.[9] For GBD 2015, we rewrote the 'wrapper' code that organizes the flow of data and settings at each level of the analytical cascade. The sequence of estimation occurs at five Levels: global, super-region, region, country and, where applicable, subnational location. The super-region priors are generated at the global Level with mixed-effects, nonlinear regression using all available data; the super-region fit, in turn, informs the region fit, and so on down the cascade. The wrapper gives analysts the choice to branch the cascade in terms of time and sex at different levels depending on data density. The default used in most models is to branch by sex after the global fit but to retain all years of data until the lowest Level in the cascade. Appendix Figure 2 summarizes the DisMod-MR process.

In updating the 'wrapper,' we consolidated the code base into a single language, Python, to make the code more transparent and efficient and to better deal with subnational estimation. The computational engine is limited to three levels of random effects; we differentiate estimates at the super-region, region and country Level. In GBD 2013, the subnational units of China, the UK and Mexico were treated as 'countries' such that a random effect was estimated for every location with contributing data. However, the lack of a hierarchy between country and subnational units meant that the fit to country data contributed as much to the estimation of a subnational unit as the fits for all other countries in the region. We found inconsistency between the country fit and the aggregation of subnational estimates when the country's epidemiology varied from the average of the region. Adding an additional level of random effects required a prohibitively comprehensive rewrite of the underlying DisMod-MR engine. Instead, we added a fifth layer to the cascade, with subnational estimation informed by the country fit and country covariates, plus an adjustment based on the average of the residuals between the subnational location's available data and its prior. This mimicked the impact of a random effect on estimates between subnationals.

In GBD 2015, we also improved how country covariates differentiate nonfatal estimates for diseases with sparse data. The coefficients for country covariates are re-estimated at each Level of the cascade. For a given location, country coefficients are calculated using both data and prior information available for that location. In the absence of data, the coefficient of its parent location is used, in order to utilize the predictive power of our covariates in data sparse situations.

For GBD 2016, the computational engine (DisMod-MR 2.1) remained substantively unchanged from GBD 2015. We changed the prediction year set to generate fits for the years 1990, 1995, 2000, 2005, 2010, and 2016. We updated the age prediction sets to include age groups 80-84, 85-89, 90-94, and 95+, to comply with changes across all functional areas of the GBD. We also expanded the set of locations where subnational units are modelled; the set now includes: Brazil, China, England, India, Indonesia, Japan, Kenya, Mexico, Saudi Arabia, South Africa, Sweden, and the United States.

In GBD 2017, we continued to use DisMod-MR 2.1, as there were no substantial changes. Updates to computation include extending the terminal prediction year to 2017 and additional subnational units in

Ethiopia, Iran, New Zealand, Norway, and Russia. Saudi Arabia was also modelled only at the national level in 2017.

The flowchart for the DisMod-MR 2.1 process can be found in Appendix Figure 2.

### DisMod-MR 2.1 likelihood estimation

Analysts have the choice of using a Gaussian, log-Gaussian, Laplace or Log-Laplace likelihood function in DisMod-MR 2.1. The default log-Gaussian equation for the data likelihood is:

$$-log\left[p\left(y_j|\Phi\right)\right] = \log\left(\sqrt{2\pi}\right) + \log\left(\delta_j + s_j\right) + \frac{1}{2}\left(\frac{\log\left(a_j + \eta_j\right) - \log\left(m_j + \eta_j\right)}{\delta_j + s_j}\right)^2$$

where, $y_j$ is a 'measurement value' (i.e., data point); Φ denotes all model random variables; $\eta_j$ is the offset value, eta, for a particular 'integrand' (prevalence, incidence, remission, excess mortality rate, with-condition mortality rate, cause-specific mortality rate, relative risk or standardized mortality ratio) and $a_j$ is the adjusted measurement for data point j, defined by:

$$a_j = e^{(-u_j - c_j)} y_j$$

where $u_j$ is the total 'area effect' (i.e., the sum of the random effects at three Levels of the cascade: super-region, region and country) and $c_j$ is the total covariate effect (i.e., the mean combined fixed effects for sex, study level and country level covariates), defined by:

$$c_j = \sum_{k=0}^{K[I(j)]-1} \beta_{I(j),k} \hat{X}_{k,j}$$

with standard deviation

$$s_j = \sum_{l=0}^{L[I(j)]-1} \zeta_{I(j),l} \hat{Z}_{k,j}$$

where k denotes the mean value of each data point in relation to a covariate (also called x-covariate); I(j) denotes a data point for a particular integrand, j; $\beta_{I(j),k}$ is the multiplier of the $k^{th}$ x-covariate for the $i^{th}$ integrand; $\hat{X}_{k,j}$ is the covariate value corresponding to the data point j for covariate k; l denotes the standard deviation of each data point in relation to a covariate (also called z-covariate); $\zeta_{I(j),k}$ is the multiplier of the $l^{th}$ z-covariate for the $i^{th}$ integrand; and $\delta_j$ is the standard deviation for adjusted measurement j, defined by:

$$\delta_j = log\left[y_j + e^{(-u_j - c_j)}\eta_j + c_j\right] - log\left[y_j + e^{(-u_j - c_j)}\eta_j\right]$$

Where $m_j$ denotes the model for the $j^{th}$ measurement, not counting effects or measurement noise and defined by:

$$m_j = \frac{1}{B(j) - A(j)} \int_{A(j)}^{B(j)} I_j(\mathrm{a})\ \mathrm{da}$$

where A(j) is the lower bound of the age range for a data point; B(j) is the upper bound of the age range for a data point; and $I_j$ denotes the function of age corresponding to the integrand for data point j.

### *Spatiotemporal Gaussian process regression*

Spatiotemporal Gaussian process regression (ST-GPR) has been used for risk factors where the data density is sufficient to estimate a very flexible time trend. The flowchart showing the analytic steps can be found in Appendix Figure 3. The approach is a stochastic modelling technique that is designed to detect signals amidst noisy data. It also serves as a powerful tool for interpolating non-linear trends.[10,11] Unlike classical linear models that assume that the trend underlying data follows a definitive functional form, GPR assumes that the specific trend of interest follows a Gaussian Process, which is defined by a mean function $m(\cdot)$ and a covariance function $Cov(\cdot)$. For example, let $p_{c,a,s,t}$ be the exposure, in normal, log, or logit space, observed in country $c$, for age group $a$, and sex $s$ at time $t$:

$$\left(p_{c,a,s,t}\right) = g_{c,a,s}(t) + \epsilon_{c,a,s,t}$$

where

$$\epsilon_{c,a,s,t} \sim Normal\left(0, \sigma_p^2\right),$$

$$g_{c,a,s}(t) \sim GP\left(m_{c,a,s}(t), Cov\left(g_{c,a,s}(t)\right)\right).$$

The derivation of the mean and covariance functions, $m_{c,a,s}(t)$ and $Cov\left(g_{c,a,s}(t)\right)$, along with a more detailed description of the error variance ($\sigma_p^2$), is described below.

### Estimating mean functions

We estimated mean functions using a two-step approach. To be more specific, $m_{c,a,s}(t)$ can be expressed, depending on the exposure transformation, as:

$$log\left(p_{c,a,s}(t)\right) = X_{c,a,s}\beta + h(r_{c,a,s,t})$$

$$logit\left(p_{c,a,s}(t)\right) = X_{c,a,s}\beta + h(r_{c,a,s,t})$$

$$p_{c,a,s}(t) = X_{c,a,s}\beta + h(r_{c,a,s,t})$$

where $X\beta$ is the summation of the components of a hierarchical mixed-effects linear regression, including the intercept and the product of covariates with their corresponding fixed effect coefficients. Some models were run as hierarchical mixed-effects linear regressions, with random effects on the levels of the geographic hierarchy. For most mixed-effects models, random effects were only used in the fit, not in the prediction. The second part of the equation, $h(r_{c,a,s,t})$, is a smoothing function for the residuals, $r_{c,a,s,t}$, derived from the linear model.[3] Descriptions of exposure transformations and which covariates were used in linear models can be found in Appendix Section 4, which described the risk-specific estimation approaches. Some models used a custom stage-1 estimate – these risks will have detailed information on their mixed-effect estimation process in the risk-specific appendix sections.

While the linear component captures the general trend in exposures over time, much of the data varability may still not be adequately accounted for. To address this, we fit a locally weighted polynomial regression (LOESS) function $h(r_{c,a,s,t})$ to systematically estimate this residual variability by

borrowing strength across time, age, and space patterns (the spatiotemporal component of ST-GPR).[12,13] The time adjustment parameter, defined by $\lambda$ , aims to borrow strength from neighboring time points (i.e. the exposure in this year is highly correlated with exposure in the previous year but less so further back in time). The age adjustment parameter, defined by ω, borrows strength from data in neighboring age groups. The space adjustment parameter, defined by $\xi$, aims to borrow strength across the hierarchy of geographical locations. This year, we further combined the spatial and temporal weights into a single space-time weight, to allow the amount of spatial weight given to a particular point $r_{c,a,s,t}$ to fluctuate given the data availability at each time *t* and location-level *l* in the location hierarchy.

Let $w_{c,a,s,t}$ be the final weight assigned to observation $r_{c,a,s,t}$ with reference to a focal observation $r_{c_0,a_0,s_0,t_0}$. We first generated a temporal weight $t.w_{c,a,s,t}$ for smoothing over time, which was based on the scaled distance along the time dimension of the two observations[14]:

$$t.w_{c,a,s,t} = \frac{1}{e^{\lambda|t-t_0|}}$$

Next, we generated a spatial weight to smooth over geography. Specifically, we defined a geospatial relationship by categorizing data based on the GBD location hierarchy (Appendix Table 7).

In previous GBD iterations, a vector of spatial weights corresponding to each level of the location hierarchy was derived as $[\xi, \xi * (1-\xi)^{n_1-1}, \ldots, \xi * (1-\xi)^{n_i-1}, (1-\xi)^{n_i}]$, where $n_i$, designated the number of location levels in between the given location and the global level and ζ was typically between .7 - .99. Under the previous spatial weighting system, all country datapoints would receive a weight of ζ, all regional datapoints a weight of ζ *(1- ζ), etc, no matter how much data was available in the country compared to the region. For example, if there was only a single datapoint for a given country and ζ was set to .7 , that lone datapoint would receive 70% of the spatial weight.

This year, we reformulated zeta to act as a scalar on a given datapoint given its proximity to the target location:

$$t.w_{c,a,s,t} = \zeta^{|c-c_0|}$$

For example, estimating a country would use the following weighting scheme:

- Country data: $\zeta^0$ = 1
- Regional data not from the country being estimated: $\zeta^1$
- Data from other regions in the same super region: $\xi^2$
- Global data from other super regions: $\zeta^3$

Under the new spatial weighting specification, typical values of ζ range from [.001, .2], where ζ can be interpreted as the amount to downweight regional datapoints compared to country datapoints for a given estimating country. For example, for a given datapoint $r_{c,a,s,t}$ and ζ = .01, a datapoint not within country *c* but within the same region *r* as $r_{c,a,s,t}$ would be assigned $\frac{1}{100}$ the weight of a datapoint within the country.

The spatial and temporal weights were then multiplied and summed across each level of the location hierarchy, and normalized for each time period *t* . This allows the space-time weight to implicitly take

into account the amount of data available at the country vs. region vs super-region level and attribute spatial weight accordingly.

Given a normalization constant,

$$K_i = \sum_{c\epsilon C} s.w_{c,t} * t.w_{c.t} + \sum_{c\epsilon R} s.w_{c,t} * t.w_{c.t} + \sum_{c\epsilon SR} s.w_{c,t} * t.w_{c.t}$$

the final space-time weight would then equal

$$w'_{c,a,s,t} = \frac{s.w_{c,t} * t.w_{c,t}}{K_i}$$

Finally, we calculated the weight $w''_{c,a,s,t}$ to smooth over age, which is based on a distance along the age dimension of two observations. For a point between the age $a$ of the observation $r_{c,a,s,t}$ and a focal observation $r_{c_0,a_0,s_0,t_0}$, the weight is defined as follows:

$$w''_{c,a,s,t} = \frac{1}{e^{\omega|a-a_0|}}$$

The final weights would then be computed by simply multiplying the space-time weights and age weights and normalizing so all weights for a given time period *t* sum to 1. A full derivation of weights for each category follow, assuming the location being estimated was a country, follows:

1) If the observation $r_{c,t}$ belongs to the same country $c_0$ of the focal observation $r_{c_0,t_0}$:

$$w_{c,a,s,t} = \frac{(w'_{c,a,s,t} w''_{c,a,s,t})}{\sum_{c=c_0}(w'_{c,a,s,t} w''_{c,a,s,t})} \qquad \forall c = c_0$$

2) If the observation $r_{c,t}$ belongs to a different country than the focal observation $r_{c_0,t_0}$, but both belong to the same region R:

$$w_{c,a,s,t} = \frac{(w'_{c,a,s,t} w''_{c,a,s,t})}{\sum_{c\neq c_0}(w'_{c,a,s,t} w''_{c,a,s,t})} \qquad \forall c \neq c_0 \cap R[c] = R[c_0]$$

3) If the observation $r_{c,t}$ belongs to the same super region SR but to a both different country $c_0$ and region $R[c_0]$ than the focal observation $r_{c_0,t_0}$:

$$w_{c,a,s,t} = \frac{(w'_{c,a,s,t} w''_{c,a,s,t})}{\sum_{c\neq c_0}(w'_{c,a,s,t} w''_{c,a,s,t})} \qquad \forall c \neq c_0 \cap R[c] \neq R[c_0] \cap SR[c] = SR[c_0]$$

4) If the observation $r_{c,t}$ is from a different super region than the focal observation $r_{c_0,t_0}$(ie. all other data currently not receiving a weight):

$$w_{c,a,s,t} = \frac{(w'_{c,a,s,t} w''_{c,a,s,t})}{\sum_{c\neq c_0}(w'_{c,a,s,t} w''_{c,a,s,t})} \qquad \forall c \neq c_0 \cap R[c] \neq R[c_0] \cap SR[c] \neq SR[c_0]$$

Observations could be downweighted by a factor of 0.1, usually because they were not geographically representative at the unit of estimation. Details of reasons for downweighting can be found in risk-specific modeling summaries. The final weights were then normalized such that the sum of weights across age, time, and geographic hierarchy for a reference group was 1.

### Estimating error variance

$\sigma_p^2$ represents the error variance in normal or transformed space including sampling variance of the estimates and prediction error from any crosswalks performed. First, variance was systematically imputed if the data extraction did not include any measure of uncertainty. When some sample sizes for data were available, missing sample sizes were imputed as the 5th percentile of available sample sizes. Missing variances were then calculated as $\sigma_p^2 = \frac{p*(1-p)}{n}$ for proportions or were predicted from the mean using a regression for continuous values. When sample sizes were entirely missing and could not be imputed, the 95th percentile of available variances at the most granular geographic level (ie, first country, then region, etc.) were used to impute missing variances. For proportions where *p*n* or *(1-p)*n* is < 20, variance was replaced using the Wilson Interval Score method.

Next, if the exposure was modelled as a log transformation, the error variance was transformed into log-space using the delta method approximation as follows,

$$\sigma_p^2 \cong \frac{\sigma_{p'}^2}{p_{c,a,s,t}^2}$$

where $\sigma_{p'}^2$ represents the error variance in normal space. If the exposure was modelled as a logit transformation, the error variance was transformed into logit-space using the delta method approximation as follows,

$$\sigma_p^2 \cong \frac{\sigma_{p'}^2}{(p_{c,a,s,t} * (1 - p_{c,a,s,t}))^2}$$

Finally, prior to GPR, an approximation of non-sampling variance was added to the error variance. Calculations of non-sampling variance were performed on normal-space variances. Non-sampling variance was calculated as the variance of inverse-variance weighted residuals from the space-time estimate at a given location level hierarchy. If there were fewer than 10 data points at a given level of the location hierarchy the non-sampling variance was replaced with that of the next highest geography level with more than 10 data points.

### Estimating the covariance function

The final input into GPR is the covariance function, which defines the shape and distribution of the trends. Here, we have chosen the Matern-Euclidian covariance function, which offers the flexibility to model a wide spectrum of trends with varying degrees of smoothness. The function is defined as follows:

$$M(t,t') = \sigma^2 \frac{2^{1-v}}{\Gamma(v)} \left(\frac{d(t,t')\sqrt{2v}}{l}\right)^v K_v\left(\frac{d(t,t')\sqrt{2v}}{l}\right)$$

where $d(\cdot)$is a distance function; $\sigma^2$, $\nu$, $l$, and $K_\nu$ are hyperparameters of the covariance function—specifically $\sigma^2$ is the marginal variance, $\nu$ is the smoothness parameter that defines the differentiability of the function, $l$ is the length scale, which roughly defines the distance between which two points become uncorrelated, and $K_\nu$ is the Bessel function. We approximated $\sigma^2$ by taking the normalized median absolute deviation $MADN(r_c')$ of the difference  which is the normalized absolute deviation of the difference of the first-stage linear regression estimate from the second-stage spatiotemporal smoothing step for each country. We then took the mean of these country-level MADN estimates for all countries with 10+ country-years of data, to ensure that differences between first- and second-stage estimates had sufficient data to truly convey meaningful information on model uncertainty. We used the parameter specifications $\nu = 2$ for all models. The scale parameter $l$ used for each risk is reported in Appendix Section 4.

### Prediction using GPR

We integrated over $g_{c,t}(t_*)$ to predict a full time series for country $c$, age $a$, sex $s$, and the prediction time $t_*$:

$$p_{c,a,s}(t_*) \sim N\left(m_{c,a,s,t}(t_*), \sigma_p^2 I + Cov\left(g_{c,a,s,t}(t_*)\right)\right)$$

Random draws of 1000 samples were obtained from the distributions above for every country for a given indicator. The final estimated mean for each country was the mean of the draws. In addition, 95% uncertainty intervals were calculated by taking the 2.5 and 97.5 percentile of the sample distribution. The linear modelling process was implemented using the lmer4 package in R, and the ST-GPR analysis was implemented through the PyMC2 package in Python.

### Subnational Scaling and Aggregation

To ensure internal consistency of the estimates between countries and their respective subnational locations, national estimates were either created by population-weighted aggregation or subnational estimates were adjusted by population-weighted scaling to the national estimates, depending on the data coverage of a given country compared to that of its subnational locations. For example, if there was better data coverage at the national level, relative to its corresponding subnational locations, for a given country and risk across age, sex, and time, estimates were rescaled to be consistent with the national level. Conversely, if there was better data coverage at the subnational level, estimates for its parent country were generated through population-weighted aggregation of subnational estimates.

This GBD iteration, we incorporated an option to scale estimates within logit space. Scaling in logit space ensures that subnational estimates of proportion models will not exceed one after being rescaled to the national estimate.

### *Fitting a distribution to exposure data*

The most informative data describing the distribution of risk factors within a population come from individual-level data; additional sources of data include reported means and variances. In cases when a risk factor also defines a disease or disease severity cut-off, such as haemoglobin level and mild, moderate, or severe anaemia or diabetes and fasting plasma glucose, the prevalence of disease is also frequently reported. To model the distribution of any particular risk factor, we seek a family of probability density functions (PDFs), a fitting method, and a model selection criterion. To make use of the most commonly available data describing most populations, we used the method of moments

(MoM); the first two empirical moments from a population, the mean and variance, were used to determine the parameters of two-parameter probability distribution families (PDF) describing the distribution of risk within any population. Exceptions to this rule are justified by context. We used the Kolmogorov-Smirnov[15] (KS) test to measure the goodness of fit (GoF), comparing the distance between the empirical and ensemble distributions, but in some cases, the GoF was based on the prediction error for the prevalence of disease.

We used an ensemble technique in which a model selection algorithm is used to choose the best model for each continuous risk factor.[15] We drew the initial set of candidate models from commonly used PDF families, including both right-skewed and left-skewed distributions. These included: beta, exponential, gamma, gumbel, inverse gamma, inverse Weibull, log-logistic, lognormal, mirrored gamma, mirrored gumbel, normal, and Weibull. We fitted each PDF candidate family to each dataset using the MoM, and used the KS test as the measure of GoF. Preliminary analysis showed that the GoF ranking of PDF families varied across datasets for any particular risk factor and that combining the predictions of differently fitted PDF families could dramatically improve the GoF for each dataset. Therefore, we developed a new model for prediction using the ensemble of candidate models, which is a weighted linear combination of all candidate models, $\{f\}$, where a set of weights $\{w\}$ is chosen such that $\sum_i w_i = 1$, and the values of the weights were determined by a second GoF criterion with its own validation process. For each risk, we pooled all available microdata and performed Nelder-Mead numeric optimisation across demographics subsets of data to derive a set of distribution-specific weights such that the average KS statistic across data sets is minimised. The details can be summarised by 1) the summary statistics for each dataset; 2) a table showing the KS statistic for each candidate model; and 3) the weights defining the final ensemble model for each dataset. We then averaged across demographic subsets and data sets to determine the final weights for modelling the distribution of any particular risk factor.

## Section 2.4: Step 3. TMREL

In this and all previous GBD studies, the counterfactual level of risk exposure used is the risk exposure that is both theoretically possible and minimizes risk in the exposed population that consequently captures the maximum population attributable burden.[2] For each risk evaluated in GBD 2017, Step 4 of the analytical flowchart describes the use of the best available epidemiological evidence from published and unpublished relative risks by level of exposure and the lowest observed level of exposure from cohorts, used to select a single level of risk exposure that minimises risk from all causes of DALYs combined to establish the TMREL. In principle, the TMREL for a given risk may vary by age, sex, and location if supported by clear evidence. Based on the available evidence, the TMREL itself can be uncertain, which is reflected in the 95% uncertainty intervals (UIs) in Appendix Table 6. An estimation of uncertainty was derived by resampling from a uniform distribution of TMRELs where evidence supporting the selection of the TMREL was uncertain (for example, elevated systolic blood pressure or cholesterol).

## Section 2.5: Step 4. Estimate population attributable fractions

Risks are categorised on the basis of how exposure was measured: dichotomous, polytomous, and continuous. High low-density lipoprotein (LDL) cholesterol is an example of a risk measured on a continuous scale. The population attributable fraction (PAF), which represents the proportion of risk

that would be reduced in a given year if the exposure to a risk factor in the past were reduced to an ideal exposure scenario, is defined for a continuous risk factor as:[16]

$$PAF_{joasgt} = \frac{\int_{x=l}^{u} RR_{joasg}(x)P_{jasgt}(x)dx - RR_{joasg}(TMREL_{jas})}{\int_{x=l}^{u} RR_{joasg}(x)P_{jasgt}(x)dx}$$

Where $PAF_{joasgt}$ is the population attributable fraction for cause $o$ due to risk factor $j$ for age group $a$, sex $s$, location $g$, and year $t$. $RR_{joasg}(x)$ is the relative risk as a function of exposure level $x$ for risk factor $j$ for cause $o$, age group $a$, sex $s$, and location $g$ with the lowest level of observed exposure as $l$ and the highest as $u$; $P_{jasgt}(x)$ is the distribution of exposure at $x$ for age group $a$, sex $s$, location $g$, and year $t$; $TMREL_{jas}$ is the TMREL for risk factor $j$, age group $a$, and sex $s$.

The $PAF_{joasgt}$ for dichotomous and polytomous risk factors for every country is defined as:

$$PAF_{joasgt} = \frac{\sum_{x=1}^{u} RR_{joast}(x)P_{jasgt}(x) - RR_{joasg}(TMRE_{jas})}{\sum_{x=1}^{u} RR_{joas}(x)P_{jasgt}(x)}$$

Where $PAF_{joasgt}$ is the population attributable fraction for cause $o$ due to risk factor $j$ for age group $a$, sex $s$, location $g$, and year $t$. $RR_{joasg}(x)$ is the relative risk as a function of exposure level $x$ for risk factor $j$ for cause $o$, age group $a$, sex $s$, and location $g$ on a plausible range of exposure levels from l to u $P_{jasgt}(x)$ is the proportion of population in risk group (prevalence), for age group $a$, sex $s$, location $g$, and year $t$; $TMREL_{jas}$ is the TMREL for risk factor $j$, age group $a$, and sex $s$.

## Section 2.6: Step 5. Estimate summary exposure values

Summary exposure value (SEV), is the relative risk-weighted prevalence of exposure, a univariate measure of risk-weighted exposure, taking the value zero when no excess risk for a population exists and the value one when the population is at the highest level of risk. We report SEVs on a scale from 0% to 100% where a decline in SEV indicates reduced exposure to a given risk factor and an increase in SEV indicates increased exposure.

We first calculate risk, $r$, and cause, $c$, specific SEVs using the following equation,

$$SEV_{rc} = \frac{\frac{PAF_{rc}}{1 - PAF_{rc}}}{RR_{max} - 1}$$

for each most-detailed age, sex, location, year, and outcome. $PAF$ is the YLL (expect for occupational noise, bullying victimization, and occupational ergonomic factors which are YLD only and thus use the YLD) PAF. $RR_{max}$ for categorical risks is the RR at the highest category of exposure. For continuous risks, this is

$$RR_{max} = RR^{\frac{TMREL - 1^{st} exposure}{RR_{scalar}}} \text{ if protective, or}$$

$$= RR^{\frac{99^{th} exposure - TMREL}{RR_{scalar}}}$$

otherwise, and for custom modelled risks like ambient particulate matter pollution, household air pollution from solid fuels (HAP), alcohol, smoking, bullying, and activity, the modeller provides draws of $RR_{max}$. Generally, RRs do not vary across time and space, however, there are exceptions – an example being those risks such as second-hand smoke (SHS) or HAP where the RR is based on the integrated exposure response [IER], curve) and in these cases the RR is averaged across location and year to ensure no time or space variation. If the PAF is negative, signifying a protective effect for that outcome, the PAF is set to 0 and the SEV is then also 0 as the SEV univariate and constrained to be a value between 0 and 1. Once we have a set of risk cause specific SEVs at the most-detailed risk, cause, age, sex, location for all years, we average across causes to produce the final risk specific $SEV_r$,

$$SEV_r = \frac{1}{N(c)} \sum_c SEV_{rc}$$

## Section 2.7: Step 6. Mediation

### Section 2.7.1: Summary

The portion of the burden of disease that is attributable to various combinations of risk factors or to all risk factors combined has been a topic of broad interest.[17] Assumptions about how one risk factor is mediated through other risk factors are needed in order to estimate the joint risk factor burden for combinations of metabolic risks and behavioural or environmental risks. To accomplish this, in Step 6 of the estimation process, for every two risk factors for an outcome, we estimated the fraction of risk that was mediated through the other risk. This resulted in a matrix of parameters containing each possible pairing of risk factors included in the GBD 2017. Using this matrix, we computed the aggregated burden of disease at each level of the GBD 2017 hierarchy and for all risk factors using the following formula:

$$PAF_{Joasgt} = 1 - \prod_{j=1}^{J} \left( 1 - PAF_{joasgt} \prod_{i=1}^{J} (1 - MF_{jio}) \right) \quad (5)$$

where $J$ is a set of risk factors for the aggregation; $PAF_{joasgt}$ is the PAF for risk $j$ for age group $a$, sex $s$, location $g$, and year $t$; and $MF_{jio}$ is the mediation factor for risk $j$ mediated through $i$ for cause $o$. Mediation factors can be found in Appendix Table 8.

### Section 2.7.2: Additional detail

In GBD 2010, we only aggregated the burden of risk factors for some clusters of risks including access to improved water and sanitation, child and maternal malnutrition, tobacco smoking, alcohol use, dietary risk factors, occupational risk factors, and sexual abuse and violence. We did not aggregate air pollution and metabolic risk factors. For GBD 2013, GBD 2015, GBD 2016, and GBD 2017, we aggregated all risk factors into three large categories: behavioural, environmental and occupational, and metabolic risks -- as well as aggregating all GBD risk factors into a single attributable fraction for each diseases and eventually for all-causes of burden.

Aggregating risk factors at different levels share three essential challenges:

1. Risk factor coexistence or aggregation: for example, metabolic risk factors often occur together or high-risk behaviours are related such as drug abuse and unsafe sex.
2. Mediation: a risk factor may effect another risk factor that lies in the physiological pathway to a disease outcome. It can be inside a cluster of risk factors such as the effect of obesity through an increase in fasting plasma glucose (FPG) and later cardiovascular disease outcomes, or between clusters of risk factors such as the effect of fibre on cholesterol.
3. The formula to calculate the aggregated PAF.

The aggregation method is conceptually applicable to other aggregations such as socioeconomic factors, education, homelessness and refugee status that are being considered for inclusion in future GBD iterations. In the next section, we explain our approach to deal with these challenges.

There are three patterns of associations between risk factors to take into consideration. The first concerns confounding; risk B affects risk A and outcome C (Pattern 1 in *Patterns of associations between risk factors*). In these cases, the relative risk (RR) for A should be adjusted for B, for example, the fruit RR is adjusted for smoking. If part of the effect of A is through B, a mediator, we do not adjust the effect of A for B. For example, we do not adjust the RR of body-mass index (BMI) for cholesterol as cholesterol lies in the biological pathway between BMI and cardiovascular outcomes (Pattern 2 in in *Patterns of associations between risk factors*). The third pattern occurs when risks A and B are proxies of a third variable Z and aggregation aims to estimate the total effect of a latent variable Z, on C. An example is child growth failure, which is measured by stunting, wasting, and underweight as proxies.

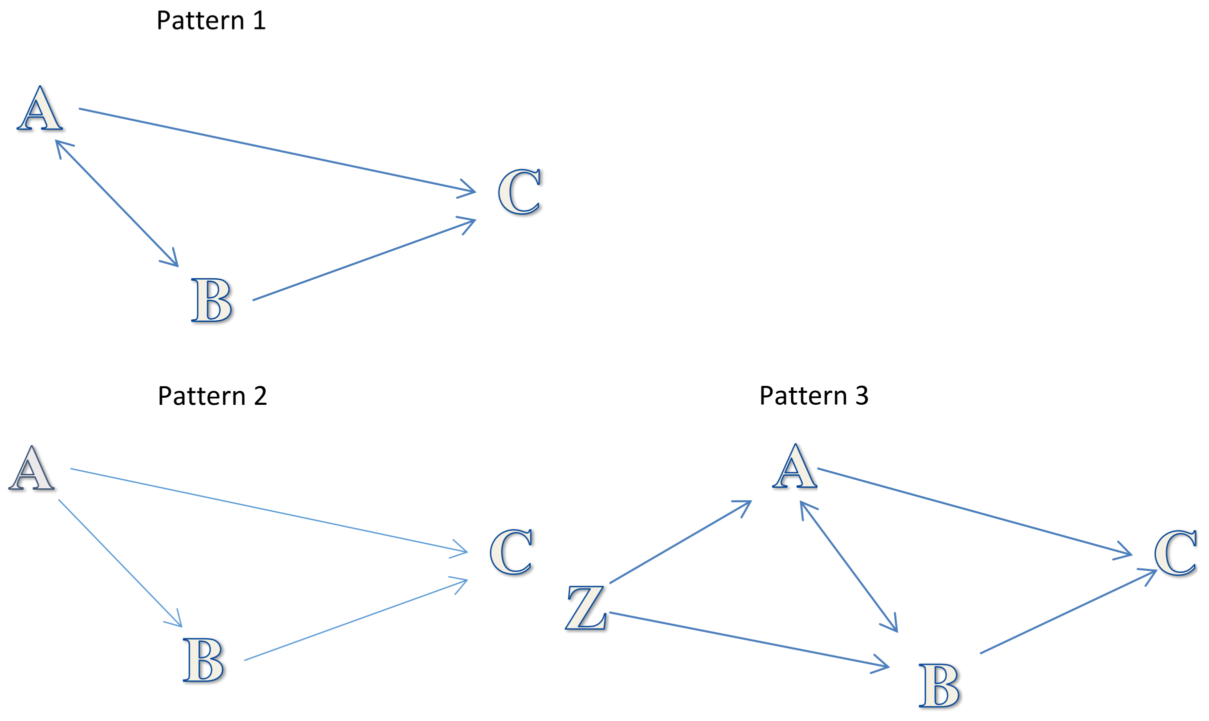


*Patterns of associations between risk factors*

### Section 2.7.3: Calculating burden of multiple risk factors

Validation studies have reported congruency between the true risk associated with multiple risk factors affecting the same outcome and a multiplicative aggregation of the population attributable fractions of the individual risk factors (formula below).[18]

$$PAF_{1..i} = 1 - \prod_{i=1}^{n}(1 - PAF_i)$$

Where *PAF* is the population attributable fraction and *i* is each individual risk factor. The same validation studies also found that the overestimation from ignoring the covariance between risk factors is small. This was important to note as there are few data sources from which we can draw information on covariance.

We endeavoured to evaluate RRs that were controlled for confounders. However, as we had to rely on the literature for many RRs we did not always have full control over the choice of confounders controlled for in each study.

### Section 2.7.4: Adjusting for mediation

When aggregating the effects of multiple risk factors, we included a mediation factor if a part of the effect of one risk factor was included in the effect estimated for in the mediator. First, we prepared a list of possible mediations especially between behavioural risks and metabolic risk factors with cardiometabolic outcomes. We did not assume any mediation effect between risk factors for cancers.

Danaei and colleagues assumed that part of the effect of BMI on ischemic heart disease (IHD) is through high systolic blood pressure (SBP), cholesterol and FPG.[19] The proportion of the BMI effect that can be explained by other metabolic risk factors is the amount of mediation. The difference between the crude RR of BMI on IHD with the RR adjusted for SBP, FPG, and cholesterol reflects the amount of BMI effect on IHD that is mediated and already included in SBP, FPG, and cholesterol:

$$MF = \frac{RR_{crude} - RR_{adjusted}}{RR_{crude} - 1}$$

We used this approach for estimating mediation factors to adjust PAFs before aggregation.

$$MF = \frac{R_c^+ - R_a^+}{R_c^+ - R_c^-}$$

$$\text{So: } R_a^+ = R_c^+ - MF * (R_c^+ - R_c^-)$$

$$PAF_c = \frac{p * (R_c^+ - R_c^-)}{p * R_c^+ + (1-p) * R_c^-} = \frac{p * (R_c^+ - R_c^-)}{R_T}$$

If $R_c^+$: crude risk of outcome in exposed population

$R_c^-$: crude risk of outcome in non-exposed population

$R_a^+$: adjusted risk of outcome in exposed population

$R_a^-$: adjusted risk of outcome in non-exposed population

$R_T$ is the overall rate of the outcome in the population. Since we are interested in the part which is from BMI but through cholesterol, the total risk in the population will be the same for the adjusted RR, so the unmediated part of the risk factor would be:

$$PAF_a = \frac{p*(R_a^+ - R_a^-)}{R_T} = \frac{p*(R_c^+ - MF*(R_c^+ - R_c^-) - R_c^-)}{R_T} = \frac{p*(R_c^+ - R_c^-)*(1-MF)}{R_T} = PAF_c * (1 - MF)$$

So for aggregating the PAF of multiple risk factors, we first calculated the part of the effect of every risk factor that is not mediated and then aggregated these assuming they are independent.

Therefore the aggregated PAF would be:

If MF is mediation factor of R2 through R1:

$$PAF_{1,2} = 1 - (1 - PAF_1) * (1 - PAF_2 * (1 - MF_{2/1}))$$

and a generalization for multiple pathways of R1 through other RFs:

$$PAF_{1..i} = 1 - \prod_{i=1}^{n} \left( 1 - PAF_i * \left( 1 - \prod_{j=1}^{n} (1 - MF_{i/j}) \right) \right)$$

For every risk factor outcome pair, the matrix of possible mediations was calculated and used.

## Section 2.7.5: Calculating mediation factor

### *1 – Comparing crude RR versus mediator-adjusted RR*

The best example is the mediation of BMI through SBP, FPG, and cholesterol reported by Danaei et al.[19] In their meta-analysis, they report the adjusted and unadjusted RR of BMI on IHD and stroke based on combined data from individual cohorts. They calculated the mediation factor using the equation below, and we used it directly as mediation factor in risk factor aggregation. Using individual level data from cohort studies, we estimated the mediation factor for other metabolic risk factors and some dietary risks.

$$MF = \frac{RR_{crude} - RR_{adjusted}}{RR_{crude} - 1}$$

### *2 – Estimating the mediation factor by pathway of the effect*

For many other risk factors, there are no data available to use the first method. Instead, we searched studies to estimate the effect of the risk factor on the mediator and finally the expected increase in IHD risk. We pooled available studies to calculate the unit increase in the mediator per unit increase in the risk factor to calculate the size of the IHD RR.

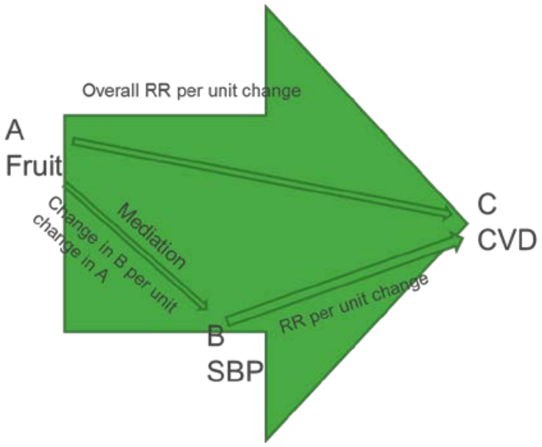


*Example of pathway between fruit, high systolic blood pressure, and cardiovascular diseases*

We have RRs for the effect of A on C and B on C in GBD from a meta-analysis of studies in the literature. The effect of A on B was estimated by analysis of diet trials.

$$RR_{ABC} = RR_{BC}{}^{\Delta_{AB}}$$

$RR_{ABC}$ is expected effect of A through B on C

$RR_{BC}$ is relative risk of each unit increase in mediator on outcome C

$\Delta_{AB}$ is change in mediator level B per each unit change in A

If $RR_{AB}$ is the overall effect of A on B then:

The mediation factor would be

$$MF = \frac{RR_{ABC} - 1}{RR_{AB} - 1}$$

We kept uncertainty of each parameter by generating and following 1000 draws of the estimates to calculate 1000 draws of the posterior distribution of the mediation factor. We did not include risk-mediator pairs if the mediation factor was not significant at 5% level (more than 50 out of 1000 draws were negative). We truncated the mediation factor distribution at 1 where the whole effect of the risk factor on the outcome would be assumed to be through the mediator pathway.

Some mediation factors equal 1 where the whole effect was calculated through other risk factor, e.g. the effect of salt through SBP, or when we assumed other risk factors are sources of the exposure, for example, fibre is provided by consuming fruit, vegetable, and whole grains and all the beneficial effect of milk on colorectal cancer is mediated through calcium.

### Dietary risk factors

For each dietary risk factor, we searched for randomised trials evaluating the effect of the diet component on metabolic risk factors and estimated the change in a given mediator per unit change in the diet component. Only salt outcomes, with the exception of stomach cancer, are mediated through BMI – all other dietary risk factors have their relative risk applied directly.

### Physical activity

We found cohort studies on the effect of physical activity on FPG. The data was more on the effect of physical activity on diabetes incidence, so we calculated the shift in FPG using the provided RR value. We used this to calculate the mediated part of effect of physical activity on cardiovascular disease (CVD).[20–26]

### Air pollution

We considered mediation for particulate matter pollution, but the evidence was not strong enough to justify inclusion of $PM_{2.5}$ on SBP, FPG, or cholesterol. There are two cohort studies which have published findings of increased risk of hypertension due to long-term exposure to ambient $PM_{2.5}$ and several studies which have found elevated SPB due to household solid fuel use. We found time series studies with different $PM_{2.5}$ lag (by day) that show very short-term and confounded effects. We decided to re-examine these outcomes when more evidence becomes available.

### Assumed mediations

For the risk factors with PAFs of 100% such as FPG and diabetes, impaired kidney function and chronic kidney disease, SBP and hypertensive heart disease, alcohol and alcohol use disorders, child underweight and protein-energy malnutrition, and child wasting and protein-energy malnutrition, and drug use and drug use disorders, no mediation is needed.

## Section 2.7.6: Piecewise aggregation (Pattern 3)

There are three anthropometric indicators that are highly correlated: child underweight, stunting, and wasting, as demonstrated in *Venn diagram demonstrating the correlation between child underweight, stunting, and wasting*. Available RRs for each indicator are not adjusted for the other two because there is a high correlation between these indicators and also interaction where the majority of the burden occurs. Estimating the total burden due to childe growth failure, a latent variable, is difficult. The three anthropometric indicators are not independent, so the covariance between them should be considered. This was the main reason that GBD 2010 only included child underweight. If covariance between these indicators is significant (as is shown in the Figure below), aggregating these indicators assuming independence would overestimate the total burden significantly.

To use the best available data, we adjusted observed RRs reported by Olofin et al for underweight, stunting and wasting by simulating the joint distribution of the three indicators using the distribution of each indicator and covariance between indicators in the countries included in the meta-analysis (extracted from Demographic and Health Survey (DHS) micro-data).[27] Based on the analysis done by McDonald et al, we assumed there is an interaction between the three indicators, and extracted the interaction terms from the corresponding analysis.[28] We calculated the adjusted RRs by minimizing the error between observed crude RRs (from meta-analysis) and expected crude RRs derived from adjusted RRs.

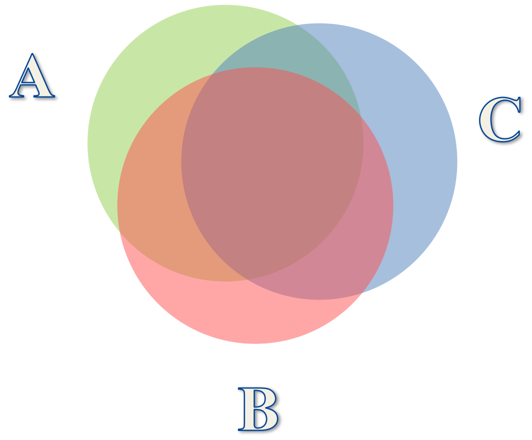


*Venn diagram demonstrating the correlation between child underweight, stunting, and wasting*

After adjusting for the three risk factors, we calculated the PAFs and aggregated underweight, stunting and wasting burden.

### Section 2.7.7: Uncertainty of aggregated and mediated PAFs

We generated 1000 draws of posterior distribution of mediation factor calculated by different methods to use beside draws of other inputs to the PAF aggregation.

### Section 2.7.8: Important assumptions in aggregating risk factors and including mediation

1 – The mediation factors or PAF adjustments are similar across countries, age, sex, and years. While it is quite likely that the size of mediation is different in different populations, there is little data to inform the covariance between different risk factors or the mediation factor amount by age and countries. For example, in some countries, the size of the mediated BMI-IHD PAF through cholesterol, calculated by the mediation factor, was even bigger than the total burden of cholesterol, indicating that less effect of BMI is mediated through cholesterol and mediation factors are not similar across countries.

2 – For many risk-mediator-outcome pairs, there are no data available, so we assumed the mediation is zero.

3 – Since the covariance between undernutrition indicators is different by location (and across time, results were not reported), and there is an interaction between these indicators, the total burden might be underestimated.

4 – It is assumed that there is no significant covariance between PAFs, which might not be true between some risk factors such as between metabolic risk factors. While this overestimation is controlled by using adjusted RRs, using crude RRs for BMI and other metabolic risk factors may cause significant overestimation of aggregated metabolic risks burden.

## Section 2.8: Step 7. Estimate attributable burden

Four key components are included in estimation of the burden attributable to a given risk factor: the metric of burden being assessed (the number of deaths, years of life lost [YLLs], years lived with disability [YLDs], or DALYs [the sum of YLLs and YLDs]); the exposure levels for a risk factor; the relative

risk of a given outcome due to exposure; and the counterfactual level of risk factor exposure. Estimates of attributable burden as DALYs for risk-outcome pairs were generated using the following model:

$$AB_{jasgt} = \sum_{o=1}^{w} DALY_{joasgt}\, PAF_{joasgt}$$

where $AB_{jasgt}$ is the attributable burden for risk factor $j$ for age group $a$, sex $s$, location $g$, and year $t$; $DALY_{joasgt}$ is total DALYs for cause $o$ (of $w$ relevant outcomes for risk factor $j$) for age group $a$, sex $s$, location $g$, and year $t$; $PAF_{joasgt}$ is the population attributable fraction (PAF) for cause $o$ due to risk factor $j$ for age group $a$, sex $s$, location $g$, and year $t$. The proportion of deaths, YLLs, or YLDs attributable to a given risk factor or risk factor cluster were analogously computed by sequentially substituting each metric in place of DALYs in the equation above.

## Section 2.9: Decomposition analysis of deaths and DALYs

We conducted a decomposition analysis of changes in DALYs from 2007 to 2017, decomposing changes in all-age cause-specific DALYs attributable to all risk factors and individual risk factors due to changes in population growth, population age structure, exposure to the given risk for a disease, and risk-deleted death and DALY rates. In this case, risk-deleted rates are the rates after removing the effect of a risk factor or combination of risk factors; in other words, observed DALY rates multiplied by one minus the PAF for the risk or set of risks. Our decomposition analyses draw from methods developed by Das Gupta[29] to provide a computationally tractable solution to isolating drivers of burden changes whereby all combinations of possible pathways are averaged across factors. Attributable burden is determined, following the methods of Das Gupta, as a product of three factors such that:

$$T_{asgt} = \left(A_{asgt}\, B_{asgt}\, C_{asgt}\right)$$

where $T_{asgt}$ represents the attributable burden at year $t$; $A_{sgt}$is the age-specific population size for a given age group $a$, sex $s$ and location $g$ at year $t$; $B_{asgt}$ is the underlying rate of the outcome unrelated to the risk factor or observed rate, multiplied by $1 - PAF$ for a given age group $a$, sex $s$ and location $g$ at year $t$; and where $C_{asgt}$ is the ratio of attributable burden to the underlying rate, which reflects the risk exposure effect for a given age group $a$, sex $s$, and location $g$ at year $t$ defined as $PAF/(1 - PAF)$ in the case of decomposing attributable burden to a risk. Risk exposure effects for individual risk factors are scaled such that they sum to the all risk exposure effect by location, age, sex, and cause accounting for mediation. This allows for aggregation of risks; the exposure for all risks for a disease can be split into exposure to metabolic, behavioural, and environmental risks. The contribution of each factor to total change in attributable burden was determined by changing the level of one factor from time $t_0$ to $t_1$ – here 2007 to 2017 – with all other factors held constant. Thus, the effect of any of the three factors, for example $A_{asgt}$on the change of attributable burden between 2007 ($A_{07}$) and 2017 ($A_{17}$) is calculated as:

$$E_A = (A_{17} - A_{07})\left(\frac{B_{07}C_{07} + B_{17}C_{17}}{3} + \frac{B_{07}C_{17} + B_{17}C_{07}}{6}\right)$$

Where $E_A$ is the proportion of change due to factor $A$, and the subscripts for each factor in the equation denote the year for each estimate. Since the effect depends on the order of entry of the factor, we calculated the average of all combinations of the three factors.[29] The proportion of change due to factor$A_{sgt}$, the age-specific population size for a given age group $a$, sex $s$ and location $g$ at year $t$, is

then further split, setting change in population growth equal to the percent change in all-age population from time $t_0$ to $t_1$ and change in population age structure to the residual, giving four factors.

This three factor decomposition method does not work for risks where the PAF, by definition, is 100% (such as high fasting plasma glucose and diabetes type 2) or where the PAF is directly estimated (such as for unsafe sex and HIV). In the cases of child underweight and protein-energy malnutrition, child wasting and protein-energy malnutrition, short gestation for birth weight and neonatal preterm birth complications, low birth weight for gestation and neonatal preterm birth complications, iron deficiency and iron-deficiency anaemia, alcohol use and liver cancer due to alcohol use, alcohol use and cirrhosis and other chronic liver diseases due to alcohol use, alcohol use and alcohol use disorders, alcohol use and alcoholic cardiomyopathy, drug use and drug use disorders, occupational particulate matter, gases, and fumes and other pneumoconiosis, occupational particulate matter, gases, and fumes and coal workers pneumoconiosis, occupational exposure to asbestos and asbestosis, and occupational exposure to silica and silicosis, we used a two factor decomposition method, which examines the contribution of population, ageing, and risk exposure. Effectively, we assume trends in these cases are driven by exposure, not change in the risk-deleted rates. Conversely, for unsafe sex and sexually transmitted diseases excluding HIV, we used a two-factor decomposition method, which examines the contribution of population, ageing, and risk-deleted death and DALY rates, assuming trends in these cases are driven by risk-deleted rates, not change in exposure. For high fasting plasma glucose and diabetes mellitus type 1 and 2, high fasting plasma glucose and chronic kidney disease due to diabetes mellitus type 1 and 2, high systolic blood pressure and hypertensive heart disease, high systolic blood pressure and chronic kidney disease due to hypertension, and impaired kidney function and chronic kidney disease, we used GBD estimates of Summary Exposure Values (SEVs) for the given risk and the case-fatality rate decompose trends into the contribution of the three factors. Similarly, for unsafe sex and cervical cancer, we used GBD estimates of the incidence of cervical cancer and the case-fatality rate to decompose trends into the contribution of the three factors. For unsafe sex and HIV, we used spectrum counterfactual and CD4 risk-weighted prevalence.

## Section 2.10: SDI Analysis

### Section 2.10.1:Development of SDI

The Socio-demographic Index (SDI) is a composite indicator of development status strongly correlated with health outcomes. In short, it is the geometric mean of 0 to 1 indices of total fertility rate under the age of 25 (TFU25), mean education for those aged 15 and older (EDU15+), and lag distributed income (LDI) per capita.

### Section 2.10.2:Development of revised SDI indicator

SDI was originally constructed for GBD 2015 using the Human Development Index (HDI) methodology, wherein a 0 to 1 index value was determined for each of the original three covariate inputs (total fertility rate in ages 15 to 49, EDU15+, and LDI per capita) using the observed minima and maxima over the estimation period to set the scales.

In response to feedback from collaborators and the evolution of the GBD, we have refined the indicator with each GBD cycle. For GBD 2017, in conjunction with our expanded estimation of age-specific fertility, we chose to replace the total fertility rate as one of the three component indices with the total fertility rate under 25 (TFU25). The TFU25 provides a better measure of women’s status in society, as it focuses on ages where childbearing disrupts the pursuit of education and entrance into the workforce.

During GBD 2016, we moved from using relative index scales to absolute scales to enhance the stability of SDI's interpretation over time, as we noticed that the measure was highly sensitive to the addition of subnational units that tended to stretch the empirical minima and maxima. We selected the minima and maxima of the scales by examining the relationships each of the inputs had with life expectancy at birth and under-5 mortality and identifying points of limiting returns at both high and low values, if they occurred prior to theoretical limits (e.g., a TFU25 of 0).

Thus, an index score of 0 represents the minimum level of each covariate input past which selected health outcomes can get no worse, while an index score of 1 represents the maximum level of each covariate input past which selected health outcomes cease to improve. As a composite, a location with an SDI of 0 would have a theoretical minimum level of development relevant to these health outcomes, while a location with an SDI of 1 would have a theoretical maximum level of development relevant to these health outcomes.

We summarize the final scales for GBD 2017 in the table below.

| Input | Lower Bound | Upper Bound |
|---|---|---|
| TFU25 | 0 | 3 |
| LDI per capita | 250 USD (5.5 log USD)[b] | 60,000 USD (11.0 log USD) |
| EDU15+ | 0 years | 17 years |

[b] The minimum for the LDI scale was originally set at the theoretical limit of 0 USD, as we did not observe an asymptotic relationship between log(LDI) and $E_0$ or 5q0 at lower values of log(LDI). Empirically, however, we also did not observe an LDI below 350 USD (5.86 log USD) for the estimation period 1970-2016. In log-space, this meant that approximately half of our scale was not being utilized, compressing the observed variation in LDI and diminishing its meaningful contribution to SDI. Accordingly, we set the lower limit on LDI to 250 USD (5.52 log USD) to ensure we were fully utilizing the range of the scale to capture its variation across space and time, as is the case with the other two inputs.

Using scales described above, we computed the index scores underlying SDI as follows:

$$I_{Cly} = {(C_{ly} - C_{low})}\Big/{(C_{high} - C_{low})}$$

Where $I_{cly}$ – the index for covariate *C*, location *l*, and year *y* – is equal to the difference between the value of that covariate in that location-year and the lower bound of the covariate divided by the difference between the upper and lower bounds for that covariate. If the values of input covariates fell outside the upper or lower bounds (e.g. LDI per capita greater than 60,000 USD), they were mapped to the respective upper or lower bounds. We also note that the index value for TFU25 was computed as $1 - I_{TFU25ly}$, as lower TFU25s correspond to higher levels of development, and thus higher index scores. For GBD 2017 we expanded the computation of SDI to 890 national and subnational locations spanning the time period 1950-2017.

The composite Socio-Demographic Index is the geometric mean of these three indices for a given location-year. The cut-off values used to determine quintiles for analysis were then computed using country-level estimates of SDI for the year 2017, excluding countries with populations less than 1 million.

### *Example calculation*

Below we present an example calculation of SDI for "Country X":

$$TFU25 = 1.09;\ Mean\ educ\ yrs\ pc = 8.23;\ lnLDI = 9.60$$

$$I_{TFU25} = 1 - \frac{1.09 - 0}{3 - 0} = .637$$

$$I_{Educ} = \frac{8.23 - 0}{17 - 0} = .484$$

$$I_{lnLDI} = \frac{9.60 - 5.52}{11.00 - 5.52} = .744$$

$$SDI = \sqrt[3]{I_{TFU25} * I_{Educ} * I_{lnLDI}} = \sqrt[3]{.637 * .484 * .744} = .611$$

$$I_{lnLDI} = \frac{9.58 - 5.52}{11.00 - 5.52} = .741$$

$$SDI = \sqrt[3]{I_{TFR} * I_{Educ} * I_{lnLDI}} = \sqrt[3]{.855 * .543 * .741} = .701$$

SDI grouping by location can be found in Appendix Table 9, and SDI values can be found in Appendix Table 10.

## Section 2.11: Epidemiological Transition

### Section 2.11.1:Derivation of expected SEVs

In order to evaluate the average relationship between SDI attributable burden, we first quantified that between SDI and population-level exposure. Using the "gam" package in R, we fit a generalized additive model (GAM) with a loess smoother on SDI by age and sex group. For age *a*, sex *s*¸and risk *r*.

$$logit(SEV_{asr}) = \beta_0 + s(SDI) + \epsilon$$

Inputs to this model were age-sex specific SEVs for all most-detailed risks in the GBD risk hierarchy corresponding to all national GBD locations and years between 1990 and 2017. The span for the loess term was set at a default of .7, and the model was fit on the middle 95% of the data to mitigate the impact of compositional bias evident in outliers very close to zero or one. Expected age and sex proportions of the population on the basis of SDI produced through an analogous modelling framework were used to generate age and sex aggregates of expected exposure.

### Section 2.11.2:Calculating attributable burden

Borrowing from forecasting methods, we generated an estimated expected risk-specific $PAF$ by back-transforming the expected $SEV$ to $PAF$, rearranging the formula for $SEV$ described in further detail in the appendix to solve to $PAF$ for a given age $a$, sex $s$, cause $c$, risk $r$, and SDI $d$,

$$PAF_{ascrd} = 1 - \frac{1}{SEV_{asrd} \times (RR_{ascr}^{max} - 1) + 1}$$

As $PAF$ estimates are derived directly from the $SEV$ which is not cause specific, but averaged across causes, we then calculated a correction factor $CF$ by comparing in logit space the empirical $PAF$ to the $SEV$ derived estimate $PAF$,

$$CF_{ascr} = logit\left(PAF_{ascrly}\right) - logit(PAF_{ascrly})$$

and apply the correction factor to the estimated expected $PAF$ to derived an adjusted expected estimated $PAF$

$$PAF_{ascrd} = expit(logit(PAF_{ascrd}) + CF_{ascr})$$

for all most-detailed risks in the hierarchy, excluding unsafe sex and occupational injuries, by age, sex, cause, and SDI, adding in risk-outcome

To estimate expected risk-attributable burden, we draw from the CRA methods (see Appendix Section 2.6), first calculating the joint adjusted expected $PAF$ for all risks for a cause using mediation factors. We then draw from the methods for observed risk-attributable burden calculation, using expected YLLs, deaths, and YLDs (see Appendix Section 2.7) to generate expected burden for a given SDI, not location year.

$$AB_{rascd} = \sum DALY_{ascd} PAF_{rascd}$$

where $AB_{rascd}$ is the expected attributable burden; $DALY_{ascd}$ is expected total; $PAF_{ascrd}$ is the expected PAF. The proportion of expected deaths, YLLs, or YLDs attributable to a given risk factor or risk factor cluster were analogously computed by sequentially substituting each measure in place of expected DALYs in the equation above. Comparisons of observed to expected attributable burden were made to identify locations exhibiting exceptional deviations relative to what would be expected based on their development status.

## Section 2.12: Additional Methods Information

### Section 2.12.1: Risk-specific comparisons to other estimates

*Low birth weight / Short Gestation:*

GBD 2017 estimates of total preterm birth prevalence are generally in line with country-specific reports[30] as well as the most recent global analysis completed by Blencowe and colleagues.[31] GBD 2017 estimates of preterm birth prevalence in 2010 are very similar, 11.3% (95% UI: 11.1% to 11.5%) versus 11.1% of live births, compared to estimates by Blencowe and colleagues. Close agreement is not surprising as most of the same data sources were used as data inputs to our modelling process, although the GBD analysis included almost eight times as many data points. Most reports, like GBD 2017, have assessed temporal trends in preterm birth in many locations to be either static or increasing. Compared to UNICEF estimates of low birth weight,[32] GBD 2017 estimates of global birth prevalence of 14.4% (14.0% to 14.8%) are similar but slightly lower than the estimate of 15.5% birth prevalence globally. The geographic variation in low birth weight largely mirrors that of the UNICEF report.

*Chewing Tobacco*

In GBD 2016, we estimated age-sex specific and aggregate current smokeless tobacco use prevalence for all countries and territories from 1990-2016 using all available data. The estimated prevalence was then

attributed to either all chewing tobacco, or all snus/snuff by country, based on input from smokeless tobacco experts.

For GBD 2017, we have changed the exposure definition from current smokeless tobacco use to current chewing tobacco use, based on the strength of evidence supporting the health effects of chewing tobacco use. By estimating chewing tobacco exposure in all countries and territories, burden is now estimated for locations previously classified as predominantly snus/snuff, but still have non-negligible use of chewing tobacco.

We compared GBD estimates to recent research by Siddiqi et al.[33] on smokeless tobacco prevalence, risk, and burden in 2015. Our methods differed from Siddiqi on some key points:

> Prevalence estimation: We estimated both age-sex specific and aggregate current smokeless tobacco use prevalence for all countries and territories included in GBD from 1990-2017 using all available data. Siddiqi et al. used only the single most recent survey available containing data on smokeless tobacco use among adults.
>
> Relative risks and attributable burden: GBD excluded hospital-based case-control studies, while Siddiqi included them. GBD calculated relative risks for chewing tobacco (and by sex for oral cancer only), while Siddiqi calculated separate relative risks by geography, and then pooled these to produce global relative risks. Siddiqi used country- or region-specific relative risks where available, and in the absence of region-specific relative risks assigned global relative risks in countries predominantly using products with moderate to high pH and tobacco-specific nitrosamine (TSNA) levels.

The main differences in attributable burden come from the relative risk exclusion criteria. GBD's exclusion of hospital-based led to very different relative risk outputs: we found significant relative risks for oral cancer and oesophageal cancer. Siddiqi found significant relative risks for oral, pharyngeal, and oesophageal cancers and ischemic heart disease, resulting in higher levels of global burden.

### *Smoking*

We compared GBD estimates to the most recent report on the global tobacco epidemic published by WHO[30]. Overall, we found marked similarities in estimates. Among the 142 countries and territories included in the WHO report and estimated in GBD, the correlation coefficient for current smoking prevalence estimates among females was 0.91 and among males was 0.85. In cases where estimates diverge, discordance can be attributed to differing modelling methods or data sources. GBD uses ST-GPR to estimate smoking prevalence, whereas WHO uses Bayesian meta-regression (DisMod MR). Additionally, the WHO model was fit on 1,175 country-year data sources, whereas the GBD model was fit on 2,870 country-year data sources. There are no comparable global estimates of the burden of disease attributable to smoking, as GBD 2015 estimates of attributable burden were used in the most recent WHO report.

### *Ambient air pollution*

In the past few years, other researchers have estimated the burden of disease due to air pollution using different data and methods. Other sources of estimates have been compared to those of the GBD. Since their introduction in GBD 2010, satellite-based estimates of $PM_{2.5}$ and the Integrated Exposure Response (IER), have been widely adopted. Recent estimates from WHO[31] of 3·0 million deaths in 2012 used a

similar exposure estimate model as that presented here, but an earlier (GBD 2013) version of the integrated exposure response (IER) and somewhat different baseline disease burden estimates. Lelieveld and colleagues[32] analysed source sector contributions to air pollution and the resulting disease burden in 2010 and estimated the burden in 2050. These estimates used an older (GBD 2010) IER. Furthermore, the coarse spatial resolution (∼100 × 100 km) of the exposure estimates introduced errors via spatial misalignment between exposure and population density compared with our estimates. Silva et al. estimated 2.23 million deaths/year due to anthropogenic $PM_{2.5}$ and 493 thousand deaths per year due to ozone,[33] while Butt et al. estimated 12.4% global attributable deaths due to $PM_{2.5}$ in the year 2009,[34] both using versions of the IER.

In GBD 2017, we added Type 2 diabetes as an outcome of ambient particulate matter pollution estimating 184 thousand attributable deaths and 10.5 million attributable DALYs globally in the year 2017. An independent group of researchers recently published work estimating the 2016 burden of diabetes attributable to ambient $PM_{2.5}$.[35] Using GBD 2015 $PM_{2.5}$ exposure estimates, GBD 2016 diabetes burden, and generating their own IER the team estimated 206 thousand attributable deaths and 8.23 million attributable DALYs globally for the year 2016.

### *Occupational*

Takala and colleagues[36] reported 2.3 million deaths attributable to occupational injury/illness in 2011. In the closest comparison year of 2007, GBD estimated nearly 1.1 million deaths. This discrepancy is largely driven by the cause-outcome pairs that GBD currently has the evidence to include based on the criteria of the CRA framework. For example, 45% of Takala's reported burden is driven by occupational circulatory disease (35%) and occupational communicable disease (10%). Circulatory diseases are linked to occupational risks like shift work and lack of control, but the GBD approach currently has insufficient evidence to estimate the variability in exposure to these factors on a global scale. Additionally, the use of a CRA approach in GBD estimates requires careful consideration of proposed counterfactual in order to derive the TMREL for a given risk. The TMREL for something like occupational lack of control is a challenging concept, and as such, these risks are still being reviewed for possible inclusion in future iterations of the GBD.

Takala also reports higher burden from occupational cancer based on the inclusion of carcinogens that are currently still out of the scope of GBD. For example, the authors use attributable fractions derived from Rushton and colleagues to attribute pairs like breast cancer and shift work or skin cancer and solar radiation. These carcinogens, which form a large part of the cancer burden in Takala/Rushton are currently not included in the GBD based on limited exposure data across the time/space that GBD estimates[38].

In terms of fatal occupational injuries, Takala reported 353 000 deaths in 2011. The GBD 2017 estimate for deaths attributable to occupation was approximately 348 000 deaths for 2007. The figures are very similar but the GBD estimates are slightly lower, again due to the selection of risk-cause pairs. The ILO estimation strategy includes some kinds of injuries, such as deaths due to intentional violence that the GBD does not attribute to occupation.

*Child growth failure (stunting, wasting, and underweight)*

UNICEF et al estimate lower proportion of stunting (height-for-age z-score < -2 standard deviations below the reference median) in children under five in 2017 than GBD 2017.[37] The geographic patterns generally agree in identifying sub-Saharan Africa and South Asia as the regions with the largest burden of stunting (prevalence and magnitude, estimated as number of stunted children in UNICEF et al, and as DALYs in GBD 2017), with additional high prevalence in Oceania (excluding Australia and New Zealand) and moderate prevalence in Latin America and the Caribbean. While UNICEF et al estimates highlight minimal or lack of progress in reducing stunting since 2000 in Africa and Oceania, GBD 2017 estimates show moderate decline in sub-Saharan Africa and North Africa and the Middle East, and a small decline in Oceania (compared to UNICEF et al's rise in stunting prevalence in Oceania). GBD 2017 estimates show a downward trend in the prevalence of underweight (weight-for-age z-score < -2 standard deviations below the reference median) among children under 5 in 2016, driven largely by populations in sub-Saharan Africa and South Asia, a trend also reflected in the UNICEF et al estimates.

*Impaired Kidney Function*

Recently published estimates from a meta-analysis of global data on exposure to impaired kidney function indicate prevalence of chronic kidney disease (CKD) stages 1-5 to be 13.4% (11.7-15.1%). (ref) These estimates are similar to the current GBD 2017 exposure estimates across all four levels of impaired kidney function, which indicate a prevalence for individuals over the age of 25 of 14.0% (13.0-15.1%).

*Household Air Pollution*

WHO estimated 4.3 million deaths and 146.5 million DALYs attributable to exposure to household air pollution globally in 2012, as compared to GBD 2017 estimates for the year 2017 of 1.6 million deaths and 59 million DALYs. Differences in attributable burden arise between the WHO estimates and GBD 2016 for a number of reasons. First, the IER curve was used for all outcomes (LRI, IHD, cerebrovascular stroke, COPD, Type II Diabetes, and lung cancer) except cataracts in our analysis, while WHO adapted relative risks for COPD based on a meta-analysis of published RR estimates. The excess relative risks for COPD used by WHO are larger than the excess relative risks used in GBD, resulting in a larger PAF. Additionally, this was the first year we included Type II Diabetes as an outcome of particulate matter pollution, including household exposure to solid fuel. WHO has not included this as an outcome in their estimates. Finally, WHO only estimates LRI deaths in children aged 0-4, while we estimate respiratory infections and pneumonia at all ages.

Additionally, by adapting the new proportional PAF approach for particulate matter pollution, beginning in GBD 2017 we are taking into account ambient pollution in the counterfactual of household. This has reduced burden estimates from previously published numbers, which we believe more accurately reflects the exposure-outcome relationship.

Another difference from the WHO estimates is the use of a database which maps solid cooking fuel use to $PM_{2.5}$ exposure, allowing us to model differences in exposure level and relative risk by location, while WHO relies on global relative risks. We also adjust this household $PM_{2.5}$ exposure by ambient air pollution levels since personal $PM_{2.5}$ exposure captures all sources of exposure. WHO makes no

adjustments for ambient air pollution exposure. In addition to the differences in data sources, we estimated the burden of cataract attributable to household air pollution (HAP) only in women while WHO estimated for both sexes.

Below is a comparison between the WHO[38] and GBD 2017 of the number of DALYs attributed to HAP contributed by each cause (GBD 2017 on right):

| | |
|---|---|
| • 48,500,000-Pneumonia/LRI | 25,400,000-Lower respiratory infections |
| • 36,900,000-Stroke | 8,010,000-stroke |
| • 30,500,000-Ischaemic heart disease | 9,170,000-IHD |
| • 22,100,000-COPD | 11,700,000-COPD |
| • 6,720,000-Lung cancer | 1,780,000-Lung cancer |
| • 1,670,000-Cataract | 1,230,000-Cataract |
| • 0-Type 2 diabetes | 830,000-Type 2 diabetes |

### *Breastfeeding*

Globally, WHO estimates that 40% of infants under six months of age are exclusively breastfed, which is consistent with the GBD 2017 estimate (40.3%). A recent WHO study estimates 73.3% continued breastfeeding at one year of age globally, where the GBD 2017 estimate of continued breastfeeding between 6 and 11 months is 88% (we expect our estimates to be higher, as our estimates measure breastfeeding for any infants age 6-11 months, while the WHO estimates focus on breastfeeding status at the age of 1 year).[39] CDC reports 25% of mothers in the United States exclusively breastfeed their child through 6 months of age, while GBD 2017 estimates that 42% of infants 0-5 months are exclusively breastfed (this difference is again explained by our estimates focusing on all infants 0-5 months, while the CDC reports infants who complete 6 full months of exclusive breastfeeding). Additionally, CDC reports 34% of mothers in the U.S. continue to breastfeed at 12 months of age. GBD estimates 44% of mothers in the U.S. continue to breastfeed from 6 to 11 months of age. Finally, in India, WHO and UNICEF estimate 55% exclusive breastfeeding in the first six months of life, comparable to the GBD 2017 estimate of 55%.

### *WaSH*

The Joint Monitoring Project[40] (JMP), which is led by WHO and UNICEF, estimates water, sanitation, and handwashing access throughout the world. Globally, JMP estimates that 91% of population had access to an improved water source in 2015, while GBD estimates 88% of the population have access to improved water. Additionally, JMP reported the global prevalence of households with piped water connection to be 57% in 2015, while GBD reports piped prevalence of 53% for that year. JMP reported 68% of population had access to improved sanitation in 2015, whereas GBD estimates improved sanitation prevalence of 74%. The slight discrepancies in these estimates at the global level can be largely attributed to differences in input data. The JMP relies almost exclusively on large-scale household surveys (DHS and Multiple Indicator Cluster Surveys [MICS]), while GBD estimates incorporate exposure data from smaller, yet still nationally representative, survey series such as Reproductive Health Survey and various country specific surveys. Due to the relative dearth of data regarding access to a handwashing facility, the JMP only generates handwashing estimates for a select

number of countries (mostly sub-Saharan Africa) where those data are actually collected. However, we model and predict handwashing facility prevalence for all locations, even in the absence of data, and estimates that 67% of the globe has access to a handwashing facility.

### *Lead*

The most recent external estimates for the burden of lead exposure were conducted by WHO in 2004 and provided disaggregated average exposures for children and adults in different regions of the world.[41] The GBD 2017 exposure estimates for the early 2000s are generally higher than these estimates, as we have observed higher estimates of exposure in GBD 2017 with changes in model covariates and covariate effects. The WHO study calculated a global burden attributable to lead exposure of 13 million DALYs, which includes 229 000 deaths. GBD 2017 estimates global burden attributable to lead exposure to be 24.4 million DALYs, including approximately 1.05 million deaths.

In addition to differences in overall attributable burden, the breakdown of burden from intellectual disability and cardiovascular disease is very different. Both attribute 2% to 3% of global CVD to lead exposure, but our estimates of CVD burden in 2007 are higher than WHO's (such that we estimate 18.1 million DALYs from CVD due to lead in 2007 compared to their estimate of 3.1 million). Additionally, our methodology for intellectual disability differs substantially from theirs. In the WHO study, they used a higher disability weight of 0.361 for intellectual disability, whereas we currently use weights ranging from 0.01 to 0.2 (depending on the severity). However, WHO's estimates of intelligence quotient (IQ) shift from lead exposure are much lower than ours, since recent studies have provided better evidence for notable effects of lead on IQ at low levels of exposure. Still, due to differences in our estimates of the underlying burden of intellectual disability, our estimate of 2.67 million DALYs from intellectual disability attributable to lead exposure in 2007 is much smaller than their estimate of 9.8 million.

### *Intimate partner violence*

WHO reports a global lifetime prevalence of physical and/or sexual intimate partner violence among ever-partnered women of 30.0% (27.8-32.2).(ref) For GBD 2017, the estimated all-age global exposure for intimate partner violence (IPV) in 2017 is 19.7% (16.1-24.3) among all women, which is a smaller estimate than the WHO estimate because WHO estimates are among only ever-partnered women, while the estimates used for GBD risk factor exposure are among all women. After making an adjustment using our model for the proportion of women who have ever been partnered, we estimate global lifetime IPV exposure as 32.5% (25.9-40.9) among ever-partnered women – an estimate that agrees with the WHO report. The regional distribution reported by WHO is in agreement with the distribution by GBD super-region; highest prevalence of IPV in North Africa and Middle East; South Asia; and sub-Saharan Africa and lower prevalence in Southeast Asia, East Asia, and Oceania; Central Europe, Eastern Europe, and Central Asia; high-income; and Latin American and Caribbean.

### *Iron deficiency*

Iron deficiency (ID) was the fourth ranked level 3 risk factor in 1990, decreasing to sixth in 2017 after increasing 15.23% over that time period in terms of attributable YLDs, almost all of which was YLDs due to dietary iron deficiency (IDA). We have not identified any other global, systematic analyses of ID as a risk factor for increased disease burden so we are not able to compare our estimates of ID-attributable health loss. There are a number of other studies that have evaluated the prevalence of ID and IDA, however.[42] The most comprehensive meta-analysis from Low and Middle-Income Countries (LMIC) estimated a much lower prevalence of ID/IDA than we have for GBD 2017. There are three aspects that

make a direct comparison with GBD 2017 difficult. First, the study by Petry and colleagues likely underestimated ID/IDA somewhat by applying a single cut-off for diagnosing ID of <12 grams per decilitre of plasma ferritin concentration, especially with the acknowledged limitation of not being able to fully account for the effect of inflammation in many of its component studies. Second, Petry and colleagues did not distinguish aetiologies of ID/IDA whereas GBD does distinguish many causes of anaemia (e.g. hookworm, gastritis) that can manifest as ID. Third, whereas Petry and colleagues made direct estimation of ID/IDA from serum measurements, the GBD approach for estimating ID/IDA is indirect and therefore does not have a directly comparable case definition. We began by first estimating overall anaemia then, after reassigning large portions to >25 other underlying causes, used fixed proportion redistribution methods to estimate IDA. The risk exposure for ID was then estimated as a counterfactual haemoglobin concentration in the absence of all the "other" causes rather than an explicit prevalence value. Unless all possible causes of anaemia are included, the GBD approach has potential to overestimate the proportion of anaemia to be redistributed to ID/IDA in places where other causes are important. We have begun work to address this in GBD 2017 by adding Cirrhosis, Crohn's Disease, Ulcerative Colitis, and others as causes of anaemia, but there are still a number of others (e.g. cancers, alpha thalassemia, intestinal infections, and other nutritional deficiencies) that have yet to be included.

# Section 3: References


1 Murray CJ, Lopez AD. Global mortality, disability, and the contribution of risk factors: Global Burden of Disease Study. *Lancet* 1997; **349**: 1436–42.

2 Murray CJ, Lopez AD. On the comparable quantification of health risks: lessons from the Global Burden of Disease Study. *Epidemiology* 1999; **10**: 594–605.

3 Forouzanfar M, Afshin A, Alexander LT, Anderson H, Bhutta Z, Murray CJL. Global, regional, and national comparative risk assessment of 79 behavioural, environmental and occupational, and metabolic risks or clusters of risks, 1990-2015: a systematic analysis for the Global Burden of Disease Study 2015. *Lancet* 2016; **388**: 1659–724.

4 Stevens GA, Alkema L, Black RE, *et al.* Guidelines for Accurate and Transparent Health Estimates Reporting: the GATHER statement. *Lancet* 2016; published online June 28.

5 Food, nutrition, physical activity and the prevention of cancer: a global perspective. Washington, D.C: World Cancer Research Fund & American Institute for Cancer Research, 2007.

6 Law MR, Morris JK, Wald NJ. Use of blood pressure lowering drugs in the prevention of cardiovascular disease: meta-analysis of 147 randomised trials in the context of expectations from prospective epidemiological studies. *BMJ* 2009; **338**. DOI:10.1136/bmj.b1665.

7 Harris PA, Taylor R, Thielke R, Payne J, Gonzalez N, Conde JG. Research Electronic Data Capture (REDCap) - A metadata-driven methodology and workflow process for providing translational research informatics support. *J Biomed Inform* 2009; **42**: 377–81.

8 GBD 2015 Diseases and Injury Incidence and prevalence Collaborators. Global, regional, and national incidence, prevalence, and years lived with disability (YLDs) for 310 acute and chronic diseases and injuries, 1990-2015: a systematic analysis for the Global Burden of Disease Study 2015. *The Lancet Under review*.

9 An Integrative Metaregression Framework for Descriptive Epidemiology (Publications on Global Health, Institute for Health Metrics and Evaluation): Abraham D. Flaxman, Theo Vos, Christopher J.L. Murray, Patricia Kiyono: 9780295991849: Amazon.com: Books. https://www.amazon.com/Integrative-Metaregression-Descriptive-Epidemiology-Publications/dp/0295991844 (accessed June 24, 2016).

10 Vasudevan S, Ramos F, Nettleton E, Durrant-Whyte H, Blair A. Gaussian Process modeling of large scale terrain. In: 2009 IEEE International Conference on Robotics and Automation. 2009: 1047–53.

11 Rasmussen CE, Williams CKI. Gaussian Processes for Machine Learning. Cambridge, Mass: The MIT Press, 2005.

12 Ng M, Freeman MK, Fleming TD, *et al.* Smoking prevalence and cigarette consumption in 187 countries, 1980-2012. *JAMA* 2014; **311**: 183–92.

13 Ng M, Fleming T, Robinson M, *et al.* Global, regional, and national prevalence of overweight and obesity in children and adults during 1980-2013: a systematic analysis for the Global Burden of Disease Study 2013. *Lancet* 2014; **384**: 766–81.

14 Ng M, Freeman MK, Fleming TD, *et al.* Smoking Prevalence and Cigarette Consumption in 187 Countries, 1980-2012. *JAMA* 2014; **311**: 183–92.

15 Massey Jr FJ. The Kolmogorov-Smirnov test for goodness of fit. *Journal of the American statistical Association* 1951; **46**: 68–78.

16 Vander Hoorn S, Ezzati M, Rodgers A, Lopez AD, Murray CJL. Estimating attributable burden of disease from exposure and hazard data. In: Comparative Quanitifcation of Health Risks: Global and regional burden of disease attribution to selected major risk factors. World Health Organisation, 2004: 2129–40.

17 Preston SH. Causes and Consequences of Mortality Declines in Less Developed Countries during the Twentieth Century. In: Population and economic change in developing countries. Chicago: Univ. of Chicago Pr, 1980: 289–360.

18 Carnahan E, Lim SS, Nelson EC, *et al.* Validation of a new predictive risk model: measuring the impact of major modifiable risks of death for patients and populations. *The Lancet* 2013; **381**: S26.

19 Danaei G, Singh GM, Paciorek CJ, *et al.* The global cardiovascular risk transition: associations of four metabolic risk factors with national income, urbanization, and Western diet in 1980 and 2008. *Circulation* 2013; **127**: 1493–502, 1502e1-8.

20 Nieman DC, Brock DW, Butterworth D, Utter AC, Nieman CC. Reducing diet and/or exercise training decreases the lipid and lipoprotein risk factors of moderately obese women. *J Am Coll Nutr* 2002; **21**: 344–50.

21 Tjønna AE, Lee SJ, Rognmo Ø, *et al.* Aerobic interval training versus continuous moderate exercise as a treatment for the metabolic syndrome: a pilot study. *Circulation* 2008; **118**: 346–54.

22 Snyder KA, Donnelly JE, Jabobsen DJ, Hertner G, Jakicic JM. The effects of long-term, moderate intensity, intermittent exercise on aerobic capacity, body composition, blood lipids, insulin and glucose in overweight females. *Int J Obes Relat Metab Disord* 1997; **21**: 1180–9.

23 Nordby P, Auerbach PL, Rosenkilde M, *et al.* Endurance training per se increases metabolic health in young, moderately overweight men. *Obesity (Silver Spring)* 2012; **20**: 2202–12.

24 Christiansen T, Paulsen SK, Bruun JM, Pedersen SB, Richelsen B. Exercise training versus diet-induced weight-loss on metabolic risk factors and inflammatory markers in obese subjects: a 12-week randomized intervention study. *Am J Physiol Endocrinol Metab* 2010; **298**: E824-831.

25 Coggan AR, Kohrt WM, Spina RJ, Bier DM, Holloszy JO. Endurance training decreases plasma glucose turnover and oxidation during moderate-intensity exercise in men. *J Appl Physiol* 1990; **68**: 990–6.

26 Arsenault BJ, Côté M, Cartier A, *et al.* Effect of exercise training on cardiometabolic risk markers among sedentary, but metabolically healthy overweight or obese post-menopausal women with elevated blood pressure. *Atherosclerosis* 2009; **207**: 530–3.

27 Olofin I, McDonald CM, Ezzati M, *et al.* Associations of suboptimal growth with all-cause and cause-specific mortality in children under five years: a pooled analysis of ten prospective studies. *PLoS ONE* 2013; **8**: e64636.

28 McDonald CM, Olofin I, Flaxman S, *et al.* The effect of multiple anthropometric deficits on child mortality: meta-analysis of individual data in 10 prospective studies from developing countries. *Am J Clin Nutr* 2013; **97**: 896–901.

29 Das Gupta P. Standardization and Decomposition of Rates: A User's Manual. Washington D.C.: U.S. Bureau of the Census, 1993.

30 WHO report on the global tobacco epidemic, 2017: monitoring tobacco use and prevention policies. Geneva: World Health Organization, 2017.

31 Global Health Observatory data: Household air pollution. WHO http://www.who.int/gho/phe/indoor_air_pollution/en/.

32 Lelieveld J, Evans J, Fnais M, Giannadaki D, Pozzer A. The contribution of outdoor air pollution sources to premature mortality on a global scale. *Nature* 2015; : 367–71.

33 Silva RA, Adelman Z, Fry MM, West JJ. The Impact of Individual Anthropogenic Emissions Sectors on the Global Burden of Human Mortality due to Ambient Air Pollution. *Environ Health Perspect* 2016; **124**: 1776–84.

34 Butt EW, Turnock ST, Rigby R, *et al.* Global and regional trends in particulate air pollution and attributable health burden over the past 50 years. *Environmental Research Letters* 2017; **12**: 104017.

35 Bowe B, Xie Y, Li T, Yan Y, Xian H, Al-Aly Z. The 2016 global and national burden of diabetes mellitus attributable to PM2·5 air pollution. *The Lancet Planetary Health* 2018; **2**: e301–12.

36 Takala J, Hamalainen P, Nenonen N, Takahashi K, Chimed-Ochir O, Rantanen J. Comparative Analysis of the Burden of Injury and Illness at Work in Selected Countries and Regions. *Central European Journal Occupational and Environmental Medicine* 2017; **23**: 6–32.

37 UNICEF, WHO, The World Bank. Joint child malnutrition estimates - Levels and trends (2017 edition). Geneva, Switzerland: World Health Organization, 2017 http://www.who.int/nutgrowthdb/estimates2016/en/ (accessed May 24, 2018). .

38 WHO. Global Health Observatory data repository. WHO. http://apps.who.int/gho/data/node.main.139?lang=en (accessed May 24, 2018).

39 Victora CG, Bahl R, Barros AJD, *et al.* Breastfeeding in the 21st century: epidemiology, mechanisms, and lifelong effect. *Lancet* 2016; **387**: 475–90.

40 2016 ANNUAL REPORT WHO/UNICEF Joint Monitoring Programme for Water Supply, Sanitation and Hygiene (JMP). UNICEF.

41 Pruss-Astun A, Fewtrell L, Landrigan PJ, Ayuso-Mateos JL. Lead Exposure. In: Comparative quantifications of health risks: Global and regional burden of disease attributable to selected major risk factors. Geneva: World Health Organization, 2004: 1496–542.

42 Petry N, Olofin I, Hurrell RF, *et al.* The Proportion of Anemia Associated with Iron Deficiency in Low, Medium, and High Human Development Index Countries: A Systematic Analysis of National Surveys. *Nutrients* 2016; **8**. DOI:10.3390/nu8110693.

# Section 4: Risk-specific estimation

The risk-specific modelling write-ups follow the order of the risk factor hierarchy for GBD 2017. In some cases, multiple risk factors are addressed in a single write-up, for example child underweight, wasting, and stunting are all included in a single detailed write-up.

# Unsafe Water Capstone Appendix

## Flowchart

Unsafe Drinking Water

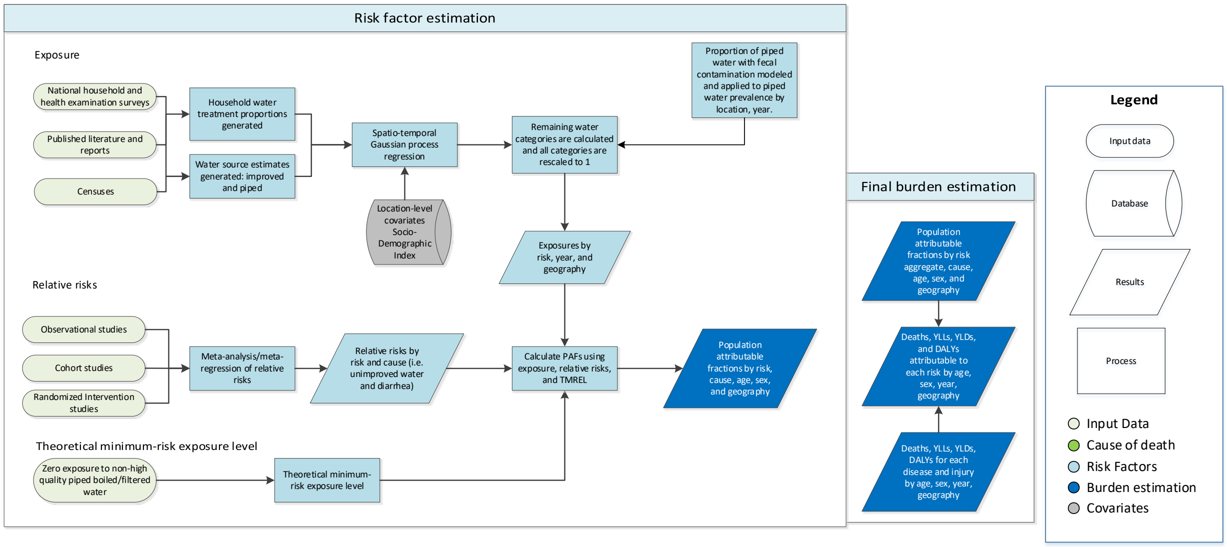


## Input data & methodological summary

### Exposure

#### *Case definition*

For GBD 2017, exposure to unsafe water was defined based on reported primary water source used by the household and use of household water treatment (HWT) to improve the quality of drinking water before consumption. Water sources were defined as “improved” based on the JMP designation,[1] which includes piped water as improved water, and households with access to piped water connection to the house, yard, or plot were defined as having access to piped water supply. Solar treatment, chlorine treatment, boiling, or the use of filters were all established as effective point-of-use household water treatments based on effect sizes calculated from network meta-analysis.

#### *Input data*

The search for usable household surveys and censuses was conducted using the Global Health Data Exchange (GHDx) database. HWT input data is primarily limited to two large survey series (DHS and MICS) due to data availability. Water source data includes censuses and nationally representative surveys such as DHS, MICS, AIS, and WHS. For each survey, household sample weights were multiplied by the number of household members to produce a weighting scheme that estimates proportion of individuals, not proportion of households, exposed to a given indicator. Surveys and censuses were then tabulated to the two water source and two water treatment categories of interest for each location.

### Modelling

Water source data is modelled using an ordinal framework, with two distinct models: prevalence of piped water and proportion of improved water (excluding piped) within the non-piped population. Both models produce results for each unique location, year combination. This ordinal framework allows us to estimate the category with the most data (piped water prevalence) and leverage that estimate to

anchor the estimates for improved and unimproved water categories. The results of the improved proportion model are multiplied by the piped water prevalence to calculate improved water prevalence. The sum of improved and piped water prevalence are subtracted from 1 to yield unimproved water prevalence.

HWT categories are estimated in a similar ordinal framework, by modelling prevalence of individuals using no water treatment methods and proportions of households that boil/filter water within the population of households that engage in treatment methods. The prevalence of individuals that boil/filter drinking water is calculated by multiplying the proportion that boil/filter modelled previously times prevalence of any water treatment (estimated by subtracting prevalence of no treatment from 1). The prevalence of individuals that treat their water using solar/chlorine methods was estimated by subtracting the sum of prevalence of no treatment estimates and prevalence of filter/boil treatment from 1. By year and location, each of the above categories are modelled using a 3-step modelling scheme of mixed effect linear regression followed by spatio-temporal Gaussian process regression (ST-GPR), which produces full time series estimates for each GBD 2017 location. Socio-demographic index (SDI), a composite metric combining education per capita, income per capita, and fertility, was set as a fixed effect in the linear regression since it proved to be a significant predictor. Random effects were set at GBD 2017 region and super-region levels to fit the models but were not used in the predictions.

The process of vetting and validating models was accomplished primarily through an examination of ST-GPR scatter plots by GBD 2017 location from 1990-2017. Any unfitting data points were re-inspected for error at the level of extraction and survey implementation, and subsequently excluded from analysis if deemed appropriate. In addition to SDI, a number of different potential fixed effects were considered, including lag-distributed income and urbanicity, but SDI proved to be the strongest predictor of the unsafe water categories. Uncertainty in the estimates was initially formed based on standard deviation by survey, then propagated through ST-GPR modelling by means of confidence intervals around each data point that reflect the point-estimate specific variance.

Once models are vetted, full time series outputs from ST-GPR modelling are then converted from proportion to prevalence by year and geography and then rescaled to form 9 mutually exclusive categories that sum up to 1. The table below provides the final result of this rescaling.

| Category | Definition |
|---|---|
| Unimproved, no HWT | Proportion of individuals that primarily use unimproved source, and *do not* use any HWT to purify their drinking water. |
| Unimproved, chlorine/solar | Proportion of individuals that primarily use unimproved source, and solar or chlorine treatment to purify their drinking water. |
| Unimproved, boil/filter | Proportion of individuals that primarily use unimproved source, and boil or filter to purify their drinking water. |
| Improved water except piped, no HWT | Proportion of individuals that primarily use improved sources other than piped water supply, and *do not* use any HWT to purify their drinking water. |

| | |
|---|---|
| Improved water except piped, chlorine/solar | Proportion of individuals that primarily use improved sources other than piped water supply, and use solar or chlorine treatment to purify their drinking water. |
| Improved water except piped, boil/filter | Proportion of individuals that primarily use improved sources other than piped water supply, and boil/filter their drinking water. |
| Basic piped water, no HWT | Proportion of individuals that primarily use basic piped water supply, and *do not* use any HWT to purify their drinking water |
| Basic piped water, chlorine/solar | Proportion of individuals that primarily use basic piped water supply, and *use* solar or chlorine water treatment to purify their drinking water. |
| Basic piped water, boil/filter | Proportion of individuals that primarily use basic piped water supply, and boil or filter to purify their drinking water |
| High-quality (HQ) piped water, boil/filter | Proportion of individuals that primarily use basic piped water supply, and boil or filter to purify their drinking water |

We modelled the microbiological quality of piped water sources primarily using data a review by Bain et al.[1] that measured proportion of piped water sources contaminated with fecal indicators. We use the value generated from this model to split the prevalence of piped water into basic piped water and high quality piped water by location, year, age, and sex.

A substantial limitation in our analysis is the paucity of data on HWT and piped water quality. The inclusion of more location-specific data on water treatment utilisation at the household level can greatly improve our estimates in future iterations.

## Theoretical minimum-risk exposure level

The theoretical minimum-risk exposure level for unsafe water is defined as all households have access to high quality piped water that has been boiled or filtered before drinking.

## Relative risks

For GBD 2017, unsafe water was paired with one outcome-diarrheal diseases-given evidence provided by relative risk studies. A meta-analysis by Wolf et al.[3] provided the bulk of the relative risk evidence for the relationship between unsafe water and diarrheal diseases. This meta-analysis was updated through a literature review that searched for related intervention studies post-2014 conducted in PubMed. Search terms used were identical to those provided by Wolf et al.[3] Relative risk values for water-source interventions and point-of-use treatment interventions were calculated using network meta-analysis approach so as to include studies that differ in control groups within the same analysis. This analysis produced distinct relative risks for each water source and water treatment category. The combined effect of a source intervention and point-of-use intervention was assumed to be multiplicative in order to match GBD 2017 exposure definitions. Please refer to appendix tables for more information on relative risk values and citations.

## References


1. "Improved and Unimproved Water Sources and Sanitation Facilities."*WHO / UNICEF Joint Monitoring Programme: Wat/san Categories*. The WHO/UNICEF, n.d. Web. 08 June 2016
2. Bain, R., Cronk, R., Wright, J., Yang, H., Slaymaker, T., & Bartram, J. (2014). Fecal Contamination of Drinking-Water in Low- and Middle-Income Countries: A Systematic Review and Meta-Analysis. PLoS Medicine, 11(5). doi:10.1371/journal.pmed.1001644
3. Wolf, Jennyfer, Annette Prüss-Ustün, Oliver Cumming, Jamie Bartram, Sophie Bonjour, Sandy Cairncross, Thomas Clasen, John M. Colford, Valerie Curtis, Jennifer De France, Lorna Fewtrell, Matthew C. Freeman, Bruce Gordon, Paul R. Hunter, Aurelie Jeandron, Richard B. Johnston, Daniel Mäusezahl, Colin Mathers, Maria Neira, and Julian P. T. Higgins. "Systematic Review: Assessing the Impact of Drinking Water and Sanitation on Diarrhoeal Disease in Low- and Middle-income Settings: Systematic Review and Meta-regression." Trop Med Int Health Tropical Medicine & International Health 19.8 (2014): 928-42. Web.

# Unsafe Sanitation Capstone Appendix

## Flowchart

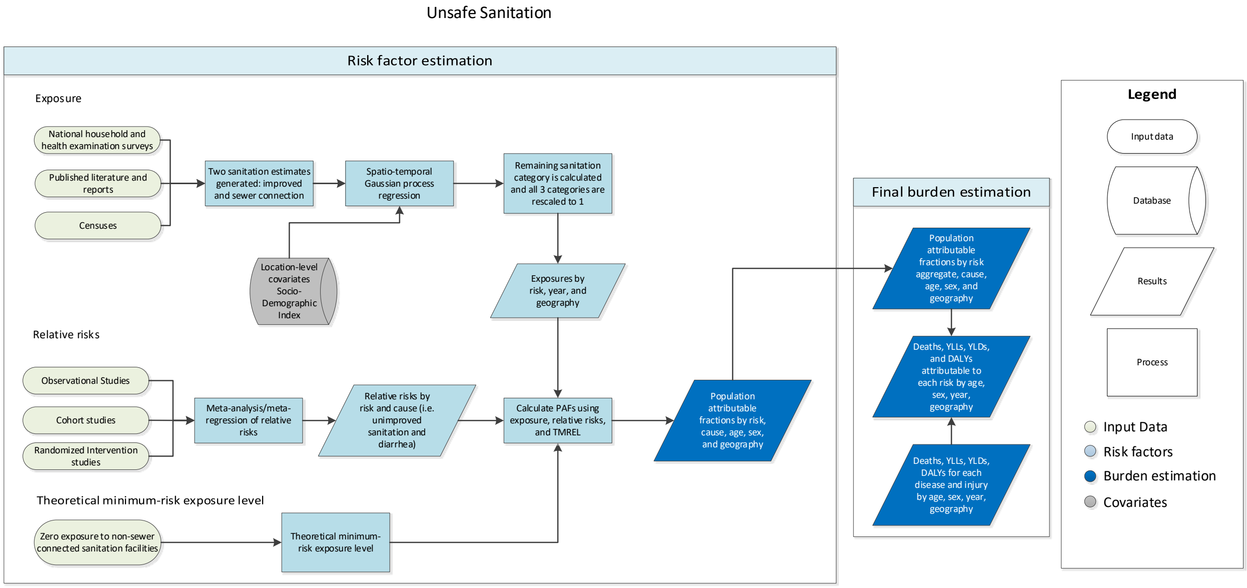


## Input data & methodological summary

### Exposure

#### *Case definition*

Exposure to unsafe sanitation is defined based on the primary toilet type used by households. Improved facilities are defined as such based on JMP designation (WHO). Sewer connection toilets included flush toilets or any toilet with connection to the sewer or septic tank.

#### *Input data*

The search for usable household surveys and censuses was conducted using the Global Health Data Exchange (GHDx) database. For each survey, household sample weights were multiplied by the number of household members to produce a weighting scheme that estimates proportion of individuals, not proportion of households, exposed to a given indicator. Surveys and censuses were then tabulated to two sanitation categories, sewer connection and improved sanitation, for each location. Data in tabulated form was lower priority to add to models and was only updated when time permitted.

### Modeling

A change made for GBD 2017 was to model sanitation categories in an ordinal framework instead of independent models. Two distinct indicators were estimated: the prevalence of individuals using sewer connection or septic tank facilities and the proportion of individuals with improved sanitation within the population not connected to sewer or septic tank. This ordinal framework allows us to estimate the category with the most data (sewer connection/septic tank prevalence) and leverage that estimate to anchor the estimates for improved and unimproved sanitation categories. The results of the improved proportion model are multiplied by the sewer connection/septic tank prevalence to calculate improved sanitation prevalence. The sum of improved and sewer connection/septic tank prevalence are subtracted from 1 to yield unimproved sanitation prevalence.

The two indicators were modeled using a 3-step modeling scheme of mixed effect linear regression followed by spatio-temporal Gaussian process regression (ST-GPR), which produced full time series estimates for each GBD 2017 location. Socio-demographic index (SDI), a composite metric combining education per capita, income per capita, and fertility, was set as a fixed effect in the linear regression since it proved to be a significant predictor. Random effects were set at GBD 2017 region and super-region levels to fit the models but were not used in the predictions.

The process of vetting and validating models was accomplished primarily through an examination of ST-GPR scatter plots by GBD 2017 location from 1990-2017. Any unfitting data points were re-inspected for error at the level of extraction and survey implementation, and subsequently excluded from analysis if deemed appropriate. In addition to SDI, a number of different potential fixed effects were considered, including lag-distributed income and urbanicity, but SDI proved to be the strongest predictor of unsafe sanitation in terms of magnitude of the coefficient. Uncertainty in the estimates was initially constructed based on standard deviation around each survey mean, then propagated through ST-GPR modeling by incorporating the variance of each data point in the Gaussian process regression step. A data point with high variance, for example, would contribute relatively less influence to the model than a data point with lower variance.

Once models are vetted, full time series outputs from ST-GPR modeling are then converted from proportion to prevalence by year and geography and then rescaled to form three mutually exclusive categories that sum up to 1. The table below provides the final result of this rescaling.

| *Category* | *Definition* |
|---|---|
| Unimproved sanitation | Proportion of individuals that use unimproved sanitation facilities. |
| Improved sanitation | Proportion of individuals with access to improved sanitation facilities, excluding sewer connection or septic tank. |
| Sanitation facilities with sewer connection or septic tank | Proportion of individuals with access to toilet facilities with sewer connection or septic tank. |

## Theoretical minimum-risk exposure level

The theoretical minimum-risk exposure level for unsafe sanitation was defined as all individuals have access to a sanitation facility with sewer connection.

## Relative risks

For GBD 2017, unsafe sanitation was only paired with one outcome, diarrheal diseases. A meta-analysis by Wolf et al. 2014 provides the bulk of the relative risk evidence for the relationship between unsafe sanitation and diarrheal diseases. This meta-analysis was updated through a literature review that searched for related intervention studies post-2014 conducted in PubMed. Search terms used were identical to those provided by Wolf et al. 2014. Please refer to appendix tables for more information on relative risk values and citations.

## References


1. "Improved and Unimproved Water Sources and Sanitation Facilities."*WHO / UNICEF Joint Monitoring Programme: Wat/san Categories*. The WHO/UNICEF, n.d. Web. 08 June 2016
2. Wolf, Jennyfer, Annette Prüss-Ustün, Oliver Cumming, Jamie Bartram, Sophie Bonjour, Sandy Cairncross, Thomas Clasen, John M. Colford, Valerie Curtis, Jennifer De France, Lorna Fewtrell, Matthew C. Freeman, Bruce Gordon, Paul R. Hunter, Aurelie Jeandron, Richard B. Johnston, Daniel Mäusezahl, Colin Mathers, Maria Neira, and Julian P. T. Higgins. "Systematic Review: Assessing the Impact of Drinking Water and Sanitation on Diarrhoeal Disease in Low- and Middle-income Settings: Systematic Review and Meta-regression." Trop Med Int Health Tropical Medicine & International Health 19.8 (2014): 928-42. Web.

# Unsafe Hygiene Capstone Appendix

## Flowchart

Unsafe Handwashing

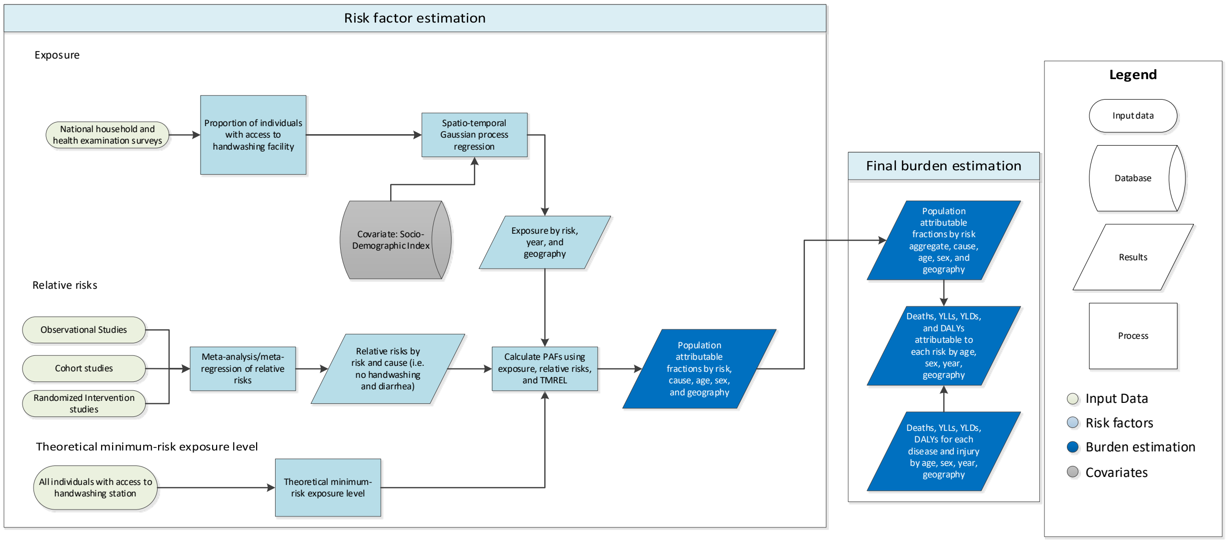


## Input data & methodological summary

### Exposure

#### *Case definition*

Unsafe hygiene is defined as lack of access to a handwashing station with available soap and water. We estimated the burden of unsafe hygiene in both developed and developing settings.

#### *Input data*

Since water and soap availability data are very limited, only country-specific Demographic Health Surveys (DHS) and Malaria Indicator Survey Series (MICS) conducted after 2006 were included as input data.

### Modelling strategy

By year and location, proportion of households with handwashing facility is modelled using a 3-step modelling scheme of mixed effect linear regression followed by spatio-temporal Gaussian process regression (ST-GPR), which outputs full time series estimates for each GBD 2017 location. Socio-demographic index (SDI), a composite index that include income per capita, education, and fertility, was set as a fixed effect in the linear regression since it proved to have significant coefficient. Random effects were set at GBD 2017 region and super-region levels to fit the model but were not used in the predictions.

The process of vetting and validating models was accomplished primarily through an examination of ST-GPR scatter plots by GBD 2016 location from 1990-2016. Any data points lacking face validity were re-inspected for error at the level of extraction and survey implementation, and subsequently excluded from analysis if deemed appropriate. In addition to SDI, a number of different potential fixed effects

were considered, including lag-distributed income and urbanicity. However, SDI proved to be the strongest predictor.

A considerable limitation for when estimating handwashing practices for over 190 independent locations around the world was data sparseness. Even when data were published on handwashing prevalence, the definition was often altered from the GBD 2017 standard definition or it may only have pertained to certain populations (such as hospital patients) and lacked representativeness at the geographic scale we required. The incorporation of questions about soap and water availability in DHS and MICS added much-needed information but there remains a large data gap to be filled if we are to become more certain in handwashing access estimates.

## Theoretical minimum-risk exposure level

The theoretical minimum-risk exposure level for unsafe hygiene is defined as all individuals with access to handwashing facility after any contact with excreta, including children's excreta.

## Relative risks

A meta-analysis by Cairncross et al.[1] provide relative risk values describing the relationship between lack of facility access and diarrheal diseases. A meta-analysis by Rabie and Curtis[2] provided relative risk evidence for the relationship between lack of facility access and lower respiratory infection. Please refer to appendix tables for more information on relative risk values and citations.

## References

1. Cairncross, S., Hunt, C., Boisson, S., Bostoen, K., Curtis, V., Fung, I. C., & Schmidt, W. P. (2010). Water, sanitation and hygiene for the prevention of diarrhoea. International Journal of Epidemiology, 39(Supplement 1), I193-I205. doi:10.1093/ije/dyq035
2. Rabie, T., & Curtis, V. (2006). Handwashing and risk of respiratory infections: a quantitative systematic review. Tropical Medicine and International Health, 11(3), 258-267. doi:10.1111/j.1365-3156.2006.01568.x

# Ambient Particulate Matter Pollution Capstone Appendix

## Flowchart

Ambient particulate matter pollution

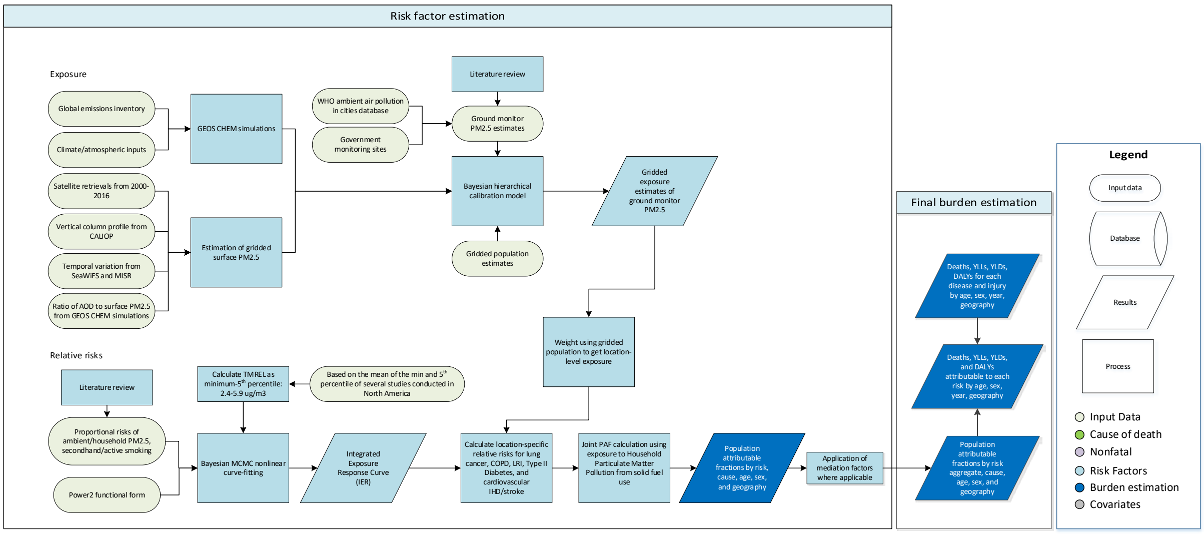


## Input data and modeling strategy

### Exposure

#### *Definition*

Exposure to ambient air pollution is defined as the population-weighted annual average mass concentration of particles with an aerodynamic diameter less than 2.5 micrometers ($PM_{2.5}$) in a cubic meter of air. This measurement is reported in µg/m$^3$.

#### *Input Data*

The data used to estimate exposure to ambient air pollution is drawn from multiple sources, including satellite observations of aerosols in the atmosphere, ground measurements, chemical transport model simulations, population estimates, and land-use data.

The following details the updates in methodology and input data used in GBD 2017.

#### $PM_{2.5}$ ground measurement database

Updates of ground measurements used for GBD 2017 include using more recent data than that used previously and the addition of data from new locations. The data from the 2018 update of the WHO Global Ambient Air Quality Database include monitor-specific measurements of concentrations of $PM_{10}$ and $PM_{2.5}$ from 9,960 ground monitors (up from 6,003 in GBD 2016) from 108 countries. The majority of measurements were recorded in 2016 (as there is a lag in reporting measurements, little data from 2017 were available). Annual averages were excluded if they were based on less than 75% coverage within a year. Collection year ranged from 2008 to 2017 in data used. If information on coverage was not available then data were included unless they were already sufficient data within a country (monitor density greater than 0.1).

For locations measuring only $PM_{10}$, $PM_{2.5}$ measurements were estimated from $PM_{10}$. This was performed using a hierarchy of conversion factors ($PM_{2.5}$/$PM_{10}$ ratios): (i) for any location a 'local'

conversation factor was used, constructed as the ratio of the average measurements (of $PM_{2.5}$ and $PM_{10}$) from within 50km and within the same country, if such were available' (ii) if there was not sufficient local information to construct a conversion factor then a country-wide conversion factor was used; and (iii) if there was no appropriate information within a country then a regional factor was used. In each case, to avoid the possible effects of outliers in the measured data (both $PM_{2.5}$ and $PM_{10}$), extreme values of the ratios were excluded (defined as being greater/lesser than the 95 and 5% quantiles of the empirical distributions of conversion factors) of the latter two cases for the country measurements were available, for both metrics. As in the GBD 2013 and GBD 2015/GBD 2016 databases, in addition to values of $PM_{2.5}$ and whether they were direct measurement or converted from $PM_{10}$, the database also included additional information, where available, related to the ground measurements such as monitor geo coordinates and monitor site type.

### Satellite-based estimates

The updated satellite-based estimates for years 1998-2016 are described in detail in van Donkelaar et al. 2016.[1] These estimates were available at 0.1°×0.1° resolution (~11 x 11 km resolution at the equator) and combine aerosol optical depth retrievals from multiple satellites with the GEOS Chem chemical transport model and land use information.

### Population data

A comprehensive set of population data on a high-resolution grid was obtained from the Gridded Population of the World (GPW) database. These estimates are adjusted to match UN2015 Population Prosepectus. These data are provided on a 0.0417°×0.0417° resolution. Aggregation to each 0.1°×0.1° grid cell comprised of summing the central 3 × 3 population cells. As this resulted in a resolution higher than necessary, it was repeated four times, each offset by one cell in a North, South, East and West direction. The average of the resulting five quantities was used as the estimated population for each grid cell. Population estimates for 2000, 2005, 2010, 2015 and 2020 were available from GPW version 4 revision 10. Populations for 2016 and 2017 were obtained by interpolation using natural splines with knots placed at 2000, 2005, 2010, 2015 and 2020. This was performed for each grid cell.

### Chemical transport model simulations

Estimates of the sum of particulate sulfate, nitrate, ammonium and organic carbon and the compositional concentrations of mineral dust simulated using the GEOS Chem chemical transport model, and a measure combining elevation and the distance to the nearest urban land surface (as described in van Donkelaar et al. 2016[1]) were available for 2000 to 2016 for each 0.1°×0.1° grid cell. These were not included within the GBD 2013 analysis.

### *Modelling strategy*

Significant advances have been made in the methodology used to estimate exposure to ambient particulate matter pollution since GBD 2013. The following is a summary of the modelling approach, known as the Data Integration Model for Air Quality (DIMAQ) used in GBD 2015, 2016, and 2017; further details can be found in Shaddick *et al.* (2017).[2]

In GBD 2010 and GBD 2013 exposure estimates were obtained using a single global function to calibrate available ground measurements to a 'fused' estimate of $PM_{2.5}$; the mean of satellite-based estimates and those from the TM5 chemical transport model, calculated for each 0.1°×0.1° grid cell. This was recognised to represent a trade-off between accuracy and computationally efficiency when utilising all the available data sources. In particular, the GBD 2013 exposure estimates were known

to underestimate ground measurements in specific locations (see discussion in Brauer et al., 2013[3]). This underestimation was largely due to the use of a single, global, calibration function, whereas in reality the relationship between ground measurements and other variables will vary spatially.

In GBD 2015 and GBD 2016, coefficients in the calibration model were estimated for each country. Where data were insufficient within a country, information can be `borrowed' from a higher aggregation (region) and if enough information is still not available from an even higher level (super-region). Individual country level estimates were therefore based on a combination of information from the country, its region and super-region. This was implemented within a Bayesian Hierarchical modelling (BHM) framework. BHMs provide an extremely useful and flexible framework in which to model complex relationships and dependencies in data. Uncertainty can also be propagated through the model allowing uncertainty arising from different components, both data sources and models, to be incorporated within estimates of uncertainty associated with the final estimates. The results of the modelling comprise a posterior distribution for each grid cell, rather than just a single point estimate, allowing a variety of summaries to be calculated. The primary outputs here are the median and 95% credible intervals for each grid cell. Based on the availability of ground measurement data, modelling and evaluation was focused on the year 2016.

The GBD 2017 model was updated to also include within country calibration variation. [4] The model used for GBD2017, henceforth referred to as DIMAQ2, provides a number of substantial improvements over the initial formulation of DIMAQ. In DIMAQ, ground measurements from different years were all assumed to have been made in the primary year of interest (i.e. 2014 for GBD2015 before extrapolation) and then regressed against values from other inputs (e.g. satellites etc.) made in that year. In the presence of changes over time therefore, and particularly in areas where no recent measurements were available, there was the possibility of mismatches between the ground measurements and other variables. In DIMAQ2, ground measurements and matched with other inputs (over time) and the possibility of the (global level) coefficients being allowed to vary over time, subject to smoothing that is induced by a second-order random walk process. In addition, the manner in which spatial variation can be incorporated within the model has developed: where there is sufficient data, the calibration equations can now vary (smoothly) both within and between countries, achieved by allowing the coefficients to follow (smooth) Gaussian processes. Where there is insufficient data within a country, to produce accurate equations, as before information is borrowed from lower down the hierarchy and it is supplemented with information from the wider region.

DIMAQ2 is used for all regions except for the North Africa-Middle East and Sub-Saharan super-regions and remote islands where there is insufficient data to allow the extra complexities of the new model to be implemented. In the North Africa-Middle East and Sub-Saharan super-regions a simplified version of DIMAQ2 is used in which the temporal component is dropped, and for remote islands the original DIMAQ is used.

Due to both the complexity of the models and the size of the data, notably the number of spatial predictions that are required, recently developed techniques that perform 'approximate' Bayesian inference based on integrated nested Laplace approximations (INLA) were used.[5] Computation was performed using the R interface to the INLA computational engine (R-INLA). Fitting the models and performing predictions for each of the ca. 1.4 million grid cells required the use of a high performance computing cluster (HPC) making use of high memory nodes.

*Model evaluation*

Model development and comparison was performed using within- and out-of-sample assessment. In the evaluation, cross validation was performed using 25 combinations of training (80%) and validation (20%) datasets. Validation sets were obtained by taking a stratified random sample, using sampling probabilities based on the cross-tabulation of $PM_{2.5}$ categories (0-24.9, 25-49.9, 50-74.9, 75-99.9, 100+ $\mu g/m^3$) and super-regions, resulting in them having the same distribution of $PM_{2.5}$ concentrations and super-regions as the overall set of sites. The following metrics were calculated for each training/evaluation set combination: for model fit - $R^2$ and deviance information criteria (DIC, a measure of model fit for Bayesian models); for predictive accuracy - root mean squared error (RMSE) and population weighted root mean squared error (PwRMSE).

All modelling was performed on the log-scale. The choice of which variables were included in the model was made based on their contribution to model fit and predictive ability. The following is a list variables and model structures that were included in DIMAQ.

Continuous explanatory variables:

- (SAT) Estimate of $PM_{2.5}$ (in $\mu gm^{-3}$) from satellite remote sensing on the log-scale.
- (POP) Estimate of population for the same year as SAT on the log-scale.
- (SNAOC) Estimate of the sum of sulfate, nitrate, ammonium and organic carbon simulated using the GEOS Chem chemical transport model.
- (DST) Estimate of compositional concentrations of mineral dust simulated using the GEOS Chem chemical transport model.
- (EDxDU) The log of the elevation difference between the elevation at the ground measurement location and the mean elevation within the GEOS Chem simulation grid cell multiplied by the inverse distance to the nearest urban land surface.

Discrete explanatory variables:

- (LOC) Binary variable indicating whether exact location of ground measurement is known.
- (TYPE) Binary variable indicating whether exact type of ground monitor is known.
- (CONV) Binary variable indicating whether ground measurement is $PM_{2.5}$ or converted from $PM_{10}$.

Random Effects:

- Grid cell random effects on the intercept to allow for multiple ground monitors in a grid cell.
- Country-region-super-region hierarchical random effects for the intercept.
- Country-region-super-region hierarchical random effects for the coefficient associated with SAT .
- Country-region-super-region hierarchical random effects for the coefficient associated with the difference between estimates from CTM and SAT.
- Country-region-super-region hierarchical random effects for the coefficient associated with POP.
- Country level random effects for population uses a neighbourhood structure allowing specific borrowing of information from neighbouring countries.
- Within a region, country level effects of SAT and the difference between SAT AND CTM are assumed to be independent and identically distributed.
- Within a super-region, region level random effects are assumed to be independent and identically distributed.

- Super-region random effects are assumed to be independent and identically distributed.

Interactions*:*

- Interactions between the binary variables and the effects of SAT and CTM.

In addition, DIMAQ2 includes

- Smoothed, spatially varying, random-effects for the intercept
- Smoothed, spatially varying, random-effects for the coefficient of coefficient associated with SAT
- Smoothed, temporally varying, random-effect for the intercept

### *Results*

The final model contained the following variables: SAT, POP, SNAOC, DST, EDxDU, LOC, TYPE, and CONV, together with interactions between SAT and each of LOC, TYPE and CONV. The model structure contained grid cell random effects on the intercept to allow for multiple ground monitors in a grid cell, country-region-super-region hierarchical random effects for intercepts and SAT and country level random effects for population using a neighbourhood structure allowing specific borrowing of information from neighbouring countries together with region-super-region hierarchical random effects for POP. Notably, and as in GBD 2015 and GBD 2016, based on the evaluation of candidate models, including estimates from the TM5 chemical transport model (CTM) used in GBD 2013 did not improve the predictive ability of the model and was therefore not included.

Compared to the model used in GBD2013, DIMAQ showed improved predictions of ground measurements in all super regions with improvements in both within-sample fit; with a global population-weighted RMSE of 12.1 µg/m$^3$ compared to 23.1 µg/m$^3$ when using the GBD 2013 approach.[1] Using the larger database available for GBD2017, with potentially more variability in measurements, DIMAQ2 shows an additional improvement on DIMAQ: overall population-weighted RMSE reduced from 9.32 to 8.11 (12.12 to 11.17 when using all data, irrespective of within-year coverage). Reductions by super-region can be seen in Figure 1. Reductions can be seen in all super-regions with particular improvement in the Southeast Asia, East Asia and Oceania super-region which is based largely on a substantial increase in accuracy in China, PwRMSE 6 vs 9 µg/m$^3$

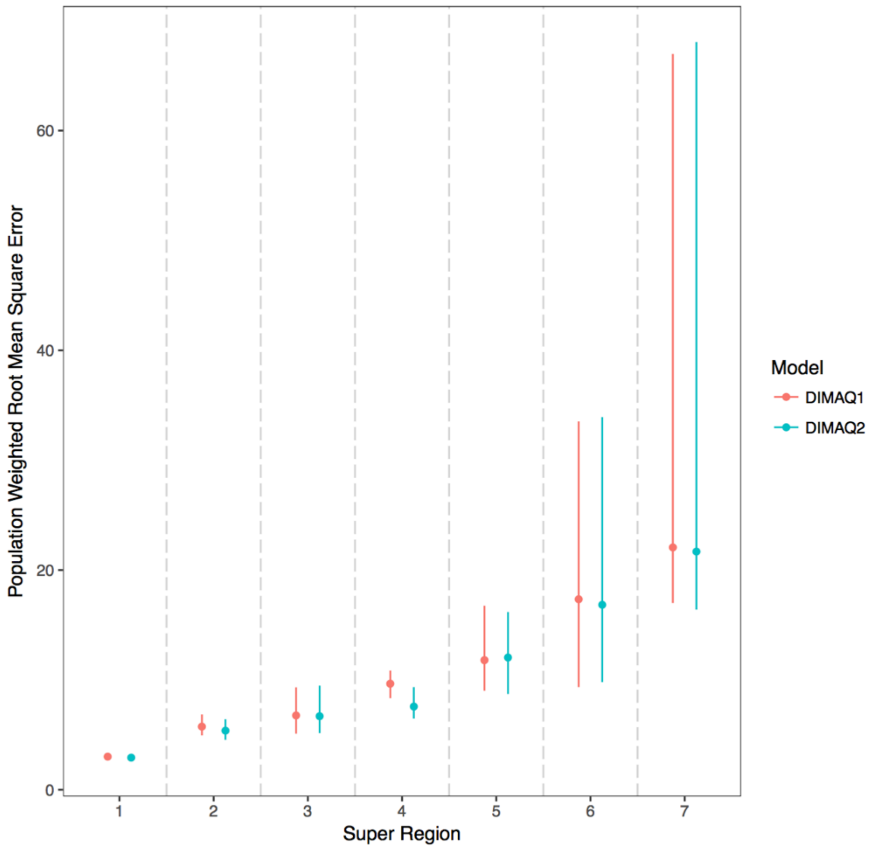

*Figure 1: Summary measures of predictive ability, globally and by super-region. Dots denote the median values of population weighted root mean squared error (μg/m³) from 25 validation sets with vertical lines showing the range of values over those sets.*

## *Estimates for other years*

In contrast to the method used previously, where estimates (of $PM_{2.5}$) were extrapolated to produce estimates for the year of interest (e.g. 2017 where data was available up to and including 2016) due to the extra complexity of the smooth spatial processes in DIMAQ2 this would not be possible in any straightforward manner. With DIMAQ2 it is the input variables that are extrapolated; this allows estimates for 2017 to be produced in the same way as other years and crucially, allows measures of uncertainty to be produced within the BHM framework rather than by using post-hoc approximations.

Satellite estimates and quantities estimated using the GEOS-Chem model were available for 1990, 1995, 2000, 2005, 2010-2016. Estimates of these input variables for 2017 were produced by extrapolating, on a cell-by-cell basis, using natural splines. Population estimates for 2000, 2005, 2010, 2015 and 2020 were availalble from GPW version 4. For 1990 and 1995 data were extracted from GPW version 3, as in GBD2013.[2] As with populations for 2015, values for each cell for 2011-2017 were obtained by interpolation using natural splines with knots placed at 2000, 2005, 2010, 2015 and 2020.

These were used as inputs to DIMAQ, enabling estimates of exposures to be obtained for each of these years respectively. For 2017, estimates of exposures were obtained from predictions from locally-varying regression models.[6] For each cell a model was fit to the values within that cell over time, with a constraint placed on the rate of change between 2016 and 2017 to avoid unrealistic and/or unjustified extrapolation of trends. Measures of uncertainty were obtained by repeating the procedure for the limits of the 95% credible intervals, again on a cell-by-cell basis.

### *Population-weighted exposure generation*

To generate a distribution of the population-weighted ambient particulate matter, we took a weighted sampling strategy, taking samples from all grid cells in a given location. For example, for a country with n grid cells, we randomly sampled 1000 values from the n (grid cells) x 1000 (samples) where the probability of being sampled was proportional to the population of that grid cell.

## Theoretical minimum-risk exposure level

The TMREL was assigned a uniform distribution with lower/upper bounds given by the average of the minimum and 5$^{th}$ percentiles of outdoor air pollution cohort studies exposure distributions conducted in North America, with the assumption that current evidence was insufficient to precisely characterise the shape of the concentration-response function below the 5$^{th}$ percentile of the exposure distributions. The TMREL was defined as a uniform distribution rather than a fixed value in order to represent the uncertainty regarding the level at which the scientific evidence was consistent with adverse effects of exposure. The specific outdoor air pollution cohort studies selected for this averaging were based on the criteria that their 5$^{th}$ percentiles were less than that of the American Cancer Society Cancer Prevention II (CPSII) cohort's 5$^{th}$ percentile of 8.2 based on Turner et al. (2016).[7] This criterion was selected since GBD 2010 used the minimum, 5.8, and 5th percentile solely from the CPS II cohort. The resulting lower/upper bounds of the distribution for GBD 2017 were 2.4 and 5.9. This has not changed since GBD 2015.

## Relative risks and population attributable fractions

We estimated the Ambient Air Pollution-attributable burden of disease based on the relation of long-term exposure to PM2.5 with Ischemic Heart Disease, stroke (ischemic and hemorrhagic), COPD, lung cancer and acute lower respiratory infection. These were also the pollutant-outcome pairs used to estimate the Ambient Air Pollution attributable burden since GBD 2010. For GBD 2017 we also added Type II Diabetes as an outcome of ambient air pollution. We used results from all cohort studies published as of July 2018 that reported cause-specific relative risk estimates based on measured or modelled PM2.5 and that adjusted for potential confounding due to other major risk factors such as tobacco smoking using data for each study participant.

Bowe et al. recently published work that assembled the evidence for the relationship between particulate matter and diabetes to generate IER curves and attributable burden estimates based on methodologies similar to those of the GBD. [8]

When generating the IER for Type II Diabetes, we included all eight of the studies summarized by Bowe et al. in addition to six other cohorts. Resulting attributable burden estimates were remarkably similar to GBD 2017 results. All citations for studies used in the fitting of the IER curve can be found using the GBD 17 Data Input Sources Tool.

### Integrated exposure response function

The Integrated Exposure Response Function (IER) was created to ascertain the shape of the dose response curve for a variety of health outcomes across a wide range of exposure to PM2.5. The IER model is fit by integrating RR information from studies of outdoor air pollution (OAP), Second hand tobacco smoke (SHS), Household Air Pollution (HAP), and Active Smoking (AS). Because OAP studies are often performed at the lower end of the ambient air pollution range, incorporating other exposures to particulate matter enables RR estimation across the global range of exposure. These methods have been described in detail elsewhere.[9,10]

Notable changes for GBD 2017 include added studies for OAP, SHS, and HAP, updated literature reviews for AS studies, and more informative priors to stabilize the shape of the IER curves.

- We added all newly published cohorts of long-term exposure to Ambient PM2.5 and incidence or mortality due to IHD, stroke, COPD, lung cancer, and LRI. One notable addition was the China Male Cohort which included mortality due to IHD, Stroke, COPD, Lung Cancer, and Diabetes (unpublished analysis).[11] This study represented a higher exposure range than most of our previously incorporated studies with 5th and 95th percentile of 15.5 and 77.1 micrograms/$m^3$. For Type II Diabetes, the new outcome included in GBD 2017, we included all cohorts which measured long-term PM2.5 exposure and incident diabetes or mortality due to diabetes.
- We did not change the SHS input studies with the exception of including all studies from a recent meta-analysis examining the relationship between SHS and Type II Diabetes.[12] We also added seven studies found from a systematic review examining SHS exposure and COPD. We had previously not included SHS in the formation of this curve.
- We added four cohort studies of HAP and any of our measured outcomes. Previously we have only included which measured levels of PM2.5 exposure. To incorporate cohort studies with binary exposure data (presence or absence of solid-fuel use for cooking) we used the PM2.5 mapping function (see Household Air Pollution Appendix for more details) to obtain a PM2.5 level attributed to solid fuel use for cooking for the location-year of the study ($Exp_{HAP}$). We also used the OAP exposure model to obtain an OAP PM2.5 level for the location-year ($Exp_{OAP}$). The study RR was used to inform the curve on the range of $Exp_{OAP}$ to ($Exp_{OAP}$ + $Exp_{HAP}$).
- For all outcomes, we used updated systematic reviews of the literature performed by the GBD smoking team for studies examining cigarettes smoked per day and the six IER outcomes to inform the high exposure range of the curve. The smoking team found that the process of systematic review and inclusion of all acceptable studies led to lower relative risks.
- To help obtain more reasonable curve fits, we added more informative priors to two of three IER function parameters in the MCMC Bayesian fitting process.

### *Limitations*

It is important to recognize the inherent limitations of the IER approach. The use of various sources to construct a risk curve assumes an equitoxicity of particles, consistent with evaluations by US EPA and WHO. However, current evidence suggests there are differences in health impact by source, size, and chemical composition. This is seen when comparing studies of ambient and household particulate matter. As this body of evidence grows, we will continue to re-examine our strategy for the integrated exposure-response curve. For now, the IER is a practical solution to fill gaps in the literature where we do not have sufficient evidence such as household air pollution exposures and ambient in highly polluted areas.

Additionally, currently the exposure concentrations used for both SHS and AS data points when fitting the IER are contrasted with the TMREL and do not take into account ambient particulate matter pollution. In future iterations of fitting the curve, we will test alternate approaches, including a similar approach to HAP, allowing each data point to inform the curve on the range of $Exp_{OAP}$ to ($Exp_{OAP}$ + $Exp_{AS/SHS}$).

## Relative risk and proportional PAF approach

For GBD 2017 we developed a new approach to use the IER for obtaining PAFs for both OAP and HAP. Previously, relative risks for both exposures were obtained from the IER as a function of exposure and relative to the same TMREL. In reality, were a country to reduce only one of these risk factors, the other would remain. We failed to consider the joint effects of particulate matter from outdoor exposure and burning solid fuels for cooking.

In GBD 2017, relative risks were still estimated from the output of the IER curve. Everyone is exposed to some level of OAP, but only a proportion of the population in each location-year use solid cooking fuel and are exposed to HAP. For the proportion of the population not exposed to HAP the relative risk was obtained by $RR_{OAP} = IER(z = Exp_{OAP})$ and used to calculate the PAF for each location based on the population-weighted exposure.

For the proportion of the population exposed to both OAP and HAP, we calculated a joint relative risk from the IER by $RR_{OAP+HAP} = IER(z = Exp_{OAP}+Exp_{HAP})$. This joint relative risk is used to calculate a joint PAF for each location. PAF calculation is detailed in the methods appendix. For each location, we proportioned the joint PAF based on the proportion of exposure due to OAP and HAP respectively. See the table below for equations used to calculate proportional PAFs.

| PAF | Population not exposed to HAP | Population exposed to HAP |
|---|---|---|
| **OAP** | $PAF_{OAP}$ | $(Exp_{OAP}/(Exp_{OAP}+Exp_{HAP}))*PAF_{OAP+HAP}$ |
| **HAP** | 0 | $(Exp_{HAP}/(Exp_{OAP}+Exp_{HAP}))*PAF_{OAP+HAP}$ |

Generally, as expected, this new strategy led to lower PAFs for both ambient and household particulate matter pollution.

## References


1. van Donkelaar, A.; Martin, R. V; Brauer, M.; Hsu, N. C.; Kahn, R. A.; Levy, R. C.; Lyapustin, A.; Sayer, A. M.; Winker, D. M. Global Estimates of Fine Particulate Matter using a Combined Geophysical-Statistical Method with Information from Satellites, Models, and Monitors. Environ. Sci. Technol. 2016, 50 (7), 3762–3772
2. Shaddick, G., Thomas, M.L., Jobling, A., Brauer, M., van Donkelaar, A., Burnett, R., Chang, H., Cohen, A., Van Dingenen, R., Dora, C. and Gumy, S., 2016. Data Integration Model for Air Quality: A Hierarchical Approach to the Global Estimation of Exposures to Ambient Air Pollution. Journal of Royal Statistical Society Series C (Applied Statistics). 2017. DOI: 10.1111/rssc.12227
3. Brauer, M.; Freedman, G.; Frostad, J.; van Donkelaar, A.; Martin, R. V; Dentener, F.; Van Dingenen, R.; Estep, K.; Amini, H.; Apte, J. S.; et al. Ambient Air Pollution Exposure Estimation for the Global Burden of Disease 2013. Environ. Sci. Technol. 2015, 50 (1), 79–88.
4. Shaddick G, Thomas M, Amini H, Broday DM, Cohen A, Frostad J, Green A, Gumy S, Liu Y, Martin RV, Prüss-Üstün A, Simpson D, van Donkelaar A, Brauer M. Data integration for the assessment of population exposure to ambient air pollution for global burden of disease assessment. Environ Sci Technol. 2018 Jun 29. doi: 10.1021/acs.est.8b02864
5. Rue, H.; Martino, S.; Chopin, N.; Approximate Bayesian inference for latent Gaussian models by using integrated nested Laplace approximations. Journal of the royal statistical society: Series b (statistical methodology). 2009;71(2):319-92.
6. Cleveland, W.S. and Devlin, S.J., 1988. Locally weighted regression: an approach to regression analysis by local fitting. Journal of the American statistical association, 83(403), pp.596-610.
7. Turner MC,Jerrett M, Pope CA 3rd, Krewski D, Gapstur SM, Diver WR, Beckerman BS, Marshall JD, Su J, Crouse DL, Burnett RT. Long-term ozone exposure and mortality in a large prospective study . Am J Respir Crit Care Med. 2016; 193(10): 1134-42.

8. Bowe B, Xie Y, Li T, Yan Y, Xian H, Al-Aly Z. The 2016 global and national burden of diabetes mellitus attributable to PM2.5 air pollution. *The Lancet Planetary Health*. 2018; 2(7): e301–12.
9. Cohen AJ, Brauer M, Burnett R, et al. Estimates and 25-year trends of the global burden of disease attributable to ambient air pollution: an analysis of data from the Global Burden of Diseases Study 2015. Lancet 2017; published online April 10. http://dx.doi.org/10.1016/S0140-6736(17)30505-6.
10. Burnett RT, Pope CA 3rd, Ezzati M, Olives C, Lim SS, Mehta S, Shin HH, Singh G, Hubbell B, Brauer M, Anderson HR, Smith KR, Balmes JR, Bruce NG, Kan H, Laden F, Prüss-Ustün A, Turner MC, Gapstur SM, Diver WR, Cohen A. An integrated risk function for estimating the global burden of disease attributable to ambient fine particulate matter exposure. *Environ Health Perspect*. 2014; 122(4): 397-403.
11. Yin P, Brauer M, Cohen A, Burnett RT, Liu J, Liu Y, Liang R, Wang W, Qi J, Wang L, Zhou M. Long-term Fine Particulate Matter Exposure and Nonaccidental and Cause-specific Mortality in a Large National Cohort of Chinese Men. *Environ Health Perspect*. 2017; 125(11): 117002.
12. Xiaomin Wei, Meng E., Sufang Yu. A meta-analysis of passive smoking and risk of developing Type 2 Diabetes Mellitus. *Diabetes Research and Clinical Practice*. 2015; 107(1): 9-14.

# Household Air Pollution Capstone Appendix

## Flowchart

Household Air Pollution from Solid Fuels

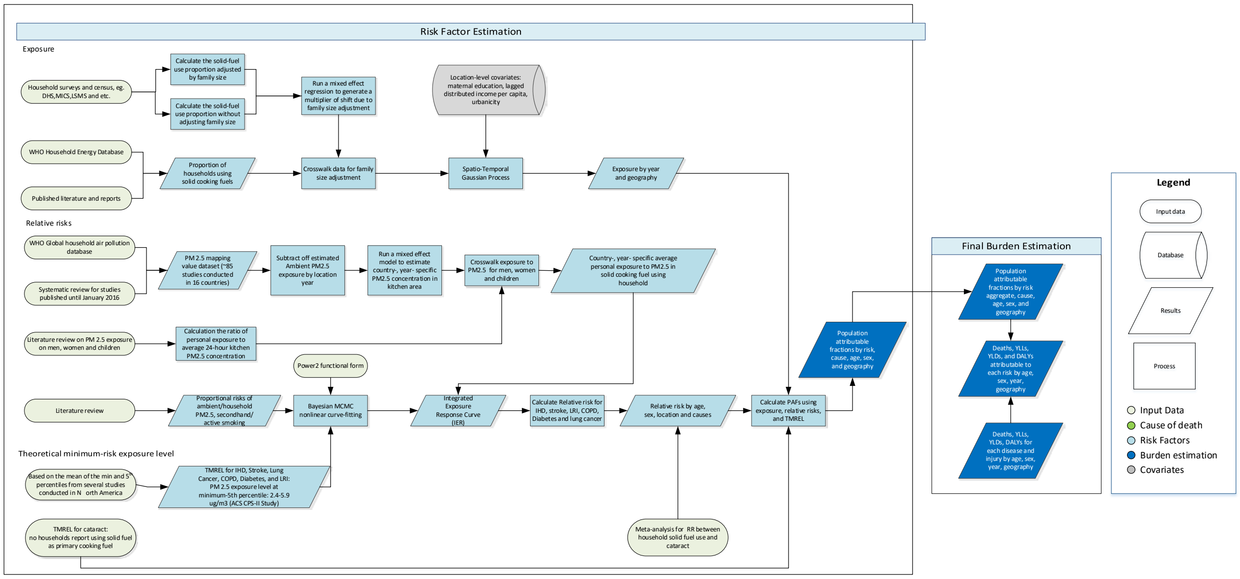


## Input Data & Methodological Summary

### Exposure

#### *Case definition*

Exposure to household air pollution from solid fuels (HAP) is defined as the proportion of households using solid cooking fuels. The definition of solid fuel in our analysis includes coal, wood, charcoal, dung, and agricultural residues.

#### *Input data*

Data were extracted from the standard multi-country survey series such as Demographic and Health Surveys (DHS), Living Standards Measurement Surveys (LSMS), Multiple Indicator Cluster Surveys (MICS), and World Health Surveys (WHS), as well as country-specific survey series such as Kenya Welfare Monitoring Survey and South Africa General Household Survey. To fill the gaps of data in surveys and censuses, we also downloaded and updated HAP estimates from WHO Energy Database and extracted from literature through systematic review. Each nationally or sub-nationally representative data point provided an estimate for the percentage of households using solid cooking fuels. Estimates for the usage of solid fuels for non-cooking purpose were excluded, i.e. primary fuels for lighting. The database, with estimates from 1980 to 2017, contained about 680 studies from 150 countries. As updates to systematic reviews are performed on an ongoing schedule across all GBD causes and risk factors, an update for household air pollution will be performed in the next 1-2 iterations.

#### *Modelling strategy*

Household air pollution was modelled at household level using a three-step modelling strategy that uses linear regression, spatiotemporal regression and Gaussian Process Regression (GPR). The first step is a mixed-effect linear regression of logit-transformed proportion of households using solid cooking fuels. The linear model contains maternal education, proportion of population living in urban areas, and

lagged-distributed income as covariates and has nested random effect by GBD region, and GBD super region respectively. The full ST-GPR process is specified elsewhere this appendix. No substantial modelling changes were made in this round compared to GBD 2016.

## Theoretical minimum-risk exposure level

For cataract, the TMREL is defined as no households using solid cooking fuel. For outcomes that utilise evidence based on the Integrated Exposure Response (IER), the TMREL is defined as uniform distribution between 2.4 and 5.9 ug/$m^3$.

## Relative risks

In addition to the previously included outcomes of lower respiratory infections (LRI), stroke, Ischemic Heart Disease (IHD), Chronic Obstructive Pulmonary Disease (COPD), lung cancer, and cataract, in GBD 2017 we added Type II Diabetes as a new outcome of household air pollution. The relative risk for cataracts was extracted from a meta-analysis and is 2.47 with 95% (1.61, 3.73).[1] GBD currently only estimates cataracts as an outcome for females.

In GBD 2017, we adopted a new approach for risk attribution using the Integrated Exposure-Response Function (IER). Updates to the IER and the new joint-estimation PAF approach is described in the Ambient Particulate Matter appendix.

## $PM_{2.5}$ mapping value

In order to use the IER curve, we must estimate the exposure to particulate matter with diameter of less than 2.5 micrometers ($PM_{2.5}$). Since GBD 2015 we have been using a mapping model relying on a database of now almost 90 studies which measures $PM_{2.5}$ exposure in households using solid cooking fuel. Using socio-demographic index and study-level factors as covariates, we predict exposure for all location-years.

In GBD 2017, we updated the model to estimate the individual exposure to $PM_{2.5}$ over and above ambient levels due to the use of solid cooking fuel. We did this by subtracting off the estimated ambient level $PM_{2.5}$ for the location-year of each study in the database before inputting them into the model. By doing this we have independent estimates for $PM_{2.5}$ exposure due to ambient and household solid fuel use.

These exposures are cross-walked to values for men, women, and children by generating the ratio of each group's mean exposure to the overall mean personal exposure. The resulting location, year, sex, and age specific $PM_{2.5}$ exposure values are used as inputs in the IER and attributable burden calculation process.

## References

1. Smith KR, Bruce N, Balakrishnan K, Adair-Rohani H, Balmes J, Chafe Z, et al. Millions Dead: How Do We Know and What Does It Mean? Methods Used in the Comparative Risk Assessment of Household Air Pollution. Annu Rev Public Health. 2014;35(1):185–206.

# Ambient Ozone Pollution Capstone Appendix

## Flowchart

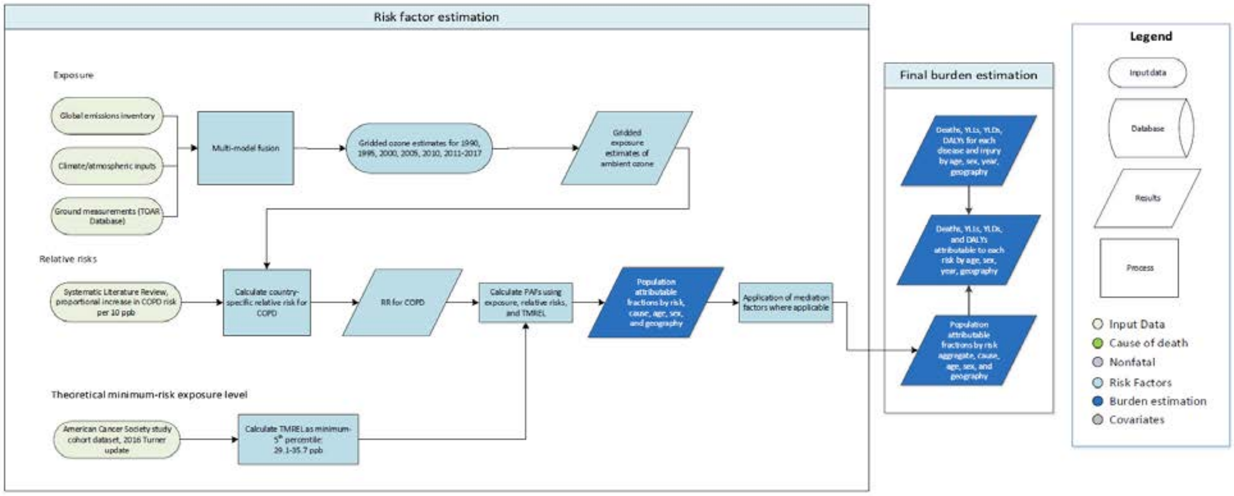


## Input data and methodological summary

### Exposure

#### *Case definition*

For GBD 2017, exposure to ozone pollution is defined as the seasonal (6 month period with highest mean) 8 hour daily maximum ozone concentrations, measured in ppb. This was an update from the previous exposure metric in accordance with an update of the American Cancer Society Cancer Prevention Study II (ACS CPS-II).[1]

#### *Input data*

Previously, exposure estimates were based on a chemical transport model with no measurement database or evaluation. In GBD 2017, exposure estimates incorporated a new comprehensive ozone measurement database (TOAR).[2] This enabled a continent-specific weighted blend of 6 chemical transport models with grid cell level bias correction. The use of ground measurements also enabled the incorporation of error estimation, where previously we had assumed a +/- 6% error. The output of this model is a global raster of ozone exposure which is a summary for the years 2008-2014.[3]

#### *Modelling strategy for trends*

To estimate ozone concentrations over time, we used the trend from the former GBD model for 1990, 2000, and 2010 and cubic splines for 1995, 2005, and 2011, after applying an adjustment for the difference in trends between the previous (1 hour daily maximum) and current (8 hour daily maximum metrics. Annualised rate of change was used to predict for the years 2012-2017.

## Theoretical minimum-risk exposure level

The TMREL of ozone was updated this year based on the exposure distribution from the updated ACS CPS-II study.[1] A uniform distribution was drawn around the minimum and 5th percentile values experienced by the cohort, defined as ~U(29.1, 35.7), in ppb.

## Relative risks

Since the inclusion of ozone in GBD 2010 the relative risk of ozone exposure for respiratory COPD mortality has been defined to be 1.029, 95% C.I. (1.01-1.048) per 10 ppb of ozone exposure. Note that this comes from one study that looked at all respiratory mortality.[4] For GBD 2017, we performed a literature review and included five cohorts from Canada, the UK, and the US which all measured COPD mortality. For cohorts with multiple analyses we chose the most recent analysis. We found a resulting relative risk of 1.06, 95% C.I. (1.02, 1.10).

## References


1. Turner MC,Jerrett M, Pope CA 3rd, Krewski D, Gapstur SM, Diver WR, Beckerman BS, Marshall JD, Su J, Crouse DL, Burnett RT. Long-term ozone exposure and mortality in a large prospective study. *Am J Respir Crit Care Med*. 2016; 193(10): 1134-42.
2. Schultz MG, Schröder S, Lyapina O, Cooper O, Galbally I, Petropavlovskikh I, et al.. Tropospheric Ozone Assessment Report: Database and Metrics Data of Global Surface Ozone Observations. Elem Sci Anth. 2017;5:58. DOI: http://doi.org/10.1525/elementa.244
3. Chang K-L, Cooper OR. A new method for combining observations and multiple model output for an improved estimate of the global surface ozone distribution. Atmospheric Chemistry and Physics Discussion, 2018.
4. Jerrett M, Burnett RT, Pope CA, et al. Long-term ozone exposure and mortality. N Engl J Med 2009; 360: 1085–95.

# Radon Exposure Capstone Appendix

## Flowchart

Radon Exposure

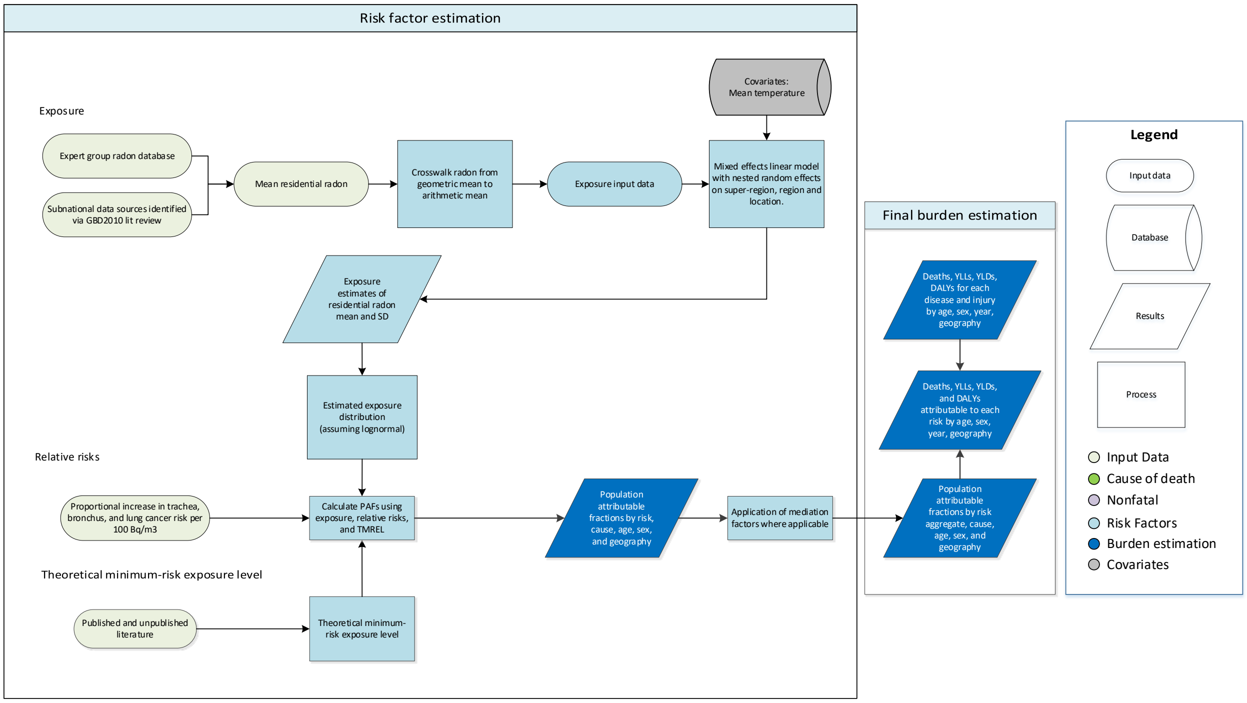


## Input data & methodological summary

### Exposure

#### *Case definition*

Radon is a radioactive gas that is produced as a byproduct of the decay chain of uranium, occurring naturally within the Earth's crust. Some fraction of this natural radon production escapes into the atmosphere, where it forms at low concentration unless build-up is caused by enclosed spaces like homes, mines, or caves. Radon exposure is expressed as average daily exposure to indoor air radon gas levels measured in Becquerels (disintegrations per second) per cubic meter (Bq/m$^3$).

#### *Input data*

Exposure to radon is determined using values curated by an expert group. These values are taken from a variety of sources including literature, government agencies, and monitoring stations. Their methodology is then inspected to determine if they are robust enough to be considered as country-level averages. 76 data points were added for GBD 2017 including several from a study which reports on subnational variation in India. Before modelling, a crosswalk is performed from studies measuring geometric to arithmetic mean.

### *Modelling strategy*

Because radon is naturally occurring and is not considered to have much temporal fluctuation,[1] we shifted from a spatio-temporal GPR model to a mixed effects linear model. The model included nested random effects on super-region, region, and location (most detailed) and one fixed effect covariate, long-term mean temperature as a proxy for adequate building ventilation.

We did not have the microdata necessary to use ensemble modelling to inform our radon exposure distribution, so for GBD 2017 we continued to assume a lognormal distribution. Arithmetic mean exposure estimates obtained were used to fit the lognormal distribution before applying relative risks.

## Theoretical minimum-risk exposure level

The TMREL was also taken directly from literature values that were not updated for GBD 2017. Given that radon is naturally occurring, zero exposure would be impossible. As such, we continue to use a TMREL of 10 $Bq/m^3$, which is equivalent to the outdoor concentration of radon.[2]

## Relative risks

The relative risk for radon exposure was extracted from literature values – a 2005 meta-analysis of case-control studies showing the association of radon with lung cancer.[3] This value was used in GBD 2010 and has not been changed since.

# References

1. Steck DJ. Annual average indoor radon variations over two decades. Health Phys. 2009;96(1):37-47.
2. Menzler S, Piller G, Gruson M, Rosario AS, Wichmann HE, Kreienbrock L. Population attributable fraction for lung cancer due to residential radon in Switzerland and Germany. Health Phys. 2008;95(2):179-89.
3. Darby S, Hill D, Auvinen A, et al. Radon in homes and risk of lung cancer: collaborative analysis of individual data from 13 European case-control studies. BMJ. 2005;330(7485):223.

# Lead Exposure Capstone Appendix

## Flowchart

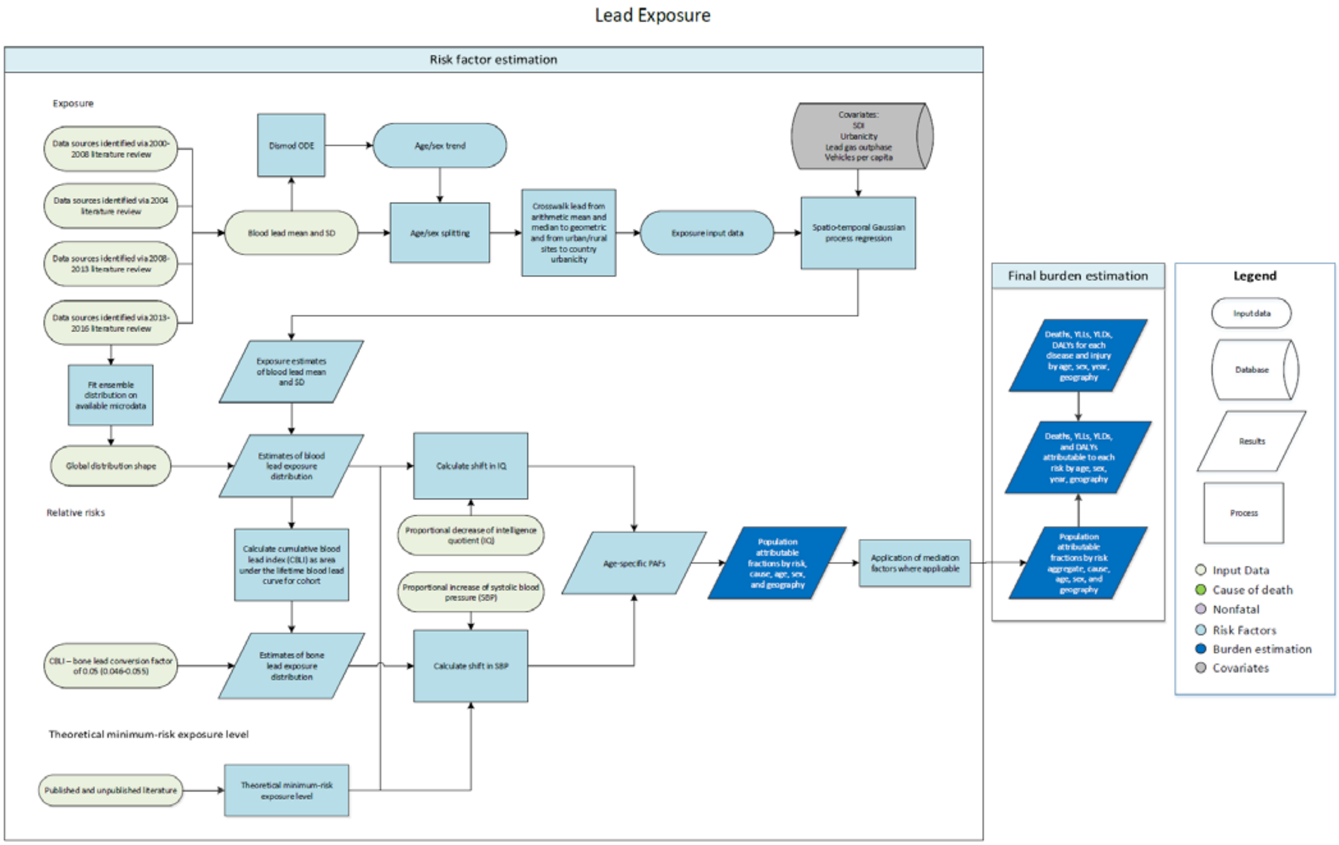


## Definitions

Exposure to lead is defined in two different ways according to the currently known pathways of health loss. Acute lead exposure, relevant to disease burden through IQ loss in children, is measured as the micrograms of lead per deciliter of blood (µg/dL). Long-term lead exposure, relevant to disease burden in adults given the manifestation of health impact through increased systolic blood pressure and hence a decline of cardiovascular health, is measured as the accumulation of lead in the bone as micrograms of lead per gram of bone (µg/g).

## Input Data

The input data for lead exposure is primarily extracted from literature regarding blood lead, in addition to a few blood lead surveys. Blood lead values are derived from studies that take blood samples and analyze them using various techniques to determine the level of lead present. Our literature review resulted in 3,183 usable data points from 554 different studies, which span the years 1970 to 2017. The database of literature values was modelled for data-sparse countries using spatio-temporal Gaussian process regression (ST-GPR). These values were used as blood lead exposure estimates. The second pathway of burden is related to bone lead exposure, which was estimated by calculating a cumulative

blood lead index for cohorts using estimated blood lead over their lifetime. The cumulative blood lead index is then used to estimate bone lead using a scalar defined in literature.[1]

## Exposure Modeling

The methodology to estimate lead exposure last underwent significant change in GBD 2013. Global exposure had been previously modelled using age-integrating Bayesian hierarchal modelling (DisMod-MR). The modelling process was updated for GBD 2013 by shifting to spatial-temporal GPR methodology. This allowed for estimates of all country-age-sex-year groups for single years instead of five year periods. This approach improved the granularity of estimates for bone lead, which requires back-estimation of previous blood lead to calculate a cumulative blood lead index.

For GBD 2017, the spatio-temporal Gaussian process regression modelling methodology was updated as detailed in the appendix specific to this analytical technique, which is common to a variety of risk factors. In order to predict blood lead in country-years with insufficient data, covariates that have been produced across time and space relevant to this analysis were used. For blood lead exposure, the covariates determined to have predictive ability were the socio-demographic index (SDI), the proportion of a location's population living in urban settings (logit transformed), the combined number of 2 and 4-wheel vehicles per capita, and a covariate indicating whether leaded gasoline had been phased out in a given country-year (smoothed over the first 5 years of phase-out to reflect its gradual implementation). ST-GPR was used to produce estimates of mean and standard deviation of blood lead for all age groups, for both sexes, and for all GBD locations from 1970 to 2017.

In earlier iterations of GBD, the distribution of lead exposure was assumed to be log-normal. Since GBD 2016, ensemble modelling techniques were used to find an optimal global distribution by fitting a variety of distributions to the available blood lead microdata. This was a common update for all continuous risk factors. The ST-GPR estimates of mean and standard deviation blood lead were used with the global distribution shape to determine distributions for blood lead exposure.

To calculate blood lead over the lifetime of a given cohort, blood lead was assumed to grow linearly from 2.0 ug/dL in 1920 (see TMREL) to the value for that cohort in 1970. Using the exposure distributions of blood lead over time and space, cohorts were constructed such that lifetime blood lead could be expressed as a curve over each year of life. The area under this curve was the cumulative blood lead index, which could be used to estimate bone lead in a given year with the aforementioned scalar.

## Estimating Attributable Burden

### Assessment of risk-outcome pairs

We included outcomes based on the strength of available evidence supporting a causal relationship. Blood lead level (a measure of acute lead exposure) is paired with idiopathic developmental intellectual disability as modeled through the impact of blood lead levels on IQ in children. Bone lead level (a measure of cumulative lead exposure) is paired with systolic blood pressure as an outcome, and subsequently to all cardiovascular outcomes to which systolic blood pressure is paired, which includes rheumatic heart disease, ischemic heart disease, ischemic stroke, intracerebral hemorrhage, hypertensive heart disease, other cardiomyopathy, atrial fibrillation and flutter, aortic aneurysm, peripheral artery disease, endocarditis, other cardiovascular and circulatory diseases, chronic kidney

disease due to hypertension, chronic kidney disease due to glomerulonephritis, and chronic kidney disease due to other and unspecified causes.

## Theoretical minimum-risk exposure level

In previous iterations of GBD, the TMREL was taken from literature estimates of pre-industrial blood lead in humans.[2] That value was estimated at 2.0 ug/dL. The decision was made that the TMREL of blood lead could not be 0 given the ambient sources of lead that would be impossible to eliminate.[3]

However, average blood lead exposures in a number of countries have fallen below 2.0 ug/dL in the past few years, suggesting that the TMREL ought to be lowered. Unfortunately, we were not able to find literature with statistically significant estimates for relative risk at such low levels of blood lead exposure. As a result, we have continued to use a TMREL of 2.0 ug/dL for GBD 2017.

## Relative Risks

Because the relative risk of IQ loss from lead exposure is specific to children, in GBD 2015 no burden of lead via IQ loss was estimated in the population aged 15 and above. To better account for the continued burden of past lead exposure on IQ in older age groups, since GBD 2016 we have constructed cohorts from the entire population. Estimates of a cohort's lead exposure in early childhood (at 24 months of age) were used to determine past IQ loss, and thus calculate burden via the impact on concurrent IQ in the older population.

Blood lead relative risks were previously taken from a 2005 pooled analysis that was first incorporated in GBD 2010.[4] For GBD 2017, blood lead relative risks have been updated with a 2013 re-analysis of the findings of that 2005 paper, providing slightly adjusted relative risk estimates specific to exposure at 24 months of age.[5] The bone lead relative risks were taken from a 2008 meta-analysis that was updated for GBD 2010.[6]

## Population Attributable Fraction

We used the standard GBD population attributable fraction (PAF) equation to calculate PAFs for bone lead exposure and each of its paired outcomes using exposure estimates and relative risks. We used a similar approach for estimating PAFs for the burden of intellectual disability attributable to blood lead, which uses the estimated distribution of intellectual disability and the modeled shifts in IQ due to blood lead levels to determine the PAF.

## References

1. Hu H, Shih R, Rothenberg S, Schwartz BS. The epidemiology of lead toxicity in adults: measuring dose and consideration of other methodologic issues. Environ Health Perspect. 2007;115(3):455-62.
2. Flegal AR, Smith DR. Lead levels in preindustrial humans. N Engl J Med. 1992;326(19):1293-4.
3. Pruss-Astun A, Fewtrell L, Landrigan PJ, Ayuso-Mateos JL. Lead Exposure. In: Ezzati M, Lopez AD, Rodgers A, Murray CJ, eds. Comparative quantifications of health risks: Global and regional burden of disease attrituable to selected major risk factors. Geneva, World Health Organization, 2004: 1496-542
4. Lanphear BP, Hornung R, Khoury J, et al. Low-level environmental lead exposure and children's intellectual function: an international pooled analysis. Environ Health Perspect. 2005;113(7):894-9.

5. Crump K, Van Landingham C, Bowers T, Cahoy D, Chandalia J. A statistical reevaluation of the data used in the Lanphear et al. () pooled-analysis that related low levels of blood lead to intellectual deficits in children. Critical Reviews in Toxicology. 2013;43(9):785-799.
6. Navas-acien A, Schwartz BS, Rothenberg SJ, Hu H, Silbergeld EK, Guallar E. Bone lead levels and blood pressure endpoints: a meta-analysis. Epidemiology. 2008;19(3):496-504.

# Occupational Risk Factors Capstone Appendix

## Exposure definitions

The following definitions were used for occupational risk factor exposures. All exposures were estimated for ages 15 and older.

| | |
|---|---|
| Occupational Asbestos | Cumulative lifetime exposure to occupational asbestos, using mesothelioma death rate as an analogue |
| Occupational Asthmagens | Proportion of the working population exposed to asthmagens, based on population distributions across nine occupational categories |
| Occupational Carcinogens (arsenic, benzene, beryllium, cadmium, chromium, diesel engine exhaust, formaldehyde, nickel, polycyclic aromatic hydrocarbons, silica, sulfuric acid, and trichloroethylene) | Proportion of the population that was ever occupationally exposed to carcinogens at high or low exposure levels, based on population distributions across seventeen economic activities |
| Occupational Ergonomic Factors | Proportion of the working population exposed to low back pain-inducing work, based on population distributions across nine occupational categories |
| Occupational Injuries | Proportion of injuries in the working-age population attributable to occupational work, based on fatal injury rates in seventeen economic activities |
| Occupational Noise | Proportion of the population occupationally exposed to 85+ decibels of noise, based on population distributions across seventeen economic activities |
| Occupational Particulates | Proportion of the population occupationally exposed to particulates, based on population distributions across seventeen economic activities |

Economic activities and occupations were coded according to the following categories:

| **Economic Activities** | **Occupations** |
|---|---|
| Agriculture, hunting, forestry | Legislators, senior officials, and managers |
| Fishing | Professionals |
| Mining and Quarrying | Technicians and associate professionals |
| Manufacturing | Clerks |
| Electricity, gas, and water | Service workers and shop/market sales workers |
| Construction | Skilled agricultural and fishery workers |
| Wholesale and retail trade/repair | Plant and machine operators and assemblers |
| Hospitality | Craft and related workers |

| | |
|---|---|
| Transport, storage, and communication | Elementary occupations |
| Financial intermediation | |
| Real estate/renting | |
| Public administration/defense; compulsory social security | |
| Education | |
| Health and social work | |
| Other community/social/personal service activities | |
| Private households | |
| Extra-territorial organisations/bodies | |

## Input data

Primary inputs were obtained from the ILO,[1-4] and included raw data on economic activity proportions, occupation proportions, fatal injury rates, and employment to population ratio estimates. A systematic web review was conducted in order to collect the underlying microdata from the ILO's estimates to aid in re-extraction at greater levels of granularity. Where freely available, survey datasets were downloaded from the survey organisations in question. Other datasets were obtained through submission of requests to agencies and through the GBD collaborator network. Microdata was tabulated in order to create survey-weighted estimates of economic activities and occupations for the GBD geographies and years. Various classification systems were crosswalked to ISIC Rev.3 (for economic activities) and ISCO 1988 (for occupations). Subnational estimates for UK and China were added to the datasets for economic activities and occupations.[5,6]

For occupational asbestos, primary inputs were obtained through GBD 2017 cause of death estimates and published studies.[7,13,14]

Uncertainty for inputs where microdata was unavailable was generated by fitting a Loess curve to the data and determining the standard deviation of the data from the fitted curve.

## Modelling strategies

A Spatio-temporal Gaussian process regression (ST-GPR) was used to generate estimates for all years and locations for the primary inputs. Study level covariates used in the prior model were education in years per capita, geological covariates (for mining models), the proportion of the population living with access to a coastline (for fishing models), the IHME socio-demographic index (SDI), the mean temperature/latitude (for agriculture models), and the proportion of the population living in urban areas. Space-time parameters were chosen by maximising out-of-sample cross-validation and minimising RMSE. For economic activity and occupation proportions, estimates from ST-GPR were then re-scaled to sum to 1 across categories by dividing each estimate by the sum of all the estimates.

The following sections describe the modelling approaches for each occupational risk's exposure prevalence.

### Occupational carcinogens, occupational noise, and occupational particulates

Prevalence of exposure to these risks was determined using the following equation:

$$Prevalence\ of\ Exposure_{c,y,s,a,r,l} = \sum_{EA} Proportion_{EA,c,y} * EAP_{c,y,s,a} * Exposure\ rate_{EA,r,l,d}$$

where:

| | | |
|---|---|---|
| EAP = economically active population | c = country | r = risk |
| EA = economic activity | d = duration | s = sex |
| a = age | l = level of exposure | y =year |

Exposure rate was provided by expert group recommendations and literature[8-11] (see table 1). The CAREX database was used in order to quantify the association between exposure by industry/carcinogen to SDI across all the countries in the database. This effect was used to predict exposure in countries that were not included in CAREX. Duration was considered for occupational carcinogens through application of occupational turnover factors[12] and for occupational noise and particulates by calculating cumulative exposure as the average exposure over the lifetime (the past 50 years) for each age/sex cohort.

## Occupational ergonomic factors and occupational asthmagens

Prevalence of exposure to these risks was determined using the following equation:

$$Prevalence\ of\ Exposure_{c,y,s,a,r} = \sum_{EA} Proportion_{OCC,c,y} * EAP_{c,y,s,a}$$

where:

| | | |
|---|---|---|
| EAP = economically active population | c = country | r = risk |
| OCC = occupation | a = age | s = sex |
| | | y = year |

## Occupational injuries

Occupational injury counts were estimated using the following equation:

$$Occupational\ fatal\ injuries_{c,y,a,s} = \sum_{EA} Injury\ rate_{EA,c,y,s} * Population_{c,y,a,s} * EAP_{c,y,s,a} * Proportion_{EA,c,y}$$

where:

| | | |
|---|---|---|
| EAP = economically active population | c = country | y = year |
| EA = economic activity | a = age | s = sex |

## Occupational asbestos

Prevalence of exposure to asbestos was estimated using the asbestos impact ratio (AIR), which is equivalent to the excess deaths due to mesothelioma observed in a population divided by excess deaths due to mesothelioma in a population heavily exposed to asbestos. Formally, this is defined using the following equation:

$$AIR = \frac{Mort_{c,y,s} - N_{c,y,s}}{Mort^{*}_{c,ys,} - N_{c,y,s}}$$

where:

Mort = Mortality rate due to mesothelioma
Mort* = Mortality rate due to mesothelioma in population highly exposed to asbestos
N = Mortality rate due to mesothelioma in population not exposed to asbestos

c = country
y = year
s = sex

Mortality rate due to mesothelioma was estimated from GBD 2017 causes of death.[7] Mortality rate due to mesothelioma in populations not exposed to asbestos was calculated using the model in Lin et al.,[13] while the mortality rate due to high exposure to asbestos was estimated in Goodman et al.[14] Asbestos exposure prevalence created using the AIR was used to estimate PAFs for all asbestos-associated causes except for mesothelioma. Custom PAFs were calculated for mesothelioma by using the ratio of the excess mortality with respect to an unexposed population (Mort – N) divided by the mortality rate in the population in question (Mort). This calculation assumes that all mesothelioma is a product of occupational asbestos exposure and could potentially over-estimate burden due to occupational asbestos exposure in populations with high non-occupational asbestos exposure.

## Theoretical minimum-risk exposure level

For all occupational risks, with the exception of occupational asbestos, the theoretical minimum-risk exposure level was assumed to be no exposure to that risk.

## Relative risk

Relative risks were obtained for all occupational risks by conducting a systematic review of published meta-analysis. The estimates used, as well as the associated studies, are reported by category group in appendix table 5.

## PAFs

For all occupational risks, with the exception of injuries (outlined below) and mesothelioma (outlined above), PAFs were calculated using the prevalences estimated above, using the PAF formula in outlined in the GBD 2017 methods appendix.

### Occupational injuries PAF

The PAFs for occupational injuries were calculated using the following formula:

$$PAF_{c,y,a,s} = \frac{Occupational\ fatal\ injuries_{c,y,a,s} - TMREL}{Fatal\ injuries_{c,y,a,s}}$$

where:

c = country
y = year

a = age
s = sex

Fatal injury totals were obtained from GBD 2017 causes of death.[7]

## References

1. International Labour Organization (ILO). International Labour Organization Database (ILOSTAT) - Employment by Sex and Economic Activity. International Labour Organization (ILO).

2. International Labour Organization (ILO). International Labour Organization Database (ILOSTAT) - Employment by Sex and Occupation. International Labour Organization (ILO).

3. International Labour Organization (ILO). International Labour Organization Database (ILOSTAT) - Fatal Injuries by Sex and Economic Activity. International Labour Organization (ILO).

4. International Labour Organization (ILO). International Labour Organization LABORSTA Economically Active Population, Estimates and Projections, October 2011. International Labour Organization (ILO), 2011.

5. Office for National Statistics (United Kingdom). Nomis Official Labor Market Statistics - Annual Population Survey. Newport, United Kingdom: Office for National Statistics (United Kingdom).

6. National Bureau of Statistics of China. China 1% National Population Sample Survey 1995. Ann Arbor, United States: China Data Center, University of Michigan.

7. GBD 2017 Mortality and Causes of Death Collaborators. Global, regional, and national life expectancy, all-cause and cause-specific mortality for 249 causes of death, 1980–2017: a systematic analysis for the Global Burden of Disease Study 2017. *Lancet Rev*.

8. Wilson DH, Walsh PG, Sanchez L, *et al.* The epidemiology of hearing impairment in an Australian adult population. *Int J Epidemiol* 1999; 28: 247–52

9. Kauppinen T, Toikkanen J, Pederson D, Young R, Kogevinas M, Ahrens W, et al. Occupational Exposure to Carcinogens in the European Union in 1990-93. Helsinki, Finland: Finnish Institute of Occupational Health; 1998.

10. Kauppinen T, Toikkanen J, Pedersen D, Young R, Ahrens W, Boffetta P, et al. Occupational exposure to carcinogens in the European Union. *Occup Environ Med* 2000; 57(1): 10–18.

11. Driscoll T, et al. The global burden of non-malignant respiratory disease due to occupational airborne exposures. *American Journal of Industrial Medicine 2005; 48*(6): 432-445.

12. Nelson, D. I., Concha-Barrientos, M., Driscoll, T., Steenland, K., Fingerhut, M., Punnett, L. & Corvalan, C. (2005). The global burden of selected occupational diseases and injury risks: Methodology and summary. *American journal of industrial medicine*, *48*(6), 400-418

13. Lin R-T, Takahashi K, Karjalainen A, *et al.* Ecological association between asbestos-related diseases and historical asbestos consumption: an international analysis. *Lancet* 2007; **369**: 844–9.

14. Goodman M, Morgan RW, Ray R, Malloy CD, Zhao K. Cancer in asbestos-exposed occupational cohorts: a meta-analysis. *Cancer Causes Control* 1999; **10**: 453–65.

# Suboptimal Breastfeeding Capstone Appendix

## Flowchart

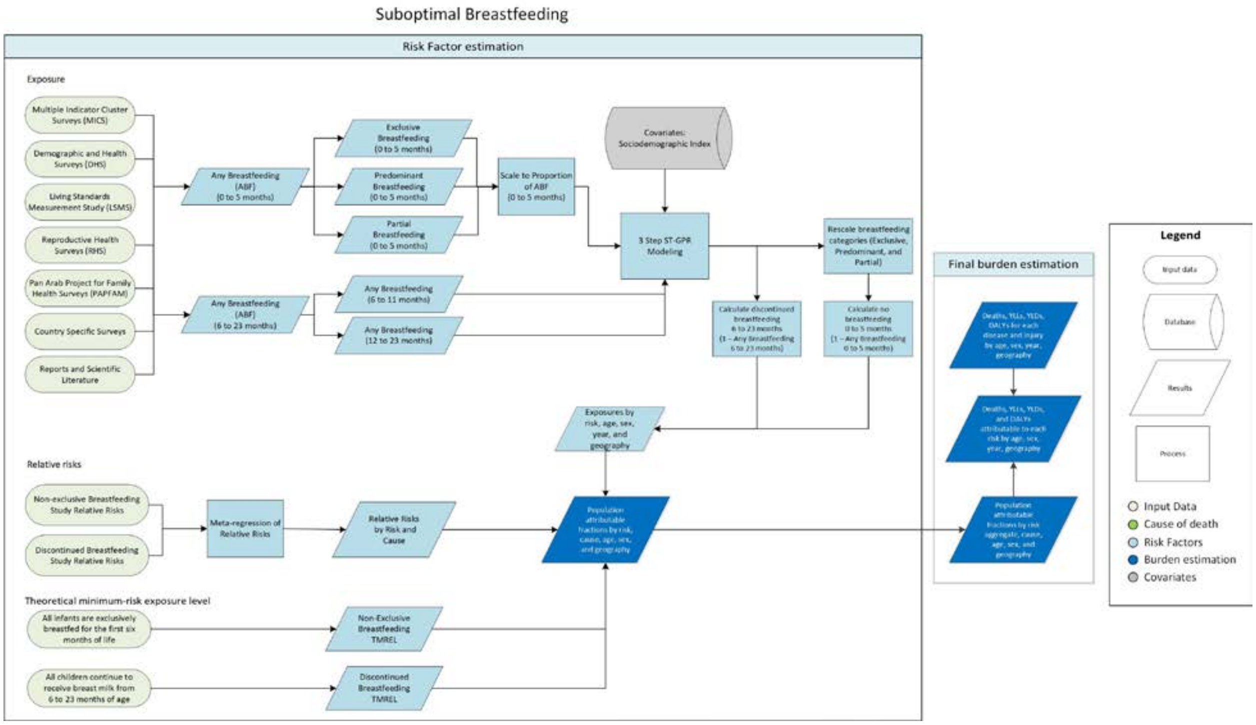


## Definitions

Exposure to suboptimal breastfeeding is composed of 2 distinct categories: non-exclusive breastfeeding and discontinued breastfeeding.

**Non-exclusive breastfeeding** is defined as the proportion of children under 6 months of age who are not exclusively breastfed. We then parse those not exclusively breastfed into 3 categories – predominant, partial, and no breastfeeding. Exclusive breastfeeding is defined as the proportion of children who receive no other food or drink except breast milk (allowing for ORS, drops, or syrups containing vitamins, minerals, or medicines). Predominant breastfeeding is the proportion of children whose predominant source of nourishment is breastmilk but also receive other liquids. Partial breastfeeding refers to those infants who receive breastmilk as well as food and liquids, including non-human milk and formula. No breastfeeding refers to infants who do not receive breast milk as a source of nourishment.

**Discontinued breastfeeding** is defined as the proportion of children between 6 to 23 months who receive no breast milk as a source of nourishment.

## Input Data

We made substantial exposure data updates for GBD 2017, including extracting identified surveys not included in previous rounds and re-extracting all surveys for new GBD 2017 subnational locations. We searched the Global Health Data Exchange (GHDx) database for sources using the keyword "Breastfeeding." Of 2,026 potential sources identified, we extracted 1,081 unique country-years of data (2,262 unique geography-years, including subnational geographies) that met our inclusion criteria. The

data used in the analysis consists mostly of processed individual-level microdata from surveys; in the cases where microdata was unavailable, we used reported tabulated data from survey reports and scientific literature. Data used to categorize type of non-exclusive breastfeeding (predominant, partial, and none) come from surveys with 24-hour dietary logs based on maternal recall.

## Exposure Modelling

Using the processed microdata and tabulated data from reports, we generated a complete time series from 1980 to 2017 for the prevalence of breastfeeding patterns for children 0 to 5 months and 6 to 23 months using a three-step spatio-temporal Gaussian process regression modelling process.

First, we estimated a robust linear regression using each geography's sociodemographic index as a covariate. The following linear model was used for the estimation of breastfeeding indicators:

$$logit(P_{x,c,t}) = \beta_0 + \beta_1 SDI_{\mathrm{c,t}} + \alpha_c + \gamma_{R[c]} + \omega_{\mathrm{SR[c]}} + \varepsilon_{c,t}$$

where $P_{x,c,t}$ is prevalence for breastfeeding category *x* in country $c$ and year $t$; $SDI_{c,t}$ is value of the Sociodemographic Index for country $c$ and year $t$; $\alpha_c$, $\gamma_{R[c]}$, and $\omega_{\mathrm{SR[c]}}$ are country, region, and super-region random intercepts, respectively.

We then followed this with a spatio-temporal regression that uses the residuals of the predictions from the linear regression to perform a locally-weighted regression that provides a greater weighting factor to those nearer in space and time. The predicted residuals from this step are then added to those created in the linear regression step.

Finally, we run a Gaussian process regression that incorporates the variance of the input data as well as the variance of the model predictions. It uses predictions from the spatio-temporal regression as the mean function and generates draws from a multinomial distribution (based on the data uncertainty in the prior) to generate the final prevalence estimates and their confidence intervals.

We estimated six models to produce each of our categories: the proportion of currently breastfeeding infants 0-5 months of age, the ratio of infants exclusively breastfed to breastfed infants 0-5 months of age, the ratio of infants predominantly breastfed to breastfed infants 0-5 months of age, the ratio of infants partially breastfed to breastfed infants 0-5 months of age, the proportion of currently breastfeeding infants 6-11 months of age, and the proportion of currently breastfeeding infants 12-23 months of age. We convert the ratios of exclusive, predominant, and partial breastfeeding to the total category prevalence proportions by multiplying each ratio by the estimates of any breastfeeding among infants aged 0-5 months. This ensures that these categories sum correctly to the "any breastfeeding 0-5 months" envelope. We calculate the proportion of infants receiving no breastmilk 0-5 months of age by subtracting the estimates of current breastfeeding from 1. We perform the same operation to estimate discontinued breastfeeding in the 6-11 months and 12-23 months categories.

## Estimating Attributable Burden

### Assessment of risk-outcome pairs

We included outcomes based on the strength of available evidence supporting a causal relationship. Studies evaluating the causal evidence for our risk-outcome pairs came primarily from articles found in a review published by the World Health Organization.[1] Non-exclusive breastfeeding was paired with diarrhea and lower-respiratory infection as diseases outcomes. Discontinued breastfeeding was paired with diarrhea as an outcome.

### Theoretical minimum-risk exposure level

For non-exclusive breastfeeding, those children that received no source of nourishment other than breastmilk ("exclusively breastfed") were considered to be at the lowest risk of any of the disease outcomes. For discontinued breastfeeding, we assumed that children aged 6 to 23 months who received any breastmilk as a source of nourishment to be at the lowest risk of disease outcome.

### Relative Risks

We estimate relative risks for both non-exclusive and discontinued breastfeeding in a meta-analysis using relative risks from studies compiled in a published review by the World Health Organization.[1]

### Population Attributable Fraction

We use the standard GBD population attributable fraction (PAF) equation to calculate PAFs for non-exclusive breastfeeding and discontinued breastfeeding and each of their paired outcomes using exposure estimates and relative risks.

## References

1. Horta, B., Voctora, C. (2013) Short-term effects of breastfeeding: a systematic review on the benefits of breastfeeding on diarrhoea and pneumonia mortality. The World Health Organization.

# Child Growth Failure Capstone Appendix

## Flowchart

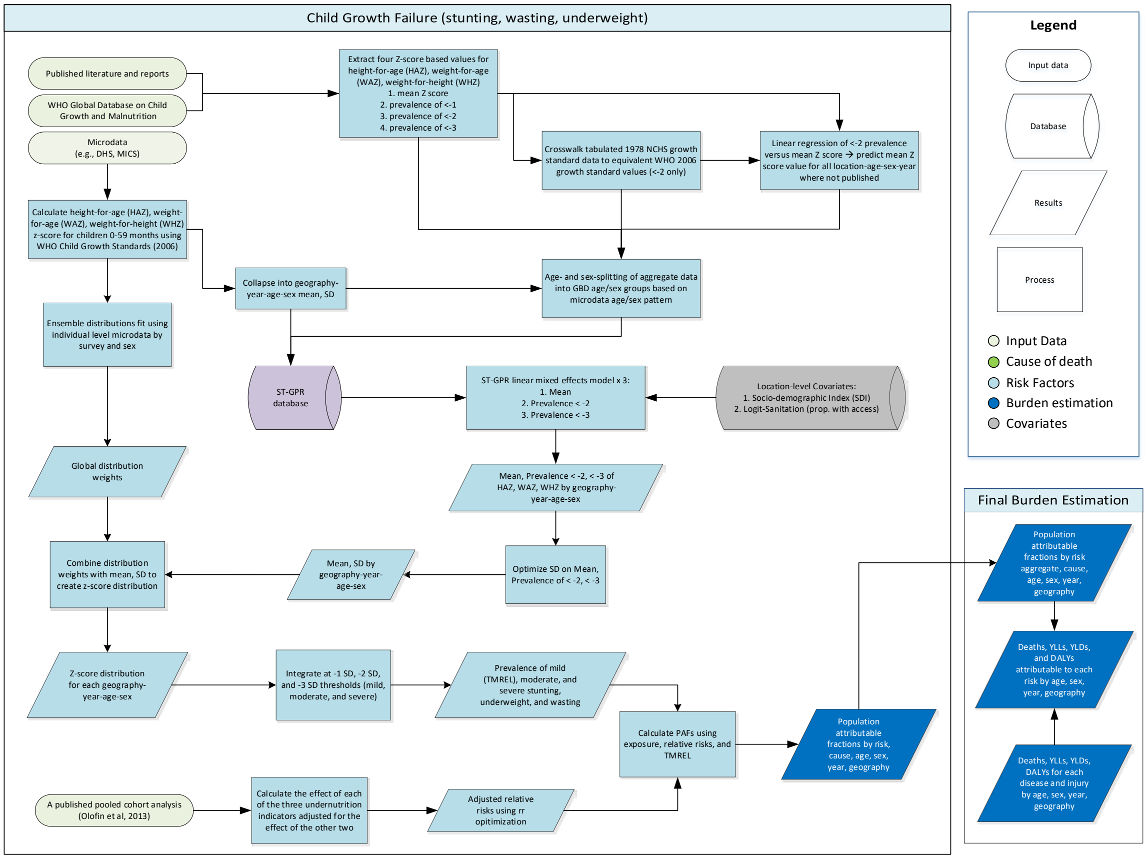


## Input data & methodological summary

## Exposure

### *Case definition*

Child growth failure is estimated using three indicators, stunting, wasting, and underweight, all of which all of which are based on categorical definitions using the WHO 2006 growth standards for children 0-59 months. Definitions are based on Z scores from the growth standards, which were derived from an international reference population. Mild, moderate, and severe categorical prevalences were estimated for each of the three indicators.

### *Input data*

There are three main inputs for the GBD child growth failure models: microdata from population surveys and tabulated data from reports, published literature, and the WHO Global Database on Child Growth and Malnutrition.[1] The primary data additions in GBD 2017 for child growth failure were from population surveys that include anthropometry. Population surveys include a variety of multi-country and country-specific survey series such as Multiple Indicator Cluster Surveys (MICS), Demographic and Health Surveys (DHS), Living Standards Measurement Surveys (LSMS), and the China Health and Nutrition Survey (CHNS), as well as other one time country specific surveys such as the Indonesia Family Life Survey and the Brazil National Demographic and Health Survey of Children and Women. These microdata contain information

about each individual child's age (from which age in weeks and age in months are calculated), as well as height and/or weight. From that information, a height-for-age z-score (HAZ), weight-for-age z-score (WAZ), and weight-for-height z-score (WHZ) are calculated using the WHO 2006 Child Growth Standards and the LMS method.[2]

All available data from the WHO Global Database on Child Growth and Malnutrition was extracted for GBD 2016 – much of which is from published studies. Exclusions included examination date prior to 1985, non-population representative studies, and those based on self-report. A systematic literature review was last completed in GBD 2010. We looked for four metrics from all sources with tabulated data: mean Z score, prevalence <-1 Z score (mild), prevalence <-2 Z score (moderate), and prevalence <-3 Z score (severe). All data for each metric was extracted for each of stunting (height-for-age Z score; HAZ), wasting (weight-for-height Z score; WHZ), and underweight (weight-for-age Z score; WAZ).

To maximise internal-consistency and comprehensiveness of the modelling dataset, we performed three data transformations. First, any data that were reported using the National Center for Health Statistics (NCHS) 1978 growth standards were crosswalked to corresponding values on the WHO 2006 Growth Standards curves based on a study that evaluated growth standard concordance.[3] Crosswalks from 1978 to 2006 growth standards were performed only on <-2 (i.e. moderate) prevalence data as that is where the concordance was most consistent. Second, for any study that lacked a measure of mean Z score for any of stunting, wasting, or underweight, we predicted a mean value for that study based on an ordinary-least squares regression of mean Z score versus <-2 prevalence for that metric from all sources where both were available. Third, any data that was presented as both sexes combined or for 0-59 months combined, we used the age and sex pattern from all data sources that included that detail to split into corresponding and age- and sex-specific data. All data was uploaded to a database and all inputs are catalogued in the Global Health Data Exchange (http://ghdx.healthdata.org). A representative dataset coverage map for moderate stunting is shown below.

*Figure 1: Number of data points in moderate stunting (<-2 HAZ) in males, 1990 to 2017*

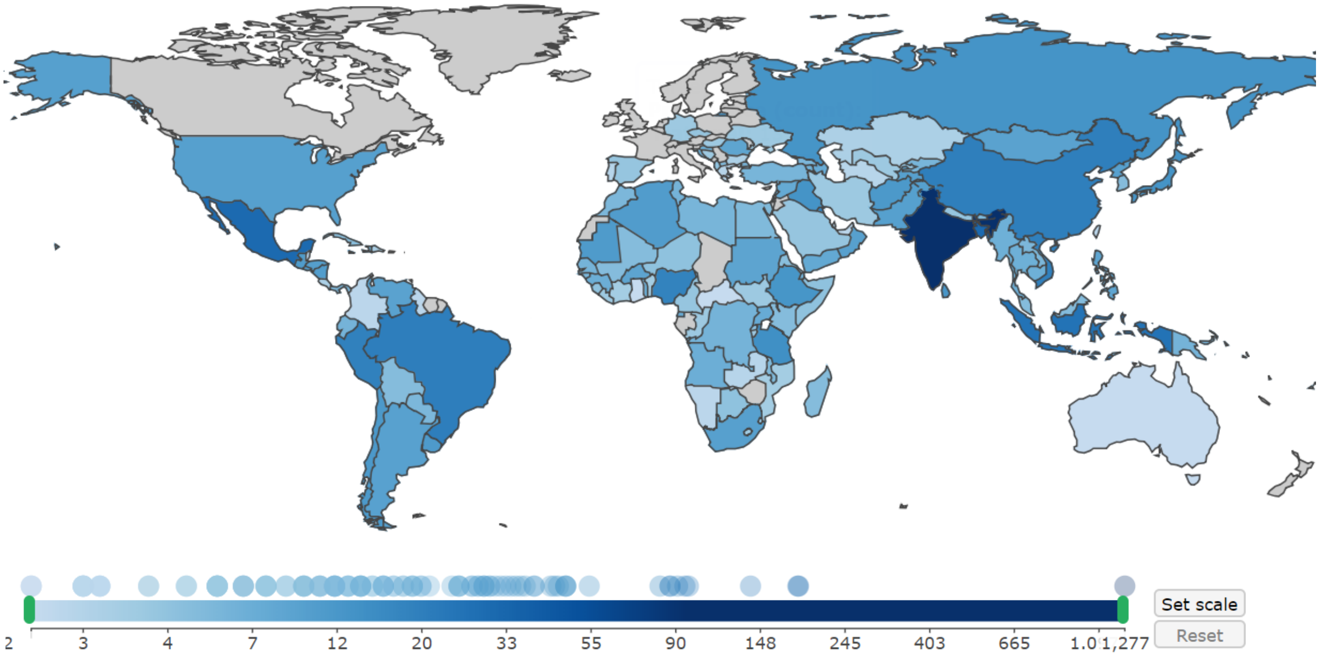

## Modelling strategy

### Exposure estimation

The following three-step modelling process was applied to each of stunting, wasting, and underweight.

First, all microdata was fit using an ensemble modelling process, a modelling framework developed for GBD 2016 that is described elsewhere in this appendix. A series of 12 individual distributions (normal, log normal, log logistic, exponential, gamma, mirror gamma, inverse gamma, gumbel, mirror gumbel, Weibull, inverse Weibull, and beta) were fit to the entire set of microdata (approximately 2.5 million individual z-scores) at the individual survey level. A weighting algorithm combined each distribution to find the optimal combination of these distributions for each survey, minimising the absolute prediction error across the entire distribution. Ensemble weights for each survey were then averaged across all surveys to produce a single set of global weights of the ensemble distributions. Weights were different for each sex, but invariant across geography, time, and age group. All component distributions that were used to derive weights were parameterised using “method of moments,” meaning that each corresponding probability density function (PDF) could be described as a function of the mean and variance of the quantity of interest.

Second, models were developed for mean Z scores and prevalence of moderate and severe growth failure. Individual level microdata were collapsed to calculate three metrics: mean z-score, moderate prevalence, and severe prevalence. These data were combined with that derived from literature, GHDx review, and the WHO Global Database on Child Growth and Malnutrition. Each of the three metrics was then modelled using spatiotemporal Gaussian process regression (ST-GPR), a common modelling framework used across GBD, generating estimates for each age-group, sex, year, and location. Location-level covariates used in all models included Socio-demographic Index (SDI) and logit-transformed proportion of households with improved sanitation.

Third, we combined estimates of mean, prevalence (moderate and severe) with ensemble weights in an optimisation framework in order to derive the variance that would best correspond to the predicted mean and prevalence. This variance was then paired with the mean and, using the method of moments equation for each of the component distributions of the ensemble, PDF of the distribution of Z-scores were calculated for each location, year, age-group, and sex. PDFs were integrated to determine the prevalence between -1 and -2 Z scores (mild), between -2 and -3 Z scores (moderate), and below -3 Z scores (severe). These were categorical exposures used for subsequent attributable risk analysis.

Ad-hoc data exclusions were limited. In some cases, we identified surveys with evidence of data entry issues (e.g. weights entered in a mixture of pounds and kilograms) that could not be corrected and these data were outliered. We initially ran all models with the complete dataset. Data plausibility inspection began with examination of time trends in stunting. If a given datum was judged to have led to a change in the prevalence of moderate stunting in 1-4 year olds of 50% or greater in 5 years or fewer, and was inconsistent with data prior to and after that year (a change considered implausible), we outliered the offending datum and reran the model. We then further visually-inspected the results of moderate stunting, wasting, and underweight in parallel to look for location-age-sex-years where the results were not internally-consistent (e.g. stunting and wasting decreasing, underweight rapidly increasing). This inspection revealed very few inconsistent data.

## Improvements from GBD 2015 to GBD 2016/ 2017

In GBD 2017, the primary changes from GBD 2016 were the 1) addition of a significant volume of new survey data, 2) crosswalking instead of down-weighting data based on NCHS 1978 growth standard, 3) utilisation of updated versions of location-level covariates, and 4) utilisation of an updated version of the ST-GPR modelling framework that empirically derives many of the modelling parameters.

There are several important differences from the GBD 2015 analysis. First, our systematic data searching efforts led to an approximately 30% increase in the number of data sources since GBD 2015, including a significant increase in data sources for Oceania, Latin America, and South Asia. Most notable was the increase in data for India through our collaboration with the India Council for Medical Research (ICMR) and Public Health Foundation of India (PHFI). Second, while GBD 2015 also used ST-GPR to model growth failure, models were completed for a single 0-5 age group, followed by application of a pooled uniform age-sex split which resulted in the implicit assumption that the age pattern of growth failure is invariant over time and geography. GBD 2016 estimates, owing to smaller sample sizes in younger age groups, do have wider uncertainty in those age groups. Third, GBD 2015, like all analyses of growth failure before it, assumed that high-income countries had zero prevalence of child growth failure. We suspended this assumption in GBD 2016 as it is not accurate and instead made explicit estimates of growth failure in all locations. Fourth, GBD 2015 did not use an ensemble approach or estimate the entire distribution of Z scores. Fifth, we changed the name of this risk factor category changed from childhood undernutrition to child growth failure to more explicitly identify the specific aspects of childhood undernutrition that are covered by the three component indicators.

## Theoretical minimum-risk exposure level

Theoretical minimum risk exposure level (TMREL) for underweight, stunting, and wasting was assigned to be greater than or equal to -1 SD of the WHO 2006 standard weight-for-age, height-for-age, and weight-for-height curves respectively. This has not changed since GBD 2010.

## Relative risks

The final list of outcomes paired with child growth failure risks included lower respiratory infections (LRI), diarrhea, measles, and protein energy malnutrition (PEM) as shown in Table 1. These were derived from a pooled cohort analysis by Olofin and colleagues.[5]

There is a high degree of correlation between stunting, wasting, and underweight. Failing to account for their covariance and assuming independence would overestimate the total burden significantly. This is the main reason that GBD 2010 only included childhood underweight. In GBD 2013, a method was developed to adjust observed RRs of Olofin and colleagues by simulating the joint distribution of the three indicators using the distribution of each indicator and covariance between indicators in the countries included in the meta-analysis (extracted from Demographic and Health Survey (DHS) micro-data).[4] Based on the analysis done by McDonald and colleagues, we assumed there is an interaction between the three indicators, and extracted the interaction terms from the corresponding analysis. We calculated the adjusted RRs by minimising the error between observed crude RRs (from meta-analysis) and expected crude RRs derived from adjusted RRs.

Of historical note, URI and otitis media were included as outcomes in the GBD 2013 risk analysis, based on the "analogy" causal criterion, assuming there is similar pathway as LRI outcome. However, closer review for GBD 2015 did not find sufficient evidence to support their inclusion and they were excluded, a decision that was carried forward into GBD 2016. We also attributed 100% of PEM to childhood wasting

and underweight but not stunting. To build on the existing literature base for GBD on risk-outcome pairs, a literature search was conducted for GBD 2017 searching for case-control studies published after January 1st, 1985; this search did not return any sources that were appropriate for this work.

*Table 1: Adjusted RRs for each risk-outcome pair for child growth failure*

| ***Outcome*** | **Stunting** | **Wasting** | **Underweight** |
|---|---|---|---|
| Diarrhea | <-1: 1.111 (1.023-1.273)<br><-2: 1.222 (1.067-1.5)<br><-3: 1.851 (1.28-2.699) | <-1: 6.601 (2.158-11.243)<br><-2: 23.261 (9.02-35.845)<br><-3: 105.759 (42.198-157.813) | <-1: 1.088 (1.046-1.134)<br><-2: 1.23 (1.163-1.314)<br><-3: 2.332 (2.076-2.802) |
| Lower respiratory infections (LRI) | <-1: 1.125 (0.998-1.655)<br><-2: 1.318 (1.014-2.165)<br><-3: 2.355 (1.15-5.114) | <-1: 5.941 (1.972-11.992)<br><-2: 20.455 (70.84-37.929)<br><-3: 47.67 (15.923-94.874) | <-1: 1.145 (1.044-1.364)<br><-2: 1.365 (1.215-1.755)<br><-3: 2.593 (1.908-4.39) |
| Measles | <-1: 1.103 (0.861-1.719)<br><-2: 1.54 (1.029-3.222)<br><-3: 2.487 (1.129-6.528) | <-1: 1.833 (0.569-8.965)<br><-2: 8.477 (1.33-42.777)<br><-3: 37.936 (5.088-199.126) | <-1: 0.995 (0.5-1.726)<br><-2: 2.458 (1.26-5.118)<br><-3: 5.668 (1.767-12.414) |
| Protein-energy malnutrition | 0% PAF | 100% PAF | 100% PAF |

# References


1 WHO | WHO Global Database on Child Growth and Malnutrition. WHO. http://www.who.int/nutgrowthdb/en/ (accessed July 30, 2018).

2 Wang Y, Chen H-J. Use of Percentiles and Z-Scores in Anthropometry. In: Preedy VR, ed. Handbook of Anthropometry. New York, NY: Springer New York, 2012: 29–48.

3 Uribe Á, Cecilia M, López Gaviria A, Estrada Restrepo A. Concordance between Z scores from WHO 2006 and the NCHS 1978 growth standards of children younger than five. Antioquia-Colombia. *Perspectivas en Nutrición Humana* 2008; **10**: 177–87.

4 McDonald CM, Olofin I, Flaxman S, *et al.* The effect of multiple anthropometric deficits on child mortality: meta-analysis of individual data in 10 prospective studies from developing countries. *Am J Clin Nutr* 2013; **97**: 896–901.

5 Olofin I, McDonald CM, Ezzati M, *et al.* Associations of Suboptimal Growth with All-Cause and Cause-Specific Mortality in Children under Five Years: A Pooled Analysis of Ten Prospective Studies. *PLOS ONE* 2013; **8**: e64636.

# Low Birth Weight and Short Gestation Capstone Appendix

## Flowchart

**Low birth weight and Short gestation Risk Factors**

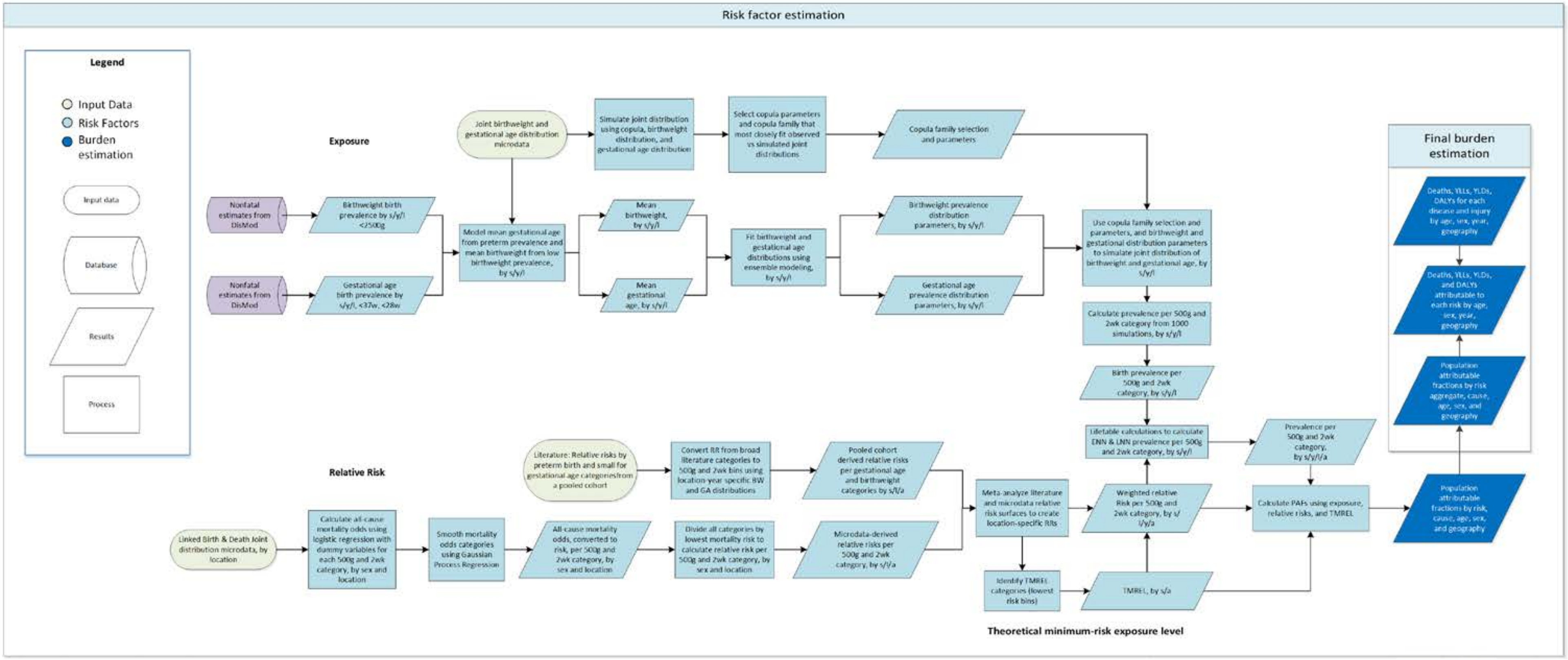

## Input data and methodological summary

The "Low Birth Weight and Short Gestation" (LBWSG) risk factor and its child risks "Low Birth Weight for Gestation" and "Short Gestation for Birth Weight" first were included as risk factors in GBD 2016.

Although low birth weight for gestation and short gestation for birth weight are separate risk factors, the exposures and relative risks for both are estimated jointly through the low birth weight and short gestation parent risk factor. As of GBD 2017, LBWSG are the only risk factors estimated jointly.

### Case definition

The meaning of the "low birth weight" and "short gestation" in GBD have subtle definitional differences compared to other usages of "low birth weight" and "short gestation" in literature. The term "low birth weight" has historically been used to refer to birth weight (BW) less than 2500 grams. However, because the goal of the GBD risk factors analysis is to quantify the entirety of attributable burden due to each risk factor, the GBD definition of "low birth weight" therefore refers to all birth weight below the Theoretical Minimum Risk Exposure Level (TMREL) for birth weight. Likewise, new-borns have been typically been classified into gestational age (GA) categories of "extremely preterm" (<28 weeks of gestation), "very preterm" (28-<32 weeks of gestation), and "moderate to late preterm" (32-<37 weeks of gestation). "Short gestation" in GBD refers to all gestational ages below the gestational age TMREL.

Exposures and relative risks for the GBD Low birth weight and short gestation risk factors are divided into joint 500-gram birth weight and 2-week gestational age combinations. The lowest risk overall 500-gram/2-week bin is the overall TMREL. The univariate TMRELs vary with GA and BW. The lowest risk GA varies by BW category and the lowest risk BWs vary with GA category. The latter are used to quantify univariate attributable risk. Under this framework, all attributable burden under the joint TMREL is referred to jointly as burden of LBWSG. All attributable burden to BWs under the TMREL for each GA category are, on aggregate, "low birth weight" and all attributable burden to GAs under the TMREL for each BW category are, on aggregate, "short gestation." Each combination of 500-grams and 2-wks is associated with a relative risk for mortality by neonatal period (early and late neonatal) and by the causes listed in Table 2 and described below, and relative to the joint TMREL.

## Exposure

### Input data

To model the joint distribution of exposure of low birth weight and short gestation for each location, year, and sex estimated in GBD 2017, three types of information are used:

- Distribution of gestational age for each location, year, and sex
- Distribution of birth weight for each location, year, and sex
- Copula family and parameters, specifying correlation between gestational age and birth weight distributions

### Modelling strategy

*Distributions of birth weight & gestational age*

To model the joint distribution of birth weight and gestational age for every location-sex-year, ensemble model methods standard to GBD risk factors (described elsewhere in the methods appendix), are first used to create separate distributions of birth weight and gestational age for every location-sex-year.

Microdata is the most ideal data source for modelling distributions; however, microdata is not widely available for birth weight and is more scarce for gestational age. Categorical prevalence data is much more readily available, and from a wider range of locations and years, for low birth weight (<2500g), extremely preterm (<28 weeks of gestation), very preterm (28-32 weeks of gestation), moderate to late preterm (32-37 weeks of gestation), and preterm birth (<37 weeks of gestation). From GBD 2010 to GBD 2015, this categorical data has been used model birth prevalence of preterm birth by gestational age (<28 weeks, 28-<32 weeks, and 32-<37 weeks) and low birth weight (<2500g) for every location, sex, and year estimated in GBD. Starting in GBD 2016 with the introduction of the LBWSG risk factors, the full distributions at birth have been modelled for gestational age and birth weight for all GBD locations, estimation years, and both sexes. The gestational age and birth weight distributions are then aggregated into the categorical estimates of <28 weeks, 28-<32 weeks, 32-<37 weeks gestation, and <2500 g birth weight.

Ensemble model methods standard to GBD are used to model the distribution at birth of gestational age and birth weight. Gestational age ensemble distribution models use the prevalence of <37 weeks gestation, the prevalence of <28 weeks gestation, and mean gestational age per each location-year-sex as inputs into the model. Birth weight distribution models use the prevalence of <2500 grams birth weight and mean birth weight per each location-year-sex. Prevalence of <37 weeks gestation and of <2500 grams birth weight was estimated for all location-year-sexes using STGPR modelling processes standard to GBD.

Low birth weight (<2500 grams) data was extracted from literature, vital registration systems, and surveys. DHS survey data were observed to have high missingness; to correct for the missingness, birth weight was imputed using the Amelia package in R. Birth weight was predicted using standard Amelia imputation methods from the following variables also in the DHS surveys: urbanicity, sex, birthweight recorded on card, birth order, maternal education, paternal education, child age, child weight, child height, mother's age at birth, mother's weight, shared toilet facility, and household water treated. Data counts for categorical prevalence models are listed in Table 1.

**Table 1: Data Counts for Categorical Prevalence Models**

| | <28 weeks | <37 weeks | <2500 grams |
|---|---|---|---|
| Site-years (total) | 1872 | 2420 | 2980 |
| Number of GBD regions with data (out of 21 regions) | 14 | 21 | 21 |
| Number of GBD super-regions with data (out of 7 super-regions) | 6 | 7 | 7 |

Global ensemble weights for gestational age were derived by using a 3 million sample of all available microdata in Table 2 to select the ensemble weights. Of the exponential, gamma, inverse gamma, Weibull, log normal, and normal distributions, the three distribution families that received the highest weights were the Weibull (87%), normal (4%), and inverse gamma (4%) distributions. Global ensemble weights for birth weight were derived using a 3 million sample of all available microdata in Table 2, in addition to birth weight microdata available primarily through the DHS and MICS surveys. Of the exponential, gamma, inverse gamma, Weibull, log normal, and normal distributions, the three

distribution families that received the highest weights were the log normal (38%), normal (32%), and Weibull (20%) distributions.

Ordinary least squares was used to model mean gestational age for all location-year-sexes by regressing mean gestational age on prevalence of <37 weeks gestation per location-year. All available microdata (Table 2) was used to fit the model. OLS was also used to model mean birth weight by regressing prevalence of <2500 g birth weight per location-year. All available joint microdata (Table 2), as well as additional birth weight microdata extracted primarily through DHS and MICS surveys, was used to fit the model. As estimates of prevalence of <37 weeks gestation and prevalence of <2500g birth weight are available for all location-year-sexes through STGPR models, mean gestational age and mean birth weight were predicted for all location-year-sexes.

### *Copula optimisation*

In order to model the joint distribution of gestational age and birth weight from separate distributions, information is needed about the correlation between the two distributions. Distributions of gestational age and birth weight are not independent; the Spearman correlation for each country where joint microdata was available (Table 2), pooling across all years of data available, ranged from 0.25-0.49. The overall Spearman correlation was 0.38, pooling across all countries in the dataset.

***Table 3: Summary of Data Inputs***

| *Location* | Years of data | Total births* | Format of data | Spearman correlation | Used in Ensemble Weight Selection | Used in Copula Parameter Selection | Used in Relative Risk Models |
|---|---|---|---|---|---|---|---|
| *BRA* | 2016 | 2,854,380 | Microdata | 0.37 | Yes | Yes | No |
| *ECU* | 2003-2015 | 2,473,039 | Microdata | 0.34 | Yes | Yes | No |
| *ESP* | 1990-2014 | 8,537,220 | Microdata | 0.42 | Yes | Yes | No |
| *JPN* | 1995-2015 | 23,644,506 | Tabulations | 0.41 | No | No | Yes |
| *MEX* | 2008-2012 | 10,256,117 | Microdata | 0.35 | Yes | Yes | No |
| *NOR* | 1990-2014 | 1,489,210 | Microdata | 0.44 | Yes | Yes | Yes |
| *NZL* | 1990-2016 | 1,600,501 | Microdata | 0.25 | Yes | Yes | Yes |
| *SGP* | 1993-2015 | 972,775 | Tabulations | 0.41 | No | No | Yes |
| *TWN* | 1998-2002 | 1,331,760 | Tabulations | 0.38 | No | No | Yes |
| *URY* | 1996-2014 | 698,622 | Microdata | 0.49 | Yes | Yes | No |
| *USA* | 1990-2014 | 81,929,879 | Microdata | 0.38 | Yes | Yes | Yes |

** Pooled across all year and sexes, excluding data missing year of birth, gestational age, or birth weight*

Copula modelling is used to model joint distributions between the birth weight and gestational age marginal distributions. The Copula and VineCopula packages in R were used to select the optimal copula family and copula parameters to model the joint distribution, using joint microdata from the country-years in Table 2. The copula family selected from the microdata was "Survival BB8", with theta parameter set to 1.75 and delta parameter set to 1.

The joint distribution of birth weight and gestational age per location-year-sex was modelled using the global copula family and parameters selected and the location-year-sex gestational age and birth weight distributions. The joint distribution was simulated 100 times to capture uncertainty. Each simulation consisted of 100,000 simulated joint birth weight and gestational age data points. Each joint distribution

was divided into 500g by 2wk bins to match the categorical bins of the relative risk surface. Birth prevalence was then calculated for each 500g by 2wk bin.

### *Estimating Early Neonatal Prevalence & Late Neonatal Prevalence from Birth Cohorts*

Early neonatal prevalence and late neonatal prevalence was estimated using life table approaches for each 500g & 2wk bin. Using the all-cause early neonatal mortality rate for each location-year-sex, births per location-year-sex-bin, and the relative risks for each location-year-sex-bin in the early neonatal period, the all-cause early neonatal mortality rate was calculated for each location-year-sex-*bin*. The early neonatal mortality rate per bin was used to calculate the number of survivors at 7 days and prevalence in the early neonatal period. Using the same process, the all-cause late neonatal mortality rate for each location-year-sex was paired with the number of survivors at 7 days and late neonatal relative risks per bin to calculate late neonatal prevalence and survivors at 28 days.

## Relative risks & theoretical minimum-risk exposure level

### Causes

The available data for deriving relative risk was only for all-cause mortality. The exception was the USA linked infant birth-death cohort data, which contained 3-digit ICD causes of death, but also had nearly 30% of deaths coded to causes that are ill-defined, or intermediate, in the GBD cause classification system. For GBD 2017, like in GBD 2016, we analysed the relative risk of all-cause mortality across all available sources and selected outcomes based on criteria of biologic plausibility. Some causes, most notably congenital birth defects, haemoglobinopathies, malaria, and HIV/AIDS, were excluded based on the criteria that reverse causality could not be excluded. The final list of outcomes included in calculating the attributable burden for LBW/SG are in Table 3.

***Table 3: Cause list of outcomes for low birth weight and short gestation***

| *Cause ID* | Cause name |
|---|---|
| *302* | Diarrheal diseases |
| *322* | Lower respiratory infections |
| *328* | Upper respiratory infections |
| *329* | Otitis media |
| *333* | Pneumococcal meningitis |
| *334* | H influenzae type B meningitis |
| *335* | Meningococcal meningitis |
| *336* | Other meningitis |
| *337* | Encephalitis |
| *381* | Neonatal preterm birth complications |
| *382* | Neonatal encephalopathy due to birth asphyxia and trauma |
| *383* | Neonatal sepsis and other neonatal infections |
| *384* | Hemolytic disease and other neonatal jaundice |
| *385* | Other neonatal disorders |
| *686* | Sudden infant death syndrome |

## Input data

In the Norway, New Zealand, and US Linked Birth/Death Cohort microdata datasets, live births are reported with gestational age, birth weight, and an indicator of death at 7 days and 28 days. For this analysis, gestational age was grouped into two-week categories, and birth weight was grouped into 500-gram categories. The Taiwan, Japan, and Singapore datasets were prepared in tabulations of joint 500-gram and two-week categories.

## Modelling strategy

For each location, data was pooled across years, and the risk of all-cause mortality at the early neonatal period and late neonatal period at joint birth weight and gestational age combinations was calculated. In all datasets except for the United States, sex-specific data were combined to maximise sample size. The United States analyses were sex-specific. To calculate relative risk at each 500g and 2wk combination, logistic regression was first used to calculate mortality odds for each joint 2-week gestational age and 500-gram birth weight category. Mortality odds were smoothed with Gaussian Process Regression, with the independent distributions of mortality odds by birth weight and mortality odds by gestational age serving as priors in the regression.

A pooled country analysis[1] of mortality risk in the early neonatal period and late neonatal period by SGA category in developing countries in Asia and Sub-Saharan Africa were also converted into 500-gram and 2-week bin mortality odds surfaces. The relative risk surfaces produced from microdata and the Asia and Africa surfaces produced from the pooled country analysis were meta-analyzed, resulting in a meta-analysed mortality odds surface for each location. The meta-analysed mortality odds surface for each location was smoothed using Gaussian Process Regression and then converted into mortality risk. To calculate mortality relative risks, the risk of each joint 2-week gestational age and 500-gram birth weight category were divided by the risk of mortality in the joint gestational age and birth weight category with the lowest mortality risk.

For each of the country-derived relative risk surfaces, the 500 g and 2-week gestational age joint bin with the lowest risk was identified. This bin differed within each country dataset. To identify the universal 500 g and 2-week gestational age category that would serve as the universal TMREL for our analysis, we chose the bins that was identified to be the TMREL in each country dataset to contribute to the universal TMREL. Therefore, the joint categories that served as our universal TMREL for the LBWSG risk factor were “38-40 weeks of gestation and 3500-4000 grams", "38-40 weeks of gestation and 4000-4500 grams", and "40-42 weeks of gestation and 4000-4500 grams". As the joint TMREL, all three categories were assigned to a relative risk equal to 1.

## PAF calculations

The total PAF for the low birth weight and short gestation joint risk factor is calculated by summing the PAF calculated from each 500g x 2wk category, with the lowest risk category among all the 500g x 2wk categories serving as the TMREL. The equation for calculating PAF for each 500g x 2wk category is:

$$PAF_{joasgt} = \frac{\sum_{x=1}^{u} RR_{joast}(x)P_{jasgt}(x) - RR_{joasg}(TMRE_{jas})}{\sum_{x=1}^{u} RR_{joas}(x)P_{jasgt}(x)}$$

To calculate the overall PAF for the short gestation for birth weight risk factor, PAF was once again calculated for each joint 500-gram and 2-week category. Unlike the joint PAF calculation, which used only one TMREL for all 500-gram and 2-week categories, the joint 500-gram and 2-week category with the lowest risk for each 500-gram birth weight grouping served as the TMREL for that 500-gram birth weight grouping. For example, the [3000, 3500) gram birth weight grouping contains five joint categories: [34, 36) weeks and [3000, 3500) grams; [36, 37) weeks and [3000, 3500) grams; [37, 38) weeks and [3000, 3500) grams; [38, 40) weeks and [3000, 3500) grams; and [40, 42) weeks and [3000, 3500) grams. The [40, 42) weeks and [3000, 3500) grams joint category has the lowest risk, and so it serves as the TMREL for the [3000, 3500) gram birth weight grouping. In the Relative Risk surface figures, a birth weight grouping is one “column” of the birth weight and gestational age matrix.

The overall PAF for the short gestation for birth weight risk factor was then calculated for all the joint 500-gram and 2-week categories using the formula below:

$$PAF_{1..i} = 1 - \prod_{i=1}^{n}(1 - PAF_i)$$

The same methodology was applied to calculate the total PAF for the low birth weight for gestation risk factor, using two-week gestational age categories (each “row” of the matrix) instead of 500-gram birth weight categories. For example, the [24, 26) weeks gestational age grouping contains three joint categories: [0, 500) grams and [24, 26) weeks; [500, 1000) grams and [24, 26) weeks; and [1000, 1500) grams and [24, 26) weeks. The [1000, 1500) grams and [24, 26) weeks joint category has the lowest risk, and so it serves as the TMREL for the [24, 26) weeks gestational age grouping.

After the short gestation for birth weight PAF and low birth weight for gestational age PAF were calculated, they were then scaled so that the sum of the short gestation for birth weight PAF and low birth weight for gestation PAF equal the low birth weight and short gestation parent PAF calculated for each location/year/sex/age group.

## References

1. Katz et al. Mortality risk in preterm and small-for-gestational-age infants in low-income and middle-income countries: a pooled country analysis. 2013. Volume 382, Issue 9890. *The Lancet.*

# Iron Deficiency Capstone Appendix

## Flowchart

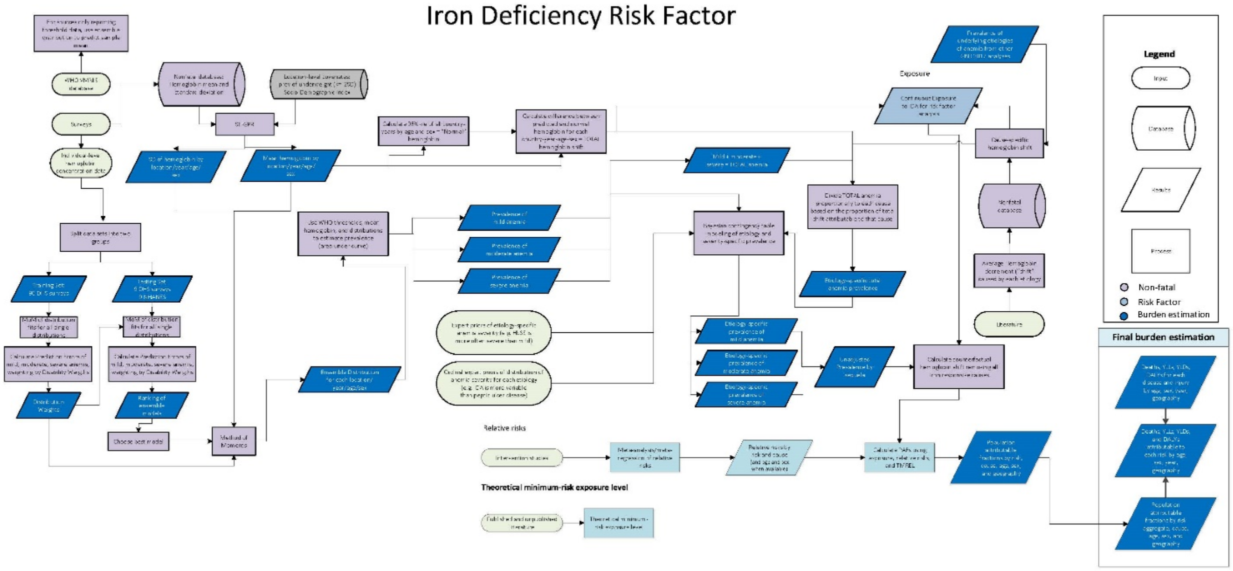


## Input data & methodological summary

### Exposure

#### *Case definition*

For GBD 2017, as with GBD 2016, the anemia model has two main steps: estimation of the anemia envelope and causal attribution. Our analytic strategy began with calculation of an anemia envelope – a determination of mean hemoglobin, as well as a sum total of anemia prevalence, by severity for each country, age group, and both sexes for each year from 1990 through 2017. The envelope approach avoids double-counting while capturing potentially different disease profiles within each population group. We defined a population group as a specific geography, sex, age-group, and year.

#### *Input data*

Iron-deficiency anemia (IDA) estimates include acute and chronic hemorrhagic states for which supplementation may be helpful, but poor nutritional intake is not the only underlying problem. A few causes in this category – hookworm, schistosomiasis, upper gastrointestinal bleeding, and gynecologic diseases – were considered separately from IDA in GBD 2016 because there was enough data from GBD prevalence estimation processes to do so. Distribution of anemia burden to IDA only after assignment to “known” causes avoided double counting of these cases. In GBD 2017, we redefined the iron deficiency risk factor to encompass all causes of anemia that would respond to iron supplementation.

For our nonfatal anemia estimates, the envelope approach to the anemia impairment utilises data from a variety of sources. Population-based surveys of hemoglobin concentration were the primary input to our analytic dataset. Examples include the Demographic and Health Survey (DHS) and Multiple Indicator Cluster Survey (MICS) series, along with other national and subnational surveys that completed hemoglobin testing. We supplemented with pertinent sources downloaded from the WHO Vitamin and

Mineral Nutrition Information System (VMNIS) available at http://www.who.int/vmnis/database/anaemia/countries/en/. A full source list is available elsewhere in this appendix. Most used a HemoCue test, adjusted for altitude, and excluded those with terminal or acute medical conditions.  Inclusion, exclusion and diagnostic criteria for other studies were similar and can be found in each study.

### *Modelling strategy*

For GBD 2017, we estimated the mean hemoglobin in g/dL among pregnant women aged 15 to 49 years of age and the implied mean hemoglobin among pregnant women in the absence of iron deficiency anemia, as the risk exposure for maternal iron deficiency anemia.

## Theoretical minimum-risk exposure level

The implied mean hemoglobin in the absence of iron deficiency anemia is the theoretical minimum risk exposure level. This was calculated by adding the iron responsive hemoglobin shift back onto the observed hemoglobin concentration for each demographic. For example, if the observed hemoglobin concentration among 30-34 year old pregnant women in Ethiopia was 132.9 g/L, and the shift was 1.6 g/L in that demographic, then the counterfactual was 134.5 g/L. The GBD 2017 anemia modelling strategy provides details on how the iron deficiency shifts were calculated.

## Relative risk

We attribute 100% of iron-deficiency anemia to iron deficiency. The other outcomes used in GBD 2016 are maternal hemorrhage, maternal sepsis and other maternal infections. In GBD 2017 we added the following additional maternal outcomes: maternal hypertensive disorders, maternal obstructed labor an uterine rupture, maternal abortion and miscarriage, ectopic pregnancy, indirect maternal deaths, late maternal deaths, maternal deaths aggravated by HIV/AIDS, and other maternal disorders. For these additional maternal outcomes, we assigned the same relative risk as that used for the maternal outcomes from 2016. Sources of evidence for these relative risks are unchanged from GBD 2013.

## References


1. Centers for Disease Control and Prevention (CDC). Iron deficiency--United States, 1999-2000. MMWR Morb Mortal Wkly Rep 2002; 51: 897–9.

2. Looker AC, Dallman PR, Carroll MD, Gunter EW, Johnson CL. Prevalence of iron deficiency in the united states. JAMA 1997; 277: 973–6.

# Vitamin A Deficiency Capstone Appendix

## Flowchart

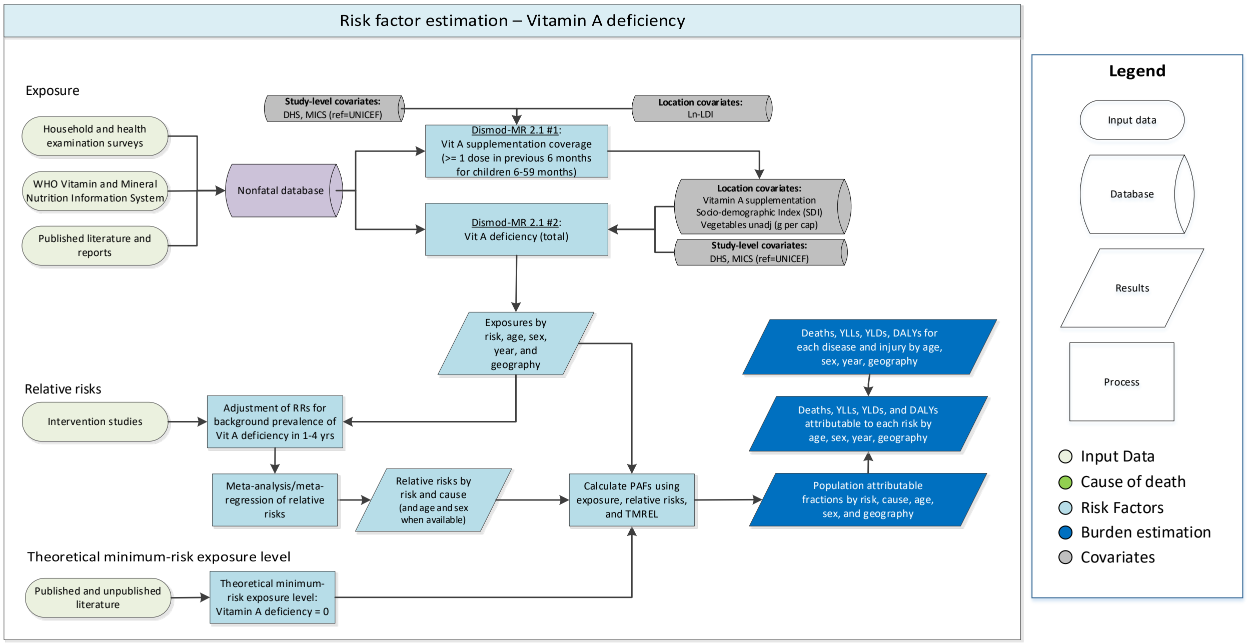


## Input data & methodological summary

### Exposure

#### *Case definition*

For GBD 2017, vitamin A deficiency is defined as serum retinol <70 µmol/L. We examined vitamin A deficiency as a risk factor in children aged 6 months to 5 years.

To ensure we were using as much information as possible, and therefore maximize the data basis of our estimates, we modeled Vitamin A deficiency sequentially. The first step was to estimate the coverage of Vitamin A supplementation. Although the typical metric on which supplementation is tracked is 2+ doses of Vitamin A in the previous 12 months for children under 5 years, most existing health surveys do not routinely provide sufficient information to calculate it. Our case definition for the supplementation model was therefore the proportion of children 6-59 months of age who received at least one dose of Vitamin A in the previous 6 months. Supplementation estimates were then used as a location-level covariate to guide exposure models of overall Vitamin A deficiency.

#### *Input data*

For GBD 2017, we used data from the WHO Vitamin and Mineral Nutrition Information System, health surveys such as DHS and MICS, and studies identified through literature review. This included updating the dataset to include all ages and all studies available in VMNIS as of April 2018. A separate systematic review was last conducted for GBD 2013. The PubMed search terms were: ((vitamin A deficiency[Title/Abstract] AND prevalence[Title/Abstract]) AND (“2009”[Date – Publication] : “2013”[Date – Publication])). The table below shows the number of data points included in the final datasets**.** Exclusion criteria were:

1. Studies that were not population-based, e.g., hospital or clinic-based studies
2. Studies that did not provide primary data on epidemiological parameters, e.g., commentaries
3. Review articles
4. Case series
5. Self-reported cases

Table 1. Geographic representation of datasets used for three stages of Vitamin A deficiency risk factor burden estimation (number of data points per geography)

| Geography | Supplementation (proportion) | Deficiency (prevalence) |
|---|---|---|
| Global | 900 | 1540 |
| East Asia | 12 | 27 |
| Southeast Asia | 102 | 212 |
| Oceania | 24 | 54 |
| Central Asia | 51 | 66 |
| Central Europe | 2 | 13 |
| Eastern Europe | | 3 |
| Australasia | | 1 |
| Western Europe | | 38 |
| Southern Latin America | | 16 |
| High-income North America | | 33 |
| Caribbean | 17 | 34 |
| Andean Latin America | 25 | 70 |
| Central Latin America | 33 | 212 |
| Tropical Latin America | 1 | 52 |
| North Africa and Middle East | 49 | 148 |
| South Asia | 61 | 96 |
| Central Sub-Saharan Africa | 60 | 8 |
| Eastern Sub-Saharan Africa | 182 | 220 |
| Southern Sub-Saharan Africa | 49 | 57 |
| Western Sub-Saharan Africa | 232 | 180 |

## Modeling strategy

All Vitamin A deficiency estimates were made using DisMod-MR 2.1. As described above, we first estimated Vitamin A supplementation coverage. Although all data was from ages 6-59 months, we assumed no difference in age pattern of supplementation coverage and used the natural log of lag-distributed income per capita (LN-LDI) as a location-level covariate to inform estimates where data was absent. DHS and MICS data was cross-walked to the reference data source, which came from UNICEF (http://data.worldbank.org/indicator/SN.ITK.VITA.ZS).

Table 2: Covariate effects for Vitamin A supplementation model

| Measure | Covariate | Type | Value | Exponentiated |
|---|---|---|---|---|
| Prevalence | MICS | Study-level | -0.6<br>(-0.76 — -0.45) | 0.55<br>(0.47 — 0.64) |
| Prevalence | DHS | Study-level | -0.09<br>(-0.2 — 0.025) | 0.91<br>(0.82 — 1.03) |
| Prevalence | LDI (I$ per capita) | Country-level | 0.013<br>(0.00033 — 0.042) | 1.01<br>(1.00 — 1.04) |

Second, we estimated the age- and sex-specific prevalence of Vitamin A deficiency (serum retinol < 0.7 µmol/L). WHO VMNIS was the primary data source for this model and was supplemented with data from DHS and other health surveys where testing was performed. We assumed the following in our model: no excess mortality, birth prevalence is possible, incidence is decreasing after age 5 and remission is increasing after age 5. Data from subnational locations was crosswalked to the reference data sources of nationally-representative data. Females were found to have 1.09 times higher Vitamin A deficiency, although the uncertainty in that ratio ranged from 0.97 to 1.24. Location-level covariates were used for Vitamin A supplementation coverage from the above model as well as GBD 2016 Socio-demographic Index (SDI) numbers.

Table 3: Covariate effects for Vitamin A deficiency model

| Measure | Covariate | Type | Value | Exponentiated |
|---|---|---|---|---|
| Prevalence | Sex | Study-level (x-cov) | -0.0091 ( -0.088 — 0.065) | 0.99 (0.92 — 1.07) |
| Prevalence | Subnational | Study-level (x-cov) | -0.28 ( -0.44 — -0.1) | 0.76 (0.64 — 0.90) |
| Prevalence | Vitamin A supplem. coverage rate | Country-level | -0.028 ( -0.1 — -0.00071) | 0.97 (0.90 — 1.00) |
| Prevalence | Socio-demographic Index | Country-level | -2.98 ( -3 — -2.92) | 0.051 (0.050 — 0.054) |
| Prevalence | vegetables unadjusted(g) | Country-level | -1.36 ( -1.53 — -1.12) | 0.26 (0.22 — 0.33) |

## Theoretical minimum-risk exposure level

The theoretical minimum risk exposure is that the prevalence of vitamin A deficiency is zero.

## Relative risks

The relative risks were updated in GBD 2017 to reflect studies included in the most recently published systematic review by Imdad and colleagues.[1] The overall estimation strategy has not changed. For each trial identified by the systematic review, we adjusted the relative risk for the background prevalence of Vitamin A deficiency in 1-4 years from the GBD 2017 model described above. This adjustment assumes the effect of supplementation is observed only in the fraction of the trial population that are Vitamin A deficient. Many studies evaluate either incidence or mortality. A subset of studies evaluated both incidence and cause-specific mortality as outcomes for the same cause. We found no statistical difference between the effect sizes of incidence and mortality in any of these studies so pooled all incidence and mortality observations as independent observations prior to meta-analysis. We then performed a fixed effects meta-analysis of all adjusted RRs to determine final outcomes to be included in GBD risk factor attribution estimates. Forest plots are shown in Figures 1-5; Final RRs are shown in Table 4. Three outcomes – diarrhea, lower respiratory infections (LRI), and measles – were found to be statistically significant after adjustment, pooling, and meta-analysis. Meningitis was non-significant. Malaria was significant, but only a single study was identified that evaluated this outcomes, which does not meet GBD causal criteria.

Table 4: Pooled relative risks for risk-outcome pairs included in GBD 2017

| Cause | GBD 2016 RR | GBD 2017 RR | Include in GBD 2017 |
|---|---|---|---|
| Diarrhea | 1.6 (1.21 - 2.02) | **2.35 (2.17 - 2.54)** | Yes |
| Measles | 2.4 (1.61 - 3.48) | **2.76 (2.01 - 3.78)** | Yes |
| Lower Respiratory Infections (LRI) | | **1.23 (1.03 - 1.48)** | Yes |
| Meningitis | | 3.2 (0.69 - 14.75) | No (not significant) |
| Malaria | | 3.65 (2.23 - 5.97) | No (only one study) |

Figure 1: Forest plot of RR of diarrhea in Vitamin A deficiency

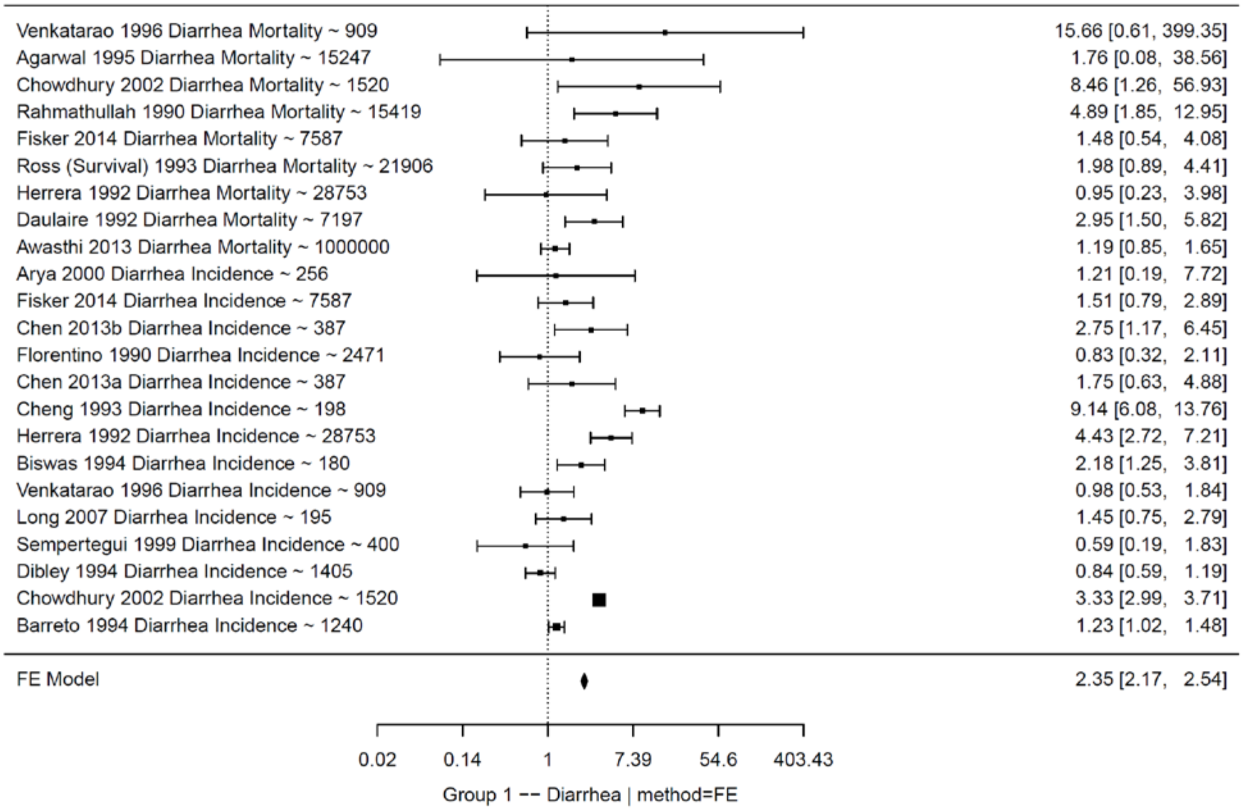

Figure 2: Forest plot of RR of measles in Vitamin A deficiency

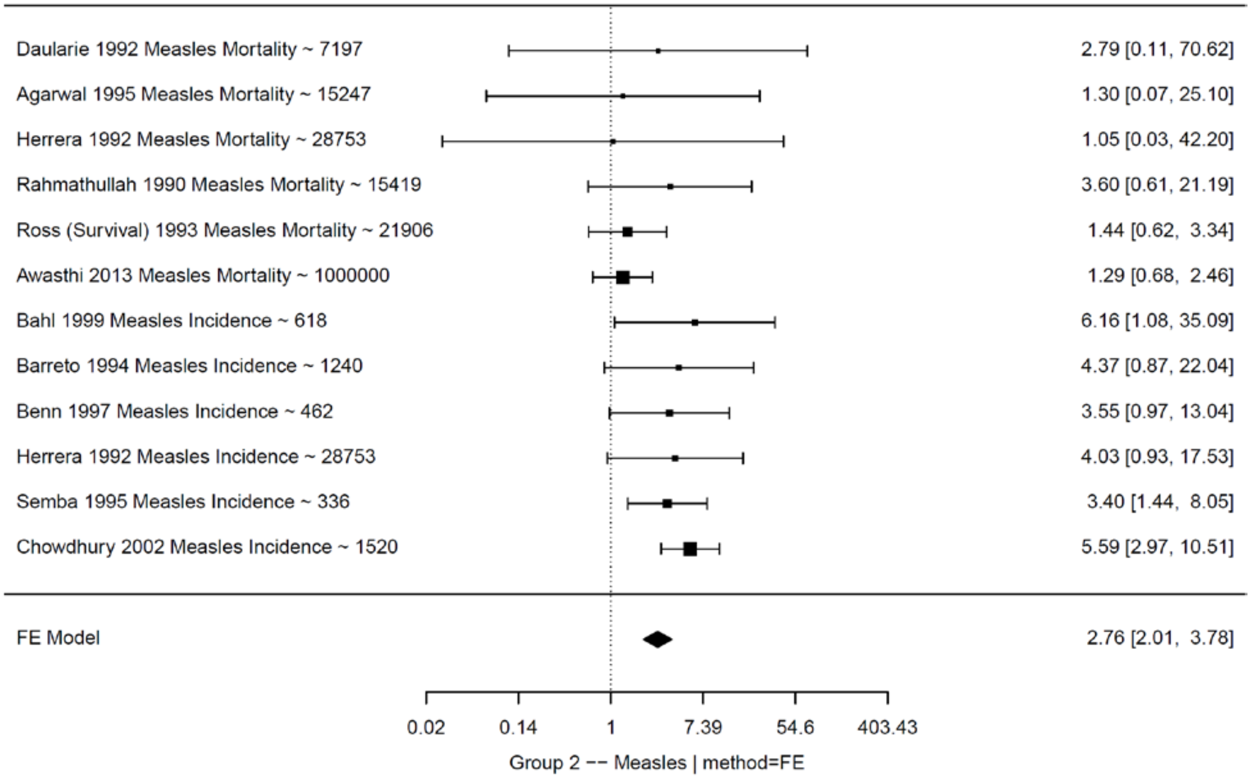


Figure 3: Forest plot of RR of LRI in Vitamin A deficiency

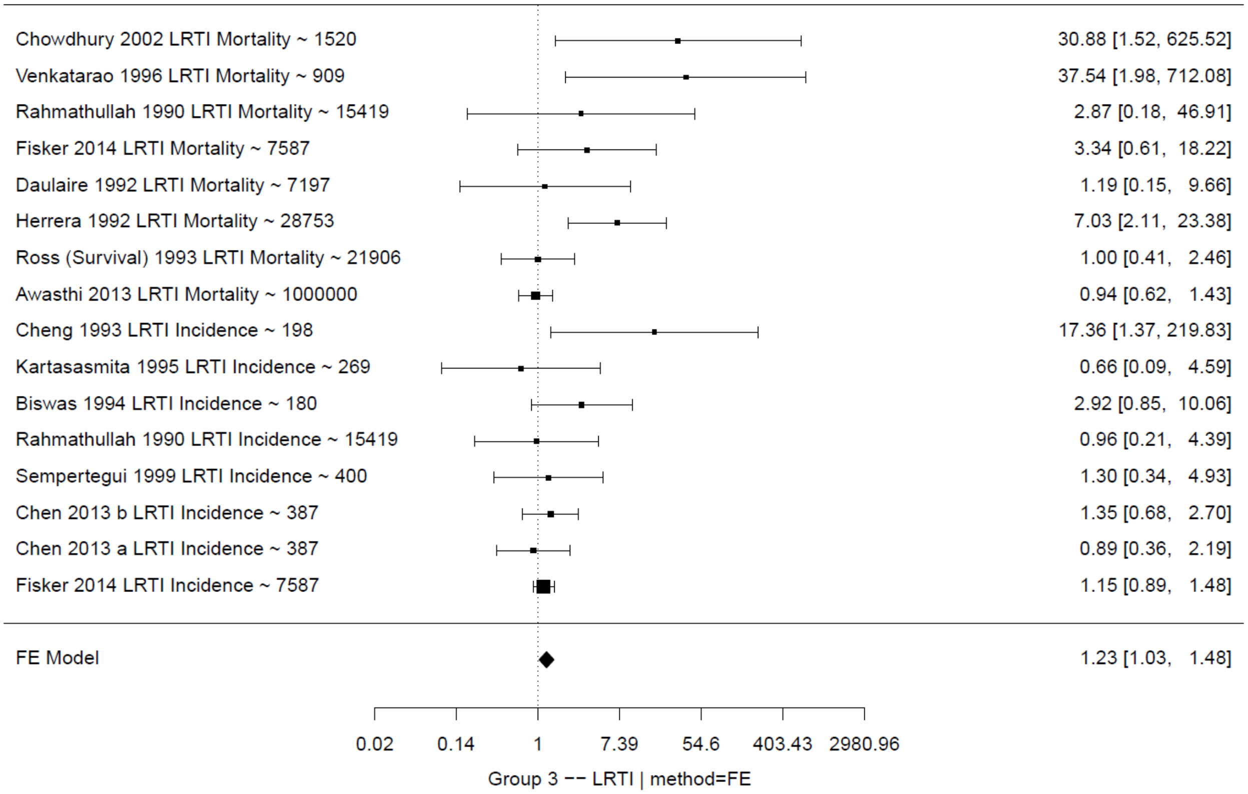

Figure 4: Forest plot of RR of meningitis in Vitamin A deficiency

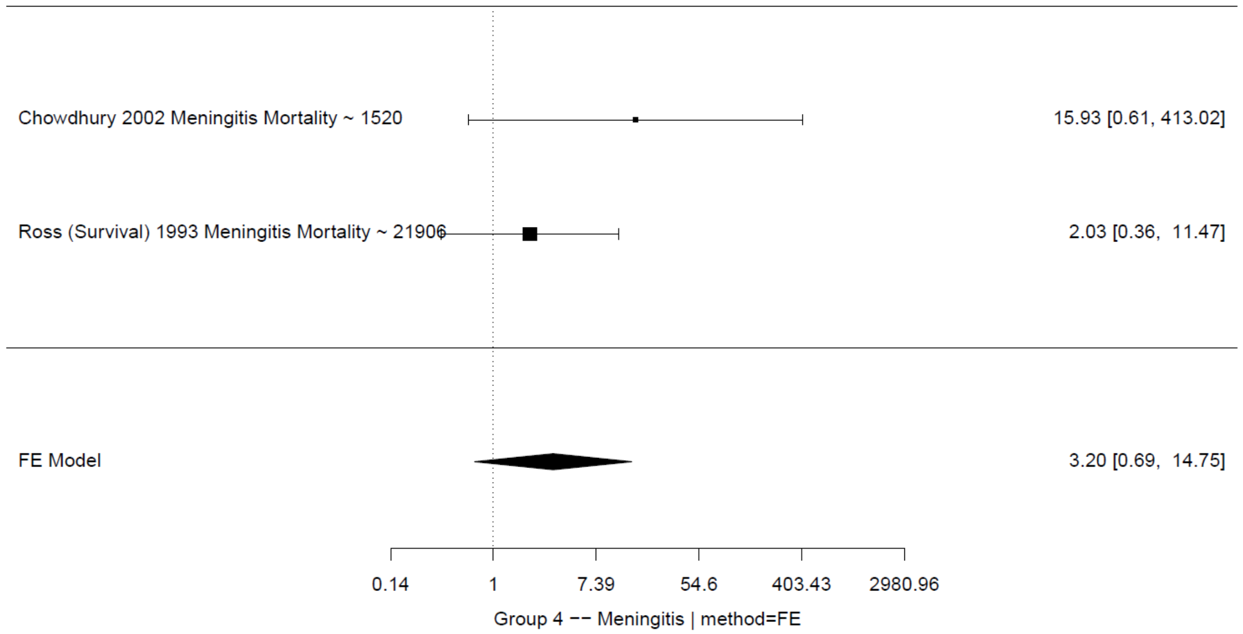


Figure 5: Forest plot of RR of malaria in Vitamin A deficiency

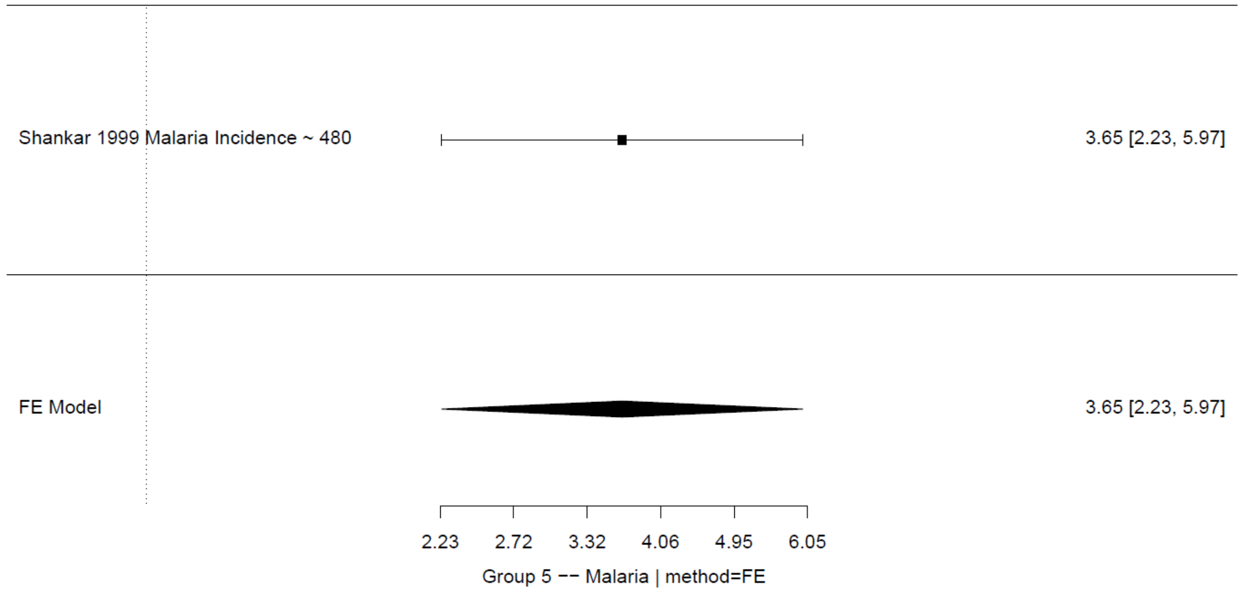


## References


1. Imdad A, Ahmed Z, Bhutta ZA. Vitamin A supplementation for the prevention of morbidity and mortality in infants one to six months of age. *Cochrane Database of Systematic Reviews* 2016; Sep 28; 9. Art. No: CD007480.

# Zinc Deficiency Capstone Appendix

## Input data & methodological summary

### Exposure

#### *Case definition*

Exposure to zinc deficiency is defined as consumption of less than 2.5 milligrams of zinc per day among children between the ages of 1 and 4 years old.

#### *Input data*

We used dietary data from nationally and sub-nationally representative nutrition surveys and United Nations FAO Supply and Utilization Accounts to estimate the mean intake of zinc at the population level.

### Modelling strategy

For GBD 2016, we first used a spatio-temporal Gaussian process regression (ST-GPR) framework to estimate the mean intake of zinc by age, sex, country, and year. To assist with estimation for locations and years without data, we used the lag-distributed income of that location-year as a covariate. We considered data from 24-hour diet recall as the gold standard, and adjusted data from other sources to the gold standard method. Using the method described in the dietary risks section, we characterised the distribution of zinc intake for children between ages of 1 and 4 years old and estimate the proportion of the children with intake of less than 2.5 milligrams of zinc per day.

### Relative risk

Relative risks used for zinc deficiency is based on the results of randomised trials that measured the effect of zinc supplementation.

### Theoretical minimum-risk exposure level

The theoretical minimum-risk exposure level for proportion zinc deficient is zero percent deficient.

# Smoking Capstone Appendix

## Flowchart

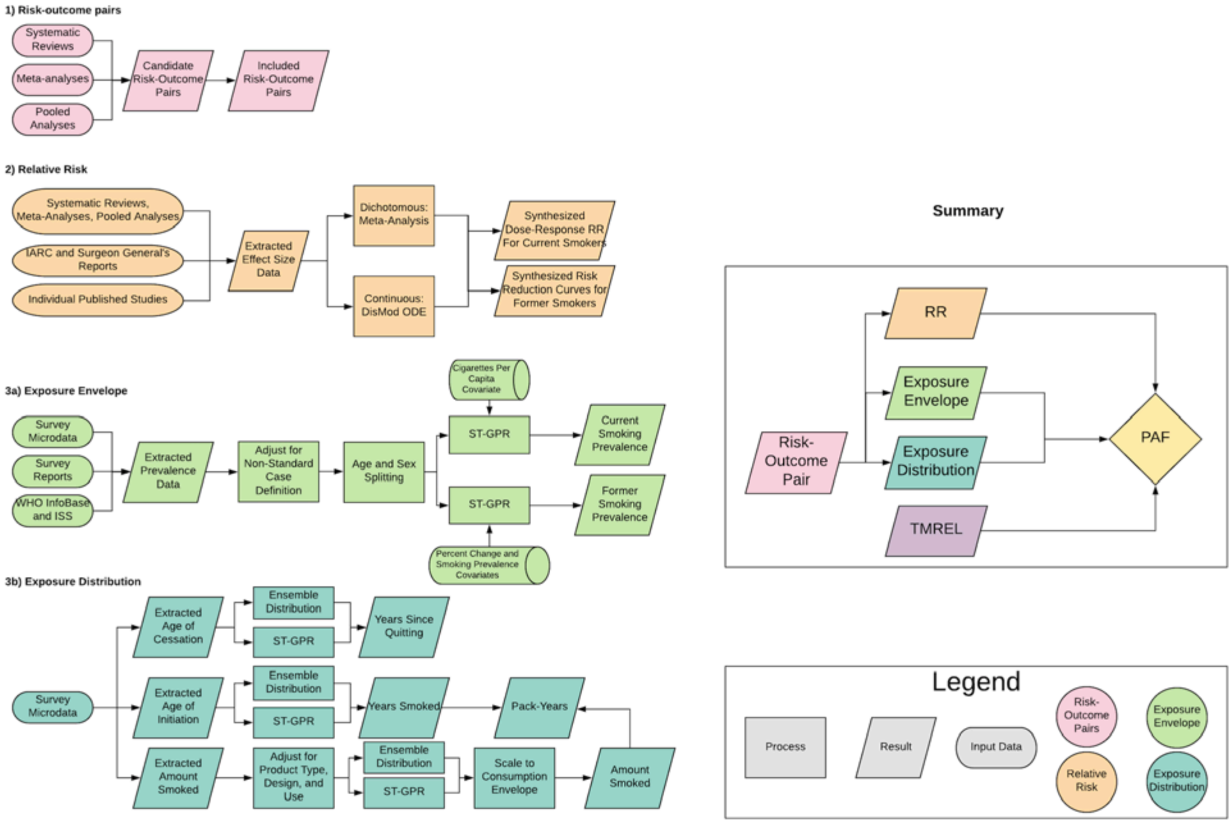


We made significant changes to the methods used to estimate smoking attributable burden in GBD 2017. In previous iterations of the GBD, we have used the Peto-Lopez (Smoking Impact Ratio) method to estimate burden attributable to cancers and chronic respiratory diseases. Although this method provides robust estimates of the burden of cancers and chronic respiratory diseases related to tobacco, it is not fully consistent with the GBD approach of estimating exposure independently of the outcomes affected by exposure. For cardiovascular diseases and all other smoking attributable health outcomes, we used five-year lagged daily smoking prevalence as the exposure. With a growing body of evidence on the association between smoking and several types of cancers and with cardiovascular disease, coupled with good estimates of the distribution of cumulative smoking exposure, direct estimation of attributable burden is possible. In GBD 2017, we have transitioned to using continuous measures of exposure that incorporate dose-response effects among daily, occasional, and former smokers for all health outcomes except fractures.

## Current and former smoking prevalence

We estimated the prevalence of current smoking and the prevalence of former smoking using data from cross-sectional nationally representative household surveys. We defined current smokers as individuals who currently use any smoked tobacco product on a daily or occasional basis. We defined former

smokers as individuals who quit using all smoked tobacco products for at least 6 months, where possible, or according to the definition used by the survey. Prior to modelling a complete time series for all demographic groups, we made adjustments for alternative case definitions as well as for data reported in non-standard age or sex groups. We modelled current and former prevalence using spatiotemporal Gaussian process regression.

### Data extraction

We extracted primary data from individual-level microdata and survey report tabulations. We extracted data on current, former, and/or ever smoked tobacco use reported as any combination of frequency of use (daily, occasional, and unspecified, which includes both daily and occasional smokers) and type of smoked tobacco used (all smoked tobacco, cigarettes, hookah, and other smoked tobacco products such as cigars or pipes), resulting in 36 possible combinations. Other variants of tobacco products, for example hand-rolled cigarettes, were grouped into the four type categories listed above based on product similarities. Only smoked tobacco products are included, smoked drugs are estimated separately as part of the drug use risk factor.

For microdata, we extracted relevant demographic information, including age, sex, location, and year, as well as survey metadata, including survey weights, primary sampling units, and strata. This information allowed us to tabulate individual-level data in the standard GBD five-year age-sex groups and produce accurate estimates of uncertainty. For survey report tabulations, we extracted data at the most granular age-sex group provided.

### Crosswalk

Our GBD smoking case definitions were current smoking of any tobacco product and former smoking of any tobacco product. All other data points were adjusted to be consistent with either of these definitions. Some sources contained information on more than one case definition and these sources were used to develop the adjustment coefficient to transform alternative case definitions to the GBD case definition. The adjustment coefficient was the beta value derived from a linear model with one predictor and no intercept.

We generated separate crosswalk coefficients for the 10-14 age group and the 15-19 age group, as we found the relationships between case definitions differed strongly in the younger age groups compared to the 20+ age groups. To account for this, we attempted to generate a global crosswalk coefficient for both the 10-14 and 15-19 age groups, using the same regression as above. Due to data limitations, none of the crosswalk coefficients met the criteria outlined above, so no data covering youths under 20 years old were crosswalked. In other words, all data from these age groups that appear in the model were asked according to our case definition in the survey.

We propagated uncertainty at the survey level from the crosswalk by incorporating both the variance of the errors and the variance of the adjustment coefficients.

For each source that needed adjusting, we assigned space weights based on GBD region and super region to the sources containing more than one case definition. Data from the same region receiving a full weight of 1, and data from the same super-region received a weight of ½. We explored using a time weight, to control for possible changes in the relationship between smokeless tobacco use behaviours over time. We found incorporating temporal information did not significantly change the estimated

coefficients but did undercut sample sizes, and chose to exclude the time weight. Crosswalk coefficients generated from fewer than 20 data sources were dropped

### Age and sex splitting

We split data reported in broader age groups than the GBD 5-year age groups or as both sexes combined by adapting the method reported in Ng et al. (http://jamanetwork.com/journals/jama/fullarticle/1812960) to split using a sex- geography- time specific reference age pattern. We separated the data into two sets: a training dataset, with data already falling into GBD sex-specific 5-year age groups, and a split dataset, which reported data in aggregated age or sex groups. We then used spatiotemporal Gaussian Process Regression (ST-GPR) to estimate sex-geography-time specific age patterns using data in the training dataset. The estimated age patterns were used to split each source in the split dataset.

The ST-GPR model used to estimate the age patterns for age-sex splitting used an age weight parameter value that minimises the effect of any age smoothing. This parameter choice allows the estimated age pattern to be driven by data, rather than being enforced by any smoothing parameters of the model. Because these age-sex split data points will be incorporated in the final ST-GPR exposure model, we do not want to doubly enforce a modelled age pattern for a given sex-location-year on a given aggregate data point.

### Smoking prevalence modelling

We used ST-GPR to model current and former smoking prevalence. Full details on the ST-GPR method are reported elsewhere in the Appendix. Briefly, the mean function input to GPR is a complete time series of estimates generated from a mixed effects hierarchical linear model plus weighted residuals smoothed across time, space and age. The linear model formula for current smoking, fit separately by sex using restricted maximum likelihood in R, is:

$$logit(p_{g,a,t}) = \beta_0 + \beta_1 CPC_{g,t} + \sum_{k=2}^{19} \beta_k I_{A[a]} + \alpha_s + \alpha_r + \alpha_g + \epsilon_{g,a,t}$$

Where $CPC_{g,t}$ is the tobacco consumption covariate by geography $g$ and time $t$, described above, $I_{A[a]}$ is a dummy variable indicating specific age group $A$ that the prevalence point $p_{g,a,t}$ captures, and $\alpha_s, \alpha_r$, and $\alpha_g$ are super region, region, and geography random intercepts, respectively. Random effects were used in model fitting but not in prediction.

The linear model formula for former smoking is:

$$logit(p_{g,a,t}) = \beta_0 + \beta_1 PctChange_{A[a],g,t} + \beta_3 CSP_{A[a],g,t} + \sum_{k=3}^{20} \beta_k I_{A[a]} + \alpha_s + \alpha_r + \alpha_g + \epsilon_{g,a,t}$$

Where $PctChange_{A[a],g,t}$ is the percent change in current smoking prevalence from the previous year, and $CSP_{A[a],g,t}$ is the current smoking prevalence by specific age group $A$, geography $g$, and time $t$ that point $p_{g,a,t}$ captures, both derived from the current smoking ST-GPR model defined above.

## Exposure among current and former smokers

We estimated exposure among current smokers for two continuous indicators: cigarettes per smoker per day and pack-years. Pack-years incorporates aspects of both duration and amount. One pack-year represents the equivalent of smoking one pack of cigarettes (assuming a 20 cigarette pack) per day for one year. Since the pack-years indicator collapses duration and intensity into a single dimension, one pack-year of exposure can reflect smoking 40 cigarettes per day for six months or smoking 10 cigarettes per day for two years.

To produce these indicators, we simulated individual smoking histories based on distributions of age of initiation and amount smoked. We informed the simulation with cross-sectional survey data capturing these indicators, modelled at the mean level for all locations, years, ages, and sexes using spatiotemporal Gaussian process regression. We rescaled estimates of cigarettes per smoker per day to an envelope of cigarette consumption based on supply-side data. We estimated pack-years of exposure by summing samples from age- and time-specific distributions of cigarettes per smoker for a birth cohort in order to capture both age trends and time trends and avoid the common assumption that the amount someone currently smokes is the amount they have smoked since they began smoking. All distributions were age-, sex-, and region- specific ensemble distributions, which were found to outperform any single distribution.

We estimated exposure among former smokers using years since cessation. We utilised spatiotemporal Gaussian process regression to model mean age of cessation using cross-sectional survey data capturing age of cessation. Using these estimates, we generated ensemble distributions of years since cessation for every location, year, age group, and sex.

## Risk-outcome pairs

We included the following risk-outcome pairs based on evidence supporting a causal relationship: tuberculosis, lower respiratory tract infections, esophageal cancer, stomach cancer, bladder cancer, liver cancer, laryngeal cancer, lung cancer, breast cancer, cervical cancer, colorectal cancer, lip and oral cancer, nasopharyngeal cancer, other pharyngeal cancer, pancreatic cancer, kidney cancer, leukemia, ischemic heart disease, ischemic stroke, hemorrhagic stroke, subarachnoid hemorrhage, atrial fibrillation and flutter, aortic aneurysm, peripheral arterial disease, chronic obstructive pulmonary disease, other chronic respiratory diseases, asthma, peptic ulcer disease, gallbladder and biliary tract diseases, Alzheimer disease and other dementias, Parkinson disease (protective), multiple sclerosis, type-II diabetes, rheumatoid arthritis, low back pain, cataracts, macular degeneration, and fracture.

## Dose-response risk curves

We conducted systematic literature reviews for all risk-outcome pairs identified as being caused by smoking. We extracted effect sizes by cigarettes per smoker per day, pack-years, and years since quitting from cohort and case-control studies. We synthesised these data to produce non-linear dose response curves using a Bayesian meta-regression model. For outcomes with significant differences in effect size by sex or age, we produced sex- or age-specific risk curves.

We estimate risk curves of former smokers compared to never smokers taking into account the rate of risk reduction among former smokers seen in the cohort and case-control studies, and the cumulative exposure among former smokers within each age, sex, location and year group.

## PAF calculation

We estimated population attributable fractions based on the following equation:

$$PAF = \frac{p(n) + p(f)\int \exp(x) * rr(x) + p(c)\int \exp(y) * rr(y) - 1}{p(n) + p(f)\int \exp(x) * rr(x) + p(c)\int \exp(y) * rr(y)}$$

where $p(n)$ is the prevalence of never smokers, $p(f)$ is the prevalence of former smokers, $p(c)$ is the prevalence of current smokers, $\exp(x)$ is a distribution of years since quitting among former smokers, $rr(x)$ is the relative risk for years since quitting, $\exp(y)$ is a distribution of cigarettes per smoker per day or pack-years, and $rr(y)$ is the relative risk for cigarettes per smoker per day or pack-years.

We used pack-years as the exposure definition for cancers and chronic respiratory diseases, and cigarettes per smoker per day for cardiovascular diseases and all other health outcomes.

# Secondhand Smoke Capstone Appendix

## Flowchart

Secondhand smoke

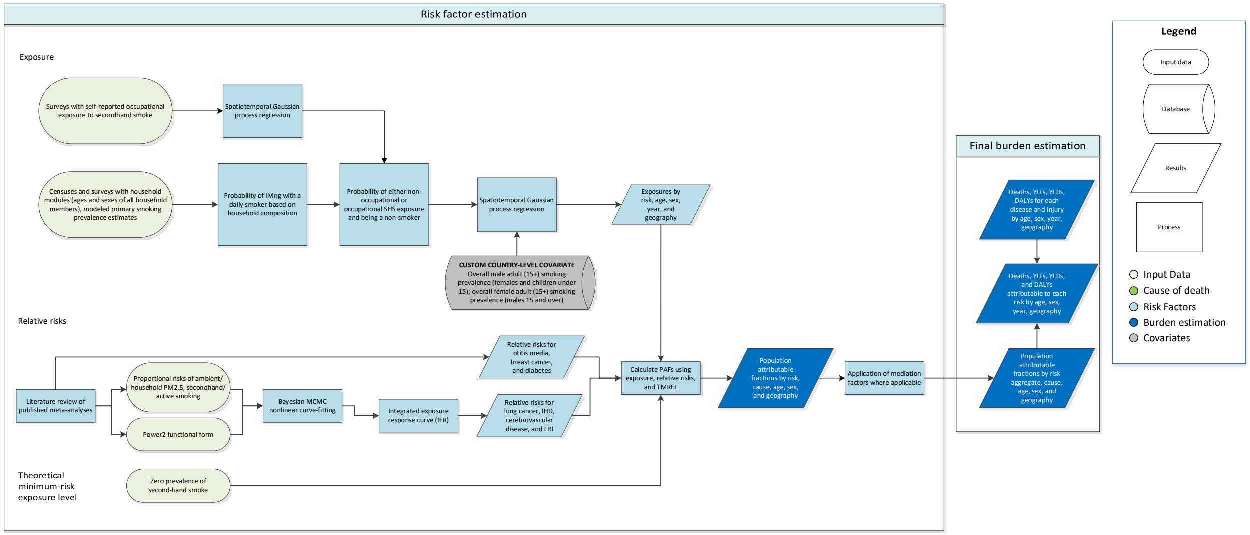


## Exposure

### Case definition

We define secondhand smoke exposure as current exposure to secondhand tobacco smoke at home, at work, or in other public places. We use household composition as a proxy for non-occupational secondhand smoke exposure and make the assumption that all persons living with a daily smoker are exposed to tobacco smoke. We use surveys to estimate the proportion of individuals exposed to secondhand smoke at work. We only consider non-smokers to be exposed to secondhand smoke. Non-smokers are defined as all persons who are not daily smokers. Ex-smokers and occasional smokers are considered non-smokers in this analysis. Exposure is evaluated for both children and adults.

### Input data

To calculate the proportion of non-smokers who live with at least one smoker, we used unit record data on household composition, which included the ages and sexes of all persons living in the same household. Our sources included representative major survey series with a household composition module, including the Demographic Health Surveys (DHS), the Multiple Indicator Cluster Surveys (MICS), and the Living Standards Measurement Surveys (LSMS); and national and subnational censuses, which included those captured in the IPUMS project and identified using the Global Health Data Exchange catalog (GHDx).

To calculate the proportion of individuals exposed to secondhand smoke at work, by age and sex, we used cross-sectional surveys that ask respondents about self-reported occupational secondhand smoke exposure. Sources include the Global Adult Tobacco Surveys, Eurobarometer Surveys, and WHO STEPS Surveys. We identified sources using the GHDx.

Estimates of primary smoking prevalence in each location were also used in our calculations. Further details on the estimation of primary smoking prevalence can be found in the Smoking methods appendix.

## Modelling strategy

We estimated the probability that each person is living with a smoker and is also a non-smoker themselves using set theory. First, household composition data were used at the individual level to capture the ages and sexes of each person in the household. Second, we analyzed surveys with both household composition data and tobacco use questions and determined that the distribution of household size, mean age of the household members, and the age distribution were not significantly different between households with and without a self-reported smoker. Since we did not find that household composition varied between smokers and non-smokers, we then used the GBD 2017 primary smoking prevalence model to calculate the probability that each household member is a smoker. Next, we used the probability of the union of sets on each individual household member to calculate the overall probability that at least one of the other household members was a smoker. We incorporated occupational exposure by modelling prevalence of current exposure to secondhand smoke at work, by age, sex, location, and year, using ST-GPR. In order to avoid double counting we calculated the probability that an individual is exposed through either non-occupational exposure or occupational exposure, given their age, sex, and household composition. Finally, we multiplied this probability of exposure by the probability that the individual is not a smoker themselves (i.e. 1 minus primary smoking prevalence for that person's location, year, age, and sex). We then collapse these individual-level probabilities to produce average probabilities of exposure by location, year, age, and sex.

These probabilities were modelled in the GBD ST-GPR framework, which generates exposure estimates from a mixed effects hierarchical linear model plus weighted residuals smoothed across time, space, and age. The linear model formula was fit separately by sex using restricted maximum likelihood in R.

We used the sex-specific overall smoking prevalence for adults (age 15 and older) as a country-level covariate in the model. The overall male adult daily smoking prevalence was used as the covariate for females of all ages and for males under age 15. The overall female adult daily smoking prevalence was used as the covariate for males age 15 and older. This was a modelling change from GBD 2015, in which we used the male age-standardised smoking prevalence for the adult female and children under 15 model, and the female age-standardised smoking prevalence for the adult male model.

All input data points from the probability calculation had a measure of uncertainty (variance and sample size) coming from the uncertainty of the primary smoking prevalence model and the sample size from the unit record data going into the modelling process. Geographic random effects were used in model fitting but were not used in prediction.

## Theoretical minimum-risk exposure level

The theoretical minimum-risk exposure level for secondhand smoke is zero exposure among non-smokers, meaning that non-smokers would not live with any primary smokers.

## Relative risks

For children ages 0-14, we estimated the burden of otitis media attributable to secondhand smoke exposure. For all ages we estimated the burden of lower respiratory infections (LRI), and for adults

greater or equal to 25 years of age we estimated the burden of lung cancer, chronic obstructive pulmonary disease (COPD), ischemic heart disease, and cerebrovascular disease attributable to secondhand smoke exposure, breast cancer, and type-II diabetes.

For lung cancer, ischemic heart disease, cerebrovascular disease, and LRI, we used country-specific relative risks created using integrated exposure response curves (IER) for PM2.5 air pollution. The relative risks for otitis media, breast cancer, and diabetes are derived from published meta-analyses.

We used the standard GBD population attributable fraction (PAF) equation to estimate burden based on exposure and relative risks.

# Chewing Tobacco Capstone Appendix

## Flowchart

Chewing Tobacco: Data and Model Flow Chart

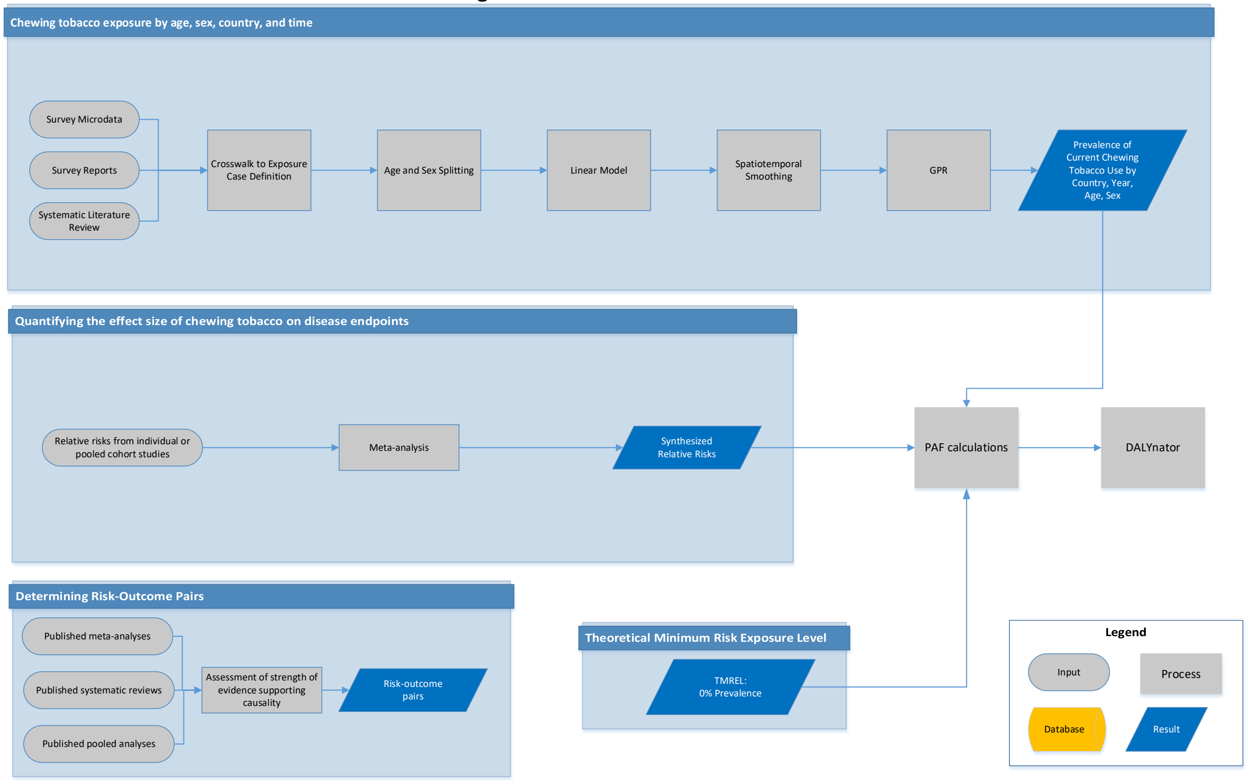


In GBD 2016, we estimated age-sex specific current smokeless tobacco use prevalence for all countries and territories from 1990-2016 using all available data. The estimated prevalence was then attributed to either all chewing tobacco, or all snus/snuff by country, based on input from smokeless tobacco experts.

For GBD 2017, we have changed the exposure definition from current smokeless tobacco use to current chewing tobacco use, based on the strength of evidence supporting the health effects of chewing tobacco use. By estimating chewing tobacco exposure in all countries and territories, burden is now estimated for locations previously classified as predominantly snus/snuff, but still have non-negligible use of chewing tobacco.

## Input data

### Inclusion criteria

We included sources that reported primary chewing tobacco use among respondents over age 10. To be eligible for inclusion, sources had to be representative for their level of estimation (ie. National sources needed to be nationally representative, subnational sources subnationally representative). We included only self-reported chewing tobacco use data and excluded data from questions asking about others' tobacco use behaviors. We included data collected between 1 January 1980 and 1 January 2018.

## Prevalence

We searched the Global Health Data Exchange (GHDx) database for primary data sources with the keyword "Tobacco Use" on January 1, 2018 to ensure all available data sources were captured. Of the 3,318 sources identified in the GHDx, 1,578 country-year sources met inclusion criteria and were included.

In addition to the primary data sources identified through the GHDx, we performed a systematic literature search on PubMed. The search was conducted on January 19, 2017 and returned 5982 hits, of which 267 were eligible for inclusion. Of these 267 sources, 200 had already been identified in the GHDx, so the Pubmed search yielded 67 additional sources overall. The search string is shown below: ("smokeless tobacco"[tiab] OR "Tobacco, Smokeless"[Mesh] OR bajjar[tiab] OR ("betel quid"[tiab] AND tobacco[tiab]) OR "chewing tobacco"[tiab] OR chimó[tiab] OR snuff[tiab] OR snuif[tiab] OR dip[tiab] OR dohra[tiab] OR gudakhu[tiab] OR gul[tiab] OR gutka[tiab] OR gutkha[tiab] OR "hnat hsey"[tiab] OR iq'mik[tiab] OR khaini[tiab] OR kharra[tiab] OR khiwam[tiab] OR khimam[tiab] OR kiwam[tiab] OR kimam[tiab] OR "lal dant manjan"[tiab] OR ("loose leaf"[tiab] AND (chew[tiab] OR tobacco[tiab])) OR mainpuri[tiab] OR maras[tiab] OR mawa[tiab] OR mshri[tiab] OR naffa[tiab] OR nas[Supplementary Concept] OR ((nas[tiab] OR nass[tiab]) AND tobacco[tiab]) OR naswar[tiab] OR nasway[tiab] OR nasvay[tiab] OR neffa[tiab] OR ((pan[tiab] OR paan[tiab]) AND tobacco[tiab]) OR (plug[tiab] AND tobacco[tiab]) OR (rapé[tiab] AND tobacco[tiab]) OR ((red[tiab] OR tobacco[tiab]) AND (toothpowder[tiab] OR toothpaste[tiab])) OR shammah[tiab] OR snus[tiab] OR taaba[tiab] OR tapkeer[tiab] OR tawa[tiab] OR tombol[tiab] OR toombak[tiab] OR tuibur[tiab] OR "tobacco water"[tiab] OR (twist[tiab] AND tobacco[tiab]) OR zarda[tiab]) AND Humans[Mesh] AND English[Language] NOT Case Reports[ptyp]

# Prevalence data preprocessing

## Data extraction

We extracted primary data from individual-level microdata and survey report tabulations. We extracted data on current, former, and/or ever chewing tobacco use as well as frequency of use (daily, occasional, and unspecified, which includes both daily and occasional smokers). Products that do not include tobacco, such as betel quid without tobacco, were excluded or estimated separately as part of the drug use risk factor, if applicable.

For microdata, we extracted relevant demographic information, including age, sex, location, and year, as well as survey metadata, including survey weights, primary sampling units, and strata. This information allowed us to tabulate individual-level data in the standard GBD five-year age-sex groups and produce accurate estimates of uncertainty. For survey report tabulations, we extracted data at the most granular age-sex group provided.

## Crosswalk

Our GBD chewing tobacco case definition is current use of chewing tobacco. All other data points measuring other types of chewing tobacco prevalence were adjusted to be consistent with this definition. Some sources contained information on more than one case definition and these sources were used to develop the adjustment coefficient to transform alternative case definitions to the GBD case definition. The adjustment coefficient was the beta value derived from a linear model with one predictor and no intercept.

We generated separate crosswalk coefficients for the 10-14 age group and the 15-19 age group, as we found the relationships between case definitions differed strongly in the younger age groups compared to the 20+ age groups. To account for this, we attempted to generate a global crosswalk coefficient for both the 10-14 and 15-19 age groups, using the same regression as above. Due to data limitations, none of the crosswalk coefficients met the criteria outlined above, so no data covering youths under 20 years old were crosswalked. In other words, all data from these age groups that appear in the model were asked according to our case definition in the survey.

We propagated uncertainty at the survey level from the crosswalk by incorporating both the variance of the errors and the variance of the adjustment coefficients.

For each source that needed adjusting, we assigned space weights based on GBD region and superregion to the sources containing more than one case definition. Data from the same region receiving a full weight of 1, and data from the same super-region received a weight of ½. We explored using a time weight, to control for possible changes in the relationship between chewing tobacco use behaviors over time. We found incorporating temporal information did not significantly change the estimated coefficients but did undercut sample sizes, and chose to exclude the time weight. Crosswalk coefficients generated from fewer than 20 data sources were dropped.

## Age and sex splitting

We split data reported in broader age groups than the GBD 5-year age groups or as both sexes combined by adapting the method reported in Ng et al. (http://jamanetwork.com/journals/jama/fullarticle/1812960) to split using a sex- geography- time specific reference age pattern. We separated the data into two sets: a training dataset, with data already falling into GBD sex-specific 5-year age groups, and a split dataset, which reported data in aggregated age or sex groups. We then used spatiotemporal Gaussian Process Regression (ST-GPR) to estimate sex-geography-time specific age patterns using data in the training dataset. The estimated age patterns were then used to split each source in the split dataset.

The ST-GPR model used to estimate the age patterns for age-sex splitting used an age weight parameter value that minimizes the effect of any age smoothing. This parameter choice allows the estimated age pattern to be driven by data, rather than being enforced by any smoothing parameters of the model. Because these age-sex split data points will be incorporated in the final ST-GPR exposure model, we do not want to doubly enforce a modelled age pattern for a given sex-location-year on a given aggregate data point.

## Chewing tobacco prevalence modelling

We used ST-GPR to model chewing tobacco prevalence. Full details on the ST-GPR method are reported elsewhere in the Appendix. Briefly, the mean function input to GPR is a complete time series of estimates generated from a mixed effects hierarchical linear model plus weighted residuals smoothed across time, space and age. The linear model formula for chewing tobacco, fit separately by sex using restricted maximum likelihood in R, is:

$$logit(p_{g,a,t}) = \beta_0 + \sum_{k=1}^{18} \beta_k I_{A[a]} + \alpha_s + \alpha_r + \alpha_g + \epsilon_{g,a,t}$$

Where $I_{A[a]}$ is a dummy variable indicating specific age group $A$ that the prevalence point $p_{g,a,t}$ captures, and $\alpha_s$, $\alpha_r$, and $\alpha_g$ are super region, region, and geography random intercepts, respectively. Random effects were used in model fitting but not in prediction.

The hyperparameters for ST-GPR were as follows: age weight = 0.87 for data density below 10 and 1.48 for data density above 10, time weight of 0.04, space weight of 0.01 for data density below 10 and 0.005 for data density above 10, and scale = 20. Amplitude was calculated at the region level. Hyperparameters were decided based on a random grid search evaluated on out of sample RMSE performed on direct smoking data, but due to computational time was not able to be applied to the chewing tobacco data.

## Estimating attributable burden

### Assessment of risk-outcome pairs

We included outcomes based on the strength of available evidence supporting a causal relationship. There was sufficient evidence to include oral cancer and oesophageal cancer as health outcomes caused by chewing tobacco use. Appendix Table 4 reports the strength of evidence for included outcomes.

### Relative risk

Relative risk estimates were derived from prospective cohort studies and population-based case-control studies. Sources used in relative risk estimation are reported in Appendix Table 4.

Appendix Table 6a reports relative risk estimates and uncertainty for the two outcomes included in the analysis, by sex. We extracted the underlying effect size estimates from prospective cohort studies and population-based case-control studies identified by performing a systematic literature review as well as by reviewing the underlying studies included in published meta-analyses. We did not include hospital-based case control studies due to concerns over representativeness. We only included sources that adequately adjusted for major confounders, especially smoking status. Summary effect size estimates were calculated in R, using the 'metafor' package. We performed a random effects meta-analysis using the DerSimonian and Laird method, which does not assume a true effect size but considers each input study as selected from a random sample of all possible sets of studies for the outcome of interest. The random-effects method allows for more variation between the studies, and incorporates this variance into the estimation process. We used an inverse-variance weighting method to determine component study weights. We found significantly different relative risks for oral cancer for males and females, and estimated relative risks separately by sex for oral cancer alone.

### Theoretical minimum risk exposure level

The theoretical minimum risk exposure level is that everyone in the population has been a lifelong non-user of chewing tobacco.

# Alcohol Use Capstone Appendix

## Flowchart

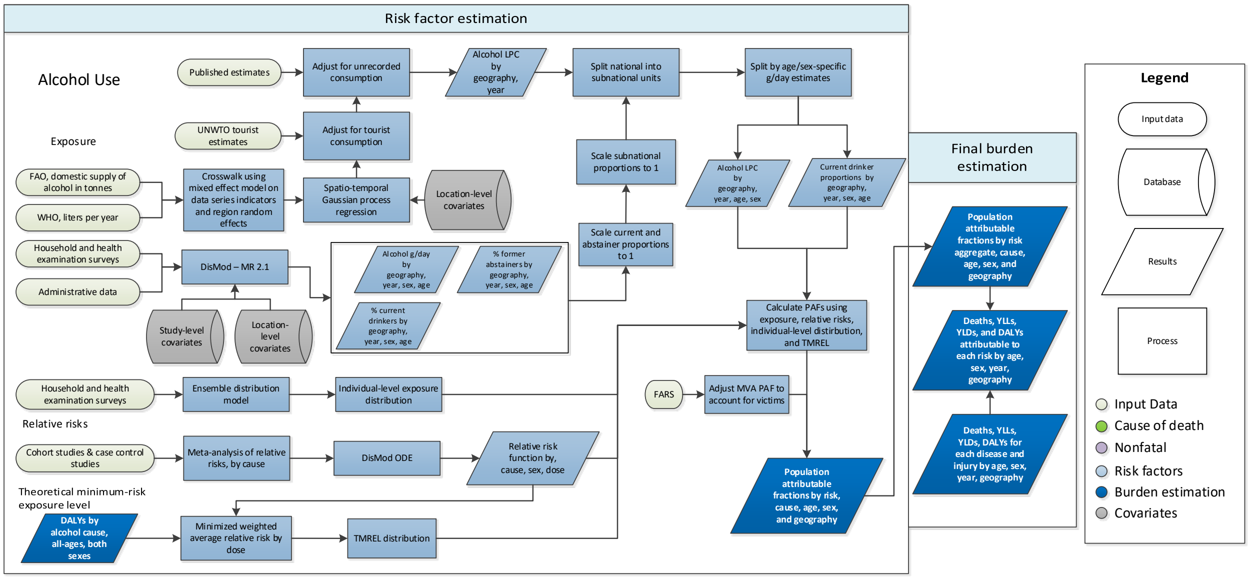


## Input data & methodological summary

### Exposure

#### *Case definition*

We defined exposure as the grams per day of pure alcohol consumed amongst drinkers. We constructed this exposure using the indicators outlined below:

1. Current drinkers, defined as the proportion of individuals who have consumed at least one alcoholic beverage (or some approximation) in a 12-month period.
2. Lifetime abstainers, defined as the proportion of individuals who have never consumed an alcoholic beverage.
3. Alcohol consumption (in grams per day), defined as grams of alcohol consumed by current drinkers, per day, over a 12-month period.
4. Alcohol liters per capita stock, defined in liters per capita of pure alcohol, over a 12-month period.

We also used three additional indicators to adjust alcohol exposure estimates to account for different types of bias:

1. Number of tourists within a location, defined as the total amount of visitors to a location within a 12 month period.
2. Tourists' duration of stay, defined as the number of days resided in a hosting country.

3. Unrecorded alcohol stock, defined as a percentage of the total alcohol stock produced outside established markets.

*Input data*

A systematic review of the literature was performed to extract data on our primary indicators. The Global Health Exchange (GHDx), IHME's online database of health-related data, was searched for population survey data containing participant-level information from which we could formulate the required alcohol use indicators on current drinkers, lifetime abstainers, alcohol consumption, and binge drinkers. Data-sources were included if they captured a sample representative of the geographic location under study. We documented relevant survey variables from each data-source in a spreadsheet and extracted using STATA 13.1 and R 3.3 . A total of 2,821 potential data-sources were available in the GHDx across countries with subnational locations, out of which 191 data-sources (corresponding 88,734 tabulated data-points by location/year/sex/age) were included across the four indicators mentioned above.

Within the grams per day, current drinkers, and abstainers model, we had a large amount of data on male drinking but not female drinking. To ensure a balanced dataset between sexes for use within DisMod MR 2.1, we imputed for missing sex observations within locations where data existed on male drinking but not female drinking. We used the following models to do so:

For grams per day:

$$y_i \sim Gamma(\mu, \phi)$$
$$\mu = log^{-1}(1 + SDI + sex + age + sex * age + (1 + sex|super\ region) + (1 + sex|region)$$

Where y is average amount of grams per day within a demographic, $\mu$ a parameter for the mean of the average amounts, and $\phi$ is a dispersion parameter

For current drinking and abstention:

$$y_i \sim Binomial(\pi_i, n)$$
$$\pi_i \sim Beta(\mu_i, \theta_i)$$
$$\mu_i = cloglog^{-1}(1 + SDI + sex + age + sex * age + (1 + sex|super\ region) + (1 + sex|region)$$

We then sampled 1000 draws from the above estimator for both sex = male & sex = female, with all other variables fixed by demographic unit. For sampling draws, we assumed the parameters were Gaussian multivariately distributed. For each demographic unit with only male observations, we multiplied male data by the ratio between the draws with sex = male & sex = female to impute for female observations.

To generate estimates of alcohol consumption in liters per capita (LPC), we obtained data from FAOSTAT, and WHO GISAH database.[1,2] To provide more stable time trends in the model, we transformed FAO sales data (which calculates stock based on primary inputs) to a lagged five-year average. Given WHO uses FAO data in locations where WHO could not find data using their own methods, we removed FAO data in the locations where WHO used FAO data in place of their own. To correct for bias in the underlying data sources, we adjusted the input data (crosswalked), by running a mixed effect model on the log average of the data with dummy variables for the data series, as well as random effects on super region, region, country, and time. We adjusted the data points using the following equation:

$$Log\ Average\ Data = D + (Super\ Region\ |D, Region\ |\ D, Country\ |D, Year|\ D)$$

$$Transformed\ data = \ data\ *\ e^{\widehat{\beta_1} + \widehat{\beta_3}}$$

where:

$D\ is\ a\ dummy\ variable\ for\ a\ data\ source$

None of the data sources on liters per capita provided estimates of uncertainty, which is a component required for our eventual modelling strategy. To generate uncertainty, we ran a Loess model on the adjusted data points and the standard deviation between the difference of the Loess smoothed model and the adjusted data points across a five-year span was used as the standard deviation of the data. (i.e., if the total stock changes more variably in a narrow time frame, we believe the data to be more uncertain).

We obtained data on the number of tourists and their duration of stay from the UNWTO.[3] We applied a crosswalk across different tourist categories, similar to the one used for the liters per capita data, to arrive at a consistent definition (i.e. visitors to a country).

We obtained estimates on unrecorded alcohol stock from six published papers,[4-9] consisting of 166 locations.

## Modelling strategy

While population-based surveys provide accurate estimates of the prevalence of lifetime abstainers and current drinkers, they typically underestimate real alcohol consumption levels.[10-12] As a result, we considered the liter per capita input to be a better estimate of overall volume of consumption. Per capita consumption, however, does not provide age- and sex-specific consumption estimates needed to compute alcohol-attributable burden of disease. Therefore, we use the age-sex pattern of consumption among drinkers modelled from the population survey data and the overall volume of consumption from FAO and GISAH to determine the total amount of alcohol consumed within a location. In the paragraphs we outline how we estimated each primary input in the alcohol exposure model, as well as how we combined these inputs to arrive at our final estimate of grams per day of pure alcohol. We estimated all models below using 1,000 draws.

For data obtained through surveys, we used DisMod-MR 2.1 to construct estimates for each country/year/age/sex. We chose to use DisMod due to its ability to leverage information across the heterogeneous age groups reported in the surveys, through age-integration, as well as the model's ability to leverage information available from data in nearby locations or time-periods.[13]

We modelled the alcohol liters per capita data, as well as the total number of tourists, using a spatio-temporal Gaussian process regression (ST-GPR). We chose parameters, as well as our final model, using out-of-sample 10-fold cross validation.

Given the heterogeneous nature of the estimates on unrecorded consumption, as well as the wide variation across countries and time-periods, we took 1,000 draws from the uniform distribution of the lowest and highest estimates available for a given country. We did this to incorporate the diffuse

uncertainty within the unrecorded estimates reported. We used these 1,000 draws in the above equation. We adjusted LPC only for countries where estimates were available.

We adjusted the alcohol LPC for unrecorded consumption using the following equation:

$$Alcohol\ LPC = \frac{Alcohol\ LPC}{(1 - \%\ Unrecorded)}$$

We then adjusted the estimates for alcohol LPC for tourist consumption by adding in the per capita rate of consumption abroad and subtracting the per capita rate of tourist consumption domestically.

$$Alcohol\ LPC_d = Unadjusted\ Alcohol\ LPC_d + Alcohol\ LPC_{Domestic\ consumption\ abroad} - Alcohol\ LPC_{Tourist\ consumption\ domestically}$$

$$Alcohol\ LPC_i = \frac{\sum_l Tourist\ Population_l * Proportion\ of\ tourists_{i,l} * Unadjusted\ Alcohol\ LPC_l * \frac{Average\ length\ of\ stay_{i,l}}{365} *}{Population_d}$$

where:

*l is the set of all locations, i is either* Domestic consumption abroad *or* Tourist consumption domestically, *and d is a domestic location*

After adjusting alcohol LPC by tourist consumption and unrecorded consumption for all location/years reported, sex-specific and age-specific estimates were generated by incorporating estimates modelled in DisMod for percentage of current drinkers within a location/year/sex/age, as well as consumption trends modelled in the DisMod g/day model. We do this by first making sure the sum of percent current drinkers and percent abstainers sum to one for a given location/year/age/sex. We then calculate the proportion of total consumption for a given location/year by age and sex, using the estimates of alcohol consumed per day, the population size, and the percentage of current drinkers. Lastly, we then multiply this proportion of total stock for a given location/year/sex/age by the total stock for a given location/year to calculate the consumption in terms of liter per capita for a given location/year/sex/age. We then convert these estimates to be in terms of grams/per day. The following equations describe these calculations:

$$\%\ Current\ drinkers_{l,y,s,a} = \frac{\%\ Current\ drinkers_{l,y,s,a}}{\%\ Current\ drinkers_{l,y,s,a} + \%\ Abstainers_{l,y,s,a}}$$

$$Proportion\ of\ total\ consumption_{l,y,s,a} = \frac{Alcohol\ \ g/day_{l,y,s,a} * Population_{l,y,s,a} * \%\ Current\ drinkers_{l,y,s,a}}{\sum_{s,a} Alcohol\ \ g/day_{l,y,s,a} * Population_{l,y,s,a} * \%\ Current\ drinkers_{l,y,s,a}}$$

$$Alcohol\ LPC_{l,y,s,a} = \frac{Alcohol\ LPC_{l,y} * Population_{l,y} * Proportion\ of\ total\ consumption_{l,y,s,a}}{\%\ Current\ drinkers_{l,y,s,a} * Population_{l,y,s,a}}$$

$$Alcohol\ g/day_{l,y,s,a} = Alcohol\ LPC_{l,y,s,a} * \frac{1000}{365}$$

where:

$l$ is a location, $y$ is a year, $s$ is a sex, and $a$ is an age group.

We then used the gamma distribution to estimate individual level variation within location, year, sex, age drinking populations, following the recommendations of other published alcohol studies.[7,8] We chose parameters of the gamma distribution based on the mean and standard deviation of the 1,000 draws of alcohol g/day exposure for a given population.

## Theoretical minimum-risk exposure level

We calculated TMREL by first calculating the overall risk attributable to alcohol. We did this by weighting each relative risk curve by the share of overall DALYs for a given cause. We then took the minimum of this overall-risk curve as the TMREL of alcohol-use. More formally,

$$TMREL = argmin\ average\ overall\ risk_{\omega}(g/day)$$

$$Average\ overall\ risk_{\omega}(g/day) = \sum_{i}^{\omega} RR_i(g/day) * \frac{DALY_i}{\sum_i^{\omega} DALY_i}$$

Where:

$\omega$ is the set of causes associated with alcohol, $i$ is a given cause from that set, $DALY$ is the global $DALY$ rate in 2010, and $RR$ is the dose response curve for a given cause and exposure level in grams per day.

In other words, we chose TMREL as being the exposure that minimises your risk of suffering burden from any given cause related to alcohol. We weight the risk for a particular cause in our aggregation by the proportion of DALYs due to that cause. (e.g. since more observed people die from IHD, we weight the risk for IHD more in the above calculation of average risk compared to, say, diabetes, even if both have the same relative risk for a given level of consumption)

## Relative risks

For GBD 2016, we performed a systematic literature review of all cohort and case-control studies reporting a relative risk, hazard ratio, or odds ratio for any risk-outcome pairs studied in GBD 2016. Studies were included if they reported a categorical or continuous dose for alcohol consumption, as well as uncertainty measures for their outcomes, and the population under study was representative. Relative risk estimates by dose can be found in Appendix Table 6c.

We then used these studies to calculate a dose-response, modelled using DisMod ODE. We chose DisMod ODE rather than a conventional mixed effect meta-regression because of its ability to estimate nonparametric splines over doses (i.e. for most alcohol causes, there is a non-linear relationship with different doses) and incorporate heterogeneous doses through dose-integration (i.e. most studies report doses categorically in wide ranges. DisMod ODE estimates specific doses when categories overlap across studies, through an integration step.) We used the results of the meta-regression to estimate a non-parametric curve for all doses between 0-150 g/day and their corresponding relative risks. For all causes, we assumed the relative risk was the same for all-ages and sexes, with the exception of ischemic heart disease, ischemic stroke, hemorrhagic stroke, and diabetes, which we estimated by sex.

Regarding injuries outcomes, we constructed relative risks based on chronic exposure rather than acute, which has a weaker relationship to the outcome, though still significant.[15,16,18-21] We decided to use chronic exposure given the lack of available data on acute exposure, as well as, the lack of cohort studies using acute exposure as a metric. Further, using chronic exposure allowed us to construct relative risks curves for unintentional injuries, interpersonal violence, motor vehicle accidents, and self-harm using the same method as reported above.

In the case of motor vehicle accidents, we adjusted the PAF to account for victims of drunk drivers that are involved in accidents. Using data from the Fatality Analysis Reporting System in the US,[17] we calculated the average number of fatalities in a car crash involving alcohol, as well as the percentage of those fatalities distributed by age and sex (figures 1 and 2). We aggregated FARS data across the years 1985-2015, given there was little variation in the data temporally and the number of cases in old age groups had too much variance when constructing estimates by year. To adjust PAFs, we multiplied attributable deaths by the average number of fatalities from FARS and redistributed the PAF amongst each population, based on the probability of being a victim to a certain drunk driver by age and sex, based on the FARS data. The following equation describes this process:

$$Adjusted\ PAF_i = \frac{\sum_d PAF_d * DALY_d * Avg\ Fatalities_d * \ P(i\ is\ a\ victim)_d}{DALY_i}$$

where:

$i$ is a population by location, year, age, sex and
$d$ is the set of all age and sex exposed groups within that location and year.

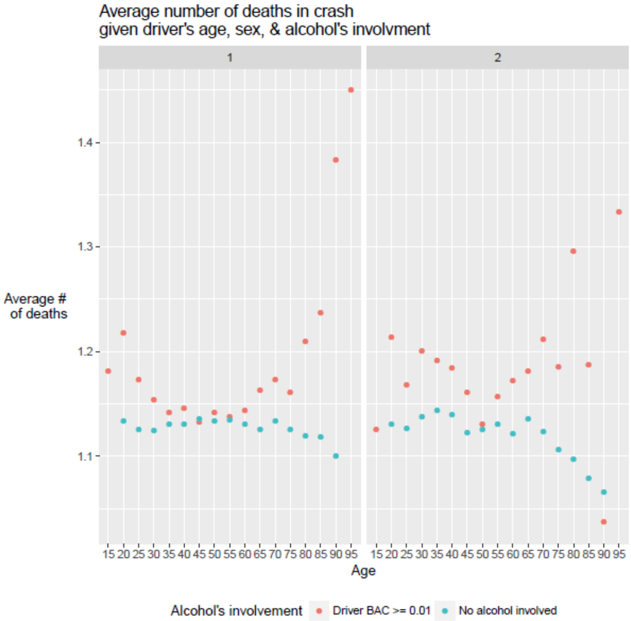


**Figure 1**

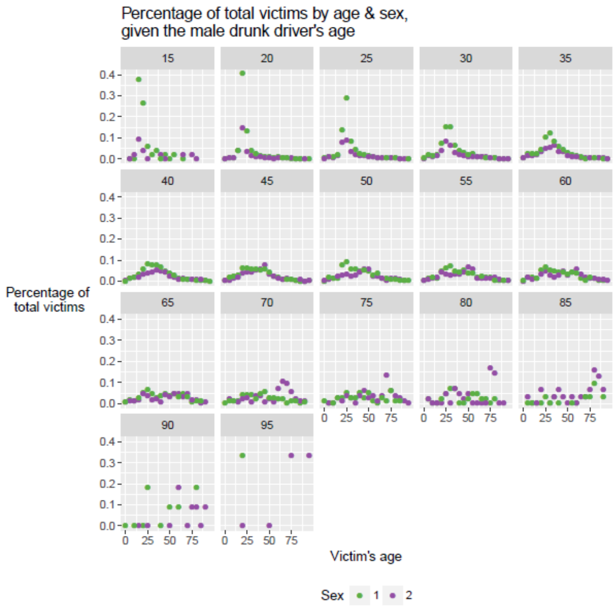


**Figure 2**

## Population attributable fraction

For all causes, we defined PAF as:

$$PAF(x) = \frac{P_A + \int_0^{150} P(x) * RR_C(x)\,dx - 1}{P_A + \int_0^{150} P(x) * RR_C(x)\,dx} \qquad P(x) = P_C * \Gamma(\boldsymbol{p})$$

where:

$P_C$ *is the prevalence of current drinkers,* $P_a$ *is the prevalence of abstainers,* $RR_c(x)$ *is the relative risk function for current drinkers, and* $\boldsymbol{p}$ *are parameters determined by the mean and sd of exposure*

We performed the above equation for 1,000 draws of the exposure and relative risk models. We then used the estimated PAF draws to calculate YLL, YLDs, and DALYs, as per the other risk factors.

## References


1. Food and Agriculture Organization of the United Nations (FAO). FAOSTAT Food Balance Sheets, October 2014. Rome, Italy: Food and Agriculture Organization of the United Nations (FAO).
2. World Health Organization (WHO). WHO Global Health Observatory - Recorded adult per capita alcohol consumption, Total per country. Geneva, Switzerland: World Health Organization (WHO).
3. UN World Tourism Organization (UNWTO). UN World Tourism Organization Compendium of Tourism Statistics 2015 [Electronic]. Madrid, Spain: UN World Tourism Organization (UNWTO), 2016.
4. Norstrom, Thor. "Estimating changes in unrecorded alcohol consumption in Norway using indicators of harm." *Addiction* 93.10 (1998): 1531-1538.
5. Macdonald, Scott. "Unrecorded alcohol consumption in Ontario, Canada: estimation procedures and research implications." *Drug and Alcohol Review* 18.1 (1999): 21-29.
6. Meier, Petra Sylvia, et al. "Adjusting for unrecorded consumption in survey and per capita sales data: quantification of impact on gender-and age-specific alcohol-attributable fractions for oral and pharyngeal cancers in Great Britain." *Alcohol and Alcoholism* 48.2 (2013): 241-249.
7. Hao, Wei, Hanhui Chen, and Zhonghua Su. "China: alcohol today." *Addiction* 100.6 (2005): 737-741.
8. Rehm, Jürgen, and Vladimir Poznyak. "On monitoring unrecorded alcohol consumption." *Alcoholism and Drug Addiction* 28.2 (2015): 79-89.
9. Probst et al. "Unrecorded Alcohol Use: A global modeling study based on Delphi assessments and survey data". Toronto, Canada: CAMH.
10. Ramstedt, Mats. "How much alcohol do you buy? A comparison of self-reported alcohol purchases with actual sales." *Addiction* 105.4 (2010): 649-654.
11. Stockwell, Tim, et al. "Under-reporting of alcohol consumption in household surveys: a comparison of quantity–frequency, graduated–frequency and recent recall." *Addiction* 99.8 (2004): 1024-1033.
12. Kerr, William C., and Thomas K. Greenfield. "Distribution of alcohol consumption and expenditures and the impact of improved measurement on coverage of alcohol sales in the 2000 National Alcohol Survey." *Alcoholism: Clinical and Experimental Research* 31.10 (2007): 1714-1722.
13. An Integrative Metaregression Framework for Descriptive Epidemiology. Abraham D. Flaxman, Theo Vos, Christopher J. L. Murray. Seattle: University of Washington Press, [2015]
14. Taylor, Bruce, et al. "The more you drink, the harder you fall: a systematic review and meta-analysis of how acute alcohol consumption and injury or collision risk increase together." *Drug and alcohol dependence* 110.1 (2010): 108-116.
15. Vinson, Daniel C., Guilherme Borges, and Cheryl J. Cherpitel. "The risk of intentional injury with acute and chronic alcohol exposures: a case-control and case-crossover study." *Journal of studies on alcohol* 64.3 (2003): 350-357.
16. Vinson, Daniel C., et al. "A population-based case-crossover and case-control study of alcohol and the risk of injury." *Journal of studies on alcohol*64.3 (2003): 358-366.
17. Fatal Accident Reporting System (FARS). National Highway Traffic Safety Administration, National Center for Statistics and Analysis Data Reporting and Information Division (NVS-424); 1985, 1990, 1995, 2000, 2005, 2010, 2015

18. Chen, Li-Hui, Susan P. Baker, and Guohua Li. "Drinking history and risk of fatal injury: comparison among specific injury causes." *Accident Analysis & Prevention* 37.2 (2005): 245-251.
19. Bell, Nicole S., et al. "Self-reported risk-taking behaviors and hospitalization for motor vehicle injury among active duty army personnel." *American journal of preventive medicine* 18.3 (2000): 85-95.
20. Margolis, Karen L., et al. "Risk factors for motor vehicle crashes in older women." *The Journals of Gerontology Series A: Biological Sciences and Medical Sciences* 57.3 (2002): M186-M191.
21. Sorock, Gary S., et al. "Alcohol-drinking history and fatal injury in older adults." *Alcohol* 40.3 (2006): 193-199.

# Injecting Drug Use Capstone Appendix

## Flowchart

Drug Use

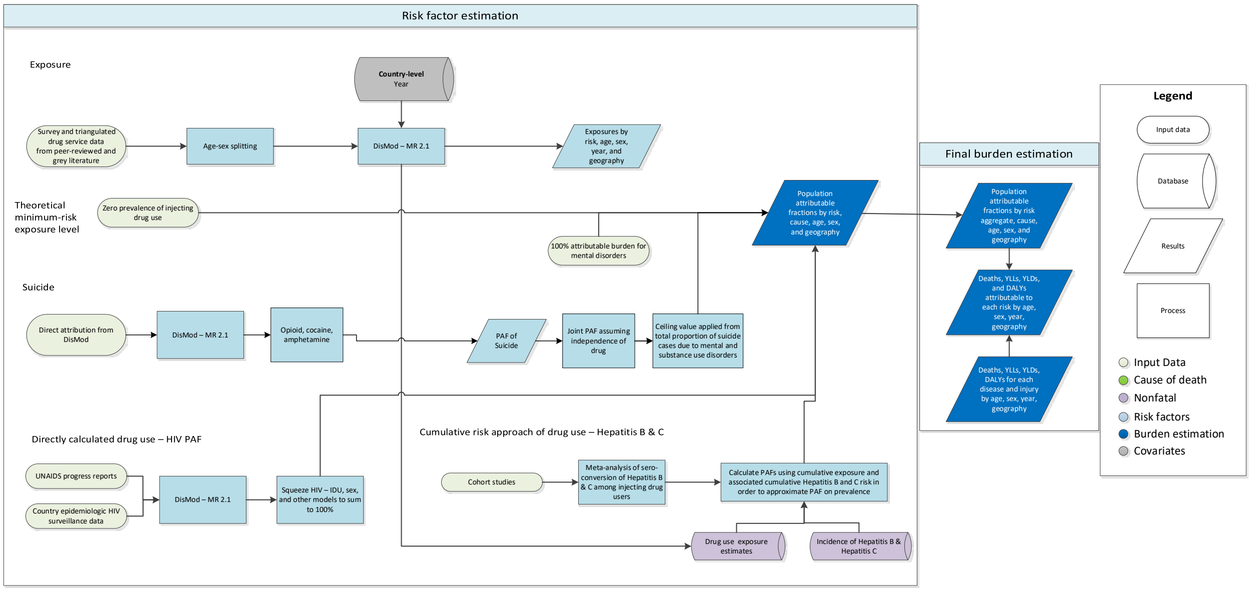


## Input data & methodological summary

### Exposure

#### *Case definition*

Injecting drug users (IDU) are at high risk from blood-borne infections, including human immunodeficiency virus (HIV) and Hepatitis B and C viruses (HBV and HCV, respectively), through the use of shared needles and injection equipment. In GBD 2010, based on the available epidemiological literature and the availability of exposure estimates[1,2] we measure the burden of disease attributable to HIV, HBV and HCV due to injecting drug use. An injecting drug user was defined as a current or recent user aged 15-64 years old.

#### Input data

The major burden of mortality from viral hepatitis is due to cirrhosis and liver cancer resulting from chronic hepatitis infection. Cirrhosis mortality was modelled with vital registration data using CODEm. Etiologic proportion models, estimated using DisMod-MR 2.1, were used to split the overarching cirrhosis mortality estimates into cases of cirrhosis attributable to hepatitis B, hepatitis C, alcohol, and other causes.[1-4]

Liver cancer mortality was modelled using cancer registry data. The incidence numbers were transformed into mortality estimates using mortality to incidence ratios. The mortality estimates from cancer registries were then combined with vital registration system data as input data into CODEm, which produced the final mortality estimates for liver cancer. As with cirrhosis mortality, etiologic

proportions for liver cancer due to hepatitis B, and C, alcohol, and other causes were generated using DisMod-MR 2.1.

To estimate the burden of HIV cases attributable to IDU, we extracted data on the proportion of notified HIV cases by transmission route – sexual intercourse, injecting drug use, commercial sex work and other -- from a number of agencies that conduct surveillance of HIV across the globe.[6-13]

The prevalence of current injecting drug use was estimated using data from a 2008 review conducted by the Reference Group to the UN on HIV and injecting drug use,[15] and a new review currently being conducted by international collaborations and experts. The reviews used a multistage process of systematic review adhering to international guidelines. It involved multiple stages of peer and expert review, with searches of the peer-reviewed literature in addition to an extensive review of online grey literature databases in the drug and alcohol and HIV fields. Additional data on the age and sex distribution of injecting drug use were sourced for this modelling exercise.

In order to generate a pooled incidence rate/absolute relative risk for viral hepatitis among people who inject drugs, we conducted a meta-analysis of longitudinal epidemiological studies that reported a hepatitis B[16-20] or hepatitis C[16-31] incidence rate among PWID. We calculated confidence intervals for the incidence rate (where no CI was reported) from a Poisson distribution around the number of cases.

We excluded studies that focused on non-representative subgroups, such as recent injectors or adolescents or because hepatitis incidence is far higher in those groups than for all people who inject drugs (e.g. Larney et al.[32]) We did not vary incidence among active injectors according the availability of blood borne virus prevention strategies (e.g. NSPs, opioid substitution therapy) because too few studies have examined different levels of incidence according to variable coverage, and we were not able to estimate coverage by country over time. In any case, in most countries, coverage of virus prevention strategies remains very low among people who inject drugs,[33] and would have been negligible in most countries until recent years.

## Modelling strategy

As part of the GBD 2017 study, we measured the burden of hepatitis B and hepatitis C (including attributable cirrhosis and liver cancer) and HIV at the country, regional, and global level for each age-sex group for the years 1990 to 2017. For HIV, hepatitis B and hepatitis C, disease-specific natural history models were used to estimate deaths and YLDs, because the three-state model in DisMod-MR 2.1(susceptible, cases, dead) did not capture the complexity of the disease processes.

### *Mortality estimation*

Mortality due to overall acute hepatitis was modelled with vital registration data using the Cause of Death Ensemble Modelling tool (CODEm), an analytical tool that tests the predictive power of hundreds of models to estimate trends in causes of death.[5] Due to poor coverage of cause of death data for each of the acute hepatitis varieties, four natural history models for hepatitis B and C were used to estimate mortality by deriving incidence from measurements of seroprevalence and then multiplying incidence by case fatality to estimate the number of deaths. These four models were then squeezed so as to fit the parent cause of death model.

We estimated HIV mortality using a modified UNAIDS Spectrum model.[2] This is a compartmental HIV progression model estimates age-specific incidence, prevalence and death rates using methods

described elsewhere.[2] This modelling approach was adapted according to epidemic type, including concentrated and generalized epidemics. For concentrated epidemics, the Spectrum models were corrected for misclassification of HIV deaths and then calibrated to align with vital registration data. For generalised HIV epidemics, we minimised a loss function to select epidemic curves that were most consistent with the prevalence and all-cause mortality data.[2]

### *Estimation of Years Lived with Disability*

For non-fatal estimation, we estimated the incidence of hepatitis B and C using seroprevalence data in DisMod-MR 2.1. For both hepatitis B and C, we use data on the seroprevalence of the hepatitis surface antigen (a marker of chronic infection in hepatitis B and a marker of ever-infection in hepatitis C), excess mortality, and remission, to estimate incidence of both hepatitis infections. Incidence of cirrhosis was also estimated in DisMod using cirrhosis hospital data and cause-specific mortality rate (CSMR) data.

Incidence of liver cancer was derived by dividing mortality by the mortality to incidence ratios, which were then used to predict liver cancer survival. Finally, we estimated prevalence as a function of incidence and survival by splitting prevalence into four phases. Each phase had different disability weights, which were used to generate YLDs for that phase.

Finally, incidence of HIV was also estimated using the UNAIDS Spectrum modelling approach described above in the mortality estimation section.

### *Burden of HIV attributable to injecting drug use*

We then estimated the proportion of HIV cases attributable to three transmission categories (sex, IDU and other) for all country-time periods using DisMod-MR 2.1. The only covariate used in the model was one that added variance to the data points derived from data sources that attributed a portion of HIV cases to “unknown” transmission sources. We scaled the proportions from each of the three transmission models (sex, IDU and other) to ensure that they fit the total HIV transmission envelope by country, year, age and sex.

### *Burden of hepatitis B and hepatitis C attributable to injecting drug use*

To estimate the relative contribution of IDU to hepatitis B and C disease burden at the country, regional and global level, we used a cohort method. We re-calibrated individuals according to history of injecting drug use, and their accumulated risk of incident hepatitis B and C due to IDU. We made use of data on prevalence of current injecting drug use, pooled in DisMod-MR 2.1; a meta-analysis of incidence rates of hepatitis B and hepatitis C among people who inject drugs; and estimates of population-level incidence of hepatitis B and C between 1990 and 2017. We used back extrapolations to estimate incidence before 1990. These steps are detailed below.

To estimate the lifetime risk of being infected with hepatitis B or C, we undertook a cohort analysis for each country, year, age, and sex category and estimated the probability of an individual having been infected in each preceding year. One of the main inputs to this cohort method was the probability of having injected drugs in a specific age cohort in a given calendar year. For example, for a cohort of 40-year-olds in 2015, the relevant probability in 2005 is the estimated prevalence of injecting drug use among 30-year-olds.

In addition to a global time series of estimated prevalence of injecting drug use, we also used the incidence of hepatitis B or C and the sero-conversion rate of hepatitis B and hepatitis C among people who inject drugs for each age-sex-country-year from 1960 to 2013 by 5-year age groups.

1. Incidence rate of Hepatitis B and C in the general population

We modelled the annual incidence rate of hepatitis B and hepatitis C using sero-prevalence data in DisMod-MR 2.1. We assumed a low remission (mean 0.015 and standard error 0.0075)[14] in the hepatitis B model to reflect the small proportion of cases who spontaneously clear the infection. We assumed zero remission for hepatitis C.

2. Prevalence of ever-injecting drug use

DisMod-MR 2.1 was used to estimate the prevalence of injecting drug use with year as a covariate to estimate the trends over time. DisMod makes an average estimate of the change in drug use over the time period from 1990-2017 and we took draws from a normal distribution of the coefficient to project IDU prevalence backward in time to 1960 from baseline level in 1990.

3. Pooled seroconversion hazard of hepatitis C and hepatitis B among people who ever injected drugs

This pooled sero-conversion hazard for both hepatitis C and hepatitis B was derived from a meta-analysis of longitudinal epidemiologic studies described above in the input data section.

## Theoretical minimum-risk exposure level

The theoretical minimum-risk exposure level is defined as zero exposure to injecting drug use.

## Relative risks

For drug use, there were not substantial changes made to the effect sizes from GBD 2015. We used a pooled absolute risk of Hepatitis C and Hepatitis B among those who have ever used injecting drugs.

In addition to assessing IDU as a risk factor for blood-borne infections, the broader category of mental and substance use disorders is assessed as risk factors for suicide. The suicide burden attributable to mental and substance use disorders is estimated by comparing the current health status with a theoretical-minimum-risk exposure defined as the counterfactual status of the absence of mental and substance use disorders (Ferrari, Norman et al 2014).

## References


1. Fitzmaurice C, Dicker D, Pain A, Hamavid H, Moradi-Lakeh M, MacIntyre MF, et al. The Global Burden of Cancer 2013. JAMA oncology. 2015;1(4):505-27.
2. Murray CJ, Ortblad KF, Guinovart C, Lim SS, Wolock TM, Roberts DA, et al. Global, regional, and national incidence and mortality for HIV, tuberculosis, and malaria during 1990-2013: a systematic analysis for the Global Burden of Disease Study 2013. Lancet. 2014;384(9947):1005-70.
3. GBD 2013 YLDs Collaborators. Global, regional, and national incidence, prevalence and YLDs for 301 acute and chronic diseases and injuries for 188 countries, 1990-2013: A systematic analysis for the Global Burden of Disease Study 2013. The Lancet. 2015;386:743-800.
4. Naghavi M, Wang H, Lozano R, Davis A, Liang X, Zhou M, et al. Global, regional, and national age-sex specific all-cause and cause-specific mortality for 240 causes of death, 1990-2013: a systematic analysis for the Global Burden of Disease Study 2013. Lancet. 2015;385(9963):117-71.
5. Foreman KJ, Lozano R, Lopez AD, Murray C. Modeling causes of death: an integrated approach using CODEm. Population Health Metrics. 2012;10(1).
6. European Centre for Disease Prevention. HIV/AIDS surveillance in Europe 2014 Solna, Sweden. http://ecdc.europa.eu/en/publications/surveillance_reports/HIV_STI_and_blood_borne_viruses/Pages/HIV_STI_and_blood_borne_viruses.aspx: ECDC, 2014.
7. Family Health International, Bureau of AIDS TB and STIs Department of Disease Control. The Asian Epidemic Model (AEM) Projections for HIV/AIDS in Thailand:2005-2025. Bangkok: Family Health International (FHI) and Bureau of AIDS, TB and STIs, Department of Disease Control, Ministry of Public Health, Thailand, 2008.
8. Kirby Institute. 2015 Annual Surveillance Report of HIV, viral hepatitis, STIs. Sydney, New South Wales. https://kirby.unsw.edu.au/surveillance/2015-annual-surveillance-report-hiv-viral-hepatitis-stis: Kirby Institute, UNSW Australia, 2015.
9. Kirby Institute. Australian NSP survey national data report 2015. Sydney, New South Wales: Kirby Institute, University of New South Wales, 2015.
10. Country reports for Global AIDS Response Progress Reporting [Internet]. UNAIDS. 2014.
11. UNAIDS. UNAIDS Country reports. Geneva: Joint United Nations Programme on HIV/AIDS. http://www.unaids.org/en/regionscountries/countries, 2015.
12. United States Center for Disease Control and Prevention. HIV/AIDS Statistics. Atlanta, Georgia: US CDC. http://www.cdc.gov/hiv/statistics/index.html, 2015.
13. Gouws E, White PJ, Stover J, Brown T. Short term estimates of adult HIV incidence by mode of transmission: Kenya and Thailand as examples. Sex Transm Infect. 2006;82 Suppl 3:iii51-5.
14. McMahon B. The natural history of chronic hepatitis B virus infection. Hepatology. 2009;49(5 Suppl):S45-S55.
15. Mathers BM, Degenhardt L, Phillips B, Wiessing L, Hickman M, Strathdee SA, et al. Global epidemiology of injecting drug use and HIV among people who inject drugs: a systematic review. Lancet. 2008;372(9651):1733-45.
16. Jackson JB, Wei L, Liping F, Aramrattana A, Celentano DD, Walshe L, et al. Prevalence and Seroincidence of Hepatitis B and Hepatitis C Infection in High Risk People Who Inject Drugs in China and Thailand. Hepatitis research and treatment. 2014;2014.

17. Månsson A-S, Moestrup T, Nordenfelt E, Widell A. Continued transmission of hepatitis B and C viruses, but no transmission of human immunodeficiency virus among intravenous drug users participating in a syringe/needle exchange program. Scandinavian Journal of Infectious Diseases. 2000;32(3):253-8.
18. Blomé MA, Björkman P, Flamholc L, Jacobsson H, Molnegren V, Widell A. Minimal transmission of HIV despite persistently high transmission of hepatitis C virus in a Swedish needle exchange program. Journal of viral hepatitis. 2011;18(12):831-9.
19. Hagan H, McGough JP, Thiede H, Weiss NS, Hopkins S, Alexander ER. Syringe exchange and risk of infection with hepatitis B and C viruses. American journal of epidemiology. 1999;149(3):203-13.
20. Crofts N, Aitken CK. Incidence of bloodborne virus infection and risk behaviours in a cohort of injecting drug users in Victoria in 1990-1995. Medical Journal of Australia. 1997;167(1):17-20.
21. Roy K, Goldberg D, Taylor A, Hutchinson S, MacDonald L, Wilson K, et al. A method to detect the incidence of hepatitis C infection among injecting drug users in Glasgow 1993–98. Journal of Infection. 2001;43(3):200-5.
22. Abou-Saleh M, Davis P, Rice P, Checinski K, Drummond C, Maxwell D, et al. The effectiveness of behavioural interventions in the primary prevention of hepatitis C amongst injecting drug users: a randomised controlled trial and lessons learned. Harm reduction journal. 2008;5(1):1.
23. Turner KM, Hutchinson S, Vickerman P, Hope V, Craine N, Palmateer N, et al. The impact of needle and syringe provision and opiate substitution therapy on the incidence of hepatitis C virus in injecting drug users: pooling of UK evidence. Addiction. 2011;106(11):1978-88.
24. Grebely J, Lima VD, Marshall BD, Milloy M, DeBeck K, Montaner J, et al. Declining incidence of hepatitis C virus infection among people who inject drugs in a Canadian setting, 1996-2012. PloS one. 2014;9(6):e97726.
25. Foley S, Abou-Saleh MT. Risk behaviors and transmission of hepatitis C in injecting drug users. Addictive Disorders & Their Treatment. 2009;8(1):13-21.
26. Craine N, Hickman M, Parry J, Smith J, Walker A, Russell D, et al. Incidence of hepatitis C in drug injectors: the role of homelessness, opiate substitution treatment, equipment sharing, and community size. Epidemiology and Infection. 2009;137(09):1255-65.
27. Villano SA, Vlahov D, Nelson KE, Lyles CM, Cohn S, Thomas DL. Incidence and risk factors for hepatitis C among injection drug users in Baltimore, Maryland. Journal of clinical microbiology. 1997;35(12):3274-7.
28. Maher L, Jalaludin B, Chant KG, Jayasuriya R, Sladden T, Kaldor JM, et al. Incidence and risk factors for hepatitis C seroconversion in injecting drug users in Australia. Addiction. 2006;101(10):1499-508.
29. Lucidarme D, Bruandet A, Ilef D, Harbonnier J, Jacob C, Decoster A, et al. Incidence and risk factors of HCV and HIV infections in a cohort of intravenous drug users in the North and East of France. Epidemiology and infection. 2004;132(04):699-708.
30. Partanen A, Malin K, Perälä R, Harju O, Holopainen A, Holmström P, et al. Riski-tutkimus 2000-2003. Pistämällä huumeita käyttävien seurantatutkimus. A-Klinikkasäätiön Raporttisarja nro 52. Helsinki: A-Klinikkasäätiön, 2006.
31. Van Den Berg C, Smit C, Van Brussel G, Coutinho R, Prins M. Full participation in harm reduction programmes is associated with decreased risk for human immunodeficiency virus and hepatitis C

virus: evidence from the Amsterdam Cohort Studies among drug users. Addiction. 2007;102(9):1454-62.

32. Larney S, Kopinski H, Beckwith CG, Zaller ND, Jarlais DD, Hagan H, et al. Incidence and prevalence of hepatitis C in prisons and other closed settings: results of a systematic review and meta-analysis. Hepatology. 2013;58(4):1215-24.
33. Degenhardt L, Mathers B, Vickerman P, Rhodes T, Latkin C, Hickman M. Prevention of HIV infection for people who inject drugs: Why individual, structural, and combination approaches are needed. The Lancet. 2010;376:285-301.

# Dietary Risks Capstone Appendix

## Flowchart

Dietary risks

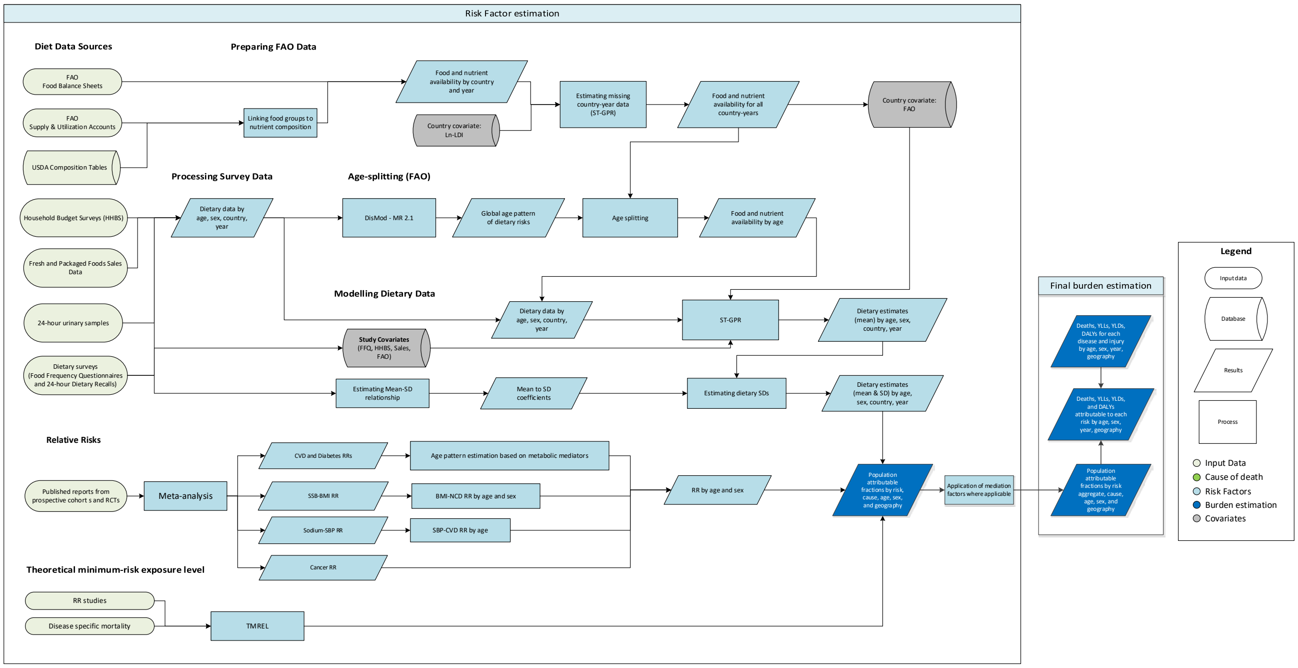


## Input data & methodological summary

### Exposure

#### *Case definition*

For GBD 2017, risk factors associated with diet include: diet low in fruits, vegetables, legumes, whole grains, nuts and seeds, fiber, seafood omega-3 fatty acids, polyunsaturated fatty acids, calcium, milk; and diet high in red meat, processed meat, sugar sweetened beverages, trans fatty acids, and sodium. Exposure to a diet low in fruits is defined as average daily consumption of less than 250 grams per day of fruits (fresh, frozen, cooked, canned, or dried, excluding fruit juices and salted or pickled fruits). Exposure to diet low in vegetables is defined as average daily consumption of less than 360 grams per day of vegetables (fresh, frozen, cooked, canned or dried vegetables excluding legumes and salted or pickled vegetables, juices, nuts and seeds, and starchy vegetables such as potatoes or corn). Exposure to a diet low in legumes is defined as average daily consumption of less than 60 grams per day of legumes. Exposure to diet low in whole grains is defined as average daily consumption of less than 125 grams per day of whole grains (bran, germ, and endosperm in their natural proportion) from breakfast cereals, bread, rice, pasta, biscuits, muffins, tortillas, pancakes and other sources. Exposure to diet low in nuts and seeds is defined as average daily consumption of less than 20.5 grams per day of nuts and seeds. Exposure to diet low in milk is defined as average daily consumption of less than 435 grams per day of milk including non-fat, low-fat, and full-fat milk, excluding soy milk and other plant derivatives. Exposure to diet low in calcium is defined as average daily consumption of less than 1.15 grams per day of calcium

from all sources, including milk, yogurt, and cheese. Exposure to diet low in fiber is defined as average daily consumption of less 23.5 grams per day of than fiber from all sources including fruits, vegetables, grains, legumes and pulses. Exposure to diet low in seafood omega-3 fatty acids is defined as average daily consumption of less than 250 milligrams per day of eicosapentaenoic acid (EPA) and docosahexaenoic acid (DHA). Exposure to diet low in polyunsaturated fatty acids is defined as average daily consumption of less than 11% of total energy intake from polyunsaturated fatty acids. This represents a change from the last round of GBD where the burden associated with a diet low in polyunsaturated fatty acids was estimated as a replacement for consumption of saturated fatty acids. Exposure to diet high in red meat is defined as average daily consumption of greater than 22.5 grams per day of red meat (beef, pork, lamb, and goat but excluding poultry, fish, eggs, and all processed meats). Exposure to diet high in processed meat is defined as average daily consumption of greater than 2 grams of meat preserved by smoking, curing, salting, or addition of chemical preservatives. Exposure to diet high in sugar sweetened beverages is defined as average daily consumption of greater than 2.5 grams per day of beverages with ≥50 kcal per 226.8 gram serving, including carbonated beverages, sodas, energy drinks, fruit drinks, but excluding 100% fruit and vegetable juices. Exposure to diet high in trans-fatty acids is defined as average daily consumption of greater than 0.5% of trans fat from all sources, mainly partially hydrogenated vegetable oils and ruminant products. Exposure to diet high in sodium is defined as average 24 hour urinary sodium greater than 3 grams per day.

### *Input data*

We used dietary data from multiple sources including nationally and sub-nationally representative nutrition surveys, household budget surveys, accounts of national sales, and United Nations FAO Food Balance Sheets and Supply and Utilization Accounts. A specific improvement for this round was a novel approach we developed that allows the incorporation of FAO-based data to assist with our estimation of whole grains consumption. This is a powerful development as it dramatically increases our data coverage across countries and through time. This was achieved through separate estimation of total grain and refined grain availability. With whole grains and refined grains representing the entirety of all grain available, we were then able to calculate the availability of whole grains by difference. Additionally, for sodium and trans-fatty acids, we used data on 24-hour urinary sodium and availability of hydrogenated vegetable oil in packaged foods, respectively. Polyunsaturated and trans-fatty acids were modelled as a percent of total dietary energy. We modelled missing country-year data from FAO using a space-time Gaussian process regression and lag-distributed country income as the covariate. For each dietary factor, we estimated the global age pattern of consumption based on nutrition surveys (i.e., 24-hour diet recall) and applied that age pattern to the FAO data. Substantive changes in input data compared to GBD 2015 are as follows: (a) re-extracting data from all nutrition surveys and standardising the definition of dietary components across sources; (b) incorporating data gathered through a systematic review of literature for each of our dietary risk factors; (c) using sales data for fruit, vegetables, legumes, processed meats, red meats, sugar-sweetened beverages, and milk.

## Modelling strategy

We used a spatio-temporal Gaussian process regression (ST-GPR) framework to estimate the intake of each dietary factor by age, sex, country, and year. In GBD 2017, for all dietary factors other than sodium, we considered data from 24-hour diet recall as the gold standard, and cross-walked other methods of assessment to the gold standard method. For sodium, the 24-hour urinary sodium was considered as the

gold standard. To estimate the 24-hour urinary sodium based on dietary sodium, we performed a crosswalk adjustment between these two types of data.

Table 1 summarises the study-level and country-level covariates used in modelling of each dietary factor.

Table 1. Types of data sources (other than 24-hour dietary recall) and covariates used in modelling of each dietary factor.

| | Data Sources | | | | Country level covariate |
|---|---|---|---|---|---|
| | Sales | FFQ[1] | HBS[2] | FAO | |
| Diet low in fruits | ● | ● | ● | ● | Lag-distributed income, total available kilocalories per person per day |
| Diet low in vegetables | ● | ● | ● | ● | Lag-distributed income, total available kilocalories per person per day |
| Diet low in legumes | ● | - | ● | ● | Lag-distributed income, total available kilocalories per person per day |
| Diet low in whole grains | - | ● | - | ● | Lag-distributed income |
| Diet low in nuts and seeds | - | - | ● | ● | Lag-distributed income, total available kilocalories per person per day |
| Diet low in milk | ● | ● | ● | ● | Lag-distributed income, total available kilocalories per person per day |
| Diet high in red meat | ● | ● | ● | ● | Lag-distributed income, total available kilocalories per person per day |
| Diet high in processed meat | ● | ● | ● | - | Lag-distributed income |
| Diet high in sugar-sweetened beverages | ● | ● | ● | - | National availability of sugar (grams/person/day), Lag-distributed income, total available kilocalories per person per day |
| Diet low in fiber | - | ● | - | ● | Lag-distributed income, total available kilocalories per person per day |
| Diet suboptimal in calcium | - | ● | - | ● | Lag-distributed income, total available kilocalories per person per day |
| Diet low in seafood omega-3 fatty acids | - | - | - | ● | Lag-distributed income |
| Diet low in polyunsaturated fatty acids | - | ● | - | ● | Lag-distributed income, total available kilocalories per person per day |
| Diet high in trans fatty acids | ● | ● | - | - | - |
| Diet high in sodium[3] | - | - | - | - | - |

[1] Food Frequency Questionnaire
[2] Household Budge Survey
[3] For sodium, we used data from the 24-hour urinary sodium and 24-hour dietary recall.

To characterise the distribution of each dietary factor at population level, we use an ensemble approach that separately fit 12 distributions for individual level microdata to specific to each data source's sampled population. The respective goodness of fit of each family was assessed and a weighting scheme was determined to optimise overall fit to the unique distribution of each risk factor. A global mean of the weights for each risk factor's data sources was created. We then determined the standard deviation of each population's consumption through a linear regression that captured the relationship between

the standard deviation and mean of intake in nationally representative nutrition surveys using 24-hour diet recalls:

$$ln\ (Standard\ deviation) = \beta_0 + \beta_1 \times ln\ (Mean_i)$$

Then we applied the coefficients of this regression to the outputs of our ST-GPR model to calculate the standard deviation of intake by age, sex, year, and country. We also quantified the within person variation in consumption of each dietary component and adjusted the standard deviations accordingly.

## Theoretical minimum-risk exposure level

In GBD 2016, to estimate the TMREL for each dietary factor, we first calculated the level of intake associated with the lowest risk of mortality from each disease endpoint based on the studies included in the meta-analyses of the dietary relative risks. Then, we calculated the TMREL as the weighted average of these numbers using the global number of deaths from each of outcome as the weight (Table 2).

Table 2. Theoretical minimum-risk exposure level for dietary factors GBD 2017.

| Dietary Factor | GBD 2017 |
|---|---|
| Fruits | 200-300 gr/day |
| Vegetables | 290-430 gr/day |
| Legumes | 50-70 gr/day |
| Whole grains | 100-150 gr/day |
| Nuts | 16-25 gr/day |
| Red meats | 18-27 gr/day |
| Processed meats | 0-4 gr/day |
| Milk | 350-520 gr/day |
| Sugar sweetened beverages | 0-5 gr/day |
| Polyunsaturated fatty acids | 9-13% of total daily energy |
| Seafood omega-3 fatty acids | 200-300 mg/day |
| Trans fatty acids | 0-1% of total daily energy |
| Dietary fiber | 19-28 gr/day |
| Dietary calcium | 1.0-1.3 gr/day |

## Relative risk

In GBD 2016, we measured the health effects of a diet high in sugar-sweetened beverages (SSBs) through how it changes a population's body-mass index (BMI). All attributed disease burden was then solely through the health outcomes associated with a high BMI. Given the more recent publication of dose-response meta-analyses that quantifies the direct effects of SSB consumption on incidence of and mortality from disease endpoints (i.e., ischemic heart disease, and type-2 diabetes), we have updated

our approach to reflect the best available evidence. In GBD 2017, consistent with other dietary risks, we have estimated the health effects of SSBs on morbidity and mortality from disease endpoints. Considering that the relative risks included in these new meta-analyses were mostly adjusted for BMI, our estimated disease burden reflects the burden of disease attributable to SSB consumption independent of its effect on BMI.

We obtained the relative risk of each disease endpoint per serving of the dietary components from recent dose-response meta-analyses of prospective observational studies, and where available randomised controlled trials. Considering the well-established age trend of the relative risks of metabolic risk factors for cardiovascular disease and diabetes, we conducted a literature review to identify the most important metabolic mediators for each dietary factor and used the age trend of the relative risk of that mediator(s) and the disease endpoint to estimate the age-specific relative risk for each dietary factors (Table 3).

Table 3. Metabolic mediators used to determine the age trend of the effect of dietary factors on cardiometabolic outcomes.

| | Body Mass Index | Total Serum Cholesterol | Fasting Plasma Glucose | Systolic Blood Pressure |
|---|---|---|---|---|
| Diet low in fruits | ● | ● | ● | ● |
| Diet low in vegetables | ● | ● | ● | ● |
| Diet low in legumes | ● | ● | ● | ● |
| Diet low in whole grains | ● | ● | ● | - |
| Diet low in nuts and seeds | ● | ● | ● | ● |
| Diet high in red meats | ● | - | ● | - |
| Diet high in processed meats | ● | - | ● | ● |
| Diet low in fiber | - | ● | - | - |
| Diet low in seafood omega-3 fatty acids | ● | - | - | ● |
| Diet low in polyunsaturated fatty acids | - | ● | ● | - |
| Diet high in trans fatty acids | ● | ● | - | - |

# Intimate Partner Violence Capstone Appendix

## Flowchart

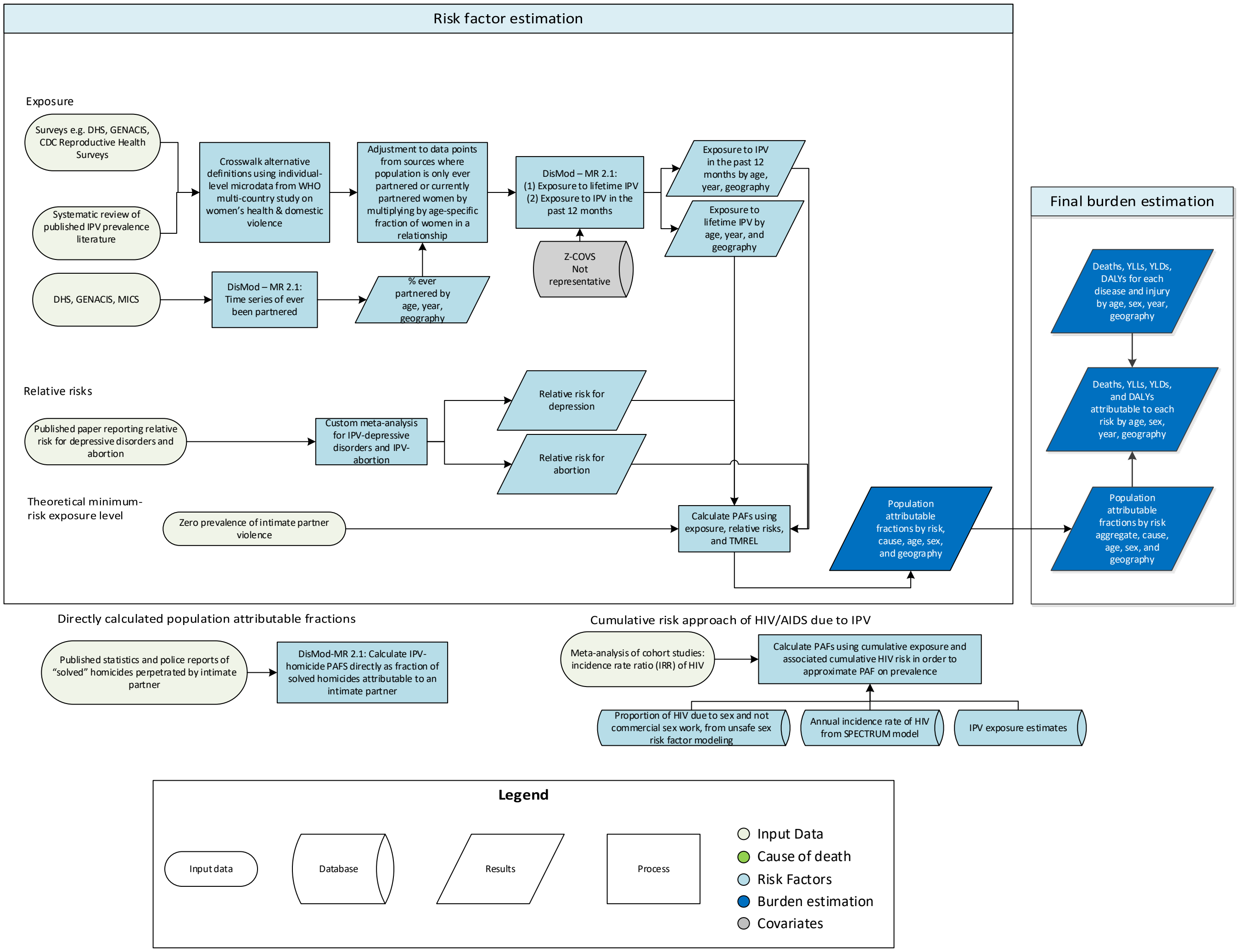


## Input data & methodological summary

### Exposure

#### *Case definition*

The case definition for intimate partner violence (IPV) is ever experienced one or more acts of physical and/or sexual violence by a current or former intimate partner since the age of 15 years. Estimated in females only because evidence of risk-outcomes for males does not meet our criteria.

- Physical violence is defined as: 'being slapped or having something thrown at you that could hurt you, being pushed or shoved, being hit with a fist or something else that could hurt, being kicked, dragged, or beaten up, being choked or burnt on purpose, and/or being threatened with or actually having a gun, knife, or other weapon used on you.'

- Sexual violence is defined as: ‘being physically forced to have intercourse when you did not want to, having sexual intercourse because you were afraid of what your partner might do, and/or being forced to do something that you found humiliating or degrading’ (the definition of humiliating and degrading may vary across studies depending on the regional and cultural setting).
- Intimate partner is defined as: ‘a partner to whom you are married or with whom you cohabit.’ In countries where people date, dating partners will also be considered (a partner with whom you have an intimate (sexual) relationship with but are not married to or cohabiting).

### *Input data*

No systematic review of the literature was completed for GBD 2017. However, a systematic review of the intimate partner violence prevalence literature was conducted in PubMed for anything published between January 2016 and January 2017 for the GBD 2016 cycle. The following search terms were used to conduct the systematic review:

((("health surveys"[MeSH Terms] AND prevalence[Title/Abstract]) OR ("sentinel surveillance"[MeSH Terms] AND prevalence[Title/Abstract]) OR ("prevalence"[Title/Abstract] AND cross sectional studies[MeSH Terms])) AND (abuse, sexual[MeSH Terms] OR domestic violence[MeSH Terms] OR abuse, partner[MeSH Terms] OR abuse, spousal[MeSH Terms] OR rape[MeSH Terms]) NOT ("comment"[Publication Type] OR "letter"[Publication Type] OR "editorial"[Publication Type]))

We get the proportion of solved homicides that were perpetrated by an intimate partner from crime statistics and police reports.

In GBD 2015, an updated systematic review was done for IPV homicide sources in PubMed through April 2016. The query used for this Pubmed search was:

((IPV[All Fields] OR ("intimate partner violence"[MeSH Terms] OR ("intimate"[All Fields] AND "partner"[All Fields] AND "violence"[All Fields]) OR "intimate partner violence"[All Fields])) AND (("homicide"[MeSH Terms] OR "homicide"[All Fields]) OR femicide[All Fields])) AND ("2013/01/01"[PDAT] : "3000/12/31"[PDAT])

These literature sources were supplemented with sources from the GHDx that were tagged with Intimate partner violence AND Homicide.

## Modelling strategy

We use three distinct approaches to estimate burden attributable to IPV, including 1) the traditional exposure and relative risk (RR) to percent attributable fraction (PAF) method for depression and abortion; 2) the direct PAF approach for estimating the proportion of homicides that are perpetrated by an intimate partner; and 3) a cumulative risk approach for estimating the burden of HIV/AIDS attributable to IPV.

### *Estimating attributable burden to IPV for depression, suicide and abortion*

We first adjusted data with variable recall periods (previous 12 months versus lifetime), type of violence (sexual, physical, or both) and severity (severe only versus all levels). To convert data to our reference definition of ever having experienced any physical or sexual IPV, we used data from the WHO Multi-country Study on Women’s Health and Domestic Violence against Women to construct crosswalk

regressions. The dependent variable in each of these regressions was ever any IPV (reference), while the key independent variable was one of the 11 alternative metrics of IPV that were represented in our dataset:

1. Physical IPV in the past 12 months
2. Sexual IPV in the past 12 months
3. Severe IPV in the past 12 months
4. Severe physical IPV in the past 12 months
5. Severe sexual IPV in the past 12 months
6. Any IPV (physical and/or sexual) in the past 12 months
7. Ever any physical IPV
8. Ever any sexual IPV
9. Ever any severe IPV
10. Ever severe physical IPV
11. Ever severe sexual IPV

For alternate metrics 1-6 we included a series of age dummies:

$$logit(REF_{ait}) = \beta_0 + \beta_1 logit(ALT_{ait}) + \beta_2 I_a + \varepsilon$$

For alternate metrics 7-11, we ran the following regression:

$$logit(REF_{it}) = \beta_0 + \beta_1 logit(ALT_{it}) + \varepsilon$$

where REF is the reference metric of IPV prevalence, ALT is the alternate metric of IPV prevalence, $I_a$ refers to the complete set of age-group indicators, *a* refers to an age-group, *i* refers to a country, and *t* refers to year. We included age-group indicators in the first six regressions because we expected the prevalence of recent IPV to vary by age. Using the intercepts, coefficients, and variance-covariance matrix from each of these eleven regressions, we were able to convert all of the alternate metrics of IPV prevalence in our dataset to estimates of "ever any IPV." We eliminated observations based on alternate metrics of IPV which came from studies that also provided estimates of IPV based on the reference definition.

After applying crosswalks to the alternate metrics of IPV in the manner described above, we made an additional adjustment to the subset of our data that was based on only ever-partnered, currently partnered women currently married women or ever married women. To adjust these values so that they reflected IPV prevalence in the entire female population, regardless of partnered status, we multiplied estimates from these studies by the age-specific fraction of women who had ever been partnered. An updated time series was generated in GBD 2015 using MICS and DHS data in a single parameter DisMod model to reflect the most recent data on proportion of women that have ever been partnered.

After these pre-DisMod crosswalks and adjustments, a single-parameter prevalence model was run in DisMod-MR 2.1 with age mesh points at 0 14 15 20 30 40 50 60 80 & 100. A study-level fixed effect on integrand variance (z-cov) to indicate whether a study was nationally representative or not was used to account for the heterogeneity introduced by studies that are not generalizable to the entire population. This covariate was first tested as an x-cov and the coefficient indicated no systematic bias.

In addition to the lifetime exposure model as described, a 12-month exposure model was also run in DisMod-MR 2.1, with data collected and processed analogously. This 12-month exposure model was used for the IPV-abortion PAF calculation to match the exposure definition in the risk evidence.

### *Direct PAF for female homicides*

The burden of homicides attributable to intimate partner violence was modelled as a direct PAF.

Input data fed into a single-parameter proportion DisMod-MR 2.1 model, which had age mesh points at 0 10 20 45 & 100. The model had a study-level covariate for sources just including police reported homicides. We also included a study-level fixed effect on integrand variance (z-cov) to indicate whether a study was nationally representative or not. This covariate was first tested as an x-cov and the coefficient indicated no systematic bias.

In GBD 2015, we added prevalence of binge drinking to the model as a country-level covariate, but it was dropped for GBD 2016 because it produced a non-significant coefficient.

### *Cumulative risk approach for PAF of HIV/AIDS due to IPV*

The third and final modelling approach that we used to assess burden attributable to intimate partner violence was a cumulative risk approach to measure the burden of HIV/AIDS attributable to IPV.

The approach itself remained the same in GBD 2017, but included updated intimate partner violence exposure numbers from the DisMod-MR 2.1 model described above, as well as revised HIV incidence numbers.

From two cohort studies (Jewkes et al, Lancet 2010 & Kouyoumdijian, et al AIDS 2013) we pooled incidence rate ratio (IRR) of HIV incidence with a random effects model. As we measure burden based on deaths and prevalence, we needed to quantify attributable fractions for prevalence and death rather than incidence. To get a PAF for prevalence we needed to consider the history of exposure to IPV and the accumulated associated risk of incident HIV due to IPV, relative to the overall risk of HIV at the population level. The ratio of cumulative IPV-attributable HIV incidence to total HIV incidence was an approximation of the relevant PAF for HIV prevalence and we assumed this PAF can also be applied to mortality.

$$\frac{Cumulative\ HIV\ incidence\ due\ to\ IPV}{Cumulative\ HIV\ incidence\ overall} = \frac{1 - \prod_{a=o}^{a=n}\left(1 - PAF_{ay} * I_{ay}\right)}{1 - \prod_{a=o}^{a=n}(1 - I_{ay})}$$

Where:

I = annual incidence rate of HIV

*a* = age (15-95)

*y* = year (1980-2016)

$$PAF_{HIV\ incidence} = \frac{[Prevalence\ of\ IPV]_{ay} * (IRR-1)}{[Prevalence\ of\ IPV]_{ay} * (IRR-1)+1}$$

## Theoretical minimum-risk exposure level

The theoretical minimum-risk exposure level is zero exposure to intimate partner violence, as defined above.

## Relative risks

We estimate burden attributable to IPV for abortion, depressive disorders, interpersonal violence (i.e. homicide) and HIV incidence. For GBD 2016, we completed a systematic review of the literature for papers reporting relative risk of IPV and our outcomes. Suicide, which was modelled as an outcome of IPV for GBD 2015, was removed from the analysis based on the availability of relative risk literature using suicide and not attempted suicide as the outcome definition. For GBD 2017, we used the same causal evidence for relative risk as was used for GBD 2016.

For HIV, we use a pooled IRR of 1.59 (95% CI 1.3-1.94) from a random effects inverse variance weighted meta-analysis of the two available prospective studies as of date.

The relative risks for depressive disorders and suicide come came from a systematic review of longitudinal studies assessing intimate partner violence and incident diagnosed major depression. A random effects inverse variance weighted meta-analysis produced a pooled relative risk and 95% confidence interval of 1.44 (1.09, 1.92).

For the relative risk for IPV-abortion, we also performed a random effects, inverse variance meta-analysis, which produced a pooled relative risk and 95% confidence interval of 1.91 (1.15, 3.16). An important methodological note with IPV-abortion is that we must apply the pooled relative risk for abortion to the current prevalence of IPV (in the previous 12 months), rather than lifetime prevalence. This is because the case definition for the relative risk component studies was physical or sexual IPV in the past year.

**Meta-analysis for IPV-depressive disorders:**

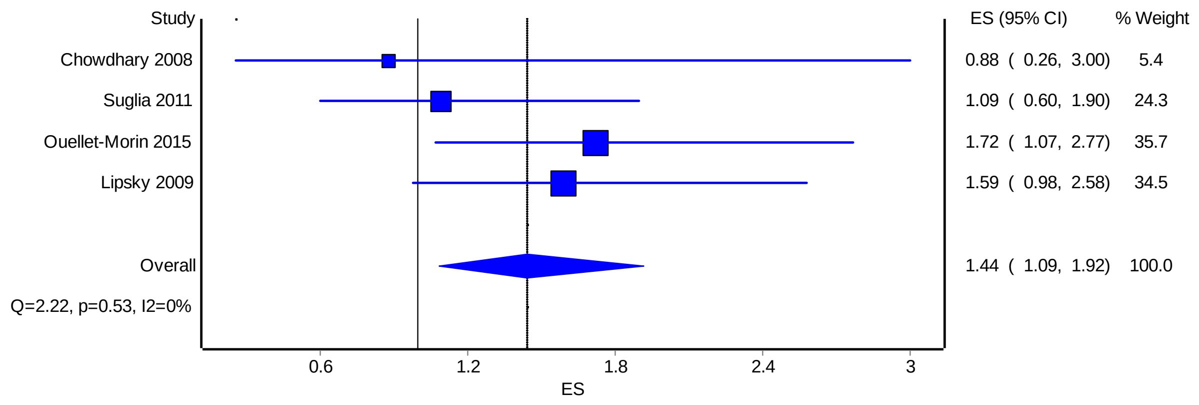

## Meta-analysis for IPV-abortion:

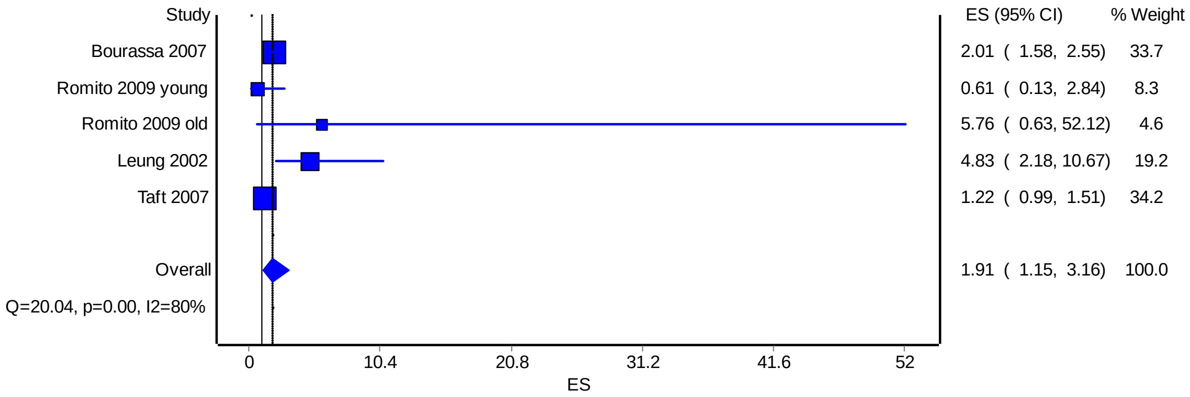


## Meta-analysis for IPV-HIV:

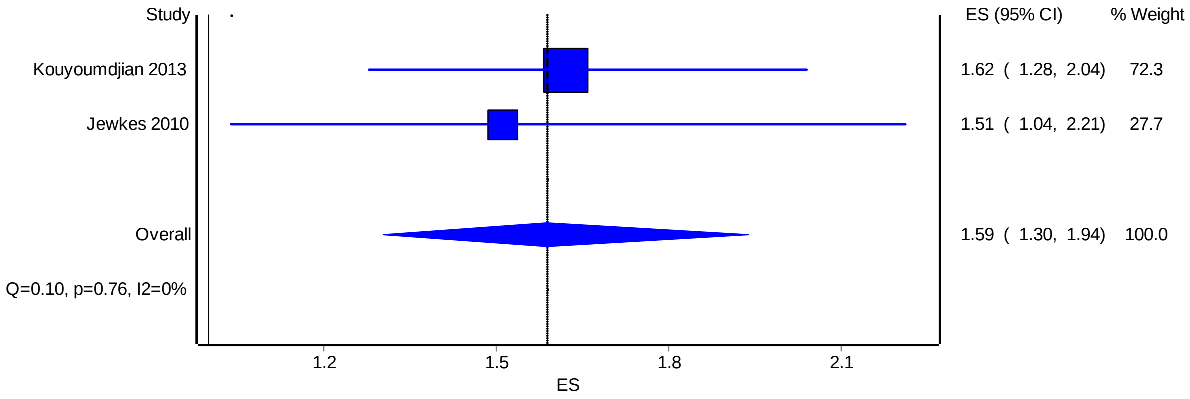

## References


1. Bourassa D, Bérubé J. The prevalence of intimate partner violence among women and teenagers seeking abortion compared with those continuing pregnancy. J Obstet Gynaecol Can. 2007; 29(5): 415-23.
2. Chowdhary N, Patel V. The effect of spousal violence on women's health: findings from the Stree Arogya Shodh in Goa, India. J Postgrad Med. 2008; 54(4): 306–12.
3. Jewkes RK, Dunkle K, Nduna M, Shai N. Intimate partner violence, relationship power inequity, and incidence of HIV infection in young women in South Africa: a cohort study. Lancet. 2010; 41-48.
4. Kouyoumdjian FG, Calzavara LM, Bondy SJ, O'Campo P, Serwadda D, Nalugoda F, Kagaayi J, Kigozi G, Wawer M, Gray R. Intimate partner violence is associated with incident HIV infection in women in Uganda. AIDS. 2013; 27(8): 1331-8.
5. Leung TW, Leung WC, Chan PL, Ho PC. A comparison of the prevalence of domestic violence between patients seeking termination of pregnancy and other general gynecology patients. Int J Gynaecol Obstet. 2002; 77(1): 47-54.
   Lipsky S, Caetano R, Roy-Byrne P. Racial and ethnic disparities in police-reported intimate partner violence and risk of hospitalization among women. Womens Health Issues. 2009; 19(2):109–118.
6. Ouellet-Morin I, Fisher HL, York-Smith M, Fincham-Campbell S, Moffitt TE, Arseneault L. Intimate partner violence and new-onset depression: a longitudinal study of women's childhood and adult histories of abuse. Depression and anxiety. 2015;32(5):316-324.
7. Romito P, Escribà-Agüir V, Pomicino L, Lucchetta C, Scrimin F, Molzan Turan J. Violence in the lives of women in Italy who have an elective abortion. Womens Health Issues. 2009; 19(5): 335-43.
8. Suglia SF, Duarte CS, Sandel MT. Housing quality, housing instability, and maternal mental health. J Urban Health. 2011; 88(6): 1105–16.
9. Taft AJ, Watson LF. Termination of pregnancy: associations with partner violence and other factors in a national cohort of young Australian women. Aust N Z J Public Health. 2007; 31(2): 135-42.

# Childhood Sexual Abuse Capstone Appendix

## Flowchart

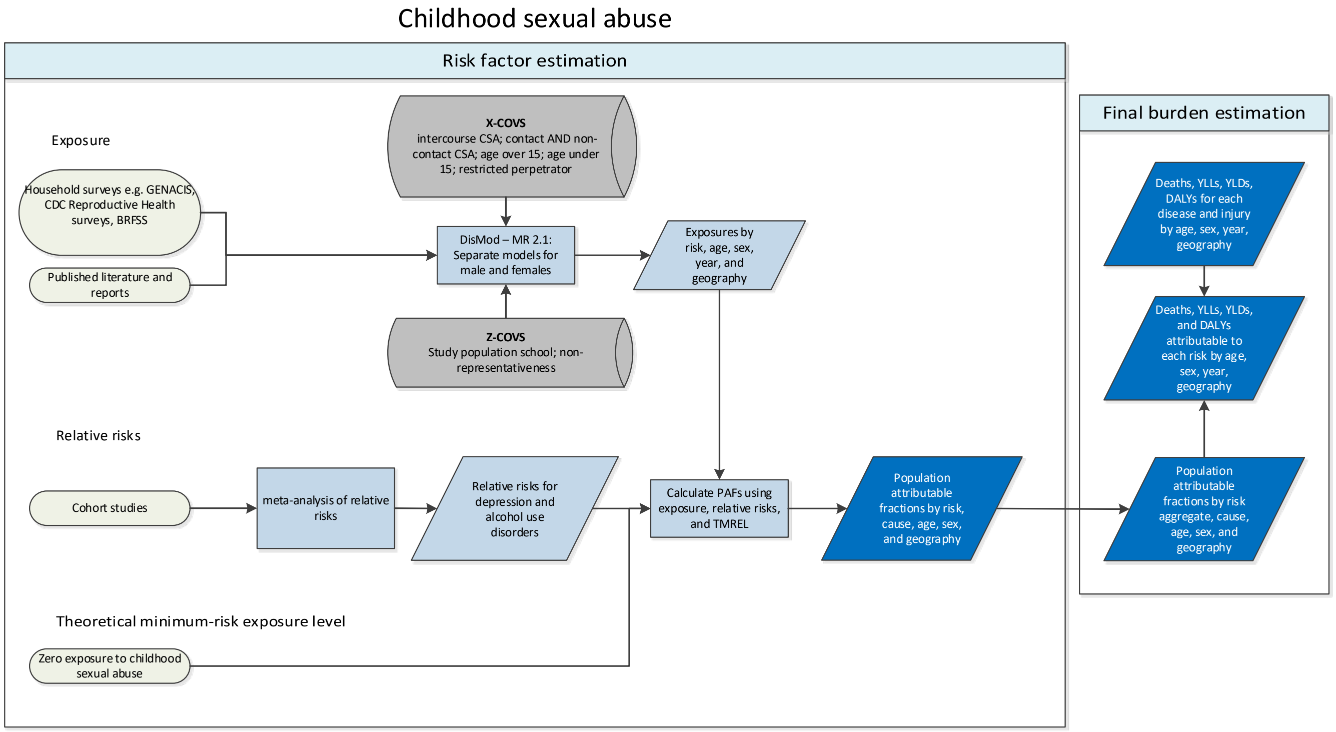


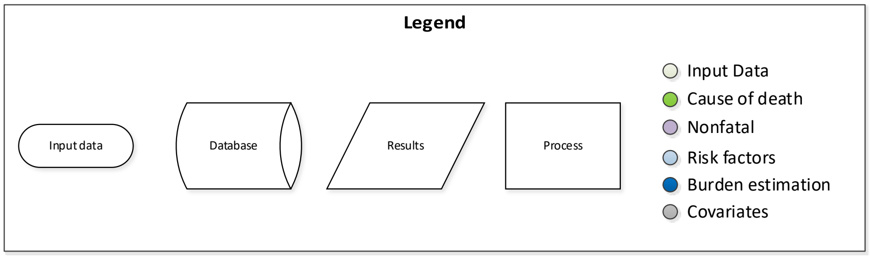


## Input data & methodological summary

## Exposure

### Case definition

The case definition for childhood sexual abuse (CSA) is ever having had the experience of intercourse or other contact abuse (i.e. fondling and other sexual touching) when aged 15 years or younger, and the perpetrator or partner was greater than five years older than the victim.

## Input data

Currently, we use self-reported survey data to measure CSA prevalence, not data from Child Protection Services (CPS) or other crime data. The reliability and comprehensiveness of CPS and crime statistics varies too much geographically to warrant inclusion.

Although no systematic review of the literature was completed for GBD 2017, an updated systematic review of CSA prevalence literature was conducted for sources published between August 2015 and January 2017 during the GBD 2016 cycle. The following search terms were used:

(((("health surveys"[MeSH Terms] AND prevalence[Title/Abstract]) OR ("sentinel surveillance"[MeSH Terms] AND prevalence[Title/Abstract]) OR ("prevalence"[Title/Abstract] AND cross sectional studies[MeSH Terms])) AND (("child abuse"[MeSH Terms] OR "child abuse, sexual"[MeSH Terms]) OR ("sex offenses"[MeSH Terms] OR "child abuse, sexual"[MeSH Terms]) OR (child*[Title/Abstract] AND sexual[Title/Abstract] AND abuse[Title/Abstract]))) NOT ("comment"[Publication Type] OR "letter"[Publication Type] OR "editorial"[Publication Type]))

We supplemented with data from relevant national health surveys and violence-specific surveys. Several survey series used include the United States Behavioral Risk Factor Surveillance System, the CDC Reproductive Health Surveys, Brazil National Alcohol and Drug Survey, and the Gender, Alcohol, and Culture International Study (GENACIS).

A number of study level covariates were also extracted that were used in the modelling process to adjust for heterogeneous definitions across sources. All crosswalks and adjustments were done in DisMod-MR 2.1.

## Modelling strategy

CSA prevalence was modeled as a single parameter prevalence model in DisMod-MR 2.1. CSA exposure is modeled separately for males and females because we observe little correlation between the prevalence of child abuse among females and males, and modeling both sexes together causes unreasonable estimates in countries where we only have data for one sex.

Three study-level covariates were used for alternate definitions of the violence.

- Study asked only about intercourse CSA
- Study asked about contact and non-contact CSA
- Study placed restrictions on the relationship between the perpetrator and the victim (e.g. only asked about CSA committed by a father)

We also included study-level fixed effects for varying age thresholds across studies.

- Study asked about recall for events before ages above 15 years (versus reference age threshold of 15)
- Study asked about recall for events before ages less than 15 years (versus reference age threshold of 15)

Two study-level covariate fixed effects on variance (z-cov) were also included in both the male and female models, including an indicator that the survey was not nationally representative, as well as whether the survey was administered in schools. These study-level covariates were tested as x-covs first, but we did not find coefficients which would indicate systematic bias. We have not included any national-level covariates to date due to lack of knowledge about a covariate (for which we have a time series for all GBD locations) that predicts CSA prevalence.

## Theoretical minimum-risk exposure level

The theoretical minimum risk exposure level is zero exposure to contact childhood sexual abuse.

## Relative risks

We estimate burden attributable to CSA for the following health outcomes: unipolar depressive disorders (major depressive disorder and dysthymia) and alcohol use disorders.

In GBD 2015, we used one twin study that compared adverse outcome risks in same-sex discordant pairs.[1] This study was deemed reliable given that environmental and contextual factors are inherently controlled for when comparing between twins, avoiding potential confounding. However, to add to the strength of the evidence for GBD 2016, we performed a systematic review and a random effects meta-analysis to produce relative risks for depressive disorders and alcohol use disorders. In a departure from GBD 2015, suicide was not used as an outcome for CSA. This decision was based on the evidence available for the relative risk of suicide given exposure to CSA – not enough studies used suicide as an outcome, but instead used attempted suicide. For GBD 2017, we used the same causal evidence as was used for GBD 2016.

The pooled relative risk figures and 95% confidence intervals were 1.63 (1.41, 1.89) for depressive disorders and 1.54 (1.19, 1.99) for alcohol use disorders. The resulting forest plots are as follows:

**CSA and depressive disorders meta-analysis**

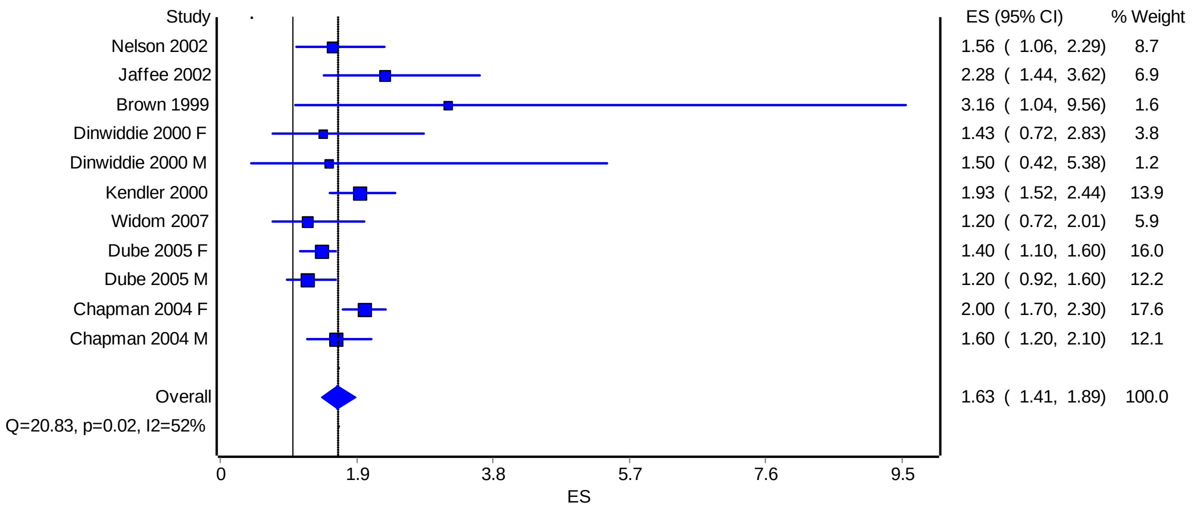

**CSA and alcohol use disorders meta-analysis**

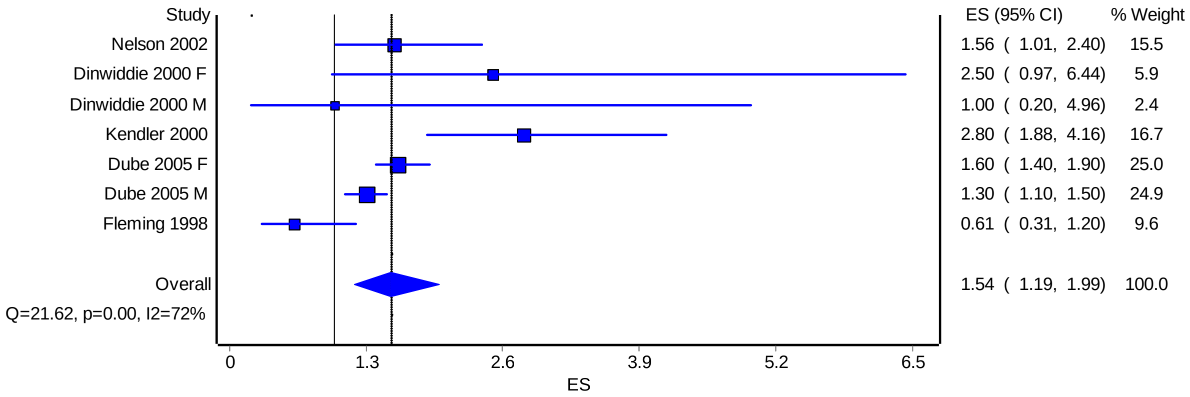


## References


1. Brown J, Cohen P, Johnson JG., and Smailes EM. Childhood abuse and neglect: specificity of effects on adolescent and young adult depression and suicidality. Journal of the American Academy of Child & Adolescent Psychiatry. 1999; 38(12): 1490-1496.
2. Chapman DP, Whitfield CL, Felitti VJ, Dube SR, Edwards VJ and Anda RF. Adverse childhood experiences and the risk of depressive disorders in adulthood. Journal of affective disorders. 2004; 82(2): 217-225.
3. Cheasty M, Clare AW and Collins C. Relation between sexual abuse in childhood and adult depression: case-control study. Bmj. 1998; 316(7126): 198-201.
4. Dinwiddie S, Heath AC, Dunne MP, Bucholz KK, Madden PA, Slutske WS, Bierut LJ, Statham DB, Martin NG. Early sexual abuse and lifetime psychopathology: a co-twin-control study. *Psychol Med*. 2000; 30(1): 41–52.
5. Dube SR, Anda RF, Whitfield CL, Brown DW, Felitti VJ, Dong M and Giles WH. Long-term consequences of childhood sexual abuse by gender of victim. American journal of preventive medicine. 2005; 28(5): 430-438.
6. Ernst C, Angst J, Földényi M. The Zurich Study. XVII. Sexual abuse in childhood. Frequency and relevance for adult morbidity data of a longitudinal epidemiological study. *Eur Arch Psychiatry Clin Neurosci*. 1993; 242(5): 293–300.
7. Fleming J, Mullen PE, Sibthorpe B, Attewell R and Bammer G. The relationship between childhood sexual abuse and alcohol abuse in women-a case-control study. Addiction. 1998; 93(12): 1787-1798.
8. Jaffee SR, Moffitt TE, Caspi A, Fombonne E, Poulton R, Martin J. Differences in early childhood risk factors for juvenile-onset and adult-onset depression. *Arch Gen Psychiatry*. 2002; 59(3): 215-22.
9. Kendler KS, Bulik CM, Silberg J, Hettema JM, Myers J, Prescott CA. Childhood sexual abuse and adult psychiatric and substance use disorders in women: an epidemiological and cotwin control analysis. *Arch Gen Psychiatry*. 2000; 57(10): 953–9.
10. Molnar BE, Buka SL and Kessler, RC. Child sexual abuse and subsequent psychopathology: results from the National Comorbidity Survey. American journal of public health. 2001; 91(5): 753.
11. Nelson EC, Heath AC, Madden PA, Cooper ML, Dinwiddie SH, Bucholz KK, Glowinski A, McLaughlin T, Dunne MP, Statham DJ, Martin NG. Association between self-reported sexual abuse and adverse psychosocial outcomes: results from a twin study. Arch Gen Psychiatry. 2002; 59(2): 139-45.

12. Peleikis DE, Mykletun A and Dahl AA. The relative influence of childhood sexual abuse and other family background risk factors on adult adversities in female outpatients treated for anxiety disorders and depression. Child Abuse & Neglect. 2004; 28(1): 61-76.
13. Sartor CE, Lynskey MT, Bucholz KK, McCutcheon VV, Nelson EC, Waldron M, Heath AC. Childhood sexual abuse and the course of alcohol dependence development: findings from a female twin sample. *Drug Alcohol Depend*. 2007; 89(2-3): 139–44.
14. Silverman AB, Reinherz HZ and Giaconia RM. The long-term sequelae of child and adolescent abuse: A longitudinal community study. Child abuse & neglect. 1996; 20(8): 709-723.
15. Widom CS, DuMont K and Czaja SJ. A prospective investigation of major depressive disorder and comorbidity in abused and neglected children grown up. Archives of general psychiatry. 2007; 64(1): 49-56.

# Bullying Victimisation Capstone Appendix

## Flowchart

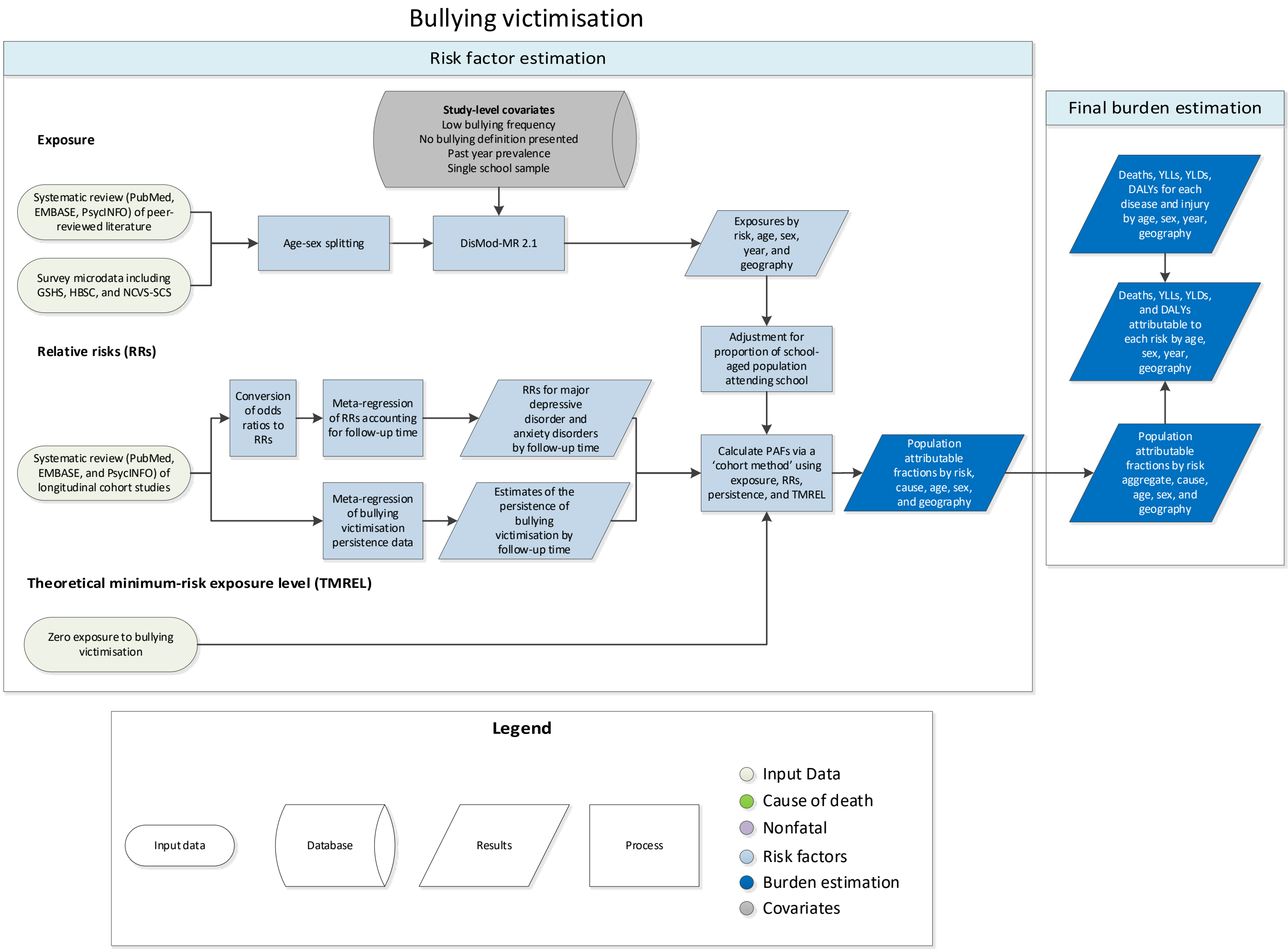


## Input data & methodological summary

## Exposure

### *Case definition*

Bullying victimisation is commonly conceptualised as the intentional and repeated harm of a less powerful individual by peers.[1] This differentiates bullying victimisation from disagreements, conflicts, or playful teasing. The case definition of bullying victimisation in the GBD context is 'bullying victimisation of children and adolescents attending school by peers.' This definition includes the global concept of bullying victimisation which incorporates combined estimates of subtypes such as physical, verbal, relational, and cyberbullying victimisation. It excludes abuse/harassment by siblings, intimate partners, and adults (e.g. teachers). While bullying can be experienced as either a victim or perpetrator,

perpetration (i.e. those who bully others) is not included in this definition although some victims will also be perpetrators.

### *Input data*

In order for a study to be included, it must report the prevalence of bullying victimisation and 1) have been published since 1980, 2) ask participants about bullying victimisation in the previous year or more recently, 3) use an appropriate frequency threshold to define bullying victimisation (approximating at least once a week or greater than 'occasionally'), 4) be representative of the general population rather than a special population (e.g. ethnic minorities), and 5) report prevalence for bullying victimisation overall rather than a subtype e.g. physical bullying victimisation.

Included studies were sourced from a systematic review of three electronic databases (PubMed, EMBASE, and PsycINFO), covering the period 1980 to 2017. No restriction was set on the language of publication. GhDx was also used to source microdata from survey series meeting the above inclusion criteria. Estimates from the Global School-based Student Health Survey (GSHS), the Health Behavior in School-aged Children (HBSC), and the National Crime Victimization Survey – School Crime Supplement (NCVS-SCS) were extracted and included in the dataset.

### *Modelling strategy*

Bullying victimisation prevalence was modelled as a single parameter prevalence model in DisMod-MR 2.1. We assumed no prevalence prior to 5 years or after 20 years of age. Four study-level covariates were included and are shown in the table below, along with their respective levels. Crosswalks for two of the covariates (low bullying frequency and no bullying definition presented) were calculated using within-study pairs of reference and non-reference estimates (n = 9 pairs and n = 3 pairs, respectively).

| **Covariate name** | **Reference** | **Non-reference** | **Exponentiated beta** |
|---|---|---|---|
| Low bullying frequency | Optimal frequency threshold used e.g. 'frequently' | Sub-optimal frequency threshold used e.g. 'sometimes + frequently' | 3.35 (3.35 – 3.35) *(n = 9 pairs)* |
| No bullying definition presented | Definition of bullying victimisation presented to participants | No definition of bullying victimisation presented to participants or not specified | 1.12 (1.12 – 1.12) *(n = 3 pairs)* |
| Recall 1 year | Asked about bullying victimisation more recently than in the past year | Asked about bullying victimisation in the past year | 1.47 (1.30 – 1.68) |
| Single school sample | Sample was a household survey or multi-school survey | Sample was from a single school | 1.21 (1.01 – 2.12) |

### *Adjustment for years of schooling*

In order to better represent the prevalence of bullying victimisation, prevalence estimates were adjusted for the proportion of children and adolescents attending school by ages 5-9, 10-14, and 15-19 years by sex, location, and year. Data on the proportion of children and adolescents attending school was sourced from the online database (http://data.uis.unesco.org/) published by the United Nations Educational, Scientific, and Culture Organization (UNESCO). The data covered 18,441 country-years for

age groups 6-11, 12-14, and 15-17 years by sex. This data was modelled in ST-GPR, with average years of education as a country-level covariate, to predict the proportion of children and adolescents attending school by these age groups. This gave estimates of the proportion of children and adolescents attending school by age, sex, year, and location.

## Theoretical minimum-risk exposure level

The theoretical minimum risk exposure level was assumed to be zero exposure to bullying victimisation.

## Relative risks (RRs)

We estimate burden attributable to bullying victimisation for major depressive disorder and anxiety disorders. Data on the association between bullying victimisation and self-harm was also reviewed but not included due to variation in the definition of 'self-harm' and only one study looking at suicide.

### *Input data for RRs*

Studies reporting the prospective longitudinal association between these outcomes and bullying victimisation were sourced from a systematic review of three electronic databases (PubMed, EMBASE, and PsycINFO), covering the period 1980 to 2017. No restriction was set on the language of publication. Studies had to report RRs, ORs, or sufficient data to calculate RRs (i.e. exposed/non-exposed cases/non-cases).

### *Meta-analysis*

The smaller number of estimates for anxiety disorders (n = 6) led to the decision to combine the major depressive disorder and anxiety disorders RR data into a single dataset. This was considered reasonable as the pooled RRs for major depressive disorder and anxiety disorders were effectively equal following an adjustment for low frequency bullying threshold studies (1.82, 95% CI: 1.62-2.04 vs 1.74, 95% CI: 1.43-2.11, respectively).

**Meta-analysis of bullying victimisation and major depressive disorder/anxiety disorders**

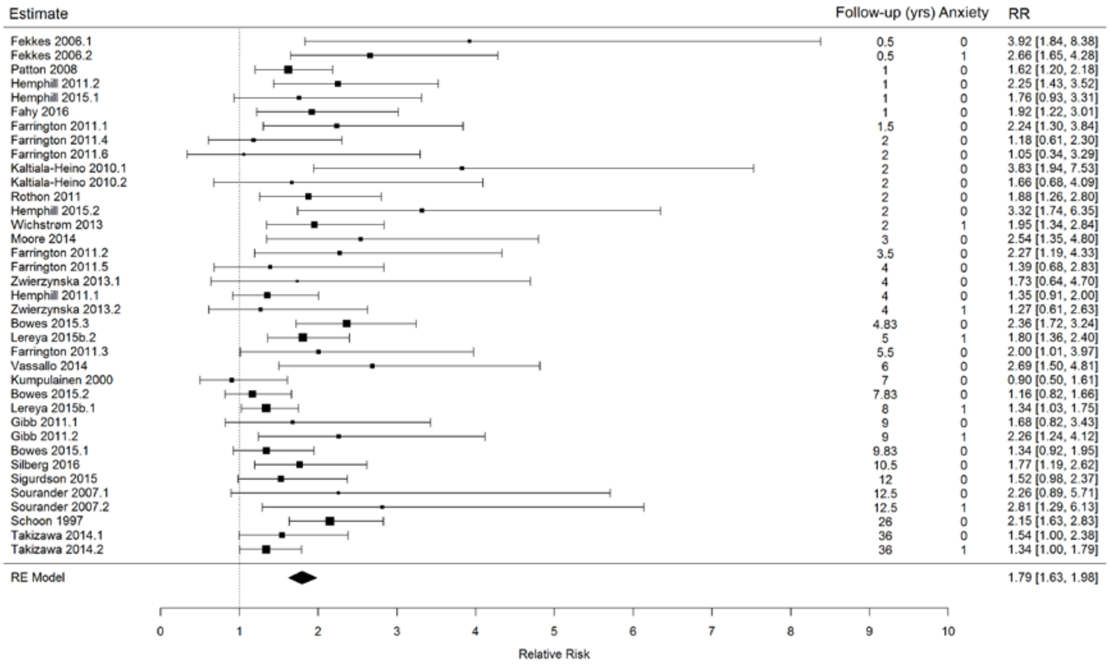

### *Meta-regression*

A meta-regression was conducted to determine the impact of follow-up time on the relationship between bullying victimisation and major depressive disorder/anxiety disorders.

Prior to this, an initial meta-regression revealed studies with a low bullying frequency threshold reported significantly lower RRs than studies utilising the optimal threshold. Six studies reported RRs for both low and optimal bullying frequency threshold and we determined that the within-study ratios from these studies would better inform an adjustment than between-study comparisons estimated as a covariate. These ratios were pooled and then applied to the low bullying frequency threshold estimates (i.e. suboptimal estimates) prior to analysis. This process could not be done for the other characteristics (i.e. symptoms vs diagnosis or no control vs control for outcome as baseline) as there were no within-study comparisons available. In the final meta-regression, these were instead controlled for using covariates.

In the final meta-regression, estimates with a follow-up time of 25 years or more were excluded due to only data from a single cohort being available to inform the effect of follow-up time after this period. In total, 19 studies were included in the analyses.[2-20] While the meta-regression demonstrated a waning effect of time, this was not statistically significant ($p$ = 0.093). However, the model still predicted plausible estimates of RR over time that matched our strong prior that the effect size would have to diminish over time. Time was also significant in a parsimonious model that did not include the non-significant covariates ($p$ = 0.042).

## PAF calculations

For bullying victimisation, the PAF calculations could not be determined by current prevalence and a single value for RR. This is due to the waning effect on outcomes over time (as demonstrated in the meta-regression) and because prevalence estimates were from surveys of young people reporting current bullying victimisation rather than estimates of past exposure at the time the outcomes occur (i.e. retrospective estimates).

A cohort method was subsequently developed to address this issue. The following steps are conducted for each point of estimation (i.e. by age, sex, location, and year), hereafter referred to as a 'cohort':

1. Pull current and past bullying victimisation prevalence for the cohort from the DisMod-MR 2.1 exposure model.
2. Adjust each bullying victimisation prevalence estimate for the proportion of  the cohort attending school in that year.
3. Divide the cohort into proportions based on time since first exposed to bullying victimisation. This equates to the incidence of bullying victimisation and is estimated using the following formula:

$$\boldsymbol{I_k = P_k - \sum_{n=0}^{k-1}(I_n \times r_{k-n})}$$

where $I$ represents incidence, $P$ represents prevalence, $r$ represents the estimate of persistence, and $k$ represents the time between the incidence estimate and the earliest possible time of exposure in the cohort. $I_k$ requires $I_0$ through to $I_{k-1}$ to first be calculated and so we complete this process by first estimating $I_0$, then $I_1$, and so on until we have estimated incidence for the latest possible year of exposure for this cohort. The persistence estimate is based on a separate meta-regression of seven studies.[11,21-26]

4. We Map RRs to the proportions of the cohort based on the time between the point of estimation and when they were first exposed to bullying victimisation and estimate PAFs via the following formula:

$$\boldsymbol{PAF} = \frac{\sum(\boldsymbol{p_t} \times \boldsymbol{RR_t}) + \sum \boldsymbol{p_{no\ exposure}} - \mathbf{1}}{\sum(\boldsymbol{p_t} \times \boldsymbol{RR_t}) + \sum \boldsymbol{p_{no\ exposure}}}$$

where $t$ is the time since first exposed to bullying victimisation, $p$ is the proportion of the cohort first exposed to bullying victimisation at time $t$ or the proportion not exposed to bullying victimisation, and $RR$ is the relative risk for depressive and anxiety disorders given $t$.

## References


1. Olweus D. Bullying at school: What we know and what we can do. MA, USA; Oxford, UK; Vic, Australia: Blackwell; 1993.
2. Bowes L, Joinson C, Wolke D, Lewis G. Peer victimisation during adolescence and its impact on depression in early adulthood: Prospective cohort study in the United Kingdom. *BMJ: British Medical Journal* 2015; **350**.
3. Fahy AE, Stansfeld SA, Smuk M, Smith NR, Cummins S, Clark C. Longitudinal associations between cyberbullying involvement and adolescent mental health. *Journal of Adolescent Health* 2016; **59**(5): 502-9.
4. Farrington DP, Loeber R, Stallings R, Ttofi MM. Bullying perpetration and victimization as predictors of delinquency and depression in the Pittsburgh Youth Study. *Journal of Aggression, Conflict and Peace Research* 2011; **3**(2): 74-81.
5. Fekkes M, Pijpers FI, Fredriks AM, Vogels T, Verloove-Vanhorick SP. Do bullied children get ill, or do ill children get bullied? A prospective cohort study on the relationship between bullying and health-related symptoms. *Pediatrics* 2006; **117**(5): 1568-74.
6. Gibb SJ, Horwood LJ, Fergusson DM. Bullying victimization/perpetration in childhood and later adjustment: Findings from a 30 year longitudinal study. *Journal of Aggression, Conflict and Peace Research* 2011; **3**(2): 82-8.
7. Hemphill SA, Kotevski A, Heerde JA. Longitudinal associations between cyber-bullying perpetration and victimization and problem behavior and mental health problems in young Australians. *International Journal of Public Health* 2015; **60**(2): 227-37.
8. Hemphill SA, Kotevski A, Herrenkohl TI, et al. Longitudinal consequences of adolescent bullying perpetration and victimisation: A study of students in Victoria, Australia. *Criminal Behaviour and Mental Health* 2011; **21**(2): 107-16.
9. Kaltiala-Heino R, Fröjd S, Marttunen M. Involvement in bullying and depression in a 2-year follow-up in middle adolescence. *European Child & Adolescent Psychiatry* 2010; **19**(1): 45-55.
10. Kumpulainen K, Rasanen E. Children involved in bullying at elementary school age: Their psychiatric symptoms and deviance in adolescence - an epidemiological sample. *Child Abuse Negl* 2000; **24**(12): 1567-77.
11. Lereya ST, Copeland WE, Zammit S, Wolke D. Bully/victims: A longitudinal, population-based cohort study of their mental health. *European Child & Adolescent Psychiatry* 2015; **24**(12): 1461-71.
12. Moore SE, Norman RE, Sly PD, Whitehouse AJO, Zubrick SR, Scott J. Adolescent peer aggression and its association with mental health and substance use in an Australian cohort. *Journal of Adolescence* 2014; **37**(1): 11-21.
13. Patton GC, Olsson C, Bond L, et al. Predicting female depression across puberty: A two-nation longitudinal study. *Journal of the American Academy of Child & Adolescent Psychiatry* 2008; **47**(12): 1424-32.

14. Rothon C, Head J, Klineberg E, Stansfeld S. Can social support protect bullied adolescents from adverse outcomes? A prospective study on the effects of bullying on the educational achievement and mental health of adolescents at secondary schools in East London. *Journal of Adolescence* 2011; **34**(3): 579-88.
15. Sigurdson JF, Undheim AM, Wallander JL, Lydersen S, Sund AM. The long-term effects of being bullied or a bully in adolescence on externalizing and internalizing mental health problems in adulthood. *Child and Adolescent Psychiatry and Mental Health* 2015; **9**(42).
16. Silberg JL, Copeland W, Linker J, Moore AA, Roberson-Nay R, York TP. Psychiatric outcomes of bullying victimization: A study of discordant monozygotic twins. *Psychological Medicine* 2016; **46**(9): 1875-83.
17. Sourander A, Jensen P, Ronning JA, et al. What is the early adulthood outcome of boys who bully or are bullied in childhood? The Finnish "From a Boy to a Man" study. *Pediatrics* 2007; **120**(2): 397-404.
18. Vassallo S, Edwards B, Renda J, Olsson CA. Bullying in early adolescence and antisocial behavior and depression six years later: What are the protective factors? *Journal of School Violence* 2014; **13**(1): 100-24.
19. Wichstrøm L, Belsky J, Berg-Nielsen TS. Preschool predictors of childhood anxiety disorders: A prospective community study. *Journal of Child Psychology and Psychiatry* 2013; **54**(12): 1327-36.
20. Zwierzynska K, Wolke D, Lereya TS. Peer victimization in childhood and internalizing problems in adolescence: A prospective longitudinal study. *Journal of Abnormal Child Psychology* 2013; **41**(2): 309-23
21. Baly MW, Cornell DG, Lovegrove P. A Longitudinal Investigation of Self- and Peer Reports of Bullying Victimization Across Middle School. *Psychology in the Schools* 2014; **51**(3): 217-40.
22. Bowes L, Maughan B, Ball H, et al. Chronic bullying victimization across school transitions: the role of genetic and environmental influences. *Development and psychopathology* 2013; **25**(2): 333-46.
23. Kumpulainen K, Rasanen E, Henttonen I. Children involved in bullying: psychological disturbance and the persistence of the involvement. *Child Abuse Negl* 1999; **23**(12): 1253-62.
24. Lien L, Welander-Vatn A. Factors Associated with the Persistence of Bullying Victimization From 10th grade to 13th Grade: A Longitudinal Study. *Clinical practice and epidemiology in mental health : CP & EMH* 2013; **9**: 243-50.
25. Sourander A, Helstela L, Helenius H, Piha J. Persistence of bullying from childhood to adolescence--a longitudinal 8-year follow-up study. *Child Abuse Negl* 2000; **24**(7): 873-81.
26. Winsper C, Lereya T, Zanarini M, Wolke D. Involvement in bullying and suicide-related behavior at 11 years: a prospective birth cohort study. *Journal of the American Academy of Child and Adolescent Psychiatry* 2012; **51**(3): 271-82.e3.

# Unsafe Sex Capstone Appendix

## Flowchart

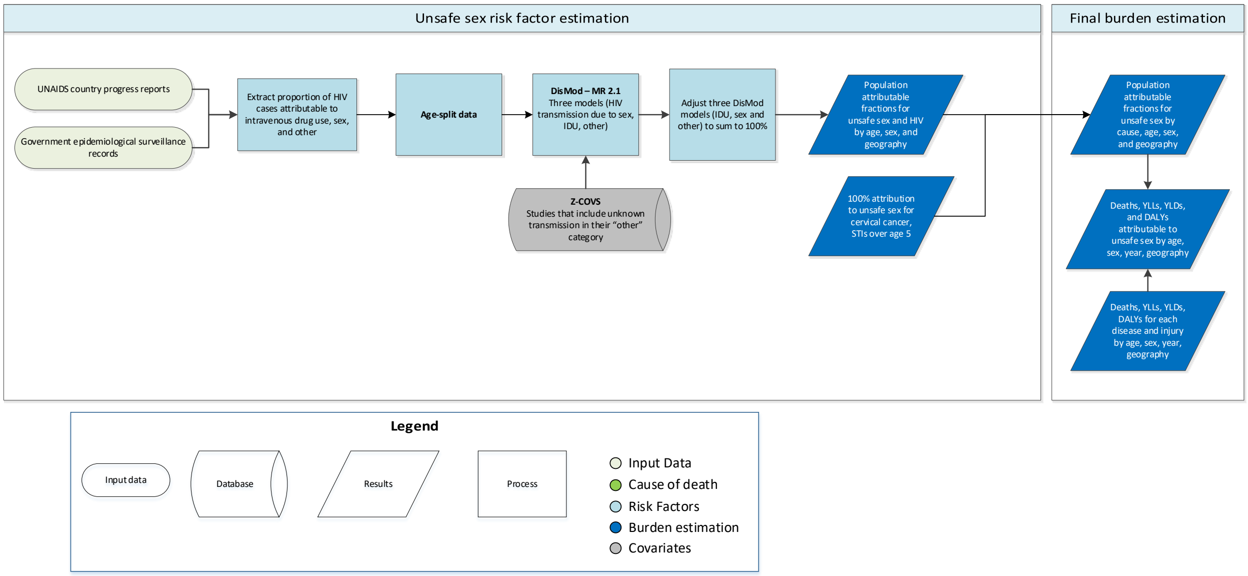


## Input data & methodological summary

### Case definition and summary of GBD approach

Unsafe sex is defined as the risk of disease due to sexual transmission. The outcomes associated with unsafe sex that we estimate for GBD include HIV, cervical cancer, and all sexually transmitted diseases (STDs) except for those in neonates from vertical transmission, including HIV, opthalmia neonatorum and neonatal syphilis. We assume 100% of cervical cancer and STDs are attributable to unsafe sex and model the proportion of HIV incidence occurring through sexual transmission to estimate the attributable burden for HIV due to unsafe sex.

### Input data

To be used in our models, sources must report HIV cases attributable to various modes of transmission. We screened all UNAIDS country progress reports and searched government epidemiological surveillance records for these data. The primary data sources we used were UNAIDS, the European CDC, and the US CDC.

We excluded all extractions where the "other" category for HIV transmissions accounted for greater than 25 percent of all cases. We believe that such high proportions raise concerns about the quality of reporting.

### Modelling strategy

We model the proportion of HIV cases attributable to unsafe sex. To do this we collect and clean data, run three DisMod models (HIV attributable to sex, HIV attributable to injection drug use, HIV

attributable to other routes of transmission), adjust results of the three DisMod models to sum to one, and prepare PAFs.

All of the DisMod models included a study-level covariate fixed effect on integrand variance (z-cov) for sources that include cases of unknown transmission in their "other" category. We assumed that the inclusion of unknown cases in the other category would impact the uncertainty around the point estimates. No country level covariates were included in the models. We tested an injection drug use covariate, an opioid use covariate in the proportion HIV due to drug use model, but found no significant coefficients so excluded them from the final model.

A new approach was introduced for GBD 2016, and used again for GBD 2017, to inform an age-pattern in these HIV transmission models. All-age data points represent the majority of the available data, so we derived an age-pattern for the HIV-IDU transmission model from the age-pattern present in the GBD 2017 population attributable fraction for hepatitis B attributable to intravenous drug use. Assuming the proportion of HIV due to other is constant over time, the age-pattern for the proportion of HIV due to sex was set to be the complement to 1 of the age-pattern for the proportion of HIV due to IDU. The all-age data were split according to these age-patterns, and the three HIV transmission DisMod models were run on the age-split data. Additional priors were set to inform an age-pattern: zero proportion HIV transmission due to IDU before age 15, zero proportion HIV transmission due to sex before age 10, and 100% transmission due to other before age 10. The results from these HIV transmission models were adjusted to sum to 100% for a given country-year-age-sex group at each of 1,000 draws.

*Squeezed global HIV transmission models by age (females, 2016):*

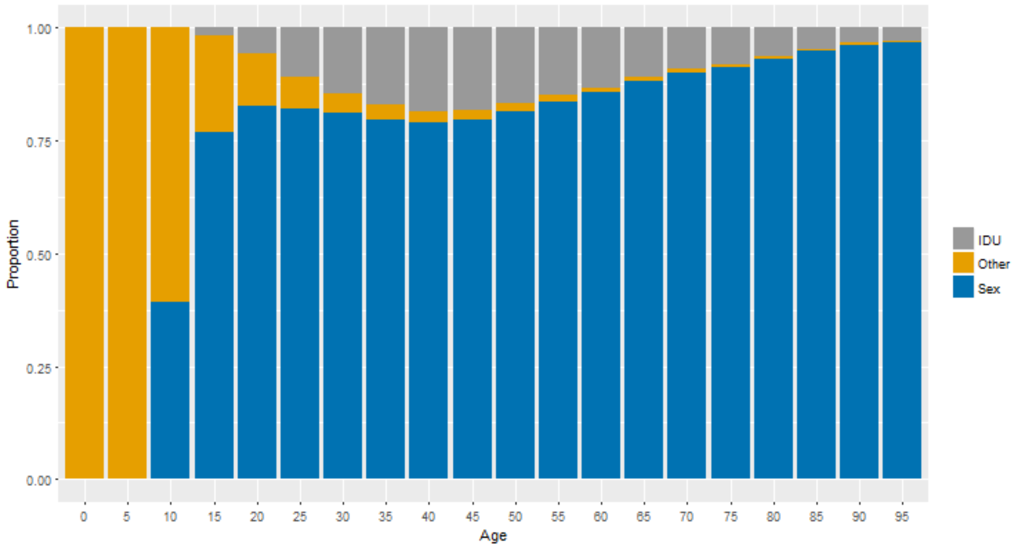

## Theoretical minimum-risk exposure level

The theoretical minimum level used for unsafe sex is the absence of disease transmission due to sexual contact.

## Population attributable fraction calculations

The outcomes associated with unsafe sex that we report on include HIV, cervical cancer, and all sexually transmitted diseases (STDs) except for those in neonates from vertical transmission, including HIV, opthalmia neonatorum and neonatal syphilis.

Based on evidence in the literature, we attribute 100% of cervical cancer to unsafe sex. These sources state that HPV infection is necessary for cervical cancer to develop and that HPV is only spread through sexual contact. The proportion of STDs attributable to unsafe sex is also 100%.

For HIV, the results from the single parameter proportion DisMod model for HIV transmission due to sex were used directly as the population attributable fraction.

# Low Physical Activity Capstone Appendix

## Flowchart

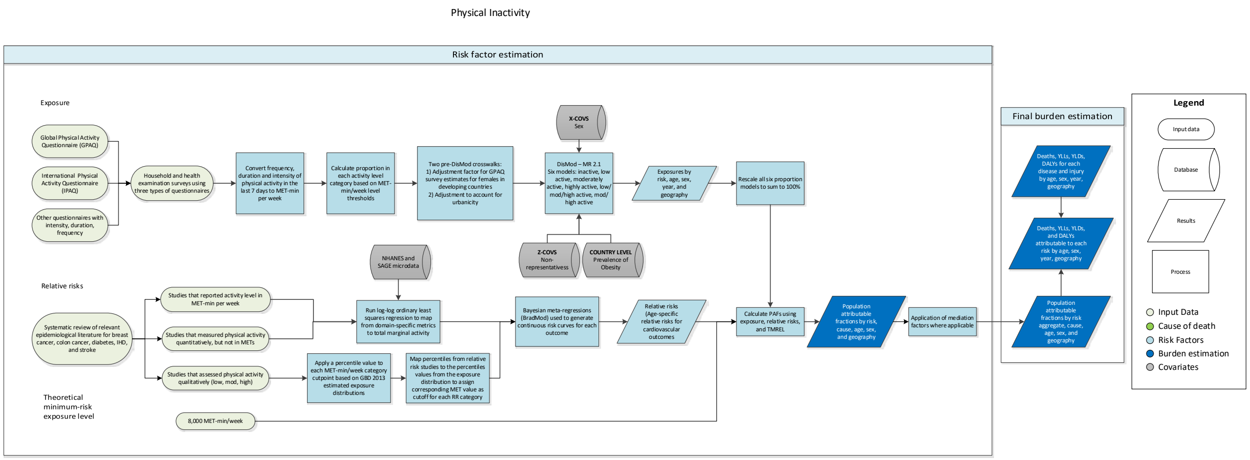


## Input data and methodological summary

## Exposure

### Case definition

We measure physical activity performed by adults greater than or equal to 25 years of age, for durations of at least ten minutes at a time, across all domains of life (leisure/recreation, work/household and transport). We use frequency, duration and intensity of activity to calculate total metabolic equivalent-minutes per week. MET (Metabolic Equivalent) is the ratio of the working metabolic rate to the resting metabolic rate. One MET is equivalent to 1 kcal/kg/hour and is equal to the energy cost of sitting quietly. A MET is also defined as the oxygen uptake in ml/kg/min with one MET equal to the oxygen cost of sitting quietly, around 3.5 ml/kl/min.

### Input data

We included surveys of the general adult population that captured self-reported physical activity in all domains of life (leisure/recreation, work/household and transport), where random sampling was used.

Data were primarily derived from two standardised questionnaires: The Global Physical Activity Questionnaire (GPAQ) and the International Physical Activity Questionnaire (IPAQ), although we included other survey instruments that asked about intensity, frequency and duration of physical activities performed across all activity domains.

Due to a lack of a consistent relationship on the individual level between activity performed in each domain and total activity, we were not able to use studies that included only recreational/leisure activities.

Physical activity level is categorised by total MET-minutes per week using four categories based on rounded values closest to the quartiles of the global distribution of total MET-minutes/week. The lower limit for the Level 1 category (600 MET-min/week) is the recommended minimum amount of physical activity to get any health benefit. We used four categories with higher thresholds rather than the GPAQ

and IPAQ recommended 3 categories to better capture any additional protective effects from higher activity levels.

- Level 0: < 600 MET-min/week (inactive)
- Level 1: 600-3999 MET-min/week (low-active)
- Level 2: 4000-7,999  MET-min/week (moderately-active)
- Level 3: ≥ 8,000 MET-min/week (highly active)

The GHDx was used to locate all surveys that use the GPAQ or IPAQ questionnaire. Although there were many other surveys that focused specifically on leisure activity, we were unable to use these sources because they did not comprise all three domains (work, transport and leisure). In addition, we excluded any surveys that did not report frequency, duration, and intensity of activity.

## Modelling strategy

### *DisMod modelling*

For this round of the GBD, we have chosen to separately model all of the GPAQ data separately from the IPAQ data. We then estimated the proportion of each country/year/age/sex subpopulation in each of the above four activity levels using 12 separate Dismod models (one for each data source). We use six categories of physical activity prevalence rather than four to accommodate the different MET-minute/week cutoffs presented in tabulated data sources where individual unit record data was not available. Since the accepted threshold/definition for inactivity is consistently <600 MET-minutes/week, the vast majority of tabulated data was broken down into proportion inactive (model A) and proportion low, moderate or highly active (model B).

| | Label | MET-min/week | Name of sequelae in online visualisation tool |
|---|---|---|---|
| A | inactive | <600 | Physical inactivity and low physical activity, inactive |
| B | low/moderately/highly active | ≥600 | Physical inactivity and low physical activity, low/moderately/highly active |
| C | low active | 600-3999 | Physical inactivity and low physical activity, low active |
| D | moderately/highly active | >4000 | Physical inactivity and low physical activity, moderately/highly active |
| E | moderately active | 4000-7999 | Physical inactivity and low physical activity, moderately active |
| F | highly active | ≥8,000 | Physical inactivity and low physical activity, highly active |

These models have mesh points at 0 15 25 35 45 55 65 75 85 100, and a study-level fixed effect on integrand variance (Z-cov) for whether a study was nationally representative or not, to account for the heterogeneity introduced boy studies that are not generalizable to the entire population.  They also have national level fixed effects on prevalence of obesity.

After DisMod, we rescale each of the 6 models specific to each data source so that the proportions sum to one. Since we have the most data for models A and B, we rescale the sum of the proportion in each category to be equal to one.  Next we rescale the sum of model C and D to be equal to the rescaled value from model B. Then we rescale the sum of models E and F to be equal to the rescaled value from model D.  After these three rescales we are left with a proportion for each of the four categories that all sum to

1. Scaled results for each data source are then hybridised to produce only one set of results for the prevalence of the four categories of physical activity.

Similar to the previous round, we have directly estimated total MET-minutes per week globally. Although, this year we made use of two specific machine learning algorithms (Random Forest & XGBoost) that were trained using data that could characterise the relationship between total MET-mins/week and each of the categorical prevalences of physical activity. This resulted in country-year-age-sex specific estimates of total physical activity in the form of MET-minutes per week.

Utilising microdata on total MET-mins per week from individual-level surveys, we characterised the distribution of activity level at the population level. We then used an ensemble approach to distribution fitting, borrowing characteristics from individual distributions to tailor a unique distribution to fit the data using a weighting scheme. We characterised the standard deviation of each population's activity through a linear regression that captured the relationship between standard deviation and mean activity levels in nationally representative IPAQ surveys:

$$ln\ (Standard\ deviation) = \beta_0 + \beta_1 \times ln\ (Mean_i)$$

We then applied the coefficients of this regression to the outputs of our estimate of total MET-minutes per week regression outputs to calculate the standard deviation by country, year, age, and sex.

## Theoretical minimum-risk exposure level

The theoretical minimum-risk exposure level for physical inactivity is 3000-4500 MET-min per week, which was calculated as the exposure at which minimal deaths across outcomes occurred.[3]

## Relative risks

We used a recently published dose-response meta-analysis of prospective cohort studies to estimate the effect size of the change in physical activity level on breast cancer, colon cancer, diabetes, ischemic heart disease and ischemic stroke.[3]

## References


1. IPAQ Research Committee. Guidelines for data processing and analysis of the International Physical Activity Questionnaire (IPAQ)–short and long forms. Retrieved September. 2005;17:2008.

2. World Health Organization. Global Physical Activity Questionnaire (GPAQ) Analysis Guide. 2011. Geneva, Switzerland: WHO Google Scholar. 2013

3. Kyu HH, Bachman VF, Alexander LT, Mumford JE, Afshin A, Estep K, Veerman JL, Delwiche K, Iannarone ML, Moyer ML, Cercy K. Physical activity and risk of breast cancer, colon cancer, diabetes, ischemic heart disease, and ischemic stroke events: systematic review and dose-response meta-analysis for the Global Burden of Disease Study 2013. bmj. 2016 Aug 9;354:i3857.

# High Fasting Plasma Glucose Capstone Appendix

## Flowchart

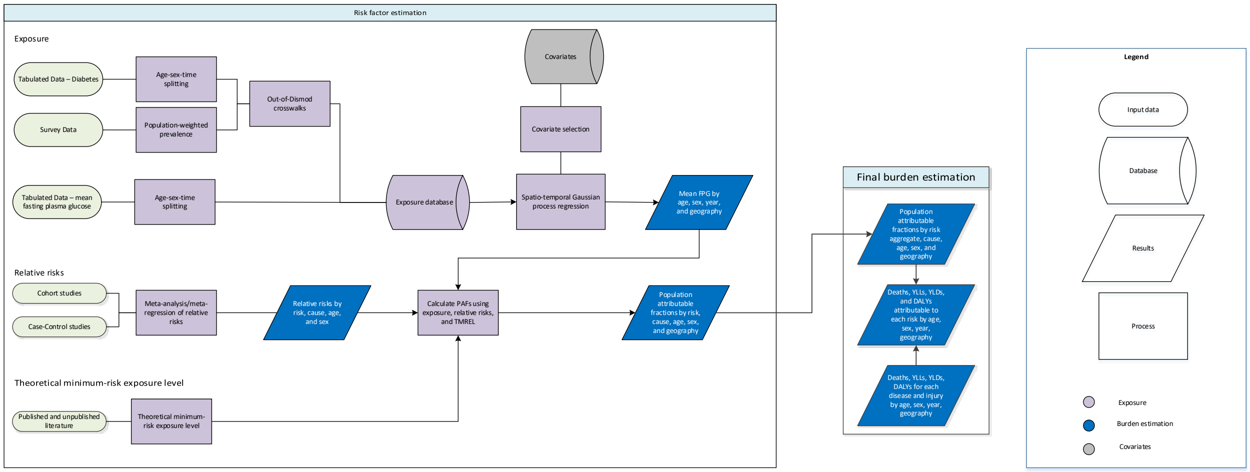


## Case definition

High fasting plasma glucose (FPG) is measured as the mean FPG in a population, where FPG is a continuous exposure in units of mmol/L.

## Data seeking

### *Exposure*

We did not conduct a systematic review for FPG in GBD 2017. However, we included data sources found in the systematic review for diabetes. Please see the diabetes mellitus appendix in *Global, regional, and national incidence, prevalence, and years lived with disability for 354 diseases and injuries for 195 countries, 1990–2017: a systematic analysis for the Global Burden of Disease Study 2017* for more information on data sources found through those efforts.

### *Data inputs*

Data inputs come from 3 sources:

- Estimates of mean FPG in a representative population
- Individual-level data of fasting plasma glucose measured from surveys
- Estimates of diabetes prevalence in a representative population

Data sources that did not report mean FPG or prevalence of diabetes are excluded from analysis. When a study reported both mean fasting plasma glucose (FPG) and prevalence of diabetes, we use the mean FPG for exposure estimates. Where possible, individual-level data supersede any data described in a study. Individual-level data are aggregated to produce estimates for each 5-year age group, sex, location, and year of a survey.

## Data processing

We perform several processing steps to the data in order to address sampling and measurement inconsistencies that will ensure the data are comparable.

1. *Small sample size*
   Estimates in a sex and age group with a sample size <30 persons is considered a small sample size. In order to avoid small sample size problems that may bias estimates, data are collapsed into the next age group in the same study till the sample size reach at least 30 persons. The intent of collapsing the data is to preserve as much granularity between age groups as possible. If the entire study sample consists of <30 persons and did not include a population-weight, the study is excluded from the modelling process.

2. *Time, Age, and Sex Splitting*
   For more details on how datapoints on mean FPG was processed, please see the diabetes mellitus appendix in *Global, regional, and national incidence, prevalence, and years lived with disability for 354 diseases and injuries for 195 countries, 1990–2017: a systematic analysis for the Global Burden of Disease Study 2017*.

3. *Crosswalks*
   We predicted mean FPG from diabetes prevalence using an ensemble distribution. We characterized the distribution of FPG using individual-level data. Details on the ensemble distribution can be found elsewhere in the Appendix. Before predicting mean FPG from prevalence of diabetes, we ensured that the prevalence of diabetes was based on the reference case definition: fasting plasma glucose (FPG) >126 mg/dL (7 mmol/L) or on treatment. For more details on how the case-definition crosswalk is conducted, please see the diabetes mellitus appendix in *Global, regional, and national incidence, prevalence, and years lived with disability for 354 diseases and injuries for 195 countries, 1990–2017: a systematic analysis for the Global Burden of Disease Study 2017*.

## Exposure modelling

Exposure estimates are produced for every year between 1980 to 2017 for each national and subnational location, sex, and for each 5-year age group starting from 25 years. As in GBD 2016, we used a Spatio-Temporal Gaussian Process Regression (ST-GPR) framework to model the mean fasting plasma glucose at the location-, year-, age-, and sex- level. Updates to the ST-GR modelling framework for GBD 2017 are detailed elsewhere in the Appendix.

Fasting plasma glucose is frequently tested or reported in surveys aiming at assessing the prevalence of diabetes mellitus. In these surveys, the case definition of diabetes may include both a glucose test and questions about treatment for diabetes. People with positive history of diabetes treatment may be excluded from the FPG test. Thus, the mean FPG in these surveys would not represent the mean FPG in the entire population. To address this limitation, using the data from the surveys reporting mean FPG in the entire population, we estimated a regression-based correction factor and adjusted the mean FPG to account for diabetics in the population. We also use an ensemble distribution to characterize the distribution of FPG in the population and estimate the standard deviation based on mean FPG and prevalence of diabetics from the non-fatal diabetes mellitus model.

To inform our estimates in data-sparse countries, we systematically tested a range of covariates and selected two covariates based on AIC and adjusted $R^2$. These included prevalence of obesity and lag-distributed income per capita (LDI).

Mean FPG iss estimated using a mixed-effects linear regression, run separately by sex:

$$\mathrm{logit}(\mathrm{FPG}_{c,a,t}) = \beta_0 + \beta_1 \log(\mathrm{LDI})_{c,t} + \beta_2 p_{\mathrm{overweight}_{c,a,t}} + \sum_{k=2}^{16} \beta_k I_{A[a]} + \alpha_s + \alpha_r + \alpha_c + \epsilon_{c,a,t}$$

where $\log(\mathrm{LDI})_{c,t}$ is the log of the lag-distributed income, $p_{\mathrm{overweight}_{c,a,t}}$ is the prevalence of overweight, $I_{A[a]}$ is an indicator variable for a fixed effect on a given 5-year age group, and $\alpha_s$ $\alpha_r$ $\alpha_c$ are random effects at the super-region, region, and country level, respectively. The estimates were then propagated through the ST-GPR framework to obtain 1000 draws for each location, year, age, and sex.

## Theoretical minimum-risk exposure level

The theoretical minimum-risk exposure level (TMREL) for FPG is 4.5-5.4 mmol/L. This was calculated by taking the person-year weighted average of the levels of FPG that were associated with the lowest risk of mortality in the pooled analyses of prospective cohort studies.[1]

## Relative risks

We estimate 15 outcomes due to high fasting plasma glucose (continuous risk) or diabetes (categorical risk).

| **Risk** | **Outcome** |
|---|---|
| Fasting plasma glucose | Ischemic heart disease |
| Fasting plasma glucose | Ischemic stroke |
| Fasting plasma glucose | Subarachnoid hemorrhage |
| Fasting plasma glucose | Intracerebral hemorrhage |
| Fasting plasma glucose | Peripheral vascular disease |
| Fasting plasma glucose | Type 1 diabetes |
| Fasting plasma glucose | Type 2 diabetes |
| Diabetes mellitus | Drug-resistant tuberculosis |
| Diabetes mellitus | Drug-susceptible tuberculosis |
| Diabetes mellitus | Multidrug-resistant tuberculosis without extensive drug resistance |
| Diabetes mellitus | Extensively drug-resistant tuberculosis |
| Diabetes mellitus | Liver cancer due to NASH |
| Diabetes mellitus | Liver cancer due to other causes |
| Diabetes mellitus | Pancreatic cancer |
| Diabetes mellitus | Ovarian cancer |
| Diabetes mellitus | Colorectal cancer |

| | |
|---|---|
| Diabetes mellitus | Bladder cancer |
| Diabetes mellitus | Lung cancer |
| Diabetes mellitus | Breast cancer |
| Diabetes mellitus | Glaucoma |
| Diabetes mellitus | Cataracts |
| Diabetes mellitus | Dementia |

Relative risks for High Fasting Plasma Glucose (continuous risk)

In GBD 2017, diabetes was further split into diabetes type 1 and diabetes type 2, and hemorrhagic stroke was further split into subarachnoid hemorrhage and intracerebral hemorrhage.

Relative risks (RR) were obtained from dose-response meta-analysis of prospective cohort studies. Please see the citation list for a full list of studies that are utilized. For cardiovascular outcomes, we estimated age-specific RRs using DisMod-MR 2.1 with log (RR) as the dependent variable and median age at event as the independent variable with an intercept at age 110. Morbidity and mortality directly caused by diabetes type 1 and diabetes type 2 is considered directly attributable to FPG.

Relative risks for Diabetes mellitus (Categorical risk)

Relative risks were obtained from meta-analysis of cohort studies. Please see the citation list for a full list of studies that are utilized.

## References

1 . Singh GM, Danaei G, Farzadfar F, *et al.* The age-specific quantitative effects of metabolic risk factors on cardiovascular diseases and diabetes: a pooled analysis. *PloS One* 2013; **8**: e65174.

# High LDL Cholesterol Capstone Appendix

## Flowchart

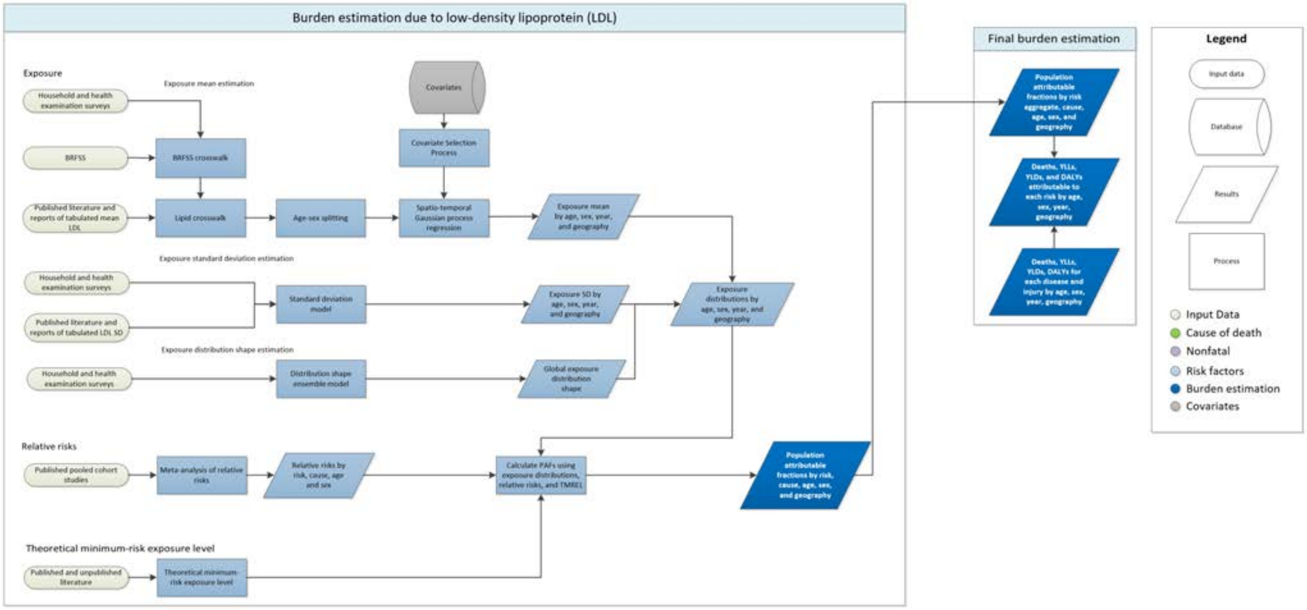


## Input Data & Methodological Summary

## Exposure

### Case Definition

In the GBD 2016 study, we estimated burden attributable to total cholesterol. For GBD 2017, we modelled blood concentration of low-density lipoprotein (LDL) in units of mmol/L.

### Input Data

We utilized data on blood low-density lipoprotein from literature and from household survey microdata and reports. Please see the appendix for a full list of included sources. For the GBD 2016 study, we carried out an updated systematic review for total cholesterol (TC), drawing from the GHDx and Medline via PubMed. For GBD 2017, we reviewed all data sources in our database that reported data on total cholesterol and re-extracted LDL data when it was available. Data on high-density lipoprotein (HDL) and triglycerides (TGL) were also extracted when available.

*Inclusion Criteria*

Studies were included if they were population-based and measured total low-density lipoprotein using a blood test or calculated using the Friedewald equation. We assumed the data is representative of the location if the geography was not related to the diseases and if it is not an outlier compared to other data in the country or region.

*Outliers*

Data was utilized in the modeling process unless an assessment of data strongly suggested that the data

was biased. A candidate source was excluded if the quality of study did not warrant a valid estimate because of selection (non-representative populations) or if the study did not provide methodological details for evaluation. In a small number of cases, data point was considered to be an outlier candidate if the level was implausibly low or high based on expert judgement and other country data.

*Data Extraction*

Where possible, individual level data on LDL estimates were extracted from survey microdata and these were collapsed across demographic groupings to produce mean estimates in the standard GBD 5-year age-sex groups. If microdata were unavailable, information from survey reports or from literature were extracted along with any available measure of uncertainty including standard error, uncertainty intervals, and sample size. Standard deviations were also extracted. Where LDL was reported split out by groups other than age, sex, location, and year (eg, by diabetes status), a weighted mean was calculated.

*Lipid crosswalk*

Total cholesterol consists of three major components: LDL, HDL, and triglycerides. LDL is often calculated for an individual using the Friedewald equation, shown below:

$$LDL = TC - \left(HDL + \frac{TGL}{2.2}\right)$$

We utilized this relationship at the individual level to impute the mean LDL for a study population when only data on total cholesterol, HDL, and TGL were available. Because studies report different combinations of TC, HDL, and TGL, we constructed a single regression to utilize al available data to evaluate the relationship between each lipid and LDL at the population level. We used the following regression:

$$LDL = ind_{tc}\beta_1 TC - \left(ind_{hdl}\beta_2 HDL + ind_{tgl}\beta_3 TGL\right) + \sum \alpha_l I_l$$

Where $ind_{tc}$, $ind_{hdl}$, and $ind_{tgl}$ are indicator variables for whether data is available for a given lipid, $I_l$ is an indicator variable a given set of available lipids $l$. $\alpha_l$ is a unique intercept for each set of available lipids. For example, for sources that only reported TC and HDL, $\alpha_{l=TC,HDL}$ should account for the missing lipid data, ie, TGL. The form of this regression allows us to estimate the betas for each lipid using all available data. As a sensitivity analysis, we also ran separate regressions for each set of available lipids and found that the single regression method had much lower root-mean squared error. A comparison of the observed vs predicted LDL for each set of available lipids is shown in Figure 1. We found almost no relationship between LDL and HDL or TGL when TC was not available, so only studies that reported TC were crosswalked to LDL.

*Figure 1. Results of the lipid crosswalk using a single regression method*

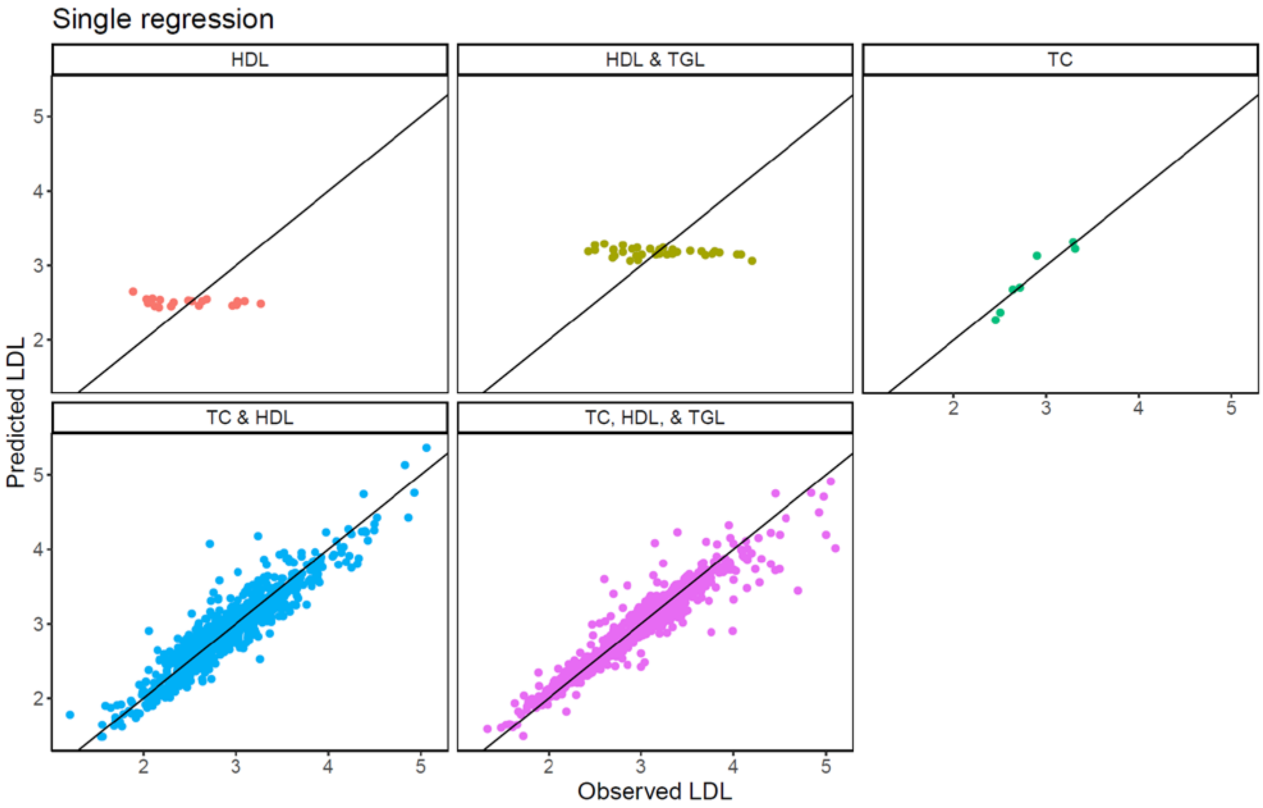


*Incorporating United States prevalence data*

Survey reports and literature often report information only about the prevalence, but not the level, of hypercholesterolemia in the population studied. These sources were not used to model LDL, with the exception of data from the Behavioral Risk Factors Surveillance System (BRFSS) because of the availability of a similarly structured exam survey covering the identical population (NHANES). BRFSS is a telephone survey conducted in the United States for all counties. It collects self-reported diagnosis of hypercholesterolemia. These self-reported values of prevalence of raised total cholesterol in each age group, sex, US state, and year were used to predict a mean total cholesterol for the same strata with a regression using data from the National Health and Nutrition Examination Survey, a nationally representative health examination survey of the US adult population. The regression was:

$$TC_{l,a,t,s} = \beta_0 + \beta_1 prev_{l,a,t,s}$$

where $TC_{l,a,t,s}$ is the location, age, time, and sex specific mean total cholesterol and $prev_{l,a,t,s}$ is the location, age, time, and sex specific prevalence of raised total cholesterol. The coefficients for both models are reported in Table 1.

*Table 2. Coefficients in the sex-specific US states TC prediction models*

| Term | Male model | Female model |
|---|---|---|
| Intercept | 4.23 | 4.36 |
| Prevalence | 6.25 | 5.22 |

Out of sample RMSE was used to quantify the predictive validity of the model. The regression was repeated 10 times for each sex, each time randomly holding out 20% of the data. The RMSEs from each holdout analysis were averaged to get the average out of sample RMSE. The results of this holdout analysis are reported in Table 2. Total cholesterol estimates were crosswalked to LDL using the lipid crosswalk reported above.

*Table 3. Out of sample RMSEs of the sex-specific US states TC prediction models*

| | Male model | Female model |
|---|---|---|
| Out of sample RMSE | 0.21 mmol/L | 0.20 mmol/L |

*Age and Sex Splitting*

Prior to modeling, data provided in age groups wider than the GBD 5-year age groups were processed using the approach outlined in Ng et al.[2] Briefly, age-sex patterns were identified using person-level microdata (58 sources), and estimate age-sex specific levels of total cholesterol from aggregated results reported in published literature or survey reports. In order to incorporate uncertainty into this process and borrow strength across age groups when constructing the age-sex pattern, we used a model with auto-regression on the change in mean LDL over age groups:

$$\mu_a = \mu_{a-1} + \omega_a$$
$$\omega_a \sim N(\omega_{a-1}, \tau)$$

Where $\mu_a$ is the mean predicted value for age group $a$, $\mu_{a-1}$ is the mean predicted value for the age group previous to age group $a$, $\omega_a$ is the difference in mean between age group *a* and age group *a-1*, $\omega_{a-1}$ is the difference between age group *a-1* and age group *a-2*, and $\tau$ is a user-input prior on how quickly the mean LDL changes for each unit increase in age. We used a $\tau$ of 0.09 mmol/L for this model. Draws of the age-sex pattern were combined with draws of the input data needing to be split in order to calculate the new variance of age-sex split data points.

## Modeling

Exposure estimates were produced from 1980 to 2017 for each national and subnational location, sex, and for each 5-year age group starting from 25+. As in GBD 2016, we used a Spatio-Temporal Gaussian Process Regression (ST-GPR) framework to model the mean LDL at the location-, year-, age-, sex- level. Updates to the ST-GR modeling framework for GBD 2017 are detailed in the appendix.

*Covariate selection*

The first step of the ST-GPR framework requires the creation of a linear model for predicting LDL the location-, year-, age-, sex- level. Covariates for this model were selected in two stages. First a list of

variables with an expected causal relationship with LDL was created based on significant association found within high-quality prospective cohort studies reported in the published scientific literature. The second stage in covariate selection was to test the predictive validity of every possible combination of covariates in the linear model, given the covariates selected above. This was done separately for each sex. Predictive validity was measured with out of sample root-mean-squared error.
In GBD 2016, the linear model with the lowest root-mean squared error for each sex was then used in the ST-GPR model. In GBD 2017, we used an ensemble model of the 50 models with the lowest root-mean squared error for each sex. This allows us to utilize covariate information from many plausible linear mixed-effects models. The 50 models were each used to predict the mean LDL for every age, sex, location, and year, and the inverse-RMSE-weighted average of this set of 50 predictions was used as the linear prior. The relative weight in 'draws' contributed by each covariate is plotted by sex in Figure 2.

*Figure 2. Results of the ensemble linear model covariate selection*

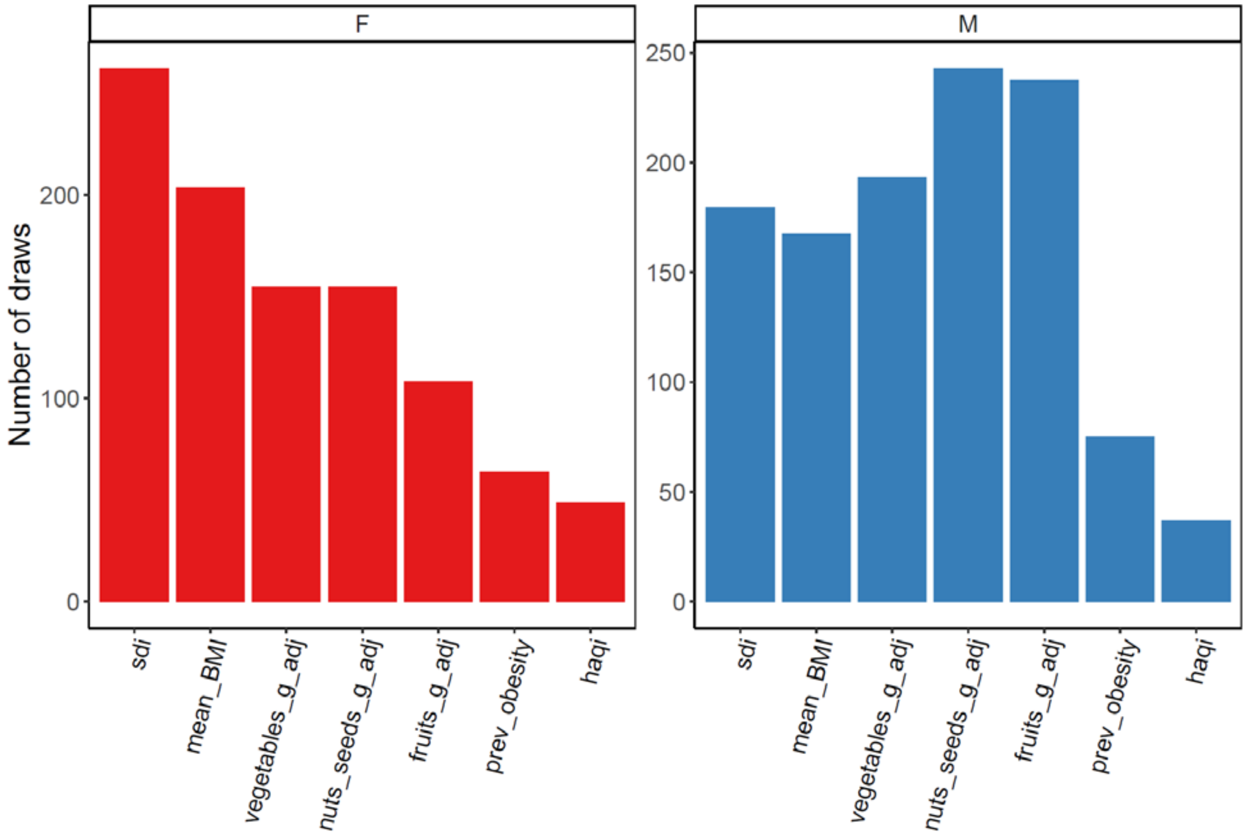


Methodological updates to ST-GPR are reported above. The result of the ST-GPR model are estimates of the mean LDL for each age, sex, location, and year.

*Estimate of standard deviation*

The standard deviation of LDL within a population was estimated for each national and subnational location, sex, and 5-year age group starting from age 25 using the standard deviation from person-level and some tabulated data sources.  Person-level microdata accounted for 3009 of the total 4001 rows of data on standard deviation.  The remaining 992 rows came from tabulated data.  Tabulated data was only used to model standard deviation if it was sex and 5-year age group specific and reported a population standard deviation LDL.  The LDL standard deviation function was estimated using a linear regression:

$$\log(SD_{c,a,t,s}) = \beta_0 + \beta_1 \log(mean\_LDL_{c,a,t,s}) + \beta_4 sex + \sum_{k=2}^{16} \beta_k I_{A[a]}$$

where $mean\_LDL_{c,a,t,s}$ is the country, age, time, and sex specific mean LDL estimate from ST-GPR, and $I_{A[a]}$ is a dummy variable for a fixed effect on a given 5-year age group,.

*Distribution shape modelling*

The shape of the distribution of LDL was estimated using all available person-level microdata sources, which was a subset of the input data into the modelling process.  The distribution shape modelling framework for GBD 2017 is detailed in the appendix.  Briefly, an ensemble distribution created from a weighted average of distribution families was fit for each individual microdata source, separately by sex. The weights for the distribution families for each individual source were then averaged and weighted to create a global ensemble distribution for each sex.

## Theoretical minimum-risk exposure level

A Meta-analysis of randomized trials has shown that outcomes can be improved even at low levels of LDL-cholesterol, below 1.3 mmol/l.[3] Recent studies of PCSK-9 inhibitors support these results.[4] We therefore used a TMREL with a uniform distribution between 0.7-1.3 mmol/l.

## Relative risks

After a systematic search, we were unable to find relative risks for LDL that were reported by age and level of LDL. Given this evidence that the relative risks for LDL and TC are very similar[5] and the strong linear correlation between TC and LDL at the individual level, we used relative risks reported for TC to approximate the relative risks for LDL. We used Dismod-MR 2.1 to pool effect sizes from included studies and generate a dose-response curve for each of the outcomes associated with LDL. The tool enabled us to incorporate random effects across studies and include data with different age ranges. RRs were used universally for all countries and produce RRs with uncertainty and covariance across ages, taking into account the uncertainty of the data points. As in GBD 2016, RRs for IHD and ischemic stroke are obtained from meta-regressions of pooled epidemiological studies: the Asia Pacific Cohort Studies Collaboration (APCSC) and the Prospective Studies Collaboration (PSC).[6]  RRs for IHD were modeled with log (RR) as the dependent variable and median age at event as the independent variable with an age intercept (RR equals 1) at age 110. For LDL and ischemic stroke, a similar approach was used, except that there was no age intercept at age 110, due to the fact that there was no statistically significant

relationship between LDL and stroke after age 70 with a mean RR less than one. We assumed that there is not a protective effect of LDL and therefore did not include an RR for ages 80+.

## References


1. Roth GA, Fihn SD, Mokdad AH, Aekplakorn W, Hasegawa T, Lim SS. High total serum cholesterol, medication coverage and therapeutic control: an analysis of national health examination survey data from eight countries. *Bull World Health Organ* 2011; **89**: 92–101.

2. Ng M, Fleming T, Robinson M, *et al.* Global, regional, and national prevalence of overweight and obesity in children and adults during 1980–2013: a systematic analysis for the Global Burden of Disease Study 2013. *The Lancet* 2014; **384**: 766–81.

3. Boekholdt SM, Hovingh GK, Mora S, *et al.* Very Low Levels of Atherogenic Lipoproteins and the Risk for Cardiovascular EventsA Meta-Analysis of Statin Trials. *J Am Coll Cardiol* 2014; **64**: 485–94.

4. Sabatine MS, Giugliano RP, Keech AC, et al. Evolocumab and Clinical Outcomes in Patients with Cardiovascular Disease. *N Engl J Med*. 2017; **376**:1713-1722.

5. Wilson PF, D'Agostino RB, Levy D, Belanger AM, Silbershatz H, Kannel WB. Prediction of Coronary Heart Disease Using Risk Factor Categories. *Circulation*. 1998; **97**:1837-1847.

6. Singh GM, Danaei G, Farzadfar F, *et al.* The age-specific quantitative effects of metabolic risk factors on cardiovascular diseases and diabetes: a pooled analysis. *PloS One* 2013; **8**: e65174.

# High Systolic Blood Pressure Capstone Appendix

## Flowchart

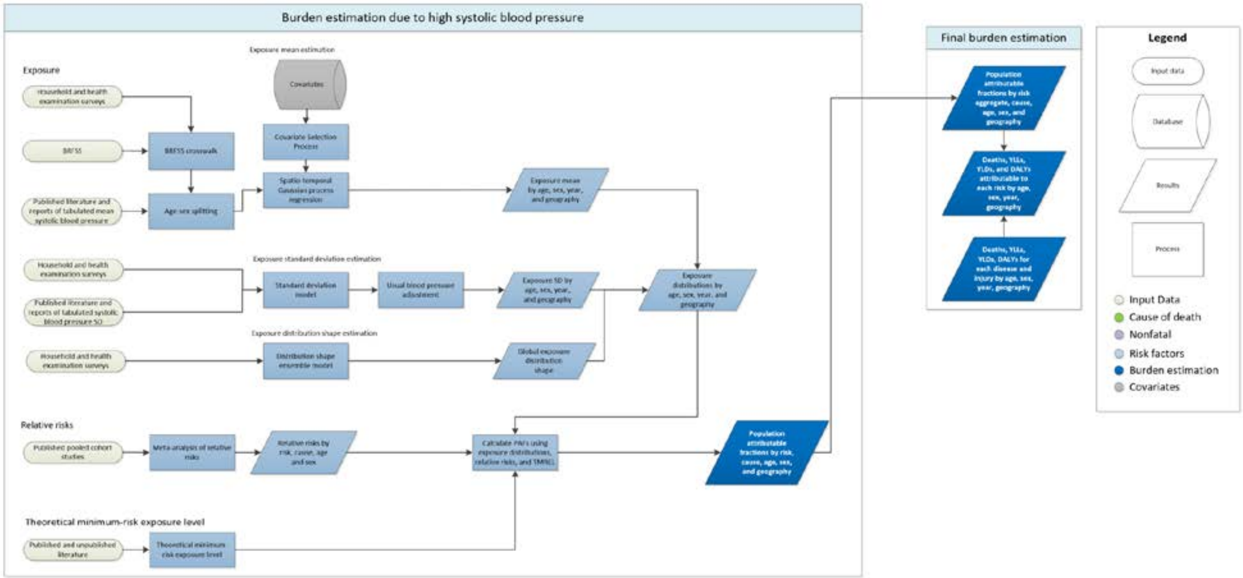


## Input Data & Methodological Summary

## Exposure

### Case Definition

Brachial systolic blood pressure in mmHg.

### Input Data

We utilised data on mean systolic blood pressure from literature and from household survey microdata and reports (e.g. STEPS, NHANES). Please see the appendix for a full list of included sources. For GBD 2017, we did not carry out a systematic review of the literature for new data. In total, we have utilised 934 sources corresponding to 49,690 unique data points.

#### *Inclusion Criteria*

Studies were included if they were population-based and measured systolic blood pressure using a blood test.  We assumed the data is representative of the location if the geography was not selected because it was related to the diseases.

#### *Outliers*

Data was utilised in the modelling process unless an assessment of data strongly suggested that the data was biased. A candidate source was excluded if the quality of study did not warrant a valid estimate because of selection (non-representative populations) or if the study did not provide methodological details for evaluation. In a small number of cases, a data point was considered to be an outlier candidate if the level was implausibly low or high based on expert judgement and data from other country data.

*Data Extraction*
Where possible, individual level data on blood pressure estimates were extracted from survey microdata and these were collapsed across individuals and collapsed across demographic groupings to produce mean estimates in the standard GBD 5-year age-sex groups. If microdata were unavailable, information from survey reports or from literature were extracted along with any available measure of uncertainty including standard error, uncertainty intervals, and sample size. Standard deviations were also extracted. Where mean systolic blood pressure was reported split out by groups other than age, sex, location, and year (eg, by hypertensive status), a weighted mean was calculated.

*Incorporating United States prevalence data*
Survey reports and literature often report information only about the prevalence, but not the level, of hypertension in the population studied. These sources were not used to model systolic blood pressure, with the exception of data from the Behavioral Risk Factors Surveillance System (BRFSS) because of the availability of a similarly structured exam survey that is representative of the same population (NHANES). BRFSS is a telephone survey conducted in the United States for all US counties. It collects self-reported diagnosis of hypertension. These self-reported values of prevalence of raised blood pressure were adjusted for self-report bias and tabulated by age group, sex, US state, and year. These prevalences were used to predict a mean systolic blood pressure for the same strata with a regression using data from the National Health and Nutrition Examination Survey, a nationally representative health examination survey of the US adult population. The regression was run separately by sex, and was specified as:

$$\mathrm{SBP}_{\mathrm{l,a,t,s}} = \beta_0 + \beta_1 \mathrm{prev}_{\mathrm{l,a,t,s}}$$

where $\mathrm{SBP}_{\mathrm{l,a,t,s}}$ is the location, age, time, and sex specific mean systolic blood pressure and $\mathrm{prev}_{\mathrm{l,a,t,s}}$ is the location, age, time, and sex specific prevalence of raised blood pressure. The coefficients for both models are reported in Table 1.

*Table 1. Coefficients in the sex-specific US states blood pressure prediction models*

| Term | Male model | Female model |
|---|---|---|
| Intercept ($\beta_0$) | 114.65 | 108.28 |
| Prevalence ($\beta_1$) | 51.86 | 68.87 |

Out of sample RMSE was used to quantify the predictive validity of the model. The regression was repeated 10 times for each sex, each time randomly holding out 20% of the data. The RMSEs from each holdout analysis were averaged to get the average out of sample RMSE. The results of this holdout analysis are reported in Table 2.

*Table 2. Out of sample RMSEs of the sex-specific US states blood pressure prediction models*

| | Male model | Female model |
|---|---|---|
| Out of sample RMSE | 2.37 mmHg | 3.27 mmHg |

*Age and Sex Splitting*

Prior to modelling, data provided in age groups wider than the GBD 5-year age groups were processed using the approach outlined in Ng et al.[2] Briefly, an age-sex pattern were identified using 115 sources of microdata with multiple age-sex groups, and these patterns were applied to estimate age-sex specific levels of mean systolic blood pressure from aggregated results reported in published literature or survey reports. In order to incorporate uncertainty into this process and borrow strength across age groups when constructing the age-sex pattern, we used a model with auto-regression on the change in mean SBP over age groups:

$$\mu_a = \mu_{a-1} + \omega_a$$
$$\omega_a \sim N(\omega_{a-1}, \tau)$$

Where $\mu_a$ is the mean predicted value for age group *a*, $\mu_{a-1}$ is the mean predicted value for the age group previous to age group *a*, $\omega_a$ is the difference in mean between age group *a* and age group *a-1*, $\omega_{a-1}$ is the difference between age group *a-1* and age group *a-2*, and $\tau$ is a user-input prior on how quickly the mean SBP changes for each unit increase in age. We used a $\tau$ of 1.5 mmHg for this model. Draws of the age-sex pattern were combined with draws of the input data needing to be split in order to calculate the new variance of age-sex split data points.

## Modelling

Exposure estimates were produced from 1980 to 2016 for each national and subnational location, sex, and for each 5-year age group starting from 25+. As in GBD 2016, we used a Spatio-Temporal Gaussian Process Regression (ST-GPR) framework to model the mean systolic blood pressure at the location-, year-, age-, sex- level.

*Covariate selection*

The first step of the ST-GPR framework requires the creation of a linear model for predicting SBP at the location-, year-, age-, sex- level. Covariates for this model were selected in two stages. First a list of variables with an expected causal relationship with SBP was created based on significant association found within high-quality prospective cohort studies reported in the published scientific literature. The second stage in covariate selection was to test the predictive validity of every possible combination of covariates in the linear model, given the covariates selected above. This was done separately for each sex. Predictive validity was measured with out of sample root-mean-squared error.

In GBD 2016, the linear model with the lowest root-mean squared error for each sex was then used in the ST-GPR model. In GBD 2017, we used an ensemble model of the 50 models with the lowest root-mean squared error for each sex. This allows us to utilise covariate information from many plausible linear mixed-effects models. The 50 models were each used to predict the mean SBP for every age, sex, location, and year, and the inverse-RMSE-weighted average of this set of 50 predictions was used as the linear prior. The relative weight in ‘draws’ contributed by each covariate is plotted by sex in Figure 2.

*Figure 2. Results of the ensemble linear model covariate selection*

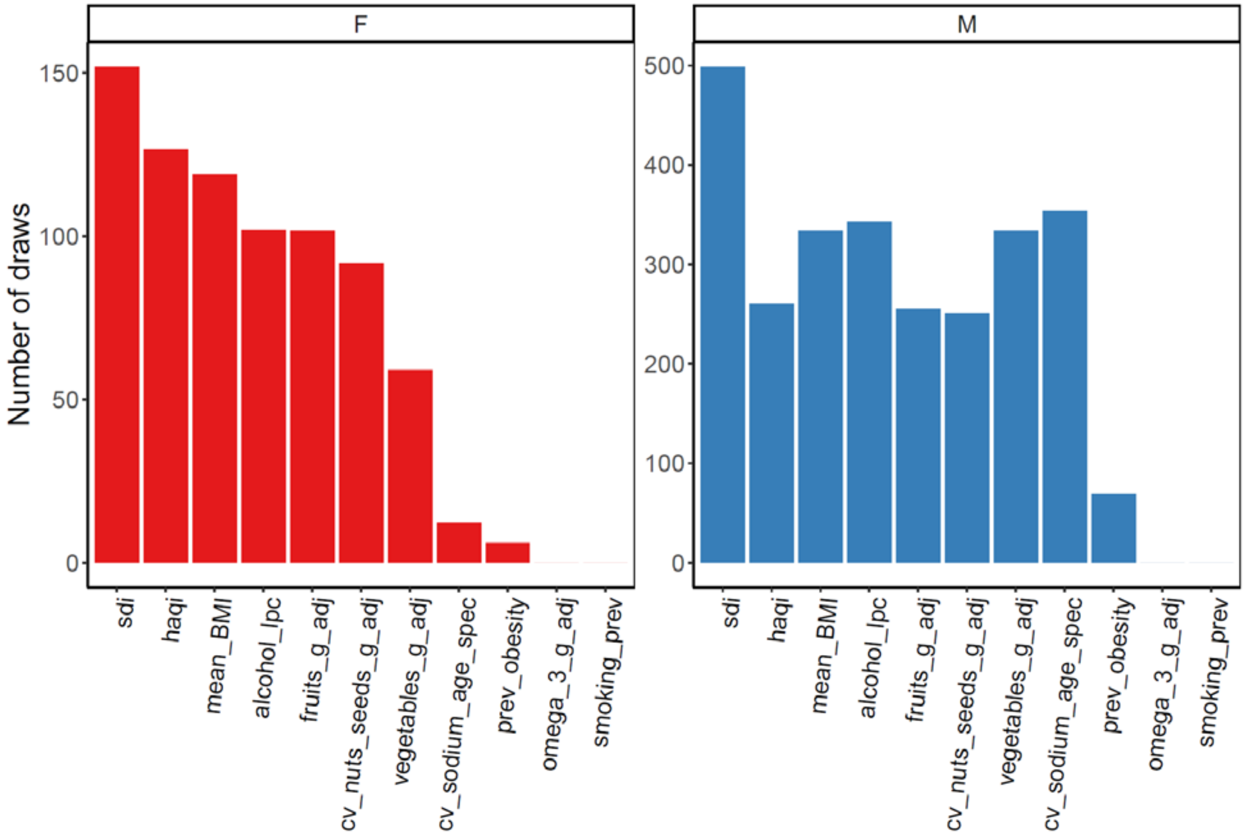


The results of the ensemble linear model were used for the first stage in an ST-GPR model. Methodological updates to ST-GPR are reported above. The result of the ST-GPR model are estimates of the mean SBP for each age, sex, location, and year.

*Estimate of Standard Deviation*

Currently, the ST-GPR model only produces an estimate of mean exposure level without standard deviation. Therefore, the standard deviation of systolic blood pressure within a population was estimated for each national and subnational location, sex, and 5-year age group starting from age 25 using the standard deviation from person-level and some tabulated data sources. Person-level microdata accounted for 10375 of the total 12570 rows of data on standard deviation. The remaining 2195 rows came from tabulated data. Tabulated data was only used to model standard deviation if it was sex and 5-year age group specific and reported a population standard deviation of systolic blood pressure. The systolic blood pressure standard deviation function was estimated using a linear regression:

$$\log\left(\mathrm{SD}_{l,a,t,s}\right) = \beta_0 + \beta_1 \log(\mathrm{mean\_SBP}_{l,a,t,s}) + \beta_4 \mathrm{sex} + \sum_{k=2}^{16} \beta_k I_A$$

where $\mathrm{mean\_SBP}_{l,a,t,s}$ is the location, age, time, and sex specific mean SBP estimate from ST-GPR, and $I_A$ is a dummy variable for a fixed effect on a given 5-year age group.

*Adjustment for Usual Levels of Blood Pressure*

To account for in-person variation in systolic blood pressure, a 'usual blood pressure' adjustment was done. The need for this adjustment has been described elsewhere.5 Briefly, measurements of a risk factor taken at a single time point may not accurately capture an individual's true long-term exposure to that risk. Blood pressure readings are highly variable over time due to measurement error as well as diurnal, seasonal, or biological variation. These sources of variation result in an over-estimation of the variation in cross-sectional studies of the distribution of SBP.
To adjust for this overestimation, we applied a correction factor to each location-, age-, time-, and sex-specific standard deviation. These correction factors were age-specific, and represented the proportion of the variation in blood pressure within a population that would be observed if there were no within-person variation across time. Four longitudinal surveys were used to estimate these factors: the China Health and Retirement Longitudinal Survey (CHRLS), the Indonesia Family Life Survey (IFLS), the National Health and Nutrition Examination Survey I Epidemiological Follow-up Study (NHANES I/EFS), and the South Africa National Income Dynamics Survey (NIDS). The sample size and number of blood pressure measurements at each measurement period for each survey is reported in Table 5.

*Table 3. Characteristics of longitudinal surveys used for the usual blood pressure adjustment*

| Source | Measurement periods | Number of measurements | Sample size |
|---|---|---|---|
| CHRLS | 2008 | 3 | 1967 |
| | 2012 | 3 | 1419 |
| IFLS | 1997 | 1 | 19418 |
| | 2000 | 1 | 16626 |
| | 2007 | 3 | 14136 |
| NIDS | 1997 | 2 | 14084 |
| | 2000 | 2 | 9612 |
| | 2007 | 2 | 9098 |
| NHANES I/EFS | 1971-1976 | 2 | 20716 |
| | 1982-1984 | 3 | 9932 |

For each survey, the following regression was created for each age group:

$$\text{SBP}_{i,a} = \beta_0 + \beta_1\text{sex}+\beta_3\text{age} + +\upsilon_i$$

where $\text{SBP}_{i,a}$ is the systolic blood pressure of an individual i at age a, sex is a dummy variable for the sex of an individual, age is a continuous variable for the age of an individual, and $\upsilon_i$ is a random intercept for each individual. Then, a blood pressure value $\widehat{\text{SBP}}_{i,b}$ was predicted for each individual i for his/her age at baseline b. The correction factor cf for each age group within each survey was calculated as variation in these predicted blood pressures was divided by the variation in the observed blood pressures at baseline, $\text{SBP}_{i,b}$:

$$\text{cf} = \sqrt{\frac{\text{var}(\widehat{\text{SBP}}_b)}{\text{var}(\text{SBP}_b)}}$$

The average of the correction factors was taken over the three surveys to get one set of age-specific correction factors, which were then multiplied by the square of the modelled standard deviations to estimate standard deviation of the 'usual blood pressure' of each age, sex, location, and year. Because of low sample sizes, the correction factors for the 75-79 age group was used for all terminal age groups. The final correction factors for each age group are reported in Table 6. Figure 1 shows the correction factors by survey and age group ID.

*Table 4. Age-specific usual blood pressure correction factors*

| Age group | Correction factor |
|---|---|
| 25-29 | .665 |
| 30-34 | .713 |
| 35-39 | .737 |
| 40-44 | .733 |
| 45-49 | .798 |
| 50-54 | .771 |
| 55-59 | .764 |
| 60-64 | .753 |
| 65-69 | .719 |
| 70-74 | .689 |
| 75+ | .678 |

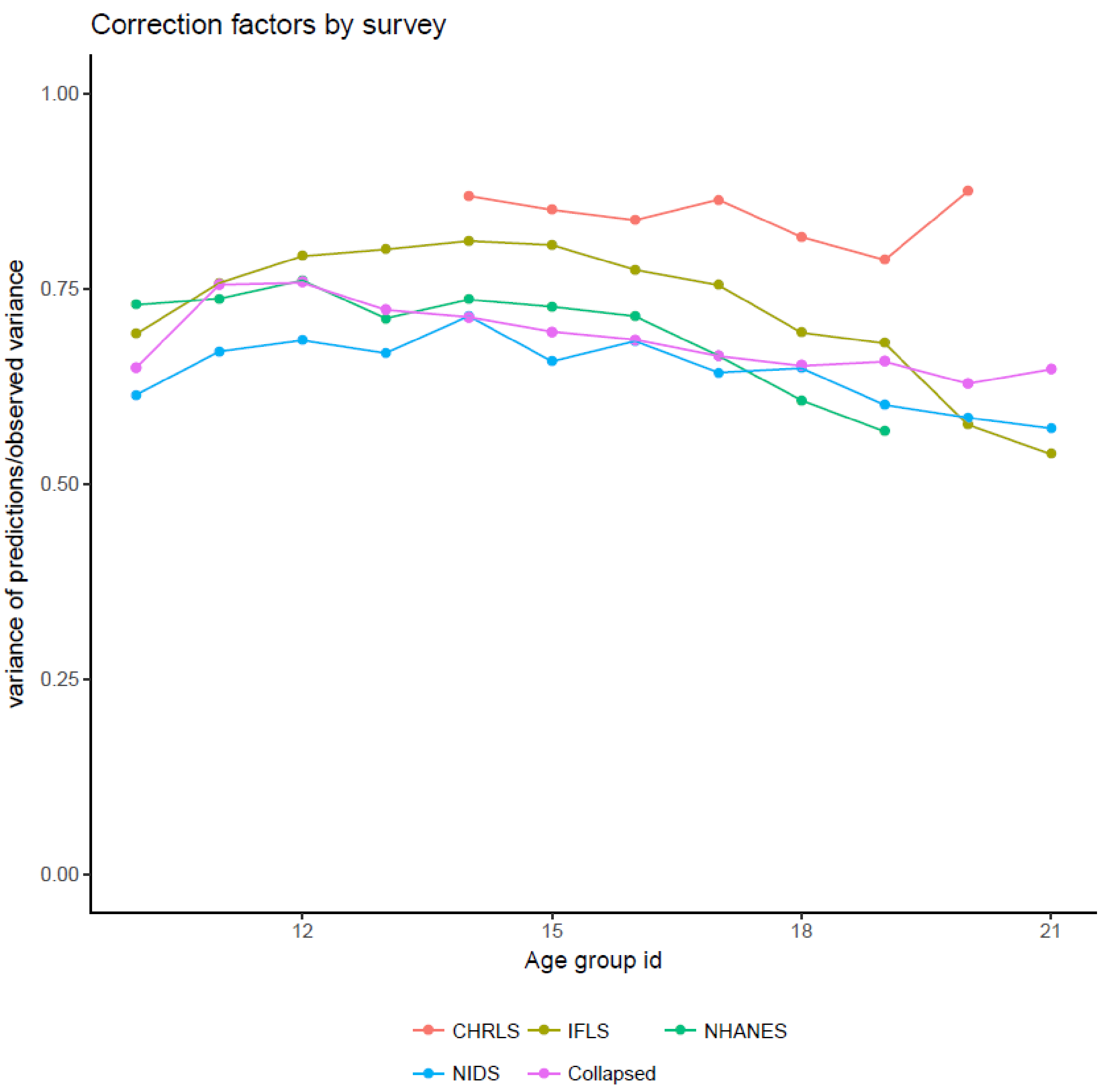


*Figure 1: Correction factor by survey and age group id. The correction factor is equal to the variance of the predictions divided by the variance of the raw dataset. In pink is the average correction factor for each age group, summarised in Table 6.*

A visualisation of how the uncorrected blood pressure measurements overestimate the 'usual' blood pressure variation is shown in Figure 1. This image shows the density of the distribution of the observed blood pressure values $\mathrm{SBP}_{i,b}$ in participants in the Indonesian Family Life Study survey in red, and the density of the predicted blood pressure values $\widehat{\mathrm{SBP}}_{i,b}$ in blue. The ratio of the variance of the blue distribution to the variance of the red distribution is an example of the scalar adjustment factor being applied to the modelled standard deviations.

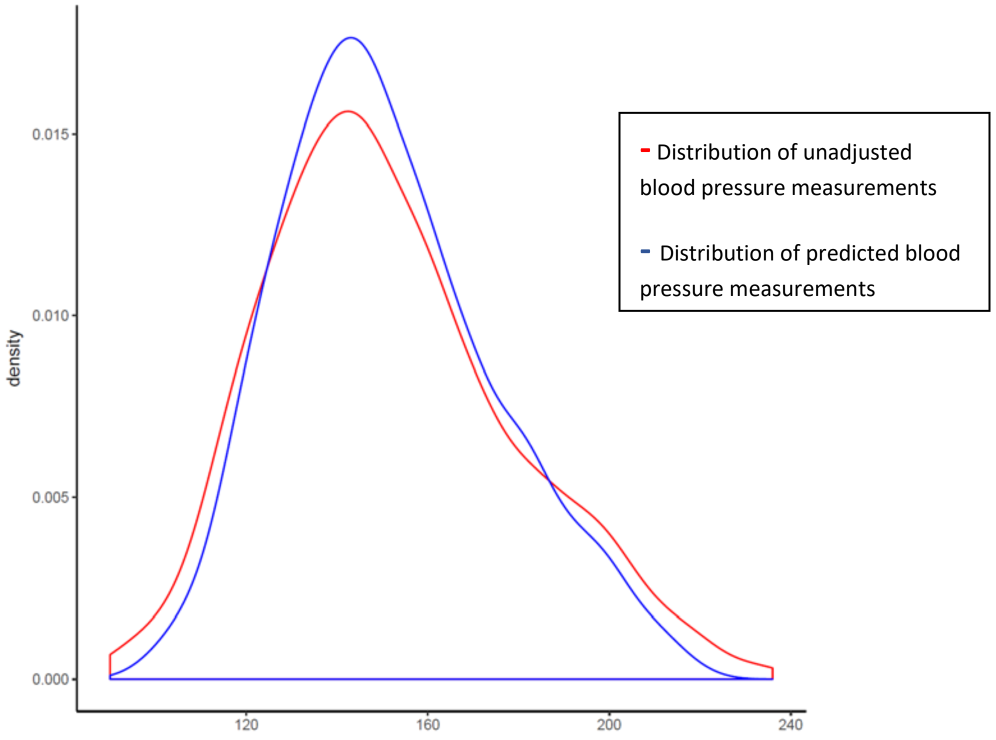


*Figure 2: Raw and predicted distributions of blood pressure in the Indonesia Family Life Survey*

*Estimating the exposure distribution shape*

The shape of the distribution of systolic blood pressure was estimated using all available person-level microdata sources, which was a subset of the input data into the modelling process. The distribution shape modelling framework for GBD 2017 is detailed in the appendix. Briefly, an ensemble distribution created from a weighted average of distribution families was fit for each individual microdata source, separately by sex. The weights for the distribution families for each individual source were then averaged and weighted to create a global ensemble distribution for each sex.

## Theoretical minimum-risk exposure level

No changes were made to TMREL used in the GBD2015 study. We estimated that the TMREL of SBP ranges from 110 to 115 mm Hg based on pooled prospective cohort studies that show risk of mortality increases for SBP above that level.[3,4] Our selection of a TMREL of 110-115 mmHg is consistent with the GBD study approach of estimating all attributable health loss that could be prevented even if current interventions do not exist that can achieve such a change in exposure level, for example a tobacco smoking prevalence of zero percent. To include the uncertainty in the TMREL, we took a random draw from the uniform distribution of the interval between 110 mm and 115 mm Hg each time the population attributable burden was calculated.

## Relative risks

No change was made to RR for blood pressure outcomes used in the GBD2016 study. RRs for chronic kidney disease are from the Renal Risk Collaboration meta-analysis of 2.7 million individuals in 106 cohorts. For other outcomes, we used data from two pooled epidemiological studies: the Asia Pacific Cohort Studies Collaboration (APCSC) and the Prospective Studies Collaboration (PSC).[4,5] Additional estimates of RR for cardiovascular outcomes were used from the CALIBER study, a health-record linkage cohort study from the UK.[6]

For cardiovascular disease, epidemiological studies have shown that the RR associated with SBP declines with age, with the log (RR) having an approximately linear relationship with age and reaching a value of 1 between the ages of 100 and 120. RRs were reported per 10 mm Hg increase in SBP above the TMREL value (115 mm Hg), calculated as in the equation below:

$$RR(x) = {RR_0}^{\frac{(x-TMREL)}{10\ mmHg}}$$

Where $RR(x)$ is the RR at exposure level x and $RR_0$ is the increase in RR for each 10 mmHg above the TMREL. We used Dismod-MR 2.1 to pool effect sizes from included studies and generate a dose-response curve for each of the outcomes associated with high SBP. The tool enabled us to incorporate random effects across studies and include data with different age ranges. RRs were used universally for all countries and the meta-regression only helped to pool the three major sources and produce RRs with uncertainty and covariance across ages taking into account the uncertainty of the data points.

**References**

1 Bangalore S, Gong Y, Cooper-DeHoff RM, Pepine CJ, Messerli FH. 2014 Eighth Joint National Committee panel recommendation for blood pressure targets revisited: results from the INVEST study. *J Am Coll Cardiol* 2014; **64**: 784–93.

2 Ng M, Fleming T, Robinson M, *et al.* Global, regional, and national prevalence of overweight and obesity in children and adults during 1980–2013: a systematic analysis for the Global Burden of Disease Study 2013. *The Lancet* 2014; **384**: 766–81.

3 Singh GM, Danaei G, Farzadfar F, *et al.* The age-specific quantitative effects of metabolic risk factors on cardiovascular diseases and diabetes: a pooled analysis. *PloS One* 2013; **8**: e65174.

4 Collaboration APCS, others. Blood pressure and cardiovascular disease in the Asia Pacific region. *J Hypertens* 2003; **21**: 707–16.

5 Prospective Studies Collaboration. Age-specific relevance of usual blood pressure to vascular mortality: a meta-analysis of individual data for one million adults in 61 prospective studies. *The Lancet* 2002; **360**: 1903–13.

6 Rapsomaniki E, Timmis A, George J, *et al.* Blood pressure and incidence of twelve cardiovascular diseases: lifetime risks, healthy life-years lost, and age-specific associations in 1·25 million people. *Lancet Lond Engl* 2014; **383**: 1899–911.

# High Body-Mass Index Capstone Appendix

## Flowchart

Adult (Ages 20+) High Body-Mass Index: Data and Model Flow Chart

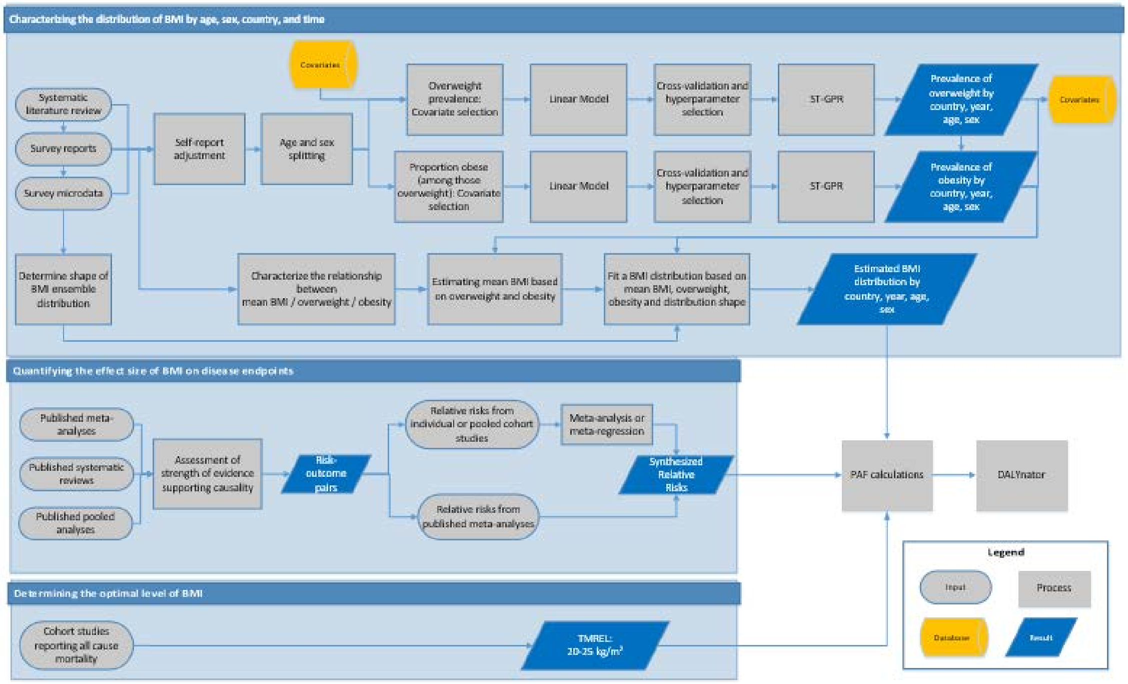


Childhood (Ages 2-19) High Body-Mass Index: Data and Model Flow Chart

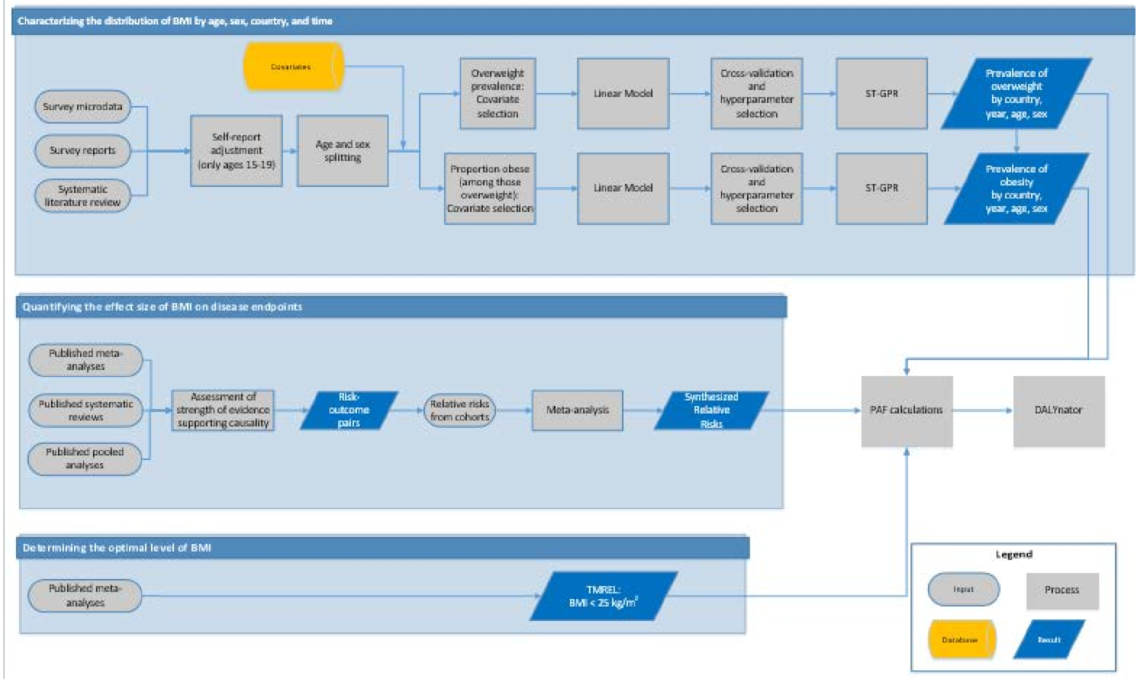

## Case definitions

High body-mass index (BMI) for adults (ages 20+) is defined as BMI greater than 20 to 25 kg/m$^2$. High BMI for children (ages 1-19) is defined as being overweight or obese based on International Obesity Task Force standards.

## Input data and methodological summary

### Data sources

We systematically searched Medline to identify studies providing nationally or subnationally representative estimates of overweight prevalence, obesity prevalence or mean body-mass index (BMI). We limited the search to literature published between January 1, 2016 and December 31, 2016 to update the systematic literature search previously performed as part of GBD 2015.

The search for adults was conducted on 4 January 2017 using the following terms:

((("Body Mass Index"[Mesh] OR "Overweight"[Mesh] OR "Obesity"[Mesh]) AND ("Geographic Locations"[Mesh] NOT "United States"[Mesh]) AND ("humans"[Mesh] AND "adult"[MeSH]) AND ("Data Collection"[Mesh] OR "Health Services Research"[Mesh] OR "Population Surveillance"[Mesh] OR "Vital statistics"[Mesh] OR "Population"[Mesh] OR "Epidemiology"[Mesh] OR "surve*"[TiAb]) NOT (Comment[ptyp] OR Case Reports[ptyp] OR "hospital"[TiAb])) AND ("2016/01/01"[Date - Publication] : "2016/12/31"[Date - Publication]))

The search for children was conducted on 4 August 2016 using the following terms:
((("Body Mass Index"[Mesh] OR "Overweight"[Mesh] OR "Obesity"[Mesh]) AND ("Geographic Locations"[Mesh] NOT "United States"[Mesh]) AND ("humans"[Mesh] AND "child"[MeSH]) AND ("Data Collection"[Mesh] OR "Health Services Research"[Mesh] OR "Population Surveillance"[Mesh] OR "Vital statistics"[Mesh] OR "Population"[Mesh] OR "Epidemiology"[Mesh] OR "surve*"[TiAb]) NOT (Comment[ptyp] OR Case Reports[ptyp] OR "hospital"[TiAb])) AND ("2016/01/01"[Date - Publication] : "2016/12/31"[Date - Publication]))

Our search for adult estimates identified 456 abstracts, of which 25 met inclusion criteria and were extracted. The search for childhood estimates identified 137 articles, of which 4 were extracted. Including sources from the previous GBD systematic literature searches, a total of 11,220 articles were identified, of which 845 were included. Additionally, we searched the Global Health Data Exchange (GHDx) database for individual-level data from major multinational survey series or country-specific surveys and identified 5,385 location-year sources meeting the inclusion criteria.

### Eligibility criteria

We included representative studies providing data on mean BMI or prevalence of overweight or obesity among adults or children. For adults, studies were included if they defined overweight as BMI≥25 kg/m$^2$ and obesity as BMI≥30 kg/m$^2$, or if estimates using those cutoffs could be back-calculated from reported categories. For children (children ages 2-18), studies were included if they used International Obesity Task Force (IOTF) standards to define overweight and obesity thresholds. We only included studies reporting data collected between January 1, 1980 and December 31, 2016. Studies were excluded if they used non-random samples (e.g. case-control studies or convenience samples), conducted among specific subpopulations (e.g. pregnant women, racial or ethnic minorities, immigrants, or individuals with specific diseases), used alternative methods to assess adiposity (e.g. waist-circumference, skin-fold thickness, or hydrodensitometry), had sample sizes of less than 20 per age-sex group, or provided inadequate

information on any of the inclusion criteria. We also excluded review articles and non-English language articles.

## Data collection process

Where individual-level survey data were available, we computed mean BMI using weight and height. We then used BMI to determine the prevalence of overweight and obesity. For individuals aged over 18 years, we considered them to be overweight if their BMI was greater than or equal to 25 kg/m$^2$, and obese if their BMI was greater than or equal to 30 kg/m$^2$. For individuals aged 2 to 18 years, we used monthly IOTF cutoffs[2] to determine overweight and obese status when age in months was available. When only age in years was available, we used the cutoff for the midpoint of that year. Obese individuals were also considered to be overweight. We excluded studies using the World Health Organization (WHO) standards or country-specific cutoffs to define childhood overweight and obesity. At the individual-level, we considered BMI<10 kg/m$^2$ and BMI>70 kg/m$^2$ to be biologically implausible and excluded those observations.

The rationale for choosing to use the IOTF cutoffs over the WHO standards has been described elsewhere.[1] Briefly, the IOTF cutoffs provide consistent child-specific standards for ages 2-18 derived surveys covering multiple countries. On the other hand, the WHO growth standards apply to children under age 5 and the WHO growth reference applies to children ages 5 to 19. The WHO growth reference for children ages 5 to 19 was derived from United States data which is less representative than the multinational data used by IOTF. Additionally, the switch between references at age 5 can produce artificial discontinuities. Given that we estimate global childhood overweight and obesity for ages 2-19 (with ages 19 using standard adult cutoffs), the IOTF cutoffs were preferable. Additionally, we found that IOTF cutoffs were more commonly used in scientific literature covering childhood obesity.

From report and literature data, we extracted data on mean BMI, prevalence of overweight, and prevalence of obesity, measures of uncertainty for each, and sample size, by the most granular age and sex groups available. Additionally, we extracted the same study-level covariates as were extracted from microdata (measurement, urbanicity, and representativeness), as well as location and year.

In addition to the primary indicators described above, we extracted relevant survey-design variables, including primary sampling unit, strata, and survey weights, which were used to tabulate individual-level microdata and produce accurate measures of uncertainty. We extracted three study-level covariates: 1) whether height and weight data were measured or self-reported; 2) whether the study was predominantly conducted in an urban area, rural area or both; and 3) the level of representativeness of the study (national or subnational).

Finally, we extracted relevant demographic indicators, including location, year, age and sex. We estimated the standard error of the mean from individual-level data, where available, and used the reported standard error of the mean for published data. When multiple data sources were available for the same country, we included all of them in our analysis. If data from the same data source were available in multiple formats such individual-level data and tabulated data, we used individual-level data.

## Self-report bias adjustment

We included both measured and self-reported data. We tested for bias in self-report data compared to measured data, which is considered to be the gold-standard. There was no clear direction of bias for children ages 2 to 14, so for these age groups we only included measured data. For individuals ages 15

and above, we adjusted self-reported data for overweight prevalence, obesity prevalence and mean BMI using the following nested hierarchical mixed-effects regression models, fit using restricted maximum likelihood separately by sex:

$$\mathrm{logit(overweight)}_{c,a,t} = \beta_0 + \beta_1 m + \sum_{k=2}^{19} \beta_k I_{A[a]} + \sum_{l=20}^{55} \beta_l I_{A[a]} I_{M[m]} + \alpha_s + \alpha_s m + \alpha_r + \alpha_r m + \alpha_c + \alpha_c m + \alpha_t + \alpha_t m + \epsilon_{c,a,t}$$

$$\mathrm{logit(obesity)}_{c,a,t} = \beta_0 + \beta_1 m + \sum_{k=2}^{19} \beta_k I_{A[a]} + \sum_{l=20}^{55} \beta_l I_{A[a]} I_{M[m]} + \alpha_s + \alpha_s m + \alpha_r + \alpha_r m + \alpha_c + \alpha_c m + \alpha_t + \alpha_t m + \epsilon_{c,a,t}$$

$$\log(\mathrm{BMI})_{c,a,t} = \beta_0 + \beta_1 m + \sum_{k=2}^{19} \beta_k I_{A[a]} + \sum_{l=20}^{55} \beta_l I_{A[a]} I_{M[m]} + \alpha_s + \alpha_s m + \alpha_r + \alpha_r m + \alpha_c + \alpha_c m + \alpha_t + \alpha_t m + \epsilon_{c,a,t}$$

Where $m$ is a fixed effect on measurement (binary, either measured (1) or self-report (0)), $I_{A[a]}$ is an indicator variable for specific age group A, $I_{A[a]} I_{M[m]}$ is an interaction term between age and measurement, $\alpha_s$, $\alpha_r$, and $\alpha_c$ are random effects at the super region, region, and country, respectively, and $\alpha_t$ is a random effect by time-period (1980-1989, 1990-1999, 2000-2009, 2010-2017). Random effects at the country level and time-period level were used to fit the models, but were taken as noise and were not used in adjustment of self-reported data. We propagated the uncertainty in the self-report adjustment model by adding the variance of each of the regression coefficients used in adjustment to the data variance in delta-transformed space. After adjustment, regressions confirmed that self-reported data was no longer significantly different from measured data.

## Age and sex splitting

Any report or literature data provided in age groups wider than the standard 5-year age groups or as both sexes combined were split using the approach used by Ng et al.[2] Briefly, age-sex patterns were identified using sources with data on multiple age-sex groups and these patterns were applied to split aggregated report and literature data. Uncertainty in the age-sex split was propagated by multiplying the standard error of the data by the square root of the number of splits performed. We did not propagate the uncertainty in the age pattern and sex pattern used to split the data as they seemed to have small effect.

## Prevalence estimation for overweight and obesity

After adjusting for self-report bias and splitting aggregated data into 5-year age-sex groups, we used spatiotemporal Gaussian process regression (ST-GPR) to estimate the prevalence of overweight and obesity. This modelling approach has been described in detail elsewhere.

The linear model, which when added to the smoothed residuals forms the mean prior for GPR is as follows:

$$\mathrm{logit(overweight)}_{c,a,t} = \beta_0 + \beta_1 \mathrm{energy}_{c,t} + \beta_2 \mathrm{SDI}_{c,t} + \beta_3 \mathrm{vehicles}_{c,t} + \beta_4 \mathrm{agriculture}_{c,t} + \sum_{k=5}^{21} \beta_k I_{A[a]} + \alpha_s + \alpha_r + \alpha_c$$

$$\mathrm{logit(obesity/overweight)}_{c,a,t} = \beta_0 + \beta_1 \mathrm{energy}_{c,t} + \beta_2 \mathrm{SDI}_{c,t} + \beta_3 \mathrm{vehicles}_{c,t} + \sum_{k=4}^{21} \beta_k I_{A[a]} + \alpha_s + \alpha_r + \alpha_c$$

where $energy$ is ten-year lag-distributed energy consumption per capita, $SDI$ is a composite index of development including lag-distributed income per capita, education, and fertility, $vehicles$ is is the number of two or four-wheel vehicles per capita, and $agriculture$ is the proportion of the population working in agriculture. $I_{A[a]}$ is a dummy variable indicating specific age group $A$ that the prevalence point

captures, and $\alpha_s$, $\alpha_r$, and $\alpha_c$ are super region, region, and country random intercepts, respectively. Random effects were used in model fitting but were not used in prediction.

We tested all combinations of the following covariates to see which performed best in terms of in-sample AIC for the overweight linear model and the obesity as a proportion of overweight linear model: ten-year lag distributed energy per capita, proportion of the population living in urban areas, SDI, lag-distributed income per capita, educational attainment (years) per capita, proportion of the population working in agriculture, grams of sugar adjusted for energy per capita, grams of sugar not adjusted for energy per capita, and the number of two or four-wheeled vehicles per capita. We selected these candidate covariates based on theory as well as reviewing covariates used in other publications. The final linear model was selected based on: 1) if the direction of covariates matched what is expected from theory, 2) all the included covariates were significant, and 3) minimising in-sample AIC. The covariate selection process was performed using the dredge package in R.

The new version of ST-GPR for GBD 2017 incorporates information about data density into the process for smoothing over space and time. Estimates in areas/years with few observations have more weight on regional observations. To specify the distribution of time weights and space weights, we used values of lambda=0.2 and zeta=0.05, respectively. We used a value of omega=1.0 for the distribution of age weights. We set the GPR scale parameter to 20, and used the default global cutoff setting for amplitude.

## Estimating mean BMI

To estimate the mean BMI for adults in each country, age, sex, and time period 1980-2017, we first used the following nested hierarchical mixed-effects model, fit using restricted maximum likelihood on data from sources containing estimates of all three indicators (prevalence of overweight, prevalence of obesity, and mean BMI), in order to characterise the relationship between overweight, obesity, and mean BMI:

$$\log(\mathrm{BMI}_{c,a,s,t}) = \beta_0 + \beta_1 \mathrm{ow}_{c,a,s,t} + \beta_2 \mathrm{ob}_{c,a,s,t} + \beta_3 \mathrm{sex} + \sum_{k=4}^{20} \beta_k \mathrm{I}_{A[a]} + \alpha_s(1 + \mathrm{ow}_{c,a,s,t} + \mathrm{ob}_{c,a,s,t}) + \alpha_r(1 + \mathrm{ow}_{c,a,s,t} + \mathrm{ob}_{c,a,s,t}) + \alpha_c(1 + \mathrm{ow}_{c,a,s,t} + \mathrm{ob}_{c,a,s,t}) + \epsilon_{c,a,s,t}$$

where $\mathrm{ow}_{c,a,s,t}$ is the prevalence of overweight in country c, age a, sex s, and year t, $\mathrm{ob}_{c,a,s,t}$ is the prevalence of obesity in country c, age a, sex s, and year t, sex is a fixed effect on sex, $I_{A[a]}$ is an indicator variable for age, and $\alpha_s$, $\alpha_r$, and $\alpha_c$ are random effects at the super region, region, and country, respectively. The model was run in Stata 13.

We applied 1,000 draws of the regression coefficients to the 1,000 draws of overweight prevalence and obesity prevalence produced through ST-GPR to estimate 1,000 draws of mean BMI for each country, year, age, and sex. This approach ensured that overweight prevalence, obesity prevalence, and mean BMI were correlated at the draw level and uncertainty was propagated.

## Estimating BMI distribution

We used the ensemble distribution approach described in the manuscript. We fit ensemble weights by source and sex, with source- and sex-specific weights averaged across all sources included to produce the final global weights. The ensemble weights were fit on measured microdata. The final ensemble weights were: exponential = 0.002, gamma = 0.028, inverse gamma = 0.085, log-logistic = 0.187, Gumbel = 0.220,

Weibull = 0.011, log-normal = 0.058, normal = 0.012, beta = 0.136, mirror gamma = 0.008, and mirror Gumbel = 0.113.

One thousand draws of BMI distributions for each location, year, age group, and sex estimated were produced by fitting an ensemble distribution using 1,000 draws of estimated mean BMI, 1,000 draws of estimated standard deviation, and the ensemble weights. Estimated standard deviation was produced by optimising a standard deviation to fit estimated overweight prevalence draws and estimated obesity prevalence draws.

## Assessment of risk-outcome pairs

Risk-outcome pairs were defined based on strength of available evidence supporting a causal effect. We performed a systematic review of published meta-analyses, pooled analyses, and systematic reviews available through PubMed using the following search string: ("Body Mass Index"[Mesh] OR "Overweight"[Mesh] OR "Obesity"[Mesh]) AND (Meta-Analysis[ptyp] OR "systematic review"[tiab] OR "pooled analysis"[tiab]). Inclusion criteria are 1) the health outcome is included in GBD, 2) at least one prospective cohort is included, and 3) that the summary effect size is statistically significant. For outcomes meeting inclusion criteria we completed causal criteria tables to evaluate the strength of evidence supporting a causal relationship (see Appendix Table 4). Gallbladder disease, cataract, multiple myeloma, gout, non-Hodgkin lymphoma, asthma, Alzheimer disease, and atrial fibrillation were added as new outcomes in GBD 2016, resulting in a total of 38 outcomes.

## Theoretical minimum risk exposure level

For adults (ages 20+), the theoretical minimum risk exposure level (TMREL) of BMI (20-25 kg/m$^2$) was determined based on the BMI level that was associated with the lowest risk of all-cause mortality in prospective cohort studies.[3]

For children (ages 2-19), the TMREL is “normal weight,” that is, not overweight or obese, based on IOTF cutoffs.

## Relative risk

The relative risk per 5-unit change in BMI for each disease endpoint was obtained from meta-analyses, and where available, pooled analyses of prospective observational studies. In cases where a relative risk per 5-unit change in BMI was not available we computed our own dose-response meta-analysis using two-step generalised least squares for time trends estimation methods.

For childhood outcomes (ages 2-19), we computed categorical relative risks for overweight and obesity using a random effects meta-analysis.

Relative risks for all 38 outcomes, by age and sex, are reported in Table 6a.

## References


1.) Cole, TJ, and T Lobstein. "Extended International (IOTF) Body Mass Index Cut-Offs for Thinness, Overweight and Obesity." Pediatric Obesity 2012; 7(4): 284–94.
2.) Ng M, Fleming T, Robinson M, et al. Global, regional, and national prevalence of overweight and obesity in children and adults during 1980–2013: a systematic analysis for the Global Burden of Disease Study 2013. The Lancet 2014; 384: 766–81.
3.) Angelantonio ED, Bhupathiraju SN, Wormser D, et al. Body-mass index and all-cause mortality: individual-participant-data meta-analysis of 239 prospective studies in four continents. The Lancet 2016; 388: 776–86.

# Bone Mineral Density Capstone Appendix

## Flowchart

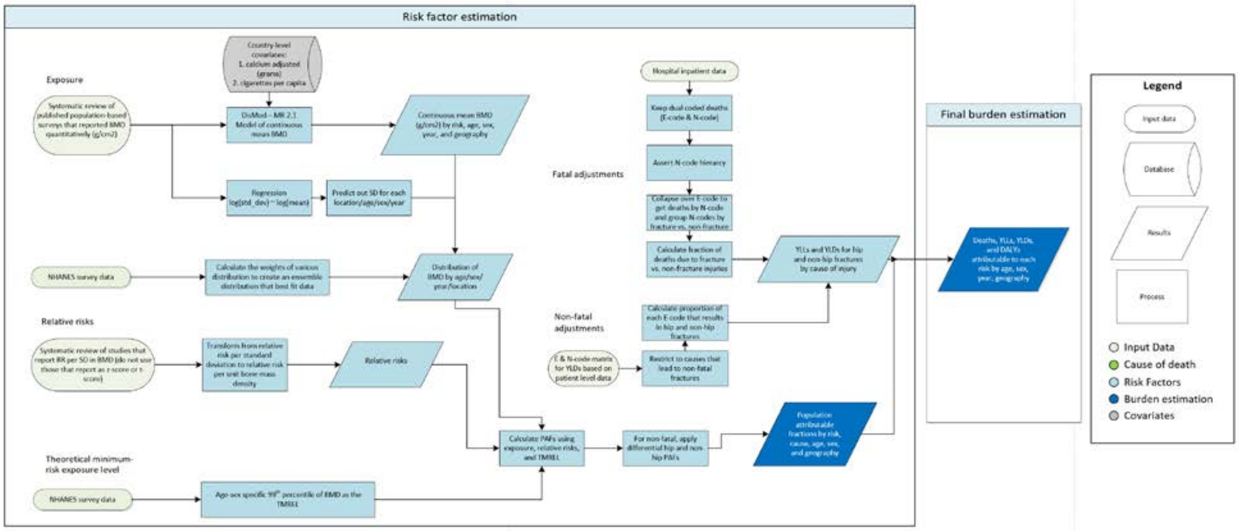


## Input data & methodological summary

### Exposure

#### *Case definition*

Bone mineral density (BMD) is a continuous variable measured by dual-x-ray-absorptiometry (DXA) at the femoral neck (FN) and is presented in g/cm$^2$ after standardizing for the brand of densitometer (sBMD). Low BMD is measured in terms of the difference between BMD of a population and the 99$^{th}$ percentile of a reference population at the same age and sex (theoretical minimum of risk exposure level, TMREL). The burden attributed to low bone mineral density is estimated for adults 20 years and older.

### Input data

A systematic review (search string at the end of document) was conducted in GBD 2015 but it was not scheduled for systematic review in GBD 2016 or 2017. Inclusion criteria that informed the search are:

- Representative, population-based surveys
- Reporting of quantitative BMD
  - measured by DXA
  - performed at the FN region
  - measured in g/cm$^2$

Mean BMD was occasionally reported in stratified groups, e.g. by fracture status but not for total sample. In these cases, the stratified means were aggregated to obtain a total mean BMD at population level for an age or sex category.

The data availability by GBD super-region is shown in table below.

| Super region | The number of data points |
|---|---|
| Southeast Asia, East Asia, and Oceania | 314 |
| Central Europe, Eastern Europe, and Central Asia | 36 |
| High-income | 682 |
| Latin America and Caribbean | 97 |
| North Africa and Middle East | 110 |
| South Asia | 39 |
| Sub-Saharan Africa | 3 |

### Modelling strategy

We modelled mean BMD in DisMod-MR 2.1 as a single 'continuous' parameter model by age and sex, and all GBD locations for years 1990 to 2017. The model had age mesh points at 0 10 20 25 30 40 50 60 70 80 90 & 100, a time window of 10 years for fitting data, and a minimum coefficient of variation of 0.1 for global, 0.06 super region and 0.08 for the region level.

The country covariates of alcohol consumption (litres per capita), tobacco consumption (cigarettes per capita), mean BMI, and adjusted calcium intake (g) were included in modelling. The country covariates total physical activity and milk consumption did not have a significant effect on BMD so we excluded them from our final model.

The uncertainty of BMD was modelled using various distributions. We tested goodness-of-fit in NHANES III data, the only survey for which we had unit record data available. We applied a weighting ensemble on those distributions. The weights were calculated in an optimisation model with an objective function that minimised Kolmogorov-Smirnov statistics. The weights of the distributions in the ensemble were calculated separately for males and females. Distribution weights are shown below.

Figure 1: Distribution of weights used for Females in GBD 2017

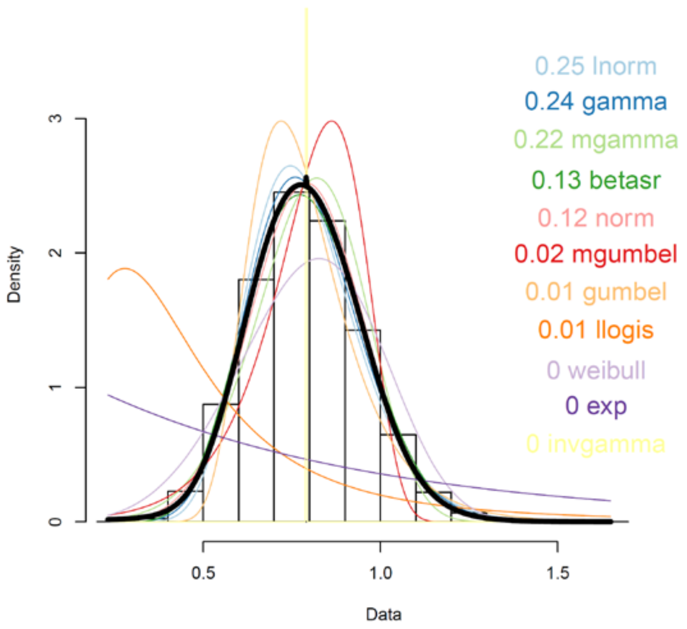


Figure 2: Distribution of weights used for Males in GBD 2017

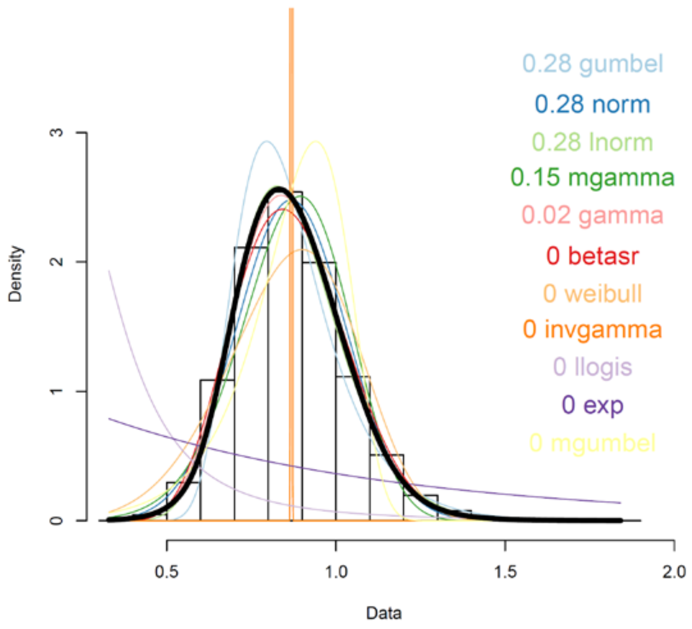


We consider the risk of fatal and non-fatal outcomes for hip non-hip fractures, separately, as relative risk data provide different estimates. Thus, there were various steps after DisMod-MR 2.1 exposure modelling to arrive at attributable fractions that can be applied to fatal and non-fatal fracture outcomes. These osteoporotic non-hip fractures include fractures of vertebrae, clavicle, scapula, humerus, skull, sternum, rib, face bone, radius or ulna, femur, patella, tibia, fibula, ankle, pelvis and vertebra.

First, we calculated the proportion of injury deaths that are due to fractures. This proportion of death caused by fracture is the envelope that we use to attribute death to BMD. In order to do this, we assumed that hip fracture and some non-hip fractures (any fractures apart from fingers and toes) are potentially fatal fractures. As cause of death data from vital registration and verbal autopsy attributes injury deaths to causes of death (e.g. fall or road injury) and not nature of injury (such as fractures), we used available hospital data to estimate the proportion of injury deaths during admission that could be ascribed to fractures. We restricted our analysis to cases that were dual-coded with both the cause of injury ("E-code") and nature of injury ("N-code"). As injury cases may have multiple forms of trauma, we applied a severity hierarchy to the fatal hospital data to determine the proportion of the deaths that could be attributed to the chosen fracture types but were not accompanied by more severe fatal trauma such as head trauma, spinal cord lesion, and intra-abdominal or thoracic organ damage. We collapsed all deaths over E-code to determine the ratio of deaths attributable to fracture versus non-fracture injuries. We applied this ratio to the YLL.

We restricted non-fatal estimates of low BMD to a list of causes that were deemed to cause osteoporotic fractures. Below is the list of injuries for which a PAF was calculated:

- Transport injuries
- Road injuries
- Pedestrian road injuries
- Cyclist road injuries
- Motorcyclist road injuries
- Motor vehicle road injuries
- Other road injuries
- Other transport injuries
- Unintentional injuries
- Falls
- Exposure to mechanical forces
- Other exposure to mechanical forces
- Non-venomous animal contact
- Interpersonal violence
- Assault by other means

We made use of the E to N-code matrix generated from dual-coded (E-code/N-code) patient level data in our injury analyses to determine the proportion of each E-code that results in a certain N-code. The hip and non-hip fracture population attributable fractions were applied to the appropriate combinations of external cause and fracture estimates of YLD and then summed together to produce a single estimate.

Figure 3: Plot of hospital fractions used by hip and non-hip

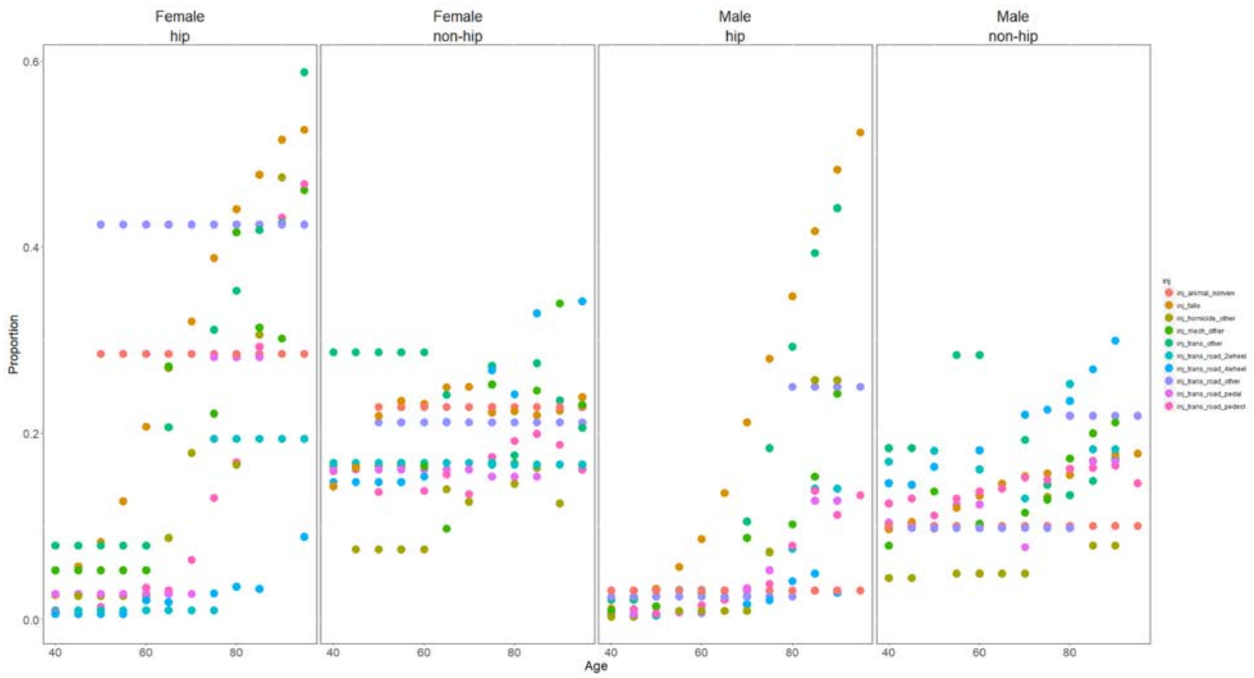


## Theoretical minimum-risk exposure level

The theoretical minimum of risk exposure level or TMREL was chosen as the age-sex specific 99th percentile of BMD from 5 cycles of NHANES study as the reference population. Below is a descriptive table of the 5 NHANES cycles used.

| **NHANES cycle** | **Age range (years)** | **Number of people tested** | **BMD range (g/cm2)** |
|---|---|---|---|
| 1988 | 20 – 90 | 14,646 | 0.23 – 1.84 |
| 2005 | 20 – 85 | 3,494 | 0.40 – 1.50 |
| 2007 | 20 – 80 | 4,726 | 0.34 – 1.46 |
| 2009 | 20 – 80 | 5,052 | 0.33 – 1.63 |
| 2013 | 40 – 80 | 3,127 | 0.39 – 1.36 |

Figure 4: Plot of 99th percentile of BMD at femoral neck in each cycle of NHANES

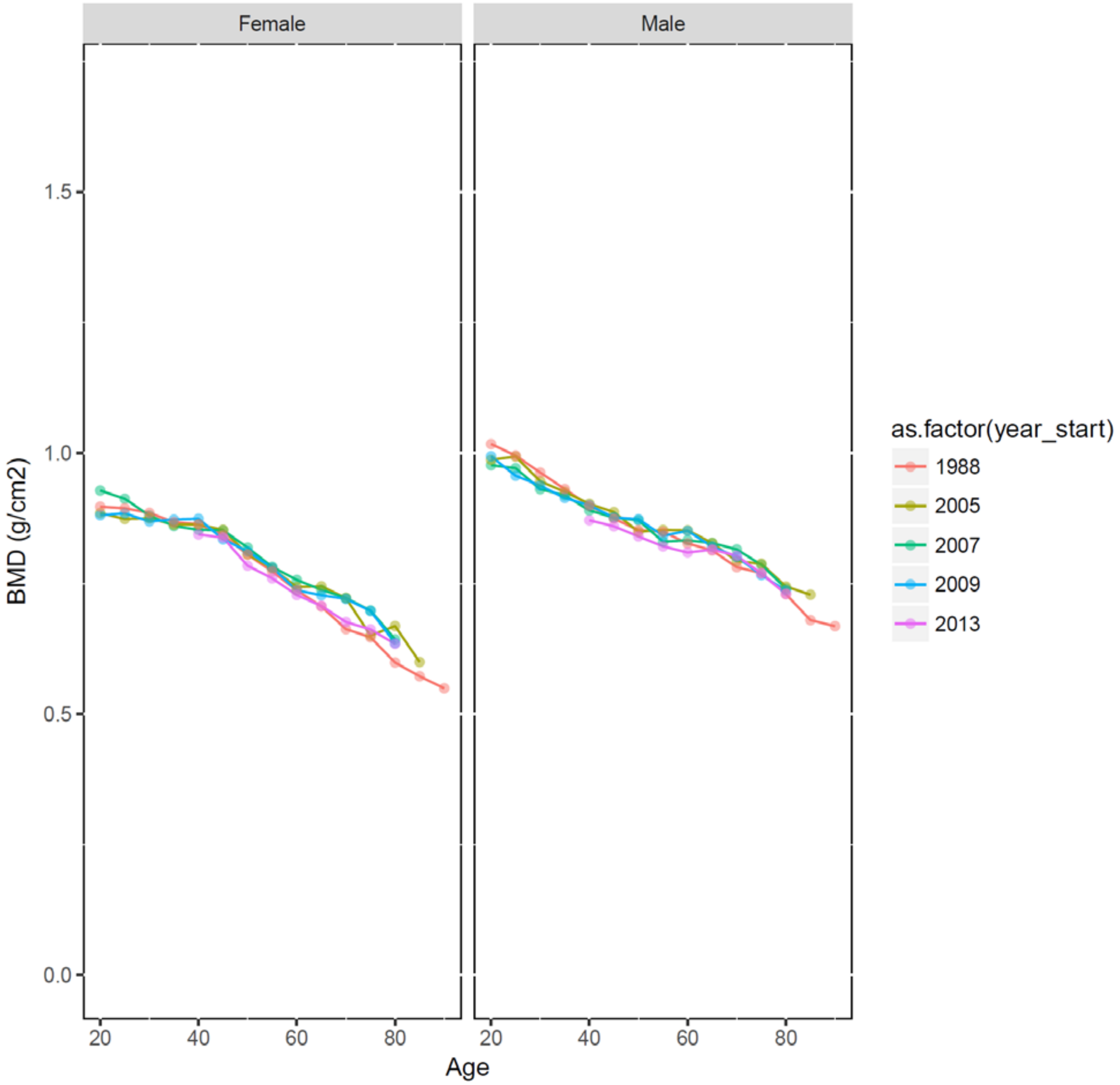


## Relative risks

Relative risks must be reported per standard deviation or per unit bone mass density in order for us to use the data. Many studies report relative risk based on a z-score or the relative risks in the osteoporotic group versus the non-osteoporotic group; neither of these relative risks are usable.

For GBD 2017, we did not update the systematic review for the RR of BMD that was done in GBD 2013. In the GBD 2013 review, twelve prospective observational studies were found, but one meta-analysis of 12 studies[1] reported the dose-response relationship between low BMD and high relative risk of hip and other fractures that are prone to osteoporosis, as shown in the below table.

| BMD z score | Any fracture | | Osteoporotic fracture | | Hip fracture | |
|---|---|---|---|---|---|---|
| | RR | 95% CI | RR | 95% CI | RR | 95% CI |
| −4 | 1.79 | 1.44–2.23 | 2.10 | 1.63–2.71 | 2.14 | 1.40–3.26 |
| −3 | 1.71 | 1.44–2.02 | 1.96 | 1.61–2.39 | 2.12 | 1.54–2.92 |
| −2 | 1.63 | 1.45–1.84 | 1.84 | 1.60–2.12 | 2.11 | 1.70–2.62 |
| −1 | 1.56 | 1.45–1.69 | 1.73 | 1.59–1.89 | 2.11 | 1.86–2.39 |
| 0 | 1.50 | 1.44–1.56 | 1.62 | 1.54–1.71 | 2.08 | 1.91–2.26 |
| 1 | 1.39 | 1.32–1.46 | 1.42 | 1.34–1.51 | 2.04 | 1.78–2.34 |
| 2 | 1.32 | 1.21–1.45 | 1.33 | 1.19–1.48 | 2.03 | 1.60–2.56 |
| 3 | 1.26 | 1.10–1.45 | 1.25 | 1.06–1.47 | 2.01 | 1.44–2.81 |
| 4 | 1.21 | 1.00–1.45 | 1.17 | 0.93–1.46 | 1.99 | 1.28–3.10 |

The z score ranged from −5.1 to +5.8.

Search string from GBD 2015 systematic review:

| Search | Query | Items found | Time |
|---|---|---|---|
| #11 | Search (#8 AND #10) Filters: Humans | 326 | 12:37:09 |
| #10 | Search ("Cross-Sectional Studies"[Mesh] OR "cross-sectional"[title/abstract] OR "Health Surveys"[Mesh] OR Survey[title/abstract] OR cohort[title/abstract] OR "Diet Surveys"[Mesh] OR "Longitudinal Studies"[Mesh] OR "Nutrition Surveys"[Mesh] OR "Surveys and Questionnaires"[Mesh]) Filters: Humans | 1324376 | 12:36:29 |
| #8 | Search (#7 AND #6) Filters: Humans | 622 | 12:33:16 |
| #7 | Search ("Absorptiometry, Photon"[Mesh] OR "dual-energy x-ray absorptiometry" OR "dual energy x-ray absorptiometry") Filters: Humans | 21368 | 12:32:34 |
| #6 | Search (#5 AND ("2010"[Date - Publication] : "3000"[Date - Publication])) Filters: Humans | 1387 | 12:30:26 |
| #5 | Search ((#1 OR #2) AND #3) Filters: Humans | 3702 | 12:29:47 |
| #4 | Search ((#1 OR #2) AND #3) | 4015 | 12:29:33 |
| #3 | Search (((("bone mineral density"[title/abstract] OR "bone mineral densities"[title/abstract]) OR "Bone Density"[Mesh]) AND (mean[title/abstract] OR average[title/abstract]))) | 12892 | 12:29:00 |
| #2 | Search ((((multinational[TIAB] OR international[TIAB] OR national[TIAB] OR nationwide[TIAB] OR nation-wide[TIAB] OR equitorial[TIAB] OR equator[TIAB] OR global[TIAB] OR globe[TIAB] OR world[TIAB] OR worldwide[TIAB] OR world-wide[TIAB] OR countrywide[TIAB] OR countries[TIAB] OR continental[TIAB] OR continent[TIAB] OR continents[TIAB] OR global burden[TIAB] OR burden of disease[TIAB] OR disease burden[TIAB] OR tropic[TIAB] OR tropics[TIAB] OR tropical[TIAB] OR Oceania[TIAB] OR South America[TIAB] OR Central America[TIAB] OR Mesoamerica[TIAB] OR Americas[TIAB] OR Latin America[TIAB] OR paho[TIAB] OR pan-american[TIAB] OR panamerican[TIAB] OR pan-america[TIAB] OR Caribbean[TIAB] OR Indies[TIAB] OR Australasia[TIAB] OR Australasian[TIAB] OR developing countries[TIAB] OR developing nations[TIAB] OR developed countries[TIAB] OR developed nations[TIAB] OR commonwealth[TIAB] OR industrialized[TIAB] OR nonindustrialized[TIAB] OR non-industrialized[TIAB] OR underdeveloped countries[TIAB] OR underdeveloped nation[TIAB] OR underdeveloped nations[TIAB] OR under-developed country[TIAB] OR under-developed countries[TIAB] OR under-developed nation[TIAB] OR under-developed nations[TIAB] OR low-income country[TIAB] OR low-income countries[TIAB] OR low-income nation[TIAB] OR low-income nations[TIAB] OR nondeveloped country[TIAB] OR nondeveloped countries[TIAB] OR nondeveloped nation[TIAB] OR nondeveloped nations[TIAB] OR non-developed country[TIAB] OR non-developed countries[TIAB] OR non-developed nation[TIAB] OR non-developed nations[TIAB] OR International Cooperation[TIAB] OR World Health Organization[TIAB] OR Asia[TIAB] OR Far East[TIAB] OR Near East[TIAB] OR Middle East[TIAB] OR Scandinavia[TIAB] OR Europe[TIAB] OR European[TIAB] OR Eastern Hemisphere[TIAB] OR Western Hemisphere[TIAB] OR Northern Hemisphere[TIAB] OR Southern Hemisphere[TIAB] OR North America[TIAB] OR island[TIAB] OR islands[TIAB] OR United Nations[TIAB] OR unesco[TIAB] OR unicef[TIAB] OR Worldbank[TIAB] OR Benelux[TIAB] OR sub-Saharan[TIAB] OR subsaharan[TIAB] OR Sahara[TIAB] OR sub-Sahara[TIAB] OR Amazon[TIAB] OR Amazonian[TIAB] OR valley[TIAB] OR river[TIAB] | 3585538 | 12:28:22 |

| | | | |
|---|---|---|---|
| | OR mountain[TIAB] OR mountains[TIAB] OR forest[TIAB] OR forests[TIAB] OR rainforest[TIAB] OR rainforests[TIAB] OR jungle[TIAB] OR jungles[TIAB] OR archipelago[TIAB] OR archipelagos[TIAB] OR archipelagoes[TIAB] OR patagonia[TIAB] OR andes[TIAB] OR mediterranean region[TIAB] OR Africa[TIAB] OR registry[TIAB] OR North Korea[TIAB] OR Timor[TIAB] OR Palestine[TIAB] OR Syrian Arab Republic[TIAB] OR Baltic[TIAB] OR Atlantic Islands[TIAB] OR Indian Ocean[TIAB] OR Pacific[TIAB] OR multicenter[TIAB] OR multi-center[TIAB] OR registry[TIAB] OR registries[TIAB] OR Algeria[TIAB] OR Egypt[TIAB] OR Libya[TIAB] OR Morocco[TIAB] OR Tunisia[TIAB] OR Cameroon[TIAB] OR Central African Republic[TIAB] OR Chad[TIAB] OR Congo[TIAB] OR Congo[TIAB] OR Equatorial Guinea[TIAB] OR Gabon[TIAB] OR Burundi[TIAB] OR Djibouti[TIAB] OR Eritrea[TIAB] OR Ethiopia[TIAB] OR Kenya[TIAB] OR Rwanda[TIAB] OR Somalia[TIAB] OR Sudan[TIAB] OR Tanzania[TIAB] OR Uganda[TIAB] OR Angola[TIAB] OR Botswana[TIAB] OR Lesotho[TIAB] OR Malawi[TIAB] OR Mozambique[TIAB] OR Namibia[TIAB] OR South Africa[TIAB] OR Swaziland[TIAB] OR Zambia[TIAB] OR Zimbabwe[TIAB] OR Benin[TIAB] OR Burkina Faso[TIAB] OR Cote d'Ivoire[TIAB] OR Gambia[TIAB] OR Ghana[TIAB] OR Guinea[TIAB] OR Guinea-Bissau[TIAB] OR Liberia[TIAB] OR Mali[TIAB] OR Mauritania[TIAB] OR Niger[TIAB] OR Nigeria[TIAB] OR Senegal[TIAB] OR Sierra Leone[TIAB] OR Togo[TIAB] OR Antigua[TIAB] OR Bahamas[TIAB] OR Barbados[TIAB] OR Cuba[TIAB] OR Dominica[TIAB] OR Dominican Republic[TIAB] OR Grenada[TIAB] OR Guadeloupe[TIAB] OR Haiti[TIAB] OR Jamaica[TIAB] OR Martinique[TIAB] OR Netherlands Antilles[TIAB] OR Puerto Rico[TIAB] OR Saint Kitts and Nevis[TIAB] OR Saint Lucia[TIAB] OR Saint Vincent[TIAB] OR Grenadines[TIAB] OR Trinidad and Tobago[TIAB] OR Virgin Islands[TIAB] OR Belize[TIAB] OR Costa Rica[TIAB] OR El Salvador[TIAB] OR Guatemala[TIAB] OR Honduras[TIAB] OR Nicaragua[TIAB] OR Panama[TIAB] OR Mexico[TIAB] OR Argentina[TIAB] OR Bolivia[TIAB] OR Brazil[TIAB] OR Chile[TIAB] OR Colombia[TIAB] OR Ecuador[TIAB] OR French Guiana[TIAB] OR French Guiana[TIAB] OR Paraguay[TIAB] OR Peru[TIAB] OR Suriname[TIAB] OR Uruguay[TIAB] OR Venezuela[TIAB] OR Kazakhstan[TIAB] OR Kyrgyzstan[TIAB] OR Tajikistan[TIAB] OR Turkmenistan[TIAB] OR Uzbekistan[TIAB] OR Borneo[TIAB] OR Cambodia[TIAB] OR Timor[TIAB] OR Indonesia[TIAB] OR Laos[TIAB] OR Malaysia[TIAB] OR Mekong Valley[TIAB] OR Myanmar[TIAB] OR Philippines[TIAB] OR Thailand[TIAB] OR Vietnam[TIAB] OR Viet Nam[TIAB] OR Bangladesh[TIAB] OR Bhutan[TIAB] OR India[TIAB] OR Afghanistan[TIAB] OR Bahrain[TIAB] OR Iran[TIAB] OR Iraq[TIAB] OR Jordan[TIAB] OR Kuwait[TIAB] OR Lebanon[TIAB] OR Oman[TIAB] OR Qatar[TIAB] OR Saudi Arabia[TIAB] OR Syria[TIAB] OR Turkey[TIAB] OR United Arab Emirates[TIAB] OR Yemen[TIAB] OR Nepal[TIAB] OR Pakistan[TIAB] OR Sri Lanka[TIAB] OR China[TIAB] OR Macao[TIAB] OR Mongolia[TIAB] OR Taiwan[TIAB] OR Azores[TIAB] OR Bermuda[TIAB] OR Falkland Islands[TIAB] OR Albania[TIAB] OR Estonia[TIAB] OR Latvia[TIAB] OR Lithuania[TIAB] OR Bosnia-Herzegovina[TIAB] OR Bulgaria[TIAB] OR Byelarus[TIAB] OR Croatia[TIAB] OR Czech Republic[TIAB] OR Hungary[TIAB] OR Macedonia[TIAB] OR Moldova[TIAB] OR Montenegro[TIAB] OR Poland[TIAB] OR Romania[TIAB] OR Russia[TIAB] OR Slovakia[TIAB] OR Slovenia[TIAB] OR Ukraine[TIAB] OR Yugoslavia[TIAB] OR Armenia[TIAB] OR Azerbaijan[TIAB] OR Georgia[TIAB] OR Comoros[TIAB] OR Madagascar[TIAB] OR Mauritius[TIAB] OR Reunion[TIAB] OR Seychelles[TIAB] OR Fiji[TIAB] OR New Caledonia[TIAB] OR Papua New Guinea[TIAB] OR Vanuatu[TIAB] OR Guam[TIAB] OR Palau[TIAB] OR Pitcairn Island[TIAB] OR Samoa[TIAB] OR Tonga[TIAB] OR Czechoslovakia[TIAB] OR East Germany[TIAB] OR New Guinea[TIAB] OR USSR[TIAB] OR Yugoslavia[TIAB] OR Ivory Coast[TIAB] OR Hong Kong[TIAB] OR china[TIAB] OR North Korea[TIAB] OR Palestine[TIAB] OR Syrian Arab Republic[TIAB]) AND (hasabstract[text] AND Humans[Mesh] AND middle age[MeSH])) OR ((International Cooperation[Mesh:noexp] OR developing countries[Mesh] OR developed countries[Mesh] OR WORLD HEALTH[Mesh] OR WORLD HEALTH ORGANIZATION[Mesh] OR AFRICA[Mesh] OR Americas[Mesh:noexp] OR Caribbean Region[Mesh] OR West Indies[Mesh] OR Central America[Mesh] OR Latin America[Mesh:noexp] OR North America[Mesh:noexp] OR South America[Mesh] OR Antarctic Regions[Mesh:noexp] OR Arctic Regions[Mesh:noexp] OR Asia[Mesh:noexp] OR Asia, Central[Mesh] OR Asia, Southeastern[Mesh:noexp] OR Asia, Western[Mesh:noexp] OR Middle East[Mesh:noexp] OR Far East[Mesh:noexp] OR Atlantic Islands[Mesh] OR Europe[Mesh:noexp] OR Europe, Eastern[Mesh] OR Scandinavia[Mesh:noexp] OR Transcaucasia[Mesh] OR Indian Ocean Islands[Mesh] OR Oceania[Mesh:noexp] OR Australasia[Mesh:noexp] OR Pacific Islands[Mesh:noexp] OR Melanesia[Mesh:noexp] OR Micronesia[Mesh:noexp] OR Polynesia[Mesh:noexp] OR Mexico[Mesh] OR Borneo[Mesh] OR Cambodia[Mesh] OR East Timor[Mesh] OR Indonesia[Mesh] OR Laos[Mesh] OR Malaysia[Mesh] OR Mekong Valley[Mesh] OR Myanmar[Mesh] OR Philippines[Mesh] OR Thailand[Mesh] OR Vietnam[Mesh] OR Bangladesh[Mesh] OR Bhutan[Mesh] OR India[Mesh] OR Afghanistan[Mesh] OR Bahrain[Mesh] OR Iran[Mesh] OR Iraq[Mesh] OR Jordan[Mesh] OR Kuwait[Mesh] OR Lebanon[Mesh] OR Oman[Mesh] OR Qatar[Mesh] OR Saudi Arabia[Mesh] OR Syria[Mesh] OR Turkey[Mesh] OR United Arab Emirates[Mesh] OR Yemen[Mesh] OR Nepal[Mesh] OR Pakistan[Mesh] OR Sri Lanka[Mesh] OR China[Mesh] OR Macao[Mesh] OR Mongolia[Mesh] OR Taiwan[Mesh] OR Multicenter Studies As Topic[Mesh] OR Multicenter Study[PT] OR Algeria[PL] OR Egypt[PL] OR Libya[PL] OR Morocco[PL] OR Tunisia[PL] OR Cameroon[PL] OR Central African Republic[PL] OR Chad[PL] OR Congo[PL] OR Congo[PL] OR Equatorial Guinea[PL] OR Gabon[PL] OR Burundi[PL] OR Djibouti[PL] OR Eritrea[PL] OR Ethiopia[PL] OR Kenya[PL] OR Rwanda[PL] OR Somalia[PL] OR Sudan[PL] OR Tanzania[PL] OR Uganda[PL] OR Angola[PL] OR Botswana[PL] OR Lesotho[PL] OR Malawi[PL] OR Mozambique[PL] OR Namibia[PL] OR South Africa[PL] OR Swaziland[PL] OR Zambia[PL] OR Zimbabwe[PL] OR Benin[PL] OR Burkina Faso[PL] OR Cote d'Ivoire[PL] OR Gambia[PL] OR Ghana[PL] OR Guinea[PL] OR Guinea-Bissau[PL] OR Liberia[PL] OR Mali[PL] OR Mauritania[PL] OR Niger[PL] OR Nigeria[PL] OR Senegal[PL] OR Sierra Leone[PL] OR Togo[PL] OR | | |

| | | | |
|---|---|---|---|
| | Antigua[PL] OR Bahamas[PL] OR Barbados[PL] OR Cuba[PL] OR Dominica[PL] OR Dominican Republic[PL] OR Grenada[PL] OR Guadeloupe[PL] OR Haiti[PL] OR Jamaica[PL] OR Martinique[PL] OR Netherlands Antilles[PL] OR Puerto Rico[PL] OR Saint Kitts and Nevis[PL] OR Saint Lucia[PL] OR Saint Vincent[PL] OR Grenadines[PL] OR Trinidad and Tobago[PL] OR Virgin Islands[PL] OR Belize[PL] OR Costa Rica[PL] OR El Salvador[PL] OR Guatemala[PL] OR Honduras[PL] OR Nicaragua[PL] OR Panama[PL] OR Mexico[PL] OR Argentina[PL] OR Bolivia[PL] OR Brazil[PL] OR Chile[PL] OR Colombia[PL] OR Ecuador[PL] OR French Guiana[PL] OR French Guiana[PL] OR Paraguay[PL] OR Peru[PL] OR Suriname[PL] OR Uruguay[PL] OR Venezuela[PL] OR Kazakhstan[PL] OR Kyrgyzstan[PL] OR Tajikistan[PL] OR Turkmenistan[PL] OR Uzbekistan[PL] OR Borneo[PL] OR Cambodia[PL] OR East Timor[PL] OR Indonesia[PL] OR Laos[PL] OR Malaysia[PL] OR Mekong Valley[PL] OR Myanmar[PL] OR Philippines[PL] OR Thailand[PL] OR Vietnam[PL] OR Bangladesh[PL] OR Bhutan[PL] OR India[PL] OR Afghanistan[PL] OR Bahrain[PL] OR Iran[PL] OR Iraq[PL] OR Jordan[PL] OR Kuwait[PL] OR Lebanon[PL] OR Oman[PL] OR Qatar[PL] OR Saudi Arabia[PL] OR Syria[PL] OR Turkey[PL] OR United Arab Emirates[PL] OR Yemen[PL] OR Nepal[PL] OR Pakistan[PL] OR Sri Lanka[PL] OR China[PL] OR Macao[PL] OR Mongolia[PL] OR Taiwan[PL] OR Azores[PL] OR Bermuda[PL] OR Falkland Islands[PL] OR Albania[PL] OR Estonia[PL] OR Latvia[PL] OR Lithuania[PL] OR Bosnia-Herzegovina[PL] OR Bulgaria[PL] OR Byelarus[PL] OR Croatia[PL] OR Czech Republic[PL] OR Hungary[PL] OR Macedonia[PL] OR Moldova[PL] OR Montenegro[PL] OR Poland[PL] OR Romania[PL] OR Russia[PL] OR Slovakia[PL] OR Slovenia[PL] OR Ukraine[PL] OR Armenia[PL] OR Azerbaijan[PL] OR Georgia[PL] OR Comoros[PL] OR Madagascar[PL] OR Mauritius[PL] OR Reunion[PL] OR Seychelles[PL] OR Fiji[PL] OR New Caledonia[PL] OR Papua New Guinea[PL] OR Vanuatu[PL] OR Guam[PL] OR Palau[PL] OR Pitcairn Island[PL] OR Samoa[PL] OR Tonga[PL] OR Czechoslovakia[PL] OR Germany, East[PL] OR New Guinea[PL] OR USSR[PL] OR Yugoslavia[PL] OR Ivory Coast[PL] OR Hong Kong[PL] OR republic of china[PL]))) | | |
| #1 | Search ((Canada[Mesh] OR Greenland[Mesh] OR United States[Mesh] OR Brunei[Mesh] OR Singapore[Mesh] OR Israel[Mesh] OR Japan[Mesh] OR Korea[Mesh] OR Australia[Mesh] OR Andorra[Mesh] OR Austria[Mesh] OR Belgium[Mesh] OR Finland[Mesh] OR France[Mesh] OR Germany[Mesh] OR Gibraltar[Mesh] OR Great Britain[Mesh] OR Greece[Mesh] OR Iceland[Mesh] OR Ireland[Mesh] OR Italy[Mesh] OR Liechtenstein[Mesh] OR Luxembourg[Mesh] OR Mediterranean Region[Mesh] OR Monaco[Mesh] OR Netherlands[Mesh] OR Portugal[Mesh] OR San Marino[Mesh] OR Scandinavia[Mesh] OR Spain[Mesh] OR Switzerland[Mesh] OR Vatican City[Mesh] OR Australia[Mesh] OR New Zealand[Mesh] OR Brunei[TIAB] OR Japan[TIAB] OR South Korea[TIAB] OR Singapore[TIAB] OR Andorra[TIAB] OR Austria[TIAB] OR Belgium[TIAB] OR Cyprus[TIAB] OR Denmark[TIAB] OR Finland[TIAB] OR France[TIAB] OR Germany[TIAB] OR Gibraltar[TIAB] OR Greece[TIAB] OR Greenland[TIAB] OR Vatican[TIAB] OR Iceland[TIAB] OR Ireland[TIAB] OR Israel[TIAB] OR Italy[TIAB] OR Liechtenstein[TIAB] OR Luxembourg[TIAB] OR Malta[TIAB] OR Monaco[TIAB] OR Netherlands[TIAB] OR Norway[TIAB] OR Portugal[TIAB] OR San Marino[TIAB] OR Spain[TIAB] OR Sweden[TIAB] OR Switzerland[TIAB] OR United Kingdom[TIAB] OR England[TIAB] OR Wales[TIAB] OR Scotland[TIAB] OR Canada[TIAB] OR United States[TIAB] OR Australia[TIAB] OR New Zealand[TIAB])) | 3182449 | 12:27:20 |

## References


1. Johnell O, Kanis JA, Oden A, Johansson H, De Laet C, Delmas P, Eisman JA, Fujiwara S, Kroger H, Mellstrom D, Meunier PJ, Melton LJ, 3rd, O'Neill T, Pols H, Reeve J, Silman A, Tenenhouse A (2005) Predictive value of BMD for hip and other fractures. J Bone Miner Res 20 (7):1185-1194

# Impaired Kidney Function Capstone Appendix

## Flowchart

Impaired Kidney Function

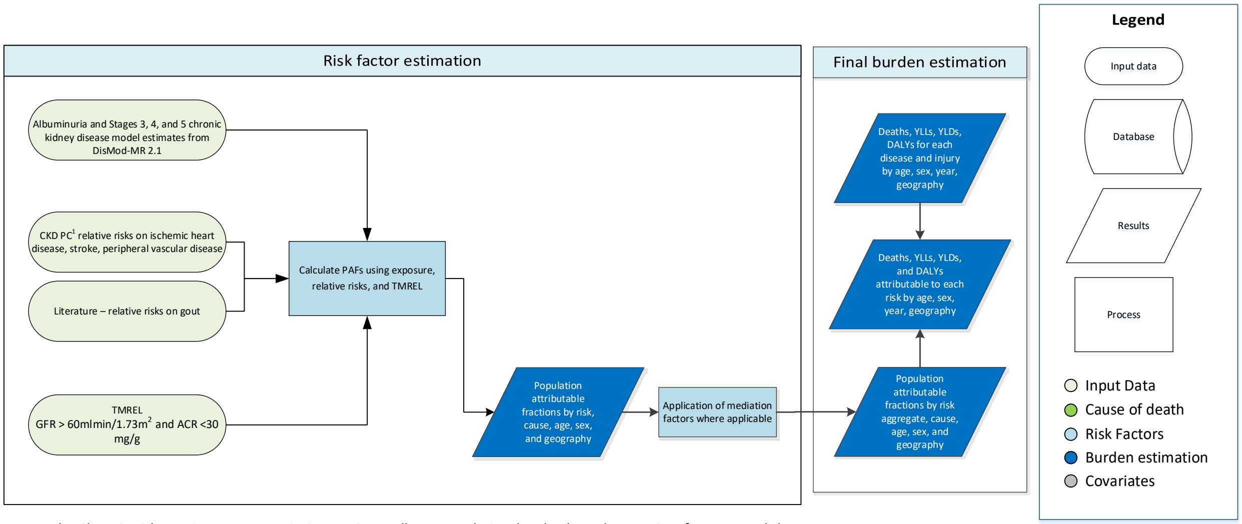


1. The Chronic Kidney Disease Prognosis Consortium collects population-level cohort data on CKD from around the world for the purpose of collective meta-analyses

## Input Data & Methodological Summary

### Exposure

#### *Case Definition*

The impaired kidney function risk factor exposure is divided into four categories of renal function defined by urinary albumin to creatinine ratio (ACR) and estimated glomerular filtration rate (GFR): albuminuria with preserved GFR (ACR >30 mg/g & GFR >=60 ml/min/1.73m$^2$), chronic kidney disease (CKD) stage 3 (GFR of 30-59 ml/min/1.73m$^2$), CKD stage 4 (GFR of 15-29 ml/min/1.73m$^2$), and CKD stage 5 (GFR <15ml/min/1.73m$^2$, not yet on renal replacement therapy). The modelling of renal function prevalence estimates is described in detail in the appendix to the GBD 2017 non-fatal capstone paper as these are also disease sequelae.

#### *Input data*

For GBD 2010, a systematic review of the prevalence of low glomerular filtration rate throughout the world was conducted. This search was updated for GBD 2013, GBD 2015, and 2016. Exclusion criteria included surveys that were not population-representative, studies not reporting on CKD by stage, and studies not reporting on albuminuria with preserved GFR (GFR >=60 ml/min/1.73m$^2$).

| Disease | Number of sources | Number of countries | Number of new sources for GBD 2016 |
|---|---|---|---|
| Albuminuria | 72 | 31 | 72 |
| CKD Stage III | 112 | 47 | 49 |
| CKD Stage IV | 94 | 40 | 45 |
| CKD Stage V | 92 | 38 | 49 |

## Modeling strategy

Estimates of exposure to albuminuria and CKD were obtained from the GBD 2017 non-fatal burden of disease analysis, which includes stage-specific prevalence estimates at the country level across twenty-three age-groups for both genders. The modeling strategy for these estimates is detailed in the appendix to the GBD 2017 non-fatal capstone paper.

Relative risks were calculated by the Chronic Kidney Disease Prognosis Consortium, a consortium composed of population-level cohorts with prospective data collection from several countries (details below). YLDs and YLLs for cardiovascular diseases and gout were obtained from the GBD 2017 Study for the same geographic, time-period, and age-groups as detailed above.

## Theoretical minimum-risk exposure level

The theoretical minimum risk is a diagnosis of albuminuria or CKD stages 3, 4, or 5. An ACR above 30 mg/g and eGFR below 60ml/min/1.73m$^2$ have been demonstrated in the literature to be the thresholds at which increased cardiovascular and gout events occur secondary to impaired kidney function. (1-10)

## Relative risk

A two-stage pooled meta-analysis was used to calculate relative risks for ischemic heart disease, stroke, and peripheral vascular disease. The relative risk of these conditions was first determined within each cohort, and then a pooled analysis of cohort-level relative risks was performed using a random effects meta-analysis approach. Uncertainty intervals largely overlapped for the relative risks of fatal and nonfatal cardiovascular events from impaired kidney function exposure. Thus, we decided to use the relative risks from the combined analysis for fatal and nonfatal cardiovascular outcomes. Gout relative risk was determined by meta-analysis of a literature review performed for GBD 2013. Search terms included “gout” and “chronic kidney disease”. Exclusion criteria for search results included special populations, reversal of exposure and outcome categories, or unclear exposure category definition. This search resulted in four eligible studies; no new studies indicated an increased risk of gout with albuminuria.

## Population Attributable Fraction

We calculated the cardiovascular and gout fatal and nonfatal burden attributable to the categorical exposure to impaired kidney function using the following equation:

$$PAF = \frac{\sum_{i=1}^{n} P_i(RR_i - 1)}{\sum_{i=1}^{n} P_i(RR_i - 1) + 1}$$

**Equation 1.** PAF based on categorical exposure

where $RR_i$ is the relative risk for exposure level *i*, $P_i$ is the proportion of the population in that exposure category, and *n* is the number of exposure categories.(11)

## References


1. Go AS, Chertow GM, Fan D, McCulloch CE, Hsu CY. Chronic kidney disease and the risks of death, cardiovascular events, and hospitalization. N Engl J Med. 2004;351(13):1296-305.
2. Ninomiya T, Kiyohara Y, Kubo M, Tanizaki Y, Doi Y, Okubo K, et al. Chronic kidney disease and cardiovascular disease in a general Japanese population: the Hisayama Study. Kidney international. 2005;68(1):228-36.
3. Shara NM, Wang H, Mete M, Al-Balha YR, Azalddin N, Lee ET, et al. Estimated GFR and incident cardiovascular disease events in American Indians: the Strong Heart Study. American journal of kidney diseases : the official journal of the National Kidney Foundation. 2012;60(5):795-803.
4. Mann JF, Gerstein HC, Pogue J, Bosch J, Yusuf S. Renal insufficiency as a predictor of cardiovascular outcomes and the impact of ramipril: the HOPE randomized trial. Annals of internal medicine. 2001;134(8):629-36.
5. Chronic Kidney Disease Prognosis C, Matsushita K, van der Velde M, Astor BC, Woodward M, Levey AS, et al. Association of estimated glomerular filtration rate and albuminuria with all-cause and cardiovascular mortality in general population cohorts: a collaborative meta-analysis. Lancet. 2010;375(9731):2073-81.
6. De Graauw J, Chonchol M, Poppert H, Etgen T, Sander D. Relationship between kidney function and risk of asymptomatic peripheral arterial disease in elderly subjects. Nephrology, dialysis, transplantation : official publication of the European Dialysis and Transplant Association - European Renal Association. 2011;26(3):927-32.
7. Wattanakit K, Folsom AR, Selvin E, Coresh J, Hirsch AT, Weatherley BD. Kidney function and risk of peripheral arterial disease: results from the Atherosclerosis Risk in Communities (ARIC) Study. Journal of the American Society of Nephrology : JASN. 2007;18(2):629-36.
8. O'Hare AM, Vittinghoff E, Hsia J, Shlipak MG. Renal insufficiency and the risk of lower extremity peripheral arterial disease: results from the Heart and Estrogen/Progestin Replacement Study (HERS). Journal of the American Society of Nephrology : JASN. 2004;15(4):1046-51.
9. Manjunath G, Tighiouart H, Coresh J, Macleod B, Salem DN, Griffith JL, et al. Level of kidney function as a risk factor for cardiovascular outcomes in the elderly. Kidney international. 2003;63(3):1121-9.
10. Manjunath G, Tighiouart H, Ibrahim H, MacLeod B, Salem DN, Griffith JL, et al. Level of kidney function as a risk factor for atherosclerotic cardiovascular outcomes in the community. Journal of the American College of Cardiology. 2003;41(1):47-55.
11. Miettinen OS. Proportion of disease caused or prevented by a given exposure, trait or intervention. American journal of epidemiology. 1974;99(5):325-32.

# Section 5: Tables and Figures

**Appendix Figure 1. Analytical flowchart of the comparative risk assessment for the estimation of population attributable fractions by geography, age, sex, and year for GBD 2017.** Ovals represent data inputs, rectangular boxes represent analytical steps, cylinders represent databases, and parallelograms represent intermediate and final results. GBD=Global Burden of Disease. SEVs=Summary exposure values. TMREL=Theoretical minimum-risk exposure level. PAFs=Population attributable fractions. YLLs=years of life lost. YLDs=years lived with disability. DALYs=disability-adjusted life-years.

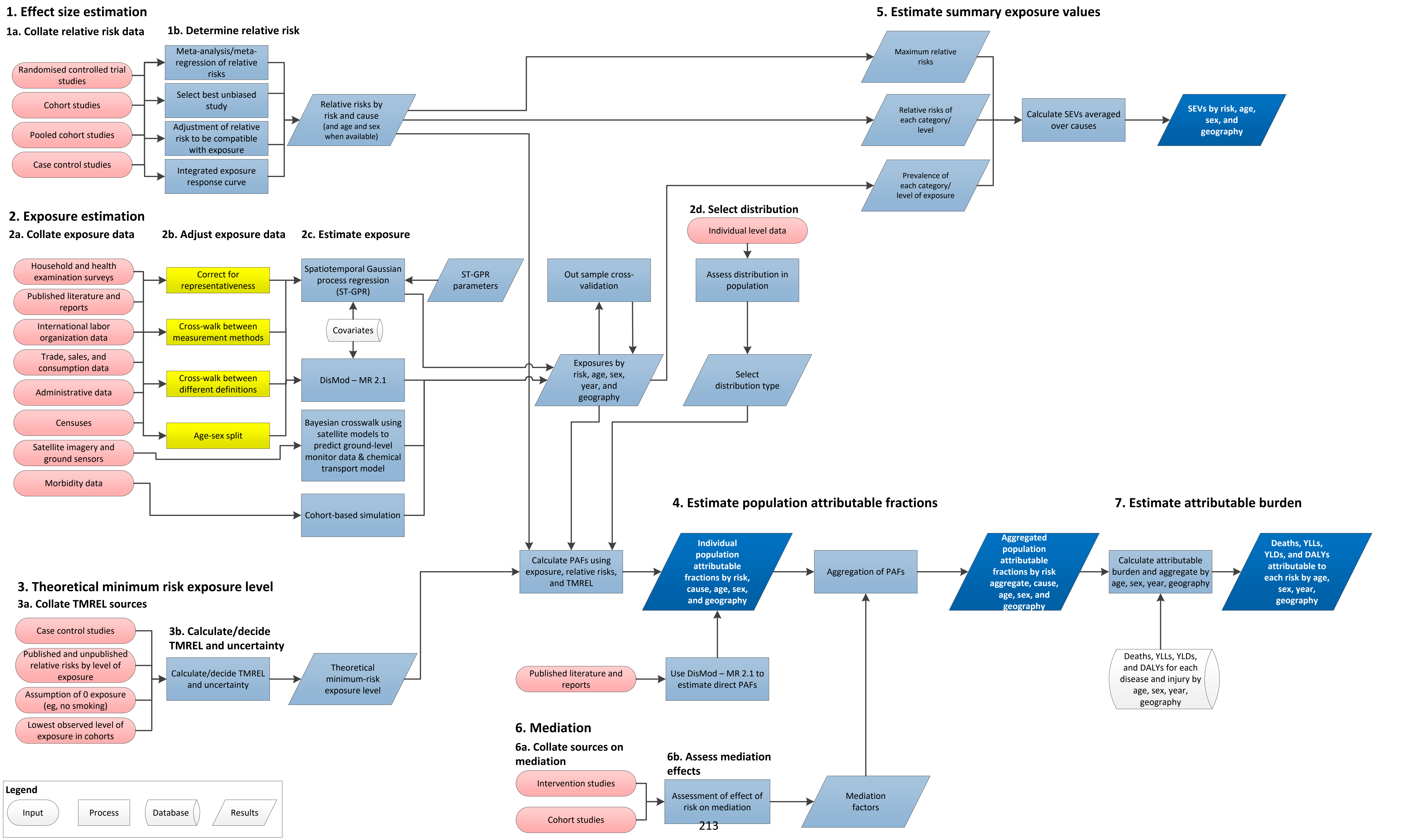

**Appendix Figure 2. GBD 2017 DisMod-MR 2.1 analytical cascade**

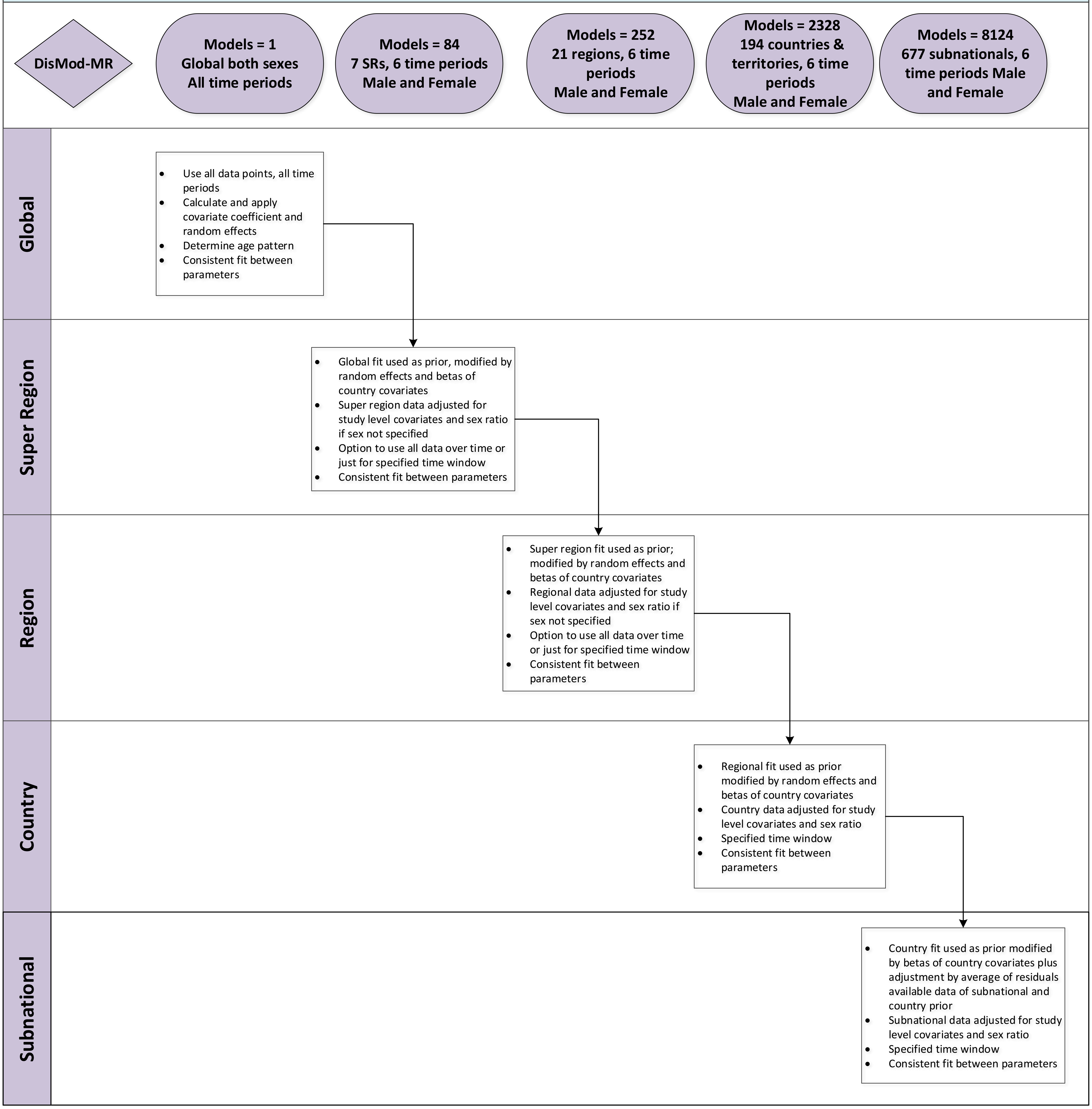

# Appendix Figure 3: Spatiotemporal Gaussian Process Regression

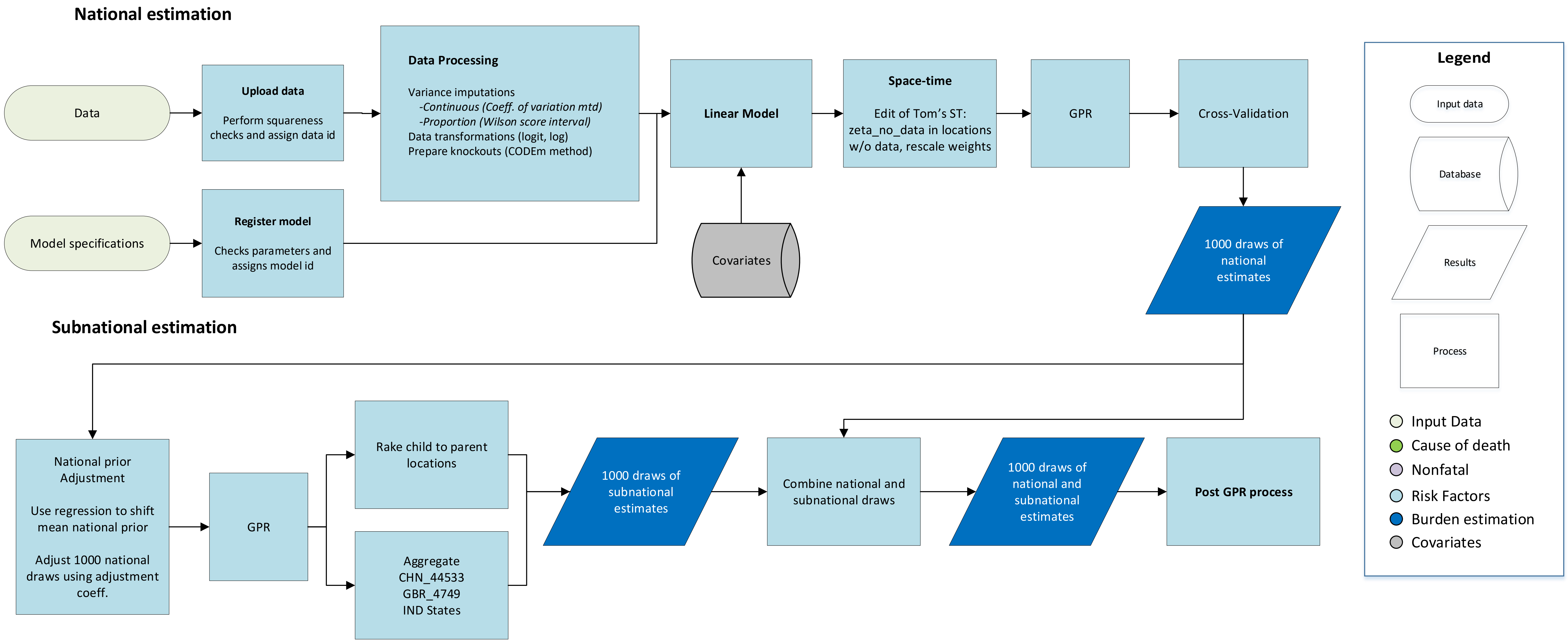

Appendix Table 1. GATHER checklist of information that should be included in reports of global health estimates, with description of compliance and location of information for GBD 2017.

| # | GATHER checklist item | Description of compliance | Reference |
|---|---|---|---|
| **Objectives and funding** | | | |
| 1 | Define the indicators, populations, and time periods for which estimates were made. | Narrative provided in paper and methods appendix describing indicators, definitions, and populations | Main text (Methods—Overview, Geographic units and time periods) and methods appendix |
| 2 | List the funding sources for the work. | Funding sources listed in paper | Summary (Funding) |
| **Data Inputs** | | | |
| *For all data inputs from multiple sources that are synthesized as part of the study:* | | | |
| 3 | Describe how the data were identified and how the data were accessed. | Narrative description of data seeking methods provided | Main text (Methods) and methods appendix |
| 4 | Specify the inclusion and exclusion criteria. Identify all ad-hoc exclusions. | Narrative about inclusion and exclusion criteria by data type provided | Main text (Methods) and methods appendix |
| 5 | Provide information on all included data sources and their main characteristics. For each data source used, report reference information or contact name/institution, population represented, data collection method, year(s) of data collection, sex and age range, diagnostic criteria or measurement method, and sample size, as relevant. | An interactive, online data source tool that provides metadata for data sources by component, geography, cause, risk, or impairment has been developed | Online data citation tools |
| 6 | Identify and describe any categories of input data that have potentially important biases (e.g., based on characteristics listed in item 5). | Summary of known biases by cause included in methods appendix | Methods appendix |
| *For data inputs that contribute to the analysis but were not synthesized as part of the study:* | | | |
| 7 | Describe and give sources for any other data inputs. | Included in online data source tool, http://ghdx.healthdata.org/gbd-2017 | Online data citation tools |
| *For all data inputs:* | | | |
| 8 | Provide all data inputs in a file format from which data can be efficiently extracted (e.g., a spreadsheet as opposed to a PDF), including all relevant meta-data listed in item 5. For any data inputs that cannot be shared due to ethical or legal reasons, such as third-party ownership, provide a contact name or the name of the institution that retains the right to the data. | Downloads of input data available through online tools, including data visualization tools and data query tools, http://ghdx.healthdata. | Online data visualization tools, data query tools, and the Global Health Data Exchange, http://ghdx.healthdata.org |

| | | | |
|---|---|---|---|
| | | org/gbd-2017; input data not available in tools will be made available upon request | |
| **Data analysis** | | | |
| 9 | Provide a conceptual overview of the data analysis method. A diagram may be helpful. | Flow diagrams of the overall methodological processes, as well as cause-specific modelling processes, have been provided | Main text (Methods) and methods appendix |
| 10 | Provide a detailed description of all steps of the analysis, including mathematical formulae. This description should cover, as relevant, data cleaning, data pre-processing, data adjustments and weighting of data sources, and mathematical or statistical model(s). | Flow diagrams and corresponding methodological write-ups for each cause, as well as the demographics and causes of death databases and modelling processes, have been provided | Main text (Methods) and methods appendix |
| 11 | Describe how candidate models were evaluated and how the final model(s) were selected. | Provided in the methodological write-ups | Methods appendix |
| 12 | Provide the results of an evaluation of model performance, if done, as well as the results of any relevant sensitivity analysis. | Provided in the methodological write-ups | Methods appendix |
| 13 | Describe methods for calculating uncertainty of the estimates. State which sources of uncertainty were, and were not, accounted for in the uncertainty analysis. | Provided in the methodological write-ups | Methods appendix |
| 14 | State how analytic or statistical source code used to generate estimates can be accessed. | Access statement provided | Code is provided in an online repository |
| **Results and Discussion** | | | |
| 15 | Provide published estimates in a file format from which data can be efficiently extracted. | Results are available through online data visualization tools, the Global Health Data Exchange, and the online data query tool (http://ghdx.healthdata.org/gbd-2017) | Main text, methods appendix, and online data tools (data visualization tools, data query tools, and the Global Health Data Exchange, http://ghdx.healthdata.org/gbd-2017) |
| 16 | Report a quantitative measure of the uncertainty of the estimates (e.g. uncertainty intervals). | Uncertainty intervals are provided with all results | Main text, methods appendix, and online data tools (data visualization tools, data query tools, and the |

| | | | |
|---|---|---|---|
| | | | Global Health Data Exchange, http://ghdx.healthdata.org/gbd-2017) |
| 17 | Interpret results in light of existing evidence. If updating a previous set of estimates, describe the reasons for changes in estimates. | Discussion of methodological changes between GBD rounds provided in the narrative of the Article and methods appendix | Main text (Methods and Discussion) and methods appendix |
| 18 | Discuss limitations of the estimates. Include a discussion of any modelling assumptions or data limitations that affect interpretation of the estimates. | Discussion of limitations provided in the narrative of the main paper, as well as in the methodological write-ups in the methods appendix | Main text (Limitations) and methods appendix |

| **Appendix Table 2. GBD 2017 risk factor hierarchy with levels, modeling strategies, and the main type of data sources used to estimate exposure levels**<br>GBD=Global Burden of Disease. | | | |
|---|---|---|---|
| **Risk factor** | **Level** | **Model type** | **Main data source for exposure** |
| **All risk factors** | **0** | | |
| **Environmental/occupational risks** | **1** | | |
| **Unsafe water, sanitation, and handwashing** | **2** | | |
| Unsafe water source | 3 | Spatiotemporal Gauissian process regression (ST-GPR) | Population surveys and censuses |
| Unsafe sanitation | 3 | ST-GPR | Population surveys and censuses |
| No handwashing with soap | 3 | ST-GPR | Population surveys, censuses, and epidemiological studies |
| **Air pollution** | **2** | | |
| Particulate matter pollution | 3 | | |
| Ambient particulate matter pollution | 4 | Regression crosswalk between grid-level fusion of satellite/chemical transport models and ground level monitoring data | Atmospheric chemical transport models, satellite measurements of aerosols in the atmosphere, data from ground-level monitoring sites |
| Household air pollution from solid fuels | 4 | ST-GPR | Population surveys and censuses |
| Ambient ozone pollution | 3 | Chemical transport model | Atmospheric chemical transport models |
| **Other environmental risks** | **2** | | |
| Residential radon | 3 | ST-GPR | Literature review |
| Lead exposure | 3 | ST-GPR | Literature review |
| **Occupational risks** | **2** | | |
| Occupational carcinogens | 3 | | |
| Occupational exposure to asbestos | 4 | Asbestos Impact Ratio approach | GBD cause-specific mortality data for mesothelioma, epidemiological studies |
| Occupational exposure to arsenic | 4 | ST-GPR | Labor force surveys, censuses, and international information system on occupational exposure to carcinogens |
| Occupational exposure to benzene | 4 | ST-GPR | Labor force surveys, censuses, and international information system on occupational exposure to carcinogens |
| Occupational exposure to beryllium | 4 | ST-GPR | Labor force surveys, censuses, and international information system on occupational exposure to carcinogens |
| Occupational exposure to cadmium | 4 | ST-GPR | Labor force surveys, censuses, and international information system on occupational exposure to carcinogens |
| Occupational exposure to chromium | 4 | ST-GPR | Labor force surveys, censuses, and international information system on occupational exposure to carcinogens |
| Occupational exposure to diesel engine exhaust | 4 | ST-GPR | Labor force surveys, censuses, and international information system on occupational exposure to carcinogens |
| Occupational exposure to formaldehyde | 4 | ST-GPR | Labor force surveys, censuses, and international information system on occupational exposure to carcinogens |
| Occupational exposure to nickel | 4 | ST-GPR | Labor force surveys, censuses, and international information system on occupational exposure to carcinogens |
| Occupational exposure to polycyclic aromatic hydrocarbons | 4 | ST-GPR | Labor force surveys, censuses, and international information system on occupational exposure to carcinogens |
| Occupational exposure to silica | 4 | ST-GPR | Labor force surveys, censuses, and international information system on occupational exposure to carcinogens |
| Occupational exposure to sulfphuric acid | 4 | ST-GPR | Labor force surveys, censuses, and international information system on occupational exposure to carcinogens |
| Occupational exposure to trichloroethylene | 4 | ST-GPR | Labor force surveys, censuses, and international information system on occupational exposure to carcinogens |
| Occupational asthmagens | 3 | ST-GPR | Labor force surveys and censuses |
| Occupational particulate matter, gases, and fumes | 3 | ST-GPR | Labor force surveys and censuses |
| Occupational noise | 3 | ST-GPR | Labor force surveys and censuses, industry-based surveys of noise exposure |
| Occupational injuries | 3 | ST-GPR | International Labor Organization injury database |
| Occupational ergonomic factors | 3 | ST-GPR | Labor force surveys and censuses |
| **Behavioural risks** | **1** | | |
| **Child and maternal malnutrition** | **2** | | |
| Suboptimal breastfeeding | 3 | | |
| Non-exclusive breastfeeding | 4 | ST-GPR | Population surveys |
| Discontinued breastfeeding | 4 | ST-GPR | Population surveys |
| Childhood undernutrition | 3 | | |
| Childhood underweight | 4 | ST-GPR | Examination surveys and epidemiological studies |
| Childhood wasting | 4 | ST-GPR | Examination surveys and epidemiological studies |
| Childhood stunting | 4 | ST-GPR | Examination surveys and epidemiological studies |
| Iron deficiency | 3 | Mixed effect regression | Examination surveys and epidemiological studies |
| Vitamin A deficiency | 3 | DisMod-MR 2.1 | Examination surveys and epidemiological studies |
| Zinc deficiency | 3 | Mixed effect regression based on stunting prevalence and dietary composition | FAO food balance sheets |
| **Tobacco smoke** | **2** | | |
| Smoking | 3 | • Smoking Impact Ratio (SIR) calculated from lung cancer mortality rates<br>• Smoking prevalence estimated using ST-GPR | SIR input data: mortality and cause of death data including vital registration and verbal autopsy<br>Smoking prevalence input data: nationally representative survey and report data |
| Second-hand smoke | 3 | DisMod-MR 2.1 | Household surveys and national health surveys |

| Risk factor | Level | Model type | Main data source for exposure |
|---|---|---|---|
| **Alcohol use** | **2** | • Alcohol consumption per capita obtained from the FAO and the WHO Global Information System on Alcohol and Health (GISAH)<br>• ST-GPR used to integrate the data and to derive coherent time series for each country<br>• Prevalence of current alcohol drinkers, lifetime abstainers, former drinkers, and binge drinkers estimated using DisMod-MR 2.1<br>• DisMod-MR 2.1 used to estimate the relative sex- and age-specific pattern of alcohol consumption in current drinkers | Population surveys, alcohol sales, production, and other economic statistics |
| **Drug Use** | **2** | DisMod-MR 2.1 | Systematic review of published literature, reports from governments and international organizations, which include data from: school surveys, population surveys, registration data, and indirect estimates of prevalence |
| **Dietary risks** | **2** | | |
| Diet low in fruits | 3 | DisMod-MR 2.1 | Nutrition and health surveys, FAO food balance sheets |
| Diet low in vegetables | 3 | DisMod-MR 2.1 | Nutrition and health surveys, FAO food balance sheets |
| Diet low in whole grains | 3 | DisMod-MR 2.1 | Nutrition and health surveys |
| Diet low in nuts and seeds | 3 | DisMod-MR 2.1 | Nutrition and health surveys, FAO food balance sheets |
| Diet low in milk | 3 | DisMod-MR 2.1 | Nutrition and health surveys, FAO food balance sheets |
| Diet high in red meat | 3 | DisMod-MR 2.1 | Nutrition and health surveys, FAO food balance sheets |
| Diet high in processed meat | 3 | DisMod-MR 2.1 | Nutrition and health surveys |
| Diet high in sugar-sweetened beverages | 3 | DisMod-MR 2.1 | Nutrition and health surveys |
| Diet low in fibre | 3 | DisMod-MR 2.1 | Nutrition and health surveys, FAO SUA/USDA |
| Diet suboptimal in calcium | 3 | DisMod-MR 2.1 | Nutrition and health surveys, FAO SUA/USDA |
| Diet low in seafood omega-3 fatty acids | 3 | DisMod-MR 2.1 | Nutrition and health surveys, FAO SUA/USDA |
| Diet low in polyunsaturated fatty acids | 3 | DisMod-MR 2.1 | Nutrition and health surveys, FAO SUA/USDA |
| Diet high in trans fatty acids | 3 | DisMod-MR 2.1 | Nutrition and health surveys |
| Diet high in sodium | 3 | DisMod-MR 2.1 | Nutrition and health surveys |
| **Intimate partner violence** | **2** | DisMod-MR 2.1 | Systematic review of published literature, national health surveys, violence-specific surveys |
| **Childhood maltreatment** | **2** | | |
| Childhood sexual abuse | 3 | DisMod-MR 2.1 | Systematic review of published literature, national health surveys, violence-specific surveys |
| Bullying victimization | 3 | DisMod-MR 2.1 | Systematic review of published literature and surveys reporting bullying victimization, including Global School-based Student Health Survey (GSHS), the Health Behavior in School-aged Children (HBSC), and the National Crime Victimization Survey – School Crime Supplement (NCVS-SCS). |
| **Unsafe sex** | 2 | DisMod-MR 2.1 | UNAIDS country progress reports, disease surveillance reports |
| **Low physical activity** | 2 | DisMod-MR 2.1 | Surveys of the adult population that capture reported frequency, duration and intensity of physical activity undertaken in the past seven days across all domains of life (work, transport, recreation or house/yard work) |
| **Metabolic risks** | **1** | | |
| High fasting plasma glucose | 2 | ST-GPR | Examination surveys and epidemiological studies |
| High LDL cholesterol | 2 | ST-GPR | Examination surveys and epidemiological studies |
| High systolic blood pressure | 2 | ST-GPR | Examination surveys and epidemiological studies |
| High body-mass index | 2 | ST-GPR | Examination surveys and epidemiological studies |
| Low bone mineral density | 2 | DisMod-MR 2.1 | Examination surveys and epidemiological studies |
| Low glomerular filtration rate | 2 | DisMod-MR 2.1 | Examination surveys and epidemiological studies |

**Appendix Table 3. Types of Comparative Risk Assessments (CRA) based on the time perspective and the nature of the counterfactual level or distribution of exposure.** The shaded box represents the type of CRA currently undertaken in GBD 2017. GBD=Global Burden of Disease.

| | **Counterfactual distributions of exposure** | | | |
|---|---|---|---|---|
| Construct | Theoretical minimum risk: level of risk with the lowest level of burden | Plausible minimum risk: level of risk with the lowest level of burden that could be imagined with current technology and knowledge | Feasible minimum risk: level of risk with the lowest level of burden that has been achieved in any population | Cost-effective minimum risk: lowest level of risk that can be achieved cost-effectively in a given population |
| **Attributable burden:** burden of disease today that would be avoided if each individual in the past had been exposed to the counterfactual level of exposure | **Currently in GBD** | | | |
| **Avoidable burden:** burden of disease in the future that would be avoided if each individual today was shifted to the counterfactual level of exposure | | | | |

**Appendix Table 4. Descriptive cataloguing of the epidemiological evidence used to assess whether each risk-outcome paper meets the causal criteria for inclusion in the Global Burden of Disease**

| Risk Level | Risk | Outcome | RCTs (n) | RCTs with significant effect in the opposite direction (%) | RCTs with null findings (%) | Prospective observational studies (n)* | Prospective observational studies with significant association in the opposite direction (%) | Case-control studies assessing the risk-outcome pair relationship (n)** | Case-control studies that show significant association in the opposite direction (%) | Lower limit of RR > 1.5 | Dose-response relationship | Biological plausibility † | Analogy ‡ |
|---|---|---|---|---|---|---|---|---|---|---|---|---|---|
| 2 | **Unsafe water, sanitation, and handwashing** | | | | | | | | | | | | |
| 3 | Unsafe water source - chlorination or solar (point of use treatment) | Diarrhoeal diseases | 25 | 0 | 44 | 6 | 0 | - | - | Yes | - | Yes | No |
| 3 | Unsafe water source - piped | Diarrhoeal diseases | 1 | 0 | 0 | 9 | 11 | - | - | Yes | - | Yes | No |
| 3 | Unsafe water source - filter | Diarrhoeal diseases | 11 | 0 | 45 | 2 | 0 | - | - | Yes | - | Yes | No |
| 3 | Unsafe water source - improved water | Diarrhoeal diseases | 0 | - | - | 5 | 0 | - | - | Yes | - | Yes | No |
| 3 | Unsafe sanitation - piped | Diarrhoeal diseases | 0 | - | - | 7 | 0 | - | - | Yes | - | Yes | No |
| 3 | Unsafe sanitation - improved sanitation | Diarrhoeal diseases | 1 | - | 100 | 11 | 0 | - | - | Yes | - | Yes | No |
| 3 | No access to handwashing facility | Diarrhoeal diseases | 19 | 0 | 42 | 0 | - | - | - | No | - | Yes | No |
| 3 | No access to handwashing facility | Lower respiratory infections | 8 | 0 | 50 | 11 | 0 | - | - | No | - | Yes | No |
| 2 | **Air pollution** | | | | | | | | | | | | |
| 3 | **Particulate matter pollution** | | | | | | | | | | | | |
| 4 | Ambient particulate matter pollution | Lower respiratory infections | 0 | - | - | 17 | 0 | - | - | No | Yes | Yes | No |
| 4 | Ambient particulate matter pollution | Tracheal, bronchus, and lung cancer | 0 | - | - | 30 | 0 | - | - | No | Yes | Yes | Yes |
| 4 | Ambient particulate matter pollution | Ischaemic heart disease | 0 | - | - | 16 | 0 | - | - | No | Yes | Yes | Yes |
| 4 | Ambient particulate matter pollution | Ischaemic stroke | 0 | - | - | 30 | 0 | - | - | No | Yes | Yes | Yes |
| 4 | Ambient particulate matter pollution | Intracerebral hemorrhage | 0 | - | - | 30 | 0 | - | - | No | Yes | Yes | Yes |
| 4 | Ambient particulate matter pollution | Subarachnoid hemorrhage | 0 | - | - | 30 | 0 | - | - | No | Yes | Yes | Yes |
| 4 | Ambient particulate matter pollution | Chronic obstructive pulmonary disease | 0 | - | - | 12 | 0 | - | - | No | Yes | Yes | Yes |
| 4 | Ambient particulate matter pollution | Diabetes mellitus type 2 | 0 | - | - | 8 | 0 | - | - | | | Yes | Yes |
| 4 | Household air pollution from solid fuels | Lower respiratory infections | 2 | 0 | 1 | 8 | 0 | 17 | 0 | No | Yes | Yes | No |
| 4 | Household air pollution from solid fuels | Tracheal, bronchus, and lung cancer | 0 | - | - | 0 | - | 28 | 0 | No | Yes | Yes | Yes |
| 4 | Household air pollution from solid fuels | Ischaemic heart disease | 0 | - | - | 2 | - | 2 | - | No | Yes | Yes | Yes |
| 4 | Household air pollution from solid fuels | Ischaemic stroke | 0 | - | - | 1 | - | - | - | No | Yes | Yes | Yes |
| 4 | Household air pollution from solid fuels | Intracerebral hemorrhage | 0 | - | - | 1 | - | - | - | No | Yes | Yes | Yes |
| 4 | Household air pollution from solid fuels | Subarachnoid hemorrhage | 0 | - | - | 1 | - | - | - | No | Yes | Yes | Yes |
| 4 | Household air pollution from solid fuels | Chronic obstructive pulmonary disease | 0 | - | - | 0 | - | 11 | 0 | No | Yes | Yes | Yes |
| 4 | Household air pollution from solid fuels | Diabetes mellitus type 2 | 0 | - | - | 1 | - | - | - | | | Yes | Yes |
| 4 | Household air pollution from solid fuels | Cataract | 0 | - | - | 0 | - | 8 | 0 | No | Yes | Yes | No |
| 3 | Ambient ozone pollution | Chronic obstructive pulmonary disease | 0 | - | - | 3 | 0 | - | - | No | Yes | Yes | No |
| 2 | **Other environmental risks** | | | | | | | | | | | | |
| 3 | Residential radon | Tracheal, bronchus, and lung cancer | 0 | - | - | 1 | 0 | 29 | 0 | No | Yes | Yes | No |
| 3 | Lead exposure | Idiopathic developmental intellectual disability | 0 | - | - | 8 | 0 | - | - | No | Yes | Yes | No |
| 3 | Lead exposure | Systolic blood pressure | 0 | - | - | 3 | 0 | 1 | 0 | No | Yes | Yes | No |
| 2 | **Occupational risks** | | | | | | | | | | | | |
| 4 | Occupational exposure to asbestos | Larynx cancer | 0 | - | - | 27 | 0 | - | - | No | - | Yes | Yes |
| 4 | Occupational exposure to asbestos | Tracheal, bronchus, and lung cancer | 0 | - | - | 18 | 0 | - | - | Yes | - | Yes | Yes |
| 4 | Occupational exposure to asbestos | Ovarian cancer | 0 | - | - | 15 | 0 | - | - | No | - | Yes | Yes |
| 4 | Occupational exposure to asbestos | Mesothelioma | 0 | - | - | 5 | 0 | - | - | Yes | - | Yes | Yes |
| 4 | Occupational exposure to arsenic | Tracheal, bronchus, and lung cancer | 0 | - | - | 9 | 0 | - | - | No | - | Yes | No |
| 4 | Occupational exposure to benzene | Leukaemia | 0 | - | - | 12 | 0 | - | - | Yes | - | Yes | No |
| 4 | Occupational exposure to beryllium | Tracheal, bronchus, and lung cancer | 0 | - | - | 3 | 0 | 2 | 0 | No | - | Yes | No |
| 4 | Occupational exposure to cadmium | Tracheal, bronchus, and lung cancer | 0 | - | - | 7 | 0 | - | - | No | - | Yes | No |
| 4 | Occupational exposure to chromium | Tracheal, bronchus, and lung cancer | 0 | - | - | 26 | 0 | - | - | No | - | Yes | No |
| 4 | Occupational exposure to diesel engine exhaust | Tracheal, bronchus, and lung cancer | 0 | - | - | 17 | 0 | - | - | No | - | Yes | No |
| 4 | Occupational exposure to formaldehyde | Nasopharynx cancer | 0 | - | - | 2 | 0 | 6 | 0 | No | - | Yes | Yes |
| 4 | Occupational exposure to formaldehyde | Leukaemia | 0 | - | - | 13 | 0 | - | - | No | - | Yes | Yes |
| 4 | Occupational exposure to nickel | Tracheal, bronchus, and lung cancer | 0 | - | - | 6 | 0 | - | - | No | - | Yes | No |
| 4 | Occupational exposure to polycyclic aromatic | Tracheal, bronchus, and lung cancer | 0 | - | - | 39 | 0 | - | - | No | - | Yes | No |
| 4 | Occupational exposure to silica | Tracheal, bronchus, and lung cancer | 0 | - | - | 17 | 0 | - | - | No | - | Yes | No |
| 4 | Occupational exposure to sulfphuric acid | Larynx cancer | 0 | - | - | 14 | 0 | - | - | Yes | - | Yes | No |
| 4 | Occupational exposure to trichloroethylene | Kidney cancer | 0 | - | - | 20 | 0 | - | - | No | - | Yes | No |
| 3 | Occupational asthmagens | Asthma | 0 | - | - | 16 | 0 | - | - | No | - | Yes | No |
| 3 | Occupational particulate matter, gases, and | Chronic obstructive pulmonary disease | 0 | - | - | 9 | 0 | - | - | No | - | Yes | No |
| 3 | Occupational noise | Age-related and other hearing loss | 0 | - | - | 5 | 0 | - | - | Yes | - | Yes | No |
| 3 | Occupational ergonomic factors | Low back pain | 0 | - | - | 10 | 0 | - | - | No | - | Yes | No |
| 2 | **Child and maternal malnutrition** | | | | | | | | | | | | |
| 4 | Non-exclusive breastfeeding | Diarrhoeal diseases | 0 | - | - | 5 | 0 | 7 | 0 | Yes | - | Yes | No |
| 4 | Non-exclusive breastfeeding | Lower respiratory infections | 0 | - | - | 6 | 0 | 8 | 0 | Yes | - | Yes | No |
| 4 | Discontinued breastfeeding | Diarrhoeal diseases | 0 | - | - | 2 | 0 | - | - | No | - | Yes | No |
| 4 | Child underweight | Diarrhoeal diseases | 0 | - | - | 7 | 0 | - | - | Yes | - | Yes | No |
| 4 | Child underweight | Lower respiratory infections | 0 | - | - | 7 | 0 | - | - | Yes | - | Yes | No |
| 4 | Child underweight | Measles | 0 | - | - | 7 | 0 | - | - | Yes | - | Yes | No |
| 4 | Child wasting | Diarrhoeal diseases | 0 | - | - | 7 | 0 | - | - | Yes | - | Yes | No |
| 4 | Child wasting | Lower respiratory infections | 0 | - | - | 7 | 0 | - | - | Yes | - | Yes | No |
| 4 | Child wasting | Measles | 0 | - | - | 7 | 0 | - | - | Yes | - | Yes | No |
| 4 | Child stunting | Diarrhoeal diseases | 0 | - | - | 7 | 0 | - | - | No | - | Yes | No |
| 4 | Child stunting | Lower respiratory infections | 0 | - | - | 7 | 0 | - | - | No | - | Yes | No |
| 4 | Child stunting | Measles | 0 | - | - | 7 | 0 | - | - | No | - | Yes | No |

| Risk Level | Risk | Outcome | RCTs (n) | RCTs with significant effect in the opposite direction (%) | RCTs with null findings (%) | Prospective observational studies (n)* | Prospective observational studies with significant association in the opposite direction (%) | Case-control studies assessing the risk-outcome pair relationship (n)** | Case-control studies that show significant association in the opposite direction (%) | Lower limit of RR > 1.5 | Dose-response relationship | Biological plausibility † | Analogy ‡ |
|---|---|---|---|---|---|---|---|---|---|---|---|---|---|
| 4 | Short gestation for birth weight | Diarrhoeal diseases | 0 | - | - | 20 | 0 | - | - | Yes | Yes | Yes | Yes |
| 4 | Short gestation for birth weight | Lower respiratory infections | 0 | - | - | 20 | 0 | - | - | Yes | Yes | Yes | Yes |
| 4 | Short gestation for birth weight | Upper respiratory infections | 0 | - | - | 20 | 0 | - | - | Yes | Yes | Yes | Yes |
| 4 | Short gestation for birth weight | Otitis media | 0 | - | - | 20 | 0 | - | - | Yes | Yes | Yes | Yes |
| 4 | Short gestation for birth weight | Pneumococcal meningitis | 0 | - | - | 20 | 0 | - | - | Yes | Yes | Yes | Yes |
| 4 | Short gestation for birth weight | H influenzae type B meningitis | 0 | - | - | 20 | 0 | - | - | Yes | Yes | Yes | Yes |
| 4 | Short gestation for birth weight | Meningococcal infection | 0 | - | - | 20 | 0 | - | - | Yes | Yes | Yes | Yes |
| 4 | Short gestation for birth weight | Other meningitis | 0 | - | - | 20 | 0 | - | - | Yes | Yes | Yes | Yes |
| 4 | Short gestation for birth weight | Encephalitis | 0 | - | - | 20 | 0 | - | - | Yes | Yes | Yes | Yes |
| 4 | Short gestation for birth weight | Neonatal preterm birth complications | 0 | - | - | 20 | 0 | - | - | Yes | Yes | Yes | Yes |
| 4 | Short gestation for birth weight | Neonatal encephalopathy due to birth | 0 | - | - | 20 | 0 | - | - | Yes | Yes | Yes | Yes |
| 4 | Short gestation for birth weight | Neonatal sepsis and other neonatal | 0 | - | - | 20 | 0 | - | - | Yes | Yes | Yes | Yes |
| 4 | Short gestation for birth weight | Hemolytic disease and other neonatal | 0 | - | - | 20 | 0 | - | - | Yes | Yes | Yes | Yes |
| 4 | Short gestation for birth weight | Other neonatal disorders | 0 | - | - | 20 | 0 | - | - | Yes | Yes | Yes | Yes |
| 4 | Short gestation for birth weight | Sudden infant death syndrome | 0 | - | - | 20 | 0 | - | - | Yes | Yes | Yes | Yes |
| 4 | Low birth weight for gestation | Diarrhoeal diseases | 0 | - | - | 20 | 0 | - | - | Yes | Yes | Yes | Yes |
| 4 | Low birth weight for gestation | Lower respiratory infections | 0 | - | - | 20 | 0 | - | - | Yes | Yes | Yes | Yes |
| 4 | Low birth weight for gestation | Upper respiratory infections | 0 | - | - | 20 | 0 | - | - | Yes | Yes | Yes | Yes |
| 4 | Low birth weight for gestation | Otitis media | 0 | - | - | 20 | 0 | - | - | Yes | Yes | Yes | Yes |
| 4 | Low birth weight for gestation | Pneumococcal meningitis | 0 | - | - | 20 | 0 | - | - | Yes | Yes | Yes | Yes |
| 4 | Low birth weight for gestation | H influenzae type B meningitis | 0 | - | - | 20 | 0 | - | - | Yes | Yes | Yes | Yes |
| 4 | Low birth weight for gestation | Meningococcal infection | 0 | - | - | 20 | 0 | - | - | Yes | Yes | Yes | Yes |
| 4 | Low birth weight for gestation | Other meningitis | 0 | - | - | 20 | 0 | - | - | Yes | Yes | Yes | Yes |
| 4 | Low birth weight for gestation | Encephalitis | 0 | - | - | 20 | 0 | - | - | Yes | Yes | Yes | Yes |
| 4 | Low birth weight for gestation | Neonatal preterm birth complications | 0 | - | - | 20 | 0 | - | - | Yes | Yes | Yes | Yes |
| 4 | Low birth weight for gestation | Neonatal encephalopathy due to birth | 0 | - | - | 20 | 0 | - | - | Yes | Yes | Yes | Yes |
| 4 | Low birth weight for gestation | Neonatal sepsis and other neonatal | 0 | - | - | 20 | 0 | - | - | Yes | Yes | Yes | Yes |
| 4 | Low birth weight for gestation | Hemolytic disease and other neonatal | 0 | - | - | 20 | 0 | - | - | Yes | Yes | Yes | Yes |
| 4 | Low birth weight for gestation | Other neonatal disorders | 0 | - | - | 20 | 0 | - | - | Yes | Yes | Yes | Yes |
| 4 | Low birth weight for gestation | Sudden infant death syndrome | 0 | - | - | 20 | 0 | - | - | Yes | Yes | Yes | Yes |
| 3 | Vitamin A deficiency | Diarrhoeal diseases | 19 | 0 | 63 | 0 | - | - | - | No | - | Yes | No |
| 3 | Vitamin A deficiency | Measles | 12 | 0 | 83 | 0 | - | - | - | Yes | - | Yes | No |
| 3 | Zinc deficiency | Diarrhoeal diseases | 14 | 0 | 29 | 0 | - | - | - | No | - | Yes | No |
| 3 | Zinc deficiency | Lower respiratory infections | 6 | 0 | 17 | 0 | - | - | - | No | - | Yes | No |
| **2** | **Tobacco** | | | | | | | | | | | | |
| 3 | Smoking | Tuberculosis | 0 | - | - | 4 | 0 | 10 | 0 | No | - | Yes | Yes |
| 3 | Smoking | Lip and oral cavity cancer | 0 | - | - | 5 | 0 | - | - | Yes | - | Yes | Yes |
| 3 | Smoking | Nasopharynx cancer | 0 | - | - | 4 | 0 | 28 | 0 | Yes | - | Yes | Yes |
| 3 | Smoking | Oesophageal cancer | 0 | - | - | 5 | 0 | - | - | Yes | - | Yes | Yes |
| 3 | Smoking | Colon and rectum cancer | 0 | - | - | 19 | 0 | - | - | No | - | Yes | Yes |
| 3 | Smoking | Liver cancer | 0 | - | - | 54 | 0 | - | - | Yes | - | Yes | Yes |
| 3 | Smoking | Gastric cancer | 0 | - | - | 19 | 0 | - | - | No | - | Yes | Yes |
| 3 | Smoking | Pancreatic cancer | 0 | - | - | 19 | 0 | - | - | Yes | - | Yes | Yes |
| 3 | Smoking | Larynx cancer | 0 | - | - | 5 | 0 | - | - | Yes | - | Yes | Yes |
| 3 | Smoking | Tracheal, bronchus, and lung cancer | 0 | - | - | 38 | 0 | - | - | Yes | - | Yes | Yes |
| 3 | Smoking | Breast cancer | 0 | - | - | 19 | 0 | - | - | No | - | Yes | Yes |
| 3 | Smoking | Cervical cancer | 0 | - | - | 15 | 0 | - | - | No | - | Yes | Yes |
| 3 | Smoking | Prostate cancer | 0 | - | - | 19 | 0 | - | - | No | - | Yes | Yes |
| 3 | Smoking | Kidney cancer | 0 | - | - | 8 | 0 | - | - | Yes | - | Yes | Yes |
| 3 | Smoking | Bladder cancer | 0 | - | - | 37 | 0 | - | - | Yes | - | Yes | Yes |
| 3 | Smoking | Leukaemia | 0 | - | - | 22 | 0 | - | - | No | - | Yes | Yes |
| 3 | Smoking | Ischaemic heart disease | 0 | - | - | 86 | - | - | - | No | - | Yes | Yes |
| 3 | Smoking | Stroke | 0 | - | - | 60 | - | - | - | No | - | Yes | Yes |
| 3 | Smoking | Atrial fibrillation and flutter | 0 | - | - | 16 | 0 | - | - | No | - | Yes | Yes |
| 3 | Smoking | Peripheral vascular disease | 0 | - | - | 10 | 0 | - | - | No | - | Yes | Yes |
| 3 | Smoking | Chronic obstructive pulmonary disease | 0 | - | - | 42 | 0 | - | - | Yes | - | Yes | Yes |
| 3 | Smoking | Asthma | 0 | - | - | 8 | 12 | - | - | No | - | Yes | Yes |
| 3 | Smoking | Peptic ulcer disease | 0 | - | - | 7 | 0 | - | - | No | - | Yes | No |
| 3 | Smoking | Gallbladder and biliary diseases | 0 | - | - | 10 | 0 | - | - | No | - | Yes | Yes |
| 3 | Smoking | Alzheimer's disease and other dementias | 0 | - | - | 13 | 8 | - | - | No | - | Yes | Yes |
| 3 | Smoking | Parkinson's disease | 0 | - | - | 8 | 0 | - | - | Yes | - | Yes | Yes |
| 3 | Smoking | Multiple sclerosis | 0 | - | - | 6 | 0 | - | - | No | - | Yes | Yes |
| 3 | Smoking | Diabetes mellitus type 2 | 0 | - | - | 88 | 0 | - | - | No | - | Yes | No |
| 3 | Smoking | Rheumatoid arthritis | 0 | - | - | 5 | 0 | - | - | No | - | Yes | No |
| 3 | Smoking | Low back pain | 0 | - | - | 13 | 0 | - | - | No | - | Yes | Yes |
| 3 | Smoking | Cataract | 0 | - | - | 13 | 0 | - | - | No | - | Yes | No |
| 3 | Smoking | Macular degeneration | 0 | - | - | 5 | 0 | - | - | No | - | Yes | No |
| 3 | Smoking | Non-Hip Fracture | 0 | - | - | 14 | 14 | - | - | No | - | Yes | Yes |
| 3 | Smoking | Abdominal aortic aneurysm | 0 | - | - | 10 | 0 | - | - | No | - | Yes | Yes |
| 3 | Smoking | Hip fracture | 0 | - | - | 15 | 20 | - | - | No | - | Yes | Yes |
| 3 | Chewing tobacco | Lip and oral cavity cancer | 0 | - | - | 4 | 0 | 21 | 5 | Yes | - | Yes | Yes |
| 3 | Chewing tobacco | Oesophageal cancer | 0 | - | - | 2 | 0 | 10 | 0 | Yes | - | Yes | Yes |

| Risk Level | Risk | Outcome | RCTs (n) | RCTs with significant effect in the opposite direction (%) | RCTs with null findings (%) | Prospective observational studies (n)* | Prospective observational studies with significant association in the opposite direction (%) | Case-control studies assessing the risk-outcome pair relationship (n)** | Case-control studies that show significant association in the opposite direction (%) | Lower limit of RR > 1.5 | Dose-response relationship | Biological plausibility † | Analogy ‡ |
|---|---|---|---|---|---|---|---|---|---|---|---|---|---|
| 3 | Second-hand smoke | Stroke | 0 | - | - | 4 | 0 | 3 | - | No | Yes | Yes | Yes |
| 3 | Second-hand smoke | Lower respiratory infections | 0 | - | - | 18 | 0 | - | - | No | Yes | Yes | Yes |
| 3 | Second-hand smoke | Otitis media | 0 | - | - | 1 | 0 | 4 | 0 | No | - | Yes | Yes |
| 3 | Second-hand smoke | Tracheal, bronchus, and lung cancer | 0 | - | - | 13 | 0 | - | - | No | Yes | Yes | Yes |
| 3 | Second-hand smoke | Breast cancer | 0 | - | - | 21 | 0 | - | - | No | - | Yes | Yes |
| 3 | Second-hand smoke | Ischaemic heart disease | 0 | - | - | 5 | 0 | - | - | No | Yes | Yes | Yes |
| 3 | Second-hand smoke | Chronic obstructive pulmonary disease | 0 | - | - | 2 | 0 | 1 | 0 | No | Yes | Yes | Yes |
| 3 | Second-hand smoke | Diabetes mellitus type 2 | 0 | - | - | 5 | 0 | - | - | No | - | Yes | Yes |
| 2 | Alcohol use | Tuberculosis | 0 | - | - | 9 | 0 | 18 | | Yes | Yes | Yes | Yes |
| 2 | Alcohol use | Lower respiratory infections | 0 | - | - | 3 | 0 | 2 | | Yes | Yes | Yes | Yes |
| 2 | Alcohol use | Lip and oral cavity cancer | 0 | - | - | 26 | 0 | - | - | Yes | Yes | Yes | Yes |
| 2 | Alcohol use | Nasopharynx cancer | 0 | - | - | 6 | 0 | - | - | Yes | Yes | Yes | Yes |
| 2 | Alcohol use | Other pharynx cancer | 0 | - | - | 19 | 0 | - | - | Yes | Yes | Yes | Yes |
| 2 | Alcohol use | Oesophageal cancer | 0 | - | - | 57 | 0 | - | - | Yes | Yes | Yes | Yes |
| 2 | Alcohol use | Colon and rectum cancer | 0 | - | - | 56 | 0 | - | - | Yes | Yes | Yes | Yes |
| 2 | Alcohol use | Liver cancer | 0 | - | - | 32 | 0 | - | - | Yes | Yes | Yes | Yes |
| 2 | Alcohol use | Larynx cancer | 0 | - | - | 35 | 0 | - | - | Yes | Yes | Yes | Yes |
| 2 | Alcohol use | Breast cancer | 0 | - | - | 123 | 0 | - | - | Yes | Yes | Yes | Yes |
| 2 | Alcohol use | Ischaemic heart disease | 0 | - | - | 93 | 0 | - | - | Yes | Yes | Yes | Yes |
| 2 | Alcohol use | Ischaemic stroke | 0 | - | - | 24 | 0 | - | - | Yes | Yes | Yes | Yes |
| 2 | Alcohol use | Haemorrhagic stroke | 0 | - | - | 21 | 0 | - | - | Yes | Yes | Yes | Yes |
| 2 | Alcohol use | Hypertensive heart disease | 0 | - | - | 13 | 0 | - | - | Yes | Yes | Yes | Yes |
| 2 | Alcohol use | Atrial fibrillation and flutter | 0 | - | - | 9 | 0 | - | - | Yes | Yes | Yes | Yes |
| 2 | Alcohol use | Cirrhosis | 0 | - | - | 14 | 0 | - | - | Yes | Yes | Yes | Yes |
| 2 | Alcohol use | Pancreatitis | 0 | - | - | 4 | 0 | 3 | 0 | Yes | Yes | Yes | No |
| 2 | Alcohol use | Epilepsy | 0 | - | - | 2 | 0 | 2 | 0 | No | Yes | Yes | No |
| 2 | Alcohol use | Diabetes mellitus type 2 | 0 | - | - | 43 | 0 | - | - | Yes | Yes | Yes | No |
| 2 | Alcohol use | Motor vehicle road injuries | 0 | - | - | 3 | 0 | - | - | Yes | Yes | Yes | Yes |
| 2 | Alcohol use | Unintentional injuries | 0 | - | - | 4 | 0 | 4 | 0 | Yes | Yes | Yes | Yes |
| 2 | Alcohol use | Self-harm | 0 | - | - | 7 | 0 | | | Yes | Yes | Yes | Yes |
| 2 | Alcohol use | Interpersonal violence | 0 | - | - | 3 | 0 | 1 | 0 | Yes | Yes | Yes | Yes |
| 2 | Drug use | Hepatitis B | 1 | - | - | 5 | 0 | - | - | No | - | Yes | Yes |
| 2 | Drug use | Hepatitis C | 2 | - | - | 10 | 0 | - | - | No | - | Yes | Yes |
| 2 | Drug use | Self-harm | 0 | - | - | 15 | 0 | 0 | 0 | No | - | Yes | No |
| **2** | **Dietary risks** | | | | | | | | | | | | |
| 3 | Diet low in fruits | Lip and oral cavity cancer | 0 | - | - | 2 | 0 | 15 | 0 | No | Yes | Yes | Yes |
| 3 | Diet low in fruits | Nasopharynx cancer | 0 | - | - | 2 | 0 | 15 | 0 | No | Yes | Yes | Yes |
| 3 | Diet low in fruits | Other pharynx cancer | 0 | - | - | 2 | 0 | 15 | 0 | No | Yes | Yes | Yes |
| 3 | Diet low in fruits | Oesophageal cancer | 0 | - | - | 5 | 0 | - | - | No | Yes | Yes | Yes |
| 3 | Diet low in fruits | Larynx cancer | 0 | - | - | 2 | 0 | 15 | 0 | No | Yes | Yes | Yes |
| 3 | Diet low in fruits | Tracheal, bronchus, and lung cancer | 0 | - | - | 22 | 0 | - | - | No | Yes | Yes | Yes |
| 3 | Diet low in fruits | Ischaemic heart disease | 0 | - | - | 9 | 0 | - | - | No | Yes | Yes | Yes |
| 3 | Diet low in fruits | Ischaemic stroke | 0 | - | - | 9 | 0 | - | - | No | Yes | Yes | Yes |
| 3 | Diet low in fruits | Diabetes mellitus | 0 | - | - | 9 | 0 | - | - | No | Yes | Yes | No |
| 3 | Diet low in vegetables | Oesophageal cancer | 0 | - | - | 5 | 0 | - | - | No | Yes | Yes | No |
| 3 | Diet low in vegetables | Ischaemic heart disease | 0 | - | - | 9 | 0 | - | - | No | Yes | Yes | Yes |
| 3 | Diet low in vegetables | Ischaemic stroke | 0 | - | - | 8 | 0 | - | - | No | Yes | Yes | Yes |
| 3 | Diet low in vegetables | Haemorrhagic stroke | 0 | - | - | 5 | 0 | - | - | No | Yes | Yes | Yes |
| 3 | Diet low in legumes | Ischaemic heart disease | 0 | - | - | 5 | 0 | - | - | No | Yes | Yes | No |
| 3 | Diet low in whole grains | Ischaemic heart disease | 0 | - | - | 7 | 0 | - | - | No | Yes | Yes | Yes |
| 3 | Diet low in whole grains | Ischaemic stroke | 0 | - | - | 6 | 0 | - | - | No | Yes | Yes | Yes |
| 3 | Diet low in whole grains | Haemorrhagic stroke | 0 | - | - | 6 | 0 | - | - | No | Yes | Yes | Yes |
| 3 | Diet low in whole grains | Diabetes mellitus | 0 | - | - | 10 | 0 | - | - | No | Yes | Yes | No |
| 3 | Diet low in nuts and seeds | Ischaemic heart disease | 1 | 0 | 100 | 6 | 0 | - | - | No | Yes | Yes | No |
| 3 | Diet low in nuts and seeds | Diabetes mellitus | 1 | 0 | 100 | 5 | 0 | - | - | No | Yes | Yes | No |
| 3 | Diet low in milk | Colon and rectum cancer | 0 | - | - | 7 | 0 | - | - | No | Yes | Yes | No |
| 3 | Diet high in red meat | Colon and rectum cancer | 0 | - | - | 8 | 0 | - | - | No | Yes | Yes | No |
| 3 | Diet high in red meat | Diabetes mellitus | 0 | - | - | 9 | 11 | - | - | No | Yes | Yes | No |
| 3 | Diet high in processed meat | Colon and rectum cancer | 0 | - | - | 9 | 11 | - | - | No | Yes | Yes | No |
| 3 | Diet high in processed meat | Ischaemic heart disease | 0 | - | - | 5 | 0 | - | - | No | Yes | Yes | No |
| 3 | Diet high in processed meat | Diabetes mellitus | 0 | - | - | 8 | 0 | - | - | No | Yes | Yes | No |
| 3 | Diet high in sugar-sweetened beverages | Diabetes mellitus type 2 | 0 | - | - | 17 | 0 | - | - | No | Yes | Yes | No |
| 3 | Diet high in sugar-sweetened beverages | Ischemic heart disease | 0 | - | - | 4 | 0 | - | - | No | Yes | Yes | No |
| 3 | Diet low in fibre | Colon and rectum cancer | 0 | - | - | 15 | 0 | - | - | No | Yes | Yes | No |
| 3 | Diet low in fibre | Ischaemic heart disease | 0 | - | - | 12 | 0 | - | - | No | Yes | Yes | No |
| 3 | Diet low in calcium | Colon and rectum cancer | 0 | - | - | 13 | 0 | - | - | No | Yes | Yes | No |
| 3 | Diet low in seafood omega-3 fatty acids | Ischaemic heart disease | 17 | 0 | 94 | 16 | 0 | - | - | No | Yes | Yes | No |
| 3 | Diet low in polyunsaturated fatty acids | Ischaemic heart disease | 8 | 0 | 75 | 11 | 0 | - | - | No | Yes | Yes | No |
| 3 | Diet high in trans fatty acids | Ischaemic heart disease | 0 | - | - | 13 | 0 | - | - | No | Yes | Yes | No |
| 3 | Diet high in sodium | Stomach cancer | 0 | - | - | 10 | 0 | - | - | No | Yes | Yes | No |
| 3 | Diet high in sodium | Systolic blood pressure | 45 | 0 | 73 | 0 | - | - | - | No | Yes | Yes | No |
| **2** | **Child maltreatment** | | | | | | | | | | | | |
| 3 | Childhood sexual abuse | Alcohol use disorders | 0 | - | - | 2 | 0 | 3 | 0 | No | - | Yes | Yes |
| 3 | Childhood sexual abuse | Depressive disorders | 0 | - | - | 7 | 0 | - | - | No | - | Yes | Yes |
| 3 | Bullying victimization | Anxiety disorders | 0 | - | - | 19 | 0 | 0 | 0 | No | Yes | Yes | No |

| Risk Level | Risk | Outcome | RCTs (n) | RCTs with significant effect in the opposite direction (%) | RCTs with null findings (%) | Prospective observational studies (n)* | Prospective observational studies with significant association in the opposite direction (%) | Case-control studies assessing the risk-outcome pair relationship (n)** | Case-control studies that show significant association in the opposite direction (%) | Lower limit of RR > 1.5 | Dose-response relationship | Biological plausibility † | Analogy ‡ |
|---|---|---|---|---|---|---|---|---|---|---|---|---|---|
| 3 | Bullying victimization | Depressive disorders | 0 | - | - | 19 | 0 | 0 | 0 | No | Yes | Yes | No |
| 2 | Intimate partner violence | HIV/AIDS | 0 | - | - | 2 | 0 | 0 | 0 | No | - | Yes | No |
| 2 | Intimate partner violence | Maternal abortion and miscarriage | 0 | - | - | 1 | 0 | 3 | 0 | Yes | - | Yes | No |
| 2 | Intimate partner violence | Depressive disorders | 0 | - | - | 4 | 0 | 0 | 0 | No | - | Yes | Yes |
| 2 | Low physical activity | Colon and rectum cancer | 0 | - | - | 20 | 15 | - | - | No | Yes | Yes | Yes |
| 2 | Low physical activity | Breast cancer | 0 | - | - | 35 | 0 | - | - | No | Yes | Yes | Yes |
| 2 | Low physical activity | Ischaemic heart disease | 0 | - | - | 45 | 9 | - | - | No | Yes | Yes | Yes |
| 2 | Low physical activity | Ischaemic stroke | 0 | - | - | 27 | 11 | - | - | No | Yes | Yes | Yes |
| 2 | Low physical activity | Diabetes mellitus | 0 | - | - | 57 | 7 | - | - | No | Yes | Yes | No |
| 2 | High fasting plasma glucose | Tuberculosis | 0 | - | - | 18 | 0 | - | - | Yes | Yes | Yes | No |
| 2 | High fasting plasma glucose | Colon and rectum cancer | 0 | - | - | 21 | 0 | - | - | No | - | - | Yes |
| 2 | High fasting plasma glucose | Liver cancer | 0 | - | - | 28 | 0 | - | - | Yes | - | - | No |
| 2 | High fasting plasma glucose | Pancreatic cancer | 0 | - | - | 35 | 0 | - | - | Yes | - | - | Yes |
| 2 | High fasting plasma glucose | Lung cancer | 0 | - | - | 16 | 6 | - | - | No | - | - | Yes |
| 2 | High fasting plasma glucose | Breast cancer | 0 | - | - | 39 | 0 | - | - | No | - | - | Yes |
| 2 | High fasting plasma glucose | Ovarian cancer | 0 | - | - | 11 | 0 | - | - | No | - | - | Yes |
| 2 | High fasting plasma glucose | Bladder cancer | 0 | - | - | 14 | 0 | - | - | No | - | - | Yes |
| 2 | High fasting plasma glucose | Ischaemic heart disease | 8 | 0 | 100 | 150 | - | - | - | Yes | Yes | Yes | Yes |
| 2 | High fasting plasma glucose | Ischaemic stroke | 9 | 0 | 100 | 150 | - | - | - | Yes | Yes | Yes | Yes |
| 2 | High fasting plasma glucose | Subarachnoid hemorrhage | 9 | 0 | 100 | 150 | - | - | - | Yes | Yes | Yes | Yes |
| 2 | High fasting plasma glucose | Intracerebral hemorrhage | 9 | 0 | 100 | 150 | - | - | - | Yes | Yes | Yes | Yes |
| 2 | High fasting plasma glucose | Alzheimer's disease and other dementias | 0 | - | - | 17 | 0 | - | - | No | - | - | No |
| 2 | High fasting plasma glucose | Peripheral vascular disease | 14 | - | - | 4 | 0 | - | - | Yes | Yes | Yes | Yes |
| 2 | High fasting plasma glucose | Chronic kidney disease | 5 | - | - | 32 | - | - | - | Yes | Yes | Yes | No |
| 2 | High fasting plasma glucose | Glaucoma | 0 | - | - | 5 | 0 | - | - | No | - | - | Yes |
| 2 | High fasting plasma glucose | Cataract | 0 | - | - | 1 | 0 | 1 | 0 | No | - | - | Yes |
| 2 | High LDL cholesterol | Ischaemic heart disease | 21 | 0 | 57 | 88 | - | - | - | Yes | Yes | Yes | Yes |
| 2 | High LDL cholesterol | Ischaemic stroke | 21 | 0 | 57 | 88 | - | - | - | Yes | Yes | Yes | Yes |
| 2 | High systolic blood pressure | Rheumatic heart disease | 0 | - | - | 62 | - | - | - | Yes | Yes | Yes | Yes |
| 2 | High systolic blood pressure | Ischaemic heart disease | 56 | 0 | - | 88 | - | - | - | Yes | Yes | Yes | Yes |
| 2 | High systolic blood pressure | Ischaemic stroke | 54 | 0 | - | 150 | - | - | - | Yes | Yes | Yes | Yes |
| 2 | High systolic blood pressure | Intracerebral hemorrhage | 54 | 0 | - | 150 | - | - | - | Yes | Yes | Yes | Yes |
| 2 | High systolic blood pressure | Subarachnoid hemorrhage | 54 | 0 | - | 150 | - | - | - | Yes | Yes | Yes | Yes |
| 2 | High systolic blood pressure | Cardiomyopathy and myocarditis | 0 | - | - | 62 | - | - | - | Yes | Yes | Yes | Yes |
| 2 | High systolic blood pressure | Other cardiomyopathy | 0 | - | - | 62 | - | - | - | Yes | Yes | Yes | Yes |
| 2 | High systolic blood pressure | Atrial fibrillation and flutter | 20 | 5 | 60 | 88 | - | - | - | Yes | Yes | Yes | Yes |
| 2 | High systolic blood pressure | Aortic aneurysm | 0 | - | - | 62 | - | - | - | Yes | Yes | Yes | Yes |
| 2 | High systolic blood pressure | Peripheral vascular disease | 0 | - | - | 88 | - | - | - | Yes | Yes | Yes | Yes |
| 2 | High systolic blood pressure | Endocarditis | 0 | - | - | 62 | - | - | - | Yes | Yes | Yes | Yes |
| 3 | High systolic blood pressure | Other cardiovascular and circulatory diseases | 0 | - | - | 88 | - | - | - | No | Yes | Yes | Yes |
| 2 | High systolic blood pressure | Non-rheumatic calcific aortic valve disease | 0 | - | - | 2 | 0 | 1 | 0 | No | Yes | Yes | Yes |
| 2 | High systolic blood pressure | Chronic kidney disease | 8 | - | - | 88 | - | - | - | Yes | Yes | Yes | No |
| 2 | High body-mass index (adult) | Oesophageal cancer | 0 | - | - | 6 | 0 | - | - | No | Yes | Yes | Yes |
| 2 | High body-mass index (adult) | Non-hodgkin lymphoma | 0 | - | - | 18 | 0 | - | - | No | Yes | Yes | Yes |
| 2 | High body-mass index (adult) | Colon and rectum cancer | 0 | - | - | 38 | 0 | - | - | No | Yes | Yes | Yes |
| 2 | High body-mass index (adult) | Liver cancer | 0 | - | - | 34 | 0 | - | - | No | Yes | Yes | Yes |
| 2 | High body-mass index (adult) | Gallbladder and biliary tract cancer | 0 | - | - | 10 | 0 | - | - | No | Yes | Yes | Yes |
| 2 | High body-mass index (adult) | Pancreatic cancer | 0 | - | - | 20 | 0 | - | - | No | Yes | Yes | Yes |
| 2 | High body-mass index (adult) | Breast cancer (pre-menopausal) | 0 | - | - | 44 | 2 | - | - | No | Yes | Yes | Yes |
| 2 | High body-mass index (adult) | Breast cancer (post-menopausal) | 0 | - | - | 25 | 8 | - | - | No | Yes | Yes | No |
| 2 | High body-mass index (adult) | Uterine cancer | 0 | - | - | 37 | 0 | - | - | No | Yes | Yes | Yes |
| 2 | High body-mass index (adult) | Ovarian cancer | 0 | - | - | 31 | 3 | - | - | No | Yes | Yes | Yes |
| 2 | High body-mass index (adult) | Kidney cancer | 0 | - | - | 28 | 0 | - | - | No | Yes | Yes | Yes |
| 2 | High body-mass index (adult) | Thyroid cancer | 0 | - | - | 16 | 0 | - | - | No | Yes | Yes | Yes |
| 2 | High body-mass index (adult) | Multiple myeloma | 0 | - | - | 20 | - | - | - | - | Yes | Yes | Yes |
| 2 | High body-mass index (adult) | Leukaemia | 0 | - | - | 17 | 0 | - | - | No | Yes | Yes | Yes |
| 2 | High body-mass index (adult) | Ischaemic heart disease | 0 | - | - | 129 | - | - | - | No | Yes | Yes | Yes |
| 2 | High body-mass index (adult) | Ischaemic stroke | 0 | - | - | 102 | - | - | - | No | Yes | Yes | Yes |
| 2 | High body-mass index (adult) | Haemorrhagic stroke | 0 | - | - | 129 | - | - | - | No | Yes | Yes | Yes |
| 2 | High body-mass index (adult) | Hypertensive heart disease | 0 | - | - | 85 | - | - | - | No | Yes | Yes | Yes |
| 2 | High body-mass index (adult) | Atrial fibrillation and flutter | 0 | - | - | 5 | 0 | - | - | - | No | Yes | Yes |
| 2 | High body-mass index (adult) | Asthma | 0 | - | - | 7 | 0 | - | - | - | Yes | Yes | No |
| 2 | High body-mass index (adult) | Alzheimer's disease and other dementias | 0 | - | - | 6 | 0 | - | - | - | No | Yes | No |
| 2 | High body-mass index (adult) | Gallbladder disease | 0 | - | - | 16 | 0 | - | - | - | Yes | Yes | Yes |
| 2 | High body-mass index (adult) | Diabetes mellitus | 0 | - | - | 85 | - | - | - | Yes | Yes | Yes | No |
| 2 | High body-mass index (adult) | Chronic kidney disease | 0 | - | - | 57 | - | - | - | No | Yes | Yes | No |
| 2 | High body-mass index (adult) | Osteoarthritis | 0 | - | - | 32 | 0 | - | - | No | Yes | Yes | Yes |
| 2 | High body-mass index (adult) | Low back pain | 0 | - | - | 5 | 0 | - | - | No | Yes | Yes | Yes |
| 2 | High body-mass index (adult) | Gout | 0 | - | - | 10 | 0 | - | - | - | Yes | Yes | No |
| 2 | High body-mass index (adult) | Cataract | 0 | - | - | 17 | 0 | - | - | - | Yes | Yes | No |
| 2 | High body-mass index (child) | Asthma | 0 | - | - | 5 | 0 | - | - | No | Yes | Yes | No |
| 2 | Low bone mineral density | Injuries | 0 | - | - | 12 | - | - | - | No | Yes | Yes | Yes |

| Risk Level | Risk | Outcome | RCTs (n) | RCTs with significant effect in the opposite direction (%) | RCTs with null findings (%) | Prospective observational studies (n)* | Prospective observational studies with significant association in the opposite direction (%) | Case-control studies assessing the risk-outcome pair relationship (n)** | Case-control studies that show significant association in the opposite direction (%) | Lower limit of RR > 1.5 | Dose-response relationship | Biological plausibility † | Analogy ‡ |
|---|---|---|---|---|---|---|---|---|---|---|---|---|---|
| 2 | Impaired kidney function | Ischaemic heart disease | 0 | - | - | 6 | 0 | - | - | Yes | - | Yes | Yes |
| 2 | Impaired kidney function | Ischaemic stroke | 0 | - | - | 6 | 0 | - | - | Yes | - | Yes | Yes |
| 2 | Impaired kidney function | Intracerebral hemorrhage | 0 | - | - | 8 | 0 | - | - | Yes | - | Yes | Yes |
| 2 | Impaired kidney function | Peripheral vascular disease | 0 | - | - | 5 | 0 | - | - | Yes | - | Yes | Yes |
| 2 | Impaired kidney function | Gout | 0 | - | - | 3 | 0 | 0 | 0 | Yes | - | Yes | No |

| **Appendix Table 5. Epidemiological evidence supporting causality between risk-outcome pairs included in the Global Burden of Disease 2017 study including A. Citations and B. Additional information<br>A. Citations** | | |
|---|---|---|
| **Risk** | **Outcome** | **Citation/Note** |
| Unsafe water | Diarrhoeal diseases | Cairncross S, Valdmanis V. Water Supply, Sanitation, and Hygiene Promotion. In: Jamison DT, Breman JG, Measham AR, et al., eds. Disease Control Priorities in Developing Countries, 2nd edn. Washington (DC): World Bank, 2006. |
| Unsafe water | Diarrhoeal diseases | Wolf J, Prüss-Ustün A, Cumming O, et al. Assessing the impact of drinking water and sanitation on diarrhoeal disease in low- and middle-income settings: systematic review and meta-regression. Trop Med Int Health 2014; 19: 928–42. |
| Unsafe water | Diarrhoeal diseases | Fewtrell, L., Kaufmann, R. B., Kay, D., Enanoria, W., Haller, L., & Colford, J. M. (2005). Water, sanitation, and hygiene interventions to reduce diarrhoea in less developed countries: a systematic review and meta-analysis. The Lancet Infectious Diseases, 5(1), 42-52. doi:10.1016/s1473-3099(04)01253-8 |
| Unsafe sanitation - improved sanitation | Diarrhoeal diseases | Wolf J, Prüss-Ustün A, Cumming O, et al. Assessing the impact of drinking water and sanitation on diarrhoeal disease in low- and middle-income settings: systematic review and meta-regression. Trop Med Int Health 2014; 19: 928–42. |
| Unsafe sanitation - improved sanitation | Diarrhoeal diseases | Fewtrell, L., Kaufmann, R. B., Kay, D., Enanoria, W., Haller, L., & Colford, J. M. (2005). Water, sanitation, and hygiene interventions to reduce diarrhoea in less developed countries: a systematic review and meta-analysis. The Lancet Infectious Diseases, 5(1), 42-52. doi:10.1016/s1473-3099(04)01253-8 |
| Unsafe sanitation - improved sanitation | Diarrhoeal diseases | Baker, K. K., O'Reilly, C. E., Levine, M. M., Kotloff, K. L., Nataro, J. P., Ayers, T. L., . . . Mintz, E. D. (2016). Sanitation and Hygiene-Specific Risk Factors for Moderate-to-Severe Diarrhea in Young Children in the Global Enteric Multicenter Study, 2007–2011: Case-Control Study. PLOS Medicine,13(5). doi:10.1371/journal.pmed.1002010 |
| Unsafe sanitation - piped | Diarrhoeal diseases | Genser, B., Strina, A., Santos, L. A., Teles, C. A., Prado, M. S., Cairncross, S., & Barreto, M. L. (2008). Impact of a city-wide sanitation intervention in a large urban centre on social, environmental and behavioural determinants of childhood diarrhoea: analysis of two cohort studies. International Journal of Epidemiology, 37(4), 831-840. doi:10.1093/ije/dyn101 |
| Unsafe sanitation - piped | Diarrhoeal diseases | Norman, G., Pedley, S., & Takkouche, B. (2010). Effects of sewerage on diarrhoea and enteric infections: a systematic review and meta-analysis. The Lancet Infectious Diseases, 10(8), 536-544. doi:10.1016/s1473-3099(10)70123-7 |
| Unsafe sanitation - piped | Diarrhoeal diseases | Wolf J, Prüss-Ustün A, Cumming O, et al. Assessing the impact of drinking water and sanitation on diarrhoeal disease in low- and middle-income settings: systematic review and meta-regression. Trop Med Int Health 2014; 19: 928–42. |
| No access to handwashing facility | Diarrhoeal diseases | Ejemot-Nwadiaro RI, Ehiri JE, Arikpo D, Meremikwu MM, Critchley JA. Hand washing promotion for preventing diarrhoea. Cochrane Database Syst Rev 2015; : CD004265. |
| No access to handwashing facility | Lower respiratory infections | Rabie T, Curtis V. Handwashing and risk of respiratory infections: a quantitative systematic review. Trop Med Int Health Tropical Medicine and International Health. 2006;11(3):258–67. |
| No access to handwashing facility | Lower respiratory infections | Aiello, A. E., Coulborn, R. M., Perez, V., & Larson, E. L. (2008). Effect of Hand Hygiene on Infectious Disease Risk in the Community Setting: A Meta-Analysis. American Journal of Public Health, 98(8), 1372-1381. doi:10.2105/ajph.2007.124610 |
| Ambient particulate matter pollution | Lower respiratory infections | Hoek G, Krishnan RM, Beelen R, et al. Long-term air pollution exposure and cardio- respiratory mortality: a review. Environ Health 2013; 12: 43. |
| Ambient particulate matter pollution | Lower respiratory infections | Mehta S, Shin H, Burnett R, North T, Cohen AJ. Ambient particulate air pollution and acute lower respiratory infections: a systematic review and implications for estimating the global burden of disease. Air Qual Atmos Health 2013; 6: 69–83. |
| Ambient particulate matter pollution | Lower respiratory infections | MacIntyre EA, Gehring U, Mölter A, Fuertes E, Klümper C, Krämer U, et al. Air Pollution and Respiratory Infections during Early Childhood: An Analysis of 10 European Birth Cohorts within the ESCAPE Project. Environ Health Perspect. 2014 Jan 1;122(1):107–13. |
| Ambient particulate matter pollution | Tracheal, bronchus and lung cancer | Raaschou-Nielsen O, Beelen R, Wang M, Hoek G, Andersen ZJ, Hoffmann B, et al. Particulate matter air pollution components and risk for lung cancer. Environ Int. 2016 Feb;87:66–73. |
| Ambient particulate matter pollution | Tracheal, bronchus and lung cancer | Huang F, Pan B, Wu J, Chen E, Chen L. Relationship between exposure to PM2.5 and lung cancer incidence and mortality: A meta-analysis. Oncotarget. 2017 Jun 27;8(26):43322–31. |
| Ambient particulate matter pollution | Ischaemic heart disease | Hoek G, Krishnan RM, Beelen R, et al. Long-term air pollution exposure and cardio- respiratory mortality: a review. Environ Health 2013; 12: 43. |
| Ambient particulate matter pollution | Ischaemic heart disease | Beelen R, Stafoggia M, Raaschou-Nielsen O, Andersen ZJ, Xun WW, Katsouyanni K, et al. Long-term exposure to air pollution and cardiovascular mortality: an analysis of 22 European cohorts. Epidemiology. 2014 May;25(3):368–78. |
| Ambient particulate matter pollution | Stroke | Beelen R, Stafoggia M, Raaschou-Nielsen O, Andersen ZJ, Xun WW, Katsouyanni K, et al. Long-term exposure to air pollution and cardiovascular mortality: an analysis of 22 European cohorts. Epidemiology. 2014 May;25(3):368–78. |
| Ambient particulate matter pollution | Stroke | Scheers H, Jacobs L, Casas L, Nemery B, Nawrot TS. Long-Term Exposure to Particulate Matter Air Pollution Is a Risk Factor for Stroke: Meta-Analytical Evidence. Stroke. 2015 Nov;46(11):3058–66. |
| Ambient particulate matter pollution | Chronic obstructive pulmonary disease | Cohen AJ, Brauer M, Burnett R, Anderson HR, Frostad J, Estep K, et al. Estimates and 25-year trends of the global burden of disease attributable to ambient air pollution: an analysis of data from the Global Burden of Diseases Study 2015. The Lancet. 2017 May 13;389(10082):1907–18. |
| Ambient particulate matter pollution | Diabetes mellitus type 2 | He D, Wu S, Zhao H, Qiu H, Fu Y, Li X, et al. Association between particulate matter 2.5 and diabetes mellitus: A meta-analysis of cohort studies. J Diabetes Investig. 2017 Sep;8(5):687–96. |
| Household air pollution from solid fuels | Lower respiratory infections | Dherani M, Pope D, Mascarenhas M, Smith KR, Weber M, Bruce N. Indoor air pollution from unprocessed solid fuel use and pneumonia risk in children aged under five years: a systematic review and meta-analysis. Bull World Health Organ 2008; 86: 390–398C. |
| Household air pollution from solid fuels | Lower respiratory infections | Jary, H., Simpson, H., Havens, D., Manda, G., Pope, D., Bruce, N., & Mortimer, K. (2016). Household Air Pollution and Acute Lower Respiratory Infections in Adults: A Systematic Review. Plos One, 11(12). doi:10.1371/journal.pone.0167656 |

**Appendix Table 5. Epidemiological evidence supporting causality between risk-outcome pairs included in the Global Burden of Disease 2017 study including A. Citations and B. Additional information**

**A. Citations**

| Risk | Outcome | Citation/Note |
|---|---|---|
| Household air pollution from solid fuels | Tracheal, bronchus and lung cancer | Kurmi OP, Arya PH, Lam K-BH, Sorahan T, Ayres JG. Lung cancer risk and solid fuel smoke exposure: a systematic review and meta-analysis. Eur Respir J. 2012 Nov;40(5):1228–37. |
| Household air pollution from solid fuels | Ischaemic heart disease | Fatmi Z, Coggon D. Coronary heart disease and household air pollution from use of solid fuel: a systematic review. Br Med Bull. 2016 Jun;118(1):91–109. |
| Household air pollution from solid fuels | Stroke | Fatmi Z, Coggon D. Coronary heart disease and household air pollution from use of solid fuel: a systematic review. Br Med Bull. 2016 Jun;118(1):91–109. |
| Household air pollution from solid fuels | Chronic obstructive pulmonary disease | Kurmi OP, Arya PH, Lam K-BH, Sorahan T, Ayres JG. Lung cancer risk and solid fuel smoke exposure: a systematic review and meta-analysis. Eur Respir J. 2012 Nov;40(5):1228–37. |
| Household air pollution from solid fuels | Diabetes mellitus type 2 | Kim C, Seow WJ, Shu X-O, Bassig BA, Rothman N, Chen BE, et al. Cooking Coal Use and All-Cause and Cause-Specific Mortality in a Prospective Cohort Study of Women in Shanghai, China. Environ Health Perspect. 2016 Sep;124(9):1384–9. |
| Household air pollution from solid fuels | Cataract | West, S., Bates, M., Lee, J., Schaumberg, D., Lee, D., Adair-Rohani, H., . . . Araj, H. (2013). Is Household Air Pollution a Risk Factor for Eye Disease? International Journal of Environmental Research and Public Health, 10(11), 5378-5398. doi:10.3390/ijerph10115378 |
| Ambient ozone pollution | Chronic obstructive pulmonary disease | Weichenthal S, Pinault LL, Burnett RT. Impact of Oxidant Gases on the Relationship between Outdoor Fine Particulate Air Pollution and Nonaccidental, Cardiovascular, and Respiratory Mortality. Scientific Reports [Internet]. 2017 Dec;7(1). |
| Ambient ozone pollution | Chronic obstructive pulmonary disease | Turner MC, Jerrett M, Pope CA, et al. Long-Term Ozone Exposure and Mortality in a Large Prospective Study. Am J Respir Crit Care Med 2016; 193: 1134–42. |
| Ambient ozone pollution | Chronic obstructive pulmonary disease | Carey IM, Atkinson RW, Kent AJ, van Staa T, Cook DG, Anderson HR. Mortality Associations with Long-Term Exposure to Outdoor Air Pollution in a National English Cohort. Am J Respir Crit Care Med. 2013 Apr 3;187(11):1226–33. |
| Residential radon | Tracheal, bronchus and lung cancer | Torres-Durán MCAD, Barros-Dios JM, Fernández-Villar A, Ruano-Ravina A. Residential radon and lung cancer in never smokers. A systematic review. Cancer Letters. 2014;345(1):21–6. |
| Residential radon | Tracheal, bronchus and lung cancer | Turner MC, Krewski D, Chen Y, Pope CA, Gapstur S, Thun MJ. Radon and Lung Cancer in the American Cancer Society Cohort. Cancer Epidemiology Biomarkers & Prevention. 2011Jun;20(3):438–48. |
| Residential radon | Tracheal, bronchus and lung cancer | Krewski D, Lubin JH, Zielinski JM, Alavanja M, Catalan VS, Field RW, et al. Residential Radon and Risk of Lung Cancer. Epidemiology. 2005;16(2):137–45. |
| Residential radon | Tracheal, bronchus and lung cancer | Darby S. Radon in homes and risk of lung cancer: collaborative analysis of individual data from 13 European case-control studies. Bmj. 2005;330(7485):223. |
| Residential radon | Tracheal, bronchus and lung cancer | Lubin JH. Studies of radon and lung cancer in North America and China. Radiation Protection Dosimetry. 2003Jan;104(4):315–9. |
| Residential radon | Tracheal, bronchus and lung cancer | Kreuzer M, Gerken M, Kreienbrock L, Wellmann J, Wichmann HE. Lung cancer in lifetime nonsmoking men – results of a case-control study in Germany. British Journal of Cancer. 2001May;84(1):134–40. |
| Residential radon | Tracheal, bronchus and lung cancer | Kreuzer M, Heinrich J, Kreienbrock L, Rosario AS, Gerken M, Wichmann HE. Risk factors for lung cancer among nonsmoking women. International Journal of Cancer. 2002;100(6):706–13. |
| Residential radon | Tracheal, bronchus and lung cancer | Field R, Steck DJ, Smith BJ, Brus CP, Fisher EL, Neuberger JS, et al. The Iowa radon lung cancer study — phase I: residential radon gas exposure and lung cancer. Science of The Total Environment. 2001;272(1-3):67–72. |
| Residential radon | Tracheal, bronchus and lung cancer | Wang Z. Residential Radon and Lung Cancer Risk in a High-exposure Area of Gansu Province, China. American Journal of Epidemiology. 2002;155(6):554–64. |
| Residential radon | Tracheal, bronchus and lung cancer | Lagarde F, Axelsson G, Damber L, Mellander H, Nyberg F, Pershagen G. Residential Radon and Lung Cancer among Never-Smokers in Sweden. Epidemiology. 2001;12(4):396–404. |
| Residential radon | Tracheal, bronchus and lung cancer | Pershagen G, Akerblom G, Axelson O, Clavensjo B, Damber L, Desai G, et al. Residential Radon Exposure and Lung Cancer in Sweden. New England Journal of Medicine. 1994;330(3):159–64. |
| Residential radon | Tracheal, bronchus and lung cancer | Alavanja MCR, Brownson RC, Lubin JH, Berger E, Chang J, Boice JD. Residential Radon Exposure and Lung Cancer Among Nonsmoking Women. JNCI Journal of the National Cancer Institute. 1994;86(24):1829–37. |
| Residential radon | Tracheal, bronchus and lung cancer | Schoenberg JB, Klotz JB, Wilcox HB, Nicholls GP, Gil-del-Real MT, Stemhagen A. Case-control study of residential radon and lung cancer among New Jersey women. Cancer Research. 1990Oct15;50(20):6520–4. |
| Lead exposure | Systolic blood pressure | Navas-Acien A, Schwartz BS, Rothenberg SJ, Hu H, Silbergeld EK, Guallar E. Bone lead levels and blood pressure endpoints: a meta-analysis. Epidemiology 2008; 19: 496–504. |
| Lead exposure | Systolic blood pressure | Glenn BS, Stewart WF, Links JM, Todd AC, Schwartz BS. The Longitudinal Association of Lead with Blood Pressure. Epidemiology. 2003;14(1):30–6. |
| Lead exposure | Systolic blood pressure | Glenn BS, Bandeen-Roche K, Lee B-K, Weaver VM, Todd AC, Schwartz BS. Changes in Systolic Blood Pressure Associated With Lead in Blood and Bone. Epidemiology. 2006;17(5):538–44. |
| Lead exposure | Systolic blood pressure | Korrick SA, Hunter DJ, Rotnitzky A, Hu H, Speizer FE. Lead and hypertension in a sample of middle-aged women. American Journal of Public Health. 1999;89(3):330–5. |
| Lead exposure | Systolic blood pressure | Cheng Y, Schwartz J, Sparrow D, Aro A, Weiss ST, Hu H. Bone Lead and Blood Lead Levels in Relation to Baseline Blood Pressure and the Prospective Development of Hypertension The Normative Aging Study. American Journal of Epidemiology. 2001;153(2):164–71. |
| Lead exposure | Idiopathic intellectual disability | Lanphear BP, Hornung R, Khoury J, et al. Low-level environmental lead exposure and children’s intellectual function: an international pooled analysis. Environ Health Perspect 2005; 113: 894–9. |
| Lead exposure | Idiopathic intellectual disability | Liu J, Li L, Wang Y, Yan C, Liu X. Impact of low blood lead concentrations on IQ and school performance in Chinese children. PLoS ONE 2013; 8: e65230. |
| Lead exposure | Idiopathic intellectual disability | Lanphear BP, Hornung R, Khoury J, et al. Low-level environmental lead exposure and children’s intellectual function: an international pooled analysis. Environ Health Perspect 2005; 113: 894–9. |

**Appendix Table 5. Epidemiological evidence supporting causality between risk-outcome pairs included in the Global Burden of Disease 2017 study including A. Citations and B. Additional information**

**A. Citations**

| Risk | Outcome | Citation/Note |
|---|---|---|
| Lead exposure | Idiopathic intellectual disability | Needleman HL, Schell A, Bellinger D, Leviton A, Allred EN. The Long-Term Effects of Exposure to Low Doses of Lead in Childhood. New England Journal of Medicine. 1990Nov;322(2):83–8. |
| Occupational exposure to asbestos | Larynx cancer | Goodman M, Morgan RW, Ray R, Malloy CD, Zhao K. Cancer in asbestos-exposed occupational cohorts: a meta-analysis. Cancer Causes Control 1999; 10: 453–65. |
| Occupational exposure to asbestos | Tracheal, bronchus and lung cancer | Lenters V, Vermeulen R, Dogger S, et al. A meta-analysis of asbestos and lung cancer: is better quality exposure assessment associated with steeper slopes of the exposure-response relationships? Environ Health Perspect 2011; 119: 1547–55. |
| Occupational exposure to asbestos | Ovarian cancer | Camargo MC, Stayner LT, Straif K, et al. Occupational exposure to asbestos and ovarian cancer: a meta-analysis. Environ Health Perspect 2011; 119: 1211–7. |
| Occupational exposure to asbestos | Mesothelioma | Bourdès V, Boffetta P, Pisani P. Environmental exposure to asbestos and risk of pleural mesothelioma: review and meta-analysis. Eur J Epidemiol 2000; 16: 411–7. |
| Occupational exposure to arsenic | Tracheal, bronchus and lung cancer | Lenters V, Vermeulen R, Dogger S, et al. A meta-analysis of asbestos and lung cancer: is better quality exposure assessment associated with steeper slopes of the exposure-response relationships? Environ Health Perspect 2011; 119: 1547–55. |
| Occupational exposure to benzene | Leukaemia | Khalade A, Jaakkola MS, Pukkala E, Jaakkola JJK. Exposure to benzene at work and the risk of leukemia: a systematic review and meta-analysis. Environ Health 2010; 9: 31. |
| Occupational exposure to beryllium | Tracheal, bronchus and lung cancer | Boffetta P, Fryzek JP, Mandel JS. Occupational exposure to beryllium and cancer risk: a review of the epidemiologic evidence. Crit Rev Toxicol 2012; 42: 107–18. |
| Occupational exposure to cadmium | Tracheal, bronchus and lung cancer | Verougstraete V, Lison D, Hotz P. Cadmium, lung and prostate cancer: a systematic review of recent epidemiological data. J Toxicol Environ Health B Crit Rev 2003; 6: 227–55. |
| Occupational exposure to chromium | Tracheal, bronchus and lung cancer | Denis Ambroise, Pascal Wild and Jean-Jacques Moulin, Scandinavian Journal of Work, Environment & Health, Vol. 32, No. 1 (February 2006), pp. 22-31 |
| Occupational exposure to diesel engine exhaust | Tracheal, bronchus and lung cancer | Lipsett M, Campleman S. Occupational exposure to diesel exhaust and lung cancer: a meta-analysis. Am J Public Health 1999; 89: 1009–17. |
| Occupational exposure to formaldehyde | Nasopharynx cancer | Hauptmann M, Lubin JH, Stewart PA, Hayes RB, Blair A. Mortality from solid cancers among workers in formaldehyde industries. Am J Epidemiol 2004; 159: 1117–30. |
| Occupational exposure to formaldehyde | Leukaemia | Collins JJ, Lineker GA. A review and meta-analysis of formaldehyde exposure and leukemia. Regul Toxicol Pharmacol 2004; 40: 81–91. |
| Occupational exposure to nickel | Tracheal, bronchus and lung cancer | Grimsrud TK, Berge SR, Haldorsen T, Andersen A. Can lung cancer risk among nickel refinery workers be explained by occupational exposures other than nickel? Epidemiology 2005; 16: 146–54. |
| Occupational exposure to polycyclic aromatic hydrocarbons | Tracheal, bronchus and lung cancer | Armstrong B, Hutchinson E, Unwin J, Fletcher T. Lung cancer risk after exposure to polycyclic aromatic hydrocarbons: a review and meta-analysis. Environ Health Perspect 2004; 112: 970–8. |
| Occupational exposure to silica | Tracheal, bronchus and lung cancer | Liu Y, Steenland K, Rong Y, Hnizdo E, Huang X, Zhang H, et al. Exposure-Response Analysis and Risk Assessment for Lung Cancer in Relationship to Silica Exposure: A 44-Year Cohort Study of 34,018 Workers. Am J Epidemiol. 2013 Nov 1;178(9):1424–33. |
| Occupational exposure to sulfuric acid | Larynx cancer | Soskolne CL, Jhangri GS, Siemiatycki J, et al. Occupational exposure to sulfuric acid in southern Ontario, Canada, in association with laryngeal cancer. Scand J Work Environ Health 1992; 18: 225–32. |
| Occupational exposure to trichloroethylene | Kidney cancer | Kelsh MA, Alexander DD, Mink PJ, Mandel JH. Occupational trichloroethylene exposure and kidney cancer: a meta-analysis. Epidemiology 2010; 21: 95–102. |
| Occupational asthmagens | Asthma | Karjalainen A, Kurppa K, Martikainen R, Klaukka T, Karjalainen J. Work is related to a substantial portion of adult-onset asthma incidence in the Finnish population. Am J Respir Crit Care Med 2001; 164: 565–8. |
| Occupational particulate matter, gases, and fumes | Chronic obstructive pulmonary disease | Blanc PD, Iribarren C, Trupin L, et al. Occupational exposures and the risk of COPD: dusty trades revisited. Thorax 2009; 64: 6–12. |
| Occupational noise | Age-related and other hearing loss | Agrawal Y, Platz EA, Niparko JK. Prevalence of hearing loss and differences by demographic characteristics among US adults: data from the National Health and Nutrition Examination Survey, 1999-2004. Arch Intern Med 2008; 168: 1522–30. |
| Occupational noise | Age-related and other hearing loss | Davis A. The prevalence of hearing impairment and reported hearing disability among adults in Great Britain. International Journal of Epidemiology 1989,18: 911-917. |
| Occupational noise | Age-related and other hearing loss | Wilson D, Walsh P, Sanchez L, Davis A, Taylor A, Tucker G, Meagher I. The epidemiology of hearing impairment in an Australian adult population. International Journal of Epidemiology 1999;28:247-252. |
| Occupational injuries | Injuries | International Labour Organization. Resolution concerning statistics of occupational injuries (resulting from occupational accidents). 1998; published online Oct. http://www.ilo.org/global/statistics-and-databases/standards-and-guidelines/resolutions-adopted-by-international-conferences-of-labour-statisticians/WCMS_087528/lang--en/index.htm. |
| Occupational injuries | Injuries | Eurostat. Accidents at work statistics. http://ec.europa.eu/eurostat/statistics-explained/index.php/Accidents_at_work_statistics. |
| Occupational ergonomic factors | Low back pain | Driscoll T, Jacklyn G, Orchard J, et al. The global burden of occupationally related low back pain: estimates from the Global Burden of Disease 2010 study. Ann Rheum Dis 2014; 73: 975–81. |
| Non-exclusive breastfeeding | Lower respiratory infections | Horta BL, Victora CG. Short-term effects of breastfeeding: a systematic review on the benefits of breastfeeding on diarrhoea and pneumonia mortality. World Health Organization, 2013 http://allattamento.sip.it/wp-content/uploads/2014/03/WHO_breve-termine.pdf. |
| Non-exclusive breastfeeding | Diarrhoeal diseases | Horta BL, Victora CG. Short-term effects of breastfeeding: a systematic review on the benefits of breastfeeding on diarrhoea and pneumonia mortality. World Health Organization, 2013 http://allattamento.sip.it/wp-content/uploads/2014/03/WHO_breve-termine.pdf. |
| Discontinued breastfeeding | Diarrhoeal diseases | Olofin I, McDonald CM, Ezzati M, et al. Associations of suboptimal growth with all-cause and cause-specific mortality in children under five years: a pooled analysis of ten prospective studies. PLoS ONE 2013; 8: e64636. |

| **Appendix Table 5. Epidemiological evidence supporting causality between risk-outcome pairs included in the Global Burden of Disease 2017 study including A. Citations and B. Additional information<br>A. Citations** | | |
|---|---|---|
| **Risk** | **Outcome** | **Citation/Note** |
| Discontinued breastfeeding | Diarrhoeal diseases | Genser, B., Strina, A., Santos, L. A., Teles, C. A., Prado, M. S., Cairncross, S., & Barreto, M. L. (2008). Impact of a city-wide sanitation intervention in a large urban centre on social, environmental and behavioural determinants of childhood diarrhoea: analysis of two cohort studies. International Journal of Epidemiology, 37(4), 831-840. doi:10.1093/ije/dyn101 |
| Childhood underweight | Diarrhoeal diseases | Olofin I, McDonald CM, Ezzati M, et al. Associations of suboptimal growth with all-cause and cause-specific mortality in children under five years: a pooled analysis of ten prospective studies. PLoS ONE 2013; 8: e64636. |
| Childhood underweight | Lower respiratory infections | Olofin I, McDonald CM, Ezzati M, et al. Associations of suboptimal growth with all-cause and cause-specific mortality in children under five years: a pooled analysis of ten prospective studies. PLoS ONE 2013; 8: e64636. |
| Childhood underweight | Measles | Olofin I, McDonald CM, Ezzati M, et al. Associations of suboptimal growth with all-cause and cause-specific mortality in children under five years: a pooled analysis of ten prospective studies. PLoS ONE 2013; 8: e64636. |
| Childhood wasting | Diarrhoeal diseases | Olofin I, McDonald CM, Ezzati M, et al. Associations of suboptimal growth with all-cause and cause-specific mortality in children under five years: a pooled analysis of ten prospective studies. PLoS ONE 2013; 8: e64636. |
| Childhood wasting | Lower respiratory infections | Olofin I, McDonald CM, Ezzati M, et al. Associations of suboptimal growth with all-cause and cause-specific mortality in children under five years: a pooled analysis of ten prospective studies. PLoS ONE 2013; 8: e64636. |
| Childhood wasting | Measles | Olofin I, McDonald CM, Ezzati M, et al. Associations of suboptimal growth with all-cause and cause-specific mortality in children under five years: a pooled analysis of ten prospective studies. PLoS ONE 2013; 8: e64636. |
| Childhood stunting | Diarrhoeal diseases | Olofin I, McDonald CM, Ezzati M, et al. Associations of suboptimal growth with all-cause and cause-specific mortality in children under five years: a pooled analysis of ten prospective studies. PLoS ONE 2013; 8: e64636. |
| Childhood stunting | Lower respiratory infections | Olofin I, McDonald CM, Ezzati M, et al. Associations of suboptimal growth with all-cause and cause-specific mortality in children under five years: a pooled analysis of ten prospective studies. PLoS ONE 2013; 8: e64636. |
| Childhood stunting | Measles | Olofin I, McDonald CM, Ezzati M, et al. Associations of suboptimal growth with all-cause and cause-specific mortality in children under five years: a pooled analysis of ten prospective studies. PLoS ONE 2013; 8: e64636. |
| Iron deficiency | Maternal hemorrhage | Murray-Kolb LE, Chen L, Chen P, Shapiro M, Caulfield L. CHERG Iron Report: Maternal Mortality, Child Mortality, Perinatal Mortality, Child Cognition, and Estimates of Prevalence of Anemia due to Iron Deficiency. Baltimore, USA: CHERG, 2012. |
| Vitamin A deficiency | Diarrhoeal diseases | Awasthi S, Peto R, Read S, et al. Vitamin A supplementation every 6 months with retinol in 1 million pre-school children in north India: DEVTA, a cluster-randomised trial. Lancet 2013; 381: 1469–77. |
| Vitamin A deficiency | Diarrhoeal diseases | Diness BR, Christoffersen D, Pedersen UB, Rodrigues A, Fischer TK, Andersen A, Whittle H, Yazdanbakhsh M, Aaby P, Benn CS. The effect of high-dose vitamin A supplementation given with bacille Calmette-Guérin vaccine at birth on infant rotavirus infection and diarrhea: a randomized prospective study from Guinea-Bissau. J Infect Dis. 2010; S243-251. |
| Vitamin A deficiency | Diarrhoeal diseases | Imdad A, Herzer K, Mayo-Wilson E, Yakoob MY, Bhutta ZA. Vitamin A supplementation for preventing morbidity and mortality in children from 6 months to 5 years of age. Cochrane Database Syst Rev. 2010; CD008524. |
| Vitamin A deficiency | Diarrhoeal diseases | Imdad A, Yakoob MY, Sudfeld C, Haider BA, Black RE, Bhutta ZA. Impact of vitamin A supplementation on infant and childhood mortality. BMC Public Health. 2011; S20. |
| Vitamin A deficiency | Measles | Awasthi S, Peto R, Read S, et al. Vitamin A supplementation every 6 months with retinol in 1 million pre-school children in north India: DEVTA, a cluster-randomised trial. Lancet 2013; 381: 1469–77. |
| Vitamin A deficiency | Measles | Imdad A, Herzer K, Mayo-Wilson E, Yakoob MY, Bhutta ZA. Vitamin A supplementation for preventing morbidity and mortality in children from 6 months to 5 years of age. Cochrane Database Syst Rev. 2010; CD008524. |
| Vitamin A deficiency | Measles | Imdad A, Yakoob MY, Sudfeld C, Haider BA, Black RE, Bhutta ZA. Impact of vitamin A supplementation on infant and childhood mortality. BMC Public Health. 2011; S20. |
| Zinc deficiency | Diarrhoeal diseases | Shankar AH, Prasad AS. Zinc and immune function: the biological basis of altered resistance to infection. Am J Clin Nutr 1998; 68: 447S–463S. |
| Zinc deficiency | Diarrhoeal diseases | Yakoob MY, Theodoratou E, Jabeen A, et al. Preventive zinc supplementation in developing countries: impact on mortality and morbidity due to diarrhea, pneumonia and malaria. BMC Public Health 2011; 11 Suppl 3: S23. |
| Zinc deficiency | Lower respiratory infections | Shankar AH, Prasad AS. Zinc and immune function: the biological basis of altered resistance to infection. Am J Clin Nutr 1998; 68: 447S–463S. |
| Zinc deficiency | Lower respiratory infections | Yakoob MY, Theodoratou E, Jabeen A, et al. Preventive zinc supplementation in developing countries: impact on mortality and morbidity due to diarrhea, pneumonia and malaria. BMC Public Health 2011; 11 Suppl 3: S23. |
| Smoking | Lower respiratory infections | Surgeon General's Report - The Health Consequences of Smoking. U.S. Department of Health & Human Services, 2004 http://www.cdc.gov/tobacco/data_statistics/sgr/2004/. |
| Smoking | Larynx cancer | Carter BD, Abnet CC, Feskanich D, et al. Smoking and mortality--beyond established causes. N Engl J Med 2015; 372: 631–40. |
| Smoking | Lip and oral cavity cancer | Carter BD, Abnet CC, Feskanich D, et al. Smoking and mortality--beyond established causes. N Engl J Med 2015; 372: 631–40. |

**Appendix Table 5. Epidemiological evidence supporting causality between risk-outcome pairs included in the Global Burden of Disease 2017 study including A. Citations and B. Additional information**

**A. Citations**

| Risk | Outcome | Citation/Note |
|---|---|---|
| Smoking | Tracheal, bronchus and lung cancer | International Agency for Research on Cancer Working Group on the Evaluation of Carcinogenic Risks to Humans. IARC monographs on the evaluation of carcinogenic risks to humans: Tobacco Smoke and Involuntary Smoking. Lyon: IARC, 2004. |
| Smoking | Breast cancer | Ordóñez-Mena JM, Schöttker B, Mons U, et al. Quantification of the smoking-associated cancer risk with rate advancement periods: meta-analysis of individual participant data from cohorts of the CHANCES consortium. BMC Medicine 2016; 14: 62. |
| Smoking | Cervical cancer | International Agency for Research on Cancer Working Group on the Evaluation of Carcinogenic Risks to Humans. IARC monographs on the evaluation of carcinogenic risks to humans: Tobacco Smoke and Involuntary Smoking. Lyon: IARC, 2004. |
| Smoking | Prostate cancer | Islami F, Moreira DM, Boffetta P, Freedland SJ. A Systematic Review and Meta-analysis of Tobacco Use and Prostate Cancer Mortality and Incidence in Prospective Cohort Studies. European Urology 2014; 66: 1054–64. |
| Smoking | Kidney cancer | International Agency for Research on Cancer Working Group on the Evaluation of Carcinogenic Risks to Humans. IARC monographs on the evaluation of carcinogenic risks to humans: Tobacco Smoke and Involuntary Smoking. Lyon: IARC, 2004. |
| Smoking | Nasopharynx cancer | Xue W-Q, Qin H-D, Ruan H-L, Shugart YY, Jia W-H. Quantitative Association of Tobacco Smoking With the Risk of Nasopharyngeal Carcinoma: A Comprehensive Meta-Analysis of Studies Conducted Between 1979 and 2011. Am J Epidemiol 2013; 178: 325–38. |
| Smoking | Bladder cancer | Cumberbatch MG, Rota M, Catto JWF, La Vecchia C. The Role of Tobacco Smoke in Bladder and Kidney Carcinogenesis: A Comparison of Exposures and Meta-analysis of Incidence and Mortality Risks. Eur Urol 2016; 70: 458–66. |
| Smoking | Leukaemia | Colamesta V, D'Aguanno S, Breccia M, Bruffa S, Cartoni C, Torre GL. Do the smoking intensity and duration, the years since quitting, the methodological quality and the year of publication of the studies affect the results of the meta-analysis on cigarette smoking and Acute Myeloid Leukemia (AML) in adults? Critical Reviews in Oncology / Hematology 2016; 99: 376–88. |
| Smoking | Oesophageal cancer | Surgeon General's Report - The Health Consequences of Smoking. U.S. Department of Health & Human Services, 2004 http://www.cdc.gov/tobacco/data_statistics/sgr/2004/. |
| Smoking | Stomach cancer | Ordóñez-Mena JM, Schöttker B, Mons U, et al. Quantification of the smoking-associated cancer risk with rate advancement periods: meta-analysis of individual participant data from cohorts of the CHANCES consortium. BMC Medicine 2016; 14: 62. |
| Smoking | Colon and rectum cancer | Surgeon General's Report - The Health Consequences of Smoking. U.S. Department of Health & Human Services, 2004 http://www.cdc.gov/tobacco/data_statistics/sgr/2004/. |
| Smoking | Liver cancer | Surgeon General's Report - The Health Consequences of Smoking. U.S. Department of Health & Human Services, 2004 http://www.cdc.gov/tobacco/data_statistics/sgr/2004/. |
| Smoking | Pancreatic cancer | Ordóñez-Mena JM, Schöttker B, Mons U, et al. Quantification of the smoking-associated cancer risk with rate advancement periods: meta-analysis of individual participant data from cohorts of the CHANCES consortium. BMC Medicine 2016; 14: 62. |
| Smoking | Ischaemic heart disease | Huxley RR, Woodward M. Cigarette smoking as a risk factor for coronary heart disease in women compared with men: a systematic review and meta-analysis of prospective cohort studies. Lancet 2011; 378: 1297–305. |
| Smoking | Cerebrovascular disease | Peters SAE, Huxley RR, Woodward M. Smoking as a risk factor for stroke in women compared with men: a systematic review and meta-analysis of 81 cohorts, including 3,980,359 individuals and 42,401 strokes. Stroke 2013; 44: 2821–8. |
| Smoking | Atrial fibrillation and flutter | Zhu W, Yuan P, Shen Y, Wan R, Hong K. Association of smoking with the risk of incident atrial fibrillation: A meta-analysis of prospective studies. Int J Cardiol 2016; 218: 259–66. |
| Smoking | Abdominal aortic aneurysm | Lederle FA, Nelson DB, Joseph AM. Smokers' relative risk for aortic aneurysm compared with other smoking-related diseases: a systematic review. J Vasc Surg 2003; 38: 329–34. |
| Smoking | Peripheral vascular disease | Lu L, Mackay DF, Pell JP. Meta-analysis of the association between cigarette smoking and peripheral arterial disease. Heart 2014; 100: 414–23. |
| Smoking | Chronic obstructive pulmonary disease | Forey BA, Thornton AJ, Lee PN. Systematic review with meta-analysis of the epidemiological evidence relating smoking to COPD, chronic bronchitis and emphysema. BMC Pulm Med 2011; 11: 36. |
| Smoking | Asthma | Jayes L, Haslam PL, Gratziou CG, et al. SmokeHaz: Systematic Reviews and Meta-analyses of the Effects of Smoking on Respiratory Health. Chest 2016; 150: 164–79. |
| Smoking | Peptic ulcer disease | Kurata JH, Nogawa AN. Meta-analysis of risk factors for peptic ulcer. Nonsteroidal antiinflammatory drugs, Helicobacter pylori, and smoking. J Clin Gastroenterol 1997; 24: 2–17. |
| Smoking | Gallbladder and biliary tract disease | Aune D, Vatten LJ, Boffetta P. Tobacco smoking and the risk of gallbladder disease. Eur J Epidemiol 2016; 31: 643–53. |
| Smoking | Alzheimer disease and other dimentias | Zhong G, Wang Y, Zhang Y, Guo JJ, Zhao Y. Smoking Is Associated with an Increased Risk of Dementia: A Meta-Analysis of Prospective Cohort Studies with Investigation of Potential Effect Modifiers. PLoS One 2015; 10. DOI:10.1371/journal.pone.0118333. |
| Smoking | Parkinson disease | Li X, Li W, Liu G, Shen X, Tang Y. Association between cigarette smoking and Parkinson's disease: A meta-analysis. Arch Gerontol Geriatr 2015; 61: 510–6. |
| Smoking | Multiple sclerosis | O'Gorman C, Broadley SA. Smoking and multiple sclerosis: evidence for latitudinal and temporal variation. J Neurol 2014; 261: 1677–83. |
| Smoking | Diabetes mellitus type 2 | Pan A, Wang Y, Talaei M, Hu FB, Wu T. Relation of active, passive, and quitting smoking with incident type 2 diabetes: a systematic review and meta-analysis. The Lancet Diabetes & Endocrinology 2015; 3: 958–67. |
| Smoking | Rheumatoid arthritis | Sugiyama D, Nishimura K, Tamaki K, et al. Impact of smoking as a risk factor for developing rheumatoid arthritis: a meta-analysis of observational studies. Ann Rheum Dis 2010; 69: 70–81. |

**Appendix Table 5. Epidemiological evidence supporting causality between risk-outcome pairs included in the Global Burden of Disease 2017 study including A. Citations and B. Additional information**

**A. Citations**

| Risk | Outcome | Citation/Note |
|---|---|---|
| Smoking | Low back pain | Shiri R, Karppinen J, Leino-Arjas P, Solovieva S, Viikari-Juntura E. The Association between Smoking and Low Back Pain: A Meta-analysis. The American Journal of Medicine 2010; 123: 87.e7-87.e35. |
| Smoking | Cataract | Ye J, He J, Wang C, et al. Smoking and risk of age-related cataract: a meta-analysis. Invest Ophthalmol Vis Sci 2012; 53: 3885–95. |
| Smoking | Macular degeneration | Chakravarthy U, Wong TY, Fletcher A, et al. Clinical risk factors for age-related macular degeneration: a systematic review and meta-analysis. BMC Ophthalmol 2010; 10: 31. |
| Smoking | Injuries | Vestergaard P, Mosekilde L. Fracture risk associated with smoking: a meta-analysis. J Intern Med 2003; 254: 572–83. |
| Chewing Tobacco | Lip and oral cavity cancer | Siddiqi K, Shah S, Abbas SM, et al. Global burden of disease due to smokeless tobacco consumption in adults: analysis of data from 113 countries. BMC Med 2015; 13: 194. |
| Chewing Tobacco | Oesophageal cancer | Siddiqi K, Shah S, Abbas SM, et al. Global burden of disease due to smokeless tobacco consumption in adults: analysis of data from 113 countries. BMC Med 2015; 13: 194. |
| Second-hand smoke | Breast cancer | Macacu A, Autier P, Boniol M, Boyle P. Active and passive smoking and risk of breast cancer: a meta-analysis. Breast Cancer Res Treat 2015; 154: 213–24. |
| Second-hand smoke | Chronic obstructive pulmonary disease | Fischer F, Kraemer A. Meta-analysis of the association between second-hand smoke exposure and ischaemic heart diseases, COPD and stroke. BMC Public Health 2015; 15: 1202. |
| Second-hand smoke | Diabetes mellitus type 2 | Zhu B, Wu X, Wang X, Zheng Q, Sun G. The association between passive smoking and type 2 diabetes: a meta-analysis. Asia Pac J Public Health 2014; 26: 226–37. |
| Second-hand smoke | Tuberculosis | Dogar OF, Pillai N, Safdar N, Shah SK, Zahid R, Siddiqi K. Second-hand smoke and the risk of tuberculosis: a systematic review and a meta-analysis. Epidemiol Infect 2015; 143: 3158–72. |
| Second-hand smoke | Lower respiratory infections | Baker RJ, Hertz-Picciotto I, Dostal M, Keller JA, Nozicka J, Kotesovec F, Dejmek J, Loomis D, Sram RJ. Coal home heating and environmental tobacco smoke in relation to lower respiratory illness in Czech children, from birth to 3 years of age. Environ Health Perspect. 2006; 1126-32. |
| Second-hand smoke | Lower respiratory infections | Blizzard L, Ponsonby A-L, Dwyer T, Venn A, Cochrane JA. Parental smoking and infant respiratory infection: how important is not smoking in the same room with the baby?. Am J Public Health. 2003; 482-8. |
| Second-hand smoke | Lower respiratory infections | Bonu S, Rani M, Jha P, Peters DH, Nguyen SN. Household tobacco and alcohol use, and child health: an exploratory study from India. Health Policy. 2004; 67-83. |
| Second-hand smoke | Lower respiratory infections | Broor S, Pandey RM, Ghosh M, Maitreyi RS, Lodha R, Singhal T, Kabra SK. Risk factors for severe acute lower respiratory tract infection in under-five children. Indian Pediatr. 2001; 1361-9. |
| Second-hand smoke | Lower respiratory infections | Chen Y, Li WX, Yu SZ, Qian WH. Chang-Ning epidemiological study of children's health: I: Passive smoking and children's respiratory diseases. Int J Epidemiol. 1988; 348-55. |
| Second-hand smoke | Lower respiratory infections | Duijts L, Jaddoe VWV, Hofman A, Steegers EAP, Mackenbach JP, de Jongste JC, Moll HA. Maternal smoking in pre-natal and early post-natal life and the risk of respiratory tract infections in infancy. The Generation R study. Eur J Epidemiol. 2008; 547-55. |
| Second-hand smoke | Lower respiratory infections | Ekwo EE, Weinberger MM, Lachenbruch PA, Huntley WH. Relationship of parental smoking and gas cooking to respiratory disease in children. Chest. 1983; 662-8. |
| Second-hand smoke | Lower respiratory infections | Etiler N, Velipasaoglu S, Aktekin M. Incidence of acute respiratory infections and the relationship with some factors in infancy in Antalya, Turkey. Pediatr Int 2002; 44: 64–9. |
| Second-hand smoke | Lower respiratory infections | Ferris BG, Ware JH, Berkey CS, Dockery DW, Spiro A, Speizer FE. Effects of passive smoking on health of children. Environ Health Perspect. 1985; 289-95. |
| Second-hand smoke | Lower respiratory infections | Forastiere F, Corbo GM, Michelozzi P, Pistelli R, Agabiti N, Brancato G, Ciappi G, Perucci CA. Effects of environment and passive smoking on the respiratory health of children. Int J Epidemiol. 1992; 66-73. |
| Second-hand smoke | Lower respiratory infections | Gardner G, Frank AL, Taber LH. Effects of social and family factors on viral respiratory infection and illness in the first year of life. J Epidemiol Community Health. 1984; 42-8. |
| Second-hand smoke | Lower respiratory infections | Hassan MK, Al-Sadoon I. Risk factors for severe pneumonia in children in Basrah. Trop Doct. 2001; 139-41. |
| Second-hand smoke | Lower respiratory infections | Koch A, Molbak K, Homoe P, Sorensen P, Hjuler T, Olesen ME, Pejl J, Pedersen FK, Olsen OR, Melbye M. Risk factors for acute respiratory tract infections in young Greenlandic children. Am J Epidemiol. 2003; 374-84. |
| Second-hand smoke | Lower respiratory infections | Kristensen IA, Olsen J. Determinants of acute respiratory infections in Soweto--a population-based birth cohort. S Afr Med J. 2006; 633-40. |
| Second-hand smoke | Lower respiratory infections | Margolis PA, Keyes LL, Greenberg RA, Bauman KE, LaVange LM. Urinary cotinine and parent history (questionnaire) as indicators of passive smoking and predictors of lower respiratory illness in infants. Pediatr Pulmonol. 1997; 417-23. |
| Second-hand smoke | Lower respiratory infections | Nuesslein TG, Beckers D, Rieger CH. Cotinine in meconium indicates risk for early respiratory tract infections. Hum Exp Toxicol. 1999; 283-90. |
| Second-hand smoke | Lower respiratory infections | Ogston SA, Florey CD, Walker CH. The Tayside infant morbidity and mortality study: effect on health of using gas for cooking. BMJ. 1985; 957-60. |
| Second-hand smoke | Lower respiratory infections | Ogston SA, Florey CD, Walker CH. Association of infant alimentary and respiratory illness with parental smoking and other environmental factors. J Epidemiol Community Health. 1987; 21-5. |
| Second-hand smoke | Lower respiratory infections | Pedreira FA, Guandolo VL, Feroli EJ, Mella GW, Weiss IP. Involuntary smoking and incidence of respiratory illness during the first year of life. Pediatrics. 1985; 594-7. |
| Second-hand smoke | Lower respiratory infections | Rylander E, Pershagen G, Eriksson M, Bermann G. Parental smoking, urinary cotinine, and wheezing bronchitis in children. Epidemiology. 1995; 289-93. |
| Second-hand smoke | Lower respiratory infections | Suzuki M, Thiem VD, Yanai H, Matsubayashi T, Yoshida LM, Tho LH, Minh TT, Anh DD, Kilgore PE, Ariyoshi K. Association of environmental tobacco smoking exposure with an increased risk of hospital admissions for pneumonia in children under 5 years of age in Vietnam. Thorax. 2009; 484-9. |

**Appendix Table 5. Epidemiological evidence supporting causality between risk-outcome pairs included in the Global Burden of Disease 2017 study including A. Citations and B. Additional information**

**A. Citations**

| Risk | Outcome | Citation/Note |
|---|---|---|
| Second-hand smoke | Lower respiratory infections | Taylor B, Wadsworth J. Maternal smoking during pregnancy and lower respiratory tract illness in early life. Arch Dis Child. 1987; 786-91. |
| Second-hand smoke | Lower respiratory infections | Victora CG, Fuchs SC, Flores JA, Fonseca W, Kirkwood B. Risk factors for pneumonia among children in a Brazilian metropolitan area. Pediatrics. 1994; 977-85. |
| Second-hand smoke | Otitis media | Jones LL, Hassanien A, Cook DG, Britton J, Leonardi-Bee J. Parental smoking and the risk of middle ear disease in children: a systematic review and meta-analysis. Arch Pediatr Adolesc Med 2012; 166: 18–27. |
| Second-hand smoke | Tracheal, bronchus, and lung cancer | Jayes L, Haslam PL, Gratziou CG, et al. SmokeHaz: Systematic Reviews and Meta-analyses of the Effects of Smoking on Respiratory Health. Chest 2016; 150: 164–79. |
| Second-hand smoke | Ischaemic heart disease | Fischer F, Kraemer A. Meta-analysis of the association between second-hand smoke exposure and ischaemic heart diseases, COPD and stroke. BMC Public Health 2015; 15: 1202. |
| Second-hand smoke | Ischaemic stroke | Oono IP, Mackay DF, Pell JP. Meta-analysis of the association between secondhand smoke exposure and stroke. J Public Health (Oxf) 2011; 33: 496–502. |
| Alcohol use | Tuberculosis | Lönnroth K, Williams BG, Stadlin S, Jaramillo E, Dye C. Alcohol use as a risk factor for tuberculosis - a systematic review. BMC Public Health 2008; 8: 289. |
| Alcohol use | Tuberculosis | Rehm J, Samokhvalov AV, Neuman MG, et al. The association between alcohol use, alcohol use disorders and tuberculosis (TB). A systematic review. BMC Public Health 2009; 9: 450. |
| Alcohol use | Tuberculosis | Hemilä H, Kaprio J, Pietinen P, Albanes D, Heinonen OP. Vitamin C and other compounds in vitamin C rich food in relation to risk of tuberculosis in male smokers. Am J Epidemiol 1999; 150: 632–41. |
| Alcohol use | Tuberculosis | Brown KE, Campbell AH. Tobacco, alcohol and tuberculosis. British Journal of Diseases of the Chest 1961; 55: 150–8. |
| Alcohol use | Tuberculosis | Buskin SE, Gale JL, Weiss NS, Nolan CM. Tuberculosis risk factors in adults in King County, Washington, 1988 through 1990. Am J Public Health 1994; 84: 1750–6. |
| Alcohol use | Tuberculosis | Crampin AC, Glynn JR, Floyd S, et al. Tuberculosis and gender: exploring the patterns in a case control study in Malawi. Int J Tuberc Lung Dis 2004; 8: 194–203. |
| Alcohol use | Tuberculosis | Lewis JG, Chamberlain DA. Alcohol consumption and smoking habits in male patients with pulmonary tuberculosis. Br J Prev Soc Med 1963; 17: 149–52. |
| Alcohol use | Tuberculosis | Rosenman KD, Hall N. Occupational risk factors for developing tuberculosis. Am J Ind Med 1996; 30: 148–54. |
| Alcohol use | Tuberculosis | Tekkel M, Rahu M, Loit HM, Baburin A. Risk factors for pulmonary tuberculosis in Estonia. Int J Tuberc Lung Dis 2002; 6: 887–94. |
| Alcohol use | Tuberculosis | Tocque K, Bellis MA, Beeching NJ, Syed Q, Remmington T, Davies PD. A case-control study of lifestyle risk factors associated with tuberculosis in Liverpool, North-West England. Eur Respir J 2001; 18: 959–64. |
| Alcohol use | Tuberculosis | Zaridze D, Brennan P, Boreham J, et al. Alcohol and cause-specific mortality in Russia: a retrospective case-control study of 48,557 adult deaths. Lancet 2009; 373: 2201–14. |
| Alcohol use | Lower respiratory infections | Samokhvalov AV, Irving HM, Rehm J. Alcohol consumption as a risk factor for pneumonia: a systematic review and meta-analysis. Epidemiol Infect 2010; 138: 1789–95. |
| Alcohol use | Lower respiratory infections | Baik I, Curhan GC, Rimm EB, Bendich A, Willett WC, Fawzi WW. A prospective study of age and lifestyle factors in relation to community-acquired pneumonia in US men and women. Arch Intern Med 2000; 160: 3082–8. |
| Alcohol use | Lower respiratory infections | Kornum JB, Due KM, Nørgaard M, et al. Alcohol drinking and risk of subsequent hospitalisation with pneumonia. Eur Respir J 2012; 39: 149–55. |
| Alcohol use | Lower respiratory infections | Shen C, Ni MY, Schooling CM, Chan WM, Lee SY, Lam TH. Alcohol use and death from respiratory disease in a prospective Chinese elderly cohort study in Hong Kong. Prev Med 2013; 57: 819–23. |
| Alcohol use | Lower respiratory infections | Almirall J, Bolíbar I, Serra-Prat M, et al. New evidence of risk factors for community-acquired pneumonia: a population-based study. Eur Respir J 2008; 31: 1274–84. |
| Alcohol use | Lower respiratory infections | Almirall J, Bolíbar I, Balanzó X, González CA. Risk factors for community-acquired pneumonia in adults: a population-based case-control study. Eur Respir J 1999; 13: 349–55. |
| Alcohol use | Lip and oral cavity cancer | Bagnardi V, Blangiardo M, La Vecchia C, Corrao G. A meta-analysis of alcohol drinking and cancer risk. Br J Cancer 2001; 85: 1700–5. |
| Alcohol use | Lip and oral cavity cancer | Jayasekara H, MacInnis RJ, Room R, English DR. Long-Term Alcohol Consumption and Breast, Upper Aero-Digestive Tract and Colorectal Cancer Risk: A Systematic Review and Meta-Analysis. Alcohol Alcohol 2016; 51: 315–30. |
| Alcohol use | Lip and oral cavity cancer | Turati F, Garavello W, Tramacere I, et al. A meta-analysis of alcohol drinking and oral and pharyngeal cancers. Part 2: results by subsites. Oral Oncol 2010; 46: 720–6. |
| Alcohol use | Lip and oral cavity cancer | Shanmugham JR, Zavras AI, Rosner BA, Giovannucci EL. Alcohol-folate interactions in the risk of oral cancer in women: a prospective cohort study. Cancer Epidemiol Biomarkers Prev 2010; 19: 2516–24. |
| Alcohol use | Lip and oral cavity cancer | Weikert C, Dietrich T, Boeing H, et al. Lifetime and baseline alcohol intake and risk of cancer of the upper aero-digestive tract in the European Prospective Investigation into Cancer and Nutrition (EPIC) study. Int J Cancer 2009; 125: 406–12. |
| Alcohol use | Lip and oral cavity cancer | Martinez I. Factors associated with cancer of the esophagus, mouth, and pharynx in Puerto Rico. J Natl Cancer Inst 1969; 42: 1069–94. |
| Alcohol use | Nasopharynx cancer | Bagnardi V, Blangiardo M, La Vecchia C, Corrao G. A meta-analysis of alcohol drinking and cancer risk. Br J Cancer 2001; 85: 1700–5. |
| Alcohol use | Other pharynx cancer | Bagnardi V, Blangiardo M, La Vecchia C, Corrao G. A meta-analysis of alcohol drinking and cancer risk. Br J Cancer 2001; 85: 1700–5. |
| Alcohol use | Oesophageal cancer | Bagnardi V, Blangiardo M, La Vecchia C, Corrao G. A meta-analysis of alcohol drinking and cancer risk. Br J Cancer 2001; 85: 1700–5. |
| Alcohol use | Oesophageal cancer | Allen NE, Beral V, Casabonne D, et al. Moderate alcohol intake and cancer incidence in women. J Natl Cancer Inst 2009; 101: 296–305. |

**Appendix Table 5. Epidemiological evidence supporting causality between risk-outcome pairs included in the Global Burden of Disease 2017 study including A. Citations and B. Additional information**
**A. Citations**

| Risk | Outcome | Citation/Note |
|---|---|---|
| Alcohol use | Oesophageal cancer | Boffetta P, Garfinkel L. Alcohol drinking and mortality among men enrolled in an American Cancer Society prospective study. Epidemiology 1990; 1: 342–8. |
| Alcohol use | Oesophageal cancer | Fan Y, Yuan J-M, Wang R, Gao Y-T, Yu MC. Alcohol, tobacco, and diet in relation to esophageal cancer: the Shanghai Cohort Study. Nutr Cancer 2008; 60: 354–63. |
| Alcohol use | Oesophageal cancer | Freedman ND, Abnet CC, Leitzmann MF, et al. A prospective study of tobacco, alcohol, and the risk of esophageal and gastric cancer subtypes. Am J Epidemiol 2007; 165: 1424–33. |
| Alcohol use | Oesophageal cancer | Ishiguro S, Sasazuki S, Inoue M, Kurahashi N, Iwasaki M, Tsugane S. Effect of alcohol consumption, cigarette smoking and flushing response on esophageal cancer risk: a population-based cohort study (JPHC study). Cancer Lett 2009; 275: 240–6. |
| Alcohol use | Oesophageal cancer | Kim MK, Ko MJ, Han JT. Alcohol consumption and mortality from all-cause and cancers among 1.34 million Koreans: the results from the Korea national health insurance corporation’s health examinee cohort in 2000. Cancer Causes Control 2010; 21: 2295–302. |
| Alcohol use | Oesophageal cancer | Kimm H, Kim S, Jee SH. The independent effects of cigarette smoking, alcohol consumption, and serum aspartate aminotransferase on the alanine aminotransferase ratio in korean men for the risk for esophageal cancer. Yonsei Med J 2010; 51: 310–7. |
| Alcohol use | Oesophageal cancer | Kono S, Ikeda M, Tokudome S, Nishizumi M, Kuratsune M. Cigarette smoking, alcohol and cancer mortality: a cohort study of male Japanese physicians. Jpn J Cancer Res 1987; 78: 1323–8. |
| Alcohol use | Oesophageal cancer | Nakaya N, Tsubono Y, Kuriyama S, et al. Alcohol consumption and the risk of cancer in Japanese men: the Miyagi cohort study. Eur J Cancer Prev 2005; 14: 169–74. |
| Alcohol use | Oesophageal cancer | Ozasa K. Alcohol use and mortality in the Japan Collaborative Cohort Study for Evaluation of Cancer (JACC). Asian Pac J Cancer Prev 2007; 8 Suppl: 81–8. |
| Alcohol use | Oesophageal cancer | Smith M, Zhou M, Whitlock G, et al. Esophageal cancer and body mass index: results from a prospective study of 220,000 men in China and a meta-analysis of published studies. Int J Cancer 2008; 122: 1604–10. |
| Alcohol use | Oesophageal cancer | Wu M, Zhang Z-F, Kampman E, et al. Does family history of cancer modify the effects of lifestyle risk factors on esophageal cancer? A population-based case-control study in China. Int J Cancer 2011; 128: 2147–57. |
| Alcohol use | Colon and rectum cancer | Bagnardi V, Blangiardo M, La Vecchia C, Corrao G. A meta-analysis of alcohol drinking and cancer risk. Br J Cancer 2001; 85: 1700–5. |
| Alcohol use | Colon and rectum cancer | Akhter M, Kuriyama S, Nakaya N, et al. Alcohol consumption is associated with an increased risk of distal colon and rectal cancer in Japanese men: the Miyagi Cohort Study. Eur J Cancer 2007; 43: 383–90. |
| Alcohol use | Colon and rectum cancer | Allen NE, Beral V, Casabonne D, et al. Moderate alcohol intake and cancer incidence in women. J Natl Cancer Inst 2009; 101: 296–305. |
| Alcohol use | Colon and rectum cancer | Breslow RA, Chen CM, Graubard BI, Mukamal KJ. Prospective study of alcohol consumption quantity and frequency and cancer-specific mortality in the US population. Am J Epidemiol 2011; 174: 1044–53. |
| Alcohol use | Colon and rectum cancer | Chen K, Jiang Q, Ma X, et al. Alcohol drinking and colorectal cancer: a population-based prospective cohort study in China. Eur J Epidemiol 2005; 20: 149–54. |
| Alcohol use | Colon and rectum cancer | Chyou PH, Nomura AM, Stemmermann GN. A prospective study of colon and rectal cancer among Hawaii Japanese men. Ann Epidemiol 1996; 6: 276–82. |
| Alcohol use | Colon and rectum cancer | Crockett SD, Long MD, Dellon ES, Martin CF, Galanko JA, Sandler RS. Inverse relationship between moderate alcohol intake and rectal cancer: analysis of the North Carolina Colon Cancer Study. Dis Colon Rectum 2011; 54: 887–94. |
| Alcohol use | Colon and rectum cancer | Ferrari P, Jenab M, Norat T, et al. Lifetime and baseline alcohol intake and risk of colon and rectal cancers in the European prospective investigation into cancer and nutrition (EPIC). Int J Cancer 2007; 121: 2065–72. |
| Alcohol use | Colon and rectum cancer | Flood A, Caprario L, Chaterjee N, Lacey JVJ, Schairer C, Schatzkin A. Folate, methionine, alcohol, and colorectal cancer in a prospective study of women in the United States. Cancer Causes Control 2002; 13: 551–61. |
| Alcohol use | Colon and rectum cancer | Gaziano JM, Gaziano TA, Glynn RJ, et al. Light-to-moderate alcohol consumption and mortality in the Physicians’ Health Study enrollment cohort. J Am Coll Cardiol 2000; 35: 96–105. |
| Alcohol use | Colon and rectum cancer | Kabat GC, Miller AB, Jain M, Rohan TE. Dietary intake of selected B vitamins in relation to risk of major cancers in women. Br J Cancer 2008; 99: 816–21. |
| Alcohol use | Colon and rectum cancer | Kim MK, Ko MJ, Han JT. Alcohol consumption and mortality from all-cause and cancers among 1.34 million Koreans: the results from the Korea national health insurance corporation’s health examinee cohort in 2000. Cancer Causes Control 2010; 21: 2295–302. |
| Alcohol use | Colon and rectum cancer | Klatsky AL, Armstrong MA, Friedman GD, Hiatt RA. The relations of alcoholic beverage use to colon and rectal cancer. Am J Epidemiol 1988; 128: 1007–15. |
| Alcohol use | Colon and rectum cancer | Kono S, Ikeda M, Tokudome S, Nishizumi M, Kuratsune M. Cigarette smoking, alcohol and cancer mortality: a cohort study of male Japanese physicians. Jpn J Cancer Res 1987; 78: 1323–8. |
| Alcohol use | Colon and rectum cancer | Lim HJ, Park BJ. [Cohort study on the association between alcohol consumption and the risk of colorectal cancer in the Korean elderly]. J Prev Med Public Health 2008; 41: 23–9. |
| Alcohol use | Colon and rectum cancer | Nakaya N, Tsubono Y, Kuriyama S, et al. Alcohol consumption and the risk of cancer in Japanese men: the Miyagi cohort study. Eur J Cancer Prev 2005; 14: 169–74. |
| Alcohol use | Colon and rectum cancer | Otani T, Iwasaki M, Yamamoto S, et al. Alcohol consumption, smoking, and subsequent risk of colorectal cancer in middle-aged and elderly Japanese men and women: Japan Public Health Center-based prospective study. Cancer Epidemiol Biomarkers Prev 2003; 12: 1492–500. |
| Alcohol use | Colon and rectum cancer | Ozasa K. Alcohol use and mortality in the Japan Collaborative Cohort Study for Evaluation of Cancer (JACC). Asian Pac J Cancer Prev 2007; 8 Suppl: 81–8. |

| **Appendix Table 5. Epidemiological evidence supporting causality between risk-outcome pairs included in the Global Burden of Disease 2017 study including A. Citations and B. Additional information** | | |
|---|---|---|
| **A. Citations** | | |
| **Risk** | **Outcome** | **Citation/Note** |
| Alcohol use | Colon and rectum cancer | Pedersen A, Johansen C, Gronbaek M. Relations between amount and type of alcohol and colon and rectal cancer in a Danish population based cohort study. Gut 2003; 52: 861–7. |
| Alcohol use | Colon and rectum cancer | Sanjoaquin MA, Appleby PN, Thorogood M, Mann JI, Key TJ. Nutrition, lifestyle and colorectal cancer incidence: a prospective investigation of 10998 vegetarians and non-vegetarians in the United Kingdom. Br J Cancer 2004; 90: 118–21. |
| Alcohol use | Colon and rectum cancer | Thun MJ, Peto R, Lopez AD, et al. Alcohol consumption and mortality among middle-aged and elderly U.S. adults. N Engl J Med 1997; 337: 1705–14. |
| Alcohol use | Colon and rectum cancer | Thygesen LC, Wu K, Gronbaek M, Fuchs CS, Willett WC, Giovannucci E. Alcohol intake and colorectal cancer: a comparison of approaches for including repeated measures of alcohol consumption. Epidemiology 2008; 19: 258–64. |
| Alcohol use | Colon and rectum cancer | Toriola AT, Kurl S, Laukanen JA, Mazengo C, Kauhanen J. Alcohol consumption and risk of colorectal cancer: the Findrink study. Eur J Epidemiol 2008; 23: 395–401. |
| Alcohol use | Colon and rectum cancer | Wakai K, Kojima M, Tamakoshi K, et al. Alcohol consumption and colorectal cancer risk: findings from the JACC Study. J Epidemiol 2005; 15 Suppl 2: S173-179. |
| Alcohol use | Colon and rectum cancer | Wu AH, Paganini-Hill A, Ross RK, Henderson BE. Alcohol, physical activity and other risk factors for colorectal cancer: a prospective study. Br J Cancer 1987; 55: 687–94. |
| Alcohol use | Colon and rectum cancer | Yamamoto S, Nakagawa T, Matsushita Y, et al. Visceral fat area and markers of insulin resistance in relation to colorectal neoplasia. Diabetes Care 2010; 33: 184–9. |
| Alcohol use | Colon and rectum cancer | Yi S-W, Sull JW, Linton JA, Nam CM, Ohrr H. Alcohol consumption and digestive cancer mortality in Koreans: the Kangwha Cohort Study. J Epidemiol 2010; 20: 204–11. |
| Alcohol use | Colon and rectum cancer | Yuan JM, Ross RK, Gao YT, Henderson BE, Yu MC. Follow up study of moderate alcohol intake and mortality among middle aged men in Shanghai, China. BMJ 1997; 314: 18–23. |
| Alcohol use | Larynx cancer | Bagnardi V, Blangiardo M, La Vecchia C, Corrao G. A meta-analysis of alcohol drinking and cancer risk. Br J Cancer 2001; 85: 1700–5. |
| Alcohol use | Larynx cancer | Allen NE, Beral V, Casabonne D, et al. Moderate alcohol intake and cancer incidence in women. J Natl Cancer Inst 2009; 101: 296–305. |
| Alcohol use | Larynx cancer | Freedman ND, Schatzkin A, Leitzmann MF, Hollenbeck AR, Abnet CC. Alcohol and head and neck cancer risk in a prospective study. Br J Cancer 2007; 96: 1469–74. |
| Alcohol use | Larynx cancer | Garavello W, Bosetti C, Gallus S, et al. Type of alcoholic beverage and the risk of laryngeal cancer. Eur J Cancer Prev 2006; 15: 69–73. |
| Alcohol use | Larynx cancer | Kim MK, Ko MJ, Han JT. Alcohol consumption and mortality from all-cause and cancers among 1.34 million Koreans: the results from the Korea national health insurance corporation’s health examinee cohort in 2000. Cancer Causes Control 2010; 21: 2295–302. |
| Alcohol use | Breast cancer | Bagnardi V, Blangiardo M, La Vecchia C, Corrao G. A meta-analysis of alcohol drinking and cancer risk. Br J Cancer 2001; 85: 1700–5. |
| Alcohol use | Breast cancer | Allen NE, Beral V, Casabonne D, et al. Moderate alcohol intake and cancer incidence in women. J Natl Cancer Inst 2009; 101: 296–305. |
| Alcohol use | Breast cancer | Baglietto L, English DR, Gertig DM, Hopper JL, Giles GG. Does dietary folate intake modify effect of alcohol consumption on breast cancer risk? Prospective cohort study. BMJ 2005; 331: 807. |
| Alcohol use | Breast cancer | Breslow RA, Chen CM, Graubard BI, Mukamal KJ. Prospective study of alcohol consumption quantity and frequency and cancer-specific mortality in the US population. Am J Epidemiol 2011; 174: 1044–53. |
| Alcohol use | Breast cancer | Chen WY, Colditz GA, Rosner B, et al. Use of postmenopausal hormones, alcohol, and risk for invasive breast cancer. Ann Intern Med 2002; 137: 798–804. |
| Alcohol use | Ischaemic heart disease | Roerecke M, Rehm J. The cardioprotective association of average alcohol consumption and ischaemic heart disease: a systematic review and meta-analysis. Addiction 2012; 107: 1246–60. |
| Alcohol use | Ischaemic heart disease | Ronksley PE, Brien SE, Turner BJ, Mukamal KJ, Ghali WA. Association of alcohol consumption with selected cardiovascular disease outcomes: a systematic review and meta-analysis. BMJ 2011; 342: d671. |
| Alcohol use | Ischaemic heart disease | Corrao G, Rubbiati L, Bagnardi V, Zambon A, Poikolainen K. Alcohol and coronary heart disease: a meta-analysis. Addiction 2000; 95: 1505–23. |
| Alcohol use | Ischaemic heart disease | Zhao J, Stockwell T, Roemer A, Naimi T, Chikritzhs T. Alcohol Consumption and Mortality From Coronary Heart Disease: An Updated Meta-Analysis of Cohort Studies. J Stud Alcohol Drugs 2017; 78: 375–86. |
| Alcohol use | Ischaemic heart disease | Zhang X-Y, Shu L, Si C-J, et al. Dietary Patterns, Alcohol Consumption and Risk of Coronary Heart Disease in Adults: A Meta-Analysis. Nutrients 2015; 7: 6582–605. |
| Alcohol use | Ischaemic heart disease | Zheng Y-L, Lian F, Shi Q, et al. Alcohol intake and associated risk of major cardiovascular outcomes in women compared with men: a systematic review and meta-analysis of prospective observational studies. BMC Public Health 2015; 15: 773. |
| Alcohol use | Stroke | Patra J, Taylor B, Irving H, et al. Alcohol consumption and the risk of morbidity and mortality for different stroke types--a systematic review and meta-analysis. BMC Public Health 2010; 10: 258. |
| Alcohol use | Hypertensive heart disease | Briasoulis A, Agarwal V, Messerli FH. Alcohol consumption and the risk of hypertension in men and women: a systematic review and meta-analysis. J Clin Hypertens (Greenwich) 2012; 14: 792–8. |
| Alcohol use | Hypertensive heart disease | Taylor B, Irving HM, Baliunas D, et al. Alcohol and hypertension: gender differences in dose-response relationships determined through systematic review and meta-analysis. Addiction 2009; 104: 1981–90. |
| Alcohol use | Atrial fibrillation and flutter | Kodama S, Saito K, Tanaka S, et al. Alcohol consumption and risk of atrial fibrillation: a meta-analysis. J Am Coll Cardiol 2011; 57: 427–36. |
| Alcohol use | Atrial fibrillation and flutter | Samokhvalov AV, Irving HM, Rehm J. Alcohol consumption as a risk factor for atrial fibrillation: a systematic review and meta-analysis. Eur J Cardiovasc Prev Rehabil 2010; 17: 706–12. |
| Alcohol use | Cirrhosis | Rehm J, Taylor B, Mohapatra S, et al. Alcohol as a risk factor for liver cirrhosis: a systematic review and meta-analysis. Drug Alcohol Rev 2010; 29: 437–45. |

**Appendix Table 5. Epidemiological evidence supporting causality between risk-outcome pairs included in the Global Burden of Disease 2017 study including A. Citations and B. Additional information**

**A. Citations**

| Risk | Outcome | Citation/Note |
|---|---|---|
| Alcohol use | Cirrhosis | Becker U, Grønbaek M, Johansen D, Sørensen TIA. Lower risk for alcohol-induced cirrhosis in wine drinkers. Hepatology 2002; 35: 868–75. |
| Alcohol use | Cirrhosis | Becker U, Deis A, Sørensen TI, et al. Prediction of risk of liver disease by alcohol intake, sex, and age: a prospective population study. Hepatology 1996; 23: 1025–9. |
| Alcohol use | Cirrhosis | Bellentani S, Saccoccio G, Costa G, et al. Drinking habits as cofactors of risk for alcohol induced liver damage. The Dionysos Study Group. Gut 1997; 41: 845–50. |
| Alcohol use | Cirrhosis | Blackwelder WC, Yano K, Rhoads GG, Kagan A, Gordon T, Palesch Y. Alcohol and mortality: the Honolulu Heart Study. Am J Med 1980; 68: 164–9. |
| Alcohol use | Cirrhosis | Boffetta P, Garfinkel L. Alcohol drinking and mortality among men enrolled in an American Cancer Society prospective study. Epidemiology 1990; 1: 342–8. |
| Alcohol use | Cirrhosis | Fuchs CS, Stampfer MJ, Colditz GA, et al. Alcohol consumption and mortality among women. N Engl J Med 1995; 332: 1245–50. |
| Alcohol use | Cirrhosis | Garfinkel L, Boffetta P, Stellman SD. Alcohol and breast cancer: a cohort study. Prev Med 1988; 17: 686–93. |
| Alcohol use | Cirrhosis | Gordon T, Doyle JT. Drinking and mortality. The Albany Study. Am J Epidemiol 1987; 125: 263–70. |
| Alcohol use | Cirrhosis | Gordon T, Kannel WB. Drinking and mortality. The Framingham Study. Am J Epidemiol 1984; 120: 97–107. |
| Alcohol use | Cirrhosis | Kono S, Ikeda M, Tokudome S, Nishizumi M, Kuratsune M. Alcohol and mortality: a cohort study of male Japanese physicians. Int J Epidemiol 1986; 15: 527–32. |
| Alcohol use | Cirrhosis | Thun MJ, Peto R, Lopez AD, et al. Alcohol consumption and mortality among middle-aged and elderly U.S. adults. N Engl J Med 1997; 337: 1705–14. |
| Alcohol use | Cirrhosis | Yuan JM, Ross RK, Gao YT, Henderson BE, Yu MC. Follow up study of moderate alcohol intake and mortality among middle aged men in Shanghai, China. BMJ 1997; 314: 18–23. |
| Alcohol use | Liver cancer | Bagnardi V, Rota M, Botteri E, et al. Alcohol consumption and site-specific cancer risk: a comprehensive dose-response meta-analysis. Br J Cancer 2015; 112: 580–93. |
| Alcohol use | Liver cancer | Allen NE, Beral V, Casabonne D, et al. Moderate alcohol intake and cancer incidence in women. J Natl Cancer Inst 2009; 101: 296–305. |
| Alcohol use | Liver cancer | Jee SH, Ohrr H, Sull JW, Samet JM. Cigarette smoking, alcohol drinking, hepatitis B, and risk for hepatocellular carcinoma in Korea. J Natl Cancer Inst 2004; 96: 1851–6. |
| Alcohol use | Liver cancer | Joshi S, Song Y-M, Kim T-H, Cho S-I. Socio-economic status and the risk of liver cancer mortality: a prospective study in Korean men. Public Health 2008; 122: 1144–51. |
| Alcohol use | Liver cancer | Kim MK, Ko MJ, Han JT. Alcohol consumption and mortality from all-cause and cancers among 1.34 million Koreans: the results from the Korea national health insurance corporation’s health examinee cohort in 2000. Cancer Causes Control 2010; 21: 2295–302. |
| Alcohol use | Liver cancer | Koh W-P, Robien K, Wang R, Govindarajan S, Yuan J-M, Yu MC. Smoking as an independent risk factor for hepatocellular carcinoma: the Singapore Chinese Health Study. Br J Cancer 2011; 105: 1430–5. |
| Alcohol use | Liver cancer | Kono S, Ikeda M, Tokudome S, Nishizumi M, Kuratsune M. Cigarette smoking, alcohol and cancer mortality: a cohort study of male Japanese physicians. Jpn J Cancer Res 1987; 78: 1323–8. |
| Alcohol use | Liver cancer | Nakaya N, Tsubono Y, Kuriyama S, et al. Alcohol consumption and the risk of cancer in Japanese men: the Miyagi cohort study. Eur J Cancer Prev 2005; 14: 169–74. |
| Alcohol use | Liver cancer | Ozasa K. Alcohol use and mortality in the Japan Collaborative Cohort Study for Evaluation of Cancer (JACC). Asian Pac J Cancer Prev 2007; 8 Suppl: 81–8. |
| Alcohol use | Liver cancer | Yi S-W, Sull JW, Linton JA, Nam CM, Ohrr H. Alcohol consumption and digestive cancer mortality in Koreans: the Kangwha Cohort Study. J Epidemiol 2010; 20: 204–11. |
| Alcohol use | Pancreatitis | Alsamarrai A, Das SLM, Windsor JA, Petrov MS. Factors that affect risk for pancreatic disease in the general population: a systematic review and meta-analysis of prospective cohort studies. Clin Gastroenterol Hepatol 2014; 12: 1635–1644.e5; quiz e103. |
| Alcohol use | Pancreatitis | Irving HM, Samokhvalov AV, Rehm J. Alcohol as a risk factor for pancreatitis. A systematic review and meta-analysis. JOP 2009; 10: 387–92. |
| Alcohol use | Pancreatitis | Samokhvalov AV, Rehm J, Roerecke M. Alcohol Consumption as a Risk Factor for Acute and Chronic Pancreatitis: A Systematic Review and a Series of Meta-analyses. EBioMedicine 2015; 2: 1996–2002. |
| Alcohol use | Epilepsy | Samokhvalov AV, Irving H, Mohapatra S, Rehm J. Alcohol consumption, unprovoked seizures, and epilepsy: a systematic review and meta-analysis. Epilepsia 2010; 51: 1177–84. |
| Alcohol use | Epilepsy | Dworetzky BA, Bromfield EB, Townsend MK, Kang JH. A prospective study of smoking, caffeine, and alcohol as risk factors for seizures or epilepsy in young adult women: data from the Nurses’ Health Study II. Epilepsia 2010; 51: 198–205. |
| Alcohol use | Diabetes mellitus type 2 | Carlsson S, Hammar N, Grill V. Alcohol consumption and type 2 diabetes Meta-analysis of epidemiological studies indicates a U-shaped relationship. Diabetologia 2005; 48: 1051–4. |
| Alcohol use | Diabetes mellitus type 2 | Li X-H, Yu F-F, Zhou Y-H, He J. Association between alcohol consumption and the risk of incident type 2 diabetes: a systematic review and dose-response meta-analysis. Am J Clin Nutr 2016; 103: 818–29. |
| Alcohol use | Unintentional Injuries | Cherpitel CJ, Ye Y, Bond J, et al. Alcohol Attributable Fraction for Injury Morbidity from the Dose-Response Relationship of Acute Alcohol Consumption: Emergency Department Data from 18 Countries. Addiction 2015; 110: 1724–32. |
| Alcohol use | Unintentional Injuries | Corrao G, Bagnardi V, Zambon A, La Vecchia C. A meta-analysis of alcohol consumption and the risk of 15 diseases. Prev Med 2004; 38: 613–9. |
| Alcohol use | Unintentional Injuries | Taylor B, Irving HM, Kanteres F, et al. The more you drink, the harder you fall: a systematic review and meta-analysis of how acute alcohol consumption and injury or collision risk increase together. Drug Alcohol Depend 2010; 110: 108–16. |

**Appendix Table 5. Epidemiological evidence supporting causality between risk-outcome pairs included in the Global Burden of Disease 2017 study including A. Citations and B. Additional information**

**A. Citations**

| Risk | Outcome | Citation/Note |
|---|---|---|
| Alcohol use | Self-harm | Beghi M, Rosenbaum JF, Cerri C, Cornaggia CM. Risk factors for fatal and nonfatal repetition of suicide attempts: a literature review. Neuropsychiatr Dis Treat2013;9: 1725–36. |
| Alcohol use | Self-harm | Borges G, Bagge CL, Cherpitel CJ, Conner KR, Orozco R, Rossow I. A meta-analysis of acute use of alcohol and the risk of suicide attempt. Psychol Med 2017; 47: 949–57. |
| Alcohol use | Self-harm | Cherpitel CJ, Borges GLG, Wilcox HC. Acute alcohol use and suicidal behavior: a review of the literature. Alcohol Clin Exp Res 2004; 28: 18S–28S. |
| Alcohol use | Self-harm | Cherpitel CJ, Ye Y, Bond J, et al. Alcohol Attributable Fraction for Injury Morbidity from the Dose-Response Relationship of Acute Alcohol Consumption: Emergency Department Data from 18 Countries. Addiction 2015; 110: 1724–32. |
| Alcohol use | Self-harm | Corrao G, Bagnardi V, Zambon A, La Vecchia C. A meta-analysis of alcohol consumption and the risk of 15 diseases. Prev Med 2004; 38: 613–9. |
| Alcohol use | Self-harm | Devries KM, Mak JY, Bacchus LJ, et al. Intimate partner violence and incident depressive symptoms and suicide attempts: a systematic review of longitudinal studies. PLoS Med 2013; 10: e1001439. |
| Alcohol use | Self-harm | Haw C, Hawton K, Casey D, Bale E, Shepherd A. Alcohol dependence, excessive drinking and deliberate self-harm: trends and patterns in Oxford, 1989-2002. Soc Psychiatry Psychiatr Epidemiol 2005; 40: 964–71. |
| Alcohol use | Self-harm | Taylor B, Irving HM, Kanteres F, et al. The more you drink, the harder you fall: a systematic review and meta-analysis of how acute alcohol consumption and injury or collision risk increase together. Drug Alcohol Depend 2010; 110: 108–16. |
| Alcohol use | Interpersonal violence | Cherpitel CJ, Ye Y, Bond J, et al. Alcohol Attributable Fraction for Injury Morbidity from the Dose-Response Relationship of Acute Alcohol Consumption: Emergency Department Data from 18 Countries. Addiction 2015; 110: 1724–32. |
| Alcohol use | Interpersonal violence | Taylor B, Irving HM, Kanteres F, et al. The more you drink, the harder you fall: a systematic review and meta-analysis of how acute alcohol consumption and injury or collision risk increase together. Drug Alcohol Depend 2010; 110: 108–16. |
| Alcohol use | Ischaemic and haemorrhagic stroke | Patra J, Taylor B, Irving H, et al. Alcohol consumption and the risk of morbidity and mortality for different stroke types--a systematic review and meta-analysis. BMC Public Health 2010; 10: 258. |
| Alcohol use | Ischaemic and haemorrhagic stroke | Ronksley PE, Brien SE, Turner BJ, Mukamal KJ, Ghali WA. Association of alcohol consumption with selected cardiovascular disease outcomes: a systematic review and meta-analysis. BMJ 2011; 342: d671. |
| Alcohol use | Ischaemic and haemorrhagic stroke | Bazzano LA, Gu D, Reynolds K, et al. Alcohol consumption and risk for stroke among Chinese men. Ann Neurol 2007; 62: 569–78. |
| Alcohol use | Ischaemic and haemorrhagic stroke | Berger K, Ajani UA, Kase CS, et al. Light-to-moderate alcohol consumption and the risk of stroke among U.S. male physicians. N Engl J Med 1999; 341: 1557–64. |
| Alcohol use | Ischaemic and haemorrhagic stroke | Chiuve SE, Rexrode KM, Spiegelman D, Logroscino G, Manson JE, Rimm EB. Primary prevention of stroke by healthy lifestyle. Circulation 2008; 118: 947–54. |
| Alcohol use | Ischaemic and haemorrhagic stroke | Djoussé L, Ellison RC, Beiser A, Scaramucci A, D'Agostino RB, Wolf PA. Alcohol consumption and risk of ischemic stroke: The Framingham Study. Stroke 2002; 33: 907–12. |
| Alcohol use | Ischaemic and haemorrhagic stroke | Donahue RP, Abbott RD, Reed DM, Yano K. Alcohol and hemorrhagic stroke. The Honolulu Heart Program. JAMA 1986; 255: 2311–4. |
| Alcohol use | Ischaemic and haemorrhagic stroke | Elkind MSV, Sciacca R, Boden-Albala B, Rundek T, Paik MC, Sacco RL. Moderate alcohol consumption reduces risk of ischemic stroke: the Northern Manhattan Study. Stroke 2006; 37: 13–9. |
| Alcohol use | Ischaemic and haemorrhagic stroke | Hansagi H, Romelsjö A, Gerhardsson de Verdier M, Andréasson S, Leifman A. Alcohol consumption and stroke mortality. 20-year follow-up of 15,077 men and women. Stroke 1995; 26: 1768–73. |
| Alcohol use | Ischaemic and haemorrhagic stroke | Higashiyama A, Wakabayashi I, Ono Y, et al. Association with serum gamma-glutamyltransferase levels and alcohol consumption on stroke and coronary artery disease: the Suita study. Stroke 2011; 42: 1764–7. |
| Alcohol use | Ischaemic and haemorrhagic stroke | Ikehara S, Iso H, Toyoshima H, et al. Alcohol consumption and mortality from stroke and coronary heart disease among Japanese men and women: the Japan collaborative cohort study. Stroke 2008; 39: 2936–42. |
| Alcohol use | Ischaemic and haemorrhagic stroke | Ikehara S, Iso H, Yamagishi K, et al. Alcohol consumption and risk of stroke and coronary heart disease among Japanese women: the Japan Public Health Center-based prospective study. Prev Med 2013; 57: 505–10. |
| Alcohol use | Ischaemic and haemorrhagic stroke | Ikehara S, Iso H, Yamagishi K, et al. Alcohol consumption, social support, and risk of stroke and coronary heart disease among Japanese men: the JPHC Study. Alcohol Clin Exp Res 2009; 33: 1025–32. |
| Alcohol use | Ischaemic and haemorrhagic stroke | Iso H, Kitamura A, Shimamoto T, et al. Alcohol intake and the risk of cardiovascular disease in middle-aged Japanese men. Stroke 1995; 26: 767–73. |
| Alcohol use | Ischaemic and haemorrhagic stroke | Jones SB, Loehr L, Avery CL, et al. Midlife Alcohol Consumption and the Risk of Stroke in the Atherosclerosis Risk in Communities Study. Stroke 2015; 46: 3124–30. |
| Alcohol use | Ischaemic and haemorrhagic stroke | Klatsky AL, Armstrong MA, Friedman GD, Sidney S. Alcohol drinking and risk of hemorrhagic stroke. Neuroepidemiology 2002; 21: 115–22. |
| Alcohol use | Ischaemic and haemorrhagic stroke | Klatsky AL, Armstrong MA, Friedman GD, Sidney S. Alcohol drinking and risk of hospitalization for ischemic stroke. Am J Cardiol 2001; 88: 703–6. |
| Alcohol use | Ischaemic and haemorrhagic stroke | Klatsky AL, Armstrong MA, Friedman GD. Alcohol use and subsequent cerebrovascular disease hospitalizations. Stroke 1989; 20: 741–6. |
| Alcohol use | Ischaemic and haemorrhagic stroke | Kono S, Ikeda M, Tokudome S, Nishizumi M, Kuratsune M. Alcohol and mortality: a cohort study of male Japanese physicians. Int J Epidemiol 1986; 15: 527–32. |
| Alcohol use | Ischaemic and haemorrhagic stroke | Leppälä JM, Paunio M, Virtamo J, et al. Alcohol consumption and stroke incidence in male smokers. Circulation 1999; 100: 1209–14. |
| Alcohol use | Ischaemic and haemorrhagic stroke | Mukamal KJ, Ascherio A, Mittleman MA, et al. Alcohol and risk for ischemic stroke in men: the role of drinking patterns and usual beverage. Ann Intern Med 2005; 142: 11–9. |

**Appendix Table 5. Epidemiological evidence supporting causality between risk-outcome pairs included in the Global Burden of Disease 2017 study including A. Citations and B. Additional information**

**A. Citations**

| Risk | Outcome | Citation/Note |
|---|---|---|
| Alcohol use | Ischaemic and haemorrhagic stroke | Mukamal KJ, Chung H, Jenny NS, et al. Alcohol use and risk of ischemic stroke among older adults: the cardiovascular health study. Stroke 2005; 36: 1830–4. |
| Alcohol use | Ischaemic and haemorrhagic stroke | Nielsen NR, Truelsen T, Barefoot JC, et al. Is the effect of alcohol on risk of stroke confined to highly stressed persons? Neuroepidemiology 2005; 25: 105–13. |
| Alcohol use | Ischaemic and haemorrhagic stroke | Sankai T, Iso H, Shimamoto T, et al. Prospective study on alcohol intake and risk of subarachnoid hemorrhage among Japanese men and women. Alcohol Clin Exp Res 2000; 24: 386–9. |
| Alcohol use | Ischaemic and haemorrhagic stroke | Stampfer MJ, Colditz GA, Willett WC, Speizer FE, Hennekens CH. A prospective study of moderate alcohol consumption and the risk of coronary disease and stroke in women. N Engl J Med 1988; 319: 267–73. |
| Alcohol use | Ischaemic and haemorrhagic stroke | Suh I, Jee SH, Kim HC, Nam CM, Kim IS, Appel LJ. Low serum cholesterol and haemorrhagic stroke in men: Korea Medical Insurance Corporation Study. Lancet 2001; 357: 922–5. |
| Alcohol use | Ischaemic and haemorrhagic stroke | Yamada S, Koizumi A, Iso H, et al. Risk factors for fatal subarachnoid hemorrhage: the Japan Collaborative Cohort Study. Stroke 2003; 34: 2781–7. |
| Drug use | Hepatitis B | Blomé MA, Björkman P, Flamholc L, Jacobsson H, Molnegren V, Widell A. Minimal transmission of HIV despite persistently high transmission of hepatitis C virus in a Swedish needle exchange program. J Viral Hepat 2011; 18: 831–9. |
| Drug use | Hepatitis B | Crofts N, Aitken CK. Incidence of bloodborne virus infection and risk behaviours in a cohort of injecting drug users in Victoria, 1990-1995. Med J Aust 1997; 167: 17–20. |
| Drug use | Hepatitis B | Hagan H, McGough JP, Thiede H, Weiss NS, Hopkins S, Alexander ER. Syringe Exchange and Risk of Infection with Hepatitis B and C Viruses, Am. J. Epi. 1999; 149(3): 203–213. |
| Drug use | Hepatitis B | Jackson JB, Wei L, Liping F, et al. Prevalence and seroincidence of hepatitis B and hepatitis C infection in high risk people who inject drugs in china and Thailand. Hepat Res Treat 2014; 2014: 296958. |
| Drug use | Hepatitis B | Månsson AS, Moestrup T, Nordenfelt E, Widell A. Continued transmission of hepatitis B and C viruses, but no transmission of human immunodeficiency virus among intravenous drug users participating in a syringe/needle exchange program. Scand J Infect Dis 2000; 32: 253–8. |
| Drug use | Hepatitis C | Abou-Saleh M, Davis P, Rice P, et al. The effectiveness of behavioural interventions in the primary prevention of hepatitis C amongst injecting drug users: a randomised controlled trial and lessons learned. Harm Reduct J 2008; 5: 25. |
| Drug use | Hepatitis C | Blomé MA, Björkman P, Flamholc L, Jacobsson H, Molnegren V, Widell A. Minimal transmission of HIV despite persistently high transmission of hepatitis C virus in a Swedish needle exchange program. J Viral Hepat 2011; 18: 831–9. |
| Drug use | Hepatitis C | Craine N, Hickman M, Parry JV, et al. Incidence of hepatitis C in drug injectors: the role of homelessness, opiate substitution treatment, equipment sharing, and community size. Epidemiol Infect 2009; 137: 1255–65. |
| Drug use | Hepatitis C | Crofts N, Aitken CK. Incidence of bloodborne virus infection and risk behaviours in a cohort of injecting drug users in Victoria, 1990-1995. Med J Aust 1997; 167: 17–20. |
| Drug use | Hepatitis C | Foley SB, Abou-Saleh MT. Risk Behaviors and Transmission of Hepatitis C in Injecting Drug Users. Addictive Disorders & Their Treatment 2009; 8: 13–21. |
| Drug use | Hepatitis C | Grebely J, Lima VD, Marshall BDL, et al. Declining incidence of hepatitis C virus infection among people who inject drugs in a Canadian setting, 1996-2012. PLoS ONE 2014; 9: e97726. |
| Drug use | Hepatitis C | Hagan H, McGough JP, Thiede H, Weiss NS, Hopkins S, Alexander ER. Syringe exchange and risk of infection with hepatitis B and C viruses. Am J Epidemiol 1999; 149: 203–13. |
| Drug use | Hepatitis C | Jackson JB, Wei L, Liping F, et al. Prevalence and seroincidence of hepatitis B and hepatitis C infection in high risk people who inject drugs in china and Thailand. Hepat Res Treat 2014; 2014: 296958. |
| Drug use | Hepatitis C | Lucidarme D, Bruandet A, Ilef D, et al. Incidence and risk factors of HCV and HIV infections in a cohort of intravenous drug users in the North and East of France. Epidemiol Infect 2004; 132: 699–708. |
| Drug use | Hepatitis C | Maher L, Jalaludin B, Chant KG, et al. Incidence and risk factors for hepatitis C seroconversion in injecting drug users in Australia. Addiction 2006; 101: 1499–508. |
| Drug use | Hepatitis C | Månsson AS, Moestrup T, Nordenfelt E, Widell A. Continued transmission of hepatitis B and C viruses, but no transmission of human immunodeficiency virus among intravenous drug users participating in a syringe/needle exchange program. Scand J Infect Dis 2000; 32: 253–8. |
| Drug use | Hepatitis C | Partanen A, Malin K, Perälä R, Harju O, Holopainen A, Holmström P, et al. Riski-tutkimus 2000-2003. Pistämällä huumeita käyttävien seurantatutkimus. A-Klinikkasäätiön Raporttisarja nro 52. Helsinki: A-Klinikkasäätiön, 2006. |
| Drug use | Hepatitis C | Roy KM, Goldberg D, Taylor A, et al. A method to detect the incidence of hepatitis C infection among injecting drug users in Glasgow 1993-98. J Infect 2001; 43: 200–5. |
| Drug use | Hepatitis C | Turner KME, Hutchinson S, Vickerman P, et al. The impact of needle and syringe provision and opiate substitution therapy on the incidence of hepatitis C virus in injecting drug users: pooling of UK evidence. Addiction 2011; 106: 1978–88. |
| Drug use | Hepatitis C | Van Den Berg C, Smit C, Van Brussel G, Coutinho R, Prins M, Amsterdam Cohort. Full participation in harm reduction programmes is associated with decreased risk for human immunodeficiency virus and hepatitis C virus: evidence from the Amsterdam Cohort Studies among drug users. Addiction 2007; 102: 1454–62. |
| Drug use | Hepatitis C | Villano SA, Vlahov D, Nelson KE, Lyles CM, Cohn S, Thomas DL. Incidence and risk factors for hepatitis C among injection drug users in Baltimore, Maryland. J Clin Microbiol 1997; 35: 3274–7. |
| Diet low in fruits | Lip and oral cavity cancer | Key TJ. Fruit and vegetables and cancer risk. British Journal of Cancer 2011; 104: 6–11. |
| Diet low in fruits | Lip and oral cavity cancer | Jin, Jian, Zhiguo Ouyang, and Zhaoyan Wang. 2014. “Association of Fruit and Vegetables with the Risk of Nasopharyngeal Cancer: Evidence from a Meta-Analysis.” Scientific Reports4 (July): srep05229. doi:10.1038/srep05229. |

**Appendix Table 5. Epidemiological evidence supporting causality between risk-outcome pairs included in the Global Burden of Disease 2017 study including A. Citations and B. Additional information**

**A. Citations**

| Risk | Outcome | Citation/Note |
|---|---|---|
| Diet low in fruits | Lip and oral cavity cancer | Pavia, Maria, Claudia Pileggi, Carmelo GA Nobile, and Italo F. Angelillo. 2006. "Association between Fruit and Vegetable Consumption and Oral Cancer: A Meta-Analysis of Observational Studies." The American Journal of Clinical Nutrition83 (5): 1126–34. |
| Diet low in fruits | Nasopharynx cancer | Key TJ. Fruit and vegetables and cancer risk. British Journal of Cancer 2011; 104: 6–11. |
| Diet low in fruits | Nasopharynx cancer | American Institute for Cancer Research, and World Cancer Research Fund, eds. 2007. Food, Nutrition, Physical Activity and the Prevention of Cancer: A Global Perspective: A Project of World Cancer Research Fund International. Washington, D.C: American Institute for Cancer Research. |
| Diet low in fruits | Nasopharynx cancer | Pavia, Maria, Claudia Pileggi, Carmelo GA Nobile, and Italo F. Angelillo. 2006. "Association between Fruit and Vegetable Consumption and Oral Cancer: A Meta-Analysis of Observational Studies." The American Journal of Clinical Nutrition83 (5): 1126–34. |
| Diet low in fruits | Other pharynx cancer | Key TJ. Fruit and vegetables and cancer risk. British Journal of Cancer 2011; 104: 6–11. |
| Diet low in fruits | Other pharynx cancer | American Institute for Cancer Research, and World Cancer Research Fund, eds. 2007. Food, Nutrition, Physical Activity and the Prevention of Cancer: A Global Perspective: A Project of World Cancer Research Fund International. Washington, D.C: American Institute for Cancer Research. |
| Diet low in fruits | Other pharynx cancer | Pavia, Maria, Claudia Pileggi, Carmelo GA Nobile, and Italo F. Angelillo. 2006. "Association between Fruit and Vegetable Consumption and Oral Cancer: A Meta-Analysis of Observational Studies." The American Journal of Clinical Nutrition83 (5): 1126–34. |
| Diet low in fruits | Larynx cancer | Key TJ. Fruit and vegetables and cancer risk. British Journal of Cancer 2011; 104: 6–11. |
| Diet low in fruits | Larynx cancer | American Institute for Cancer Research, and World Cancer Research Fund, eds. 2007. Food, Nutrition, Physical Activity and the Prevention of Cancer: A Global Perspective: A Project of World Cancer Research Fund International. Washington, D.C: American Institute for Cancer Research. |
| Diet low in fruits | Larynx cancer | Pavia, Maria, Claudia Pileggi, Carmelo GA Nobile, and Italo F. Angelillo. 2006. "Association between Fruit and Vegetable Consumption and Oral Cancer: A Meta-Analysis of Observational Studies." The American Journal of Clinical Nutrition83 (5): 1126–34. |
| Diet low in fruits | Oesophageal cancer | Liu J, Wang J, Leng Y, Lv C. Intake of fruit and vegetables and risk of esophageal squamous cell carcinoma: a meta-analysis of observational studies. Int J Cancer 2013; 133: 473–85. |
| Diet low in fruits | Tracheal, bronchus and lung cancer | Vieira AR, Abar L, Vingeliene S, et al. Fruits, vegetables and lung cancer risk: a systematic review and meta-analysis. Ann Oncol 2016; 27: 81–96. |
| Diet low in fruits | Ischaemic heart disease | Wang X, Ouyang Y, Liu J, et al. Fruit and vegetable consumption and mortality from all causes, cardiovascular disease, and cancer: systematic review and dose-response meta-analysis of prospective cohort studies. BMJ 2014; 349: g4490. |
| Diet low in fruits | Ischaemic stroke | Hu D, Huang J, Wang Y, Zhang D, Qu Y. Fruits and vegetables consumption and risk of stroke: a meta-analysis of prospective cohort studies. Stroke 2014; 45: 1613–9. |
| Diet low in fruits | Hemorrhagic stroke | Hu D, Huang J, Wang Y, Zhang D, Qu Y. Fruits and vegetables consumption and risk of stroke: a meta-analysis of prospective cohort studies. Stroke 2014; 45: 1613–9. |
| Diet low in fruits | Diabetes mellitus | Li M, Fan Y, Zhang X, Hou W, Tang Z. Fruit and vegetable intake and risk of type 2 diabetes mellitus: meta-analysis of prospective cohort studies. BMJ open 2014; 4(11): e005497. |
| Diet low in vegetables | Oesophageal cancer | Liu J, Wang J, Leng Y, Lv C. Intake of fruit and vegetables and risk of esophageal squamous cell carcinoma: a meta-analysis of observational studies. Int J Cancer 2013; 133: 473–85. |
| Diet low in vegetables | Ischaemic heart disease | Wang X, Ouyang Y, Liu J, et al. Fruit and vegetable consumption and mortality from all causes, cardiovascular disease, and cancer: systematic review and dose-response meta-analysis of prospective cohort studies. BMJ 2014; 349: g4490. |
| Diet low in vegetables | Ischaemic stroke | Hu D, Huang J, Wang Y, Zhang D, Qu Y. Fruits and vegetables consumption and risk of stroke: a meta-analysis of prospective cohort studies. Stroke 2014; 45: 1613–9. |
| Diet low in vegetables | Hemorrhagic stroke | Hu D, Huang J, Wang Y, Zhang D, Qu Y. Fruits and vegetables consumption and risk of stroke: a meta-analysis of prospective cohort studies. Stroke 2014; 45: 1613–9. |
| Diet low in legumes | Ischaemic heart disease | Afshin A, Micha R, Khatibzadeh S, Mozaffarian D. Consumption of nuts and legumes and risk of incident ischemic heart disease, stroke, and diabetes: a systematic review and meta-analysis. Am J Clin Nutr 2014; 100: 278–88. |
| Diet low in whole grains | Diabetes mellitus | Aune D, Norat T, Romundstad P, Vatten LJ. Whole grain and refined grain consumption and the risk of type 2 diabetes: a systematic review and dose-response meta-analysis of cohort studies. Eur J Epidemiol 2013; 28: 845–58. |
| Diet low in whole grains | Ischaemic heart disease | Aune D, Keum N, Giovannucci E, et al. Whole grain consumption and risk of cardiovascular disease, cancer, and all cause and cause specific mortality: systematic review and dose-response meta-analysis of prospective studies. BMJ 2016; 353: i2716. |
| Diet low in nuts and seeds | Ischaemic heart disease | Afshin A, Micha R, Khatibzadeh S, Mozaffarian D. Consumption of nuts and legumes and risk of incident ischemic heart disease, stroke, and diabetes: a systematic review and meta-analysis. Am J Clin Nutr 2014; 100: 278–88. |
| Diet low in nuts and seeds | Diabetes mellitus | Afshin A, Micha R, Khatibzadeh S, Mozaffarian D. Consumption of nuts and legumes and risk of incident ischemic heart disease, stroke, and diabetes: a systematic review and meta-analysis. Am J Clin Nutr 2014; 100: 278–88. |
| Diet low in milk | Colon and rectum cancer | World Cancer Research Fund, American Institute for Cancer Research, Imperial College London. WCRF/AICR Systematic Literature Review Continuous Update Project Report: The Associations between Food, Nutrition and Physical Activity and the Risk of Colorectal Cancer. Oct 2010. |
| Diet high in red meat | Colon and rectum cancer | World Cancer Research Fund, American Institute for Cancer Research, Imperial College London. WCRF/AICR Systematic Literature Review Continuous Update Project Report: The Associations between Food, Nutrition and Physical Activity and the Risk of Colorectal Cancer. Oct 2010. |
| Diet high in red meat | Diabetes mellitus | Pan A, Sun Q, Bernstein AM, et al. Red meat consumption and risk of type 2 diabetes: 3 cohorts of US adults and an updated meta-analysis. Am J Clin Nutr 2011; 94: 1088–96. |

**Appendix Table 5. Epidemiological evidence supporting causality between risk-outcome pairs included in the Global Burden of Disease 2017 study including A. Citations and B. Additional information**

**A. Citations**

| Risk | Outcome | Citation/Note |
|---|---|---|
| Diet high in processed meat | Colon and rectum cancer | World Cancer Research Fund, American Institute for Cancer Research, Imperial College London. WCRF/AICR Systematic Literature Review Continuous Update Project Report: The Associations between Food, Nutrition and Physical Activity and the Risk of Colorectal Cancer. Oct 2010. |
| Diet high in processed meat | Ischaemic heart disease | Micha R, Wallace SK, Mozaffarian D. Red and processed meat consumption and risk of incident coronary heart disease, stroke, and diabetes mellitus: a systematic review and meta-analysis. Circulation 2010; 121: 2271–83. |
| Diet high in processed meat | Diabetes mellitus | Pan A, Sun Q, Bernstein AM, et al. Red meat consumption and risk of type 2 diabetes: 3 cohorts of US adults and an updated meta-analysis. Am J Clin Nutr 2011; 94: 1088–96. |
| Diet high in sugar-sweetened beverages and high body-mass index | n/a | Malik VS, Pan A, Willett WC, Hu FB. Sugar-sweetened beverages and weight gain in children and adults: a systematic review and meta-analysis. Am J Clin Nutr 2013; 98: 1084–102. |
| Diet low fibre | Colon and rectum cancer | World Cancer Research Fund, American Institute for Cancer Research, Imperial College London. WCRF/AICR Systematic Literature Review Continuous Update Project Report: The Associations between Food, Nutrition and Physical Activity and the Risk of Colorectal Cancer. Oct 2010. |
| Diet low fibre | Ischaemic heart disease | Threapleton DE, Greenwood DC, Evans CE, et al. Dietary fibre intake and risk of cardiovascular disease: systematic review and meta-analysis. BMJ (Clinical research ed) 2013; 347: f6879. |
| Diet low in calcium | Colon and rectum cancer | World Cancer Research Fund, American Institute for Cancer Research, Imperial College London. WCRF/AICR Systematic Literature Review Continuous Update Project Report: The Associations between Food, Nutrition and Physical Activity and the Risk of Colorectal Cancer. Oct 2010. |
| Diet low in seafood omega-3 fats | Ischaemic heart disease | Chowdhury R, Stevens S, Gorman D, et al. Association between fish consumption, long chain omega 3 fatty acids, and risk of cerebrovascular disease: systematic review and meta-analysis. BMJ (Clinical research ed) 2012; 345: e6698. |
| Diet low in polyunsaturated fats | Ischaemic heart disease | Farvid MS, Ding M, Pan A, et al. Dietary linoleic acid and risk of coronary heart disease: a systematic review and meta-analysis of prospective cohort studies. Circulation 2014; 130: 1568–78. |
| Diet low in polyunsaturated fats | Ischaemic heart disease | Mozaffarian D, Micha R, Wallace S. Effects on coronary heart disease of increasing polyunsaturated fat in place of saturated fat: a systematic review and meta-analysis of randomized controlled trials. PLoS Med 2010; 7: e1000252. |
| Diet high in trans fats | Ischaemic heart disease | Mozaffarian D, Clarke R. Quantitative effects on cardiovascular risk factors and coronary heart disease risk of replacing partially hydrogenated vegetable oils with other fats and oils. Eur J Clin Nutr. 2009; 63(Suppl 2): S22-33. |
| Diet high in trans fats | Ischaemic heart disease | http://www.bmj.com/content/bmj/suppl/2015/08/11/bmj.h3978.DC1/sour025275.ww2_default.pdf; pg. 44 |
| Diet high in sodium and high systolic blood pressure | n/a | Aburto NJ, Ziolkovska A, Hooper L, Elliott P, Cappuccio FP, Meerpohl JJ. Effect of lower sodium intake on health: systematic review and meta-analyses. BMJ 2013; 346: f1326. |
| Diet high in sodium | Stomach cancer | World Cancer Research Fund, American Institute for Cancer Research. Food, Nutrition, Physical Activity, and the Prevention of Cancer: a Global Perspective. Washington DC: AICR, 2007. |
| Diet high in sodium | Stomach cancer | D'Elia, Lanfranco, Giovanni Rossi, Renato Ippolito, Francesco P. Cappuccio, and Pasquale Strazzullo. 2012. "Habitual Salt Intake and Risk of Gastric Cancer: A Meta-Analysis of Prospective Studies." Clinical Nutrition31 (4): 489–98. doi:10.1016/j.clnu.2012.01.003. |
| Childhood sexual abuse | Depressive disorders | Brown J, Cohen P, Johnson JG, Smailes EM. Childhood abuse and neglect: specificity of effects on adolescent and young adult depression and suicidality. J Am Acad Child Adolesc Psychiatry 1999; 38: 1490–6. |
| Childhood sexual abuse | Depressive disorders | Chapman, D.P., Whitfield, C.L., Felitti, V.J., Dube, S.R., Edwards, V.J. and Anda, R.F., 2004. Adverse childhood experiences and the risk of depressive disorders in adulthood. Journal of affective disorders, 82(2), pp.217-225. |
| Childhood sexual abuse | Depressive disorders | Cheasty, M., Clare, A.W. and Collins, C., 1998. Relation between sexual abuse in childhood and adult depression: case-control study. Bmj, 316(7126), pp.198-201. |
| Childhood sexual abuse | Depressive disorders | Dinwiddie S, Heath AC, Dunne MP, Bucholz KK, Madden PA, Slutske WS, Bierut LJ, Statham DB, Martin NG. Early sexual abuse and lifetime psychopathology: a co-twin-control study. Psychol Med. 2000; 30(1): 41–52. |
| Childhood sexual abuse | Depressive disorders | Dube, S.R., Anda, R.F., Whitfield, C.L., Brown, D.W., Felitti, V.J., Dong, M. and Giles, W.H., 2005. Long-term consequences of childhood sexual abuse by gender of victim. American journal of preventive medicine, 28(5), pp.430-438. |
| Childhood sexual abuse | Depressive disorders | Ernst C, Angst J, Földényi M. The Zurich Study. XVII. Sexual abuse in childhood. Frequency and relevance for adult morbidity data of a longitudinal epidemiological study. Eur Arch Psychiatry Clin Neurosci. 1993; 242(5): 293–300. |
| Childhood sexual abuse | Depressive disorders | Jaffee SR, Moffitt TE, Caspi A, Fombonne E, Poulton R, Martin J. Differences in early childhood risk factors for juvenile-onset and adult-onset depression. Arch Gen Psychiatry. 2002; 59(3): 215-22. |
| Childhood sexual abuse | Depressive disorders | Kendler KS, Bulik CM, Silberg J, Hettema JM, Myers J, Prescott CA. Childhood sexual abuse and adult psychiatric and substance use disorders in women: an epidemiological and cotwin control analysis. Arch Gen Psychiatry. 2000; 57(10): 953–9. |
| Childhood sexual abuse | Depressive disorders | Molnar, B.E., Buka, S.L. and Kessler, R.C., 2001. Child sexual abuse and subsequent psychopathology: results from the National Comorbidity Survey. American journal of public health, 91(5), p.753. |
| Childhood sexual abuse | Depressive disorders | Nelson EC, Heath AC, Madden PA, Cooper ML, Dinwiddie SH, Bucholz KK, Glowinski A, McLaughlin T, Dunne MP, Statham DJ, Martin NG. Association between self-reported sexual abuse and adverse psychosocial outcomes: results from a twin study. Arch Gen Psychiatry. 2002; 59(2): 139-45. |
| Childhood sexual abuse | Depressive disorders | Peleikis, D.E., Mykletun, A. and Dahl, A.A., 2004. The relative influence of childhood sexual abuse and other family background risk factors on adult adversities in female outpatients treated for anxiety disorders and depression. Child Abuse & Neglect, 28(1), pp.61-76. |

**Appendix Table 5. Epidemiological evidence supporting causality between risk-outcome pairs included in the Global Burden of Disease 2017 study including A. Citations and B. Additional information**

**A. Citations**

| Risk | Outcome | Citation/Note |
|---|---|---|
| Childhood sexual abuse | Depressive disorders | Silverman, A.B., Reinherz, H.Z. and Giaconia, R.M., 1996. The long-term sequelae of child and adolescent abuse: A longitudinal community study. Child abuse & neglect, 20(8), pp.709-723. |
| Childhood sexual abuse | Depressive disorders | Widom, C.S., DuMont, K. and Czaja, S.J., 2007. A prospective investigation of major depressive disorder and comorbidity in abused and neglected children grown up. Archives of general psychiatry, 64(1), pp.49-56. |
| Childhood sexual abuse | Alcohol use disorders | Dinwiddie S, Heath AC, Dunne MP, Bucholz KK, Madden PA, Slutske WS, Bierut LJ, Statham DB, Martin NG. Early sexual abuse and lifetime psychopathology: a co-twin-control study. Psychol Med. 2000; 30(1): 41–52. |
| Childhood sexual abuse | Alcohol use disorders | Dube, S.R., Anda, R.F., Whitfield, C.L., Brown, D.W., Felitti, V.J., Dong, M. and Giles, W.H., 2005. Long-term consequences of childhood sexual abuse by gender of victim. American journal of preventive medicine, 28(5), pp.430-438. |
| Childhood sexual abuse | Alcohol use disorders | Fleming, J., Mullen, P.E., Sibthorpe, B., Attewell, R. and Bammer, G., 1998. The relationship between childhood sexual abuse and alcohol abuse in women-a case-control study. Addiction, 93(12), pp.1787-1798. |
| Childhood sexual abuse | Alcohol use disorders | Kendler KS, Bulik CM, Silberg J, Hettema JM, Myers J, Prescott CA. Childhood sexual abuse and adult psychiatric and substance use disorders in women: an epidemiological and cotwin control analysis. Arch Gen Psychiatry. 2000; 57(10): 953–9. |
| Childhood sexual abuse | Alcohol use disorders | Molnar, B.E., Buka, S.L. and Kessler, R.C., 2001. Child sexual abuse and subsequent psychopathology: results from the National Comorbidity Survey. American journal of public health, 91(5), p.753. |
| Childhood sexual abuse | Alcohol use disorders | Nelson EC, Heath AC, Madden PA, Cooper ML, Dinwiddie SH, Bucholz KK, Glowinski A, McLaughlin T, Dunne MP, Statham DJ, Martin NG. Association between self-reported sexual abuse and adverse psychosocial outcomes: results from a twin study. Arch Gen Psychiatry. 2002; 59(2): 139-45. |
| Childhood sexual abuse | Alcohol use disorders | Sartor CE, Lynskey MT, Bucholz KK, McCutcheon VV, Nelson EC, Waldron M, Heath AC. Childhood sexual abuse and the course of alcohol dependence development: findings from a female twin sample. Drug Alcohol Depend. 2007; 89(2-3): 139–44. |
| Childhood sexual abuse | Alcohol use disorders | Silverman, A.B., Reinherz, H.Z. and Giaconia, R.M., 1996. The long-term sequelae of child and adolescent abuse: A longitudinal community study. Child abuse & neglect, 20(8), pp.709-723. |
| Bullying victimization | Depressive disorders | Bowes L, Joinson C, Wolke D, Lewis G. Peer victimisation during adolescence and its impact on depression in early adulthood: prospective cohort study in the United Kingdom. <i>BMJ</i>. 2015; 350: h2469. |
| Bullying victimization | Depressive disorders | Fahy AE, Stansfeld SA, Smuk M, Smith NR, Cummins S, Clark C. Longitudinal Associations Between Cyberbullying Involvement and Adolescent Mental Health. <i>J Adolesc Health</i>. 2016; 59(5): 502–9. |
| Bullying victimization | Depressive disorders | Farrington DP, Loeber R, Stallings R, Ttofi MM. Bullying perpetration and victimization as predictors of delinquency and depression in the Pittsburgh Youth Study. <i>Journal of Aggression Conflict and Peace Research</i>. 2011; 3(2): 74–81. |
| Bullying victimization | Depressive disorders | Fekkes M, Pijpers FIM, Fredriks AM, Vogels T, Verloove-Vanhorick SP. Do bullied children get ill, or do ill children get bullied? A prospective cohort study on the relationship between bullying and health-related symptoms. <i>Pediatrics</i>. 2006; 117(5): 1568–74. |
| Bullying victimization | Depressive disorders | Gibb SJ, Horwood LJ, Fergusson DM. Bullying victimization/perpetration in childhood and later adjustment: findings from a 30 year longitudinal study. <i>Jnl Aggress Conflict Peace Res</i>. 2011; 3(2): 82–8. |
| Bullying victimization | Depressive disorders | Hemphill SA, Kotevski A, Heerde JA. Longitudinal associations between cyber-bullying perpetration and victimization and problem behavior and mental health problems in young Australians. <i>Int J Public Health</i>. 2015; 60(2): 227–37. |
| Bullying victimization | Depressive disorders | Hemphill SA, Kotevski A, Herrenkohl TI, Bond L, Kim MJ, Toumbourou JW, Catalano RF. Longitudinal consequences of adolescent bullying perpetration and victimisation: A study of students in Victoria, Australia. <i>Crim Behav Ment Health</i>. 2011; 21(2): 107–16. |
| Bullying victimization | Depressive disorders | Kaltiala-Heino R, Fröjd S, Marttunen M. Involvement in bullying and depression in a 2-year follow-up in middle adolescence. <i>Eur Child Adolesc Psychiatry</i>. 2010; 19(1): 45–55. |
| Bullying victimization | Depressive disorders | Kumpulainen K, Räsänen E. Children involved in bullying at elementary school age: their psychiatric symptoms and deviance in adolescence: An epidemiological sample. <i>Child Abuse Negl</i>. 2000; 24(12): 1567–77. |
| Bullying victimization | Depressive disorders | Lereya ST, Copeland WE, Zammit S, Wolke D. Bully/victims: a longitudinal, population-based cohort study of their mental health. <i>Eur Child Adolesc Psychiatry</i>. 2015; 24(12): 1461–71. |
| Bullying victimization | Depressive disorders | Moore SE, Norman RE, Sly PD, Whitehouse AJO, Zubrick SR, Scott J. Adolescent peer aggression and its association with mental health and substance use in an Australian cohort. <i>J Adolesc</i>. 2014; 37(1): 11–21. |
| Bullying victimization | Depressive disorders | Patton GC, Olsson C, Bond L, Toumbourou JW, Carlin JB, Hemphill SA, Catalano RF. Predicting female depression across puberty: a two-nation longitudinal study. <i>J Am Acad Child Adolesc Psychiatry</i>. 2008; 47(12): 1424–32. |
| Bullying victimization | Depressive disorders | Rothon C, Head J, Klineberg E, Stansfeld S. Can social support protect bullied adolescents from adverse outcomes? A prospective study on the effects of bullying on the educational achievement and mental health of adolescents at secondary schools in East London. <i>J Adolesc</i>. 2011; 34(3): 579–88. |
| Bullying victimization | Depressive disorders | Sigurdson JF, Undheim AM, Wallander JL, Lydersen S, Sund AM. The long-term effects of being bullied or a bully in adolescence on externalizing and internalizing mental health problems in adulthood. <i>Child Adolesc Psychiatry Ment Health</i>. 2015; 9(1): 1-13. |
| Bullying victimization | Depressive disorders | Silberg JL, Copeland W, Linker J, Moore AA, Roberson-Nay R, York TP. Psychiatric outcomes of bullying victimization: a study of discordant monozygotic twins. <i>Psychol Med</i>. 2016; 46(9): 1875–83. |

| Appendix Table 5. Epidemiological evidence supporting causality between risk-outcome pairs included in the Global Burden of Disease 2017 study including A. Citations and B. Additional information<br>A. Citations | | |
|---|---|---|
| **Risk** | **Outcome** | **Citation/Note** |
| Bullying victimization | Depressive disorders | Sourander A, Jensen P, Rönning JA, Niemelä S, Helenius H, Sillanmäki L, Kumpulainen K, Piha J, Tamminen T, Moilanen I, Almqvist F. What is the early adulthood outcome of boys who bully or are bullied in childhood? The Finnish "From a Boy to a Man" study. <i>Pediatrics</i>. 2007; 120(2): 397–404. |
| Bullying victimization | Depressive disorders | Vassallo S, Edwards B, Renda J, Olsson CA. Bullying in Early Adolescence and Antisocial Behavior and Depression Six Years Later: What Are the Protective Factors?. <i>J Sch Violence</i>. 2014; 13(1): 100–24. |
| Bullying victimization | Depressive disorders | Wichstrøm L, Belsky J, Berg-Nielsen TS. Preschool predictors of childhood anxiety disorders: a prospective community study. <i>Journal of Child Psychology and Psychiatry</i>. 2013; 54(12): 1327–36. |
| Bullying victimization | Depressive disorders | Zwierzynska K, Wolke D, Lereya TS. Peer victimization in childhood and internalizing problems in adolescence: a prospective longitudinal study. <i>J Abnorm Child Psychol</i>. 2013; 41(2): 309–23. |
| Bullying victimization | Anxiety disorders | Bowes L, Joinson C, Wolke D, Lewis G. Peer victimisation during adolescence and its impact on depression in early adulthood: prospective cohort study in the United Kingdom. <i>BMJ</i>. 2015; 350: h2469. |
| Bullying victimization | Anxiety disorders | Fahy AE, Stansfeld SA, Smuk M, Smith NR, Cummins S, Clark C. Longitudinal Associations Between Cyberbullying Involvement and Adolescent Mental Health. <i>J Adolesc Health</i>. 2016; 59(5): 502–9. |
| Bullying victimization | Anxiety disorders | Farrington DP, Loeber R, Stallings R, Ttofi MM. Bullying perpetration and victimization as predictors of delinquency and depression in the Pittsburgh Youth Study. <i>Journal of Aggression Conflict and Peace Research</i>. 2011; 3(2): 74–81. |
| Bullying victimization | Anxiety disorders | Fekkes M, Pijpers FIM, Fredriks AM, Vogels T, Verloove-Vanhorick SP. Do bullied children get ill, or do ill children get bullied? A prospective cohort study on the relationship between bullying and health-related symptoms. <i>Pediatrics</i>. 2006; 117(5): 1568–74. |
| Bullying victimization | Anxiety disorders | Gibb SJ, Horwood LJ, Fergusson DM. Bullying victimization/perpetration in childhood and later adjustment: findings from a 30 year longitudinal study. <i>Jnl Aggress Conflict Peace Res</i>. 2011; 3(2): 82–8. |
| Bullying victimization | Anxiety disorders | Hemphill SA, Kotevski A, Heerde JA. Longitudinal associations between cyber-bullying perpetration and victimization and problem behavior and mental health problems in young Australians. <i>Int J Public Health</i>. 2015; 60(2): 227–37. |
| Bullying victimization | Anxiety disorders | Hemphill SA, Kotevski A, Herrenkohl TI, Bond L, Kim MJ, Toumbourou JW, Catalano RF. Longitudinal consequences of adolescent bullying perpetration and victimisation: A study of students in Victoria, Australia. <i>Crim Behav Ment Health</i>. 2011; 21(2): 107–16. |
| Bullying victimization | Anxiety disorders | Kaltiala-Heino R, Fröjd S, Marttunen M. Involvement in bullying and depression in a 2-year follow-up in middle adolescence. <i>Eur Child Adolesc Psychiatry</i>. 2010; 19(1): 45–55. |
| Bullying victimization | Anxiety disorders | Kumpulainen K, Räsänen E. Children involved in bullying at elementary school age: their psychiatric symptoms and deviance in adolescence: An epidemiological sample. <i>Child Abuse Negl</i>. 2000; 24(12): 1567–77. |
| Bullying victimization | Anxiety disorders | Lereya ST, Copeland WE, Zammit S, Wolke D. Bully/victims: a longitudinal, population-based cohort study of their mental health. <i>Eur Child Adolesc Psychiatry</i>. 2015; 24(12): 1461–71. |
| Bullying victimization | Anxiety disorders | Moore SE, Norman RE, Sly PD, Whitehouse AJO, Zubrick SR, Scott J. Adolescent peer aggression and its association with mental health and substance use in an Australian cohort. <i>J Adolesc</i>. 2014; 37(1): 11–21. |
| Bullying victimization | Anxiety disorders | Patton GC, Olsson C, Bond L, Toumbourou JW, Carlin JB, Hemphill SA, Catalano RF. Predicting female depression across puberty: a two-nation longitudinal study. <i>J Am Acad Child Adolesc Psychiatry</i>. 2008; 47(12): 1424–32. |
| Bullying victimization | Anxiety disorders | Rothon C, Head J, Klineberg E, Stansfeld S. Can social support protect bullied adolescents from adverse outcomes? A prospective study on the effects of bullying on the educational achievement and mental health of adolescents at secondary schools in East London. <i>J Adolesc</i>. 2011; 34(3): 579–88. |
| Bullying victimization | Anxiety disorders | Sigurdson JF, Undheim AM, Wallander JL, Lydersen S, Sund AM. The long-term effects of being bullied or a bully in adolescence on externalizing and internalizing mental health problems in adulthood. <i>Child Adolesc Psychiatry Ment Health</i>. 2015; 9(1): 1-13. |
| Bullying victimization | Anxiety disorders | Silberg JL, Copeland W, Linker J, Moore AA, Roberson-Nay R, York TP. Psychiatric outcomes of bullying victimization: a study of discordant monozygotic twins. <i>Psychol Med</i>. 2016; 46(9): 1875–83. |
| Bullying victimization | Anxiety disorders | Sourander A, Jensen P, Rönning JA, Niemelä S, Helenius H, Sillanmäki L, Kumpulainen K, Piha J, Tamminen T, Moilanen I, Almqvist F. What is the early adulthood outcome of boys who bully or are bullied in childhood? The Finnish "From a Boy to a Man" study. <i>Pediatrics</i>. 2007; 120(2): 397–404. |
| Bullying victimization | Anxiety disorders | Vassallo S, Edwards B, Renda J, Olsson CA. Bullying in Early Adolescence and Antisocial Behavior and Depression Six Years Later: What Are the Protective Factors?. <i>J Sch Violence</i>. 2014; 13(1): 100–24. |
| Bullying victimization | Anxiety disorders | Wichstrøm L, Belsky J, Berg-Nielsen TS. Preschool predictors of childhood anxiety disorders: a prospective community study. <i>Journal of Child Psychology and Psychiatry</i>. 2013; 54(12): 1327–36. |
| Bullying victimization | Anxiety disorders | Zwierzynska K, Wolke D, Lereya TS. Peer victimization in childhood and internalizing problems in adolescence: a prospective longitudinal study. <i>J Abnorm Child Psychol</i>. 2013; 41(2): 309–23. |
| Intimate partner violence | HIV/AIDS | Jewkes RK, Dunkle K, Nduna M, Shai N. Intimate partner violence, relationship power inequity, and incidence of HIV infection in young women in South Africa: a cohort study. Lancet 2010; 376: 41–8. |

| Appendix Table 5. Epidemiological evidence supporting causality between risk-outcome pairs included in the Global Burden of Disease 2017 study including A. Citations and B. Additional information<br>A. Citations | | |
|---|---|---|
| **Risk** | **Outcome** | **Citation/Note** |
| Intimate partner violence | HIV/AIDS | Kouyoumdjian FG, Calzavara LM, Bondy SJ, et al. Intimate partner violence is associated with incident HIV infection in women in Uganda. AIDS 2013; 27: 1331–8. |
| Intimate partner violence | Depressive disorders | Chowdhary N, Patel V. The effect of spousal violence on women's health: findings from the Stree Arogya Shodh in Goa, India. J Postgrad Med. 2008; 54(4): 306–12. |
| Intimate partner violence | Depressive disorders | Lipsky S, Caetano R, Roy-Byrne P. Racial and ethnic disparities in police-reported intimate partner violence and risk of hospitalization among women. Womens Health Issues. 2009; 19(2):109–118. |
| Intimate partner violence | Depressive disorders | Ouellet-Morin I, Fisher HL, York-Smith M, Fincham-Campbell S, Moffitt TE, Arseneault L. Intimate partner violence and new-onset depression: a longitudinal study of women's childhood and adult histories of abuse. Depression and anxiety. 2015;32(5):316-324. |
| Intimate partner violence | Depressive disorders | Suglia SF, Duarte CS, Sandel MT. Housing quality, housing instability, and maternal mental health. J Urban Health. 2011; 88(6): 1105–16. |
| Intimate partner violence | Maternal abortion and miscarriage | Bourassa D, Bérubé J. The prevalence of intimate partner violence among women and teenagers seeking abortion compared with those continuing pregnancy. J Obstet Gynaecol Can 2007; 29: 415–23. |
| Intimate partner violence | Maternal abortion and miscarriage | Leung TW, Leung WC, Chan PL, Ho PC. A comparison of the prevalence of domestic violence between patients seeking termination of pregnancy and other general gynecology patients. Int J Gynaecol Obstet 2002; 77: 47–54. |
| Intimate partner violence | Maternal abortion and miscarriage | Romito P, Escribà-Agüir V, Pomicino L, Lucchetta C, Scrimin F, Molzan Turan J. Violence in the lives of women in Italy who have an elective abortion. Womens Health Issues 2009; 19: 335–43. |
| Intimate partner violence | Maternal abortion and miscarriage | Taft AJ, Watson LF. Termination of pregnancy: associations with partner violence and other factors in a national cohort of young Australian women. Aust N Z J Public Health 2007; 31: 135–42. |
| Low physical activity | Colon and rectum cancer | Bostick RM, Potter JD, Kushi LH, et al. Sugar, meat, and fat intake, and non-dietary risk factors for colon cancer incidence in Iowa women (United States). Cancer Causes Control 1994; 5: 38–52. |
| Low physical activity | Colon and rectum cancer | Calton BA, Lacey JV, Schatzkin A, et al. Physical activity and the risk of colon cancer among women: a prospective cohort study (United States). Int J Cancer 2006; 119: 385–91. |
| Low physical activity | Colon and rectum cancer | Chao A, Connell CJ, Jacobs EJ, et al. Amount, type, and timing of recreational physical activity in relation to colon and rectal cancer in older adults: the Cancer Prevention Study II Nutrition Cohort. Cancer Epidemiol Biomarkers Prev 2004; 13: 2187–95. |
| Low physical activity | Colon and rectum cancer | Colbert LH, Hartman TJ, Malila N, et al. Physical activity in relation to cancer of the colon and rectum in a cohort of male smokers. Cancer Epidemiol Biomarkers Prev 2001; 10: 265–8. |
| Low physical activity | Colon and rectum cancer | Fraser G, Pearce N. Occupational physical activity and risk of cancer of the colon and rectum in New Zealand males. Cancer Causes Control 1993; 4: 45–50. |
| Low physical activity | Colon and rectum cancer | Friedenreich C, Norat T, Steindorf K, et al. Physical activity and risk of colon and rectal cancers: the European prospective investigation into cancer and nutrition. Cancer Epidemiol Biomarkers Prev 2006; 15: 2398–407. |
| Low physical activity | Colon and rectum cancer | Garabrant DH, Peters JM, Mack TM, Bernstein L. Job activity and colon cancer risk. Am J Epidemiol 1984; 119: 1005–14. |
| Low physical activity | Colon and rectum cancer | Gerhardsson M, Norell SE, Kiviranta H, Pedersen NL, Ahlbom A. Sedentary jobs and colon cancer. Am J Epidemiol 1986; 123: 775–80. |
| Low physical activity | Colon and rectum cancer | Giovannucci E, Ascherio A, Rimm EB, Colditz GA, Stampfer MJ, Willett WC. Physical activity, obesity, and risk for colon cancer and adenoma in men. Ann Intern Med 1995; 122: 327–34. |
| Low physical activity | Colon and rectum cancer | Howard RA, Freedman DM, Park Y, Hollenbeck A, Schatzkin A, Leitzmann MF. Physical activity, sedentary behavior, and the risk of colon and rectal cancer in the NIH-AARP Diet and Health Study. Cancer Causes Control 2008; 19: 939–53. |
| Low physical activity | Colon and rectum cancer | Larsson SC, Rutegård J, Bergkvist L, Wolk A. Physical activity, obesity, and risk of colon and rectal cancer in a cohort of Swedish men. Eur J Cancer 2006; 42: 2590–7. |
| Low physical activity | Colon and rectum cancer | Lee IM, Manson JE, Ajani U, Paffenbarger RS, Hennekens CH, Buring JE. Physical activity and risk of colon cancer: the Physicians' Health Study (United States). Cancer Causes Control 1997; 8: 568–74. |
| Low physical activity | Colon and rectum cancer | Lee IM, Paffenbarger RS. Physical activity and its relation to cancer risk: a prospective study of college alumni. Med Sci Sports Exerc 1994; 26: 831–7. |
| Low physical activity | Colon and rectum cancer | Lee K-J, Inoue M, Otani T, et al. Physical activity and risk of colorectal cancer in Japanese men and women: the Japan Public Health Center-based prospective study. Cancer Causes Control 2007; 18: 199–209. |
| Low physical activity | Colon and rectum cancer | Mai PL, Sullivan-Halley J, Ursin G, et al. Physical activity and colon cancer risk among women in the California Teachers Study. Cancer Epidemiol Biomarkers Prev 2007; 16: 517–25. |
| Low physical activity | Colon and rectum cancer | Moradi T, Gridley G, Björk J, et al. Occupational physical activity and risk for cancer of the colon and rectum in Sweden among men and women by anatomic subsite. Eur J Cancer Prev 2008; 17: 201–8. |
| Low physical activity | Colon and rectum cancer | Nilsen TIL, Romundstad PR, Petersen H, Gunnell D, Vatten LJ. Recreational physical activity and cancer risk in subsites of the colon (the Nord-Trøndelag Health Study). Cancer Epidemiol Biomarkers Prev 2008; 17: 183–8. |
| Low physical activity | Colon and rectum cancer | Severson RK, Nomura AM, Grove JS, Stemmermann GN. A prospective analysis of physical activity and cancer. Am J Epidemiol 1989; 130: 522–9. |
| Low physical activity | Colon and rectum cancer | Thune I, Lund E. Physical activity and risk of colorectal cancer in men and women. Br J Cancer 1996; 73: 1134–40. |
| Low physical activity | Colon and rectum cancer | Wolin KY, Lee I-M, Colditz GA, Glynn RJ, Fuchs C, Giovannucci E. Leisure-time physical activity patterns and risk of colon cancer in women. Int J Cancer 2007; 121: 2776–81. |
| Low physical activity | Breast cancer | Bardia A, Hartmann LC, Vachon CM, et al. Recreational physical activity and risk of postmenopausal breast cancer based on hormone receptor status. Arch Intern Med 2006; 166: 2478–83. |
| Low physical activity | Breast cancer | Borch KB, Lund E, Braaten T, Weiderpass E. Physical activity and the risk of postmenopausal breast cancer - the Norwegian Women and Cancer Study. J Negat Results Biomed 2014; 13: 3. |

**Appendix Table 5. Epidemiological evidence supporting causality between risk-outcome pairs included in the Global Burden of Disease 2017 study including A. Citations and B. Additional information**

**A. Citations**

| Risk | Outcome | Citation/Note |
|---|---|---|
| Low physical activity | Breast cancer | Breslow RA, Ballard-Barbash R, Munoz K, Graubard BI. Long-term recreational physical activity and breast cancer in the National Health and Nutrition Examination Survey I epidemiologic follow-up study. Cancer Epidemiol Biomarkers Prev 2001; 10: 805–8. |
| Low physical activity | Breast cancer | Cerhan JR, Chiu BC, Wallace RB, et al. Physical activity, physical function, and the risk of breast cancer in a prospective study among elderly women. J Gerontol A Biol Sci Med Sci 1998; 53: M251-256. |
| Low physical activity | Breast cancer | Chang S-C, Ziegler RG, Dunn B, et al. Association of energy intake and energy balance with postmenopausal breast cancer in the prostate, lung, colorectal, and ovarian cancer screening trial. Cancer Epidemiol Biomarkers Prev 2006; 15: 334–41. |
| Low physical activity | Breast cancer | Colditz GA, Feskanich D, Chen WY, Hunter DJ, Willett WC. Physical activity and risk of breast cancer in premenopausal women. Br J Cancer 2003; 89: 847–51. |
| Low physical activity | Breast cancer | Dallal CM, Sullivan-Halley J, Ross RK, et al. Long-term recreational physical activity and risk of invasive and in situ breast cancer: the California teachers study. Arch Intern Med 2007; 167: 408–15. |
| Low physical activity | Breast cancer | Dorgan JF, Brown C, Barrett M, et al. Physical activity and risk of breast cancer in the Framingham Heart Study. Am J Epidemiol 1994; 139: 662–9. |
| Low physical activity | Breast cancer | Eliassen AH, Hankinson SE, Rosner B, Holmes MD, Willett WC. Physical activity and risk of breast cancer among postmenopausal women. Arch Intern Med 2010; 170: 1758–64. |
| Low physical activity | Breast cancer | Frisch RE, Wyshak G, Witschi J, Albright NL, Albright TE, Schiff I. Lower lifetime occurrence of breast cancer and cancers of the reproductive system among former college athletes. Int J Fertil 1987; 32: 217–25. |
| Low physical activity | Breast cancer | Hastert TA, Beresford SAA, Patterson RE, Kristal AR, White E. Adherence to WCRF/AICR cancer prevention recommendations and risk of postmenopausal breast cancer. Cancer Epidemiol Biomarkers Prev 2013; 22: 1498–508. |
| Low physical activity | Breast cancer | Hildebrand JS, Gapstur SM, Campbell PT, Gaudet MM, Patel AV. Recreational physical activity and leisure-time sitting in relation to postmenopausal breast cancer risk. Cancer Epidemiol Biomarkers Prev 2013; 22: 1906–12. |
| Low physical activity | Breast cancer | Howard RA, Leitzmann MF, Linet MS, Freedman DM. Physical activity and breast cancer risk among pre- and postmenopausal women in the U.S. Radiologic Technologists cohort. Cancer Causes Control 2009; 20: 323–33. |
| Low physical activity | Breast cancer | Leitzmann MF, Moore SC, Peters TM, et al. Prospective study of physical activity and risk of postmenopausal breast cancer. Breast Cancer Res 2008; 10: R92. |
| Low physical activity | Breast cancer | Luoto R, Latikka P, Pukkala E, Hakulinen T, Vihko V. The effect of physical activity on breast cancer risk: a cohort study of 30,548 women. Eur J Epidemiol 2000; 16: 973–80. |
| Low physical activity | Breast cancer | Margolis KL, Mucci L, Braaten T, et al. Physical activity in different periods of life and the risk of breast cancer: the Norwegian-Swedish Women's Lifestyle and Health cohort study. Cancer Epidemiol Biomarkers Prev 2005; 14: 27–32. |
| Low physical activity | Breast cancer | Mertens AJ, Sweeney C, Shahar E, Rosamond WD, Folsom AR. Physical activity and breast cancer incidence in middle-aged women: a prospective cohort study. Breast Cancer Res Treat 2006; 97: 209–14. |
| Low physical activity | Breast cancer | Ministry of Health (Benin), National Institute of Statistics and Economic Analysis (INSAE) (Benin). Benin Health Statistical Yearbook 2005. Porto-Novo, Benin: Ministry of Health (Benin), 2006. |
| Low physical activity | Breast cancer | Ministry of Health (Burkina Faso). Burkina Faso Health Statistical Yearbook 2007. Ouagadougou, Burkina Faso: Ministry of Health (Burkina Faso), 2008. |
| Low physical activity | Breast cancer | Ministry of Health (Burkina Faso). Burkina Faso Health Statistical Yearbook 2008. Ouagadougou, Burkina Faso: Ministry of Health (Burkina Faso), 2009. |
| Low physical activity | Breast cancer | Moradi T, Adami HO, Bergström R, et al. Occupational physical activity and risk for breast cancer in a nationwide cohort study in Sweden. Cancer Causes Control 1999; 10: 423–30. |
| Low physical activity | Breast cancer | Moradi T, Adami H-O, Ekbom A, et al. Physical activity and risk for breast cancer a prospective cohort study among Swedish twins. Int J Cancer 2002; 100: 76–81. |
| Low physical activity | Breast cancer | Peters TM, Schatzkin A, Gierach GL, et al. Physical activity and postmenopausal breast cancer risk in the NIH-AARP diet and health study. Cancer Epidemiol Biomarkers Prev 2009; 18: 289–96. |
| Low physical activity | Breast cancer | Pronk A, Ji B-T, Shu X-O, et al. Physical activity and breast cancer risk in Chinese women. Br J Cancer 2011; 105: 1443–50. |
| Low physical activity | Breast cancer | Rintala by PE, Pukkala E, Paakkulainen HT, Vihko VJ. Self-experienced physical workload and risk of breast cancer. Scand J Work Environ Health 2002; 28: 158–62. |
| Low physical activity | Breast cancer | Rockhill B, Willett WC, Hunter DJ, Manson JE, Hankinson SE, Colditz GA. A prospective study of recreational physical activity and breast cancer risk. Arch Intern Med 1999; 159: 2290–6. |
| Low physical activity | Breast cancer | Rosenberg L, Palmer JR, Bethea TN, Ban Y, Kipping-Ruane K, Adams-Campbell LL. A prospective study of physical activity and breast cancer incidence in African-American women. Cancer Epidemiol Biomarkers Prev 2014; 23: 2522–31. |
| Low physical activity | Breast cancer | Sesso HD, Paffenbarger RS, Lee IM. Physical activity and breast cancer risk in the College Alumni Health Study (United States). Cancer Causes Control 1998; 9: 433–9. |
| Low physical activity | Breast cancer | Silvera SAN, Jain M, Howe GR, Miller AB, Rohan TE. Energy balance and breast cancer risk: a prospective cohort study. Breast Cancer Res Treat 2006; 97: 97–106. |
| Low physical activity | Breast cancer | Suzuki R, Iwasaki M, Yamamoto S, et al. Leisure-time physical activity and breast cancer risk defined by estrogen and progesterone receptor status--the Japan Public Health Center-based Prospective Study. Prev Med 2011; 52: 227–33. |
| Low physical activity | Breast cancer | Suzuki S, Kojima M, Tokudome S, et al. Effect of physical activity on breast cancer risk: findings of the Japan collaborative cohort study. Cancer Epidemiol Biomarkers Prev 2008; 17: 3396–401. |
| Low physical activity | Breast cancer | Thune I, Brenn T, Lund E, Gaard M. Physical activity and the risk of breast cancer. N Engl J Med 1997; 336: 1269–75. |

**Appendix Table 5. Epidemiological evidence supporting causality between risk-outcome pairs included in the Global Burden of Disease 2017 study including A. Citations and B. Additional information**

**A. Citations**

| Risk | Outcome | Citation/Note |
|---|---|---|
| Low physical activity | Breast cancer | Wyrwich KW, Wolinsky FD. Physical activity, disability, and the risk of hospitalization for breast cancer among older women. J Gerontol A Biol Sci Med Sci 2000; 55: M418-421. |
| Low physical activity | Breast cancer | Wyshak G, Frisch RE. Breast cancer among former college athletes compared to non-athletes: a 15-year follow-up. Br J Cancer 2000; 82: 726–30. |
| Low physical activity | Ischaemic stroke | Abbott RD, Rodriguez BL, Burchfiel CM, Curb JD. Physical activity in older middle-aged men and reduced risk of stroke: the Honolulu Heart Program. Am J Epidemiol 1994; 139: 881–93. |
| Low physical activity | Ischaemic stroke | Agnarsson U, Thorgeirsson G, Sigvaldason H, Sigfusson N. Effects of leisure-time physical activity and ventilatory function on risk for stroke in men: the Reykjavík Study. Ann Intern Med 1999; 130: 987–90. |
| Low physical activity | Ischaemic stroke | Autenrieth CS, Evenson KR, Yatsuya H, Shahar E, Baggett C, Rosamond WD. Association between physical activity and risk of stroke subtypes: the atherosclerosis risk in communities study. Neuroepidemiology 2013; 40: 109–16. |
| Low physical activity | Ischaemic stroke | Bijnen FC, Caspersen CJ, Feskens EJ, Saris WH, Mosterd WL, Kromhout D. Physical activity and 10-year mortality from cardiovascular diseases and all causes: The Zutphen Elderly Study. Arch Intern Med 1998; 158: 1499–505. |
| Low physical activity | Ischaemic stroke | Calling S, Hedblad B, Engström G, Berglund G, Janzon L. Effects of body fatness and physical activity on cardiovascular risk: risk prediction using the bioelectrical impedance method. Scand J Public Health 2006; 34: 568–75. |
| Low physical activity | Ischaemic stroke | Chiuve SE, Rexrode KM, Spiegelman D, Logroscino G, Manson JE, Rimm EB. Primary prevention of stroke by healthy lifestyle. Circulation 2008; 118: 947–54. |
| Low physical activity | Ischaemic stroke | Ellekjaer H, Holmen J, Ellekjaer E, Vatten L. Physical activity and stroke mortality in women. Ten-year follow-up of the Nord-Trondelag health survey, 1984-1986. Stroke 2000; 31: 14–8. |
| Low physical activity | Ischaemic stroke | Gulsvik AK, Thelle DS, Samuelsen SO, Myrstad M, Mowé M, Wyller TB. Ageing, physical activity and mortality--a 42-year follow-up study. Int J Epidemiol 2012; 41: 521–30. |
| Low physical activity | Ischaemic stroke | Håheim LL, Holme I, Hjermann I, Leren P. Risk factors of stroke incidence and mortality. A 12-year follow-up of the Oslo Study. Stroke 1993; 24: 1484–9. |
| Low physical activity | Ischaemic stroke | Hu FB, Stampfer MJ, Colditz GA, et al. Physical activity and risk of stroke in women. JAMA 2000; 283: 2961–7. |
| Low physical activity | Ischaemic stroke | Hu G, Sarti C, Jousilahti P, Silventoinen K, Barengo NC, Tuomilehto J. Leisure time, occupational, and commuting physical activity and the risk of stroke. Stroke 2005; 36: 1994–9. |
| Low physical activity | Ischaemic stroke | Lapidus L, Bengtsson C. Socioeconomic factors and physical activity in relation to cardiovascular disease and death. A 12 year follow up of participants in a population study of women in Gothenburg, Sweden. Br Heart J 1986; 55: 295–301. |
| Low physical activity | Ischaemic stroke | Lee IM, Hennekens CH, Berger K, Buring JE, Manson JE. Exercise and risk of stroke in male physicians. Stroke 1999; 30: 1–6. |
| Low physical activity | Ischaemic stroke | Lee IM, Paffenbarger RS. Physical activity and stroke incidence: the Harvard Alumni Health Study. Stroke 1998; 29: 2049–54. |
| Low physical activity | Ischaemic stroke | Lindenstrøm E, Boysen G, Nyboe J. Lifestyle factors and risk of cerebrovascular disease in women. The Copenhagen City Heart Study. Stroke 1993; 24: 1468–72. |
| Low physical activity | Ischaemic stroke | Myint PK, Luben RN, Wareham NJ, et al. Combined work and leisure physical activity and risk of stroke in men and women in the European prospective investigation into Cancer-Norfolk Prospective Population Study. Neuroepidemiology 2006; 27: 122–9. |
| Low physical activity | Ischaemic stroke | Okada H, Horibe H, Yoshiyuki O, Hayakawa N, Aoki N. A prospective study of cerebrovascular disease in Japanese rural communities, Akabane and Asahi. Part 1: evaluation of risk factors in the occurrence of cerebral hemorrhage and thrombosis. Stroke 1976; 7: 599–607. |
| Low physical activity | Ischaemic stroke | Paffenbarger RS, Brand RJ, Sholtz RI, Jung DL. Energy expenditure, cigarette smoking, and blood pressure level as related to death from specific diseases. Am J Epidemiol 1978; 108: 12–8. |
| Low physical activity | Ischaemic stroke | Paganini-Hill A, Perez Barreto M. Stroke risk in older men and women: aspirin, estrogen, exercise, vitamins, and other factors. J Gend Specif Med 2001; 4: 18–28. |
| Low physical activity | Ischaemic stroke | Salonen JT, Puska P, Tuomilehto J. Physical activity and risk of myocardial infarction, cerebral stroke and death: a longitudinal study in Eastern Finland. Am J Epidemiol 1982; 115: 526–37. |
| Low physical activity | Ischaemic stroke | Sattelmair JR, Kurth T, Buring JE, Lee I-M. Physical activity and risk of stroke in women. Stroke 2010; 41: 1243–50. |
| Low physical activity | Ischaemic stroke | Simonsick EM, Lafferty ME, Phillips CL, et al. Risk due to inactivity in physically capable older adults. Am J Public Health 1993; 83: 1443–50. |
| Low physical activity | Ischaemic stroke | Wannamethee G, Shaper AG. Physical activity and stroke in British middle aged men. BMJ 1992; 304: 597–601. |
| Low physical activity | Ischaemic stroke | Willey JZ, Moon YP, Paik MC, Boden-Albala B, Sacco RL, Elkind MSV. Physical activity and risk of ischemic stroke in the Northern Manhattan Study. Neurology 2009; 73: 1774–9. |
| Low physical activity | Ischaemic stroke | Zhang Q, Zhou Y, Gao X, et al. Ideal cardiovascular health metrics and the risks of ischemic and intracerebral hemorrhagic stroke. Stroke 2013; 44: 2451–6. |
| Low physical activity | Diabetes mellitus | Baan CA, Stolk RP, Grobbee DE, Witteman JC, Feskens EJ. Physical activity in elderly subjects with impaired glucose tolerance and newly diagnosed diabetes mellitus. Am J Epidemiol 1999; 149: 219–27. |
| Low physical activity | Diabetes mellitus | Bonora E, Kiechl S, Willeit J, et al. Population-based incidence rates and risk factors for type 2 diabetes in white individuals: the Bruneck study. Diabetes 2004; 53: 1782–9. |
| Low physical activity | Diabetes mellitus | Burchfiel CM, Sharp DS, Curb JD, et al. Physical activity and incidence of diabetes: the Honolulu Heart Program. Am J Epidemiol 1995; 141: 360–8. |
| Low physical activity | Diabetes mellitus | Carlsson S, Ahlbom A, Lichtenstein P, Andersson T. Shared genetic influence of BMI, physical activity and type 2 diabetes: a twin study. Diabetologia 2013; 56: 1031–5. |

**Appendix Table 5. Epidemiological evidence supporting causality between risk-outcome pairs included in the Global Burden of Disease 2017 study including A. Citations and B. Additional information**

**A. Citations**

| Risk | Outcome | Citation/Note |
|---|---|---|
| Low physical activity | Diabetes mellitus | Carlsson S, Midthjell K, Tesfamarian MY, Grill V. Age, overweight and physical inactivity increase the risk of latent autoimmune diabetes in adults: results from the Nord-Trøndelag health study. Diabetologia 2007; 50: 55–8. |
| Low physical activity | Diabetes mellitus | Chien K-L, Chen M-F, Hsu H-C, Su T-C, Lee Y-T. Sports activity and risk of type 2 diabetes in Chinese. Diabetes Res Clin Pract 2009; 84: 311–8. |
| Low physical activity | Diabetes mellitus | Demakakos P, Hamer M, Stamatakis E, Steptoe A. Low-intensity physical activity is associated with reduced risk of incident type 2 diabetes in older adults: evidence from the English Longitudinal Study of Ageing. Diabetologia 2010; 53: 1877–85. |
| Low physical activity | Diabetes mellitus | Doi Y, Ninomiya T, Hata J, et al. Two risk score models for predicting incident Type 2 diabetes in Japan. Diabet Med 2012; 29: 107–14. |
| Low physical activity | Diabetes mellitus | Dotevall A, Johansson S, Wilhelmsen L, Rosengren A. Increased levels of triglycerides, BMI and blood pressure and low physical activity increase the risk of diabetes in Swedish women. A prospective 18-year follow-up of the BEDA study. Diabet Med 2004; 21: 615–22. |
| Low physical activity | Diabetes mellitus | Elwood P, Galante J, Pickering J, et al. Healthy lifestyles reduce the incidence of chronic diseases and dementia: evidence from the Caerphilly cohort study. PLoS ONE 2013; 8: e81877. |
| Low physical activity | Diabetes mellitus | Fan S, Chen J, Huang J, et al. Physical activity level and incident type 2 diabetes among Chinese adults. Med Sci Sports Exerc 2015; 47: 751–6. |
| Low physical activity | Diabetes mellitus | Folsom AR, Kushi LH, Hong CP. Physical activity and incident diabetes mellitus in postmenopausal women. Am J Public Health 2000; 90: 134–8. |
| Low physical activity | Diabetes mellitus | Fretts AM, Howard BV, Kriska AM, et al. Physical activity and incident diabetes in American Indians: the Strong Heart Study. Am J Epidemiol 2009; 170: 632–9. |
| Low physical activity | Diabetes mellitus | Grøntved A, Pan A, Mekary RA, et al. Muscle-strengthening and conditioning activities and risk of type 2 diabetes: a prospective study in two cohorts of US women. PLoS Med 2014; 11: e1001587. |
| Low physical activity | Diabetes mellitus | Gurwitz JH, Field TS, Glynn RJ, et al. Risk factors for non-insulin-dependent diabetes mellitus requiring treatment in the elderly. J Am Geriatr Soc 1994; 42: 1235–40. |
| Low physical activity | Diabetes mellitus | Haapanen N, Miilunpalo S, Vuori I, Oja P, Pasanen M. Association of leisure time physical activity with the risk of coronary heart disease, hypertension and diabetes in middle-aged men and women. Int J Epidemiol 1997; 26: 739–47. |
| Low physical activity | Diabetes mellitus | Helmrich SP, Ragland DR, Paffenbarger RS. Prevention of non-insulin-dependent diabetes mellitus with physical activity. Med Sci Sports Exerc 1994; 26: 824–30. |
| Low physical activity | Diabetes mellitus | Holme I, Tonstad S, Sogaard AJ, Larsen PGL, Haheim LL. Leisure time physical activity in middle age predicts the metabolic syndrome in old age: results of a 28-year follow-up of men in the Oslo study. BMC Public Health 2007; 7: 154. |
| Low physical activity | Diabetes mellitus | Hsia J, Wu L, Allen C, et al. Physical activity and diabetes risk in postmenopausal women. Am J Prev Med 2005; 28: 19–25. |
| Low physical activity | Diabetes mellitus | Hu FB, Leitzmann MF, Stampfer MJ, Colditz GA, Willett WC, Rimm EB. Physical activity and television watching in relation to risk for type 2 diabetes mellitus in men. Arch Intern Med 2001; 161: 1542–8. |
| Low physical activity | Diabetes mellitus | Hu FB, Sigal RJ, Rich-Edwards JW, et al. Walking compared with vigorous physical activity and risk of type 2 diabetes in women: a prospective study. JAMA 1999; 282: 1433–9. |
| Low physical activity | Diabetes mellitus | Hu G, Qiao Q, Silventoinen K, et al. Occupational, commuting, and leisure-time physical activity in relation to risk for Type 2 diabetes in middle-aged Finnish men and women. Diabetologia 2003; 46: 322–9. |
| Low physical activity | Diabetes mellitus | James SA, Jamjoum L, Raghunathan TE, Strogatz DS, Furth ED, Khazanie PG. Physical activity and NIDDM in African-Americans. The Pitt County Study. Diabetes Care 1998; 21: 555–62. |
| Low physical activity | Diabetes mellitus | Jefferis BJ, Whincup PH, Lennon L, Wannamethee SG. Longitudinal associations between changes in physical activity and onset of type 2 diabetes in older British men: the influence of adiposity. Diabetes Care 2012; 35: 1876–83. |
| Low physical activity | Diabetes mellitus | Joseph J, Svartberg J, Njølstad I, Schirmer H. Incidence of and risk factors for type-2 diabetes in a general population: the Tromsø Study. Scand J Public Health 2010; 38: 768–75. |
| Low physical activity | Diabetes mellitus | Koloverou E, Panagiotakos DB, Pitsavos C, et al. 10-year incidence of diabetes and associated risk factors in Greece: the ATTICA study (2002-2012). Rev Diabet Stud 2014; 11: 181–9. |
| Low physical activity | Diabetes mellitus | Krishnan S, Rosenberg L, Palmer JR. Physical activity and television watching in relation to risk of type 2 diabetes: the Black Women’s Health Study. Am J Epidemiol 2009; 169: 428–34. |
| Low physical activity | Diabetes mellitus | Laaksonen MA, Knekt P, Rissanen H, et al. The relative importance of modifiable potential risk factors of type 2 diabetes: a meta-analysis of two cohorts. Eur J Epidemiol 2010; 25: 115–24. |
| Low physical activity | Diabetes mellitus | Lee D, Park I, Jun T-W, et al. Physical activity and body mass index and their associations with the development of type 2 diabetes in korean men. Am J Epidemiol 2012; 176: 43–51. |
| Low physical activity | Diabetes mellitus | Longo-Mbenza B, On’kin JBKL, Okwe AN, Kabangu NK, Fuele SM. Metabolic syndrome, aging, physical inactivity, and incidence of type 2 diabetes in general African population. Diab Vasc Dis Res 2010; 7: 28–39. |
| Low physical activity | Diabetes mellitus | Lucke J, Waters B, Hockey R, et al. Trends in women’s risk factors and chronic conditions: findings from the Australian Longitudinal Study on Women’s Health. Womens Health (Lond) 2007; 3: 423–32. |
| Low physical activity | Diabetes mellitus | Magliano DJ, Barr ELM, Zimmet PZ, et al. Glucose indices, health behaviors, and incidence of diabetes in Australia: the Australian Diabetes, Obesity and Lifestyle Study. Diabetes Care 2008; 31: 267–72. |
| Low physical activity | Diabetes mellitus | Manson JE, Nathan DM, Krolewski AS, Stampfer MJ, Willett WC, Hennekens CH. A prospective study of exercise and incidence of diabetes among US male physicians. JAMA 1992; 268: 63–7. |
| Low physical activity | Diabetes mellitus | Manson JE, Rimm EB, Stampfer MJ, et al. Physical activity and incidence of non-insulin-dependent diabetes mellitus in women. Lancet 1991; 338: 774–8. |

| Appendix Table 5. Epidemiological evidence supporting causality between risk-outcome pairs included in the Global Burden of Disease 2017 study including A. Citations and B. Additional information<br>A. Citations | | |
|---|---|---|
| **Risk** | **Outcome** | **Citation/Note** |
| Low physical activity | Diabetes mellitus | Meisinger C, Löwel H, Thorand B, Döring A. Leisure time physical activity and the risk of type 2 diabetes in men and women from the general population. The MONICA/KORA Augsburg Cohort Study. Diabetologia 2005; 48: 27–34. |
| Low physical activity | Diabetes mellitus | Mozaffarian D, Kamineni A, Carnethon M, Djoussé L, Mukamal KJ, Siscovick D. Lifestyle risk factors and new-onset diabetes mellitus in older adults: the cardiovascular health study. Arch Intern Med 2009; 169: 798–807. |
| Low physical activity | Diabetes mellitus | Okada K, Hayashi T, Tsumura K, Suematsu C, Endo G, Fujii S. Leisure-time physical activity at weekends and the risk of Type 2 diabetes mellitus in Japanese men: the Osaka Health Survey. Diabet Med 2000; 17: 53–8. |
| Low physical activity | Diabetes mellitus | Panagiotakos DB, Pitsavos C, Skoumas Y, Lentzas Y, Stefanadis C. Five-year incidence of type 2 diabetes mellitus among cardiovascular disease-free Greek adults: findings from the ATTICA study. Vasc Health Risk Manag 2008; 4: 691–8. |
| Low physical activity | Diabetes mellitus | Rathmann W, Strassburger K, Heier M, et al. Incidence of Type 2 diabetes in the elderly German population and the effect of clinical and lifestyle risk factors: KORA S4/F4 cohort study. Diabet Med 2009; 26: 1212–9. |
| Low physical activity | Diabetes mellitus | Reis JP, Loria CM, Sorlie PD, Park Y, Hollenbeck A, Schatzkin A. Lifestyle factors and risk for new-onset diabetes: a population-based cohort study. Ann Intern Med 2011; 155: 292–9. |
| Low physical activity | Diabetes mellitus | Shi L, Shu X-O, Li H, et al. Physical activity, smoking, and alcohol consumption in association with incidence of type 2 diabetes among middle-aged and elderly Chinese men. PLoS ONE 2013; 8: e77919. |
| Low physical activity | Diabetes mellitus | Siegel LC, Sesso HD, Bowman TS, Lee I-M, Manson JE, Gaziano JM. Physical activity, body mass index, and diabetes risk in men: a prospective study. Am J Med 2009; 122: 1115–21. |
| Low physical activity | Diabetes mellitus | Simonsick EM, Lafferty ME, Phillips CL, et al. Risk due to inactivity in physically capable older adults. Am J Public Health 1993; 83: 1443–50. |
| Low physical activity | Diabetes mellitus | Steinbrecher A, Erber E, Grandinetti A, Nigg C, Kolonel LN, Maskarinec G. Physical activity and risk of type 2 diabetes among Native Hawaiians, Japanese Americans, and Caucasians: the Multiethnic Cohort. J Phys Act Health 2012; 9: 634–41. |
| Low physical activity | Diabetes mellitus | Stringhini S, Tabak AG, Akbaraly TN, et al. Contribution of modifiable risk factors to social inequalities in type 2 diabetes: prospective Whitehall II cohort study. BMJ 2012; 345: e5452. |
| Low physical activity | Diabetes mellitus | Sun F, Tao Q, Zhan S. An accurate risk score for estimation 5-year risk of type 2 diabetes based on a health screening population in Taiwan. Diabetes Res Clin Pract 2009; 85: 228–34. |
| Low physical activity | Diabetes mellitus | Tsai AC, Lee S-H. Determinants of new-onset diabetes in older adults—Results of a national cohort study. Clin Nutr 2015; 34: 937–42. |
| Low physical activity | Diabetes mellitus | Villegas R, Shu X-O, Li H, et al. Physical activity and the incidence of type 2 diabetes in the Shanghai women's health study. Int J Epidemiol 2006; 35: 1553–62. |
| Low physical activity | Diabetes mellitus | Waki K, Noda M, Sasaki S, et al. Alcohol consumption and other risk factors for self-reported diabetes among middle-aged Japanese: a population-based prospective study in the JPHC study cohort I. Diabet Med 2005; 22: 323–31. |
| Low physical activity | Diabetes mellitus | Waller K, Kaprio J, Lehtovirta M, Silventoinen K, Koskenvuo M, Kujala UM. Leisure-time physical activity and type 2 diabetes during a 28 year follow-up in twins. Diabetologia 2010; 53: 2531–7. |
| Low physical activity | Diabetes mellitus | Wannamethee SG, Shaper AG, Alberti KG. Physical activity, metabolic factors, and the incidence of coronary heart disease and type 2 diabetes. Arch Intern Med 2000; 160: 2108–16. |
| Low physical activity | Diabetes mellitus | Weinstein AR, Sesso HD, Lee IM, et al. Relationship of physical activity vs body mass index with type 2 diabetes in women. JAMA 2004; 292: 1188–94. |
| Low physical activity | Diabetes mellitus | Williams PT, Thompson PD. Walking versus running for hypertension, cholesterol, and diabetes mellitus risk reduction. Arterioscler Thromb Vasc Biol 2013; 33: 1085–91. |
| Low physical activity | Diabetes mellitus | Xu F, Ware RS, Tse LA, et al. Joint associations of physical activity and hypertension with the development of type 2 diabetes among urban men and women in Mainland China. PLoS ONE 2014; 9: e88719. |
| High fasting plasma glucose | Ischaemic heart disease | Boussageon R, Bejan-Angoulvant T, Saadatian-Elahi M, et al. Effect of intensive glucose lowering treatment on all cause mortality, cardiovascular death, and microvascular events in type 2 diabetes: meta-analysis of randomised controlled trials. BMJ 2011; 343: d4169. |
| High fasting plasma glucose | Ischaemic heart disease | Singh GM, Danaei G, Farzadfar F, et al. The age-specific quantitative effects of metabolic risk factors on cardiovascular diseases and diabetes: a pooled analysis. PLoS ONE 2013; 8: e65174. |
| High fasting plasma glucose | Ischaemic stroke | Singh GM, Danaei G, Farzadfar F, et al. The age-specific quantitative effects of metabolic risk factors on cardiovascular diseases and diabetes: a pooled analysis. PLoS ONE 2013; 8: e65174. |
| High fasting plasma glucose | Ischaemic stroke | Zhang C, Zhou Y-H, Xu C-L, Chi F-L, Ju H-N. Efficacy of intensive control of glucose in stroke prevention: a meta-analysis of data from 59,197 participants in 9 randomized controlled trials. PloS One 2013; 8: e54465. |
| High fasting plasma glucose | Hemorrhagic stroke | Singh GM, Danaei G, Farzadfar F, et al. The age-specific quantitative effects of metabolic risk factors on cardiovascular diseases and diabetes: a pooled analysis. PLoS ONE 2013; 8: e65174. |
| High fasting plasma glucose | Hemorrhagic stroke | Zhang C, Zhou Y-H, Xu C-L, Chi F-L, Ju H-N. Efficacy of intensive control of glucose in stroke prevention: a meta-analysis of data from 59,197 participants in 9 randomized controlled trials. PloS One 2013; 8: e54465. |
| High fasting plasma glucose | Chronic kidney disease due to diabetes mellitus | Coca SG, Ismail-Beigi F, Haq N, Krumholz HM, Parikh CR. Role of intensive glucose control in development of renal end points in type 2 diabetes mellitus: systematic review and meta-analysis intensive glucose control in type 2 diabetes. Arch Intern Med 2012; 172: 761–9. |
| High fasting plasma glucose | Chronic kidney disease due to diabetes mellitus | O'Seaghdha CM, Perkovic V, Lam TH, et al. Blood Pressure Is a Major Risk Factor for Renal Death An Analysis of 560 352 Participants From the Asia-Pacific Region. Hypertension 2009; 54: 509–15. |

| **Appendix Table 5. Epidemiological evidence supporting causality between risk-outcome pairs included in the Global Burden of Disease 2017 study including A. Citations and B. Additional information<br>A. Citations** | | |
|---|---|---|
| **Risk** | **Outcome** | **Citation/Note** |
| High fasting plasma glucose | Chronic kidney disease due to hypertension | Coca SG, Ismail-Beigi F, Haq N, Krumholz HM, Parikh CR. Role of intensive glucose control in development of renal end points in type 2 diabetes mellitus: systematic review and meta-analysis intensive glucose control in type 2 diabetes. Arch Intern Med 2012; 172: 761–9. |
| High fasting plasma glucose | Chronic kidney disease due to hypertension | O'Seaghdha CM, Perkovic V, Lam TH, et al. Blood Pressure Is a Major Risk Factor for Renal Death An Analysis of 560 352 Participants From the Asia-Pacific Region. Hypertension 2009; 54: 509–15. |
| High fasting plasma glucose | Chronic kidney disease due to glomerulonephritis | Coca SG, Ismail-Beigi F, Haq N, Krumholz HM, Parikh CR. Role of intensive glucose control in development of renal end points in type 2 diabetes mellitus: systematic review and meta-analysis intensive glucose control in type 2 diabetes. Arch Intern Med 2012; 172: 761–9. |
| High fasting plasma glucose | Chronic kidney disease due to glomerulonephritis | O'Seaghdha CM, Perkovic V, Lam TH, et al. Blood Pressure Is a Major Risk Factor for Renal Death An Analysis of 560 352 Participants From the Asia-Pacific Region. Hypertension 2009; 54: 509–15. |
| High fasting plasma glucose | Chronic kidney disease due to other causes | Coca SG, Ismail-Beigi F, Haq N, Krumholz HM, Parikh CR. Role of intensive glucose control in development of renal end points in type 2 diabetes mellitus: systematic review and meta-analysis intensive glucose control in type 2 diabetes. Arch Intern Med 2012; 172: 761–9. |
| High fasting plasma glucose | Chronic kidney disease due to other causes | O'Seaghdha CM, Perkovic V, Lam TH, et al. Blood Pressure Is a Major Risk Factor for Renal Death An Analysis of 560 352 Participants From the Asia-Pacific Region. Hypertension 2009; 54: 509–15. |
| High fasting plasma glucose | Tuberculosis | Young F, Wotton CJ, Critchley JA, Unwin NC, Goldacre MJ. Increased risk of tuberculosis disease in people with diabetes mellitus: record-linkage study in a UK population. J Epidemiol Community Health. 2012; 66(6): 519-23. |
| High fasting plasma glucose | Tuberculosis | Kuo MC, Lin SH, Lin CH, Mao IC, Chang SJ, Hsieh MC. Type 2 diabetes: an independent risk factor for tuberculosis: a nationwide population-based study. PLoS One. 2013; 8((11):e78924). |
| High fasting plasma glucose | Tuberculosis | Dobler CC, Flack JR, Marks GB. Risk of tuberculosis among people with diabetes mellitus: an Australian nationwide cohort study. BMJ Open. 2((1):e000666). |
| High fasting plasma glucose | Tuberculosis | Baker MA, Lin HH, Chang HY, Murray MB. The risk of tuberculosis disease among persons with diabetes mellitus: a prospective cohort study. Clin Infect Dis. 2012; 54(6): 818-25. |
| High fasting plasma glucose | Tuberculosis | Leung CC, Lam TH, Chan WM, Yew WW, Ho KS, Leung GM, Law WS, Tam CM, Chan CK, Chang KC. Diabetic control and risk of tuberculosis: a cohort study. Am J Epidemiol. 2008; 167(12): 1486-94. |
| High fasting plasma glucose | Tuberculosis | Kim SJ, Hong YP, Lew WJ, Yang SC, Lee EG. Incidence of pulmonary tuberculosis among diabetics. Tuber Lung Dis. 1995; 76(6): 529-33. |
| High fasting plasma glucose | Tuberculosis | Pealing L, Wing K, Mathur R, Prieto-Merino D, Smeeth L, Moore DA. Risk of tuberculosis in patients with diabetes: population based cohort study using the UK Clinical Practice Research Datalink. BMC Med. 2015; 13(135). |
| High fasting plasma glucose | Dementia/Alzheimer's | Zhang J, Chen C, Hua S, Liao H, Wang M, Xiong Y, Cao F. An updated meta-analysis of cohort studies: diabetes and risk of Alzheimer's disease. Diabetes Res Clin Pract. 2017; Feb (124): 41-47. |
| High fasting plasma glucose | Cataracts | Li L, Wan XH, Zhao GH. Meta-analysis of the risk of cataract in type 2 diabetes. BMC Ophthalmol. 2014; 14(94). |
| High fasting plasma glucose | Glaucoma | Zhao D, Cho J, Kim MH, Friedman DS, Guallar E. Diabetes, fasting glucose, and the risk of glaucoma: a meta-analysis. Ophthalmology. 2015; 122(1): 72-8. |
| High fasting plasma glucose | Lung cancer | Zhu L, Cao H, Zhang T, Shen H, Dong W, Wang L, Du J. The effect of diabetes mellitus on lung cancer prognosis: a PRISMA-compliant meta-analysis of cohort studies. Medicine (Baltimore). 2016; 95(17): :e3528. |
| High fasting plasma glucose | Breast cancer | Boyle P, Boniol M, Koechlin A, et al. Diabetes and breast cancer risk: a meta-analysis. Br J Cancer 2012; 107: 1608–17. |
| High fasting plasma glucose | Bladder cancer | Fang H, Yao B, Yan Y, Xu H, Liu Y, Tang H, Zhou J, Cao L, Wang W, Zhang J, Zhao L, Chen X, Zhang F, Zhao Y. Diabetes mellitus increases the risk of bladder cancer: an updated meta-analysis. Diabetes Technol Ther. 2013; 15(11): 914-22. |
| High fasting plasma glucose | Colon cancer | Shi J, Xiong L, Li J, Cao H, Jiang W, Liu B, Chen X, Liu C, Liu K, Wang G, Cai K . A linear dose-response relationship between fasting plasma glucose and colorectal cancer risk: systematic review and meta-analysis. Sci Rep. 2015; 5(17591). |
| High fasting plasma glucose | Ovarian cancer | Lee JY1, Jeon I, Kim JW, Song YS, Yoon JM, Park SM.. Diabetes mellitus and ovarian cancer risk: a systematic review and meta-analysis of observational studies. Int J Gynaecol Obstet. 2013; 23(3): 402-12. |
| High fasting plasma glucose | Pancreatic cancer | Ben Q, Xu M, Ning X, Liu J, Hong S, Huang W, Zhang H, Li Z. Diabetes mellitus and risk of pancreatic cancer: A meta-analysis of cohort studies. Eur J Cancer. 2011; 47(13): 1928-1937. |
| High fasting plasma glucose | Liver cancer | Yang WS, Va P, Bray F, Gao S, Gao J, Li HL, Xiang YB. The role of pre-existing diabetes mellitus on hepatocellular carcinoma occurrence and prognosis: a meta-analysis of prospective cohort studies. PLoS One. 2011; 6(12): :e27326. |
| High fasting plasma glucose | Peripheral vascular disease | Rapsomaniki E, Timmis A, George J, Pujades-Rodriguez M, Shah AD, Denaxas S, White IR, Caulfield MJ, Deanfield JE, Smeeth L, Williams B, Hingorani A, Hemingway H. Blood pressure and incidence of twelve cardiovascular diseases: lifetime risks, healthy life-years lost, and age-specific associations in 1·25 million people. Lancet. 2014; 31(383): 1899-911. |
| High fasting plasma glucose | Ischaemic stroke | Rapsomaniki E, Timmis A, George J, Pujades-Rodriguez M, Shah AD, Denaxas S, White IR, Caulfield MJ, Deanfield JE, Smeeth L, Williams B, Hingorani A, Hemingway H. Blood pressure and incidence of twelve cardiovascular diseases: lifetime risks, healthy life-years lost, and age-specific associations in 1·25 million people. Lancet. 2014; 31(383): 1899-911. |
| High fasting plasma glucose | Hemorrhagic stroke | Rapsomaniki E, Timmis A, George J, Pujades-Rodriguez M, Shah AD, Denaxas S, White IR, Caulfield MJ, Deanfield JE, Smeeth L, Williams B, Hingorani A, Hemingway H. Blood pressure and incidence of twelve cardiovascular diseases: lifetime risks, healthy life-years lost, and age-specific associations in 1·25 million people. Lancet. 2014; 31(383): 1899-911. |

**Appendix Table 5. Epidemiological evidence supporting causality between risk-outcome pairs included in the Global Burden of Disease 2017 study including A. Citations and B. Additional information**

**A. Citations**

| Risk | Outcome | Citation/Note |
|---|---|---|
| High LDL cholesterol | Ischaemic heart disease | Cholesterol Treatment Trialists' (CTT) Collaboration, Baigent C, Blackwell L, et al. Efficacy and safety of more intensive lowering of LDL cholesterol: a meta-analysis of data from 170,000 participants in 26 randomised trials. Lancet Lond Engl 2010; 376: 1670–81. |
| High LDL cholesterol | Ischaemic heart disease | Singh GM, Danaei G, Farzadfar F, et al. The age-specific quantitative effects of metabolic risk factors on cardiovascular diseases and diabetes: a pooled analysis. PLoS ONE 2013; 8: e65174. |
| High LDL cholesterol | Ischaemic stroke | Cholesterol Treatment Trialists' (CTT) Collaboration, Baigent C, Blackwell L, et al. Efficacy and safety of more intensive lowering of LDL cholesterol: a meta-analysis of data from 170,000 participants in 26 randomised trials. Lancet Lond Engl 2010; 376: 1670–81. |
| High LDL cholesterol | Ischaemic stroke | Singh GM, Danaei G, Farzadfar F, et al. The age-specific quantitative effects of metabolic risk factors on cardiovascular diseases and diabetes: a pooled analysis. PLoS ONE 2013; 8: e65174. |
| High systolic blood pressure | Rheumatic heart disease | Singh GM, Danaei G, Farzadfar F, et al. The age-specific quantitative effects of metabolic risk factors on cardiovascular diseases and diabetes: a pooled analysis. PLoS ONE 2013; 8: e65174. |
| High systolic blood pressure | Ischaemic heart disease | Singh GM, Danaei G, Farzadfar F, et al. The age-specific quantitative effects of metabolic risk factors on cardiovascular diseases and diabetes: a pooled analysis. PLoS ONE 2013; 8: e65174. |
| High systolic blood pressure | Ischaemic heart disease | Thomopoulos C, Parati G, Zanchetti A. Effects of blood pressure lowering on outcome incidence in hypertension. 1. Overview, meta-analyses, and meta-regression analyses of randomized trials. J Hypertens 2014; 32: 2285–95. |
| High systolic blood pressure | Ischaemic stroke | Singh GM, Danaei G, Farzadfar F, et al. The age-specific quantitative effects of metabolic risk factors on cardiovascular diseases and diabetes: a pooled analysis. PLoS ONE 2013; 8: e65174. |
| High systolic blood pressure | Ischaemic stroke | Thomopoulos C, Parati G, Zanchetti A. Effects of blood pressure lowering on outcome incidence in hypertension. 1. Overview, meta-analyses, and meta-regression analyses of randomized trials. J Hypertens 2014; 32: 2285–95. |
| High systolic blood pressure | Hemorrhagic stroke | Singh GM, Danaei G, Farzadfar F, et al. The age-specific quantitative effects of metabolic risk factors on cardiovascular diseases and diabetes: a pooled analysis. PLoS ONE 2013; 8: e65174. |
| High systolic blood pressure | Hemorrhagic stroke | Thomopoulos C, Parati G, Zanchetti A. Effects of blood pressure lowering on outcome incidence in hypertension. 1. Overview, meta-analyses, and meta-regression analyses of randomized trials. J Hypertens 2014; 32: 2285–95. |
| High systolic blood pressure | Hypertensive heart disease | Singh GM, Danaei G, Farzadfar F, et al. The age-specific quantitative effects of metabolic risk factors on cardiovascular diseases and diabetes: a pooled analysis. PLoS ONE 2013; 8: e65174. |
| High systolic blood pressure | Hypertensive heart disease | Thomopoulos C, Parati G, Zanchetti A. Effects of blood pressure lowering on outcome incidence in hypertension. 1. Overview, meta-analyses, and meta-regression analyses of randomized trials. J Hypertens 2014; 32: 2285–95. |
| High systolic blood pressure | Cardiomyopathy and myocarditis | Singh GM, Danaei G, Farzadfar F, et al. The age-specific quantitative effects of metabolic risk factors on cardiovascular diseases and diabetes: a pooled analysis. PLoS ONE 2013; 8: e65174. |
| High systolic blood pressure | Atrial fibrillation and flutter | Emdin CA, Callender T, Cao J, Rahimi K. Effect of antihypertensive agents on risk of atrial fibrillation: a meta-analysis of large-scale randomized trials. Eur Eur Pacing Arrhythm Card Electrophysiol J Work Groups Card Pacing Arrhythm Card Cell Electrophysiol Eur Soc Cardiol 2015; 17: 701–10. |
| High systolic blood pressure | Atrial fibrillation and flutter | Singh GM, Danaei G, Farzadfar F, et al. The age-specific quantitative effects of metabolic risk factors on cardiovascular diseases and diabetes: a pooled analysis. PLoS ONE 2013; 8: e65174. |
| High systolic blood pressure | Aortic aneurysm | Singh GM, Danaei G, Farzadfar F, et al. The age-specific quantitative effects of metabolic risk factors on cardiovascular diseases and diabetes: a pooled analysis. PLoS ONE 2013; 8: e65174. |
| High systolic blood pressure | Peripheral vascular disease | Singh GM, Danaei G, Farzadfar F, et al. The age-specific quantitative effects of metabolic risk factors on cardiovascular diseases and diabetes: a pooled analysis. PLoS ONE 2013; 8: e65174. |
| High systolic blood pressure | Endocarditis | Singh GM, Danaei G, Farzadfar F, et al. The age-specific quantitative effects of metabolic risk factors on cardiovascular diseases and diabetes: a pooled analysis. PLoS ONE 2013; 8: e65174. |
| High systolic blood pressure | Other cardiovascular and circulatory diseases | Singh GM, Danaei G, Farzadfar F, et al. The age-specific quantitative effects of metabolic risk factors on cardiovascular diseases and diabetes: a pooled analysis. PLoS ONE 2013; 8: e65174. |
| High systolic blood pressure | Chronic kidney disease due to diabetes mellitus | The Renal Risk Collaboration, Foote C, Lin J, et al. The effect of Blood Pressure on Kidney Failure: a systematic review and meta-analysis in 2.7 million participants (unpublished). |
| High systolic blood pressure | Chronic kidney disease due to diabetes mellitus | Xie X, Atkins E, Lv J, et al. Effects of intensive blood pressure lowering on cardiovascular and renal outcomes: updated systematic review and meta-analysis. Lancet Lond Engl 2016; 387: 435–43. |
| High systolic blood pressure | Chronic kidney disease due to hypertension | The Renal Risk Collaboration, Foote C, Lin J, et al. The effect of Blood Pressure on Kidney Failure: a systematic review and meta-analysis in 2.7 million participants (unpublished). |
| High systolic blood pressure | Chronic kidney disease due to hypertension | Xie X, Atkins E, Lv J, et al. Effects of intensive blood pressure lowering on cardiovascular and renal outcomes: updated systematic review and meta-analysis. Lancet Lond Engl 2016; 387: 435–43. |
| High systolic blood pressure | Chronic kidney disease due to glomerulonephritis | The Renal Risk Collaboration, Foote C, Lin J, et al. The effect of Blood Pressure on Kidney Failure: a systematic review and meta-analysis in 2.7 million participants (unpublished). |
| High systolic blood pressure | Chronic kidney disease due to glomerulonephritis | Xie X, Atkins E, Lv J, et al. Effects of intensive blood pressure lowering on cardiovascular and renal outcomes: updated systematic review and meta-analysis. Lancet Lond Engl 2016; 387: 435–43. |
| High systolic blood pressure | Chronic kidney disease due to other causes | The Renal Risk Collaboration, Foote C, Lin J, et al. The effect of Blood Pressure on Kidney Failure: a systematic review and meta-analysis in 2.7 million participants (unpublished). |
| High systolic blood pressure | Chronic kidney disease due to other causes | Xie X, Atkins E, Lv J, et al. Effects of intensive blood pressure lowering on cardiovascular and renal outcomes: updated systematic review and meta-analysis. Lancet Lond Engl 2016; 387: 435–43. |
| High systolic blood pressure | Non-rheumatic calcific aortic valve disease | Bahler RC, Desser DR, Finkelhor RS, Brener SJ, Youssefi M. Factors leading to progression of valvular aortic stenosis. Am J Cardiol. 1999;84(9):1044-8. |
| High systolic blood pressure | Non-rheumatic calcific aortic valve disease | Hoagland PM, Cook EF, Flatley M, Walker C, Goldman L. Case-control analysis of risk factors for presence of aortic stenosis in adults (age 50 years or older). Am J Cardiol. 1985;55(6):744-7. |
| High systolic blood pressure | Non-rheumatic calcific aortic valve disease | Stewart BF, Siscovick D, Lind BK, et al. Clinical factors associated with calcific aortic valve disease. Cardiovascular Health Study. J Am Coll Cardiol. 1997;29(3):630-4. |

**Appendix Table 5. Epidemiological evidence supporting causality between risk-outcome pairs included in the Global Burden of Disease 2017 study including A. Citations and B. Additional information**
**A. Citations**

| Risk | Outcome | Citation/Note |
|---|---|---|
| High body-mass index | Colon and rectum cancer | Schlesinger S, Lieb W, Koch M, et al. Body weight gain and risk of colorectal cancer: a systematic review and meta-analysis of observational studies. Obes Rev 2015; 16: 607–19. |
| High body-mass index | Liver cancer | Chen Y, Wang X, Wang J, Yan Z, Luo J. Excess body weight and the risk of primary liver cancer: an updated meta-analysis of prospective studies. Eur J Cancer 2012; 48: 2137–45. |
| High body-mass index | Liver cancer | Renehan AG, Tyson M, Egger M, Heller RF, Zwahlen M. Body-mass index and incidence of cancer: a systematic review and meta-analysis of prospective observational studies. Lancet 2008; 371: 569–78. |
| High body-mass index | Liver cancer | Rui R, Lou J, Zou L, et al. Excess body mass index and risk of liver cancer: a nonlinear dose-response meta-analysis of prospective studies. PLoS ONE 2012; 7: e44522. |
| High body-mass index | Liver cancer | Tanaka K, Tsuji I, Tamakoshi A, et al. Obesity and liver cancer risk: an evaluation based on a systematic review of epidemiologic evidence among the Japanese population. Jpn J Clin Oncol 2012; 42: 212–21. |
| High body-mass index | Liver cancer | Wang Y, Wang B, Shen F, Fan J, Cao H. Body mass index and risk of primary liver cancer: a meta-analysis of prospective studies. Oncologist 2012; 17: 1461–8. |
| High body-mass index | Gallbladder and biliary tract cancer | Park M, Song DY, Je Y, Lee JE. Body mass index and biliary tract disease: a systematic review and meta-analysis of prospective studies. Prev Med 2014; 65: 13–22. |
| High body-mass index | Gallbladder and biliary tract cancer | Renehan AG, Tyson M, Egger M, Heller RF, Zwahlen M. Body-mass index and incidence of cancer: a systematic review and meta-analysis of prospective observational studies. Lancet 2008; 371: 569–78. |
| High body-mass index | Pancreatic cancer | Alsamarrai A, Das SLM, Windsor JA, Petrov MS. Factors that affect risk for pancreatic disease in the general population: a systematic review and meta-analysis of prospective cohort studies. Clin Gastroenterol Hepatol 2014; 12: 1635–1644.e5; quiz e103. |
| High body-mass index | Pancreatic cancer | Renehan AG, Tyson M, Egger M, Heller RF, Zwahlen M. Body-mass index and incidence of cancer: a systematic review and meta-analysis of prospective observational studies. Lancet 2008; 371: 569–78. |
| High body-mass index | Breast cancer (Pre-menopause) | Renehan AG, Tyson M, Egger M, Heller RF, Zwahlen M. Body-mass index and incidence of cancer: a systematic review and meta-analysis of prospective observational studies. Lancet 2008; 371: 569–78. |
| High body-mass index | Breast cancer (Pre-menopause) | Xia X, Chen W, Li J, et al. Body mass index and risk of breast cancer: a nonlinear dose-response meta-analysis of prospective studies. Sci Rep 2014; 4: 7480. |
| High body-mass index | Breast cancer (Post-menopause) | Renehan AG, Tyson M, Egger M, Heller RF, Zwahlen M. Body-mass index and incidence of cancer: a systematic review and meta-analysis of prospective observational studies. Lancet 2008; 371: 569–78. |
| High body-mass index | Breast cancer (Post-menopause) | Xia X, Chen W, Li J, et al. Body mass index and risk of breast cancer: a nonlinear dose-response meta-analysis of prospective studies. Sci Rep 2014; 4: 7480. |
| High body-mass index | Uterine cancer | Aune D, Greenwood DC, Chan DSM, et al. Body mass index, abdominal fatness and pancreatic cancer risk: a systematic review and non-linear dose-response meta-analysis of prospective studies. Ann Oncol 2012; 23: 843–52. |
| High body-mass index | Uterine cancer | Jenabi E, Poorolajal J. The effect of body mass index on endometrial cancer: a meta-analysis. Public Health 2015; 129: 872–80. |
| High body-mass index | Uterine cancer | Renehan AG, Tyson M, Egger M, Heller RF, Zwahlen M. Body-mass index and incidence of cancer: a systematic review and meta-analysis of prospective observational studies. Lancet 2008; 371: 569–78. |
| High body-mass index | Ovarian cancer | Aune D, Navarro Rosenblatt DA, Chan DSM, et al. Anthropometric factors and ovarian cancer risk: a systematic review and nonlinear dose-response meta-analysis of prospective studies. Int J Cancer 2015; 136: 1888–98. |
| High body-mass index | Ovarian cancer | Collaborative Group on Epidemiological Studies of Ovarian Cancer. Ovarian cancer and body size: individual participant meta-analysis including 25,157 women with ovarian cancer from 47 epidemiological studies. PLoS Med 2012; 9: e1001200. |
| High body-mass index | Ovarian cancer | Liu Z, Zhang T-T, Zhao J-J, et al. The association between overweight, obesity and ovarian cancer: a meta-analysis. Jpn J Clin Oncol 2015; 45: 1107–15. |
| High body-mass index | Ovarian cancer | Renehan AG, Tyson M, Egger M, Heller RF, Zwahlen M. Body-mass index and incidence of cancer: a systematic review and meta-analysis of prospective observational studies. Lancet 2008; 371: 569–78. |
| High body-mass index | Kidney cancer | Renehan AG, Tyson M, Egger M, Heller RF, Zwahlen M. Body-mass index and incidence of cancer: a systematic review and meta-analysis of prospective observational studies. Lancet 2008; 371: 569–78. |
| High body-mass index | Kidney cancer | Wang F, Xu Y. Body mass index and risk of renal cell cancer: a dose-response meta-analysis of published cohort studies. Int J Cancer 2014; 135: 1673–86. |
| High body-mass index | Thyroid cancer | Ma J, Huang M, Wang L, Ye W, Tong Y, Wang H. Obesity and risk of thyroid cancer: evidence from a meta-analysis of 21 observational studies. Med Sci Monit 2015; 21: 283–91. |
| High body-mass index | Thyroid cancer | Renehan AG, Tyson M, Egger M, Heller RF, Zwahlen M. Body-mass index and incidence of cancer: a systematic review and meta-analysis of prospective observational studies. Lancet. 2008; 371(9612): 569-78. |
| High body-mass index | Non-hodgkin lymphoma | Larsson SC, Wolk A. Body mass index and risk of non-Hodgkin's and Hodgkin's lymphoma: A meta-analysis of prospective studies. European Journal of Cancer 2011; 47: 2422–30. |
| High body-mass index | Leukaemia | Castillo JJ, Reagan JL, Ingham RR, et al. Obesity but not overweight increases the incidence and mortality of leukemia in adults: a meta-analysis of prospective cohort studies. Leuk Res 2012; 36: 868–75. |
| High body-mass index | Leukaemia | Renehan AG, Tyson M, Egger M, Heller RF, Zwahlen M. Body-mass index and incidence of cancer: a systematic review and meta-analysis of prospective observational studies. Lancet. 2008; 371(9612): 569-78. |
| High body-mass index | Multiple myeloma | Teras LR, Kitahara CM, Birmann BM, et al. Body Size and Multiple Myeloma Mortality: a pooled analysis of 20 prospective studies. Br J Haematol 2014; 166: 667–76. |
| High body-mass index | Ischaemic heart disease | Singh GM, Danaei G, Farzadfar F, et al. The age-specific quantitative effects of metabolic risk factors on cardiovascular diseases and diabetes: a pooled analysis. PLoS ONE 2013; 8: e65174. |
| High body-mass index | Cerebrovascular disease | Singh GM, Danaei G, Farzadfar F, et al. The age-specific quantitative effects of metabolic risk factors on cardiovascular diseases and diabetes: a pooled analysis. PLoS ONE 2013; 8: e65174. |

**Appendix Table 5. Epidemiological evidence supporting causality between risk-outcome pairs included in the Global Burden of Disease 2017 study including A. Citations and B. Additional information**

**A. Citations**

| Risk | Outcome | Citation/Note |
|---|---|---|
| High body-mass index | Hypertensive heart disease | Singh GM, Danaei G, Farzadfar F, et al. The age-specific quantitative effects of metabolic risk factors on cardiovascular diseases and diabetes: a pooled analysis. PLoS ONE 2013; 8: e65174. |
| High body-mass index | Atrial fibrillation and flutter | Wanahita N, Messerli FH, Bangalore S, Gami AS, Somers VK, Steinberg JS. Atrial fibrillation and obesity—results of a meta-analysis. American Heart Journal 2008; 155: 310–5. |
| High body-mass index | Asthma | Beuther DA, Sutherland ER. Overweight, Obesity, and Incident Asthma. Am J Respir Crit Care Med 2007; 175: 661–6. |
| High body-mass index | Gallbladder diseases | Aune D, Norat T, Vatten LJ. Body mass index, abdominal fatness and the risk of gallbladder disease. Eur J Epidemiol 2015; 30: 1009–19. |
| High body-mass index | Alzheimer disease and other dementias | Profenno LA, Porsteinsson AP, Faraone SV. Meta-Analysis of Alzheimer's Disease Risk with Obesity, Diabetes, and Related Disorders. Biological Psychiatry 2010; 67: 505–12. |
| High body-mass index | Diabetes mellitus | Singh GM, Danaei G, Farzadfar F, et al. The age-specific quantitative effects of metabolic risk factors on cardiovascular diseases and diabetes: a pooled analysis. PLoS ONE 2013; 8: e65174. |
| High body-mass index | Chronic kidney disease | Emerging Risk Factors Collaboration, Wormser D, Kaptoge S, et al. Separate and combined associations of body-mass index and abdominal adiposity with cardiovascular disease: collaborative analysis of 58 prospective studies. Lancet 2011; 377: 1085–95. |
| High body-mass index | Chronic kidney disease | Ni Mhurchu C, Rodgers A, Pan WH, Gu DF, Woodward M, Asia Pacific Cohort Studies Collaboration. Body mass index and cardiovascular disease in the Asia-Pacific Region: an overview of 33 cohorts involving 310 000 participants. Int J Epidemiol 2004; 33: 751–8. |
| High body-mass index | Chronic kidney disease | Prospective Studies Collaboration, Whitlock G, Lewington S, et al. Body-mass index and cause-specific mortality in 900 000 adults: collaborative analyses of 57 prospective studies. Lancet 2009; 373: 1083–96. |
| High body-mass index | Osteoarthritis | Jiang L, Rong J, Wang Y, et al. The relationship between body mass index and hip osteoarthritis: a systematic review and meta-analysis. Joint Bone Spine 2011; 78: 150–5. |
| High body-mass index | Osteoarthritis | Jiang L, Tian W, Wang Y, et al. Body mass index and susceptibility to knee osteoarthritis: a systematic review and meta-analysis. Joint Bone Spine 2012; 79: 291–7. |
| High body-mass index | Osteoarthritis | Silverwood V, Blagojevic-Bucknall M, Jinks C, Jordan JL, Protheroe J, Jordan KP. Current evidence on risk factors for knee osteoarthritis in older adults: a systematic review and meta-analysis. Osteoarthr Cartil 2015; 23: 507–15. |
| High body-mass index | Low back pain | Shiri R, Karppinen J, Leino-Arjas P, Solovieva S, Viikari-Juntura E. The association between obesity and low back pain: a meta-analysis. Am J Epidemiol 2010; 171: 135–54. |
| High body-mass index | Gout | Aune D, Norat T, Vatten LJ. Body mass index and the risk of gout: a systematic review and dose-response meta-analysis of prospective studies. Eur J Nutr 2014; 53: 1591–601. |
| High body-mass index | Cataract | Ye J, Lou L-X, He J-J, Xu Y-F. Body Mass Index and Risk of Age-Related Cataract: A Meta-Analysis of Prospective Cohort Studies. PLOS ONE 2014; 9: e89923. |
| High body-mass index (child) | Asthma | Mebrahtu TF, Feltbower RG, Greenwood DC, Parslow RC. Childhood body mass index and wheezing disorders: a systematic review and meta-analysis. Pediatr Allergy Immunol 2015; 26: 62–72. |
| Low bone mineral density | Injuries | Johnell O, Kanis JA, Oden A, et al. Predictive value of BMD for hip and other fractures. J Bone Miner Res 2005; 20: 1185–94. |
| Impaired kidney function | Ischaemic heart disease | Chronic Kidney Disease Prognosis Consortium (CKD-PC). Chronic Kidney Disease Prognosis Consortium GBD 2016 Impaired Kidney Function Relative Risk Meta-Analysis. |
| Impaired kidney function | Ischaemic heart disease | National Heart, Lung, and Blood Institute, National Institutes of Health (NIH). United States Atherosclerosis Risk in Communities Study. Bethesda, United States: National Heart, Lung, and Blood Institute, National Institutes of Health (NIH). |
| Impaired kidney function | Ischaemic heart disease | International Diabetes Institute (IDI). Australia Diabetes, Obesity and Lifestyle Study 1999-2000. Melbourne, Australia: International Diabetes Institute (IDI). |
| Impaired kidney function | Ischaemic heart disease | Boston University, School of Medicine, Framingham Heart Study, National Heart, Lung, and Blood Institute, National Institutes of Health (NIH). United States Framingham Heart Study . |
| Impaired kidney function | Ischaemic heart disease | Association for Cardiac Research, Rome (Italy). The Gubbio Population Study. |
| Impaired kidney function | Ischaemic heart disease | National Heart, Lung, and Blood Institute, National Institutes of Health, University of California, Los Angeles (UCLA), University of Minnesota. United States Multi-Ethnic Study of Atherosclerosis First Examination 2000-2002. Bethesda, United States: National Heart, Lung, and Blood Institute, National Institutes of Health. |
| Impaired kidney function | Ischaemic heart disease | Uppsala University. Sweden Uppsala Longitudinal Study of Adult Men. |
| Impaired kidney function | Cerebrovascular disease | Chronic Kidney Disease Prognosis Consortium (CKD-PC). Chronic Kidney Disease Prognosis Consortium GBD 2016 Impaired Kidney Function Relative Risk Meta-Analysis. |
| Impaired kidney function | Cerebrovascular disease | National Heart, Lung, and Blood Institute, National Institutes of Health (NIH). United States Atherosclerosis Risk in Communities Study. Bethesda, United States: National Heart, Lung, and Blood Institute, National Institutes of Health (NIH). |
| Impaired kidney function | Cerebrovascular disease | International Diabetes Institute (IDI). Australia Diabetes, Obesity and Lifestyle Study 1999-2000. Melbourne, Australia: International Diabetes Institute (IDI). |
| Impaired kidney function | Cerebrovascular disease | National Heart, Lung, and Blood Institute, National Institutes of Health, University of California, Los Angeles (UCLA), University of Minnesota. United States Multi-Ethnic Study of Atherosclerosis First Examination 2000-2002. Bethesda, United States: National Heart, Lung, and Blood Institute, National Institutes of Health. |
| Impaired kidney function | Cerebrovascular disease | Uppsala University. Sweden Uppsala Longitudinal Study of Adult Men. |
| Impaired kidney function | Peripheral vascular disease | Chronic Kidney Disease Prognosis Consortium (CKD-PC). Chronic Kidney Disease Prognosis Consortium GBD 2016 Impaired Kidney Function Relative Risk Meta-Analysis. |
| Impaired kidney function | Peripheral vascular disease | National Heart, Lung, and Blood Institute, National Institutes of Health (NIH). United States Atherosclerosis Risk in Communities Study. Bethesda, United States: National Heart, Lung, and Blood Institute, National Institutes of Health (NIH). |

**Appendix Table 5. Epidemiological evidence supporting causality between risk-outcome pairs included in the Global Burden of Disease 2017 study including A. Citations and B. Additional information**
**A. Citations**

| Risk | Outcome | Citation/Note |
|---|---|---|
| Impaired kidney function | Peripheral vascular disease | Association for Cardiac Research, Rome (Italy). The Gubbio Population Study. |
| Impaired kidney function | Peripheral vascular disease | National Heart, Lung, and Blood Institute, National Institutes of Health, University of California, Los Angeles (UCLA), University of Minnesota. United States Multi-Ethnic Study of Atherosclerosis First Examination 2000-2002. Bethesda, United States: National Heart, Lung, and Blood Institute, National Institutes of Health. |
| Impaired kidney function | Gout | Cea Soriano L, Rothenbacher D, Choi HK, García Rodríguez LA. Contemporary epidemiology of gout in the UK general population. Arthritis Res Ther 2011; 13: R39. |
| Impaired kidney function | Gout | Krishnan E. Chronic kidney disease and the risk of incident gout among middle-aged men: a seven-year prospective observational study. Arthritis Rheum 2013; 65: 3271–8. |
| Impaired kidney function | Gout | McAdams-DeMarco MA, Maynard JW, Baer AN, Coresh J. Hypertension and the risk of incident gout in a population-based study: the atherosclerosis risk in communities cohort. J Clin Hypertens (Greenwich) 2012; 14: 675–9. |
| Impaired kidney function | Gout | Trifirò G, Morabito P, Cavagna L, et al. Epidemiology of gout and hyperuricaemia in Italy during the years 2005-2009: a nationwide population-based study. Ann Rheum Dis 2013; 72: 694–700. |
| Short gestation for birth weight | Diarrheal diseases | Katz J, Lee AC, Kozuki N, Lawn JE, Cousens S, Blencowe H, Ezzati M, Bhutta ZA, Marchant T, Willey BA, Adair L, Barros F, Baqui AH, Christian P, Fawzi W, Gonzalez R, Humphrey J, Huybregts L, Kolsteren P, Mongkolchati A, Mullany LC, Ndyomugyenyi R, Nien JK, Osrin D, Roberfroid D, Sania A, Schmiegelow C, Silveira MF, Tielsch J, Vaidya A, Velaphi SC, Victora CG, Watson-Jones D, Black RE; CHERG Small-for-Gestational-Age-Preterm Birth Working Group. Mortality risk in preterm and small-for-gestational-age infants in low-income and middle-income countries: a pooled country analysis. Lancet. 2013; 382(9890): 417-25. |
| Short gestation for birth weight | Lower respiratory infections | Katz J, Lee AC, Kozuki N, Lawn JE, Cousens S, Blencowe H, Ezzati M, Bhutta ZA, Marchant T, Willey BA, Adair L, Barros F, Baqui AH, Christian P, Fawzi W, Gonzalez R, Humphrey J, Huybregts L, Kolsteren P, Mongkolchati A, Mullany LC, Ndyomugyenyi R, Nien JK, Osrin D, Roberfroid D, Sania A, Schmiegelow C, Silveira MF, Tielsch J, Vaidya A, Velaphi SC, Victora CG, Watson-Jones D, Black RE; CHERG Small-for-Gestational-Age-Preterm Birth Working Group. Mortality risk in preterm and small-for-gestational-age infants in low-income and middle-income countries: a pooled country analysis. Lancet. 2013; 382(9890): 417-25. |
| Short gestation for birth weight | Upper respiratory infections | Katz J, Lee AC, Kozuki N, Lawn JE, Cousens S, Blencowe H, Ezzati M, Bhutta ZA, Marchant T, Willey BA, Adair L, Barros F, Baqui AH, Christian P, Fawzi W, Gonzalez R, Humphrey J, Huybregts L, Kolsteren P, Mongkolchati A, Mullany LC, Ndyomugyenyi R, Nien JK, Osrin D, Roberfroid D, Sania A, Schmiegelow C, Silveira MF, Tielsch J, Vaidya A, Velaphi SC, Victora CG, Watson-Jones D, Black RE; CHERG Small-for-Gestational-Age-Preterm Birth Working Group. Mortality risk in preterm and small-for-gestational-age infants in low-income and middle-income countries: a pooled country analysis. Lancet. 2013; 382(9890): 417-25. |
| Short gestation for birth weight | Otitis media | Katz J, Lee AC, Kozuki N, Lawn JE, Cousens S, Blencowe H, Ezzati M, Bhutta ZA, Marchant T, Willey BA, Adair L, Barros F, Baqui AH, Christian P, Fawzi W, Gonzalez R, Humphrey J, Huybregts L, Kolsteren P, Mongkolchati A, Mullany LC, Ndyomugyenyi R, Nien JK, Osrin D, Roberfroid D, Sania A, Schmiegelow C, Silveira MF, Tielsch J, Vaidya A, Velaphi SC, Victora CG, Watson-Jones D, Black RE; CHERG Small-for-Gestational-Age-Preterm Birth Working Group. Mortality risk in preterm and small-for-gestational-age infants in low-income and middle-income countries: a pooled country analysis. Lancet. 2013; 382(9890): 417-25. |
| Short gestation for birth weight | Pneumococcal meningitis | Katz J, Lee AC, Kozuki N, Lawn JE, Cousens S, Blencowe H, Ezzati M, Bhutta ZA, Marchant T, Willey BA, Adair L, Barros F, Baqui AH, Christian P, Fawzi W, Gonzalez R, Humphrey J, Huybregts L, Kolsteren P, Mongkolchati A, Mullany LC, Ndyomugyenyi R, Nien JK, Osrin D, Roberfroid D, Sania A, Schmiegelow C, Silveira MF, Tielsch J, Vaidya A, Velaphi SC, Victora CG, Watson-Jones D, Black RE; CHERG Small-for-Gestational-Age-Preterm Birth Working Group. Mortality risk in preterm and small-for-gestational-age infants in low-income and middle-income countries: a pooled country analysis. Lancet. 2013; 382(9890): 417-25. |
| Short gestation for birth weight | H influenzae type B meningitis | Katz J, Lee AC, Kozuki N, Lawn JE, Cousens S, Blencowe H, Ezzati M, Bhutta ZA, Marchant T, Willey BA, Adair L, Barros F, Baqui AH, Christian P, Fawzi W, Gonzalez R, Humphrey J, Huybregts L, Kolsteren P, Mongkolchati A, Mullany LC, Ndyomugyenyi R, Nien JK, Osrin D, Roberfroid D, Sania A, Schmiegelow C, Silveira MF, Tielsch J, Vaidya A, Velaphi SC, Victora CG, Watson-Jones D, Black RE; CHERG Small-for-Gestational-Age-Preterm Birth Working Group. Mortality risk in preterm and small-for-gestational-age infants in low-income and middle-income countries: a pooled country analysis. Lancet. 2013; 382(9890): 417-25. |
| Short gestation for birth weight | Meningococcal meningitis | Katz J, Lee AC, Kozuki N, Lawn JE, Cousens S, Blencowe H, Ezzati M, Bhutta ZA, Marchant T, Willey BA, Adair L, Barros F, Baqui AH, Christian P, Fawzi W, Gonzalez R, Humphrey J, Huybregts L, Kolsteren P, Mongkolchati A, Mullany LC, Ndyomugyenyi R, Nien JK, Osrin D, Roberfroid D, Sania A, Schmiegelow C, Silveira MF, Tielsch J, Vaidya A, Velaphi SC, Victora CG, Watson-Jones D, Black RE; CHERG Small-for-Gestational-Age-Preterm Birth Working Group. Mortality risk in preterm and small-for-gestational-age infants in low-income and middle-income countries: a pooled country analysis. Lancet. 2013; 382(9890): 417-25. |
| Short gestation for birth weight | Other meningitis | Katz J, Lee AC, Kozuki N, Lawn JE, Cousens S, Blencowe H, Ezzati M, Bhutta ZA, Marchant T, Willey BA, Adair L, Barros F, Baqui AH, Christian P, Fawzi W, Gonzalez R, Humphrey J, Huybregts L, Kolsteren P, Mongkolchati A, Mullany LC, Ndyomugyenyi R, Nien JK, Osrin D, Roberfroid D, Sania A, Schmiegelow C, Silveira MF, Tielsch J, Vaidya A, Velaphi SC, Victora CG, Watson-Jones D, Black RE; CHERG Small-for-Gestational-Age-Preterm Birth Working Group. Mortality risk in preterm and small-for-gestational-age infants in low-income and middle-income countries: a pooled country analysis. Lancet. 2013; 382(9890): 417-25. |

**Appendix Table 5. Epidemiological evidence supporting causality between risk-outcome pairs included in the Global Burden of Disease 2017 study including A. Citations and B. Additional information**
**A. Citations**

| Risk | Outcome | Citation/Note |
|---|---|---|
| Short gestation for birth weight | Encephalitis | Katz J, Lee AC, Kozuki N, Lawn JE, Cousens S, Blencowe H, Ezzati M, Bhutta ZA, Marchant T, Willey BA, Adair L, Barros F, Baqui AH, Christian P, Fawzi W, Gonzalez R, Humphrey J, Huybregts L, Kolsteren P, Mongkolchati A, Mullany LC, Ndyomugyenyi R, Nien JK, Osrin D, Roberfroid D, Sania A, Schmiegelow C, Silveira MF, Tielsch J, Vaidya A, Velaphi SC, Victora CG, Watson-Jones D, Black RE; CHERG Small-for-Gestational-Age-Preterm Birth Working Group. Mortality risk in preterm and small-for-gestational-age infants in low-income and middle-income countries: a pooled country analysis. Lancet. 2013; 382(9890): 417-25. |
| Short gestation for birth weight | Neonatal preterm birth complications | Katz J, Lee AC, Kozuki N, Lawn JE, Cousens S, Blencowe H, Ezzati M, Bhutta ZA, Marchant T, Willey BA, Adair L, Barros F, Baqui AH, Christian P, Fawzi W, Gonzalez R, Humphrey J, Huybregts L, Kolsteren P, Mongkolchati A, Mullany LC, Ndyomugyenyi R, Nien JK, Osrin D, Roberfroid D, Sania A, Schmiegelow C, Silveira MF, Tielsch J, Vaidya A, Velaphi SC, Victora CG, Watson-Jones D, Black RE; CHERG Small-for-Gestational-Age-Preterm Birth Working Group. Mortality risk in preterm and small-for-gestational-age infants in low-income and middle-income countries: a pooled country analysis. Lancet. 2013; 382(9890): 417-25. |
| Short gestation for birth weight | Neonatal encephalopathy due to birth asphyxia and trauma | Katz J, Lee AC, Kozuki N, Lawn JE, Cousens S, Blencowe H, Ezzati M, Bhutta ZA, Marchant T, Willey BA, Adair L, Barros F, Baqui AH, Christian P, Fawzi W, Gonzalez R, Humphrey J, Huybregts L, Kolsteren P, Mongkolchati A, Mullany LC, Ndyomugyenyi R, Nien JK, Osrin D, Roberfroid D, Sania A, Schmiegelow C, Silveira MF, Tielsch J, Vaidya A, Velaphi SC, Victora CG, Watson-Jones D, Black RE; CHERG Small-for-Gestational-Age-Preterm Birth Working Group. Mortality risk in preterm and small-for-gestational-age infants in low-income and middle-income countries: a pooled country analysis. Lancet. 2013; 382(9890): 417-25. |
| Short gestation for birth weight | Neonatal sepsis and other neonatal infections | Katz J, Lee AC, Kozuki N, Lawn JE, Cousens S, Blencowe H, Ezzati M, Bhutta ZA, Marchant T, Willey BA, Adair L, Barros F, Baqui AH, Christian P, Fawzi W, Gonzalez R, Humphrey J, Huybregts L, Kolsteren P, Mongkolchati A, Mullany LC, Ndyomugyenyi R, Nien JK, Osrin D, Roberfroid D, Sania A, Schmiegelow C, Silveira MF, Tielsch J, Vaidya A, Velaphi SC, Victora CG, Watson-Jones D, Black RE; CHERG Small-for-Gestational-Age-Preterm Birth Working Group. Mortality risk in preterm and small-for-gestational-age infants in low-income and middle-income countries: a pooled country analysis. Lancet. 2013; 382(9890): 417-25. |
| Short gestation for birth weight | Hemolytic disease and other neonatal jaundice | Katz J, Lee AC, Kozuki N, Lawn JE, Cousens S, Blencowe H, Ezzati M, Bhutta ZA, Marchant T, Willey BA, Adair L, Barros F, Baqui AH, Christian P, Fawzi W, Gonzalez R, Humphrey J, Huybregts L, Kolsteren P, Mongkolchati A, Mullany LC, Ndyomugyenyi R, Nien JK, Osrin D, Roberfroid D, Sania A, Schmiegelow C, Silveira MF, Tielsch J, Vaidya A, Velaphi SC, Victora CG, Watson-Jones D, Black RE; CHERG Small-for-Gestational-Age-Preterm Birth Working Group. Mortality risk in preterm and small-for-gestational-age infants in low-income and middle-income countries: a pooled country analysis. Lancet. 2013; 382(9890): 417-25. |
| Short gestation for birth weight | Other neonatal disorders | Katz J, Lee AC, Kozuki N, Lawn JE, Cousens S, Blencowe H, Ezzati M, Bhutta ZA, Marchant T, Willey BA, Adair L, Barros F, Baqui AH, Christian P, Fawzi W, Gonzalez R, Humphrey J, Huybregts L, Kolsteren P, Mongkolchati A, Mullany LC, Ndyomugyenyi R, Nien JK, Osrin D, Roberfroid D, Sania A, Schmiegelow C, Silveira MF, Tielsch J, Vaidya A, Velaphi SC, Victora CG, Watson-Jones D, Black RE; CHERG Small-for-Gestational-Age-Preterm Birth Working Group. Mortality risk in preterm and small-for-gestational-age infants in low-income and middle-income countries: a pooled country analysis. Lancet. 2013; 382(9890): 417-25. |
| Short gestation for birth weight | Sudden infant death syndrome | Katz J, Lee AC, Kozuki N, Lawn JE, Cousens S, Blencowe H, Ezzati M, Bhutta ZA, Marchant T, Willey BA, Adair L, Barros F, Baqui AH, Christian P, Fawzi W, Gonzalez R, Humphrey J, Huybregts L, Kolsteren P, Mongkolchati A, Mullany LC, Ndyomugyenyi R, Nien JK, Osrin D, Roberfroid D, Sania A, Schmiegelow C, Silveira MF, Tielsch J, Vaidya A, Velaphi SC, Victora CG, Watson-Jones D, Black RE; CHERG Small-for-Gestational-Age-Preterm Birth Working Group. Mortality risk in preterm and small-for-gestational-age infants in low-income and middle-income countries: a pooled country analysis. Lancet. 2013; 382(9890): 417-25. |
| Low birth weight for gestation | Diarrheal diseases | Katz J, Lee AC, Kozuki N, Lawn JE, Cousens S, Blencowe H, Ezzati M, Bhutta ZA, Marchant T, Willey BA, Adair L, Barros F, Baqui AH, Christian P, Fawzi W, Gonzalez R, Humphrey J, Huybregts L, Kolsteren P, Mongkolchati A, Mullany LC, Ndyomugyenyi R, Nien JK, Osrin D, Roberfroid D, Sania A, Schmiegelow C, Silveira MF, Tielsch J, Vaidya A, Velaphi SC, Victora CG, Watson-Jones D, Black RE; CHERG Small-for-Gestational-Age-Preterm Birth Working Group. Mortality risk in preterm and small-for-gestational-age infants in low-income and middle-income countries: a pooled country analysis. Lancet. 2013; 382(9890): 417-25. |
| Low birth weight for gestation | Lower respiratory infections | Katz J, Lee AC, Kozuki N, Lawn JE, Cousens S, Blencowe H, Ezzati M, Bhutta ZA, Marchant T, Willey BA, Adair L, Barros F, Baqui AH, Christian P, Fawzi W, Gonzalez R, Humphrey J, Huybregts L, Kolsteren P, Mongkolchati A, Mullany LC, Ndyomugyenyi R, Nien JK, Osrin D, Roberfroid D, Sania A, Schmiegelow C, Silveira MF, Tielsch J, Vaidya A, Velaphi SC, Victora CG, Watson-Jones D, Black RE; CHERG Small-for-Gestational-Age-Preterm Birth Working Group. Mortality risk in preterm and small-for-gestational-age infants in low-income and middle-income countries: a pooled country analysis. Lancet. 2013; 382(9890): 417-25. |
| Low birth weight for gestation | Upper respiratory infections | Katz J, Lee AC, Kozuki N, Lawn JE, Cousens S, Blencowe H, Ezzati M, Bhutta ZA, Marchant T, Willey BA, Adair L, Barros F, Baqui AH, Christian P, Fawzi W, Gonzalez R, Humphrey J, Huybregts L, Kolsteren P, Mongkolchati A, Mullany LC, Ndyomugyenyi R, Nien JK, Osrin D, Roberfroid D, Sania A, Schmiegelow C, Silveira MF, Tielsch J, Vaidya A, Velaphi SC, Victora CG, Watson-Jones D, Black RE; CHERG Small-for-Gestational-Age-Preterm Birth Working Group. Mortality risk in preterm and small-for-gestational-age infants in low-income and middle-income countries: a pooled country analysis. Lancet. 2013; 382(9890): 417-25. |

| Appendix Table 5. Epidemiological evidence supporting causality between risk-outcome pairs included in the Global Burden of Disease 2017 study including A. Citations and B. Additional information<br>A. Citations | | |
|---|---|---|
| **Risk** | **Outcome** | **Citation/Note** |
| Low birth weight for gestation | Otitis media | Katz J, Lee AC, Kozuki N, Lawn JE, Cousens S, Blencowe H, Ezzati M, Bhutta ZA, Marchant T, Willey BA, Adair L, Barros F, Baqui AH, Christian P, Fawzi W, Gonzalez R, Humphrey J, Huybregts L, Kolsteren P, Mongkolchati A, Mullany LC, Ndyomugyenyi R, Nien JK, Osrin D, Roberfroid D, Sania A, Schmiegelow C, Silveira MF, Tielsch J, Vaidya A, Velaphi SC, Victora CG, Watson-Jones D, Black RE; CHERG Small-for-Gestational-Age-Preterm Birth Working Group. Mortality risk in preterm and small-for-gestational-age infants in low-income and middle-income countries: a pooled country analysis. Lancet. 2013; 382(9890): 417-25. |
| Low birth weight for gestation | Pneumococcal meningitis | Katz J, Lee AC, Kozuki N, Lawn JE, Cousens S, Blencowe H, Ezzati M, Bhutta ZA, Marchant T, Willey BA, Adair L, Barros F, Baqui AH, Christian P, Fawzi W, Gonzalez R, Humphrey J, Huybregts L, Kolsteren P, Mongkolchati A, Mullany LC, Ndyomugyenyi R, Nien JK, Osrin D, Roberfroid D, Sania A, Schmiegelow C, Silveira MF, Tielsch J, Vaidya A, Velaphi SC, Victora CG, Watson-Jones D, Black RE; CHERG Small-for-Gestational-Age-Preterm Birth Working Group. Mortality risk in preterm and small-for-gestational-age infants in low-income and middle-income countries: a pooled country analysis. Lancet. 2013; 382(9890): 417-25. |
| Low birth weight for gestation | H influenzae type B meningitis | Katz J, Lee AC, Kozuki N, Lawn JE, Cousens S, Blencowe H, Ezzati M, Bhutta ZA, Marchant T, Willey BA, Adair L, Barros F, Baqui AH, Christian P, Fawzi W, Gonzalez R, Humphrey J, Huybregts L, Kolsteren P, Mongkolchati A, Mullany LC, Ndyomugyenyi R, Nien JK, Osrin D, Roberfroid D, Sania A, Schmiegelow C, Silveira MF, Tielsch J, Vaidya A, Velaphi SC, Victora CG, Watson-Jones D, Black RE; CHERG Small-for-Gestational-Age-Preterm Birth Working Group. Mortality risk in preterm and small-for-gestational-age infants in low-income and middle-income countries: a pooled country analysis. Lancet. 2013; 382(9890): 417-25. |
| Low birth weight for gestation | Meningococcal meningitis | Katz J, Lee AC, Kozuki N, Lawn JE, Cousens S, Blencowe H, Ezzati M, Bhutta ZA, Marchant T, Willey BA, Adair L, Barros F, Baqui AH, Christian P, Fawzi W, Gonzalez R, Humphrey J, Huybregts L, Kolsteren P, Mongkolchati A, Mullany LC, Ndyomugyenyi R, Nien JK, Osrin D, Roberfroid D, Sania A, Schmiegelow C, Silveira MF, Tielsch J, Vaidya A, Velaphi SC, Victora CG, Watson-Jones D, Black RE; CHERG Small-for-Gestational-Age-Preterm Birth Working Group. Mortality risk in preterm and small-for-gestational-age infants in low-income and middle-income countries: a pooled country analysis. Lancet. 2013; 382(9890): 417-25. |
| Low birth weight for gestation | Other meningitis | Katz J, Lee AC, Kozuki N, Lawn JE, Cousens S, Blencowe H, Ezzati M, Bhutta ZA, Marchant T, Willey BA, Adair L, Barros F, Baqui AH, Christian P, Fawzi W, Gonzalez R, Humphrey J, Huybregts L, Kolsteren P, Mongkolchati A, Mullany LC, Ndyomugyenyi R, Nien JK, Osrin D, Roberfroid D, Sania A, Schmiegelow C, Silveira MF, Tielsch J, Vaidya A, Velaphi SC, Victora CG, Watson-Jones D, Black RE; CHERG Small-for-Gestational-Age-Preterm Birth Working Group. Mortality risk in preterm and small-for-gestational-age infants in low-income and middle-income countries: a pooled country analysis. Lancet. 2013; 382(9890): 417-25. |
| Low birth weight for gestation | Encephalitis | Katz J, Lee AC, Kozuki N, Lawn JE, Cousens S, Blencowe H, Ezzati M, Bhutta ZA, Marchant T, Willey BA, Adair L, Barros F, Baqui AH, Christian P, Fawzi W, Gonzalez R, Humphrey J, Huybregts L, Kolsteren P, Mongkolchati A, Mullany LC, Ndyomugyenyi R, Nien JK, Osrin D, Roberfroid D, Sania A, Schmiegelow C, Silveira MF, Tielsch J, Vaidya A, Velaphi SC, Victora CG, Watson-Jones D, Black RE; CHERG Small-for-Gestational-Age-Preterm Birth Working Group. Mortality risk in preterm and small-for-gestational-age infants in low-income and middle-income countries: a pooled country analysis. Lancet. 2013; 382(9890): 417-25. |
| Low birth weight for gestation | Neonatal preterm birth complications | Katz J, Lee AC, Kozuki N, Lawn JE, Cousens S, Blencowe H, Ezzati M, Bhutta ZA, Marchant T, Willey BA, Adair L, Barros F, Baqui AH, Christian P, Fawzi W, Gonzalez R, Humphrey J, Huybregts L, Kolsteren P, Mongkolchati A, Mullany LC, Ndyomugyenyi R, Nien JK, Osrin D, Roberfroid D, Sania A, Schmiegelow C, Silveira MF, Tielsch J, Vaidya A, Velaphi SC, Victora CG, Watson-Jones D, Black RE; CHERG Small-for-Gestational-Age-Preterm Birth Working Group. Mortality risk in preterm and small-for-gestational-age infants in low-income and middle-income countries: a pooled country analysis. Lancet. 2013; 382(9890): 417-25. |
| Low birth weight for gestation | Neonatal encephalopathy due to birth asphyxia and trauma | Katz J, Lee AC, Kozuki N, Lawn JE, Cousens S, Blencowe H, Ezzati M, Bhutta ZA, Marchant T, Willey BA, Adair L, Barros F, Baqui AH, Christian P, Fawzi W, Gonzalez R, Humphrey J, Huybregts L, Kolsteren P, Mongkolchati A, Mullany LC, Ndyomugyenyi R, Nien JK, Osrin D, Roberfroid D, Sania A, Schmiegelow C, Silveira MF, Tielsch J, Vaidya A, Velaphi SC, Victora CG, Watson-Jones D, Black RE; CHERG Small-for-Gestational-Age-Preterm Birth Working Group. Mortality risk in preterm and small-for-gestational-age infants in low-income and middle-income countries: a pooled country analysis. Lancet. 2013; 382(9890): 417-25. |
| Low birth weight for gestation | Neonatal sepsis and other neonatal infections | Katz J, Lee AC, Kozuki N, Lawn JE, Cousens S, Blencowe H, Ezzati M, Bhutta ZA, Marchant T, Willey BA, Adair L, Barros F, Baqui AH, Christian P, Fawzi W, Gonzalez R, Humphrey J, Huybregts L, Kolsteren P, Mongkolchati A, Mullany LC, Ndyomugyenyi R, Nien JK, Osrin D, Roberfroid D, Sania A, Schmiegelow C, Silveira MF, Tielsch J, Vaidya A, Velaphi SC, Victora CG, Watson-Jones D, Black RE; CHERG Small-for-Gestational-Age-Preterm Birth Working Group. Mortality risk in preterm and small-for-gestational-age infants in low-income and middle-income countries: a pooled country analysis. Lancet. 2013; 382(9890): 417-25. |
| Low birth weight for gestation | Hemolytic disease and other neonatal jaundice | Katz J, Lee AC, Kozuki N, Lawn JE, Cousens S, Blencowe H, Ezzati M, Bhutta ZA, Marchant T, Willey BA, Adair L, Barros F, Baqui AH, Christian P, Fawzi W, Gonzalez R, Humphrey J, Huybregts L, Kolsteren P, Mongkolchati A, Mullany LC, Ndyomugyenyi R, Nien JK, Osrin D, Roberfroid D, Sania A, Schmiegelow C, Silveira MF, Tielsch J, Vaidya A, Velaphi SC, Victora CG, Watson-Jones D, Black RE; CHERG Small-for-Gestational-Age-Preterm Birth Working Group. Mortality risk in preterm and small-for-gestational-age infants in low-income and middle-income countries: a pooled country analysis. Lancet. 2013; 382(9890): 417-25. |

**Appendix Table 5. Epidemiological evidence supporting causality between risk-outcome pairs included in the Global Burden of Disease 2017 study including A. Citations and B. Additional information**
**A. Citations**

| Risk | Outcome | Citation/Note |
|---|---|---|
| Low birth weight for gestation | Other neonatal disorders | Katz J, Lee AC, Kozuki N, Lawn JE, Cousens S, Blencowe H, Ezzati M, Bhutta ZA, Marchant T, Willey BA, Adair L, Barros F, Baqui AH, Christian P, Fawzi W, Gonzalez R, Humphrey J, Huybregts L, Kolsteren P, Mongkolchati A, Mullany LC, Ndyomugyenyi R, Nien JK, Osrin D, Roberfroid D, Sania A, Schmiegelow C, Silveira MF, Tielsch J, Vaidya A, Velaphi SC, Victora CG, Watson-Jones D, Black RE; CHERG Small-for-Gestational-Age-Preterm Birth Working Group. Mortality risk in preterm and small-for-gestational-age infants in low-income and middle-income countries: a pooled country analysis. Lancet. 2013; 382(9890): 417-25. |
| Low birth weight for gestation | Sudden infant death syndrome | Katz J, Lee AC, Kozuki N, Lawn JE, Cousens S, Blencowe H, Ezzati M, Bhutta ZA, Marchant T, Willey BA, Adair L, Barros F, Baqui AH, Christian P, Fawzi W, Gonzalez R, Humphrey J, Huybregts L, Kolsteren P, Mongkolchati A, Mullany LC, Ndyomugyenyi R, Nien JK, Osrin D, Roberfroid D, Sania A, Schmiegelow C, Silveira MF, Tielsch J, Vaidya A, Velaphi SC, Victora CG, Watson-Jones D, Black RE; CHERG Small-for-Gestational-Age-Preterm Birth Working Group. Mortality risk in preterm and small-for-gestational-age infants in low-income and middle-income countries: a pooled country analysis. Lancet. 2013; 382(9890): 417-25. |
| Low birth weight and short gestation | Diarrheal diseases | Katz J, Lee AC, Kozuki N, Lawn JE, Cousens S, Blencowe H, Ezzati M, Bhutta ZA, Marchant T, Willey BA, Adair L, Barros F, Baqui AH, Christian P, Fawzi W, Gonzalez R, Humphrey J, Huybregts L, Kolsteren P, Mongkolchati A, Mullany LC, Ndyomugyenyi R, Nien JK, Osrin D, Roberfroid D, Sania A, Schmiegelow C, Silveira MF, Tielsch J, Vaidya A, Velaphi SC, Victora CG, Watson-Jones D, Black RE; CHERG Small-for-Gestational-Age-Preterm Birth Working Group. Mortality risk in preterm and small-for-gestational-age infants in low-income and middle-income countries: a pooled country analysis. Lancet. 2013; 382(9890): 417-25. |
| Low birth weight and short gestation | Lower respiratory infections | Katz J, Lee AC, Kozuki N, Lawn JE, Cousens S, Blencowe H, Ezzati M, Bhutta ZA, Marchant T, Willey BA, Adair L, Barros F, Baqui AH, Christian P, Fawzi W, Gonzalez R, Humphrey J, Huybregts L, Kolsteren P, Mongkolchati A, Mullany LC, Ndyomugyenyi R, Nien JK, Osrin D, Roberfroid D, Sania A, Schmiegelow C, Silveira MF, Tielsch J, Vaidya A, Velaphi SC, Victora CG, Watson-Jones D, Black RE; CHERG Small-for-Gestational-Age-Preterm Birth Working Group. Mortality risk in preterm and small-for-gestational-age infants in low-income and middle-income countries: a pooled country analysis. Lancet. 2013; 382(9890): 417-25. |
| Low birth weight and short gestation | Upper respiratory infections | Katz J, Lee AC, Kozuki N, Lawn JE, Cousens S, Blencowe H, Ezzati M, Bhutta ZA, Marchant T, Willey BA, Adair L, Barros F, Baqui AH, Christian P, Fawzi W, Gonzalez R, Humphrey J, Huybregts L, Kolsteren P, Mongkolchati A, Mullany LC, Ndyomugyenyi R, Nien JK, Osrin D, Roberfroid D, Sania A, Schmiegelow C, Silveira MF, Tielsch J, Vaidya A, Velaphi SC, Victora CG, Watson-Jones D, Black RE; CHERG Small-for-Gestational-Age-Preterm Birth Working Group. Mortality risk in preterm and small-for-gestational-age infants in low-income and middle-income countries: a pooled country analysis. Lancet. 2013; 382(9890): 417-25. |
| Low birth weight and short gestation | Otitis media | Katz J, Lee AC, Kozuki N, Lawn JE, Cousens S, Blencowe H, Ezzati M, Bhutta ZA, Marchant T, Willey BA, Adair L, Barros F, Baqui AH, Christian P, Fawzi W, Gonzalez R, Humphrey J, Huybregts L, Kolsteren P, Mongkolchati A, Mullany LC, Ndyomugyenyi R, Nien JK, Osrin D, Roberfroid D, Sania A, Schmiegelow C, Silveira MF, Tielsch J, Vaidya A, Velaphi SC, Victora CG, Watson-Jones D, Black RE; CHERG Small-for-Gestational-Age-Preterm Birth Working Group. Mortality risk in preterm and small-for-gestational-age infants in low-income and middle-income countries: a pooled country analysis. Lancet. 2013; 382(9890): 417-25. |
| Low birth weight and short gestation | Pneumococcal meningitis | Katz J, Lee AC, Kozuki N, Lawn JE, Cousens S, Blencowe H, Ezzati M, Bhutta ZA, Marchant T, Willey BA, Adair L, Barros F, Baqui AH, Christian P, Fawzi W, Gonzalez R, Humphrey J, Huybregts L, Kolsteren P, Mongkolchati A, Mullany LC, Ndyomugyenyi R, Nien JK, Osrin D, Roberfroid D, Sania A, Schmiegelow C, Silveira MF, Tielsch J, Vaidya A, Velaphi SC, Victora CG, Watson-Jones D, Black RE; CHERG Small-for-Gestational-Age-Preterm Birth Working Group. Mortality risk in preterm and small-for-gestational-age infants in low-income and middle-income countries: a pooled country analysis. Lancet. 2013; 382(9890): 417-25. |
| Low birth weight and short gestation | H influenzae type B meningitis | Katz J, Lee AC, Kozuki N, Lawn JE, Cousens S, Blencowe H, Ezzati M, Bhutta ZA, Marchant T, Willey BA, Adair L, Barros F, Baqui AH, Christian P, Fawzi W, Gonzalez R, Humphrey J, Huybregts L, Kolsteren P, Mongkolchati A, Mullany LC, Ndyomugyenyi R, Nien JK, Osrin D, Roberfroid D, Sania A, Schmiegelow C, Silveira MF, Tielsch J, Vaidya A, Velaphi SC, Victora CG, Watson-Jones D, Black RE; CHERG Small-for-Gestational-Age-Preterm Birth Working Group. Mortality risk in preterm and small-for-gestational-age infants in low-income and middle-income countries: a pooled country analysis. Lancet. 2013; 382(9890): 417-25. |
| Low birth weight and short gestation | Meningococcal meningitis | Katz J, Lee AC, Kozuki N, Lawn JE, Cousens S, Blencowe H, Ezzati M, Bhutta ZA, Marchant T, Willey BA, Adair L, Barros F, Baqui AH, Christian P, Fawzi W, Gonzalez R, Humphrey J, Huybregts L, Kolsteren P, Mongkolchati A, Mullany LC, Ndyomugyenyi R, Nien JK, Osrin D, Roberfroid D, Sania A, Schmiegelow C, Silveira MF, Tielsch J, Vaidya A, Velaphi SC, Victora CG, Watson-Jones D, Black RE; CHERG Small-for-Gestational-Age-Preterm Birth Working Group. Mortality risk in preterm and small-for-gestational-age infants in low-income and middle-income countries: a pooled country analysis. Lancet. 2013; 382(9890): 417-25. |
| Low birth weight and short gestation | Other meningitis | Katz J, Lee AC, Kozuki N, Lawn JE, Cousens S, Blencowe H, Ezzati M, Bhutta ZA, Marchant T, Willey BA, Adair L, Barros F, Baqui AH, Christian P, Fawzi W, Gonzalez R, Humphrey J, Huybregts L, Kolsteren P, Mongkolchati A, Mullany LC, Ndyomugyenyi R, Nien JK, Osrin D, Roberfroid D, Sania A, Schmiegelow C, Silveira MF, Tielsch J, Vaidya A, Velaphi SC, Victora CG, Watson-Jones D, Black RE; CHERG Small-for-Gestational-Age-Preterm Birth Working Group. Mortality risk in preterm and small-for-gestational-age infants in low-income and middle-income countries: a pooled country analysis. Lancet. 2013; 382(9890): 417-25. |

**Appendix Table 5. Epidemiological evidence supporting causality between risk-outcome pairs included in the Global Burden of Disease 2017 study including A. Citations and B. Additional information**

**A. Citations**

| Risk | Outcome | Citation/Note |
|---|---|---|
| Low birth weight and short gestation | Encephalitis | Katz J, Lee AC, Kozuki N, Lawn JE, Cousens S, Blencowe H, Ezzati M, Bhutta ZA, Marchant T, Willey BA, Adair L, Barros F, Baqui AH, Christian P, Fawzi W, Gonzalez R, Humphrey J, Huybregts L, Kolsteren P, Mongkolchati A, Mullany LC, Ndyomugyenyi R, Nien JK, Osrin D, Roberfroid D, Sania A, Schmiegelow C, Silveira MF, Tielsch J, Vaidya A, Velaphi SC, Victora CG, Watson-Jones D, Black RE; CHERG Small-for-Gestational-Age-Preterm Birth Working Group. Mortality risk in preterm and small-for-gestational-age infants in low-income and middle-income countries: a pooled country analysis. Lancet. 2013; 382(9890): 417-25. |
| Low birth weight and short gestation | Neonatal preterm birth complications | Katz J, Lee AC, Kozuki N, Lawn JE, Cousens S, Blencowe H, Ezzati M, Bhutta ZA, Marchant T, Willey BA, Adair L, Barros F, Baqui AH, Christian P, Fawzi W, Gonzalez R, Humphrey J, Huybregts L, Kolsteren P, Mongkolchati A, Mullany LC, Ndyomugyenyi R, Nien JK, Osrin D, Roberfroid D, Sania A, Schmiegelow C, Silveira MF, Tielsch J, Vaidya A, Velaphi SC, Victora CG, Watson-Jones D, Black RE; CHERG Small-for-Gestational-Age-Preterm Birth Working Group. Mortality risk in preterm and small-for-gestational-age infants in low-income and middle-income countries: a pooled country analysis. Lancet. 2013; 382(9890): 417-25. |
| Low birth weight and short gestation | Neonatal encephalopathy due to birth asphyxia and trauma | Katz J, Lee AC, Kozuki N, Lawn JE, Cousens S, Blencowe H, Ezzati M, Bhutta ZA, Marchant T, Willey BA, Adair L, Barros F, Baqui AH, Christian P, Fawzi W, Gonzalez R, Humphrey J, Huybregts L, Kolsteren P, Mongkolchati A, Mullany LC, Ndyomugyenyi R, Nien JK, Osrin D, Roberfroid D, Sania A, Schmiegelow C, Silveira MF, Tielsch J, Vaidya A, Velaphi SC, Victora CG, Watson-Jones D, Black RE; CHERG Small-for-Gestational-Age-Preterm Birth Working Group. Mortality risk in preterm and small-for-gestational-age infants in low-income and middle-income countries: a pooled country analysis. Lancet. 2013; 382(9890): 417-25. |
| Low birth weight and short gestation | Neonatal sepsis and other neonatal infections | Katz J, Lee AC, Kozuki N, Lawn JE, Cousens S, Blencowe H, Ezzati M, Bhutta ZA, Marchant T, Willey BA, Adair L, Barros F, Baqui AH, Christian P, Fawzi W, Gonzalez R, Humphrey J, Huybregts L, Kolsteren P, Mongkolchati A, Mullany LC, Ndyomugyenyi R, Nien JK, Osrin D, Roberfroid D, Sania A, Schmiegelow C, Silveira MF, Tielsch J, Vaidya A, Velaphi SC, Victora CG, Watson-Jones D, Black RE; CHERG Small-for-Gestational-Age-Preterm Birth Working Group. Mortality risk in preterm and small-for-gestational-age infants in low-income and middle-income countries: a pooled country analysis. Lancet. 2013; 382(9890): 417-25. |
| Low birth weight and short gestation | Hemolytic disease and other neonatal jaundice | Katz J, Lee AC, Kozuki N, Lawn JE, Cousens S, Blencowe H, Ezzati M, Bhutta ZA, Marchant T, Willey BA, Adair L, Barros F, Baqui AH, Christian P, Fawzi W, Gonzalez R, Humphrey J, Huybregts L, Kolsteren P, Mongkolchati A, Mullany LC, Ndyomugyenyi R, Nien JK, Osrin D, Roberfroid D, Sania A, Schmiegelow C, Silveira MF, Tielsch J, Vaidya A, Velaphi SC, Victora CG, Watson-Jones D, Black RE; CHERG Small-for-Gestational-Age-Preterm Birth Working Group. Mortality risk in preterm and small-for-gestational-age infants in low-income and middle-income countries: a pooled country analysis. Lancet. 2013; 382(9890): 417-25. |
| Low birth weight and short gestation | Other neonatal disorders | Katz J, Lee AC, Kozuki N, Lawn JE, Cousens S, Blencowe H, Ezzati M, Bhutta ZA, Marchant T, Willey BA, Adair L, Barros F, Baqui AH, Christian P, Fawzi W, Gonzalez R, Humphrey J, Huybregts L, Kolsteren P, Mongkolchati A, Mullany LC, Ndyomugyenyi R, Nien JK, Osrin D, Roberfroid D, Sania A, Schmiegelow C, Silveira MF, Tielsch J, Vaidya A, Velaphi SC, Victora CG, Watson-Jones D, Black RE; CHERG Small-for-Gestational-Age-Preterm Birth Working Group. Mortality risk in preterm and small-for-gestational-age infants in low-income and middle-income countries: a pooled country analysis. Lancet. 2013; 382(9890): 417-25. |
| Low birth weight and short gestation | Sudden infant death syndrome | Katz J, Lee AC, Kozuki N, Lawn JE, Cousens S, Blencowe H, Ezzati M, Bhutta ZA, Marchant T, Willey BA, Adair L, Barros F, Baqui AH, Christian P, Fawzi W, Gonzalez R, Humphrey J, Huybregts L, Kolsteren P, Mongkolchati A, Mullany LC, Ndyomugyenyi R, Nien JK, Osrin D, Roberfroid D, Sania A, Schmiegelow C, Silveira MF, Tielsch J, Vaidya A, Velaphi SC, Victora CG, Watson-Jones D, Black RE; CHERG Small-for-Gestational-Age-Preterm Birth Working Group. Mortality risk in preterm and small-for-gestational-age infants in low-income and middle-income countries: a pooled country analysis. Lancet. 2013; 382(9890): 417-25. |

**Appendix Table 5. Epidemiological evidence supporting causality between risk-outcome pairs included in the Global Burden of Disease 2017 study including A. Citations and B. Additional information**
**A. Citations**

| Risk | Outcome | Citation/Note |
|---|---|---|

**6B. Supplemental information**

“RCTs (Number)” represents the total number of independent randomized controlled trials evaluating the relationship of each risk-outcome pair. “RCTs with significant effect in the opposite direction (%)” represents the percentage of randomized controlled trials showing a significant effect in the opposite direction. “Prospective observational studies (Number)” shows the total number of independent prospective cohort studies or non-randomized interventions evaluating the relationship of the risk-outcome pair. “Prospective observational studies with significant association in the opposite direction (%)” represents the percentage of prospective cohort studies or non-randomized interventions reporting a significant association in the opposite direction. “Lower limit of RR > 1.5” shows whether the lower limit of the 95% confidence interval for the relative risk of the risk-outcome pair is greater than 1.5. “Dose-response relationship” shows whether there is any evidence of linear or non-linear dose-response relationship between the risk and the outcome. “Biologic plausibility” shows whether there is any biologic or mechanistic pathway that could potentially explain the relationship of the risk-outcome pair. “Analogy” shows whether the risk is associated with another outcome from the same category and there is evidence that it can cause the current outcome through the same pathway. The numbers in the table represent the independent RCTs and prospective observational studies evaluated the relationship between each risk-outcome pairs. If there were multiple reports from one study, they were counted as one study. Dose-response relationship was only assessed for continuous risks. To evaluate the magnitude of the effect size for continuous risks, we evaluated the RR comparing the 75th percentile to the 25th percentile of the exposure distribution at the global level .

| | | |
|---|---|---|
| Unsafe water, sanitation, and handwashing | Typhoid and paratyphoid fever | Typhoid and paratyphoid were included as outcome for unsafe water and sanitation by analogy to diarrhoeal diseases |
| Household air pollution from solid fuels | Cataract | Evidence on the relationship between household air pollution and cataract was from 6 case-control and 1 cross-sectional studies |
| Air pollution | -- | The relationships of cerebrovascular disease, chronic obstructive pulmonary disease, ischaemic heart disease, and lung cancer with ambient air pollution, second-hand smoke, and active smoking were used to interpolate their relationship with household air pollution. We considered the biological pathway for health impact of all four sources to be PM2.5 exposure, with the effect size being a function of the level of PM2.5. As such, we presented data from cohorts reporting on ambient PM2.5 and the outcome was used to inform the strength of evidence for household air pollution. |
| Other environmental risks and dietary risks | Cardiovascular diseases and chronic kidney disease | The health effects of lead and sodium on cardiovascular outcomes and chronic kidney disease were assessed through systolic blood pressure and the health effects of sugar sweetened beverages were assessed through body mass index. |
| Residential Radon | Tracheal, bronchus, and lung cancer | In evaluation of evidence on the relationship of residential radon and lung cancer, we excluded evidence from cohorts of miners as they were not from a representative population. Evidence on this risk-outcome pair mostly comes from case-control studies |
| Occupational injuries | Injuries | Evidence from International Labour Organization Safety and Health and Eurostat Safety and Health was used to establish causality between occupational injuries and injuries |
| Child and maternal malnutrition | -- | Evidence on the causal relationship of childhood stunting, underweight, and wasting was from a pooled analysis of 7 prospective cohorts |
| Child and maternal malnutrition | -- | For the following risk-outcome pairs, the risk factor was considered as the necessary cause: childhood underweight and protein-energy malnutrition; childhood wasting and protein-energy malnutrition; vitamin A deficiency and vitamin A deficiency; alcohol use and cirrhosis due to alcohol use; alcohol use and alcohol use disorders; alcohol use and liver cancer due to alcohol use; drug use and amphetamine use disorders; drug use and cannabis use disorders; drug use and cocaine use disorders; drug use and opioid use disorders; drug use and other drug use disorders; iron deficiency and iron-deficiency anemia; unsafe sex and cervical cancer; unsafe sex and syphilis; unsafe sex and chlamydial infection; unsafe sex and gonococcal infection; unsafe sex and trichomoniasis; unsafe sex and genital herpes; unsafe sex and other sexually transmitted diseases; high systolic blood pressure and hypertensive heart disease; high systolic blood pressure and chronic kidney disease due to hypertension; high fasting plasma glucose and chronic kidney disease due to diabetes mellitus; high fasting plasma glucose and diabetes mellitus; low glomerular filtration rate and chronic kidney disease |
| Iron deficiency | Maternal haemorrhage | Evidence on the relationship of iron deficiency with maternal haemorrhage and maternal sepsis mainly came 10 observational studies evaluating the association between low hemoglobin and maternal mortality using hospital records |
| Smoking, alcohol use, and high body mass index | -- | For smoking, alcohol use, and high body mass index evidence from risk reduction trials has not been included |
| Smoking, alcohol use, and high body mass index | Liver cancer | Liver cancer included liver cancer due to alcohol use, hepatitis B, hepatitis C, and other causes |
| Smoking | Lower respiratory infections | Evidence on the relationship between smoking and lower respiratory infections comes 10 case-control or cross-sectional studies |
| Smoking, alcohol use | Nasopharynx cancer | The evidence on causal relationship of alcohol and smoking with nasopharynx cancer was from the studies evaluating oral cavity and pharyngeal cancers as outcome |
| Smoking | Bladder cancer | The evidence on causal relationship of smoking and bladder cancer was based on the studies evaluating the lower urinary tract as outcome |
| Smoking | Asbestosis | Asbestosis, coal workers pneumoconiosis, other pneumoconiosis, silicosis were included as outcomes for smoking as they were included in the other chronic respiratory diseases category |
| Alcohol use | Ischaemic heart disease, cerebrovascular disease, hypertensive heart disease, and diabetes mellitus | Alcohol was included as both a protective and harmful risk factor for ischaemic heart disease, cerebrovascular disease, hypertensive heart disease, and diabetes mellitus |
| Alcohol use | Cirrhosis | Cirrhosis included cirrhosis due to alcohol use, hepatitis B, hepatitis C, and other causes |
| Alcohol use | Self-harm | Self-harm was included as an outcome for alcohol use by analogy to injury |
| Alcohol use | Injuries | Injuries included pedestrian road injuries, cyclist road injuries, motorcyclist road injuries, motor vehicle road injuries, drowning, falls, fire, heat, hot substances, poisonings, unintentional firearm injuries, unintentional suffocation, other exposure to mechanical forces |
| Alcohol use | Interpersonal violence | Interpersonal violence included assault by firearm, sharp object, other means |
| Diet low in nuts and seeds | Ischaemic heart disease and diabetes mellitus | Experimental evidence on the relationship of nuts with ischaemic heart disease and diabetes mellitus come from the PREDIMED trial; a randomized trial consisting of three arms: a Mediterranean diet with extra-virgin olive oil, a Mediterranean diet with nuts, and a control diet. Given that the intake of dietary factors other than nuts changed in the intervention arms of this trial, the observed effect might be fully attributable to nuts. |

**Appendix Table 5. Epidemiological evidence supporting causality between risk-outcome pairs included in the Global Burden of Disease 2017 study including A. Citations and B. Additional information**

**A. Citations**

| Risk | Outcome | Citation/Note |
|---|---|---|
| Diet high in sugar sweetened beverages and body mass index | -- | Evidence on the relationship between sugar-sweetened beverages and body mass index comes from the interventional and prospective observational studies evaluating the relationship of sugar-sweetened beverages with weight change among children and adults. |
| Diet high in sodium | Cardiovascular diseases | Evidence on the direct effect of sodium on cardiovascular disease mainly comes from prospective cohort studies. Considering that, in GBD, we have only evaluated the effect of sodium mediated through systolic blood pressure, we did not present epidemiologic evidence on the direct effect of sodium on cardiovascular disease in this table. Evidence on the effect of sodium on systolic blood pressure mostly comes from randomized controlled trials. While some cohort studies evaluated the relationship between sodium and systolic blood pressure, we did not identify a systematic evaluation of these studies. |
| Drug use | Hepatitis B and C | We included liver cancer due to Hepatitis B and Hepatitis C and cirrhosis due to Hepatitis B and Hepatitis C as outcomes for drug use because these were considered secondary outcomes of Hepatitis B and Hepatitis C. |
| Drug use, unsafe sex | HIV/AIDS | For the following risk-outcome pairs, the risk factor was considered as the sufficient cause: drug use and HIV/AIDS and unsafe sex and HIV/AIDS |
| Metabolic risks | Chronic kidney disease | Chronic kidney disease included chronic kidney disease due to diabetes mellitus, hypertension, glomerulonephritis, or other causes |
| High fasting plasma glucose | Cerebrovascular disease, chronic kidney disease, ischaemic heart disease | Evidence on the relationship of high fasting plasma glucose with stroke (DECODE, APCSC, ERFC); chronic kidney disease (APCSC), and ischaemic heart disease (DECODE, APCSC, ERFC) was from pooled analysis of cohorts |
| High systolic blood pressure | Atrial fibrillation and flutter, peripheral vascular disease | Evidence on the relationship of high systolic blood pressure with atrial fibrillation and peripheral vascular disease was from two pooled cohort analysis (APCSC and PSC) |
| High systolic blood pressure | Rheumatic heart disease, cardiomyopathy and myocarditis, aortic aneurysm, endocarditis, and other cardiovascular diseases | Evidence on the relationship of high systolic blood pressure with rheumatic heart disease, cardiomyopathy and myocarditis, aortic aneurysm, endocarditis, and other cardiovascular diseases came from a pooled cohort analysis (PSC) |
| High body-mass index | Ischaemic heart disease | Evidence on the relationship of high body-mass index with ischaemic heart disease (APCSC, ERFC, PSC) and stroke (ischaemic: APCSC, ERFC, PSC; hemorrhagic: PSC and ERFC) came from three pooled cohort analysis |
| High body-mass index | Diabetes mellitus, hypertensive heart disease | Evidence on the relationship of high body-mass index with diabetes mellitus and hypertensive heart disease came from two pooled cohort analysis (APCSC and PSC) |
| High body-mass index | Chronic kidney disease | Evidence on the relationship of high body-mass index with chronic kidney disease was from a pooled cohort analysis (PSC) |
| High LDL cholesterol | Ischaemic heart disease, ischaemic stroke | Evidence on the relationship of high LDL cholesterol with ischaemic heart disease and ischaemic stroke came from two pooled cohort analysis (APCSC and PSC) |
| Impaired kidney function | Tuberculosis | Glycemic Control and the Risk of Tuberculosis: A Cohort Study. |

**Appendix Table 6a. Relative risks used by age and sex for each outcome for all risk factors except for ambient air pollution alcohol, and smoking.**

| | | | | | Ages | | | | | | | | | | | | | | | | | | | | | | |
|---|---|---|---|---|---|---|---|---|---|---|---|---|---|---|---|---|---|---|---|---|---|---|---|---|---|---|---|
| Risk - Outcome | Category / Units | Morbidity / Mortality | Sex | All-age | 0-6 days | 7-27 days | 28-364 days | 1-4 years | 5-9 years | 10-14 years | 15-19 years | 20-24 years | 25-29 years | 30-34 years | 35-39 years | 40-44 years | 45-49 years | 50-54 years | 55-59 years | 60-64 years | 65-69 years | 70-74 years | 75-79 years | 80-84 years | 85-89 years | 90-94 years | 95+ years |
| **Unsafe water source** | | | | | | | | | | | | | | | | | | | | | | | | | | | |
| Diarrhoeal diseases | Unimproved, untreated | Both | Both | | 11.501 (2.761 to 31.282) | 11.501 (2.761 to 31.282) | 11.501 (2.761 to 31.282) | 11.501 (2.761 to 31.282) | 11.501 (2.761 to 31.282) | 11.501 (2.761 to 31.282) | 11.501 (2.761 to 31.282) | 11.501 (2.761 to 31.282) | 11.501 (2.761 to 31.282) | 11.501 (2.761 to 31.282) | 11.501 (2.761 to 31.282) | 11.501 (2.761 to 31.282) | 11.501 (2.761 to 31.282) | 11.501 (2.761 to 31.282) | 11.501 (2.761 to 31.282) | 11.501 (2.761 to 31.282) | 11.501 (2.761 to 31.282) | 11.501 (2.761 to 31.282) | 11.501 (2.761 to 31.282) | 11.501 (2.761 to 31.282) | 11.501 (2.761 to 31.282) | 11.501 (2.761 to 31.282) | 11.501 (2.761 to 31.282) |
| Diarrhoeal diseases | Unimproved, chlorinated | Both | Both | | 7.914 (1.971 to 21.188) | 7.914 (1.971 to 21.188) | 7.914 (1.971 to 21.188) | 7.914 (1.971 to 21.188) | 7.914 (1.971 to 21.188) | 7.914 (1.971 to 21.188) | 7.914 (1.971 to 21.188) | 7.914 (1.971 to 21.188) | 7.914 (1.971 to 21.188) | 7.914 (1.971 to 21.188) | 7.914 (1.971 to 21.188) | 7.914 (1.971 to 21.188) | 7.914 (1.971 to 21.188) | 7.914 (1.971 to 21.188) | 7.914 (1.971 to 21.188) | 7.914 (1.971 to 21.188) | 7.914 (1.971 to 21.188) | 7.914 (1.971 to 21.188) | 7.914 (1.971 to 21.188) | 7.914 (1.971 to 21.188) | 7.914 (1.971 to 21.188) | 7.914 (1.971 to 21.188) | 7.914 (1.971 to 21.188) |
| Diarrhoeal diseases | Unimproved, filter | Both | Both | | 4.789 (1.204 to 12.752) | 4.789 (1.204 to 12.752) | 4.789 (1.204 to 12.752) | 4.789 (1.204 to 12.752) | 4.789 (1.204 to 12.752) | 4.789 (1.204 to 12.752) | 4.789 (1.204 to 12.752) | 4.789 (1.204 to 12.752) | 4.789 (1.204 to 12.752) | 4.789 (1.204 to 12.752) | 4.789 (1.204 to 12.752) | 4.789 (1.204 to 12.752) | 4.789 (1.204 to 12.752) | 4.789 (1.204 to 12.752) | 4.789 (1.204 to 12.752) | 4.789 (1.204 to 12.752) | 4.789 (1.204 to 12.752) | 4.789 (1.204 to 12.752) | 4.789 (1.204 to 12.752) | 4.789 (1.204 to 12.752) | 4.789 (1.204 to 12.752) | 4.789 (1.204 to 12.752) | 4.789 (1.204 to 12.752) |
| Diarrhoeal diseases | Improved, untreated | Both | Both | | 9.428 (2.782 to 23.028) | 9.428 (2.782 to 23.028) | 9.428 (2.782 to 23.028) | 9.428 (2.782 to 23.028) | 9.428 (2.782 to 23.028) | 9.428 (2.782 to 23.028) | 9.428 (2.782 to 23.028) | 9.428 (2.782 to 23.028) | 9.428 (2.782 to 23.028) | 9.428 (2.782 to 23.028) | 9.428 (2.782 to 23.028) | 9.428 (2.782 to 23.028) | 9.428 (2.782 to 23.028) | 9.428 (2.782 to 23.028) | 9.428 (2.782 to 23.028) | 9.428 (2.782 to 23.028) | 9.428 (2.782 to 23.028) | 9.428 (2.782 to 23.028) | 9.428 (2.782 to 23.028) | 9.428 (2.782 to 23.028) | 9.428 (2.782 to 23.028) | 9.428 (2.782 to 23.028) | 9.428 (2.782 to 23.028) |
| Diarrhoeal diseases | Improved, chlorinated | Both | Both | | 6.488 (1.997 to 15.677) | 6.488 (1.997 to 15.677) | 6.488 (1.997 to 15.677) | 6.488 (1.997 to 15.677) | 6.488 (1.997 to 15.677) | 6.488 (1.997 to 15.677) | 6.488 (1.997 to 15.677) | 6.488 (1.997 to 15.677) | 6.488 (1.997 to 15.677) | 6.488 (1.997 to 15.677) | 6.488 (1.997 to 15.677) | 6.488 (1.997 to 15.677) | 6.488 (1.997 to 15.677) | 6.488 (1.997 to 15.677) | 6.488 (1.997 to 15.677) | 6.488 (1.997 to 15.677) | 6.488 (1.997 to 15.677) | 6.488 (1.997 to 15.677) | 6.488 (1.997 to 15.677) | 6.488 (1.997 to 15.677) | 6.488 (1.997 to 15.677) | 6.488 (1.997 to 15.677) | 6.488 (1.997 to 15.677) |
| Diarrhoeal diseases | Improved, filtered | Both | Both | | 3.926 (1.22 to 9.401) | 3.926 (1.22 to 9.401) | 3.926 (1.22 to 9.401) | 3.926 (1.22 to 9.401) | 3.926 (1.22 to 9.401) | 3.926 (1.22 to 9.401) | 3.926 (1.22 to 9.401) | 3.926 (1.22 to 9.401) | 3.926 (1.22 to 9.401) | 3.926 (1.22 to 9.401) | 3.926 (1.22 to 9.401) | 3.926 (1.22 to 9.401) | 3.926 (1.22 to 9.401) | 3.926 (1.22 to 9.401) | 3.926 (1.22 to 9.401) | 3.926 (1.22 to 9.401) | 3.926 (1.22 to 9.401) | 3.926 (1.22 to 9.401) | 3.926 (1.22 to 9.401) | 3.926 (1.22 to 9.401) | 3.926 (1.22 to 9.401) | 3.926 (1.22 to 9.401) | 3.926 (1.22 to 9.401) |
| Diarrhoeal diseases | Piped, untreated | Both | Both | | 8.431 (2.533 to 20.446) | 8.431 (2.533 to 20.446) | 8.431 (2.533 to 20.446) | 8.431 (2.533 to 20.446) | 8.431 (2.533 to 20.446) | 8.431 (2.533 to 20.446) | 8.431 (2.533 to 20.446) | 8.431 (2.533 to 20.446) | 8.431 (2.533 to 20.446) | 8.431 (2.533 to 20.446) | 8.431 (2.533 to 20.446) | 8.431 (2.533 to 20.446) | 8.431 (2.533 to 20.446) | 8.431 (2.533 to 20.446) | 8.431 (2.533 to 20.446) | 8.431 (2.533 to 20.446) | 8.431 (2.533 to 20.446) | 8.431 (2.533 to 20.446) | 8.431 (2.533 to 20.446) | 8.431 (2.533 to 20.446) | 8.431 (2.533 to 20.446) | 8.431 (2.533 to 20.446) | 8.431 (2.533 to 20.446) |
| Diarrhoeal diseases | Piped, chlorinated | Both | Both | | 5.802 (1.807 to 13.843) | 5.802 (1.807 to 13.843) | 5.802 (1.807 to 13.843) | 5.802 (1.807 to 13.843) | 5.802 (1.807 to 13.843) | 5.802 (1.807 to 13.843) | 5.802 (1.807 to 13.843) | 5.802 (1.807 to 13.843) | 5.802 (1.807 to 13.843) | 5.802 (1.807 to 13.843) | 5.802 (1.807 to 13.843) | 5.802 (1.807 to 13.843) | 5.802 (1.807 to 13.843) | 5.802 (1.807 to 13.843) | 5.802 (1.807 to 13.843) | 5.802 (1.807 to 13.843) | 5.802 (1.807 to 13.843) | 5.802 (1.807 to 13.843) | 5.802 (1.807 to 13.843) | 5.802 (1.807 to 13.843) | 5.802 (1.807 to 13.843) | 5.802 (1.807 to 13.843) | 5.802 (1.807 to 13.843) |
| Diarrhoeal diseases | Piped, filtered | Both | Both | | 3.511 (1.107 to 8.331) | 3.511 (1.107 to 8.331) | 3.511 (1.107 to 8.331) | 3.511 (1.107 to 8.331) | 3.511 (1.107 to 8.331) | 3.511 (1.107 to 8.331) | 3.511 (1.107 to 8.331) | 3.511 (1.107 to 8.331) | 3.511 (1.107 to 8.331) | 3.511 (1.107 to 8.331) | 3.511 (1.107 to 8.331) | 3.511 (1.107 to 8.331) | 3.511 (1.107 to 8.331) | 3.511 (1.107 to 8.331) | 3.511 (1.107 to 8.331) | 3.511 (1.107 to 8.331) | 3.511 (1.107 to 8.331) | 3.511 (1.107 to 8.331) | 3.511 (1.107 to 8.331) | 3.511 (1.107 to 8.331) | 3.511 (1.107 to 8.331) | 3.511 (1.107 to 8.331) | 3.511 (1.107 to 8.331) |
| Diarrhoeal diseases | High quality (HQ) piped, untreated | Both | Both | | 2.401 (2.037 to 2.818) | 2.401 (2.037 to 2.818) | 2.401 (2.037 to 2.818) | 2.401 (2.037 to 2.818) | 2.401 (2.037 to 2.818) | 2.401 (2.037 to 2.818) | 2.401 (2.037 to 2.818) | 2.401 (2.037 to 2.818) | 2.401 (2.037 to 2.818) | 2.401 (2.037 to 2.818) | 2.401 (2.037 to 2.818) | 2.401 (2.037 to 2.818) | 2.401 (2.037 to 2.818) | 2.401 (2.037 to 2.818) | 2.401 (2.037 to 2.818) | 2.401 (2.037 to 2.818) | 2.401 (2.037 to 2.818) | 2.401 (2.037 to 2.818) | 2.401 (2.037 to 2.818) | 2.401 (2.037 to 2.818) | 2.401 (2.037 to 2.818) | 2.401 (2.037 to 2.818) | 2.401 (2.037 to 2.818) |
| Diarrhoeal diseases | HQ piped, chlorinated | Both | Both | | 1.653 (1.56 to 1.748) | 1.653 (1.56 to 1.748) | 1.653 (1.56 to 1.748) | 1.653 (1.56 to 1.748) | 1.653 (1.56 to 1.748) | 1.653 (1.56 to 1.748) | 1.653 (1.56 to 1.748) | 1.653 (1.56 to 1.748) | 1.653 (1.56 to 1.748) | 1.653 (1.56 to 1.748) | 1.653 (1.56 to 1.748) | 1.653 (1.56 to 1.748) | 1.653 (1.56 to 1.748) | 1.653 (1.56 to 1.748) | 1.653 (1.56 to 1.748) | 1.653 (1.56 to 1.748) | 1.653 (1.56 to 1.748) | 1.653 (1.56 to 1.748) | 1.653 (1.56 to 1.748) | 1.653 (1.56 to 1.748) | 1.653 (1.56 to 1.748) | 1.653 (1.56 to 1.748) | 1.653 (1.56 to 1.748) |
| Diarrhoeal diseases | HQ piped, filtered | Both | Both | | 1.0 (1.0 to 1.0) | 1.0 (1.0 to 1.0) | 1.0 (1.0 to 1.0) | 1.0 (1.0 to 1.0) | 1.0 (1.0 to 1.0) | 1.0 (1.0 to 1.0) | 1.0 (1.0 to 1.0) | 1.0 (1.0 to 1.0) | 1.0 (1.0 to 1.0) | 1.0 (1.0 to 1.0) | 1.0 (1.0 to 1.0) | 1.0 (1.0 to 1.0) | 1.0 (1.0 to 1.0) | 1.0 (1.0 to 1.0) | 1.0 (1.0 to 1.0) | 1.0 (1.0 to 1.0) | 1.0 (1.0 to 1.0) | 1.0 (1.0 to 1.0) | 1.0 (1.0 to 1.0) | 1.0 (1.0 to 1.0) | 1.0 (1.0 to 1.0) | 1.0 (1.0 to 1.0) | 1.0 (1.0 to 1.0) |
| **Unsafe sanitation** | | | | | | | | | | | | | | | | | | | | | | | | | | | |
| Diarrhoeal diseases | Unimproved & untreated | Both | Both | | 3.242 (2.528 to 4.067) | 3.242 (2.528 to 4.067) | 3.242 (2.528 to 4.067) | 3.242 (2.528 to 4.067) | 3.242 (2.528 to 4.067) | 3.242 (2.528 to 4.067) | 3.242 (2.528 to 4.067) | 3.242 (2.528 to 4.067) | 3.242 (2.528 to 4.067) | 3.242 (2.528 to 4.067) | 3.242 (2.528 to 4.067) | 3.242 (2.528 to 4.067) | 3.242 (2.528 to 4.067) | 3.242 (2.528 to 4.067) | 3.242 (2.528 to 4.067) | 3.242 (2.528 to 4.067) | 3.242 (2.528 to 4.067) | 3.242 (2.528 to 4.067) | 3.242 (2.528 to 4.067) | 3.242 (2.528 to 4.067) | 3.242 (2.528 to 4.067) | 3.242 (2.528 to 4.067) | 3.242 (2.528 to 4.067) |
| Diarrhoeal diseases | Improved | Both | Both | | 2.595 (2.044 to 3.285) | 2.595 (2.044 to 3.285) | 2.595 (2.044 to 3.285) | 2.595 (2.044 to 3.285) | 2.595 (2.044 to 3.285) | 2.595 (2.044 to 3.285) | 2.595 (2.044 to 3.285) | 2.595 (2.044 to 3.285) | 2.595 (2.044 to 3.285) | 2.595 (2.044 to 3.285) | 2.595 (2.044 to 3.285) | 2.595 (2.044 to 3.285) | 2.595 (2.044 to 3.285) | 2.595 (2.044 to 3.285) | 2.595 (2.044 to 3.285) | 2.595 (2.044 to 3.285) | 2.595 (2.044 to 3.285) | 2.595 (2.044 to 3.285) | 2.595 (2.044 to 3.285) | 2.595 (2.044 to 3.285) | 2.595 (2.044 to 3.285) | 2.595 (2.044 to 3.285) | 2.595 (2.044 to 3.285) |
| Diarrhoeal diseases | Sewer | Both | Both | | 1.0 (1.0 to 1.0) | 1.0 (1.0 to 1.0) | 1.0 (1.0 to 1.0) | 1.0 (1.0 to 1.0) | 1.0 (1.0 to 1.0) | 1.0 (1.0 to 1.0) | 1.0 (1.0 to 1.0) | 1.0 (1.0 to 1.0) | 1.0 (1.0 to 1.0) | 1.0 (1.0 to 1.0) | 1.0 (1.0 to 1.0) | 1.0 (1.0 to 1.0) | 1.0 (1.0 to 1.0) | 1.0 (1.0 to 1.0) | 1.0 (1.0 to 1.0) | 1.0 (1.0 to 1.0) | 1.0 (1.0 to 1.0) | 1.0 (1.0 to 1.0) | 1.0 (1.0 to 1.0) | 1.0 (1.0 to 1.0) | 1.0 (1.0 to 1.0) | 1.0 (1.0 to 1.0) | 1.0 (1.0 to 1.0) |
| **No access to handwashing facility** | | | | | | | | | | | | | | | | | | | | | | | | | | | |
| Lower respiratory infections | No handwashing w/soap & water | Both | Both | | 1.191 (1.119 to 1.266) | 1.191 (1.119 to 1.266) | 1.191 (1.119 to 1.266) | 1.191 (1.119 to 1.266) | 1.191 (1.119 to 1.266) | 1.191 (1.119 to 1.266) | 1.191 (1.119 to 1.266) | 1.191 (1.119 to 1.266) | 1.191 (1.119 to 1.266) | 1.191 (1.119 to 1.266) | 1.191 (1.119 to 1.266) | 1.191 (1.119 to 1.266) | 1.191 (1.119 to 1.266) | 1.191 (1.119 to 1.266) | 1.191 (1.119 to 1.266) | 1.191 (1.119 to 1.266) | 1.191 (1.119 to 1.266) | 1.191 (1.119 to 1.266) | 1.191 (1.119 to 1.266) | 1.191 (1.119 to 1.266) | 1.191 (1.119 to 1.266) | 1.191 (1.119 to 1.266) | 1.191 (1.119 to 1.266) |
| Lower respiratory infections | Handwashing w/soap & water | Both | Both | | 1.0 (1.0 to 1.0) | 1.0 (1.0 to 1.0) | 1.0 (1.0 to 1.0) | 1.0 (1.0 to 1.0) | 1.0 (1.0 to 1.0) | 1.0 (1.0 to 1.0) | 1.0 (1.0 to 1.0) | 1.0 (1.0 to 1.0) | 1.0 (1.0 to 1.0) | 1.0 (1.0 to 1.0) | 1.0 (1.0 to 1.0) | 1.0 (1.0 to 1.0) | 1.0 (1.0 to 1.0) | 1.0 (1.0 to 1.0) | 1.0 (1.0 to 1.0) | 1.0 (1.0 to 1.0) | 1.0 (1.0 to 1.0) | 1.0 (1.0 to 1.0) | 1.0 (1.0 to 1.0) | 1.0 (1.0 to 1.0) | 1.0 (1.0 to 1.0) | 1.0 (1.0 to 1.0) | 1.0 (1.0 to 1.0) |
| Diarrhoeal diseases | No handwashing w/soap & water | Both | Both | | 1.908 (1.32 to 2.668) | 1.908 (1.32 to 2.668) | 1.908 (1.32 to 2.668) | 1.908 (1.32 to 2.668) | 1.908 (1.32 to 2.668) | 1.908 (1.32 to 2.668) | 1.908 (1.32 to 2.668) | 1.908 (1.32 to 2.668) | 1.908 (1.32 to 2.668) | 1.908 (1.32 to 2.668) | 1.908 (1.32 to 2.668) | 1.908 (1.32 to 2.668) | 1.908 (1.32 to 2.668) | 1.908 (1.32 to 2.668) | 1.908 (1.32 to 2.668) | 1.908 (1.32 to 2.668) | 1.908 (1.32 to 2.668) | 1.908 (1.32 to 2.668) | 1.908 (1.32 to 2.668) | 1.908 (1.32 to 2.668) | 1.908 (1.32 to 2.668) | 1.908 (1.32 to 2.668) | 1.908 (1.32 to 2.668) |
| Diarrhoeal diseases | Handwashing w/soap & water | Both | Both | | 1.0 (1.0 to 1.0) | 1.0 (1.0 to 1.0) | 1.0 (1.0 to 1.0) | 1.0 (1.0 to 1.0) | 1.0 (1.0 to 1.0) | 1.0 (1.0 to 1.0) | 1.0 (1.0 to 1.0) | 1.0 (1.0 to 1.0) | 1.0 (1.0 to 1.0) | 1.0 (1.0 to 1.0) | 1.0 (1.0 to 1.0) | 1.0 (1.0 to 1.0) | 1.0 (1.0 to 1.0) | 1.0 (1.0 to 1.0) | 1.0 (1.0 to 1.0) | 1.0 (1.0 to 1.0) | 1.0 (1.0 to 1.0) | 1.0 (1.0 to 1.0) | 1.0 (1.0 to 1.0) | 1.0 (1.0 to 1.0) | 1.0 (1.0 to 1.0) | 1.0 (1.0 to 1.0) | 1.0 (1.0 to 1.0) |
| **Household air pollution from solid fuels** | | | | | | | | | | | | | | | | | | | | | | | | | | | |
| Cataract | Exposed | morbidity | Both | 2.47 (1.61 to 3.73) | | | | | | | | | | | | | | | | | | | | | | | |
| **Ambient ozone pollution** | | | | | | | | | | | | | | | | | | | | | | | | | | | |
| Chronic obstructive pulmonary disease | 10 ppb | Mortality | Both | | | | | | | | | | 1.06 (1.017 to 1.101) | 1.06 (1.017 to 1.101) | 1.06 (1.017 to 1.101) | 1.06 (1.017 to 1.101) | 1.06 (1.017 to 1.101) | 1.06 (1.017 to 1.101) | 1.06 (1.017 to 1.101) | 1.06 (1.017 to 1.101) | 1.06 (1.017 to 1.101) | 1.06 (1.017 to 1.101) | 1.06 (1.017 to 1.101) | 1.06 (1.017 to 1.101) | 1.06 (1.017 to 1.101) | 1.06 (1.017 to 1.101) | 1.06 (1.017 to 1.101) |
| **Residential radon** | | | | | | | | | | | | | | | | | | | | | | | | | | | |
| Tracheal, bronchus, and lung cancer | Bq/m3 | Both | Both | | 1.002 (1.0 to 1.003) | 1.002 (1.0 to 1.003) | 1.002 (1.0 to 1.003) | 1.002 (1.0 to 1.003) | 1.002 (1.0 to 1.003) | 1.002 (1.0 to 1.003) | 1.002 (1.0 to 1.003) | 1.002 (1.0 to 1.003) | 1.002 (1.0 to 1.003) | 1.002 (1.0 to 1.003) | 1.002 (1.0 to 1.003) | 1.002 (1.0 to 1.003) | 1.002 (1.0 to 1.003) | 1.002 (1.0 to 1.003) | 1.002 (1.0 to 1.003) | 1.002 (1.0 to 1.003) | 1.002 (1.0 to 1.003) | 1.002 (1.0 to 1.003) | 1.002 (1.0 to 1.003) | 1.002 (1.0 to 1.003) | 1.002 (1.0 to 1.003) | 1.002 (1.0 to 1.003) | 1.002 (1.0 to 1.003) |
| **Lead exposure in blood** | | | | | | | | | | | | | | | | | | | | | | | | | | | |
| IQ shift | 10 µg/dL | Morbidity | Both | 4.688 (1.719 to 7.656) | | | | | | | | | | | | | | | | | | | | | | | |
| IQ shift | 12 µg/dL | Morbidity | Both | 5.014 (1.839 to 8.19) | | | | | | | | | | | | | | | | | | | | | | | |
| IQ shift | 15 µg/dL | Morbidity | Both | 5.42 (1.988 to 8.853) | | | | | | | | | | | | | | | | | | | | | | | |
| IQ shift | 2 µg/dL | Morbidity | Both | 0.0 (0.0 to 0.0) | | | | | | | | | | | | | | | | | | | | | | | |
| IQ shift | 20 µg/dL | Morbidity | Both | 5.952 (2.183 to 9.721) | | | | | | | | | | | | | | | | | | | | | | | |
| IQ shift | 25 µg/dL | Morbidity | Both | 6.37 (2.336 to 10.403) | | | | | | | | | | | | | | | | | | | | | | | |
| IQ shift | 30 µg/dL | Morbidity | Both | 6.713 (2.462 to 10.965) | | | | | | | | | | | | | | | | | | | | | | | |
| IQ shift | 35 µg/dL | Morbidity | Both | 7.006 (2.569 to 11.442) | | | | | | | | | | | | | | | | | | | | | | | |
| IQ shift | 4 µg/dL | Morbidity | Both | 3.146 (1.154 to 5.139) | | | | | | | | | | | | | | | | | | | | | | | |
| IQ shift | 40 µg/dL | Morbidity | Both | 7.26 (2.662 to 11.857) | | | | | | | | | | | | | | | | | | | | | | | |
| IQ shift | 6 µg/dL | Morbidity | Both | 3.804 (1.395 to 6.213) | | | | | | | | | | | | | | | | | | | | | | | |
| IQ shift | 8 µg/dL | Morbidity | Both | 4.296 (1.575 to 7.016) | | | | | | | | | | | | | | | | | | | | | | | |
| **Lead exposure in bone** | | | | | | | | | | | | | | | | | | | | | | | | | | | |
| Rheumatic heart disease | 10 µg/g | Both | Both | | | | | | | | | | 1.03 (1.01 to 1.052) | 1.024 (1.01 to 1.04) | 1.017 (1.008 to 1.028) | 1.013 (1.005 to 1.022) | 1.012 (1.006 to 1.019) | 1.011 (1.006 to 1.017) | 1.01 (1.006 to 1.016) | 1.009 (1.005 to 1.014) | 1.008 (1.003 to 1.014) | 1.007 (1.003 to 1.013) | 1.007 (1.004 to 1.013) | 1.006 (1.002 to 1.015) | 1.006 (1.002 to 1.015) | 1.006 (1.002 to 1.015) | 1.006 (1.002 to 1.015) |
| Ischaemic heart disease | 10 µg/g | Both | Both | | | | | | | | | | 1.042 (1.022 to 1.06) | 1.037 (1.023 to 1.049) | 1.032 (1.023 to 1.04) | 1.028 (1.021 to 1.036) | 1.026 (1.02 to 1.033) | 1.024 (1.02 to 1.03) | 1.023 (1.019 to 1.026) | 1.021 (1.018 to 1.025) | 1.019 (1.014 to 1.023) | 1.018 (1.012 to 1.022) | 1.016 (1.012 to 1.021) | 1.014 (1.008 to 1.022) | 1.014 (1.008 to 1.022) | 1.014 (1.008 to 1.022) | 1.014 (1.008 to 1.022) |
| Ischaemic stroke | 10 µg/g | Both | Both | | | | | | | | | | 1.038 (1.021 to 1.06) | 1.036 (1.022 to 1.051) | 1.033 (1.021 to 1.044) | 1.03 (1.019 to 1.042) | 1.028 (1.019 to 1.037) | 1.026 (1.019 to 1.033) | 1.024 (1.018 to 1.029) | 1.021 (1.016 to 1.026) | 1.019 (1.012 to 1.025) | 1.017 (1.01 to 1.023) | 1.015 (1.01 to 1.02) | 1.011 (1.006 to 1.019) | 1.011 (1.006 to 1.019) | 1.011 (1.006 to 1.019) | 1.011 (1.006 to 1.019) |
| Intracerebral hemorrhage | 10 µg/g | Both | Both | | | | | | | | | | 1.047 (1.027 to 1.068) | 1.045 (1.029 to 1.061) | 1.042 (1.029 to 1.057) | 1.039 (1.025 to 1.052) | 1.036 (1.024 to 1.047) | 1.032 (1.023 to 1.041) | 1.028 (1.021 to 1.035) | 1.024 (1.018 to 1.03) | 1.02 (1.012 to 1.027) | 1.017 (1.009 to 1.025) | 1.017 (1.011 to 1.023) | 1.015 (1.007 to 1.026) | 1.015 (1.007 to 1.026) | 1.015 (1.007 to 1.026) | 1.015 (1.007 to 1.026) |
| Subarachnoid hemorrhage | 10 µg/g | Both | Both | | | | | | | | | | 1.047 (1.027 to 1.068) | 1.045 (1.029 to 1.061) | 1.042 (1.029 to 1.057) | 1.039 (1.025 to 1.052) | 1.036 (1.024 to 1.047) | 1.032 (1.023 to 1.041) | 1.028 (1.021 to 1.035) | 1.024 (1.018 to 1.03) | 1.02 (1.012 to 1.027) | 1.017 (1.009 to 1.025) | 1.017 (1.011 to 1.023) | 1.015 (1.007 to 1.026) | 1.015 (1.007 to 1.026) | 1.015 (1.007 to 1.026) | 1.015 (1.007 to 1.026) |
| Hypertensive heart disease | 10 µg/g | Both | Both | | | | | | | | | | 1.066 (1.038 to 1.09) | 1.066 (1.038 to 1.091) | 1.065 (1.037 to 1.094) | 1.063 (1.035 to 1.091) | 1.058 (1.037 to 1.084) | 1.052 (1.036 to 1.079) | 1.046 (1.031 to 1.076) | 1.04 (1.023 to 1.073) | 1.033 (1.011 to 1.074) | 1.03 (1.003 to 1.072) | 1.031 (1.005 to 1.071) | 1.033 (1.006 to 1.075) | 1.033 (1.006 to 1.075) | 1.033 (1.006 to 1.075) | 1.033 (1.006 to 1.075) |
| Non-rheumatic calcific aortic valve disease | 10 µg/g | Both | Both | | | | | | | | | | 1.035 (1.015 to 1.055) | 1.029 (1.016 to 1.044) | 1.023 (1.015 to 1.031) | 1.019 (1.013 to 1.025) | 1.018 (1.012 to 1.023) | 1.016 (1.012 to 1.02) | 1.015 (1.012 to 1.018) | 1.014 (1.01 to 1.016) | 1.012 (1.008 to 1.015) | 1.011 (1.007 to 1.014) | 1.01 (1.007 to 1.013) | 1.007 (1.004 to 1.013) | 1.007 (1.004 to 1.013) | 1.007 (1.004 to 1.013) | 1.007 (1.004 to 1.013) |
| Other cardiomyopathy | 10 µg/g | Both | Both | | | | | | | | | | 1.035 (1.015 to 1.055) | 1.029 (1.016 to 1.044) | 1.023 (1.015 to 1.031) | 1.019 (1.013 to 1.025) | 1.018 (1.012 to 1.023) | 1.016 (1.012 to 1.02) | 1.015 (1.012 to 1.018) | 1.014 (1.01 to 1.016) | 1.012 (1.008 to 1.015) | 1.011 (1.007 to 1.014) | 1.01 (1.007 to 1.013) | 1.007 (1.004 to 1.013) | 1.007 (1.004 to 1.013) | 1.007 (1.004 to 1.013) | 1.007 (1.004 to 1.013) |
| Atrial fibrillation and flutter | 10 µg/g | Both | Both | | | | | | | | | | 1.035 (1.018 to 1.056) | 1.03 (1.02 to 1.044) | 1.025 (1.021 to 1.031) | 1.022 (1.018 to 1.025) | 1.02 (1.017 to 1.023) | 1.019 (1.017 to 1.021) | 1.018 (1.016 to 1.019) | 1.016 (1.014 to 1.018) | 1.015 (1.013 to 1.017) | 1.013 (1.011 to 1.015) | 1.012 (1.01 to 1.013) | 1.008 (1.005 to 1.01) | 1.008 (1.005 to 1.01) | 1.008 (1.005 to 1.01) | 1.008 (1.005 to 1.01) |
| Aortic aneurysm | 10 µg/g | Both | Both | | | | | | | | | | 1.027 (1.014 to 1.048) | 1.024 (1.016 to 1.037) | 1.02 (1.016 to 1.026) | 1.018 (1.013 to 1.023) | 1.017 (1.013 to 1.021) | 1.016 (1.013 to 1.019) | 1.015 (1.012 to 1.017) | 1.014 (1.011 to 1.016) | 1.012 (1.009 to 1.015) | 1.011 (1.008 to 1.014) | 1.01 (1.007 to 1.013) | 1.007 (1.004 to 1.01) | 1.007 (1.004 to 1.01) | 1.007 (1.004 to 1.01) | 1.007 (1.004 to 1.01) |
| Peripheral vascular disease | 10 µg/g | Both | Both | | | | | | | | | | 1.034 (1.011 to 1.056) | 1.025 (1.012 to 1.039) | 1.014 (1.01 to 1.018) | 1.008 (1.001 to 1.014) | 1.008 (1.003 to 1.013) | 1.008 (1.004 to 1.012) | 1.009 (1.005 to 1.012) | 1.009 (1.006 to 1.011) | 1.009 (1.007 to 1.012) | 1.009 (1.006 to 1.011) | 1.008 (1.006 to 1.01) | 1.006 (1.003 to 1.009) | 1.006 (1.003 to 1.009) | 1.006 (1.003 to 1.009) | 1.006 (1.003 to 1.009) |
| Endocarditis | 10 µg/g | Both | Both | | | | | | | | | | 1.035 (1.015 to 1.055) | 1.029 (1.016 to 1.044) | 1.023 (1.015 to 1.031) | 1.019 (1.013 to 1.025) | 1.018 (1.012 to 1.023) | 1.016 (1.012 to 1.02) | 1.015 (1.012 to 1.018) | 1.014 (1.01 to 1.016) | 1.012 (1.008 to 1.015) | 1.011 (1.007 to 1.014) | 1.01 (1.007 to 1.013) | 1.007 (1.004 to 1.013) | 1.007 (1.004 to 1.013) | 1.007 (1.004 to 1.013) | 1.007 (1.004 to 1.013) |
| Other cardiovascular and circulatory diseases | 10 µg/g | Both | Both | | | | | | | | | | 1.034 (1.018 to 1.055) | 1.03 (1.02 to 1.043) | 1.025 (1.021 to 1.03) | 1.022 (1.019 to 1.025) | 1.021 (1.018 to 1.023) | 1.019 (1.017 to 1.021) | 1.018 (1.016 to 1.019) | 1.016 (1.015 to 1.018) | 1.014 (1.013 to 1.016) | 1.013 (1.011 to 1.015) | 1.012 (1.01 to 1.013) | 1.008 (1.006 to 1.011) | 1.008 (1.006 to 1.011) | 1.008 (1.006 to 1.011) | 1.008 (1.006 to 1.011) |
| Chronic kidney disease due to diabetes mellitus type 1 | 10 µg/g | Both | Both | | | | | | | | | | 1.015 (1.01 to 1.021) | 1.015 (1.01 to 1.021) | 1.015 (1.01 to 1.021) | 1.015 (1.01 to 1.021) | 1.015 (1.01 to 1.021) | 1.015 (1.01 to 1.021) | 1.015 (1.01 to 1.021) | 1.015 (1.01 to 1.021) | 1.015 (1.01 to 1.021) | 1.015 (1.01 to 1.021) | 1.015 (1.01 to 1.021) | 1.015 (1.01 to 1.021) | 1.015 (1.01 to 1.021) | 1.015 (1.01 to 1.021) | 1.015 (1.01 to 1.021) |
| Chronic kidney disease due to diabetes mellitus type 2 | 10 µg/g | Both | Both | | | | | | | | | | 1.015 (1.01 to 1.021) | 1.015 (1.01 to 1.021) | 1.015 (1.01 to 1.021) | 1.015 (1.01 to 1.021) | 1.015 (1.01 to 1.021) | 1.015 (1.01 to 1.021) | 1.015 (1.01 to 1.021) | 1.015 (1.01 to 1.021) | 1.015 (1.01 to 1.021) | 1.015 (1.01 to 1.021) | 1.015 (1.01 to 1.021) | 1.015 (1.01 to 1.021) | 1.015 (1.01 to 1.021) | 1.015 (1.01 to 1.021) | 1.015 (1.01 to 1.021) |

**Appendix Table 6a. Relative risks used by age and sex for each outcome for all risk factors except for ambient air pollution alcohol, and smoking.**

| | | | | Ages | | | | | | | | | | | | | | | | | | | | | | | |
|---|---|---|---|---|---|---|---|---|---|---|---|---|---|---|---|---|---|---|---|---|---|---|---|---|---|---|---|
| Risk - Outcome | Category / Units | Morbidity / Mortality | Sex | All-age | 0-6 days | 7-27 days | 28-364 days | 1-4 years | 5-9 years | 10-14 years | 15-19 years | 20-24 years | 25-29 years | 30-34 years | 35-39 years | 40-44 years | 45-49 years | 50-54 years | 55-59 years | 60-64 years | 65-69 years | 70-74 years | 75-79 years | 80-84 years | 85-89 years | 90-94 years | 95+ years |
| Chronic kidney disease due to hypertension | 10 μg/g | Both | Both | | | | | | | | | | 1.015 (1.01 to 1.02) | 1.015 (1.01 to 1.02) | 1.015 (1.01 to 1.02) | 1.015 (1.01 to 1.02) | 1.015 (1.01 to 1.02) | 1.015 (1.01 to 1.02) | 1.015 (1.01 to 1.02) | 1.015 (1.01 to 1.02) | 1.015 (1.01 to 1.02) | 1.015 (1.01 to 1.02) | 1.015 (1.01 to 1.02) | 1.015 (1.01 to 1.02) | 1.015 (1.01 to 1.02) | 1.015 (1.01 to 1.02) | 1.015 (1.01 to 1.02) |
| Chronic kidney disease due to glomerulonephritis | 10 μg/g | Both | Both | | | | | | | | | | 1.015 (1.01 to 1.02) | 1.015 (1.01 to 1.02) | 1.015 (1.01 to 1.02) | 1.015 (1.01 to 1.02) | 1.015 (1.01 to 1.02) | 1.015 (1.01 to 1.02) | 1.015 (1.01 to 1.02) | 1.015 (1.01 to 1.02) | 1.015 (1.01 to 1.02) | 1.015 (1.01 to 1.02) | 1.015 (1.01 to 1.02) | 1.015 (1.01 to 1.02) | 1.015 (1.01 to 1.02) | 1.015 (1.01 to 1.02) | 1.015 (1.01 to 1.02) |
| Chronic kidney disease due to other and unspecified causes | 10 μg/g | Both | Both | | | | | | | | | | 1.015 (1.01 to 1.021) | 1.015 (1.01 to 1.021) | 1.015 (1.01 to 1.021) | 1.015 (1.01 to 1.021) | 1.015 (1.01 to 1.021) | 1.015 (1.01 to 1.021) | 1.015 (1.01 to 1.021) | 1.015 (1.01 to 1.021) | 1.015 (1.01 to 1.021) | 1.015 (1.01 to 1.021) | 1.015 (1.01 to 1.021) | 1.015 (1.01 to 1.021) | 1.015 (1.01 to 1.021) | 1.015 (1.01 to 1.021) | 1.015 (1.01 to 1.021) |
| **Occupational exposure to asbestos** | | | | | | | | | | | | | | | | | | | | | | | | | | | |
| Larynx cancer | High exposure | Both | Males | | | | | | | | 1.38 (1.188 to 1.612) | 1.38 (1.188 to 1.612) | 1.38 (1.188 to 1.612) | 1.38 (1.188 to 1.612) | 1.38 (1.188 to 1.612) | 1.38 (1.188 to 1.612) | 1.38 (1.188 to 1.612) | 1.38 (1.188 to 1.612) | 1.38 (1.188 to 1.612) | 1.38 (1.188 to 1.612) | 1.38 (1.188 to 1.612) | 1.38 (1.188 to 1.612) | 1.38 (1.188 to 1.612) | 1.38 (1.188 to 1.612) | 1.38 (1.188 to 1.612) | 1.38 (1.188 to 1.612) | 1.38 (1.188 to 1.612) |
| Larynx cancer | High exposure | Both | Females | | | | | | | | 1.385 (1.187 to 1.598) | 1.385 (1.187 to 1.598) | 1.385 (1.187 to 1.598) | 1.385 (1.187 to 1.598) | 1.385 (1.187 to 1.598) | 1.385 (1.187 to 1.598) | 1.385 (1.187 to 1.598) | 1.385 (1.187 to 1.598) | 1.385 (1.187 to 1.598) | 1.385 (1.187 to 1.598) | 1.385 (1.187 to 1.598) | 1.385 (1.187 to 1.598) | 1.385 (1.187 to 1.598) | 1.385 (1.187 to 1.598) | 1.385 (1.187 to 1.598) | 1.385 (1.187 to 1.598) | 1.385 (1.187 to 1.598) |
| Larynx cancer | Low exposure | Both | Males | | | | | | | | 1.0 (1.0 to 1.0) | 1.0 (1.0 to 1.0) | 1.0 (1.0 to 1.0) | 1.0 (1.0 to 1.0) | 1.0 (1.0 to 1.0) | 1.0 (1.0 to 1.0) | 1.0 (1.0 to 1.0) | 1.0 (1.0 to 1.0) | 1.0 (1.0 to 1.0) | 1.0 (1.0 to 1.0) | 1.0 (1.0 to 1.0) | 1.0 (1.0 to 1.0) | 1.0 (1.0 to 1.0) | 1.0 (1.0 to 1.0) | 1.0 (1.0 to 1.0) | 1.0 (1.0 to 1.0) | 1.0 (1.0 to 1.0) |
| Larynx cancer | Low exposure | Both | Females | | | | | | | | 1.0 (1.0 to 1.0) | 1.0 (1.0 to 1.0) | 1.0 (1.0 to 1.0) | 1.0 (1.0 to 1.0) | 1.0 (1.0 to 1.0) | 1.0 (1.0 to 1.0) | 1.0 (1.0 to 1.0) | 1.0 (1.0 to 1.0) | 1.0 (1.0 to 1.0) | 1.0 (1.0 to 1.0) | 1.0 (1.0 to 1.0) | 1.0 (1.0 to 1.0) | 1.0 (1.0 to 1.0) | 1.0 (1.0 to 1.0) | 1.0 (1.0 to 1.0) | 1.0 (1.0 to 1.0) | 1.0 (1.0 to 1.0) |
| Tracheal, bronchus, and lung cancer | High exposure | Both | Males | | | | | | | | 2.279 (1.741 to 2.936) | 2.279 (1.741 to 2.936) | 2.279 (1.741 to 2.936) | 2.279 (1.741 to 2.936) | 2.279 (1.741 to 2.936) | 2.279 (1.741 to 2.936) | 2.279 (1.741 to 2.936) | 2.279 (1.741 to 2.936) | 2.279 (1.741 to 2.936) | 2.279 (1.741 to 2.936) | 2.279 (1.741 to 2.936) | 2.279 (1.741 to 2.936) | 2.279 (1.741 to 2.936) | 2.279 (1.741 to 2.936) | 2.279 (1.741 to 2.936) | 2.279 (1.741 to 2.936) | 2.279 (1.741 to 2.936) |
| Tracheal, bronchus, and lung cancer | High exposure | Both | Females | | | | | | | | 1.875 (1.589 to 2.208) | 1.875 (1.589 to 2.208) | 1.875 (1.589 to 2.208) | 1.875 (1.589 to 2.208) | 1.875 (1.589 to 2.208) | 1.875 (1.589 to 2.208) | 1.875 (1.589 to 2.208) | 1.875 (1.589 to 2.208) | 1.875 (1.589 to 2.208) | 1.875 (1.589 to 2.208) | 1.875 (1.589 to 2.208) | 1.875 (1.589 to 2.208) | 1.875 (1.589 to 2.208) | 1.875 (1.589 to 2.208) | 1.875 (1.589 to 2.208) | 1.875 (1.589 to 2.208) | 1.875 (1.589 to 2.208) |
| Tracheal, bronchus, and lung cancer | Low exposure | Both | Males | | | | | | | | 1.0 (1.0 to 1.0) | 1.0 (1.0 to 1.0) | 1.0 (1.0 to 1.0) | 1.0 (1.0 to 1.0) | 1.0 (1.0 to 1.0) | 1.0 (1.0 to 1.0) | 1.0 (1.0 to 1.0) | 1.0 (1.0 to 1.0) | 1.0 (1.0 to 1.0) | 1.0 (1.0 to 1.0) | 1.0 (1.0 to 1.0) | 1.0 (1.0 to 1.0) | 1.0 (1.0 to 1.0) | 1.0 (1.0 to 1.0) | 1.0 (1.0 to 1.0) | 1.0 (1.0 to 1.0) | 1.0 (1.0 to 1.0) |
| Tracheal, bronchus, and lung cancer | Low exposure | Both | Females | | | | | | | | 1.0 (1.0 to 1.0) | 1.0 (1.0 to 1.0) | 1.0 (1.0 to 1.0) | 1.0 (1.0 to 1.0) | 1.0 (1.0 to 1.0) | 1.0 (1.0 to 1.0) | 1.0 (1.0 to 1.0) | 1.0 (1.0 to 1.0) | 1.0 (1.0 to 1.0) | 1.0 (1.0 to 1.0) | 1.0 (1.0 to 1.0) | 1.0 (1.0 to 1.0) | 1.0 (1.0 to 1.0) | 1.0 (1.0 to 1.0) | 1.0 (1.0 to 1.0) | 1.0 (1.0 to 1.0) | 1.0 (1.0 to 1.0) |
| Ovarian cancer | High exposure | Both | Both | | | | | | | | 1.811 (1.385 to 2.306) | 1.811 (1.385 to 2.306) | 1.811 (1.385 to 2.306) | 1.811 (1.385 to 2.306) | 1.811 (1.385 to 2.306) | 1.811 (1.385 to 2.306) | 1.811 (1.385 to 2.306) | 1.811 (1.385 to 2.306) | 1.811 (1.385 to 2.306) | 1.811 (1.385 to 2.306) | 1.811 (1.385 to 2.306) | 1.811 (1.385 to 2.306) | 1.811 (1.385 to 2.306) | 1.811 (1.385 to 2.306) | 1.811 (1.385 to 2.306) | 1.811 (1.385 to 2.306) | 1.811 (1.385 to 2.306) |
| Ovarian cancer | Low exposure | Both | Both | | | | | | | | 1.0 (1.0 to 1.0) | 1.0 (1.0 to 1.0) | 1.0 (1.0 to 1.0) | 1.0 (1.0 to 1.0) | 1.0 (1.0 to 1.0) | 1.0 (1.0 to 1.0) | 1.0 (1.0 to 1.0) | 1.0 (1.0 to 1.0) | 1.0 (1.0 to 1.0) | 1.0 (1.0 to 1.0) | 1.0 (1.0 to 1.0) | 1.0 (1.0 to 1.0) | 1.0 (1.0 to 1.0) | 1.0 (1.0 to 1.0) | 1.0 (1.0 to 1.0) | 1.0 (1.0 to 1.0) | 1.0 (1.0 to 1.0) |
| **Occupational exposure to arsenic** | | | | | | | | | | | | | | | | | | | | | | | | | | | |
| Tracheal, bronchus, and lung cancer | High exposure | Both | Both | | | | | | | | 2.061 (1.521 to 2.553) | 2.061 (1.521 to 2.553) | 2.061 (1.521 to 2.553) | 2.061 (1.521 to 2.553) | 2.061 (1.521 to 2.553) | 2.061 (1.521 to 2.553) | 2.061 (1.521 to 2.553) | 2.061 (1.521 to 2.553) | 2.061 (1.521 to 2.553) | 2.061 (1.521 to 2.553) | 2.061 (1.521 to 2.553) | 2.061 (1.521 to 2.553) | 2.061 (1.521 to 2.553) | 2.061 (1.521 to 2.553) | 2.061 (1.521 to 2.553) | 2.061 (1.521 to 2.553) | 2.061 (1.521 to 2.553) |
| Tracheal, bronchus, and lung cancer | Low exposure | Both | Both | | | | | | | | 1.749 (0.698 to 2.775) | 1.749 (0.698 to 2.775) | 1.749 (0.698 to 2.775) | 1.749 (0.698 to 2.775) | 1.749 (0.698 to 2.775) | 1.749 (0.698 to 2.775) | 1.749 (0.698 to 2.775) | 1.749 (0.698 to 2.775) | 1.749 (0.698 to 2.775) | 1.749 (0.698 to 2.775) | 1.749 (0.698 to 2.775) | 1.749 (0.698 to 2.775) | 1.749 (0.698 to 2.775) | 1.749 (0.698 to 2.775) | 1.749 (0.698 to 2.775) | 1.749 (0.698 to 2.775) | 1.749 (0.698 to 2.775) |
| Tracheal, bronchus, and lung cancer | No exposure | Both | Both | | | | | | | | 1.0 (1.0 to 1.0) | 1.0 (1.0 to 1.0) | 1.0 (1.0 to 1.0) | 1.0 (1.0 to 1.0) | 1.0 (1.0 to 1.0) | 1.0 (1.0 to 1.0) | 1.0 (1.0 to 1.0) | 1.0 (1.0 to 1.0) | 1.0 (1.0 to 1.0) | 1.0 (1.0 to 1.0) | 1.0 (1.0 to 1.0) | 1.0 (1.0 to 1.0) | 1.0 (1.0 to 1.0) | 1.0 (1.0 to 1.0) | 1.0 (1.0 to 1.0) | 1.0 (1.0 to 1.0) | 1.0 (1.0 to 1.0) |
| **Occupational exposure to benzene** | | | | | | | | | | | | | | | | | | | | | | | | | | | |
| Acute lymphoid leukaemia | High exposure | Both | Both | | | | | | | | 2.623 (1.222 to 3.975) | 2.623 (1.222 to 3.975) | 2.623 (1.222 to 3.975) | 2.623 (1.222 to 3.975) | 2.623 (1.222 to 3.975) | 2.623 (1.222 to 3.975) | 2.623 (1.222 to 3.975) | 2.623 (1.222 to 3.975) | 2.623 (1.222 to 3.975) | 2.623 (1.222 to 3.975) | 2.623 (1.222 to 3.975) | 2.623 (1.222 to 3.975) | 2.623 (1.222 to 3.975) | 2.623 (1.222 to 3.975) | 2.623 (1.222 to 3.975) | 2.623 (1.222 to 3.975) | 2.623 (1.222 to 3.975) |
| Acute lymphoid leukaemia | Low exposure | Both | Both | | | | | | | | 1.626 (0.998 to 2.256) | 1.626 (0.998 to 2.256) | 1.626 (0.998 to 2.256) | 1.626 (0.998 to 2.256) | 1.626 (0.998 to 2.256) | 1.626 (0.998 to 2.256) | 1.626 (0.998 to 2.256) | 1.626 (0.998 to 2.256) | 1.626 (0.998 to 2.256) | 1.626 (0.998 to 2.256) | 1.626 (0.998 to 2.256) | 1.626 (0.998 to 2.256) | 1.626 (0.998 to 2.256) | 1.626 (0.998 to 2.256) | 1.626 (0.998 to 2.256) | 1.626 (0.998 to 2.256) | 1.626 (0.998 to 2.256) |
| Acute lymphoid leukaemia | No exposure | Both | Both | | | | | | | | 1.0 (1.0 to 1.0) | 1.0 (1.0 to 1.0) | 1.0 (1.0 to 1.0) | 1.0 (1.0 to 1.0) | 1.0 (1.0 to 1.0) | 1.0 (1.0 to 1.0) | 1.0 (1.0 to 1.0) | 1.0 (1.0 to 1.0) | 1.0 (1.0 to 1.0) | 1.0 (1.0 to 1.0) | 1.0 (1.0 to 1.0) | 1.0 (1.0 to 1.0) | 1.0 (1.0 to 1.0) | 1.0 (1.0 to 1.0) | 1.0 (1.0 to 1.0) | 1.0 (1.0 to 1.0) | 1.0 (1.0 to 1.0) |
| Chronic lymphoid leukaemia | High exposure | Both | Both | | | | | | | | 2.623 (1.222 to 3.975) | 2.623 (1.222 to 3.975) | 2.623 (1.222 to 3.975) | 2.623 (1.222 to 3.975) | 2.623 (1.222 to 3.975) | 2.623 (1.222 to 3.975) | 2.623 (1.222 to 3.975) | 2.623 (1.222 to 3.975) | 2.623 (1.222 to 3.975) | 2.623 (1.222 to 3.975) | 2.623 (1.222 to 3.975) | 2.623 (1.222 to 3.975) | 2.623 (1.222 to 3.975) | 2.623 (1.222 to 3.975) | 2.623 (1.222 to 3.975) | 2.623 (1.222 to 3.975) | 2.623 (1.222 to 3.975) |
| Chronic lymphoid leukaemia | Low exposure | Both | Both | | | | | | | | 1.626 (0.998 to 2.256) | 1.626 (0.998 to 2.256) | 1.626 (0.998 to 2.256) | 1.626 (0.998 to 2.256) | 1.626 (0.998 to 2.256) | 1.626 (0.998 to 2.256) | 1.626 (0.998 to 2.256) | 1.626 (0.998 to 2.256) | 1.626 (0.998 to 2.256) | 1.626 (0.998 to 2.256) | 1.626 (0.998 to 2.256) | 1.626 (0.998 to 2.256) | 1.626 (0.998 to 2.256) | 1.626 (0.998 to 2.256) | 1.626 (0.998 to 2.256) | 1.626 (0.998 to 2.256) | 1.626 (0.998 to 2.256) |
| Chronic lymphoid leukaemia | No exposure | Both | Both | | | | | | | | 1.0 (1.0 to 1.0) | 1.0 (1.0 to 1.0) | 1.0 (1.0 to 1.0) | 1.0 (1.0 to 1.0) | 1.0 (1.0 to 1.0) | 1.0 (1.0 to 1.0) | 1.0 (1.0 to 1.0) | 1.0 (1.0 to 1.0) | 1.0 (1.0 to 1.0) | 1.0 (1.0 to 1.0) | 1.0 (1.0 to 1.0) | 1.0 (1.0 to 1.0) | 1.0 (1.0 to 1.0) | 1.0 (1.0 to 1.0) | 1.0 (1.0 to 1.0) | 1.0 (1.0 to 1.0) | 1.0 (1.0 to 1.0) |
| Acute myeloid leukaemia | High exposure | Both | Both | | | | | | | | 2.623 (1.222 to 3.975) | 2.623 (1.222 to 3.975) | 2.623 (1.222 to 3.975) | 2.623 (1.222 to 3.975) | 2.623 (1.222 to 3.975) | 2.623 (1.222 to 3.975) | 2.623 (1.222 to 3.975) | 2.623 (1.222 to 3.975) | 2.623 (1.222 to 3.975) | 2.623 (1.222 to 3.975) | 2.623 (1.222 to 3.975) | 2.623 (1.222 to 3.975) | 2.623 (1.222 to 3.975) | 2.623 (1.222 to 3.975) | 2.623 (1.222 to 3.975) | 2.623 (1.222 to 3.975) | 2.623 (1.222 to 3.975) |
| Acute myeloid leukaemia | Low exposure | Both | Both | | | | | | | | 1.626 (0.998 to 2.256) | 1.626 (0.998 to 2.256) | 1.626 (0.998 to 2.256) | 1.626 (0.998 to 2.256) | 1.626 (0.998 to 2.256) | 1.626 (0.998 to 2.256) | 1.626 (0.998 to 2.256) | 1.626 (0.998 to 2.256) | 1.626 (0.998 to 2.256) | 1.626 (0.998 to 2.256) | 1.626 (0.998 to 2.256) | 1.626 (0.998 to 2.256) | 1.626 (0.998 to 2.256) | 1.626 (0.998 to 2.256) | 1.626 (0.998 to 2.256) | 1.626 (0.998 to 2.256) | 1.626 (0.998 to 2.256) |
| Acute myeloid leukaemia | No exposure | Both | Both | | | | | | | | 1.0 (1.0 to 1.0) | 1.0 (1.0 to 1.0) | 1.0 (1.0 to 1.0) | 1.0 (1.0 to 1.0) | 1.0 (1.0 to 1.0) | 1.0 (1.0 to 1.0) | 1.0 (1.0 to 1.0) | 1.0 (1.0 to 1.0) | 1.0 (1.0 to 1.0) | 1.0 (1.0 to 1.0) | 1.0 (1.0 to 1.0) | 1.0 (1.0 to 1.0) | 1.0 (1.0 to 1.0) | 1.0 (1.0 to 1.0) | 1.0 (1.0 to 1.0) | 1.0 (1.0 to 1.0) | 1.0 (1.0 to 1.0) |
| Chronic myeloid leukaemia | High exposure | Both | Both | | | | | | | | 2.623 (1.222 to 3.975) | 2.623 (1.222 to 3.975) | 2.623 (1.222 to 3.975) | 2.623 (1.222 to 3.975) | 2.623 (1.222 to 3.975) | 2.623 (1.222 to 3.975) | 2.623 (1.222 to 3.975) | 2.623 (1.222 to 3.975) | 2.623 (1.222 to 3.975) | 2.623 (1.222 to 3.975) | 2.623 (1.222 to 3.975) | 2.623 (1.222 to 3.975) | 2.623 (1.222 to 3.975) | 2.623 (1.222 to 3.975) | 2.623 (1.222 to 3.975) | 2.623 (1.222 to 3.975) | 2.623 (1.222 to 3.975) |
| Chronic myeloid leukaemia | Low exposure | Both | Both | | | | | | | | 1.626 (0.998 to 2.256) | 1.626 (0.998 to 2.256) | 1.626 (0.998 to 2.256) | 1.626 (0.998 to 2.256) | 1.626 (0.998 to 2.256) | 1.626 (0.998 to 2.256) | 1.626 (0.998 to 2.256) | 1.626 (0.998 to 2.256) | 1.626 (0.998 to 2.256) | 1.626 (0.998 to 2.256) | 1.626 (0.998 to 2.256) | 1.626 (0.998 to 2.256) | 1.626 (0.998 to 2.256) | 1.626 (0.998 to 2.256) | 1.626 (0.998 to 2.256) | 1.626 (0.998 to 2.256) | 1.626 (0.998 to 2.256) |
| Chronic myeloid leukaemia | No exposure | Both | Both | | | | | | | | 1.0 (1.0 to 1.0) | 1.0 (1.0 to 1.0) | 1.0 (1.0 to 1.0) | 1.0 (1.0 to 1.0) | 1.0 (1.0 to 1.0) | 1.0 (1.0 to 1.0) | 1.0 (1.0 to 1.0) | 1.0 (1.0 to 1.0) | 1.0 (1.0 to 1.0) | 1.0 (1.0 to 1.0) | 1.0 (1.0 to 1.0) | 1.0 (1.0 to 1.0) | 1.0 (1.0 to 1.0) | 1.0 (1.0 to 1.0) | 1.0 (1.0 to 1.0) | 1.0 (1.0 to 1.0) | 1.0 (1.0 to 1.0) |
| Other leukaemia | High exposure | Both | Both | | | | | | | | 2.623 (1.222 to 3.975) | 2.623 (1.222 to 3.975) | 2.623 (1.222 to 3.975) | 2.623 (1.222 to 3.975) | 2.623 (1.222 to 3.975) | 2.623 (1.222 to 3.975) | 2.623 (1.222 to 3.975) | 2.623 (1.222 to 3.975) | 2.623 (1.222 to 3.975) | 2.623 (1.222 to 3.975) | 2.623 (1.222 to 3.975) | 2.623 (1.222 to 3.975) | 2.623 (1.222 to 3.975) | 2.623 (1.222 to 3.975) | 2.623 (1.222 to 3.975) | 2.623 (1.222 to 3.975) | 2.623 (1.222 to 3.975) |
| Other leukaemia | Low exposure | Both | Both | | | | | | | | 1.626 (0.998 to 2.256) | 1.626 (0.998 to 2.256) | 1.626 (0.998 to 2.256) | 1.626 (0.998 to 2.256) | 1.626 (0.998 to 2.256) | 1.626 (0.998 to 2.256) | 1.626 (0.998 to 2.256) | 1.626 (0.998 to 2.256) | 1.626 (0.998 to 2.256) | 1.626 (0.998 to 2.256) | 1.626 (0.998 to 2.256) | 1.626 (0.998 to 2.256) | 1.626 (0.998 to 2.256) | 1.626 (0.998 to 2.256) | 1.626 (0.998 to 2.256) | 1.626 (0.998 to 2.256) | 1.626 (0.998 to 2.256) |
| Other leukaemia | No exposure | Both | Both | | | | | | | | 1.0 (1.0 to 1.0) | 1.0 (1.0 to 1.0) | 1.0 (1.0 to 1.0) | 1.0 (1.0 to 1.0) | 1.0 (1.0 to 1.0) | 1.0 (1.0 to 1.0) | 1.0 (1.0 to 1.0) | 1.0 (1.0 to 1.0) | 1.0 (1.0 to 1.0) | 1.0 (1.0 to 1.0) | 1.0 (1.0 to 1.0) | 1.0 (1.0 to 1.0) | 1.0 (1.0 to 1.0) | 1.0 (1.0 to 1.0) | 1.0 (1.0 to 1.0) | 1.0 (1.0 to 1.0) | 1.0 (1.0 to 1.0) |
| **Occupational exposure to beryllium** | | | | | | | | | | | | | | | | | | | | | | | | | | | |
| Tracheal, bronchus, and lung cancer | High exposure | Both | Males | | | | | | | | 1.174 (1.086 to 1.269) | 1.169 (1.065 to 1.276) | 1.17 (1.073 to 1.274) | 1.169 (1.073 to 1.269) | 1.172 (1.081 to 1.271) | 1.168 (1.07 to 1.276) | 1.171 (1.071 to 1.274) | 1.171 (1.075 to 1.273) | 1.17 (1.073 to 1.271) | 1.171 (1.072 to 1.271) | 1.172 (1.081 to 1.276) | 1.171 (1.076 to 1.27) | 1.174 (1.079 to 1.276) | 1.171 (1.072 to 1.273) | 1.171 (1.072 to 1.273) | 1.171 (1.072 to 1.273) | 1.171 (1.072 to 1.273) |
| Tracheal, bronchus, and lung cancer | High exposure | Both | Females | | | | | | | | 1.17 (1.082 to 1.262) | 1.17 (1.076 to 1.277) | 1.169 (1.072 to 1.277) | 1.17 (1.077 to 1.275) | 1.172 (1.075 to 1.274) | 1.171 (1.078 to 1.276) | 1.173 (1.074 to 1.276) | 1.172 (1.074 to 1.273) | 1.173 (1.081 to 1.277) | 1.169 (1.074 to 1.269) | 1.173 (1.077 to 1.282) | 1.167 (1.069 to 1.269) | 1.169 (1.073 to 1.274) | 1.171 (1.068 to 1.279) | 1.171 (1.068 to 1.279) | 1.171 (1.068 to 1.279) | 1.171 (1.068 to 1.279) |
| Tracheal, bronchus, and lung cancer | Low exposure | Both | Males | | | | | | | | 1.0 (1.0 to 1.0) | 1.0 (1.0 to 1.0) | 1.0 (1.0 to 1.0) | 1.0 (1.0 to 1.0) | 1.0 (1.0 to 1.0) | 1.0 (1.0 to 1.0) | 1.0 (1.0 to 1.0) | 1.0 (1.0 to 1.0) | 1.0 (1.0 to 1.0) | 1.0 (1.0 to 1.0) | 1.0 (1.0 to 1.0) | 1.0 (1.0 to 1.0) | 1.0 (1.0 to 1.0) | 1.0 (1.0 to 1.0) | 1.0 (1.0 to 1.0) | 1.0 (1.0 to 1.0) | 1.0 (1.0 to 1.0) |
| Tracheal, bronchus, and lung cancer | Low exposure | Both | Females | | | | | | | | 1.0 (1.0 to 1.0) | 1.0 (1.0 to 1.0) | 1.0 (1.0 to 1.0) | 1.0 (1.0 to 1.0) | 1.0 (1.0 to 1.0) | 1.0 (1.0 to 1.0) | 1.0 (1.0 to 1.0) | 1.0 (1.0 to 1.0) | 1.0 (1.0 to 1.0) | 1.0 (1.0 to 1.0) | 1.0 (1.0 to 1.0) | 1.0 (1.0 to 1.0) | 1.0 (1.0 to 1.0) | 1.0 (1.0 to 1.0) | 1.0 (1.0 to 1.0) | 1.0 (1.0 to 1.0) | 1.0 (1.0 to 1.0) |
| Tracheal, bronchus, and lung cancer | No exposure | Both | Males | | | | | | | | 1.0 (1.0 to 1.0) | 1.0 (1.0 to 1.0) | 1.0 (1.0 to 1.0) | 1.0 (1.0 to 1.0) | 1.0 (1.0 to 1.0) | 1.0 (1.0 to 1.0) | 1.0 (1.0 to 1.0) | 1.0 (1.0 to 1.0) | 1.0 (1.0 to 1.0) | 1.0 (1.0 to 1.0) | 1.0 (1.0 to 1.0) | 1.0 (1.0 to 1.0) | 1.0 (1.0 to 1.0) | 1.0 (1.0 to 1.0) | 1.0 (1.0 to 1.0) | 1.0 (1.0 to 1.0) | 1.0 (1.0 to 1.0) |
| Tracheal, bronchus, and lung cancer | No exposure | Both | Females | | | | | | | | 1.0 (1.0 to 1.0) | 1.0 (1.0 to 1.0) | 1.0 (1.0 to 1.0) | 1.0 (1.0 to 1.0) | 1.0 (1.0 to 1.0) | 1.0 (1.0 to 1.0) | 1.0 (1.0 to 1.0) | 1.0 (1.0 to 1.0) | 1.0 (1.0 to 1.0) | 1.0 (1.0 to 1.0) | 1.0 (1.0 to 1.0) | 1.0 (1.0 to 1.0) | 1.0 (1.0 to 1.0) | 1.0 (1.0 to 1.0) | 1.0 (1.0 to 1.0) | 1.0 (1.0 to 1.0) | 1.0 (1.0 to 1.0) |
| **Occupational exposure to cadmium** | | | | | | | | | | | | | | | | | | | | | | | | | | | |
| Tracheal, bronchus, and lung cancer | High exposure | Both | Males | | | | | | | | 1.192 (1.097 to 1.292) | 1.188 (1.083 to 1.287) | 1.19 (1.102 to 1.295) | 1.191 (1.092 to 1.304) | 1.19 (1.091 to 1.298) | 1.19 (1.092 to 1.297) | 1.192 (1.096 to 1.298) | 1.193 (1.096 to 1.296) | 1.19 (1.089 to 1.293) | 1.192 (1.089 to 1.297) | 1.193 (1.088 to 1.3) | 1.187 (1.095 to 1.29) | 1.191 (1.095 to 1.289) | 1.19 (1.088 to 1.296) | 1.19 (1.088 to 1.296) | 1.19 (1.088 to 1.296) | 1.19 (1.088 to 1.296) |
| Tracheal, bronchus, and lung cancer | High exposure | Both | Females | | | | | | | | 1.191 (1.087 to 1.295) | 1.191 (1.099 to 1.29) | 1.188 (1.093 to 1.293) | 1.19 (1.092 to 1.302) | 1.191 (1.089 to 1.292) | 1.194 (1.098 to 1.297) | 1.188 (1.091 to 1.3) | 1.19 (1.095 to 1.291) | 1.19 (1.096 to 1.294) | 1.189 (1.095 to 1.29) | 1.192 (1.095 to 1.296) | 1.192 (1.09 to 1.298) | 1.193 (1.097 to 1.297) | 1.193 (1.1 to 1.301) | 1.193 (1.1 to 1.301) | 1.193 (1.1 to 1.301) | 1.193 (1.1 to 1.301) |
| Tracheal, bronchus, and lung cancer | Low exposure | Both | Males | | | | | | | | 1.0 (1.0 to 1.0) | 1.0 (1.0 to 1.0) | 1.0 (1.0 to 1.0) | 1.0 (1.0 to 1.0) | 1.0 (1.0 to 1.0) | 1.0 (1.0 to 1.0) | 1.0 (1.0 to 1.0) | 1.0 (1.0 to 1.0) | 1.0 (1.0 to 1.0) | 1.0 (1.0 to 1.0) | 1.0 (1.0 to 1.0) | 1.0 (1.0 to 1.0) | 1.0 (1.0 to 1.0) | 1.0 (1.0 to 1.0) | 1.0 (1.0 to 1.0) | 1.0 (1.0 to 1.0) | 1.0 (1.0 to 1.0) |
| Tracheal, bronchus, and lung cancer | Low exposure | Both | Females | | | | | | | | 1.0 (1.0 to 1.0) | 1.0 (1.0 to 1.0) | 1.0 (1.0 to 1.0) | 1.0 (1.0 to 1.0) | 1.0 (1.0 to 1.0) | 1.0 (1.0 to 1.0) | 1.0 (1.0 to 1.0) | 1.0 (1.0 to 1.0) | 1.0 (1.0 to 1.0) | 1.0 (1.0 to 1.0) | 1.0 (1.0 to 1.0) | 1.0 (1.0 to 1.0) | 1.0 (1.0 to 1.0) | 1.0 (1.0 to 1.0) | 1.0 (1.0 to 1.0) | 1.0 (1.0 to 1.0) | 1.0 (1.0 to 1.0) |
| Tracheal, bronchus, and lung cancer | No exposure | Both | Males | | | | | | | | 1.0 (1.0 to 1.0) | 1.0 (1.0 to 1.0) | 1.0 (1.0 to 1.0) | 1.0 (1.0 to 1.0) | 1.0 (1.0 to 1.0) | 1.0 (1.0 to 1.0) | 1.0 (1.0 to 1.0) | 1.0 (1.0 to 1.0) | 1.0 (1.0 to 1.0) | 1.0 (1.0 to 1.0) | 1.0 (1.0 to 1.0) | 1.0 (1.0 to 1.0) | 1.0 (1.0 to 1.0) | 1.0 (1.0 to 1.0) | 1.0 (1.0 to 1.0) | 1.0 (1.0 to 1.0) | 1.0 (1.0 to 1.0) |
| Tracheal, bronchus, and lung cancer | No exposure | Both | Females | | | | | | | | 1.0 (1.0 to 1.0) | 1.0 (1.0 to 1.0) | 1.0 (1.0 to 1.0) | 1.0 (1.0 to 1.0) | 1.0 (1.0 to 1.0) | 1.0 (1.0 to 1.0) | 1.0 (1.0 to 1.0) | 1.0 (1.0 to 1.0) | 1.0 (1.0 to 1.0) | 1.0 (1.0 to 1.0) | 1.0 (1.0 to 1.0) | 1.0 (1.0 to 1.0) | 1.0 (1.0 to 1.0) | 1.0 (1.0 to 1.0) | 1.0 (1.0 to 1.0) | 1.0 (1.0 to 1.0) | 1.0 (1.0 to 1.0) |
| **Occupational exposure to chromium** | | | | | | | | | | | | | | | | | | | | | | | | | | | |
| Tracheal, bronchus, and lung cancer | High exposure | Both | Males | | | | | | | | 1.179 (1.114 to 1.245) | 1.181 (1.117 to 1.25) | 1.181 (1.118 to 1.249) | 1.18 (1.117 to 1.244) | 1.183 (1.117 to 1.249) | 1.18 (1.117 to 1.239) | 1.18 (1.117 to 1.249) | 1.182 (1.12 to 1.246) | 1.181 (1.119 to 1.246) | 1.179 (1.115 to 1.242) | 1.182 (1.121 to 1.247) | 1.18 (1.118 to 1.248) | 1.18 (1.113 to 1.245) | 1.181 (1.117 to 1.244) | 1.181 (1.117 to 1.244) | 1.181 (1.117 to 1.244) | 1.181 (1.117 to 1.244) |
| Tracheal, bronchus, and lung cancer | High exposure | Both | Females | | | | | | | | 1.179 (1.116 to 1.247) | 1.18 (1.115 to 1.248) | 1.18 (1.115 to 1.244) | 1.179 (1.118 to 1.243) | 1.18 (1.116 to 1.248) | 1.181 (1.115 to 1.245) | 1.181 (1.119 to 1.25) | 1.181 (1.118 to 1.245) | 1.181 (1.117 to 1.248) | 1.181 (1.117 to 1.244) | 1.179 (1.121 to 1.245) | 1.181 (1.116 to 1.254) | 1.181 (1.116 to 1.245) | 1.179 (1.113 to 1.247) | 1.179 (1.113 to 1.247) | 1.179 (1.113 to 1.247) | 1.179 (1.113 to 1.247) |
| Tracheal, bronchus, and lung cancer | Low exposure | Both | Males | | | | | | | | 1.0 (1.0 to 1.0) | 1.0 (1.0 to 1.0) | 1.0 (1.0 to 1.0) | 1.0 (1.0 to 1.0) | 1.0 (1.0 to 1.0) | 1.0 (1.0 to 1.0) | 1.0 (1.0 to 1.0) | 1.0 (1.0 to 1.0) | 1.0 (1.0 to 1.0) | 1.0 (1.0 to 1.0) | 1.0 (1.0 to 1.0) | 1.0 (1.0 to 1.0) | 1.0 (1.0 to 1.0) | 1.0 (1.0 to 1.0) | 1.0 (1.0 to 1.0) | 1.0 (1.0 to 1.0) | 1.0 (1.0 to 1.0) |
| Tracheal, bronchus, and lung cancer | Low exposure | Both | Females | | | | | | | | 1.0 (1.0 to 1.0) | 1.0 (1.0 to 1.0) | 1.0 (1.0 to 1.0) | 1.0 (1.0 to 1.0) | 1.0 (1.0 to 1.0) | 1.0 (1.0 to 1.0) | 1.0 (1.0 to 1.0) | 1.0 (1.0 to 1.0) | 1.0 (1.0 to 1.0) | 1.0 (1.0 to 1.0) | 1.0 (1.0 to 1.0) | 1.0 (1.0 to 1.0) | 1.0 (1.0 to 1.0) | 1.0 (1.0 to 1.0) | 1.0 (1.0 to 1.0) | 1.0 (1.0 to 1.0) | 1.0 (1.0 to 1.0) |
| Tracheal, bronchus, and lung cancer | No exposure | Both | Males | | | | | | | | 1.0 (1.0 to 1.0) | 1.0 (1.0 to 1.0) | 1.0 (1.0 to 1.0) | 1.0 (1.0 to 1.0) | 1.0 (1.0 to 1.0) | 1.0 (1.0 to 1.0) | 1.0 (1.0 to 1.0) | 1.0 (1.0 to 1.0) | 1.0 (1.0 to 1.0) | 1.0 (1.0 to 1.0) | 1.0 (1.0 to 1.0) | 1.0 (1.0 to 1.0) | 1.0 (1.0 to 1.0) | 1.0 (1.0 to 1.0) | 1.0 (1.0 to 1.0) | 1.0 (1.0 to 1.0) | 1.0 (1.0 to 1.0) |
| Tracheal, bronchus, and lung cancer | No exposure | Both | Females | | | | | | | | 1.0 (1.0 to 1.0) | 1.0 (1.0 to 1.0) | 1.0 (1.0 to 1.0) | 1.0 (1.0 to 1.0) | 1.0 (1.0 to 1.0) | 1.0 (1.0 to 1.0) | 1.0 (1.0 to 1.0) | 1.0 (1.0 to 1.0) | 1.0 (1.0 to 1.0) | 1.0 (1.0 to 1.0) | 1.0 (1.0 to 1.0) | 1.0 (1.0 to 1.0) | 1.0 (1.0 to 1.0) | 1.0 (1.0 to 1.0) | 1.0 (1.0 to 1.0) | 1.0 (1.0 to 1.0) | 1.0 (1.0 to 1.0) |
| **Occupational exposure to diesel engine exhaust** | | | | | | | | | | | | | | | | | | | | | | | | | | | |
| Tracheal, bronchus, and lung cancer | High exposure | Both | Males | | | | | | | | 1.469 (1.294 to 1.658) | 1.477 (1.301 to 1.665) | 1.473 (1.29 to 1.668) | 1.474 (1.293 to 1.675) | 1.472 (1.301 to 1.669) | 1.47 (1.294 to 1.662) | 1.475 (1.3 to 1.667) | 1.469 (1.301 to 1.644) | 1.474 (1.295 to 1.676) | 1.477 (1.292 to 1.671) | 1.477 (1.311 to 1.68) | 1.476 (1.302 to 1.669) | 1.473 (1.287 to 1.669) | 1.477 (1.302 to 1.666) | 1.477 (1.302 to 1.666) | 1.477 (1.302 to 1.666) | 1.477 (1.302 to 1.666) |

Appendix Table 6a. Relative risks used by age and sex for each outcome for all risk factors except for ambient air pollution alcohol, and smoking.

| Risk - Outcome | Category / Units | Morbidity / Mortality | Sex | All-age | 0-6 days | 7-27 days | 28-364 days | 1-4 years | 5-9 years | 10-14 years | 15-19 years | 20-24 years | 25-29 years | 30-34 years | 35-39 years | 40-44 years | 45-49 years | 50-54 years | 55-59 years | 60-64 years | 65-69 years | 70-74 years | 75-79 years | 80-84 years | 85-89 years | 90-94 years | 95+ years |
|---|---|---|---|---|---|---|---|---|---|---|---|---|---|---|---|---|---|---|---|---|---|---|---|---|---|---|---|
| Tracheal, bronchus, and lung cancer | High exposure | Both | Females | | | | | | | | 1.473 (1.287 to 1.682) | 1.476 (1.303 to 1.681) | 1.469 (1.288 to 1.67) | 1.467 (1.282 to 1.67) | 1.473 (1.288 to 1.66) | 1.475 (1.294 to 1.68) | 1.477 (1.3 to 1.679) | 1.475 (1.302 to 1.674) | 1.476 (1.292 to 1.68) | 1.481 (1.309 to 1.681) | 1.478 (1.295 to 1.683) | 1.473 (1.291 to 1.661) | 1.473 (1.29 to 1.676) | 1.476 (1.289 to 1.662) | 1.476 (1.289 to 1.662) | 1.476 (1.289 to 1.662) | 1.476 (1.289 to 1.662) |
| Tracheal, bronchus, and lung cancer | Low exposure | Both | Males | | | | | | | | 1.0 (1.0 to 1.0) | 1.0 (1.0 to 1.0) | 1.0 (1.0 to 1.0) | 1.0 (1.0 to 1.0) | 1.0 (1.0 to 1.0) | 1.0 (1.0 to 1.0) | 1.0 (1.0 to 1.0) | 1.0 (1.0 to 1.0) | 1.0 (1.0 to 1.0) | 1.0 (1.0 to 1.0) | 1.0 (1.0 to 1.0) | 1.0 (1.0 to 1.0) | 1.0 (1.0 to 1.0) | 1.0 (1.0 to 1.0) | 1.0 (1.0 to 1.0) | 1.0 (1.0 to 1.0) | 1.0 (1.0 to 1.0) |
| Tracheal, bronchus, and lung cancer | Low exposure | Both | Females | | | | | | | | 1.0 (1.0 to 1.0) | 1.0 (1.0 to 1.0) | 1.0 (1.0 to 1.0) | 1.0 (1.0 to 1.0) | 1.0 (1.0 to 1.0) | 1.0 (1.0 to 1.0) | 1.0 (1.0 to 1.0) | 1.0 (1.0 to 1.0) | 1.0 (1.0 to 1.0) | 1.0 (1.0 to 1.0) | 1.0 (1.0 to 1.0) | 1.0 (1.0 to 1.0) | 1.0 (1.0 to 1.0) | 1.0 (1.0 to 1.0) | 1.0 (1.0 to 1.0) | 1.0 (1.0 to 1.0) | 1.0 (1.0 to 1.0) |
| Tracheal, bronchus, and lung cancer | No exposure | Both | Males | | | | | | | | 1.0 (1.0 to 1.0) | 1.0 (1.0 to 1.0) | 1.0 (1.0 to 1.0) | 1.0 (1.0 to 1.0) | 1.0 (1.0 to 1.0) | 1.0 (1.0 to 1.0) | 1.0 (1.0 to 1.0) | 1.0 (1.0 to 1.0) | 1.0 (1.0 to 1.0) | 1.0 (1.0 to 1.0) | 1.0 (1.0 to 1.0) | 1.0 (1.0 to 1.0) | 1.0 (1.0 to 1.0) | 1.0 (1.0 to 1.0) | 1.0 (1.0 to 1.0) | 1.0 (1.0 to 1.0) | 1.0 (1.0 to 1.0) |
| Tracheal, bronchus, and lung cancer | No exposure | Both | Females | | | | | | | | 1.0 (1.0 to 1.0) | 1.0 (1.0 to 1.0) | 1.0 (1.0 to 1.0) | 1.0 (1.0 to 1.0) | 1.0 (1.0 to 1.0) | 1.0 (1.0 to 1.0) | 1.0 (1.0 to 1.0) | 1.0 (1.0 to 1.0) | 1.0 (1.0 to 1.0) | 1.0 (1.0 to 1.0) | 1.0 (1.0 to 1.0) | 1.0 (1.0 to 1.0) | 1.0 (1.0 to 1.0) | 1.0 (1.0 to 1.0) | 1.0 (1.0 to 1.0) | 1.0 (1.0 to 1.0) | 1.0 (1.0 to 1.0) |
| **Occupational exposure to formaldehyde** | | | | | | | | | | | | | | | | | | | | | | | | | | | |
| Nasopharynx cancer | High exposure | Both | Males | | | | | | | | 2.222 (1.026 to 4.233) | 2.294 (1.056 to 4.409) | 2.204 (1.023 to 4.036) | 2.211 (0.994 to 4.221) | 2.23 (1.042 to 4.13) | 2.241 (1.077 to 4.068) | 2.265 (1.056 to 4.33) | 2.198 (1.041 to 4.081) | 2.232 (1.104 to 4.091) | 2.215 (1.022 to 4.191) | 2.251 (1.056 to 4.236) | 2.233 (1.022 to 4.329) | 2.234 (1.02 to 4.325) | 2.204 (1.064 to 4.14) | 2.204 (1.064 to 4.14) | 2.204 (1.064 to 4.14) | 2.204 (1.064 to 4.14) |
| Nasopharynx cancer | High exposure | Both | Females | | | | | | | | 2.202 (1.04 to 4.059) | 2.246 (1.039 to 4.184) | 2.227 (1.05 to 4.207) | 2.269 (1.084 to 4.182) | 2.264 (1.069 to 4.385) | 2.217 (1.041 to 4.21) | 2.201 (1.035 to 4.162) | 2.221 (1.035 to 4.234) | 2.225 (1.025 to 4.212) | 2.276 (1.072 to 4.204) | 2.261 (1.04 to 4.208) | 2.258 (1.042 to 4.371) | 2.241 (1.031 to 4.156) | 2.229 (1.025 to 4.237) | 2.229 (1.025 to 4.237) | 2.229 (1.025 to 4.237) | 2.229 (1.025 to 4.237) |
| Nasopharynx cancer | Low exposure | Both | Males | | | | | | | | 1.0 (1.0 to 1.0) | 1.0 (1.0 to 1.0) | 1.0 (1.0 to 1.0) | 1.0 (1.0 to 1.0) | 1.0 (1.0 to 1.0) | 1.0 (1.0 to 1.0) | 1.0 (1.0 to 1.0) | 1.0 (1.0 to 1.0) | 1.0 (1.0 to 1.0) | 1.0 (1.0 to 1.0) | 1.0 (1.0 to 1.0) | 1.0 (1.0 to 1.0) | 1.0 (1.0 to 1.0) | 1.0 (1.0 to 1.0) | 1.0 (1.0 to 1.0) | 1.0 (1.0 to 1.0) | 1.0 (1.0 to 1.0) |
| Nasopharynx cancer | Low exposure | Both | Females | | | | | | | | 1.0 (1.0 to 1.0) | 1.0 (1.0 to 1.0) | 1.0 (1.0 to 1.0) | 1.0 (1.0 to 1.0) | 1.0 (1.0 to 1.0) | 1.0 (1.0 to 1.0) | 1.0 (1.0 to 1.0) | 1.0 (1.0 to 1.0) | 1.0 (1.0 to 1.0) | 1.0 (1.0 to 1.0) | 1.0 (1.0 to 1.0) | 1.0 (1.0 to 1.0) | 1.0 (1.0 to 1.0) | 1.0 (1.0 to 1.0) | 1.0 (1.0 to 1.0) | 1.0 (1.0 to 1.0) | 1.0 (1.0 to 1.0) |
| Nasopharynx cancer | No exposure | Both | Males | | | | | | | | 1.0 (1.0 to 1.0) | 1.0 (1.0 to 1.0) | 1.0 (1.0 to 1.0) | 1.0 (1.0 to 1.0) | 1.0 (1.0 to 1.0) | 1.0 (1.0 to 1.0) | 1.0 (1.0 to 1.0) | 1.0 (1.0 to 1.0) | 1.0 (1.0 to 1.0) | 1.0 (1.0 to 1.0) | 1.0 (1.0 to 1.0) | 1.0 (1.0 to 1.0) | 1.0 (1.0 to 1.0) | 1.0 (1.0 to 1.0) | 1.0 (1.0 to 1.0) | 1.0 (1.0 to 1.0) | 1.0 (1.0 to 1.0) |
| Nasopharynx cancer | No exposure | Both | Females | | | | | | | | 1.0 (1.0 to 1.0) | 1.0 (1.0 to 1.0) | 1.0 (1.0 to 1.0) | 1.0 (1.0 to 1.0) | 1.0 (1.0 to 1.0) | 1.0 (1.0 to 1.0) | 1.0 (1.0 to 1.0) | 1.0 (1.0 to 1.0) | 1.0 (1.0 to 1.0) | 1.0 (1.0 to 1.0) | 1.0 (1.0 to 1.0) | 1.0 (1.0 to 1.0) | 1.0 (1.0 to 1.0) | 1.0 (1.0 to 1.0) | 1.0 (1.0 to 1.0) | 1.0 (1.0 to 1.0) | 1.0 (1.0 to 1.0) |
| Acute lymphoid leukaemia | High exposure | Both | Males | | | | | | | | 1.483 (1.191 to 1.818) | 1.479 (1.183 to 1.83) | 1.474 (1.182 to 1.815) | 1.48 (1.193 to 1.827) | 1.479 (1.197 to 1.804) | 1.467 (1.174 to 1.843) | 1.481 (1.178 to 1.839) | 1.49 (1.199 to 1.831) | 1.47 (1.197 to 1.842) | 1.487 (1.2 to 1.846) | 1.485 (1.188 to 1.803) | 1.48 (1.18 to 1.836) | 1.482 (1.202 to 1.839) | 1.488 (1.198 to 1.827) | 1.488 (1.198 to 1.827) | 1.488 (1.198 to 1.827) | 1.488 (1.198 to 1.827) |
| Acute lymphoid leukaemia | High exposure | Both | Females | | | | | | | | 1.485 (1.199 to 1.845) | 1.485 (1.183 to 1.855) | 1.479 (1.184 to 1.844) | 1.471 (1.181 to 1.774) | 1.469 (1.183 to 1.819) | 1.486 (1.196 to 1.823) | 1.485 (1.19 to 1.848) | 1.481 (1.191 to 1.856) | 1.48 (1.199 to 1.814) | 1.47 (1.184 to 1.812) | 1.473 (1.19 to 1.814) | 1.471 (1.194 to 1.789) | 1.49 (1.209 to 1.859) | 1.464 (1.18 to 1.788) | 1.464 (1.18 to 1.788) | 1.464 (1.18 to 1.788) | 1.464 (1.18 to 1.788) |
| Acute lymphoid leukaemia | Low exposure | Both | Males | | | | | | | | 1.0 (1.0 to 1.0) | 1.0 (1.0 to 1.0) | 1.0 (1.0 to 1.0) | 1.0 (1.0 to 1.0) | 1.0 (1.0 to 1.0) | 1.0 (1.0 to 1.0) | 1.0 (1.0 to 1.0) | 1.0 (1.0 to 1.0) | 1.0 (1.0 to 1.0) | 1.0 (1.0 to 1.0) | 1.0 (1.0 to 1.0) | 1.0 (1.0 to 1.0) | 1.0 (1.0 to 1.0) | 1.0 (1.0 to 1.0) | 1.0 (1.0 to 1.0) | 1.0 (1.0 to 1.0) | 1.0 (1.0 to 1.0) |
| Acute lymphoid leukaemia | Low exposure | Both | Females | | | | | | | | 1.0 (1.0 to 1.0) | 1.0 (1.0 to 1.0) | 1.0 (1.0 to 1.0) | 1.0 (1.0 to 1.0) | 1.0 (1.0 to 1.0) | 1.0 (1.0 to 1.0) | 1.0 (1.0 to 1.0) | 1.0 (1.0 to 1.0) | 1.0 (1.0 to 1.0) | 1.0 (1.0 to 1.0) | 1.0 (1.0 to 1.0) | 1.0 (1.0 to 1.0) | 1.0 (1.0 to 1.0) | 1.0 (1.0 to 1.0) | 1.0 (1.0 to 1.0) | 1.0 (1.0 to 1.0) | 1.0 (1.0 to 1.0) |
| Acute lymphoid leukaemia | No exposure | Both | Males | | | | | | | | 1.0 (1.0 to 1.0) | 1.0 (1.0 to 1.0) | 1.0 (1.0 to 1.0) | 1.0 (1.0 to 1.0) | 1.0 (1.0 to 1.0) | 1.0 (1.0 to 1.0) | 1.0 (1.0 to 1.0) | 1.0 (1.0 to 1.0) | 1.0 (1.0 to 1.0) | 1.0 (1.0 to 1.0) | 1.0 (1.0 to 1.0) | 1.0 (1.0 to 1.0) | 1.0 (1.0 to 1.0) | 1.0 (1.0 to 1.0) | 1.0 (1.0 to 1.0) | 1.0 (1.0 to 1.0) | 1.0 (1.0 to 1.0) |
| Acute lymphoid leukaemia | No exposure | Both | Females | | | | | | | | 1.0 (1.0 to 1.0) | 1.0 (1.0 to 1.0) | 1.0 (1.0 to 1.0) | 1.0 (1.0 to 1.0) | 1.0 (1.0 to 1.0) | 1.0 (1.0 to 1.0) | 1.0 (1.0 to 1.0) | 1.0 (1.0 to 1.0) | 1.0 (1.0 to 1.0) | 1.0 (1.0 to 1.0) | 1.0 (1.0 to 1.0) | 1.0 (1.0 to 1.0) | 1.0 (1.0 to 1.0) | 1.0 (1.0 to 1.0) | 1.0 (1.0 to 1.0) | 1.0 (1.0 to 1.0) | 1.0 (1.0 to 1.0) |
| Chronic lymphoid leukaemia | High exposure | Both | Males | | | | | | | | 1.483 (1.191 to 1.818) | 1.479 (1.183 to 1.83) | 1.474 (1.182 to 1.815) | 1.48 (1.193 to 1.827) | 1.479 (1.197 to 1.804) | 1.467 (1.174 to 1.843) | 1.481 (1.178 to 1.839) | 1.49 (1.199 to 1.831) | 1.47 (1.197 to 1.842) | 1.487 (1.2 to 1.846) | 1.485 (1.188 to 1.803) | 1.48 (1.18 to 1.836) | 1.482 (1.202 to 1.839) | 1.488 (1.198 to 1.827) | 1.488 (1.198 to 1.827) | 1.488 (1.198 to 1.827) | 1.488 (1.198 to 1.827) |
| Chronic lymphoid leukaemia | High exposure | Both | Females | | | | | | | | 1.485 (1.199 to 1.845) | 1.485 (1.183 to 1.855) | 1.479 (1.184 to 1.844) | 1.471 (1.181 to 1.774) | 1.469 (1.183 to 1.819) | 1.486 (1.196 to 1.823) | 1.485 (1.19 to 1.848) | 1.481 (1.191 to 1.856) | 1.48 (1.199 to 1.814) | 1.47 (1.184 to 1.812) | 1.473 (1.19 to 1.814) | 1.471 (1.194 to 1.789) | 1.49 (1.209 to 1.859) | 1.464 (1.18 to 1.788) | 1.464 (1.18 to 1.788) | 1.464 (1.18 to 1.788) | 1.464 (1.18 to 1.788) |
| Chronic lymphoid leukaemia | Low exposure | Both | Males | | | | | | | | 1.0 (1.0 to 1.0) | 1.0 (1.0 to 1.0) | 1.0 (1.0 to 1.0) | 1.0 (1.0 to 1.0) | 1.0 (1.0 to 1.0) | 1.0 (1.0 to 1.0) | 1.0 (1.0 to 1.0) | 1.0 (1.0 to 1.0) | 1.0 (1.0 to 1.0) | 1.0 (1.0 to 1.0) | 1.0 (1.0 to 1.0) | 1.0 (1.0 to 1.0) | 1.0 (1.0 to 1.0) | 1.0 (1.0 to 1.0) | 1.0 (1.0 to 1.0) | 1.0 (1.0 to 1.0) | 1.0 (1.0 to 1.0) |
| Chronic lymphoid leukaemia | Low exposure | Both | Females | | | | | | | | 1.0 (1.0 to 1.0) | 1.0 (1.0 to 1.0) | 1.0 (1.0 to 1.0) | 1.0 (1.0 to 1.0) | 1.0 (1.0 to 1.0) | 1.0 (1.0 to 1.0) | 1.0 (1.0 to 1.0) | 1.0 (1.0 to 1.0) | 1.0 (1.0 to 1.0) | 1.0 (1.0 to 1.0) | 1.0 (1.0 to 1.0) | 1.0 (1.0 to 1.0) | 1.0 (1.0 to 1.0) | 1.0 (1.0 to 1.0) | 1.0 (1.0 to 1.0) | 1.0 (1.0 to 1.0) | 1.0 (1.0 to 1.0) |
| Chronic lymphoid leukaemia | No exposure | Both | Males | | | | | | | | 1.0 (1.0 to 1.0) | 1.0 (1.0 to 1.0) | 1.0 (1.0 to 1.0) | 1.0 (1.0 to 1.0) | 1.0 (1.0 to 1.0) | 1.0 (1.0 to 1.0) | 1.0 (1.0 to 1.0) | 1.0 (1.0 to 1.0) | 1.0 (1.0 to 1.0) | 1.0 (1.0 to 1.0) | 1.0 (1.0 to 1.0) | 1.0 (1.0 to 1.0) | 1.0 (1.0 to 1.0) | 1.0 (1.0 to 1.0) | 1.0 (1.0 to 1.0) | 1.0 (1.0 to 1.0) | 1.0 (1.0 to 1.0) |
| Chronic lymphoid leukaemia | No exposure | Both | Females | | | | | | | | 1.0 (1.0 to 1.0) | 1.0 (1.0 to 1.0) | 1.0 (1.0 to 1.0) | 1.0 (1.0 to 1.0) | 1.0 (1.0 to 1.0) | 1.0 (1.0 to 1.0) | 1.0 (1.0 to 1.0) | 1.0 (1.0 to 1.0) | 1.0 (1.0 to 1.0) | 1.0 (1.0 to 1.0) | 1.0 (1.0 to 1.0) | 1.0 (1.0 to 1.0) | 1.0 (1.0 to 1.0) | 1.0 (1.0 to 1.0) | 1.0 (1.0 to 1.0) | 1.0 (1.0 to 1.0) | 1.0 (1.0 to 1.0) |
| Acute myeloid leukaemia | High exposure | Both | Males | | | | | | | | 1.483 (1.191 to 1.818) | 1.479 (1.183 to 1.83) | 1.474 (1.182 to 1.815) | 1.48 (1.193 to 1.827) | 1.479 (1.197 to 1.804) | 1.467 (1.174 to 1.843) | 1.481 (1.178 to 1.839) | 1.49 (1.199 to 1.831) | 1.47 (1.197 to 1.842) | 1.487 (1.2 to 1.846) | 1.485 (1.188 to 1.803) | 1.48 (1.18 to 1.836) | 1.482 (1.202 to 1.839) | 1.488 (1.198 to 1.827) | 1.488 (1.198 to 1.827) | 1.488 (1.198 to 1.827) | 1.488 (1.198 to 1.827) |
| Acute myeloid leukaemia | High exposure | Both | Females | | | | | | | | 1.485 (1.199 to 1.845) | 1.485 (1.183 to 1.855) | 1.479 (1.184 to 1.844) | 1.471 (1.181 to 1.774) | 1.469 (1.183 to 1.819) | 1.486 (1.196 to 1.823) | 1.485 (1.19 to 1.848) | 1.481 (1.191 to 1.856) | 1.48 (1.199 to 1.814) | 1.47 (1.184 to 1.812) | 1.473 (1.19 to 1.814) | 1.471 (1.194 to 1.789) | 1.49 (1.209 to 1.859) | 1.464 (1.18 to 1.788) | 1.464 (1.18 to 1.788) | 1.464 (1.18 to 1.788) | 1.464 (1.18 to 1.788) |
| Acute myeloid leukaemia | Low exposure | Both | Males | | | | | | | | 1.0 (1.0 to 1.0) | 1.0 (1.0 to 1.0) | 1.0 (1.0 to 1.0) | 1.0 (1.0 to 1.0) | 1.0 (1.0 to 1.0) | 1.0 (1.0 to 1.0) | 1.0 (1.0 to 1.0) | 1.0 (1.0 to 1.0) | 1.0 (1.0 to 1.0) | 1.0 (1.0 to 1.0) | 1.0 (1.0 to 1.0) | 1.0 (1.0 to 1.0) | 1.0 (1.0 to 1.0) | 1.0 (1.0 to 1.0) | 1.0 (1.0 to 1.0) | 1.0 (1.0 to 1.0) | 1.0 (1.0 to 1.0) |
| Acute myeloid leukaemia | Low exposure | Both | Females | | | | | | | | 1.0 (1.0 to 1.0) | 1.0 (1.0 to 1.0) | 1.0 (1.0 to 1.0) | 1.0 (1.0 to 1.0) | 1.0 (1.0 to 1.0) | 1.0 (1.0 to 1.0) | 1.0 (1.0 to 1.0) | 1.0 (1.0 to 1.0) | 1.0 (1.0 to 1.0) | 1.0 (1.0 to 1.0) | 1.0 (1.0 to 1.0) | 1.0 (1.0 to 1.0) | 1.0 (1.0 to 1.0) | 1.0 (1.0 to 1.0) | 1.0 (1.0 to 1.0) | 1.0 (1.0 to 1.0) | 1.0 (1.0 to 1.0) |
| Acute myeloid leukaemia | No exposure | Both | Males | | | | | | | | 1.0 (1.0 to 1.0) | 1.0 (1.0 to 1.0) | 1.0 (1.0 to 1.0) | 1.0 (1.0 to 1.0) | 1.0 (1.0 to 1.0) | 1.0 (1.0 to 1.0) | 1.0 (1.0 to 1.0) | 1.0 (1.0 to 1.0) | 1.0 (1.0 to 1.0) | 1.0 (1.0 to 1.0) | 1.0 (1.0 to 1.0) | 1.0 (1.0 to 1.0) | 1.0 (1.0 to 1.0) | 1.0 (1.0 to 1.0) | 1.0 (1.0 to 1.0) | 1.0 (1.0 to 1.0) | 1.0 (1.0 to 1.0) |
| Acute myeloid leukaemia | No exposure | Both | Females | | | | | | | | 1.0 (1.0 to 1.0) | 1.0 (1.0 to 1.0) | 1.0 (1.0 to 1.0) | 1.0 (1.0 to 1.0) | 1.0 (1.0 to 1.0) | 1.0 (1.0 to 1.0) | 1.0 (1.0 to 1.0) | 1.0 (1.0 to 1.0) | 1.0 (1.0 to 1.0) | 1.0 (1.0 to 1.0) | 1.0 (1.0 to 1.0) | 1.0 (1.0 to 1.0) | 1.0 (1.0 to 1.0) | 1.0 (1.0 to 1.0) | 1.0 (1.0 to 1.0) | 1.0 (1.0 to 1.0) | 1.0 (1.0 to 1.0) |
| Chronic myeloid leukaemia | High exposure | Both | Males | | | | | | | | 1.483 (1.191 to 1.818) | 1.479 (1.183 to 1.83) | 1.474 (1.182 to 1.815) | 1.48 (1.193 to 1.827) | 1.479 (1.197 to 1.804) | 1.467 (1.174 to 1.843) | 1.481 (1.178 to 1.839) | 1.49 (1.199 to 1.831) | 1.47 (1.197 to 1.842) | 1.487 (1.2 to 1.846) | 1.485 (1.188 to 1.803) | 1.48 (1.18 to 1.836) | 1.482 (1.202 to 1.839) | 1.488 (1.198 to 1.827) | 1.488 (1.198 to 1.827) | 1.488 (1.198 to 1.827) | 1.488 (1.198 to 1.827) |
| Chronic myeloid leukaemia | High exposure | Both | Females | | | | | | | | 1.485 (1.199 to 1.845) | 1.485 (1.183 to 1.855) | 1.479 (1.184 to 1.844) | 1.471 (1.181 to 1.774) | 1.469 (1.183 to 1.819) | 1.486 (1.196 to 1.823) | 1.485 (1.19 to 1.848) | 1.481 (1.191 to 1.856) | 1.48 (1.199 to 1.814) | 1.47 (1.184 to 1.812) | 1.473 (1.19 to 1.814) | 1.471 (1.194 to 1.789) | 1.49 (1.209 to 1.859) | 1.464 (1.18 to 1.788) | 1.464 (1.18 to 1.788) | 1.464 (1.18 to 1.788) | 1.464 (1.18 to 1.788) |
| Chronic myeloid leukaemia | Low exposure | Both | Males | | | | | | | | 1.0 (1.0 to 1.0) | 1.0 (1.0 to 1.0) | 1.0 (1.0 to 1.0) | 1.0 (1.0 to 1.0) | 1.0 (1.0 to 1.0) | 1.0 (1.0 to 1.0) | 1.0 (1.0 to 1.0) | 1.0 (1.0 to 1.0) | 1.0 (1.0 to 1.0) | 1.0 (1.0 to 1.0) | 1.0 (1.0 to 1.0) | 1.0 (1.0 to 1.0) | 1.0 (1.0 to 1.0) | 1.0 (1.0 to 1.0) | 1.0 (1.0 to 1.0) | 1.0 (1.0 to 1.0) | 1.0 (1.0 to 1.0) |
| Chronic myeloid leukaemia | Low exposure | Both | Females | | | | | | | | 1.0 (1.0 to 1.0) | 1.0 (1.0 to 1.0) | 1.0 (1.0 to 1.0) | 1.0 (1.0 to 1.0) | 1.0 (1.0 to 1.0) | 1.0 (1.0 to 1.0) | 1.0 (1.0 to 1.0) | 1.0 (1.0 to 1.0) | 1.0 (1.0 to 1.0) | 1.0 (1.0 to 1.0) | 1.0 (1.0 to 1.0) | 1.0 (1.0 to 1.0) | 1.0 (1.0 to 1.0) | 1.0 (1.0 to 1.0) | 1.0 (1.0 to 1.0) | 1.0 (1.0 to 1.0) | 1.0 (1.0 to 1.0) |
| Chronic myeloid leukaemia | No exposure | Both | Males | | | | | | | | 1.0 (1.0 to 1.0) | 1.0 (1.0 to 1.0) | 1.0 (1.0 to 1.0) | 1.0 (1.0 to 1.0) | 1.0 (1.0 to 1.0) | 1.0 (1.0 to 1.0) | 1.0 (1.0 to 1.0) | 1.0 (1.0 to 1.0) | 1.0 (1.0 to 1.0) | 1.0 (1.0 to 1.0) | 1.0 (1.0 to 1.0) | 1.0 (1.0 to 1.0) | 1.0 (1.0 to 1.0) | 1.0 (1.0 to 1.0) | 1.0 (1.0 to 1.0) | 1.0 (1.0 to 1.0) | 1.0 (1.0 to 1.0) |
| Chronic myeloid leukaemia | No exposure | Both | Females | | | | | | | | 1.0 (1.0 to 1.0) | 1.0 (1.0 to 1.0) | 1.0 (1.0 to 1.0) | 1.0 (1.0 to 1.0) | 1.0 (1.0 to 1.0) | 1.0 (1.0 to 1.0) | 1.0 (1.0 to 1.0) | 1.0 (1.0 to 1.0) | 1.0 (1.0 to 1.0) | 1.0 (1.0 to 1.0) | 1.0 (1.0 to 1.0) | 1.0 (1.0 to 1.0) | 1.0 (1.0 to 1.0) | 1.0 (1.0 to 1.0) | 1.0 (1.0 to 1.0) | 1.0 (1.0 to 1.0) | 1.0 (1.0 to 1.0) |
| Other leukaemia | High exposure | Both | Males | | | | | | | | 1.483 (1.191 to 1.818) | 1.479 (1.183 to 1.83) | 1.474 (1.182 to 1.815) | 1.48 (1.193 to 1.827) | 1.479 (1.197 to 1.804) | 1.467 (1.174 to 1.843) | 1.481 (1.178 to 1.839) | 1.49 (1.199 to 1.831) | 1.47 (1.197 to 1.842) | 1.487 (1.2 to 1.846) | 1.485 (1.188 to 1.803) | 1.48 (1.18 to 1.836) | 1.482 (1.202 to 1.839) | 1.488 (1.198 to 1.827) | 1.488 (1.198 to 1.827) | 1.488 (1.198 to 1.827) | 1.488 (1.198 to 1.827) |
| Other leukaemia | High exposure | Both | Females | | | | | | | | 1.485 (1.199 to 1.845) | 1.485 (1.183 to 1.855) | 1.479 (1.184 to 1.844) | 1.471 (1.181 to 1.774) | 1.469 (1.183 to 1.819) | 1.486 (1.196 to 1.823) | 1.485 (1.19 to 1.848) | 1.481 (1.191 to 1.856) | 1.48 (1.199 to 1.814) | 1.47 (1.184 to 1.812) | 1.473 (1.19 to 1.814) | 1.471 (1.194 to 1.789) | 1.49 (1.209 to 1.859) | 1.464 (1.18 to 1.788) | 1.464 (1.18 to 1.788) | 1.464 (1.18 to 1.788) | 1.464 (1.18 to 1.788) |
| Other leukaemia | Low exposure | Both | Males | | | | | | | | 1.0 (1.0 to 1.0) | 1.0 (1.0 to 1.0) | 1.0 (1.0 to 1.0) | 1.0 (1.0 to 1.0) | 1.0 (1.0 to 1.0) | 1.0 (1.0 to 1.0) | 1.0 (1.0 to 1.0) | 1.0 (1.0 to 1.0) | 1.0 (1.0 to 1.0) | 1.0 (1.0 to 1.0) | 1.0 (1.0 to 1.0) | 1.0 (1.0 to 1.0) | 1.0 (1.0 to 1.0) | 1.0 (1.0 to 1.0) | 1.0 (1.0 to 1.0) | 1.0 (1.0 to 1.0) | 1.0 (1.0 to 1.0) |
| Other leukaemia | Low exposure | Both | Females | | | | | | | | 1.0 (1.0 to 1.0) | 1.0 (1.0 to 1.0) | 1.0 (1.0 to 1.0) | 1.0 (1.0 to 1.0) | 1.0 (1.0 to 1.0) | 1.0 (1.0 to 1.0) | 1.0 (1.0 to 1.0) | 1.0 (1.0 to 1.0) | 1.0 (1.0 to 1.0) | 1.0 (1.0 to 1.0) | 1.0 (1.0 to 1.0) | 1.0 (1.0 to 1.0) | 1.0 (1.0 to 1.0) | 1.0 (1.0 to 1.0) | 1.0 (1.0 to 1.0) | 1.0 (1.0 to 1.0) | 1.0 (1.0 to 1.0) |
| Other leukaemia | No exposure | Both | Males | | | | | | | | 1.0 (1.0 to 1.0) | 1.0 (1.0 to 1.0) | 1.0 (1.0 to 1.0) | 1.0 (1.0 to 1.0) | 1.0 (1.0 to 1.0) | 1.0 (1.0 to 1.0) | 1.0 (1.0 to 1.0) | 1.0 (1.0 to 1.0) | 1.0 (1.0 to 1.0) | 1.0 (1.0 to 1.0) | 1.0 (1.0 to 1.0) | 1.0 (1.0 to 1.0) | 1.0 (1.0 to 1.0) | 1.0 (1.0 to 1.0) | 1.0 (1.0 to 1.0) | 1.0 (1.0 to 1.0) | 1.0 (1.0 to 1.0) |
| Other leukaemia | No exposure | Both | Females | | | | | | | | 1.0 (1.0 to 1.0) | 1.0 (1.0 to 1.0) | 1.0 (1.0 to 1.0) | 1.0 (1.0 to 1.0) | 1.0 (1.0 to 1.0) | 1.0 (1.0 to 1.0) | 1.0 (1.0 to 1.0) | 1.0 (1.0 to 1.0) | 1.0 (1.0 to 1.0) | 1.0 (1.0 to 1.0) | 1.0 (1.0 to 1.0) | 1.0 (1.0 to 1.0) | 1.0 (1.0 to 1.0) | 1.0 (1.0 to 1.0) | 1.0 (1.0 to 1.0) | 1.0 (1.0 to 1.0) | 1.0 (1.0 to 1.0) |
| **Occupational exposure to nickel** | | | | | | | | | | | | | | | | | | | | | | | | | | | |
| Tracheal, bronchus, and lung cancer | High exposure | Both | Both | | | | | | | | 2.148 (1.296 to 3.297) | 2.148 (1.296 to 3.297) | 2.148 (1.296 to 3.297) | 2.148 (1.296 to 3.297) | 2.148 (1.296 to 3.297) | 2.148 (1.296 to 3.297) | 2.148 (1.296 to 3.297) | 2.148 (1.296 to 3.297) | 2.148 (1.296 to 3.297) | 2.148 (1.296 to 3.297) | 2.148 (1.296 to 3.297) | 2.148 (1.296 to 3.297) | 2.148 (1.296 to 3.297) | 2.148 (1.296 to 3.297) | 2.148 (1.296 to 3.297) | 2.148 (1.296 to 3.297) | 2.148 (1.296 to 3.297) |
| Tracheal, bronchus, and lung cancer | Low exposure | Both | Both | | | | | | | | 1.538 (0.606 to 3.355) | 1.538 (0.606 to 3.355) | 1.538 (0.606 to 3.355) | 1.538 (0.606 to 3.355) | 1.538 (0.606 to 3.355) | 1.538 (0.606 to 3.355) | 1.538 (0.606 to 3.355) | 1.538 (0.606 to 3.355) | 1.538 (0.606 to 3.355) | 1.538 (0.606 to 3.355) | 1.538 (0.606 to 3.355) | 1.538 (0.606 to 3.355) | 1.538 (0.606 to 3.355) | 1.538 (0.606 to 3.355) | 1.538 (0.606 to 3.355) | 1.538 (0.606 to 3.355) | 1.538 (0.606 to 3.355) |
| Tracheal, bronchus, and lung cancer | No exposure | Both | Both | | | | | | | | 1.0 (1.0 to 1.0) | 1.0 (1.0 to 1.0) | 1.0 (1.0 to 1.0) | 1.0 (1.0 to 1.0) | 1.0 (1.0 to 1.0) | 1.0 (1.0 to 1.0) | 1.0 (1.0 to 1.0) | 1.0 (1.0 to 1.0) | 1.0 (1.0 to 1.0) | 1.0 (1.0 to 1.0) | 1.0 (1.0 to 1.0) | 1.0 (1.0 to 1.0) | 1.0 (1.0 to 1.0) | 1.0 (1.0 to 1.0) | 1.0 (1.0 to 1.0) | 1.0 (1.0 to 1.0) | 1.0 (1.0 to 1.0) |
| **Occupational exposure to polycyclic aromatic hydrocarbons** | | | | | | | | | | | | | | | | | | | | | | | | | | | |
| Tracheal, bronchus, and lung cancer | High exposure | Both | Males | | | | | | | | 1.31 (1.165 to 1.468) | 1.304 (1.146 to 1.466) | 1.313 (1.164 to 1.485) | 1.314 (1.167 to 1.477) | 1.315 (1.17 to 1.474) | 1.314 (1.16 to 1.475) | 1.315 (1.154 to 1.477) | 1.312 (1.16 to 1.477) | 1.318 (1.166 to 1.483) | 1.311 (1.158 to 1.479) | 1.313 (1.166 to 1.485) | 1.314 (1.154 to 1.483) | 1.313 (1.161 to 1.478) | 1.309 (1.176 to 1.477) | 1.309 (1.176 to 1.477) | 1.309 (1.176 to 1.477) | 1.309 (1.176 to 1.477) |
| Tracheal, bronchus, and lung cancer | High exposure | Both | Females | | | | | | | | 1.31 (1.154 to 1.486) | 1.311 (1.157 to 1.469) | 1.313 (1.154 to 1.472) | 1.313 (1.155 to 1.469) | 1.316 (1.162 to 1.483) | 1.313 (1.156 to 1.476) | 1.314 (1.155 to 1.483) | 1.314 (1.171 to 1.483) | 1.311 (1.146 to 1.477) | 1.312 (1.163 to 1.481) | 1.309 (1.147 to 1.489) | 1.316 (1.168 to 1.475) | 1.315 (1.15 to 1.479) | 1.315 (1.166 to 1.481) | 1.315 (1.166 to 1.481) | 1.315 (1.166 to 1.481) | 1.315 (1.166 to 1.481) |
| Tracheal, bronchus, and lung cancer | Low exposure | Both | Males | | | | | | | | 1.0 (1.0 to 1.0) | 1.0 (1.0 to 1.0) | 1.0 (1.0 to 1.0) | 1.0 (1.0 to 1.0) | 1.0 (1.0 to 1.0) | 1.0 (1.0 to 1.0) | 1.0 (1.0 to 1.0) | 1.0 (1.0 to 1.0) | 1.0 (1.0 to 1.0) | 1.0 (1.0 to 1.0) | 1.0 (1.0 to 1.0) | 1.0 (1.0 to 1.0) | 1.0 (1.0 to 1.0) | 1.0 (1.0 to 1.0) | 1.0 (1.0 to 1.0) | 1.0 (1.0 to 1.0) | 1.0 (1.0 to 1.0) |
| Tracheal, bronchus, and lung cancer | Low exposure | Both | Females | | | | | | | | 1.0 (1.0 to 1.0) | 1.0 (1.0 to 1.0) | 1.0 (1.0 to 1.0) | 1.0 (1.0 to 1.0) | 1.0 (1.0 to 1.0) | 1.0 (1.0 to 1.0) | 1.0 (1.0 to 1.0) | 1.0 (1.0 to 1.0) | 1.0 (1.0 to 1.0) | 1.0 (1.0 to 1.0) | 1.0 (1.0 to 1.0) | 1.0 (1.0 to 1.0) | 1.0 (1.0 to 1.0) | 1.0 (1.0 to 1.0) | 1.0 (1.0 to 1.0) | 1.0 (1.0 to 1.0) | 1.0 (1.0 to 1.0) |
| Tracheal, bronchus, and lung cancer | No exposure | Both | Males | | | | | | | | 1.0 (1.0 to 1.0) | 1.0 (1.0 to 1.0) | 1.0 (1.0 to 1.0) | 1.0 (1.0 to 1.0) | 1.0 (1.0 to 1.0) | 1.0 (1.0 to 1.0) | 1.0 (1.0 to 1.0) | 1.0 (1.0 to 1.0) | 1.0 (1.0 to 1.0) | 1.0 (1.0 to 1.0) | 1.0 (1.0 to 1.0) | 1.0 (1.0 to 1.0) | 1.0 (1.0 to 1.0) | 1.0 (1.0 to 1.0) | 1.0 (1.0 to 1.0) | 1.0 (1.0 to 1.0) | 1.0 (1.0 to 1.0) |
| Tracheal, bronchus, and lung cancer | No exposure | Both | Females | | | | | | | | 1.0 (1.0 to 1.0) | 1.0 (1.0 to 1.0) | 1.0 (1.0 to 1.0) | 1.0 (1.0 to 1.0) | 1.0 (1.0 to 1.0) | 1.0 (1.0 to 1.0) | 1.0 (1.0 to 1.0) | 1.0 (1.0 to 1.0) | 1.0 (1.0 to 1.0) | 1.0 (1.0 to 1.0) | 1.0 (1.0 to 1.0) | 1.0 (1.0 to 1.0) | 1.0 (1.0 to 1.0) | 1.0 (1.0 to 1.0) | 1.0 (1.0 to 1.0) | 1.0 (1.0 to 1.0) | 1.0 (1.0 to 1.0) |
| **Occupational exposure to silica** | | | | | | | | | | | | | | | | | | | | | | | | | | | |
| Tracheal, bronchus, and lung cancer | High exposure | Both | Both | | | | | | | | 1.698 (1.164 to 2.259) | 1.698 (1.164 to 2.259) | 1.698 (1.164 to 2.259) | 1.698 (1.164 to 2.259) | 1.698 (1.164 to 2.259) | 1.698 (1.164 to 2.259) | 1.698 (1.164 to 2.259) | 1.698 (1.164 to 2.259) | 1.698 (1.164 to 2.259) | 1.698 (1.164 to 2.259) | 1.698 (1.164 to 2.259) | 1.698 (1.164 to 2.259) | 1.698 (1.164 to 2.259) | 1.698 (1.164 to 2.259) | 1.698 (1.164 to 2.259) | 1.698 (1.164 to 2.259) | 1.698 (1.164 to 2.259) |
| Tracheal, bronchus, and lung cancer | Low exposure | Both | Both | | | | | | | | 1.537 (1.063 to 1.986) | 1.537 (1.063 to 1.986) | 1.537 (1.063 to 1.986) | 1.537 (1.063 to 1.986) | 1.537 (1.063 to 1.986) | 1.537 (1.063 to 1.986) | 1.537 (1.063 to 1.986) | 1.537 (1.063 to 1.986) | 1.537 (1.063 to 1.986) | 1.537 (1.063 to 1.986) | 1.537 (1.063 to 1.986) | 1.537 (1.063 to 1.986) | 1.537 (1.063 to 1.986) | 1.537 (1.063 to 1.986) | 1.537 (1.063 to 1.986) | 1.537 (1.063 to 1.986) | 1.537 (1.063 to 1.986) |
| Tracheal, bronchus, and lung cancer | No exposure | Both | Both | | | | | | | | 1.0 (1.0 to 1.0) | 1.0 (1.0 to 1.0) | 1.0 (1.0 to 1.0) | 1.0 (1.0 to 1.0) | 1.0 (1.0 to 1.0) | 1.0 (1.0 to 1.0) | 1.0 (1.0 to 1.0) | 1.0 (1.0 to 1.0) | 1.0 (1.0 to 1.0) | 1.0 (1.0 to 1.0) | 1.0 (1.0 to 1.0) | 1.0 (1.0 to 1.0) | 1.0 (1.0 to 1.0) | 1.0 (1.0 to 1.0) | 1.0 (1.0 to 1.0) | 1.0 (1.0 to 1.0) | 1.0 (1.0 to 1.0) |

**Appendix Table 6a. Relative risks used by age and sex for each outcome for all risk factors except for ambient air pollution alcohol, and smoking.**

| | | | | Ages | | | | | | | | | | | | | | | | | | | | |
|---|---|---|---|---|---|---|---|---|---|---|---|---|---|---|---|---|---|---|---|---|---|---|---|---|
| **Risk - Outcome** | **Category / Units** | **Morbidity / Mortality** | **Sex** | **All-age** | **0-6 days** | **7-27 days** | **28-364 days** | **1-4 years** | **5-9 years** | **10-14 years** | **15-19 years** | **20-24 years** | **25-29 years** | **30-34 years** | **35-39 years** | **40-44 years** | **45-49 years** | **50-54 years** | **55-59 years** | **60-64 years** | **65-69 years** | **70-74 years** | **75-79 years** | **80-84 years** | **85-89 years** | **90-94 years** | **95+ years** |
| **Occupational exposure to sulphuric acid** | | | | | | | | | | | | | | | | | | | | | | | | | | | |
| Larynx cancer | High exposure | Both | Both | | | | | | | | 4.566 (2.122 to 8.328) | 4.566 (2.122 to 8.328) | 4.566 (2.122 to 8.328) | 4.566 (2.122 to 8.328) | 4.566 (2.122 to 8.328) | 4.566 (2.122 to 8.328) | 4.566 (2.122 to 8.328) | 4.566 (2.122 to 8.328) | 4.566 (2.122 to 8.328) | 4.566 (2.122 to 8.328) | 4.566 (2.122 to 8.328) | 4.566 (2.122 to 8.328) | 4.566 (2.122 to 8.328) | 4.566 (2.122 to 8.328) | 4.566 (2.122 to 8.328) | 4.566 (2.122 to 8.328) | 4.566 (2.122 to 8.328) |
| Larynx cancer | Low exposure | Both | Both | | | | | | | | 2.024 (0.944 to 3.782) | 2.024 (0.944 to 3.782) | 2.024 (0.944 to 3.782) | 2.024 (0.944 to 3.782) | 2.024 (0.944 to 3.782) | 2.024 (0.944 to 3.782) | 2.024 (0.944 to 3.782) | 2.024 (0.944 to 3.782) | 2.024 (0.944 to 3.782) | 2.024 (0.944 to 3.782) | 2.024 (0.944 to 3.782) | 2.024 (0.944 to 3.782) | 2.024 (0.944 to 3.782) | 2.024 (0.944 to 3.782) | 2.024 (0.944 to 3.782) | 2.024 (0.944 to 3.782) | 2.024 (0.944 to 3.782) |
| Larynx cancer | No exposure | Both | Both | | | | | | | | 1.0 (1.0 to 1.0) | 1.0 (1.0 to 1.0) | 1.0 (1.0 to 1.0) | 1.0 (1.0 to 1.0) | 1.0 (1.0 to 1.0) | 1.0 (1.0 to 1.0) | 1.0 (1.0 to 1.0) | 1.0 (1.0 to 1.0) | 1.0 (1.0 to 1.0) | 1.0 (1.0 to 1.0) | 1.0 (1.0 to 1.0) | 1.0 (1.0 to 1.0) | 1.0 (1.0 to 1.0) | 1.0 (1.0 to 1.0) | 1.0 (1.0 to 1.0) | 1.0 (1.0 to 1.0) | 1.0 (1.0 to 1.0) |
| **Occupational exposure to trichloroethylene** | | | | | | | | | | | | | | | | | | | | | | | | | | | |
| Kidney cancer | High exposure | Both | Both | | | | | | | | 1.245 (1.054 to 1.456) | 1.245 (1.054 to 1.456) | 1.245 (1.054 to 1.456) | 1.245 (1.054 to 1.456) | 1.245 (1.054 to 1.456) | 1.245 (1.054 to 1.456) | 1.245 (1.054 to 1.456) | 1.245 (1.054 to 1.456) | 1.245 (1.054 to 1.456) | 1.245 (1.054 to 1.456) | 1.245 (1.054 to 1.456) | 1.245 (1.054 to 1.456) | 1.245 (1.054 to 1.456) | 1.245 (1.054 to 1.456) | 1.245 (1.054 to 1.456) | 1.245 (1.054 to 1.456) | 1.245 (1.054 to 1.456) |
| Kidney cancer | Low exposure | Both | Both | | | | | | | | 1.0 (1.0 to 1.0) | 1.0 (1.0 to 1.0) | 1.0 (1.0 to 1.0) | 1.0 (1.0 to 1.0) | 1.0 (1.0 to 1.0) | 1.0 (1.0 to 1.0) | 1.0 (1.0 to 1.0) | 1.0 (1.0 to 1.0) | 1.0 (1.0 to 1.0) | 1.0 (1.0 to 1.0) | 1.0 (1.0 to 1.0) | 1.0 (1.0 to 1.0) | 1.0 (1.0 to 1.0) | 1.0 (1.0 to 1.0) | 1.0 (1.0 to 1.0) | 1.0 (1.0 to 1.0) | 1.0 (1.0 to 1.0) |
| Kidney cancer | No exposure | Both | Both | | | | | | | | 1.0 (1.0 to 1.0) | 1.0 (1.0 to 1.0) | 1.0 (1.0 to 1.0) | 1.0 (1.0 to 1.0) | 1.0 (1.0 to 1.0) | 1.0 (1.0 to 1.0) | 1.0 (1.0 to 1.0) | 1.0 (1.0 to 1.0) | 1.0 (1.0 to 1.0) | 1.0 (1.0 to 1.0) | 1.0 (1.0 to 1.0) | 1.0 (1.0 to 1.0) | 1.0 (1.0 to 1.0) | 1.0 (1.0 to 1.0) | 1.0 (1.0 to 1.0) | 1.0 (1.0 to 1.0) | 1.0 (1.0 to 1.0) |
| **Occupational asthmagens** | | | | | | | | | | | | | | | | | | | | | | | | | | | |
| Asthma | Admin | Both | Males | | | | | | | | 1.0 (1.0 to 1.0) | 1.0 (1.0 to 1.0) | 1.0 (1.0 to 1.0) | 1.0 (1.0 to 1.0) | 1.0 (1.0 to 1.0) | 1.0 (1.0 to 1.0) | 1.0 (1.0 to 1.0) | 1.0 (1.0 to 1.0) | 1.0 (1.0 to 1.0) | 1.0 (1.0 to 1.0) | 1.0 (1.0 to 1.0) | 1.0 (1.0 to 1.0) | 1.0 (1.0 to 1.0) | 1.0 (1.0 to 1.0) | | | |
| Asthma | Admin | Both | Females | | | | | | | | 1.0 (1.0 to 1.0) | 1.0 (1.0 to 1.0) | 1.0 (1.0 to 1.0) | 1.0 (1.0 to 1.0) | 1.0 (1.0 to 1.0) | 1.0 (1.0 to 1.0) | 1.0 (1.0 to 1.0) | 1.0 (1.0 to 1.0) | 1.0 (1.0 to 1.0) | 1.0 (1.0 to 1.0) | 1.0 (1.0 to 1.0) | 1.0 (1.0 to 1.0) | 1.0 (1.0 to 1.0) | 1.0 (1.0 to 1.0) | | | |
| Asthma | Technical | Both | Males | | | | | | | | 1.05 (0.977 to 1.125) | 1.051 (0.979 to 1.122) | 1.051 (0.982 to 1.124) | 1.051 (0.983 to 1.118) | 1.05 (0.977 to 1.12) | 1.05 (0.981 to 1.122) | 1.051 (0.98 to 1.121) | 1.051 (0.986 to 1.121) | 1.05 (0.976 to 1.125) | 1.05 (0.987 to 1.118) | 1.051 (0.984 to 1.121) | 1.051 (0.982 to 1.122) | 1.051 (0.981 to 1.122) | 1.051 (0.981 to 1.122) | | | |
| Asthma | Technical | Both | Females | | | | | | | | 1.06 (1.028 to 1.095) | 1.06 (1.024 to 1.099) | 1.059 (1.025 to 1.095) | 1.061 (1.026 to 1.099) | 1.06 (1.027 to 1.093) | 1.06 (1.026 to 1.094) | 1.059 (1.024 to 1.093) | 1.06 (1.028 to 1.094) | 1.06 (1.027 to 1.092) | 1.061 (1.026 to 1.095) | 1.06 (1.027 to 1.096) | 1.061 (1.023 to 1.096) | 1.06 (1.027 to 1.096) | 1.06 (1.027 to 1.096) | | | |
| Asthma | Sales | Both | Males | | | | | | | | 1.14 (1.047 to 1.237) | 1.14 (1.049 to 1.234) | 1.144 (1.055 to 1.233) | 1.142 (1.048 to 1.234) | 1.14 (1.046 to 1.234) | 1.141 (1.053 to 1.235) | 1.143 (1.052 to 1.238) | 1.14 (1.047 to 1.236) | 1.141 (1.056 to 1.235) | 1.138 (1.046 to 1.236) | 1.139 (1.057 to 1.226) | 1.141 (1.052 to 1.243) | 1.14 (1.058 to 1.239) | 1.14 (1.058 to 1.239) | | | |
| Asthma | Sales | Both | Females | | | | | | | | 1.131 (1.083 to 1.182) | 1.13 (1.083 to 1.178) | 1.129 (1.083 to 1.178) | 1.13 (1.081 to 1.182) | 1.131 (1.084 to 1.18) | 1.13 (1.08 to 1.181) | 1.13 (1.083 to 1.181) | 1.13 (1.079 to 1.178) | 1.131 (1.077 to 1.184) | 1.13 (1.081 to 1.182) | 1.129 (1.082 to 1.181) | 1.131 (1.083 to 1.184) | 1.131 (1.083 to 1.183) | 1.131 (1.083 to 1.183) | | | |
| Asthma | Agriculture | Both | Males | | | | | | | | 1.519 (1.1 to 2.029) | 1.527 (1.119 to 2.063) | 1.524 (1.103 to 2.019) | 1.513 (1.127 to 1.999) | 1.523 (1.108 to 2.023) | 1.516 (1.083 to 2.043) | 1.522 (1.106 to 2.038) | 1.52 (1.122 to 2.023) | 1.519 (1.103 to 2.011) | 1.531 (1.122 to 2.047) | 1.52 (1.106 to 2.041) | 1.508 (1.083 to 2.019) | 1.498 (1.081 to 1.965) | 1.498 (1.081 to 1.965) | | | |
| Asthma | Agriculture | Both | Females | | | | | | | | 1.52 (1.125 to 2.022) | 1.506 (1.099 to 1.997) | 1.52 (1.115 to 2.05) | 1.514 (1.109 to 2.048) | 1.509 (1.101 to 1.969) | 1.513 (1.128 to 2.026) | 1.508 (1.108 to 2.0) | 1.526 (1.108 to 2.07) | 1.519 (1.117 to 1.981) | 1.53 (1.105 to 2.017) | 1.519 (1.107 to 2.024) | 1.518 (1.115 to 2.018) | 1.502 (1.094 to 2.03) | 1.502 (1.094 to 2.03) | | | |
| Asthma | Mining | Both | Males | | | | | | | | 1.959 (1.576 to 2.413) | 1.959 (1.568 to 2.381) | 1.971 (1.602 to 2.414) | 1.966 (1.571 to 2.396) | 1.963 (1.601 to 2.395) | 1.959 (1.588 to 2.385) | 1.956 (1.595 to 2.406) | 1.964 (1.57 to 2.397) | 1.969 (1.616 to 2.417) | 1.954 (1.574 to 2.406) | 1.965 (1.558 to 2.393) | 1.955 (1.577 to 2.39) | 1.953 (1.551 to 2.418) | 1.953 (1.551 to 2.418) | | | |
| Asthma | Mining | Both | Females | | | | | | | | 1.956 (1.58 to 2.408) | 1.961 (1.574 to 2.395) | 1.967 (1.586 to 2.417) | 1.952 (1.567 to 2.371) | 1.959 (1.575 to 2.416) | 1.959 (1.567 to 2.417) | 1.962 (1.585 to 2.386) | 1.955 (1.591 to 2.393) | 1.948 (1.57 to 2.381) | 1.964 (1.597 to 2.409) | 1.965 (1.59 to 2.414) | 1.959 (1.588 to 2.39) | 1.973 (1.586 to 2.392) | 1.973 (1.586 to 2.392) | | | |
| Asthma | Transport | Both | Males | | | | | | | | 1.313 (1.22 to 1.402) | 1.311 (1.222 to 1.406) | 1.312 (1.225 to 1.399) | 1.31 (1.218 to 1.398) | 1.312 (1.225 to 1.404) | 1.311 (1.225 to 1.401) | 1.313 (1.226 to 1.402) | 1.31 (1.223 to 1.398) | 1.314 (1.221 to 1.396) | 1.313 (1.224 to 1.408) | 1.311 (1.228 to 1.397) | 1.312 (1.225 to 1.409) | 1.311 (1.226 to 1.407) | 1.311 (1.226 to 1.407) | | | |
| Asthma | Transport | Both | Females | | | | | | | | 1.221 (1.132 to 1.312) | 1.22 (1.138 to 1.31) | 1.217 (1.136 to 1.303) | 1.221 (1.138 to 1.313) | 1.221 (1.137 to 1.317) | 1.221 (1.132 to 1.314) | 1.22 (1.132 to 1.312) | 1.22 (1.134 to 1.313) | 1.221 (1.14 to 1.315) | 1.223 (1.139 to 1.309) | 1.221 (1.133 to 1.312) | 1.224 (1.135 to 1.316) | 1.22 (1.133 to 1.313) | 1.22 (1.133 to 1.313) | | | |
| Asthma | Manufact | Both | Males | | | | | | | | 1.559 (1.477 to 1.647) | 1.561 (1.474 to 1.652) | 1.56 (1.474 to 1.657) | 1.562 (1.472 to 1.658) | 1.561 (1.473 to 1.65) | 1.561 (1.471 to 1.655) | 1.56 (1.471 to 1.649) | 1.562 (1.473 to 1.654) | 1.558 (1.461 to 1.655) | 1.562 (1.479 to 1.655) | 1.559 (1.475 to 1.652) | 1.562 (1.476 to 1.657) | 1.561 (1.474 to 1.654) | 1.561 (1.474 to 1.654) | | | |
| Asthma | Manufact | Both | Females | | | | | | | | 1.33 (1.272 to 1.392) | 1.331 (1.27 to 1.39) | 1.33 (1.271 to 1.39) | 1.329 (1.271 to 1.388) | 1.331 (1.268 to 1.392) | 1.329 (1.269 to 1.39) | 1.33 (1.269 to 1.391) | 1.332 (1.272 to 1.394) | 1.331 (1.273 to 1.389) | 1.331 (1.272 to 1.391) | 1.33 (1.268 to 1.392) | 1.33 (1.268 to 1.394) | 1.331 (1.272 to 1.39) | 1.331 (1.272 to 1.39) | | | |
| Asthma | Services | Both | Males | | | | | | | | 1.531 (1.415 to 1.646) | 1.531 (1.416 to 1.652) | 1.529 (1.409 to 1.649) | 1.532 (1.413 to 1.653) | 1.529 (1.424 to 1.648) | 1.529 (1.416 to 1.645) | 1.533 (1.418 to 1.646) | 1.535 (1.427 to 1.649) | 1.531 (1.416 to 1.655) | 1.528 (1.411 to 1.651) | 1.53 (1.416 to 1.652) | 1.53 (1.411 to 1.658) | 1.531 (1.415 to 1.655) | 1.531 (1.415 to 1.655) | | | |
| Asthma | Services | Both | Females | | | | | | | | 1.41 (1.352 to 1.467) | 1.41 (1.357 to 1.469) | 1.412 (1.357 to 1.467) | 1.41 (1.356 to 1.464) | 1.409 (1.353 to 1.463) | 1.409 (1.354 to 1.467) | 1.41 (1.354 to 1.467) | 1.411 (1.357 to 1.464) | 1.409 (1.357 to 1.46) | 1.411 (1.355 to 1.466) | 1.41 (1.354 to 1.465) | 1.41 (1.357 to 1.467) | 1.411 (1.357 to 1.464) | 1.411 (1.357 to 1.464) | | | |
| Asthma | Other | Both | Males | | | | | | | | 1.0 (1.0 to 1.0) | 1.0 (1.0 to 1.0) | 1.0 (1.0 to 1.0) | 1.0 (1.0 to 1.0) | 1.0 (1.0 to 1.0) | 1.0 (1.0 to 1.0) | 1.0 (1.0 to 1.0) | 1.0 (1.0 to 1.0) | 1.0 (1.0 to 1.0) | 1.0 (1.0 to 1.0) | 1.0 (1.0 to 1.0) | 1.0 (1.0 to 1.0) | 1.0 (1.0 to 1.0) | 1.0 (1.0 to 1.0) | | | |
| Asthma | Other | Both | Females | | | | | | | | 1.0 (1.0 to 1.0) | 1.0 (1.0 to 1.0) | 1.0 (1.0 to 1.0) | 1.0 (1.0 to 1.0) | 1.0 (1.0 to 1.0) | 1.0 (1.0 to 1.0) | 1.0 (1.0 to 1.0) | 1.0 (1.0 to 1.0) | 1.0 (1.0 to 1.0) | 1.0 (1.0 to 1.0) | 1.0 (1.0 to 1.0) | 1.0 (1.0 to 1.0) | 1.0 (1.0 to 1.0) | 1.0 (1.0 to 1.0) | | | |
| **Occupational particulate matter, gases, and fumes** | | | | | | | | | | | | | | | | | | | | | | | | | | | |
| Chronic obstructive pulmonary disease | High | Both | Males | | | | | | | | 2.364 (1.463 to 3.64) | 2.341 (1.426 to 3.555) | 2.37 (1.41 to 3.596) | 2.391 (1.455 to 3.756) | 2.387 (1.409 to 3.791) | 2.379 (1.4 to 3.672) | 2.409 (1.439 to 3.833) | 2.361 (1.447 to 3.684) | 2.367 (1.454 to 3.654) | 2.373 (1.375 to 3.64) | 2.357 (1.438 to 3.685) | 2.402 (1.434 to 3.763) | 2.398 (1.441 to 3.813) | 2.359 (1.429 to 3.695) | 2.359 (1.429 to 3.695) | 2.359 (1.429 to 3.695) | 2.359 (1.429 to 3.695) |
| Chronic obstructive pulmonary disease | High | Both | Females | | | | | | | | 2.371 (1.459 to 3.719) | 2.395 (1.483 to 3.669) | 2.364 (1.473 to 3.694) | 2.377 (1.431 to 3.763) | 2.375 (1.446 to 3.698) | 2.326 (1.438 to 3.576) | 2.35 (1.432 to 3.702) | 2.35 (1.425 to 3.609) | 2.364 (1.461 to 3.677) | 2.364 (1.475 to 3.64) | 2.395 (1.476 to 3.715) | 2.336 (1.434 to 3.673) | 2.363 (1.431 to 3.741) | 2.404 (1.462 to 3.619) | 2.404 (1.462 to 3.619) | 2.404 (1.462 to 3.619) | 2.404 (1.462 to 3.619) |
| Chronic obstructive pulmonary disease | Low | Both | Males | | | | | | | | 1.462 (1.057 to 1.915) | 1.457 (1.052 to 1.935) | 1.464 (1.085 to 1.91) | 1.45 (1.067 to 1.947) | 1.452 (1.06 to 1.963) | 1.454 (1.075 to 1.96) | 1.446 (1.055 to 1.929) | 1.457 (1.076 to 1.933) | 1.453 (1.052 to 1.903) | 1.46 (1.067 to 1.954) | 1.443 (1.057 to 1.916) | 1.462 (1.097 to 1.954) | 1.464 (1.079 to 1.952) | 1.462 (1.096 to 1.965) | 1.462 (1.096 to 1.965) | 1.462 (1.096 to 1.965) | 1.462 (1.096 to 1.965) |
| Chronic obstructive pulmonary disease | Low | Both | Females | | | | | | | | 1.446 (1.063 to 1.904) | 1.459 (1.09 to 1.954) | 1.453 (1.055 to 2.003) | 1.459 (1.072 to 1.931) | 1.456 (1.056 to 1.968) | 1.47 (1.089 to 1.96) | 1.44 (1.046 to 1.932) | 1.457 (1.097 to 1.925) | 1.455 (1.061 to 1.921) | 1.451 (1.082 to 1.912) | 1.459 (1.102 to 1.941) | 1.448 (1.077 to 1.929) | 1.467 (1.072 to 1.971) | 1.456 (1.056 to 1.927) | 1.456 (1.056 to 1.927) | 1.456 (1.056 to 1.927) | 1.456 (1.056 to 1.927) |
| Chronic obstructive pulmonary disease | None | Both | Males | | | | | | | | 1.0 (1.0 to 1.0) | 1.0 (1.0 to 1.0) | 1.0 (1.0 to 1.0) | 1.0 (1.0 to 1.0) | 1.0 (1.0 to 1.0) | 1.0 (1.0 to 1.0) | 1.0 (1.0 to 1.0) | 1.0 (1.0 to 1.0) | 1.0 (1.0 to 1.0) | 1.0 (1.0 to 1.0) | 1.0 (1.0 to 1.0) | 1.0 (1.0 to 1.0) | 1.0 (1.0 to 1.0) | 1.0 (1.0 to 1.0) | 1.0 (1.0 to 1.0) | 1.0 (1.0 to 1.0) | 1.0 (1.0 to 1.0) |
| Chronic obstructive pulmonary disease | None | Both | Females | | | | | | | | 1.0 (1.0 to 1.0) | 1.0 (1.0 to 1.0) | 1.0 (1.0 to 1.0) | 1.0 (1.0 to 1.0) | 1.0 (1.0 to 1.0) | 1.0 (1.0 to 1.0) | 1.0 (1.0 to 1.0) | 1.0 (1.0 to 1.0) | 1.0 (1.0 to 1.0) | 1.0 (1.0 to 1.0) | 1.0 (1.0 to 1.0) | 1.0 (1.0 to 1.0) | 1.0 (1.0 to 1.0) | 1.0 (1.0 to 1.0) | 1.0 (1.0 to 1.0) | 1.0 (1.0 to 1.0) | 1.0 (1.0 to 1.0) |
| **Occupational noise *** | | | | | | | | | | | | | | | | | | | | | | | | | | | |
| Mild hearing loss due to age-related and other hearing loss | High exposure, >90dB | Morbidity | Both | | | | | | | | 7.477 (5.049 to 11.132) | 7.423 (4.787 to 10.956) | 5.504 (4.293 to 6.843) | 5.456 (4.339 to 6.828) | 3.07 (2.692 to 3.51) | 3.081 (2.715 to 3.498) | 2.546 (2.332 to 2.774) | 2.554 (2.367 to 2.752) | 1.849 (1.712 to 1.995) | 1.85 (1.707 to 1.995) | 1.45 (1.372 to 1.526) | 1.454 (1.378 to 1.529) | 1.131 (1.048 to 1.211) | 1.131 (1.048 to 1.211) | 1.131 (1.048 to 1.211) | 1.131 (1.048 to 1.211) | 1.131 (1.048 to 1.211) |
| Mild hearing loss with ringing due to age-related and other hearing loss | High exposure, >90dB | Morbidity | Both | | | | | | | | 7.477 (5.049 to 11.132) | 7.423 (4.787 to 10.956) | 5.504 (4.293 to 6.843) | 5.456 (4.339 to 6.828) | 3.07 (2.692 to 3.51) | 3.081 (2.715 to 3.498) | 2.546 (2.332 to 2.774) | 2.554 (2.367 to 2.752) | 1.849 (1.712 to 1.995) | 1.85 (1.707 to 1.995) | 1.45 (1.372 to 1.526) | 1.454 (1.378 to 1.529) | 1.131 (1.048 to 1.211) | 1.131 (1.048 to 1.211) | 1.131 (1.048 to 1.211) | 1.131 (1.048 to 1.211) | 1.131 (1.048 to 1.211) |
| Moderate hearing loss due to age-related and other hearing loss | High exposure, >90dB | Morbidity | Both | | | | | | | | 8.175 (4.62 to 13.221) | 8.31 (4.828 to 13.462) | 6.71 (4.785 to 9.239) | 6.756 (4.705 to 9.168) | 5.992 (4.34 to 8.307) | 5.987 (4.304 to 8.107) | 5.624 (3.954 to 7.821) | 5.61 (3.96 to 7.69) | 3.643 (2.576 to 5.019) | 3.601 (2.494 to 4.936) | 2.17 (1.597 to 2.84) | 2.146 (1.656 to 2.862) | 1.291 (1.06 to 1.539) | 1.291 (1.06 to 1.539) | 1.291 (1.06 to 1.539) | 1.291 (1.06 to 1.539) | 1.291 (1.06 to 1.539) |
| Moderate hearing loss with ringing due to age-related and other hearing loss | High exposure, >90dB | Morbidity | Both | | | | | | | | 8.175 (4.62 to 13.221) | 8.31 (4.828 to 13.462) | 6.71 (4.785 to 9.239) | 6.756 (4.705 to 9.168) | 5.992 (4.34 to 8.307) | 5.987 (4.304 to 8.107) | 5.624 (3.954 to 7.821) | 5.61 (3.96 to 7.69) | 3.643 (2.576 to 5.019) | 3.601 (2.494 to 4.936) | 2.17 (1.597 to 2.84) | 2.146 (1.656 to 2.862) | 1.291 (1.06 to 1.539) | 1.291 (1.06 to 1.539) | 1.291 (1.06 to 1.539) | 1.291 (1.06 to 1.539) | 1.291 (1.06 to 1.539) |
| Moderately severe hearing loss due to age-related and other hearing loss | High exposure, >90dB | Morbidity | Both | | | | | | | | 8.27 (4.807 to 13.224) | 8.233 (4.709 to 13.126) | 6.764 (4.814 to 9.271) | 6.726 (4.762 to 9.201) | 6.007 (4.114 to 8.374) | 6.072 (4.34 to 8.475) | 5.638 (3.943 to 7.822) | 5.704 (4.007 to 8.022) | 3.638 (2.576 to 4.959) | 3.591 (2.583 to 4.981) | 2.162 (1.64 to 2.816) | 2.168 (1.631 to 2.827) | 1.297 (1.06 to 1.561) | 1.297 (1.06 to 1.561) | 1.297 (1.06 to 1.561) | 1.297 (1.06 to 1.561) | 1.297 (1.06 to 1.561) |
| Moderately severe hearing loss with ringing due to age-related and other hearing loss | High exposure, >90dB | Morbidity | Both | | | | | | | | 8.27 (4.807 to 13.224) | 8.233 (4.709 to 13.126) | 6.764 (4.814 to 9.271) | 6.726 (4.762 to 9.201) | 6.007 (4.114 to 8.374) | 6.072 (4.34 to 8.475) | 5.638 (3.943 to 7.822) | 5.704 (4.007 to 8.022) | 3.638 (2.576 to 4.959) | 3.591 (2.583 to 4.981) | 2.162 (1.64 to 2.816) | 2.168 (1.631 to 2.827) | 1.297 (1.06 to 1.561) | 1.297 (1.06 to 1.561) | 1.297 (1.06 to 1.561) | 1.297 (1.06 to 1.561) | 1.297 (1.06 to 1.561) |
| Severe hearing loss with ringing due to age-related and other hearing loss | High exposure, >90dB | Morbidity | Both | | | | | | | | 8.249 (4.72 to 13.18) | 8.292 (4.754 to 13.144) | 6.707 (4.73 to 9.34) | 6.687 (4.739 to 9.181) | 6.075 (4.315 to 8.372) | 5.983 (4.247 to 8.221) | 5.66 (4.039 to 7.875) | 5.61 (3.946 to 7.534) | 3.62 (2.541 to 5.01) | 3.583 (2.513 to 5.117) | 2.138 (1.606 to 2.802) | 2.173 (1.645 to 2.807) | 1.294 (1.059 to 1.569) | 1.294 (1.059 to 1.569) | 1.294 (1.059 to 1.569) | 1.294 (1.059 to 1.569) | 1.294 (1.059 to 1.569) |
| Severe hearing loss due to age-related and other hearing loss | High exposure, >90dB | Morbidity | Both | | | | | | | | 8.249 (4.72 to 13.18) | 8.292 (4.754 to 13.144) | 6.707 (4.73 to 9.34) | 6.687 (4.739 to 9.181) | 6.075 (4.315 to 8.372) | 5.983 (4.247 to 8.221) | 5.66 (4.039 to 7.875) | 5.61 (3.946 to 7.534) | 3.62 (2.541 to 5.01) | 3.583 (2.513 to 5.117) | 2.138 (1.606 to 2.802) | 2.173 (1.645 to 2.807) | 1.294 (1.059 to 1.569) | 1.294 (1.059 to 1.569) | 1.294 (1.059 to 1.569) | 1.294 (1.059 to 1.569) | 1.294 (1.059 to 1.569) |
| Profound hearing loss due to age-related and other hearing loss | High exposure, >90dB | Morbidity | Both | | | | | | | | 8.33 (4.77 to 13.62) | 8.437 (4.945 to 13.539) | 6.69 (4.739 to 9.26) | 6.734 (4.811 to 9.238) | 5.974 (4.17 to 8.13) | 5.981 (4.273 to 8.413) | 5.629 (3.965 to 7.879) | 5.589 (3.838 to 7.693) | 3.591 (2.489 to 5.087) | 3.607 (2.48 to 5.048) | 2.144 (1.619 to 2.83) | 2.173 (1.638 to 2.822) | 1.294 (1.081 to 1.562) | 1.294 (1.081 to 1.562) | 1.294 (1.081 to 1.562) | 1.294 (1.081 to 1.562) | 1.294 (1.081 to 1.562) |
| Profound hearing loss with ringing due to age-related and other hearing loss | High exposure, >90dB | Morbidity | Both | | | | | | | | 8.33 (4.77 to 13.62) | 8.437 (4.945 to 13.539) | 6.69 (4.739 to 9.26) | 6.734 (4.811 to 9.238) | 5.974 (4.17 to 8.13) | 5.981 (4.273 to 8.413) | 5.629 (3.965 to 7.879) | 5.589 (3.838 to 7.693) | 3.591 (2.489 to 5.087) | 3.607 (2.48 to 5.048) | 2.144 (1.619 to 2.83) | 2.173 (1.638 to 2.822) | 1.294 (1.081 to 1.562) | 1.294 (1.081 to 1.562) | 1.294 (1.081 to 1.562) | 1.294 (1.081 to 1.562) | 1.294 (1.081 to 1.562) |
| Complete hearing loss due to age-related and other hearing loss | High exposure, >90dB | Morbidity | Both | | | | | | | | 8.242 (4.856 to 13.285) | 8.371 (4.712 to 13.317) | 6.771 (4.783 to 9.41) | 6.703 (4.728 to 9.419) | 5.932 (4.209 to 8.003) | 5.941 (4.176 to 8.222) | 5.62 (3.963 to 7.916) | 5.628 (4.002 to 7.742) | 3.625 (2.498 to 5.132) | 3.625 (2.558 to 5.036) | 2.17 (1.614 to 2.802) | 2.17 (1.602 to 2.881) | 1.291 (1.06 to 1.548) | 1.291 (1.06 to 1.548) | 1.291 (1.06 to 1.548) | 1.291 (1.06 to 1.548) | 1.291 (1.06 to 1.548) |
| Complete hearing loss with ringing due to age-related and other hearing loss | High exposure, >90dB | Morbidity | Both | | | | | | | | 8.242 (4.856 to 13.285) | 8.371 (4.712 to 13.317) | 6.771 (4.783 to 9.41) | 6.703 (4.728 to 9.419) | 5.932 (4.209 to 8.003) | 5.941 (4.176 to 8.222) | 5.62 (3.963 to 7.916) | 5.628 (4.002 to 7.742) | 3.625 (2.498 to 5.132) | 3.625 (2.558 to 5.036) | 2.17 (1.614 to 2.802) | 2.17 (1.602 to 2.881) | 1.291 (1.06 to 1.548) | 1.291 (1.06 to 1.548) | 1.291 (1.06 to 1.548) | 1.291 (1.06 to 1.548) | 1.291 (1.06 to 1.548) |
| Mild hearing loss due to age-related and other hearing loss | Low exposure, 85-90dB | Morbidity | Both | | | | | | | | 2.807 (1.872 to 3.963) | 2.764 (1.846 to 4.054) | 2.953 (2.342 to 3.708) | 2.934 (2.322 to 3.605) | 2.176 (1.917 to 2.47) | 2.172 (1.929 to 2.475) | 1.988 (1.831 to 2.153) | 1.991 (1.826 to 2.156) | 1.551 (1.432 to 1.675) | 1.553 (1.432 to 1.686) | 1.301 (1.231 to 1.37) | 1.3 (1.233 to 1.37) | 1.091 (1.017 to 1.172) | 1.091 (1.017 to 1.172) | 1.091 (1.017 to 1.172) | 1.091 (1.017 to 1.172) | 1.091 (1.017 to 1.172) |
| Mild hearing loss with ringing due to age-related and other hearing loss | Low exposure, 85-90dB | Morbidity | Both | | | | | | | | 2.807 (1.872 to 3.963) | 2.764 (1.846 to 4.054) | 2.953 (2.342 to 3.708) | 2.934 (2.322 to 3.605) | 2.176 (1.917 to 2.47) | 2.172 (1.929 to 2.475) | 1.988 (1.831 to 2.153) | 1.991 (1.826 to 2.156) | 1.551 (1.432 to 1.675) | 1.553 (1.432 to 1.686) | 1.301 (1.231 to 1.37) | 1.3 (1.233 to 1.37) | 1.091 (1.017 to 1.172) | 1.091 (1.017 to 1.172) | 1.091 (1.017 to 1.172) | 1.091 (1.017 to 1.172) | 1.091 (1.017 to 1.172) |
| Moderate hearing loss due to age-related and other hearing loss | Low exposure, 85-90dB | Morbidity | Both | | | | | | | | 3.072 (1.768 to 4.925) | 3.037 (1.78 to 4.813) | 3.471 (2.441 to 4.807) | 3.454 (2.449 to 4.718) | 3.888 (2.724 to 5.326) | 3.855 (2.701 to 5.4) | 3.93 (2.791 to 5.397) | 3.925 (2.715 to 5.487) | 2.719 (1.933 to 3.726) | 2.689 (1.863 to 3.673) | 1.815 (1.363 to 2.404) | 1.817 (1.363 to 2.435) | 1.228 (1.022 to 1.468) | 1.228 (1.022 to 1.468) | 1.228 (1.022 to 1.468) | 1.228 (1.022 to 1.468) | 1.228 (1.022 to 1.468) |
| Moderate hearing loss with ringing due to age-related and other hearing loss | Low exposure, 85-90dB | Morbidity | Both | | | | | | | | 3.072 (1.768 to 4.925) | 3.037 (1.78 to 4.813) | 3.471 (2.441 to 4.807) | 3.454 (2.449 to 4.718) | 3.888 (2.724 to 5.326) | 3.855 (2.701 to 5.4) | 3.93 (2.791 to 5.397) | 3.925 (2.715 to 5.487) | 2.719 (1.933 to 3.726) | 2.689 (1.863 to 3.673) | 1.815 (1.363 to 2.404) | 1.817 (1.363 to 2.435) | 1.228 (1.022 to 1.468) | 1.228 (1.022 to 1.468) | 1.228 (1.022 to 1.468) | 1.228 (1.022 to 1.468) | 1.228 (1.022 to 1.468) |
| Moderately severe hearing loss due to age-related and other hearing loss | Low exposure, 85-90dB | Morbidity | Both | | | | | | | | 3.043 (1.733 to 5.008) | 3.008 (1.821 to 4.959) | 3.472 (2.48 to 4.825) | 3.45 (2.427 to 4.851) | 3.84 (2.68 to 5.285) | 3.809 (2.686 to 5.308) | 3.952 (2.757 to 5.596) | 3.931 (2.788 to 5.437) | 2.732 (1.891 to 3.782) | 2.709 (1.9 to 3.742) | 1.837 (1.33 to 2.433) | 1.807 (1.37 to 2.364) | 1.219 (1.017 to 1.455) | 1.219 (1.017 to 1.455) | 1.219 (1.017 to 1.455) | 1.219 (1.017 to 1.455) | 1.219 (1.017 to 1.455) |
| Moderately severe hearing loss with ringing due to age-related and other hearing loss | Low exposure, 85-90dB | Morbidity | Both | | | | | | | | 3.043 (1.733 to 5.008) | 3.008 (1.821 to 4.959) | 3.472 (2.48 to 4.825) | 3.45 (2.427 to 4.851) | 3.84 (2.68 to 5.285) | 3.809 (2.686 to 5.308) | 3.952 (2.757 to 5.596) | 3.931 (2.788 to 5.437) | 2.732 (1.891 to 3.782) | 2.709 (1.9 to 3.742) | 1.837 (1.33 to 2.433) | 1.807 (1.37 to 2.364) | 1.219 (1.017 to 1.455) | 1.219 (1.017 to 1.455) | 1.219 (1.017 to 1.455) | 1.219 (1.017 to 1.455) | 1.219 (1.017 to 1.455) |
| Severe hearing loss with ringing due to age-related and other hearing loss | Low exposure, 85-90dB | Morbidity | Both | | | | | | | | 3.023 (1.794 to 4.956) | 2.972 (1.758 to 4.883) | 3.444 (2.425 to 4.739) | 3.482 (2.463 to 4.764) | 3.837 (2.732 to 5.263) | 3.867 (2.71 to 5.354) | 3.98 (2.746 to 5.531) | 3.943 (2.754 to 5.548) | 2.711 (1.904 to 3.803) | 2.693 (1.929 to 3.736) | 1.83 (1.387 to 2.422) | 1.825 (1.347 to 2.402) | 1.218 (1.0 to 1.475) | 1.218 (1.0 to 1.475) | 1.218 (1.0 to 1.475) | 1.218 (1.0 to 1.475) | 1.218 (1.0 to 1.475) |
| Severe hearing loss due to age-related and other hearing loss | Low exposure, 85-90dB | Morbidity | Both | | | | | | | | 3.023 (1.794 to 4.956) | 2.972 (1.758 to 4.883) | 3.444 (2.425 to 4.739) | 3.482 (2.463 to 4.764) | 3.837 (2.732 to 5.263) | 3.867 (2.71 to 5.354) | 3.98 (2.746 to 5.531) | 3.943 (2.754 to 5.548) | 2.711 (1.904 to 3.803) | 2.693 (1.929 to 3.736) | 1.83 (1.387 to 2.422) | 1.825 (1.347 to 2.402) | 1.218 (1.0 to 1.475) | 1.218 (1.0 to 1.475) | 1.218 (1.0 to 1.475) | 1.218 (1.0 to 1.475) | 1.218 (1.0 to 1.475) |
| Profound hearing loss due to age-related and other hearing loss | Low exposure, 85-90dB | Morbidity | Both | | | | | | | | 3.023 (1.718 to 5.041) | 3.018 (1.749 to 4.908) | 3.455 (2.448 to 4.681) | 3.446 (2.457 to 4.727) | 3.875 (2.755 to 5.323) | 3.848 (2.721 to 5.132) | 3.943 (2.697 to 5.393) | 3.944 (2.769 to 5.398) | 2.697 (1.935 to 3.775) | 2.69 (1.888 to 3.733) | 1.833 (1.374 to 2.406) | 1.813 (1.34 to 2.386) | 1.222 (0.998 to 1.479) | 1.222 (0.998 to 1.479) | 1.222 (0.998 to 1.479) | 1.222 (0.998 to 1.479) | 1.222 (0.998 to 1.479) |
| Profound hearing loss with ringing due to age-related and other hearing loss | Low exposure, 85-90dB | Morbidity | Both | | | | | | | | 3.023 (1.718 to 5.041) | 3.018 (1.749 to 4.908) | 3.455 (2.448 to 4.681) | 3.446 (2.457 to 4.727) | 3.875 (2.755 to 5.323) | 3.848 (2.721 to 5.132) | 3.943 (2.697 to 5.393) | 3.944 (2.769 to 5.398) | 2.697 (1.935 to 3.775) | 2.69 (1.888 to 3.733) | 1.833 (1.374 to 2.406) | 1.813 (1.34 to 2.386) | 1.222 (0.998 to 1.479) | 1.222 (0.998 to 1.479) | 1.222 (0.998 to 1.479) | 1.222 (0.998 to 1.479) | 1.222 (0.998 to 1.479) |

**Appendix Table 6a. Relative risks used by age and sex for each outcome for all risk factors except for ambient air pollution alcohol, and smoking.**

| | | | | Ages | | | | | | | | | | | | | | | | | | | | | | | |
|---|---|---|---|---|---|---|---|---|---|---|---|---|---|---|---|---|---|---|---|---|---|---|---|---|---|---|---|
| Risk - Outcome | Category / Units | Morbidity / Mortality | Sex | All-age | 0-6 days | 7-27 days | 28-364 days | 1-4 years | 5-9 years | 10-14 years | 15-19 years | 20-24 years | 25-29 years | 30-34 years | 35-39 years | 40-44 years | 45-49 years | 50-54 years | 55-59 years | 60-64 years | 65-69 years | 70-74 years | 75-79 years | 80-84 years | 85-89 years | 90-94 years | 95+ years |
| Complete hearing loss due to age-related and other hearing loss | Low exposure, 85-90dB | Morbidity | Both | | | | | | | | 3.004 (1.775 to 4.913) | 2.986 (1.804 to 4.882) | 3.482 (2.473 to 4.715) | 3.478 (2.449 to 4.702) | 3.858 (2.702 to 5.284) | 3.813 (2.745 to 5.341) | 3.917 (2.805 to 5.383) | 3.985 (2.826 to 5.477) | 2.701 (1.874 to 3.658) | 2.695 (1.885 to 3.731) | 1.812 (1.376 to 2.371) | 1.824 (1.359 to 2.407) | 1.222 (1.015 to 1.475) | 1.222 (1.015 to 1.475) | 1.222 (1.015 to 1.475) | 1.222 (1.015 to 1.475) | 1.222 (1.015 to 1.475) |
| Complete hearing loss with ringing due to age-related and other hearing loss | Low exposure, 85-90dB | Morbidity | Both | | | | | | | | 3.004 (1.775 to 4.913) | 2.986 (1.804 to 4.882) | 3.482 (2.473 to 4.715) | 3.478 (2.449 to 4.702) | 3.858 (2.702 to 5.284) | 3.813 (2.745 to 5.341) | 3.917 (2.805 to 5.383) | 3.985 (2.826 to 5.477) | 2.701 (1.874 to 3.658) | 2.695 (1.885 to 3.731) | 1.812 (1.376 to 2.371) | 1.824 (1.359 to 2.407) | 1.222 (1.015 to 1.475) | 1.222 (1.015 to 1.475) | 1.222 (1.015 to 1.475) | 1.222 (1.015 to 1.475) | 1.222 (1.015 to 1.475) |
| Mild hearing loss due to age-related and other hearing loss | No exposure | Morbidity | Both | | | | | | | | 1.0 (1.0 to 1.0) | 1.0 (1.0 to 1.0) | 1.0 (1.0 to 1.0) | 1.0 (1.0 to 1.0) | 1.0 (1.0 to 1.0) | 1.0 (1.0 to 1.0) | 1.0 (1.0 to 1.0) | 1.0 (1.0 to 1.0) | 1.0 (1.0 to 1.0) | 1.0 (1.0 to 1.0) | 1.0 (1.0 to 1.0) | 1.0 (1.0 to 1.0) | 1.0 (1.0 to 1.0) | 1.0 (1.0 to 1.0) | 1.0 (1.0 to 1.0) | 1.0 (1.0 to 1.0) | 1.0 (1.0 to 1.0) |
| Mild hearing loss with ringing due to age-related and other hearing loss | No exposure | Morbidity | Both | | | | | | | | 1.0 (1.0 to 1.0) | 1.0 (1.0 to 1.0) | 1.0 (1.0 to 1.0) | 1.0 (1.0 to 1.0) | 1.0 (1.0 to 1.0) | 1.0 (1.0 to 1.0) | 1.0 (1.0 to 1.0) | 1.0 (1.0 to 1.0) | 1.0 (1.0 to 1.0) | 1.0 (1.0 to 1.0) | 1.0 (1.0 to 1.0) | 1.0 (1.0 to 1.0) | 1.0 (1.0 to 1.0) | 1.0 (1.0 to 1.0) | 1.0 (1.0 to 1.0) | 1.0 (1.0 to 1.0) | 1.0 (1.0 to 1.0) |
| Moderate hearing loss due to age-related and other hearing loss | No exposure | Morbidity | Both | | | | | | | | 1.0 (1.0 to 1.0) | 1.0 (1.0 to 1.0) | 1.0 (1.0 to 1.0) | 1.0 (1.0 to 1.0) | 1.0 (1.0 to 1.0) | 1.0 (1.0 to 1.0) | 1.0 (1.0 to 1.0) | 1.0 (1.0 to 1.0) | 1.0 (1.0 to 1.0) | 1.0 (1.0 to 1.0) | 1.0 (1.0 to 1.0) | 1.0 (1.0 to 1.0) | 1.0 (1.0 to 1.0) | 1.0 (1.0 to 1.0) | 1.0 (1.0 to 1.0) | 1.0 (1.0 to 1.0) | 1.0 (1.0 to 1.0) |
| Moderate hearing loss with ringing due to age-related and other hearing loss | No exposure | Morbidity | Both | | | | | | | | 1.0 (1.0 to 1.0) | 1.0 (1.0 to 1.0) | 1.0 (1.0 to 1.0) | 1.0 (1.0 to 1.0) | 1.0 (1.0 to 1.0) | 1.0 (1.0 to 1.0) | 1.0 (1.0 to 1.0) | 1.0 (1.0 to 1.0) | 1.0 (1.0 to 1.0) | 1.0 (1.0 to 1.0) | 1.0 (1.0 to 1.0) | 1.0 (1.0 to 1.0) | 1.0 (1.0 to 1.0) | 1.0 (1.0 to 1.0) | 1.0 (1.0 to 1.0) | 1.0 (1.0 to 1.0) | 1.0 (1.0 to 1.0) |
| Moderately severe hearing loss due to age-related and other hearing loss | No exposure | Morbidity | Both | | | | | | | | 1.0 (1.0 to 1.0) | 1.0 (1.0 to 1.0) | 1.0 (1.0 to 1.0) | 1.0 (1.0 to 1.0) | 1.0 (1.0 to 1.0) | 1.0 (1.0 to 1.0) | 1.0 (1.0 to 1.0) | 1.0 (1.0 to 1.0) | 1.0 (1.0 to 1.0) | 1.0 (1.0 to 1.0) | 1.0 (1.0 to 1.0) | 1.0 (1.0 to 1.0) | 1.0 (1.0 to 1.0) | 1.0 (1.0 to 1.0) | 1.0 (1.0 to 1.0) | 1.0 (1.0 to 1.0) | 1.0 (1.0 to 1.0) |
| Moderately severe hearing loss with ringing due to age-related and other hearing loss | No exposure | Morbidity | Both | | | | | | | | 1.0 (1.0 to 1.0) | 1.0 (1.0 to 1.0) | 1.0 (1.0 to 1.0) | 1.0 (1.0 to 1.0) | 1.0 (1.0 to 1.0) | 1.0 (1.0 to 1.0) | 1.0 (1.0 to 1.0) | 1.0 (1.0 to 1.0) | 1.0 (1.0 to 1.0) | 1.0 (1.0 to 1.0) | 1.0 (1.0 to 1.0) | 1.0 (1.0 to 1.0) | 1.0 (1.0 to 1.0) | 1.0 (1.0 to 1.0) | 1.0 (1.0 to 1.0) | 1.0 (1.0 to 1.0) | 1.0 (1.0 to 1.0) |
| Severe hearing loss with ringing due to age-related and other hearing loss | No exposure | Morbidity | Both | | | | | | | | 1.0 (1.0 to 1.0) | 1.0 (1.0 to 1.0) | 1.0 (1.0 to 1.0) | 1.0 (1.0 to 1.0) | 1.0 (1.0 to 1.0) | 1.0 (1.0 to 1.0) | 1.0 (1.0 to 1.0) | 1.0 (1.0 to 1.0) | 1.0 (1.0 to 1.0) | 1.0 (1.0 to 1.0) | 1.0 (1.0 to 1.0) | 1.0 (1.0 to 1.0) | 1.0 (1.0 to 1.0) | 1.0 (1.0 to 1.0) | 1.0 (1.0 to 1.0) | 1.0 (1.0 to 1.0) | 1.0 (1.0 to 1.0) |
| Severe hearing loss due to age-related and other hearing loss | No exposure | Morbidity | Both | | | | | | | | 1.0 (1.0 to 1.0) | 1.0 (1.0 to 1.0) | 1.0 (1.0 to 1.0) | 1.0 (1.0 to 1.0) | 1.0 (1.0 to 1.0) | 1.0 (1.0 to 1.0) | 1.0 (1.0 to 1.0) | 1.0 (1.0 to 1.0) | 1.0 (1.0 to 1.0) | 1.0 (1.0 to 1.0) | 1.0 (1.0 to 1.0) | 1.0 (1.0 to 1.0) | 1.0 (1.0 to 1.0) | 1.0 (1.0 to 1.0) | 1.0 (1.0 to 1.0) | 1.0 (1.0 to 1.0) | 1.0 (1.0 to 1.0) |
| Profound hearing loss due to age-related and other hearing loss | No exposure | Morbidity | Both | | | | | | | | 1.0 (1.0 to 1.0) | 1.0 (1.0 to 1.0) | 1.0 (1.0 to 1.0) | 1.0 (1.0 to 1.0) | 1.0 (1.0 to 1.0) | 1.0 (1.0 to 1.0) | 1.0 (1.0 to 1.0) | 1.0 (1.0 to 1.0) | 1.0 (1.0 to 1.0) | 1.0 (1.0 to 1.0) | 1.0 (1.0 to 1.0) | 1.0 (1.0 to 1.0) | 1.0 (1.0 to 1.0) | 1.0 (1.0 to 1.0) | 1.0 (1.0 to 1.0) | 1.0 (1.0 to 1.0) | 1.0 (1.0 to 1.0) |
| Profound hearing loss with ringing due to age-related and other hearing loss | No exposure | Morbidity | Both | | | | | | | | 1.0 (1.0 to 1.0) | 1.0 (1.0 to 1.0) | 1.0 (1.0 to 1.0) | 1.0 (1.0 to 1.0) | 1.0 (1.0 to 1.0) | 1.0 (1.0 to 1.0) | 1.0 (1.0 to 1.0) | 1.0 (1.0 to 1.0) | 1.0 (1.0 to 1.0) | 1.0 (1.0 to 1.0) | 1.0 (1.0 to 1.0) | 1.0 (1.0 to 1.0) | 1.0 (1.0 to 1.0) | 1.0 (1.0 to 1.0) | 1.0 (1.0 to 1.0) | 1.0 (1.0 to 1.0) | 1.0 (1.0 to 1.0) |
| Complete hearing loss due to age-related and other hearing loss | No exposure | Morbidity | Both | | | | | | | | 1.0 (1.0 to 1.0) | 1.0 (1.0 to 1.0) | 1.0 (1.0 to 1.0) | 1.0 (1.0 to 1.0) | 1.0 (1.0 to 1.0) | 1.0 (1.0 to 1.0) | 1.0 (1.0 to 1.0) | 1.0 (1.0 to 1.0) | 1.0 (1.0 to 1.0) | 1.0 (1.0 to 1.0) | 1.0 (1.0 to 1.0) | 1.0 (1.0 to 1.0) | 1.0 (1.0 to 1.0) | 1.0 (1.0 to 1.0) | 1.0 (1.0 to 1.0) | 1.0 (1.0 to 1.0) | 1.0 (1.0 to 1.0) |
| Complete hearing loss with ringing due to age-related and other hearing loss | No exposure | Morbidity | Both | | | | | | | | 1.0 (1.0 to 1.0) | 1.0 (1.0 to 1.0) | 1.0 (1.0 to 1.0) | 1.0 (1.0 to 1.0) | 1.0 (1.0 to 1.0) | 1.0 (1.0 to 1.0) | 1.0 (1.0 to 1.0) | 1.0 (1.0 to 1.0) | 1.0 (1.0 to 1.0) | 1.0 (1.0 to 1.0) | 1.0 (1.0 to 1.0) | 1.0 (1.0 to 1.0) | 1.0 (1.0 to 1.0) | 1.0 (1.0 to 1.0) | 1.0 (1.0 to 1.0) | 1.0 (1.0 to 1.0) | 1.0 (1.0 to 1.0) |
| **Occupational ergonomic factors** | | | | | | | | | | | | | | | | | | | | | | | | | | | |
| Low back pain | Professional, technical and related workers | Morbidity | Both | | | | | | | | 1.173 (1.066 to 1.282) | 1.172 (1.062 to 1.283) | 1.169 (1.065 to 1.283) | 1.17 (1.062 to 1.284) | 1.17 (1.062 to 1.285) | 1.172 (1.062 to 1.283) | 1.171 (1.071 to 1.27) | 1.169 (1.063 to 1.288) | 1.171 (1.059 to 1.281) | 1.17 (1.058 to 1.286) | 1.17 (1.07 to 1.279) | 1.172 (1.065 to 1.287) | 1.172 (1.07 to 1.283) | 1.172 (1.07 to 1.283) | | | |
| Low back pain | Administrative and managerial workers | Morbidity | Both | | | | | | | | 1.211 (0.964 to 1.508) | 1.21 (0.964 to 1.492) | 1.209 (0.965 to 1.487) | 1.209 (0.963 to 1.524) | 1.207 (0.976 to 1.496) | 1.207 (0.965 to 1.5) | 1.205 (0.946 to 1.489) | 1.205 (0.967 to 1.472) | 1.205 (0.961 to 1.509) | 1.203 (0.948 to 1.515) | 1.209 (0.976 to 1.479) | 1.21 (0.964 to 1.49) | 1.203 (0.961 to 1.501) | 1.203 (0.961 to 1.501) | | | |
| Low back pain | Clerical and related workers | Morbidity | Both | | | | | | | | 1.0 (1.0 to 1.0) | 1.0 (1.0 to 1.0) | 1.0 (1.0 to 1.0) | 1.0 (1.0 to 1.0) | 1.0 (1.0 to 1.0) | 1.0 (1.0 to 1.0) | 1.0 (1.0 to 1.0) | 1.0 (1.0 to 1.0) | 1.0 (1.0 to 1.0) | 1.0 (1.0 to 1.0) | 1.0 (1.0 to 1.0) | 1.0 (1.0 to 1.0) | 1.0 (1.0 to 1.0) | 1.0 (1.0 to 1.0) | | | |
| Low back pain | Sales workers | Morbidity | Both | | | | | | | | 1.22 (1.029 to 1.434) | 1.21 (1.018 to 1.418) | 1.213 (1.028 to 1.434) | 1.214 (1.004 to 1.448) | 1.207 (1.017 to 1.445) | 1.218 (1.016 to 1.455) | 1.212 (1.012 to 1.444) | 1.216 (1.01 to 1.448) | 1.219 (1.019 to 1.45) | 1.211 (1.014 to 1.444) | 1.213 (1.015 to 1.455) | 1.21 (1.007 to 1.423) | 1.214 (1.017 to 1.446) | 1.214 (1.017 to 1.446) | | | |
| Low back pain | Service workers | Morbidity | Both | | | | | | | | 1.472 (1.385 to 1.568) | 1.472 (1.383 to 1.569) | 1.471 (1.372 to 1.563) | 1.472 (1.382 to 1.571) | 1.469 (1.378 to 1.567) | 1.472 (1.377 to 1.57) | 1.469 (1.375 to 1.568) | 1.47 (1.378 to 1.57) | 1.472 (1.379 to 1.575) | 1.472 (1.381 to 1.572) | 1.474 (1.386 to 1.571) | 1.47 (1.377 to 1.568) | 1.472 (1.38 to 1.571) | 1.472 (1.38 to 1.571) | | | |
| Low back pain | Agriculture, animal husbandry and forestry workers, fishermen and hunters | Morbidity | Both | | | | | | | | 3.789 (2.58 to 5.376) | 3.762 (2.621 to 5.284) | 3.869 (2.642 to 5.486) | 3.775 (2.569 to 5.369) | 3.774 (2.606 to 5.314) | 3.771 (2.532 to 5.317) | 3.793 (2.632 to 5.361) | 3.785 (2.556 to 5.333) | 3.776 (2.645 to 5.157) | 3.792 (2.536 to 5.421) | 3.802 (2.684 to 5.428) | 3.746 (2.609 to 5.175) | 3.77 (2.635 to 5.151) | 3.77 (2.635 to 5.151) | | | |
| Low back pain | Production and related workers, transport equipment operators and labourers | Morbidity | Both | | | | | | | | 1.543 (1.409 to 1.679) | 1.54 (1.406 to 1.676) | 1.542 (1.415 to 1.677) | 1.543 (1.413 to 1.695) | 1.542 (1.416 to 1.685) | 1.543 (1.418 to 1.685) | 1.541 (1.402 to 1.684) | 1.542 (1.41 to 1.684) | 1.541 (1.404 to 1.683) | 1.54 (1.414 to 1.677) | 1.54 (1.408 to 1.683) | 1.538 (1.408 to 1.673) | 1.541 (1.41 to 1.677) | 1.541 (1.41 to 1.677) | | | |
| Low back pain | Background | Morbidity | Both | | | | | | | | 1.0 (1.0 to 1.0) | 1.0 (1.0 to 1.0) | 1.0 (1.0 to 1.0) | 1.0 (1.0 to 1.0) | 1.0 (1.0 to 1.0) | 1.0 (1.0 to 1.0) | 1.0 (1.0 to 1.0) | 1.0 (1.0 to 1.0) | 1.0 (1.0 to 1.0) | 1.0 (1.0 to 1.0) | 1.0 (1.0 to 1.0) | 1.0 (1.0 to 1.0) | 1.0 (1.0 to 1.0) | 1.0 (1.0 to 1.0) | | | |
| **Non-exclusive breastfeeding** | | | | | | | | | | | | | | | | | | | | | | | | | | | |
| Lower respiratory infections | None | Both | Both | | | 1.739 (1.493 to 2.025) | 1.739 (1.493 to 2.025) | | | | | | | | | | | | | | | | | | | | |
| Lower respiratory infections | Partial | Both | Both | | | 1.483 (1.206 to 1.792) | 1.483 (1.206 to 1.792) | | | | | | | | | | | | | | | | | | | | |
| Lower respiratory infections | Predominant | Both | Both | | | 1.369 (1.055 to 1.8) | 1.369 (1.055 to 1.8) | | | | | | | | | | | | | | | | | | | | |
| Lower respiratory infections | Exclusive | Both | Both | | | 1.0 (1.0 to 1.0) | 1.0 (1.0 to 1.0) | | | | | | | | | | | | | | | | | | | | |
| Diarrhoeal diseases | None | Both | Both | | | 3.605 (2.716 to 4.703) | 3.605 (2.716 to 4.703) | | | | | | | | | | | | | | | | | | | | |
| Diarrhoeal diseases | Partial | Both | Both | | | 2.633 (1.942 to 3.481) | 2.633 (1.942 to 3.481) | | | | | | | | | | | | | | | | | | | | |
| Diarrhoeal diseases | Predominant | Both | Both | | | 2.346 (1.667 to 3.234) | 2.346 (1.667 to 3.234) | | | | | | | | | | | | | | | | | | | | |
| Diarrhoeal diseases | Exclusive | Both | Both | | | 1.0 (1.0 to 1.0) | 1.0 (1.0 to 1.0) | | | | | | | | | | | | | | | | | | | | |
| **Discontinued breastfeeding** | | | | | | | | | | | | | | | | | | | | | | | | | | | |
| Diarrhoeal diseases | Continued | Both | Both | | | | 1.313 (1.111 to 1.549) | 1.313 (1.111 to 1.549) | | | | | | | | | | | | | | | | | | | |
| Diarrhoeal diseases | Not continued | Both | Both | | | | 1.0 (1.0 to 1.0) | 1.0 (1.0 to 1.0) | | | | | | | | | | | | | | | | | | | |
| **Child underweight** | | | | | | | | | | | | | | | | | | | | | | | | | | | |
| Lower respiratory infections | <-3 sd | Both | Both | | | | 2.593 (1.908 to 4.39) | 2.593 (1.908 to 4.39) | | | | | | | | | | | | | | | | | | | |
| Lower respiratory infections | <-2 sd | Both | Both | | | | 1.365 (1.215 to 1.755) | 1.365 (1.215 to 1.755) | | | | | | | | | | | | | | | | | | | |
| Lower respiratory infections | <-1 sd | Both | Both | | | | 1.145 (1.044 to 1.364) | 1.145 (1.044 to 1.364) | | | | | | | | | | | | | | | | | | | |
| Lower respiratory infections | -1 sd and above | Both | Both | | | | 1.0 (1.0 to 1.0) | 1.0 (1.0 to 1.0) | | | | | | | | | | | | | | | | | | | |
| Diarrhoeal diseases | <-3 sd | Both | Both | | | | 2.332 (2.076 to 2.802) | 2.332 (2.076 to 2.802) | | | | | | | | | | | | | | | | | | | |
| Diarrhoeal diseases | <-2 sd | Both | Both | | | | 1.23 (1.163 to 1.314) | 1.23 (1.163 to 1.314) | | | | | | | | | | | | | | | | | | | |
| Diarrhoeal diseases | <-1 sd | Both | Both | | | | 1.088 (1.046 to 1.134) | 1.088 (1.046 to 1.134) | | | | | | | | | | | | | | | | | | | |
| Diarrhoeal diseases | -1 sd and above | Both | Both | | | | 1.0 (1.0 to 1.0) | 1.0 (1.0 to 1.0) | | | | | | | | | | | | | | | | | | | |
| Measles | <-3 sd | Both | Both | | | | 5.668 (1.767 to 12.414) | 5.668 (1.767 to 12.414) | | | | | | | | | | | | | | | | | | | |
| Measles | <-2 sd | Both | Both | | | | 2.458 (1.26 to 5.118) | 2.458 (1.26 to 5.118) | | | | | | | | | | | | | | | | | | | |
| Measles | <-1 sd | Both | Both | | | | 0.995 (0.5 to 1.726) | 0.995 (0.5 to 1.726) | | | | | | | | | | | | | | | | | | | |
| Measles | -1 sd and above | Both | Both | | | | 1.0 (1.0 to 1.0) | 1.0 (1.0 to 1.0) | | | | | | | | | | | | | | | | | | | |
| **Child wasting** | | | | | | | | | | | | | | | | | | | | | | | | | | | |
| Lower respiratory infections | <-3 sd | Both | Both | | | | 47.67 (15.923 to 94.874) | 47.67 (15.923 to 94.874) | | | | | | | | | | | | | | | | | | | |
| Lower respiratory infections | <-2 sd | Both | Both | | | | 20.455 (7.084 to 37.929) | 20.455 (7.084 to 37.929) | | | | | | | | | | | | | | | | | | | |
| Lower respiratory infections | <-1 sd | Both | Both | | | | 5.941 (1.972 to 11.992) | 5.941 (1.972 to 11.992) | | | | | | | | | | | | | | | | | | | |
| Lower respiratory infections | -1 sd and above | Both | Both | | | | 1.0 (1.0 to 1.0) | 1.0 (1.0 to 1.0) | | | | | | | | | | | | | | | | | | | |
| Diarrhoeal diseases | <-3 sd | Both | Both | | | | 105.759 (42.198 to 157.813) | 105.759 (42.198 to 157.813) | | | | | | | | | | | | | | | | | | | |
| Diarrhoeal diseases | <-2 sd | Both | Both | | | | 23.261 (9.02 to 35.845) | 23.261 (9.02 to 35.845) | | | | | | | | | | | | | | | | | | | |
| Diarrhoeal diseases | <-1 sd | Both | Both | | | | 6.601 (2.158 to 11.243) | 6.601 (2.158 to 11.243) | | | | | | | | | | | | | | | | | | | |
| Diarrhoeal diseases | -1 sd and above | Both | Both | | | | 1.0 (1.0 to 1.0) | 1.0 (1.0 to 1.0) | | | | | | | | | | | | | | | | | | | |

**Appendix Table 6a. Relative risks used by age and sex for each outcome for all risk factors except for ambient air pollution alcohol, and smoking.**

| | | | | Ages | | | | | | | | | | | | | | | | | | | | | | | |
|---|---|---|---|---|---|---|---|---|---|---|---|---|---|---|---|---|---|---|---|---|---|---|---|---|---|---|---|
| Risk - Outcome | Category / Units | Morbidity / Mortality | Sex | All-age | 0-6 days | 7-27 days | 28-364 days | 1-4 years | 5-9 years | 10-14 years | 15-19 years | 20-24 years | 25-29 years | 30-34 years | 35-39 years | 40-44 years | 45-49 years | 50-54 years | 55-59 years | 60-64 years | 65-69 years | 70-74 years | 75-79 years | 80-84 years | 85-89 years | 90-94 years | 95+ years |
| Measles | <-3 sd | Both | Both | | | | 37.936 (5.088 to 199.126) | 37.936 (5.088 to 199.126) | | | | | | | | | | | | | | | | | | | |
| Measles | <-2 sd | Both | Both | | | | 8.477 (1.33 to 42.777) | 8.477 (1.33 to 42.777) | | | | | | | | | | | | | | | | | | | |
| Measles | <-1 sd | Both | Both | | | | 1.833 (0.569 to 8.985) | 1.833 (0.569 to 8.985) | | | | | | | | | | | | | | | | | | | |
| Measles | -1 sd and above | Both | Both | | | | 1.0 (1.0 to 1.0) | 1.0 (1.0 to 1.0) | | | | | | | | | | | | | | | | | | | |
| **Child stunting** | | | | | | | | | | | | | | | | | | | | | | | | | | | |
| Lower respiratory infections | <-3 sd | Both | Both | | | | 2.355 (1.15 to 5.114) | 2.355 (1.15 to 5.114) | | | | | | | | | | | | | | | | | | | |
| Lower respiratory infections | <-2 sd | Both | Both | | | | 1.318 (1.014 to 2.165) | 1.318 (1.014 to 2.165) | | | | | | | | | | | | | | | | | | | |
| Lower respiratory infections | <-1 sd | Both | Both | | | | 1.158 (0.998 to 1.655) | 1.158 (0.998 to 1.655) | | | | | | | | | | | | | | | | | | | |
| Lower respiratory infections | -1 sd and above | Both | Both | | | | 1.0 (1.0 to 1.0) | 1.0 (1.0 to 1.0) | | | | | | | | | | | | | | | | | | | |
| Diarrhoeal diseases | <-3 sd | Both | Both | | | | 1.851 (1.28 to 2.699) | 1.851 (1.28 to 2.699) | | | | | | | | | | | | | | | | | | | |
| Diarrhoeal diseases | <-2 sd | Both | Both | | | | 1.222 (1.067 to 1.5) | 1.222 (1.067 to 1.5) | | | | | | | | | | | | | | | | | | | |
| Diarrhoeal diseases | <-1 sd | Both | Both | | | | 1.111 (1.023 to 1.273) | 1.111 (1.023 to 1.273) | | | | | | | | | | | | | | | | | | | |
| Diarrhoeal diseases | -1 sd and above | Both | Both | | | | 1.0 (1.0 to 1.0) | 1.0 (1.0 to 1.0) | | | | | | | | | | | | | | | | | | | |
| Measles | <-3 sd | Both | Both | | | | 2.487 (1.129 to 6.528) | 2.487 (1.129 to 6.528) | | | | | | | | | | | | | | | | | | | |
| Measles | <-2 sd | Both | Both | | | | 1.54 (1.029 to 3.222) | 1.54 (1.029 to 3.222) | | | | | | | | | | | | | | | | | | | |
| Measles | <-1 sd | Both | Both | | | | 1.103 (0.861 to 1.719) | 1.103 (0.861 to 1.719) | | | | | | | | | | | | | | | | | | | |
| Measles | -1 sd and above | Both | Both | | | | 1.0 (1.0 to 1.0) | 1.0 (1.0 to 1.0) | | | | | | | | | | | | | | | | | | | |
| **Low birth weight and short gestation** | | | | | | | | | | | | | | | | | | | | | | | | | | | |
| Lower respiratory infections | Birth prevalence - [0, 24) wks, [0, 500) g | Mortality | Males | | 1564.792 (1056.542 to [illegible] | 618.595 (458.842 to 812.921) | | | | | | | | | | | | | | | | | | | | | |
| Lower respiratory infections | Birth prevalence - [0, 24) wks, [0, 500) g | Mortality | Females | | 1600.122 (1050.664 to [illegible] | 713.571 (526.178 to 921.018) | | | | | | | | | | | | | | | | | | | | | |
| Lower respiratory infections | Birth prevalence - [0, 24) wks, [500, 1000) g | Mortality | Males | | 1155.815 (825.412 to 1506.837) | 457.5 (352.552 to 573.483) | | | | | | | | | | | | | | | | | | | | | |
| Lower respiratory infections | Birth prevalence - [0, 24) wks, [500, 1000) g | Mortality | Females | | 1169.123 (802.003 to 1617.979) | 515.406 (396.713 to 641.541) | | | | | | | | | | | | | | | | | | | | | |
| Lower respiratory infections | Birth prevalence - [24, 26) wks, [500, 1000) g | Mortality | Males | | 955.583 (723.748 to 1244.265) | 443.357 (363.03 to 534.695) | | | | | | | | | | | | | | | | | | | | | |
| Lower respiratory infections | Birth prevalence - [24, 26) wks, [500, 1000) g | Mortality | Females | | 947.143 (702.662 to 1237.093) | 487.549 (387.307 to 603.498) | | | | | | | | | | | | | | | | | | | | | |
| Lower respiratory infections | Birth prevalence - [26, 28) wks, [500, 1000) g | Mortality | Males | | 497.817 (377.617 to 648.547) | 330.886 (261.438 to 401.709) | | | | | | | | | | | | | | | | | | | | | |
| Lower respiratory infections | Birth prevalence - [26, 28) wks, [500, 1000) g | Mortality | Females | | 483.682 (354.946 to 629.517) | 344.618 (274.427 to 419.864) | | | | | | | | | | | | | | | | | | | | | |
| Lower respiratory infections | Birth prevalence - [30, 32) wks, [500, 1000) g | Mortality | Males | | 236.614 (163.821 to 324.502) | 149.995 (117.866 to 188.368) | | | | | | | | | | | | | | | | | | | | | |
| Lower respiratory infections | Birth prevalence - [30, 32) wks, [500, 1000) g | Mortality | Females | | 229.197 (157.606 to 317.194) | 152.117 (120.779 to 190.583) | | | | | | | | | | | | | | | | | | | | | |
| Lower respiratory infections | Birth prevalence - [28, 30) wks, [500, 1000) g | Mortality | Males | | 297.629 (214.953 to 396.586) | 216.995 (173.321 to 271.466) | | | | | | | | | | | | | | | | | | | | | |
| Lower respiratory infections | Birth prevalence - [28, 30) wks, [500, 1000) g | Mortality | Females | | 281.056 (198.176 to 386.635) | 219.884 (174.264 to 272.704) | | | | | | | | | | | | | | | | | | | | | |
| Lower respiratory infections | Birth prevalence - [26, 28) wks, [1000, 1500) g | Mortality | Males | | 267.91 (210.177 to 332.92) | 164.167 (132.898 to 200.569) | | | | | | | | | | | | | | | | | | | | | |
| Lower respiratory infections | Birth prevalence - [26, 28) wks, [1000, 1500) g | Mortality | Females | | 266.509 (197.461 to 346.932) | 174.222 (137.431 to 217.349) | | | | | | | | | | | | | | | | | | | | | |
| Lower respiratory infections | Birth prevalence - [34, 36) wks, [1000, 1500) g | Mortality | Males | | 142.056 (98.086 to 197.774) | 52.86 (42.914 to 64.617) | | | | | | | | | | | | | | | | | | | | | |
| Lower respiratory infections | Birth prevalence - [34, 36) wks, [1000, 1500) g | Mortality | Females | | 141.899 (95.864 to 197.656) | 57.421 (46.452 to 71.339) | | | | | | | | | | | | | | | | | | | | | |
| Lower respiratory infections | Birth prevalence - [28, 30) wks, [1500, 2000) g | Mortality | Males | | 127.966 (97.178 to 167.026) | 50.018 (40.539 to 61.919) | | | | | | | | | | | | | | | | | | | | | |
| Lower respiratory infections | Birth prevalence - [28, 30) wks, [1500, 2000) g | Mortality | Females | | 130.924 (96.513 to 172.188) | 57.275 (46.36 to 70.038) | | | | | | | | | | | | | | | | | | | | | |
| Lower respiratory infections | Birth prevalence - [28, 30) wks, [1000, 1500) g | Mortality | Males | | 158.563 (120.99 to 204.947) | 103.32 (83.486 to 127.144) | | | | | | | | | | | | | | | | | | | | | |
| Lower respiratory infections | Birth prevalence - [28, 30) wks, [1000, 1500) g | Mortality | Females | | 153.905 (112.327 to 200.786) | 107.529 (86.954 to 131.78) | | | | | | | | | | | | | | | | | | | | | |
| Lower respiratory infections | Birth prevalence - [32, 34) wks, [1000, 1500) g | Mortality | Males | | 117.142 (81.354 to 161.101) | 53.185 (43.049 to 66.274) | | | | | | | | | | | | | | | | | | | | | |
| Lower respiratory infections | Birth prevalence - [32, 34) wks, [1000, 1500) g | Mortality | Females | | 115.171 (79.363 to 159.206) | 56.034 (45.982 to 68.36) | | | | | | | | | | | | | | | | | | | | | |
| Lower respiratory infections | Birth prevalence - [30, 32) wks, [1000, 1500) g | Mortality | Males | | 119.308 (87.769 to 160.885) | 67.163 (54.863 to 82.638) | | | | | | | | | | | | | | | | | | | | | |
| Lower respiratory infections | Birth prevalence - [30, 32) wks, [1000, 1500) g | Mortality | Females | | 115.448 (84.272 to 156.425) | 69.14 (55.873 to 85.012) | | | | | | | | | | | | | | | | | | | | | |
| Lower respiratory infections | Birth prevalence - [37, 38) wks, [1500, 2000) g | Mortality | Males | | 62.972 (46.159 to 83.484) | 24.148 (20.066 to 29.406) | | | | | | | | | | | | | | | | | | | | | |
| Lower respiratory infections | Birth prevalence - [37, 38) wks, [1500, 2000) g | Mortality | Females | | 59.988 (43.974 to 79.053) | 26.719 (21.746 to 32.816) | | | | | | | | | | | | | | | | | | | | | |
| Lower respiratory infections | Birth prevalence - [36, 37) wks, [1500, 2000) g | Mortality | Males | | 60.218 (43.669 to 82.48) | 23.031 (18.793 to 28.483) | | | | | | | | | | | | | | | | | | | | | |
| Lower respiratory infections | Birth prevalence - [36, 37) wks, [1500, 2000) g | Mortality | Females | | 58.527 (42.172 to 80.557) | 25.143 (20.331 to 30.566) | | | | | | | | | | | | | | | | | | | | | |
| Lower respiratory infections | Birth prevalence - [30, 32) wks, [2000, 2500) g | Mortality | Males | | 67.971 (50.354 to 88.935) | 18.03 (14.621 to 22.103) | | | | | | | | | | | | | | | | | | | | | |
| Lower respiratory infections | Birth prevalence - [30, 32) wks, [2000, 2500) g | Mortality | Females | | 69.383 (49.108 to 94.583) | 22.069 (17.836 to 27.163) | | | | | | | | | | | | | | | | | | | | | |
| Lower respiratory infections | Birth prevalence - [30, 32) wks, [1500, 2000) g | Mortality | Males | | 77.369 (59.702 to 99.232) | 31.079 (25.786 to 36.724) | | | | | | | | | | | | | | | | | | | | | |
| Lower respiratory infections | Birth prevalence - [30, 32) wks, [1500, 2000) g | Mortality | Females | | 76.134 (56.885 to 100.996) | 34.756 (28.764 to 41.849) | | | | | | | | | | | | | | | | | | | | | |
| Lower respiratory infections | Birth prevalence - [34, 36) wks, [1500, 2000) g | Mortality | Males | | 55.555 (39.553 to 75.104) | 21.346 (17.677 to 26.143) | | | | | | | | | | | | | | | | | | | | | |
| Lower respiratory infections | Birth prevalence - [34, 36) wks, [1500, 2000) g | Mortality | Females | | 54.335 (38.617 to 75.24) | 23.046 (18.743 to 28.287) | | | | | | | | | | | | | | | | | | | | | |
| Lower respiratory infections | Birth prevalence - [32, 34) wks, [1500, 2000) g | Mortality | Males | | 57.155 (42.484 to 73.651) | 23.114 (19.028 to 27.915) | | | | | | | | | | | | | | | | | | | | | |
| Lower respiratory infections | Birth prevalence - [32, 34) wks, [1500, 2000) g | Mortality | Females | | 56.101 (39.794 to 76.295) | 25.149 (20.615 to 30.388) | | | | | | | | | | | | | | | | | | | | | |
| Lower respiratory infections | Birth prevalence - [32, 34) wks, [2000, 2500) g | Mortality | Males | | 37.444 (29.026 to 48.227) | 12.233 (10.252 to 14.477) | | | | | | | | | | | | | | | | | | | | | |
| Lower respiratory infections | Birth prevalence - [32, 34) wks, [2000, 2500) g | Mortality | Females | | 36.874 (26.658 to 49.653) | 14.384 (12.095 to 17.03) | | | | | | | | | | | | | | | | | | | | | |
| Lower respiratory infections | Birth prevalence - [40, 42) wks, [2000, 2500) g | Mortality | Males | | 18.092 (13.292 to 23.719) | 9.23 (7.037 to 11.454) | | | | | | | | | | | | | | | | | | | | | |

Appendix Table 6a. Relative risks used by age and sex for each outcome for all risk factors except for ambient air pollution alcohol, and smoking.

| | | | | Ages | | | | | | | | | | | | | | | | | | | | | | | |
|---|---|---|---|---|---|---|---|---|---|---|---|---|---|---|---|---|---|---|---|---|---|---|---|---|---|---|---|
| Risk - Outcome | Category / Units | Morbidity / Mortality | Sex | All-age | 0-6 days | 7-27 days | 28-364 days | 1-4 years | 5-9 years | 10-14 years | 15-19 years | 20-24 years | 25-29 years | 30-34 years | 35-39 years | 40-44 years | 45-49 years | 50-54 years | 55-59 years | 60-64 years | 65-69 years | 70-74 years | 75-79 years | 80-84 years | 85-89 years | 90-94 years | 95+ years |
| Lower respiratory infections | Birth prevalence - [40, 42) wks, [2000, 2500) g | Mortality | Females | | 15.574 (11.516 to 20.778) | 9.975 (7.82 to 12.46) | | | | | | | | | | | | | | | | | | | | | |
| Lower respiratory infections | Birth prevalence - [38, 40) wks, [2000, 2500) g | Mortality | Males | | 13.104 (9.829 to 16.99) | 8.198 (6.786 to 9.959) | | | | | | | | | | | | | | | | | | | | | |
| Lower respiratory infections | Birth prevalence - [38, 40) wks, [2000, 2500) g | Mortality | Females | | 11.308 (8.389 to 14.38) | 8.577 (7.04 to 10.449) | | | | | | | | | | | | | | | | | | | | | |
| Lower respiratory infections | Birth prevalence - [32, 34) wks, [2500, 3000) g | Mortality | Males | | 33.063 (24.393 to 43.503) | 8.441 (6.822 to 10.431) | | | | | | | | | | | | | | | | | | | | | |
| Lower respiratory infections | Birth prevalence - [32, 34) wks, [2500, 3000) g | Mortality | Females | | 32.812 (23.439 to 45.567) | 10.398 (8.227 to 13.042) | | | | | | | | | | | | | | | | | | | | | |
| Lower respiratory infections | Birth prevalence - [34, 36) wks, [2000, 2500) g | Mortality | Males | | 21.925 (16.305 to 29.433) | 9.367 (7.859 to 11.112) | | | | | | | | | | | | | | | | | | | | | |
| Lower respiratory infections | Birth prevalence - [34, 36) wks, [2000, 2500) g | Mortality | Females | | 21.297 (15.657 to 28.761) | 10.295 (8.548 to 12.273) | | | | | | | | | | | | | | | | | | | | | |
| Lower respiratory infections | Birth prevalence - [37, 38) wks, [2000, 2500) g | Mortality | Males | | 13.0 (10.102 to 16.456) | 8.096 (6.724 to 9.676) | | | | | | | | | | | | | | | | | | | | | |
| Lower respiratory infections | Birth prevalence - [37, 38) wks, [2000, 2500) g | Mortality | Females | | 11.563 (8.805 to 15.11) | 8.467 (6.994 to 10.342) | | | | | | | | | | | | | | | | | | | | | |
| Lower respiratory infections | Birth prevalence - [36, 37) wks, [2000, 2500) g | Mortality | Males | | 14.401 (10.789 to 18.654) | 8.221 (6.917 to 9.923) | | | | | | | | | | | | | | | | | | | | | |
| Lower respiratory infections | Birth prevalence - [36, 37) wks, [2000, 2500) g | Mortality | Females | | 13.513 (9.817 to 17.942) | 8.654 (7.215 to 10.369) | | | | | | | | | | | | | | | | | | | | | |
| Lower respiratory infections | Birth prevalence - [34, 36) wks, [2500, 3000) g | Mortality | Males | | 13.419 (10.387 to 16.819) | 5.562 (4.646 to 6.696) | | | | | | | | | | | | | | | | | | | | | |
| Lower respiratory infections | Birth prevalence - [34, 36) wks, [2500, 3000) g | Mortality | Females | | 13.266 (9.666 to 17.689) | 6.395 (5.292 to 7.606) | | | | | | | | | | | | | | | | | | | | | |
| Lower respiratory infections | Birth prevalence - [34, 36) wks, [4000, 4500) g | Mortality | Males | | 23.096 (14.708 to 35.098) | 2.895 (2.245 to 3.716) | | | | | | | | | | | | | | | | | | | | | |
| Lower respiratory infections | Birth prevalence - [34, 36) wks, [4000, 4500) g | Mortality | Females | | 25.038 (15.763 to 37.255) | 3.778 (2.855 to 4.925) | | | | | | | | | | | | | | | | | | | | | |
| Lower respiratory infections | Birth prevalence - [34, 36) wks, [3000, 3500) g | Mortality | Males | | 14.006 (10.222 to 18.478) | 4.322 (3.449 to 5.338) | | | | | | | | | | | | | | | | | | | | | |
| Lower respiratory infections | Birth prevalence - [34, 36) wks, [3000, 3500) g | Mortality | Females | | 14.375 (10.269 to 20.114) | 5.265 (4.145 to 6.564) | | | | | | | | | | | | | | | | | | | | | |
| Lower respiratory infections | Birth prevalence - [36, 37) wks, [2500, 3000) g | Mortality | Males | | 4.874 (4.014 to 5.713) | 3.699 (3.134 to 4.374) | | | | | | | | | | | | | | | | | | | | | |
| Lower respiratory infections | Birth prevalence - [36, 37) wks, [2500, 3000) g | Mortality | Females | | 4.609 (3.731 to 5.61) | 3.898 (3.235 to 4.67) | | | | | | | | | | | | | | | | | | | | | |
| Lower respiratory infections | Birth prevalence - [34, 36) wks, [3500, 4000) g | Mortality | Males | | 18.024 (12.279 to 25.547) | 3.657 (2.838 to 4.675) | | | | | | | | | | | | | | | | | | | | | |
| Lower respiratory infections | Birth prevalence - [34, 36) wks, [3500, 4000) g | Mortality | Females | | 19.263 (12.567 to 27.924) | 4.634 (3.576 to 5.989) | | | | | | | | | | | | | | | | | | | | | |
| Lower respiratory infections | Birth prevalence - [37, 38) wks, [2500, 3000) g | Mortality | Males | | 3.306 (2.82 to 3.843) | 3.194 (2.691 to 3.803) | | | | | | | | | | | | | | | | | | | | | |
| Lower respiratory infections | Birth prevalence - [37, 38) wks, [2500, 3000) g | Mortality | Females | | 2.991 (2.496 to 3.61) | 3.242 (2.745 to 3.817) | | | | | | | | | | | | | | | | | | | | | |
| Lower respiratory infections | Birth prevalence - [40, 42) wks, [2500, 3000) g | Mortality | Males | | 3.771 (3.002 to 4.693) | 3.175 (2.56 to 3.923) | | | | | | | | | | | | | | | | | | | | | |
| Lower respiratory infections | Birth prevalence - [40, 42) wks, [2500, 3000) g | Mortality | Females | | 3.244 (2.486 to 4.159) | 3.228 (2.605 to 3.985) | | | | | | | | | | | | | | | | | | | | | |
| Lower respiratory infections | Birth prevalence - [38, 40) wks, [2500, 3000) g | Mortality | Males | | 2.755 (2.274 to 3.309) | 2.944 (2.44 to 3.548) | | | | | | | | | | | | | | | | | | | | | |
| Lower respiratory infections | Birth prevalence - [38, 40) wks, [2500, 3000) g | Mortality | Females | | 2.376 (1.91 to 2.886) | 2.938 (2.434 to 3.503) | | | | | | | | | | | | | | | | | | | | | |
| Lower respiratory infections | Birth prevalence - [36, 37) wks, [3000, 3500) g | Mortality | Males | | 3.774 (3.094 to 4.497) | 2.466 (2.058 to 2.929) | | | | | | | | | | | | | | | | | | | | | |
| Lower respiratory infections | Birth prevalence - [36, 37) wks, [3000, 3500) g | Mortality | Females | | 3.73 (2.981 to 4.646) | 2.715 (2.277 to 3.218) | | | | | | | | | | | | | | | | | | | | | |
| Lower respiratory infections | Birth prevalence - [36, 37) wks, [4000, 4500) g | Mortality | Males | | 6.826 (5.212 to 9.045) | 1.77 (1.491 to 2.082) | | | | | | | | | | | | | | | | | | | | | |
| Lower respiratory infections | Birth prevalence - [36, 37) wks, [4000, 4500) g | Mortality | Females | | 7.269 (5.144 to 9.821) | 2.177 (1.786 to 2.607) | | | | | | | | | | | | | | | | | | | | | |
| Lower respiratory infections | Birth prevalence - [36, 37) wks, [3500, 4000) g | Mortality | Males | | 4.544 (3.64 to 5.622) | 2.057 (1.74 to 2.44) | | | | | | | | | | | | | | | | | | | | | |
| Lower respiratory infections | Birth prevalence - [36, 37) wks, [3500, 4000) g | Mortality | Females | | 4.662 (3.577 to 6.014) | 2.398 (1.98 to 2.864) | | | | | | | | | | | | | | | | | | | | | |
| Lower respiratory infections | Birth prevalence - [37, 38) wks, [3000, 3500) g | Mortality | Males | | 2.007 (1.759 to 2.293) | 1.888 (1.613 to 2.224) | | | | | | | | | | | | | | | | | | | | | |
| Lower respiratory infections | Birth prevalence - [37, 38) wks, [3000, 3500) g | Mortality | Females | | 1.925 (1.582 to 2.328) | 1.972 (1.669 to 2.313) | | | | | | | | | | | | | | | | | | | | | |
| Lower respiratory infections | Birth prevalence - [37, 38) wks, [4000, 4500) g | Mortality | Males | | 3.28 (2.596 to 4.133) | 1.335 (1.171 to 1.532) | | | | | | | | | | | | | | | | | | | | | |
| Lower respiratory infections | Birth prevalence - [37, 38) wks, [4000, 4500) g | Mortality | Females | | 3.521 (2.649 to 4.5) | 1.559 (1.333 to 1.835) | | | | | | | | | | | | | | | | | | | | | |
| Lower respiratory infections | Birth prevalence - [37, 38) wks, [3500, 4000) g | Mortality | Males | | 2.128 (1.833 to 2.466) | 1.505 (1.299 to 1.76) | | | | | | | | | | | | | | | | | | | | | |
| Lower respiratory infections | Birth prevalence - [37, 38) wks, [3500, 4000) g | Mortality | Females | | 2.142 (1.694 to 2.67) | 1.661 (1.411 to 1.961) | | | | | | | | | | | | | | | | | | | | | |
| Lower respiratory infections | Birth prevalence - [40, 42) wks, [3000, 3500) g | Mortality | Males | | 1.436 (1.245 to 1.65) | 1.47 (1.199 to 1.8) | | | | | | | | | | | | | | | | | | | | | |
| Lower respiratory infections | Birth prevalence - [40, 42) wks, [3000, 3500) g | Mortality | Females | | 1.326 (1.069 to 1.614) | 1.465 (1.188 to 1.775) | | | | | | | | | | | | | | | | | | | | | |
| Lower respiratory infections | Birth prevalence - [38, 40) wks, [3000, 3500) g | Mortality | Males | | 1.33 (1.155 to 1.53) | 1.559 (1.305 to 1.851) | | | | | | | | | | | | | | | | | | | | | |
| Lower respiratory infections | Birth prevalence - [38, 40) wks, [3000, 3500) g | Mortality | Females | | 1.224 (1.0 to 1.492) | 1.564 (1.304 to 1.847) | | | | | | | | | | | | | | | | | | | | | |
| Lower respiratory infections | Birth prevalence - [38, 40) wks, [4000, 4500) g | Mortality | Males | | 1.787 (1.453 to 2.182) | 1.175 (1.005 to 1.371) | | | | | | | | | | | | | | | | | | | | | |
| Lower respiratory infections | Birth prevalence - [38, 40) wks, [4000, 4500) g | Mortality | Females | | 1.877 (1.467 to 2.388) | 1.224 (1.022 to 1.465) | | | | | | | | | | | | | | | | | | | | | |
| Lower respiratory infections | Birth prevalence - [38, 40) wks, [3500, 4000) g | Mortality | Males | | 1.785 (1.478 to 2.147) | 1.173 (1.0 to 1.377) | | | | | | | | | | | | | | | | | | | | | |
| Lower respiratory infections | Birth prevalence - [38, 40) wks, [3500, 4000) g | Mortality | Females | | 1.892 (1.481 to 2.352) | 1.23 (1.03 to 1.46) | | | | | | | | | | | | | | | | | | | | | |
| Lower respiratory infections | Birth prevalence - [40, 42) wks, [3500, 4000) g | Mortality | Males | | 1.0 (1.0 to 1.0) | 1.003 (1.0 to 1.046) | | | | | | | | | | | | | | | | | | | | | |
| Lower respiratory infections | Birth prevalence - [40, 42) wks, [3500, 4000) g | Mortality | Females | | 1.002 (1.0 to 1.013) | 1.001 (1.0 to 1.006) | | | | | | | | | | | | | | | | | | | | | |
| Lower respiratory infections | Birth prevalence - [40, 42) wks, [4000, 4500) g | Mortality | Males | | 1.0 (1.0 to 1.0) | 1.0 (1.0 to 1.0) | | | | | | | | | | | | | | | | | | | | | |
| Lower respiratory infections | Birth prevalence - [40, 42) wks, [4000, 4500) g | Mortality | Females | | 1.0 (1.0 to 1.0) | 1.0 (1.0 to 1.0) | | | | | | | | | | | | | | | | | | | | | |
| Lower respiratory infections | Birth prevalence - [28, 30) wks, [2000, 2500) g | Mortality | Males | | 117.172 (83.895 to 158.056) | 27.726 (21.877 to 34.972) | | | | | | | | | | | | | | | | | | | | | |
| Lower respiratory infections | Birth prevalence - [28, 30) wks, [2000, 2500) g | Mortality | Females | | 121.682 (84.349 to 171.375) | 33.983 (26.101 to 43.404) | | | | | | | | | | | | | | | | | | | | | |
| Lower respiratory infections | Birth prevalence - [28, 30) wks, [2500, 3000) g | Mortality | Males | | 77.948 (54.687 to 105.047) | 16.608 (12.653 to 21.188) | | | | | | | | | | | | | | | | | | | | | |
| Lower respiratory infections | Birth prevalence - [28, 30) wks, [2500, 3000) g | Mortality | Females | | 79.193 (54.099 to 112.236) | 20.387 (15.089 to 26.434) | | | | | | | | | | | | | | | | | | | | | |
| Lower respiratory infections | Birth prevalence - [28, 30) wks, [3000, 3500) g | Mortality | Males | | 42.199 (29.891 to 57.227) | 10.082 (7.777 to 13.056) | | | | | | | | | | | | | | | | | | | | | |
| Lower respiratory infections | Birth prevalence - [28, 30) wks, [3000, 3500) g | Mortality | Females | | 42.551 (28.25 to 63.209) | 11.989 (9.084 to 15.544) | | | | | | | | | | | | | | | | | | | | | |

**Appendix Table 6a. Relative risks used by age and sex for each outcome for all risk factors except for ambient air pollution alcohol, and smoking.**

| | | | | Ages | | | | | | | | | | | | | | | | | | | | | | | |
|---|---|---|---|---|---|---|---|---|---|---|---|---|---|---|---|---|---|---|---|---|---|---|---|---|---|---|---|
| Risk - Outcome | Category / Units | Morbidity / Mortality | Sex | All-age | 0-6 days | 7-27 days | 28-364 days | 1-4 years | 5-9 years | 10-14 years | 15-19 years | 20-24 years | 25-29 years | 30-34 years | 35-39 years | 40-44 years | 45-49 years | 50-54 years | 55-59 years | 60-64 years | 65-69 years | 70-74 years | 75-79 years | 80-84 years | 85-89 years | 90-94 years | 95+ years |
| Lower respiratory infections | Birth prevalence - [30, 32) wks, [2500, 3000) g | Mortality | Males | | 58.722 (42.419 to 78.873) | 12.115 (9.518 to 15.521) | | | | | | | | | | | | | | | | | | | | | |
| Lower respiratory infections | Birth prevalence - [30, 32) wks, [2500, 3000) g | Mortality | Females | | 59.522 (42.058 to 82.793) | 15.364 (11.936 to 19.581) | | | | | | | | | | | | | | | | | | | | | |
| Lower respiratory infections | Birth prevalence - [30, 32) wks, [3000, 3500) g | Mortality | Males | | 45.67 (32.014 to 65.531) | 8.381 (6.362 to 10.842) | | | | | | | | | | | | | | | | | | | | | |
| Lower respiratory infections | Birth prevalence - [30, 32) wks, [3000, 3500) g | Mortality | Females | | 46.104 (30.243 to 66.207) | 10.506 (8.041 to 13.513) | | | | | | | | | | | | | | | | | | | | | |
| Lower respiratory infections | Birth prevalence - [30, 32) wks, [3500, 4000) g | Mortality | Males | | 36.334 (21.558 to 54.813) | 5.698 (4.293 to 7.349) | | | | | | | | | | | | | | | | | | | | | |
| Lower respiratory infections | Birth prevalence - [30, 32) wks, [3500, 4000) g | Mortality | Females | | 37.931 (22.692 to 61.276) | 6.892 (5.175 to 9.058) | | | | | | | | | | | | | | | | | | | | | |
| Lower respiratory infections | Birth prevalence - [32, 34) wks, [3000, 3500) g | Mortality | Males | | 34.016 (23.515 to 48.37) | 6.577 (4.934 to 8.436) | | | | | | | | | | | | | | | | | | | | | |
| Lower respiratory infections | Birth prevalence - [32, 34) wks, [3000, 3500) g | Mortality | Females | | 34.585 (22.909 to 49.754) | 8.314 (6.362 to 10.789) | | | | | | | | | | | | | | | | | | | | | |
| Lower respiratory infections | Birth prevalence - [32, 34) wks, [3500, 4000) g | Mortality | Males | | 36.248 (23.158 to 54.67) | 5.068 (3.761 to 6.741) | | | | | | | | | | | | | | | | | | | | | |
| Lower respiratory infections | Birth prevalence - [32, 34) wks, [3500, 4000) g | Mortality | Females | | 38.098 (23.301 to 59.429) | 6.476 (4.666 to 8.689) | | | | | | | | | | | | | | | | | | | | | |
| Lower respiratory infections | Birth prevalence - [36, 37) wks, [1000, 1500) g | Mortality | Males | | 166.686 (118.487 to 222.581) | 57.535 (45.999 to 71.742) | | | | | | | | | | | | | | | | | | | | | |
| Lower respiratory infections | Birth prevalence - [36, 37) wks, [1000, 1500) g | Mortality | Females | | 169.725 (119.017 to 229.008) | 63.564 (50.068 to 80.703) | | | | | | | | | | | | | | | | | | | | | |
| Lower respiratory infections | Birth prevalence - [38, 40) wks, [1000, 1500) g | Mortality | Males | | 174.066 (125.125 to 232.507) | 57.966 (44.393 to 73.241) | | | | | | | | | | | | | | | | | | | | | |
| Lower respiratory infections | Birth prevalence - [38, 40) wks, [1000, 1500) g | Mortality | Females | | 171.557 (121.585 to 237.047) | 65.208 (48.821 to 84.308) | | | | | | | | | | | | | | | | | | | | | |
| Lower respiratory infections | Birth prevalence - [38, 40) wks, [1500, 2000) g | Mortality | Males | | 67.302 (49.547 to 89.055) | 25.206 (20.365 to 31.168) | | | | | | | | | | | | | | | | | | | | | |
| Lower respiratory infections | Birth prevalence - [38, 40) wks, [1500, 2000) g | Mortality | Females | | 62.19 (45.884 to 83.445) | 28.05 (22.625 to 35.139) | | | | | | | | | | | | | | | | | | | | | |
| Lower respiratory infections | Birth prevalence - [40, 42) wks, [1500, 2000) g | Mortality | Males | | 76.673 (56.177 to 102.468) | 25.785 (19.387 to 34.168) | | | | | | | | | | | | | | | | | | | | | |
| Lower respiratory infections | Birth prevalence - [40, 42) wks, [1500, 2000) g | Mortality | Females | | 70.411 (49.221 to 97.952) | 29.113 (21.355 to 38.272) | | | | | | | | | | | | | | | | | | | | | |
| Upper respiratory infections | Birth prevalence - [0, 24) wks, [0, 500) g | Mortality | Males | | 1564.792 (1056.542 to [illegible] | 618.595 (458.842 to 812.921) | | | | | | | | | | | | | | | | | | | | | |
| Upper respiratory infections | Birth prevalence - [0, 24) wks, [0, 500) g | Mortality | Females | | 1600.122 (1050.664 to [illegible] | 713.571 (526.178 to 921.018) | | | | | | | | | | | | | | | | | | | | | |
| Upper respiratory infections | Birth prevalence - [0, 24) wks, [500, 1000) g | Mortality | Males | | 1155.815 (825.412 to 1506.837) | 457.5 (352.552 to 573.483) | | | | | | | | | | | | | | | | | | | | | |
| Upper respiratory infections | Birth prevalence - [0, 24) wks, [500, 1000) g | Mortality | Females | | 1169.123 (802.003 to 1617.979) | 515.406 (396.713 to 641.541) | | | | | | | | | | | | | | | | | | | | | |
| Upper respiratory infections | Birth prevalence - [24, 26) wks, [500, 1000) g | Mortality | Males | | 955.583 (723.748 to 1244.265) | 443.357 (363.03 to 534.695) | | | | | | | | | | | | | | | | | | | | | |
| Upper respiratory infections | Birth prevalence - [24, 26) wks, [500, 1000) g | Mortality | Females | | 947.143 (702.662 to 1237.093) | 487.549 (387.307 to 603.498) | | | | | | | | | | | | | | | | | | | | | |
| Upper respiratory infections | Birth prevalence - [26, 28) wks, [500, 1000) g | Mortality | Males | | 497.817 (377.617 to 648.547) | 330.886 (261.438 to 401.709) | | | | | | | | | | | | | | | | | | | | | |
| Upper respiratory infections | Birth prevalence - [26, 28) wks, [500, 1000) g | Mortality | Females | | 483.682 (354.946 to 629.517) | 344.618 (274.427 to 419.864) | | | | | | | | | | | | | | | | | | | | | |
| Upper respiratory infections | Birth prevalence - [30, 32) wks, [500, 1000) g | Mortality | Males | | 236.614 (163.821 to 324.502) | 149.995 (117.866 to 188.368) | | | | | | | | | | | | | | | | | | | | | |
| Upper respiratory infections | Birth prevalence - [30, 32) wks, [500, 1000) g | Mortality | Females | | 229.197 (157.606 to 317.194) | 152.117 (120.779 to 190.583) | | | | | | | | | | | | | | | | | | | | | |
| Upper respiratory infections | Birth prevalence - [28, 30) wks, [500, 1000) g | Mortality | Males | | 297.629 (214.953 to 396.586) | 216.995 (173.321 to 271.466) | | | | | | | | | | | | | | | | | | | | | |
| Upper respiratory infections | Birth prevalence - [28, 30) wks, [500, 1000) g | Mortality | Females | | 281.056 (198.176 to 386.635) | 219.884 (174.264 to 272.704) | | | | | | | | | | | | | | | | | | | | | |
| Upper respiratory infections | Birth prevalence - [26, 28) wks, [1000, 1500) g | Mortality | Males | | 267.91 (210.177 to 332.92) | 164.167 (132.898 to 200.569) | | | | | | | | | | | | | | | | | | | | | |
| Upper respiratory infections | Birth prevalence - [26, 28) wks, [1000, 1500) g | Mortality | Females | | 266.509 (197.461 to 346.932) | 174.222 (137.431 to 217.349) | | | | | | | | | | | | | | | | | | | | | |
| Upper respiratory infections | Birth prevalence - [34, 36) wks, [1000, 1500) g | Mortality | Males | | 142.056 (98.086 to 197.774) | 52.86 (42.914 to 64.617) | | | | | | | | | | | | | | | | | | | | | |
| Upper respiratory infections | Birth prevalence - [34, 36) wks, [1000, 1500) g | Mortality | Females | | 141.899 (95.864 to 197.656) | 57.421 (46.452 to 71.339) | | | | | | | | | | | | | | | | | | | | | |
| Upper respiratory infections | Birth prevalence - [28, 30) wks, [1500, 2000) g | Mortality | Males | | 127.966 (97.178 to 167.026) | 50.018 (40.539 to 61.919) | | | | | | | | | | | | | | | | | | | | | |
| Upper respiratory infections | Birth prevalence - [28, 30) wks, [1500, 2000) g | Mortality | Females | | 130.924 (96.513 to 172.188) | 57.275 (46.36 to 70.038) | | | | | | | | | | | | | | | | | | | | | |
| Upper respiratory infections | Birth prevalence - [28, 30) wks, [1000, 1500) g | Mortality | Males | | 158.563 (120.99 to 204.947) | 103.32 (83.486 to 127.144) | | | | | | | | | | | | | | | | | | | | | |
| Upper respiratory infections | Birth prevalence - [28, 30) wks, [1000, 1500) g | Mortality | Females | | 153.905 (112.327 to 200.786) | 107.529 (86.954 to 131.78) | | | | | | | | | | | | | | | | | | | | | |
| Upper respiratory infections | Birth prevalence - [32, 34) wks, [1000, 1500) g | Mortality | Males | | 117.142 (81.354 to 161.101) | 53.185 (43.049 to 66.274) | | | | | | | | | | | | | | | | | | | | | |
| Upper respiratory infections | Birth prevalence - [32, 34) wks, [1000, 1500) g | Mortality | Females | | 115.171 (79.363 to 159.206) | 56.034 (45.982 to 68.36) | | | | | | | | | | | | | | | | | | | | | |
| Upper respiratory infections | Birth prevalence - [30, 32) wks, [1000, 1500) g | Mortality | Males | | 119.308 (87.769 to 160.885) | 67.163 (54.863 to 82.638) | | | | | | | | | | | | | | | | | | | | | |
| Upper respiratory infections | Birth prevalence - [30, 32) wks, [1000, 1500) g | Mortality | Females | | 115.448 (84.272 to 156.425) | 69.14 (55.873 to 85.012) | | | | | | | | | | | | | | | | | | | | | |
| Upper respiratory infections | Birth prevalence - [37, 38) wks, [1500, 2000) g | Mortality | Males | | 62.972 (46.159 to 83.484) | 24.148 (20.066 to 29.406) | | | | | | | | | | | | | | | | | | | | | |
| Upper respiratory infections | Birth prevalence - [37, 38) wks, [1500, 2000) g | Mortality | Females | | 59.988 (43.974 to 79.053) | 26.719 (21.746 to 32.816) | | | | | | | | | | | | | | | | | | | | | |
| Upper respiratory infections | Birth prevalence - [36, 37) wks, [1500, 2000) g | Mortality | Males | | 60.218 (43.669 to 82.48) | 23.031 (18.793 to 28.483) | | | | | | | | | | | | | | | | | | | | | |
| Upper respiratory infections | Birth prevalence - [36, 37) wks, [1500, 2000) g | Mortality | Females | | 58.527 (42.172 to 80.557) | 25.143 (20.331 to 30.566) | | | | | | | | | | | | | | | | | | | | | |
| Upper respiratory infections | Birth prevalence - [30, 32) wks, [2000, 2500) g | Mortality | Males | | 67.971 (50.354 to 88.935) | 18.03 (14.621 to 22.103) | | | | | | | | | | | | | | | | | | | | | |
| Upper respiratory infections | Birth prevalence - [30, 32) wks, [2000, 2500) g | Mortality | Females | | 69.383 (49.108 to 94.583) | 22.069 (17.836 to 27.163) | | | | | | | | | | | | | | | | | | | | | |
| Upper respiratory infections | Birth prevalence - [30, 32) wks, [1500, 2000) g | Mortality | Males | | 77.369 (59.702 to 99.232) | 31.079 (25.786 to 36.724) | | | | | | | | | | | | | | | | | | | | | |
| Upper respiratory infections | Birth prevalence - [30, 32) wks, [1500, 2000) g | Mortality | Females | | 76.134 (56.885 to 100.996) | 34.756 (28.764 to 41.849) | | | | | | | | | | | | | | | | | | | | | |
| Upper respiratory infections | Birth prevalence - [34, 36) wks, [1500, 2000) g | Mortality | Males | | 55.555 (39.553 to 75.104) | 21.346 (17.677 to 26.143) | | | | | | | | | | | | | | | | | | | | | |
| Upper respiratory infections | Birth prevalence - [34, 36) wks, [1500, 2000) g | Mortality | Females | | 54.335 (38.617 to 75.24) | 23.046 (18.743 to 28.287) | | | | | | | | | | | | | | | | | | | | | |
| Upper respiratory infections | Birth prevalence - [32, 34) wks, [1500, 2000) g | Mortality | Males | | 57.155 (42.484 to 73.651) | 23.114 (19.028 to 27.915) | | | | | | | | | | | | | | | | | | | | | |
| Upper respiratory infections | Birth prevalence - [32, 34) wks, [1500, 2000) g | Mortality | Females | | 56.101 (39.794 to 76.295) | 25.149 (20.615 to 30.388) | | | | | | | | | | | | | | | | | | | | | |
| Upper respiratory infections | Birth prevalence - [32, 34) wks, [2000, 2500) g | Mortality | Males | | 37.444 (29.026 to 48.227) | 12.233 (10.252 to 14.477) | | | | | | | | | | | | | | | | | | | | | |
| Upper respiratory infections | Birth prevalence - [32, 34) wks, [2000, 2500) g | Mortality | Females | | 36.874 (26.658 to 49.653) | 14.384 (12.095 to 17.03) | | | | | | | | | | | | | | | | | | | | | |
| Upper respiratory infections | Birth prevalence - [40, 42) wks, [2000, 2500) g | Mortality | Males | | 18.092 (13.292 to 23.719) | 9.23 (7.037 to 11.454) | | | | | | | | | | | | | | | | | | | | | |

**Appendix Table 6a. Relative risks used by age and sex for each outcome for all risk factors except for ambient air pollution alcohol, and smoking.**

| | | | | Ages | | | | | | | | | | | | | | | | | | | | | | | |
|---|---|---|---|---|---|---|---|---|---|---|---|---|---|---|---|---|---|---|---|---|---|---|---|---|---|---|---|
| Risk - Outcome | Category / Units | Morbidity / Mortality | Sex | All-age | 0-6 days | 7-27 days | 28-364 days | 1-4 years | 5-9 years | 10-14 years | 15-19 years | 20-24 years | 25-29 years | 30-34 years | 35-39 years | 40-44 years | 45-49 years | 50-54 years | 55-59 years | 60-64 years | 65-69 years | 70-74 years | 75-79 years | 80-84 years | 85-89 years | 90-94 years | 95+ years |
| Upper respiratory infections | Birth prevalence - [40, 42) wks, [2000, 2500) g | Mortality | Females | | 15.574 (11.516 to 20.778) | 9.975 (7.82 to 12.46) | | | | | | | | | | | | | | | | | | | | | |
| Upper respiratory infections | Birth prevalence - [38, 40) wks, [2000, 2500) g | Mortality | Males | | 13.104 (9.829 to 16.99) | 8.198 (6.786 to 9.959) | | | | | | | | | | | | | | | | | | | | | |
| Upper respiratory infections | Birth prevalence - [38, 40) wks, [2000, 2500) g | Mortality | Females | | 11.308 (8.389 to 14.38) | 8.577 (7.04 to 10.449) | | | | | | | | | | | | | | | | | | | | | |
| Upper respiratory infections | Birth prevalence - [32, 34) wks, [2500, 3000) g | Mortality | Males | | 33.063 (24.393 to 43.503) | 8.441 (6.822 to 10.431) | | | | | | | | | | | | | | | | | | | | | |
| Upper respiratory infections | Birth prevalence - [32, 34) wks, [2500, 3000) g | Mortality | Females | | 32.812 (23.439 to 45.567) | 10.398 (8.227 to 13.042) | | | | | | | | | | | | | | | | | | | | | |
| Upper respiratory infections | Birth prevalence - [34, 36) wks, [2000, 2500) g | Mortality | Males | | 21.925 (16.305 to 29.433) | 9.367 (7.859 to 11.112) | | | | | | | | | | | | | | | | | | | | | |
| Upper respiratory infections | Birth prevalence - [34, 36) wks, [2000, 2500) g | Mortality | Females | | 21.297 (15.657 to 28.761) | 10.295 (8.548 to 12.273) | | | | | | | | | | | | | | | | | | | | | |
| Upper respiratory infections | Birth prevalence - [37, 38) wks, [2000, 2500) g | Mortality | Males | | 13.0 (10.102 to 16.456) | 8.096 (6.724 to 9.676) | | | | | | | | | | | | | | | | | | | | | |
| Upper respiratory infections | Birth prevalence - [37, 38) wks, [2000, 2500) g | Mortality | Females | | 11.563 (8.805 to 15.11) | 8.467 (6.994 to 10.342) | | | | | | | | | | | | | | | | | | | | | |
| Upper respiratory infections | Birth prevalence - [36, 37) wks, [2000, 2500) g | Mortality | Males | | 14.401 (10.789 to 18.654) | 8.221 (6.917 to 9.923) | | | | | | | | | | | | | | | | | | | | | |
| Upper respiratory infections | Birth prevalence - [36, 37) wks, [2000, 2500) g | Mortality | Females | | 13.513 (9.817 to 17.942) | 8.654 (7.215 to 10.369) | | | | | | | | | | | | | | | | | | | | | |
| Upper respiratory infections | Birth prevalence - [34, 36) wks, [2500, 3000) g | Mortality | Males | | 13.419 (10.387 to 16.819) | 5.562 (4.646 to 6.696) | | | | | | | | | | | | | | | | | | | | | |
| Upper respiratory infections | Birth prevalence - [34, 36) wks, [2500, 3000) g | Mortality | Females | | 13.266 (9.666 to 17.689) | 6.395 (5.292 to 7.606) | | | | | | | | | | | | | | | | | | | | | |
| Upper respiratory infections | Birth prevalence - [34, 36) wks, [4000, 4500) g | Mortality | Males | | 23.096 (14.708 to 35.098) | 2.895 (2.245 to 3.716) | | | | | | | | | | | | | | | | | | | | | |
| Upper respiratory infections | Birth prevalence - [34, 36) wks, [4000, 4500) g | Mortality | Females | | 25.038 (15.763 to 37.255) | 3.778 (2.855 to 4.925) | | | | | | | | | | | | | | | | | | | | | |
| Upper respiratory infections | Birth prevalence - [34, 36) wks, [3000, 3500) g | Mortality | Males | | 14.006 (10.222 to 18.478) | 4.322 (3.449 to 5.338) | | | | | | | | | | | | | | | | | | | | | |
| Upper respiratory infections | Birth prevalence - [34, 36) wks, [3000, 3500) g | Mortality | Females | | 14.375 (10.269 to 20.114) | 5.265 (4.145 to 6.564) | | | | | | | | | | | | | | | | | | | | | |
| Upper respiratory infections | Birth prevalence - [36, 37) wks, [2500, 3000) g | Mortality | Males | | 4.874 (4.014 to 5.713) | 3.699 (3.134 to 4.374) | | | | | | | | | | | | | | | | | | | | | |
| Upper respiratory infections | Birth prevalence - [36, 37) wks, [2500, 3000) g | Mortality | Females | | 4.609 (3.731 to 5.61) | 3.898 (3.235 to 4.67) | | | | | | | | | | | | | | | | | | | | | |
| Upper respiratory infections | Birth prevalence - [34, 36) wks, [3500, 4000) g | Mortality | Males | | 18.024 (12.279 to 25.547) | 3.657 (2.838 to 4.675) | | | | | | | | | | | | | | | | | | | | | |
| Upper respiratory infections | Birth prevalence - [34, 36) wks, [3500, 4000) g | Mortality | Females | | 19.263 (12.567 to 27.924) | 4.634 (3.576 to 5.989) | | | | | | | | | | | | | | | | | | | | | |
| Upper respiratory infections | Birth prevalence - [37, 38) wks, [2500, 3000) g | Mortality | Males | | 3.306 (2.82 to 3.843) | 3.194 (2.691 to 3.803) | | | | | | | | | | | | | | | | | | | | | |
| Upper respiratory infections | Birth prevalence - [37, 38) wks, [2500, 3000) g | Mortality | Females | | 2.991 (2.496 to 3.61) | 3.242 (2.745 to 3.817) | | | | | | | | | | | | | | | | | | | | | |
| Upper respiratory infections | Birth prevalence - [40, 42) wks, [2500, 3000) g | Mortality | Males | | 3.771 (3.002 to 4.693) | 3.175 (2.56 to 3.923) | | | | | | | | | | | | | | | | | | | | | |
| Upper respiratory infections | Birth prevalence - [40, 42) wks, [2500, 3000) g | Mortality | Females | | 3.244 (2.486 to 4.159) | 3.228 (2.605 to 3.985) | | | | | | | | | | | | | | | | | | | | | |
| Upper respiratory infections | Birth prevalence - [38, 40) wks, [2500, 3000) g | Mortality | Males | | 2.755 (2.274 to 3.309) | 2.944 (2.44 to 3.548) | | | | | | | | | | | | | | | | | | | | | |
| Upper respiratory infections | Birth prevalence - [38, 40) wks, [2500, 3000) g | Mortality | Females | | 2.376 (1.91 to 2.886) | 2.938 (2.434 to 3.503) | | | | | | | | | | | | | | | | | | | | | |
| Upper respiratory infections | Birth prevalence - [36, 37) wks, [3000, 3500) g | Mortality | Males | | 3.774 (3.094 to 4.497) | 2.466 (2.058 to 2.929) | | | | | | | | | | | | | | | | | | | | | |
| Upper respiratory infections | Birth prevalence - [36, 37) wks, [3000, 3500) g | Mortality | Females | | 3.73 (2.981 to 4.646) | 2.715 (2.277 to 3.218) | | | | | | | | | | | | | | | | | | | | | |
| Upper respiratory infections | Birth prevalence - [36, 37) wks, [4000, 4500) g | Mortality | Males | | 6.826 (5.212 to 9.045) | 1.77 (1.491 to 2.082) | | | | | | | | | | | | | | | | | | | | | |
| Upper respiratory infections | Birth prevalence - [36, 37) wks, [4000, 4500) g | Mortality | Females | | 7.269 (5.144 to 9.821) | 2.177 (1.786 to 2.607) | | | | | | | | | | | | | | | | | | | | | |
| Upper respiratory infections | Birth prevalence - [36, 37) wks, [3500, 4000) g | Mortality | Males | | 4.544 (3.64 to 5.622) | 2.057 (1.74 to 2.44) | | | | | | | | | | | | | | | | | | | | | |
| Upper respiratory infections | Birth prevalence - [36, 37) wks, [3500, 4000) g | Mortality | Females | | 4.662 (3.577 to 6.014) | 2.398 (1.98 to 2.864) | | | | | | | | | | | | | | | | | | | | | |
| Upper respiratory infections | Birth prevalence - [37, 38) wks, [3000, 3500) g | Mortality | Males | | 2.007 (1.759 to 2.293) | 1.888 (1.613 to 2.224) | | | | | | | | | | | | | | | | | | | | | |
| Upper respiratory infections | Birth prevalence - [37, 38) wks, [3000, 3500) g | Mortality | Females | | 1.925 (1.582 to 2.328) | 1.972 (1.669 to 2.313) | | | | | | | | | | | | | | | | | | | | | |
| Upper respiratory infections | Birth prevalence - [37, 38) wks, [4000, 4500) g | Mortality | Males | | 3.28 (2.596 to 4.133) | 1.335 (1.171 to 1.532) | | | | | | | | | | | | | | | | | | | | | |
| Upper respiratory infections | Birth prevalence - [37, 38) wks, [4000, 4500) g | Mortality | Females | | 3.521 (2.649 to 4.5) | 1.559 (1.333 to 1.835) | | | | | | | | | | | | | | | | | | | | | |
| Upper respiratory infections | Birth prevalence - [37, 38) wks, [3500, 4000) g | Mortality | Males | | 2.128 (1.833 to 2.466) | 1.505 (1.299 to 1.76) | | | | | | | | | | | | | | | | | | | | | |
| Upper respiratory infections | Birth prevalence - [37, 38) wks, [3500, 4000) g | Mortality | Females | | 2.142 (1.694 to 2.67) | 1.661 (1.411 to 1.961) | | | | | | | | | | | | | | | | | | | | | |
| Upper respiratory infections | Birth prevalence - [40, 42) wks, [3000, 3500) g | Mortality | Males | | 1.436 (1.245 to 1.65) | 1.47 (1.199 to 1.8) | | | | | | | | | | | | | | | | | | | | | |
| Upper respiratory infections | Birth prevalence - [40, 42) wks, [3000, 3500) g | Mortality | Females | | 1.326 (1.069 to 1.614) | 1.465 (1.188 to 1.775) | | | | | | | | | | | | | | | | | | | | | |
| Upper respiratory infections | Birth prevalence - [38, 40) wks, [3000, 3500) g | Mortality | Males | | 1.33 (1.155 to 1.53) | 1.559 (1.305 to 1.851) | | | | | | | | | | | | | | | | | | | | | |
| Upper respiratory infections | Birth prevalence - [38, 40) wks, [3000, 3500) g | Mortality | Females | | 1.224 (1.0 to 1.492) | 1.564 (1.304 to 1.847) | | | | | | | | | | | | | | | | | | | | | |
| Upper respiratory infections | Birth prevalence - [38, 40) wks, [4000, 4500) g | Mortality | Males | | 1.787 (1.453 to 2.182) | 1.175 (1.005 to 1.371) | | | | | | | | | | | | | | | | | | | | | |
| Upper respiratory infections | Birth prevalence - [38, 40) wks, [4000, 4500) g | Mortality | Females | | 1.877 (1.467 to 2.388) | 1.224 (1.022 to 1.465) | | | | | | | | | | | | | | | | | | | | | |
| Upper respiratory infections | Birth prevalence - [38, 40) wks, [3500, 4000) g | Mortality | Males | | 1.785 (1.478 to 2.147) | 1.173 (1.0 to 1.377) | | | | | | | | | | | | | | | | | | | | | |
| Upper respiratory infections | Birth prevalence - [38, 40) wks, [3500, 4000) g | Mortality | Females | | 1.892 (1.481 to 2.352) | 1.23 (1.03 to 1.46) | | | | | | | | | | | | | | | | | | | | | |
| Upper respiratory infections | Birth prevalence - [40, 42) wks, [3500, 4000) g | Mortality | Males | | 1.0 (1.0 to 1.0) | 1.003 (1.0 to 1.046) | | | | | | | | | | | | | | | | | | | | | |
| Upper respiratory infections | Birth prevalence - [40, 42) wks, [3500, 4000) g | Mortality | Females | | 1.002 (1.0 to 1.013) | 1.001 (1.0 to 1.006) | | | | | | | | | | | | | | | | | | | | | |
| Upper respiratory infections | Birth prevalence - [40, 42) wks, [4000, 4500) g | Mortality | Males | | 1.0 (1.0 to 1.0) | 1.0 (1.0 to 1.0) | | | | | | | | | | | | | | | | | | | | | |
| Upper respiratory infections | Birth prevalence - [40, 42) wks, [4000, 4500) g | Mortality | Females | | 1.0 (1.0 to 1.0) | 1.0 (1.0 to 1.0) | | | | | | | | | | | | | | | | | | | | | |
| Upper respiratory infections | Birth prevalence - [28, 30) wks, [2000, 2500) g | Mortality | Males | | 117.172 (83.895 to 158.056) | 27.726 (21.877 to 34.972) | | | | | | | | | | | | | | | | | | | | | |
| Upper respiratory infections | Birth prevalence - [28, 30) wks, [2000, 2500) g | Mortality | Females | | 121.682 (84.349 to 171.375) | 33.983 (26.101 to 43.404) | | | | | | | | | | | | | | | | | | | | | |
| Upper respiratory infections | Birth prevalence - [28, 30) wks, [2500, 3000) g | Mortality | Males | | 77.948 (54.687 to 105.047) | 16.608 (12.653 to 21.188) | | | | | | | | | | | | | | | | | | | | | |
| Upper respiratory infections | Birth prevalence - [28, 30) wks, [2500, 3000) g | Mortality | Females | | 79.193 (54.099 to 112.236) | 20.387 (15.089 to 26.434) | | | | | | | | | | | | | | | | | | | | | |
| Upper respiratory infections | Birth prevalence - [28, 30) wks, [3000, 3500) g | Mortality | Males | | 42.199 (29.891 to 57.227) | 10.082 (7.777 to 13.056) | | | | | | | | | | | | | | | | | | | | | |
| Upper respiratory infections | Birth prevalence - [28, 30) wks, [3000, 3500) g | Mortality | Females | | 42.551 (28.25 to 63.209) | 11.989 (9.084 to 15.544) | | | | | | | | | | | | | | | | | | | | | |

**Appendix Table 6a. Relative risks used by age and sex for each outcome for all risk factors except for ambient air pollution alcohol, and smoking.**

| | | | | Ages | | | | | | | | | | | | | | | | | | | | |
|---|---|---|---|---|---|---|---|---|---|---|---|---|---|---|---|---|---|---|---|---|---|---|---|---|
| Risk - Outcome | Category / Units | Morbidity / Mortality | Sex | All-age | 0-6 days | 7-27 days | 28-364 days | 1-4 years | 5-9 years | 10-14 years | 15-19 years | 20-24 years | 25-29 years | 30-34 years | 35-39 years | 40-44 years | 45-49 years | 50-54 years | 55-59 years | 60-64 years | 65-69 years | 70-74 years | 75-79 years | 80-84 years | 85-89 years | 90-94 years | 95+ years |
| Upper respiratory infections | Birth prevalence - [30, 32) wks, [2500, 3000) g | Mortality | Males | | 58.722 (42.419 to 78.873) | 12.115 (9.518 to 15.521) | | | | | | | | | | | | | | | | | | | | | |
| Upper respiratory infections | Birth prevalence - [30, 32) wks, [2500, 3000) g | Mortality | Females | | 59.522 (42.058 to 82.793) | 15.364 (11.936 to 19.581) | | | | | | | | | | | | | | | | | | | | | |
| Upper respiratory infections | Birth prevalence - [30, 32) wks, [3000, 3500) g | Mortality | Males | | 45.67 (32.014 to 65.531) | 8.381 (6.362 to 10.842) | | | | | | | | | | | | | | | | | | | | | |
| Upper respiratory infections | Birth prevalence - [30, 32) wks, [3000, 3500) g | Mortality | Females | | 46.104 (30.243 to 66.207) | 10.506 (8.041 to 13.513) | | | | | | | | | | | | | | | | | | | | | |
| Upper respiratory infections | Birth prevalence - [30, 32) wks, [3500, 4000) g | Mortality | Males | | 36.334 (21.558 to 54.813) | 5.698 (4.293 to 7.349) | | | | | | | | | | | | | | | | | | | | | |
| Upper respiratory infections | Birth prevalence - [30, 32) wks, [3500, 4000) g | Mortality | Females | | 37.931 (22.692 to 61.276) | 6.892 (5.175 to 9.058) | | | | | | | | | | | | | | | | | | | | | |
| Upper respiratory infections | Birth prevalence - [32, 34) wks, [3000, 3500) g | Mortality | Males | | 34.016 (23.515 to 48.37) | 6.577 (4.934 to 8.436) | | | | | | | | | | | | | | | | | | | | | |
| Upper respiratory infections | Birth prevalence - [32, 34) wks, [3000, 3500) g | Mortality | Females | | 34.585 (22.909 to 49.754) | 8.314 (6.362 to 10.789) | | | | | | | | | | | | | | | | | | | | | |
| Upper respiratory infections | Birth prevalence - [32, 34) wks, [3500, 4000) g | Mortality | Males | | 36.248 (23.158 to 54.67) | 5.068 (3.761 to 6.741) | | | | | | | | | | | | | | | | | | | | | |
| Upper respiratory infections | Birth prevalence - [32, 34) wks, [3500, 4000) g | Mortality | Females | | 38.098 (23.301 to 59.429) | 6.476 (4.666 to 8.689) | | | | | | | | | | | | | | | | | | | | | |
| Upper respiratory infections | Birth prevalence - [36, 37) wks, [1000, 1500) g | Mortality | Males | | 166.686 (118.487 to 222.581) | 57.535 (45.999 to 71.742) | | | | | | | | | | | | | | | | | | | | | |
| Upper respiratory infections | Birth prevalence - [36, 37) wks, [1000, 1500) g | Mortality | Females | | 169.725 (119.017 to 229.008) | 63.564 (50.068 to 80.703) | | | | | | | | | | | | | | | | | | | | | |
| Upper respiratory infections | Birth prevalence - [38, 40) wks, [1000, 1500) g | Mortality | Males | | 174.066 (125.125 to 232.507) | 57.966 (44.393 to 73.241) | | | | | | | | | | | | | | | | | | | | | |
| Upper respiratory infections | Birth prevalence - [38, 40) wks, [1000, 1500) g | Mortality | Females | | 171.557 (121.585 to 237.047) | 65.208 (48.821 to 84.308) | | | | | | | | | | | | | | | | | | | | | |
| Upper respiratory infections | Birth prevalence - [38, 40) wks, [1500, 2000) g | Mortality | Males | | 67.302 (49.547 to 89.055) | 25.206 (20.365 to 31.168) | | | | | | | | | | | | | | | | | | | | | |
| Upper respiratory infections | Birth prevalence - [38, 40) wks, [1500, 2000) g | Mortality | Females | | 62.19 (45.884 to 83.445) | 28.05 (22.625 to 35.139) | | | | | | | | | | | | | | | | | | | | | |
| Upper respiratory infections | Birth prevalence - [40, 42) wks, [1500, 2000) g | Mortality | Males | | 76.673 (56.177 to 102.468) | 25.785 (19.387 to 34.168) | | | | | | | | | | | | | | | | | | | | | |
| Upper respiratory infections | Birth prevalence - [40, 42) wks, [1500, 2000) g | Mortality | Females | | 70.411 (49.221 to 97.952) | 29.113 (21.355 to 38.272) | | | | | | | | | | | | | | | | | | | | | |
| Otitis media | Birth prevalence - [0, 24) wks, [0, 500) g | Mortality | Males | | 1564.792 (1056.542 to [illegible] | 618.595 (458.842 to 812.921) | | | | | | | | | | | | | | | | | | | | | |
| Otitis media | Birth prevalence - [0, 24) wks, [0, 500) g | Mortality | Females | | 1600.122 (1050.664 to [illegible] | 713.571 (526.178 to 921.018) | | | | | | | | | | | | | | | | | | | | | |
| Otitis media | Birth prevalence - [0, 24) wks, [500, 1000) g | Mortality | Males | | 1155.815 (825.412 to 1506.837) | 457.5 (352.552 to 573.483) | | | | | | | | | | | | | | | | | | | | | |
| Otitis media | Birth prevalence - [0, 24) wks, [500, 1000) g | Mortality | Females | | 1169.123 (802.003 to 1617.979) | 515.406 (396.713 to 641.541) | | | | | | | | | | | | | | | | | | | | | |
| Otitis media | Birth prevalence - [24, 26) wks, [500, 1000) g | Mortality | Males | | 955.583 (723.748 to 1244.265) | 443.357 (363.03 to 534.695) | | | | | | | | | | | | | | | | | | | | | |
| Otitis media | Birth prevalence - [24, 26) wks, [500, 1000) g | Mortality | Females | | 947.143 (702.662 to 1237.093) | 487.549 (387.307 to 603.498) | | | | | | | | | | | | | | | | | | | | | |
| Otitis media | Birth prevalence - [26, 28) wks, [500, 1000) g | Mortality | Males | | 497.817 (377.617 to 648.547) | 330.886 (261.438 to 401.709) | | | | | | | | | | | | | | | | | | | | | |
| Otitis media | Birth prevalence - [26, 28) wks, [500, 1000) g | Mortality | Females | | 483.682 (354.946 to 629.517) | 344.618 (274.427 to 419.864) | | | | | | | | | | | | | | | | | | | | | |
| Otitis media | Birth prevalence - [30, 32) wks, [500, 1000) g | Mortality | Males | | 236.614 (163.821 to 324.502) | 149.995 (117.866 to 188.368) | | | | | | | | | | | | | | | | | | | | | |
| Otitis media | Birth prevalence - [30, 32) wks, [500, 1000) g | Mortality | Females | | 229.197 (157.606 to 317.194) | 152.117 (120.779 to 190.583) | | | | | | | | | | | | | | | | | | | | | |
| Otitis media | Birth prevalence - [28, 30) wks, [500, 1000) g | Mortality | Males | | 297.629 (214.953 to 396.586) | 216.995 (173.321 to 271.466) | | | | | | | | | | | | | | | | | | | | | |
| Otitis media | Birth prevalence - [28, 30) wks, [500, 1000) g | Mortality | Females | | 281.056 (198.176 to 386.635) | 219.884 (174.264 to 272.704) | | | | | | | | | | | | | | | | | | | | | |
| Otitis media | Birth prevalence - [26, 28) wks, [1000, 1500) g | Mortality | Males | | 267.91 (210.177 to 332.92) | 164.167 (132.898 to 200.569) | | | | | | | | | | | | | | | | | | | | | |
| Otitis media | Birth prevalence - [26, 28) wks, [1000, 1500) g | Mortality | Females | | 266.509 (197.461 to 346.932) | 174.222 (137.431 to 217.349) | | | | | | | | | | | | | | | | | | | | | |
| Otitis media | Birth prevalence - [34, 36) wks, [1000, 1500) g | Mortality | Males | | 142.056 (98.086 to 197.774) | 52.86 (42.914 to 64.617) | | | | | | | | | | | | | | | | | | | | | |
| Otitis media | Birth prevalence - [34, 36) wks, [1000, 1500) g | Mortality | Females | | 141.899 (95.864 to 197.656) | 57.421 (46.452 to 71.339) | | | | | | | | | | | | | | | | | | | | | |
| Otitis media | Birth prevalence - [28, 30) wks, [1500, 2000) g | Mortality | Males | | 127.966 (97.178 to 167.026) | 50.018 (40.539 to 61.919) | | | | | | | | | | | | | | | | | | | | | |
| Otitis media | Birth prevalence - [28, 30) wks, [1500, 2000) g | Mortality | Females | | 130.924 (96.513 to 172.188) | 57.275 (46.36 to 70.038) | | | | | | | | | | | | | | | | | | | | | |
| Otitis media | Birth prevalence - [28, 30) wks, [1000, 1500) g | Mortality | Males | | 158.563 (120.99 to 204.947) | 103.32 (83.486 to 127.144) | | | | | | | | | | | | | | | | | | | | | |
| Otitis media | Birth prevalence - [28, 30) wks, [1000, 1500) g | Mortality | Females | | 153.905 (112.327 to 200.786) | 107.529 (86.954 to 131.78) | | | | | | | | | | | | | | | | | | | | | |
| Otitis media | Birth prevalence - [32, 34) wks, [1000, 1500) g | Mortality | Males | | 117.142 (81.354 to 161.101) | 53.185 (43.049 to 66.274) | | | | | | | | | | | | | | | | | | | | | |
| Otitis media | Birth prevalence - [32, 34) wks, [1000, 1500) g | Mortality | Females | | 115.171 (79.363 to 159.206) | 56.034 (45.982 to 68.36) | | | | | | | | | | | | | | | | | | | | | |
| Otitis media | Birth prevalence - [30, 32) wks, [1000, 1500) g | Mortality | Males | | 119.308 (87.769 to 160.885) | 67.163 (54.863 to 82.638) | | | | | | | | | | | | | | | | | | | | | |
| Otitis media | Birth prevalence - [30, 32) wks, [1000, 1500) g | Mortality | Females | | 115.448 (84.272 to 156.425) | 69.14 (55.873 to 85.012) | | | | | | | | | | | | | | | | | | | | | |
| Otitis media | Birth prevalence - [37, 38) wks, [1500, 2000) g | Mortality | Males | | 62.972 (46.159 to 83.484) | 24.148 (20.066 to 29.406) | | | | | | | | | | | | | | | | | | | | | |
| Otitis media | Birth prevalence - [37, 38) wks, [1500, 2000) g | Mortality | Females | | 59.988 (43.974 to 79.053) | 26.719 (21.746 to 32.816) | | | | | | | | | | | | | | | | | | | | | |
| Otitis media | Birth prevalence - [36, 37) wks, [1500, 2000) g | Mortality | Males | | 60.218 (43.669 to 82.48) | 23.031 (18.793 to 28.483) | | | | | | | | | | | | | | | | | | | | | |
| Otitis media | Birth prevalence - [36, 37) wks, [1500, 2000) g | Mortality | Females | | 58.527 (42.172 to 80.557) | 25.143 (20.331 to 30.566) | | | | | | | | | | | | | | | | | | | | | |
| Otitis media | Birth prevalence - [30, 32) wks, [2000, 2500) g | Mortality | Males | | 67.971 (50.354 to 88.935) | 18.03 (14.621 to 22.103) | | | | | | | | | | | | | | | | | | | | | |
| Otitis media | Birth prevalence - [30, 32) wks, [2000, 2500) g | Mortality | Females | | 69.383 (49.108 to 94.583) | 22.069 (17.836 to 27.163) | | | | | | | | | | | | | | | | | | | | | |
| Otitis media | Birth prevalence - [30, 32) wks, [1500, 2000) g | Mortality | Males | | 77.369 (59.702 to 99.232) | 31.079 (25.786 to 36.724) | | | | | | | | | | | | | | | | | | | | | |
| Otitis media | Birth prevalence - [30, 32) wks, [1500, 2000) g | Mortality | Females | | 76.134 (56.885 to 100.996) | 34.756 (28.764 to 41.849) | | | | | | | | | | | | | | | | | | | | | |
| Otitis media | Birth prevalence - [34, 36) wks, [1500, 2000) g | Mortality | Males | | 55.555 (39.553 to 75.104) | 21.346 (17.677 to 26.143) | | | | | | | | | | | | | | | | | | | | | |
| Otitis media | Birth prevalence - [34, 36) wks, [1500, 2000) g | Mortality | Females | | 54.335 (38.617 to 75.24) | 23.046 (18.743 to 28.287) | | | | | | | | | | | | | | | | | | | | | |
| Otitis media | Birth prevalence - [32, 34) wks, [1500, 2000) g | Mortality | Males | | 57.155 (42.484 to 73.651) | 23.114 (19.028 to 27.915) | | | | | | | | | | | | | | | | | | | | | |
| Otitis media | Birth prevalence - [32, 34) wks, [1500, 2000) g | Mortality | Females | | 56.101 (39.794 to 76.295) | 25.149 (20.615 to 30.388) | | | | | | | | | | | | | | | | | | | | | |
| Otitis media | Birth prevalence - [32, 34) wks, [2000, 2500) g | Mortality | Males | | 37.444 (29.026 to 48.227) | 12.233 (10.252 to 14.477) | | | | | | | | | | | | | | | | | | | | | |
| Otitis media | Birth prevalence - [32, 34) wks, [2000, 2500) g | Mortality | Females | | 36.874 (26.658 to 49.653) | 14.384 (12.095 to 17.03) | | | | | | | | | | | | | | | | | | | | | |
| Otitis media | Birth prevalence - [40, 42) wks, [2000, 2500) g | Mortality | Males | | 18.092 (13.292 to 23.719) | 9.23 (7.037 to 11.454) | | | | | | | | | | | | | | | | | | | | | |

**Appendix Table 6a. Relative risks used by age and sex for each outcome for all risk factors except for ambient air pollution alcohol, and smoking.**

| | | | | Ages | | | | | | | | | | | | | | | | | | | | | | | |
|---|---|---|---|---|---|---|---|---|---|---|---|---|---|---|---|---|---|---|---|---|---|---|---|---|---|---|---|
| Risk - Outcome | Category / Units | Morbidity / Mortality | Sex | All-age | 0-6 days | 7-27 days | 28-364 days | 1-4 years | 5-9 years | 10-14 years | 15-19 years | 20-24 years | 25-29 years | 30-34 years | 35-39 years | 40-44 years | 45-49 years | 50-54 years | 55-59 years | 60-64 years | 65-69 years | 70-74 years | 75-79 years | 80-84 years | 85-89 years | 90-94 years | 95+ years |
| Otitis media | Birth prevalence - [40, 42) wks, [2000, 2500) g | Mortality | Females | | 15.574 (11.516 to 20.778) | 9.975 (7.82 to 12.46) | | | | | | | | | | | | | | | | | | | | | |
| Otitis media | Birth prevalence - [38, 40) wks, [2000, 2500) g | Mortality | Males | | 13.104 (9.829 to 16.99) | 8.198 (6.786 to 9.959) | | | | | | | | | | | | | | | | | | | | | |
| Otitis media | Birth prevalence - [38, 40) wks, [2000, 2500) g | Mortality | Females | | 11.308 (8.389 to 14.38) | 8.577 (7.04 to 10.449) | | | | | | | | | | | | | | | | | | | | | |
| Otitis media | Birth prevalence - [32, 34) wks, [2500, 3000) g | Mortality | Males | | 33.063 (24.393 to 43.503) | 8.441 (6.822 to 10.431) | | | | | | | | | | | | | | | | | | | | | |
| Otitis media | Birth prevalence - [32, 34) wks, [2500, 3000) g | Mortality | Females | | 32.812 (23.439 to 45.567) | 10.398 (8.227 to 13.042) | | | | | | | | | | | | | | | | | | | | | |
| Otitis media | Birth prevalence - [34, 36) wks, [2000, 2500) g | Mortality | Males | | 21.925 (16.305 to 29.433) | 9.367 (7.859 to 11.112) | | | | | | | | | | | | | | | | | | | | | |
| Otitis media | Birth prevalence - [34, 36) wks, [2000, 2500) g | Mortality | Females | | 21.297 (15.657 to 28.761) | 10.295 (8.548 to 12.273) | | | | | | | | | | | | | | | | | | | | | |
| Otitis media | Birth prevalence - [37, 38) wks, [2000, 2500) g | Mortality | Males | | 13.0 (10.102 to 16.456) | 8.096 (6.724 to 9.676) | | | | | | | | | | | | | | | | | | | | | |
| Otitis media | Birth prevalence - [37, 38) wks, [2000, 2500) g | Mortality | Females | | 11.563 (8.805 to 15.11) | 8.467 (6.994 to 10.342) | | | | | | | | | | | | | | | | | | | | | |
| Otitis media | Birth prevalence - [36, 37) wks, [2000, 2500) g | Mortality | Males | | 14.401 (10.789 to 18.654) | 8.221 (6.917 to 9.923) | | | | | | | | | | | | | | | | | | | | | |
| Otitis media | Birth prevalence - [36, 37) wks, [2000, 2500) g | Mortality | Females | | 13.513 (9.817 to 17.942) | 8.654 (7.215 to 10.369) | | | | | | | | | | | | | | | | | | | | | |
| Otitis media | Birth prevalence - [34, 36) wks, [2500, 3000) g | Mortality | Males | | 13.419 (10.387 to 16.819) | 5.562 (4.646 to 6.696) | | | | | | | | | | | | | | | | | | | | | |
| Otitis media | Birth prevalence - [34, 36) wks, [2500, 3000) g | Mortality | Females | | 13.266 (9.666 to 17.689) | 6.395 (5.292 to 7.606) | | | | | | | | | | | | | | | | | | | | | |
| Otitis media | Birth prevalence - [34, 36) wks, [4000, 4500) g | Mortality | Males | | 23.096 (14.708 to 35.098) | 2.895 (2.245 to 3.716) | | | | | | | | | | | | | | | | | | | | | |
| Otitis media | Birth prevalence - [34, 36) wks, [4000, 4500) g | Mortality | Females | | 25.038 (15.763 to 37.255) | 3.778 (2.855 to 4.925) | | | | | | | | | | | | | | | | | | | | | |
| Otitis media | Birth prevalence - [34, 36) wks, [3000, 3500) g | Mortality | Males | | 14.006 (10.222 to 18.478) | 4.322 (3.449 to 5.338) | | | | | | | | | | | | | | | | | | | | | |
| Otitis media | Birth prevalence - [34, 36) wks, [3000, 3500) g | Mortality | Females | | 14.375 (10.269 to 20.114) | 5.265 (4.145 to 6.564) | | | | | | | | | | | | | | | | | | | | | |
| Otitis media | Birth prevalence - [36, 37) wks, [2500, 3000) g | Mortality | Males | | 4.874 (4.014 to 5.713) | 3.699 (3.134 to 4.374) | | | | | | | | | | | | | | | | | | | | | |
| Otitis media | Birth prevalence - [36, 37) wks, [2500, 3000) g | Mortality | Females | | 4.609 (3.731 to 5.61) | 3.898 (3.235 to 4.67) | | | | | | | | | | | | | | | | | | | | | |
| Otitis media | Birth prevalence - [34, 36) wks, [3500, 4000) g | Mortality | Males | | 18.024 (12.279 to 25.547) | 3.657 (2.838 to 4.675) | | | | | | | | | | | | | | | | | | | | | |
| Otitis media | Birth prevalence - [34, 36) wks, [3500, 4000) g | Mortality | Females | | 19.263 (12.567 to 27.924) | 4.634 (3.576 to 5.989) | | | | | | | | | | | | | | | | | | | | | |
| Otitis media | Birth prevalence - [37, 38) wks, [2500, 3000) g | Mortality | Males | | 3.306 (2.82 to 3.843) | 3.194 (2.691 to 3.803) | | | | | | | | | | | | | | | | | | | | | |
| Otitis media | Birth prevalence - [37, 38) wks, [2500, 3000) g | Mortality | Females | | 2.991 (2.496 to 3.61) | 3.242 (2.745 to 3.817) | | | | | | | | | | | | | | | | | | | | | |
| Otitis media | Birth prevalence - [40, 42) wks, [2500, 3000) g | Mortality | Males | | 3.771 (3.002 to 4.693) | 3.175 (2.56 to 3.923) | | | | | | | | | | | | | | | | | | | | | |
| Otitis media | Birth prevalence - [40, 42) wks, [2500, 3000) g | Mortality | Females | | 3.244 (2.486 to 4.159) | 3.228 (2.605 to 3.985) | | | | | | | | | | | | | | | | | | | | | |
| Otitis media | Birth prevalence - [38, 40) wks, [2500, 3000) g | Mortality | Males | | 2.755 (2.274 to 3.309) | 2.944 (2.44 to 3.548) | | | | | | | | | | | | | | | | | | | | | |
| Otitis media | Birth prevalence - [38, 40) wks, [2500, 3000) g | Mortality | Females | | 2.376 (1.91 to 2.886) | 2.938 (2.434 to 3.503) | | | | | | | | | | | | | | | | | | | | | |
| Otitis media | Birth prevalence - [36, 37) wks, [3000, 3500) g | Mortality | Males | | 3.774 (3.094 to 4.497) | 2.466 (2.058 to 2.929) | | | | | | | | | | | | | | | | | | | | | |
| Otitis media | Birth prevalence - [36, 37) wks, [3000, 3500) g | Mortality | Females | | 3.73 (2.981 to 4.646) | 2.715 (2.277 to 3.218) | | | | | | | | | | | | | | | | | | | | | |
| Otitis media | Birth prevalence - [36, 37) wks, [4000, 4500) g | Mortality | Males | | 6.826 (5.212 to 9.045) | 1.77 (1.491 to 2.082) | | | | | | | | | | | | | | | | | | | | | |
| Otitis media | Birth prevalence - [36, 37) wks, [4000, 4500) g | Mortality | Females | | 7.269 (5.144 to 9.821) | 2.177 (1.786 to 2.607) | | | | | | | | | | | | | | | | | | | | | |
| Otitis media | Birth prevalence - [36, 37) wks, [3500, 4000) g | Mortality | Males | | 4.544 (3.64 to 5.622) | 2.057 (1.74 to 2.44) | | | | | | | | | | | | | | | | | | | | | |
| Otitis media | Birth prevalence - [36, 37) wks, [3500, 4000) g | Mortality | Females | | 4.662 (3.577 to 6.014) | 2.398 (1.98 to 2.864) | | | | | | | | | | | | | | | | | | | | | |
| Otitis media | Birth prevalence - [37, 38) wks, [3000, 3500) g | Mortality | Males | | 2.007 (1.759 to 2.293) | 1.888 (1.613 to 2.224) | | | | | | | | | | | | | | | | | | | | | |
| Otitis media | Birth prevalence - [37, 38) wks, [3000, 3500) g | Mortality | Females | | 1.925 (1.582 to 2.328) | 1.972 (1.669 to 2.313) | | | | | | | | | | | | | | | | | | | | | |
| Otitis media | Birth prevalence - [37, 38) wks, [4000, 4500) g | Mortality | Males | | 3.28 (2.596 to 4.133) | 1.335 (1.171 to 1.532) | | | | | | | | | | | | | | | | | | | | | |
| Otitis media | Birth prevalence - [37, 38) wks, [4000, 4500) g | Mortality | Females | | 3.521 (2.649 to 4.5) | 1.559 (1.333 to 1.835) | | | | | | | | | | | | | | | | | | | | | |
| Otitis media | Birth prevalence - [37, 38) wks, [3500, 4000) g | Mortality | Males | | 2.128 (1.833 to 2.466) | 1.505 (1.299 to 1.76) | | | | | | | | | | | | | | | | | | | | | |
| Otitis media | Birth prevalence - [37, 38) wks, [3500, 4000) g | Mortality | Females | | 2.142 (1.694 to 2.67) | 1.661 (1.411 to 1.961) | | | | | | | | | | | | | | | | | | | | | |
| Otitis media | Birth prevalence - [40, 42) wks, [3000, 3500) g | Mortality | Males | | 1.436 (1.245 to 1.65) | 1.47 (1.199 to 1.8) | | | | | | | | | | | | | | | | | | | | | |
| Otitis media | Birth prevalence - [40, 42) wks, [3000, 3500) g | Mortality | Females | | 1.326 (1.069 to 1.614) | 1.465 (1.188 to 1.775) | | | | | | | | | | | | | | | | | | | | | |
| Otitis media | Birth prevalence - [38, 40) wks, [3000, 3500) g | Mortality | Males | | 1.33 (1.155 to 1.53) | 1.559 (1.305 to 1.851) | | | | | | | | | | | | | | | | | | | | | |
| Otitis media | Birth prevalence - [38, 40) wks, [3000, 3500) g | Mortality | Females | | 1.224 (1.0 to 1.492) | 1.564 (1.304 to 1.847) | | | | | | | | | | | | | | | | | | | | | |
| Otitis media | Birth prevalence - [38, 40) wks, [4000, 4500) g | Mortality | Males | | 1.787 (1.453 to 2.182) | 1.175 (1.005 to 1.371) | | | | | | | | | | | | | | | | | | | | | |
| Otitis media | Birth prevalence - [38, 40) wks, [4000, 4500) g | Mortality | Females | | 1.877 (1.467 to 2.388) | 1.224 (1.022 to 1.465) | | | | | | | | | | | | | | | | | | | | | |
| Otitis media | Birth prevalence - [38, 40) wks, [3500, 4000) g | Mortality | Males | | 1.785 (1.478 to 2.147) | 1.173 (1.0 to 1.377) | | | | | | | | | | | | | | | | | | | | | |
| Otitis media | Birth prevalence - [38, 40) wks, [3500, 4000) g | Mortality | Females | | 1.892 (1.481 to 2.352) | 1.23 (1.03 to 1.46) | | | | | | | | | | | | | | | | | | | | | |
| Otitis media | Birth prevalence - [40, 42) wks, [3500, 4000) g | Mortality | Males | | 1.0 (1.0 to 1.0) | 1.003 (1.0 to 1.046) | | | | | | | | | | | | | | | | | | | | | |
| Otitis media | Birth prevalence - [40, 42) wks, [3500, 4000) g | Mortality | Females | | 1.002 (1.0 to 1.013) | 1.001 (1.0 to 1.006) | | | | | | | | | | | | | | | | | | | | | |
| Otitis media | Birth prevalence - [40, 42) wks, [4000, 4500) g | Mortality | Males | | 1.0 (1.0 to 1.0) | 1.0 (1.0 to 1.0) | | | | | | | | | | | | | | | | | | | | | |
| Otitis media | Birth prevalence - [40, 42) wks, [4000, 4500) g | Mortality | Females | | 1.0 (1.0 to 1.0) | 1.0 (1.0 to 1.0) | | | | | | | | | | | | | | | | | | | | | |
| Otitis media | Birth prevalence - [28, 30) wks, [2000, 2500) g | Mortality | Males | | 117.172 (83.895 to 158.056) | 27.726 (21.877 to 34.972) | | | | | | | | | | | | | | | | | | | | | |
| Otitis media | Birth prevalence - [28, 30) wks, [2000, 2500) g | Mortality | Females | | 121.682 (84.349 to 171.375) | 33.983 (26.101 to 43.404) | | | | | | | | | | | | | | | | | | | | | |
| Otitis media | Birth prevalence - [28, 30) wks, [2500, 3000) g | Mortality | Males | | 77.948 (54.687 to 105.047) | 16.608 (12.653 to 21.188) | | | | | | | | | | | | | | | | | | | | | |
| Otitis media | Birth prevalence - [28, 30) wks, [2500, 3000) g | Mortality | Females | | 79.193 (54.099 to 112.236) | 20.387 (15.089 to 26.434) | | | | | | | | | | | | | | | | | | | | | |
| Otitis media | Birth prevalence - [28, 30) wks, [3000, 3500) g | Mortality | Males | | 42.199 (29.891 to 57.227) | 10.082 (7.777 to 13.056) | | | | | | | | | | | | | | | | | | | | | |
| Otitis media | Birth prevalence - [28, 30) wks, [3000, 3500) g | Mortality | Females | | 42.551 (28.25 to 63.209) | 11.989 (9.084 to 15.544) | | | | | | | | | | | | | | | | | | | | | |

**Appendix Table 6a. Relative risks used by age and sex for each outcome for all risk factors except for ambient air pollution alcohol, and smoking.**

| Risk - Outcome | Category / Units | Morbidity / Mortality | Sex | Ages | | | | | | | | | | | | | | | | | | | | | | | |
|---|---|---|---|---|---|---|---|---|---|---|---|---|---|---|---|---|---|---|---|---|---|---|---|---|---|---|---|
| | | | | All-age | 0-6 days | 7-27 days | 28-364 days | 1-4 years | 5-9 years | 10-14 years | 15-19 years | 20-24 years | 25-29 years | 30-34 years | 35-39 years | 40-44 years | 45-49 years | 50-54 years | 55-59 years | 60-64 years | 65-69 years | 70-74 years | 75-79 years | 80-84 years | 85-89 years | 90-94 years | 95+ years |
| Otitis media | Birth prevalence - [30, 32) wks, [2500, 3000) g | Mortality | Males | | 58.722 (42.419 to 78.873) | 12.115 (9.518 to 15.521) | | | | | | | | | | | | | | | | | | | | | |
| Otitis media | Birth prevalence - [30, 32) wks, [2500, 3000) g | Mortality | Females | | 59.522 (42.058 to 82.793) | 15.364 (11.936 to 19.581) | | | | | | | | | | | | | | | | | | | | | |
| Otitis media | Birth prevalence - [30, 32) wks, [3000, 3500) g | Mortality | Males | | 45.67 (32.014 to 65.531) | 8.381 (6.362 to 10.842) | | | | | | | | | | | | | | | | | | | | | |
| Otitis media | Birth prevalence - [30, 32) wks, [3000, 3500) g | Mortality | Females | | 46.104 (30.243 to 66.207) | 10.506 (8.041 to 13.513) | | | | | | | | | | | | | | | | | | | | | |
| Otitis media | Birth prevalence - [30, 32) wks, [3500, 4000) g | Mortality | Males | | 36.334 (21.558 to 54.813) | 5.698 (4.293 to 7.349) | | | | | | | | | | | | | | | | | | | | | |
| Otitis media | Birth prevalence - [30, 32) wks, [3500, 4000) g | Mortality | Females | | 37.931 (22.692 to 61.276) | 6.892 (5.175 to 9.058) | | | | | | | | | | | | | | | | | | | | | |
| Otitis media | Birth prevalence - [32, 34) wks, [3000, 3500) g | Mortality | Males | | 34.016 (23.515 to 48.37) | 6.577 (4.934 to 8.436) | | | | | | | | | | | | | | | | | | | | | |
| Otitis media | Birth prevalence - [32, 34) wks, [3000, 3500) g | Mortality | Females | | 34.585 (22.909 to 49.754) | 8.314 (6.362 to 10.789) | | | | | | | | | | | | | | | | | | | | | |
| Otitis media | Birth prevalence - [32, 34) wks, [3500, 4000) g | Mortality | Males | | 36.248 (23.158 to 54.67) | 5.068 (3.761 to 6.741) | | | | | | | | | | | | | | | | | | | | | |
| Otitis media | Birth prevalence - [32, 34) wks, [3500, 4000) g | Mortality | Females | | 38.098 (23.301 to 59.429) | 6.476 (4.666 to 8.689) | | | | | | | | | | | | | | | | | | | | | |
| Otitis media | Birth prevalence - [36, 37) wks, [1000, 1500) g | Mortality | Males | | 166.686 (118.487 to 222.581) | 57.535 (45.999 to 71.742) | | | | | | | | | | | | | | | | | | | | | |
| Otitis media | Birth prevalence - [36, 37) wks, [1000, 1500) g | Mortality | Females | | 169.725 (119.017 to 229.008) | 63.564 (50.068 to 80.703) | | | | | | | | | | | | | | | | | | | | | |
| Otitis media | Birth prevalence - [38, 40) wks, [1000, 1500) g | Mortality | Males | | 174.066 (125.125 to 232.507) | 57.966 (44.393 to 73.241) | | | | | | | | | | | | | | | | | | | | | |
| Otitis media | Birth prevalence - [38, 40) wks, [1000, 1500) g | Mortality | Females | | 171.557 (121.585 to 237.047) | 65.208 (48.821 to 84.308) | | | | | | | | | | | | | | | | | | | | | |
| Otitis media | Birth prevalence - [38, 40) wks, [1500, 2000) g | Mortality | Males | | 67.302 (49.547 to 89.055) | 25.206 (20.365 to 31.168) | | | | | | | | | | | | | | | | | | | | | |
| Otitis media | Birth prevalence - [38, 40) wks, [1500, 2000) g | Mortality | Females | | 62.19 (45.884 to 83.445) | 28.05 (22.625 to 35.139) | | | | | | | | | | | | | | | | | | | | | |
| Otitis media | Birth prevalence - [40, 42) wks, [1500, 2000) g | Mortality | Males | | 76.673 (56.177 to 102.468) | 25.785 (19.387 to 34.168) | | | | | | | | | | | | | | | | | | | | | |
| Otitis media | Birth prevalence - [40, 42) wks, [1500, 2000) g | Mortality | Females | | 70.411 (49.221 to 97.952) | 29.113 (21.355 to 38.272) | | | | | | | | | | | | | | | | | | | | | |
| Diarrhoeal diseases | Birth prevalence - [0, 24) wks, [0, 500) g | Mortality | Males | | 1564.792 (1056.542 to [illegible] | 618.595 (458.842 to 812.921) | | | | | | | | | | | | | | | | | | | | | |
| Diarrhoeal diseases | Birth prevalence - [0, 24) wks, [0, 500) g | Mortality | Females | | 1600.122 (1050.664 to [illegible] | 713.571 (526.178 to 921.018) | | | | | | | | | | | | | | | | | | | | | |
| Diarrhoeal diseases | Birth prevalence - [0, 24) wks, [500, 1000) g | Mortality | Males | | 1155.815 (825.412 to 1506.837) | 457.5 (352.552 to 573.483) | | | | | | | | | | | | | | | | | | | | | |
| Diarrhoeal diseases | Birth prevalence - [0, 24) wks, [500, 1000) g | Mortality | Females | | 1169.123 (802.003 to 1617.979) | 515.406 (396.713 to 641.541) | | | | | | | | | | | | | | | | | | | | | |
| Diarrhoeal diseases | Birth prevalence - [24, 26) wks, [500, 1000) g | Mortality | Males | | 955.583 (723.748 to 1244.265) | 443.357 (363.03 to 534.695) | | | | | | | | | | | | | | | | | | | | | |
| Diarrhoeal diseases | Birth prevalence - [24, 26) wks, [500, 1000) g | Mortality | Females | | 947.143 (702.662 to 1237.093) | 487.549 (387.307 to 603.498) | | | | | | | | | | | | | | | | | | | | | |
| Diarrhoeal diseases | Birth prevalence - [26, 28) wks, [500, 1000) g | Mortality | Males | | 497.817 (377.617 to 648.547) | 330.886 (261.438 to 401.709) | | | | | | | | | | | | | | | | | | | | | |
| Diarrhoeal diseases | Birth prevalence - [26, 28) wks, [500, 1000) g | Mortality | Females | | 483.682 (354.946 to 629.517) | 344.618 (274.427 to 419.864) | | | | | | | | | | | | | | | | | | | | | |
| Diarrhoeal diseases | Birth prevalence - [30, 32) wks, [500, 1000) g | Mortality | Males | | 236.614 (163.821 to 324.502) | 149.995 (117.866 to 188.368) | | | | | | | | | | | | | | | | | | | | | |
| Diarrhoeal diseases | Birth prevalence - [30, 32) wks, [500, 1000) g | Mortality | Females | | 229.197 (157.606 to 317.194) | 152.117 (120.779 to 190.583) | | | | | | | | | | | | | | | | | | | | | |
| Diarrhoeal diseases | Birth prevalence - [28, 30) wks, [500, 1000) g | Mortality | Males | | 297.629 (214.953 to 396.586) | 216.995 (173.321 to 271.466) | | | | | | | | | | | | | | | | | | | | | |
| Diarrhoeal diseases | Birth prevalence - [28, 30) wks, [500, 1000) g | Mortality | Females | | 281.056 (198.176 to 386.635) | 219.884 (174.264 to 272.704) | | | | | | | | | | | | | | | | | | | | | |
| Diarrhoeal diseases | Birth prevalence - [26, 28) wks, [1000, 1500) g | Mortality | Males | | 267.91 (210.177 to 332.92) | 164.167 (132.898 to 200.569) | | | | | | | | | | | | | | | | | | | | | |
| Diarrhoeal diseases | Birth prevalence - [26, 28) wks, [1000, 1500) g | Mortality | Females | | 266.509 (197.461 to 346.932) | 174.222 (137.431 to 217.349) | | | | | | | | | | | | | | | | | | | | | |
| Diarrhoeal diseases | Birth prevalence - [34, 36) wks, [1000, 1500) g | Mortality | Males | | 142.056 (98.086 to 197.774) | 52.86 (42.914 to 64.617) | | | | | | | | | | | | | | | | | | | | | |
| Diarrhoeal diseases | Birth prevalence - [34, 36) wks, [1000, 1500) g | Mortality | Females | | 141.899 (95.864 to 197.656) | 57.421 (46.452 to 71.339) | | | | | | | | | | | | | | | | | | | | | |
| Diarrhoeal diseases | Birth prevalence - [28, 30) wks, [1500, 2000) g | Mortality | Males | | 127.966 (97.178 to 167.026) | 50.018 (40.539 to 61.919) | | | | | | | | | | | | | | | | | | | | | |
| Diarrhoeal diseases | Birth prevalence - [28, 30) wks, [1500, 2000) g | Mortality | Females | | 130.924 (96.513 to 172.188) | 57.275 (46.36 to 70.038) | | | | | | | | | | | | | | | | | | | | | |
| Diarrhoeal diseases | Birth prevalence - [28, 30) wks, [1000, 1500) g | Mortality | Males | | 158.563 (120.99 to 204.947) | 103.32 (83.486 to 127.144) | | | | | | | | | | | | | | | | | | | | | |
| Diarrhoeal diseases | Birth prevalence - [28, 30) wks, [1000, 1500) g | Mortality | Females | | 153.905 (112.327 to 200.786) | 107.529 (86.954 to 131.78) | | | | | | | | | | | | | | | | | | | | | |
| Diarrhoeal diseases | Birth prevalence - [32, 34) wks, [1000, 1500) g | Mortality | Males | | 117.142 (81.354 to 161.101) | 53.185 (43.049 to 66.274) | | | | | | | | | | | | | | | | | | | | | |
| Diarrhoeal diseases | Birth prevalence - [32, 34) wks, [1000, 1500) g | Mortality | Females | | 115.171 (79.363 to 159.206) | 56.034 (45.982 to 68.36) | | | | | | | | | | | | | | | | | | | | | |
| Diarrhoeal diseases | Birth prevalence - [30, 32) wks, [1000, 1500) g | Mortality | Males | | 119.308 (87.769 to 160.885) | 67.163 (54.863 to 82.638) | | | | | | | | | | | | | | | | | | | | | |
| Diarrhoeal diseases | Birth prevalence - [30, 32) wks, [1000, 1500) g | Mortality | Females | | 115.448 (84.272 to 156.425) | 69.14 (55.873 to 85.012) | | | | | | | | | | | | | | | | | | | | | |
| Diarrhoeal diseases | Birth prevalence - [37, 38) wks, [1500, 2000) g | Mortality | Males | | 62.972 (46.159 to 83.484) | 24.148 (20.066 to 29.406) | | | | | | | | | | | | | | | | | | | | | |
| Diarrhoeal diseases | Birth prevalence - [37, 38) wks, [1500, 2000) g | Mortality | Females | | 59.988 (43.974 to 79.053) | 26.719 (21.746 to 32.816) | | | | | | | | | | | | | | | | | | | | | |
| Diarrhoeal diseases | Birth prevalence - [36, 37) wks, [1500, 2000) g | Mortality | Males | | 60.218 (43.669 to 82.48) | 23.031 (18.793 to 28.483) | | | | | | | | | | | | | | | | | | | | | |
| Diarrhoeal diseases | Birth prevalence - [36, 37) wks, [1500, 2000) g | Mortality | Females | | 58.527 (42.172 to 80.557) | 25.143 (20.331 to 30.566) | | | | | | | | | | | | | | | | | | | | | |
| Diarrhoeal diseases | Birth prevalence - [30, 32) wks, [2000, 2500) g | Mortality | Males | | 67.971 (50.354 to 88.935) | 18.03 (14.621 to 22.103) | | | | | | | | | | | | | | | | | | | | | |
| Diarrhoeal diseases | Birth prevalence - [30, 32) wks, [2000, 2500) g | Mortality | Females | | 69.383 (49.108 to 94.583) | 22.069 (17.836 to 27.163) | | | | | | | | | | | | | | | | | | | | | |
| Diarrhoeal diseases | Birth prevalence - [30, 32) wks, [1500, 2000) g | Mortality | Males | | 77.369 (59.702 to 99.232) | 31.079 (25.786 to 36.724) | | | | | | | | | | | | | | | | | | | | | |
| Diarrhoeal diseases | Birth prevalence - [30, 32) wks, [1500, 2000) g | Mortality | Females | | 76.134 (56.885 to 100.996) | 34.756 (28.764 to 41.849) | | | | | | | | | | | | | | | | | | | | | |
| Diarrhoeal diseases | Birth prevalence - [34, 36) wks, [1500, 2000) g | Mortality | Males | | 55.555 (39.553 to 75.104) | 21.346 (17.677 to 26.143) | | | | | | | | | | | | | | | | | | | | | |
| Diarrhoeal diseases | Birth prevalence - [34, 36) wks, [1500, 2000) g | Mortality | Females | | 54.335 (38.617 to 75.24) | 23.046 (18.743 to 28.287) | | | | | | | | | | | | | | | | | | | | | |
| Diarrhoeal diseases | Birth prevalence - [32, 34) wks, [1500, 2000) g | Mortality | Males | | 57.155 (42.484 to 73.651) | 23.114 (19.028 to 27.915) | | | | | | | | | | | | | | | | | | | | | |
| Diarrhoeal diseases | Birth prevalence - [32, 34) wks, [1500, 2000) g | Mortality | Females | | 56.101 (39.794 to 76.295) | 25.149 (20.615 to 30.388) | | | | | | | | | | | | | | | | | | | | | |
| Diarrhoeal diseases | Birth prevalence - [32, 34) wks, [2000, 2500) g | Mortality | Males | | 37.444 (29.026 to 48.227) | 12.233 (10.252 to 14.477) | | | | | | | | | | | | | | | | | | | | | |
| Diarrhoeal diseases | Birth prevalence - [32, 34) wks, [2000, 2500) g | Mortality | Females | | 36.874 (26.658 to 49.653) | 14.384 (12.095 to 17.03) | | | | | | | | | | | | | | | | | | | | | |
| Diarrhoeal diseases | Birth prevalence - [40, 42) wks, [2000, 2500) g | Mortality | Males | | 18.092 (13.292 to 23.719) | 9.23 (7.037 to 11.454) | | | | | | | | | | | | | | | | | | | | | |

Appendix Table 6a. Relative risks used by age and sex for each outcome for all risk factors except for ambient air pollution alcohol, and smoking.

| | | | | Ages | | | | | | | | | | | | | | | | | | | | | | | |
|---|---|---|---|---|---|---|---|---|---|---|---|---|---|---|---|---|---|---|---|---|---|---|---|---|---|---|---|
| Risk - Outcome | Category / Units | Morbidity / Mortality | Sex | All-age | 0-6 days | 7-27 days | 28-364 days | 1-4 years | 5-9 years | 10-14 years | 15-19 years | 20-24 years | 25-29 years | 30-34 years | 35-39 years | 40-44 years | 45-49 years | 50-54 years | 55-59 years | 60-64 years | 65-69 years | 70-74 years | 75-79 years | 80-84 years | 85-89 years | 90-94 years | 95+ years |
| Diarrhoeal diseases | Birth prevalence - [40, 42) wks, [2000, 2500) g | Mortality | Females | | 15.574 (11.516 to 20.778) | 9.975 (7.82 to 12.46) | | | | | | | | | | | | | | | | | | | | | |
| Diarrhoeal diseases | Birth prevalence - [38, 40) wks, [2000, 2500) g | Mortality | Males | | 13.104 (9.829 to 16.99) | 8.198 (6.786 to 9.959) | | | | | | | | | | | | | | | | | | | | | |
| Diarrhoeal diseases | Birth prevalence - [38, 40) wks, [2000, 2500) g | Mortality | Females | | 11.308 (8.389 to 14.38) | 8.577 (7.04 to 10.449) | | | | | | | | | | | | | | | | | | | | | |
| Diarrhoeal diseases | Birth prevalence - [32, 34) wks, [2500, 3000) g | Mortality | Males | | 33.063 (24.393 to 43.503) | 8.441 (6.822 to 10.431) | | | | | | | | | | | | | | | | | | | | | |
| Diarrhoeal diseases | Birth prevalence - [32, 34) wks, [2500, 3000) g | Mortality | Females | | 32.812 (23.439 to 45.567) | 10.398 (8.227 to 13.042) | | | | | | | | | | | | | | | | | | | | | |
| Diarrhoeal diseases | Birth prevalence - [34, 36) wks, [2000, 2500) g | Mortality | Males | | 21.925 (16.305 to 29.433) | 9.367 (7.859 to 11.112) | | | | | | | | | | | | | | | | | | | | | |
| Diarrhoeal diseases | Birth prevalence - [34, 36) wks, [2000, 2500) g | Mortality | Females | | 21.297 (15.657 to 28.761) | 10.295 (8.548 to 12.273) | | | | | | | | | | | | | | | | | | | | | |
| Diarrhoeal diseases | Birth prevalence - [37, 38) wks, [2000, 2500) g | Mortality | Males | | 13.0 (10.102 to 16.456) | 8.096 (6.724 to 9.676) | | | | | | | | | | | | | | | | | | | | | |
| Diarrhoeal diseases | Birth prevalence - [37, 38) wks, [2000, 2500) g | Mortality | Females | | 11.563 (8.805 to 15.11) | 8.467 (6.994 to 10.342) | | | | | | | | | | | | | | | | | | | | | |
| Diarrhoeal diseases | Birth prevalence - [36, 37) wks, [2000, 2500) g | Mortality | Males | | 14.401 (10.789 to 18.654) | 8.221 (6.917 to 9.923) | | | | | | | | | | | | | | | | | | | | | |
| Diarrhoeal diseases | Birth prevalence - [36, 37) wks, [2000, 2500) g | Mortality | Females | | 13.513 (9.817 to 17.942) | 8.654 (7.215 to 10.369) | | | | | | | | | | | | | | | | | | | | | |
| Diarrhoeal diseases | Birth prevalence - [34, 36) wks, [2500, 3000) g | Mortality | Males | | 13.419 (10.387 to 16.819) | 5.562 (4.646 to 6.696) | | | | | | | | | | | | | | | | | | | | | |
| Diarrhoeal diseases | Birth prevalence - [34, 36) wks, [2500, 3000) g | Mortality | Females | | 13.266 (9.666 to 17.689) | 6.395 (5.292 to 7.606) | | | | | | | | | | | | | | | | | | | | | |
| Diarrhoeal diseases | Birth prevalence - [34, 36) wks, [4000, 4500) g | Mortality | Males | | 23.096 (14.708 to 35.098) | 2.895 (2.245 to 3.716) | | | | | | | | | | | | | | | | | | | | | |
| Diarrhoeal diseases | Birth prevalence - [34, 36) wks, [4000, 4500) g | Mortality | Females | | 25.038 (15.763 to 37.255) | 3.778 (2.855 to 4.925) | | | | | | | | | | | | | | | | | | | | | |
| Diarrhoeal diseases | Birth prevalence - [34, 36) wks, [3000, 3500) g | Mortality | Males | | 14.006 (10.222 to 18.478) | 4.322 (3.449 to 5.338) | | | | | | | | | | | | | | | | | | | | | |
| Diarrhoeal diseases | Birth prevalence - [34, 36) wks, [3000, 3500) g | Mortality | Females | | 14.375 (10.269 to 20.114) | 5.265 (4.145 to 6.564) | | | | | | | | | | | | | | | | | | | | | |
| Diarrhoeal diseases | Birth prevalence - [36, 37) wks, [2500, 3000) g | Mortality | Males | | 4.874 (4.014 to 5.713) | 3.699 (3.134 to 4.374) | | | | | | | | | | | | | | | | | | | | | |
| Diarrhoeal diseases | Birth prevalence - [36, 37) wks, [2500, 3000) g | Mortality | Females | | 4.609 (3.731 to 5.61) | 3.898 (3.235 to 4.67) | | | | | | | | | | | | | | | | | | | | | |
| Diarrhoeal diseases | Birth prevalence - [34, 36) wks, [3500, 4000) g | Mortality | Males | | 18.024 (12.279 to 25.547) | 3.657 (2.838 to 4.675) | | | | | | | | | | | | | | | | | | | | | |
| Diarrhoeal diseases | Birth prevalence - [34, 36) wks, [3500, 4000) g | Mortality | Females | | 19.263 (12.567 to 27.924) | 4.634 (3.576 to 5.989) | | | | | | | | | | | | | | | | | | | | | |
| Diarrhoeal diseases | Birth prevalence - [37, 38) wks, [2500, 3000) g | Mortality | Males | | 3.306 (2.82 to 3.843) | 3.194 (2.691 to 3.803) | | | | | | | | | | | | | | | | | | | | | |
| Diarrhoeal diseases | Birth prevalence - [37, 38) wks, [2500, 3000) g | Mortality | Females | | 2.991 (2.496 to 3.61) | 3.242 (2.745 to 3.817) | | | | | | | | | | | | | | | | | | | | | |
| Diarrhoeal diseases | Birth prevalence - [40, 42) wks, [2500, 3000) g | Mortality | Males | | 3.771 (3.002 to 4.693) | 3.175 (2.56 to 3.923) | | | | | | | | | | | | | | | | | | | | | |
| Diarrhoeal diseases | Birth prevalence - [40, 42) wks, [2500, 3000) g | Mortality | Females | | 3.244 (2.486 to 4.159) | 3.228 (2.605 to 3.985) | | | | | | | | | | | | | | | | | | | | | |
| Diarrhoeal diseases | Birth prevalence - [38, 40) wks, [2500, 3000) g | Mortality | Males | | 2.755 (2.274 to 3.309) | 2.944 (2.44 to 3.548) | | | | | | | | | | | | | | | | | | | | | |
| Diarrhoeal diseases | Birth prevalence - [38, 40) wks, [2500, 3000) g | Mortality | Females | | 2.376 (1.91 to 2.886) | 2.938 (2.434 to 3.503) | | | | | | | | | | | | | | | | | | | | | |
| Diarrhoeal diseases | Birth prevalence - [36, 37) wks, [3000, 3500) g | Mortality | Males | | 3.774 (3.094 to 4.497) | 2.466 (2.058 to 2.929) | | | | | | | | | | | | | | | | | | | | | |
| Diarrhoeal diseases | Birth prevalence - [36, 37) wks, [3000, 3500) g | Mortality | Females | | 3.73 (2.981 to 4.646) | 2.715 (2.277 to 3.218) | | | | | | | | | | | | | | | | | | | | | |
| Diarrhoeal diseases | Birth prevalence - [36, 37) wks, [4000, 4500) g | Mortality | Males | | 6.826 (5.212 to 9.045) | 1.77 (1.491 to 2.082) | | | | | | | | | | | | | | | | | | | | | |
| Diarrhoeal diseases | Birth prevalence - [36, 37) wks, [4000, 4500) g | Mortality | Females | | 7.269 (5.144 to 9.821) | 2.177 (1.786 to 2.607) | | | | | | | | | | | | | | | | | | | | | |
| Diarrhoeal diseases | Birth prevalence - [36, 37) wks, [3500, 4000) g | Mortality | Males | | 4.544 (3.64 to 5.622) | 2.057 (1.74 to 2.44) | | | | | | | | | | | | | | | | | | | | | |
| Diarrhoeal diseases | Birth prevalence - [36, 37) wks, [3500, 4000) g | Mortality | Females | | 4.662 (3.577 to 6.014) | 2.398 (1.98 to 2.864) | | | | | | | | | | | | | | | | | | | | | |
| Diarrhoeal diseases | Birth prevalence - [37, 38) wks, [3000, 3500) g | Mortality | Males | | 2.007 (1.759 to 2.293) | 1.888 (1.613 to 2.224) | | | | | | | | | | | | | | | | | | | | | |
| Diarrhoeal diseases | Birth prevalence - [37, 38) wks, [3000, 3500) g | Mortality | Females | | 1.925 (1.582 to 2.328) | 1.972 (1.669 to 2.313) | | | | | | | | | | | | | | | | | | | | | |
| Diarrhoeal diseases | Birth prevalence - [37, 38) wks, [4000, 4500) g | Mortality | Males | | 3.28 (2.596 to 4.133) | 1.335 (1.171 to 1.532) | | | | | | | | | | | | | | | | | | | | | |
| Diarrhoeal diseases | Birth prevalence - [37, 38) wks, [4000, 4500) g | Mortality | Females | | 3.521 (2.649 to 4.5) | 1.559 (1.333 to 1.835) | | | | | | | | | | | | | | | | | | | | | |
| Diarrhoeal diseases | Birth prevalence - [37, 38) wks, [3500, 4000) g | Mortality | Males | | 2.128 (1.833 to 2.466) | 1.505 (1.299 to 1.76) | | | | | | | | | | | | | | | | | | | | | |
| Diarrhoeal diseases | Birth prevalence - [37, 38) wks, [3500, 4000) g | Mortality | Females | | 2.142 (1.694 to 2.67) | 1.661 (1.411 to 1.961) | | | | | | | | | | | | | | | | | | | | | |
| Diarrhoeal diseases | Birth prevalence - [40, 42) wks, [3000, 3500) g | Mortality | Males | | 1.436 (1.245 to 1.65) | 1.47 (1.199 to 1.8) | | | | | | | | | | | | | | | | | | | | | |
| Diarrhoeal diseases | Birth prevalence - [40, 42) wks, [3000, 3500) g | Mortality | Females | | 1.326 (1.069 to 1.614) | 1.465 (1.188 to 1.775) | | | | | | | | | | | | | | | | | | | | | |
| Diarrhoeal diseases | Birth prevalence - [38, 40) wks, [3000, 3500) g | Mortality | Males | | 1.33 (1.155 to 1.53) | 1.559 (1.305 to 1.851) | | | | | | | | | | | | | | | | | | | | | |
| Diarrhoeal diseases | Birth prevalence - [38, 40) wks, [3000, 3500) g | Mortality | Females | | 1.224 (1.0 to 1.492) | 1.564 (1.304 to 1.847) | | | | | | | | | | | | | | | | | | | | | |
| Diarrhoeal diseases | Birth prevalence - [38, 40) wks, [4000, 4500) g | Mortality | Males | | 1.787 (1.453 to 2.182) | 1.175 (1.005 to 1.371) | | | | | | | | | | | | | | | | | | | | | |
| Diarrhoeal diseases | Birth prevalence - [38, 40) wks, [4000, 4500) g | Mortality | Females | | 1.877 (1.467 to 2.388) | 1.224 (1.022 to 1.465) | | | | | | | | | | | | | | | | | | | | | |
| Diarrhoeal diseases | Birth prevalence - [38, 40) wks, [3500, 4000) g | Mortality | Males | | 1.785 (1.478 to 2.147) | 1.173 (1.0 to 1.377) | | | | | | | | | | | | | | | | | | | | | |
| Diarrhoeal diseases | Birth prevalence - [38, 40) wks, [3500, 4000) g | Mortality | Females | | 1.892 (1.481 to 2.352) | 1.23 (1.03 to 1.46) | | | | | | | | | | | | | | | | | | | | | |
| Diarrhoeal diseases | Birth prevalence - [40, 42) wks, [3500, 4000) g | Mortality | Males | | 1.0 (1.0 to 1.0) | 1.003 (1.0 to 1.046) | | | | | | | | | | | | | | | | | | | | | |
| Diarrhoeal diseases | Birth prevalence - [40, 42) wks, [3500, 4000) g | Mortality | Females | | 1.002 (1.0 to 1.013) | 1.001 (1.0 to 1.006) | | | | | | | | | | | | | | | | | | | | | |
| Diarrhoeal diseases | Birth prevalence - [40, 42) wks, [4000, 4500) g | Mortality | Males | | 1.0 (1.0 to 1.0) | 1.0 (1.0 to 1.0) | | | | | | | | | | | | | | | | | | | | | |
| Diarrhoeal diseases | Birth prevalence - [40, 42) wks, [4000, 4500) g | Mortality | Females | | 1.0 (1.0 to 1.0) | 1.0 (1.0 to 1.0) | | | | | | | | | | | | | | | | | | | | | |
| Diarrhoeal diseases | Birth prevalence - [28, 30) wks, [2000, 2500) g | Mortality | Males | | 117.172 (83.895 to 158.056) | 27.726 (21.877 to 34.972) | | | | | | | | | | | | | | | | | | | | | |
| Diarrhoeal diseases | Birth prevalence - [28, 30) wks, [2000, 2500) g | Mortality | Females | | 121.682 (84.349 to 171.375) | 33.983 (26.101 to 43.404) | | | | | | | | | | | | | | | | | | | | | |
| Diarrhoeal diseases | Birth prevalence - [28, 30) wks, [2500, 3000) g | Mortality | Males | | 77.948 (54.687 to 105.047) | 16.608 (12.653 to 21.188) | | | | | | | | | | | | | | | | | | | | | |
| Diarrhoeal diseases | Birth prevalence - [28, 30) wks, [2500, 3000) g | Mortality | Females | | 79.193 (54.099 to 112.236) | 20.387 (15.089 to 26.434) | | | | | | | | | | | | | | | | | | | | | |
| Diarrhoeal diseases | Birth prevalence - [28, 30) wks, [3000, 3500) g | Mortality | Males | | 42.199 (29.891 to 57.227) | 10.082 (7.777 to 13.056) | | | | | | | | | | | | | | | | | | | | | |
| Diarrhoeal diseases | Birth prevalence - [28, 30) wks, [3000, 3500) g | Mortality | Females | | 42.551 (28.25 to 63.209) | 11.989 (9.084 to 15.544) | | | | | | | | | | | | | | | | | | | | | |

**Appendix Table 6a. Relative risks used by age and sex for each outcome for all risk factors except for ambient air pollution alcohol, and smoking.**

| | | | | Ages | | | | | | | | | | | | | | | | | | | | | | | |
|---|---|---|---|---|---|---|---|---|---|---|---|---|---|---|---|---|---|---|---|---|---|---|---|---|---|---|---|
| Risk - Outcome | Category / Units | Morbidity / Mortality | Sex | All-age | 0-6 days | 7-27 days | 28-364 days | 1-4 years | 5-9 years | 10-14 years | 15-19 years | 20-24 years | 25-29 years | 30-34 years | 35-39 years | 40-44 years | 45-49 years | 50-54 years | 55-59 years | 60-64 years | 65-69 years | 70-74 years | 75-79 years | 80-84 years | 85-89 years | 90-94 years | 95+ years |
| Diarrhoeal diseases | Birth prevalence - [30, 32) wks, [2500, 3000) g | Mortality | Males | | 58.722 (42.419 to 78.873) | 12.115 (9.518 to 15.521) | | | | | | | | | | | | | | | | | | | | | |
| Diarrhoeal diseases | Birth prevalence - [30, 32) wks, [2500, 3000) g | Mortality | Females | | 59.522 (42.058 to 82.793) | 15.364 (11.936 to 19.581) | | | | | | | | | | | | | | | | | | | | | |
| Diarrhoeal diseases | Birth prevalence - [30, 32) wks, [3000, 3500) g | Mortality | Males | | 45.67 (32.014 to 65.531) | 8.381 (6.362 to 10.842) | | | | | | | | | | | | | | | | | | | | | |
| Diarrhoeal diseases | Birth prevalence - [30, 32) wks, [3000, 3500) g | Mortality | Females | | 46.104 (30.243 to 66.207) | 10.506 (8.041 to 13.513) | | | | | | | | | | | | | | | | | | | | | |
| Diarrhoeal diseases | Birth prevalence - [30, 32) wks, [3500, 4000) g | Mortality | Males | | 36.334 (21.558 to 54.813) | 5.698 (4.293 to 7.349) | | | | | | | | | | | | | | | | | | | | | |
| Diarrhoeal diseases | Birth prevalence - [30, 32) wks, [3500, 4000) g | Mortality | Females | | 37.931 (22.692 to 61.276) | 6.892 (5.175 to 9.058) | | | | | | | | | | | | | | | | | | | | | |
| Diarrhoeal diseases | Birth prevalence - [32, 34) wks, [3000, 3500) g | Mortality | Males | | 34.016 (23.515 to 48.37) | 6.577 (4.934 to 8.436) | | | | | | | | | | | | | | | | | | | | | |
| Diarrhoeal diseases | Birth prevalence - [32, 34) wks, [3000, 3500) g | Mortality | Females | | 34.585 (22.909 to 49.754) | 8.314 (6.362 to 10.789) | | | | | | | | | | | | | | | | | | | | | |
| Diarrhoeal diseases | Birth prevalence - [32, 34) wks, [3500, 4000) g | Mortality | Males | | 36.248 (23.158 to 54.67) | 5.068 (3.761 to 6.741) | | | | | | | | | | | | | | | | | | | | | |
| Diarrhoeal diseases | Birth prevalence - [32, 34) wks, [3500, 4000) g | Mortality | Females | | 38.098 (23.301 to 59.429) | 6.476 (4.666 to 8.689) | | | | | | | | | | | | | | | | | | | | | |
| Diarrhoeal diseases | Birth prevalence - [36, 37) wks, [1000, 1500) g | Mortality | Males | | 166.686 (118.487 to 222.581) | 57.535 (45.999 to 71.742) | | | | | | | | | | | | | | | | | | | | | |
| Diarrhoeal diseases | Birth prevalence - [36, 37) wks, [1000, 1500) g | Mortality | Females | | 169.725 (119.017 to 229.008) | 63.564 (50.068 to 80.703) | | | | | | | | | | | | | | | | | | | | | |
| Diarrhoeal diseases | Birth prevalence - [38, 40) wks, [1000, 1500) g | Mortality | Males | | 174.066 (125.125 to 232.507) | 57.966 (44.393 to 73.241) | | | | | | | | | | | | | | | | | | | | | |
| Diarrhoeal diseases | Birth prevalence - [38, 40) wks, [1000, 1500) g | Mortality | Females | | 171.557 (121.585 to 237.047) | 65.208 (48.821 to 84.308) | | | | | | | | | | | | | | | | | | | | | |
| Diarrhoeal diseases | Birth prevalence - [38, 40) wks, [1500, 2000) g | Mortality | Males | | 67.302 (49.547 to 89.055) | 25.206 (20.365 to 31.168) | | | | | | | | | | | | | | | | | | | | | |
| Diarrhoeal diseases | Birth prevalence - [38, 40) wks, [1500, 2000) g | Mortality | Females | | 62.19 (45.884 to 83.445) | 28.05 (22.625 to 35.139) | | | | | | | | | | | | | | | | | | | | | |
| Diarrhoeal diseases | Birth prevalence - [40, 42) wks, [1500, 2000) g | Mortality | Males | | 76.673 (56.177 to 102.468) | 25.785 (19.387 to 34.168) | | | | | | | | | | | | | | | | | | | | | |
| Diarrhoeal diseases | Birth prevalence - [40, 42) wks, [1500, 2000) g | Mortality | Females | | 70.411 (49.221 to 97.952) | 29.113 (21.355 to 38.272) | | | | | | | | | | | | | | | | | | | | | |
| Pneumococcal meningitis | Birth prevalence - [0, 24) wks, [0, 500) g | Mortality | Males | | 1564.792 (1056.542 to [illegible] | 618.595 (458.842 to 812.921) | | | | | | | | | | | | | | | | | | | | | |
| Pneumococcal meningitis | Birth prevalence - [0, 24) wks, [0, 500) g | Mortality | Females | | 1600.122 (1050.664 to [illegible] | 713.571 (526.178 to 921.018) | | | | | | | | | | | | | | | | | | | | | |
| Pneumococcal meningitis | Birth prevalence - [0, 24) wks, [500, 1000) g | Mortality | Males | | 1155.815 (825.412 to 1506.837) | 457.5 (352.552 to 573.483) | | | | | | | | | | | | | | | | | | | | | |
| Pneumococcal meningitis | Birth prevalence - [0, 24) wks, [500, 1000) g | Mortality | Females | | 1169.123 (802.003 to 1617.979) | 515.406 (396.713 to 641.541) | | | | | | | | | | | | | | | | | | | | | |
| Pneumococcal meningitis | Birth prevalence - [24, 26) wks, [500, 1000) g | Mortality | Males | | 955.583 (723.748 to 1244.265) | 443.357 (363.03 to 534.695) | | | | | | | | | | | | | | | | | | | | | |
| Pneumococcal meningitis | Birth prevalence - [24, 26) wks, [500, 1000) g | Mortality | Females | | 947.143 (702.662 to 1237.093) | 487.549 (387.307 to 603.498) | | | | | | | | | | | | | | | | | | | | | |
| Pneumococcal meningitis | Birth prevalence - [26, 28) wks, [500, 1000) g | Mortality | Males | | 497.817 (377.617 to 648.547) | 330.886 (261.438 to 401.709) | | | | | | | | | | | | | | | | | | | | | |
| Pneumococcal meningitis | Birth prevalence - [26, 28) wks, [500, 1000) g | Mortality | Females | | 483.682 (354.946 to 629.517) | 344.618 (274.427 to 419.864) | | | | | | | | | | | | | | | | | | | | | |
| Pneumococcal meningitis | Birth prevalence - [30, 32) wks, [500, 1000) g | Mortality | Males | | 236.614 (163.821 to 324.502) | 149.995 (117.866 to 188.368) | | | | | | | | | | | | | | | | | | | | | |
| Pneumococcal meningitis | Birth prevalence - [30, 32) wks, [500, 1000) g | Mortality | Females | | 229.197 (157.606 to 317.194) | 152.117 (120.779 to 190.583) | | | | | | | | | | | | | | | | | | | | | |
| Pneumococcal meningitis | Birth prevalence - [28, 30) wks, [500, 1000) g | Mortality | Males | | 297.629 (214.953 to 396.586) | 216.995 (173.321 to 271.466) | | | | | | | | | | | | | | | | | | | | | |
| Pneumococcal meningitis | Birth prevalence - [28, 30) wks, [500, 1000) g | Mortality | Females | | 281.056 (198.176 to 386.635) | 219.884 (174.264 to 272.704) | | | | | | | | | | | | | | | | | | | | | |
| Pneumococcal meningitis | Birth prevalence - [26, 28) wks, [1000, 1500) g | Mortality | Males | | 267.91 (210.177 to 332.92) | 164.167 (132.898 to 200.569) | | | | | | | | | | | | | | | | | | | | | |
| Pneumococcal meningitis | Birth prevalence - [26, 28) wks, [1000, 1500) g | Mortality | Females | | 266.509 (197.461 to 346.932) | 174.222 (137.431 to 217.349) | | | | | | | | | | | | | | | | | | | | | |
| Pneumococcal meningitis | Birth prevalence - [34, 36) wks, [1000, 1500) g | Mortality | Males | | 142.056 (98.086 to 197.774) | 52.86 (42.914 to 64.617) | | | | | | | | | | | | | | | | | | | | | |
| Pneumococcal meningitis | Birth prevalence - [34, 36) wks, [1000, 1500) g | Mortality | Females | | 141.899 (95.864 to 197.656) | 57.421 (46.452 to 71.339) | | | | | | | | | | | | | | | | | | | | | |
| Pneumococcal meningitis | Birth prevalence - [28, 30) wks, [1500, 2000) g | Mortality | Males | | 127.966 (97.178 to 167.026) | 50.018 (40.539 to 61.919) | | | | | | | | | | | | | | | | | | | | | |
| Pneumococcal meningitis | Birth prevalence - [28, 30) wks, [1500, 2000) g | Mortality | Females | | 130.924 (96.513 to 172.188) | 57.275 (46.36 to 70.038) | | | | | | | | | | | | | | | | | | | | | |
| Pneumococcal meningitis | Birth prevalence - [28, 30) wks, [1000, 1500) g | Mortality | Males | | 158.563 (120.99 to 204.947) | 103.32 (83.486 to 127.144) | | | | | | | | | | | | | | | | | | | | | |
| Pneumococcal meningitis | Birth prevalence - [28, 30) wks, [1000, 1500) g | Mortality | Females | | 153.905 (112.327 to 200.786) | 107.529 (86.954 to 131.78) | | | | | | | | | | | | | | | | | | | | | |
| Pneumococcal meningitis | Birth prevalence - [32, 34) wks, [1000, 1500) g | Mortality | Males | | 117.142 (81.354 to 161.101) | 53.185 (43.049 to 66.274) | | | | | | | | | | | | | | | | | | | | | |
| Pneumococcal meningitis | Birth prevalence - [32, 34) wks, [1000, 1500) g | Mortality | Females | | 115.171 (79.363 to 159.206) | 56.034 (45.982 to 68.36) | | | | | | | | | | | | | | | | | | | | | |
| Pneumococcal meningitis | Birth prevalence - [30, 32) wks, [1000, 1500) g | Mortality | Males | | 119.308 (87.769 to 160.885) | 67.163 (54.863 to 82.638) | | | | | | | | | | | | | | | | | | | | | |
| Pneumococcal meningitis | Birth prevalence - [30, 32) wks, [1000, 1500) g | Mortality | Females | | 115.448 (84.272 to 156.425) | 69.14 (55.873 to 85.012) | | | | | | | | | | | | | | | | | | | | | |
| Pneumococcal meningitis | Birth prevalence - [37, 38) wks, [1500, 2000) g | Mortality | Males | | 62.972 (46.159 to 83.484) | 24.148 (20.066 to 29.406) | | | | | | | | | | | | | | | | | | | | | |
| Pneumococcal meningitis | Birth prevalence - [37, 38) wks, [1500, 2000) g | Mortality | Females | | 59.988 (43.974 to 79.053) | 26.719 (21.746 to 32.816) | | | | | | | | | | | | | | | | | | | | | |
| Pneumococcal meningitis | Birth prevalence - [36, 37) wks, [1500, 2000) g | Mortality | Males | | 60.218 (43.669 to 82.48) | 23.031 (18.793 to 28.483) | | | | | | | | | | | | | | | | | | | | | |
| Pneumococcal meningitis | Birth prevalence - [36, 37) wks, [1500, 2000) g | Mortality | Females | | 58.527 (42.172 to 80.557) | 25.143 (20.331 to 30.566) | | | | | | | | | | | | | | | | | | | | | |
| Pneumococcal meningitis | Birth prevalence - [30, 32) wks, [2000, 2500) g | Mortality | Males | | 67.971 (50.354 to 88.935) | 18.03 (14.621 to 22.103) | | | | | | | | | | | | | | | | | | | | | |
| Pneumococcal meningitis | Birth prevalence - [30, 32) wks, [2000, 2500) g | Mortality | Females | | 69.383 (49.108 to 94.583) | 22.069 (17.836 to 27.163) | | | | | | | | | | | | | | | | | | | | | |
| Pneumococcal meningitis | Birth prevalence - [30, 32) wks, [1500, 2000) g | Mortality | Males | | 77.369 (59.702 to 99.232) | 31.079 (25.786 to 36.724) | | | | | | | | | | | | | | | | | | | | | |
| Pneumococcal meningitis | Birth prevalence - [30, 32) wks, [1500, 2000) g | Mortality | Females | | 76.134 (56.885 to 100.996) | 34.756 (28.764 to 41.849) | | | | | | | | | | | | | | | | | | | | | |
| Pneumococcal meningitis | Birth prevalence - [34, 36) wks, [1500, 2000) g | Mortality | Males | | 55.555 (39.553 to 75.104) | 21.346 (17.677 to 26.143) | | | | | | | | | | | | | | | | | | | | | |
| Pneumococcal meningitis | Birth prevalence - [34, 36) wks, [1500, 2000) g | Mortality | Females | | 54.335 (38.617 to 75.24) | 23.046 (18.743 to 28.287) | | | | | | | | | | | | | | | | | | | | | |
| Pneumococcal meningitis | Birth prevalence - [32, 34) wks, [1500, 2000) g | Mortality | Males | | 57.155 (42.484 to 73.651) | 23.114 (19.028 to 27.915) | | | | | | | | | | | | | | | | | | | | | |
| Pneumococcal meningitis | Birth prevalence - [32, 34) wks, [1500, 2000) g | Mortality | Females | | 56.101 (39.794 to 76.295) | 25.149 (20.615 to 30.388) | | | | | | | | | | | | | | | | | | | | | |
| Pneumococcal meningitis | Birth prevalence - [32, 34) wks, [2000, 2500) g | Mortality | Males | | 37.444 (29.026 to 48.227) | 12.233 (10.252 to 14.477) | | | | | | | | | | | | | | | | | | | | | |
| Pneumococcal meningitis | Birth prevalence - [32, 34) wks, [2000, 2500) g | Mortality | Females | | 36.874 (26.658 to 49.653) | 14.384 (12.095 to 17.03) | | | | | | | | | | | | | | | | | | | | | |
| Pneumococcal meningitis | Birth prevalence - [40, 42) wks, [2000, 2500) g | Mortality | Males | | 18.092 (13.292 to 23.719) | 9.23 (7.037 to 11.454) | | | | | | | | | | | | | | | | | | | | | |

Appendix Table 6a. Relative risks used by age and sex for each outcome for all risk factors except for ambient air pollution alcohol, and smoking.

| | | | | Ages | | | | | | | | | | | | | | | | | | | | | | | |
|---|---|---|---|---|---|---|---|---|---|---|---|---|---|---|---|---|---|---|---|---|---|---|---|---|---|---|---|
| Risk - Outcome | Category / Units | Morbidity / Mortality | Sex | All-age | 0-6 days | 7-27 days | 28-364 days | 1-4 years | 5-9 years | 10-14 years | 15-19 years | 20-24 years | 25-29 years | 30-34 years | 35-39 years | 40-44 years | 45-49 years | 50-54 years | 55-59 years | 60-64 years | 65-69 years | 70-74 years | 75-79 years | 80-84 years | 85-89 years | 90-94 years | 95+ years |
| Pneumococcal meningitis | Birth prevalence - [40, 42) wks, [2000, 2500) g | Mortality | Females | | 15.574 (11.516 to 20.778) | 9.975 (7.82 to 12.46) | | | | | | | | | | | | | | | | | | | | | |
| Pneumococcal meningitis | Birth prevalence - [38, 40) wks, [2000, 2500) g | Mortality | Males | | 13.104 (9.829 to 16.99) | 8.198 (6.786 to 9.959) | | | | | | | | | | | | | | | | | | | | | |
| Pneumococcal meningitis | Birth prevalence - [38, 40) wks, [2000, 2500) g | Mortality | Females | | 11.308 (8.389 to 14.38) | 8.577 (7.04 to 10.449) | | | | | | | | | | | | | | | | | | | | | |
| Pneumococcal meningitis | Birth prevalence - [32, 34) wks, [2500, 3000) g | Mortality | Males | | 33.063 (24.393 to 43.503) | 8.441 (6.822 to 10.431) | | | | | | | | | | | | | | | | | | | | | |
| Pneumococcal meningitis | Birth prevalence - [32, 34) wks, [2500, 3000) g | Mortality | Females | | 32.812 (23.439 to 45.567) | 10.398 (8.227 to 13.042) | | | | | | | | | | | | | | | | | | | | | |
| Pneumococcal meningitis | Birth prevalence - [34, 36) wks, [2000, 2500) g | Mortality | Males | | 21.925 (16.305 to 29.433) | 9.367 (7.859 to 11.112) | | | | | | | | | | | | | | | | | | | | | |
| Pneumococcal meningitis | Birth prevalence - [34, 36) wks, [2000, 2500) g | Mortality | Females | | 21.297 (15.657 to 28.761) | 10.295 (8.548 to 12.273) | | | | | | | | | | | | | | | | | | | | | |
| Pneumococcal meningitis | Birth prevalence - [37, 38) wks, [2000, 2500) g | Mortality | Males | | 13.0 (10.102 to 16.456) | 8.096 (6.724 to 9.676) | | | | | | | | | | | | | | | | | | | | | |
| Pneumococcal meningitis | Birth prevalence - [37, 38) wks, [2000, 2500) g | Mortality | Females | | 11.563 (8.805 to 15.11) | 8.467 (6.994 to 10.342) | | | | | | | | | | | | | | | | | | | | | |
| Pneumococcal meningitis | Birth prevalence - [36, 37) wks, [2000, 2500) g | Mortality | Males | | 14.401 (10.789 to 18.654) | 8.221 (6.917 to 9.923) | | | | | | | | | | | | | | | | | | | | | |
| Pneumococcal meningitis | Birth prevalence - [36, 37) wks, [2000, 2500) g | Mortality | Females | | 13.513 (9.817 to 17.942) | 8.654 (7.215 to 10.369) | | | | | | | | | | | | | | | | | | | | | |
| Pneumococcal meningitis | Birth prevalence - [34, 36) wks, [2500, 3000) g | Mortality | Males | | 13.419 (10.387 to 16.819) | 5.562 (4.646 to 6.696) | | | | | | | | | | | | | | | | | | | | | |
| Pneumococcal meningitis | Birth prevalence - [34, 36) wks, [2500, 3000) g | Mortality | Females | | 13.266 (9.666 to 17.689) | 6.395 (5.292 to 7.606) | | | | | | | | | | | | | | | | | | | | | |
| Pneumococcal meningitis | Birth prevalence - [34, 36) wks, [4000, 4500) g | Mortality | Males | | 23.096 (14.708 to 35.098) | 2.895 (2.245 to 3.716) | | | | | | | | | | | | | | | | | | | | | |
| Pneumococcal meningitis | Birth prevalence - [34, 36) wks, [4000, 4500) g | Mortality | Females | | 25.038 (15.763 to 37.255) | 3.778 (2.855 to 4.925) | | | | | | | | | | | | | | | | | | | | | |
| Pneumococcal meningitis | Birth prevalence - [34, 36) wks, [3000, 3500) g | Mortality | Males | | 14.006 (10.222 to 18.478) | 4.322 (3.449 to 5.338) | | | | | | | | | | | | | | | | | | | | | |
| Pneumococcal meningitis | Birth prevalence - [34, 36) wks, [3000, 3500) g | Mortality | Females | | 14.375 (10.269 to 20.114) | 5.265 (4.145 to 6.564) | | | | | | | | | | | | | | | | | | | | | |
| Pneumococcal meningitis | Birth prevalence - [36, 37) wks, [2500, 3000) g | Mortality | Males | | 4.874 (4.014 to 5.713) | 3.699 (3.134 to 4.374) | | | | | | | | | | | | | | | | | | | | | |
| Pneumococcal meningitis | Birth prevalence - [36, 37) wks, [2500, 3000) g | Mortality | Females | | 4.609 (3.731 to 5.61) | 3.898 (3.235 to 4.67) | | | | | | | | | | | | | | | | | | | | | |
| Pneumococcal meningitis | Birth prevalence - [34, 36) wks, [3500, 4000) g | Mortality | Males | | 18.024 (12.279 to 25.547) | 3.657 (2.838 to 4.675) | | | | | | | | | | | | | | | | | | | | | |
| Pneumococcal meningitis | Birth prevalence - [34, 36) wks, [3500, 4000) g | Mortality | Females | | 19.263 (12.567 to 27.924) | 4.634 (3.576 to 5.989) | | | | | | | | | | | | | | | | | | | | | |
| Pneumococcal meningitis | Birth prevalence - [37, 38) wks, [2500, 3000) g | Mortality | Males | | 3.306 (2.82 to 3.843) | 3.194 (2.691 to 3.803) | | | | | | | | | | | | | | | | | | | | | |
| Pneumococcal meningitis | Birth prevalence - [37, 38) wks, [2500, 3000) g | Mortality | Females | | 2.991 (2.496 to 3.61) | 3.242 (2.745 to 3.817) | | | | | | | | | | | | | | | | | | | | | |
| Pneumococcal meningitis | Birth prevalence - [40, 42) wks, [2500, 3000) g | Mortality | Males | | 3.771 (3.002 to 4.693) | 3.175 (2.56 to 3.923) | | | | | | | | | | | | | | | | | | | | | |
| Pneumococcal meningitis | Birth prevalence - [40, 42) wks, [2500, 3000) g | Mortality | Females | | 3.244 (2.486 to 4.159) | 3.228 (2.605 to 3.985) | | | | | | | | | | | | | | | | | | | | | |
| Pneumococcal meningitis | Birth prevalence - [38, 40) wks, [2500, 3000) g | Mortality | Males | | 2.755 (2.274 to 3.309) | 2.944 (2.44 to 3.548) | | | | | | | | | | | | | | | | | | | | | |
| Pneumococcal meningitis | Birth prevalence - [38, 40) wks, [2500, 3000) g | Mortality | Females | | 2.376 (1.91 to 2.886) | 2.938 (2.434 to 3.503) | | | | | | | | | | | | | | | | | | | | | |
| Pneumococcal meningitis | Birth prevalence - [36, 37) wks, [3000, 3500) g | Mortality | Males | | 3.774 (3.094 to 4.497) | 2.466 (2.058 to 2.929) | | | | | | | | | | | | | | | | | | | | | |
| Pneumococcal meningitis | Birth prevalence - [36, 37) wks, [3000, 3500) g | Mortality | Females | | 3.73 (2.981 to 4.646) | 2.715 (2.277 to 3.218) | | | | | | | | | | | | | | | | | | | | | |
| Pneumococcal meningitis | Birth prevalence - [36, 37) wks, [4000, 4500) g | Mortality | Males | | 6.826 (5.212 to 9.045) | 1.77 (1.491 to 2.082) | | | | | | | | | | | | | | | | | | | | | |
| Pneumococcal meningitis | Birth prevalence - [36, 37) wks, [4000, 4500) g | Mortality | Females | | 7.269 (5.144 to 9.821) | 2.177 (1.786 to 2.607) | | | | | | | | | | | | | | | | | | | | | |
| Pneumococcal meningitis | Birth prevalence - [36, 37) wks, [3500, 4000) g | Mortality | Males | | 4.544 (3.64 to 5.622) | 2.057 (1.74 to 2.44) | | | | | | | | | | | | | | | | | | | | | |
| Pneumococcal meningitis | Birth prevalence - [36, 37) wks, [3500, 4000) g | Mortality | Females | | 4.662 (3.577 to 6.014) | 2.398 (1.98 to 2.864) | | | | | | | | | | | | | | | | | | | | | |
| Pneumococcal meningitis | Birth prevalence - [37, 38) wks, [3000, 3500) g | Mortality | Males | | 2.007 (1.759 to 2.293) | 1.888 (1.613 to 2.224) | | | | | | | | | | | | | | | | | | | | | |
| Pneumococcal meningitis | Birth prevalence - [37, 38) wks, [3000, 3500) g | Mortality | Females | | 1.925 (1.582 to 2.328) | 1.972 (1.669 to 2.313) | | | | | | | | | | | | | | | | | | | | | |
| Pneumococcal meningitis | Birth prevalence - [37, 38) wks, [4000, 4500) g | Mortality | Males | | 3.28 (2.596 to 4.133) | 1.335 (1.171 to 1.532) | | | | | | | | | | | | | | | | | | | | | |
| Pneumococcal meningitis | Birth prevalence - [37, 38) wks, [4000, 4500) g | Mortality | Females | | 3.521 (2.649 to 4.5) | 1.559 (1.333 to 1.835) | | | | | | | | | | | | | | | | | | | | | |
| Pneumococcal meningitis | Birth prevalence - [37, 38) wks, [3500, 4000) g | Mortality | Males | | 2.128 (1.833 to 2.466) | 1.505 (1.299 to 1.76) | | | | | | | | | | | | | | | | | | | | | |
| Pneumococcal meningitis | Birth prevalence - [37, 38) wks, [3500, 4000) g | Mortality | Females | | 2.142 (1.694 to 2.67) | 1.661 (1.411 to 1.961) | | | | | | | | | | | | | | | | | | | | | |
| Pneumococcal meningitis | Birth prevalence - [40, 42) wks, [3000, 3500) g | Mortality | Males | | 1.436 (1.245 to 1.65) | 1.47 (1.199 to 1.8) | | | | | | | | | | | | | | | | | | | | | |
| Pneumococcal meningitis | Birth prevalence - [40, 42) wks, [3000, 3500) g | Mortality | Females | | 1.326 (1.069 to 1.614) | 1.465 (1.188 to 1.775) | | | | | | | | | | | | | | | | | | | | | |
| Pneumococcal meningitis | Birth prevalence - [38, 40) wks, [3000, 3500) g | Mortality | Males | | 1.33 (1.155 to 1.53) | 1.559 (1.305 to 1.851) | | | | | | | | | | | | | | | | | | | | | |
| Pneumococcal meningitis | Birth prevalence - [38, 40) wks, [3000, 3500) g | Mortality | Females | | 1.224 (1.0 to 1.492) | 1.564 (1.304 to 1.847) | | | | | | | | | | | | | | | | | | | | | |
| Pneumococcal meningitis | Birth prevalence - [38, 40) wks, [4000, 4500) g | Mortality | Males | | 1.787 (1.453 to 2.182) | 1.175 (1.005 to 1.371) | | | | | | | | | | | | | | | | | | | | | |
| Pneumococcal meningitis | Birth prevalence - [38, 40) wks, [4000, 4500) g | Mortality | Females | | 1.877 (1.467 to 2.388) | 1.224 (1.022 to 1.465) | | | | | | | | | | | | | | | | | | | | | |
| Pneumococcal meningitis | Birth prevalence - [38, 40) wks, [3500, 4000) g | Mortality | Males | | 1.785 (1.478 to 2.147) | 1.173 (1.0 to 1.377) | | | | | | | | | | | | | | | | | | | | | |
| Pneumococcal meningitis | Birth prevalence - [38, 40) wks, [3500, 4000) g | Mortality | Females | | 1.892 (1.481 to 2.352) | 1.23 (1.03 to 1.46) | | | | | | | | | | | | | | | | | | | | | |
| Pneumococcal meningitis | Birth prevalence - [40, 42) wks, [3500, 4000) g | Mortality | Males | | 1.0 (1.0 to 1.0) | 1.003 (1.0 to 1.046) | | | | | | | | | | | | | | | | | | | | | |
| Pneumococcal meningitis | Birth prevalence - [40, 42) wks, [3500, 4000) g | Mortality | Females | | 1.002 (1.0 to 1.013) | 1.001 (1.0 to 1.006) | | | | | | | | | | | | | | | | | | | | | |
| Pneumococcal meningitis | Birth prevalence - [40, 42) wks, [4000, 4500) g | Mortality | Males | | 1.0 (1.0 to 1.0) | 1.0 (1.0 to 1.0) | | | | | | | | | | | | | | | | | | | | | |
| Pneumococcal meningitis | Birth prevalence - [40, 42) wks, [4000, 4500) g | Mortality | Females | | 1.0 (1.0 to 1.0) | 1.0 (1.0 to 1.0) | | | | | | | | | | | | | | | | | | | | | |
| Pneumococcal meningitis | Birth prevalence - [28, 30) wks, [2000, 2500) g | Mortality | Males | | 117.172 (83.895 to 158.056) | 27.726 (21.877 to 34.972) | | | | | | | | | | | | | | | | | | | | | |
| Pneumococcal meningitis | Birth prevalence - [28, 30) wks, [2000, 2500) g | Mortality | Females | | 121.682 (84.349 to 171.375) | 33.983 (26.101 to 43.404) | | | | | | | | | | | | | | | | | | | | | |
| Pneumococcal meningitis | Birth prevalence - [28, 30) wks, [2500, 3000) g | Mortality | Males | | 77.948 (54.687 to 105.047) | 16.608 (12.653 to 21.188) | | | | | | | | | | | | | | | | | | | | | |
| Pneumococcal meningitis | Birth prevalence - [28, 30) wks, [2500, 3000) g | Mortality | Females | | 79.193 (54.099 to 112.236) | 20.387 (15.089 to 26.434) | | | | | | | | | | | | | | | | | | | | | |
| Pneumococcal meningitis | Birth prevalence - [28, 30) wks, [3000, 3500) g | Mortality | Males | | 42.199 (29.891 to 57.227) | 10.082 (7.777 to 13.056) | | | | | | | | | | | | | | | | | | | | | |
| Pneumococcal meningitis | Birth prevalence - [28, 30) wks, [3000, 3500) g | Mortality | Females | | 42.551 (28.25 to 63.209) | 11.989 (9.084 to 15.544) | | | | | | | | | | | | | | | | | | | | | |

**Appendix Table 6a. Relative risks used by age and sex for each outcome for all risk factors except for ambient air pollution alcohol, and smoking.**

| Risk - Outcome | Category / Units | Morbidity / Mortality | Sex | All-age | 0-6 days | 7-27 days | 28-364 days | 1-4 years | 5-9 years | 10-14 years | 15-19 years | 20-24 years | 25-29 years | 30-34 years | 35-39 years | 40-44 years | 45-49 years | 50-54 years | 55-59 years | 60-64 years | 65-69 years | 70-74 years | 75-79 years | 80-84 years | 85-89 years | 90-94 years | 95+ years |
|---|---|---|---|---|---|---|---|---|---|---|---|---|---|---|---|---|---|---|---|---|---|---|---|---|---|---|---|
| Pneumococcal meningitis | Birth prevalence - [30, 32) wks, [2500, 3000) g | Mortality | Males | | 58.722 (42.419 to 78.873) | 12.115 (9.518 to 15.521) | | | | | | | | | | | | | | | | | | | | | |
| Pneumococcal meningitis | Birth prevalence - [30, 32) wks, [2500, 3000) g | Mortality | Females | | 59.522 (42.058 to 82.793) | 15.364 (11.936 to 19.581) | | | | | | | | | | | | | | | | | | | | | |
| Pneumococcal meningitis | Birth prevalence - [30, 32) wks, [3000, 3500) g | Mortality | Males | | 45.67 (32.014 to 65.531) | 8.381 (6.362 to 10.842) | | | | | | | | | | | | | | | | | | | | | |
| Pneumococcal meningitis | Birth prevalence - [30, 32) wks, [3000, 3500) g | Mortality | Females | | 46.104 (30.243 to 66.207) | 10.506 (8.041 to 13.513) | | | | | | | | | | | | | | | | | | | | | |
| Pneumococcal meningitis | Birth prevalence - [30, 32) wks, [3500, 4000) g | Mortality | Males | | 36.334 (21.558 to 54.813) | 5.698 (4.293 to 7.349) | | | | | | | | | | | | | | | | | | | | | |
| Pneumococcal meningitis | Birth prevalence - [30, 32) wks, [3500, 4000) g | Mortality | Females | | 37.931 (22.692 to 61.276) | 6.892 (5.175 to 9.058) | | | | | | | | | | | | | | | | | | | | | |
| Pneumococcal meningitis | Birth prevalence - [32, 34) wks, [3000, 3500) g | Mortality | Males | | 34.016 (23.515 to 48.37) | 6.577 (4.934 to 8.436) | | | | | | | | | | | | | | | | | | | | | |
| Pneumococcal meningitis | Birth prevalence - [32, 34) wks, [3000, 3500) g | Mortality | Females | | 34.585 (22.909 to 49.754) | 8.314 (6.362 to 10.789) | | | | | | | | | | | | | | | | | | | | | |
| Pneumococcal meningitis | Birth prevalence - [32, 34) wks, [3500, 4000) g | Mortality | Males | | 36.248 (23.158 to 54.67) | 5.068 (3.761 to 6.741) | | | | | | | | | | | | | | | | | | | | | |
| Pneumococcal meningitis | Birth prevalence - [32, 34) wks, [3500, 4000) g | Mortality | Females | | 38.098 (23.301 to 59.429) | 6.476 (4.666 to 8.689) | | | | | | | | | | | | | | | | | | | | | |
| Pneumococcal meningitis | Birth prevalence - [36, 37) wks, [1000, 1500) g | Mortality | Males | | 166.686 (118.487 to 222.581) | 57.535 (45.999 to 71.742) | | | | | | | | | | | | | | | | | | | | | |
| Pneumococcal meningitis | Birth prevalence - [36, 37) wks, [1000, 1500) g | Mortality | Females | | 169.725 (119.017 to 229.008) | 63.564 (50.068 to 80.703) | | | | | | | | | | | | | | | | | | | | | |
| Pneumococcal meningitis | Birth prevalence - [38, 40) wks, [1000, 1500) g | Mortality | Males | | 174.066 (125.125 to 232.507) | 57.966 (44.393 to 73.241) | | | | | | | | | | | | | | | | | | | | | |
| Pneumococcal meningitis | Birth prevalence - [38, 40) wks, [1000, 1500) g | Mortality | Females | | 171.557 (121.585 to 237.047) | 65.208 (48.821 to 84.308) | | | | | | | | | | | | | | | | | | | | | |
| Pneumococcal meningitis | Birth prevalence - [38, 40) wks, [1500, 2000) g | Mortality | Males | | 67.302 (49.547 to 89.055) | 25.206 (20.365 to 31.168) | | | | | | | | | | | | | | | | | | | | | |
| Pneumococcal meningitis | Birth prevalence - [38, 40) wks, [1500, 2000) g | Mortality | Females | | 62.19 (45.884 to 83.445) | 28.05 (22.625 to 35.139) | | | | | | | | | | | | | | | | | | | | | |
| Pneumococcal meningitis | Birth prevalence - [40, 42) wks, [1500, 2000) g | Mortality | Males | | 76.673 (56.177 to 102.468) | 25.785 (19.387 to 34.168) | | | | | | | | | | | | | | | | | | | | | |
| Pneumococcal meningitis | Birth prevalence - [40, 42) wks, [1500, 2000) g | Mortality | Females | | 70.411 (49.221 to 97.952) | 29.113 (21.355 to 38.272) | | | | | | | | | | | | | | | | | | | | | |
| H influenzae type B meningitis | Birth prevalence - [0, 24) wks, [0, 500) g | Mortality | Males | | 1564.792 (1056.542 to [illegible] | 618.595 (458.842 to 812.921) | | | | | | | | | | | | | | | | | | | | | |
| H influenzae type B meningitis | Birth prevalence - [0, 24) wks, [0, 500) g | Mortality | Females | | 1600.122 (1050.664 to [illegible] | 713.571 (526.178 to 921.018) | | | | | | | | | | | | | | | | | | | | | |
| H influenzae type B meningitis | Birth prevalence - [0, 24) wks, [500, 1000) g | Mortality | Males | | 1155.815 (825.412 to 1506.837) | 457.5 (352.552 to 573.483) | | | | | | | | | | | | | | | | | | | | | |
| H influenzae type B meningitis | Birth prevalence - [0, 24) wks, [500, 1000) g | Mortality | Females | | 1169.123 (802.003 to 1617.979) | 515.406 (396.713 to 641.541) | | | | | | | | | | | | | | | | | | | | | |
| H influenzae type B meningitis | Birth prevalence - [24, 26) wks, [500, 1000) g | Mortality | Males | | 955.583 (723.748 to 1244.265) | 443.357 (363.03 to 534.695) | | | | | | | | | | | | | | | | | | | | | |
| H influenzae type B meningitis | Birth prevalence - [24, 26) wks, [500, 1000) g | Mortality | Females | | 947.143 (702.662 to 1237.093) | 487.549 (387.307 to 603.498) | | | | | | | | | | | | | | | | | | | | | |
| H influenzae type B meningitis | Birth prevalence - [26, 28) wks, [500, 1000) g | Mortality | Males | | 497.817 (377.617 to 648.547) | 330.886 (261.438 to 401.709) | | | | | | | | | | | | | | | | | | | | | |
| H influenzae type B meningitis | Birth prevalence - [26, 28) wks, [500, 1000) g | Mortality | Females | | 483.682 (354.946 to 629.517) | 344.618 (274.427 to 419.864) | | | | | | | | | | | | | | | | | | | | | |
| H influenzae type B meningitis | Birth prevalence - [30, 32) wks, [500, 1000) g | Mortality | Males | | 236.614 (163.821 to 324.502) | 149.995 (117.866 to 188.368) | | | | | | | | | | | | | | | | | | | | | |
| H influenzae type B meningitis | Birth prevalence - [30, 32) wks, [500, 1000) g | Mortality | Females | | 229.197 (157.606 to 317.194) | 152.117 (120.779 to 190.583) | | | | | | | | | | | | | | | | | | | | | |
| H influenzae type B meningitis | Birth prevalence - [28, 30) wks, [500, 1000) g | Mortality | Males | | 297.629 (214.953 to 396.586) | 216.995 (173.321 to 271.466) | | | | | | | | | | | | | | | | | | | | | |
| H influenzae type B meningitis | Birth prevalence - [28, 30) wks, [500, 1000) g | Mortality | Females | | 281.056 (198.176 to 386.635) | 219.884 (174.264 to 272.704) | | | | | | | | | | | | | | | | | | | | | |
| H influenzae type B meningitis | Birth prevalence - [26, 28) wks, [1000, 1500) g | Mortality | Males | | 267.91 (210.177 to 332.92) | 164.167 (132.898 to 200.569) | | | | | | | | | | | | | | | | | | | | | |
| H influenzae type B meningitis | Birth prevalence - [26, 28) wks, [1000, 1500) g | Mortality | Females | | 266.509 (197.461 to 346.932) | 174.222 (137.431 to 217.349) | | | | | | | | | | | | | | | | | | | | | |
| H influenzae type B meningitis | Birth prevalence - [34, 36) wks, [1000, 1500) g | Mortality | Males | | 142.056 (98.086 to 197.774) | 52.86 (42.914 to 64.617) | | | | | | | | | | | | | | | | | | | | | |
| H influenzae type B meningitis | Birth prevalence - [34, 36) wks, [1000, 1500) g | Mortality | Females | | 141.899 (95.864 to 197.656) | 57.421 (46.452 to 71.339) | | | | | | | | | | | | | | | | | | | | | |
| H influenzae type B meningitis | Birth prevalence - [28, 30) wks, [1500, 2000) g | Mortality | Males | | 127.966 (97.178 to 167.026) | 50.018 (40.539 to 61.919) | | | | | | | | | | | | | | | | | | | | | |
| H influenzae type B meningitis | Birth prevalence - [28, 30) wks, [1500, 2000) g | Mortality | Females | | 130.924 (96.513 to 172.188) | 57.275 (46.36 to 70.038) | | | | | | | | | | | | | | | | | | | | | |
| H influenzae type B meningitis | Birth prevalence - [28, 30) wks, [1000, 1500) g | Mortality | Males | | 158.563 (120.99 to 204.947) | 103.32 (83.486 to 127.144) | | | | | | | | | | | | | | | | | | | | | |
| H influenzae type B meningitis | Birth prevalence - [28, 30) wks, [1000, 1500) g | Mortality | Females | | 153.905 (112.327 to 200.786) | 107.529 (86.954 to 131.78) | | | | | | | | | | | | | | | | | | | | | |
| H influenzae type B meningitis | Birth prevalence - [32, 34) wks, [1000, 1500) g | Mortality | Males | | 117.142 (81.354 to 161.101) | 53.185 (43.049 to 66.274) | | | | | | | | | | | | | | | | | | | | | |
| H influenzae type B meningitis | Birth prevalence - [32, 34) wks, [1000, 1500) g | Mortality | Females | | 115.171 (79.363 to 159.206) | 56.034 (45.982 to 68.36) | | | | | | | | | | | | | | | | | | | | | |
| H influenzae type B meningitis | Birth prevalence - [30, 32) wks, [1000, 1500) g | Mortality | Males | | 119.308 (87.769 to 160.885) | 67.163 (54.863 to 82.638) | | | | | | | | | | | | | | | | | | | | | |
| H influenzae type B meningitis | Birth prevalence - [30, 32) wks, [1000, 1500) g | Mortality | Females | | 115.448 (84.272 to 156.425) | 69.14 (55.873 to 85.012) | | | | | | | | | | | | | | | | | | | | | |
| H influenzae type B meningitis | Birth prevalence - [37, 38) wks, [1500, 2000) g | Mortality | Males | | 62.972 (46.159 to 83.484) | 24.148 (20.066 to 29.406) | | | | | | | | | | | | | | | | | | | | | |
| H influenzae type B meningitis | Birth prevalence - [37, 38) wks, [1500, 2000) g | Mortality | Females | | 59.988 (43.974 to 79.053) | 26.719 (21.746 to 32.816) | | | | | | | | | | | | | | | | | | | | | |
| H influenzae type B meningitis | Birth prevalence - [36, 37) wks, [1500, 2000) g | Mortality | Males | | 60.218 (43.669 to 82.48) | 23.031 (18.793 to 28.483) | | | | | | | | | | | | | | | | | | | | | |
| H influenzae type B meningitis | Birth prevalence - [36, 37) wks, [1500, 2000) g | Mortality | Females | | 58.527 (42.172 to 80.557) | 25.143 (20.331 to 30.566) | | | | | | | | | | | | | | | | | | | | | |
| H influenzae type B meningitis | Birth prevalence - [30, 32) wks, [2000, 2500) g | Mortality | Males | | 67.971 (50.354 to 88.935) | 18.03 (14.621 to 22.103) | | | | | | | | | | | | | | | | | | | | | |
| H influenzae type B meningitis | Birth prevalence - [30, 32) wks, [2000, 2500) g | Mortality | Females | | 69.383 (49.108 to 94.583) | 22.069 (17.836 to 27.163) | | | | | | | | | | | | | | | | | | | | | |
| H influenzae type B meningitis | Birth prevalence - [30, 32) wks, [1500, 2000) g | Mortality | Males | | 77.369 (59.702 to 99.232) | 31.079 (25.786 to 36.724) | | | | | | | | | | | | | | | | | | | | | |
| H influenzae type B meningitis | Birth prevalence - [30, 32) wks, [1500, 2000) g | Mortality | Females | | 76.134 (56.885 to 100.996) | 34.756 (28.764 to 41.849) | | | | | | | | | | | | | | | | | | | | | |
| H influenzae type B meningitis | Birth prevalence - [34, 36) wks, [1500, 2000) g | Mortality | Males | | 55.555 (39.553 to 75.104) | 21.346 (17.677 to 26.143) | | | | | | | | | | | | | | | | | | | | | |
| H influenzae type B meningitis | Birth prevalence - [34, 36) wks, [1500, 2000) g | Mortality | Females | | 54.335 (38.617 to 75.24) | 23.046 (18.743 to 28.287) | | | | | | | | | | | | | | | | | | | | | |
| H influenzae type B meningitis | Birth prevalence - [32, 34) wks, [1500, 2000) g | Mortality | Males | | 57.155 (42.484 to 73.651) | 23.114 (19.028 to 27.915) | | | | | | | | | | | | | | | | | | | | | |
| H influenzae type B meningitis | Birth prevalence - [32, 34) wks, [1500, 2000) g | Mortality | Females | | 56.101 (39.794 to 76.295) | 25.149 (20.615 to 30.388) | | | | | | | | | | | | | | | | | | | | | |
| H influenzae type B meningitis | Birth prevalence - [32, 34) wks, [2000, 2500) g | Mortality | Males | | 37.444 (29.026 to 48.227) | 12.233 (10.252 to 14.477) | | | | | | | | | | | | | | | | | | | | | |
| H influenzae type B meningitis | Birth prevalence - [32, 34) wks, [2000, 2500) g | Mortality | Females | | 36.874 (26.658 to 49.653) | 14.384 (12.095 to 17.03) | | | | | | | | | | | | | | | | | | | | | |
| H influenzae type B meningitis | Birth prevalence - [40, 42) wks, [2000, 2500) g | Mortality | Males | | 18.092 (13.292 to 23.719) | 9.23 (7.037 to 11.454) | | | | | | | | | | | | | | | | | | | | | |

**Appendix Table 6a. Relative risks used by age and sex for each outcome for all risk factors except for ambient air pollution alcohol, and smoking.**

| | | | | Ages | | | | | | | | | | | | | | | | | | | | | | | |
|---|---|---|---|---|---|---|---|---|---|---|---|---|---|---|---|---|---|---|---|---|---|---|---|---|---|---|---|
| Risk - Outcome | Category / Units | Morbidity / Mortality | Sex | All-age | 0-6 days | 7-27 days | 28-364 days | 1-4 years | 5-9 years | 10-14 years | 15-19 years | 20-24 years | 25-29 years | 30-34 years | 35-39 years | 40-44 years | 45-49 years | 50-54 years | 55-59 years | 60-64 years | 65-69 years | 70-74 years | 75-79 years | 80-84 years | 85-89 years | 90-94 years | 95+ years |
| H influenzae type B meningitis | Birth prevalence - [40, 42) wks, [2000, 2500) g | Mortality | Females | | 15.574 (11.516 to 20.778) | 9.975 (7.82 to 12.46) | | | | | | | | | | | | | | | | | | | | | |
| H influenzae type B meningitis | Birth prevalence - [38, 40) wks, [2000, 2500) g | Mortality | Males | | 13.104 (9.829 to 16.99) | 8.198 (6.786 to 9.959) | | | | | | | | | | | | | | | | | | | | | |
| H influenzae type B meningitis | Birth prevalence - [38, 40) wks, [2000, 2500) g | Mortality | Females | | 11.308 (8.389 to 14.38) | 8.577 (7.04 to 10.449) | | | | | | | | | | | | | | | | | | | | | |
| H influenzae type B meningitis | Birth prevalence - [32, 34) wks, [2500, 3000) g | Mortality | Males | | 33.063 (24.393 to 43.503) | 8.441 (6.822 to 10.431) | | | | | | | | | | | | | | | | | | | | | |
| H influenzae type B meningitis | Birth prevalence - [32, 34) wks, [2500, 3000) g | Mortality | Females | | 32.812 (23.439 to 45.567) | 10.398 (8.227 to 13.042) | | | | | | | | | | | | | | | | | | | | | |
| H influenzae type B meningitis | Birth prevalence - [34, 36) wks, [2000, 2500) g | Mortality | Males | | 21.925 (16.305 to 29.433) | 9.367 (7.859 to 11.112) | | | | | | | | | | | | | | | | | | | | | |
| H influenzae type B meningitis | Birth prevalence - [34, 36) wks, [2000, 2500) g | Mortality | Females | | 21.297 (15.657 to 28.761) | 10.295 (8.548 to 12.273) | | | | | | | | | | | | | | | | | | | | | |
| H influenzae type B meningitis | Birth prevalence - [37, 38) wks, [2000, 2500) g | Mortality | Males | | 13.0 (10.102 to 16.456) | 8.096 (6.724 to 9.676) | | | | | | | | | | | | | | | | | | | | | |
| H influenzae type B meningitis | Birth prevalence - [37, 38) wks, [2000, 2500) g | Mortality | Females | | 11.563 (8.805 to 15.11) | 8.467 (6.994 to 10.342) | | | | | | | | | | | | | | | | | | | | | |
| H influenzae type B meningitis | Birth prevalence - [36, 37) wks, [2000, 2500) g | Mortality | Males | | 14.401 (10.789 to 18.654) | 8.221 (6.917 to 9.923) | | | | | | | | | | | | | | | | | | | | | |
| H influenzae type B meningitis | Birth prevalence - [36, 37) wks, [2000, 2500) g | Mortality | Females | | 13.513 (9.817 to 17.942) | 8.654 (7.215 to 10.369) | | | | | | | | | | | | | | | | | | | | | |
| H influenzae type B meningitis | Birth prevalence - [34, 36) wks, [2500, 3000) g | Mortality | Males | | 13.419 (10.387 to 16.819) | 5.562 (4.646 to 6.696) | | | | | | | | | | | | | | | | | | | | | |
| H influenzae type B meningitis | Birth prevalence - [34, 36) wks, [2500, 3000) g | Mortality | Females | | 13.266 (9.666 to 17.689) | 6.395 (5.292 to 7.606) | | | | | | | | | | | | | | | | | | | | | |
| H influenzae type B meningitis | Birth prevalence - [34, 36) wks, [4000, 4500) g | Mortality | Males | | 23.096 (14.708 to 35.098) | 2.895 (2.245 to 3.716) | | | | | | | | | | | | | | | | | | | | | |
| H influenzae type B meningitis | Birth prevalence - [34, 36) wks, [4000, 4500) g | Mortality | Females | | 25.038 (15.763 to 37.255) | 3.778 (2.855 to 4.925) | | | | | | | | | | | | | | | | | | | | | |
| H influenzae type B meningitis | Birth prevalence - [34, 36) wks, [3000, 3500) g | Mortality | Males | | 14.006 (10.222 to 18.478) | 4.322 (3.449 to 5.338) | | | | | | | | | | | | | | | | | | | | | |
| H influenzae type B meningitis | Birth prevalence - [34, 36) wks, [3000, 3500) g | Mortality | Females | | 14.375 (10.269 to 20.114) | 5.265 (4.145 to 6.564) | | | | | | | | | | | | | | | | | | | | | |
| H influenzae type B meningitis | Birth prevalence - [36, 37) wks, [2500, 3000) g | Mortality | Males | | 4.874 (4.014 to 5.713) | 3.699 (3.134 to 4.374) | | | | | | | | | | | | | | | | | | | | | |
| H influenzae type B meningitis | Birth prevalence - [36, 37) wks, [2500, 3000) g | Mortality | Females | | 4.609 (3.731 to 5.61) | 3.898 (3.235 to 4.67) | | | | | | | | | | | | | | | | | | | | | |
| H influenzae type B meningitis | Birth prevalence - [34, 36) wks, [3500, 4000) g | Mortality | Males | | 18.024 (12.279 to 25.547) | 3.657 (2.838 to 4.675) | | | | | | | | | | | | | | | | | | | | | |
| H influenzae type B meningitis | Birth prevalence - [34, 36) wks, [3500, 4000) g | Mortality | Females | | 19.263 (12.567 to 27.924) | 4.634 (3.576 to 5.989) | | | | | | | | | | | | | | | | | | | | | |
| H influenzae type B meningitis | Birth prevalence - [37, 38) wks, [2500, 3000) g | Mortality | Males | | 3.306 (2.82 to 3.843) | 3.194 (2.691 to 3.803) | | | | | | | | | | | | | | | | | | | | | |
| H influenzae type B meningitis | Birth prevalence - [37, 38) wks, [2500, 3000) g | Mortality | Females | | 2.991 (2.496 to 3.61) | 3.242 (2.745 to 3.817) | | | | | | | | | | | | | | | | | | | | | |
| H influenzae type B meningitis | Birth prevalence - [40, 42) wks, [2500, 3000) g | Mortality | Males | | 3.771 (3.002 to 4.693) | 3.175 (2.56 to 3.923) | | | | | | | | | | | | | | | | | | | | | |
| H influenzae type B meningitis | Birth prevalence - [40, 42) wks, [2500, 3000) g | Mortality | Females | | 3.244 (2.486 to 4.159) | 3.228 (2.605 to 3.985) | | | | | | | | | | | | | | | | | | | | | |
| H influenzae type B meningitis | Birth prevalence - [38, 40) wks, [2500, 3000) g | Mortality | Males | | 2.755 (2.274 to 3.309) | 2.944 (2.44 to 3.548) | | | | | | | | | | | | | | | | | | | | | |
| H influenzae type B meningitis | Birth prevalence - [38, 40) wks, [2500, 3000) g | Mortality | Females | | 2.376 (1.91 to 2.886) | 2.938 (2.434 to 3.503) | | | | | | | | | | | | | | | | | | | | | |
| H influenzae type B meningitis | Birth prevalence - [36, 37) wks, [3000, 3500) g | Mortality | Males | | 3.774 (3.094 to 4.497) | 2.466 (2.058 to 2.929) | | | | | | | | | | | | | | | | | | | | | |
| H influenzae type B meningitis | Birth prevalence - [36, 37) wks, [3000, 3500) g | Mortality | Females | | 3.73 (2.981 to 4.646) | 2.715 (2.277 to 3.218) | | | | | | | | | | | | | | | | | | | | | |
| H influenzae type B meningitis | Birth prevalence - [36, 37) wks, [4000, 4500) g | Mortality | Males | | 6.826 (5.212 to 9.045) | 1.77 (1.491 to 2.082) | | | | | | | | | | | | | | | | | | | | | |
| H influenzae type B meningitis | Birth prevalence - [36, 37) wks, [4000, 4500) g | Mortality | Females | | 7.269 (5.144 to 9.821) | 2.177 (1.786 to 2.607) | | | | | | | | | | | | | | | | | | | | | |
| H influenzae type B meningitis | Birth prevalence - [36, 37) wks, [3500, 4000) g | Mortality | Males | | 4.544 (3.64 to 5.622) | 2.057 (1.74 to 2.44) | | | | | | | | | | | | | | | | | | | | | |
| H influenzae type B meningitis | Birth prevalence - [36, 37) wks, [3500, 4000) g | Mortality | Females | | 4.662 (3.577 to 6.014) | 2.398 (1.98 to 2.864) | | | | | | | | | | | | | | | | | | | | | |
| H influenzae type B meningitis | Birth prevalence - [37, 38) wks, [3000, 3500) g | Mortality | Males | | 2.007 (1.759 to 2.293) | 1.888 (1.613 to 2.224) | | | | | | | | | | | | | | | | | | | | | |
| H influenzae type B meningitis | Birth prevalence - [37, 38) wks, [3000, 3500) g | Mortality | Females | | 1.925 (1.582 to 2.328) | 1.972 (1.669 to 2.313) | | | | | | | | | | | | | | | | | | | | | |
| H influenzae type B meningitis | Birth prevalence - [37, 38) wks, [4000, 4500) g | Mortality | Males | | 3.28 (2.596 to 4.133) | 1.335 (1.171 to 1.532) | | | | | | | | | | | | | | | | | | | | | |
| H influenzae type B meningitis | Birth prevalence - [37, 38) wks, [4000, 4500) g | Mortality | Females | | 3.521 (2.649 to 4.5) | 1.559 (1.333 to 1.835) | | | | | | | | | | | | | | | | | | | | | |
| H influenzae type B meningitis | Birth prevalence - [37, 38) wks, [3500, 4000) g | Mortality | Males | | 2.128 (1.833 to 2.466) | 1.505 (1.299 to 1.76) | | | | | | | | | | | | | | | | | | | | | |
| H influenzae type B meningitis | Birth prevalence - [37, 38) wks, [3500, 4000) g | Mortality | Females | | 2.142 (1.694 to 2.67) | 1.661 (1.411 to 1.961) | | | | | | | | | | | | | | | | | | | | | |
| H influenzae type B meningitis | Birth prevalence - [40, 42) wks, [3000, 3500) g | Mortality | Males | | 1.436 (1.245 to 1.65) | 1.47 (1.199 to 1.8) | | | | | | | | | | | | | | | | | | | | | |
| H influenzae type B meningitis | Birth prevalence - [40, 42) wks, [3000, 3500) g | Mortality | Females | | 1.326 (1.069 to 1.614) | 1.465 (1.188 to 1.775) | | | | | | | | | | | | | | | | | | | | | |
| H influenzae type B meningitis | Birth prevalence - [38, 40) wks, [3000, 3500) g | Mortality | Males | | 1.33 (1.155 to 1.53) | 1.559 (1.305 to 1.851) | | | | | | | | | | | | | | | | | | | | | |
| H influenzae type B meningitis | Birth prevalence - [38, 40) wks, [3000, 3500) g | Mortality | Females | | 1.224 (1.0 to 1.492) | 1.564 (1.304 to 1.847) | | | | | | | | | | | | | | | | | | | | | |
| H influenzae type B meningitis | Birth prevalence - [38, 40) wks, [4000, 4500) g | Mortality | Males | | 1.787 (1.453 to 2.182) | 1.175 (1.005 to 1.371) | | | | | | | | | | | | | | | | | | | | | |
| H influenzae type B meningitis | Birth prevalence - [38, 40) wks, [4000, 4500) g | Mortality | Females | | 1.877 (1.467 to 2.388) | 1.224 (1.022 to 1.465) | | | | | | | | | | | | | | | | | | | | | |
| H influenzae type B meningitis | Birth prevalence - [38, 40) wks, [3500, 4000) g | Mortality | Males | | 1.785 (1.478 to 2.147) | 1.173 (1.0 to 1.377) | | | | | | | | | | | | | | | | | | | | | |
| H influenzae type B meningitis | Birth prevalence - [38, 40) wks, [3500, 4000) g | Mortality | Females | | 1.892 (1.481 to 2.352) | 1.23 (1.03 to 1.46) | | | | | | | | | | | | | | | | | | | | | |
| H influenzae type B meningitis | Birth prevalence - [40, 42) wks, [3500, 4000) g | Mortality | Males | | 1.0 (1.0 to 1.0) | 1.003 (1.0 to 1.046) | | | | | | | | | | | | | | | | | | | | | |
| H influenzae type B meningitis | Birth prevalence - [40, 42) wks, [3500, 4000) g | Mortality | Females | | 1.002 (1.0 to 1.013) | 1.001 (1.0 to 1.006) | | | | | | | | | | | | | | | | | | | | | |
| H influenzae type B meningitis | Birth prevalence - [40, 42) wks, [4000, 4500) g | Mortality | Males | | 1.0 (1.0 to 1.0) | 1.0 (1.0 to 1.0) | | | | | | | | | | | | | | | | | | | | | |
| H influenzae type B meningitis | Birth prevalence - [40, 42) wks, [4000, 4500) g | Mortality | Females | | 1.0 (1.0 to 1.0) | 1.0 (1.0 to 1.0) | | | | | | | | | | | | | | | | | | | | | |
| H influenzae type B meningitis | Birth prevalence - [28, 30) wks, [2000, 2500) g | Mortality | Males | | 117.172 (83.895 to 158.056) | 27.726 (21.877 to 34.972) | | | | | | | | | | | | | | | | | | | | | |
| H influenzae type B meningitis | Birth prevalence - [28, 30) wks, [2000, 2500) g | Mortality | Females | | 121.682 (84.349 to 171.375) | 33.983 (26.101 to 43.404) | | | | | | | | | | | | | | | | | | | | | |
| H influenzae type B meningitis | Birth prevalence - [28, 30) wks, [2500, 3000) g | Mortality | Males | | 77.948 (54.687 to 105.047) | 16.608 (12.653 to 21.188) | | | | | | | | | | | | | | | | | | | | | |
| H influenzae type B meningitis | Birth prevalence - [28, 30) wks, [2500, 3000) g | Mortality | Females | | 79.193 (54.099 to 112.236) | 20.387 (15.089 to 26.434) | | | | | | | | | | | | | | | | | | | | | |
| H influenzae type B meningitis | Birth prevalence - [28, 30) wks, [3000, 3500) g | Mortality | Males | | 42.199 (29.891 to 57.227) | 10.082 (7.777 to 13.056) | | | | | | | | | | | | | | | | | | | | | |
| H influenzae type B meningitis | Birth prevalence - [28, 30) wks, [3000, 3500) g | Mortality | Females | | 42.551 (28.25 to 63.209) | 11.989 (9.084 to 15.544) | | | | | | | | | | | | | | | | | | | | | |

Appendix Table 6a. Relative risks used by age and sex for each outcome for all risk factors except for ambient air pollution alcohol, and smoking.

| | | | | Ages | | | | | | | | | | | | | | | | | | | | | | | |
|---|---|---|---|---|---|---|---|---|---|---|---|---|---|---|---|---|---|---|---|---|---|---|---|---|---|---|---|
| Risk - Outcome | Category / Units | Morbidity / Mortality | Sex | All-age | 0-6 days | 7-27 days | 28-364 days | 1-4 years | 5-9 years | 10-14 years | 15-19 years | 20-24 years | 25-29 years | 30-34 years | 35-39 years | 40-44 years | 45-49 years | 50-54 years | 55-59 years | 60-64 years | 65-69 years | 70-74 years | 75-79 years | 80-84 years | 85-89 years | 90-94 years | 95+ years |
| H influenzae type B meningitis | Birth prevalence - [30, 32) wks, [2500, 3000) g | Mortality | Males | | 58.722 (42.419 to 78.873) | 12.115 (9.518 to 15.521) | | | | | | | | | | | | | | | | | | | | | |
| H influenzae type B meningitis | Birth prevalence - [30, 32) wks, [2500, 3000) g | Mortality | Females | | 59.522 (42.058 to 82.793) | 15.364 (11.936 to 19.581) | | | | | | | | | | | | | | | | | | | | | |
| H influenzae type B meningitis | Birth prevalence - [30, 32) wks, [3000, 3500) g | Mortality | Males | | 45.67 (32.014 to 65.531) | 8.381 (6.362 to 10.842) | | | | | | | | | | | | | | | | | | | | | |
| H influenzae type B meningitis | Birth prevalence - [30, 32) wks, [3000, 3500) g | Mortality | Females | | 46.104 (30.243 to 66.207) | 10.506 (8.041 to 13.513) | | | | | | | | | | | | | | | | | | | | | |
| H influenzae type B meningitis | Birth prevalence - [30, 32) wks, [3500, 4000) g | Mortality | Males | | 36.334 (21.558 to 54.813) | 5.698 (4.293 to 7.349) | | | | | | | | | | | | | | | | | | | | | |
| H influenzae type B meningitis | Birth prevalence - [30, 32) wks, [3500, 4000) g | Mortality | Females | | 37.931 (22.692 to 61.276) | 6.892 (5.175 to 9.058) | | | | | | | | | | | | | | | | | | | | | |
| H influenzae type B meningitis | Birth prevalence - [32, 34) wks, [3000, 3500) g | Mortality | Males | | 34.016 (23.515 to 48.37) | 6.577 (4.934 to 8.436) | | | | | | | | | | | | | | | | | | | | | |
| H influenzae type B meningitis | Birth prevalence - [32, 34) wks, [3000, 3500) g | Mortality | Females | | 34.585 (22.909 to 49.754) | 8.314 (6.362 to 10.789) | | | | | | | | | | | | | | | | | | | | | |
| H influenzae type B meningitis | Birth prevalence - [32, 34) wks, [3500, 4000) g | Mortality | Males | | 36.248 (23.158 to 54.67) | 5.068 (3.761 to 6.741) | | | | | | | | | | | | | | | | | | | | | |
| H influenzae type B meningitis | Birth prevalence - [32, 34) wks, [3500, 4000) g | Mortality | Females | | 38.098 (23.301 to 59.429) | 6.476 (4.666 to 8.689) | | | | | | | | | | | | | | | | | | | | | |
| H influenzae type B meningitis | Birth prevalence - [36, 37) wks, [1000, 1500) g | Mortality | Males | | 166.686 (118.487 to 222.581) | 57.535 (45.999 to 71.742) | | | | | | | | | | | | | | | | | | | | | |
| H influenzae type B meningitis | Birth prevalence - [36, 37) wks, [1000, 1500) g | Mortality | Females | | 169.725 (119.017 to 229.008) | 63.564 (50.068 to 80.703) | | | | | | | | | | | | | | | | | | | | | |
| H influenzae type B meningitis | Birth prevalence - [38, 40) wks, [1000, 1500) g | Mortality | Males | | 174.066 (125.125 to 232.507) | 57.966 (44.393 to 73.241) | | | | | | | | | | | | | | | | | | | | | |
| H influenzae type B meningitis | Birth prevalence - [38, 40) wks, [1000, 1500) g | Mortality | Females | | 171.557 (121.585 to 237.047) | 65.208 (48.821 to 84.308) | | | | | | | | | | | | | | | | | | | | | |
| H influenzae type B meningitis | Birth prevalence - [38, 40) wks, [1500, 2000) g | Mortality | Males | | 67.302 (49.547 to 89.055) | 25.206 (20.365 to 31.168) | | | | | | | | | | | | | | | | | | | | | |
| H influenzae type B meningitis | Birth prevalence - [38, 40) wks, [1500, 2000) g | Mortality | Females | | 62.19 (45.884 to 83.445) | 28.05 (22.625 to 35.139) | | | | | | | | | | | | | | | | | | | | | |
| H influenzae type B meningitis | Birth prevalence - [40, 42) wks, [1500, 2000) g | Mortality | Males | | 76.673 (56.177 to 102.468) | 25.785 (19.387 to 34.168) | | | | | | | | | | | | | | | | | | | | | |
| H influenzae type B meningitis | Birth prevalence - [40, 42) wks, [1500, 2000) g | Mortality | Females | | 70.411 (49.221 to 97.952) | 29.113 (21.355 to 38.272) | | | | | | | | | | | | | | | | | | | | | |
| Meningococcal infection | Birth prevalence - [0, 24) wks, [0, 500) g | Mortality | Males | | 1564.792 (1056.542 to [illegible] | 618.595 (458.842 to 812.921) | | | | | | | | | | | | | | | | | | | | | |
| Meningococcal infection | Birth prevalence - [0, 24) wks, [0, 500) g | Mortality | Females | | 1600.122 (1050.664 to [illegible] | 713.571 (526.178 to 921.018) | | | | | | | | | | | | | | | | | | | | | |
| Meningococcal infection | Birth prevalence - [0, 24) wks, [500, 1000) g | Mortality | Males | | 1155.815 (825.412 to 1506.837) | 457.5 (352.552 to 573.483) | | | | | | | | | | | | | | | | | | | | | |
| Meningococcal infection | Birth prevalence - [0, 24) wks, [500, 1000) g | Mortality | Females | | 1169.123 (802.003 to 1617.979) | 515.406 (396.713 to 641.541) | | | | | | | | | | | | | | | | | | | | | |
| Meningococcal infection | Birth prevalence - [24, 26) wks, [500, 1000) g | Mortality | Males | | 955.583 (723.748 to 1244.265) | 443.357 (363.03 to 534.695) | | | | | | | | | | | | | | | | | | | | | |
| Meningococcal infection | Birth prevalence - [24, 26) wks, [500, 1000) g | Mortality | Females | | 947.143 (702.662 to 1237.093) | 487.549 (387.307 to 603.498) | | | | | | | | | | | | | | | | | | | | | |
| Meningococcal infection | Birth prevalence - [26, 28) wks, [500, 1000) g | Mortality | Males | | 497.817 (377.617 to 648.547) | 330.886 (261.438 to 401.709) | | | | | | | | | | | | | | | | | | | | | |
| Meningococcal infection | Birth prevalence - [26, 28) wks, [500, 1000) g | Mortality | Females | | 483.682 (354.946 to 629.517) | 344.618 (274.427 to 419.864) | | | | | | | | | | | | | | | | | | | | | |
| Meningococcal infection | Birth prevalence - [30, 32) wks, [500, 1000) g | Mortality | Males | | 236.614 (163.821 to 324.502) | 149.995 (117.866 to 188.368) | | | | | | | | | | | | | | | | | | | | | |
| Meningococcal infection | Birth prevalence - [30, 32) wks, [500, 1000) g | Mortality | Females | | 229.197 (157.606 to 317.194) | 152.117 (120.779 to 190.583) | | | | | | | | | | | | | | | | | | | | | |
| Meningococcal infection | Birth prevalence - [28, 30) wks, [500, 1000) g | Mortality | Males | | 297.629 (214.953 to 396.586) | 216.995 (173.321 to 271.466) | | | | | | | | | | | | | | | | | | | | | |
| Meningococcal infection | Birth prevalence - [28, 30) wks, [500, 1000) g | Mortality | Females | | 281.056 (198.176 to 386.635) | 219.884 (174.264 to 272.704) | | | | | | | | | | | | | | | | | | | | | |
| Meningococcal infection | Birth prevalence - [26, 28) wks, [1000, 1500) g | Mortality | Males | | 267.91 (210.177 to 332.92) | 164.167 (132.898 to 200.569) | | | | | | | | | | | | | | | | | | | | | |
| Meningococcal infection | Birth prevalence - [26, 28) wks, [1000, 1500) g | Mortality | Females | | 266.509 (197.461 to 346.932) | 174.222 (137.431 to 217.349) | | | | | | | | | | | | | | | | | | | | | |
| Meningococcal infection | Birth prevalence - [34, 36) wks, [1000, 1500) g | Mortality | Males | | 142.056 (98.086 to 197.774) | 52.86 (42.914 to 64.617) | | | | | | | | | | | | | | | | | | | | | |
| Meningococcal infection | Birth prevalence - [34, 36) wks, [1000, 1500) g | Mortality | Females | | 141.899 (95.864 to 197.656) | 57.421 (46.452 to 71.339) | | | | | | | | | | | | | | | | | | | | | |
| Meningococcal infection | Birth prevalence - [28, 30) wks, [1500, 2000) g | Mortality | Males | | 127.966 (97.178 to 167.026) | 50.018 (40.539 to 61.919) | | | | | | | | | | | | | | | | | | | | | |
| Meningococcal infection | Birth prevalence - [28, 30) wks, [1500, 2000) g | Mortality | Females | | 130.924 (96.513 to 172.188) | 57.275 (46.36 to 70.038) | | | | | | | | | | | | | | | | | | | | | |
| Meningococcal infection | Birth prevalence - [28, 30) wks, [1000, 1500) g | Mortality | Males | | 158.563 (120.99 to 204.947) | 103.32 (83.486 to 127.144) | | | | | | | | | | | | | | | | | | | | | |
| Meningococcal infection | Birth prevalence - [28, 30) wks, [1000, 1500) g | Mortality | Females | | 153.905 (112.327 to 200.786) | 107.529 (86.954 to 131.78) | | | | | | | | | | | | | | | | | | | | | |
| Meningococcal infection | Birth prevalence - [32, 34) wks, [1000, 1500) g | Mortality | Males | | 117.142 (81.354 to 161.101) | 53.185 (43.049 to 66.274) | | | | | | | | | | | | | | | | | | | | | |
| Meningococcal infection | Birth prevalence - [32, 34) wks, [1000, 1500) g | Mortality | Females | | 115.171 (79.363 to 159.206) | 56.034 (45.982 to 68.36) | | | | | | | | | | | | | | | | | | | | | |
| Meningococcal infection | Birth prevalence - [30, 32) wks, [1000, 1500) g | Mortality | Males | | 119.308 (87.769 to 160.885) | 67.163 (54.863 to 82.638) | | | | | | | | | | | | | | | | | | | | | |
| Meningococcal infection | Birth prevalence - [30, 32) wks, [1000, 1500) g | Mortality | Females | | 115.448 (84.272 to 156.425) | 69.14 (55.873 to 85.012) | | | | | | | | | | | | | | | | | | | | | |
| Meningococcal infection | Birth prevalence - [37, 38) wks, [1500, 2000) g | Mortality | Males | | 62.972 (46.159 to 83.484) | 24.148 (20.066 to 29.406) | | | | | | | | | | | | | | | | | | | | | |
| Meningococcal infection | Birth prevalence - [37, 38) wks, [1500, 2000) g | Mortality | Females | | 59.988 (43.974 to 79.053) | 26.719 (21.746 to 32.816) | | | | | | | | | | | | | | | | | | | | | |
| Meningococcal infection | Birth prevalence - [36, 37) wks, [1500, 2000) g | Mortality | Males | | 60.218 (43.669 to 82.48) | 23.031 (18.793 to 28.483) | | | | | | | | | | | | | | | | | | | | | |
| Meningococcal infection | Birth prevalence - [36, 37) wks, [1500, 2000) g | Mortality | Females | | 58.527 (42.172 to 80.557) | 25.143 (20.331 to 30.566) | | | | | | | | | | | | | | | | | | | | | |
| Meningococcal infection | Birth prevalence - [30, 32) wks, [2000, 2500) g | Mortality | Males | | 67.971 (50.354 to 88.935) | 18.03 (14.621 to 22.103) | | | | | | | | | | | | | | | | | | | | | |
| Meningococcal infection | Birth prevalence - [30, 32) wks, [2000, 2500) g | Mortality | Females | | 69.383 (49.108 to 94.583) | 22.069 (17.836 to 27.163) | | | | | | | | | | | | | | | | | | | | | |
| Meningococcal infection | Birth prevalence - [30, 32) wks, [1500, 2000) g | Mortality | Males | | 77.369 (59.702 to 99.232) | 31.079 (25.786 to 36.724) | | | | | | | | | | | | | | | | | | | | | |
| Meningococcal infection | Birth prevalence - [30, 32) wks, [1500, 2000) g | Mortality | Females | | 76.134 (56.885 to 100.996) | 34.756 (28.764 to 41.849) | | | | | | | | | | | | | | | | | | | | | |
| Meningococcal infection | Birth prevalence - [34, 36) wks, [1500, 2000) g | Mortality | Males | | 55.555 (39.553 to 75.104) | 21.346 (17.677 to 26.143) | | | | | | | | | | | | | | | | | | | | | |
| Meningococcal infection | Birth prevalence - [34, 36) wks, [1500, 2000) g | Mortality | Females | | 54.335 (38.617 to 75.24) | 23.046 (18.743 to 28.287) | | | | | | | | | | | | | | | | | | | | | |
| Meningococcal infection | Birth prevalence - [32, 34) wks, [1500, 2000) g | Mortality | Males | | 57.155 (42.484 to 73.651) | 23.114 (19.028 to 27.915) | | | | | | | | | | | | | | | | | | | | | |
| Meningococcal infection | Birth prevalence - [32, 34) wks, [1500, 2000) g | Mortality | Females | | 56.101 (39.794 to 76.295) | 25.149 (20.615 to 30.388) | | | | | | | | | | | | | | | | | | | | | |
| Meningococcal infection | Birth prevalence - [32, 34) wks, [2000, 2500) g | Mortality | Males | | 37.444 (29.026 to 48.227) | 12.233 (10.252 to 14.477) | | | | | | | | | | | | | | | | | | | | | |
| Meningococcal infection | Birth prevalence - [32, 34) wks, [2000, 2500) g | Mortality | Females | | 36.874 (26.658 to 49.653) | 14.384 (12.095 to 17.03) | | | | | | | | | | | | | | | | | | | | | |
| Meningococcal infection | Birth prevalence - [40, 42) wks, [2000, 2500) g | Mortality | Males | | 18.092 (13.292 to 23.719) | 9.23 (7.037 to 11.454) | | | | | | | | | | | | | | | | | | | | | |

**Appendix Table 6a. Relative risks used by age and sex for each outcome for all risk factors except for ambient air pollution alcohol, and smoking.**

| | | | | Ages | | | | | | | | | | | | | | | | | | | | | | | |
|---|---|---|---|---|---|---|---|---|---|---|---|---|---|---|---|---|---|---|---|---|---|---|---|---|---|---|---|
| **Risk - Outcome** | **Category / Units** | **Morbidity / Mortality** | **Sex** | **All-age** | **0-6 days** | **7-27 days** | **28-364 days** | **1-4 years** | **5-9 years** | **10-14 years** | **15-19 years** | **20-24 years** | **25-29 years** | **30-34 years** | **35-39 years** | **40-44 years** | **45-49 years** | **50-54 years** | **55-59 years** | **60-64 years** | **65-69 years** | **70-74 years** | **75-79 years** | **80-84 years** | **85-89 years** | **90-94 years** | **95+ years** |
| Meningococcal infection | Birth prevalence - [40, 42) wks, [2000, 2500) g | Mortality | Females | | 15.574 (11.516 to 20.778) | 9.975 (7.82 to 12.46) | | | | | | | | | | | | | | | | | | | | | |
| Meningococcal infection | Birth prevalence - [38, 40) wks, [2000, 2500) g | Mortality | Males | | 13.104 (9.829 to 16.99) | 8.198 (6.786 to 9.959) | | | | | | | | | | | | | | | | | | | | | |
| Meningococcal infection | Birth prevalence - [38, 40) wks, [2000, 2500) g | Mortality | Females | | 11.308 (8.389 to 14.38) | 8.577 (7.04 to 10.449) | | | | | | | | | | | | | | | | | | | | | |
| Meningococcal infection | Birth prevalence - [32, 34) wks, [2500, 3000) g | Mortality | Males | | 33.063 (24.393 to 43.503) | 8.441 (6.822 to 10.431) | | | | | | | | | | | | | | | | | | | | | |
| Meningococcal infection | Birth prevalence - [32, 34) wks, [2500, 3000) g | Mortality | Females | | 32.812 (23.439 to 45.567) | 10.398 (8.227 to 13.042) | | | | | | | | | | | | | | | | | | | | | |
| Meningococcal infection | Birth prevalence - [34, 36) wks, [2000, 2500) g | Mortality | Males | | 21.925 (16.305 to 29.433) | 9.367 (7.859 to 11.112) | | | | | | | | | | | | | | | | | | | | | |
| Meningococcal infection | Birth prevalence - [34, 36) wks, [2000, 2500) g | Mortality | Females | | 21.297 (15.657 to 28.761) | 10.295 (8.548 to 12.273) | | | | | | | | | | | | | | | | | | | | | |
| Meningococcal infection | Birth prevalence - [37, 38) wks, [2000, 2500) g | Mortality | Males | | 13.0 (10.102 to 16.456) | 8.096 (6.724 to 9.676) | | | | | | | | | | | | | | | | | | | | | |
| Meningococcal infection | Birth prevalence - [37, 38) wks, [2000, 2500) g | Mortality | Females | | 11.563 (8.805 to 15.11) | 8.467 (6.994 to 10.342) | | | | | | | | | | | | | | | | | | | | | |
| Meningococcal infection | Birth prevalence - [36, 37) wks, [2000, 2500) g | Mortality | Males | | 14.401 (10.789 to 18.654) | 8.221 (6.917 to 9.923) | | | | | | | | | | | | | | | | | | | | | |
| Meningococcal infection | Birth prevalence - [36, 37) wks, [2000, 2500) g | Mortality | Females | | 13.513 (9.817 to 17.942) | 8.654 (7.215 to 10.369) | | | | | | | | | | | | | | | | | | | | | |
| Meningococcal infection | Birth prevalence - [34, 36) wks, [2500, 3000) g | Mortality | Males | | 13.419 (10.387 to 16.819) | 5.562 (4.646 to 6.696) | | | | | | | | | | | | | | | | | | | | | |
| Meningococcal infection | Birth prevalence - [34, 36) wks, [2500, 3000) g | Mortality | Females | | 13.266 (9.666 to 17.689) | 6.395 (5.292 to 7.606) | | | | | | | | | | | | | | | | | | | | | |
| Meningococcal infection | Birth prevalence - [34, 36) wks, [4000, 4500) g | Mortality | Males | | 23.096 (14.708 to 35.098) | 2.895 (2.245 to 3.716) | | | | | | | | | | | | | | | | | | | | | |
| Meningococcal infection | Birth prevalence - [34, 36) wks, [4000, 4500) g | Mortality | Females | | 25.038 (15.763 to 37.255) | 3.778 (2.855 to 4.925) | | | | | | | | | | | | | | | | | | | | | |
| Meningococcal infection | Birth prevalence - [34, 36) wks, [3000, 3500) g | Mortality | Males | | 14.006 (10.222 to 18.478) | 4.322 (3.449 to 5.338) | | | | | | | | | | | | | | | | | | | | | |
| Meningococcal infection | Birth prevalence - [34, 36) wks, [3000, 3500) g | Mortality | Females | | 14.375 (10.269 to 20.114) | 5.265 (4.145 to 6.564) | | | | | | | | | | | | | | | | | | | | | |
| Meningococcal infection | Birth prevalence - [36, 37) wks, [2500, 3000) g | Mortality | Males | | 4.874 (4.014 to 5.713) | 3.699 (3.134 to 4.374) | | | | | | | | | | | | | | | | | | | | | |
| Meningococcal infection | Birth prevalence - [36, 37) wks, [2500, 3000) g | Mortality | Females | | 4.609 (3.731 to 5.61) | 3.898 (3.235 to 4.67) | | | | | | | | | | | | | | | | | | | | | |
| Meningococcal infection | Birth prevalence - [34, 36) wks, [3500, 4000) g | Mortality | Males | | 18.024 (12.279 to 25.547) | 3.657 (2.838 to 4.675) | | | | | | | | | | | | | | | | | | | | | |
| Meningococcal infection | Birth prevalence - [34, 36) wks, [3500, 4000) g | Mortality | Females | | 19.263 (12.567 to 27.924) | 4.634 (3.576 to 5.989) | | | | | | | | | | | | | | | | | | | | | |
| Meningococcal infection | Birth prevalence - [37, 38) wks, [2500, 3000) g | Mortality | Males | | 3.306 (2.82 to 3.843) | 3.194 (2.691 to 3.803) | | | | | | | | | | | | | | | | | | | | | |
| Meningococcal infection | Birth prevalence - [37, 38) wks, [2500, 3000) g | Mortality | Females | | 2.991 (2.496 to 3.61) | 3.242 (2.745 to 3.817) | | | | | | | | | | | | | | | | | | | | | |
| Meningococcal infection | Birth prevalence - [40, 42) wks, [2500, 3000) g | Mortality | Males | | 3.771 (3.002 to 4.693) | 3.175 (2.56 to 3.923) | | | | | | | | | | | | | | | | | | | | | |
| Meningococcal infection | Birth prevalence - [40, 42) wks, [2500, 3000) g | Mortality | Females | | 3.244 (2.486 to 4.159) | 3.228 (2.605 to 3.985) | | | | | | | | | | | | | | | | | | | | | |
| Meningococcal infection | Birth prevalence - [38, 40) wks, [2500, 3000) g | Mortality | Males | | 2.755 (2.274 to 3.309) | 2.944 (2.44 to 3.548) | | | | | | | | | | | | | | | | | | | | | |
| Meningococcal infection | Birth prevalence - [38, 40) wks, [2500, 3000) g | Mortality | Females | | 2.376 (1.91 to 2.886) | 2.938 (2.434 to 3.503) | | | | | | | | | | | | | | | | | | | | | |
| Meningococcal infection | Birth prevalence - [36, 37) wks, [3000, 3500) g | Mortality | Males | | 3.774 (3.094 to 4.497) | 2.466 (2.058 to 2.929) | | | | | | | | | | | | | | | | | | | | | |
| Meningococcal infection | Birth prevalence - [36, 37) wks, [3000, 3500) g | Mortality | Females | | 3.73 (2.981 to 4.646) | 2.715 (2.277 to 3.218) | | | | | | | | | | | | | | | | | | | | | |
| Meningococcal infection | Birth prevalence - [36, 37) wks, [4000, 4500) g | Mortality | Males | | 6.826 (5.212 to 9.045) | 1.77 (1.491 to 2.082) | | | | | | | | | | | | | | | | | | | | | |
| Meningococcal infection | Birth prevalence - [36, 37) wks, [4000, 4500) g | Mortality | Females | | 7.269 (5.144 to 9.821) | 2.177 (1.786 to 2.607) | | | | | | | | | | | | | | | | | | | | | |
| Meningococcal infection | Birth prevalence - [36, 37) wks, [3500, 4000) g | Mortality | Males | | 4.544 (3.64 to 5.622) | 2.057 (1.74 to 2.44) | | | | | | | | | | | | | | | | | | | | | |
| Meningococcal infection | Birth prevalence - [36, 37) wks, [3500, 4000) g | Mortality | Females | | 4.662 (3.577 to 6.014) | 2.398 (1.98 to 2.864) | | | | | | | | | | | | | | | | | | | | | |
| Meningococcal infection | Birth prevalence - [37, 38) wks, [3000, 3500) g | Mortality | Males | | 2.007 (1.759 to 2.293) | 1.888 (1.613 to 2.224) | | | | | | | | | | | | | | | | | | | | | |
| Meningococcal infection | Birth prevalence - [37, 38) wks, [3000, 3500) g | Mortality | Females | | 1.925 (1.582 to 2.328) | 1.972 (1.669 to 2.313) | | | | | | | | | | | | | | | | | | | | | |
| Meningococcal infection | Birth prevalence - [37, 38) wks, [4000, 4500) g | Mortality | Males | | 3.28 (2.596 to 4.133) | 1.335 (1.171 to 1.532) | | | | | | | | | | | | | | | | | | | | | |
| Meningococcal infection | Birth prevalence - [37, 38) wks, [4000, 4500) g | Mortality | Females | | 3.521 (2.649 to 4.5) | 1.559 (1.333 to 1.835) | | | | | | | | | | | | | | | | | | | | | |
| Meningococcal infection | Birth prevalence - [37, 38) wks, [3500, 4000) g | Mortality | Males | | 2.128 (1.833 to 2.466) | 1.505 (1.299 to 1.76) | | | | | | | | | | | | | | | | | | | | | |
| Meningococcal infection | Birth prevalence - [37, 38) wks, [3500, 4000) g | Mortality | Females | | 2.142 (1.694 to 2.67) | 1.661 (1.411 to 1.961) | | | | | | | | | | | | | | | | | | | | | |
| Meningococcal infection | Birth prevalence - [40, 42) wks, [3000, 3500) g | Mortality | Males | | 1.436 (1.245 to 1.65) | 1.47 (1.199 to 1.8) | | | | | | | | | | | | | | | | | | | | | |
| Meningococcal infection | Birth prevalence - [40, 42) wks, [3000, 3500) g | Mortality | Females | | 1.326 (1.069 to 1.614) | 1.465 (1.188 to 1.775) | | | | | | | | | | | | | | | | | | | | | |
| Meningococcal infection | Birth prevalence - [38, 40) wks, [3000, 3500) g | Mortality | Males | | 1.33 (1.155 to 1.53) | 1.559 (1.305 to 1.851) | | | | | | | | | | | | | | | | | | | | | |
| Meningococcal infection | Birth prevalence - [38, 40) wks, [3000, 3500) g | Mortality | Females | | 1.224 (1.0 to 1.492) | 1.564 (1.304 to 1.847) | | | | | | | | | | | | | | | | | | | | | |
| Meningococcal infection | Birth prevalence - [38, 40) wks, [4000, 4500) g | Mortality | Males | | 1.787 (1.453 to 2.182) | 1.175 (1.005 to 1.371) | | | | | | | | | | | | | | | | | | | | | |
| Meningococcal infection | Birth prevalence - [38, 40) wks, [4000, 4500) g | Mortality | Females | | 1.877 (1.467 to 2.388) | 1.224 (1.022 to 1.465) | | | | | | | | | | | | | | | | | | | | | |
| Meningococcal infection | Birth prevalence - [38, 40) wks, [3500, 4000) g | Mortality | Males | | 1.785 (1.478 to 2.147) | 1.173 (1.0 to 1.377) | | | | | | | | | | | | | | | | | | | | | |
| Meningococcal infection | Birth prevalence - [38, 40) wks, [3500, 4000) g | Mortality | Females | | 1.892 (1.481 to 2.352) | 1.23 (1.03 to 1.46) | | | | | | | | | | | | | | | | | | | | | |
| Meningococcal infection | Birth prevalence - [40, 42) wks, [3500, 4000) g | Mortality | Males | | 1.0 (1.0 to 1.0) | 1.003 (1.0 to 1.046) | | | | | | | | | | | | | | | | | | | | | |
| Meningococcal infection | Birth prevalence - [40, 42) wks, [3500, 4000) g | Mortality | Females | | 1.002 (1.0 to 1.013) | 1.001 (1.0 to 1.006) | | | | | | | | | | | | | | | | | | | | | |
| Meningococcal infection | Birth prevalence - [40, 42) wks, [4000, 4500) g | Mortality | Males | | 1.0 (1.0 to 1.0) | 1.0 (1.0 to 1.0) | | | | | | | | | | | | | | | | | | | | | |
| Meningococcal infection | Birth prevalence - [40, 42) wks, [4000, 4500) g | Mortality | Females | | 1.0 (1.0 to 1.0) | 1.0 (1.0 to 1.0) | | | | | | | | | | | | | | | | | | | | | |
| Meningococcal infection | Birth prevalence - [28, 30) wks, [2000, 2500) g | Mortality | Males | | 117.172 (83.895 to 158.056) | 27.726 (21.877 to 34.972) | | | | | | | | | | | | | | | | | | | | | |
| Meningococcal infection | Birth prevalence - [28, 30) wks, [2000, 2500) g | Mortality | Females | | 121.682 (84.349 to 171.375) | 33.983 (26.101 to 43.404) | | | | | | | | | | | | | | | | | | | | | |
| Meningococcal infection | Birth prevalence - [28, 30) wks, [2500, 3000) g | Mortality | Males | | 77.948 (54.687 to 105.047) | 16.608 (12.653 to 21.188) | | | | | | | | | | | | | | | | | | | | | |
| Meningococcal infection | Birth prevalence - [28, 30) wks, [2500, 3000) g | Mortality | Females | | 79.193 (54.099 to 112.236) | 20.387 (15.089 to 26.434) | | | | | | | | | | | | | | | | | | | | | |
| Meningococcal infection | Birth prevalence - [28, 30) wks, [3000, 3500) g | Mortality | Males | | 42.199 (29.891 to 57.227) | 10.082 (7.777 to 13.056) | | | | | | | | | | | | | | | | | | | | | |
| Meningococcal infection | Birth prevalence - [28, 30) wks, [3000, 3500) g | Mortality | Females | | 42.551 (28.25 to 63.209) | 11.989 (9.084 to 15.544) | | | | | | | | | | | | | | | | | | | | | |

**Appendix Table 6a. Relative risks used by age and sex for each outcome for all risk factors except for ambient air pollution alcohol, and smoking.**

| | | | | Ages | | | | | | | | | | | | | | | | | | | | | | | |
|---|---|---|---|---|---|---|---|---|---|---|---|---|---|---|---|---|---|---|---|---|---|---|---|---|---|---|---|
| **Risk - Outcome** | **Category / Units** | **Morbidity / Mortality** | **Sex** | **All-age** | **0-6 days** | **7-27 days** | **28-364 days** | **1-4 years** | **5-9 years** | **10-14 years** | **15-19 years** | **20-24 years** | **25-29 years** | **30-34 years** | **35-39 years** | **40-44 years** | **45-49 years** | **50-54 years** | **55-59 years** | **60-64 years** | **65-69 years** | **70-74 years** | **75-79 years** | **80-84 years** | **85-89 years** | **90-94 years** | **95+ years** |
| Meningococcal infection | Birth prevalence - [30, 32) wks, [2500, 3000) g | Mortality | Males | | 58.722 (42.419 to 78.873) | 12.115 (9.518 to 15.521) | | | | | | | | | | | | | | | | | | | | | |
| Meningococcal infection | Birth prevalence - [30, 32) wks, [2500, 3000) g | Mortality | Females | | 59.522 (42.058 to 82.793) | 15.364 (11.936 to 19.581) | | | | | | | | | | | | | | | | | | | | | |
| Meningococcal infection | Birth prevalence - [30, 32) wks, [3000, 3500) g | Mortality | Males | | 45.67 (32.014 to 65.531) | 8.381 (6.362 to 10.842) | | | | | | | | | | | | | | | | | | | | | |
| Meningococcal infection | Birth prevalence - [30, 32) wks, [3000, 3500) g | Mortality | Females | | 46.104 (30.243 to 66.207) | 10.506 (8.041 to 13.513) | | | | | | | | | | | | | | | | | | | | | |
| Meningococcal infection | Birth prevalence - [30, 32) wks, [3500, 4000) g | Mortality | Males | | 36.334 (21.558 to 54.813) | 5.698 (4.293 to 7.349) | | | | | | | | | | | | | | | | | | | | | |
| Meningococcal infection | Birth prevalence - [30, 32) wks, [3500, 4000) g | Mortality | Females | | 37.931 (22.692 to 61.276) | 6.892 (5.175 to 9.058) | | | | | | | | | | | | | | | | | | | | | |
| Meningococcal infection | Birth prevalence - [32, 34) wks, [3000, 3500) g | Mortality | Males | | 34.016 (23.515 to 48.37) | 6.577 (4.934 to 8.436) | | | | | | | | | | | | | | | | | | | | | |
| Meningococcal infection | Birth prevalence - [32, 34) wks, [3000, 3500) g | Mortality | Females | | 34.585 (22.909 to 49.754) | 8.314 (6.362 to 10.789) | | | | | | | | | | | | | | | | | | | | | |
| Meningococcal infection | Birth prevalence - [32, 34) wks, [3500, 4000) g | Mortality | Males | | 36.248 (23.158 to 54.67) | 5.068 (3.761 to 6.741) | | | | | | | | | | | | | | | | | | | | | |
| Meningococcal infection | Birth prevalence - [32, 34) wks, [3500, 4000) g | Mortality | Females | | 38.098 (23.301 to 59.429) | 6.476 (4.666 to 8.689) | | | | | | | | | | | | | | | | | | | | | |
| Meningococcal infection | Birth prevalence - [36, 37) wks, [1000, 1500) g | Mortality | Males | | 166.686 (118.487 to 222.581) | 57.535 (45.999 to 71.742) | | | | | | | | | | | | | | | | | | | | | |
| Meningococcal infection | Birth prevalence - [36, 37) wks, [1000, 1500) g | Mortality | Females | | 169.725 (119.017 to 229.008) | 63.564 (50.068 to 80.703) | | | | | | | | | | | | | | | | | | | | | |
| Meningococcal infection | Birth prevalence - [38, 40) wks, [1000, 1500) g | Mortality | Males | | 174.066 (125.125 to 232.507) | 57.966 (44.393 to 73.241) | | | | | | | | | | | | | | | | | | | | | |
| Meningococcal infection | Birth prevalence - [38, 40) wks, [1000, 1500) g | Mortality | Females | | 171.557 (121.585 to 237.047) | 65.208 (48.821 to 84.308) | | | | | | | | | | | | | | | | | | | | | |
| Meningococcal infection | Birth prevalence - [38, 40) wks, [1500, 2000) g | Mortality | Males | | 67.302 (49.547 to 89.055) | 25.206 (20.365 to 31.168) | | | | | | | | | | | | | | | | | | | | | |
| Meningococcal infection | Birth prevalence - [38, 40) wks, [1500, 2000) g | Mortality | Females | | 62.19 (45.884 to 83.445) | 28.05 (22.625 to 35.139) | | | | | | | | | | | | | | | | | | | | | |
| Meningococcal infection | Birth prevalence - [40, 42) wks, [1500, 2000) g | Mortality | Males | | 76.673 (56.177 to 102.468) | 25.785 (19.387 to 34.168) | | | | | | | | | | | | | | | | | | | | | |
| Meningococcal infection | Birth prevalence - [40, 42) wks, [1500, 2000) g | Mortality | Females | | 70.411 (49.221 to 97.952) | 29.113 (21.355 to 38.272) | | | | | | | | | | | | | | | | | | | | | |
| Other meningitis | Birth prevalence - [0, 24) wks, [0, 500) g | Mortality | Males | | 1564.792 (1056.542 to [illegible] | 618.595 (458.842 to 812.921) | | | | | | | | | | | | | | | | | | | | | |
| Other meningitis | Birth prevalence - [0, 24) wks, [0, 500) g | Mortality | Females | | 1600.122 (1050.664 to [illegible] | 713.571 (526.178 to 921.018) | | | | | | | | | | | | | | | | | | | | | |
| Other meningitis | Birth prevalence - [0, 24) wks, [500, 1000) g | Mortality | Males | | 1155.815 (825.412 to 1506.837) | 457.5 (352.552 to 573.483) | | | | | | | | | | | | | | | | | | | | | |
| Other meningitis | Birth prevalence - [0, 24) wks, [500, 1000) g | Mortality | Females | | 1169.123 (802.003 to 1617.979) | 515.406 (396.713 to 641.541) | | | | | | | | | | | | | | | | | | | | | |
| Other meningitis | Birth prevalence - [24, 26) wks, [500, 1000) g | Mortality | Males | | 955.583 (723.748 to 1244.265) | 443.357 (363.03 to 534.695) | | | | | | | | | | | | | | | | | | | | | |
| Other meningitis | Birth prevalence - [24, 26) wks, [500, 1000) g | Mortality | Females | | 947.143 (702.662 to 1237.093) | 487.549 (387.307 to 603.498) | | | | | | | | | | | | | | | | | | | | | |
| Other meningitis | Birth prevalence - [26, 28) wks, [500, 1000) g | Mortality | Males | | 497.817 (377.617 to 648.547) | 330.886 (261.438 to 401.709) | | | | | | | | | | | | | | | | | | | | | |
| Other meningitis | Birth prevalence - [26, 28) wks, [500, 1000) g | Mortality | Females | | 483.682 (354.946 to 629.517) | 344.618 (274.427 to 419.864) | | | | | | | | | | | | | | | | | | | | | |
| Other meningitis | Birth prevalence - [30, 32) wks, [500, 1000) g | Mortality | Males | | 236.614 (163.821 to 324.502) | 149.995 (117.866 to 188.368) | | | | | | | | | | | | | | | | | | | | | |
| Other meningitis | Birth prevalence - [30, 32) wks, [500, 1000) g | Mortality | Females | | 229.197 (157.606 to 317.194) | 152.117 (120.779 to 190.583) | | | | | | | | | | | | | | | | | | | | | |
| Other meningitis | Birth prevalence - [28, 30) wks, [500, 1000) g | Mortality | Males | | 297.629 (214.953 to 396.586) | 216.995 (173.321 to 271.466) | | | | | | | | | | | | | | | | | | | | | |
| Other meningitis | Birth prevalence - [28, 30) wks, [500, 1000) g | Mortality | Females | | 281.056 (198.176 to 386.635) | 219.884 (174.264 to 272.704) | | | | | | | | | | | | | | | | | | | | | |
| Other meningitis | Birth prevalence - [26, 28) wks, [1000, 1500) g | Mortality | Males | | 267.91 (210.177 to 332.92) | 164.167 (132.898 to 200.569) | | | | | | | | | | | | | | | | | | | | | |
| Other meningitis | Birth prevalence - [26, 28) wks, [1000, 1500) g | Mortality | Females | | 266.509 (197.461 to 346.932) | 174.222 (137.431 to 217.349) | | | | | | | | | | | | | | | | | | | | | |
| Other meningitis | Birth prevalence - [34, 36) wks, [1000, 1500) g | Mortality | Males | | 142.056 (98.086 to 197.774) | 52.86 (42.914 to 64.617) | | | | | | | | | | | | | | | | | | | | | |
| Other meningitis | Birth prevalence - [34, 36) wks, [1000, 1500) g | Mortality | Females | | 141.899 (95.864 to 197.656) | 57.421 (46.452 to 71.339) | | | | | | | | | | | | | | | | | | | | | |
| Other meningitis | Birth prevalence - [28, 30) wks, [1500, 2000) g | Mortality | Males | | 127.966 (97.178 to 167.026) | 50.018 (40.539 to 61.919) | | | | | | | | | | | | | | | | | | | | | |
| Other meningitis | Birth prevalence - [28, 30) wks, [1500, 2000) g | Mortality | Females | | 130.924 (96.513 to 172.188) | 57.275 (46.36 to 70.038) | | | | | | | | | | | | | | | | | | | | | |
| Other meningitis | Birth prevalence - [28, 30) wks, [1000, 1500) g | Mortality | Males | | 158.563 (120.99 to 204.947) | 103.32 (83.486 to 127.144) | | | | | | | | | | | | | | | | | | | | | |
| Other meningitis | Birth prevalence - [28, 30) wks, [1000, 1500) g | Mortality | Females | | 153.905 (112.327 to 200.786) | 107.529 (86.954 to 131.78) | | | | | | | | | | | | | | | | | | | | | |
| Other meningitis | Birth prevalence - [32, 34) wks, [1000, 1500) g | Mortality | Males | | 117.142 (81.354 to 161.101) | 53.185 (43.049 to 66.274) | | | | | | | | | | | | | | | | | | | | | |
| Other meningitis | Birth prevalence - [32, 34) wks, [1000, 1500) g | Mortality | Females | | 115.171 (79.363 to 159.206) | 56.034 (45.982 to 68.36) | | | | | | | | | | | | | | | | | | | | | |
| Other meningitis | Birth prevalence - [30, 32) wks, [1000, 1500) g | Mortality | Males | | 119.308 (87.769 to 160.885) | 67.163 (54.863 to 82.638) | | | | | | | | | | | | | | | | | | | | | |
| Other meningitis | Birth prevalence - [30, 32) wks, [1000, 1500) g | Mortality | Females | | 115.448 (84.272 to 156.425) | 69.14 (55.873 to 85.012) | | | | | | | | | | | | | | | | | | | | | |
| Other meningitis | Birth prevalence - [37, 38) wks, [1500, 2000) g | Mortality | Males | | 62.972 (46.159 to 83.484) | 24.148 (20.066 to 29.406) | | | | | | | | | | | | | | | | | | | | | |
| Other meningitis | Birth prevalence - [37, 38) wks, [1500, 2000) g | Mortality | Females | | 59.988 (43.974 to 79.053) | 26.719 (21.746 to 32.816) | | | | | | | | | | | | | | | | | | | | | |
| Other meningitis | Birth prevalence - [36, 37) wks, [1500, 2000) g | Mortality | Males | | 60.218 (43.669 to 82.48) | 23.031 (18.793 to 28.483) | | | | | | | | | | | | | | | | | | | | | |
| Other meningitis | Birth prevalence - [36, 37) wks, [1500, 2000) g | Mortality | Females | | 58.527 (42.172 to 80.557) | 25.143 (20.331 to 30.566) | | | | | | | | | | | | | | | | | | | | | |
| Other meningitis | Birth prevalence - [30, 32) wks, [2000, 2500) g | Mortality | Males | | 67.971 (50.354 to 88.935) | 18.03 (14.621 to 22.103) | | | | | | | | | | | | | | | | | | | | | |
| Other meningitis | Birth prevalence - [30, 32) wks, [2000, 2500) g | Mortality | Females | | 69.383 (49.108 to 94.583) | 22.069 (17.836 to 27.163) | | | | | | | | | | | | | | | | | | | | | |
| Other meningitis | Birth prevalence - [30, 32) wks, [1500, 2000) g | Mortality | Males | | 77.369 (59.702 to 99.232) | 31.079 (25.786 to 36.724) | | | | | | | | | | | | | | | | | | | | | |
| Other meningitis | Birth prevalence - [30, 32) wks, [1500, 2000) g | Mortality | Females | | 76.134 (56.885 to 100.996) | 34.756 (28.764 to 41.849) | | | | | | | | | | | | | | | | | | | | | |
| Other meningitis | Birth prevalence - [34, 36) wks, [1500, 2000) g | Mortality | Males | | 55.555 (39.553 to 75.104) | 21.346 (17.677 to 26.143) | | | | | | | | | | | | | | | | | | | | | |
| Other meningitis | Birth prevalence - [34, 36) wks, [1500, 2000) g | Mortality | Females | | 54.335 (38.617 to 75.24) | 23.046 (18.743 to 28.287) | | | | | | | | | | | | | | | | | | | | | |
| Other meningitis | Birth prevalence - [32, 34) wks, [1500, 2000) g | Mortality | Males | | 57.155 (42.484 to 73.651) | 23.114 (19.028 to 27.915) | | | | | | | | | | | | | | | | | | | | | |
| Other meningitis | Birth prevalence - [32, 34) wks, [1500, 2000) g | Mortality | Females | | 56.101 (39.794 to 76.295) | 25.149 (20.615 to 30.388) | | | | | | | | | | | | | | | | | | | | | |
| Other meningitis | Birth prevalence - [32, 34) wks, [2000, 2500) g | Mortality | Males | | 37.444 (29.026 to 48.227) | 12.233 (10.252 to 14.477) | | | | | | | | | | | | | | | | | | | | | |
| Other meningitis | Birth prevalence - [32, 34) wks, [2000, 2500) g | Mortality | Females | | 36.874 (26.658 to 49.653) | 14.384 (12.095 to 17.03) | | | | | | | | | | | | | | | | | | | | | |
| Other meningitis | Birth prevalence - [40, 42) wks, [2000, 2500) g | Mortality | Males | | 18.092 (13.292 to 23.719) | 9.23 (7.037 to 11.454) | | | | | | | | | | | | | | | | | | | | | |

**Appendix Table 6a. Relative risks used by age and sex for each outcome for all risk factors except for ambient air pollution alcohol, and smoking.**

| | | | | Ages | | | | | | | | | | | | | | | | | | | | | | | |
|---|---|---|---|---|---|---|---|---|---|---|---|---|---|---|---|---|---|---|---|---|---|---|---|---|---|---|---|
| Risk - Outcome | Category / Units | Morbidity / Mortality | Sex | All-age | 0-6 days | 7-27 days | 28-364 days | 1-4 years | 5-9 years | 10-14 years | 15-19 years | 20-24 years | 25-29 years | 30-34 years | 35-39 years | 40-44 years | 45-49 years | 50-54 years | 55-59 years | 60-64 years | 65-69 years | 70-74 years | 75-79 years | 80-84 years | 85-89 years | 90-94 years | 95+ years |
| Other meningitis | Birth prevalence - [40, 42) wks, [2000, 2500) g | Mortality | Females | | 15.574 (11.516 to 20.778) | 9.975 (7.82 to 12.46) | | | | | | | | | | | | | | | | | | | | | |
| Other meningitis | Birth prevalence - [38, 40) wks, [2000, 2500) g | Mortality | Males | | 13.104 (9.829 to 16.99) | 8.198 (6.786 to 9.959) | | | | | | | | | | | | | | | | | | | | | |
| Other meningitis | Birth prevalence - [38, 40) wks, [2000, 2500) g | Mortality | Females | | 11.308 (8.389 to 14.38) | 8.577 (7.04 to 10.449) | | | | | | | | | | | | | | | | | | | | | |
| Other meningitis | Birth prevalence - [32, 34) wks, [2500, 3000) g | Mortality | Males | | 33.063 (24.393 to 43.503) | 8.441 (6.822 to 10.431) | | | | | | | | | | | | | | | | | | | | | |
| Other meningitis | Birth prevalence - [32, 34) wks, [2500, 3000) g | Mortality | Females | | 32.812 (23.439 to 45.567) | 10.398 (8.227 to 13.042) | | | | | | | | | | | | | | | | | | | | | |
| Other meningitis | Birth prevalence - [34, 36) wks, [2000, 2500) g | Mortality | Males | | 21.925 (16.305 to 29.433) | 9.367 (7.859 to 11.112) | | | | | | | | | | | | | | | | | | | | | |
| Other meningitis | Birth prevalence - [34, 36) wks, [2000, 2500) g | Mortality | Females | | 21.297 (15.657 to 28.761) | 10.295 (8.548 to 12.273) | | | | | | | | | | | | | | | | | | | | | |
| Other meningitis | Birth prevalence - [37, 38) wks, [2000, 2500) g | Mortality | Males | | 13.0 (10.102 to 16.456) | 8.096 (6.724 to 9.676) | | | | | | | | | | | | | | | | | | | | | |
| Other meningitis | Birth prevalence - [37, 38) wks, [2000, 2500) g | Mortality | Females | | 11.563 (8.805 to 15.11) | 8.467 (6.994 to 10.342) | | | | | | | | | | | | | | | | | | | | | |
| Other meningitis | Birth prevalence - [36, 37) wks, [2000, 2500) g | Mortality | Males | | 14.401 (10.789 to 18.654) | 8.221 (6.917 to 9.923) | | | | | | | | | | | | | | | | | | | | | |
| Other meningitis | Birth prevalence - [36, 37) wks, [2000, 2500) g | Mortality | Females | | 13.513 (9.817 to 17.942) | 8.654 (7.215 to 10.369) | | | | | | | | | | | | | | | | | | | | | |
| Other meningitis | Birth prevalence - [34, 36) wks, [2500, 3000) g | Mortality | Males | | 13.419 (10.387 to 16.819) | 5.562 (4.646 to 6.696) | | | | | | | | | | | | | | | | | | | | | |
| Other meningitis | Birth prevalence - [34, 36) wks, [2500, 3000) g | Mortality | Females | | 13.266 (9.666 to 17.689) | 6.395 (5.292 to 7.606) | | | | | | | | | | | | | | | | | | | | | |
| Other meningitis | Birth prevalence - [34, 36) wks, [4000, 4500) g | Mortality | Males | | 23.096 (14.708 to 35.098) | 2.895 (2.245 to 3.716) | | | | | | | | | | | | | | | | | | | | | |
| Other meningitis | Birth prevalence - [34, 36) wks, [4000, 4500) g | Mortality | Females | | 25.038 (15.763 to 37.255) | 3.778 (2.855 to 4.925) | | | | | | | | | | | | | | | | | | | | | |
| Other meningitis | Birth prevalence - [34, 36) wks, [3000, 3500) g | Mortality | Males | | 14.006 (10.222 to 18.478) | 4.322 (3.449 to 5.338) | | | | | | | | | | | | | | | | | | | | | |
| Other meningitis | Birth prevalence - [34, 36) wks, [3000, 3500) g | Mortality | Females | | 14.375 (10.269 to 20.114) | 5.265 (4.145 to 6.564) | | | | | | | | | | | | | | | | | | | | | |
| Other meningitis | Birth prevalence - [36, 37) wks, [2500, 3000) g | Mortality | Males | | 4.874 (4.014 to 5.713) | 3.699 (3.134 to 4.374) | | | | | | | | | | | | | | | | | | | | | |
| Other meningitis | Birth prevalence - [36, 37) wks, [2500, 3000) g | Mortality | Females | | 4.609 (3.731 to 5.61) | 3.898 (3.235 to 4.67) | | | | | | | | | | | | | | | | | | | | | |
| Other meningitis | Birth prevalence - [34, 36) wks, [3500, 4000) g | Mortality | Males | | 18.024 (12.279 to 25.547) | 3.657 (2.838 to 4.675) | | | | | | | | | | | | | | | | | | | | | |
| Other meningitis | Birth prevalence - [34, 36) wks, [3500, 4000) g | Mortality | Females | | 19.263 (12.567 to 27.924) | 4.634 (3.576 to 5.989) | | | | | | | | | | | | | | | | | | | | | |
| Other meningitis | Birth prevalence - [37, 38) wks, [2500, 3000) g | Mortality | Males | | 3.306 (2.82 to 3.843) | 3.194 (2.691 to 3.803) | | | | | | | | | | | | | | | | | | | | | |
| Other meningitis | Birth prevalence - [37, 38) wks, [2500, 3000) g | Mortality | Females | | 2.991 (2.496 to 3.61) | 3.242 (2.745 to 3.817) | | | | | | | | | | | | | | | | | | | | | |
| Other meningitis | Birth prevalence - [40, 42) wks, [2500, 3000) g | Mortality | Males | | 3.771 (3.002 to 4.693) | 3.175 (2.56 to 3.923) | | | | | | | | | | | | | | | | | | | | | |
| Other meningitis | Birth prevalence - [40, 42) wks, [2500, 3000) g | Mortality | Females | | 3.244 (2.486 to 4.159) | 3.228 (2.605 to 3.985) | | | | | | | | | | | | | | | | | | | | | |
| Other meningitis | Birth prevalence - [38, 40) wks, [2500, 3000) g | Mortality | Males | | 2.755 (2.274 to 3.309) | 2.944 (2.44 to 3.548) | | | | | | | | | | | | | | | | | | | | | |
| Other meningitis | Birth prevalence - [38, 40) wks, [2500, 3000) g | Mortality | Females | | 2.376 (1.91 to 2.886) | 2.938 (2.434 to 3.503) | | | | | | | | | | | | | | | | | | | | | |
| Other meningitis | Birth prevalence - [36, 37) wks, [3000, 3500) g | Mortality | Males | | 3.774 (3.094 to 4.497) | 2.466 (2.058 to 2.929) | | | | | | | | | | | | | | | | | | | | | |
| Other meningitis | Birth prevalence - [36, 37) wks, [3000, 3500) g | Mortality | Females | | 3.73 (2.981 to 4.646) | 2.715 (2.277 to 3.218) | | | | | | | | | | | | | | | | | | | | | |
| Other meningitis | Birth prevalence - [36, 37) wks, [4000, 4500) g | Mortality | Males | | 6.826 (5.212 to 9.045) | 1.77 (1.491 to 2.082) | | | | | | | | | | | | | | | | | | | | | |
| Other meningitis | Birth prevalence - [36, 37) wks, [4000, 4500) g | Mortality | Females | | 7.269 (5.144 to 9.821) | 2.177 (1.786 to 2.607) | | | | | | | | | | | | | | | | | | | | | |
| Other meningitis | Birth prevalence - [36, 37) wks, [3500, 4000) g | Mortality | Males | | 4.544 (3.64 to 5.622) | 2.057 (1.74 to 2.44) | | | | | | | | | | | | | | | | | | | | | |
| Other meningitis | Birth prevalence - [36, 37) wks, [3500, 4000) g | Mortality | Females | | 4.662 (3.577 to 6.014) | 2.398 (1.98 to 2.864) | | | | | | | | | | | | | | | | | | | | | |
| Other meningitis | Birth prevalence - [37, 38) wks, [3000, 3500) g | Mortality | Males | | 2.007 (1.759 to 2.293) | 1.888 (1.613 to 2.224) | | | | | | | | | | | | | | | | | | | | | |
| Other meningitis | Birth prevalence - [37, 38) wks, [3000, 3500) g | Mortality | Females | | 1.925 (1.582 to 2.328) | 1.972 (1.669 to 2.313) | | | | | | | | | | | | | | | | | | | | | |
| Other meningitis | Birth prevalence - [37, 38) wks, [4000, 4500) g | Mortality | Males | | 3.28 (2.596 to 4.133) | 1.335 (1.171 to 1.532) | | | | | | | | | | | | | | | | | | | | | |
| Other meningitis | Birth prevalence - [37, 38) wks, [4000, 4500) g | Mortality | Females | | 3.521 (2.649 to 4.5) | 1.559 (1.333 to 1.835) | | | | | | | | | | | | | | | | | | | | | |
| Other meningitis | Birth prevalence - [37, 38) wks, [3500, 4000) g | Mortality | Males | | 2.128 (1.833 to 2.466) | 1.505 (1.299 to 1.76) | | | | | | | | | | | | | | | | | | | | | |
| Other meningitis | Birth prevalence - [37, 38) wks, [3500, 4000) g | Mortality | Females | | 2.142 (1.694 to 2.67) | 1.661 (1.411 to 1.961) | | | | | | | | | | | | | | | | | | | | | |
| Other meningitis | Birth prevalence - [40, 42) wks, [3000, 3500) g | Mortality | Males | | 1.436 (1.245 to 1.65) | 1.47 (1.199 to 1.8) | | | | | | | | | | | | | | | | | | | | | |
| Other meningitis | Birth prevalence - [40, 42) wks, [3000, 3500) g | Mortality | Females | | 1.326 (1.069 to 1.614) | 1.465 (1.188 to 1.775) | | | | | | | | | | | | | | | | | | | | | |
| Other meningitis | Birth prevalence - [38, 40) wks, [3000, 3500) g | Mortality | Males | | 1.33 (1.155 to 1.53) | 1.559 (1.305 to 1.851) | | | | | | | | | | | | | | | | | | | | | |
| Other meningitis | Birth prevalence - [38, 40) wks, [3000, 3500) g | Mortality | Females | | 1.224 (1.0 to 1.492) | 1.564 (1.304 to 1.847) | | | | | | | | | | | | | | | | | | | | | |
| Other meningitis | Birth prevalence - [38, 40) wks, [4000, 4500) g | Mortality | Males | | 1.787 (1.453 to 2.182) | 1.175 (1.005 to 1.371) | | | | | | | | | | | | | | | | | | | | | |
| Other meningitis | Birth prevalence - [38, 40) wks, [4000, 4500) g | Mortality | Females | | 1.877 (1.467 to 2.388) | 1.224 (1.022 to 1.465) | | | | | | | | | | | | | | | | | | | | | |
| Other meningitis | Birth prevalence - [38, 40) wks, [3500, 4000) g | Mortality | Males | | 1.785 (1.478 to 2.147) | 1.173 (1.0 to 1.377) | | | | | | | | | | | | | | | | | | | | | |
| Other meningitis | Birth prevalence - [38, 40) wks, [3500, 4000) g | Mortality | Females | | 1.892 (1.481 to 2.352) | 1.23 (1.03 to 1.46) | | | | | | | | | | | | | | | | | | | | | |
| Other meningitis | Birth prevalence - [40, 42) wks, [3500, 4000) g | Mortality | Males | | 1.0 (1.0 to 1.0) | 1.003 (1.0 to 1.046) | | | | | | | | | | | | | | | | | | | | | |
| Other meningitis | Birth prevalence - [40, 42) wks, [3500, 4000) g | Mortality | Females | | 1.002 (1.0 to 1.013) | 1.001 (1.0 to 1.006) | | | | | | | | | | | | | | | | | | | | | |
| Other meningitis | Birth prevalence - [40, 42) wks, [4000, 4500) g | Mortality | Males | | 1.0 (1.0 to 1.0) | 1.0 (1.0 to 1.0) | | | | | | | | | | | | | | | | | | | | | |
| Other meningitis | Birth prevalence - [40, 42) wks, [4000, 4500) g | Mortality | Females | | 1.0 (1.0 to 1.0) | 1.0 (1.0 to 1.0) | | | | | | | | | | | | | | | | | | | | | |
| Other meningitis | Birth prevalence - [28, 30) wks, [2000, 2500) g | Mortality | Males | | 117.172 (83.895 to 158.056) | 27.726 (21.877 to 34.972) | | | | | | | | | | | | | | | | | | | | | |
| Other meningitis | Birth prevalence - [28, 30) wks, [2000, 2500) g | Mortality | Females | | 121.682 (84.349 to 171.375) | 33.983 (26.101 to 43.404) | | | | | | | | | | | | | | | | | | | | | |
| Other meningitis | Birth prevalence - [28, 30) wks, [2500, 3000) g | Mortality | Males | | 77.948 (54.687 to 105.047) | 16.608 (12.653 to 21.188) | | | | | | | | | | | | | | | | | | | | | |
| Other meningitis | Birth prevalence - [28, 30) wks, [2500, 3000) g | Mortality | Females | | 79.193 (54.099 to 112.236) | 20.387 (15.089 to 26.434) | | | | | | | | | | | | | | | | | | | | | |
| Other meningitis | Birth prevalence - [28, 30) wks, [3000, 3500) g | Mortality | Males | | 42.199 (29.891 to 57.227) | 10.082 (7.777 to 13.056) | | | | | | | | | | | | | | | | | | | | | |
| Other meningitis | Birth prevalence - [28, 30) wks, [3000, 3500) g | Mortality | Females | | 42.551 (28.25 to 63.209) | 11.989 (9.084 to 15.544) | | | | | | | | | | | | | | | | | | | | | |

Appendix Table 6a. Relative risks used by age and sex for each outcome for all risk factors except for ambient air pollution alcohol, and smoking.

| Risk - Outcome | Category / Units | Morbidity / Mortality | Sex | Ages | | | | | | | | | | | | | | | | | | | | | | | |
|---|---|---|---|---|---|---|---|---|---|---|---|---|---|---|---|---|---|---|---|---|---|---|---|---|---|---|---|
| | | | | All-age | 0-6 days | 7-27 days | 28-364 days | 1-4 years | 5-9 years | 10-14 years | 15-19 years | 20-24 years | 25-29 years | 30-34 years | 35-39 years | 40-44 years | 45-49 years | 50-54 years | 55-59 years | 60-64 years | 65-69 years | 70-74 years | 75-79 years | 80-84 years | 85-89 years | 90-94 years | 95+ years |
| Other meningitis | Birth prevalence - [30, 32) wks, [2500, 3000) g | Mortality | Males | | 58.722 (42.419 to 78.873) | 12.115 (9.518 to 15.521) | | | | | | | | | | | | | | | | | | | | | |
| Other meningitis | Birth prevalence - [30, 32) wks, [2500, 3000) g | Mortality | Females | | 59.522 (42.058 to 82.793) | 15.364 (11.936 to 19.581) | | | | | | | | | | | | | | | | | | | | | |
| Other meningitis | Birth prevalence - [30, 32) wks, [3000, 3500) g | Mortality | Males | | 45.67 (32.014 to 65.531) | 8.381 (6.362 to 10.842) | | | | | | | | | | | | | | | | | | | | | |
| Other meningitis | Birth prevalence - [30, 32) wks, [3000, 3500) g | Mortality | Females | | 46.104 (30.243 to 66.207) | 10.506 (8.041 to 13.513) | | | | | | | | | | | | | | | | | | | | | |
| Other meningitis | Birth prevalence - [30, 32) wks, [3500, 4000) g | Mortality | Males | | 36.334 (21.558 to 54.813) | 5.698 (4.293 to 7.349) | | | | | | | | | | | | | | | | | | | | | |
| Other meningitis | Birth prevalence - [30, 32) wks, [3500, 4000) g | Mortality | Females | | 37.931 (22.692 to 61.276) | 6.892 (5.175 to 9.058) | | | | | | | | | | | | | | | | | | | | | |
| Other meningitis | Birth prevalence - [32, 34) wks, [3000, 3500) g | Mortality | Males | | 34.016 (23.515 to 48.37) | 6.577 (4.934 to 8.436) | | | | | | | | | | | | | | | | | | | | | |
| Other meningitis | Birth prevalence - [32, 34) wks, [3000, 3500) g | Mortality | Females | | 34.585 (22.909 to 49.754) | 8.314 (6.362 to 10.789) | | | | | | | | | | | | | | | | | | | | | |
| Other meningitis | Birth prevalence - [32, 34) wks, [3500, 4000) g | Mortality | Males | | 36.248 (23.158 to 54.67) | 5.068 (3.761 to 6.741) | | | | | | | | | | | | | | | | | | | | | |
| Other meningitis | Birth prevalence - [32, 34) wks, [3500, 4000) g | Mortality | Females | | 38.098 (23.301 to 59.429) | 6.476 (4.666 to 8.689) | | | | | | | | | | | | | | | | | | | | | |
| Other meningitis | Birth prevalence - [36, 37) wks, [1000, 1500) g | Mortality | Males | | 166.686 (118.487 to 222.581) | 57.535 (45.999 to 71.742) | | | | | | | | | | | | | | | | | | | | | |
| Other meningitis | Birth prevalence - [36, 37) wks, [1000, 1500) g | Mortality | Females | | 169.725 (119.017 to 229.008) | 63.564 (50.068 to 80.703) | | | | | | | | | | | | | | | | | | | | | |
| Other meningitis | Birth prevalence - [38, 40) wks, [1000, 1500) g | Mortality | Males | | 174.066 (125.125 to 232.507) | 57.966 (44.393 to 73.241) | | | | | | | | | | | | | | | | | | | | | |
| Other meningitis | Birth prevalence - [38, 40) wks, [1000, 1500) g | Mortality | Females | | 171.557 (121.585 to 237.047) | 65.208 (48.821 to 84.308) | | | | | | | | | | | | | | | | | | | | | |
| Other meningitis | Birth prevalence - [38, 40) wks, [1500, 2000) g | Mortality | Males | | 67.302 (49.547 to 89.055) | 25.206 (20.365 to 31.168) | | | | | | | | | | | | | | | | | | | | | |
| Other meningitis | Birth prevalence - [38, 40) wks, [1500, 2000) g | Mortality | Females | | 62.19 (45.884 to 83.445) | 28.05 (22.625 to 35.139) | | | | | | | | | | | | | | | | | | | | | |
| Other meningitis | Birth prevalence - [40, 42) wks, [1500, 2000) g | Mortality | Males | | 76.673 (56.177 to 102.468) | 25.785 (19.387 to 34.168) | | | | | | | | | | | | | | | | | | | | | |
| Other meningitis | Birth prevalence - [40, 42) wks, [1500, 2000) g | Mortality | Females | | 70.411 (49.221 to 97.952) | 29.113 (21.355 to 38.272) | | | | | | | | | | | | | | | | | | | | | |
| Encephalitis | Birth prevalence - [0, 24) wks, [0, 500) g | Mortality | Males | | 1564.792 (1056.542 to [illegible] | 618.595 (458.842 to 812.921) | | | | | | | | | | | | | | | | | | | | | |
| Encephalitis | Birth prevalence - [0, 24) wks, [0, 500) g | Mortality | Females | | 1600.122 (1050.664 to [illegible] | 713.571 (526.178 to 921.018) | | | | | | | | | | | | | | | | | | | | | |
| Encephalitis | Birth prevalence - [0, 24) wks, [500, 1000) g | Mortality | Males | | 1155.815 (825.412 to 1506.837) | 457.5 (352.552 to 573.483) | | | | | | | | | | | | | | | | | | | | | |
| Encephalitis | Birth prevalence - [0, 24) wks, [500, 1000) g | Mortality | Females | | 1169.123 (802.003 to 1617.979) | 515.406 (396.713 to 641.541) | | | | | | | | | | | | | | | | | | | | | |
| Encephalitis | Birth prevalence - [24, 26) wks, [500, 1000) g | Mortality | Males | | 955.583 (723.748 to 1244.265) | 443.357 (363.03 to 534.695) | | | | | | | | | | | | | | | | | | | | | |
| Encephalitis | Birth prevalence - [24, 26) wks, [500, 1000) g | Mortality | Females | | 947.143 (702.662 to 1237.093) | 487.549 (387.307 to 603.498) | | | | | | | | | | | | | | | | | | | | | |
| Encephalitis | Birth prevalence - [26, 28) wks, [500, 1000) g | Mortality | Males | | 497.817 (377.617 to 648.547) | 330.886 (261.438 to 401.709) | | | | | | | | | | | | | | | | | | | | | |
| Encephalitis | Birth prevalence - [26, 28) wks, [500, 1000) g | Mortality | Females | | 483.682 (354.946 to 629.517) | 344.618 (274.427 to 419.864) | | | | | | | | | | | | | | | | | | | | | |
| Encephalitis | Birth prevalence - [30, 32) wks, [500, 1000) g | Mortality | Males | | 236.614 (163.821 to 324.502) | 149.995 (117.866 to 188.368) | | | | | | | | | | | | | | | | | | | | | |
| Encephalitis | Birth prevalence - [30, 32) wks, [500, 1000) g | Mortality | Females | | 229.197 (157.606 to 317.194) | 152.117 (120.779 to 190.583) | | | | | | | | | | | | | | | | | | | | | |
| Encephalitis | Birth prevalence - [28, 30) wks, [500, 1000) g | Mortality | Males | | 297.629 (214.953 to 396.586) | 216.995 (173.321 to 271.466) | | | | | | | | | | | | | | | | | | | | | |
| Encephalitis | Birth prevalence - [28, 30) wks, [500, 1000) g | Mortality | Females | | 281.056 (198.176 to 386.635) | 219.884 (174.264 to 272.704) | | | | | | | | | | | | | | | | | | | | | |
| Encephalitis | Birth prevalence - [26, 28) wks, [1000, 1500) g | Mortality | Males | | 267.91 (210.177 to 332.92) | 164.167 (132.898 to 200.569) | | | | | | | | | | | | | | | | | | | | | |
| Encephalitis | Birth prevalence - [26, 28) wks, [1000, 1500) g | Mortality | Females | | 266.509 (197.461 to 346.932) | 174.222 (137.431 to 217.349) | | | | | | | | | | | | | | | | | | | | | |
| Encephalitis | Birth prevalence - [34, 36) wks, [1000, 1500) g | Mortality | Males | | 142.056 (98.086 to 197.774) | 52.86 (42.914 to 64.617) | | | | | | | | | | | | | | | | | | | | | |
| Encephalitis | Birth prevalence - [34, 36) wks, [1000, 1500) g | Mortality | Females | | 141.899 (95.864 to 197.656) | 57.421 (46.452 to 71.339) | | | | | | | | | | | | | | | | | | | | | |
| Encephalitis | Birth prevalence - [28, 30) wks, [1500, 2000) g | Mortality | Males | | 127.966 (97.178 to 167.026) | 50.018 (40.539 to 61.919) | | | | | | | | | | | | | | | | | | | | | |
| Encephalitis | Birth prevalence - [28, 30) wks, [1500, 2000) g | Mortality | Females | | 130.924 (96.513 to 172.188) | 57.275 (46.36 to 70.038) | | | | | | | | | | | | | | | | | | | | | |
| Encephalitis | Birth prevalence - [28, 30) wks, [1000, 1500) g | Mortality | Males | | 158.563 (120.99 to 204.947) | 103.32 (83.486 to 127.144) | | | | | | | | | | | | | | | | | | | | | |
| Encephalitis | Birth prevalence - [28, 30) wks, [1000, 1500) g | Mortality | Females | | 153.905 (112.327 to 200.786) | 107.529 (86.954 to 131.78) | | | | | | | | | | | | | | | | | | | | | |
| Encephalitis | Birth prevalence - [32, 34) wks, [1000, 1500) g | Mortality | Males | | 117.142 (81.354 to 161.101) | 53.185 (43.049 to 66.274) | | | | | | | | | | | | | | | | | | | | | |
| Encephalitis | Birth prevalence - [32, 34) wks, [1000, 1500) g | Mortality | Females | | 115.171 (79.363 to 159.206) | 56.034 (45.982 to 68.36) | | | | | | | | | | | | | | | | | | | | | |
| Encephalitis | Birth prevalence - [30, 32) wks, [1000, 1500) g | Mortality | Males | | 119.308 (87.769 to 160.885) | 67.163 (54.863 to 82.638) | | | | | | | | | | | | | | | | | | | | | |
| Encephalitis | Birth prevalence - [30, 32) wks, [1000, 1500) g | Mortality | Females | | 115.448 (84.272 to 156.425) | 69.14 (55.873 to 85.012) | | | | | | | | | | | | | | | | | | | | | |
| Encephalitis | Birth prevalence - [37, 38) wks, [1500, 2000) g | Mortality | Males | | 62.972 (46.159 to 83.484) | 24.148 (20.066 to 29.406) | | | | | | | | | | | | | | | | | | | | | |
| Encephalitis | Birth prevalence - [37, 38) wks, [1500, 2000) g | Mortality | Females | | 59.988 (43.974 to 79.053) | 26.719 (21.746 to 32.816) | | | | | | | | | | | | | | | | | | | | | |
| Encephalitis | Birth prevalence - [36, 37) wks, [1500, 2000) g | Mortality | Males | | 60.218 (43.669 to 82.48) | 23.031 (18.793 to 28.483) | | | | | | | | | | | | | | | | | | | | | |
| Encephalitis | Birth prevalence - [36, 37) wks, [1500, 2000) g | Mortality | Females | | 58.527 (42.172 to 80.557) | 25.143 (20.331 to 30.566) | | | | | | | | | | | | | | | | | | | | | |
| Encephalitis | Birth prevalence - [30, 32) wks, [2000, 2500) g | Mortality | Males | | 67.971 (50.354 to 88.935) | 18.03 (14.621 to 22.103) | | | | | | | | | | | | | | | | | | | | | |
| Encephalitis | Birth prevalence - [30, 32) wks, [2000, 2500) g | Mortality | Females | | 69.383 (49.108 to 94.583) | 22.069 (17.836 to 27.163) | | | | | | | | | | | | | | | | | | | | | |
| Encephalitis | Birth prevalence - [30, 32) wks, [1500, 2000) g | Mortality | Males | | 77.369 (59.702 to 99.232) | 31.079 (25.786 to 36.724) | | | | | | | | | | | | | | | | | | | | | |
| Encephalitis | Birth prevalence - [30, 32) wks, [1500, 2000) g | Mortality | Females | | 76.134 (56.885 to 100.996) | 34.756 (28.764 to 41.849) | | | | | | | | | | | | | | | | | | | | | |
| Encephalitis | Birth prevalence - [34, 36) wks, [1500, 2000) g | Mortality | Males | | 55.555 (39.553 to 75.104) | 21.346 (17.677 to 26.143) | | | | | | | | | | | | | | | | | | | | | |
| Encephalitis | Birth prevalence - [34, 36) wks, [1500, 2000) g | Mortality | Females | | 54.335 (38.617 to 75.24) | 23.046 (18.743 to 28.287) | | | | | | | | | | | | | | | | | | | | | |
| Encephalitis | Birth prevalence - [32, 34) wks, [1500, 2000) g | Mortality | Males | | 57.155 (42.484 to 73.651) | 23.114 (19.028 to 27.915) | | | | | | | | | | | | | | | | | | | | | |
| Encephalitis | Birth prevalence - [32, 34) wks, [1500, 2000) g | Mortality | Females | | 56.101 (39.794 to 76.295) | 25.149 (20.615 to 30.388) | | | | | | | | | | | | | | | | | | | | | |
| Encephalitis | Birth prevalence - [32, 34) wks, [2000, 2500) g | Mortality | Males | | 37.444 (29.026 to 48.227) | 12.233 (10.252 to 14.477) | | | | | | | | | | | | | | | | | | | | | |
| Encephalitis | Birth prevalence - [32, 34) wks, [2000, 2500) g | Mortality | Females | | 36.874 (26.658 to 49.653) | 14.384 (12.095 to 17.03) | | | | | | | | | | | | | | | | | | | | | |
| Encephalitis | Birth prevalence - [40, 42) wks, [2000, 2500) g | Mortality | Males | | 18.092 (13.292 to 23.719) | 9.23 (7.037 to 11.454) | | | | | | | | | | | | | | | | | | | | | |

Appendix Table 6a. Relative risks used by age and sex for each outcome for all risk factors except for ambient air pollution alcohol, and smoking.

| | | | | Ages | | | | | | | | | | | | | | | | | | | | | | | |
|---|---|---|---|---|---|---|---|---|---|---|---|---|---|---|---|---|---|---|---|---|---|---|---|---|---|---|---|
| Risk - Outcome | Category / Units | Morbidity / Mortality | Sex | All-age | 0-6 days | 7-27 days | 28-364 days | 1-4 years | 5-9 years | 10-14 years | 15-19 years | 20-24 years | 25-29 years | 30-34 years | 35-39 years | 40-44 years | 45-49 years | 50-54 years | 55-59 years | 60-64 years | 65-69 years | 70-74 years | 75-79 years | 80-84 years | 85-89 years | 90-94 years | 95+ years |
| Encephalitis | Birth prevalence - [40, 42) wks, [2000, 2500) g | Mortality | Females | | 15.574 (11.516 to 20.778) | 9.975 (7.82 to 12.46) | | | | | | | | | | | | | | | | | | | | | |
| Encephalitis | Birth prevalence - [38, 40) wks, [2000, 2500) g | Mortality | Males | | 13.104 (9.829 to 16.99) | 8.198 (6.786 to 9.959) | | | | | | | | | | | | | | | | | | | | | |
| Encephalitis | Birth prevalence - [38, 40) wks, [2000, 2500) g | Mortality | Females | | 11.308 (8.389 to 14.38) | 8.577 (7.04 to 10.449) | | | | | | | | | | | | | | | | | | | | | |
| Encephalitis | Birth prevalence - [32, 34) wks, [2500, 3000) g | Mortality | Males | | 33.063 (24.393 to 43.503) | 8.441 (6.822 to 10.431) | | | | | | | | | | | | | | | | | | | | | |
| Encephalitis | Birth prevalence - [32, 34) wks, [2500, 3000) g | Mortality | Females | | 32.812 (23.439 to 45.567) | 10.398 (8.227 to 13.042) | | | | | | | | | | | | | | | | | | | | | |
| Encephalitis | Birth prevalence - [34, 36) wks, [2000, 2500) g | Mortality | Males | | 21.925 (16.305 to 29.433) | 9.367 (7.859 to 11.112) | | | | | | | | | | | | | | | | | | | | | |
| Encephalitis | Birth prevalence - [34, 36) wks, [2000, 2500) g | Mortality | Females | | 21.297 (15.657 to 28.761) | 10.295 (8.548 to 12.273) | | | | | | | | | | | | | | | | | | | | | |
| Encephalitis | Birth prevalence - [37, 38) wks, [2000, 2500) g | Mortality | Males | | 13.0 (10.102 to 16.456) | 8.096 (6.724 to 9.676) | | | | | | | | | | | | | | | | | | | | | |
| Encephalitis | Birth prevalence - [37, 38) wks, [2000, 2500) g | Mortality | Females | | 11.563 (8.805 to 15.11) | 8.467 (6.994 to 10.342) | | | | | | | | | | | | | | | | | | | | | |
| Encephalitis | Birth prevalence - [36, 37) wks, [2000, 2500) g | Mortality | Males | | 14.401 (10.789 to 18.654) | 8.221 (6.917 to 9.923) | | | | | | | | | | | | | | | | | | | | | |
| Encephalitis | Birth prevalence - [36, 37) wks, [2000, 2500) g | Mortality | Females | | 13.513 (9.817 to 17.942) | 8.654 (7.215 to 10.369) | | | | | | | | | | | | | | | | | | | | | |
| Encephalitis | Birth prevalence - [34, 36) wks, [2500, 3000) g | Mortality | Males | | 13.419 (10.387 to 16.819) | 5.562 (4.646 to 6.696) | | | | | | | | | | | | | | | | | | | | | |
| Encephalitis | Birth prevalence - [34, 36) wks, [2500, 3000) g | Mortality | Females | | 13.266 (9.666 to 17.689) | 6.395 (5.292 to 7.606) | | | | | | | | | | | | | | | | | | | | | |
| Encephalitis | Birth prevalence - [34, 36) wks, [4000, 4500) g | Mortality | Males | | 23.096 (14.708 to 35.098) | 2.895 (2.245 to 3.716) | | | | | | | | | | | | | | | | | | | | | |
| Encephalitis | Birth prevalence - [34, 36) wks, [4000, 4500) g | Mortality | Females | | 25.038 (15.763 to 37.255) | 3.778 (2.855 to 4.925) | | | | | | | | | | | | | | | | | | | | | |
| Encephalitis | Birth prevalence - [34, 36) wks, [3000, 3500) g | Mortality | Males | | 14.006 (10.222 to 18.478) | 4.322 (3.449 to 5.338) | | | | | | | | | | | | | | | | | | | | | |
| Encephalitis | Birth prevalence - [34, 36) wks, [3000, 3500) g | Mortality | Females | | 14.375 (10.269 to 20.114) | 5.265 (4.145 to 6.564) | | | | | | | | | | | | | | | | | | | | | |
| Encephalitis | Birth prevalence - [36, 37) wks, [2500, 3000) g | Mortality | Males | | 4.874 (4.014 to 5.713) | 3.699 (3.134 to 4.374) | | | | | | | | | | | | | | | | | | | | | |
| Encephalitis | Birth prevalence - [36, 37) wks, [2500, 3000) g | Mortality | Females | | 4.609 (3.731 to 5.61) | 3.898 (3.235 to 4.67) | | | | | | | | | | | | | | | | | | | | | |
| Encephalitis | Birth prevalence - [34, 36) wks, [3500, 4000) g | Mortality | Males | | 18.024 (12.279 to 25.547) | 3.657 (2.838 to 4.675) | | | | | | | | | | | | | | | | | | | | | |
| Encephalitis | Birth prevalence - [34, 36) wks, [3500, 4000) g | Mortality | Females | | 19.263 (12.567 to 27.924) | 4.634 (3.576 to 5.989) | | | | | | | | | | | | | | | | | | | | | |
| Encephalitis | Birth prevalence - [37, 38) wks, [2500, 3000) g | Mortality | Males | | 3.306 (2.82 to 3.843) | 3.194 (2.691 to 3.803) | | | | | | | | | | | | | | | | | | | | | |
| Encephalitis | Birth prevalence - [37, 38) wks, [2500, 3000) g | Mortality | Females | | 2.991 (2.496 to 3.61) | 3.242 (2.745 to 3.817) | | | | | | | | | | | | | | | | | | | | | |
| Encephalitis | Birth prevalence - [40, 42) wks, [2500, 3000) g | Mortality | Males | | 3.771 (3.002 to 4.693) | 3.175 (2.56 to 3.923) | | | | | | | | | | | | | | | | | | | | | |
| Encephalitis | Birth prevalence - [40, 42) wks, [2500, 3000) g | Mortality | Females | | 3.244 (2.486 to 4.159) | 3.228 (2.605 to 3.985) | | | | | | | | | | | | | | | | | | | | | |
| Encephalitis | Birth prevalence - [38, 40) wks, [2500, 3000) g | Mortality | Males | | 2.755 (2.274 to 3.309) | 2.944 (2.44 to 3.548) | | | | | | | | | | | | | | | | | | | | | |
| Encephalitis | Birth prevalence - [38, 40) wks, [2500, 3000) g | Mortality | Females | | 2.376 (1.91 to 2.886) | 2.938 (2.434 to 3.503) | | | | | | | | | | | | | | | | | | | | | |
| Encephalitis | Birth prevalence - [36, 37) wks, [3000, 3500) g | Mortality | Males | | 3.774 (3.094 to 4.497) | 2.466 (2.058 to 2.929) | | | | | | | | | | | | | | | | | | | | | |
| Encephalitis | Birth prevalence - [36, 37) wks, [3000, 3500) g | Mortality | Females | | 3.73 (2.981 to 4.646) | 2.715 (2.277 to 3.218) | | | | | | | | | | | | | | | | | | | | | |
| Encephalitis | Birth prevalence - [36, 37) wks, [4000, 4500) g | Mortality | Males | | 6.826 (5.212 to 9.045) | 1.77 (1.491 to 2.082) | | | | | | | | | | | | | | | | | | | | | |
| Encephalitis | Birth prevalence - [36, 37) wks, [4000, 4500) g | Mortality | Females | | 7.269 (5.144 to 9.821) | 2.177 (1.786 to 2.607) | | | | | | | | | | | | | | | | | | | | | |
| Encephalitis | Birth prevalence - [36, 37) wks, [3500, 4000) g | Mortality | Males | | 4.544 (3.64 to 5.622) | 2.057 (1.74 to 2.44) | | | | | | | | | | | | | | | | | | | | | |
| Encephalitis | Birth prevalence - [36, 37) wks, [3500, 4000) g | Mortality | Females | | 4.662 (3.577 to 6.014) | 2.398 (1.98 to 2.864) | | | | | | | | | | | | | | | | | | | | | |
| Encephalitis | Birth prevalence - [37, 38) wks, [3000, 3500) g | Mortality | Males | | 2.007 (1.759 to 2.293) | 1.888 (1.613 to 2.224) | | | | | | | | | | | | | | | | | | | | | |
| Encephalitis | Birth prevalence - [37, 38) wks, [3000, 3500) g | Mortality | Females | | 1.925 (1.582 to 2.328) | 1.972 (1.669 to 2.313) | | | | | | | | | | | | | | | | | | | | | |
| Encephalitis | Birth prevalence - [37, 38) wks, [4000, 4500) g | Mortality | Males | | 3.28 (2.596 to 4.133) | 1.335 (1.171 to 1.532) | | | | | | | | | | | | | | | | | | | | | |
| Encephalitis | Birth prevalence - [37, 38) wks, [4000, 4500) g | Mortality | Females | | 3.521 (2.649 to 4.5) | 1.559 (1.333 to 1.835) | | | | | | | | | | | | | | | | | | | | | |
| Encephalitis | Birth prevalence - [37, 38) wks, [3500, 4000) g | Mortality | Males | | 2.128 (1.833 to 2.466) | 1.505 (1.299 to 1.76) | | | | | | | | | | | | | | | | | | | | | |
| Encephalitis | Birth prevalence - [37, 38) wks, [3500, 4000) g | Mortality | Females | | 2.142 (1.694 to 2.67) | 1.661 (1.411 to 1.961) | | | | | | | | | | | | | | | | | | | | | |
| Encephalitis | Birth prevalence - [40, 42) wks, [3000, 3500) g | Mortality | Males | | 1.436 (1.245 to 1.65) | 1.47 (1.199 to 1.8) | | | | | | | | | | | | | | | | | | | | | |
| Encephalitis | Birth prevalence - [40, 42) wks, [3000, 3500) g | Mortality | Females | | 1.326 (1.069 to 1.614) | 1.465 (1.188 to 1.775) | | | | | | | | | | | | | | | | | | | | | |
| Encephalitis | Birth prevalence - [38, 40) wks, [3000, 3500) g | Mortality | Males | | 1.33 (1.155 to 1.53) | 1.559 (1.305 to 1.851) | | | | | | | | | | | | | | | | | | | | | |
| Encephalitis | Birth prevalence - [38, 40) wks, [3000, 3500) g | Mortality | Females | | 1.224 (1.0 to 1.492) | 1.564 (1.304 to 1.847) | | | | | | | | | | | | | | | | | | | | | |
| Encephalitis | Birth prevalence - [38, 40) wks, [4000, 4500) g | Mortality | Males | | 1.787 (1.453 to 2.182) | 1.175 (1.005 to 1.371) | | | | | | | | | | | | | | | | | | | | | |
| Encephalitis | Birth prevalence - [38, 40) wks, [4000, 4500) g | Mortality | Females | | 1.877 (1.467 to 2.388) | 1.224 (1.022 to 1.465) | | | | | | | | | | | | | | | | | | | | | |
| Encephalitis | Birth prevalence - [38, 40) wks, [3500, 4000) g | Mortality | Males | | 1.785 (1.478 to 2.147) | 1.173 (1.0 to 1.377) | | | | | | | | | | | | | | | | | | | | | |
| Encephalitis | Birth prevalence - [38, 40) wks, [3500, 4000) g | Mortality | Females | | 1.892 (1.481 to 2.352) | 1.23 (1.03 to 1.46) | | | | | | | | | | | | | | | | | | | | | |
| Encephalitis | Birth prevalence - [40, 42) wks, [3500, 4000) g | Mortality | Males | | 1.0 (1.0 to 1.0) | 1.003 (1.0 to 1.046) | | | | | | | | | | | | | | | | | | | | | |
| Encephalitis | Birth prevalence - [40, 42) wks, [3500, 4000) g | Mortality | Females | | 1.002 (1.0 to 1.013) | 1.001 (1.0 to 1.006) | | | | | | | | | | | | | | | | | | | | | |
| Encephalitis | Birth prevalence - [40, 42) wks, [4000, 4500) g | Mortality | Males | | 1.0 (1.0 to 1.0) | 1.0 (1.0 to 1.0) | | | | | | | | | | | | | | | | | | | | | |
| Encephalitis | Birth prevalence - [40, 42) wks, [4000, 4500) g | Mortality | Females | | 1.0 (1.0 to 1.0) | 1.0 (1.0 to 1.0) | | | | | | | | | | | | | | | | | | | | | |
| Encephalitis | Birth prevalence - [28, 30) wks, [2000, 2500) g | Mortality | Males | | 117.172 (83.895 to 158.056) | 27.726 (21.877 to 34.972) | | | | | | | | | | | | | | | | | | | | | |
| Encephalitis | Birth prevalence - [28, 30) wks, [2000, 2500) g | Mortality | Females | | 121.682 (84.349 to 171.375) | 33.983 (26.101 to 43.404) | | | | | | | | | | | | | | | | | | | | | |
| Encephalitis | Birth prevalence - [28, 30) wks, [2500, 3000) g | Mortality | Males | | 77.948 (54.687 to 105.047) | 16.608 (12.653 to 21.188) | | | | | | | | | | | | | | | | | | | | | |
| Encephalitis | Birth prevalence - [28, 30) wks, [2500, 3000) g | Mortality | Females | | 79.193 (54.099 to 112.236) | 20.387 (15.089 to 26.434) | | | | | | | | | | | | | | | | | | | | | |
| Encephalitis | Birth prevalence - [28, 30) wks, [3000, 3500) g | Mortality | Males | | 42.199 (29.891 to 57.227) | 10.082 (7.777 to 13.056) | | | | | | | | | | | | | | | | | | | | | |
| Encephalitis | Birth prevalence - [28, 30) wks, [3000, 3500) g | Mortality | Females | | 42.551 (28.25 to 63.209) | 11.989 (9.084 to 15.544) | | | | | | | | | | | | | | | | | | | | | |

**Appendix Table 6a. Relative risks used by age and sex for each outcome for all risk factors except for ambient air pollution alcohol, and smoking.**

| | | | | Ages | | | | | | | | | | | | | | | | | | | | | | | |
|---|---|---|---|---|---|---|---|---|---|---|---|---|---|---|---|---|---|---|---|---|---|---|---|---|---|---|---|
| Risk - Outcome | Category / Units | Morbidity / Mortality | Sex | All-age | 0-6 days | 7-27 days | 28-364 days | 1-4 years | 5-9 years | 10-14 years | 15-19 years | 20-24 years | 25-29 years | 30-34 years | 35-39 years | 40-44 years | 45-49 years | 50-54 years | 55-59 years | 60-64 years | 65-69 years | 70-74 years | 75-79 years | 80-84 years | 85-89 years | 90-94 years | 95+ years |
| Encephalitis | Birth prevalence - [30, 32) wks, [2500, 3000) g | Mortality | Males | | 58.722 (42.419 to 78.873) | 12.115 (9.518 to 15.521) | | | | | | | | | | | | | | | | | | | | | |
| Encephalitis | Birth prevalence - [30, 32) wks, [2500, 3000) g | Mortality | Females | | 59.522 (42.058 to 82.793) | 15.364 (11.936 to 19.581) | | | | | | | | | | | | | | | | | | | | | |
| Encephalitis | Birth prevalence - [30, 32) wks, [3000, 3500) g | Mortality | Males | | 45.67 (32.014 to 65.531) | 8.381 (6.362 to 10.842) | | | | | | | | | | | | | | | | | | | | | |
| Encephalitis | Birth prevalence - [30, 32) wks, [3000, 3500) g | Mortality | Females | | 46.104 (30.243 to 66.207) | 10.506 (8.041 to 13.513) | | | | | | | | | | | | | | | | | | | | | |
| Encephalitis | Birth prevalence - [30, 32) wks, [3500, 4000) g | Mortality | Males | | 36.334 (21.558 to 54.813) | 5.698 (4.293 to 7.349) | | | | | | | | | | | | | | | | | | | | | |
| Encephalitis | Birth prevalence - [30, 32) wks, [3500, 4000) g | Mortality | Females | | 37.931 (22.692 to 61.276) | 6.892 (5.175 to 9.058) | | | | | | | | | | | | | | | | | | | | | |
| Encephalitis | Birth prevalence - [32, 34) wks, [3000, 3500) g | Mortality | Males | | 34.016 (23.515 to 48.37) | 6.577 (4.934 to 8.436) | | | | | | | | | | | | | | | | | | | | | |
| Encephalitis | Birth prevalence - [32, 34) wks, [3000, 3500) g | Mortality | Females | | 34.585 (22.909 to 49.754) | 8.314 (6.362 to 10.789) | | | | | | | | | | | | | | | | | | | | | |
| Encephalitis | Birth prevalence - [32, 34) wks, [3500, 4000) g | Mortality | Males | | 36.248 (23.158 to 54.67) | 5.068 (3.761 to 6.741) | | | | | | | | | | | | | | | | | | | | | |
| Encephalitis | Birth prevalence - [32, 34) wks, [3500, 4000) g | Mortality | Females | | 38.098 (23.301 to 59.429) | 6.476 (4.666 to 8.689) | | | | | | | | | | | | | | | | | | | | | |
| Encephalitis | Birth prevalence - [36, 37) wks, [1000, 1500) g | Mortality | Males | | 166.686 (118.487 to 222.581) | 57.535 (45.999 to 71.742) | | | | | | | | | | | | | | | | | | | | | |
| Encephalitis | Birth prevalence - [36, 37) wks, [1000, 1500) g | Mortality | Females | | 169.725 (119.017 to 229.008) | 63.564 (50.068 to 80.703) | | | | | | | | | | | | | | | | | | | | | |
| Encephalitis | Birth prevalence - [38, 40) wks, [1000, 1500) g | Mortality | Males | | 174.066 (125.125 to 232.507) | 57.966 (44.393 to 73.241) | | | | | | | | | | | | | | | | | | | | | |
| Encephalitis | Birth prevalence - [38, 40) wks, [1000, 1500) g | Mortality | Females | | 171.557 (121.585 to 237.047) | 65.208 (48.821 to 84.308) | | | | | | | | | | | | | | | | | | | | | |
| Encephalitis | Birth prevalence - [38, 40) wks, [1500, 2000) g | Mortality | Males | | 67.302 (49.547 to 89.055) | 25.206 (20.365 to 31.168) | | | | | | | | | | | | | | | | | | | | | |
| Encephalitis | Birth prevalence - [38, 40) wks, [1500, 2000) g | Mortality | Females | | 62.19 (45.884 to 83.445) | 28.05 (22.625 to 35.139) | | | | | | | | | | | | | | | | | | | | | |
| Encephalitis | Birth prevalence - [40, 42) wks, [1500, 2000) g | Mortality | Males | | 76.673 (56.177 to 102.468) | 25.785 (19.387 to 34.168) | | | | | | | | | | | | | | | | | | | | | |
| Encephalitis | Birth prevalence - [40, 42) wks, [1500, 2000) g | Mortality | Females | | 70.411 (49.221 to 97.952) | 29.113 (21.355 to 38.272) | | | | | | | | | | | | | | | | | | | | | |
| Neonatal preterm birth | Birth prevalence - [0, 24) wks, [0, 500) g | Mortality | Males | | 1564.792 (1056.542 to [illegible] | 618.595 (458.842 to 812.921) | | | | | | | | | | | | | | | | | | | | | |
| Neonatal preterm birth | Birth prevalence - [0, 24) wks, [0, 500) g | Mortality | Females | | 1600.122 (1050.664 to [illegible] | 713.571 (526.178 to 921.018) | | | | | | | | | | | | | | | | | | | | | |
| Neonatal preterm birth | Birth prevalence - [0, 24) wks, [500, 1000) g | Mortality | Males | | 1155.815 (825.412 to 1506.837) | 457.5 (352.552 to 573.483) | | | | | | | | | | | | | | | | | | | | | |
| Neonatal preterm birth | Birth prevalence - [0, 24) wks, [500, 1000) g | Mortality | Females | | 1169.123 (802.003 to 1617.979) | 515.406 (396.713 to 641.541) | | | | | | | | | | | | | | | | | | | | | |
| Neonatal preterm birth | Birth prevalence - [24, 26) wks, [500, 1000) g | Mortality | Males | | 955.583 (723.748 to 1244.265) | 443.357 (363.03 to 534.695) | | | | | | | | | | | | | | | | | | | | | |
| Neonatal preterm birth | Birth prevalence - [24, 26) wks, [500, 1000) g | Mortality | Females | | 947.143 (702.662 to 1237.093) | 487.549 (387.307 to 603.498) | | | | | | | | | | | | | | | | | | | | | |
| Neonatal preterm birth | Birth prevalence - [26, 28) wks, [500, 1000) g | Mortality | Males | | 497.817 (377.617 to 648.547) | 330.886 (261.438 to 401.709) | | | | | | | | | | | | | | | | | | | | | |
| Neonatal preterm birth | Birth prevalence - [26, 28) wks, [500, 1000) g | Mortality | Females | | 483.682 (354.946 to 629.517) | 344.618 (274.427 to 419.864) | | | | | | | | | | | | | | | | | | | | | |
| Neonatal preterm birth | Birth prevalence - [30, 32) wks, [500, 1000) g | Mortality | Males | | 236.614 (163.821 to 324.502) | 149.995 (117.866 to 188.368) | | | | | | | | | | | | | | | | | | | | | |
| Neonatal preterm birth | Birth prevalence - [30, 32) wks, [500, 1000) g | Mortality | Females | | 229.197 (157.606 to 317.194) | 152.117 (120.779 to 190.583) | | | | | | | | | | | | | | | | | | | | | |
| Neonatal preterm birth | Birth prevalence - [28, 30) wks, [500, 1000) g | Mortality | Males | | 297.629 (214.953 to 396.586) | 216.995 (173.321 to 271.466) | | | | | | | | | | | | | | | | | | | | | |
| Neonatal preterm birth | Birth prevalence - [28, 30) wks, [500, 1000) g | Mortality | Females | | 281.056 (198.176 to 386.635) | 219.884 (174.264 to 272.704) | | | | | | | | | | | | | | | | | | | | | |
| Neonatal preterm birth | Birth prevalence - [26, 28) wks, [1000, 1500) g | Mortality | Males | | 267.91 (210.177 to 332.92) | 164.167 (132.898 to 200.569) | | | | | | | | | | | | | | | | | | | | | |
| Neonatal preterm birth | Birth prevalence - [26, 28) wks, [1000, 1500) g | Mortality | Females | | 266.509 (197.461 to 346.932) | 174.222 (137.431 to 217.349) | | | | | | | | | | | | | | | | | | | | | |
| Neonatal preterm birth | Birth prevalence - [34, 36) wks, [1000, 1500) g | Mortality | Males | | 142.056 (98.086 to 197.774) | 52.86 (42.914 to 64.617) | | | | | | | | | | | | | | | | | | | | | |
| Neonatal preterm birth | Birth prevalence - [34, 36) wks, [1000, 1500) g | Mortality | Females | | 141.899 (95.864 to 197.656) | 57.421 (46.452 to 71.339) | | | | | | | | | | | | | | | | | | | | | |
| Neonatal preterm birth | Birth prevalence - [28, 30) wks, [1500, 2000) g | Mortality | Males | | 127.966 (97.178 to 167.026) | 50.018 (40.539 to 61.919) | | | | | | | | | | | | | | | | | | | | | |
| Neonatal preterm birth | Birth prevalence - [28, 30) wks, [1500, 2000) g | Mortality | Females | | 130.924 (96.513 to 172.188) | 57.275 (46.36 to 70.038) | | | | | | | | | | | | | | | | | | | | | |
| Neonatal preterm birth | Birth prevalence - [28, 30) wks, [1000, 1500) g | Mortality | Males | | 158.563 (120.99 to 204.947) | 103.32 (83.486 to 127.144) | | | | | | | | | | | | | | | | | | | | | |
| Neonatal preterm birth | Birth prevalence - [28, 30) wks, [1000, 1500) g | Mortality | Females | | 153.905 (112.327 to 200.786) | 107.529 (86.954 to 131.78) | | | | | | | | | | | | | | | | | | | | | |
| Neonatal preterm birth | Birth prevalence - [32, 34) wks, [1000, 1500) g | Mortality | Males | | 117.142 (81.354 to 161.101) | 53.185 (43.049 to 66.274) | | | | | | | | | | | | | | | | | | | | | |
| Neonatal preterm birth | Birth prevalence - [32, 34) wks, [1000, 1500) g | Mortality | Females | | 115.171 (79.363 to 159.206) | 56.034 (45.982 to 68.36) | | | | | | | | | | | | | | | | | | | | | |
| Neonatal preterm birth | Birth prevalence - [30, 32) wks, [1000, 1500) g | Mortality | Males | | 119.308 (87.769 to 160.885) | 67.163 (54.863 to 82.638) | | | | | | | | | | | | | | | | | | | | | |
| Neonatal preterm birth | Birth prevalence - [30, 32) wks, [1000, 1500) g | Mortality | Females | | 115.448 (84.272 to 156.425) | 69.14 (55.873 to 85.012) | | | | | | | | | | | | | | | | | | | | | |
| Neonatal preterm birth | Birth prevalence - [37, 38) wks, [1500, 2000) g | Mortality | Males | | 62.972 (46.159 to 83.484) | 24.148 (20.066 to 29.406) | | | | | | | | | | | | | | | | | | | | | |
| Neonatal preterm birth | Birth prevalence - [37, 38) wks, [1500, 2000) g | Mortality | Females | | 59.988 (43.974 to 79.053) | 26.719 (21.746 to 32.816) | | | | | | | | | | | | | | | | | | | | | |
| Neonatal preterm birth | Birth prevalence - [36, 37) wks, [1500, 2000) g | Mortality | Males | | 60.218 (43.669 to 82.48) | 23.031 (18.793 to 28.483) | | | | | | | | | | | | | | | | | | | | | |
| Neonatal preterm birth | Birth prevalence - [36, 37) wks, [1500, 2000) g | Mortality | Females | | 58.527 (42.172 to 80.557) | 25.143 (20.331 to 30.566) | | | | | | | | | | | | | | | | | | | | | |
| Neonatal preterm birth | Birth prevalence - [30, 32) wks, [2000, 2500) g | Mortality | Males | | 67.971 (50.354 to 88.935) | 18.03 (14.621 to 22.103) | | | | | | | | | | | | | | | | | | | | | |
| Neonatal preterm birth | Birth prevalence - [30, 32) wks, [2000, 2500) g | Mortality | Females | | 69.383 (49.108 to 94.583) | 22.069 (17.836 to 27.163) | | | | | | | | | | | | | | | | | | | | | |
| Neonatal preterm birth | Birth prevalence - [30, 32) wks, [1500, 2000) g | Mortality | Males | | 77.369 (59.702 to 99.232) | 31.079 (25.786 to 36.724) | | | | | | | | | | | | | | | | | | | | | |
| Neonatal preterm birth | Birth prevalence - [30, 32) wks, [1500, 2000) g | Mortality | Females | | 76.134 (56.885 to 100.996) | 34.756 (28.764 to 41.849) | | | | | | | | | | | | | | | | | | | | | |
| Neonatal preterm birth | Birth prevalence - [34, 36) wks, [1500, 2000) g | Mortality | Males | | 55.555 (39.553 to 75.104) | 21.346 (17.677 to 26.143) | | | | | | | | | | | | | | | | | | | | | |
| Neonatal preterm birth | Birth prevalence - [34, 36) wks, [1500, 2000) g | Mortality | Females | | 54.335 (38.617 to 75.24) | 23.046 (18.743 to 28.287) | | | | | | | | | | | | | | | | | | | | | |
| Neonatal preterm birth | Birth prevalence - [32, 34) wks, [1500, 2000) g | Mortality | Males | | 57.155 (42.484 to 73.651) | 23.114 (19.028 to 27.915) | | | | | | | | | | | | | | | | | | | | | |
| Neonatal preterm birth | Birth prevalence - [32, 34) wks, [1500, 2000) g | Mortality | Females | | 56.101 (39.794 to 76.295) | 25.149 (20.615 to 30.388) | | | | | | | | | | | | | | | | | | | | | |
| Neonatal preterm birth | Birth prevalence - [32, 34) wks, [2000, 2500) g | Mortality | Males | | 37.444 (29.026 to 48.227) | 12.233 (10.252 to 14.477) | | | | | | | | | | | | | | | | | | | | | |
| Neonatal preterm birth | Birth prevalence - [32, 34) wks, [2000, 2500) g | Mortality | Females | | 36.874 (26.658 to 49.653) | 14.384 (12.095 to 17.03) | | | | | | | | | | | | | | | | | | | | | |
| Neonatal preterm birth | Birth prevalence - [40, 42) wks, [2000, 2500) g | Mortality | Males | | 18.092 (13.292 to 23.719) | 9.23 (7.037 to 11.454) | | | | | | | | | | | | | | | | | | | | | |

**Appendix Table 6a. Relative risks used by age and sex for each outcome for all risk factors except for ambient air pollution alcohol, and smoking.**

| | | | | Ages | | | | | | | | | | | | | | | | | | | | | | | |
|---|---|---|---|---|---|---|---|---|---|---|---|---|---|---|---|---|---|---|---|---|---|---|---|---|---|---|---|
| Risk - Outcome | Category / Units | Morbidity / Mortality | Sex | All-age | 0-6 days | 7-27 days | 28-364 days | 1-4 years | 5-9 years | 10-14 years | 15-19 years | 20-24 years | 25-29 years | 30-34 years | 35-39 years | 40-44 years | 45-49 years | 50-54 years | 55-59 years | 60-64 years | 65-69 years | 70-74 years | 75-79 years | 80-84 years | 85-89 years | 90-94 years | 95+ years |
| Neonatal preterm birth | Birth prevalence - [40, 42) wks, [2000, 2500) g | Mortality | Females | | 15.574 (11.516 to 20.778) | 9.975 (7.82 to 12.46) | | | | | | | | | | | | | | | | | | | | | |
| Neonatal preterm birth | Birth prevalence - [38, 40) wks, [2000, 2500) g | Mortality | Males | | 13.104 (9.829 to 16.99) | 8.198 (6.786 to 9.959) | | | | | | | | | | | | | | | | | | | | | |
| Neonatal preterm birth | Birth prevalence - [38, 40) wks, [2000, 2500) g | Mortality | Females | | 11.308 (8.389 to 14.38) | 8.577 (7.04 to 10.449) | | | | | | | | | | | | | | | | | | | | | |
| Neonatal preterm birth | Birth prevalence - [32, 34) wks, [2500, 3000) g | Mortality | Males | | 33.063 (24.393 to 43.503) | 8.441 (6.822 to 10.431) | | | | | | | | | | | | | | | | | | | | | |
| Neonatal preterm birth | Birth prevalence - [32, 34) wks, [2500, 3000) g | Mortality | Females | | 32.812 (23.439 to 45.567) | 10.398 (8.227 to 13.042) | | | | | | | | | | | | | | | | | | | | | |
| Neonatal preterm birth | Birth prevalence - [34, 36) wks, [2000, 2500) g | Mortality | Males | | 21.925 (16.305 to 29.433) | 9.367 (7.859 to 11.112) | | | | | | | | | | | | | | | | | | | | | |
| Neonatal preterm birth | Birth prevalence - [34, 36) wks, [2000, 2500) g | Mortality | Females | | 21.297 (15.657 to 28.761) | 10.295 (8.548 to 12.273) | | | | | | | | | | | | | | | | | | | | | |
| Neonatal preterm birth | Birth prevalence - [37, 38) wks, [2000, 2500) g | Mortality | Males | | 13.0 (10.102 to 16.456) | 8.096 (6.724 to 9.676) | | | | | | | | | | | | | | | | | | | | | |
| Neonatal preterm birth | Birth prevalence - [37, 38) wks, [2000, 2500) g | Mortality | Females | | 11.563 (8.805 to 15.11) | 8.467 (6.994 to 10.342) | | | | | | | | | | | | | | | | | | | | | |
| Neonatal preterm birth | Birth prevalence - [36, 37) wks, [2000, 2500) g | Mortality | Males | | 14.401 (10.789 to 18.654) | 8.221 (6.917 to 9.923) | | | | | | | | | | | | | | | | | | | | | |
| Neonatal preterm birth | Birth prevalence - [36, 37) wks, [2000, 2500) g | Mortality | Females | | 13.513 (9.817 to 17.942) | 8.654 (7.215 to 10.369) | | | | | | | | | | | | | | | | | | | | | |
| Neonatal preterm birth | Birth prevalence - [34, 36) wks, [2500, 3000) g | Mortality | Males | | 13.419 (10.387 to 16.819) | 5.562 (4.646 to 6.696) | | | | | | | | | | | | | | | | | | | | | |
| Neonatal preterm birth | Birth prevalence - [34, 36) wks, [2500, 3000) g | Mortality | Females | | 13.266 (9.666 to 17.689) | 6.395 (5.292 to 7.606) | | | | | | | | | | | | | | | | | | | | | |
| Neonatal preterm birth | Birth prevalence - [34, 36) wks, [4000, 4500) g | Mortality | Males | | 23.096 (14.708 to 35.098) | 2.895 (2.245 to 3.716) | | | | | | | | | | | | | | | | | | | | | |
| Neonatal preterm birth | Birth prevalence - [34, 36) wks, [4000, 4500) g | Mortality | Females | | 25.038 (15.763 to 37.255) | 3.778 (2.855 to 4.925) | | | | | | | | | | | | | | | | | | | | | |
| Neonatal preterm birth | Birth prevalence - [34, 36) wks, [3000, 3500) g | Mortality | Males | | 14.006 (10.222 to 18.478) | 4.322 (3.449 to 5.338) | | | | | | | | | | | | | | | | | | | | | |
| Neonatal preterm birth | Birth prevalence - [34, 36) wks, [3000, 3500) g | Mortality | Females | | 14.375 (10.269 to 20.114) | 5.265 (4.145 to 6.564) | | | | | | | | | | | | | | | | | | | | | |
| Neonatal preterm birth | Birth prevalence - [36, 37) wks, [2500, 3000) g | Mortality | Males | | 4.874 (4.014 to 5.713) | 3.699 (3.134 to 4.374) | | | | | | | | | | | | | | | | | | | | | |
| Neonatal preterm birth | Birth prevalence - [36, 37) wks, [2500, 3000) g | Mortality | Females | | 4.609 (3.731 to 5.61) | 3.898 (3.235 to 4.67) | | | | | | | | | | | | | | | | | | | | | |
| Neonatal preterm birth | Birth prevalence - [34, 36) wks, [3500, 4000) g | Mortality | Males | | 18.024 (12.279 to 25.547) | 3.657 (2.838 to 4.675) | | | | | | | | | | | | | | | | | | | | | |
| Neonatal preterm birth | Birth prevalence - [34, 36) wks, [3500, 4000) g | Mortality | Females | | 19.263 (12.567 to 27.924) | 4.634 (3.576 to 5.989) | | | | | | | | | | | | | | | | | | | | | |
| Neonatal preterm birth | Birth prevalence - [37, 38) wks, [2500, 3000) g | Mortality | Males | | 3.306 (2.82 to 3.843) | 3.194 (2.691 to 3.803) | | | | | | | | | | | | | | | | | | | | | |
| Neonatal preterm birth | Birth prevalence - [37, 38) wks, [2500, 3000) g | Mortality | Females | | 2.991 (2.496 to 3.61) | 3.242 (2.745 to 3.817) | | | | | | | | | | | | | | | | | | | | | |
| Neonatal preterm birth | Birth prevalence - [40, 42) wks, [2500, 3000) g | Mortality | Males | | 3.771 (3.002 to 4.693) | 3.175 (2.56 to 3.923) | | | | | | | | | | | | | | | | | | | | | |
| Neonatal preterm birth | Birth prevalence - [40, 42) wks, [2500, 3000) g | Mortality | Females | | 3.244 (2.486 to 4.159) | 3.228 (2.605 to 3.985) | | | | | | | | | | | | | | | | | | | | | |
| Neonatal preterm birth | Birth prevalence - [38, 40) wks, [2500, 3000) g | Mortality | Males | | 2.755 (2.274 to 3.309) | 2.944 (2.44 to 3.548) | | | | | | | | | | | | | | | | | | | | | |
| Neonatal preterm birth | Birth prevalence - [38, 40) wks, [2500, 3000) g | Mortality | Females | | 2.376 (1.91 to 2.886) | 2.938 (2.434 to 3.503) | | | | | | | | | | | | | | | | | | | | | |
| Neonatal preterm birth | Birth prevalence - [36, 37) wks, [3000, 3500) g | Mortality | Males | | 3.774 (3.094 to 4.497) | 2.466 (2.058 to 2.929) | | | | | | | | | | | | | | | | | | | | | |
| Neonatal preterm birth | Birth prevalence - [36, 37) wks, [3000, 3500) g | Mortality | Females | | 3.73 (2.981 to 4.646) | 2.715 (2.277 to 3.218) | | | | | | | | | | | | | | | | | | | | | |
| Neonatal preterm birth | Birth prevalence - [36, 37) wks, [4000, 4500) g | Mortality | Males | | 6.826 (5.212 to 9.045) | 1.77 (1.491 to 2.082) | | | | | | | | | | | | | | | | | | | | | |
| Neonatal preterm birth | Birth prevalence - [36, 37) wks, [4000, 4500) g | Mortality | Females | | 7.269 (5.144 to 9.821) | 2.177 (1.786 to 2.607) | | | | | | | | | | | | | | | | | | | | | |
| Neonatal preterm birth | Birth prevalence - [36, 37) wks, [3500, 4000) g | Mortality | Males | | 4.544 (3.64 to 5.622) | 2.057 (1.74 to 2.44) | | | | | | | | | | | | | | | | | | | | | |
| Neonatal preterm birth | Birth prevalence - [36, 37) wks, [3500, 4000) g | Mortality | Females | | 4.662 (3.577 to 6.014) | 2.398 (1.98 to 2.864) | | | | | | | | | | | | | | | | | | | | | |
| Neonatal preterm birth | Birth prevalence - [37, 38) wks, [3000, 3500) g | Mortality | Males | | 2.007 (1.759 to 2.293) | 1.888 (1.613 to 2.224) | | | | | | | | | | | | | | | | | | | | | |
| Neonatal preterm birth | Birth prevalence - [37, 38) wks, [3000, 3500) g | Mortality | Females | | 1.925 (1.582 to 2.328) | 1.972 (1.669 to 2.313) | | | | | | | | | | | | | | | | | | | | | |
| Neonatal preterm birth | Birth prevalence - [37, 38) wks, [4000, 4500) g | Mortality | Males | | 3.28 (2.596 to 4.133) | 1.335 (1.171 to 1.532) | | | | | | | | | | | | | | | | | | | | | |
| Neonatal preterm birth | Birth prevalence - [37, 38) wks, [4000, 4500) g | Mortality | Females | | 3.521 (2.649 to 4.5) | 1.559 (1.333 to 1.835) | | | | | | | | | | | | | | | | | | | | | |
| Neonatal preterm birth | Birth prevalence - [37, 38) wks, [3500, 4000) g | Mortality | Males | | 2.128 (1.833 to 2.466) | 1.505 (1.299 to 1.76) | | | | | | | | | | | | | | | | | | | | | |
| Neonatal preterm birth | Birth prevalence - [37, 38) wks, [3500, 4000) g | Mortality | Females | | 2.142 (1.694 to 2.67) | 1.661 (1.411 to 1.961) | | | | | | | | | | | | | | | | | | | | | |
| Neonatal preterm birth | Birth prevalence - [40, 42) wks, [3000, 3500) g | Mortality | Males | | 1.436 (1.245 to 1.65) | 1.47 (1.199 to 1.8) | | | | | | | | | | | | | | | | | | | | | |
| Neonatal preterm birth | Birth prevalence - [40, 42) wks, [3000, 3500) g | Mortality | Females | | 1.326 (1.069 to 1.614) | 1.465 (1.188 to 1.775) | | | | | | | | | | | | | | | | | | | | | |
| Neonatal preterm birth | Birth prevalence - [38, 40) wks, [3000, 3500) g | Mortality | Males | | 1.33 (1.155 to 1.53) | 1.559 (1.305 to 1.851) | | | | | | | | | | | | | | | | | | | | | |
| Neonatal preterm birth | Birth prevalence - [38, 40) wks, [3000, 3500) g | Mortality | Females | | 1.224 (1.0 to 1.492) | 1.564 (1.304 to 1.847) | | | | | | | | | | | | | | | | | | | | | |
| Neonatal preterm birth | Birth prevalence - [38, 40) wks, [4000, 4500) g | Mortality | Males | | 1.787 (1.453 to 2.182) | 1.175 (1.005 to 1.371) | | | | | | | | | | | | | | | | | | | | | |
| Neonatal preterm birth | Birth prevalence - [38, 40) wks, [4000, 4500) g | Mortality | Females | | 1.877 (1.467 to 2.388) | 1.224 (1.022 to 1.465) | | | | | | | | | | | | | | | | | | | | | |
| Neonatal preterm birth | Birth prevalence - [38, 40) wks, [3500, 4000) g | Mortality | Males | | 1.785 (1.478 to 2.147) | 1.173 (1.0 to 1.377) | | | | | | | | | | | | | | | | | | | | | |
| Neonatal preterm birth | Birth prevalence - [38, 40) wks, [3500, 4000) g | Mortality | Females | | 1.892 (1.481 to 2.352) | 1.23 (1.03 to 1.46) | | | | | | | | | | | | | | | | | | | | | |
| Neonatal preterm birth | Birth prevalence - [40, 42) wks, [3500, 4000) g | Mortality | Males | | 1.0 (1.0 to 1.0) | 1.003 (1.0 to 1.046) | | | | | | | | | | | | | | | | | | | | | |
| Neonatal preterm birth | Birth prevalence - [40, 42) wks, [3500, 4000) g | Mortality | Females | | 1.002 (1.0 to 1.013) | 1.001 (1.0 to 1.006) | | | | | | | | | | | | | | | | | | | | | |
| Neonatal preterm birth | Birth prevalence - [40, 42) wks, [4000, 4500) g | Mortality | Males | | 1.0 (1.0 to 1.0) | 1.0 (1.0 to 1.0) | | | | | | | | | | | | | | | | | | | | | |
| Neonatal preterm birth | Birth prevalence - [40, 42) wks, [4000, 4500) g | Mortality | Females | | 1.0 (1.0 to 1.0) | 1.0 (1.0 to 1.0) | | | | | | | | | | | | | | | | | | | | | |
| Neonatal preterm birth | Birth prevalence - [28, 30) wks, [2000, 2500) g | Mortality | Males | | 117.172 (83.895 to 158.056) | 27.726 (21.877 to 34.972) | | | | | | | | | | | | | | | | | | | | | |
| Neonatal preterm birth | Birth prevalence - [28, 30) wks, [2000, 2500) g | Mortality | Females | | 121.682 (84.349 to 171.375) | 33.983 (26.101 to 43.404) | | | | | | | | | | | | | | | | | | | | | |
| Neonatal preterm birth | Birth prevalence - [28, 30) wks, [2500, 3000) g | Mortality | Males | | 77.948 (54.687 to 105.047) | 16.608 (12.653 to 21.188) | | | | | | | | | | | | | | | | | | | | | |
| Neonatal preterm birth | Birth prevalence - [28, 30) wks, [2500, 3000) g | Mortality | Females | | 79.193 (54.099 to 112.236) | 20.387 (15.089 to 26.434) | | | | | | | | | | | | | | | | | | | | | |
| Neonatal preterm birth | Birth prevalence - [28, 30) wks, [3000, 3500) g | Mortality | Males | | 42.199 (29.891 to 57.227) | 10.082 (7.777 to 13.056) | | | | | | | | | | | | | | | | | | | | | |
| Neonatal preterm birth | Birth prevalence - [28, 30) wks, [3000, 3500) g | Mortality | Females | | 42.551 (28.25 to 63.209) | 11.989 (9.084 to 15.544) | | | | | | | | | | | | | | | | | | | | | |

**Appendix Table 6a. Relative risks used by age and sex for each outcome for all risk factors except for ambient air pollution alcohol, and smoking.**

| | | | | Ages | | | | | | | | | | | | | | | | | | | | | | | |
|---|---|---|---|---|---|---|---|---|---|---|---|---|---|---|---|---|---|---|---|---|---|---|---|---|---|---|---|
| **Risk - Outcome** | **Category / Units** | **Morbidity / Mortality** | **Sex** | **All-age** | **0-6 days** | **7-27 days** | **28-364 days** | **1-4 years** | **5-9 years** | **10-14 years** | **15-19 years** | **20-24 years** | **25-29 years** | **30-34 years** | **35-39 years** | **40-44 years** | **45-49 years** | **50-54 years** | **55-59 years** | **60-64 years** | **65-69 years** | **70-74 years** | **75-79 years** | **80-84 years** | **85-89 years** | **90-94 years** | **95+ years** |
| Neonatal preterm birth | Birth prevalence - [30, 32) wks, [2500, 3000) g | Mortality | Males | | 58.722 (42.419 to 78.873) | 12.115 (9.518 to 15.521) | | | | | | | | | | | | | | | | | | | | | |
| Neonatal preterm birth | Birth prevalence - [30, 32) wks, [2500, 3000) g | Mortality | Females | | 59.522 (42.058 to 82.793) | 15.364 (11.936 to 19.581) | | | | | | | | | | | | | | | | | | | | | |
| Neonatal preterm birth | Birth prevalence - [30, 32) wks, [3000, 3500) g | Mortality | Males | | 45.67 (32.014 to 65.531) | 8.381 (6.362 to 10.842) | | | | | | | | | | | | | | | | | | | | | |
| Neonatal preterm birth | Birth prevalence - [30, 32) wks, [3000, 3500) g | Mortality | Females | | 46.104 (30.243 to 66.207) | 10.506 (8.041 to 13.513) | | | | | | | | | | | | | | | | | | | | | |
| Neonatal preterm birth | Birth prevalence - [30, 32) wks, [3500, 4000) g | Mortality | Males | | 36.334 (21.558 to 54.813) | 5.698 (4.293 to 7.349) | | | | | | | | | | | | | | | | | | | | | |
| Neonatal preterm birth | Birth prevalence - [30, 32) wks, [3500, 4000) g | Mortality | Females | | 37.931 (22.692 to 61.276) | 6.892 (5.175 to 9.058) | | | | | | | | | | | | | | | | | | | | | |
| Neonatal preterm birth | Birth prevalence - [32, 34) wks, [3000, 3500) g | Mortality | Males | | 34.016 (23.515 to 48.37) | 6.577 (4.934 to 8.436) | | | | | | | | | | | | | | | | | | | | | |
| Neonatal preterm birth | Birth prevalence - [32, 34) wks, [3000, 3500) g | Mortality | Females | | 34.585 (22.909 to 49.754) | 8.314 (6.362 to 10.789) | | | | | | | | | | | | | | | | | | | | | |
| Neonatal preterm birth | Birth prevalence - [32, 34) wks, [3500, 4000) g | Mortality | Males | | 36.248 (23.158 to 54.67) | 5.068 (3.761 to 6.741) | | | | | | | | | | | | | | | | | | | | | |
| Neonatal preterm birth | Birth prevalence - [32, 34) wks, [3500, 4000) g | Mortality | Females | | 38.098 (23.301 to 59.429) | 6.476 (4.666 to 8.689) | | | | | | | | | | | | | | | | | | | | | |
| Neonatal preterm birth | Birth prevalence - [36, 37) wks, [1000, 1500) g | Mortality | Males | | 166.686 (118.487 to 222.581) | 57.535 (45.999 to 71.742) | | | | | | | | | | | | | | | | | | | | | |
| Neonatal preterm birth | Birth prevalence - [36, 37) wks, [1000, 1500) g | Mortality | Females | | 169.725 (119.017 to 229.008) | 63.564 (50.068 to 80.703) | | | | | | | | | | | | | | | | | | | | | |
| Neonatal preterm birth | Birth prevalence - [38, 40) wks, [1000, 1500) g | Mortality | Males | | 174.066 (125.125 to 232.507) | 57.966 (44.393 to 73.241) | | | | | | | | | | | | | | | | | | | | | |
| Neonatal preterm birth | Birth prevalence - [38, 40) wks, [1000, 1500) g | Mortality | Females | | 171.557 (121.585 to 237.047) | 65.208 (48.821 to 84.308) | | | | | | | | | | | | | | | | | | | | | |
| Neonatal preterm birth | Birth prevalence - [38, 40) wks, [1500, 2000) g | Mortality | Males | | 67.302 (49.547 to 89.055) | 25.206 (20.365 to 31.168) | | | | | | | | | | | | | | | | | | | | | |
| Neonatal preterm birth | Birth prevalence - [38, 40) wks, [1500, 2000) g | Mortality | Females | | 62.19 (45.884 to 83.445) | 28.05 (22.625 to 35.139) | | | | | | | | | | | | | | | | | | | | | |
| Neonatal preterm birth | Birth prevalence - [40, 42) wks, [1500, 2000) g | Mortality | Males | | 76.673 (56.177 to 102.468) | 25.785 (19.387 to 34.168) | | | | | | | | | | | | | | | | | | | | | |
| Neonatal preterm birth | Birth prevalence - [40, 42) wks, [1500, 2000) g | Mortality | Females | | 70.411 (49.221 to 97.952) | 29.113 (21.355 to 38.272) | | | | | | | | | | | | | | | | | | | | | |
| Neonatal encephalopathy due to birth asphyxia and trauma | Birth prevalence - [0, 24) wks, [0, 500) g | Mortality | Males | | 1564.792 (1056.542 to 2116.062) | 618.595 (458.842 to 812.921) | | | | | | | | | | | | | | | | | | | | | |
| Neonatal encephalopathy due to birth asphyxia and trauma | Birth prevalence - [0, 24) wks, [0, 500) g | Mortality | Females | | 1600.122 (1050.664 to 2211.877) | 713.571 (526.178 to 921.018) | | | | | | | | | | | | | | | | | | | | | |
| Neonatal encephalopathy due to birth asphyxia and trauma | Birth prevalence - [0, 24) wks, [500, 1000) g | Mortality | Males | | 1155.815 (825.412 to 1506.837) | 457.5 (352.552 to 573.483) | | | | | | | | | | | | | | | | | | | | | |
| Neonatal encephalopathy due to birth asphyxia and trauma | Birth prevalence - [0, 24) wks, [500, 1000) g | Mortality | Females | | 1169.123 (802.003 to 1617.979) | 515.406 (396.713 to 641.541) | | | | | | | | | | | | | | | | | | | | | |
| Neonatal encephalopathy due to birth asphyxia and trauma | Birth prevalence - [24, 26) wks, [500, 1000) g | Mortality | Males | | 955.583 (723.748 to 1244.265) | 443.357 (363.03 to 534.695) | | | | | | | | | | | | | | | | | | | | | |
| Neonatal encephalopathy due to birth asphyxia and trauma | Birth prevalence - [24, 26) wks, [500, 1000) g | Mortality | Females | | 947.143 (702.662 to 1237.093) | 487.549 (387.307 to 603.498) | | | | | | | | | | | | | | | | | | | | | |
| Neonatal encephalopathy due to birth asphyxia and trauma | Birth prevalence - [26, 28) wks, [500, 1000) g | Mortality | Males | | 497.817 (377.617 to 648.547) | 330.886 (261.438 to 401.709) | | | | | | | | | | | | | | | | | | | | | |
| Neonatal encephalopathy due to birth asphyxia and trauma | Birth prevalence - [26, 28) wks, [500, 1000) g | Mortality | Females | | 483.682 (354.946 to 629.517) | 344.618 (274.427 to 419.864) | | | | | | | | | | | | | | | | | | | | | |
| Neonatal encephalopathy due to birth asphyxia and trauma | Birth prevalence - [30, 32) wks, [500, 1000) g | Mortality | Males | | 236.614 (163.821 to 324.502) | 149.995 (117.866 to 188.368) | | | | | | | | | | | | | | | | | | | | | |
| Neonatal encephalopathy due to birth asphyxia and trauma | Birth prevalence - [30, 32) wks, [500, 1000) g | Mortality | Females | | 229.197 (157.606 to 317.194) | 152.117 (120.779 to 190.583) | | | | | | | | | | | | | | | | | | | | | |
| Neonatal encephalopathy due to birth asphyxia and trauma | Birth prevalence - [28, 30) wks, [500, 1000) g | Mortality | Males | | 297.629 (214.953 to 396.586) | 216.995 (173.321 to 271.466) | | | | | | | | | | | | | | | | | | | | | |
| Neonatal encephalopathy due to birth asphyxia and trauma | Birth prevalence - [28, 30) wks, [500, 1000) g | Mortality | Females | | 281.056 (198.176 to 386.635) | 219.884 (174.264 to 272.704) | | | | | | | | | | | | | | | | | | | | | |
| Neonatal encephalopathy due to birth asphyxia and trauma | Birth prevalence - [26, 28) wks, [1000, 1500) g | Mortality | Males | | 267.91 (210.177 to 332.92) | 164.167 (132.898 to 200.569) | | | | | | | | | | | | | | | | | | | | | |
| Neonatal encephalopathy due to birth asphyxia and trauma | Birth prevalence - [26, 28) wks, [1000, 1500) g | Mortality | Females | | 266.509 (197.461 to 346.932) | 174.222 (137.431 to 217.349) | | | | | | | | | | | | | | | | | | | | | |
| Neonatal encephalopathy due to birth asphyxia and trauma | Birth prevalence - [34, 36) wks, [1000, 1500) g | Mortality | Males | | 142.056 (98.086 to 197.774) | 52.86 (42.914 to 64.617) | | | | | | | | | | | | | | | | | | | | | |
| Neonatal encephalopathy due to birth asphyxia and trauma | Birth prevalence - [34, 36) wks, [1000, 1500) g | Mortality | Females | | 141.899 (95.864 to 197.656) | 57.421 (46.452 to 71.339) | | | | | | | | | | | | | | | | | | | | | |
| Neonatal encephalopathy due to birth asphyxia and trauma | Birth prevalence - [28, 30) wks, [1500, 2000) g | Mortality | Males | | 127.966 (97.178 to 167.026) | 50.018 (40.539 to 61.919) | | | | | | | | | | | | | | | | | | | | | |
| Neonatal encephalopathy due to birth asphyxia and trauma | Birth prevalence - [28, 30) wks, [1500, 2000) g | Mortality | Females | | 130.924 (96.513 to 172.188) | 57.275 (46.36 to 70.038) | | | | | | | | | | | | | | | | | | | | | |
| Neonatal encephalopathy due to birth asphyxia and trauma | Birth prevalence - [28, 30) wks, [1000, 1500) g | Mortality | Males | | 158.563 (120.99 to 204.947) | 103.32 (83.486 to 127.144) | | | | | | | | | | | | | | | | | | | | | |
| Neonatal encephalopathy due to birth asphyxia and trauma | Birth prevalence - [28, 30) wks, [1000, 1500) g | Mortality | Females | | 153.905 (112.327 to 200.786) | 107.529 (86.954 to 131.78) | | | | | | | | | | | | | | | | | | | | | |
| Neonatal encephalopathy due to birth asphyxia and trauma | Birth prevalence - [32, 34) wks, [1000, 1500) g | Mortality | Males | | 117.142 (81.354 to 161.101) | 53.185 (43.049 to 66.274) | | | | | | | | | | | | | | | | | | | | | |
| Neonatal encephalopathy due to birth asphyxia and trauma | Birth prevalence - [32, 34) wks, [1000, 1500) g | Mortality | Females | | 115.171 (79.363 to 159.206) | 56.034 (45.982 to 68.36) | | | | | | | | | | | | | | | | | | | | | |
| Neonatal encephalopathy due to birth asphyxia and trauma | Birth prevalence - [30, 32) wks, [1000, 1500) g | Mortality | Males | | 119.308 (87.769 to 160.885) | 67.163 (54.863 to 82.638) | | | | | | | | | | | | | | | | | | | | | |
| Neonatal encephalopathy due to birth asphyxia and trauma | Birth prevalence - [30, 32) wks, [1000, 1500) g | Mortality | Females | | 115.448 (84.272 to 156.425) | 69.14 (55.873 to 85.012) | | | | | | | | | | | | | | | | | | | | | |
| Neonatal encephalopathy due to birth asphyxia and trauma | Birth prevalence - [37, 38) wks, [1500, 2000) g | Mortality | Males | | 62.972 (46.159 to 83.484) | 24.148 (20.066 to 29.406) | | | | | | | | | | | | | | | | | | | | | |
| Neonatal encephalopathy due to birth asphyxia and trauma | Birth prevalence - [37, 38) wks, [1500, 2000) g | Mortality | Females | | 59.988 (43.974 to 79.053) | 26.719 (21.746 to 32.816) | | | | | | | | | | | | | | | | | | | | | |
| Neonatal encephalopathy due to birth asphyxia and trauma | Birth prevalence - [36, 37) wks, [1500, 2000) g | Mortality | Males | | 60.218 (43.669 to 82.48) | 23.031 (18.793 to 28.483) | | | | | | | | | | | | | | | | | | | | | |
| Neonatal encephalopathy due to birth asphyxia and trauma | Birth prevalence - [36, 37) wks, [1500, 2000) g | Mortality | Females | | 58.527 (42.172 to 80.557) | 25.143 (20.331 to 30.566) | | | | | | | | | | | | | | | | | | | | | |
| Neonatal encephalopathy due to birth asphyxia and trauma | Birth prevalence - [30, 32) wks, [2000, 2500) g | Mortality | Males | | 67.971 (50.354 to 88.935) | 18.03 (14.621 to 22.103) | | | | | | | | | | | | | | | | | | | | | |
| Neonatal encephalopathy due to birth asphyxia and trauma | Birth prevalence - [30, 32) wks, [2000, 2500) g | Mortality | Females | | 69.383 (49.108 to 94.583) | 22.069 (17.836 to 27.163) | | | | | | | | | | | | | | | | | | | | | |
| Neonatal encephalopathy due to birth asphyxia and trauma | Birth prevalence - [30, 32) wks, [1500, 2000) g | Mortality | Males | | 77.369 (59.702 to 99.232) | 31.079 (25.786 to 36.724) | | | | | | | | | | | | | | | | | | | | | |
| Neonatal encephalopathy due to birth asphyxia and trauma | Birth prevalence - [30, 32) wks, [1500, 2000) g | Mortality | Females | | 76.134 (56.885 to 100.996) | 34.756 (28.764 to 41.849) | | | | | | | | | | | | | | | | | | | | | |
| Neonatal encephalopathy due to birth asphyxia and trauma | Birth prevalence - [34, 36) wks, [1500, 2000) g | Mortality | Males | | 55.555 (39.553 to 75.104) | 21.346 (17.677 to 26.143) | | | | | | | | | | | | | | | | | | | | | |
| Neonatal encephalopathy due to birth asphyxia and trauma | Birth prevalence - [34, 36) wks, [1500, 2000) g | Mortality | Females | | 54.335 (38.617 to 75.24) | 23.046 (18.743 to 28.287) | | | | | | | | | | | | | | | | | | | | | |
| Neonatal encephalopathy due to birth asphyxia and trauma | Birth prevalence - [32, 34) wks, [1500, 2000) g | Mortality | Males | | 57.155 (42.484 to 73.651) | 23.114 (19.028 to 27.915) | | | | | | | | | | | | | | | | | | | | | |
| Neonatal encephalopathy due to birth asphyxia and trauma | Birth prevalence - [32, 34) wks, [1500, 2000) g | Mortality | Females | | 56.101 (39.794 to 76.295) | 25.149 (20.615 to 30.388) | | | | | | | | | | | | | | | | | | | | | |
| Neonatal encephalopathy due to birth asphyxia and trauma | Birth prevalence - [32, 34) wks, [2000, 2500) g | Mortality | Males | | 37.444 (29.026 to 48.227) | 12.233 (10.252 to 14.477) | | | | | | | | | | | | | | | | | | | | | |
| Neonatal encephalopathy due to birth asphyxia and trauma | Birth prevalence - [32, 34) wks, [2000, 2500) g | Mortality | Females | | 36.874 (26.658 to 49.653) | 14.384 (12.095 to 17.03) | | | | | | | | | | | | | | | | | | | | | |
| Neonatal encephalopathy due to birth asphyxia and trauma | Birth prevalence - [40, 42) wks, [2000, 2500) g | Mortality | Males | | 18.092 (13.292 to 23.719) | 9.23 (7.037 to 11.454) | | | | | | | | | | | | | | | | | | | | | |

**Appendix Table 6a. Relative risks used by age and sex for each outcome for all risk factors except for ambient air pollution alcohol, and smoking.**

| | | | | Ages | | | | | | | | | | | | | | | | | | | | | | | |
|---|---|---|---|---|---|---|---|---|---|---|---|---|---|---|---|---|---|---|---|---|---|---|---|---|---|---|---|
| Risk - Outcome | Category / Units | Morbidity / Mortality | Sex | All-age | 0-6 days | 7-27 days | 28-364 days | 1-4 years | 5-9 years | 10-14 years | 15-19 years | 20-24 years | 25-29 years | 30-34 years | 35-39 years | 40-44 years | 45-49 years | 50-54 years | 55-59 years | 60-64 years | 65-69 years | 70-74 years | 75-79 years | 80-84 years | 85-89 years | 90-94 years | 95+ years |
| Neonatal encephalopathy due to birth asphyxia and trauma | Birth prevalence - [40, 42) wks, [2000, 2500) g | Mortality | Females | | 15.574 (11.516 to 20.778) | 9.975 (7.82 to 12.46) | | | | | | | | | | | | | | | | | | | | | |
| Neonatal encephalopathy due to birth asphyxia and trauma | Birth prevalence - [38, 40) wks, [2000, 2500) g | Mortality | Males | | 13.104 (9.829 to 16.99) | 8.198 (6.786 to 9.959) | | | | | | | | | | | | | | | | | | | | | |
| Neonatal encephalopathy due to birth asphyxia and trauma | Birth prevalence - [38, 40) wks, [2000, 2500) g | Mortality | Females | | 11.308 (8.389 to 14.38) | 8.577 (7.04 to 10.449) | | | | | | | | | | | | | | | | | | | | | |
| Neonatal encephalopathy due to birth asphyxia and trauma | Birth prevalence - [32, 34) wks, [2500, 3000) g | Mortality | Males | | 33.063 (24.393 to 43.503) | 8.441 (6.822 to 10.431) | | | | | | | | | | | | | | | | | | | | | |
| Neonatal encephalopathy due to birth asphyxia and trauma | Birth prevalence - [32, 34) wks, [2500, 3000) g | Mortality | Females | | 32.812 (23.439 to 45.567) | 10.398 (8.227 to 13.042) | | | | | | | | | | | | | | | | | | | | | |
| Neonatal encephalopathy due to birth asphyxia and trauma | Birth prevalence - [34, 36) wks, [2000, 2500) g | Mortality | Males | | 21.925 (16.305 to 29.433) | 9.367 (7.859 to 11.112) | | | | | | | | | | | | | | | | | | | | | |
| Neonatal encephalopathy due to birth asphyxia and trauma | Birth prevalence - [34, 36) wks, [2000, 2500) g | Mortality | Females | | 21.297 (15.657 to 28.761) | 10.295 (8.548 to 12.273) | | | | | | | | | | | | | | | | | | | | | |
| Neonatal encephalopathy due to birth asphyxia and trauma | Birth prevalence - [37, 38) wks, [2000, 2500) g | Mortality | Males | | 13.0 (10.102 to 16.456) | 8.096 (6.724 to 9.676) | | | | | | | | | | | | | | | | | | | | | |
| Neonatal encephalopathy due to birth asphyxia and trauma | Birth prevalence - [37, 38) wks, [2000, 2500) g | Mortality | Females | | 11.563 (8.805 to 15.11) | 8.467 (6.994 to 10.342) | | | | | | | | | | | | | | | | | | | | | |
| Neonatal encephalopathy due to birth asphyxia and trauma | Birth prevalence - [36, 37) wks, [2000, 2500) g | Mortality | Males | | 14.401 (10.789 to 18.654) | 8.221 (6.917 to 9.923) | | | | | | | | | | | | | | | | | | | | | |
| Neonatal encephalopathy due to birth asphyxia and trauma | Birth prevalence - [36, 37) wks, [2000, 2500) g | Mortality | Females | | 13.513 (9.817 to 17.942) | 8.654 (7.215 to 10.369) | | | | | | | | | | | | | | | | | | | | | |
| Neonatal encephalopathy due to birth asphyxia and trauma | Birth prevalence - [34, 36) wks, [2500, 3000) g | Mortality | Males | | 13.419 (10.387 to 16.819) | 5.562 (4.646 to 6.696) | | | | | | | | | | | | | | | | | | | | | |
| Neonatal encephalopathy due to birth asphyxia and trauma | Birth prevalence - [34, 36) wks, [2500, 3000) g | Mortality | Females | | 13.266 (9.666 to 17.689) | 6.395 (5.292 to 7.606) | | | | | | | | | | | | | | | | | | | | | |
| Neonatal encephalopathy due to birth asphyxia and trauma | Birth prevalence - [34, 36) wks, [4000, 4500) g | Mortality | Males | | 23.096 (14.708 to 35.098) | 2.895 (2.245 to 3.716) | | | | | | | | | | | | | | | | | | | | | |
| Neonatal encephalopathy due to birth asphyxia and trauma | Birth prevalence - [34, 36) wks, [4000, 4500) g | Mortality | Females | | 25.038 (15.763 to 37.255) | 3.778 (2.855 to 4.925) | | | | | | | | | | | | | | | | | | | | | |
| Neonatal encephalopathy due to birth asphyxia and trauma | Birth prevalence - [34, 36) wks, [3000, 3500) g | Mortality | Males | | 14.006 (10.222 to 18.478) | 4.322 (3.449 to 5.338) | | | | | | | | | | | | | | | | | | | | | |
| Neonatal encephalopathy due to birth asphyxia and trauma | Birth prevalence - [34, 36) wks, [3000, 3500) g | Mortality | Females | | 14.375 (10.269 to 20.114) | 5.265 (4.145 to 6.564) | | | | | | | | | | | | | | | | | | | | | |
| Neonatal encephalopathy due to birth asphyxia and trauma | Birth prevalence - [36, 37) wks, [2500, 3000) g | Mortality | Males | | 4.874 (4.014 to 5.713) | 3.699 (3.134 to 4.374) | | | | | | | | | | | | | | | | | | | | | |
| Neonatal encephalopathy due to birth asphyxia and trauma | Birth prevalence - [36, 37) wks, [2500, 3000) g | Mortality | Females | | 4.609 (3.731 to 5.61) | 3.898 (3.235 to 4.67) | | | | | | | | | | | | | | | | | | | | | |
| Neonatal encephalopathy due to birth asphyxia and trauma | Birth prevalence - [34, 36) wks, [3500, 4000) g | Mortality | Males | | 18.024 (12.279 to 25.547) | 3.657 (2.838 to 4.675) | | | | | | | | | | | | | | | | | | | | | |
| Neonatal encephalopathy due to birth asphyxia and trauma | Birth prevalence - [34, 36) wks, [3500, 4000) g | Mortality | Females | | 19.263 (12.567 to 27.924) | 4.634 (3.576 to 5.989) | | | | | | | | | | | | | | | | | | | | | |
| Neonatal encephalopathy due to birth asphyxia and trauma | Birth prevalence - [37, 38) wks, [2500, 3000) g | Mortality | Males | | 3.306 (2.82 to 3.843) | 3.194 (2.691 to 3.803) | | | | | | | | | | | | | | | | | | | | | |
| Neonatal encephalopathy due to birth asphyxia and trauma | Birth prevalence - [37, 38) wks, [2500, 3000) g | Mortality | Females | | 2.991 (2.496 to 3.61) | 3.242 (2.745 to 3.817) | | | | | | | | | | | | | | | | | | | | | |
| Neonatal encephalopathy due to birth asphyxia and trauma | Birth prevalence - [40, 42) wks, [2500, 3000) g | Mortality | Males | | 3.771 (3.002 to 4.693) | 3.175 (2.56 to 3.923) | | | | | | | | | | | | | | | | | | | | | |
| Neonatal encephalopathy due to birth asphyxia and trauma | Birth prevalence - [40, 42) wks, [2500, 3000) g | Mortality | Females | | 3.244 (2.486 to 4.159) | 3.228 (2.605 to 3.985) | | | | | | | | | | | | | | | | | | | | | |
| Neonatal encephalopathy due to birth asphyxia and trauma | Birth prevalence - [38, 40) wks, [2500, 3000) g | Mortality | Males | | 2.755 (2.274 to 3.309) | 2.944 (2.44 to 3.548) | | | | | | | | | | | | | | | | | | | | | |
| Neonatal encephalopathy due to birth asphyxia and trauma | Birth prevalence - [38, 40) wks, [2500, 3000) g | Mortality | Females | | 2.376 (1.91 to 2.886) | 2.938 (2.434 to 3.503) | | | | | | | | | | | | | | | | | | | | | |
| Neonatal encephalopathy due to birth asphyxia and trauma | Birth prevalence - [36, 37) wks, [3000, 3500) g | Mortality | Males | | 3.774 (3.094 to 4.497) | 2.466 (2.058 to 2.929) | | | | | | | | | | | | | | | | | | | | | |
| Neonatal encephalopathy due to birth asphyxia and trauma | Birth prevalence - [36, 37) wks, [3000, 3500) g | Mortality | Females | | 3.73 (2.981 to 4.646) | 2.715 (2.277 to 3.218) | | | | | | | | | | | | | | | | | | | | | |
| Neonatal encephalopathy due to birth asphyxia and trauma | Birth prevalence - [36, 37) wks, [4000, 4500) g | Mortality | Males | | 6.826 (5.212 to 9.045) | 1.77 (1.491 to 2.082) | | | | | | | | | | | | | | | | | | | | | |
| Neonatal encephalopathy due to birth asphyxia and trauma | Birth prevalence - [36, 37) wks, [4000, 4500) g | Mortality | Females | | 7.269 (5.144 to 9.821) | 2.177 (1.786 to 2.607) | | | | | | | | | | | | | | | | | | | | | |
| Neonatal encephalopathy due to birth asphyxia and trauma | Birth prevalence - [36, 37) wks, [3500, 4000) g | Mortality | Males | | 4.544 (3.64 to 5.622) | 2.057 (1.74 to 2.44) | | | | | | | | | | | | | | | | | | | | | |
| Neonatal encephalopathy due to birth asphyxia and trauma | Birth prevalence - [36, 37) wks, [3500, 4000) g | Mortality | Females | | 4.662 (3.577 to 6.014) | 2.398 (1.98 to 2.864) | | | | | | | | | | | | | | | | | | | | | |
| Neonatal encephalopathy due to birth asphyxia and trauma | Birth prevalence - [37, 38) wks, [3000, 3500) g | Mortality | Males | | 2.007 (1.759 to 2.293) | 1.888 (1.613 to 2.224) | | | | | | | | | | | | | | | | | | | | | |
| Neonatal encephalopathy due to birth asphyxia and trauma | Birth prevalence - [37, 38) wks, [3000, 3500) g | Mortality | Females | | 1.925 (1.582 to 2.328) | 1.972 (1.669 to 2.313) | | | | | | | | | | | | | | | | | | | | | |
| Neonatal encephalopathy due to birth asphyxia and trauma | Birth prevalence - [37, 38) wks, [4000, 4500) g | Mortality | Males | | 3.28 (2.596 to 4.133) | 1.335 (1.171 to 1.532) | | | | | | | | | | | | | | | | | | | | | |
| Neonatal encephalopathy due to birth asphyxia and trauma | Birth prevalence - [37, 38) wks, [4000, 4500) g | Mortality | Females | | 3.521 (2.649 to 4.5) | 1.559 (1.333 to 1.835) | | | | | | | | | | | | | | | | | | | | | |
| Neonatal encephalopathy due to birth asphyxia and trauma | Birth prevalence - [37, 38) wks, [3500, 4000) g | Mortality | Males | | 2.128 (1.833 to 2.466) | 1.505 (1.299 to 1.76) | | | | | | | | | | | | | | | | | | | | | |
| Neonatal encephalopathy due to birth asphyxia and trauma | Birth prevalence - [37, 38) wks, [3500, 4000) g | Mortality | Females | | 2.142 (1.694 to 2.67) | 1.661 (1.411 to 1.961) | | | | | | | | | | | | | | | | | | | | | |
| Neonatal encephalopathy due to birth asphyxia and trauma | Birth prevalence - [40, 42) wks, [3000, 3500) g | Mortality | Males | | 1.436 (1.245 to 1.65) | 1.47 (1.199 to 1.8) | | | | | | | | | | | | | | | | | | | | | |
| Neonatal encephalopathy due to birth asphyxia and trauma | Birth prevalence - [40, 42) wks, [3000, 3500) g | Mortality | Females | | 1.326 (1.069 to 1.614) | 1.465 (1.188 to 1.775) | | | | | | | | | | | | | | | | | | | | | |
| Neonatal encephalopathy due to birth asphyxia and trauma | Birth prevalence - [38, 40) wks, [3000, 3500) g | Mortality | Males | | 1.33 (1.155 to 1.53) | 1.559 (1.305 to 1.851) | | | | | | | | | | | | | | | | | | | | | |
| Neonatal encephalopathy due to birth asphyxia and trauma | Birth prevalence - [38, 40) wks, [3000, 3500) g | Mortality | Females | | 1.224 (1.0 to 1.492) | 1.564 (1.304 to 1.847) | | | | | | | | | | | | | | | | | | | | | |
| Neonatal encephalopathy due to birth asphyxia and trauma | Birth prevalence - [38, 40) wks, [4000, 4500) g | Mortality | Males | | 1.787 (1.453 to 2.182) | 1.175 (1.005 to 1.371) | | | | | | | | | | | | | | | | | | | | | |
| Neonatal encephalopathy due to birth asphyxia and trauma | Birth prevalence - [38, 40) wks, [4000, 4500) g | Mortality | Females | | 1.877 (1.467 to 2.388) | 1.224 (1.022 to 1.465) | | | | | | | | | | | | | | | | | | | | | |
| Neonatal encephalopathy due to birth asphyxia and trauma | Birth prevalence - [38, 40) wks, [3500, 4000) g | Mortality | Males | | 1.785 (1.478 to 2.147) | 1.173 (1.0 to 1.377) | | | | | | | | | | | | | | | | | | | | | |
| Neonatal encephalopathy due to birth asphyxia and trauma | Birth prevalence - [38, 40) wks, [3500, 4000) g | Mortality | Females | | 1.892 (1.481 to 2.352) | 1.23 (1.03 to 1.46) | | | | | | | | | | | | | | | | | | | | | |
| Neonatal encephalopathy due to birth asphyxia and trauma | Birth prevalence - [40, 42) wks, [3500, 4000) g | Mortality | Males | | 1.0 (1.0 to 1.0) | 1.003 (1.0 to 1.046) | | | | | | | | | | | | | | | | | | | | | |
| Neonatal encephalopathy due to birth asphyxia and trauma | Birth prevalence - [40, 42) wks, [3500, 4000) g | Mortality | Females | | 1.002 (1.0 to 1.013) | 1.001 (1.0 to 1.006) | | | | | | | | | | | | | | | | | | | | | |
| Neonatal encephalopathy due to birth asphyxia and trauma | Birth prevalence - [40, 42) wks, [4000, 4500) g | Mortality | Males | | 1.0 (1.0 to 1.0) | 1.0 (1.0 to 1.0) | | | | | | | | | | | | | | | | | | | | | |
| Neonatal encephalopathy due to birth asphyxia and trauma | Birth prevalence - [40, 42) wks, [4000, 4500) g | Mortality | Females | | 1.0 (1.0 to 1.0) | 1.0 (1.0 to 1.0) | | | | | | | | | | | | | | | | | | | | | |
| Neonatal encephalopathy due to birth asphyxia and trauma | Birth prevalence - [28, 30) wks, [2000, 2500) g | Mortality | Males | | 117.172 (83.895 to 158.056) | 27.726 (21.877 to 34.972) | | | | | | | | | | | | | | | | | | | | | |
| Neonatal encephalopathy due to birth asphyxia and trauma | Birth prevalence - [28, 30) wks, [2000, 2500) g | Mortality | Females | | 121.682 (84.349 to 171.375) | 33.983 (26.101 to 43.404) | | | | | | | | | | | | | | | | | | | | | |
| Neonatal encephalopathy due to birth asphyxia and trauma | Birth prevalence - [28, 30) wks, [2500, 3000) g | Mortality | Males | | 77.948 (54.687 to 105.047) | 16.608 (12.653 to 21.188) | | | | | | | | | | | | | | | | | | | | | |
| Neonatal encephalopathy due to birth asphyxia and trauma | Birth prevalence - [28, 30) wks, [2500, 3000) g | Mortality | Females | | 79.193 (54.099 to 112.236) | 20.387 (15.089 to 26.434) | | | | | | | | | | | | | | | | | | | | | |
| Neonatal encephalopathy due to birth asphyxia and trauma | Birth prevalence - [28, 30) wks, [3000, 3500) g | Mortality | Males | | 42.199 (29.891 to 57.227) | 10.082 (7.777 to 13.056) | | | | | | | | | | | | | | | | | | | | | |
| Neonatal encephalopathy due to birth asphyxia and trauma | Birth prevalence - [28, 30) wks, [3000, 3500) g | Mortality | Females | | 42.551 (28.25 to 63.209) | 11.989 (9.084 to 15.544) | | | | | | | | | | | | | | | | | | | | | |

Appendix Table 6a. Relative risks used by age and sex for each outcome for all risk factors except for ambient air pollution alcohol, and smoking.

| | | | | Ages | | | | | | | | | | | | | | | | | | | | | | | |
|---|---|---|---|---|---|---|---|---|---|---|---|---|---|---|---|---|---|---|---|---|---|---|---|---|---|---|---|
| Risk - Outcome | Category / Units | Morbidity / Mortality | Sex | All-age | 0-6 days | 7-27 days | 28-364 days | 1-4 years | 5-9 years | 10-14 years | 15-19 years | 20-24 years | 25-29 years | 30-34 years | 35-39 years | 40-44 years | 45-49 years | 50-54 years | 55-59 years | 60-64 years | 65-69 years | 70-74 years | 75-79 years | 80-84 years | 85-89 years | 90-94 years | 95+ years |
| Neonatal encephalopathy due to birth asphyxia and trauma | Birth prevalence - [30, 32) wks, [2500, 3000) g | Mortality | Males | | 58.722 (42.419 to 78.873) | 12.115 (9.518 to 15.521) | | | | | | | | | | | | | | | | | | | | | |
| Neonatal encephalopathy due to birth asphyxia and trauma | Birth prevalence - [30, 32) wks, [2500, 3000) g | Mortality | Females | | 59.522 (42.058 to 82.793) | 15.364 (11.936 to 19.581) | | | | | | | | | | | | | | | | | | | | | |
| Neonatal encephalopathy due to birth asphyxia and trauma | Birth prevalence - [30, 32) wks, [3000, 3500) g | Mortality | Males | | 45.67 (32.014 to 65.531) | 8.381 (6.362 to 10.842) | | | | | | | | | | | | | | | | | | | | | |
| Neonatal encephalopathy due to birth asphyxia and trauma | Birth prevalence - [30, 32) wks, [3000, 3500) g | Mortality | Females | | 46.104 (30.243 to 66.207) | 10.506 (8.041 to 13.513) | | | | | | | | | | | | | | | | | | | | | |
| Neonatal encephalopathy due to birth asphyxia and trauma | Birth prevalence - [30, 32) wks, [3500, 4000) g | Mortality | Males | | 36.334 (21.558 to 54.813) | 5.698 (4.293 to 7.349) | | | | | | | | | | | | | | | | | | | | | |
| Neonatal encephalopathy due to birth asphyxia and trauma | Birth prevalence - [30, 32) wks, [3500, 4000) g | Mortality | Females | | 37.931 (22.692 to 61.276) | 6.892 (5.175 to 9.058) | | | | | | | | | | | | | | | | | | | | | |
| Neonatal encephalopathy due to birth asphyxia and trauma | Birth prevalence - [32, 34) wks, [3000, 3500) g | Mortality | Males | | 34.016 (23.515 to 48.37) | 6.577 (4.934 to 8.436) | | | | | | | | | | | | | | | | | | | | | |
| Neonatal encephalopathy due to birth asphyxia and trauma | Birth prevalence - [32, 34) wks, [3000, 3500) g | Mortality | Females | | 34.585 (22.909 to 49.754) | 8.314 (6.362 to 10.789) | | | | | | | | | | | | | | | | | | | | | |
| Neonatal encephalopathy due to birth asphyxia and trauma | Birth prevalence - [32, 34) wks, [3500, 4000) g | Mortality | Males | | 36.248 (23.158 to 54.67) | 5.068 (3.761 to 6.741) | | | | | | | | | | | | | | | | | | | | | |
| Neonatal encephalopathy due to birth asphyxia and trauma | Birth prevalence - [32, 34) wks, [3500, 4000) g | Mortality | Females | | 38.098 (23.301 to 59.429) | 6.476 (4.666 to 8.689) | | | | | | | | | | | | | | | | | | | | | |
| Neonatal encephalopathy due to birth asphyxia and trauma | Birth prevalence - [36, 37) wks, [1000, 1500) g | Mortality | Males | | 166.686 (118.487 to 222.581) | 57.535 (45.999 to 71.742) | | | | | | | | | | | | | | | | | | | | | |
| Neonatal encephalopathy due to birth asphyxia and trauma | Birth prevalence - [36, 37) wks, [1000, 1500) g | Mortality | Females | | 169.725 (119.017 to 229.008) | 63.564 (50.068 to 80.703) | | | | | | | | | | | | | | | | | | | | | |
| Neonatal encephalopathy due to birth asphyxia and trauma | Birth prevalence - [38, 40) wks, [1000, 1500) g | Mortality | Males | | 174.066 (125.125 to 232.507) | 57.966 (44.393 to 73.241) | | | | | | | | | | | | | | | | | | | | | |
| Neonatal encephalopathy due to birth asphyxia and trauma | Birth prevalence - [38, 40) wks, [1000, 1500) g | Mortality | Females | | 171.557 (121.585 to 237.047) | 65.208 (48.821 to 84.308) | | | | | | | | | | | | | | | | | | | | | |
| Neonatal encephalopathy due to birth asphyxia and trauma | Birth prevalence - [38, 40) wks, [1500, 2000) g | Mortality | Males | | 67.302 (49.547 to 89.055) | 25.206 (20.365 to 31.168) | | | | | | | | | | | | | | | | | | | | | |
| Neonatal encephalopathy due to birth asphyxia and trauma | Birth prevalence - [38, 40) wks, [1500, 2000) g | Mortality | Females | | 62.19 (45.884 to 83.445) | 28.05 (22.625 to 35.139) | | | | | | | | | | | | | | | | | | | | | |
| Neonatal encephalopathy due to birth asphyxia and trauma | Birth prevalence - [40, 42) wks, [1500, 2000) g | Mortality | Males | | 76.673 (56.177 to 102.468) | 25.785 (19.387 to 34.168) | | | | | | | | | | | | | | | | | | | | | |
| Neonatal encephalopathy due to birth asphyxia and trauma | Birth prevalence - [40, 42) wks, [1500, 2000) g | Mortality | Females | | 70.411 (49.221 to 97.952) | 29.113 (21.355 to 38.272) | | | | | | | | | | | | | | | | | | | | | |
| Neonatal sepsis and other neonatal infections | Birth prevalence - [0, 24) wks, [0, 500) g | Mortality | Males | | 1564.792 (1056.542 to [illegible] | 618.595 (458.842 to 812.921) | | | | | | | | | | | | | | | | | | | | | |
| Neonatal sepsis and other neonatal infections | Birth prevalence - [0, 24) wks, [0, 500) g | Mortality | Females | | 1600.122 (1050.664 to [illegible] | 713.571 (526.178 to 921.018) | | | | | | | | | | | | | | | | | | | | | |
| Neonatal sepsis and other neonatal infections | Birth prevalence - [0, 24) wks, [500, 1000) g | Mortality | Males | | 1155.815 (825.412 to 1506.837) | 457.5 (352.552 to 573.483) | | | | | | | | | | | | | | | | | | | | | |
| Neonatal sepsis and other neonatal infections | Birth prevalence - [0, 24) wks, [500, 1000) g | Mortality | Females | | 1169.123 (802.003 to 1617.979) | 515.406 (396.713 to 641.541) | | | | | | | | | | | | | | | | | | | | | |
| Neonatal sepsis and other neonatal infections | Birth prevalence - [24, 26) wks, [500, 1000) g | Mortality | Males | | 955.583 (723.748 to 1244.265) | 443.357 (363.03 to 534.695) | | | | | | | | | | | | | | | | | | | | | |
| Neonatal sepsis and other neonatal infections | Birth prevalence - [24, 26) wks, [500, 1000) g | Mortality | Females | | 947.143 (702.662 to 1237.093) | 487.549 (387.307 to 603.498) | | | | | | | | | | | | | | | | | | | | | |
| Neonatal sepsis and other neonatal infections | Birth prevalence - [26, 28) wks, [500, 1000) g | Mortality | Males | | 497.817 (377.617 to 648.547) | 330.886 (261.438 to 401.709) | | | | | | | | | | | | | | | | | | | | | |
| Neonatal sepsis and other neonatal infections | Birth prevalence - [26, 28) wks, [500, 1000) g | Mortality | Females | | 483.682 (354.946 to 629.517) | 344.618 (274.427 to 419.864) | | | | | | | | | | | | | | | | | | | | | |
| Neonatal sepsis and other neonatal infections | Birth prevalence - [30, 32) wks, [500, 1000) g | Mortality | Males | | 236.614 (163.821 to 324.502) | 149.995 (117.866 to 188.368) | | | | | | | | | | | | | | | | | | | | | |
| Neonatal sepsis and other neonatal infections | Birth prevalence - [30, 32) wks, [500, 1000) g | Mortality | Females | | 229.197 (157.606 to 317.194) | 152.117 (120.779 to 190.583) | | | | | | | | | | | | | | | | | | | | | |
| Neonatal sepsis and other neonatal infections | Birth prevalence - [28, 30) wks, [500, 1000) g | Mortality | Males | | 297.629 (214.953 to 396.586) | 216.995 (173.321 to 271.466) | | | | | | | | | | | | | | | | | | | | | |
| Neonatal sepsis and other neonatal infections | Birth prevalence - [28, 30) wks, [500, 1000) g | Mortality | Females | | 281.056 (198.176 to 386.635) | 219.884 (174.264 to 272.704) | | | | | | | | | | | | | | | | | | | | | |
| Neonatal sepsis and other neonatal infections | Birth prevalence - [26, 28) wks, [1000, 1500) g | Mortality | Males | | 267.91 (210.177 to 332.92) | 164.167 (132.898 to 200.569) | | | | | | | | | | | | | | | | | | | | | |
| Neonatal sepsis and other neonatal infections | Birth prevalence - [26, 28) wks, [1000, 1500) g | Mortality | Females | | 266.509 (197.461 to 346.932) | 174.222 (137.431 to 217.349) | | | | | | | | | | | | | | | | | | | | | |
| Neonatal sepsis and other neonatal infections | Birth prevalence - [34, 36) wks, [1000, 1500) g | Mortality | Males | | 142.056 (98.086 to 197.774) | 52.86 (42.914 to 64.617) | | | | | | | | | | | | | | | | | | | | | |
| Neonatal sepsis and other neonatal infections | Birth prevalence - [34, 36) wks, [1000, 1500) g | Mortality | Females | | 141.899 (95.864 to 197.656) | 57.421 (46.452 to 71.339) | | | | | | | | | | | | | | | | | | | | | |
| Neonatal sepsis and other neonatal infections | Birth prevalence - [28, 30) wks, [1500, 2000) g | Mortality | Males | | 127.966 (97.178 to 167.026) | 50.018 (40.539 to 61.919) | | | | | | | | | | | | | | | | | | | | | |
| Neonatal sepsis and other neonatal infections | Birth prevalence - [28, 30) wks, [1500, 2000) g | Mortality | Females | | 130.924 (96.513 to 172.188) | 57.275 (46.36 to 70.038) | | | | | | | | | | | | | | | | | | | | | |
| Neonatal sepsis and other neonatal infections | Birth prevalence - [28, 30) wks, [1000, 1500) g | Mortality | Males | | 158.563 (120.99 to 204.947) | 103.32 (83.486 to 127.144) | | | | | | | | | | | | | | | | | | | | | |
| Neonatal sepsis and other neonatal infections | Birth prevalence - [28, 30) wks, [1000, 1500) g | Mortality | Females | | 153.905 (112.327 to 200.786) | 107.529 (86.954 to 131.78) | | | | | | | | | | | | | | | | | | | | | |
| Neonatal sepsis and other neonatal infections | Birth prevalence - [32, 34) wks, [1000, 1500) g | Mortality | Males | | 117.142 (81.354 to 161.101) | 53.185 (43.049 to 66.274) | | | | | | | | | | | | | | | | | | | | | |
| Neonatal sepsis and other neonatal infections | Birth prevalence - [32, 34) wks, [1000, 1500) g | Mortality | Females | | 115.171 (79.363 to 159.206) | 56.034 (45.982 to 68.36) | | | | | | | | | | | | | | | | | | | | | |
| Neonatal sepsis and other neonatal infections | Birth prevalence - [30, 32) wks, [1000, 1500) g | Mortality | Males | | 119.308 (87.769 to 160.885) | 67.163 (54.863 to 82.638) | | | | | | | | | | | | | | | | | | | | | |
| Neonatal sepsis and other neonatal infections | Birth prevalence - [30, 32) wks, [1000, 1500) g | Mortality | Females | | 115.448 (84.272 to 156.425) | 69.14 (55.873 to 85.012) | | | | | | | | | | | | | | | | | | | | | |
| Neonatal sepsis and other neonatal infections | Birth prevalence - [37, 38) wks, [1500, 2000) g | Mortality | Males | | 62.972 (46.159 to 83.484) | 24.148 (20.066 to 29.406) | | | | | | | | | | | | | | | | | | | | | |
| Neonatal sepsis and other neonatal infections | Birth prevalence - [37, 38) wks, [1500, 2000) g | Mortality | Females | | 59.988 (43.974 to 79.053) | 26.719 (21.746 to 32.816) | | | | | | | | | | | | | | | | | | | | | |
| Neonatal sepsis and other neonatal infections | Birth prevalence - [36, 37) wks, [1500, 2000) g | Mortality | Males | | 60.218 (43.669 to 82.48) | 23.031 (18.793 to 28.483) | | | | | | | | | | | | | | | | | | | | | |
| Neonatal sepsis and other neonatal infections | Birth prevalence - [36, 37) wks, [1500, 2000) g | Mortality | Females | | 58.527 (42.172 to 80.557) | 25.143 (20.331 to 30.566) | | | | | | | | | | | | | | | | | | | | | |
| Neonatal sepsis and other neonatal infections | Birth prevalence - [30, 32) wks, [2000, 2500) g | Mortality | Males | | 67.971 (50.354 to 88.935) | 18.03 (14.621 to 22.103) | | | | | | | | | | | | | | | | | | | | | |
| Neonatal sepsis and other neonatal infections | Birth prevalence - [30, 32) wks, [2000, 2500) g | Mortality | Females | | 69.383 (49.108 to 94.583) | 22.069 (17.836 to 27.163) | | | | | | | | | | | | | | | | | | | | | |
| Neonatal sepsis and other neonatal infections | Birth prevalence - [30, 32) wks, [1500, 2000) g | Mortality | Males | | 77.369 (59.702 to 99.232) | 31.079 (25.786 to 36.724) | | | | | | | | | | | | | | | | | | | | | |
| Neonatal sepsis and other neonatal infections | Birth prevalence - [30, 32) wks, [1500, 2000) g | Mortality | Females | | 76.134 (56.885 to 100.996) | 34.756 (28.764 to 41.849) | | | | | | | | | | | | | | | | | | | | | |
| Neonatal sepsis and other neonatal infections | Birth prevalence - [34, 36) wks, [1500, 2000) g | Mortality | Males | | 55.555 (39.553 to 75.104) | 21.346 (17.677 to 26.143) | | | | | | | | | | | | | | | | | | | | | |
| Neonatal sepsis and other neonatal infections | Birth prevalence - [34, 36) wks, [1500, 2000) g | Mortality | Females | | 54.335 (38.617 to 75.24) | 23.046 (18.743 to 28.287) | | | | | | | | | | | | | | | | | | | | | |
| Neonatal sepsis and other neonatal infections | Birth prevalence - [32, 34) wks, [1500, 2000) g | Mortality | Males | | 57.155 (42.484 to 73.651) | 23.114 (19.028 to 27.915) | | | | | | | | | | | | | | | | | | | | | |
| Neonatal sepsis and other neonatal infections | Birth prevalence - [32, 34) wks, [1500, 2000) g | Mortality | Females | | 56.101 (39.794 to 76.295) | 25.149 (20.615 to 30.388) | | | | | | | | | | | | | | | | | | | | | |
| Neonatal sepsis and other neonatal infections | Birth prevalence - [32, 34) wks, [2000, 2500) g | Mortality | Males | | 37.444 (29.026 to 48.227) | 12.233 (10.252 to 14.477) | | | | | | | | | | | | | | | | | | | | | |
| Neonatal sepsis and other neonatal infections | Birth prevalence - [32, 34) wks, [2000, 2500) g | Mortality | Females | | 36.874 (26.658 to 49.653) | 14.384 (12.095 to 17.03) | | | | | | | | | | | | | | | | | | | | | |
| Neonatal sepsis and other neonatal infections | Birth prevalence - [40, 42) wks, [2000, 2500) g | Mortality | Males | | 18.092 (13.292 to 23.719) | 9.23 (7.037 to 11.454) | | | | | | | | | | | | | | | | | | | | | |

**Appendix Table 6a. Relative risks used by age and sex for each outcome for all risk factors except for ambient air pollution alcohol, and smoking.**

| | | | | Ages | | | | | | | | | | | | | | | | | | | | | | | |
|---|---|---|---|---|---|---|---|---|---|---|---|---|---|---|---|---|---|---|---|---|---|---|---|---|---|---|---|
| Risk - Outcome | Category / Units | Morbidity / Mortality | Sex | All-age | 0-6 days | 7-27 days | 28-364 days | 1-4 years | 5-9 years | 10-14 years | 15-19 years | 20-24 years | 25-29 years | 30-34 years | 35-39 years | 40-44 years | 45-49 years | 50-54 years | 55-59 years | 60-64 years | 65-69 years | 70-74 years | 75-79 years | 80-84 years | 85-89 years | 90-94 years | 95+ years |
| Neonatal sepsis and other neonatal infections | Birth prevalence - [40, 42) wks, [2000, 2500) g | Mortality | Females | | 15.574 (11.516 to 20.778) | 9.975 (7.82 to 12.46) | | | | | | | | | | | | | | | | | | | | | |
| Neonatal sepsis and other neonatal infections | Birth prevalence - [38, 40) wks, [2000, 2500) g | Mortality | Males | | 13.104 (9.829 to 16.99) | 8.198 (6.786 to 9.959) | | | | | | | | | | | | | | | | | | | | | |
| Neonatal sepsis and other neonatal infections | Birth prevalence - [38, 40) wks, [2000, 2500) g | Mortality | Females | | 11.308 (8.389 to 14.38) | 8.577 (7.04 to 10.449) | | | | | | | | | | | | | | | | | | | | | |
| Neonatal sepsis and other neonatal infections | Birth prevalence - [32, 34) wks, [2500, 3000) g | Mortality | Males | | 33.063 (24.393 to 43.503) | 8.441 (6.822 to 10.431) | | | | | | | | | | | | | | | | | | | | | |
| Neonatal sepsis and other neonatal infections | Birth prevalence - [32, 34) wks, [2500, 3000) g | Mortality | Females | | 32.812 (23.439 to 45.567) | 10.398 (8.227 to 13.042) | | | | | | | | | | | | | | | | | | | | | |
| Neonatal sepsis and other neonatal infections | Birth prevalence - [34, 36) wks, [2000, 2500) g | Mortality | Males | | 21.925 (16.305 to 29.433) | 9.367 (7.859 to 11.112) | | | | | | | | | | | | | | | | | | | | | |
| Neonatal sepsis and other neonatal infections | Birth prevalence - [34, 36) wks, [2000, 2500) g | Mortality | Females | | 21.297 (15.657 to 28.761) | 10.295 (8.548 to 12.273) | | | | | | | | | | | | | | | | | | | | | |
| Neonatal sepsis and other neonatal infections | Birth prevalence - [37, 38) wks, [2000, 2500) g | Mortality | Males | | 13.0 (10.102 to 16.456) | 8.096 (6.724 to 9.676) | | | | | | | | | | | | | | | | | | | | | |
| Neonatal sepsis and other neonatal infections | Birth prevalence - [37, 38) wks, [2000, 2500) g | Mortality | Females | | 11.563 (8.805 to 15.11) | 8.467 (6.994 to 10.342) | | | | | | | | | | | | | | | | | | | | | |
| Neonatal sepsis and other neonatal infections | Birth prevalence - [36, 37) wks, [2000, 2500) g | Mortality | Males | | 14.401 (10.789 to 18.654) | 8.221 (6.917 to 9.923) | | | | | | | | | | | | | | | | | | | | | |
| Neonatal sepsis and other neonatal infections | Birth prevalence - [36, 37) wks, [2000, 2500) g | Mortality | Females | | 13.513 (9.817 to 17.942) | 8.654 (7.215 to 10.369) | | | | | | | | | | | | | | | | | | | | | |
| Neonatal sepsis and other neonatal infections | Birth prevalence - [34, 36) wks, [2500, 3000) g | Mortality | Males | | 13.419 (10.387 to 16.819) | 5.562 (4.646 to 6.696) | | | | | | | | | | | | | | | | | | | | | |
| Neonatal sepsis and other neonatal infections | Birth prevalence - [34, 36) wks, [2500, 3000) g | Mortality | Females | | 13.266 (9.666 to 17.689) | 6.395 (5.292 to 7.606) | | | | | | | | | | | | | | | | | | | | | |
| Neonatal sepsis and other neonatal infections | Birth prevalence - [34, 36) wks, [4000, 4500) g | Mortality | Males | | 23.096 (14.708 to 35.098) | 2.895 (2.245 to 3.716) | | | | | | | | | | | | | | | | | | | | | |
| Neonatal sepsis and other neonatal infections | Birth prevalence - [34, 36) wks, [4000, 4500) g | Mortality | Females | | 25.038 (15.763 to 37.255) | 3.778 (2.855 to 4.925) | | | | | | | | | | | | | | | | | | | | | |
| Neonatal sepsis and other neonatal infections | Birth prevalence - [34, 36) wks, [3000, 3500) g | Mortality | Males | | 14.006 (10.222 to 18.478) | 4.322 (3.449 to 5.338) | | | | | | | | | | | | | | | | | | | | | |
| Neonatal sepsis and other neonatal infections | Birth prevalence - [34, 36) wks, [3000, 3500) g | Mortality | Females | | 14.375 (10.269 to 20.114) | 5.265 (4.145 to 6.564) | | | | | | | | | | | | | | | | | | | | | |
| Neonatal sepsis and other neonatal infections | Birth prevalence - [36, 37) wks, [2500, 3000) g | Mortality | Males | | 4.874 (4.014 to 5.713) | 3.699 (3.134 to 4.374) | | | | | | | | | | | | | | | | | | | | | |
| Neonatal sepsis and other neonatal infections | Birth prevalence - [36, 37) wks, [2500, 3000) g | Mortality | Females | | 4.609 (3.731 to 5.61) | 3.898 (3.235 to 4.67) | | | | | | | | | | | | | | | | | | | | | |
| Neonatal sepsis and other neonatal infections | Birth prevalence - [34, 36) wks, [3500, 4000) g | Mortality | Males | | 18.024 (12.279 to 25.547) | 3.657 (2.838 to 4.675) | | | | | | | | | | | | | | | | | | | | | |
| Neonatal sepsis and other neonatal infections | Birth prevalence - [34, 36) wks, [3500, 4000) g | Mortality | Females | | 19.263 (12.567 to 27.924) | 4.634 (3.576 to 5.989) | | | | | | | | | | | | | | | | | | | | | |
| Neonatal sepsis and other neonatal infections | Birth prevalence - [37, 38) wks, [2500, 3000) g | Mortality | Males | | 3.306 (2.82 to 3.843) | 3.194 (2.691 to 3.803) | | | | | | | | | | | | | | | | | | | | | |
| Neonatal sepsis and other neonatal infections | Birth prevalence - [37, 38) wks, [2500, 3000) g | Mortality | Females | | 2.991 (2.496 to 3.61) | 3.242 (2.745 to 3.817) | | | | | | | | | | | | | | | | | | | | | |
| Neonatal sepsis and other neonatal infections | Birth prevalence - [40, 42) wks, [2500, 3000) g | Mortality | Males | | 3.771 (3.002 to 4.693) | 3.175 (2.56 to 3.923) | | | | | | | | | | | | | | | | | | | | | |
| Neonatal sepsis and other neonatal infections | Birth prevalence - [40, 42) wks, [2500, 3000) g | Mortality | Females | | 3.244 (2.486 to 4.159) | 3.228 (2.605 to 3.985) | | | | | | | | | | | | | | | | | | | | | |
| Neonatal sepsis and other neonatal infections | Birth prevalence - [38, 40) wks, [2500, 3000) g | Mortality | Males | | 2.755 (2.274 to 3.309) | 2.944 (2.44 to 3.548) | | | | | | | | | | | | | | | | | | | | | |
| Neonatal sepsis and other neonatal infections | Birth prevalence - [38, 40) wks, [2500, 3000) g | Mortality | Females | | 2.376 (1.91 to 2.886) | 2.938 (2.434 to 3.503) | | | | | | | | | | | | | | | | | | | | | |
| Neonatal sepsis and other neonatal infections | Birth prevalence - [36, 37) wks, [3000, 3500) g | Mortality | Males | | 3.774 (3.094 to 4.497) | 2.466 (2.058 to 2.929) | | | | | | | | | | | | | | | | | | | | | |
| Neonatal sepsis and other neonatal infections | Birth prevalence - [36, 37) wks, [3000, 3500) g | Mortality | Females | | 3.73 (2.981 to 4.646) | 2.715 (2.277 to 3.218) | | | | | | | | | | | | | | | | | | | | | |
| Neonatal sepsis and other neonatal infections | Birth prevalence - [36, 37) wks, [4000, 4500) g | Mortality | Males | | 6.826 (5.212 to 9.045) | 1.77 (1.491 to 2.082) | | | | | | | | | | | | | | | | | | | | | |
| Neonatal sepsis and other neonatal infections | Birth prevalence - [36, 37) wks, [4000, 4500) g | Mortality | Females | | 7.269 (5.144 to 9.821) | 2.177 (1.786 to 2.607) | | | | | | | | | | | | | | | | | | | | | |
| Neonatal sepsis and other neonatal infections | Birth prevalence - [36, 37) wks, [3500, 4000) g | Mortality | Males | | 4.544 (3.64 to 5.622) | 2.057 (1.74 to 2.44) | | | | | | | | | | | | | | | | | | | | | |
| Neonatal sepsis and other neonatal infections | Birth prevalence - [36, 37) wks, [3500, 4000) g | Mortality | Females | | 4.662 (3.577 to 6.014) | 2.398 (1.98 to 2.864) | | | | | | | | | | | | | | | | | | | | | |
| Neonatal sepsis and other neonatal infections | Birth prevalence - [37, 38) wks, [3000, 3500) g | Mortality | Males | | 2.007 (1.759 to 2.293) | 1.888 (1.613 to 2.224) | | | | | | | | | | | | | | | | | | | | | |
| Neonatal sepsis and other neonatal infections | Birth prevalence - [37, 38) wks, [3000, 3500) g | Mortality | Females | | 1.925 (1.582 to 2.328) | 1.972 (1.669 to 2.313) | | | | | | | | | | | | | | | | | | | | | |
| Neonatal sepsis and other neonatal infections | Birth prevalence - [37, 38) wks, [4000, 4500) g | Mortality | Males | | 3.28 (2.596 to 4.133) | 1.335 (1.171 to 1.532) | | | | | | | | | | | | | | | | | | | | | |
| Neonatal sepsis and other neonatal infections | Birth prevalence - [37, 38) wks, [4000, 4500) g | Mortality | Females | | 3.521 (2.649 to 4.5) | 1.559 (1.333 to 1.835) | | | | | | | | | | | | | | | | | | | | | |
| Neonatal sepsis and other neonatal infections | Birth prevalence - [37, 38) wks, [3500, 4000) g | Mortality | Males | | 2.128 (1.833 to 2.466) | 1.505 (1.299 to 1.76) | | | | | | | | | | | | | | | | | | | | | |
| Neonatal sepsis and other neonatal infections | Birth prevalence - [37, 38) wks, [3500, 4000) g | Mortality | Females | | 2.142 (1.694 to 2.67) | 1.661 (1.411 to 1.961) | | | | | | | | | | | | | | | | | | | | | |
| Neonatal sepsis and other neonatal infections | Birth prevalence - [40, 42) wks, [3000, 3500) g | Mortality | Males | | 1.436 (1.245 to 1.65) | 1.47 (1.199 to 1.8) | | | | | | | | | | | | | | | | | | | | | |
| Neonatal sepsis and other neonatal infections | Birth prevalence - [40, 42) wks, [3000, 3500) g | Mortality | Females | | 1.326 (1.069 to 1.614) | 1.465 (1.188 to 1.775) | | | | | | | | | | | | | | | | | | | | | |
| Neonatal sepsis and other neonatal infections | Birth prevalence - [38, 40) wks, [3000, 3500) g | Mortality | Males | | 1.33 (1.155 to 1.53) | 1.559 (1.305 to 1.851) | | | | | | | | | | | | | | | | | | | | | |
| Neonatal sepsis and other neonatal infections | Birth prevalence - [38, 40) wks, [3000, 3500) g | Mortality | Females | | 1.224 (1.0 to 1.492) | 1.564 (1.304 to 1.847) | | | | | | | | | | | | | | | | | | | | | |
| Neonatal sepsis and other neonatal infections | Birth prevalence - [38, 40) wks, [4000, 4500) g | Mortality | Males | | 1.787 (1.453 to 2.182) | 1.175 (1.005 to 1.371) | | | | | | | | | | | | | | | | | | | | | |
| Neonatal sepsis and other neonatal infections | Birth prevalence - [38, 40) wks, [4000, 4500) g | Mortality | Females | | 1.877 (1.467 to 2.388) | 1.224 (1.022 to 1.465) | | | | | | | | | | | | | | | | | | | | | |
| Neonatal sepsis and other neonatal infections | Birth prevalence - [38, 40) wks, [3500, 4000) g | Mortality | Males | | 1.785 (1.478 to 2.147) | 1.173 (1.0 to 1.377) | | | | | | | | | | | | | | | | | | | | | |
| Neonatal sepsis and other neonatal infections | Birth prevalence - [38, 40) wks, [3500, 4000) g | Mortality | Females | | 1.892 (1.481 to 2.352) | 1.23 (1.03 to 1.46) | | | | | | | | | | | | | | | | | | | | | |
| Neonatal sepsis and other neonatal infections | Birth prevalence - [40, 42) wks, [3500, 4000) g | Mortality | Males | | 1.0 (1.0 to 1.0) | 1.003 (1.0 to 1.046) | | | | | | | | | | | | | | | | | | | | | |
| Neonatal sepsis and other neonatal infections | Birth prevalence - [40, 42) wks, [3500, 4000) g | Mortality | Females | | 1.002 (1.0 to 1.013) | 1.001 (1.0 to 1.006) | | | | | | | | | | | | | | | | | | | | | |
| Neonatal sepsis and other neonatal infections | Birth prevalence - [40, 42) wks, [4000, 4500) g | Mortality | Males | | 1.0 (1.0 to 1.0) | 1.0 (1.0 to 1.0) | | | | | | | | | | | | | | | | | | | | | |
| Neonatal sepsis and other neonatal infections | Birth prevalence - [40, 42) wks, [4000, 4500) g | Mortality | Females | | 1.0 (1.0 to 1.0) | 1.0 (1.0 to 1.0) | | | | | | | | | | | | | | | | | | | | | |
| Neonatal sepsis and other neonatal infections | Birth prevalence - [28, 30) wks, [2000, 2500) g | Mortality | Males | | 117.172 (83.895 to 158.056) | 27.726 (21.877 to 34.972) | | | | | | | | | | | | | | | | | | | | | |
| Neonatal sepsis and other neonatal infections | Birth prevalence - [28, 30) wks, [2000, 2500) g | Mortality | Females | | 121.682 (84.349 to 171.375) | 33.983 (26.101 to 43.404) | | | | | | | | | | | | | | | | | | | | | |
| Neonatal sepsis and other neonatal infections | Birth prevalence - [28, 30) wks, [2500, 3000) g | Mortality | Males | | 77.948 (54.687 to 105.047) | 16.608 (12.653 to 21.188) | | | | | | | | | | | | | | | | | | | | | |
| Neonatal sepsis and other neonatal infections | Birth prevalence - [28, 30) wks, [2500, 3000) g | Mortality | Females | | 79.193 (54.099 to 112.236) | 20.387 (15.089 to 26.434) | | | | | | | | | | | | | | | | | | | | | |
| Neonatal sepsis and other neonatal infections | Birth prevalence - [28, 30) wks, [3000, 3500) g | Mortality | Males | | 42.199 (29.891 to 57.227) | 10.082 (7.777 to 13.056) | | | | | | | | | | | | | | | | | | | | | |
| Neonatal sepsis and other neonatal infections | Birth prevalence - [28, 30) wks, [3000, 3500) g | Mortality | Females | | 42.551 (28.25 to 63.209) | 11.989 (9.084 to 15.544) | | | | | | | | | | | | | | | | | | | | | |

**Appendix Table 6a. Relative risks used by age and sex for each outcome for all risk factors except for ambient air pollution alcohol, and smoking.**

| | | | | Ages | | | | | | | | | | | | | | | | | | | | | | | |
|---|---|---|---|---|---|---|---|---|---|---|---|---|---|---|---|---|---|---|---|---|---|---|---|---|---|---|---|
| Risk - Outcome | Category / Units | Morbidity / Mortality | Sex | All-age | 0-6 days | 7-27 days | 28-364 days | 1-4 years | 5-9 years | 10-14 years | 15-19 years | 20-24 years | 25-29 years | 30-34 years | 35-39 years | 40-44 years | 45-49 years | 50-54 years | 55-59 years | 60-64 years | 65-69 years | 70-74 years | 75-79 years | 80-84 years | 85-89 years | 90-94 years | 95+ years |
| Neonatal sepsis and other neonatal infections | Birth prevalence - [30, 32) wks, [2500, 3000) g | Mortality | Males | | 58.722 (42.419 to 78.873) | 12.115 (9.518 to 15.521) | | | | | | | | | | | | | | | | | | | | | |
| Neonatal sepsis and other neonatal infections | Birth prevalence - [30, 32) wks, [2500, 3000) g | Mortality | Females | | 59.522 (42.058 to 82.793) | 15.364 (11.936 to 19.581) | | | | | | | | | | | | | | | | | | | | | |
| Neonatal sepsis and other neonatal infections | Birth prevalence - [30, 32) wks, [3000, 3500) g | Mortality | Males | | 45.67 (32.014 to 65.531) | 8.381 (6.362 to 10.842) | | | | | | | | | | | | | | | | | | | | | |
| Neonatal sepsis and other neonatal infections | Birth prevalence - [30, 32) wks, [3000, 3500) g | Mortality | Females | | 46.104 (30.243 to 66.207) | 10.506 (8.041 to 13.513) | | | | | | | | | | | | | | | | | | | | | |
| Neonatal sepsis and other neonatal infections | Birth prevalence - [30, 32) wks, [3500, 4000) g | Mortality | Males | | 36.334 (21.558 to 54.813) | 5.698 (4.293 to 7.349) | | | | | | | | | | | | | | | | | | | | | |
| Neonatal sepsis and other neonatal infections | Birth prevalence - [30, 32) wks, [3500, 4000) g | Mortality | Females | | 37.931 (22.692 to 61.276) | 6.892 (5.175 to 9.058) | | | | | | | | | | | | | | | | | | | | | |
| Neonatal sepsis and other neonatal infections | Birth prevalence - [32, 34) wks, [3000, 3500) g | Mortality | Males | | 34.016 (23.515 to 48.37) | 6.577 (4.934 to 8.436) | | | | | | | | | | | | | | | | | | | | | |
| Neonatal sepsis and other neonatal infections | Birth prevalence - [32, 34) wks, [3000, 3500) g | Mortality | Females | | 34.585 (22.909 to 49.754) | 8.314 (6.362 to 10.789) | | | | | | | | | | | | | | | | | | | | | |
| Neonatal sepsis and other neonatal infections | Birth prevalence - [32, 34) wks, [3500, 4000) g | Mortality | Males | | 36.248 (23.158 to 54.67) | 5.068 (3.761 to 6.741) | | | | | | | | | | | | | | | | | | | | | |
| Neonatal sepsis and other neonatal infections | Birth prevalence - [32, 34) wks, [3500, 4000) g | Mortality | Females | | 38.098 (23.301 to 59.429) | 6.476 (4.666 to 8.689) | | | | | | | | | | | | | | | | | | | | | |
| Neonatal sepsis and other neonatal infections | Birth prevalence - [36, 37) wks, [1000, 1500) g | Mortality | Males | | 166.686 (118.487 to 222.581) | 57.535 (45.999 to 71.742) | | | | | | | | | | | | | | | | | | | | | |
| Neonatal sepsis and other neonatal infections | Birth prevalence - [36, 37) wks, [1000, 1500) g | Mortality | Females | | 169.725 (119.017 to 229.008) | 63.564 (50.068 to 80.703) | | | | | | | | | | | | | | | | | | | | | |
| Neonatal sepsis and other neonatal infections | Birth prevalence - [38, 40) wks, [1000, 1500) g | Mortality | Males | | 174.066 (125.125 to 232.507) | 57.966 (44.393 to 73.241) | | | | | | | | | | | | | | | | | | | | | |
| Neonatal sepsis and other neonatal infections | Birth prevalence - [38, 40) wks, [1000, 1500) g | Mortality | Females | | 171.557 (121.585 to 237.047) | 65.208 (48.821 to 84.308) | | | | | | | | | | | | | | | | | | | | | |
| Neonatal sepsis and other neonatal infections | Birth prevalence - [38, 40) wks, [1500, 2000) g | Mortality | Males | | 67.302 (49.547 to 89.055) | 25.206 (20.365 to 31.168) | | | | | | | | | | | | | | | | | | | | | |
| Neonatal sepsis and other neonatal infections | Birth prevalence - [38, 40) wks, [1500, 2000) g | Mortality | Females | | 62.19 (45.884 to 83.445) | 28.05 (22.625 to 35.139) | | | | | | | | | | | | | | | | | | | | | |
| Neonatal sepsis and other neonatal infections | Birth prevalence - [40, 42) wks, [1500, 2000) g | Mortality | Males | | 76.673 (56.177 to 102.468) | 25.785 (19.387 to 34.168) | | | | | | | | | | | | | | | | | | | | | |
| Neonatal sepsis and other neonatal infections | Birth prevalence - [40, 42) wks, [1500, 2000) g | Mortality | Females | | 70.411 (49.221 to 97.952) | 29.113 (21.355 to 38.272) | | | | | | | | | | | | | | | | | | | | | |
| Hemolytic disease and other neonatal jaundice | Birth prevalence - [0, 24) wks, [0, 500) g | Mortality | Males | | 1564.792 (1056.542 to [illegible] | 618.595 (458.842 to 812.921) | | | | | | | | | | | | | | | | | | | | | |
| Hemolytic disease and other neonatal jaundice | Birth prevalence - [0, 24) wks, [0, 500) g | Mortality | Females | | 1600.122 (1050.664 to [illegible] | 713.571 (526.178 to 921.018) | | | | | | | | | | | | | | | | | | | | | |
| Hemolytic disease and other neonatal jaundice | Birth prevalence - [0, 24) wks, [500, 1000) g | Mortality | Males | | 1155.815 (825.412 to 1506.837) | 457.5 (352.552 to 573.483) | | | | | | | | | | | | | | | | | | | | | |
| Hemolytic disease and other neonatal jaundice | Birth prevalence - [0, 24) wks, [500, 1000) g | Mortality | Females | | 1169.123 (802.003 to 1617.979) | 515.406 (396.713 to 641.541) | | | | | | | | | | | | | | | | | | | | | |
| Hemolytic disease and other neonatal jaundice | Birth prevalence - [24, 26) wks, [500, 1000) g | Mortality | Males | | 955.583 (723.748 to 1244.265) | 443.357 (363.03 to 534.695) | | | | | | | | | | | | | | | | | | | | | |
| Hemolytic disease and other neonatal jaundice | Birth prevalence - [24, 26) wks, [500, 1000) g | Mortality | Females | | 947.143 (702.662 to 1237.093) | 487.549 (387.307 to 603.498) | | | | | | | | | | | | | | | | | | | | | |
| Hemolytic disease and other neonatal jaundice | Birth prevalence - [26, 28) wks, [500, 1000) g | Mortality | Males | | 497.817 (377.617 to 648.547) | 330.886 (261.438 to 401.709) | | | | | | | | | | | | | | | | | | | | | |
| Hemolytic disease and other neonatal jaundice | Birth prevalence - [26, 28) wks, [500, 1000) g | Mortality | Females | | 483.682 (354.946 to 629.517) | 344.618 (274.427 to 419.864) | | | | | | | | | | | | | | | | | | | | | |
| Hemolytic disease and other neonatal jaundice | Birth prevalence - [30, 32) wks, [500, 1000) g | Mortality | Males | | 236.614 (163.821 to 324.502) | 149.995 (117.866 to 188.368) | | | | | | | | | | | | | | | | | | | | | |
| Hemolytic disease and other neonatal jaundice | Birth prevalence - [30, 32) wks, [500, 1000) g | Mortality | Females | | 229.197 (157.606 to 317.194) | 152.117 (120.779 to 190.583) | | | | | | | | | | | | | | | | | | | | | |
| Hemolytic disease and other neonatal jaundice | Birth prevalence - [28, 30) wks, [500, 1000) g | Mortality | Males | | 297.629 (214.953 to 396.586) | 216.995 (173.321 to 271.466) | | | | | | | | | | | | | | | | | | | | | |
| Hemolytic disease and other neonatal jaundice | Birth prevalence - [28, 30) wks, [500, 1000) g | Mortality | Females | | 281.056 (198.176 to 386.635) | 219.884 (174.264 to 272.704) | | | | | | | | | | | | | | | | | | | | | |
| Hemolytic disease and other neonatal jaundice | Birth prevalence - [26, 28) wks, [1000, 1500) g | Mortality | Males | | 267.91 (210.177 to 332.92) | 164.167 (132.898 to 200.569) | | | | | | | | | | | | | | | | | | | | | |
| Hemolytic disease and other neonatal jaundice | Birth prevalence - [26, 28) wks, [1000, 1500) g | Mortality | Females | | 266.509 (197.461 to 346.932) | 174.222 (137.431 to 217.349) | | | | | | | | | | | | | | | | | | | | | |
| Hemolytic disease and other neonatal jaundice | Birth prevalence - [34, 36) wks, [1000, 1500) g | Mortality | Males | | 142.056 (98.086 to 197.774) | 52.86 (42.914 to 64.617) | | | | | | | | | | | | | | | | | | | | | |
| Hemolytic disease and other neonatal jaundice | Birth prevalence - [34, 36) wks, [1000, 1500) g | Mortality | Females | | 141.899 (95.864 to 197.656) | 57.421 (46.452 to 71.339) | | | | | | | | | | | | | | | | | | | | | |
| Hemolytic disease and other neonatal jaundice | Birth prevalence - [28, 30) wks, [1500, 2000) g | Mortality | Males | | 127.966 (97.178 to 167.026) | 50.018 (40.539 to 61.919) | | | | | | | | | | | | | | | | | | | | | |
| Hemolytic disease and other neonatal jaundice | Birth prevalence - [28, 30) wks, [1500, 2000) g | Mortality | Females | | 130.924 (96.513 to 172.188) | 57.275 (46.36 to 70.038) | | | | | | | | | | | | | | | | | | | | | |
| Hemolytic disease and other neonatal jaundice | Birth prevalence - [28, 30) wks, [1000, 1500) g | Mortality | Males | | 158.563 (120.99 to 204.947) | 103.32 (83.486 to 127.144) | | | | | | | | | | | | | | | | | | | | | |
| Hemolytic disease and other neonatal jaundice | Birth prevalence - [28, 30) wks, [1000, 1500) g | Mortality | Females | | 153.905 (112.327 to 200.786) | 107.529 (86.954 to 131.78) | | | | | | | | | | | | | | | | | | | | | |
| Hemolytic disease and other neonatal jaundice | Birth prevalence - [32, 34) wks, [1000, 1500) g | Mortality | Males | | 117.142 (81.354 to 161.101) | 53.185 (43.049 to 66.274) | | | | | | | | | | | | | | | | | | | | | |
| Hemolytic disease and other neonatal jaundice | Birth prevalence - [32, 34) wks, [1000, 1500) g | Mortality | Females | | 115.171 (79.363 to 159.206) | 56.034 (45.982 to 68.36) | | | | | | | | | | | | | | | | | | | | | |
| Hemolytic disease and other neonatal jaundice | Birth prevalence - [30, 32) wks, [1000, 1500) g | Mortality | Males | | 119.308 (87.769 to 160.885) | 67.163 (54.863 to 82.638) | | | | | | | | | | | | | | | | | | | | | |
| Hemolytic disease and other neonatal jaundice | Birth prevalence - [30, 32) wks, [1000, 1500) g | Mortality | Females | | 115.448 (84.272 to 156.425) | 69.14 (55.873 to 85.012) | | | | | | | | | | | | | | | | | | | | | |
| Hemolytic disease and other neonatal jaundice | Birth prevalence - [37, 38) wks, [1500, 2000) g | Mortality | Males | | 62.972 (46.159 to 83.484) | 24.148 (20.066 to 29.406) | | | | | | | | | | | | | | | | | | | | | |
| Hemolytic disease and other neonatal jaundice | Birth prevalence - [37, 38) wks, [1500, 2000) g | Mortality | Females | | 59.988 (43.974 to 79.053) | 26.719 (21.746 to 32.816) | | | | | | | | | | | | | | | | | | | | | |
| Hemolytic disease and other neonatal jaundice | Birth prevalence - [36, 37) wks, [1500, 2000) g | Mortality | Males | | 60.218 (43.669 to 82.48) | 23.031 (18.793 to 28.483) | | | | | | | | | | | | | | | | | | | | | |
| Hemolytic disease and other neonatal jaundice | Birth prevalence - [36, 37) wks, [1500, 2000) g | Mortality | Females | | 58.527 (42.172 to 80.557) | 25.143 (20.331 to 30.566) | | | | | | | | | | | | | | | | | | | | | |
| Hemolytic disease and other neonatal jaundice | Birth prevalence - [30, 32) wks, [2000, 2500) g | Mortality | Males | | 67.971 (50.354 to 88.935) | 18.03 (14.621 to 22.103) | | | | | | | | | | | | | | | | | | | | | |
| Hemolytic disease and other neonatal jaundice | Birth prevalence - [30, 32) wks, [2000, 2500) g | Mortality | Females | | 69.383 (49.108 to 94.583) | 22.069 (17.836 to 27.163) | | | | | | | | | | | | | | | | | | | | | |
| Hemolytic disease and other neonatal jaundice | Birth prevalence - [30, 32) wks, [1500, 2000) g | Mortality | Males | | 77.369 (59.702 to 99.232) | 31.079 (25.786 to 36.724) | | | | | | | | | | | | | | | | | | | | | |
| Hemolytic disease and other neonatal jaundice | Birth prevalence - [30, 32) wks, [1500, 2000) g | Mortality | Females | | 76.134 (56.885 to 100.996) | 34.756 (28.764 to 41.849) | | | | | | | | | | | | | | | | | | | | | |
| Hemolytic disease and other neonatal jaundice | Birth prevalence - [34, 36) wks, [1500, 2000) g | Mortality | Males | | 55.555 (39.553 to 75.104) | 21.346 (17.677 to 26.143) | | | | | | | | | | | | | | | | | | | | | |
| Hemolytic disease and other neonatal jaundice | Birth prevalence - [34, 36) wks, [1500, 2000) g | Mortality | Females | | 54.335 (38.617 to 75.24) | 23.046 (18.743 to 28.287) | | | | | | | | | | | | | | | | | | | | | |
| Hemolytic disease and other neonatal jaundice | Birth prevalence - [32, 34) wks, [1500, 2000) g | Mortality | Males | | 57.155 (42.484 to 73.651) | 23.114 (19.028 to 27.915) | | | | | | | | | | | | | | | | | | | | | |
| Hemolytic disease and other neonatal jaundice | Birth prevalence - [32, 34) wks, [1500, 2000) g | Mortality | Females | | 56.101 (39.794 to 76.295) | 25.149 (20.615 to 30.388) | | | | | | | | | | | | | | | | | | | | | |
| Hemolytic disease and other neonatal jaundice | Birth prevalence - [32, 34) wks, [2000, 2500) g | Mortality | Males | | 37.444 (29.026 to 48.227) | 12.233 (10.252 to 14.477) | | | | | | | | | | | | | | | | | | | | | |
| Hemolytic disease and other neonatal jaundice | Birth prevalence - [32, 34) wks, [2000, 2500) g | Mortality | Females | | 36.874 (26.658 to 49.653) | 14.384 (12.095 to 17.03) | | | | | | | | | | | | | | | | | | | | | |
| Hemolytic disease and other neonatal jaundice | Birth prevalence - [40, 42) wks, [2000, 2500) g | Mortality | Males | | 18.092 (13.292 to 23.719) | 9.23 (7.037 to 11.454) | | | | | | | | | | | | | | | | | | | | | |

Appendix Table 6a. Relative risks used by age and sex for each outcome for all risk factors except for ambient air pollution alcohol, and smoking.

| | | | | Ages | | | | | | | | | | | | | | | | | | | | | | | |
|---|---|---|---|---|---|---|---|---|---|---|---|---|---|---|---|---|---|---|---|---|---|---|---|---|---|---|---|
| Risk - Outcome | Category / Units | Morbidity / Mortality | Sex | All-age | 0-6 days | 7-27 days | 28-364 days | 1-4 years | 5-9 years | 10-14 years | 15-19 years | 20-24 years | 25-29 years | 30-34 years | 35-39 years | 40-44 years | 45-49 years | 50-54 years | 55-59 years | 60-64 years | 65-69 years | 70-74 years | 75-79 years | 80-84 years | 85-89 years | 90-94 years | 95+ years |
| Hemolytic disease and other neonatal jaundice | Birth prevalence - [40, 42) wks, [2000, 2500) g | Mortality | Females | | 15.574 (11.516 to 20.778) | 9.975 (7.82 to 12.46) | | | | | | | | | | | | | | | | | | | | | |
| Hemolytic disease and other neonatal jaundice | Birth prevalence - [38, 40) wks, [2000, 2500) g | Mortality | Males | | 13.104 (9.829 to 16.99) | 8.198 (6.786 to 9.959) | | | | | | | | | | | | | | | | | | | | | |
| Hemolytic disease and other neonatal jaundice | Birth prevalence - [38, 40) wks, [2000, 2500) g | Mortality | Females | | 11.308 (8.389 to 14.38) | 8.577 (7.04 to 10.449) | | | | | | | | | | | | | | | | | | | | | |
| Hemolytic disease and other neonatal jaundice | Birth prevalence - [32, 34) wks, [2500, 3000) g | Mortality | Males | | 33.063 (24.393 to 43.503) | 8.441 (6.822 to 10.431) | | | | | | | | | | | | | | | | | | | | | |
| Hemolytic disease and other neonatal jaundice | Birth prevalence - [32, 34) wks, [2500, 3000) g | Mortality | Females | | 32.812 (23.439 to 45.567) | 10.398 (8.227 to 13.042) | | | | | | | | | | | | | | | | | | | | | |
| Hemolytic disease and other neonatal jaundice | Birth prevalence - [34, 36) wks, [2000, 2500) g | Mortality | Males | | 21.925 (16.305 to 29.433) | 9.367 (7.859 to 11.112) | | | | | | | | | | | | | | | | | | | | | |
| Hemolytic disease and other neonatal jaundice | Birth prevalence - [34, 36) wks, [2000, 2500) g | Mortality | Females | | 21.297 (15.657 to 28.761) | 10.295 (8.548 to 12.273) | | | | | | | | | | | | | | | | | | | | | |
| Hemolytic disease and other neonatal jaundice | Birth prevalence - [37, 38) wks, [2000, 2500) g | Mortality | Males | | 13.0 (10.102 to 16.456) | 8.096 (6.724 to 9.676) | | | | | | | | | | | | | | | | | | | | | |
| Hemolytic disease and other neonatal jaundice | Birth prevalence - [37, 38) wks, [2000, 2500) g | Mortality | Females | | 11.563 (8.805 to 15.11) | 8.467 (6.994 to 10.342) | | | | | | | | | | | | | | | | | | | | | |
| Hemolytic disease and other neonatal jaundice | Birth prevalence - [36, 37) wks, [2000, 2500) g | Mortality | Males | | 14.401 (10.789 to 18.654) | 8.221 (6.917 to 9.923) | | | | | | | | | | | | | | | | | | | | | |
| Hemolytic disease and other neonatal jaundice | Birth prevalence - [36, 37) wks, [2000, 2500) g | Mortality | Females | | 13.513 (9.817 to 17.942) | 8.654 (7.215 to 10.369) | | | | | | | | | | | | | | | | | | | | | |
| Hemolytic disease and other neonatal jaundice | Birth prevalence - [34, 36) wks, [2500, 3000) g | Mortality | Males | | 13.419 (10.387 to 16.819) | 5.562 (4.646 to 6.696) | | | | | | | | | | | | | | | | | | | | | |
| Hemolytic disease and other neonatal jaundice | Birth prevalence - [34, 36) wks, [2500, 3000) g | Mortality | Females | | 13.266 (9.666 to 17.689) | 6.395 (5.292 to 7.606) | | | | | | | | | | | | | | | | | | | | | |
| Hemolytic disease and other neonatal jaundice | Birth prevalence - [34, 36) wks, [4000, 4500) g | Mortality | Males | | 23.096 (14.708 to 35.098) | 2.895 (2.245 to 3.716) | | | | | | | | | | | | | | | | | | | | | |
| Hemolytic disease and other neonatal jaundice | Birth prevalence - [34, 36) wks, [4000, 4500) g | Mortality | Females | | 25.038 (15.763 to 37.255) | 3.778 (2.855 to 4.925) | | | | | | | | | | | | | | | | | | | | | |
| Hemolytic disease and other neonatal jaundice | Birth prevalence - [34, 36) wks, [3000, 3500) g | Mortality | Males | | 14.006 (10.222 to 18.478) | 4.322 (3.449 to 5.338) | | | | | | | | | | | | | | | | | | | | | |
| Hemolytic disease and other neonatal jaundice | Birth prevalence - [34, 36) wks, [3000, 3500) g | Mortality | Females | | 14.375 (10.269 to 20.114) | 5.265 (4.145 to 6.564) | | | | | | | | | | | | | | | | | | | | | |
| Hemolytic disease and other neonatal jaundice | Birth prevalence - [36, 37) wks, [2500, 3000) g | Mortality | Males | | 4.874 (4.014 to 5.713) | 3.699 (3.134 to 4.374) | | | | | | | | | | | | | | | | | | | | | |
| Hemolytic disease and other neonatal jaundice | Birth prevalence - [36, 37) wks, [2500, 3000) g | Mortality | Females | | 4.609 (3.731 to 5.61) | 3.898 (3.235 to 4.67) | | | | | | | | | | | | | | | | | | | | | |
| Hemolytic disease and other neonatal jaundice | Birth prevalence - [34, 36) wks, [3500, 4000) g | Mortality | Males | | 18.024 (12.279 to 25.547) | 3.657 (2.838 to 4.675) | | | | | | | | | | | | | | | | | | | | | |
| Hemolytic disease and other neonatal jaundice | Birth prevalence - [34, 36) wks, [3500, 4000) g | Mortality | Females | | 19.263 (12.567 to 27.924) | 4.634 (3.576 to 5.989) | | | | | | | | | | | | | | | | | | | | | |
| Hemolytic disease and other neonatal jaundice | Birth prevalence - [37, 38) wks, [2500, 3000) g | Mortality | Males | | 3.306 (2.82 to 3.843) | 3.194 (2.691 to 3.803) | | | | | | | | | | | | | | | | | | | | | |
| Hemolytic disease and other neonatal jaundice | Birth prevalence - [37, 38) wks, [2500, 3000) g | Mortality | Females | | 2.991 (2.496 to 3.61) | 3.242 (2.745 to 3.817) | | | | | | | | | | | | | | | | | | | | | |
| Hemolytic disease and other neonatal jaundice | Birth prevalence - [40, 42) wks, [2500, 3000) g | Mortality | Males | | 3.771 (3.002 to 4.693) | 3.175 (2.56 to 3.923) | | | | | | | | | | | | | | | | | | | | | |
| Hemolytic disease and other neonatal jaundice | Birth prevalence - [40, 42) wks, [2500, 3000) g | Mortality | Females | | 3.244 (2.486 to 4.159) | 3.228 (2.605 to 3.985) | | | | | | | | | | | | | | | | | | | | | |
| Hemolytic disease and other neonatal jaundice | Birth prevalence - [38, 40) wks, [2500, 3000) g | Mortality | Males | | 2.755 (2.274 to 3.309) | 2.944 (2.44 to 3.548) | | | | | | | | | | | | | | | | | | | | | |
| Hemolytic disease and other neonatal jaundice | Birth prevalence - [38, 40) wks, [2500, 3000) g | Mortality | Females | | 2.376 (1.91 to 2.886) | 2.938 (2.434 to 3.503) | | | | | | | | | | | | | | | | | | | | | |
| Hemolytic disease and other neonatal jaundice | Birth prevalence - [36, 37) wks, [3000, 3500) g | Mortality | Males | | 3.774 (3.094 to 4.497) | 2.466 (2.058 to 2.929) | | | | | | | | | | | | | | | | | | | | | |
| Hemolytic disease and other neonatal jaundice | Birth prevalence - [36, 37) wks, [3000, 3500) g | Mortality | Females | | 3.73 (2.981 to 4.646) | 2.715 (2.277 to 3.218) | | | | | | | | | | | | | | | | | | | | | |
| Hemolytic disease and other neonatal jaundice | Birth prevalence - [36, 37) wks, [4000, 4500) g | Mortality | Males | | 6.826 (5.212 to 9.045) | 1.77 (1.491 to 2.082) | | | | | | | | | | | | | | | | | | | | | |
| Hemolytic disease and other neonatal jaundice | Birth prevalence - [36, 37) wks, [4000, 4500) g | Mortality | Females | | 7.269 (5.144 to 9.821) | 2.177 (1.786 to 2.607) | | | | | | | | | | | | | | | | | | | | | |
| Hemolytic disease and other neonatal jaundice | Birth prevalence - [36, 37) wks, [3500, 4000) g | Mortality | Males | | 4.544 (3.64 to 5.622) | 2.057 (1.74 to 2.44) | | | | | | | | | | | | | | | | | | | | | |
| Hemolytic disease and other neonatal jaundice | Birth prevalence - [36, 37) wks, [3500, 4000) g | Mortality | Females | | 4.662 (3.577 to 6.014) | 2.398 (1.98 to 2.864) | | | | | | | | | | | | | | | | | | | | | |
| Hemolytic disease and other neonatal jaundice | Birth prevalence - [37, 38) wks, [3000, 3500) g | Mortality | Males | | 2.007 (1.759 to 2.293) | 1.888 (1.613 to 2.224) | | | | | | | | | | | | | | | | | | | | | |
| Hemolytic disease and other neonatal jaundice | Birth prevalence - [37, 38) wks, [3000, 3500) g | Mortality | Females | | 1.925 (1.582 to 2.328) | 1.972 (1.669 to 2.313) | | | | | | | | | | | | | | | | | | | | | |
| Hemolytic disease and other neonatal jaundice | Birth prevalence - [37, 38) wks, [4000, 4500) g | Mortality | Males | | 3.28 (2.596 to 4.133) | 1.335 (1.171 to 1.532) | | | | | | | | | | | | | | | | | | | | | |
| Hemolytic disease and other neonatal jaundice | Birth prevalence - [37, 38) wks, [4000, 4500) g | Mortality | Females | | 3.521 (2.649 to 4.5) | 1.559 (1.333 to 1.835) | | | | | | | | | | | | | | | | | | | | | |
| Hemolytic disease and other neonatal jaundice | Birth prevalence - [37, 38) wks, [3500, 4000) g | Mortality | Males | | 2.128 (1.833 to 2.466) | 1.505 (1.299 to 1.76) | | | | | | | | | | | | | | | | | | | | | |
| Hemolytic disease and other neonatal jaundice | Birth prevalence - [37, 38) wks, [3500, 4000) g | Mortality | Females | | 2.142 (1.694 to 2.67) | 1.661 (1.411 to 1.961) | | | | | | | | | | | | | | | | | | | | | |
| Hemolytic disease and other neonatal jaundice | Birth prevalence - [40, 42) wks, [3000, 3500) g | Mortality | Males | | 1.436 (1.245 to 1.65) | 1.47 (1.199 to 1.8) | | | | | | | | | | | | | | | | | | | | | |
| Hemolytic disease and other neonatal jaundice | Birth prevalence - [40, 42) wks, [3000, 3500) g | Mortality | Females | | 1.326 (1.069 to 1.614) | 1.465 (1.188 to 1.775) | | | | | | | | | | | | | | | | | | | | | |
| Hemolytic disease and other neonatal jaundice | Birth prevalence - [38, 40) wks, [3000, 3500) g | Mortality | Males | | 1.33 (1.155 to 1.53) | 1.559 (1.305 to 1.851) | | | | | | | | | | | | | | | | | | | | | |
| Hemolytic disease and other neonatal jaundice | Birth prevalence - [38, 40) wks, [3000, 3500) g | Mortality | Females | | 1.224 (1.0 to 1.492) | 1.564 (1.304 to 1.847) | | | | | | | | | | | | | | | | | | | | | |
| Hemolytic disease and other neonatal jaundice | Birth prevalence - [38, 40) wks, [4000, 4500) g | Mortality | Males | | 1.787 (1.453 to 2.182) | 1.175 (1.005 to 1.371) | | | | | | | | | | | | | | | | | | | | | |
| Hemolytic disease and other neonatal jaundice | Birth prevalence - [38, 40) wks, [4000, 4500) g | Mortality | Females | | 1.877 (1.467 to 2.388) | 1.224 (1.022 to 1.465) | | | | | | | | | | | | | | | | | | | | | |
| Hemolytic disease and other neonatal jaundice | Birth prevalence - [38, 40) wks, [3500, 4000) g | Mortality | Males | | 1.785 (1.478 to 2.147) | 1.173 (1.0 to 1.377) | | | | | | | | | | | | | | | | | | | | | |
| Hemolytic disease and other neonatal jaundice | Birth prevalence - [38, 40) wks, [3500, 4000) g | Mortality | Females | | 1.892 (1.481 to 2.352) | 1.23 (1.03 to 1.46) | | | | | | | | | | | | | | | | | | | | | |
| Hemolytic disease and other neonatal jaundice | Birth prevalence - [40, 42) wks, [3500, 4000) g | Mortality | Males | | 1.0 (1.0 to 1.0) | 1.003 (1.0 to 1.046) | | | | | | | | | | | | | | | | | | | | | |
| Hemolytic disease and other neonatal jaundice | Birth prevalence - [40, 42) wks, [3500, 4000) g | Mortality | Females | | 1.002 (1.0 to 1.013) | 1.001 (1.0 to 1.006) | | | | | | | | | | | | | | | | | | | | | |
| Hemolytic disease and other neonatal jaundice | Birth prevalence - [40, 42) wks, [4000, 4500) g | Mortality | Males | | 1.0 (1.0 to 1.0) | 1.0 (1.0 to 1.0) | | | | | | | | | | | | | | | | | | | | | |
| Hemolytic disease and other neonatal jaundice | Birth prevalence - [40, 42) wks, [4000, 4500) g | Mortality | Females | | 1.0 (1.0 to 1.0) | 1.0 (1.0 to 1.0) | | | | | | | | | | | | | | | | | | | | | |
| Hemolytic disease and other neonatal jaundice | Birth prevalence - [28, 30) wks, [2000, 2500) g | Mortality | Males | | 117.172 (83.895 to 158.056) | 27.726 (21.877 to 34.972) | | | | | | | | | | | | | | | | | | | | | |
| Hemolytic disease and other neonatal jaundice | Birth prevalence - [28, 30) wks, [2000, 2500) g | Mortality | Females | | 121.682 (84.349 to 171.375) | 33.983 (26.101 to 43.404) | | | | | | | | | | | | | | | | | | | | | |
| Hemolytic disease and other neonatal jaundice | Birth prevalence - [28, 30) wks, [2500, 3000) g | Mortality | Males | | 77.948 (54.687 to 105.047) | 16.608 (12.653 to 21.188) | | | | | | | | | | | | | | | | | | | | | |
| Hemolytic disease and other neonatal jaundice | Birth prevalence - [28, 30) wks, [2500, 3000) g | Mortality | Females | | 79.193 (54.099 to 112.236) | 20.387 (15.089 to 26.434) | | | | | | | | | | | | | | | | | | | | | |
| Hemolytic disease and other neonatal jaundice | Birth prevalence - [28, 30) wks, [3000, 3500) g | Mortality | Males | | 42.199 (29.891 to 57.227) | 10.082 (7.777 to 13.056) | | | | | | | | | | | | | | | | | | | | | |
| Hemolytic disease and other neonatal jaundice | Birth prevalence - [28, 30) wks, [3000, 3500) g | Mortality | Females | | 42.551 (28.25 to 63.209) | 11.989 (9.084 to 15.544) | | | | | | | | | | | | | | | | | | | | | |

**Appendix Table 6a. Relative risks used by age and sex for each outcome for all risk factors except for ambient air pollution alcohol, and smoking.**

| | | | | Ages | | | | | | | | | | | | | | | | | | | | | | | |
|---|---|---|---|---|---|---|---|---|---|---|---|---|---|---|---|---|---|---|---|---|---|---|---|---|---|---|---|
| Risk - Outcome | Category / Units | Morbidity / Mortality | Sex | All-age | 0-6 days | 7-27 days | 28-364 days | 1-4 years | 5-9 years | 10-14 years | 15-19 years | 20-24 years | 25-29 years | 30-34 years | 35-39 years | 40-44 years | 45-49 years | 50-54 years | 55-59 years | 60-64 years | 65-69 years | 70-74 years | 75-79 years | 80-84 years | 85-89 years | 90-94 years | 95+ years |
| Hemolytic disease and other neonatal jaundice | Birth prevalence - [30, 32) wks, [2500, 3000) g | Mortality | Males | | 58.722 (42.419 to 78.873) | 12.115 (9.518 to 15.521) | | | | | | | | | | | | | | | | | | | | | |
| Hemolytic disease and other neonatal jaundice | Birth prevalence - [30, 32) wks, [2500, 3000) g | Mortality | Females | | 59.522 (42.058 to 82.793) | 15.364 (11.936 to 19.581) | | | | | | | | | | | | | | | | | | | | | |
| Hemolytic disease and other neonatal jaundice | Birth prevalence - [30, 32) wks, [3000, 3500) g | Mortality | Males | | 45.67 (32.014 to 65.531) | 8.381 (6.362 to 10.842) | | | | | | | | | | | | | | | | | | | | | |
| Hemolytic disease and other neonatal jaundice | Birth prevalence - [30, 32) wks, [3000, 3500) g | Mortality | Females | | 46.104 (30.243 to 66.207) | 10.506 (8.041 to 13.513) | | | | | | | | | | | | | | | | | | | | | |
| Hemolytic disease and other neonatal jaundice | Birth prevalence - [30, 32) wks, [3500, 4000) g | Mortality | Males | | 36.334 (21.558 to 54.813) | 5.698 (4.293 to 7.349) | | | | | | | | | | | | | | | | | | | | | |
| Hemolytic disease and other neonatal jaundice | Birth prevalence - [30, 32) wks, [3500, 4000) g | Mortality | Females | | 37.931 (22.692 to 61.276) | 6.892 (5.175 to 9.058) | | | | | | | | | | | | | | | | | | | | | |
| Hemolytic disease and other neonatal jaundice | Birth prevalence - [32, 34) wks, [3000, 3500) g | Mortality | Males | | 34.016 (23.515 to 48.37) | 6.577 (4.934 to 8.436) | | | | | | | | | | | | | | | | | | | | | |
| Hemolytic disease and other neonatal jaundice | Birth prevalence - [32, 34) wks, [3000, 3500) g | Mortality | Females | | 34.585 (22.909 to 49.754) | 8.314 (6.362 to 10.789) | | | | | | | | | | | | | | | | | | | | | |
| Hemolytic disease and other neonatal jaundice | Birth prevalence - [32, 34) wks, [3500, 4000) g | Mortality | Males | | 36.248 (23.158 to 54.67) | 5.068 (3.761 to 6.741) | | | | | | | | | | | | | | | | | | | | | |
| Hemolytic disease and other neonatal jaundice | Birth prevalence - [32, 34) wks, [3500, 4000) g | Mortality | Females | | 38.098 (23.301 to 59.429) | 6.476 (4.666 to 8.689) | | | | | | | | | | | | | | | | | | | | | |
| Hemolytic disease and other neonatal jaundice | Birth prevalence - [36, 37) wks, [1000, 1500) g | Mortality | Males | | 166.686 (118.487 to 222.581) | 57.535 (45.999 to 71.742) | | | | | | | | | | | | | | | | | | | | | |
| Hemolytic disease and other neonatal jaundice | Birth prevalence - [36, 37) wks, [1000, 1500) g | Mortality | Females | | 169.725 (119.017 to 229.008) | 63.564 (50.068 to 80.703) | | | | | | | | | | | | | | | | | | | | | |
| Hemolytic disease and other neonatal jaundice | Birth prevalence - [38, 40) wks, [1000, 1500) g | Mortality | Males | | 174.066 (125.125 to 232.507) | 57.966 (44.393 to 73.241) | | | | | | | | | | | | | | | | | | | | | |
| Hemolytic disease and other neonatal jaundice | Birth prevalence - [38, 40) wks, [1000, 1500) g | Mortality | Females | | 171.557 (121.585 to 237.047) | 65.208 (48.821 to 84.308) | | | | | | | | | | | | | | | | | | | | | |
| Hemolytic disease and other neonatal jaundice | Birth prevalence - [38, 40) wks, [1500, 2000) g | Mortality | Males | | 67.302 (49.547 to 89.055) | 25.206 (20.365 to 31.168) | | | | | | | | | | | | | | | | | | | | | |
| Hemolytic disease and other neonatal jaundice | Birth prevalence - [38, 40) wks, [1500, 2000) g | Mortality | Females | | 62.19 (45.884 to 83.445) | 28.05 (22.625 to 35.139) | | | | | | | | | | | | | | | | | | | | | |
| Hemolytic disease and other neonatal jaundice | Birth prevalence - [40, 42) wks, [1500, 2000) g | Mortality | Males | | 76.673 (56.177 to 102.468) | 25.785 (19.387 to 34.168) | | | | | | | | | | | | | | | | | | | | | |
| Hemolytic disease and other neonatal jaundice | Birth prevalence - [40, 42) wks, [1500, 2000) g | Mortality | Females | | 70.411 (49.221 to 97.952) | 29.113 (21.355 to 38.272) | | | | | | | | | | | | | | | | | | | | | |
| Other neonatal disorders | Birth prevalence - [0, 24) wks, [0, 500) g | Mortality | Males | | 1564.792 (1056.542 to [illegible] | 618.595 (458.842 to 812.921) | | | | | | | | | | | | | | | | | | | | | |
| Other neonatal disorders | Birth prevalence - [0, 24) wks, [0, 500) g | Mortality | Females | | 1600.122 (1050.664 to [illegible] | 713.571 (526.178 to 921.018) | | | | | | | | | | | | | | | | | | | | | |
| Other neonatal disorders | Birth prevalence - [0, 24) wks, [500, 1000) g | Mortality | Males | | 1155.815 (825.412 to 1506.837) | 457.5 (352.552 to 573.483) | | | | | | | | | | | | | | | | | | | | | |
| Other neonatal disorders | Birth prevalence - [0, 24) wks, [500, 1000) g | Mortality | Females | | 1169.123 (802.003 to 1617.979) | 515.406 (396.713 to 641.541) | | | | | | | | | | | | | | | | | | | | | |
| Other neonatal disorders | Birth prevalence - [24, 26) wks, [500, 1000) g | Mortality | Males | | 955.583 (723.748 to 1244.265) | 443.357 (363.03 to 534.695) | | | | | | | | | | | | | | | | | | | | | |
| Other neonatal disorders | Birth prevalence - [24, 26) wks, [500, 1000) g | Mortality | Females | | 947.143 (702.662 to 1237.093) | 487.549 (387.307 to 603.498) | | | | | | | | | | | | | | | | | | | | | |
| Other neonatal disorders | Birth prevalence - [26, 28) wks, [500, 1000) g | Mortality | Males | | 497.817 (377.617 to 648.547) | 330.886 (261.438 to 401.709) | | | | | | | | | | | | | | | | | | | | | |
| Other neonatal disorders | Birth prevalence - [26, 28) wks, [500, 1000) g | Mortality | Females | | 483.682 (354.946 to 629.517) | 344.618 (274.427 to 419.864) | | | | | | | | | | | | | | | | | | | | | |
| Other neonatal disorders | Birth prevalence - [30, 32) wks, [500, 1000) g | Mortality | Males | | 236.614 (163.821 to 324.502) | 149.995 (117.866 to 188.368) | | | | | | | | | | | | | | | | | | | | | |
| Other neonatal disorders | Birth prevalence - [30, 32) wks, [500, 1000) g | Mortality | Females | | 229.197 (157.606 to 317.194) | 152.117 (120.779 to 190.583) | | | | | | | | | | | | | | | | | | | | | |
| Other neonatal disorders | Birth prevalence - [28, 30) wks, [500, 1000) g | Mortality | Males | | 297.629 (214.953 to 396.586) | 216.995 (173.321 to 271.466) | | | | | | | | | | | | | | | | | | | | | |
| Other neonatal disorders | Birth prevalence - [28, 30) wks, [500, 1000) g | Mortality | Females | | 281.056 (198.176 to 386.635) | 219.884 (174.264 to 272.704) | | | | | | | | | | | | | | | | | | | | | |
| Other neonatal disorders | Birth prevalence - [26, 28) wks, [1000, 1500) g | Mortality | Males | | 267.91 (210.177 to 332.92) | 164.167 (132.898 to 200.569) | | | | | | | | | | | | | | | | | | | | | |
| Other neonatal disorders | Birth prevalence - [26, 28) wks, [1000, 1500) g | Mortality | Females | | 266.509 (197.461 to 346.932) | 174.222 (137.431 to 217.349) | | | | | | | | | | | | | | | | | | | | | |
| Other neonatal disorders | Birth prevalence - [34, 36) wks, [1000, 1500) g | Mortality | Males | | 142.056 (98.086 to 197.774) | 52.86 (42.914 to 64.617) | | | | | | | | | | | | | | | | | | | | | |
| Other neonatal disorders | Birth prevalence - [34, 36) wks, [1000, 1500) g | Mortality | Females | | 141.899 (95.864 to 197.656) | 57.421 (46.452 to 71.339) | | | | | | | | | | | | | | | | | | | | | |
| Other neonatal disorders | Birth prevalence - [28, 30) wks, [1500, 2000) g | Mortality | Males | | 127.966 (97.178 to 167.026) | 50.018 (40.539 to 61.919) | | | | | | | | | | | | | | | | | | | | | |
| Other neonatal disorders | Birth prevalence - [28, 30) wks, [1500, 2000) g | Mortality | Females | | 130.924 (96.513 to 172.188) | 57.275 (46.36 to 70.038) | | | | | | | | | | | | | | | | | | | | | |
| Other neonatal disorders | Birth prevalence - [28, 30) wks, [1000, 1500) g | Mortality | Males | | 158.563 (120.99 to 204.947) | 103.32 (83.486 to 127.144) | | | | | | | | | | | | | | | | | | | | | |
| Other neonatal disorders | Birth prevalence - [28, 30) wks, [1000, 1500) g | Mortality | Females | | 153.905 (112.327 to 200.786) | 107.529 (86.954 to 131.78) | | | | | | | | | | | | | | | | | | | | | |
| Other neonatal disorders | Birth prevalence - [32, 34) wks, [1000, 1500) g | Mortality | Males | | 117.142 (81.354 to 161.101) | 53.185 (43.049 to 66.274) | | | | | | | | | | | | | | | | | | | | | |
| Other neonatal disorders | Birth prevalence - [32, 34) wks, [1000, 1500) g | Mortality | Females | | 115.171 (79.363 to 159.206) | 56.034 (45.982 to 68.36) | | | | | | | | | | | | | | | | | | | | | |
| Other neonatal disorders | Birth prevalence - [30, 32) wks, [1000, 1500) g | Mortality | Males | | 119.308 (87.769 to 160.885) | 67.163 (54.863 to 82.638) | | | | | | | | | | | | | | | | | | | | | |
| Other neonatal disorders | Birth prevalence - [30, 32) wks, [1000, 1500) g | Mortality | Females | | 115.448 (84.272 to 156.425) | 69.14 (55.873 to 85.012) | | | | | | | | | | | | | | | | | | | | | |
| Other neonatal disorders | Birth prevalence - [37, 38) wks, [1500, 2000) g | Mortality | Males | | 62.972 (46.159 to 83.484) | 24.148 (20.066 to 29.406) | | | | | | | | | | | | | | | | | | | | | |
| Other neonatal disorders | Birth prevalence - [37, 38) wks, [1500, 2000) g | Mortality | Females | | 59.988 (43.974 to 79.053) | 26.719 (21.746 to 32.816) | | | | | | | | | | | | | | | | | | | | | |
| Other neonatal disorders | Birth prevalence - [36, 37) wks, [1500, 2000) g | Mortality | Males | | 60.218 (43.669 to 82.48) | 23.031 (18.793 to 28.483) | | | | | | | | | | | | | | | | | | | | | |
| Other neonatal disorders | Birth prevalence - [36, 37) wks, [1500, 2000) g | Mortality | Females | | 58.527 (42.172 to 80.557) | 25.143 (20.331 to 30.566) | | | | | | | | | | | | | | | | | | | | | |
| Other neonatal disorders | Birth prevalence - [30, 32) wks, [2000, 2500) g | Mortality | Males | | 67.971 (50.354 to 88.935) | 18.03 (14.621 to 22.103) | | | | | | | | | | | | | | | | | | | | | |
| Other neonatal disorders | Birth prevalence - [30, 32) wks, [2000, 2500) g | Mortality | Females | | 69.383 (49.108 to 94.583) | 22.069 (17.836 to 27.163) | | | | | | | | | | | | | | | | | | | | | |
| Other neonatal disorders | Birth prevalence - [30, 32) wks, [1500, 2000) g | Mortality | Males | | 77.369 (59.702 to 99.232) | 31.079 (25.786 to 36.724) | | | | | | | | | | | | | | | | | | | | | |
| Other neonatal disorders | Birth prevalence - [30, 32) wks, [1500, 2000) g | Mortality | Females | | 76.134 (56.885 to 100.996) | 34.756 (28.764 to 41.849) | | | | | | | | | | | | | | | | | | | | | |
| Other neonatal disorders | Birth prevalence - [34, 36) wks, [1500, 2000) g | Mortality | Males | | 55.555 (39.553 to 75.104) | 21.346 (17.677 to 26.143) | | | | | | | | | | | | | | | | | | | | | |
| Other neonatal disorders | Birth prevalence - [34, 36) wks, [1500, 2000) g | Mortality | Females | | 54.335 (38.617 to 75.24) | 23.046 (18.743 to 28.287) | | | | | | | | | | | | | | | | | | | | | |
| Other neonatal disorders | Birth prevalence - [32, 34) wks, [1500, 2000) g | Mortality | Males | | 57.155 (42.484 to 73.651) | 23.114 (19.028 to 27.915) | | | | | | | | | | | | | | | | | | | | | |
| Other neonatal disorders | Birth prevalence - [32, 34) wks, [1500, 2000) g | Mortality | Females | | 56.101 (39.794 to 76.295) | 25.149 (20.615 to 30.388) | | | | | | | | | | | | | | | | | | | | | |
| Other neonatal disorders | Birth prevalence - [32, 34) wks, [2000, 2500) g | Mortality | Males | | 37.444 (29.026 to 48.227) | 12.233 (10.252 to 14.477) | | | | | | | | | | | | | | | | | | | | | |
| Other neonatal disorders | Birth prevalence - [32, 34) wks, [2000, 2500) g | Mortality | Females | | 36.874 (26.658 to 49.653) | 14.384 (12.095 to 17.03) | | | | | | | | | | | | | | | | | | | | | |
| Other neonatal disorders | Birth prevalence - [40, 42) wks, [2000, 2500) g | Mortality | Males | | 18.092 (13.292 to 23.719) | 9.23 (7.037 to 11.454) | | | | | | | | | | | | | | | | | | | | | |

**Appendix Table 6a. Relative risks used by age and sex for each outcome for all risk factors except for ambient air pollution alcohol, and smoking.**

| | | | | Ages | | | | | | | | | | | | | | | | | | | | | | | |
|---|---|---|---|---|---|---|---|---|---|---|---|---|---|---|---|---|---|---|---|---|---|---|---|---|---|---|---|
| Risk - Outcome | Category / Units | Morbidity / Mortality | Sex | All-age | 0-6 days | 7-27 days | 28-364 days | 1-4 years | 5-9 years | 10-14 years | 15-19 years | 20-24 years | 25-29 years | 30-34 years | 35-39 years | 40-44 years | 45-49 years | 50-54 years | 55-59 years | 60-64 years | 65-69 years | 70-74 years | 75-79 years | 80-84 years | 85-89 years | 90-94 years | 95+ years |
| Other neonatal disorders | Birth prevalence - [40, 42) wks, [2000, 2500) g | Mortality | Females | | 15.574 (11.516 to 20.778) | 9.975 (7.82 to 12.46) | | | | | | | | | | | | | | | | | | | | | |
| Other neonatal disorders | Birth prevalence - [38, 40) wks, [2000, 2500) g | Mortality | Males | | 13.104 (9.829 to 16.99) | 8.198 (6.786 to 9.959) | | | | | | | | | | | | | | | | | | | | | |
| Other neonatal disorders | Birth prevalence - [38, 40) wks, [2000, 2500) g | Mortality | Females | | 11.308 (8.389 to 14.38) | 8.577 (7.04 to 10.449) | | | | | | | | | | | | | | | | | | | | | |
| Other neonatal disorders | Birth prevalence - [32, 34) wks, [2500, 3000) g | Mortality | Males | | 33.063 (24.393 to 43.503) | 8.441 (6.822 to 10.431) | | | | | | | | | | | | | | | | | | | | | |
| Other neonatal disorders | Birth prevalence - [32, 34) wks, [2500, 3000) g | Mortality | Females | | 32.812 (23.439 to 45.567) | 10.398 (8.227 to 13.042) | | | | | | | | | | | | | | | | | | | | | |
| Other neonatal disorders | Birth prevalence - [34, 36) wks, [2000, 2500) g | Mortality | Males | | 21.925 (16.305 to 29.433) | 9.367 (7.859 to 11.112) | | | | | | | | | | | | | | | | | | | | | |
| Other neonatal disorders | Birth prevalence - [34, 36) wks, [2000, 2500) g | Mortality | Females | | 21.297 (15.657 to 28.761) | 10.295 (8.548 to 12.273) | | | | | | | | | | | | | | | | | | | | | |
| Other neonatal disorders | Birth prevalence - [37, 38) wks, [2000, 2500) g | Mortality | Males | | 13.0 (10.102 to 16.456) | 8.096 (6.724 to 9.676) | | | | | | | | | | | | | | | | | | | | | |
| Other neonatal disorders | Birth prevalence - [37, 38) wks, [2000, 2500) g | Mortality | Females | | 11.563 (8.805 to 15.11) | 8.467 (6.994 to 10.342) | | | | | | | | | | | | | | | | | | | | | |
| Other neonatal disorders | Birth prevalence - [36, 37) wks, [2000, 2500) g | Mortality | Males | | 14.401 (10.789 to 18.654) | 8.221 (6.917 to 9.923) | | | | | | | | | | | | | | | | | | | | | |
| Other neonatal disorders | Birth prevalence - [36, 37) wks, [2000, 2500) g | Mortality | Females | | 13.513 (9.817 to 17.942) | 8.654 (7.215 to 10.369) | | | | | | | | | | | | | | | | | | | | | |
| Other neonatal disorders | Birth prevalence - [34, 36) wks, [2500, 3000) g | Mortality | Males | | 13.419 (10.387 to 16.819) | 5.562 (4.646 to 6.696) | | | | | | | | | | | | | | | | | | | | | |
| Other neonatal disorders | Birth prevalence - [34, 36) wks, [2500, 3000) g | Mortality | Females | | 13.266 (9.666 to 17.689) | 6.395 (5.292 to 7.606) | | | | | | | | | | | | | | | | | | | | | |
| Other neonatal disorders | Birth prevalence - [34, 36) wks, [4000, 4500) g | Mortality | Males | | 23.096 (14.708 to 35.098) | 2.895 (2.245 to 3.716) | | | | | | | | | | | | | | | | | | | | | |
| Other neonatal disorders | Birth prevalence - [34, 36) wks, [4000, 4500) g | Mortality | Females | | 25.038 (15.763 to 37.255) | 3.778 (2.855 to 4.925) | | | | | | | | | | | | | | | | | | | | | |
| Other neonatal disorders | Birth prevalence - [34, 36) wks, [3000, 3500) g | Mortality | Males | | 14.006 (10.222 to 18.478) | 4.322 (3.449 to 5.338) | | | | | | | | | | | | | | | | | | | | | |
| Other neonatal disorders | Birth prevalence - [34, 36) wks, [3000, 3500) g | Mortality | Females | | 14.375 (10.269 to 20.114) | 5.265 (4.145 to 6.564) | | | | | | | | | | | | | | | | | | | | | |
| Other neonatal disorders | Birth prevalence - [36, 37) wks, [2500, 3000) g | Mortality | Males | | 4.874 (4.014 to 5.713) | 3.699 (3.134 to 4.374) | | | | | | | | | | | | | | | | | | | | | |
| Other neonatal disorders | Birth prevalence - [36, 37) wks, [2500, 3000) g | Mortality | Females | | 4.609 (3.731 to 5.61) | 3.898 (3.235 to 4.67) | | | | | | | | | | | | | | | | | | | | | |
| Other neonatal disorders | Birth prevalence - [34, 36) wks, [3500, 4000) g | Mortality | Males | | 18.024 (12.279 to 25.547) | 3.657 (2.838 to 4.675) | | | | | | | | | | | | | | | | | | | | | |
| Other neonatal disorders | Birth prevalence - [34, 36) wks, [3500, 4000) g | Mortality | Females | | 19.263 (12.567 to 27.924) | 4.634 (3.576 to 5.989) | | | | | | | | | | | | | | | | | | | | | |
| Other neonatal disorders | Birth prevalence - [37, 38) wks, [2500, 3000) g | Mortality | Males | | 3.306 (2.82 to 3.843) | 3.194 (2.691 to 3.803) | | | | | | | | | | | | | | | | | | | | | |
| Other neonatal disorders | Birth prevalence - [37, 38) wks, [2500, 3000) g | Mortality | Females | | 2.991 (2.496 to 3.61) | 3.242 (2.745 to 3.817) | | | | | | | | | | | | | | | | | | | | | |
| Other neonatal disorders | Birth prevalence - [40, 42) wks, [2500, 3000) g | Mortality | Males | | 3.771 (3.002 to 4.693) | 3.175 (2.56 to 3.923) | | | | | | | | | | | | | | | | | | | | | |
| Other neonatal disorders | Birth prevalence - [40, 42) wks, [2500, 3000) g | Mortality | Females | | 3.244 (2.486 to 4.159) | 3.228 (2.605 to 3.985) | | | | | | | | | | | | | | | | | | | | | |
| Other neonatal disorders | Birth prevalence - [38, 40) wks, [2500, 3000) g | Mortality | Males | | 2.755 (2.274 to 3.309) | 2.944 (2.44 to 3.548) | | | | | | | | | | | | | | | | | | | | | |
| Other neonatal disorders | Birth prevalence - [38, 40) wks, [2500, 3000) g | Mortality | Females | | 2.376 (1.91 to 2.886) | 2.938 (2.434 to 3.503) | | | | | | | | | | | | | | | | | | | | | |
| Other neonatal disorders | Birth prevalence - [36, 37) wks, [3000, 3500) g | Mortality | Males | | 3.774 (3.094 to 4.497) | 2.466 (2.058 to 2.929) | | | | | | | | | | | | | | | | | | | | | |
| Other neonatal disorders | Birth prevalence - [36, 37) wks, [3000, 3500) g | Mortality | Females | | 3.73 (2.981 to 4.646) | 2.715 (2.277 to 3.218) | | | | | | | | | | | | | | | | | | | | | |
| Other neonatal disorders | Birth prevalence - [36, 37) wks, [4000, 4500) g | Mortality | Males | | 6.826 (5.212 to 9.045) | 1.77 (1.491 to 2.082) | | | | | | | | | | | | | | | | | | | | | |
| Other neonatal disorders | Birth prevalence - [36, 37) wks, [4000, 4500) g | Mortality | Females | | 7.269 (5.144 to 9.821) | 2.177 (1.786 to 2.607) | | | | | | | | | | | | | | | | | | | | | |
| Other neonatal disorders | Birth prevalence - [36, 37) wks, [3500, 4000) g | Mortality | Males | | 4.544 (3.64 to 5.622) | 2.057 (1.74 to 2.44) | | | | | | | | | | | | | | | | | | | | | |
| Other neonatal disorders | Birth prevalence - [36, 37) wks, [3500, 4000) g | Mortality | Females | | 4.662 (3.577 to 6.014) | 2.398 (1.98 to 2.864) | | | | | | | | | | | | | | | | | | | | | |
| Other neonatal disorders | Birth prevalence - [37, 38) wks, [3000, 3500) g | Mortality | Males | | 2.007 (1.759 to 2.293) | 1.888 (1.613 to 2.224) | | | | | | | | | | | | | | | | | | | | | |
| Other neonatal disorders | Birth prevalence - [37, 38) wks, [3000, 3500) g | Mortality | Females | | 1.925 (1.582 to 2.328) | 1.972 (1.669 to 2.313) | | | | | | | | | | | | | | | | | | | | | |
| Other neonatal disorders | Birth prevalence - [37, 38) wks, [4000, 4500) g | Mortality | Males | | 3.28 (2.596 to 4.133) | 1.335 (1.171 to 1.532) | | | | | | | | | | | | | | | | | | | | | |
| Other neonatal disorders | Birth prevalence - [37, 38) wks, [4000, 4500) g | Mortality | Females | | 3.521 (2.649 to 4.5) | 1.559 (1.333 to 1.835) | | | | | | | | | | | | | | | | | | | | | |
| Other neonatal disorders | Birth prevalence - [37, 38) wks, [3500, 4000) g | Mortality | Males | | 2.128 (1.833 to 2.466) | 1.505 (1.299 to 1.76) | | | | | | | | | | | | | | | | | | | | | |
| Other neonatal disorders | Birth prevalence - [37, 38) wks, [3500, 4000) g | Mortality | Females | | 2.142 (1.694 to 2.67) | 1.661 (1.411 to 1.961) | | | | | | | | | | | | | | | | | | | | | |
| Other neonatal disorders | Birth prevalence - [40, 42) wks, [3000, 3500) g | Mortality | Males | | 1.436 (1.245 to 1.65) | 1.47 (1.199 to 1.8) | | | | | | | | | | | | | | | | | | | | | |
| Other neonatal disorders | Birth prevalence - [40, 42) wks, [3000, 3500) g | Mortality | Females | | 1.326 (1.069 to 1.614) | 1.465 (1.188 to 1.775) | | | | | | | | | | | | | | | | | | | | | |
| Other neonatal disorders | Birth prevalence - [38, 40) wks, [3000, 3500) g | Mortality | Males | | 1.33 (1.155 to 1.53) | 1.559 (1.305 to 1.851) | | | | | | | | | | | | | | | | | | | | | |
| Other neonatal disorders | Birth prevalence - [38, 40) wks, [3000, 3500) g | Mortality | Females | | 1.224 (1.0 to 1.492) | 1.564 (1.304 to 1.847) | | | | | | | | | | | | | | | | | | | | | |
| Other neonatal disorders | Birth prevalence - [38, 40) wks, [4000, 4500) g | Mortality | Males | | 1.787 (1.453 to 2.182) | 1.175 (1.005 to 1.371) | | | | | | | | | | | | | | | | | | | | | |
| Other neonatal disorders | Birth prevalence - [38, 40) wks, [4000, 4500) g | Mortality | Females | | 1.877 (1.467 to 2.388) | 1.224 (1.022 to 1.465) | | | | | | | | | | | | | | | | | | | | | |
| Other neonatal disorders | Birth prevalence - [38, 40) wks, [3500, 4000) g | Mortality | Males | | 1.785 (1.478 to 2.147) | 1.173 (1.0 to 1.377) | | | | | | | | | | | | | | | | | | | | | |
| Other neonatal disorders | Birth prevalence - [38, 40) wks, [3500, 4000) g | Mortality | Females | | 1.892 (1.481 to 2.352) | 1.23 (1.03 to 1.46) | | | | | | | | | | | | | | | | | | | | | |
| Other neonatal disorders | Birth prevalence - [40, 42) wks, [3500, 4000) g | Mortality | Males | | 1.0 (1.0 to 1.0) | 1.003 (1.0 to 1.046) | | | | | | | | | | | | | | | | | | | | | |
| Other neonatal disorders | Birth prevalence - [40, 42) wks, [3500, 4000) g | Mortality | Females | | 1.002 (1.0 to 1.013) | 1.001 (1.0 to 1.006) | | | | | | | | | | | | | | | | | | | | | |
| Other neonatal disorders | Birth prevalence - [40, 42) wks, [4000, 4500) g | Mortality | Males | | 1.0 (1.0 to 1.0) | 1.0 (1.0 to 1.0) | | | | | | | | | | | | | | | | | | | | | |
| Other neonatal disorders | Birth prevalence - [40, 42) wks, [4000, 4500) g | Mortality | Females | | 1.0 (1.0 to 1.0) | 1.0 (1.0 to 1.0) | | | | | | | | | | | | | | | | | | | | | |
| Other neonatal disorders | Birth prevalence - [28, 30) wks, [2000, 2500) g | Mortality | Males | | 117.172 (83.895 to 158.056) | 27.726 (21.877 to 34.972) | | | | | | | | | | | | | | | | | | | | | |
| Other neonatal disorders | Birth prevalence - [28, 30) wks, [2000, 2500) g | Mortality | Females | | 121.682 (84.349 to 171.375) | 33.983 (26.101 to 43.404) | | | | | | | | | | | | | | | | | | | | | |
| Other neonatal disorders | Birth prevalence - [28, 30) wks, [2500, 3000) g | Mortality | Males | | 77.948 (54.687 to 105.047) | 16.608 (12.653 to 21.188) | | | | | | | | | | | | | | | | | | | | | |
| Other neonatal disorders | Birth prevalence - [28, 30) wks, [2500, 3000) g | Mortality | Females | | 79.193 (54.099 to 112.236) | 20.387 (15.089 to 26.434) | | | | | | | | | | | | | | | | | | | | | |
| Other neonatal disorders | Birth prevalence - [28, 30) wks, [3000, 3500) g | Mortality | Males | | 42.199 (29.891 to 57.227) | 10.082 (7.777 to 13.056) | | | | | | | | | | | | | | | | | | | | | |
| Other neonatal disorders | Birth prevalence - [28, 30) wks, [3000, 3500) g | Mortality | Females | | 42.551 (28.25 to 63.209) | 11.989 (9.084 to 15.544) | | | | | | | | | | | | | | | | | | | | | |

**Appendix Table 6a. Relative risks used by age and sex for each outcome for all risk factors except for ambient air pollution alcohol, and smoking.**

| Risk - Outcome | Category / Units | Morbidity / Mortality | Sex | Ages | | | | | | | | | | | | | | | | | | | | | | | |
|---|---|---|---|---|---|---|---|---|---|---|---|---|---|---|---|---|---|---|---|---|---|---|---|---|---|---|---|
| | | | | All-age | 0-6 days | 7-27 days | 28-364 days | 1-4 years | 5-9 years | 10-14 years | 15-19 years | 20-24 years | 25-29 years | 30-34 years | 35-39 years | 40-44 years | 45-49 years | 50-54 years | 55-59 years | 60-64 years | 65-69 years | 70-74 years | 75-79 years | 80-84 years | 85-89 years | 90-94 years | 95+ years |
| Other neonatal disorders | Birth prevalence - [30, 32) wks, [2500, 3000) g | Mortality | Males | | 58.722 (42.419 to 78.873) | 12.115 (9.518 to 15.521) | | | | | | | | | | | | | | | | | | | | | |
| Other neonatal disorders | Birth prevalence - [30, 32) wks, [2500, 3000) g | Mortality | Females | | 59.522 (42.058 to 82.793) | 15.364 (11.936 to 19.581) | | | | | | | | | | | | | | | | | | | | | |
| Other neonatal disorders | Birth prevalence - [30, 32) wks, [3000, 3500) g | Mortality | Males | | 45.67 (32.014 to 65.531) | 8.381 (6.362 to 10.842) | | | | | | | | | | | | | | | | | | | | | |
| Other neonatal disorders | Birth prevalence - [30, 32) wks, [3000, 3500) g | Mortality | Females | | 46.104 (30.243 to 66.207) | 10.506 (8.041 to 13.513) | | | | | | | | | | | | | | | | | | | | | |
| Other neonatal disorders | Birth prevalence - [30, 32) wks, [3500, 4000) g | Mortality | Males | | 36.334 (21.558 to 54.813) | 5.698 (4.293 to 7.349) | | | | | | | | | | | | | | | | | | | | | |
| Other neonatal disorders | Birth prevalence - [30, 32) wks, [3500, 4000) g | Mortality | Females | | 37.931 (22.692 to 61.276) | 6.892 (5.175 to 9.058) | | | | | | | | | | | | | | | | | | | | | |
| Other neonatal disorders | Birth prevalence - [32, 34) wks, [3000, 3500) g | Mortality | Males | | 34.016 (23.515 to 48.37) | 6.577 (4.934 to 8.436) | | | | | | | | | | | | | | | | | | | | | |
| Other neonatal disorders | Birth prevalence - [32, 34) wks, [3000, 3500) g | Mortality | Females | | 34.585 (22.909 to 49.754) | 8.314 (6.362 to 10.789) | | | | | | | | | | | | | | | | | | | | | |
| Other neonatal disorders | Birth prevalence - [32, 34) wks, [3500, 4000) g | Mortality | Males | | 36.248 (23.158 to 54.67) | 5.068 (3.761 to 6.741) | | | | | | | | | | | | | | | | | | | | | |
| Other neonatal disorders | Birth prevalence - [32, 34) wks, [3500, 4000) g | Mortality | Females | | 38.098 (23.301 to 59.429) | 6.476 (4.666 to 8.689) | | | | | | | | | | | | | | | | | | | | | |
| Other neonatal disorders | Birth prevalence - [36, 37) wks, [1000, 1500) g | Mortality | Males | | 166.686 (118.487 to 222.581) | 57.535 (45.999 to 71.742) | | | | | | | | | | | | | | | | | | | | | |
| Other neonatal disorders | Birth prevalence - [36, 37) wks, [1000, 1500) g | Mortality | Females | | 169.725 (119.017 to 229.008) | 63.564 (50.068 to 80.703) | | | | | | | | | | | | | | | | | | | | | |
| Other neonatal disorders | Birth prevalence - [38, 40) wks, [1000, 1500) g | Mortality | Males | | 174.066 (125.125 to 232.507) | 57.966 (44.393 to 73.241) | | | | | | | | | | | | | | | | | | | | | |
| Other neonatal disorders | Birth prevalence - [38, 40) wks, [1000, 1500) g | Mortality | Females | | 171.557 (121.585 to 237.047) | 65.208 (48.821 to 84.308) | | | | | | | | | | | | | | | | | | | | | |
| Other neonatal disorders | Birth prevalence - [38, 40) wks, [1500, 2000) g | Mortality | Males | | 67.302 (49.547 to 89.055) | 25.206 (20.365 to 31.168) | | | | | | | | | | | | | | | | | | | | | |
| Other neonatal disorders | Birth prevalence - [38, 40) wks, [1500, 2000) g | Mortality | Females | | 62.19 (45.884 to 83.445) | 28.05 (22.625 to 35.139) | | | | | | | | | | | | | | | | | | | | | |
| Other neonatal disorders | Birth prevalence - [40, 42) wks, [1500, 2000) g | Mortality | Males | | 76.673 (56.177 to 102.468) | 25.785 (19.387 to 34.168) | | | | | | | | | | | | | | | | | | | | | |
| Other neonatal disorders | Birth prevalence - [40, 42) wks, [1500, 2000) g | Mortality | Females | | 70.411 (49.221 to 97.952) | 29.113 (21.355 to 38.272) | | | | | | | | | | | | | | | | | | | | | |
| Sudden infant death syndrome | Birth prevalence - [0, 24) wks, [0, 500) g | Mortality | Males | | 1564.792 (1056.542 to [illegible] | 618.595 (458.842 to 812.921) | | | | | | | | | | | | | | | | | | | | | |
| Sudden infant death syndrome | Birth prevalence - [0, 24) wks, [0, 500) g | Mortality | Females | | 1600.122 (1050.664 to [illegible] | 713.571 (526.178 to 921.018) | | | | | | | | | | | | | | | | | | | | | |
| Sudden infant death syndrome | Birth prevalence - [0, 24) wks, [500, 1000) g | Mortality | Males | | 1155.815 (825.412 to 1506.837) | 457.5 (352.552 to 573.483) | | | | | | | | | | | | | | | | | | | | | |
| Sudden infant death syndrome | Birth prevalence - [0, 24) wks, [500, 1000) g | Mortality | Females | | 1169.123 (802.003 to 1617.979) | 515.406 (396.713 to 641.541) | | | | | | | | | | | | | | | | | | | | | |
| Sudden infant death syndrome | Birth prevalence - [24, 26) wks, [500, 1000) g | Mortality | Males | | 955.583 (723.748 to 1244.265) | 443.357 (363.03 to 534.695) | | | | | | | | | | | | | | | | | | | | | |
| Sudden infant death syndrome | Birth prevalence - [24, 26) wks, [500, 1000) g | Mortality | Females | | 947.143 (702.662 to 1237.093) | 487.549 (387.307 to 603.498) | | | | | | | | | | | | | | | | | | | | | |
| Sudden infant death syndrome | Birth prevalence - [26, 28) wks, [500, 1000) g | Mortality | Males | | 497.817 (377.617 to 648.547) | 330.886 (261.438 to 401.709) | | | | | | | | | | | | | | | | | | | | | |
| Sudden infant death syndrome | Birth prevalence - [26, 28) wks, [500, 1000) g | Mortality | Females | | 483.682 (354.946 to 629.517) | 344.618 (274.427 to 419.864) | | | | | | | | | | | | | | | | | | | | | |
| Sudden infant death syndrome | Birth prevalence - [30, 32) wks, [500, 1000) g | Mortality | Males | | 236.614 (163.821 to 324.502) | 149.995 (117.866 to 188.368) | | | | | | | | | | | | | | | | | | | | | |
| Sudden infant death syndrome | Birth prevalence - [30, 32) wks, [500, 1000) g | Mortality | Females | | 229.197 (157.606 to 317.194) | 152.117 (120.779 to 190.583) | | | | | | | | | | | | | | | | | | | | | |
| Sudden infant death syndrome | Birth prevalence - [28, 30) wks, [500, 1000) g | Mortality | Males | | 297.629 (214.953 to 396.586) | 216.995 (173.321 to 271.466) | | | | | | | | | | | | | | | | | | | | | |
| Sudden infant death syndrome | Birth prevalence - [28, 30) wks, [500, 1000) g | Mortality | Females | | 281.056 (198.176 to 386.635) | 219.884 (174.264 to 272.704) | | | | | | | | | | | | | | | | | | | | | |
| Sudden infant death syndrome | Birth prevalence - [26, 28) wks, [1000, 1500) g | Mortality | Males | | 267.91 (210.177 to 332.92) | 164.167 (132.898 to 200.569) | | | | | | | | | | | | | | | | | | | | | |
| Sudden infant death syndrome | Birth prevalence - [26, 28) wks, [1000, 1500) g | Mortality | Females | | 266.509 (197.461 to 346.932) | 174.222 (137.431 to 217.349) | | | | | | | | | | | | | | | | | | | | | |
| Sudden infant death syndrome | Birth prevalence - [34, 36) wks, [1000, 1500) g | Mortality | Males | | 142.056 (98.086 to 197.774) | 52.86 (42.914 to 64.617) | | | | | | | | | | | | | | | | | | | | | |
| Sudden infant death syndrome | Birth prevalence - [34, 36) wks, [1000, 1500) g | Mortality | Females | | 141.899 (95.864 to 197.656) | 57.421 (46.452 to 71.339) | | | | | | | | | | | | | | | | | | | | | |
| Sudden infant death syndrome | Birth prevalence - [28, 30) wks, [1500, 2000) g | Mortality | Males | | 127.966 (97.178 to 167.026) | 50.018 (40.539 to 61.919) | | | | | | | | | | | | | | | | | | | | | |
| Sudden infant death syndrome | Birth prevalence - [28, 30) wks, [1500, 2000) g | Mortality | Females | | 130.924 (96.513 to 172.188) | 57.275 (46.36 to 70.038) | | | | | | | | | | | | | | | | | | | | | |
| Sudden infant death syndrome | Birth prevalence - [28, 30) wks, [1000, 1500) g | Mortality | Males | | 158.563 (120.99 to 204.947) | 103.32 (83.486 to 127.144) | | | | | | | | | | | | | | | | | | | | | |
| Sudden infant death syndrome | Birth prevalence - [28, 30) wks, [1000, 1500) g | Mortality | Females | | 153.905 (112.327 to 200.786) | 107.529 (86.954 to 131.78) | | | | | | | | | | | | | | | | | | | | | |
| Sudden infant death syndrome | Birth prevalence - [32, 34) wks, [1000, 1500) g | Mortality | Males | | 117.142 (81.354 to 161.101) | 53.185 (43.049 to 66.274) | | | | | | | | | | | | | | | | | | | | | |
| Sudden infant death syndrome | Birth prevalence - [32, 34) wks, [1000, 1500) g | Mortality | Females | | 115.171 (79.363 to 159.206) | 56.034 (45.982 to 68.36) | | | | | | | | | | | | | | | | | | | | | |
| Sudden infant death syndrome | Birth prevalence - [30, 32) wks, [1000, 1500) g | Mortality | Males | | 119.308 (87.769 to 160.885) | 67.163 (54.863 to 82.638) | | | | | | | | | | | | | | | | | | | | | |
| Sudden infant death syndrome | Birth prevalence - [30, 32) wks, [1000, 1500) g | Mortality | Females | | 115.448 (84.272 to 156.425) | 69.14 (55.873 to 85.012) | | | | | | | | | | | | | | | | | | | | | |
| Sudden infant death syndrome | Birth prevalence - [37, 38) wks, [1500, 2000) g | Mortality | Males | | 62.972 (46.159 to 83.484) | 24.148 (20.066 to 29.406) | | | | | | | | | | | | | | | | | | | | | |
| Sudden infant death syndrome | Birth prevalence - [37, 38) wks, [1500, 2000) g | Mortality | Females | | 59.988 (43.974 to 79.053) | 26.719 (21.746 to 32.816) | | | | | | | | | | | | | | | | | | | | | |
| Sudden infant death syndrome | Birth prevalence - [36, 37) wks, [1500, 2000) g | Mortality | Males | | 60.218 (43.669 to 82.48) | 23.031 (18.793 to 28.483) | | | | | | | | | | | | | | | | | | | | | |
| Sudden infant death syndrome | Birth prevalence - [36, 37) wks, [1500, 2000) g | Mortality | Females | | 58.527 (42.172 to 80.557) | 25.143 (20.331 to 30.566) | | | | | | | | | | | | | | | | | | | | | |
| Sudden infant death syndrome | Birth prevalence - [30, 32) wks, [2000, 2500) g | Mortality | Males | | 67.971 (50.354 to 88.935) | 18.03 (14.621 to 22.103) | | | | | | | | | | | | | | | | | | | | | |
| Sudden infant death syndrome | Birth prevalence - [30, 32) wks, [2000, 2500) g | Mortality | Females | | 69.383 (49.108 to 94.583) | 22.069 (17.836 to 27.163) | | | | | | | | | | | | | | | | | | | | | |
| Sudden infant death syndrome | Birth prevalence - [30, 32) wks, [1500, 2000) g | Mortality | Males | | 77.369 (59.702 to 99.232) | 31.079 (25.786 to 36.724) | | | | | | | | | | | | | | | | | | | | | |
| Sudden infant death syndrome | Birth prevalence - [30, 32) wks, [1500, 2000) g | Mortality | Females | | 76.134 (56.885 to 100.996) | 34.756 (28.764 to 41.849) | | | | | | | | | | | | | | | | | | | | | |
| Sudden infant death syndrome | Birth prevalence - [34, 36) wks, [1500, 2000) g | Mortality | Males | | 55.555 (39.553 to 75.104) | 21.346 (17.677 to 26.143) | | | | | | | | | | | | | | | | | | | | | |
| Sudden infant death syndrome | Birth prevalence - [34, 36) wks, [1500, 2000) g | Mortality | Females | | 54.335 (38.617 to 75.24) | 23.046 (18.743 to 28.287) | | | | | | | | | | | | | | | | | | | | | |
| Sudden infant death syndrome | Birth prevalence - [32, 34) wks, [1500, 2000) g | Mortality | Males | | 57.155 (42.484 to 73.651) | 23.114 (19.028 to 27.915) | | | | | | | | | | | | | | | | | | | | | |
| Sudden infant death syndrome | Birth prevalence - [32, 34) wks, [1500, 2000) g | Mortality | Females | | 56.101 (39.794 to 76.295) | 25.149 (20.615 to 30.388) | | | | | | | | | | | | | | | | | | | | | |
| Sudden infant death syndrome | Birth prevalence - [32, 34) wks, [2000, 2500) g | Mortality | Males | | 37.444 (29.026 to 48.227) | 12.233 (10.252 to 14.477) | | | | | | | | | | | | | | | | | | | | | |
| Sudden infant death syndrome | Birth prevalence - [32, 34) wks, [2000, 2500) g | Mortality | Females | | 36.874 (26.658 to 49.653) | 14.384 (12.095 to 17.03) | | | | | | | | | | | | | | | | | | | | | |
| Sudden infant death syndrome | Birth prevalence - [40, 42) wks, [2000, 2500) g | Mortality | Males | | 18.092 (13.292 to 23.719) | 9.23 (7.037 to 11.454) | | | | | | | | | | | | | | | | | | | | | |

**Appendix Table 6a. Relative risks used by age and sex for each outcome for all risk factors except for ambient air pollution alcohol, and smoking.**

| Risk - Outcome | Category / Units | Morbidity / Mortality | Sex | All-age | 0-6 days | 7-27 days | 28-364 days | 1-4 years | 5-9 years | 10-14 years | 15-19 years | 20-24 years | 25-29 years | 30-34 years | 35-39 years | 40-44 years | 45-49 years | 50-54 years | 55-59 years | 60-64 years | 65-69 years | 70-74 years | 75-79 years | 80-84 years | 85-89 years | 90-94 years | 95+ years |
|---|---|---|---|---|---|---|---|---|---|---|---|---|---|---|---|---|---|---|---|---|---|---|---|---|---|---|---|
| Sudden infant death syndrome | Birth prevalence - [40, 42) wks, [2000, 2500) g | Mortality | Females | | 15.574 (11.516 to 20.778) | 9.975 (7.82 to 12.46) | | | | | | | | | | | | | | | | | | | | | |
| Sudden infant death syndrome | Birth prevalence - [38, 40) wks, [2000, 2500) g | Mortality | Males | | 13.104 (9.829 to 16.99) | 8.198 (6.786 to 9.959) | | | | | | | | | | | | | | | | | | | | | |
| Sudden infant death syndrome | Birth prevalence - [38, 40) wks, [2000, 2500) g | Mortality | Females | | 11.308 (8.389 to 14.38) | 8.577 (7.04 to 10.449) | | | | | | | | | | | | | | | | | | | | | |
| Sudden infant death syndrome | Birth prevalence - [32, 34) wks, [2500, 3000) g | Mortality | Males | | 33.063 (24.393 to 43.503) | 8.441 (6.822 to 10.431) | | | | | | | | | | | | | | | | | | | | | |
| Sudden infant death syndrome | Birth prevalence - [32, 34) wks, [2500, 3000) g | Mortality | Females | | 32.812 (23.439 to 45.567) | 10.398 (8.227 to 13.042) | | | | | | | | | | | | | | | | | | | | | |
| Sudden infant death syndrome | Birth prevalence - [34, 36) wks, [2000, 2500) g | Mortality | Males | | 21.925 (16.305 to 29.433) | 9.367 (7.859 to 11.112) | | | | | | | | | | | | | | | | | | | | | |
| Sudden infant death syndrome | Birth prevalence - [34, 36) wks, [2000, 2500) g | Mortality | Females | | 21.297 (15.657 to 28.761) | 10.295 (8.548 to 12.273) | | | | | | | | | | | | | | | | | | | | | |
| Sudden infant death syndrome | Birth prevalence - [37, 38) wks, [2000, 2500) g | Mortality | Males | | 13.0 (10.102 to 16.456) | 8.096 (6.724 to 9.676) | | | | | | | | | | | | | | | | | | | | | |
| Sudden infant death syndrome | Birth prevalence - [37, 38) wks, [2000, 2500) g | Mortality | Females | | 11.563 (8.805 to 15.11) | 8.467 (6.994 to 10.342) | | | | | | | | | | | | | | | | | | | | | |
| Sudden infant death syndrome | Birth prevalence - [36, 37) wks, [2000, 2500) g | Mortality | Males | | 14.401 (10.789 to 18.654) | 8.221 (6.917 to 9.923) | | | | | | | | | | | | | | | | | | | | | |
| Sudden infant death syndrome | Birth prevalence - [36, 37) wks, [2000, 2500) g | Mortality | Females | | 13.513 (9.817 to 17.942) | 8.654 (7.215 to 10.369) | | | | | | | | | | | | | | | | | | | | | |
| Sudden infant death syndrome | Birth prevalence - [34, 36) wks, [2500, 3000) g | Mortality | Males | | 13.419 (10.387 to 16.819) | 5.562 (4.646 to 6.696) | | | | | | | | | | | | | | | | | | | | | |
| Sudden infant death syndrome | Birth prevalence - [34, 36) wks, [2500, 3000) g | Mortality | Females | | 13.266 (9.666 to 17.689) | 6.395 (5.292 to 7.606) | | | | | | | | | | | | | | | | | | | | | |
| Sudden infant death syndrome | Birth prevalence - [34, 36) wks, [4000, 4500) g | Mortality | Males | | 23.096 (14.708 to 35.098) | 2.895 (2.245 to 3.716) | | | | | | | | | | | | | | | | | | | | | |
| Sudden infant death syndrome | Birth prevalence - [34, 36) wks, [4000, 4500) g | Mortality | Females | | 25.038 (15.763 to 37.255) | 3.778 (2.855 to 4.925) | | | | | | | | | | | | | | | | | | | | | |
| Sudden infant death syndrome | Birth prevalence - [34, 36) wks, [3000, 3500) g | Mortality | Males | | 14.006 (10.222 to 18.478) | 4.322 (3.449 to 5.338) | | | | | | | | | | | | | | | | | | | | | |
| Sudden infant death syndrome | Birth prevalence - [34, 36) wks, [3000, 3500) g | Mortality | Females | | 14.375 (10.269 to 20.114) | 5.265 (4.145 to 6.564) | | | | | | | | | | | | | | | | | | | | | |
| Sudden infant death syndrome | Birth prevalence - [36, 37) wks, [2500, 3000) g | Mortality | Males | | 4.874 (4.014 to 5.713) | 3.699 (3.134 to 4.374) | | | | | | | | | | | | | | | | | | | | | |
| Sudden infant death syndrome | Birth prevalence - [36, 37) wks, [2500, 3000) g | Mortality | Females | | 4.609 (3.731 to 5.61) | 3.898 (3.235 to 4.67) | | | | | | | | | | | | | | | | | | | | | |
| Sudden infant death syndrome | Birth prevalence - [34, 36) wks, [3500, 4000) g | Mortality | Males | | 18.024 (12.279 to 25.547) | 3.657 (2.838 to 4.675) | | | | | | | | | | | | | | | | | | | | | |
| Sudden infant death syndrome | Birth prevalence - [34, 36) wks, [3500, 4000) g | Mortality | Females | | 19.263 (12.567 to 27.924) | 4.634 (3.576 to 5.989) | | | | | | | | | | | | | | | | | | | | | |
| Sudden infant death syndrome | Birth prevalence - [37, 38) wks, [2500, 3000) g | Mortality | Males | | 3.306 (2.82 to 3.843) | 3.194 (2.691 to 3.803) | | | | | | | | | | | | | | | | | | | | | |
| Sudden infant death syndrome | Birth prevalence - [37, 38) wks, [2500, 3000) g | Mortality | Females | | 2.991 (2.496 to 3.61) | 3.242 (2.745 to 3.817) | | | | | | | | | | | | | | | | | | | | | |
| Sudden infant death syndrome | Birth prevalence - [40, 42) wks, [2500, 3000) g | Mortality | Males | | 3.771 (3.002 to 4.693) | 3.175 (2.56 to 3.923) | | | | | | | | | | | | | | | | | | | | | |
| Sudden infant death syndrome | Birth prevalence - [40, 42) wks, [2500, 3000) g | Mortality | Females | | 3.244 (2.486 to 4.159) | 3.228 (2.605 to 3.985) | | | | | | | | | | | | | | | | | | | | | |
| Sudden infant death syndrome | Birth prevalence - [38, 40) wks, [2500, 3000) g | Mortality | Males | | 2.755 (2.274 to 3.309) | 2.944 (2.44 to 3.548) | | | | | | | | | | | | | | | | | | | | | |
| Sudden infant death syndrome | Birth prevalence - [38, 40) wks, [2500, 3000) g | Mortality | Females | | 2.376 (1.91 to 2.886) | 2.938 (2.434 to 3.503) | | | | | | | | | | | | | | | | | | | | | |
| Sudden infant death syndrome | Birth prevalence - [36, 37) wks, [3000, 3500) g | Mortality | Males | | 3.774 (3.094 to 4.497) | 2.466 (2.058 to 2.929) | | | | | | | | | | | | | | | | | | | | | |
| Sudden infant death syndrome | Birth prevalence - [36, 37) wks, [3000, 3500) g | Mortality | Females | | 3.73 (2.981 to 4.646) | 2.715 (2.277 to 3.218) | | | | | | | | | | | | | | | | | | | | | |
| Sudden infant death syndrome | Birth prevalence - [36, 37) wks, [4000, 4500) g | Mortality | Males | | 6.826 (5.212 to 9.045) | 1.77 (1.491 to 2.082) | | | | | | | | | | | | | | | | | | | | | |
| Sudden infant death syndrome | Birth prevalence - [36, 37) wks, [4000, 4500) g | Mortality | Females | | 7.269 (5.144 to 9.821) | 2.177 (1.786 to 2.607) | | | | | | | | | | | | | | | | | | | | | |
| Sudden infant death syndrome | Birth prevalence - [36, 37) wks, [3500, 4000) g | Mortality | Males | | 4.544 (3.64 to 5.622) | 2.057 (1.74 to 2.44) | | | | | | | | | | | | | | | | | | | | | |
| Sudden infant death syndrome | Birth prevalence - [36, 37) wks, [3500, 4000) g | Mortality | Females | | 4.662 (3.577 to 6.014) | 2.398 (1.98 to 2.864) | | | | | | | | | | | | | | | | | | | | | |
| Sudden infant death syndrome | Birth prevalence - [37, 38) wks, [3000, 3500) g | Mortality | Males | | 2.007 (1.759 to 2.293) | 1.888 (1.613 to 2.224) | | | | | | | | | | | | | | | | | | | | | |
| Sudden infant death syndrome | Birth prevalence - [37, 38) wks, [3000, 3500) g | Mortality | Females | | 1.925 (1.582 to 2.328) | 1.972 (1.669 to 2.313) | | | | | | | | | | | | | | | | | | | | | |
| Sudden infant death syndrome | Birth prevalence - [37, 38) wks, [4000, 4500) g | Mortality | Males | | 3.28 (2.596 to 4.133) | 1.335 (1.171 to 1.532) | | | | | | | | | | | | | | | | | | | | | |
| Sudden infant death syndrome | Birth prevalence - [37, 38) wks, [4000, 4500) g | Mortality | Females | | 3.521 (2.649 to 4.5) | 1.559 (1.333 to 1.835) | | | | | | | | | | | | | | | | | | | | | |
| Sudden infant death syndrome | Birth prevalence - [37, 38) wks, [3500, 4000) g | Mortality | Males | | 2.128 (1.833 to 2.466) | 1.505 (1.299 to 1.76) | | | | | | | | | | | | | | | | | | | | | |
| Sudden infant death syndrome | Birth prevalence - [37, 38) wks, [3500, 4000) g | Mortality | Females | | 2.142 (1.694 to 2.67) | 1.661 (1.411 to 1.961) | | | | | | | | | | | | | | | | | | | | | |
| Sudden infant death syndrome | Birth prevalence - [40, 42) wks, [3000, 3500) g | Mortality | Males | | 1.436 (1.245 to 1.65) | 1.47 (1.199 to 1.8) | | | | | | | | | | | | | | | | | | | | | |
| Sudden infant death syndrome | Birth prevalence - [40, 42) wks, [3000, 3500) g | Mortality | Females | | 1.326 (1.069 to 1.614) | 1.465 (1.188 to 1.775) | | | | | | | | | | | | | | | | | | | | | |
| Sudden infant death syndrome | Birth prevalence - [38, 40) wks, [3000, 3500) g | Mortality | Males | | 1.33 (1.155 to 1.53) | 1.559 (1.305 to 1.851) | | | | | | | | | | | | | | | | | | | | | |
| Sudden infant death syndrome | Birth prevalence - [38, 40) wks, [3000, 3500) g | Mortality | Females | | 1.224 (1.0 to 1.492) | 1.564 (1.304 to 1.847) | | | | | | | | | | | | | | | | | | | | | |
| Sudden infant death syndrome | Birth prevalence - [38, 40) wks, [4000, 4500) g | Mortality | Males | | 1.787 (1.453 to 2.182) | 1.175 (1.005 to 1.371) | | | | | | | | | | | | | | | | | | | | | |
| Sudden infant death syndrome | Birth prevalence - [38, 40) wks, [4000, 4500) g | Mortality | Females | | 1.877 (1.467 to 2.388) | 1.224 (1.022 to 1.465) | | | | | | | | | | | | | | | | | | | | | |
| Sudden infant death syndrome | Birth prevalence - [38, 40) wks, [3500, 4000) g | Mortality | Males | | 1.785 (1.478 to 2.147) | 1.173 (1.0 to 1.377) | | | | | | | | | | | | | | | | | | | | | |
| Sudden infant death syndrome | Birth prevalence - [38, 40) wks, [3500, 4000) g | Mortality | Females | | 1.892 (1.481 to 2.352) | 1.23 (1.03 to 1.46) | | | | | | | | | | | | | | | | | | | | | |
| Sudden infant death syndrome | Birth prevalence - [40, 42) wks, [3500, 4000) g | Mortality | Males | | 1.0 (1.0 to 1.0) | 1.003 (1.0 to 1.046) | | | | | | | | | | | | | | | | | | | | | |
| Sudden infant death syndrome | Birth prevalence - [40, 42) wks, [3500, 4000) g | Mortality | Females | | 1.002 (1.0 to 1.013) | 1.001 (1.0 to 1.006) | | | | | | | | | | | | | | | | | | | | | |
| Sudden infant death syndrome | Birth prevalence - [40, 42) wks, [4000, 4500) g | Mortality | Males | | 1.0 (1.0 to 1.0) | 1.0 (1.0 to 1.0) | | | | | | | | | | | | | | | | | | | | | |
| Sudden infant death syndrome | Birth prevalence - [40, 42) wks, [4000, 4500) g | Mortality | Females | | 1.0 (1.0 to 1.0) | 1.0 (1.0 to 1.0) | | | | | | | | | | | | | | | | | | | | | |
| Sudden infant death syndrome | Birth prevalence - [28, 30) wks, [2000, 2500) g | Mortality | Males | | 117.172 (83.895 to 158.056) | 27.726 (21.877 to 34.972) | | | | | | | | | | | | | | | | | | | | | |
| Sudden infant death syndrome | Birth prevalence - [28, 30) wks, [2000, 2500) g | Mortality | Females | | 121.682 (84.349 to 171.375) | 33.983 (26.101 to 43.404) | | | | | | | | | | | | | | | | | | | | | |
| Sudden infant death syndrome | Birth prevalence - [28, 30) wks, [2500, 3000) g | Mortality | Males | | 77.948 (54.687 to 105.047) | 16.608 (12.653 to 21.188) | | | | | | | | | | | | | | | | | | | | | |
| Sudden infant death syndrome | Birth prevalence - [28, 30) wks, [2500, 3000) g | Mortality | Females | | 79.193 (54.099 to 112.236) | 20.387 (15.089 to 26.434) | | | | | | | | | | | | | | | | | | | | | |
| Sudden infant death syndrome | Birth prevalence - [28, 30) wks, [3000, 3500) g | Mortality | Males | | 42.199 (29.891 to 57.227) | 10.082 (7.777 to 13.056) | | | | | | | | | | | | | | | | | | | | | |
| Sudden infant death syndrome | Birth prevalence - [28, 30) wks, [3000, 3500) g | Mortality | Females | | 42.551 (28.25 to 63.209) | 11.989 (9.084 to 15.544) | | | | | | | | | | | | | | | | | | | | | |

**Appendix Table 6a. Relative risks used by age and sex for each outcome for all risk factors except for ambient air pollution alcohol, and smoking.**

| | | | | Ages | | | | | | | | | | | | | | | | | | | | | | | |
|---|---|---|---|---|---|---|---|---|---|---|---|---|---|---|---|---|---|---|---|---|---|---|---|---|---|---|---|
| Risk - Outcome | Category / Units | Morbidity / Mortality | Sex | All-age | 0-6 days | 7-27 days | 28-364 days | 1-4 years | 5-9 years | 10-14 years | 15-19 years | 20-24 years | 25-29 years | 30-34 years | 35-39 years | 40-44 years | 45-49 years | 50-54 years | 55-59 years | 60-64 years | 65-69 years | 70-74 years | 75-79 years | 80-84 years | 85-89 years | 90-94 years | 95+ years |
| Sudden infant death syndrome | Birth prevalence - [30, 32) wks, [2500, 3000) g | Mortality | Males | | 58.722 (42.419 to 78.873) | 12.115 (9.518 to 15.521) | | | | | | | | | | | | | | | | | | | | | |
| Sudden infant death syndrome | Birth prevalence - [30, 32) wks, [2500, 3000) g | Mortality | Females | | 59.522 (42.058 to 82.793) | 15.364 (11.936 to 19.581) | | | | | | | | | | | | | | | | | | | | | |
| Sudden infant death syndrome | Birth prevalence - [30, 32) wks, [3000, 3500) g | Mortality | Males | | 45.67 (32.014 to 65.531) | 8.381 (6.362 to 10.842) | | | | | | | | | | | | | | | | | | | | | |
| Sudden infant death syndrome | Birth prevalence - [30, 32) wks, [3000, 3500) g | Mortality | Females | | 46.104 (30.243 to 66.207) | 10.506 (8.041 to 13.513) | | | | | | | | | | | | | | | | | | | | | |
| Sudden infant death syndrome | Birth prevalence - [30, 32) wks, [3500, 4000) g | Mortality | Males | | 36.334 (21.558 to 54.813) | 5.698 (4.293 to 7.349) | | | | | | | | | | | | | | | | | | | | | |
| Sudden infant death syndrome | Birth prevalence - [30, 32) wks, [3500, 4000) g | Mortality | Females | | 37.931 (22.692 to 61.276) | 6.892 (5.175 to 9.058) | | | | | | | | | | | | | | | | | | | | | |
| Sudden infant death syndrome | Birth prevalence - [32, 34) wks, [3000, 3500) g | Mortality | Males | | 34.016 (23.515 to 48.37) | 6.577 (4.934 to 8.436) | | | | | | | | | | | | | | | | | | | | | |
| Sudden infant death syndrome | Birth prevalence - [32, 34) wks, [3000, 3500) g | Mortality | Females | | 34.585 (22.909 to 49.754) | 8.314 (6.362 to 10.789) | | | | | | | | | | | | | | | | | | | | | |
| Sudden infant death syndrome | Birth prevalence - [32, 34) wks, [3500, 4000) g | Mortality | Males | | 36.248 (23.158 to 54.67) | 5.068 (3.761 to 6.741) | | | | | | | | | | | | | | | | | | | | | |
| Sudden infant death syndrome | Birth prevalence - [32, 34) wks, [3500, 4000) g | Mortality | Females | | 38.098 (23.301 to 59.429) | 6.476 (4.666 to 8.689) | | | | | | | | | | | | | | | | | | | | | |
| Sudden infant death syndrome | Birth prevalence - [36, 37) wks, [1000, 1500) g | Mortality | Males | | 166.686 (118.487 to 222.581) | 57.535 (45.999 to 71.742) | | | | | | | | | | | | | | | | | | | | | |
| Sudden infant death syndrome | Birth prevalence - [36, 37) wks, [1000, 1500) g | Mortality | Females | | 169.725 (119.017 to 229.008) | 63.564 (50.068 to 80.703) | | | | | | | | | | | | | | | | | | | | | |
| Sudden infant death syndrome | Birth prevalence - [38, 40) wks, [1000, 1500) g | Mortality | Males | | 174.066 (125.125 to 232.507) | 57.966 (44.393 to 73.241) | | | | | | | | | | | | | | | | | | | | | |
| Sudden infant death syndrome | Birth prevalence - [38, 40) wks, [1000, 1500) g | Mortality | Females | | 171.557 (121.585 to 237.047) | 65.208 (48.821 to 84.308) | | | | | | | | | | | | | | | | | | | | | |
| Sudden infant death syndrome | Birth prevalence - [38, 40) wks, [1500, 2000) g | Mortality | Males | | 67.302 (49.547 to 89.055) | 25.206 (20.365 to 31.168) | | | | | | | | | | | | | | | | | | | | | |
| Sudden infant death syndrome | Birth prevalence - [38, 40) wks, [1500, 2000) g | Mortality | Females | | 62.19 (45.884 to 83.445) | 28.05 (22.625 to 35.139) | | | | | | | | | | | | | | | | | | | | | |
| Sudden infant death syndrome | Birth prevalence - [40, 42) wks, [1500, 2000) g | Mortality | Males | | 76.673 (56.177 to 102.468) | 25.785 (19.387 to 34.168) | | | | | | | | | | | | | | | | | | | | | |
| Sudden infant death syndrome | Birth prevalence - [40, 42) wks, [1500, 2000) g | Mortality | Females | | 70.411 (49.221 to 97.952) | 29.113 (21.355 to 38.272) | | | | | | | | | | | | | | | | | | | | | |
| **Iron deficiency** | | | | | | | | | | | | | | | | | | | | | | | | | | | |
| Maternal haemorrhage | 1 g/dL | Both | Both | | | | | | | 1.252 (1.087 to 1.425) | 1.252 (1.087 to 1.425) | 1.252 (1.087 to 1.425) | 1.252 (1.087 to 1.425) | 1.252 (1.087 to 1.425) | 1.252 (1.087 to 1.425) | 1.252 (1.087 to 1.425) | 1.252 (1.087 to 1.425) | 1.252 (1.087 to 1.425) | | | | | | | | | |
| Maternal sepsis and other pregnancy related infections | 1 g/dL | Both | Both | | | | | | | 1.252 (1.087 to 1.425) | 1.252 (1.087 to 1.425) | 1.252 (1.087 to 1.425) | 1.252 (1.087 to 1.425) | 1.252 (1.087 to 1.425) | 1.252 (1.087 to 1.425) | 1.252 (1.087 to 1.425) | 1.252 (1.087 to 1.425) | 1.252 (1.087 to 1.425) | | | | | | | | | |
| Maternal hypertensive disorders | 1 g/dL | Both | Both | | | | | | | 1.252 (1.087 to 1.425) | 1.252 (1.087 to 1.425) | 1.252 (1.087 to 1.425) | 1.252 (1.087 to 1.425) | 1.252 (1.087 to 1.425) | 1.252 (1.087 to 1.425) | 1.252 (1.087 to 1.425) | 1.252 (1.087 to 1.425) | 1.252 (1.087 to 1.425) | | | | | | | | | |
| Maternal obstructed labour and uterine rupture | 1 g/dL | Both | Both | | | | | | | 1.252 (1.087 to 1.425) | 1.252 (1.087 to 1.425) | 1.252 (1.087 to 1.425) | 1.252 (1.087 to 1.425) | 1.252 (1.087 to 1.425) | 1.252 (1.087 to 1.425) | 1.252 (1.087 to 1.425) | 1.252 (1.087 to 1.425) | 1.252 (1.087 to 1.425) | | | | | | | | | |
| Maternal abortive outcome | 1 g/dL | Both | Both | | | | | | | 1.252 (1.087 to 1.425) | 1.252 (1.087 to 1.425) | 1.252 (1.087 to 1.425) | 1.252 (1.087 to 1.425) | 1.252 (1.087 to 1.425) | 1.252 (1.087 to 1.425) | 1.252 (1.087 to 1.425) | 1.252 (1.087 to 1.425) | 1.252 (1.087 to 1.425) | | | | | | | | | |
| Ectopic pregnancy | 1 g/dL | Both | Both | | | | | | | 1.252 (1.087 to 1.425) | 1.252 (1.087 to 1.425) | 1.252 (1.087 to 1.425) | 1.252 (1.087 to 1.425) | 1.252 (1.087 to 1.425) | 1.252 (1.087 to 1.425) | 1.252 (1.087 to 1.425) | 1.252 (1.087 to 1.425) | 1.252 (1.087 to 1.425) | | | | | | | | | |
| Indirect maternal deaths | 1 g/dL | Both | Both | | | | | | | 1.252 (1.087 to 1.425) | 1.252 (1.087 to 1.425) | 1.252 (1.087 to 1.425) | 1.252 (1.087 to 1.425) | 1.252 (1.087 to 1.425) | 1.252 (1.087 to 1.425) | 1.252 (1.087 to 1.425) | 1.252 (1.087 to 1.425) | 1.252 (1.087 to 1.425) | | | | | | | | | |
| Late maternal deaths | 1 g/dL | Both | Both | | | | | | | 1.252 (1.087 to 1.425) | 1.252 (1.087 to 1.425) | 1.252 (1.087 to 1.425) | 1.252 (1.087 to 1.425) | 1.252 (1.087 to 1.425) | 1.252 (1.087 to 1.425) | 1.252 (1.087 to 1.425) | 1.252 (1.087 to 1.425) | 1.252 (1.087 to 1.425) | | | | | | | | | |
| Maternal deaths aggravated by HIV/AIDS | 1 g/dL | Both | Both | | | | | | | 1.252 (1.087 to 1.425) | 1.252 (1.087 to 1.425) | 1.252 (1.087 to 1.425) | 1.252 (1.087 to 1.425) | 1.252 (1.087 to 1.425) | 1.252 (1.087 to 1.425) | 1.252 (1.087 to 1.425) | 1.252 (1.087 to 1.425) | 1.252 (1.087 to 1.425) | | | | | | | | | |
| Other maternal disorders | 1 g/dL | Both | Both | | | | | | | 1.252 (1.087 to 1.425) | 1.252 (1.087 to 1.425) | 1.252 (1.087 to 1.425) | 1.252 (1.087 to 1.425) | 1.252 (1.087 to 1.425) | 1.252 (1.087 to 1.425) | 1.252 (1.087 to 1.425) | 1.252 (1.087 to 1.425) | 1.252 (1.087 to 1.425) | | | | | | | | | |
| **Vitamin A deficiency** | | | | | | | | | | | | | | | | | | | | | | | | | | | |
| Lower respiratory infections | Vitamin A deficient | Both | Both | | | | 1.33 (1.114 to 1.585) | 1.33 (1.114 to 1.585) | | | | | | | | | | | | | | | | | | | |
| Lower respiratory infections | Not deficient | Both | Both | | | | 1.0 (1.0 to 1.0) | 1.0 (1.0 to 1.0) | | | | | | | | | | | | | | | | | | | |
| Diarrhoeal diseases | Vitamin A deficient | Both | Both | | | | 2.444 (2.268 to 2.626) | 2.444 (2.268 to 2.626) | | | | | | | | | | | | | | | | | | | |
| Diarrhoeal diseases | Not deficient | Both | Both | | | | 1.0 (1.0 to 1.0) | 1.0 (1.0 to 1.0) | | | | | | | | | | | | | | | | | | | |
| Measles | Vitamin A deficient | Both | Both | | | | 3.515 (2.526 to 4.671) | 3.515 (2.526 to 4.671) | | | | | | | | | | | | | | | | | | | |
| Measles | Not deficient | Both | Both | | | | 1.0 (1.0 to 1.0) | 1.0 (1.0 to 1.0) | | | | | | | | | | | | | | | | | | | |
| **Zinc deficiency** | | | | | | | | | | | | | | | | | | | | | | | | | | | |
| Lower respiratory infections | Zinc deficient | Morbidity | Both | | | | | 1.837 (1.275 to 2.523) | | | | | | | | | | | | | | | | | | | |
| Lower respiratory infections | Zinc deficient | Mortality | Both | | | | | 1.672 (0.458 to 4.135) | | | | | | | | | | | | | | | | | | | |
| Lower respiratory infections | Not deficient | Both | Both | | | | | 1.0 (1.0 to 1.0) | | | | | | | | | | | | | | | | | | | |
| Diarrhoeal diseases | Zinc deficient | Morbidity | Both | | | | | 1.903 (1.517 to 2.335) | | | | | | | | | | | | | | | | | | | |
| Diarrhoeal diseases | Zinc deficient | Mortality | Both | | | | | 1.951 (0.905 to 3.909) | | | | | | | | | | | | | | | | | | | |
| Diarrhoeal diseases | Not deficient | Both | Both | | | | | 1.0 (1.0 to 1.0) | | | | | | | | | | | | | | | | | | | |
| **Chewing tobacco** | | | | | | | | | | | | | | | | | | | | | | | | | | | |
| Lip and oral cavity cancer | Exposed | Both | Males | | | | | | | | | | | 3.021 (2.015 to 4.47) | 3.021 (2.015 to 4.47) | 3.021 (2.015 to 4.47) | 3.021 (2.015 to 4.47) | 3.021 (2.015 to 4.47) | 3.021 (2.015 to 4.47) | 3.021 (2.015 to 4.47) | 3.021 (2.015 to 4.47) | 3.021 (2.015 to 4.47) | 3.021 (2.015 to 4.47) | 3.021 (2.015 to 4.47) | 3.021 (2.015 to 4.47) | 3.021 (2.015 to 4.47) | 3.021 (2.015 to 4.47) |
| Lip and oral cavity cancer | Exposed | Both | Females | | | | | | | | | | | 8.09 (5.463 to 11.767) | 8.09 (5.463 to 11.767) | 8.09 (5.463 to 11.767) | 8.09 (5.463 to 11.767) | 8.09 (5.463 to 11.767) | 8.09 (5.463 to 11.767) | 8.09 (5.463 to 11.767) | 8.09 (5.463 to 11.767) | 8.09 (5.463 to 11.767) | 8.09 (5.463 to 11.767) | 8.09 (5.463 to 11.767) | 8.09 (5.463 to 11.767) | 8.09 (5.463 to 11.767) | 8.09 (5.463 to 11.767) |
| Oesophageal cancer | Exposed | Both | Both | | | | | | | | | | | 2.571 (1.854 to 3.416) | 2.571 (1.854 to 3.416) | 2.571 (1.854 to 3.416) | 2.571 (1.854 to 3.416) | 2.571 (1.854 to 3.416) | 2.571 (1.854 to 3.416) | 2.571 (1.854 to 3.416) | 2.571 (1.854 to 3.416) | 2.571 (1.854 to 3.416) | 2.571 (1.854 to 3.416) | 2.571 (1.854 to 3.416) | 2.571 (1.854 to 3.416) | 2.571 (1.854 to 3.416) | 2.571 (1.854 to 3.416) |
| **Second-hand smoke** | | | | | | | | | | | | | | | | | | | | | | | | | | | |
| Otitis media | Exposed | Morbidity | Both | | 1.37 (1.238 to 1.493) | 1.37 (1.238 to 1.493) | 1.37 (1.238 to 1.493) | 1.37 (1.238 to 1.493) | 1.37 (1.238 to 1.493) | 1.37 (1.238 to 1.493) | | | | | | | | | | | | | | | | | |
| Otitis media | Exposed | Mortality | Both | | 1.37 (1.238 to 1.493) | 1.37 (1.238 to 1.493) | 1.37 (1.238 to 1.493) | 1.37 (1.238 to 1.493) | 1.37 (1.238 to 1.493) | 1.37 (1.238 to 1.493) | | | | | | | | | | | | | | | | | |
| Otitis media | Not exposed | Morbidity | Both | | 1.0 (1.0 to 1.0) | 1.0 (1.0 to 1.0) | 1.0 (1.0 to 1.0) | 1.0 (1.0 to 1.0) | 1.0 (1.0 to 1.0) | 1.0 (1.0 to 1.0) | | | | | | | | | | | | | | | | | |
| Otitis media | Not exposed | Mortality | Both | | 1.0 (1.0 to 1.0) | 1.0 (1.0 to 1.0) | 1.0 (1.0 to 1.0) | 1.0 (1.0 to 1.0) | 1.0 (1.0 to 1.0) | 1.0 (1.0 to 1.0) | | | | | | | | | | | | | | | | | |
| Breast cancer | Exposed | Morbidity | Both | | | | | | | | | | 1.072 (1.017 to 1.126) | 1.072 (1.017 to 1.126) | 1.072 (1.017 to 1.126) | 1.072 (1.017 to 1.126) | 1.072 (1.017 to 1.126) | 1.072 (1.017 to 1.126) | 1.072 (1.017 to 1.126) | 1.072 (1.017 to 1.126) | 1.072 (1.017 to 1.126) | 1.072 (1.017 to 1.126) | 1.072 (1.017 to 1.126) | 1.072 (1.017 to 1.126) | 1.072 (1.017 to 1.126) | 1.072 (1.017 to 1.126) | 1.072 (1.017 to 1.126) |
| Breast cancer | Exposed | Mortality | Both | | | | | | | | | | 1.072 (1.017 to 1.126) | 1.072 (1.017 to 1.126) | 1.072 (1.017 to 1.126) | 1.072 (1.017 to 1.126) | 1.072 (1.017 to 1.126) | 1.072 (1.017 to 1.126) | 1.072 (1.017 to 1.126) | 1.072 (1.017 to 1.126) | 1.072 (1.017 to 1.126) | 1.072 (1.017 to 1.126) | 1.072 (1.017 to 1.126) | 1.072 (1.017 to 1.126) | 1.072 (1.017 to 1.126) | 1.072 (1.017 to 1.126) | 1.072 (1.017 to 1.126) |
| Breast cancer | Not exposed | Morbidity | Both | | | | | | | | | | 1.0 (1.0 to 1.0) | 1.0 (1.0 to 1.0) | 1.0 (1.0 to 1.0) | 1.0 (1.0 to 1.0) | 1.0 (1.0 to 1.0) | 1.0 (1.0 to 1.0) | 1.0 (1.0 to 1.0) | 1.0 (1.0 to 1.0) | 1.0 (1.0 to 1.0) | 1.0 (1.0 to 1.0) | 1.0 (1.0 to 1.0) | 1.0 (1.0 to 1.0) | 1.0 (1.0 to 1.0) | 1.0 (1.0 to 1.0) | 1.0 (1.0 to 1.0) |
| Breast cancer | Not exposed | Mortality | Both | | | | | | | | | | 1.0 (1.0 to 1.0) | 1.0 (1.0 to 1.0) | 1.0 (1.0 to 1.0) | 1.0 (1.0 to 1.0) | 1.0 (1.0 to 1.0) | 1.0 (1.0 to 1.0) | 1.0 (1.0 to 1.0) | 1.0 (1.0 to 1.0) | 1.0 (1.0 to 1.0) | 1.0 (1.0 to 1.0) | 1.0 (1.0 to 1.0) | 1.0 (1.0 to 1.0) | 1.0 (1.0 to 1.0) | 1.0 (1.0 to 1.0) | 1.0 (1.0 to 1.0) |
| **Diet low in fruits** | | | | | | | | | | | | | | | | | | | | | | | | | | | |

**Appendix Table 6a. Relative risks used by age and sex for each outcome for all risk factors except for ambient air pollution alcohol, and smoking.**

| Risk - Outcome | Category / Units | Morbidity / Mortality | Sex | Ages: All-age | 0-6 days | 7-27 days | 28-364 days | 1-4 years | 5-9 years | 10-14 years | 15-19 years | 20-24 years | 25-29 years | 30-34 years | 35-39 years | 40-44 years | 45-49 years | 50-54 years | 55-59 years | 60-64 years | 65-69 years | 70-74 years | 75-79 years | 80-84 years | 85-89 years | 90-94 years | 95+ years |
|---|---|---|---|---|---|---|---|---|---|---|---|---|---|---|---|---|---|---|---|---|---|---|---|---|---|---|---|
| Lip and oral cavity cancer | 100 g/day | Both | Both | | | | | | | | | | 1.042 (0.994 to 1.091) | 1.042 (0.994 to 1.091) | 1.042 (0.994 to 1.091) | 1.042 (0.994 to 1.091) | 1.042 (0.994 to 1.091) | 1.042 (0.994 to 1.091) | 1.042 (0.994 to 1.091) | 1.042 (0.994 to 1.091) | 1.042 (0.994 to 1.091) | 1.042 (0.994 to 1.091) | 1.042 (0.994 to 1.091) | 1.042 (0.994 to 1.091) | 1.042 (0.994 to 1.091) | 1.042 (0.994 to 1.091) | 1.042 (0.994 to 1.091) |
| Nasopharynx cancer | 100 g/day | Both | Both | | | | | | | | | | 1.043 (0.992 to 1.092) | 1.043 (0.992 to 1.092) | 1.043 (0.992 to 1.092) | 1.043 (0.992 to 1.092) | 1.043 (0.992 to 1.092) | 1.043 (0.992 to 1.092) | 1.043 (0.992 to 1.092) | 1.043 (0.992 to 1.092) | 1.043 (0.992 to 1.092) | 1.043 (0.992 to 1.092) | 1.043 (0.992 to 1.092) | 1.043 (0.992 to 1.092) | 1.043 (0.992 to 1.092) | 1.043 (0.992 to 1.092) | 1.043 (0.992 to 1.092) |
| Other pharynx cancer | 100 g/day | Both | Both | | | | | | | | | | 1.042 (0.996 to 1.095) | 1.042 (0.996 to 1.095) | 1.042 (0.996 to 1.095) | 1.042 (0.996 to 1.095) | 1.042 (0.996 to 1.095) | 1.042 (0.996 to 1.095) | 1.042 (0.996 to 1.095) | 1.042 (0.996 to 1.095) | 1.042 (0.996 to 1.095) | 1.042 (0.996 to 1.095) | 1.042 (0.996 to 1.095) | 1.042 (0.996 to 1.095) | 1.042 (0.996 to 1.095) | 1.042 (0.996 to 1.095) | 1.042 (0.996 to 1.095) |
| Oesophageal cancer | 100 g/day | Both | Both | | | | | | | | | | 1.153 (1.033 to 1.288) | 1.153 (1.033 to 1.288) | 1.153 (1.033 to 1.288) | 1.153 (1.033 to 1.288) | 1.153 (1.033 to 1.288) | 1.153 (1.033 to 1.288) | 1.153 (1.033 to 1.288) | 1.153 (1.033 to 1.288) | 1.153 (1.033 to 1.288) | 1.153 (1.033 to 1.288) | 1.153 (1.033 to 1.288) | 1.153 (1.033 to 1.288) | 1.153 (1.033 to 1.288) | 1.153 (1.033 to 1.288) | 1.153 (1.033 to 1.288) |
| Larynx cancer | 100 g/day | Both | Both | | | | | | | | | | 1.042 (0.995 to 1.095) | 1.042 (0.995 to 1.095) | 1.042 (0.995 to 1.095) | 1.042 (0.995 to 1.095) | 1.042 (0.995 to 1.095) | 1.042 (0.995 to 1.095) | 1.042 (0.995 to 1.095) | 1.042 (0.995 to 1.095) | 1.042 (0.995 to 1.095) | 1.042 (0.995 to 1.095) | 1.042 (0.995 to 1.095) | 1.042 (0.995 to 1.095) | 1.042 (0.995 to 1.095) | 1.042 (0.995 to 1.095) | 1.042 (0.995 to 1.095) |
| Tracheal, bronchus, and lung cancer | 100 g/day | Both | Both | | | | | | | | | | 1.076 (1.031 to 1.123) | 1.076 (1.031 to 1.123) | 1.076 (1.031 to 1.123) | 1.076 (1.031 to 1.123) | 1.076 (1.031 to 1.123) | 1.076 (1.031 to 1.123) | 1.076 (1.031 to 1.123) | 1.076 (1.031 to 1.123) | 1.076 (1.031 to 1.123) | 1.076 (1.031 to 1.123) | 1.076 (1.031 to 1.123) | 1.076 (1.031 to 1.123) | 1.076 (1.031 to 1.123) | 1.076 (1.031 to 1.123) | 1.076 (1.031 to 1.123) |
| Ischaemic heart disease | 100 g/day | Both | Both | | | | | | | | | | 1.254 (1.083 to 1.442) | 1.209 (1.07 to 1.361) | 1.159 (1.054 to 1.271) | 1.131 (1.045 to 1.221) | 1.125 (1.043 to 1.211) | 1.114 (1.039 to 1.193) | 1.099 (1.034 to 1.167) | 1.087 (1.03 to 1.146) | 1.078 (1.027 to 1.13) | 1.07 (1.025 to 1.117) | 1.064 (1.022 to 1.106) | 1.057 (1.02 to 1.095) | 1.057 (1.02 to 1.095) | 1.057 (1.02 to 1.095) | 1.057 (1.02 to 1.095) |
| Ischaemic stroke | 100 g/day | Both | Both | | | | | | | | | | 2.024 (1.465 to 2.818) | 1.834 (1.39 to 2.444) | 1.621 (1.301 to 2.043) | 1.48 (1.239 to 1.787) | 1.403 (1.204 to 1.653) | 1.333 (1.171 to 1.533) | 1.272 (1.142 to 1.432) | 1.222 (1.116 to 1.348) | 1.181 (1.096 to 1.283) | 1.145 (1.078 to 1.225) | 1.114 (1.061 to 1.175) | 1.054 (1.029 to 1.082) | 1.054 (1.029 to 1.082) | 1.054 (1.029 to 1.082) | 1.054 (1.029 to 1.082) |
| Intracerebral hemorrhage | 100 g/day | Both | Both | | | | | | | | | | 1.688 (1.319 to 2.182) | 1.576 (1.273 to 1.972) | 1.444 (1.215 to 1.732) | 1.365 (1.18 to 1.595) | 1.336 (1.167 to 1.544) | 1.3 (1.15 to 1.483) | 1.26 (1.131 to 1.415) | 1.226 (1.115 to 1.358) | 1.193 (1.099 to 1.305) | 1.164 (1.084 to 1.256) | 1.133 (1.069 to 1.207) | 1.065 (1.034 to 1.1) | 1.065 (1.034 to 1.1) | 1.065 (1.034 to 1.1) | 1.065 (1.034 to 1.1) |
| Subarachnoid hemorrhage | 100 g/day | Both | Both | | | | | | | | | | 1.688 (1.319 to 2.182) | 1.576 (1.273 to 1.972) | 1.444 (1.215 to 1.732) | 1.365 (1.18 to 1.595) | 1.336 (1.167 to 1.544) | 1.3 (1.15 to 1.483) | 1.26 (1.131 to 1.415) | 1.226 (1.115 to 1.358) | 1.193 (1.099 to 1.305) | 1.164 (1.084 to 1.256) | 1.133 (1.069 to 1.207) | 1.065 (1.034 to 1.1) | 1.065 (1.034 to 1.1) | 1.065 (1.034 to 1.1) | 1.065 (1.034 to 1.1) |
| Diabetes mellitus type 2 | 100 g/day | Both | Both | | | | | | | | | | 1.125 (1.027 to 1.238) | 1.122 (1.026 to 1.232) | 1.119 (1.026 to 1.226) | 1.113 (1.024 to 1.214) | 1.102 (1.022 to 1.194) | 1.093 (1.02 to 1.176) | 1.085 (1.019 to 1.16) | 1.076 (1.017 to 1.143) | 1.068 (1.015 to 1.128) | 1.061 (1.014 to 1.114) | 1.052 (1.012 to 1.098) | 1.036 (1.008 to 1.066) | 1.036 (1.008 to 1.066) | 1.036 (1.008 to 1.066) | 1.036 (1.008 to 1.066) |
| **Diet low in vegetables** | | | | | | | | | | | | | | | | | | | | | | | | | | | |
| Ischaemic heart disease | 100 g/day | Both | Both | | | | | | | | | | 1.249 (1.089 to 1.446) | 1.205 (1.074 to 1.362) | 1.154 (1.057 to 1.269) | 1.126 (1.047 to 1.219) | 1.121 (1.045 to 1.21) | 1.111 (1.042 to 1.193) | 1.098 (1.037 to 1.168) | 1.086 (1.032 to 1.148) | 1.077 (1.029 to 1.133) | 1.07 (1.027 to 1.12) | 1.064 (1.024 to 1.109) | 1.057 (1.022 to 1.097) | 1.057 (1.022 to 1.097) | 1.057 (1.022 to 1.097) | 1.057 (1.022 to 1.097) |
| Ischaemic stroke | 100 g/day | Both | Both | | | | | | | | | | 1.249 (1.049 to 1.463) | 1.211 (1.042 to 1.388) | 1.165 (1.033 to 1.3) | 1.132 (1.027 to 1.238) | 1.113 (1.023 to 1.203) | 1.095 (1.02 to 1.17) | 1.079 (1.017 to 1.141) | 1.065 (1.014 to 1.116) | 1.054 (1.012 to 1.096) | 1.044 (1.009 to 1.077) | 1.035 (1.007 to 1.061) | 1.017 (1.004 to 1.029) | 1.017 (1.004 to 1.029) | 1.017 (1.004 to 1.029) | 1.017 (1.004 to 1.029) |
| Intracerebral hemorrhage | 100 g/day | Both | Both | | | | | | | | | | 1.177 (1.046 to 1.326) | 1.153 (1.04 to 1.278) | 1.122 (1.032 to 1.22) | 1.102 (1.027 to 1.184) | 1.095 (1.025 to 1.17) | 1.086 (1.023 to 1.153) | 1.075 (1.02 to 1.134) | 1.066 (1.018 to 1.117) | 1.057 (1.015 to 1.101) | 1.049 (1.013 to 1.086) | 1.04 (1.011 to 1.071) | 1.02 (1.005 to 1.035) | 1.02 (1.005 to 1.035) | 1.02 (1.005 to 1.035) | 1.02 (1.005 to 1.035) |
| Subarachnoid hemorrhage | 100 g/day | Both | Both | | | | | | | | | | 1.177 (1.046 to 1.326) | 1.153 (1.04 to 1.278) | 1.122 (1.032 to 1.22) | 1.102 (1.027 to 1.184) | 1.095 (1.025 to 1.17) | 1.086 (1.023 to 1.153) | 1.075 (1.02 to 1.134) | 1.066 (1.018 to 1.117) | 1.057 (1.015 to 1.101) | 1.049 (1.013 to 1.086) | 1.04 (1.011 to 1.071) | 1.02 (1.005 to 1.035) | 1.02 (1.005 to 1.035) | 1.02 (1.005 to 1.035) | 1.02 (1.005 to 1.035) |
| **Diet low in legumes** | | | | | | | | | | | | | | | | | | | | | | | | | | | |
| Ischaemic heart disease | 50 g/day | Both | Both | | | | | | | | | | 1.499 (1.18 to 1.89) | 1.453 (1.166 to 1.801) | 1.388 (1.144 to 1.677) | 1.332 (1.125 to 1.573) | 1.287 (1.11 to 1.49) | 1.237 (1.092 to 1.401) | 1.181 (1.071 to 1.303) | 1.139 (1.055 to 1.23) | 1.111 (1.045 to 1.183) | 1.089 (1.036 to 1.146) | 1.074 (1.03 to 1.12) | 1.101 (1.041 to 1.165) | 1.101 (1.041 to 1.165) | 1.101 (1.041 to 1.165) | 1.101 (1.041 to 1.165) |
| **Diet low in whole grains** | | | | | | | | | | | | | | | | | | | | | | | | | | | |
| Ischaemic heart disease | 50 g/day | Both | Both | | | | | | | | | | 1.478 (1.274 to 1.722) | 1.387 (1.225 to 1.578) | 1.285 (1.168 to 1.418) | 1.228 (1.136 to 1.333) | 1.216 (1.129 to 1.313) | 1.194 (1.117 to 1.281) | 1.165 (1.1 to 1.238) | 1.141 (1.086 to 1.203) | 1.125 (1.076 to 1.179) | 1.112 (1.068 to 1.16) | 1.102 (1.062 to 1.145) | 1.097 (1.059 to 1.138) | 1.097 (1.059 to 1.138) | 1.097 (1.059 to 1.138) | 1.097 (1.059 to 1.138) |
| Ischaemic stroke | 50 g/day | Both | Both | | | | | | | | | | 2.075 (1.669 to 2.517) | 1.863 (1.548 to 2.199) | 1.624 (1.406 to 1.849) | 1.466 (1.309 to 1.625) | 1.38 (1.255 to 1.505) | 1.304 (1.206 to 1.401) | 1.241 (1.165 to 1.316) | 1.189 (1.13 to 1.247) | 1.15 (1.104 to 1.195) | 1.117 (1.081 to 1.151) | 1.09 (1.063 to 1.116) | 1.041 (1.029 to 1.053) | 1.041 (1.029 to 1.053) | 1.041 (1.029 to 1.053) | 1.041 (1.029 to 1.053) |
| Intracerebral hemorrhage | 50 g/day | Both | Both | | | | | | | | | | 1.596 (1.406 to 1.825) | 1.484 (1.333 to 1.662) | 1.349 (1.244 to 1.471) | 1.276 (1.194 to 1.369) | 1.258 (1.182 to 1.344) | 1.232 (1.165 to 1.309) | 1.201 (1.143 to 1.267) | 1.176 (1.126 to 1.233) | 1.15 (1.108 to 1.198) | 1.128 (1.092 to 1.169) | 1.106 (1.076 to 1.139) | 1.05 (1.036 to 1.065) | 1.05 (1.036 to 1.065) | 1.05 (1.036 to 1.065) | 1.05 (1.036 to 1.065) |
| Subarachnoid hemorrhage | 50 g/day | Both | Both | | | | | | | | | | 1.596 (1.406 to 1.825) | 1.484 (1.333 to 1.662) | 1.349 (1.244 to 1.471) | 1.276 (1.194 to 1.369) | 1.258 (1.182 to 1.344) | 1.232 (1.165 to 1.309) | 1.201 (1.143 to 1.267) | 1.176 (1.126 to 1.233) | 1.15 (1.108 to 1.198) | 1.128 (1.092 to 1.169) | 1.106 (1.076 to 1.139) | 1.05 (1.036 to 1.065) | 1.05 (1.036 to 1.065) | 1.05 (1.036 to 1.065) | 1.05 (1.036 to 1.065) |
| Diabetes mellitus type 2 | 50 g/day | Both | Both | | | | | | | | | | 1.231 (1.125 to 1.349) | 1.226 (1.122 to 1.341) | 1.22 (1.119 to 1.331) | 1.208 (1.113 to 1.313) | 1.189 (1.103 to 1.283) | 1.172 (1.094 to 1.256) | 1.156 (1.085 to 1.232) | 1.139 (1.077 to 1.207) | 1.125 (1.069 to 1.184) | 1.111 (1.061 to 1.163) | 1.095 (1.053 to 1.14) | 1.064 (1.036 to 1.094) | 1.064 (1.036 to 1.094) | 1.064 (1.036 to 1.094) | 1.064 (1.036 to 1.094) |
| **Diet low in nuts and seeds** | | | | | | | | | | | | | | | | | | | | | | | | | | | |
| Ischaemic heart disease | 4.05 g/day | Morbidity | Both | | | | | | | | | | 1.176 (1.055 to 1.322) | 1.143 (1.045 to 1.259) | 1.105 (1.033 to 1.188) | 1.084 (1.027 to 1.15) | 1.081 (1.026 to 1.144) | 1.074 (1.024 to 1.132) | 1.064 (1.021 to 1.114) | 1.056 (1.018 to 1.099) | 1.05 (1.016 to 1.089) | 1.046 (1.015 to 1.081) | 1.042 (1.014 to 1.075) | 1.039 (1.013 to 1.069) | 1.039 (1.013 to 1.069) | 1.039 (1.013 to 1.069) | 1.039 (1.013 to 1.069) |
| Ischaemic heart disease | 4.05 g/day | Mortality | Both | | | | | | | | | | 1.209 (1.128 to 1.296) | 1.169 (1.105 to 1.239) | 1.124 (1.077 to 1.174) | 1.099 (1.062 to 1.138) | 1.095 (1.06 to 1.133) | 1.088 (1.055 to 1.122) | 1.076 (1.048 to 1.105) | 1.066 (1.042 to 1.092) | 1.059 (1.037 to 1.082) | 1.054 (1.034 to 1.075) | 1.05 (1.032 to 1.069) | 1.046 (1.029 to 1.064) | 1.046 (1.029 to 1.064) | 1.046 (1.029 to 1.064) | 1.046 (1.029 to 1.064) |
| Diabetes mellitus type 2 | 4.05 g/day | Both | Both | | | | | | | | | | 1.05 (1.025 to 1.075) | 1.049 (1.025 to 1.073) | 1.048 (1.024 to 1.071) | 1.045 (1.023 to 1.068) | 1.041 (1.021 to 1.062) | 1.038 (1.019 to 1.056) | 1.035 (1.018 to 1.052) | 1.031 (1.016 to 1.046) | 1.028 (1.014 to 1.042) | 1.025 (1.013 to 1.037) | 1.022 (1.011 to 1.032) | 1.015 (1.007 to 1.022) | 1.015 (1.007 to 1.022) | 1.015 (1.007 to 1.022) | 1.015 (1.007 to 1.022) |
| **Diet low in milk** | | | | | | | | | | | | | | | | | | | | | | | | | | | |
| Colon and rectum cancer | 226.8 g/day | Both | Both | | | | | | | | | | 1.113 (1.039 to 1.202) | 1.113 (1.039 to 1.202) | 1.113 (1.039 to 1.202) | 1.113 (1.039 to 1.202) | 1.113 (1.039 to 1.202) | 1.113 (1.039 to 1.202) | 1.113 (1.039 to 1.202) | 1.113 (1.039 to 1.202) | 1.113 (1.039 to 1.202) | 1.113 (1.039 to 1.202) | 1.113 (1.039 to 1.202) | 1.113 (1.039 to 1.202) | 1.113 (1.039 to 1.202) | 1.113 (1.039 to 1.202) | 1.113 (1.039 to 1.202) |
| **Diet high in red meat** | | | | | | | | | | | | | | | | | | | | | | | | | | | |
| Colon and rectum cancer | 100 g/day | Both | Both | | | | | | | | | | 1.167 (1.033 to 1.309) | 1.167 (1.033 to 1.309) | 1.167 (1.033 to 1.309) | 1.167 (1.033 to 1.309) | 1.167 (1.033 to 1.309) | 1.167 (1.033 to 1.309) | 1.167 (1.033 to 1.309) | 1.167 (1.033 to 1.309) | 1.167 (1.033 to 1.309) | 1.167 (1.033 to 1.309) | 1.167 (1.033 to 1.309) | 1.167 (1.033 to 1.309) | 1.167 (1.033 to 1.309) | 1.167 (1.033 to 1.309) | 1.167 (1.033 to 1.309) |
| Diabetes mellitus type 2 | 100 g/day | Both | Both | | | | | | | | | | 1.322 (1.037 to 1.603) | 1.314 (1.036 to 1.588) | 1.305 (1.035 to 1.569) | 1.288 (1.034 to 1.536) | 1.26 (1.031 to 1.48) | 1.236 (1.028 to 1.433) | 1.213 (1.026 to 1.389) | 1.19 (1.023 to 1.345) | 1.169 (1.021 to 1.306) | 1.15 (1.019 to 1.269) | 1.128 (1.016 to 1.229) | 1.086 (1.011 to 1.152) | 1.086 (1.011 to 1.152) | 1.086 (1.011 to 1.152) | 1.086 (1.011 to 1.152) |
| **Diet high in processed meat** | | | | | | | | | | | | | | | | | | | | | | | | | | | |
| Colon and rectum cancer | 50 g/day | Both | Both | | | | | | | | | | 1.179 (1.093 to 1.267) | 1.179 (1.093 to 1.267) | 1.179 (1.093 to 1.267) | 1.179 (1.093 to 1.267) | 1.179 (1.093 to 1.267) | 1.179 (1.093 to 1.267) | 1.179 (1.093 to 1.267) | 1.179 (1.093 to 1.267) | 1.179 (1.093 to 1.267) | 1.179 (1.093 to 1.267) | 1.179 (1.093 to 1.267) | 1.179 (1.093 to 1.267) | 1.179 (1.093 to 1.267) | 1.179 (1.093 to 1.267) | 1.179 (1.093 to 1.267) |
| Ischaemic heart disease | 50 g/day | Both | Both | | | | | | | | | | 2.568 (1.047 to 4.657) | 2.124 (1.038 to 3.478) | 1.72 (1.028 to 2.489) | 1.545 (1.022 to 2.093) | 1.547 (1.022 to 2.097) | 1.52 (1.022 to 2.037) | 1.467 (1.02 to 1.922) | 1.422 (1.018 to 1.826) | 1.386 (1.017 to 1.75) | 1.354 (1.016 to 1.683) | 1.325 (1.015 to 1.622) | 1.252 (1.012 to 1.475) | 1.252 (1.012 to 1.475) | 1.252 (1.012 to 1.475) | 1.252 (1.012 to 1.475) |
| Diabetes mellitus type 2 | 50 g/day | Both | Both | | | | | | | | | | 1.94 (1.395 to 2.545) | 1.913 (1.386 to 2.496) | 1.881 (1.375 to 2.439) | 1.824 (1.354 to 2.337) | 1.731 (1.319 to 2.173) | 1.653 (1.289 to 2.038) | 1.583 (1.261 to 1.918) | 1.512 (1.233 to 1.798) | 1.45 (1.207 to 1.696) | 1.393 (1.183 to 1.603) | 1.332 (1.157 to 1.505) | 1.216 (1.105 to 1.323) | 1.216 (1.105 to 1.323) | 1.216 (1.105 to 1.323) | 1.216 (1.105 to 1.323) |
| **Diet high in sugar-sweetened beverages** | | | | | | | | | | | | | | | | | | | | | | | | | | | |
| Ischaemic heart disease | 2.5 g/day | Both | Both | | | | | | | | | | 1.377 (0.933 to 1.883) | 1.311 (0.943 to 1.717) | 1.232 (0.955 to 1.521) | 1.195 (0.961 to 1.436) | 1.186 (0.963 to 1.413) | 1.172 (0.965 to 1.381) | 1.156 (0.968 to 1.343) | 1.14 (0.971 to 1.306) | 1.124 (0.974 to 1.27) | 1.11 (0.977 to 1.238) | 1.095 (0.98 to 1.205) | 1.067 (0.985 to 1.143) | 1.067 (0.985 to 1.143) | 1.067 (0.985 to 1.143) | 1.067 (0.985 to 1.143) |
| Diabetes mellitus type 2 | 2.5 g/day | Both | Both | | | | | | | | | | 1.263 (1.129 to 1.4) | 1.257 (1.126 to 1.39) | 1.25 (1.123 to 1.379) | 1.237 (1.117 to 1.358) | 1.214 (1.106 to 1.322) | 1.195 (1.097 to 1.292) | 1.177 (1.088 to 1.264) | 1.158 (1.079 to 1.235) | 1.141 (1.071 to 1.209) | 1.125 (1.063 to 1.185) | 1.107 (1.055 to 1.158) | 1.08 (1.041 to 1.118) | 1.08 (1.041 to 1.118) | 1.08 (1.041 to 1.118) | 1.08 (1.041 to 1.118) |
| **Diet low in fibre** | | | | | | | | | | | | | | | | | | | | | | | | | | | |
| Colon and rectum cancer | 20 g/day | Both | Both | | | | | | | | | | 1.236 (1.133 to 1.35) | 1.236 (1.133 to 1.35) | 1.236 (1.133 to 1.35) | 1.236 (1.133 to 1.35) | 1.236 (1.133 to 1.35) | 1.236 (1.133 to 1.35) | 1.236 (1.133 to 1.35) | 1.236 (1.133 to 1.35) | 1.236 (1.133 to 1.35) | 1.236 (1.133 to 1.35) | 1.236 (1.133 to 1.35) | 1.236 (1.133 to 1.35) | 1.236 (1.133 to 1.35) | 1.236 (1.133 to 1.35) | 1.236 (1.133 to 1.35) |
| Ischaemic heart disease | 20 g/day | Both | Both | | | | | | | | | | 1.688 (1.415 to 2.028) | 1.622 (1.379 to 1.922) | 1.529 (1.326 to 1.776) | 1.45 (1.28 to 1.654) | 1.387 (1.243 to 1.558) | 1.318 (1.202 to 1.455) | 1.242 (1.156 to 1.342) | 1.184 (1.119 to 1.258) | 1.147 (1.096 to 1.205) | 1.118 (1.077 to 1.163) | 1.097 (1.064 to 1.135) | 1.133 (1.087 to 1.185) | 1.133 (1.087 to 1.185) | 1.133 (1.087 to 1.185) | 1.133 (1.087 to 1.185) |
| **Diet low in calcium** | | | | | | | | | | | | | | | | | | | | | | | | | | | |
| Colon and rectum cancer | 1 g/day | Both | Both | | | | | | | | | | 1.372 (1.268 to 1.485) | 1.372 (1.268 to 1.485) | 1.372 (1.268 to 1.485) | 1.372 (1.268 to 1.485) | 1.372 (1.268 to 1.485) | 1.372 (1.268 to 1.485) | 1.372 (1.268 to 1.485) | 1.372 (1.268 to 1.485) | 1.372 (1.268 to 1.485) | 1.372 (1.268 to 1.485) | 1.372 (1.268 to 1.485) | 1.372 (1.268 to 1.485) | 1.372 (1.268 to 1.485) | 1.372 (1.268 to 1.485) | 1.372 (1.268 to 1.485) |
| **Diet low in seafood omega-3 fatty acids** | | | | | | | | | | | | | | | | | | | | | | | | | | | |
| Ischaemic heart disease | 100 mg/day | Morbidity | Both | | | | | | | | | | 1.0 (1.0 to 1.0) | 1.0 (1.0 to 1.0) | 1.0 (1.0 to 1.0) | 1.0 (1.0 to 1.0) | 1.0 (1.0 to 1.0) | 1.0 (1.0 to 1.0) | 1.0 (1.0 to 1.0) | 1.0 (1.0 to 1.0) | 1.0 (1.0 to 1.0) | 1.0 (1.0 to 1.0) | 1.0 (1.0 to 1.0) | 1.0 (1.0 to 1.0) | 1.0 (1.0 to 1.0) | 1.0 (1.0 to 1.0) | 1.0 (1.0 to 1.0) |
| Ischaemic heart disease | 100 mg/day | Mortality | Both | | | | | | | | | | 1.291 (1.109 to 1.505) | 1.249 (1.094 to 1.428) | 1.199 (1.077 to 1.338) | 1.173 (1.067 to 1.293) | 1.165 (1.064 to 1.279) | 1.154 (1.06 to 1.26) | 1.14 (1.055 to 1.235) | 1.126 (1.05 to 1.211) | 1.113 (1.045 to 1.189) | 1.101 (1.04 to 1.167) | 1.088 (1.035 to 1.145) | 1.062 (1.025 to 1.102) | 1.062 (1.025 to 1.102) | 1.062 (1.025 to 1.102) | 1.062 (1.025 to 1.102) |
| **Diet low in polyunsaturated fatty acids** | | | | | | | | | | | | | | | | | | | | | | | | | | | |
| Ischaemic heart disease | 5% energy/day | Both | Both | | | | | | | | | | 1.267 (1.098 to 1.452) | 1.211 (1.079 to 1.352) | 1.148 (1.056 to 1.244) | 1.114 (1.044 to 1.186) | 1.111 (1.043 to 1.181) | 1.101 (1.039 to 1.165) | 1.086 (1.033 to 1.14) | 1.075 (1.029 to 1.121) | 1.068 (1.026 to 1.11) | 1.063 (1.025 to 1.102) | 1.06 (1.024 to 1.097) | 1.063 (1.025 to 1.102) | 1.063 (1.025 to 1.102) | 1.063 (1.025 to 1.102) | 1.063 (1.025 to 1.102) |
| **Diet high in trans fatty acids** | | | | | | | | | | | | | | | | | | | | | | | | | | | |
| Ischaemic heart disease | 2% energy/day | Both | Both | | | | | | | | | | 1.901 (1.591 to 2.275) | 1.775 (1.514 to 2.085) | 1.615 (1.415 to 1.848) | 1.517 (1.352 to 1.707) | 1.461 (1.316 to 1.627) | 1.396 (1.274 to 1.535) | 1.323 (1.225 to 1.433) | 1.264 (1.186 to 1.352) | 1.222 (1.157 to 1.294) | 1.186 (1.132 to 1.246) | 1.158 (1.112 to 1.207) | 1.15 (1.107 to 1.197) | 1.15 (1.107 to 1.197) | 1.15 (1.107 to 1.197) | 1.15 (1.107 to 1.197) |
| **Diet high in sodium **** | | | | | | | | | | | | | | | | | | | | | | | | | | | |
| | Non-Black, Non-Hypertensive | Both | Both | | | | | | | | | | -1.366 (-1.937 to -0.795) | -1.882 (-2.434 to -1.330) | -2.397 (-2.967 to -1.828) | -2.913 (-3.533 to -2.292) | -3.428 (-4.126 to -2.730) | -3.944 (-4.738 to -3.150) | -4.459 (-5.362 to -3.556) | -4.975 (-5.995 to -3.954) | -5.490 (-6.634 to -4.347) | -5.490 (-6.634 to -4.347) | -5.490 (-6.634 to -4.347) | -5.490 (-6.634 to -4.347) | -5.490 (-6.634 to -4.347) | -5.490 (-6.634 to -4.347) | -5.490 (-6.634 to -4.347) |
| | Non-Black, Hypertensive | Both | Both | | | | | | | | | | -3.300 (-4.147 to -2.454) | -3.816 (-4.547 to -3.085) | -4.331 (-4.959 to -3.704) | -4.847 (-5.389 to -4.305) | -5.363 (-5.848 to -4.877) | -5.878 (-6.346 to -5.411) | -6.394 (-6.886 to -5.901) | -6.909 (-7.464 to -6.354) | -7.425 (-8.069 to -6.781) | -7.425 (-8.069 to -6.781) | -7.425 (-8.069 to -6.781) | -7.425 (-8.069 to -6.781) | -7.425 (-8.069 to -6.781) | -7.425 (-8.069 to -6.781) | -7.425 (-8.069 to -6.781) |
| | Black, Non-Hypertensive | Both | Both | | | | | | | | | | -3.910 (-5.065 to -2.755) | -4.426 (-5.564 to -3.287) | -4.941 (-6.081 to -3.802) | -5.457 (-6.616 to -4.298) | -5.972 (-7.168 to -4.777) | -6.488 (-7.735 to -5.241) | -7.004 (-8.316 to -5.691) | -7.519 (-8.909 to -6.129) | -8.035 (-9.512 to -6.557) | -8.035 (-9.512 to -6.557) | -8.035 (-9.512 to -6.557) | -8.035 (-9.512 to -6.557) | -8.035 (-9.512 to -6.557) | -8.035 (-9.512 to -6.557) | -8.035 (-9.512 to -6.557) |
| | Black, Hypertensive | Both | Both | | | | | | | | | | -5.844 (-7.222 to -4.467) | -6.360 (-7.663 to -5.057) | -6.876 (-8.117 to -5.635) | -7.391 (-8.584 to -6.198) | -7.907 (-9.068 to -6.745) | -8.422 (-9.569 to -7.275) | -8.938 (-10.088 to -7.788) | -9.453 (-10.624 to -8.282) | -9.969 (-11.178 to -8.760) | -9.969 (-11.178 to -8.760) | -9.969 (-11.178 to -8.760) | -9.969 (-11.178 to -8.760) | -9.969 (-11.178 to -8.760) | -9.969 (-11.178 to -8.760) | -9.969 (-11.178 to -8.760) |

**Appendix Table 6a. Relative risks used by age and sex for each outcome for all risk factors except for ambient air pollution alcohol, and smoking.**

| | | | | Ages | | | | | | | | | | | | | | | | | | | | | | | |
|---|---|---|---|---|---|---|---|---|---|---|---|---|---|---|---|---|---|---|---|---|---|---|---|---|---|---|---|
| **Risk - Outcome** | **Category / Units** | **Morbidity / Mortality** | **Sex** | **All-age** | **0-6 days** | **7-27 days** | **28-364 days** | **1-4 years** | **5-9 years** | **10-14 years** | **15-19 years** | **20-24 years** | **25-29 years** | **30-34 years** | **35-39 years** | **40-44 years** | **45-49 years** | **50-54 years** | **55-59 years** | **60-64 years** | **65-69 years** | **70-74 years** | **75-79 years** | **80-84 years** | **85-89 years** | **90-94 years** | **95+ years** |
| Stomach cancer | 1 g/day | Both | Both | | | | | | | | | | 1.199 (0.987 to 1.443) | 1.205 (1.008 to 1.428) | 1.205 (0.989 to 1.459) | 1.202 (0.996 to 1.443) | 1.209 (0.996 to 1.448) | 1.198 (0.984 to 1.429) | 1.204 (1.008 to 1.428) | 1.2 (0.982 to 1.457) | 1.206 (1.003 to 1.431) | 1.21 (0.995 to 1.444) | 1.203 (0.987 to 1.433) | 1.205 (0.984 to 1.459) | 1.205 (0.984 to 1.459) | 1.205 (0.984 to 1.459) | 1.205 (0.984 to 1.459) |
| **Intimate partner violence (HIV PAF approach)** | | | | | | | | | | | | | | | | | | | | | | | | | | | |
| HIV/AIDS - Drug-susceptible Tuberculosis | Exposed | morbidity | Both | 1.59 (1.3 to 1.94) | | | | | | | | | | | | | | | | | | | | | | | |
| HIV/AIDS - Drug-susceptible Tuberculosis | Exposed | mortality | Both | 1.59 (1.3 to 1.94) | | | | | | | | | | | | | | | | | | | | | | | |
| HIV/AIDS - Drug-susceptible Tuberculosis | Not exposed | morbidity | Both | 1.0 (1.0 to 1.0) | | | | | | | | | | | | | | | | | | | | | | | |
| HIV/AIDS - Drug-susceptible Tuberculosis | Not exposed | mortality | Both | 1.0 (1.0 to 1.0) | | | | | | | | | | | | | | | | | | | | | | | |
| HIV/AIDS - Multidrug-resistant Tuberculosis without extensive drug resistance | Exposed | morbidity | Both | 1.59 (1.3 to 1.94) | | | | | | | | | | | | | | | | | | | | | | | |
| HIV/AIDS - Multidrug-resistant Tuberculosis without extensive drug resistance | Exposed | mortality | Both | 1.59 (1.3 to 1.94) | | | | | | | | | | | | | | | | | | | | | | | |
| HIV/AIDS - Multidrug-resistant Tuberculosis without extensive drug resistance | Not exposed | morbidity | Both | 1.0 (1.0 to 1.0) | | | | | | | | | | | | | | | | | | | | | | | |
| HIV/AIDS - Multidrug-resistant Tuberculosis without extensive drug resistance | Not exposed | mortality | Both | 1.0 (1.0 to 1.0) | | | | | | | | | | | | | | | | | | | | | | | |
| HIV/AIDS - Extensively drug-resistant Tuberculosis | Exposed | morbidity | Both | 1.59 (1.3 to 1.94) | | | | | | | | | | | | | | | | | | | | | | | |
| HIV/AIDS - Extensively drug-resistant Tuberculosis | Exposed | mortality | Both | 1.59 (1.3 to 1.94) | | | | | | | | | | | | | | | | | | | | | | | |
| HIV/AIDS - Extensively drug-resistant Tuberculosis | Not exposed | morbidity | Both | 1.0 (1.0 to 1.0) | | | | | | | | | | | | | | | | | | | | | | | |
| HIV/AIDS - Extensively drug-resistant Tuberculosis | Not exposed | mortality | Both | 1.0 (1.0 to 1.0) | | | | | | | | | | | | | | | | | | | | | | | |
| HIV/AIDS resulting in other diseases | Exposed | morbidity | Both | 1.59 (1.3 to 1.94) | | | | | | | | | | | | | | | | | | | | | | | |
| HIV/AIDS resulting in other diseases | Exposed | mortality | Both | 1.59 (1.3 to 1.94) | | | | | | | | | | | | | | | | | | | | | | | |
| HIV/AIDS resulting in other diseases | Not exposed | morbidity | Both | 1.0 (1.0 to 1.0) | | | | | | | | | | | | | | | | | | | | | | | |
| HIV/AIDS resulting in other diseases | Not exposed | mortality | Both | 1.0 (1.0 to 1.0) | | | | | | | | | | | | | | | | | | | | | | | |
| **Intimate partner violence (exposure approach)** | | | | | | | | | | | | | | | | | | | | | | | | | | | |
| Maternal abortive outcome | Exposed | Both | Both | | 1.971 (1.154 to 3.098) | 1.96 (1.145 to 3.167) | 1.949 (1.169 to 3.094) | 1.981 (1.151 to 3.204) | 1.981 (1.137 to 3.211) | 1.967 (1.144 to 3.144) | 1.945 (1.168 to 3.019) | 1.997 (1.124 to 3.269) | 1.977 (1.161 to 3.187) | 1.994 (1.154 to 3.119) | 1.944 (1.128 to 3.04) | 2.0 (1.175 to 3.159) | 1.965 (1.175 to 3.202) | 1.971 (1.156 to 3.107) | 1.957 (1.132 to 3.217) | 1.975 (1.164 to 3.09) | 1.995 (1.146 to 3.147) | 1.975 (1.154 to 3.189) | 1.969 (1.188 to 3.11) | 1.978 (1.176 to 3.148) | 1.969 (1.192 to 3.065) | 1.975 (1.149 to 3.087) | 1.976 (1.136 to 3.152) |
| Maternal abortive outcome | Not exposed | Both | Both | | 1.0 (1.0 to 1.0) | 1.0 (1.0 to 1.0) | 1.0 (1.0 to 1.0) | 1.0 (1.0 to 1.0) | 1.0 (1.0 to 1.0) | 1.0 (1.0 to 1.0) | 1.0 (1.0 to 1.0) | 1.0 (1.0 to 1.0) | 1.0 (1.0 to 1.0) | 1.0 (1.0 to 1.0) | 1.0 (1.0 to 1.0) | 1.0 (1.0 to 1.0) | 1.0 (1.0 to 1.0) | 1.0 (1.0 to 1.0) | 1.0 (1.0 to 1.0) | 1.0 (1.0 to 1.0) | 1.0 (1.0 to 1.0) | 1.0 (1.0 to 1.0) | 1.0 (1.0 to 1.0) | 1.0 (1.0 to 1.0) | 1.0 (1.0 to 1.0) | 1.0 (1.0 to 1.0) | 1.0 (1.0 to 1.0) |
| Major depressive disorder | Exposed | Morbidity | Both | | 1.474 (1.099 to 1.949) | 1.456 (1.051 to 1.898) | 1.464 (1.099 to 1.914) | 1.454 (1.103 to 1.922) | 1.457 (1.088 to 1.917) | 1.46 (1.106 to 1.92) | 1.454 (1.094 to 1.872) | 1.455 (1.098 to 1.92) | 1.459 (1.091 to 1.917) | 1.447 (1.079 to 1.885) | 1.458 (1.099 to 1.921) | 1.442 (1.068 to 1.924) | 1.449 (1.061 to 1.942) | 1.448 (1.104 to 1.875) | 1.458 (1.111 to 1.905) | 1.461 (1.083 to 1.92) | 1.458 (1.094 to 1.928) | 1.456 (1.09 to 1.912) | 1.462 (1.097 to 1.914) | 1.47 (1.116 to 1.892) | 1.447 (1.061 to 1.898) | 1.448 (1.064 to 1.879) | 1.45 (1.081 to 1.915) |
| Major depressive disorder | Not exposed | Morbidity | Both | | 1.0 (1.0 to 1.0) | 1.0 (1.0 to 1.0) | 1.0 (1.0 to 1.0) | 1.0 (1.0 to 1.0) | 1.0 (1.0 to 1.0) | 1.0 (1.0 to 1.0) | 1.0 (1.0 to 1.0) | 1.0 (1.0 to 1.0) | 1.0 (1.0 to 1.0) | 1.0 (1.0 to 1.0) | 1.0 (1.0 to 1.0) | 1.0 (1.0 to 1.0) | 1.0 (1.0 to 1.0) | 1.0 (1.0 to 1.0) | 1.0 (1.0 to 1.0) | 1.0 (1.0 to 1.0) | 1.0 (1.0 to 1.0) | 1.0 (1.0 to 1.0) | 1.0 (1.0 to 1.0) | 1.0 (1.0 to 1.0) | 1.0 (1.0 to 1.0) | 1.0 (1.0 to 1.0) | 1.0 (1.0 to 1.0) |
| **Childhood sexual abuse** | | | | | | | | | | | | | | | | | | | | | | | | | | | |
| Major depressive disorder | Exposed | Morbidity | Both | | 1.631 (1.405 to 1.877) | 1.631 (1.422 to 1.881) | 1.633 (1.409 to 1.893) | 1.63 (1.404 to 1.896) | 1.627 (1.396 to 1.863) | 1.63 (1.407 to 1.881) | 1.635 (1.401 to 1.894) | 1.637 (1.41 to 1.879) | 1.638 (1.42 to 1.888) | 1.633 (1.416 to 1.876) | 1.633 (1.416 to 1.874) | 1.634 (1.417 to 1.882) | 1.632 (1.411 to 1.91) | 1.631 (1.416 to 1.889) | 1.634 (1.402 to 1.883) | 1.634 (1.408 to 1.866) | 1.634 (1.422 to 1.886) | 1.634 (1.412 to 1.878) | 1.633 (1.406 to 1.886) | 1.634 (1.419 to 1.899) | 1.637 (1.412 to 1.856) | 1.636 (1.401 to 1.877) | 1.635 (1.402 to 1.903) |
| Major depressive disorder | Not exposed | Morbidity | Both | | 1.0 (1.0 to 1.0) | 1.0 (1.0 to 1.0) | 1.0 (1.0 to 1.0) | 1.0 (1.0 to 1.0) | 1.0 (1.0 to 1.0) | 1.0 (1.0 to 1.0) | 1.0 (1.0 to 1.0) | 1.0 (1.0 to 1.0) | 1.0 (1.0 to 1.0) | 1.0 (1.0 to 1.0) | 1.0 (1.0 to 1.0) | 1.0 (1.0 to 1.0) | 1.0 (1.0 to 1.0) | 1.0 (1.0 to 1.0) | 1.0 (1.0 to 1.0) | 1.0 (1.0 to 1.0) | 1.0 (1.0 to 1.0) | 1.0 (1.0 to 1.0) | 1.0 (1.0 to 1.0) | 1.0 (1.0 to 1.0) | 1.0 (1.0 to 1.0) | 1.0 (1.0 to 1.0) | 1.0 (1.0 to 1.0) |
| Alcohol use disorders | Exposed | Both | Both | | 1.55 (1.186 to 1.952) | 1.549 (1.194 to 2.024) | 1.551 (1.208 to 1.983) | 1.55 (1.184 to 1.941) | 1.551 (1.172 to 2.013) | 1.55 (1.202 to 1.965) | 1.553 (1.182 to 2.008) | 1.545 (1.194 to 1.939) | 1.557 (1.202 to 1.994) | 1.54 (1.167 to 2.047) | 1.541 (1.184 to 1.953) | 1.554 (1.187 to 1.97) | 1.552 (1.196 to 1.997) | 1.549 (1.188 to 2.0) | 1.55 (1.183 to 1.971) | 1.562 (1.206 to 1.992) | 1.546 (1.206 to 1.973) | 1.556 (1.19 to 1.971) | 1.562 (1.179 to 1.982) | 1.545 (1.187 to 1.961) | 1.549 (1.2 to 1.967) | 1.545 (1.211 to 1.967) | 1.549 (1.19 to 1.944) |
| Alcohol use disorders | Not exposed | Both | Both | | 1.0 (1.0 to 1.0) | 1.0 (1.0 to 1.0) | 1.0 (1.0 to 1.0) | 1.0 (1.0 to 1.0) | 1.0 (1.0 to 1.0) | 1.0 (1.0 to 1.0) | 1.0 (1.0 to 1.0) | 1.0 (1.0 to 1.0) | 1.0 (1.0 to 1.0) | 1.0 (1.0 to 1.0) | 1.0 (1.0 to 1.0) | 1.0 (1.0 to 1.0) | 1.0 (1.0 to 1.0) | 1.0 (1.0 to 1.0) | 1.0 (1.0 to 1.0) | 1.0 (1.0 to 1.0) | 1.0 (1.0 to 1.0) | 1.0 (1.0 to 1.0) | 1.0 (1.0 to 1.0) | 1.0 (1.0 to 1.0) | 1.0 (1.0 to 1.0) | 1.0 (1.0 to 1.0) | 1.0 (1.0 to 1.0) |
| **Bullying victimization** | | | | | | | | | | | | | | | | | | | | | | | | | | | |
| Major depressive disorder | First exposed to bullying in current year | morbidity | Both | 2.016 ( 1.488 to 2.731) | | | | | | | | | | | | | | | | | | | | | | | |
| Major depressive disorder | First exposed to bullying 1 year prior | morbidity | Both | 1.955 (1.481 to 2.579) | | | | | | | | | | | | | | | | | | | | | | | |
| Major depressive disorder | First exposed to bullying 2 years prior | morbidity | Both | 1.895 (1.471 to 2.441) | | | | | | | | | | | | | | | | | | | | | | | |
| Major depressive disorder | First exposed to bullying 3 years prior | morbidity | Both | 1.838 (1.457 to 2.318) | | | | | | | | | | | | | | | | | | | | | | | |
| Major depressive disorder | First exposed to bullying 4 years prior | morbidity | Both | 1.782 (1.437 to 2.21) | | | | | | | | | | | | | | | | | | | | | | | |
| Major depressive disorder | First exposed to bullying 5 years prior | morbidity | Both | 1.728 (1.41 to 2.117) | | | | | | | | | | | | | | | | | | | | | | | |
| Major depressive disorder | First exposed to bullying 6 years prior | morbidity | Both | 1.675 (1.376 to 2.04) | | | | | | | | | | | | | | | | | | | | | | | |
| Major depressive disorder | First exposed to bullying 7 years prior | morbidity | Both | 1.624 (1.333 to 1.979) | | | | | | | | | | | | | | | | | | | | | | | |
| Major depressive disorder | First exposed to bullying 8 years prior | morbidity | Both | 1.575 (1.284 to 1.932) | | | | | | | | | | | | | | | | | | | | | | | |
| Major depressive disorder | First exposed to bullying 9 years prior | morbidity | Both | 1.527 (1.229 to 1.898) | | | | | | | | | | | | | | | | | | | | | | | |
| Major depressive disorder | First exposed to bullying 10 years prior | morbidity | Both | 1.481 (1.171 to 1.873) | | | | | | | | | | | | | | | | | | | | | | | |
| Major depressive disorder | First exposed to bullying 11 years prior | morbidity | Both | 1.436 (1.112 to 1.855) | | | | | | | | | | | | | | | | | | | | | | | |
| Major depressive disorder | First exposed to bullying 12 years prior | morbidity | Both | 1.392 (1.052 to 1.843) | | | | | | | | | | | | | | | | | | | | | | | |
| Major depressive disorder | First exposed to bullying 13 years prior | morbidity | Both | 1.35 (0.993 to 1.836) | | | | | | | | | | | | | | | | | | | | | | | |
| Major depressive disorder | First exposed to bullying 14 years prior | morbidity | Both | 1.309 (0.936 to 1.832) | | | | | | | | | | | | | | | | | | | | | | | |
| Major depressive disorder | First exposed to bullying 15 years prior | morbidity | Both | 1.269 (0.881 to 1.83) | | | | | | | | | | | | | | | | | | | | | | | |
| Major depressive disorder | First exposed to bullying 16 years prior | morbidity | Both | 1.231 (0.828 to 1.83) | | | | | | | | | | | | | | | | | | | | | | | |
| Major depressive disorder | First exposed to bullying 17 years prior | morbidity | Both | 1.193 (0.778 to 1.831) | | | | | | | | | | | | | | | | | | | | | | | |
| Major depressive disorder | First exposed to bullying 18 years prior | morbidity | Both | 1.157 (0.73 to 1.834) | | | | | | | | | | | | | | | | | | | | | | | |
| Major depressive disorder | First exposed to bullying 19 years prior | morbidity | Both | 1.122 (0.685 to 1.837) | | | | | | | | | | | | | | | | | | | | | | | |
| Major depressive disorder | First exposed to bullying 20 years prior | morbidity | Both | 1.088 (0.643 to 1.842) | | | | | | | | | | | | | | | | | | | | | | | |
| Major depressive disorder | First exposed to bullying 21 years prior | morbidity | Both | 1.055 (0.603 to 1.847) | | | | | | | | | | | | | | | | | | | | | | | |
| Major depressive disorder | First exposed to bullying 22 years prior | morbidity | Both | 1.023 (0.565 to 1.852) | | | | | | | | | | | | | | | | | | | | | | | |
| Major depressive disorder | Never exposed to bullying or first exposed over 22 years prior | morbidity | Both | 1.0 (1.0 to 1.0) | | | | | | | | | | | | | | | | | | | | | | | |
| Anxiety disorders | First exposed to bullying in current year | morbidity | Both | 2.016 (1.488 to 2.731) | | | | | | | | | | | | | | | | | | | | | | | |
| Anxiety disorders | First exposed to bullying 1 year prior | morbidity | Both | 1.955 (1.481 to 2.579) | | | | | | | | | | | | | | | | | | | | | | | |
| Anxiety disorders | First exposed to bullying 2 years prior | morbidity | Both | 1.895 (1.471 to 2.441) | | | | | | | | | | | | | | | | | | | | | | | |
| Anxiety disorders | First exposed to bullying 3 years prior | morbidity | Both | 1.838 (1.457 to 2.318) | | | | | | | | | | | | | | | | | | | | | | | |

**Appendix Table 6a. Relative risks used by age and sex for each outcome for all risk factors except for ambient air pollution alcohol, and smoking.**

| | | | | Ages | | | | | | | | | | | | | | | | | | | | | | | |
|---|---|---|---|---|---|---|---|---|---|---|---|---|---|---|---|---|---|---|---|---|---|---|---|---|---|---|---|
| **Risk - Outcome** | **Category / Units** | **Morbidity / Mortality** | **Sex** | **All-age** | **0-6 days** | **7-27 days** | **28-364 days** | **1-4 years** | **5-9 years** | **10-14 years** | **15-19 years** | **20-24 years** | **25-29 years** | **30-34 years** | **35-39 years** | **40-44 years** | **45-49 years** | **50-54 years** | **55-59 years** | **60-64 years** | **65-69 years** | **70-74 years** | **75-79 years** | **80-84 years** | **85-89 years** | **90-94 years** | **95+ years** |
| Anxiety disorders | First exposed to bullying 4 years prior | morbidity | Both | 1.782 (1.437 to 2.21) | | | | | | | | | | | | | | | | | | | | | | | |
| Anxiety disorders | First exposed to bullying 5 years prior | morbidity | Both | 1.728 (1.41 to 2.117) | | | | | | | | | | | | | | | | | | | | | | | |
| Anxiety disorders | First exposed to bullying 6 years prior | morbidity | Both | 1.675 (1.376 to 2.04) | | | | | | | | | | | | | | | | | | | | | | | |
| Anxiety disorders | First exposed to bullying 7 years prior | morbidity | Both | 1.624 (1.333 to 1.979) | | | | | | | | | | | | | | | | | | | | | | | |
| Anxiety disorders | First exposed to bullying 8 years prior | morbidity | Both | 1.575 (1.284 to 1.932) | | | | | | | | | | | | | | | | | | | | | | | |
| Anxiety disorders | First exposed to bullying 9 years prior | morbidity | Both | 1.527 (1.229 to 1.898) | | | | | | | | | | | | | | | | | | | | | | | |
| Anxiety disorders | First exposed to bullying 10 years prior | morbidity | Both | 1.481 (1.171 to 1.873) | | | | | | | | | | | | | | | | | | | | | | | |
| Anxiety disorders | First exposed to bullying 11 years prior | morbidity | Both | 1.436 (1.112 to 1.855) | | | | | | | | | | | | | | | | | | | | | | | |
| Anxiety disorders | First exposed to bullying 12 years prior | morbidity | Both | 1.392 (1.052 to 1.843) | | | | | | | | | | | | | | | | | | | | | | | |
| Anxiety disorders | First exposed to bullying 13 years prior | morbidity | Both | 1.35 (0.993 to 1.836) | | | | | | | | | | | | | | | | | | | | | | | |
| Anxiety disorders | First exposed to bullying 14 years prior | morbidity | Both | 1.309 (0.936 to 1.832) | | | | | | | | | | | | | | | | | | | | | | | |
| Anxiety disorders | First exposed to bullying 15 years prior | morbidity | Both | 1.269 (0.881 to 1.83) | | | | | | | | | | | | | | | | | | | | | | | |
| Anxiety disorders | First exposed to bullying 16 years prior | morbidity | Both | 1.231 (0.828 to 1.83) | | | | | | | | | | | | | | | | | | | | | | | |
| Anxiety disorders | First exposed to bullying 17 years prior | morbidity | Both | 1.193 (0.778 to 1.831) | | | | | | | | | | | | | | | | | | | | | | | |
| Anxiety disorders | First exposed to bullying 18 years prior | morbidity | Both | 1.157 (0.73 to 1.834) | | | | | | | | | | | | | | | | | | | | | | | |
| Anxiety disorders | First exposed to bullying 19 years prior | morbidity | Both | 1.122 (0.685 to 1.837) | | | | | | | | | | | | | | | | | | | | | | | |
| Anxiety disorders | First exposed to bullying 20 years prior | morbidity | Both | 1.088 (0.643 to 1.842) | | | | | | | | | | | | | | | | | | | | | | | |
| Anxiety disorders | First exposed to bullying 21 years prior | morbidity | Both | 1.055 (0.603 to 1.847) | | | | | | | | | | | | | | | | | | | | | | | |
| Anxiety disorders | First exposed to bullying 22 years prior | morbidity | Both | 1.023 (0.565 to 1.852) | | | | | | | | | | | | | | | | | | | | | | | |
| Anxiety disorders | Never exposed to bullying or first exposed over 22 years prior | morbidity | Both | 1.0 (1.0 to 1.0) | | | | | | | | | | | | | | | | | | | | | | | |
| **Low physical activity** | | | | | | | | | | | | | | | | | | | | | | | | | | | |
| Colon and rectum cancer | 0 METs | Both | Both | 1.0 (1.0 to 1.0) | | | | | | | | | | | | | | | | | | | | | | | |
| Colon and rectum cancer | 10200 METs | Both | Both | 0.811 (0.758 to 0.864) | | | | | | | | | | | | | | | | | | | | | | | |
| Colon and rectum cancer | 10800 METs | Both | Both | 0.809 (0.757 to 0.862) | | | | | | | | | | | | | | | | | | | | | | | |
| Colon and rectum cancer | 11400 METs | Both | Both | 0.807 (0.756 to 0.86) | | | | | | | | | | | | | | | | | | | | | | | |
| Colon and rectum cancer | 1200 METs | Both | Both | 0.956 (0.88 to 1.032) | | | | | | | | | | | | | | | | | | | | | | | |
| Colon and rectum cancer | 12000 METs | Both | Both | 0.805 (0.754 to 0.858) | | | | | | | | | | | | | | | | | | | | | | | |
| Colon and rectum cancer | 12600 METs | Both | Both | 0.804 (0.753 to 0.856) | | | | | | | | | | | | | | | | | | | | | | | |
| Colon and rectum cancer | 13200 METs | Both | Both | 0.802 (0.752 to 0.855) | | | | | | | | | | | | | | | | | | | | | | | |
| Colon and rectum cancer | 13800 METs | Both | Both | 0.8 (0.75 to 0.854) | | | | | | | | | | | | | | | | | | | | | | | |
| Colon and rectum cancer | 14400 METs | Both | Both | 0.798 (0.749 to 0.851) | | | | | | | | | | | | | | | | | | | | | | | |
| Colon and rectum cancer | 15000 METs | Both | Both | 0.796 (0.747 to 0.85) | | | | | | | | | | | | | | | | | | | | | | | |
| Colon and rectum cancer | 15600 METs | Both | Both | 0.795 (0.744 to 0.849) | | | | | | | | | | | | | | | | | | | | | | | |
| Colon and rectum cancer | 16200 METs | Both | Both | 0.793 (0.742 to 0.849) | | | | | | | | | | | | | | | | | | | | | | | |
| Colon and rectum cancer | 16800 METs | Both | Both | 0.791 (0.739 to 0.848) | | | | | | | | | | | | | | | | | | | | | | | |
| Colon and rectum cancer | 17400 METs | Both | Both | 0.789 (0.736 to 0.847) | | | | | | | | | | | | | | | | | | | | | | | |
| Colon and rectum cancer | 1800 METs | Both | Both | 0.933 (0.821 to 1.048) | | | | | | | | | | | | | | | | | | | | | | | |
| Colon and rectum cancer | 18000 METs | Both | Both | 0.787 (0.733 to 0.847) | | | | | | | | | | | | | | | | | | | | | | | |
| Colon and rectum cancer | 18600 METs | Both | Both | 0.786 (0.729 to 0.846) | | | | | | | | | | | | | | | | | | | | | | | |
| Colon and rectum cancer | 19200 METs | Both | Both | 0.784 (0.726 to 0.846) | | | | | | | | | | | | | | | | | | | | | | | |
| Colon and rectum cancer | 19800 METs | Both | Both | 0.782 (0.723 to 0.845) | | | | | | | | | | | | | | | | | | | | | | | |
| Colon and rectum cancer | 20400 METs | Both | Both | 0.78 (0.72 to 0.845) | | | | | | | | | | | | | | | | | | | | | | | |
| Colon and rectum cancer | 21000 METs | Both | Both | 0.778 (0.717 to 0.845) | | | | | | | | | | | | | | | | | | | | | | | |
| Colon and rectum cancer | 21600 METs | Both | Both | 0.777 (0.713 to 0.846) | | | | | | | | | | | | | | | | | | | | | | | |
| Colon and rectum cancer | 22200 METs | Both | Both | 0.775 (0.709 to 0.847) | | | | | | | | | | | | | | | | | | | | | | | |
| Colon and rectum cancer | 22800 METs | Both | Both | 0.773 (0.706 to 0.848) | | | | | | | | | | | | | | | | | | | | | | | |
| Colon and rectum cancer | 23400 METs | Both | Both | 0.771 (0.702 to 0.849) | | | | | | | | | | | | | | | | | | | | | | | |
| Colon and rectum cancer | 2400 METs | Both | Both | 0.883 (0.806 to 0.958) | | | | | | | | | | | | | | | | | | | | | | | |
| Colon and rectum cancer | 24000 METs | Both | Both | 0.769 (0.698 to 0.849) | | | | | | | | | | | | | | | | | | | | | | | |
| Colon and rectum cancer | 24600 METs | Both | Both | 0.767 (0.694 to 0.85) | | | | | | | | | | | | | | | | | | | | | | | |
| Colon and rectum cancer | 25200 METs | Both | Both | 0.766 (0.689 to 0.85) | | | | | | | | | | | | | | | | | | | | | | | |
| Colon and rectum cancer | 25800 METs | Both | Both | 0.764 (0.685 to 0.849) | | | | | | | | | | | | | | | | | | | | | | | |
| Colon and rectum cancer | 26400 METs | Both | Both | 0.762 (0.679 to 0.851) | | | | | | | | | | | | | | | | | | | | | | | |
| Colon and rectum cancer | 27000 METs | Both | Both | 0.76 (0.675 to 0.853) | | | | | | | | | | | | | | | | | | | | | | | |
| Colon and rectum cancer | 27600 METs | Both | Both | 0.758 (0.671 to 0.853) | | | | | | | | | | | | | | | | | | | | | | | |
| Colon and rectum cancer | 28200 METs | Both | Both | 0.757 (0.668 to 0.856) | | | | | | | | | | | | | | | | | | | | | | | |
| Colon and rectum cancer | 28800 METs | Both | Both | 0.755 (0.664 to 0.856) | | | | | | | | | | | | | | | | | | | | | | | |

**Appendix Table 6a. Relative risks used by age and sex for each outcome for all risk factors except for ambient air pollution alcohol, and smoking.**

| | | | | Ages | | | | | | | | | | | | | | | | | | | | | | | |
|---|---|---|---|---|---|---|---|---|---|---|---|---|---|---|---|---|---|---|---|---|---|---|---|---|---|---|---|
| Risk - Outcome | Category / Units | Morbidity / Mortality | Sex | All-age | 0-6 days | 7-27 days | 28-364 days | 1-4 years | 5-9 years | 10-14 years | 15-19 years | 20-24 years | 25-29 years | 30-34 years | 35-39 years | 40-44 years | 45-49 years | 50-54 years | 55-59 years | 60-64 years | 65-69 years | 70-74 years | 75-79 years | 80-84 years | 85-89 years | 90-94 years | 95+ years |
| Colon and rectum cancer | 29400 METs | Both | Both | 0.753 (0.659 to 0.856) | | | | | | | | | | | | | | | | | | | | | | | |
| Colon and rectum cancer | 3000 METs | Both | Both | 0.833 (0.76 to 0.907) | | | | | | | | | | | | | | | | | | | | | | | |
| Colon and rectum cancer | 30000 METs | Both | Both | 0.751 (0.654 to 0.856) | | | | | | | | | | | | | | | | | | | | | | | |
| Colon and rectum cancer | 30600 METs | Both | Both | 0.749 (0.649 to 0.857) | | | | | | | | | | | | | | | | | | | | | | | |
| Colon and rectum cancer | 31200 METs | Both | Both | 0.748 (0.644 to 0.858) | | | | | | | | | | | | | | | | | | | | | | | |
| Colon and rectum cancer | 31800 METs | Both | Both | 0.746 (0.64 to 0.859) | | | | | | | | | | | | | | | | | | | | | | | |
| Colon and rectum cancer | 32400 METs | Both | Both | 0.744 (0.635 to 0.86) | | | | | | | | | | | | | | | | | | | | | | | |
| Colon and rectum cancer | 33000 METs | Both | Both | 0.743 (0.632 to 0.86) | | | | | | | | | | | | | | | | | | | | | | | |
| Colon and rectum cancer | 3600 METs | Both | Both | 0.831 (0.761 to 0.903) | | | | | | | | | | | | | | | | | | | | | | | |
| Colon and rectum cancer | 4200 METs | Both | Both | 0.829 (0.761 to 0.898) | | | | | | | | | | | | | | | | | | | | | | | |
| Colon and rectum cancer | 4800 METs | Both | Both | 0.827 (0.761 to 0.893) | | | | | | | | | | | | | | | | | | | | | | | |
| Colon and rectum cancer | 5400 METs | Both | Both | 0.825 (0.763 to 0.89) | | | | | | | | | | | | | | | | | | | | | | | |
| Colon and rectum cancer | 600 METs | Both | Both | 0.978 (0.94 to 1.016) | | | | | | | | | | | | | | | | | | | | | | | |
| Colon and rectum cancer | 6000 METs | Both | Both | 0.824 (0.763 to 0.887) | | | | | | | | | | | | | | | | | | | | | | | |
| Colon and rectum cancer | 6600 METs | Both | Both | 0.822 (0.762 to 0.884) | | | | | | | | | | | | | | | | | | | | | | | |
| Colon and rectum cancer | 7200 METs | Both | Both | 0.82 (0.762 to 0.88) | | | | | | | | | | | | | | | | | | | | | | | |
| Colon and rectum cancer | 7800 METs | Both | Both | 0.818 (0.761 to 0.877) | | | | | | | | | | | | | | | | | | | | | | | |
| Colon and rectum cancer | 8400 METs | Both | Both | 0.816 (0.76 to 0.872) | | | | | | | | | | | | | | | | | | | | | | | |
| Colon and rectum cancer | 9000 METs | Both | Both | 0.815 (0.758 to 0.871) | | | | | | | | | | | | | | | | | | | | | | | |
| Colon and rectum cancer | 9600 METs | Both | Both | 0.813 (0.758 to 0.868) | | | | | | | | | | | | | | | | | | | | | | | |
| Breast cancer | 0 METs | Both | Both | 1.0 (1.0 to 1.0) | | | | | | | | | | | | | | | | | | | | | | | |
| Breast cancer | 10200 METs | Both | Both | 0.908 (0.861 to 0.961) | | | | | | | | | | | | | | | | | | | | | | | |
| Breast cancer | 10800 METs | Both | Both | 0.904 (0.859 to 0.952) | | | | | | | | | | | | | | | | | | | | | | | |
| Breast cancer | 11400 METs | Both | Both | 0.9 (0.855 to 0.948) | | | | | | | | | | | | | | | | | | | | | | | |
| Breast cancer | 1200 METs | Both | Both | 0.974 (0.942 to 1.005) | | | | | | | | | | | | | | | | | | | | | | | |
| Breast cancer | 12000 METs | Both | Both | 0.895 (0.85 to 0.946) | | | | | | | | | | | | | | | | | | | | | | | |
| Breast cancer | 12600 METs | Both | Both | 0.891 (0.842 to 0.946) | | | | | | | | | | | | | | | | | | | | | | | |
| Breast cancer | 13200 METs | Both | Both | 0.887 (0.832 to 0.948) | | | | | | | | | | | | | | | | | | | | | | | |
| Breast cancer | 13800 METs | Both | Both | 0.883 (0.819 to 0.949) | | | | | | | | | | | | | | | | | | | | | | | |
| Breast cancer | 14400 METs | Both | Both | 0.879 (0.807 to 0.952) | | | | | | | | | | | | | | | | | | | | | | | |
| Breast cancer | 15000 METs | Both | Both | 0.874 (0.796 to 0.957) | | | | | | | | | | | | | | | | | | | | | | | |
| Breast cancer | 15600 METs | Both | Both | 0.872 (0.797 to 0.951) | | | | | | | | | | | | | | | | | | | | | | | |
| Breast cancer | 16200 METs | Both | Both | 0.87 (0.799 to 0.946) | | | | | | | | | | | | | | | | | | | | | | | |
| Breast cancer | 16800 METs | Both | Both | 0.868 (0.801 to 0.941) | | | | | | | | | | | | | | | | | | | | | | | |
| Breast cancer | 17400 METs | Both | Both | 0.865 (0.804 to 0.933) | | | | | | | | | | | | | | | | | | | | | | | |
| Breast cancer | 1800 METs | Both | Both | 0.96 (0.914 to 1.008) | | | | | | | | | | | | | | | | | | | | | | | |
| Breast cancer | 18000 METs | Both | Both | 0.863 (0.807 to 0.927) | | | | | | | | | | | | | | | | | | | | | | | |
| Breast cancer | 18600 METs | Both | Both | 0.861 (0.807 to 0.919) | | | | | | | | | | | | | | | | | | | | | | | |
| Breast cancer | 19200 METs | Both | Both | 0.859 (0.808 to 0.912) | | | | | | | | | | | | | | | | | | | | | | | |
| Breast cancer | 19800 METs | Both | Both | 0.856 (0.809 to 0.909) | | | | | | | | | | | | | | | | | | | | | | | |
| Breast cancer | 20400 METs | Both | Both | 0.854 (0.809 to 0.906) | | | | | | | | | | | | | | | | | | | | | | | |
| Breast cancer | 21000 METs | Both | Both | 0.852 (0.809 to 0.9) | | | | | | | | | | | | | | | | | | | | | | | |
| Breast cancer | 21600 METs | Both | Both | 0.85 (0.808 to 0.896) | | | | | | | | | | | | | | | | | | | | | | | |
| Breast cancer | 22200 METs | Both | Both | 0.847 (0.805 to 0.893) | | | | | | | | | | | | | | | | | | | | | | | |
| Breast cancer | 22800 METs | Both | Both | 0.845 (0.804 to 0.891) | | | | | | | | | | | | | | | | | | | | | | | |
| Breast cancer | 23400 METs | Both | Both | 0.843 (0.801 to 0.888) | | | | | | | | | | | | | | | | | | | | | | | |
| Breast cancer | 2400 METs | Both | Both | 0.957 (0.914 to 1.001) | | | | | | | | | | | | | | | | | | | | | | | |
| Breast cancer | 24000 METs | Both | Both | 0.841 (0.797 to 0.888) | | | | | | | | | | | | | | | | | | | | | | | |
| Breast cancer | 24600 METs | Both | Both | 0.839 (0.793 to 0.889) | | | | | | | | | | | | | | | | | | | | | | | |
| Breast cancer | 25200 METs | Both | Both | 0.836 (0.789 to 0.891) | | | | | | | | | | | | | | | | | | | | | | | |
| Breast cancer | 25800 METs | Both | Both | 0.834 (0.784 to 0.892) | | | | | | | | | | | | | | | | | | | | | | | |
| Breast cancer | 26400 METs | Both | Both | 0.832 (0.779 to 0.892) | | | | | | | | | | | | | | | | | | | | | | | |
| Breast cancer | 27000 METs | Both | Both | 0.83 (0.773 to 0.892) | | | | | | | | | | | | | | | | | | | | | | | |
| Breast cancer | 27600 METs | Both | Both | 0.827 (0.77 to 0.894) | | | | | | | | | | | | | | | | | | | | | | | |
| Breast cancer | 28200 METs | Both | Both | 0.825 (0.766 to 0.897) | | | | | | | | | | | | | | | | | | | | | | | |
| Breast cancer | 28800 METs | Both | Both | 0.823 (0.759 to 0.9) | | | | | | | | | | | | | | | | | | | | | | | |
| Breast cancer | 29400 METs | Both | Both | 0.821 (0.752 to 0.903) | | | | | | | | | | | | | | | | | | | | | | | |

Appendix Table 6a. Relative risks used by age and sex for each outcome for all risk factors except for ambient air pollution alcohol, and smoking.

| | | | | Ages | | | | | | | | | | | | | | | | | | | | | | | |
|---|---|---|---|---|---|---|---|---|---|---|---|---|---|---|---|---|---|---|---|---|---|---|---|---|---|---|---|
| Risk - Outcome | Category / Units | Morbidity / Mortality | Sex | All-age | 0-6 days | 7-27 days | 28-364 days | 1-4 years | 5-9 years | 10-14 years | 15-19 years | 20-24 years | 25-29 years | 30-34 years | 35-39 years | 40-44 years | 45-49 years | 50-54 years | 55-59 years | 60-64 years | 65-69 years | 70-74 years | 75-79 years | 80-84 years | 85-89 years | 90-94 years | 95+ years |
| Breast cancer | 3000 METs | Both | Both | 0.953 (0.913 to 0.994) | | | | | | | | | | | | | | | | | | | | | | | |
| Breast cancer | 30000 METs | Both | Both | 0.818 (0.745 to 0.904) | | | | | | | | | | | | | | | | | | | | | | | |
| Breast cancer | 30600 METs | Both | Both | 0.816 (0.738 to 0.906) | | | | | | | | | | | | | | | | | | | | | | | |
| Breast cancer | 31200 METs | Both | Both | 0.814 (0.731 to 0.907) | | | | | | | | | | | | | | | | | | | | | | | |
| Breast cancer | 31800 METs | Both | Both | 0.812 (0.724 to 0.909) | | | | | | | | | | | | | | | | | | | | | | | |
| Breast cancer | 32400 METs | Both | Both | 0.809 (0.717 to 0.911) | | | | | | | | | | | | | | | | | | | | | | | |
| Breast cancer | 33000 METs | Both | Both | 0.808 (0.713 to 0.912) | | | | | | | | | | | | | | | | | | | | | | | |
| Breast cancer | 3600 METs | Both | Both | 0.949 (0.912 to 0.99) | | | | | | | | | | | | | | | | | | | | | | | |
| Breast cancer | 4200 METs | Both | Both | 0.946 (0.909 to 0.985) | | | | | | | | | | | | | | | | | | | | | | | |
| Breast cancer | 4800 METs | Both | Both | 0.942 (0.907 to 0.982) | | | | | | | | | | | | | | | | | | | | | | | |
| Breast cancer | 5400 METs | Both | Both | 0.938 (0.903 to 0.979) | | | | | | | | | | | | | | | | | | | | | | | |
| Breast cancer | 600 METs | Both | Both | 0.987 (0.971 to 1.003) | | | | | | | | | | | | | | | | | | | | | | | |
| Breast cancer | 6000 METs | Both | Both | 0.935 (0.897 to 0.979) | | | | | | | | | | | | | | | | | | | | | | | |
| Breast cancer | 6600 METs | Both | Both | 0.931 (0.889 to 0.978) | | | | | | | | | | | | | | | | | | | | | | | |
| Breast cancer | 7200 METs | Both | Both | 0.928 (0.883 to 0.978) | | | | | | | | | | | | | | | | | | | | | | | |
| Breast cancer | 7800 METs | Both | Both | 0.924 (0.875 to 0.979) | | | | | | | | | | | | | | | | | | | | | | | |
| Breast cancer | 8400 METs | Both | Both | 0.92 (0.867 to 0.979) | | | | | | | | | | | | | | | | | | | | | | | |
| Breast cancer | 9000 METs | Both | Both | 0.917 (0.858 to 0.979) | | | | | | | | | | | | | | | | | | | | | | | |
| Breast cancer | 9600 METs | Both | Both | 0.912 (0.862 to 0.97) | | | | | | | | | | | | | | | | | | | | | | | |
| Ischaemic heart disease | 0 METs | Both | Both | 1.0 (1.0 to 1.0) | | | | | | | | | | | | | | | | | | | | | | | |
| Ischaemic heart disease | 10200 METs | Both | Both | 0.73 (0.668 to 0.793) | | | | | | | | | | | | | | | | | | | | | | | |
| Ischaemic heart disease | 10800 METs | Both | Both | 0.73 (0.669 to 0.791) | | | | | | | | | | | | | | | | | | | | | | | |
| Ischaemic heart disease | 11400 METs | Both | Both | 0.729 (0.671 to 0.789) | | | | | | | | | | | | | | | | | | | | | | | |
| Ischaemic heart disease | 1200 METs | Both | Both | 0.819 (0.714 to 0.928) | | | | | | | | | | | | | | | | | | | | | | | |
| Ischaemic heart disease | 12000 METs | Both | Both | 0.728 (0.672 to 0.787) | | | | | | | | | | | | | | | | | | | | | | | |
| Ischaemic heart disease | 12600 METs | Both | Both | 0.728 (0.672 to 0.786) | | | | | | | | | | | | | | | | | | | | | | | |
| Ischaemic heart disease | 13200 METs | Both | Both | 0.727 (0.671 to 0.785) | | | | | | | | | | | | | | | | | | | | | | | |
| Ischaemic heart disease | 13800 METs | Both | Both | 0.727 (0.67 to 0.784) | | | | | | | | | | | | | | | | | | | | | | | |
| Ischaemic heart disease | 14400 METs | Both | Both | 0.726 (0.671 to 0.782) | | | | | | | | | | | | | | | | | | | | | | | |
| Ischaemic heart disease | 15000 METs | Both | Both | 0.725 (0.67 to 0.781) | | | | | | | | | | | | | | | | | | | | | | | |
| Ischaemic heart disease | 15600 METs | Both | Both | 0.725 (0.671 to 0.781) | | | | | | | | | | | | | | | | | | | | | | | |
| Ischaemic heart disease | 16200 METs | Both | Both | 0.724 (0.671 to 0.779) | | | | | | | | | | | | | | | | | | | | | | | |
| Ischaemic heart disease | 16800 METs | Both | Both | 0.724 (0.669 to 0.779) | | | | | | | | | | | | | | | | | | | | | | | |
| Ischaemic heart disease | 17400 METs | Both | Both | 0.723 (0.669 to 0.78) | | | | | | | | | | | | | | | | | | | | | | | |
| Ischaemic heart disease | 1800 METs | Both | Both | 0.808 (0.739 to 0.886) | | | | | | | | | | | | | | | | | | | | | | | |
| Ischaemic heart disease | 18000 METs | Both | Both | 0.722 (0.668 to 0.78) | | | | | | | | | | | | | | | | | | | | | | | |
| Ischaemic heart disease | 18600 METs | Both | Both | 0.722 (0.667 to 0.781) | | | | | | | | | | | | | | | | | | | | | | | |
| Ischaemic heart disease | 19200 METs | Both | Both | 0.721 (0.666 to 0.781) | | | | | | | | | | | | | | | | | | | | | | | |
| Ischaemic heart disease | 19800 METs | Both | Both | 0.721 (0.665 to 0.781) | | | | | | | | | | | | | | | | | | | | | | | |
| Ischaemic heart disease | 20400 METs | Both | Both | 0.72 (0.664 to 0.781) | | | | | | | | | | | | | | | | | | | | | | | |
| Ischaemic heart disease | 21000 METs | Both | Both | 0.72 (0.663 to 0.782) | | | | | | | | | | | | | | | | | | | | | | | |
| Ischaemic heart disease | 21600 METs | Both | Both | 0.719 (0.662 to 0.782) | | | | | | | | | | | | | | | | | | | | | | | |
| Ischaemic heart disease | 22200 METs | Both | Both | 0.718 (0.66 to 0.783) | | | | | | | | | | | | | | | | | | | | | | | |
| Ischaemic heart disease | 22800 METs | Both | Both | 0.718 (0.659 to 0.784) | | | | | | | | | | | | | | | | | | | | | | | |
| Ischaemic heart disease | 23400 METs | Both | Both | 0.717 (0.657 to 0.786) | | | | | | | | | | | | | | | | | | | | | | | |
| Ischaemic heart disease | 2400 METs | Both | Both | 0.796 (0.711 to 0.895) | | | | | | | | | | | | | | | | | | | | | | | |
| Ischaemic heart disease | 24000 METs | Both | Both | 0.717 (0.655 to 0.787) | | | | | | | | | | | | | | | | | | | | | | | |
| Ischaemic heart disease | 24600 METs | Both | Both | 0.716 (0.653 to 0.788) | | | | | | | | | | | | | | | | | | | | | | | |
| Ischaemic heart disease | 25200 METs | Both | Both | 0.715 (0.65 to 0.789) | | | | | | | | | | | | | | | | | | | | | | | |
| Ischaemic heart disease | 25800 METs | Both | Both | 0.715 (0.648 to 0.79) | | | | | | | | | | | | | | | | | | | | | | | |
| Ischaemic heart disease | 26400 METs | Both | Both | 0.714 (0.646 to 0.792) | | | | | | | | | | | | | | | | | | | | | | | |
| Ischaemic heart disease | 27000 METs | Both | Both | 0.714 (0.643 to 0.794) | | | | | | | | | | | | | | | | | | | | | | | |
| Ischaemic heart disease | 27600 METs | Both | Both | 0.713 (0.64 to 0.796) | | | | | | | | | | | | | | | | | | | | | | | |
| Ischaemic heart disease | 28200 METs | Both | Both | 0.712 (0.636 to 0.798) | | | | | | | | | | | | | | | | | | | | | | | |
| Ischaemic heart disease | 28800 METs | Both | Both | 0.712 (0.633 to 0.8) | | | | | | | | | | | | | | | | | | | | | | | |
| Ischaemic heart disease | 29400 METs | Both | Both | 0.711 (0.63 to 0.801) | | | | | | | | | | | | | | | | | | | | | | | |
| Ischaemic heart disease | 3000 METs | Both | Both | 0.776 (0.713 to 0.844) | | | | | | | | | | | | | | | | | | | | | | | |

**Appendix Table 6a. Relative risks used by age and sex for each outcome for all risk factors except for ambient air pollution alcohol, and smoking.**

| | | | | Ages | | | | | | | | | | | | | | | | | | | | |
|---|---|---|---|---|---|---|---|---|---|---|---|---|---|---|---|---|---|---|---|---|---|---|---|---|
| Risk - Outcome | Category / Units | Morbidity / Mortality | Sex | All-age | 0-6 days | 7-27 days | 28-364 days | 1-4 years | 5-9 years | 10-14 years | 15-19 years | 20-24 years | 25-29 years | 30-34 years | 35-39 years | 40-44 years | 45-49 years | 50-54 years | 55-59 years | 60-64 years | 65-69 years | 70-74 years | 75-79 years | 80-84 years | 85-89 years | 90-94 years | 95+ years |
| Ischaemic heart disease | 30000 METs | Both | Both | 0.711 (0.626 to 0.801) | | | | | | | | | | | | | | | | | | | | | | | |
| Ischaemic heart disease | 30600 METs | Both | Both | 0.71 (0.623 to 0.802) | | | | | | | | | | | | | | | | | | | | | | | |
| Ischaemic heart disease | 31200 METs | Both | Both | 0.709 (0.62 to 0.804) | | | | | | | | | | | | | | | | | | | | | | | |
| Ischaemic heart disease | 31800 METs | Both | Both | 0.709 (0.616 to 0.806) | | | | | | | | | | | | | | | | | | | | | | | |
| Ischaemic heart disease | 32400 METs | Both | Both | 0.708 (0.615 to 0.808) | | | | | | | | | | | | | | | | | | | | | | | |
| Ischaemic heart disease | 33000 METs | Both | Both | 0.708 (0.613 to 0.81) | | | | | | | | | | | | | | | | | | | | | | | |
| Ischaemic heart disease | 3600 METs | Both | Both | 0.756 (0.692 to 0.816) | | | | | | | | | | | | | | | | | | | | | | | |
| Ischaemic heart disease | 4200 METs | Both | Both | 0.736 (0.652 to 0.821) | | | | | | | | | | | | | | | | | | | | | | | |
| Ischaemic heart disease | 4800 METs | Both | Both | 0.736 (0.653 to 0.818) | | | | | | | | | | | | | | | | | | | | | | | |
| Ischaemic heart disease | 5400 METs | Both | Both | 0.735 (0.654 to 0.815) | | | | | | | | | | | | | | | | | | | | | | | |
| Ischaemic heart disease | 600 METs | Both | Both | 0.909 (0.857 to 0.964) | | | | | | | | | | | | | | | | | | | | | | | |
| Ischaemic heart disease | 6000 METs | Both | Both | 0.734 (0.656 to 0.811) | | | | | | | | | | | | | | | | | | | | | | | |
| Ischaemic heart disease | 6600 METs | Both | Both | 0.734 (0.657 to 0.808) | | | | | | | | | | | | | | | | | | | | | | | |
| Ischaemic heart disease | 7200 METs | Both | Both | 0.733 (0.659 to 0.806) | | | | | | | | | | | | | | | | | | | | | | | |
| Ischaemic heart disease | 7800 METs | Both | Both | 0.733 (0.66 to 0.803) | | | | | | | | | | | | | | | | | | | | | | | |
| Ischaemic heart disease | 8400 METs | Both | Both | 0.732 (0.662 to 0.801) | | | | | | | | | | | | | | | | | | | | | | | |
| Ischaemic heart disease | 9000 METs | Both | Both | 0.731 (0.664 to 0.799) | | | | | | | | | | | | | | | | | | | | | | | |
| Ischaemic heart disease | 9600 METs | Both | Both | 0.731 (0.666 to 0.796) | | | | | | | | | | | | | | | | | | | | | | | |
| Ischaemic stroke | 0 METs | Both | Both | 1.0 (1.0 to 1.0) | | | | | | | | | | | | | | | | | | | | | | | |
| Ischaemic stroke | 10200 METs | Both | Both | 0.753 (0.653 to 0.864) | | | | | | | | | | | | | | | | | | | | | | | |
| Ischaemic stroke | 10800 METs | Both | Both | 0.75 (0.653 to 0.858) | | | | | | | | | | | | | | | | | | | | | | | |
| Ischaemic stroke | 11400 METs | Both | Both | 0.747 (0.653 to 0.851) | | | | | | | | | | | | | | | | | | | | | | | |
| Ischaemic stroke | 1200 METs | Both | Both | 0.819 (0.662 to 0.999) | | | | | | | | | | | | | | | | | | | | | | | |
| Ischaemic stroke | 12000 METs | Both | Both | 0.744 (0.651 to 0.845) | | | | | | | | | | | | | | | | | | | | | | | |
| Ischaemic stroke | 12600 METs | Both | Both | 0.741 (0.65 to 0.84) | | | | | | | | | | | | | | | | | | | | | | | |
| Ischaemic stroke | 13200 METs | Both | Both | 0.738 (0.65 to 0.833) | | | | | | | | | | | | | | | | | | | | | | | |
| Ischaemic stroke | 13800 METs | Both | Both | 0.735 (0.65 to 0.828) | | | | | | | | | | | | | | | | | | | | | | | |
| Ischaemic stroke | 14400 METs | Both | Both | 0.732 (0.647 to 0.825) | | | | | | | | | | | | | | | | | | | | | | | |
| Ischaemic stroke | 15000 METs | Both | Both | 0.729 (0.644 to 0.82) | | | | | | | | | | | | | | | | | | | | | | | |
| Ischaemic stroke | 15600 METs | Both | Both | 0.727 (0.642 to 0.813) | | | | | | | | | | | | | | | | | | | | | | | |
| Ischaemic stroke | 16200 METs | Both | Both | 0.724 (0.639 to 0.809) | | | | | | | | | | | | | | | | | | | | | | | |
| Ischaemic stroke | 16800 METs | Both | Both | 0.721 (0.639 to 0.806) | | | | | | | | | | | | | | | | | | | | | | | |
| Ischaemic stroke | 17400 METs | Both | Both | 0.718 (0.636 to 0.802) | | | | | | | | | | | | | | | | | | | | | | | |
| Ischaemic stroke | 1800 METs | Both | Both | 0.802 (0.702 to 0.915) | | | | | | | | | | | | | | | | | | | | | | | |
| Ischaemic stroke | 18000 METs | Both | Both | 0.715 (0.633 to 0.797) | | | | | | | | | | | | | | | | | | | | | | | |
| Ischaemic stroke | 18600 METs | Both | Both | 0.712 (0.63 to 0.793) | | | | | | | | | | | | | | | | | | | | | | | |
| Ischaemic stroke | 19200 METs | Both | Both | 0.709 (0.626 to 0.791) | | | | | | | | | | | | | | | | | | | | | | | |
| Ischaemic stroke | 19800 METs | Both | Both | 0.706 (0.623 to 0.789) | | | | | | | | | | | | | | | | | | | | | | | |
| Ischaemic stroke | 20400 METs | Both | Both | 0.703 (0.62 to 0.79) | | | | | | | | | | | | | | | | | | | | | | | |
| Ischaemic stroke | 21000 METs | Both | Both | 0.7 (0.615 to 0.787) | | | | | | | | | | | | | | | | | | | | | | | |
| Ischaemic stroke | 21600 METs | Both | Both | 0.697 (0.611 to 0.787) | | | | | | | | | | | | | | | | | | | | | | | |
| Ischaemic stroke | 22200 METs | Both | Both | 0.694 (0.606 to 0.787) | | | | | | | | | | | | | | | | | | | | | | | |
| Ischaemic stroke | 22800 METs | Both | Both | 0.691 (0.602 to 0.785) | | | | | | | | | | | | | | | | | | | | | | | |
| Ischaemic stroke | 23400 METs | Both | Both | 0.688 (0.595 to 0.783) | | | | | | | | | | | | | | | | | | | | | | | |
| Ischaemic stroke | 2400 METs | Both | Both | 0.785 (0.643 to 0.943) | | | | | | | | | | | | | | | | | | | | | | | |
| Ischaemic stroke | 24000 METs | Both | Both | 0.685 (0.593 to 0.78) | | | | | | | | | | | | | | | | | | | | | | | |
| Ischaemic stroke | 24600 METs | Both | Both | 0.682 (0.586 to 0.779) | | | | | | | | | | | | | | | | | | | | | | | |
| Ischaemic stroke | 25200 METs | Both | Both | 0.679 (0.579 to 0.778) | | | | | | | | | | | | | | | | | | | | | | | |
| Ischaemic stroke | 25800 METs | Both | Both | 0.677 (0.572 to 0.776) | | | | | | | | | | | | | | | | | | | | | | | |
| Ischaemic stroke | 26400 METs | Both | Both | 0.674 (0.566 to 0.777) | | | | | | | | | | | | | | | | | | | | | | | |
| Ischaemic stroke | 27000 METs | Both | Both | 0.671 (0.56 to 0.779) | | | | | | | | | | | | | | | | | | | | | | | |
| Ischaemic stroke | 27600 METs | Both | Both | 0.668 (0.554 to 0.781) | | | | | | | | | | | | | | | | | | | | | | | |
| Ischaemic stroke | 28200 METs | Both | Both | 0.665 (0.547 to 0.781) | | | | | | | | | | | | | | | | | | | | | | | |
| Ischaemic stroke | 28800 METs | Both | Both | 0.662 (0.541 to 0.783) | | | | | | | | | | | | | | | | | | | | | | | |
| Ischaemic stroke | 29400 METs | Both | Both | 0.659 (0.534 to 0.785) | | | | | | | | | | | | | | | | | | | | | | | |
| Ischaemic stroke | 3000 METs | Both | Both | 0.784 (0.701 to 0.888) | | | | | | | | | | | | | | | | | | | | | | | |
| Ischaemic stroke | 30000 METs | Both | Both | 0.656 (0.527 to 0.787) | | | | | | | | | | | | | | | | | | | | | | | |

**Appendix Table 6a. Relative risks used by age and sex for each outcome for all risk factors except for ambient air pollution alcohol, and smoking.**

| | | | | Ages | | | | | | | | | | | | | | | | | | | | | | | |
|---|---|---|---|---|---|---|---|---|---|---|---|---|---|---|---|---|---|---|---|---|---|---|---|---|---|---|---|
| **Risk - Outcome** | **Category / Units** | **Morbidity / Mortality** | **Sex** | **All-age** | **0-6 days** | **7-27 days** | **28-364 days** | **1-4 years** | **5-9 years** | **10-14 years** | **15-19 years** | **20-24 years** | **25-29 years** | **30-34 years** | **35-39 years** | **40-44 years** | **45-49 years** | **50-54 years** | **55-59 years** | **60-64 years** | **65-69 years** | **70-74 years** | **75-79 years** | **80-84 years** | **85-89 years** | **90-94 years** | **95+ years** |
| Ischaemic stroke | 30600 METs | Both | Both | 0.653<br>(0.521 to 0.789) | | | | | | | | | | | | | | | | | | | | | | | |
| Ischaemic stroke | 31200 METs | Both | Both | 0.65<br>(0.514 to 0.791) | | | | | | | | | | | | | | | | | | | | | | | |
| Ischaemic stroke | 31800 METs | Both | Both | 0.647<br>(0.506 to 0.792) | | | | | | | | | | | | | | | | | | | | | | | |
| Ischaemic stroke | 32400 METs | Both | Both | 0.644<br>(0.498 to 0.792) | | | | | | | | | | | | | | | | | | | | | | | |
| Ischaemic stroke | 33000 METs | Both | Both | 0.643<br>(0.493 to 0.792) | | | | | | | | | | | | | | | | | | | | | | | |
| Ischaemic stroke | 3600 METs | Both | Both | 0.783<br>(0.695 to 0.883) | | | | | | | | | | | | | | | | | | | | | | | |
| Ischaemic stroke | 4200 METs | Both | Both | 0.782<br>(0.644 to 0.934) | | | | | | | | | | | | | | | | | | | | | | | |
| Ischaemic stroke | 4800 METs | Both | Both | 0.779<br>(0.647 to 0.926) | | | | | | | | | | | | | | | | | | | | | | | |
| Ischaemic stroke | 5400 METs | Both | Both | 0.776<br>(0.648 to 0.918) | | | | | | | | | | | | | | | | | | | | | | | |
| Ischaemic stroke | 600 METs | Both | Both | 0.91<br>(0.831 to 0.999) | | | | | | | | | | | | | | | | | | | | | | | |
| Ischaemic stroke | 6000 METs | Both | Both | 0.774<br>(0.649 to 0.913) | | | | | | | | | | | | | | | | | | | | | | | |
| Ischaemic stroke | 6600 METs | Both | Both | 0.771<br>(0.65 to 0.905) | | | | | | | | | | | | | | | | | | | | | | | |
| Ischaemic stroke | 7200 METs | Both | Both | 0.768<br>(0.652 to 0.898) | | | | | | | | | | | | | | | | | | | | | | | |
| Ischaemic stroke | 7800 METs | Both | Both | 0.765<br>(0.65 to 0.891) | | | | | | | | | | | | | | | | | | | | | | | |
| Ischaemic stroke | 8400 METs | Both | Both | 0.762<br>(0.651 to 0.884) | | | | | | | | | | | | | | | | | | | | | | | |
| Ischaemic stroke | 9000 METs | Both | Both | 0.759<br>(0.652 to 0.878) | | | | | | | | | | | | | | | | | | | | | | | |
| Ischaemic stroke | 9600 METs | Both | Both | 0.756<br>(0.652 to 0.87) | | | | | | | | | | | | | | | | | | | | | | | |
| Diabetes mellitus type 2 | 0 METs | Both | Both | 1.0<br>(1.0 to 1.0) | | | | | | | | | | | | | | | | | | | | | | | |
| Diabetes mellitus type 2 | 10200 METs | Both | Both | 0.727<br>(0.684 to 0.774) | | | | | | | | | | | | | | | | | | | | | | | |
| Diabetes mellitus type 2 | 10800 METs | Both | Both | 0.726<br>(0.683 to 0.772) | | | | | | | | | | | | | | | | | | | | | | | |
| Diabetes mellitus type 2 | 11400 METs | Both | Both | 0.725<br>(0.682 to 0.77) | | | | | | | | | | | | | | | | | | | | | | | |
| Diabetes mellitus type 2 | 1200 METs | Both | Both | 0.961<br>(0.933 to 0.992) | | | | | | | | | | | | | | | | | | | | | | | |
| Diabetes mellitus type 2 | 12000 METs | Both | Both | 0.723<br>(0.682 to 0.771) | | | | | | | | | | | | | | | | | | | | | | | |
| Diabetes mellitus type 2 | 12600 METs | Both | Both | 0.722<br>(0.682 to 0.768) | | | | | | | | | | | | | | | | | | | | | | | |
| Diabetes mellitus type 2 | 13200 METs | Both | Both | 0.721<br>(0.681 to 0.766) | | | | | | | | | | | | | | | | | | | | | | | |
| Diabetes mellitus type 2 | 13800 METs | Both | Both | 0.72<br>(0.68 to 0.765) | | | | | | | | | | | | | | | | | | | | | | | |
| Diabetes mellitus type 2 | 14400 METs | Both | Both | 0.719<br>(0.678 to 0.764) | | | | | | | | | | | | | | | | | | | | | | | |
| Diabetes mellitus type 2 | 15000 METs | Both | Both | 0.718<br>(0.678 to 0.762) | | | | | | | | | | | | | | | | | | | | | | | |
| Diabetes mellitus type 2 | 15600 METs | Both | Both | 0.717<br>(0.677 to 0.761) | | | | | | | | | | | | | | | | | | | | | | | |
| Diabetes mellitus type 2 | 16200 METs | Both | Both | 0.716<br>(0.676 to 0.76) | | | | | | | | | | | | | | | | | | | | | | | |
| Diabetes mellitus type 2 | 16800 METs | Both | Both | 0.715<br>(0.674 to 0.759) | | | | | | | | | | | | | | | | | | | | | | | |
| Diabetes mellitus type 2 | 17400 METs | Both | Both | 0.714<br>(0.673 to 0.759) | | | | | | | | | | | | | | | | | | | | | | | |
| Diabetes mellitus type 2 | 1800 METs | Both | Both | 0.921<br>(0.867 to 0.985) | | | | | | | | | | | | | | | | | | | | | | | |
| Diabetes mellitus type 2 | 18000 METs | Both | Both | 0.713<br>(0.67 to 0.759) | | | | | | | | | | | | | | | | | | | | | | | |
| Diabetes mellitus type 2 | 18600 METs | Both | Both | 0.712<br>(0.668 to 0.759) | | | | | | | | | | | | | | | | | | | | | | | |
| Diabetes mellitus type 2 | 19200 METs | Both | Both | 0.711<br>(0.665 to 0.758) | | | | | | | | | | | | | | | | | | | | | | | |
| Diabetes mellitus type 2 | 19800 METs | Both | Both | 0.709<br>(0.663 to 0.758) | | | | | | | | | | | | | | | | | | | | | | | |
| Diabetes mellitus type 2 | 20400 METs | Both | Both | 0.708<br>(0.66 to 0.759) | | | | | | | | | | | | | | | | | | | | | | | |
| Diabetes mellitus type 2 | 21000 METs | Both | Both | 0.707<br>(0.658 to 0.759) | | | | | | | | | | | | | | | | | | | | | | | |
| Diabetes mellitus type 2 | 21600 METs | Both | Both | 0.706<br>(0.656 to 0.759) | | | | | | | | | | | | | | | | | | | | | | | |
| Diabetes mellitus type 2 | 22200 METs | Both | Both | 0.705<br>(0.654 to 0.759) | | | | | | | | | | | | | | | | | | | | | | | |
| Diabetes mellitus type 2 | 22800 METs | Both | Both | 0.704<br>(0.651 to 0.759) | | | | | | | | | | | | | | | | | | | | | | | |
| Diabetes mellitus type 2 | 23400 METs | Both | Both | 0.703<br>(0.65 to 0.759) | | | | | | | | | | | | | | | | | | | | | | | |
| Diabetes mellitus type 2 | 2400 METs | Both | Both | 0.882<br>(0.8 to 0.977) | | | | | | | | | | | | | | | | | | | | | | | |
| Diabetes mellitus type 2 | 24000 METs | Both | Both | 0.702<br>(0.648 to 0.76) | | | | | | | | | | | | | | | | | | | | | | | |
| Diabetes mellitus type 2 | 24600 METs | Both | Both | 0.701<br>(0.646 to 0.76) | | | | | | | | | | | | | | | | | | | | | | | |
| Diabetes mellitus type 2 | 25200 METs | Both | Both | 0.7<br>(0.643 to 0.76) | | | | | | | | | | | | | | | | | | | | | | | |
| Diabetes mellitus type 2 | 25800 METs | Both | Both | 0.699<br>(0.64 to 0.76) | | | | | | | | | | | | | | | | | | | | | | | |
| Diabetes mellitus type 2 | 26400 METs | Both | Both | 0.698<br>(0.638 to 0.76) | | | | | | | | | | | | | | | | | | | | | | | |
| Diabetes mellitus type 2 | 27000 METs | Both | Both | 0.697<br>(0.635 to 0.76) | | | | | | | | | | | | | | | | | | | | | | | |
| Diabetes mellitus type 2 | 27600 METs | Both | Both | 0.696<br>(0.631 to 0.761) | | | | | | | | | | | | | | | | | | | | | | | |
| Diabetes mellitus type 2 | 28200 METs | Both | Both | 0.694<br>(0.628 to 0.762) | | | | | | | | | | | | | | | | | | | | | | | |
| Diabetes mellitus type 2 | 28800 METs | Both | Both | 0.693<br>(0.624 to 0.762) | | | | | | | | | | | | | | | | | | | | | | | |
| Diabetes mellitus type 2 | 29400 METs | Both | Both | 0.692<br>(0.621 to 0.763) | | | | | | | | | | | | | | | | | | | | | | | |
| Diabetes mellitus type 2 | 3000 METs | Both | Both | 0.834<br>(0.773 to 0.904) | | | | | | | | | | | | | | | | | | | | | | | |
| Diabetes mellitus type 2 | 30000 METs | Both | Both | 0.691<br>(0.618 to 0.764) | | | | | | | | | | | | | | | | | | | | | | | |
| Diabetes mellitus type 2 | 30600 METs | Both | Both | 0.69<br>(0.615 to 0.765) | | | | | | | | | | | | | | | | | | | | | | | |

**Appendix Table 6a. Relative risks used by age and sex for each outcome for all risk factors except for ambient air pollution alcohol, and smoking.**

| | | | | Ages | | | | | | | | | | | | | | | | | | | | | | | |
|---|---|---|---|---|---|---|---|---|---|---|---|---|---|---|---|---|---|---|---|---|---|---|---|---|---|---|---|
| **Risk - Outcome** | **Category / Units** | **Morbidity / Mortality** | **Sex** | **All-age** | **0-6 days** | **7-27 days** | **28-364 days** | **1-4 years** | **5-9 years** | **10-14 years** | **15-19 years** | **20-24 years** | **25-29 years** | **30-34 years** | **35-39 years** | **40-44 years** | **45-49 years** | **50-54 years** | **55-59 years** | **60-64 years** | **65-69 years** | **70-74 years** | **75-79 years** | **80-84 years** | **85-89 years** | **90-94 years** | **95+ years** |
| Diabetes mellitus type 2 | 31200 METs | Both | Both | 0.689 (0.612 to 0.766) | | | | | | | | | | | | | | | | | | | | | | | |
| Diabetes mellitus type 2 | 31800 METs | Both | Both | 0.688 (0.609 to 0.768) | | | | | | | | | | | | | | | | | | | | | | | |
| Diabetes mellitus type 2 | 32400 METs | Both | Both | 0.687 (0.607 to 0.768) | | | | | | | | | | | | | | | | | | | | | | | |
| Diabetes mellitus type 2 | 33000 METs | Both | Both | 0.686 (0.605 to 0.769) | | | | | | | | | | | | | | | | | | | | | | | |
| Diabetes mellitus type 2 | 3600 METs | Both | Both | 0.786 (0.735 to 0.841) | | | | | | | | | | | | | | | | | | | | | | | |
| Diabetes mellitus type 2 | 4200 METs | Both | Both | 0.737 (0.684 to 0.794) | | | | | | | | | | | | | | | | | | | | | | | |
| Diabetes mellitus type 2 | 4800 METs | Both | Both | 0.736 (0.685 to 0.791) | | | | | | | | | | | | | | | | | | | | | | | |
| Diabetes mellitus type 2 | 5400 METs | Both | Both | 0.735 (0.684 to 0.789) | | | | | | | | | | | | | | | | | | | | | | | |
| Diabetes mellitus type 2 | 600 METs | Both | Both | 0.98 (0.967 to 0.996) | | | | | | | | | | | | | | | | | | | | | | | |
| Diabetes mellitus type 2 | 6000 METs | Both | Both | 0.734 (0.684 to 0.786) | | | | | | | | | | | | | | | | | | | | | | | |
| Diabetes mellitus type 2 | 6600 METs | Both | Both | 0.733 (0.685 to 0.784) | | | | | | | | | | | | | | | | | | | | | | | |
| Diabetes mellitus type 2 | 7200 METs | Both | Both | 0.732 (0.685 to 0.782) | | | | | | | | | | | | | | | | | | | | | | | |
| Diabetes mellitus type 2 | 7800 METs | Both | Both | 0.731 (0.684 to 0.78) | | | | | | | | | | | | | | | | | | | | | | | |
| Diabetes mellitus type 2 | 8400 METs | Both | Both | 0.73 (0.684 to 0.779) | | | | | | | | | | | | | | | | | | | | | | | |
| Diabetes mellitus type 2 | 9000 METs | Both | Both | 0.729 (0.684 to 0.777) | | | | | | | | | | | | | | | | | | | | | | | |
| Diabetes mellitus type 2 | 9600 METs | Both | Both | 0.728 (0.684 to 0.776) | | | | | | | | | | | | | | | | | | | | | | | |
| **High fasting plasma glucose (continuous)** | | | | | | | | | | | | | | | | | | | | | | | | | | | |
| Ischaemic heart disease | mmol/L | Both | Both | | | | | | | | | | 1.471 (1.147 to 2.099) | 1.373 (1.153 to 1.741) | 1.274 (1.13 to 1.451) | 1.22 (1.085 to 1.365) | 1.211 (1.111 to 1.327) | 1.201 (1.121 to 1.297) | 1.192 (1.123 to 1.269) | 1.182 (1.111 to 1.267) | 1.173 (1.085 to 1.271) | 1.168 (1.08 to 1.273) | 1.168 (1.097 to 1.264) | 1.169 (1.071 to 1.316) | 1.169 (1.071 to 1.316) | 1.169 (1.071 to 1.316) | 1.169 (1.071 to 1.316) |
| Ischaemic stroke | mmol/L | Both | Both | | | | | | | | | | 1.526 (1.11 to 2.227) | 1.4 (1.101 to 1.85) | 1.275 (1.081 to 1.561) | 1.21 (1.044 to 1.441) | 1.204 (1.075 to 1.383) | 1.199 (1.095 to 1.334) | 1.194 (1.108 to 1.302) | 1.188 (1.101 to 1.295) | 1.183 (1.069 to 1.308) | 1.174 (1.056 to 1.309) | 1.162 (1.075 to 1.295) | 1.133 (1.055 to 1.332) | 1.133 (1.055 to 1.332) | 1.133 (1.055 to 1.332) | 1.133 (1.055 to 1.332) |
| Intracerebral hemorrhage | mmol/L | Both | Both | | | | | | | | | | 1.506 (1.112 to 2.221) | 1.382 (1.111 to 1.843) | 1.258 (1.085 to 1.487) | 1.196 (1.053 to 1.371) | 1.193 (1.082 to 1.333) | 1.191 (1.106 to 1.298) | 1.189 (1.115 to 1.262) | 1.187 (1.115 to 1.252) | 1.184 (1.097 to 1.264) | 1.175 (1.087 to 1.263) | 1.158 (1.09 to 1.233) | 1.116 (1.056 to 1.25) | 1.116 (1.056 to 1.25) | 1.116 (1.056 to 1.25) | 1.116 (1.056 to 1.25) |
| Subarachnoid hemorrhage | mmol/L | Both | Both | | | | | | | | | | 1.506 (1.112 to 2.221) | 1.382 (1.111 to 1.843) | 1.258 (1.085 to 1.487) | 1.196 (1.053 to 1.371) | 1.193 (1.082 to 1.333) | 1.191 (1.106 to 1.298) | 1.189 (1.115 to 1.262) | 1.187 (1.115 to 1.252) | 1.184 (1.097 to 1.264) | 1.175 (1.087 to 1.263) | 1.158 (1.09 to 1.233) | 1.116 (1.056 to 1.25) | 1.116 (1.056 to 1.25) | 1.116 (1.056 to 1.25) | 1.116 (1.056 to 1.25) |
| Chronic kidney disease due to hypertension | mmol/L | Both | Both | | | | | | | | | | 1.388 (1.272 to 1.512) | 1.388 (1.272 to 1.512) | 1.388 (1.272 to 1.512) | 1.388 (1.272 to 1.512) | 1.388 (1.272 to 1.512) | 1.388 (1.272 to 1.512) | 1.388 (1.272 to 1.512) | 1.388 (1.272 to 1.512) | 1.388 (1.272 to 1.512) | 1.388 (1.272 to 1.512) | 1.388 (1.272 to 1.512) | 1.388 (1.272 to 1.512) | 1.388 (1.272 to 1.512) | 1.388 (1.272 to 1.512) | 1.388 (1.272 to 1.512) |
| Chronic kidney disease due to glomerulonephritis | mmol/L | Both | Both | | | | | | | | | | 1.388 (1.272 to 1.512) | 1.388 (1.272 to 1.512) | 1.388 (1.272 to 1.512) | 1.388 (1.272 to 1.512) | 1.388 (1.272 to 1.512) | 1.388 (1.272 to 1.512) | 1.388 (1.272 to 1.512) | 1.388 (1.272 to 1.512) | 1.388 (1.272 to 1.512) | 1.388 (1.272 to 1.512) | 1.388 (1.272 to 1.512) | 1.388 (1.272 to 1.512) | 1.388 (1.272 to 1.512) | 1.388 (1.272 to 1.512) | 1.388 (1.272 to 1.512) |
| Chronic kidney disease due to other and unspecified causes | mmol/L | Both | Both | | | | | | | | | | 1.388 (1.272 to 1.512) | 1.388 (1.272 to 1.512) | 1.388 (1.272 to 1.512) | 1.388 (1.272 to 1.512) | 1.388 (1.272 to 1.512) | 1.388 (1.272 to 1.512) | 1.388 (1.272 to 1.512) | 1.388 (1.272 to 1.512) | 1.388 (1.272 to 1.512) | 1.388 (1.272 to 1.512) | 1.388 (1.272 to 1.512) | 1.388 (1.272 to 1.512) | 1.388 (1.272 to 1.512) | 1.388 (1.272 to 1.512) | 1.388 (1.272 to 1.512) |
| **High fasting plasma glucose (categorical)** | | | | | | | | | | | | | | | | | | | | | | | | | | | |
| Latent tuberculosis infection | Diabetic | Both | Both | | | | | | | | | | 2.73 (1.973 to 3.602) | 2.801 (2.056 to 3.658) | 2.871 (2.047 to 3.698) | 2.798 (1.97 to 3.63) | 2.581 (1.91 to 3.268) | 2.364 (1.817 to 2.943) | 2.147 (1.692 to 2.673) | 1.93 (1.485 to 2.44) | 1.713 (1.231 to 2.317) | 1.598 (1.125 to 2.239) | 1.587 (1.184 to 2.115) | 1.559 (1.182 to 2.174) | 1.559 (1.182 to 2.174) | 1.559 (1.182 to 2.174) | 1.559 (1.182 to 2.174) |
| Latent tuberculosis infection | Not diabetic | Both | Both | | | | | | | | | | 1.0 (1.0 to 1.0) | 1.0 (1.0 to 1.0) | 1.0 (1.0 to 1.0) | 1.0 (1.0 to 1.0) | 1.0 (1.0 to 1.0) | 1.0 (1.0 to 1.0) | 1.0 (1.0 to 1.0) | 1.0 (1.0 to 1.0) | 1.0 (1.0 to 1.0) | 1.0 (1.0 to 1.0) | 1.0 (1.0 to 1.0) | 1.0 (1.0 to 1.0) | 1.0 (1.0 to 1.0) | 1.0 (1.0 to 1.0) | 1.0 (1.0 to 1.0) |
| Drug-susceptible tuberculosis | Diabetic | Both | Both | | | | | | | | | | 2.73 (1.973 to 3.602) | 2.801 (2.056 to 3.658) | 2.871 (2.047 to 3.698) | 2.798 (1.97 to 3.63) | 2.581 (1.91 to 3.268) | 2.364 (1.817 to 2.943) | 2.147 (1.692 to 2.673) | 1.93 (1.485 to 2.44) | 1.713 (1.231 to 2.317) | 1.598 (1.125 to 2.239) | 1.587 (1.184 to 2.115) | 1.559 (1.182 to 2.174) | 1.559 (1.182 to 2.174) | 1.559 (1.182 to 2.174) | 1.559 (1.182 to 2.174) |
| Drug-susceptible tuberculosis | Not diabetic | Both | Both | | | | | | | | | | 1.0 (1.0 to 1.0) | 1.0 (1.0 to 1.0) | 1.0 (1.0 to 1.0) | 1.0 (1.0 to 1.0) | 1.0 (1.0 to 1.0) | 1.0 (1.0 to 1.0) | 1.0 (1.0 to 1.0) | 1.0 (1.0 to 1.0) | 1.0 (1.0 to 1.0) | 1.0 (1.0 to 1.0) | 1.0 (1.0 to 1.0) | 1.0 (1.0 to 1.0) | 1.0 (1.0 to 1.0) | 1.0 (1.0 to 1.0) | 1.0 (1.0 to 1.0) |
| Multidrug-resistant tuberculosis without extensive drug resistance | Diabetic | Both | Both | | | | | | | | | | 2.73 (1.973 to 3.602) | 2.801 (2.056 to 3.658) | 2.871 (2.047 to 3.698) | 2.798 (1.97 to 3.63) | 2.581 (1.91 to 3.268) | 2.364 (1.817 to 2.943) | 2.147 (1.692 to 2.673) | 1.93 (1.485 to 2.44) | 1.713 (1.231 to 2.317) | 1.598 (1.125 to 2.239) | 1.587 (1.184 to 2.115) | 1.559 (1.182 to 2.174) | 1.559 (1.182 to 2.174) | 1.559 (1.182 to 2.174) | 1.559 (1.182 to 2.174) |
| Multidrug-resistant tuberculosis without extensive drug resistance | Not diabetic | Both | Both | | | | | | | | | | 1.0 (1.0 to 1.0) | 1.0 (1.0 to 1.0) | 1.0 (1.0 to 1.0) | 1.0 (1.0 to 1.0) | 1.0 (1.0 to 1.0) | 1.0 (1.0 to 1.0) | 1.0 (1.0 to 1.0) | 1.0 (1.0 to 1.0) | 1.0 (1.0 to 1.0) | 1.0 (1.0 to 1.0) | 1.0 (1.0 to 1.0) | 1.0 (1.0 to 1.0) | 1.0 (1.0 to 1.0) | 1.0 (1.0 to 1.0) | 1.0 (1.0 to 1.0) |
| Extensively drug-resistant tuberculosis | Diabetic | Both | Both | | | | | | | | | | 2.73 (1.973 to 3.602) | 2.801 (2.056 to 3.658) | 2.871 (2.047 to 3.698) | 2.798 (1.97 to 3.63) | 2.581 (1.91 to 3.268) | 2.364 (1.817 to 2.943) | 2.147 (1.692 to 2.673) | 1.93 (1.485 to 2.44) | 1.713 (1.231 to 2.317) | 1.598 (1.125 to 2.239) | 1.587 (1.184 to 2.115) | 1.559 (1.182 to 2.174) | 1.559 (1.182 to 2.174) | 1.559 (1.182 to 2.174) | 1.559 (1.182 to 2.174) |
| Extensively drug-resistant tuberculosis | Not diabetic | Both | Both | | | | | | | | | | 1.0 (1.0 to 1.0) | 1.0 (1.0 to 1.0) | 1.0 (1.0 to 1.0) | 1.0 (1.0 to 1.0) | 1.0 (1.0 to 1.0) | 1.0 (1.0 to 1.0) | 1.0 (1.0 to 1.0) | 1.0 (1.0 to 1.0) | 1.0 (1.0 to 1.0) | 1.0 (1.0 to 1.0) | 1.0 (1.0 to 1.0) | 1.0 (1.0 to 1.0) | 1.0 (1.0 to 1.0) | 1.0 (1.0 to 1.0) | 1.0 (1.0 to 1.0) |
| Colon and rectum cancer | Diabetic | Both | Males | | | | | | | | | | 1.527 (1.081 to 2.304) | 1.527 (1.081 to 2.304) | 1.527 (1.081 to 2.304) | 1.527 (1.081 to 2.304) | 1.527 (1.081 to 2.304) | 1.527 (1.081 to 2.304) | 1.527 (1.081 to 2.304) | 1.527 (1.081 to 2.304) | 1.527 (1.081 to 2.304) | 1.527 (1.081 to 2.304) | 1.527 (1.081 to 2.304) | 1.527 (1.081 to 2.304) | 1.527 (1.081 to 2.304) | 1.527 (1.081 to 2.304) | 1.527 (1.081 to 2.304) |
| Colon and rectum cancer | Diabetic | Both | Females | | | | | | | | | | 1.527 (1.086 to 2.315) | 1.527 (1.086 to 2.315) | 1.527 (1.086 to 2.315) | 1.527 (1.086 to 2.315) | 1.527 (1.086 to 2.315) | 1.527 (1.086 to 2.315) | 1.527 (1.086 to 2.315) | 1.527 (1.086 to 2.315) | 1.527 (1.086 to 2.315) | 1.527 (1.086 to 2.315) | 1.527 (1.086 to 2.315) | 1.527 (1.086 to 2.315) | 1.527 (1.086 to 2.315) | 1.527 (1.086 to 2.315) | 1.527 (1.086 to 2.315) |
| Colon and rectum cancer | Not diabetic | Both | Males | | | | | | | | | | 1.0 (1.0 to 1.0) | 1.0 (1.0 to 1.0) | 1.0 (1.0 to 1.0) | 1.0 (1.0 to 1.0) | 1.0 (1.0 to 1.0) | 1.0 (1.0 to 1.0) | 1.0 (1.0 to 1.0) | 1.0 (1.0 to 1.0) | 1.0 (1.0 to 1.0) | 1.0 (1.0 to 1.0) | 1.0 (1.0 to 1.0) | 1.0 (1.0 to 1.0) | 1.0 (1.0 to 1.0) | 1.0 (1.0 to 1.0) | 1.0 (1.0 to 1.0) |
| Colon and rectum cancer | Not diabetic | Both | Females | | | | | | | | | | 1.0 (1.0 to 1.0) | 1.0 (1.0 to 1.0) | 1.0 (1.0 to 1.0) | 1.0 (1.0 to 1.0) | 1.0 (1.0 to 1.0) | 1.0 (1.0 to 1.0) | 1.0 (1.0 to 1.0) | 1.0 (1.0 to 1.0) | 1.0 (1.0 to 1.0) | 1.0 (1.0 to 1.0) | 1.0 (1.0 to 1.0) | 1.0 (1.0 to 1.0) | 1.0 (1.0 to 1.0) | 1.0 (1.0 to 1.0) | 1.0 (1.0 to 1.0) |
| Liver cancer due to NASH | Diabetic | Both | Males | | | | | | | | | | 1.523 (1.093 to 2.3) | 1.523 (1.093 to 2.3) | 1.523 (1.093 to 2.3) | 1.523 (1.093 to 2.3) | 1.523 (1.093 to 2.3) | 1.523 (1.093 to 2.3) | 1.523 (1.093 to 2.3) | 1.523 (1.093 to 2.3) | 1.523 (1.093 to 2.3) | 1.523 (1.093 to 2.3) | 1.523 (1.093 to 2.3) | 1.523 (1.093 to 2.3) | 1.523 (1.093 to 2.3) | 1.523 (1.093 to 2.3) | 1.523 (1.093 to 2.3) |
| Liver cancer due to NASH | Diabetic | Both | Females | | | | | | | | | | 1.512 (1.083 to 2.293) | 1.512 (1.083 to 2.293) | 1.512 (1.083 to 2.293) | 1.512 (1.083 to 2.293) | 1.512 (1.083 to 2.293) | 1.512 (1.083 to 2.293) | 1.512 (1.083 to 2.293) | 1.512 (1.083 to 2.293) | 1.512 (1.083 to 2.293) | 1.512 (1.083 to 2.293) | 1.512 (1.083 to 2.293) | 1.512 (1.083 to 2.293) | 1.512 (1.083 to 2.293) | 1.512 (1.083 to 2.293) | 1.512 (1.083 to 2.293) |
| Liver cancer due to NASH | Not diabetic | Both | Males | | | | | | | | | | 1.0 (1.0 to 1.0) | 1.0 (1.0 to 1.0) | 1.0 (1.0 to 1.0) | 1.0 (1.0 to 1.0) | 1.0 (1.0 to 1.0) | 1.0 (1.0 to 1.0) | 1.0 (1.0 to 1.0) | 1.0 (1.0 to 1.0) | 1.0 (1.0 to 1.0) | 1.0 (1.0 to 1.0) | 1.0 (1.0 to 1.0) | 1.0 (1.0 to 1.0) | 1.0 (1.0 to 1.0) | 1.0 (1.0 to 1.0) | 1.0 (1.0 to 1.0) |
| Liver cancer due to NASH | Not diabetic | Both | Females | | | | | | | | | | 1.0 (1.0 to 1.0) | 1.0 (1.0 to 1.0) | 1.0 (1.0 to 1.0) | 1.0 (1.0 to 1.0) | 1.0 (1.0 to 1.0) | 1.0 (1.0 to 1.0) | 1.0 (1.0 to 1.0) | 1.0 (1.0 to 1.0) | 1.0 (1.0 to 1.0) | 1.0 (1.0 to 1.0) | 1.0 (1.0 to 1.0) | 1.0 (1.0 to 1.0) | 1.0 (1.0 to 1.0) | 1.0 (1.0 to 1.0) | 1.0 (1.0 to 1.0) |
| Liver cancer due to other causes | Diabetic | Both | Males | | | | | | | | | | 1.523 (1.093 to 2.3) | 1.523 (1.093 to 2.3) | 1.523 (1.093 to 2.3) | 1.523 (1.093 to 2.3) | 1.523 (1.093 to 2.3) | 1.523 (1.093 to 2.3) | 1.523 (1.093 to 2.3) | 1.523 (1.093 to 2.3) | 1.523 (1.093 to 2.3) | 1.523 (1.093 to 2.3) | 1.523 (1.093 to 2.3) | 1.523 (1.093 to 2.3) | 1.523 (1.093 to 2.3) | 1.523 (1.093 to 2.3) | 1.523 (1.093 to 2.3) |
| Liver cancer due to other causes | Diabetic | Both | Females | | | | | | | | | | 1.512 (1.083 to 2.293) | 1.512 (1.083 to 2.293) | 1.512 (1.083 to 2.293) | 1.512 (1.083 to 2.293) | 1.512 (1.083 to 2.293) | 1.512 (1.083 to 2.293) | 1.512 (1.083 to 2.293) | 1.512 (1.083 to 2.293) | 1.512 (1.083 to 2.293) | 1.512 (1.083 to 2.293) | 1.512 (1.083 to 2.293) | 1.512 (1.083 to 2.293) | 1.512 (1.083 to 2.293) | 1.512 (1.083 to 2.293) | 1.512 (1.083 to 2.293) |
| Liver cancer due to other causes | Not diabetic | Both | Males | | | | | | | | | | 1.0 (1.0 to 1.0) | 1.0 (1.0 to 1.0) | 1.0 (1.0 to 1.0) | 1.0 (1.0 to 1.0) | 1.0 (1.0 to 1.0) | 1.0 (1.0 to 1.0) | 1.0 (1.0 to 1.0) | 1.0 (1.0 to 1.0) | 1.0 (1.0 to 1.0) | 1.0 (1.0 to 1.0) | 1.0 (1.0 to 1.0) | 1.0 (1.0 to 1.0) | 1.0 (1.0 to 1.0) | 1.0 (1.0 to 1.0) | 1.0 (1.0 to 1.0) |
| Liver cancer due to other causes | Not diabetic | Both | Females | | | | | | | | | | 1.0 (1.0 to 1.0) | 1.0 (1.0 to 1.0) | 1.0 (1.0 to 1.0) | 1.0 (1.0 to 1.0) | 1.0 (1.0 to 1.0) | 1.0 (1.0 to 1.0) | 1.0 (1.0 to 1.0) | 1.0 (1.0 to 1.0) | 1.0 (1.0 to 1.0) | 1.0 (1.0 to 1.0) | 1.0 (1.0 to 1.0) | 1.0 (1.0 to 1.0) | 1.0 (1.0 to 1.0) | 1.0 (1.0 to 1.0) | 1.0 (1.0 to 1.0) |
| Pancreatic cancer | Diabetic | Both | Males | | | | | | | | | | 1.517 (1.085 to 2.314) | 1.517 (1.085 to 2.314) | 1.517 (1.085 to 2.314) | 1.517 (1.085 to 2.314) | 1.517 (1.085 to 2.314) | 1.517 (1.085 to 2.314) | 1.517 (1.085 to 2.314) | 1.517 (1.085 to 2.314) | 1.517 (1.085 to 2.314) | 1.517 (1.085 to 2.314) | 1.517 (1.085 to 2.314) | 1.517 (1.085 to 2.314) | 1.517 (1.085 to 2.314) | 1.517 (1.085 to 2.314) | 1.517 (1.085 to 2.314) |
| Pancreatic cancer | Diabetic | Both | Females | | | | | | | | | | 1.518 (1.075 to 2.311) | 1.518 (1.075 to 2.311) | 1.518 (1.075 to 2.311) | 1.518 (1.075 to 2.311) | 1.518 (1.075 to 2.311) | 1.518 (1.075 to 2.311) | 1.518 (1.075 to 2.311) | 1.518 (1.075 to 2.311) | 1.518 (1.075 to 2.311) | 1.518 (1.075 to 2.311) | 1.518 (1.075 to 2.311) | 1.518 (1.075 to 2.311) | 1.518 (1.075 to 2.311) | 1.518 (1.075 to 2.311) | 1.518 (1.075 to 2.311) |
| Pancreatic cancer | Not diabetic | Both | Males | | | | | | | | | | 1.0 (1.0 to 1.0) | 1.0 (1.0 to 1.0) | 1.0 (1.0 to 1.0) | 1.0 (1.0 to 1.0) | 1.0 (1.0 to 1.0) | 1.0 (1.0 to 1.0) | 1.0 (1.0 to 1.0) | 1.0 (1.0 to 1.0) | 1.0 (1.0 to 1.0) | 1.0 (1.0 to 1.0) | 1.0 (1.0 to 1.0) | 1.0 (1.0 to 1.0) | 1.0 (1.0 to 1.0) | 1.0 (1.0 to 1.0) | 1.0 (1.0 to 1.0) |
| Pancreatic cancer | Not diabetic | Both | Females | | | | | | | | | | 1.0 (1.0 to 1.0) | 1.0 (1.0 to 1.0) | 1.0 (1.0 to 1.0) | 1.0 (1.0 to 1.0) | 1.0 (1.0 to 1.0) | 1.0 (1.0 to 1.0) | 1.0 (1.0 to 1.0) | 1.0 (1.0 to 1.0) | 1.0 (1.0 to 1.0) | 1.0 (1.0 to 1.0) | 1.0 (1.0 to 1.0) | 1.0 (1.0 to 1.0) | 1.0 (1.0 to 1.0) | 1.0 (1.0 to 1.0) | 1.0 (1.0 to 1.0) |
| Tracheal, bronchus, and lung cancer | Diabetic | Both | Males | | | | | | | | | | 1.515 (1.076 to 2.309) | 1.515 (1.076 to 2.309) | 1.515 (1.076 to 2.309) | 1.515 (1.076 to 2.309) | 1.515 (1.076 to 2.309) | 1.515 (1.076 to 2.309) | 1.515 (1.076 to 2.309) | 1.515 (1.076 to 2.309) | 1.515 (1.076 to 2.309) | 1.515 (1.076 to 2.309) | 1.515 (1.076 to 2.309) | 1.515 (1.076 to 2.309) | 1.515 (1.076 to 2.309) | 1.515 (1.076 to 2.309) | 1.515 (1.076 to 2.309) |
| Tracheal, bronchus, and lung cancer | Diabetic | Both | Females | | | | | | | | | | 1.512 (1.087 to 2.299) | 1.512 (1.087 to 2.299) | 1.512 (1.087 to 2.299) | 1.512 (1.087 to 2.299) | 1.512 (1.087 to 2.299) | 1.512 (1.087 to 2.299) | 1.512 (1.087 to 2.299) | 1.512 (1.087 to 2.299) | 1.512 (1.087 to 2.299) | 1.512 (1.087 to 2.299) | 1.512 (1.087 to 2.299) | 1.512 (1.087 to 2.299) | 1.512 (1.087 to 2.299) | 1.512 (1.087 to 2.299) | 1.512 (1.087 to 2.299) |
| Tracheal, bronchus, and lung cancer | Not diabetic | Both | Males | | | | | | | | | | 1.0 (1.0 to 1.0) | 1.0 (1.0 to 1.0) | 1.0 (1.0 to 1.0) | 1.0 (1.0 to 1.0) | 1.0 (1.0 to 1.0) | 1.0 (1.0 to 1.0) | 1.0 (1.0 to 1.0) | 1.0 (1.0 to 1.0) | 1.0 (1.0 to 1.0) | 1.0 (1.0 to 1.0) | 1.0 (1.0 to 1.0) | 1.0 (1.0 to 1.0) | 1.0 (1.0 to 1.0) | 1.0 (1.0 to 1.0) | 1.0 (1.0 to 1.0) |
| Tracheal, bronchus, and lung cancer | Not diabetic | Both | Females | | | | | | | | | | 1.0 (1.0 to 1.0) | 1.0 (1.0 to 1.0) | 1.0 (1.0 to 1.0) | 1.0 (1.0 to 1.0) | 1.0 (1.0 to 1.0) | 1.0 (1.0 to 1.0) | 1.0 (1.0 to 1.0) | 1.0 (1.0 to 1.0) | 1.0 (1.0 to 1.0) | 1.0 (1.0 to 1.0) | 1.0 (1.0 to 1.0) | 1.0 (1.0 to 1.0) | 1.0 (1.0 to 1.0) | 1.0 (1.0 to 1.0) | 1.0 (1.0 to 1.0) |
| Breast cancer | Diabetic | Both | Both | | | | | | | | | | 1.513 (1.087 to 2.206) | 1.513 (1.087 to 2.206) | 1.513 (1.087 to 2.206) | 1.513 (1.087 to 2.206) | 1.513 (1.087 to 2.206) | 1.513 (1.087 to 2.206) | 1.513 (1.087 to 2.206) | 1.513 (1.087 to 2.206) | 1.513 (1.087 to 2.206) | 1.513 (1.087 to 2.206) | 1.513 (1.087 to 2.206) | 1.513 (1.087 to 2.206) | 1.513 (1.087 to 2.206) | 1.513 (1.087 to 2.206) | 1.513 (1.087 to 2.206) |
| Breast cancer | Not diabetic | Both | Both | | | | | | | | | | 1.0 (1.0 to 1.0) | 1.0 (1.0 to 1.0) | 1.0 (1.0 to 1.0) | 1.0 (1.0 to 1.0) | 1.0 (1.0 to 1.0) | 1.0 (1.0 to 1.0) | 1.0 (1.0 to 1.0) | 1.0 (1.0 to 1.0) | 1.0 (1.0 to 1.0) | 1.0 (1.0 to 1.0) | 1.0 (1.0 to 1.0) | 1.0 (1.0 to 1.0) | 1.0 (1.0 to 1.0) | 1.0 (1.0 to 1.0) | 1.0 (1.0 to 1.0) |
| Ovarian cancer | Diabetic | Both | Both | | | | | | | | | | 1.522 (1.092 to 2.32) | 1.522 (1.092 to 2.32) | 1.522 (1.092 to 2.32) | 1.522 (1.092 to 2.32) | 1.522 (1.092 to 2.32) | 1.522 (1.092 to 2.32) | 1.522 (1.092 to 2.32) | 1.522 (1.092 to 2.32) | 1.522 (1.092 to 2.32) | 1.522 (1.092 to 2.32) | 1.522 (1.092 to 2.32) | 1.522 (1.092 to 2.32) | 1.522 (1.092 to 2.32) | 1.522 (1.092 to 2.32) | 1.522 (1.092 to 2.32) |
| Ovarian cancer | Not diabetic | Both | Both | | | | | | | | | | 1.0 (1.0 to 1.0) | 1.0 (1.0 to 1.0) | 1.0 (1.0 to 1.0) | 1.0 (1.0 to 1.0) | 1.0 (1.0 to 1.0) | 1.0 (1.0 to 1.0) | 1.0 (1.0 to 1.0) | 1.0 (1.0 to 1.0) | 1.0 (1.0 to 1.0) | 1.0 (1.0 to 1.0) | 1.0 (1.0 to 1.0) | 1.0 (1.0 to 1.0) | 1.0 (1.0 to 1.0) | 1.0 (1.0 to 1.0) | 1.0 (1.0 to 1.0) |

**Appendix Table 6a. Relative risks used by age and sex for each outcome for all risk factors except for ambient air pollution alcohol, and smoking.**

| Risk - Outcome | Category / Units | Morbidity / Mortality | Sex | Ages | | | | | | | | | | | | | | | | | | | | | | | |
|---|---|---|---|---|---|---|---|---|---|---|---|---|---|---|---|---|---|---|---|---|---|---|---|---|---|---|---|
| | | | | All-age | 0-6 days | 7-27 days | 28-364 days | 1-4 years | 5-9 years | 10-14 years | 15-19 years | 20-24 years | 25-29 years | 30-34 years | 35-39 years | 40-44 years | 45-49 years | 50-54 years | 55-59 years | 60-64 years | 65-69 years | 70-74 years | 75-79 years | 80-84 years | 85-89 years | 90-94 years | 95+ years |
| Bladder cancer | Diabetic | Both | Males | | | | | | | | | | 1.514 (1.076 to 2.256) | 1.514 (1.076 to 2.256) | 1.514 (1.076 to 2.256) | 1.514 (1.076 to 2.256) | 1.514 (1.076 to 2.256) | 1.514 (1.076 to 2.256) | 1.514 (1.076 to 2.256) | 1.514 (1.076 to 2.256) | 1.514 (1.076 to 2.256) | 1.514 (1.076 to 2.256) | 1.514 (1.076 to 2.256) | 1.514 (1.076 to 2.256) | 1.514 (1.076 to 2.256) | 1.514 (1.076 to 2.256) | 1.514 (1.076 to 2.256) |
| Bladder cancer | Diabetic | Both | Females | | | | | | | | | | 1.511 (1.082 to 2.253) | 1.511 (1.082 to 2.253) | 1.511 (1.082 to 2.253) | 1.511 (1.082 to 2.253) | 1.511 (1.082 to 2.253) | 1.511 (1.082 to 2.253) | 1.511 (1.082 to 2.253) | 1.511 (1.082 to 2.253) | 1.511 (1.082 to 2.253) | 1.511 (1.082 to 2.253) | 1.511 (1.082 to 2.253) | 1.511 (1.082 to 2.253) | 1.511 (1.082 to 2.253) | 1.511 (1.082 to 2.253) | 1.511 (1.082 to 2.253) |
| Bladder cancer | Not diabetic | Both | Males | | | | | | | | | | 1.0 (1.0 to 1.0) | 1.0 (1.0 to 1.0) | 1.0 (1.0 to 1.0) | 1.0 (1.0 to 1.0) | 1.0 (1.0 to 1.0) | 1.0 (1.0 to 1.0) | 1.0 (1.0 to 1.0) | 1.0 (1.0 to 1.0) | 1.0 (1.0 to 1.0) | 1.0 (1.0 to 1.0) | 1.0 (1.0 to 1.0) | 1.0 (1.0 to 1.0) | 1.0 (1.0 to 1.0) | 1.0 (1.0 to 1.0) | 1.0 (1.0 to 1.0) |
| Bladder cancer | Not diabetic | Both | Females | | | | | | | | | | 1.0 (1.0 to 1.0) | 1.0 (1.0 to 1.0) | 1.0 (1.0 to 1.0) | 1.0 (1.0 to 1.0) | 1.0 (1.0 to 1.0) | 1.0 (1.0 to 1.0) | 1.0 (1.0 to 1.0) | 1.0 (1.0 to 1.0) | 1.0 (1.0 to 1.0) | 1.0 (1.0 to 1.0) | 1.0 (1.0 to 1.0) | 1.0 (1.0 to 1.0) | 1.0 (1.0 to 1.0) | 1.0 (1.0 to 1.0) | 1.0 (1.0 to 1.0) |
| Peripheral vascular disease | Diabetic | Both | Both | | | | | | | | | | 8.264 (6.044 to 9.303) | 6.651 (5.375 to 7.452) | 5.039 (4.436 to 5.673) | 4.138 (3.561 to 4.76) | 3.947 (3.447 to 4.504) | 3.756 (3.347 to 4.232) | 3.565 (3.214 to 3.972) | 3.374 (3.079 to 3.714) | 3.183 (2.92 to 3.466) | 2.992 (2.76 to 3.25) | 2.801 (2.558 to 3.05) | 2.324 (1.977 to 2.671) | 2.324 (1.977 to 2.671) | 2.324 (1.977 to 2.671) | 2.324 (1.977 to 2.671) |
| Peripheral vascular disease | Not diabetic | Both | Both | | | | | | | | | | 1.0 (1.0 to 1.0) | 1.0 (1.0 to 1.0) | 1.0 (1.0 to 1.0) | 1.0 (1.0 to 1.0) | 1.0 (1.0 to 1.0) | 1.0 (1.0 to 1.0) | 1.0 (1.0 to 1.0) | 1.0 (1.0 to 1.0) | 1.0 (1.0 to 1.0) | 1.0 (1.0 to 1.0) | 1.0 (1.0 to 1.0) | 1.0 (1.0 to 1.0) | 1.0 (1.0 to 1.0) | 1.0 (1.0 to 1.0) | 1.0 (1.0 to 1.0) |
| Alzheimer's disease and other dementias | Diabetic | Both | Males | | | | | | | | | | 1.516 (1.084 to 2.295) | 1.516 (1.084 to 2.295) | 1.516 (1.084 to 2.295) | 1.516 (1.084 to 2.295) | 1.516 (1.084 to 2.295) | 1.516 (1.084 to 2.295) | 1.516 (1.084 to 2.295) | 1.516 (1.084 to 2.295) | 1.516 (1.084 to 2.295) | 1.516 (1.084 to 2.295) | 1.516 (1.084 to 2.295) | 1.516 (1.084 to 2.295) | 1.516 (1.084 to 2.295) | 1.516 (1.084 to 2.295) | 1.516 (1.084 to 2.295) |
| Alzheimer's disease and other dementias | Diabetic | Both | Females | | | | | | | | | | 1.52 (1.08 to 2.301) | 1.52 (1.08 to 2.301) | 1.52 (1.08 to 2.301) | 1.52 (1.08 to 2.301) | 1.52 (1.08 to 2.301) | 1.52 (1.08 to 2.301) | 1.52 (1.08 to 2.301) | 1.52 (1.08 to 2.301) | 1.52 (1.08 to 2.301) | 1.52 (1.08 to 2.301) | 1.52 (1.08 to 2.301) | 1.52 (1.08 to 2.301) | 1.52 (1.08 to 2.301) | 1.52 (1.08 to 2.301) | 1.52 (1.08 to 2.301) |
| Alzheimer's disease and other dementias | Not diabetic | Both | Males | | | | | | | | | | 1.0 (1.0 to 1.0) | 1.0 (1.0 to 1.0) | 1.0 (1.0 to 1.0) | 1.0 (1.0 to 1.0) | 1.0 (1.0 to 1.0) | 1.0 (1.0 to 1.0) | 1.0 (1.0 to 1.0) | 1.0 (1.0 to 1.0) | 1.0 (1.0 to 1.0) | 1.0 (1.0 to 1.0) | 1.0 (1.0 to 1.0) | 1.0 (1.0 to 1.0) | 1.0 (1.0 to 1.0) | 1.0 (1.0 to 1.0) | 1.0 (1.0 to 1.0) |
| Alzheimer's disease and other dementias | Not diabetic | Both | Females | | | | | | | | | | 1.0 (1.0 to 1.0) | 1.0 (1.0 to 1.0) | 1.0 (1.0 to 1.0) | 1.0 (1.0 to 1.0) | 1.0 (1.0 to 1.0) | 1.0 (1.0 to 1.0) | 1.0 (1.0 to 1.0) | 1.0 (1.0 to 1.0) | 1.0 (1.0 to 1.0) | 1.0 (1.0 to 1.0) | 1.0 (1.0 to 1.0) | 1.0 (1.0 to 1.0) | 1.0 (1.0 to 1.0) | 1.0 (1.0 to 1.0) | 1.0 (1.0 to 1.0) |
| Glaucoma | Diabetic | Both | Males | | | | | | | | | | 1.52 (1.098 to 2.325) | 1.52 (1.098 to 2.325) | 1.52 (1.098 to 2.325) | 1.52 (1.098 to 2.325) | 1.52 (1.098 to 2.325) | 1.52 (1.098 to 2.325) | 1.52 (1.098 to 2.325) | 1.52 (1.098 to 2.325) | 1.52 (1.098 to 2.325) | 1.52 (1.098 to 2.325) | 1.52 (1.098 to 2.325) | 1.52 (1.098 to 2.325) | 1.52 (1.098 to 2.325) | 1.52 (1.098 to 2.325) | 1.52 (1.098 to 2.325) |
| Glaucoma | Diabetic | Both | Females | | | | | | | | | | 1.516 (1.08 to 2.328) | 1.516 (1.08 to 2.328) | 1.516 (1.08 to 2.328) | 1.516 (1.08 to 2.328) | 1.516 (1.08 to 2.328) | 1.516 (1.08 to 2.328) | 1.516 (1.08 to 2.328) | 1.516 (1.08 to 2.328) | 1.516 (1.08 to 2.328) | 1.516 (1.08 to 2.328) | 1.516 (1.08 to 2.328) | 1.516 (1.08 to 2.328) | 1.516 (1.08 to 2.328) | 1.516 (1.08 to 2.328) | 1.516 (1.08 to 2.328) |
| Glaucoma | Not diabetic | Both | Males | | | | | | | | | | 1.0 (1.0 to 1.0) | 1.0 (1.0 to 1.0) | 1.0 (1.0 to 1.0) | 1.0 (1.0 to 1.0) | 1.0 (1.0 to 1.0) | 1.0 (1.0 to 1.0) | 1.0 (1.0 to 1.0) | 1.0 (1.0 to 1.0) | 1.0 (1.0 to 1.0) | 1.0 (1.0 to 1.0) | 1.0 (1.0 to 1.0) | 1.0 (1.0 to 1.0) | 1.0 (1.0 to 1.0) | 1.0 (1.0 to 1.0) | 1.0 (1.0 to 1.0) |
| Glaucoma | Not diabetic | Both | Females | | | | | | | | | | 1.0 (1.0 to 1.0) | 1.0 (1.0 to 1.0) | 1.0 (1.0 to 1.0) | 1.0 (1.0 to 1.0) | 1.0 (1.0 to 1.0) | 1.0 (1.0 to 1.0) | 1.0 (1.0 to 1.0) | 1.0 (1.0 to 1.0) | 1.0 (1.0 to 1.0) | 1.0 (1.0 to 1.0) | 1.0 (1.0 to 1.0) | 1.0 (1.0 to 1.0) | 1.0 (1.0 to 1.0) | 1.0 (1.0 to 1.0) | 1.0 (1.0 to 1.0) |
| Cataract | Diabetic | Both | Males | | | | | | | | | | 1.52 (1.09 to 2.259) | 1.52 (1.09 to 2.259) | 1.52 (1.09 to 2.259) | 1.52 (1.09 to 2.259) | 1.52 (1.09 to 2.259) | 1.52 (1.09 to 2.259) | 1.52 (1.09 to 2.259) | 1.52 (1.09 to 2.259) | 1.52 (1.09 to 2.259) | 1.52 (1.09 to 2.259) | 1.52 (1.09 to 2.259) | 1.52 (1.09 to 2.259) | 1.52 (1.09 to 2.259) | 1.52 (1.09 to 2.259) | 1.52 (1.09 to 2.259) |
| Cataract | Diabetic | Both | Females | | | | | | | | | | 1.522 (1.094 to 2.289) | 1.522 (1.094 to 2.289) | 1.522 (1.094 to 2.289) | 1.522 (1.094 to 2.289) | 1.522 (1.094 to 2.289) | 1.522 (1.094 to 2.289) | 1.522 (1.094 to 2.289) | 1.522 (1.094 to 2.289) | 1.522 (1.094 to 2.289) | 1.522 (1.094 to 2.289) | 1.522 (1.094 to 2.289) | 1.522 (1.094 to 2.289) | 1.522 (1.094 to 2.289) | 1.522 (1.094 to 2.289) | 1.522 (1.094 to 2.289) |
| Cataract | Not diabetic | Both | Males | | | | | | | | | | 1.0 (1.0 to 1.0) | 1.0 (1.0 to 1.0) | 1.0 (1.0 to 1.0) | 1.0 (1.0 to 1.0) | 1.0 (1.0 to 1.0) | 1.0 (1.0 to 1.0) | 1.0 (1.0 to 1.0) | 1.0 (1.0 to 1.0) | 1.0 (1.0 to 1.0) | 1.0 (1.0 to 1.0) | 1.0 (1.0 to 1.0) | 1.0 (1.0 to 1.0) | 1.0 (1.0 to 1.0) | 1.0 (1.0 to 1.0) | 1.0 (1.0 to 1.0) |
| Cataract | Not diabetic | Both | Females | | | | | | | | | | 1.0 (1.0 to 1.0) | 1.0 (1.0 to 1.0) | 1.0 (1.0 to 1.0) | 1.0 (1.0 to 1.0) | 1.0 (1.0 to 1.0) | 1.0 (1.0 to 1.0) | 1.0 (1.0 to 1.0) | 1.0 (1.0 to 1.0) | 1.0 (1.0 to 1.0) | 1.0 (1.0 to 1.0) | 1.0 (1.0 to 1.0) | 1.0 (1.0 to 1.0) | 1.0 (1.0 to 1.0) | 1.0 (1.0 to 1.0) | 1.0 (1.0 to 1.0) |
| **High LDL cholesterol** | | | | | | | | | | | | | | | | | | | | | | | | | | | |
| Ischaemic heart disease | mmol/L | Both | Both | | | | | | | | | | 2.016 (1.684 to 2.544) | 2.027 (1.768 to 2.354) | 2.038 (1.831 to 2.273) | 1.971 (1.775 to 2.191) | 1.828 (1.676 to 2.004) | 1.685 (1.561 to 1.815) | 1.541 (1.446 to 1.648) | 1.398 (1.306 to 1.494) | 1.254 (1.141 to 1.372) | 1.193 (1.088 to 1.312) | 1.213 (1.124 to 1.321) | 1.262 (1.11 to 1.465) | 1.262 (1.11 to 1.465) | 1.262 (1.11 to 1.465) | 1.262 (1.11 to 1.465) |
| Ischaemic stroke | mmol/L | Both | Both | | | | | | | | | | 1.67 (1.334 to 2.339) | 1.626 (1.352 to 2.041) | 1.583 (1.342 to 1.849) | 1.518 (1.287 to 1.76) | 1.434 (1.242 to 1.636) | 1.35 (1.212 to 1.514) | 1.265 (1.164 to 1.391) | 1.181 (1.109 to 1.299) | 1.096 (1.043 to 1.223) | 1.062 (1.008 to 1.193) | 1.077 (1.012 to 1.216) | 1.116 (1.014 to 1.344) | 1.116 (1.014 to 1.344) | 1.116 (1.014 to 1.344) | 1.116 (1.014 to 1.344) |
| **High systolic blood pressure** | | | | | | | | | | | | | | | | | | | | | | | | | | | |
| Rheumatic heart disease | 10 mmHg | Both | Both | | | | | | | | | | 1.631 (1.174 to 2.306) | 1.474 (1.17 to 1.898) | 1.317 (1.144 to 1.575) | 1.229 (1.089 to 1.422) | 1.211 (1.101 to 1.367) | 1.193 (1.107 to 1.328) | 1.175 (1.101 to 1.287) | 1.157 (1.086 to 1.265) | 1.139 (1.055 to 1.248) | 1.127 (1.048 to 1.241) | 1.12 (1.06 to 1.238) | 1.104 (1.04 to 1.28) | 1.104 (1.04 to 1.28) | 1.104 (1.04 to 1.28) | 1.104 (1.04 to 1.28) |
| Ischaemic heart disease | 10 mmHg | Both | Both | | | | | | | | | | 1.972 (1.44 to 2.596) | 1.818 (1.458 to 2.207) | 1.665 (1.461 to 1.911) | 1.568 (1.398 to 1.799) | 1.527 (1.393 to 1.705) | 1.487 (1.385 to 1.619) | 1.446 (1.368 to 1.535) | 1.405 (1.332 to 1.488) | 1.364 (1.257 to 1.456) | 1.33 (1.224 to 1.424) | 1.303 (1.225 to 1.404) | 1.266 (1.134 to 1.437) | 1.266 (1.134 to 1.437) | 1.266 (1.134 to 1.437) | 1.266 (1.134 to 1.437) |
| Ischaemic stroke | 10 mmHg | Both | Both | | | | | | | | | | 1.854 (1.395 to 2.588) | 1.774 (1.427 to 2.252) | 1.694 (1.404 to 2.035) | 1.628 (1.354 to 1.95) | 1.574 (1.36 to 1.823) | 1.521 (1.361 to 1.698) | 1.468 (1.344 to 1.596) | 1.414 (1.302 to 1.524) | 1.361 (1.214 to 1.49) | 1.318 (1.168 to 1.451) | 1.284 (1.179 to 1.389) | 1.201 (1.109 to 1.37) | 1.201 (1.109 to 1.37) | 1.201 (1.109 to 1.37) | 1.201 (1.109 to 1.37) |
| Intracerebral hemorrhage | 10 mmHg | Both | Both | | | | | | | | | | 2.134 (1.555 to 2.919) | 2.05 (1.593 to 2.648) | 1.966 (1.589 to 2.465) | 1.874 (1.492 to 2.302) | 1.775 (1.484 to 2.114) | 1.676 (1.446 to 1.932) | 1.577 (1.402 to 1.754) | 1.478 (1.331 to 1.619) | 1.379 (1.207 to 1.54) | 1.323 (1.162 to 1.495) | 1.311 (1.193 to 1.45) | 1.279 (1.126 to 1.515) | 1.279 (1.126 to 1.515) | 1.279 (1.126 to 1.515) | 1.279 (1.126 to 1.515) |
| Subarachnoid hemorrhage | 10 mmHg | Both | Both | | | | | | | | | | 2.134 (1.555 to 2.919) | 2.05 (1.593 to 2.648) | 1.966 (1.589 to 2.465) | 1.874 (1.492 to 2.302) | 1.775 (1.484 to 2.114) | 1.676 (1.446 to 1.932) | 1.577 (1.402 to 1.754) | 1.478 (1.331 to 1.619) | 1.379 (1.207 to 1.54) | 1.323 (1.162 to 1.495) | 1.311 (1.193 to 1.45) | 1.279 (1.126 to 1.515) | 1.279 (1.126 to 1.515) | 1.279 (1.126 to 1.515) | 1.279 (1.126 to 1.515) |
| Hypertensive heart disease | 10 mmHg | Both | Both | | | | | | | | | | 2.862 (1.829 to 4.108) | 2.838 (1.857 to 4.187) | 2.814 (1.802 to 4.337) | 2.703 (1.762 to 4.186) | 2.504 (1.804 to 3.758) | 2.304 (1.772 to 3.464) | 2.105 (1.645 to 3.336) | 1.905 (1.447 to 3.171) | 1.706 (1.188 to 3.215) | 1.619 (1.053 to 3.136) | 1.644 (1.078 to 3.091) | 1.708 (1.103 to 3.258) | 1.708 (1.103 to 3.258) | 1.708 (1.103 to 3.258) | 1.708 (1.103 to 3.258) |
| Non-rheumatic calcific aortic valve disease | 10 mmHg | Both | Both | | | | | | | | | | 1.755 (1.266 to 2.423) | 1.605 (1.293 to 2.011) | 1.455 (1.278 to 1.642) | 1.365 (1.232 to 1.51) | 1.335 (1.222 to 1.449) | 1.306 (1.219 to 1.394) | 1.276 (1.212 to 1.342) | 1.247 (1.183 to 1.303) | 1.217 (1.131 to 1.284) | 1.193 (1.116 to 1.263) | 1.175 (1.12 to 1.237) | 1.128 (1.071 to 1.235) | 1.128 (1.071 to 1.235) | 1.128 (1.071 to 1.235) | 1.128 (1.071 to 1.235) |
| Other cardiomyopathy | 10 mmHg | Both | Both | | | | | | | | | | 1.755 (1.266 to 2.423) | 1.605 (1.293 to 2.011) | 1.455 (1.278 to 1.642) | 1.365 (1.232 to 1.51) | 1.335 (1.222 to 1.449) | 1.306 (1.219 to 1.394) | 1.276 (1.212 to 1.342) | 1.247 (1.183 to 1.303) | 1.217 (1.131 to 1.284) | 1.193 (1.116 to 1.263) | 1.175 (1.12 to 1.237) | 1.128 (1.071 to 1.235) | 1.128 (1.071 to 1.235) | 1.128 (1.071 to 1.235) | 1.128 (1.071 to 1.235) |
| Atrial fibrillation and flutter | 10 mmHg | Both | Both | | | | | | | | | | 1.76 (1.336 to 2.43) | 1.631 (1.379 to 2.026) | 1.503 (1.396 to 1.644) | 1.423 (1.34 to 1.505) | 1.392 (1.328 to 1.457) | 1.361 (1.313 to 1.411) | 1.33 (1.293 to 1.369) | 1.299 (1.265 to 1.333) | 1.268 (1.233 to 1.308) | 1.237 (1.202 to 1.277) | 1.208 (1.177 to 1.238) | 1.134 (1.092 to 1.185) | 1.134 (1.092 to 1.185) | 1.134 (1.092 to 1.185) | 1.134 (1.092 to 1.185) |
| Aortic aneurysm | 10 mmHg | Both | Both | | | | | | | | | | 1.544 (1.259 to 2.164) | 1.469 (1.29 to 1.816) | 1.394 (1.3 to 1.535) | 1.345 (1.227 to 1.451) | 1.321 (1.233 to 1.405) | 1.296 (1.229 to 1.362) | 1.272 (1.218 to 1.327) | 1.248 (1.191 to 1.299) | 1.223 (1.16 to 1.286) | 1.2 (1.137 to 1.261) | 1.177 (1.126 to 1.229) | 1.119 (1.071 to 1.184) | 1.119 (1.071 to 1.184) | 1.119 (1.071 to 1.184) | 1.119 (1.071 to 1.184) |
| Peripheral vascular disease | 10 mmHg | Both | Both | | | | | | | | | | 1.728 (1.203 to 2.428) | 1.491 (1.206 to 1.87) | 1.254 (1.182 to 1.329) | 1.138 (1.019 to 1.263) | 1.142 (1.047 to 1.243) | 1.146 (1.071 to 1.224) | 1.15 (1.094 to 1.208) | 1.154 (1.11 to 1.199) | 1.159 (1.113 to 1.207) | 1.152 (1.104 to 1.201) | 1.136 (1.098 to 1.176) | 1.095 (1.054 to 1.154) | 1.095 (1.054 to 1.154) | 1.095 (1.054 to 1.154) | 1.095 (1.054 to 1.154) |
| Endocarditis | 10 mmHg | Both | Both | | | | | | | | | | 1.755 (1.266 to 2.423) | 1.605 (1.293 to 2.011) | 1.455 (1.278 to 1.642) | 1.365 (1.232 to 1.51) | 1.335 (1.222 to 1.449) | 1.306 (1.219 to 1.394) | 1.276 (1.212 to 1.342) | 1.247 (1.183 to 1.303) | 1.217 (1.131 to 1.284) | 1.193 (1.116 to 1.263) | 1.175 (1.12 to 1.237) | 1.128 (1.071 to 1.235) | 1.128 (1.071 to 1.235) | 1.128 (1.071 to 1.235) | 1.128 (1.071 to 1.235) |
| Other cardiovascular and circulatory diseases | 10 mmHg | Both | Both | | | | | | | | | | 1.744 (1.339 to 2.396) | 1.624 (1.384 to 2.006) | 1.504 (1.405 to 1.626) | 1.427 (1.354 to 1.498) | 1.395 (1.336 to 1.452) | 1.363 (1.318 to 1.406) | 1.33 (1.296 to 1.365) | 1.298 (1.266 to 1.332) | 1.265 (1.231 to 1.303) | 1.235 (1.201 to 1.27) | 1.207 (1.177 to 1.235) | 1.137 (1.095 to 1.187) | 1.137 (1.095 to 1.187) | 1.137 (1.095 to 1.187) | 1.137 (1.095 to 1.187) |
| Chronic kidney disease due to diabetes mellitus type 1 | 10 mmHg | Both | Both | | | | | | | | | | 1.283 (1.186 to 1.397) | 1.283 (1.186 to 1.397) | 1.283 (1.186 to 1.397) | 1.283 (1.186 to 1.397) | 1.283 (1.186 to 1.397) | 1.283 (1.186 to 1.397) | 1.283 (1.186 to 1.397) | 1.283 (1.186 to 1.397) | 1.283 (1.186 to 1.397) | 1.283 (1.186 to 1.397) | 1.283 (1.186 to 1.397) | 1.283 (1.186 to 1.397) | 1.283 (1.186 to 1.397) | 1.283 (1.186 to 1.397) | 1.283 (1.186 to 1.397) |
| Chronic kidney disease due to diabetes mellitus type 2 | 10 mmHg | Both | Both | | | | | | | | | | 1.283 (1.186 to 1.397) | 1.283 (1.186 to 1.397) | 1.283 (1.186 to 1.397) | 1.283 (1.186 to 1.397) | 1.283 (1.186 to 1.397) | 1.283 (1.186 to 1.397) | 1.283 (1.186 to 1.397) | 1.283 (1.186 to 1.397) | 1.283 (1.186 to 1.397) | 1.283 (1.186 to 1.397) | 1.283 (1.186 to 1.397) | 1.283 (1.186 to 1.397) | 1.283 (1.186 to 1.397) | 1.283 (1.186 to 1.397) | 1.283 (1.186 to 1.397) |
| Chronic kidney disease due to hypertension | 10 mmHg | Both | Both | | | | | | | | | | 1.281 (1.18 to 1.385) | 1.281 (1.18 to 1.385) | 1.281 (1.18 to 1.385) | 1.281 (1.18 to 1.385) | 1.281 (1.18 to 1.385) | 1.281 (1.18 to 1.385) | 1.281 (1.18 to 1.385) | 1.281 (1.18 to 1.385) | 1.281 (1.18 to 1.385) | 1.281 (1.18 to 1.385) | 1.281 (1.18 to 1.385) | 1.281 (1.18 to 1.385) | 1.281 (1.18 to 1.385) | 1.281 (1.18 to 1.385) | 1.281 (1.18 to 1.385) |
| Chronic kidney disease due to glomerulonephritis | 10 mmHg | Both | Both | | | | | | | | | | 1.281 (1.182 to 1.383) | 1.281 (1.182 to 1.383) | 1.281 (1.182 to 1.383) | 1.281 (1.182 to 1.383) | 1.281 (1.182 to 1.383) | 1.281 (1.182 to 1.383) | 1.281 (1.182 to 1.383) | 1.281 (1.182 to 1.383) | 1.281 (1.182 to 1.383) | 1.281 (1.182 to 1.383) | 1.281 (1.182 to 1.383) | 1.281 (1.182 to 1.383) | 1.281 (1.182 to 1.383) | 1.281 (1.182 to 1.383) | 1.281 (1.182 to 1.383) |
| Chronic kidney disease due to other and unspecified causes | 10 mmHg | Both | Both | | | | | | | | | | 1.282 (1.181 to 1.395) | 1.282 (1.181 to 1.395) | 1.282 (1.181 to 1.395) | 1.282 (1.181 to 1.395) | 1.282 (1.181 to 1.395) | 1.282 (1.181 to 1.395) | 1.282 (1.181 to 1.395) | 1.282 (1.181 to 1.395) | 1.282 (1.181 to 1.395) | 1.282 (1.181 to 1.395) | 1.282 (1.181 to 1.395) | 1.282 (1.181 to 1.395) | 1.282 (1.181 to 1.395) | 1.282 (1.181 to 1.395) | 1.282 (1.181 to 1.395) |
| **High body-mass index in adults** | | | | | | | | | | | | | | | | | | | | | | | | | | | |
| Oesophageal cancer | 5 kg/m2 | Both | Males | | | | | | | | | 1.391 (1.077 to 1.754) | 1.391 (1.077 to 1.754) | 1.391 (1.077 to 1.754) | 1.391 (1.077 to 1.754) | 1.391 (1.077 to 1.754) | 1.391 (1.077 to 1.754) | 1.391 (1.077 to 1.754) | 1.391 (1.077 to 1.754) | 1.391 (1.077 to 1.754) | 1.391 (1.077 to 1.754) | 1.391 (1.077 to 1.754) | 1.391 (1.077 to 1.754) | 1.391 (1.077 to 1.754) | 1.391 (1.077 to 1.754) | 1.391 (1.077 to 1.754) | 1.391 (1.077 to 1.754) |
| Oesophageal cancer | 5 kg/m2 | Both | Females | | | | | | | | | 1.351 (1.012 to 1.745) | 1.351 (1.012 to 1.745) | 1.351 (1.012 to 1.745) | 1.351 (1.012 to 1.745) | 1.351 (1.012 to 1.745) | 1.351 (1.012 to 1.745) | 1.351 (1.012 to 1.745) | 1.351 (1.012 to 1.745) | 1.351 (1.012 to 1.745) | 1.351 (1.012 to 1.745) | 1.351 (1.012 to 1.745) | 1.351 (1.012 to 1.745) | 1.351 (1.012 to 1.745) | 1.351 (1.012 to 1.745) | 1.351 (1.012 to 1.745) | 1.351 (1.012 to 1.745) |
| Colon and rectum cancer | 5 kg/m2 | Both | Males | | | | | | | | | 1.177 (1.145 to 1.208) | 1.177 (1.145 to 1.208) | 1.177 (1.145 to 1.208) | 1.177 (1.145 to 1.208) | 1.177 (1.145 to 1.208) | 1.177 (1.145 to 1.208) | 1.177 (1.145 to 1.208) | 1.177 (1.145 to 1.208) | 1.177 (1.145 to 1.208) | 1.177 (1.145 to 1.208) | 1.177 (1.145 to 1.208) | 1.177 (1.145 to 1.208) | 1.177 (1.145 to 1.208) | 1.177 (1.145 to 1.208) | 1.177 (1.145 to 1.208) | 1.177 (1.145 to 1.208) |
| Colon and rectum cancer | 5 kg/m2 | Both | Females | | | | | | | | | 1.059 (1.031 to 1.083) | 1.059 (1.031 to 1.083) | 1.059 (1.031 to 1.083) | 1.059 (1.031 to 1.083) | 1.059 (1.031 to 1.083) | 1.059 (1.031 to 1.083) | 1.059 (1.031 to 1.083) | 1.059 (1.031 to 1.083) | 1.059 (1.031 to 1.083) | 1.059 (1.031 to 1.083) | 1.059 (1.031 to 1.083) | 1.059 (1.031 to 1.083) | 1.059 (1.031 to 1.083) | 1.059 (1.031 to 1.083) | 1.059 (1.031 to 1.083) | 1.059 (1.031 to 1.083) |
| Liver cancer due to hepatitis B | 5 kg/m2 | Both | Males | | | | | | | | | 1.289 (1.109 to 1.491) | 1.289 (1.109 to 1.491) | 1.289 (1.109 to 1.491) | 1.289 (1.109 to 1.491) | 1.289 (1.109 to 1.491) | 1.289 (1.109 to 1.491) | 1.289 (1.109 to 1.491) | 1.289 (1.109 to 1.491) | 1.289 (1.109 to 1.491) | 1.289 (1.109 to 1.491) | 1.289 (1.109 to 1.491) | 1.289 (1.109 to 1.491) | 1.289 (1.109 to 1.491) | 1.289 (1.109 to 1.491) | 1.289 (1.109 to 1.491) | 1.289 (1.109 to 1.491) |
| Liver cancer due to hepatitis B | 5 kg/m2 | Both | Females | | | | | | | | | 1.176 (1.03 to 1.334) | 1.176 (1.03 to 1.334) | 1.176 (1.03 to 1.334) | 1.176 (1.03 to 1.334) | 1.176 (1.03 to 1.334) | 1.176 (1.03 to 1.334) | 1.176 (1.03 to 1.334) | 1.176 (1.03 to 1.334) | 1.176 (1.03 to 1.334) | 1.176 (1.03 to 1.334) | 1.176 (1.03 to 1.334) | 1.176 (1.03 to 1.334) | 1.176 (1.03 to 1.334) | 1.176 (1.03 to 1.334) | 1.176 (1.03 to 1.334) | 1.176 (1.03 to 1.334) |
| Liver cancer due to hepatitis C | 5 kg/m2 | Both | Males | | | | | | | | | 1.289 (1.109 to 1.491) | 1.289 (1.109 to 1.491) | 1.289 (1.109 to 1.491) | 1.289 (1.109 to 1.491) | 1.289 (1.109 to 1.491) | 1.289 (1.109 to 1.491) | 1.289 (1.109 to 1.491) | 1.289 (1.109 to 1.491) | 1.289 (1.109 to 1.491) | 1.289 (1.109 to 1.491) | 1.289 (1.109 to 1.491) | 1.289 (1.109 to 1.491) | 1.289 (1.109 to 1.491) | 1.289 (1.109 to 1.491) | 1.289 (1.109 to 1.491) | 1.289 (1.109 to 1.491) |
| Liver cancer due to hepatitis C | 5 kg/m2 | Both | Females | | | | | | | | | 1.176 (1.03 to 1.334) | 1.176 (1.03 to 1.334) | 1.176 (1.03 to 1.334) | 1.176 (1.03 to 1.334) | 1.176 (1.03 to 1.334) | 1.176 (1.03 to 1.334) | 1.176 (1.03 to 1.334) | 1.176 (1.03 to 1.334) | 1.176 (1.03 to 1.334) | 1.176 (1.03 to 1.334) | 1.176 (1.03 to 1.334) | 1.176 (1.03 to 1.334) | 1.176 (1.03 to 1.334) | 1.176 (1.03 to 1.334) | 1.176 (1.03 to 1.334) | 1.176 (1.03 to 1.334) |
| Liver cancer due to alcohol use | 5 kg/m2 | Both | Males | | | | | | | | | 1.289 (1.109 to 1.491) | 1.289 (1.109 to 1.491) | 1.289 (1.109 to 1.491) | 1.289 (1.109 to 1.491) | 1.289 (1.109 to 1.491) | 1.289 (1.109 to 1.491) | 1.289 (1.109 to 1.491) | 1.289 (1.109 to 1.491) | 1.289 (1.109 to 1.491) | 1.289 (1.109 to 1.491) | 1.289 (1.109 to 1.491) | 1.289 (1.109 to 1.491) | 1.289 (1.109 to 1.491) | 1.289 (1.109 to 1.491) | 1.289 (1.109 to 1.491) | 1.289 (1.109 to 1.491) |
| Liver cancer due to alcohol use | 5 kg/m2 | Both | Females | | | | | | | | | 1.176 (1.03 to 1.334) | 1.176 (1.03 to 1.334) | 1.176 (1.03 to 1.334) | 1.176 (1.03 to 1.334) | 1.176 (1.03 to 1.334) | 1.176 (1.03 to 1.334) | 1.176 (1.03 to 1.334) | 1.176 (1.03 to 1.334) | 1.176 (1.03 to 1.334) | 1.176 (1.03 to 1.334) | 1.176 (1.03 to 1.334) | 1.176 (1.03 to 1.334) | 1.176 (1.03 to 1.334) | 1.176 (1.03 to 1.334) | 1.176 (1.03 to 1.334) | 1.176 (1.03 to 1.334) |
| Liver cancer due to other causes | 5 kg/m2 | Both | Males | | | | | | | | | 1.289 (1.109 to 1.491) | 1.289 (1.109 to 1.491) | 1.289 (1.109 to 1.491) | 1.289 (1.109 to 1.491) | 1.289 (1.109 to 1.491) | 1.289 (1.109 to 1.491) | 1.289 (1.109 to 1.491) | 1.289 (1.109 to 1.491) | 1.289 (1.109 to 1.491) | 1.289 (1.109 to 1.491) | 1.289 (1.109 to 1.491) | 1.289 (1.109 to 1.491) | 1.289 (1.109 to 1.491) | 1.289 (1.109 to 1.491) | 1.289 (1.109 to 1.491) | 1.289 (1.109 to 1.491) |
| Liver cancer due to other causes | 5 kg/m2 | Both | Females | | | | | | | | | 1.176 (1.03 to 1.334) | 1.176 (1.03 to 1.334) | 1.176 (1.03 to 1.334) | 1.176 (1.03 to 1.334) | 1.176 (1.03 to 1.334) | 1.176 (1.03 to 1.334) | 1.176 (1.03 to 1.334) | 1.176 (1.03 to 1.334) | 1.176 (1.03 to 1.334) | 1.176 (1.03 to 1.334) | 1.176 (1.03 to 1.334) | 1.176 (1.03 to 1.334) | 1.176 (1.03 to 1.334) | 1.176 (1.03 to 1.334) | 1.176 (1.03 to 1.334) | 1.176 (1.03 to 1.334) |
| Gallbladder and biliary tract cancer | 5 kg/m2 | Both | Males | | | | | | | | | 1.155 (1.033 to 1.281) | 1.155 (1.033 to 1.281) | 1.155 (1.033 to 1.281) | 1.155 (1.033 to 1.281) | 1.155 (1.033 to 1.281) | 1.155 (1.033 to 1.281) | 1.155 (1.033 to 1.281) | 1.155 (1.033 to 1.281) | 1.155 (1.033 to 1.281) | 1.155 (1.033 to 1.281) | 1.155 (1.033 to 1.281) | 1.155 (1.033 to 1.281) | 1.155 (1.033 to 1.281) | 1.155 (1.033 to 1.281) | 1.155 (1.033 to 1.281) | 1.155 (1.033 to 1.281) |
| Gallbladder and biliary tract cancer | 5 kg/m2 | Both | Females | | | | | | | | | 1.344 (1.223 to 1.477) | 1.344 (1.223 to 1.477) | 1.344 (1.223 to 1.477) | 1.344 (1.223 to 1.477) | 1.344 (1.223 to 1.477) | 1.344 (1.223 to 1.477) | 1.344 (1.223 to 1.477) | 1.344 (1.223 to 1.477) | 1.344 (1.223 to 1.477) | 1.344 (1.223 to 1.477) | 1.344 (1.223 to 1.477) | 1.344 (1.223 to 1.477) | 1.344 (1.223 to 1.477) | 1.344 (1.223 to 1.477) | 1.344 (1.223 to 1.477) | 1.344 (1.223 to 1.477) |
| Pancreatic cancer | 5 kg/m2 | Both | Males | | | | | | | | | 1.071 (0.999 to 1.153) | 1.071 (0.999 to 1.153) | 1.071 (0.999 to 1.153) | 1.071 (0.999 to 1.153) | 1.071 (0.999 to 1.153) | 1.071 (0.999 to 1.153) | 1.071 (0.999 to 1.153) | 1.071 (0.999 to 1.153) | 1.071 (0.999 to 1.153) | 1.071 (0.999 to 1.153) | 1.071 (0.999 to 1.153) | 1.071 (0.999 to 1.153) | 1.071 (0.999 to 1.153) | 1.071 (0.999 to 1.153) | 1.071 (0.999 to 1.153) | 1.071 (0.999 to 1.153) |
| Pancreatic cancer | 5 kg/m2 | Both | Females | | | | | | | | | 1.092 (1.037 to 1.144) | 1.092 (1.037 to 1.144) | 1.092 (1.037 to 1.144) | 1.092 (1.037 to 1.144) | 1.092 (1.037 to 1.144) | 1.092 (1.037 to 1.144) | 1.092 (1.037 to 1.144) | 1.092 (1.037 to 1.144) | 1.092 (1.037 to 1.144) | 1.092 (1.037 to 1.144) | 1.092 (1.037 to 1.144) | 1.092 (1.037 to 1.144) | 1.092 (1.037 to 1.144) | 1.092 (1.037 to 1.144) | 1.092 (1.037 to 1.144) | 1.092 (1.037 to 1.144) |

**Appendix Table 6a. Relative risks used by age and sex for each outcome for all risk factors except for ambient air pollution alcohol, and smoking.**

| | | | | Ages | | | | | | | | | | | | | | | | | | | | | | | |
|---|---|---|---|---|---|---|---|---|---|---|---|---|---|---|---|---|---|---|---|---|---|---|---|---|---|---|---|
| Risk - Outcome | Category / Units | Morbidity / Mortality | Sex | All-age | 0-6 days | 7-27 days | 28-364 days | 1-4 years | 5-9 years | 10-14 years | 15-19 years | 20-24 years | 25-29 years | 30-34 years | 35-39 years | 40-44 years | 45-49 years | 50-54 years | 55-59 years | 60-64 years | 65-69 years | 70-74 years | 75-79 years | 80-84 years | 85-89 years | 90-94 years | 95+ years |
| Breast cancer (premenopausal) | 5 kg/m2 | Both | Both | | | | | | | | | 0.89 (0.869 to 0.914) | 0.89 (0.869 to 0.914) | 0.89 (0.869 to 0.914) | 0.89 (0.869 to 0.914) | 0.89 (0.869 to 0.914) | 0.89 (0.869 to 0.914) | | | | | | | | | | |
| Breast cancer (postmenopausal) | 5 kg/m2 | Both | Both | | | | | | | | | | | | | | | 1.089 (1.037 to 1.14) | 1.089 (1.037 to 1.14) | 1.089 (1.037 to 1.14) | 1.089 (1.037 to 1.14) | 1.089 (1.037 to 1.14) | 1.089 (1.037 to 1.14) | 1.089 (1.037 to 1.14) | 1.089 (1.037 to 1.14) | 1.089 (1.037 to 1.14) | 1.089 (1.037 to 1.14) |
| Uterine cancer | 5 kg/m2 | Both | Both | | | | | | | | | 1.613 (1.543 to 1.681) | 1.613 (1.543 to 1.681) | 1.613 (1.543 to 1.681) | 1.613 (1.543 to 1.681) | 1.613 (1.543 to 1.681) | 1.613 (1.543 to 1.681) | 1.613 (1.543 to 1.681) | 1.613 (1.543 to 1.681) | 1.613 (1.543 to 1.681) | 1.613 (1.543 to 1.681) | 1.613 (1.543 to 1.681) | 1.613 (1.543 to 1.681) | 1.613 (1.543 to 1.681) | 1.613 (1.543 to 1.681) | 1.613 (1.543 to 1.681) | 1.613 (1.543 to 1.681) |
| Ovarian cancer | 5 kg/m2 | Both | Both | | | | | | | | | 1.038 (0.999 to 1.077) | 1.038 (0.999 to 1.077) | 1.038 (0.999 to 1.077) | 1.038 (0.999 to 1.077) | 1.038 (0.999 to 1.077) | 1.038 (0.999 to 1.077) | 1.038 (0.999 to 1.077) | 1.038 (0.999 to 1.077) | 1.038 (0.999 to 1.077) | 1.038 (0.999 to 1.077) | 1.038 (0.999 to 1.077) | 1.038 (0.999 to 1.077) | 1.038 (0.999 to 1.077) | 1.038 (0.999 to 1.077) | 1.038 (0.999 to 1.077) | 1.038 (0.999 to 1.077) |
| Kidney cancer | 5 kg/m2 | Both | Males | | | | | | | | | 1.24 (1.171 to 1.313) | 1.24 (1.171 to 1.313) | 1.24 (1.171 to 1.313) | 1.24 (1.171 to 1.313) | 1.24 (1.171 to 1.313) | 1.24 (1.171 to 1.313) | 1.24 (1.171 to 1.313) | 1.24 (1.171 to 1.313) | 1.24 (1.171 to 1.313) | 1.24 (1.171 to 1.313) | 1.24 (1.171 to 1.313) | 1.24 (1.171 to 1.313) | 1.24 (1.171 to 1.313) | 1.24 (1.171 to 1.313) | 1.24 (1.171 to 1.313) | 1.24 (1.171 to 1.313) |
| Kidney cancer | 5 kg/m2 | Both | Females | | | | | | | | | 1.32 (1.254 to 1.394) | 1.32 (1.254 to 1.394) | 1.32 (1.254 to 1.394) | 1.32 (1.254 to 1.394) | 1.32 (1.254 to 1.394) | 1.32 (1.254 to 1.394) | 1.32 (1.254 to 1.394) | 1.32 (1.254 to 1.394) | 1.32 (1.254 to 1.394) | 1.32 (1.254 to 1.394) | 1.32 (1.254 to 1.394) | 1.32 (1.254 to 1.394) | 1.32 (1.254 to 1.394) | 1.32 (1.254 to 1.394) | 1.32 (1.254 to 1.394) | 1.32 (1.254 to 1.394) |
| Thyroid cancer | 5 kg/m2 | Both | Males | | | | | | | | | 1.221 (1.068 to 1.381) | 1.221 (1.068 to 1.381) | 1.221 (1.068 to 1.381) | 1.221 (1.068 to 1.381) | 1.221 (1.068 to 1.381) | 1.221 (1.068 to 1.381) | 1.221 (1.068 to 1.381) | 1.221 (1.068 to 1.381) | 1.221 (1.068 to 1.381) | 1.221 (1.068 to 1.381) | 1.221 (1.068 to 1.381) | 1.221 (1.068 to 1.381) | 1.221 (1.068 to 1.381) | 1.221 (1.068 to 1.381) | 1.221 (1.068 to 1.381) | 1.221 (1.068 to 1.381) |
| Thyroid cancer | 5 kg/m2 | Both | Females | | | | | | | | | 1.136 (1.094 to 1.178) | 1.136 (1.094 to 1.178) | 1.136 (1.094 to 1.178) | 1.136 (1.094 to 1.178) | 1.136 (1.094 to 1.178) | 1.136 (1.094 to 1.178) | 1.136 (1.094 to 1.178) | 1.136 (1.094 to 1.178) | 1.136 (1.094 to 1.178) | 1.136 (1.094 to 1.178) | 1.136 (1.094 to 1.178) | 1.136 (1.094 to 1.178) | 1.136 (1.094 to 1.178) | 1.136 (1.094 to 1.178) | 1.136 (1.094 to 1.178) | 1.136 (1.094 to 1.178) |
| Non-Hodgkin's lymphoma | 5 kg/m2 | Both | Males | | | | | | | | | 1.089 (1.038 to 1.143) | 1.089 (1.038 to 1.143) | 1.089 (1.038 to 1.143) | 1.089 (1.038 to 1.143) | 1.089 (1.038 to 1.143) | 1.089 (1.038 to 1.143) | 1.089 (1.038 to 1.143) | 1.089 (1.038 to 1.143) | 1.089 (1.038 to 1.143) | 1.089 (1.038 to 1.143) | 1.089 (1.038 to 1.143) | 1.089 (1.038 to 1.143) | 1.089 (1.038 to 1.143) | 1.089 (1.038 to 1.143) | 1.089 (1.038 to 1.143) | 1.089 (1.038 to 1.143) |
| Non-Hodgkin's lymphoma | 5 kg/m2 | Both | Females | | | | | | | | | 1.068 (1.01 to 1.125) | 1.068 (1.01 to 1.125) | 1.068 (1.01 to 1.125) | 1.068 (1.01 to 1.125) | 1.068 (1.01 to 1.125) | 1.068 (1.01 to 1.125) | 1.068 (1.01 to 1.125) | 1.068 (1.01 to 1.125) | 1.068 (1.01 to 1.125) | 1.068 (1.01 to 1.125) | 1.068 (1.01 to 1.125) | 1.068 (1.01 to 1.125) | 1.068 (1.01 to 1.125) | 1.068 (1.01 to 1.125) | 1.068 (1.01 to 1.125) | 1.068 (1.01 to 1.125) |
| Multiple myeloma | 5 kg/m2 | Both | Males | | | | | | | | | 1.089 (1.027 to 1.153) | 1.089 (1.027 to 1.153) | 1.089 (1.027 to 1.153) | 1.089 (1.027 to 1.153) | 1.089 (1.027 to 1.153) | 1.089 (1.027 to 1.153) | 1.089 (1.027 to 1.153) | 1.089 (1.027 to 1.153) | 1.089 (1.027 to 1.153) | 1.089 (1.027 to 1.153) | 1.089 (1.027 to 1.153) | 1.089 (1.027 to 1.153) | 1.089 (1.027 to 1.153) | 1.089 (1.027 to 1.153) | 1.089 (1.027 to 1.153) | 1.089 (1.027 to 1.153) |
| Multiple myeloma | 5 kg/m2 | Both | Females | | | | | | | | | 1.092 (1.034 to 1.157) | 1.092 (1.034 to 1.157) | 1.092 (1.034 to 1.157) | 1.092 (1.034 to 1.157) | 1.092 (1.034 to 1.157) | 1.092 (1.034 to 1.157) | 1.092 (1.034 to 1.157) | 1.092 (1.034 to 1.157) | 1.092 (1.034 to 1.157) | 1.092 (1.034 to 1.157) | 1.092 (1.034 to 1.157) | 1.092 (1.034 to 1.157) | 1.092 (1.034 to 1.157) | 1.092 (1.034 to 1.157) | 1.092 (1.034 to 1.157) | 1.092 (1.034 to 1.157) |
| Acute lymphoid leukaemia | 5 kg/m2 | Both | Males | | | | | | | | | 1.086 (1.053 to 1.119) | 1.086 (1.053 to 1.119) | 1.086 (1.053 to 1.119) | 1.086 (1.053 to 1.119) | 1.086 (1.053 to 1.119) | 1.086 (1.053 to 1.119) | 1.086 (1.053 to 1.119) | 1.086 (1.053 to 1.119) | 1.086 (1.053 to 1.119) | 1.086 (1.053 to 1.119) | 1.086 (1.053 to 1.119) | 1.086 (1.053 to 1.119) | 1.086 (1.053 to 1.119) | 1.086 (1.053 to 1.119) | 1.086 (1.053 to 1.119) | 1.086 (1.053 to 1.119) |
| Acute lymphoid leukaemia | 5 kg/m2 | Both | Females | | | | | | | | | 1.131 (1.061 to 1.208) | 1.131 (1.061 to 1.208) | 1.131 (1.061 to 1.208) | 1.131 (1.061 to 1.208) | 1.131 (1.061 to 1.208) | 1.131 (1.061 to 1.208) | 1.131 (1.061 to 1.208) | 1.131 (1.061 to 1.208) | 1.131 (1.061 to 1.208) | 1.131 (1.061 to 1.208) | 1.131 (1.061 to 1.208) | 1.131 (1.061 to 1.208) | 1.131 (1.061 to 1.208) | 1.131 (1.061 to 1.208) | 1.131 (1.061 to 1.208) | 1.131 (1.061 to 1.208) |
| Chronic lymphoid leukaemia | 5 kg/m2 | Both | Males | | | | | | | | | 1.086 (1.053 to 1.119) | 1.086 (1.053 to 1.119) | 1.086 (1.053 to 1.119) | 1.086 (1.053 to 1.119) | 1.086 (1.053 to 1.119) | 1.086 (1.053 to 1.119) | 1.086 (1.053 to 1.119) | 1.086 (1.053 to 1.119) | 1.086 (1.053 to 1.119) | 1.086 (1.053 to 1.119) | 1.086 (1.053 to 1.119) | 1.086 (1.053 to 1.119) | 1.086 (1.053 to 1.119) | 1.086 (1.053 to 1.119) | 1.086 (1.053 to 1.119) | 1.086 (1.053 to 1.119) |
| Chronic lymphoid leukaemia | 5 kg/m2 | Both | Females | | | | | | | | | 1.131 (1.061 to 1.208) | 1.131 (1.061 to 1.208) | 1.131 (1.061 to 1.208) | 1.131 (1.061 to 1.208) | 1.131 (1.061 to 1.208) | 1.131 (1.061 to 1.208) | 1.131 (1.061 to 1.208) | 1.131 (1.061 to 1.208) | 1.131 (1.061 to 1.208) | 1.131 (1.061 to 1.208) | 1.131 (1.061 to 1.208) | 1.131 (1.061 to 1.208) | 1.131 (1.061 to 1.208) | 1.131 (1.061 to 1.208) | 1.131 (1.061 to 1.208) | 1.131 (1.061 to 1.208) |
| Acute myeloid leukaemia | 5 kg/m2 | Both | Males | | | | | | | | | 1.086 (1.053 to 1.119) | 1.086 (1.053 to 1.119) | 1.086 (1.053 to 1.119) | 1.086 (1.053 to 1.119) | 1.086 (1.053 to 1.119) | 1.086 (1.053 to 1.119) | 1.086 (1.053 to 1.119) | 1.086 (1.053 to 1.119) | 1.086 (1.053 to 1.119) | 1.086 (1.053 to 1.119) | 1.086 (1.053 to 1.119) | 1.086 (1.053 to 1.119) | 1.086 (1.053 to 1.119) | 1.086 (1.053 to 1.119) | 1.086 (1.053 to 1.119) | 1.086 (1.053 to 1.119) |
| Acute myeloid leukaemia | 5 kg/m2 | Both | Females | | | | | | | | | 1.131 (1.061 to 1.208) | 1.131 (1.061 to 1.208) | 1.131 (1.061 to 1.208) | 1.131 (1.061 to 1.208) | 1.131 (1.061 to 1.208) | 1.131 (1.061 to 1.208) | 1.131 (1.061 to 1.208) | 1.131 (1.061 to 1.208) | 1.131 (1.061 to 1.208) | 1.131 (1.061 to 1.208) | 1.131 (1.061 to 1.208) | 1.131 (1.061 to 1.208) | 1.131 (1.061 to 1.208) | 1.131 (1.061 to 1.208) | 1.131 (1.061 to 1.208) | 1.131 (1.061 to 1.208) |
| Chronic myeloid leukaemia | 5 kg/m2 | Both | Males | | | | | | | | | 1.086 (1.053 to 1.119) | 1.086 (1.053 to 1.119) | 1.086 (1.053 to 1.119) | 1.086 (1.053 to 1.119) | 1.086 (1.053 to 1.119) | 1.086 (1.053 to 1.119) | 1.086 (1.053 to 1.119) | 1.086 (1.053 to 1.119) | 1.086 (1.053 to 1.119) | 1.086 (1.053 to 1.119) | 1.086 (1.053 to 1.119) | 1.086 (1.053 to 1.119) | 1.086 (1.053 to 1.119) | 1.086 (1.053 to 1.119) | 1.086 (1.053 to 1.119) | 1.086 (1.053 to 1.119) |
| Chronic myeloid leukaemia | 5 kg/m2 | Both | Females | | | | | | | | | 1.131 (1.061 to 1.208) | 1.131 (1.061 to 1.208) | 1.131 (1.061 to 1.208) | 1.131 (1.061 to 1.208) | 1.131 (1.061 to 1.208) | 1.131 (1.061 to 1.208) | 1.131 (1.061 to 1.208) | 1.131 (1.061 to 1.208) | 1.131 (1.061 to 1.208) | 1.131 (1.061 to 1.208) | 1.131 (1.061 to 1.208) | 1.131 (1.061 to 1.208) | 1.131 (1.061 to 1.208) | 1.131 (1.061 to 1.208) | 1.131 (1.061 to 1.208) | 1.131 (1.061 to 1.208) |
| Other leukaemia | 5 kg/m2 | Both | Males | | | | | | | | | 1.086 (1.053 to 1.119) | 1.086 (1.053 to 1.119) | 1.086 (1.053 to 1.119) | 1.086 (1.053 to 1.119) | 1.086 (1.053 to 1.119) | 1.086 (1.053 to 1.119) | 1.086 (1.053 to 1.119) | 1.086 (1.053 to 1.119) | 1.086 (1.053 to 1.119) | 1.086 (1.053 to 1.119) | 1.086 (1.053 to 1.119) | 1.086 (1.053 to 1.119) | 1.086 (1.053 to 1.119) | 1.086 (1.053 to 1.119) | 1.086 (1.053 to 1.119) | 1.086 (1.053 to 1.119) |
| Other leukaemia | 5 kg/m2 | Both | Females | | | | | | | | | 1.131 (1.061 to 1.208) | 1.131 (1.061 to 1.208) | 1.131 (1.061 to 1.208) | 1.131 (1.061 to 1.208) | 1.131 (1.061 to 1.208) | 1.131 (1.061 to 1.208) | 1.131 (1.061 to 1.208) | 1.131 (1.061 to 1.208) | 1.131 (1.061 to 1.208) | 1.131 (1.061 to 1.208) | 1.131 (1.061 to 1.208) | 1.131 (1.061 to 1.208) | 1.131 (1.061 to 1.208) | 1.131 (1.061 to 1.208) | 1.131 (1.061 to 1.208) | 1.131 (1.061 to 1.208) |
| Ischaemic heart disease | 5 kg/m2 | Both | Both | | | | | | | | | 2.274 (1.259 to 3.683) | 2.274 (1.259 to 3.683) | 2.018 (1.3 to 3.099) | 1.724 (1.533 to 1.93) | 1.599 (1.418 to 1.784) | 1.567 (1.458 to 1.68) | 1.52 (1.417 to 1.631) | 1.466 (1.372 to 1.557) | 1.414 (1.325 to 1.504) | 1.364 (1.287 to 1.448) | 1.319 (1.242 to 1.4) | 1.274 (1.187 to 1.365) | 1.17 (1.091 to 1.252) | 1.17 (1.091 to 1.252) | 1.17 (1.091 to 1.252) | 1.17 (1.091 to 1.252) |
| Ischaemic stroke | 5 kg/m2 | Both | Both | | | | | | | | | 2.472 (1.4 to 3.975) | 2.472 (1.4 to 3.975) | 2.235 (1.457 to 3.329) | 1.979 (1.699 to 2.313) | 1.826 (1.6 to 2.075) | 1.733 (1.581 to 1.898) | 1.635 (1.479 to 1.795) | 1.543 (1.441 to 1.653) | 1.455 (1.345 to 1.566) | 1.38 (1.31 to 1.458) | 1.304 (1.234 to 1.376) | 1.228 (1.16 to 1.304) | 1.068 (0.992 to 1.143) | 1.068 (0.992 to 1.143) | 1.068 (0.992 to 1.143) | 1.068 (0.992 to 1.143) |
| Intracerebral hemorrhage | 5 kg/m2 | Both | Both | | | | | | | | | 3.066 (1.751 to 5.334) | 3.066 (1.751 to 5.334) | 2.913 (1.862 to 4.395) | 2.597 (1.974 to 3.375) | 2.389 (1.87 to 2.998) | 2.199 (1.822 to 2.672) | 1.996 (1.626 to 2.416) | 1.805 (1.574 to 2.06) | 1.665 (1.438 to 1.932) | 1.523 (1.377 to 1.683) | 1.41 (1.266 to 1.57) | 1.295 (1.162 to 1.438) | 1.07 (0.928 to 1.219) | 1.07 (0.928 to 1.219) | 1.07 (0.928 to 1.219) | 1.07 (0.928 to 1.219) |
| Subarachnoid hemorrhage | 5 kg/m2 | Both | Both | | | | | | | | | 3.066 (1.751 to 5.334) | 3.066 (1.751 to 5.334) | 2.913 (1.862 to 4.395) | 2.597 (1.974 to 3.375) | 2.389 (1.87 to 2.998) | 2.199 (1.822 to 2.672) | 1.996 (1.626 to 2.416) | 1.805 (1.574 to 2.06) | 1.665 (1.438 to 1.932) | 1.523 (1.377 to 1.683) | 1.41 (1.266 to 1.57) | 1.295 (1.162 to 1.438) | 1.07 (0.928 to 1.219) | 1.07 (0.928 to 1.219) | 1.07 (0.928 to 1.219) | 1.07 (0.928 to 1.219) |
| Hypertensive heart disease | 5 kg/m2 | Both | Both | | | | | | | | | 3.122 (1.588 to 5.498) | 3.122 (1.588 to 5.498) | 3.0 (1.783 to 4.902) | 2.769 (1.816 to 4.211) | 2.573 (1.743 to 3.636) | 2.407 (1.717 to 3.293) | 2.281 (1.602 to 3.188) | 2.159 (1.505 to 3.036) | 2.035 (1.452 to 2.822) | 1.955 (1.343 to 2.698) | 1.86 (1.296 to 2.617) | 1.792 (1.17 to 2.553) | 1.697 (1.069 to 2.618) | 1.697 (1.069 to 2.618) | 1.697 (1.069 to 2.618) | 1.697 (1.069 to 2.618) |
| Atrial fibrillation and flutter | 5 kg/m2 | Both | Males | | | | | | | | | 1.344 (1.231 to 1.473) | 1.344 (1.231 to 1.473) | 1.344 (1.231 to 1.473) | 1.344 (1.231 to 1.473) | 1.344 (1.231 to 1.473) | 1.344 (1.231 to 1.473) | 1.344 (1.231 to 1.473) | 1.344 (1.231 to 1.473) | 1.344 (1.231 to 1.473) | 1.344 (1.231 to 1.473) | 1.344 (1.231 to 1.473) | 1.344 (1.231 to 1.473) | 1.344 (1.231 to 1.473) | 1.344 (1.231 to 1.473) | 1.344 (1.231 to 1.473) | 1.344 (1.231 to 1.473) |
| Atrial fibrillation and flutter | 5 kg/m2 | Both | Females | | | | | | | | | 1.346 (1.22 to 1.475) | 1.346 (1.22 to 1.475) | 1.346 (1.22 to 1.475) | 1.346 (1.22 to 1.475) | 1.346 (1.22 to 1.475) | 1.346 (1.22 to 1.475) | 1.346 (1.22 to 1.475) | 1.346 (1.22 to 1.475) | 1.346 (1.22 to 1.475) | 1.346 (1.22 to 1.475) | 1.346 (1.22 to 1.475) | 1.346 (1.22 to 1.475) | 1.346 (1.22 to 1.475) | 1.346 (1.22 to 1.475) | 1.346 (1.22 to 1.475) | 1.346 (1.22 to 1.475) |
| Asthma | 5 kg/m2 | Both | Males | | | | | | | | | 1.409 (1.29 to 1.545) | 1.409 (1.29 to 1.545) | 1.409 (1.29 to 1.545) | 1.409 (1.29 to 1.545) | 1.409 (1.29 to 1.545) | 1.409 (1.29 to 1.545) | 1.409 (1.29 to 1.545) | 1.409 (1.29 to 1.545) | 1.409 (1.29 to 1.545) | 1.409 (1.29 to 1.545) | 1.409 (1.29 to 1.545) | 1.409 (1.29 to 1.545) | 1.409 (1.29 to 1.545) | 1.409 (1.29 to 1.545) | 1.409 (1.29 to 1.545) | 1.409 (1.29 to 1.545) |
| Asthma | 5 kg/m2 | Both | Females | | | | | | | | | 1.402 (1.275 to 1.532) | 1.402 (1.275 to 1.532) | 1.402 (1.275 to 1.532) | 1.402 (1.275 to 1.532) | 1.402 (1.275 to 1.532) | 1.402 (1.275 to 1.532) | 1.402 (1.275 to 1.532) | 1.402 (1.275 to 1.532) | 1.402 (1.275 to 1.532) | 1.402 (1.275 to 1.532) | 1.402 (1.275 to 1.532) | 1.402 (1.275 to 1.532) | 1.402 (1.275 to 1.532) | 1.402 (1.275 to 1.532) | 1.402 (1.275 to 1.532) | 1.402 (1.275 to 1.532) |
| Gallbladder and biliary diseases | 5 kg/m2 | Both | Males | | | | | | | | | 1.464 (1.291 to 1.64) | 1.464 (1.291 to 1.64) | 1.464 (1.291 to 1.64) | 1.464 (1.291 to 1.64) | 1.464 (1.291 to 1.64) | 1.464 (1.291 to 1.64) | 1.464 (1.291 to 1.64) | 1.464 (1.291 to 1.64) | 1.464 (1.291 to 1.64) | 1.464 (1.291 to 1.64) | 1.464 (1.291 to 1.64) | 1.464 (1.291 to 1.64) | 1.464 (1.291 to 1.64) | 1.464 (1.291 to 1.64) | 1.464 (1.291 to 1.64) | 1.464 (1.291 to 1.64) |
| Gallbladder and biliary diseases | 5 kg/m2 | Both | Females | | | | | | | | | 1.729 (1.571 to 1.893) | 1.729 (1.571 to 1.893) | 1.729 (1.571 to 1.893) | 1.729 (1.571 to 1.893) | 1.729 (1.571 to 1.893) | 1.729 (1.571 to 1.893) | 1.729 (1.571 to 1.893) | 1.729 (1.571 to 1.893) | 1.729 (1.571 to 1.893) | 1.729 (1.571 to 1.893) | 1.729 (1.571 to 1.893) | 1.729 (1.571 to 1.893) | 1.729 (1.571 to 1.893) | 1.729 (1.571 to 1.893) | 1.729 (1.571 to 1.893) | 1.729 (1.571 to 1.893) |
| Alzheimer's disease and other dementias | 5 kg/m2 | Both | Males | | | | | | | | | 1.218 (1.054 to 1.409) | 1.218 (1.054 to 1.409) | 1.218 (1.054 to 1.409) | 1.218 (1.054 to 1.409) | 1.218 (1.054 to 1.409) | 1.218 (1.054 to 1.409) | 1.218 (1.054 to 1.409) | 1.218 (1.054 to 1.409) | 1.218 (1.054 to 1.409) | 1.218 (1.054 to 1.409) | 1.218 (1.054 to 1.409) | 1.218 (1.054 to 1.409) | 1.218 (1.054 to 1.409) | 1.218 (1.054 to 1.409) | 1.218 (1.054 to 1.409) | 1.218 (1.054 to 1.409) |
| Alzheimer's disease and other dementias | 5 kg/m2 | Both | Females | | | | | | | | | 1.214 (1.047 to 1.404) | 1.214 (1.047 to 1.404) | 1.214 (1.047 to 1.404) | 1.214 (1.047 to 1.404) | 1.214 (1.047 to 1.404) | 1.214 (1.047 to 1.404) | 1.214 (1.047 to 1.404) | 1.214 (1.047 to 1.404) | 1.214 (1.047 to 1.404) | 1.214 (1.047 to 1.404) | 1.214 (1.047 to 1.404) | 1.214 (1.047 to 1.404) | 1.214 (1.047 to 1.404) | 1.214 (1.047 to 1.404) | 1.214 (1.047 to 1.404) | 1.214 (1.047 to 1.404) |
| Diabetes mellitus type 2 | 5 kg/m2 | Both | Both | | | | | | | | | 3.547 (2.314 to 5.219) | 3.547 (2.314 to 5.219) | 3.455 (2.516 to 4.691) | 3.349 (2.803 to 3.918) | 3.16 (2.697 to 3.699) | 2.864 (2.453 to 3.312) | 2.624 (2.224 to 3.038) | 2.417 (2.089 to 2.779) | 2.215 (1.868 to 2.606) | 2.046 (1.724 to 2.379) | 1.896 (1.597 to 2.228) | 1.74 (1.446 to 2.074) | 1.461 (1.207 to 1.758) | 1.461 (1.207 to 1.758) | 1.461 (1.207 to 1.758) | 1.461 (1.207 to 1.758) |
| Chronic kidney disease due to diabetes mellitus type 2 | 5 kg/m2 | Both | Both | | | | | | | | | | | | 1.746 (1.054 to 2.746) | 1.746 (1.054 to 2.746) | 1.746 (1.054 to 2.746) | 1.746 (1.054 to 2.746) | 1.746 (1.054 to 2.746) | 2.036 (1.298 to 3.044) | 2.036 (1.298 to 3.044) | 1.621 (1.063 to 2.378) | 1.621 (1.063 to 2.378) | 1.431 (0.802 to 2.396) | 1.431 (0.802 to 2.396) | 1.431 (0.802 to 2.396) | 1.431 (0.802 to 2.396) |
| Chronic kidney disease due to hypertension | 5 kg/m2 | Both | Both | | | | | | | | | | | | 1.763 (1.09 to 2.755) | 1.763 (1.09 to 2.755) | 1.763 (1.09 to 2.755) | 1.763 (1.09 to 2.755) | 1.763 (1.09 to 2.755) | 2.044 (1.305 to 3.082) | 2.044 (1.305 to 3.082) | 1.605 (1.067 to 2.31) | 1.605 (1.067 to 2.31) | 1.437 (0.829 to 2.415) | 1.437 (0.829 to 2.415) | 1.437 (0.829 to 2.415) | 1.437 (0.829 to 2.415) |
| Chronic kidney disease due to glomerulonephritis | 5 kg/m2 | Both | Both | | | | | | | | | | | | 1.742 (1.021 to 2.775) | 1.742 (1.021 to 2.775) | 1.742 (1.021 to 2.775) | 1.742 (1.021 to 2.775) | 1.742 (1.021 to 2.775) | 2.044 (1.254 to 3.154) | 2.044 (1.254 to 3.154) | 1.604 (1.109 to 2.254) | 1.604 (1.109 to 2.254) | 1.452 (0.851 to 2.345) | 1.452 (0.851 to 2.345) | 1.452 (0.851 to 2.345) | 1.452 (0.851 to 2.345) |
| Chronic kidney disease due to other and unspecified causes | 5 kg/m2 | Both | Both | | | | | | | | | | | | 1.732 (1.052 to 2.681) | 1.732 (1.052 to 2.681) | 1.732 (1.052 to 2.681) | 1.732 (1.052 to 2.681) | 1.732 (1.052 to 2.681) | 2.032 (1.216 to 3.101) | 2.032 (1.216 to 3.101) | 1.625 (1.068 to 2.365) | 1.625 (1.068 to 2.365) | 1.433 (0.778 to 2.344) | 1.433 (0.778 to 2.344) | 1.433 (0.778 to 2.344) | 1.433 (0.778 to 2.344) |
| Cataract | 5 kg/m2 | Both | Males | | | | | | | | | 1.104 (1.052 to 1.157) | 1.104 (1.052 to 1.157) | 1.104 (1.052 to 1.157) | 1.104 (1.052 to 1.157) | 1.104 (1.052 to 1.157) | 1.104 (1.052 to 1.157) | 1.104 (1.052 to 1.157) | 1.104 (1.052 to 1.157) | 1.104 (1.052 to 1.157) | 1.104 (1.052 to 1.157) | 1.104 (1.052 to 1.157) | 1.104 (1.052 to 1.157) | 1.104 (1.052 to 1.157) | 1.104 (1.052 to 1.157) | 1.104 (1.052 to 1.157) | 1.104 (1.052 to 1.157) |
| Cataract | 5 kg/m2 | Both | Females | | | | | | | | | 1.104 (1.051 to 1.156) | 1.104 (1.051 to 1.156) | 1.104 (1.051 to 1.156) | 1.104 (1.051 to 1.156) | 1.104 (1.051 to 1.156) | 1.104 (1.051 to 1.156) | 1.104 (1.051 to 1.156) | 1.104 (1.051 to 1.156) | 1.104 (1.051 to 1.156) | 1.104 (1.051 to 1.156) | 1.104 (1.051 to 1.156) | 1.104 (1.051 to 1.156) | 1.104 (1.051 to 1.156) | 1.104 (1.051 to 1.156) | 1.104 (1.051 to 1.156) | 1.104 (1.051 to 1.156) |
| Low back pain | 5 kg/m2 | Morbidity | Both | | | | | | | | | 1.1 (1.073 to 1.126) | 1.1 (1.073 to 1.126) | 1.1 (1.073 to 1.127) | 1.101 (1.076 to 1.128) | 1.1 (1.074 to 1.126) | 1.099 (1.075 to 1.123) | 1.1 (1.075 to 1.128) | 1.1 (1.075 to 1.126) | 1.101 (1.077 to 1.126) | 1.1 (1.075 to 1.126) | 1.1 (1.076 to 1.124) | 1.1 (1.075 to 1.124) | 1.1 (1.074 to 1.125) | 1.1 (1.074 to 1.125) | 1.1 (1.074 to 1.125) | 1.1 (1.074 to 1.125) |
| Gout | 5 kg/m2 | Both | Males | | | | | | | | | 1.628 (1.34 to 1.964) | 1.628 (1.34 to 1.964) | 1.628 (1.34 to 1.964) | 1.628 (1.34 to 1.964) | 1.628 (1.34 to 1.964) | 1.628 (1.34 to 1.964) | 1.628 (1.34 to 1.964) | 1.628 (1.34 to 1.964) | 1.628 (1.34 to 1.964) | 1.628 (1.34 to 1.964) | 1.628 (1.34 to 1.964) | 1.628 (1.34 to 1.964) | 1.628 (1.34 to 1.964) | 1.628 (1.34 to 1.964) | 1.628 (1.34 to 1.964) | 1.628 (1.34 to 1.964) |
| Gout | 5 kg/m2 | Both | Females | | | | | | | | | 1.493 (1.322 to 1.677) | 1.493 (1.322 to 1.677) | 1.493 (1.322 to 1.677) | 1.493 (1.322 to 1.677) | 1.493 (1.322 to 1.677) | 1.493 (1.322 to 1.677) | 1.493 (1.322 to 1.677) | 1.493 (1.322 to 1.677) | 1.493 (1.322 to 1.677) | 1.493 (1.322 to 1.677) | 1.493 (1.322 to 1.677) | 1.493 (1.322 to 1.677) | 1.493 (1.322 to 1.677) | 1.493 (1.322 to 1.677) | 1.493 (1.322 to 1.677) | 1.493 (1.322 to 1.677) |
| Osteoarthritis of the hip | 5 kg/m2 | Morbidity | Males | | | | | | | | | 1.11 (1.06 to 1.157) | 1.11 (1.06 to 1.157) | 1.11 (1.06 to 1.157) | 1.11 (1.06 to 1.157) | 1.11 (1.06 to 1.157) | 1.11 (1.06 to 1.157) | 1.11 (1.06 to 1.157) | 1.11 (1.06 to 1.157) | 1.11 (1.06 to 1.157) | 1.11 (1.06 to 1.157) | 1.11 (1.06 to 1.157) | 1.11 (1.06 to 1.157) | 1.11 (1.06 to 1.157) | 1.11 (1.06 to 1.157) | 1.11 (1.06 to 1.157) | 1.11 (1.06 to 1.157) |
| Osteoarthritis of the knee | 5 kg/m2 | Morbidity | Males | | | | | | | | | 1.37 (1.201 to 1.538) | 1.37 (1.201 to 1.538) | 1.37 (1.201 to 1.538) | 1.37 (1.201 to 1.538) | 1.37 (1.201 to 1.538) | 1.37 (1.201 to 1.538) | 1.37 (1.201 to 1.538) | 1.37 (1.201 to 1.538) | 1.37 (1.201 to 1.538) | 1.37 (1.201 to 1.538) | 1.37 (1.201 to 1.538) | 1.37 (1.201 to 1.538) | 1.37 (1.201 to 1.538) | 1.37 (1.201 to 1.538) | 1.37 (1.201 to 1.538) | 1.37 (1.201 to 1.538) |
| Osteoarthritis of the hip | 5 kg/m2 | Morbidity | Females | | | | | | | | | 1.112 (1.062 to 1.16) | 1.112 (1.062 to 1.16) | 1.112 (1.062 to 1.16) | 1.112 (1.062 to 1.16) | 1.112 (1.062 to 1.16) | 1.112 (1.062 to 1.16) | 1.112 (1.062 to 1.16) | 1.112 (1.062 to 1.16) | 1.112 (1.062 to 1.16) | 1.112 (1.062 to 1.16) | 1.112 (1.062 to 1.16) | 1.112 (1.062 to 1.16) | 1.112 (1.062 to 1.16) | 1.112 (1.062 to 1.16) | 1.112 (1.062 to 1.16) | 1.112 (1.062 to 1.16) |
| Osteoarthritis of the knee | 5 kg/m2 | Morbidity | Females | | | | | | | | | 1.375 (1.188 to 1.559) | 1.375 (1.188 to 1.559) | 1.375 (1.188 to 1.559) | 1.375 (1.188 to 1.559) | 1.375 (1.188 to 1.559) | 1.375 (1.188 to 1.559) | 1.375 (1.188 to 1.559) | 1.375 (1.188 to 1.559) | 1.375 (1.188 to 1.559) | 1.375 (1.188 to 1.559) | 1.375 (1.188 to 1.559) | 1.375 (1.188 to 1.559) | 1.375 (1.188 to 1.559) | 1.375 (1.188 to 1.559) | 1.375 (1.188 to 1.559) | 1.375 (1.188 to 1.559) |
| **High body-mass index in children** | | | | | | | | | | | | | | | | | | | | | | | | | | | |
| Asthma | Obese | Both | Males | | | | | 1.315 (1.098 to 1.515) | 1.315 (1.095 to 1.513) | 1.316 (1.096 to 1.522) | 1.321 (1.103 to 1.531) | | | | | | | | | | | | | | | | |
| Asthma | Obese | Both | Females | | | | | 1.313 (1.098 to 1.522) | 1.316 (1.102 to 1.524) | 1.317 (1.091 to 1.522) | 1.316 (1.102 to 1.516) | | | | | | | | | | | | | | | | |
| Asthma | Overweight | Both | Males | | | | | 1.312 (1.1 to 1.512) | 1.316 (1.105 to 1.508) | 1.316 (1.094 to 1.525) | 1.319 (1.11 to 1.526) | | | | | | | | | | | | | | | | |
| Asthma | Overweight | Both | Females | | | | | 1.315 (1.096 to 1.518) | 1.318 (1.1 to 1.519) | 1.316 (1.106 to 1.519) | 1.316 (1.091 to 1.521) | | | | | | | | | | | | | | | | |
| **Low bone mineral density** | | | | | | | | | | | | | | | | | | | | | | | | | | | |
| Non-hip fractures | 0.1 g/cm2 | Both | Males | | | | | | | | | | | | | 1.077 (1.074 to 1.08) | 1.114 (1.112 to 1.115) | 1.151 (1.057 to 1.258) | 1.182 (1.1 to 1.264) | 1.214 (1.148 to 1.284) | 1.247 (1.186 to 1.309) | 1.297 (1.241 to 1.353) | 1.339 (1.278 to 1.399) | 1.37 (1.297 to 1.448) | 1.37 (1.297 to 1.448) | 1.37 (1.297 to 1.448) | 1.37 (1.297 to 1.448) |
| Hip fractures | 0.1 g/cm2 | Both | Males | | | | | | | | | | | | | 2.945 (2.121 to 3.906) | 2.85 (2.133 to 3.819) | 2.614 (2.019 to 3.326) | 2.438 (2.002 to 2.966) | 2.286 (1.964 to 2.662) | 2.184 (1.912 to 2.473) | 2.102 (1.888 to 2.322) | 1.921 (1.786 to 2.084) | 1.732 (1.631 to 1.84) | 1.732 (1.631 to 1.84) | 1.732 (1.631 to 1.84) | 1.732 (1.631 to 1.84) |

**Appendix Table 6a. Relative risks used by age and sex for each outcome for all risk factors except for ambient air pollution alcohol, and smoking.**

| Risk - Outcome | Category / Units | Morbidity / Mortality | Sex | Ages | | | | | | | | | | | | | | | | | | | | | | | |
|---|---|---|---|---|---|---|---|---|---|---|---|---|---|---|---|---|---|---|---|---|---|---|---|---|---|---|---|
| | | | | All-age | 0-6 days | 7-27 days | 28-364 days | 1-4 years | 5-9 years | 10-14 years | 15-19 years | 20-24 years | 25-29 years | 30-34 years | 35-39 years | 40-44 years | 45-49 years | 50-54 years | 55-59 years | 60-64 years | 65-69 years | 70-74 years | 75-79 years | 80-84 years | 85-89 years | 90-94 years | 95+ years |
| Non-hip fractures | 0.1 g/cm2 | Both | Females | | | | | | | | | | | | | 1.083 (1.08 to 1.087) | 1.118 (1.116 to 1.12) | 1.163 (1.065 to 1.273) | 1.203 (1.119 to 1.294) | 1.239 (1.161 to 1.317) | 1.287 (1.216 to 1.361) | 1.343 (1.273 to 1.418) | 1.401 (1.33 to 1.479) | 1.437 (1.352 to 1.524) | 1.437 (1.352 to 1.524) | 1.437 (1.352 to 1.524) | 1.437 (1.352 to 1.524) |
| Hip fractures | 0.1 g/cm2 | Both | Females | | | | | | | | | | | | | 3.255 (2.261 to 4.486) | 2.94 (2.146 to 3.899) | 2.713 (2.074 to 3.427) | 2.642 (2.094 to 3.271) | 2.474 (2.061 to 2.95) | 2.412 (2.058 to 2.768) | 2.32 (2.077 to 2.571) | 2.118 (1.938 to 2.299) | 1.876 (1.748 to 2.001) | 1.876 (1.748 to 2.001) | 1.876 (1.748 to 2.001) | 1.876 (1.748 to 2.001) |
| **Impaired kidney function** | | | | | | | | | | | | | | | | | | | | | | | | | | | |
| Ischaemic heart disease | Stage 5 CKD | Both | Both | | | | | | | | | | 6.403 (1.632 to 17.373) | 6.579 (1.642 to 17.922) | 6.222 (1.812 to 16.832) | 6.454 (1.486 to 18.425) | 6.576 (1.712 to 19.892) | 6.638 (1.619 to 18.398) | 6.447 (1.575 to 17.801) | 3.682 (2.162 to 6.067) | 3.674 (2.083 to 6.132) | 3.058 (1.883 to 4.917) | 3.066 (1.895 to 4.796) | 2.611 (1.17 to 5.088) | 2.546 (1.085 to 4.94) | 2.593 (1.177 to 5.083) | 2.545 (1.026 to 4.924) |
| Ischaemic heart disease | Stage 4 CKD | Both | Both | | | | | | | | | | 4.187 (1.636 to 8.618) | 4.138 (1.626 to 9.02) | 4.158 (1.576 to 9.128) | 4.096 (1.662 to 8.682) | 4.106 (1.554 to 8.24) | 4.15 (1.564 to 9.113) | 4.15 (1.579 to 9.015) | 2.596 (1.79 to 3.639) | 2.54 (1.751 to 3.555) | 2.263 (1.621 to 3.112) | 2.272 (1.624 to 3.038) | 2.01 (1.167 to 3.381) | 2.01 (1.18 to 3.267) | 2.041 (1.192 to 3.214) | 2.029 (1.172 to 3.309) |
| Ischaemic heart disease | Stage 3 CKD | Both | Both | | | | | | | | | | 1.508 (1.187 to 1.905) | 1.51 (1.183 to 1.902) | 1.509 (1.195 to 1.905) | 1.509 (1.195 to 1.893) | 1.519 (1.201 to 1.94) | 1.506 (1.169 to 1.856) | 1.507 (1.182 to 1.887) | 1.406 (1.233 to 1.597) | 1.403 (1.227 to 1.615) | 1.375 (1.205 to 1.564) | 1.381 (1.23 to 1.544) | 1.316 (1.12 to 1.534) | 1.321 (1.146 to 1.532) | 1.312 (1.129 to 1.525) | 1.312 (1.125 to 1.522) |
| Ischaemic heart disease | Albuminira | Both | Both | | | | | | | | | | 1.627 (1.249 to 2.107) | 1.64 (1.252 to 2.097) | 1.63 (1.216 to 2.103) | 1.638 (1.26 to 2.081) | 1.636 (1.255 to 2.08) | 1.632 (1.246 to 2.074) | 1.628 (1.253 to 2.071) | 1.172 (1.072 to 1.272) | 1.173 (1.067 to 1.287) | 1.041 (0.918 to 1.178) | 1.041 (0.914 to 1.173) | 0.923 (0.749 to 1.107) | 0.927 (0.762 to 1.115) | 0.923 (0.754 to 1.125) | 0.921 (0.75 to 1.122) |
| Ischaemic heart disease | None | Both | Both | | | | | | | | | | 1.0 (1.0 to 1.0) | 1.0 (1.0 to 1.0) | 1.0 (1.0 to 1.0) | 1.0 (1.0 to 1.0) | 1.0 (1.0 to 1.0) | 1.0 (1.0 to 1.0) | 1.0 (1.0 to 1.0) | 1.0 (1.0 to 1.0) | 1.0 (1.0 to 1.0) | 1.0 (1.0 to 1.0) | 1.0 (1.0 to 1.0) | 1.0 (1.0 to 1.0) | 1.0 (1.0 to 1.0) | 1.0 (1.0 to 1.0) | 1.0 (1.0 to 1.0) |
| Ischaemic stroke | Stage 5 CKD | Both | Both | | | | | | | | | | 6.665 (0.633 to 28.506) | 6.631 (0.586 to 26.898) | 6.571 (0.671 to 25.958) | 6.66 (0.529 to 28.335) | 6.568 (0.674 to 27.459) | 7.071 (0.699 to 27.677) | 6.859 (0.656 to 28.559) | 3.305 (1.133 to 7.402) | 3.34 (1.183 to 7.005) | 2.175 (1.085 to 4.053) | 2.154 (1.054 to 4.058) | 1.608 (0.478 to 3.837) | 1.567 (0.488 to 3.819) | 1.539 (0.499 to 3.799) | 1.544 (0.454 to 3.893) |
| Ischaemic stroke | Stage 4 CKD | Both | Both | | | | | | | | | | 3.642 (0.769 to 10.195) | 3.665 (0.716 to 11.442) | 3.493 (0.721 to 10.629) | 3.634 (0.744 to 10.727) | 3.518 (0.743 to 9.943) | 3.579 (0.832 to 10.234) | 3.576 (0.774 to 10.582) | 2.291 (1.209 to 3.935) | 2.287 (1.13 to 4.048) | 1.708 (1.023 to 2.625) | 1.67 (1.026 to 2.52) | 1.298 (0.628 to 2.387) | 1.331 (0.65 to 2.537) | 1.323 (0.61 to 2.458) | 1.301 (0.624 to 2.507) |
| Ischaemic stroke | Stage 3 CKD | Both | Both | | | | | | | | | | 1.063 (0.699 to 1.513) | 1.073 (0.733 to 1.534) | 1.071 (0.73 to 1.524) | 1.072 (0.717 to 1.565) | 1.081 (0.721 to 1.571) | 1.077 (0.725 to 1.55) | 1.077 (0.724 to 1.542) | 1.324 (1.072 to 1.619) | 1.323 (1.069 to 1.629) | 1.255 (1.064 to 1.462) | 1.254 (1.08 to 1.46) | 1.155 (0.925 to 1.405) | 1.148 (0.936 to 1.393) | 1.147 (0.931 to 1.385) | 1.156 (0.932 to 1.41) |
| Ischaemic stroke | Albuminira | Both | Both | | | | | | | | | | 2.09 (1.406 to 3.11) | 2.077 (1.425 to 2.946) | 2.074 (1.405 to 2.996) | 2.093 (1.413 to 3.032) | 2.092 (1.365 to 3.032) | 2.085 (1.391 to 2.983) | 2.079 (1.4 to 2.988) | 1.478 (1.289 to 1.683) | 1.487 (1.319 to 1.692) | 1.272 (1.093 to 1.49) | 1.27 (1.076 to 1.497) | 1.11 (0.854 to 1.433) | 1.102 (0.834 to 1.419) | 1.107 (0.835 to 1.446) | 1.109 (0.822 to 1.429) |
| Ischaemic stroke | None | Both | Both | | | | | | | | | | 1.0 (1.0 to 1.0) | 1.0 (1.0 to 1.0) | 1.0 (1.0 to 1.0) | 1.0 (1.0 to 1.0) | 1.0 (1.0 to 1.0) | 1.0 (1.0 to 1.0) | 1.0 (1.0 to 1.0) | 1.0 (1.0 to 1.0) | 1.0 (1.0 to 1.0) | 1.0 (1.0 to 1.0) | 1.0 (1.0 to 1.0) | 1.0 (1.0 to 1.0) | 1.0 (1.0 to 1.0) | 1.0 (1.0 to 1.0) | 1.0 (1.0 to 1.0) |
| Intracerebral hemorrhage | Stage 5 CKD | Both | Both | | | | | | | | | | 6.665 (0.633 to 28.506) | 6.631 (0.586 to 26.898) | 6.571 (0.671 to 25.958) | 6.66 (0.529 to 28.335) | 6.568 (0.674 to 27.459) | 7.071 (0.699 to 27.677) | 6.859 (0.656 to 28.559) | 3.305 (1.133 to 7.402) | 3.34 (1.183 to 7.005) | 2.175 (1.085 to 4.053) | 2.154 (1.054 to 4.058) | 1.608 (0.478 to 3.837) | 1.567 (0.488 to 3.819) | 1.539 (0.499 to 3.799) | 1.544 (0.454 to 3.893) |
| Intracerebral hemorrhage | Stage 4 CKD | Both | Both | | | | | | | | | | 3.642 (0.769 to 10.195) | 3.665 (0.716 to 11.442) | 3.493 (0.721 to 10.629) | 3.634 (0.744 to 10.727) | 3.518 (0.743 to 9.943) | 3.579 (0.832 to 10.234) | 3.576 (0.774 to 10.582) | 2.291 (1.209 to 3.935) | 2.287 (1.13 to 4.048) | 1.708 (1.023 to 2.625) | 1.67 (1.026 to 2.52) | 1.298 (0.628 to 2.387) | 1.331 (0.65 to 2.537) | 1.323 (0.61 to 2.458) | 1.301 (0.624 to 2.507) |
| Intracerebral hemorrhage | Stage 3 CKD | Both | Both | | | | | | | | | | 1.063 (0.699 to 1.513) | 1.073 (0.733 to 1.534) | 1.071 (0.73 to 1.524) | 1.072 (0.717 to 1.565) | 1.081 (0.721 to 1.571) | 1.077 (0.725 to 1.55) | 1.077 (0.724 to 1.542) | 1.324 (1.072 to 1.619) | 1.323 (1.069 to 1.629) | 1.255 (1.064 to 1.462) | 1.254 (1.08 to 1.46) | 1.155 (0.925 to 1.405) | 1.148 (0.936 to 1.393) | 1.147 (0.931 to 1.385) | 1.156 (0.932 to 1.41) |
| Intracerebral hemorrhage | Albuminira | Both | Both | | | | | | | | | | 2.09 (1.406 to 3.11) | 2.077 (1.425 to 2.946) | 2.074 (1.405 to 2.996) | 2.093 (1.413 to 3.032) | 2.092 (1.365 to 3.032) | 2.085 (1.391 to 2.983) | 2.079 (1.4 to 2.988) | 1.478 (1.289 to 1.683) | 1.487 (1.319 to 1.692) | 1.272 (1.093 to 1.49) | 1.27 (1.076 to 1.497) | 1.11 (0.854 to 1.433) | 1.102 (0.834 to 1.419) | 1.107 (0.835 to 1.446) | 1.109 (0.822 to 1.429) |
| Intracerebral hemorrhage | None | Both | Both | | | | | | | | | | 1.0 (1.0 to 1.0) | 1.0 (1.0 to 1.0) | 1.0 (1.0 to 1.0) | 1.0 (1.0 to 1.0) | 1.0 (1.0 to 1.0) | 1.0 (1.0 to 1.0) | 1.0 (1.0 to 1.0) | 1.0 (1.0 to 1.0) | 1.0 (1.0 to 1.0) | 1.0 (1.0 to 1.0) | 1.0 (1.0 to 1.0) | 1.0 (1.0 to 1.0) | 1.0 (1.0 to 1.0) | 1.0 (1.0 to 1.0) | 1.0 (1.0 to 1.0) |
| Peripheral vascular disease | Stage 5 CKD | Both | Both | | | | | | | | | | 207.753 (30.856 to 704.06) | 200.617 (34.114 to 695.696) | 211.239 (32.055 to 800.634) | 205.926 (31.084 to 751.759) | 200.856 (33.786 to 732.241) | 207.342 (30.718 to 650.978) | 202.164 (32.222 to 701.663) | 17.647 (7.939 to 32.318) | 17.353 (8.097 to 31.55) | 5.347 (2.334 to 10.628) | 5.427 (2.272 to 10.749) | 1.895 (0.429 to 5.39) | 1.852 (0.444 to 5.291) | 1.856 (0.437 to 5.035) | 1.925 (0.427 to 5.377) |
| Peripheral vascular disease | Stage 4 CKD | Both | Both | | | | | | | | | | 58.362 (15.568 to 149.001) | 54.062 (15.177 to 136.039) | 54.638 (14.697 to 139.549) | 55.201 (16.279 to 139.398) | 54.066 (14.955 to 142.728) | 55.805 (15.506 to 137.121) | 55.87 (15.838 to 139.768) | 7.713 (4.647 to 11.89) | 7.845 (4.686 to 12.511) | 3.141 (1.836 to 5.218) | 3.213 (1.87 to 5.196) | 1.464 (0.558 to 3.037) | 1.47 (0.601 to 3.006) | 1.462 (0.599 to 3.073) | 1.488 (0.593 to 3.038) |
| Peripheral vascular disease | Stage 3 CKD | Both | Both | | | | | | | | | | 3.083 (1.826 to 4.774) | 3.102 (1.839 to 4.864) | 3.045 (1.792 to 4.748) | 3.114 (1.906 to 4.86) | 3.039 (1.817 to 4.807) | 3.117 (1.883 to 4.881) | 3.113 (1.839 to 4.781) | 2.055 (1.57 to 2.665) | 2.05 (1.584 to 2.635) | 1.525 (1.216 to 1.891) | 1.531 (1.221 to 1.92) | 1.142 (0.868 to 1.461) | 1.158 (0.875 to 1.513) | 1.147 (0.871 to 1.476) | 1.158 (0.879 to 1.484) |
| Peripheral vascular disease | Albuminira | Both | Both | | | | | | | | | | 3.542 (1.986 to 5.721) | 3.572 (1.98 to 5.768) | 3.57 (1.977 to 5.944) | 3.569 (1.991 to 5.91) | 3.543 (1.934 to 5.923) | 3.607 (1.98 to 6.203) | 3.552 (1.902 to 5.879) | 1.758 (1.442 to 2.135) | 1.759 (1.451 to 2.101) | 1.336 (1.033 to 1.695) | 1.327 (1.018 to 1.727) | 1.037 (0.664 to 1.542) | 1.029 (0.687 to 1.478) | 1.033 (0.646 to 1.496) | 1.038 (0.68 to 1.548) |
| Peripheral vascular disease | None | Both | Both | | | | | | | | | | 1.0 (1.0 to 1.0) | 1.0 (1.0 to 1.0) | 1.0 (1.0 to 1.0) | 1.0 (1.0 to 1.0) | 1.0 (1.0 to 1.0) | 1.0 (1.0 to 1.0) | 1.0 (1.0 to 1.0) | 1.0 (1.0 to 1.0) | 1.0 (1.0 to 1.0) | 1.0 (1.0 to 1.0) | 1.0 (1.0 to 1.0) | 1.0 (1.0 to 1.0) | 1.0 (1.0 to 1.0) | 1.0 (1.0 to 1.0) | 1.0 (1.0 to 1.0) |
| Gout | Stage 5 CKD | Both | Both | | | | | | | | | | 2.749 (2.491 to 3.017) | 2.747 (2.47 to 3.035) | 2.747 (2.482 to 3.035) | 2.736 (2.466 to 3.014) | 2.741 (2.48 to 3.053) | 2.729 (2.47 to 3.007) | 2.74 (2.479 to 3.038) | 2.747 (2.485 to 3.049) | 2.742 (2.479 to 3.021) | 2.742 (2.496 to 3.039) | 2.74 (2.48 to 3.042) | 2.736 (2.468 to 3.013) | 2.736 (2.468 to 3.013) | 2.736 (2.468 to 3.013) | 2.736 (2.468 to 3.013) |
| Gout | Stage 4 CKD | Both | Both | | | | | | | | | | 2.745 (2.473 to 3.032) | 2.743 (2.484 to 3.014) | 2.742 (2.473 to 3.034) | 2.742 (2.442 to 3.029) | 2.746 (2.494 to 3.045) | 2.748 (2.483 to 3.045) | 2.744 (2.462 to 3.029) | 2.749 (2.477 to 3.018) | 2.755 (2.495 to 3.038) | 2.747 (2.473 to 3.034) | 2.738 (2.463 to 3.041) | 2.744 (2.478 to 3.021) | 2.744 (2.478 to 3.021) | 2.744 (2.478 to 3.021) | 2.744 (2.478 to 3.021) |
| Gout | Stage 3 CKD | Both | Both | | | | | | | | | | 2.748 (2.457 to 3.035) | 2.753 (2.48 to 3.038) | 2.743 (2.465 to 3.024) | 2.748 (2.491 to 3.029) | 2.743 (2.467 to 3.051) | 2.749 (2.473 to 3.042) | 2.744 (2.475 to 3.017) | 2.741 (2.464 to 3.034) | 2.745 (2.478 to 3.037) | 2.739 (2.465 to 3.006) | 2.745 (2.473 to 3.029) | 2.747 (2.475 to 3.028) | 2.747 (2.475 to 3.028) | 2.747 (2.475 to 3.028) | 2.747 (2.475 to 3.028) |
| Gout | Albuminira | Both | Both | | | | | | | | | | 1.0 (1.0 to 1.0) | 1.0 (1.0 to 1.0) | 1.0 (1.0 to 1.0) | 1.0 (1.0 to 1.0) | 1.0 (1.0 to 1.0) | 1.0 (1.0 to 1.0) | 1.0 (1.0 to 1.0) | 1.0 (1.0 to 1.0) | 1.0 (1.0 to 1.0) | 1.0 (1.0 to 1.0) | 1.0 (1.0 to 1.0) | 1.0 (1.0 to 1.0) | 1.0 (1.0 to 1.0) | 1.0 (1.0 to 1.0) | 1.0 (1.0 to 1.0) |
| Gout | None | Both | Both | | | | | | | | | | 1.0 (1.0 to 1.0) | 1.0 (1.0 to 1.0) | 1.0 (1.0 to 1.0) | 1.0 (1.0 to 1.0) | 1.0 (1.0 to 1.0) | 1.0 (1.0 to 1.0) | 1.0 (1.0 to 1.0) | 1.0 (1.0 to 1.0) | 1.0 (1.0 to 1.0) | 1.0 (1.0 to 1.0) | 1.0 (1.0 to 1.0) | 1.0 (1.0 to 1.0) | 1.0 (1.0 to 1.0) | 1.0 (1.0 to 1.0) | 1.0 (1.0 to 1.0) |

* Occupational noise relative risk values are for sequela

** Shifts are reported for diet high in sodium as the estimation is based on mediation through high systolic blood pressure.

**Risk-outcome pairs with 100% attribution**

**Alcohol use**
Liver cancer due to alcohol use
Cirrhosis due to alcohol use
Alcohol use disorders
**Childhood underweight**
Protein-energy malnutrition
**Childhood wasting**
Protein-energy malnutrition
**Drug use**
Opioid use disorders
Cocaine use disorders
Amphetamine use disorders
Cannabis use disorders
Other drug use disorders

**High fasting plasma glucose**
Diabetes mellitus
Chronic kidney disease due to diabetes mellitus
**High systolic blood pressure**
Hypertensive heart disease
Chronic kidney disease due to hypertension
**Iron deficiency**
Iron-deficiency anaemia
**Low glomerular filtration rate**
Chronic kidney disease due to diabetes mellitus
Chronic kidney disease due to hypertension
Chronic kidney disease due to glomerulonephritis
Chronic kidney disease due to other causes

**Occupational particulate matter, gases, and fumes**
Coal workers pneumoconiosis
**Unsafe sex**
Syphilis
Chlamydial infection
Gonococcal infection
Trichomoniasis
Genital herpes
Other sexually transmitted diseases
Cervical cancer
Sexually transmitted diseases excluding HIV
**Vitamin A deficiency**
Vitamin A deficiency

| Appendix Table 6b. Relative risks used by age and sex for each outcome for the particulate matter integrated exposure response curve. | | | | | | | | | | | | | | | | | | | |
|---|---|---|---|---|---|---|---|---|---|---|---|---|---|---|---|---|---|---|---|
| Risk - Outcome | Category / Units | Morbidity / Mortality | Sex | Age | | | | | | | | | | | | | | | |
| | | | | All ages | 25-29 years | 30-34 years | 35-39 years | 40-44 years | 45-49 years | 50-54 years | 55-59 years | 60-64 years | 65-69 years | 70-74 years | 75-79 years | 80-84 years | 85-89 years | 90-94 years | 95+ years |
| **Ambient particulate matter pollution (PM2.5)** | | | | | | | | | | | | | | | | | | | |
| Lower respiratory infections | 600 µg/m$^3$ | Both | Both | 2.38 (1.968 to 2.776) | | | | | | | | | | | | | | | |
| Lower respiratory infections | 500 µg/m$^3$ | Both | Both | 2.347 (1.936 to 2.735) | | | | | | | | | | | | | | | |
| Lower respiratory infections | 400 µg/m$^3$ | Both | Both | 2.297 (1.883 to 2.687) | | | | | | | | | | | | | | | |
| Lower respiratory infections | 300 µg/m$^3$ | Both | Both | 2.213 (1.809 to 2.615) | | | | | | | | | | | | | | | |
| Lower respiratory infections | 200 µg/m$^3$ | Both | Both | 2.062 (1.702 to 2.44) | | | | | | | | | | | | | | | |
| Lower respiratory infections | 150 µg/m$^3$ | Both | Both | 1.938 (1.629 to 2.281) | | | | | | | | | | | | | | | |
| Lower respiratory infections | 135 µg/m$^3$ | Both | Both | 1.891 (1.6 to 2.209) | | | | | | | | | | | | | | | |
| Lower respiratory infections | 120 µg/m$^3$ | Both | Both | 1.838 (1.571 to 2.129) | | | | | | | | | | | | | | | |
| Lower respiratory infections | 105 µg/m$^3$ | Both | Both | 1.778 (1.54 to 2.05) | | | | | | | | | | | | | | | |
| Lower respiratory infections | 90 µg/m$^3$ | Both | Both | 1.711 (1.505 to 1.945) | | | | | | | | | | | | | | | |
| Lower respiratory infections | 75 µg/m$^3$ | Both | Both | 1.634 (1.455 to 1.827) | | | | | | | | | | | | | | | |
| Lower respiratory infections | 60 µg/m$^3$ | Both | Both | 1.546 (1.4 to 1.711) | | | | | | | | | | | | | | | |
| Lower respiratory infections | 45 µg/m$^3$ | Both | Both | 1.443 (1.323 to 1.576) | | | | | | | | | | | | | | | |
| Lower respiratory infections | 30 µg/m$^3$ | Both | Both | 1.322 (1.225 to 1.428) | | | | | | | | | | | | | | | |
| Lower respiratory infections | 25 µg/m$^3$ | Both | Both | 1.276 (1.184 to 1.379) | | | | | | | | | | | | | | | |
| Lower respiratory infections | 20 µg/m$^3$ | Both | Both | 1.226 (1.14 to 1.335) | | | | | | | | | | | | | | | |
| Lower respiratory infections | 15 µg/m$^3$ | Both | Both | 1.171 (1.093 to 1.282) | | | | | | | | | | | | | | | |
| Lower respiratory infections | 10 µg/m$^3$ | Both | Both | 1.108 (1.046 to 1.219) | | | | | | | | | | | | | | | |
| Lower respiratory infections | 5 µg/m$^3$ | Both | Both | 1.025 (1.0 to 1.119) | | | | | | | | | | | | | | | |
| Lower respiratory infections | 0 µg/m$^3$ | Both | Both | 1.0 (1.0 to 1.0) | | | | | | | | | | | | | | | |
| Tracheal, bronchus, and lung cancer | 600 µg/m$^3$ | Both | Both | 2.541 (2.222 to 2.868) | | | | | | | | | | | | | | | |
| Tracheal, bronchus, and lung cancer | 500 µg/m$^3$ | Both | Both | 2.369 (2.074 to 2.675) | | | | | | | | | | | | | | | |
| Tracheal, bronchus, and lung cancer | 400 µg/m$^3$ | Both | Both | 2.185 (1.919 to 2.469) | | | | | | | | | | | | | | | |
| Tracheal, bronchus, and lung cancer | 300 µg/m$^3$ | Both | Both | 1.982 (1.748 to 2.229) | | | | | | | | | | | | | | | |
| Tracheal, bronchus, and lung cancer | 200 µg/m$^3$ | Both | Both | 1.753 (1.56 to 1.958) | | | | | | | | | | | | | | | |
| Tracheal, bronchus, and lung cancer | 150 µg/m$^3$ | Both | Both | 1.622 (1.454 to 1.802) | | | | | | | | | | | | | | | |
| Tracheal, bronchus, and lung cancer | 135 µg/m$^3$ | Both | Both | 1.58 (1.421 to 1.754) | | | | | | | | | | | | | | | |
| Tracheal, bronchus, and lung cancer | 120 µg/m$^3$ | Both | Both | 1.536 (1.385 to 1.701) | | | | | | | | | | | | | | | |
| Tracheal, bronchus, and lung cancer | 105 µg/m$^3$ | Both | Both | 1.49 (1.348 to 1.648) | | | | | | | | | | | | | | | |

**Appendix Table 6b. Relative risks used by age and sex for each outcome for the particulate matter integrated exposure response curve.**

| Risk - Outcome | Category / Units | Morbidity / Mortality | Sex | Age | | | | | | | | | | | | | | | |
|---|---|---|---|---|---|---|---|---|---|---|---|---|---|---|---|---|---|---|---|
| | | | | All ages | 25-29 years | 30-34 years | 35-39 years | 40-44 years | 45-49 years | 50-54 years | 55-59 years | 60-64 years | 65-69 years | 70-74 years | 75-79 years | 80-84 years | 85-89 years | 90-94 years | 95+ years |
| **Ambient particulate matter pollution (PM2.5)** | | | | | | | | | | | | | | | | | | | |
| Tracheal, bronchus, and lung cancer | 90 $\mu g/m^3$ | Both | Both | 1.442 (1.309 to 1.588) | | | | | | | | | | | | | | | |
| Tracheal, bronchus, and lung cancer | 75 $\mu g/m^3$ | Both | Both | 1.39 (1.269 to 1.526) | | | | | | | | | | | | | | | |
| Tracheal, bronchus, and lung cancer | 60 $\mu g/m^3$ | Both | Both | 1.335 (1.227 to 1.458) | | | | | | | | | | | | | | | |
| Tracheal, bronchus, and lung cancer | 45 $\mu g/m^3$ | Both | Both | 1.273 (1.18 to 1.383) | | | | | | | | | | | | | | | |
| Tracheal, bronchus, and lung cancer | 30 $\mu g/m^3$ | Both | Both | 1.203 (1.128 to 1.295) | | | | | | | | | | | | | | | |
| Tracheal, bronchus, and lung cancer | 25 $\mu g/m^3$ | Both | Both | 1.177 (1.109 to 1.261) | | | | | | | | | | | | | | | |
| Tracheal, bronchus, and lung cancer | 20 $\mu g/m^3$ | Both | Both | 1.148 (1.089 to 1.223) | | | | | | | | | | | | | | | |
| Tracheal, bronchus, and lung cancer | 15 $\mu g/m^3$ | Both | Both | 1.116 (1.067 to 1.178) | | | | | | | | | | | | | | | |
| Tracheal, bronchus, and lung cancer | 10 $\mu g/m^3$ | Both | Both | 1.077 (1.041 to 1.126) | | | | | | | | | | | | | | | |
| Tracheal, bronchus, and lung cancer | 5 $\mu g/m^3$ | Both | Both | 1.02 (1.0 to 1.056) | | | | | | | | | | | | | | | |
| Tracheal, bronchus, and lung cancer | 0 $\mu g/m^3$ | Both | Both | 1.0 (1.0 to 1.0) | | | | | | | | | | | | | | | |
| Ischaemic heart disease | 600 $\mu g/m^3$ | Both | Both | | 2.244 (1.922 to 2.608) | 2.142 (1.848 to 2.464) | 2.033 (1.78 to 2.343) | 1.917 (1.671 to 2.195) | 1.831 (1.624 to 2.079) | 1.743 (1.557 to 1.943) | 1.661 (1.5 to 1.837) | 1.583 (1.437 to 1.739) | 1.506 (1.384 to 1.639) | 1.434 (1.329 to 1.545) | 1.371 (1.287 to 1.462) | 1.307 (1.231 to 1.384) | 1.249 (1.191 to 1.309) | 1.193 (1.151 to 1.241) | 1.141 (1.108 to 1.174) |
| Ischaemic heart disease | 500 $\mu g/m^3$ | Both | Both | | 2.166 (1.852 to 2.523) | 2.073 (1.784 to 2.395) | 1.971 (1.726 to 2.279) | 1.863 (1.618 to 2.133) | 1.783 (1.581 to 2.031) | 1.701 (1.52 to 1.898) | 1.625 (1.465 to 1.8) | 1.551 (1.406 to 1.705) | 1.479 (1.358 to 1.608) | 1.412 (1.307 to 1.52) | 1.352 (1.269 to 1.442) | 1.292 (1.216 to 1.368) | 1.237 (1.179 to 1.297) | 1.184 (1.142 to 1.232) | 1.134 (1.101 to 1.168) |
| Ischaemic heart disease | 400 $\mu g/m^3$ | Both | Both | | 2.076 (1.77 to 2.427) | 1.993 (1.708 to 2.317) | 1.901 (1.663 to 2.201) | 1.8 (1.559 to 2.069) | 1.727 (1.53 to 1.977) | 1.652 (1.475 to 1.843) | 1.582 (1.425 to 1.755) | 1.515 (1.371 to 1.665) | 1.448 (1.329 to 1.576) | 1.386 (1.282 to 1.496) | 1.331 (1.249 to 1.419) | 1.274 (1.199 to 1.351) | 1.223 (1.165 to 1.282) | 1.174 (1.131 to 1.222) | 1.127 (1.094 to 1.161) |
| Ischaemic heart disease | 300 $\mu g/m^3$ | Both | Both | | 1.971 (1.678 to 2.305) | 1.898 (1.622 to 2.217) | 1.816 (1.585 to 2.11) | 1.725 (1.492 to 1.981) | 1.662 (1.469 to 1.903) | 1.594 (1.42 to 1.782) | 1.532 (1.38 to 1.703) | 1.471 (1.332 to 1.618) | 1.41 (1.295 to 1.535) | 1.354 (1.251 to 1.463) | 1.304 (1.224 to 1.394) | 1.253 (1.179 to 1.329) | 1.206 (1.149 to 1.264) | 1.161 (1.119 to 1.208) | 1.117 (1.085 to 1.152) |
| Ischaemic heart disease | 200 $\mu g/m^3$ | Both | Both | | 1.838 (1.562 to 2.163) | 1.779 (1.515 to 2.08) | 1.71 (1.493 to 1.989) | 1.631 (1.413 to 1.879) | 1.579 (1.393 to 1.809) | 1.521 (1.356 to 1.704) | 1.467 (1.325 to 1.634) | 1.416 (1.282 to 1.561) | 1.363 (1.253 to 1.488) | 1.314 (1.215 to 1.423) | 1.271 (1.192 to 1.362) | 1.225 (1.154 to 1.302) | 1.184 (1.128 to 1.241) | 1.144 (1.103 to 1.19) | 1.106 (1.073 to 1.139) |
| Ischaemic heart disease | 150 $\mu g/m^3$ | Both | Both | | 1.754 (1.492 to 2.069) | 1.703 (1.452 to 1.992) | 1.643 (1.433 to 1.914) | 1.572 (1.362 to 1.807) | 1.526 (1.346 to 1.75) | 1.474 (1.313 to 1.652) | 1.426 (1.29 to 1.591) | 1.38 (1.253 to 1.52) | 1.332 (1.226 to 1.455) | 1.288 (1.192 to 1.397) | 1.249 (1.172 to 1.339) | 1.207 (1.138 to 1.283) | 1.17 (1.115 to 1.226) | 1.133 (1.094 to 1.179) | 1.098 (1.066 to 1.132) |
| Ischaemic heart disease | 135 $\mu g/m^3$ | Both | Both | | 1.726 (1.466 to 2.036) | 1.677 (1.43 to 1.965) | 1.62 (1.413 to 1.889) | 1.551 (1.345 to 1.782) | 1.507 (1.33 to 1.732) | 1.457 (1.299 to 1.634) | 1.412 (1.278 to 1.576) | 1.367 (1.243 to 1.507) | 1.321 (1.217 to 1.443) | 1.279 (1.184 to 1.387) | 1.241 (1.165 to 1.331) | 1.201 (1.132 to 1.277) | 1.165 (1.11 to 1.221) | 1.129 (1.09 to 1.176) | 1.095 (1.064 to 1.129) |
| Ischaemic heart disease | 120 $\mu g/m^3$ | Both | Both | | 1.695 (1.439 to 2.0) | 1.649 (1.408 to 1.935) | 1.595 (1.394 to 1.86) | 1.529 (1.326 to 1.759) | 1.487 (1.313 to 1.71) | 1.44 (1.284 to 1.614) | 1.396 (1.266 to 1.559) | 1.354 (1.232 to 1.491) | 1.31 (1.207 to 1.431) | 1.269 (1.176 to 1.376) | 1.233 (1.158 to 1.321) | 1.194 (1.127 to 1.27) | 1.16 (1.106 to 1.215) | 1.125 (1.086 to 1.171) | 1.092 (1.061 to 1.126) |
| Ischaemic heart disease | 105 $\mu g/m^3$ | Both | Both | | 1.661 (1.413 to 1.963) | 1.619 (1.383 to 1.904) | 1.567 (1.373 to 1.829) | 1.505 (1.306 to 1.731) | 1.466 (1.295 to 1.686) | 1.421 (1.268 to 1.593) | 1.38 (1.253 to 1.542) | 1.339 (1.22 to 1.474) | 1.297 (1.196 to 1.418) | 1.258 (1.167 to 1.363) | 1.224 (1.15 to 1.312) | 1.187 (1.121 to 1.262) | 1.154 (1.101 to 1.209) | 1.121 (1.082 to 1.167) | 1.089 (1.058 to 1.122) |
| Ischaemic heart disease | 90 $\mu g/m^3$ | Both | Both | | 1.624 (1.382 to 1.921) | 1.585 (1.356 to 1.86) | 1.537 (1.347 to 1.794) | 1.478 (1.284 to 1.7) | 1.442 (1.276 to 1.66) | 1.399 (1.251 to 1.568) | 1.361 (1.238 to 1.518) | 1.323 (1.206 to 1.457) | 1.283 (1.185 to 1.402) | 1.246 (1.157 to 1.35) | 1.214 (1.141 to 1.3) | 1.179 (1.113 to 1.252) | 1.147 (1.094 to 1.202) | 1.115 (1.078 to 1.161) | 1.085 (1.055 to 1.119) |
| Ischaemic heart disease | 75 $\mu g/m^3$ | Both | Both | | 1.583 (1.345 to 1.871) | 1.547 (1.325 to 1.813) | 1.503 (1.32 to 1.755) | 1.448 (1.259 to 1.665) | 1.415 (1.254 to 1.628) | 1.376 (1.232 to 1.542) | 1.34 (1.22 to 1.495) | 1.305 (1.19 to 1.437) | 1.267 (1.171 to 1.383) | 1.233 (1.146 to 1.335) | 1.202 (1.131 to 1.287) | 1.169 (1.106 to 1.241) | 1.14 (1.088 to 1.193) | 1.11 (1.073 to 1.155) | 1.081 (1.051 to 1.114) |
| Ischaemic heart disease | 60 $\mu g/m^3$ | Both | Both | | 1.535 (1.308 to 1.813) | 1.504 (1.291 to 1.761) | 1.464 (1.287 to 1.709) | 1.413 (1.232 to 1.621) | 1.384 (1.229 to 1.591) | 1.348 (1.21 to 1.511) | 1.315 (1.2 to 1.463) | 1.283 (1.172 to 1.412) | 1.249 (1.156 to 1.361) | 1.217 (1.134 to 1.317) | 1.189 (1.12 to 1.272) | 1.158 (1.096 to 1.229) | 1.131 (1.081 to 1.183) | 1.103 (1.067 to 1.148) | 1.076 (1.047 to 1.109) |
| Ischaemic heart disease | 45 $\mu g/m^3$ | Both | Both | | 1.479 (1.268 to 1.741) | 1.452 (1.251 to 1.695) | 1.417 (1.25 to 1.652) | 1.372 (1.2 to 1.573) | 1.347 (1.202 to 1.546) | 1.314 (1.184 to 1.473) | 1.286 (1.176 to 1.431) | 1.257 (1.152 to 1.381) | 1.226 (1.139 to 1.337) | 1.198 (1.118 to 1.295) | 1.173 (1.107 to 1.253) | 1.145 (1.086 to 1.214) | 1.12 (1.072 to 1.171) | 1.095 (1.06 to 1.138) | 1.07 (1.042 to 1.102) |
| Ischaemic heart disease | 30 $\mu g/m^3$ | Both | Both | | 1.406 (1.219 to 1.651) | 1.385 (1.205 to 1.609) | 1.357 (1.205 to 1.578) | 1.318 (1.163 to 1.506) | 1.299 (1.167 to 1.492) | 1.271 (1.15 to 1.423) | 1.247 (1.146 to 1.387) | 1.224 (1.126 to 1.342) | 1.197 (1.116 to 1.303) | 1.173 (1.098 to 1.265) | 1.152 (1.09 to 1.229) | 1.128 (1.071 to 1.193) | 1.106 (1.061 to 1.156) | 1.084 (1.05 to 1.126) | 1.062 (1.036 to 1.093) |
| Ischaemic heart disease | 25 $\mu g/m^3$ | Both | Both | | 1.376 (1.198 to 1.614) | 1.357 (1.187 to 1.574) | 1.332 (1.187 to 1.546) | 1.296 (1.149 to 1.479) | 1.278 (1.152 to 1.464) | 1.253 (1.137 to 1.402) | 1.231 (1.133 to 1.365) | 1.209 (1.115 to 1.323) | 1.185 (1.105 to 1.289) | 1.163 (1.09 to 1.253) | 1.143 (1.082 to 1.219) | 1.12 (1.066 to 1.184) | 1.1 (1.056 to 1.149) | 1.079 (1.047 to 1.121) | 1.059 (1.033 to 1.089) |
| Ischaemic heart disease | 20 $\mu g/m^3$ | Both | Both | | 1.341 (1.175 to 1.566) | 1.324 (1.166 to 1.533) | 1.303 (1.166 to 1.506) | 1.27 (1.132 to 1.446) | 1.254 (1.134 to 1.43) | 1.232 (1.122 to 1.374) | 1.212 (1.119 to 1.342) | 1.192 (1.103 to 1.302) | 1.17 (1.095 to 1.272) | 1.15 (1.081 to 1.237) | 1.132 (1.074 to 1.206) | 1.111 (1.06 to 1.173) | 1.093 (1.051 to 1.141) | 1.073 (1.042 to 1.114) | 1.055 (1.03 to 1.084) |
| Ischaemic heart disease | 15 $\mu g/m^3$ | Both | Both | | 1.298 (1.143 to 1.508) | 1.284 (1.139 to 1.476) | 1.266 (1.14 to 1.458) | 1.237 (1.112 to 1.404) | 1.225 (1.113 to 1.386) | 1.205 (1.103 to 1.341) | 1.188 (1.102 to 1.315) | 1.171 (1.088 to 1.276) | 1.152 (1.081 to 1.249) | 1.134 (1.07 to 1.219) | 1.118 (1.064 to 1.188) | 1.1 (1.052 to 1.159) | 1.083 (1.044 to 1.13) | 1.066 (1.037 to 1.107) | 1.05 (1.026 to 1.078) |
| Ischaemic heart disease | 10 $\mu g/m^3$ | Both | Both | | 1.238 (1.105 to 1.425) | 1.229 (1.101 to 1.402) | 1.215 (1.102 to 1.385) | 1.192 (1.083 to 1.339) | 1.183 (1.086 to 1.325) | 1.168 (1.077 to 1.293) | 1.155 (1.078 to 1.268) | 1.141 (1.068 to 1.241) | 1.125 (1.062 to 1.217) | 1.112 (1.053 to 1.19) | 1.099 (1.049 to 1.162) | 1.084 (1.041 to 1.139) | 1.07 (1.036 to 1.113) | 1.056 (1.03 to 1.095) | 1.042 (1.021 to 1.069) |

**Appendix Table 6b. Relative risks used by age and sex for each outcome for the particulate matter integrated exposure response curve.**

| Risk - Outcome | Category / Units | Morbidity / Mortality | Sex | Age | | | | | | | | | | | | | | | |
|---|---|---|---|---|---|---|---|---|---|---|---|---|---|---|---|---|---|---|---|
| | | | | All ages | 25-29 years | 30-34 years | 35-39 years | 40-44 years | 45-49 years | 50-54 years | 55-59 years | 60-64 years | 65-69 years | 70-74 years | 75-79 years | 80-84 years | 85-89 years | 90-94 years | 95+ years |
| **Ambient particulate matter pollution (PM2.5)** | | | | | | | | | | | | | | | | | | | |
| Ischaemic heart disease | 5 µg/m³ | Both | Both | | 1.097 (1.0 to 1.284) | 1.095 (1.0 to 1.266) | 1.088 (1.0 to 1.253) | 1.079 (1.0 to 1.228) | 1.077 (1.0 to 1.209) | 1.071 (1.0 to 1.188) | 1.067 (1.0 to 1.181) | 1.062 (1.0 to 1.166) | 1.055 (1.0 to 1.145) | 1.049 (1.0 to 1.126) | 1.045 (1.0 to 1.114) | 1.038 (1.0 to 1.098) | 1.032 (1.0 to 1.081) | 1.026 (1.0 to 1.067) | 1.02 (1.0 to 1.051) |
| Ischaemic heart disease | 0 µg/m³ | Both | Both | | 1.0 (1.0 to 1.0) | 1.0 (1.0 to 1.0) | 1.0 (1.0 to 1.0) | 1.0 (1.0 to 1.0) | 1.0 (1.0 to 1.0) | 1.0 (1.0 to 1.0) | 1.0 (1.0 to 1.0) | 1.0 (1.0 to 1.0) | 1.0 (1.0 to 1.0) | 1.0 (1.0 to 1.0) | 1.0 (1.0 to 1.0) | 1.0 (1.0 to 1.0) | 1.0 (1.0 to 1.0) | 1.0 (1.0 to 1.0) | 1.0 (1.0 to 1.0) |
| Stroke | 600 µg/m³ | Both | Both | | 1.704 (1.392 to 1.966) | 1.641 (1.36 to 1.902) | 1.587 (1.303 to 1.823) | 1.535 (1.297 to 1.749) | 1.481 (1.269 to 1.674) | 1.436 (1.245 to 1.606) | 1.39 (1.225 to 1.552) | 1.347 (1.201 to 1.489) | 1.306 (1.186 to 1.427) | 1.267 (1.159 to 1.367) | 1.228 (1.136 to 1.313) | 1.191 (1.117 to 1.256) | 1.158 (1.097 to 1.215) | 1.123 (1.074 to 1.167) | 1.091 (1.058 to 1.127) |
| Stroke | 500 µg/m³ | Both | Both | | 1.664 (1.355 to 1.925) | 1.606 (1.328 to 1.862) | 1.555 (1.275 to 1.791) | 1.506 (1.271 to 1.719) | 1.455 (1.244 to 1.651) | 1.413 (1.223 to 1.585) | 1.37 (1.207 to 1.529) | 1.329 (1.184 to 1.473) | 1.291 (1.17 to 1.412) | 1.254 (1.146 to 1.354) | 1.216 (1.125 to 1.302) | 1.181 (1.109 to 1.247) | 1.15 (1.089 to 1.208) | 1.117 (1.068 to 1.161) | 1.087 (1.053 to 1.123) |
| Stroke | 400 µg/m³ | Both | Both | | 1.619 (1.314 to 1.881) | 1.565 (1.293 to 1.822) | 1.518 (1.244 to 1.755) | 1.472 (1.242 to 1.68) | 1.426 (1.217 to 1.623) | 1.387 (1.199 to 1.559) | 1.346 (1.186 to 1.505) | 1.308 (1.165 to 1.452) | 1.273 (1.153 to 1.394) | 1.238 (1.131 to 1.34) | 1.203 (1.113 to 1.29) | 1.171 (1.098 to 1.237) | 1.141 (1.081 to 1.2) | 1.11 (1.061 to 1.155) | 1.082 (1.049 to 1.117) |
| Stroke | 300 µg/m³ | Both | Both | | 1.565 (1.27 to 1.825) | 1.516 (1.249 to 1.773) | 1.474 (1.208 to 1.709) | 1.432 (1.21 to 1.641) | 1.39 (1.185 to 1.587) | 1.355 (1.174 to 1.525) | 1.318 (1.162 to 1.479) | 1.283 (1.143 to 1.425) | 1.251 (1.133 to 1.373) | 1.22 (1.114 to 1.322) | 1.187 (1.098 to 1.275) | 1.157 (1.086 to 1.224) | 1.131 (1.072 to 1.189) | 1.102 (1.054 to 1.147) | 1.076 (1.043 to 1.111) |
| Stroke | 200 µg/m³ | Both | Both | | 1.496 (1.219 to 1.757) | 1.454 (1.203 to 1.712) | 1.418 (1.166 to 1.646) | 1.381 (1.17 to 1.591) | 1.345 (1.148 to 1.542) | 1.314 (1.142 to 1.483) | 1.282 (1.132 to 1.445) | 1.251 (1.117 to 1.394) | 1.223 (1.108 to 1.343) | 1.196 (1.094 to 1.299) | 1.167 (1.081 to 1.255) | 1.14 (1.072 to 1.207) | 1.117 (1.06 to 1.175) | 1.092 (1.046 to 1.137) | 1.068 (1.036 to 1.104) |
| Stroke | 150 µg/m³ | Both | Both | | 1.453 (1.189 to 1.705) | 1.415 (1.175 to 1.667) | 1.382 (1.141 to 1.605) | 1.348 (1.146 to 1.558) | 1.315 (1.125 to 1.509) | 1.288 (1.123 to 1.453) | 1.259 (1.115 to 1.42) | 1.231 (1.102 to 1.375) | 1.205 (1.094 to 1.325) | 1.181 (1.081 to 1.283) | 1.154 (1.07 to 1.24) | 1.13 (1.063 to 1.195) | 1.108 (1.053 to 1.166) | 1.085 (1.04 to 1.13) | 1.063 (1.032 to 1.098) |
| Stroke | 135 µg/m³ | Both | Both | | 1.438 (1.179 to 1.69) | 1.401 (1.166 to 1.653) | 1.37 (1.133 to 1.591) | 1.337 (1.138 to 1.545) | 1.305 (1.118 to 1.498) | 1.279 (1.117 to 1.443) | 1.251 (1.109 to 1.412) | 1.224 (1.096 to 1.366) | 1.199 (1.089 to 1.318) | 1.175 (1.077 to 1.277) | 1.15 (1.067 to 1.236) | 1.126 (1.06 to 1.191) | 1.105 (1.05 to 1.163) | 1.082 (1.038 to 1.127) | 1.062 (1.031 to 1.096) |
| Stroke | 120 µg/m³ | Both | Both | | 1.421 (1.168 to 1.672) | 1.386 (1.156 to 1.637) | 1.357 (1.125 to 1.576) | 1.325 (1.13 to 1.531) | 1.295 (1.111 to 1.486) | 1.269 (1.111 to 1.433) | 1.243 (1.103 to 1.403) | 1.216 (1.091 to 1.355) | 1.192 (1.083 to 1.311) | 1.169 (1.073 to 1.271) | 1.145 (1.063 to 1.231) | 1.122 (1.057 to 1.186) | 1.102 (1.047 to 1.16) | 1.08 (1.036 to 1.125) | 1.06 (1.029 to 1.094) |
| Stroke | 105 µg/m³ | Both | Both | | 1.404 (1.157 to 1.65) | 1.37 (1.146 to 1.619) | 1.342 (1.115 to 1.559) | 1.312 (1.121 to 1.517) | 1.283 (1.104 to 1.472) | 1.258 (1.103 to 1.421) | 1.233 (1.097 to 1.393) | 1.208 (1.085 to 1.342) | 1.185 (1.078 to 1.304) | 1.163 (1.068 to 1.265) | 1.139 (1.059 to 1.226) | 1.117 (1.053 to 1.181) | 1.098 (1.044 to 1.156) | 1.077 (1.034 to 1.122) | 1.058 (1.027 to 1.093) |
| Stroke | 90 µg/m³ | Both | Both | | 1.384 (1.144 to 1.632) | 1.352 (1.135 to 1.599) | 1.326 (1.105 to 1.539) | 1.297 (1.112 to 1.501) | 1.269 (1.096 to 1.457) | 1.246 (1.095 to 1.41) | 1.222 (1.09 to 1.381) | 1.198 (1.078 to 1.332) | 1.177 (1.072 to 1.293) | 1.156 (1.062 to 1.258) | 1.133 (1.055 to 1.219) | 1.112 (1.049 to 1.176) | 1.094 (1.041 to 1.151) | 1.074 (1.032 to 1.118) | 1.055 (1.026 to 1.09) |
| Stroke | 75 µg/m³ | Both | Both | | 1.362 (1.131 to 1.608) | 1.332 (1.122 to 1.574) | 1.307 (1.094 to 1.52) | 1.28 (1.102 to 1.482) | 1.254 (1.086 to 1.439) | 1.233 (1.086 to 1.398) | 1.211 (1.082 to 1.368) | 1.188 (1.071 to 1.321) | 1.167 (1.065 to 1.283) | 1.148 (1.056 to 1.249) | 1.126 (1.05 to 1.212) | 1.106 (1.045 to 1.17) | 1.089 (1.038 to 1.146) | 1.07 (1.029 to 1.114) | 1.053 (1.024 to 1.087) |
| Stroke | 60 µg/m³ | Both | Both | | 1.336 (1.116 to 1.574) | 1.309 (1.108 to 1.542) | 1.286 (1.083 to 1.492) | 1.261 (1.09 to 1.461) | 1.237 (1.076 to 1.418) | 1.217 (1.074 to 1.377) | 1.197 (1.073 to 1.352) | 1.175 (1.063 to 1.306) | 1.157 (1.059 to 1.27) | 1.138 (1.05 to 1.239) | 1.118 (1.045 to 1.203) | 1.1 (1.041 to 1.163) | 1.084 (1.035 to 1.14) | 1.066 (1.026 to 1.109) | 1.049 (1.021 to 1.083) |
| Stroke | 45 µg/m³ | Both | Both | | 1.305 (1.099 to 1.532) | 1.281 (1.093 to 1.502) | 1.261 (1.069 to 1.458) | 1.237 (1.077 to 1.433) | 1.216 (1.064 to 1.392) | 1.198 (1.062 to 1.354) | 1.18 (1.063 to 1.331) | 1.161 (1.054 to 1.288) | 1.144 (1.051 to 1.254) | 1.127 (1.044 to 1.226) | 1.109 (1.039 to 1.192) | 1.091 (1.036 to 1.153) | 1.077 (1.03 to 1.132) | 1.061 (1.023 to 1.103) | 1.046 (1.019 to 1.079) |
| Stroke | 30 µg/m³ | Both | Both | | 1.265 (1.077 to 1.48) | 1.244 (1.073 to 1.455) | 1.227 (1.054 to 1.415) | 1.207 (1.061 to 1.396) | 1.189 (1.05 to 1.357) | 1.174 (1.05 to 1.326) | 1.158 (1.05 to 1.303) | 1.141 (1.043 to 1.264) | 1.126 (1.041 to 1.233) | 1.112 (1.035 to 1.208) | 1.096 (1.031 to 1.175) | 1.081 (1.029 to 1.141) | 1.069 (1.025 to 1.122) | 1.054 (1.019 to 1.095) | 1.041 (1.016 to 1.073) |
| Stroke | 25 µg/m³ | Both | Both | | 1.248 (1.069 to 1.46) | 1.229 (1.066 to 1.436) | 1.213 (1.047 to 1.396) | 1.194 (1.054 to 1.38) | 1.177 (1.045 to 1.342) | 1.163 (1.045 to 1.312) | 1.149 (1.045 to 1.291) | 1.133 (1.039 to 1.253) | 1.119 (1.037 to 1.223) | 1.106 (1.031 to 1.2) | 1.091 (1.028 to 1.168) | 1.076 (1.027 to 1.136) | 1.065 (1.022 to 1.117) | 1.051 (1.018 to 1.091) | 1.038 (1.014 to 1.07) |
| Stroke | 20 µg/m³ | Both | Both | | 1.227 (1.06 to 1.43) | 1.211 (1.056 to 1.409) | 1.196 (1.041 to 1.373) | 1.179 (1.047 to 1.359) | 1.164 (1.039 to 1.32) | 1.151 (1.039 to 1.297) | 1.137 (1.04 to 1.275) | 1.123 (1.034 to 1.24) | 1.11 (1.032 to 1.213) | 1.098 (1.027 to 1.189) | 1.084 (1.024 to 1.16) | 1.071 (1.024 to 1.129) | 1.06 (1.02 to 1.112) | 1.047 (1.016 to 1.087) | 1.036 (1.012 to 1.067) |
| Stroke | 15 µg/m³ | Both | Both | | 1.202 (1.05 to 1.396) | 1.188 (1.046 to 1.381) | 1.176 (1.033 to 1.346) | 1.16 (1.039 to 1.334) | 1.146 (1.033 to 1.295) | 1.135 (1.031 to 1.277) | 1.123 (1.033 to 1.256) | 1.11 (1.028 to 1.222) | 1.099 (1.027 to 1.196) | 1.088 (1.023 to 1.175) | 1.076 (1.02 to 1.148) | 1.064 (1.02 to 1.12) | 1.055 (1.017 to 1.104) | 1.043 (1.013 to 1.081) | 1.033 (1.01 to 1.064) |
| Stroke | 10 µg/m³ | Both | Both | | 1.167 (1.036 to 1.347) | 1.155 (1.032 to 1.333) | 1.146 (1.024 to 1.303) | 1.133 (1.027 to 1.297) | 1.122 (1.025 to 1.261) | 1.113 (1.023 to 1.248) | 1.104 (1.024 to 1.228) | 1.093 (1.021 to 1.196) | 1.083 (1.019 to 1.174) | 1.075 (1.017 to 1.157) | 1.064 (1.015 to 1.133) | 1.054 (1.015 to 1.107) | 1.047 (1.012 to 1.094) | 1.037 (1.01 to 1.073) | 1.028 (1.008 to 1.057) |
| Stroke | 5 µg/m³ | Both | Both | | 1.073 (1.0 to 1.236) | 1.069 (1.0 to 1.224) | 1.066 (1.0 to 1.211) | 1.059 (1.0 to 1.193) | 1.054 (1.0 to 1.184) | 1.051 (1.0 to 1.171) | 1.046 (1.0 to 1.15) | 1.043 (1.0 to 1.135) | 1.039 (1.0 to 1.123) | 1.035 (1.0 to 1.11) | 1.03 (1.0 to 1.093) | 1.025 (1.0 to 1.075) | 1.022 (1.0 to 1.07) | 1.017 (1.0 to 1.051) | 1.013 (1.0 to 1.042) |
| Stroke | 0 µg/m³ | Both | Both | | 1.0 (1.0 to 1.0) | 1.0 (1.0 to 1.0) | 1.0 (1.0 to 1.0) | 1.0 (1.0 to 1.0) | 1.0 (1.0 to 1.0) | 1.0 (1.0 to 1.0) | 1.0 (1.0 to 1.0) | 1.0 (1.0 to 1.0) | 1.0 (1.0 to 1.0) | 1.0 (1.0 to 1.0) | 1.0 (1.0 to 1.0) | 1.0 (1.0 to 1.0) | 1.0 (1.0 to 1.0) | 1.0 (1.0 to 1.0) | 1.0 (1.0 to 1.0) |
| Chronic obstructive pulmonary disease | 600 µg/m³ | Both | Both | 2.335 (1.956 to 2.701) | | | | | | | | | | | | | | | |
| Chronic obstructive pulmonary disease | 500 µg/m³ | Both | Both | 2.231 (1.864 to 2.585) | | | | | | | | | | | | | | | |
| Chronic obstructive pulmonary disease | 400 µg/m³ | Both | Both | 2.114 (1.767 to 2.447) | | | | | | | | | | | | | | | |
| Chronic obstructive pulmonary disease | 300 µg/m³ | Both | Both | 1.979 (1.653 to 2.292) | | | | | | | | | | | | | | | |
| Chronic obstructive pulmonary disease | 200 µg/m³ | Both | Both | 1.815 (1.52 to 2.099) | | | | | | | | | | | | | | | |
| Chronic obstructive pulmonary disease | 150 µg/m³ | Both | Both | 1.714 (1.441 to 1.976) | | | | | | | | | | | | | | | |
| Chronic obstructive pulmonary disease | 135 µg/m³ | Both | Both | 1.681 (1.415 to 1.94) | | | | | | | | | | | | | | | |

**Appendix Table 6b. Relative risks used by age and sex for each outcome for the particulate matter integrated exposure response curve.**

| Risk - Outcome | Category / Units | Morbidity / Mortality | Sex | Age | | | | | | | | | | | | | | | | |
|---|---|---|---|---|---|---|---|---|---|---|---|---|---|---|---|---|---|---|---|---|
| | | | | All ages | 25-29 years | 30-34 years | 35-39 years | 40-44 years | 45-49 years | 50-54 years | 55-59 years | 60-64 years | 65-69 years | 70-74 years | 75-79 years | 80-84 years | 85-89 years | 90-94 years | 95+ years |
| **Ambient particulate matter pollution (PM2.5)** | | | | | | | | | | | | | | | | | | | | |
| Chronic obstructive pulmonary disease | 120 µg/m³ | Both | Both | 1.645 (1.388 to 1.899) | | | | | | | | | | | | | | | | |
| Chronic obstructive pulmonary disease | 105 µg/m³ | Both | Both | 1.606 (1.359 to 1.854) | | | | | | | | | | | | | | | | |
| Chronic obstructive pulmonary disease | 90 µg/m³ | Both | Both | 1.564 (1.328 to 1.804) | | | | | | | | | | | | | | | | |
| Chronic obstructive pulmonary disease | 75 µg/m³ | Both | Both | 1.518 (1.294 to 1.745) | | | | | | | | | | | | | | | | |
| Chronic obstructive pulmonary disease | 60 µg/m³ | Both | Both | 1.466 (1.258 to 1.68) | | | | | | | | | | | | | | | | |
| Chronic obstructive pulmonary disease | 45 µg/m³ | Both | Both | 1.405 (1.217 to 1.607) | | | | | | | | | | | | | | | | |
| Chronic obstructive pulmonary disease | 30 µg/m³ | Both | Both | 1.33 (1.168 to 1.512) | | | | | | | | | | | | | | | | |
| Chronic obstructive pulmonary disease | 25 µg/m³ | Both | Both | 1.3 (1.149 to 1.473) | | | | | | | | | | | | | | | | |
| Chronic obstructive pulmonary disease | 20 µg/m³ | Both | Both | 1.266 (1.126 to 1.426) | | | | | | | | | | | | | | | | |
| Chronic obstructive pulmonary disease | 15 µg/m³ | Both | Both | 1.224 (1.101 to 1.371) | | | | | | | | | | | | | | | | |
| Chronic obstructive pulmonary disease | 10 µg/m³ | Both | Both | 1.17 (1.07 to 1.296) | | | | | | | | | | | | | | | | |
| Chronic obstructive pulmonary disease | 5 µg/m³ | Both | Both | 1.06 (1.0 to 1.17) | | | | | | | | | | | | | | | | |
| Chronic obstructive pulmonary disease | 0 µg/m³ | Both | Both | 1.0 (1.0 to 1.0) | | | | | | | | | | | | | | | | |
| Diabetes mellitus type 2 | 600 µg/m³ | Both | Both | 1.448 (1.33 to 1.545) | | | | | | | | | | | | | | | | |
| Diabetes mellitus type 2 | 500 µg/m³ | Both | Both | 1.447 (1.324 to 1.545) | | | | | | | | | | | | | | | | |
| Diabetes mellitus type 2 | 400 µg/m³ | Both | Both | 1.446 (1.317 to 1.545) | | | | | | | | | | | | | | | | |
| Diabetes mellitus type 2 | 300 µg/m³ | Both | Both | 1.445 (1.306 to 1.545) | | | | | | | | | | | | | | | | |
| Diabetes mellitus type 2 | 200 µg/m³ | Both | Both | 1.443 (1.292 to 1.545) | | | | | | | | | | | | | | | | |
| Diabetes mellitus type 2 | 150 µg/m³ | Both | Both | 1.441 (1.281 to 1.545) | | | | | | | | | | | | | | | | |
| Diabetes mellitus type 2 | 135 µg/m³ | Both | Both | 1.44 (1.276 to 1.545) | | | | | | | | | | | | | | | | |
| Diabetes mellitus type 2 | 120 µg/m³ | Both | Both | 1.439 (1.272 to 1.544) | | | | | | | | | | | | | | | | |
| Diabetes mellitus type 2 | 105 µg/m³ | Both | Both | 1.438 (1.266 to 1.538) | | | | | | | | | | | | | | | | |
| Diabetes mellitus type 2 | 90 µg/m³ | Both | Both | 1.436 (1.26 to 1.533) | | | | | | | | | | | | | | | | |
| Diabetes mellitus type 2 | 75 µg/m³ | Both | Both | 1.433 (1.253 to 1.53) | | | | | | | | | | | | | | | | |
| Diabetes mellitus type 2 | 60 µg/m³ | Both | Both | 1.429 (1.245 to 1.526) | | | | | | | | | | | | | | | | |
| Diabetes mellitus type 2 | 45 µg/m³ | Both | Both | 1.422 (1.234 to 1.52) | | | | | | | | | | | | | | | | |
| Diabetes mellitus type 2 | 30 µg/m³ | Both | Both | 1.405 (1.219 to 1.504) | | | | | | | | | | | | | | | | |
| Diabetes mellitus type 2 | 25 µg/m³ | Both | Both | 1.393 (1.213 to 1.501) | | | | | | | | | | | | | | | | |
| Diabetes mellitus type 2 | 20 µg/m³ | Both | Both | 1.375 (1.197 to 1.494) | | | | | | | | | | | | | | | | |
| Diabetes mellitus type 2 | 15 µg/m³ | Both | Both | 1.345 (1.177 to 1.489) | | | | | | | | | | | | | | | | |
| Diabetes mellitus type 2 | 10 µg/m³ | Both | Both | 1.282 (1.116 to 1.466) | | | | | | | | | | | | | | | | |
| Diabetes mellitus type 2 | 5 µg/m³ | Both | Both | 1.089 (1.0 to 1.346) | | | | | | | | | | | | | | | | |
| Diabetes mellitus type 2 | 0 µg/m³ | Both | Both | 1.0 (1.0 to 1.0) | | | | | | | | | | | | | | | | |

| Appendix Table 6c. Relative risks used by age and sex for each outcome for alcohol use globally. | | | | |
|---|---|---|---|---|
| Risk - Outcome | Category / Units | Morbidity / Mortality | Sex | All ages |
| Alcohol use | | | | |
| Tuberculosis | 72 g/day | Both | Both | 3.507 (2.596 to 4.474) |
| Tuberculosis | 60 g/day | Both | Both | 2.994 (1.972 to 4.204) |
| Tuberculosis | 48 g/day | Both | Both | 2.535 (1.701 to 3.51) |
| Tuberculosis | 36 g/day | Both | Both | 2.058 (1.485 to 2.795) |
| Tuberculosis | 24 g/day | Both | Both | 1.531 (1.165 to 1.98) |
| Tuberculosis | 12 g/day | Both | Both | 1.101 (0.815 to 1.425) |
| Tuberculosis | 0 g/day | Both | Both | 1.0 (1.0 to 1.0) |
| Lower respiratory infections | 72 g/day | Both | Both | 1.357 (1.113 to 1.648) |
| Lower respiratory infections | 60 g/day | Both | Both | 1.226 (1.036 to 1.423) |
| Lower respiratory infections | 48 g/day | Both | Both | 1.127 (0.936 to 1.327) |
| Lower respiratory infections | 36 g/day | Both | Both | 1.064 (0.928 to 1.219) |
| Lower respiratory infections | 24 g/day | Both | Both | 1.026 (0.901 to 1.167) |
| Lower respiratory infections | 12 g/day | Both | Both | 1.013 (0.951 to 1.084) |
| Lower respiratory infections | 0 g/day | Both | Both | 1.0 (1.0 to 1.0) |
| Oesophageal cancer | 72 g/day | Both | Both | 2.669 (2.074 to 3.348) |
| Oesophageal cancer | 60 g/day | Both | Both | 2.452 (1.905 to 3.094) |
| Oesophageal cancer | 48 g/day | Both | Both | 2.202 (1.73 to 2.703) |
| Oesophageal cancer | 36 g/day | Both | Both | 1.815 (1.468 to 2.222) |
| Oesophageal cancer | 24 g/day | Both | Both | 1.466 (1.209 to 1.764) |
| Oesophageal cancer | 12 g/day | Both | Both | 1.212 (1.031 to 1.439) |
| Oesophageal cancer | 0 g/day | Both | Both | 1.0 (1.0 to 1.0) |
| Liver cancer due to alcohol use | 72 g/day | Both | Both | 1.424 (1.088 to 1.855) |
| Liver cancer due to alcohol use | 60 g/day | Both | Both | 1.372 (1.093 to 1.692) |
| Liver cancer due to alcohol use | 48 g/day | Both | Both | 1.31 (1.036 to 1.639) |
| Liver cancer due to alcohol use | 36 g/day | Both | Both | 1.225 (1.009 to 1.455) |
| Liver cancer due to alcohol use | 24 g/day | Both | Both | 1.14 (0.934 to 1.359) |
| Liver cancer due to alcohol use | 12 g/day | Both | Both | 1.067 (0.936 to 1.207) |
| Liver cancer due to alcohol use | 0 g/day | Both | Both | 1.0 (1.0 to 1.0) |
| Larynx cancer | 72 g/day | Both | Both | 2.461 (1.758 to 3.228) |
| Larynx cancer | 60 g/day | Both | Both | 2.144 (1.46 to 2.935) |
| Larynx cancer | 48 g/day | Both | Both | 1.813 (1.3 to 2.421) |

| Appendix Table 6c. Relative risks used by age and sex for each outcome for alcohol use globally. | | | | |
|---|---|---|---|---|
| **Risk - Outcome** | **Category / Units** | **Morbidity / Mortality** | **Sex** | **All ages** |
| **Alcohol use** | | | | |
| Larynx cancer | 36 g/day | Both | Both | 1.531 (1.126 to 2.061) |
| Larynx cancer | 24 g/day | Both | Both | 1.304 (1.006 to 1.659) |
| Larynx cancer | 12 g/day | Both | Both | 1.12 (0.903 to 1.386) |
| Larynx cancer | 0 g/day | Both | Both | 1.0 (1.0 to 1.0) |
| Breast cancer | 72 g/day | Both | Both | 1.476 (1.282 to 1.691) |
| Breast cancer | 60 g/day | Both | Both | 1.452 (1.312 to 1.599) |
| Breast cancer | 48 g/day | Both | Both | 1.443 (1.348 to 1.542) |
| Breast cancer | 36 g/day | Both | Both | 1.433 (1.311 to 1.551) |
| Breast cancer | 24 g/day | Both | Both | 1.329 (1.237 to 1.419) |
| Breast cancer | 12 g/day | Both | Both | 1.17 (1.081 to 1.265) |
| Breast cancer | 0 g/day | Both | Both | 1.0 (1.0 to 1.0) |
| Colon and rectum cancer | 72 g/day | Both | Both | 1.616 (1.38 to 1.861) |
| Colon and rectum cancer | 60 g/day | Both | Both | 1.468 (1.329 to 1.615) |
| Colon and rectum cancer | 48 g/day | Both | Both | 1.323 (1.156 to 1.501) |
| Colon and rectum cancer | 36 g/day | Both | Both | 1.237 (1.148 to 1.336) |
| Colon and rectum cancer | 24 g/day | Both | Both | 1.156 (1.067 to 1.248) |
| Colon and rectum cancer | 12 g/day | Both | Both | 1.078 (1.034 to 1.124) |
| Colon and rectum cancer | 0 g/day | Both | Both | 1.0 (1.0 to 1.0) |
| Lip and oral cavity cancer | 72 g/day | Both | Both | 4.858 (3.74 to 6.076) |
| Lip and oral cavity cancer | 60 g/day | Both | Both | 3.766 (2.839 to 4.9) |
| Lip and oral cavity cancer | 48 g/day | Both | Both | 2.991 (2.283 to 3.896) |
| Lip and oral cavity cancer | 36 g/day | Both | Both | 2.311 (1.757 to 2.929) |
| Lip and oral cavity cancer | 24 g/day | Both | Both | 1.738 (1.383 to 2.161) |
| Lip and oral cavity cancer | 12 g/day | Both | Both | 1.293 (1.076 to 1.551) |
| Lip and oral cavity cancer | 0 g/day | Both | Both | 1.0 (1.0 to 1.0) |
| Nasopharynx cancer | 72 g/day | Both | Both | 4.545 (4.1 to 4.982) |
| Nasopharynx cancer | 60 g/day | Both | Both | 3.803 (3.509 to 4.102) |
| Nasopharynx cancer | 48 g/day | Both | Both | 3.062 (2.873 to 3.258) |
| Nasopharynx cancer | 36 g/day | Both | Both | 2.385 (2.25 to 2.552) |
| Nasopharynx cancer | 24 g/day | Both | Both | 1.839 (1.77 to 1.907) |
| Nasopharynx cancer | 12 g/day | Both | Both | 1.371 (1.341 to 1.398) |

| Appendix Table 6c. Relative risks used by age and sex for each outcome for alcohol use globally. | | | | |
|---|---|---|---|---|
| **Risk - Outcome** | **Category / Units** | **Morbidity / Mortality** | **Sex** | **All ages** |
| **Alcohol use** | | | | |
| Nasopharynx cancer | 0 g/day | Both | Both | 1.0 (1.0 to 1.0) |
| Other pharynx cancer | 72 g/day | Both | Both | 4.764 (3.315 to 6.576) |
| Other pharynx cancer | 60 g/day | Both | Both | 3.972 (2.813 to 5.354) |
| Other pharynx cancer | 48 g/day | Both | Both | 3.199 (2.202 to 4.407) |
| Other pharynx cancer | 36 g/day | Both | Both | 2.519 (1.843 to 3.299) |
| Other pharynx cancer | 24 g/day | Both | Both | 1.943 (1.467 to 2.484) |
| Other pharynx cancer | 12 g/day | Both | Both | 1.472 (1.234 to 1.742) |
| Other pharynx cancer | 0 g/day | Both | Both | 1.0 (1.0 to 1.0) |
| Ischaemic heart disease | 72 g/day | Both | Male | 1.091 (0.933 to 1.271) |
| Ischaemic heart disease | 60 g/day | Both | Male | 0.993 (0.883 to 1.105) |
| Ischaemic heart disease | 48 g/day | Both | Male | 0.906 (0.797 to 1.035) |
| Ischaemic heart disease | 36 g/day | Both | Male | 0.871 (0.788 to 0.964) |
| Ischaemic heart disease | 24 g/day | Both | Male | 0.857 (0.779 to 0.943) |
| Ischaemic heart disease | 12 g/day | Both | Male | 0.865 (0.79 to 0.948) |
| Ischaemic heart disease | 0 g/day | Both | Male | 1.0 (1.0 to 1.0) |
| Ischaemic heart disease | 72 g/day | Both | Female | 1.107 (0.894 to 1.341) |
| Ischaemic heart disease | 60 g/day | Both | Female | 1.012 (0.869 to 1.174) |
| Ischaemic heart disease | 48 g/day | Both | Female | 0.932 (0.786 to 1.113) |
| Ischaemic heart disease | 36 g/day | Both | Female | 0.882 (0.781 to 0.997) |
| Ischaemic heart disease | 24 g/day | Both | Female | 0.846 (0.749 to 0.948) |
| Ischaemic heart disease | 12 g/day | Both | Female | 0.823 (0.733 to 0.926) |
| Ischaemic heart disease | 0 g/day | Both | Female | 1.0 (1.0 to 1.0) |
| Ischaemic stroke | 72 g/day | Both | Male | 1.451 (1.228 to 1.69) |
| Ischaemic stroke | 60 g/day | Both | Male | 1.312 (1.167 to 1.471) |
| Ischaemic stroke | 48 g/day | Both | Male | 1.159 (0.98 to 1.353) |
| Ischaemic stroke | 36 g/day | Both | Male | 1.057 (0.931 to 1.192) |
| Ischaemic stroke | 24 g/day | Both | Male | 0.97 (0.862 to 1.088) |
| Ischaemic stroke | 12 g/day | Both | Male | 0.938 (0.83 to 1.054) |
| Ischaemic stroke | 0 g/day | Both | Male | 1.0 (1.0 to 1.0) |
| Ischaemic stroke | 72 g/day | Both | Female | 1.43 (1.147 to 1.771) |
| Ischaemic stroke | 60 g/day | Both | Female | 1.3 (1.121 to 1.496) |

| Appendix Table 6c. Relative risks used by age and sex for each outcome for alcohol use globally. | | | | |
|---|---|---|---|---|
| Risk - Outcome | Category / Units | Morbidity / Mortality | Sex | All ages |
| Alcohol use | | | | |
| Ischaemic stroke | 48 g/day | Both | Female | 1.145 (0.946 to 1.359) |
| Ischaemic stroke | 36 g/day | Both | Female | 0.985 (0.834 to 1.149) |
| Ischaemic stroke | 24 g/day | Both | Female | 0.85 (0.726 to 0.985) |
| Ischaemic stroke | 12 g/day | Both | Female | 0.824 (0.718 to 0.939) |
| Ischaemic stroke | 0 g/day | Both | Female | 1.0 (1.0 to 1.0) |
| Intracerebral hemorrhage | 72 g/day | Both | Male | 1.971 (1.663 to 2.316) |
| Intracerebral hemorrhage | 60 g/day | Both | Male | 1.705 (1.45 to 1.991) |
| Intracerebral hemorrhage | 48 g/day | Both | Male | 1.458 (1.182 to 1.768) |
| Intracerebral hemorrhage | 36 g/day | Both | Male | 1.31 (1.105 to 1.539) |
| Intracerebral hemorrhage | 24 g/day | Both | Male | 1.162 (0.973 to 1.385) |
| Intracerebral hemorrhage | 12 g/day | Both | Male | 1.068 (0.945 to 1.214) |
| Intracerebral hemorrhage | 0 g/day | Both | Male | 1.0 (1.0 to 1.0) |
| Intracerebral hemorrhage | 72 g/day | Both | Female | 2.276 (1.701 to 2.934) |
| Intracerebral hemorrhage | 60 g/day | Both | Female | 1.964 (1.536 to 2.464) |
| Intracerebral hemorrhage | 48 g/day | Both | Female | 1.614 (1.245 to 2.048) |
| Intracerebral hemorrhage | 36 g/day | Both | Female | 1.337 (1.065 to 1.664) |
| Intracerebral hemorrhage | 24 g/day | Both | Female | 1.11 (0.884 to 1.367) |
| Intracerebral hemorrhage | 12 g/day | Both | Female | 1.031 (0.897 to 1.18) |
| Intracerebral hemorrhage | 0 g/day | Both | Female | 1.0 (1.0 to 1.0) |
| Hypertensive heart disease | 72 g/day | Both | Both | 1.86 (1.445 to 2.358) |
| Hypertensive heart disease | 60 g/day | Both | Both | 1.705 (1.297 to 2.175) |
| Hypertensive heart disease | 48 g/day | Both | Both | 1.614 (1.25 to 2.049) |
| Hypertensive heart disease | 36 g/day | Both | Both | 1.479 (1.232 to 1.759) |
| Hypertensive heart disease | 24 g/day | Both | Both | 1.315 (1.136 to 1.526) |
| Hypertensive heart disease | 12 g/day | Both | Both | 1.046 (0.913 to 1.198) |
| Hypertensive heart disease | 0 g/day | Both | Both | 1.0 (1.0 to 1.0) |
| Atrial fibrillation and flutter | 72 g/day | Both | Both | 1.535 (1.348 to 1.728) |
| Atrial fibrillation and flutter | 60 g/day | Both | Both | 1.411 (1.26 to 1.569) |
| Atrial fibrillation and flutter | 48 g/day | Both | Both | 1.312 (1.218 to 1.407) |
| Atrial fibrillation and flutter | 36 g/day | Both | Both | 1.214 (1.145 to 1.29) |
| Atrial fibrillation and flutter | 24 g/day | Both | Both | 1.131 (1.067 to 1.204) |

| Appendix Table 6c. Relative risks used by age and sex for each outcome for alcohol use globally. | | | | |
|---|---|---|---|---|
| **Risk - Outcome** | **Category / Units** | **Morbidity / Mortality** | **Sex** | **All ages** |
| **Alcohol use** | | | | |
| Atrial fibrillation and flutter | 12 g/day | Both | Both | 1.066 (1.034 to 1.102) |
| Atrial fibrillation and flutter | 0 g/day | Both | Both | 1.0 (1.0 to 1.0) |
| Cirrhosis and other chronic liver diseases due to alcohol use | 72 g/day | Both | Both | 9.427 (6.131 to 13.804) |
| Cirrhosis and other chronic liver diseases due to alcohol use | 60 g/day | Both | Both | 6.274 (3.958 to 9.319) |
| Cirrhosis and other chronic liver diseases due to alcohol use | 48 g/day | Both | Both | 4.673 (3.25 to 6.717) |
| Cirrhosis and other chronic liver diseases due to alcohol use | 36 g/day | Both | Both | 3.274 (2.309 to 4.485) |
| Cirrhosis and other chronic liver diseases due to alcohol use | 24 g/day | Both | Both | 2.055 (1.521 to 2.688) |
| Cirrhosis and other chronic liver diseases due to alcohol use | 12 g/day | Both | Both | 1.243 (0.943 to 1.611) |
| Cirrhosis and other chronic liver diseases due to alcohol use | 0 g/day | Both | Both | 1.0 (1.0 to 1.0) |
| Pancreatitis | 72 g/day | Both | Both | 3.298 (2.473 to 4.458) |
| Pancreatitis | 60 g/day | Both | Both | 2.217 (1.415 to 3.389) |
| Pancreatitis | 48 g/day | Both | Both | 1.717 (1.199 to 2.477) |
| Pancreatitis | 36 g/day | Both | Both | 1.471 (1.062 to 2.021) |
| Pancreatitis | 24 g/day | Both | Both | 1.228 (0.874 to 1.67) |
| Pancreatitis | 12 g/day | Both | Both | 1.073 (0.791 to 1.481) |
| Pancreatitis | 0 g/day | Both | Both | 1.0 (1.0 to 1.0) |
| Epilepsy | 72 g/day | Both | Both | 2.48 (1.929 to 3.144) |
| Epilepsy | 60 g/day | Both | Both | 2.186 (1.781 to 2.622) |
| Epilepsy | 48 g/day | Both | Both | 1.872 (1.438 to 2.369) |
| Epilepsy | 36 g/day | Both | Both | 1.585 (1.303 to 1.898) |
| Epilepsy | 24 g/day | Both | Both | 1.353 (1.118 to 1.633) |
| Epilepsy | 12 g/day | Both | Both | 1.177 (1.059 to 1.316) |
| Epilepsy | 0 g/day | Both | Both | 1.0 (1.0 to 1.0) |
| Diabetes mellitus | 72 g/day | Both | Male | 1.198 (1.065 to 1.337) |
| Diabetes mellitus | 60 g/day | Both | Male | 1.165 (0.998 to 1.342) |
| Diabetes mellitus | 48 g/day | Both | Male | 1.084 (0.933 to 1.239) |
| Diabetes mellitus | 36 g/day | Both | Male | 1.0 (0.891 to 1.119) |
| Diabetes mellitus | 24 g/day | Both | Male | 0.932 (0.841 to 1.03) |
| Diabetes mellitus | 12 g/day | Both | Male | 0.921 (0.833 to 1.015) |
| Diabetes mellitus | 0 g/day | Both | Male | 1.0 (1.0 to 1.0) |
| Diabetes mellitus | 72 g/day | Both | Female | 1.172 (0.81 to 1.652) |

| Appendix Table 6c. Relative risks used by age and sex for each outcome for alcohol use globally. | | | | |
|---|---|---|---|---|
| Risk - Outcome | Category / Units | Morbidity / Mortality | Sex | All ages |
| Alcohol use | | | | |
| Diabetes mellitus | 60 g/day | Both | Female | 1.074 (0.765 to 1.443) |
| Diabetes mellitus | 48 g/day | Both | Female | 0.945 (0.737 to 1.173) |
| Diabetes mellitus | 36 g/day | Both | Female | 0.836 (0.702 to 0.981) |
| Diabetes mellitus | 24 g/day | Both | Female | 0.76 (0.66 to 0.872) |
| Diabetes mellitus | 12 g/day | Both | Female | 0.733 (0.658 to 0.826) |
| Diabetes mellitus | 0 g/day | Both | Female | 1.0 (1.0 to 1.0) |
| Transport injuries | 72 g/day | Both | Both | 1.552 (1.201 to 2.032) |
| Transport injuries | 60 g/day | Both | Both | 1.456 (1.186 to 1.818) |
| Transport injuries | 48 g/day | Both | Both | 1.366 (1.101 to 1.692) |
| Transport injuries | 36 g/day | Both | Both | 1.288 (1.089 to 1.534) |
| Transport injuries | 24 g/day | Both | Both | 1.22 (1.062 to 1.4) |
| Transport injuries | 12 g/day | Both | Both | 1.163 (1.021 to 1.346) |
| Transport injuries | 0 g/day | Both | Both | 1.0 (1.0 to 1.0) |
| Unintentional injuries | 72 g/day | Both | Both | 1.266 (1.063 to 1.555) |
| Unintentional injuries | 60 g/day | Both | Both | 1.221 (1.059 to 1.46) |
| Unintentional injuries | 48 g/day | Both | Both | 1.182 (1.024 to 1.428) |
| Unintentional injuries | 36 g/day | Both | Both | 1.168 (1.054 to 1.347) |
| Unintentional injuries | 24 g/day | Both | Both | 1.154 (1.046 to 1.319) |
| Unintentional injuries | 12 g/day | Both | Both | 1.09 (1.016 to 1.187) |
| Unintentional injuries | 0 g/day | Both | Both | 1.0 (1.0 to 1.0) |
| Self-harm | 72 g/day | Both | Both | 1.927 (1.398 to 2.665) |
| Self-harm | 60 g/day | Both | Both | 1.734 (1.29 to 2.308) |
| Self-harm | 48 g/day | Both | Both | 1.545 (1.132 to 2.048) |
| Self-harm | 36 g/day | Both | Both | 1.376 (1.05 to 1.751) |
| Self-harm | 24 g/day | Both | Both | 1.23 (0.972 to 1.533) |
| Self-harm | 12 g/day | Both | Both | 1.107 (0.908 to 1.343) |
| Self-harm | 0 g/day | Both | Both | 1.0 (1.0 to 1.0) |
| Interpersonal violence | 72 g/day | Both | Both | 1.516 (1.255 to 1.867) |
| Interpersonal violence | 60 g/day | Both | Both | 1.452 (1.215 to 1.719) |
| Interpersonal violence | 48 g/day | Both | Both | 1.396 (1.118 to 1.739) |
| Interpersonal violence | 36 g/day | Both | Both | 1.345 (1.14 to 1.585) |

**Appendix Table 6c. Relative risks used by age and sex for each outcome for alcohol use globally.**

| Risk - Outcome | Category / Units | Morbidity / Mortality | Sex | All ages |
|---|---|---|---|---|
| **Alcohol use** | | | | |
| Interpersonal violence | 24 g/day | Both | Both | 1.256 (1.055 to 1.46) |
| Interpersonal violence | 12 g/day | Both | Both | 1.129 (0.963 to 1.317) |
| Interpersonal violence | 0 g/day | Both | Both | 1.0 (1.0 to 1.0) |

| Appendix Table 6d. Relative risks used by age and sex for each outcome for smoking globally. | | | | | | | | | | | | | | | | | |
|---|---|---|---|---|---|---|---|---|---|---|---|---|---|---|---|---|---|
| **Risk - Outcome** | **Category / Units** | **Morbidity / Mortality** | **Sex** | **Age** | | | | | | | | | | | | | |
| | | | | **30 to 34** | **35 to 39** | **40 to 44** | **45 to 49** | **50 to 54** | **55 to 59** | **60 to 64** | **65 to 69** | **70 to 74** | **75 to 79** | **80 to 84** | **85 to 89** | **90 to 94** | **95 plus** |
| **Smoking** | | | | | | | | | | | | | | | | | |
| Latent tuberculosis infection | 0 Cigarettes Per Smoker Per Day | Both | Male | 1.0 (1.0 to 1.0) | 1.0 (1.0 to 1.0) | 1.0 (1.0 to 1.0) | 1.0 (1.0 to 1.0) | 1.0 (1.0 to 1.0) | 1.0 (1.0 to 1.0) | 1.0 (1.0 to 1.0) | 1.0 (1.0 to 1.0) | 1.0 (1.0 to 1.0) | 1.0 (1.0 to 1.0) | 1.0 (1.0 to 1.0) | 1.0 (1.0 to 1.0) | 1.0 (1.0 to 1.0) | 1.0 (1.0 to 1.0) |
| Latent tuberculosis infection | 0 Cigarettes Per Smoker Per Day | Both | Female | 1.0 (1.0 to 1.0) | 1.0 (1.0 to 1.0) | 1.0 (1.0 to 1.0) | 1.0 (1.0 to 1.0) | 1.0 (1.0 to 1.0) | 1.0 (1.0 to 1.0) | 1.0 (1.0 to 1.0) | 1.0 (1.0 to 1.0) | 1.0 (1.0 to 1.0) | 1.0 (1.0 to 1.0) | 1.0 (1.0 to 1.0) | 1.0 (1.0 to 1.0) | 1.0 (1.0 to 1.0) | 1.0 (1.0 to 1.0) |
| Latent tuberculosis infection | 9 Cigarettes Per Smoker Per Day | Both | Male | 2.073 (1.605 to 2.549) | 2.073 (1.605 to 2.549) | 2.073 (1.605 to 2.549) | 2.073 (1.605 to 2.549) | 2.073 (1.605 to 2.549) | 2.073 (1.605 to 2.549) | 2.073 (1.605 to 2.549) | 2.073 (1.605 to 2.549) | 2.073 (1.605 to 2.549) | 2.073 (1.605 to 2.549) | 2.073 (1.605 to 2.549) | 2.073 (1.605 to 2.549) | 2.073 (1.605 to 2.549) | 2.073 (1.605 to 2.549) |
| Latent tuberculosis infection | 9 Cigarettes Per Smoker Per Day | Both | Female | 2.073 (1.605 to 2.549) | 2.073 (1.605 to 2.549) | 2.073 (1.605 to 2.549) | 2.073 (1.605 to 2.549) | 2.073 (1.605 to 2.549) | 2.073 (1.605 to 2.549) | 2.073 (1.605 to 2.549) | 2.073 (1.605 to 2.549) | 2.073 (1.605 to 2.549) | 2.073 (1.605 to 2.549) | 2.073 (1.605 to 2.549) | 2.073 (1.605 to 2.549) | 2.073 (1.605 to 2.549) | 2.073 (1.605 to 2.549) |
| Latent tuberculosis infection | 18 Cigarettes Per Smoker Per Day | Both | Male | 2.311 (1.808 to 2.921) | 2.311 (1.808 to 2.921) | 2.311 (1.808 to 2.921) | 2.311 (1.808 to 2.921) | 2.311 (1.808 to 2.921) | 2.311 (1.808 to 2.921) | 2.311 (1.808 to 2.921) | 2.311 (1.808 to 2.921) | 2.311 (1.808 to 2.921) | 2.311 (1.808 to 2.921) | 2.311 (1.808 to 2.921) | 2.311 (1.808 to 2.921) | 2.311 (1.808 to 2.921) | 2.311 (1.808 to 2.921) |
| Latent tuberculosis infection | 18 Cigarettes Per Smoker Per Day | Both | Female | 2.311 (1.808 to 2.921) | 2.311 (1.808 to 2.921) | 2.311 (1.808 to 2.921) | 2.311 (1.808 to 2.921) | 2.311 (1.808 to 2.921) | 2.311 (1.808 to 2.921) | 2.311 (1.808 to 2.921) | 2.311 (1.808 to 2.921) | 2.311 (1.808 to 2.921) | 2.311 (1.808 to 2.921) | 2.311 (1.808 to 2.921) | 2.311 (1.808 to 2.921) | 2.311 (1.808 to 2.921) | 2.311 (1.808 to 2.921) |
| Latent tuberculosis infection | 27 Cigarettes Per Smoker Per Day | Both | Male | 3.029 (2.315 to 3.969) | 3.029 (2.315 to 3.969) | 3.029 (2.315 to 3.969) | 3.029 (2.315 to 3.969) | 3.029 (2.315 to 3.969) | 3.029 (2.315 to 3.969) | 3.029 (2.315 to 3.969) | 3.029 (2.315 to 3.969) | 3.029 (2.315 to 3.969) | 3.029 (2.315 to 3.969) | 3.029 (2.315 to 3.969) | 3.029 (2.315 to 3.969) | 3.029 (2.315 to 3.969) | 3.029 (2.315 to 3.969) |
| Latent tuberculosis infection | 27 Cigarettes Per Smoker Per Day | Both | Female | 3.029 (2.315 to 3.969) | 3.029 (2.315 to 3.969) | 3.029 (2.315 to 3.969) | 3.029 (2.315 to 3.969) | 3.029 (2.315 to 3.969) | 3.029 (2.315 to 3.969) | 3.029 (2.315 to 3.969) | 3.029 (2.315 to 3.969) | 3.029 (2.315 to 3.969) | 3.029 (2.315 to 3.969) | 3.029 (2.315 to 3.969) | 3.029 (2.315 to 3.969) | 3.029 (2.315 to 3.969) | 3.029 (2.315 to 3.969) |
| Latent tuberculosis infection | 36 Cigarettes Per Smoker Per Day | Both | Male | 4.013 (2.49 to 5.991) | 4.013 (2.49 to 5.991) | 4.013 (2.49 to 5.991) | 4.013 (2.49 to 5.991) | 4.013 (2.49 to 5.991) | 4.013 (2.49 to 5.991) | 4.013 (2.49 to 5.991) | 4.013 (2.49 to 5.991) | 4.013 (2.49 to 5.991) | 4.013 (2.49 to 5.991) | 4.013 (2.49 to 5.991) | 4.013 (2.49 to 5.991) | 4.013 (2.49 to 5.991) | 4.013 (2.49 to 5.991) |
| Latent tuberculosis infection | 36 Cigarettes Per Smoker Per Day | Both | Female | 4.013 (2.49 to 5.991) | 4.013 (2.49 to 5.991) | 4.013 (2.49 to 5.991) | 4.013 (2.49 to 5.991) | 4.013 (2.49 to 5.991) | 4.013 (2.49 to 5.991) | 4.013 (2.49 to 5.991) | 4.013 (2.49 to 5.991) | 4.013 (2.49 to 5.991) | 4.013 (2.49 to 5.991) | 4.013 (2.49 to 5.991) | 4.013 (2.49 to 5.991) | 4.013 (2.49 to 5.991) | 4.013 (2.49 to 5.991) |
| Drug-susceptible tuberculosis | 0 Cigarettes Per Smoker Per Day | Both | Male | 1.0 (1.0 to 1.0) | 1.0 (1.0 to 1.0) | 1.0 (1.0 to 1.0) | 1.0 (1.0 to 1.0) | 1.0 (1.0 to 1.0) | 1.0 (1.0 to 1.0) | 1.0 (1.0 to 1.0) | 1.0 (1.0 to 1.0) | 1.0 (1.0 to 1.0) | 1.0 (1.0 to 1.0) | 1.0 (1.0 to 1.0) | 1.0 (1.0 to 1.0) | 1.0 (1.0 to 1.0) | 1.0 (1.0 to 1.0) |
| Drug-susceptible tuberculosis | 0 Cigarettes Per Smoker Per Day | Both | Female | 1.0 (1.0 to 1.0) | 1.0 (1.0 to 1.0) | 1.0 (1.0 to 1.0) | 1.0 (1.0 to 1.0) | 1.0 (1.0 to 1.0) | 1.0 (1.0 to 1.0) | 1.0 (1.0 to 1.0) | 1.0 (1.0 to 1.0) | 1.0 (1.0 to 1.0) | 1.0 (1.0 to 1.0) | 1.0 (1.0 to 1.0) | 1.0 (1.0 to 1.0) | 1.0 (1.0 to 1.0) | 1.0 (1.0 to 1.0) |
| Drug-susceptible tuberculosis | 9 Cigarettes Per Smoker Per Day | Both | Male | 2.073 (1.605 to 2.549) | 2.073 (1.605 to 2.549) | 2.073 (1.605 to 2.549) | 2.073 (1.605 to 2.549) | 2.073 (1.605 to 2.549) | 2.073 (1.605 to 2.549) | 2.073 (1.605 to 2.549) | 2.073 (1.605 to 2.549) | 2.073 (1.605 to 2.549) | 2.073 (1.605 to 2.549) | 2.073 (1.605 to 2.549) | 2.073 (1.605 to 2.549) | 2.073 (1.605 to 2.549) | 2.073 (1.605 to 2.549) |
| Drug-susceptible tuberculosis | 9 Cigarettes Per Smoker Per Day | Both | Female | 2.073 (1.605 to 2.549) | 2.073 (1.605 to 2.549) | 2.073 (1.605 to 2.549) | 2.073 (1.605 to 2.549) | 2.073 (1.605 to 2.549) | 2.073 (1.605 to 2.549) | 2.073 (1.605 to 2.549) | 2.073 (1.605 to 2.549) | 2.073 (1.605 to 2.549) | 2.073 (1.605 to 2.549) | 2.073 (1.605 to 2.549) | 2.073 (1.605 to 2.549) | 2.073 (1.605 to 2.549) | 2.073 (1.605 to 2.549) |
| Drug-susceptible tuberculosis | 18 Cigarettes Per Smoker Per Day | Both | Male | 2.311 (1.808 to 2.921) | 2.311 (1.808 to 2.921) | 2.311 (1.808 to 2.921) | 2.311 (1.808 to 2.921) | 2.311 (1.808 to 2.921) | 2.311 (1.808 to 2.921) | 2.311 (1.808 to 2.921) | 2.311 (1.808 to 2.921) | 2.311 (1.808 to 2.921) | 2.311 (1.808 to 2.921) | 2.311 (1.808 to 2.921) | 2.311 (1.808 to 2.921) | 2.311 (1.808 to 2.921) | 2.311 (1.808 to 2.921) |
| Drug-susceptible tuberculosis | 18 Cigarettes Per Smoker Per Day | Both | Female | 2.311 (1.808 to 2.921) | 2.311 (1.808 to 2.921) | 2.311 (1.808 to 2.921) | 2.311 (1.808 to 2.921) | 2.311 (1.808 to 2.921) | 2.311 (1.808 to 2.921) | 2.311 (1.808 to 2.921) | 2.311 (1.808 to 2.921) | 2.311 (1.808 to 2.921) | 2.311 (1.808 to 2.921) | 2.311 (1.808 to 2.921) | 2.311 (1.808 to 2.921) | 2.311 (1.808 to 2.921) | 2.311 (1.808 to 2.921) |
| Drug-susceptible tuberculosis | 27 Cigarettes Per Smoker Per Day | Both | Male | 3.029 (2.315 to 3.969) | 3.029 (2.315 to 3.969) | 3.029 (2.315 to 3.969) | 3.029 (2.315 to 3.969) | 3.029 (2.315 to 3.969) | 3.029 (2.315 to 3.969) | 3.029 (2.315 to 3.969) | 3.029 (2.315 to 3.969) | 3.029 (2.315 to 3.969) | 3.029 (2.315 to 3.969) | 3.029 (2.315 to 3.969) | 3.029 (2.315 to 3.969) | 3.029 (2.315 to 3.969) | 3.029 (2.315 to 3.969) |
| Drug-susceptible tuberculosis | 27 Cigarettes Per Smoker Per Day | Both | Female | 3.029 (2.315 to 3.969) | 3.029 (2.315 to 3.969) | 3.029 (2.315 to 3.969) | 3.029 (2.315 to 3.969) | 3.029 (2.315 to 3.969) | 3.029 (2.315 to 3.969) | 3.029 (2.315 to 3.969) | 3.029 (2.315 to 3.969) | 3.029 (2.315 to 3.969) | 3.029 (2.315 to 3.969) | 3.029 (2.315 to 3.969) | 3.029 (2.315 to 3.969) | 3.029 (2.315 to 3.969) | 3.029 (2.315 to 3.969) |
| Drug-susceptible tuberculosis | 36 Cigarettes Per Smoker Per Day | Both | Male | 4.013 (2.49 to 5.991) | 4.013 (2.49 to 5.991) | 4.013 (2.49 to 5.991) | 4.013 (2.49 to 5.991) | 4.013 (2.49 to 5.991) | 4.013 (2.49 to 5.991) | 4.013 (2.49 to 5.991) | 4.013 (2.49 to 5.991) | 4.013 (2.49 to 5.991) | 4.013 (2.49 to 5.991) | 4.013 (2.49 to 5.991) | 4.013 (2.49 to 5.991) | 4.013 (2.49 to 5.991) | 4.013 (2.49 to 5.991) |
| Drug-susceptible tuberculosis | 36 Cigarettes Per Smoker Per Day | Both | Female | 4.013 (2.49 to 5.991) | 4.013 (2.49 to 5.991) | 4.013 (2.49 to 5.991) | 4.013 (2.49 to 5.991) | 4.013 (2.49 to 5.991) | 4.013 (2.49 to 5.991) | 4.013 (2.49 to 5.991) | 4.013 (2.49 to 5.991) | 4.013 (2.49 to 5.991) | 4.013 (2.49 to 5.991) | 4.013 (2.49 to 5.991) | 4.013 (2.49 to 5.991) | 4.013 (2.49 to 5.991) | 4.013 (2.49 to 5.991) |
| Multidrug-resistant tuberculosis without extensive drug resistance | 0 Cigarettes Per Smoker Per Day | Both | Male | 1.0 (1.0 to 1.0) | 1.0 (1.0 to 1.0) | 1.0 (1.0 to 1.0) | 1.0 (1.0 to 1.0) | 1.0 (1.0 to 1.0) | 1.0 (1.0 to 1.0) | 1.0 (1.0 to 1.0) | 1.0 (1.0 to 1.0) | 1.0 (1.0 to 1.0) | 1.0 (1.0 to 1.0) | 1.0 (1.0 to 1.0) | 1.0 (1.0 to 1.0) | 1.0 (1.0 to 1.0) | 1.0 (1.0 to 1.0) |
| Multidrug-resistant tuberculosis without extensive drug resistance | 0 Cigarettes Per Smoker Per Day | Both | Female | 1.0 (1.0 to 1.0) | 1.0 (1.0 to 1.0) | 1.0 (1.0 to 1.0) | 1.0 (1.0 to 1.0) | 1.0 (1.0 to 1.0) | 1.0 (1.0 to 1.0) | 1.0 (1.0 to 1.0) | 1.0 (1.0 to 1.0) | 1.0 (1.0 to 1.0) | 1.0 (1.0 to 1.0) | 1.0 (1.0 to 1.0) | 1.0 (1.0 to 1.0) | 1.0 (1.0 to 1.0) | 1.0 (1.0 to 1.0) |
| Multidrug-resistant tuberculosis without extensive drug resistance | 9 Cigarettes Per Smoker Per Day | Both | Male | 2.073 (1.605 to 2.549) | 2.073 (1.605 to 2.549) | 2.073 (1.605 to 2.549) | 2.073 (1.605 to 2.549) | 2.073 (1.605 to 2.549) | 2.073 (1.605 to 2.549) | 2.073 (1.605 to 2.549) | 2.073 (1.605 to 2.549) | 2.073 (1.605 to 2.549) | 2.073 (1.605 to 2.549) | 2.073 (1.605 to 2.549) | 2.073 (1.605 to 2.549) | 2.073 (1.605 to 2.549) | 2.073 (1.605 to 2.549) |
| Multidrug-resistant tuberculosis without extensive drug resistance | 9 Cigarettes Per Smoker Per Day | Both | Female | 2.073 (1.605 to 2.549) | 2.073 (1.605 to 2.549) | 2.073 (1.605 to 2.549) | 2.073 (1.605 to 2.549) | 2.073 (1.605 to 2.549) | 2.073 (1.605 to 2.549) | 2.073 (1.605 to 2.549) | 2.073 (1.605 to 2.549) | 2.073 (1.605 to 2.549) | 2.073 (1.605 to 2.549) | 2.073 (1.605 to 2.549) | 2.073 (1.605 to 2.549) | 2.073 (1.605 to 2.549) | 2.073 (1.605 to 2.549) |
| Multidrug-resistant tuberculosis without extensive drug resistance | 18 Cigarettes Per Smoker Per Day | Both | Male | 2.311 (1.808 to 2.921) | 2.311 (1.808 to 2.921) | 2.311 (1.808 to 2.921) | 2.311 (1.808 to 2.921) | 2.311 (1.808 to 2.921) | 2.311 (1.808 to 2.921) | 2.311 (1.808 to 2.921) | 2.311 (1.808 to 2.921) | 2.311 (1.808 to 2.921) | 2.311 (1.808 to 2.921) | 2.311 (1.808 to 2.921) | 2.311 (1.808 to 2.921) | 2.311 (1.808 to 2.921) | 2.311 (1.808 to 2.921) |
| Multidrug-resistant tuberculosis without extensive drug resistance | 18 Cigarettes Per Smoker Per Day | Both | Female | 2.311 (1.808 to 2.921) | 2.311 (1.808 to 2.921) | 2.311 (1.808 to 2.921) | 2.311 (1.808 to 2.921) | 2.311 (1.808 to 2.921) | 2.311 (1.808 to 2.921) | 2.311 (1.808 to 2.921) | 2.311 (1.808 to 2.921) | 2.311 (1.808 to 2.921) | 2.311 (1.808 to 2.921) | 2.311 (1.808 to 2.921) | 2.311 (1.808 to 2.921) | 2.311 (1.808 to 2.921) | 2.311 (1.808 to 2.921) |
| Multidrug-resistant tuberculosis without extensive drug resistance | 27 Cigarettes Per Smoker Per Day | Both | Male | 3.029 (2.315 to 3.969) | 3.029 (2.315 to 3.969) | 3.029 (2.315 to 3.969) | 3.029 (2.315 to 3.969) | 3.029 (2.315 to 3.969) | 3.029 (2.315 to 3.969) | 3.029 (2.315 to 3.969) | 3.029 (2.315 to 3.969) | 3.029 (2.315 to 3.969) | 3.029 (2.315 to 3.969) | 3.029 (2.315 to 3.969) | 3.029 (2.315 to 3.969) | 3.029 (2.315 to 3.969) | 3.029 (2.315 to 3.969) |
| Multidrug-resistant tuberculosis without extensive drug resistance | 27 Cigarettes Per Smoker Per Day | Both | Female | 3.029 (2.315 to 3.969) | 3.029 (2.315 to 3.969) | 3.029 (2.315 to 3.969) | 3.029 (2.315 to 3.969) | 3.029 (2.315 to 3.969) | 3.029 (2.315 to 3.969) | 3.029 (2.315 to 3.969) | 3.029 (2.315 to 3.969) | 3.029 (2.315 to 3.969) | 3.029 (2.315 to 3.969) | 3.029 (2.315 to 3.969) | 3.029 (2.315 to 3.969) | 3.029 (2.315 to 3.969) | 3.029 (2.315 to 3.969) |
| Multidrug-resistant tuberculosis without extensive drug resistance | 36 Cigarettes Per Smoker Per Day | Both | Male | 4.013 (2.49 to 5.991) | 4.013 (2.49 to 5.991) | 4.013 (2.49 to 5.991) | 4.013 (2.49 to 5.991) | 4.013 (2.49 to 5.991) | 4.013 (2.49 to 5.991) | 4.013 (2.49 to 5.991) | 4.013 (2.49 to 5.991) | 4.013 (2.49 to 5.991) | 4.013 (2.49 to 5.991) | 4.013 (2.49 to 5.991) | 4.013 (2.49 to 5.991) | 4.013 (2.49 to 5.991) | 4.013 (2.49 to 5.991) |
| Multidrug-resistant tuberculosis without extensive drug resistance | 36 Cigarettes Per Smoker Per Day | Both | Female | 4.013 (2.49 to 5.991) | 4.013 (2.49 to 5.991) | 4.013 (2.49 to 5.991) | 4.013 (2.49 to 5.991) | 4.013 (2.49 to 5.991) | 4.013 (2.49 to 5.991) | 4.013 (2.49 to 5.991) | 4.013 (2.49 to 5.991) | 4.013 (2.49 to 5.991) | 4.013 (2.49 to 5.991) | 4.013 (2.49 to 5.991) | 4.013 (2.49 to 5.991) | 4.013 (2.49 to 5.991) | 4.013 (2.49 to 5.991) |
| Extensively drug-resistant tuberculosis | 0 Cigarettes Per Smoker Per Day | Both | Male | 1.0 (1.0 to 1.0) | 1.0 (1.0 to 1.0) | 1.0 (1.0 to 1.0) | 1.0 (1.0 to 1.0) | 1.0 (1.0 to 1.0) | 1.0 (1.0 to 1.0) | 1.0 (1.0 to 1.0) | 1.0 (1.0 to 1.0) | 1.0 (1.0 to 1.0) | 1.0 (1.0 to 1.0) | 1.0 (1.0 to 1.0) | 1.0 (1.0 to 1.0) | 1.0 (1.0 to 1.0) | 1.0 (1.0 to 1.0) |
| Extensively drug-resistant tuberculosis | 0 Cigarettes Per Smoker Per Day | Both | Female | 1.0 (1.0 to 1.0) | 1.0 (1.0 to 1.0) | 1.0 (1.0 to 1.0) | 1.0 (1.0 to 1.0) | 1.0 (1.0 to 1.0) | 1.0 (1.0 to 1.0) | 1.0 (1.0 to 1.0) | 1.0 (1.0 to 1.0) | 1.0 (1.0 to 1.0) | 1.0 (1.0 to 1.0) | 1.0 (1.0 to 1.0) | 1.0 (1.0 to 1.0) | 1.0 (1.0 to 1.0) | 1.0 (1.0 to 1.0) |
| Extensively drug-resistant tuberculosis | 9 Cigarettes Per Smoker Per Day | Both | Male | 2.073 (1.605 to 2.549) | 2.073 (1.605 to 2.549) | 2.073 (1.605 to 2.549) | 2.073 (1.605 to 2.549) | 2.073 (1.605 to 2.549) | 2.073 (1.605 to 2.549) | 2.073 (1.605 to 2.549) | 2.073 (1.605 to 2.549) | 2.073 (1.605 to 2.549) | 2.073 (1.605 to 2.549) | 2.073 (1.605 to 2.549) | 2.073 (1.605 to 2.549) | 2.073 (1.605 to 2.549) | 2.073 (1.605 to 2.549) |
| Extensively drug-resistant tuberculosis | 9 Cigarettes Per Smoker Per Day | Both | Female | 2.073 (1.605 to 2.549) | 2.073 (1.605 to 2.549) | 2.073 (1.605 to 2.549) | 2.073 (1.605 to 2.549) | 2.073 (1.605 to 2.549) | 2.073 (1.605 to 2.549) | 2.073 (1.605 to 2.549) | 2.073 (1.605 to 2.549) | 2.073 (1.605 to 2.549) | 2.073 (1.605 to 2.549) | 2.073 (1.605 to 2.549) | 2.073 (1.605 to 2.549) | 2.073 (1.605 to 2.549) | 2.073 (1.605 to 2.549) |
| Extensively drug-resistant tuberculosis | 18 Cigarettes Per Smoker Per Day | Both | Male | 2.311 (1.808 to 2.921) | 2.311 (1.808 to 2.921) | 2.311 (1.808 to 2.921) | 2.311 (1.808 to 2.921) | 2.311 (1.808 to 2.921) | 2.311 (1.808 to 2.921) | 2.311 (1.808 to 2.921) | 2.311 (1.808 to 2.921) | 2.311 (1.808 to 2.921) | 2.311 (1.808 to 2.921) | 2.311 (1.808 to 2.921) | 2.311 (1.808 to 2.921) | 2.311 (1.808 to 2.921) | 2.311 (1.808 to 2.921) |
| Extensively drug-resistant tuberculosis | 18 Cigarettes Per Smoker Per Day | Both | Female | 2.311 (1.808 to 2.921) | 2.311 (1.808 to 2.921) | 2.311 (1.808 to 2.921) | 2.311 (1.808 to 2.921) | 2.311 (1.808 to 2.921) | 2.311 (1.808 to 2.921) | 2.311 (1.808 to 2.921) | 2.311 (1.808 to 2.921) | 2.311 (1.808 to 2.921) | 2.311 (1.808 to 2.921) | 2.311 (1.808 to 2.921) | 2.311 (1.808 to 2.921) | 2.311 (1.808 to 2.921) | 2.311 (1.808 to 2.921) |
| Extensively drug-resistant tuberculosis | 27 Cigarettes Per Smoker Per Day | Both | Male | 3.029 (2.315 to 3.969) | 3.029 (2.315 to 3.969) | 3.029 (2.315 to 3.969) | 3.029 (2.315 to 3.969) | 3.029 (2.315 to 3.969) | 3.029 (2.315 to 3.969) | 3.029 (2.315 to 3.969) | 3.029 (2.315 to 3.969) | 3.029 (2.315 to 3.969) | 3.029 (2.315 to 3.969) | 3.029 (2.315 to 3.969) | 3.029 (2.315 to 3.969) | 3.029 (2.315 to 3.969) | 3.029 (2.315 to 3.969) |

| Appendix Table 6d. Relative risks used by age and sex for each outcome for smoking globally. | | | | | | | | | | | | | | | | | |
|---|---|---|---|---|---|---|---|---|---|---|---|---|---|---|---|---|---|
| Risk - Outcome | Category / Units | Morbidity / Mortality | Sex | Age | | | | | | | | | | | | | |
| | | | | 30 to 34 | 35 to 39 | 40 to 44 | 45 to 49 | 50 to 54 | 55 to 59 | 60 to 64 | 65 to 69 | 70 to 74 | 75 to 79 | 80 to 84 | 85 to 89 | 90 to 94 | 95 plus |
| Smoking | | | | | | | | | | | | | | | | | |
| Extensively drug-resistant tuberculosis | 27 Cigarettes Per Smoker Per Day | Both | Female | 3.029 (2.315 to 3.969) | 3.029 (2.315 to 3.969) | 3.029 (2.315 to 3.969) | 3.029 (2.315 to 3.969) | 3.029 (2.315 to 3.969) | 3.029 (2.315 to 3.969) | 3.029 (2.315 to 3.969) | 3.029 (2.315 to 3.969) | 3.029 (2.315 to 3.969) | 3.029 (2.315 to 3.969) | 3.029 (2.315 to 3.969) | 3.029 (2.315 to 3.969) | 3.029 (2.315 to 3.969) | 3.029 (2.315 to 3.969) |
| Extensively drug-resistant tuberculosis | 36 Cigarettes Per Smoker Per Day | Both | Male | 4.013 (2.49 to 5.991) | 4.013 (2.49 to 5.991) | 4.013 (2.49 to 5.991) | 4.013 (2.49 to 5.991) | 4.013 (2.49 to 5.991) | 4.013 (2.49 to 5.991) | 4.013 (2.49 to 5.991) | 4.013 (2.49 to 5.991) | 4.013 (2.49 to 5.991) | 4.013 (2.49 to 5.991) | 4.013 (2.49 to 5.991) | 4.013 (2.49 to 5.991) | 4.013 (2.49 to 5.991) | 4.013 (2.49 to 5.991) |
| Extensively drug-resistant tuberculosis | 36 Cigarettes Per Smoker Per Day | Both | Female | 4.013 (2.49 to 5.991) | 4.013 (2.49 to 5.991) | 4.013 (2.49 to 5.991) | 4.013 (2.49 to 5.991) | 4.013 (2.49 to 5.991) | 4.013 (2.49 to 5.991) | 4.013 (2.49 to 5.991) | 4.013 (2.49 to 5.991) | 4.013 (2.49 to 5.991) | 4.013 (2.49 to 5.991) | 4.013 (2.49 to 5.991) | 4.013 (2.49 to 5.991) | 4.013 (2.49 to 5.991) | 4.013 (2.49 to 5.991) |
| Lower respiratory infections | 0 Cigarettes Per Smoker Per Day | Both | Male | 1.0 (1.0 to 1.0) | 1.0 (1.0 to 1.0) | 1.0 (1.0 to 1.0) | 1.0 (1.0 to 1.0) | 1.0 (1.0 to 1.0) | 1.0 (1.0 to 1.0) | 1.0 (1.0 to 1.0) | 1.0 (1.0 to 1.0) | 1.0 (1.0 to 1.0) | 1.0 (1.0 to 1.0) | 1.0 (1.0 to 1.0) | 1.0 (1.0 to 1.0) | 1.0 (1.0 to 1.0) | 1.0 (1.0 to 1.0) |
| Lower respiratory infections | 0 Cigarettes Per Smoker Per Day | Both | Female | 1.0 (1.0 to 1.0) | 1.0 (1.0 to 1.0) | 1.0 (1.0 to 1.0) | 1.0 (1.0 to 1.0) | 1.0 (1.0 to 1.0) | 1.0 (1.0 to 1.0) | 1.0 (1.0 to 1.0) | 1.0 (1.0 to 1.0) | 1.0 (1.0 to 1.0) | 1.0 (1.0 to 1.0) | 1.0 (1.0 to 1.0) | 1.0 (1.0 to 1.0) | 1.0 (1.0 to 1.0) | 1.0 (1.0 to 1.0) |
| Lower respiratory infections | 1.5 Cigarettes Per Smoker Per Day | Both | Male | 1.318 (1.0 to 1.785) | 1.318 (1.0 to 1.785) | 1.318 (1.0 to 1.785) | 1.318 (1.0 to 1.785) | 1.318 (1.0 to 1.785) | 1.318 (1.0 to 1.785) | 1.318 (1.0 to 1.785) | 1.318 (1.0 to 1.785) | 1.318 (1.0 to 1.785) | 1.318 (1.0 to 1.785) | 1.318 (1.0 to 1.785) | 1.318 (1.0 to 1.785) | 1.318 (1.0 to 1.785) | 1.318 (1.0 to 1.785) |
| Lower respiratory infections | 1.5 Cigarettes Per Smoker Per Day | Both | Female | 1.318 (1.0 to 1.785) | 1.318 (1.0 to 1.785) | 1.318 (1.0 to 1.785) | 1.318 (1.0 to 1.785) | 1.318 (1.0 to 1.785) | 1.318 (1.0 to 1.785) | 1.318 (1.0 to 1.785) | 1.318 (1.0 to 1.785) | 1.318 (1.0 to 1.785) | 1.318 (1.0 to 1.785) | 1.318 (1.0 to 1.785) | 1.318 (1.0 to 1.785) | 1.318 (1.0 to 1.785) | 1.318 (1.0 to 1.785) |
| Lower respiratory infections | 10 Cigarettes Per Smoker Per Day | Both | Male | 1.958 (1.356 to 2.731) | 1.958 (1.356 to 2.731) | 1.958 (1.356 to 2.731) | 1.958 (1.356 to 2.731) | 1.958 (1.356 to 2.731) | 1.958 (1.356 to 2.731) | 1.958 (1.356 to 2.731) | 1.958 (1.356 to 2.731) | 1.958 (1.356 to 2.731) | 1.958 (1.356 to 2.731) | 1.958 (1.356 to 2.731) | 1.958 (1.356 to 2.731) | 1.958 (1.356 to 2.731) | 1.958 (1.356 to 2.731) |
| Lower respiratory infections | 10 Cigarettes Per Smoker Per Day | Both | Female | 1.958 (1.356 to 2.731) | 1.958 (1.356 to 2.731) | 1.958 (1.356 to 2.731) | 1.958 (1.356 to 2.731) | 1.958 (1.356 to 2.731) | 1.958 (1.356 to 2.731) | 1.958 (1.356 to 2.731) | 1.958 (1.356 to 2.731) | 1.958 (1.356 to 2.731) | 1.958 (1.356 to 2.731) | 1.958 (1.356 to 2.731) | 1.958 (1.356 to 2.731) | 1.958 (1.356 to 2.731) | 1.958 (1.356 to 2.731) |
| Lower respiratory infections | 19.5 Cigarettes Per Smoker Per Day | Both | Male | 2.434 (1.738 to 3.307) | 2.434 (1.738 to 3.307) | 2.434 (1.738 to 3.307) | 2.434 (1.738 to 3.307) | 2.434 (1.738 to 3.307) | 2.434 (1.738 to 3.307) | 2.434 (1.738 to 3.307) | 2.434 (1.738 to 3.307) | 2.434 (1.738 to 3.307) | 2.434 (1.738 to 3.307) | 2.434 (1.738 to 3.307) | 2.434 (1.738 to 3.307) | 2.434 (1.738 to 3.307) | 2.434 (1.738 to 3.307) |
| Lower respiratory infections | 19.5 Cigarettes Per Smoker Per Day | Both | Female | 2.434 (1.738 to 3.307) | 2.434 (1.738 to 3.307) | 2.434 (1.738 to 3.307) | 2.434 (1.738 to 3.307) | 2.434 (1.738 to 3.307) | 2.434 (1.738 to 3.307) | 2.434 (1.738 to 3.307) | 2.434 (1.738 to 3.307) | 2.434 (1.738 to 3.307) | 2.434 (1.738 to 3.307) | 2.434 (1.738 to 3.307) | 2.434 (1.738 to 3.307) | 2.434 (1.738 to 3.307) | 2.434 (1.738 to 3.307) |
| Lower respiratory infections | 31.2 Cigarettes Per Smoker Per Day | Both | Male | 3.158 (2.286 to 4.195) | 3.158 (2.286 to 4.195) | 3.158 (2.286 to 4.195) | 3.158 (2.286 to 4.195) | 3.158 (2.286 to 4.195) | 3.158 (2.286 to 4.195) | 3.158 (2.286 to 4.195) | 3.158 (2.286 to 4.195) | 3.158 (2.286 to 4.195) | 3.158 (2.286 to 4.195) | 3.158 (2.286 to 4.195) | 3.158 (2.286 to 4.195) | 3.158 (2.286 to 4.195) | 3.158 (2.286 to 4.195) |
| Lower respiratory infections | 31.2 Cigarettes Per Smoker Per Day | Both | Female | 3.158 (2.286 to 4.195) | 3.158 (2.286 to 4.195) | 3.158 (2.286 to 4.195) | 3.158 (2.286 to 4.195) | 3.158 (2.286 to 4.195) | 3.158 (2.286 to 4.195) | 3.158 (2.286 to 4.195) | 3.158 (2.286 to 4.195) | 3.158 (2.286 to 4.195) | 3.158 (2.286 to 4.195) | 3.158 (2.286 to 4.195) | 3.158 (2.286 to 4.195) | 3.158 (2.286 to 4.195) | 3.158 (2.286 to 4.195) |
| Lip and oral cavity cancer | 0 Pack-Years | Both | Male | 1.0 (1.0 to 1.0) | 1.0 (1.0 to 1.0) | 1.0 (1.0 to 1.0) | 1.0 (1.0 to 1.0) | 1.0 (1.0 to 1.0) | 1.0 (1.0 to 1.0) | 1.0 (1.0 to 1.0) | 1.0 (1.0 to 1.0) | 1.0 (1.0 to 1.0) | 1.0 (1.0 to 1.0) | 1.0 (1.0 to 1.0) | 1.0 (1.0 to 1.0) | 1.0 (1.0 to 1.0) | 1.0 (1.0 to 1.0) |
| Lip and oral cavity cancer | 0 Pack-Years | Both | Female | 1.0 (1.0 to 1.0) | 1.0 (1.0 to 1.0) | 1.0 (1.0 to 1.0) | 1.0 (1.0 to 1.0) | 1.0 (1.0 to 1.0) | 1.0 (1.0 to 1.0) | 1.0 (1.0 to 1.0) | 1.0 (1.0 to 1.0) | 1.0 (1.0 to 1.0) | 1.0 (1.0 to 1.0) | 1.0 (1.0 to 1.0) | 1.0 (1.0 to 1.0) | 1.0 (1.0 to 1.0) | 1.0 (1.0 to 1.0) |
| Lip and oral cavity cancer | 17.7 Pack-Years | Both | Male | 3.19 (2.162 to 4.362) | 3.19 (2.162 to 4.362) | 3.19 (2.162 to 4.362) | 3.19 (2.162 to 4.362) | 3.19 (2.162 to 4.362) | 3.19 (2.162 to 4.362) | 3.19 (2.162 to 4.362) | 3.19 (2.162 to 4.362) | 3.19 (2.162 to 4.362) | 3.19 (2.162 to 4.362) | 3.19 (2.162 to 4.362) | 3.19 (2.162 to 4.362) | 3.19 (2.162 to 4.362) | 3.19 (2.162 to 4.362) |
| Lip and oral cavity cancer | 17.7 Pack-Years | Both | Female | 3.19 (2.162 to 4.362) | 3.19 (2.162 to 4.362) | 3.19 (2.162 to 4.362) | 3.19 (2.162 to 4.362) | 3.19 (2.162 to 4.362) | 3.19 (2.162 to 4.362) | 3.19 (2.162 to 4.362) | 3.19 (2.162 to 4.362) | 3.19 (2.162 to 4.362) | 3.19 (2.162 to 4.362) | 3.19 (2.162 to 4.362) | 3.19 (2.162 to 4.362) | 3.19 (2.162 to 4.362) | 3.19 (2.162 to 4.362) |
| Lip and oral cavity cancer | 35.4 Pack-Years | Both | Male | 3.754 (2.335 to 5.44) | 3.754 (2.335 to 5.44) | 3.754 (2.335 to 5.44) | 3.754 (2.335 to 5.44) | 3.754 (2.335 to 5.44) | 3.754 (2.335 to 5.44) | 3.754 (2.335 to 5.44) | 3.754 (2.335 to 5.44) | 3.754 (2.335 to 5.44) | 3.754 (2.335 to 5.44) | 3.754 (2.335 to 5.44) | 3.754 (2.335 to 5.44) | 3.754 (2.335 to 5.44) | 3.754 (2.335 to 5.44) |
| Lip and oral cavity cancer | 35.4 Pack-Years | Both | Female | 3.754 (2.335 to 5.44) | 3.754 (2.335 to 5.44) | 3.754 (2.335 to 5.44) | 3.754 (2.335 to 5.44) | 3.754 (2.335 to 5.44) | 3.754 (2.335 to 5.44) | 3.754 (2.335 to 5.44) | 3.754 (2.335 to 5.44) | 3.754 (2.335 to 5.44) | 3.754 (2.335 to 5.44) | 3.754 (2.335 to 5.44) | 3.754 (2.335 to 5.44) | 3.754 (2.335 to 5.44) | 3.754 (2.335 to 5.44) |
| Lip and oral cavity cancer | 53.1 Pack-Years | Both | Male | 4.017 (2.376 to 6.004) | 4.017 (2.376 to 6.004) | 4.017 (2.376 to 6.004) | 4.017 (2.376 to 6.004) | 4.017 (2.376 to 6.004) | 4.017 (2.376 to 6.004) | 4.017 (2.376 to 6.004) | 4.017 (2.376 to 6.004) | 4.017 (2.376 to 6.004) | 4.017 (2.376 to 6.004) | 4.017 (2.376 to 6.004) | 4.017 (2.376 to 6.004) | 4.017 (2.376 to 6.004) | 4.017 (2.376 to 6.004) |
| Lip and oral cavity cancer | 53.1 Pack-Years | Both | Female | 4.017 (2.376 to 6.004) | 4.017 (2.376 to 6.004) | 4.017 (2.376 to 6.004) | 4.017 (2.376 to 6.004) | 4.017 (2.376 to 6.004) | 4.017 (2.376 to 6.004) | 4.017 (2.376 to 6.004) | 4.017 (2.376 to 6.004) | 4.017 (2.376 to 6.004) | 4.017 (2.376 to 6.004) | 4.017 (2.376 to 6.004) | 4.017 (2.376 to 6.004) | 4.017 (2.376 to 6.004) | 4.017 (2.376 to 6.004) |
| Lip and oral cavity cancer | 70.8 Pack-Years | Both | Male | 5.517 (2.574 to 9.804) | 5.517 (2.574 to 9.804) | 5.517 (2.574 to 9.804) | 5.517 (2.574 to 9.804) | 5.517 (2.574 to 9.804) | 5.517 (2.574 to 9.804) | 5.517 (2.574 to 9.804) | 5.517 (2.574 to 9.804) | 5.517 (2.574 to 9.804) | 5.517 (2.574 to 9.804) | 5.517 (2.574 to 9.804) | 5.517 (2.574 to 9.804) | 5.517 (2.574 to 9.804) | 5.517 (2.574 to 9.804) |
| Lip and oral cavity cancer | 70.8 Pack-Years | Both | Female | 5.517 (2.574 to 9.804) | 5.517 (2.574 to 9.804) | 5.517 (2.574 to 9.804) | 5.517 (2.574 to 9.804) | 5.517 (2.574 to 9.804) | 5.517 (2.574 to 9.804) | 5.517 (2.574 to 9.804) | 5.517 (2.574 to 9.804) | 5.517 (2.574 to 9.804) | 5.517 (2.574 to 9.804) | 5.517 (2.574 to 9.804) | 5.517 (2.574 to 9.804) | 5.517 (2.574 to 9.804) | 5.517 (2.574 to 9.804) |
| Lip and oral cavity cancer | 88.5 Pack-Years | Both | Male | 7.27 (2.599 to 14.741) | 7.27 (2.599 to 14.741) | 7.27 (2.599 to 14.741) | 7.27 (2.599 to 14.741) | 7.27 (2.599 to 14.741) | 7.27 (2.599 to 14.741) | 7.27 (2.599 to 14.741) | 7.27 (2.599 to 14.741) | 7.27 (2.599 to 14.741) | 7.27 (2.599 to 14.741) | 7.27 (2.599 to 14.741) | 7.27 (2.599 to 14.741) | 7.27 (2.599 to 14.741) | 7.27 (2.599 to 14.741) |
| Lip and oral cavity cancer | 88.5 Pack-Years | Both | Female | 7.27 (2.599 to 14.741) | 7.27 (2.599 to 14.741) | 7.27 (2.599 to 14.741) | 7.27 (2.599 to 14.741) | 7.27 (2.599 to 14.741) | 7.27 (2.599 to 14.741) | 7.27 (2.599 to 14.741) | 7.27 (2.599 to 14.741) | 7.27 (2.599 to 14.741) | 7.27 (2.599 to 14.741) | 7.27 (2.599 to 14.741) | 7.27 (2.599 to 14.741) | 7.27 (2.599 to 14.741) | 7.27 (2.599 to 14.741) |
| Nasopharynx cancer | 0 Pack-Years | Both | Male | 1.0 (1.0 to 1.0) | 1.0 (1.0 to 1.0) | 1.0 (1.0 to 1.0) | 1.0 (1.0 to 1.0) | 1.0 (1.0 to 1.0) | 1.0 (1.0 to 1.0) | 1.0 (1.0 to 1.0) | 1.0 (1.0 to 1.0) | 1.0 (1.0 to 1.0) | 1.0 (1.0 to 1.0) | 1.0 (1.0 to 1.0) | 1.0 (1.0 to 1.0) | 1.0 (1.0 to 1.0) | 1.0 (1.0 to 1.0) |
| Nasopharynx cancer | 0 Pack-Years | Both | Female | 1.0 (1.0 to 1.0) | 1.0 (1.0 to 1.0) | 1.0 (1.0 to 1.0) | 1.0 (1.0 to 1.0) | 1.0 (1.0 to 1.0) | 1.0 (1.0 to 1.0) | 1.0 (1.0 to 1.0) | 1.0 (1.0 to 1.0) | 1.0 (1.0 to 1.0) | 1.0 (1.0 to 1.0) | 1.0 (1.0 to 1.0) | 1.0 (1.0 to 1.0) | 1.0 (1.0 to 1.0) | 1.0 (1.0 to 1.0) |
| Nasopharynx cancer | 10.7 Pack-Years | Both | Male | 1.879 (1.34 to 2.522) | 1.879 (1.34 to 2.522) | 1.879 (1.34 to 2.522) | 1.879 (1.34 to 2.522) | 1.879 (1.34 to 2.522) | 1.879 (1.34 to 2.522) | 1.879 (1.34 to 2.522) | 1.879 (1.34 to 2.522) | 1.879 (1.34 to 2.522) | 1.879 (1.34 to 2.522) | 1.879 (1.34 to 2.522) | 1.879 (1.34 to 2.522) | 1.879 (1.34 to 2.522) | 1.879 (1.34 to 2.522) |
| Nasopharynx cancer | 10.7 Pack-Years | Both | Female | 1.879 (1.34 to 2.522) | 1.879 (1.34 to 2.522) | 1.879 (1.34 to 2.522) | 1.879 (1.34 to 2.522) | 1.879 (1.34 to 2.522) | 1.879 (1.34 to 2.522) | 1.879 (1.34 to 2.522) | 1.879 (1.34 to 2.522) | 1.879 (1.34 to 2.522) | 1.879 (1.34 to 2.522) | 1.879 (1.34 to 2.522) | 1.879 (1.34 to 2.522) | 1.879 (1.34 to 2.522) | 1.879 (1.34 to 2.522) |
| Nasopharynx cancer | 21.4 Pack-Years | Both | Male | 1.992 (1.322 to 2.865) | 1.992 (1.322 to 2.865) | 1.992 (1.322 to 2.865) | 1.992 (1.322 to 2.865) | 1.992 (1.322 to 2.865) | 1.992 (1.322 to 2.865) | 1.992 (1.322 to 2.865) | 1.992 (1.322 to 2.865) | 1.992 (1.322 to 2.865) | 1.992 (1.322 to 2.865) | 1.992 (1.322 to 2.865) | 1.992 (1.322 to 2.865) | 1.992 (1.322 to 2.865) | 1.992 (1.322 to 2.865) |
| Nasopharynx cancer | 21.4 Pack-Years | Both | Female | 1.992 (1.322 to 2.865) | 1.992 (1.322 to 2.865) | 1.992 (1.322 to 2.865) | 1.992 (1.322 to 2.865) | 1.992 (1.322 to 2.865) | 1.992 (1.322 to 2.865) | 1.992 (1.322 to 2.865) | 1.992 (1.322 to 2.865) | 1.992 (1.322 to 2.865) | 1.992 (1.322 to 2.865) | 1.992 (1.322 to 2.865) | 1.992 (1.322 to 2.865) | 1.992 (1.322 to 2.865) | 1.992 (1.322 to 2.865) |
| Nasopharynx cancer | 32.1 Pack-Years | Both | Male | 2.297 (1.448 to 3.32) | 2.297 (1.448 to 3.32) | 2.297 (1.448 to 3.32) | 2.297 (1.448 to 3.32) | 2.297 (1.448 to 3.32) | 2.297 (1.448 to 3.32) | 2.297 (1.448 to 3.32) | 2.297 (1.448 to 3.32) | 2.297 (1.448 to 3.32) | 2.297 (1.448 to 3.32) | 2.297 (1.448 to 3.32) | 2.297 (1.448 to 3.32) | 2.297 (1.448 to 3.32) | 2.297 (1.448 to 3.32) |
| Nasopharynx cancer | 32.1 Pack-Years | Both | Female | 2.297 (1.448 to 3.32) | 2.297 (1.448 to 3.32) | 2.297 (1.448 to 3.32) | 2.297 (1.448 to 3.32) | 2.297 (1.448 to 3.32) | 2.297 (1.448 to 3.32) | 2.297 (1.448 to 3.32) | 2.297 (1.448 to 3.32) | 2.297 (1.448 to 3.32) | 2.297 (1.448 to 3.32) | 2.297 (1.448 to 3.32) | 2.297 (1.448 to 3.32) | 2.297 (1.448 to 3.32) | 2.297 (1.448 to 3.32) |
| Nasopharynx cancer | 42.9 Pack-Years | Both | Male | 2.662 (1.646 to 4.007) | 2.662 (1.646 to 4.007) | 2.662 (1.646 to 4.007) | 2.662 (1.646 to 4.007) | 2.662 (1.646 to 4.007) | 2.662 (1.646 to 4.007) | 2.662 (1.646 to 4.007) | 2.662 (1.646 to 4.007) | 2.662 (1.646 to 4.007) | 2.662 (1.646 to 4.007) | 2.662 (1.646 to 4.007) | 2.662 (1.646 to 4.007) | 2.662 (1.646 to 4.007) | 2.662 (1.646 to 4.007) |
| Nasopharynx cancer | 42.9 Pack-Years | Both | Female | 2.662 (1.646 to 4.007) | 2.662 (1.646 to 4.007) | 2.662 (1.646 to 4.007) | 2.662 (1.646 to 4.007) | 2.662 (1.646 to 4.007) | 2.662 (1.646 to 4.007) | 2.662 (1.646 to 4.007) | 2.662 (1.646 to 4.007) | 2.662 (1.646 to 4.007) | 2.662 (1.646 to 4.007) | 2.662 (1.646 to 4.007) | 2.662 (1.646 to 4.007) | 2.662 (1.646 to 4.007) | 2.662 (1.646 to 4.007) |
| Nasopharynx cancer | 53.6 Pack-Years | Both | Male | 3.377 (2.01 to 5.041) | 3.377 (2.01 to 5.041) | 3.377 (2.01 to 5.041) | 3.377 (2.01 to 5.041) | 3.377 (2.01 to 5.041) | 3.377 (2.01 to 5.041) | 3.377 (2.01 to 5.041) | 3.377 (2.01 to 5.041) | 3.377 (2.01 to 5.041) | 3.377 (2.01 to 5.041) | 3.377 (2.01 to 5.041) | 3.377 (2.01 to 5.041) | 3.377 (2.01 to 5.041) | 3.377 (2.01 to 5.041) |

**Appendix Table 6d. Relative risks used by age and sex for each outcome for smoking globally.**

| Risk - Outcome | Category / Units | Morbidity / Mortality | Sex | Age | | | | | | | | | | | | | |
|---|---|---|---|---|---|---|---|---|---|---|---|---|---|---|---|---|---|
| | | | | 30 to 34 | 35 to 39 | 40 to 44 | 45 to 49 | 50 to 54 | 55 to 59 | 60 to 64 | 65 to 69 | 70 to 74 | 75 to 79 | 80 to 84 | 85 to 89 | 90 to 94 | 95 plus |
| **Smoking** | | | | | | | | | | | | | | | | | |
| Nasopharynx cancer | 53.6 Pack-Years | Both | Female | 3.377 (2.01 to 5.041) | 3.377 (2.01 to 5.041) | 3.377 (2.01 to 5.041) | 3.377 (2.01 to 5.041) | 3.377 (2.01 to 5.041) | 3.377 (2.01 to 5.041) | 3.377 (2.01 to 5.041) | 3.377 (2.01 to 5.041) | 3.377 (2.01 to 5.041) | 3.377 (2.01 to 5.041) | 3.377 (2.01 to 5.041) | 3.377 (2.01 to 5.041) | 3.377 (2.01 to 5.041) | 3.377 (2.01 to 5.041) |
| Nasopharynx cancer | 64.3 Pack-Years | Both | Male | 4.466 (2.643 to 6.831) | 4.466 (2.643 to 6.831) | 4.466 (2.643 to 6.831) | 4.466 (2.643 to 6.831) | 4.466 (2.643 to 6.831) | 4.466 (2.643 to 6.831) | 4.466 (2.643 to 6.831) | 4.466 (2.643 to 6.831) | 4.466 (2.643 to 6.831) | 4.466 (2.643 to 6.831) | 4.466 (2.643 to 6.831) | 4.466 (2.643 to 6.831) | 4.466 (2.643 to 6.831) | 4.466 (2.643 to 6.831) |
| Nasopharynx cancer | 64.3 Pack-Years | Both | Female | 4.466 (2.643 to 6.831) | 4.466 (2.643 to 6.831) | 4.466 (2.643 to 6.831) | 4.466 (2.643 to 6.831) | 4.466 (2.643 to 6.831) | 4.466 (2.643 to 6.831) | 4.466 (2.643 to 6.831) | 4.466 (2.643 to 6.831) | 4.466 (2.643 to 6.831) | 4.466 (2.643 to 6.831) | 4.466 (2.643 to 6.831) | 4.466 (2.643 to 6.831) | 4.466 (2.643 to 6.831) | 4.466 (2.643 to 6.831) |
| Other pharynx cancer | 0 Pack-Years | Both | Male | 1.0 (1.0 to 1.0) | 1.0 (1.0 to 1.0) | 1.0 (1.0 to 1.0) | 1.0 (1.0 to 1.0) | 1.0 (1.0 to 1.0) | 1.0 (1.0 to 1.0) | 1.0 (1.0 to 1.0) | 1.0 (1.0 to 1.0) | 1.0 (1.0 to 1.0) | 1.0 (1.0 to 1.0) | 1.0 (1.0 to 1.0) | 1.0 (1.0 to 1.0) | 1.0 (1.0 to 1.0) | 1.0 (1.0 to 1.0) |
| Other pharynx cancer | 0 Pack-Years | Both | Female | 1.0 (1.0 to 1.0) | 1.0 (1.0 to 1.0) | 1.0 (1.0 to 1.0) | 1.0 (1.0 to 1.0) | 1.0 (1.0 to 1.0) | 1.0 (1.0 to 1.0) | 1.0 (1.0 to 1.0) | 1.0 (1.0 to 1.0) | 1.0 (1.0 to 1.0) | 1.0 (1.0 to 1.0) | 1.0 (1.0 to 1.0) | 1.0 (1.0 to 1.0) | 1.0 (1.0 to 1.0) | 1.0 (1.0 to 1.0) |
| Other pharynx cancer | 17.7 Pack-Years | Both | Male | 4.792 (3.267 to 6.498) | 4.792 (3.267 to 6.498) | 4.792 (3.267 to 6.498) | 4.792 (3.267 to 6.498) | 4.792 (3.267 to 6.498) | 4.792 (3.267 to 6.498) | 4.792 (3.267 to 6.498) | 4.792 (3.267 to 6.498) | 4.792 (3.267 to 6.498) | 4.792 (3.267 to 6.498) | 4.792 (3.267 to 6.498) | 4.792 (3.267 to 6.498) | 4.792 (3.267 to 6.498) | 4.792 (3.267 to 6.498) |
| Other pharynx cancer | 17.7 Pack-Years | Both | Female | 4.792 (3.267 to 6.498) | 4.792 (3.267 to 6.498) | 4.792 (3.267 to 6.498) | 4.792 (3.267 to 6.498) | 4.792 (3.267 to 6.498) | 4.792 (3.267 to 6.498) | 4.792 (3.267 to 6.498) | 4.792 (3.267 to 6.498) | 4.792 (3.267 to 6.498) | 4.792 (3.267 to 6.498) | 4.792 (3.267 to 6.498) | 4.792 (3.267 to 6.498) | 4.792 (3.267 to 6.498) | 4.792 (3.267 to 6.498) |
| Other pharynx cancer | 35.4 Pack-Years | Both | Male | 5.255 (3.541 to 7.384) | 5.255 (3.541 to 7.384) | 5.255 (3.541 to 7.384) | 5.255 (3.541 to 7.384) | 5.255 (3.541 to 7.384) | 5.255 (3.541 to 7.384) | 5.255 (3.541 to 7.384) | 5.255 (3.541 to 7.384) | 5.255 (3.541 to 7.384) | 5.255 (3.541 to 7.384) | 5.255 (3.541 to 7.384) | 5.255 (3.541 to 7.384) | 5.255 (3.541 to 7.384) | 5.255 (3.541 to 7.384) |
| Other pharynx cancer | 35.4 Pack-Years | Both | Female | 5.255 (3.541 to 7.384) | 5.255 (3.541 to 7.384) | 5.255 (3.541 to 7.384) | 5.255 (3.541 to 7.384) | 5.255 (3.541 to 7.384) | 5.255 (3.541 to 7.384) | 5.255 (3.541 to 7.384) | 5.255 (3.541 to 7.384) | 5.255 (3.541 to 7.384) | 5.255 (3.541 to 7.384) | 5.255 (3.541 to 7.384) | 5.255 (3.541 to 7.384) | 5.255 (3.541 to 7.384) | 5.255 (3.541 to 7.384) |
| Other pharynx cancer | 53.1 Pack-Years | Both | Male | 6.588 (4.406 to 8.892) | 6.588 (4.406 to 8.892) | 6.588 (4.406 to 8.892) | 6.588 (4.406 to 8.892) | 6.588 (4.406 to 8.892) | 6.588 (4.406 to 8.892) | 6.588 (4.406 to 8.892) | 6.588 (4.406 to 8.892) | 6.588 (4.406 to 8.892) | 6.588 (4.406 to 8.892) | 6.588 (4.406 to 8.892) | 6.588 (4.406 to 8.892) | 6.588 (4.406 to 8.892) | 6.588 (4.406 to 8.892) |
| Other pharynx cancer | 53.1 Pack-Years | Both | Female | 6.588 (4.406 to 8.892) | 6.588 (4.406 to 8.892) | 6.588 (4.406 to 8.892) | 6.588 (4.406 to 8.892) | 6.588 (4.406 to 8.892) | 6.588 (4.406 to 8.892) | 6.588 (4.406 to 8.892) | 6.588 (4.406 to 8.892) | 6.588 (4.406 to 8.892) | 6.588 (4.406 to 8.892) | 6.588 (4.406 to 8.892) | 6.588 (4.406 to 8.892) | 6.588 (4.406 to 8.892) | 6.588 (4.406 to 8.892) |
| Other pharynx cancer | 70.8 Pack-Years | Both | Male | 7.21 (3.973 to 11.61) | 7.21 (3.973 to 11.61) | 7.21 (3.973 to 11.61) | 7.21 (3.973 to 11.61) | 7.21 (3.973 to 11.61) | 7.21 (3.973 to 11.61) | 7.21 (3.973 to 11.61) | 7.21 (3.973 to 11.61) | 7.21 (3.973 to 11.61) | 7.21 (3.973 to 11.61) | 7.21 (3.973 to 11.61) | 7.21 (3.973 to 11.61) | 7.21 (3.973 to 11.61) | 7.21 (3.973 to 11.61) |
| Other pharynx cancer | 70.8 Pack-Years | Both | Female | 7.21 (3.973 to 11.61) | 7.21 (3.973 to 11.61) | 7.21 (3.973 to 11.61) | 7.21 (3.973 to 11.61) | 7.21 (3.973 to 11.61) | 7.21 (3.973 to 11.61) | 7.21 (3.973 to 11.61) | 7.21 (3.973 to 11.61) | 7.21 (3.973 to 11.61) | 7.21 (3.973 to 11.61) | 7.21 (3.973 to 11.61) | 7.21 (3.973 to 11.61) | 7.21 (3.973 to 11.61) | 7.21 (3.973 to 11.61) |
| Other pharynx cancer | 88.5 Pack-Years | Both | Male | 11.149 (5.54 to 18.571) | 11.149 (5.54 to 18.571) | 11.149 (5.54 to 18.571) | 11.149 (5.54 to 18.571) | 11.149 (5.54 to 18.571) | 11.149 (5.54 to 18.571) | 11.149 (5.54 to 18.571) | 11.149 (5.54 to 18.571) | 11.149 (5.54 to 18.571) | 11.149 (5.54 to 18.571) | 11.149 (5.54 to 18.571) | 11.149 (5.54 to 18.571) | 11.149 (5.54 to 18.571) | 11.149 (5.54 to 18.571) |
| Other pharynx cancer | 88.5 Pack-Years | Both | Female | 11.149 (5.54 to 18.571) | 11.149 (5.54 to 18.571) | 11.149 (5.54 to 18.571) | 11.149 (5.54 to 18.571) | 11.149 (5.54 to 18.571) | 11.149 (5.54 to 18.571) | 11.149 (5.54 to 18.571) | 11.149 (5.54 to 18.571) | 11.149 (5.54 to 18.571) | 11.149 (5.54 to 18.571) | 11.149 (5.54 to 18.571) | 11.149 (5.54 to 18.571) | 11.149 (5.54 to 18.571) | 11.149 (5.54 to 18.571) |
| Oesophageal cancer | 0 Pack-Years | Both | Male | 1.0 (1.0 to 1.0) | 1.0 (1.0 to 1.0) | 1.0 (1.0 to 1.0) | 1.0 (1.0 to 1.0) | 1.0 (1.0 to 1.0) | 1.0 (1.0 to 1.0) | 1.0 (1.0 to 1.0) | 1.0 (1.0 to 1.0) | 1.0 (1.0 to 1.0) | 1.0 (1.0 to 1.0) | 1.0 (1.0 to 1.0) | 1.0 (1.0 to 1.0) | 1.0 (1.0 to 1.0) | 1.0 (1.0 to 1.0) |
| Oesophageal cancer | 0 Pack-Years | Both | Female | 1.0 (1.0 to 1.0) | 1.0 (1.0 to 1.0) | 1.0 (1.0 to 1.0) | 1.0 (1.0 to 1.0) | 1.0 (1.0 to 1.0) | 1.0 (1.0 to 1.0) | 1.0 (1.0 to 1.0) | 1.0 (1.0 to 1.0) | 1.0 (1.0 to 1.0) | 1.0 (1.0 to 1.0) | 1.0 (1.0 to 1.0) | 1.0 (1.0 to 1.0) | 1.0 (1.0 to 1.0) | 1.0 (1.0 to 1.0) |
| Oesophageal cancer | 15 Pack-Years | Both | Male | 3.038 (2.318 to 3.817) | 3.038 (2.318 to 3.817) | 3.038 (2.318 to 3.817) | 3.038 (2.318 to 3.817) | 3.038 (2.318 to 3.817) | 3.038 (2.318 to 3.817) | 3.038 (2.318 to 3.817) | 3.038 (2.318 to 3.817) | 3.038 (2.318 to 3.817) | 3.038 (2.318 to 3.817) | 3.038 (2.318 to 3.817) | 3.038 (2.318 to 3.817) | 3.038 (2.318 to 3.817) | 3.038 (2.318 to 3.817) |
| Oesophageal cancer | 15 Pack-Years | Both | Female | 3.038 (2.318 to 3.817) | 3.038 (2.318 to 3.817) | 3.038 (2.318 to 3.817) | 3.038 (2.318 to 3.817) | 3.038 (2.318 to 3.817) | 3.038 (2.318 to 3.817) | 3.038 (2.318 to 3.817) | 3.038 (2.318 to 3.817) | 3.038 (2.318 to 3.817) | 3.038 (2.318 to 3.817) | 3.038 (2.318 to 3.817) | 3.038 (2.318 to 3.817) | 3.038 (2.318 to 3.817) | 3.038 (2.318 to 3.817) |
| Oesophageal cancer | 30 Pack-Years | Both | Male | 2.778 (1.884 to 3.796) | 2.778 (1.884 to 3.796) | 2.778 (1.884 to 3.796) | 2.778 (1.884 to 3.796) | 2.778 (1.884 to 3.796) | 2.778 (1.884 to 3.796) | 2.778 (1.884 to 3.796) | 2.778 (1.884 to 3.796) | 2.778 (1.884 to 3.796) | 2.778 (1.884 to 3.796) | 2.778 (1.884 to 3.796) | 2.778 (1.884 to 3.796) | 2.778 (1.884 to 3.796) | 2.778 (1.884 to 3.796) |
| Oesophageal cancer | 30 Pack-Years | Both | Female | 2.778 (1.884 to 3.796) | 2.778 (1.884 to 3.796) | 2.778 (1.884 to 3.796) | 2.778 (1.884 to 3.796) | 2.778 (1.884 to 3.796) | 2.778 (1.884 to 3.796) | 2.778 (1.884 to 3.796) | 2.778 (1.884 to 3.796) | 2.778 (1.884 to 3.796) | 2.778 (1.884 to 3.796) | 2.778 (1.884 to 3.796) | 2.778 (1.884 to 3.796) | 2.778 (1.884 to 3.796) | 2.778 (1.884 to 3.796) |
| Oesophageal cancer | 45 Pack-Years | Both | Male | 4.471 (3.099 to 5.83) | 4.471 (3.099 to 5.83) | 4.471 (3.099 to 5.83) | 4.471 (3.099 to 5.83) | 4.471 (3.099 to 5.83) | 4.471 (3.099 to 5.83) | 4.471 (3.099 to 5.83) | 4.471 (3.099 to 5.83) | 4.471 (3.099 to 5.83) | 4.471 (3.099 to 5.83) | 4.471 (3.099 to 5.83) | 4.471 (3.099 to 5.83) | 4.471 (3.099 to 5.83) | 4.471 (3.099 to 5.83) |
| Oesophageal cancer | 45 Pack-Years | Both | Female | 4.471 (3.099 to 5.83) | 4.471 (3.099 to 5.83) | 4.471 (3.099 to 5.83) | 4.471 (3.099 to 5.83) | 4.471 (3.099 to 5.83) | 4.471 (3.099 to 5.83) | 4.471 (3.099 to 5.83) | 4.471 (3.099 to 5.83) | 4.471 (3.099 to 5.83) | 4.471 (3.099 to 5.83) | 4.471 (3.099 to 5.83) | 4.471 (3.099 to 5.83) | 4.471 (3.099 to 5.83) | 4.471 (3.099 to 5.83) |
| Oesophageal cancer | 60 Pack-Years | Both | Male | 5.327 (2.865 to 8.227) | 5.327 (2.865 to 8.227) | 5.327 (2.865 to 8.227) | 5.327 (2.865 to 8.227) | 5.327 (2.865 to 8.227) | 5.327 (2.865 to 8.227) | 5.327 (2.865 to 8.227) | 5.327 (2.865 to 8.227) | 5.327 (2.865 to 8.227) | 5.327 (2.865 to 8.227) | 5.327 (2.865 to 8.227) | 5.327 (2.865 to 8.227) | 5.327 (2.865 to 8.227) | 5.327 (2.865 to 8.227) |
| Oesophageal cancer | 60 Pack-Years | Both | Female | 5.327 (2.865 to 8.227) | 5.327 (2.865 to 8.227) | 5.327 (2.865 to 8.227) | 5.327 (2.865 to 8.227) | 5.327 (2.865 to 8.227) | 5.327 (2.865 to 8.227) | 5.327 (2.865 to 8.227) | 5.327 (2.865 to 8.227) | 5.327 (2.865 to 8.227) | 5.327 (2.865 to 8.227) | 5.327 (2.865 to 8.227) | 5.327 (2.865 to 8.227) | 5.327 (2.865 to 8.227) | 5.327 (2.865 to 8.227) |
| Oesophageal cancer | 75 Pack-Years | Both | Male | 7.326 (3.276 to 12.451) | 7.326 (3.276 to 12.451) | 7.326 (3.276 to 12.451) | 7.326 (3.276 to 12.451) | 7.326 (3.276 to 12.451) | 7.326 (3.276 to 12.451) | 7.326 (3.276 to 12.451) | 7.326 (3.276 to 12.451) | 7.326 (3.276 to 12.451) | 7.326 (3.276 to 12.451) | 7.326 (3.276 to 12.451) | 7.326 (3.276 to 12.451) | 7.326 (3.276 to 12.451) | 7.326 (3.276 to 12.451) |
| Oesophageal cancer | 75 Pack-Years | Both | Female | 7.326 (3.276 to 12.451) | 7.326 (3.276 to 12.451) | 7.326 (3.276 to 12.451) | 7.326 (3.276 to 12.451) | 7.326 (3.276 to 12.451) | 7.326 (3.276 to 12.451) | 7.326 (3.276 to 12.451) | 7.326 (3.276 to 12.451) | 7.326 (3.276 to 12.451) | 7.326 (3.276 to 12.451) | 7.326 (3.276 to 12.451) | 7.326 (3.276 to 12.451) | 7.326 (3.276 to 12.451) | 7.326 (3.276 to 12.451) |
| Oesophageal cancer | 90 Pack-Years | Both | Male | 9.593 (4.866 to 15.62) | 9.593 (4.866 to 15.62) | 9.593 (4.866 to 15.62) | 9.593 (4.866 to 15.62) | 9.593 (4.866 to 15.62) | 9.593 (4.866 to 15.62) | 9.593 (4.866 to 15.62) | 9.593 (4.866 to 15.62) | 9.593 (4.866 to 15.62) | 9.593 (4.866 to 15.62) | 9.593 (4.866 to 15.62) | 9.593 (4.866 to 15.62) | 9.593 (4.866 to 15.62) | 9.593 (4.866 to 15.62) |
| Oesophageal cancer | 90 Pack-Years | Both | Female | 9.593 (4.866 to 15.62) | 9.593 (4.866 to 15.62) | 9.593 (4.866 to 15.62) | 9.593 (4.866 to 15.62) | 9.593 (4.866 to 15.62) | 9.593 (4.866 to 15.62) | 9.593 (4.866 to 15.62) | 9.593 (4.866 to 15.62) | 9.593 (4.866 to 15.62) | 9.593 (4.866 to 15.62) | 9.593 (4.866 to 15.62) | 9.593 (4.866 to 15.62) | 9.593 (4.866 to 15.62) | 9.593 (4.866 to 15.62) |
| Stomach cancer | 0 Pack-Years | Both | Male | 1.0 (1.0 to 1.0) | 1.0 (1.0 to 1.0) | 1.0 (1.0 to 1.0) | 1.0 (1.0 to 1.0) | 1.0 (1.0 to 1.0) | 1.0 (1.0 to 1.0) | 1.0 (1.0 to 1.0) | 1.0 (1.0 to 1.0) | 1.0 (1.0 to 1.0) | 1.0 (1.0 to 1.0) | 1.0 (1.0 to 1.0) | 1.0 (1.0 to 1.0) | 1.0 (1.0 to 1.0) | 1.0 (1.0 to 1.0) |
| Stomach cancer | 0 Pack-Years | Both | Female | 1.0 (1.0 to 1.0) | 1.0 (1.0 to 1.0) | 1.0 (1.0 to 1.0) | 1.0 (1.0 to 1.0) | 1.0 (1.0 to 1.0) | 1.0 (1.0 to 1.0) | 1.0 (1.0 to 1.0) | 1.0 (1.0 to 1.0) | 1.0 (1.0 to 1.0) | 1.0 (1.0 to 1.0) | 1.0 (1.0 to 1.0) | 1.0 (1.0 to 1.0) | 1.0 (1.0 to 1.0) | 1.0 (1.0 to 1.0) |
| Stomach cancer | 12.5 Pack-Years | Both | Male | 1.427 (1.184 to 1.713) | 1.427 (1.184 to 1.713) | 1.427 (1.184 to 1.713) | 1.427 (1.184 to 1.713) | 1.427 (1.184 to 1.713) | 1.427 (1.184 to 1.713) | 1.427 (1.184 to 1.713) | 1.427 (1.184 to 1.713) | 1.427 (1.184 to 1.713) | 1.427 (1.184 to 1.713) | 1.427 (1.184 to 1.713) | 1.427 (1.184 to 1.713) | 1.427 (1.184 to 1.713) | 1.427 (1.184 to 1.713) |
| Stomach cancer | 12.5 Pack-Years | Both | Female | 1.427 (1.184 to 1.713) | 1.427 (1.184 to 1.713) | 1.427 (1.184 to 1.713) | 1.427 (1.184 to 1.713) | 1.427 (1.184 to 1.713) | 1.427 (1.184 to 1.713) | 1.427 (1.184 to 1.713) | 1.427 (1.184 to 1.713) | 1.427 (1.184 to 1.713) | 1.427 (1.184 to 1.713) | 1.427 (1.184 to 1.713) | 1.427 (1.184 to 1.713) | 1.427 (1.184 to 1.713) | 1.427 (1.184 to 1.713) |
| Stomach cancer | 25 Pack-Years | Both | Male | 1.607 (1.286 to 1.991) | 1.607 (1.286 to 1.991) | 1.607 (1.286 to 1.991) | 1.607 (1.286 to 1.991) | 1.607 (1.286 to 1.991) | 1.607 (1.286 to 1.991) | 1.607 (1.286 to 1.991) | 1.607 (1.286 to 1.991) | 1.607 (1.286 to 1.991) | 1.607 (1.286 to 1.991) | 1.607 (1.286 to 1.991) | 1.607 (1.286 to 1.991) | 1.607 (1.286 to 1.991) | 1.607 (1.286 to 1.991) |
| Stomach cancer | 25 Pack-Years | Both | Female | 1.607 (1.286 to 1.991) | 1.607 (1.286 to 1.991) | 1.607 (1.286 to 1.991) | 1.607 (1.286 to 1.991) | 1.607 (1.286 to 1.991) | 1.607 (1.286 to 1.991) | 1.607 (1.286 to 1.991) | 1.607 (1.286 to 1.991) | 1.607 (1.286 to 1.991) | 1.607 (1.286 to 1.991) | 1.607 (1.286 to 1.991) | 1.607 (1.286 to 1.991) | 1.607 (1.286 to 1.991) | 1.607 (1.286 to 1.991) |
| Stomach cancer | 37.5 Pack-Years | Both | Male | 1.907 (1.47 to 2.407) | 1.907 (1.47 to 2.407) | 1.907 (1.47 to 2.407) | 1.907 (1.47 to 2.407) | 1.907 (1.47 to 2.407) | 1.907 (1.47 to 2.407) | 1.907 (1.47 to 2.407) | 1.907 (1.47 to 2.407) | 1.907 (1.47 to 2.407) | 1.907 (1.47 to 2.407) | 1.907 (1.47 to 2.407) | 1.907 (1.47 to 2.407) | 1.907 (1.47 to 2.407) | 1.907 (1.47 to 2.407) |

**Appendix Table 6d. Relative risks used by age and sex for each outcome for smoking globally.**

| Risk - Outcome | Category / Units | Morbidity / Mortality | Sex | Age: 30 to 34 | 35 to 39 | 40 to 44 | 45 to 49 | 50 to 54 | 55 to 59 | 60 to 64 | 65 to 69 | 70 to 74 | 75 to 79 | 80 to 84 | 85 to 89 | 90 to 94 | 95 plus |
|---|---|---|---|---|---|---|---|---|---|---|---|---|---|---|---|---|---|
| **Smoking** | | | | | | | | | | | | | | | | | |
| Stomach cancer | 37.5 Pack-Years | Both | Female | 1.907 (1.47 to 2.407) | 1.907 (1.47 to 2.407) | 1.907 (1.47 to 2.407) | 1.907 (1.47 to 2.407) | 1.907 (1.47 to 2.407) | 1.907 (1.47 to 2.407) | 1.907 (1.47 to 2.407) | 1.907 (1.47 to 2.407) | 1.907 (1.47 to 2.407) | 1.907 (1.47 to 2.407) | 1.907 (1.47 to 2.407) | 1.907 (1.47 to 2.407) | 1.907 (1.47 to 2.407) | 1.907 (1.47 to 2.407) |
| Stomach cancer | 50 Pack-Years | Both | Male | 2.055 (1.558 to 2.544) | 2.055 (1.558 to 2.544) | 2.055 (1.558 to 2.544) | 2.055 (1.558 to 2.544) | 2.055 (1.558 to 2.544) | 2.055 (1.558 to 2.544) | 2.055 (1.558 to 2.544) | 2.055 (1.558 to 2.544) | 2.055 (1.558 to 2.544) | 2.055 (1.558 to 2.544) | 2.055 (1.558 to 2.544) | 2.055 (1.558 to 2.544) | 2.055 (1.558 to 2.544) | 2.055 (1.558 to 2.544) |
| Stomach cancer | 50 Pack-Years | Both | Female | 2.055 (1.558 to 2.544) | 2.055 (1.558 to 2.544) | 2.055 (1.558 to 2.544) | 2.055 (1.558 to 2.544) | 2.055 (1.558 to 2.544) | 2.055 (1.558 to 2.544) | 2.055 (1.558 to 2.544) | 2.055 (1.558 to 2.544) | 2.055 (1.558 to 2.544) | 2.055 (1.558 to 2.544) | 2.055 (1.558 to 2.544) | 2.055 (1.558 to 2.544) | 2.055 (1.558 to 2.544) | 2.055 (1.558 to 2.544) |
| Stomach cancer | 75 Pack-Years | Both | Male | 2.122 (1.547 to 2.928) | 2.122 (1.547 to 2.928) | 2.122 (1.547 to 2.928) | 2.122 (1.547 to 2.928) | 2.122 (1.547 to 2.928) | 2.122 (1.547 to 2.928) | 2.122 (1.547 to 2.928) | 2.122 (1.547 to 2.928) | 2.122 (1.547 to 2.928) | 2.122 (1.547 to 2.928) | 2.122 (1.547 to 2.928) | 2.122 (1.547 to 2.928) | 2.122 (1.547 to 2.928) | 2.122 (1.547 to 2.928) |
| Stomach cancer | 75 Pack-Years | Both | Female | 2.122 (1.547 to 2.928) | 2.122 (1.547 to 2.928) | 2.122 (1.547 to 2.928) | 2.122 (1.547 to 2.928) | 2.122 (1.547 to 2.928) | 2.122 (1.547 to 2.928) | 2.122 (1.547 to 2.928) | 2.122 (1.547 to 2.928) | 2.122 (1.547 to 2.928) | 2.122 (1.547 to 2.928) | 2.122 (1.547 to 2.928) | 2.122 (1.547 to 2.928) | 2.122 (1.547 to 2.928) | 2.122 (1.547 to 2.928) |
| Colon and rectum cancer | 0 Pack-Years | Both | Male | 1.0 (1.0 to 1.0) | 1.0 (1.0 to 1.0) | 1.0 (1.0 to 1.0) | 1.0 (1.0 to 1.0) | 1.0 (1.0 to 1.0) | 1.0 (1.0 to 1.0) | 1.0 (1.0 to 1.0) | 1.0 (1.0 to 1.0) | 1.0 (1.0 to 1.0) | 1.0 (1.0 to 1.0) | 1.0 (1.0 to 1.0) | 1.0 (1.0 to 1.0) | 1.0 (1.0 to 1.0) | 1.0 (1.0 to 1.0) |
| Colon and rectum cancer | 0 Pack-Years | Both | Female | 1.0 (1.0 to 1.0) | 1.0 (1.0 to 1.0) | 1.0 (1.0 to 1.0) | 1.0 (1.0 to 1.0) | 1.0 (1.0 to 1.0) | 1.0 (1.0 to 1.0) | 1.0 (1.0 to 1.0) | 1.0 (1.0 to 1.0) | 1.0 (1.0 to 1.0) | 1.0 (1.0 to 1.0) | 1.0 (1.0 to 1.0) | 1.0 (1.0 to 1.0) | 1.0 (1.0 to 1.0) | 1.0 (1.0 to 1.0) |
| Colon and rectum cancer | 18.8 Pack-Years | Both | Male | 1.505 (1.144 to 1.852) | 1.505 (1.144 to 1.852) | 1.505 (1.144 to 1.852) | 1.505 (1.144 to 1.852) | 1.505 (1.144 to 1.852) | 1.505 (1.144 to 1.852) | 1.505 (1.144 to 1.852) | 1.505 (1.144 to 1.852) | 1.505 (1.144 to 1.852) | 1.505 (1.144 to 1.852) | 1.505 (1.144 to 1.852) | 1.505 (1.144 to 1.852) | 1.505 (1.144 to 1.852) | 1.505 (1.144 to 1.852) |
| Colon and rectum cancer | 18.8 Pack-Years | Both | Female | 1.505 (1.144 to 1.852) | 1.505 (1.144 to 1.852) | 1.505 (1.144 to 1.852) | 1.505 (1.144 to 1.852) | 1.505 (1.144 to 1.852) | 1.505 (1.144 to 1.852) | 1.505 (1.144 to 1.852) | 1.505 (1.144 to 1.852) | 1.505 (1.144 to 1.852) | 1.505 (1.144 to 1.852) | 1.505 (1.144 to 1.852) | 1.505 (1.144 to 1.852) | 1.505 (1.144 to 1.852) | 1.505 (1.144 to 1.852) |
| Colon and rectum cancer | 37.5 Pack-Years | Both | Male | 1.607 (1.235 to 2.055) | 1.607 (1.235 to 2.055) | 1.607 (1.235 to 2.055) | 1.607 (1.235 to 2.055) | 1.607 (1.235 to 2.055) | 1.607 (1.235 to 2.055) | 1.607 (1.235 to 2.055) | 1.607 (1.235 to 2.055) | 1.607 (1.235 to 2.055) | 1.607 (1.235 to 2.055) | 1.607 (1.235 to 2.055) | 1.607 (1.235 to 2.055) | 1.607 (1.235 to 2.055) | 1.607 (1.235 to 2.055) |
| Colon and rectum cancer | 37.5 Pack-Years | Both | Female | 1.607 (1.235 to 2.055) | 1.607 (1.235 to 2.055) | 1.607 (1.235 to 2.055) | 1.607 (1.235 to 2.055) | 1.607 (1.235 to 2.055) | 1.607 (1.235 to 2.055) | 1.607 (1.235 to 2.055) | 1.607 (1.235 to 2.055) | 1.607 (1.235 to 2.055) | 1.607 (1.235 to 2.055) | 1.607 (1.235 to 2.055) | 1.607 (1.235 to 2.055) | 1.607 (1.235 to 2.055) | 1.607 (1.235 to 2.055) |
| Colon and rectum cancer | 56.2 Pack-Years | Both | Male | 1.583 (1.155 to 2.103) | 1.583 (1.155 to 2.103) | 1.583 (1.155 to 2.103) | 1.583 (1.155 to 2.103) | 1.583 (1.155 to 2.103) | 1.583 (1.155 to 2.103) | 1.583 (1.155 to 2.103) | 1.583 (1.155 to 2.103) | 1.583 (1.155 to 2.103) | 1.583 (1.155 to 2.103) | 1.583 (1.155 to 2.103) | 1.583 (1.155 to 2.103) | 1.583 (1.155 to 2.103) | 1.583 (1.155 to 2.103) |
| Colon and rectum cancer | 56.2 Pack-Years | Both | Female | 1.583 (1.155 to 2.103) | 1.583 (1.155 to 2.103) | 1.583 (1.155 to 2.103) | 1.583 (1.155 to 2.103) | 1.583 (1.155 to 2.103) | 1.583 (1.155 to 2.103) | 1.583 (1.155 to 2.103) | 1.583 (1.155 to 2.103) | 1.583 (1.155 to 2.103) | 1.583 (1.155 to 2.103) | 1.583 (1.155 to 2.103) | 1.583 (1.155 to 2.103) | 1.583 (1.155 to 2.103) | 1.583 (1.155 to 2.103) |
| Liver cancer due to hepatitis B | 0 Pack-Years | Both | Male | 1.0 (1.0 to 1.0) | 1.0 (1.0 to 1.0) | 1.0 (1.0 to 1.0) | 1.0 (1.0 to 1.0) | 1.0 (1.0 to 1.0) | 1.0 (1.0 to 1.0) | 1.0 (1.0 to 1.0) | 1.0 (1.0 to 1.0) | 1.0 (1.0 to 1.0) | 1.0 (1.0 to 1.0) | 1.0 (1.0 to 1.0) | 1.0 (1.0 to 1.0) | 1.0 (1.0 to 1.0) | 1.0 (1.0 to 1.0) |
| Liver cancer due to hepatitis B | 0 Pack-Years | Both | Female | 1.0 (1.0 to 1.0) | 1.0 (1.0 to 1.0) | 1.0 (1.0 to 1.0) | 1.0 (1.0 to 1.0) | 1.0 (1.0 to 1.0) | 1.0 (1.0 to 1.0) | 1.0 (1.0 to 1.0) | 1.0 (1.0 to 1.0) | 1.0 (1.0 to 1.0) | 1.0 (1.0 to 1.0) | 1.0 (1.0 to 1.0) | 1.0 (1.0 to 1.0) | 1.0 (1.0 to 1.0) | 1.0 (1.0 to 1.0) |
| Liver cancer due to hepatitis B | 14.6 Pack-Years | Both | Male | 1.512 (1.04 to 2.038) | 1.512 (1.04 to 2.038) | 1.512 (1.04 to 2.038) | 1.512 (1.04 to 2.038) | 1.512 (1.04 to 2.038) | 1.512 (1.04 to 2.038) | 1.512 (1.04 to 2.038) | 1.512 (1.04 to 2.038) | 1.512 (1.04 to 2.038) | 1.512 (1.04 to 2.038) | 1.512 (1.04 to 2.038) | 1.512 (1.04 to 2.038) | 1.512 (1.04 to 2.038) | 1.512 (1.04 to 2.038) |
| Liver cancer due to hepatitis B | 14.6 Pack-Years | Both | Female | 1.512 (1.04 to 2.038) | 1.512 (1.04 to 2.038) | 1.512 (1.04 to 2.038) | 1.512 (1.04 to 2.038) | 1.512 (1.04 to 2.038) | 1.512 (1.04 to 2.038) | 1.512 (1.04 to 2.038) | 1.512 (1.04 to 2.038) | 1.512 (1.04 to 2.038) | 1.512 (1.04 to 2.038) | 1.512 (1.04 to 2.038) | 1.512 (1.04 to 2.038) | 1.512 (1.04 to 2.038) | 1.512 (1.04 to 2.038) |
| Liver cancer due to hepatitis B | 29.2 Pack-Years | Both | Male | 1.638 (1.099 to 2.256) | 1.638 (1.099 to 2.256) | 1.638 (1.099 to 2.256) | 1.638 (1.099 to 2.256) | 1.638 (1.099 to 2.256) | 1.638 (1.099 to 2.256) | 1.638 (1.099 to 2.256) | 1.638 (1.099 to 2.256) | 1.638 (1.099 to 2.256) | 1.638 (1.099 to 2.256) | 1.638 (1.099 to 2.256) | 1.638 (1.099 to 2.256) | 1.638 (1.099 to 2.256) | 1.638 (1.099 to 2.256) |
| Liver cancer due to hepatitis B | 29.2 Pack-Years | Both | Female | 1.638 (1.099 to 2.256) | 1.638 (1.099 to 2.256) | 1.638 (1.099 to 2.256) | 1.638 (1.099 to 2.256) | 1.638 (1.099 to 2.256) | 1.638 (1.099 to 2.256) | 1.638 (1.099 to 2.256) | 1.638 (1.099 to 2.256) | 1.638 (1.099 to 2.256) | 1.638 (1.099 to 2.256) | 1.638 (1.099 to 2.256) | 1.638 (1.099 to 2.256) | 1.638 (1.099 to 2.256) | 1.638 (1.099 to 2.256) |
| Liver cancer due to hepatitis B | 43.8 Pack-Years | Both | Male | 1.83 (1.224 to 2.606) | 1.83 (1.224 to 2.606) | 1.83 (1.224 to 2.606) | 1.83 (1.224 to 2.606) | 1.83 (1.224 to 2.606) | 1.83 (1.224 to 2.606) | 1.83 (1.224 to 2.606) | 1.83 (1.224 to 2.606) | 1.83 (1.224 to 2.606) | 1.83 (1.224 to 2.606) | 1.83 (1.224 to 2.606) | 1.83 (1.224 to 2.606) | 1.83 (1.224 to 2.606) | 1.83 (1.224 to 2.606) |
| Liver cancer due to hepatitis B | 43.8 Pack-Years | Both | Female | 1.83 (1.224 to 2.606) | 1.83 (1.224 to 2.606) | 1.83 (1.224 to 2.606) | 1.83 (1.224 to 2.606) | 1.83 (1.224 to 2.606) | 1.83 (1.224 to 2.606) | 1.83 (1.224 to 2.606) | 1.83 (1.224 to 2.606) | 1.83 (1.224 to 2.606) | 1.83 (1.224 to 2.606) | 1.83 (1.224 to 2.606) | 1.83 (1.224 to 2.606) | 1.83 (1.224 to 2.606) | 1.83 (1.224 to 2.606) |
| Liver cancer due to hepatitis B | 58.3 Pack-Years | Both | Male | 1.786 (1.139 to 2.722) | 1.786 (1.139 to 2.722) | 1.786 (1.139 to 2.722) | 1.786 (1.139 to 2.722) | 1.786 (1.139 to 2.722) | 1.786 (1.139 to 2.722) | 1.786 (1.139 to 2.722) | 1.786 (1.139 to 2.722) | 1.786 (1.139 to 2.722) | 1.786 (1.139 to 2.722) | 1.786 (1.139 to 2.722) | 1.786 (1.139 to 2.722) | 1.786 (1.139 to 2.722) | 1.786 (1.139 to 2.722) |
| Liver cancer due to hepatitis B | 58.3 Pack-Years | Both | Female | 1.786 (1.139 to 2.722) | 1.786 (1.139 to 2.722) | 1.786 (1.139 to 2.722) | 1.786 (1.139 to 2.722) | 1.786 (1.139 to 2.722) | 1.786 (1.139 to 2.722) | 1.786 (1.139 to 2.722) | 1.786 (1.139 to 2.722) | 1.786 (1.139 to 2.722) | 1.786 (1.139 to 2.722) | 1.786 (1.139 to 2.722) | 1.786 (1.139 to 2.722) | 1.786 (1.139 to 2.722) | 1.786 (1.139 to 2.722) |
| Liver cancer due to hepatitis B | 72.9 Pack-Years | Both | Male | 1.937 (1.147 to 3.012) | 1.937 (1.147 to 3.012) | 1.937 (1.147 to 3.012) | 1.937 (1.147 to 3.012) | 1.937 (1.147 to 3.012) | 1.937 (1.147 to 3.012) | 1.937 (1.147 to 3.012) | 1.937 (1.147 to 3.012) | 1.937 (1.147 to 3.012) | 1.937 (1.147 to 3.012) | 1.937 (1.147 to 3.012) | 1.937 (1.147 to 3.012) | 1.937 (1.147 to 3.012) | 1.937 (1.147 to 3.012) |
| Liver cancer due to hepatitis B | 72.9 Pack-Years | Both | Female | 1.937 (1.147 to 3.012) | 1.937 (1.147 to 3.012) | 1.937 (1.147 to 3.012) | 1.937 (1.147 to 3.012) | 1.937 (1.147 to 3.012) | 1.937 (1.147 to 3.012) | 1.937 (1.147 to 3.012) | 1.937 (1.147 to 3.012) | 1.937 (1.147 to 3.012) | 1.937 (1.147 to 3.012) | 1.937 (1.147 to 3.012) | 1.937 (1.147 to 3.012) | 1.937 (1.147 to 3.012) | 1.937 (1.147 to 3.012) |
| Liver cancer due to hepatitis C | 0 Pack-Years | Both | Male | 1.0 (1.0 to 1.0) | 1.0 (1.0 to 1.0) | 1.0 (1.0 to 1.0) | 1.0 (1.0 to 1.0) | 1.0 (1.0 to 1.0) | 1.0 (1.0 to 1.0) | 1.0 (1.0 to 1.0) | 1.0 (1.0 to 1.0) | 1.0 (1.0 to 1.0) | 1.0 (1.0 to 1.0) | 1.0 (1.0 to 1.0) | 1.0 (1.0 to 1.0) | 1.0 (1.0 to 1.0) | 1.0 (1.0 to 1.0) |
| Liver cancer due to hepatitis C | 0 Pack-Years | Both | Female | 1.0 (1.0 to 1.0) | 1.0 (1.0 to 1.0) | 1.0 (1.0 to 1.0) | 1.0 (1.0 to 1.0) | 1.0 (1.0 to 1.0) | 1.0 (1.0 to 1.0) | 1.0 (1.0 to 1.0) | 1.0 (1.0 to 1.0) | 1.0 (1.0 to 1.0) | 1.0 (1.0 to 1.0) | 1.0 (1.0 to 1.0) | 1.0 (1.0 to 1.0) | 1.0 (1.0 to 1.0) | 1.0 (1.0 to 1.0) |
| Liver cancer due to hepatitis C | 14.6 Pack-Years | Both | Male | 1.512 (1.04 to 2.038) | 1.512 (1.04 to 2.038) | 1.512 (1.04 to 2.038) | 1.512 (1.04 to 2.038) | 1.512 (1.04 to 2.038) | 1.512 (1.04 to 2.038) | 1.512 (1.04 to 2.038) | 1.512 (1.04 to 2.038) | 1.512 (1.04 to 2.038) | 1.512 (1.04 to 2.038) | 1.512 (1.04 to 2.038) | 1.512 (1.04 to 2.038) | 1.512 (1.04 to 2.038) | 1.512 (1.04 to 2.038) |
| Liver cancer due to hepatitis C | 14.6 Pack-Years | Both | Female | 1.512 (1.04 to 2.038) | 1.512 (1.04 to 2.038) | 1.512 (1.04 to 2.038) | 1.512 (1.04 to 2.038) | 1.512 (1.04 to 2.038) | 1.512 (1.04 to 2.038) | 1.512 (1.04 to 2.038) | 1.512 (1.04 to 2.038) | 1.512 (1.04 to 2.038) | 1.512 (1.04 to 2.038) | 1.512 (1.04 to 2.038) | 1.512 (1.04 to 2.038) | 1.512 (1.04 to 2.038) | 1.512 (1.04 to 2.038) |
| Liver cancer due to hepatitis C | 29.2 Pack-Years | Both | Male | 1.638 (1.099 to 2.256) | 1.638 (1.099 to 2.256) | 1.638 (1.099 to 2.256) | 1.638 (1.099 to 2.256) | 1.638 (1.099 to 2.256) | 1.638 (1.099 to 2.256) | 1.638 (1.099 to 2.256) | 1.638 (1.099 to 2.256) | 1.638 (1.099 to 2.256) | 1.638 (1.099 to 2.256) | 1.638 (1.099 to 2.256) | 1.638 (1.099 to 2.256) | 1.638 (1.099 to 2.256) | 1.638 (1.099 to 2.256) |
| Liver cancer due to hepatitis C | 29.2 Pack-Years | Both | Female | 1.638 (1.099 to 2.256) | 1.638 (1.099 to 2.256) | 1.638 (1.099 to 2.256) | 1.638 (1.099 to 2.256) | 1.638 (1.099 to 2.256) | 1.638 (1.099 to 2.256) | 1.638 (1.099 to 2.256) | 1.638 (1.099 to 2.256) | 1.638 (1.099 to 2.256) | 1.638 (1.099 to 2.256) | 1.638 (1.099 to 2.256) | 1.638 (1.099 to 2.256) | 1.638 (1.099 to 2.256) | 1.638 (1.099 to 2.256) |
| Liver cancer due to hepatitis C | 43.8 Pack-Years | Both | Male | 1.83 (1.224 to 2.606) | 1.83 (1.224 to 2.606) | 1.83 (1.224 to 2.606) | 1.83 (1.224 to 2.606) | 1.83 (1.224 to 2.606) | 1.83 (1.224 to 2.606) | 1.83 (1.224 to 2.606) | 1.83 (1.224 to 2.606) | 1.83 (1.224 to 2.606) | 1.83 (1.224 to 2.606) | 1.83 (1.224 to 2.606) | 1.83 (1.224 to 2.606) | 1.83 (1.224 to 2.606) | 1.83 (1.224 to 2.606) |
| Liver cancer due to hepatitis C | 43.8 Pack-Years | Both | Female | 1.83 (1.224 to 2.606) | 1.83 (1.224 to 2.606) | 1.83 (1.224 to 2.606) | 1.83 (1.224 to 2.606) | 1.83 (1.224 to 2.606) | 1.83 (1.224 to 2.606) | 1.83 (1.224 to 2.606) | 1.83 (1.224 to 2.606) | 1.83 (1.224 to 2.606) | 1.83 (1.224 to 2.606) | 1.83 (1.224 to 2.606) | 1.83 (1.224 to 2.606) | 1.83 (1.224 to 2.606) | 1.83 (1.224 to 2.606) |
| Liver cancer due to hepatitis C | 58.3 Pack-Years | Both | Male | 1.786 (1.139 to 2.722) | 1.786 (1.139 to 2.722) | 1.786 (1.139 to 2.722) | 1.786 (1.139 to 2.722) | 1.786 (1.139 to 2.722) | 1.786 (1.139 to 2.722) | 1.786 (1.139 to 2.722) | 1.786 (1.139 to 2.722) | 1.786 (1.139 to 2.722) | 1.786 (1.139 to 2.722) | 1.786 (1.139 to 2.722) | 1.786 (1.139 to 2.722) | 1.786 (1.139 to 2.722) | 1.786 (1.139 to 2.722) |
| Liver cancer due to hepatitis C | 58.3 Pack-Years | Both | Female | 1.786 (1.139 to 2.722) | 1.786 (1.139 to 2.722) | 1.786 (1.139 to 2.722) | 1.786 (1.139 to 2.722) | 1.786 (1.139 to 2.722) | 1.786 (1.139 to 2.722) | 1.786 (1.139 to 2.722) | 1.786 (1.139 to 2.722) | 1.786 (1.139 to 2.722) | 1.786 (1.139 to 2.722) | 1.786 (1.139 to 2.722) | 1.786 (1.139 to 2.722) | 1.786 (1.139 to 2.722) | 1.786 (1.139 to 2.722) |
| Liver cancer due to hepatitis C | 72.9 Pack-Years | Both | Male | 1.937 (1.147 to 3.012) | 1.937 (1.147 to 3.012) | 1.937 (1.147 to 3.012) | 1.937 (1.147 to 3.012) | 1.937 (1.147 to 3.012) | 1.937 (1.147 to 3.012) | 1.937 (1.147 to 3.012) | 1.937 (1.147 to 3.012) | 1.937 (1.147 to 3.012) | 1.937 (1.147 to 3.012) | 1.937 (1.147 to 3.012) | 1.937 (1.147 to 3.012) | 1.937 (1.147 to 3.012) | 1.937 (1.147 to 3.012) |
| Liver cancer due to hepatitis C | 72.9 Pack-Years | Both | Female | 1.937 (1.147 to 3.012) | 1.937 (1.147 to 3.012) | 1.937 (1.147 to 3.012) | 1.937 (1.147 to 3.012) | 1.937 (1.147 to 3.012) | 1.937 (1.147 to 3.012) | 1.937 (1.147 to 3.012) | 1.937 (1.147 to 3.012) | 1.937 (1.147 to 3.012) | 1.937 (1.147 to 3.012) | 1.937 (1.147 to 3.012) | 1.937 (1.147 to 3.012) | 1.937 (1.147 to 3.012) | 1.937 (1.147 to 3.012) |

**Appendix Table 6d. Relative risks used by age and sex for each outcome for smoking globally.**

| Risk - Outcome | Category / Units | Morbidity / Mortality | Sex | Age | | | | | | | | | | | | | |
|---|---|---|---|---|---|---|---|---|---|---|---|---|---|---|---|---|---|
| | | | | 30 to 34 | 35 to 39 | 40 to 44 | 45 to 49 | 50 to 54 | 55 to 59 | 60 to 64 | 65 to 69 | 70 to 74 | 75 to 79 | 80 to 84 | 85 to 89 | 90 to 94 | 95 plus |
| Smoking | | | | | | | | | | | | | | | | | |
| Liver cancer due to alcohol use | 0 Pack-Years | Both | Male | 1.0 (1.0 to 1.0) | 1.0 (1.0 to 1.0) | 1.0 (1.0 to 1.0) | 1.0 (1.0 to 1.0) | 1.0 (1.0 to 1.0) | 1.0 (1.0 to 1.0) | 1.0 (1.0 to 1.0) | 1.0 (1.0 to 1.0) | 1.0 (1.0 to 1.0) | 1.0 (1.0 to 1.0) | 1.0 (1.0 to 1.0) | 1.0 (1.0 to 1.0) | 1.0 (1.0 to 1.0) | 1.0 (1.0 to 1.0) |
| Liver cancer due to alcohol use | 0 Pack-Years | Both | Female | 1.0 (1.0 to 1.0) | 1.0 (1.0 to 1.0) | 1.0 (1.0 to 1.0) | 1.0 (1.0 to 1.0) | 1.0 (1.0 to 1.0) | 1.0 (1.0 to 1.0) | 1.0 (1.0 to 1.0) | 1.0 (1.0 to 1.0) | 1.0 (1.0 to 1.0) | 1.0 (1.0 to 1.0) | 1.0 (1.0 to 1.0) | 1.0 (1.0 to 1.0) | 1.0 (1.0 to 1.0) | 1.0 (1.0 to 1.0) |
| Liver cancer due to alcohol use | 14.6 Pack-Years | Both | Male | 1.512 (1.04 to 2.038) | 1.512 (1.04 to 2.038) | 1.512 (1.04 to 2.038) | 1.512 (1.04 to 2.038) | 1.512 (1.04 to 2.038) | 1.512 (1.04 to 2.038) | 1.512 (1.04 to 2.038) | 1.512 (1.04 to 2.038) | 1.512 (1.04 to 2.038) | 1.512 (1.04 to 2.038) | 1.512 (1.04 to 2.038) | 1.512 (1.04 to 2.038) | 1.512 (1.04 to 2.038) | 1.512 (1.04 to 2.038) |
| Liver cancer due to alcohol use | 14.6 Pack-Years | Both | Female | 1.512 (1.04 to 2.038) | 1.512 (1.04 to 2.038) | 1.512 (1.04 to 2.038) | 1.512 (1.04 to 2.038) | 1.512 (1.04 to 2.038) | 1.512 (1.04 to 2.038) | 1.512 (1.04 to 2.038) | 1.512 (1.04 to 2.038) | 1.512 (1.04 to 2.038) | 1.512 (1.04 to 2.038) | 1.512 (1.04 to 2.038) | 1.512 (1.04 to 2.038) | 1.512 (1.04 to 2.038) | 1.512 (1.04 to 2.038) |
| Liver cancer due to alcohol use | 29.2 Pack-Years | Both | Male | 1.638 (1.099 to 2.256) | 1.638 (1.099 to 2.256) | 1.638 (1.099 to 2.256) | 1.638 (1.099 to 2.256) | 1.638 (1.099 to 2.256) | 1.638 (1.099 to 2.256) | 1.638 (1.099 to 2.256) | 1.638 (1.099 to 2.256) | 1.638 (1.099 to 2.256) | 1.638 (1.099 to 2.256) | 1.638 (1.099 to 2.256) | 1.638 (1.099 to 2.256) | 1.638 (1.099 to 2.256) | 1.638 (1.099 to 2.256) |
| Liver cancer due to alcohol use | 29.2 Pack-Years | Both | Female | 1.638 (1.099 to 2.256) | 1.638 (1.099 to 2.256) | 1.638 (1.099 to 2.256) | 1.638 (1.099 to 2.256) | 1.638 (1.099 to 2.256) | 1.638 (1.099 to 2.256) | 1.638 (1.099 to 2.256) | 1.638 (1.099 to 2.256) | 1.638 (1.099 to 2.256) | 1.638 (1.099 to 2.256) | 1.638 (1.099 to 2.256) | 1.638 (1.099 to 2.256) | 1.638 (1.099 to 2.256) | 1.638 (1.099 to 2.256) |
| Liver cancer due to alcohol use | 43.8 Pack-Years | Both | Male | 1.83 (1.224 to 2.606) | 1.83 (1.224 to 2.606) | 1.83 (1.224 to 2.606) | 1.83 (1.224 to 2.606) | 1.83 (1.224 to 2.606) | 1.83 (1.224 to 2.606) | 1.83 (1.224 to 2.606) | 1.83 (1.224 to 2.606) | 1.83 (1.224 to 2.606) | 1.83 (1.224 to 2.606) | 1.83 (1.224 to 2.606) | 1.83 (1.224 to 2.606) | 1.83 (1.224 to 2.606) | 1.83 (1.224 to 2.606) |
| Liver cancer due to alcohol use | 43.8 Pack-Years | Both | Female | 1.83 (1.224 to 2.606) | 1.83 (1.224 to 2.606) | 1.83 (1.224 to 2.606) | 1.83 (1.224 to 2.606) | 1.83 (1.224 to 2.606) | 1.83 (1.224 to 2.606) | 1.83 (1.224 to 2.606) | 1.83 (1.224 to 2.606) | 1.83 (1.224 to 2.606) | 1.83 (1.224 to 2.606) | 1.83 (1.224 to 2.606) | 1.83 (1.224 to 2.606) | 1.83 (1.224 to 2.606) | 1.83 (1.224 to 2.606) |
| Liver cancer due to alcohol use | 58.3 Pack-Years | Both | Male | 1.786 (1.139 to 2.722) | 1.786 (1.139 to 2.722) | 1.786 (1.139 to 2.722) | 1.786 (1.139 to 2.722) | 1.786 (1.139 to 2.722) | 1.786 (1.139 to 2.722) | 1.786 (1.139 to 2.722) | 1.786 (1.139 to 2.722) | 1.786 (1.139 to 2.722) | 1.786 (1.139 to 2.722) | 1.786 (1.139 to 2.722) | 1.786 (1.139 to 2.722) | 1.786 (1.139 to 2.722) | 1.786 (1.139 to 2.722) |
| Liver cancer due to alcohol use | 58.3 Pack-Years | Both | Female | 1.786 (1.139 to 2.722) | 1.786 (1.139 to 2.722) | 1.786 (1.139 to 2.722) | 1.786 (1.139 to 2.722) | 1.786 (1.139 to 2.722) | 1.786 (1.139 to 2.722) | 1.786 (1.139 to 2.722) | 1.786 (1.139 to 2.722) | 1.786 (1.139 to 2.722) | 1.786 (1.139 to 2.722) | 1.786 (1.139 to 2.722) | 1.786 (1.139 to 2.722) | 1.786 (1.139 to 2.722) | 1.786 (1.139 to 2.722) |
| Liver cancer due to alcohol use | 72.9 Pack-Years | Both | Male | 1.937 (1.147 to 3.012) | 1.937 (1.147 to 3.012) | 1.937 (1.147 to 3.012) | 1.937 (1.147 to 3.012) | 1.937 (1.147 to 3.012) | 1.937 (1.147 to 3.012) | 1.937 (1.147 to 3.012) | 1.937 (1.147 to 3.012) | 1.937 (1.147 to 3.012) | 1.937 (1.147 to 3.012) | 1.937 (1.147 to 3.012) | 1.937 (1.147 to 3.012) | 1.937 (1.147 to 3.012) | 1.937 (1.147 to 3.012) |
| Liver cancer due to alcohol use | 72.9 Pack-Years | Both | Female | 1.937 (1.147 to 3.012) | 1.937 (1.147 to 3.012) | 1.937 (1.147 to 3.012) | 1.937 (1.147 to 3.012) | 1.937 (1.147 to 3.012) | 1.937 (1.147 to 3.012) | 1.937 (1.147 to 3.012) | 1.937 (1.147 to 3.012) | 1.937 (1.147 to 3.012) | 1.937 (1.147 to 3.012) | 1.937 (1.147 to 3.012) | 1.937 (1.147 to 3.012) | 1.937 (1.147 to 3.012) | 1.937 (1.147 to 3.012) |
| Liver cancer due to NASH | 0 Pack-Years | Both | Male | 1.0 (1.0 to 1.0) | 1.0 (1.0 to 1.0) | 1.0 (1.0 to 1.0) | 1.0 (1.0 to 1.0) | 1.0 (1.0 to 1.0) | 1.0 (1.0 to 1.0) | 1.0 (1.0 to 1.0) | 1.0 (1.0 to 1.0) | 1.0 (1.0 to 1.0) | 1.0 (1.0 to 1.0) | 1.0 (1.0 to 1.0) | 1.0 (1.0 to 1.0) | 1.0 (1.0 to 1.0) | 1.0 (1.0 to 1.0) |
| Liver cancer due to NASH | 0 Pack-Years | Both | Female | 1.0 (1.0 to 1.0) | 1.0 (1.0 to 1.0) | 1.0 (1.0 to 1.0) | 1.0 (1.0 to 1.0) | 1.0 (1.0 to 1.0) | 1.0 (1.0 to 1.0) | 1.0 (1.0 to 1.0) | 1.0 (1.0 to 1.0) | 1.0 (1.0 to 1.0) | 1.0 (1.0 to 1.0) | 1.0 (1.0 to 1.0) | 1.0 (1.0 to 1.0) | 1.0 (1.0 to 1.0) | 1.0 (1.0 to 1.0) |
| Liver cancer due to NASH | 14.6 Pack-Years | Both | Male | 1.512 (1.04 to 2.038) | 1.512 (1.04 to 2.038) | 1.512 (1.04 to 2.038) | 1.512 (1.04 to 2.038) | 1.512 (1.04 to 2.038) | 1.512 (1.04 to 2.038) | 1.512 (1.04 to 2.038) | 1.512 (1.04 to 2.038) | 1.512 (1.04 to 2.038) | 1.512 (1.04 to 2.038) | 1.512 (1.04 to 2.038) | 1.512 (1.04 to 2.038) | 1.512 (1.04 to 2.038) | 1.512 (1.04 to 2.038) |
| Liver cancer due to NASH | 14.6 Pack-Years | Both | Female | 1.512 (1.04 to 2.038) | 1.512 (1.04 to 2.038) | 1.512 (1.04 to 2.038) | 1.512 (1.04 to 2.038) | 1.512 (1.04 to 2.038) | 1.512 (1.04 to 2.038) | 1.512 (1.04 to 2.038) | 1.512 (1.04 to 2.038) | 1.512 (1.04 to 2.038) | 1.512 (1.04 to 2.038) | 1.512 (1.04 to 2.038) | 1.512 (1.04 to 2.038) | 1.512 (1.04 to 2.038) | 1.512 (1.04 to 2.038) |
| Liver cancer due to NASH | 29.2 Pack-Years | Both | Male | 1.638 (1.099 to 2.256) | 1.638 (1.099 to 2.256) | 1.638 (1.099 to 2.256) | 1.638 (1.099 to 2.256) | 1.638 (1.099 to 2.256) | 1.638 (1.099 to 2.256) | 1.638 (1.099 to 2.256) | 1.638 (1.099 to 2.256) | 1.638 (1.099 to 2.256) | 1.638 (1.099 to 2.256) | 1.638 (1.099 to 2.256) | 1.638 (1.099 to 2.256) | 1.638 (1.099 to 2.256) | 1.638 (1.099 to 2.256) |
| Liver cancer due to NASH | 29.2 Pack-Years | Both | Female | 1.638 (1.099 to 2.256) | 1.638 (1.099 to 2.256) | 1.638 (1.099 to 2.256) | 1.638 (1.099 to 2.256) | 1.638 (1.099 to 2.256) | 1.638 (1.099 to 2.256) | 1.638 (1.099 to 2.256) | 1.638 (1.099 to 2.256) | 1.638 (1.099 to 2.256) | 1.638 (1.099 to 2.256) | 1.638 (1.099 to 2.256) | 1.638 (1.099 to 2.256) | 1.638 (1.099 to 2.256) | 1.638 (1.099 to 2.256) |
| Liver cancer due to NASH | 43.8 Pack-Years | Both | Male | 1.83 (1.224 to 2.606) | 1.83 (1.224 to 2.606) | 1.83 (1.224 to 2.606) | 1.83 (1.224 to 2.606) | 1.83 (1.224 to 2.606) | 1.83 (1.224 to 2.606) | 1.83 (1.224 to 2.606) | 1.83 (1.224 to 2.606) | 1.83 (1.224 to 2.606) | 1.83 (1.224 to 2.606) | 1.83 (1.224 to 2.606) | 1.83 (1.224 to 2.606) | 1.83 (1.224 to 2.606) | 1.83 (1.224 to 2.606) |
| Liver cancer due to NASH | 43.8 Pack-Years | Both | Female | 1.83 (1.224 to 2.606) | 1.83 (1.224 to 2.606) | 1.83 (1.224 to 2.606) | 1.83 (1.224 to 2.606) | 1.83 (1.224 to 2.606) | 1.83 (1.224 to 2.606) | 1.83 (1.224 to 2.606) | 1.83 (1.224 to 2.606) | 1.83 (1.224 to 2.606) | 1.83 (1.224 to 2.606) | 1.83 (1.224 to 2.606) | 1.83 (1.224 to 2.606) | 1.83 (1.224 to 2.606) | 1.83 (1.224 to 2.606) |
| Liver cancer due to NASH | 58.3 Pack-Years | Both | Male | 1.786 (1.139 to 2.722) | 1.786 (1.139 to 2.722) | 1.786 (1.139 to 2.722) | 1.786 (1.139 to 2.722) | 1.786 (1.139 to 2.722) | 1.786 (1.139 to 2.722) | 1.786 (1.139 to 2.722) | 1.786 (1.139 to 2.722) | 1.786 (1.139 to 2.722) | 1.786 (1.139 to 2.722) | 1.786 (1.139 to 2.722) | 1.786 (1.139 to 2.722) | 1.786 (1.139 to 2.722) | 1.786 (1.139 to 2.722) |
| Liver cancer due to NASH | 58.3 Pack-Years | Both | Female | 1.786 (1.139 to 2.722) | 1.786 (1.139 to 2.722) | 1.786 (1.139 to 2.722) | 1.786 (1.139 to 2.722) | 1.786 (1.139 to 2.722) | 1.786 (1.139 to 2.722) | 1.786 (1.139 to 2.722) | 1.786 (1.139 to 2.722) | 1.786 (1.139 to 2.722) | 1.786 (1.139 to 2.722) | 1.786 (1.139 to 2.722) | 1.786 (1.139 to 2.722) | 1.786 (1.139 to 2.722) | 1.786 (1.139 to 2.722) |
| Liver cancer due to NASH | 72.9 Pack-Years | Both | Male | 1.937 (1.147 to 3.012) | 1.937 (1.147 to 3.012) | 1.937 (1.147 to 3.012) | 1.937 (1.147 to 3.012) | 1.937 (1.147 to 3.012) | 1.937 (1.147 to 3.012) | 1.937 (1.147 to 3.012) | 1.937 (1.147 to 3.012) | 1.937 (1.147 to 3.012) | 1.937 (1.147 to 3.012) | 1.937 (1.147 to 3.012) | 1.937 (1.147 to 3.012) | 1.937 (1.147 to 3.012) | 1.937 (1.147 to 3.012) |
| Liver cancer due to NASH | 72.9 Pack-Years | Both | Female | 1.937 (1.147 to 3.012) | 1.937 (1.147 to 3.012) | 1.937 (1.147 to 3.012) | 1.937 (1.147 to 3.012) | 1.937 (1.147 to 3.012) | 1.937 (1.147 to 3.012) | 1.937 (1.147 to 3.012) | 1.937 (1.147 to 3.012) | 1.937 (1.147 to 3.012) | 1.937 (1.147 to 3.012) | 1.937 (1.147 to 3.012) | 1.937 (1.147 to 3.012) | 1.937 (1.147 to 3.012) | 1.937 (1.147 to 3.012) |
| Liver cancer due to other causes | 0 Pack-Years | Both | Male | 1.0 (1.0 to 1.0) | 1.0 (1.0 to 1.0) | 1.0 (1.0 to 1.0) | 1.0 (1.0 to 1.0) | 1.0 (1.0 to 1.0) | 1.0 (1.0 to 1.0) | 1.0 (1.0 to 1.0) | 1.0 (1.0 to 1.0) | 1.0 (1.0 to 1.0) | 1.0 (1.0 to 1.0) | 1.0 (1.0 to 1.0) | 1.0 (1.0 to 1.0) | 1.0 (1.0 to 1.0) | 1.0 (1.0 to 1.0) |
| Liver cancer due to other causes | 0 Pack-Years | Both | Female | 1.0 (1.0 to 1.0) | 1.0 (1.0 to 1.0) | 1.0 (1.0 to 1.0) | 1.0 (1.0 to 1.0) | 1.0 (1.0 to 1.0) | 1.0 (1.0 to 1.0) | 1.0 (1.0 to 1.0) | 1.0 (1.0 to 1.0) | 1.0 (1.0 to 1.0) | 1.0 (1.0 to 1.0) | 1.0 (1.0 to 1.0) | 1.0 (1.0 to 1.0) | 1.0 (1.0 to 1.0) | 1.0 (1.0 to 1.0) |
| Liver cancer due to other causes | 14.6 Pack-Years | Both | Male | 1.512 (1.04 to 2.038) | 1.512 (1.04 to 2.038) | 1.512 (1.04 to 2.038) | 1.512 (1.04 to 2.038) | 1.512 (1.04 to 2.038) | 1.512 (1.04 to 2.038) | 1.512 (1.04 to 2.038) | 1.512 (1.04 to 2.038) | 1.512 (1.04 to 2.038) | 1.512 (1.04 to 2.038) | 1.512 (1.04 to 2.038) | 1.512 (1.04 to 2.038) | 1.512 (1.04 to 2.038) | 1.512 (1.04 to 2.038) |
| Liver cancer due to other causes | 14.6 Pack-Years | Both | Female | 1.512 (1.04 to 2.038) | 1.512 (1.04 to 2.038) | 1.512 (1.04 to 2.038) | 1.512 (1.04 to 2.038) | 1.512 (1.04 to 2.038) | 1.512 (1.04 to 2.038) | 1.512 (1.04 to 2.038) | 1.512 (1.04 to 2.038) | 1.512 (1.04 to 2.038) | 1.512 (1.04 to 2.038) | 1.512 (1.04 to 2.038) | 1.512 (1.04 to 2.038) | 1.512 (1.04 to 2.038) | 1.512 (1.04 to 2.038) |
| Liver cancer due to other causes | 29.2 Pack-Years | Both | Male | 1.638 (1.099 to 2.256) | 1.638 (1.099 to 2.256) | 1.638 (1.099 to 2.256) | 1.638 (1.099 to 2.256) | 1.638 (1.099 to 2.256) | 1.638 (1.099 to 2.256) | 1.638 (1.099 to 2.256) | 1.638 (1.099 to 2.256) | 1.638 (1.099 to 2.256) | 1.638 (1.099 to 2.256) | 1.638 (1.099 to 2.256) | 1.638 (1.099 to 2.256) | 1.638 (1.099 to 2.256) | 1.638 (1.099 to 2.256) |
| Liver cancer due to other causes | 29.2 Pack-Years | Both | Female | 1.638 (1.099 to 2.256) | 1.638 (1.099 to 2.256) | 1.638 (1.099 to 2.256) | 1.638 (1.099 to 2.256) | 1.638 (1.099 to 2.256) | 1.638 (1.099 to 2.256) | 1.638 (1.099 to 2.256) | 1.638 (1.099 to 2.256) | 1.638 (1.099 to 2.256) | 1.638 (1.099 to 2.256) | 1.638 (1.099 to 2.256) | 1.638 (1.099 to 2.256) | 1.638 (1.099 to 2.256) | 1.638 (1.099 to 2.256) |
| Liver cancer due to other causes | 43.8 Pack-Years | Both | Male | 1.83 (1.224 to 2.606) | 1.83 (1.224 to 2.606) | 1.83 (1.224 to 2.606) | 1.83 (1.224 to 2.606) | 1.83 (1.224 to 2.606) | 1.83 (1.224 to 2.606) | 1.83 (1.224 to 2.606) | 1.83 (1.224 to 2.606) | 1.83 (1.224 to 2.606) | 1.83 (1.224 to 2.606) | 1.83 (1.224 to 2.606) | 1.83 (1.224 to 2.606) | 1.83 (1.224 to 2.606) | 1.83 (1.224 to 2.606) |
| Liver cancer due to other causes | 43.8 Pack-Years | Both | Female | 1.83 (1.224 to 2.606) | 1.83 (1.224 to 2.606) | 1.83 (1.224 to 2.606) | 1.83 (1.224 to 2.606) | 1.83 (1.224 to 2.606) | 1.83 (1.224 to 2.606) | 1.83 (1.224 to 2.606) | 1.83 (1.224 to 2.606) | 1.83 (1.224 to 2.606) | 1.83 (1.224 to 2.606) | 1.83 (1.224 to 2.606) | 1.83 (1.224 to 2.606) | 1.83 (1.224 to 2.606) | 1.83 (1.224 to 2.606) |
| Liver cancer due to other causes | 58.3 Pack-Years | Both | Male | 1.786 (1.139 to 2.722) | 1.786 (1.139 to 2.722) | 1.786 (1.139 to 2.722) | 1.786 (1.139 to 2.722) | 1.786 (1.139 to 2.722) | 1.786 (1.139 to 2.722) | 1.786 (1.139 to 2.722) | 1.786 (1.139 to 2.722) | 1.786 (1.139 to 2.722) | 1.786 (1.139 to 2.722) | 1.786 (1.139 to 2.722) | 1.786 (1.139 to 2.722) | 1.786 (1.139 to 2.722) | 1.786 (1.139 to 2.722) |
| Liver cancer due to other causes | 58.3 Pack-Years | Both | Female | 1.786 (1.139 to 2.722) | 1.786 (1.139 to 2.722) | 1.786 (1.139 to 2.722) | 1.786 (1.139 to 2.722) | 1.786 (1.139 to 2.722) | 1.786 (1.139 to 2.722) | 1.786 (1.139 to 2.722) | 1.786 (1.139 to 2.722) | 1.786 (1.139 to 2.722) | 1.786 (1.139 to 2.722) | 1.786 (1.139 to 2.722) | 1.786 (1.139 to 2.722) | 1.786 (1.139 to 2.722) | 1.786 (1.139 to 2.722) |
| Liver cancer due to other causes | 72.9 Pack-Years | Both | Male | 1.937 (1.147 to 3.012) | 1.937 (1.147 to 3.012) | 1.937 (1.147 to 3.012) | 1.937 (1.147 to 3.012) | 1.937 (1.147 to 3.012) | 1.937 (1.147 to 3.012) | 1.937 (1.147 to 3.012) | 1.937 (1.147 to 3.012) | 1.937 (1.147 to 3.012) | 1.937 (1.147 to 3.012) | 1.937 (1.147 to 3.012) | 1.937 (1.147 to 3.012) | 1.937 (1.147 to 3.012) | 1.937 (1.147 to 3.012) |
| Liver cancer due to other causes | 72.9 Pack-Years | Both | Female | 1.937 (1.147 to 3.012) | 1.937 (1.147 to 3.012) | 1.937 (1.147 to 3.012) | 1.937 (1.147 to 3.012) | 1.937 (1.147 to 3.012) | 1.937 (1.147 to 3.012) | 1.937 (1.147 to 3.012) | 1.937 (1.147 to 3.012) | 1.937 (1.147 to 3.012) | 1.937 (1.147 to 3.012) | 1.937 (1.147 to 3.012) | 1.937 (1.147 to 3.012) | 1.937 (1.147 to 3.012) | 1.937 (1.147 to 3.012) |
| Pancreatic cancer | 0 Pack-Years | Both | Male | 1.0 (1.0 to 1.0) | 1.0 (1.0 to 1.0) | 1.0 (1.0 to 1.0) | 1.0 (1.0 to 1.0) | 1.0 (1.0 to 1.0) | 1.0 (1.0 to 1.0) | 1.0 (1.0 to 1.0) | 1.0 (1.0 to 1.0) | 1.0 (1.0 to 1.0) | 1.0 (1.0 to 1.0) | 1.0 (1.0 to 1.0) | 1.0 (1.0 to 1.0) | 1.0 (1.0 to 1.0) | 1.0 (1.0 to 1.0) |

**Appendix Table 6d. Relative risks used by age and sex for each outcome for smoking globally.**

| Risk - Outcome | Category / Units | Morbidity / Mortality | Sex | Age | | | | | | | | | | | | | |
|---|---|---|---|---|---|---|---|---|---|---|---|---|---|---|---|---|---|
| | | | | 30 to 34 | 35 to 39 | 40 to 44 | 45 to 49 | 50 to 54 | 55 to 59 | 60 to 64 | 65 to 69 | 70 to 74 | 75 to 79 | 80 to 84 | 85 to 89 | 90 to 94 | 95 plus |
| **Smoking** | | | | | | | | | | | | | | | | | |
| Pancreatic cancer | 0 Pack-Years | Both | Female | 1.0 (1.0 to 1.0) | 1.0 (1.0 to 1.0) | 1.0 (1.0 to 1.0) | 1.0 (1.0 to 1.0) | 1.0 (1.0 to 1.0) | 1.0 (1.0 to 1.0) | 1.0 (1.0 to 1.0) | 1.0 (1.0 to 1.0) | 1.0 (1.0 to 1.0) | 1.0 (1.0 to 1.0) | 1.0 (1.0 to 1.0) | 1.0 (1.0 to 1.0) | 1.0 (1.0 to 1.0) | 1.0 (1.0 to 1.0) |
| Pancreatic cancer | 15.8 Pack-Years | Both | Male | 1.653 (1.332 to 1.98) | 1.653 (1.332 to 1.98) | 1.653 (1.332 to 1.98) | 1.653 (1.332 to 1.98) | 1.653 (1.332 to 1.98) | 1.653 (1.332 to 1.98) | 1.653 (1.332 to 1.98) | 1.653 (1.332 to 1.98) | 1.653 (1.332 to 1.98) | 1.653 (1.332 to 1.98) | 1.653 (1.332 to 1.98) | 1.653 (1.332 to 1.98) | 1.653 (1.332 to 1.98) | 1.653 (1.332 to 1.98) |
| Pancreatic cancer | 15.8 Pack-Years | Both | Female | 2.361 (1.902 to 2.846) | 2.361 (1.902 to 2.846) | 2.361 (1.902 to 2.846) | 2.361 (1.902 to 2.846) | 2.361 (1.902 to 2.846) | 2.361 (1.902 to 2.846) | 2.361 (1.902 to 2.846) | 2.361 (1.902 to 2.846) | 2.361 (1.902 to 2.846) | 2.361 (1.902 to 2.846) | 2.361 (1.902 to 2.846) | 2.361 (1.902 to 2.846) | 2.361 (1.902 to 2.846) | 2.361 (1.902 to 2.846) |
| Pancreatic cancer | 31.5 Pack-Years | Both | Male | 1.799 (1.448 to 2.184) | 1.799 (1.448 to 2.184) | 1.799 (1.448 to 2.184) | 1.799 (1.448 to 2.184) | 1.799 (1.448 to 2.184) | 1.799 (1.448 to 2.184) | 1.799 (1.448 to 2.184) | 1.799 (1.448 to 2.184) | 1.799 (1.448 to 2.184) | 1.799 (1.448 to 2.184) | 1.799 (1.448 to 2.184) | 1.799 (1.448 to 2.184) | 1.799 (1.448 to 2.184) | 1.799 (1.448 to 2.184) |
| Pancreatic cancer | 31.5 Pack-Years | Both | Female | 2.745 (2.099 to 3.457) | 2.745 (2.099 to 3.457) | 2.745 (2.099 to 3.457) | 2.745 (2.099 to 3.457) | 2.745 (2.099 to 3.457) | 2.745 (2.099 to 3.457) | 2.745 (2.099 to 3.457) | 2.745 (2.099 to 3.457) | 2.745 (2.099 to 3.457) | 2.745 (2.099 to 3.457) | 2.745 (2.099 to 3.457) | 2.745 (2.099 to 3.457) | 2.745 (2.099 to 3.457) | 2.745 (2.099 to 3.457) |
| Pancreatic cancer | 47.3 Pack-Years | Both | Male | 2.173 (1.75 to 2.605) | 2.173 (1.75 to 2.605) | 2.173 (1.75 to 2.605) | 2.173 (1.75 to 2.605) | 2.173 (1.75 to 2.605) | 2.173 (1.75 to 2.605) | 2.173 (1.75 to 2.605) | 2.173 (1.75 to 2.605) | 2.173 (1.75 to 2.605) | 2.173 (1.75 to 2.605) | 2.173 (1.75 to 2.605) | 2.173 (1.75 to 2.605) | 2.173 (1.75 to 2.605) | 2.173 (1.75 to 2.605) |
| Pancreatic cancer | 47.3 Pack-Years | Both | Female | 3.281 (2.36 to 4.34) | 3.281 (2.36 to 4.34) | 3.281 (2.36 to 4.34) | 3.281 (2.36 to 4.34) | 3.281 (2.36 to 4.34) | 3.281 (2.36 to 4.34) | 3.281 (2.36 to 4.34) | 3.281 (2.36 to 4.34) | 3.281 (2.36 to 4.34) | 3.281 (2.36 to 4.34) | 3.281 (2.36 to 4.34) | 3.281 (2.36 to 4.34) | 3.281 (2.36 to 4.34) | 3.281 (2.36 to 4.34) |
| Pancreatic cancer | 63 Pack-Years | Both | Male | 2.429 (1.793 to 3.211) | 2.429 (1.793 to 3.211) | 2.429 (1.793 to 3.211) | 2.429 (1.793 to 3.211) | 2.429 (1.793 to 3.211) | 2.429 (1.793 to 3.211) | 2.429 (1.793 to 3.211) | 2.429 (1.793 to 3.211) | 2.429 (1.793 to 3.211) | 2.429 (1.793 to 3.211) | 2.429 (1.793 to 3.211) | 2.429 (1.793 to 3.211) | 2.429 (1.793 to 3.211) | 2.429 (1.793 to 3.211) |
| Pancreatic cancer | 63 Pack-Years | Both | Female | 3.843 (2.059 to 6.055) | 3.843 (2.059 to 6.055) | 3.843 (2.059 to 6.055) | 3.843 (2.059 to 6.055) | 3.843 (2.059 to 6.055) | 3.843 (2.059 to 6.055) | 3.843 (2.059 to 6.055) | 3.843 (2.059 to 6.055) | 3.843 (2.059 to 6.055) | 3.843 (2.059 to 6.055) | 3.843 (2.059 to 6.055) | 3.843 (2.059 to 6.055) | 3.843 (2.059 to 6.055) | 3.843 (2.059 to 6.055) |
| Larynx cancer | 0 Pack-Years | Both | Male | 1.0 (1.0 to 1.0) | 1.0 (1.0 to 1.0) | 1.0 (1.0 to 1.0) | 1.0 (1.0 to 1.0) | 1.0 (1.0 to 1.0) | 1.0 (1.0 to 1.0) | 1.0 (1.0 to 1.0) | 1.0 (1.0 to 1.0) | 1.0 (1.0 to 1.0) | 1.0 (1.0 to 1.0) | 1.0 (1.0 to 1.0) | 1.0 (1.0 to 1.0) | 1.0 (1.0 to 1.0) | 1.0 (1.0 to 1.0) |
| Larynx cancer | 0 Pack-Years | Both | Female | 1.0 (1.0 to 1.0) | 1.0 (1.0 to 1.0) | 1.0 (1.0 to 1.0) | 1.0 (1.0 to 1.0) | 1.0 (1.0 to 1.0) | 1.0 (1.0 to 1.0) | 1.0 (1.0 to 1.0) | 1.0 (1.0 to 1.0) | 1.0 (1.0 to 1.0) | 1.0 (1.0 to 1.0) | 1.0 (1.0 to 1.0) | 1.0 (1.0 to 1.0) | 1.0 (1.0 to 1.0) | 1.0 (1.0 to 1.0) |
| Larynx cancer | 15 Pack-Years | Both | Male | 4.593 (2.791 to 6.823) | 4.593 (2.791 to 6.823) | 4.593 (2.791 to 6.823) | 4.593 (2.791 to 6.823) | 4.593 (2.791 to 6.823) | 4.593 (2.791 to 6.823) | 4.593 (2.791 to 6.823) | 4.593 (2.791 to 6.823) | 4.593 (2.791 to 6.823) | 4.593 (2.791 to 6.823) | 4.593 (2.791 to 6.823) | 4.593 (2.791 to 6.823) | 4.593 (2.791 to 6.823) | 4.593 (2.791 to 6.823) |
| Larynx cancer | 15 Pack-Years | Both | Female | 4.593 (2.791 to 6.823) | 4.593 (2.791 to 6.823) | 4.593 (2.791 to 6.823) | 4.593 (2.791 to 6.823) | 4.593 (2.791 to 6.823) | 4.593 (2.791 to 6.823) | 4.593 (2.791 to 6.823) | 4.593 (2.791 to 6.823) | 4.593 (2.791 to 6.823) | 4.593 (2.791 to 6.823) | 4.593 (2.791 to 6.823) | 4.593 (2.791 to 6.823) | 4.593 (2.791 to 6.823) | 4.593 (2.791 to 6.823) |
| Larynx cancer | 30 Pack-Years | Both | Male | 8.448 (5.061 to 12.894) | 8.448 (5.061 to 12.894) | 8.448 (5.061 to 12.894) | 8.448 (5.061 to 12.894) | 8.448 (5.061 to 12.894) | 8.448 (5.061 to 12.894) | 8.448 (5.061 to 12.894) | 8.448 (5.061 to 12.894) | 8.448 (5.061 to 12.894) | 8.448 (5.061 to 12.894) | 8.448 (5.061 to 12.894) | 8.448 (5.061 to 12.894) | 8.448 (5.061 to 12.894) | 8.448 (5.061 to 12.894) |
| Larynx cancer | 30 Pack-Years | Both | Female | 8.448 (5.061 to 12.894) | 8.448 (5.061 to 12.894) | 8.448 (5.061 to 12.894) | 8.448 (5.061 to 12.894) | 8.448 (5.061 to 12.894) | 8.448 (5.061 to 12.894) | 8.448 (5.061 to 12.894) | 8.448 (5.061 to 12.894) | 8.448 (5.061 to 12.894) | 8.448 (5.061 to 12.894) | 8.448 (5.061 to 12.894) | 8.448 (5.061 to 12.894) | 8.448 (5.061 to 12.894) | 8.448 (5.061 to 12.894) |
| Larynx cancer | 45 Pack-Years | Both | Male | 16.162 (9.234 to 25.714) | 16.162 (9.234 to 25.714) | 16.162 (9.234 to 25.714) | 16.162 (9.234 to 25.714) | 16.162 (9.234 to 25.714) | 16.162 (9.234 to 25.714) | 16.162 (9.234 to 25.714) | 16.162 (9.234 to 25.714) | 16.162 (9.234 to 25.714) | 16.162 (9.234 to 25.714) | 16.162 (9.234 to 25.714) | 16.162 (9.234 to 25.714) | 16.162 (9.234 to 25.714) | 16.162 (9.234 to 25.714) |
| Larynx cancer | 45 Pack-Years | Both | Female | 16.162 (9.234 to 25.714) | 16.162 (9.234 to 25.714) | 16.162 (9.234 to 25.714) | 16.162 (9.234 to 25.714) | 16.162 (9.234 to 25.714) | 16.162 (9.234 to 25.714) | 16.162 (9.234 to 25.714) | 16.162 (9.234 to 25.714) | 16.162 (9.234 to 25.714) | 16.162 (9.234 to 25.714) | 16.162 (9.234 to 25.714) | 16.162 (9.234 to 25.714) | 16.162 (9.234 to 25.714) | 16.162 (9.234 to 25.714) |
| Larynx cancer | 60 Pack-Years | Both | Male | 21.948 (10.569 to 36.711) | 21.948 (10.569 to 36.711) | 21.948 (10.569 to 36.711) | 21.948 (10.569 to 36.711) | 21.948 (10.569 to 36.711) | 21.948 (10.569 to 36.711) | 21.948 (10.569 to 36.711) | 21.948 (10.569 to 36.711) | 21.948 (10.569 to 36.711) | 21.948 (10.569 to 36.711) | 21.948 (10.569 to 36.711) | 21.948 (10.569 to 36.711) | 21.948 (10.569 to 36.711) | 21.948 (10.569 to 36.711) |
| Larynx cancer | 60 Pack-Years | Both | Female | 21.948 (10.569 to 36.711) | 21.948 (10.569 to 36.711) | 21.948 (10.569 to 36.711) | 21.948 (10.569 to 36.711) | 21.948 (10.569 to 36.711) | 21.948 (10.569 to 36.711) | 21.948 (10.569 to 36.711) | 21.948 (10.569 to 36.711) | 21.948 (10.569 to 36.711) | 21.948 (10.569 to 36.711) | 21.948 (10.569 to 36.711) | 21.948 (10.569 to 36.711) | 21.948 (10.569 to 36.711) | 21.948 (10.569 to 36.711) |
| Larynx cancer | 75 Pack-Years | Both | Male | 26.147 (9.619 to 56.862) | 26.147 (9.619 to 56.862) | 26.147 (9.619 to 56.862) | 26.147 (9.619 to 56.862) | 26.147 (9.619 to 56.862) | 26.147 (9.619 to 56.862) | 26.147 (9.619 to 56.862) | 26.147 (9.619 to 56.862) | 26.147 (9.619 to 56.862) | 26.147 (9.619 to 56.862) | 26.147 (9.619 to 56.862) | 26.147 (9.619 to 56.862) | 26.147 (9.619 to 56.862) | 26.147 (9.619 to 56.862) |
| Larynx cancer | 75 Pack-Years | Both | Female | 26.147 (9.619 to 56.862) | 26.147 (9.619 to 56.862) | 26.147 (9.619 to 56.862) | 26.147 (9.619 to 56.862) | 26.147 (9.619 to 56.862) | 26.147 (9.619 to 56.862) | 26.147 (9.619 to 56.862) | 26.147 (9.619 to 56.862) | 26.147 (9.619 to 56.862) | 26.147 (9.619 to 56.862) | 26.147 (9.619 to 56.862) | 26.147 (9.619 to 56.862) | 26.147 (9.619 to 56.862) | 26.147 (9.619 to 56.862) |
| Tracheal, bronchus, and lung cancer | 0 Pack-Years | Both | Male | 1.0 (1.0 to 1.0) | 1.0 (1.0 to 1.0) | 1.0 (1.0 to 1.0) | 1.0 (1.0 to 1.0) | 1.0 (1.0 to 1.0) | 1.0 (1.0 to 1.0) | 1.0 (1.0 to 1.0) | 1.0 (1.0 to 1.0) | 1.0 (1.0 to 1.0) | 1.0 (1.0 to 1.0) | 1.0 (1.0 to 1.0) | 1.0 (1.0 to 1.0) | 1.0 (1.0 to 1.0) | 1.0 (1.0 to 1.0) |
| Tracheal, bronchus, and lung cancer | 0 Pack-Years | Both | Female | 1.0 (1.0 to 1.0) | 1.0 (1.0 to 1.0) | 1.0 (1.0 to 1.0) | 1.0 (1.0 to 1.0) | 1.0 (1.0 to 1.0) | 1.0 (1.0 to 1.0) | 1.0 (1.0 to 1.0) | 1.0 (1.0 to 1.0) | 1.0 (1.0 to 1.0) | 1.0 (1.0 to 1.0) | 1.0 (1.0 to 1.0) | 1.0 (1.0 to 1.0) | 1.0 (1.0 to 1.0) | 1.0 (1.0 to 1.0) |
| Tracheal, bronchus, and lung cancer | 5 Pack-Years | Both | Male | 1.758 (1.215 to 2.45) | 1.758 (1.215 to 2.45) | 1.758 (1.215 to 2.45) | 1.758 (1.215 to 2.45) | 1.758 (1.215 to 2.45) | 1.758 (1.215 to 2.45) | 1.758 (1.215 to 2.45) | 1.758 (1.215 to 2.45) | 1.758 (1.215 to 2.45) | 1.758 (1.215 to 2.45) | 1.758 (1.215 to 2.45) | 1.758 (1.215 to 2.45) | 1.758 (1.215 to 2.45) | 1.758 (1.215 to 2.45) |
| Tracheal, bronchus, and lung cancer | 5 Pack-Years | Both | Female | 1.758 (1.215 to 2.45) | 1.758 (1.215 to 2.45) | 1.758 (1.215 to 2.45) | 1.758 (1.215 to 2.45) | 1.758 (1.215 to 2.45) | 1.758 (1.215 to 2.45) | 1.758 (1.215 to 2.45) | 1.758 (1.215 to 2.45) | 1.758 (1.215 to 2.45) | 1.758 (1.215 to 2.45) | 1.758 (1.215 to 2.45) | 1.758 (1.215 to 2.45) | 1.758 (1.215 to 2.45) | 1.758 (1.215 to 2.45) |
| Tracheal, bronchus, and lung cancer | 14.3 Pack-Years | Both | Male | 4.86 (3.917 to 5.866) | 4.86 (3.917 to 5.866) | 4.86 (3.917 to 5.866) | 4.86 (3.917 to 5.866) | 4.86 (3.917 to 5.866) | 4.86 (3.917 to 5.866) | 4.86 (3.917 to 5.866) | 4.86 (3.917 to 5.866) | 4.86 (3.917 to 5.866) | 4.86 (3.917 to 5.866) | 4.86 (3.917 to 5.866) | 4.86 (3.917 to 5.866) | 4.86 (3.917 to 5.866) | 4.86 (3.917 to 5.866) |
| Tracheal, bronchus, and lung cancer | 14.3 Pack-Years | Both | Female | 4.86 (3.917 to 5.866) | 4.86 (3.917 to 5.866) | 4.86 (3.917 to 5.866) | 4.86 (3.917 to 5.866) | 4.86 (3.917 to 5.866) | 4.86 (3.917 to 5.866) | 4.86 (3.917 to 5.866) | 4.86 (3.917 to 5.866) | 4.86 (3.917 to 5.866) | 4.86 (3.917 to 5.866) | 4.86 (3.917 to 5.866) | 4.86 (3.917 to 5.866) | 4.86 (3.917 to 5.866) | 4.86 (3.917 to 5.866) |
| Tracheal, bronchus, and lung cancer | 28.6 Pack-Years | Both | Male | 8.899 (7.377 to 10.562) | 8.899 (7.377 to 10.562) | 8.899 (7.377 to 10.562) | 8.899 (7.377 to 10.562) | 8.899 (7.377 to 10.562) | 8.899 (7.377 to 10.562) | 8.899 (7.377 to 10.562) | 8.899 (7.377 to 10.562) | 8.899 (7.377 to 10.562) | 8.899 (7.377 to 10.562) | 8.899 (7.377 to 10.562) | 8.899 (7.377 to 10.562) | 8.899 (7.377 to 10.562) | 8.899 (7.377 to 10.562) |
| Tracheal, bronchus, and lung cancer | 28.6 Pack-Years | Both | Female | 8.899 (7.377 to 10.562) | 8.899 (7.377 to 10.562) | 8.899 (7.377 to 10.562) | 8.899 (7.377 to 10.562) | 8.899 (7.377 to 10.562) | 8.899 (7.377 to 10.562) | 8.899 (7.377 to 10.562) | 8.899 (7.377 to 10.562) | 8.899 (7.377 to 10.562) | 8.899 (7.377 to 10.562) | 8.899 (7.377 to 10.562) | 8.899 (7.377 to 10.562) | 8.899 (7.377 to 10.562) | 8.899 (7.377 to 10.562) |
| Tracheal, bronchus, and lung cancer | 42.9 Pack-Years | Both | Male | 13.509 (10.479 to 16.81) | 13.509 (10.479 to 16.81) | 13.509 (10.479 to 16.81) | 13.509 (10.479 to 16.81) | 13.509 (10.479 to 16.81) | 13.509 (10.479 to 16.81) | 13.509 (10.479 to 16.81) | 13.509 (10.479 to 16.81) | 13.509 (10.479 to 16.81) | 13.509 (10.479 to 16.81) | 13.509 (10.479 to 16.81) | 13.509 (10.479 to 16.81) | 13.509 (10.479 to 16.81) | 13.509 (10.479 to 16.81) |
| Tracheal, bronchus, and lung cancer | 42.9 Pack-Years | Both | Female | 13.509 (10.479 to 16.81) | 13.509 (10.479 to 16.81) | 13.509 (10.479 to 16.81) | 13.509 (10.479 to 16.81) | 13.509 (10.479 to 16.81) | 13.509 (10.479 to 16.81) | 13.509 (10.479 to 16.81) | 13.509 (10.479 to 16.81) | 13.509 (10.479 to 16.81) | 13.509 (10.479 to 16.81) | 13.509 (10.479 to 16.81) | 13.509 (10.479 to 16.81) | 13.509 (10.479 to 16.81) | 13.509 (10.479 to 16.81) |

**Appendix Table 6d. Relative risks used by age and sex for each outcome for smoking globally.**

| Risk - Outcome | Category / Units | Morbidity / Mortality | Sex | Age | | | | | | | | | | | | | |
|---|---|---|---|---|---|---|---|---|---|---|---|---|---|---|---|---|---|
| | | | | 30 to 34 | 35 to 39 | 40 to 44 | 45 to 49 | 50 to 54 | 55 to 59 | 60 to 64 | 65 to 69 | 70 to 74 | 75 to 79 | 80 to 84 | 85 to 89 | 90 to 94 | 95 plus |
| **Smoking** | | | | | | | | | | | | | | | | | |
| Tracheal, bronchus, and lung cancer | 57.1 Pack-Years | Both | Male | 14.83 (10.64 to 19.371) | 14.83 (10.64 to 19.371) | 14.83 (10.64 to 19.371) | 14.83 (10.64 to 19.371) | 14.83 (10.64 to 19.371) | 14.83 (10.64 to 19.371) | 14.83 (10.64 to 19.371) | 14.83 (10.64 to 19.371) | 14.83 (10.64 to 19.371) | 14.83 (10.64 to 19.371) | 14.83 (10.64 to 19.371) | 14.83 (10.64 to 19.371) | 14.83 (10.64 to 19.371) | 14.83 (10.64 to 19.371) |
| Tracheal, bronchus, and lung cancer | 57.1 Pack-Years | Both | Female | 14.83 (10.64 to 19.371) | 14.83 (10.64 to 19.371) | 14.83 (10.64 to 19.371) | 14.83 (10.64 to 19.371) | 14.83 (10.64 to 19.371) | 14.83 (10.64 to 19.371) | 14.83 (10.64 to 19.371) | 14.83 (10.64 to 19.371) | 14.83 (10.64 to 19.371) | 14.83 (10.64 to 19.371) | 14.83 (10.64 to 19.371) | 14.83 (10.64 to 19.371) | 14.83 (10.64 to 19.371) | 14.83 (10.64 to 19.371) |
| Tracheal, bronchus, and lung cancer | 71.4 Pack-Years | Both | Male | 18.644 (12.787 to 25.573) | 18.644 (12.787 to 25.573) | 18.644 (12.787 to 25.573) | 18.644 (12.787 to 25.573) | 18.644 (12.787 to 25.573) | 18.644 (12.787 to 25.573) | 18.644 (12.787 to 25.573) | 18.644 (12.787 to 25.573) | 18.644 (12.787 to 25.573) | 18.644 (12.787 to 25.573) | 18.644 (12.787 to 25.573) | 18.644 (12.787 to 25.573) | 18.644 (12.787 to 25.573) | 18.644 (12.787 to 25.573) |
| Tracheal, bronchus, and lung cancer | 71.4 Pack-Years | Both | Female | 18.644 (12.787 to 25.573) | 18.644 (12.787 to 25.573) | 18.644 (12.787 to 25.573) | 18.644 (12.787 to 25.573) | 18.644 (12.787 to 25.573) | 18.644 (12.787 to 25.573) | 18.644 (12.787 to 25.573) | 18.644 (12.787 to 25.573) | 18.644 (12.787 to 25.573) | 18.644 (12.787 to 25.573) | 18.644 (12.787 to 25.573) | 18.644 (12.787 to 25.573) | 18.644 (12.787 to 25.573) | 18.644 (12.787 to 25.573) |
| Tracheal, bronchus, and lung cancer | 85.7 Pack-Years | Both | Male | 21.525 (13.899 to 30.415) | 21.525 (13.899 to 30.415) | 21.525 (13.899 to 30.415) | 21.525 (13.899 to 30.415) | 21.525 (13.899 to 30.415) | 21.525 (13.899 to 30.415) | 21.525 (13.899 to 30.415) | 21.525 (13.899 to 30.415) | 21.525 (13.899 to 30.415) | 21.525 (13.899 to 30.415) | 21.525 (13.899 to 30.415) | 21.525 (13.899 to 30.415) | 21.525 (13.899 to 30.415) | 21.525 (13.899 to 30.415) |
| Tracheal, bronchus, and lung cancer | 85.7 Pack-Years | Both | Female | 21.525 (13.899 to 30.415) | 21.525 (13.899 to 30.415) | 21.525 (13.899 to 30.415) | 21.525 (13.899 to 30.415) | 21.525 (13.899 to 30.415) | 21.525 (13.899 to 30.415) | 21.525 (13.899 to 30.415) | 21.525 (13.899 to 30.415) | 21.525 (13.899 to 30.415) | 21.525 (13.899 to 30.415) | 21.525 (13.899 to 30.415) | 21.525 (13.899 to 30.415) | 21.525 (13.899 to 30.415) | 21.525 (13.899 to 30.415) |
| Breast cancer | 0 Pack-Years | Both | Female | 1.0 (1.0 to 1.0) | 1.0 (1.0 to 1.0) | 1.0 (1.0 to 1.0) | 1.0 (1.0 to 1.0) | 1.0 (1.0 to 1.0) | 1.0 (1.0 to 1.0) | 1.0 (1.0 to 1.0) | 1.0 (1.0 to 1.0) | 1.0 (1.0 to 1.0) | 1.0 (1.0 to 1.0) | 1.0 (1.0 to 1.0) | 1.0 (1.0 to 1.0) | 1.0 (1.0 to 1.0) | 1.0 (1.0 to 1.0) |
| Breast cancer | 12.8 Pack-Years | Both | Female | 1.207 (1.094 to 1.322) | 1.207 (1.094 to 1.322) | 1.207 (1.094 to 1.322) | 1.207 (1.094 to 1.322) | 1.207 (1.094 to 1.322) | 1.207 (1.094 to 1.322) | 1.207 (1.094 to 1.322) | 1.207 (1.094 to 1.322) | 1.207 (1.094 to 1.322) | 1.207 (1.094 to 1.322) | 1.207 (1.094 to 1.322) | 1.207 (1.094 to 1.322) | 1.207 (1.094 to 1.322) | 1.207 (1.094 to 1.322) |
| Breast cancer | 25.5 Pack-Years | Both | Female | 1.31 (1.205 to 1.431) | 1.31 (1.205 to 1.431) | 1.31 (1.205 to 1.431) | 1.31 (1.205 to 1.431) | 1.31 (1.205 to 1.431) | 1.31 (1.205 to 1.431) | 1.31 (1.205 to 1.431) | 1.31 (1.205 to 1.431) | 1.31 (1.205 to 1.431) | 1.31 (1.205 to 1.431) | 1.31 (1.205 to 1.431) | 1.31 (1.205 to 1.431) | 1.31 (1.205 to 1.431) | 1.31 (1.205 to 1.431) |
| Breast cancer | 38.2 Pack-Years | Both | Female | 1.242 (1.095 to 1.383) | 1.242 (1.095 to 1.383) | 1.242 (1.095 to 1.383) | 1.242 (1.095 to 1.383) | 1.242 (1.095 to 1.383) | 1.242 (1.095 to 1.383) | 1.242 (1.095 to 1.383) | 1.242 (1.095 to 1.383) | 1.242 (1.095 to 1.383) | 1.242 (1.095 to 1.383) | 1.242 (1.095 to 1.383) | 1.242 (1.095 to 1.383) | 1.242 (1.095 to 1.383) | 1.242 (1.095 to 1.383) |
| Breast cancer | 63.8 Pack-Years | Both | Female | 1.274 (1.083 to 1.492) | 1.274 (1.083 to 1.492) | 1.274 (1.083 to 1.492) | 1.274 (1.083 to 1.492) | 1.274 (1.083 to 1.492) | 1.274 (1.083 to 1.492) | 1.274 (1.083 to 1.492) | 1.274 (1.083 to 1.492) | 1.274 (1.083 to 1.492) | 1.274 (1.083 to 1.492) | 1.274 (1.083 to 1.492) | 1.274 (1.083 to 1.492) | 1.274 (1.083 to 1.492) | 1.274 (1.083 to 1.492) |
| Cervical cancer | 0 Pack-Years | Both | Female | 1.0 (1.0 to 1.0) | 1.0 (1.0 to 1.0) | 1.0 (1.0 to 1.0) | 1.0 (1.0 to 1.0) | 1.0 (1.0 to 1.0) | 1.0 (1.0 to 1.0) | 1.0 (1.0 to 1.0) | 1.0 (1.0 to 1.0) | 1.0 (1.0 to 1.0) | 1.0 (1.0 to 1.0) | 1.0 (1.0 to 1.0) | 1.0 (1.0 to 1.0) | 1.0 (1.0 to 1.0) | 1.0 (1.0 to 1.0) |
| Cervical cancer | 5 Pack-Years | Both | Female | 1.785 (1.101 to 2.751) | 1.785 (1.101 to 2.751) | 1.785 (1.101 to 2.751) | 1.785 (1.101 to 2.751) | 1.785 (1.101 to 2.751) | 1.785 (1.101 to 2.751) | 1.785 (1.101 to 2.751) | 1.785 (1.101 to 2.751) | 1.785 (1.101 to 2.751) | 1.785 (1.101 to 2.751) | 1.785 (1.101 to 2.751) | 1.785 (1.101 to 2.751) | 1.785 (1.101 to 2.751) | 1.785 (1.101 to 2.751) |
| Cervical cancer | 7.4 Pack-Years | Both | Female | 1.941 (1.17 to 2.981) | 1.941 (1.17 to 2.981) | 1.941 (1.17 to 2.981) | 1.941 (1.17 to 2.981) | 1.941 (1.17 to 2.981) | 1.941 (1.17 to 2.981) | 1.941 (1.17 to 2.981) | 1.941 (1.17 to 2.981) | 1.941 (1.17 to 2.981) | 1.941 (1.17 to 2.981) | 1.941 (1.17 to 2.981) | 1.941 (1.17 to 2.981) | 1.941 (1.17 to 2.981) | 1.941 (1.17 to 2.981) |
| Cervical cancer | 14.8 Pack-Years | Both | Female | 2.245 (1.257 to 4.099) | 2.245 (1.257 to 4.099) | 2.245 (1.257 to 4.099) | 2.245 (1.257 to 4.099) | 2.245 (1.257 to 4.099) | 2.245 (1.257 to 4.099) | 2.245 (1.257 to 4.099) | 2.245 (1.257 to 4.099) | 2.245 (1.257 to 4.099) | 2.245 (1.257 to 4.099) | 2.245 (1.257 to 4.099) | 2.245 (1.257 to 4.099) | 2.245 (1.257 to 4.099) | 2.245 (1.257 to 4.099) |
| Cervical cancer | 22.1 Pack-Years | Both | Female | 3.355 (1.72 to 6.124) | 3.355 (1.72 to 6.124) | 3.355 (1.72 to 6.124) | 3.355 (1.72 to 6.124) | 3.355 (1.72 to 6.124) | 3.355 (1.72 to 6.124) | 3.355 (1.72 to 6.124) | 3.355 (1.72 to 6.124) | 3.355 (1.72 to 6.124) | 3.355 (1.72 to 6.124) | 3.355 (1.72 to 6.124) | 3.355 (1.72 to 6.124) | 3.355 (1.72 to 6.124) | 3.355 (1.72 to 6.124) |
| Cervical cancer | 29.5 Pack-Years | Both | Female | 4.207 (1.745 to 8.534) | 4.207 (1.745 to 8.534) | 4.207 (1.745 to 8.534) | 4.207 (1.745 to 8.534) | 4.207 (1.745 to 8.534) | 4.207 (1.745 to 8.534) | 4.207 (1.745 to 8.534) | 4.207 (1.745 to 8.534) | 4.207 (1.745 to 8.534) | 4.207 (1.745 to 8.534) | 4.207 (1.745 to 8.534) | 4.207 (1.745 to 8.534) | 4.207 (1.745 to 8.534) | 4.207 (1.745 to 8.534) |
| Prostate cancer | 0 Cigarettes Per Smoker Per Day | Both | Male | 1.0 (1.0 to 1.0) | 1.0 (1.0 to 1.0) | 1.0 (1.0 to 1.0) | 1.0 (1.0 to 1.0) | 1.0 (1.0 to 1.0) | 1.0 (1.0 to 1.0) | 1.0 (1.0 to 1.0) | 1.0 (1.0 to 1.0) | 1.0 (1.0 to 1.0) | 1.0 (1.0 to 1.0) | 1.0 (1.0 to 1.0) | 1.0 (1.0 to 1.0) | 1.0 (1.0 to 1.0) | 1.0 (1.0 to 1.0) |
| Prostate cancer | 7.5 Cigarettes Per Smoker Per Day | Both | Male | 1.192 (1.008 to 1.382) | 1.192 (1.008 to 1.382) | 1.192 (1.008 to 1.382) | 1.192 (1.008 to 1.382) | 1.192 (1.008 to 1.382) | 1.192 (1.008 to 1.382) | 1.192 (1.008 to 1.382) | 1.192 (1.008 to 1.382) | 1.192 (1.008 to 1.382) | 1.192 (1.008 to 1.382) | 1.192 (1.008 to 1.382) | 1.192 (1.008 to 1.382) | 1.192 (1.008 to 1.382) | 1.192 (1.008 to 1.382) |
| Prostate cancer | 15 Cigarettes Per Smoker Per Day | Both | Male | 1.173 (1.004 to 1.371) | 1.173 (1.004 to 1.371) | 1.173 (1.004 to 1.371) | 1.173 (1.004 to 1.371) | 1.173 (1.004 to 1.371) | 1.173 (1.004 to 1.371) | 1.173 (1.004 to 1.371) | 1.173 (1.004 to 1.371) | 1.173 (1.004 to 1.371) | 1.173 (1.004 to 1.371) | 1.173 (1.004 to 1.371) | 1.173 (1.004 to 1.371) | 1.173 (1.004 to 1.371) | 1.173 (1.004 to 1.371) |
| Prostate cancer | 22.5 Cigarettes Per Smoker Per Day | Both | Male | 1.166 (1.001 to 1.359) | 1.166 (1.001 to 1.359) | 1.166 (1.001 to 1.359) | 1.166 (1.001 to 1.359) | 1.166 (1.001 to 1.359) | 1.166 (1.001 to 1.359) | 1.166 (1.001 to 1.359) | 1.166 (1.001 to 1.359) | 1.166 (1.001 to 1.359) | 1.166 (1.001 to 1.359) | 1.166 (1.001 to 1.359) | 1.166 (1.001 to 1.359) | 1.166 (1.001 to 1.359) | 1.166 (1.001 to 1.359) |
| Prostate cancer | 30 Cigarettes Per Smoker Per Day | Both | Male | 1.235 (1.006 to 1.511) | 1.235 (1.006 to 1.511) | 1.235 (1.006 to 1.511) | 1.235 (1.006 to 1.511) | 1.235 (1.006 to 1.511) | 1.235 (1.006 to 1.511) | 1.235 (1.006 to 1.511) | 1.235 (1.006 to 1.511) | 1.235 (1.006 to 1.511) | 1.235 (1.006 to 1.511) | 1.235 (1.006 to 1.511) | 1.235 (1.006 to 1.511) | 1.235 (1.006 to 1.511) | 1.235 (1.006 to 1.511) |
| Prostate cancer | 37.5 Cigarettes Per Smoker Per Day | Both | Male | 1.33 (1.0 to 1.783) | 1.33 (1.0 to 1.783) | 1.33 (1.0 to 1.783) | 1.33 (1.0 to 1.783) | 1.33 (1.0 to 1.783) | 1.33 (1.0 to 1.783) | 1.33 (1.0 to 1.783) | 1.33 (1.0 to 1.783) | 1.33 (1.0 to 1.783) | 1.33 (1.0 to 1.783) | 1.33 (1.0 to 1.783) | 1.33 (1.0 to 1.783) | 1.33 (1.0 to 1.783) | 1.33 (1.0 to 1.783) |
| Kidney cancer | 0 Pack-Years | Both | Male | 1.0 (1.0 to 1.0) | 1.0 (1.0 to 1.0) | 1.0 (1.0 to 1.0) | 1.0 (1.0 to 1.0) | 1.0 (1.0 to 1.0) | 1.0 (1.0 to 1.0) | 1.0 (1.0 to 1.0) | 1.0 (1.0 to 1.0) | 1.0 (1.0 to 1.0) | 1.0 (1.0 to 1.0) | 1.0 (1.0 to 1.0) | 1.0 (1.0 to 1.0) | 1.0 (1.0 to 1.0) | 1.0 (1.0 to 1.0) |
| Kidney cancer | 0 Pack-Years | Both | Female | 1.0 (1.0 to 1.0) | 1.0 (1.0 to 1.0) | 1.0 (1.0 to 1.0) | 1.0 (1.0 to 1.0) | 1.0 (1.0 to 1.0) | 1.0 (1.0 to 1.0) | 1.0 (1.0 to 1.0) | 1.0 (1.0 to 1.0) | 1.0 (1.0 to 1.0) | 1.0 (1.0 to 1.0) | 1.0 (1.0 to 1.0) | 1.0 (1.0 to 1.0) | 1.0 (1.0 to 1.0) | 1.0 (1.0 to 1.0) |
| Kidney cancer | 12.3 Pack-Years | Both | Male | 1.366 (1.046 to 1.748) | 1.366 (1.046 to 1.748) | 1.366 (1.046 to 1.748) | 1.366 (1.046 to 1.748) | 1.366 (1.046 to 1.748) | 1.366 (1.046 to 1.748) | 1.366 (1.046 to 1.748) | 1.366 (1.046 to 1.748) | 1.366 (1.046 to 1.748) | 1.366 (1.046 to 1.748) | 1.366 (1.046 to 1.748) | 1.366 (1.046 to 1.748) | 1.366 (1.046 to 1.748) | 1.366 (1.046 to 1.748) |
| Kidney cancer | 12.3 Pack-Years | Both | Female | 1.366 (1.046 to 1.748) | 1.366 (1.046 to 1.748) | 1.366 (1.046 to 1.748) | 1.366 (1.046 to 1.748) | 1.366 (1.046 to 1.748) | 1.366 (1.046 to 1.748) | 1.366 (1.046 to 1.748) | 1.366 (1.046 to 1.748) | 1.366 (1.046 to 1.748) | 1.366 (1.046 to 1.748) | 1.366 (1.046 to 1.748) | 1.366 (1.046 to 1.748) | 1.366 (1.046 to 1.748) | 1.366 (1.046 to 1.748) |
| Kidney cancer | 24.6 Pack-Years | Both | Male | 1.73 (1.34 to 2.164) | 1.73 (1.34 to 2.164) | 1.73 (1.34 to 2.164) | 1.73 (1.34 to 2.164) | 1.73 (1.34 to 2.164) | 1.73 (1.34 to 2.164) | 1.73 (1.34 to 2.164) | 1.73 (1.34 to 2.164) | 1.73 (1.34 to 2.164) | 1.73 (1.34 to 2.164) | 1.73 (1.34 to 2.164) | 1.73 (1.34 to 2.164) | 1.73 (1.34 to 2.164) | 1.73 (1.34 to 2.164) |
| Kidney cancer | 24.6 Pack-Years | Both | Female | 1.73 (1.34 to 2.164) | 1.73 (1.34 to 2.164) | 1.73 (1.34 to 2.164) | 1.73 (1.34 to 2.164) | 1.73 (1.34 to 2.164) | 1.73 (1.34 to 2.164) | 1.73 (1.34 to 2.164) | 1.73 (1.34 to 2.164) | 1.73 (1.34 to 2.164) | 1.73 (1.34 to 2.164) | 1.73 (1.34 to 2.164) | 1.73 (1.34 to 2.164) | 1.73 (1.34 to 2.164) | 1.73 (1.34 to 2.164) |
| Kidney cancer | 36.9 Pack-Years | Both | Male | 1.757 (1.296 to 2.309) | 1.757 (1.296 to 2.309) | 1.757 (1.296 to 2.309) | 1.757 (1.296 to 2.309) | 1.757 (1.296 to 2.309) | 1.757 (1.296 to 2.309) | 1.757 (1.296 to 2.309) | 1.757 (1.296 to 2.309) | 1.757 (1.296 to 2.309) | 1.757 (1.296 to 2.309) | 1.757 (1.296 to 2.309) | 1.757 (1.296 to 2.309) | 1.757 (1.296 to 2.309) | 1.757 (1.296 to 2.309) |
| Kidney cancer | 36.9 Pack-Years | Both | Female | 1.757 (1.296 to 2.309) | 1.757 (1.296 to 2.309) | 1.757 (1.296 to 2.309) | 1.757 (1.296 to 2.309) | 1.757 (1.296 to 2.309) | 1.757 (1.296 to 2.309) | 1.757 (1.296 to 2.309) | 1.757 (1.296 to 2.309) | 1.757 (1.296 to 2.309) | 1.757 (1.296 to 2.309) | 1.757 (1.296 to 2.309) | 1.757 (1.296 to 2.309) | 1.757 (1.296 to 2.309) | 1.757 (1.296 to 2.309) |
| Kidney cancer | 49.2 Pack-Years | Both | Male | 1.892 (1.429 to 2.44) | 1.892 (1.429 to 2.44) | 1.892 (1.429 to 2.44) | 1.892 (1.429 to 2.44) | 1.892 (1.429 to 2.44) | 1.892 (1.429 to 2.44) | 1.892 (1.429 to 2.44) | 1.892 (1.429 to 2.44) | 1.892 (1.429 to 2.44) | 1.892 (1.429 to 2.44) | 1.892 (1.429 to 2.44) | 1.892 (1.429 to 2.44) | 1.892 (1.429 to 2.44) | 1.892 (1.429 to 2.44) |
| Kidney cancer | 49.2 Pack-Years | Both | Female | 1.892 (1.429 to 2.44) | 1.892 (1.429 to 2.44) | 1.892 (1.429 to 2.44) | 1.892 (1.429 to 2.44) | 1.892 (1.429 to 2.44) | 1.892 (1.429 to 2.44) | 1.892 (1.429 to 2.44) | 1.892 (1.429 to 2.44) | 1.892 (1.429 to 2.44) | 1.892 (1.429 to 2.44) | 1.892 (1.429 to 2.44) | 1.892 (1.429 to 2.44) | 1.892 (1.429 to 2.44) | 1.892 (1.429 to 2.44) |
| Kidney cancer | 61.5 Pack-Years | Both | Male | 2.006 (1.254 to 3.032) | 2.006 (1.254 to 3.032) | 2.006 (1.254 to 3.032) | 2.006 (1.254 to 3.032) | 2.006 (1.254 to 3.032) | 2.006 (1.254 to 3.032) | 2.006 (1.254 to 3.032) | 2.006 (1.254 to 3.032) | 2.006 (1.254 to 3.032) | 2.006 (1.254 to 3.032) | 2.006 (1.254 to 3.032) | 2.006 (1.254 to 3.032) | 2.006 (1.254 to 3.032) | 2.006 (1.254 to 3.032) |

**Appendix Table 6d. Relative risks used by age and sex for each outcome for smoking globally.**

| Risk - Outcome | Category / Units | Morbidity / Mortality | Sex | Age | | | | | | | | | | | | | |
|---|---|---|---|---|---|---|---|---|---|---|---|---|---|---|---|---|---|
| | | | | 30 to 34 | 35 to 39 | 40 to 44 | 45 to 49 | 50 to 54 | 55 to 59 | 60 to 64 | 65 to 69 | 70 to 74 | 75 to 79 | 80 to 84 | 85 to 89 | 90 to 94 | 95 plus |
| **Smoking** | | | | | | | | | | | | | | | | | |
| Kidney cancer | 61.5 Pack-Years | Both | Female | 2.006 (1.254 to 3.032) | 2.006 (1.254 to 3.032) | 2.006 (1.254 to 3.032) | 2.006 (1.254 to 3.032) | 2.006 (1.254 to 3.032) | 2.006 (1.254 to 3.032) | 2.006 (1.254 to 3.032) | 2.006 (1.254 to 3.032) | 2.006 (1.254 to 3.032) | 2.006 (1.254 to 3.032) | 2.006 (1.254 to 3.032) | 2.006 (1.254 to 3.032) | 2.006 (1.254 to 3.032) | 2.006 (1.254 to 3.032) |
| Bladder cancer | 0 Pack-Years | Both | Male | 1.0 (1.0 to 1.0) | 1.0 (1.0 to 1.0) | 1.0 (1.0 to 1.0) | 1.0 (1.0 to 1.0) | 1.0 (1.0 to 1.0) | 1.0 (1.0 to 1.0) | 1.0 (1.0 to 1.0) | 1.0 (1.0 to 1.0) | 1.0 (1.0 to 1.0) | 1.0 (1.0 to 1.0) | 1.0 (1.0 to 1.0) | 1.0 (1.0 to 1.0) | 1.0 (1.0 to 1.0) | 1.0 (1.0 to 1.0) |
| Bladder cancer | 0 Pack-Years | Both | Female | 1.0 (1.0 to 1.0) | 1.0 (1.0 to 1.0) | 1.0 (1.0 to 1.0) | 1.0 (1.0 to 1.0) | 1.0 (1.0 to 1.0) | 1.0 (1.0 to 1.0) | 1.0 (1.0 to 1.0) | 1.0 (1.0 to 1.0) | 1.0 (1.0 to 1.0) | 1.0 (1.0 to 1.0) | 1.0 (1.0 to 1.0) | 1.0 (1.0 to 1.0) | 1.0 (1.0 to 1.0) | 1.0 (1.0 to 1.0) |
| Bladder cancer | 15 Pack-Years | Both | Male | 2.804 (1.695 to 4.161) | 2.804 (1.695 to 4.161) | 2.804 (1.695 to 4.161) | 2.804 (1.695 to 4.161) | 2.804 (1.695 to 4.161) | 2.804 (1.695 to 4.161) | 2.804 (1.695 to 4.161) | 2.804 (1.695 to 4.161) | 2.804 (1.695 to 4.161) | 2.804 (1.695 to 4.161) | 2.804 (1.695 to 4.161) | 2.804 (1.695 to 4.161) | 2.804 (1.695 to 4.161) | 2.804 (1.695 to 4.161) |
| Bladder cancer | 15 Pack-Years | Both | Female | 2.804 (1.695 to 4.161) | 2.804 (1.695 to 4.161) | 2.804 (1.695 to 4.161) | 2.804 (1.695 to 4.161) | 2.804 (1.695 to 4.161) | 2.804 (1.695 to 4.161) | 2.804 (1.695 to 4.161) | 2.804 (1.695 to 4.161) | 2.804 (1.695 to 4.161) | 2.804 (1.695 to 4.161) | 2.804 (1.695 to 4.161) | 2.804 (1.695 to 4.161) | 2.804 (1.695 to 4.161) | 2.804 (1.695 to 4.161) |
| Bladder cancer | 30 Pack-Years | Both | Male | 3.328 (2.093 to 4.997) | 3.328 (2.093 to 4.997) | 3.328 (2.093 to 4.997) | 3.328 (2.093 to 4.997) | 3.328 (2.093 to 4.997) | 3.328 (2.093 to 4.997) | 3.328 (2.093 to 4.997) | 3.328 (2.093 to 4.997) | 3.328 (2.093 to 4.997) | 3.328 (2.093 to 4.997) | 3.328 (2.093 to 4.997) | 3.328 (2.093 to 4.997) | 3.328 (2.093 to 4.997) | 3.328 (2.093 to 4.997) |
| Bladder cancer | 30 Pack-Years | Both | Female | 3.328 (2.093 to 4.997) | 3.328 (2.093 to 4.997) | 3.328 (2.093 to 4.997) | 3.328 (2.093 to 4.997) | 3.328 (2.093 to 4.997) | 3.328 (2.093 to 4.997) | 3.328 (2.093 to 4.997) | 3.328 (2.093 to 4.997) | 3.328 (2.093 to 4.997) | 3.328 (2.093 to 4.997) | 3.328 (2.093 to 4.997) | 3.328 (2.093 to 4.997) | 3.328 (2.093 to 4.997) | 3.328 (2.093 to 4.997) |
| Bladder cancer | 45 Pack-Years | Both | Male | 4.239 (2.45 to 6.526) | 4.239 (2.45 to 6.526) | 4.239 (2.45 to 6.526) | 4.239 (2.45 to 6.526) | 4.239 (2.45 to 6.526) | 4.239 (2.45 to 6.526) | 4.239 (2.45 to 6.526) | 4.239 (2.45 to 6.526) | 4.239 (2.45 to 6.526) | 4.239 (2.45 to 6.526) | 4.239 (2.45 to 6.526) | 4.239 (2.45 to 6.526) | 4.239 (2.45 to 6.526) | 4.239 (2.45 to 6.526) |
| Bladder cancer | 45 Pack-Years | Both | Female | 4.239 (2.45 to 6.526) | 4.239 (2.45 to 6.526) | 4.239 (2.45 to 6.526) | 4.239 (2.45 to 6.526) | 4.239 (2.45 to 6.526) | 4.239 (2.45 to 6.526) | 4.239 (2.45 to 6.526) | 4.239 (2.45 to 6.526) | 4.239 (2.45 to 6.526) | 4.239 (2.45 to 6.526) | 4.239 (2.45 to 6.526) | 4.239 (2.45 to 6.526) | 4.239 (2.45 to 6.526) | 4.239 (2.45 to 6.526) |
| Bladder cancer | 60 Pack-Years | Both | Male | 4.553 (2.544 to 7.643) | 4.553 (2.544 to 7.643) | 4.553 (2.544 to 7.643) | 4.553 (2.544 to 7.643) | 4.553 (2.544 to 7.643) | 4.553 (2.544 to 7.643) | 4.553 (2.544 to 7.643) | 4.553 (2.544 to 7.643) | 4.553 (2.544 to 7.643) | 4.553 (2.544 to 7.643) | 4.553 (2.544 to 7.643) | 4.553 (2.544 to 7.643) | 4.553 (2.544 to 7.643) | 4.553 (2.544 to 7.643) |
| Bladder cancer | 60 Pack-Years | Both | Female | 4.553 (2.544 to 7.643) | 4.553 (2.544 to 7.643) | 4.553 (2.544 to 7.643) | 4.553 (2.544 to 7.643) | 4.553 (2.544 to 7.643) | 4.553 (2.544 to 7.643) | 4.553 (2.544 to 7.643) | 4.553 (2.544 to 7.643) | 4.553 (2.544 to 7.643) | 4.553 (2.544 to 7.643) | 4.553 (2.544 to 7.643) | 4.553 (2.544 to 7.643) | 4.553 (2.544 to 7.643) | 4.553 (2.544 to 7.643) |
| Acute lymphoid leukaemia | 0 Pack-Years | Both | Male | 1.0 (1.0 to 1.0) | 1.0 (1.0 to 1.0) | 1.0 (1.0 to 1.0) | 1.0 (1.0 to 1.0) | 1.0 (1.0 to 1.0) | 1.0 (1.0 to 1.0) | 1.0 (1.0 to 1.0) | 1.0 (1.0 to 1.0) | 1.0 (1.0 to 1.0) | 1.0 (1.0 to 1.0) | 1.0 (1.0 to 1.0) | 1.0 (1.0 to 1.0) | 1.0 (1.0 to 1.0) | 1.0 (1.0 to 1.0) |
| Acute lymphoid leukaemia | 0 Pack-Years | Both | Female | 1.0 (1.0 to 1.0) | 1.0 (1.0 to 1.0) | 1.0 (1.0 to 1.0) | 1.0 (1.0 to 1.0) | 1.0 (1.0 to 1.0) | 1.0 (1.0 to 1.0) | 1.0 (1.0 to 1.0) | 1.0 (1.0 to 1.0) | 1.0 (1.0 to 1.0) | 1.0 (1.0 to 1.0) | 1.0 (1.0 to 1.0) | 1.0 (1.0 to 1.0) | 1.0 (1.0 to 1.0) | 1.0 (1.0 to 1.0) |
| Acute lymphoid leukaemia | 12.4 Pack-Years | Both | Male | 2.011 (1.0 to 3.609) | 2.011 (1.0 to 3.609) | 2.011 (1.0 to 3.609) | 2.011 (1.0 to 3.609) | 2.011 (1.0 to 3.609) | 2.011 (1.0 to 3.609) | 2.011 (1.0 to 3.609) | 2.011 (1.0 to 3.609) | 2.011 (1.0 to 3.609) | 2.011 (1.0 to 3.609) | 2.011 (1.0 to 3.609) | 2.011 (1.0 to 3.609) | 2.011 (1.0 to 3.609) | 2.011 (1.0 to 3.609) |
| Acute lymphoid leukaemia | 12.4 Pack-Years | Both | Female | 2.011 (1.0 to 3.609) | 2.011 (1.0 to 3.609) | 2.011 (1.0 to 3.609) | 2.011 (1.0 to 3.609) | 2.011 (1.0 to 3.609) | 2.011 (1.0 to 3.609) | 2.011 (1.0 to 3.609) | 2.011 (1.0 to 3.609) | 2.011 (1.0 to 3.609) | 2.011 (1.0 to 3.609) | 2.011 (1.0 to 3.609) | 2.011 (1.0 to 3.609) | 2.011 (1.0 to 3.609) | 2.011 (1.0 to 3.609) |
| Acute lymphoid leukaemia | 24.8 Pack-Years | Both | Male | 2.253 (1.071 to 3.961) | 2.253 (1.071 to 3.961) | 2.253 (1.071 to 3.961) | 2.253 (1.071 to 3.961) | 2.253 (1.071 to 3.961) | 2.253 (1.071 to 3.961) | 2.253 (1.071 to 3.961) | 2.253 (1.071 to 3.961) | 2.253 (1.071 to 3.961) | 2.253 (1.071 to 3.961) | 2.253 (1.071 to 3.961) | 2.253 (1.071 to 3.961) | 2.253 (1.071 to 3.961) | 2.253 (1.071 to 3.961) |
| Acute lymphoid leukaemia | 24.8 Pack-Years | Both | Female | 2.253 (1.071 to 3.961) | 2.253 (1.071 to 3.961) | 2.253 (1.071 to 3.961) | 2.253 (1.071 to 3.961) | 2.253 (1.071 to 3.961) | 2.253 (1.071 to 3.961) | 2.253 (1.071 to 3.961) | 2.253 (1.071 to 3.961) | 2.253 (1.071 to 3.961) | 2.253 (1.071 to 3.961) | 2.253 (1.071 to 3.961) | 2.253 (1.071 to 3.961) | 2.253 (1.071 to 3.961) | 2.253 (1.071 to 3.961) |
| Acute lymphoid leukaemia | 37.1 Pack-Years | Both | Male | 2.64 (1.483 to 4.386) | 2.64 (1.483 to 4.386) | 2.64 (1.483 to 4.386) | 2.64 (1.483 to 4.386) | 2.64 (1.483 to 4.386) | 2.64 (1.483 to 4.386) | 2.64 (1.483 to 4.386) | 2.64 (1.483 to 4.386) | 2.64 (1.483 to 4.386) | 2.64 (1.483 to 4.386) | 2.64 (1.483 to 4.386) | 2.64 (1.483 to 4.386) | 2.64 (1.483 to 4.386) | 2.64 (1.483 to 4.386) |
| Acute lymphoid leukaemia | 37.1 Pack-Years | Both | Female | 2.64 (1.483 to 4.386) | 2.64 (1.483 to 4.386) | 2.64 (1.483 to 4.386) | 2.64 (1.483 to 4.386) | 2.64 (1.483 to 4.386) | 2.64 (1.483 to 4.386) | 2.64 (1.483 to 4.386) | 2.64 (1.483 to 4.386) | 2.64 (1.483 to 4.386) | 2.64 (1.483 to 4.386) | 2.64 (1.483 to 4.386) | 2.64 (1.483 to 4.386) | 2.64 (1.483 to 4.386) | 2.64 (1.483 to 4.386) |
| Chronic lymphoid leukaemia | 0 Pack-Years | Both | Male | 1.0 (1.0 to 1.0) | 1.0 (1.0 to 1.0) | 1.0 (1.0 to 1.0) | 1.0 (1.0 to 1.0) | 1.0 (1.0 to 1.0) | 1.0 (1.0 to 1.0) | 1.0 (1.0 to 1.0) | 1.0 (1.0 to 1.0) | 1.0 (1.0 to 1.0) | 1.0 (1.0 to 1.0) | 1.0 (1.0 to 1.0) | 1.0 (1.0 to 1.0) | 1.0 (1.0 to 1.0) | 1.0 (1.0 to 1.0) |
| Chronic lymphoid leukaemia | 0 Pack-Years | Both | Female | 1.0 (1.0 to 1.0) | 1.0 (1.0 to 1.0) | 1.0 (1.0 to 1.0) | 1.0 (1.0 to 1.0) | 1.0 (1.0 to 1.0) | 1.0 (1.0 to 1.0) | 1.0 (1.0 to 1.0) | 1.0 (1.0 to 1.0) | 1.0 (1.0 to 1.0) | 1.0 (1.0 to 1.0) | 1.0 (1.0 to 1.0) | 1.0 (1.0 to 1.0) | 1.0 (1.0 to 1.0) | 1.0 (1.0 to 1.0) |
| Chronic lymphoid leukaemia | 12.4 Pack-Years | Both | Male | 2.011 (1.0 to 3.609) | 2.011 (1.0 to 3.609) | 2.011 (1.0 to 3.609) | 2.011 (1.0 to 3.609) | 2.011 (1.0 to 3.609) | 2.011 (1.0 to 3.609) | 2.011 (1.0 to 3.609) | 2.011 (1.0 to 3.609) | 2.011 (1.0 to 3.609) | 2.011 (1.0 to 3.609) | 2.011 (1.0 to 3.609) | 2.011 (1.0 to 3.609) | 2.011 (1.0 to 3.609) | 2.011 (1.0 to 3.609) |
| Chronic lymphoid leukaemia | 12.4 Pack-Years | Both | Female | 2.011 (1.0 to 3.609) | 2.011 (1.0 to 3.609) | 2.011 (1.0 to 3.609) | 2.011 (1.0 to 3.609) | 2.011 (1.0 to 3.609) | 2.011 (1.0 to 3.609) | 2.011 (1.0 to 3.609) | 2.011 (1.0 to 3.609) | 2.011 (1.0 to 3.609) | 2.011 (1.0 to 3.609) | 2.011 (1.0 to 3.609) | 2.011 (1.0 to 3.609) | 2.011 (1.0 to 3.609) | 2.011 (1.0 to 3.609) |
| Chronic lymphoid leukaemia | 24.8 Pack-Years | Both | Male | 2.253 (1.071 to 3.961) | 2.253 (1.071 to 3.961) | 2.253 (1.071 to 3.961) | 2.253 (1.071 to 3.961) | 2.253 (1.071 to 3.961) | 2.253 (1.071 to 3.961) | 2.253 (1.071 to 3.961) | 2.253 (1.071 to 3.961) | 2.253 (1.071 to 3.961) | 2.253 (1.071 to 3.961) | 2.253 (1.071 to 3.961) | 2.253 (1.071 to 3.961) | 2.253 (1.071 to 3.961) | 2.253 (1.071 to 3.961) |
| Chronic lymphoid leukaemia | 24.8 Pack-Years | Both | Female | 2.253 (1.071 to 3.961) | 2.253 (1.071 to 3.961) | 2.253 (1.071 to 3.961) | 2.253 (1.071 to 3.961) | 2.253 (1.071 to 3.961) | 2.253 (1.071 to 3.961) | 2.253 (1.071 to 3.961) | 2.253 (1.071 to 3.961) | 2.253 (1.071 to 3.961) | 2.253 (1.071 to 3.961) | 2.253 (1.071 to 3.961) | 2.253 (1.071 to 3.961) | 2.253 (1.071 to 3.961) | 2.253 (1.071 to 3.961) |
| Chronic lymphoid leukaemia | 37.1 Pack-Years | Both | Male | 2.64 (1.483 to 4.386) | 2.64 (1.483 to 4.386) | 2.64 (1.483 to 4.386) | 2.64 (1.483 to 4.386) | 2.64 (1.483 to 4.386) | 2.64 (1.483 to 4.386) | 2.64 (1.483 to 4.386) | 2.64 (1.483 to 4.386) | 2.64 (1.483 to 4.386) | 2.64 (1.483 to 4.386) | 2.64 (1.483 to 4.386) | 2.64 (1.483 to 4.386) | 2.64 (1.483 to 4.386) | 2.64 (1.483 to 4.386) |
| Chronic lymphoid leukaemia | 37.1 Pack-Years | Both | Female | 2.64 (1.483 to 4.386) | 2.64 (1.483 to 4.386) | 2.64 (1.483 to 4.386) | 2.64 (1.483 to 4.386) | 2.64 (1.483 to 4.386) | 2.64 (1.483 to 4.386) | 2.64 (1.483 to 4.386) | 2.64 (1.483 to 4.386) | 2.64 (1.483 to 4.386) | 2.64 (1.483 to 4.386) | 2.64 (1.483 to 4.386) | 2.64 (1.483 to 4.386) | 2.64 (1.483 to 4.386) | 2.64 (1.483 to 4.386) |
| Acute myeloid leukaemia | 0 Pack-Years | Both | Male | 1.0 (1.0 to 1.0) | 1.0 (1.0 to 1.0) | 1.0 (1.0 to 1.0) | 1.0 (1.0 to 1.0) | 1.0 (1.0 to 1.0) | 1.0 (1.0 to 1.0) | 1.0 (1.0 to 1.0) | 1.0 (1.0 to 1.0) | 1.0 (1.0 to 1.0) | 1.0 (1.0 to 1.0) | 1.0 (1.0 to 1.0) | 1.0 (1.0 to 1.0) | 1.0 (1.0 to 1.0) | 1.0 (1.0 to 1.0) |
| Acute myeloid leukaemia | 0 Pack-Years | Both | Female | 1.0 (1.0 to 1.0) | 1.0 (1.0 to 1.0) | 1.0 (1.0 to 1.0) | 1.0 (1.0 to 1.0) | 1.0 (1.0 to 1.0) | 1.0 (1.0 to 1.0) | 1.0 (1.0 to 1.0) | 1.0 (1.0 to 1.0) | 1.0 (1.0 to 1.0) | 1.0 (1.0 to 1.0) | 1.0 (1.0 to 1.0) | 1.0 (1.0 to 1.0) | 1.0 (1.0 to 1.0) | 1.0 (1.0 to 1.0) |
| Acute myeloid leukaemia | 12.4 Pack-Years | Both | Male | 2.011 | 2.011 | 2.011 | 2.011 | 2.011 | 2.011 | 2.011 | 2.011 | 2.011 | 2.011 | 2.011 | 2.011 | 2.011 | 2.011 |
| Acute myeloid leukaemia | 12.4 Pack-Years | Both | Female | 2.011 (1.0 to 3.609) | 2.011 (1.0 to 3.609) | 2.011 (1.0 to 3.609) | 2.011 (1.0 to 3.609) | 2.011 (1.0 to 3.609) | 2.011 (1.0 to 3.609) | 2.011 (1.0 to 3.609) | 2.011 (1.0 to 3.609) | 2.011 (1.0 to 3.609) | 2.011 (1.0 to 3.609) | 2.011 (1.0 to 3.609) | 2.011 (1.0 to 3.609) | 2.011 (1.0 to 3.609) | 2.011 (1.0 to 3.609) |
| Acute myeloid leukaemia | 24.8 Pack-Years | Both | Male | 2.253 (1.071 to 3.961) | 2.253 (1.071 to 3.961) | 2.253 (1.071 to 3.961) | 2.253 (1.071 to 3.961) | 2.253 (1.071 to 3.961) | 2.253 (1.071 to 3.961) | 2.253 (1.071 to 3.961) | 2.253 (1.071 to 3.961) | 2.253 (1.071 to 3.961) | 2.253 (1.071 to 3.961) | 2.253 (1.071 to 3.961) | 2.253 (1.071 to 3.961) | 2.253 (1.071 to 3.961) | 2.253 (1.071 to 3.961) |
| Acute myeloid leukaemia | 24.8 Pack-Years | Both | Female | 2.253 (1.071 to 3.961) | 2.253 (1.071 to 3.961) | 2.253 (1.071 to 3.961) | 2.253 (1.071 to 3.961) | 2.253 (1.071 to 3.961) | 2.253 (1.071 to 3.961) | 2.253 (1.071 to 3.961) | 2.253 (1.071 to 3.961) | 2.253 (1.071 to 3.961) | 2.253 (1.071 to 3.961) | 2.253 (1.071 to 3.961) | 2.253 (1.071 to 3.961) | 2.253 (1.071 to 3.961) | 2.253 (1.071 to 3.961) |
| Acute myeloid leukaemia | 37.1 Pack-Years | Both | Male | 2.64 (1.483 to 4.386) | 2.64 (1.483 to 4.386) | 2.64 (1.483 to 4.386) | 2.64 (1.483 to 4.386) | 2.64 (1.483 to 4.386) | 2.64 (1.483 to 4.386) | 2.64 (1.483 to 4.386) | 2.64 (1.483 to 4.386) | 2.64 (1.483 to 4.386) | 2.64 (1.483 to 4.386) | 2.64 (1.483 to 4.386) | 2.64 (1.483 to 4.386) | 2.64 (1.483 to 4.386) | 2.64 (1.483 to 4.386) |
| Acute myeloid leukaemia | 37.1 Pack-Years | Both | Female | 2.64 (1.483 to 4.386) | 2.64 (1.483 to 4.386) | 2.64 (1.483 to 4.386) | 2.64 (1.483 to 4.386) | 2.64 (1.483 to 4.386) | 2.64 (1.483 to 4.386) | 2.64 (1.483 to 4.386) | 2.64 (1.483 to 4.386) | 2.64 (1.483 to 4.386) | 2.64 (1.483 to 4.386) | 2.64 (1.483 to 4.386) | 2.64 (1.483 to 4.386) | 2.64 (1.483 to 4.386) | 2.64 (1.483 to 4.386) |
| Chronic myeloid leukaemia | 0 Pack-Years | Both | Male | 1.0 (1.0 to 1.0) | 1.0 (1.0 to 1.0) | 1.0 (1.0 to 1.0) | 1.0 (1.0 to 1.0) | 1.0 (1.0 to 1.0) | 1.0 (1.0 to 1.0) | 1.0 (1.0 to 1.0) | 1.0 (1.0 to 1.0) | 1.0 (1.0 to 1.0) | 1.0 (1.0 to 1.0) | 1.0 (1.0 to 1.0) | 1.0 (1.0 to 1.0) | 1.0 (1.0 to 1.0) | 1.0 (1.0 to 1.0) |
| Chronic myeloid leukaemia | 0 Pack-Years | Both | Female | 1.0 (1.0 to 1.0) | 1.0 (1.0 to 1.0) | 1.0 (1.0 to 1.0) | 1.0 (1.0 to 1.0) | 1.0 (1.0 to 1.0) | 1.0 (1.0 to 1.0) | 1.0 (1.0 to 1.0) | 1.0 (1.0 to 1.0) | 1.0 (1.0 to 1.0) | 1.0 (1.0 to 1.0) | 1.0 (1.0 to 1.0) | 1.0 (1.0 to 1.0) | 1.0 (1.0 to 1.0) | 1.0 (1.0 to 1.0) |
| Chronic myeloid leukaemia | 12.4 Pack-Years | Both | Male | 2.011 (1.0 to 3.609) | 2.011 (1.0 to 3.609) | 2.011 (1.0 to 3.609) | 2.011 (1.0 to 3.609) | 2.011 (1.0 to 3.609) | 2.011 (1.0 to 3.609) | 2.011 (1.0 to 3.609) | 2.011 (1.0 to 3.609) | 2.011 (1.0 to 3.609) | 2.011 (1.0 to 3.609) | 2.011 (1.0 to 3.609) | 2.011 (1.0 to 3.609) | 2.011 (1.0 to 3.609) | 2.011 (1.0 to 3.609) |

| Appendix Table 6d. Relative risks used by age and sex for each outcome for smoking globally. | | | | | | | | | | | | | | | | | |
|---|---|---|---|---|---|---|---|---|---|---|---|---|---|---|---|---|---|
| Risk - Outcome | Category / Units | Morbidity / Mortality | Sex | Age | | | | | | | | | | | | | |
| | | | | 30 to 34 | 35 to 39 | 40 to 44 | 45 to 49 | 50 to 54 | 55 to 59 | 60 to 64 | 65 to 69 | 70 to 74 | 75 to 79 | 80 to 84 | 85 to 89 | 90 to 94 | 95 plus |
| Smoking | | | | | | | | | | | | | | | | | |
| Chronic myeloid leukaemia | 12.4 Pack-Years | Both | Female | 2.011 (1.0 to 3.609) | 2.011 (1.0 to 3.609) | 2.011 (1.0 to 3.609) | 2.011 (1.0 to 3.609) | 2.011 (1.0 to 3.609) | 2.011 (1.0 to 3.609) | 2.011 (1.0 to 3.609) | 2.011 (1.0 to 3.609) | 2.011 (1.0 to 3.609) | 2.011 (1.0 to 3.609) | 2.011 (1.0 to 3.609) | 2.011 (1.0 to 3.609) | 2.011 (1.0 to 3.609) | 2.011 (1.0 to 3.609) |
| Chronic myeloid leukaemia | 24.8 Pack-Years | Both | Male | 2.253 (1.071 to 3.961) | 2.253 (1.071 to 3.961) | 2.253 (1.071 to 3.961) | 2.253 (1.071 to 3.961) | 2.253 (1.071 to 3.961) | 2.253 (1.071 to 3.961) | 2.253 (1.071 to 3.961) | 2.253 (1.071 to 3.961) | 2.253 (1.071 to 3.961) | 2.253 (1.071 to 3.961) | 2.253 (1.071 to 3.961) | 2.253 (1.071 to 3.961) | 2.253 (1.071 to 3.961) | 2.253 (1.071 to 3.961) |
| Chronic myeloid leukaemia | 24.8 Pack-Years | Both | Female | 2.253 (1.071 to 3.961) | 2.253 (1.071 to 3.961) | 2.253 (1.071 to 3.961) | 2.253 (1.071 to 3.961) | 2.253 (1.071 to 3.961) | 2.253 (1.071 to 3.961) | 2.253 (1.071 to 3.961) | 2.253 (1.071 to 3.961) | 2.253 (1.071 to 3.961) | 2.253 (1.071 to 3.961) | 2.253 (1.071 to 3.961) | 2.253 (1.071 to 3.961) | 2.253 (1.071 to 3.961) | 2.253 (1.071 to 3.961) |
| Chronic myeloid leukaemia | 37.1 Pack-Years | Both | Male | 2.64 (1.483 to 4.386) | 2.64 (1.483 to 4.386) | 2.64 (1.483 to 4.386) | 2.64 (1.483 to 4.386) | 2.64 (1.483 to 4.386) | 2.64 (1.483 to 4.386) | 2.64 (1.483 to 4.386) | 2.64 (1.483 to 4.386) | 2.64 (1.483 to 4.386) | 2.64 (1.483 to 4.386) | 2.64 (1.483 to 4.386) | 2.64 (1.483 to 4.386) | 2.64 (1.483 to 4.386) | 2.64 (1.483 to 4.386) |
| Chronic myeloid leukaemia | 37.1 Pack-Years | Both | Female | 2.64 (1.483 to 4.386) | 2.64 (1.483 to 4.386) | 2.64 (1.483 to 4.386) | 2.64 (1.483 to 4.386) | 2.64 (1.483 to 4.386) | 2.64 (1.483 to 4.386) | 2.64 (1.483 to 4.386) | 2.64 (1.483 to 4.386) | 2.64 (1.483 to 4.386) | 2.64 (1.483 to 4.386) | 2.64 (1.483 to 4.386) | 2.64 (1.483 to 4.386) | 2.64 (1.483 to 4.386) | 2.64 (1.483 to 4.386) |
| Other leukaemia | 0 Pack-Years | Both | Male | 1.0 (1.0 to 1.0) | 1.0 (1.0 to 1.0) | 1.0 (1.0 to 1.0) | 1.0 (1.0 to 1.0) | 1.0 (1.0 to 1.0) | 1.0 (1.0 to 1.0) | 1.0 (1.0 to 1.0) | 1.0 (1.0 to 1.0) | 1.0 (1.0 to 1.0) | 1.0 (1.0 to 1.0) | 1.0 (1.0 to 1.0) | 1.0 (1.0 to 1.0) | 1.0 (1.0 to 1.0) | 1.0 (1.0 to 1.0) |
| Other leukaemia | 0 Pack-Years | Both | Female | 1.0 (1.0 to 1.0) | 1.0 (1.0 to 1.0) | 1.0 (1.0 to 1.0) | 1.0 (1.0 to 1.0) | 1.0 (1.0 to 1.0) | 1.0 (1.0 to 1.0) | 1.0 (1.0 to 1.0) | 1.0 (1.0 to 1.0) | 1.0 (1.0 to 1.0) | 1.0 (1.0 to 1.0) | 1.0 (1.0 to 1.0) | 1.0 (1.0 to 1.0) | 1.0 (1.0 to 1.0) | 1.0 (1.0 to 1.0) |
| Other leukaemia | 12.4 Pack-Years | Both | Male | 2.011 (1.0 to 3.609) | 2.011 (1.0 to 3.609) | 2.011 (1.0 to 3.609) | 2.011 (1.0 to 3.609) | 2.011 (1.0 to 3.609) | 2.011 (1.0 to 3.609) | 2.011 (1.0 to 3.609) | 2.011 (1.0 to 3.609) | 2.011 (1.0 to 3.609) | 2.011 (1.0 to 3.609) | 2.011 (1.0 to 3.609) | 2.011 (1.0 to 3.609) | 2.011 (1.0 to 3.609) | 2.011 (1.0 to 3.609) |
| Other leukaemia | 12.4 Pack-Years | Both | Female | 2.011 (1.0 to 3.609) | 2.011 (1.0 to 3.609) | 2.011 (1.0 to 3.609) | 2.011 (1.0 to 3.609) | 2.011 (1.0 to 3.609) | 2.011 (1.0 to 3.609) | 2.011 (1.0 to 3.609) | 2.011 (1.0 to 3.609) | 2.011 (1.0 to 3.609) | 2.011 (1.0 to 3.609) | 2.011 (1.0 to 3.609) | 2.011 (1.0 to 3.609) | 2.011 (1.0 to 3.609) | 2.011 (1.0 to 3.609) |
| Other leukaemia | 24.8 Pack-Years | Both | Male | 2.253 (1.071 to 3.961) | 2.253 (1.071 to 3.961) | 2.253 (1.071 to 3.961) | 2.253 (1.071 to 3.961) | 2.253 (1.071 to 3.961) | 2.253 (1.071 to 3.961) | 2.253 (1.071 to 3.961) | 2.253 (1.071 to 3.961) | 2.253 (1.071 to 3.961) | 2.253 (1.071 to 3.961) | 2.253 (1.071 to 3.961) | 2.253 (1.071 to 3.961) | 2.253 (1.071 to 3.961) | 2.253 (1.071 to 3.961) |
| Other leukaemia | 24.8 Pack-Years | Both | Female | 2.253 (1.071 to 3.961) | 2.253 (1.071 to 3.961) | 2.253 (1.071 to 3.961) | 2.253 (1.071 to 3.961) | 2.253 (1.071 to 3.961) | 2.253 (1.071 to 3.961) | 2.253 (1.071 to 3.961) | 2.253 (1.071 to 3.961) | 2.253 (1.071 to 3.961) | 2.253 (1.071 to 3.961) | 2.253 (1.071 to 3.961) | 2.253 (1.071 to 3.961) | 2.253 (1.071 to 3.961) | 2.253 (1.071 to 3.961) |
| Other leukaemia | 37.1 Pack-Years | Both | Male | 2.64 (1.483 to 4.386) | 2.64 (1.483 to 4.386) | 2.64 (1.483 to 4.386) | 2.64 (1.483 to 4.386) | 2.64 (1.483 to 4.386) | 2.64 (1.483 to 4.386) | 2.64 (1.483 to 4.386) | 2.64 (1.483 to 4.386) | 2.64 (1.483 to 4.386) | 2.64 (1.483 to 4.386) | 2.64 (1.483 to 4.386) | 2.64 (1.483 to 4.386) | 2.64 (1.483 to 4.386) | 2.64 (1.483 to 4.386) |
| Other leukaemia | 37.1 Pack-Years | Both | Female | 2.64 (1.483 to 4.386) | 2.64 (1.483 to 4.386) | 2.64 (1.483 to 4.386) | 2.64 (1.483 to 4.386) | 2.64 (1.483 to 4.386) | 2.64 (1.483 to 4.386) | 2.64 (1.483 to 4.386) | 2.64 (1.483 to 4.386) | 2.64 (1.483 to 4.386) | 2.64 (1.483 to 4.386) | 2.64 (1.483 to 4.386) | 2.64 (1.483 to 4.386) | 2.64 (1.483 to 4.386) | 2.64 (1.483 to 4.386) |
| Ischaemic heart disease | 0 Cigarettes Per Smoker Per Day | Both | Male | 1.0 (1.0 to 1.0) | 1.0 (1.0 to 1.0) | 1.0 (1.0 to 1.0) | 1.0 (1.0 to 1.0) | 1.0 (1.0 to 1.0) | 1.0 (1.0 to 1.0) | 1.0 (1.0 to 1.0) | 1.0 (1.0 to 1.0) | 1.0 (1.0 to 1.0) | 1.0 (1.0 to 1.0) | 1.0 (1.0 to 1.0) | 1.0 (1.0 to 1.0) | 1.0 (1.0 to 1.0) | 1.0 (1.0 to 1.0) |
| Ischaemic heart disease | 0 Cigarettes Per Smoker Per Day | Both | Female | 1.0 (1.0 to 1.0) | 1.0 (1.0 to 1.0) | 1.0 (1.0 to 1.0) | 1.0 (1.0 to 1.0) | 1.0 (1.0 to 1.0) | 1.0 (1.0 to 1.0) | 1.0 (1.0 to 1.0) | 1.0 (1.0 to 1.0) | 1.0 (1.0 to 1.0) | 1.0 (1.0 to 1.0) | 1.0 (1.0 to 1.0) | 1.0 (1.0 to 1.0) | 1.0 (1.0 to 1.0) | 1.0 (1.0 to 1.0) |
| Ischaemic heart disease | 11.2 Cigarettes Per Smoker Per Day | Both | Male | 2.965 (2.571 to 3.372) | 2.965 (2.571 to 3.372) | 2.965 (2.571 to 3.372) | 2.526 (2.229 to 2.813) | 2.526 (2.229 to 2.813) | 2.125 (1.907 to 2.346) | 2.125 (1.907 to 2.346) | 1.773 (1.641 to 1.908) | 1.773 (1.641 to 1.908) | 1.46 (1.375 to 1.558) | 1.46 (1.375 to 1.558) | 1.187 (1.132 to 1.247) | 1.187 (1.132 to 1.247) | 1.002 (1.0 to 1.01) |
| Ischaemic heart disease | 11.2 Cigarettes Per Smoker Per Day | Both | Female | 3.783 (2.956 to 4.813) | 3.783 (2.956 to 4.813) | 3.783 (2.956 to 4.813) | 3.183 (2.593 to 3.822) | 3.183 (2.593 to 3.822) | 2.608 (2.201 to 3.027) | 2.608 (2.201 to 3.027) | 2.131 (1.906 to 2.365) | 2.131 (1.906 to 2.365) | 1.68 (1.559 to 1.792) | 1.68 (1.559 to 1.792) | 1.277 (1.224 to 1.332) | 1.277 (1.224 to 1.332) | 1.004 (1.0 to 1.013) |
| Ischaemic heart disease | 22.5 Cigarettes Per Smoker Per Day | Both | Male | 3.29 (2.805 to 3.847) | 3.29 (2.805 to 3.847) | 3.29 (2.805 to 3.847) | 2.671 (2.327 to 3.056) | 2.671 (2.327 to 3.056) | 2.188 (1.945 to 2.427) | 2.188 (1.945 to 2.427) | 1.784 (1.626 to 1.964) | 1.784 (1.626 to 1.964) | 1.457 (1.369 to 1.557) | 1.457 (1.369 to 1.557) | 1.188 (1.147 to 1.234) | 1.188 (1.147 to 1.234) | 1.001 (1.0 to 1.007) |
| Ischaemic heart disease | 22.5 Cigarettes Per Smoker Per Day | Both | Female | 6.684 (5.11 to 8.413) | 6.684 (5.11 to 8.413) | 6.684 (5.11 to 8.413) | 4.917 (3.923 to 5.931) | 4.917 (3.923 to 5.931) | 3.569 (3.012 to 4.161) | 3.569 (3.012 to 4.161) | 2.572 (2.258 to 2.875) | 2.572 (2.258 to 2.875) | 1.85 (1.709 to 1.999) | 1.85 (1.709 to 1.999) | 1.33 (1.279 to 1.386) | 1.33 (1.279 to 1.386) | 1.003 (1.0 to 1.012) |
| Ischaemic heart disease | 33.8 Cigarettes Per Smoker Per Day | Both | Male | 4.276 (3.407 to 5.103) | 4.276 (3.407 to 5.103) | 4.276 (3.407 to 5.103) | 3.388 (2.792 to 4.008) | 3.388 (2.792 to 4.008) | 2.634 (2.256 to 3.038) | 2.634 (2.256 to 3.038) | 2.054 (1.821 to 2.296) | 2.054 (1.821 to 2.296) | 1.599 (1.458 to 1.731) | 1.599 (1.458 to 1.731) | 1.236 (1.169 to 1.311) | 1.236 (1.169 to 1.311) | 1.002 (1.0 to 1.013) |
| Ischaemic heart disease | 33.8 Cigarettes Per Smoker Per Day | Both | Female | 9.744 (7.026 to 12.903) | 9.744 (7.026 to 12.903) | 9.744 (7.026 to 12.903) | 6.798 (5.18 to 8.755) | 6.798 (5.18 to 8.755) | 4.678 (3.713 to 5.73) | 4.678 (3.713 to 5.73) | 3.246 (2.742 to 3.794) | 3.246 (2.742 to 3.794) | 2.178 (1.938 to 2.425) | 2.178 (1.938 to 2.425) | 1.452 (1.369 to 1.536) | 1.452 (1.369 to 1.536) | 1.004 (1.0 to 1.014) |
| Ischaemic heart disease | 45 Cigarettes Per Smoker Per Day | Both | Male | 5.45 (3.718 to 7.541) | 5.45 (3.718 to 7.541) | 5.45 (3.718 to 7.541) | 4.139 (2.953 to 5.545) | 4.139 (2.953 to 5.545) | 3.075 (2.296 to 3.931) | 3.075 (2.296 to 3.931) | 2.325 (1.746 to 2.927) | 2.325 (1.746 to 2.927) | 1.737 (1.39 to 2.104) | 1.737 (1.39 to 2.104) | 1.303 (1.132 to 1.484) | 1.303 (1.132 to 1.484) | 1.008 (1.0 to 1.038) |
| Ischaemic heart disease | 45 Cigarettes Per Smoker Per Day | Both | Female | 11.771 (7.222 to 17.611) | 11.771 (7.222 to 17.611) | 11.771 (7.222 to 17.611) | 7.729 (4.893 to 11.42) | 7.729 (4.893 to 11.42) | 5.097 (3.524 to 7.021) | 5.097 (3.524 to 7.021) | 3.414 (2.463 to 4.466) | 3.414 (2.463 to 4.466) | 2.239 (1.754 to 2.79) | 2.239 (1.754 to 2.79) | 1.436 (1.236 to 1.651) | 1.436 (1.236 to 1.651) | 1.006 (1.0 to 1.036) |
| Ischaemic heart disease | 56.2 Cigarettes Per Smoker Per Day | Both | Male | 5.574 (3.774 to 7.899) | 5.574 (3.774 to 7.899) | 5.574 (3.774 to 7.899) | 4.182 (2.877 to 5.614) | 4.182 (2.877 to 5.614) | 3.016 (2.218 to 3.956) | 3.016 (2.218 to 3.956) | 2.227 (1.65 to 2.849) | 2.227 (1.65 to 2.849) | 1.647 (1.286 to 2.018) | 1.647 (1.286 to 2.018) | 1.229 (1.022 to 1.441) | 1.229 (1.022 to 1.441) | 1.008 (1.0 to 1.05) |
| Ischaemic heart disease | 56.2 Cigarettes Per Smoker Per Day | Both | Female | 13.641 (7.463 to 22.811) | 13.641 (7.463 to 22.811) | 13.641 (7.463 to 22.811) | 8.813 (4.958 to 14.032) | 8.813 (4.958 to 14.032) | 5.612 (3.445 to 8.338) | 5.612 (3.445 to 8.338) | 3.69 (2.602 to 5.039) | 3.69 (2.602 to 5.039) | 2.405 (1.788 to 3.044) | 2.405 (1.788 to 3.044) | 1.58 (1.33 to 1.849) | 1.58 (1.33 to 1.849) | 1.018 (1.0 to 1.077) |
| Ischaemic stroke | 0 Cigarettes Per Smoker Per Day | Both | Male | 1.0 (1.0 to 1.0) | 1.0 (1.0 to 1.0) | 1.0 (1.0 to 1.0) | 1.0 (1.0 to 1.0) | 1.0 (1.0 to 1.0) | 1.0 (1.0 to 1.0) | 1.0 (1.0 to 1.0) | 1.0 (1.0 to 1.0) | 1.0 (1.0 to 1.0) | 1.0 (1.0 to 1.0) | 1.0 (1.0 to 1.0) | 1.0 (1.0 to 1.0) | 1.0 (1.0 to 1.0) | 1.0 (1.0 to 1.0) |
| Ischaemic stroke | 0 Cigarettes Per Smoker Per Day | Both | Female | 1.0 (1.0 to 1.0) | 1.0 (1.0 to 1.0) | 1.0 (1.0 to 1.0) | 1.0 (1.0 to 1.0) | 1.0 (1.0 to 1.0) | 1.0 (1.0 to 1.0) | 1.0 (1.0 to 1.0) | 1.0 (1.0 to 1.0) | 1.0 (1.0 to 1.0) | 1.0 (1.0 to 1.0) | 1.0 (1.0 to 1.0) | 1.0 (1.0 to 1.0) | 1.0 (1.0 to 1.0) | 1.0 (1.0 to 1.0) |
| Ischaemic stroke | 10 Cigarettes Per Smoker Per Day | Both | Male | 2.363 (1.976 to 2.74) | 2.363 (1.976 to 2.74) | 2.363 (1.976 to 2.74) | 2.112 (1.84 to 2.402) | 2.112 (1.84 to 2.402) | 1.843 (1.639 to 2.061) | 1.843 (1.639 to 2.061) | 1.606 (1.461 to 1.755) | 1.606 (1.461 to 1.755) | 1.386 (1.289 to 1.48) | 1.386 (1.289 to 1.48) | 1.153 (1.101 to 1.204) | 1.153 (1.101 to 1.204) | 1.011 (1.0 to 1.041) |
| Ischaemic stroke | 10 Cigarettes Per Smoker Per Day | Both | Female | 2.334 (1.689 to 3.166) | 2.334 (1.689 to 3.166) | 2.334 (1.689 to 3.166) | 2.101 (1.605 to 2.723) | 2.101 (1.605 to 2.723) | 1.916 (1.509 to 2.33) | 1.916 (1.509 to 2.33) | 1.721 (1.435 to 2.014) | 1.721 (1.435 to 2.014) | 1.479 (1.317 to 1.639) | 1.479 (1.317 to 1.639) | 1.204 (1.116 to 1.301) | 1.204 (1.116 to 1.301) | 1.007 (1.0 to 1.027) |
| Ischaemic stroke | 20 Cigarettes Per Smoker Per Day | Both | Male | 2.319 (1.923 to 2.747) | 2.319 (1.923 to 2.747) | 2.319 (1.923 to 2.747) | 1.971 (1.703 to 2.277) | 1.971 (1.703 to 2.277) | 1.662 (1.454 to 1.884) | 1.662 (1.454 to 1.884) | 1.415 (1.285 to 1.56) | 1.415 (1.285 to 1.56) | 1.222 (1.143 to 1.307) | 1.222 (1.143 to 1.307) | 1.08 (1.045 to 1.119) | 1.08 (1.045 to 1.119) | 1.004 (1.0 to 1.016) |
| Ischaemic stroke | 20 Cigarettes Per Smoker Per Day | Both | Female | 3.737 (2.688 to 4.979) | 3.737 (2.688 to 4.979) | 3.737 (2.688 to 4.979) | 3.04 (2.299 to 3.938) | 3.04 (2.299 to 3.938) | 2.443 (1.876 to 3.059) | 2.443 (1.876 to 3.059) | 1.94 (1.598 to 2.277) | 1.94 (1.598 to 2.277) | 1.527 (1.326 to 1.723) | 1.527 (1.326 to 1.723) | 1.194 (1.127 to 1.274) | 1.194 (1.127 to 1.274) | 1.003 (1.0 to 1.014) |
| Ischaemic stroke | 30 Cigarettes Per Smoker Per Day | Both | Male | 3.178 (2.653 to 3.77) | 3.178 (2.653 to 3.77) | 3.178 (2.653 to 3.77) | 2.666 (2.265 to 3.069) | 2.666 (2.265 to 3.069) | 2.224 (1.945 to 2.515) | 2.224 (1.945 to 2.515) | 1.849 (1.663 to 2.037) | 1.849 (1.663 to 2.037) | 1.518 (1.397 to 1.64) | 1.518 (1.397 to 1.64) | 1.209 (1.15 to 1.263) | 1.209 (1.15 to 1.263) | 1.009 (1.0 to 1.034) |
| Ischaemic stroke | 30 Cigarettes Per Smoker Per Day | Both | Female | 5.491 (3.813 to 7.703) | 5.491 (3.813 to 7.703) | 5.491 (3.813 to 7.703) | 4.206 (2.989 to 5.642) | 4.206 (2.989 to 5.642) | 3.136 (2.326 to 4.018) | 3.136 (2.326 to 4.018) | 2.3 (1.87 to 2.815) | 2.3 (1.87 to 2.815) | 1.721 (1.474 to 2.006) | 1.721 (1.474 to 2.006) | 1.292 (1.172 to 1.413) | 1.292 (1.172 to 1.413) | 1.007 (1.0 to 1.025) |
| Ischaemic stroke | 40 Cigarettes Per Smoker Per Day | Both | Male | 3.431 (2.454 to 4.606) | 3.431 (2.454 to 4.606) | 3.431 (2.454 to 4.606) | 2.734 (2.011 to 3.596) | 2.734 (2.011 to 3.596) | 2.117 (1.619 to 2.739) | 2.117 (1.619 to 2.739) | 1.659 (1.313 to 2.061) | 1.659 (1.313 to 2.061) | 1.337 (1.123 to 1.585) | 1.337 (1.123 to 1.585) | 1.119 (1.021 to 1.22) | 1.119 (1.021 to 1.22) | 1.011 (1.0 to 1.038) |
| Ischaemic stroke | 40 Cigarettes Per Smoker Per Day | Both | Female | 7.127 (4.104 to 11.541) | 7.127 (4.104 to 11.541) | 7.127 (4.104 to 11.541) | 5.103 (3.045 to 7.724) | 5.103 (3.045 to 7.724) | 3.636 (2.299 to 5.219) | 3.636 (2.299 to 5.219) | 2.593 (1.808 to 3.589) | 2.593 (1.808 to 3.589) | 1.781 (1.325 to 2.376) | 1.781 (1.325 to 2.376) | 1.209 (1.0 to 1.43) | 1.209 (1.0 to 1.43) | 1.008 (1.0 to 1.036) |

**Appendix Table 6d. Relative risks used by age and sex for each outcome for smoking globally.**

| Risk - Outcome | Category / Units | Morbidity / Mortality | Sex | Age | | | | | | | | | | | | | |
|---|---|---|---|---|---|---|---|---|---|---|---|---|---|---|---|---|---|
| | | | | 30 to 34 | 35 to 39 | 40 to 44 | 45 to 49 | 50 to 54 | 55 to 59 | 60 to 64 | 65 to 69 | 70 to 74 | 75 to 79 | 80 to 84 | 85 to 89 | 90 to 94 | 95 plus |
| **Smoking** | | | | | | | | | | | | | | | | | |
| Ischaemic stroke | 50 Cigarettes Per Smoker Per Day | Both | Male | 4.456 (2.943 to 6.299) | 4.456 (2.943 to 6.299) | 4.456 (2.943 to 6.299) | 3.491 (2.396 to 4.743) | 3.491 (2.396 to 4.743) | 2.659 (1.915 to 3.56) | 2.659 (1.915 to 3.56) | 2.048 (1.526 to 2.611) | 2.048 (1.526 to 2.611) | 1.579 (1.203 to 1.976) | 1.579 (1.203 to 1.976) | 1.211 (1.0 to 1.464) | 1.211 (1.0 to 1.464) | 1.035 (1.0 to 1.144) |
| Ischaemic stroke | 50 Cigarettes Per Smoker Per Day | Both | Female | 9.851 (5.013 to 17.851) | 9.851 (5.013 to 17.851) | 9.851 (5.013 to 17.851) | 6.922 (3.959 to 11.353) | 6.922 (3.959 to 11.353) | 5.0 (3.058 to 7.745) | 5.0 (3.058 to 7.745) | 3.569 (2.397 to 4.973) | 3.569 (2.397 to 4.973) | 2.352 (1.697 to 3.091) | 2.352 (1.697 to 3.091) | 1.453 (1.136 to 1.809) | 1.453 (1.136 to 1.809) | 1.026 (1.0 to 1.112) |
| Ischaemic stroke | 60 Cigarettes Per Smoker Per Day | Both | Male | 5.317 (2.793 to 8.673) | 5.317 (2.793 to 8.673) | 5.317 (2.793 to 8.673) | 4.155 (2.304 to 6.585) | 4.155 (2.304 to 6.585) | 3.175 (1.804 to 4.855) | 3.175 (1.804 to 4.855) | 2.395 (1.464 to 3.507) | 2.395 (1.464 to 3.507) | 1.844 (1.18 to 2.676) | 1.844 (1.18 to 2.676) | 1.385 (1.0 to 1.885) | 1.385 (1.0 to 1.885) | 1.101 (1.0 to 1.366) |
| Ischaemic stroke | 60 Cigarettes Per Smoker Per Day | Both | Female | 11.722 (4.845 to 23.87) | 11.722 (4.845 to 23.87) | 11.722 (4.845 to 23.87) | 8.28 (3.608 to 16.396) | 8.28 (3.608 to 16.396) | 5.942 (3.01 to 11.062) | 5.942 (3.01 to 11.062) | 4.286 (2.319 to 7.079) | 4.286 (2.319 to 7.079) | 2.783 (1.643 to 4.284) | 2.783 (1.643 to 4.284) | 1.689 (1.093 to 2.378) | 1.689 (1.093 to 2.378) | 1.043 (1.0 to 1.234) |
| Intracerebral hemorrhage | 0 Cigarettes Per Smoker Per Day | Both | Male | 1.0 (1.0 to 1.0) | 1.0 (1.0 to 1.0) | 1.0 (1.0 to 1.0) | 1.0 (1.0 to 1.0) | 1.0 (1.0 to 1.0) | 1.0 (1.0 to 1.0) | 1.0 (1.0 to 1.0) | 1.0 (1.0 to 1.0) | 1.0 (1.0 to 1.0) | 1.0 (1.0 to 1.0) | 1.0 (1.0 to 1.0) | 1.0 (1.0 to 1.0) | 1.0 (1.0 to 1.0) | 1.0 (1.0 to 1.0) |
| Intracerebral hemorrhage | 0 Cigarettes Per Smoker Per Day | Both | Female | 1.0 (1.0 to 1.0) | 1.0 (1.0 to 1.0) | 1.0 (1.0 to 1.0) | 1.0 (1.0 to 1.0) | 1.0 (1.0 to 1.0) | 1.0 (1.0 to 1.0) | 1.0 (1.0 to 1.0) | 1.0 (1.0 to 1.0) | 1.0 (1.0 to 1.0) | 1.0 (1.0 to 1.0) | 1.0 (1.0 to 1.0) | 1.0 (1.0 to 1.0) | 1.0 (1.0 to 1.0) | 1.0 (1.0 to 1.0) |
| Intracerebral hemorrhage | 10 Cigarettes Per Smoker Per Day | Both | Male | 2.363 (1.976 to 2.74) | 2.363 (1.976 to 2.74) | 2.363 (1.976 to 2.74) | 2.112 (1.84 to 2.402) | 2.112 (1.84 to 2.402) | 1.843 (1.639 to 2.061) | 1.843 (1.639 to 2.061) | 1.606 (1.461 to 1.755) | 1.606 (1.461 to 1.755) | 1.386 (1.289 to 1.48) | 1.386 (1.289 to 1.48) | 1.153 (1.101 to 1.204) | 1.153 (1.101 to 1.204) | 1.011 (1.0 to 1.041) |
| Intracerebral hemorrhage | 10 Cigarettes Per Smoker Per Day | Both | Female | 2.334 (1.689 to 3.166) | 2.334 (1.689 to 3.166) | 2.334 (1.689 to 3.166) | 2.101 (1.605 to 2.723) | 2.101 (1.605 to 2.723) | 1.916 (1.509 to 2.33) | 1.916 (1.509 to 2.33) | 1.721 (1.435 to 2.014) | 1.721 (1.435 to 2.014) | 1.479 (1.317 to 1.639) | 1.479 (1.317 to 1.639) | 1.204 (1.116 to 1.301) | 1.204 (1.116 to 1.301) | 1.007 (1.0 to 1.027) |
| Intracerebral hemorrhage | 20 Cigarettes Per Smoker Per Day | Both | Male | 2.319 (1.923 to 2.747) | 2.319 (1.923 to 2.747) | 2.319 (1.923 to 2.747) | 1.971 (1.703 to 2.277) | 1.971 (1.703 to 2.277) | 1.662 (1.454 to 1.884) | 1.662 (1.454 to 1.884) | 1.415 (1.285 to 1.56) | 1.415 (1.285 to 1.56) | 1.222 (1.143 to 1.307) | 1.222 (1.143 to 1.307) | 1.08 (1.045 to 1.119) | 1.08 (1.045 to 1.119) | 1.004 (1.0 to 1.016) |
| Intracerebral hemorrhage | 20 Cigarettes Per Smoker Per Day | Both | Female | 3.737 (2.688 to 4.979) | 3.737 (2.688 to 4.979) | 3.737 (2.688 to 4.979) | 3.04 (2.299 to 3.938) | 3.04 (2.299 to 3.938) | 2.443 (1.876 to 3.059) | 2.443 (1.876 to 3.059) | 1.94 (1.598 to 2.277) | 1.94 (1.598 to 2.277) | 1.527 (1.326 to 1.723) | 1.527 (1.326 to 1.723) | 1.194 (1.127 to 1.274) | 1.194 (1.127 to 1.274) | 1.003 (1.0 to 1.014) |
| Intracerebral hemorrhage | 30 Cigarettes Per Smoker Per Day | Both | Male | 3.178 (2.653 to 3.77) | 3.178 (2.653 to 3.77) | 3.178 (2.653 to 3.77) | 2.666 (2.265 to 3.069) | 2.666 (2.265 to 3.069) | 2.224 (1.945 to 2.515) | 2.224 (1.945 to 2.515) | 1.849 (1.663 to 2.037) | 1.849 (1.663 to 2.037) | 1.518 (1.397 to 1.64) | 1.518 (1.397 to 1.64) | 1.209 (1.15 to 1.263) | 1.209 (1.15 to 1.263) | 1.009 (1.0 to 1.034) |
| Intracerebral hemorrhage | 30 Cigarettes Per Smoker Per Day | Both | Female | 5.491 (3.813 to 7.703) | 5.491 (3.813 to 7.703) | 5.491 (3.813 to 7.703) | 4.206 (2.989 to 5.642) | 4.206 (2.989 to 5.642) | 3.136 (2.326 to 4.018) | 3.136 (2.326 to 4.018) | 2.3 (1.87 to 2.815) | 2.3 (1.87 to 2.815) | 1.721 (1.474 to 2.006) | 1.721 (1.474 to 2.006) | 1.292 (1.172 to 1.413) | 1.292 (1.172 to 1.413) | 1.007 (1.0 to 1.025) |
| Intracerebral hemorrhage | 40 Cigarettes Per Smoker Per Day | Both | Male | 3.431 (2.454 to 4.606) | 3.431 (2.454 to 4.606) | 3.431 (2.454 to 4.606) | 2.734 (2.011 to 3.596) | 2.734 (2.011 to 3.596) | 2.117 (1.619 to 2.739) | 2.117 (1.619 to 2.739) | 1.659 (1.313 to 2.061) | 1.659 (1.313 to 2.061) | 1.337 (1.123 to 1.585) | 1.337 (1.123 to 1.585) | 1.119 (1.021 to 1.22) | 1.119 (1.021 to 1.22) | 1.011 (1.0 to 1.038) |
| Intracerebral hemorrhage | 40 Cigarettes Per Smoker Per Day | Both | Female | 7.127 (4.104 to 11.541) | 7.127 (4.104 to 11.541) | 7.127 (4.104 to 11.541) | 5.103 (3.045 to 7.724) | 5.103 (3.045 to 7.724) | 3.636 (2.299 to 5.219) | 3.636 (2.299 to 5.219) | 2.593 (1.808 to 3.589) | 2.593 (1.808 to 3.589) | 1.781 (1.325 to 2.376) | 1.781 (1.325 to 2.376) | 1.209 (1.0 to 1.43) | 1.209 (1.0 to 1.43) | 1.008 (1.0 to 1.036) |
| Intracerebral hemorrhage | 50 Cigarettes Per Smoker Per Day | Both | Male | 4.456 (2.943 to 6.299) | 4.456 (2.943 to 6.299) | 4.456 (2.943 to 6.299) | 3.491 (2.396 to 4.743) | 3.491 (2.396 to 4.743) | 2.659 (1.915 to 3.56) | 2.659 (1.915 to 3.56) | 2.048 (1.526 to 2.611) | 2.048 (1.526 to 2.611) | 1.579 (1.203 to 1.976) | 1.579 (1.203 to 1.976) | 1.211 (1.0 to 1.464) | 1.211 (1.0 to 1.464) | 1.035 (1.0 to 1.144) |
| Intracerebral hemorrhage | 50 Cigarettes Per Smoker Per Day | Both | Female | 9.851 (5.013 to 17.851) | 9.851 (5.013 to 17.851) | 9.851 (5.013 to 17.851) | 6.922 (3.959 to 11.353) | 6.922 (3.959 to 11.353) | 5.0 (3.058 to 7.745) | 5.0 (3.058 to 7.745) | 3.569 (2.397 to 4.973) | 3.569 (2.397 to 4.973) | 2.352 (1.697 to 3.091) | 2.352 (1.697 to 3.091) | 1.453 (1.136 to 1.809) | 1.453 (1.136 to 1.809) | 1.026 (1.0 to 1.112) |
| Intracerebral hemorrhage | 60 Cigarettes Per Smoker Per Day | Both | Male | 5.317 (2.793 to 8.673) | 5.317 (2.793 to 8.673) | 5.317 (2.793 to 8.673) | 4.155 (2.304 to 6.585) | 4.155 (2.304 to 6.585) | 3.175 (1.804 to 4.855) | 3.175 (1.804 to 4.855) | 2.395 (1.464 to 3.507) | 2.395 (1.464 to 3.507) | 1.844 (1.18 to 2.676) | 1.844 (1.18 to 2.676) | 1.385 (1.0 to 1.885) | 1.385 (1.0 to 1.885) | 1.101 (1.0 to 1.366) |
| Intracerebral hemorrhage | 60 Cigarettes Per Smoker Per Day | Both | Female | 11.722 (4.845 to 23.87) | 11.722 (4.845 to 23.87) | 11.722 (4.845 to 23.87) | 8.28 (3.608 to 16.396) | 8.28 (3.608 to 16.396) | 5.942 (3.01 to 11.062) | 5.942 (3.01 to 11.062) | 4.286 (2.319 to 7.079) | 4.286 (2.319 to 7.079) | 2.783 (1.643 to 4.284) | 2.783 (1.643 to 4.284) | 1.689 (1.093 to 2.378) | 1.689 (1.093 to 2.378) | 1.043 (1.0 to 1.234) |
| Subarachnoid hemorrhage | 0 Cigarettes Per Smoker Per Day | Both | Male | 1.0 (1.0 to 1.0) | 1.0 (1.0 to 1.0) | 1.0 (1.0 to 1.0) | 1.0 (1.0 to 1.0) | 1.0 (1.0 to 1.0) | 1.0 (1.0 to 1.0) | 1.0 (1.0 to 1.0) | 1.0 (1.0 to 1.0) | 1.0 (1.0 to 1.0) | 1.0 (1.0 to 1.0) | 1.0 (1.0 to 1.0) | 1.0 (1.0 to 1.0) | 1.0 (1.0 to 1.0) | 1.0 (1.0 to 1.0) |
| Subarachnoid hemorrhage | 0 Cigarettes Per Smoker Per Day | Both | Female | 1.0 (1.0 to 1.0) | 1.0 (1.0 to 1.0) | 1.0 (1.0 to 1.0) | 1.0 (1.0 to 1.0) | 1.0 (1.0 to 1.0) | 1.0 (1.0 to 1.0) | 1.0 (1.0 to 1.0) | 1.0 (1.0 to 1.0) | 1.0 (1.0 to 1.0) | 1.0 (1.0 to 1.0) | 1.0 (1.0 to 1.0) | 1.0 (1.0 to 1.0) | 1.0 (1.0 to 1.0) | 1.0 (1.0 to 1.0) |
| Subarachnoid hemorrhage | 10 Cigarettes Per Smoker Per Day | Both | Male | 2.363 (1.976 to 2.74) | 2.363 (1.976 to 2.74) | 2.363 (1.976 to 2.74) | 2.112 (1.84 to 2.402) | 2.112 (1.84 to 2.402) | 1.843 (1.639 to 2.061) | 1.843 (1.639 to 2.061) | 1.606 (1.461 to 1.755) | 1.606 (1.461 to 1.755) | 1.386 (1.289 to 1.48) | 1.386 (1.289 to 1.48) | 1.153 (1.101 to 1.204) | 1.153 (1.101 to 1.204) | 1.011 (1.0 to 1.041) |
| Subarachnoid hemorrhage | 10 Cigarettes Per Smoker Per Day | Both | Female | 2.334 (1.689 to 3.166) | 2.334 (1.689 to 3.166) | 2.334 (1.689 to 3.166) | 2.101 (1.605 to 2.723) | 2.101 (1.605 to 2.723) | 1.916 (1.509 to 2.33) | 1.916 (1.509 to 2.33) | 1.721 (1.435 to 2.014) | 1.721 (1.435 to 2.014) | 1.479 (1.317 to 1.639) | 1.479 (1.317 to 1.639) | 1.204 (1.116 to 1.301) | 1.204 (1.116 to 1.301) | 1.007 (1.0 to 1.027) |
| Subarachnoid hemorrhage | 20 Cigarettes Per Smoker Per Day | Both | Male | 2.319 (1.923 to 2.747) | 2.319 (1.923 to 2.747) | 2.319 (1.923 to 2.747) | 1.971 (1.703 to 2.277) | 1.971 (1.703 to 2.277) | 1.662 (1.454 to 1.884) | 1.662 (1.454 to 1.884) | 1.415 (1.285 to 1.56) | 1.415 (1.285 to 1.56) | 1.222 (1.143 to 1.307) | 1.222 (1.143 to 1.307) | 1.08 (1.045 to 1.119) | 1.08 (1.045 to 1.119) | 1.004 (1.0 to 1.016) |
| Subarachnoid hemorrhage | 20 Cigarettes Per Smoker Per Day | Both | Female | 3.737 (2.688 to 4.979) | 3.737 (2.688 to 4.979) | 3.737 (2.688 to 4.979) | 3.04 (2.299 to 3.938) | 3.04 (2.299 to 3.938) | 2.443 (1.876 to 3.059) | 2.443 (1.876 to 3.059) | 1.94 (1.598 to 2.277) | 1.94 (1.598 to 2.277) | 1.527 (1.326 to 1.723) | 1.527 (1.326 to 1.723) | 1.194 (1.127 to 1.274) | 1.194 (1.127 to 1.274) | 1.003 (1.0 to 1.014) |
| Subarachnoid hemorrhage | 30 Cigarettes Per Smoker Per Day | Both | Male | 3.178 (2.653 to 3.77) | 3.178 (2.653 to 3.77) | 3.178 (2.653 to 3.77) | 2.666 (2.265 to 3.069) | 2.666 (2.265 to 3.069) | 2.224 (1.945 to 2.515) | 2.224 (1.945 to 2.515) | 1.849 (1.663 to 2.037) | 1.849 (1.663 to 2.037) | 1.518 (1.397 to 1.64) | 1.518 (1.397 to 1.64) | 1.209 (1.15 to 1.263) | 1.209 (1.15 to 1.263) | 1.009 (1.0 to 1.034) |
| Subarachnoid hemorrhage | 30 Cigarettes Per Smoker Per Day | Both | Female | 5.491 (3.813 to 7.703) | 5.491 (3.813 to 7.703) | 5.491 (3.813 to 7.703) | 4.206 (2.989 to 5.642) | 4.206 (2.989 to 5.642) | 3.136 (2.326 to 4.018) | 3.136 (2.326 to 4.018) | 2.3 (1.87 to 2.815) | 2.3 (1.87 to 2.815) | 1.721 (1.474 to 2.006) | 1.721 (1.474 to 2.006) | 1.292 (1.172 to 1.413) | 1.292 (1.172 to 1.413) | 1.007 (1.0 to 1.025) |
| Subarachnoid hemorrhage | 40 Cigarettes Per Smoker Per Day | Both | Male | 3.431 (2.454 to 4.606) | 3.431 (2.454 to 4.606) | 3.431 (2.454 to 4.606) | 2.734 (2.011 to 3.596) | 2.734 (2.011 to 3.596) | 2.117 (1.619 to 2.739) | 2.117 (1.619 to 2.739) | 1.659 (1.313 to 2.061) | 1.659 (1.313 to 2.061) | 1.337 (1.123 to 1.585) | 1.337 (1.123 to 1.585) | 1.119 (1.021 to 1.22) | 1.119 (1.021 to 1.22) | 1.011 (1.0 to 1.038) |
| Subarachnoid hemorrhage | 40 Cigarettes Per Smoker Per Day | Both | Female | 7.127 (4.104 to 11.541) | 7.127 (4.104 to 11.541) | 7.127 (4.104 to 11.541) | 5.103 (3.045 to 7.724) | 5.103 (3.045 to 7.724) | 3.636 (2.299 to 5.219) | 3.636 (2.299 to 5.219) | 2.593 (1.808 to 3.589) | 2.593 (1.808 to 3.589) | 1.781 (1.325 to 2.376) | 1.781 (1.325 to 2.376) | 1.209 (1.0 to 1.43) | 1.209 (1.0 to 1.43) | 1.008 (1.0 to 1.036) |
| Subarachnoid hemorrhage | 50 Cigarettes Per Smoker Per Day | Both | Male | 4.456 (2.943 to 6.299) | 4.456 (2.943 to 6.299) | 4.456 (2.943 to 6.299) | 3.491 (2.396 to 4.743) | 3.491 (2.396 to 4.743) | 2.659 (1.915 to 3.56) | 2.659 (1.915 to 3.56) | 2.048 (1.526 to 2.611) | 2.048 (1.526 to 2.611) | 1.579 (1.203 to 1.976) | 1.579 (1.203 to 1.976) | 1.211 (1.0 to 1.464) | 1.211 (1.0 to 1.464) | 1.035 (1.0 to 1.144) |
| Subarachnoid hemorrhage | 50 Cigarettes Per Smoker Per Day | Both | Female | 9.851 (5.013 to 17.851) | 9.851 (5.013 to 17.851) | 9.851 (5.013 to 17.851) | 6.922 (3.959 to 11.353) | 6.922 (3.959 to 11.353) | 5.0 (3.058 to 7.745) | 5.0 (3.058 to 7.745) | 3.569 (2.397 to 4.973) | 3.569 (2.397 to 4.973) | 2.352 (1.697 to 3.091) | 2.352 (1.697 to 3.091) | 1.453 (1.136 to 1.809) | 1.453 (1.136 to 1.809) | 1.026 (1.0 to 1.112) |
| Subarachnoid hemorrhage | 60 Cigarettes Per Smoker Per Day | Both | Male | 5.317 (2.793 to 8.673) | 5.317 (2.793 to 8.673) | 5.317 (2.793 to 8.673) | 4.155 (2.304 to 6.585) | 4.155 (2.304 to 6.585) | 3.175 (1.804 to 4.855) | 3.175 (1.804 to 4.855) | 2.395 (1.464 to 3.507) | 2.395 (1.464 to 3.507) | 1.844 (1.18 to 2.676) | 1.844 (1.18 to 2.676) | 1.385 (1.0 to 1.885) | 1.385 (1.0 to 1.885) | 1.101 (1.0 to 1.366) |
| Subarachnoid hemorrhage | 60 Cigarettes Per Smoker Per Day | Both | Female | 11.722 (4.845 to 23.87) | 11.722 (4.845 to 23.87) | 11.722 (4.845 to 23.87) | 8.28 (3.608 to 16.396) | 8.28 (3.608 to 16.396) | 5.942 (3.01 to 11.062) | 5.942 (3.01 to 11.062) | 4.286 (2.319 to 7.079) | 4.286 (2.319 to 7.079) | 2.783 (1.643 to 4.284) | 2.783 (1.643 to 4.284) | 1.689 (1.093 to 2.378) | 1.689 (1.093 to 2.378) | 1.043 (1.0 to 1.234) |
| Atrial fibrillation and flutter | 0 Cigarettes Per Smoker Per Day | Both | Male | 1.0 (1.0 to 1.0) | 1.0 (1.0 to 1.0) | 1.0 (1.0 to 1.0) | 1.0 (1.0 to 1.0) | 1.0 (1.0 to 1.0) | 1.0 (1.0 to 1.0) | 1.0 (1.0 to 1.0) | 1.0 (1.0 to 1.0) | 1.0 (1.0 to 1.0) | 1.0 (1.0 to 1.0) | 1.0 (1.0 to 1.0) | 1.0 (1.0 to 1.0) | 1.0 (1.0 to 1.0) | 1.0 (1.0 to 1.0) |

**Appendix Table 6d. Relative risks used by age and sex for each outcome for smoking globally.**

| Risk - Outcome | Category / Units | Morbidity / Mortality | Sex | Age 30 to 34 | 35 to 39 | 40 to 44 | 45 to 49 | 50 to 54 | 55 to 59 | 60 to 64 | 65 to 69 | 70 to 74 | 75 to 79 | 80 to 84 | 85 to 89 | 90 to 94 | 95 plus |
|---|---|---|---|---|---|---|---|---|---|---|---|---|---|---|---|---|---|
| **Smoking** | | | | | | | | | | | | | | | | | |
| Atrial fibrillation and flutter | 0 Cigarettes Per Smoker Per Day | Both | Female | 1.0 (1.0 to 1.0) | 1.0 (1.0 to 1.0) | 1.0 (1.0 to 1.0) | 1.0 (1.0 to 1.0) | 1.0 (1.0 to 1.0) | 1.0 (1.0 to 1.0) | 1.0 (1.0 to 1.0) | 1.0 (1.0 to 1.0) | 1.0 (1.0 to 1.0) | 1.0 (1.0 to 1.0) | 1.0 (1.0 to 1.0) | 1.0 (1.0 to 1.0) | 1.0 (1.0 to 1.0) | 1.0 (1.0 to 1.0) |
| Atrial fibrillation and flutter | 6.3 Cigarettes Per Smoker Per Day | Both | Male | 1.532 (1.044 to 2.151) | 1.532 (1.044 to 2.151) | 1.532 (1.044 to 2.151) | 1.507 (1.048 to 2.051) | 1.507 (1.048 to 2.051) | 1.474 (1.035 to 1.935) | 1.474 (1.035 to 1.935) | 1.429 (1.039 to 1.787) | 1.429 (1.039 to 1.787) | 1.324 (1.052 to 1.59) | 1.324 (1.052 to 1.59) | 1.182 (1.0 to 1.358) | 1.182 (1.0 to 1.358) | 1.008 (1.0 to 1.047) |
| Atrial fibrillation and flutter | 6.3 Cigarettes Per Smoker Per Day | Both | Female | 1.532 (1.044 to 2.151) | 1.532 (1.044 to 2.151) | 1.532 (1.044 to 2.151) | 1.507 (1.048 to 2.051) | 1.507 (1.048 to 2.051) | 1.474 (1.035 to 1.935) | 1.474 (1.035 to 1.935) | 1.429 (1.039 to 1.787) | 1.429 (1.039 to 1.787) | 1.324 (1.052 to 1.59) | 1.324 (1.052 to 1.59) | 1.182 (1.0 to 1.358) | 1.182 (1.0 to 1.358) | 1.008 (1.0 to 1.047) |
| Atrial fibrillation and flutter | 12.6 Cigarettes Per Smoker Per Day | Both | Male | 1.895 (1.239 to 2.794) | 1.895 (1.239 to 2.794) | 1.895 (1.239 to 2.794) | 1.774 (1.21 to 2.55) | 1.774 (1.21 to 2.55) | 1.632 (1.154 to 2.241) | 1.632 (1.154 to 2.241) | 1.459 (1.101 to 1.896) | 1.459 (1.101 to 1.896) | 1.283 (1.024 to 1.607) | 1.283 (1.024 to 1.607) | 1.129 (1.0 to 1.32) | 1.129 (1.0 to 1.32) | 1.021 (1.0 to 1.111) |
| Atrial fibrillation and flutter | 12.6 Cigarettes Per Smoker Per Day | Both | Female | 1.895 (1.239 to 2.794) | 1.895 (1.239 to 2.794) | 1.895 (1.239 to 2.794) | 1.774 (1.21 to 2.55) | 1.774 (1.21 to 2.55) | 1.632 (1.154 to 2.241) | 1.632 (1.154 to 2.241) | 1.459 (1.101 to 1.896) | 1.459 (1.101 to 1.896) | 1.283 (1.024 to 1.607) | 1.283 (1.024 to 1.607) | 1.129 (1.0 to 1.32) | 1.129 (1.0 to 1.32) | 1.021 (1.0 to 1.111) |
| Atrial fibrillation and flutter | 18.9 Cigarettes Per Smoker Per Day | Both | Male | 2.222 (1.43 to 3.334) | 2.222 (1.43 to 3.334) | 2.222 (1.43 to 3.334) | 1.997 (1.35 to 2.909) | 1.997 (1.35 to 2.909) | 1.766 (1.249 to 2.477) | 1.766 (1.249 to 2.477) | 1.518 (1.132 to 2.048) | 1.518 (1.132 to 2.048) | 1.299 (1.003 to 1.669) | 1.299 (1.003 to 1.669) | 1.124 (1.0 to 1.381) | 1.124 (1.0 to 1.381) | 1.028 (1.0 to 1.159) |
| Atrial fibrillation and flutter | 18.9 Cigarettes Per Smoker Per Day | Both | Female | 2.222 (1.43 to 3.334) | 2.222 (1.43 to 3.334) | 2.222 (1.43 to 3.334) | 1.997 (1.35 to 2.909) | 1.997 (1.35 to 2.909) | 1.766 (1.249 to 2.477) | 1.766 (1.249 to 2.477) | 1.518 (1.132 to 2.048) | 1.518 (1.132 to 2.048) | 1.299 (1.003 to 1.669) | 1.299 (1.003 to 1.669) | 1.124 (1.0 to 1.381) | 1.124 (1.0 to 1.381) | 1.028 (1.0 to 1.159) |
| Atrial fibrillation and flutter | 25.2 Cigarettes Per Smoker Per Day | Both | Male | 2.588 (1.542 to 4.076) | 2.588 (1.542 to 4.076) | 2.588 (1.542 to 4.076) | 2.261 (1.451 to 3.404) | 2.261 (1.451 to 3.404) | 1.952 (1.347 to 2.865) | 1.952 (1.347 to 2.865) | 1.629 (1.227 to 2.188) | 1.629 (1.227 to 2.188) | 1.37 (1.104 to 1.74) | 1.37 (1.104 to 1.74) | 1.157 (1.0 to 1.366) | 1.157 (1.0 to 1.366) | 1.012 (1.0 to 1.09) |
| Atrial fibrillation and flutter | 25.2 Cigarettes Per Smoker Per Day | Both | Female | 2.588 (1.542 to 4.076) | 2.588 (1.542 to 4.076) | 2.588 (1.542 to 4.076) | 2.261 (1.451 to 3.404) | 2.261 (1.451 to 3.404) | 1.952 (1.347 to 2.865) | 1.952 (1.347 to 2.865) | 1.629 (1.227 to 2.188) | 1.629 (1.227 to 2.188) | 1.37 (1.104 to 1.74) | 1.37 (1.104 to 1.74) | 1.157 (1.0 to 1.366) | 1.157 (1.0 to 1.366) | 1.012 (1.0 to 1.09) |
| Aortic aneurysm | 0 Cigarettes Per Smoker Per Day | Both | Male | 1.0 (1.0 to 1.0) | 1.0 (1.0 to 1.0) | 1.0 (1.0 to 1.0) | 1.0 (1.0 to 1.0) | 1.0 (1.0 to 1.0) | 1.0 (1.0 to 1.0) | 1.0 (1.0 to 1.0) | 1.0 (1.0 to 1.0) | 1.0 (1.0 to 1.0) | 1.0 (1.0 to 1.0) | 1.0 (1.0 to 1.0) | 1.0 (1.0 to 1.0) | 1.0 (1.0 to 1.0) | 1.0 (1.0 to 1.0) |
| Aortic aneurysm | 0 Cigarettes Per Smoker Per Day | Both | Female | 1.0 (1.0 to 1.0) | 1.0 (1.0 to 1.0) | 1.0 (1.0 to 1.0) | 1.0 (1.0 to 1.0) | 1.0 (1.0 to 1.0) | 1.0 (1.0 to 1.0) | 1.0 (1.0 to 1.0) | 1.0 (1.0 to 1.0) | 1.0 (1.0 to 1.0) | 1.0 (1.0 to 1.0) | 1.0 (1.0 to 1.0) | 1.0 (1.0 to 1.0) | 1.0 (1.0 to 1.0) | 1.0 (1.0 to 1.0) |
| Aortic aneurysm | 12 Cigarettes Per Smoker Per Day | Both | Male | 5.046 (3.685 to 6.516) | 5.046 (3.685 to 6.516) | 5.046 (3.685 to 6.516) | 4.201 (3.207 to 5.298) | 4.201 (3.207 to 5.298) | 3.404 (2.713 to 4.162) | 3.404 (2.713 to 4.162) | 2.669 (2.263 to 3.044) | 2.669 (2.263 to 3.044) | 2.014 (1.79 to 2.242) | 2.014 (1.79 to 2.242) | 1.441 (1.327 to 1.563) | 1.441 (1.327 to 1.563) | 1.005 (1.0 to 1.03) |
| Aortic aneurysm | 12 Cigarettes Per Smoker Per Day | Both | Female | 5.046 (3.685 to 6.516) | 5.046 (3.685 to 6.516) | 5.046 (3.685 to 6.516) | 4.201 (3.207 to 5.298) | 4.201 (3.207 to 5.298) | 3.404 (2.713 to 4.162) | 3.404 (2.713 to 4.162) | 2.669 (2.263 to 3.044) | 2.669 (2.263 to 3.044) | 2.014 (1.79 to 2.242) | 2.014 (1.79 to 2.242) | 1.441 (1.327 to 1.563) | 1.441 (1.327 to 1.563) | 1.005 (1.0 to 1.03) |
| Aortic aneurysm | 24 Cigarettes Per Smoker Per Day | Both | Male | 9.532 (6.977 to 12.48) | 9.532 (6.977 to 12.48) | 9.532 (6.977 to 12.48) | 6.722 (5.101 to 8.596) | 6.722 (5.101 to 8.596) | 4.62 (3.71 to 5.762) | 4.62 (3.71 to 5.762) | 3.116 (2.577 to 3.724) | 3.116 (2.577 to 3.724) | 2.076 (1.797 to 2.395) | 2.076 (1.797 to 2.395) | 1.387 (1.242 to 1.547) | 1.387 (1.242 to 1.547) | 1.004 (1.0 to 1.021) |
| Aortic aneurysm | 24 Cigarettes Per Smoker Per Day | Both | Female | 9.532 (6.977 to 12.48) | 9.532 (6.977 to 12.48) | 9.532 (6.977 to 12.48) | 6.722 (5.101 to 8.596) | 6.722 (5.101 to 8.596) | 4.62 (3.71 to 5.762) | 4.62 (3.71 to 5.762) | 3.116 (2.577 to 3.724) | 3.116 (2.577 to 3.724) | 2.076 (1.797 to 2.395) | 2.076 (1.797 to 2.395) | 1.387 (1.242 to 1.547) | 1.387 (1.242 to 1.547) | 1.004 (1.0 to 1.021) |
| Aortic aneurysm | 36 Cigarettes Per Smoker Per Day | Both | Male | 12.382 (7.774 to 18.221) | 12.382 (7.774 to 18.221) | 12.382 (7.774 to 18.221) | 8.505 (5.659 to 11.932) | 8.505 (5.659 to 11.932) | 5.626 (3.935 to 7.702) | 5.626 (3.935 to 7.702) | 3.697 (2.779 to 4.795) | 3.697 (2.779 to 4.795) | 2.455 (1.897 to 3.038) | 2.455 (1.897 to 3.038) | 1.593 (1.306 to 1.875) | 1.593 (1.306 to 1.875) | 1.006 (1.0 to 1.03) |
| Aortic aneurysm | 36 Cigarettes Per Smoker Per Day | Both | Female | 12.382 (7.774 to 18.221) | 12.382 (7.774 to 18.221) | 12.382 (7.774 to 18.221) | 8.505 (5.659 to 11.932) | 8.505 (5.659 to 11.932) | 5.626 (3.935 to 7.702) | 5.626 (3.935 to 7.702) | 3.697 (2.779 to 4.795) | 3.697 (2.779 to 4.795) | 2.455 (1.897 to 3.038) | 2.455 (1.897 to 3.038) | 1.593 (1.306 to 1.875) | 1.593 (1.306 to 1.875) | 1.006 (1.0 to 1.03) |
| Aortic aneurysm | 48 Cigarettes Per Smoker Per Day | Both | Male | 14.479 (7.446 to 24.32) | 14.479 (7.446 to 24.32) | 14.479 (7.446 to 24.32) | 9.77 (5.928 to 15.073) | 9.77 (5.928 to 15.073) | 6.258 (4.093 to 9.24) | 6.258 (4.093 to 9.24) | 3.954 (2.756 to 5.443) | 3.954 (2.756 to 5.443) | 2.503 (1.879 to 3.295) | 2.503 (1.879 to 3.295) | 1.562 (1.216 to 1.946) | 1.562 (1.216 to 1.946) | 1.034 (1.0 to 1.156) |
| Aortic aneurysm | 48 Cigarettes Per Smoker Per Day | Both | Female | 14.479 (7.446 to 24.32) | 14.479 (7.446 to 24.32) | 14.479 (7.446 to 24.32) | 9.77 (5.928 to 15.073) | 9.77 (5.928 to 15.073) | 6.258 (4.093 to 9.24) | 6.258 (4.093 to 9.24) | 3.954 (2.756 to 5.443) | 3.954 (2.756 to 5.443) | 2.503 (1.879 to 3.295) | 2.503 (1.879 to 3.295) | 1.562 (1.216 to 1.946) | 1.562 (1.216 to 1.946) | 1.034 (1.0 to 1.156) |
| Peripheral vascular disease | 0 Cigarettes Per Smoker Per Day | Both | Male | 1.0 (1.0 to 1.0) | 1.0 (1.0 to 1.0) | 1.0 (1.0 to 1.0) | 1.0 (1.0 to 1.0) | 1.0 (1.0 to 1.0) | 1.0 (1.0 to 1.0) | 1.0 (1.0 to 1.0) | 1.0 (1.0 to 1.0) | 1.0 (1.0 to 1.0) | 1.0 (1.0 to 1.0) | 1.0 (1.0 to 1.0) | 1.0 (1.0 to 1.0) | 1.0 (1.0 to 1.0) | 1.0 (1.0 to 1.0) |
| Peripheral vascular disease | 0 Cigarettes Per Smoker Per Day | Both | Female | 1.0 (1.0 to 1.0) | 1.0 (1.0 to 1.0) | 1.0 (1.0 to 1.0) | 1.0 (1.0 to 1.0) | 1.0 (1.0 to 1.0) | 1.0 (1.0 to 1.0) | 1.0 (1.0 to 1.0) | 1.0 (1.0 to 1.0) | 1.0 (1.0 to 1.0) | 1.0 (1.0 to 1.0) | 1.0 (1.0 to 1.0) | 1.0 (1.0 to 1.0) | 1.0 (1.0 to 1.0) | 1.0 (1.0 to 1.0) |
| Peripheral vascular disease | 9.3 Cigarettes Per Smoker Per Day | Both | Male | 3.033 (1.979 to 4.598) | 3.033 (1.979 to 4.598) | 3.033 (1.979 to 4.598) | 2.78 (1.756 to 4.24) | 2.78 (1.756 to 4.24) | 3.077 (1.721 to 4.737) | 3.077 (1.721 to 4.737) | 2.978 (2.177 to 3.749) | 2.978 (2.177 to 3.749) | 2.194 (1.724 to 2.64) | 2.194 (1.724 to 2.64) | 1.535 (1.238 to 1.832) | 1.535 (1.238 to 1.832) | 1.042 (1.0 to 1.183) |
| Peripheral vascular disease | 9.3 Cigarettes Per Smoker Per Day | Both | Female | 3.033 (1.979 to 4.598) | 3.033 (1.979 to 4.598) | 3.033 (1.979 to 4.598) | 2.78 (1.756 to 4.24) | 2.78 (1.756 to 4.24) | 3.077 (1.721 to 4.737) | 3.077 (1.721 to 4.737) | 2.978 (2.177 to 3.749) | 2.978 (2.177 to 3.749) | 2.194 (1.724 to 2.64) | 2.194 (1.724 to 2.64) | 1.535 (1.238 to 1.832) | 1.535 (1.238 to 1.832) | 1.042 (1.0 to 1.183) |
| Peripheral vascular disease | 18.6 Cigarettes Per Smoker Per Day | Both | Male | 6.374 (3.686 to 10.26) | 6.374 (3.686 to 10.26) | 6.374 (3.686 to 10.26) | 5.44 (3.032 to 8.936) | 5.44 (3.032 to 8.936) | 5.689 (2.725 to 8.816) | 5.689 (2.725 to 8.816) | 4.868 (3.556 to 6.076) | 4.868 (3.556 to 6.076) | 3.01 (2.439 to 3.56) | 3.01 (2.439 to 3.56) | 1.75 (1.446 to 2.077) | 1.75 (1.446 to 2.077) | 1.033 (1.0 to 1.167) |
| Peripheral vascular disease | 18.6 Cigarettes Per Smoker Per Day | Both | Female | 6.374 (3.686 to 10.26) | 6.374 (3.686 to 10.26) | 6.374 (3.686 to 10.26) | 5.44 (3.032 to 8.936) | 5.44 (3.032 to 8.936) | 5.689 (2.725 to 8.816) | 5.689 (2.725 to 8.816) | 4.868 (3.556 to 6.076) | 4.868 (3.556 to 6.076) | 3.01 (2.439 to 3.56) | 3.01 (2.439 to 3.56) | 1.75 (1.446 to 2.077) | 1.75 (1.446 to 2.077) | 1.033 (1.0 to 1.167) |
| Peripheral vascular disease | 27.9 Cigarettes Per Smoker Per Day | Both | Male | 8.997 (4.62 to 15.851) | 8.997 (4.62 to 15.851) | 8.997 (4.62 to 15.851) | 7.43 (3.684 to 13.161) | 7.43 (3.684 to 13.161) | 7.069 (3.105 to 11.181) | 7.069 (3.105 to 11.181) | 5.123 (3.758 to 6.66) | 5.123 (3.758 to 6.66) | 2.887 (2.243 to 3.712) | 2.887 (2.243 to 3.712) | 1.651 (1.297 to 2.064) | 1.651 (1.297 to 2.064) | 1.047 (1.0 to 1.217) |
| Peripheral vascular disease | 27.9 Cigarettes Per Smoker Per Day | Both | Female | 8.997 (4.62 to 15.851) | 8.997 (4.62 to 15.851) | 8.997 (4.62 to 15.851) | 7.43 (3.684 to 13.161) | 7.43 (3.684 to 13.161) | 7.069 (3.105 to 11.181) | 7.069 (3.105 to 11.181) | 5.123 (3.758 to 6.66) | 5.123 (3.758 to 6.66) | 2.887 (2.243 to 3.712) | 2.887 (2.243 to 3.712) | 1.651 (1.297 to 2.064) | 1.651 (1.297 to 2.064) | 1.047 (1.0 to 1.217) |
| Peripheral vascular disease | 37.2 Cigarettes Per Smoker Per Day | Both | Male | 10.787 (4.702 to 20.954) | 10.787 (4.702 to 20.954) | 10.787 (4.702 to 20.954) | 8.838 (3.845 to 18.062) | 8.838 (3.845 to 18.062) | 8.201 (3.609 to 14.687) | 8.201 (3.609 to 14.687) | 5.967 (3.758 to 9.089) | 5.967 (3.758 to 9.089) | 3.335 (2.184 to 4.946) | 3.335 (2.184 to 4.946) | 1.894 (1.247 to 2.825) | 1.894 (1.247 to 2.825) | 1.16 (1.0 to 1.68) |
| Peripheral vascular disease | 37.2 Cigarettes Per Smoker Per Day | Both | Female | 10.787 (4.702 to 20.954) | 10.787 (4.702 to 20.954) | 10.787 (4.702 to 20.954) | 8.838 (3.845 to 18.062) | 8.838 (3.845 to 18.062) | 8.201 (3.609 to 14.687) | 8.201 (3.609 to 14.687) | 5.967 (3.758 to 9.089) | 5.967 (3.758 to 9.089) | 3.335 (2.184 to 4.946) | 3.335 (2.184 to 4.946) | 1.894 (1.247 to 2.825) | 1.894 (1.247 to 2.825) | 1.16 (1.0 to 1.68) |
| Peripheral vascular disease | 46.5 Cigarettes Per Smoker Per Day | Both | Male | 11.975 (4.66 to 25.273) | 11.975 (4.66 to 25.273) | 11.975 (4.66 to 25.273) | 9.778 (3.819 to 20.981) | 9.778 (3.819 to 20.981) | 9.03 (3.611 to 18.958) | 9.03 (3.611 to 18.958) | 6.528 (3.542 to 11.14) | 6.528 (3.542 to 11.14) | 3.629 (1.986 to 6.135) | 3.629 (1.986 to 6.135) | 2.061 (1.118 to 3.541) | 2.061 (1.118 to 3.541) | 1.25 (1.0 to 2.038) |
| Peripheral vascular disease | 46.5 Cigarettes Per Smoker Per Day | Both | Female | 11.975 (4.66 to 25.273) | 11.975 (4.66 to 25.273) | 11.975 (4.66 to 25.273) | 9.778 (3.819 to 20.981) | 9.778 (3.819 to 20.981) | 9.03 (3.611 to 18.958) | 9.03 (3.611 to 18.958) | 6.528 (3.542 to 11.14) | 6.528 (3.542 to 11.14) | 3.629 (1.986 to 6.135) | 3.629 (1.986 to 6.135) | 2.061 (1.118 to 3.541) | 2.061 (1.118 to 3.541) | 1.25 (1.0 to 2.038) |
| Chronic obstructive pulmonary disease | 0 Pack-Years | Both | Male | 1.0 (1.0 to 1.0) | 1.0 (1.0 to 1.0) | 1.0 (1.0 to 1.0) | 1.0 (1.0 to 1.0) | 1.0 (1.0 to 1.0) | 1.0 (1.0 to 1.0) | 1.0 (1.0 to 1.0) | 1.0 (1.0 to 1.0) | 1.0 (1.0 to 1.0) | 1.0 (1.0 to 1.0) | 1.0 (1.0 to 1.0) | 1.0 (1.0 to 1.0) | 1.0 (1.0 to 1.0) | 1.0 (1.0 to 1.0) |

**Appendix Table 6d. Relative risks used by age and sex for each outcome for smoking globally.**

| Risk - Outcome | Category / Units | Morbidity / Mortality | Sex | Age | | | | | | | | | | | | | |
|---|---|---|---|---|---|---|---|---|---|---|---|---|---|---|---|---|---|
| | | | | 30 to 34 | 35 to 39 | 40 to 44 | 45 to 49 | 50 to 54 | 55 to 59 | 60 to 64 | 65 to 69 | 70 to 74 | 75 to 79 | 80 to 84 | 85 to 89 | 90 to 94 | 95 plus |
| **Smoking** | | | | | | | | | | | | | | | | | |
| Chronic obstructive pulmonary disease | 0 Pack-Years | Both | Female | 1.0 (1.0 to 1.0) | 1.0 (1.0 to 1.0) | 1.0 (1.0 to 1.0) | 1.0 (1.0 to 1.0) | 1.0 (1.0 to 1.0) | 1.0 (1.0 to 1.0) | 1.0 (1.0 to 1.0) | 1.0 (1.0 to 1.0) | 1.0 (1.0 to 1.0) | 1.0 (1.0 to 1.0) | 1.0 (1.0 to 1.0) | 1.0 (1.0 to 1.0) | 1.0 (1.0 to 1.0) | 1.0 (1.0 to 1.0) |
| Chronic obstructive pulmonary disease | 10 Pack-Years | Both | Male | 2.512 (1.692 to 3.297) | 2.512 (1.692 to 3.297) | 2.512 (1.692 to 3.297) | 2.512 (1.692 to 3.297) | 2.512 (1.692 to 3.297) | 2.512 (1.692 to 3.297) | 2.512 (1.692 to 3.297) | 2.512 (1.692 to 3.297) | 2.512 (1.692 to 3.297) | 2.512 (1.692 to 3.297) | 2.512 (1.692 to 3.297) | 2.512 (1.692 to 3.297) | 2.512 (1.692 to 3.297) | 2.512 (1.692 to 3.297) |
| Chronic obstructive pulmonary disease | 10 Pack-Years | Both | Female | 2.512 (1.692 to 3.297) | 2.512 (1.692 to 3.297) | 2.512 (1.692 to 3.297) | 2.512 (1.692 to 3.297) | 2.512 (1.692 to 3.297) | 2.512 (1.692 to 3.297) | 2.512 (1.692 to 3.297) | 2.512 (1.692 to 3.297) | 2.512 (1.692 to 3.297) | 2.512 (1.692 to 3.297) | 2.512 (1.692 to 3.297) | 2.512 (1.692 to 3.297) | 2.512 (1.692 to 3.297) | 2.512 (1.692 to 3.297) |
| Chronic obstructive pulmonary disease | 20 Pack-Years | Both | Male | 3.942 (3.079 to 4.753) | 3.942 (3.079 to 4.753) | 3.942 (3.079 to 4.753) | 3.942 (3.079 to 4.753) | 3.942 (3.079 to 4.753) | 3.942 (3.079 to 4.753) | 3.942 (3.079 to 4.753) | 3.942 (3.079 to 4.753) | 3.942 (3.079 to 4.753) | 3.942 (3.079 to 4.753) | 3.942 (3.079 to 4.753) | 3.942 (3.079 to 4.753) | 3.942 (3.079 to 4.753) | 3.942 (3.079 to 4.753) |
| Chronic obstructive pulmonary disease | 20 Pack-Years | Both | Female | 3.942 (3.079 to 4.753) | 3.942 (3.079 to 4.753) | 3.942 (3.079 to 4.753) | 3.942 (3.079 to 4.753) | 3.942 (3.079 to 4.753) | 3.942 (3.079 to 4.753) | 3.942 (3.079 to 4.753) | 3.942 (3.079 to 4.753) | 3.942 (3.079 to 4.753) | 3.942 (3.079 to 4.753) | 3.942 (3.079 to 4.753) | 3.942 (3.079 to 4.753) | 3.942 (3.079 to 4.753) | 3.942 (3.079 to 4.753) |
| Chronic obstructive pulmonary disease | 30 Pack-Years | Both | Male | 4.619 (3.761 to 5.569) | 4.619 (3.761 to 5.569) | 4.619 (3.761 to 5.569) | 4.619 (3.761 to 5.569) | 4.619 (3.761 to 5.569) | 4.619 (3.761 to 5.569) | 4.619 (3.761 to 5.569) | 4.619 (3.761 to 5.569) | 4.619 (3.761 to 5.569) | 4.619 (3.761 to 5.569) | 4.619 (3.761 to 5.569) | 4.619 (3.761 to 5.569) | 4.619 (3.761 to 5.569) | 4.619 (3.761 to 5.569) |
| Chronic obstructive pulmonary disease | 30 Pack-Years | Both | Female | 4.619 (3.761 to 5.569) | 4.619 (3.761 to 5.569) | 4.619 (3.761 to 5.569) | 4.619 (3.761 to 5.569) | 4.619 (3.761 to 5.569) | 4.619 (3.761 to 5.569) | 4.619 (3.761 to 5.569) | 4.619 (3.761 to 5.569) | 4.619 (3.761 to 5.569) | 4.619 (3.761 to 5.569) | 4.619 (3.761 to 5.569) | 4.619 (3.761 to 5.569) | 4.619 (3.761 to 5.569) | 4.619 (3.761 to 5.569) |
| Chronic obstructive pulmonary disease | 40 Pack-Years | Both | Male | 5.255 (4.289 to 6.601) | 5.255 (4.289 to 6.601) | 5.255 (4.289 to 6.601) | 5.255 (4.289 to 6.601) | 5.255 (4.289 to 6.601) | 5.255 (4.289 to 6.601) | 5.255 (4.289 to 6.601) | 5.255 (4.289 to 6.601) | 5.255 (4.289 to 6.601) | 5.255 (4.289 to 6.601) | 5.255 (4.289 to 6.601) | 5.255 (4.289 to 6.601) | 5.255 (4.289 to 6.601) | 5.255 (4.289 to 6.601) |
| Chronic obstructive pulmonary disease | 40 Pack-Years | Both | Female | 5.255 (4.289 to 6.601) | 5.255 (4.289 to 6.601) | 5.255 (4.289 to 6.601) | 5.255 (4.289 to 6.601) | 5.255 (4.289 to 6.601) | 5.255 (4.289 to 6.601) | 5.255 (4.289 to 6.601) | 5.255 (4.289 to 6.601) | 5.255 (4.289 to 6.601) | 5.255 (4.289 to 6.601) | 5.255 (4.289 to 6.601) | 5.255 (4.289 to 6.601) | 5.255 (4.289 to 6.601) | 5.255 (4.289 to 6.601) |
| Chronic obstructive pulmonary disease | 50 Pack-Years | Both | Male | 5.967 (4.774 to 7.471) | 5.967 (4.774 to 7.471) | 5.967 (4.774 to 7.471) | 5.967 (4.774 to 7.471) | 5.967 (4.774 to 7.471) | 5.967 (4.774 to 7.471) | 5.967 (4.774 to 7.471) | 5.967 (4.774 to 7.471) | 5.967 (4.774 to 7.471) | 5.967 (4.774 to 7.471) | 5.967 (4.774 to 7.471) | 5.967 (4.774 to 7.471) | 5.967 (4.774 to 7.471) | 5.967 (4.774 to 7.471) |
| Chronic obstructive pulmonary disease | 50 Pack-Years | Both | Female | 5.967 (4.774 to 7.471) | 5.967 (4.774 to 7.471) | 5.967 (4.774 to 7.471) | 5.967 (4.774 to 7.471) | 5.967 (4.774 to 7.471) | 5.967 (4.774 to 7.471) | 5.967 (4.774 to 7.471) | 5.967 (4.774 to 7.471) | 5.967 (4.774 to 7.471) | 5.967 (4.774 to 7.471) | 5.967 (4.774 to 7.471) | 5.967 (4.774 to 7.471) | 5.967 (4.774 to 7.471) | 5.967 (4.774 to 7.471) |
| Chronic obstructive pulmonary disease | 60 Pack-Years | Both | Male | 6.995 (5.423 to 9.578) | 6.995 (5.423 to 9.578) | 6.995 (5.423 to 9.578) | 6.995 (5.423 to 9.578) | 6.995 (5.423 to 9.578) | 6.995 (5.423 to 9.578) | 6.995 (5.423 to 9.578) | 6.995 (5.423 to 9.578) | 6.995 (5.423 to 9.578) | 6.995 (5.423 to 9.578) | 6.995 (5.423 to 9.578) | 6.995 (5.423 to 9.578) | 6.995 (5.423 to 9.578) | 6.995 (5.423 to 9.578) |
| Chronic obstructive pulmonary disease | 60 Pack-Years | Both | Female | 6.995 (5.423 to 9.578) | 6.995 (5.423 to 9.578) | 6.995 (5.423 to 9.578) | 6.995 (5.423 to 9.578) | 6.995 (5.423 to 9.578) | 6.995 (5.423 to 9.578) | 6.995 (5.423 to 9.578) | 6.995 (5.423 to 9.578) | 6.995 (5.423 to 9.578) | 6.995 (5.423 to 9.578) | 6.995 (5.423 to 9.578) | 6.995 (5.423 to 9.578) | 6.995 (5.423 to 9.578) | 6.995 (5.423 to 9.578) |
| Chronic obstructive pulmonary disease | 70 Pack-Years | Both | Male | 8.274 (6.17 to 11.785) | 8.274 (6.17 to 11.785) | 8.274 (6.17 to 11.785) | 8.274 (6.17 to 11.785) | 8.274 (6.17 to 11.785) | 8.274 (6.17 to 11.785) | 8.274 (6.17 to 11.785) | 8.274 (6.17 to 11.785) | 8.274 (6.17 to 11.785) | 8.274 (6.17 to 11.785) | 8.274 (6.17 to 11.785) | 8.274 (6.17 to 11.785) | 8.274 (6.17 to 11.785) | 8.274 (6.17 to 11.785) |
| Chronic obstructive pulmonary disease | 70 Pack-Years | Both | Female | 8.274 (6.17 to 11.785) | 8.274 (6.17 to 11.785) | 8.274 (6.17 to 11.785) | 8.274 (6.17 to 11.785) | 8.274 (6.17 to 11.785) | 8.274 (6.17 to 11.785) | 8.274 (6.17 to 11.785) | 8.274 (6.17 to 11.785) | 8.274 (6.17 to 11.785) | 8.274 (6.17 to 11.785) | 8.274 (6.17 to 11.785) | 8.274 (6.17 to 11.785) | 8.274 (6.17 to 11.785) | 8.274 (6.17 to 11.785) |
| Chronic obstructive pulmonary disease | 80 Pack-Years | Both | Male | 9.838 (6.933 to 15.001) | 9.838 (6.933 to 15.001) | 9.838 (6.933 to 15.001) | 9.838 (6.933 to 15.001) | 9.838 (6.933 to 15.001) | 9.838 (6.933 to 15.001) | 9.838 (6.933 to 15.001) | 9.838 (6.933 to 15.001) | 9.838 (6.933 to 15.001) | 9.838 (6.933 to 15.001) | 9.838 (6.933 to 15.001) | 9.838 (6.933 to 15.001) | 9.838 (6.933 to 15.001) | 9.838 (6.933 to 15.001) |
| Chronic obstructive pulmonary disease | 80 Pack-Years | Both | Female | 9.838 (6.933 to 15.001) | 9.838 (6.933 to 15.001) | 9.838 (6.933 to 15.001) | 9.838 (6.933 to 15.001) | 9.838 (6.933 to 15.001) | 9.838 (6.933 to 15.001) | 9.838 (6.933 to 15.001) | 9.838 (6.933 to 15.001) | 9.838 (6.933 to 15.001) | 9.838 (6.933 to 15.001) | 9.838 (6.933 to 15.001) | 9.838 (6.933 to 15.001) | 9.838 (6.933 to 15.001) | 9.838 (6.933 to 15.001) |
| Chronic obstructive pulmonary disease | 90 Pack-Years | Both | Male | 12.905 (8.309 to 21.862) | 12.905 (8.309 to 21.862) | 12.905 (8.309 to 21.862) | 12.905 (8.309 to 21.862) | 12.905 (8.309 to 21.862) | 12.905 (8.309 to 21.862) | 12.905 (8.309 to 21.862) | 12.905 (8.309 to 21.862) | 12.905 (8.309 to 21.862) | 12.905 (8.309 to 21.862) | 12.905 (8.309 to 21.862) | 12.905 (8.309 to 21.862) | 12.905 (8.309 to 21.862) | 12.905 (8.309 to 21.862) |
| Chronic obstructive pulmonary disease | 90 Pack-Years | Both | Female | 12.905 (8.309 to 21.862) | 12.905 (8.309 to 21.862) | 12.905 (8.309 to 21.862) | 12.905 (8.309 to 21.862) | 12.905 (8.309 to 21.862) | 12.905 (8.309 to 21.862) | 12.905 (8.309 to 21.862) | 12.905 (8.309 to 21.862) | 12.905 (8.309 to 21.862) | 12.905 (8.309 to 21.862) | 12.905 (8.309 to 21.862) | 12.905 (8.309 to 21.862) | 12.905 (8.309 to 21.862) | 12.905 (8.309 to 21.862) |
| Asthma | 0 Cigarettes Per Smoker Per Day | Both | Male | 1.0 (1.0 to 1.0) | 1.0 (1.0 to 1.0) | 1.0 (1.0 to 1.0) | 1.0 (1.0 to 1.0) | 1.0 (1.0 to 1.0) | 1.0 (1.0 to 1.0) | 1.0 (1.0 to 1.0) | 1.0 (1.0 to 1.0) | 1.0 (1.0 to 1.0) | 1.0 (1.0 to 1.0) | 1.0 (1.0 to 1.0) | 1.0 (1.0 to 1.0) | 1.0 (1.0 to 1.0) | 1.0 (1.0 to 1.0) |
| Asthma | 0 Cigarettes Per Smoker Per Day | Both | Female | 1.0 (1.0 to 1.0) | 1.0 (1.0 to 1.0) | 1.0 (1.0 to 1.0) | 1.0 (1.0 to 1.0) | 1.0 (1.0 to 1.0) | 1.0 (1.0 to 1.0) | 1.0 (1.0 to 1.0) | 1.0 (1.0 to 1.0) | 1.0 (1.0 to 1.0) | 1.0 (1.0 to 1.0) | 1.0 (1.0 to 1.0) | 1.0 (1.0 to 1.0) | 1.0 (1.0 to 1.0) | 1.0 (1.0 to 1.0) |
| Asthma | 2.5 Cigarettes Per Smoker Per Day | Both | Male | 1.236 (1.0 to 1.521) | 1.236 (1.0 to 1.521) | 1.236 (1.0 to 1.521) | 1.236 (1.0 to 1.521) | 1.236 (1.0 to 1.521) | 1.236 (1.0 to 1.521) | 1.236 (1.0 to 1.521) | 1.236 (1.0 to 1.521) | 1.236 (1.0 to 1.521) | 1.236 (1.0 to 1.521) | 1.236 (1.0 to 1.521) | 1.236 (1.0 to 1.521) | 1.236 (1.0 to 1.521) | 1.236 (1.0 to 1.521) |
| Asthma | 2.5 Cigarettes Per Smoker Per Day | Both | Female | 1.236 (1.0 to 1.521) | 1.236 (1.0 to 1.521) | 1.236 (1.0 to 1.521) | 1.236 (1.0 to 1.521) | 1.236 (1.0 to 1.521) | 1.236 (1.0 to 1.521) | 1.236 (1.0 to 1.521) | 1.236 (1.0 to 1.521) | 1.236 (1.0 to 1.521) | 1.236 (1.0 to 1.521) | 1.236 (1.0 to 1.521) | 1.236 (1.0 to 1.521) | 1.236 (1.0 to 1.521) | 1.236 (1.0 to 1.521) |
| Asthma | 7.5 Cigarettes Per Smoker Per Day | Both | Male | 1.659 (1.19 to 2.18) | 1.659 (1.19 to 2.18) | 1.659 (1.19 to 2.18) | 1.659 (1.19 to 2.18) | 1.659 (1.19 to 2.18) | 1.659 (1.19 to 2.18) | 1.659 (1.19 to 2.18) | 1.659 (1.19 to 2.18) | 1.659 (1.19 to 2.18) | 1.659 (1.19 to 2.18) | 1.659 (1.19 to 2.18) | 1.659 (1.19 to 2.18) | 1.659 (1.19 to 2.18) | 1.659 (1.19 to 2.18) |
| Asthma | 7.5 Cigarettes Per Smoker Per Day | Both | Female | 1.659 (1.19 to 2.18) | 1.659 (1.19 to 2.18) | 1.659 (1.19 to 2.18) | 1.659 (1.19 to 2.18) | 1.659 (1.19 to 2.18) | 1.659 (1.19 to 2.18) | 1.659 (1.19 to 2.18) | 1.659 (1.19 to 2.18) | 1.659 (1.19 to 2.18) | 1.659 (1.19 to 2.18) | 1.659 (1.19 to 2.18) | 1.659 (1.19 to 2.18) | 1.659 (1.19 to 2.18) | 1.659 (1.19 to 2.18) |
| Asthma | 15 Cigarettes Per Smoker Per Day | Both | Male | 1.858 (1.323 to 2.404) | 1.858 (1.323 to 2.404) | 1.858 (1.323 to 2.404) | 1.858 (1.323 to 2.404) | 1.858 (1.323 to 2.404) | 1.858 (1.323 to 2.404) | 1.858 (1.323 to 2.404) | 1.858 (1.323 to 2.404) | 1.858 (1.323 to 2.404) | 1.858 (1.323 to 2.404) | 1.858 (1.323 to 2.404) | 1.858 (1.323 to 2.404) | 1.858 (1.323 to 2.404) | 1.858 (1.323 to 2.404) |
| Asthma | 15 Cigarettes Per Smoker Per Day | Both | Female | 1.858 (1.323 to 2.404) | 1.858 (1.323 to 2.404) | 1.858 (1.323 to 2.404) | 1.858 (1.323 to 2.404) | 1.858 (1.323 to 2.404) | 1.858 (1.323 to 2.404) | 1.858 (1.323 to 2.404) | 1.858 (1.323 to 2.404) | 1.858 (1.323 to 2.404) | 1.858 (1.323 to 2.404) | 1.858 (1.323 to 2.404) | 1.858 (1.323 to 2.404) | 1.858 (1.323 to 2.404) | 1.858 (1.323 to 2.404) |
| Asthma | 22.5 Cigarettes Per Smoker Per Day | Both | Male | 1.987 (1.373 to 2.875) | 1.987 (1.373 to 2.875) | 1.987 (1.373 to 2.875) | 1.987 (1.373 to 2.875) | 1.987 (1.373 to 2.875) | 1.987 (1.373 to 2.875) | 1.987 (1.373 to 2.875) | 1.987 (1.373 to 2.875) | 1.987 (1.373 to 2.875) | 1.987 (1.373 to 2.875) | 1.987 (1.373 to 2.875) | 1.987 (1.373 to 2.875) | 1.987 (1.373 to 2.875) | 1.987 (1.373 to 2.875) |
| Asthma | 22.5 Cigarettes Per Smoker Per Day | Both | Female | 1.987 (1.373 to 2.875) | 1.987 (1.373 to 2.875) | 1.987 (1.373 to 2.875) | 1.987 (1.373 to 2.875) | 1.987 (1.373 to 2.875) | 1.987 (1.373 to 2.875) | 1.987 (1.373 to 2.875) | 1.987 (1.373 to 2.875) | 1.987 (1.373 to 2.875) | 1.987 (1.373 to 2.875) | 1.987 (1.373 to 2.875) | 1.987 (1.373 to 2.875) | 1.987 (1.373 to 2.875) | 1.987 (1.373 to 2.875) |
| Asthma | 30 Cigarettes Per Smoker Per Day | Both | Male | 2.366 (1.453 to 3.847) | 2.366 (1.453 to 3.847) | 2.366 (1.453 to 3.847) | 2.366 (1.453 to 3.847) | 2.366 (1.453 to 3.847) | 2.366 (1.453 to 3.847) | 2.366 (1.453 to 3.847) | 2.366 (1.453 to 3.847) | 2.366 (1.453 to 3.847) | 2.366 (1.453 to 3.847) | 2.366 (1.453 to 3.847) | 2.366 (1.453 to 3.847) | 2.366 (1.453 to 3.847) | 2.366 (1.453 to 3.847) |
| Asthma | 30 Cigarettes Per Smoker Per Day | Both | Female | 2.366 (1.453 to 3.847) | 2.366 (1.453 to 3.847) | 2.366 (1.453 to 3.847) | 2.366 (1.453 to 3.847) | 2.366 (1.453 to 3.847) | 2.366 (1.453 to 3.847) | 2.366 (1.453 to 3.847) | 2.366 (1.453 to 3.847) | 2.366 (1.453 to 3.847) | 2.366 (1.453 to 3.847) | 2.366 (1.453 to 3.847) | 2.366 (1.453 to 3.847) | 2.366 (1.453 to 3.847) | 2.366 (1.453 to 3.847) |
| Peptic ulcer disease | 0 Cigarettes Per Smoker Per Day | Both | Male | 1.0 (1.0 to 1.0) | 1.0 (1.0 to 1.0) | 1.0 (1.0 to 1.0) | 1.0 (1.0 to 1.0) | 1.0 (1.0 to 1.0) | 1.0 (1.0 to 1.0) | 1.0 (1.0 to 1.0) | 1.0 (1.0 to 1.0) | 1.0 (1.0 to 1.0) | 1.0 (1.0 to 1.0) | 1.0 (1.0 to 1.0) | 1.0 (1.0 to 1.0) | 1.0 (1.0 to 1.0) | 1.0 (1.0 to 1.0) |
| Peptic ulcer disease | 0 Cigarettes Per Smoker Per Day | Both | Female | 1.0 (1.0 to 1.0) | 1.0 (1.0 to 1.0) | 1.0 (1.0 to 1.0) | 1.0 (1.0 to 1.0) | 1.0 (1.0 to 1.0) | 1.0 (1.0 to 1.0) | 1.0 (1.0 to 1.0) | 1.0 (1.0 to 1.0) | 1.0 (1.0 to 1.0) | 1.0 (1.0 to 1.0) | 1.0 (1.0 to 1.0) | 1.0 (1.0 to 1.0) | 1.0 (1.0 to 1.0) | 1.0 (1.0 to 1.0) |
| Peptic ulcer disease | 6.5 Cigarettes Per Smoker Per Day | Both | Male | 1.824 (1.46 to 2.2) | 1.824 (1.46 to 2.2) | 1.824 (1.46 to 2.2) | 1.824 (1.46 to 2.2) | 1.824 (1.46 to 2.2) | 1.824 (1.46 to 2.2) | 1.824 (1.46 to 2.2) | 1.824 (1.46 to 2.2) | 1.824 (1.46 to 2.2) | 1.824 (1.46 to 2.2) | 1.824 (1.46 to 2.2) | 1.824 (1.46 to 2.2) | 1.824 (1.46 to 2.2) | 1.824 (1.46 to 2.2) |
| Peptic ulcer disease | 6.5 Cigarettes Per Smoker Per Day | Both | Female | 1.824 (1.46 to 2.2) | 1.824 (1.46 to 2.2) | 1.824 (1.46 to 2.2) | 1.824 (1.46 to 2.2) | 1.824 (1.46 to 2.2) | 1.824 (1.46 to 2.2) | 1.824 (1.46 to 2.2) | 1.824 (1.46 to 2.2) | 1.824 (1.46 to 2.2) | 1.824 (1.46 to 2.2) | 1.824 (1.46 to 2.2) | 1.824 (1.46 to 2.2) | 1.824 (1.46 to 2.2) | 1.824 (1.46 to 2.2) |

**Appendix Table 6d. Relative risks used by age and sex for each outcome for smoking globally.**

| Risk - Outcome | Category / Units | Morbidity / Mortality | Sex | Age | | | | | | | | | | | | | |
|---|---|---|---|---|---|---|---|---|---|---|---|---|---|---|---|---|---|
| | | | | 30 to 34 | 35 to 39 | 40 to 44 | 45 to 49 | 50 to 54 | 55 to 59 | 60 to 64 | 65 to 69 | 70 to 74 | 75 to 79 | 80 to 84 | 85 to 89 | 90 to 94 | 95 plus |
| **Smoking** | | | | | | | | | | | | | | | | | |
| Peptic ulcer disease | 13 Cigarettes Per Smoker Per Day | Both | Male | 2.156 (1.764 to 2.568) | 2.156 (1.764 to 2.568) | 2.156 (1.764 to 2.568) | 2.156 (1.764 to 2.568) | 2.156 (1.764 to 2.568) | 2.156 (1.764 to 2.568) | 2.156 (1.764 to 2.568) | 2.156 (1.764 to 2.568) | 2.156 (1.764 to 2.568) | 2.156 (1.764 to 2.568) | 2.156 (1.764 to 2.568) | 2.156 (1.764 to 2.568) | 2.156 (1.764 to 2.568) | 2.156 (1.764 to 2.568) |
| Peptic ulcer disease | 13 Cigarettes Per Smoker Per Day | Both | Female | 2.156 (1.764 to 2.568) | 2.156 (1.764 to 2.568) | 2.156 (1.764 to 2.568) | 2.156 (1.764 to 2.568) | 2.156 (1.764 to 2.568) | 2.156 (1.764 to 2.568) | 2.156 (1.764 to 2.568) | 2.156 (1.764 to 2.568) | 2.156 (1.764 to 2.568) | 2.156 (1.764 to 2.568) | 2.156 (1.764 to 2.568) | 2.156 (1.764 to 2.568) | 2.156 (1.764 to 2.568) | 2.156 (1.764 to 2.568) |
| Peptic ulcer disease | 19.5 Cigarettes Per Smoker Per Day | Both | Male | 2.519 (2.066 to 2.994) | 2.519 (2.066 to 2.994) | 2.519 (2.066 to 2.994) | 2.519 (2.066 to 2.994) | 2.519 (2.066 to 2.994) | 2.519 (2.066 to 2.994) | 2.519 (2.066 to 2.994) | 2.519 (2.066 to 2.994) | 2.519 (2.066 to 2.994) | 2.519 (2.066 to 2.994) | 2.519 (2.066 to 2.994) | 2.519 (2.066 to 2.994) | 2.519 (2.066 to 2.994) | 2.519 (2.066 to 2.994) |
| Peptic ulcer disease | 19.5 Cigarettes Per Smoker Per Day | Both | Female | 2.519 (2.066 to 2.994) | 2.519 (2.066 to 2.994) | 2.519 (2.066 to 2.994) | 2.519 (2.066 to 2.994) | 2.519 (2.066 to 2.994) | 2.519 (2.066 to 2.994) | 2.519 (2.066 to 2.994) | 2.519 (2.066 to 2.994) | 2.519 (2.066 to 2.994) | 2.519 (2.066 to 2.994) | 2.519 (2.066 to 2.994) | 2.519 (2.066 to 2.994) | 2.519 (2.066 to 2.994) | 2.519 (2.066 to 2.994) |
| Peptic ulcer disease | 26 Cigarettes Per Smoker Per Day | Both | Male | 2.552 (1.952 to 3.262) | 2.552 (1.952 to 3.262) | 2.552 (1.952 to 3.262) | 2.552 (1.952 to 3.262) | 2.552 (1.952 to 3.262) | 2.552 (1.952 to 3.262) | 2.552 (1.952 to 3.262) | 2.552 (1.952 to 3.262) | 2.552 (1.952 to 3.262) | 2.552 (1.952 to 3.262) | 2.552 (1.952 to 3.262) | 2.552 (1.952 to 3.262) | 2.552 (1.952 to 3.262) | 2.552 (1.952 to 3.262) |
| Peptic ulcer disease | 26 Cigarettes Per Smoker Per Day | Both | Female | 2.552 (1.952 to 3.262) | 2.552 (1.952 to 3.262) | 2.552 (1.952 to 3.262) | 2.552 (1.952 to 3.262) | 2.552 (1.952 to 3.262) | 2.552 (1.952 to 3.262) | 2.552 (1.952 to 3.262) | 2.552 (1.952 to 3.262) | 2.552 (1.952 to 3.262) | 2.552 (1.952 to 3.262) | 2.552 (1.952 to 3.262) | 2.552 (1.952 to 3.262) | 2.552 (1.952 to 3.262) | 2.552 (1.952 to 3.262) |
| Peptic ulcer disease | 32.5 Cigarettes Per Smoker Per Day | Both | Male | 2.888 (2.15 to 3.741) | 2.888 (2.15 to 3.741) | 2.888 (2.15 to 3.741) | 2.888 (2.15 to 3.741) | 2.888 (2.15 to 3.741) | 2.888 (2.15 to 3.741) | 2.888 (2.15 to 3.741) | 2.888 (2.15 to 3.741) | 2.888 (2.15 to 3.741) | 2.888 (2.15 to 3.741) | 2.888 (2.15 to 3.741) | 2.888 (2.15 to 3.741) | 2.888 (2.15 to 3.741) | 2.888 (2.15 to 3.741) |
| Peptic ulcer disease | 32.5 Cigarettes Per Smoker Per Day | Both | Female | 2.888 (2.15 to 3.741) | 2.888 (2.15 to 3.741) | 2.888 (2.15 to 3.741) | 2.888 (2.15 to 3.741) | 2.888 (2.15 to 3.741) | 2.888 (2.15 to 3.741) | 2.888 (2.15 to 3.741) | 2.888 (2.15 to 3.741) | 2.888 (2.15 to 3.741) | 2.888 (2.15 to 3.741) | 2.888 (2.15 to 3.741) | 2.888 (2.15 to 3.741) | 2.888 (2.15 to 3.741) | 2.888 (2.15 to 3.741) |
| Gallbladder and biliary diseases | 0 Cigarettes Per Smoker Per Day | Both | Male | 1.0 (1.0 to 1.0) | 1.0 (1.0 to 1.0) | 1.0 (1.0 to 1.0) | 1.0 (1.0 to 1.0) | 1.0 (1.0 to 1.0) | 1.0 (1.0 to 1.0) | 1.0 (1.0 to 1.0) | 1.0 (1.0 to 1.0) | 1.0 (1.0 to 1.0) | 1.0 (1.0 to 1.0) | 1.0 (1.0 to 1.0) | 1.0 (1.0 to 1.0) | 1.0 (1.0 to 1.0) | 1.0 (1.0 to 1.0) |
| Gallbladder and biliary diseases | 0 Cigarettes Per Smoker Per Day | Both | Female | 1.0 (1.0 to 1.0) | 1.0 (1.0 to 1.0) | 1.0 (1.0 to 1.0) | 1.0 (1.0 to 1.0) | 1.0 (1.0 to 1.0) | 1.0 (1.0 to 1.0) | 1.0 (1.0 to 1.0) | 1.0 (1.0 to 1.0) | 1.0 (1.0 to 1.0) | 1.0 (1.0 to 1.0) | 1.0 (1.0 to 1.0) | 1.0 (1.0 to 1.0) | 1.0 (1.0 to 1.0) | 1.0 (1.0 to 1.0) |
| Gallbladder and biliary diseases | 8.8 Cigarettes Per Smoker Per Day | Both | Male | 1.36 (1.197 to 1.53) | 1.36 (1.197 to 1.53) | 1.36 (1.197 to 1.53) | 1.36 (1.197 to 1.53) | 1.36 (1.197 to 1.53) | 1.36 (1.197 to 1.53) | 1.36 (1.197 to 1.53) | 1.36 (1.197 to 1.53) | 1.36 (1.197 to 1.53) | 1.36 (1.197 to 1.53) | 1.36 (1.197 to 1.53) | 1.36 (1.197 to 1.53) | 1.36 (1.197 to 1.53) | 1.36 (1.197 to 1.53) |
| Gallbladder and biliary diseases | 8.8 Cigarettes Per Smoker Per Day | Both | Female | 1.36 (1.197 to 1.53) | 1.36 (1.197 to 1.53) | 1.36 (1.197 to 1.53) | 1.36 (1.197 to 1.53) | 1.36 (1.197 to 1.53) | 1.36 (1.197 to 1.53) | 1.36 (1.197 to 1.53) | 1.36 (1.197 to 1.53) | 1.36 (1.197 to 1.53) | 1.36 (1.197 to 1.53) | 1.36 (1.197 to 1.53) | 1.36 (1.197 to 1.53) | 1.36 (1.197 to 1.53) | 1.36 (1.197 to 1.53) |
| Gallbladder and biliary diseases | 17.5 Cigarettes Per Smoker Per Day | Both | Male | 1.303 (1.178 to 1.429) | 1.303 (1.178 to 1.429) | 1.303 (1.178 to 1.429) | 1.303 (1.178 to 1.429) | 1.303 (1.178 to 1.429) | 1.303 (1.178 to 1.429) | 1.303 (1.178 to 1.429) | 1.303 (1.178 to 1.429) | 1.303 (1.178 to 1.429) | 1.303 (1.178 to 1.429) | 1.303 (1.178 to 1.429) | 1.303 (1.178 to 1.429) | 1.303 (1.178 to 1.429) | 1.303 (1.178 to 1.429) |
| Gallbladder and biliary diseases | 17.5 Cigarettes Per Smoker Per Day | Both | Female | 1.303 (1.178 to 1.429) | 1.303 (1.178 to 1.429) | 1.303 (1.178 to 1.429) | 1.303 (1.178 to 1.429) | 1.303 (1.178 to 1.429) | 1.303 (1.178 to 1.429) | 1.303 (1.178 to 1.429) | 1.303 (1.178 to 1.429) | 1.303 (1.178 to 1.429) | 1.303 (1.178 to 1.429) | 1.303 (1.178 to 1.429) | 1.303 (1.178 to 1.429) | 1.303 (1.178 to 1.429) | 1.303 (1.178 to 1.429) |
| Gallbladder and biliary diseases | 26.2 Cigarettes Per Smoker Per Day | Both | Male | 1.398 (1.238 to 1.56) | 1.398 (1.238 to 1.56) | 1.398 (1.238 to 1.56) | 1.398 (1.238 to 1.56) | 1.398 (1.238 to 1.56) | 1.398 (1.238 to 1.56) | 1.398 (1.238 to 1.56) | 1.398 (1.238 to 1.56) | 1.398 (1.238 to 1.56) | 1.398 (1.238 to 1.56) | 1.398 (1.238 to 1.56) | 1.398 (1.238 to 1.56) | 1.398 (1.238 to 1.56) | 1.398 (1.238 to 1.56) |
| Gallbladder and biliary diseases | 26.2 Cigarettes Per Smoker Per Day | Both | Female | 1.398 (1.238 to 1.56) | 1.398 (1.238 to 1.56) | 1.398 (1.238 to 1.56) | 1.398 (1.238 to 1.56) | 1.398 (1.238 to 1.56) | 1.398 (1.238 to 1.56) | 1.398 (1.238 to 1.56) | 1.398 (1.238 to 1.56) | 1.398 (1.238 to 1.56) | 1.398 (1.238 to 1.56) | 1.398 (1.238 to 1.56) | 1.398 (1.238 to 1.56) | 1.398 (1.238 to 1.56) | 1.398 (1.238 to 1.56) |
| Gallbladder and biliary diseases | 35 Cigarettes Per Smoker Per Day | Both | Male | 1.612 (1.295 to 1.96) | 1.612 (1.295 to 1.96) | 1.612 (1.295 to 1.96) | 1.612 (1.295 to 1.96) | 1.612 (1.295 to 1.96) | 1.612 (1.295 to 1.96) | 1.612 (1.295 to 1.96) | 1.612 (1.295 to 1.96) | 1.612 (1.295 to 1.96) | 1.612 (1.295 to 1.96) | 1.612 (1.295 to 1.96) | 1.612 (1.295 to 1.96) | 1.612 (1.295 to 1.96) | 1.612 (1.295 to 1.96) |
| Gallbladder and biliary diseases | 35 Cigarettes Per Smoker Per Day | Both | Female | 1.612 (1.295 to 1.96) | 1.612 (1.295 to 1.96) | 1.612 (1.295 to 1.96) | 1.612 (1.295 to 1.96) | 1.612 (1.295 to 1.96) | 1.612 (1.295 to 1.96) | 1.612 (1.295 to 1.96) | 1.612 (1.295 to 1.96) | 1.612 (1.295 to 1.96) | 1.612 (1.295 to 1.96) | 1.612 (1.295 to 1.96) | 1.612 (1.295 to 1.96) | 1.612 (1.295 to 1.96) | 1.612 (1.295 to 1.96) |
| Gallbladder and biliary diseases | 43.8 Cigarettes Per Smoker Per Day | Both | Male | 1.81 (1.451 to 2.164) | 1.81 (1.451 to 2.164) | 1.81 (1.451 to 2.164) | 1.81 (1.451 to 2.164) | 1.81 (1.451 to 2.164) | 1.81 (1.451 to 2.164) | 1.81 (1.451 to 2.164) | 1.81 (1.451 to 2.164) | 1.81 (1.451 to 2.164) | 1.81 (1.451 to 2.164) | 1.81 (1.451 to 2.164) | 1.81 (1.451 to 2.164) | 1.81 (1.451 to 2.164) | 1.81 (1.451 to 2.164) |
| Gallbladder and biliary diseases | 43.8 Cigarettes Per Smoker Per Day | Both | Female | 1.81 (1.451 to 2.164) | 1.81 (1.451 to 2.164) | 1.81 (1.451 to 2.164) | 1.81 (1.451 to 2.164) | 1.81 (1.451 to 2.164) | 1.81 (1.451 to 2.164) | 1.81 (1.451 to 2.164) | 1.81 (1.451 to 2.164) | 1.81 (1.451 to 2.164) | 1.81 (1.451 to 2.164) | 1.81 (1.451 to 2.164) | 1.81 (1.451 to 2.164) | 1.81 (1.451 to 2.164) | 1.81 (1.451 to 2.164) |
| Alzheimer's disease and other dementias | 0 Cigarettes Per Smoker Per Day | Both | Male | 1.0 (1.0 to 1.0) | 1.0 (1.0 to 1.0) | 1.0 (1.0 to 1.0) | 1.0 (1.0 to 1.0) | 1.0 (1.0 to 1.0) | 1.0 (1.0 to 1.0) | 1.0 (1.0 to 1.0) | 1.0 (1.0 to 1.0) | 1.0 (1.0 to 1.0) | 1.0 (1.0 to 1.0) | 1.0 (1.0 to 1.0) | 1.0 (1.0 to 1.0) | 1.0 (1.0 to 1.0) | 1.0 (1.0 to 1.0) |
| Alzheimer's disease and other dementias | 0 Cigarettes Per Smoker Per Day | Both | Female | 1.0 (1.0 to 1.0) | 1.0 (1.0 to 1.0) | 1.0 (1.0 to 1.0) | 1.0 (1.0 to 1.0) | 1.0 (1.0 to 1.0) | 1.0 (1.0 to 1.0) | 1.0 (1.0 to 1.0) | 1.0 (1.0 to 1.0) | 1.0 (1.0 to 1.0) | 1.0 (1.0 to 1.0) | 1.0 (1.0 to 1.0) | 1.0 (1.0 to 1.0) | 1.0 (1.0 to 1.0) | 1.0 (1.0 to 1.0) |
| Alzheimer's disease and other dementias | 12 Cigarettes Per Smoker Per Day | Both | Male | 2.078 (1.396 to 2.844) | 2.078 (1.396 to 2.844) | 2.078 (1.396 to 2.844) | 2.078 (1.396 to 2.844) | 2.078 (1.396 to 2.844) | 2.078 (1.396 to 2.844) | 2.078 (1.396 to 2.844) | 2.078 (1.396 to 2.844) | 2.078 (1.396 to 2.844) | 2.078 (1.396 to 2.844) | 2.078 (1.396 to 2.844) | 2.078 (1.396 to 2.844) | 2.078 (1.396 to 2.844) | 2.078 (1.396 to 2.844) |
| Alzheimer's disease and other dementias | 12 Cigarettes Per Smoker Per Day | Both | Female | 2.078 (1.396 to 2.844) | 2.078 (1.396 to 2.844) | 2.078 (1.396 to 2.844) | 2.078 (1.396 to 2.844) | 2.078 (1.396 to 2.844) | 2.078 (1.396 to 2.844) | 2.078 (1.396 to 2.844) | 2.078 (1.396 to 2.844) | 2.078 (1.396 to 2.844) | 2.078 (1.396 to 2.844) | 2.078 (1.396 to 2.844) | 2.078 (1.396 to 2.844) | 2.078 (1.396 to 2.844) | 2.078 (1.396 to 2.844) |
| Alzheimer's disease and other dementias | 24 Cigarettes Per Smoker Per Day | Both | Male | 2.936 (1.782 to 4.472) | 2.936 (1.782 to 4.472) | 2.936 (1.782 to 4.472) | 2.936 (1.782 to 4.472) | 2.936 (1.782 to 4.472) | 2.936 (1.782 to 4.472) | 2.936 (1.782 to 4.472) | 2.936 (1.782 to 4.472) | 2.936 (1.782 to 4.472) | 2.936 (1.782 to 4.472) | 2.936 (1.782 to 4.472) | 2.936 (1.782 to 4.472) | 2.936 (1.782 to 4.472) | 2.936 (1.782 to 4.472) |
| Alzheimer's disease and other dementias | 24 Cigarettes Per Smoker Per Day | Both | Female | 2.936 (1.782 to 4.472) | 2.936 (1.782 to 4.472) | 2.936 (1.782 to 4.472) | 2.936 (1.782 to 4.472) | 2.936 (1.782 to 4.472) | 2.936 (1.782 to 4.472) | 2.936 (1.782 to 4.472) | 2.936 (1.782 to 4.472) | 2.936 (1.782 to 4.472) | 2.936 (1.782 to 4.472) | 2.936 (1.782 to 4.472) | 2.936 (1.782 to 4.472) | 2.936 (1.782 to 4.472) | 2.936 (1.782 to 4.472) |
| Alzheimer's disease and other dementias | 36 Cigarettes Per Smoker Per Day | Both | Male | 3.737 (1.972 to 6.019) | 3.737 (1.972 to 6.019) | 3.737 (1.972 to 6.019) | 3.737 (1.972 to 6.019) | 3.737 (1.972 to 6.019) | 3.737 (1.972 to 6.019) | 3.737 (1.972 to 6.019) | 3.737 (1.972 to 6.019) | 3.737 (1.972 to 6.019) | 3.737 (1.972 to 6.019) | 3.737 (1.972 to 6.019) | 3.737 (1.972 to 6.019) | 3.737 (1.972 to 6.019) | 3.737 (1.972 to 6.019) |
| Alzheimer's disease and other dementias | 36 Cigarettes Per Smoker Per Day | Both | Female | 3.737 (1.972 to 6.019) | 3.737 (1.972 to 6.019) | 3.737 (1.972 to 6.019) | 3.737 (1.972 to 6.019) | 3.737 (1.972 to 6.019) | 3.737 (1.972 to 6.019) | 3.737 (1.972 to 6.019) | 3.737 (1.972 to 6.019) | 3.737 (1.972 to 6.019) | 3.737 (1.972 to 6.019) | 3.737 (1.972 to 6.019) | 3.737 (1.972 to 6.019) | 3.737 (1.972 to 6.019) | 3.737 (1.972 to 6.019) |
| Alzheimer's disease and other dementias | 48 Cigarettes Per Smoker Per Day | Both | Male | 4.103 (2.075 to 7.232) | 4.103 (2.075 to 7.232) | 4.103 (2.075 to 7.232) | 4.103 (2.075 to 7.232) | 4.103 (2.075 to 7.232) | 4.103 (2.075 to 7.232) | 4.103 (2.075 to 7.232) | 4.103 (2.075 to 7.232) | 4.103 (2.075 to 7.232) | 4.103 (2.075 to 7.232) | 4.103 (2.075 to 7.232) | 4.103 (2.075 to 7.232) | 4.103 (2.075 to 7.232) | 4.103 (2.075 to 7.232) |
| Alzheimer's disease and other dementias | 48 Cigarettes Per Smoker Per Day | Both | Female | 4.103 (2.075 to 7.232) | 4.103 (2.075 to 7.232) | 4.103 (2.075 to 7.232) | 4.103 (2.075 to 7.232) | 4.103 (2.075 to 7.232) | 4.103 (2.075 to 7.232) | 4.103 (2.075 to 7.232) | 4.103 (2.075 to 7.232) | 4.103 (2.075 to 7.232) | 4.103 (2.075 to 7.232) | 4.103 (2.075 to 7.232) | 4.103 (2.075 to 7.232) | 4.103 (2.075 to 7.232) | 4.103 (2.075 to 7.232) |
| Parkinson's disease | 0 Cigarettes Per Smoker Per Day | Both | Male | 1.0 (1.0 to 1.0) | 1.0 (1.0 to 1.0) | 1.0 (1.0 to 1.0) | 1.0 (1.0 to 1.0) | 1.0 (1.0 to 1.0) | 1.0 (1.0 to 1.0) | 1.0 (1.0 to 1.0) | 1.0 (1.0 to 1.0) | 1.0 (1.0 to 1.0) | 1.0 (1.0 to 1.0) | 1.0 (1.0 to 1.0) | 1.0 (1.0 to 1.0) | 1.0 (1.0 to 1.0) | 1.0 (1.0 to 1.0) |
| Parkinson's disease | 0 Cigarettes Per Smoker Per Day | Both | Female | 1.0 (1.0 to 1.0) | 1.0 (1.0 to 1.0) | 1.0 (1.0 to 1.0) | 1.0 (1.0 to 1.0) | 1.0 (1.0 to 1.0) | 1.0 (1.0 to 1.0) | 1.0 (1.0 to 1.0) | 1.0 (1.0 to 1.0) | 1.0 (1.0 to 1.0) | 1.0 (1.0 to 1.0) | 1.0 (1.0 to 1.0) | 1.0 (1.0 to 1.0) | 1.0 (1.0 to 1.0) | 1.0 (1.0 to 1.0) |
| Parkinson's disease | 7.5 Cigarettes Per Smoker Per Day | Both | Male | 0.835 (0.644 to 0.989) | 0.835 (0.644 to 0.989) | 0.835 (0.644 to 0.989) | 0.835 (0.644 to 0.989) | 0.835 (0.644 to 0.989) | 0.835 (0.644 to 0.989) | 0.835 (0.644 to 0.989) | 0.835 (0.644 to 0.989) | 0.835 (0.644 to 0.989) | 0.835 (0.644 to 0.989) | 0.835 (0.644 to 0.989) | 0.835 (0.644 to 0.989) | 0.835 (0.644 to 0.989) | 0.835 (0.644 to 0.989) |
| Parkinson's disease | 7.5 Cigarettes Per Smoker Per Day | Both | Female | 0.835 (0.644 to 0.989) | 0.835 (0.644 to 0.989) | 0.835 (0.644 to 0.989) | 0.835 (0.644 to 0.989) | 0.835 (0.644 to 0.989) | 0.835 (0.644 to 0.989) | 0.835 (0.644 to 0.989) | 0.835 (0.644 to 0.989) | 0.835 (0.644 to 0.989) | 0.835 (0.644 to 0.989) | 0.835 (0.644 to 0.989) | 0.835 (0.644 to 0.989) | 0.835 (0.644 to 0.989) | 0.835 (0.644 to 0.989) |
| Parkinson's disease | 15 Cigarettes Per Smoker Per Day | Both | Male | 0.689 (0.531 to 0.869) | 0.689 (0.531 to 0.869) | 0.689 (0.531 to 0.869) | 0.689 (0.531 to 0.869) | 0.689 (0.531 to 0.869) | 0.689 (0.531 to 0.869) | 0.689 (0.531 to 0.869) | 0.689 (0.531 to 0.869) | 0.689 (0.531 to 0.869) | 0.689 (0.531 to 0.869) | 0.689 (0.531 to 0.869) | 0.689 (0.531 to 0.869) | 0.689 (0.531 to 0.869) | 0.689 (0.531 to 0.869) |
| Parkinson's disease | 15 Cigarettes Per Smoker Per Day | Both | Female | 0.689 (0.531 to 0.869) | 0.689 (0.531 to 0.869) | 0.689 (0.531 to 0.869) | 0.689 (0.531 to 0.869) | 0.689 (0.531 to 0.869) | 0.689 (0.531 to 0.869) | 0.689 (0.531 to 0.869) | 0.689 (0.531 to 0.869) | 0.689 (0.531 to 0.869) | 0.689 (0.531 to 0.869) | 0.689 (0.531 to 0.869) | 0.689 (0.531 to 0.869) | 0.689 (0.531 to 0.869) | 0.689 (0.531 to 0.869) |
| Parkinson's disease | 22.5 Cigarettes Per Smoker Per Day | Both | Male | 0.604 (0.44 to 0.789) | 0.604 (0.44 to 0.789) | 0.604 (0.44 to 0.789) | 0.604 (0.44 to 0.789) | 0.604 (0.44 to 0.789) | 0.604 (0.44 to 0.789) | 0.604 (0.44 to 0.789) | 0.604 (0.44 to 0.789) | 0.604 (0.44 to 0.789) | 0.604 (0.44 to 0.789) | 0.604 (0.44 to 0.789) | 0.604 (0.44 to 0.789) | 0.604 (0.44 to 0.789) | 0.604 (0.44 to 0.789) |

**Appendix Table 6d. Relative risks used by age and sex for each outcome for smoking globally.**

| Risk - Outcome | Category / Units | Morbidity / Mortality | Sex | Age 30 to 34 | 35 to 39 | 40 to 44 | 45 to 49 | 50 to 54 | 55 to 59 | 60 to 64 | 65 to 69 | 70 to 74 | 75 to 79 | 80 to 84 | 85 to 89 | 90 to 94 | 95 plus |
|---|---|---|---|---|---|---|---|---|---|---|---|---|---|---|---|---|---|
| **Smoking** | | | | | | | | | | | | | | | | | |
| Parkinson's disease | 22.5 Cigarettes Per Smoker Per Day | Both | Female | 0.604 (0.44 to 0.789) | 0.604 (0.44 to 0.789) | 0.604 (0.44 to 0.789) | 0.604 (0.44 to 0.789) | 0.604 (0.44 to 0.789) | 0.604 (0.44 to 0.789) | 0.604 (0.44 to 0.789) | 0.604 (0.44 to 0.789) | 0.604 (0.44 to 0.789) | 0.604 (0.44 to 0.789) | 0.604 (0.44 to 0.789) | 0.604 (0.44 to 0.789) | 0.604 (0.44 to 0.789) | 0.604 (0.44 to 0.789) |
| Parkinson's disease | 30 Cigarettes Per Smoker Per Day | Both | Male | 0.52 (0.346 to 0.71) | 0.52 (0.346 to 0.71) | 0.52 (0.346 to 0.71) | 0.52 (0.346 to 0.71) | 0.52 (0.346 to 0.71) | 0.52 (0.346 to 0.71) | 0.52 (0.346 to 0.71) | 0.52 (0.346 to 0.71) | 0.52 (0.346 to 0.71) | 0.52 (0.346 to 0.71) | 0.52 (0.346 to 0.71) | 0.52 (0.346 to 0.71) | 0.52 (0.346 to 0.71) | 0.52 (0.346 to 0.71) |
| Parkinson's disease | 30 Cigarettes Per Smoker Per Day | Both | Female | 0.52 (0.346 to 0.71) | 0.52 (0.346 to 0.71) | 0.52 (0.346 to 0.71) | 0.52 (0.346 to 0.71) | 0.52 (0.346 to 0.71) | 0.52 (0.346 to 0.71) | 0.52 (0.346 to 0.71) | 0.52 (0.346 to 0.71) | 0.52 (0.346 to 0.71) | 0.52 (0.346 to 0.71) | 0.52 (0.346 to 0.71) | 0.52 (0.346 to 0.71) | 0.52 (0.346 to 0.71) | 0.52 (0.346 to 0.71) |
| Parkinson's disease | 37.5 Cigarettes Per Smoker Per Day | Both | Male | 0.432 (0.238 to 0.644) | 0.432 (0.238 to 0.644) | 0.432 (0.238 to 0.644) | 0.432 (0.238 to 0.644) | 0.432 (0.238 to 0.644) | 0.432 (0.238 to 0.644) | 0.432 (0.238 to 0.644) | 0.432 (0.238 to 0.644) | 0.432 (0.238 to 0.644) | 0.432 (0.238 to 0.644) | 0.432 (0.238 to 0.644) | 0.432 (0.238 to 0.644) | 0.432 (0.238 to 0.644) | 0.432 (0.238 to 0.644) |
| Parkinson's disease | 37.5 Cigarettes Per Smoker Per Day | Both | Female | 0.432 (0.238 to 0.644) | 0.432 (0.238 to 0.644) | 0.432 (0.238 to 0.644) | 0.432 (0.238 to 0.644) | 0.432 (0.238 to 0.644) | 0.432 (0.238 to 0.644) | 0.432 (0.238 to 0.644) | 0.432 (0.238 to 0.644) | 0.432 (0.238 to 0.644) | 0.432 (0.238 to 0.644) | 0.432 (0.238 to 0.644) | 0.432 (0.238 to 0.644) | 0.432 (0.238 to 0.644) | 0.432 (0.238 to 0.644) |
| Parkinson's disease | 45 Cigarettes Per Smoker Per Day | Both | Male | 0.323 (0.127 to 0.561) | 0.323 (0.127 to 0.561) | 0.323 (0.127 to 0.561) | 0.323 (0.127 to 0.561) | 0.323 (0.127 to 0.561) | 0.323 (0.127 to 0.561) | 0.323 (0.127 to 0.561) | 0.323 (0.127 to 0.561) | 0.323 (0.127 to 0.561) | 0.323 (0.127 to 0.561) | 0.323 (0.127 to 0.561) | 0.323 (0.127 to 0.561) | 0.323 (0.127 to 0.561) | 0.323 (0.127 to 0.561) |
| Parkinson's disease | 45 Cigarettes Per Smoker Per Day | Both | Female | 0.323 (0.127 to 0.561) | 0.323 (0.127 to 0.561) | 0.323 (0.127 to 0.561) | 0.323 (0.127 to 0.561) | 0.323 (0.127 to 0.561) | 0.323 (0.127 to 0.561) | 0.323 (0.127 to 0.561) | 0.323 (0.127 to 0.561) | 0.323 (0.127 to 0.561) | 0.323 (0.127 to 0.561) | 0.323 (0.127 to 0.561) | 0.323 (0.127 to 0.561) | 0.323 (0.127 to 0.561) | 0.323 (0.127 to 0.561) |
| Multiple sclerosis | 0 Cigarettes Per Smoker Per Day | Both | Male | 1.0 (1.0 to 1.0) | 1.0 (1.0 to 1.0) | 1.0 (1.0 to 1.0) | 1.0 (1.0 to 1.0) | 1.0 (1.0 to 1.0) | 1.0 (1.0 to 1.0) | 1.0 (1.0 to 1.0) | 1.0 (1.0 to 1.0) | 1.0 (1.0 to 1.0) | 1.0 (1.0 to 1.0) | 1.0 (1.0 to 1.0) | 1.0 (1.0 to 1.0) | 1.0 (1.0 to 1.0) | 1.0 (1.0 to 1.0) |
| Multiple sclerosis | 0 Cigarettes Per Smoker Per Day | Both | Female | 1.0 (1.0 to 1.0) | 1.0 (1.0 to 1.0) | 1.0 (1.0 to 1.0) | 1.0 (1.0 to 1.0) | 1.0 (1.0 to 1.0) | 1.0 (1.0 to 1.0) | 1.0 (1.0 to 1.0) | 1.0 (1.0 to 1.0) | 1.0 (1.0 to 1.0) | 1.0 (1.0 to 1.0) | 1.0 (1.0 to 1.0) | 1.0 (1.0 to 1.0) | 1.0 (1.0 to 1.0) | 1.0 (1.0 to 1.0) |
| Multiple sclerosis | 5.6 Cigarettes Per Smoker Per Day | Both | Male | 1.377 (1.152 to 1.617) | 1.377 (1.152 to 1.617) | 1.377 (1.152 to 1.617) | 1.377 (1.152 to 1.617) | 1.377 (1.152 to 1.617) | 1.377 (1.152 to 1.617) | 1.377 (1.152 to 1.617) | 1.377 (1.152 to 1.617) | 1.377 (1.152 to 1.617) | 1.377 (1.152 to 1.617) | 1.377 (1.152 to 1.617) | 1.377 (1.152 to 1.617) | 1.377 (1.152 to 1.617) | 1.377 (1.152 to 1.617) |
| Multiple sclerosis | 5.6 Cigarettes Per Smoker Per Day | Both | Female | 1.377 (1.152 to 1.617) | 1.377 (1.152 to 1.617) | 1.377 (1.152 to 1.617) | 1.377 (1.152 to 1.617) | 1.377 (1.152 to 1.617) | 1.377 (1.152 to 1.617) | 1.377 (1.152 to 1.617) | 1.377 (1.152 to 1.617) | 1.377 (1.152 to 1.617) | 1.377 (1.152 to 1.617) | 1.377 (1.152 to 1.617) | 1.377 (1.152 to 1.617) | 1.377 (1.152 to 1.617) | 1.377 (1.152 to 1.617) |
| Multiple sclerosis | 11.2 Cigarettes Per Smoker Per Day | Both | Male | 1.756 (1.49 to 2.048) | 1.756 (1.49 to 2.048) | 1.756 (1.49 to 2.048) | 1.756 (1.49 to 2.048) | 1.756 (1.49 to 2.048) | 1.756 (1.49 to 2.048) | 1.756 (1.49 to 2.048) | 1.756 (1.49 to 2.048) | 1.756 (1.49 to 2.048) | 1.756 (1.49 to 2.048) | 1.756 (1.49 to 2.048) | 1.756 (1.49 to 2.048) | 1.756 (1.49 to 2.048) | 1.756 (1.49 to 2.048) |
| Multiple sclerosis | 11.2 Cigarettes Per Smoker Per Day | Both | Female | 1.756 (1.49 to 2.048) | 1.756 (1.49 to 2.048) | 1.756 (1.49 to 2.048) | 1.756 (1.49 to 2.048) | 1.756 (1.49 to 2.048) | 1.756 (1.49 to 2.048) | 1.756 (1.49 to 2.048) | 1.756 (1.49 to 2.048) | 1.756 (1.49 to 2.048) | 1.756 (1.49 to 2.048) | 1.756 (1.49 to 2.048) | 1.756 (1.49 to 2.048) | 1.756 (1.49 to 2.048) | 1.756 (1.49 to 2.048) |
| Multiple sclerosis | 16.9 Cigarettes Per Smoker Per Day | Both | Male | 1.965 (1.646 to 2.291) | 1.965 (1.646 to 2.291) | 1.965 (1.646 to 2.291) | 1.965 (1.646 to 2.291) | 1.965 (1.646 to 2.291) | 1.965 (1.646 to 2.291) | 1.965 (1.646 to 2.291) | 1.965 (1.646 to 2.291) | 1.965 (1.646 to 2.291) | 1.965 (1.646 to 2.291) | 1.965 (1.646 to 2.291) | 1.965 (1.646 to 2.291) | 1.965 (1.646 to 2.291) | 1.965 (1.646 to 2.291) |
| Multiple sclerosis | 16.9 Cigarettes Per Smoker Per Day | Both | Female | 1.965 (1.646 to 2.291) | 1.965 (1.646 to 2.291) | 1.965 (1.646 to 2.291) | 1.965 (1.646 to 2.291) | 1.965 (1.646 to 2.291) | 1.965 (1.646 to 2.291) | 1.965 (1.646 to 2.291) | 1.965 (1.646 to 2.291) | 1.965 (1.646 to 2.291) | 1.965 (1.646 to 2.291) | 1.965 (1.646 to 2.291) | 1.965 (1.646 to 2.291) | 1.965 (1.646 to 2.291) | 1.965 (1.646 to 2.291) |
| Multiple sclerosis | 22.5 Cigarettes Per Smoker Per Day | Both | Male | 2.04 (1.58 to 2.545) | 2.04 (1.58 to 2.545) | 2.04 (1.58 to 2.545) | 2.04 (1.58 to 2.545) | 2.04 (1.58 to 2.545) | 2.04 (1.58 to 2.545) | 2.04 (1.58 to 2.545) | 2.04 (1.58 to 2.545) | 2.04 (1.58 to 2.545) | 2.04 (1.58 to 2.545) | 2.04 (1.58 to 2.545) | 2.04 (1.58 to 2.545) | 2.04 (1.58 to 2.545) | 2.04 (1.58 to 2.545) |
| Multiple sclerosis | 22.5 Cigarettes Per Smoker Per Day | Both | Female | 2.04 (1.58 to 2.545) | 2.04 (1.58 to 2.545) | 2.04 (1.58 to 2.545) | 2.04 (1.58 to 2.545) | 2.04 (1.58 to 2.545) | 2.04 (1.58 to 2.545) | 2.04 (1.58 to 2.545) | 2.04 (1.58 to 2.545) | 2.04 (1.58 to 2.545) | 2.04 (1.58 to 2.545) | 2.04 (1.58 to 2.545) | 2.04 (1.58 to 2.545) | 2.04 (1.58 to 2.545) | 2.04 (1.58 to 2.545) |
| Diabetes mellitus type 2 | 0 Cigarettes Per Smoker Per Day | Both | Male | 1.0 (1.0 to 1.0) | 1.0 (1.0 to 1.0) | 1.0 (1.0 to 1.0) | 1.0 (1.0 to 1.0) | 1.0 (1.0 to 1.0) | 1.0 (1.0 to 1.0) | 1.0 (1.0 to 1.0) | 1.0 (1.0 to 1.0) | 1.0 (1.0 to 1.0) | 1.0 (1.0 to 1.0) | 1.0 (1.0 to 1.0) | 1.0 (1.0 to 1.0) | 1.0 (1.0 to 1.0) | 1.0 (1.0 to 1.0) |
| Diabetes mellitus type 2 | 0 Cigarettes Per Smoker Per Day | Both | Female | 1.0 (1.0 to 1.0) | 1.0 (1.0 to 1.0) | 1.0 (1.0 to 1.0) | 1.0 (1.0 to 1.0) | 1.0 (1.0 to 1.0) | 1.0 (1.0 to 1.0) | 1.0 (1.0 to 1.0) | 1.0 (1.0 to 1.0) | 1.0 (1.0 to 1.0) | 1.0 (1.0 to 1.0) | 1.0 (1.0 to 1.0) | 1.0 (1.0 to 1.0) | 1.0 (1.0 to 1.0) | 1.0 (1.0 to 1.0) |
| Diabetes mellitus type 2 | 6.5 Cigarettes Per Smoker Per Day | Both | Male | 1.439 (1.213 to 1.653) | 1.439 (1.213 to 1.653) | 1.439 (1.213 to 1.653) | 1.439 (1.213 to 1.653) | 1.439 (1.213 to 1.653) | 1.439 (1.213 to 1.653) | 1.439 (1.213 to 1.653) | 1.439 (1.213 to 1.653) | 1.439 (1.213 to 1.653) | 1.439 (1.213 to 1.653) | 1.439 (1.213 to 1.653) | 1.439 (1.213 to 1.653) | 1.439 (1.213 to 1.653) | 1.439 (1.213 to 1.653) |
| Diabetes mellitus type 2 | 6.5 Cigarettes Per Smoker Per Day | Both | Female | 1.439 (1.213 to 1.653) | 1.439 (1.213 to 1.653) | 1.439 (1.213 to 1.653) | 1.439 (1.213 to 1.653) | 1.439 (1.213 to 1.653) | 1.439 (1.213 to 1.653) | 1.439 (1.213 to 1.653) | 1.439 (1.213 to 1.653) | 1.439 (1.213 to 1.653) | 1.439 (1.213 to 1.653) | 1.439 (1.213 to 1.653) | 1.439 (1.213 to 1.653) | 1.439 (1.213 to 1.653) | 1.439 (1.213 to 1.653) |
| Diabetes mellitus type 2 | 12.9 Cigarettes Per Smoker Per Day | Both | Male | 1.43 (1.131 to 1.756) | 1.43 (1.131 to 1.756) | 1.43 (1.131 to 1.756) | 1.43 (1.131 to 1.756) | 1.43 (1.131 to 1.756) | 1.43 (1.131 to 1.756) | 1.43 (1.131 to 1.756) | 1.43 (1.131 to 1.756) | 1.43 (1.131 to 1.756) | 1.43 (1.131 to 1.756) | 1.43 (1.131 to 1.756) | 1.43 (1.131 to 1.756) | 1.43 (1.131 to 1.756) | 1.43 (1.131 to 1.756) |
| Diabetes mellitus type 2 | 12.9 Cigarettes Per Smoker Per Day | Both | Female | 1.43 (1.131 to 1.756) | 1.43 (1.131 to 1.756) | 1.43 (1.131 to 1.756) | 1.43 (1.131 to 1.756) | 1.43 (1.131 to 1.756) | 1.43 (1.131 to 1.756) | 1.43 (1.131 to 1.756) | 1.43 (1.131 to 1.756) | 1.43 (1.131 to 1.756) | 1.43 (1.131 to 1.756) | 1.43 (1.131 to 1.756) | 1.43 (1.131 to 1.756) | 1.43 (1.131 to 1.756) | 1.43 (1.131 to 1.756) |
| Diabetes mellitus type 2 | 19.4 Cigarettes Per Smoker Per Day | Both | Male | 1.637 (1.25 to 2.031) | 1.637 (1.25 to 2.031) | 1.637 (1.25 to 2.031) | 1.637 (1.25 to 2.031) | 1.637 (1.25 to 2.031) | 1.637 (1.25 to 2.031) | 1.637 (1.25 to 2.031) | 1.637 (1.25 to 2.031) | 1.637 (1.25 to 2.031) | 1.637 (1.25 to 2.031) | 1.637 (1.25 to 2.031) | 1.637 (1.25 to 2.031) | 1.637 (1.25 to 2.031) | 1.637 (1.25 to 2.031) |
| Diabetes mellitus type 2 | 19.4 Cigarettes Per Smoker Per Day | Both | Female | 1.637 (1.25 to 2.031) | 1.637 (1.25 to 2.031) | 1.637 (1.25 to 2.031) | 1.637 (1.25 to 2.031) | 1.637 (1.25 to 2.031) | 1.637 (1.25 to 2.031) | 1.637 (1.25 to 2.031) | 1.637 (1.25 to 2.031) | 1.637 (1.25 to 2.031) | 1.637 (1.25 to 2.031) | 1.637 (1.25 to 2.031) | 1.637 (1.25 to 2.031) | 1.637 (1.25 to 2.031) | 1.637 (1.25 to 2.031) |
| Diabetes mellitus type 2 | 25.8 Cigarettes Per Smoker Per Day | Both | Male | 1.665 (1.355 to 1.992) | 1.665 (1.355 to 1.992) | 1.665 (1.355 to 1.992) | 1.665 (1.355 to 1.992) | 1.665 (1.355 to 1.992) | 1.665 (1.355 to 1.992) | 1.665 (1.355 to 1.992) | 1.665 (1.355 to 1.992) | 1.665 (1.355 to 1.992) | 1.665 (1.355 to 1.992) | 1.665 (1.355 to 1.992) | 1.665 (1.355 to 1.992) | 1.665 (1.355 to 1.992) | 1.665 (1.355 to 1.992) |
| Diabetes mellitus type 2 | 25.8 Cigarettes Per Smoker Per Day | Both | Female | 1.665 (1.355 to 1.992) | 1.665 (1.355 to 1.992) | 1.665 (1.355 to 1.992) | 1.665 (1.355 to 1.992) | 1.665 (1.355 to 1.992) | 1.665 (1.355 to 1.992) | 1.665 (1.355 to 1.992) | 1.665 (1.355 to 1.992) | 1.665 (1.355 to 1.992) | 1.665 (1.355 to 1.992) | 1.665 (1.355 to 1.992) | 1.665 (1.355 to 1.992) | 1.665 (1.355 to 1.992) | 1.665 (1.355 to 1.992) |
| Diabetes mellitus type 2 | 32.3 Cigarettes Per Smoker Per Day | Both | Male | 1.841 (1.221 to 2.458) | 1.841 (1.221 to 2.458) | 1.841 (1.221 to 2.458) | 1.841 (1.221 to 2.458) | 1.841 (1.221 to 2.458) | 1.841 (1.221 to 2.458) | 1.841 (1.221 to 2.458) | 1.841 (1.221 to 2.458) | 1.841 (1.221 to 2.458) | 1.841 (1.221 to 2.458) | 1.841 (1.221 to 2.458) | 1.841 (1.221 to 2.458) | 1.841 (1.221 to 2.458) | 1.841 (1.221 to 2.458) |
| Diabetes mellitus type 2 | 32.3 Cigarettes Per Smoker Per Day | Both | Female | 1.841 (1.221 to 2.458) | 1.841 (1.221 to 2.458) | 1.841 (1.221 to 2.458) | 1.841 (1.221 to 2.458) | 1.841 (1.221 to 2.458) | 1.841 (1.221 to 2.458) | 1.841 (1.221 to 2.458) | 1.841 (1.221 to 2.458) | 1.841 (1.221 to 2.458) | 1.841 (1.221 to 2.458) | 1.841 (1.221 to 2.458) | 1.841 (1.221 to 2.458) | 1.841 (1.221 to 2.458) | 1.841 (1.221 to 2.458) |
| Diabetes mellitus type 2 | 38.8 Cigarettes Per Smoker Per Day | Both | Male | 2.163 (1.161 to 3.354) | 2.163 (1.161 to 3.354) | 2.163 (1.161 to 3.354) | 2.163 (1.161 to 3.354) | 2.163 (1.161 to 3.354) | 2.163 (1.161 to 3.354) | 2.163 (1.161 to 3.354) | 2.163 (1.161 to 3.354) | 2.163 (1.161 to 3.354) | 2.163 (1.161 to 3.354) | 2.163 (1.161 to 3.354) | 2.163 (1.161 to 3.354) | 2.163 (1.161 to 3.354) | 2.163 (1.161 to 3.354) |
| Diabetes mellitus type 2 | 38.8 Cigarettes Per Smoker Per Day | Both | Female | 2.163 (1.161 to 3.354) | 2.163 (1.161 to 3.354) | 2.163 (1.161 to 3.354) | 2.163 (1.161 to 3.354) | 2.163 (1.161 to 3.354) | 2.163 (1.161 to 3.354) | 2.163 (1.161 to 3.354) | 2.163 (1.161 to 3.354) | 2.163 (1.161 to 3.354) | 2.163 (1.161 to 3.354) | 2.163 (1.161 to 3.354) | 2.163 (1.161 to 3.354) | 2.163 (1.161 to 3.354) | 2.163 (1.161 to 3.354) |
| Cataract | 0 Cigarettes Per Smoker Per Day | Both | Male | 1.0 (1.0 to 1.0) | 1.0 (1.0 to 1.0) | 1.0 (1.0 to 1.0) | 1.0 (1.0 to 1.0) | 1.0 (1.0 to 1.0) | 1.0 (1.0 to 1.0) | 1.0 (1.0 to 1.0) | 1.0 (1.0 to 1.0) | 1.0 (1.0 to 1.0) | 1.0 (1.0 to 1.0) | 1.0 (1.0 to 1.0) | 1.0 (1.0 to 1.0) | 1.0 (1.0 to 1.0) | 1.0 (1.0 to 1.0) |
| Cataract | 0 Cigarettes Per Smoker Per Day | Both | Female | 1.0 (1.0 to 1.0) | 1.0 (1.0 to 1.0) | 1.0 (1.0 to 1.0) | 1.0 (1.0 to 1.0) | 1.0 (1.0 to 1.0) | 1.0 (1.0 to 1.0) | 1.0 (1.0 to 1.0) | 1.0 (1.0 to 1.0) | 1.0 (1.0 to 1.0) | 1.0 (1.0 to 1.0) | 1.0 (1.0 to 1.0) | 1.0 (1.0 to 1.0) | 1.0 (1.0 to 1.0) | 1.0 (1.0 to 1.0) |
| Cataract | 9 Cigarettes Per Smoker Per Day | Both | Male | 1.438 (1.288 to 1.608) | 1.438 (1.288 to 1.608) | 1.438 (1.288 to 1.608) | 1.438 (1.288 to 1.608) | 1.438 (1.288 to 1.608) | 1.438 (1.288 to 1.608) | 1.438 (1.288 to 1.608) | 1.438 (1.288 to 1.608) | 1.438 (1.288 to 1.608) | 1.438 (1.288 to 1.608) | 1.438 (1.288 to 1.608) | 1.438 (1.288 to 1.608) | 1.438 (1.288 to 1.608) | 1.438 (1.288 to 1.608) |
| Cataract | 9 Cigarettes Per Smoker Per Day | Both | Female | 1.438 (1.288 to 1.608) | 1.438 (1.288 to 1.608) | 1.438 (1.288 to 1.608) | 1.438 (1.288 to 1.608) | 1.438 (1.288 to 1.608) | 1.438 (1.288 to 1.608) | 1.438 (1.288 to 1.608) | 1.438 (1.288 to 1.608) | 1.438 (1.288 to 1.608) | 1.438 (1.288 to 1.608) | 1.438 (1.288 to 1.608) | 1.438 (1.288 to 1.608) | 1.438 (1.288 to 1.608) | 1.438 (1.288 to 1.608) |
| Cataract | 18 Cigarettes Per Smoker Per Day | Both | Male | 1.479 (1.288 to 1.694) | 1.479 (1.288 to 1.694) | 1.479 (1.288 to 1.694) | 1.479 (1.288 to 1.694) | 1.479 (1.288 to 1.694) | 1.479 (1.288 to 1.694) | 1.479 (1.288 to 1.694) | 1.479 (1.288 to 1.694) | 1.479 (1.288 to 1.694) | 1.479 (1.288 to 1.694) | 1.479 (1.288 to 1.694) | 1.479 (1.288 to 1.694) | 1.479 (1.288 to 1.694) | 1.479 (1.288 to 1.694) |
| Cataract | 18 Cigarettes Per Smoker Per Day | Both | Female | 1.479 (1.288 to 1.694) | 1.479 (1.288 to 1.694) | 1.479 (1.288 to 1.694) | 1.479 (1.288 to 1.694) | 1.479 (1.288 to 1.694) | 1.479 (1.288 to 1.694) | 1.479 (1.288 to 1.694) | 1.479 (1.288 to 1.694) | 1.479 (1.288 to 1.694) | 1.479 (1.288 to 1.694) | 1.479 (1.288 to 1.694) | 1.479 (1.288 to 1.694) | 1.479 (1.288 to 1.694) | 1.479 (1.288 to 1.694) |

**Appendix Table 6d. Relative risks used by age and sex for each outcome for smoking globally.**

| Risk - Outcome | Category / Units | Morbidity / Mortality | Sex | Age | | | | | | | | | | | | | |
|---|---|---|---|---|---|---|---|---|---|---|---|---|---|---|---|---|---|
| | | | | 30 to 34 | 35 to 39 | 40 to 44 | 45 to 49 | 50 to 54 | 55 to 59 | 60 to 64 | 65 to 69 | 70 to 74 | 75 to 79 | 80 to 84 | 85 to 89 | 90 to 94 | 95 plus |
| **Smoking** | | | | | | | | | | | | | | | | | |
| Cataract | 27 Cigarettes Per Smoker Per Day | Both | Male | 1.786 (1.356 to 2.266) | 1.786 (1.356 to 2.266) | 1.786 (1.356 to 2.266) | 1.786 (1.356 to 2.266) | 1.786 (1.356 to 2.266) | 1.786 (1.356 to 2.266) | 1.786 (1.356 to 2.266) | 1.786 (1.356 to 2.266) | 1.786 (1.356 to 2.266) | 1.786 (1.356 to 2.266) | 1.786 (1.356 to 2.266) | 1.786 (1.356 to 2.266) | 1.786 (1.356 to 2.266) | 1.786 (1.356 to 2.266) |
| Cataract | 27 Cigarettes Per Smoker Per Day | Both | Female | 1.786 (1.356 to 2.266) | 1.786 (1.356 to 2.266) | 1.786 (1.356 to 2.266) | 1.786 (1.356 to 2.266) | 1.786 (1.356 to 2.266) | 1.786 (1.356 to 2.266) | 1.786 (1.356 to 2.266) | 1.786 (1.356 to 2.266) | 1.786 (1.356 to 2.266) | 1.786 (1.356 to 2.266) | 1.786 (1.356 to 2.266) | 1.786 (1.356 to 2.266) | 1.786 (1.356 to 2.266) | 1.786 (1.356 to 2.266) |
| Cataract | 36 Cigarettes Per Smoker Per Day | Both | Male | 1.882 (1.21 to 2.734) | 1.882 (1.21 to 2.734) | 1.882 (1.21 to 2.734) | 1.882 (1.21 to 2.734) | 1.882 (1.21 to 2.734) | 1.882 (1.21 to 2.734) | 1.882 (1.21 to 2.734) | 1.882 (1.21 to 2.734) | 1.882 (1.21 to 2.734) | 1.882 (1.21 to 2.734) | 1.882 (1.21 to 2.734) | 1.882 (1.21 to 2.734) | 1.882 (1.21 to 2.734) | 1.882 (1.21 to 2.734) |
| Cataract | 36 Cigarettes Per Smoker Per Day | Both | Female | 1.882 (1.21 to 2.734) | 1.882 (1.21 to 2.734) | 1.882 (1.21 to 2.734) | 1.882 (1.21 to 2.734) | 1.882 (1.21 to 2.734) | 1.882 (1.21 to 2.734) | 1.882 (1.21 to 2.734) | 1.882 (1.21 to 2.734) | 1.882 (1.21 to 2.734) | 1.882 (1.21 to 2.734) | 1.882 (1.21 to 2.734) | 1.882 (1.21 to 2.734) | 1.882 (1.21 to 2.734) | 1.882 (1.21 to 2.734) |
| Age-related macular degeneration | 0 Cigarettes Per Smoker Per Day | Both | Male | 1.0 (1.0 to 1.0) | 1.0 (1.0 to 1.0) | 1.0 (1.0 to 1.0) | 1.0 (1.0 to 1.0) | 1.0 (1.0 to 1.0) | 1.0 (1.0 to 1.0) | 1.0 (1.0 to 1.0) | 1.0 (1.0 to 1.0) | 1.0 (1.0 to 1.0) | 1.0 (1.0 to 1.0) | 1.0 (1.0 to 1.0) | 1.0 (1.0 to 1.0) | 1.0 (1.0 to 1.0) | 1.0 (1.0 to 1.0) |
| Age-related macular degeneration | 0 Cigarettes Per Smoker Per Day | Both | Female | 1.0 (1.0 to 1.0) | 1.0 (1.0 to 1.0) | 1.0 (1.0 to 1.0) | 1.0 (1.0 to 1.0) | 1.0 (1.0 to 1.0) | 1.0 (1.0 to 1.0) | 1.0 (1.0 to 1.0) | 1.0 (1.0 to 1.0) | 1.0 (1.0 to 1.0) | 1.0 (1.0 to 1.0) | 1.0 (1.0 to 1.0) | 1.0 (1.0 to 1.0) | 1.0 (1.0 to 1.0) | 1.0 (1.0 to 1.0) |
| Age-related macular degeneration | 7.5 Cigarettes Per Smoker Per Day | Both | Male | 1.347 (1.0 to 1.769) | 1.347 (1.0 to 1.769) | 1.347 (1.0 to 1.769) | 1.347 (1.0 to 1.769) | 1.347 (1.0 to 1.769) | 1.347 (1.0 to 1.769) | 1.347 (1.0 to 1.769) | 1.347 (1.0 to 1.769) | 1.347 (1.0 to 1.769) | 1.347 (1.0 to 1.769) | 1.347 (1.0 to 1.769) | 1.347 (1.0 to 1.769) | 1.347 (1.0 to 1.769) | 1.347 (1.0 to 1.769) |
| Age-related macular degeneration | 7.5 Cigarettes Per Smoker Per Day | Both | Female | 1.347 (1.0 to 1.769) | 1.347 (1.0 to 1.769) | 1.347 (1.0 to 1.769) | 1.347 (1.0 to 1.769) | 1.347 (1.0 to 1.769) | 1.347 (1.0 to 1.769) | 1.347 (1.0 to 1.769) | 1.347 (1.0 to 1.769) | 1.347 (1.0 to 1.769) | 1.347 (1.0 to 1.769) | 1.347 (1.0 to 1.769) | 1.347 (1.0 to 1.769) | 1.347 (1.0 to 1.769) | 1.347 (1.0 to 1.769) |
| Age-related macular degeneration | 15 Cigarettes Per Smoker Per Day | Both | Male | 1.67 (1.152 to 2.349) | 1.67 (1.152 to 2.349) | 1.67 (1.152 to 2.349) | 1.67 (1.152 to 2.349) | 1.67 (1.152 to 2.349) | 1.67 (1.152 to 2.349) | 1.67 (1.152 to 2.349) | 1.67 (1.152 to 2.349) | 1.67 (1.152 to 2.349) | 1.67 (1.152 to 2.349) | 1.67 (1.152 to 2.349) | 1.67 (1.152 to 2.349) | 1.67 (1.152 to 2.349) | 1.67 (1.152 to 2.349) |
| Age-related macular degeneration | 15 Cigarettes Per Smoker Per Day | Both | Female | 1.67 (1.152 to 2.349) | 1.67 (1.152 to 2.349) | 1.67 (1.152 to 2.349) | 1.67 (1.152 to 2.349) | 1.67 (1.152 to 2.349) | 1.67 (1.152 to 2.349) | 1.67 (1.152 to 2.349) | 1.67 (1.152 to 2.349) | 1.67 (1.152 to 2.349) | 1.67 (1.152 to 2.349) | 1.67 (1.152 to 2.349) | 1.67 (1.152 to 2.349) | 1.67 (1.152 to 2.349) | 1.67 (1.152 to 2.349) |
| Age-related macular degeneration | 22.5 Cigarettes Per Smoker Per Day | Both | Male | 2.024 (1.375 to 2.775) | 2.024 (1.375 to 2.775) | 2.024 (1.375 to 2.775) | 2.024 (1.375 to 2.775) | 2.024 (1.375 to 2.775) | 2.024 (1.375 to 2.775) | 2.024 (1.375 to 2.775) | 2.024 (1.375 to 2.775) | 2.024 (1.375 to 2.775) | 2.024 (1.375 to 2.775) | 2.024 (1.375 to 2.775) | 2.024 (1.375 to 2.775) | 2.024 (1.375 to 2.775) | 2.024 (1.375 to 2.775) |
| Age-related macular degeneration | 22.5 Cigarettes Per Smoker Per Day | Both | Female | 2.024 (1.375 to 2.775) | 2.024 (1.375 to 2.775) | 2.024 (1.375 to 2.775) | 2.024 (1.375 to 2.775) | 2.024 (1.375 to 2.775) | 2.024 (1.375 to 2.775) | 2.024 (1.375 to 2.775) | 2.024 (1.375 to 2.775) | 2.024 (1.375 to 2.775) | 2.024 (1.375 to 2.775) | 2.024 (1.375 to 2.775) | 2.024 (1.375 to 2.775) | 2.024 (1.375 to 2.775) | 2.024 (1.375 to 2.775) |
| Age-related macular degeneration | 30 Cigarettes Per Smoker Per Day | Both | Male | 2.42 (1.624 to 3.354) | 2.42 (1.624 to 3.354) | 2.42 (1.624 to 3.354) | 2.42 (1.624 to 3.354) | 2.42 (1.624 to 3.354) | 2.42 (1.624 to 3.354) | 2.42 (1.624 to 3.354) | 2.42 (1.624 to 3.354) | 2.42 (1.624 to 3.354) | 2.42 (1.624 to 3.354) | 2.42 (1.624 to 3.354) | 2.42 (1.624 to 3.354) | 2.42 (1.624 to 3.354) | 2.42 (1.624 to 3.354) |
| Age-related macular degeneration | 30 Cigarettes Per Smoker Per Day | Both | Female | 2.42 (1.624 to 3.354) | 2.42 (1.624 to 3.354) | 2.42 (1.624 to 3.354) | 2.42 (1.624 to 3.354) | 2.42 (1.624 to 3.354) | 2.42 (1.624 to 3.354) | 2.42 (1.624 to 3.354) | 2.42 (1.624 to 3.354) | 2.42 (1.624 to 3.354) | 2.42 (1.624 to 3.354) | 2.42 (1.624 to 3.354) | 2.42 (1.624 to 3.354) | 2.42 (1.624 to 3.354) | 2.42 (1.624 to 3.354) |
| Rheumatoid arthritis | 0 Cigarettes Per Smoker Per Day | Both | Male | 1.0 (1.0 to 1.0) | 1.0 (1.0 to 1.0) | 1.0 (1.0 to 1.0) | 1.0 (1.0 to 1.0) | 1.0 (1.0 to 1.0) | 1.0 (1.0 to 1.0) | 1.0 (1.0 to 1.0) | 1.0 (1.0 to 1.0) | 1.0 (1.0 to 1.0) | 1.0 (1.0 to 1.0) | 1.0 (1.0 to 1.0) | 1.0 (1.0 to 1.0) | 1.0 (1.0 to 1.0) | 1.0 (1.0 to 1.0) |
| Rheumatoid arthritis | 0 Cigarettes Per Smoker Per Day | Both | Female | 1.0 (1.0 to 1.0) | 1.0 (1.0 to 1.0) | 1.0 (1.0 to 1.0) | 1.0 (1.0 to 1.0) | 1.0 (1.0 to 1.0) | 1.0 (1.0 to 1.0) | 1.0 (1.0 to 1.0) | 1.0 (1.0 to 1.0) | 1.0 (1.0 to 1.0) | 1.0 (1.0 to 1.0) | 1.0 (1.0 to 1.0) | 1.0 (1.0 to 1.0) | 1.0 (1.0 to 1.0) | 1.0 (1.0 to 1.0) |
| Rheumatoid arthritis | 7.5 Cigarettes Per Smoker Per Day | Both | Male | 1.406 (1.0 to 1.965) | 1.406 (1.0 to 1.965) | 1.406 (1.0 to 1.965) | 1.406 (1.0 to 1.965) | 1.406 (1.0 to 1.965) | 1.406 (1.0 to 1.965) | 1.406 (1.0 to 1.965) | 1.406 (1.0 to 1.965) | 1.406 (1.0 to 1.965) | 1.406 (1.0 to 1.965) | 1.406 (1.0 to 1.965) | 1.406 (1.0 to 1.965) | 1.406 (1.0 to 1.965) | 1.406 (1.0 to 1.965) |
| Rheumatoid arthritis | 7.5 Cigarettes Per Smoker Per Day | Both | Female | 1.406 (1.0 to 1.965) | 1.406 (1.0 to 1.965) | 1.406 (1.0 to 1.965) | 1.406 (1.0 to 1.965) | 1.406 (1.0 to 1.965) | 1.406 (1.0 to 1.965) | 1.406 (1.0 to 1.965) | 1.406 (1.0 to 1.965) | 1.406 (1.0 to 1.965) | 1.406 (1.0 to 1.965) | 1.406 (1.0 to 1.965) | 1.406 (1.0 to 1.965) | 1.406 (1.0 to 1.965) | 1.406 (1.0 to 1.965) |
| Rheumatoid arthritis | 15 Cigarettes Per Smoker Per Day | Both | Male | 1.742 (1.101 to 2.633) | 1.742 (1.101 to 2.633) | 1.742 (1.101 to 2.633) | 1.742 (1.101 to 2.633) | 1.742 (1.101 to 2.633) | 1.742 (1.101 to 2.633) | 1.742 (1.101 to 2.633) | 1.742 (1.101 to 2.633) | 1.742 (1.101 to 2.633) | 1.742 (1.101 to 2.633) | 1.742 (1.101 to 2.633) | 1.742 (1.101 to 2.633) | 1.742 (1.101 to 2.633) | 1.742 (1.101 to 2.633) |
| Rheumatoid arthritis | 15 Cigarettes Per Smoker Per Day | Both | Female | 1.742 (1.101 to 2.633) | 1.742 (1.101 to 2.633) | 1.742 (1.101 to 2.633) | 1.742 (1.101 to 2.633) | 1.742 (1.101 to 2.633) | 1.742 (1.101 to 2.633) | 1.742 (1.101 to 2.633) | 1.742 (1.101 to 2.633) | 1.742 (1.101 to 2.633) | 1.742 (1.101 to 2.633) | 1.742 (1.101 to 2.633) | 1.742 (1.101 to 2.633) | 1.742 (1.101 to 2.633) | 1.742 (1.101 to 2.633) |
| Rheumatoid arthritis | 22.5 Cigarettes Per Smoker Per Day | Both | Male | 2.0 (1.167 to 3.187) | 2.0 (1.167 to 3.187) | 2.0 (1.167 to 3.187) | 2.0 (1.167 to 3.187) | 2.0 (1.167 to 3.187) | 2.0 (1.167 to 3.187) | 2.0 (1.167 to 3.187) | 2.0 (1.167 to 3.187) | 2.0 (1.167 to 3.187) | 2.0 (1.167 to 3.187) | 2.0 (1.167 to 3.187) | 2.0 (1.167 to 3.187) | 2.0 (1.167 to 3.187) | 2.0 (1.167 to 3.187) |
| Rheumatoid arthritis | 22.5 Cigarettes Per Smoker Per Day | Both | Female | 2.0 (1.167 to 3.187) | 2.0 (1.167 to 3.187) | 2.0 (1.167 to 3.187) | 2.0 (1.167 to 3.187) | 2.0 (1.167 to 3.187) | 2.0 (1.167 to 3.187) | 2.0 (1.167 to 3.187) | 2.0 (1.167 to 3.187) | 2.0 (1.167 to 3.187) | 2.0 (1.167 to 3.187) | 2.0 (1.167 to 3.187) | 2.0 (1.167 to 3.187) | 2.0 (1.167 to 3.187) | 2.0 (1.167 to 3.187) |
| Rheumatoid arthritis | 30 Cigarettes Per Smoker Per Day | Both | Male | 2.256 (1.147 to 3.913) | 2.256 (1.147 to 3.913) | 2.256 (1.147 to 3.913) | 2.256 (1.147 to 3.913) | 2.256 (1.147 to 3.913) | 2.256 (1.147 to 3.913) | 2.256 (1.147 to 3.913) | 2.256 (1.147 to 3.913) | 2.256 (1.147 to 3.913) | 2.256 (1.147 to 3.913) | 2.256 (1.147 to 3.913) | 2.256 (1.147 to 3.913) | 2.256 (1.147 to 3.913) | 2.256 (1.147 to 3.913) |
| Rheumatoid arthritis | 30 Cigarettes Per Smoker Per Day | Both | Female | 2.256 (1.147 to 3.913) | 2.256 (1.147 to 3.913) | 2.256 (1.147 to 3.913) | 2.256 (1.147 to 3.913) | 2.256 (1.147 to 3.913) | 2.256 (1.147 to 3.913) | 2.256 (1.147 to 3.913) | 2.256 (1.147 to 3.913) | 2.256 (1.147 to 3.913) | 2.256 (1.147 to 3.913) | 2.256 (1.147 to 3.913) | 2.256 (1.147 to 3.913) | 2.256 (1.147 to 3.913) | 2.256 (1.147 to 3.913) |
| Rheumatoid arthritis | 37.5 Cigarettes Per Smoker Per Day | Both | Male | 2.438 (1.15 to 4.713) | 2.438 (1.15 to 4.713) | 2.438 (1.15 to 4.713) | 2.438 (1.15 to 4.713) | 2.438 (1.15 to 4.713) | 2.438 (1.15 to 4.713) | 2.438 (1.15 to 4.713) | 2.438 (1.15 to 4.713) | 2.438 (1.15 to 4.713) | 2.438 (1.15 to 4.713) | 2.438 (1.15 to 4.713) | 2.438 (1.15 to 4.713) | 2.438 (1.15 to 4.713) | 2.438 (1.15 to 4.713) |
| Rheumatoid arthritis | 37.5 Cigarettes Per Smoker Per Day | Both | Female | 2.438 (1.15 to 4.713) | 2.438 (1.15 to 4.713) | 2.438 (1.15 to 4.713) | 2.438 (1.15 to 4.713) | 2.438 (1.15 to 4.713) | 2.438 (1.15 to 4.713) | 2.438 (1.15 to 4.713) | 2.438 (1.15 to 4.713) | 2.438 (1.15 to 4.713) | 2.438 (1.15 to 4.713) | 2.438 (1.15 to 4.713) | 2.438 (1.15 to 4.713) | 2.438 (1.15 to 4.713) | 2.438 (1.15 to 4.713) |
| Low back pain | 0 Cigarettes Per Smoker Per Day | Both | Male | 1.0 (1.0 to 1.0) | 1.0 (1.0 to 1.0) | 1.0 (1.0 to 1.0) | 1.0 (1.0 to 1.0) | 1.0 (1.0 to 1.0) | 1.0 (1.0 to 1.0) | 1.0 (1.0 to 1.0) | 1.0 (1.0 to 1.0) | 1.0 (1.0 to 1.0) | 1.0 (1.0 to 1.0) | 1.0 (1.0 to 1.0) | 1.0 (1.0 to 1.0) | 1.0 (1.0 to 1.0) | 1.0 (1.0 to 1.0) |
| Low back pain | 0 Cigarettes Per Smoker Per Day | Both | Female | 1.0 (1.0 to 1.0) | 1.0 (1.0 to 1.0) | 1.0 (1.0 to 1.0) | 1.0 (1.0 to 1.0) | 1.0 (1.0 to 1.0) | 1.0 (1.0 to 1.0) | 1.0 (1.0 to 1.0) | 1.0 (1.0 to 1.0) | 1.0 (1.0 to 1.0) | 1.0 (1.0 to 1.0) | 1.0 (1.0 to 1.0) | 1.0 (1.0 to 1.0) | 1.0 (1.0 to 1.0) | 1.0 (1.0 to 1.0) |
| Low back pain | 2.5 Cigarettes Per Smoker Per Day | Both | Male | 1.315 (1.104 to 1.564) | 1.315 (1.104 to 1.564) | 1.315 (1.104 to 1.564) | 1.315 (1.104 to 1.564) | 1.315 (1.104 to 1.564) | 1.315 (1.104 to 1.564) | 1.315 (1.104 to 1.564) | 1.315 (1.104 to 1.564) | 1.315 (1.104 to 1.564) | 1.315 (1.104 to 1.564) | 1.315 (1.104 to 1.564) | 1.315 (1.104 to 1.564) | 1.315 (1.104 to 1.564) | 1.315 (1.104 to 1.564) |
| Low back pain | 2.5 Cigarettes Per Smoker Per Day | Both | Female | 1.315 (1.104 to 1.564) | 1.315 (1.104 to 1.564) | 1.315 (1.104 to 1.564) | 1.315 (1.104 to 1.564) | 1.315 (1.104 to 1.564) | 1.315 (1.104 to 1.564) | 1.315 (1.104 to 1.564) | 1.315 (1.104 to 1.564) | 1.315 (1.104 to 1.564) | 1.315 (1.104 to 1.564) | 1.315 (1.104 to 1.564) | 1.315 (1.104 to 1.564) | 1.315 (1.104 to 1.564) | 1.315 (1.104 to 1.564) |
| Low back pain | 5 Cigarettes Per Smoker Per Day | Both | Male | 1.666 (1.387 to 1.975) | 1.666 (1.387 to 1.975) | 1.666 (1.387 to 1.975) | 1.666 (1.387 to 1.975) | 1.666 (1.387 to 1.975) | 1.666 (1.387 to 1.975) | 1.666 (1.387 to 1.975) | 1.666 (1.387 to 1.975) | 1.666 (1.387 to 1.975) | 1.666 (1.387 to 1.975) | 1.666 (1.387 to 1.975) | 1.666 (1.387 to 1.975) | 1.666 (1.387 to 1.975) | 1.666 (1.387 to 1.975) |
| Low back pain | 5 Cigarettes Per Smoker Per Day | Both | Female | 1.666 (1.387 to 1.975) | 1.666 (1.387 to 1.975) | 1.666 (1.387 to 1.975) | 1.666 (1.387 to 1.975) | 1.666 (1.387 to 1.975) | 1.666 (1.387 to 1.975) | 1.666 (1.387 to 1.975) | 1.666 (1.387 to 1.975) | 1.666 (1.387 to 1.975) | 1.666 (1.387 to 1.975) | 1.666 (1.387 to 1.975) | 1.666 (1.387 to 1.975) | 1.666 (1.387 to 1.975) | 1.666 (1.387 to 1.975) |
| Low back pain | 10 Cigarettes Per Smoker Per Day | Both | Male | 2.161 (1.746 to 2.636) | 2.161 (1.746 to 2.636) | 2.161 (1.746 to 2.636) | 2.161 (1.746 to 2.636) | 2.161 (1.746 to 2.636) | 2.161 (1.746 to 2.636) | 2.161 (1.746 to 2.636) | 2.161 (1.746 to 2.636) | 2.161 (1.746 to 2.636) | 2.161 (1.746 to 2.636) | 2.161 (1.746 to 2.636) | 2.161 (1.746 to 2.636) | 2.161 (1.746 to 2.636) | 2.161 (1.746 to 2.636) |
| Low back pain | 10 Cigarettes Per Smoker Per Day | Both | Female | 2.161 (1.746 to 2.636) | 2.161 (1.746 to 2.636) | 2.161 (1.746 to 2.636) | 2.161 (1.746 to 2.636) | 2.161 (1.746 to 2.636) | 2.161 (1.746 to 2.636) | 2.161 (1.746 to 2.636) | 2.161 (1.746 to 2.636) | 2.161 (1.746 to 2.636) | 2.161 (1.746 to 2.636) | 2.161 (1.746 to 2.636) | 2.161 (1.746 to 2.636) | 2.161 (1.746 to 2.636) | 2.161 (1.746 to 2.636) |
| Low back pain | 15 Cigarettes Per Smoker Per Day | Both | Male | 2.244 (1.799 to 2.806) | 2.244 (1.799 to 2.806) | 2.244 (1.799 to 2.806) | 2.244 (1.799 to 2.806) | 2.244 (1.799 to 2.806) | 2.244 (1.799 to 2.806) | 2.244 (1.799 to 2.806) | 2.244 (1.799 to 2.806) | 2.244 (1.799 to 2.806) | 2.244 (1.799 to 2.806) | 2.244 (1.799 to 2.806) | 2.244 (1.799 to 2.806) | 2.244 (1.799 to 2.806) | 2.244 (1.799 to 2.806) |
| Low back pain | 15 Cigarettes Per Smoker Per Day | Both | Female | 2.244 (1.799 to 2.806) | 2.244 (1.799 to 2.806) | 2.244 (1.799 to 2.806) | 2.244 (1.799 to 2.806) | 2.244 (1.799 to 2.806) | 2.244 (1.799 to 2.806) | 2.244 (1.799 to 2.806) | 2.244 (1.799 to 2.806) | 2.244 (1.799 to 2.806) | 2.244 (1.799 to 2.806) | 2.244 (1.799 to 2.806) | 2.244 (1.799 to 2.806) | 2.244 (1.799 to 2.806) | 2.244 (1.799 to 2.806) |
| Low back pain | 20 Cigarettes Per Smoker Per Day | Both | Male | 2.353 (1.614 to 3.307) | 2.353 (1.614 to 3.307) | 2.353 (1.614 to 3.307) | 2.353 (1.614 to 3.307) | 2.353 (1.614 to 3.307) | 2.353 (1.614 to 3.307) | 2.353 (1.614 to 3.307) | 2.353 (1.614 to 3.307) | 2.353 (1.614 to 3.307) | 2.353 (1.614 to 3.307) | 2.353 (1.614 to 3.307) | 2.353 (1.614 to 3.307) | 2.353 (1.614 to 3.307) | 2.353 (1.614 to 3.307) |

| Appendix Table 6d. Relative risks used by age and sex for each outcome for smoking globally. | | | | | | | | | | | | | | | | | |
|---|---|---|---|---|---|---|---|---|---|---|---|---|---|---|---|---|---|
| Risk - Outcome | Category / Units | Morbidity / Mortality | Sex | Age | | | | | | | | | | | | | |
| | | | | 30 to 34 | 35 to 39 | 40 to 44 | 45 to 49 | 50 to 54 | 55 to 59 | 60 to 64 | 65 to 69 | 70 to 74 | 75 to 79 | 80 to 84 | 85 to 89 | 90 to 94 | 95 plus |
| Smoking | | | | | | | | | | | | | | | | | |
| Low back pain | 20 Cigarettes Per Smoker Per Day | Both | Female | 2.353 (1.614 to 3.307) | 2.353 (1.614 to 3.307) | 2.353 (1.614 to 3.307) | 2.353 (1.614 to 3.307) | 2.353 (1.614 to 3.307) | 2.353 (1.614 to 3.307) | 2.353 (1.614 to 3.307) | 2.353 (1.614 to 3.307) | 2.353 (1.614 to 3.307) | 2.353 (1.614 to 3.307) | 2.353 (1.614 to 3.307) | 2.353 (1.614 to 3.307) | 2.353 (1.614 to 3.307) | 2.353 (1.614 to 3.307) |
| Hip fractures | 1 Prevalence | Both | Male | 1.85 (1.518 to 2.249) | 1.85 (1.518 to 2.249) | 1.85 (1.518 to 2.249) | 1.85 (1.518 to 2.249) | 1.85 (1.518 to 2.249) | 1.85 (1.518 to 2.249) | 1.85 (1.518 to 2.249) | 1.85 (1.518 to 2.249) | 1.85 (1.518 to 2.249) | 1.85 (1.518 to 2.249) | 1.85 (1.518 to 2.249) | 1.85 (1.518 to 2.249) | 1.85 (1.518 to 2.249) | 1.85 (1.518 to 2.249) |
| Hip fractures | 1 Prevalence | Both | Female | 1.85 (1.518 to 2.249) | 1.85 (1.518 to 2.249) | 1.85 (1.518 to 2.249) | 1.85 (1.518 to 2.249) | 1.85 (1.518 to 2.249) | 1.85 (1.518 to 2.249) | 1.85 (1.518 to 2.249) | 1.85 (1.518 to 2.249) | 1.85 (1.518 to 2.249) | 1.85 (1.518 to 2.249) | 1.85 (1.518 to 2.249) | 1.85 (1.518 to 2.249) | 1.85 (1.518 to 2.249) | 1.85 (1.518 to 2.249) |
| Non-hip fractures | 1 Prevalence | Both | Male | 1.25 (1.153 to 1.358) | 1.25 (1.153 to 1.358) | 1.25 (1.153 to 1.358) | 1.25 (1.153 to 1.358) | 1.25 (1.153 to 1.358) | 1.25 (1.153 to 1.358) | 1.25 (1.153 to 1.358) | 1.25 (1.153 to 1.358) | 1.25 (1.153 to 1.358) | 1.25 (1.153 to 1.358) | 1.25 (1.153 to 1.358) | 1.25 (1.153 to 1.358) | 1.25 (1.153 to 1.358) | 1.25 (1.153 to 1.358) |
| Non-hip fractures | 1 Prevalence | Both | Female | 1.25 (1.153 to 1.358) | 1.25 (1.153 to 1.358) | 1.25 (1.153 to 1.358) | 1.25 (1.153 to 1.358) | 1.25 (1.153 to 1.358) | 1.25 (1.153 to 1.358) | 1.25 (1.153 to 1.358) | 1.25 (1.153 to 1.358) | 1.25 (1.153 to 1.358) | 1.25 (1.153 to 1.358) | 1.25 (1.153 to 1.358) | 1.25 (1.153 to 1.358) | 1.25 (1.153 to 1.358) | 1.25 (1.153 to 1.358) |

**Appendix Table 7. GBD location hierarchy with levels**

| Geography | Level |
|---|---|
| Global | 0 |
| Low SDI | 1 |
| Low-middle SDI | 1 |
| Middle SDI | 1 |
| High-middle SDI | 1 |
| High SDI | 1 |
| Central Europe, Eastern Europe, and Central Asia | 1 |
| Central Asia | 2 |
| Armenia | 3 |
| Azerbaijan | 3 |
| Georgia | 3 |
| Kazakhstan | 3 |
| Kyrgyzstan | 3 |
| Mongolia | 3 |
| Tajikistan | 3 |
| Turkmenistan | 3 |
| Uzbekistan | 3 |
| Central Europe | 2 |
| Albania | 3 |
| Bosnia and Herzegovina | 3 |
| Bulgaria | 3 |
| Croatia | 3 |
| Czech Republic | 3 |
| Hungary | 3 |
| Macedonia | 3 |
| Montenegro | 3 |
| Poland | 3 |
| Romania | 3 |
| Serbia | 3 |
| Slovakia | 3 |
| Slovenia | 3 |
| Eastern Europe | 2 |
| Belarus | 3 |
| Estonia | 3 |
| Latvia | 3 |
| Lithuania | 3 |
| Moldova | 3 |
| Russian Federation | 3 |
| Ukraine | 3 |
| High-income | 1 |
| Australasia | 2 |
| Australia | 3 |
| New Zealand | 3 |
| High-income Asia-Pacific | 2 |
| Brunei | 3 |

| Appendix Table 7. GBD location hierarchy with levels | |
|---|---|
| **Geography** | **Level** |
| Japan | 3 |
| Aichi | 4 |
| Akita | 4 |
| Aomori | 4 |
| Chiba | 4 |
| Ehime | 4 |
| Fukui | 4 |
| Fukuoka | 4 |
| Fukushima | 4 |
| Gifu | 4 |
| Gunma | 4 |
| Hiroshima | 4 |
| Hokkaidō | 4 |
| Hyōgo | 4 |
| Ibaraki | 4 |
| Ishikawa | 4 |
| Iwate | 4 |
| Kagawa | 4 |
| Kagoshima | 4 |
| Kanagawa | 4 |
| Kōchi | 4 |
| Kumamoto | 4 |
| Kyōto | 4 |
| Mie | 4 |
| Miyagi | 4 |
| Miyazaki | 4 |
| Nagano | 4 |
| Nagasaki | 4 |
| Nara | 4 |
| Niigata | 4 |
| Ōita | 4 |
| Okayama | 4 |
| Okinawa | 4 |
| Ōsaka | 4 |
| Saga | 4 |
| Saitama | 4 |
| Shiga | 4 |
| Shimane | 4 |
| Shizuoka | 4 |
| Tochigi | 4 |
| Tokushima | 4 |
| Tōkyō | 4 |
| Tottori | 4 |
| Toyama | 4 |
| Wakayama | 4 |

| Appendix Table 7. GBD location hierarchy with levels | |
|---|---|
| **Geography** | **Level** |
| Yamagata | 4 |
| Yamaguchi | 4 |
| Yamanashi | 4 |
| South Korea | 3 |
| Singapore | 3 |
| High-income North America | 2 |
| Canada | 3 |
| Greenland | 3 |
| USA | 3 |
| Alabama | 4 |
| Alaska | 4 |
| Arizona | 4 |
| Arkansas | 4 |
| California | 4 |
| Colorado | 4 |
| Connecticut | 4 |
| Delaware | 4 |
| Washington, DC | 4 |
| Florida | 4 |
| Georgia | 4 |
| Hawaii | 4 |
| Idaho | 4 |
| Illinois | 4 |
| Indiana | 4 |
| Iowa | 4 |
| Kansas | 4 |
| Kentucky | 4 |
| Louisiana | 4 |
| Maine | 4 |
| Maryland | 4 |
| Massachusetts | 4 |
| Michigan | 4 |
| Minnesota | 4 |
| Mississippi | 4 |
| Missouri | 4 |
| Montana | 4 |
| Nebraska | 4 |
| Nevada | 4 |
| New Hampshire | 4 |
| New Jersey | 4 |
| New Mexico | 4 |
| New York | 4 |
| North Carolina | 4 |
| North Dakota | 4 |
| Ohio | 4 |

**Appendix Table 7. GBD location hierarchy with levels**

| Geography | Level |
|---|---|
| Oklahoma | 4 |
| Oregon | 4 |
| Pennsylvania | 4 |
| Rhode Island | 4 |
| South Carolina | 4 |
| South Dakota | 4 |
| Tennessee | 4 |
| Texas | 4 |
| Utah | 4 |
| Vermont | 4 |
| Virginia | 4 |
| Washington | 4 |
| West Virginia | 4 |
| Wisconsin | 4 |
| Wyoming | 4 |
| Southern Latin America | 2 |
| Argentina | 3 |
| Chile | 3 |
| Uruguay | 3 |
| Western Europe | 2 |
| Andorra | 3 |
| Austria | 3 |
| Belgium | 3 |
| Cyprus | 3 |
| Denmark | 3 |
| Finland | 3 |
| France | 3 |
| Germany | 3 |
| Greece | 3 |
| Iceland | 3 |
| Ireland | 3 |
| Israel | 3 |
| Italy | 3 |
| Luxembourg | 3 |
| Malta | 3 |
| Netherlands | 3 |
| Norway | 3 |
| Portugal | 3 |
| Spain | 3 |
| Sweden | 3 |
| Stockholm | 4 |
| Sweden except Stockholm | 4 |
| Switzerland | 3 |
| United Kingdom | 3 |
| England | 4 |

## Appendix Table 7. GBD location hierarchy with levels

| Geography | Level |
|---|---|
| East Midlands | 5 |
| Derby | 6 |
| Derbyshire | 6 |
| Leicester | 6 |
| Leicestershire | 6 |
| Lincolnshire | 6 |
| Northamptonshire | 6 |
| Nottingham | 6 |
| Nottinghamshire | 6 |
| Rutland | 6 |
| East of England | 5 |
| Bedford | 6 |
| Cambridgeshire | 6 |
| Central Bedfordshire | 6 |
| Essex | 6 |
| Hertfordshire | 6 |
| Luton | 6 |
| Norfolk | 6 |
| Peterborough | 6 |
| Southend-on-Sea | 6 |
| Suffolk | 6 |
| Thurrock | 6 |
| Greater London | 5 |
| Barking and Dagenham | 6 |
| Barnet | 6 |
| Bexley | 6 |
| Brent | 6 |
| Bromley | 6 |
| Camden | 6 |
| Croydon | 6 |
| Ealing | 6 |
| Enfield | 6 |
| Greenwich | 6 |
| Hackney | 6 |
| Hammersmith and Fulham | 6 |
| Haringey | 6 |
| Harrow | 6 |
| Havering | 6 |
| Hillingdon | 6 |
| Hounslow | 6 |
| Islington | 6 |
| Kensington and Chelsea | 6 |
| Kingston upon Thames | 6 |
| Lambeth | 6 |
| Lewisham | 6 |

**Appendix Table 7. GBD location hierarchy with levels**

| Geography | Level |
|---|---|
| Merton | 6 |
| Newham | 6 |
| Redbridge | 6 |
| Richmond upon Thames | 6 |
| Southwark | 6 |
| Sutton | 6 |
| Tower Hamlets | 6 |
| Waltham Forest | 6 |
| Wandsworth | 6 |
| Westminster | 6 |
| North East England | 5 |
| County Durham | 6 |
| Darlington | 6 |
| Gateshead | 6 |
| Hartlepool | 6 |
| Middlesbrough | 6 |
| Newcastle upon Tyne | 6 |
| North Tyneside | 6 |
| Northumberland | 6 |
| Redcar and Cleveland | 6 |
| South Tyneside | 6 |
| Stockton-on-Tees | 6 |
| Sunderland | 6 |
| North West England | 5 |
| Blackburn with Darwen | 6 |
| Blackpool | 6 |
| Bolton | 6 |
| Bury | 6 |
| Cheshire East | 6 |
| Cheshire West and Chester | 6 |
| Cumbria | 6 |
| Halton | 6 |
| Knowsley | 6 |
| Lancashire | 6 |
| Liverpool | 6 |
| Manchester | 6 |
| Oldham | 6 |
| Rochdale | 6 |
| Salford | 6 |
| Sefton | 6 |
| St Helens | 6 |
| Stockport | 6 |
| Tameside | 6 |
| Trafford | 6 |
| Warrington | 6 |

| Appendix Table 7. GBD location hierarchy with levels | |
|---|---|
| **Geography** | **Level** |
| Wigan | 6 |
| Wirral | 6 |
| South East England | 5 |
| Bracknell Forest | 6 |
| Brighton and Hove | 6 |
| Buckinghamshire | 6 |
| East Sussex | 6 |
| Hampshire | 6 |
| Isle of Wight | 6 |
| Kent | 6 |
| Medway | 6 |
| Milton Keynes | 6 |
| Oxfordshire | 6 |
| Portsmouth | 6 |
| Reading | 6 |
| Slough | 6 |
| Southampton | 6 |
| Surrey | 6 |
| West Berkshire | 6 |
| West Sussex | 6 |
| Windsor and Maidenhead | 6 |
| Wokingham | 6 |
| South West England | 5 |
| Bath and North East Somerset | 6 |
| Bournemouth | 6 |
| Bristol, City of | 6 |
| Cornwall | 6 |
| Devon | 6 |
| Dorset | 6 |
| Gloucestershire | 6 |
| North Somerset | 6 |
| Plymouth | 6 |
| Poole | 6 |
| Somerset | 6 |
| South Gloucestershire | 6 |
| Swindon | 6 |
| Torbay | 6 |
| Wiltshire | 6 |
| West Midlands | 5 |
| Birmingham | 6 |
| Coventry | 6 |
| Dudley | 6 |
| Herefordshire, County of | 6 |
| Sandwell | 6 |
| Shropshire | 6 |

| Appendix Table 7. GBD location hierarchy with levels | |
|---|---|
| Geography | Level |
| Solihull | 6 |
| Staffordshire | 6 |
| Stoke-on-Trent | 6 |
| Telford and Wrekin | 6 |
| Walsall | 6 |
| Warwickshire | 6 |
| Wolverhampton | 6 |
| Worcestershire | 6 |
| Yorkshire and the Humber | 5 |
| Barnsley | 6 |
| Bradford | 6 |
| Calderdale | 6 |
| Doncaster | 6 |
| East Riding of Yorkshire | 6 |
| Kingston upon Hull, City of | 6 |
| Kirklees | 6 |
| Leeds | 6 |
| North East Lincolnshire | 6 |
| North Lincolnshire | 6 |
| North Yorkshire | 6 |
| Rotherham | 6 |
| Sheffield | 6 |
| Wakefield | 6 |
| York | 6 |
| Northern Ireland | 4 |
| Scotland | 4 |
| Wales | 4 |
| Latin America and Caribbean | 1 |
| Andean Latin America | 2 |
| Bolivia | 3 |
| Ecuador | 3 |
| Peru | 3 |
| Caribbean | 2 |
| Antigua and Barbuda | 3 |
| The Bahamas | 3 |
| Barbados | 3 |
| Belize | 3 |
| Bermuda | 3 |
| Cuba | 3 |
| Dominica | 3 |
| Dominican Republic | 3 |
| Grenada | 3 |
| Guyana | 3 |
| Haiti | 3 |
| Jamaica | 3 |

## Appendix Table 7. GBD location hierarchy with levels

| Geography | Level |
|---|---|
| Puerto Rico | 3 |
| Saint Lucia | 3 |
| Saint Vincent and the Grenadines | 3 |
| Suriname | 3 |
| Trinidad and Tobago | 3 |
| Virgin Islands | 3 |
| Central Latin America | 2 |
| Colombia | 3 |
| Costa Rica | 3 |
| El Salvador | 3 |
| Guatemala | 3 |
| Honduras | 3 |
| Mexico | 3 |
| Aguascalientes | 4 |
| Baja California | 4 |
| Baja California Sur | 4 |
| Campeche | 4 |
| Chiapas | 4 |
| Chihuahua | 4 |
| Coahuila | 4 |
| Colima | 4 |
| Mexico City | 4 |
| Durango | 4 |
| Guanajuato | 4 |
| Guerrero | 4 |
| Hidalgo | 4 |
| Jalisco | 4 |
| México | 4 |
| Michoacán de Ocampo | 4 |
| Morelos | 4 |
| Nayarit | 4 |
| Nuevo León | 4 |
| Oaxaca | 4 |
| Puebla | 4 |
| Querétaro | 4 |
| Quintana Roo | 4 |
| San Luis Potosí | 4 |
| Sinaloa | 4 |
| Sonora | 4 |
| Tabasco | 4 |
| Tamaulipas | 4 |
| Tlaxcala | 4 |
| Veracruz de Ignacio de la Llave | 4 |
| Yucatán | 4 |
| Zacatecas | 4 |

| Appendix Table 7. GBD location hierarchy with levels | |
|---|---|
| **Geography** | **Level** |
| Nicaragua | 3 |
| Panama | 3 |
| Venezuela | 3 |
| Tropical Latin America | 2 |
| Brazil | 3 |
| Acre | 4 |
| Alagoas | 4 |
| Amapá | 4 |
| Amazonas | 4 |
| Bahia | 4 |
| Ceará | 4 |
| Distrito Federal | 4 |
| Espírito Santo | 4 |
| Goiás | 4 |
| Maranhão | 4 |
| Mato Grosso | 4 |
| Mato Grosso do Sul | 4 |
| Minas Gerais | 4 |
| Pará | 4 |
| Paraíba | 4 |
| Paraná | 4 |
| Pernambuco | 4 |
| Piauí | 4 |
| Rio de Janeiro | 4 |
| Rio Grande do Norte | 4 |
| Rio Grande do Sul | 4 |
| Rondônia | 4 |
| Roraima | 4 |
| Santa Catarina | 4 |
| São Paulo | 4 |
| Sergipe | 4 |
| Tocantins | 4 |
| Paraguay | 3 |
| North Africa and Middle East | 1 |
| North Africa and Middle East | 2 |
| Afghanistan | 3 |
| Algeria | 3 |
| Bahrain | 3 |
| Egypt | 3 |
| Iran | 3 |
| Iraq | 3 |
| Jordan | 3 |
| Kuwait | 3 |
| Lebanon | 3 |
| Libya | 3 |

| Appendix Table 7. GBD location hierarchy with levels | |
|---|---|
| **Geography** | **Level** |
| Morocco | 3 |
| Palestine | 3 |
| Oman | 3 |
| Qatar | 3 |
| Saudi Arabia | 3 |
| Sudan | 3 |
| Syria | 3 |
| Tunisia | 3 |
| Turkey | 3 |
| United Arab Emirates | 3 |
| Yemen | 3 |
| South Asia | 1 |
| South Asia | 2 |
| Bangladesh | 3 |
| Bhutan | 3 |
| India | 3 |
| Andhra Pradesh | 4 |
| Arunachal Pradesh | 4 |
| Assam | 4 |
| Bihar | 4 |
| Chhattisgarh | 4 |
| Delhi | 4 |
| Goa | 4 |
| Gujarat | 4 |
| Haryana | 4 |
| Himachal Pradesh | 4 |
| Jammu and Kashmir | 4 |
| Jharkhand | 4 |
| Karnataka | 4 |
| Kerala | 4 |
| Madhya Pradesh | 4 |
| Maharashtra | 4 |
| Manipur | 4 |
| Meghalaya | 4 |
| Mizoram | 4 |
| Nagaland | 4 |
| Odisha | 4 |
| Punjab | 4 |
| Rajasthan | 4 |
| Sikkim | 4 |
| Tamil Nadu | 4 |
| Telangana | 4 |
| Tripura | 4 |
| Uttar Pradesh | 4 |
| Uttarakhand | 4 |

**Appendix Table 7. GBD location hierarchy with levels**

| Geography | Level |
|---|---|
| West Bengal | 4 |
| Union Territories other than Delhi | 4 |
| Nepal | 3 |
| Pakistan | 3 |
| Southeast Asia, East Asia, and Oceania | 1 |
| East Asia | 2 |
| China | 3 |
| North Korea | 3 |
| Taiwan (Province of China) | 3 |
| Oceania | 2 |
| American Samoa | 3 |
| Federated States of Micronesia | 3 |
| Fiji | 3 |
| Guam | 3 |
| Kiribati | 3 |
| Marshall Islands | 3 |
| Northern Mariana Islands | 3 |
| Papua New Guinea | 3 |
| Samoa | 3 |
| Solomon Islands | 3 |
| Tonga | 3 |
| Vanuatu | 3 |
| Southeast Asia | 2 |
| Cambodia | 3 |
| Indonesia | 3 |
| Laos | 3 |
| Malaysia | 3 |
| Maldives | 3 |
| Mauritius | 3 |
| Myanmar | 3 |
| Philippines | 3 |
| Sri Lanka | 3 |
| Seychelles | 3 |
| Thailand | 3 |
| Timor-Leste | 3 |
| Vietnam | 3 |
| Sub-Saharan Africa | 1 |
| Central sub-Saharan Africa | 2 |
| Angola | 3 |
| Central African Republic | 3 |
| Congo (Brazzaville) | 3 |
| DR Congo | 3 |
| Equatorial Guinea | 3 |
| Gabon | 3 |
| Eastern sub-Saharan Africa | 2 |

**Appendix Table 7. GBD location hierarchy with levels**

| Geography | Level |
|---|---|
| Burundi | 3 |
| Comoros | 3 |
| Djibouti | 3 |
| Eritrea | 3 |
| Ethiopia | 3 |
| Kenya | 3 |
| Baringo | 4 |
| Bomet | 4 |
| Bungoma | 4 |
| Busia | 4 |
| Elgeyo Marakwet | 4 |
| Embu | 4 |
| Garissa | 4 |
| Homa Bay | 4 |
| Isiolo | 4 |
| Kajiado | 4 |
| Kakamega | 4 |
| Kericho | 4 |
| Kiambu | 4 |
| Kilifi | 4 |
| Kirinyaga | 4 |
| Kisii | 4 |
| Kisumu | 4 |
| Kitui | 4 |
| Kwale | 4 |
| Laikipia | 4 |
| Lamu | 4 |
| Machakos | 4 |
| Makueni | 4 |
| Mandera | 4 |
| Marsabit | 4 |
| Meru | 4 |
| Migori | 4 |
| Mombasa | 4 |
| Murang’a | 4 |
| Nairobi | 4 |
| Nakuru | 4 |
| Nandi | 4 |
| Narok | 4 |
| Nyamira | 4 |
| Nyandarua | 4 |
| Nyeri | 4 |
| Samburu | 4 |
| Siaya | 4 |
| Taita Taveta | 4 |

| Appendix Table 7. GBD location hierarchy with levels | |
|---|---|
| **Geography** | **Level** |
| Tana River | 4 |
| Tharaka Nithi | 4 |
| Trans Nzoia | 4 |
| Turkana | 4 |
| Uasin Gishu | 4 |
| Vihiga | 4 |
| Wajir | 4 |
| West Pokot | 4 |
| Madagascar | 3 |
| Malawi | 3 |
| Mozambique | 3 |
| Rwanda | 3 |
| Somalia | 3 |
| South Sudan | 3 |
| Tanzania | 3 |
| Uganda | 3 |
| Zambia | 3 |
| Southern sub-Saharan Africa | 2 |
| Botswana | 3 |
| Lesotho | 3 |
| Namibia | 3 |
| South Africa | 3 |
| Swaziland | 3 |
| Zimbabwe | 3 |
| Western sub-Saharan Africa | 2 |
| Benin | 3 |
| Burkina Faso | 3 |
| Cameroon | 3 |
| Cape Verde | 3 |
| Chad | 3 |
| Cote d'Ivoire | 3 |
| The Gambia | 3 |
| Ghana | 3 |
| Guinea | 3 |
| Guinea-Bissau | 3 |
| Liberia | 3 |
| Mali | 3 |
| Mauritania | 3 |
| Niger | 3 |
| Nigeria | 3 |
| Sao Tome and Principe | 3 |
| Senegal | 3 |
| Sierra Leone | 3 |
| Togo | 3 |

**Appendix Table 8. Mediation factors**

For IHD, stroke, and diabetes we pooled all available cohorts and estimated relative risks with and without adjustment across all combinations of metabolic risk factors. We then computed the excess attenuated risk for each mediation-risk-cause set.

| Risk Factor | Mediator | Cause | Mediation Factor |
|---|---|---|---|
| Lead exposure in bone | High systolic blood pressure | Rheumatic heart disease | 1<br>(1 to 1) |
| Lead exposure in bone | High systolic blood pressure | Ischaemic heart disease | 1<br>(1 to 1) |
| Lead exposure in bone | High systolic blood pressure | Ischaemic stroke | 1<br>(1 to 1) |
| Lead exposure in bone | High systolic blood pressure | Intracerebral hemorrhage | 1<br>(1 to 1) |
| Lead exposure in bone | High systolic blood pressure | Subarachnoid hemorrhage | 1<br>(1 to 1) |
| Lead exposure in bone | High systolic blood pressure | Hypertensive heart disease | 1<br>(1 to 1) |
| Lead exposure in bone | High systolic blood pressure | Other cardiomyopathy | 1<br>(1 to 1) |
| Lead exposure in bone | High systolic blood pressure | Atrial fibrillation and flutter | 1<br>(1 to 1) |
| Lead exposure in bone | High systolic blood pressure | Aortic aneurysm | 1<br>(1 to 1) |
| Lead exposure in bone | High systolic blood pressure | Peripheral vascular disease | 1<br>(1 to 1) |
| Lead exposure in bone | High systolic blood pressure | Other cardiovascular and circulatory diseases | 1<br>(1 to 1) |
| Lead exposure in bone | High systolic blood pressure | Chronic kidney disease due to diabetes mellitus type 1 | 1<br>(1 to 1) |
| Lead exposure in bone | High systolic blood pressure | Chronic kidney disease due to diabetes mellitus type 2 | 1<br>(1 to 1) |
| Lead exposure in bone | High systolic blood pressure | Chronic kidney disease due to hypertension | 1<br>(1 to 1) |
| Lead exposure in bone | High systolic blood pressure | Chronic kidney disease due to glomerulonephritis | 1<br>(1 to 1) |
| Lead exposure in bone | High systolic blood pressure | Chronic kidney disease due to other and unspecified causes | 1<br>(1 to 1) |
| Lead exposure in bone | Impaired kidney function | Chronic kidney disease due to diabetes mellitus type 1 | 1<br>(1 to 1) |
| Lead exposure in bone | Impaired kidney function | Chronic kidney disease due to diabetes mellitus type 2 | 1<br>(1 to 1) |
| Lead exposure in bone | Impaired kidney function | Chronic kidney disease due to hypertension | 1<br>(1 to 1) |
| Lead exposure in bone | Impaired kidney function | Chronic kidney disease due to glomerulonephritis | 1<br>(1 to 1) |
| Lead exposure in bone | Impaired kidney function | Chronic kidney disease due to other and unspecified causes | 1<br>(1 to 1) |
| Smoking | High fasting plasma glucose | Diabetes mellitus type 2 | 1<br>(1 to 1) |
| Smoking | Low bone mineral density | Pedestrian road injuries | 1<br>(1 to 1) |
| Smoking | Low bone mineral density | Cyclist road injuries | 1<br>(1 to 1) |
| Smoking | Low bone mineral density | Motor vehicle road injuries | 1<br>(1 to 1) |
| Smoking | Low bone mineral density | Other road injuries | 1<br>(1 to 1) |
| Smoking | Low bone mineral density | Other transport injuries | 1<br>(1 to 1) |
| Smoking | Low bone mineral density | Falls | 1<br>(1 to 1) |
| Smoking | Low bone mineral density | Other exposure to mechanical forces | 1<br>(1 to 1) |
| Smoking | Low bone mineral density | Non-venomous animal contact | 1<br>(1 to 1) |
| Smoking | Low bone mineral density | Assault by other means | 1<br>(1 to 1) |
| Diet low in fruits | High fasting plasma glucose | Ischaemic stroke | 0.05<br>(0.04 to 0.06) |

**Appendix Table 8. Mediation factors**

For IHD, stroke, and diabetes we pooled all available cohorts and estimated relative risks with and without adjustment across all combinations of metabolic risk factors. We then computed the excess attenuated risk for each mediation-risk-cause set.

| Risk Factor | Mediator | Cause | Mediation Factor |
|---|---|---|---|
| Diet low in fruits | High fasting plasma glucose | Diabetes mellitus type 2 | 0.99 (0.99 to 0.99) |
| Diet low in fruits | High LDL cholesterol | Ischaemic heart disease | 0.06 (0.05 to 0.08) |
| Diet low in fruits | High LDL cholesterol | Ischaemic stroke | 0.05 (0.04 to 0.06) |
| Diet low in fruits | High systolic blood pressure | Ischaemic heart disease | 0.06 (0.05 to 0.08) |
| Diet low in fruits | High systolic blood pressure | Ischaemic stroke | 0.05 (0.04 to 0.06) |
| Diet low in fruits | High systolic blood pressure | Intracerebral hemorrhage | 0.02 (0.02 to 0.03) |
| Diet low in fruits | High systolic blood pressure | Subarachnoid hemorrhage | 0.02 (0.02 to 0.03) |
| Diet low in vegetables | High fasting plasma glucose | Ischaemic heart disease | 0.06 (0.01 to 0.2) |
| Diet low in vegetables | High fasting plasma glucose | Ischaemic stroke | 0.08 (0.04 to 0.16) |
| Diet low in vegetables | High fasting plasma glucose | Intracerebral hemorrhage | 0.08 (0.04 to 0.16) |
| Diet low in vegetables | High fasting plasma glucose | Subarachnoid hemorrhage | 0.08 (0.04 to 0.16) |
| Diet low in vegetables | High LDL cholesterol | Ischaemic heart disease | 0.04 (0.03 to 0.05) |
| Diet low in vegetables | High LDL cholesterol | Ischaemic stroke | 0.09 (0.04 to 0.16) |
| Diet low in vegetables | High systolic blood pressure | Ischaemic heart disease | 0.04 (0.03 to 0.05) |
| Diet low in vegetables | High systolic blood pressure | Ischaemic stroke | 0.03 (0.02 to 0.04) |
| Diet low in vegetables | High systolic blood pressure | Intracerebral hemorrhage | 0.04 (0.02 to 0.05) |
| Diet low in vegetables | High systolic blood pressure | Subarachnoid hemorrhage | 0.04 (0.02 to 0.05) |
| Diet low in whole grains | High fasting plasma glucose | Diabetes mellitus type 2 | 1 (1 to 1) |
| Diet low in whole grains | High LDL cholesterol | Ischaemic heart disease | 0.39 (0.17 to 0.54) |
| Diet low in whole grains | High LDL cholesterol | Ischaemic stroke | 0.16 (0.05 to 0.37) |
| Diet low in nuts and seeds | High fasting plasma glucose | Ischaemic heart disease | 0.03 (0.02 to 0.06) |
| Diet low in nuts and seeds | High fasting plasma glucose | Diabetes mellitus type 2 | 0.99 (0.99 to 0.99) |
| Diet low in nuts and seeds | High LDL cholesterol | Ischaemic heart disease | 0.28 (0.01 to 1.62) |
| Diet low in nuts and seeds | High systolic blood pressure | Ischaemic heart disease | 0.34 (0.24 to 0.47) |
| Diet low in milk | Diet low in calcium | Colon and rectum cancer | 1 (1 to 1) |
| Diet high in red meat | High fasting plasma glucose | Diabetes mellitus type 2 | 1 (1 to 1) |
| Diet high in processed meat | High fasting plasma glucose | Ischaemic heart disease | 0.01 (0.01 to 0.02) |
| Diet high in processed meat | High fasting plasma glucose | Diabetes mellitus type 2 | 1 (1 to 1) |
| Diet high in sugar-sweetened beverages | High fasting plasma glucose | Ischaemic heart disease | 0.15 (0.1 to 0.2) |
| Diet high in sugar-sweetened beverages | High fasting plasma glucose | Diabetes mellitus type 2 | 1 (1 to 1) |
| Diet high in sugar-sweetened beverages | High LDL cholesterol | Ischaemic heart disease | 0.1 (0.05 to 0.15) |
| Diet high in sugar-sweetened beverages | High systolic blood pressure | Ischaemic heart disease | 0.31 (0.28 to 0.34) |

**Appendix Table 8. Mediation factors**

For IHD, stroke, and diabetes we pooled all available cohorts and estimated relative risks with and without adjustment across all combinations of metabolic risk factors. We then computed the excess attenuated risk for each mediation-risk-cause set.

| Risk Factor | Mediator | Cause | Mediation Factor |
|---|---|---|---|
| Diet high in sugar-sweetened beverages | High body-mass index | Ischaemic heart disease | 1 (1 to 1) |
| Diet high in sugar-sweetened beverages | High body-mass index | Diabetes mellitus type 2 | 1 (1 to 1) |
| Diet low in fibre | Diet low in fruits | Ischaemic heart disease | 1 (1 to 1) |
| Diet low in fibre | Diet low in vegetables | Ischaemic heart disease | 1 (1 to 1) |
| Diet low in fibre | Diet low in whole grains | Ischaemic heart disease | 1 (1 to 1) |
| Diet low in seafood omega-3 fatty acids | High systolic blood pressure | Ischaemic heart disease | 0.01 (0 to 0.02) |
| Diet low in polyunsaturated fatty acids | High fasting plasma glucose | Ischaemic heart disease | 0.57 (0.39 to 0.77) |
| Diet low in polyunsaturated fatty acids | High systolic blood pressure | Ischaemic heart disease | 0.72 (0.57 to 0.89) |
| Diet high in trans fatty acids | High LDL cholesterol | Ischaemic heart disease | 0.15 (0.02 to 0.24) |
| Diet high in trans fatty acids | High systolic blood pressure | Ischaemic heart disease | 0.15 (0.02 to 0.24) |
| Diet high in sodium | High systolic blood pressure | Rheumatic heart disease | 1 (1 to 1) |
| Diet high in sodium | High systolic blood pressure | Ischaemic heart disease | 1 (1 to 1) |
| Diet high in sodium | High systolic blood pressure | Ischaemic stroke | 1 (1 to 1) |
| Diet high in sodium | High systolic blood pressure | Intracerebral hemorrhage | 1 (1 to 1) |
| Diet high in sodium | High systolic blood pressure | Subarachnoid hemorrhage | 1 (1 to 1) |
| Diet high in sodium | High systolic blood pressure | Hypertensive heart disease | 1 (1 to 1) |
| Diet high in sodium | High systolic blood pressure | Other cardiomyopathy | 1 (1 to 1) |
| Diet high in sodium | High systolic blood pressure | Atrial fibrillation and flutter | 1 (1 to 1) |
| Diet high in sodium | High systolic blood pressure | Aortic aneurysm | 1 (1 to 1) |
| Diet high in sodium | High systolic blood pressure | Peripheral vascular disease | 1 (1 to 1) |
| Diet high in sodium | High systolic blood pressure | Other cardiovascular and circulatory diseases | 1 (1 to 1) |
| Diet high in sodium | High systolic blood pressure | Chronic kidney disease due to diabetes mellitus type 1 | 1 (1 to 1) |
| Diet high in sodium | High systolic blood pressure | Chronic kidney disease due to diabetes mellitus type 2 | 1 (1 to 1) |
| Diet high in sodium | High systolic blood pressure | Chronic kidney disease due to hypertension | 1 (1 to 1) |
| Diet high in sodium | High systolic blood pressure | Chronic kidney disease due to glomerulonephritis | 1 (1 to 1) |
| Diet high in sodium | High systolic blood pressure | Chronic kidney disease due to other and unspecified causes | 1 (1 to 1) |
| Diet high in sodium | Impaired kidney function | Chronic kidney disease due to diabetes mellitus type 1 | 1 (1 to 1) |
| Diet high in sodium | Impaired kidney function | Chronic kidney disease due to diabetes mellitus type 2 | 1 (1 to 1) |
| Diet high in sodium | Impaired kidney function | Chronic kidney disease due to hypertension | 1 (1 to 1) |
| Diet high in sodium | Impaired kidney function | Chronic kidney disease due to glomerulonephritis | 1 (1 to 1) |
| Diet high in sodium | Impaired kidney function | Chronic kidney disease due to other and unspecified causes | 1 (1 to 1) |
| Childhood sexual abuse | Alcohol use | Alcohol use disorders | 1 (1 to 1) |

**Appendix Table 8. Mediation factors**

For IHD, stroke, and diabetes we pooled all available cohorts and estimated relative risks with and without adjustment across all combinations of metabolic risk factors. We then computed the excess attenuated risk for each mediation-risk-cause set.

| Risk Factor | Mediator | Cause | Mediation Factor |
|---|---|---|---|
| Low physical activity | High fasting plasma glucose | Ischaemic heart disease | 0.14<br>(0.11 to 0.18) |
| Low physical activity | High fasting plasma glucose | Ischaemic stroke | 0.08<br>(0.03 to 0.14) |
| Low physical activity | High fasting plasma glucose | Diabetes mellitus type 2 | 1<br>(1 to 1) |
| High fasting plasma glucose | High LDL cholesterol | Ischaemic heart disease | 0.04<br>(0.02 to 0.05) |
| High fasting plasma glucose | High LDL cholesterol | Ischaemic stroke | 0.04<br>(0.03 to 0.06) |
| High fasting plasma glucose | High systolic blood pressure | Ischaemic heart disease | 0.1<br>(0.08 to 0.11) |
| High fasting plasma glucose | High systolic blood pressure | Ischaemic stroke | 0.15<br>(0.14 to 0.17) |
| High fasting plasma glucose | High systolic blood pressure | Intracerebral hemorrhage | 0.15<br>(0.14 to 0.17) |
| High fasting plasma glucose | High systolic blood pressure | Subarachnoid hemorrhage | 0.15<br>(0.14 to 0.17) |
| High fasting plasma glucose | Impaired kidney function | Chronic kidney disease due to diabetes mellitus type 1 | 1<br>(1 to 1) |
| High fasting plasma glucose | Impaired kidney function | Chronic kidney disease due to diabetes mellitus type 2 | 1<br>(1 to 1) |
| High fasting plasma glucose | Impaired kidney function | Chronic kidney disease due to hypertension | 1<br>(1 to 1) |
| High fasting plasma glucose | Impaired kidney function | Chronic kidney disease due to glomerulonephritis | 1<br>(1 to 1) |
| High fasting plasma glucose | Impaired kidney function | Chronic kidney disease due to other and unspecified causes | 1<br>(1 to 1) |
| High LDL cholesterol | High systolic blood pressure | Ischaemic heart disease | 0.09<br>(0.07 to 0.11) |
| High LDL cholesterol | High systolic blood pressure | Ischaemic stroke | 0.16<br>(0.14 to 0.18) |
| High systolic blood pressure | Impaired kidney function | Chronic kidney disease due to diabetes mellitus type 1 | 1<br>(1 to 1) |
| High systolic blood pressure | Impaired kidney function | Chronic kidney disease due to diabetes mellitus type 2 | 1<br>(1 to 1) |
| High systolic blood pressure | Impaired kidney function | Chronic kidney disease due to hypertension | 1<br>(1 to 1) |
| High systolic blood pressure | Impaired kidney function | Chronic kidney disease due to glomerulonephritis | 1<br>(1 to 1) |
| High systolic blood pressure | Impaired kidney function | Chronic kidney disease due to other and unspecified causes | 1<br>(1 to 1) |
| High body-mass index | High fasting plasma glucose | Ischaemic heart disease | 0.15<br>(0.1 to 0.2) |
| High body-mass index | High fasting plasma glucose | Ischaemic stroke | 0.22<br>(0.12 to 0.31) |
| High body-mass index | High fasting plasma glucose | Intracerebral hemorrhage | 0.22<br>(0.13 to 0.32) |
| High body-mass index | High fasting plasma glucose | Subarachnoid hemorrhage | 0.22<br>(0.13 to 0.32) |
| High body-mass index | High fasting plasma glucose | Diabetes mellitus type 2 | 1<br>(1 to 1) |
| High body-mass index | High LDL cholesterol | Ischaemic heart disease | 0.1<br>(0.05 to 0.15) |
| High body-mass index | High LDL cholesterol | Ischaemic stroke | 0.03<br>(0 to 0.08) |
| High body-mass index | High systolic blood pressure | Ischaemic heart disease | 0.31<br>(0.28 to 0.34) |
| High body-mass index | High systolic blood pressure | Ischaemic stroke | 0.65<br>(0.57 to 0.72) |
| High body-mass index | High systolic blood pressure | Intracerebral hemorrhage | 0.65<br>(0.58 to 0.73) |
| High body-mass index | High systolic blood pressure | Subarachnoid hemorrhage | 0.65<br>(0.58 to 0.73) |

**Appendix Table 8. Mediation factors**

For IHD, stroke, and diabetes we pooled all available cohorts and estimated relative risks with and without adjustment across all combinations of metabolic risk factors. We then computed the excess attenuated risk for each mediation-risk-cause set.

| Risk Factor | Mediator | Cause | Mediation Factor |
|---|---|---|---|
| High body-mass index | High systolic blood pressure | Hypertensive heart disease | 1<br>(1 to 1) |
| High body-mass index | High systolic blood pressure | Atrial fibrillation and flutter | 0.31<br>(0.28 to 0.34) |
| High body-mass index | Impaired kidney function | Chronic kidney disease due to diabetes mellitus type 2 | 1<br>(1 to 1) |
| High body-mass index | Impaired kidney function | Chronic kidney disease due to hypertension | 1<br>(1 to 1) |
| High body-mass index | Impaired kidney function | Chronic kidney disease due to glomerulonephritis | 1<br>(1 to 1) |
| High body-mass index | Impaired kidney function | Chronic kidney disease due to other and unspecified causes | 1<br>(1 to 1) |

| Appendix Table 9. Socio-Demographic Index groupings by geography, based on 2017 values | | |
|---|---|---|
| **Geography** | **2017 SDI** | **SDI Quintile** |
| Global | 0.652 | |
| Central Europe, Eastern Europe, and Central Asia | 0.766 | |
| Central Asia | 0.673 | |
| Armenia | 0.702 | High-middle SDI |
| Azerbaijan | 0.701 | High-middle SDI |
| Georgia | 0.7 | High-middle SDI |
| Kazakhstan | 0.735 | High-middle SDI |
| Kyrgyzstan | 0.607 | Low-middle SDI |
| Mongolia | 0.662 | Middle SDI |
| Tajikistan | 0.523 | Low-middle SDI |
| Turkmenistan | 0.696 | Middle SDI |
| Uzbekistan | 0.63 | Middle SDI |
| Central Europe | 0.814 | |
| Albania | 0.685 | Middle SDI |
| Bosnia and Herzegovina | 0.713 | High-middle SDI |
| Bulgaria | 0.792 | High-middle SDI |
| Croatia | 0.825 | High SDI |
| Czech Republic | 0.851 | High SDI |
| Hungary | 0.817 | High-middle SDI |
| Macedonia | 0.754 | High-middle SDI |
| Montenegro | 0.788 | High-middle SDI |
| Poland | 0.844 | High SDI |
| Romania | 0.784 | High-middle SDI |
| Serbia | 0.752 | High-middle SDI |
| Slovakia | 0.842 | High SDI |
| Slovenia | 0.86 | High SDI |
| Eastern Europe | 0.785 | |
| Belarus | 0.773 | High-middle SDI |
| Estonia | 0.858 | High SDI |
| Latvia | 0.825 | High SDI |
| Lithuania | 0.841 | High SDI |
| Moldova | 0.676 | Middle SDI |
| Russian Federation | 0.792 | High-middle SDI |
| Ukraine | 0.74 | High-middle SDI |
| High-income | 0.854 | |
| Australasia | 0.869 | |
| Australia | 0.873 | High SDI |
| New Zealand | 0.842 | High SDI |
| High-income Asia-Pacific | 0.869 | |
| Brunei | 0.856 | High SDI |
| Japan | 0.865 | High SDI |
| Aichi | 0.875 | High SDI |
| Akita | 0.829 | High SDI |
| Aomori | 0.825 | High SDI |
| Chiba | 0.859 | High SDI |

| Appendix Table 9. Socio-Demographic Index groupings by geography, based on 2017 values | | |
|---|---|---|
| **Geography** | **2017 SDI** | **SDI Quintile** |
| Ehime | 0.838 | High SDI |
| Fukui | 0.852 | High SDI |
| Fukuoka | 0.855 | High SDI |
| Fukushima | 0.831 | High SDI |
| Gifu | 0.849 | High SDI |
| Gunma | 0.851 | High SDI |
| Hiroshima | 0.863 | High SDI |
| Hokkaidō | 0.842 | High SDI |
| Hyōgo | 0.86 | High SDI |
| Ibaraki | 0.851 | High SDI |
| Ishikawa | 0.856 | High SDI |
| Iwate | 0.825 | High SDI |
| Kagawa | 0.85 | High SDI |
| Kagoshima | 0.83 | High SDI |
| Kanagawa | 0.875 | High SDI |
| Kōchi | 0.825 | High SDI |
| Kumamoto | 0.832 | High SDI |
| Kyōto | 0.873 | High SDI |
| Mie | 0.854 | High SDI |
| Miyagi | 0.85 | High SDI |
| Miyazaki | 0.823 | High SDI |
| Nagano | 0.851 | High SDI |
| Nagasaki | 0.826 | High SDI |
| Nara | 0.848 | High SDI |
| Niigata | 0.843 | High SDI |
| Ōita | 0.846 | High SDI |
| Okayama | 0.856 | High SDI |
| Okinawa | 0.818 | High SDI |
| Ōsaka | 0.872 | High SDI |
| Saga | 0.834 | High SDI |
| Saitama | 0.852 | High SDI |
| Shiga | 0.871 | High SDI |
| Shimane | 0.831 | High SDI |
| Shizuoka | 0.859 | High SDI |
| Tochigi | 0.853 | High SDI |
| Tokushima | 0.845 | High SDI |
| Tōkyō | 0.924 | High SDI |
| Tottori | 0.834 | High SDI |
| Toyama | 0.86 | High SDI |
| Wakayama | 0.84 | High SDI |
| Yamagata | 0.832 | High SDI |
| Yamaguchi | 0.849 | High SDI |
| Yamanashi | 0.854 | High SDI |
| South Korea | 0.872 | High SDI |
| Singapore | 0.872 | High SDI |

| Appendix Table 9. Socio-Demographic Index groupings by geography, based on 2017 values | | |
|---|---|---|
| **Geography** | **2017 SDI** | **SDI Quintile** |
| High-income North America | 0.868 | |
| Canada | 0.882 | High SDI |
| Greenland | 0.76 | High-middle SDI |
| USA | 0.867 | High SDI |
| Alabama | 0.837 | High SDI |
| Alaska | 0.861 | High SDI |
| Arizona | 0.845 | High SDI |
| Arkansas | 0.826 | High SDI |
| California | 0.872 | High SDI |
| Colorado | 0.882 | High SDI |
| Connecticut | 0.906 | High SDI |
| Delaware | 0.874 | High SDI |
| Washington, DC | 0.89 | High SDI |
| Florida | 0.864 | High SDI |
| Georgia | 0.848 | High SDI |
| Hawaii | 0.872 | High SDI |
| Idaho | 0.841 | High SDI |
| Illinois | 0.879 | High SDI |
| Indiana | 0.848 | High SDI |
| Iowa | 0.87 | High SDI |
| Kansas | 0.864 | High SDI |
| Kentucky | 0.831 | High SDI |
| Louisiana | 0.835 | High SDI |
| Maine | 0.872 | High SDI |
| Maryland | 0.896 | High SDI |
| Massachusetts | 0.913 | High SDI |
| Michigan | 0.868 | High SDI |
| Minnesota | 0.893 | High SDI |
| Mississippi | 0.819 | High SDI |
| Missouri | 0.853 | High SDI |
| Montana | 0.863 | High SDI |
| Nebraska | 0.873 | High SDI |
| Nevada | 0.847 | High SDI |
| New Hampshire | 0.904 | High SDI |
| New Jersey | 0.899 | High SDI |
| New Mexico | 0.835 | High SDI |
| New York | 0.893 | High SDI |
| North Carolina | 0.85 | High SDI |
| North Dakota | 0.88 | High SDI |
| Ohio | 0.858 | High SDI |
| Oklahoma | 0.838 | High SDI |
| Oregon | 0.871 | High SDI |
| Pennsylvania | 0.879 | High SDI |
| Rhode Island | 0.89 | High SDI |
| South Carolina | 0.846 | High SDI |

| Appendix Table 9. Socio-Demographic Index groupings by geography, based on 2017 values | | |
|---|---|---|
| **Geography** | **2017 SDI** | **SDI Quintile** |
| South Dakota | 0.86 | High SDI |
| Tennessee | 0.837 | High SDI |
| Texas | 0.838 | High SDI |
| Utah | 0.856 | High SDI |
| Vermont | 0.896 | High SDI |
| Virginia | 0.885 | High SDI |
| Washington | 0.884 | High SDI |
| West Virginia | 0.825 | High SDI |
| Wisconsin | 0.878 | High SDI |
| Wyoming | 0.869 | High SDI |
| Southern Latin America | 0.72 | |
| Argentina | 0.71 | High-middle SDI |
| Chile | 0.748 | High-middle SDI |
| Uruguay | 0.707 | High-middle SDI |
| Western Europe | 0.857 | |
| Andorra | 0.902 | High SDI |
| Austria | 0.866 | High SDI |
| Belgium | 0.886 | High SDI |
| Cyprus | 0.865 | High SDI |
| Denmark | 0.918 | High SDI |
| Finland | 0.893 | High SDI |
| France | 0.865 | High SDI |
| Germany | 0.87 | High SDI |
| Greece | 0.817 | High SDI |
| Iceland | 0.907 | High SDI |
| Ireland | 0.882 | High SDI |
| Israel | 0.816 | High-middle SDI |
| Italy | 0.843 | High SDI |
| Luxembourg | 0.916 | High SDI |
| Malta | 0.836 | High SDI |
| Netherlands | 0.912 | High SDI |
| Norway | 0.911 | High SDI |
| Portugal | 0.778 | High-middle SDI |
| Spain | 0.825 | High SDI |
| Sweden | 0.883 | High SDI |
| Stockholm | 0.914 | High SDI |
| Sweden except Stockholm | 0.873 | High SDI |
| Switzerland | 0.889 | High SDI |
| United Kingdom | 0.843 | High SDI |
| England | 0.849 | High SDI |
| East Midlands | 0.83 | High SDI |
| Derby | 0.846 | High SDI |
| Derbyshire | 0.817 | High SDI |
| Leicester | 0.839 | High SDI |
| Leicestershire | 0.846 | High SDI |

| Appendix Table 9. Socio-Demographic Index groupings by geography, based on 2017 values | | |
|---|---|---|
| **Geography** | **2017 SDI** | **SDI Quintile** |
| Lincolnshire | 0.812 | High SDI |
| Northamptonshire | 0.829 | High SDI |
| Nottingham | 0.863 | High SDI |
| Nottinghamshire | 0.814 | High SDI |
| Rutland | 0.833 | High SDI |
| East of England | 0.84 | High SDI |
| Bedford | 0.838 | High SDI |
| Cambridgeshire | 0.871 | High SDI |
| Central Bedfordshire | 0.834 | High SDI |
| Essex | 0.832 | High SDI |
| Hertfordshire | 0.87 | High SDI |
| Luton | 0.833 | High SDI |
| Norfolk | 0.826 | High SDI |
| Peterborough | 0.818 | High SDI |
| Southend-on-Sea | 0.811 | High SDI |
| Suffolk | 0.821 | High SDI |
| Thurrock | 0.807 | High SDI |
| Greater London | 0.894 | High SDI |
| Barking and Dagenham | 0.802 | High SDI |
| Barnet | 0.865 | High SDI |
| Bexley | 0.826 | High SDI |
| Brent | 0.849 | High SDI |
| Bromley | 0.848 | High SDI |
| Camden | 0.93 | High SDI |
| Croydon | 0.833 | High SDI |
| Ealing | 0.865 | High SDI |
| Enfield | 0.839 | High SDI |
| Greenwich | 0.833 | High SDI |
| Hackney | 0.887 | High SDI |
| Hammersmith and Fulham | 0.927 | High SDI |
| Haringey | 0.854 | High SDI |
| Harrow | 0.848 | High SDI |
| Havering | 0.824 | High SDI |
| Hillingdon | 0.882 | High SDI |
| Hounslow | 0.879 | High SDI |
| Islington | 0.922 | High SDI |
| Kensington and Chelsea | 0.932 | High SDI |
| Kingston upon Thames | 0.89 | High SDI |
| Lambeth | 0.9 | High SDI |
| Lewisham | 0.843 | High SDI |
| Merton | 0.873 | High SDI |
| Newham | 0.838 | High SDI |
| Redbridge | 0.831 | High SDI |
| Richmond upon Thames | 0.902 | High SDI |
| Southwark | 0.912 | High SDI |

| Appendix Table 9. Socio-Demographic Index groupings by geography, based on 2017 values | | |
|---|---|---|
| **Geography** | **2017 SDI** | **SDI Quintile** |
| Sutton | 0.843 | High SDI |
| Tower Hamlets | 0.905 | High SDI |
| Waltham Forest | 0.819 | High SDI |
| Wandsworth | 0.911 | High SDI |
| Westminster | 0.927 | High SDI |
| North East England | 0.821 | High SDI |
| County Durham | 0.81 | High SDI |
| Darlington | 0.825 | High SDI |
| Gateshead | 0.826 | High SDI |
| Hartlepool | 0.793 | High SDI |
| Middlesbrough | 0.808 | High SDI |
| Newcastle upon Tyne | 0.872 | High SDI |
| North Tyneside | 0.825 | High SDI |
| Northumberland | 0.808 | High SDI |
| Redcar and Cleveland | 0.79 | High SDI |
| South Tyneside | 0.794 | High SDI |
| Stockton-on-Tees | 0.823 | High SDI |
| Sunderland | 0.815 | High SDI |
| North West England | 0.834 | High SDI |
| Blackburn with Darwen | 0.802 | High SDI |
| Blackpool | 0.781 | High SDI |
| Bolton | 0.805 | High SDI |
| Bury | 0.815 | High SDI |
| Cheshire East | 0.864 | High SDI |
| Cheshire West and Chester | 0.855 | High SDI |
| Cumbria | 0.828 | High SDI |
| Halton | 0.824 | High SDI |
| Knowsley | 0.816 | High SDI |
| Lancashire | 0.831 | High SDI |
| Liverpool | 0.852 | High SDI |
| Manchester | 0.885 | High SDI |
| Oldham | 0.79 | High SDI |
| Rochdale | 0.795 | High SDI |
| Salford | 0.838 | High SDI |
| Sefton | 0.812 | High SDI |
| St Helens | 0.803 | High SDI |
| Stockport | 0.843 | High SDI |
| Tameside | 0.797 | High SDI |
| Trafford | 0.873 | High SDI |
| Warrington | 0.86 | High SDI |
| Wigan | 0.798 | High SDI |
| Wirral | 0.803 | High SDI |
| South East England | 0.856 | High SDI |
| Bracknell Forest | 0.869 | High SDI |
| Brighton and Hove | 0.885 | High SDI |

| Appendix Table 9. Socio-Demographic Index groupings by geography, based on 2017 values | | |
|---|---|---|
| **Geography** | **2017 SDI** | **SDI Quintile** |
| Buckinghamshire | 0.865 | High SDI |
| East Sussex | 0.814 | High SDI |
| Hampshire | 0.85 | High SDI |
| Isle of Wight | 0.814 | High SDI |
| Kent | 0.828 | High SDI |
| Medway | 0.809 | High SDI |
| Milton Keynes | 0.86 | High SDI |
| Oxfordshire | 0.879 | High SDI |
| Portsmouth | 0.86 | High SDI |
| Reading | 0.895 | High SDI |
| Slough | 0.859 | High SDI |
| Southampton | 0.858 | High SDI |
| Surrey | 0.883 | High SDI |
| West Berkshire | 0.872 | High SDI |
| West Sussex | 0.843 | High SDI |
| Windsor and Maidenhead | 0.889 | High SDI |
| Wokingham | 0.885 | High SDI |
| South West England | 0.841 | High SDI |
| Bath and North East Somerset | 0.875 | High SDI |
| Bournemouth | 0.858 | High SDI |
| Bristol, City of | 0.884 | High SDI |
| Cornwall | 0.817 | High SDI |
| Devon | 0.837 | High SDI |
| Dorset | 0.825 | High SDI |
| Gloucestershire | 0.85 | High SDI |
| North Somerset | 0.832 | High SDI |
| Plymouth | 0.836 | High SDI |
| Poole | 0.842 | High SDI |
| Somerset | 0.816 | High SDI |
| South Gloucestershire | 0.867 | High SDI |
| Swindon | 0.847 | High SDI |
| Torbay | 0.79 | High SDI |
| Wiltshire | 0.829 | High SDI |
| West Midlands | 0.829 | High SDI |
| Birmingham | 0.84 | High SDI |
| Coventry | 0.848 | High SDI |
| Dudley | 0.799 | High SDI |
| Herefordshire, County of | 0.828 | High SDI |
| Sandwell | 0.797 | High SDI |
| Shropshire | 0.832 | High SDI |
| Solihull | 0.855 | High SDI |
| Staffordshire | 0.826 | High SDI |
| Stoke-on-Trent | 0.804 | High SDI |
| Telford and Wrekin | 0.822 | High SDI |
| Walsall | 0.791 | High SDI |

| Appendix Table 9. Socio-Demographic Index groupings by geography, based on 2017 values | | |
|---|---|---|
| **Geography** | **2017 SDI** | **SDI Quintile** |
| Warwickshire | 0.857 | High SDI |
| Wolverhampton | 0.811 | High SDI |
| Worcestershire | 0.833 | High SDI |
| Yorkshire and the Humber | 0.83 | High SDI |
| Barnsley | 0.787 | High SDI |
| Bradford | 0.807 | High SDI |
| Calderdale | 0.827 | High SDI |
| Doncaster | 0.791 | High SDI |
| East Riding of Yorkshire | 0.822 | High SDI |
| Kingston upon Hull, City of | 0.813 | High SDI |
| Kirklees | 0.816 | High SDI |
| Leeds | 0.868 | High SDI |
| North East Lincolnshire | 0.804 | High SDI |
| North Lincolnshire | 0.811 | High SDI |
| North Yorkshire | 0.839 | High SDI |
| Rotherham | 0.796 | High SDI |
| Sheffield | 0.853 | High SDI |
| Wakefield | 0.806 | High SDI |
| York | 0.879 | High SDI |
| Northern Ireland | 0.835 | High SDI |
| Scotland | 0.805 | High SDI |
| Wales | 0.806 | High SDI |
| Latin America and Caribbean | 0.64 | |
| Andean Latin America | 0.628 | |
| Bolivia | 0.587 | Low-middle SDI |
| Ecuador | 0.636 | Middle SDI |
| Peru | 0.636 | Middle SDI |
| Caribbean | 0.638 | |
| Antigua and Barbuda | 0.715 | High-middle SDI |
| The Bahamas | 0.756 | High-middle SDI |
| Barbados | 0.739 | High-middle SDI |
| Belize | 0.602 | Low-middle SDI |
| Bermuda | 0.805 | High-middle SDI |
| Cuba | 0.688 | Middle SDI |
| Dominica | 0.687 | Middle SDI |
| Dominican Republic | 0.593 | Low-middle SDI |
| Grenada | 0.64 | Middle SDI |
| Guyana | 0.584 | Low-middle SDI |
| Haiti | 0.442 | Low SDI |
| Jamaica | 0.679 | Middle SDI |
| Puerto Rico | 0.813 | High-middle SDI |
| Saint Lucia | 0.653 | Middle SDI |
| Saint Vincent and the Grenadines | 0.608 | Middle SDI |
| Suriname | 0.641 | Middle SDI |
| Trinidad and Tobago | 0.698 | Middle SDI |

**Appendix Table 9. Socio-Demographic Index groupings by geography, based on 2017 values**

| Geography | 2017 SDI | SDI Quintile |
| --- | --- | --- |
| Virgin Islands | 0.807 | High-middle SDI |
| Central Latin America | 0.623 | |
| Colombia | 0.634 | Middle SDI |
| Costa Rica | 0.662 | Middle SDI |
| El Salvador | 0.593 | Low-middle SDI |
| Guatemala | 0.524 | Low-middle SDI |
| Honduras | 0.512 | Low-middle SDI |
| Mexico | 0.628 | Middle SDI |
| Aguascalientes | 0.659 | Middle SDI |
| Baja California | 0.657 | Middle SDI |
| Baja California Sur | 0.659 | Middle SDI |
| Campeche | 0.616 | Middle SDI |
| Chiapas | 0.533 | Middle SDI |
| Chihuahua | 0.639 | Middle SDI |
| Coahuila | 0.645 | Middle SDI |
| Colima | 0.654 | Middle SDI |
| Mexico City | 0.716 | Middle SDI |
| Durango | 0.624 | Middle SDI |
| Guanajuato | 0.621 | Middle SDI |
| Guerrero | 0.562 | Middle SDI |
| Hidalgo | 0.587 | Middle SDI |
| Jalisco | 0.649 | Middle SDI |
| México | 0.635 | Middle SDI |
| Michoacán de Ocampo | 0.586 | Middle SDI |
| Morelos | 0.635 | Middle SDI |
| Nayarit | 0.62 | Middle SDI |
| Nuevo León | 0.677 | Middle SDI |
| Oaxaca | 0.561 | Middle SDI |
| Puebla | 0.584 | Middle SDI |
| Querétaro | 0.639 | Middle SDI |
| Quintana Roo | 0.626 | Middle SDI |
| San Luis Potosí | 0.621 | Middle SDI |
| Sinaloa | 0.649 | Middle SDI |
| Sonora | 0.65 | Middle SDI |
| Tabasco | 0.611 | Middle SDI |
| Tamaulipas | 0.647 | Middle SDI |
| Tlaxcala | 0.604 | Middle SDI |
| Veracruz de Ignacio de la Llave | 0.592 | Middle SDI |
| Yucatán | 0.63 | Middle SDI |
| Zacatecas | 0.608 | Middle SDI |
| Nicaragua | 0.53 | Low-middle SDI |
| Panama | 0.677 | Middle SDI |
| Venezuela | 0.655 | Middle SDI |
| Tropical Latin America | 0.662 | |
| Brazil | 0.663 | Middle SDI |

| Appendix Table 9. Socio-Demographic Index groupings by geography, based on 2017 values | | |
|---|---|---|
| **Geography** | **2017 SDI** | **SDI Quintile** |
| Acre | 0.602 | Low-middle SDI |
| Alagoas | 0.556 | Low-middle SDI |
| Amapá | 0.659 | Middle SDI |
| Amazonas | 0.629 | Middle SDI |
| Bahia | 0.591 | Low-middle SDI |
| Ceará | 0.6 | Low-middle SDI |
| Distrito Federal | 0.792 | High-middle SDI |
| Espírito Santo | 0.677 | Middle SDI |
| Goiás | 0.65 | Middle SDI |
| Maranhão | 0.507 | Low-middle SDI |
| Mato Grosso | 0.662 | Middle SDI |
| Mato Grosso do Sul | 0.65 | Middle SDI |
| Minas Gerais | 0.661 | Middle SDI |
| Pará | 0.579 | Low-middle SDI |
| Paraíba | 0.574 | Low-middle SDI |
| Paraná | 0.682 | Middle SDI |
| Pernambuco | 0.594 | Low-middle SDI |
| Piauí | 0.552 | Low-middle SDI |
| Rio de Janeiro | 0.709 | High-middle SDI |
| Rio Grande do Norte | 0.605 | Low-middle SDI |
| Rio Grande do Sul | 0.693 | Middle SDI |
| Rondônia | 0.622 | Middle SDI |
| Roraima | 0.646 | Middle SDI |
| Santa Catarina | 0.702 | High-middle SDI |
| São Paulo | 0.72 | High-middle SDI |
| Sergipe | 0.616 | Middle SDI |
| Tocantins | 0.611 | Middle SDI |
| Paraguay | 0.619 | Middle SDI |
| North Africa and Middle East | 0.639 | |
| North Africa and Middle East | 0.639 | |
| Afghanistan | 0.29 | Low SDI |
| Algeria | 0.696 | Middle SDI |
| Bahrain | 0.712 | High-middle SDI |
| Egypt | 0.604 | Low-middle SDI |
| Iran | 0.7 | High-middle SDI |
| Iraq | 0.585 | Low-middle SDI |
| Jordan | 0.697 | Middle SDI |
| Kuwait | 0.786 | High-middle SDI |
| Lebanon | 0.73 | High-middle SDI |
| Libya | 0.761 | High-middle SDI |
| Morocco | 0.579 | Low-middle SDI |
| Palestine | 0.541 | Low-middle SDI |
| Oman | 0.744 | High-middle SDI |
| Qatar | 0.766 | High-middle SDI |
| Saudi Arabia | 0.779 | High-middle SDI |

| Appendix Table 9. Socio-Demographic Index groupings by geography, based on 2017 values | | |
|---|---|---|
| **Geography** | **2017 SDI** | **SDI Quintile** |
| Sudan | 0.478 | Low-middle SDI |
| Syria | 0.611 | Middle SDI |
| Tunisia | 0.675 | Middle SDI |
| Turkey | 0.729 | High-middle SDI |
| United Arab Emirates | 0.795 | High-middle SDI |
| Yemen | 0.43 | Low SDI |
| South Asia | 0.534 | |
| South Asia | 0.534 | |
| Bangladesh | 0.458 | Low SDI |
| Bhutan | 0.57 | Low-middle SDI |
| India | 0.55 | Low-middle SDI |
| Andhra Pradesh | 0.536 | Low-middle SDI |
| Arunachal Pradesh | 0.556 | Low-middle SDI |
| Assam | 0.53 | Low-middle SDI |
| Bihar | 0.433 | Low SDI |
| Chhattisgarh | 0.512 | Low-middle SDI |
| Delhi | 0.715 | High-middle SDI |
| Goa | 0.74 | High-middle SDI |
| Gujarat | 0.584 | Low-middle SDI |
| Haryana | 0.6 | Low-middle SDI |
| Himachal Pradesh | 0.633 | Middle SDI |
| Jammu and Kashmir | 0.59 | Low-middle SDI |
| Jharkhand | 0.487 | Low-middle SDI |
| Karnataka | 0.574 | Low-middle SDI |
| Kerala | 0.659 | Middle SDI |
| Madhya Pradesh | 0.487 | Low-middle SDI |
| Maharashtra | 0.618 | Middle SDI |
| Manipur | 0.59 | Low-middle SDI |
| Meghalaya | 0.565 | Low-middle SDI |
| Mizoram | 0.616 | Middle SDI |
| Nagaland | 0.633 | Middle SDI |
| Odisha | 0.524 | Low-middle SDI |
| Punjab | 0.622 | Middle SDI |
| Rajasthan | 0.492 | Low-middle SDI |
| Sikkim | 0.628 | Middle SDI |
| Tamil Nadu | 0.615 | Middle SDI |
| Telangana | 0.575 | Low-middle SDI |
| Tripura | 0.543 | Low-middle SDI |
| Uttar Pradesh | 0.488 | Low-middle SDI |
| Uttarakhand | 0.607 | Middle SDI |
| West Bengal | 0.538 | Low-middle SDI |
| Union Territories other than Delhi | 0.653 | Middle SDI |
| Nepal | 0.429 | Low SDI |
| Pakistan | 0.492 | Low-middle SDI |
| Southeast Asia, East Asia, and Oceania | 0.685 | |

## Appendix Table 9. Socio-Demographic Index groupings by geography, based on 2017 values

| Geography | 2017 SDI | SDI Quintile |
|---|---|---|
| East Asia | 0.709 | |
| China | 0.707 | High-middle SDI |
| North Korea | 0.538 | Low-middle SDI |
| Taiwan (Province of China) | 0.864 | High SDI |
| Oceania | 0.471 | |
| American Samoa | 0.702 | High-middle SDI |
| Federated States of Micronesia | 0.575 | Low-middle SDI |
| Fiji | 0.641 | Middle SDI |
| Guam | 0.794 | High-middle SDI |
| Kiribati | 0.427 | Low SDI |
| Marshall Islands | 0.55 | Low-middle SDI |
| Northern Mariana Islands | 0.758 | High-middle SDI |
| Papua New Guinea | 0.419 | Low SDI |
| Samoa | 0.576 | Low-middle SDI |
| Solomon Islands | 0.425 | Low SDI |
| Tonga | 0.625 | Middle SDI |
| Vanuatu | 0.475 | Low-middle SDI |
| Southeast Asia | 0.641 | |
| Cambodia | 0.482 | Low-middle SDI |
| Indonesia | 0.648 | Middle SDI |
| Laos | 0.519 | Low-middle SDI |
| Malaysia | 0.759 | High-middle SDI |
| Maldives | 0.655 | Middle SDI |
| Mauritius | 0.72 | High-middle SDI |
| Myanmar | 0.556 | Low-middle SDI |
| Philippines | 0.617 | Middle SDI |
| Sri Lanka | 0.68 | Middle SDI |
| Seychelles | 0.692 | Middle SDI |
| Thailand | 0.684 | Middle SDI |
| Timor-Leste | 0.505 | Low-middle SDI |
| Vietnam | 0.607 | Middle SDI |
| Sub-Saharan Africa | 0.446 | |
| Central sub-Saharan Africa | 0.457 | |
| Angola | 0.461 | Low-middle SDI |
| Central African Republic | 0.334 | Low SDI |
| Congo (Brazzaville) | 0.574 | Low-middle SDI |
| DR Congo | 0.364 | Low SDI |
| Equatorial Guinea | 0.625 | Middle SDI |
| Gabon | 0.651 | Middle SDI |
| Eastern sub-Saharan Africa | 0.387 | |
| Burundi | 0.31 | Low SDI |
| Comoros | 0.434 | Low SDI |
| Djibouti | 0.485 | Low-middle SDI |
| Eritrea | 0.409 | Low SDI |
| Ethiopia | 0.334 | Low SDI |

**Appendix Table 9. Socio-Demographic Index groupings by geography, based on 2017 values**

| Geography | 2017 SDI | SDI Quintile |
|---|---|---|
| Kenya | 0.499 | Low-middle SDI |
| Baringo | 0.444 | Low-middle SDI |
| Bomet | 0.496 | Low-middle SDI |
| Bungoma | 0.463 | Low-middle SDI |
| Busia | 0.438 | Low-middle SDI |
| Elgeyo Marakwet | 0.496 | Low-middle SDI |
| Embu | 0.533 | Low-middle SDI |
| Garissa | 0.334 | Low-middle SDI |
| Homa Bay | 0.425 | Low-middle SDI |
| Isiolo | 0.385 | Low-middle SDI |
| Kajiado | 0.534 | Low-middle SDI |
| Kakamega | 0.45 | Low-middle SDI |
| Kericho | 0.5 | Low-middle SDI |
| Kiambu | 0.58 | Low-middle SDI |
| Kilifi | 0.456 | Low-middle SDI |
| Kirinyaga | 0.533 | Low-middle SDI |
| Kisii | 0.522 | Low-middle SDI |
| Kisumu | 0.503 | Low-middle SDI |
| Kitui | 0.461 | Low-middle SDI |
| Kwale | 0.457 | Low-middle SDI |
| Laikipia | 0.556 | Low-middle SDI |
| Lamu | 0.453 | Low-middle SDI |
| Machakos | 0.518 | Low-middle SDI |
| Makueni | 0.469 | Low-middle SDI |
| Mandera | 0.295 | Low-middle SDI |
| Marsabit | 0.34 | Low-middle SDI |
| Meru | 0.508 | Low-middle SDI |
| Migori | 0.419 | Low-middle SDI |
| Mombasa | 0.568 | Low-middle SDI |
| Murang’a | 0.528 | Low-middle SDI |
| Nairobi | 0.674 | Low-middle SDI |
| Nakuru | 0.545 | Low-middle SDI |
| Nandi | 0.501 | Low-middle SDI |
| Narok | 0.402 | Low-middle SDI |
| Nyamira | 0.544 | Low-middle SDI |
| Nyandarua | 0.534 | Low-middle SDI |
| Nyeri | 0.554 | Low-middle SDI |
| Samburu | 0.308 | Low-middle SDI |
| Siaya | 0.46 | Low-middle SDI |
| Taita Taveta | 0.529 | Low-middle SDI |
| Tana River | 0.379 | Low-middle SDI |
| Tharaka Nithi | 0.528 | Low-middle SDI |
| Trans Nzoia | 0.496 | Low-middle SDI |
| Turkana | 0.295 | Low-middle SDI |
| Uasin Gishu | 0.545 | Low-middle SDI |

| Appendix Table 9. Socio-Demographic Index groupings by geography, based on 2017 values | | |
|---|---|---|
| **Geography** | **2017 SDI** | **SDI Quintile** |
| Vihiga | 0.477 | Low-middle SDI |
| Wajir | 0.243 | Low-middle SDI |
| West Pokot | 0.382 | Low-middle SDI |
| Madagascar | 0.331 | Low SDI |
| Malawi | 0.349 | Low SDI |
| Mozambique | 0.34 | Low SDI |
| Rwanda | 0.407 | Low SDI |
| Somalia | 0.235 | Low SDI |
| South Sudan | 0.275 | Low SDI |
| Tanzania | 0.412 | Low SDI |
| Uganda | 0.388 | Low SDI |
| Zambia | 0.472 | Low-middle SDI |
| Southern sub-Saharan Africa | 0.64 | |
| Botswana | 0.663 | Middle SDI |
| Lesotho | 0.493 | Low-middle SDI |
| Namibia | 0.616 | Middle SDI |
| South Africa | 0.677 | Middle SDI |
| Swaziland | 0.578 | Low-middle SDI |
| Zimbabwe | 0.463 | Low-middle SDI |
| Western sub-Saharan Africa | 0.441 | |
| Benin | 0.373 | Low SDI |
| Burkina Faso | 0.284 | Low SDI |
| Cameroon | 0.482 | Low-middle SDI |
| Cape Verde | 0.549 | Low-middle SDI |
| Chad | 0.253 | Low SDI |
| Cote d'Ivoire | 0.412 | Low SDI |
| The Gambia | 0.405 | Low SDI |
| Ghana | 0.537 | Low-middle SDI |
| Guinea | 0.325 | Low SDI |
| Guinea-Bissau | 0.349 | Low SDI |
| Liberia | 0.328 | Low SDI |
| Mali | 0.267 | Low SDI |
| Mauritania | 0.471 | Low-middle SDI |
| Niger | 0.191 | Low SDI |
| Nigeria | 0.493 | Low-middle SDI |
| Sao Tome and Principe | 0.488 | Low-middle SDI |
| Senegal | 0.373 | Low SDI |
| Sierra Leone | 0.357 | Low SDI |
| Togo | 0.413 | Low SDI |

**Appendix Table 10: Socio-Demographic Index values for all estimated GBD 2017 locations, 1990-2017**

| Location | 1990 | 1991 | 1992 | 1993 | 1994 | 1995 | 1996 | 1997 | 1998 | 1999 | 2000 | 2001 | 2002 | 2003 | 2004 | 2005 | 2006 | 2007 | 2008 | 2009 | 2010 | 2011 | 2012 | 2013 | 2014 | 2015 | 2016 | 2017 |
|---|---|---|---|---|---|---|---|---|---|---|---|---|---|---|---|---|---|---|---|---|---|---|---|---|---|---|---|---|
| Global | 0.523 | 0.529 | 0.534 | 0.539 | 0.543 | 0.548 | 0.553 | 0.557 | 0.561 | 0.566 | 0.571 | 0.576 | 0.581 | 0.585 | 0.59 | 0.595 | 0.601 | 0.606 | 0.611 | 0.616 | 0.62 | 0.624 | 0.628 | 0.633 | 0.639 | 0.644 | 0.647 | 0.652 |
| Central Europe, Eastern Europe, and Central Asia | 0.656 | 0.662 | 0.67 | 0.674 | 0.677 | 0.682 | 0.686 | 0.689 | 0.691 | 0.694 | 0.698 | 0.701 | 0.705 | 0.709 | 0.715 | 0.72 | 0.725 | 0.73 | 0.735 | 0.739 | 0.743 | 0.747 | 0.75 | 0.753 | 0.757 | 0.76 | 0.763 | 0.766 |
| Central Asia | 0.563 | 0.567 | 0.57 | 0.573 | 0.575 | 0.577 | 0.578 | 0.579 | 0.58 | 0.582 | 0.585 | 0.588 | 0.593 | 0.598 | 0.603 | 0.609 | 0.615 | 0.621 | 0.627 | 0.633 | 0.639 | 0.644 | 0.649 | 0.654 | 0.659 | 0.664 | 0.669 | 0.673 |
| Armenia | 0.555 | 0.559 | 0.56 | 0.562 | 0.565 | 0.567 | 0.57 | 0.573 | 0.577 | 0.581 | 0.586 | 0.592 | 0.6 | 0.61 | 0.619 | 0.629 | 0.639 | 0.65 | 0.66 | 0.667 | 0.673 | 0.678 | 0.683 | 0.687 | 0.691 | 0.695 | 0.699 | 0.702 |
| Azerbaijan | 0.611 | 0.614 | 0.616 | 0.617 | 0.616 | 0.613 | 0.61 | 0.607 | 0.604 | 0.601 | 0.6 | 0.6 | 0.602 | 0.605 | 0.608 | 0.615 | 0.625 | 0.635 | 0.645 | 0.654 | 0.664 | 0.672 | 0.678 | 0.684 | 0.689 | 0.694 | 0.698 | 0.701 |
| Georgia | 0.654 | 0.66 | 0.661 | 0.658 | 0.653 | 0.645 | 0.639 | 0.634 | 0.63 | 0.625 | 0.621 | 0.62 | 0.622 | 0.624 | 0.628 | 0.633 | 0.638 | 0.644 | 0.649 | 0.654 | 0.659 | 0.665 | 0.671 | 0.676 | 0.682 | 0.688 | 0.695 | 0.7 |
| Kazakhstan | 0.613 | 0.615 | 0.619 | 0.625 | 0.632 | 0.638 | 0.643 | 0.645 | 0.646 | 0.647 | 0.651 | 0.656 | 0.661 | 0.666 | 0.671 | 0.677 | 0.683 | 0.689 | 0.696 | 0.702 | 0.705 | 0.707 | 0.708 | 0.711 | 0.716 | 0.723 | 0.73 | 0.735 |
| Kyrgyzstan | 0.565 | 0.571 | 0.576 | 0.578 | 0.577 | 0.572 | 0.569 | 0.567 | 0.564 | 0.562 | 0.56 | 0.559 | 0.56 | 0.562 | 0.565 | 0.566 | 0.567 | 0.569 | 0.572 | 0.575 | 0.576 | 0.581 | 0.584 | 0.589 | 0.594 | 0.598 | 0.603 | 0.607 |
| Mongolia | 0.537 | 0.545 | 0.55 | 0.555 | 0.559 | 0.564 | 0.569 | 0.573 | 0.577 | 0.581 | 0.585 | 0.589 | 0.594 | 0.598 | 0.603 | 0.608 | 0.614 | 0.619 | 0.624 | 0.628 | 0.632 | 0.636 | 0.641 | 0.646 | 0.65 | 0.654 | 0.658 | 0.662 |
| Tajikistan | 0.474 | 0.481 | 0.485 | 0.487 | 0.486 | 0.481 | 0.474 | 0.468 | 0.463 | 0.459 | 0.455 | 0.454 | 0.456 | 0.462 | 0.465 | 0.466 | 0.472 | 0.479 | 0.483 | 0.488 | 0.494 | 0.501 | 0.506 | 0.51 | 0.514 | 0.517 | 0.52 | 0.523 |
| Turkmenistan | 0.588 | 0.592 | 0.594 | 0.599 | 0.602 | 0.604 | 0.606 | 0.606 | 0.606 | 0.607 | 0.61 | 0.613 | 0.617 | 0.622 | 0.628 | 0.635 | 0.638 | 0.641 | 0.644 | 0.647 | 0.651 | 0.657 | 0.663 | 0.669 | 0.678 | 0.685 | 0.691 | 0.696 |
| Uzbekistan | 0.481 | 0.484 | 0.487 | 0.493 | 0.497 | 0.502 | 0.508 | 0.513 | 0.52 | 0.526 | 0.532 | 0.537 | 0.543 | 0.549 | 0.555 | 0.56 | 0.565 | 0.57 | 0.575 | 0.581 | 0.587 | 0.592 | 0.598 | 0.604 | 0.611 | 0.618 | 0.624 | 0.63 |
| Central Europe | 0.665 | 0.671 | 0.677 | 0.683 | 0.69 | 0.698 | 0.705 | 0.711 | 0.717 | 0.723 | 0.731 | 0.738 | 0.745 | 0.751 | 0.757 | 0.762 | 0.767 | 0.772 | 0.776 | 0.782 | 0.788 | 0.793 | 0.797 | 0.802 | 0.805 | 0.808 | 0.811 | 0.814 |
| Albania | 0.548 | 0.545 | 0.542 | 0.541 | 0.542 | 0.546 | 0.552 | 0.558 | 0.566 | 0.577 | 0.584 | 0.593 | 0.602 | 0.611 | 0.619 | 0.627 | 0.635 | 0.642 | 0.648 | 0.653 | 0.658 | 0.661 | 0.665 | 0.668 | 0.672 | 0.676 | 0.681 | 0.685 |
| Bosnia and Herzegovina | 0.497 | 0.499 | 0.5 | 0.5 | 0.501 | 0.507 | 0.525 | 0.549 | 0.571 | 0.592 | 0.607 | 0.619 | 0.63 | 0.639 | 0.647 | 0.654 | 0.66 | 0.667 | 0.673 | 0.679 | 0.685 | 0.69 | 0.694 | 0.699 | 0.703 | 0.706 | 0.71 | 0.713 |
| Bulgaria | 0.658 | 0.668 | 0.676 | 0.684 | 0.693 | 0.699 | 0.705 | 0.706 | 0.704 | 0.703 | 0.708 | 0.715 | 0.721 | 0.726 | 0.731 | 0.736 | 0.741 | 0.746 | 0.751 | 0.757 | 0.765 | 0.771 | 0.775 | 0.778 | 0.781 | 0.784 | 0.788 | 0.792 |
| Croatia | 0.725 | 0.73 | 0.732 | 0.732 | 0.731 | 0.731 | 0.732 | 0.737 | 0.743 | 0.749 | 0.755 | 0.762 | 0.768 | 0.773 | 0.778 | 0.782 | 0.787 | 0.792 | 0.797 | 0.801 | 0.805 | 0.809 | 0.813 | 0.816 | 0.818 | 0.821 | 0.823 | 0.825 |
| Czech Republic | 0.711 | 0.717 | 0.726 | 0.74 | 0.757 | 0.769 | 0.777 | 0.783 | 0.788 | 0.794 | 0.799 | 0.804 | 0.809 | 0.814 | 0.819 | 0.823 | 0.827 | 0.83 | 0.833 | 0.836 | 0.84 | 0.843 | 0.846 | 0.847 | 0.848 | 0.848 | 0.849 | 0.851 |
| Hungary | 0.678 | 0.683 | 0.691 | 0.699 | 0.707 | 0.716 | 0.724 | 0.732 | 0.739 | 0.745 | 0.751 | 0.758 | 0.764 | 0.77 | 0.776 | 0.781 | 0.786 | 0.791 | 0.795 | 0.799 | 0.803 | 0.806 | 0.807 | 0.808 | 0.809 | 0.811 | 0.814 | 0.817 |
| Macedonia | 0.626 | 0.629 | 0.63 | 0.631 | 0.632 | 0.635 | 0.64 | 0.647 | 0.654 | 0.661 | 0.665 | 0.67 | 0.677 | 0.685 | 0.693 | 0.699 | 0.704 | 0.709 | 0.715 | 0.719 | 0.724 | 0.729 | 0.734 | 0.739 | 0.744 | 0.748 | 0.751 | 0.754 |
| Montenegro | 0.705 | 0.706 | 0.705 | 0.701 | 0.698 | 0.696 | 0.696 | 0.698 | 0.7 | 0.703 | 0.706 | 0.711 | 0.716 | 0.721 | 0.726 | 0.731 | 0.737 | 0.743 | 0.75 | 0.756 | 0.761 | 0.767 | 0.771 | 0.775 | 0.779 | 0.782 | 0.785 | 0.788 |
| Poland | 0.662 | 0.668 | 0.678 | 0.686 | 0.697 | 0.707 | 0.714 | 0.724 | 0.733 | 0.741 | 0.75 | 0.759 | 0.767 | 0.773 | 0.779 | 0.784 | 0.789 | 0.792 | 0.797 | 0.804 | 0.811 | 0.818 | 0.823 | 0.829 | 0.833 | 0.837 | 0.841 | 0.844 |
| Romania | 0.652 | 0.66 | 0.663 | 0.666 | 0.671 | 0.678 | 0.682 | 0.685 | 0.689 | 0.694 | 0.7 | 0.707 | 0.713 | 0.718 | 0.724 | 0.73 | 0.734 | 0.739 | 0.745 | 0.751 | 0.758 | 0.763 | 0.768 | 0.772 | 0.774 | 0.777 | 0.78 | 0.784 |
| Serbia | 0.632 | 0.638 | 0.643 | 0.642 | 0.641 | 0.641 | 0.643 | 0.648 | 0.653 | 0.655 | 0.661 | 0.665 | 0.669 | 0.675 | 0.684 | 0.692 | 0.699 | 0.705 | 0.709 | 0.713 | 0.718 | 0.723 | 0.729 | 0.736 | 0.742 | 0.747 | 0.75 | 0.752 |
| Slovakia | 0.684 | 0.69 | 0.699 | 0.71 | 0.722 | 0.732 | 0.74 | 0.748 | 0.756 | 0.764 | 0.772 | 0.779 | 0.784 | 0.788 | 0.793 | 0.798 | 0.804 | 0.809 | 0.814 | 0.818 | 0.823 | 0.828 | 0.832 | 0.834 | 0.836 | 0.838 | 0.839 | 0.842 |
| Slovenia | 0.741 | 0.747 | 0.753 | 0.759 | 0.764 | 0.769 | 0.775 | 0.781 | 0.788 | 0.794 | 0.801 | 0.808 | 0.814 | 0.819 | 0.824 | 0.828 | 0.833 | 0.837 | 0.841 | 0.843 | 0.846 | 0.848 | 0.85 | 0.852 | 0.854 | 0.856 | 0.858 | 0.86 |
| Eastern Europe | 0.678 | 0.685 | 0.694 | 0.698 | 0.7 | 0.704 | 0.708 | 0.708 | 0.71 | 0.711 | 0.712 | 0.713 | 0.715 | 0.72 | 0.727 | 0.734 | 0.739 | 0.745 | 0.751 | 0.756 | 0.761 | 0.764 | 0.767 | 0.772 | 0.776 | 0.779 | 0.783 | 0.785 |
| Belarus | 0.625 | 0.631 | 0.636 | 0.641 | 0.645 | 0.647 | 0.65 | 0.654 | 0.657 | 0.661 | 0.665 | 0.67 | 0.676 | 0.682 | 0.689 | 0.696 | 0.704 | 0.712 | 0.72 | 0.727 | 0.733 | 0.74 | 0.747 | 0.753 | 0.759 | 0.764 | 0.769 | 0.773 |
| Estonia | 0.711 | 0.719 | 0.728 | 0.736 | 0.742 | 0.746 | 0.75 | 0.755 | 0.761 | 0.766 | 0.772 | 0.778 | 0.783 | 0.788 | 0.794 | 0.799 | 0.806 | 0.813 | 0.82 | 0.826 | 0.832 | 0.838 | 0.843 | 0.847 | 0.851 | 0.854 | 0.856 | 0.858 |
| Latvia | 0.696 | 0.703 | 0.712 | 0.721 | 0.727 | 0.731 | 0.733 | 0.734 | 0.735 | 0.738 | 0.741 | 0.745 | 0.75 | 0.757 | 0.763 | 0.769 | 0.776 | 0.783 | 0.792 | 0.8 | 0.806 | 0.81 | 0.814 | 0.816 | 0.817 | 0.819 | 0.822 | 0.825 |
| Lithuania | 0.707 | 0.71 | 0.717 | 0.725 | 0.728 | 0.731 | 0.733 | 0.736 | 0.74 | 0.746 | 0.753 | 0.76 | 0.765 | 0.772 | 0.779 | 0.785 | 0.79 | 0.796 | 0.802 | 0.808 | 0.815 | 0.822 | 0.828 | 0.833 | 0.836 | 0.838 | 0.839 | 0.841 |
| Moldova | 0.575 | 0.578 | 0.58 | 0.582 | 0.583 | 0.584 | 0.584 | 0.582 | 0.58 | 0.577 | 0.574 | 0.574 | 0.577 | 0.582 | 0.588 | 0.595 | 0.602 | 0.61 | 0.618 | 0.624 | 0.632 | 0.64 | 0.647 | 0.654 | 0.66 | 0.666 | 0.671 | 0.676 |
| Russian Federation | 0.683 | 0.692 | 0.704 | 0.708 | 0.708 | 0.714 | 0.718 | 0.719 | 0.72 | 0.722 | 0.722 | 0.722 | 0.724 | 0.728 | 0.734 | 0.742 | 0.747 | 0.752 | 0.757 | 0.763 | 0.768 | 0.77 | 0.772 | 0.777 | 0.781 | 0.785 | 0.789 | 0.792 |
| Ukraine | 0.664 | 0.667 | 0.67 | 0.673 | 0.675 | 0.676 | 0.676 | 0.675 | 0.673 | 0.672 | 0.672 | 0.673 | 0.675 | 0.68 | 0.687 | 0.694 | 0.7 | 0.707 | 0.714 | 0.717 | 0.721 | 0.725 | 0.729 | 0.732 | 0.735 | 0.736 | 0.738 | 0.74 |
| High-income | 0.769 | 0.774 | 0.779 | 0.783 | 0.787 | 0.792 | 0.796 | 0.798 | 0.801 | 0.804 | 0.807 | 0.811 | 0.814 | 0.817 | 0.82 | 0.822 | 0.823 | 0.826 | 0.829 | 0.832 | 0.836 | 0.839 | 0.842 | 0.845 | 0.848 | 0.851 | 0.853 | 0.854 |
| Australasia | 0.783 | 0.786 | 0.79 | 0.794 | 0.797 | 0.801 | 0.805 | 0.809 | 0.813 | 0.817 | 0.821 | 0.825 | 0.828 | 0.832 | 0.835 | 0.837 | 0.838 | 0.84 | 0.842 | 0.845 | 0.848 | 0.851 | 0.855 | 0.859 | 0.862 | 0.864 | 0.867 | 0.869 |
| Australia | 0.786 | 0.79 | 0.793 | 0.797 | 0.801 | 0.805 | 0.81 | 0.814 | 0.818 | 0.822 | 0.825 | 0.829 | 0.833 | 0.837 | 0.84 | 0.843 | 0.844 | 0.845 | 0.848 | 0.851 | 0.854 | 0.856 | 0.86 | 0.864 | 0.867 | 0.869 | 0.871 | 0.873 |
| New Zealand | 0.765 | 0.768 | 0.771 | 0.774 | 0.777 | 0.78 | 0.783 | 0.786 | 0.79 | 0.794 | 0.798 | 0.802 | 0.805 | 0.807 | 0.809 | 0.811 | 0.811 | 0.811 | 0.813 | 0.816 | 0.819 | 0.823 | 0.828 | 0.832 | 0.835 | 0.838 | 0.84 | 0.842 |
| High-income Asia-Pacific | 0.783 | 0.789 | 0.794 | 0.799 | 0.804 | 0.809 | 0.813 | 0.817 | 0.82 | 0.823 | 0.826 | 0.83 | 0.833 | 0.836 | 0.839 | 0.842 | 0.844 | 0.846 | 0.849 | 0.851 | 0.853 | 0.856 | 0.858 | 0.861 | 0.863 | 0.865 | 0.867 | 0.869 |
| Brunei | 0.728 | 0.733 | 0.739 | 0.745 | 0.751 | 0.757 | 0.763 | 0.769 | 0.774 | 0.779 | 0.784 | 0.789 | 0.795 | 0.802 | 0.808 | 0.814 | 0.819 | 0.824 | 0.828 | 0.831 | 0.835 | 0.838 | 0.842 | 0.845 | 0.848 | 0.851 | 0.854 | 0.856 |
| Japan | 0.803 | 0.807 | 0.812 | 0.816 | 0.82 | 0.823 | 0.826 | 0.829 | 0.831 | 0.833 | 0.834 | 0.836 | 0.838 | 0.84 | 0.842 | 0.844 | 0.846 | 0.847 | 0.849 | 0.851 | 0.853 | 0.855 | 0.857 | 0.859 | 0.861 | 0.862 | 0.863 | 0.865 |
| Aichi | 0.812 | 0.816 | 0.821 | 0.825 | 0.829 | 0.833 | 0.836 | 0.839 | 0.841 | 0.843 | 0.844 | 0.846 | 0.847 | 0.85 | 0.852 | 0.854 | 0.855 | 0.856 | 0.858 | 0.86 | 0.862 | 0.864 | 0.866 | 0.869 | 0.871 | 0.872 | 0.873 | 0.875 |
| Akita | 0.766 | 0.77 | 0.775 | 0.778 | 0.781 | 0.785 | 0.788 | 0.791 | 0.793 | 0.795 | 0.796 | 0.798 | 0.8 | 0.802 | 0.805 | 0.806 | 0.808 | 0.81 | 0.812 | 0.814 | 0.816 | 0.818 | 0.821 | 0.823 | 0.825 | 0.826 | 0.827 | 0.829 |
| Aomori | 0.761 | 0.765 | 0.77 | 0.773 | 0.777 | 0.78 | 0.783 | 0.786 | 0.788 | 0.79 | 0.791 | 0.793 | 0.795 | 0.798 | 0.801 | 0.803 | 0.805 | 0.807 | 0.809 | 0.81 | 0.812 | 0.815 | 0.817 | 0.819 | 0.821 | 0.822 | 0.823 | 0.825 |
| Chiba | 0.803 | 0.807 | 0.812 | 0.816 | 0.82 | 0.823 | 0.827 | 0.829 | 0.831 | 0.832 | 0.833 | 0.834 | 0.836 | 0.837 | 0.839 | 0.841 | 0.842 | 0.843 | 0.845 | 0.846 | 0.848 | 0.85 | 0.852 | 0.854 | 0.856 | 0.857 | 0.858 | 0.859 |
| Ehime | 0.776 | 0.78 | 0.785 | 0.788 | 0.792 | 0.796 | 0.799 | 0.802 | 0.804 | 0.806 | 0.808 | 0.809 | 0.811 | 0.814 | 0.816 | 0.818 | 0.819 | 0.82 | 0.822 | 0.824 | 0.825 | 0.828 | 0.83 | 0.832 | 0.834 | 0.835 | 0.836 | 0.838 |
| Fukui | 0.784 | 0.789 | 0.794 | 0.798 | 0.802 | 0.806 | 0.81 | 0.813 | 0.815 | 0.818 | 0.819 | 0.821 | 0.823 | 0.826 | 0.828 | 0.83 | 0.832 | 0.834 | 0.836 | 0.837 | 0.839 | 0.842 | 0.844 | 0.846 | 0.848 | 0.849 | 0.85 | 0.852 |
| Fukuoka | 0.797 | 0.801 | 0.806 | 0.809 | 0.813 | 0.816 | 0.819 | 0.822 | 0.824 | 0.826 | 0.828 | 0.83 | 0.832 | 0.834 | 0.836 | 0.837 | 0.838 | 0.839 | 0.841 | 0.842 | 0.843 | 0.845 | 0.847 | 0.849 | 0.851 | 0.853 | 0.854 | 0.855 |
| Fukushima | 0.769 | 0.773 | 0.778 | 0.781 | 0.784 | 0.788 | 0.791 | 0.793 | 0.794 | 0.795 | 0.797 | 0.798 | 0.8 | 0.803 | 0.807 | 0.809 | 0.811 | 0.813 | 0.814 | 0.816 | 0.819 | 0.821 | 0.823 | 0.825 | 0.827 | 0.828 | 0.829 | 0.831 |
| Gifu | 0.786 | 0.79 | 0.795 | 0.799 | 0.803 | 0.807 | 0.81 | 0.813 | 0.815 | 0.817 | 0.818 | 0.82 | 0.821 | 0.824 | 0.826 | 0.828 | 0.829 | 0.831 | 0.833 | 0.835 | 0.837 | 0.839 | 0.841 | 0.843 | 0.845 | 0.846 | 0.847 | 0.849 |
| Gunma | 0.787 | 0.791 | 0.796 | 0.8 | 0.803 | 0.807 | 0.81 | 0.813 | 0.815 | 0.816 | 0.818 | 0.819 | 0.821 | 0.824 | 0.827 | 0.829 | 0.831 | 0.833 | 0.835 | 0.837 | 0.839 | 0.841 | 0.843 | 0.845 | 0.847 | 0.848 | 0.849 | 0.851 |
| Hiroshima | 0.8 | 0.805 | 0.809 | 0.813 | 0.817 | 0.821 | 0.825 | 0.828 | 0.83 | 0.832 | 0.833 | 0.835 | 0.836 | 0.839 | 0.841 | 0.842 | 0.843 | 0.844 | 0.846 | 0.847 | 0.849 | 0.852 | 0.854 | 0.856 | 0.858 | 0.86 | 0.861 | 0.863 |
| Hokkaidō | 0.783 | 0.788 | 0.792 | 0.796 | 0.799 | 0.803 | 0.806 | 0.808 | 0.809 | 0.811 | 0.812 | 0.813 | 0.815 | 0.818 | 0.82 | 0.822 | 0.824 | 0.825 | 0.827 | 0.828 | 0.83 | 0.832 | 0.834 | 0.836 | 0.838 | 0.839 | 0.84 | 0.842 |
| Hyōgo | 0.798 | 0.803 | 0.807 | 0.811 | 0.815 | 0.819 | 0.822 | 0.825 | 0.827 | 0.829 | 0.831 | 0.833 | 0.835 | 0.837 | 0.839 | 0.841 | 0.842 | 0.843 | 0.845 | 0.846 | 0.848 | 0.85 | 0.852 | 0.854 | 0.856 | 0.857 | 0.858 | 0.86 |
| Ibaraki | 0.789 | 0.793 | 0.798 | 0.802 | 0.805 | 0.809 | 0.813 | 0.815 | 0.817 | 0.819 | 0.82 | 0.821 | 0.823 | 0.826 | 0.828 | 0.83 | 0.831 | 0.833 | 0.835 | 0.837 | 0.839 | 0.841 | 0.843 | 0.845 | 0.847 | 0.848 | 0.849 | 0.851 |
| Ishikawa | 0.789 | 0.795 | 0.8 | 0.804 | 0.808 | 0.812 | 0.815 | 0.818 | 0.82 | 0.822 | 0.824 | 0.826 | 0.828 | 0.831 | 0.833 | 0.835 | 0.837 | 0.839 | 0.84 | 0.842 | 0.844 | 0.846 | 0.848 | 0.85 | 0.852 | 0.853 | 0.854 | 0.856 |
| Iwate | 0.76 | 0.764 | 0.768 | 0.772 | 0.775 | 0.779 | 0.782 | 0.785 | 0.787 | 0.788 | 0.79 | 0.791 | 0.793 | 0.796 | 0.799 | 0.801 | 0.803 | 0.806 | 0.808 | 0.81 | 0.812 | 0.815 | 0.817 | 0.819 | 0.821 | 0.823 | 0.823 | 0.825 |
| Kagawa | 0.787 | 0.792 | 0.797 | 0.801 | 0.804 | 0.808 | 0.811 | 0.814 | 0.816 | 0.817 | 0.819 | 0.82 | 0.822 | 0.825 | 0.827 | 0.829 | 0.831 | 0.832 | 0.834 | 0.835 | 0.837 | 0.839 | 0.842 | 0.844 | 0.846 | 0.847 | 0.848 | 0.85 |
| Kagoshima | 0.768 | 0.772 | 0.777 | 0.781 | 0.784 | 0.788 | 0.791 | 0.793 | 0.795 | 0.797 | 0.798 | 0.799 | 0.801 | 0.804 | 0.806 | 0.808 | 0.809 | 0.81 | 0.812 | 0.814 | 0.816 | 0.818 | 0.82 | 0.823 | 0.825 | 0.827 | 0.828 | 0.83 |
| Kanagawa | 0.818 | 0.823 | 0.827 | 0.831 | 0.835 | 0.838 | 0.842 | 0.844 | 0.846 | 0.848 | 0.849 | 0.85 | 0.852 | 0.854 | 0.856 | 0.857 | 0.859 | 0.86 | 0.862 | 0.863 | 0.865 | 0.866 | 0.868 | 0.87 | 0.871 | 0.873 | 0.873 | 0.875 |
| Kōchi | 0.759 | 0.763 | 0.767 | 0.771 | 0.775 | 0.779 | 0.783 | 0.786 | 0.788 | 0.791 | 0.793 | 0.795 | 0.797 | 0.8 | 0.803 | 0.805 | 0.806 | 0.808 | 0.81 | 0.812 | 0.813 | 0.816 | 0.818 | 0.82 | 0.822 | 0.823 | 0.824 | 0.825 |
| Kumamoto | 0.77 | 0.774 | 0.778 | 0.782 | 0.785 | 0.789 | 0.792 | 0.794 | 0.796 | 0.798 | 0.799 | 0.801 | 0.803 | 0.805 | 0.808 | 0.81 | 0.811 | 0.812 | 0.814 | 0.815 | 0.817 | 0.82 | 0.822 | 0.825 | 0.827 | 0.829 | 0.83 | 0.832 |
| Kyōto | 0.813 | 0.817 | 0.822 | 0.826 | 0.83 | 0.833 | 0.837 | 0.839 | 0.841 | 0.843 | 0.845 | 0.847 | 0.849 | 0.851 | 0.853 | 0.854 | 0.856 | 0.857 | 0.859 | 0.86 | 0.862 | 0.863 | 0.865 | 0.867 | 0.869 | 0.87 | 0.871 | 0.873 |
| Mie | 0.787 | 0.792 | 0.797 | 0.801 | 0.805 | 0.81 | 0.813 | 0.815 | 0.818 | 0.819 | 0.821 | 0.823 | 0.825 | 0.827 | 0.83 | 0.832 | 0.833 | 0.835 | 0.836 | 0.838 | 0.84 | 0.842 | 0.845 | 0.847 | 0.849 | 0.851 | 0.852 | 0.854 |
| Miyagi | 0.788 | 0.793 | 0.798 | 0.801 | 0.805 | 0.809 | 0.812 | 0.814 | 0.816 | 0.817 | 0.818 | 0.819 | 0.821 | 0.824 | 0.827 | 0.829 | 0.831 | 0.833 | 0.835 | 0.836 | 0.838 | 0.841 | 0.843 | 0.845 | 0.847 | 0.848 | 0.849 | 0.85 |
| Miyazaki | 0.764 | 0.768 | 0.772 | 0.775 | 0.778 | 0.781 | 0.784 | 0.786 | 0.788 | 0.79 | 0.791 | 0.793 | 0.795 | 0.797 | 0.8 | 0.801 | 0.802 | 0.804 | 0.805 | 0.807 | 0.809 | 0.812 | 0.814 | 0.816 | 0.819 | 0.82 | 0.821 | 0.823 |
| Nagano | 0.792 | 0.797 | 0.801 | 0.805 | 0.808 | 0.811 | 0.814 | 0.817 | 0.818 | 0.82 | 0.821 | 0.823 | 0.824 | 0.827 | 0.829 | 0.831 | 0.832 | 0.834 | 0.836 | 0.838 | 0.839 | 0.842 | 0.844 | 0.845 | 0.847 | 0.849 | 0.849 | 0.851 |
| Nagasaki | 0.766 | 0.77 | 0.774 | 0.777 | 0.781 | 0.784 | 0.787 | 0.79 | 0.792 | 0.794 | 0.795 | 0.797 | 0.799 | 0.801 | 0.803 | 0.805 | 0.806 | 0.808 | 0.809 | 0.811 | 0.813 | 0.815 | 0.817 | 0.82 | 0.822 | 0.823 | 0.824 | 0.826 |
| Nara | 0.789 | 0.794 | 0.799 | 0.803 | 0.807 | 0.811 | 0.814 | 0.817 | 0.819 | 0.821 | 0.822 | 0.824 | 0.825 | 0.827 | 0.829 | 0.831 | 0.832 | 0.834 | 0.835 | 0.836 | 0.838 | 0.839 | 0.841 | 0.843 | 0.844 | 0.845 | 0.846 | 0.848 |

**Appendix Table 10: Socio-Demographic Index values for all estimated GBD 2017 locations, 1990-2017**

| Location | 1990 | 1991 | 1992 | 1993 | 1994 | 1995 | 1996 | 1997 | 1998 | 1999 | 2000 | 2001 | 2002 | 2003 | 2004 | 2005 | 2006 | 2007 | 2008 | 2009 | 2010 | 2011 | 2012 | 2013 | 2014 | 2015 | 2016 | 2017 |
|---|---|---|---|---|---|---|---|---|---|---|---|---|---|---|---|---|---|---|---|---|---|---|---|---|---|---|---|---|
| Niigata | 0.776 | 0.78 | 0.784 | 0.788 | 0.792 | 0.795 | 0.799 | 0.801 | 0.804 | 0.806 | 0.808 | 0.81 | 0.813 | 0.815 | 0.818 | 0.82 | 0.822 | 0.824 | 0.826 | 0.828 | 0.83 | 0.832 | 0.835 | 0.837 | 0.839 | 0.84 | 0.842 | 0.843 |
| Ōita | 0.785 | 0.789 | 0.795 | 0.802 | 0.808 | 0.809 | 0.809 | 0.81 | 0.812 | 0.813 | 0.815 | 0.816 | 0.819 | 0.821 | 0.824 | 0.825 | 0.827 | 0.828 | 0.83 | 0.831 | 0.833 | 0.835 | 0.838 | 0.84 | 0.842 | 0.843 | 0.844 | 0.846 |
| Okayama | 0.79 | 0.794 | 0.799 | 0.803 | 0.807 | 0.811 | 0.814 | 0.817 | 0.819 | 0.821 | 0.823 | 0.824 | 0.827 | 0.829 | 0.832 | 0.834 | 0.836 | 0.837 | 0.839 | 0.841 | 0.843 | 0.846 | 0.848 | 0.85 | 0.852 | 0.853 | 0.854 | 0.856 |
| Okinawa | 0.753 | 0.757 | 0.764 | 0.772 | 0.778 | 0.779 | 0.777 | 0.777 | 0.778 | 0.78 | 0.781 | 0.783 | 0.786 | 0.789 | 0.793 | 0.795 | 0.797 | 0.799 | 0.8 | 0.802 | 0.804 | 0.807 | 0.809 | 0.812 | 0.814 | 0.815 | 0.816 | 0.818 |
| Ōsaka | 0.815 | 0.819 | 0.823 | 0.827 | 0.831 | 0.834 | 0.837 | 0.84 | 0.842 | 0.844 | 0.845 | 0.847 | 0.849 | 0.851 | 0.853 | 0.854 | 0.855 | 0.856 | 0.858 | 0.859 | 0.861 | 0.863 | 0.865 | 0.867 | 0.868 | 0.87 | 0.871 | 0.872 |
| Saga | 0.773 | 0.777 | 0.782 | 0.785 | 0.788 | 0.792 | 0.795 | 0.797 | 0.799 | 0.8 | 0.801 | 0.803 | 0.805 | 0.808 | 0.81 | 0.812 | 0.814 | 0.815 | 0.817 | 0.819 | 0.821 | 0.823 | 0.825 | 0.827 | 0.83 | 0.831 | 0.832 | 0.834 |
| Saitama | 0.793 | 0.798 | 0.802 | 0.806 | 0.81 | 0.814 | 0.818 | 0.82 | 0.822 | 0.824 | 0.825 | 0.826 | 0.827 | 0.829 | 0.831 | 0.833 | 0.835 | 0.836 | 0.838 | 0.839 | 0.841 | 0.843 | 0.845 | 0.847 | 0.848 | 0.849 | 0.85 | 0.852 |
| Shiga | 0.804 | 0.809 | 0.814 | 0.819 | 0.823 | 0.827 | 0.83 | 0.833 | 0.836 | 0.838 | 0.84 | 0.841 | 0.843 | 0.846 | 0.848 | 0.85 | 0.852 | 0.853 | 0.855 | 0.857 | 0.858 | 0.861 | 0.863 | 0.865 | 0.867 | 0.868 | 0.869 | 0.871 |
| Shimane | 0.762 | 0.766 | 0.771 | 0.775 | 0.779 | 0.783 | 0.787 | 0.79 | 0.793 | 0.795 | 0.797 | 0.798 | 0.801 | 0.803 | 0.806 | 0.808 | 0.81 | 0.812 | 0.814 | 0.816 | 0.818 | 0.82 | 0.823 | 0.825 | 0.827 | 0.828 | 0.829 | 0.831 |
| Shizuoka | 0.798 | 0.802 | 0.807 | 0.81 | 0.814 | 0.818 | 0.821 | 0.823 | 0.825 | 0.826 | 0.827 | 0.828 | 0.83 | 0.833 | 0.835 | 0.837 | 0.838 | 0.84 | 0.842 | 0.843 | 0.846 | 0.848 | 0.85 | 0.853 | 0.855 | 0.856 | 0.857 | 0.859 |
| Tochigi | 0.787 | 0.792 | 0.797 | 0.801 | 0.805 | 0.809 | 0.813 | 0.815 | 0.817 | 0.819 | 0.82 | 0.821 | 0.823 | 0.826 | 0.829 | 0.831 | 0.832 | 0.834 | 0.836 | 0.838 | 0.84 | 0.842 | 0.845 | 0.847 | 0.849 | 0.85 | 0.851 | 0.853 |
| Tokushima | 0.776 | 0.781 | 0.786 | 0.79 | 0.794 | 0.798 | 0.802 | 0.805 | 0.807 | 0.809 | 0.812 | 0.814 | 0.816 | 0.819 | 0.822 | 0.824 | 0.826 | 0.828 | 0.83 | 0.831 | 0.833 | 0.835 | 0.837 | 0.839 | 0.841 | 0.842 | 0.843 | 0.845 |
| Tōkyō | 0.87 | 0.875 | 0.879 | 0.883 | 0.887 | 0.892 | 0.896 | 0.899 | 0.901 | 0.903 | 0.905 | 0.906 | 0.908 | 0.91 | 0.911 | 0.913 | 0.914 | 0.915 | 0.916 | 0.917 | 0.918 | 0.919 | 0.92 | 0.921 | 0.922 | 0.923 | 0.923 | 0.924 |
| Tottori | 0.77 | 0.775 | 0.78 | 0.783 | 0.787 | 0.791 | 0.794 | 0.797 | 0.799 | 0.801 | 0.802 | 0.804 | 0.806 | 0.808 | 0.811 | 0.813 | 0.814 | 0.816 | 0.818 | 0.819 | 0.821 | 0.824 | 0.826 | 0.828 | 0.831 | 0.832 | 0.833 | 0.834 |
| Toyama | 0.79 | 0.795 | 0.8 | 0.805 | 0.808 | 0.812 | 0.816 | 0.818 | 0.821 | 0.823 | 0.825 | 0.827 | 0.829 | 0.832 | 0.835 | 0.837 | 0.839 | 0.841 | 0.843 | 0.845 | 0.847 | 0.849 | 0.852 | 0.854 | 0.856 | 0.857 | 0.858 | 0.86 |
| Wakayama | 0.775 | 0.78 | 0.785 | 0.789 | 0.792 | 0.796 | 0.8 | 0.802 | 0.805 | 0.806 | 0.808 | 0.81 | 0.812 | 0.815 | 0.817 | 0.819 | 0.821 | 0.822 | 0.824 | 0.825 | 0.827 | 0.829 | 0.831 | 0.833 | 0.836 | 0.837 | 0.838 | 0.84 |
| Yamagata | 0.766 | 0.77 | 0.775 | 0.778 | 0.782 | 0.785 | 0.788 | 0.791 | 0.792 | 0.794 | 0.795 | 0.796 | 0.799 | 0.802 | 0.805 | 0.807 | 0.809 | 0.812 | 0.814 | 0.816 | 0.819 | 0.821 | 0.824 | 0.826 | 0.828 | 0.829 | 0.83 | 0.832 |
| Yamaguchi | 0.79 | 0.794 | 0.799 | 0.802 | 0.806 | 0.809 | 0.812 | 0.815 | 0.817 | 0.819 | 0.82 | 0.822 | 0.824 | 0.826 | 0.828 | 0.83 | 0.831 | 0.832 | 0.834 | 0.835 | 0.837 | 0.839 | 0.841 | 0.843 | 0.846 | 0.847 | 0.848 | 0.849 |
| Yamanashi | 0.791 | 0.796 | 0.8 | 0.804 | 0.808 | 0.812 | 0.815 | 0.818 | 0.82 | 0.822 | 0.823 | 0.825 | 0.827 | 0.829 | 0.832 | 0.834 | 0.836 | 0.837 | 0.839 | 0.841 | 0.842 | 0.844 | 0.846 | 0.848 | 0.85 | 0.851 | 0.853 | 0.854 |
| South Korea | 0.713 | 0.724 | 0.733 | 0.742 | 0.751 | 0.76 | 0.768 | 0.777 | 0.783 | 0.79 | 0.799 | 0.806 | 0.814 | 0.82 | 0.825 | 0.83 | 0.834 | 0.839 | 0.843 | 0.846 | 0.85 | 0.853 | 0.857 | 0.86 | 0.864 | 0.867 | 0.869 | 0.872 |
| Singapore | 0.736 | 0.744 | 0.75 | 0.758 | 0.765 | 0.772 | 0.78 | 0.786 | 0.79 | 0.79 | 0.797 | 0.808 | 0.815 | 0.82 | 0.824 | 0.827 | 0.832 | 0.838 | 0.844 | 0.849 | 0.854 | 0.857 | 0.86 | 0.863 | 0.865 | 0.868 | 0.87 | 0.872 |
| High-income North America | 0.784 | 0.786 | 0.789 | 0.793 | 0.796 | 0.8 | 0.805 | 0.807 | 0.809 | 0.812 | 0.815 | 0.82 | 0.824 | 0.827 | 0.829 | 0.83 | 0.829 | 0.832 | 0.837 | 0.843 | 0.848 | 0.852 | 0.856 | 0.859 | 0.861 | 0.865 | 0.867 | 0.868 |
| Canada | 0.802 | 0.805 | 0.808 | 0.811 | 0.814 | 0.818 | 0.823 | 0.828 | 0.832 | 0.836 | 0.841 | 0.846 | 0.85 | 0.853 | 0.857 | 0.859 | 0.861 | 0.862 | 0.864 | 0.867 | 0.871 | 0.874 | 0.877 | 0.878 | 0.879 | 0.88 | 0.881 | 0.882 |
| Greenland | 0.671 | 0.67 | 0.67 | 0.671 | 0.672 | 0.675 | 0.679 | 0.683 | 0.687 | 0.691 | 0.695 | 0.699 | 0.703 | 0.708 | 0.713 | 0.717 | 0.722 | 0.726 | 0.731 | 0.737 | 0.743 | 0.747 | 0.751 | 0.754 | 0.756 | 0.757 | 0.759 | 0.76 |
| USA | 0.781 | 0.784 | 0.787 | 0.79 | 0.793 | 0.798 | 0.803 | 0.804 | 0.806 | 0.809 | 0.812 | 0.817 | 0.821 | 0.823 | 0.826 | 0.826 | 0.826 | 0.829 | 0.834 | 0.84 | 0.846 | 0.85 | 0.853 | 0.857 | 0.859 | 0.863 | 0.866 | 0.867 |
| Alabama | 0.745 | 0.749 | 0.753 | 0.757 | 0.76 | 0.765 | 0.769 | 0.769 | 0.771 | 0.773 | 0.777 | 0.783 | 0.787 | 0.791 | 0.794 | 0.794 | 0.793 | 0.796 | 0.801 | 0.809 | 0.816 | 0.82 | 0.824 | 0.827 | 0.83 | 0.834 | 0.837 | 0.837 |
| Alaska | 0.755 | 0.757 | 0.761 | 0.767 | 0.772 | 0.781 | 0.788 | 0.79 | 0.793 | 0.795 | 0.799 | 0.804 | 0.808 | 0.811 | 0.814 | 0.813 | 0.811 | 0.813 | 0.818 | 0.826 | 0.833 | 0.838 | 0.843 | 0.847 | 0.851 | 0.857 | 0.86 | 0.861 |
| Arizona | 0.751 | 0.753 | 0.755 | 0.758 | 0.76 | 0.764 | 0.769 | 0.77 | 0.771 | 0.772 | 0.775 | 0.781 | 0.784 | 0.787 | 0.79 | 0.791 | 0.791 | 0.797 | 0.806 | 0.815 | 0.823 | 0.828 | 0.832 | 0.836 | 0.839 | 0.842 | 0.845 | 0.845 |
| Arkansas | 0.723 | 0.727 | 0.733 | 0.738 | 0.741 | 0.746 | 0.751 | 0.751 | 0.753 | 0.754 | 0.757 | 0.763 | 0.767 | 0.77 | 0.773 | 0.772 | 0.77 | 0.774 | 0.78 | 0.789 | 0.797 | 0.802 | 0.807 | 0.811 | 0.816 | 0.821 | 0.825 | 0.826 |
| California | 0.771 | 0.771 | 0.773 | 0.776 | 0.78 | 0.787 | 0.794 | 0.798 | 0.803 | 0.807 | 0.812 | 0.819 | 0.823 | 0.826 | 0.829 | 0.83 | 0.831 | 0.835 | 0.84 | 0.846 | 0.852 | 0.856 | 0.859 | 0.863 | 0.865 | 0.869 | 0.871 | 0.872 |
| Colorado | 0.798 | 0.801 | 0.804 | 0.807 | 0.81 | 0.815 | 0.818 | 0.819 | 0.82 | 0.821 | 0.824 | 0.83 | 0.833 | 0.836 | 0.839 | 0.84 | 0.84 | 0.844 | 0.849 | 0.855 | 0.861 | 0.865 | 0.869 | 0.872 | 0.875 | 0.879 | 0.881 | 0.882 |
| Connecticut | 0.841 | 0.844 | 0.847 | 0.85 | 0.853 | 0.856 | 0.859 | 0.861 | 0.863 | 0.866 | 0.869 | 0.874 | 0.877 | 0.879 | 0.881 | 0.881 | 0.881 | 0.883 | 0.886 | 0.89 | 0.894 | 0.896 | 0.899 | 0.901 | 0.903 | 0.905 | 0.906 | 0.906 |
| Delaware | 0.801 | 0.804 | 0.808 | 0.811 | 0.813 | 0.816 | 0.819 | 0.819 | 0.82 | 0.822 | 0.825 | 0.83 | 0.833 | 0.836 | 0.838 | 0.839 | 0.838 | 0.841 | 0.846 | 0.852 | 0.858 | 0.861 | 0.864 | 0.866 | 0.868 | 0.871 | 0.873 | 0.874 |
| Washington, DC | 0.797 | 0.801 | 0.806 | 0.812 | 0.818 | 0.826 | 0.834 | 0.839 | 0.845 | 0.85 | 0.855 | 0.861 | 0.865 | 0.868 | 0.869 | 0.868 | 0.866 | 0.866 | 0.868 | 0.871 | 0.874 | 0.876 | 0.877 | 0.88 | 0.883 | 0.887 | 0.89 | 0.89 |
| Florida | 0.774 | 0.778 | 0.782 | 0.786 | 0.789 | 0.794 | 0.799 | 0.8 | 0.802 | 0.804 | 0.808 | 0.813 | 0.817 | 0.82 | 0.823 | 0.824 | 0.825 | 0.828 | 0.834 | 0.84 | 0.846 | 0.85 | 0.853 | 0.855 | 0.858 | 0.861 | 0.863 | 0.864 |
| Georgia | 0.755 | 0.759 | 0.764 | 0.769 | 0.772 | 0.778 | 0.782 | 0.783 | 0.784 | 0.785 | 0.788 | 0.793 | 0.796 | 0.798 | 0.801 | 0.801 | 0.8 | 0.804 | 0.81 | 0.818 | 0.825 | 0.83 | 0.834 | 0.837 | 0.841 | 0.845 | 0.848 | 0.848 |
| Hawaii | 0.781 | 0.784 | 0.788 | 0.792 | 0.797 | 0.805 | 0.811 | 0.813 | 0.815 | 0.817 | 0.82 | 0.824 | 0.828 | 0.83 | 0.833 | 0.833 | 0.832 | 0.834 | 0.839 | 0.845 | 0.851 | 0.854 | 0.858 | 0.861 | 0.864 | 0.868 | 0.871 | 0.872 |
| Idaho | 0.756 | 0.76 | 0.764 | 0.769 | 0.773 | 0.778 | 0.783 | 0.783 | 0.784 | 0.785 | 0.787 | 0.791 | 0.794 | 0.796 | 0.799 | 0.798 | 0.797 | 0.801 | 0.807 | 0.814 | 0.821 | 0.824 | 0.827 | 0.83 | 0.832 | 0.836 | 0.84 | 0.841 |
| Illinois | 0.787 | 0.789 | 0.792 | 0.796 | 0.799 | 0.805 | 0.81 | 0.812 | 0.816 | 0.818 | 0.823 | 0.828 | 0.833 | 0.837 | 0.84 | 0.841 | 0.841 | 0.845 | 0.85 | 0.855 | 0.861 | 0.864 | 0.867 | 0.87 | 0.873 | 0.876 | 0.879 | 0.879 |
| Indiana | 0.772 | 0.774 | 0.778 | 0.781 | 0.783 | 0.787 | 0.791 | 0.791 | 0.793 | 0.794 | 0.797 | 0.802 | 0.806 | 0.808 | 0.81 | 0.809 | 0.807 | 0.809 | 0.813 | 0.819 | 0.825 | 0.829 | 0.832 | 0.836 | 0.839 | 0.844 | 0.847 | 0.848 |
| Iowa | 0.794 | 0.797 | 0.8 | 0.803 | 0.806 | 0.809 | 0.813 | 0.814 | 0.816 | 0.818 | 0.821 | 0.825 | 0.828 | 0.829 | 0.832 | 0.831 | 0.83 | 0.832 | 0.837 | 0.843 | 0.848 | 0.852 | 0.856 | 0.86 | 0.863 | 0.867 | 0.87 | 0.87 |
| Kansas | 0.782 | 0.785 | 0.788 | 0.791 | 0.794 | 0.798 | 0.802 | 0.803 | 0.804 | 0.806 | 0.809 | 0.814 | 0.817 | 0.819 | 0.821 | 0.819 | 0.817 | 0.819 | 0.823 | 0.83 | 0.837 | 0.842 | 0.847 | 0.852 | 0.856 | 0.861 | 0.864 | 0.864 |
| Kentucky | 0.743 | 0.747 | 0.751 | 0.755 | 0.759 | 0.763 | 0.768 | 0.769 | 0.77 | 0.772 | 0.776 | 0.782 | 0.786 | 0.789 | 0.791 | 0.79 | 0.787 | 0.789 | 0.793 | 0.799 | 0.806 | 0.81 | 0.814 | 0.817 | 0.821 | 0.827 | 0.83 | 0.831 |
| Louisiana | 0.735 | 0.739 | 0.743 | 0.748 | 0.752 | 0.758 | 0.762 | 0.764 | 0.765 | 0.767 | 0.771 | 0.777 | 0.782 | 0.786 | 0.789 | 0.788 | 0.787 | 0.79 | 0.796 | 0.803 | 0.811 | 0.815 | 0.819 | 0.823 | 0.826 | 0.831 | 0.835 | 0.835 |
| Maine | 0.789 | 0.795 | 0.8 | 0.805 | 0.809 | 0.814 | 0.817 | 0.819 | 0.821 | 0.823 | 0.826 | 0.83 | 0.834 | 0.837 | 0.84 | 0.84 | 0.84 | 0.842 | 0.846 | 0.851 | 0.856 | 0.859 | 0.861 | 0.864 | 0.866 | 0.869 | 0.871 | 0.872 |
| Maryland | 0.813 | 0.817 | 0.821 | 0.825 | 0.828 | 0.833 | 0.836 | 0.838 | 0.839 | 0.841 | 0.845 | 0.85 | 0.854 | 0.857 | 0.86 | 0.861 | 0.861 | 0.864 | 0.868 | 0.874 | 0.879 | 0.883 | 0.886 | 0.889 | 0.891 | 0.894 | 0.896 | 0.896 |
| Massachusetts | 0.843 | 0.847 | 0.85 | 0.853 | 0.856 | 0.86 | 0.864 | 0.866 | 0.869 | 0.871 | 0.875 | 0.88 | 0.883 | 0.886 | 0.888 | 0.889 | 0.89 | 0.893 | 0.896 | 0.899 | 0.902 | 0.904 | 0.907 | 0.909 | 0.91 | 0.912 | 0.913 | 0.913 |
| Michigan | 0.788 | 0.791 | 0.795 | 0.799 | 0.803 | 0.808 | 0.813 | 0.815 | 0.817 | 0.82 | 0.823 | 0.829 | 0.832 | 0.835 | 0.838 | 0.838 | 0.837 | 0.839 | 0.841 | 0.845 | 0.849 | 0.852 | 0.854 | 0.857 | 0.86 | 0.864 | 0.867 | 0.868 |
| Minnesota | 0.818 | 0.821 | 0.825 | 0.828 | 0.83 | 0.834 | 0.837 | 0.839 | 0.841 | 0.843 | 0.845 | 0.849 | 0.852 | 0.855 | 0.857 | 0.858 | 0.858 | 0.86 | 0.865 | 0.87 | 0.874 | 0.877 | 0.881 | 0.884 | 0.886 | 0.89 | 0.892 | 0.893 |
| Mississippi | 0.719 | 0.722 | 0.727 | 0.732 | 0.736 | 0.741 | 0.746 | 0.747 | 0.748 | 0.75 | 0.753 | 0.758 | 0.762 | 0.764 | 0.765 | 0.764 | 0.76 | 0.763 | 0.77 | 0.779 | 0.789 | 0.794 | 0.8 | 0.804 | 0.809 | 0.814 | 0.818 | 0.819 |
| Missouri | 0.77 | 0.773 | 0.777 | 0.781 | 0.784 | 0.789 | 0.793 | 0.795 | 0.797 | 0.799 | 0.802 | 0.806 | 0.81 | 0.812 | 0.813 | 0.812 | 0.811 | 0.813 | 0.818 | 0.825 | 0.831 | 0.835 | 0.839 | 0.842 | 0.846 | 0.85 | 0.853 | 0.853 |
| Montana | 0.775 | 0.779 | 0.783 | 0.789 | 0.793 | 0.799 | 0.804 | 0.805 | 0.807 | 0.808 | 0.81 | 0.814 | 0.816 | 0.819 | 0.822 | 0.822 | 0.821 | 0.823 | 0.827 | 0.833 | 0.839 | 0.843 | 0.847 | 0.851 | 0.855 | 0.859 | 0.862 | 0.863 |
| Nebraska | 0.795 | 0.798 | 0.802 | 0.805 | 0.808 | 0.813 | 0.817 | 0.817 | 0.819 | 0.819 | 0.821 | 0.824 | 0.827 | 0.829 | 0.831 | 0.831 | 0.83 | 0.832 | 0.836 | 0.842 | 0.848 | 0.853 | 0.857 | 0.861 | 0.865 | 0.869 | 0.872 | 0.873 |
| Nevada | 0.76 | 0.761 | 0.762 | 0.765 | 0.767 | 0.773 | 0.779 | 0.781 | 0.784 | 0.787 | 0.791 | 0.798 | 0.802 | 0.805 | 0.808 | 0.809 | 0.81 | 0.814 | 0.82 | 0.827 | 0.833 | 0.836 | 0.838 | 0.84 | 0.842 | 0.845 | 0.847 | 0.847 |
| New Hampshire | 0.827 | 0.832 | 0.835 | 0.838 | 0.84 | 0.844 | 0.847 | 0.849 | 0.852 | 0.856 | 0.86 | 0.865 | 0.869 | 0.872 | 0.874 | 0.875 | 0.876 | 0.878 | 0.881 | 0.885 | 0.889 | 0.891 | 0.894 | 0.897 | 0.899 | 0.902 | 0.904 | 0.904 |
| New Jersey | 0.828 | 0.831 | 0.834 | 0.838 | 0.841 | 0.845 | 0.849 | 0.85 | 0.852 | 0.854 | 0.857 | 0.861 | 0.864 | 0.867 | 0.87 | 0.87 | 0.871 | 0.873 | 0.876 | 0.881 | 0.885 | 0.888 | 0.89 | 0.893 | 0.895 | 0.897 | 0.899 | 0.899 |
| New Mexico | 0.733 | 0.735 | 0.739 | 0.743 | 0.748 | 0.755 | 0.76 | 0.762 | 0.764 | 0.765 | 0.767 | 0.773 | 0.777 | 0.779 | 0.782 | 0.781 | 0.779 | 0.783 | 0.79 | 0.799 | 0.808 | 0.814 | 0.819 | 0.823 | 0.828 | 0.832 | 0.835 | 0.835 |
| New York | 0.816 | 0.819 | 0.822 | 0.825 | 0.828 | 0.832 | 0.836 | 0.838 | 0.841 | 0.843 | 0.847 | 0.852 | 0.855 | 0.858 | 0.86 | 0.861 | 0.861 | 0.864 | 0.868 | 0.872 | 0.877 | 0.88 | 0.883 | 0.886 | 0.889 | 0.892 | 0.893 | 0.893 |
| North Carolina | 0.763 | 0.766 | 0.77 | 0.774 | 0.777 | 0.781 | 0.785 | 0.785 | 0.786 | 0.788 | 0.791 | 0.796 | 0.8 | 0.802 | 0.805 | 0.805 | 0.805 | 0.809 | 0.815 | 0.822 | 0.829 | 0.833 | 0.836 | 0.84 | 0.843 | 0.846 | 0.849 | 0.85 |
| North Dakota | 0.79 | 0.792 | 0.796 | 0.799 | 0.802 | 0.807 | 0.813 | 0.816 | 0.82 | 0.823 | 0.827 | 0.83 | 0.833 | 0.834 | 0.835 | 0.835 | 0.833 | 0.835 | 0.839 | 0.844 | 0.85 | 0.854 | 0.859 | 0.864 | 0.87 | 0.875 | 0.879 | 0.88 |
| Ohio | 0.78 | 0.783 | 0.787 | 0.79 | 0.793 | 0.797 | 0.801 | 0.802 | 0.804 | 0.806 | 0.809 | 0.814 | 0.818 | 0.82 | 0.822 | 0.822 | 0.821 | 0.822 | 0.826 | 0.832 | 0.837 | 0.841 | 0.844 | 0.847 | 0.85 | 0.854 | 0.857 | 0.858 |
| Oklahoma | 0.749 | 0.751 | 0.755 | 0.758 | 0.76 | 0.764 | 0.768 | 0.768 | 0.768 | 0.769 | 0.772 | 0.777 | 0.781 | 0.784 | 0.786 | 0.785 | 0.784 | 0.786 | 0.792 | 0.8 | 0.808 | 0.813 | 0.818 | 0.824 | 0.829 | 0.835 | 0.838 | 0.838 |
| Oregon | 0.785 | 0.788 | 0.791 | 0.794 | 0.797 | 0.802 | 0.806 | 0.808 | 0.811 | 0.814 | 0.818 | 0.824 | 0.827 | 0.83 | 0.833 | 0.833 | 0.833 | 0.836 | 0.841 | 0.847 | 0.852 | 0.855 | 0.858 | 0.861 | 0.864 | 0.867 | 0.87 | 0.871 |
| Pennsylvania | 0.8 | 0.804 | 0.808 | 0.812 | 0.816 | 0.821 | 0.824 | 0.826 | 0.828 | 0.83 | 0.833 | 0.837 | 0.84 | 0.842 | 0.845 | 0.845 | 0.845 | 0.847 | 0.851 | 0.856 | 0.86 | 0.863 | 0.866 | 0.869 | 0.871 | 0.875 | 0.878 | 0.879 |
| Rhode Island | 0.815 | 0.818 | 0.822 | 0.825 | 0.827 | 0.831 | 0.834 | 0.835 | 0.838 | 0.84 | 0.843 | 0.848 | 0.851 | 0.854 | 0.857 | 0.858 | 0.859 | 0.862 | 0.866 | 0.87 | 0.875 | 0.877 | 0.88 | 0.882 | 0.885 | 0.887 | 0.889 | 0.89 |
| South Carolina | 0.752 | 0.757 | 0.762 | 0.768 | 0.772 | 0.777 | 0.781 | 0.782 | 0.783 | 0.785 | 0.787 | 0.793 | 0.796 | 0.799 | 0.801 | 0.8 | 0.799 | 0.802 | 0.808 | 0.815 | 0.822 | 0.826 | 0.83 | 0.834 | 0.838 | 0.842 | 0.845 | 0.846 |
| South Dakota | 0.769 | 0.772 | 0.777 | 0.783 | 0.788 | 0.794 | 0.799 | 0.801 | 0.804 | 0.805 | 0.808 | 0.811 | 0.813 | 0.814 | 0.816 | 0.814 | 0.812 | 0.814 | 0.819 | 0.826 | 0.833 | 0.838 | 0.842 | 0.847 | 0.851 | 0.856 | 0.859 | 0.86 |
| Tennessee | 0.749 | 0.752 | 0.757 | 0.761 | 0.765 | 0.77 | 0.774 | 0.775 | 0.777 | 0.779 | 0.781 | 0.786 | 0.788 | 0.789 | 0.79 | 0.789 | 0.786 | 0.789 | 0.795 | 0.803 | 0.81 | 0.815 | 0.819 | 0.823 | 0.827 | 0.832 | 0.836 | 0.837 |
| Texas | 0.743 | 0.745 | 0.747 | 0.75 | 0.752 | 0.757 | 0.761 | 0.763 | 0.764 | 0.766 | 0.769 | 0.775 | 0.779 | 0.782 | 0.784 | 0.783 | 0.782 | 0.785 | 0.792 | 0.801 | 0.809 | 0.815 | 0.82 | 0.824 | 0.829 | 0.834 | 0.837 | 0.838 |

**Appendix Table 10: Socio-Demographic Index values for all estimated GBD 2017 locations, 1990-2017**

| Location | 1990 | 1991 | 1992 | 1993 | 1994 | 1995 | 1996 | 1997 | 1998 | 1999 | 2000 | 2001 | 2002 | 2003 | 2004 | 2005 | 2006 | 2007 | 2008 | 2009 | 2010 | 2011 | 2012 | 2013 | 2014 | 2015 | 2016 | 2017 |
|---|---|---|---|---|---|---|---|---|---|---|---|---|---|---|---|---|---|---|---|---|---|---|---|---|---|---|---|---|
| Utah | 0.761 | 0.765 | 0.769 | 0.773 | 0.776 | 0.781 | 0.786 | 0.787 | 0.79 | 0.792 | 0.795 | 0.8 | 0.803 | 0.806 | 0.808 | 0.808 | 0.807 | 0.811 | 0.817 | 0.825 | 0.832 | 0.837 | 0.841 | 0.845 | 0.848 | 0.852 | 0.855 | 0.856 |
| Vermont | 0.815 | 0.819 | 0.823 | 0.827 | 0.83 | 0.833 | 0.837 | 0.839 | 0.841 | 0.844 | 0.848 | 0.853 | 0.857 | 0.86 | 0.864 | 0.866 | 0.866 | 0.869 | 0.872 | 0.876 | 0.88 | 0.882 | 0.885 | 0.887 | 0.89 | 0.893 | 0.895 | 0.896 |
| Virginia | 0.8 | 0.803 | 0.807 | 0.81 | 0.814 | 0.818 | 0.822 | 0.823 | 0.824 | 0.826 | 0.829 | 0.834 | 0.838 | 0.841 | 0.845 | 0.846 | 0.847 | 0.851 | 0.856 | 0.862 | 0.867 | 0.871 | 0.874 | 0.877 | 0.88 | 0.883 | 0.885 | 0.885 |
| Washington | 0.797 | 0.8 | 0.804 | 0.807 | 0.811 | 0.816 | 0.821 | 0.823 | 0.827 | 0.83 | 0.834 | 0.84 | 0.844 | 0.846 | 0.849 | 0.848 | 0.848 | 0.85 | 0.855 | 0.86 | 0.865 | 0.868 | 0.871 | 0.874 | 0.877 | 0.881 | 0.883 | 0.884 |
| West Virginia | 0.749 | 0.752 | 0.756 | 0.761 | 0.764 | 0.769 | 0.773 | 0.774 | 0.775 | 0.776 | 0.778 | 0.783 | 0.786 | 0.787 | 0.789 | 0.787 | 0.784 | 0.784 | 0.787 | 0.793 | 0.799 | 0.802 | 0.806 | 0.81 | 0.814 | 0.82 | 0.824 | 0.825 |
| Wisconsin | 0.801 | 0.804 | 0.808 | 0.812 | 0.815 | 0.819 | 0.823 | 0.825 | 0.826 | 0.828 | 0.831 | 0.835 | 0.839 | 0.841 | 0.843 | 0.843 | 0.843 | 0.845 | 0.849 | 0.853 | 0.858 | 0.862 | 0.865 | 0.868 | 0.871 | 0.874 | 0.877 | 0.878 |
| Wyoming | 0.766 | 0.771 | 0.777 | 0.782 | 0.786 | 0.792 | 0.796 | 0.797 | 0.799 | 0.801 | 0.804 | 0.809 | 0.813 | 0.816 | 0.818 | 0.819 | 0.819 | 0.823 | 0.831 | 0.838 | 0.846 | 0.851 | 0.855 | 0.858 | 0.862 | 0.866 | 0.869 | 0.869 |
| Southern Latin America | 0.594 | 0.6 | 0.607 | 0.613 | 0.619 | 0.626 | 0.632 | 0.638 | 0.643 | 0.648 | 0.652 | 0.655 | 0.658 | 0.662 | 0.667 | 0.673 | 0.677 | 0.679 | 0.682 | 0.685 | 0.69 | 0.695 | 0.7 | 0.704 | 0.707 | 0.713 | 0.717 | 0.72 |
| Argentina | 0.59 | 0.595 | 0.604 | 0.61 | 0.617 | 0.624 | 0.63 | 0.635 | 0.64 | 0.644 | 0.647 | 0.649 | 0.65 | 0.653 | 0.658 | 0.665 | 0.669 | 0.672 | 0.675 | 0.677 | 0.681 | 0.686 | 0.691 | 0.693 | 0.696 | 0.702 | 0.707 | 0.71 |
| Chile | 0.6 | 0.608 | 0.615 | 0.62 | 0.626 | 0.633 | 0.64 | 0.647 | 0.654 | 0.661 | 0.667 | 0.674 | 0.681 | 0.687 | 0.692 | 0.696 | 0.698 | 0.701 | 0.704 | 0.708 | 0.714 | 0.721 | 0.727 | 0.732 | 0.738 | 0.742 | 0.746 | 0.748 |
| Uruguay | 0.592 | 0.597 | 0.6 | 0.602 | 0.606 | 0.609 | 0.613 | 0.618 | 0.625 | 0.632 | 0.637 | 0.64 | 0.643 | 0.647 | 0.652 | 0.656 | 0.659 | 0.661 | 0.663 | 0.666 | 0.671 | 0.675 | 0.68 | 0.685 | 0.691 | 0.697 | 0.702 | 0.707 |
| Western Europe | 0.764 | 0.77 | 0.776 | 0.782 | 0.787 | 0.791 | 0.795 | 0.798 | 0.801 | 0.805 | 0.809 | 0.813 | 0.817 | 0.82 | 0.822 | 0.825 | 0.828 | 0.83 | 0.833 | 0.836 | 0.838 | 0.842 | 0.845 | 0.848 | 0.851 | 0.853 | 0.855 | 0.857 |
| Andorra | 0.85 | 0.854 | 0.856 | 0.857 | 0.858 | 0.859 | 0.86 | 0.863 | 0.866 | 0.868 | 0.871 | 0.873 | 0.875 | 0.878 | 0.881 | 0.883 | 0.885 | 0.886 | 0.888 | 0.89 | 0.891 | 0.894 | 0.896 | 0.897 | 0.899 | 0.9 | 0.901 | 0.902 |
| Austria | 0.776 | 0.778 | 0.78 | 0.785 | 0.79 | 0.795 | 0.8 | 0.805 | 0.809 | 0.813 | 0.818 | 0.822 | 0.825 | 0.828 | 0.831 | 0.834 | 0.838 | 0.841 | 0.845 | 0.847 | 0.85 | 0.854 | 0.857 | 0.859 | 0.862 | 0.863 | 0.865 | 0.866 |
| Belgium | 0.803 | 0.808 | 0.813 | 0.818 | 0.822 | 0.826 | 0.829 | 0.832 | 0.835 | 0.837 | 0.84 | 0.843 | 0.847 | 0.849 | 0.852 | 0.854 | 0.856 | 0.858 | 0.861 | 0.863 | 0.867 | 0.871 | 0.875 | 0.879 | 0.882 | 0.884 | 0.885 | 0.886 |
| Cyprus | 0.724 | 0.73 | 0.74 | 0.75 | 0.758 | 0.765 | 0.771 | 0.778 | 0.784 | 0.789 | 0.795 | 0.803 | 0.81 | 0.817 | 0.824 | 0.83 | 0.837 | 0.842 | 0.847 | 0.851 | 0.854 | 0.857 | 0.859 | 0.861 | 0.862 | 0.863 | 0.864 | 0.865 |
| Denmark | 0.846 | 0.849 | 0.852 | 0.855 | 0.858 | 0.862 | 0.866 | 0.87 | 0.874 | 0.877 | 0.881 | 0.884 | 0.888 | 0.891 | 0.893 | 0.895 | 0.897 | 0.898 | 0.9 | 0.902 | 0.904 | 0.907 | 0.91 | 0.912 | 0.914 | 0.915 | 0.916 | 0.918 |
| Finland | 0.813 | 0.813 | 0.814 | 0.817 | 0.821 | 0.825 | 0.828 | 0.831 | 0.835 | 0.84 | 0.844 | 0.847 | 0.851 | 0.854 | 0.857 | 0.859 | 0.862 | 0.865 | 0.869 | 0.871 | 0.875 | 0.878 | 0.881 | 0.884 | 0.887 | 0.889 | 0.891 | 0.893 |
| France | 0.769 | 0.776 | 0.783 | 0.79 | 0.793 | 0.795 | 0.802 | 0.806 | 0.808 | 0.813 | 0.816 | 0.819 | 0.824 | 0.827 | 0.83 | 0.833 | 0.836 | 0.838 | 0.84 | 0.842 | 0.845 | 0.848 | 0.851 | 0.854 | 0.857 | 0.86 | 0.863 | 0.865 |
| Germany | 0.787 | 0.796 | 0.801 | 0.805 | 0.809 | 0.811 | 0.812 | 0.813 | 0.813 | 0.814 | 0.818 | 0.823 | 0.827 | 0.829 | 0.832 | 0.835 | 0.838 | 0.842 | 0.846 | 0.848 | 0.851 | 0.855 | 0.858 | 0.861 | 0.864 | 0.866 | 0.868 | 0.87 |
| Greece | 0.717 | 0.723 | 0.731 | 0.738 | 0.744 | 0.75 | 0.755 | 0.761 | 0.767 | 0.773 | 0.778 | 0.782 | 0.787 | 0.792 | 0.796 | 0.8 | 0.803 | 0.806 | 0.809 | 0.812 | 0.815 | 0.818 | 0.819 | 0.82 | 0.819 | 0.818 | 0.817 | 0.817 |
| Iceland | 0.814 | 0.818 | 0.821 | 0.825 | 0.828 | 0.83 | 0.833 | 0.835 | 0.839 | 0.843 | 0.848 | 0.854 | 0.859 | 0.862 | 0.865 | 0.869 | 0.872 | 0.876 | 0.88 | 0.883 | 0.886 | 0.889 | 0.892 | 0.895 | 0.899 | 0.902 | 0.905 | 0.907 |
| Ireland | 0.756 | 0.762 | 0.768 | 0.774 | 0.779 | 0.785 | 0.79 | 0.795 | 0.802 | 0.808 | 0.814 | 0.821 | 0.827 | 0.834 | 0.84 | 0.844 | 0.846 | 0.849 | 0.851 | 0.855 | 0.858 | 0.862 | 0.865 | 0.867 | 0.87 | 0.874 | 0.878 | 0.882 |
| Israel | 0.734 | 0.738 | 0.743 | 0.748 | 0.752 | 0.757 | 0.76 | 0.764 | 0.768 | 0.772 | 0.776 | 0.78 | 0.783 | 0.786 | 0.789 | 0.793 | 0.796 | 0.798 | 0.798 | 0.799 | 0.801 | 0.803 | 0.805 | 0.808 | 0.81 | 0.812 | 0.814 | 0.816 |
| Italy | 0.767 | 0.772 | 0.778 | 0.783 | 0.788 | 0.793 | 0.797 | 0.8 | 0.804 | 0.807 | 0.81 | 0.814 | 0.817 | 0.819 | 0.821 | 0.823 | 0.825 | 0.827 | 0.829 | 0.83 | 0.832 | 0.834 | 0.836 | 0.838 | 0.839 | 0.841 | 0.842 | 0.843 |
| Luxembourg | 0.845 | 0.849 | 0.851 | 0.854 | 0.858 | 0.862 | 0.866 | 0.869 | 0.873 | 0.876 | 0.878 | 0.88 | 0.881 | 0.883 | 0.885 | 0.888 | 0.891 | 0.894 | 0.896 | 0.899 | 0.901 | 0.904 | 0.906 | 0.909 | 0.912 | 0.913 | 0.915 | 0.916 |
| Malta | 0.729 | 0.733 | 0.737 | 0.743 | 0.748 | 0.752 | 0.756 | 0.761 | 0.766 | 0.773 | 0.779 | 0.784 | 0.788 | 0.792 | 0.796 | 0.799 | 0.802 | 0.805 | 0.808 | 0.811 | 0.814 | 0.817 | 0.82 | 0.823 | 0.826 | 0.829 | 0.833 | 0.836 |
| Netherlands | 0.827 | 0.832 | 0.837 | 0.841 | 0.845 | 0.849 | 0.852 | 0.855 | 0.858 | 0.862 | 0.866 | 0.87 | 0.873 | 0.876 | 0.879 | 0.882 | 0.885 | 0.887 | 0.89 | 0.892 | 0.895 | 0.898 | 0.901 | 0.904 | 0.906 | 0.908 | 0.91 | 0.912 |
| Norway | 0.811 | 0.816 | 0.821 | 0.827 | 0.831 | 0.835 | 0.84 | 0.846 | 0.85 | 0.855 | 0.86 | 0.866 | 0.87 | 0.873 | 0.876 | 0.878 | 0.88 | 0.882 | 0.885 | 0.888 | 0.892 | 0.896 | 0.9 | 0.903 | 0.906 | 0.909 | 0.91 | 0.911 |
| Portugal | 0.642 | 0.65 | 0.659 | 0.667 | 0.675 | 0.682 | 0.688 | 0.694 | 0.699 | 0.705 | 0.71 | 0.716 | 0.722 | 0.727 | 0.732 | 0.736 | 0.741 | 0.744 | 0.748 | 0.751 | 0.755 | 0.76 | 0.764 | 0.768 | 0.771 | 0.773 | 0.775 | 0.778 |
| Spain | 0.715 | 0.723 | 0.731 | 0.738 | 0.745 | 0.752 | 0.758 | 0.763 | 0.768 | 0.773 | 0.778 | 0.782 | 0.786 | 0.79 | 0.794 | 0.797 | 0.799 | 0.802 | 0.805 | 0.809 | 0.812 | 0.815 | 0.818 | 0.819 | 0.82 | 0.822 | 0.823 | 0.825 |
| Sweden | 0.784 | 0.789 | 0.795 | 0.802 | 0.808 | 0.815 | 0.82 | 0.825 | 0.831 | 0.835 | 0.838 | 0.841 | 0.844 | 0.847 | 0.85 | 0.853 | 0.855 | 0.857 | 0.86 | 0.862 | 0.865 | 0.868 | 0.871 | 0.874 | 0.876 | 0.879 | 0.881 | 0.883 |
| Stockholm | 0.825 | 0.83 | 0.835 | 0.84 | 0.845 | 0.85 | 0.854 | 0.859 | 0.864 | 0.867 | 0.871 | 0.873 | 0.876 | 0.879 | 0.882 | 0.885 | 0.888 | 0.891 | 0.893 | 0.896 | 0.899 | 0.902 | 0.904 | 0.907 | 0.909 | 0.911 | 0.913 | 0.914 |
| Sweden except Stockholm | 0.773 | 0.778 | 0.785 | 0.792 | 0.798 | 0.805 | 0.811 | 0.816 | 0.821 | 0.825 | 0.829 | 0.832 | 0.834 | 0.838 | 0.841 | 0.843 | 0.845 | 0.848 | 0.85 | 0.852 | 0.854 | 0.858 | 0.86 | 0.863 | 0.866 | 0.868 | 0.87 | 0.873 |
| Switzerland | 0.841 | 0.842 | 0.844 | 0.846 | 0.847 | 0.848 | 0.849 | 0.85 | 0.851 | 0.852 | 0.854 | 0.857 | 0.859 | 0.86 | 0.862 | 0.863 | 0.865 | 0.868 | 0.871 | 0.873 | 0.875 | 0.877 | 0.88 | 0.882 | 0.884 | 0.886 | 0.887 | 0.889 |
| United Kingdom | 0.723 | 0.729 | 0.736 | 0.743 | 0.749 | 0.754 | 0.758 | 0.762 | 0.767 | 0.774 | 0.78 | 0.786 | 0.791 | 0.794 | 0.797 | 0.801 | 0.804 | 0.807 | 0.81 | 0.813 | 0.817 | 0.821 | 0.826 | 0.832 | 0.836 | 0.839 | 0.841 | 0.843 |
| England | 0.732 | 0.738 | 0.745 | 0.752 | 0.758 | 0.763 | 0.766 | 0.77 | 0.776 | 0.782 | 0.788 | 0.794 | 0.798 | 0.801 | 0.804 | 0.807 | 0.81 | 0.813 | 0.816 | 0.819 | 0.823 | 0.827 | 0.832 | 0.838 | 0.842 | 0.845 | 0.847 | 0.849 |
| East Midlands | 0.71 | 0.716 | 0.723 | 0.73 | 0.737 | 0.741 | 0.745 | 0.75 | 0.755 | 0.762 | 0.768 | 0.773 | 0.778 | 0.781 | 0.785 | 0.788 | 0.791 | 0.794 | 0.797 | 0.8 | 0.804 | 0.808 | 0.813 | 0.819 | 0.823 | 0.826 | 0.828 | 0.83 |
| Derby | 0.715 | 0.722 | 0.73 | 0.738 | 0.745 | 0.75 | 0.754 | 0.758 | 0.764 | 0.771 | 0.778 | 0.785 | 0.79 | 0.793 | 0.797 | 0.801 | 0.803 | 0.806 | 0.809 | 0.811 | 0.815 | 0.82 | 0.826 | 0.832 | 0.837 | 0.841 | 0.844 | 0.846 |
| Derbyshire | 0.698 | 0.703 | 0.71 | 0.716 | 0.723 | 0.727 | 0.731 | 0.735 | 0.74 | 0.747 | 0.753 | 0.758 | 0.762 | 0.765 | 0.768 | 0.771 | 0.774 | 0.777 | 0.779 | 0.782 | 0.785 | 0.79 | 0.796 | 0.803 | 0.808 | 0.812 | 0.815 | 0.817 |
| Leicester | 0.708 | 0.715 | 0.723 | 0.73 | 0.737 | 0.743 | 0.747 | 0.751 | 0.756 | 0.763 | 0.769 | 0.775 | 0.78 | 0.785 | 0.79 | 0.794 | 0.798 | 0.802 | 0.805 | 0.809 | 0.813 | 0.817 | 0.822 | 0.828 | 0.832 | 0.835 | 0.837 | 0.839 |
| Leicestershire | 0.727 | 0.733 | 0.74 | 0.748 | 0.754 | 0.759 | 0.763 | 0.767 | 0.773 | 0.778 | 0.784 | 0.789 | 0.794 | 0.798 | 0.802 | 0.806 | 0.81 | 0.814 | 0.817 | 0.82 | 0.823 | 0.827 | 0.831 | 0.836 | 0.839 | 0.842 | 0.844 | 0.846 |
| Lincolnshire | 0.699 | 0.705 | 0.711 | 0.718 | 0.724 | 0.729 | 0.732 | 0.737 | 0.742 | 0.748 | 0.754 | 0.76 | 0.764 | 0.768 | 0.77 | 0.772 | 0.774 | 0.776 | 0.779 | 0.781 | 0.784 | 0.788 | 0.793 | 0.8 | 0.804 | 0.808 | 0.81 | 0.812 |
| Northamptonshire | 0.714 | 0.72 | 0.728 | 0.735 | 0.741 | 0.746 | 0.749 | 0.753 | 0.757 | 0.764 | 0.77 | 0.775 | 0.779 | 0.781 | 0.784 | 0.787 | 0.79 | 0.792 | 0.796 | 0.798 | 0.802 | 0.806 | 0.812 | 0.818 | 0.822 | 0.825 | 0.828 | 0.829 |
| Nottingham | 0.735 | 0.742 | 0.749 | 0.757 | 0.763 | 0.769 | 0.774 | 0.78 | 0.786 | 0.793 | 0.8 | 0.806 | 0.812 | 0.817 | 0.822 | 0.826 | 0.829 | 0.832 | 0.835 | 0.839 | 0.842 | 0.847 | 0.852 | 0.856 | 0.859 | 0.861 | 0.862 | 0.863 |
| Nottinghamshire | 0.696 | 0.702 | 0.708 | 0.715 | 0.722 | 0.726 | 0.73 | 0.734 | 0.74 | 0.747 | 0.754 | 0.76 | 0.765 | 0.768 | 0.771 | 0.775 | 0.778 | 0.781 | 0.783 | 0.785 | 0.788 | 0.792 | 0.797 | 0.803 | 0.807 | 0.81 | 0.812 | 0.814 |
| Rutland | 0.73 | 0.735 | 0.741 | 0.746 | 0.752 | 0.756 | 0.759 | 0.762 | 0.767 | 0.772 | 0.777 | 0.781 | 0.784 | 0.787 | 0.79 | 0.793 | 0.797 | 0.8 | 0.804 | 0.806 | 0.81 | 0.814 | 0.818 | 0.823 | 0.826 | 0.829 | 0.831 | 0.833 |
| East of England | 0.724 | 0.73 | 0.737 | 0.744 | 0.75 | 0.755 | 0.759 | 0.763 | 0.768 | 0.774 | 0.78 | 0.786 | 0.79 | 0.793 | 0.796 | 0.799 | 0.802 | 0.805 | 0.808 | 0.811 | 0.814 | 0.818 | 0.824 | 0.83 | 0.834 | 0.837 | 0.839 | 0.84 |
| Bedford | 0.73 | 0.736 | 0.743 | 0.749 | 0.756 | 0.76 | 0.764 | 0.768 | 0.774 | 0.78 | 0.785 | 0.79 | 0.794 | 0.797 | 0.8 | 0.803 | 0.805 | 0.807 | 0.81 | 0.812 | 0.816 | 0.82 | 0.825 | 0.83 | 0.833 | 0.836 | 0.837 | 0.838 |
| Cambridgeshire | 0.75 | 0.757 | 0.764 | 0.771 | 0.777 | 0.782 | 0.786 | 0.791 | 0.797 | 0.803 | 0.809 | 0.815 | 0.82 | 0.823 | 0.827 | 0.83 | 0.833 | 0.836 | 0.84 | 0.842 | 0.846 | 0.85 | 0.855 | 0.86 | 0.864 | 0.867 | 0.869 | 0.871 |
| Central Bedfordshire | 0.723 | 0.729 | 0.736 | 0.743 | 0.749 | 0.753 | 0.757 | 0.76 | 0.765 | 0.771 | 0.776 | 0.781 | 0.785 | 0.787 | 0.79 | 0.793 | 0.795 | 0.797 | 0.8 | 0.803 | 0.806 | 0.81 | 0.816 | 0.822 | 0.827 | 0.83 | 0.833 | 0.834 |
| Essex | 0.713 | 0.719 | 0.726 | 0.734 | 0.74 | 0.745 | 0.748 | 0.753 | 0.758 | 0.764 | 0.77 | 0.775 | 0.78 | 0.783 | 0.787 | 0.79 | 0.793 | 0.796 | 0.8 | 0.802 | 0.806 | 0.81 | 0.816 | 0.821 | 0.825 | 0.828 | 0.83 | 0.832 |
| Hertfordshire | 0.749 | 0.755 | 0.762 | 0.769 | 0.776 | 0.781 | 0.784 | 0.789 | 0.795 | 0.802 | 0.81 | 0.816 | 0.821 | 0.825 | 0.829 | 0.832 | 0.835 | 0.837 | 0.84 | 0.843 | 0.846 | 0.85 | 0.855 | 0.86 | 0.863 | 0.866 | 0.868 | 0.87 |
| Luton | 0.71 | 0.716 | 0.723 | 0.731 | 0.737 | 0.742 | 0.745 | 0.749 | 0.754 | 0.76 | 0.766 | 0.771 | 0.775 | 0.778 | 0.781 | 0.785 | 0.79 | 0.795 | 0.8 | 0.805 | 0.809 | 0.814 | 0.819 | 0.825 | 0.828 | 0.83 | 0.832 | 0.833 |
| Norfolk | 0.712 | 0.717 | 0.724 | 0.731 | 0.737 | 0.741 | 0.745 | 0.749 | 0.754 | 0.761 | 0.767 | 0.772 | 0.776 | 0.779 | 0.781 | 0.784 | 0.787 | 0.79 | 0.792 | 0.795 | 0.798 | 0.803 | 0.808 | 0.814 | 0.819 | 0.822 | 0.824 | 0.826 |
| Peterborough | 0.713 | 0.718 | 0.724 | 0.731 | 0.737 | 0.741 | 0.743 | 0.746 | 0.751 | 0.757 | 0.761 | 0.765 | 0.768 | 0.77 | 0.772 | 0.774 | 0.776 | 0.778 | 0.779 | 0.781 | 0.784 | 0.788 | 0.795 | 0.803 | 0.808 | 0.813 | 0.816 | 0.818 |
| Southend-on-Sea | 0.696 | 0.701 | 0.707 | 0.714 | 0.72 | 0.724 | 0.727 | 0.731 | 0.736 | 0.743 | 0.75 | 0.755 | 0.761 | 0.765 | 0.768 | 0.77 | 0.773 | 0.775 | 0.778 | 0.781 | 0.785 | 0.79 | 0.795 | 0.801 | 0.805 | 0.808 | 0.81 | 0.811 |
| Suffolk | 0.713 | 0.719 | 0.726 | 0.732 | 0.738 | 0.742 | 0.745 | 0.748 | 0.753 | 0.758 | 0.763 | 0.767 | 0.771 | 0.773 | 0.775 | 0.777 | 0.78 | 0.782 | 0.785 | 0.788 | 0.792 | 0.797 | 0.803 | 0.81 | 0.814 | 0.817 | 0.819 | 0.821 |
| Thurrock | 0.71 | 0.716 | 0.723 | 0.73 | 0.737 | 0.741 | 0.745 | 0.749 | 0.754 | 0.759 | 0.763 | 0.767 | 0.769 | 0.77 | 0.772 | 0.774 | 0.775 | 0.776 | 0.778 | 0.781 | 0.784 | 0.789 | 0.793 | 0.799 | 0.802 | 0.804 | 0.806 | 0.807 |
| Greater London | 0.78 | 0.786 | 0.793 | 0.8 | 0.806 | 0.81 | 0.814 | 0.818 | 0.823 | 0.83 | 0.836 | 0.842 | 0.846 | 0.849 | 0.852 | 0.855 | 0.858 | 0.861 | 0.863 | 0.866 | 0.87 | 0.875 | 0.88 | 0.885 | 0.889 | 0.891 | 0.893 | 0.894 |
| Barking and Dagenham | 0.704 | 0.709 | 0.716 | 0.723 | 0.73 | 0.734 | 0.737 | 0.741 | 0.746 | 0.752 | 0.756 | 0.76 | 0.762 | 0.762 | 0.763 | 0.763 | 0.763 | 0.763 | 0.764 | 0.765 | 0.768 | 0.773 | 0.78 | 0.787 | 0.793 | 0.797 | 0.8 | 0.802 |
| Barnet | 0.759 | 0.765 | 0.771 | 0.777 | 0.783 | 0.788 | 0.792 | 0.796 | 0.802 | 0.809 | 0.814 | 0.82 | 0.824 | 0.827 | 0.829 | 0.831 | 0.834 | 0.835 | 0.838 | 0.84 | 0.843 | 0.847 | 0.852 | 0.856 | 0.859 | 0.862 | 0.864 | 0.865 |
| Bexley | 0.721 | 0.727 | 0.734 | 0.74 | 0.746 | 0.75 | 0.753 | 0.757 | 0.761 | 0.767 | 0.772 | 0.777 | 0.781 | 0.784 | 0.787 | 0.789 | 0.791 | 0.793 | 0.796 | 0.798 | 0.801 | 0.804 | 0.809 | 0.815 | 0.819 | 0.822 | 0.824 | 0.826 |
| Brent | 0.753 | 0.758 | 0.764 | 0.771 | 0.777 | 0.781 | 0.785 | 0.789 | 0.795 | 0.801 | 0.806 | 0.811 | 0.814 | 0.816 | 0.818 | 0.819 | 0.821 | 0.821 | 0.822 | 0.823 | 0.825 | 0.828 | 0.833 | 0.839 | 0.842 | 0.845 | 0.847 | 0.849 |
| Bromley | 0.751 | 0.756 | 0.762 | 0.768 | 0.774 | 0.778 | 0.782 | 0.786 | 0.791 | 0.798 | 0.804 | 0.809 | 0.814 | 0.817 | 0.82 | 0.822 | 0.825 | 0.827 | 0.829 | 0.83 | 0.831 | 0.833 | 0.837 | 0.84 | 0.843 | 0.845 | 0.847 | 0.848 |
| Camden | 0.838 | 0.843 | 0.848 | 0.854 | 0.858 | 0.862 | 0.866 | 0.869 | 0.873 | 0.88 | 0.885 | 0.889 | 0.892 | 0.895 | 0.898 | 0.901 | 0.904 | 0.906 | 0.908 | 0.911 | 0.913 | 0.917 | 0.92 | 0.924 | 0.926 | 0.927 | 0.929 | 0.93 |
| Croydon | 0.741 | 0.747 | 0.754 | 0.761 | 0.767 | 0.771 | 0.774 | 0.777 | 0.782 | 0.788 | 0.794 | 0.799 | 0.803 | 0.805 | 0.808 | 0.81 | 0.812 | 0.813 | 0.815 | 0.816 | 0.818 | 0.82 | 0.824 | 0.827 | 0.83 | 0.831 | 0.832 | 0.833 |
| Ealing | 0.764 | 0.77 | 0.776 | 0.783 | 0.789 | 0.794 | 0.797 | 0.801 | 0.807 | 0.814 | 0.82 | 0.825 | 0.828 | 0.831 | 0.833 | 0.834 | 0.835 | 0.836 | 0.837 | 0.837 | 0.839 | 0.843 | 0.848 | 0.853 | 0.857 | 0.86 | 0.863 | 0.865 |
| Enfield | 0.737 | 0.742 | 0.749 | 0.755 | 0.761 | 0.765 | 0.768 | 0.772 | 0.777 | 0.783 | 0.788 | 0.793 | 0.797 | 0.799 | 0.801 | 0.802 | 0.804 | 0.806 | 0.809 | 0.811 | 0.814 | 0.818 | 0.824 | 0.829 | 0.833 | 0.836 | 0.838 | 0.839 |

**Appendix Table 10: Socio-Demographic Index values for all estimated GBD 2017 locations, 1990-2017**

| Location | 1990 | 1991 | 1992 | 1993 | 1994 | 1995 | 1996 | 1997 | 1998 | 1999 | 2000 | 2001 | 2002 | 2003 | 2004 | 2005 | 2006 | 2007 | 2008 | 2009 | 2010 | 2011 | 2012 | 2013 | 2014 | 2015 | 2016 | 2017 |
|---|---|---|---|---|---|---|---|---|---|---|---|---|---|---|---|---|---|---|---|---|---|---|---|---|---|---|---|---|
| Greenwich | 0.725 | 0.732 | 0.74 | 0.747 | 0.754 | 0.758 | 0.762 | 0.766 | 0.771 | 0.777 | 0.783 | 0.788 | 0.791 | 0.793 | 0.795 | 0.797 | 0.799 | 0.801 | 0.803 | 0.805 | 0.809 | 0.813 | 0.819 | 0.824 | 0.828 | 0.83 | 0.832 | 0.833 |
| Hackney | 0.767 | 0.774 | 0.781 | 0.789 | 0.795 | 0.798 | 0.8 | 0.801 | 0.805 | 0.811 | 0.817 | 0.822 | 0.825 | 0.828 | 0.831 | 0.834 | 0.838 | 0.843 | 0.848 | 0.853 | 0.858 | 0.864 | 0.871 | 0.877 | 0.882 | 0.884 | 0.886 | 0.887 |
| Hammersmith and Fulham | 0.825 | 0.833 | 0.839 | 0.845 | 0.851 | 0.856 | 0.86 | 0.864 | 0.869 | 0.875 | 0.879 | 0.883 | 0.887 | 0.889 | 0.892 | 0.895 | 0.898 | 0.901 | 0.904 | 0.907 | 0.91 | 0.914 | 0.917 | 0.921 | 0.923 | 0.924 | 0.925 | 0.927 |
| Haringey | 0.755 | 0.761 | 0.766 | 0.773 | 0.778 | 0.782 | 0.785 | 0.788 | 0.792 | 0.798 | 0.804 | 0.809 | 0.813 | 0.815 | 0.818 | 0.821 | 0.823 | 0.825 | 0.828 | 0.829 | 0.832 | 0.835 | 0.84 | 0.845 | 0.848 | 0.851 | 0.852 | 0.854 |
| Harrow | 0.747 | 0.754 | 0.76 | 0.767 | 0.773 | 0.777 | 0.78 | 0.784 | 0.79 | 0.796 | 0.802 | 0.808 | 0.812 | 0.815 | 0.819 | 0.821 | 0.824 | 0.825 | 0.827 | 0.829 | 0.83 | 0.832 | 0.835 | 0.839 | 0.842 | 0.844 | 0.846 | 0.848 |
| Havering | 0.719 | 0.725 | 0.731 | 0.737 | 0.743 | 0.747 | 0.75 | 0.754 | 0.759 | 0.765 | 0.77 | 0.775 | 0.78 | 0.783 | 0.786 | 0.789 | 0.792 | 0.794 | 0.797 | 0.8 | 0.802 | 0.805 | 0.809 | 0.814 | 0.817 | 0.82 | 0.823 | 0.824 |
| Hillingdon | 0.781 | 0.787 | 0.794 | 0.801 | 0.807 | 0.811 | 0.815 | 0.818 | 0.822 | 0.828 | 0.832 | 0.836 | 0.84 | 0.842 | 0.845 | 0.846 | 0.848 | 0.85 | 0.852 | 0.854 | 0.857 | 0.862 | 0.867 | 0.872 | 0.876 | 0.879 | 0.881 | 0.882 |
| Hounslow | 0.769 | 0.775 | 0.781 | 0.788 | 0.793 | 0.797 | 0.801 | 0.805 | 0.811 | 0.818 | 0.824 | 0.83 | 0.834 | 0.837 | 0.84 | 0.842 | 0.844 | 0.845 | 0.848 | 0.849 | 0.852 | 0.856 | 0.862 | 0.868 | 0.872 | 0.875 | 0.877 | 0.879 |
| Islington | 0.812 | 0.818 | 0.825 | 0.832 | 0.838 | 0.843 | 0.847 | 0.851 | 0.856 | 0.862 | 0.867 | 0.872 | 0.876 | 0.879 | 0.882 | 0.885 | 0.888 | 0.891 | 0.895 | 0.898 | 0.902 | 0.906 | 0.91 | 0.914 | 0.917 | 0.919 | 0.921 | 0.922 |
| Kensington and Chelsea | 0.839 | 0.845 | 0.851 | 0.857 | 0.861 | 0.865 | 0.869 | 0.873 | 0.877 | 0.884 | 0.889 | 0.894 | 0.897 | 0.899 | 0.902 | 0.905 | 0.907 | 0.909 | 0.912 | 0.915 | 0.918 | 0.921 | 0.924 | 0.927 | 0.929 | 0.93 | 0.931 | 0.932 |
| Kingston upon Thames | 0.787 | 0.794 | 0.8 | 0.807 | 0.812 | 0.817 | 0.821 | 0.826 | 0.831 | 0.838 | 0.844 | 0.849 | 0.854 | 0.858 | 0.861 | 0.864 | 0.867 | 0.869 | 0.872 | 0.874 | 0.876 | 0.879 | 0.882 | 0.885 | 0.887 | 0.888 | 0.889 | 0.89 |
| Lambeth | 0.775 | 0.783 | 0.791 | 0.797 | 0.804 | 0.808 | 0.812 | 0.816 | 0.821 | 0.828 | 0.835 | 0.84 | 0.845 | 0.848 | 0.852 | 0.856 | 0.86 | 0.864 | 0.869 | 0.873 | 0.877 | 0.882 | 0.887 | 0.892 | 0.895 | 0.897 | 0.899 | 0.9 |
| Lewisham | 0.733 | 0.74 | 0.746 | 0.753 | 0.759 | 0.763 | 0.766 | 0.77 | 0.775 | 0.781 | 0.787 | 0.793 | 0.797 | 0.799 | 0.802 | 0.804 | 0.806 | 0.808 | 0.811 | 0.813 | 0.816 | 0.821 | 0.826 | 0.832 | 0.836 | 0.839 | 0.841 | 0.843 |
| Merton | 0.758 | 0.765 | 0.771 | 0.779 | 0.785 | 0.79 | 0.795 | 0.799 | 0.805 | 0.812 | 0.819 | 0.824 | 0.828 | 0.83 | 0.833 | 0.835 | 0.837 | 0.839 | 0.841 | 0.843 | 0.845 | 0.849 | 0.855 | 0.86 | 0.865 | 0.868 | 0.871 | 0.873 |
| Newham | 0.716 | 0.722 | 0.728 | 0.734 | 0.74 | 0.743 | 0.746 | 0.748 | 0.753 | 0.76 | 0.765 | 0.771 | 0.775 | 0.777 | 0.78 | 0.781 | 0.783 | 0.785 | 0.788 | 0.792 | 0.799 | 0.806 | 0.815 | 0.823 | 0.829 | 0.833 | 0.836 | 0.838 |
| Redbridge | 0.728 | 0.733 | 0.739 | 0.746 | 0.751 | 0.756 | 0.759 | 0.763 | 0.768 | 0.774 | 0.78 | 0.785 | 0.789 | 0.791 | 0.794 | 0.796 | 0.798 | 0.801 | 0.803 | 0.805 | 0.807 | 0.811 | 0.816 | 0.821 | 0.825 | 0.827 | 0.83 | 0.831 |
| Richmond upon Thames | 0.794 | 0.801 | 0.807 | 0.813 | 0.819 | 0.824 | 0.827 | 0.832 | 0.837 | 0.844 | 0.85 | 0.856 | 0.86 | 0.864 | 0.867 | 0.87 | 0.872 | 0.874 | 0.876 | 0.878 | 0.88 | 0.883 | 0.887 | 0.891 | 0.895 | 0.897 | 0.9 | 0.902 |
| Southwark | 0.793 | 0.8 | 0.807 | 0.815 | 0.821 | 0.827 | 0.83 | 0.835 | 0.84 | 0.847 | 0.852 | 0.857 | 0.86 | 0.863 | 0.866 | 0.869 | 0.872 | 0.875 | 0.879 | 0.883 | 0.887 | 0.893 | 0.898 | 0.904 | 0.907 | 0.909 | 0.911 | 0.912 |
| Sutton | 0.735 | 0.74 | 0.747 | 0.754 | 0.761 | 0.765 | 0.769 | 0.774 | 0.78 | 0.787 | 0.793 | 0.798 | 0.802 | 0.805 | 0.808 | 0.809 | 0.811 | 0.813 | 0.815 | 0.817 | 0.819 | 0.823 | 0.828 | 0.833 | 0.836 | 0.839 | 0.841 | 0.843 |
| Tower Hamlets | 0.766 | 0.773 | 0.78 | 0.788 | 0.795 | 0.801 | 0.805 | 0.81 | 0.817 | 0.825 | 0.832 | 0.839 | 0.844 | 0.848 | 0.852 | 0.857 | 0.861 | 0.866 | 0.871 | 0.876 | 0.881 | 0.886 | 0.891 | 0.896 | 0.9 | 0.902 | 0.904 | 0.905 |
| Waltham Forest | 0.712 | 0.718 | 0.724 | 0.731 | 0.738 | 0.742 | 0.745 | 0.749 | 0.754 | 0.76 | 0.765 | 0.77 | 0.773 | 0.775 | 0.777 | 0.778 | 0.78 | 0.781 | 0.783 | 0.784 | 0.787 | 0.792 | 0.798 | 0.805 | 0.81 | 0.814 | 0.817 | 0.819 |
| Wandsworth | 0.793 | 0.8 | 0.807 | 0.814 | 0.82 | 0.825 | 0.83 | 0.835 | 0.84 | 0.848 | 0.855 | 0.862 | 0.867 | 0.87 | 0.873 | 0.876 | 0.879 | 0.881 | 0.885 | 0.887 | 0.889 | 0.893 | 0.897 | 0.901 | 0.905 | 0.907 | 0.909 | 0.911 |
| Westminster | 0.831 | 0.837 | 0.843 | 0.849 | 0.854 | 0.858 | 0.862 | 0.865 | 0.87 | 0.875 | 0.881 | 0.885 | 0.889 | 0.891 | 0.895 | 0.898 | 0.901 | 0.903 | 0.905 | 0.907 | 0.91 | 0.913 | 0.916 | 0.92 | 0.922 | 0.924 | 0.925 | 0.927 |
| North East England | 0.697 | 0.703 | 0.711 | 0.718 | 0.725 | 0.73 | 0.733 | 0.738 | 0.744 | 0.75 | 0.757 | 0.763 | 0.767 | 0.771 | 0.774 | 0.778 | 0.781 | 0.784 | 0.787 | 0.79 | 0.794 | 0.798 | 0.804 | 0.81 | 0.814 | 0.817 | 0.819 | 0.821 |
| County Durham | 0.69 | 0.696 | 0.703 | 0.71 | 0.717 | 0.721 | 0.725 | 0.73 | 0.735 | 0.742 | 0.748 | 0.754 | 0.758 | 0.762 | 0.765 | 0.767 | 0.77 | 0.772 | 0.776 | 0.778 | 0.782 | 0.786 | 0.792 | 0.798 | 0.802 | 0.806 | 0.808 | 0.81 |
| Darlington | 0.703 | 0.709 | 0.716 | 0.723 | 0.73 | 0.734 | 0.737 | 0.74 | 0.745 | 0.752 | 0.758 | 0.764 | 0.769 | 0.773 | 0.777 | 0.78 | 0.783 | 0.785 | 0.788 | 0.791 | 0.795 | 0.8 | 0.806 | 0.812 | 0.817 | 0.821 | 0.823 | 0.825 |
| Gateshead | 0.7 | 0.706 | 0.713 | 0.72 | 0.727 | 0.732 | 0.735 | 0.739 | 0.745 | 0.751 | 0.758 | 0.764 | 0.769 | 0.773 | 0.777 | 0.781 | 0.784 | 0.787 | 0.791 | 0.794 | 0.798 | 0.803 | 0.808 | 0.814 | 0.818 | 0.821 | 0.824 | 0.826 |
| Hartlepool | 0.677 | 0.683 | 0.69 | 0.697 | 0.703 | 0.707 | 0.71 | 0.714 | 0.719 | 0.726 | 0.732 | 0.737 | 0.74 | 0.742 | 0.744 | 0.747 | 0.749 | 0.752 | 0.755 | 0.758 | 0.762 | 0.767 | 0.773 | 0.78 | 0.785 | 0.788 | 0.791 | 0.793 |
| Middlesbrough | 0.688 | 0.695 | 0.702 | 0.709 | 0.716 | 0.72 | 0.723 | 0.727 | 0.732 | 0.74 | 0.746 | 0.751 | 0.755 | 0.758 | 0.762 | 0.766 | 0.769 | 0.772 | 0.775 | 0.778 | 0.781 | 0.786 | 0.792 | 0.797 | 0.801 | 0.804 | 0.806 | 0.808 |
| Newcastle upon Tyne | 0.736 | 0.743 | 0.751 | 0.759 | 0.765 | 0.771 | 0.776 | 0.781 | 0.787 | 0.794 | 0.801 | 0.808 | 0.814 | 0.82 | 0.825 | 0.831 | 0.835 | 0.84 | 0.843 | 0.846 | 0.85 | 0.854 | 0.858 | 0.863 | 0.866 | 0.869 | 0.87 | 0.872 |
| North Tyneside | 0.696 | 0.703 | 0.71 | 0.718 | 0.724 | 0.729 | 0.733 | 0.737 | 0.743 | 0.749 | 0.756 | 0.762 | 0.768 | 0.772 | 0.776 | 0.78 | 0.784 | 0.787 | 0.79 | 0.793 | 0.797 | 0.802 | 0.807 | 0.813 | 0.817 | 0.821 | 0.823 | 0.825 |
| Northumberland | 0.697 | 0.703 | 0.709 | 0.716 | 0.722 | 0.727 | 0.73 | 0.735 | 0.74 | 0.746 | 0.752 | 0.757 | 0.761 | 0.764 | 0.767 | 0.77 | 0.773 | 0.776 | 0.779 | 0.781 | 0.784 | 0.787 | 0.793 | 0.798 | 0.802 | 0.805 | 0.807 | 0.808 |
| Redcar and Cleveland | 0.67 | 0.675 | 0.682 | 0.689 | 0.696 | 0.701 | 0.704 | 0.708 | 0.714 | 0.721 | 0.727 | 0.732 | 0.736 | 0.738 | 0.741 | 0.743 | 0.745 | 0.747 | 0.75 | 0.752 | 0.756 | 0.76 | 0.767 | 0.775 | 0.78 | 0.784 | 0.787 | 0.79 |
| South Tyneside | 0.664 | 0.671 | 0.678 | 0.685 | 0.692 | 0.697 | 0.7 | 0.705 | 0.711 | 0.718 | 0.725 | 0.731 | 0.737 | 0.74 | 0.744 | 0.748 | 0.751 | 0.754 | 0.757 | 0.759 | 0.762 | 0.767 | 0.773 | 0.78 | 0.785 | 0.789 | 0.792 | 0.794 |
| Stockton-on-Tees | 0.705 | 0.712 | 0.72 | 0.728 | 0.735 | 0.74 | 0.744 | 0.748 | 0.754 | 0.761 | 0.767 | 0.772 | 0.775 | 0.777 | 0.779 | 0.781 | 0.784 | 0.785 | 0.788 | 0.791 | 0.794 | 0.798 | 0.804 | 0.811 | 0.815 | 0.818 | 0.821 | 0.823 |
| Sunderland | 0.689 | 0.696 | 0.704 | 0.713 | 0.72 | 0.725 | 0.729 | 0.733 | 0.739 | 0.746 | 0.752 | 0.758 | 0.763 | 0.766 | 0.77 | 0.773 | 0.776 | 0.779 | 0.782 | 0.784 | 0.787 | 0.791 | 0.797 | 0.803 | 0.808 | 0.811 | 0.813 | 0.815 |
| North West England | 0.716 | 0.723 | 0.729 | 0.736 | 0.742 | 0.747 | 0.751 | 0.755 | 0.76 | 0.766 | 0.772 | 0.778 | 0.782 | 0.786 | 0.789 | 0.792 | 0.795 | 0.798 | 0.801 | 0.804 | 0.807 | 0.812 | 0.817 | 0.823 | 0.827 | 0.83 | 0.832 | 0.834 |
| Blackburn with Darwen | 0.691 | 0.695 | 0.701 | 0.707 | 0.713 | 0.717 | 0.719 | 0.723 | 0.728 | 0.734 | 0.74 | 0.745 | 0.748 | 0.75 | 0.753 | 0.756 | 0.758 | 0.761 | 0.764 | 0.766 | 0.769 | 0.774 | 0.78 | 0.788 | 0.793 | 0.797 | 0.8 | 0.802 |
| Blackpool | 0.68 | 0.685 | 0.691 | 0.698 | 0.703 | 0.707 | 0.71 | 0.713 | 0.718 | 0.724 | 0.729 | 0.733 | 0.737 | 0.738 | 0.741 | 0.743 | 0.745 | 0.747 | 0.748 | 0.75 | 0.752 | 0.756 | 0.762 | 0.768 | 0.773 | 0.776 | 0.779 | 0.781 |
| Bolton | 0.701 | 0.707 | 0.714 | 0.72 | 0.726 | 0.73 | 0.733 | 0.736 | 0.741 | 0.746 | 0.751 | 0.756 | 0.759 | 0.761 | 0.763 | 0.765 | 0.768 | 0.769 | 0.772 | 0.774 | 0.777 | 0.781 | 0.787 | 0.794 | 0.798 | 0.801 | 0.803 | 0.805 |
| Bury | 0.703 | 0.709 | 0.716 | 0.723 | 0.73 | 0.734 | 0.738 | 0.742 | 0.747 | 0.752 | 0.758 | 0.762 | 0.766 | 0.768 | 0.771 | 0.774 | 0.777 | 0.779 | 0.781 | 0.783 | 0.786 | 0.791 | 0.797 | 0.803 | 0.807 | 0.81 | 0.813 | 0.815 |
| Cheshire East | 0.748 | 0.754 | 0.76 | 0.767 | 0.773 | 0.778 | 0.782 | 0.787 | 0.792 | 0.798 | 0.803 | 0.809 | 0.813 | 0.817 | 0.82 | 0.823 | 0.826 | 0.829 | 0.832 | 0.834 | 0.836 | 0.84 | 0.845 | 0.851 | 0.856 | 0.859 | 0.861 | 0.864 |
| Cheshire West and Chester | 0.74 | 0.747 | 0.754 | 0.761 | 0.768 | 0.772 | 0.776 | 0.78 | 0.785 | 0.792 | 0.797 | 0.803 | 0.807 | 0.811 | 0.815 | 0.818 | 0.822 | 0.825 | 0.828 | 0.831 | 0.834 | 0.838 | 0.842 | 0.847 | 0.85 | 0.852 | 0.854 | 0.855 |
| Cumbria | 0.719 | 0.724 | 0.731 | 0.737 | 0.743 | 0.748 | 0.751 | 0.755 | 0.76 | 0.765 | 0.77 | 0.774 | 0.777 | 0.779 | 0.782 | 0.784 | 0.787 | 0.789 | 0.792 | 0.794 | 0.798 | 0.802 | 0.808 | 0.815 | 0.819 | 0.823 | 0.826 | 0.828 |
| Halton | 0.702 | 0.708 | 0.715 | 0.723 | 0.729 | 0.734 | 0.737 | 0.74 | 0.745 | 0.75 | 0.756 | 0.762 | 0.766 | 0.769 | 0.772 | 0.775 | 0.778 | 0.78 | 0.784 | 0.787 | 0.791 | 0.797 | 0.803 | 0.81 | 0.815 | 0.819 | 0.822 | 0.824 |
| Knowsley | 0.691 | 0.696 | 0.702 | 0.708 | 0.714 | 0.718 | 0.722 | 0.726 | 0.731 | 0.737 | 0.743 | 0.749 | 0.754 | 0.757 | 0.76 | 0.764 | 0.767 | 0.77 | 0.774 | 0.777 | 0.782 | 0.787 | 0.793 | 0.8 | 0.806 | 0.81 | 0.813 | 0.816 |
| Lancashire | 0.72 | 0.726 | 0.734 | 0.741 | 0.747 | 0.752 | 0.756 | 0.76 | 0.765 | 0.77 | 0.775 | 0.78 | 0.784 | 0.786 | 0.789 | 0.792 | 0.794 | 0.797 | 0.799 | 0.802 | 0.805 | 0.809 | 0.815 | 0.82 | 0.824 | 0.827 | 0.829 | 0.831 |
| Liverpool | 0.721 | 0.728 | 0.735 | 0.742 | 0.748 | 0.753 | 0.757 | 0.762 | 0.768 | 0.775 | 0.783 | 0.789 | 0.795 | 0.801 | 0.806 | 0.811 | 0.815 | 0.819 | 0.823 | 0.828 | 0.831 | 0.835 | 0.84 | 0.844 | 0.847 | 0.849 | 0.851 | 0.852 |
| Manchester | 0.746 | 0.753 | 0.76 | 0.768 | 0.775 | 0.78 | 0.785 | 0.79 | 0.796 | 0.804 | 0.812 | 0.819 | 0.825 | 0.83 | 0.835 | 0.84 | 0.844 | 0.849 | 0.853 | 0.856 | 0.86 | 0.864 | 0.869 | 0.874 | 0.878 | 0.88 | 0.883 | 0.885 |
| Oldham | 0.685 | 0.69 | 0.697 | 0.703 | 0.708 | 0.711 | 0.713 | 0.715 | 0.719 | 0.724 | 0.729 | 0.733 | 0.737 | 0.739 | 0.741 | 0.744 | 0.746 | 0.749 | 0.752 | 0.754 | 0.758 | 0.763 | 0.77 | 0.778 | 0.782 | 0.786 | 0.788 | 0.79 |
| Rochdale | 0.686 | 0.692 | 0.698 | 0.705 | 0.711 | 0.715 | 0.717 | 0.721 | 0.726 | 0.732 | 0.738 | 0.742 | 0.746 | 0.748 | 0.75 | 0.753 | 0.755 | 0.757 | 0.76 | 0.762 | 0.765 | 0.77 | 0.777 | 0.783 | 0.787 | 0.791 | 0.793 | 0.795 |
| Salford | 0.708 | 0.714 | 0.721 | 0.728 | 0.735 | 0.74 | 0.743 | 0.747 | 0.753 | 0.761 | 0.768 | 0.775 | 0.781 | 0.785 | 0.79 | 0.794 | 0.797 | 0.8 | 0.803 | 0.806 | 0.809 | 0.814 | 0.819 | 0.826 | 0.83 | 0.834 | 0.836 | 0.838 |
| Sefton | 0.706 | 0.713 | 0.72 | 0.727 | 0.733 | 0.738 | 0.741 | 0.746 | 0.751 | 0.757 | 0.763 | 0.768 | 0.773 | 0.776 | 0.78 | 0.783 | 0.786 | 0.788 | 0.79 | 0.792 | 0.794 | 0.796 | 0.8 | 0.803 | 0.806 | 0.809 | 0.81 | 0.812 |
| St Helens | 0.684 | 0.69 | 0.696 | 0.703 | 0.709 | 0.713 | 0.716 | 0.72 | 0.725 | 0.731 | 0.738 | 0.744 | 0.749 | 0.753 | 0.756 | 0.759 | 0.762 | 0.765 | 0.768 | 0.771 | 0.774 | 0.779 | 0.785 | 0.791 | 0.795 | 0.798 | 0.801 | 0.803 |
| Stockport | 0.727 | 0.734 | 0.741 | 0.748 | 0.755 | 0.759 | 0.763 | 0.767 | 0.772 | 0.779 | 0.785 | 0.791 | 0.796 | 0.799 | 0.803 | 0.806 | 0.809 | 0.812 | 0.814 | 0.817 | 0.82 | 0.823 | 0.828 | 0.833 | 0.837 | 0.839 | 0.841 | 0.843 |
| Tameside | 0.691 | 0.697 | 0.703 | 0.71 | 0.716 | 0.721 | 0.724 | 0.728 | 0.734 | 0.74 | 0.746 | 0.751 | 0.755 | 0.758 | 0.76 | 0.762 | 0.763 | 0.764 | 0.766 | 0.768 | 0.77 | 0.774 | 0.779 | 0.785 | 0.79 | 0.793 | 0.795 | 0.797 |
| Trafford | 0.751 | 0.757 | 0.764 | 0.771 | 0.778 | 0.782 | 0.786 | 0.79 | 0.795 | 0.802 | 0.809 | 0.815 | 0.82 | 0.824 | 0.829 | 0.832 | 0.836 | 0.839 | 0.842 | 0.844 | 0.848 | 0.852 | 0.856 | 0.862 | 0.865 | 0.868 | 0.871 | 0.873 |
| Warrington | 0.739 | 0.745 | 0.752 | 0.759 | 0.765 | 0.769 | 0.773 | 0.776 | 0.782 | 0.788 | 0.795 | 0.801 | 0.807 | 0.811 | 0.814 | 0.818 | 0.822 | 0.825 | 0.827 | 0.83 | 0.833 | 0.838 | 0.843 | 0.849 | 0.853 | 0.856 | 0.858 | 0.86 |
| Wigan | 0.691 | 0.697 | 0.703 | 0.71 | 0.716 | 0.72 | 0.723 | 0.727 | 0.731 | 0.737 | 0.742 | 0.747 | 0.75 | 0.753 | 0.755 | 0.757 | 0.76 | 0.762 | 0.764 | 0.766 | 0.769 | 0.774 | 0.78 | 0.786 | 0.79 | 0.793 | 0.796 | 0.798 |
| Wirral | 0.695 | 0.701 | 0.708 | 0.714 | 0.721 | 0.725 | 0.728 | 0.732 | 0.737 | 0.743 | 0.749 | 0.753 | 0.757 | 0.761 | 0.763 | 0.766 | 0.768 | 0.77 | 0.773 | 0.775 | 0.777 | 0.781 | 0.786 | 0.792 | 0.796 | 0.799 | 0.801 | 0.803 |
| South East England | 0.749 | 0.755 | 0.761 | 0.767 | 0.773 | 0.777 | 0.781 | 0.784 | 0.789 | 0.795 | 0.801 | 0.806 | 0.809 | 0.812 | 0.815 | 0.818 | 0.821 | 0.823 | 0.826 | 0.828 | 0.831 | 0.836 | 0.841 | 0.846 | 0.85 | 0.852 | 0.855 | 0.856 |
| Bracknell Forest | 0.759 | 0.764 | 0.77 | 0.776 | 0.781 | 0.785 | 0.788 | 0.791 | 0.797 | 0.804 | 0.811 | 0.817 | 0.822 | 0.825 | 0.829 | 0.832 | 0.835 | 0.838 | 0.841 | 0.843 | 0.845 | 0.849 | 0.854 | 0.859 | 0.862 | 0.865 | 0.867 | 0.869 |
| Brighton and Hove | 0.766 | 0.772 | 0.779 | 0.785 | 0.791 | 0.796 | 0.801 | 0.806 | 0.811 | 0.816 | 0.822 | 0.827 | 0.833 | 0.838 | 0.843 | 0.847 | 0.851 | 0.855 | 0.859 | 0.863 | 0.865 | 0.868 | 0.872 | 0.876 | 0.879 | 0.881 | 0.883 | 0.885 |
| Buckinghamshire | 0.764 | 0.769 | 0.775 | 0.782 | 0.788 | 0.792 | 0.795 | 0.799 | 0.804 | 0.81 | 0.815 | 0.82 | 0.824 | 0.826 | 0.829 | 0.832 | 0.834 | 0.836 | 0.838 | 0.84 | 0.842 | 0.846 | 0.851 | 0.855 | 0.859 | 0.861 | 0.863 | 0.865 |
| East Sussex | 0.712 | 0.718 | 0.724 | 0.73 | 0.736 | 0.74 | 0.742 | 0.745 | 0.749 | 0.754 | 0.759 | 0.763 | 0.766 | 0.769 | 0.771 | 0.773 | 0.776 | 0.778 | 0.781 | 0.783 | 0.787 | 0.791 | 0.797 | 0.803 | 0.807 | 0.81 | 0.812 | 0.814 |
| Hampshire | 0.744 | 0.75 | 0.756 | 0.762 | 0.768 | 0.772 | 0.775 | 0.778 | 0.782 | 0.788 | 0.793 | 0.798 | 0.802 | 0.804 | 0.807 | 0.81 | 0.812 | 0.815 | 0.818 | 0.82 | 0.824 | 0.828 | 0.834 | 0.839 | 0.843 | 0.846 | 0.848 | 0.85 |
| Isle of Wight | 0.704 | 0.709 | 0.715 | 0.722 | 0.727 | 0.732 | 0.735 | 0.739 | 0.744 | 0.749 | 0.756 | 0.761 | 0.765 | 0.767 | 0.768 | 0.771 | 0.773 | 0.776 | 0.779 | 0.781 | 0.784 | 0.788 | 0.794 | 0.801 | 0.806 | 0.809 | 0.812 | 0.814 |
| Kent | 0.723 | 0.728 | 0.734 | 0.74 | 0.746 | 0.75 | 0.752 | 0.756 | 0.76 | 0.765 | 0.77 | 0.774 | 0.777 | 0.779 | 0.782 | 0.785 | 0.787 | 0.79 | 0.793 | 0.796 | 0.8 | 0.805 | 0.811 | 0.817 | 0.822 | 0.824 | 0.826 | 0.828 |
| Medway | 0.703 | 0.709 | 0.715 | 0.722 | 0.728 | 0.731 | 0.734 | 0.737 | 0.742 | 0.747 | 0.752 | 0.756 | 0.76 | 0.762 | 0.765 | 0.768 | 0.771 | 0.773 | 0.776 | 0.778 | 0.781 | 0.785 | 0.791 | 0.797 | 0.802 | 0.805 | 0.807 | 0.809 |

**Appendix Table 10: Socio-Demographic Index values for all estimated GBD 2017 locations, 1990-2017**

| Location | 1990 | 1991 | 1992 | 1993 | 1994 | 1995 | 1996 | 1997 | 1998 | 1999 | 2000 | 2001 | 2002 | 2003 | 2004 | 2005 | 2006 | 2007 | 2008 | 2009 | 2010 | 2011 | 2012 | 2013 | 2014 | 2015 | 2016 | 2017 |
|---|---|---|---|---|---|---|---|---|---|---|---|---|---|---|---|---|---|---|---|---|---|---|---|---|---|---|---|---|
| Milton Keynes | 0.754 | 0.76 | 0.767 | 0.774 | 0.78 | 0.784 | 0.786 | 0.789 | 0.793 | 0.798 | 0.802 | 0.806 | 0.81 | 0.812 | 0.815 | 0.817 | 0.819 | 0.821 | 0.823 | 0.825 | 0.829 | 0.834 | 0.84 | 0.847 | 0.852 | 0.856 | 0.859 | 0.86 |
| Oxfordshire | 0.769 | 0.775 | 0.781 | 0.788 | 0.794 | 0.798 | 0.801 | 0.805 | 0.81 | 0.816 | 0.822 | 0.827 | 0.831 | 0.835 | 0.838 | 0.841 | 0.844 | 0.847 | 0.849 | 0.852 | 0.855 | 0.859 | 0.864 | 0.869 | 0.872 | 0.875 | 0.878 | 0.879 |
| Portsmouth | 0.75 | 0.756 | 0.763 | 0.77 | 0.776 | 0.781 | 0.785 | 0.79 | 0.795 | 0.8 | 0.805 | 0.81 | 0.815 | 0.818 | 0.822 | 0.824 | 0.827 | 0.829 | 0.832 | 0.835 | 0.838 | 0.842 | 0.846 | 0.851 | 0.854 | 0.857 | 0.858 | 0.86 |
| Reading | 0.785 | 0.791 | 0.797 | 0.803 | 0.809 | 0.813 | 0.817 | 0.821 | 0.827 | 0.834 | 0.84 | 0.847 | 0.852 | 0.856 | 0.86 | 0.864 | 0.866 | 0.868 | 0.87 | 0.872 | 0.874 | 0.877 | 0.882 | 0.887 | 0.89 | 0.892 | 0.894 | 0.895 |
| Slough | 0.764 | 0.77 | 0.777 | 0.784 | 0.79 | 0.793 | 0.796 | 0.799 | 0.802 | 0.806 | 0.81 | 0.813 | 0.815 | 0.816 | 0.817 | 0.818 | 0.819 | 0.82 | 0.822 | 0.825 | 0.829 | 0.834 | 0.841 | 0.848 | 0.852 | 0.855 | 0.858 | 0.859 |
| Southampton | 0.752 | 0.758 | 0.765 | 0.772 | 0.779 | 0.784 | 0.789 | 0.794 | 0.8 | 0.805 | 0.81 | 0.815 | 0.819 | 0.823 | 0.826 | 0.829 | 0.831 | 0.834 | 0.836 | 0.837 | 0.839 | 0.842 | 0.845 | 0.849 | 0.852 | 0.855 | 0.856 | 0.858 |
| Surrey | 0.773 | 0.779 | 0.785 | 0.791 | 0.797 | 0.801 | 0.805 | 0.809 | 0.814 | 0.821 | 0.827 | 0.833 | 0.838 | 0.841 | 0.845 | 0.847 | 0.85 | 0.853 | 0.856 | 0.858 | 0.861 | 0.864 | 0.868 | 0.873 | 0.876 | 0.879 | 0.881 | 0.883 |
| West Berkshire | 0.774 | 0.78 | 0.786 | 0.793 | 0.799 | 0.803 | 0.805 | 0.808 | 0.813 | 0.819 | 0.824 | 0.829 | 0.832 | 0.835 | 0.836 | 0.836 | 0.837 | 0.838 | 0.84 | 0.842 | 0.846 | 0.851 | 0.857 | 0.863 | 0.867 | 0.869 | 0.871 | 0.872 |
| West Sussex | 0.74 | 0.745 | 0.751 | 0.757 | 0.763 | 0.767 | 0.77 | 0.773 | 0.777 | 0.783 | 0.788 | 0.793 | 0.796 | 0.799 | 0.802 | 0.804 | 0.807 | 0.809 | 0.812 | 0.814 | 0.818 | 0.822 | 0.827 | 0.833 | 0.837 | 0.84 | 0.842 | 0.843 |
| Windsor and Maidenhead | 0.778 | 0.783 | 0.789 | 0.795 | 0.8 | 0.805 | 0.808 | 0.811 | 0.816 | 0.823 | 0.829 | 0.835 | 0.839 | 0.843 | 0.847 | 0.851 | 0.854 | 0.857 | 0.86 | 0.863 | 0.866 | 0.87 | 0.874 | 0.88 | 0.883 | 0.885 | 0.887 | 0.889 |
| Wokingham | 0.778 | 0.784 | 0.79 | 0.797 | 0.802 | 0.806 | 0.81 | 0.814 | 0.82 | 0.826 | 0.832 | 0.837 | 0.842 | 0.845 | 0.849 | 0.853 | 0.856 | 0.858 | 0.861 | 0.863 | 0.865 | 0.868 | 0.871 | 0.876 | 0.879 | 0.882 | 0.883 | 0.885 |
| South West England | 0.729 | 0.735 | 0.741 | 0.748 | 0.754 | 0.758 | 0.762 | 0.766 | 0.771 | 0.777 | 0.783 | 0.788 | 0.792 | 0.796 | 0.799 | 0.802 | 0.805 | 0.807 | 0.81 | 0.813 | 0.816 | 0.82 | 0.825 | 0.831 | 0.835 | 0.838 | 0.84 | 0.841 |
| Bath and North East Somerset | 0.752 | 0.758 | 0.764 | 0.77 | 0.777 | 0.782 | 0.786 | 0.79 | 0.796 | 0.803 | 0.809 | 0.816 | 0.822 | 0.828 | 0.833 | 0.838 | 0.842 | 0.846 | 0.85 | 0.853 | 0.856 | 0.859 | 0.863 | 0.867 | 0.869 | 0.872 | 0.874 | 0.875 |
| Bournemouth | 0.736 | 0.743 | 0.75 | 0.757 | 0.763 | 0.768 | 0.773 | 0.778 | 0.784 | 0.79 | 0.797 | 0.803 | 0.808 | 0.812 | 0.816 | 0.821 | 0.824 | 0.828 | 0.831 | 0.834 | 0.836 | 0.839 | 0.843 | 0.848 | 0.851 | 0.853 | 0.856 | 0.858 |
| Bristol, City of | 0.763 | 0.77 | 0.777 | 0.784 | 0.791 | 0.796 | 0.8 | 0.805 | 0.81 | 0.817 | 0.823 | 0.828 | 0.833 | 0.836 | 0.839 | 0.843 | 0.846 | 0.849 | 0.853 | 0.856 | 0.859 | 0.863 | 0.868 | 0.873 | 0.877 | 0.88 | 0.882 | 0.884 |
| Cornwall | 0.7 | 0.706 | 0.713 | 0.721 | 0.727 | 0.731 | 0.734 | 0.738 | 0.743 | 0.749 | 0.755 | 0.76 | 0.764 | 0.768 | 0.771 | 0.774 | 0.777 | 0.78 | 0.783 | 0.786 | 0.789 | 0.793 | 0.799 | 0.806 | 0.81 | 0.813 | 0.815 | 0.817 |
| Devon | 0.72 | 0.726 | 0.733 | 0.74 | 0.746 | 0.75 | 0.753 | 0.757 | 0.762 | 0.769 | 0.775 | 0.78 | 0.785 | 0.789 | 0.793 | 0.796 | 0.8 | 0.803 | 0.806 | 0.808 | 0.811 | 0.816 | 0.821 | 0.826 | 0.83 | 0.833 | 0.835 | 0.837 |
| Dorset | 0.716 | 0.721 | 0.727 | 0.734 | 0.74 | 0.744 | 0.747 | 0.751 | 0.756 | 0.762 | 0.769 | 0.773 | 0.777 | 0.779 | 0.781 | 0.783 | 0.786 | 0.788 | 0.791 | 0.793 | 0.797 | 0.802 | 0.808 | 0.814 | 0.818 | 0.821 | 0.823 | 0.825 |
| Gloucestershire | 0.735 | 0.741 | 0.747 | 0.754 | 0.76 | 0.765 | 0.768 | 0.772 | 0.777 | 0.783 | 0.79 | 0.795 | 0.8 | 0.804 | 0.808 | 0.811 | 0.813 | 0.816 | 0.818 | 0.82 | 0.824 | 0.828 | 0.833 | 0.839 | 0.843 | 0.846 | 0.848 | 0.85 |
| North Somerset | 0.714 | 0.72 | 0.727 | 0.733 | 0.739 | 0.743 | 0.746 | 0.75 | 0.755 | 0.76 | 0.766 | 0.771 | 0.776 | 0.78 | 0.783 | 0.786 | 0.789 | 0.792 | 0.795 | 0.798 | 0.801 | 0.806 | 0.813 | 0.819 | 0.824 | 0.827 | 0.83 | 0.832 |
| Plymouth | 0.724 | 0.73 | 0.737 | 0.744 | 0.75 | 0.754 | 0.758 | 0.762 | 0.767 | 0.772 | 0.778 | 0.783 | 0.787 | 0.79 | 0.793 | 0.796 | 0.799 | 0.802 | 0.805 | 0.807 | 0.81 | 0.814 | 0.819 | 0.825 | 0.829 | 0.832 | 0.834 | 0.836 |
| Poole | 0.727 | 0.733 | 0.74 | 0.746 | 0.753 | 0.758 | 0.761 | 0.765 | 0.771 | 0.777 | 0.783 | 0.789 | 0.793 | 0.796 | 0.798 | 0.801 | 0.804 | 0.806 | 0.809 | 0.811 | 0.814 | 0.818 | 0.824 | 0.83 | 0.835 | 0.838 | 0.84 | 0.842 |
| Somerset | 0.713 | 0.718 | 0.724 | 0.731 | 0.737 | 0.741 | 0.744 | 0.748 | 0.752 | 0.757 | 0.763 | 0.767 | 0.77 | 0.772 | 0.775 | 0.777 | 0.78 | 0.782 | 0.785 | 0.787 | 0.789 | 0.794 | 0.799 | 0.805 | 0.809 | 0.812 | 0.814 | 0.816 |
| South Gloucestershire | 0.747 | 0.752 | 0.758 | 0.765 | 0.771 | 0.775 | 0.779 | 0.783 | 0.789 | 0.796 | 0.802 | 0.808 | 0.813 | 0.817 | 0.821 | 0.824 | 0.827 | 0.831 | 0.834 | 0.837 | 0.84 | 0.844 | 0.849 | 0.855 | 0.859 | 0.862 | 0.865 | 0.867 |
| Swindon | 0.747 | 0.753 | 0.76 | 0.767 | 0.773 | 0.776 | 0.778 | 0.781 | 0.786 | 0.792 | 0.797 | 0.801 | 0.805 | 0.806 | 0.807 | 0.809 | 0.811 | 0.813 | 0.815 | 0.818 | 0.82 | 0.825 | 0.831 | 0.837 | 0.841 | 0.844 | 0.846 | 0.847 |
| Torbay | 0.699 | 0.705 | 0.711 | 0.717 | 0.723 | 0.727 | 0.73 | 0.733 | 0.736 | 0.741 | 0.745 | 0.749 | 0.75 | 0.751 | 0.753 | 0.755 | 0.757 | 0.759 | 0.761 | 0.762 | 0.764 | 0.767 | 0.773 | 0.779 | 0.783 | 0.786 | 0.789 | 0.79 |
| Wiltshire | 0.726 | 0.731 | 0.737 | 0.743 | 0.749 | 0.753 | 0.756 | 0.759 | 0.763 | 0.769 | 0.775 | 0.78 | 0.784 | 0.786 | 0.789 | 0.791 | 0.793 | 0.795 | 0.797 | 0.799 | 0.803 | 0.808 | 0.813 | 0.819 | 0.823 | 0.826 | 0.828 | 0.829 |
| West Midlands | 0.707 | 0.713 | 0.72 | 0.727 | 0.734 | 0.739 | 0.743 | 0.747 | 0.752 | 0.759 | 0.765 | 0.771 | 0.776 | 0.779 | 0.783 | 0.786 | 0.789 | 0.792 | 0.795 | 0.798 | 0.802 | 0.806 | 0.812 | 0.818 | 0.822 | 0.825 | 0.828 | 0.829 |
| Birmingham | 0.707 | 0.714 | 0.721 | 0.728 | 0.735 | 0.739 | 0.743 | 0.747 | 0.753 | 0.76 | 0.766 | 0.773 | 0.778 | 0.782 | 0.787 | 0.791 | 0.794 | 0.798 | 0.803 | 0.807 | 0.811 | 0.817 | 0.823 | 0.829 | 0.834 | 0.837 | 0.839 | 0.84 |
| Coventry | 0.722 | 0.729 | 0.737 | 0.745 | 0.751 | 0.757 | 0.762 | 0.766 | 0.772 | 0.779 | 0.785 | 0.791 | 0.796 | 0.8 | 0.803 | 0.807 | 0.81 | 0.812 | 0.815 | 0.818 | 0.821 | 0.825 | 0.831 | 0.837 | 0.841 | 0.844 | 0.847 | 0.848 |
| Dudley | 0.692 | 0.698 | 0.704 | 0.71 | 0.716 | 0.721 | 0.724 | 0.728 | 0.733 | 0.739 | 0.746 | 0.751 | 0.756 | 0.759 | 0.762 | 0.765 | 0.767 | 0.77 | 0.772 | 0.774 | 0.776 | 0.779 | 0.784 | 0.789 | 0.793 | 0.795 | 0.797 | 0.799 |
| Herefordshire, County of | 0.706 | 0.712 | 0.719 | 0.726 | 0.733 | 0.738 | 0.742 | 0.747 | 0.752 | 0.759 | 0.766 | 0.771 | 0.776 | 0.779 | 0.783 | 0.786 | 0.789 | 0.792 | 0.795 | 0.798 | 0.801 | 0.806 | 0.812 | 0.818 | 0.822 | 0.825 | 0.827 | 0.828 |
| Sandwell | 0.682 | 0.688 | 0.695 | 0.702 | 0.708 | 0.712 | 0.715 | 0.719 | 0.724 | 0.729 | 0.735 | 0.74 | 0.744 | 0.747 | 0.75 | 0.752 | 0.754 | 0.756 | 0.759 | 0.761 | 0.765 | 0.769 | 0.776 | 0.783 | 0.788 | 0.792 | 0.795 | 0.797 |
| Shropshire | 0.708 | 0.714 | 0.721 | 0.728 | 0.734 | 0.739 | 0.743 | 0.747 | 0.752 | 0.759 | 0.766 | 0.771 | 0.776 | 0.781 | 0.785 | 0.789 | 0.793 | 0.795 | 0.799 | 0.802 | 0.806 | 0.811 | 0.816 | 0.821 | 0.825 | 0.828 | 0.83 | 0.832 |
| Solihull | 0.733 | 0.74 | 0.747 | 0.755 | 0.762 | 0.767 | 0.771 | 0.776 | 0.783 | 0.789 | 0.795 | 0.801 | 0.807 | 0.811 | 0.815 | 0.818 | 0.822 | 0.825 | 0.828 | 0.83 | 0.832 | 0.835 | 0.84 | 0.845 | 0.848 | 0.851 | 0.853 | 0.855 |
| Staffordshire | 0.708 | 0.714 | 0.722 | 0.729 | 0.736 | 0.74 | 0.744 | 0.749 | 0.755 | 0.761 | 0.767 | 0.772 | 0.777 | 0.78 | 0.783 | 0.787 | 0.79 | 0.793 | 0.796 | 0.799 | 0.802 | 0.806 | 0.811 | 0.816 | 0.819 | 0.822 | 0.824 | 0.826 |
| Stoke-on-Trent | 0.689 | 0.696 | 0.703 | 0.71 | 0.716 | 0.721 | 0.724 | 0.728 | 0.733 | 0.738 | 0.743 | 0.747 | 0.75 | 0.751 | 0.753 | 0.754 | 0.756 | 0.757 | 0.76 | 0.763 | 0.767 | 0.772 | 0.78 | 0.788 | 0.794 | 0.798 | 0.801 | 0.804 |
| Telford and Wrekin | 0.713 | 0.719 | 0.726 | 0.733 | 0.74 | 0.745 | 0.749 | 0.754 | 0.759 | 0.764 | 0.769 | 0.773 | 0.776 | 0.777 | 0.779 | 0.781 | 0.783 | 0.784 | 0.787 | 0.789 | 0.791 | 0.796 | 0.802 | 0.809 | 0.814 | 0.817 | 0.82 | 0.822 |
| Walsall | 0.681 | 0.686 | 0.692 | 0.698 | 0.704 | 0.707 | 0.709 | 0.712 | 0.717 | 0.723 | 0.729 | 0.734 | 0.738 | 0.74 | 0.743 | 0.746 | 0.749 | 0.752 | 0.755 | 0.757 | 0.761 | 0.766 | 0.772 | 0.779 | 0.783 | 0.787 | 0.789 | 0.791 |
| Warwickshire | 0.732 | 0.738 | 0.746 | 0.753 | 0.76 | 0.765 | 0.769 | 0.774 | 0.779 | 0.786 | 0.792 | 0.798 | 0.802 | 0.806 | 0.809 | 0.812 | 0.816 | 0.819 | 0.823 | 0.826 | 0.83 | 0.834 | 0.84 | 0.845 | 0.85 | 0.853 | 0.855 | 0.857 |
| Wolverhampton | 0.691 | 0.697 | 0.704 | 0.712 | 0.719 | 0.723 | 0.727 | 0.731 | 0.737 | 0.744 | 0.751 | 0.757 | 0.761 | 0.764 | 0.768 | 0.771 | 0.774 | 0.776 | 0.779 | 0.781 | 0.784 | 0.788 | 0.794 | 0.8 | 0.804 | 0.807 | 0.809 | 0.811 |
| Worcestershire | 0.707 | 0.713 | 0.72 | 0.727 | 0.733 | 0.738 | 0.742 | 0.747 | 0.754 | 0.762 | 0.771 | 0.778 | 0.782 | 0.786 | 0.791 | 0.794 | 0.798 | 0.8 | 0.803 | 0.805 | 0.808 | 0.811 | 0.816 | 0.822 | 0.826 | 0.829 | 0.831 | 0.833 |
| Yorkshire and the Humber | 0.711 | 0.717 | 0.724 | 0.731 | 0.737 | 0.742 | 0.745 | 0.75 | 0.755 | 0.762 | 0.768 | 0.773 | 0.778 | 0.781 | 0.785 | 0.788 | 0.791 | 0.794 | 0.797 | 0.8 | 0.803 | 0.808 | 0.813 | 0.819 | 0.823 | 0.826 | 0.828 | 0.83 |
| Barnsley | 0.675 | 0.68 | 0.686 | 0.692 | 0.698 | 0.702 | 0.705 | 0.709 | 0.715 | 0.721 | 0.726 | 0.731 | 0.735 | 0.738 | 0.741 | 0.743 | 0.746 | 0.748 | 0.75 | 0.752 | 0.755 | 0.759 | 0.765 | 0.772 | 0.778 | 0.781 | 0.785 | 0.787 |
| Bradford | 0.693 | 0.699 | 0.705 | 0.712 | 0.718 | 0.721 | 0.723 | 0.725 | 0.729 | 0.736 | 0.741 | 0.747 | 0.751 | 0.753 | 0.756 | 0.759 | 0.762 | 0.765 | 0.768 | 0.771 | 0.775 | 0.78 | 0.787 | 0.794 | 0.799 | 0.803 | 0.805 | 0.807 |
| Calderdale | 0.708 | 0.714 | 0.721 | 0.729 | 0.735 | 0.739 | 0.743 | 0.746 | 0.751 | 0.758 | 0.763 | 0.768 | 0.771 | 0.774 | 0.777 | 0.78 | 0.783 | 0.785 | 0.788 | 0.791 | 0.795 | 0.8 | 0.807 | 0.814 | 0.819 | 0.823 | 0.825 | 0.827 |
| Doncaster | 0.674 | 0.679 | 0.685 | 0.692 | 0.697 | 0.701 | 0.704 | 0.707 | 0.712 | 0.718 | 0.724 | 0.729 | 0.733 | 0.736 | 0.74 | 0.743 | 0.746 | 0.749 | 0.752 | 0.755 | 0.759 | 0.764 | 0.771 | 0.778 | 0.783 | 0.786 | 0.789 | 0.791 |
| East Riding of Yorkshire | 0.714 | 0.72 | 0.727 | 0.734 | 0.741 | 0.745 | 0.749 | 0.753 | 0.758 | 0.764 | 0.769 | 0.774 | 0.778 | 0.781 | 0.784 | 0.787 | 0.79 | 0.792 | 0.794 | 0.797 | 0.799 | 0.802 | 0.807 | 0.812 | 0.815 | 0.818 | 0.82 | 0.822 |
| Kingston upon Hull, City of | 0.689 | 0.695 | 0.702 | 0.71 | 0.717 | 0.722 | 0.726 | 0.73 | 0.736 | 0.742 | 0.749 | 0.755 | 0.76 | 0.763 | 0.767 | 0.77 | 0.773 | 0.776 | 0.779 | 0.782 | 0.785 | 0.789 | 0.795 | 0.801 | 0.806 | 0.809 | 0.811 | 0.813 |
| Kirklees | 0.703 | 0.709 | 0.716 | 0.723 | 0.73 | 0.734 | 0.737 | 0.741 | 0.746 | 0.752 | 0.758 | 0.763 | 0.767 | 0.77 | 0.773 | 0.776 | 0.779 | 0.781 | 0.783 | 0.785 | 0.788 | 0.793 | 0.798 | 0.805 | 0.809 | 0.812 | 0.814 | 0.816 |
| Leeds | 0.739 | 0.746 | 0.753 | 0.761 | 0.768 | 0.773 | 0.778 | 0.783 | 0.789 | 0.797 | 0.803 | 0.81 | 0.816 | 0.82 | 0.825 | 0.83 | 0.834 | 0.837 | 0.841 | 0.843 | 0.846 | 0.849 | 0.854 | 0.858 | 0.862 | 0.864 | 0.867 | 0.868 |
| North East Lincolnshire | 0.698 | 0.705 | 0.712 | 0.72 | 0.727 | 0.731 | 0.733 | 0.737 | 0.741 | 0.745 | 0.749 | 0.753 | 0.755 | 0.757 | 0.758 | 0.761 | 0.763 | 0.767 | 0.77 | 0.775 | 0.779 | 0.784 | 0.79 | 0.796 | 0.799 | 0.802 | 0.803 | 0.804 |
| North Lincolnshire | 0.702 | 0.707 | 0.714 | 0.72 | 0.727 | 0.731 | 0.734 | 0.737 | 0.742 | 0.748 | 0.752 | 0.757 | 0.76 | 0.762 | 0.764 | 0.766 | 0.768 | 0.77 | 0.773 | 0.776 | 0.78 | 0.785 | 0.792 | 0.799 | 0.803 | 0.807 | 0.809 | 0.811 |
| North Yorkshire | 0.724 | 0.73 | 0.737 | 0.743 | 0.75 | 0.754 | 0.758 | 0.762 | 0.767 | 0.774 | 0.78 | 0.785 | 0.79 | 0.794 | 0.798 | 0.801 | 0.803 | 0.806 | 0.808 | 0.81 | 0.813 | 0.817 | 0.822 | 0.827 | 0.831 | 0.834 | 0.836 | 0.839 |
| Rotherham | 0.681 | 0.687 | 0.693 | 0.7 | 0.707 | 0.711 | 0.714 | 0.717 | 0.722 | 0.728 | 0.734 | 0.739 | 0.744 | 0.747 | 0.75 | 0.753 | 0.756 | 0.759 | 0.761 | 0.763 | 0.766 | 0.77 | 0.776 | 0.782 | 0.787 | 0.791 | 0.794 | 0.796 |
| Sheffield | 0.727 | 0.733 | 0.74 | 0.747 | 0.753 | 0.758 | 0.762 | 0.767 | 0.773 | 0.78 | 0.786 | 0.792 | 0.798 | 0.803 | 0.807 | 0.811 | 0.815 | 0.819 | 0.823 | 0.826 | 0.83 | 0.834 | 0.839 | 0.843 | 0.847 | 0.849 | 0.852 | 0.853 |
| Wakefield | 0.692 | 0.698 | 0.705 | 0.712 | 0.718 | 0.722 | 0.726 | 0.729 | 0.734 | 0.741 | 0.747 | 0.752 | 0.755 | 0.758 | 0.76 | 0.763 | 0.765 | 0.766 | 0.768 | 0.769 | 0.772 | 0.777 | 0.784 | 0.791 | 0.797 | 0.801 | 0.804 | 0.806 |
| York | 0.762 | 0.769 | 0.776 | 0.783 | 0.789 | 0.795 | 0.799 | 0.804 | 0.81 | 0.816 | 0.822 | 0.829 | 0.834 | 0.839 | 0.844 | 0.848 | 0.851 | 0.854 | 0.857 | 0.859 | 0.862 | 0.865 | 0.868 | 0.872 | 0.874 | 0.876 | 0.878 | 0.879 |
| Northern Ireland | 0.71 | 0.718 | 0.726 | 0.735 | 0.742 | 0.747 | 0.751 | 0.755 | 0.761 | 0.767 | 0.774 | 0.78 | 0.786 | 0.79 | 0.795 | 0.798 | 0.801 | 0.805 | 0.808 | 0.812 | 0.815 | 0.818 | 0.822 | 0.825 | 0.828 | 0.831 | 0.833 | 0.835 |
| Scotland | 0.672 | 0.678 | 0.686 | 0.693 | 0.701 | 0.706 | 0.711 | 0.716 | 0.722 | 0.73 | 0.737 | 0.743 | 0.748 | 0.752 | 0.757 | 0.761 | 0.764 | 0.768 | 0.772 | 0.777 | 0.781 | 0.785 | 0.789 | 0.793 | 0.797 | 0.8 | 0.803 | 0.805 |
| Wales | 0.642 | 0.65 | 0.659 | 0.668 | 0.676 | 0.683 | 0.688 | 0.694 | 0.702 | 0.711 | 0.719 | 0.727 | 0.734 | 0.739 | 0.745 | 0.75 | 0.755 | 0.76 | 0.765 | 0.77 | 0.774 | 0.78 | 0.786 | 0.792 | 0.797 | 0.8 | 0.803 | 0.806 |
| Latin America and Caribbean | 0.497 | 0.502 | 0.508 | 0.514 | 0.521 | 0.527 | 0.534 | 0.54 | 0.546 | 0.552 | 0.557 | 0.563 | 0.567 | 0.571 | 0.576 | 0.581 | 0.587 | 0.592 | 0.598 | 0.603 | 0.608 | 0.613 | 0.618 | 0.624 | 0.628 | 0.633 | 0.637 | 0.64 |
| Andean Latin America | 0.483 | 0.486 | 0.49 | 0.495 | 0.501 | 0.507 | 0.512 | 0.518 | 0.526 | 0.533 | 0.539 | 0.546 | 0.552 | 0.558 | 0.563 | 0.567 | 0.572 | 0.577 | 0.581 | 0.585 | 0.59 | 0.595 | 0.601 | 0.606 | 0.612 | 0.618 | 0.624 | 0.628 |
| Bolivia | 0.403 | 0.408 | 0.416 | 0.424 | 0.431 | 0.438 | 0.447 | 0.456 | 0.466 | 0.475 | 0.485 | 0.494 | 0.504 | 0.513 | 0.52 | 0.526 | 0.532 | 0.536 | 0.539 | 0.543 | 0.547 | 0.551 | 0.555 | 0.559 | 0.565 | 0.573 | 0.581 | 0.587 |
| Ecuador | 0.506 | 0.51 | 0.515 | 0.52 | 0.524 | 0.524 | 0.522 | 0.521 | 0.526 | 0.532 | 0.539 | 0.548 | 0.557 | 0.564 | 0.57 | 0.575 | 0.581 | 0.585 | 0.588 | 0.59 | 0.594 | 0.603 | 0.611 | 0.618 | 0.623 | 0.628 | 0.632 | 0.636 |
| Peru | 0.493 | 0.495 | 0.497 | 0.501 | 0.509 | 0.517 | 0.526 | 0.534 | 0.542 | 0.549 | 0.555 | 0.56 | 0.564 | 0.568 | 0.571 | 0.575 | 0.578 | 0.584 | 0.59 | 0.594 | 0.6 | 0.604 | 0.608 | 0.614 | 0.62 | 0.626 | 0.632 | 0.636 |
| Caribbean | 0.531 | 0.536 | 0.539 | 0.543 | 0.546 | 0.549 | 0.552 | 0.555 | 0.559 | 0.563 | 0.568 | 0.573 | 0.578 | 0.583 | 0.589 | 0.596 | 0.602 | 0.607 | 0.61 | 0.613 | 0.617 | 0.621 | 0.624 | 0.626 | 0.628 | 0.631 | 0.635 | 0.638 |
| Antigua and Barbuda | 0.61 | 0.616 | 0.622 | 0.627 | 0.631 | 0.633 | 0.636 | 0.64 | 0.644 | 0.649 | 0.653 | 0.657 | 0.661 | 0.666 | 0.67 | 0.675 | 0.68 | 0.685 | 0.69 | 0.694 | 0.696 | 0.698 | 0.7 | 0.703 | 0.706 | 0.709 | 0.712 | 0.715 |
| The Bahamas | 0.674 | 0.669 | 0.665 | 0.667 | 0.671 | 0.676 | 0.681 | 0.687 | 0.695 | 0.703 | 0.71 | 0.715 | 0.718 | 0.721 | 0.723 | 0.727 | 0.732 | 0.737 | 0.742 | 0.746 | 0.751 | 0.753 | 0.755 | 0.755 | 0.755 | 0.755 | 0.755 | 0.756 |

**Appendix Table 10: Socio-Demographic Index values for all estimated GBD 2017 locations, 1990-2017**

| Location | 1990 | 1991 | 1992 | 1993 | 1994 | 1995 | 1996 | 1997 | 1998 | 1999 | 2000 | 2001 | 2002 | 2003 | 2004 | 2005 | 2006 | 2007 | 2008 | 2009 | 2010 | 2011 | 2012 | 2013 | 2014 | 2015 | 2016 | 2017 |
|---|---|---|---|---|---|---|---|---|---|---|---|---|---|---|---|---|---|---|---|---|---|---|---|---|---|---|---|---|
| Barbados | 0.65 | 0.655 | 0.661 | 0.667 | 0.672 | 0.675 | 0.678 | 0.68 | 0.682 | 0.683 | 0.686 | 0.689 | 0.693 | 0.698 | 0.703 | 0.707 | 0.71 | 0.713 | 0.717 | 0.72 | 0.723 | 0.726 | 0.729 | 0.732 | 0.734 | 0.736 | 0.738 | 0.739 |
| Belize | 0.402 | 0.414 | 0.429 | 0.445 | 0.458 | 0.471 | 0.482 | 0.49 | 0.496 | 0.501 | 0.506 | 0.511 | 0.518 | 0.525 | 0.533 | 0.541 | 0.549 | 0.557 | 0.563 | 0.569 | 0.576 | 0.581 | 0.586 | 0.59 | 0.594 | 0.598 | 0.6 | 0.602 |
| Bermuda | 0.73 | 0.732 | 0.734 | 0.737 | 0.738 | 0.74 | 0.741 | 0.743 | 0.746 | 0.749 | 0.752 | 0.754 | 0.757 | 0.761 | 0.765 | 0.769 | 0.773 | 0.777 | 0.782 | 0.786 | 0.79 | 0.794 | 0.797 | 0.8 | 0.803 | 0.804 | 0.805 | 0.805 |
| Cuba | 0.592 | 0.6 | 0.604 | 0.605 | 0.604 | 0.603 | 0.601 | 0.6 | 0.601 | 0.604 | 0.607 | 0.611 | 0.617 | 0.623 | 0.629 | 0.636 | 0.644 | 0.646 | 0.647 | 0.649 | 0.652 | 0.656 | 0.663 | 0.668 | 0.671 | 0.676 | 0.683 | 0.688 |
| Dominica | 0.534 | 0.535 | 0.539 | 0.546 | 0.555 | 0.563 | 0.57 | 0.577 | 0.584 | 0.592 | 0.599 | 0.606 | 0.614 | 0.621 | 0.63 | 0.636 | 0.641 | 0.645 | 0.65 | 0.656 | 0.662 | 0.668 | 0.673 | 0.677 | 0.68 | 0.682 | 0.684 | 0.687 |
| Dominican Republic | 0.442 | 0.443 | 0.445 | 0.449 | 0.454 | 0.459 | 0.464 | 0.469 | 0.475 | 0.48 | 0.484 | 0.49 | 0.495 | 0.499 | 0.511 | 0.525 | 0.536 | 0.547 | 0.555 | 0.562 | 0.568 | 0.572 | 0.575 | 0.576 | 0.578 | 0.583 | 0.589 | 0.593 |
| Grenada | 0.46 | 0.472 | 0.485 | 0.497 | 0.508 | 0.519 | 0.529 | 0.54 | 0.55 | 0.558 | 0.566 | 0.573 | 0.578 | 0.584 | 0.589 | 0.594 | 0.599 | 0.604 | 0.608 | 0.612 | 0.615 | 0.618 | 0.62 | 0.624 | 0.628 | 0.632 | 0.637 | 0.64 |
| Guyana | 0.442 | 0.446 | 0.45 | 0.454 | 0.458 | 0.465 | 0.472 | 0.481 | 0.489 | 0.497 | 0.503 | 0.51 | 0.516 | 0.521 | 0.526 | 0.53 | 0.533 | 0.537 | 0.542 | 0.547 | 0.552 | 0.557 | 0.563 | 0.568 | 0.573 | 0.576 | 0.58 | 0.584 |
| Haiti | 0.328 | 0.332 | 0.336 | 0.339 | 0.342 | 0.345 | 0.35 | 0.355 | 0.361 | 0.367 | 0.373 | 0.379 | 0.384 | 0.389 | 0.393 | 0.397 | 0.401 | 0.405 | 0.409 | 0.413 | 0.416 | 0.42 | 0.423 | 0.427 | 0.431 | 0.435 | 0.439 | 0.442 |
| Jamaica | 0.547 | 0.553 | 0.56 | 0.565 | 0.571 | 0.578 | 0.585 | 0.591 | 0.596 | 0.6 | 0.607 | 0.615 | 0.621 | 0.626 | 0.63 | 0.635 | 0.639 | 0.644 | 0.649 | 0.653 | 0.658 | 0.663 | 0.667 | 0.671 | 0.673 | 0.676 | 0.677 | 0.679 |
| Puerto Rico | 0.684 | 0.689 | 0.693 | 0.698 | 0.703 | 0.708 | 0.714 | 0.719 | 0.724 | 0.729 | 0.736 | 0.745 | 0.752 | 0.756 | 0.759 | 0.762 | 0.766 | 0.77 | 0.774 | 0.778 | 0.782 | 0.786 | 0.791 | 0.797 | 0.805 | 0.811 | 0.813 | 0.813 |
| Saint Lucia | 0.509 | 0.516 | 0.524 | 0.532 | 0.54 | 0.549 | 0.558 | 0.566 | 0.573 | 0.58 | 0.585 | 0.589 | 0.593 | 0.598 | 0.603 | 0.606 | 0.609 | 0.613 | 0.618 | 0.623 | 0.628 | 0.633 | 0.636 | 0.64 | 0.643 | 0.646 | 0.65 | 0.653 |
| Saint Vincent and the Grenadines | 0.465 | 0.472 | 0.479 | 0.486 | 0.492 | 0.499 | 0.506 | 0.512 | 0.518 | 0.524 | 0.53 | 0.537 | 0.543 | 0.549 | 0.553 | 0.557 | 0.561 | 0.566 | 0.571 | 0.575 | 0.579 | 0.584 | 0.588 | 0.593 | 0.597 | 0.601 | 0.605 | 0.608 |
| Suriname | 0.529 | 0.534 | 0.538 | 0.541 | 0.543 | 0.543 | 0.544 | 0.547 | 0.551 | 0.555 | 0.56 | 0.568 | 0.576 | 0.585 | 0.592 | 0.597 | 0.601 | 0.604 | 0.608 | 0.612 | 0.616 | 0.62 | 0.625 | 0.628 | 0.632 | 0.636 | 0.639 | 0.641 |
| Trinidad and Tobago | 0.601 | 0.604 | 0.608 | 0.611 | 0.615 | 0.619 | 0.624 | 0.629 | 0.634 | 0.64 | 0.646 | 0.652 | 0.657 | 0.663 | 0.669 | 0.675 | 0.68 | 0.685 | 0.689 | 0.691 | 0.693 | 0.694 | 0.695 | 0.696 | 0.697 | 0.698 | 0.698 | 0.698 |
| Virgin Islands | 0.673 | 0.683 | 0.691 | 0.699 | 0.706 | 0.713 | 0.719 | 0.725 | 0.73 | 0.734 | 0.737 | 0.74 | 0.748 | 0.755 | 0.762 | 0.768 | 0.774 | 0.78 | 0.786 | 0.79 | 0.795 | 0.799 | 0.802 | 0.804 | 0.805 | 0.806 | 0.806 | 0.807 |
| Central Latin America | 0.492 | 0.496 | 0.501 | 0.507 | 0.515 | 0.521 | 0.528 | 0.534 | 0.54 | 0.545 | 0.551 | 0.557 | 0.561 | 0.564 | 0.567 | 0.571 | 0.577 | 0.583 | 0.588 | 0.593 | 0.597 | 0.601 | 0.606 | 0.61 | 0.614 | 0.617 | 0.621 | 0.623 |
| Colombia | 0.482 | 0.483 | 0.485 | 0.49 | 0.496 | 0.503 | 0.511 | 0.518 | 0.524 | 0.528 | 0.533 | 0.537 | 0.541 | 0.544 | 0.548 | 0.553 | 0.558 | 0.565 | 0.573 | 0.581 | 0.588 | 0.596 | 0.604 | 0.611 | 0.617 | 0.624 | 0.629 | 0.634 |
| Costa Rica | 0.524 | 0.53 | 0.536 | 0.54 | 0.545 | 0.55 | 0.555 | 0.56 | 0.566 | 0.573 | 0.581 | 0.589 | 0.594 | 0.598 | 0.603 | 0.607 | 0.609 | 0.612 | 0.616 | 0.623 | 0.629 | 0.634 | 0.64 | 0.645 | 0.649 | 0.654 | 0.658 | 0.662 |
| El Salvador | 0.406 | 0.409 | 0.413 | 0.418 | 0.424 | 0.431 | 0.44 | 0.449 | 0.459 | 0.47 | 0.481 | 0.491 | 0.5 | 0.508 | 0.515 | 0.523 | 0.531 | 0.539 | 0.545 | 0.55 | 0.556 | 0.561 | 0.567 | 0.573 | 0.578 | 0.584 | 0.589 | 0.593 |
| Guatemala | 0.313 | 0.318 | 0.326 | 0.338 | 0.353 | 0.363 | 0.371 | 0.381 | 0.397 | 0.411 | 0.42 | 0.427 | 0.433 | 0.44 | 0.449 | 0.458 | 0.465 | 0.47 | 0.476 | 0.482 | 0.489 | 0.496 | 0.501 | 0.507 | 0.513 | 0.517 | 0.521 | 0.524 |
| Honduras | 0.34 | 0.344 | 0.347 | 0.352 | 0.358 | 0.365 | 0.372 | 0.381 | 0.39 | 0.4 | 0.41 | 0.419 | 0.428 | 0.437 | 0.445 | 0.452 | 0.459 | 0.465 | 0.47 | 0.475 | 0.481 | 0.486 | 0.49 | 0.495 | 0.499 | 0.504 | 0.508 | 0.512 |
| Mexico | 0.513 | 0.517 | 0.521 | 0.529 | 0.537 | 0.543 | 0.549 | 0.555 | 0.561 | 0.566 | 0.572 | 0.577 | 0.581 | 0.586 | 0.59 | 0.595 | 0.598 | 0.602 | 0.606 | 0.608 | 0.611 | 0.613 | 0.616 | 0.619 | 0.622 | 0.624 | 0.626 | 0.628 |
| Aguascalientes | 0.558 | 0.561 | 0.565 | 0.571 | 0.577 | 0.583 | 0.591 | 0.597 | 0.603 | 0.606 | 0.609 | 0.612 | 0.615 | 0.621 | 0.626 | 0.632 | 0.636 | 0.64 | 0.643 | 0.644 | 0.645 | 0.647 | 0.649 | 0.651 | 0.653 | 0.655 | 0.657 | 0.659 |
| Baja California | 0.57 | 0.572 | 0.575 | 0.579 | 0.582 | 0.585 | 0.589 | 0.594 | 0.599 | 0.604 | 0.608 | 0.611 | 0.615 | 0.618 | 0.622 | 0.626 | 0.63 | 0.634 | 0.639 | 0.642 | 0.644 | 0.646 | 0.649 | 0.651 | 0.653 | 0.654 | 0.656 | 0.657 |
| Baja California Sur | 0.569 | 0.573 | 0.577 | 0.582 | 0.586 | 0.589 | 0.593 | 0.598 | 0.602 | 0.605 | 0.609 | 0.612 | 0.614 | 0.617 | 0.621 | 0.624 | 0.628 | 0.633 | 0.637 | 0.641 | 0.644 | 0.647 | 0.649 | 0.652 | 0.654 | 0.656 | 0.657 | 0.659 |
| Campeche | 0.485 | 0.49 | 0.496 | 0.504 | 0.511 | 0.517 | 0.525 | 0.534 | 0.543 | 0.551 | 0.558 | 0.564 | 0.569 | 0.573 | 0.577 | 0.581 | 0.585 | 0.589 | 0.593 | 0.595 | 0.597 | 0.599 | 0.602 | 0.605 | 0.608 | 0.611 | 0.614 | 0.616 |
| Chiapas | 0.403 | 0.407 | 0.413 | 0.422 | 0.43 | 0.436 | 0.444 | 0.452 | 0.459 | 0.465 | 0.47 | 0.475 | 0.48 | 0.484 | 0.489 | 0.494 | 0.498 | 0.503 | 0.508 | 0.512 | 0.514 | 0.517 | 0.52 | 0.522 | 0.525 | 0.528 | 0.531 | 0.533 |
| Chihuahua | 0.546 | 0.549 | 0.552 | 0.555 | 0.558 | 0.559 | 0.562 | 0.566 | 0.57 | 0.574 | 0.577 | 0.581 | 0.585 | 0.589 | 0.594 | 0.599 | 0.604 | 0.61 | 0.615 | 0.619 | 0.622 | 0.625 | 0.628 | 0.63 | 0.633 | 0.635 | 0.637 | 0.639 |
| Coahuila | 0.549 | 0.555 | 0.561 | 0.567 | 0.573 | 0.576 | 0.581 | 0.587 | 0.592 | 0.597 | 0.601 | 0.604 | 0.607 | 0.611 | 0.616 | 0.62 | 0.624 | 0.628 | 0.631 | 0.633 | 0.634 | 0.635 | 0.637 | 0.638 | 0.64 | 0.642 | 0.644 | 0.645 |
| Colima | 0.538 | 0.543 | 0.549 | 0.556 | 0.561 | 0.565 | 0.571 | 0.577 | 0.583 | 0.588 | 0.594 | 0.598 | 0.603 | 0.607 | 0.611 | 0.616 | 0.621 | 0.626 | 0.631 | 0.634 | 0.637 | 0.64 | 0.643 | 0.645 | 0.648 | 0.65 | 0.652 | 0.654 |
| Mexico City | 0.602 | 0.606 | 0.61 | 0.617 | 0.629 | 0.64 | 0.65 | 0.656 | 0.661 | 0.666 | 0.671 | 0.676 | 0.681 | 0.685 | 0.689 | 0.693 | 0.696 | 0.698 | 0.7 | 0.701 | 0.702 | 0.704 | 0.706 | 0.708 | 0.71 | 0.712 | 0.714 | 0.716 |
| Durango | 0.506 | 0.508 | 0.511 | 0.516 | 0.52 | 0.523 | 0.528 | 0.535 | 0.542 | 0.55 | 0.556 | 0.562 | 0.568 | 0.574 | 0.581 | 0.586 | 0.592 | 0.597 | 0.602 | 0.605 | 0.607 | 0.609 | 0.612 | 0.614 | 0.617 | 0.619 | 0.622 | 0.624 |
| Guanajuato | 0.494 | 0.499 | 0.504 | 0.511 | 0.519 | 0.527 | 0.534 | 0.541 | 0.546 | 0.551 | 0.558 | 0.565 | 0.571 | 0.575 | 0.579 | 0.583 | 0.587 | 0.591 | 0.595 | 0.598 | 0.601 | 0.604 | 0.607 | 0.611 | 0.614 | 0.616 | 0.619 | 0.621 |
| Guerrero | 0.426 | 0.429 | 0.433 | 0.44 | 0.445 | 0.45 | 0.456 | 0.465 | 0.473 | 0.48 | 0.487 | 0.492 | 0.498 | 0.505 | 0.511 | 0.517 | 0.523 | 0.529 | 0.535 | 0.539 | 0.542 | 0.545 | 0.548 | 0.552 | 0.555 | 0.558 | 0.56 | 0.562 |
| Hidalgo | 0.417 | 0.424 | 0.433 | 0.444 | 0.454 | 0.462 | 0.471 | 0.481 | 0.491 | 0.501 | 0.511 | 0.52 | 0.527 | 0.531 | 0.535 | 0.538 | 0.543 | 0.548 | 0.554 | 0.558 | 0.562 | 0.567 | 0.572 | 0.576 | 0.58 | 0.583 | 0.585 | 0.587 |
| Jalisco | 0.532 | 0.538 | 0.545 | 0.552 | 0.558 | 0.563 | 0.569 | 0.576 | 0.582 | 0.588 | 0.593 | 0.598 | 0.602 | 0.606 | 0.611 | 0.615 | 0.619 | 0.624 | 0.628 | 0.63 | 0.632 | 0.635 | 0.637 | 0.64 | 0.642 | 0.645 | 0.647 | 0.649 |
| México | 0.529 | 0.524 | 0.525 | 0.539 | 0.558 | 0.568 | 0.574 | 0.577 | 0.58 | 0.585 | 0.59 | 0.596 | 0.6 | 0.603 | 0.607 | 0.611 | 0.613 | 0.614 | 0.614 | 0.614 | 0.615 | 0.617 | 0.621 | 0.625 | 0.628 | 0.631 | 0.633 | 0.635 |
| Michoacán de Ocampo | 0.464 | 0.47 | 0.476 | 0.483 | 0.489 | 0.494 | 0.501 | 0.508 | 0.515 | 0.522 | 0.527 | 0.532 | 0.537 | 0.542 | 0.546 | 0.551 | 0.555 | 0.559 | 0.563 | 0.566 | 0.568 | 0.571 | 0.573 | 0.576 | 0.579 | 0.581 | 0.584 | 0.586 |
| Morelos | 0.525 | 0.532 | 0.539 | 0.547 | 0.553 | 0.558 | 0.564 | 0.571 | 0.577 | 0.582 | 0.587 | 0.591 | 0.595 | 0.598 | 0.601 | 0.605 | 0.608 | 0.612 | 0.615 | 0.617 | 0.619 | 0.622 | 0.624 | 0.627 | 0.629 | 0.631 | 0.634 | 0.635 |
| Nayarit | 0.495 | 0.5 | 0.506 | 0.512 | 0.518 | 0.523 | 0.529 | 0.537 | 0.545 | 0.553 | 0.56 | 0.566 | 0.571 | 0.576 | 0.581 | 0.585 | 0.59 | 0.594 | 0.598 | 0.6 | 0.602 | 0.604 | 0.607 | 0.61 | 0.613 | 0.615 | 0.618 | 0.62 |
| Nuevo León | 0.598 | 0.602 | 0.606 | 0.611 | 0.615 | 0.617 | 0.62 | 0.625 | 0.63 | 0.635 | 0.64 | 0.644 | 0.647 | 0.65 | 0.653 | 0.656 | 0.659 | 0.662 | 0.665 | 0.666 | 0.667 | 0.669 | 0.67 | 0.672 | 0.673 | 0.675 | 0.676 | 0.677 |
| Oaxaca | 0.431 | 0.435 | 0.441 | 0.447 | 0.452 | 0.455 | 0.459 | 0.466 | 0.473 | 0.479 | 0.485 | 0.49 | 0.496 | 0.501 | 0.507 | 0.512 | 0.517 | 0.523 | 0.529 | 0.533 | 0.537 | 0.541 | 0.545 | 0.549 | 0.552 | 0.555 | 0.558 | 0.561 |
| Puebla | 0.456 | 0.463 | 0.47 | 0.478 | 0.487 | 0.493 | 0.496 | 0.497 | 0.498 | 0.499 | 0.504 | 0.509 | 0.516 | 0.524 | 0.532 | 0.538 | 0.544 | 0.549 | 0.554 | 0.558 | 0.563 | 0.567 | 0.571 | 0.575 | 0.577 | 0.58 | 0.582 | 0.584 |
| Querétaro | 0.497 | 0.502 | 0.508 | 0.518 | 0.53 | 0.543 | 0.556 | 0.566 | 0.575 | 0.583 | 0.591 | 0.597 | 0.602 | 0.606 | 0.609 | 0.612 | 0.616 | 0.619 | 0.623 | 0.624 | 0.626 | 0.629 | 0.631 | 0.633 | 0.635 | 0.636 | 0.638 | 0.639 |
| Quintana Roo | 0.52 | 0.526 | 0.533 | 0.541 | 0.548 | 0.555 | 0.562 | 0.57 | 0.577 | 0.582 | 0.587 | 0.591 | 0.594 | 0.596 | 0.599 | 0.601 | 0.605 | 0.608 | 0.612 | 0.614 | 0.616 | 0.618 | 0.62 | 0.621 | 0.623 | 0.624 | 0.625 | 0.626 |
| San Luis Potosí | 0.482 | 0.486 | 0.492 | 0.5 | 0.508 | 0.514 | 0.522 | 0.53 | 0.538 | 0.545 | 0.551 | 0.556 | 0.561 | 0.566 | 0.571 | 0.575 | 0.58 | 0.586 | 0.591 | 0.595 | 0.599 | 0.602 | 0.606 | 0.61 | 0.613 | 0.616 | 0.619 | 0.621 |
| Sinaloa | 0.523 | 0.528 | 0.533 | 0.539 | 0.544 | 0.549 | 0.555 | 0.562 | 0.57 | 0.577 | 0.583 | 0.589 | 0.594 | 0.599 | 0.604 | 0.609 | 0.614 | 0.619 | 0.623 | 0.627 | 0.63 | 0.633 | 0.636 | 0.639 | 0.642 | 0.644 | 0.646 | 0.649 |
| Sonora | 0.553 | 0.557 | 0.562 | 0.566 | 0.57 | 0.573 | 0.578 | 0.583 | 0.588 | 0.593 | 0.597 | 0.601 | 0.605 | 0.608 | 0.612 | 0.616 | 0.621 | 0.625 | 0.629 | 0.632 | 0.635 | 0.637 | 0.64 | 0.643 | 0.645 | 0.647 | 0.649 | 0.65 |
| Tabasco | 0.474 | 0.479 | 0.486 | 0.493 | 0.5 | 0.507 | 0.515 | 0.524 | 0.533 | 0.541 | 0.548 | 0.553 | 0.558 | 0.563 | 0.568 | 0.573 | 0.578 | 0.583 | 0.588 | 0.591 | 0.594 | 0.596 | 0.599 | 0.602 | 0.604 | 0.607 | 0.609 | 0.611 |
| Tamaulipas | 0.548 | 0.553 | 0.558 | 0.564 | 0.568 | 0.571 | 0.574 | 0.579 | 0.586 | 0.592 | 0.598 | 0.602 | 0.606 | 0.609 | 0.613 | 0.616 | 0.62 | 0.624 | 0.628 | 0.63 | 0.633 | 0.635 | 0.637 | 0.64 | 0.642 | 0.643 | 0.645 | 0.647 |
| Tlaxcala | 0.478 | 0.482 | 0.487 | 0.495 | 0.506 | 0.515 | 0.524 | 0.531 | 0.536 | 0.541 | 0.545 | 0.55 | 0.556 | 0.561 | 0.567 | 0.573 | 0.578 | 0.583 | 0.587 | 0.59 | 0.591 | 0.594 | 0.596 | 0.598 | 0.6 | 0.601 | 0.603 | 0.604 |
| Veracruz de Ignacio de la Llave | 0.461 | 0.463 | 0.467 | 0.472 | 0.477 | 0.48 | 0.485 | 0.492 | 0.5 | 0.509 | 0.517 | 0.523 | 0.529 | 0.534 | 0.54 | 0.546 | 0.551 | 0.557 | 0.563 | 0.567 | 0.571 | 0.574 | 0.578 | 0.581 | 0.584 | 0.587 | 0.59 | 0.592 |
| Yucatán | 0.497 | 0.502 | 0.508 | 0.516 | 0.524 | 0.531 | 0.54 | 0.548 | 0.556 | 0.562 | 0.569 | 0.575 | 0.581 | 0.587 | 0.592 | 0.596 | 0.599 | 0.602 | 0.605 | 0.608 | 0.61 | 0.613 | 0.617 | 0.62 | 0.623 | 0.626 | 0.628 | 0.63 |
| Zacatecas | 0.483 | 0.489 | 0.495 | 0.502 | 0.509 | 0.514 | 0.52 | 0.527 | 0.534 | 0.54 | 0.546 | 0.551 | 0.555 | 0.56 | 0.564 | 0.568 | 0.572 | 0.577 | 0.581 | 0.584 | 0.586 | 0.589 | 0.592 | 0.595 | 0.598 | 0.602 | 0.605 | 0.608 |
| Nicaragua | 0.357 | 0.363 | 0.368 | 0.374 | 0.381 | 0.389 | 0.397 | 0.406 | 0.415 | 0.424 | 0.432 | 0.439 | 0.446 | 0.453 | 0.46 | 0.466 | 0.47 | 0.475 | 0.481 | 0.486 | 0.492 | 0.499 | 0.504 | 0.509 | 0.514 | 0.52 | 0.525 | 0.53 |
| Panama | 0.542 | 0.546 | 0.55 | 0.555 | 0.56 | 0.565 | 0.569 | 0.573 | 0.578 | 0.583 | 0.589 | 0.595 | 0.6 | 0.604 | 0.608 | 0.611 | 0.614 | 0.618 | 0.622 | 0.626 | 0.63 | 0.635 | 0.641 | 0.648 | 0.656 | 0.664 | 0.671 | 0.677 |
| Venezuela | 0.528 | 0.536 | 0.553 | 0.559 | 0.566 | 0.578 | 0.587 | 0.592 | 0.591 | 0.587 | 0.591 | 0.602 | 0.602 | 0.588 | 0.576 | 0.579 | 0.594 | 0.61 | 0.621 | 0.625 | 0.629 | 0.633 | 0.64 | 0.646 | 0.648 | 0.651 | 0.654 | 0.655 |
| Tropical Latin America | 0.494 | 0.5 | 0.507 | 0.514 | 0.521 | 0.529 | 0.537 | 0.544 | 0.55 | 0.556 | 0.561 | 0.566 | 0.571 | 0.577 | 0.582 | 0.588 | 0.594 | 0.601 | 0.608 | 0.614 | 0.621 | 0.628 | 0.635 | 0.642 | 0.648 | 0.654 | 0.659 | 0.662 |
| Brazil | 0.494 | 0.501 | 0.508 | 0.515 | 0.522 | 0.53 | 0.537 | 0.545 | 0.551 | 0.556 | 0.562 | 0.567 | 0.572 | 0.577 | 0.583 | 0.589 | 0.595 | 0.602 | 0.608 | 0.615 | 0.622 | 0.629 | 0.636 | 0.643 | 0.649 | 0.655 | 0.66 | 0.663 |
| Acre | 0.376 | 0.386 | 0.395 | 0.405 | 0.415 | 0.424 | 0.435 | 0.445 | 0.453 | 0.46 | 0.466 | 0.472 | 0.479 | 0.485 | 0.492 | 0.5 | 0.508 | 0.517 | 0.527 | 0.536 | 0.546 | 0.556 | 0.565 | 0.575 | 0.583 | 0.591 | 0.597 | 0.602 |
| Alagoas | 0.355 | 0.363 | 0.371 | 0.379 | 0.387 | 0.395 | 0.404 | 0.412 | 0.419 | 0.425 | 0.431 | 0.436 | 0.442 | 0.448 | 0.455 | 0.462 | 0.47 | 0.478 | 0.487 | 0.496 | 0.505 | 0.514 | 0.523 | 0.531 | 0.539 | 0.546 | 0.552 | 0.556 |
| Amapá | 0.467 | 0.475 | 0.483 | 0.491 | 0.5 | 0.508 | 0.517 | 0.526 | 0.534 | 0.54 | 0.546 | 0.552 | 0.558 | 0.564 | 0.57 | 0.576 | 0.583 | 0.591 | 0.598 | 0.605 | 0.613 | 0.621 | 0.629 | 0.636 | 0.643 | 0.65 | 0.655 | 0.659 |
| Amazonas | 0.438 | 0.447 | 0.457 | 0.466 | 0.475 | 0.483 | 0.492 | 0.499 | 0.505 | 0.51 | 0.514 | 0.519 | 0.523 | 0.528 | 0.533 | 0.539 | 0.546 | 0.553 | 0.561 | 0.568 | 0.577 | 0.585 | 0.594 | 0.602 | 0.611 | 0.618 | 0.625 | 0.629 |
| Bahia | 0.402 | 0.41 | 0.419 | 0.427 | 0.435 | 0.443 | 0.451 | 0.459 | 0.465 | 0.47 | 0.475 | 0.48 | 0.485 | 0.491 | 0.496 | 0.503 | 0.51 | 0.518 | 0.526 | 0.534 | 0.542 | 0.551 | 0.559 | 0.567 | 0.575 | 0.582 | 0.587 | 0.591 |
| Ceará | 0.411 | 0.419 | 0.426 | 0.433 | 0.44 | 0.448 | 0.455 | 0.463 | 0.469 | 0.475 | 0.48 | 0.486 | 0.492 | 0.498 | 0.505 | 0.512 | 0.52 | 0.528 | 0.536 | 0.544 | 0.553 | 0.561 | 0.569 | 0.577 | 0.584 | 0.591 | 0.596 | 0.6 |
| Distrito Federal | 0.63 | 0.636 | 0.642 | 0.649 | 0.656 | 0.663 | 0.671 | 0.679 | 0.685 | 0.691 | 0.696 | 0.702 | 0.707 | 0.713 | 0.719 | 0.725 | 0.731 | 0.738 | 0.744 | 0.75 | 0.756 | 0.763 | 0.769 | 0.775 | 0.78 | 0.785 | 0.789 | 0.792 |
| Espírito Santo | 0.499 | 0.507 | 0.515 | 0.524 | 0.532 | 0.54 | 0.549 | 0.557 | 0.564 | 0.57 | 0.576 | 0.582 | 0.588 | 0.593 | 0.599 | 0.606 | 0.612 | 0.618 | 0.625 | 0.631 | 0.638 | 0.644 | 0.651 | 0.657 | 0.663 | 0.669 | 0.673 | 0.677 |
| Goiás | 0.46 | 0.468 | 0.476 | 0.484 | 0.493 | 0.501 | 0.51 | 0.518 | 0.526 | 0.532 | 0.538 | 0.545 | 0.551 | 0.558 | 0.564 | 0.571 | 0.579 | 0.586 | 0.594 | 0.601 | 0.608 | 0.616 | 0.623 | 0.63 | 0.636 | 0.642 | 0.647 | 0.65 |

**Appendix Table 10: Socio-Demographic Index values for all estimated GBD 2017 locations, 1990-2017**

| Location | 1990 | 1991 | 1992 | 1993 | 1994 | 1995 | 1996 | 1997 | 1998 | 1999 | 2000 | 2001 | 2002 | 2003 | 2004 | 2005 | 2006 | 2007 | 2008 | 2009 | 2010 | 2011 | 2012 | 2013 | 2014 | 2015 | 2016 | 2017 |
|---|---|---|---|---|---|---|---|---|---|---|---|---|---|---|---|---|---|---|---|---|---|---|---|---|---|---|---|---|
| Maranhão | 0.313 | 0.322 | 0.33 | 0.339 | 0.347 | 0.355 | 0.364 | 0.371 | 0.377 | 0.38 | 0.383 | 0.386 | 0.389 | 0.392 | 0.396 | 0.402 | 0.409 | 0.418 | 0.427 | 0.436 | 0.446 | 0.456 | 0.467 | 0.477 | 0.486 | 0.495 | 0.502 | 0.507 |
| Mato Grosso | 0.475 | 0.484 | 0.492 | 0.501 | 0.509 | 0.518 | 0.527 | 0.535 | 0.543 | 0.548 | 0.554 | 0.559 | 0.564 | 0.57 | 0.576 | 0.582 | 0.589 | 0.596 | 0.604 | 0.611 | 0.618 | 0.626 | 0.633 | 0.641 | 0.648 | 0.654 | 0.659 | 0.662 |
| Mato Grosso do Sul | 0.465 | 0.473 | 0.481 | 0.489 | 0.497 | 0.506 | 0.515 | 0.523 | 0.531 | 0.537 | 0.543 | 0.549 | 0.555 | 0.56 | 0.566 | 0.573 | 0.58 | 0.587 | 0.594 | 0.6 | 0.607 | 0.614 | 0.622 | 0.629 | 0.636 | 0.642 | 0.647 | 0.65 |
| Minas Gerais | 0.491 | 0.498 | 0.506 | 0.513 | 0.521 | 0.53 | 0.538 | 0.545 | 0.551 | 0.557 | 0.562 | 0.567 | 0.573 | 0.579 | 0.585 | 0.591 | 0.598 | 0.604 | 0.611 | 0.618 | 0.624 | 0.631 | 0.637 | 0.643 | 0.649 | 0.654 | 0.658 | 0.661 |
| Pará | 0.41 | 0.418 | 0.425 | 0.432 | 0.44 | 0.447 | 0.454 | 0.461 | 0.466 | 0.47 | 0.473 | 0.476 | 0.48 | 0.483 | 0.488 | 0.493 | 0.499 | 0.506 | 0.514 | 0.521 | 0.529 | 0.538 | 0.546 | 0.554 | 0.562 | 0.569 | 0.575 | 0.579 |
| Paraíba | 0.399 | 0.406 | 0.413 | 0.42 | 0.427 | 0.434 | 0.441 | 0.447 | 0.453 | 0.457 | 0.461 | 0.465 | 0.469 | 0.474 | 0.48 | 0.486 | 0.493 | 0.501 | 0.509 | 0.517 | 0.526 | 0.535 | 0.543 | 0.551 | 0.559 | 0.565 | 0.571 | 0.574 |
| Paraná | 0.513 | 0.519 | 0.525 | 0.532 | 0.539 | 0.548 | 0.556 | 0.564 | 0.572 | 0.578 | 0.585 | 0.591 | 0.597 | 0.603 | 0.609 | 0.615 | 0.622 | 0.628 | 0.634 | 0.64 | 0.646 | 0.652 | 0.658 | 0.664 | 0.67 | 0.675 | 0.679 | 0.682 |
| Pernambuco | 0.416 | 0.423 | 0.43 | 0.437 | 0.444 | 0.451 | 0.458 | 0.466 | 0.472 | 0.477 | 0.481 | 0.486 | 0.492 | 0.497 | 0.503 | 0.51 | 0.517 | 0.525 | 0.533 | 0.54 | 0.548 | 0.556 | 0.564 | 0.572 | 0.579 | 0.585 | 0.59 | 0.594 |
| Piauí | 0.365 | 0.372 | 0.379 | 0.386 | 0.393 | 0.4 | 0.408 | 0.415 | 0.42 | 0.425 | 0.429 | 0.434 | 0.439 | 0.444 | 0.45 | 0.457 | 0.465 | 0.473 | 0.482 | 0.491 | 0.5 | 0.51 | 0.518 | 0.527 | 0.535 | 0.542 | 0.548 | 0.552 |
| Rio de Janeiro | 0.576 | 0.581 | 0.585 | 0.59 | 0.595 | 0.601 | 0.608 | 0.614 | 0.619 | 0.624 | 0.628 | 0.632 | 0.637 | 0.641 | 0.645 | 0.65 | 0.655 | 0.66 | 0.665 | 0.67 | 0.675 | 0.681 | 0.686 | 0.692 | 0.697 | 0.702 | 0.706 | 0.709 |
| Rio Grande do Norte | 0.415 | 0.422 | 0.429 | 0.436 | 0.444 | 0.451 | 0.46 | 0.467 | 0.474 | 0.48 | 0.485 | 0.491 | 0.497 | 0.503 | 0.509 | 0.516 | 0.524 | 0.532 | 0.541 | 0.549 | 0.558 | 0.567 | 0.575 | 0.583 | 0.59 | 0.597 | 0.602 | 0.605 |
| Rio Grande do Sul | 0.543 | 0.549 | 0.555 | 0.561 | 0.567 | 0.574 | 0.581 | 0.587 | 0.593 | 0.598 | 0.603 | 0.608 | 0.614 | 0.619 | 0.624 | 0.63 | 0.635 | 0.641 | 0.647 | 0.653 | 0.659 | 0.665 | 0.67 | 0.676 | 0.681 | 0.686 | 0.69 | 0.693 |
| Rondônia | 0.423 | 0.433 | 0.441 | 0.45 | 0.458 | 0.467 | 0.475 | 0.484 | 0.491 | 0.497 | 0.502 | 0.508 | 0.515 | 0.521 | 0.528 | 0.535 | 0.543 | 0.551 | 0.559 | 0.567 | 0.575 | 0.584 | 0.592 | 0.599 | 0.606 | 0.613 | 0.618 | 0.622 |
| Roraima | 0.428 | 0.438 | 0.447 | 0.456 | 0.465 | 0.474 | 0.483 | 0.492 | 0.499 | 0.504 | 0.509 | 0.514 | 0.521 | 0.527 | 0.534 | 0.543 | 0.552 | 0.562 | 0.572 | 0.581 | 0.591 | 0.601 | 0.611 | 0.62 | 0.628 | 0.636 | 0.642 | 0.646 |
| Santa Catarina | 0.541 | 0.548 | 0.554 | 0.56 | 0.567 | 0.574 | 0.582 | 0.589 | 0.595 | 0.601 | 0.606 | 0.612 | 0.618 | 0.623 | 0.629 | 0.635 | 0.641 | 0.647 | 0.653 | 0.659 | 0.665 | 0.672 | 0.678 | 0.684 | 0.69 | 0.695 | 0.699 | 0.702 |
| São Paulo | 0.558 | 0.565 | 0.572 | 0.579 | 0.587 | 0.595 | 0.603 | 0.611 | 0.618 | 0.624 | 0.63 | 0.636 | 0.641 | 0.646 | 0.652 | 0.657 | 0.663 | 0.669 | 0.674 | 0.68 | 0.685 | 0.691 | 0.697 | 0.703 | 0.708 | 0.713 | 0.717 | 0.72 |
| Sergipe | 0.425 | 0.433 | 0.441 | 0.448 | 0.456 | 0.464 | 0.473 | 0.481 | 0.488 | 0.494 | 0.5 | 0.506 | 0.512 | 0.518 | 0.524 | 0.531 | 0.538 | 0.546 | 0.554 | 0.562 | 0.57 | 0.578 | 0.586 | 0.594 | 0.601 | 0.607 | 0.612 | 0.616 |
| Tocantins | 0.396 | 0.404 | 0.412 | 0.42 | 0.428 | 0.436 | 0.445 | 0.453 | 0.46 | 0.466 | 0.471 | 0.477 | 0.484 | 0.491 | 0.498 | 0.507 | 0.517 | 0.527 | 0.537 | 0.547 | 0.558 | 0.568 | 0.577 | 0.586 | 0.594 | 0.601 | 0.607 | 0.611 |
| Paraguay | 0.467 | 0.471 | 0.475 | 0.48 | 0.485 | 0.491 | 0.497 | 0.504 | 0.512 | 0.519 | 0.525 | 0.532 | 0.538 | 0.544 | 0.548 | 0.553 | 0.558 | 0.562 | 0.566 | 0.569 | 0.573 | 0.577 | 0.582 | 0.589 | 0.598 | 0.606 | 0.613 | 0.619 |
| North Africa and Middle East | 0.456 | 0.464 | 0.472 | 0.478 | 0.485 | 0.492 | 0.499 | 0.506 | 0.513 | 0.521 | 0.531 | 0.539 | 0.547 | 0.553 | 0.561 | 0.568 | 0.574 | 0.582 | 0.589 | 0.596 | 0.602 | 0.607 | 0.612 | 0.617 | 0.622 | 0.628 | 0.635 | 0.639 |
| North Africa and Middle East | 0.456 | 0.464 | 0.472 | 0.478 | 0.485 | 0.492 | 0.499 | 0.506 | 0.513 | 0.521 | 0.531 | 0.539 | 0.547 | 0.553 | 0.561 | 0.568 | 0.574 | 0.582 | 0.589 | 0.596 | 0.602 | 0.607 | 0.612 | 0.617 | 0.622 | 0.628 | 0.635 | 0.639 |
| Afghanistan | 0.149 | 0.15 | 0.151 | 0.152 | 0.151 | 0.151 | 0.152 | 0.152 | 0.154 | 0.155 | 0.157 | 0.159 | 0.164 | 0.171 | 0.177 | 0.184 | 0.191 | 0.199 | 0.208 | 0.218 | 0.228 | 0.238 | 0.248 | 0.258 | 0.267 | 0.276 | 0.284 | 0.29 |
| Algeria | 0.495 | 0.504 | 0.513 | 0.522 | 0.531 | 0.54 | 0.549 | 0.558 | 0.567 | 0.574 | 0.583 | 0.591 | 0.599 | 0.608 | 0.617 | 0.625 | 0.633 | 0.641 | 0.648 | 0.653 | 0.659 | 0.665 | 0.67 | 0.675 | 0.68 | 0.685 | 0.691 | 0.696 |
| Bahrain | 0.612 | 0.618 | 0.624 | 0.63 | 0.635 | 0.639 | 0.644 | 0.648 | 0.653 | 0.657 | 0.662 | 0.668 | 0.673 | 0.677 | 0.68 | 0.684 | 0.687 | 0.69 | 0.693 | 0.695 | 0.696 | 0.697 | 0.698 | 0.7 | 0.702 | 0.706 | 0.709 | 0.712 |
| Egypt | 0.441 | 0.451 | 0.461 | 0.47 | 0.477 | 0.485 | 0.493 | 0.5 | 0.507 | 0.514 | 0.52 | 0.526 | 0.532 | 0.537 | 0.542 | 0.547 | 0.55 | 0.553 | 0.556 | 0.559 | 0.563 | 0.565 | 0.564 | 0.566 | 0.572 | 0.584 | 0.596 | 0.604 |
| Iran | 0.503 | 0.513 | 0.522 | 0.532 | 0.541 | 0.548 | 0.553 | 0.556 | 0.56 | 0.572 | 0.589 | 0.604 | 0.616 | 0.626 | 0.634 | 0.641 | 0.649 | 0.656 | 0.662 | 0.667 | 0.672 | 0.677 | 0.68 | 0.683 | 0.687 | 0.691 | 0.696 | 0.7 |
| Iraq | 0.433 | 0.433 | 0.437 | 0.441 | 0.444 | 0.446 | 0.45 | 0.455 | 0.463 | 0.473 | 0.481 | 0.488 | 0.494 | 0.496 | 0.502 | 0.507 | 0.512 | 0.517 | 0.522 | 0.528 | 0.535 | 0.542 | 0.551 | 0.559 | 0.566 | 0.572 | 0.58 | 0.585 |
| Jordan | 0.552 | 0.556 | 0.561 | 0.566 | 0.57 | 0.574 | 0.578 | 0.584 | 0.589 | 0.594 | 0.597 | 0.6 | 0.603 | 0.607 | 0.612 | 0.616 | 0.621 | 0.626 | 0.633 | 0.643 | 0.653 | 0.662 | 0.671 | 0.679 | 0.685 | 0.69 | 0.694 | 0.697 |
| Kuwait | 0.656 | 0.651 | 0.642 | 0.637 | 0.633 | 0.632 | 0.638 | 0.651 | 0.665 | 0.674 | 0.681 | 0.683 | 0.685 | 0.687 | 0.693 | 0.701 | 0.711 | 0.721 | 0.73 | 0.737 | 0.743 | 0.748 | 0.754 | 0.762 | 0.769 | 0.776 | 0.781 | 0.786 |
| Lebanon | 0.519 | 0.528 | 0.536 | 0.544 | 0.552 | 0.56 | 0.569 | 0.578 | 0.588 | 0.597 | 0.604 | 0.611 | 0.618 | 0.626 | 0.635 | 0.643 | 0.65 | 0.658 | 0.667 | 0.676 | 0.686 | 0.694 | 0.702 | 0.711 | 0.717 | 0.722 | 0.726 | 0.73 |
| Libya | 0.645 | 0.657 | 0.669 | 0.679 | 0.688 | 0.696 | 0.705 | 0.713 | 0.72 | 0.725 | 0.729 | 0.732 | 0.736 | 0.742 | 0.747 | 0.753 | 0.759 | 0.765 | 0.771 | 0.777 | 0.783 | 0.781 | 0.784 | 0.783 | 0.779 | 0.773 | 0.766 | 0.761 |
| Morocco | 0.389 | 0.397 | 0.404 | 0.411 | 0.419 | 0.426 | 0.434 | 0.442 | 0.449 | 0.456 | 0.463 | 0.47 | 0.477 | 0.484 | 0.49 | 0.496 | 0.503 | 0.51 | 0.517 | 0.525 | 0.532 | 0.54 | 0.547 | 0.554 | 0.561 | 0.567 | 0.574 | 0.579 |
| Palestine | 0.362 | 0.366 | 0.371 | 0.377 | 0.384 | 0.39 | 0.397 | 0.405 | 0.413 | 0.421 | 0.427 | 0.431 | 0.433 | 0.435 | 0.439 | 0.444 | 0.448 | 0.453 | 0.46 | 0.468 | 0.479 | 0.491 | 0.503 | 0.513 | 0.521 | 0.528 | 0.536 | 0.541 |
| Oman | 0.473 | 0.485 | 0.497 | 0.509 | 0.521 | 0.533 | 0.546 | 0.558 | 0.57 | 0.582 | 0.594 | 0.606 | 0.618 | 0.629 | 0.639 | 0.649 | 0.659 | 0.668 | 0.678 | 0.686 | 0.695 | 0.703 | 0.711 | 0.72 | 0.727 | 0.734 | 0.739 | 0.744 |
| Qatar | 0.603 | 0.615 | 0.626 | 0.637 | 0.647 | 0.656 | 0.665 | 0.674 | 0.681 | 0.688 | 0.695 | 0.701 | 0.706 | 0.712 | 0.717 | 0.721 | 0.725 | 0.728 | 0.731 | 0.735 | 0.738 | 0.743 | 0.747 | 0.751 | 0.755 | 0.759 | 0.762 | 0.766 |
| Saudi Arabia | 0.436 | 0.457 | 0.477 | 0.496 | 0.514 | 0.53 | 0.545 | 0.56 | 0.573 | 0.586 | 0.598 | 0.61 | 0.621 | 0.633 | 0.645 | 0.658 | 0.67 | 0.682 | 0.695 | 0.706 | 0.716 | 0.728 | 0.738 | 0.748 | 0.757 | 0.765 | 0.773 | 0.779 |
| Sudan | 0.221 | 0.228 | 0.236 | 0.244 | 0.252 | 0.26 | 0.267 | 0.275 | 0.283 | 0.291 | 0.3 | 0.308 | 0.317 | 0.326 | 0.336 | 0.346 | 0.357 | 0.367 | 0.378 | 0.389 | 0.401 | 0.414 | 0.425 | 0.437 | 0.448 | 0.46 | 0.47 | 0.478 |
| Syria | 0.389 | 0.394 | 0.401 | 0.409 | 0.419 | 0.429 | 0.439 | 0.448 | 0.458 | 0.466 | 0.474 | 0.481 | 0.489 | 0.496 | 0.506 | 0.518 | 0.527 | 0.536 | 0.545 | 0.556 | 0.566 | 0.576 | 0.584 | 0.589 | 0.595 | 0.601 | 0.606 | 0.611 |
| Tunisia | 0.453 | 0.463 | 0.473 | 0.482 | 0.491 | 0.501 | 0.511 | 0.521 | 0.532 | 0.543 | 0.553 | 0.563 | 0.572 | 0.582 | 0.592 | 0.6 | 0.609 | 0.617 | 0.625 | 0.632 | 0.639 | 0.645 | 0.65 | 0.656 | 0.661 | 0.666 | 0.671 | 0.675 |
| Turkey | 0.507 | 0.516 | 0.524 | 0.533 | 0.54 | 0.548 | 0.556 | 0.564 | 0.572 | 0.579 | 0.587 | 0.594 | 0.601 | 0.608 | 0.616 | 0.625 | 0.633 | 0.643 | 0.652 | 0.66 | 0.669 | 0.678 | 0.688 | 0.697 | 0.706 | 0.715 | 0.723 | 0.729 |
| United Arab Emirates | 0.621 | 0.637 | 0.651 | 0.663 | 0.675 | 0.686 | 0.696 | 0.707 | 0.716 | 0.726 | 0.734 | 0.742 | 0.749 | 0.755 | 0.76 | 0.765 | 0.77 | 0.776 | 0.781 | 0.784 | 0.788 | 0.79 | 0.791 | 0.793 | 0.791 | 0.794 | 0.794 | 0.795 |
| Yemen | 0.203 | 0.208 | 0.214 | 0.22 | 0.228 | 0.238 | 0.247 | 0.256 | 0.266 | 0.275 | 0.284 | 0.294 | 0.304 | 0.313 | 0.324 | 0.336 | 0.349 | 0.362 | 0.373 | 0.382 | 0.393 | 0.401 | 0.408 | 0.415 | 0.422 | 0.426 | 0.429 | 0.43 |
| South Asia | 0.312 | 0.317 | 0.324 | 0.331 | 0.338 | 0.345 | 0.352 | 0.358 | 0.363 | 0.371 | 0.38 | 0.388 | 0.395 | 0.403 | 0.412 | 0.421 | 0.43 | 0.438 | 0.446 | 0.455 | 0.462 | 0.468 | 0.476 | 0.489 | 0.504 | 0.517 | 0.526 | 0.534 |
| South Asia | 0.312 | 0.317 | 0.324 | 0.331 | 0.338 | 0.345 | 0.352 | 0.358 | 0.363 | 0.371 | 0.38 | 0.388 | 0.395 | 0.403 | 0.412 | 0.421 | 0.43 | 0.438 | 0.446 | 0.455 | 0.462 | 0.468 | 0.476 | 0.489 | 0.504 | 0.517 | 0.526 | 0.534 |
| Bangladesh | 0.256 | 0.264 | 0.272 | 0.281 | 0.289 | 0.298 | 0.306 | 0.314 | 0.322 | 0.329 | 0.335 | 0.34 | 0.345 | 0.35 | 0.356 | 0.362 | 0.369 | 0.377 | 0.385 | 0.393 | 0.401 | 0.41 | 0.418 | 0.427 | 0.435 | 0.443 | 0.451 | 0.458 |
| Bhutan | 0.324 | 0.333 | 0.343 | 0.352 | 0.362 | 0.372 | 0.382 | 0.392 | 0.402 | 0.412 | 0.422 | 0.432 | 0.442 | 0.452 | 0.461 | 0.47 | 0.479 | 0.489 | 0.498 | 0.507 | 0.516 | 0.525 | 0.534 | 0.542 | 0.549 | 0.557 | 0.564 | 0.57 |
| India | 0.32 | 0.324 | 0.331 | 0.339 | 0.346 | 0.353 | 0.36 | 0.365 | 0.37 | 0.378 | 0.387 | 0.395 | 0.403 | 0.412 | 0.422 | 0.431 | 0.44 | 0.448 | 0.457 | 0.465 | 0.472 | 0.477 | 0.485 | 0.5 | 0.517 | 0.532 | 0.542 | 0.55 |
| Andhra Pradesh | 0.28 | 0.286 | 0.294 | 0.303 | 0.312 | 0.322 | 0.331 | 0.337 | 0.344 | 0.352 | 0.363 | 0.374 | 0.384 | 0.395 | 0.406 | 0.415 | 0.424 | 0.434 | 0.442 | 0.451 | 0.459 | 0.466 | 0.473 | 0.487 | 0.503 | 0.517 | 0.528 | 0.536 |
| Arunachal Pradesh | 0.311 | 0.317 | 0.323 | 0.33 | 0.336 | 0.345 | 0.354 | 0.36 | 0.366 | 0.374 | 0.381 | 0.388 | 0.395 | 0.405 | 0.416 | 0.424 | 0.432 | 0.44 | 0.447 | 0.458 | 0.467 | 0.473 | 0.479 | 0.498 | 0.52 | 0.536 | 0.548 | 0.556 |
| Assam | 0.327 | 0.332 | 0.339 | 0.345 | 0.351 | 0.358 | 0.363 | 0.367 | 0.371 | 0.379 | 0.389 | 0.397 | 0.406 | 0.415 | 0.424 | 0.431 | 0.439 | 0.446 | 0.453 | 0.459 | 0.464 | 0.467 | 0.472 | 0.485 | 0.5 | 0.513 | 0.523 | 0.53 |
| Bihar | 0.29 | 0.293 | 0.297 | 0.298 | 0.298 | 0.298 | 0.298 | 0.297 | 0.292 | 0.291 | 0.292 | 0.294 | 0.296 | 0.299 | 0.303 | 0.308 | 0.316 | 0.324 | 0.334 | 0.342 | 0.349 | 0.355 | 0.365 | 0.382 | 0.4 | 0.414 | 0.425 | 0.433 |
| Chhattisgarh | 0.292 | 0.294 | 0.298 | 0.303 | 0.306 | 0.311 | 0.316 | 0.319 | 0.322 | 0.328 | 0.336 | 0.344 | 0.352 | 0.362 | 0.372 | 0.382 | 0.392 | 0.403 | 0.413 | 0.421 | 0.427 | 0.431 | 0.438 | 0.455 | 0.474 | 0.49 | 0.503 | 0.512 |
| Delhi | 0.486 | 0.493 | 0.504 | 0.516 | 0.522 | 0.526 | 0.531 | 0.535 | 0.537 | 0.545 | 0.555 | 0.565 | 0.575 | 0.584 | 0.593 | 0.602 | 0.612 | 0.62 | 0.628 | 0.637 | 0.644 | 0.649 | 0.656 | 0.67 | 0.686 | 0.7 | 0.709 | 0.715 |
| Goa | 0.499 | 0.505 | 0.512 | 0.519 | 0.526 | 0.534 | 0.542 | 0.55 | 0.56 | 0.571 | 0.58 | 0.588 | 0.597 | 0.606 | 0.616 | 0.626 | 0.634 | 0.643 | 0.655 | 0.665 | 0.674 | 0.683 | 0.691 | 0.702 | 0.714 | 0.724 | 0.733 | 0.74 |
| Gujarat | 0.35 | 0.353 | 0.361 | 0.37 | 0.379 | 0.388 | 0.396 | 0.402 | 0.409 | 0.418 | 0.426 | 0.433 | 0.438 | 0.447 | 0.457 | 0.469 | 0.478 | 0.487 | 0.496 | 0.506 | 0.515 | 0.521 | 0.528 | 0.541 | 0.556 | 0.569 | 0.577 | 0.584 |
| Haryana | 0.342 | 0.348 | 0.356 | 0.364 | 0.372 | 0.38 | 0.388 | 0.394 | 0.398 | 0.405 | 0.414 | 0.423 | 0.431 | 0.44 | 0.451 | 0.462 | 0.472 | 0.481 | 0.491 | 0.5 | 0.508 | 0.512 | 0.521 | 0.54 | 0.564 | 0.582 | 0.593 | 0.6 |
| Himachal Pradesh | 0.34 | 0.347 | 0.356 | 0.366 | 0.376 | 0.386 | 0.397 | 0.408 | 0.418 | 0.431 | 0.445 | 0.457 | 0.468 | 0.479 | 0.491 | 0.502 | 0.513 | 0.524 | 0.534 | 0.544 | 0.553 | 0.562 | 0.571 | 0.585 | 0.601 | 0.614 | 0.625 | 0.633 |
| Jammu and Kashmir | 0.334 | 0.337 | 0.341 | 0.349 | 0.356 | 0.363 | 0.37 | 0.38 | 0.389 | 0.402 | 0.417 | 0.43 | 0.442 | 0.454 | 0.466 | 0.476 | 0.485 | 0.493 | 0.503 | 0.511 | 0.519 | 0.527 | 0.535 | 0.547 | 0.562 | 0.574 | 0.582 | 0.59 |
| Jharkhand | 0.281 | 0.283 | 0.286 | 0.29 | 0.293 | 0.297 | 0.3 | 0.304 | 0.307 | 0.312 | 0.319 | 0.325 | 0.333 | 0.342 | 0.353 | 0.362 | 0.371 | 0.38 | 0.388 | 0.397 | 0.405 | 0.411 | 0.419 | 0.434 | 0.451 | 0.466 | 0.478 | 0.487 |
| Karnataka | 0.323 | 0.331 | 0.339 | 0.349 | 0.357 | 0.366 | 0.375 | 0.38 | 0.386 | 0.396 | 0.406 | 0.415 | 0.424 | 0.434 | 0.444 | 0.454 | 0.463 | 0.472 | 0.481 | 0.49 | 0.498 | 0.504 | 0.512 | 0.526 | 0.542 | 0.556 | 0.566 | 0.574 |
| Kerala | 0.404 | 0.408 | 0.418 | 0.429 | 0.438 | 0.447 | 0.456 | 0.463 | 0.472 | 0.483 | 0.496 | 0.506 | 0.515 | 0.525 | 0.536 | 0.548 | 0.557 | 0.565 | 0.574 | 0.581 | 0.587 | 0.592 | 0.599 | 0.612 | 0.629 | 0.642 | 0.652 | 0.659 |
| Madhya Pradesh | 0.284 | 0.288 | 0.293 | 0.299 | 0.303 | 0.309 | 0.315 | 0.317 | 0.319 | 0.326 | 0.334 | 0.342 | 0.348 | 0.355 | 0.363 | 0.369 | 0.373 | 0.378 | 0.384 | 0.39 | 0.394 | 0.398 | 0.406 | 0.425 | 0.447 | 0.465 | 0.477 | 0.487 |
| Maharashtra | 0.363 | 0.369 | 0.379 | 0.39 | 0.399 | 0.41 | 0.419 | 0.426 | 0.431 | 0.44 | 0.451 | 0.459 | 0.468 | 0.478 | 0.489 | 0.5 | 0.51 | 0.52 | 0.529 | 0.538 | 0.546 | 0.552 | 0.559 | 0.572 | 0.588 | 0.602 | 0.611 | 0.618 |
| Manipur | 0.381 | 0.387 | 0.393 | 0.4 | 0.405 | 0.411 | 0.417 | 0.421 | 0.427 | 0.436 | 0.445 | 0.454 | 0.461 | 0.469 | 0.479 | 0.49 | 0.498 | 0.505 | 0.512 | 0.52 | 0.526 | 0.532 | 0.539 | 0.549 | 0.561 | 0.572 | 0.582 | 0.59 |
| Meghalaya | 0.334 | 0.339 | 0.345 | 0.352 | 0.357 | 0.364 | 0.37 | 0.376 | 0.382 | 0.39 | 0.401 | 0.411 | 0.42 | 0.43 | 0.441 | 0.45 | 0.46 | 0.469 | 0.478 | 0.487 | 0.495 | 0.501 | 0.507 | 0.52 | 0.534 | 0.547 | 0.557 | 0.565 |
| Mizoram | 0.392 | 0.399 | 0.408 | 0.417 | 0.424 | 0.433 | 0.443 | 0.449 | 0.456 | 0.465 | 0.475 | 0.483 | 0.492 | 0.502 | 0.511 | 0.52 | 0.526 | 0.531 | 0.537 | 0.545 | 0.55 | 0.552 | 0.557 | 0.57 | 0.587 | 0.6 | 0.609 | 0.616 |
| Nagaland | 0.395 | 0.399 | 0.406 | 0.414 | 0.421 | 0.429 | 0.435 | 0.441 | 0.445 | 0.452 | 0.462 | 0.472 | 0.482 | 0.492 | 0.503 | 0.513 | 0.522 | 0.532 | 0.543 | 0.552 | 0.56 | 0.567 | 0.574 | 0.587 | 0.602 | 0.614 | 0.625 | 0.633 |
| Odisha | 0.283 | 0.287 | 0.292 | 0.299 | 0.305 | 0.314 | 0.32 | 0.325 | 0.331 | 0.34 | 0.349 | 0.357 | 0.364 | 0.373 | 0.384 | 0.393 | 0.404 | 0.415 | 0.425 | 0.434 | 0.441 | 0.447 | 0.456 | 0.472 | 0.49 | 0.504 | 0.515 | 0.524 |
| Punjab | 0.383 | 0.388 | 0.396 | 0.404 | 0.413 | 0.422 | 0.429 | 0.435 | 0.441 | 0.449 | 0.459 | 0.468 | 0.475 | 0.483 | 0.492 | 0.501 | 0.511 | 0.52 | 0.53 | 0.539 | 0.547 | 0.554 | 0.563 | 0.576 | 0.592 | 0.605 | 0.614 | 0.622 |

**Appendix Table 10: Socio-Demographic Index values for all estimated GBD 2017 locations, 1990-2017**

| Location | 1990 | 1991 | 1992 | 1993 | 1994 | 1995 | 1996 | 1997 | 1998 | 1999 | 2000 | 2001 | 2002 | 2003 | 2004 | 2005 | 2006 | 2007 | 2008 | 2009 | 2010 | 2011 | 2012 | 2013 | 2014 | 2015 | 2016 | 2017 |
|---|---|---|---|---|---|---|---|---|---|---|---|---|---|---|---|---|---|---|---|---|---|---|---|---|---|---|---|---|
| Rajasthan | 0.263 | 0.268 | 0.274 | 0.28 | 0.287 | 0.295 | 0.303 | 0.309 | 0.314 | 0.322 | 0.332 | 0.342 | 0.349 | 0.359 | 0.369 | 0.377 | 0.385 | 0.391 | 0.398 | 0.404 | 0.41 | 0.415 | 0.421 | 0.438 | 0.458 | 0.473 | 0.484 | 0.492 |
| Sikkim | 0.343 | 0.349 | 0.355 | 0.362 | 0.368 | 0.375 | 0.382 | 0.388 | 0.395 | 0.403 | 0.413 | 0.421 | 0.431 | 0.442 | 0.453 | 0.463 | 0.473 | 0.482 | 0.492 | 0.512 | 0.528 | 0.541 | 0.555 | 0.574 | 0.593 | 0.608 | 0.619 | 0.628 |
| Tamil Nadu | 0.35 | 0.354 | 0.363 | 0.373 | 0.383 | 0.391 | 0.399 | 0.406 | 0.414 | 0.424 | 0.436 | 0.446 | 0.455 | 0.465 | 0.475 | 0.486 | 0.497 | 0.506 | 0.514 | 0.522 | 0.529 | 0.535 | 0.543 | 0.559 | 0.579 | 0.595 | 0.607 | 0.615 |
| Telangana | 0.288 | 0.294 | 0.3 | 0.307 | 0.315 | 0.323 | 0.331 | 0.337 | 0.344 | 0.352 | 0.363 | 0.373 | 0.383 | 0.395 | 0.407 | 0.418 | 0.429 | 0.441 | 0.454 | 0.465 | 0.478 | 0.488 | 0.5 | 0.517 | 0.536 | 0.552 | 0.565 | 0.575 |
| Tripura | 0.333 | 0.336 | 0.34 | 0.346 | 0.35 | 0.356 | 0.363 | 0.368 | 0.373 | 0.385 | 0.398 | 0.409 | 0.419 | 0.43 | 0.44 | 0.449 | 0.457 | 0.462 | 0.469 | 0.476 | 0.48 | 0.483 | 0.488 | 0.5 | 0.515 | 0.527 | 0.536 | 0.543 |
| Uttar Pradesh | 0.272 | 0.276 | 0.281 | 0.286 | 0.291 | 0.297 | 0.303 | 0.307 | 0.31 | 0.317 | 0.325 | 0.333 | 0.34 | 0.348 | 0.358 | 0.366 | 0.374 | 0.382 | 0.39 | 0.398 | 0.404 | 0.41 | 0.419 | 0.436 | 0.454 | 0.469 | 0.48 | 0.488 |
| Uttarakhand | 0.304 | 0.306 | 0.31 | 0.315 | 0.321 | 0.329 | 0.336 | 0.341 | 0.347 | 0.355 | 0.367 | 0.378 | 0.391 | 0.405 | 0.42 | 0.434 | 0.449 | 0.463 | 0.478 | 0.494 | 0.509 | 0.522 | 0.535 | 0.552 | 0.57 | 0.586 | 0.598 | 0.607 |
| West Bengal | 0.321 | 0.322 | 0.329 | 0.338 | 0.345 | 0.353 | 0.36 | 0.366 | 0.371 | 0.38 | 0.389 | 0.398 | 0.406 | 0.416 | 0.425 | 0.434 | 0.441 | 0.448 | 0.455 | 0.462 | 0.467 | 0.471 | 0.476 | 0.49 | 0.506 | 0.519 | 0.53 | 0.538 |
| Union Territories other than Delhi | 0.406 | 0.408 | 0.412 | 0.417 | 0.423 | 0.43 | 0.439 | 0.446 | 0.453 | 0.463 | 0.474 | 0.486 | 0.498 | 0.511 | 0.524 | 0.537 | 0.548 | 0.559 | 0.569 | 0.579 | 0.586 | 0.591 | 0.597 | 0.61 | 0.624 | 0.636 | 0.646 | 0.653 |
| Nepal | 0.216 | 0.222 | 0.228 | 0.235 | 0.242 | 0.249 | 0.257 | 0.265 | 0.273 | 0.281 | 0.29 | 0.298 | 0.307 | 0.315 | 0.323 | 0.331 | 0.339 | 0.347 | 0.355 | 0.364 | 0.373 | 0.382 | 0.39 | 0.399 | 0.407 | 0.415 | 0.422 | 0.429 |
| Pakistan | 0.305 | 0.31 | 0.314 | 0.319 | 0.324 | 0.33 | 0.337 | 0.343 | 0.35 | 0.359 | 0.367 | 0.375 | 0.382 | 0.389 | 0.396 | 0.404 | 0.412 | 0.42 | 0.428 | 0.436 | 0.443 | 0.449 | 0.458 | 0.466 | 0.474 | 0.48 | 0.486 | 0.492 |
| Southeast Asia, East Asia, and Oceania | 0.468 | 0.479 | 0.489 | 0.498 | 0.506 | 0.517 | 0.528 | 0.538 | 0.546 | 0.553 | 0.561 | 0.568 | 0.574 | 0.58 | 0.588 | 0.596 | 0.606 | 0.615 | 0.624 | 0.632 | 0.641 | 0.649 | 0.656 | 0.662 | 0.67 | 0.673 | 0.675 | 0.685 |
| East Asia | 0.463 | 0.476 | 0.486 | 0.495 | 0.504 | 0.516 | 0.528 | 0.54 | 0.55 | 0.559 | 0.568 | 0.577 | 0.584 | 0.591 | 0.599 | 0.61 | 0.621 | 0.632 | 0.642 | 0.652 | 0.663 | 0.672 | 0.679 | 0.685 | 0.694 | 0.696 | 0.696 | 0.709 |
| China | 0.456 | 0.469 | 0.479 | 0.489 | 0.498 | 0.51 | 0.523 | 0.535 | 0.545 | 0.554 | 0.563 | 0.573 | 0.58 | 0.587 | 0.595 | 0.606 | 0.618 | 0.63 | 0.64 | 0.65 | 0.661 | 0.671 | 0.678 | 0.683 | 0.693 | 0.695 | 0.694 | 0.707 |
| North Korea | 0.512 | 0.517 | 0.52 | 0.524 | 0.524 | 0.522 | 0.519 | 0.515 | 0.51 | 0.506 | 0.503 | 0.5 | 0.498 | 0.498 | 0.499 | 0.501 | 0.503 | 0.506 | 0.508 | 0.511 | 0.514 | 0.517 | 0.52 | 0.524 | 0.527 | 0.531 | 0.535 | 0.538 |
| Taiwan (Province of China) | 0.691 | 0.7 | 0.708 | 0.715 | 0.723 | 0.731 | 0.739 | 0.75 | 0.759 | 0.764 | 0.77 | 0.778 | 0.786 | 0.793 | 0.8 | 0.807 | 0.813 | 0.821 | 0.827 | 0.833 | 0.838 | 0.842 | 0.846 | 0.85 | 0.854 | 0.858 | 0.861 | 0.864 |
| Oceania | 0.406 | 0.408 | 0.41 | 0.412 | 0.415 | 0.418 | 0.421 | 0.423 | 0.426 | 0.428 | 0.43 | 0.431 | 0.432 | 0.433 | 0.435 | 0.436 | 0.438 | 0.44 | 0.442 | 0.444 | 0.446 | 0.449 | 0.452 | 0.455 | 0.459 | 0.463 | 0.467 | 0.471 |
| American Samoa | 0.609 | 0.612 | 0.615 | 0.62 | 0.624 | 0.629 | 0.633 | 0.636 | 0.639 | 0.643 | 0.647 | 0.651 | 0.655 | 0.659 | 0.662 | 0.666 | 0.668 | 0.671 | 0.673 | 0.675 | 0.678 | 0.682 | 0.687 | 0.691 | 0.694 | 0.697 | 0.7 | 0.702 |
| Federated States of Micronesia | 0.462 | 0.469 | 0.476 | 0.483 | 0.489 | 0.496 | 0.501 | 0.505 | 0.509 | 0.514 | 0.518 | 0.523 | 0.527 | 0.532 | 0.536 | 0.54 | 0.543 | 0.547 | 0.549 | 0.552 | 0.555 | 0.559 | 0.562 | 0.565 | 0.567 | 0.57 | 0.573 | 0.575 |
| Fiji | 0.533 | 0.537 | 0.542 | 0.547 | 0.552 | 0.558 | 0.563 | 0.568 | 0.574 | 0.58 | 0.585 | 0.589 | 0.594 | 0.598 | 0.602 | 0.604 | 0.607 | 0.61 | 0.611 | 0.613 | 0.615 | 0.617 | 0.619 | 0.623 | 0.627 | 0.632 | 0.637 | 0.641 |
| Guam | 0.698 | 0.695 | 0.693 | 0.694 | 0.698 | 0.703 | 0.709 | 0.717 | 0.727 | 0.739 | 0.75 | 0.759 | 0.765 | 0.768 | 0.77 | 0.77 | 0.769 | 0.77 | 0.771 | 0.774 | 0.776 | 0.78 | 0.784 | 0.788 | 0.792 | 0.794 | 0.794 | 0.794 |
| Kiribati | 0.355 | 0.357 | 0.359 | 0.361 | 0.363 | 0.365 | 0.368 | 0.37 | 0.373 | 0.376 | 0.379 | 0.382 | 0.385 | 0.388 | 0.39 | 0.393 | 0.395 | 0.397 | 0.398 | 0.399 | 0.401 | 0.403 | 0.406 | 0.41 | 0.414 | 0.418 | 0.423 | 0.427 |
| Marshall Islands | 0.413 | 0.421 | 0.429 | 0.436 | 0.444 | 0.451 | 0.457 | 0.462 | 0.465 | 0.469 | 0.473 | 0.477 | 0.482 | 0.485 | 0.489 | 0.493 | 0.498 | 0.503 | 0.507 | 0.512 | 0.518 | 0.523 | 0.528 | 0.533 | 0.538 | 0.542 | 0.547 | 0.55 |
| Northern Mariana Islands | 0.738 | 0.744 | 0.748 | 0.75 | 0.752 | 0.754 | 0.756 | 0.757 | 0.758 | 0.759 | 0.763 | 0.766 | 0.767 | 0.767 | 0.767 | 0.766 | 0.765 | 0.764 | 0.763 | 0.761 | 0.759 | 0.758 | 0.757 | 0.756 | 0.756 | 0.756 | 0.757 | 0.758 |
| Papua New Guinea | 0.318 | 0.321 | 0.324 | 0.33 | 0.335 | 0.34 | 0.344 | 0.348 | 0.351 | 0.354 | 0.356 | 0.358 | 0.36 | 0.361 | 0.363 | 0.365 | 0.368 | 0.371 | 0.375 | 0.379 | 0.383 | 0.388 | 0.393 | 0.398 | 0.404 | 0.409 | 0.415 | 0.419 |
| Samoa | 0.538 | 0.539 | 0.538 | 0.536 | 0.535 | 0.536 | 0.536 | 0.537 | 0.538 | 0.54 | 0.542 | 0.544 | 0.546 | 0.548 | 0.55 | 0.552 | 0.554 | 0.556 | 0.558 | 0.559 | 0.561 | 0.563 | 0.56 | 0.558 | 0.562 | 0.567 | 0.572 | 0.576 |
| Solomon Islands | 0.316 | 0.32 | 0.326 | 0.331 | 0.337 | 0.344 | 0.35 | 0.355 | 0.36 | 0.364 | 0.365 | 0.366 | 0.366 | 0.366 | 0.367 | 0.369 | 0.371 | 0.375 | 0.38 | 0.384 | 0.388 | 0.394 | 0.4 | 0.406 | 0.411 | 0.416 | 0.421 | 0.425 |
| Tonga | 0.522 | 0.528 | 0.533 | 0.538 | 0.544 | 0.549 | 0.553 | 0.557 | 0.56 | 0.564 | 0.566 | 0.569 | 0.573 | 0.577 | 0.581 | 0.584 | 0.587 | 0.589 | 0.592 | 0.595 | 0.599 | 0.603 | 0.607 | 0.61 | 0.614 | 0.617 | 0.621 | 0.625 |
| Vanuatu | 0.38 | 0.384 | 0.388 | 0.391 | 0.395 | 0.398 | 0.402 | 0.406 | 0.409 | 0.413 | 0.416 | 0.419 | 0.421 | 0.423 | 0.425 | 0.428 | 0.431 | 0.435 | 0.44 | 0.445 | 0.449 | 0.453 | 0.458 | 0.462 | 0.465 | 0.469 | 0.472 | 0.475 |
| Southeast Asia | 0.467 | 0.477 | 0.486 | 0.495 | 0.504 | 0.513 | 0.521 | 0.529 | 0.534 | 0.538 | 0.543 | 0.548 | 0.552 | 0.557 | 0.562 | 0.567 | 0.572 | 0.578 | 0.584 | 0.589 | 0.595 | 0.602 | 0.609 | 0.616 | 0.622 | 0.629 | 0.635 | 0.641 |
| Cambodia | 0.259 | 0.266 | 0.273 | 0.279 | 0.286 | 0.294 | 0.302 | 0.31 | 0.317 | 0.326 | 0.335 | 0.344 | 0.353 | 0.362 | 0.372 | 0.382 | 0.392 | 0.401 | 0.411 | 0.42 | 0.427 | 0.435 | 0.443 | 0.451 | 0.459 | 0.467 | 0.475 | 0.482 |
| Indonesia | 0.465 | 0.476 | 0.487 | 0.499 | 0.51 | 0.52 | 0.53 | 0.537 | 0.542 | 0.546 | 0.55 | 0.554 | 0.558 | 0.562 | 0.566 | 0.571 | 0.576 | 0.581 | 0.587 | 0.594 | 0.6 | 0.608 | 0.615 | 0.622 | 0.629 | 0.636 | 0.642 | 0.648 |
| Laos | 0.307 | 0.313 | 0.32 | 0.326 | 0.332 | 0.339 | 0.345 | 0.352 | 0.358 | 0.364 | 0.371 | 0.378 | 0.385 | 0.392 | 0.4 | 0.407 | 0.416 | 0.425 | 0.434 | 0.444 | 0.453 | 0.463 | 0.473 | 0.483 | 0.493 | 0.502 | 0.511 | 0.519 |
| Malaysia | 0.57 | 0.575 | 0.581 | 0.589 | 0.598 | 0.606 | 0.616 | 0.626 | 0.638 | 0.654 | 0.661 | 0.664 | 0.671 | 0.679 | 0.684 | 0.689 | 0.697 | 0.704 | 0.709 | 0.714 | 0.721 | 0.727 | 0.733 | 0.739 | 0.744 | 0.749 | 0.754 | 0.759 |
| Maldives | 0.386 | 0.399 | 0.412 | 0.424 | 0.435 | 0.446 | 0.46 | 0.477 | 0.493 | 0.507 | 0.518 | 0.528 | 0.537 | 0.545 | 0.554 | 0.56 | 0.567 | 0.575 | 0.584 | 0.593 | 0.602 | 0.611 | 0.62 | 0.629 | 0.636 | 0.643 | 0.65 | 0.655 |
| Mauritius | 0.554 | 0.561 | 0.568 | 0.576 | 0.585 | 0.595 | 0.603 | 0.609 | 0.613 | 0.616 | 0.621 | 0.627 | 0.633 | 0.639 | 0.646 | 0.651 | 0.657 | 0.663 | 0.669 | 0.675 | 0.68 | 0.687 | 0.694 | 0.701 | 0.708 | 0.713 | 0.717 | 0.72 |
| Myanmar | 0.33 | 0.333 | 0.337 | 0.341 | 0.347 | 0.353 | 0.36 | 0.367 | 0.375 | 0.383 | 0.392 | 0.402 | 0.412 | 0.423 | 0.434 | 0.446 | 0.458 | 0.47 | 0.481 | 0.492 | 0.501 | 0.51 | 0.518 | 0.527 | 0.535 | 0.542 | 0.549 | 0.556 |
| Philippines | 0.511 | 0.516 | 0.521 | 0.525 | 0.53 | 0.534 | 0.539 | 0.542 | 0.545 | 0.547 | 0.55 | 0.553 | 0.555 | 0.557 | 0.559 | 0.561 | 0.563 | 0.566 | 0.568 | 0.569 | 0.572 | 0.579 | 0.586 | 0.593 | 0.599 | 0.605 | 0.612 | 0.617 |
| Sri Lanka | 0.49 | 0.495 | 0.501 | 0.508 | 0.516 | 0.524 | 0.532 | 0.54 | 0.547 | 0.553 | 0.559 | 0.565 | 0.571 | 0.578 | 0.584 | 0.59 | 0.597 | 0.604 | 0.611 | 0.618 | 0.626 | 0.634 | 0.642 | 0.65 | 0.658 | 0.666 | 0.673 | 0.68 |
| Seychelles | 0.549 | 0.557 | 0.565 | 0.573 | 0.582 | 0.589 | 0.597 | 0.605 | 0.613 | 0.62 | 0.626 | 0.631 | 0.636 | 0.64 | 0.643 | 0.646 | 0.65 | 0.653 | 0.656 | 0.658 | 0.66 | 0.663 | 0.667 | 0.671 | 0.675 | 0.68 | 0.686 | 0.692 |
| Thailand | 0.502 | 0.514 | 0.525 | 0.534 | 0.542 | 0.552 | 0.561 | 0.567 | 0.569 | 0.572 | 0.579 | 0.587 | 0.594 | 0.6 | 0.605 | 0.61 | 0.616 | 0.623 | 0.629 | 0.635 | 0.641 | 0.647 | 0.654 | 0.66 | 0.667 | 0.673 | 0.679 | 0.684 |
| Timor-Leste | 0.276 | 0.283 | 0.29 | 0.296 | 0.302 | 0.307 | 0.314 | 0.321 | 0.325 | 0.321 | 0.32 | 0.325 | 0.332 | 0.345 | 0.362 | 0.379 | 0.4 | 0.419 | 0.437 | 0.449 | 0.46 | 0.471 | 0.481 | 0.49 | 0.495 | 0.5 | 0.504 | 0.505 |
| Vietnam | 0.406 | 0.413 | 0.42 | 0.427 | 0.435 | 0.444 | 0.452 | 0.461 | 0.469 | 0.477 | 0.483 | 0.49 | 0.497 | 0.504 | 0.511 | 0.518 | 0.525 | 0.532 | 0.54 | 0.547 | 0.554 | 0.562 | 0.57 | 0.578 | 0.585 | 0.593 | 0.6 | 0.607 |
| Sub-Saharan Africa | 0.304 | 0.307 | 0.311 | 0.314 | 0.317 | 0.32 | 0.324 | 0.328 | 0.332 | 0.335 | 0.339 | 0.343 | 0.348 | 0.353 | 0.359 | 0.365 | 0.371 | 0.379 | 0.386 | 0.393 | 0.4 | 0.407 | 0.414 | 0.421 | 0.428 | 0.435 | 0.441 | 0.446 |
| Central sub-Saharan Africa | 0.298 | 0.303 | 0.307 | 0.309 | 0.311 | 0.313 | 0.316 | 0.318 | 0.32 | 0.323 | 0.325 | 0.328 | 0.332 | 0.336 | 0.341 | 0.348 | 0.355 | 0.364 | 0.373 | 0.382 | 0.391 | 0.402 | 0.413 | 0.423 | 0.433 | 0.443 | 0.452 | 0.457 |
| Angola | 0.235 | 0.24 | 0.245 | 0.249 | 0.253 | 0.258 | 0.263 | 0.269 | 0.276 | 0.282 | 0.288 | 0.293 | 0.299 | 0.305 | 0.312 | 0.32 | 0.329 | 0.34 | 0.351 | 0.363 | 0.375 | 0.389 | 0.401 | 0.414 | 0.428 | 0.441 | 0.453 | 0.461 |
| Central African Republic | 0.22 | 0.225 | 0.228 | 0.232 | 0.236 | 0.24 | 0.242 | 0.245 | 0.249 | 0.254 | 0.257 | 0.261 | 0.265 | 0.268 | 0.271 | 0.275 | 0.28 | 0.285 | 0.29 | 0.296 | 0.304 | 0.313 | 0.323 | 0.325 | 0.328 | 0.33 | 0.333 | 0.334 |
| Congo (Brazzaville) | 0.382 | 0.39 | 0.398 | 0.405 | 0.41 | 0.416 | 0.421 | 0.426 | 0.43 | 0.434 | 0.439 | 0.444 | 0.449 | 0.455 | 0.46 | 0.467 | 0.475 | 0.482 | 0.49 | 0.499 | 0.509 | 0.52 | 0.531 | 0.542 | 0.552 | 0.561 | 0.569 | 0.574 |
| DR Congo | 0.293 | 0.296 | 0.298 | 0.298 | 0.296 | 0.294 | 0.291 | 0.288 | 0.283 | 0.279 | 0.274 | 0.269 | 0.265 | 0.263 | 0.262 | 0.264 | 0.264 | 0.265 | 0.267 | 0.27 | 0.278 | 0.288 | 0.3 | 0.315 | 0.33 | 0.344 | 0.356 | 0.364 |
| Equatorial Guinea | 0.2 | 0.204 | 0.212 | 0.22 | 0.229 | 0.241 | 0.26 | 0.292 | 0.316 | 0.339 | 0.363 | 0.388 | 0.41 | 0.429 | 0.449 | 0.467 | 0.483 | 0.499 | 0.516 | 0.53 | 0.544 | 0.559 | 0.573 | 0.587 | 0.599 | 0.61 | 0.62 | 0.625 |
| Gabon | 0.433 | 0.443 | 0.453 | 0.462 | 0.472 | 0.481 | 0.49 | 0.498 | 0.506 | 0.514 | 0.522 | 0.529 | 0.535 | 0.542 | 0.549 | 0.556 | 0.562 | 0.569 | 0.576 | 0.582 | 0.589 | 0.598 | 0.607 | 0.616 | 0.625 | 0.634 | 0.644 | 0.651 |
| Eastern sub-Saharan Africa | 0.23 | 0.233 | 0.236 | 0.239 | 0.241 | 0.245 | 0.249 | 0.254 | 0.259 | 0.262 | 0.266 | 0.271 | 0.276 | 0.282 | 0.288 | 0.294 | 0.301 | 0.308 | 0.316 | 0.324 | 0.332 | 0.34 | 0.348 | 0.356 | 0.365 | 0.373 | 0.381 | 0.387 |
| Burundi | 0.247 | 0.252 | 0.257 | 0.258 | 0.263 | 0.265 | 0.265 | 0.266 | 0.268 | 0.268 | 0.268 | 0.267 | 0.268 | 0.269 | 0.271 | 0.272 | 0.274 | 0.276 | 0.278 | 0.282 | 0.286 | 0.29 | 0.295 | 0.299 | 0.303 | 0.306 | 0.308 | 0.31 |
| Comoros | 0.272 | 0.279 | 0.286 | 0.293 | 0.298 | 0.303 | 0.306 | 0.31 | 0.314 | 0.319 | 0.325 | 0.331 | 0.338 | 0.344 | 0.351 | 0.358 | 0.365 | 0.372 | 0.378 | 0.384 | 0.39 | 0.396 | 0.403 | 0.41 | 0.417 | 0.423 | 0.429 | 0.434 |
| Djibouti | 0.313 | 0.317 | 0.32 | 0.322 | 0.325 | 0.329 | 0.333 | 0.337 | 0.339 | 0.341 | 0.342 | 0.347 | 0.359 | 0.374 | 0.388 | 0.4 | 0.407 | 0.412 | 0.419 | 0.425 | 0.432 | 0.439 | 0.446 | 0.454 | 0.462 | 0.47 | 0.478 | 0.485 |
| Eritrea | 0.202 | 0.214 | 0.223 | 0.234 | 0.247 | 0.26 | 0.272 | 0.285 | 0.296 | 0.306 | 0.315 | 0.323 | 0.331 | 0.337 | 0.343 | 0.348 | 0.353 | 0.357 | 0.36 | 0.364 | 0.368 | 0.372 | 0.378 | 0.383 | 0.39 | 0.396 | 0.403 | 0.409 |
| Ethiopia | 0.138 | 0.141 | 0.143 | 0.146 | 0.148 | 0.15 | 0.155 | 0.161 | 0.166 | 0.169 | 0.172 | 0.177 | 0.183 | 0.189 | 0.195 | 0.202 | 0.21 | 0.221 | 0.233 | 0.245 | 0.257 | 0.268 | 0.28 | 0.292 | 0.303 | 0.314 | 0.325 | 0.334 |
| Kenya | 0.341 | 0.349 | 0.357 | 0.364 | 0.372 | 0.377 | 0.382 | 0.387 | 0.392 | 0.398 | 0.401 | 0.403 | 0.406 | 0.411 | 0.416 | 0.42 | 0.425 | 0.432 | 0.438 | 0.445 | 0.452 | 0.459 | 0.465 | 0.473 | 0.481 | 0.488 | 0.494 | 0.499 |
| Baringo | 0.254 | 0.266 | 0.278 | 0.289 | 0.299 | 0.307 | 0.313 | 0.319 | 0.326 | 0.333 | 0.338 | 0.341 | 0.345 | 0.352 | 0.358 | 0.362 | 0.368 | 0.376 | 0.384 | 0.393 | 0.401 | 0.408 | 0.414 | 0.421 | 0.428 | 0.434 | 0.439 | 0.444 |
| Bomet | 0.306 | 0.315 | 0.325 | 0.333 | 0.341 | 0.347 | 0.351 | 0.355 | 0.361 | 0.367 | 0.371 | 0.373 | 0.378 | 0.385 | 0.392 | 0.398 | 0.406 | 0.414 | 0.423 | 0.433 | 0.442 | 0.449 | 0.456 | 0.465 | 0.475 | 0.483 | 0.49 | 0.496 |
| Bungoma | 0.316 | 0.325 | 0.333 | 0.341 | 0.348 | 0.353 | 0.357 | 0.36 | 0.365 | 0.37 | 0.373 | 0.373 | 0.376 | 0.38 | 0.384 | 0.387 | 0.391 | 0.397 | 0.403 | 0.41 | 0.417 | 0.423 | 0.429 | 0.436 | 0.445 | 0.451 | 0.458 | 0.463 |
| Busia | 0.297 | 0.304 | 0.312 | 0.32 | 0.327 | 0.332 | 0.336 | 0.339 | 0.344 | 0.349 | 0.352 | 0.353 | 0.356 | 0.361 | 0.367 | 0.37 | 0.375 | 0.381 | 0.386 | 0.393 | 0.4 | 0.404 | 0.409 | 0.415 | 0.423 | 0.428 | 0.434 | 0.438 |
| Elgeyo Marakwet | 0.292 | 0.302 | 0.312 | 0.321 | 0.329 | 0.336 | 0.342 | 0.348 | 0.355 | 0.362 | 0.368 | 0.372 | 0.378 | 0.386 | 0.394 | 0.4 | 0.408 | 0.417 | 0.425 | 0.435 | 0.443 | 0.451 | 0.458 | 0.467 | 0.475 | 0.483 | 0.49 | 0.496 |
| Embu | 0.375 | 0.384 | 0.393 | 0.4 | 0.407 | 0.413 | 0.417 | 0.422 | 0.427 | 0.431 | 0.434 | 0.437 | 0.44 | 0.444 | 0.449 | 0.452 | 0.458 | 0.464 | 0.47 | 0.478 | 0.486 | 0.493 | 0.499 | 0.507 | 0.514 | 0.521 | 0.527 | 0.533 |
| Garissa | 0.153 | 0.16 | 0.168 | 0.177 | 0.184 | 0.19 | 0.195 | 0.201 | 0.207 | 0.213 | 0.217 | 0.219 | 0.223 | 0.228 | 0.233 | 0.237 | 0.242 | 0.249 | 0.255 | 0.263 | 0.272 | 0.28 | 0.288 | 0.298 | 0.309 | 0.318 | 0.326 | 0.334 |
| Homa Bay | 0.214 | 0.222 | 0.232 | 0.243 | 0.253 | 0.26 | 0.265 | 0.271 | 0.279 | 0.288 | 0.292 | 0.293 | 0.297 | 0.305 | 0.313 | 0.319 | 0.328 | 0.338 | 0.346 | 0.356 | 0.366 | 0.374 | 0.382 | 0.392 | 0.403 | 0.411 | 0.419 | 0.425 |
| Isiolo | 0.264 | 0.27 | 0.276 | 0.282 | 0.288 | 0.292 | 0.295 | 0.298 | 0.301 | 0.305 | 0.307 | 0.308 | 0.31 | 0.314 | 0.318 | 0.321 | 0.326 | 0.331 | 0.337 | 0.343 | 0.35 | 0.355 | 0.36 | 0.365 | 0.372 | 0.377 | 0.381 | 0.385 |
| Kajiado | 0.384 | 0.392 | 0.4 | 0.407 | 0.414 | 0.42 | 0.425 | 0.429 | 0.434 | 0.438 | 0.442 | 0.445 | 0.448 | 0.452 | 0.456 | 0.46 | 0.464 | 0.47 | 0.475 | 0.481 | 0.486 | 0.492 | 0.498 | 0.506 | 0.514 | 0.521 | 0.528 | 0.534 |
| Kakamega | 0.295 | 0.303 | 0.311 | 0.319 | 0.326 | 0.332 | 0.337 | 0.342 | 0.348 | 0.356 | 0.36 | 0.361 | 0.365 | 0.37 | 0.375 | 0.378 | 0.383 | 0.389 | 0.394 | 0.4 | 0.407 | 0.412 | 0.417 | 0.425 | 0.433 | 0.439 | 0.445 | 0.45 |

**Appendix Table 10: Socio-Demographic Index values for all estimated GBD 2017 locations, 1990-2017**

| Location | 1990 | 1991 | 1992 | 1993 | 1994 | 1995 | 1996 | 1997 | 1998 | 1999 | 2000 | 2001 | 2002 | 2003 | 2004 | 2005 | 2006 | 2007 | 2008 | 2009 | 2010 | 2011 | 2012 | 2013 | 2014 | 2015 | 2016 | 2017 |
|---|---|---|---|---|---|---|---|---|---|---|---|---|---|---|---|---|---|---|---|---|---|---|---|---|---|---|---|---|
| Kericho | 0.266 | 0.277 | 0.288 | 0.299 | 0.309 | 0.317 | 0.324 | 0.331 | 0.339 | 0.348 | 0.353 | 0.356 | 0.362 | 0.37 | 0.378 | 0.385 | 0.394 | 0.404 | 0.414 | 0.425 | 0.436 | 0.445 | 0.454 | 0.464 | 0.475 | 0.485 | 0.493 | 0.5 |
| Kiambu | 0.435 | 0.443 | 0.45 | 0.457 | 0.464 | 0.469 | 0.473 | 0.476 | 0.48 | 0.484 | 0.487 | 0.489 | 0.492 | 0.496 | 0.5 | 0.504 | 0.509 | 0.516 | 0.521 | 0.528 | 0.535 | 0.541 | 0.548 | 0.555 | 0.562 | 0.569 | 0.575 | 0.58 |
| Kilifi | 0.292 | 0.3 | 0.307 | 0.314 | 0.321 | 0.327 | 0.331 | 0.336 | 0.34 | 0.346 | 0.348 | 0.349 | 0.352 | 0.357 | 0.361 | 0.365 | 0.371 | 0.378 | 0.385 | 0.392 | 0.4 | 0.408 | 0.415 | 0.424 | 0.434 | 0.442 | 0.45 | 0.456 |
| Kirinyaga | 0.389 | 0.396 | 0.402 | 0.407 | 0.411 | 0.415 | 0.418 | 0.422 | 0.425 | 0.429 | 0.432 | 0.434 | 0.437 | 0.442 | 0.447 | 0.451 | 0.457 | 0.464 | 0.471 | 0.479 | 0.486 | 0.493 | 0.5 | 0.507 | 0.514 | 0.521 | 0.527 | 0.533 |
| Kisii | 0.34 | 0.35 | 0.36 | 0.368 | 0.377 | 0.383 | 0.389 | 0.395 | 0.401 | 0.407 | 0.411 | 0.414 | 0.418 | 0.424 | 0.429 | 0.434 | 0.44 | 0.448 | 0.454 | 0.463 | 0.471 | 0.479 | 0.486 | 0.495 | 0.503 | 0.51 | 0.517 | 0.522 |
| Kisumu | 0.315 | 0.325 | 0.334 | 0.342 | 0.349 | 0.355 | 0.36 | 0.364 | 0.37 | 0.376 | 0.381 | 0.384 | 0.388 | 0.395 | 0.402 | 0.407 | 0.415 | 0.424 | 0.432 | 0.442 | 0.451 | 0.459 | 0.466 | 0.475 | 0.484 | 0.491 | 0.497 | 0.503 |
| Kitui | 0.28 | 0.288 | 0.297 | 0.304 | 0.311 | 0.317 | 0.322 | 0.327 | 0.332 | 0.338 | 0.343 | 0.346 | 0.351 | 0.357 | 0.363 | 0.369 | 0.376 | 0.383 | 0.391 | 0.399 | 0.408 | 0.416 | 0.423 | 0.432 | 0.44 | 0.448 | 0.455 | 0.461 |
| Kwale | 0.294 | 0.301 | 0.308 | 0.314 | 0.321 | 0.326 | 0.33 | 0.334 | 0.338 | 0.342 | 0.344 | 0.346 | 0.348 | 0.352 | 0.357 | 0.36 | 0.366 | 0.374 | 0.381 | 0.39 | 0.399 | 0.407 | 0.414 | 0.424 | 0.433 | 0.442 | 0.45 | 0.457 |
| Laikipia | 0.346 | 0.354 | 0.361 | 0.368 | 0.375 | 0.38 | 0.385 | 0.389 | 0.395 | 0.401 | 0.405 | 0.409 | 0.415 | 0.423 | 0.433 | 0.442 | 0.451 | 0.462 | 0.472 | 0.483 | 0.494 | 0.502 | 0.511 | 0.521 | 0.531 | 0.54 | 0.549 | 0.556 |
| Lamu | 0.295 | 0.303 | 0.312 | 0.321 | 0.328 | 0.334 | 0.338 | 0.343 | 0.347 | 0.352 | 0.355 | 0.356 | 0.359 | 0.364 | 0.369 | 0.373 | 0.378 | 0.384 | 0.39 | 0.397 | 0.405 | 0.411 | 0.417 | 0.425 | 0.434 | 0.441 | 0.448 | 0.453 |
| Machakos | 0.352 | 0.362 | 0.372 | 0.38 | 0.389 | 0.396 | 0.403 | 0.411 | 0.418 | 0.426 | 0.431 | 0.435 | 0.44 | 0.444 | 0.449 | 0.452 | 0.456 | 0.461 | 0.465 | 0.471 | 0.476 | 0.482 | 0.487 | 0.493 | 0.5 | 0.507 | 0.512 | 0.518 |
| Makueni | 0.3 | 0.307 | 0.314 | 0.322 | 0.328 | 0.333 | 0.336 | 0.339 | 0.343 | 0.348 | 0.35 | 0.351 | 0.354 | 0.36 | 0.367 | 0.373 | 0.381 | 0.391 | 0.401 | 0.413 | 0.423 | 0.432 | 0.439 | 0.447 | 0.454 | 0.46 | 0.465 | 0.469 |
| Mandera | 0.102 | 0.109 | 0.118 | 0.126 | 0.135 | 0.142 | 0.149 | 0.156 | 0.164 | 0.172 | 0.178 | 0.181 | 0.187 | 0.194 | 0.201 | 0.206 | 0.212 | 0.218 | 0.224 | 0.231 | 0.238 | 0.244 | 0.251 | 0.261 | 0.271 | 0.28 | 0.288 | 0.295 |
| Marsabit | 0.22 | 0.223 | 0.227 | 0.231 | 0.236 | 0.239 | 0.241 | 0.244 | 0.247 | 0.252 | 0.255 | 0.256 | 0.259 | 0.264 | 0.268 | 0.271 | 0.276 | 0.281 | 0.286 | 0.293 | 0.299 | 0.304 | 0.309 | 0.315 | 0.323 | 0.329 | 0.335 | 0.34 |
| Meru | 0.338 | 0.346 | 0.354 | 0.362 | 0.369 | 0.376 | 0.381 | 0.387 | 0.393 | 0.399 | 0.403 | 0.407 | 0.411 | 0.417 | 0.423 | 0.428 | 0.434 | 0.441 | 0.447 | 0.454 | 0.462 | 0.469 | 0.475 | 0.482 | 0.49 | 0.496 | 0.503 | 0.508 |
| Migori | 0.229 | 0.237 | 0.246 | 0.255 | 0.262 | 0.267 | 0.27 | 0.274 | 0.279 | 0.285 | 0.287 | 0.286 | 0.289 | 0.296 | 0.303 | 0.307 | 0.315 | 0.325 | 0.333 | 0.344 | 0.355 | 0.364 | 0.372 | 0.383 | 0.395 | 0.404 | 0.412 | 0.419 |
| Mombasa | 0.396 | 0.405 | 0.414 | 0.422 | 0.43 | 0.437 | 0.443 | 0.449 | 0.455 | 0.461 | 0.465 | 0.468 | 0.471 | 0.475 | 0.478 | 0.481 | 0.486 | 0.491 | 0.496 | 0.502 | 0.51 | 0.517 | 0.525 | 0.534 | 0.543 | 0.552 | 0.561 | 0.568 |
| Murang'a | 0.381 | 0.389 | 0.396 | 0.402 | 0.408 | 0.413 | 0.418 | 0.424 | 0.429 | 0.436 | 0.441 | 0.444 | 0.449 | 0.454 | 0.46 | 0.465 | 0.47 | 0.476 | 0.482 | 0.488 | 0.494 | 0.499 | 0.504 | 0.51 | 0.515 | 0.52 | 0.524 | 0.528 |
| Nairobi | 0.499 | 0.506 | 0.513 | 0.519 | 0.525 | 0.53 | 0.534 | 0.539 | 0.544 | 0.549 | 0.553 | 0.556 | 0.56 | 0.566 | 0.572 | 0.577 | 0.585 | 0.593 | 0.602 | 0.611 | 0.619 | 0.627 | 0.634 | 0.643 | 0.652 | 0.66 | 0.667 | 0.674 |
| Nakuru | 0.337 | 0.347 | 0.357 | 0.367 | 0.375 | 0.383 | 0.389 | 0.394 | 0.399 | 0.405 | 0.409 | 0.413 | 0.417 | 0.423 | 0.43 | 0.436 | 0.445 | 0.454 | 0.464 | 0.474 | 0.484 | 0.493 | 0.502 | 0.511 | 0.521 | 0.53 | 0.538 | 0.545 |
| Nandi | 0.323 | 0.333 | 0.344 | 0.354 | 0.362 | 0.369 | 0.375 | 0.381 | 0.388 | 0.394 | 0.399 | 0.402 | 0.406 | 0.412 | 0.418 | 0.422 | 0.428 | 0.435 | 0.441 | 0.449 | 0.456 | 0.462 | 0.468 | 0.475 | 0.483 | 0.49 | 0.496 | 0.501 |
| Narok | 0.217 | 0.225 | 0.234 | 0.242 | 0.25 | 0.255 | 0.259 | 0.264 | 0.272 | 0.28 | 0.284 | 0.285 | 0.289 | 0.297 | 0.305 | 0.31 | 0.318 | 0.326 | 0.333 | 0.342 | 0.35 | 0.356 | 0.362 | 0.371 | 0.381 | 0.389 | 0.396 | 0.402 |
| Nyamira | 0.361 | 0.37 | 0.379 | 0.387 | 0.395 | 0.401 | 0.406 | 0.41 | 0.414 | 0.419 | 0.421 | 0.423 | 0.426 | 0.431 | 0.437 | 0.443 | 0.451 | 0.46 | 0.468 | 0.478 | 0.488 | 0.496 | 0.504 | 0.513 | 0.523 | 0.531 | 0.538 | 0.544 |
| Nyandarua | 0.353 | 0.361 | 0.369 | 0.376 | 0.382 | 0.388 | 0.392 | 0.397 | 0.402 | 0.409 | 0.413 | 0.416 | 0.421 | 0.428 | 0.436 | 0.442 | 0.449 | 0.458 | 0.466 | 0.475 | 0.484 | 0.491 | 0.498 | 0.506 | 0.514 | 0.521 | 0.528 | 0.534 |
| Nyeri | 0.407 | 0.414 | 0.421 | 0.427 | 0.434 | 0.439 | 0.444 | 0.449 | 0.454 | 0.459 | 0.463 | 0.466 | 0.47 | 0.475 | 0.479 | 0.484 | 0.489 | 0.496 | 0.502 | 0.509 | 0.516 | 0.522 | 0.527 | 0.533 | 0.539 | 0.544 | 0.549 | 0.554 |
| Samburu | 0.205 | 0.21 | 0.215 | 0.221 | 0.225 | 0.228 | 0.23 | 0.232 | 0.235 | 0.239 | 0.24 | 0.239 | 0.24 | 0.243 | 0.247 | 0.249 | 0.253 | 0.258 | 0.262 | 0.267 | 0.273 | 0.277 | 0.28 | 0.287 | 0.294 | 0.299 | 0.304 | 0.308 |
| Siaya | 0.227 | 0.236 | 0.246 | 0.255 | 0.265 | 0.272 | 0.279 | 0.287 | 0.294 | 0.303 | 0.309 | 0.313 | 0.318 | 0.326 | 0.334 | 0.34 | 0.349 | 0.359 | 0.368 | 0.379 | 0.39 | 0.4 | 0.409 | 0.421 | 0.432 | 0.443 | 0.452 | 0.46 |
| Taita Taveta | 0.352 | 0.362 | 0.371 | 0.38 | 0.389 | 0.396 | 0.403 | 0.409 | 0.416 | 0.422 | 0.427 | 0.43 | 0.434 | 0.438 | 0.443 | 0.447 | 0.452 | 0.458 | 0.464 | 0.472 | 0.48 | 0.487 | 0.494 | 0.502 | 0.509 | 0.516 | 0.523 | 0.529 |
| Tana River | 0.231 | 0.237 | 0.244 | 0.25 | 0.255 | 0.26 | 0.263 | 0.267 | 0.27 | 0.274 | 0.276 | 0.276 | 0.278 | 0.281 | 0.285 | 0.288 | 0.292 | 0.298 | 0.304 | 0.311 | 0.319 | 0.326 | 0.333 | 0.342 | 0.353 | 0.362 | 0.371 | 0.379 |
| Tharaka Nithi | 0.342 | 0.35 | 0.357 | 0.364 | 0.371 | 0.377 | 0.382 | 0.387 | 0.392 | 0.397 | 0.402 | 0.405 | 0.41 | 0.417 | 0.423 | 0.429 | 0.437 | 0.445 | 0.453 | 0.463 | 0.472 | 0.48 | 0.488 | 0.497 | 0.506 | 0.514 | 0.522 | 0.528 |
| Trans Nzoia | 0.318 | 0.328 | 0.337 | 0.346 | 0.353 | 0.359 | 0.363 | 0.368 | 0.373 | 0.38 | 0.384 | 0.388 | 0.392 | 0.399 | 0.406 | 0.411 | 0.418 | 0.426 | 0.433 | 0.441 | 0.448 | 0.454 | 0.46 | 0.468 | 0.477 | 0.484 | 0.491 | 0.496 |
| Turkana | 0.211 | 0.216 | 0.221 | 0.226 | 0.231 | 0.234 | 0.237 | 0.239 | 0.242 | 0.245 | 0.247 | 0.247 | 0.248 | 0.25 | 0.252 | 0.252 | 0.253 | 0.255 | 0.257 | 0.26 | 0.264 | 0.266 | 0.269 | 0.274 | 0.281 | 0.286 | 0.291 | 0.295 |
| Uasin Gishu | 0.363 | 0.373 | 0.382 | 0.391 | 0.399 | 0.405 | 0.41 | 0.416 | 0.421 | 0.428 | 0.433 | 0.436 | 0.441 | 0.447 | 0.453 | 0.458 | 0.465 | 0.472 | 0.48 | 0.488 | 0.496 | 0.503 | 0.51 | 0.517 | 0.526 | 0.533 | 0.539 | 0.545 |
| Vihiga | 0.328 | 0.337 | 0.346 | 0.355 | 0.362 | 0.367 | 0.371 | 0.374 | 0.376 | 0.378 | 0.378 | 0.378 | 0.379 | 0.382 | 0.387 | 0.392 | 0.399 | 0.408 | 0.417 | 0.427 | 0.437 | 0.444 | 0.45 | 0.457 | 0.464 | 0.469 | 0.474 | 0.477 |
| Wajir | 0.104 | 0.111 | 0.12 | 0.128 | 0.136 | 0.142 | 0.146 | 0.151 | 0.156 | 0.162 | 0.165 | 0.166 | 0.168 | 0.172 | 0.176 | 0.178 | 0.181 | 0.185 | 0.189 | 0.195 | 0.201 | 0.206 | 0.21 | 0.218 | 0.226 | 0.233 | 0.238 | 0.243 |
| West Pokot | 0.213 | 0.219 | 0.226 | 0.233 | 0.24 | 0.245 | 0.249 | 0.253 | 0.258 | 0.265 | 0.268 | 0.27 | 0.274 | 0.28 | 0.286 | 0.291 | 0.298 | 0.305 | 0.312 | 0.319 | 0.327 | 0.333 | 0.34 | 0.349 | 0.359 | 0.368 | 0.376 | 0.382 |
| Madagascar | 0.262 | 0.264 | 0.265 | 0.265 | 0.265 | 0.265 | 0.266 | 0.268 | 0.27 | 0.272 | 0.274 | 0.278 | 0.282 | 0.286 | 0.291 | 0.294 | 0.295 | 0.295 | 0.298 | 0.3 | 0.302 | 0.303 | 0.306 | 0.31 | 0.315 | 0.321 | 0.326 | 0.331 |
| Malawi | 0.199 | 0.202 | 0.204 | 0.206 | 0.208 | 0.212 | 0.216 | 0.221 | 0.225 | 0.23 | 0.234 | 0.238 | 0.244 | 0.249 | 0.256 | 0.262 | 0.27 | 0.279 | 0.288 | 0.296 | 0.305 | 0.313 | 0.32 | 0.327 | 0.333 | 0.339 | 0.345 | 0.349 |
| Mozambique | 0.133 | 0.135 | 0.136 | 0.138 | 0.14 | 0.142 | 0.147 | 0.154 | 0.162 | 0.169 | 0.175 | 0.182 | 0.189 | 0.197 | 0.206 | 0.215 | 0.224 | 0.235 | 0.246 | 0.257 | 0.268 | 0.279 | 0.29 | 0.301 | 0.311 | 0.322 | 0.332 | 0.34 |
| Rwanda | 0.267 | 0.269 | 0.272 | 0.274 | 0.268 | 0.267 | 0.269 | 0.272 | 0.276 | 0.28 | 0.284 | 0.29 | 0.297 | 0.304 | 0.311 | 0.319 | 0.327 | 0.334 | 0.342 | 0.349 | 0.356 | 0.363 | 0.371 | 0.378 | 0.386 | 0.393 | 0.401 | 0.407 |
| Somalia | 0.153 | 0.156 | 0.157 | 0.159 | 0.16 | 0.161 | 0.162 | 0.163 | 0.163 | 0.164 | 0.165 | 0.167 | 0.169 | 0.172 | 0.176 | 0.179 | 0.183 | 0.188 | 0.193 | 0.198 | 0.203 | 0.208 | 0.212 | 0.217 | 0.222 | 0.227 | 0.231 | 0.235 |
| South Sudan | 0.179 | 0.18 | 0.182 | 0.183 | 0.184 | 0.186 | 0.187 | 0.189 | 0.191 | 0.194 | 0.197 | 0.2 | 0.204 | 0.207 | 0.211 | 0.215 | 0.22 | 0.225 | 0.229 | 0.234 | 0.239 | 0.244 | 0.249 | 0.254 | 0.259 | 0.265 | 0.27 | 0.275 |
| Tanzania | 0.275 | 0.28 | 0.285 | 0.289 | 0.291 | 0.294 | 0.297 | 0.3 | 0.304 | 0.307 | 0.311 | 0.315 | 0.32 | 0.325 | 0.33 | 0.334 | 0.339 | 0.344 | 0.35 | 0.355 | 0.36 | 0.366 | 0.373 | 0.381 | 0.388 | 0.397 | 0.405 | 0.412 |
| Uganda | 0.163 | 0.164 | 0.166 | 0.169 | 0.173 | 0.178 | 0.185 | 0.193 | 0.202 | 0.212 | 0.222 | 0.232 | 0.243 | 0.254 | 0.265 | 0.276 | 0.287 | 0.298 | 0.31 | 0.32 | 0.33 | 0.339 | 0.348 | 0.357 | 0.366 | 0.374 | 0.382 | 0.388 |
| Zambia | 0.312 | 0.314 | 0.316 | 0.319 | 0.321 | 0.324 | 0.326 | 0.329 | 0.332 | 0.335 | 0.338 | 0.343 | 0.348 | 0.353 | 0.358 | 0.365 | 0.373 | 0.382 | 0.392 | 0.402 | 0.412 | 0.421 | 0.431 | 0.44 | 0.449 | 0.458 | 0.466 | 0.472 |
| Southern sub-Saharan Africa | 0.521 | 0.525 | 0.53 | 0.535 | 0.539 | 0.543 | 0.546 | 0.55 | 0.555 | 0.56 | 0.565 | 0.569 | 0.574 | 0.578 | 0.582 | 0.585 | 0.59 | 0.596 | 0.603 | 0.607 | 0.612 | 0.616 | 0.619 | 0.622 | 0.627 | 0.633 | 0.637 | 0.64 |
| Botswana | 0.463 | 0.473 | 0.483 | 0.492 | 0.501 | 0.509 | 0.517 | 0.524 | 0.532 | 0.539 | 0.546 | 0.553 | 0.56 | 0.567 | 0.574 | 0.581 | 0.588 | 0.597 | 0.605 | 0.612 | 0.619 | 0.626 | 0.633 | 0.639 | 0.646 | 0.652 | 0.658 | 0.663 |
| Lesotho | 0.333 | 0.34 | 0.347 | 0.354 | 0.362 | 0.369 | 0.376 | 0.383 | 0.389 | 0.396 | 0.402 | 0.407 | 0.413 | 0.417 | 0.422 | 0.426 | 0.431 | 0.436 | 0.441 | 0.446 | 0.452 | 0.458 | 0.464 | 0.47 | 0.476 | 0.482 | 0.488 | 0.493 |
| Namibia | 0.454 | 0.459 | 0.465 | 0.471 | 0.477 | 0.483 | 0.489 | 0.495 | 0.501 | 0.507 | 0.512 | 0.516 | 0.521 | 0.525 | 0.53 | 0.534 | 0.54 | 0.546 | 0.552 | 0.558 | 0.565 | 0.572 | 0.579 | 0.587 | 0.595 | 0.603 | 0.61 | 0.616 |
| South Africa | 0.557 | 0.56 | 0.565 | 0.57 | 0.574 | 0.576 | 0.579 | 0.583 | 0.587 | 0.593 | 0.598 | 0.602 | 0.607 | 0.611 | 0.615 | 0.619 | 0.624 | 0.631 | 0.639 | 0.644 | 0.649 | 0.653 | 0.655 | 0.658 | 0.664 | 0.669 | 0.674 | 0.677 |
| Swaziland | 0.426 | 0.435 | 0.444 | 0.452 | 0.459 | 0.466 | 0.473 | 0.478 | 0.483 | 0.489 | 0.494 | 0.499 | 0.503 | 0.508 | 0.513 | 0.518 | 0.523 | 0.528 | 0.533 | 0.538 | 0.543 | 0.547 | 0.552 | 0.557 | 0.563 | 0.569 | 0.574 | 0.578 |
| Zimbabwe | 0.408 | 0.417 | 0.424 | 0.43 | 0.436 | 0.44 | 0.446 | 0.45 | 0.453 | 0.456 | 0.457 | 0.459 | 0.458 | 0.456 | 0.453 | 0.449 | 0.443 | 0.438 | 0.43 | 0.426 | 0.424 | 0.425 | 0.43 | 0.436 | 0.444 | 0.451 | 0.458 | 0.463 |
| Western sub-Saharan Africa | 0.293 | 0.296 | 0.299 | 0.303 | 0.306 | 0.309 | 0.313 | 0.316 | 0.32 | 0.324 | 0.328 | 0.333 | 0.337 | 0.343 | 0.35 | 0.357 | 0.365 | 0.373 | 0.381 | 0.389 | 0.396 | 0.403 | 0.411 | 0.417 | 0.424 | 0.43 | 0.437 | 0.441 |
| Benin | 0.218 | 0.223 | 0.228 | 0.233 | 0.238 | 0.243 | 0.248 | 0.253 | 0.258 | 0.263 | 0.268 | 0.273 | 0.278 | 0.284 | 0.29 | 0.296 | 0.301 | 0.307 | 0.313 | 0.32 | 0.326 | 0.332 | 0.338 | 0.345 | 0.353 | 0.36 | 0.367 | 0.373 |
| Burkina Faso | 0.139 | 0.143 | 0.147 | 0.151 | 0.154 | 0.158 | 0.163 | 0.168 | 0.173 | 0.179 | 0.184 | 0.19 | 0.195 | 0.201 | 0.208 | 0.214 | 0.22 | 0.226 | 0.232 | 0.237 | 0.242 | 0.248 | 0.253 | 0.259 | 0.266 | 0.272 | 0.279 | 0.284 |
| Cameroon | 0.332 | 0.338 | 0.343 | 0.347 | 0.35 | 0.352 | 0.355 | 0.357 | 0.359 | 0.36 | 0.361 | 0.361 | 0.361 | 0.362 | 0.366 | 0.373 | 0.38 | 0.388 | 0.396 | 0.405 | 0.414 | 0.423 | 0.433 | 0.443 | 0.453 | 0.464 | 0.474 | 0.482 |
| Cape Verde | 0.307 | 0.315 | 0.322 | 0.329 | 0.337 | 0.347 | 0.357 | 0.367 | 0.377 | 0.387 | 0.399 | 0.41 | 0.42 | 0.431 | 0.441 | 0.451 | 0.461 | 0.472 | 0.482 | 0.491 | 0.5 | 0.509 | 0.517 | 0.524 | 0.531 | 0.538 | 0.544 | 0.549 |
| Chad | 0.119 | 0.123 | 0.128 | 0.131 | 0.135 | 0.138 | 0.141 | 0.144 | 0.146 | 0.148 | 0.15 | 0.152 | 0.154 | 0.156 | 0.161 | 0.166 | 0.171 | 0.178 | 0.185 | 0.192 | 0.2 | 0.207 | 0.216 | 0.224 | 0.232 | 0.24 | 0.248 | 0.253 |
| Cote d'Ivoire | 0.273 | 0.28 | 0.287 | 0.292 | 0.297 | 0.301 | 0.306 | 0.312 | 0.317 | 0.322 | 0.326 | 0.33 | 0.334 | 0.338 | 0.342 | 0.346 | 0.349 | 0.353 | 0.357 | 0.361 | 0.367 | 0.371 | 0.377 | 0.384 | 0.391 | 0.398 | 0.406 | 0.412 |
| The Gambia | 0.245 | 0.251 | 0.257 | 0.263 | 0.269 | 0.275 | 0.282 | 0.288 | 0.294 | 0.3 | 0.307 | 0.314 | 0.319 | 0.325 | 0.332 | 0.338 | 0.343 | 0.349 | 0.355 | 0.361 | 0.367 | 0.372 | 0.378 | 0.384 | 0.389 | 0.395 | 0.401 | 0.405 |
| Ghana | 0.38 | 0.386 | 0.392 | 0.396 | 0.4 | 0.403 | 0.408 | 0.413 | 0.416 | 0.418 | 0.422 | 0.425 | 0.43 | 0.435 | 0.439 | 0.443 | 0.45 | 0.459 | 0.467 | 0.474 | 0.482 | 0.49 | 0.498 | 0.505 | 0.513 | 0.521 | 0.53 | 0.537 |
| Guinea | 0.164 | 0.168 | 0.171 | 0.175 | 0.178 | 0.183 | 0.188 | 0.194 | 0.2 | 0.206 | 0.211 | 0.217 | 0.222 | 0.228 | 0.234 | 0.24 | 0.246 | 0.252 | 0.26 | 0.266 | 0.273 | 0.281 | 0.288 | 0.295 | 0.303 | 0.31 | 0.318 | 0.325 |
| Guinea-Bissau | 0.183 | 0.189 | 0.194 | 0.199 | 0.205 | 0.211 | 0.217 | 0.224 | 0.229 | 0.235 | 0.241 | 0.246 | 0.252 | 0.257 | 0.263 | 0.268 | 0.274 | 0.28 | 0.286 | 0.293 | 0.3 | 0.308 | 0.315 | 0.322 | 0.329 | 0.336 | 0.343 | 0.349 |
| Liberia | 0.207 | 0.209 | 0.21 | 0.206 | 0.199 | 0.19 | 0.178 | 0.174 | 0.175 | 0.183 | 0.198 | 0.214 | 0.228 | 0.234 | 0.239 | 0.245 | 0.251 | 0.257 | 0.264 | 0.27 | 0.276 | 0.284 | 0.292 | 0.301 | 0.31 | 0.318 | 0.324 | 0.328 |
| Mali | 0.13 | 0.134 | 0.137 | 0.141 | 0.145 | 0.148 | 0.152 | 0.157 | 0.161 | 0.166 | 0.171 | 0.176 | 0.181 | 0.187 | 0.192 | 0.198 | 0.203 | 0.209 | 0.215 | 0.22 | 0.226 | 0.232 | 0.238 | 0.243 | 0.249 | 0.255 | 0.262 | 0.267 |
| Mauritania | 0.299 | 0.307 | 0.314 | 0.321 | 0.328 | 0.335 | 0.341 | 0.346 | 0.351 | 0.356 | 0.359 | 0.363 | 0.367 | 0.371 | 0.377 | 0.382 | 0.39 | 0.398 | 0.406 | 0.412 | 0.42 | 0.427 | 0.435 | 0.442 | 0.45 | 0.458 | 0.465 | 0.471 |
| Niger | 0.092 | 0.097 | 0.101 | 0.105 | 0.108 | 0.111 | 0.113 | 0.115 | 0.118 | 0.121 | 0.123 | 0.125 | 0.127 | 0.13 | 0.134 | 0.137 | 0.14 | 0.144 | 0.147 | 0.151 | 0.156 | 0.16 | 0.165 | 0.17 | 0.176 | 0.181 | 0.186 | 0.191 |
| Nigeria | 0.344 | 0.346 | 0.347 | 0.349 | 0.352 | 0.355 | 0.358 | 0.362 | 0.365 | 0.369 | 0.374 | 0.379 | 0.385 | 0.391 | 0.4 | 0.41 | 0.419 | 0.429 | 0.437 | 0.445 | 0.452 | 0.459 | 0.466 | 0.473 | 0.478 | 0.484 | 0.49 | 0.493 |
| Sao Tome and Principe | 0.287 | 0.292 | 0.296 | 0.3 | 0.305 | 0.309 | 0.314 | 0.32 | 0.326 | 0.332 | 0.338 | 0.345 | 0.353 | 0.362 | 0.371 | 0.381 | 0.39 | 0.399 | 0.408 | 0.416 | 0.426 | 0.435 | 0.444 | 0.453 | 0.463 | 0.472 | 0.481 | 0.488 |

**Appendix Table 10: Socio-Demographic Index values for all estimated GBD 2017 locations, 1990-2017**

| Location | 1990 | 1991 | 1992 | 1993 | 1994 | 1995 | 1996 | 1997 | 1998 | 1999 | 2000 | 2001 | 2002 | 2003 | 2004 | 2005 | 2006 | 2007 | 2008 | 2009 | 2010 | 2011 | 2012 | 2013 | 2014 | 2015 | 2016 | 2017 |
|---|---|---|---|---|---|---|---|---|---|---|---|---|---|---|---|---|---|---|---|---|---|---|---|---|---|---|---|---|
| Senegal | 0.244 | 0.25 | 0.257 | 0.262 | 0.267 | 0.272 | 0.277 | 0.281 | 0.286 | 0.291 | 0.295 | 0.299 | 0.303 | 0.307 | 0.311 | 0.316 | 0.319 | 0.324 | 0.328 | 0.333 | 0.338 | 0.342 | 0.347 | 0.352 | 0.357 | 0.362 | 0.368 | 0.373 |
| Sierra Leone | 0.202 | 0.205 | 0.207 | 0.211 | 0.216 | 0.219 | 0.222 | 0.225 | 0.226 | 0.228 | 0.23 | 0.233 | 0.238 | 0.244 | 0.249 | 0.256 | 0.262 | 0.27 | 0.278 | 0.286 | 0.294 | 0.302 | 0.312 | 0.325 | 0.336 | 0.344 | 0.351 | 0.357 |
| Togo | 0.263 | 0.27 | 0.276 | 0.281 | 0.286 | 0.292 | 0.297 | 0.303 | 0.307 | 0.311 | 0.315 | 0.318 | 0.321 | 0.325 | 0.329 | 0.333 | 0.337 | 0.343 | 0.348 | 0.353 | 0.36 | 0.366 | 0.373 | 0.381 | 0.39 | 0.398 | 0.406 | 0.413 |
