# Supplementary material for: Global, regional, and national comparative risk assessment of 84 behavioural, environmental and occupational, and metabolic risks or clusters of risks for 195 countries and territories, 1990–2017: a systematic analysis for the Global Burden of Disease Study 2017
Source: Lancet. 2018 Nov 10;392(10159):1923–94. doi: 10.1016/S0140-6736(18)32225-6 (PMC6227755; doi:10.1016/S0140-6736(18)32225-6)
Supplement: Supplementary appendix 2 [file mmc2.pdf]

THE LANCET

Supplementary appendix 2

This appendix formed part of the original submission and has been peer reviewed. We post it as supplied by the authors.

Supplement to: GBD 2017 Risk Factor Collaborators. Global, regional, and national comparative risk assessment of 84 behavioural, environmental and occupational, and metabolic risks or clusters of risks for 195 countries and territories, 1990–2017: a systematic analysis for the Global Burden of Disease Study 2017. *Lancet* 2018; **392:** 1923–45.

Supplementary results appendix for Global, regional, and national comparative risk assessment of 84 behavioural, environmental and occupational, and metabolic risks or clusters of risks for 195 countries and territories, 1990-2017: a systematic analysis for the Global Burden of Disease Study 2017

Table of Contents

Supplementary Results Table 1. Age-standardised SEVs for Level 4 risk factors and percentage change, by sex, 1990, 2007, and 2017, by geography. SEV=summary exposure value. To download the data in this table, please visit the Global Health Data Exchange (GHDx) at http://ghdx.healthdata.org/gbd-2017 1

Supplementary Results Table 2. All-age PAFs attributable to the joint distribution of all risk factors, both sexes combined for each GBD Level 1, Level 2, and Level 3 cause and all causes in 2017, by geography. Results are shown for deaths, YLLs, YLDs and DALYs. Data in parentheses are 95% uncertainty intervals. PAFs=population attributable fractions. YLLs=years of life lost. YLDs=years lived with disability. DALYs=disability-adjusted life-years. To download the data in this table, please visit the Global Health Data Exchange (GHDx) at http://ghdx.healthdata.org/gbd-2017 1,259

Supplementary Results Table 3. All-age deaths, death PAFs, DALYs, and DALY PAFs, and percentage change for each risk factor and outcome for both sexes combined in 2007 and 2017, by geography. PAFs=population attributable fractions. DALYs=disability-adjusted life-years. To download the data in this table, please visit the Global Health Data Exchange (GHDx) at http://ghdx.healthdata.org/gbd-2017 5,033

Supplementary Results Figure 1. Relationship between age-standardised SEVs and SDI for all Level 4 risk factors not included in main text figure 1. Each point corresponds to the values in a country for males (blue), females (red), or both sexes (purple) for SEVs that are not sex-specific. Points depict all country-years, 1990 through 2017. Lines show the expected SEV by SDI for each sex. Note that the y-axis scales differ by risk to correspond to the range of observed SEVs. SEV=summary exposure value. SDI=Socio-demographic Index. Handwashing=no access to handwashing facility. Ozone=ambient ozone pollution. Radon=residential radon exposure. Occupational PAH=polycyclic aromatic hydrocarbons. Short gestation=short gestation for birth weight. Low fruit=diet low in fruit. Low vegetables=diet low in vegetables. Low legumes=diet low in legumes. Low whole grains=diet low in whole grains. Low nuts and seeds=diet low in nuts and seeds. Low milk=diet low in milk. High red meat=diet high in red meat. High processed meat=diet high in processed meat. High sweetened beverages=diet high in sugar-sweetened beverages. Low fibre=diet low in fibre. Low calcium=diet low in calcium. Low omega-3=diet low in seafood omega-3 fatty acids. Low PUFA=diet low polyunsaturated fatty acids. High trans fat=diet high in trans fatty acids. High sodium=diet high in sodium. Bullying=bullying victimisation. High LDL= high low-density lipoprotein cholesterol 10,065

Supplementary Results Figure 2. Leading 20 Level 4 risk factors for DALYs by SDI level and sex, 1990, 2007, and 2017. Risk factors are connected by lines between time periods; solid lines are increases and dashed lines are decreases. Environmental and occupational risk factors are shown in green, behavioural risk factors in red, and metabolic risk factors in blue. For the time period of 1990 to 2007 and for 2007 to 2017, three measures of change are shown: percentage change in the number of DALYs, percentage change in the all-age DALY rate, and percentage change in the age-standardised DALY rate. Statistically significant increases or decreases are shown in bold. DALYs=disability-adjusted life-years. SDI=Socio-demographic Index. Handwashing=no access to handwashing facility. High LDL=high low-density lipoprotein cholesterol. Particulate matter=particulate matter pollution........10,072

Supplementary Results Figure 3. Leading 10 Level 3 risk factors for deaths in 2017 by location. Each risk factor is coloured by the percentage change in all-age deaths from 2007 to 2017……10,077

Supplementary Results Figure 4. Leading 10 Level 3 risk factors for DALYs in 2017 by location. Each risk factor is coloured by the percentage change in all-age DALYs from 2007 to 2017. DALYs=disability-adjusted life-years……10,094

Supplementary Results Figure 5. Percentage change in the absolute number of global risk-attributable DALYs, 2007-2017, due to population growth, population ageing, changes in exposure to each GBD Level 1 risk factor, and changes in risk-deleted DALY rates for males (A) and females (B), by Level 2 causes and all causes combined. DALYs=disability-adjusted life-years……10,111

Supplementary Results Figure 6: Leading five risk factors for DALYs with the ratio of observed to expected DALYs based on SDI, by location and sex, in 2017. The ratios of observed to expected DALYs based on SDI are provided in brackets for each risk factor and cells are colour-coded by ratio ranges. Shades of blue represent lower observed DALYs than expected based on SDI, whereas red shows observed DALYs that exceed expected levels. Observed to expected ratios are based on age-standardised DALY rates. DALYs=disability-adjusted life-years. SDI=Socio-demographic Index. Alcohol=alcohol use. Ambient PM=ambient particulate matter pollution. BMI=high body-mass index. Drugs=drug use. Ergonomic=Occupational ergonomic factors. Fruits=diet low in fruits. High LDL=high low-density lipoprotein cholesterol. Household air=household air pollution from solid fuels. IPV=intimate partner violence. Iron=iron deficiency. Kidney=impaired kidney function. Low birth weight=low birth weight for gestation. Nuts and Seeds=diet low in nuts and seeds. FPG=high fasting plasma glucose. SBP=high systolic blood pressure. Sanitation=unsafe sanitation. Short gestation=short gestation for birth weight. Sodium=diet high in sodium. Underweight=child underweight. Wasting=child wasting. Water=unsafe water source. Whole grains=Diet low in whole grains……10,112

Supplementary Results Table 1. Global age-standardised Summary Exposure Values (SEVs) for Level 4 risk factors and percentage change, by sex, 1990, 2007, and 2017.Data in parentheses are 95% uncertainty intervals.

	Male					Female				
	**1990**	**2007**	**2017**	**Percentage change 1990-2007**	**Percentage change 2007-2017**	**1990**	**2007**	**2017**	**Percentage change 1990-2007**	**Percentage change 2007-2017**
Unsafe water source	43.2 (41.2 to 44.8)	36.1 (33.7 to 37.9)	33.6 (30.8 to 35.6)	-16.5 (-19.3 to -14.0)	-6.9 (-9.7 to -3.9)	42.5 (40.4 to 44.0)	35.9 (33.6 to 37.7)	33.9 (31.1 to 35.9)	-15.4 (-18.2 to -12.8)	-5.8 (-8.5 to -2.8)
Unsafe sanitation	58.1 (53.4 to 64.5)	40.9 (36.5 to 47.0)	29.9 (25.7 to 35.4)	-29.6 (-33.2 to -26.1)	-26.9 (-31.8 to -21.9)	57.2 (52.5 to 63.7)	40.8 (36.4 to 46.9)	30.3 (26.0 to 35.9)	-28.6 (-32.2 to -25.2)	-25.8 (-30.6 to -21.0)
No access to handwashing facility	37.8 (36.8 to 38.8)	34.5 (33.4 to 35.6)	32.5 (31.4 to 33.6)	-8.7 (-11.6 to -5.6)	-5.8 (-8.3 to -3.1)	37.3 (36.3 to 38.3)	34.6 (33.5 to 35.6)	33.0 (31.9 to 34.1)	-7.4 (-10.2 to -4.4)	-4.4 (-6.9 to -1.8)
Ambient particulate matter pollution	30.1 (23.5 to 38.6)	39.9 (31.9 to 50.7)	41.9 (33.9 to 52.7)	32.7 (25.6 to 40.4)	5.0 (-0.6 to 10.9)	26.8 (21.0 to 34.7)	36.1 (28.6 to 46.3)	38.5 (30.9 to 49.1)	34.7 (27.6 to 42.8)	6.5 (0.7 to 13.2)
Household air pollution from solid fuels	45.6 (39.2 to 54.2)	31.6 (26.4 to 39.1)	23.9 (19.5 to 30.2)	-30.7 (-34.4 to -26.7)	-24.3 (-28.4 to -20.0)	46.0 (39.7 to 54.6)	33.1 (27.9 to 40.7)	25.7 (21.0 to 32.0)	-28.0 (-31.5 to -24.3)	-22.5 (-26.6 to -18.3)
Ambient ozone pollution	41.7 (18.1 to 51.1)	41.9 (18.1 to 51.4)	42.9 (18.9 to 52.3)	0.4 (0.2 to 0.6)	2.4 (1.8 to 4.1)	41.2 (17.7 to 51.0)	41.5 (17.9 to 51.3)	42.6 (18.7 to 52.3)	0.6 (0.4 to 0.9)	2.6 (1.9 to 4.4)
Residential radon	23.7 (14.8 to 33.9)	23.6 (14.8 to 34.0)	23.7 (14.7 to 34.2)	-0.4 (-3.4 to 2.8)	0.2 (-2.0 to 2.3)	23.7 (14.8 to 33.6)	23.6 (14.8 to 33.9)	23.7 (14.8 to 34.1)	-0.3 (-3.6 to 3.2)	0.1 (-2.3 to 2.4)
Lead exposure	15.7 (11.5 to 19.4)	13.9 (10.0 to 17.6)	11.1 (7.5 to 14.7)	-10.9 (-13.4 to -8.8)	-20.1 (-24.4 to -16.2)	9.6 (5.9 to 13.2)	8.8 (5.3 to 12.4)	7.1 (4.1 to 10.6)	-8.1 (-10.4 to -6.1)	-18.7 (-23.4 to -14.2)
Occupational exposure to asbestos	2.7 (2.3 to 3.1)	2.5 (2.4 to 2.6)	2.4 (2.3 to 2.5)	-7.7 (-15.8 to 3.7)	-4.2 (-8.9 to -0.3)	1.0 (0.8 to 1.3)	0.8 (0.7 to 0.9)	0.7 (0.7 to 0.8)	-18.6 (-31.0 to -2.3)	-6.6 (-13.5 to -0.9)
Occupational exposure to arsenic	0.3 (0.1 to 0.6)	0.3 (0.1 to 0.6)	0.3 (0.1 to 0.6)	2.1 (-1.6 to 13.8)	3.0 (0.3 to 8.7)	0.3 (0.1 to 0.6)	0.3 (0.1 to 0.6)	0.3 (0.1 to 0.6)	3.1 (-1.8 to 18.4)	2.1 (-1.9 to 9.2)
Occupational exposure to benzene	0.5 (0.3 to 1.1)	0.6 (0.3 to 1.2)	0.7 (0.3 to 1.3)	11.1 (7.2 to 19.4)	7.9 (5.7 to 11.9)	0.5 (0.2 to 1.1)	0.6 (0.3 to 1.3)	0.7 (0.3 to 1.4)	18.2 (13.6 to 28.2)	12.4 (8.9 to 18.8)
Occupational exposure to beryllium	0.1 (0.1 to 0.1)	0.1 (0.1 to 0.1)	0.1 (0.1 to 0.1)	8.5 (6.8 to 10.3)	4.8 (3.5 to 6.1)	0.1 (0.1 to 0.1)	0.1 (0.1 to 0.1)	0.1 (0.1 to 0.1)	13.4 (10.8 to 16.0)	6.4 (4.6 to 8.2)
Occupational exposure to cadmium	0.1 (0.1 to 0.1)	0.1 (0.1 to 0.1)	0.1 (0.1 to 0.2)	10.9 (7.8 to 14.2)	7.0 (4.2 to 9.5)	0.1 (0.1 to 0.1)	0.1 (0.1 to 0.1)	0.1 (0.1 to 0.1)	13.7 (8.9 to 18.8)	7.0 (2.9 to 11.7)
Occupational exposure to chromium	0.3 (0.3 to 0.3)	0.3 (0.3 to 0.3)	0.3 (0.3 to 0.3)	14.8 (11.6 to 18.2)	9.6 (6.9 to 12.1)	0.2 (0.2 to 0.2)	0.3 (0.3 to 0.3)	0.3 (0.3 to 0.3)	17.5 (12.3 to 22.8)	10.0 (5.7 to 15.0)
Occupational exposure to diesel engine exhaust	1.5 (1.5 to 1.5)	1.8 (1.8 to 1.9)	2.1 (2.0 to 2.1)	21.6 (18.8 to 24.5)	13.0 (10.7 to 15.2)	0.9 (0.9 to 1.0)	1.1 (1.1 to 1.1)	1.3 (1.2 to 1.3)	19.1 (15.6 to 22.6)	11.9 (8.8 to 15.0)
Occupational exposure to formaldehyde	0.6 (0.6 to 0.6)	0.7 (0.6 to 0.7)	0.7 (0.7 to 0.7)	12.4 (9.0 to 16.1)	8.1 (5.1 to 10.9)	0.5 (0.5 to 0.5)	0.6 (0.5 to 0.6)	0.6 (0.6 to 0.6)	12.8 (7.2 to 18.5)	8.0 (3.5 to 13.0)
Occupational exposure to nickel	0.4 (0.1 to 1.1)	0.4 (0.1 to 1.1)	0.4 (0.1 to 1.1)	0.8 (-3.9 to 11.9)	2.4 (-0.4 to 8.2)	0.3 (0.1 to 0.9)	0.3 (0.1 to 0.8)	0.3 (0.1 to 0.8)	-0.3 (-5.5 to 14.2)	0.2 (-3.5 to 8.5)
Occupational exposure to polycyclic aromatic hydrocarbons	0.6 (0.5 to 0.6)	0.6 (0.6 to 0.7)	0.7 (0.7 to 0.7)	14.8 (11.9 to 18.0)	9.7 (7.2 to 12.0)	0.5 (0.5 to 0.5)	0.6 (0.5 to 0.6)	0.6 (0.6 to 0.6)	17.5 (12.5 to 22.5)	10.5 (6.2 to 15.1)
Occupational exposure to silica	3.7 (1.5 to 9.3)	3.9 (1.7 to 9.3)	4.0 (1.9 to 9.6)	4.0 (0.3 to 11.5)	4.8 (2.2 to 8.8)	2.5 (1.0 to 6.3)	2.4 (1.0 to 5.6)	2.3 (1.0 to 5.5)	-5.4 (-9.1 to 2.2)	-2.1 (-5.2 to 2.8)
Occupational exposure to sulphuric acid	0.7 (0.4 to 1.3)	0.7 (0.4 to 1.4)	0.7 (0.4 to 1.3)	4.2 (0.1 to 10.2)	2.3 (-0.4 to 5.6)	0.6 (0.3 to 1.2)	0.6 (0.4 to 1.2)	0.6 (0.4 to 1.2)	5.3 (0.2 to 12.1)	1.2 (-2.5 to 6.1)
Occupational exposure to trichloroethylene	0.2 (0.2 to 0.2)	0.2 (0.2 to 0.2)	0.2 (0.2 to 0.2)	16.8 (14.3 to 19.7)	10.2 (8.2 to 12.2)	0.1 (0.1 to 0.1)	0.2 (0.2 to 0.2)	0.2 (0.2 to 0.2)	19.6 (15.6 to 23.8)	10.7 (7.4 to 14.6)
Occupational asthmagens	16.1 (13.4 to 19.4)	15.6 (13.0 to 18.8)	15.4 (12.9 to 18.4)	-3.3 (-7.3 to 0.7)	-1.3 (-4.6 to 1.7)	8.5 (6.8 to 10.6)	8.3 (6.7 to 10.2)	8.0 (6.6 to 9.7)	-2.8 (-9.2 to 4.0)	-2.7 (-7.6 to 2.7)
Occupational particulate matter, gases, and fumes	8.5 (6.4 to 11.4)	8.5 (6.5 to 11.4)	8.5 (6.6 to 11.3)	0.5 (-0.7 to 2.1)	-0.2 (-1.2 to 0.8)	5.0 (3.8 to 6.7)	5.2 (4.0 to 7.0)	5.2 (4.0 to 6.9)	4.3 (2.5 to 6.6)	-0.3 (-1.6 to 1.2)
Occupational noise	8.6 (8.2 to 9.1)	8.8 (8.4 to 9.3)	8.9 (8.6 to 9.4)	2.3 (1.3 to 3.3)	1.3 (0.7 to 1.9)	5.2 (5.0 to 5.5)	5.6 (5.3 to 5.9)	5.7 (5.5 to 6.1)	7.2 (5.6 to 8.9)	2.9 (1.9 to 3.8)
Occupational ergonomic factors	17.1 (15.9 to 18.4)	15.9 (14.7 to 17.4)	14.7 (13.5 to 16.1)	-6.9 (-11.2 to -2.8)	-7.5 (-10.6 to -4.4)	11.2 (10.4 to 12.2)	10.5 (9.7 to 11.5)	9.7 (8.7 to 10.7)	-6.3 (-12.8 to 0.5)	-8.4 (-13.3 to -3.4)
Non-exclusive breastfeeding	0.4 (0.3 to 0.6)	0.4 (0.3 to 0.6)	0.4 (0.3 to 0.5)	-7.1 (-9.0 to -5.2)	-4.2 (-6.9 to -1.1)	0.4 (0.3 to 0.6)	0.4 (0.3 to 0.6)	0.4 (0.3 to 0.5)	-6.8 (-8.6 to -5.0)	-4.3 (-6.9 to -1.2)
Discontinued breastfeeding	1.2 (1.1 to 1.2)	1.1 (1.1 to 1.1)	1.1 (1.1 to 1.2)	-6.7 (-8.6 to -4.8)	5.9 (4.4 to 7.4)	1.2 (1.1 to 1.2)	1.1 (1.1 to 1.1)	1.1 (1.1 to 1.2)	-7.3 (-9.0 to -5.4)	6.1 (4.7 to 7.5)
Child underweight	1.6 (1.5 to 1.8)	1.2 (1.1 to 1.4)	0.9 (0.8 to 1.1)	-26.0 (-30.0 to -22.7)	-23.1 (-25.4 to -21.6)	1.6 (1.4 to 1.8)	1.1 (1.0 to 1.3)	0.9 (0.7 to 1.0)	-28.4 (-32.9 to -24.9)	-24.2 (-26.3 to -22.4)
Child wasting	0.6 (0.5 to 0.6)	0.5 (0.4 to 0.6)	0.4 (0.4 to 0.5)	-11.9 (-16.4 to -8.3)	-11.3 (-13.2 to -9.4)	0.5 (0.4 to 0.6)	0.5 (0.4 to 0.5)	0.4 (0.3 to 0.5)	-11.7 (-16.1 to -8.3)	-13.2 (-15.0 to -11.4)
Child stunting	2.7 (1.9 to 3.1)	2.4 (1.6 to 2.6)	1.8 (1.3 to 2.1)	-14.3 (-18.9 to -10.0)	-23.0 (-26.7 to -20.0)	2.7 (1.9 to 3.0)	2.2 (1.6 to 2.5)	1.7 (1.2 to 1.9)	-16.9 (-22.0 to -12.8)	-26.0 (-30.1 to -22.8)
Short gestation for birth weight	0.0 (0.0 to 0.0)	0.0 (0.0 to 0.0)	0.0 (0.0 to 0.0)	18.6 (14.9 to 22.6)	7.4 (3.8 to 10.3)	0.0 (0.0 to 0.0)	0.0 (0.0 to 0.0)	0.0 (0.0 to 0.0)	16.5 (13.1 to 19.7)	5.1 (2.4 to 7.7)
Low birth weight for gestation	0.0 (0.0 to 0.0)	0.0 (0.0 to 0.0)	0.0 (0.0 to 0.0)	2.7 (-2.4 to 8.2)	3.5 (0.6 to 7.0)	0.0 (0.0 to 0.0)	0.0 (0.0 to 0.0)	0.0 (0.0 to 0.0)	2.3 (-1.9 to 7.3)	3.3 (0.7 to 5.6)
Iron deficiency	-	-	-	-	-	12.7 (10.5 to 15.3)	10.8 (8.7 to 13.3)	9.7 (7.7 to 12.2)	-15.0 (-18.6 to -12.0)	-10.0 (-13.0 to -7.3)
Vitamin A deficiency	2.6 (2.2 to 3.0)	2.4 (2.1 to 2.8)	2.2 (1.9 to 2.5)	-5.9 (-7.3 to -4.6)	-10.1 (-12.4 to -7.7)	2.5 (2.2 to 2.9)	2.4 (2.0 to 2.7)	2.1 (1.8 to 2.5)	-5.3 (-6.8 to -4.0)	-9.6 (-12.2 to -7.3)
Zinc deficiency	0.9 (0.3 to 1.8)	0.8 (0.2 to 1.5)	0.6 (0.2 to 1.3)	-18.0 (-23.6 to -8.4)	-14.8 (-22.5 to -8.7)	0.9 (0.3 to 1.8)	0.8 (0.2 to 1.4)	0.6 (0.2 to 1.3)	-18.3 (-23.9 to -9.5)	-14.8 (-22.9 to -8.5)
Smoking	11.3 (10.0 to 12.7)	9.9 (8.7 to 11.1)	8.7 (7.7 to 9.8)	-12.6 (-15.2 to -10.0)	-11.8 (-13.4 to -10.0)	3.0 (2.7 to 3.4)	2.1 (1.9 to 2.4)	1.8 (1.5 to 2.0)	-29.4 (-32.1 to -26.7)	-17.7 (-19.4 to -16.2)
Chewing tobacco	3.9 (3.5 to 4.3)	3.8 (3.6 to 4.1)	3.7 (3.5 to 4.0)	-0.7 (-10.2 to 10.9)	-2.4 (-8.9 to 5.1)	2.3 (2.0 to 2.6)	2.5 (2.3 to 2.8)	2.5 (2.3 to 2.8)	11.0 (-3.1 to 26.4)	0.0 (-9.1 to 10.8)
Second-hand smoke	37.7 (36.7 to 38.7)	31.6 (31.0 to 32.3)	30.3 (29.5 to 31.0)	-16.1 (-18.8 to -13.4)	-4.3 (-6.1 to -2.5)	55.7 (54.8 to 56.7)	46.2 (45.6 to 46.8)	43.1 (42.3 to 43.7)	-17.0 (-18.8 to -15.2)	-6.9 (-7.9 to -5.8)
Alcohol use	14.7 (10.9 to 19.1)	15.6 (11.7 to 19.9)	16.2 (12.1 to 20.6)	6.2 (1.4 to 12.0)	4.0 (-3.3 to 12.1)	5.2 (3.3 to 8.1)	4.8 (3.0 to 7.4)	4.6 (3.0 to 7.2)	-8.3 (-11.6 to -4.8)	-2.6 (-7.8 to 4.0)
Drug use	0.8 (0.7 to 1.0)	0.8 (0.7 to 1.0)	0.9 (0.7 to 1.0)	1.6 (-2.6 to 5.4)	6.5 (2.6 to 10.6)	0.4 (0.3 to 0.5)	0.4 (0.3 to 0.5)	0.4 (0.3 to 0.5)	-2.1 (-6.2 to 1.4)	4.6 (1.9 to 7.4)
Diet low in fruits	41.2 (35.9 to 43.9)	37.8 (32.4 to 41.0)	34.7 (29.0 to 38.3)	-8.2 (-10.8 to -6.4)	-8.1 (-10.7 to -6.1)	39.0 (33.8 to 42.0)	35.4 (29.9 to 38.9)	32.1 (26.6 to 36.1)	-9.3 (-12.2 to -7.1)	-9.1 (-12.0 to -6.8)
Diet low in vegetables	35.0 (31.6 to 38.2)	29.4 (25.2 to 33.3)	25.6 (21.2 to 29.7)	-16.0 (-21.0 to -12.1)	-12.9 (-17.1 to -9.5)	36.4 (33.1 to 39.4)	31.2 (27.0 to 34.9)	27.5 (23.1 to 31.6)	-14.4 (-19.0 to -10.8)	-11.8 (-15.6 to -8.6)
Diet low in legumes	21.1 (17.4 to 24.3)	21.1 (18.1 to 23.9)	19.9 (16.9 to 22.6)	0.3 (-3.8 to 5.8)	-5.9 (-8.6 to -3.7)	24.8 (21.3 to 28.0)	24.5 (21.4 to 27.3)	23.2 (20.2 to 25.8)	-1.1 (-4.0 to 2.8)	-5.5 (-7.4 to -3.5)
Diet low in whole grains	39.2 (36.8 to 41.3)	39.2 (36.8 to 41.3)	38.5 (35.9 to 40.6)	-0.0 (-0.7 to 0.6)	-1.9 (-2.8 to -1.2)	40.2 (37.9 to 42.1)	40.1 (37.8 to 42.0)	39.4 (37.0 to 41.4)	-0.1 (-0.8 to 0.5)	-1.8 (-2.6 to -1.2)
Diet low in nuts and seeds	50.9 (50.1 to 51.6)	48.7 (47.5 to 49.7)	46.7 (45.0 to 48.0)	-4.3 (-5.3 to -3.5)	-4.2 (-5.3 to -3.3)	51.1 (50.3 to 51.7)	49.0 (47.8 to 50.0)	47.1 (45.5 to 48.4)	-4.0 (-4.9 to -3.3)	-3.9 (-5.0 to -3.1)
Diet low in milk	45.5 (43.7 to 47.1)	45.6 (43.7 to 47.1)	45.3 (43.5 to 47.0)	0.1 (-0.2 to 0.3)	-0.5 (-0.8 to -0.2)	45.6 (43.7 to 47.1)	45.7 (43.9 to 47.2)	45.6 (43.8 to 47.1)	0.3 (0.0 to 0.5)	-0.2 (-0.5 to -0.0)
Diet high in red meat	11.9 (10.6 to 13.6)	13.2 (10.8 to 15.3)	15.1 (12.7 to 17.2)	10.7 (-1.3 to 23.0)	14.5 (4.2 to 25.7)	5.7 (4.6 to 7.0)	5.5 (3.8 to 7.3)	6.8 (4.7 to 8.8)	-3.4 (-18.8 to 13.1)	23.7 (7.4 to 42.8)
Diet high in processed meat	5.1 (4.2 to 6.4)	5.0 (4.2 to 6.5)	4.7 (3.6 to 6.3)	-1.9 (-7.7 to 3.4)	-6.9 (-14.1 to -1.1)	3.7 (2.7 to 4.9)	3.6 (2.7 to 4.9)	3.3 (2.4 to 4.8)	-3.1 (-9.8 to 3.2)	-7.0 (-15.2 to -0.5)
Diet high in sugar-sweetened beverages	5.4 (2.8 to 6.1)	5.8 (3.0 to 6.5)	6.6 (3.4 to 7.5)	7.1 (-0.8 to 16.9)	13.8 (7.2 to 22.0)	4.3 (2.2 to 4.9)	4.3 (2.2 to 4.9)	4.7 (2.4 to 5.5)	1.5 (-8.1 to 12.7)	9.7 (2.5 to 18.1)
Diet low in fibre	33.3 (29.0 to 36.8)	31.6 (27.1 to 35.3)	29.9 (25.3 to 33.8)	-5.0 (-7.5 to -3.1)	-5.5 (-8.1 to -3.6)	37.4 (33.8 to 40.3)	36.0 (32.3 to 39.1)	34.5 (30.4 to 37.8)	-3.8 (-5.6 to -2.4)	-4.2 (-6.1 to -2.8)
Diet low in calcium	40.3 (37.5 to 42.6)	38.3 (35.2 to 40.9)	36.2 (32.6 to 39.2)	-4.8 (-6.3 to -3.7)	-5.5 (-7.3 to -4.1)	41.7 (39.1 to 43.8)	40.2 (37.4 to 42.6)	38.5 (35.4 to 41.2)	-3.5 (-4.6 to -2.7)	-4.2 (-5.6 to -3.2)
Diet low in seafood omega-3 fatty acids	44.0 (42.4 to 45.6)	42.1 (40.1 to 44.1)	40.6 (38.2 to 42.8)	-4.4 (-5.5 to -3.4)	-3.6 (-4.8 to -2.6)	44.8 (43.3 to 46.3)	43.4 (41.6 to 45.2)	42.2 (40.1 to 44.3)	-3.2 (-4.0 to -2.4)	-2.7 (-3.7 to -1.9)
Diet low in polyunsaturated fatty acids	39.1 (36.7 to 41.4)	35.3 (32.3 to 38.1)	33.8 (30.6 to 36.8)	-9.8 (-12.8 to -7.2)	-4.2 (-5.8 to -3.0)	39.1 (36.6 to 41.4)	35.6 (32.5 to 38.3)	34.3 (31.1 to 37.2)	-8.9 (-11.8 to -6.6)	-3.7 (-5.2 to -2.6)
Diet high in trans fatty acids	4.3 (2.4 to 6.9)	2.9 (1.2 to 5.4)	2.3 (0.8 to 4.7)	-33.1 (-49.6 to -22.6)	-20.6 (-37.7 to -12.3)	5.7 (3.6 to 8.6)	3.9 (2.0 to 6.7)	3.1 (1.3 to 5.7)	-31.8 (-45.7 to -22.3)	-21.3 (-35.6 to -13.4)
Diet high in sodium	17.3 (12.5 to 22.3)	16.8 (11.8 to 22.0)	16.6 (11.7 to 21.8)	-2.4 (-10.6 to 4.0)	-1.7 (-6.1 to 2.6)	16.9 (12.3 to 21.9)	15.6 (10.8 to 20.9)	14.6 (9.9 to 19.9)	-7.5 (-16.4 to -2.4)	-6.4 (-11.9 to -2.6)
Intimate partner violence	-	-	-	-	-	6.8 (5.7 to 7.9)	6.5 (5.6 to 7.4)	6.5 (5.5 to 7.4)	-4.8 (-8.1 to -1.0)	-0.2 (-2.2 to 1.5)
Childhood sexual abuse	6.2 (5.1 to 7.6)	6.4 (5.3 to 7.8)	6.8 (5.6 to 8.3)	3.6 (1.2 to 6.0)	6.2 (4.2 to 8.1)	6.8 (5.5 to 8.2)	6.5 (5.4 to 7.8)	6.9 (5.6 to 8.3)	-3.9 (-5.8 to -1.9)	5.2 (2.7 to 7.9)

Supplementary Results Table 1. Global age-standardised Summary Exposure Values (SEVs) for Level 4 risk factors and percentage change, by sex, 1990, 2007, and 2017.Data in parentheses are 95% uncertainty intervals.

	Male					Female				
	**1990**	**2007**	**2017**	**Percentage change 1990-2007**	**Percentage change 2007-2017**	**1990**	**2007**	**2017**	**Percentage change 1990-2007**	**Percentage change 2007-2017**
Bullying victimization	5.9 (2.6 to 11.9)	7.1 (3.2 to 14.3)	7.6 (3.4 to 15.3)	20.1 (14.3 to 27.5)	6.4 (4.7 to 8.4)	4.3 (1.9 to 8.7)	5.5 (2.4 to 10.7)	6.0 (2.7 to 11.9)	25.9 (19.7 to 34.5)	9.7 (8.0 to 11.8)
Low physical activity	0.4 (0.2 to 0.7)	0.4 (0.2 to 0.8)	0.4 (0.2 to 0.8)	0.3 (-0.0 to 0.6)	0.7 (0.3 to 1.0)	0.4 (0.2 to 0.7)	0.4 (0.2 to 0.7)	0.4 (0.2 to 0.7)	-1.3 (-2.0 to -0.5)	0.4 (0.2 to 0.7)
High fasting plasma glucose	1.9 (1.5 to 2.5)	2.4 (1.8 to 3.2)	2.7 (2.1 to 3.5)	26.7 (20.0 to 33.7)	11.4 (4.8 to 19.4)	1.8 (1.3 to 2.3)	2.2 (1.6 to 2.9)	2.4 (1.8 to 3.1)	24.5 (17.3 to 31.7)	7.3 (-0.1 to 15.5)
High LDL cholesterol	11.2 (9.8 to 12.8)	10.3 (8.9 to 11.9)	10.2 (8.9 to 11.8)	-8.0 (-9.3 to -6.8)	-0.9 (-1.3 to -0.5)	11.7 (10.4 to 13.2)	11.1 (9.7 to 12.6)	11.2 (9.8 to 12.7)	-5.7 (-6.8 to -4.8)	1.2 (0.8 to 1.6)
High systolic blood pressure	7.1 (6.5 to 7.8)	7.2 (6.5 to 7.9)	7.4 (6.7 to 8.1)	1.3 (-0.7 to 3.1)	2.5 (1.0 to 4.2)	7.2 (6.6 to 7.7)	6.8 (6.3 to 7.4)	6.8 (6.2 to 7.3)	-5.2 (-7.2 to -3.1)	-0.6 (-2.4 to 1.3)
High body-mass index	5.7 (4.0 to 8.1)	8.2 (6.2 to 11.1)	10.0 (7.8 to 13.3)	43.0 (34.2 to 53.9)	22.1 (18.4 to 26.3)	6.5 (4.8 to 8.8)	9.1 (7.0 to 12.0)	10.9 (8.5 to 14.2)	40.2 (32.7 to 49.6)	19.4 (16.0 to 23.2)
Low bone mineral density	5.3 (4.5 to 6.1)	5.2 (4.4 to 5.9)	5.1 (4.3 to 5.9)	-2.9 (-4.1 to -1.8)	-1.0 (-2.4 to 0.2)	6.9 (6.1 to 7.8)	6.9 (6.0 to 7.8)	6.9 (5.9 to 7.9)	-0.6 (-1.7 to 0.6)	0.0 (-1.6 to 1.8)
Impaired kidney function	2.5 (1.1 to 6.1)	2.5 (1.1 to 6.2)	2.5 (1.1 to 6.2)	0.9 (-1.3 to 3.1)	0.3 (-1.4 to 2.5)	2.9 (1.4 to 6.8)	2.9 (1.4 to 6.9)	3.0 (1.4 to 7.0)	0.3 (-2.4 to 2.6)	1.7 (-0.0 to 4.0)

Supplementary Results Table 1. Low SDI age-standardised Summary Exposure Values (SEVs) for Level 4 risk factors and percentage change, by sex, 1990, 2007, and 2017.Data in parentheses are 95% uncertainty intervals.

	Male					Female				
	1990	2007	2017	Percentage change 1990-2007	Percentage change 2007-2017	1990	2007	2017	Percentage change 1990-2007	Percentage change 2007-2017
Unsafe water source	81.5 (76.4 to 88.0)	75.7 (69.7 to 82.8)	72.9 (66.4 to 80.8)	-7.2 (-9.1 to -5.7)	-3.6 (-5.4 to -1.8)	81.6 (76.5 to 87.9)	75.7 (69.8 to 82.8)	72.8 (66.3 to 80.6)	-7.2 (-9.1 to -5.6)	-3.8 (-5.6 to -2.0)
Unsafe sanitation	89.4 (83.2 to 97.3)	81.1 (74.2 to 90.8)	66.6 (58.5 to 77.2)	-9.2 (-11.8 to -6.5)	-17.9 (-22.9 to -13.0)	89.3 (83.0 to 97.4)	81.2 (74.2 to 91.1)	67.0 (58.8 to 77.6)	-9.1 (-11.6 to -6.3)	-17.5 (-22.3 to -12.8)
No access to handwashing facility	79.5 (76.7 to 82.6)	77.4 (74.5 to 80.3)	74.7 (71.7 to 77.8)	-2.7 (-6.7 to 1.4)	-3.4 (-6.4 to -0.2)	80.1 (77.5 to 83.0)	77.8 (75.1 to 80.6)	75.1 (72.2 to 78.0)	-2.9 (-6.6 to 0.9)	-3.5 (-6.3 to -0.4)
Ambient particulate matter pollution	23.0 (15.9 to 32.3)	29.4 (21.7 to 40.1)	36.0 (26.8 to 47.6)	27.8 (1.6 to 61.0)	22.4 (0.9 to 49.3)	17.9 (12.1 to 26.1)	23.6 (16.7 to 33.0)	30.1 (21.8 to 41.0)	31.8 (3.3 to 68.2)	27.3 (3.5 to 58.0)
Household air pollution from solid fuels	82.8 (73.2 to 92.5)	73.8 (64.6 to 84.5)	61.5 (52.7 to 72.5)	-10.9 (-18.1 to -3.1)	-16.7 (-23.1 to -9.9)	84.3 (75.7 to 93.4)	76.3 (67.4 to 86.4)	64.3 (55.5 to 74.7)	-9.5 (-16.3 to -2.8)	-15.8 (-21.9 to -9.4)
Ambient ozone pollution	42.0 (18.0 to 51.1)	42.0 (17.6 to 51.5)	42.7 (17.7 to 52.5)	-0.2 (-2.5 to 0.8)	1.7 (0.3 to 2.6)	41.3 (17.5 to 50.9)	41.7 (17.4 to 51.5)	42.4 (17.5 to 52.5)	0.9 (-0.9 to 2.0)	1.9 (0.5 to 2.8)
Residential radon	28.1 (15.0 to 43.3)	27.7 (14.9 to 42.6)	27.5 (14.8 to 42.1)	-1.4 (-3.3 to 0.7)	-0.8 (-2.0 to 0.7)	27.7 (14.8 to 42.3)	27.4 (14.8 to 41.9)	27.2 (14.8 to 41.4)	-0.8 (-2.1 to 0.6)	-0.8 (-1.7 to 0.4)
Lead exposure	24.7 (20.1 to 28.9)	24.1 (19.7 to 27.9)	20.4 (16.3 to 24.1)	-2.5 (-4.4 to -0.6)	-15.2 (-18.2 to -12.7)	16.3 (11.9 to 20.2)	16.3 (12.0 to 20.1)	13.9 (9.9 to 17.6)	0.0 (-2.1 to 2.5)	-14.7 (-18.3 to -11.7)
Occupational exposure to asbestos	0.7 (0.4 to 1.4)	0.7 (0.5 to 1.2)	0.9 (0.6 to 1.2)	3.6 (-17.3 to 32.9)	16.2 (-3.0 to 42.9)	0.7 (0.3 to 1.3)	0.5 (0.3 to 0.9)	0.5 (0.3 to 0.8)	-32.6 (-41.5 to -12.4)	3.2 (-10.5 to 34.0)
Occupational exposure to arsenic	0.3 (0.1 to 0.4)	0.3 (0.1 to 0.5)	0.3 (0.1 to 0.5)	8.8 (5.3 to 12.5)	5.9 (2.8 to 9.2)	0.2 (0.1 to 0.3)	0.2 (0.1 to 0.3)	0.2 (0.1 to 0.3)	4.2 (-1.5 to 10.0)	13.7 (9.2 to 18.5)
Occupational exposure to benzene	0.5 (0.3 to 0.9)	0.6 (0.3 to 1.0)	0.6 (0.4 to 1.1)	12.5 (9.8 to 16.0)	8.5 (6.3 to 11.2)	0.3 (0.2 to 0.5)	0.4 (0.2 to 0.6)	0.4 (0.3 to 0.8)	21.7 (16.8 to 27.7)	23.8 (20.0 to 28.7)
Occupational exposure to beryllium	0.1 (0.1 to 0.1)	0.1 (0.1 to 0.1)	0.1 (0.1 to 0.1)	5.6 (3.2 to 8.3)	1.6 (-0.1 to 3.3)	0.1 (0.1 to 0.1)	0.1 (0.1 to 0.1)	0.1 (0.1 to 0.1)	7.0 (2.2 to 11.4)	10.2 (7.0 to 13.3)
Occupational exposure to cadmium	0.1 (0.1 to 0.1)	0.1 (0.1 to 0.2)	0.2 (0.2 to 0.2)	9.1 (5.8 to 12.5)	5.9 (3.1 to 8.9)	0.1 (0.1 to 0.1)	0.1 (0.1 to 0.1)	0.1 (0.1 to 0.1)	5.3 (-0.2 to 10.7)	13.9 (9.7 to 18.4)
Occupational exposure to chromium	0.3 (0.3 to 0.3)	0.3 (0.3 to 0.3)	0.3 (0.3 to 0.4)	14.3 (10.7 to 18.2)	10.0 (7.0 to 13.2)	0.2 (0.2 to 0.2)	0.2 (0.2 to 0.2)	0.2 (0.2 to 0.2)	8.8 (3.0 to 14.8)	18.3 (13.8 to 23.1)
Occupational exposure to diesel engine exhaust	1.8 (1.7 to 1.8)	2.2 (2.2 to 2.3)	2.5 (2.4 to 2.6)	25.1 (21.0 to 29.6)	12.9 (9.2 to 16.7)	0.8 (0.8 to 0.9)	0.9 (0.9 to 1.0)	1.1 (1.0 to 1.1)	14.0 (8.9 to 18.8)	15.6 (12.2 to 19.1)
Occupational exposure to formaldehyde	0.6 (0.5 to 0.6)	0.6 (0.6 to 0.7)	0.7 (0.7 to 0.7)	11.9 (7.9 to 16.1)	9.6 (6.0 to 13.3)	0.4 (0.3 to 0.4)	0.4 (0.4 to 0.4)	0.5 (0.4 to 0.5)	6.1 (-0.4 to 12.6)	18.3 (13.6 to 23.2)
Occupational exposure to nickel	0.3 (0.1 to 0.8)	0.4 (0.1 to 0.9)	0.4 (0.1 to 1.0)	10.6 (6.9 to 14.4)	4.2 (0.9 to 7.7)	0.2 (0.1 to 0.5)	0.2 (0.1 to 0.5)	0.2 (0.1 to 0.6)	0.8 (-4.6 to 6.1)	8.3 (4.3 to 12.7)
Occupational exposure to polycyclic aromatic hydrocarbons	0.6 (0.6 to 0.6)	0.7 (0.7 to 0.7)	0.7 (0.7 to 0.8)	15.0 (11.3 to 18.7)	9.7 (6.8 to 12.8)	0.4 (0.3 to 0.4)	0.4 (0.4 to 0.4)	0.5 (0.4 to 0.5)	9.0 (3.3 to 14.7)	17.7 (13.4 to 22.2)
Occupational exposure to silica	3.7 (1.9 to 8.2)	4.4 (2.3 to 9.6)	4.8 (2.5 to 10.4)	18.5 (13.5 to 24.9)	7.4 (2.4 to 12.8)	2.0 (1.0 to 4.2)	1.9 (1.0 to 4.3)	2.0 (1.0 to 4.4)	-1.5 (-6.6 to 3.7)	3.3 (-0.6 to 7.6)
Occupational exposure to sulphuric acid	0.6 (0.5 to 1.1)	0.7 (0.5 to 1.2)	0.7 (0.5 to 1.2)	6.2 (3.2 to 9.2)	2.8 (0.4 to 5.2)	0.4 (0.3 to 0.7)	0.4 (0.3 to 0.8)	0.5 (0.3 to 0.8)	4.7 (-0.5 to 9.6)	10.4 (6.6 to 14.3)
Occupational exposure to trichloroethylene	0.2 (0.2 to 0.2)	0.2 (0.2 to 0.2)	0.2 (0.2 to 0.2)	15.0 (11.9 to 18.3)	10.3 (7.9 to 12.8)	0.1 (0.1 to 0.1)	0.1 (0.1 to 0.1)	0.1 (0.1 to 0.1)	12.0 (6.6 to 17.6)	18.6 (14.8 to 22.6)
Occupational asthmagens	17.6 (13.5 to 22.4)	17.9 (14.0 to 22.4)	17.0 (13.6 to 21.0)	1.8 (-4.4 to 8.7)	-5.1 (-10.6 to 0.2)	7.9 (6.0 to 10.4)	8.3 (6.3 to 10.8)	8.2 (6.4 to 10.5)	4.6 (-3.0 to 12.6)	-1.1 (-7.0 to 4.9)
Occupational particulate matter, gases, and fumes	10.2 (7.8 to 13.6)	10.4 (7.9 to 13.8)	10.3 (8.0 to 13.7)	1.6 (0.0 to 3.2)	-0.4 (-1.5 to 0.7)	4.5 (3.4 to 6.3)	4.9 (3.7 to 6.8)	5.1 (3.9 to 6.9)	8.6 (5.0 to 12.5)	3.7 (1.5 to 5.7)
Occupational noise	10.6 (10.1 to 11.2)	10.8 (10.4 to 11.4)	10.9 (10.5 to 11.4)	2.1 (0.7 to 3.4)	0.3 (-0.4 to 1.1)	4.5 (4.2 to 4.9)	5.0 (4.7 to 5.3)	5.2 (5.0 to 5.6)	9.9 (6.7 to 12.9)	5.5 (3.5 to 7.2)
Occupational ergonomic factors	27.0 (25.3 to 28.7)	26.0 (24.6 to 27.6)	23.1 (21.6 to 24.7)	-3.8 (-9.3 to 2.1)	-11.2 (-15.4 to -6.7)	13.6 (12.7 to 14.6)	13.8 (13.0 to 14.6)	13.0 (12.1 to 13.9)	0.9 (-5.5 to 7.5)	-5.6 (-10.0 to -1.1)
Non-exclusive breastfeeding	0.4 (0.2 to 0.6)	0.3 (0.2 to 0.5)	0.3 (0.2 to 0.5)	-5.6 (-8.4 to -3.1)	-5.0 (-8.9 to 1.0)	0.4 (0.2 to 0.6)	0.3 (0.2 to 0.5)	0.3 (0.2 to 0.5)	-5.5 (-8.2 to -2.9)	-5.1 (-8.9 to 0.8)
Discontinued breastfeeding	0.5 (0.5 to 0.5)	0.5 (0.5 to 0.5)	0.6 (0.6 to 0.6)	-6.7 (-10.2 to -3.2)	26.8 (23.1 to 30.6)	0.5 (0.5 to 0.5)	0.5 (0.5 to 0.5)	0.6 (0.6 to 0.6)	-6.9 (-10.4 to -3.6)	26.3 (22.6 to 30.0)
Child underweight	3.2 (2.9 to 3.5)	2.1 (1.9 to 2.3)	1.5 (1.3 to 1.7)	-33.6 (-39.1 to -29.3)	-27.9 (-30.9 to -25.4)	3.0 (2.7 to 3.4)	2.0 (1.7 to 2.2)	1.4 (1.2 to 1.6)	-34.8 (-40.6 to -29.9)	-29.9 (-33.3 to -27.0)
Child wasting	0.9 (0.8 to 1.1)	0.7 (0.6 to 0.8)	0.6 (0.5 to 0.7)	-29.3 (-34.6 to -25.0)	-7.1 (-10.3 to -3.4)	0.8 (0.7 to 0.9)	0.6 (0.5 to 0.7)	0.5 (0.4 to 0.6)	-27.5 (-32.9 to -22.6)	-12.8 (-16.0 to -9.3)
Child stunting	4.6 (3.1 to 4.9)	3.6 (2.5 to 3.9)	2.8 (1.9 to 3.1)	-20.7 (-25.3 to -16.7)	-22.4 (-26.6 to -18.6)	4.4 (3.0 to 4.8)	3.4 (2.4 to 3.7)	2.6 (1.8 to 2.9)	-22.5 (-27.1 to -18.4)	-25.6 (-30.1 to -21.3)
Short gestation for birth weight	0.0 (0.0 to 0.0)	0.0 (0.0 to 0.0)	0.0 (0.0 to 0.0)	3.1 (-1.4 to 7.9)	4.9 (-0.5 to 9.1)	0.0 (0.0 to 0.0)	0.0 (0.0 to 0.0)	0.0 (0.0 to 0.0)	2.3 (-1.8 to 7.9)	2.7 (-1.7 to 7.2)
Low birth weight for gestation	0.0 (0.0 to 0.0)	0.0 (0.0 to 0.0)	0.0 (0.0 to 0.0)	10.8 (6.2 to 16.6)	5.6 (0.9 to 11.8)	0.0 (0.0 to 0.0)	0.0 (0.0 to 0.0)	0.0 (0.0 to 0.0)	12.5 (7.6 to 17.5)	5.8 (1.1 to 10.9)
Iron deficiency	-	-	-	-	-	20.6 (17.9 to 23.6)	17.8 (14.9 to 21.0)	15.0 (12.3 to 18.1)	-13.5 (-18.4 to -9.5)	-15.5 (-19.3 to -12.3)
Vitamin A deficiency	3.6 (3.1 to 4.2)	3.4 (2.9 to 3.9)	2.9 (2.5 to 3.4)	-6.8 (-9.2 to -3.7)	-14.6 (-17.8 to -11.3)	3.5 (3.0 to 4.1)	3.3 (2.8 to 3.8)	2.8 (2.4 to 3.3)	-6.5 (-9.1 to -3.8)	-14.1 (-17.8 to -10.7)
Zinc deficiency	1.2 (0.4 to 2.2)	1.1 (0.4 to 1.9)	0.9 (0.3 to 1.7)	-5.9 (-18.4 to 19.1)	-20.5 (-37.4 to -7.2)	1.2 (0.4 to 2.1)	1.1 (0.4 to 1.9)	0.9 (0.3 to 1.7)	-6.3 (-18.4 to 17.6)	-19.8 (-37.9 to -6.0)
Smoking	6.2 (5.1 to 7.5)	5.4 (4.5 to 6.4)	4.8 (4.0 to 5.7)	-13.1 (-18.8 to -6.7)	-11.0 (-15.2 to -6.6)	0.7 (0.6 to 0.9)	0.7 (0.6 to 0.8)	0.6 (0.5 to 0.7)	-3.2 (-17.7 to 12.9)	-11.1 (-20.5 to -0.8)
Chewing tobacco	12.7 (11.4 to 14.1)	12.3 (11.7 to 13.0)	10.8 (10.1 to 11.6)	-3.4 (-13.2 to 7.9)	-11.9 (-18.2 to -4.8)	6.2 (5.4 to 7.2)	6.4 (5.8 to 7.0)	6.2 (5.5 to 6.9)	2.8 (-11.1 to 18.6)	-3.0 (-13.6 to 8.5)
Second-hand smoke	33.7 (32.8 to 34.7)	29.1 (28.5 to 29.6)	28.4 (27.3 to 29.4)	-13.8 (-17.3 to -10.5)	-2.4 (-5.3 to 0.7)	47.0 (46.1 to 48.1)	39.9 (39.4 to 40.4)	38.4 (37.5 to 39.3)	-15.1 (-17.8 to -12.7)	-3.8 (-5.6 to -2.1)
Alcohol use	7.3 (5.3 to 9.4)	8.5 (6.5 to 10.5)	9.0 (6.7 to 11.3)	16.0 (6.5 to 30.8)	6.3 (-3.6 to 15.8)	2.3 (1.5 to 3.4)	2.3 (1.5 to 3.5)	2.3 (1.5 to 3.6)	3.9 (-3.8 to 10.1)	0.4 (-8.2 to 7.5)
Drug use	0.4 (0.3 to 0.5)	0.4 (0.3 to 0.5)	0.5 (0.4 to 0.6)	11.4 (7.8 to 15.2)	9.5 (6.1 to 13.3)	0.3 (0.2 to 0.3)	0.3 (0.2 to 0.4)	0.3 (0.2 to 0.4)	5.0 (2.4 to 7.5)	3.0 (0.4 to 5.4)
Diet low in fruits	46.6 (42.1 to 48.6)	46.4 (41.7 to 48.4)	44.3 (39.4 to 46.7)	-0.5 (-1.2 to 0.2)	-4.5 (-6.0 to -3.4)	45.3 (40.6 to 47.4)	44.9 (40.2 to 47.1)	42.6 (37.6 to 45.2)	-0.9 (-1.8 to -0.1)	-5.1 (-6.7 to -3.8)
Diet low in vegetables	46.3 (44.5 to 47.9)	44.7 (42.6 to 46.6)	40.6 (37.6 to 43.3)	-3.5 (-5.2 to -2.0)	-9.1 (-12.0 to -6.7)	47.1 (45.6 to 48.6)	45.8 (43.9 to 47.5)	42.1 (39.4 to 44.5)	-2.8 (-4.1 to -1.5)	-8.1 (-10.8 to -6.0)
Diet low in legumes	9.5 (7.5 to 11.5)	8.9 (7.1 to 10.6)	7.9 (6.0 to 9.6)	-6.0 (-8.7 to -3.1)	-11.8 (-17.3 to -8.0)	11.6 (9.3 to 14.0)	10.8 (8.8 to 12.8)	9.8 (7.9 to 11.5)	-6.9 (-10.2 to -4.0)	-9.5 (-12.6 to -6.7)
Diet low in whole grains	36.3 (33.4 to 38.7)	36.8 (34.0 to 39.2)	36.0 (33.0 to 38.6)	1.5 (0.0 to 3.0)	-2.2 (-3.5 to -0.9)	37.5 (34.8 to 39.8)	37.9 (35.2 to 40.1)	36.9 (34.1 to 39.3)	1.0 (-0.3 to 2.3)	-2.5 (-3.9 to -1.3)
Diet low in nuts and seeds	52.7 (52.3 to 53.1)	52.1 (51.6 to 52.6)	50.9 (50.1 to 51.6)	-1.1 (-1.4 to -0.8)	-2.4 (-3.0 to -1.9)	52.8 (52.4 to 53.1)	52.3 (51.7 to 52.7)	51.1 (50.3 to 51.8)	-1.0 (-1.3 to -0.7)	-2.3 (-2.9 to -1.8)
Diet low in milk	51.5 (50.9 to 52.1)	51.1 (50.3 to 51.7)	50.5 (49.6 to 51.2)	-0.8 (-1.2 to -0.5)	-1.2 (-1.7 to -0.8)	51.8 (51.1 to 52.3)	51.4 (50.6 to 52.0)	50.8 (50.0 to 51.5)	-0.8 (-1.1 to -0.5)	-1.1 (-1.4 to -0.7)
Diet high in red meat	0.0 (0.0 to 0.1)	0.0 (0.0 to 0.1)	0.1 (0.0 to 0.1)	62.8 (-4.1 to 3136.9)	38.2 (-29.2 to 129.5)	0.0 (0.0 to 0.0)	0.0 (0.0 to 0.0)	0.0 (0.0 to 0.0)	850.7 (-25.3 to [illegible]	39.8 (-92.6 to 4906.8)
Diet high in processed meat	0.1 (0.0 to 0.9)	0.2 (0.0 to 1.0)	0.3 (0.0 to 1.3)	18.9 (-91.4 to 179.7)	51.3 (24.9 to 7819.6)	0.1 (0.0 to 0.7)	0.1 (0.0 to 0.8)	0.2 (0.0 to 1.0)	19.1 (-100.0 to 126.3)	52.0 (26.1 to [illegible]
Diet high in sugar-sweetened beverages	0.5 (0.3 to 0.7)	0.6 (0.3 to 0.8)	0.8 (0.4 to 1.1)	7.0 (-9.6 to 26.2)	41.1 (23.4 to 64.5)	0.4 (0.2 to 0.6)	0.5 (0.2 to 0.7)	0.6 (0.3 to 0.9)	5.8 (-12.9 to 28.1)	34.2 (17.2 to 56.6)
Diet low in fibre	33.3 (29.2 to 36.7)	32.2 (28.1 to 35.7)	29.5 (25.1 to 33.3)	-3.2 (-5.6 to -1.3)	-8.5 (-11.9 to -6.0)	36.8 (33.3 to 39.8)	36.3 (32.7 to 39.4)	34.0 (30.1 to 37.4)	-1.5 (-3.1 to 0.2)	-6.4 (-9.1 to -4.3)
Diet low in calcium	49.7 (48.6 to 50.5)	48.8 (47.5 to 49.8)	46.5 (44.8 to 47.9)	-1.8 (-2.4 to -1.4)	-4.6 (-5.9 to -3.7)	50.8 (50.0 to 51.5)	50.0 (49.1 to 50.8)	48.0 (46.6 to 49.1)	-1.6 (-2.1 to -1.2)	-4.0 (-5.0 to -3.2)
Diet low in seafood omega-3 fatty acids	52.1 (51.6 to 52.7)	51.9 (51.4 to 52.5)	51.4 (50.8 to 52.1)	-0.3 (-0.4 to -0.1)	-1.0 (-1.3 to -0.8)	52.4 (52.0 to 52.9)	52.3 (51.9 to 52.8)	51.9 (51.3 to 52.5)	-0.2 (-0.3 to -0.1)	-0.9 (-1.1 to -0.7)
Diet low in polyunsaturated fatty acids	50.0 (48.9 to 51.0)	46.8 (45.3 to 48.1)	46.1 (44.5 to 47.6)	-6.4 (-8.1 to -4.4)	-1.5 (-2.4 to -0.5)	50.2 (49.1 to 51.1)	47.0 (45.6 to 48.3)	46.2 (44.6 to 47.6)	-6.3 (-7.9 to -4.5)	-1.7 (-2.6 to -0.9)
Diet high in trans fatty acids	3.3 (1.1 to 6.1)	2.7 (0.8 to 5.5)	2.4 (0.6 to 5.0)	-16.4 (-29.9 to -10.6)	-12.6 (-24.8 to -7.7)	4.2 (1.8 to 7.3)	3.6 (1.4 to 6.6)	3.2 (1.1 to 6.0)	-14.7 (-25.8 to -9.4)	-12.1 (-21.9 to -7.6)
Diet high in sodium	4.0 (0.2 to 9.6)	3.8 (0.2 to 9.3)	4.4 (0.4 to 9.8)	-3.6 (-37.9 to 34.9)	14.4 (3.4 to 172.3)	4.8 (0.4 to 10.7)	4.3 (0.3 to 10.1)	4.7 (0.6 to 10.4)	-10.1 (-43.2 to 10.1)	9.4 (1.4 to 153.8)
Intimate partner violence	-	-	-	-	-	13.8 (11.7 to 15.8)	12.8 (11.2 to 14.3)	11.9 (10.2 to 13.7)	-7.3 (-12.1 to -1.8)	-6.6 (-10.2 to -3.5)
Childhood sexual abuse	10.8 (8.9 to 13.2)	10.5 (8.9 to 12.4)	10.5 (8.8 to 12.5)	-3.2 (-7.6 to 1.1)	0.1 (-1.7 to 2.1)	9.1 (7.4 to 10.8)	8.8 (7.3 to 10.5)	9.0 (7.3 to 10.9)	-2.6 (-5.5 to 0.3)	1.7 (-1.7 to 4.5)

Supplementary Results Table 1. Low SDI age-standardised Summary Exposure Values (SEVs) for Level 4 risk factors and percentage change, by sex, 1990, 2007, and 2017.Data in parentheses are 95% uncertainty intervals.										
	**Male**					**Female**				
	**1990**	**2007**	**2017**	**Percentage change 1990-2007**	**Percentage change 2007-2017**	**1990**	**2007**	**2017**	**Percentage change 1990-2007**	**Percentage change 2007-2017**
Bullying victimization	5.9 (2.5 to 11.6)	6.9 (3.0 to 13.7)	7.6 (3.3 to 15.4)	16.8 (9.7 to 25.0)	10.2 (7.1 to 13.5)	3.9 (1.7 to 8.0)	5.2 (2.2 to 10.4)	6.0 (2.6 to 12.3)	31.1 (22.0 to 41.7)	17.4 (14.0 to 21.2)
Low physical activity	0.4 (0.2 to 0.7)	0.4 (0.2 to 0.7)	0.4 (0.2 to 0.7)	1.6 (0.7 to 2.5)	1.2 (0.5 to 1.9)	0.3 (0.1 to 0.6)	0.3 (0.1 to 0.6)	0.3 (0.1 to 0.6)	0.0 (-0.5 to 0.6)	1.1 (0.5 to 1.8)
High fasting plasma glucose	2.0 (1.5 to 2.8)	2.5 (1.8 to 3.4)	2.4 (1.8 to 3.2)	24.8 (10.9 to 39.6)	-3.8 (-15.4 to 9.1)	1.7 (1.2 to 2.3)	2.1 (1.5 to 2.9)	2.3 (1.7 to 3.0)	24.6 (12.5 to 38.5)	9.0 (-1.7 to 21.9)
High LDL cholesterol	5.8 (4.7 to 7.1)	6.1 (4.9 to 7.4)	6.3 (5.1 to 7.6)	5.3 (3.9 to 7.0)	2.6 (1.5 to 3.8)	6.3 (5.1 to 7.5)	6.7 (5.5 to 8.0)	7.1 (5.9 to 8.4)	7.2 (5.8 to 9.0)	5.8 (4.6 to 7.1)
High systolic blood pressure	5.7 (5.0 to 6.3)	6.1 (5.5 to 6.7)	6.4 (5.7 to 7.0)	7.8 (5.4 to 10.2)	4.2 (2.5 to 5.9)	6.2 (5.7 to 6.8)	6.5 (5.9 to 7.1)	6.5 (5.9 to 7.1)	4.3 (1.7 to 6.8)	0.5 (-1.6 to 2.6)
High body-mass index	2.6 (1.7 to 4.0)	3.5 (2.5 to 5.3)	4.5 (3.3 to 6.6)	38.4 (27.2 to 51.5)	27.3 (21.5 to 35.0)	3.0 (2.0 to 4.4)	4.5 (3.2 to 6.3)	5.8 (4.3 to 8.1)	48.1 (35.8 to 64.2)	29.2 (22.9 to 37.0)
Low bone mineral density	6.0 (5.1 to 6.9)	5.9 (5.1 to 6.7)	5.8 (4.9 to 6.7)	-2.1 (-6.0 to 1.4)	-1.6 (-5.4 to 2.6)	7.9 (6.9 to 9.2)	7.7 (6.7 to 8.9)	7.7 (6.7 to 8.9)	-2.3 (-5.4 to 0.9)	-0.4 (-3.8 to 3.1)
Impaired kidney function	2.4 (1.1 to 6.1)	2.5 (1.2 to 6.3)	2.5 (1.2 to 6.3)	3.9 (1.1 to 6.7)	0.5 (-2.0 to 2.9)	2.8 (1.4 to 6.7)	2.9 (1.4 to 6.8)	2.9 (1.5 to 6.8)	1.4 (-1.2 to 4.3)	2.6 (0.1 to 6.0)

Supplementary Results Table 1. Low-middle SDI age-standardised Summary Exposure Values (SEVs) for Level 4 risk factors and percentage change, by sex, 1990, 2007, and 2017.Data in parentheses are 95% uncertainty intervals.

	Male					Female				
	**1990**	**2007**	**2017**	**Percentage change 1990-2007**	**Percentage change 2007-2017**	**1990**	**2007**	**2017**	**Percentage change 1990-2007**	**Percentage change 2007-2017**
Unsafe water source	63.6 (60.4 to 66.0)	52.5 (49.1 to 55.2)	50.1 (45.9 to 53.6)	-17.5 (-19.6 to -15.6)	-4.5 (-7.0 to -1.7)	63.4 (60.3 to 65.8)	52.5 (49.1 to 55.1)	50.5 (46.3 to 54.0)	-17.2 (-19.3 to -15.3)	-3.9 (-6.4 to -0.9)
Unsafe sanitation	78.3 (72.8 to 86.1)	61.0 (54.8 to 69.8)	41.5 (35.8 to 49.1)	-22.2 (-25.7 to -18.4)	-32.0 (-37.0 to -27.1)	78.2 (72.6 to 86.0)	61.2 (54.9 to 70.2)	41.9 (36.2 to 49.7)	-21.7 (-25.3 to -17.9)	-31.5 (-36.5 to -26.7)
No access to handwashing facility	59.0 (56.4 to 61.4)	51.7 (49.3 to 54.0)	47.4 (45.3 to 49.5)	-12.4 (-16.3 to -8.3)	-8.3 (-11.4 to -4.8)	58.9 (56.4 to 61.3)	51.8 (49.5 to 54.0)	48.2 (46.1 to 50.2)	-12.1 (-15.9 to -8.1)	-7.0 (-10.2 to -3.7)
Ambient particulate matter pollution	25.0 (18.5 to 34.2)	34.7 (26.6 to 45.5)	40.9 (31.7 to 54.3)	38.7 (16.3 to 64.4)	17.9 (-0.3 to 40.4)	21.0 (15.2 to 29.3)	29.9 (22.5 to 39.6)	36.2 (27.3 to 48.8)	42.5 (17.7 to 70.3)	21.1 (1.4 to 47.3)
Household air pollution from solid fuels	70.7 (61.7 to 80.3)	50.4 (42.4 to 62.7)	35.5 (29.2 to 45.1)	-28.7 (-34.7 to -20.8)	-29.6 (-36.8 to -22.2)	71.9 (62.7 to 81.3)	52.4 (44.6 to 63.7)	37.8 (31.0 to 47.4)	-27.0 (-32.6 to -19.5)	-27.9 (-35.0 to -21.1)
Ambient ozone pollution	41.6 (17.1 to 51.4)	42.1 (17.3 to 51.8)	43.4 (18.2 to 52.7)	1.1 (0.5 to 1.7)	3.1 (1.6 to 5.6)	41.2 (16.9 to 51.1)	41.7 (17.1 to 51.6)	43.0 (17.9 to 52.7)	1.2 (0.7 to 1.7)	3.1 (1.8 to 5.2)
Residential radon	26.4 (15.7 to 39.6)	26.0 (15.5 to 38.9)	25.7 (15.0 to 38.5)	-1.7 (-4.1 to 0.5)	-1.2 (-3.0 to 0.7)	26.2 (15.7 to 39.5)	25.8 (15.4 to 38.9)	25.4 (14.9 to 38.2)	-1.7 (-4.3 to 0.8)	-1.7 (-3.9 to 0.4)
Lead exposure	19.2 (14.8 to 23.1)	17.8 (13.5 to 21.6)	14.5 (10.5 to 18.3)	-7.6 (-9.7 to -6.0)	-18.2 (-22.4 to -14.8)	13.6 (9.3 to 17.5)	12.7 (8.7 to 16.5)	10.4 (6.7 to 14.1)	-6.5 (-8.2 to -4.9)	-18.4 (-23.0 to -14.2)
Occupational exposure to asbestos	0.7 (0.5 to 1.1)	0.9 (0.8 to 1.3)	1.1 (1.0 to 1.3)	39.8 (15.7 to 69.0)	17.4 (1.2 to 31.5)	0.8 (0.5 to 1.3)	0.6 (0.4 to 0.9)	0.6 (0.5 to 0.7)	-23.2 (-37.2 to 2.4)	-9.0 (-22.4 to 9.4)
Occupational exposure to arsenic	0.3 (0.1 to 0.5)	0.3 (0.1 to 0.5)	0.3 (0.1 to 0.5)	7.1 (3.6 to 10.8)	6.1 (3.0 to 9.3)	0.2 (0.1 to 0.3)	0.2 (0.1 to 0.3)	0.2 (0.1 to 0.4)	13.8 (8.2 to 19.7)	8.4 (4.2 to 12.8)
Occupational exposure to benzene	0.5 (0.3 to 1.0)	0.6 (0.4 to 1.1)	0.7 (0.4 to 1.2)	12.8 (9.9 to 16.0)	11.1 (9.0 to 13.6)	0.3 (0.2 to 0.6)	0.4 (0.3 to 0.7)	0.5 (0.3 to 0.9)	28.6 (23.5 to 34.7)	23.3 (19.8 to 27.5)
Occupational exposure to beryllium	0.1 (0.1 to 0.1)	0.1 (0.1 to 0.1)	0.1 (0.1 to 0.1)	5.4 (3.0 to 7.7)	3.1 (1.6 to 4.6)	0.0 (0.0 to 0.1)	0.1 (0.1 to 0.1)	0.1 (0.1 to 0.1)	15.7 (10.6 to 21.0)	7.4 (4.3 to 10.6)
Occupational exposure to cadmium	0.1 (0.1 to 0.1)	0.2 (0.2 to 0.2)	0.2 (0.2 to 0.2)	7.6 (4.4 to 11.0)	6.3 (3.6 to 9.2)	0.1 (0.1 to 0.1)	0.1 (0.1 to 0.1)	0.1 (0.1 to 0.1)	14.6 (9.2 to 20.1)	8.9 (5.0 to 13.1)
Occupational exposure to chromium	0.3 (0.3 to 0.3)	0.3 (0.3 to 0.3)	0.4 (0.4 to 0.4)	11.7 (8.2 to 15.2)	10.1 (7.3 to 13.0)	0.2 (0.2 to 0.2)	0.2 (0.2 to 0.2)	0.2 (0.2 to 0.3)	17.4 (12.1 to 22.9)	13.3 (9.3 to 17.7)
Occupational exposure to diesel engine exhaust	1.8 (1.8 to 1.9)	2.2 (2.1 to 2.2)	2.5 (2.4 to 2.6)	18.3 (14.7 to 22.2)	15.1 (12.1 to 18.2)	0.8 (0.8 to 0.9)	0.9 (0.9 to 1.0)	1.1 (1.1 to 1.1)	15.8 (10.6 to 21.2)	14.8 (11.2 to 18.4)
Occupational exposure to formaldehyde	0.6 (0.6 to 0.7)	0.7 (0.7 to 0.7)	0.8 (0.7 to 0.8)	9.5 (5.7 to 13.6)	9.5 (6.2 to 13.0)	0.4 (0.4 to 0.4)	0.4 (0.4 to 0.5)	0.5 (0.5 to 0.5)	14.8 (9.3 to 20.6)	13.6 (9.4 to 18.4)
Occupational exposure to nickel	0.3 (0.1 to 0.9)	0.3 (0.1 to 0.9)	0.4 (0.1 to 0.9)	4.7 (1.4 to 8.2)	5.3 (2.5 to 8.5)	0.2 (0.1 to 0.5)	0.2 (0.1 to 0.5)	0.2 (0.1 to 0.5)	8.3 (2.9 to 14.3)	3.9 (-0.1 to 8.5)
Occupational exposure to polycyclic aromatic hydrocarbons	0.6 (0.6 to 0.7)	0.7 (0.7 to 0.7)	0.8 (0.8 to 0.8)	11.5 (8.3 to 14.8)	10.2 (7.6 to 12.9)	0.4 (0.4 to 0.4)	0.5 (0.4 to 0.5)	0.5 (0.5 to 0.5)	17.6 (12.1 to 23.1)	13.7 (9.8 to 17.9)
Occupational exposure to silica	3.6 (1.9 to 8.0)	4.0 (2.1 to 8.8)	4.3 (2.3 to 9.6)	8.8 (4.6 to 14.2)	9.5 (5.8 to 14.1)	1.7 (0.9 to 3.7)	1.8 (0.9 to 3.8)	1.8 (0.9 to 3.8)	2.1 (-3.1 to 8.1)	-0.0 (-4.2 to 4.4)
Occupational exposure to sulphuric acid	0.6 (0.5 to 1.1)	0.7 (0.5 to 1.2)	0.7 (0.5 to 1.2)	5.6 (2.7 to 8.5)	3.2 (0.9 to 5.6)	0.4 (0.3 to 0.7)	0.4 (0.3 to 0.8)	0.5 (0.3 to 0.8)	13.0 (7.8 to 18.5)	5.0 (1.3 to 8.9)
Occupational exposure to trichloroethylene	0.2 (0.2 to 0.2)	0.2 (0.2 to 0.2)	0.2 (0.2 to 0.2)	13.8 (10.8 to 16.9)	10.7 (8.5 to 13.1)	0.1 (0.1 to 0.1)	0.1 (0.1 to 0.1)	0.1 (0.1 to 0.1)	19.4 (14.2 to 24.6)	14.2 (10.9 to 18.2)
Occupational asthmagens	16.1 (12.8 to 20.0)	16.4 (13.2 to 20.1)	16.1 (13.2 to 19.6)	1.6 (-4.7 to 8.3)	-1.8 (-7.2 to 3.4)	6.5 (5.0 to 8.4)	7.0 (5.5 to 8.9)	7.0 (5.6 to 8.8)	8.1 (-0.3 to 17.1)	0.3 (-6.4 to 7.1)
Occupational particulate matter, gases, and fumes	9.0 (7.0 to 12.0)	9.3 (7.2 to 12.3)	9.3 (7.2 to 12.2)	2.4 (0.8 to 4.4)	0.1 (-0.9 to 1.1)	3.9 (3.0 to 5.3)	4.2 (3.2 to 5.6)	4.3 (3.3 to 5.8)	6.5 (3.0 to 10.9)	2.2 (0.1 to 4.4)
Occupational noise	9.6 (9.2 to 10.2)	9.9 (9.5 to 10.4)	10.0 (9.6 to 10.5)	3.1 (1.8 to 4.6)	1.2 (0.4 to 2.0)	4.2 (3.9 to 4.5)	4.5 (4.2 to 4.8)	4.7 (4.5 to 5.0)	8.0 (5.1 to 11.1)	5.0 (3.1 to 6.9)
Occupational ergonomic factors	21.2 (19.7 to 22.7)	20.6 (19.3 to 22.1)	18.4 (17.0 to 20.0)	-2.5 (-8.5 to 4.1)	-10.9 (-15.4 to -6.5)	10.0 (9.2 to 10.9)	10.6 (9.9 to 11.4)	9.8 (9.1 to 10.6)	6.3 (-1.6 to 14.7)	-7.9 (-13.4 to -2.5)
Non-exclusive breastfeeding	0.4 (0.3 to 0.6)	0.4 (0.3 to 0.6)	0.4 (0.3 to 0.6)	-6.8 (-9.5 to -3.5)	-3.3 (-6.5 to 0.7)	0.4 (0.3 to 0.6)	0.4 (0.3 to 0.6)	0.4 (0.3 to 0.6)	-6.5 (-9.2 to -3.1)	-3.4 (-6.5 to 0.6)
Discontinued breastfeeding	0.9 (0.9 to 0.9)	0.9 (0.8 to 0.9)	0.9 (0.9 to 0.9)	-2.0 (-4.6 to 0.6)	8.0 (5.6 to 10.7)	0.9 (0.9 to 0.9)	0.9 (0.8 to 0.9)	0.9 (0.9 to 0.9)	-2.3 (-4.8 to 0.4)	7.8 (5.3 to 10.4)
Child underweight	2.5 (2.3 to 2.7)	1.6 (1.4 to 1.8)	1.3 (1.1 to 1.4)	-37.3 (-41.8 to -33.3)	-19.3 (-21.5 to -17.3)	2.4 (2.2 to 2.6)	1.5 (1.2 to 1.6)	1.2 (1.0 to 1.3)	-39.8 (-45.4 to -35.3)	-18.3 (-20.6 to -15.5)
Child wasting	0.8 (0.7 to 0.9)	0.7 (0.6 to 0.8)	0.6 (0.5 to 0.7)	-13.3 (-17.9 to -9.1)	-15.3 (-18.4 to -12.3)	0.7 (0.6 to 0.8)	0.6 (0.5 to 0.7)	0.5 (0.4 to 0.6)	-14.9 (-19.7 to -10.7)	-16.8 (-19.8 to -14.0)
Child stunting	3.8 (2.6 to 4.1)	3.1 (2.2 to 3.4)	2.3 (1.6 to 2.6)	-18.6 (-23.1 to -14.5)	-27.0 (-31.0 to -23.0)	3.7 (2.6 to 4.1)	2.9 (2.0 to 3.2)	2.1 (1.4 to 2.3)	-21.4 (-26.5 to -16.9)	-30.0 (-34.3 to -26.1)
Short gestation for birth weight	0.0 (0.0 to 0.0)	0.0 (0.0 to 0.0)	0.0 (0.0 to 0.0)	11.1 (5.7 to 17.5)	13.5 (7.9 to 19.5)	0.0 (0.0 to 0.0)	0.0 (0.0 to 0.0)	0.0 (0.0 to 0.0)	7.6 (2.3 to 14.0)	10.0 (5.3 to 16.2)
Low birth weight for gestation	0.0 (0.0 to 0.0)	0.0 (0.0 to 0.0)	0.0 (0.0 to 0.0)	8.2 (2.5 to 15.2)	1.2 (-5.0 to 8.7)	0.0 (0.0 to 0.0)	0.0 (0.0 to 0.0)	0.0 (0.0 to 0.0)	8.8 (2.9 to 14.7)	1.0 (-5.5 to 6.9)
Iron deficiency	-	-	-	-	-	19.7 (17.2 to 22.6)	15.7 (13.1 to 18.8)	13.3 (10.7 to 16.5)	-20.3 (-25.7 to -16.0)	-15.2 (-18.6 to -11.6)
Vitamin A deficiency	3.1 (2.7 to 3.6)	2.6 (2.3 to 3.0)	2.3 (2.0 to 2.7)	-16.1 (-18.7 to -13.5)	-10.8 (-14.2 to -7.6)	3.0 (2.6 to 3.5)	2.5 (2.2 to 2.9)	2.3 (2.0 to 2.7)	-15.5 (-18.4 to -12.9)	-10.2 (-13.4 to -6.7)
Zinc deficiency	1.2 (0.4 to 2.1)	0.8 (0.2 to 1.6)	0.7 (0.2 to 1.3)	-32.0 (-45.2 to -22.8)	-16.6 (-25.7 to -8.3)	1.2 (0.4 to 2.1)	0.8 (0.2 to 1.6)	0.6 (0.2 to 1.3)	-32.3 (-45.1 to -23.1)	-16.9 (-25.8 to -8.4)
Smoking	8.1 (7.0 to 9.3)	7.4 (6.5 to 8.4)	6.5 (5.7 to 7.4)	-8.9 (-13.4 to -4.2)	-12.3 (-14.9 to -9.3)	1.0 (0.9 to 1.2)	0.9 (0.8 to 1.1)	0.8 (0.7 to 0.9)	-10.5 (-18.3 to -1.8)	-16.9 (-21.9 to -12.2)
Chewing tobacco	8.4 (7.4 to 9.5)	7.8 (7.2 to 8.4)	7.1 (6.6 to 7.7)	-7.4 (-18.1 to 6.7)	-8.3 (-16.1 to 0.2)	5.9 (5.1 to 6.8)	5.8 (5.3 to 6.4)	5.3 (4.7 to 6.0)	-1.0 (-15.2 to 15.7)	-8.8 (-18.3 to 3.0)
Second-hand smoke	35.1 (34.2 to 36.0)	29.9 (29.3 to 30.5)	28.9 (28.0 to 29.8)	-14.7 (-17.7 to -11.7)	-3.4 (-5.7 to -1.0)	51.6 (50.8 to 52.5)	43.1 (42.5 to 43.6)	39.9 (39.0 to 40.8)	-16.5 (-18.6 to -14.5)	-7.4 (-8.7 to -5.9)
Alcohol use	9.3 (6.9 to 12.0)	11.2 (8.7 to 13.9)	11.2 (8.6 to 13.9)	20.0 (9.0 to 35.3)	-0.1 (-8.0 to 8.1)	1.9 (1.2 to 3.0)	2.3 (1.5 to 3.4)	2.3 (1.5 to 3.4)	16.9 (7.7 to 29.6)	0.1 (-7.9 to 9.2)
Drug use	0.6 (0.5 to 0.8)	0.6 (0.5 to 0.8)	0.7 (0.5 to 0.8)	2.3 (-1.4 to 5.8)	6.0 (1.8 to 9.7)	0.3 (0.3 to 0.4)	0.3 (0.3 to 0.4)	0.3 (0.3 to 0.4)	-3.6 (-6.0 to -1.5)	2.1 (-0.6 to 4.6)
Diet low in fruits	45.0 (40.2 to 47.2)	41.7 (36.6 to 44.4)	38.7 (33.5 to 41.7)	-7.2 (-9.1 to -5.8)	-7.4 (-9.4 to -5.7)	43.6 (38.7 to 46.0)	40.1 (34.8 to 42.9)	36.8 (31.5 to 40.0)	-8.2 (-10.2 to -6.6)	-8.2 (-10.6 to -6.3)
Diet low in vegetables	38.8 (36.1 to 41.5)	32.9 (29.3 to 36.2)	28.6 (24.3 to 32.5)	-15.3 (-19.1 to -12.3)	-13.0 (-17.5 to -9.5)	40.1 (37.4 to 42.5)	34.7 (31.2 to 37.9)	30.7 (26.5 to 34.4)	-13.3 (-16.7 to -10.7)	-11.6 (-16.0 to -8.4)
Diet low in legumes	13.0 (9.4 to 16.4)	8.1 (5.9 to 10.3)	7.4 (5.5 to 9.3)	-37.9 (-40.7 to -34.8)	-8.1 (-12.0 to -2.4)	16.4 (12.7 to 19.8)	11.1 (8.3 to 14.0)	9.8 (7.4 to 12.3)	-32.3 (-36.7 to -27.9)	-11.6 (-15.3 to -7.3)
Diet low in whole grains	40.3 (38.0 to 42.3)	40.1 (37.8 to 42.1)	39.5 (37.1 to 41.6)	-0.3 (-1.4 to 0.8)	-1.5 (-2.5 to -0.5)	40.8 (38.7 to 42.7)	40.7 (38.5 to 42.7)	40.2 (37.9 to 42.2)	-0.3 (-1.2 to 0.8)	-1.3 (-2.2 to -0.4)
Diet low in nuts and seeds	51.8 (51.2 to 52.3)	49.7 (48.7 to 50.6)	47.3 (45.7 to 48.6)	-4.0 (-5.0 to -3.2)	-5.0 (-6.3 to -3.9)	52.0 (51.4 to 52.5)	50.0 (49.0 to 50.8)	47.5 (46.0 to 48.8)	-3.8 (-4.7 to -3.0)	-5.0 (-6.3 to -3.9)
Diet low in milk	50.7 (49.8 to 51.4)	49.8 (48.8 to 50.7)	49.3 (48.2 to 50.3)	-1.6 (-2.1 to -1.2)	-1.0 (-1.3 to -0.7)	50.9 (50.1 to 51.6)	50.1 (49.2 to 50.9)	49.7 (48.6 to 50.6)	-1.6 (-2.0 to -1.2)	-0.9 (-1.3 to -0.6)
Diet high in red meat	0.8 (0.5 to 1.2)	1.9 (1.3 to 2.5)	2.5 (1.8 to 3.1)	147.9 (81.1 to 268.6)	28.0 (6.2 to 54.7)	0.2 (0.1 to 0.4)	0.6 (0.3 to 1.0)	1.0 (0.5 to 1.4)	217.8 (85.5 to 686.2)	46.9 (8.1 to 113.4)
Diet high in processed meat	0.2 (0.0 to 1.1)	0.3 (0.0 to 1.4)	0.5 (0.0 to 1.8)	52.3 (27.7 to 1191.6)	46.4 (20.5 to 165.8)	0.1 (0.0 to 0.8)	0.2 (0.0 to 1.0)	0.3 (0.0 to 1.3)	47.5 (25.1 to 3570.4)	48.5 (23.0 to 228.9)
Diet high in sugar-sweetened beverages	1.3 (0.6 to 1.6)	1.7 (0.9 to 2.1)	2.5 (1.2 to 3.1)	36.8 (12.7 to 65.4)	42.6 (25.7 to 61.7)	1.0 (0.5 to 1.3)	1.4 (0.7 to 1.8)	1.9 (1.0 to 2.4)	39.1 (16.3 to 69.6)	35.2 (17.8 to 55.9)
Diet low in fibre	31.7 (27.3 to 35.4)	29.0 (24.6 to 32.8)	26.8 (22.1 to 31.0)	-8.4 (-11.0 to -6.4)	-7.5 (-11.2 to -5.0)	36.4 (32.7 to 39.5)	33.9 (29.9 to 37.2)	31.7 (27.4 to 35.4)	-7.0 (-9.3 to -5.2)	-6.4 (-9.2 to -4.3)
Diet low in calcium	46.8 (45.2 to 48.1)	43.8 (41.6 to 45.6)	41.0 (38.2 to 43.3)	-6.4 (-8.0 to -5.1)	-6.5 (-8.3 to -5.1)	48.2 (46.9 to 49.3)	45.6 (43.7 to 47.1)	43.1 (40.7 to 45.0)	-5.3 (-6.7 to -4.3)	-5.5 (-7.0 to -4.4)
Diet low in seafood omega-3 fatty acids	50.4 (49.5 to 51.2)	48.4 (47.2 to 49.6)	47.0 (45.5 to 48.5)	-3.8 (-4.7 to -3.1)	-2.9 (-3.5 to -2.3)	50.9 (50.2 to 51.7)	49.3 (48.2 to 50.4)	48.1 (46.8 to 49.4)	-3.3 (-4.0 to -2.6)	-2.4 (-3.0 to -1.9)
Diet low in polyunsaturated fatty acids	47.3 (46.0 to 48.5)	43.3 (41.3 to 45.0)	41.7 (39.5 to 43.6)	-8.5 (-10.6 to -6.4)	-3.8 (-4.9 to -2.8)	47.0 (45.7 to 48.3)	43.4 (41.4 to 45.1)	42.0 (39.8 to 43.9)	-7.8 (-9.9 to -5.8)	-3.2 (-4.3 to -2.4)
Diet high in trans fatty acids	5.3 (2.8 to 8.3)	3.8 (1.6 to 6.7)	3.1 (1.1 to 5.8)	-28.7 (-45.8 to -17.7)	-18.7 (-35.0 to -9.9)	6.9 (4.3 to 9.9)	5.2 (2.7 to 8.4)	4.1 (1.8 to 7.1)	-24.7 (-40.9 to -14.1)	-20.5 (-35.6 to -11.9)
Diet high in sodium	8.9 (4.5 to 14.4)	8.8 (4.1 to 14.7)	8.8 (3.9 to 14.8)	-0.6 (-13.8 to 6.1)	-0.0 (-8.9 to 5.2)	9.4 (4.6 to 15.1)	8.1 (3.3 to 14.0)	7.4 (2.7 to 13.3)	-13.5 (-30.9 to -6.4)	-9.1 (-21.9 to -4.3)
Intimate partner violence	-	-	-	-	-	9.3 (7.8 to 10.9)	8.4 (7.3 to 9.6)	8.1 (6.9 to 9.3)	-9.4 (-14.4 to -3.9)	-3.9 (-6.3 to -1.7)
Childhood sexual abuse	8.2 (6.7 to 10.0)	8.2 (6.8 to 9.9)	8.5 (6.9 to 10.4)	0.3 (-2.8 to 3.5)	3.4 (0.2 to 6.5)	8.9 (7.2 to 10.7)	8.6 (7.1 to 10.3)	9.0 (7.4 to 10.9)	-3.4 (-6.6 to -0.1)	4.4 (0.7 to 8.7)

Supplementary Results Table 1. Low-middle SDI age-standardised Summary Exposure Values (SEVs) for Level 4 risk factors and percentage change, by sex, 1990, 2007, and 2017.Data in parentheses are 95% uncertainty intervals.										
	**Male**					**Female**				
	**1990**	**2007**	**2017**	**Percentage change 1990-2007**	**Percentage change 2007-2017**	**1990**	**2007**	**2017**	**Percentage change 1990-2007**	**Percentage change 2007-2017**
Bullying victimization	6.4 (2.8 to 12.9)	7.8 (3.5 to 15.6)	8.5 (3.8 to 17.1)	21.1 (12.8 to 32.3)	9.4 (5.9 to 13.7)	4.4 (1.9 to 8.8)	5.8 (2.6 to 11.6)	6.7 (3.0 to 13.4)	32.8 (21.9 to 47.1)	14.9 (11.2 to 19.2)
Low physical activity	0.4 (0.2 to 0.7)	0.4 (0.2 to 0.7)	0.4 (0.2 to 0.7)	1.6 (0.9 to 2.4)	1.5 (0.8 to 2.1)	0.3 (0.2 to 0.6)	0.3 (0.2 to 0.6)	0.3 (0.2 to 0.6)	0.3 (-0.2 to 0.9)	1.4 (0.7 to 2.2)
High fasting plasma glucose	1.9 (1.4 to 2.5)	2.5 (1.8 to 3.4)	2.7 (2.0 to 3.6)	33.2 (21.3 to 46.3)	9.5 (-0.2 to 20.4)	1.9 (1.4 to 2.6)	2.5 (1.8 to 3.3)	2.7 (2.0 to 3.6)	27.7 (17.4 to 39.8)	7.9 (-2.0 to 18.6)
High LDL cholesterol	7.7 (6.4 to 9.1)	8.2 (6.9 to 9.7)	8.4 (7.1 to 10.0)	6.7 (5.5 to 8.3)	3.1 (2.3 to 4.0)	8.5 (7.2 to 9.9)	9.3 (8.0 to 10.7)	9.7 (8.4 to 11.2)	9.0 (7.7 to 10.7)	5.0 (4.1 to 6.1)
High systolic blood pressure	6.3 (5.7 to 7.0)	7.3 (6.6 to 8.0)	7.7 (7.0 to 8.4)	14.5 (12.5 to 16.8)	5.9 (4.5 to 7.3)	6.9 (6.4 to 7.5)	7.6 (7.0 to 8.2)	7.8 (7.2 to 8.4)	9.0 (7.0 to 11.1)	3.1 (1.4 to 4.8)
High body-mass index	4.2 (2.9 to 6.3)	6.1 (4.6 to 8.6)	8.0 (6.2 to 10.8)	47.2 (34.4 to 61.8)	31.5 (25.2 to 39.2)	5.0 (3.5 to 7.0)	7.7 (5.9 to 10.3)	9.8 (7.7 to 12.9)	55.0 (41.0 to 71.0)	26.5 (20.7 to 33.5)
Low bone mineral density	5.7 (4.9 to 6.5)	5.5 (4.8 to 6.3)	5.4 (4.6 to 6.2)	-3.4 (-6.0 to -0.9)	-2.2 (-4.8 to 0.5)	7.5 (6.5 to 8.7)	7.3 (6.3 to 8.4)	7.3 (6.3 to 8.4)	-2.8 (-5.2 to -0.2)	-0.4 (-2.9 to 2.3)
Impaired kidney function	2.5 (1.2 to 6.3)	2.7 (1.3 to 6.5)	2.7 (1.3 to 6.4)	4.3 (1.4 to 6.9)	0.5 (-1.8 to 2.7)	3.0 (1.5 to 6.9)	3.1 (1.5 to 7.0)	3.1 (1.5 to 7.0)	2.5 (0.0 to 5.2)	2.2 (0.2 to 4.9)

Supplementary Results Table 1. Middle SDI age-standardised Summary Exposure Values (SEVs) for Level 4 risk factors and percentage change, by sex, 1990, 2007, and 2017.Data in parentheses are 95% uncertainty intervals.										
	Male					Female				
	1990	2007	2017	Percentage change 1990-2007	Percentage change 2007-2017	1990	2007	2017	Percentage change 1990-2007	Percentage change 2007-2017
Unsafe water source	46.3 (43.3 to 48.9)	31.9 (29.0 to 34.5)	25.3 (22.5 to 27.8)	-31.0 (-34.8 to -27.0)	-20.9 (-25.7 to -16.2)	46.1 (43.0 to 48.7)	31.9 (28.9 to 34.5)	25.4 (22.6 to 27.9)	-30.8 (-34.5 to -26.8)	-20.5 (-25.1 to -15.9)
Unsafe sanitation	65.2 (59.6 to 73.0)	35.2 (30.4 to 41.4)	20.7 (16.7 to 26.1)	-46.0 (-50.9 to -40.9)	-41.4 (-49.0 to -33.0)	64.8 (59.2 to 72.7)	35.0 (30.3 to 41.3)	20.7 (16.7 to 26.1)	-45.9 (-50.7 to -41.0)	-41.1 (-48.5 to -32.9)
No access to handwashing facility	36.9 (35.7 to 38.1)	27.5 (26.4 to 28.5)	21.9 (20.9 to 22.8)	-25.6 (-28.6 to -22.5)	-20.3 (-22.9 to -17.6)	36.9 (35.8 to 38.1)	27.6 (26.6 to 28.6)	22.1 (21.1 to 23.1)	-25.2 (-28.1 to -22.0)	-19.9 (-22.5 to -17.3)
Ambient particulate matter pollution	28.4 (22.0 to 36.9)	43.5 (34.4 to 55.3)	44.7 (35.8 to 57.0)	53.4 (39.4 to 68.7)	2.8 (-4.0 to 10.7)	23.7 (18.1 to 31.4)	38.6 (30.0 to 49.8)	41.0 (32.4 to 52.5)	62.6 (48.0 to 79.2)	6.4 (-1.4 to 15.2)
Household air pollution from solid fuels	52.5 (44.5 to 64.3)	26.5 (21.1 to 34.6)	16.0 (12.2 to 21.2)	-49.5 (-54.1 to -44.3)	-39.8 (-44.7 to -32.9)	54.1 (46.3 to 66.0)	28.7 (23.0 to 36.3)	17.7 (13.7 to 23.4)	-46.9 (-51.5 to -42.2)	-38.2 (-43.4 to -31.2)
Ambient ozone pollution	38.7 (17.2 to 48.2)	38.6 (17.0 to 48.7)	39.7 (17.7 to 50.1)	-0.1 (-2.0 to 1.3)	2.8 (1.8 to 4.0)	37.9 (16.8 to 47.8)	38.1 (16.7 to 48.4)	39.4 (17.6 to 50.0)	0.5 (-1.1 to 1.6)	3.5 (2.6 to 4.9)
Residential radon	20.5 (11.7 to 30.7)	21.0 (12.3 to 31.5)	21.5 (12.7 to 32.0)	2.5 (-4.4 to 7.5)	2.3 (-0.3 to 4.9)	20.3 (11.6 to 30.6)	20.9 (12.3 to 31.4)	21.4 (12.6 to 31.9)	3.2 (-3.6 to 8.3)	2.5 (0.1 to 5.1)
Lead exposure	19.6 (15.1 to 23.5)	16.4 (12.2 to 20.2)	12.6 (8.8 to 16.3)	-16.4 (-20.1 to -13.3)	-23.2 (-27.8 to -18.7)	11.9 (7.8 to 15.8)	10.1 (6.2 to 13.9)	7.8 (4.5 to 11.4)	-15.5 (-20.2 to -11.8)	-22.3 (-27.9 to -17.0)
Occupational exposure to asbestos	0.9 (0.7 to 1.1)	1.0 (0.9 to 1.1)	1.1 (1.0 to 1.1)	12.6 (-4.2 to 29.0)	8.0 (-2.8 to 15.6)	0.5 (0.4 to 0.7)	0.4 (0.4 to 0.4)	0.4 (0.4 to 0.4)	-21.7 (-36.9 to -1.0)	4.3 (-6.3 to 15.2)
Occupational exposure to arsenic	0.3 (0.1 to 0.5)	0.3 (0.1 to 0.5)	0.3 (0.1 to 0.6)	4.7 (0.8 to 8.7)	3.1 (-0.3 to 6.4)	0.3 (0.1 to 0.5)	0.3 (0.1 to 0.5)	0.3 (0.1 to 0.5)	5.9 (0.8 to 11.6)	2.5 (-1.9 to 7.8)
Occupational exposure to benzene	0.6 (0.3 to 1.0)	0.6 (0.4 to 1.1)	0.7 (0.4 to 1.2)	12.5 (10.1 to 15.8)	8.0 (6.0 to 10.5)	0.5 (0.3 to 0.9)	0.6 (0.4 to 1.1)	0.7 (0.5 to 1.3)	23.1 (19.6 to 27.6)	16.7 (13.4 to 20.9)
Occupational exposure to beryllium	0.1 (0.1 to 0.1)	0.1 (0.1 to 0.1)	0.1 (0.1 to 0.1)	2.4 (0.4 to 4.5)	1.1 (-0.5 to 2.7)	0.1 (0.1 to 0.1)	0.1 (0.1 to 0.1)	0.1 (0.1 to 0.1)	4.9 (2.2 to 7.7)	1.9 (-0.1 to 3.8)
Occupational exposure to cadmium	0.2 (0.2 to 0.2)	0.2 (0.2 to 0.2)	0.2 (0.2 to 0.2)	5.4 (1.8 to 9.1)	3.4 (0.3 to 6.2)	0.2 (0.1 to 0.2)	0.2 (0.2 to 0.2)	0.2 (0.2 to 0.2)	6.9 (2.1 to 11.9)	3.1 (-1.1 to 7.7)
Occupational exposure to chromium	0.3 (0.3 to 0.4)	0.4 (0.4 to 0.4)	0.4 (0.4 to 0.4)	9.8 (6.1 to 13.6)	6.0 (2.9 to 8.8)	0.3 (0.3 to 0.3)	0.4 (0.3 to 0.4)	0.4 (0.4 to 0.4)	12.1 (7.0 to 17.7)	7.0 (2.6 to 12.0)
Occupational exposure to diesel engine exhaust	2.0 (1.9 to 2.0)	2.3 (2.3 to 2.4)	2.5 (2.4 to 2.6)	16.5 (13.2 to 19.9)	8.2 (5.7 to 10.5)	1.3 (1.3 to 1.4)	1.5 (1.5 to 1.6)	1.7 (1.6 to 1.7)	13.7 (9.9 to 17.6)	9.5 (5.9 to 13.1)
Occupational exposure to formaldehyde	0.7 (0.7 to 0.8)	0.8 (0.8 to 0.8)	0.8 (0.8 to 0.9)	8.3 (4.3 to 12.4)	5.0 (1.6 to 8.3)	0.6 (0.6 to 0.7)	0.7 (0.7 to 0.7)	0.7 (0.7 to 0.8)	10.3 (4.7 to 16.3)	6.7 (1.9 to 12.0)
Occupational exposure to nickel	0.4 (0.1 to 0.9)	0.4 (0.1 to 1.0)	0.4 (0.1 to 1.0)	1.0 (-2.3 to 4.8)	0.8 (-2.0 to 3.8)	0.3 (0.1 to 0.8)	0.3 (0.1 to 0.8)	0.3 (0.1 to 0.8)	-0.9 (-5.2 to 4.3)	-1.0 (-5.2 to 4.2)
Occupational exposure to polycyclic aromatic hydrocarbons	0.7 (0.7 to 0.7)	0.8 (0.8 to 0.8)	0.8 (0.8 to 0.9)	9.6 (6.2 to 13.2)	6.0 (3.2 to 8.7)	0.6 (0.6 to 0.7)	0.7 (0.7 to 0.8)	0.8 (0.7 to 0.8)	11.8 (6.9 to 16.8)	7.8 (3.5 to 12.4)
Occupational exposure to silica	3.6 (1.9 to 7.9)	3.8 (2.0 to 8.3)	3.9 (2.1 to 8.5)	5.3 (1.6 to 9.6)	3.1 (0.1 to 6.4)	2.7 (1.4 to 5.8)	2.5 (1.3 to 5.3)	2.4 (1.2 to 5.2)	-7.3 (-11.0 to -2.7)	-3.3 (-7.5 to 1.6)
Occupational exposure to sulphuric acid	0.7 (0.5 to 1.2)	0.7 (0.5 to 1.3)	0.7 (0.5 to 1.3)	3.1 (0.2 to 6.3)	1.2 (-1.5 to 3.8)	0.7 (0.5 to 1.1)	0.7 (0.5 to 1.2)	0.7 (0.5 to 1.2)	2.1 (-1.7 to 6.5)	-0.7 (-4.7 to 3.4)
Occupational exposure to trichloroethylene	0.2 (0.2 to 0.2)	0.2 (0.2 to 0.2)	0.2 (0.2 to 0.3)	12.1 (9.3 to 15.4)	6.6 (4.3 to 8.9)	0.2 (0.2 to 0.2)	0.2 (0.2 to 0.2)	0.2 (0.2 to 0.2)	14.3 (10.2 to 18.7)	7.7 (4.2 to 11.6)
Occupational asthmagens	16.3 (13.3 to 19.8)	15.5 (12.9 to 18.6)	15.3 (12.9 to 18.1)	-5.0 (-11.3 to 1.5)	-1.3 (-5.9 to 3.7)	9.4 (7.3 to 12.2)	8.6 (6.8 to 10.7)	8.1 (6.5 to 10.0)	-8.9 (-18.3 to 1.4)	-5.4 (-13.4 to 2.8)
Occupational particulate matter, gases, and fumes	9.3 (7.2 to 12.3)	9.2 (7.2 to 12.1)	9.0 (7.1 to 11.8)	-1.3 (-3.0 to 0.6)	-1.7 (-2.8 to -0.6)	6.3 (4.8 to 8.4)	6.3 (4.9 to 8.3)	6.1 (4.7 to 8.0)	-0.7 (-3.3 to 2.3)	-3.4 (-4.9 to -1.7)
Occupational noise	9.8 (9.3 to 10.4)	9.8 (9.3 to 10.3)	9.8 (9.3 to 10.3)	0.0 (-1.3 to 1.6)	-0.3 (-1.1 to 0.5)	6.7 (6.3 to 7.1)	6.8 (6.5 to 7.2)	6.8 (6.5 to 7.2)	2.0 (-0.1 to 4.3)	0.1 (-1.1 to 1.2)
Occupational ergonomic factors	18.9 (17.4 to 20.4)	16.3 (15.0 to 17.9)	14.4 (13.1 to 16.1)	-14.1 (-19.8 to -7.9)	-11.1 (-15.2 to -6.5)	14.4 (13.2 to 15.7)	12.0 (10.9 to 13.2)	10.1 (8.9 to 11.5)	-16.8 (-24.9 to -7.6)	-15.4 (-22.6 to -8.0)
Non-exclusive breastfeeding	0.5 (0.3 to 0.6)	0.4 (0.3 to 0.6)	0.4 (0.3 to 0.5)	-6.7 (-9.5 to -3.5)	-4.5 (-7.6 to -1.1)	0.5 (0.3 to 0.6)	0.4 (0.3 to 0.6)	0.4 (0.3 to 0.5)	-6.2 (-9.0 to -3.3)	-4.5 (-7.6 to -1.1)
Discontinued breastfeeding	1.3 (1.3 to 1.3)	1.3 (1.3 to 1.3)	1.3 (1.3 to 1.4)	-1.1 (-4.3 to 2.2)	5.5 (3.2 to 8.1)	1.3 (1.3 to 1.3)	1.3 (1.2 to 1.3)	1.3 (1.3 to 1.4)	-1.7 (-4.7 to 1.5)	6.0 (3.7 to 8.5)
Child underweight	1.2 (1.0 to 1.4)	0.8 (0.7 to 0.9)	0.6 (0.5 to 0.7)	-32.9 (-37.9 to -28.3)	-28.0 (-30.8 to -25.5)	1.2 (1.0 to 1.4)	0.8 (0.6 to 0.9)	0.5 (0.4 to 0.7)	-35.2 (-40.9 to -30.3)	-29.4 (-32.5 to -26.6)
Child wasting	0.5 (0.4 to 0.6)	0.4 (0.4 to 0.5)	0.4 (0.3 to 0.4)	-12.1 (-19.9 to -5.6)	-15.7 (-18.9 to -12.1)	0.5 (0.4 to 0.5)	0.4 (0.3 to 0.5)	0.4 (0.3 to 0.4)	-11.3 (-18.7 to -5.0)	-11.9 (-15.0 to -8.7)
Child stunting	2.4 (1.6 to 2.9)	1.7 (1.2 to 2.0)	1.3 (0.9 to 1.6)	-27.0 (-35.7 to -18.9)	-24.0 (-30.0 to -18.9)	2.4 (1.7 to 2.8)	1.7 (1.2 to 2.0)	1.2 (0.8 to 1.5)	-29.1 (-38.8 to -21.2)	-26.4 (-32.4 to -20.9)
Short gestation for birth weight	0.0 (0.0 to 0.0)	0.0 (0.0 to 0.0)	0.0 (0.0 to 0.0)	27.3 (22.1 to 32.6)	2.3 (-1.0 to 6.1)	0.0 (0.0 to 0.0)	0.0 (0.0 to 0.0)	0.0 (0.0 to 0.0)	26.3 (21.2 to 33.2)	1.5 (-1.6 to 5.3)
Low birth weight for gestation	0.0 (0.0 to 0.0)	0.0 (0.0 to 0.0)	0.0 (0.0 to 0.0)	0.1 (-6.3 to 7.0)	6.1 (2.4 to 10.1)	0.0 (0.0 to 0.0)	0.0 (0.0 to 0.0)	0.0 (0.0 to 0.0)	-0.8 (-7.0 to 6.0)	5.5 (2.2 to 8.7)
Iron deficiency	-	-	-	-	-	11.8 (9.6 to 14.4)	9.2 (7.2 to 11.5)	8.1 (6.3 to 10.2)	-22.3 (-26.2 to -18.4)	-11.5 (-14.8 to -8.3)
Vitamin A deficiency	2.5 (2.1 to 2.8)	2.2 (1.9 to 2.6)	2.1 (1.8 to 2.4)	-9.4 (-11.6 to -7.2)	-7.6 (-10.0 to -5.3)	2.4 (2.1 to 2.8)	2.2 (1.9 to 2.5)	2.0 (1.7 to 2.4)	-9.2 (-11.5 to -6.8)	-7.1 (-9.9 to -4.4)
Zinc deficiency	0.9 (0.2 to 1.8)	0.6 (0.2 to 1.3)	0.5 (0.2 to 1.2)	-29.9 (-35.6 to -22.9)	-12.3 (-16.3 to -1.3)	0.9 (0.2 to 1.8)	0.6 (0.2 to 1.3)	0.5 (0.2 to 1.2)	-30.3 (-36.1 to -23.1)	-12.5 (-16.5 to -1.7)
Smoking	10.6 (9.1 to 12.2)	10.5 (9.2 to 12.0)	9.4 (8.3 to 10.7)	-0.4 (-5.9 to 5.0)	-10.2 (-13.0 to -7.1)	1.2 (1.0 to 1.4)	1.0 (0.9 to 1.2)	0.8 (0.7 to 1.0)	-13.0 (-19.1 to -6.6)	-19.7 (-23.0 to -16.3)
Chewing tobacco	2.8 (2.5 to 3.2)	2.9 (2.7 to 3.2)	2.9 (2.6 to 3.1)	3.0 (-9.9 to 18.2)	-1.6 (-10.8 to 8.4)	2.3 (2.0 to 2.7)	2.5 (2.2 to 2.8)	2.4 (2.1 to 2.7)	6.7 (-7.7 to 24.0)	-3.8 (-14.1 to 8.5)
Second-hand smoke	38.4 (37.2 to 39.8)	33.2 (32.2 to 34.2)	31.9 (30.8 to 33.0)	-13.5 (-16.7 to -10.2)	-3.9 (-6.5 to -1.5)	64.4 (63.3 to 65.5)	53.8 (52.9 to 54.6)	50.2 (49.2 to 51.1)	-16.5 (-18.3 to -14.8)	-6.6 (-7.9 to -5.3)
Alcohol use	12.3 (8.9 to 16.2)	15.1 (11.5 to 19.1)	17.1 (12.8 to 21.5)	23.5 (12.9 to 37.9)	13.0 (2.7 to 25.5)	2.1 (1.3 to 3.4)	2.5 (1.6 to 3.9)	2.8 (1.8 to 4.1)	18.8 (10.0 to 30.5)	10.5 (2.9 to 21.1)
Drug use	0.9 (0.7 to 1.1)	0.8 (0.6 to 1.0)	0.8 (0.6 to 1.0)	-10.4 (-13.1 to -7.6)	2.8 (-0.4 to 6.2)	0.4 (0.3 to 0.5)	0.4 (0.3 to 0.5)	0.4 (0.3 to 0.5)	-13.5 (-16.4 to -10.6)	1.9 (-0.3 to 4.3)
Diet low in fruits	43.9 (39.0 to 46.3)	38.5 (33.1 to 41.7)	33.8 (28.2 to 37.6)	-12.3 (-15.7 to -9.7)	-12.2 (-16.2 to -9.3)	42.1 (37.2 to 44.7)	35.9 (30.5 to 39.5)	30.8 (25.2 to 35.0)	-14.6 (-18.8 to -11.5)	-14.3 (-18.9 to -10.8)
Diet low in vegetables	42.2 (39.4 to 44.6)	33.2 (28.9 to 37.0)	27.3 (22.6 to 31.6)	-21.3 (-27.4 to -16.1)	-17.8 (-23.9 to -13.0)	43.5 (41.1 to 45.8)	34.9 (30.7 to 38.6)	29.0 (24.1 to 33.3)	-19.8 (-25.6 to -15.0)	-16.9 (-22.6 to -12.4)
Diet low in legumes	20.1 (15.9 to 24.0)	23.1 (19.4 to 26.4)	21.3 (17.9 to 24.6)	15.0 (6.9 to 27.3)	-7.9 (-11.5 to -4.8)	23.6 (19.5 to 27.3)	26.5 (22.9 to 29.7)	24.9 (21.5 to 28.1)	12.3 (6.0 to 21.6)	-5.8 (-8.7 to -3.1)
Diet low in whole grains	36.4 (33.4 to 38.8)	36.1 (33.2 to 38.5)	35.0 (32.0 to 37.7)	-0.8 (-2.0 to 0.5)	-2.8 (-4.2 to -1.7)	37.2 (34.4 to 39.5)	37.0 (34.3 to 39.4)	36.2 (33.3 to 38.7)	-0.4 (-1.5 to 0.8)	-2.2 (-3.4 to -1.1)
Diet low in nuts and seeds	53.3 (53.0 to 53.6)	51.8 (51.2 to 52.4)	49.9 (48.8 to 50.8)	-2.8 (-3.5 to -2.3)	-3.8 (-4.7 to -3.0)	53.4 (53.1 to 53.6)	51.9 (51.3 to 52.4)	50.0 (48.9 to 50.9)	-2.8 (-3.5 to -2.2)	-3.6 (-4.6 to -2.8)
Diet low in milk	50.8 (50.0 to 51.5)	49.9 (48.9 to 50.7)	48.9 (47.7 to 49.9)	-1.8 (-2.3 to -1.4)	-2.0 (-2.6 to -1.6)	51.0 (50.2 to 51.7)	50.0 (49.1 to 50.9)	49.1 (47.9 to 50.0)	-1.9 (-2.4 to -1.5)	-2.0 (-2.5 to -1.5)
Diet high in red meat	3.3 (1.7 to 5.4)	8.4 (5.1 to 11.4)	11.9 (8.3 to 14.9)	153.0 (69.1 to 304.4)	42.9 (15.0 to 84.9)	0.8 (0.3 to 1.8)	2.6 (1.1 to 4.6)	4.6 (2.4 to 7.1)	222.7 (73.9 to 616.5)	74.7 (27.1 to 160.7)
Diet high in processed meat	0.6 (0.2 to 1.5)	0.9 (0.4 to 2.2)	1.3 (0.5 to 2.9)	62.5 (38.1 to 106.6)	43.8 (23.6 to 68.6)	0.5 (0.2 to 1.2)	0.8 (0.4 to 1.8)	1.1 (0.5 to 2.4)	63.0 (39.4 to 113.1)	36.4 (16.1 to 55.6)
Diet high in sugar-sweetened beverages	3.0 (1.5 to 3.6)	4.2 (2.1 to 4.9)	5.7 (2.9 to 6.7)	40.4 (21.8 to 62.7)	35.8 (23.0 to 50.8)	2.4 (1.2 to 3.0)	3.4 (1.7 to 4.0)	4.3 (2.2 to 5.2)	40.1 (20.9 to 66.9)	29.2 (14.9 to 44.8)
Diet low in fibre	37.7 (34.1 to 40.8)	35.4 (31.4 to 38.8)	33.0 (28.7 to 36.7)	-6.1 (-9.4 to -3.3)	-6.8 (-9.9 to -4.4)	41.7 (38.8 to 44.1)	39.6 (36.4 to 42.2)	37.5 (33.8 to 40.5)	-5.1 (-7.7 to -3.2)	-5.3 (-7.7 to -3.4)
Diet low in calcium	48.4 (47.1 to 49.5)	44.5 (42.4 to 46.2)	41.3 (38.5 to 43.5)	-8.1 (-10.2 to -6.5)	-7.2 (-9.2 to -5.7)	49.6 (48.6 to 50.5)	46.1 (44.4 to 47.6)	43.2 (40.9 to 45.2)	-7.1 (-8.8 to -5.7)	-6.2 (-7.9 to -4.9)
Diet low in seafood omega-3 fatty acids	49.3 (48.3 to 50.4)	45.9 (44.2 to 47.5)	43.5 (41.3 to 45.6)	-7.0 (-8.6 to -5.6)	-5.3 (-6.7 to -4.1)	50.0 (49.0 to 50.9)	46.9 (45.4 to 48.4)	44.9 (42.9 to 46.7)	-6.1 (-7.5 to -4.8)	-4.4 (-5.6 to -3.4)
Diet low in polyunsaturated fatty acids	43.3 (41.2 to 45.2)	38.4 (35.6 to 41.0)	36.1 (33.0 to 39.0)	-11.2 (-14.6 to -8.3)	-6.0 (-8.3 to -4.2)	43.6 (41.6 to 45.4)	38.9 (36.0 to 41.4)	36.6 (33.3 to 39.5)	-10.9 (-14.3 to -8.0)	-5.8 (-7.9 to -4.2)
Diet high in trans fatty acids	3.7 (2.2 to 6.1)	2.5 (1.1 to 4.9)	1.9 (0.6 to 4.2)	-33.4 (-51.4 to -20.8)	-22.4 (-43.5 to -12.7)	4.9 (3.2 to 7.4)	3.6 (1.9 to 6.3)	2.7 (1.1 to 5.2)	-26.8 (-42.6 to -15.2)	-24.7 (-41.5 to -14.6)
Diet high in sodium	24.9 (19.9 to 29.6)	22.9 (17.9 to 27.7)	21.3 (16.5 to 26.3)	-8.0 (-16.4 to -0.3)	-6.9 (-12.2 to -1.9)	25.1 (20.2 to 29.7)	21.9 (16.9 to 26.9)	19.6 (14.7 to 24.7)	-12.5 (-21.0 to -6.2)	-10.6 (-16.3 to -6.4)
Intimate partner violence	-	-	-	-	-	6.3 (5.3 to 7.4)	5.5 (4.7 to 6.3)	5.2 (4.4 to 6.1)	-13.1 (-15.0 to -10.9)	-4.6 (-5.8 to -3.1)
Childhood sexual abuse	5.1 (4.2 to 6.3)	5.4 (4.4 to 6.6)	5.7 (4.7 to 7.0)	4.2 (1.4 to 7.1)	6.4 (3.8 to 9.0)	4.7 (3.8 to 5.6)	4.8 (4.0 to 5.8)	5.3 (4.3 to 6.5)	3.8 (1.6 to 6.0)	10.2 (6.7 to 13.6)

Supplementary Results Table 1. Middle SDI age-standardised Summary Exposure Values (SEVs) for Level 4 risk factors and percentage change, by sex, 1990, 2007, and 2017.Data in parentheses are 95% uncertainty intervals.										
	**Male**					**Female**				
	**1990**	**2007**	**2017**	**Percentage change 1990-2007**	**Percentage change 2007-2017**	**1990**	**2007**	**2017**	**Percentage change 1990-2007**	**Percentage change 2007-2017**
Bullying victimization	5.8 (2.5 to 11.7)	6.8 (3.0 to 13.7)	7.0 (3.1 to 14.2)	18.0 (11.6 to 25.7)	2.7 (0.4 to 5.2)	4.5 (1.9 to 9.2)	5.5 (2.4 to 10.5)	5.7 (2.5 to 11.2)	21.0 (14.2 to 30.0)	4.4 (2.1 to 6.9)
Low physical activity	0.4 (0.2 to 0.6)	0.4 (0.2 to 0.7)	0.4 (0.2 to 0.7)	2.1 (1.2 to 2.8)	1.5 (0.9 to 2.0)	0.3 (0.1 to 0.6)	0.3 (0.1 to 0.6)	0.3 (0.2 to 0.6)	2.3 (1.4 to 3.0)	1.9 (1.1 to 2.6)
High fasting plasma glucose	1.8 (1.4 to 2.4)	2.3 (1.7 to 3.0)	2.7 (2.1 to 3.4)	27.0 (18.4 to 36.3)	15.6 (6.1 to 28.0)	1.8 (1.4 to 2.4)	2.2 (1.7 to 3.0)	2.4 (1.9 to 3.2)	23.8 (14.4 to 33.3)	9.2 (-0.1 to 19.6)
High LDL cholesterol	9.7 (8.3 to 11.3)	9.9 (8.5 to 11.5)	10.1 (8.7 to 11.7)	1.9 (1.1 to 2.8)	2.2 (1.5 to 2.9)	10.0 (8.6 to 11.5)	10.8 (9.4 to 12.3)	11.2 (9.9 to 12.8)	7.6 (6.4 to 9.0)	4.6 (3.8 to 5.4)
High systolic blood pressure	6.1 (5.5 to 6.8)	6.9 (6.2 to 7.6)	7.3 (6.6 to 8.1)	12.4 (8.6 to 16.7)	5.9 (3.4 to 8.5)	6.2 (5.6 to 6.7)	6.7 (6.1 to 7.3)	6.9 (6.3 to 7.5)	9.0 (4.9 to 13.5)	2.8 (-0.2 to 5.8)
High body-mass index	4.5 (3.1 to 6.7)	7.4 (5.6 to 10.2)	10.0 (7.9 to 13.6)	63.0 (48.3 to 79.2)	35.0 (28.7 to 42.1)	5.2 (3.7 to 7.4)	8.4 (6.4 to 11.2)	10.9 (8.5 to 14.2)	60.6 (47.3 to 76.8)	29.3 (23.8 to 35.2)
Low bone mineral density	5.6 (4.9 to 6.5)	5.4 (4.6 to 6.2)	5.2 (4.5 to 6.0)	-4.7 (-6.8 to -2.4)	-2.4 (-4.7 to -0.2)	7.7 (6.7 to 8.7)	7.5 (6.6 to 8.5)	7.3 (6.4 to 8.3)	-2.9 (-5.2 to -0.6)	-2.5 (-4.6 to -0.4)
Impaired kidney function	2.6 (1.2 to 6.4)	2.6 (1.2 to 6.4)	2.6 (1.2 to 6.5)	1.5 (-1.0 to 3.9)	1.3 (-0.3 to 3.5)	3.0 (1.4 to 6.9)	3.0 (1.4 to 7.0)	3.1 (1.4 to 7.1)	1.2 (-1.8 to 4.2)	2.8 (0.9 to 5.1)

Supplementary Results Table 1. High-middle SDI age-standardised Summary Exposure Values (SEVs) for Level 4 risk factors and percentage change, by sex, 1990, 2007, and 2017.Data in parentheses are 95% uncertainty intervals.										
	Male					Female				
	1990	2007	2017	Percentage change 1990-2007	Percentage change 2007-2017	1990	2007	2017	Percentage change 1990-2007	Percentage change 2007-2017
Unsafe water source	33.2 (30.7 to 35.5)	20.4 (18.2 to 22.5)	13.5 (11.3 to 15.8)	-38.7 (-44.4 to -32.5)	-33.8 (-41.9 to -24.5)	32.4 (29.9 to 34.8)	20.0 (17.9 to 22.1)	13.4 (11.3 to 15.6)	-38.2 (-43.9 to -32.0)	-33.2 (-41.2 to -24.1)
Unsafe sanitation	56.7 (50.9 to 64.6)	25.7 (21.2 to 31.5)	15.6 (11.8 to 20.1)	-54.8 (-61.1 to -48.1)	-39.4 (-50.1 to -25.7)	55.9 (50.0 to 63.6)	25.7 (21.4 to 31.4)	15.7 (12.0 to 20.2)	-54.0 (-60.3 to -47.3)	-38.8 (-49.2 to -25.5)
No access to handwashing facility	24.2 (23.2 to 25.2)	16.0 (15.3 to 16.7)	11.5 (11.0 to 12.1)	-33.7 (-36.7 to -30.5)	-28.0 (-30.8 to -25.3)	23.6 (22.7 to 24.6)	15.8 (15.1 to 16.5)	11.4 (10.9 to 12.0)	-33.3 (-36.2 to -30.1)	-27.4 (-30.1 to -24.7)
Ambient particulate matter pollution	37.3 (28.8 to 48.5)	52.3 (41.0 to 66.5)	53.0 (41.8 to 67.8)	40.3 (28.4 to 54.5)	1.4 (-5.7 to 8.7)	33.8 (26.2 to 44.7)	48.8 (38.3 to 62.4)	50.3 (39.5 to 64.9)	44.2 (31.7 to 58.6)	3.1 (-4.8 to 11.0)
Household air pollution from solid fuels	29.8 (24.1 to 37.8)	10.7 (8.1 to 15.0)	5.3 (3.8 to 8.8)	-64.2 (-69.3 to -55.3)	-50.4 (-58.0 to -37.5)	30.2 (24.7 to 38.3)	11.9 (9.2 to 16.5)	6.3 (4.5 to 10.2)	-60.5 (-66.0 to -51.9)	-47.6 (-55.9 to -33.8)
Ambient ozone pollution	43.6 (20.2 to 52.7)	44.3 (20.7 to 53.2)	45.3 (21.7 to 53.9)	1.7 (0.9 to 2.8)	2.2 (1.3 to 4.3)	42.9 (19.6 to 52.6)	43.8 (20.2 to 53.2)	44.8 (21.2 to 53.9)	2.1 (0.9 to 3.7)	2.5 (1.4 to 4.8)
Residential radon	28.1 (17.3 to 40.1)	27.0 (16.5 to 38.5)	26.3 (16.0 to 37.5)	-3.9 (-7.5 to 0.2)	-2.3 (-4.9 to 0.6)	28.6 (17.5 to 40.9)	27.5 (17.0 to 39.1)	27.0 (16.5 to 38.4)	-3.9 (-7.6 to 0.1)	-1.9 (-4.3 to 0.6)
Lead exposure	14.0 (10.2 to 17.6)	12.5 (8.8 to 16.1)	9.7 (6.4 to 13.1)	-10.9 (-14.6 to -8.2)	-22.6 (-27.0 to -18.1)	7.8 (4.6 to 11.4)	7.2 (4.0 to 10.8)	5.6 (2.9 to 9.0)	-8.5 (-13.3 to -5.7)	-21.6 (-26.9 to -16.3)
Occupational exposure to asbestos	1.0 (0.9 to 1.3)	1.4 (1.3 to 1.4)	1.4 (1.3 to 1.5)	30.6 (6.9 to 52.6)	0.8 (-8.6 to 8.9)	0.9 (0.7 to 1.2)	0.7 (0.6 to 0.7)	0.6 (0.6 to 0.7)	-27.4 (-42.6 to -5.3)	-5.8 (-15.5 to 2.7)
Occupational exposure to arsenic	0.3 (0.1 to 0.6)	0.3 (0.1 to 0.6)	0.3 (0.1 to 0.6)	5.1 (-1.2 to 17.6)	3.4 (-1.2 to 10.7)	0.4 (0.1 to 0.7)	0.4 (0.1 to 0.7)	0.4 (0.1 to 0.7)	7.0 (-1.8 to 25.4)	2.0 (-5.0 to 11.4)
Occupational exposure to benzene	0.5 (0.3 to 1.0)	0.6 (0.3 to 1.1)	0.6 (0.3 to 1.2)	13.0 (8.5 to 21.1)	8.4 (5.7 to 12.5)	0.6 (0.3 to 1.2)	0.7 (0.3 to 1.4)	0.8 (0.4 to 1.5)	17.3 (12.0 to 28.1)	11.6 (8.5 to 17.3)
Occupational exposure to beryllium	0.1 (0.1 to 0.1)	0.1 (0.1 to 0.1)	0.1 (0.1 to 0.1)	8.8 (5.6 to 12.2)	4.5 (2.0 to 7.0)	0.1 (0.1 to 0.1)	0.1 (0.1 to 0.1)	0.1 (0.1 to 0.1)	13.1 (9.8 to 16.7)	4.5 (1.9 to 7.1)
Occupational exposure to cadmium	0.1 (0.1 to 0.1)	0.2 (0.1 to 0.2)	0.2 (0.2 to 0.2)	14.2 (8.1 to 21.5)	6.6 (1.8 to 11.8)	0.1 (0.1 to 0.2)	0.2 (0.2 to 0.2)	0.2 (0.2 to 0.2)	18.1 (8.5 to 27.8)	5.8 (-1.7 to 14.7)
Occupational exposure to chromium	0.3 (0.3 to 0.3)	0.3 (0.3 to 0.4)	0.4 (0.4 to 0.4)	17.9 (11.7 to 25.0)	8.8 (3.7 to 13.9)	0.3 (0.3 to 0.3)	0.4 (0.3 to 0.4)	0.4 (0.4 to 0.4)	21.7 (11.4 to 32.1)	8.3 (0.6 to 17.6)
Occupational exposure to diesel engine exhaust	1.5 (1.4 to 1.5)	1.8 (1.7 to 1.8)	2.0 (1.9 to 2.0)	18.5 (13.8 to 23.7)	11.1 (7.4 to 14.8)	1.1 (1.0 to 1.1)	1.2 (1.2 to 1.3)	1.4 (1.3 to 1.4)	16.8 (11.0 to 23.0)	9.7 (4.3 to 15.0)
Occupational exposure to formaldehyde	0.6 (0.6 to 0.7)	0.7 (0.7 to 0.8)	0.8 (0.7 to 0.9)	14.7 (7.9 to 22.5)	7.2 (1.9 to 12.7)	0.7 (0.6 to 0.7)	0.8 (0.7 to 0.8)	0.8 (0.7 to 0.9)	16.1 (4.8 to 27.4)	5.4 (-3.1 to 15.6)
Occupational exposure to nickel	0.4 (0.1 to 1.1)	0.4 (0.1 to 1.1)	0.4 (0.1 to 1.1)	0.4 (-6.5 to 15.5)	1.8 (-2.2 to 9.4)	0.4 (0.1 to 1.1)	0.4 (0.1 to 1.1)	0.4 (0.1 to 1.0)	2.8 (-5.2 to 22.0)	-0.2 (-6.5 to 11.7)
Occupational exposure to polycyclic aromatic hydrocarbons	0.6 (0.6 to 0.6)	0.7 (0.7 to 0.7)	0.8 (0.7 to 0.8)	17.7 (11.8 to 24.3)	8.9 (4.0 to 13.7)	0.6 (0.6 to 0.6)	0.8 (0.7 to 0.8)	0.8 (0.8 to 0.9)	21.1 (11.4 to 31.1)	8.8 (1.4 to 17.3)
Occupational exposure to silica	3.7 (1.5 to 9.1)	3.7 (1.7 to 8.7)	3.8 (1.7 to 8.8)	-0.1 (-5.3 to 9.8)	3.0 (-0.5 to 8.8)	3.0 (1.2 to 7.8)	2.9 (1.3 to 6.7)	2.8 (1.3 to 6.6)	-4.6 (-10.8 to 6.3)	-3.0 (-8.7 to 4.6)
Occupational exposure to sulphuric acid	0.7 (0.4 to 1.4)	0.7 (0.5 to 1.4)	0.7 (0.5 to 1.4)	5.3 (-0.8 to 14.0)	2.3 (-1.7 to 7.1)	0.7 (0.4 to 1.5)	0.8 (0.5 to 1.5)	0.8 (0.5 to 1.5)	6.0 (-1.7 to 16.5)	0.7 (-5.2 to 8.1)
Occupational exposure to trichloroethylene	0.2 (0.2 to 0.2)	0.2 (0.2 to 0.2)	0.2 (0.2 to 0.2)	18.8 (14.0 to 24.5)	9.3 (5.5 to 13.7)	0.2 (0.2 to 0.2)	0.2 (0.2 to 0.2)	0.2 (0.2 to 0.2)	21.9 (14.4 to 30.3)	8.8 (3.0 to 16.0)
Occupational asthmagens	14.9 (12.5 to 17.9)	14.1 (11.9 to 16.8)	14.0 (11.8 to 16.5)	-5.6 (-12.9 to 1.1)	-1.0 (-5.6 to 3.7)	9.9 (7.8 to 12.5)	8.9 (7.3 to 11.0)	8.5 (6.9 to 10.4)	-9.8 (-20.4 to 0.7)	-5.3 (-13.2 to 3.3)
Occupational particulate matter, gases, and fumes	8.3 (6.4 to 11.2)	8.2 (6.5 to 11.1)	8.2 (6.5 to 10.8)	-0.6 (-2.9 to 1.9)	-0.9 (-2.5 to 0.8)	6.1 (4.7 to 8.2)	6.4 (5.0 to 8.4)	6.3 (5.0 to 8.3)	4.6 (1.7 to 7.9)	-1.4 (-3.4 to 0.8)
Occupational noise	8.4 (8.0 to 8.9)	8.5 (8.1 to 9.0)	8.5 (8.1 to 9.1)	1.1 (-0.5 to 2.9)	0.7 (-0.4 to 1.8)	6.2 (5.9 to 6.7)	6.7 (6.3 to 7.1)	6.8 (6.4 to 7.2)	6.5 (4.4 to 8.8)	1.5 (0.3 to 2.9)
Occupational ergonomic factors	14.7 (13.3 to 16.2)	12.3 (10.9 to 13.9)	11.1 (9.8 to 12.8)	-16.7 (-24.3 to -9.0)	-9.4 (-15.2 to -3.6)	13.0 (11.7 to 14.3)	10.7 (9.4 to 12.1)	9.2 (7.9 to 10.8)	-17.6 (-27.0 to -7.6)	-13.7 (-22.1 to -4.9)
Non-exclusive breastfeeding	0.5 (0.3 to 0.6)	0.4 (0.3 to 0.6)	0.4 (0.3 to 0.6)	-6.1 (-10.7 to -1.0)	-3.5 (-7.0 to 0.7)	0.5 (0.3 to 0.6)	0.4 (0.3 to 0.6)	0.4 (0.3 to 0.6)	-5.7 (-10.2 to -0.8)	-3.5 (-7.0 to 0.6)
Discontinued breastfeeding	1.5 (1.5 to 1.6)	1.7 (1.7 to 1.7)	1.7 (1.7 to 1.8)	10.0 (6.5 to 13.8)	2.6 (0.4 to 5.1)	1.5 (1.5 to 1.6)	1.7 (1.6 to 1.7)	1.7 (1.7 to 1.8)	9.4 (6.1 to 13.0)	3.0 (0.8 to 5.3)
Child underweight	0.4 (0.3 to 0.5)	0.2 (0.2 to 0.3)	0.2 (0.1 to 0.3)	-48.2 (-55.5 to -42.4)	-16.1 (-19.8 to -12.7)	0.5 (0.3 to 0.6)	0.2 (0.2 to 0.3)	0.2 (0.1 to 0.2)	-50.2 (-57.5 to -44.2)	-21.2 (-25.8 to -17.5)
Child wasting	0.3 (0.2 to 0.3)	0.2 (0.2 to 0.3)	0.2 (0.1 to 0.2)	-23.0 (-33.2 to -14.3)	-11.2 (-16.9 to -5.4)	0.3 (0.2 to 0.3)	0.2 (0.1 to 0.2)	0.2 (0.1 to 0.2)	-24.4 (-34.2 to -16.0)	-12.9 (-17.9 to -8.0)
Child stunting	1.3 (0.9 to 1.7)	0.8 (0.6 to 1.1)	0.7 (0.5 to 0.9)	-35.7 (-48.0 to -22.9)	-17.7 (-28.2 to -7.0)	1.3 (0.9 to 1.7)	0.8 (0.5 to 1.0)	0.6 (0.4 to 0.8)	-40.9 (-53.1 to -29.6)	-20.4 (-30.8 to -10.1)
Short gestation for birth weight	0.0 (0.0 to 0.0)	0.0 (0.0 to 0.0)	0.0 (0.0 to 0.0)	29.6 (19.6 to 38.5)	5.4 (-0.7 to 12.9)	0.0 (0.0 to 0.0)	0.0 (0.0 to 0.0)	0.0 (0.0 to 0.0)	24.7 (16.4 to 34.3)	3.4 (-4.0 to 11.0)
Low birth weight for gestation	0.0 (0.0 to 0.0)	0.0 (0.0 to 0.0)	0.0 (0.0 to 0.0)	6.8 (-0.3 to 15.2)	4.3 (-1.5 to 12.0)	0.0 (0.0 to 0.0)	0.0 (0.0 to 0.0)	0.0 (0.0 to 0.0)	3.7 (-2.8 to 10.7)	3.0 (-2.3 to 8.1)
Iron deficiency	-	-	-	-	-	8.9 (6.8 to 11.3)	6.7 (5.0 to 8.8)	5.6 (4.1 to 7.4)	-24.3 (-29.5 to -19.5)	-17.0 (-21.3 to -12.7)
Vitamin A deficiency	1.7 (1.4 to 2.0)	1.4 (1.2 to 1.7)	1.4 (1.1 to 1.6)	-14.7 (-18.2 to -11.2)	-4.8 (-8.4 to -1.2)	1.6 (1.4 to 1.9)	1.4 (1.2 to 1.6)	1.3 (1.1 to 1.6)	-14.9 (-18.9 to -11.2)	-3.7 (-7.2 to -0.4)
Zinc deficiency	0.6 (0.2 to 1.4)	0.5 (0.1 to 1.0)	0.5 (0.1 to 0.9)	-22.1 (-28.0 to -10.2)	-8.6 (-18.7 to 5.9)	0.6 (0.2 to 1.3)	0.5 (0.1 to 1.0)	0.5 (0.1 to 0.9)	-22.0 (-28.3 to -9.9)	-9.4 (-19.1 to 5.4)
Smoking	13.1 (11.5 to 14.7)	12.6 (11.1 to 14.1)	11.6 (10.3 to 12.9)	-3.7 (-8.0 to 0.5)	-8.1 (-10.8 to -5.3)	2.5 (2.1 to 2.9)	2.1 (1.8 to 2.4)	1.9 (1.7 to 2.3)	-15.2 (-20.6 to -9.4)	-6.4 (-9.7 to -3.3)
Chewing tobacco	1.3 (1.2 to 1.5)	1.3 (1.1 to 1.4)	1.4 (1.2 to 1.6)	-6.7 (-20.8 to 9.2)	9.9 (-2.3 to 23.1)	1.1 (0.9 to 1.4)	1.2 (1.0 to 1.5)	1.2 (0.9 to 1.5)	11.6 (-16.2 to 50.9)	-1.0 (-19.3 to 18.6)
Second-hand smoke	39.1 (37.6 to 40.5)	35.8 (34.4 to 37.2)	34.1 (32.6 to 35.5)	-8.4 (-12.0 to -4.5)	-4.8 (-7.9 to -1.9)	62.3 (61.1 to 63.5)	55.6 (54.4 to 56.7)	51.4 (50.2 to 52.6)	-10.7 (-12.7 to -8.6)	-7.5 (-9.2 to -5.8)
Alcohol use	16.5 (12.2 to 21.5)	18.4 (13.7 to 23.3)	20.2 (14.5 to 26.0)	11.1 (2.1 to 22.9)	9.9 (-3.1 to 23.8)	5.1 (3.1 to 8.2)	5.3 (3.3 to 8.1)	5.5 (3.5 to 8.4)	3.3 (-4.6 to 13.8)	5.5 (-4.9 to 19.1)
Drug use	0.9 (0.8 to 1.1)	1.1 (0.9 to 1.3)	1.2 (1.0 to 1.4)	12.5 (8.1 to 16.8)	9.8 (5.2 to 14.8)	0.4 (0.3 to 0.5)	0.4 (0.3 to 0.5)	0.4 (0.4 to 0.5)	1.0 (-2.8 to 4.6)	6.2 (3.1 to 9.0)
Diet low in fruits	42.7 (37.7 to 45.3)	36.9 (31.2 to 40.4)	31.7 (25.9 to 36.0)	-13.6 (-18.0 to -10.3)	-14.1 (-19.3 to -10.2)	41.2 (36.2 to 44.1)	34.8 (29.4 to 38.6)	29.2 (23.6 to 33.7)	-15.6 (-20.5 to -11.8)	-16.1 (-21.6 to -11.5)
Diet low in vegetables	32.0 (27.9 to 35.6)	21.3 (16.0 to 26.3)	16.1 (11.2 to 21.1)	-33.2 (-43.6 to -24.6)	-24.7 (-34.3 to -17.4)	33.8 (30.0 to 37.2)	23.5 (17.8 to 28.3)	17.8 (12.4 to 23.0)	-30.7 (-40.5 to -22.9)	-24.0 (-33.5 to -17.0)
Diet low in legumes	27.2 (23.1 to 30.7)	29.9 (26.4 to 32.8)	28.6 (25.1 to 31.7)	9.9 (3.9 to 18.4)	-4.2 (-8.3 to -0.9)	31.0 (27.4 to 34.3)	33.5 (30.3 to 36.3)	32.6 (29.3 to 35.3)	7.9 (3.2 to 14.0)	-2.8 (-5.4 to -0.1)
Diet low in whole grains	43.7 (41.9 to 45.3)	42.9 (41.0 to 44.6)	41.9 (39.8 to 43.7)	-1.8 (-2.9 to -0.8)	-2.3 (-3.5 to -1.2)	44.7 (43.1 to 46.1)	43.7 (41.9 to 45.3)	42.7 (40.7 to 44.4)	-2.1 (-3.2 to -1.1)	-2.3 (-3.4 to -1.4)
Diet low in nuts and seeds	51.5 (50.9 to 52.1)	48.9 (47.7 to 50.0)	45.0 (42.9 to 46.8)	-5.0 (-6.4 to -4.0)	-8.0 (-10.3 to -6.2)	51.7 (51.1 to 52.3)	49.5 (48.3 to 50.4)	45.9 (43.8 to 47.5)	-4.4 (-5.6 to -3.4)	-7.3 (-9.4 to -5.6)
Diet low in milk	46.3 (44.6 to 47.8)	46.7 (45.1 to 48.1)	45.9 (44.1 to 47.4)	0.8 (0.3 to 1.4)	-1.8 (-2.4 to -1.3)	46.2 (44.4 to 47.7)	46.8 (45.2 to 48.2)	46.0 (44.2 to 47.5)	1.2 (0.7 to 1.8)	-1.7 (-2.3 to -1.2)
Diet high in red meat	13.1 (10.3 to 16.2)	17.6 (12.9 to 21.6)	23.5 (18.8 to 27.4)	34.8 (10.0 to 64.0)	33.4 (15.7 to 58.3)	5.3 (3.5 to 7.4)	7.3 (4.3 to 10.6)	11.7 (7.4 to 15.7)	38.9 (3.6 to 87.8)	58.9 (28.7 to 106.5)
Diet high in processed meat	3.4 (2.2 to 4.9)	2.5 (1.4 to 4.4)	3.0 (1.5 to 5.0)	-24.4 (-45.2 to -6.4)	17.3 (0.6 to 29.7)	2.6 (1.5 to 4.0)	1.8 (0.9 to 3.5)	2.1 (0.9 to 4.0)	-29.3 (-50.8 to -10.7)	16.3 (-3.5 to 28.0)
Diet high in sugar-sweetened beverages	4.2 (2.1 to 5.1)	5.3 (2.7 to 6.5)	7.7 (4.0 to 9.8)	26.5 (7.3 to 53.6)	46.0 (27.9 to 69.0)	3.0 (1.5 to 3.7)	3.7 (1.9 to 4.6)	5.2 (2.7 to 6.7)	24.9 (3.8 to 51.0)	41.3 (24.1 to 65.1)
Diet low in fibre	32.6 (28.1 to 36.5)	31.4 (26.6 to 35.5)	29.3 (24.2 to 33.6)	-3.7 (-7.9 to -0.3)	-6.7 (-11.0 to -3.4)	36.9 (33.0 to 40.0)	36.0 (32.0 to 39.4)	34.2 (29.7 to 37.8)	-2.2 (-5.3 to 0.3)	-5.1 (-8.2 to -2.7)
Diet low in calcium	39.9 (36.8 to 42.3)	35.8 (31.9 to 38.8)	31.9 (27.3 to 35.6)	-10.3 (-13.6 to -7.9)	-10.8 (-14.5 to -8.1)	41.3 (38.6 to 43.4)	37.8 (34.4 to 40.6)	34.4 (30.3 to 37.7)	-8.4 (-11.0 to -6.4)	-9.1 (-12.2 to -6.8)
Diet low in seafood omega-3 fatty acids	44.1 (42.1 to 46.0)	40.7 (38.0 to 43.2)	36.4 (33.0 to 39.6)	-7.7 (-9.9 to -5.9)	-10.4 (-13.5 to -7.9)	45.1 (43.3 to 46.9)	42.4 (40.0 to 44.6)	38.7 (35.6 to 41.6)	-5.9 (-7.8 to -4.5)	-8.7 (-11.3 to -6.6)
Diet low in polyunsaturated fatty acids	38.1 (35.2 to 40.7)	32.5 (28.7 to 35.9)	29.1 (24.8 to 33.0)	-14.7 (-19.8 to -10.7)	-10.4 (-14.9 to -7.1)	38.4 (35.6 to 40.9)	33.0 (29.2 to 36.4)	29.6 (25.1 to 33.5)	-14.1 (-19.6 to -10.0)	-10.3 (-14.6 to -7.1)
Diet high in trans fatty acids	1.8 (0.6 to 4.0)	1.4 (0.4 to 3.4)	1.2 (0.3 to 3.1)	-23.0 (-50.9 to -11.0)	-15.3 (-42.3 to -5.7)	2.5 (1.0 to 4.9)	1.9 (0.6 to 4.2)	1.6 (0.4 to 3.9)	-22.4 (-47.1 to -9.9)	-13.9 (-37.9 to -3.9)
Diet high in sodium	25.7 (20.9 to 30.0)	25.9 (20.8 to 30.4)	26.7 (21.9 to 31.4)	0.7 (-7.6 to 9.6)	3.4 (-1.7 to 9.8)	24.0 (19.7 to 28.5)	23.7 (18.9 to 28.4)	23.7 (18.9 to 28.3)	-1.6 (-9.4 to 5.1)	0.0 (-5.3 to 5.9)
Intimate partner violence	-	-	-	-	-	5.9 (5.0 to 6.8)	5.2 (4.4 to 6.0)	4.9 (4.2 to 5.7)	-11.6 (-12.9 to -10.4)	-5.5 (-6.7 to -4.1)
Childhood sexual abuse	4.8 (3.9 to 5.9)	5.0 (4.1 to 6.1)	5.5 (4.5 to 6.7)	4.0 (1.2 to 7.0)	9.2 (6.1 to 12.3)	4.9 (3.9 to 6.0)	4.4 (3.6 to 5.4)	4.3 (3.5 to 5.3)	-10.0 (-12.5 to -7.7)	-1.8 (-3.4 to 0.0)

Supplementary Results Table 1. High-middle SDI age-standardised Summary Exposure Values (SEVs) for Level 4 risk factors and percentage change, by sex, 1990, 2007, and 2017.Data in parentheses are 95% uncertainty intervals.

	Male					Female				
	**1990**	**2007**	**2017**	**Percentage change 1990-2007**	**Percentage change 2007-2017**	**1990**	**2007**	**2017**	**Percentage change 1990-2007**	**Percentage change 2007-2017**
Bullying victimization	5.9 (2.5 to 12.1)	7.1 (3.1 to 14.2)	7.2 (3.2 to 14.4)	20.3 (13.6 to 30.6)	1.3 (-0.8 to 3.2)	4.3 (1.9 to 8.8)	5.2 (2.3 to 10.3)	5.2 (2.3 to 10.3)	20.4 (13.1 to 30.7)	-0.1 (-2.3 to 1.9)
Low physical activity	0.4 (0.2 to 0.8)	0.4 (0.2 to 0.8)	0.4 (0.2 to 0.8)	-0.5 (-1.2 to 0.2)	2.5 (1.3 to 3.5)	0.4 (0.2 to 0.7)	0.4 (0.2 to 0.7)	0.4 (0.2 to 0.7)	-1.3 (-2.2 to -0.3)	1.9 (0.9 to 2.7)
High fasting plasma glucose	2.0 (1.5 to 2.6)	2.3 (1.8 to 3.0)	2.4 (1.9 to 3.1)	17.1 (6.4 to 28.6)	4.4 (-4.0 to 14.0)	1.8 (1.3 to 2.3)	2.1 (1.6 to 2.8)	2.1 (1.5 to 2.7)	20.9 (7.7 to 34.1)	-1.8 (-13.6 to 10.3)
High LDL cholesterol	12.0 (10.5 to 13.6)	11.8 (10.3 to 13.5)	11.7 (10.2 to 13.4)	-1.5 (-2.5 to -0.4)	-0.8 (-1.7 to 0.0)	12.4 (11.0 to 13.9)	12.5 (11.1 to 14.1)	12.6 (11.2 to 14.3)	0.8 (-0.4 to 1.9)	1.4 (0.5 to 2.2)
High systolic blood pressure	7.2 (6.5 to 7.9)	8.0 (7.2 to 8.7)	8.1 (7.3 to 8.8)	10.6 (6.8 to 14.7)	1.1 (-2.0 to 4.3)	7.4 (6.9 to 8.0)	7.5 (6.9 to 8.2)	7.2 (6.5 to 7.8)	1.6 (-2.4 to 5.6)	-4.7 (-7.8 to -1.1)
High body-mass index	6.5 (4.7 to 9.5)	10.4 (8.0 to 14.3)	13.8 (10.9 to 18.4)	59.1 (47.1 to 73.1)	32.4 (27.2 to 38.9)	7.7 (5.7 to 10.5)	11.0 (8.4 to 14.6)	13.9 (10.8 to 18.1)	42.8 (34.2 to 53.2)	26.0 (21.8 to 30.9)
Low bone mineral density	5.1 (4.4 to 5.9)	5.1 (4.3 to 5.9)	5.0 (4.2 to 5.8)	-0.8 (-3.3 to 1.7)	-1.3 (-3.8 to 1.3)	6.6 (5.8 to 7.7)	6.7 (5.9 to 7.7)	6.6 (5.7 to 7.6)	1.3 (-1.4 to 4.4)	-1.7 (-5.0 to 1.5)
Impaired kidney function	2.6 (1.2 to 6.5)	2.6 (1.1 to 6.4)	2.5 (1.1 to 6.3)	-2.8 (-5.8 to -0.6)	-1.3 (-3.8 to 1.1)	3.3 (1.6 to 7.3)	3.2 (1.5 to 7.3)	3.2 (1.5 to 7.3)	-1.9 (-5.6 to 0.9)	0.7 (-1.7 to 3.2)

Supplementary Results Table 1. High SDI age-standardised Summary Exposure Values (SEVs) for Level 4 risk factors and percentage change, by sex, 1990, 2007, and 2017.Data in parentheses are 95% uncertainty intervals.

	Male					Female				
	**1990**	**2007**	**2017**	**Percentage change 1990-2007**	**Percentage change 2007-2017**	**1990**	**2007**	**2017**	**Percentage change 1990-2007**	**Percentage change 2007-2017**
Unsafe water source	3.2 (2.8 to 3.6)	1.7 (1.5 to 2.0)	1.3 (1.1 to 1.5)	-46.9 (-54.5 to -37.6)	-23.9 (-33.4 to -11.3)	3.2 (2.8 to 3.6)	1.7 (1.4 to 1.9)	1.3 (1.1 to 1.5)	-47.0 (-54.6 to -37.8)	-23.6 (-32.9 to -11.5)
Unsafe sanitation	7.1 (6.0 to 8.4)	3.1 (2.4 to 4.1)	2.2 (1.7 to 3.0)	-56.0 (-65.0 to -44.3)	-29.0 (-43.0 to -10.1)	7.0 (6.0 to 8.3)	3.1 (2.4 to 4.1)	2.2 (1.7 to 2.9)	-55.6 (-64.9 to -43.8)	-28.7 (-42.7 to -10.0)
No access to handwashing facility	4.7 (4.4 to 5.1)	4.2 (3.9 to 4.5)	3.9 (3.6 to 4.2)	-11.7 (-17.8 to -4.9)	-7.9 (-13.0 to -2.7)	4.7 (4.4 to 5.0)	4.2 (3.9 to 4.5)	3.8 (3.6 to 4.1)	-11.6 (-17.7 to -4.7)	-7.8 (-12.9 to -2.5)
Ambient particulate matter pollution	33.0 (25.0 to 44.7)	34.0 (25.6 to 46.2)	29.3 (21.8 to 41.3)	3.0 (-3.2 to 9.6)	-13.9 (-20.2 to -8.6)	32.2 (24.2 to 43.9)	33.5 (25.1 to 45.8)	28.9 (21.4 to 40.9)	4.2 (-2.3 to 11.4)	-13.9 (-20.3 to -8.4)
Household air pollution from solid fuels	3.0 (2.1 to 5.2)	0.9 (0.6 to 2.0)	0.6 (0.3 to 1.5)	-68.7 (-76.4 to -57.8)	-33.9 (-49.0 to -18.4)	3.5 (2.4 to 7.3)	1.3 (0.7 to 4.0)	0.9 (0.5 to 3.3)	-64.7 (-74.1 to -47.4)	-30.3 (-47.4 to -13.4)
Ambient ozone pollution	43.3 (17.7 to 52.8)	43.6 (18.0 to 52.9)	44.6 (19.0 to 53.3)	0.6 (0.2 to 1.7)	2.4 (0.9 to 5.7)	43.3 (17.6 to 52.8)	43.5 (17.9 to 52.8)	44.5 (18.9 to 53.3)	0.4 (0.0 to 1.4)	2.4 (0.9 to 5.8)
Residential radon	19.4 (10.7 to 29.6)	19.0 (10.6 to 29.2)	18.9 (10.5 to 29.0)	-1.7 (-3.6 to 0.7)	-1.0 (-2.2 to 0.3)	19.4 (10.8 to 29.8)	19.0 (10.6 to 29.2)	18.8 (10.4 to 29.0)	-2.0 (-4.0 to 0.4)	-1.2 (-2.4 to 0.0)
Lead exposure	7.1 (3.5 to 11.0)	5.3 (2.4 to 8.8)	3.8 (1.5 to 6.9)	-25.6 (-32.8 to -19.2)	-29.0 (-37.1 to -22.1)	4.4 (1.5 to 8.0)	3.5 (1.1 to 6.7)	2.5 (0.7 to 5.3)	-21.1 (-28.0 to -15.2)	-28.0 (-37.2 to -20.7)
Occupational exposure to asbestos	6.4 (5.5 to 6.9)	5.6 (5.5 to 5.7)	5.2 (5.0 to 5.4)	-12.4 (-19.5 to 1.0)	-6.9 (-10.4 to -2.9)	1.3 (1.1 to 1.5)	1.2 (1.2 to 1.3)	1.2 (1.1 to 1.2)	-4.0 (-17.7 to 13.4)	-5.2 (-11.0 to -0.2)
Occupational exposure to arsenic	0.4 (0.0 to 0.9)	0.4 (0.0 to 0.9)	0.4 (0.0 to 0.9)	-5.4 (-7.9 to 27.3)	0.8 (-1.7 to 27.7)	0.3 (0.0 to 0.8)	0.3 (0.0 to 0.8)	0.3 (0.0 to 0.8)	-2.4 (-5.9 to 23.3)	1.1 (-2.0 to 22.7)
Occupational exposure to benzene	0.6 (0.1 to 1.6)	0.6 (0.1 to 1.6)	0.6 (0.1 to 1.6)	4.2 (2.6 to 18.3)	1.7 (0.2 to 11.7)	0.7 (0.1 to 1.9)	0.9 (0.1 to 2.4)	0.9 (0.1 to 2.5)	22.8 (20.9 to 34.9)	7.3 (5.7 to 17.4)
Occupational exposure to beryllium	0.0 (0.0 to 0.0)	0.0 (0.0 to 0.0)	0.0 (0.0 to 0.0)	4.8 (3.6 to 6.1)	6.7 (5.4 to 8.2)	0.0 (0.0 to 0.0)	0.0 (0.0 to 0.0)	0.0 (0.0 to 0.0)	15.2 (13.8 to 16.6)	9.8 (8.5 to 11.3)
Occupational exposure to cadmium	0.1 (0.1 to 0.1)	0.1 (0.1 to 0.1)	0.1 (0.1 to 0.1)	1.3 (-1.4 to 4.0)	6.7 (3.9 to 9.7)	0.1 (0.1 to 0.1)	0.1 (0.1 to 0.1)	0.1 (0.1 to 0.1)	6.0 (1.9 to 10.1)	7.8 (4.0 to 12.1)
Occupational exposure to chromium	0.1 (0.1 to 0.1)	0.1 (0.1 to 0.1)	0.1 (0.1 to 0.1)	3.5 (1.0 to 5.9)	6.9 (4.5 to 9.5)	0.1 (0.1 to 0.1)	0.1 (0.1 to 0.1)	0.2 (0.1 to 0.2)	10.0 (6.3 to 13.7)	8.3 (5.1 to 12.0)
Occupational exposure to diesel engine exhaust	0.6 (0.6 to 0.6)	0.7 (0.6 to 0.7)	0.7 (0.7 to 0.7)	12.3 (10.3 to 14.4)	7.3 (5.3 to 9.3)	0.5 (0.5 to 0.5)	0.6 (0.6 to 0.6)	0.7 (0.7 to 0.7)	24.3 (21.0 to 27.7)	9.1 (6.5 to 11.8)
Occupational exposure to formaldehyde	0.3 (0.3 to 0.3)	0.3 (0.3 to 0.3)	0.3 (0.3 to 0.3)	0.6 (-2.6 to 3.6)	5.2 (2.2 to 8.5)	0.3 (0.3 to 0.3)	0.3 (0.3 to 0.3)	0.3 (0.3 to 0.3)	7.4 (2.8 to 12.1)	7.2 (3.1 to 11.6)
Occupational exposure to nickel	0.4 (0.0 to 1.6)	0.3 (0.0 to 1.4)	0.3 (0.0 to 1.4)	-7.2 (-9.5 to 33.7)	0.5 (-1.7 to 31.3)	0.3 (0.0 to 1.2)	0.3 (0.0 to 1.1)	0.3 (0.0 to 1.1)	-5.8 (-9.3 to 26.9)	0.5 (-2.4 to 29.4)
Occupational exposure to polycyclic aromatic hydrocarbons	0.3 (0.2 to 0.3)	0.3 (0.3 to 0.3)	0.3 (0.3 to 0.3)	4.0 (1.7 to 6.3)	6.7 (4.4 to 9.1)	0.3 (0.3 to 0.3)	0.3 (0.3 to 0.3)	0.3 (0.3 to 0.3)	11.9 (8.6 to 15.2)	8.7 (5.7 to 11.9)
Occupational exposure to silica	3.8 (0.6 to 12.2)	3.6 (0.6 to 11.4)	3.6 (0.7 to 11.3)	-5.2 (-7.3 to 0.5)	0.4 (-1.5 to 4.7)	2.5 (0.4 to 7.6)	2.3 (0.4 to 6.9)	2.3 (0.4 to 6.7)	-7.8 (-11.4 to -1.0)	-0.3 (-3.2 to 5.0)
Occupational exposure to sulphuric acid	0.6 (0.1 to 1.7)	0.6 (0.1 to 1.7)	0.6 (0.2 to 1.7)	-1.6 (-4.3 to 4.0)	0.3 (-2.1 to 6.0)	0.5 (0.1 to 1.5)	0.5 (0.1 to 1.6)	0.5 (0.2 to 1.6)	4.3 (0.6 to 10.1)	0.5 (-3.2 to 7.1)
Occupational exposure to trichloroethylene	0.1 (0.1 to 0.1)	0.1 (0.1 to 0.1)	0.1 (0.1 to 0.1)	6.3 (4.5 to 8.2)	7.2 (5.5 to 9.3)	0.1 (0.1 to 0.1)	0.1 (0.1 to 0.1)	0.1 (0.1 to 0.1)	14.4 (11.7 to 17.2)	8.8 (6.5 to 11.4)
Occupational asthmagens	16.8 (14.5 to 19.8)	15.4 (13.3 to 18.1)	15.2 (13.0 to 17.8)	-8.3 (-11.0 to -5.7)	-1.2 (-3.7 to 1.4)	8.1 (6.9 to 9.5)	8.3 (7.1 to 9.8)	8.5 (7.2 to 10.0)	3.3 (-0.2 to 6.6)	1.7 (-1.1 to 4.7)
Occupational particulate matter, gases, and fumes	6.7 (4.8 to 9.5)	6.3 (4.5 to 8.8)	6.0 (4.4 to 8.5)	-6.1 (-7.7 to -4.6)	-4.0 (-5.0 to -3.1)	3.6 (2.6 to 5.2)	3.5 (2.6 to 5.0)	3.4 (2.6 to 4.8)	-2.1 (-4.6 to 0.0)	-2.8 (-4.5 to -1.1)
Occupational noise	6.1 (5.8 to 6.6)	5.9 (5.6 to 6.4)	5.8 (5.5 to 6.2)	-3.7 (-4.3 to -3.1)	-2.4 (-2.8 to -2.0)	3.8 (3.6 to 4.1)	4.0 (3.9 to 4.3)	4.2 (4.0 to 4.5)	5.6 (4.8 to 6.5)	3.2 (2.7 to 3.7)
Occupational ergonomic factors	9.2 (8.0 to 10.6)	8.1 (7.0 to 9.5)	7.8 (6.8 to 9.2)	-11.5 (-13.6 to -9.6)	-3.2 (-5.0 to -1.6)	5.5 (4.8 to 6.4)	5.6 (4.8 to 6.6)	5.8 (4.9 to 6.9)	2.6 (-0.7 to 5.9)	3.1 (0.2 to 5.9)
Non-exclusive breastfeeding	0.5 (0.4 to 0.6)	0.5 (0.4 to 0.6)	0.5 (0.4 to 0.6)	0.0 (-3.4 to 3.6)	-3.6 (-6.3 to -1.0)	0.5 (0.4 to 0.6)	0.5 (0.4 to 0.6)	0.5 (0.4 to 0.6)	0.0 (-3.4 to 3.6)	-3.6 (-6.4 to -1.1)
Discontinued breastfeeding	2.0 (2.0 to 2.0)	2.1 (2.0 to 2.1)	2.1 (2.0 to 2.1)	2.1 (-0.0 to 4.3)	0.9 (-0.8 to 2.8)	2.0 (2.0 to 2.0)	2.1 (2.0 to 2.1)	2.1 (2.0 to 2.1)	2.1 (0.0 to 4.3)	0.8 (-0.9 to 2.7)
Child underweight	0.1 (0.1 to 0.2)	0.1 (0.1 to 0.2)	0.1 (0.1 to 0.1)	-14.9 (-19.6 to -11.1)	-8.2 (-11.0 to -5.9)	0.1 (0.1 to 0.2)	0.1 (0.1 to 0.1)	0.1 (0.1 to 0.1)	-16.7 (-21.8 to -13.1)	-5.9 (-8.5 to -3.6)
Child wasting	0.1 (0.1 to 0.1)	0.1 (0.1 to 0.1)	0.1 (0.1 to 0.1)	-12.9 (-18.6 to -7.9)	-3.2 (-6.5 to 0.2)	0.1 (0.1 to 0.1)	0.1 (0.1 to 0.1)	0.1 (0.1 to 0.1)	-11.7 (-16.8 to -6.9)	-3.9 (-7.2 to -0.2)
Child stunting	0.3 (0.2 to 0.5)	0.3 (0.2 to 0.4)	0.3 (0.1 to 0.4)	-20.0 (-31.3 to -9.0)	-5.8 (-13.6 to 2.2)	0.4 (0.2 to 0.5)	0.3 (0.2 to 0.4)	0.3 (0.1 to 0.4)	-22.7 (-33.1 to -13.8)	-3.5 (-10.4 to 3.7)
Short gestation for birth weight	0.0 (0.0 to 0.0)	0.0 (0.0 to 0.0)	0.0 (0.0 to 0.0)	7.1 (2.6 to 13.3)	2.2 (-1.8 to 6.5)	0.0 (0.0 to 0.0)	0.0 (0.0 to 0.0)	0.0 (0.0 to 0.0)	7.1 (1.8 to 13.3)	1.5 (-2.7 to 5.9)
Low birth weight for gestation	0.0 (0.0 to 0.0)	0.0 (0.0 to 0.0)	0.0 (0.0 to 0.0)	5.3 (-0.5 to 11.2)	0.4 (-4.6 to 5.1)	0.0 (0.0 to 0.0)	0.0 (0.0 to 0.0)	0.0 (0.0 to 0.0)	3.4 (-1.4 to 8.7)	0.6 (-3.1 to 5.7)
Iron deficiency	-	-	-	-	-	6.3 (4.6 to 8.4)	5.4 (3.8 to 7.2)	4.9 (3.5 to 6.6)	-15.4 (-20.9 to -10.3)	-8.4 (-13.4 to -2.9)
Vitamin A deficiency	1.0 (0.8 to 1.2)	0.9 (0.7 to 1.0)	0.9 (0.7 to 1.0)	-13.9 (-18.9 to -10.2)	-1.2 (-4.2 to 1.9)	1.0 (0.8 to 1.2)	0.9 (0.7 to 1.1)	0.9 (0.7 to 1.1)	-9.0 (-13.7 to -5.5)	-2.4 (-5.1 to 0.5)
Zinc deficiency	0.5 (0.2 to 1.0)	0.4 (0.1 to 0.7)	0.4 (0.1 to 0.8)	-19.9 (-31.1 to 5.7)	5.4 (-11.6 to 25.2)	0.5 (0.2 to 1.0)	0.4 (0.1 to 0.7)	0.4 (0.1 to 0.8)	-21.5 (-32.4 to 5.7)	6.3 (-11.9 to 27.2)
Smoking	14.4 (13.0 to 15.9)	10.1 (9.0 to 11.3)	8.4 (7.5 to 9.5)	-29.9 (-32.0 to -27.8)	-16.2 (-18.0 to -14.4)	7.4 (6.5 to 8.3)	5.2 (4.5 to 6.0)	4.4 (3.8 to 5.1)	-29.6 (-33.1 to -26.2)	-14.3 (-16.5 to -12.1)
Chewing tobacco	0.9 (0.9 to 1.0)	0.9 (0.8 to 1.0)	0.9 (0.7 to 1.0)	-8.0 (-18.8 to 5.6)	-0.2 (-12.6 to 14.4)	0.2 (0.2 to 0.2)	0.2 (0.2 to 0.2)	0.2 (0.2 to 0.3)	17.9 (-1.1 to 41.6)	11.2 (-4.5 to 28.7)
Second-hand smoke	43.3 (41.8 to 45.0)	32.8 (32.0 to 33.5)	31.5 (30.6 to 32.3)	-24.4 (-28.3 to -20.7)	-4.0 (-6.2 to -1.5)	50.2 (48.6 to 51.9)	36.8 (36.2 to 37.4)	34.7 (34.1 to 35.3)	-26.6 (-30.0 to -23.4)	-5.8 (-7.5 to -4.2)
Alcohol use	25.0 (18.3 to 32.4)	24.1 (16.6 to 32.5)	25.3 (17.4 to 34.6)	-3.5 (-11.5 to 3.6)	4.8 (-4.3 to 16.1)	13.7 (8.7 to 20.7)	12.9 (8.1 to 19.8)	13.4 (8.2 to 20.7)	-5.8 (-12.8 to 0.5)	4.2 (-5.8 to 15.8)
Drug use	1.0 (0.7 to 1.4)	1.2 (0.9 to 1.6)	1.4 (1.1 to 1.9)	22.5 (14.6 to 32.9)	20.0 (15.3 to 25.7)	0.6 (0.5 to 0.9)	0.8 (0.6 to 1.0)	0.9 (0.7 to 1.2)	27.9 (19.8 to 38.0)	18.3 (13.4 to 23.6)
Diet low in fruits	31.9 (26.1 to 35.8)	30.2 (24.4 to 34.3)	30.0 (24.3 to 34.3)	-5.4 (-8.2 to -3.6)	-0.4 (-2.4 to 1.5)	28.8 (23.0 to 33.2)	26.9 (21.0 to 31.5)	26.8 (21.0 to 31.5)	-6.4 (-9.9 to -4.2)	-0.5 (-3.1 to 1.9)
Diet low in vegetables	22.4 (17.1 to 27.2)	21.8 (16.3 to 26.8)	21.8 (16.5 to 26.5)	-2.8 (-6.2 to -0.5)	-0.0 (-2.6 to 3.5)	24.8 (19.7 to 29.5)	23.9 (18.5 to 28.6)	23.8 (18.5 to 28.5)	-3.8 (-6.9 to -1.6)	-0.3 (-2.7 to 2.7)
Diet low in legumes	26.6 (23.1 to 29.8)	26.2 (22.5 to 29.6)	27.2 (23.5 to 30.5)	-1.4 (-3.8 to 0.6)	3.7 (1.4 to 6.4)	30.3 (26.8 to 33.3)	30.0 (26.6 to 33.1)	31.1 (27.6 to 34.2)	-0.8 (-2.3 to 0.9)	3.6 (1.8 to 5.6)
Diet low in whole grains	38.7 (36.2 to 40.9)	40.1 (37.8 to 42.1)	40.0 (37.7 to 42.0)	3.6 (2.6 to 4.9)	-0.2 (-1.0 to 0.6)	39.6 (37.3 to 41.7)	41.0 (38.8 to 42.8)	41.0 (38.8 to 42.9)	3.5 (2.5 to 4.7)	-0.1 (-0.8 to 0.6)
Diet low in nuts and seeds	46.3 (44.5 to 47.7)	41.4 (38.9 to 43.6)	40.3 (37.3 to 42.7)	-10.4 (-13.0 to -8.4)	-2.7 (-4.6 to -1.2)	46.8 (45.1 to 48.2)	42.1 (39.7 to 44.2)	41.0 (38.2 to 43.3)	-10.0 (-12.5 to -8.1)	-2.7 (-4.5 to -1.4)
Diet low in milk	32.9 (28.6 to 36.6)	31.9 (27.5 to 35.7)	32.1 (27.5 to 35.8)	-3.1 (-5.0 to -1.8)	0.5 (-0.7 to 1.9)	33.9 (29.7 to 37.3)	32.3 (27.8 to 36.1)	32.4 (27.9 to 36.0)	-4.6 (-6.8 to -3.1)	0.1 (-1.1 to 1.4)
Diet high in red meat	34.0 (32.7 to 35.7)	33.0 (31.1 to 34.9)	33.8 (31.6 to 35.9)	-3.1 (-6.1 to -0.3)	2.4 (-1.2 to 6.2)	17.6 (15.3 to 20.0)	15.3 (12.6 to 17.9)	16.0 (12.9 to 18.8)	-13.4 (-19.9 to -7.6)	4.6 (-3.0 to 12.7)
Diet high in processed meat	17.8 (15.4 to 20.0)	20.6 (18.5 to 22.5)	19.6 (16.8 to 21.8)	15.6 (9.2 to 23.4)	-4.9 (-9.9 to -0.7)	11.8 (9.1 to 14.1)	14.0 (11.6 to 16.4)	13.5 (10.9 to 16.0)	19.2 (10.6 to 31.8)	-3.7 (-9.5 to 1.8)
Diet high in sugar-sweetened beverages	15.0 (7.7 to 17.3)	15.9 (8.2 to 17.3)	15.8 (8.1 to 17.6)	6.2 (-4.6 to 19.3)	-0.9 (-8.2 to 6.6)	11.8 (6.0 to 13.7)	11.7 (6.0 to 12.9)	11.4 (5.8 to 13.0)	-0.9 (-13.0 to 15.8)	-2.4 (-11.2 to 7.5)
Diet low in fibre	30.1 (25.1 to 34.1)	28.6 (23.4 to 32.8)	29.3 (24.1 to 33.5)	-5.1 (-7.7 to -3.1)	2.4 (0.5 to 4.5)	34.4 (30.3 to 37.8)	32.9 (28.4 to 36.5)	33.6 (29.2 to 37.1)	-4.5 (-6.7 to -2.8)	2.1 (0.6 to 4.0)
Diet low in calcium	23.0 (17.1 to 27.8)	22.2 (16.4 to 27.1)	22.0 (16.2 to 27.0)	-3.3 (-5.4 to -1.9)	-0.7 (-2.5 to 0.9)	26.3 (20.8 to 30.8)	25.4 (19.8 to 30.0)	25.3 (19.6 to 30.0)	-3.5 (-5.8 to -2.0)	-0.5 (-1.8 to 0.8)
Diet low in seafood omega-3 fatty acids	30.3 (27.1 to 33.3)	27.9 (24.6 to 31.1)	27.8 (24.3 to 31.2)	-7.9 (-9.6 to -6.6)	-0.3 (-1.7 to 0.8)	32.7 (29.8 to 35.4)	30.6 (27.5 to 33.5)	30.5 (27.3 to 33.6)	-6.3 (-7.8 to -5.1)	-0.2 (-1.3 to 0.6)
Diet low in polyunsaturated fatty acids	25.3 (21.1 to 29.3)	21.0 (16.7 to 25.2)	20.0 (15.9 to 24.0)	-16.7 (-22.3 to -12.2)	-5.0 (-7.0 to -2.9)	25.9 (21.8 to 29.8)	21.7 (17.2 to 25.8)	20.6 (16.5 to 24.6)	-16.4 (-22.1 to -12.2)	-4.9 (-6.9 to -2.8)
Diet high in trans fatty acids	6.8 (4.3 to 10.0)	4.2 (2.1 to 7.0)	3.2 (1.3 to 5.9)	-38.2 (-51.6 to -28.8)	-23.4 (-38.0 to -15.4)	9.5 (6.5 to 13.7)	5.6 (3.3 to 8.7)	4.3 (2.1 to 7.2)	-40.4 (-50.9 to -31.4)	-23.0 (-34.3 to -16.1)
Diet high in sodium	11.7 (5.9 to 17.8)	12.1 (5.6 to 18.2)	12.4 (5.7 to 18.6)	3.1 (-10.0 to 11.0)	2.5 (-3.3 to 10.0)	10.8 (5.9 to 16.8)	10.1 (4.5 to 16.4)	9.8 (4.1 to 16.0)	-5.8 (-25.9 to 1.7)	-3.3 (-11.9 to 1.6)
Intimate partner violence	-	-	-	-	-	2.2 (1.9 to 2.6)	2.0 (1.7 to 2.3)	2.0 (1.7 to 2.3)	-10.9 (-11.8 to -10.0)	0.0 (-1.0 to 1.0)
Childhood sexual abuse	5.0 (4.1 to 6.1)	5.0 (4.1 to 6.0)	5.0 (4.1 to 6.1)	-1.3 (-3.0 to 0.6)	0.4 (-1.2 to 2.1)	8.9 (7.3 to 10.8)	8.5 (7.1 to 10.2)	8.6 (7.1 to 10.4)	-4.8 (-7.6 to -2.0)	1.1 (-0.7 to 3.2)

Supplementary Results Table 1. High SDI age-standardised Summary Exposure Values (SEVs) for Level 4 risk factors and percentage change, by sex, 1990, 2007, and 2017.Data in parentheses are 95% uncertainty intervals.

	Male					Female				
	**1990**	**2007**	**2017**	**Percentage change 1990-2007**	**Percentage change 2007-2017**	**1990**	**2007**	**2017**	**Percentage change 1990-2007**	**Percentage change 2007-2017**
Bullying victimization	5.6 (2.5 to 11.5)	6.9 (3.2 to 13.6)	7.1 (3.3 to 14.1)	22.2 (12.8 to 33.6)	2.8 (0.1 to 5.9)	4.3 (1.9 to 8.7)	5.5 (2.5 to 10.9)	5.7 (2.7 to 11.2)	27.0 (17.7 to 40.0)	4.4 (1.2 to 8.3)
Low physical activity	0.4 (0.2 to 0.8)	0.4 (0.2 to 0.9)	0.4 (0.2 to 0.8)	1.0 (0.3 to 1.7)	-1.2 (-1.8 to -0.6)	0.5 (0.2 to 0.9)	0.5 (0.2 to 0.9)	0.5 (0.2 to 0.9)	0.2 (-0.3 to 0.8)	0.2 (-0.2 to 0.5)
High fasting plasma glucose	1.9 (1.4 to 2.5)	2.5 (1.8 to 3.3)	2.9 (2.2 to 3.8)	31.5 (20.8 to 46.2)	17.2 (6.9 to 30.1)	1.7 (1.3 to 2.2)	2.1 (1.6 to 2.7)	2.2 (1.7 to 2.9)	25.7 (16.4 to 38.0)	7.9 (-2.2 to 21.4)
High LDL cholesterol	16.6 (15.0 to 18.3)	13.1 (11.5 to 14.8)	12.7 (11.2 to 14.3)	-21.1 (-23.4 to -18.9)	-3.1 (-4.1 to -2.2)	16.3 (14.7 to 18.0)	13.1 (11.6 to 14.8)	12.9 (11.4 to 14.5)	-19.5 (-21.7 to -17.6)	-1.7 (-2.6 to -0.8)
High systolic blood pressure	8.9 (8.2 to 9.6)	7.3 (6.6 to 8.0)	7.1 (6.4 to 7.8)	-18.6 (-20.4 to -16.8)	-2.2 (-3.8 to -0.7)	8.0 (7.4 to 8.6)	5.7 (5.2 to 6.3)	5.4 (4.9 to 6.0)	-28.2 (-30.2 to -26.1)	-5.3 (-7.5 to -3.2)
High body-mass index	10.9 (8.1 to 15.1)	16.0 (12.7 to 21.0)	17.2 (13.8 to 22.3)	47.3 (38.3 to 59.5)	7.5 (4.2 to 10.9)	10.7 (8.1 to 14.4)	15.4 (12.2 to 19.8)	16.5 (13.2 to 21.1)	43.1 (34.9 to 52.7)	7.6 (4.1 to 11.4)
Low bone mineral density	4.7 (4.1 to 5.4)	4.5 (3.9 to 5.2)	4.6 (3.9 to 5.3)	-4.3 (-6.4 to -2.2)	1.3 (-1.3 to 3.6)	6.0 (5.3 to 6.7)	5.9 (5.2 to 6.6)	6.0 (5.2 to 6.9)	-2.1 (-3.9 to -0.1)	2.1 (-1.1 to 5.4)
Impaired kidney function	2.1 (1.0 to 5.4)	2.0 (0.9 to 5.2)	2.0 (0.9 to 5.2)	-3.1 (-5.2 to -1.2)	-1.6 (-4.5 to 1.4)	2.6 (1.2 to 6.3)	2.5 (1.2 to 6.2)	2.5 (1.2 to 6.2)	-2.8 (-5.0 to -1.3)	-0.1 (-2.5 to 2.2)

Supplementary Results Table 1. Central Europe, Eastern Europe, and Central Asia age-standardised Summary Exposure Values (SEVs) for Level 4 risk factors and percentage change, by sex, 1990, 2007, and 2017.Data in parentheses are 95% uncertainty										
	Male					Female				
	1990	2007	2017	Percentage change 1990-2007	Percentage change 2007-2017	1990	2007	2017	Percentage change 1990-2007	Percentage change 2007-2017
Unsafe water source	16.7 (14.4 to 19.1)	11.2 (8.8 to 13.0)	9.2 (7.2 to 11.0)	-32.7 (-43.2 to -22.3)	-17.7 (-25.6 to -9.2)	16.6 (14.3 to 19.2)	11.3 (8.9 to 13.0)	9.3 (7.2 to 11.0)	-32.3 (-42.8 to -21.8)	-17.8 (-25.8 to -9.1)
Unsafe sanitation	38.7 (31.2 to 49.8)	29.9 (24.3 to 38.4)	25.2 (20.0 to 32.2)	-22.6 (-30.9 to -13.1)	-15.9 (-23.4 to -6.4)	39.0 (31.4 to 50.2)	30.2 (24.6 to 38.7)	25.3 (20.1 to 32.4)	-22.4 (-30.8 to -12.8)	-16.2 (-23.8 to -6.4)
No access to handwashing facility	11.7 (10.8 to 12.7)	9.8 (9.1 to 10.6)	8.1 (7.5 to 8.8)	-16.5 (-23.1 to -9.3)	-16.9 (-22.1 to -11.8)	11.8 (10.9 to 12.8)	9.8 (9.1 to 10.7)	8.2 (7.6 to 8.8)	-16.6 (-23.3 to -9.3)	-16.9 (-22.1 to -11.6)
Ambient particulate matter pollution	37.2 (28.5 to 48.7)	38.5 (29.1 to 50.6)	35.4 (26.3 to 47.4)	3.7 (-9.7 to 18.3)	-8.0 (-21.0 to 5.3)	35.4 (27.0 to 46.6)	37.0 (27.9 to 49.1)	34.2 (25.2 to 46.0)	4.7 (-9.4 to 20.5)	-7.6 (-21.3 to 6.6)
Household air pollution from solid fuels	12.8 (9.8 to 17.4)	7.7 (5.6 to 11.3)	5.6 (3.9 to 8.9)	-40.2 (-49.3 to -28.9)	-27.0 (-38.4 to -14.1)	13.5 (10.3 to 18.1)	8.4 (6.1 to 12.5)	6.3 (4.4 to 10.0)	-37.5 (-46.6 to -26.6)	-25.7 (-36.7 to -13.9)
Ambient ozone pollution	39.5 (14.2 to 54.1)	40.0 (14.4 to 54.4)	41.4 (15.2 to 54.6)	1.2 (0.4 to 1.9)	3.5 (0.4 to 6.0)	39.3 (14.1 to 54.1)	39.8 (14.3 to 54.3)	41.2 (15.1 to 54.6)	1.2 (0.4 to 2.1)	3.6 (0.4 to 6.1)
Residential radon	36.7 (22.5 to 51.6)	36.5 (22.3 to 51.4)	36.4 (22.2 to 51.2)	-0.5 (-2.9 to 2.2)	-0.3 (-2.5 to 2.1)	36.7 (22.4 to 51.8)	36.5 (22.2 to 51.6)	36.4 (22.0 to 51.4)	-0.4 (-2.7 to 2.3)	-0.3 (-2.5 to 2.0)
Lead exposure	3.8 (1.1 to 7.4)	3.8 (1.2 to 7.3)	3.1 (0.9 to 6.4)	0.7 (-2.1 to 10.8)	-18.1 (-24.8 to -12.7)	1.8 (0.2 to 4.9)	1.9 (0.2 to 5.0)	1.6 (0.2 to 4.4)	3.8 (-0.3 to 28.9)	-16.1 (-21.7 to -11.1)
Occupational exposure to asbestos	1.0 (0.8 to 1.1)	1.5 (1.4 to 1.6)	1.5 (1.4 to 1.6)	54.0 (28.6 to 88.8)	-2.2 (-8.9 to 7.4)	0.7 (0.6 to 0.8)	0.8 (0.8 to 0.9)	0.8 (0.7 to 0.8)	20.2 (-1.5 to 49.1)	-8.6 (-16.9 to 0.4)
Occupational exposure to arsenic	0.3 (0.0 to 0.6)	0.3 (0.0 to 0.6)	0.3 (0.0 to 0.6)	-6.3 (-9.8 to 18.7)	-0.1 (-3.3 to 24.2)	0.3 (0.0 to 0.7)	0.3 (0.0 to 0.7)	0.3 (0.0 to 0.7)	-6.8 (-12.3 to 23.6)	-0.5 (-4.5 to 27.9)
Occupational exposure to benzene	0.4 (0.1 to 1.2)	0.5 (0.1 to 1.2)	0.5 (0.1 to 1.3)	6.4 (3.2 to 21.7)	6.6 (3.5 to 24.6)	0.6 (0.1 to 1.7)	0.7 (0.1 to 2.0)	0.8 (0.2 to 2.1)	14.8 (10.7 to 26.9)	10.8 (7.5 to 29.9)
Occupational exposure to beryllium	0.0 (0.0 to 0.0)	0.0 (0.0 to 0.0)	0.0 (0.0 to 0.0)	4.2 (2.2 to 6.2)	8.9 (7.7 to 10.2)	0.0 (0.0 to 0.0)	0.0 (0.0 to 0.0)	0.0 (0.0 to 0.0)	9.6 (6.9 to 12.5)	11.6 (9.9 to 13.4)
Occupational exposure to cadmium	0.0 (0.0 to 0.0)	0.0 (0.0 to 0.0)	0.0 (0.0 to 0.1)	1.8 (-0.9 to 4.6)	8.4 (6.2 to 10.6)	0.0 (0.0 to 0.1)	0.1 (0.0 to 0.1)	0.1 (0.1 to 0.1)	2.3 (-2.0 to 6.5)	8.7 (6.0 to 11.7)
Occupational exposure to chromium	0.1 (0.1 to 0.1)	0.1 (0.1 to 0.1)	0.1 (0.1 to 0.1)	3.9 (1.2 to 6.6)	10.2 (8.1 to 12.3)	0.1 (0.1 to 0.1)	0.1 (0.1 to 0.1)	0.1 (0.1 to 0.1)	5.2 (1.2 to 9.3)	10.9 (8.3 to 13.5)
Occupational exposure to diesel engine exhaust	0.7 (0.6 to 0.7)	0.7 (0.7 to 0.7)	0.8 (0.8 to 0.8)	4.8 (1.4 to 8.3)	11.7 (9.5 to 13.8)	0.5 (0.5 to 0.5)	0.6 (0.6 to 0.6)	0.7 (0.6 to 0.7)	11.2 (7.4 to 15.6)	12.7 (9.9 to 15.6)
Occupational exposure to formaldehyde	0.2 (0.2 to 0.2)	0.2 (0.2 to 0.2)	0.3 (0.3 to 0.3)	3.8 (0.7 to 7.0)	11.2 (8.6 to 13.8)	0.2 (0.2 to 0.3)	0.3 (0.2 to 0.3)	0.3 (0.3 to 0.3)	5.4 (1.0 to 9.9)	12.7 (9.8 to 15.7)
Occupational exposure to nickel	0.3 (0.0 to 1.3)	0.3 (0.0 to 1.2)	0.3 (0.0 to 1.1)	-11.0 (-14.2 to 9.6)	-1.3 (-4.0 to 18.9)	0.3 (0.0 to 1.2)	0.3 (0.0 to 1.1)	0.2 (0.0 to 1.0)	-10.3 (-15.1 to 16.5)	-2.6 (-6.3 to 21.8)
Occupational exposure to polycyclic aromatic hydrocarbons	0.2 (0.2 to 0.2)	0.2 (0.2 to 0.2)	0.2 (0.2 to 0.3)	4.3 (1.8 to 6.7)	10.3 (8.5 to 12.2)	0.2 (0.2 to 0.3)	0.3 (0.3 to 0.3)	0.3 (0.3 to 0.3)	6.7 (2.8 to 10.4)	11.6 (9.2 to 14.0)
Occupational exposure to silica	3.9 (0.8 to 11.9)	3.5 (0.7 to 10.7)	3.5 (0.8 to 10.5)	-9.9 (-13.5 to -4.4)	0.0 (-2.8 to 6.9)	2.7 (0.5 to 8.6)	2.4 (0.5 to 7.3)	2.3 (0.5 to 6.7)	-12.6 (-17.3 to -5.8)	-4.1 (-8.0 to 2.4)
Occupational exposure to sulphuric acid	0.5 (0.1 to 1.5)	0.5 (0.2 to 1.4)	0.5 (0.2 to 1.3)	-4.3 (-8.1 to 3.6)	-0.4 (-3.6 to 6.7)	0.5 (0.1 to 1.5)	0.5 (0.1 to 1.4)	0.5 (0.2 to 1.4)	-4.8 (-9.5 to 3.3)	-0.3 (-4.0 to 6.6)
Occupational exposure to trichloroethylene	0.1 (0.1 to 0.1)	0.1 (0.1 to 0.1)	0.1 (0.1 to 0.1)	6.9 (4.6 to 9.4)	11.4 (9.7 to 13.2)	0.1 (0.1 to 0.1)	0.1 (0.1 to 0.1)	0.1 (0.1 to 0.1)	8.8 (5.3 to 12.5)	12.2 (10.1 to 14.6)
Occupational asthmagens	14.2 (12.3 to 16.7)	13.7 (11.8 to 16.1)	13.6 (11.8 to 16.1)	-3.5 (-7.3 to -0.1)	-0.5 (-3.3 to 2.3)	8.5 (7.1 to 10.3)	8.3 (6.9 to 9.8)	8.4 (7.0 to 10.0)	-3.3 (-7.9 to 2.1)	1.6 (-2.6 to 6.2)
Occupational particulate matter, gases, and fumes	6.5 (4.8 to 9.3)	6.2 (4.5 to 8.8)	6.1 (4.5 to 8.6)	-4.3 (-6.2 to -2.6)	-1.9 (-3.0 to -0.9)	3.8 (2.8 to 5.4)	3.6 (2.7 to 5.1)	3.5 (2.6 to 5.0)	-5.3 (-8.0 to -2.6)	-4.3 (-6.1 to -2.5)
Occupational noise	6.3 (6.0 to 6.8)	6.1 (5.9 to 6.6)	6.1 (5.8 to 6.6)	-2.7 (-3.9 to -1.5)	-0.4 (-1.1 to 0.4)	4.1 (3.9 to 4.5)	4.1 (3.9 to 4.5)	4.2 (4.0 to 4.6)	-0.2 (-2.0 to 1.7)	1.1 (0.1 to 2.2)
Occupational ergonomic factors	11.1 (10.1 to 12.4)	10.4 (9.3 to 11.6)	10.0 (8.9 to 11.3)	-6.7 (-9.7 to -3.7)	-4.0 (-6.5 to -1.4)	8.5 (7.7 to 9.4)	7.9 (7.1 to 8.8)	7.7 (6.8 to 8.7)	-6.9 (-11.9 to -1.9)	-2.5 (-6.5 to 1.6)
Non-exclusive breastfeeding	0.5 (0.4 to 0.7)	0.5 (0.4 to 0.7)	0.5 (0.4 to 0.7)	-0.3 (-3.7 to 3.4)	-3.9 (-6.5 to -0.8)	0.5 (0.4 to 0.7)	0.5 (0.4 to 0.7)	0.5 (0.4 to 0.7)	-0.2 (-3.7 to 3.5)	-3.9 (-6.5 to -0.8)
Discontinued breastfeeding	1.8 (1.7 to 1.8)	1.9 (1.9 to 1.9)	1.9 (1.8 to 1.9)	6.8 (4.4 to 9.4)	-0.6 (-2.5 to 1.3)	1.8 (1.7 to 1.8)	1.9 (1.9 to 1.9)	1.9 (1.8 to 1.9)	6.9 (4.4 to 9.5)	-0.5 (-2.4 to 1.5)
Child underweight	0.3 (0.2 to 0.4)	0.2 (0.1 to 0.2)	0.1 (0.1 to 0.2)	-35.9 (-42.9 to -30.3)	-18.3 (-23.7 to -14.2)	0.3 (0.2 to 0.4)	0.2 (0.1 to 0.2)	0.1 (0.1 to 0.2)	-36.0 (-44.0 to -30.2)	-17.0 (-23.0 to -12.6)
Child wasting	0.3 (0.2 to 0.3)	0.2 (0.2 to 0.3)	0.2 (0.1 to 0.3)	-13.8 (-23.6 to -2.6)	-11.4 (-18.8 to -2.6)	0.2 (0.2 to 0.3)	0.2 (0.2 to 0.3)	0.2 (0.1 to 0.2)	-12.7 (-23.7 to -0.0)	-12.1 (-20.1 to -3.7)
Child stunting	1.5 (1.0 to 1.7)	1.0 (0.7 to 1.2)	0.8 (0.5 to 1.1)	-32.4 (-42.5 to -22.9)	-16.8 (-27.2 to -6.2)	1.4 (1.0 to 1.7)	0.9 (0.6 to 1.1)	0.7 (0.5 to 1.0)	-35.5 (-45.6 to -25.8)	-18.8 (-29.1 to -8.0)
Short gestation for birth weight	0.0 (0.0 to 0.0)	0.0 (0.0 to 0.0)	0.0 (0.0 to 0.0)	8.0 (1.8 to 16.3)	4.8 (-0.8 to 10.4)	0.0 (0.0 to 0.0)	0.0 (0.0 to 0.0)	0.0 (0.0 to 0.0)	5.5 (-0.9 to 12.3)	2.7 (-2.3 to 11.4)
Low birth weight for gestation	0.0 (0.0 to 0.0)	0.0 (0.0 to 0.0)	0.0 (0.0 to 0.0)	4.9 (-1.3 to 10.4)	2.5 (-1.1 to 7.4)	0.0 (0.0 to 0.0)	0.0 (0.0 to 0.0)	0.0 (0.0 to 0.0)	4.4 (-0.5 to 9.3)	2.9 (-1.3 to 7.0)
Iron deficiency	-	-	-	-	-	7.7 (5.8 to 9.9)	7.2 (5.3 to 9.4)	6.5 (4.8 to 8.6)	-6.0 (-12.2 to -0.1)	-9.3 (-14.1 to -4.3)
Vitamin A deficiency	1.4 (1.2 to 1.7)	1.3 (1.1 to 1.5)	1.1 (0.9 to 1.4)	-11.0 (-15.5 to -6.3)	-11.2 (-15.9 to -7.1)	1.4 (1.2 to 1.7)	1.3 (1.1 to 1.5)	1.1 (1.0 to 1.4)	-10.0 (-14.7 to -5.4)	-9.8 (-14.3 to -6.2)
Zinc deficiency	0.6 (0.2 to 1.1)	0.5 (0.1 to 1.1)	0.5 (0.1 to 0.9)	-6.4 (-23.4 to 12.3)	-11.8 (-27.2 to 10.1)	0.6 (0.2 to 1.1)	0.5 (0.1 to 1.1)	0.5 (0.1 to 0.9)	-6.2 (-22.4 to 12.3)	-12.4 (-28.4 to 8.3)
Smoking	15.7 (14.0 to 17.5)	14.2 (12.7 to 15.9)	13.5 (12.1 to 15.1)	-9.5 (-12.1 to -6.8)	-5.0 (-7.1 to -3.1)	3.9 (3.3 to 4.6)	3.5 (3.0 to 4.0)	3.8 (3.2 to 4.4)	-10.6 (-16.3 to -4.2)	8.3 (4.8 to 11.8)
Chewing tobacco	0.1 (0.1 to 0.1)	0.1 (0.1 to 0.1)	0.1 (0.1 to 0.2)	8.6 (-2.7 to 20.5)	7.1 (-1.8 to 16.9)	0.1 (0.0 to 0.1)	0.1 (0.0 to 0.1)	0.1 (0.0 to 0.1)	-0.1 (-13.4 to 15.4)	0.5 (-9.6 to 11.2)
Second-hand smoke	37.2 (36.2 to 38.1)	36.0 (35.4 to 36.5)	33.9 (33.0 to 34.9)	-3.2 (-5.9 to -0.3)	-5.8 (-7.8 to -3.7)	56.2 (55.2 to 57.1)	51.5 (50.9 to 52.1)	47.0 (46.0 to 47.9)	-8.3 (-10.0 to -6.4)	-8.9 (-10.2 to -7.4)
Alcohol use	24.1 (17.7 to 31.3)	29.1 (21.9 to 37.0)	30.2 (21.9 to 39.3)	20.6 (10.5 to 32.6)	3.7 (-5.4 to 13.8)	9.9 (5.9 to 16.0)	12.0 (7.7 to 18.3)	13.0 (8.1 to 19.4)	21.7 (10.3 to 37.7)	7.8 (-3.3 to 22.9)
Drug use	0.6 (0.5 to 0.8)	0.7 (0.6 to 0.9)	0.6 (0.5 to 0.9)	20.8 (17.2 to 24.5)	-9.5 (-13.0 to -6.0)	0.2 (0.2 to 0.3)	0.3 (0.2 to 0.4)	0.2 (0.2 to 0.3)	9.7 (5.5 to 13.9)	-5.5 (-9.1 to -2.2)
Diet low in fruits	43.6 (38.6 to 46.0)	41.4 (36.2 to 44.1)	38.1 (32.8 to 41.4)	-5.1 (-7.0 to -3.6)	-7.8 (-10.5 to -5.6)	42.2 (37.1 to 44.9)	39.5 (34.3 to 42.5)	35.9 (30.4 to 39.4)	-6.3 (-8.5 to -4.5)	-9.2 (-12.5 to -6.7)
Diet low in vegetables	26.9 (21.6 to 31.4)	19.0 (13.3 to 24.2)	16.2 (11.2 to 20.9)	-29.3 (-38.7 to -22.3)	-14.8 (-19.5 to -10.6)	29.5 (24.5 to 33.7)	21.6 (16.0 to 26.7)	18.1 (13.0 to 22.9)	-26.7 (-35.7 to -20.4)	-16.3 (-22.0 to -12.5)
Diet low in legumes	42.1 (39.8 to 44.0)	43.3 (41.3 to 45.0)	43.1 (41.2 to 44.9)	2.9 (2.0 to 3.9)	-0.3 (-1.1 to 0.4)	44.3 (42.5 to 45.9)	45.4 (43.8 to 46.8)	45.4 (43.7 to 46.8)	2.5 (1.7 to 3.3)	-0.0 (-0.7 to 0.6)
Diet low in whole grains	49.0 (48.0 to 49.8)	46.6 (45.3 to 47.7)	45.4 (43.9 to 46.7)	-4.9 (-5.8 to -4.0)	-2.5 (-3.2 to -1.8)	49.3 (48.4 to 50.0)	47.1 (45.9 to 48.2)	46.1 (44.7 to 47.3)	-4.4 (-5.2 to -3.6)	-2.2 (-2.9 to -1.7)
Diet low in nuts and seeds	51.4 (50.7 to 52.0)	50.9 (50.0 to 51.6)	48.8 (47.5 to 49.9)	-1.1 (-1.5 to -0.7)	-4.0 (-5.1 to -3.2)	51.6 (51.0 to 52.2)	51.1 (50.3 to 51.8)	49.2 (48.0 to 50.2)	-1.0 (-1.4 to -0.6)	-3.8 (-4.8 to -3.0)
Diet low in milk	37.6 (34.2 to 40.5)	39.5 (36.5 to 42.1)	38.5 (35.2 to 41.2)	5.2 (3.6 to 7.5)	-2.6 (-4.0 to -1.6)	38.5 (35.2 to 41.3)	40.3 (37.4 to 42.7)	39.1 (35.9 to 41.8)	4.5 (3.0 to 6.5)	-2.8 (-4.2 to -1.8)
Diet high in red meat	24.3 (21.7 to 26.8)	16.1 (13.5 to 18.7)	21.8 (19.0 to 24.6)	-33.7 (-40.1 to -28.1)	35.8 (24.6 to 49.0)	9.7 (7.0 to 12.5)	5.1 (3.2 to 7.1)	7.9 (5.2 to 10.4)	-47.8 (-58.8 to -38.9)	55.0 (33.2 to 92.6)
Diet high in processed meat	8.2 (5.7 to 10.8)	6.3 (4.1 to 8.8)	6.8 (4.4 to 9.4)	-23.0 (-36.9 to -10.7)	8.5 (-0.5 to 19.5)	5.7 (3.5 to 8.0)	4.2 (2.2 to 6.5)	4.7 (2.5 to 7.1)	-25.7 (-43.8 to -13.2)	10.6 (-0.4 to 24.9)
Diet high in sugar-sweetened beverages	6.0 (3.0 to 7.6)	5.8 (3.0 to 6.8)	8.2 (4.2 to 9.6)	-3.3 (-20.3 to 15.6)	40.4 (25.5 to 57.5)	4.0 (2.0 to 5.2)	3.9 (2.0 to 4.8)	5.4 (2.8 to 6.5)	-2.6 (-21.4 to 18.3)	38.1 (21.2 to 56.7)
Diet low in fibre	27.3 (21.9 to 31.7)	28.3 (23.1 to 32.6)	26.6 (21.1 to 31.3)	3.5 (1.7 to 6.3)	-5.9 (-9.8 to -3.2)	32.6 (28.1 to 36.3)	33.6 (29.2 to 37.1)	32.0 (27.2 to 35.9)	2.8 (1.2 to 5.2)	-4.6 (-7.3 to -2.6)
Diet low in calcium	28.2 (22.8 to 32.4)	25.5 (19.9 to 30.1)	22.7 (16.7 to 27.6)	-9.5 (-13.5 to -6.9)	-11.0 (-16.1 to -8.0)	31.4 (26.7 to 35.2)	28.9 (23.8 to 33.0)	26.2 (20.6 to 30.6)	-8.0 (-11.3 to -5.8)	-9.5 (-13.7 to -6.9)
Diet low in seafood omega-3 fatty acids	38.5 (35.4 to 41.3)	38.7 (35.7 to 41.5)	35.6 (32.0 to 38.9)	0.6 (0.2 to 1.1)	-8.0 (-10.2 to -6.4)	40.6 (37.9 to 43.1)	40.8 (38.2 to 43.3)	38.1 (35.0 to 41.0)	0.6 (0.3 to 1.0)	-6.8 (-8.5 to -5.3)
Diet low in polyunsaturated fatty acids	41.3 (38.8 to 43.6)	37.6 (34.4 to 40.3)	35.9 (32.3 to 38.8)	-9.1 (-12.1 to -6.5)	-4.5 (-6.5 to -3.0)	41.8 (39.3 to 44.1)	38.1 (34.9 to 40.7)	36.3 (32.9 to 39.3)	-8.9 (-11.9 to -6.5)	-4.5 (-6.5 to -3.0)
Diet high in trans fatty acids	1.8 (0.5 to 4.1)	1.4 (0.2 to 3.5)	1.2 (0.2 to 3.3)	-22.8 (-49.4 to -12.0)	-13.9 (-32.2 to -5.9)	2.4 (0.8 to 5.0)	1.9 (0.4 to 4.4)	1.6 (0.3 to 3.9)	-21.8 (-45.5 to -11.1)	-13.3 (-30.2 to -5.4)
Diet high in sodium	12.6 (6.7 to 18.8)	8.7 (2.2 to 15.4)	9.4 (2.6 to 16.2)	-31.4 (-67.3 to -16.8)	8.3 (1.5 to 27.8)	8.8 (3.7 to 15.0)	5.2 (0.4 to 11.8)	4.7 (0.2 to 11.2)	-40.6 (-88.2 to -20.8)	-10.0 (-54.4 to -2.6)
Intimate partner violence	-	-	-	-	-	4.1 (3.4 to 4.7)	3.9 (3.3 to 4.5)	3.8 (3.2 to 4.4)	-3.9 (-6.1 to -1.7)	-3.0 (-5.2 to -0.7)
Childhood sexual abuse	5.1 (4.2 to 6.2)	4.9 (4.1 to 6.0)	4.8 (4.0 to 5.9)	-3.0 (-3.8 to -2.1)	-2.6 (-3.3 to -2.1)	8.3 (6.6 to 10.3)	8.0 (6.4 to 9.9)	7.7 (6.1 to 9.6)	-3.2 (-6.6 to 0.5)	-3.6 (-4.6 to -2.5)

Supplementary Results Table 1. Central Europe, Eastern Europe, and Central Asia age-standardised Summary Exposure Values (SEVs) for Level 4 risk factors and percentage change, by sex, 1990, 2007, and 2017.Data in parentheses are 95% uncertainty										
	**Male**					**Female**				
	**1990**	**2007**	**2017**	**Percentage change 1990-2007**	**Percentage change 2007-2017**	**1990**	**2007**	**2017**	**Percentage change 1990-2007**	**Percentage change 2007-2017**
Bullying victimization	4.6 (2.0 to 9.6)	5.2 (2.3 to 10.7)	5.1 (2.3 to 10.4)	12.2 (5.7 to 20.6)	-1.8 (-5.2 to 2.1)	4.0 (1.7 to 8.1)	4.4 (2.0 to 8.9)	4.3 (1.9 to 8.6)	10.7 (3.7 to 19.7)	-1.5 (-4.6 to 2.2)
Low physical activity	0.5 (0.2 to 0.9)	0.4 (0.2 to 0.8)	0.4 (0.2 to 0.8)	-4.3 (-6.5 to -1.5)	0.9 (0.3 to 1.5)	0.4 (0.2 to 0.8)	0.4 (0.2 to 0.8)	0.4 (0.2 to 0.8)	-4.1 (-6.2 to -1.4)	0.6 (0.0 to 1.2)
High fasting plasma glucose	2.6 (1.9 to 3.4)	2.9 (2.2 to 3.9)	3.3 (2.4 to 4.4)	14.3 (-1.6 to 32.2)	10.7 (-1.9 to 27.4)	1.9 (1.4 to 2.6)	2.2 (1.6 to 2.9)	2.5 (1.8 to 3.2)	12.0 (-1.6 to 28.1)	13.8 (-0.2 to 32.8)
High LDL cholesterol	14.0 (12.4 to 15.6)	13.4 (11.9 to 15.1)	13.2 (11.7 to 15.0)	-4.0 (-5.8 to -2.1)	-1.5 (-3.1 to 0.0)	14.9 (13.4 to 16.5)	14.4 (13.0 to 16.2)	14.4 (12.9 to 16.1)	-2.9 (-4.7 to -0.8)	-0.4 (-2.1 to 1.1)
High systolic blood pressure	10.0 (9.2 to 10.8)	10.1 (9.4 to 10.9)	10.0 (9.2 to 10.8)	1.5 (0.1 to 3.0)	-1.6 (-2.9 to -0.4)	10.0 (9.3 to 10.6)	9.3 (8.7 to 10.0)	8.8 (8.2 to 9.5)	-6.6 (-8.4 to -4.9)	-5.4 (-6.9 to -3.9)
High body-mass index	9.4 (7.0 to 13.2)	11.9 (9.1 to 16.3)	14.0 (10.9 to 18.8)	26.5 (18.9 to 35.8)	17.7 (12.4 to 23.2)	11.0 (8.4 to 14.8)	13.0 (10.2 to 17.0)	15.0 (11.8 to 19.5)	18.1 (12.0 to 25.7)	15.2 (10.9 to 19.9)
Low bone mineral density	4.0 (3.3 to 4.6)	3.9 (3.2 to 4.6)	3.8 (3.2 to 4.6)	-1.3 (-4.7 to 1.9)	-1.9 (-5.1 to 1.6)	5.6 (4.8 to 6.4)	5.4 (4.7 to 6.3)	5.4 (4.6 to 6.2)	-2.2 (-5.4 to 0.7)	-1.2 (-4.4 to 1.8)
Impaired kidney function	2.8 (1.3 to 6.7)	2.8 (1.3 to 6.6)	2.8 (1.3 to 6.6)	-1.2 (-3.2 to 0.9)	0.9 (-1.3 to 3.5)	3.4 (1.7 to 7.6)	3.4 (1.7 to 7.6)	3.5 (1.7 to 7.7)	-0.6 (-2.6 to 1.0)	2.7 (0.7 to 6.0)

Supplementary Results Table 1. Central Asia age-standardised Summary Exposure Values (SEVs) for Level 4 risk factors and percentage change, by sex, 1990, 2007, and 2017.Data in parentheses are 95% uncertainty intervals.

	Male					Female				
	1990	2007	2017	Percentage change 1990-2007	Percentage change 2007-2017	1990	2007	2017	Percentage change 1990-2007	Percentage change 2007-2017
Unsafe water source	18.6 (11.2 to 22.6)	17.0 (9.7 to 20.7)	13.4 (7.6 to 16.6)	-8.8 (-16.3 to -0.8)	-21.3 (-27.5 to -14.9)	18.6 (11.2 to 22.6)	16.9 (9.6 to 20.6)	13.3 (7.6 to 16.6)	-9.1 (-16.8 to -1.2)	-21.2 (-27.4 to -14.9)
Unsafe sanitation	63.4 (51.2 to 81.7)	59.2 (48.2 to 75.7)	50.8 (40.2 to 65.8)	-6.7 (-11.4 to -1.7)	-14.2 (-20.0 to -9.1)	63.3 (51.1 to 81.6)	59.1 (48.2 to 75.4)	50.8 (40.3 to 65.6)	-6.7 (-11.5 to -1.6)	-14.1 (-20.0 to -8.9)
No access to handwashing facility	23.3 (21.4 to 25.4)	20.2 (18.8 to 21.9)	14.6 (13.4 to 15.8)	-13.2 (-20.7 to -5.0)	-28.0 (-32.8 to -22.5)	23.3 (21.3 to 25.3)	20.2 (18.7 to 21.8)	14.5 (13.4 to 15.7)	-13.4 (-20.8 to -5.1)	-27.9 (-32.7 to -22.5)
Ambient particulate matter pollution	29.2 (18.5 to 43.1)	35.3 (23.9 to 49.1)	36.2 (23.5 to 52.8)	21.0 (-20.7 to 86.9)	2.4 (-32.6 to 54.2)	25.3 (15.7 to 37.9)	31.6 (20.7 to 45.0)	33.2 (21.0 to 49.4)	24.9 (-20.9 to 96.6)	4.9 (-32.5 to 63.5)
Household air pollution from solid fuels	30.7 (23.2 to 40.3)	19.3 (14.2 to 26.4)	13.2 (9.0 to 18.9)	-37.1 (-50.5 to -21.9)	-31.6 (-47.1 to -15.2)	32.3 (25.0 to 41.5)	21.2 (16.0 to 28.5)	14.8 (10.5 to 20.5)	-34.4 (-46.6 to -20.8)	-29.9 (-44.8 to -14.8)
Ambient ozone pollution	44.9 (16.8 to 54.8)	45.2 (17.0 to 54.8)	46.1 (17.7 to 54.8)	0.6 (-0.1 to 2.0)	1.9 (-0.0 to 4.8)	44.8 (16.8 to 54.8)	45.1 (16.9 to 54.8)	46.0 (17.7 to 54.8)	0.6 (-0.1 to 2.1)	2.0 (0.0 to 4.9)
Residential radon	38.4 (19.3 to 58.9)	37.6 (18.6 to 58.5)	37.3 (17.9 to 58.6)	-2.1 (-10.1 to 5.7)	-0.8 (-4.2 to 1.7)	38.5 (19.3 to 59.0)	37.7 (18.7 to 58.7)	37.4 (17.9 to 58.9)	-1.9 (-9.4 to 5.8)	-0.9 (-4.7 to 2.0)
Lead exposure	6.0 (2.7 to 9.8)	5.8 (2.7 to 9.4)	4.8 (2.1 to 8.2)	-3.4 (-6.4 to 1.6)	-16.7 (-21.9 to -12.3)	3.2 (0.7 to 6.7)	3.3 (0.8 to 6.7)	2.8 (0.7 to 6.0)	0.7 (-2.9 to 15.6)	-14.2 (-19.2 to -10.0)
Occupational exposure to asbestos	0.6 (0.5 to 0.8)	0.8 (0.7 to 0.9)	0.8 (0.7 to 0.9)	32.8 (-0.0 to 73.5)	-4.1 (-14.4 to 12.4)	0.5 (0.4 to 0.7)	0.8 (0.7 to 0.9)	0.6 (0.6 to 0.7)	50.6 (24.7 to 90.2)	-23.2 (-33.1 to -12.5)
Occupational exposure to arsenic	0.2 (0.1 to 0.4)	0.2 (0.1 to 0.4)	0.2 (0.1 to 0.4)	-3.7 (-6.8 to -0.5)	3.5 (1.1 to 6.3)	0.2 (0.1 to 0.4)	0.2 (0.1 to 0.4)	0.2 (0.1 to 0.4)	-0.4 (-5.1 to 4.4)	5.2 (1.8 to 8.6)
Occupational exposure to benzene	0.6 (0.3 to 1.0)	0.6 (0.4 to 1.1)	0.7 (0.4 to 1.2)	6.7 (3.1 to 10.6)	8.3 (5.7 to 11.7)	0.7 (0.4 to 1.2)	0.8 (0.5 to 1.3)	0.9 (0.5 to 1.5)	13.9 (8.2 to 20.7)	14.2 (10.5 to 19.1)
Occupational exposure to beryllium	0.1 (0.1 to 0.1)	0.1 (0.1 to 0.1)	0.1 (0.1 to 0.1)	-2.5 (-5.1 to 0.3)	0.1 (-1.7 to 1.8)	0.1 (0.1 to 0.1)	0.1 (0.1 to 0.1)	0.1 (0.1 to 0.1)	3.2 (-0.7 to 7.6)	2.9 (0.3 to 5.5)
Occupational exposure to cadmium	0.1 (0.1 to 0.1)	0.1 (0.1 to 0.1)	0.1 (0.1 to 0.1)	-2.7 (-5.7 to 0.2)	3.6 (1.3 to 6.2)	0.1 (0.1 to 0.1)	0.1 (0.1 to 0.1)	0.1 (0.1 to 0.1)	0.8 (-3.8 to 5.5)	5.4 (2.2 to 8.5)
Occupational exposure to chromium	0.3 (0.3 to 0.3)	0.3 (0.3 to 0.3)	0.3 (0.3 to 0.3)	-0.6 (-3.8 to 2.4)	6.4 (4.0 to 8.9)	0.3 (0.3 to 0.3)	0.3 (0.3 to 0.3)	0.3 (0.3 to 0.3)	3.4 (-1.4 to 8.6)	8.6 (5.1 to 11.9)
Occupational exposure to diesel engine exhaust	2.4 (2.3 to 2.5)	2.3 (2.2 to 2.4)	2.3 (2.3 to 2.4)	-4.3 (-9.1 to 0.7)	3.5 (0.4 to 6.5)	1.6 (1.6 to 1.7)	1.7 (1.6 to 1.8)	1.7 (1.7 to 1.8)	2.1 (-3.3 to 8.3)	4.2 (0.6 to 7.8)
Occupational exposure to formaldehyde	0.6 (0.5 to 0.6)	0.6 (0.5 to 0.6)	0.6 (0.6 to 0.6)	-1.2 (-4.6 to 2.3)	7.4 (4.8 to 10.4)	0.5 (0.5 to 0.6)	0.6 (0.5 to 0.6)	0.6 (0.6 to 0.7)	4.0 (-1.2 to 9.5)	10.9 (7.0 to 14.8)
Occupational exposure to nickel	0.4 (0.1 to 0.9)	0.3 (0.1 to 0.8)	0.3 (0.1 to 0.8)	-13.8 (-18.5 to -8.7)	-2.8 (-6.1 to 0.6)	0.3 (0.1 to 0.7)	0.2 (0.1 to 0.6)	0.2 (0.1 to 0.6)	-8.3 (-12.8 to -3.0)	-0.8 (-4.2 to 2.9)
Occupational exposure to polycyclic aromatic hydrocarbons	0.6 (0.6 to 0.7)	0.6 (0.6 to 0.6)	0.7 (0.6 to 0.7)	-1.0 (-4.1 to 2.1)	5.8 (3.5 to 8.2)	0.6 (0.6 to 0.7)	0.6 (0.6 to 0.7)	0.7 (0.7 to 0.7)	3.7 (-1.1 to 8.9)	8.7 (5.4 to 12.0)
Occupational exposure to silica	4.3 (2.2 to 9.4)	3.8 (1.9 to 8.4)	3.7 (1.9 to 8.3)	-11.6 (-17.5 to -5.5)	-0.9 (-4.9 to 3.7)	2.4 (1.3 to 5.3)	2.2 (1.1 to 4.7)	2.0 (1.0 to 4.5)	-11.9 (-17.0 to -5.6)	-4.9 (-8.5 to -1.2)
Occupational exposure to sulphuric acid	0.7 (0.5 to 1.2)	0.6 (0.5 to 1.1)	0.6 (0.5 to 1.1)	-3.6 (-7.1 to -0.0)	-0.8 (-3.7 to 2.1)	0.6 (0.4 to 1.0)	0.6 (0.4 to 1.0)	0.6 (0.4 to 1.0)	-1.8 (-6.2 to 3.0)	-0.4 (-3.4 to 2.7)
Occupational exposure to trichloroethylene	0.2 (0.2 to 0.2)	0.2 (0.2 to 0.2)	0.2 (0.2 to 0.2)	2.3 (-0.8 to 5.2)	6.6 (4.3 to 8.9)	0.2 (0.2 to 0.2)	0.2 (0.2 to 0.2)	0.2 (0.2 to 0.2)	5.9 (1.2 to 11.3)	7.9 (4.8 to 11.2)
Occupational asthmagens	16.7 (13.7 to 20.4)	15.0 (12.3 to 18.5)	14.3 (11.8 to 17.5)	-10.1 (-15.5 to -4.7)	-4.6 (-9.1 to 0.4)	9.8 (7.6 to 12.4)	9.3 (7.3 to 11.7)	8.9 (7.0 to 11.2)	-5.0 (-12.8 to 4.0)	-4.3 (-11.2 to 3.0)
Occupational particulate matter, gases, and fumes	8.1 (6.3 to 10.9)	7.8 (6.1 to 10.4)	7.6 (5.9 to 10.2)	-3.9 (-6.0 to -2.0)	-2.3 (-3.6 to -1.1)	4.8 (3.7 to 6.5)	4.6 (3.5 to 6.3)	4.6 (3.5 to 6.2)	-3.0 (-5.9 to -0.1)	-2.1 (-4.0 to 0.0)
Occupational noise	9.1 (8.7 to 9.7)	8.9 (8.5 to 9.5)	8.8 (8.4 to 9.4)	-2.1 (-3.8 to -0.4)	-1.1 (-2.1 to -0.0)	5.8 (5.4 to 6.3)	5.8 (5.5 to 6.3)	5.9 (5.6 to 6.4)	0.7 (-1.8 to 3.4)	1.6 (-0.1 to 3.3)
Occupational ergonomic factors	18.6 (17.4 to 20.0)	17.1 (15.8 to 18.3)	15.2 (13.7 to 16.7)	-8.4 (-14.6 to -2.1)	-11.1 (-16.0 to -5.9)	13.7 (12.4 to 14.9)	12.9 (11.7 to 14.1)	11.6 (10.3 to 12.9)	-6.0 (-14.2 to 2.7)	-10.4 (-17.5 to -3.6)
Non-exclusive breastfeeding	0.5 (0.3 to 0.7)	0.5 (0.3 to 0.7)	0.4 (0.3 to 0.6)	-6.5 (-9.1 to -3.1)	-5.1 (-8.1 to -1.5)	0.5 (0.3 to 0.7)	0.5 (0.3 to 0.7)	0.4 (0.3 to 0.6)	-6.5 (-9.1 to -3.2)	-5.1 (-8.1 to -1.5)
Discontinued breastfeeding	1.3 (1.2 to 1.3)	1.3 (1.3 to 1.3)	1.3 (1.3 to 1.4)	1.7 (-2.1 to 5.9)	2.1 (-1.3 to 5.5)	1.3 (1.2 to 1.3)	1.3 (1.3 to 1.3)	1.3 (1.3 to 1.3)	1.4 (-2.5 to 5.5)	2.6 (-0.8 to 6.1)
Child underweight	0.6 (0.4 to 0.7)	0.3 (0.2 to 0.4)	0.2 (0.2 to 0.3)	-44.1 (-52.6 to -36.1)	-26.7 (-34.6 to -20.7)	0.5 (0.4 to 0.7)	0.3 (0.2 to 0.4)	0.2 (0.2 to 0.3)	-42.2 (-51.8 to -33.6)	-24.4 (-32.4 to -17.6)
Child wasting	0.4 (0.3 to 0.4)	0.3 (0.2 to 0.4)	0.2 (0.2 to 0.3)	-22.9 (-38.6 to -4.8)	-12.7 (-23.2 to -1.6)	0.3 (0.3 to 0.4)	0.3 (0.2 to 0.3)	0.2 (0.2 to 0.3)	-20.7 (-37.9 to -2.0)	-13.2 (-23.9 to -1.4)
Child stunting	2.4 (1.7 to 2.8)	1.5 (1.0 to 1.7)	1.1 (0.7 to 1.4)	-38.2 (-48.7 to -28.2)	-28.0 (-39.9 to -17.1)	2.1 (1.4 to 2.5)	1.4 (0.9 to 1.6)	1.0 (0.6 to 1.3)	-34.1 (-47.0 to -22.9)	-26.1 (-38.1 to -14.0)
Short gestation for birth weight	0.0 (0.0 to 0.0)	0.0 (0.0 to 0.0)	0.0 (0.0 to 0.0)	-1.6 (-13.9 to 13.6)	4.6 (-6.4 to 17.0)	0.0 (0.0 to 0.0)	0.0 (0.0 to 0.0)	0.0 (0.0 to 0.0)	1.2 (-9.7 to 15.4)	3.3 (-6.8 to 21.8)
Low birth weight for gestation	0.0 (0.0 to 0.0)	0.0 (0.0 to 0.0)	0.0 (0.0 to 0.0)	4.8 (-3.4 to 14.9)	5.4 (-2.0 to 13.1)	0.0 (0.0 to 0.0)	0.0 (0.0 to 0.0)	0.0 (0.0 to 0.0)	5.6 (-4.4 to 15.3)	5.3 (-5.6 to 13.5)
Iron deficiency	-	-	-	-	-	14.2 (11.4 to 17.4)	13.1 (10.4 to 16.3)	11.4 (8.8 to 14.5)	-7.7 (-14.3 to -0.9)	-13.1 (-18.7 to -7.4)
Vitamin A deficiency	1.8 (1.5 to 2.2)	1.7 (1.4 to 2.0)	1.5 (1.2 to 1.9)	-6.5 (-14.5 to 2.2)	-11.0 (-19.1 to -3.4)	2.0 (1.7 to 2.4)	1.9 (1.6 to 2.2)	1.7 (1.5 to 2.0)	-6.3 (-12.7 to -0.4)	-8.0 (-14.0 to -3.1)
Zinc deficiency	0.8 (0.2 to 1.6)	0.7 (0.2 to 1.4)	0.5 (0.1 to 1.2)	-17.1 (-42.3 to 13.6)	-17.9 (-38.0 to 4.5)	0.8 (0.2 to 1.6)	0.7 (0.2 to 1.4)	0.5 (0.2 to 1.1)	-16.3 (-40.8 to 15.0)	-18.2 (-37.1 to 5.7)
Smoking	10.8 (9.6 to 12.3)	9.9 (8.7 to 11.2)	9.9 (8.7 to 11.2)	-8.5 (-12.6 to -4.4)	-0.3 (-3.4 to 2.9)	0.7 (0.6 to 0.9)	0.7 (0.6 to 0.8)	0.8 (0.7 to 1.0)	-4.1 (-16.9 to 11.7)	18.8 (9.9 to 28.2)
Chewing tobacco	0.3 (0.3 to 0.4)	0.3 (0.3 to 0.4)	0.4 (0.3 to 0.4)	1.6 (-14.4 to 21.3)	9.9 (-4.5 to 26.5)	0.1 (0.1 to 0.1)	0.1 (0.1 to 0.1)	0.1 (0.1 to 0.1)	-1.1 (-14.6 to 15.3)	0.4 (-9.3 to 11.6)
Second-hand smoke	33.5 (32.6 to 34.4)	31.9 (31.3 to 32.5)	30.4 (29.4 to 31.4)	-4.8 (-8.0 to -1.4)	-4.7 (-7.5 to -2.0)	53.2 (52.3 to 54.0)	48.9 (48.2 to 49.5)	45.6 (44.5 to 46.6)	-8.0 (-9.9 to -6.3)	-6.8 (-8.5 to -5.0)
Alcohol use	18.7 (12.9 to 25.3)	19.0 (13.1 to 26.0)	19.4 (13.0 to 27.1)	1.6 (-9.5 to 14.9)	2.1 (-10.8 to 15.2)	6.9 (4.1 to 11.0)	6.9 (4.2 to 11.2)	7.1 (4.2 to 11.3)	0.1 (-14.6 to 13.8)	1.7 (-12.2 to 15.8)
Drug use	0.4 (0.3 to 0.5)	0.4 (0.4 to 0.6)	0.4 (0.4 to 0.6)	16.4 (10.4 to 23.9)	1.0 (-3.4 to 5.2)	0.2 (0.1 to 0.2)	0.2 (0.1 to 0.2)	0.2 (0.1 to 0.2)	6.5 (2.8 to 10.7)	0.6 (-2.9 to 4.1)
Diet low in fruits	46.3 (41.6 to 48.4)	45.1 (40.3 to 47.3)	40.9 (35.7 to 43.9)	-2.6 (-3.9 to -1.6)	-9.3 (-12.0 to -7.1)	45.0 (40.2 to 47.3)	43.7 (38.8 to 46.2)	38.9 (33.4 to 42.1)	-3.0 (-4.5 to -1.8)	-10.9 (-14.2 to -8.4)
Diet low in vegetables	27.1 (21.9 to 31.6)	12.5 (6.6 to 18.5)	5.6 (3.4 to 8.6)	-53.8 (-69.9 to -41.1)	-55.1 (-60.2 to -46.8)	29.4 (24.5 to 33.7)	15.3 (8.9 to 21.1)	7.0 (4.0 to 10.8)	-48.1 (-63.9 to -36.7)	-54.4 (-60.8 to -47.8)
Diet low in legumes	54.2 (54.0 to 54.3)	53.0 (52.6 to 53.3)	52.7 (52.3 to 53.1)	-2.2 (-2.7 to -1.8)	-0.5 (-0.7 to -0.2)	54.3 (54.2 to 54.4)	53.3 (53.0 to 53.5)	53.2 (52.9 to 53.5)	-1.8 (-2.2 to -1.5)	-0.1 (-0.3 to 0.0)
Diet low in whole grains	52.5 (52.1 to 52.8)	52.5 (52.1 to 52.8)	52.4 (52.0 to 52.7)	0.1 (-0.1 to 0.2)	-0.1 (-0.3 to 0.0)	52.4 (52.0 to 52.7)	52.5 (52.1 to 52.8)	52.5 (52.1 to 52.8)	0.2 (-0.0 to 0.4)	0.1 (-0.2 to 0.3)
Diet low in nuts and seeds	49.6 (48.5 to 50.6)	50.3 (49.4 to 51.2)	46.9 (45.1 to 48.4)	1.5 (0.7 to 2.4)	-6.8 (-8.8 to -5.3)	49.9 (48.7 to 50.8)	50.6 (49.6 to 51.4)	47.3 (45.6 to 48.7)	1.5 (0.7 to 2.4)	-6.5 (-8.4 to -5.0)
Diet low in milk	40.5 (37.5 to 42.9)	42.3 (39.7 to 44.4)	41.2 (38.5 to 43.6)	4.6 (3.1 to 6.5)	-2.6 (-3.7 to -1.7)	41.1 (38.3 to 43.4)	42.8 (40.4 to 44.9)	41.7 (39.0 to 44.0)	4.2 (2.8 to 6.0)	-2.6 (-3.7 to -1.8)
Diet high in red meat	11.8 (9.3 to 14.2)	9.2 (7.1 to 11.5)	15.7 (12.4 to 18.7)	-21.7 (-30.9 to -13.0)	70.7 (48.4 to 100.2)	4.0 (2.3 to 5.6)	2.7 (1.5 to 4.1)	5.7 (3.6 to 8.0)	-31.4 (-47.1 to -15.9)	110.9 (69.3 to 192.8)
Diet high in processed meat	1.4 (0.1 to 3.4)	1.0 (0.1 to 2.8)	2.0 (0.5 to 4.2)	-28.9 (-65.3 to -15.6)	103.7 (47.3 to 691.8)	0.8 (0.0 to 2.6)	0.6 (0.0 to 2.1)	1.3 (0.2 to 3.2)	-28.9 (-75.7 to -14.7)	111.1 (49.4 to 1806.2)
Diet high in sugar-sweetened beverages	4.6 (2.2 to 6.3)	3.9 (1.9 to 5.3)	6.6 (3.1 to 8.8)	-14.7 (-37.3 to 17.7)	68.7 (34.9 to 109.0)	3.2 (1.6 to 4.6)	2.8 (1.4 to 4.0)	4.6 (2.3 to 6.2)	-13.0 (-35.8 to 20.9)	62.0 (30.0 to 104.5)
Diet low in fibre	35.0 (30.9 to 38.5)	32.2 (27.5 to 36.0)	27.8 (22.3 to 32.3)	-8.1 (-11.8 to -5.5)	-13.8 (-19.5 to -9.6)	39.4 (36.1 to 42.1)	37.0 (33.2 to 40.1)	33.2 (28.6 to 36.9)	-5.9 (-8.5 to -4.0)	-10.3 (-14.3 to -7.4)
Diet low in calcium	33.3 (28.9 to 36.7)	29.5 (24.5 to 33.4)	24.4 (18.9 to 29.0)	-11.4 (-15.5 to -8.6)	-17.3 (-23.2 to -13.3)	35.9 (32.1 to 39.0)	32.2 (27.7 to 35.8)	27.4 (22.2 to 31.7)	-10.3 (-13.9 to -7.8)	-15.1 (-20.1 to -11.5)
Diet low in seafood omega-3 fatty acids	44.5 (42.5 to 46.4)	45.0 (43.1 to 46.9)	41.2 (38.5 to 43.6)	1.3 (0.9 to 1.7)	-8.6 (-10.8 to -6.9)	45.8 (44.1 to 47.5)	46.3 (44.7 to 47.9)	42.9 (40.6 to 45.1)	1.0 (0.7 to 1.4)	-7.3 (-9.1 to -5.8)
Diet low in polyunsaturated fatty acids	45.3 (43.4 to 47.0)	42.4 (40.1 to 44.4)	40.3 (37.6 to 42.7)	-6.4 (-8.6 to -4.0)	-5.1 (-6.7 to -3.7)	45.5 (43.7 to 47.2)	42.7 (40.4 to 44.6)	40.5 (37.9 to 42.8)	-6.3 (-8.4 to -4.1)	-5.0 (-6.7 to -3.6)
Diet high in trans fatty acids	1.7 (0.5 to 3.7)	1.5 (0.4 to 3.5)	1.3 (0.3 to 3.2)	-12.8 (-30.9 to -3.3)	-11.7 (-29.9 to -3.5)	2.3 (0.8 to 4.6)	2.0 (0.6 to 4.2)	1.7 (0.5 to 3.9)	-14.6 (-33.4 to -5.3)	-10.8 (-27.0 to -1.7)
Diet high in sodium	14.6 (7.8 to 21.0)	8.3 (1.6 to 15.3)	9.2 (2.2 to 16.3)	-43.2 (-79.6 to -25.3)	10.6 (1.8 to 41.9)	13.1 (6.2 to 19.5)	7.0 (1.1 to 13.8)	5.9 (0.6 to 12.7)	-46.5 (-83.5 to -28.0)	-15.9 (-48.8 to -6.9)
Intimate partner violence	-	-	-	-	-	4.1 (3.3 to 4.9)	3.9 (3.2 to 4.6)	3.7 (3.0 to 4.3)	-5.7 (-8.5 to -2.7)	-5.2 (-7.5 to -2.7)
Childhood sexual abuse	1.6 (1.3 to 2.0)	1.6 (1.3 to 2.0)	1.6 (1.3 to 2.0)	-0.2 (-1.4 to 1.3)	0.2 (-1.3 to 1.4)	2.2 (1.7 to 2.7)	2.2 (1.7 to 2.7)	2.2 (1.7 to 2.7)	-0.7 (-2.0 to 0.8)	0.7 (-0.8 to 2.0)

Supplementary Results Table 1. Central Asia age-standardised Summary Exposure Values (SEVs) for Level 4 risk factors and percentage change, by sex, 1990, 2007, and 2017.Data in parentheses are 95% uncertainty intervals.

	Male					Female				
	1990	2007	2017	Percentage change 1990-2007	Percentage change 2007-2017	1990	2007	2017	Percentage change 1990-2007	Percentage change 2007-2017
Bullying victimization	2.5 (1.0 to 5.0)	2.6 (1.1 to 5.2)	2.5 (1.1 to 5.0)	4.2 (0.7 to 8.1)	-2.9 (-4.5 to -1.3)	1.5 (0.6 to 2.9)	1.6 (0.7 to 3.1)	1.5 (0.6 to 3.0)	4.8 (0.8 to 9.2)	-2.7 (-4.4 to -0.9)
Low physical activity	0.3 (0.2 to 0.6)	0.3 (0.2 to 0.6)	0.3 (0.2 to 0.6)	0.4 (-0.5 to 1.4)	1.8 (0.9 to 2.9)	0.3 (0.1 to 0.5)	0.3 (0.1 to 0.5)	0.3 (0.1 to 0.5)	0.1 (-0.7 to 1.0)	1.1 (0.3 to 2.1)
High fasting plasma glucose	3.9 (2.9 to 5.6)	4.3 (3.2 to 6.3)	4.5 (3.3 to 6.4)	10.2 (-0.5 to 22.7)	4.2 (-8.1 to 17.7)	2.9 (2.1 to 4.3)	3.6 (2.6 to 5.1)	3.7 (2.7 to 5.3)	23.8 (10.2 to 42.7)	2.1 (-10.4 to 16.5)
High LDL cholesterol	9.9 (8.5 to 11.5)	10.1 (8.6 to 11.6)	10.3 (8.8 to 11.9)	1.7 (-0.4 to 3.7)	1.8 (0.4 to 3.3)	10.5 (9.2 to 12.1)	11.1 (9.7 to 12.7)	11.5 (10.1 to 13.1)	5.3 (3.4 to 7.3)	4.0 (2.4 to 5.8)
High systolic blood pressure	8.0 (7.3 to 8.8)	8.2 (7.4 to 8.9)	8.9 (8.1 to 9.7)	1.6 (-1.4 to 4.8)	8.6 (5.9 to 11.5)	7.0 (6.4 to 7.7)	7.3 (6.7 to 8.0)	7.7 (7.0 to 8.4)	3.9 (-0.0 to 8.1)	5.2 (2.0 to 8.6)
High body-mass index	9.2 (6.7 to 13.3)	11.0 (8.2 to 15.5)	13.7 (10.6 to 18.6)	19.6 (11.0 to 29.4)	24.6 (16.9 to 32.8)	11.2 (8.3 to 15.2)	13.0 (9.9 to 17.3)	16.0 (12.5 to 21.1)	16.8 (9.1 to 25.7)	22.9 (16.7 to 30.6)
Low bone mineral density	4.1 (3.4 to 4.8)	4.0 (3.3 to 4.6)	3.9 (3.2 to 4.6)	-2.6 (-8.6 to 4.3)	-2.0 (-8.7 to 4.9)	5.6 (4.8 to 6.5)	5.5 (4.7 to 6.4)	5.5 (4.6 to 6.4)	-1.6 (-7.0 to 5.1)	-1.1 (-6.8 to 4.7)
Impaired kidney function	2.7 (1.2 to 6.6)	2.8 (1.3 to 6.8)	2.8 (1.3 to 6.8)	5.1 (1.8 to 7.7)	1.2 (-0.6 to 3.4)	3.2 (1.5 to 7.2)	3.3 (1.6 to 7.5)	3.4 (1.7 to 7.6)	5.4 (2.1 to 8.2)	2.6 (0.5 to 5.0)

Supplementary Results Table 1. Armenia age-standardised Summary Exposure Values (SEVs) for Level 4 risk factors and percentage change, by sex, 1990, 2007, and 2017.Data in parentheses are 95% uncertainty intervals.										
	Male					Female				
	1990	2007	2017	Percentage change 1990-2007	Percentage change 2007-2017	1990	2007	2017	Percentage change 1990-2007	Percentage change 2007-2017
Unsafe water source	8.7 (4.8 to 12.1)	4.9 (2.8 to 6.5)	3.1 (1.7 to 4.5)	-43.7 (-60.3 to -19.2)	-36.2 (-52.5 to -9.7)	8.7 (4.8 to 12.1)	4.9 (2.8 to 6.5)	3.1 (1.7 to 4.5)	-43.7 (-60.3 to -19.2)	-36.2 (-52.5 to -9.7)
Unsafe sanitation	43.9 (31.7 to 60.5)	22.9 (17.4 to 30.5)	22.6 (16.7 to 30.8)	-47.8 (-59.4 to -31.0)	-1.3 (-19.2 to 20.0)	43.9 (31.7 to 60.5)	22.9 (17.4 to 30.5)	22.6 (16.7 to 30.8)	-47.8 (-59.4 to -31.0)	-1.3 (-19.2 to 20.0)
No access to handwashing facility	20.0 (16.5 to 23.6)	14.7 (12.4 to 17.3)	12.2 (9.8 to 14.5)	-26.3 (-39.7 to -9.3)	-17.6 (-29.8 to -4.6)	20.0 (16.5 to 23.6)	14.7 (12.4 to 17.3)	12.2 (9.8 to 14.5)	-26.3 (-39.7 to -9.3)	-17.6 (-29.8 to -4.6)
Ambient particulate matter pollution	34.5 (19.4 to 54.5)	45.0 (30.3 to 64.3)	46.8 (28.2 to 69.8)	30.6 (-15.6 to 114.8)	3.8 (-34.1 to 61.4)	29.8 (16.3 to 48.3)	41.0 (26.7 to 59.7)	43.9 (25.1 to 67.4)	37.7 (-14.7 to 133.3)	7.2 (-34.3 to 70.9)
Household air pollution from solid fuels	32.0 (20.5 to 45.7)	16.4 (9.8 to 25.2)	9.2 (4.4 to 16.2)	-49.0 (-67.6 to -22.6)	-43.7 (-68.6 to -10.5)	34.3 (22.7 to 47.4)	18.6 (11.6 to 27.0)	10.9 (5.7 to 18.2)	-45.8 (-64.3 to -21.2)	-41.5 (-66.3 to -9.9)
Ambient ozone pollution	46.8 (17.7 to 54.8)	46.0 (17.6 to 54.8)	46.6 (18.2 to 54.8)	-1.8 (-7.3 to 0.0)	1.3 (-1.3 to 7.3)	46.8 (17.7 to 54.8)	46.0 (17.6 to 54.8)	46.6 (18.2 to 54.8)	-1.8 (-7.3 to 0.0)	1.3 (-1.3 to 7.3)
Residential radon	48.2 (14.7 to 71.2)	48.2 (14.7 to 71.2)	48.2 (14.7 to 71.2)	0.0 (0.0 to 0.0)	0.0 (0.0 to 0.0)	48.2 (14.7 to 71.2)	48.2 (14.7 to 71.2)	48.2 (14.7 to 71.2)	0.0 (0.0 to 0.0)	0.0 (0.0 to 0.0)
Lead exposure	6.2 (2.6 to 10.2)	6.2 (2.8 to 10.1)	5.1 (2.2 to 8.7)	0.1 (-6.9 to 15.0)	-17.7 (-25.3 to -12.3)	2.9 (0.4 to 6.5)	3.0 (0.6 to 6.5)	2.6 (0.5 to 5.8)	3.1 (-4.4 to 35.4)	-14.7 (-23.2 to -9.4)
Occupational exposure to asbestos	2.1 (1.7 to 2.7)	2.7 (2.5 to 2.9)	2.5 (2.3 to 2.7)	29.2 (3.2 to 61.1)	-7.2 (-17.6 to 4.2)	2.4 (1.8 to 3.3)	2.6 (2.3 to 2.9)	2.0 (1.8 to 2.2)	10.6 (-16.7 to 43.2)	-24.3 (-34.6 to -13.2)
Occupational exposure to arsenic	0.2 (0.1 to 0.4)	0.2 (0.1 to 0.4)	0.3 (0.1 to 0.5)	2.8 (-6.8 to 13.9)	13.3 (4.6 to 22.9)	0.2 (0.1 to 0.3)	0.2 (0.1 to 0.4)	0.3 (0.1 to 0.5)	21.1 (6.0 to 40.1)	19.0 (6.2 to 31.4)
Occupational exposure to benzene	0.5 (0.3 to 0.9)	0.6 (0.3 to 1.0)	0.6 (0.4 to 1.1)	13.1 (4.2 to 24.6)	13.0 (5.7 to 22.8)	0.5 (0.3 to 0.9)	0.7 (0.4 to 1.2)	0.8 (0.5 to 1.4)	34.6 (19.0 to 56.0)	18.9 (7.9 to 32.1)
Occupational exposure to beryllium	0.1 (0.1 to 0.1)	0.1 (0.1 to 0.1)	0.1 (0.1 to 0.1)	1.6 (-5.4 to 9.4)	5.9 (1.2 to 10.8)	0.1 (0.1 to 0.1)	0.1 (0.1 to 0.1)	0.1 (0.1 to 0.1)	12.9 (2.1 to 25.5)	8.8 (1.6 to 15.8)
Occupational exposure to cadmium	0.1 (0.1 to 0.1)	0.1 (0.1 to 0.1)	0.1 (0.1 to 0.2)	3.2 (-5.9 to 13.4)	12.5 (4.6 to 21.1)	0.1 (0.1 to 0.1)	0.1 (0.1 to 0.1)	0.2 (0.1 to 0.2)	20.3 (6.1 to 38.2)	17.9 (6.1 to 28.9)
Occupational exposure to chromium	0.3 (0.2 to 0.3)	0.3 (0.3 to 0.3)	0.3 (0.3 to 0.3)	6.6 (-2.9 to 17.2)	14.7 (6.6 to 22.9)	0.2 (0.2 to 0.3)	0.3 (0.3 to 0.3)	0.3 (0.3 to 0.4)	24.7 (10.0 to 43.2)	20.5 (8.4 to 32.2)
Occupational exposure to diesel engine exhaust	1.7 (1.5 to 1.9)	1.8 (1.8 to 1.9)	2.0 (1.9 to 2.1)	9.2 (-1.2 to 20.4)	8.7 (1.6 to 16.4)	1.0 (0.8 to 1.1)	1.0 (1.0 to 1.1)	1.1 (1.1 to 1.2)	7.8 (-4.3 to 21.2)	8.1 (0.0 to 16.6)
Occupational exposure to formaldehyde	0.6 (0.5 to 0.6)	0.6 (0.6 to 0.6)	0.7 (0.6 to 0.7)	6.7 (-4.1 to 19.1)	16.7 (7.3 to 26.9)	0.4 (0.4 to 0.5)	0.6 (0.5 to 0.6)	0.7 (0.7 to 0.8)	30.9 (14.6 to 52.3)	23.7 (10.5 to 37.3)
Occupational exposure to nickel	0.3 (0.1 to 0.7)	0.3 (0.1 to 0.8)	0.3 (0.1 to 0.8)	-0.0 (-9.3 to 10.8)	9.1 (1.7 to 17.6)	0.2 (0.1 to 0.5)	0.2 (0.1 to 0.6)	0.3 (0.1 to 0.7)	14.1 (0.6 to 32.2)	15.2 (2.8 to 27.7)
Occupational exposure to polycyclic aromatic hydrocarbons	0.6 (0.5 to 0.6)	0.6 (0.6 to 0.7)	0.7 (0.7 to 0.8)	8.1 (-1.0 to 18.0)	13.4 (6.3 to 20.6)	0.5 (0.4 to 0.6)	0.6 (0.6 to 0.7)	0.7 (0.7 to 0.8)	25.6 (11.2 to 42.9)	19.7 (8.5 to 30.5)
Occupational exposure to silica	3.2 (1.7 to 6.9)	3.3 (1.7 to 7.4)	3.6 (1.9 to 8.0)	3.9 (-7.1 to 17.4)	7.5 (-1.3 to 17.1)	1.7 (0.8 to 3.7)	1.7 (0.9 to 3.8)	1.9 (1.0 to 4.2)	5.0 (-7.3 to 21.1)	9.0 (-1.7 to 20.6)
Occupational exposure to sulphuric acid	0.7 (0.5 to 1.2)	0.7 (0.5 to 1.3)	0.8 (0.6 to 1.4)	8.0 (-2.1 to 19.1)	5.3 (-2.8 to 13.9)	0.5 (0.3 to 0.9)	0.6 (0.4 to 1.0)	0.6 (0.4 to 1.1)	13.6 (0.7 to 29.4)	10.6 (0.6 to 20.7)
Occupational exposure to trichloroethylene	0.2 (0.1 to 0.2)	0.2 (0.2 to 0.2)	0.2 (0.2 to 0.2)	7.3 (-1.0 to 16.8)	12.8 (6.2 to 20.1)	0.1 (0.1 to 0.1)	0.2 (0.1 to 0.2)	0.2 (0.2 to 0.2)	20.2 (7.0 to 36.3)	16.9 (6.6 to 26.7)
Occupational asthmagens	14.7 (11.3 to 18.8)	14.3 (11.4 to 17.6)	14.9 (12.1 to 18.4)	-2.8 (-14.4 to 11.8)	4.1 (-6.0 to 15.3)	9.0 (6.5 to 12.3)	9.4 (7.2 to 12.2)	9.8 (7.6 to 12.6)	4.2 (-11.4 to 25.2)	4.8 (-8.4 to 20.1)
Occupational particulate matter, gases, and fumes	7.4 (5.6 to 10.0)	7.3 (5.6 to 9.7)	7.3 (5.6 to 9.6)	-1.6 (-5.7 to 2.8)	-0.3 (-3.1 to 2.5)	4.1 (3.0 to 5.6)	4.1 (3.1 to 5.7)	4.2 (3.2 to 5.8)	1.7 (-4.7 to 9.3)	2.1 (-2.6 to 7.2)
Occupational noise	8.0 (7.2 to 8.9)	8.0 (7.4 to 8.6)	8.0 (7.6 to 8.6)	-0.8 (-4.2 to 3.4)	0.8 (-1.7 to 3.3)	4.7 (4.0 to 5.4)	4.9 (4.4 to 5.4)	5.1 (4.8 to 5.6)	3.7 (-2.2 to 10.8)	4.7 (0.7 to 9.2)
Occupational ergonomic factors	17.8 (15.1 to 20.4)	16.1 (14.3 to 17.9)	15.2 (13.2 to 16.9)	-9.3 (-21.2 to 6.7)	-5.9 (-17.4 to 5.9)	13.0 (10.9 to 15.2)	12.9 (11.5 to 14.3)	12.7 (11.2 to 14.3)	-0.7 (-16.2 to 20.2)	-1.6 (-15.7 to 14.8)
Non-exclusive breastfeeding	0.4 (0.3 to 0.6)	0.4 (0.3 to 0.6)	0.4 (0.3 to 0.5)	-2.5 (-11.0 to 8.7)	-7.0 (-14.3 to 2.1)	0.4 (0.3 to 0.6)	0.4 (0.3 to 0.6)	0.4 (0.3 to 0.5)	-2.1 (-10.7 to 9.2)	-7.0 (-14.3 to 2.1)
Discontinued breastfeeding	1.8 (1.7 to 1.9)	1.9 (1.9 to 2.0)	1.9 (1.8 to 2.0)	8.6 (1.8 to 16.0)	-1.2 (-6.4 to 4.1)	1.8 (1.7 to 1.9)	1.9 (1.9 to 2.0)	1.9 (1.8 to 2.0)	9.7 (2.8 to 17.2)	-1.7 (-6.9 to 3.5)
Child underweight	0.2 (0.1 to 0.3)	0.1 (0.1 to 0.2)	0.1 (0.1 to 0.2)	-28.8 (-44.4 to -8.9)	-18.2 (-28.2 to -9.0)	0.2 (0.1 to 0.3)	0.2 (0.1 to 0.2)	0.1 (0.1 to 0.2)	-17.3 (-34.9 to 0.5)	-18.5 (-29.3 to -8.6)
Child wasting	0.1 (0.1 to 0.2)	0.2 (0.1 to 0.2)	0.2 (0.1 to 0.2)	22.6 (-8.6 to 63.8)	-4.9 (-25.4 to 20.1)	0.1 (0.1 to 0.2)	0.2 (0.1 to 0.2)	0.2 (0.1 to 0.3)	36.3 (6.7 to 76.5)	19.1 (-8.8 to 53.7)
Child stunting	1.2 (0.7 to 1.6)	1.2 (0.8 to 1.4)	0.8 (0.5 to 1.0)	-0.1 (-25.0 to 36.5)	-35.0 (-49.5 to -20.2)	1.0 (0.6 to 1.4)	1.1 (0.7 to 1.4)	0.7 (0.4 to 1.0)	6.1 (-22.3 to 48.5)	-32.8 (-50.5 to -15.4)
Short gestation for birth weight	0.0 (0.0 to 0.0)	0.0 (0.0 to 0.0)	0.0 (0.0 to 0.0)	11.4 (-22.2 to 57.2)	0.8 (-21.1 to 42.8)	0.0 (0.0 to 0.0)	0.0 (0.0 to 0.0)	0.0 (0.0 to 0.0)	15.1 (-21.3 to 53.5)	-0.7 (-29.6 to 50.6)
Low birth weight for gestation	0.0 (0.0 to 0.0)	0.0 (0.0 to 0.0)	0.0 (0.0 to 0.0)	8.7 (-10.9 to 36.4)	5.6 (-20.4 to 25.9)	0.0 (0.0 to 0.0)	0.0 (0.0 to 0.0)	0.0 (0.0 to 0.0)	6.3 (-15.1 to 38.7)	5.2 (-23.6 to 37.3)
Iron deficiency	-	-	-	-	-	6.6 (4.7 to 9.0)	7.0 (5.0 to 9.4)	6.2 (4.2 to 8.5)	5.0 (-14.2 to 28.3)	-11.5 (-26.9 to 4.3)
Vitamin A deficiency	0.2 (0.1 to 0.3)	0.1 (0.1 to 0.2)	0.1 (0.1 to 0.2)	-16.8 (-31.2 to 2.6)	-0.5 (-28.0 to 24.9)	0.2 (0.1 to 0.3)	0.1 (0.1 to 0.2)	0.1 (0.1 to 0.2)	-26.8 (-40.3 to -13.3)	-7.3 (-23.3 to 14.1)
Zinc deficiency	1.2 (0.3 to 2.6)	0.6 (0.1 to 1.3)	0.4 (0.1 to 0.9)	-53.1 (-75.7 to -23.8)	-27.2 (-62.4 to 0.3)	1.2 (0.3 to 2.6)	0.6 (0.1 to 1.3)	0.4 (0.1 to 0.9)	-53.7 (-74.7 to -24.7)	-24.6 (-59.3 to 2.9)
Smoking	18.1 (15.9 to 20.4)	16.0 (14.1 to 18.0)	15.8 (13.8 to 17.7)	-11.7 (-18.3 to -4.1)	-1.3 (-7.4 to 5.3)	0.5 (0.4 to 0.7)	0.6 (0.4 to 0.7)	0.6 (0.5 to 0.8)	5.2 (-20.6 to 40.7)	7.5 (-12.7 to 31.6)
Chewing tobacco	0.1 (0.1 to 0.1)	0.1 (0.1 to 0.1)	0.1 (0.1 to 0.1)	-2.0 (-27.5 to 35.8)	-5.3 (-24.6 to 20.8)	0.1 (0.0 to 0.1)	0.1 (0.0 to 0.1)	0.1 (0.0 to 0.1)	-0.1 (-28.2 to 40.9)	0.4 (-20.8 to 26.9)
Second-hand smoke	40.9 (38.9 to 43.1)	39.2 (38.4 to 40.1)	38.9 (37.3 to 40.5)	-4.1 (-9.0 to 1.1)	-0.9 (-5.3 to 3.6)	73.3 (71.4 to 75.0)	68.5 (67.8 to 69.2)	66.9 (65.3 to 68.3)	-6.6 (-8.7 to -4.2)	-2.3 (-4.5 to -0.2)
Alcohol use	12.2 (6.5 to 20.7)	12.9 (7.1 to 22.0)	14.9 (7.8 to 24.3)	6.3 (-15.9 to 36.4)	14.9 (-9.5 to 51.9)	5.5 (3.1 to 9.0)	5.7 (3.4 to 9.2)	6.6 (3.7 to 10.8)	4.0 (-19.1 to 40.0)	16.1 (-11.2 to 58.2)
Drug use	0.4 (0.3 to 0.5)	0.4 (0.3 to 0.5)	0.4 (0.4 to 0.6)	3.6 (-3.2 to 11.8)	13.0 (6.5 to 19.8)	0.2 (0.1 to 0.2)	0.2 (0.1 to 0.2)	0.2 (0.1 to 0.2)	1.4 (-4.3 to 7.8)	5.1 (-0.7 to 11.7)
Diet low in fruits	40.1 (34.6 to 43.5)	36.8 (31.0 to 40.6)	31.8 (25.8 to 36.7)	-8.2 (-13.8 to -2.8)	-13.6 (-20.9 to -8.1)	38.0 (32.5 to 41.8)	34.2 (28.6 to 38.3)	28.6 (22.1 to 33.8)	-10.0 (-16.7 to -4.3)	-16.4 (-26.6 to -9.5)
Diet low in vegetables	15.3 (7.2 to 22.5)	0.5 (0.0 to 2.3)	0.1 (0.0 to 0.4)	-96.8 (-100.0 to -88.9)	-87.9 (-100.0 to 7.3)	18.6 (11.0 to 25.2)	1.2 (0.0 to 4.7)	0.2 (0.0 to 0.9)	-93.4 (-99.9 to -79.9)	-87.2 (-100.0 to -38.8)
Diet low in legumes	54.8 (54.8 to 54.8)	54.8 (54.8 to 54.8)	54.8 (54.8 to 54.8)	0.0 (0.0 to 0.0)	-0.0 (0.0 to 0.0)	52.9 (52.4 to 53.4)	52.1 (51.5 to 52.6)	54.8 (54.7 to 54.8)	-1.6 (-2.6 to -0.5)	5.2 (4.1 to 6.4)
Diet low in whole grains	52.3 (51.8 to 52.6)	52.3 (51.9 to 52.7)	52.2 (51.8 to 52.6)	0.0 (-0.3 to 0.3)	-0.1 (-0.4 to 0.1)	52.2 (51.7 to 52.6)	52.3 (51.9 to 52.7)	52.3 (51.9 to 52.7)	0.2 (-0.1 to 0.6)	0.0 (-0.3 to 0.3)
Diet low in nuts and seeds	52.8 (52.2 to 53.3)	52.3 (51.6 to 52.8)	50.0 (48.6 to 51.1)	-1.0 (-1.8 to -0.3)	-4.4 (-6.0 to -3.0)	52.9 (52.4 to 53.3)	52.4 (51.8 to 53.0)	50.3 (48.9 to 51.4)	-0.9 (-1.6 to -0.2)	-4.1 (-5.7 to -2.8)
Diet low in milk	43.9 (41.5 to 45.8)	38.4 (35.0 to 41.3)	34.5 (30.2 to 38.2)	-12.4 (-16.4 to -9.2)	-10.1 (-14.6 to -6.6)	44.3 (42.0 to 46.2)	39.3 (36.0 to 42.0)	35.5 (31.4 to 39.0)	-11.5 (-15.1 to -8.5)	-9.5 (-13.5 to -6.3)
Diet high in red meat	1.0 (0.0 to 3.3)	2.4 (0.3 to 5.4)	8.5 (3.3 to 13.6)	140.3 (-26.8 to 5755.8)	255.9 (96.8 to 2049.2)	0.0 (0.0 to 0.3)	0.1 (0.0 to 0.5)	1.2 (0.0 to 3.7)	102.1 (-100.0 to [illegible]	1899.6 (342.5 to [illegible]
Diet high in processed meat	0.3 (0.0 to 1.5)	0.3 (0.0 to 1.6)	0.5 (0.0 to 2.1)	2.8 (-73.3 to 85.9)	67.3 (26.0 to 29050.3)	0.2 (0.0 to 1.2)	0.2 (0.0 to 1.2)	0.3 (0.0 to 1.5)	3.6 (-100.0 to 60.6)	67.4 (29.0 to [illegible]
Diet high in sugar-sweetened beverages	3.1 (1.3 to 5.8)	3.3 (1.5 to 6.2)	5.1 (2.4 to 9.5)	7.6 (-41.7 to 104.6)	55.5 (2.5 to 137.3)	2.2 (1.0 to 4.0)	2.4 (1.0 to 4.6)	3.6 (1.6 to 6.7)	8.9 (-42.6 to 113.2)	51.1 (0.6 to 130.8)
Diet low in fibre	35.1 (30.6 to 38.7)	30.8 (25.6 to 34.9)	27.5 (21.8 to 32.4)	-12.2 (-18.7 to -7.5)	-10.6 (-18.0 to -4.7)	39.5 (36.1 to 42.5)	35.9 (31.5 to 39.4)	33.0 (27.9 to 37.4)	-9.3 (-14.7 to -5.2)	-7.9 (-13.8 to -3.2)
Diet low in calcium	36.7 (32.9 to 39.8)	26.4 (20.7 to 31.0)	18.3 (11.2 to 24.0)	-28.1 (-37.6 to -21.6)	-30.9 (-46.5 to -21.7)	39.1 (35.9 to 41.8)	29.9 (24.8 to 33.9)	22.3 (15.7 to 27.7)	-23.6 (-31.3 to -18.3)	-25.2 (-37.2 to -17.9)
Diet low in seafood omega-3 fatty acids	47.3 (45.7 to 48.7)	40.3 (37.5 to 43.0)	36.7 (33.2 to 40.0)	-14.7 (-18.1 to -11.8)	-9.0 (-12.3 to -6.6)	48.3 (47.0 to 49.6)	42.4 (40.0 to 44.7)	39.1 (36.1 to 42.0)	-12.2 (-15.1 to -9.7)	-7.8 (-10.4 to -5.6)
Diet low in polyunsaturated fatty acids	48.3 (46.5 to 49.7)	39.5 (36.5 to 42.1)	33.3 (29.2 to 37.0)	-18.3 (-23.1 to -13.5)	-15.6 (-21.3 to -11.0)	48.6 (46.8 to 50.0)	40.0 (37.1 to 42.4)	33.9 (29.8 to 37.5)	-17.8 (-22.2 to -13.5)	-15.1 (-20.5 to -10.8)
Diet high in trans fatty acids	1.5 (0.1 to 4.1)	1.3 (0.1 to 3.6)	1.1 (0.0 to 3.3)	-18.6 (-52.8 to -5.8)	-14.9 (-49.6 to -2.6)	2.1 (0.3 to 5.0)	1.7 (0.2 to 4.4)	1.5 (0.1 to 3.9)	-18.4 (-47.4 to -4.1)	-14.9 (-45.4 to -2.5)
Diet high in sodium	13.5 (5.8 to 20.4)	8.0 (1.1 to 15.2)	8.5 (1.3 to 15.7)	-40.2 (-83.7 to -16.8)	5.9 (-21.0 to 62.7)	12.7 (5.2 to 19.7)	6.9 (0.8 to 14.1)	5.6 (0.4 to 12.3)	-45.6 (-86.7 to -24.4)	-18.8 (-58.8 to -5.3)
Intimate partner violence	-	-	-	-	-	3.8 (2.9 to 4.7)	3.6 (2.7 to 4.4)	3.3 (2.6 to 4.1)	-5.7 (-11.7 to 0.7)	-7.9 (-13.5 to -1.8)
Childhood sexual abuse	1.6 (1.3 to 2.0)	1.6 (1.3 to 2.0)	1.6 (1.3 to 2.0)	0.0 (0.0 to 0.0)	0.0 (0.0 to 0.0)	2.2 (1.7 to 2.7)	2.2 (1.7 to 2.7)	2.2 (1.7 to 2.7)	0.0 (0.0 to 0.0)	0.0 (0.0 to 0.0)

Supplementary Results Table 1. Armenia age-standardised Summary Exposure Values (SEVs) for Level 4 risk factors and percentage change, by sex, 1990, 2007, and 2017.Data in parentheses are 95% uncertainty intervals.

	Male					Female				
	**1990**	**2007**	**2017**	**Percentage change 1990-2007**	**Percentage change 2007-2017**	**1990**	**2007**	**2017**	**Percentage change 1990-2007**	**Percentage change 2007-2017**
Bullying victimization	2.0 (0.8 to 4.3)	2.2 (0.9 to 4.5)	2.2 (0.9 to 4.4)	10.5 (-8.0 to 38.3)	-2.3 (-7.7 to 3.5)	0.8 (0.3 to 1.7)	0.9 (0.4 to 1.8)	0.8 (0.4 to 1.7)	10.8 (-9.0 to 33.8)	-3.1 (-8.6 to 3.0)
Low physical activity	0.3 (0.2 to 0.6)	0.3 (0.2 to 0.6)	0.3 (0.2 to 0.6)	1.4 (-0.5 to 3.5)	1.8 (-0.1 to 3.8)	0.3 (0.1 to 0.5)	0.3 (0.1 to 0.5)	0.3 (0.1 to 0.5)	1.2 (-0.6 to 3.3)	1.2 (-0.5 to 3.4)
High fasting plasma glucose	4.3 (3.1 to 6.3)	5.0 (3.6 to 7.4)	4.9 (3.5 to 7.3)	16.8 (-3.8 to 42.6)	-3.0 (-22.0 to 17.0)	3.6 (2.5 to 5.2)	4.3 (3.1 to 6.2)	4.1 (3.0 to 6.0)	20.6 (0.5 to 48.8)	-4.2 (-20.8 to 13.0)
High LDL cholesterol	7.8 (6.5 to 9.4)	7.8 (6.5 to 9.2)	8.0 (6.7 to 9.4)	-1.1 (-5.3 to 3.1)	2.6 (-0.9 to 6.1)	8.4 (7.1 to 9.9)	8.7 (7.4 to 10.2)	9.1 (7.7 to 10.6)	3.4 (-1.1 to 8.0)	3.9 (0.9 to 7.5)
High systolic blood pressure	10.3 (9.4 to 11.4)	10.4 (9.5 to 11.4)	10.3 (9.4 to 11.2)	1.3 (-5.0 to 8.3)	-1.7 (-6.6 to 3.2)	10.0 (9.0 to 11.1)	10.1 (9.2 to 11.1)	8.8 (8.0 to 9.5)	0.7 (-7.8 to 10.2)	-13.3 (-18.6 to -7.6)
High body-mass index	8.0 (5.5 to 12.1)	11.6 (8.6 to 16.6)	14.6 (10.8 to 20.1)	46.2 (25.5 to 73.2)	25.8 (13.6 to 39.8)	9.3 (6.5 to 13.5)	13.4 (9.9 to 18.2)	16.3 (12.0 to 22.1)	43.4 (22.5 to 70.5)	21.6 (10.3 to 35.1)
Low bone mineral density	4.2 (3.4 to 5.1)	4.0 (3.3 to 4.8)	4.0 (3.1 to 4.8)	-3.6 (-16.5 to 12.5)	-2.1 (-17.1 to 14.6)	5.8 (4.7 to 6.8)	5.6 (4.7 to 6.7)	5.5 (4.5 to 6.6)	-2.2 (-14.8 to 12.5)	-1.6 (-14.7 to 12.0)
Impaired kidney function	2.8 (1.3 to 6.8)	3.0 (1.4 to 7.2)	3.1 (1.4 to 7.2)	6.8 (1.7 to 12.6)	1.0 (-2.5 to 4.8)	3.3 (1.6 to 7.4)	3.6 (1.7 to 7.8)	3.7 (1.8 to 8.0)	7.5 (1.8 to 14.0)	3.0 (-0.0 to 7.0)

Supplementary Results Table 1. Azerbaijan age-standardised Summary Exposure Values (SEVs) for Level 4 risk factors and percentage change, by sex, 1990, 2007, and 2017.Data in parentheses are 95% uncertainty intervals.

	Male					Female				
	**1990**	**2007**	**2017**	**Percentage change 1990-2007**	**Percentage change 2007-2017**	**1990**	**2007**	**2017**	**Percentage change 1990-2007**	**Percentage change 2007-2017**
Unsafe water source	22.6 (16.1 to 27.7)	18.6 (13.5 to 22.1)	10.3 (6.6 to 13.8)	-17.6 (-32.7 to 2.4)	-44.7 (-57.9 to -29.4)	22.6 (16.1 to 27.7)	18.6 (13.5 to 22.1)	10.3 (6.6 to 13.8)	-17.6 (-32.7 to 2.4)	-44.7 (-57.9 to -29.4)
Unsafe sanitation	57.6 (43.7 to 77.2)	51.2 (38.7 to 68.2)	37.6 (26.4 to 51.1)	-11.0 (-28.7 to 9.7)	-26.5 (-43.4 to -6.2)	57.6 (43.7 to 77.2)	51.2 (38.7 to 68.2)	37.6 (26.4 to 51.1)	-11.0 (-28.7 to 9.7)	-26.5 (-43.4 to -6.2)
No access to handwashing facility	21.9 (18.0 to 25.8)	16.4 (13.5 to 20.0)	9.2 (7.4 to 11.3)	-24.9 (-38.7 to -7.5)	-43.9 (-52.0 to -34.3)	21.9 (18.0 to 25.8)	16.4 (13.5 to 20.0)	9.2 (7.4 to 11.3)	-24.9 (-38.7 to -7.5)	-43.9 (-52.0 to -34.3)
Ambient particulate matter pollution	28.5 (12.4 to 52.4)	33.9 (19.6 to 54.5)	33.6 (14.8 to 58.8)	19.3 (-44.5 to 185.4)	-1.1 (-57.6 to 95.4)	24.6 (10.1 to 47.6)	30.4 (16.6 to 50.8)	30.8 (13.3 to 56.0)	23.8 (-46.6 to 221.2)	1.2 (-59.0 to 112.0)
Household air pollution from solid fuels	27.6 (16.5 to 40.5)	15.8 (9.0 to 24.4)	11.1 (5.4 to 19.1)	-42.7 (-67.1 to -4.2)	-29.8 (-62.8 to 15.5)	29.4 (18.9 to 41.5)	17.7 (10.7 to 26.1)	12.8 (6.5 to 20.8)	-39.6 (-62.7 to -5.9)	-27.9 (-58.0 to 11.5)
Ambient ozone pollution	43.4 (15.7 to 54.8)	43.1 (15.3 to 54.8)	43.9 (16.1 to 54.8)	-0.6 (-3.0 to 1.3)	1.9 (0.0 to 6.4)	43.4 (15.7 to 54.8)	43.1 (15.3 to 54.8)	43.9 (16.1 to 54.8)	-0.6 (-3.0 to 1.3)	1.9 (0.0 to 6.4)
Residential radon	32.6 (5.4 to 71.2)	32.6 (5.4 to 71.2)	32.6 (5.4 to 71.2)	0.0 (0.0 to 0.0)	0.0 (0.0 to 0.0)	32.6 (5.4 to 71.2)	32.6 (5.4 to 71.2)	32.6 (5.4 to 71.2)	0.0 (0.0 to 0.0)	0.0 (0.0 to 0.0)
Lead exposure	4.9 (1.7 to 8.8)	4.9 (1.9 to 8.6)	4.1 (1.5 to 7.6)	-0.1 (-7.3 to 15.2)	-16.9 (-25.1 to -11.6)	2.5 (0.3 to 5.9)	2.6 (0.4 to 5.9)	2.2 (0.3 to 5.3)	2.6 (-5.3 to 35.1)	-14.4 (-23.3 to -9.5)
Occupational exposure to asbestos	0.3 (0.2 to 0.4)	0.4 (0.3 to 0.5)	0.4 (0.3 to 0.5)	46.4 (7.8 to 112.2)	-10.7 (-31.5 to 16.0)	0.3 (0.2 to 0.3)	0.5 (0.4 to 0.6)	0.3 (0.2 to 0.4)	90.3 (30.8 to 178.5)	-32.6 (-48.8 to -14.2)
Occupational exposure to arsenic	0.2 (0.1 to 0.4)	0.2 (0.1 to 0.3)	0.2 (0.1 to 0.4)	-12.0 (-18.6 to -4.5)	4.6 (-1.5 to 11.1)	0.3 (0.1 to 0.5)	0.2 (0.1 to 0.4)	0.3 (0.1 to 0.4)	-14.9 (-22.5 to -6.5)	5.9 (-0.9 to 13.3)
Occupational exposure to benzene	0.6 (0.4 to 1.0)	0.7 (0.4 to 1.2)	0.7 (0.4 to 1.3)	12.7 (4.6 to 23.2)	9.4 (3.8 to 16.5)	0.7 (0.4 to 1.3)	0.8 (0.5 to 1.5)	1.0 (0.6 to 1.7)	16.7 (6.3 to 31.1)	14.6 (7.1 to 24.9)
Occupational exposure to beryllium	0.1 (0.1 to 0.1)	0.1 (0.1 to 0.1)	0.1 (0.1 to 0.1)	0.9 (-4.5 to 6.8)	1.8 (-1.7 to 5.3)	0.1 (0.1 to 0.1)	0.1 (0.1 to 0.1)	0.1 (0.1 to 0.1)	2.4 (-4.8 to 10.3)	6.7 (2.2 to 11.5)
Occupational exposure to cadmium	0.1 (0.1 to 0.1)	0.1 (0.1 to 0.1)	0.1 (0.1 to 0.1)	-10.0 (-16.4 to -3.2)	4.7 (-0.9 to 10.4)	0.2 (0.1 to 0.2)	0.1 (0.1 to 0.1)	0.1 (0.1 to 0.2)	-12.1 (-19.7 to -4.2)	6.3 (0.2 to 12.9)
Occupational exposure to chromium	0.3 (0.3 to 0.3)	0.3 (0.3 to 0.3)	0.3 (0.3 to 0.3)	-7.5 (-14.1 to -0.8)	7.6 (2.1 to 13.3)	0.3 (0.3 to 0.4)	0.3 (0.3 to 0.3)	0.3 (0.3 to 0.4)	-9.1 (-17.0 to -0.2)	8.5 (2.1 to 15.2)
Occupational exposure to diesel engine exhaust	1.9 (1.7 to 2.0)	2.0 (1.9 to 2.0)	2.1 (2.0 to 2.2)	5.2 (-2.3 to 13.3)	7.5 (1.9 to 13.6)	1.6 (1.5 to 1.8)	1.7 (1.6 to 1.8)	1.8 (1.7 to 2.0)	4.6 (-5.3 to 15.3)	7.4 (0.8 to 14.5)
Occupational exposure to formaldehyde	0.6 (0.5 to 0.6)	0.5 (0.5 to 0.6)	0.6 (0.6 to 0.6)	-9.0 (-16.2 to -1.1)	8.3 (1.8 to 15.0)	0.7 (0.6 to 0.8)	0.6 (0.6 to 0.6)	0.7 (0.6 to 0.7)	-10.7 (-18.8 to -1.0)	9.8 (2.4 to 17.5)
Occupational exposure to nickel	0.3 (0.1 to 0.7)	0.3 (0.1 to 0.7)	0.3 (0.1 to 0.7)	-5.3 (-12.2 to 2.2)	2.1 (-3.5 to 8.5)	0.3 (0.1 to 0.7)	0.2 (0.1 to 0.6)	0.2 (0.1 to 0.6)	-15.5 (-23.0 to -7.1)	3.6 (-3.2 to 11.0)
Occupational exposure to polycyclic aromatic hydrocarbons	0.6 (0.6 to 0.7)	0.6 (0.6 to 0.6)	0.6 (0.6 to 0.7)	-4.4 (-10.7 to 2.1)	7.5 (2.4 to 12.7)	0.7 (0.7 to 0.8)	0.7 (0.7 to 0.7)	0.7 (0.7 to 0.8)	-6.3 (-14.3 to 2.5)	9.3 (2.8 to 15.7)
Occupational exposure to silica	3.0 (1.6 to 6.7)	3.1 (1.6 to 6.9)	3.3 (1.7 to 7.3)	4.2 (-4.9 to 15.0)	4.3 (-2.8 to 12.7)	2.3 (1.2 to 5.0)	2.0 (1.0 to 4.5)	2.0 (1.0 to 4.3)	-13.9 (-21.2 to -5.2)	2.3 (-4.4 to 10.1)
Occupational exposure to sulphuric acid	0.6 (0.5 to 1.1)	0.5 (0.4 to 0.9)	0.5 (0.4 to 0.9)	-15.8 (-22.0 to -9.1)	-0.8 (-6.3 to 5.2)	0.7 (0.5 to 1.1)	0.6 (0.4 to 1.0)	0.6 (0.4 to 1.0)	-14.5 (-21.5 to -6.6)	1.2 (-4.4 to 7.4)
Occupational exposure to trichloroethylene	0.2 (0.2 to 0.2)	0.2 (0.2 to 0.2)	0.2 (0.2 to 0.2)	-5.3 (-11.2 to 1.0)	7.3 (2.5 to 12.4)	0.2 (0.2 to 0.2)	0.2 (0.2 to 0.2)	0.2 (0.2 to 0.2)	-4.0 (-12.1 to 5.7)	8.1 (2.5 to 14.4)
Occupational asthmagens	13.5 (10.7 to 17.2)	13.5 (10.9 to 16.6)	15.2 (12.2 to 18.6)	-0.4 (-12.2 to 12.6)	12.7 (3.4 to 22.8)	8.7 (6.5 to 11.7)	8.4 (6.6 to 10.7)	9.6 (7.5 to 12.5)	-3.2 (-20.7 to 20.7)	14.6 (-0.2 to 32.9)
Occupational particulate matter, gases, and fumes	7.1 (5.4 to 9.5)	6.7 (5.1 to 9.0)	6.6 (5.1 to 8.8)	-6.0 (-9.6 to -2.1)	-1.0 (-3.7 to 2.2)	5.4 (4.0 to 7.5)	5.0 (3.8 to 6.8)	4.9 (3.7 to 6.7)	-7.4 (-12.2 to -1.9)	-1.4 (-5.1 to 3.0)
Occupational noise	8.0 (7.3 to 8.8)	7.8 (7.2 to 8.5)	8.0 (7.5 to 8.5)	-2.5 (-5.7 to 1.0)	1.5 (-0.7 to 4.1)	6.4 (5.7 to 7.1)	6.3 (5.7 to 6.9)	6.5 (6.1 to 7.0)	-1.6 (-5.8 to 3.1)	3.5 (0.3 to 7.0)
Occupational ergonomic factors	14.9 (12.7 to 17.3)	14.9 (13.6 to 16.2)	15.7 (13.9 to 17.7)	0.1 (-14.0 to 15.8)	5.4 (-5.1 to 16.9)	11.8 (9.4 to 14.2)	11.8 (10.3 to 13.2)	12.8 (11.0 to 14.7)	0.1 (-19.0 to 26.7)	9.0 (-7.0 to 27.9)
Non-exclusive breastfeeding	0.6 (0.4 to 0.8)	0.6 (0.4 to 0.8)	0.5 (0.4 to 0.8)	2.2 (-3.0 to 10.0)	-3.9 (-8.7 to 1.2)	0.6 (0.4 to 0.8)	0.6 (0.4 to 0.8)	0.5 (0.4 to 0.8)	2.5 (-2.8 to 10.2)	-3.9 (-8.7 to 1.1)
Discontinued breastfeeding	1.8 (1.7 to 1.9)	2.0 (1.9 to 2.1)	1.9 (1.8 to 2.0)	10.3 (3.2 to 18.2)	-6.4 (-11.7 to -0.9)	1.8 (1.7 to 1.9)	2.0 (1.9 to 2.1)	1.9 (1.8 to 2.0)	10.8 (3.7 to 18.8)	-5.5 (-10.9 to -0.0)
Child underweight	0.5 (0.3 to 0.6)	0.3 (0.2 to 0.4)	0.2 (0.1 to 0.3)	-38.3 (-53.1 to -22.8)	-27.1 (-38.5 to -15.9)	0.5 (0.3 to 0.6)	0.3 (0.2 to 0.4)	0.2 (0.1 to 0.3)	-35.2 (-50.0 to -18.8)	-28.0 (-41.0 to -14.8)
Child wasting	0.2 (0.2 to 0.3)	0.2 (0.2 to 0.3)	0.2 (0.1 to 0.3)	-4.4 (-30.9 to 38.0)	-0.1 (-23.7 to 26.9)	0.2 (0.1 to 0.3)	0.2 (0.1 to 0.3)	0.2 (0.1 to 0.3)	-0.7 (-30.1 to 36.2)	0.4 (-23.0 to 27.7)
Child stunting	1.6 (1.1 to 2.1)	1.5 (1.0 to 1.7)	0.9 (0.6 to 1.3)	-9.9 (-32.9 to 20.0)	-35.0 (-52.6 to -17.5)	1.6 (1.0 to 2.1)	1.3 (0.9 to 1.6)	0.9 (0.5 to 1.2)	-15.1 (-35.8 to 16.5)	-32.2 (-50.4 to -12.4)
Short gestation for birth weight	0.0 (0.0 to 0.0)	0.0 (0.0 to 0.0)	0.0 (0.0 to 0.0)	-5.4 (-29.1 to 24.4)	-7.5 (-29.1 to 34.6)	0.0 (0.0 to 0.0)	0.0 (0.0 to 0.0)	0.0 (0.0 to 0.0)	-6.1 (-38.3 to 22.1)	-1.6 (-23.5 to 21.9)
Low birth weight for gestation	0.0 (0.0 to 0.0)	0.0 (0.0 to 0.0)	0.0 (0.0 to 0.0)	4.6 (-15.5 to 31.0)	9.2 (-14.2 to 31.5)	0.0 (0.0 to 0.0)	0.0 (0.0 to 0.0)	0.0 (0.0 to 0.0)	7.0 (-9.3 to 41.0)	8.3 (-7.5 to 28.1)
Iron deficiency	-	-	-	-	-	12.3 (9.5 to 15.5)	12.4 (9.5 to 15.7)	10.4 (7.7 to 13.6)	0.5 (-13.0 to 15.8)	-15.5 (-26.0 to -4.9)
Vitamin A deficiency	1.6 (1.1 to 2.0)	1.6 (1.2 to 1.9)	1.2 (0.9 to 1.6)	-0.9 (-18.7 to 26.7)	-20.3 (-33.8 to -5.5)	1.8 (1.5 to 2.2)	1.7 (1.4 to 2.1)	1.5 (1.2 to 1.9)	-6.4 (-17.4 to 4.7)	-11.0 (-24.0 to 1.3)
Zinc deficiency	2.3 (0.5 to 4.5)	0.8 (0.2 to 1.7)	0.5 (0.1 to 1.1)	-63.6 (-84.4 to -21.2)	-40.8 (-61.2 to -14.7)	2.2 (0.5 to 4.5)	0.8 (0.2 to 1.6)	0.5 (0.1 to 1.1)	-63.4 (-84.8 to -20.2)	-40.8 (-59.9 to -13.9)
Smoking	13.6 (11.0 to 16.1)	12.1 (10.4 to 14.0)	12.6 (11.0 to 14.4)	-11.3 (-20.6 to 0.6)	4.7 (-1.7 to 11.8)	0.3 (0.2 to 0.4)	0.3 (0.2 to 0.4)	0.4 (0.3 to 0.5)	12.1 (-8.9 to 37.8)	37.1 (20.2 to 57.0)
Chewing tobacco	0.1 (0.1 to 0.1)	0.1 (0.1 to 0.1)	0.1 (0.1 to 0.1)	0.8 (-28.2 to 43.5)	0.4 (-21.5 to 31.1)	0.1 (0.0 to 0.1)	0.1 (0.0 to 0.1)	0.1 (0.0 to 0.1)	-1.5 (-30.1 to 37.8)	0.7 (-21.0 to 28.8)
Second-hand smoke	38.1 (35.8 to 40.3)	35.8 (34.5 to 37.1)	36.3 (34.0 to 38.8)	-5.9 (-12.0 to 1.2)	1.4 (-4.4 to 6.9)	62.1 (60.2 to 63.9)	59.4 (58.2 to 60.5)	59.5 (57.1 to 61.8)	-4.4 (-7.7 to -1.1)	0.3 (-2.8 to 3.3)
Alcohol use	19.9 (12.0 to 28.6)	23.1 (14.8 to 31.3)	21.4 (12.0 to 31.5)	16.0 (-10.4 to 61.1)	-7.4 (-33.7 to 20.5)	4.9 (2.6 to 8.5)	5.8 (3.2 to 9.5)	5.8 (2.9 to 9.7)	17.7 (-3.5 to 61.7)	-0.9 (-23.5 to 26.8)
Drug use	0.4 (0.3 to 0.5)	0.4 (0.3 to 0.5)	0.4 (0.3 to 0.6)	0.8 (-4.8 to 6.9)	10.7 (3.9 to 17.7)	0.2 (0.1 to 0.2)	0.2 (0.1 to 0.2)	0.2 (0.1 to 0.2)	0.7 (-5.6 to 7.1)	5.3 (-1.3 to 12.4)
Diet low in fruits	34.8 (28.6 to 38.9)	38.8 (33.4 to 42.2)	34.0 (28.0 to 38.4)	11.7 (5.1 to 20.3)	-12.4 (-18.7 to -7.5)	31.8 (25.6 to 36.3)	36.7 (31.1 to 40.5)	31.5 (25.6 to 36.4)	15.6 (7.7 to 27.0)	-14.3 (-22.0 to -8.7)
Diet low in vegetables	33.6 (28.7 to 37.7)	10.0 (2.7 to 16.8)	2.8 (0.2 to 7.8)	-70.2 (-91.1 to -53.8)	-71.7 (-95.2 to -50.0)	35.2 (30.6 to 38.9)	13.1 (5.5 to 20.0)	4.4 (0.5 to 10.4)	-62.7 (-82.6 to -47.6)	-66.3 (-93.0 to -44.2)
Diet low in legumes	54.8 (54.8 to 54.8)	54.8 (54.8 to 54.8)	54.8 (54.8 to 54.8)	0.0 (0.0 to 0.0)	0.0 (0.0 to 0.0)	54.8 (54.8 to 54.8)	54.8 (54.8 to 54.8)	54.8 (54.8 to 54.8)	0.0 (0.0 to 0.0)	0.0 (0.0 to 0.0)
Diet low in whole grains	52.8 (52.4 to 53.1)	52.4 (52.0 to 52.8)	52.2 (51.7 to 52.5)	-0.7 (-1.2 to -0.3)	-0.5 (-0.9 to -0.3)	52.7 (52.4 to 53.1)	52.5 (52.1 to 52.9)	52.3 (51.9 to 52.7)	-0.4 (-0.9 to -0.0)	-0.3 (-0.6 to -0.1)
Diet low in nuts and seeds	45.3 (42.8 to 47.4)	47.3 (45.2 to 48.9)	40.0 (35.5 to 43.3)	4.4 (1.0 to 8.2)	-15.3 (-21.8 to -10.9)	45.8 (43.3 to 47.8)	47.7 (45.8 to 49.3)	40.8 (36.7 to 44.0)	4.1 (1.1 to 7.7)	-14.5 (-20.7 to -10.1)
Diet low in milk	43.9 (41.5 to 45.9)	44.2 (42.0 to 46.1)	41.2 (38.2 to 43.6)	0.8 (-0.7 to 2.6)	-6.9 (-9.4 to -4.8)	44.4 (42.1 to 46.3)	44.7 (42.5 to 46.5)	41.7 (38.8 to 44.1)	0.7 (-0.8 to 2.3)	-6.8 (-9.4 to -4.7)
Diet high in red meat	0.0 (0.0 to 0.1)	0.1 (0.0 to 0.5)	3.0 (0.5 to 6.5)	369.8 (-81.2 to [illegible]	4432.4 (994.9 to [illegible]	0.0 (0.0 to 0.0)	0.0 (0.0 to 0.0)	0.2 (0.0 to 1.0)	197.7 (-94.4 to [illegible]	1074158.4 (-11.3 to [illegible]
Diet high in processed meat	1.5 (0.1 to 3.8)	1.1 (0.0 to 3.2)	2.8 (0.6 to 5.5)	-28.1 (-91.2 to -2.6)	156.9 (61.8 to 7282.3)	0.9 (0.0 to 2.9)	0.6 (0.0 to 2.4)	1.8 (0.1 to 4.3)	-29.4 (-97.4 to -7.3)	174.0 (68.6 to 75139.5)
Diet high in sugar-sweetened beverages	5.0 (2.3 to 9.4)	4.4 (2.0 to 8.2)	9.3 (4.4 to 15.5)	-12.8 (-51.3 to 56.5)	111.9 (37.8 to 235.9)	3.6 (1.6 to 6.6)	3.2 (1.4 to 6.0)	6.3 (2.9 to 11.5)	-10.6 (-49.6 to 68.7)	97.6 (27.5 to 210.9)
Diet low in fibre	38.1 (34.1 to 41.2)	27.6 (21.9 to 32.3)	22.5 (16.2 to 28.1)	-27.4 (-36.9 to -20.2)	-18.7 (-30.0 to -10.5)	41.9 (38.8 to 44.4)	33.3 (28.6 to 37.1)	28.6 (22.5 to 33.6)	-20.7 (-27.4 to -15.1)	-14.1 (-23.0 to -7.9)
Diet low in calcium	40.2 (37.2 to 42.7)	35.8 (32.0 to 38.9)	27.8 (22.3 to 32.2)	-10.9 (-14.8 to -8.2)	-22.3 (-30.7 to -16.7)	42.3 (39.6 to 44.3)	38.4 (35.0 to 41.1)	31.1 (26.1 to 35.0)	-9.2 (-12.2 to -6.9)	-19.1 (-25.6 to -14.6)
Diet low in seafood omega-3 fatty acids	48.1 (46.7 to 49.4)	46.7 (45.1 to 48.3)	43.2 (40.9 to 45.4)	-2.7 (-3.5 to -2.0)	-7.6 (-9.7 to -5.9)	49.0 (47.9 to 50.2)	47.9 (46.5 to 49.2)	44.9 (42.9 to 46.8)	-2.3 (-3.0 to -1.7)	-6.3 (-8.0 to -4.9)
Diet low in polyunsaturated fatty acids	47.5 (45.6 to 49.2)	45.0 (43.1 to 46.6)	45.9 (44.1 to 47.4)	-5.3 (-8.1 to -1.7)	1.9 (0.6 to 3.4)	47.8 (45.7 to 49.3)	45.3 (43.4 to 47.0)	46.1 (44.2 to 47.7)	-5.2 (-7.9 to -1.4)	1.7 (-0.8 to 4.1)
Diet high in trans fatty acids	7.6 (3.9 to 11.7)	6.2 (2.8 to 10.0)	5.3 (2.1 to 9.1)	-18.2 (-37.9 to -2.2)	-13.8 (-33.1 to 1.7)	10.3 (6.2 to 14.5)	8.4 (4.6 to 12.8)	7.3 (3.5 to 11.3)	-18.5 (-38.9 to -1.0)	-12.4 (-30.6 to 3.2)
Diet high in sodium	15.3 (7.4 to 22.6)	8.9 (1.7 to 16.5)	10.3 (2.4 to 17.9)	-42.2 (-81.0 to -20.3)	16.2 (-0.7 to 87.8)	13.2 (5.9 to 20.2)	7.2 (1.0 to 14.4)	6.2 (0.5 to 13.3)	-45.9 (-86.4 to -24.5)	-14.1 (-53.6 to 0.0)
Intimate partner violence	-	-	-	-	-	4.1 (3.5 to 4.8)	3.6 (3.2 to 4.2)	3.3 (2.8 to 3.9)	-12.0 (-18.1 to -5.0)	-8.3 (-15.3 to -1.5)
Childhood sexual abuse	1.6 (1.3 to 2.0)	1.6 (1.3 to 2.0)	1.6 (1.3 to 2.0)	0.0 (0.0 to 0.0)	0.0 (0.0 to 0.0)	2.2 (1.7 to 2.7)	2.2 (1.7 to 2.7)	2.2 (1.7 to 2.7)	0.0 (0.0 to 0.0)	0.0 (0.0 to 0.0)

Supplementary Results Table 1. Azerbaijan age-standardised Summary Exposure Values (SEVs) for Level 4 risk factors and percentage change, by sex, 1990, 2007, and 2017.Data in parentheses are 95% uncertainty intervals.

	Male					Female				
	1990	2007	2017	Percentage change 1990-2007	Percentage change 2007-2017	1990	2007	2017	Percentage change 1990-2007	Percentage change 2007-2017
Bullying victimization	2.4 (1.0 to 4.8)	2.5 (1.1 to 5.0)	2.4 (1.0 to 4.9)	6.6 (0.7 to 14.2)	-3.8 (-7.6 to 0.3)	1.4 (0.6 to 2.9)	1.5 (0.6 to 2.9)	1.4 (0.6 to 2.9)	6.0 (-0.4 to 14.0)	-4.2 (-8.6 to -0.1)
Low physical activity	0.3 (0.2 to 0.6)	0.3 (0.2 to 0.6)	0.3 (0.2 to 0.6)	1.5 (-0.5 to 3.6)	2.3 (0.4 to 4.4)	0.3 (0.1 to 0.5)	0.3 (0.1 to 0.5)	0.3 (0.1 to 0.5)	1.2 (-0.6 to 3.3)	1.7 (-0.2 to 3.9)
High fasting plasma glucose	4.2 (3.0 to 6.2)	4.8 (3.4 to 7.2)	5.0 (3.5 to 7.4)	13.5 (-6.9 to 43.6)	4.2 (-18.1 to 25.7)	3.2 (2.1 to 4.8)	4.1 (2.9 to 5.9)	4.0 (2.8 to 5.7)	30.1 (3.5 to 75.3)	-3.7 (-23.5 to 17.0)
High LDL cholesterol	9.6 (8.2 to 11.2)	9.5 (8.1 to 11.2)	9.8 (8.4 to 11.4)	-0.7 (-5.1 to 3.5)	3.0 (-0.3 to 6.3)	10.5 (9.2 to 12.1)	10.6 (9.2 to 12.2)	11.0 (9.6 to 12.6)	0.8 (-3.2 to 5.0)	3.9 (0.7 to 7.3)
High systolic blood pressure	8.7 (7.8 to 9.7)	8.8 (7.9 to 9.6)	9.1 (8.1 to 10.0)	1.1 (-5.8 to 8.4)	3.3 (-2.4 to 9.1)	8.3 (7.4 to 9.3)	8.5 (7.7 to 9.4)	8.5 (7.6 to 9.4)	3.1 (-6.2 to 13.9)	-0.4 (-7.3 to 7.1)
High body-mass index	7.6 (5.3 to 11.3)	11.3 (8.3 to 16.3)	15.2 (11.4 to 20.8)	49.1 (28.4 to 76.7)	34.3 (20.2 to 49.1)	10.1 (7.3 to 13.9)	13.3 (10.1 to 18.2)	17.7 (13.5 to 23.8)	31.5 (13.0 to 53.7)	33.1 (21.5 to 47.5)
Low bone mineral density	4.2 (3.4 to 5.0)	4.0 (3.3 to 4.8)	4.0 (3.2 to 4.9)	-3.0 (-16.4 to 13.6)	-1.8 (-16.6 to 16.0)	5.7 (4.7 to 6.9)	5.6 (4.7 to 6.7)	5.5 (4.6 to 6.7)	-1.5 (-14.0 to 14.6)	-1.3 (-15.4 to 14.5)
Impaired kidney function	2.8 (1.3 to 6.7)	2.9 (1.3 to 7.0)	3.0 (1.4 to 7.0)	6.4 (1.9 to 11.4)	1.2 (-2.5 to 5.5)	3.2 (1.5 to 7.3)	3.5 (1.7 to 7.7)	3.6 (1.7 to 7.7)	7.3 (2.5 to 12.4)	2.3 (-1.0 to 6.5)

Supplementary Results Table 1. Georgia age-standardised Summary Exposure Values (SEVs) for Level 4 risk factors and percentage change, by sex, 1990, 2007, and 2017.Data in parentheses are 95% uncertainty intervals.

	Male					Female				
	**1990**	**2007**	**2017**	**Percentage change 1990-2007**	**Percentage change 2007-2017**	**1990**	**2007**	**2017**	**Percentage change 1990-2007**	**Percentage change 2007-2017**
Unsafe water source	10.9 (5.7 to 15.2)	11.8 (7.0 to 14.9)	9.8 (5.4 to 13.2)	8.5 (-17.7 to 49.1)	-17.3 (-36.8 to 6.7)	10.9 (5.7 to 15.2)	11.8 (7.0 to 14.9)	9.8 (5.4 to 13.2)	8.5 (-17.7 to 49.1)	-17.3 (-36.8 to 6.7)
Unsafe sanitation	43.1 (30.0 to 58.6)	46.6 (35.8 to 60.0)	34.5 (23.5 to 48.0)	8.2 (-16.5 to 41.2)	-26.0 (-43.6 to -7.2)	43.1 (30.0 to 58.6)	46.6 (35.8 to 60.0)	34.5 (23.5 to 48.0)	8.2 (-16.5 to 41.2)	-26.0 (-43.6 to -7.2)
No access to handwashing facility	13.8 (11.4 to 16.8)	12.8 (10.4 to 15.6)	10.2 (8.3 to 12.6)	-7.1 (-25.0 to 15.3)	-19.9 (-32.6 to -4.1)	13.8 (11.4 to 16.8)	12.8 (10.4 to 15.6)	10.2 (8.3 to 12.6)	-7.1 (-25.0 to 15.3)	-19.9 (-32.6 to -4.1)
Ambient particulate matter pollution	28.7 (16.2 to 46.4)	28.6 (18.4 to 42.9)	29.2 (16.6 to 45.3)	-0.0 (-40.4 to 75.3)	1.9 (-42.5 to 72.5)	24.6 (13.2 to 40.2)	24.6 (15.0 to 37.9)	25.4 (14.1 to 41.0)	-0.1 (-43.4 to 82.5)	3.6 (-43.6 to 82.4)
Household air pollution from solid fuels	32.7 (21.9 to 46.9)	31.4 (21.8 to 43.7)	24.0 (15.1 to 35.8)	-4.2 (-31.6 to 39.9)	-23.6 (-51.1 to 9.1)	34.9 (24.5 to 48.3)	33.5 (24.5 to 45.1)	26.1 (17.5 to 37.8)	-4.1 (-28.5 to 32.3)	-22.2 (-45.5 to 5.0)
Ambient ozone pollution	43.9 (15.9 to 54.8)	43.5 (15.7 to 54.8)	44.1 (16.1 to 54.8)	-0.9 (-3.8 to 0.7)	1.5 (-0.0 to 5.5)	43.9 (15.9 to 54.8)	43.5 (15.7 to 54.8)	44.1 (16.1 to 54.8)	-0.9 (-3.8 to 0.7)	1.5 (-0.0 to 5.5)
Residential radon	44.9 (14.5 to 71.2)	44.9 (14.5 to 71.2)	44.9 (14.5 to 71.2)	0.0 (0.0 to 0.0)	0.0 (0.0 to 0.0)	44.9 (14.5 to 71.2)	44.9 (14.5 to 71.2)	44.9 (14.5 to 71.2)	0.0 (0.0 to 0.0)	0.0 (0.0 to 0.0)
Lead exposure	3.1 (0.7 to 6.6)	3.5 (0.9 to 6.9)	3.0 (0.7 to 6.2)	11.1 (1.9 to 44.0)	-13.0 (-20.4 to -8.3)	1.6 (0.1 to 4.6)	1.8 (0.2 to 4.9)	1.6 (0.2 to 4.5)	11.3 (2.4 to 58.5)	-10.6 (-18.1 to -6.1)
Occupational exposure to asbestos	0.2 (0.1 to 0.2)	0.4 (0.3 to 0.4)	2.2 (1.9 to 2.4)	130.2 (65.3 to 294.9)	508.8 (411.0 to 640.1)	0.3 (0.2 to 0.4)	1.1 (1.0 to 1.2)	1.1 (1.0 to 1.3)	234.8 (148.5 to 383.2)	3.1 (-11.6 to 19.3)
Occupational exposure to arsenic	0.2 (0.1 to 0.4)	0.2 (0.1 to 0.4)	0.3 (0.1 to 0.4)	-1.3 (-9.1 to 7.8)	6.1 (-0.3 to 12.7)	0.2 (0.1 to 0.4)	0.2 (0.1 to 0.4)	0.2 (0.1 to 0.4)	-6.0 (-14.2 to 2.9)	7.4 (0.8 to 15.0)
Occupational exposure to benzene	0.6 (0.4 to 1.1)	0.6 (0.4 to 1.1)	0.7 (0.4 to 1.2)	-2.7 (-10.6 to 6.8)	13.6 (6.7 to 22.7)	0.8 (0.5 to 1.4)	0.8 (0.5 to 1.4)	1.0 (0.6 to 1.8)	-2.3 (-11.9 to 11.9)	24.2 (14.6 to 36.4)
Occupational exposure to beryllium	0.1 (0.1 to 0.1)	0.1 (0.1 to 0.1)	0.1 (0.1 to 0.1)	4.2 (-1.3 to 10.9)	4.0 (-0.2 to 8.2)	0.1 (0.1 to 0.1)	0.1 (0.1 to 0.1)	0.1 (0.1 to 0.1)	-1.5 (-8.7 to 6.2)	6.7 (0.8 to 12.4)
Occupational exposure to cadmium	0.1 (0.1 to 0.1)	0.1 (0.1 to 0.1)	0.1 (0.1 to 0.1)	-0.7 (-7.8 to 7.9)	6.4 (0.5 to 12.5)	0.1 (0.1 to 0.1)	0.1 (0.1 to 0.1)	0.1 (0.1 to 0.1)	-5.4 (-13.3 to 3.1)	8.0 (1.7 to 15.2)
Occupational exposure to chromium	0.3 (0.3 to 0.3)	0.3 (0.3 to 0.3)	0.3 (0.3 to 0.3)	-2.3 (-9.5 to 5.9)	9.9 (3.7 to 16.3)	0.3 (0.3 to 0.3)	0.3 (0.3 to 0.3)	0.3 (0.3 to 0.3)	-6.3 (-14.4 to 2.3)	13.7 (7.0 to 21.8)
Occupational exposure to diesel engine exhaust	1.7 (1.6 to 1.9)	1.8 (1.7 to 1.9)	2.0 (1.9 to 2.2)	4.2 (-3.6 to 12.4)	12.1 (5.8 to 18.3)	1.3 (1.2 to 1.5)	1.3 (1.2 to 1.4)	1.5 (1.4 to 1.6)	-4.2 (-12.3 to 4.7)	12.4 (4.9 to 20.4)
Occupational exposure to formaldehyde	0.6 (0.6 to 0.7)	0.6 (0.5 to 0.6)	0.6 (0.6 to 0.7)	-4.5 (-12.4 to 4.4)	10.4 (3.1 to 17.9)	0.6 (0.5 to 0.6)	0.5 (0.5 to 0.6)	0.6 (0.6 to 0.7)	-6.5 (-15.3 to 2.8)	16.6 (9.1 to 25.6)
Occupational exposure to nickel	0.2 (0.1 to 0.6)	0.3 (0.1 to 0.7)	0.3 (0.1 to 0.7)	2.5 (-5.3 to 11.7)	4.9 (-1.5 to 11.2)	0.2 (0.1 to 0.6)	0.2 (0.1 to 0.6)	0.2 (0.1 to 0.6)	-5.2 (-13.4 to 4.8)	2.6 (-3.9 to 9.9)
Occupational exposure to polycyclic aromatic hydrocarbons	0.6 (0.6 to 0.7)	0.6 (0.6 to 0.6)	0.7 (0.6 to 0.7)	-1.9 (-8.7 to 5.9)	10.3 (4.5 to 16.4)	0.7 (0.6 to 0.7)	0.6 (0.6 to 0.6)	0.7 (0.7 to 0.8)	-5.7 (-13.5 to 3.0)	15.3 (8.3 to 23.4)
Occupational exposure to silica	2.5 (1.3 to 5.4)	2.7 (1.4 to 6.0)	2.8 (1.5 to 6.3)	8.1 (-0.5 to 18.7)	6.5 (-1.3 to 15.1)	1.8 (0.9 to 3.8)	1.7 (0.9 to 3.6)	1.6 (0.8 to 3.6)	-4.0 (-12.3 to 5.6)	-2.5 (-8.9 to 4.9)
Occupational exposure to sulphuric acid	0.7 (0.5 to 1.2)	0.7 (0.5 to 1.2)	0.7 (0.5 to 1.3)	-0.6 (-9.1 to 8.3)	5.0 (-1.4 to 11.3)	0.6 (0.4 to 1.0)	0.6 (0.4 to 1.0)	0.6 (0.4 to 1.0)	-4.6 (-12.5 to 4.0)	3.0 (-3.3 to 9.6)
Occupational exposure to trichloroethylene	0.2 (0.2 to 0.2)	0.2 (0.2 to 0.2)	0.2 (0.2 to 0.2)	-0.6 (-7.2 to 7.0)	9.8 (4.5 to 15.7)	0.2 (0.2 to 0.2)	0.2 (0.2 to 0.2)	0.2 (0.2 to 0.2)	-5.2 (-12.7 to 3.1)	12.7 (6.2 to 20.3)
Occupational asthmagens	17.2 (13.6 to 21.8)	16.3 (12.8 to 20.3)	13.0 (10.5 to 16.1)	-5.6 (-16.7 to 7.9)	-20.0 (-29.0 to -9.3)	11.9 (8.7 to 15.8)	11.1 (8.4 to 14.6)	7.9 (5.8 to 10.6)	-7.0 (-22.1 to 11.7)	-28.6 (-41.1 to -14.5)
Occupational particulate matter, gases, and fumes	7.7 (5.8 to 10.6)	7.6 (5.8 to 10.5)	7.6 (5.7 to 10.3)	-1.2 (-4.9 to 2.7)	-1.1 (-3.8 to 1.4)	5.3 (3.8 to 7.4)	5.1 (3.7 to 7.1)	4.8 (3.5 to 6.8)	-3.4 (-9.0 to 2.7)	-6.0 (-10.4 to -2.0)
Occupational noise	8.7 (8.0 to 9.5)	8.7 (8.1 to 9.3)	8.6 (8.2 to 9.2)	-0.6 (-3.5 to 2.7)	-0.3 (-2.1 to 1.6)	6.5 (5.8 to 7.3)	6.4 (5.9 to 7.0)	6.3 (5.9 to 6.8)	-0.8 (-5.2 to 4.2)	-2.7 (-5.2 to 0.2)
Occupational ergonomic factors	20.6 (18.0 to 23.2)	20.9 (19.0 to 22.8)	14.3 (12.1 to 16.7)	1.5 (-12.0 to 18.0)	-31.9 (-40.4 to -22.1)	17.7 (14.7 to 20.5)	17.1 (15.4 to 18.8)	11.3 (8.9 to 14.0)	-3.1 (-19.5 to 18.5)	-34.0 (-46.9 to -20.0)
Non-exclusive breastfeeding	0.5 (0.4 to 0.7)	0.5 (0.4 to 0.7)	0.5 (0.4 to 0.6)	-2.6 (-10.3 to 6.2)	-6.5 (-13.2 to 0.7)	0.5 (0.4 to 0.7)	0.5 (0.4 to 0.7)	0.5 (0.4 to 0.6)	-2.5 (-10.1 to 6.4)	-6.5 (-13.2 to 0.7)
Discontinued breastfeeding	1.9 (1.8 to 1.9)	2.0 (1.9 to 2.0)	1.9 (1.8 to 2.0)	6.3 (-0.2 to 13.0)	-3.9 (-8.7 to 1.3)	1.8 (1.8 to 1.9)	2.0 (1.9 to 2.0)	1.9 (1.8 to 2.0)	6.1 (-0.4 to 12.8)	-3.6 (-8.4 to 1.6)
Child underweight	0.1 (0.1 to 0.2)	0.1 (0.1 to 0.2)	0.1 (0.1 to 0.2)	-10.3 (-23.4 to 4.4)	-13.4 (-22.4 to -4.5)	0.1 (0.1 to 0.2)	0.1 (0.1 to 0.2)	0.1 (0.1 to 0.1)	-3.0 (-15.5 to 12.0)	-10.6 (-18.4 to -2.4)
Child wasting	0.2 (0.1 to 0.2)	0.1 (0.1 to 0.2)	0.1 (0.1 to 0.2)	-9.0 (-32.9 to 20.3)	-8.9 (-22.1 to 7.9)	0.1 (0.1 to 0.2)	0.1 (0.1 to 0.2)	0.1 (0.1 to 0.2)	-4.1 (-26.4 to 23.5)	-11.6 (-25.1 to 4.5)
Child stunting	1.0 (0.6 to 1.4)	0.9 (0.6 to 1.2)	0.7 (0.4 to 1.1)	-5.4 (-37.4 to 44.9)	-19.4 (-42.6 to 7.5)	0.9 (0.5 to 1.3)	0.9 (0.5 to 1.1)	0.7 (0.4 to 1.1)	-4.6 (-38.2 to 50.4)	-18.8 (-43.3 to 7.2)
Short gestation for birth weight	0.0 (0.0 to 0.0)	0.0 (0.0 to 0.0)	0.0 (0.0 to 0.0)	6.4 (-16.0 to 39.9)	10.2 (-10.8 to 52.2)	0.0 (0.0 to 0.0)	0.0 (0.0 to 0.0)	0.0 (0.0 to 0.0)	13.0 (-12.6 to 40.2)	13.0 (-8.6 to 35.7)
Low birth weight for gestation	0.0 (0.0 to 0.0)	0.0 (0.0 to 0.0)	0.0 (0.0 to 0.0)	5.1 (-11.1 to 24.6)	10.3 (-8.5 to 27.9)	0.0 (0.0 to 0.0)	0.0 (0.0 to 0.0)	0.0 (0.0 to 0.0)	8.3 (-6.3 to 28.1)	2.9 (-11.6 to 17.6)
Iron deficiency	-	-	-	-	-	9.0 (6.4 to 11.8)	9.0 (6.8 to 11.7)	7.5 (5.4 to 10.0)	0.9 (-16.4 to 21.2)	-17.5 (-28.9 to -5.4)
Vitamin A deficiency	1.4 (1.0 to 1.8)	1.4 (1.0 to 1.8)	1.1 (0.8 to 1.6)	-0.8 (-16.8 to 25.2)	-17.1 (-30.2 to -1.1)	1.7 (1.4 to 2.0)	1.6 (1.3 to 1.9)	1.5 (1.2 to 1.9)	-3.0 (-14.8 to 9.5)	-6.9 (-23.7 to 9.0)
Zinc deficiency	0.6 (0.2 to 1.5)	0.6 (0.1 to 1.4)	0.5 (0.1 to 1.2)	-0.9 (-27.8 to 41.6)	-16.8 (-37.6 to 14.9)	0.7 (0.2 to 1.5)	0.6 (0.2 to 1.5)	0.5 (0.1 to 1.2)	-3.8 (-29.8 to 36.8)	-17.3 (-36.3 to 9.3)
Smoking	10.4 (8.6 to 12.5)	11.4 (9.7 to 13.3)	12.1 (10.4 to 14.0)	9.6 (-1.3 to 22.9)	5.5 (-2.0 to 13.7)	0.8 (0.6 to 1.1)	1.0 (0.8 to 1.3)	1.2 (1.0 to 1.5)	24.2 (-3.4 to 62.0)	17.2 (-0.5 to 40.5)
Chewing tobacco	0.2 (0.1 to 0.2)	0.2 (0.1 to 0.2)	0.2 (0.1 to 0.3)	2.6 (-27.3 to 42.3)	20.7 (-6.2 to 56.4)	0.1 (0.0 to 0.1)	0.1 (0.0 to 0.1)	0.1 (0.0 to 0.1)	-1.5 (-28.6 to 35.6)	0.2 (-22.4 to 29.5)
Second-hand smoke	35.9 (33.1 to 38.7)	38.7 (37.5 to 39.9)	41.3 (38.4 to 43.9)	7.8 (-0.9 to 17.0)	6.5 (0.3 to 12.1)	56.1 (53.8 to 58.5)	63.2 (62.2 to 64.3)	67.7 (65.0 to 70.5)	12.6 (8.0 to 17.5)	7.1 (3.7 to 10.7)
Alcohol use	20.9 (12.6 to 32.4)	22.7 (13.9 to 34.1)	28.5 (16.8 to 42.1)	8.8 (-16.7 to 44.0)	25.5 (-1.6 to 73.8)	2.9 (1.2 to 5.6)	2.9 (1.2 to 5.4)	3.7 (1.5 to 7.4)	-1.4 (-23.8 to 28.7)	29.2 (-4.3 to 72.0)
Drug use	0.4 (0.3 to 0.5)	0.4 (0.3 to 0.5)	0.4 (0.4 to 0.6)	4.7 (-1.6 to 12.2)	12.9 (6.5 to 20.1)	0.2 (0.1 to 0.2)	0.2 (0.1 to 0.2)	0.2 (0.1 to 0.3)	1.7 (-4.1 to 7.6)	5.1 (-1.1 to 11.9)
Diet low in fruits	36.9 (31.3 to 40.8)	43.8 (38.6 to 46.5)	42.1 (36.7 to 45.2)	18.5 (11.7 to 27.6)	-3.9 (-7.4 to -1.1)	34.5 (28.9 to 38.7)	42.3 (37.2 to 45.3)	40.4 (34.9 to 43.9)	22.5 (14.7 to 34.1)	-4.5 (-8.5 to -1.3)
Diet low in vegetables	28.9 (23.3 to 33.6)	34.5 (30.0 to 38.2)	32.4 (27.1 to 36.7)	19.1 (9.6 to 33.4)	-6.0 (-13.4 to 0.0)	30.9 (25.3 to 35.4)	36.1 (32.1 to 39.6)	34.0 (28.5 to 38.1)	16.7 (7.9 to 29.5)	-5.9 (-12.3 to -0.5)
Diet low in legumes	54.8 (54.7 to 54.8)	54.8 (54.8 to 54.8)	54.6 (54.5 to 54.7)	0.0 (0.0 to 0.1)	-0.3 (-0.5 to -0.1)	54.8 (54.8 to 54.8)	54.8 (54.8 to 54.8)	54.8 (54.7 to 54.8)	0.0 (0.0 to 0.0)	-0.0 (-0.1 to 0.0)
Diet low in whole grains	52.0 (51.5 to 52.4)	52.4 (52.0 to 52.7)	52.2 (51.8 to 52.6)	0.7 (0.4 to 1.0)	-0.3 (-0.5 to -0.1)	51.9 (51.4 to 52.3)	52.4 (52.0 to 52.8)	52.3 (51.9 to 52.7)	1.0 (0.7 to 1.4)	-0.2 (-0.4 to 0.0)
Diet low in nuts and seeds	39.1 (34.9 to 42.6)	47.1 (45.0 to 48.8)	43.1 (39.7 to 45.8)	20.6 (12.9 to 31.7)	-8.5 (-12.9 to -5.2)	39.9 (35.8 to 43.4)	47.5 (45.6 to 49.1)	43.8 (40.7 to 46.2)	19.0 (12.0 to 29.5)	-8.0 (-11.8 to -4.8)
Diet low in milk	41.8 (38.9 to 44.2)	45.8 (43.7 to 47.5)	45.5 (43.3 to 47.3)	9.6 (6.6 to 13.7)	-0.7 (-2.0 to 0.4)	42.5 (39.8 to 44.8)	46.4 (44.5 to 48.0)	46.0 (44.0 to 47.7)	9.3 (6.6 to 13.1)	-0.9 (-2.1 to 0.2)
Diet high in red meat	4.8 (1.4 to 8.9)	0.6 (0.0 to 2.1)	1.4 (0.0 to 4.1)	-88.3 (-99.9 to -68.5)	153.2 (-25.1 to 8447.4)	0.5 (0.0 to 1.9)	0.0 (0.0 to 0.1)	0.1 (0.0 to 0.7)	-98.4 (-100.0 to -85.5)	1263.0 (-75.1 to [illegible]
Diet high in processed meat	1.6 (0.1 to 3.9)	0.7 (0.0 to 2.6)	1.6 (0.1 to 3.9)	-52.8 (-99.5 to -30.7)	119.9 (49.5 to 17762.6)	0.9 (0.0 to 2.9)	0.4 (0.0 to 1.9)	1.0 (0.0 to 3.0)	-54.4 (-100.0 to -30.6)	125.3 (53.5 to [illegible]
Diet high in sugar-sweetened beverages	5.3 (2.2 to 10.1)	3.1 (1.4 to 5.7)	5.5 (2.6 to 10.3)	-41.7 (-68.0 to 8.6)	78.2 (14.3 to 193.8)	3.6 (1.7 to 6.4)	2.2 (1.0 to 4.0)	3.8 (1.8 to 6.9)	-38.2 (-66.4 to 9.8)	72.6 (16.2 to 162.3)
Diet low in fibre	27.5 (21.7 to 32.4)	30.2 (25.0 to 34.4)	29.5 (23.6 to 34.2)	9.6 (2.6 to 20.4)	-2.1 (-8.5 to 3.7)	33.0 (28.0 to 37.0)	35.3 (30.9 to 38.8)	34.5 (29.7 to 38.4)	6.9 (1.3 to 14.3)	-2.0 (-6.6 to 2.1)
Diet low in calcium	33.0 (28.5 to 36.6)	31.0 (26.0 to 34.8)	25.9 (19.9 to 30.6)	-6.2 (-9.9 to -3.4)	-16.4 (-24.4 to -11.6)	35.7 (31.8 to 38.9)	34.0 (29.7 to 37.4)	29.2 (24.0 to 33.6)	-4.8 (-8.0 to -2.4)	-14.0 (-20.1 to -9.8)
Diet low in seafood omega-3 fatty acids	45.8 (44.0 to 47.5)	44.6 (42.6 to 46.6)	42.4 (40.0 to 44.8)	-2.5 (-3.6 to -1.7)	-5.0 (-6.8 to -3.4)	47.1 (45.5 to 48.6)	46.1 (44.4 to 47.8)	44.2 (42.0 to 46.2)	-2.1 (-2.9 to -1.3)	-4.2 (-5.7 to -3.0)
Diet low in polyunsaturated fatty acids	47.7 (45.7 to 49.2)	39.2 (36.0 to 41.8)	35.8 (32.1 to 39.0)	-17.8 (-22.7 to -13.0)	-8.7 (-13.1 to -5.0)	47.9 (46.1 to 49.4)	39.6 (36.7 to 42.1)	36.3 (32.8 to 39.4)	-17.3 (-21.8 to -13.2)	-8.4 (-12.6 to -5.0)
Diet high in trans fatty acids	2.2 (0.3 to 5.1)	2.1 (0.3 to 4.9)	2.1 (0.3 to 4.9)	-4.5 (-28.9 to 19.1)	-1.7 (-19.5 to 19.7)	3.1 (0.7 to 6.3)	2.9 (0.6 to 6.1)	2.9 (0.6 to 6.1)	-4.7 (-28.4 to 19.4)	-0.7 (-18.4 to 20.1)
Diet high in sodium	15.4 (7.4 to 22.6)	7.9 (1.1 to 15.0)	8.9 (1.5 to 16.3)	-49.0 (-87.2 to -27.4)	12.5 (-7.1 to 92.7)	13.4 (6.0 to 20.4)	6.8 (0.7 to 13.9)	5.8 (0.4 to 12.8)	-49.0 (-88.2 to -28.2)	-15.4 (-56.5 to -1.2)
Intimate partner violence	-	-	-	-	-	2.0 (1.7 to 2.3)	1.8 (1.5 to 2.1)	1.9 (1.6 to 2.2)	-7.5 (-14.9 to -1.1)	3.3 (-4.0 to 12.4)
Childhood sexual abuse	1.6 (1.3 to 2.0)	1.6 (1.3 to 2.0)	1.6 (1.3 to 2.0)	0.0 (0.0 to 0.0)	0.0 (0.0 to 0.0)	2.2 (1.7 to 2.7)	2.2 (1.7 to 2.7)	2.2 (1.7 to 2.7)	0.0 (0.0 to 0.0)	0.0 (0.0 to 0.0)

Supplementary Results Table 1. Georgia age-standardised Summary Exposure Values (SEVs) for Level 4 risk factors and percentage change, by sex, 1990, 2007, and 2017.Data in parentheses are 95% uncertainty intervals.

	Male					Female				
	**1990**	**2007**	**2017**	**Percentage change 1990-2007**	**Percentage change 2007-2017**	**1990**	**2007**	**2017**	**Percentage change 1990-2007**	**Percentage change 2007-2017**
Bullying victimization	2.4 (1.0 to 5.0)	2.5 (1.0 to 5.3)	2.5 (1.0 to 5.4)	2.2 (-2.7 to 8.1)	2.9 (0.2 to 5.9)	1.4 (0.6 to 2.9)	1.5 (0.6 to 3.0)	1.5 (0.6 to 3.1)	4.9 (-0.8 to 13.4)	2.4 (-0.9 to 6.1)
Low physical activity	0.3 (0.2 to 0.6)	0.3 (0.2 to 0.6)	0.3 (0.2 to 0.6)	1.4 (-0.2 to 3.3)	1.4 (-0.2 to 3.0)	0.3 (0.1 to 0.5)	0.3 (0.1 to 0.5)	0.3 (0.1 to 0.5)	1.3 (-0.6 to 3.5)	0.6 (-1.2 to 2.4)
High fasting plasma glucose	4.2 (3.0 to 6.2)	5.0 (3.5 to 7.4)	5.8 (4.1 to 8.6)	19.2 (-2.6 to 53.1)	17.1 (-6.2 to 46.9)	2.6 (1.6 to 4.0)	3.4 (2.4 to 5.0)	4.2 (2.9 to 6.0)	31.1 (0.8 to 91.8)	21.4 (-1.3 to 54.4)
High LDL cholesterol	7.9 (6.6 to 9.4)	8.5 (7.1 to 10.0)	8.7 (7.3 to 10.3)	6.9 (2.2 to 11.9)	2.5 (-0.8 to 6.1)	9.7 (8.3 to 11.3)	10.1 (8.8 to 11.6)	10.5 (9.0 to 12.1)	4.7 (0.5 to 9.1)	3.5 (0.6 to 6.7)
High systolic blood pressure	11.7 (10.7 to 12.8)	10.8 (10.0 to 11.7)	11.3 (10.3 to 12.4)	-7.4 (-13.2 to -1.2)	5.0 (-0.0 to 10.5)	8.5 (7.6 to 9.4)	8.1 (7.3 to 8.9)	8.3 (7.4 to 9.2)	-4.6 (-12.6 to 4.5)	2.7 (-4.1 to 10.1)
High body-mass index	13.5 (10.0 to 18.9)	16.4 (12.4 to 22.0)	17.6 (13.4 to 23.8)	20.8 (7.8 to 38.0)	7.7 (-1.3 to 17.8)	13.6 (10.4 to 18.4)	16.9 (13.3 to 22.1)	18.0 (13.8 to 23.6)	23.6 (8.8 to 42.3)	6.7 (-2.8 to 17.1)
Low bone mineral density	4.1 (3.3 to 4.9)	4.0 (3.3 to 4.8)	4.0 (3.2 to 4.8)	-1.1 (-13.7 to 16.2)	-0.6 (-15.1 to 16.1)	5.6 (4.7 to 6.7)	5.6 (4.7 to 6.6)	5.5 (4.6 to 6.6)	-0.3 (-13.6 to 15.3)	-0.6 (-14.2 to 15.5)
Impaired kidney function	2.7 (1.2 to 6.6)	2.8 (1.3 to 6.8)	2.9 (1.3 to 6.9)	5.3 (0.9 to 11.5)	3.2 (-0.1 to 7.4)	3.1 (1.5 to 7.3)	3.4 (1.6 to 7.5)	3.5 (1.7 to 7.7)	6.7 (1.8 to 13.1)	4.4 (1.1 to 8.9)

Supplementary Results Table 1. Kazakhstan age-standardised Summary Exposure Values (SEVs) for Level 4 risk factors and percentage change, by sex, 1990, 2007, and 2017.Data in parentheses are 95% uncertainty intervals.

	Male					Female				
	1990	2007	2017	Percentage change 1990-2007	Percentage change 2007-2017	1990	2007	2017	Percentage change 1990-2007	Percentage change 2007-2017
Unsafe water source	18.2 (10.7 to 22.8)	13.9 (7.5 to 17.3)	10.0 (5.4 to 13.1)	-23.5 (-37.5 to -6.4)	-27.8 (-41.2 to -11.6)	18.2 (10.7 to 22.8)	13.9 (7.5 to 17.3)	10.0 (5.4 to 13.1)	-23.5 (-37.5 to -6.4)	-27.8 (-41.2 to -11.6)
Unsafe sanitation	62.7 (51.7 to 76.1)	50.8 (45.4 to 58.0)	37.6 (32.3 to 43.9)	-19.0 (-28.5 to -5.5)	-26.1 (-36.6 to -15.2)	62.7 (51.7 to 76.1)	50.8 (45.4 to 58.0)	37.6 (32.3 to 43.9)	-19.0 (-28.5 to -5.5)	-26.1 (-36.6 to -15.2)
No access to handwashing facility	22.3 (18.4 to 26.9)	15.9 (13.0 to 19.2)	10.4 (8.4 to 12.7)	-28.9 (-42.4 to -12.3)	-34.4 (-44.7 to -23.5)	22.3 (18.4 to 26.9)	15.9 (13.0 to 19.2)	10.4 (8.4 to 12.7)	-28.9 (-42.4 to -12.3)	-34.4 (-44.7 to -23.5)
Ambient particulate matter pollution	22.2 (4.9 to 49.7)	25.8 (8.6 to 51.0)	25.5 (4.0 to 57.8)	16.2 (-68.7 to 454.3)	-1.1 (-84.7 to 228.5)	19.1 (4.2 to 44.3)	22.9 (7.2 to 47.5)	23.4 (3.4 to 56.3)	20.1 (-70.0 to 509.9)	2.3 (-84.8 to 270.7)
Household air pollution from solid fuels	24.2 (14.4 to 36.1)	14.7 (8.4 to 23.6)	8.6 (3.9 to 16.3)	-39.3 (-66.2 to 1.1)	-41.2 (-69.9 to 1.1)	25.6 (15.7 to 36.1)	16.3 (9.4 to 24.7)	10.0 (4.7 to 17.6)	-36.1 (-61.9 to -1.7)	-38.8 (-67.8 to -0.6)
Ambient ozone pollution	41.1 (14.6 to 54.8)	41.3 (14.5 to 54.8)	42.6 (15.3 to 54.8)	0.3 (-1.7 to 2.9)	3.4 (0.0 to 7.9)	41.1 (14.6 to 54.8)	41.3 (14.5 to 54.8)	42.6 (15.3 to 54.8)	0.3 (-1.7 to 2.9)	3.4 (0.0 to 7.9)
Residential radon	41.4 (8.7 to 71.2)	41.4 (8.7 to 71.2)	41.4 (8.7 to 71.2)	0.0 (0.0 to 0.0)	0.0 (0.0 to 0.0)	41.4 (8.7 to 71.2)	41.4 (8.7 to 71.2)	41.4 (8.7 to 71.2)	0.0 (0.0 to 0.0)	0.0 (0.0 to 0.0)
Lead exposure	5.2 (1.8 to 9.0)	4.8 (1.7 to 8.5)	3.8 (1.2 to 7.1)	-7.5 (-14.4 to 2.1)	-21.4 (-30.8 to -15.1)	2.6 (0.3 to 6.1)	2.5 (0.4 to 5.8)	2.1 (0.3 to 5.0)	-3.8 (-10.6 to 28.8)	-19.1 (-29.8 to -13.0)
Occupational exposure to asbestos	1.2 (1.0 to 1.5)	1.8 (1.4 to 2.0)	1.1 (1.0 to 1.3)	42.4 (-2.9 to 91.2)	-34.7 (-45.9 to -11.6)	0.7 (0.6 to 0.9)	1.1 (0.9 to 1.3)	0.8 (0.7 to 0.9)	58.5 (18.7 to 118.6)	-29.6 (-44.8 to -5.4)
Occupational exposure to arsenic	0.3 (0.1 to 0.5)	0.3 (0.1 to 0.5)	0.3 (0.1 to 0.5)	2.2 (-5.3 to 10.1)	0.9 (-4.5 to 6.4)	0.3 (0.1 to 0.5)	0.3 (0.1 to 0.5)	0.3 (0.1 to 0.5)	2.4 (-7.7 to 13.2)	4.5 (-2.0 to 11.4)
Occupational exposure to benzene	0.6 (0.4 to 1.1)	0.7 (0.4 to 1.3)	0.7 (0.5 to 1.3)	10.5 (2.7 to 19.8)	5.3 (-0.1 to 11.5)	0.8 (0.5 to 1.5)	1.0 (0.6 to 1.8)	1.1 (0.7 to 2.0)	20.4 (8.5 to 36.5)	10.5 (4.1 to 18.3)
Occupational exposure to beryllium	0.1 (0.1 to 0.1)	0.1 (0.1 to 0.1)	0.1 (0.1 to 0.1)	0.1 (-5.0 to 5.5)	-1.3 (-4.9 to 2.2)	0.1 (0.1 to 0.1)	0.1 (0.1 to 0.1)	0.1 (0.1 to 0.1)	6.1 (-1.9 to 14.0)	2.9 (-1.7 to 7.3)
Occupational exposure to cadmium	0.1 (0.1 to 0.2)	0.2 (0.1 to 0.2)	0.2 (0.1 to 0.2)	2.7 (-4.4 to 10.0)	0.8 (-4.1 to 5.8)	0.2 (0.1 to 0.2)	0.2 (0.1 to 0.2)	0.2 (0.2 to 0.2)	3.6 (-6.1 to 13.6)	4.7 (-1.4 to 10.8)
Occupational exposure to chromium	0.3 (0.3 to 0.4)	0.4 (0.3 to 0.4)	0.4 (0.3 to 0.4)	5.5 (-1.7 to 12.9)	3.1 (-1.8 to 8.0)	0.4 (0.3 to 0.4)	0.4 (0.4 to 0.4)	0.4 (0.4 to 0.4)	7.4 (-2.8 to 17.6)	7.1 (0.9 to 13.0)
Occupational exposure to diesel engine exhaust	2.6 (2.4 to 2.8)	2.6 (2.5 to 2.7)	2.7 (2.6 to 2.9)	2.3 (-5.6 to 10.7)	3.9 (-1.5 to 9.2)	2.1 (1.9 to 2.3)	2.2 (2.1 to 2.4)	2.3 (2.2 to 2.5)	7.8 (-2.1 to 18.2)	5.1 (-1.5 to 11.9)
Occupational exposure to formaldehyde	0.7 (0.6 to 0.7)	0.7 (0.7 to 0.8)	0.7 (0.7 to 0.8)	6.2 (-1.8 to 14.8)	3.6 (-1.9 to 9.4)	0.7 (0.6 to 0.8)	0.7 (0.7 to 0.8)	0.8 (0.7 to 0.8)	8.8 (-2.6 to 20.4)	8.4 (1.7 to 15.2)
Occupational exposure to nickel	0.4 (0.1 to 1.0)	0.3 (0.1 to 0.9)	0.4 (0.1 to 0.9)	-6.4 (-14.6 to 2.6)	0.4 (-5.2 to 6.0)	0.3 (0.1 to 0.8)	0.3 (0.1 to 0.7)	0.3 (0.1 to 0.7)	-4.0 (-13.8 to 6.4)	1.0 (-5.9 to 7.9)
Occupational exposure to polycyclic aromatic hydrocarbons	0.7 (0.7 to 0.8)	0.8 (0.7 to 0.8)	0.8 (0.8 to 0.8)	5.0 (-1.9 to 11.9)	3.1 (-1.7 to 7.6)	0.8 (0.7 to 0.8)	0.8 (0.8 to 0.9)	0.9 (0.8 to 0.9)	8.4 (-1.3 to 18.7)	7.1 (1.2 to 12.8)
Occupational exposure to silica	4.3 (2.2 to 9.6)	4.1 (2.1 to 9.0)	4.2 (2.2 to 9.7)	-4.3 (-13.7 to 7.0)	3.0 (-3.8 to 10.4)	2.7 (1.4 to 6.1)	2.6 (1.3 to 5.6)	2.6 (1.3 to 5.7)	-5.2 (-15.8 to 5.8)	-0.5 (-8.2 to 7.1)
Occupational exposure to sulphuric acid	0.8 (0.6 to 1.4)	0.8 (0.6 to 1.4)	0.7 (0.5 to 1.3)	-0.5 (-8.4 to 8.4)	-5.3 (-10.7 to 0.3)	0.7 (0.5 to 1.2)	0.7 (0.5 to 1.2)	0.7 (0.5 to 1.2)	-0.4 (-9.7 to 9.2)	-1.9 (-8.2 to 4.2)
Occupational exposure to trichloroethylene	0.2 (0.2 to 0.2)	0.2 (0.2 to 0.2)	0.2 (0.2 to 0.2)	6.9 (0.3 to 14.0)	2.6 (-1.8 to 7.1)	0.2 (0.2 to 0.2)	0.2 (0.2 to 0.2)	0.3 (0.2 to 0.3)	9.1 (-0.5 to 19.0)	6.6 (0.5 to 12.5)
Occupational asthmagens	15.5 (12.6 to 18.8)	14.4 (12.2 to 17.1)	14.3 (12.1 to 17.0)	-6.9 (-17.2 to 5.1)	-0.7 (-7.9 to 7.2)	9.0 (6.9 to 11.5)	8.5 (7.0 to 10.4)	8.5 (7.0 to 10.4)	-4.7 (-20.5 to 15.0)	-0.4 (-11.6 to 11.5)
Occupational particulate matter, gases, and fumes	8.9 (7.0 to 12.0)	8.7 (6.8 to 11.6)	8.5 (6.7 to 11.4)	-3.1 (-6.2 to 0.4)	-1.2 (-3.3 to 1.3)	5.1 (3.8 to 7.2)	5.0 (3.8 to 6.8)	4.9 (3.7 to 6.6)	-3.3 (-8.3 to 2.8)	-2.3 (-5.7 to 1.7)
Occupational noise	10.0 (9.2 to 10.8)	9.8 (9.2 to 10.5)	9.8 (9.3 to 10.4)	-1.8 (-4.5 to 1.6)	-0.0 (-1.8 to 2.0)	6.5 (5.8 to 7.3)	6.7 (6.1 to 7.2)	6.8 (6.4 to 7.3)	1.9 (-2.5 to 7.0)	2.2 (-0.7 to 5.3)
Occupational ergonomic factors	16.1 (13.8 to 18.8)	14.4 (13.1 to 15.9)	12.6 (10.9 to 14.3)	-10.7 (-22.0 to 2.8)	-12.6 (-20.2 to -4.4)	12.0 (9.8 to 14.4)	10.6 (9.6 to 11.7)	9.2 (7.8 to 10.9)	-11.6 (-26.1 to 7.7)	-12.8 (-24.0 to -0.5)
Non-exclusive breastfeeding	0.5 (0.3 to 0.7)	0.5 (0.3 to 0.7)	0.4 (0.3 to 0.6)	-0.2 (-6.9 to 7.9)	-13.7 (-19.9 to -6.8)	0.5 (0.3 to 0.7)	0.5 (0.3 to 0.7)	0.4 (0.3 to 0.6)	-0.1 (-6.8 to 8.0)	-13.7 (-19.9 to -6.8)
Discontinued breastfeeding	1.4 (1.3 to 1.5)	1.6 (1.5 to 1.7)	1.5 (1.4 to 1.5)	11.9 (3.4 to 20.9)	-8.7 (-14.9 to -2.5)	1.4 (1.3 to 1.5)	1.6 (1.5 to 1.7)	1.5 (1.4 to 1.5)	11.6 (3.2 to 20.6)	-8.5 (-14.7 to -2.3)
Child underweight	0.4 (0.3 to 0.5)	0.2 (0.1 to 0.3)	0.1 (0.1 to 0.2)	-54.2 (-66.8 to -42.5)	-22.3 (-33.1 to -12.6)	0.3 (0.2 to 0.5)	0.2 (0.1 to 0.2)	0.1 (0.1 to 0.2)	-50.2 (-63.1 to -37.4)	-16.6 (-27.4 to -6.0)
Child wasting	0.2 (0.1 to 0.3)	0.2 (0.1 to 0.3)	0.1 (0.1 to 0.2)	-18.0 (-40.6 to 13.7)	-19.8 (-33.4 to -4.0)	0.2 (0.1 to 0.2)	0.2 (0.1 to 0.2)	0.1 (0.1 to 0.2)	-0.0 (-26.4 to 29.2)	-19.4 (-33.7 to -4.9)
Child stunting	1.4 (0.9 to 1.8)	1.0 (0.7 to 1.2)	0.6 (0.4 to 0.9)	-28.3 (-44.8 to -8.9)	-36.5 (-53.8 to -20.5)	1.2 (0.8 to 1.6)	0.9 (0.6 to 1.2)	0.6 (0.4 to 0.9)	-19.0 (-36.7 to 7.1)	-31.8 (-48.0 to -12.7)
Short gestation for birth weight	0.0 (0.0 to 0.0)	0.0 (0.0 to 0.0)	0.0 (0.0 to 0.0)	-10.6 (-37.2 to 10.4)	8.6 (-14.8 to 41.2)	0.0 (0.0 to 0.0)	0.0 (0.0 to 0.0)	0.0 (0.0 to 0.0)	-12.0 (-35.2 to 16.3)	3.3 (-17.9 to 26.1)
Low birth weight for gestation	0.0 (0.0 to 0.0)	0.0 (0.0 to 0.0)	0.0 (0.0 to 0.0)	10.4 (-6.6 to 38.2)	10.6 (-9.3 to 31.9)	0.0 (0.0 to 0.0)	0.0 (0.0 to 0.0)	0.0 (0.0 to 0.0)	11.6 (-6.7 to 33.4)	9.9 (-4.2 to 27.5)
Iron deficiency	-	-	-	-	-	14.1 (10.9 to 17.8)	11.6 (8.8 to 14.9)	9.9 (7.3 to 13.1)	-17.8 (-30.3 to -3.7)	-14.8 (-26.8 to -2.6)
Vitamin A deficiency	2.4 (2.0 to 2.9)	2.2 (2.0 to 2.5)	2.0 (1.6 to 2.4)	-6.4 (-15.6 to 4.1)	-11.9 (-23.1 to -1.8)	2.5 (2.1 to 3.0)	2.3 (2.0 to 2.6)	2.0 (1.7 to 2.4)	-7.5 (-16.7 to 2.8)	-12.0 (-21.4 to -2.9)
Zinc deficiency	0.4 (0.1 to 0.9)	0.4 (0.1 to 0.8)	0.6 (0.1 to 1.2)	-7.7 (-56.3 to 130.6)	48.7 (-50.6 to 307.4)	0.4 (0.1 to 0.9)	0.4 (0.1 to 0.8)	0.6 (0.1 to 1.3)	-8.0 (-54.2 to 116.9)	47.3 (-49.7 to 326.0)
Smoking	14.2 (12.4 to 16.1)	12.3 (10.8 to 13.9)	12.7 (11.2 to 14.4)	-13.3 (-19.7 to -6.4)	3.4 (-2.6 to 9.7)	1.4 (1.0 to 1.9)	1.4 (1.1 to 1.7)	1.7 (1.4 to 2.1)	-0.0 (-21.2 to 30.4)	25.4 (8.1 to 45.7)
Chewing tobacco	0.2 (0.1 to 0.2)	0.2 (0.1 to 0.2)	0.2 (0.1 to 0.3)	1.9 (-31.1 to 45.7)	21.4 (-6.6 to 56.8)	0.1 (0.0 to 0.1)	0.1 (0.0 to 0.1)	0.1 (0.0 to 0.1)	-1.9 (-29.5 to 40.4)	-0.3 (-21.9 to 26.1)
Second-hand smoke	36.9 (35.0 to 38.8)	35.8 (34.9 to 36.8)	33.4 (31.4 to 35.5)	-2.9 (-8.6 to 2.8)	-6.8 (-11.9 to -1.6)	61.9 (60.1 to 63.6)	56.8 (55.7 to 57.9)	52.4 (50.2 to 54.4)	-8.3 (-11.3 to -5.2)	-7.8 (-11.0 to -4.5)
Alcohol use	28.7 (18.8 to 38.5)	26.7 (18.3 to 36.1)	26.2 (15.8 to 37.7)	-7.0 (-25.3 to 15.6)	-1.9 (-27.5 to 22.0)	14.2 (8.3 to 22.5)	13.7 (8.4 to 21.1)	13.8 (7.7 to 21.9)	-3.5 (-25.9 to 20.1)	0.9 (-21.8 to 28.7)
Drug use	0.4 (0.3 to 0.6)	0.7 (0.6 to 0.8)	0.6 (0.5 to 0.7)	54.2 (39.2 to 73.9)	-12.7 (-20.7 to -4.4)	0.2 (0.1 to 0.2)	0.2 (0.2 to 0.3)	0.2 (0.2 to 0.3)	20.3 (10.9 to 32.2)	-7.6 (-14.6 to -0.3)
Diet low in fruits	51.9 (47.6 to 53.3)	48.4 (43.8 to 50.4)	43.9 (38.8 to 46.8)	-6.6 (-8.6 to -4.8)	-9.3 (-12.8 to -6.6)	51.4 (46.9 to 52.9)	47.4 (42.9 to 49.5)	42.3 (37.0 to 45.4)	-7.7 (-9.8 to -5.9)	-10.8 (-14.7 to -7.7)
Diet low in vegetables	36.4 (32.3 to 39.9)	9.9 (2.7 to 16.9)	1.8 (0.1 to 5.7)	-72.8 (-92.0 to -56.6)	-82.3 (-98.7 to -64.4)	37.8 (33.9 to 41.1)	13.2 (5.6 to 20.1)	3.1 (0.2 to 8.2)	-65.1 (-84.1 to -49.9)	-76.3 (-96.8 to -56.1)
Diet low in legumes	53.9 (53.6 to 54.1)	53.2 (52.8 to 53.5)	53.1 (52.7 to 53.5)	-1.2 (-1.8 to -0.7)	-0.1 (-0.6 to 0.4)	54.3 (54.1 to 54.5)	53.7 (53.4 to 54.0)	53.7 (53.4 to 54.0)	-1.0 (-1.5 to -0.6)	-0.1 (-0.4 to 0.3)
Diet low in whole grains	52.3 (51.9 to 52.7)	52.2 (51.8 to 52.6)	52.1 (51.7 to 52.5)	-0.2 (-0.3 to 0.0)	-0.2 (-0.4 to -0.0)	52.3 (51.9 to 52.7)	52.3 (51.9 to 52.6)	52.4 (52.0 to 52.7)	-0.0 (-0.3 to 0.2)	0.1 (-0.0 to 0.3)
Diet low in nuts and seeds	52.8 (52.2 to 53.2)	51.2 (50.3 to 52.0)	46.8 (44.5 to 48.6)	-3.0 (-4.3 to -2.0)	-8.5 (-11.8 to -6.2)	52.9 (52.3 to 53.3)	51.4 (50.4 to 52.1)	47.3 (45.1 to 49.0)	-2.8 (-4.0 to -1.9)	-7.9 (-10.9 to -5.6)
Diet low in milk	36.7 (32.8 to 40.0)	36.6 (32.8 to 39.8)	35.3 (31.2 to 38.8)	-0.2 (-3.6 to 3.6)	-3.6 (-6.7 to -0.8)	37.6 (33.9 to 40.8)	37.6 (34.1 to 40.6)	36.1 (32.1 to 39.5)	-0.2 (-3.6 to 3.1)	-3.8 (-7.0 to -1.0)
Diet high in red meat	24.7 (19.9 to 28.7)	21.1 (17.1 to 24.5)	31.8 (25.5 to 37.5)	-14.4 (-26.3 to -1.3)	50.4 (25.7 to 82.6)	8.5 (4.2 to 12.6)	6.7 (3.1 to 10.3)	13.5 (8.1 to 18.5)	-21.1 (-49.8 to 9.3)	100.4 (42.0 to 252.3)
Diet high in processed meat	2.3 (0.4 to 4.8)	2.0 (0.2 to 4.5)	3.8 (1.2 to 6.6)	-10.8 (-57.1 to 28.7)	87.3 (38.6 to 500.5)	1.4 (0.1 to 3.6)	1.2 (0.0 to 3.4)	2.4 (0.4 to 5.0)	-11.2 (-72.0 to 28.3)	99.4 (41.9 to 1660.9)
Diet high in sugar-sweetened beverages	7.2 (3.2 to 12.6)	7.8 (3.3 to 13.6)	12.3 (5.4 to 20.9)	7.4 (-37.9 to 99.6)	58.6 (3.8 to 137.9)	5.0 (2.2 to 9.3)	5.3 (2.4 to 9.5)	8.3 (4.0 to 14.4)	6.6 (-41.9 to 98.2)	56.3 (3.5 to 145.4)
Diet low in fibre	31.6 (26.7 to 35.8)	31.8 (26.9 to 35.8)	28.0 (22.3 to 32.9)	0.4 (-4.6 to 5.2)	-11.9 (-18.9 to -6.7)	36.6 (32.6 to 39.9)	36.7 (32.7 to 39.8)	33.5 (28.8 to 37.4)	0.0 (-3.6 to 3.9)	-8.8 (-14.0 to -4.7)
Diet low in calcium	29.3 (24.1 to 33.5)	16.6 (9.6 to 22.4)	10.8 (5.0 to 16.6)	-43.5 (-60.4 to -32.4)	-35.0 (-49.8 to -25.1)	32.4 (27.8 to 36.0)	20.6 (13.9 to 26.1)	14.3 (7.5 to 20.4)	-36.2 (-50.3 to -27.0)	-30.7 (-46.7 to -20.8)
Diet low in seafood omega-3 fatty acids	38.0 (34.9 to 41.0)	39.7 (36.8 to 42.5)	33.1 (29.0 to 36.8)	4.4 (3.0 to 6.2)	-16.6 (-21.5 to -12.9)	40.4 (37.7 to 43.0)	41.9 (39.3 to 44.3)	36.0 (32.4 to 39.4)	3.6 (2.4 to 5.1)	-13.9 (-18.1 to -10.8)
Diet low in polyunsaturated fatty acids	37.9 (34.5 to 40.8)	34.0 (30.1 to 37.2)	29.3 (24.6 to 33.3)	-10.3 (-15.7 to -5.1)	-13.7 (-19.4 to -9.4)	38.4 (35.1 to 41.4)	34.6 (30.8 to 37.9)	30.0 (25.4 to 34.0)	-10.0 (-15.1 to -5.2)	-13.2 (-19.3 to -7.7)
Diet high in trans fatty acids	0.4 (0.0 to 1.6)	0.3 (0.0 to 1.6)	0.3 (0.0 to 1.5)	-9.7 (-61.5 to 0.1)	-6.0 (-62.4 to 6.4)	0.5 (0.0 to 2.0)	0.4 (0.0 to 1.9)	0.4 (0.0 to 1.8)	-10.0 (-51.0 to 3.8)	-5.9 (-42.2 to 7.8)
Diet high in sodium	16.6 (8.3 to 23.7)	10.4 (2.4 to 18.1)	11.2 (2.9 to 18.9)	-37.6 (-75.3 to -16.5)	7.9 (-11.2 to 54.1)	13.8 (6.1 to 20.7)	7.6 (1.2 to 14.7)	6.3 (0.6 to 13.2)	-44.7 (-83.6 to -24.8)	-16.7 (-54.1 to -3.4)
Intimate partner violence	-	-	-	-	-	3.0 (2.5 to 3.5)	2.9 (2.5 to 3.3)	2.6 (2.2 to 3.0)	-4.8 (-12.1 to 2.6)	-8.0 (-15.1 to -0.1)
Childhood sexual abuse	1.6 (1.3 to 2.0)	1.6 (1.3 to 1.9)	1.6 (1.3 to 2.0)	-1.0 (-6.7 to 6.4)	1.0 (-6.0 to 7.2)	2.2 (1.7 to 2.7)	2.1 (1.7 to 2.6)	2.2 (1.7 to 2.7)	-3.3 (-9.7 to 3.9)	3.4 (-3.7 to 10.8)

Supplementary Results Table 1. Kazakhstan age-standardised Summary Exposure Values (SEVs) for Level 4 risk factors and percentage change, by sex, 1990, 2007, and 2017.Data in parentheses are 95% uncertainty intervals.

	Male					Female				
	**1990**	**2007**	**2017**	**Percentage change 1990-2007**	**Percentage change 2007-2017**	**1990**	**2007**	**2017**	**Percentage change 1990-2007**	**Percentage change 2007-2017**
Bullying victimization	2.7 (1.1 to 5.5)	2.8 (1.1 to 5.7)	2.6 (1.0 to 5.3)	3.3 (0.9 to 5.9)	-5.0 (-7.9 to -2.3)	1.6 (0.6 to 3.4)	1.7 (0.6 to 3.5)	1.6 (0.6 to 3.3)	2.2 (0.3 to 4.4)	-5.4 (-8.1 to -2.5)
Low physical activity	0.3 (0.2 to 0.6)	0.3 (0.2 to 0.6)	0.4 (0.2 to 0.6)	0.1 (-1.8 to 2.1)	2.1 (0.3 to 4.2)	0.3 (0.2 to 0.6)	0.3 (0.2 to 0.6)	0.3 (0.2 to 0.6)	0.5 (-1.3 to 2.6)	0.8 (-0.9 to 2.7)
High fasting plasma glucose	3.6 (2.4 to 5.3)	3.6 (2.4 to 5.2)	3.2 (2.0 to 4.9)	0.8 (-25.9 to 33.9)	-9.5 (-40.1 to 25.8)	2.7 (1.6 to 4.1)	2.9 (1.9 to 4.2)	2.8 (1.7 to 4.2)	7.2 (-23.2 to 61.5)	-3.2 (-34.7 to 36.0)
High LDL cholesterol	10.6 (9.1 to 12.2)	10.5 (9.1 to 12.2)	10.6 (9.1 to 12.2)	-0.5 (-4.6 to 4.0)	0.1 (-2.9 to 3.3)	10.0 (8.7 to 11.5)	10.4 (9.0 to 12.0)	10.6 (9.2 to 12.2)	3.7 (-0.4 to 8.2)	2.7 (-0.5 to 6.0)
High systolic blood pressure	8.3 (7.4 to 9.3)	8.4 (7.5 to 9.4)	8.5 (7.6 to 9.5)	1.4 (-5.6 to 9.7)	1.0 (-5.0 to 7.7)	6.8 (6.0 to 7.7)	6.9 (6.1 to 7.7)	6.7 (5.9 to 7.5)	0.8 (-9.2 to 11.6)	-3.3 (-11.3 to 5.0)
High body-mass index	9.2 (6.5 to 13.1)	13.1 (9.8 to 18.1)	16.0 (12.2 to 21.9)	42.7 (25.9 to 66.2)	22.2 (10.4 to 34.2)	12.7 (9.4 to 17.3)	16.2 (12.3 to 21.6)	18.5 (14.1 to 24.2)	27.4 (12.7 to 45.0)	13.7 (4.1 to 25.0)
Low bone mineral density	4.1 (3.3 to 5.0)	4.0 (3.3 to 4.8)	3.9 (3.1 to 4.8)	-2.8 (-17.4 to 13.9)	-2.1 (-17.2 to 14.4)	5.7 (4.7 to 6.7)	5.5 (4.6 to 6.5)	5.5 (4.6 to 6.5)	-2.1 (-14.6 to 14.1)	-1.2 (-13.0 to 13.6)
Impaired kidney function	2.7 (1.2 to 6.6)	2.7 (1.3 to 6.7)	2.7 (1.2 to 6.6)	2.2 (-2.0 to 7.1)	-1.0 (-5.2 to 2.9)	3.2 (1.6 to 7.2)	3.3 (1.6 to 7.4)	3.3 (1.6 to 7.3)	2.1 (-1.6 to 6.8)	1.0 (-2.9 to 5.3)

Supplementary Results Table 1. Kyrgyzstan age-standardised Summary Exposure Values (SEVs) for Level 4 risk factors and percentage change, by sex, 1990, 2007, and 2017.Data in parentheses are 95% uncertainty intervals.										
	Male					Female				
	**1990**	**2007**	**2017**	**Percentage change 1990-2007**	**Percentage change 2007-2017**	**1990**	**2007**	**2017**	**Percentage change 1990-2007**	**Percentage change 2007-2017**
Unsafe water source	19.9 (12.3 to 24.8)	18.2 (11.5 to 22.3)	15.7 (10.1 to 19.6)	-8.3 (-24.1 to 12.4)	-13.7 (-28.1 to 3.4)	19.9 (12.3 to 24.8)	18.2 (11.5 to 22.3)	15.7 (10.1 to 19.6)	-8.3 (-24.1 to 12.4)	-13.7 (-28.1 to 3.4)
Unsafe sanitation	64.0 (46.6 to 88.7)	72.7 (61.6 to 87.4)	74.3 (64.9 to 85.7)	13.5 (-3.0 to 36.8)	2.1 (-6.9 to 12.6)	64.0 (46.6 to 88.7)	72.7 (61.6 to 87.4)	74.3 (64.9 to 85.7)	13.5 (-3.0 to 36.8)	2.1 (-6.9 to 12.6)
No access to handwashing facility	21.0 (17.2 to 25.2)	17.0 (14.3 to 20.0)	13.3 (11.0 to 15.8)	-19.1 (-33.4 to -0.6)	-21.6 (-33.2 to -8.9)	21.0 (17.2 to 25.2)	17.0 (14.3 to 20.0)	13.3 (11.0 to 15.8)	-19.1 (-33.4 to -0.6)	-21.6 (-33.2 to -8.9)
Ambient particulate matter pollution	25.6 (9.5 to 57.0)	29.1 (12.3 to 55.4)	28.9 (10.8 to 60.7)	13.7 (-62.8 to 261.8)	-0.6 (-66.8 to 169.7)	21.8 (7.7 to 50.5)	25.0 (10.1 to 50.6)	25.3 (8.7 to 56.3)	15.0 (-65.1 to 298.9)	1.3 (-68.6 to 192.7)
Household air pollution from solid fuels	37.1 (22.2 to 54.4)	30.0 (19.1 to 42.4)	22.4 (12.5 to 33.2)	-19.2 (-50.1 to 36.0)	-25.4 (-56.8 to 16.4)	38.6 (25.4 to 54.1)	31.8 (21.5 to 43.8)	24.1 (14.5 to 34.9)	-17.7 (-44.7 to 26.4)	-24.2 (-52.3 to 10.6)
Ambient ozone pollution	47.4 (18.4 to 54.8)	47.5 (19.2 to 54.8)	47.9 (19.1 to 54.8)	0.2 (-3.1 to 4.0)	1.0 (-1.3 to 6.1)	47.4 (18.4 to 54.8)	47.5 (19.2 to 54.8)	47.9 (19.1 to 54.8)	0.2 (-3.1 to 4.0)	1.0 (-1.3 to 6.1)
Residential radon	43.1 (9.3 to 71.2)	43.1 (9.3 to 71.2)	43.1 (9.3 to 71.2)	0.0 (0.0 to 0.0)	0.0 (0.0 to 0.0)	43.1 (9.3 to 71.2)	43.1 (9.3 to 71.2)	43.1 (9.3 to 71.2)	0.0 (0.0 to 0.0)	0.0 (0.0 to 0.0)
Lead exposure	7.8 (3.7 to 11.9)	7.7 (4.0 to 11.5)	6.8 (3.3 to 10.5)	-1.5 (-8.1 to 9.3)	-11.7 (-17.7 to -7.5)	4.0 (0.9 to 7.7)	4.0 (1.1 to 7.7)	3.7 (1.0 to 7.2)	2.0 (-5.0 to 26.7)	-9.3 (-15.9 to -4.9)
Occupational exposure to asbestos	0.3 (0.2 to 0.4)	0.4 (0.4 to 0.5)	0.5 (0.4 to 0.6)	55.6 (19.4 to 132.1)	9.4 (-10.1 to 43.5)	0.1 (0.1 to 0.2)	0.2 (0.2 to 0.3)	0.2 (0.2 to 0.3)	82.6 (33.2 to 169.1)	-11.1 (-30.0 to 15.3)
Occupational exposure to arsenic	0.3 (0.1 to 0.5)	0.2 (0.1 to 0.4)	0.3 (0.1 to 0.4)	-10.4 (-16.9 to -2.9)	2.3 (-3.7 to 8.2)	0.3 (0.1 to 0.5)	0.3 (0.1 to 0.4)	0.3 (0.1 to 0.4)	-4.6 (-14.1 to 5.5)	0.9 (-7.7 to 9.3)
Occupational exposure to benzene	0.6 (0.4 to 1.1)	0.6 (0.4 to 1.1)	0.7 (0.4 to 1.2)	1.5 (-5.1 to 10.2)	2.3 (-2.5 to 7.9)	0.8 (0.5 to 1.3)	0.8 (0.5 to 1.3)	0.8 (0.5 to 1.4)	0.2 (-9.2 to 13.7)	4.4 (-3.0 to 12.5)
Occupational exposure to beryllium	0.1 (0.1 to 0.1)	0.1 (0.1 to 0.1)	0.1 (0.1 to 0.1)	1.2 (-3.9 to 6.9)	1.3 (-2.0 to 4.8)	0.1 (0.1 to 0.1)	0.1 (0.1 to 0.1)	0.1 (0.1 to 0.1)	0.7 (-7.0 to 9.2)	-2.5 (-6.8 to 2.4)
Occupational exposure to cadmium	0.1 (0.1 to 0.2)	0.1 (0.1 to 0.1)	0.1 (0.1 to 0.1)	-8.3 (-14.6 to -1.2)	2.7 (-2.6 to 7.9)	0.1 (0.1 to 0.2)	0.1 (0.1 to 0.1)	0.1 (0.1 to 0.2)	-3.1 (-12.1 to 6.5)	0.9 (-6.9 to 8.5)
Occupational exposure to chromium	0.3 (0.3 to 0.4)	0.3 (0.3 to 0.3)	0.3 (0.3 to 0.3)	-5.9 (-12.6 to 1.3)	5.7 (0.1 to 11.1)	0.3 (0.3 to 0.4)	0.3 (0.3 to 0.3)	0.3 (0.3 to 0.4)	-1.0 (-10.0 to 9.2)	3.8 (-3.9 to 11.5)
Occupational exposure to diesel engine exhaust	2.0 (1.9 to 2.2)	2.4 (2.3 to 2.5)	2.7 (2.6 to 2.8)	15.9 (6.9 to 24.5)	14.5 (8.0 to 21.2)	1.4 (1.2 to 1.5)	1.6 (1.5 to 1.7)	1.6 (1.5 to 1.7)	14.9 (3.9 to 27.5)	4.7 (-2.1 to 12.9)
Occupational exposure to formaldehyde	0.7 (0.6 to 0.8)	0.6 (0.6 to 0.7)	0.6 (0.6 to 0.7)	-10.9 (-18.0 to -2.5)	4.1 (-2.6 to 10.4)	0.7 (0.6 to 0.7)	0.6 (0.6 to 0.7)	0.7 (0.6 to 0.7)	-3.9 (-13.5 to 6.9)	4.5 (-4.2 to 12.9)
Occupational exposure to nickel	0.3 (0.1 to 0.9)	0.3 (0.1 to 0.8)	0.3 (0.1 to 0.9)	-4.2 (-11.4 to 3.4)	5.2 (-0.9 to 11.1)	0.3 (0.1 to 0.7)	0.2 (0.1 to 0.6)	0.2 (0.1 to 0.6)	-7.5 (-16.4 to 2.4)	-2.2 (-10.1 to 6.4)
Occupational exposure to polycyclic aromatic hydrocarbons	0.7 (0.7 to 0.8)	0.7 (0.7 to 0.7)	0.7 (0.7 to 0.8)	-3.8 (-10.4 to 3.3)	5.4 (0.5 to 10.2)	0.7 (0.6 to 0.8)	0.7 (0.7 to 0.7)	0.7 (0.7 to 0.8)	-2.4 (-11.0 to 7.3)	2.9 (-4.5 to 10.0)
Occupational exposure to silica	3.7 (1.9 to 8.2)	4.0 (2.1 to 9.0)	4.4 (2.3 to 9.8)	7.4 (-3.0 to 18.7)	12.0 (3.2 to 21.9)	2.2 (1.1 to 4.7)	2.0 (1.0 to 4.3)	1.9 (1.0 to 4.1)	-8.6 (-17.6 to 0.7)	-5.1 (-12.3 to 3.0)
Occupational exposure to sulphuric acid	0.7 (0.5 to 1.2)	0.7 (0.5 to 1.2)	0.7 (0.5 to 1.2)	-2.0 (-8.6 to 5.9)	-2.0 (-7.0 to 3.2)	0.6 (0.4 to 1.1)	0.6 (0.4 to 1.0)	0.6 (0.4 to 1.0)	-1.6 (-10.2 to 7.4)	-4.7 (-11.3 to 2.1)
Occupational exposure to trichloroethylene	0.2 (0.2 to 0.2)	0.2 (0.2 to 0.2)	0.2 (0.2 to 0.2)	-0.2 (-6.5 to 7.0)	7.1 (2.2 to 12.1)	0.2 (0.2 to 0.2)	0.2 (0.2 to 0.2)	0.2 (0.2 to 0.2)	5.1 (-3.8 to 16.0)	4.0 (-2.5 to 11.2)
Occupational asthmagens	17.3 (13.6 to 21.6)	16.3 (13.0 to 20.2)	15.0 (12.2 to 18.6)	-6.2 (-16.0 to 5.3)	-7.8 (-15.6 to 1.2)	9.8 (7.1 to 13.1)	9.6 (7.5 to 12.6)	8.8 (6.9 to 11.2)	-2.0 (-17.5 to 19.5)	-8.2 (-18.9 to 3.5)
Occupational particulate matter, gases, and fumes	9.0 (6.9 to 11.9)	8.7 (6.7 to 11.5)	8.7 (6.8 to 11.5)	-3.3 (-6.9 to 0.5)	-0.3 (-2.6 to 2.3)	5.0 (3.8 to 6.9)	4.9 (3.8 to 6.7)	4.8 (3.7 to 6.5)	-2.1 (-7.7 to 4.3)	-2.8 (-6.5 to 0.8)
Occupational noise	9.8 (9.0 to 10.7)	9.7 (9.1 to 10.4)	9.7 (9.2 to 10.3)	-1.4 (-4.3 to 1.7)	0.6 (-1.4 to 2.7)	6.0 (5.3 to 6.8)	6.1 (5.6 to 6.7)	6.1 (5.7 to 6.6)	1.2 (-3.4 to 6.8)	-0.3 (-3.2 to 2.9)
Occupational ergonomic factors	21.8 (19.2 to 24.5)	20.6 (19.2 to 22.0)	17.6 (15.7 to 19.5)	-5.6 (-15.5 to 7.3)	-14.7 (-22.5 to -5.9)	14.8 (12.1 to 17.4)	13.6 (12.7 to 14.6)	11.2 (9.8 to 12.7)	-8.4 (-22.4 to 13.0)	-17.9 (-27.9 to -7.5)
Non-exclusive breastfeeding	0.4 (0.2 to 0.6)	0.3 (0.2 to 0.5)	0.3 (0.2 to 0.5)	-11.9 (-20.3 to -1.6)	-5.5 (-13.4 to 3.4)	0.4 (0.2 to 0.6)	0.3 (0.2 to 0.5)	0.3 (0.2 to 0.5)	-11.8 (-20.2 to -1.4)	-5.5 (-13.4 to 3.5)
Discontinued breastfeeding	1.2 (1.1 to 1.3)	1.2 (1.2 to 1.3)	1.3 (1.2 to 1.3)	-0.4 (-9.2 to 9.9)	3.5 (-4.3 to 12.5)	1.2 (1.1 to 1.3)	1.2 (1.1 to 1.3)	1.3 (1.2 to 1.3)	-0.4 (-9.2 to 10.0)	4.1 (-3.8 to 13.2)
Child underweight	0.3 (0.2 to 0.5)	0.2 (0.1 to 0.3)	0.2 (0.1 to 0.2)	-35.5 (-49.9 to -19.9)	-22.6 (-33.1 to -12.2)	0.3 (0.2 to 0.4)	0.2 (0.1 to 0.3)	0.2 (0.1 to 0.2)	-25.7 (-42.7 to -7.0)	-19.5 (-31.6 to -6.5)
Child wasting	0.1 (0.1 to 0.2)	0.1 (0.1 to 0.2)	0.1 (0.1 to 0.2)	-10.4 (-28.8 to 11.6)	-6.0 (-19.0 to 9.1)	0.1 (0.1 to 0.2)	0.1 (0.1 to 0.2)	0.1 (0.1 to 0.2)	-6.2 (-25.9 to 16.9)	-0.7 (-15.2 to 13.6)
Child stunting	2.2 (1.4 to 2.9)	1.3 (0.9 to 1.6)	1.0 (0.6 to 1.3)	-40.4 (-54.5 to -22.6)	-24.2 (-40.3 to -8.7)	1.9 (1.2 to 2.7)	1.3 (0.9 to 1.6)	0.9 (0.6 to 1.2)	-32.9 (-52.2 to -7.4)	-28.5 (-45.6 to -11.3)
Short gestation for birth weight	0.0 (0.0 to 0.0)	0.0 (0.0 to 0.0)	0.0 (0.0 to 0.0)	7.6 (-28.5 to 45.2)	6.6 (-21.4 to 52.2)	0.0 (0.0 to 0.0)	0.0 (0.0 to 0.0)	0.0 (0.0 to 0.0)	4.8 (-19.4 to 34.7)	4.4 (-17.5 to 34.2)
Low birth weight for gestation	0.0 (0.0 to 0.0)	0.0 (0.0 to 0.0)	0.0 (0.0 to 0.0)	3.6 (-16.4 to 35.6)	4.2 (-17.5 to 23.7)	0.0 (0.0 to 0.0)	0.0 (0.0 to 0.0)	0.0 (0.0 to 0.0)	2.7 (-12.5 to 23.1)	8.4 (-7.7 to 24.2)
Iron deficiency	-	-	-	-	-	13.7 (10.7 to 17.0)	13.2 (10.2 to 16.6)	11.9 (9.1 to 15.3)	-3.4 (-17.6 to 13.2)	-9.9 (-21.1 to 1.6)
Vitamin A deficiency	1.7 (1.3 to 2.1)	1.6 (1.3 to 2.0)	1.5 (1.1 to 2.0)	-6.1 (-20.3 to 12.9)	-8.6 (-26.6 to 10.8)	1.9 (1.5 to 2.2)	1.8 (1.5 to 2.1)	1.7 (1.4 to 2.1)	-3.6 (-14.9 to 10.2)	-4.1 (-14.5 to 7.4)
Zinc deficiency	0.6 (0.2 to 1.4)	0.5 (0.1 to 1.0)	0.4 (0.1 to 0.9)	-17.5 (-36.8 to 6.5)	-11.4 (-54.8 to 7.8)	0.6 (0.2 to 1.3)	0.5 (0.1 to 1.1)	0.4 (0.1 to 0.9)	-17.5 (-36.8 to 4.3)	-11.9 (-57.6 to 13.8)
Smoking	7.7 (6.2 to 9.7)	8.6 (6.9 to 10.4)	8.8 (7.0 to 10.9)	10.6 (-4.3 to 25.7)	3.4 (-6.3 to 13.5)	0.3 (0.2 to 0.5)	0.5 (0.4 to 0.7)	0.8 (0.6 to 1.0)	60.3 (19.5 to 115.6)	50.4 (24.2 to 80.3)
Chewing tobacco	3.1 (2.3 to 4.1)	3.1 (2.5 to 3.8)	3.3 (2.7 to 4.0)	-0.7 (-25.0 to 34.5)	5.3 (-16.0 to 31.4)	0.1 (0.0 to 0.1)	0.1 (0.0 to 0.1)	0.1 (0.0 to 0.1)	-0.5 (-27.7 to 39.7)	0.1 (-21.5 to 26.2)
Second-hand smoke	35.0 (33.0 to 36.9)	34.8 (33.8 to 35.7)	32.9 (31.6 to 34.3)	-0.5 (-6.3 to 6.0)	-5.4 (-10.0 to -0.5)	57.9 (55.8 to 59.7)	53.5 (52.5 to 54.4)	52.8 (51.3 to 54.2)	-7.5 (-10.8 to -4.1)	-1.4 (-4.5 to 2.0)
Alcohol use	23.8 (17.1 to 30.4)	21.7 (14.6 to 29.2)	20.9 (12.5 to 29.5)	-8.6 (-26.8 to 8.6)	-3.7 (-29.9 to 21.4)	7.4 (4.3 to 11.7)	6.9 (4.1 to 11.1)	6.9 (3.8 to 11.3)	-6.4 (-26.5 to 14.0)	-0.2 (-24.3 to 28.3)
Drug use	0.4 (0.3 to 0.5)	0.5 (0.4 to 0.6)	0.5 (0.4 to 0.6)	20.5 (9.6 to 33.3)	-7.5 (-16.3 to 1.5)	0.2 (0.1 to 0.2)	0.2 (0.2 to 0.3)	0.2 (0.1 to 0.3)	10.1 (2.3 to 18.3)	-4.2 (-10.2 to 2.6)
Diet low in fruits	49.8 (45.2 to 51.6)	47.5 (42.7 to 49.6)	45.7 (40.6 to 48.1)	-4.6 (-6.7 to -2.8)	-3.8 (-6.1 to -1.9)	49.0 (44.5 to 51.0)	46.3 (41.5 to 48.5)	44.2 (39.1 to 46.9)	-5.7 (-8.2 to -3.6)	-4.5 (-7.2 to -2.4)
Diet low in vegetables	32.3 (27.1 to 36.5)	13.2 (5.7 to 19.9)	8.7 (2.4 to 15.9)	-59.0 (-79.9 to -43.8)	-34.6 (-67.8 to -11.9)	34.0 (29.2 to 37.9)	16.4 (8.9 to 22.7)	11.3 (3.8 to 18.9)	-51.6 (-70.3 to -38.4)	-31.1 (-63.2 to -11.1)
Diet low in legumes	51.6 (50.7 to 52.2)	38.0 (34.9 to 40.7)	39.5 (36.3 to 42.2)	-26.4 (-31.6 to -21.8)	4.0 (0.7 to 7.6)	52.4 (51.8 to 53.0)	41.0 (38.4 to 43.3)	42.1 (39.6 to 44.4)	-21.7 (-26.4 to -18.0)	2.7 (0.2 to 5.4)
Diet low in whole grains	52.1 (51.6 to 52.5)	52.2 (51.7 to 52.5)	51.9 (51.5 to 52.4)	0.1 (-0.1 to 0.4)	-0.4 (-0.7 to -0.2)	52.0 (51.5 to 52.4)	52.1 (51.6 to 52.4)	52.0 (51.5 to 52.4)	0.2 (-0.0 to 0.4)	-0.1 (-0.4 to 0.1)
Diet low in nuts and seeds	49.2 (47.6 to 50.5)	51.1 (50.1 to 51.9)	49.3 (47.6 to 50.5)	3.8 (2.1 to 6.1)	-3.5 (-5.5 to -2.1)	49.5 (48.1 to 50.7)	51.3 (50.4 to 52.1)	49.6 (48.1 to 50.8)	3.6 (2.0 to 5.6)	-3.3 (-5.0 to -2.0)
Diet low in milk	36.1 (32.1 to 39.3)	35.3 (31.2 to 38.6)	34.1 (29.8 to 37.8)	-2.3 (-6.0 to 1.1)	-3.3 (-7.0 to -0.2)	36.9 (33.0 to 40.2)	36.4 (32.5 to 39.6)	35.2 (30.7 to 38.8)	-1.4 (-4.8 to 1.8)	-3.3 (-6.8 to -0.5)
Diet high in red meat	21.3 (16.0 to 25.8)	8.3 (4.4 to 11.9)	8.8 (4.1 to 13.8)	-61.0 (-76.7 to -46.8)	6.2 (-29.6 to 58.8)	6.9 (2.7 to 11.2)	1.0 (0.0 to 2.9)	1.6 (0.1 to 4.3)	-86.1 (-99.5 to -68.3)	67.2 (-47.6 to 1762.7)
Diet high in processed meat	1.0 (0.0 to 2.9)	0.4 (0.0 to 1.8)	0.7 (0.0 to 2.4)	-58.6 (-99.5 to -35.1)	60.0 (25.5 to 1530.8)	0.6 (0.0 to 2.2)	0.2 (0.0 to 1.3)	0.4 (0.0 to 1.8)	-59.0 (-100.0 to -37.3)	58.4 (26.0 to [illegible]
Diet high in sugar-sweetened beverages	3.2 (1.5 to 5.9)	1.7 (0.7 to 3.1)	2.3 (1.0 to 4.1)	-47.8 (-72.2 to -5.0)	36.8 (-11.2 to 119.7)	2.3 (1.0 to 4.1)	1.3 (0.6 to 2.3)	1.7 (0.8 to 3.0)	-44.8 (-71.1 to -3.1)	31.4 (-14.0 to 103.2)
Diet low in fibre	36.2 (31.8 to 39.8)	26.7 (20.9 to 31.7)	24.1 (17.8 to 29.7)	-26.2 (-36.2 to -18.9)	-9.8 (-19.1 to -3.2)	40.4 (37.0 to 43.1)	32.5 (27.4 to 36.5)	30.0 (24.2 to 34.7)	-19.6 (-27.4 to -13.9)	-7.5 (-14.4 to -2.3)
Diet low in calcium	27.7 (22.2 to 32.2)	23.2 (16.9 to 28.3)	19.8 (12.8 to 25.4)	-16.3 (-25.1 to -10.8)	-15.0 (-26.0 to -8.7)	30.9 (26.1 to 34.8)	26.9 (21.2 to 31.4)	23.7 (17.2 to 28.7)	-13.0 (-19.4 to -8.6)	-12.0 (-19.6 to -7.3)
Diet low in seafood omega-3 fatty acids	45.8 (44.0 to 47.6)	46.9 (45.3 to 48.4)	46.3 (44.5 to 47.9)	2.4 (1.7 to 3.2)	-1.3 (-2.2 to -0.6)	47.1 (45.6 to 48.6)	48.0 (46.7 to 49.4)	47.5 (46.0 to 48.9)	2.0 (1.4 to 2.6)	-1.1 (-1.8 to -0.5)
Diet low in polyunsaturated fatty acids	43.1 (40.5 to 45.4)	47.2 (45.6 to 48.4)	44.5 (42.4 to 46.3)	9.5 (5.7 to 14.1)	-5.7 (-7.8 to -3.9)	43.5 (41.0 to 45.7)	47.4 (45.9 to 48.7)	44.8 (42.9 to 46.6)	9.1 (5.3 to 13.3)	-5.4 (-7.2 to -3.9)
Diet high in trans fatty acids	1.6 (0.1 to 4.1)	1.3 (0.1 to 3.6)	1.1 (0.0 to 3.3)	-18.8 (-51.6 to -5.1)	-15.1 (-47.7 to -2.6)	2.1 (0.3 to 5.1)	1.7 (0.2 to 4.4)	1.5 (0.1 to 4.0)	-18.0 (-48.3 to -3.2)	-14.9 (-45.4 to -1.8)
Diet high in sodium	13.9 (6.0 to 21.2)	6.8 (0.6 to 14.0)	7.0 (0.6 to 14.4)	-51.0 (-91.4 to -28.5)	2.4 (-29.4 to 47.4)	12.8 (5.6 to 20.2)	6.5 (0.7 to 13.5)	5.3 (0.3 to 12.1)	-49.4 (-89.1 to -28.2)	-18.7 (-60.5 to -4.8)
Intimate partner violence	-	-	-	-	-	6.7 (5.6 to 7.8)	6.5 (5.6 to 7.4)	6.3 (5.4 to 7.1)	-3.0 (-8.0 to 2.8)	-3.3 (-8.9 to 3.2)
Childhood sexual abuse	1.6 (1.3 to 2.0)	1.6 (1.3 to 2.0)	1.6 (1.3 to 2.0)	0.0 (0.0 to 0.0)	0.0 (0.0 to 0.0)	2.2 (1.7 to 2.7)	2.2 (1.7 to 2.7)	2.2 (1.7 to 2.7)	0.0 (0.0 to 0.0)	0.0 (0.0 to 0.0)

Supplementary Results Table 1. Kyrgyzstan age-standardised Summary Exposure Values (SEVs) for Level 4 risk factors and percentage change, by sex, 1990, 2007, and 2017.Data in parentheses are 95% uncertainty intervals.										
	**Male**					**Female**				
	**1990**	**2007**	**2017**	**Percentage change 1990-2007**	**Percentage change 2007-2017**	**1990**	**2007**	**2017**	**Percentage change 1990-2007**	**Percentage change 2007-2017**
Bullying victimization	2.5 (1.0 to 5.1)	2.6 (1.1 to 5.3)	2.5 (1.1 to 5.1)	4.8 (0.6 to 9.8)	-3.2 (-6.0 to -0.6)	1.5 (0.6 to 3.1)	1.6 (0.7 to 3.2)	1.5 (0.6 to 3.1)	4.8 (0.8 to 10.2)	-3.3 (-6.1 to -0.8)
Low physical activity	0.3 (0.2 to 0.6)	0.3 (0.2 to 0.6)	0.3 (0.2 to 0.6)	0.7 (-1.4 to 2.7)	1.2 (-0.6 to 3.3)	0.3 (0.1 to 0.5)	0.3 (0.1 to 0.5)	0.3 (0.1 to 0.5)	0.6 (-1.4 to 2.7)	0.9 (-1.0 to 3.1)
High fasting plasma glucose	3.7 (2.6 to 5.5)	3.7 (2.6 to 5.5)	3.5 (2.3 to 5.1)	-0.6 (-19.7 to 27.2)	-5.3 (-28.2 to 19.2)	2.6 (1.7 to 4.0)	2.6 (1.7 to 3.9)	2.5 (1.4 to 4.0)	-0.5 (-30.2 to 35.9)	-4.0 (-36.8 to 36.2)
High LDL cholesterol	9.6 (8.2 to 11.2)	9.2 (7.8 to 10.8)	9.5 (8.0 to 11.1)	-3.7 (-7.9 to 0.6)	2.7 (-0.6 to 6.2)	10.1 (8.8 to 11.7)	10.0 (8.6 to 11.5)	10.2 (8.8 to 11.7)	-1.8 (-5.7 to 2.3)	1.9 (-1.4 to 5.3)
High systolic blood pressure	6.4 (5.7 to 7.2)	6.0 (5.3 to 6.8)	6.1 (5.4 to 6.9)	-6.3 (-13.7 to 1.0)	1.7 (-5.0 to 8.3)	5.9 (5.2 to 6.6)	5.6 (4.9 to 6.3)	5.6 (4.9 to 6.4)	-4.2 (-14.1 to 6.8)	0.2 (-8.2 to 8.9)
High body-mass index	8.7 (5.9 to 13.3)	8.7 (6.1 to 13.1)	9.6 (6.9 to 14.1)	0.4 (-15.2 to 16.5)	10.5 (-0.6 to 23.0)	10.0 (7.0 to 14.2)	10.5 (7.5 to 14.7)	11.3 (8.0 to 15.5)	5.2 (-9.6 to 22.5)	7.4 (-3.9 to 18.7)
Low bone mineral density	4.0 (3.2 to 4.8)	4.0 (3.3 to 4.8)	3.9 (3.1 to 4.7)	-1.1 (-15.0 to 15.6)	-2.2 (-16.9 to 13.4)	5.5 (4.5 to 6.7)	5.5 (4.6 to 6.6)	5.4 (4.5 to 6.5)	-0.6 (-13.2 to 15.2)	-0.9 (-14.1 to 14.5)
Impaired kidney function	2.6 (1.2 to 6.4)	2.6 (1.2 to 6.5)	2.5 (1.1 to 6.4)	1.1 (-3.0 to 5.4)	-2.4 (-6.3 to 1.1)	3.0 (1.5 to 7.1)	3.1 (1.5 to 7.1)	3.1 (1.5 to 7.1)	1.7 (-1.8 to 5.9)	-0.2 (-4.1 to 4.1)

Supplementary Results Table 1. Mongolia age-standardised Summary Exposure Values (SEVs) for Level 4 risk factors and percentage change, by sex, 1990, 2007, and 2017.Data in parentheses are 95% uncertainty intervals.										
	Male					Female				
	1990	2007	2017	Percentage change 1990-2007	Percentage change 2007-2017	1990	2007	2017	Percentage change 1990-2007	Percentage change 2007-2017
Unsafe water source	30.9 (18.9 to 37.5)	31.2 (18.5 to 38.1)	30.1 (18.5 to 36.3)	1.1 (-7.9 to 9.8)	-3.6 (-10.4 to 5.8)	30.9 (18.9 to 37.5)	31.2 (18.5 to 38.1)	30.1 (18.5 to 36.3)	1.1 (-7.9 to 9.8)	-3.6 (-10.4 to 5.8)
Unsafe sanitation	81.8 (70.9 to 96.2)	73.6 (66.5 to 82.7)	68.0 (58.5 to 78.8)	-9.9 (-16.0 to -1.4)	-7.7 (-16.8 to 1.6)	81.8 (70.9 to 96.2)	73.6 (66.5 to 82.7)	68.0 (58.5 to 78.8)	-9.9 (-16.0 to -1.4)	-7.7 (-16.8 to 1.6)
No access to handwashing facility	30.6 (25.7 to 35.3)	35.7 (31.7 to 39.9)	29.3 (25.6 to 33.2)	16.7 (-0.2 to 37.3)	-17.8 (-27.7 to -6.4)	30.6 (25.7 to 35.3)	35.7 (31.7 to 39.9)	29.3 (25.6 to 33.2)	16.7 (-0.2 to 37.3)	-17.8 (-27.7 to -6.4)
Ambient particulate matter pollution	26.6 (6.7 to 49.8)	35.2 (13.6 to 58.9)	39.7 (12.2 to 65.7)	32.3 (-48.3 to 425.0)	12.9 (-57.7 to 170.5)	22.4 (5.3 to 43.3)	30.5 (11.2 to 53.3)	35.2 (10.2 to 59.6)	36.3 (-49.8 to 466.0)	15.5 (-59.6 to 187.3)
Household air pollution from solid fuels	51.2 (35.0 to 72.4)	35.5 (22.4 to 52.3)	26.4 (15.5 to 41.8)	-30.6 (-54.5 to 0.5)	-25.6 (-55.2 to 13.7)	53.0 (37.6 to 72.6)	38.0 (25.6 to 54.4)	28.8 (18.0 to 43.2)	-28.4 (-49.8 to -1.1)	-24.1 (-50.8 to 8.6)
Ambient ozone pollution	47.4 (18.4 to 54.8)	47.3 (18.6 to 54.8)	47.7 (18.8 to 54.8)	-0.3 (-2.1 to 1.1)	1.0 (-0.7 to 5.5)	47.4 (18.4 to 54.8)	47.3 (18.6 to 54.8)	47.7 (18.8 to 54.8)	-0.3 (-2.1 to 1.1)	1.0 (-0.7 to 5.5)
Residential radon	50.9 (11.3 to 71.2)	50.9 (11.3 to 71.2)	50.9 (11.3 to 71.2)	0.0 (0.0 to 0.0)	0.0 (0.0 to 0.0)	50.9 (11.3 to 71.2)	50.9 (11.3 to 71.2)	50.9 (11.3 to 71.2)	0.0 (0.0 to 0.0)	0.0 (0.0 to 0.0)
Lead exposure	8.9 (4.8 to 12.9)	7.8 (4.2 to 11.7)	6.2 (3.1 to 9.8)	-12.3 (-19.1 to -6.6)	-20.3 (-27.6 to -14.6)	4.5 (1.3 to 8.3)	4.1 (1.2 to 7.7)	3.3 (0.9 to 6.8)	-8.6 (-15.3 to 3.3)	-18.0 (-26.7 to -12.2)
Occupational exposure to asbestos	0.7 (0.5 to 0.8)	0.9 (0.8 to 1.1)	0.7 (0.6 to 0.9)	40.3 (5.1 to 86.9)	-22.0 (-39.6 to 2.0)	1.7 (1.1 to 2.3)	0.5 (0.4 to 0.6)	0.2 (0.2 to 0.3)	-70.6 (-78.8 to -55.5)	-54.7 (-68.4 to -37.6)
Occupational exposure to arsenic	0.2 (0.1 to 0.4)	0.2 (0.1 to 0.4)	0.2 (0.1 to 0.4)	0.5 (-8.3 to 10.9)	3.5 (-3.2 to 11.0)	0.2 (0.1 to 0.4)	0.3 (0.1 to 0.4)	0.3 (0.1 to 0.5)	8.1 (-3.4 to 22.7)	3.9 (-3.9 to 12.8)
Occupational exposure to benzene	0.5 (0.3 to 1.0)	0.6 (0.4 to 1.1)	0.6 (0.4 to 1.1)	10.8 (2.2 to 23.1)	7.4 (1.2 to 14.6)	0.6 (0.4 to 1.1)	0.8 (0.5 to 1.4)	0.9 (0.5 to 1.6)	27.5 (14.5 to 43.9)	13.7 (6.4 to 22.8)
Occupational exposure to beryllium	0.1 (0.1 to 0.1)	0.1 (0.1 to 0.1)	0.1 (0.1 to 0.1)	-0.9 (-7.4 to 7.1)	-0.7 (-5.0 to 3.7)	0.1 (0.1 to 0.1)	0.1 (0.1 to 0.1)	0.1 (0.1 to 0.1)	7.9 (-0.6 to 18.2)	0.4 (-4.4 to 5.1)
Occupational exposure to cadmium	0.1 (0.1 to 0.1)	0.1 (0.1 to 0.1)	0.1 (0.1 to 0.1)	1.4 (-6.8 to 11.4)	3.4 (-2.5 to 10.2)	0.1 (0.1 to 0.1)	0.1 (0.1 to 0.2)	0.2 (0.1 to 0.2)	9.4 (-1.4 to 22.5)	4.1 (-2.9 to 12.1)
Occupational exposure to chromium	0.3 (0.2 to 0.3)	0.3 (0.3 to 0.3)	0.3 (0.3 to 0.3)	7.0 (-1.7 to 17.3)	6.8 (1.0 to 13.3)	0.3 (0.3 to 0.3)	0.3 (0.3 to 0.4)	0.4 (0.3 to 0.4)	16.2 (4.5 to 30.4)	8.4 (1.2 to 16.4)
Occupational exposure to diesel engine exhaust	1.9 (1.7 to 2.1)	2.2 (2.1 to 2.3)	2.4 (2.2 to 2.5)	12.5 (1.3 to 24.9)	8.2 (1.0 to 15.6)	1.7 (1.5 to 1.9)	2.0 (1.9 to 2.2)	2.2 (2.0 to 2.3)	19.1 (7.0 to 33.8)	6.6 (-0.8 to 14.3)
Occupational exposure to formaldehyde	0.5 (0.5 to 0.6)	0.6 (0.5 to 0.6)	0.6 (0.6 to 0.6)	6.1 (-3.7 to 17.5)	7.6 (0.9 to 15.5)	0.6 (0.5 to 0.6)	0.7 (0.6 to 0.7)	0.7 (0.7 to 0.8)	16.5 (3.4 to 31.7)	10.2 (2.0 to 19.4)
Occupational exposure to nickel	0.3 (0.1 to 0.8)	0.3 (0.1 to 0.8)	0.3 (0.1 to 0.8)	-1.9 (-12.2 to 10.7)	2.6 (-5.4 to 11.2)	0.3 (0.1 to 0.7)	0.3 (0.1 to 0.7)	0.3 (0.1 to 0.7)	0.2 (-10.1 to 14.2)	-2.1 (-9.1 to 6.9)
Occupational exposure to polycyclic aromatic hydrocarbons	0.6 (0.5 to 0.6)	0.6 (0.6 to 0.6)	0.7 (0.6 to 0.7)	5.9 (-2.2 to 15.8)	6.8 (1.2 to 12.6)	0.6 (0.6 to 0.7)	0.7 (0.7 to 0.8)	0.8 (0.7 to 0.8)	15.2 (4.0 to 28.4)	7.9 (1.2 to 15.2)
Occupational exposure to silica	3.5 (1.8 to 7.6)	3.5 (1.8 to 7.9)	3.7 (1.9 to 8.3)	1.9 (-10.5 to 16.7)	5.9 (-3.6 to 17.0)	2.7 (1.4 to 5.8)	2.6 (1.4 to 5.7)	2.5 (1.3 to 5.4)	-3.5 (-13.7 to 10.0)	-5.0 (-12.6 to 3.2)
Occupational exposure to sulphuric acid	0.7 (0.5 to 1.2)	0.6 (0.4 to 1.1)	0.6 (0.4 to 1.0)	-7.4 (-15.1 to 1.9)	-1.2 (-7.7 to 5.3)	0.6 (0.4 to 1.1)	0.6 (0.5 to 1.1)	0.6 (0.4 to 1.1)	0.1 (-9.5 to 11.5)	-3.8 (-9.9 to 3.2)
Occupational exposure to trichloroethylene	0.2 (0.1 to 0.2)	0.2 (0.2 to 0.2)	0.2 (0.2 to 0.2)	9.4 (1.7 to 19.4)	6.3 (1.1 to 12.1)	0.2 (0.2 to 0.2)	0.2 (0.2 to 0.2)	0.2 (0.2 to 0.2)	19.4 (8.1 to 33.5)	8.3 (1.9 to 15.5)
Occupational asthmagens	17.3 (13.7 to 21.8)	16.3 (13.4 to 20.0)	16.0 (13.4 to 19.4)	-5.5 (-16.1 to 5.5)	-1.7 (-9.6 to 7.7)	11.1 (8.2 to 14.8)	10.7 (8.6 to 13.4)	10.4 (8.4 to 13.0)	-3.4 (-17.0 to 13.9)	-2.8 (-13.2 to 8.0)
Occupational particulate matter, gases, and fumes	7.7 (5.8 to 10.5)	7.3 (5.7 to 9.9)	7.2 (5.6 to 9.7)	-4.7 (-8.9 to -0.0)	-1.0 (-3.9 to 2.4)	4.9 (3.6 to 6.8)	4.9 (3.7 to 6.8)	4.8 (3.7 to 6.5)	0.0 (-5.9 to 7.2)	-2.9 (-6.2 to 0.7)
Occupational noise	8.5 (7.7 to 9.4)	8.3 (7.7 to 9.0)	8.4 (7.9 to 8.9)	-2.6 (-6.4 to 1.5)	0.9 (-1.7 to 3.8)	5.6 (4.9 to 6.4)	5.9 (5.4 to 6.5)	6.1 (5.7 to 6.6)	5.1 (-0.5 to 11.5)	2.7 (-0.2 to 6.1)
Occupational ergonomic factors	20.8 (18.6 to 23.3)	17.1 (16.0 to 18.2)	14.3 (12.9 to 16.0)	-18.1 (-26.6 to -8.9)	-16.0 (-24.1 to -7.4)	15.4 (13.3 to 17.7)	13.3 (12.4 to 14.3)	11.1 (9.7 to 12.5)	-13.7 (-25.3 to 1.3)	-16.6 (-26.8 to -6.0)
Non-exclusive breastfeeding	0.3 (0.2 to 0.4)	0.3 (0.2 to 0.4)	0.3 (0.2 to 0.4)	-5.2 (-15.7 to 6.7)	5.2 (-6.0 to 17.6)	0.3 (0.2 to 0.4)	0.3 (0.2 to 0.4)	0.3 (0.2 to 0.4)	-5.2 (-15.6 to 6.7)	5.2 (-5.9 to 17.7)
Discontinued breastfeeding	0.6 (0.5 to 0.6)	0.6 (0.6 to 0.7)	0.7 (0.6 to 0.8)	12.3 (-2.6 to 30.5)	12.3 (-2.2 to 28.1)	0.6 (0.5 to 0.6)	0.6 (0.6 to 0.7)	0.7 (0.6 to 0.8)	12.3 (-2.7 to 30.4)	12.4 (-2.1 to 28.2)
Child underweight	0.8 (0.6 to 1.0)	0.3 (0.2 to 0.3)	0.2 (0.1 to 0.2)	-68.5 (-77.5 to -60.1)	-35.6 (-46.5 to -25.7)	0.8 (0.6 to 1.1)	0.3 (0.2 to 0.4)	0.2 (0.1 to 0.3)	-68.7 (-77.9 to -59.2)	-30.8 (-41.4 to -21.1)
Child wasting	0.3 (0.2 to 0.4)	0.1 (0.1 to 0.2)	0.1 (0.1 to 0.2)	-50.1 (-65.1 to -32.7)	-19.2 (-30.1 to -7.3)	0.3 (0.2 to 0.4)	0.1 (0.1 to 0.2)	0.1 (0.1 to 0.1)	-50.5 (-65.1 to -30.0)	-23.5 (-32.8 to -13.5)
Child stunting	2.8 (1.8 to 4.2)	1.5 (1.1 to 1.9)	1.0 (0.6 to 1.3)	-44.8 (-63.2 to -25.6)	-35.8 (-51.0 to -21.3)	2.8 (1.8 to 4.7)	1.3 (0.9 to 1.6)	0.8 (0.5 to 1.2)	-53.2 (-71.7 to -34.9)	-35.9 (-51.5 to -18.7)
Short gestation for birth weight	0.0 (0.0 to 0.0)	0.0 (0.0 to 0.0)	0.0 (0.0 to 0.0)	0.7 (-19.2 to 21.0)	2.1 (-18.5 to 27.1)	0.0 (0.0 to 0.0)	0.0 (0.0 to 0.0)	0.0 (0.0 to 0.0)	8.7 (-18.0 to 39.5)	7.0 (-14.7 to 37.1)
Low birth weight for gestation	0.0 (0.0 to 0.0)	0.0 (0.0 to 0.0)	0.0 (0.0 to 0.0)	6.8 (-6.2 to 25.0)	5.3 (-9.7 to 20.6)	0.0 (0.0 to 0.0)	0.0 (0.0 to 0.0)	0.0 (0.0 to 0.0)	4.1 (-11.5 to 23.8)	4.2 (-11.5 to 20.4)
Iron deficiency	-	-	-	-	-	13.5 (10.9 to 16.4)	10.7 (8.4 to 13.4)	9.4 (7.0 to 12.1)	-20.7 (-33.2 to -7.0)	-12.0 (-23.8 to -0.6)
Vitamin A deficiency	2.0 (1.7 to 2.4)	1.4 (1.2 to 1.7)	1.5 (1.2 to 1.9)	-27.9 (-38.2 to -16.2)	5.1 (-9.7 to 21.1)	2.9 (2.4 to 3.4)	2.2 (1.9 to 2.5)	2.3 (1.9 to 2.7)	-24.1 (-33.0 to -13.8)	3.2 (-7.3 to 14.7)
Zinc deficiency	0.5 (0.1 to 1.0)	0.4 (0.1 to 0.9)	0.4 (0.1 to 1.1)	-10.9 (-51.5 to 3.1)	-1.3 (-60.0 to 236.2)	0.5 (0.1 to 1.0)	0.4 (0.1 to 0.9)	0.4 (0.1 to 1.1)	-11.0 (-51.2 to 5.7)	-1.1 (-61.1 to 194.1)
Smoking	10.0 (8.2 to 11.8)	9.3 (7.7 to 11.1)	9.7 (8.1 to 11.6)	-7.2 (-16.7 to 3.5)	4.8 (-3.4 to 14.0)	0.9 (0.7 to 1.2)	0.9 (0.7 to 1.1)	1.1 (0.8 to 1.3)	-2.8 (-24.2 to 26.7)	20.5 (0.9 to 44.1)
Chewing tobacco	0.2 (0.1 to 0.2)	0.2 (0.1 to 0.2)	0.2 (0.1 to 0.3)	2.8 (-26.4 to 44.1)	20.6 (-7.3 to 55.8)	0.1 (0.0 to 0.1)	0.1 (0.0 to 0.1)	0.1 (0.0 to 0.1)	-2.1 (-30.3 to 37.1)	1.1 (-21.3 to 29.6)
Second-hand smoke	35.1 (32.8 to 37.3)	34.4 (33.5 to 35.3)	33.8 (32.1 to 35.4)	-1.9 (-8.5 to 5.6)	-1.8 (-6.8 to 3.3)	58.7 (56.5 to 60.7)	58.0 (56.9 to 59.0)	57.9 (56.4 to 59.4)	-1.2 (-4.9 to 2.7)	-0.0 (-2.8 to 2.7)
Alcohol use	12.1 (7.0 to 19.6)	16.6 (10.1 to 25.1)	18.6 (10.8 to 29.4)	36.4 (11.4 to 81.3)	12.5 (-11.8 to 50.0)	4.2 (2.0 to 8.1)	6.6 (3.4 to 12.1)	7.3 (3.7 to 13.0)	57.1 (30.2 to 106.6)	10.3 (-12.0 to 43.5)
Drug use	0.4 (0.3 to 0.5)	0.4 (0.3 to 0.5)	0.4 (0.3 to 0.6)	10.8 (2.7 to 20.0)	5.0 (-2.1 to 12.5)	0.2 (0.1 to 0.2)	0.2 (0.1 to 0.2)	0.2 (0.1 to 0.2)	3.6 (-3.0 to 9.6)	4.6 (-0.9 to 11.4)
Diet low in fruits	53.7 (49.7 to 54.8)	50.5 (46.0 to 52.2)	47.1 (42.1 to 49.4)	-6.0 (-7.7 to -4.6)	-6.8 (-9.0 to -4.9)	53.6 (49.6 to 54.7)	49.7 (45.2 to 51.5)	45.8 (40.9 to 48.4)	-7.3 (-9.3 to -5.8)	-7.9 (-10.7 to -5.6)
Diet low in vegetables	53.0 (52.6 to 53.6)	46.8 (45.2 to 48.4)	39.4 (35.6 to 42.7)	-11.7 (-14.4 to -9.3)	-15.9 (-21.4 to -11.5)	53.4 (53.1 to 54.0)	47.8 (46.4 to 49.2)	40.9 (37.8 to 43.8)	-10.6 (-13.1 to -8.5)	-14.4 (-19.0 to -10.4)
Diet low in legumes	51.4 (50.7 to 52.0)	54.1 (53.9 to 54.2)	53.6 (53.3 to 53.9)	5.2 (4.3 to 6.4)	-0.8 (-1.2 to -0.4)	52.3 (51.8 to 52.7)	54.5 (54.3 to 54.6)	54.1 (53.9 to 54.3)	4.1 (3.4 to 5.1)	-0.6 (-0.9 to -0.3)
Diet low in whole grains	52.7 (52.3 to 53.0)	52.7 (52.3 to 53.0)	52.3 (51.8 to 52.6)	-0.0 (-0.5 to 0.4)	-0.8 (-1.3 to -0.5)	52.7 (52.2 to 53.1)	52.7 (52.3 to 53.0)	52.4 (52.0 to 52.7)	0.1 (-0.4 to 0.6)	-0.6 (-1.0 to -0.3)
Diet low in nuts and seeds	52.9 (52.4 to 53.3)	51.4 (50.3 to 52.1)	47.1 (44.6 to 48.9)	-2.9 (-4.3 to -1.9)	-8.4 (-11.7 to -6.0)	53.0 (52.4 to 53.4)	51.6 (50.6 to 52.3)	47.5 (45.3 to 49.2)	-2.7 (-3.8 to -1.7)	-7.9 (-10.9 to -5.6)
Diet low in milk	44.1 (41.9 to 46.0)	40.2 (37.1 to 42.9)	37.7 (34.0 to 40.9)	-8.8 (-11.9 to -6.3)	-6.3 (-9.7 to -3.8)	44.7 (42.5 to 46.5)	41.0 (38.0 to 43.4)	38.5 (34.8 to 41.4)	-8.2 (-11.2 to -5.9)	-6.2 (-9.3 to -3.8)
Diet high in red meat	53.2 (52.8 to 54.2)	43.1 (38.2 to 47.2)	46.5 (39.6 to 51.2)	-19.1 (-26.8 to -11.8)	8.1 (-2.9 to 19.5)	37.3 (32.0 to 41.9)	21.9 (16.0 to 27.3)	27.2 (18.8 to 34.7)	-41.4 (-54.7 to -26.5)	24.2 (-2.4 to 53.6)
Diet high in processed meat	1.2 (0.2 to 2.9)	1.1 (0.1 to 2.9)	2.3 (0.6 to 4.5)	-10.1 (-58.9 to 12.9)	110.9 (49.2 to 584.2)	0.8 (0.1 to 2.2)	0.7 (0.0 to 2.2)	1.5 (0.2 to 3.4)	-13.0 (-70.4 to 8.3)	119.7 (54.8 to 967.5)
Diet high in sugar-sweetened beverages	2.8 (1.3 to 5.1)	3.6 (1.7 to 6.5)	6.5 (3.0 to 11.8)	27.1 (-31.6 to 135.1)	81.5 (19.7 to 182.2)	2.1 (0.9 to 4.0)	2.6 (1.1 to 4.5)	4.4 (2.0 to 8.0)	22.3 (-28.9 to 118.6)	72.5 (10.6 to 163.2)
Diet low in fibre	49.4 (48.0 to 50.5)	45.6 (43.5 to 47.2)	41.8 (38.7 to 44.3)	-7.7 (-10.2 to -5.7)	-8.3 (-12.0 to -5.6)	51.8 (50.9 to 52.5)	48.6 (47.1 to 49.8)	45.4 (42.9 to 47.3)	-6.1 (-8.1 to -4.4)	-6.6 (-9.4 to -4.4)
Diet low in calcium	40.2 (37.2 to 42.7)	32.9 (28.5 to 36.5)	28.0 (22.4 to 32.5)	-18.1 (-23.7 to -14.2)	-15.1 (-22.1 to -10.6)	42.3 (39.7 to 44.4)	35.7 (31.7 to 38.7)	31.1 (26.1 to 35.0)	-15.7 (-20.5 to -12.4)	-12.8 (-18.1 to -9.2)
Diet low in seafood omega-3 fatty acids	44.8 (42.8 to 46.6)	47.5 (46.0 to 48.9)	45.0 (42.9 to 46.9)	5.9 (4.5 to 7.6)	-5.1 (-6.6 to -3.8)	46.2 (44.5 to 47.9)	48.5 (47.3 to 49.8)	46.4 (44.7 to 48.1)	4.9 (3.9 to 6.4)	-4.3 (-5.6 to -3.1)
Diet low in polyunsaturated fatty acids	52.8 (50.5 to 53.5)	38.8 (35.6 to 41.6)	37.6 (34.0 to 40.5)	-26.6 (-32.2 to -21.2)	-3.1 (-7.2 to 0.9)	52.9 (50.6 to 53.6)	39.3 (36.3 to 41.9)	38.0 (34.6 to 40.9)	-25.7 (-31.2 to -20.5)	-3.3 (-6.9 to -0.1)
Diet high in trans fatty acids	1.5 (0.1 to 4.0)	1.3 (0.1 to 3.6)	1.1 (0.0 to 3.3)	-18.0 (-51.1 to -4.0)	-15.0 (-50.0 to -3.6)	2.1 (0.3 to 5.0)	1.7 (0.2 to 4.4)	1.5 (0.1 to 4.0)	-19.0 (-49.8 to -4.6)	-14.9 (-45.1 to -1.8)
Diet high in sodium	13.1 (5.5 to 20.0)	8.4 (1.2 to 15.7)	9.3 (1.9 to 17.0)	-36.4 (-80.4 to -13.2)	11.5 (-9.1 to 75.2)	12.5 (5.2 to 19.5)	7.0 (0.9 to 14.2)	5.9 (0.4 to 12.9)	-44.4 (-86.0 to -23.5)	-15.8 (-56.6 to -2.1)
Intimate partner violence	-	-	-	-	-	5.0 (3.9 to 6.2)	4.5 (3.4 to 5.5)	4.2 (3.2 to 5.2)	-11.4 (-16.1 to -6.0)	-5.6 (-10.5 to 0.0)
Childhood sexual abuse	1.6 (1.3 to 2.0)	1.6 (1.3 to 2.0)	1.6 (1.3 to 2.0)	0.0 (0.0 to 0.0)	0.0 (0.0 to 0.0)	2.2 (1.7 to 2.7)	2.2 (1.7 to 2.7)	2.2 (1.7 to 2.7)	0.0 (0.0 to 0.0)	0.0 (0.0 to 0.0)

Supplementary Results Table 1. Mongolia age-standardised Summary Exposure Values (SEVs) for Level 4 risk factors and percentage change, by sex, 1990, 2007, and 2017.Data in parentheses are 95% uncertainty intervals.										
	**Male**					**Female**				
	**1990**	**2007**	**2017**	**Percentage change 1990-2007**	**Percentage change 2007-2017**	**1990**	**2007**	**2017**	**Percentage change 1990-2007**	**Percentage change 2007-2017**
Bullying victimization	5.2 (2.1 to 10.6)	5.7 (2.5 to 11.0)	6.0 (2.6 to 11.5)	10.4 (-6.3 to 31.8)	4.4 (-6.1 to 13.5)	3.9 (1.5 to 8.2)	4.2 (1.7 to 8.2)	4.2 (1.8 to 8.1)	7.6 (-9.0 to 26.9)	0.8 (-10.0 to 9.7)
Low physical activity	0.3 (0.2 to 0.6)	0.3 (0.2 to 0.6)	0.3 (0.2 to 0.6)	1.0 (-0.8 to 3.0)	1.2 (-0.6 to 3.3)	0.3 (0.1 to 0.5)	0.3 (0.1 to 0.5)	0.3 (0.1 to 0.5)	1.0 (-0.7 to 2.8)	0.8 (-0.9 to 2.6)
High fasting plasma glucose	3.0 (2.0 to 4.6)	3.4 (2.4 to 5.0)	3.5 (2.4 to 5.2)	14.5 (-8.1 to 53.1)	2.0 (-20.6 to 26.1)	2.0 (1.2 to 3.2)	2.1 (1.4 to 3.2)	2.3 (1.4 to 3.5)	7.4 (-19.4 to 51.2)	9.4 (-18.4 to 44.6)
High LDL cholesterol	11.6 (10.0 to 13.2)	12.1 (10.5 to 13.8)	12.2 (10.7 to 13.9)	4.4 (-0.2 to 9.1)	1.2 (-2.2 to 4.7)	11.8 (10.3 to 13.4)	12.6 (11.2 to 14.3)	13.0 (11.4 to 14.7)	7.5 (3.1 to 12.3)	2.7 (-0.6 to 6.0)
High systolic blood pressure	9.3 (8.4 to 10.3)	10.2 (9.3 to 11.2)	10.3 (9.3 to 11.3)	9.3 (2.2 to 17.0)	0.9 (-4.4 to 5.9)	8.2 (7.3 to 9.1)	8.6 (7.7 to 9.4)	8.5 (7.5 to 9.4)	4.8 (-4.1 to 15.0)	-1.2 (-7.8 to 5.8)
High body-mass index	7.7 (5.4 to 11.7)	10.0 (7.3 to 14.4)	12.6 (9.4 to 17.3)	29.1 (12.2 to 54.0)	25.9 (13.5 to 41.3)	9.7 (6.7 to 13.9)	12.1 (8.7 to 16.8)	14.4 (10.5 to 19.9)	24.5 (7.4 to 47.1)	19.0 (7.2 to 33.5)
Low bone mineral density	4.1 (3.3 to 5.0)	4.0 (3.3 to 4.7)	4.0 (3.2 to 4.8)	-2.8 (-15.4 to 12.2)	-1.1 (-14.3 to 14.6)	5.7 (4.7 to 6.9)	5.6 (4.6 to 6.5)	5.6 (4.6 to 6.6)	-2.2 (-14.6 to 12.5)	-0.2 (-12.7 to 13.0)
Impaired kidney function	2.7 (1.3 to 6.7)	2.8 (1.3 to 6.6)	2.7 (1.2 to 6.5)	2.3 (-2.1 to 8.8)	-3.7 (-8.5 to -0.4)	3.3 (1.7 to 7.4)	3.3 (1.6 to 7.3)	3.2 (1.6 to 7.3)	-1.7 (-7.4 to 3.4)	-1.6 (-5.7 to 1.9)

Supplementary Results Table 1. Tajikistan age-standardised Summary Exposure Values (SEVs) for Level 4 risk factors and percentage change, by sex, 1990, 2007, and 2017.Data in parentheses are 95% uncertainty intervals.										
	Male					Female				
	**1990**	**2007**	**2017**	**Percentage change 1990-2007**	**Percentage change 2007-2017**	**1990**	**2007**	**2017**	**Percentage change 1990-2007**	**Percentage change 2007-2017**
Unsafe water source	26.1 (16.2 to 32.6)	27.1 (17.3 to 32.4)	23.6 (14.7 to 29.2)	3.9 (-10.0 to 21.6)	-13.0 (-24.9 to -1.3)	26.1 (16.2 to 32.6)	27.1 (17.3 to 32.4)	23.6 (14.7 to 29.2)	3.9 (-10.0 to 21.6)	-13.0 (-24.9 to -1.3)
Unsafe sanitation	65.3 (45.0 to 94.6)	61.5 (41.5 to 91.6)	63.0 (42.1 to 96.6)	-5.8 (-14.8 to 4.4)	2.5 (-5.8 to 10.0)	65.3 (45.0 to 94.6)	61.5 (41.5 to 91.6)	63.0 (42.1 to 96.6)	-5.8 (-14.8 to 4.4)	2.5 (-5.8 to 10.0)
No access to handwashing facility	25.9 (21.6 to 30.6)	30.5 (26.3 to 34.8)	25.0 (21.7 to 28.9)	17.8 (-1.1 to 41.7)	-17.9 (-28.3 to -6.5)	25.9 (21.6 to 30.6)	30.5 (26.3 to 34.8)	25.0 (21.7 to 28.9)	17.8 (-1.1 to 41.7)	-17.9 (-28.3 to -6.5)
Ambient particulate matter pollution	32.0 (11.3 to 59.7)	38.5 (19.2 to 64.2)	41.2 (15.3 to 76.7)	20.4 (-48.6 to 239.6)	7.0 (-58.5 to 130.4)	26.9 (9.3 to 52.7)	33.1 (15.4 to 56.4)	36.3 (12.4 to 71.0)	23.0 (-51.5 to 265.3)	9.5 (-60.4 to 152.8)
Household air pollution from solid fuels	51.2 (32.6 to 70.6)	35.8 (23.0 to 51.1)	27.7 (14.7 to 42.7)	-30.1 (-54.5 to 10.0)	-22.5 (-56.3 to 23.1)	53.4 (35.9 to 71.7)	38.2 (25.7 to 52.4)	30.1 (17.4 to 43.7)	-28.4 (-51.0 to 5.0)	-21.3 (-52.1 to 16.0)
Ambient ozone pollution	49.3 (20.4 to 54.8)	49.3 (20.4 to 54.8)	49.6 (21.1 to 54.8)	-0.1 (-2.8 to 2.5)	0.7 (-0.7 to 5.3)	49.3 (20.4 to 54.8)	49.3 (20.4 to 54.8)	49.6 (21.1 to 54.8)	-0.1 (-2.8 to 2.5)	0.7 (-0.7 to 5.3)
Residential radon	34.9 (6.2 to 71.2)	34.9 (6.2 to 71.2)	34.9 (6.2 to 71.2)	0.0 (0.0 to 0.0)	0.0 (0.0 to 0.0)	34.9 (6.2 to 71.2)	34.9 (6.2 to 71.2)	34.9 (6.2 to 71.2)	0.0 (0.0 to 0.0)	0.0 (0.0 to 0.0)
Lead exposure	14.6 (9.9 to 19.2)	13.5 (9.0 to 17.6)	11.5 (7.5 to 15.5)	-7.8 (-13.8 to -2.5)	-14.5 (-19.8 to -10.5)	7.2 (3.2 to 11.4)	6.9 (3.2 to 10.9)	6.1 (2.7 to 10.0)	-4.6 (-11.3 to 5.8)	-11.8 (-18.2 to -7.5)
Occupational exposure to asbestos	0.3 (0.2 to 0.5)	0.5 (0.4 to 0.7)	0.5 (0.4 to 0.6)	63.1 (7.7 to 162.8)	-0.6 (-22.1 to 30.8)	0.6 (0.4 to 0.9)	0.7 (0.5 to 0.8)	0.6 (0.5 to 0.7)	12.3 (-23.4 to 74.2)	-10.6 (-31.5 to 17.9)
Occupational exposure to arsenic	0.2 (0.1 to 0.3)	0.2 (0.1 to 0.3)	0.2 (0.1 to 0.3)	-11.1 (-20.3 to -1.1)	13.2 (4.7 to 22.6)	0.2 (0.1 to 0.3)	0.2 (0.1 to 0.3)	0.2 (0.1 to 0.3)	-10.2 (-20.8 to 2.1)	3.7 (-5.2 to 13.9)
Occupational exposure to benzene	0.5 (0.3 to 0.8)	0.5 (0.3 to 0.8)	0.5 (0.3 to 0.9)	1.2 (-8.5 to 13.1)	14.3 (6.1 to 24.9)	0.5 (0.3 to 0.8)	0.5 (0.3 to 0.9)	0.6 (0.4 to 1.1)	8.0 (-5.4 to 25.4)	14.4 (3.3 to 28.4)
Occupational exposure to beryllium	0.1 (0.1 to 0.1)	0.1 (0.1 to 0.1)	0.1 (0.1 to 0.1)	-8.7 (-16.2 to -0.7)	10.7 (4.4 to 17.5)	0.1 (0.1 to 0.1)	0.1 (0.1 to 0.1)	0.1 (0.1 to 0.1)	-5.9 (-14.9 to 4.7)	2.5 (-4.8 to 10.5)
Occupational exposure to cadmium	0.1 (0.1 to 0.1)	0.1 (0.1 to 0.1)	0.1 (0.1 to 0.1)	-10.5 (-19.2 to -1.4)	13.2 (5.2 to 21.9)	0.1 (0.1 to 0.1)	0.1 (0.1 to 0.1)	0.1 (0.1 to 0.1)	-9.3 (-19.4 to 2.2)	4.0 (-4.6 to 13.6)
Occupational exposure to chromium	0.2 (0.2 to 0.2)	0.2 (0.2 to 0.2)	0.2 (0.2 to 0.3)	-7.8 (-16.7 to 1.9)	15.5 (7.3 to 24.1)	0.2 (0.2 to 0.2)	0.2 (0.2 to 0.2)	0.2 (0.2 to 0.2)	-7.1 (-17.8 to 5.3)	7.7 (-1.6 to 18.0)
Occupational exposure to diesel engine exhaust	1.8 (1.6 to 2.0)	1.7 (1.6 to 1.9)	2.0 (1.8 to 2.2)	-3.8 (-13.4 to 7.1)	16.2 (7.0 to 25.6)	1.1 (0.9 to 1.2)	1.0 (0.9 to 1.1)	1.0 (0.9 to 1.1)	-7.7 (-18.0 to 4.3)	5.5 (-3.8 to 14.6)
Occupational exposure to formaldehyde	0.4 (0.4 to 0.5)	0.4 (0.4 to 0.4)	0.5 (0.4 to 0.5)	-7.7 (-17.6 to 3.4)	15.3 (6.1 to 25.0)	0.4 (0.4 to 0.5)	0.4 (0.4 to 0.4)	0.4 (0.4 to 0.5)	-5.6 (-17.5 to 8.3)	9.7 (-0.8 to 20.8)
Occupational exposure to nickel	0.2 (0.1 to 0.6)	0.2 (0.1 to 0.6)	0.3 (0.1 to 0.7)	-8.9 (-17.9 to 1.9)	11.7 (3.3 to 21.6)	0.2 (0.1 to 0.6)	0.2 (0.1 to 0.5)	0.2 (0.1 to 0.5)	-12.0 (-21.3 to 0.4)	0.3 (-8.3 to 10.3)
Occupational exposure to polycyclic aromatic hydrocarbons	0.5 (0.5 to 0.6)	0.5 (0.4 to 0.5)	0.5 (0.5 to 0.6)	-5.8 (-14.7 to 4.3)	15.0 (7.1 to 23.1)	0.5 (0.4 to 0.5)	0.4 (0.4 to 0.5)	0.5 (0.4 to 0.5)	-5.1 (-15.6 to 7.1)	8.2 (-1.2 to 18.3)
Occupational exposure to silica	3.4 (1.8 to 7.7)	3.3 (1.7 to 7.6)	3.7 (1.9 to 8.4)	-4.3 (-16.2 to 10.5)	12.5 (1.6 to 26.1)	1.9 (1.0 to 4.2)	1.7 (0.9 to 3.7)	1.6 (0.8 to 3.6)	-12.9 (-22.5 to -1.4)	-3.2 (-11.5 to 6.0)
Occupational exposure to sulphuric acid	0.5 (0.4 to 0.9)	0.4 (0.3 to 0.8)	0.5 (0.4 to 0.9)	-11.9 (-20.4 to -2.2)	12.8 (4.1 to 21.6)	0.5 (0.4 to 0.9)	0.4 (0.3 to 0.8)	0.5 (0.3 to 0.8)	-11.3 (-20.6 to -0.6)	0.9 (-7.6 to 9.7)
Occupational exposure to trichloroethylene	0.1 (0.1 to 0.1)	0.1 (0.1 to 0.1)	0.1 (0.1 to 0.2)	-7.9 (-16.3 to 1.5)	15.8 (8.5 to 23.7)	0.1 (0.1 to 0.1)	0.1 (0.1 to 0.1)	0.1 (0.1 to 0.1)	-7.0 (-16.8 to 5.3)	7.1 (-1.8 to 16.7)
Occupational asthmagens	9.8 (7.5 to 12.8)	8.6 (6.8 to 11.0)	9.7 (7.6 to 12.3)	-12.2 (-26.5 to 4.8)	12.0 (-1.4 to 28.4)	9.0 (6.3 to 12.3)	8.5 (6.2 to 11.3)	8.6 (6.2 to 11.5)	-5.1 (-20.3 to 15.0)	1.1 (-14.3 to 18.5)
Occupational particulate matter, gases, and fumes	5.8 (4.3 to 8.1)	5.4 (4.1 to 7.4)	5.3 (4.1 to 7.2)	-6.7 (-11.4 to -1.1)	-2.5 (-6.5 to 2.1)	4.0 (2.9 to 5.5)	4.0 (2.9 to 5.5)	3.9 (2.9 to 5.4)	0.0 (-6.6 to 8.0)	-2.2 (-7.5 to 3.2)
Occupational noise	6.5 (5.7 to 7.2)	6.2 (5.6 to 6.8)	6.1 (5.6 to 6.7)	-4.4 (-8.7 to 0.5)	-0.7 (-4.2 to 3.1)	4.4 (3.8 to 5.1)	4.6 (4.1 to 5.1)	4.6 (4.2 to 5.1)	2.9 (-3.5 to 10.8)	0.8 (-3.9 to 6.0)
Occupational ergonomic factors	13.2 (11.1 to 15.6)	11.0 (9.4 to 12.7)	11.7 (9.6 to 13.7)	-16.5 (-29.8 to -0.6)	5.7 (-7.9 to 22.3)	14.7 (12.3 to 17.1)	13.9 (12.3 to 15.5)	13.7 (11.5 to 16.0)	-5.5 (-19.6 to 13.0)	-1.4 (-15.7 to 13.3)
Non-exclusive breastfeeding	0.4 (0.3 to 0.7)	0.4 (0.2 to 0.6)	0.4 (0.2 to 0.6)	-7.7 (-14.6 to 0.2)	-1.4 (-8.8 to 7.7)	0.4 (0.3 to 0.7)	0.4 (0.2 to 0.6)	0.4 (0.2 to 0.6)	-7.7 (-14.6 to 0.2)	-1.4 (-8.8 to 7.8)
Discontinued breastfeeding	1.0 (0.9 to 1.1)	1.0 (1.0 to 1.1)	1.1 (1.0 to 1.2)	1.4 (-8.2 to 13.5)	8.3 (-1.2 to 18.6)	1.0 (0.9 to 1.1)	1.0 (1.0 to 1.1)	1.1 (1.0 to 1.2)	2.0 (-7.6 to 14.3)	8.4 (-1.1 to 18.7)
Child underweight	0.8 (0.6 to 1.0)	0.7 (0.5 to 0.9)	0.6 (0.4 to 0.7)	-15.3 (-29.5 to 0.5)	-18.2 (-30.8 to -5.9)	0.9 (0.6 to 1.1)	0.7 (0.5 to 0.9)	0.6 (0.4 to 0.8)	-18.4 (-33.3 to -2.4)	-11.6 (-25.9 to 2.7)
Child wasting	0.4 (0.3 to 0.6)	0.5 (0.4 to 0.6)	0.6 (0.4 to 0.7)	14.4 (-9.4 to 45.1)	14.8 (-2.2 to 35.3)	0.4 (0.3 to 0.5)	0.5 (0.4 to 0.6)	0.6 (0.5 to 0.7)	18.4 (-5.4 to 55.3)	18.6 (-0.4 to 38.9)
Child stunting	2.6 (1.8 to 3.6)	2.3 (1.6 to 2.8)	1.7 (1.1 to 2.3)	-12.4 (-35.5 to 14.1)	-24.5 (-43.1 to -5.4)	2.3 (1.5 to 3.2)	2.1 (1.5 to 2.7)	1.7 (1.1 to 2.5)	-8.7 (-31.5 to 23.8)	-20.0 (-40.3 to 10.8)
Short gestation for birth weight	0.0 (0.0 to 0.0)	0.0 (0.0 to 0.0)	0.0 (0.0 to 0.0)	-6.3 (-29.2 to 34.0)	0.6 (-29.9 to 37.3)	0.0 (0.0 to 0.0)	0.0 (0.0 to 0.0)	0.0 (0.0 to 0.0)	0.5 (-25.5 to 37.7)	-1.9 (-26.0 to 40.0)
Low birth weight for gestation	0.0 (0.0 to 0.0)	0.0 (0.0 to 0.0)	0.0 (0.0 to 0.0)	2.6 (-14.8 to 22.9)	0.7 (-15.1 to 25.6)	0.0 (0.0 to 0.0)	0.0 (0.0 to 0.0)	0.0 (0.0 to 0.0)	2.7 (-16.7 to 32.3)	2.3 (-20.5 to 20.0)
Iron deficiency	-	-	-	-	-	13.4 (10.3 to 16.8)	12.0 (9.4 to 15.0)	10.4 (7.8 to 13.5)	-10.1 (-22.7 to 4.6)	-13.6 (-23.6 to -2.3)
Vitamin A deficiency	2.2 (1.8 to 2.6)	2.3 (2.1 to 2.6)	2.0 (1.7 to 2.4)	6.9 (-5.1 to 20.9)	-14.3 (-25.0 to -3.4)	2.2 (1.9 to 2.6)	2.3 (2.0 to 2.6)	2.1 (1.8 to 2.5)	2.1 (-8.4 to 13.6)	-7.4 (-16.1 to 2.2)
Zinc deficiency	0.7 (0.2 to 1.8)	1.0 (0.2 to 3.0)	0.8 (0.2 to 1.9)	38.2 (-23.9 to 256.8)	-26.2 (-64.1 to 27.1)	0.7 (0.2 to 1.8)	1.0 (0.2 to 3.2)	0.7 (0.2 to 1.9)	43.8 (-21.8 to 270.1)	-27.6 (-64.2 to 30.6)
Smoking	14.8 (13.1 to 16.7)	9.9 (8.6 to 11.2)	8.0 (6.7 to 9.3)	-33.2 (-39.8 to -25.7)	-19.0 (-26.8 to -10.6)	0.6 (0.4 to 0.8)	0.5 (0.4 to 0.6)	0.5 (0.4 to 0.7)	-17.4 (-34.9 to 4.4)	5.4 (-11.2 to 26.0)
Chewing tobacco	0.2 (0.1 to 0.2)	0.2 (0.1 to 0.2)	0.2 (0.1 to 0.3)	2.0 (-27.1 to 43.6)	20.0 (-5.7 to 51.5)	0.1 (0.1 to 0.1)	0.1 (0.1 to 0.1)	0.1 (0.1 to 0.1)	1.3 (-28.6 to 39.0)	0.0 (-22.9 to 28.4)
Second-hand smoke	27.0 (24.5 to 29.6)	19.2 (18.4 to 20.1)	19.0 (17.6 to 20.5)	-28.9 (-35.9 to -20.9)	-0.9 (-8.4 to 6.1)	36.0 (33.5 to 38.5)	22.3 (21.4 to 23.2)	22.1 (20.7 to 23.6)	-38.1 (-43.0 to -32.8)	-0.9 (-6.6 to 5.1)
Alcohol use	10.3 (5.6 to 17.9)	8.6 (4.4 to 15.6)	8.1 (4.0 to 15.1)	-16.4 (-41.1 to 6.2)	-5.8 (-28.9 to 16.9)	3.3 (1.5 to 6.6)	2.7 (1.2 to 5.4)	2.5 (1.1 to 5.0)	-17.2 (-41.3 to 14.9)	-6.6 (-31.0 to 23.4)
Drug use	0.4 (0.3 to 0.5)	0.4 (0.3 to 0.5)	0.4 (0.3 to 0.5)	0.3 (-5.0 to 6.5)	10.4 (4.0 to 17.2)	0.2 (0.1 to 0.2)	0.2 (0.1 to 0.2)	0.2 (0.1 to 0.2)	1.2 (-4.5 to 7.3)	4.7 (-0.8 to 11.0)
Diet low in fruits	47.2 (42.4 to 49.4)	49.8 (45.3 to 51.5)	48.1 (43.5 to 50.2)	5.4 (3.5 to 7.8)	-3.3 (-4.9 to -2.0)	46.1 (41.3 to 48.4)	49.0 (44.4 to 50.8)	47.1 (42.3 to 49.4)	6.4 (4.2 to 9.4)	-3.8 (-5.7 to -2.3)
Diet low in vegetables	22.4 (15.5 to 28.0)	20.6 (13.8 to 26.3)	9.4 (2.6 to 16.2)	-7.8 (-21.0 to 3.5)	-54.6 (-81.4 to -35.2)	24.8 (18.5 to 30.2)	23.3 (16.6 to 28.8)	12.3 (4.7 to 19.7)	-6.2 (-17.2 to 6.0)	-47.2 (-73.0 to -28.8)
Diet low in legumes	54.7 (54.6 to 54.8)	50.6 (49.8 to 51.3)	47.7 (46.1 to 48.9)	-7.5 (-9.0 to -6.2)	-5.8 (-7.5 to -4.3)	54.8 (54.7 to 54.8)	51.6 (51.0 to 52.2)	49.3 (48.1 to 50.4)	-5.7 (-6.9 to -4.7)	-4.5 (-6.0 to -3.3)
Diet low in whole grains	52.5 (52.1 to 52.8)	52.7 (52.3 to 53.0)	52.7 (52.3 to 53.1)	0.4 (-0.0 to 0.9)	0.0 (-0.5 to 0.5)	52.4 (52.0 to 52.8)	52.5 (52.1 to 52.9)	52.7 (52.3 to 53.0)	0.2 (-0.3 to 0.8)	0.3 (-0.3 to 0.9)
Diet low in nuts and seeds	49.7 (48.4 to 50.9)	52.3 (51.6 to 52.8)	51.5 (50.5 to 52.2)	5.1 (3.4 to 7.3)	-1.5 (-2.5 to -0.8)	50.0 (48.7 to 51.0)	52.4 (51.8 to 52.9)	51.7 (50.8 to 52.4)	4.8 (3.1 to 6.8)	-1.4 (-2.4 to -0.8)
Diet low in milk	46.9 (45.2 to 48.4)	49.3 (48.1 to 50.3)	48.5 (47.1 to 49.7)	5.0 (3.5 to 6.7)	-1.5 (-2.4 to -0.8)	47.3 (45.7 to 48.7)	49.6 (48.4 to 50.5)	48.8 (47.5 to 50.0)	4.8 (3.5 to 6.5)	-1.5 (-2.3 to -0.7)
Diet high in red meat	0.0 (0.0 to 0.2)	0.0 (0.0 to 0.0)	0.0 (0.0 to 0.0)	-100.0 (-100.0 to 114.6)	2249576.3 (-61.7 to [illegible]	0.0 (0.0 to 0.0)	0.0 (0.0 to 0.0)	0.0 (0.0 to 0.0)	-100.0 (-100.0 to 311.8)	67295666.9 (-76.2 to 366.1)
Diet high in processed meat	0.9 (0.0 to 2.8)	0.3 (0.0 to 1.6)	0.5 (0.0 to 2.2)	-65.3 (-100.0 to -41.3)	74.9 (32.2 to [illegible]	0.5 (0.0 to 2.1)	0.2 (0.0 to 1.2)	0.3 (0.0 to 1.6)	-65.1 (-100.0 to -42.4)	73.3 (-34.7 to [illegible]
Diet high in sugar-sweetened beverages	2.9 (1.3 to 5.3)	1.3 (0.5 to 2.4)	1.8 (0.7 to 3.5)	-57.2 (-77.7 to -20.0)	45.3 (-3.9 to 131.5)	2.2 (1.0 to 4.2)	1.0 (0.4 to 1.9)	1.4 (0.6 to 2.7)	-54.5 (-76.2 to -15.8)	42.1 (-9.2 to 132.9)
Diet low in fibre	41.1 (38.0 to 43.7)	38.8 (35.3 to 41.6)	35.6 (30.9 to 39.2)	-5.5 (-8.9 to -2.8)	-8.3 (-13.4 to -4.3)	44.7 (42.3 to 46.6)	42.9 (40.2 to 45.1)	40.2 (36.6 to 43.1)	-4.0 (-6.7 to -1.5)	-6.4 (-9.8 to -3.3)
Diet low in calcium	42.7 (40.2 to 44.8)	45.6 (43.6 to 47.1)	43.1 (40.7 to 45.1)	6.7 (4.9 to 9.2)	-5.4 (-7.2 to -4.1)	44.5 (42.4 to 46.3)	47.2 (45.6 to 48.4)	44.9 (42.9 to 46.6)	5.9 (4.3 to 7.9)	-4.7 (-6.3 to -3.5)
Diet low in seafood omega-3 fatty acids	50.5 (49.7 to 51.4)	50.9 (50.1 to 51.7)	47.6 (46.2 to 49.1)	0.7 (0.4 to 1.0)	-6.3 (-7.9 to -4.9)	51.1 (50.4 to 51.9)	51.4 (50.7 to 52.1)	48.7 (47.4 to 49.9)	0.6 (0.3 to 0.9)	-5.3 (-6.6 to -4.2)
Diet low in polyunsaturated fatty acids	48.6 (46.8 to 49.9)	41.4 (38.8 to 43.6)	41.0 (38.2 to 43.4)	-14.9 (-18.8 to -11.0)	-1.0 (-3.7 to 1.8)	48.9 (47.3 to 50.1)	41.8 (39.4 to 44.1)	41.5 (38.7 to 43.8)	-14.4 (-18.0 to -10.9)	-0.9 (-3.4 to 1.5)
Diet high in trans fatty acids	1.5 (0.1 to 4.1)	1.3 (0.1 to 3.6)	1.1 (0.0 to 3.2)	-18.4 (-51.3 to -5.1)	-14.9 (-49.1 to -2.7)	2.1 (0.3 to 5.0)	1.7 (0.2 to 4.3)	1.5 (0.1 to 3.9)	-18.2 (-51.2 to -3.6)	-14.7 (-45.5 to -1.2)
Diet high in sodium	13.5 (6.0 to 20.8)	6.3 (0.6 to 13.4)	6.6 (0.6 to 14.0)	-53.2 (-92.2 to -29.0)	5.0 (-23.6 to 65.7)	12.7 (5.3 to 20.0)	6.3 (0.7 to 13.2)	5.2 (0.3 to 11.9)	-50.7 (-89.3 to -28.3)	-17.5 (-62.9 to -3.3)
Intimate partner violence	-	-	-	-	-	6.5 (5.5 to 7.5)	5.9 (5.0 to 6.7)	5.6 (4.9 to 6.4)	-9.1 (-13.7 to -3.3)	-4.8 (-10.6 to 1.2)
Childhood sexual abuse	1.6 (1.3 to 2.0)	1.6 (1.3 to 2.0)	1.6 (1.3 to 2.0)	0.0 (0.0 to 0.0)	0.0 (0.0 to 0.0)	2.2 (1.7 to 2.7)	2.2 (1.7 to 2.7)	2.2 (1.7 to 2.7)	0.0 (0.0 to 0.0)	0.0 (0.0 to 0.0)

Supplementary Results Table 1. Tajikistan age-standardised Summary Exposure Values (SEVs) for Level 4 risk factors and percentage change, by sex, 1990, 2007, and 2017.Data in parentheses are 95% uncertainty intervals.

	Male					Female				
	**1990**	**2007**	**2017**	**Percentage change 1990-2007**	**Percentage change 2007-2017**	**1990**	**2007**	**2017**	**Percentage change 1990-2007**	**Percentage change 2007-2017**
Bullying victimization	1.2 (0.5 to 2.6)	1.3 (0.6 to 2.7)	1.3 (0.5 to 2.7)	8.4 (-9.7 to 35.1)	-1.1 (-7.9 to 6.0)	0.8 (0.3 to 1.7)	0.9 (0.4 to 1.8)	0.9 (0.4 to 1.9)	7.9 (-22.0 to 50.3)	1.6 (-4.3 to 9.0)
Low physical activity	0.3 (0.2 to 0.6)	0.3 (0.2 to 0.6)	0.3 (0.2 to 0.6)	0.7 (-1.4 to 3.0)	1.1 (-0.9 to 3.2)	0.3 (0.1 to 0.5)	0.3 (0.1 to 0.5)	0.3 (0.1 to 0.5)	0.5 (-1.5 to 2.6)	0.7 (-1.3 to 2.7)
High fasting plasma glucose	4.3 (3.0 to 6.6)	4.6 (3.3 to 7.3)	5.2 (3.6 to 8.1)	7.7 (-11.3 to 39.3)	11.8 (-9.0 to 37.1)	3.2 (2.1 to 4.8)	3.7 (2.6 to 5.5)	4.1 (2.9 to 6.1)	16.7 (-8.6 to 59.0)	12.7 (-7.0 to 42.9)
High LDL cholesterol	9.0 (7.6 to 10.6)	9.0 (7.6 to 10.7)	9.1 (7.7 to 10.7)	0.3 (-4.3 to 4.8)	1.0 (-2.3 to 4.3)	9.3 (8.0 to 10.9)	9.4 (8.0 to 10.9)	9.5 (8.2 to 11.0)	0.5 (-3.5 to 4.9)	1.5 (-1.8 to 5.0)
High systolic blood pressure	7.8 (6.9 to 8.7)	8.0 (7.1 to 9.0)	8.4 (7.5 to 9.4)	3.2 (-4.5 to 11.6)	4.8 (-1.4 to 11.2)	7.2 (6.3 to 8.1)	7.5 (6.7 to 8.4)	7.8 (6.9 to 8.6)	4.4 (-5.9 to 16.4)	3.3 (-4.3 to 12.2)
High body-mass index	5.5 (3.6 to 8.4)	6.2 (4.4 to 9.3)	7.3 (5.1 to 10.7)	14.4 (-1.0 to 37.1)	17.1 (4.5 to 32.3)	6.8 (4.6 to 9.7)	7.4 (5.2 to 10.6)	8.9 (6.4 to 12.4)	9.1 (-7.8 to 29.4)	19.7 (5.9 to 38.1)
Low bone mineral density	4.3 (3.5 to 5.2)	4.1 (3.4 to 4.9)	4.0 (3.3 to 4.9)	-5.0 (-16.8 to 10.2)	-1.0 (-15.9 to 14.7)	5.9 (4.9 to 7.1)	5.7 (4.9 to 6.7)	5.7 (4.7 to 6.8)	-3.4 (-15.3 to 12.2)	-0.3 (-13.1 to 14.9)
Impaired kidney function	2.7 (1.2 to 6.6)	2.7 (1.3 to 6.6)	2.7 (1.3 to 6.7)	1.8 (-1.6 to 6.7)	-0.1 (-4.1 to 4.0)	3.2 (1.6 to 7.3)	3.3 (1.6 to 7.4)	3.3 (1.6 to 7.4)	2.0 (-1.3 to 5.9)	1.7 (-2.0 to 6.3)

Supplementary Results Table 1. Turkmenistan age-standardised Summary Exposure Values (SEVs) for Level 4 risk factors and percentage change, by sex, 1990, 2007, and 2017.Data in parentheses are 95% uncertainty intervals.										
	Male					Female				
	1990	2007	2017	Percentage change 1990-2007	Percentage change 2007-2017	1990	2007	2017	Percentage change 1990-2007	Percentage change 2007-2017
Unsafe water source	26.8 (20.3 to 32.7)	23.7 (18.4 to 27.7)	19.2 (13.7 to 23.1)	-11.4 (-27.4 to 7.6)	-19.0 (-31.5 to -3.7)	26.8 (20.3 to 32.7)	23.7 (18.4 to 27.7)	19.2 (13.7 to 23.1)	-11.4 (-27.4 to 7.6)	-19.0 (-31.5 to -3.7)
Unsafe sanitation	62.2 (43.3 to 87.9)	53.7 (35.8 to 81.4)	44.0 (26.4 to 71.0)	-13.7 (-28.4 to 0.9)	-18.1 (-34.1 to -3.0)	62.2 (43.3 to 87.9)	53.7 (35.8 to 81.4)	44.0 (26.4 to 71.0)	-13.7 (-28.4 to 0.9)	-18.1 (-34.1 to -3.0)
No access to handwashing facility	23.1 (18.8 to 27.4)	21.1 (17.4 to 24.8)	15.4 (12.5 to 18.8)	-8.9 (-25.4 to 11.5)	-26.8 (-37.2 to -14.1)	23.1 (18.8 to 27.4)	21.1 (17.4 to 24.8)	15.4 (12.5 to 18.8)	-8.9 (-25.4 to 11.5)	-26.8 (-37.2 to -14.1)
Ambient particulate matter pollution	45.1 (19.6 to 79.9)	45.2 (24.5 to 71.3)	42.3 (17.6 to 72.4)	0.4 (-50.2 to 138.4)	-6.5 (-58.4 to 66.8)	44.5 (18.8 to 79.2)	44.9 (23.9 to 71.1)	42.2 (17.4 to 72.3)	1.1 (-50.7 to 149.9)	-6.1 (-58.5 to 69.1)
Household air pollution from solid fuels	1.4 (0.5 to 3.7)	0.7 (0.3 to 1.9)	0.3 (0.1 to 0.7)	-50.4 (-79.5 to 13.0)	-59.3 (-83.5 to -14.5)	1.9 (0.7 to 7.6)	1.0 (0.4 to 4.9)	0.5 (0.1 to 2.6)	-47.5 (-79.2 to 17.6)	-55.4 (-83.2 to -10.0)
Ambient ozone pollution	44.2 (16.1 to 54.8)	44.3 (16.3 to 54.8)	45.2 (16.9 to 54.8)	0.3 (-2.0 to 3.0)	2.0 (0.0 to 6.7)	44.2 (16.1 to 54.8)	44.3 (16.3 to 54.8)	45.2 (16.9 to 54.8)	0.3 (-2.0 to 3.0)	2.0 (0.0 to 6.7)
Residential radon	29.6 (4.8 to 71.2)	29.6 (4.8 to 71.2)	29.6 (4.8 to 71.2)	0.0 (0.0 to 0.0)	0.0 (0.0 to 0.0)	29.6 (4.8 to 71.2)	29.6 (4.8 to 71.2)	29.6 (4.8 to 71.2)	0.0 (0.0 to 0.0)	0.0 (0.0 to 0.0)
Lead exposure	4.7 (1.5 to 8.5)	4.6 (1.6 to 8.3)	3.7 (1.2 to 7.2)	-2.6 (-9.3 to 12.7)	-18.3 (-26.5 to -12.7)	2.6 (0.3 to 6.1)	2.7 (0.4 to 6.1)	2.2 (0.3 to 5.4)	1.1 (-6.3 to 34.3)	-16.0 (-25.6 to -10.6)
Occupational exposure to asbestos	0.3 (0.2 to 0.3)	0.5 (0.4 to 0.6)	0.4 (0.3 to 0.6)	103.0 (58.5 to 193.9)	-14.9 (-31.7 to 4.9)	0.4 (0.3 to 0.5)	0.5 (0.4 to 0.6)	0.4 (0.3 to 0.5)	45.7 (10.8 to 93.4)	-23.2 (-39.8 to -3.6)
Occupational exposure to arsenic	0.2 (0.1 to 0.4)	0.2 (0.1 to 0.4)	0.2 (0.1 to 0.4)	1.8 (-7.0 to 12.5)	4.4 (-2.6 to 11.8)	0.2 (0.1 to 0.4)	0.2 (0.1 to 0.4)	0.2 (0.1 to 0.4)	4.8 (-7.8 to 20.2)	4.9 (-4.6 to 15.7)
Occupational exposure to benzene	0.5 (0.3 to 1.0)	0.6 (0.4 to 1.0)	0.7 (0.4 to 1.2)	9.1 (0.6 to 20.0)	10.8 (3.9 to 19.3)	0.6 (0.4 to 1.1)	0.7 (0.4 to 1.3)	0.9 (0.5 to 1.6)	20.4 (6.1 to 40.1)	19.7 (9.5 to 32.5)
Occupational exposure to beryllium	0.1 (0.1 to 0.1)	0.1 (0.1 to 0.1)	0.1 (0.1 to 0.1)	2.0 (-4.4 to 8.7)	1.5 (-2.7 to 6.1)	0.1 (0.1 to 0.1)	0.1 (0.1 to 0.1)	0.1 (0.1 to 0.1)	7.4 (-2.5 to 18.3)	3.8 (-2.1 to 9.9)
Occupational exposure to cadmium	0.1 (0.1 to 0.1)	0.1 (0.1 to 0.1)	0.1 (0.1 to 0.1)	2.4 (-6.0 to 12.0)	4.5 (-1.8 to 11.2)	0.1 (0.1 to 0.1)	0.1 (0.1 to 0.1)	0.1 (0.1 to 0.2)	5.9 (-6.0 to 19.4)	5.4 (-3.5 to 14.8)
Occupational exposure to chromium	0.3 (0.2 to 0.3)	0.3 (0.3 to 0.3)	0.3 (0.3 to 0.3)	5.6 (-3.4 to 16.0)	8.3 (2.0 to 15.3)	0.3 (0.2 to 0.3)	0.3 (0.3 to 0.3)	0.3 (0.3 to 0.4)	9.6 (-2.8 to 24.4)	10.1 (1.0 to 20.0)
Occupational exposure to diesel engine exhaust	1.7 (1.6 to 1.8)	1.9 (1.7 to 2.1)	2.1 (1.9 to 2.3)	11.5 (2.0 to 21.5)	11.6 (4.7 to 19.0)	1.3 (1.2 to 1.5)	1.5 (1.3 to 1.7)	1.6 (1.4 to 1.8)	12.5 (-0.3 to 28.0)	9.8 (1.4 to 19.6)
Occupational exposure to formaldehyde	0.5 (0.5 to 0.6)	0.6 (0.5 to 0.6)	0.6 (0.5 to 0.7)	4.8 (-5.3 to 16.7)	9.0 (1.5 to 16.9)	0.5 (0.5 to 0.6)	0.6 (0.5 to 0.7)	0.6 (0.6 to 0.7)	10.3 (-3.4 to 27.0)	12.4 (2.2 to 23.6)
Occupational exposure to nickel	0.3 (0.1 to 0.6)	0.3 (0.1 to 0.7)	0.3 (0.1 to 0.7)	1.8 (-7.1 to 12.7)	4.3 (-2.3 to 12.0)	0.2 (0.1 to 0.6)	0.2 (0.1 to 0.6)	0.2 (0.1 to 0.6)	0.8 (-10.8 to 15.3)	0.9 (-8.2 to 11.7)
Occupational exposure to polycyclic aromatic hydrocarbons	0.6 (0.5 to 0.6)	0.6 (0.5 to 0.7)	0.7 (0.6 to 0.7)	6.2 (-1.8 to 16.1)	8.7 (2.7 to 15.4)	0.6 (0.5 to 0.7)	0.6 (0.6 to 0.7)	0.7 (0.6 to 0.8)	10.4 (-1.7 to 24.8)	11.2 (2.5 to 20.4)
Occupational exposure to silica	2.8 (1.4 to 6.3)	3.0 (1.5 to 6.7)	3.2 (1.7 to 7.4)	6.1 (-5.0 to 20.6)	8.3 (-0.3 to 19.3)	1.9 (1.0 to 4.2)	1.9 (1.0 to 4.1)	1.8 (0.9 to 4.0)	-1.8 (-12.9 to 11.3)	-1.9 (-10.4 to 7.8)
Occupational exposure to sulphuric acid	0.6 (0.4 to 1.1)	0.6 (0.4 to 1.1)	0.6 (0.4 to 1.1)	1.1 (-8.1 to 10.6)	0.6 (-5.7 to 7.5)	0.5 (0.4 to 0.9)	0.6 (0.4 to 1.0)	0.6 (0.4 to 1.0)	2.8 (-8.0 to 15.4)	-0.7 (-9.0 to 7.6)
Occupational exposure to trichloroethylene	0.2 (0.1 to 0.2)	0.2 (0.2 to 0.2)	0.2 (0.2 to 0.2)	6.7 (-1.1 to 16.3)	8.0 (2.6 to 14.1)	0.2 (0.1 to 0.2)	0.2 (0.2 to 0.2)	0.2 (0.2 to 0.2)	11.1 (-0.5 to 25.7)	9.5 (1.8 to 18.7)
Occupational asthmagens	15.2 (11.4 to 19.7)	14.5 (11.1 to 18.6)	14.1 (11.0 to 17.6)	-4.8 (-17.4 to 11.0)	-2.8 (-15.2 to 9.8)	9.5 (6.7 to 13.1)	9.1 (6.4 to 12.6)	8.6 (6.3 to 11.8)	-3.8 (-23.5 to 22.2)	-5.8 (-21.3 to 14.0)
Occupational particulate matter, gases, and fumes	7.9 (5.9 to 11.0)	7.6 (5.8 to 10.5)	7.5 (5.7 to 10.3)	-3.2 (-7.5 to 1.4)	-1.7 (-4.7 to 1.5)	4.7 (3.4 to 6.5)	4.6 (3.3 to 6.5)	4.5 (3.3 to 6.4)	-0.9 (-7.7 to 7.0)	-2.4 (-7.1 to 2.4)
Occupational noise	8.7 (7.9 to 9.4)	8.5 (7.8 to 9.3)	8.5 (7.9 to 9.3)	-1.4 (-5.1 to 2.5)	-0.2 (-2.7 to 2.6)	5.5 (4.7 to 6.3)	5.7 (5.0 to 6.4)	5.8 (5.1 to 6.4)	3.4 (-2.3 to 10.5)	1.9 (-1.9 to 6.0)
Occupational ergonomic factors	20.2 (17.4 to 23.2)	18.2 (15.3 to 21.1)	15.8 (13.1 to 18.5)	-10.3 (-23.7 to 5.8)	-13.1 (-25.1 to -0.9)	14.4 (11.6 to 17.1)	13.3 (10.4 to 16.3)	11.6 (8.9 to 14.6)	-7.6 (-26.6 to 17.1)	-12.9 (-27.6 to 5.2)
Non-exclusive breastfeeding	0.5 (0.3 to 0.8)	0.4 (0.3 to 0.7)	0.3 (0.2 to 0.5)	-5.3 (-11.2 to 1.8)	-28.7 (-34.0 to -22.5)	0.5 (0.3 to 0.8)	0.4 (0.3 to 0.7)	0.3 (0.2 to 0.5)	-5.3 (-11.2 to 1.8)	-28.7 (-34.0 to -22.4)
Discontinued breastfeeding	1.1 (1.0 to 1.2)	1.1 (1.1 to 1.2)	1.2 (1.2 to 1.3)	1.2 (-7.7 to 12.4)	9.3 (1.6 to 17.9)	1.1 (1.0 to 1.2)	1.1 (1.1 to 1.2)	1.2 (1.2 to 1.3)	1.4 (-7.6 to 12.6)	9.2 (1.4 to 17.7)
Child underweight	0.6 (0.5 to 0.8)	0.4 (0.2 to 0.5)	0.1 (0.1 to 0.2)	-43.3 (-55.4 to -31.8)	-58.8 (-68.3 to -51.7)	0.6 (0.4 to 0.8)	0.3 (0.2 to 0.5)	0.1 (0.1 to 0.2)	-42.0 (-54.9 to -28.4)	-59.2 (-68.7 to -52.3)
Child wasting	0.3 (0.3 to 0.5)	0.3 (0.3 to 0.4)	0.2 (0.1 to 0.2)	-1.8 (-22.9 to 24.5)	-51.9 (-60.2 to -44.1)	0.3 (0.2 to 0.4)	0.3 (0.3 to 0.4)	0.2 (0.1 to 0.2)	3.2 (-18.0 to 33.4)	-53.1 (-61.1 to -44.6)
Child stunting	1.9 (1.2 to 2.5)	1.1 (0.8 to 1.4)	0.7 (0.5 to 1.0)	-39.2 (-55.7 to -16.1)	-36.4 (-50.2 to -22.7)	1.8 (1.1 to 2.4)	1.1 (0.8 to 1.4)	0.7 (0.4 to 1.0)	-37.7 (-55.9 to -11.9)	-36.4 (-50.4 to -23.0)
Short gestation for birth weight	0.0 (0.0 to 0.0)	0.0 (0.0 to 0.0)	0.0 (0.0 to 0.0)	1.4 (-18.7 to 29.4)	3.2 (-18.1 to 29.7)	0.0 (0.0 to 0.0)	0.0 (0.0 to 0.0)	0.0 (0.0 to 0.0)	0.9 (-24.6 to 35.6)	2.8 (-25.8 to 42.1)
Low birth weight for gestation	0.0 (0.0 to 0.0)	0.0 (0.0 to 0.0)	0.0 (0.0 to 0.0)	6.8 (-4.7 to 20.2)	4.1 (-9.6 to 20.7)	0.0 (0.0 to 0.0)	0.0 (0.0 to 0.0)	0.0 (0.0 to 0.0)	4.8 (-9.4 to 22.3)	9.4 (-7.8 to 31.5)
Iron deficiency	-	-	-	-	-	10.8 (7.9 to 13.9)	9.6 (7.0 to 12.6)	8.2 (5.7 to 11.1)	-10.5 (-25.9 to 7.6)	-14.7 (-27.4 to -0.9)
Vitamin A deficiency	1.6 (1.2 to 2.0)	1.5 (1.2 to 1.9)	1.2 (0.8 to 1.6)	-3.6 (-18.9 to 13.2)	-23.0 (-42.2 to -6.3)	1.8 (1.5 to 2.2)	1.7 (1.4 to 2.0)	1.5 (1.2 to 1.9)	-6.2 (-16.4 to 5.4)	-11.4 (-27.4 to 2.2)
Zinc deficiency	0.9 (0.2 to 1.9)	0.6 (0.2 to 1.3)	0.5 (0.1 to 1.0)	-31.4 (-53.2 to 0.3)	-23.7 (-40.3 to -2.0)	0.9 (0.2 to 1.9)	0.6 (0.2 to 1.3)	0.5 (0.1 to 1.0)	-31.5 (-53.9 to 4.0)	-24.6 (-46.8 to -2.0)
Smoking	10.8 (8.5 to 13.2)	9.3 (7.3 to 11.3)	8.9 (7.0 to 10.8)	-14.4 (-25.2 to -1.8)	-4.2 (-12.5 to 5.1)	0.7 (0.5 to 1.0)	0.8 (0.5 to 1.0)	0.8 (0.6 to 1.1)	0.5 (-22.6 to 29.1)	9.1 (-9.4 to 30.4)
Chewing tobacco	0.2 (0.1 to 0.2)	0.2 (0.1 to 0.2)	0.2 (0.1 to 0.3)	3.1 (-25.3 to 45.2)	21.1 (-4.9 to 55.8)	0.1 (0.0 to 0.1)	0.1 (0.0 to 0.1)	0.1 (0.0 to 0.1)	-1.2 (-29.7 to 38.5)	0.1 (-21.7 to 27.1)
Second-hand smoke	40.4 (36.9 to 43.8)	36.8 (34.8 to 38.8)	35.2 (32.6 to 37.8)	-8.8 (-16.9 to -0.2)	-4.5 (-10.2 to 1.2)	58.9 (55.7 to 62.0)	53.1 (51.0 to 55.1)	50.3 (47.8 to 52.8)	-9.9 (-15.2 to -4.5)	-5.2 (-8.7 to -1.6)
Alcohol use	12.8 (7.1 to 21.4)	17.1 (10.1 to 26.3)	18.1 (9.8 to 29.5)	33.1 (1.0 to 90.3)	5.7 (-19.5 to 40.0)	4.3 (1.9 to 8.4)	5.9 (3.0 to 10.7)	5.9 (2.8 to 10.6)	38.2 (7.3 to 99.6)	-0.6 (-21.5 to 22.9)
Drug use	0.4 (0.3 to 0.5)	0.4 (0.3 to 0.5)	0.4 (0.3 to 0.6)	16.3 (5.9 to 29.3)	4.5 (-2.9 to 12.5)	0.2 (0.1 to 0.2)	0.2 (0.1 to 0.2)	0.2 (0.1 to 0.2)	6.8 (0.0 to 14.3)	2.4 (-3.6 to 9.0)
Diet low in fruits	46.2 (41.1 to 48.6)	40.7 (35.4 to 43.8)	34.9 (29.2 to 39.2)	-11.9 (-16.4 to -8.3)	-14.3 (-21.1 to -9.0)	45.0 (40.0 to 47.5)	38.5 (33.1 to 42.1)	31.9 (25.6 to 36.7)	-14.4 (-19.2 to -10.2)	-17.3 (-25.6 to -11.1)
Diet low in vegetables	25.3 (19.1 to 30.4)	12.0 (4.4 to 18.6)	3.9 (0.3 to 9.7)	-52.3 (-77.8 to -36.7)	-67.8 (-94.0 to -45.5)	27.5 (21.7 to 32.2)	15.2 (7.5 to 21.4)	5.8 (0.7 to 12.4)	-44.8 (-66.2 to -31.4)	-61.6 (-91.0 to -39.1)
Diet low in legumes	53.1 (52.6 to 53.5)	53.2 (52.7 to 53.5)	52.8 (52.2 to 53.3)	0.2 (-0.5 to 0.8)	-0.8 (-1.4 to -0.2)	53.6 (53.3 to 53.9)	53.7 (53.4 to 54.0)	53.4 (53.0 to 53.8)	0.1 (-0.4 to 0.6)	-0.5 (-1.0 to -0.1)
Diet low in whole grains	52.7 (52.4 to 53.1)	52.5 (52.1 to 52.9)	52.3 (51.8 to 52.7)	-0.4 (-0.9 to 0.0)	-0.5 (-0.8 to -0.2)	52.7 (52.3 to 53.0)	52.6 (52.2 to 52.9)	52.5 (52.1 to 52.8)	-0.2 (-0.7 to 0.2)	-0.2 (-0.6 to 0.0)
Diet low in nuts and seeds	53.1 (52.7 to 53.5)	53.4 (53.1 to 53.7)	52.1 (51.4 to 52.7)	0.6 (0.2 to 1.2)	-2.5 (-3.3 to -1.8)	53.2 (52.7 to 53.5)	53.5 (53.2 to 53.8)	52.2 (51.5 to 52.8)	0.6 (0.1 to 1.1)	-2.4 (-3.3 to -1.7)
Diet low in milk	41.0 (38.0 to 43.5)	39.7 (36.5 to 42.3)	36.5 (32.6 to 39.8)	-3.1 (-5.8 to -1.0)	-8.2 (-11.8 to -5.3)	41.7 (38.9 to 44.0)	40.5 (37.6 to 43.0)	37.2 (33.6 to 40.4)	-2.8 (-5.2 to -0.7)	-8.0 (-11.5 to -5.3)
Diet high in red meat	10.7 (5.7 to 15.4)	23.0 (18.1 to 27.5)	40.0 (31.7 to 46.6)	114.5 (53.8 to 243.4)	74.1 (45.6 to 111.1)	2.3 (0.2 to 5.4)	7.3 (3.1 to 11.5)	18.0 (10.8 to 25.2)	218.1 (59.6 to 2059.6)	147.3 (68.6 to 381.0)
Diet high in processed meat	1.6 (0.1 to 3.8)	1.2 (0.1 to 3.3)	3.0 (0.9 to 5.6)	-19.9 (-58.0 to 16.7)	142.7 (64.0 to 1243.1)	0.9 (0.0 to 2.8)	0.7 (0.0 to 2.5)	1.9 (0.4 to 4.2)	-18.4 (-58.3 to 36.1)	159.3 (65.8 to 3073.3)
Diet high in sugar-sweetened beverages	5.0 (2.4 to 9.4)	4.6 (2.1 to 8.6)	9.5 (4.2 to 16.1)	-9.3 (-52.3 to 72.0)	106.6 (36.0 to 221.4)	3.5 (1.7 to 6.2)	3.2 (1.4 to 6.0)	6.3 (3.0 to 11.3)	-7.4 (-48.9 to 68.1)	93.5 (29.0 to 205.5)
Diet low in fibre	39.6 (36.2 to 42.5)	35.2 (30.9 to 38.6)	32.6 (27.2 to 36.7)	-11.2 (-16.1 to -7.7)	-7.4 (-13.0 to -3.4)	43.4 (40.8 to 45.5)	39.7 (36.3 to 42.4)	37.3 (33.2 to 40.4)	-8.5 (-12.1 to -5.5)	-6.2 (-10.3 to -2.9)
Diet low in calcium	34.2 (30.1 to 37.7)	32.1 (27.5 to 35.7)	26.8 (21.0 to 31.4)	-6.3 (-9.9 to -4.0)	-16.5 (-23.7 to -11.3)	36.8 (33.1 to 39.8)	34.9 (30.9 to 38.2)	30.0 (24.9 to 34.0)	-5.2 (-7.9 to -3.2)	-14.1 (-20.0 to -10.2)
Diet low in seafood omega-3 fatty acids	46.7 (45.1 to 48.3)	44.2 (42.1 to 46.2)	39.9 (37.0 to 42.7)	-5.5 (-7.0 to -4.2)	-9.7 (-12.5 to -7.4)	47.9 (46.5 to 49.3)	45.7 (43.9 to 47.5)	42.0 (39.4 to 44.3)	-4.6 (-5.8 to -3.5)	-8.1 (-10.4 to -6.2)
Diet low in polyunsaturated fatty acids	53.0 (51.1 to 53.5)	51.3 (50.5 to 51.9)	50.1 (49.1 to 50.9)	-3.2 (-4.4 to 0.4)	-2.3 (-3.3 to -1.5)	53.0 (51.2 to 53.5)	51.4 (50.7 to 52.0)	50.3 (49.3 to 51.1)	-3.0 (-4.2 to 0.7)	-2.3 (-3.2 to -1.4)
Diet high in trans fatty acids	1.5 (0.1 to 4.0)	1.3 (0.1 to 3.6)	1.1 (0.0 to 3.3)	-19.0 (-54.2 to -4.6)	-14.5 (-45.4 to -3.1)	2.1 (0.3 to 5.0)	1.7 (0.2 to 4.4)	1.5 (0.1 to 3.9)	-17.7 (-49.3 to -1.6)	-15.1 (-44.7 to -1.3)
Diet high in sodium	15.1 (7.2 to 22.3)	8.9 (1.6 to 16.4)	10.3 (2.4 to 18.2)	-40.9 (-81.4 to -19.4)	15.9 (-3.0 to 95.7)	13.2 (5.6 to 20.1)	7.2 (0.9 to 14.2)	6.2 (0.6 to 13.1)	-45.8 (-85.8 to -24.8)	-13.7 (-52.4 to 0.5)
Intimate partner violence	-	-	-	-	-	4.6 (3.5 to 5.6)	3.9 (3.0 to 4.8)	3.5 (2.7 to 4.3)	-14.1 (-18.6 to -9.6)	-10.8 (-15.5 to -6.6)
Childhood sexual abuse	1.6 (1.3 to 2.0)	1.6 (1.3 to 2.0)	1.6 (1.3 to 2.0)	0.0 (0.0 to 0.0)	0.0 (0.0 to 0.0)	2.2 (1.7 to 2.7)	2.2 (1.7 to 2.7)	2.2 (1.7 to 2.7)	0.0 (0.0 to 0.0)	0.0 (0.0 to 0.0)

Supplementary Results Table 1. Turkmenistan age-standardised Summary Exposure Values (SEVs) for Level 4 risk factors and percentage change, by sex, 1990, 2007, and 2017.Data in parentheses are 95% uncertainty intervals.

	Male					Female				
	**1990**	**2007**	**2017**	**Percentage change 1990-2007**	**Percentage change 2007-2017**	**1990**	**2007**	**2017**	**Percentage change 1990-2007**	**Percentage change 2007-2017**
Bullying victimization	2.7 (1.1 to 5.7)	2.8 (1.2 to 5.9)	2.7 (1.1 to 5.6)	4.0 (1.4 to 6.6)	-4.3 (-6.8 to -1.9)	1.6 (0.7 to 3.3)	1.7 (0.7 to 3.5)	1.6 (0.7 to 3.3)	4.7 (2.0 to 8.1)	-3.9 (-6.4 to -1.7)
Low physical activity	0.3 (0.2 to 0.6)	0.3 (0.2 to 0.6)	0.3 (0.2 to 0.6)	2.5 (0.4 to 4.8)	2.0 (0.2 to 4.1)	0.3 (0.1 to 0.5)	0.3 (0.1 to 0.5)	0.3 (0.1 to 0.5)	2.2 (0.2 to 4.6)	1.6 (-0.4 to 3.6)
High fasting plasma glucose	4.2 (2.9 to 6.2)	4.6 (3.2 to 6.8)	4.8 (3.4 to 7.3)	10.2 (-10.9 to 37.6)	5.8 (-17.0 to 30.5)	3.1 (2.0 to 4.8)	3.8 (2.6 to 5.5)	4.0 (2.7 to 5.8)	21.3 (-6.8 to 65.6)	5.3 (-21.3 to 37.8)
High LDL cholesterol	9.7 (8.3 to 11.3)	9.8 (8.4 to 11.4)	10.1 (8.6 to 11.8)	0.3 (-4.0 to 4.7)	3.0 (-0.3 to 6.6)	10.4 (9.0 to 12.0)	10.8 (9.4 to 12.4)	11.1 (9.7 to 12.7)	3.3 (-0.8 to 7.8)	3.1 (-0.2 to 6.5)
High systolic blood pressure	8.2 (7.3 to 9.1)	8.8 (7.9 to 9.8)	9.4 (8.4 to 10.3)	7.6 (0.0 to 16.1)	6.2 (-0.2 to 12.2)	7.8 (6.9 to 8.7)	8.2 (7.3 to 9.1)	8.4 (7.5 to 9.4)	5.1 (-5.2 to 16.8)	2.6 (-5.0 to 11.0)
High body-mass index	8.6 (6.1 to 12.7)	12.1 (9.0 to 17.3)	15.0 (11.3 to 20.9)	40.6 (22.3 to 66.6)	23.4 (10.8 to 36.8)	10.9 (7.8 to 15.6)	14.1 (10.6 to 19.7)	16.7 (12.5 to 22.0)	29.3 (10.7 to 51.4)	18.3 (7.0 to 31.9)
Low bone mineral density	4.1 (3.3 to 5.0)	4.0 (3.3 to 4.7)	3.9 (3.1 to 4.7)	-4.0 (-17.6 to 13.7)	-1.9 (-15.9 to 12.6)	5.7 (4.7 to 6.8)	5.5 (4.6 to 6.5)	5.4 (4.5 to 6.6)	-3.9 (-16.8 to 11.9)	-0.8 (-14.3 to 14.1)
Impaired kidney function	2.7 (1.2 to 6.6)	2.9 (1.3 to 6.9)	2.9 (1.4 to 6.9)	6.0 (1.5 to 11.7)	1.5 (-2.6 to 5.9)	3.2 (1.6 to 7.3)	3.4 (1.7 to 7.5)	3.5 (1.7 to 7.6)	6.0 (1.7 to 11.5)	2.7 (-1.3 to 7.1)

Supplementary Results Table 1. Uzbekistan age-standardised Summary Exposure Values (SEVs) for Level 4 risk factors and percentage change, by sex, 1990, 2007, and 2017.Data in parentheses are 95% uncertainty intervals.										
	Male					Female				
	1990	2007	2017	Percentage change 1990-2007	Percentage change 2007-2017	1990	2007	2017	Percentage change 1990-2007	Percentage change 2007-2017
Unsafe water source	16.7 (7.1 to 22.4)	15.1 (6.0 to 20.1)	11.6 (4.7 to 16.6)	-9.6 (-25.1 to 11.3)	-22.9 (-37.5 to -5.6)	16.7 (7.1 to 22.4)	15.1 (6.0 to 20.1)	11.6 (4.7 to 16.6)	-9.6 (-25.1 to 11.3)	-22.9 (-37.5 to -5.6)
Unsafe sanitation	73.1 (57.6 to 94.8)	69.1 (54.2 to 90.4)	58.3 (42.9 to 79.3)	-5.4 (-14.1 to 3.7)	-15.6 (-28.1 to -4.9)	73.1 (57.6 to 94.8)	69.1 (54.2 to 90.4)	58.3 (42.9 to 79.3)	-5.4 (-14.1 to 3.7)	-15.6 (-28.1 to -4.9)
No access to handwashing facility	27.2 (22.6 to 32.2)	22.1 (18.6 to 26.0)	15.0 (12.2 to 18.1)	-18.7 (-32.8 to -1.9)	-32.3 (-42.7 to -20.7)	27.2 (22.6 to 32.2)	22.1 (18.6 to 26.0)	15.0 (12.2 to 18.1)	-18.7 (-32.8 to -1.9)	-32.3 (-42.7 to -20.7)
Ambient particulate matter pollution	32.2 (9.3 to 62.6)	39.8 (17.4 to 67.8)	41.2 (14.1 to 74.0)	23.8 (-54.0 to 315.9)	3.4 (-61.0 to 144.3)	27.7 (7.4 to 56.2)	36.0 (14.4 to 63.4)	38.3 (12.2 to 70.6)	29.9 (-55.7 to 375.9)	6.4 (-63.5 to 166.2)
Household air pollution from solid fuels	32.6 (18.2 to 47.9)	16.4 (8.8 to 26.4)	10.2 (4.8 to 18.4)	-49.6 (-74.0 to -3.9)	-37.6 (-68.7 to 12.7)	34.3 (21.1 to 48.9)	18.4 (10.7 to 27.8)	11.9 (5.9 to 19.8)	-46.4 (-69.7 to -7.4)	-35.4 (-65.6 to 6.6)
Ambient ozone pollution	47.7 (18.8 to 54.8)	47.4 (18.4 to 54.8)	47.9 (19.0 to 54.8)	-0.5 (-3.3 to 1.0)	1.1 (0.0 to 4.7)	47.7 (18.8 to 54.8)	47.4 (18.4 to 54.8)	47.9 (19.0 to 54.8)	-0.5 (-3.3 to 1.0)	1.1 (0.0 to 4.7)
Residential radon	33.7 (5.4 to 71.2)	33.7 (5.4 to 71.2)	33.7 (5.4 to 71.2)	0.0 (0.0 to 0.0)	0.0 (0.0 to 0.0)	33.7 (5.4 to 71.2)	33.7 (5.4 to 71.2)	33.7 (5.4 to 71.2)	0.0 (0.0 to 0.0)	0.0 (0.0 to 0.0)
Lead exposure	6.0 (2.5 to 9.9)	5.4 (2.3 to 9.1)	4.3 (1.7 to 7.7)	-9.0 (-15.8 to -0.8)	-20.9 (-29.8 to -14.7)	4.0 (1.0 to 7.8)	3.8 (1.1 to 7.4)	3.1 (0.8 to 6.4)	-5.5 (-12.1 to 10.3)	-18.3 (-27.0 to -12.6)
Occupational exposure to asbestos	0.3 (0.2 to 0.5)	0.3 (0.2 to 0.4)	0.3 (0.2 to 0.3)	-15.3 (-47.6 to 35.9)	-12.1 (-33.1 to 13.8)	0.2 (0.1 to 0.3)	0.3 (0.2 to 0.3)	0.2 (0.2 to 0.3)	43.5 (-15.1 to 157.6)	-11.5 (-33.8 to 17.9)
Occupational exposure to arsenic	0.2 (0.1 to 0.3)	0.2 (0.1 to 0.3)	0.2 (0.1 to 0.4)	3.2 (-3.9 to 11.6)	5.4 (-0.5 to 11.8)	0.2 (0.1 to 0.3)	0.2 (0.1 to 0.3)	0.2 (0.1 to 0.4)	16.8 (4.9 to 32.1)	8.5 (-0.0 to 17.7)
Occupational exposure to benzene	0.5 (0.3 to 0.9)	0.6 (0.3 to 1.0)	0.6 (0.4 to 1.1)	11.0 (2.2 to 22.6)	10.3 (4.1 to 19.2)	0.5 (0.3 to 0.9)	0.6 (0.4 to 1.1)	0.8 (0.4 to 1.4)	28.6 (12.5 to 50.4)	20.8 (10.8 to 35.1)
Occupational exposure to beryllium	0.1 (0.1 to 0.1)	0.1 (0.1 to 0.1)	0.1 (0.1 to 0.1)	-6.6 (-12.5 to 0.0)	-1.3 (-5.5 to 2.8)	0.1 (0.1 to 0.1)	0.1 (0.1 to 0.1)	0.1 (0.1 to 0.1)	8.9 (-1.7 to 22.1)	3.6 (-3.2 to 10.8)
Occupational exposure to cadmium	0.1 (0.1 to 0.1)	0.1 (0.1 to 0.1)	0.1 (0.1 to 0.1)	3.7 (-3.1 to 11.6)	5.4 (-0.2 to 11.1)	0.1 (0.1 to 0.1)	0.1 (0.1 to 0.1)	0.1 (0.1 to 0.1)	17.0 (5.4 to 31.6)	8.5 (0.1 to 17.0)
Occupational exposure to chromium	0.2 (0.2 to 0.3)	0.3 (0.2 to 0.3)	0.3 (0.3 to 0.3)	5.5 (-2.4 to 13.9)	8.2 (2.2 to 14.4)	0.2 (0.2 to 0.2)	0.2 (0.2 to 0.3)	0.3 (0.2 to 0.3)	21.0 (7.7 to 36.5)	12.8 (3.9 to 22.0)
Occupational exposure to diesel engine exhaust	3.1 (2.8 to 3.5)	2.5 (2.2 to 2.7)	2.3 (2.1 to 2.6)	-21.7 (-31.1 to -10.4)	-4.8 (-11.1 to 2.1)	1.7 (1.5 to 1.9)	1.6 (1.4 to 1.8)	1.6 (1.4 to 1.8)	-5.1 (-17.2 to 10.4)	0.4 (-8.1 to 10.0)
Occupational exposure to formaldehyde	0.4 (0.4 to 0.5)	0.5 (0.4 to 0.5)	0.5 (0.5 to 0.6)	8.7 (0.1 to 18.1)	10.6 (3.9 to 17.9)	0.4 (0.3 to 0.4)	0.4 (0.4 to 0.5)	0.5 (0.5 to 0.6)	25.6 (11.1 to 43.1)	17.1 (7.4 to 27.5)
Occupational exposure to nickel	0.5 (0.2 to 1.2)	0.3 (0.1 to 0.9)	0.3 (0.1 to 0.7)	-30.9 (-40.1 to -19.3)	-12.8 (-19.4 to -5.2)	0.3 (0.1 to 0.7)	0.2 (0.1 to 0.6)	0.2 (0.1 to 0.6)	-13.5 (-24.2 to 0.9)	-4.8 (-12.6 to 3.6)
Occupational exposure to polycyclic aromatic hydrocarbons	0.6 (0.5 to 0.6)	0.6 (0.5 to 0.6)	0.6 (0.6 to 0.7)	-0.1 (-7.4 to 8.5)	6.0 (-0.1 to 11.9)	0.5 (0.4 to 0.5)	0.5 (0.5 to 0.6)	0.6 (0.5 to 0.7)	17.7 (4.8 to 33.0)	12.8 (4.2 to 21.7)
Occupational exposure to silica	6.3 (3.3 to 14.0)	4.2 (2.2 to 9.6)	3.7 (1.9 to 8.0)	-32.6 (-42.8 to -20.3)	-13.3 (-21.1 to -4.0)	2.9 (1.5 to 6.7)	2.2 (1.1 to 4.8)	1.9 (1.0 to 4.2)	-24.5 (-35.3 to -10.2)	-12.9 (-21.2 to -4.6)
Occupational exposure to sulphuric acid	0.6 (0.4 to 1.0)	0.6 (0.4 to 1.1)	0.6 (0.4 to 1.1)	6.2 (-2.6 to 15.6)	2.1 (-4.5 to 8.7)	0.4 (0.3 to 0.7)	0.5 (0.3 to 0.9)	0.5 (0.4 to 0.9)	14.5 (2.6 to 28.5)	2.7 (-5.0 to 10.9)
Occupational exposure to trichloroethylene	0.1 (0.1 to 0.2)	0.2 (0.1 to 0.2)	0.2 (0.2 to 0.2)	11.7 (4.1 to 19.8)	9.1 (3.8 to 15.0)	0.1 (0.1 to 0.1)	0.2 (0.1 to 0.2)	0.2 (0.2 to 0.2)	22.8 (9.8 to 39.9)	11.2 (2.6 to 20.3)
Occupational asthmagens	21.1 (17.1 to 26.0)	16.8 (13.3 to 21.4)	14.9 (11.7 to 19.1)	-20.3 (-31.2 to -8.6)	-11.5 (-20.7 to -1.3)	10.7 (7.8 to 14.1)	9.8 (7.1 to 13.0)	8.8 (6.4 to 11.8)	-9.0 (-25.7 to 11.7)	-9.4 (-24.1 to 8.0)
Occupational particulate matter, gases, and fumes	8.6 (6.5 to 11.4)	8.3 (6.4 to 11.1)	8.0 (6.1 to 10.7)	-3.2 (-7.4 to 1.2)	-3.8 (-6.4 to -1.0)	4.3 (3.1 to 6.1)	4.3 (3.2 to 6.1)	4.3 (3.2 to 6.1)	1.3 (-5.9 to 10.0)	-0.8 (-5.6 to 4.7)
Occupational noise	9.8 (8.9 to 10.7)	9.6 (8.8 to 10.4)	9.3 (8.6 to 10.1)	-1.7 (-5.5 to 2.3)	-3.1 (-5.3 to -0.8)	5.1 (4.4 to 6.0)	5.4 (4.7 to 6.1)	5.5 (4.9 to 6.2)	5.1 (-1.8 to 12.9)	2.6 (-1.8 to 7.2)
Occupational ergonomic factors	22.2 (19.5 to 25.0)	19.3 (16.3 to 22.1)	16.8 (14.0 to 19.7)	-13.0 (-25.4 to 0.4)	-12.8 (-22.9 to -1.1)	14.5 (11.8 to 17.1)	13.6 (10.6 to 16.5)	11.9 (9.2 to 14.8)	-6.2 (-24.3 to 16.4)	-12.1 (-27.9 to 5.6)
Non-exclusive breastfeeding	0.5 (0.3 to 0.8)	0.5 (0.3 to 0.7)	0.5 (0.3 to 0.7)	-10.8 (-15.2 to -5.5)	3.0 (-2.4 to 9.8)	0.5 (0.3 to 0.8)	0.5 (0.3 to 0.7)	0.5 (0.3 to 0.7)	-10.3 (-14.8 to -5.1)	3.0 (-2.4 to 9.8)
Discontinued breastfeeding	1.1 (1.0 to 1.1)	1.0 (1.0 to 1.1)	1.2 (1.1 to 1.3)	-2.1 (-11.2 to 7.4)	12.9 (4.0 to 22.2)	1.0 (1.0 to 1.1)	1.0 (1.0 to 1.1)	1.2 (1.1 to 1.2)	-2.0 (-11.1 to 7.5)	13.0 (4.1 to 22.3)
Child underweight	0.7 (0.5 to 0.9)	0.3 (0.2 to 0.4)	0.2 (0.1 to 0.3)	-57.5 (-68.7 to -43.8)	-30.2 (-43.1 to -17.4)	0.7 (0.5 to 0.9)	0.3 (0.2 to 0.4)	0.2 (0.1 to 0.3)	-56.5 (-69.4 to -43.0)	-31.8 (-46.2 to -18.5)
Child wasting	0.5 (0.4 to 0.7)	0.3 (0.2 to 0.5)	0.2 (0.1 to 0.4)	-44.7 (-64.4 to -16.0)	-24.3 (-43.3 to -1.0)	0.6 (0.4 to 0.8)	0.3 (0.2 to 0.5)	0.2 (0.1 to 0.4)	-44.2 (-63.4 to -16.2)	-26.4 (-45.5 to -3.7)
Child stunting	3.5 (2.4 to 4.6)	1.6 (1.0 to 2.0)	1.2 (0.7 to 1.7)	-55.3 (-68.4 to -40.7)	-24.1 (-45.0 to -0.5)	2.9 (1.9 to 4.0)	1.4 (0.9 to 1.9)	1.1 (0.6 to 1.6)	-51.3 (-68.2 to -32.8)	-23.1 (-44.7 to 2.8)
Short gestation for birth weight	0.0 (0.0 to 0.0)	0.0 (0.0 to 0.0)	0.0 (0.0 to 0.0)	-2.4 (-29.2 to 24.7)	5.6 (-19.6 to 31.8)	0.0 (0.0 to 0.0)	0.0 (0.0 to 0.0)	0.0 (0.0 to 0.0)	4.8 (-18.9 to 39.8)	5.3 (-19.0 to 37.9)
Low birth weight for gestation	0.0 (0.0 to 0.0)	0.0 (0.0 to 0.0)	0.0 (0.0 to 0.0)	3.9 (-11.6 to 30.0)	4.4 (-9.7 to 19.2)	0.0 (0.0 to 0.0)	0.0 (0.0 to 0.0)	0.0 (0.0 to 0.0)	4.9 (-14.2 to 25.2)	3.1 (-15.2 to 21.3)
Iron deficiency	-	-	-	-	-	18.9 (15.1 to 23.1)	16.5 (13.0 to 20.9)	14.1 (10.5 to 18.2)	-12.3 (-23.6 to -0.3)	-14.4 (-24.5 to -3.5)
Vitamin A deficiency	1.8 (1.4 to 2.2)	1.5 (1.1 to 1.9)	1.4 (0.9 to 1.8)	-15.0 (-30.4 to 0.8)	-10.4 (-31.9 to 10.1)	2.0 (1.6 to 2.3)	1.8 (1.4 to 2.1)	1.6 (1.2 to 1.9)	-10.4 (-24.1 to 1.6)	-10.3 (-24.5 to 1.4)
Zinc deficiency	0.6 (0.2 to 1.4)	0.7 (0.2 to 1.5)	0.5 (0.1 to 1.1)	7.4 (-20.6 to 49.1)	-25.4 (-42.3 to -0.0)	0.6 (0.2 to 1.4)	0.7 (0.2 to 1.5)	0.5 (0.1 to 1.1)	7.8 (-19.6 to 45.9)	-25.6 (-43.6 to -0.2)
Smoking	4.4 (3.4 to 5.6)	6.5 (5.2 to 7.9)	6.7 (5.5 to 8.0)	46.8 (23.5 to 74.8)	3.6 (-6.2 to 14.8)	0.2 (0.2 to 0.3)	0.3 (0.2 to 0.4)	0.4 (0.3 to 0.5)	31.5 (2.0 to 67.1)	22.0 (4.3 to 41.3)
Chewing tobacco	0.2 (0.1 to 0.2)	0.2 (0.1 to 0.2)	0.2 (0.1 to 0.3)	2.0 (-28.7 to 44.4)	21.0 (-5.7 to 57.3)	0.1 (0.0 to 0.1)	0.1 (0.0 to 0.1)	0.1 (0.0 to 0.1)	-1.4 (-28.0 to 40.1)	0.8 (-22.0 to 29.4)
Second-hand smoke	29.0 (26.6 to 31.3)	30.2 (28.7 to 31.6)	27.7 (25.4 to 30.1)	4.1 (-5.7 to 15.1)	-8.0 (-14.7 to -1.9)	40.6 (38.4 to 42.8)	41.6 (40.1 to 43.0)	36.6 (34.4 to 38.8)	2.3 (-3.8 to 8.9)	-12.1 (-16.2 to -7.4)
Alcohol use	12.3 (6.8 to 20.6)	15.8 (9.5 to 24.4)	17.6 (10.0 to 28.0)	28.8 (-3.4 to 81.3)	11.3 (-13.1 to 49.1)	4.0 (1.9 to 7.9)	5.1 (2.7 to 9.3)	5.5 (2.8 to 9.8)	28.5 (-0.9 to 85.4)	7.3 (-15.2 to 40.6)
Drug use	0.4 (0.3 to 0.5)	0.4 (0.3 to 0.5)	0.4 (0.3 to 0.5)	7.6 (0.0 to 16.2)	8.4 (1.6 to 15.3)	0.2 (0.1 to 0.2)	0.2 (0.1 to 0.2)	0.2 (0.1 to 0.2)	4.5 (-1.8 to 11.3)	4.1 (-2.6 to 10.2)
Diet low in fruits	48.9 (44.2 to 50.8)	45.6 (40.7 to 47.9)	40.3 (34.7 to 43.8)	-6.9 (-9.3 to -4.7)	-11.5 (-15.7 to -8.0)	48.0 (43.3 to 50.0)	44.1 (39.0 to 46.6)	38.0 (32.1 to 41.9)	-8.2 (-10.9 to -5.8)	-13.8 (-19.2 to -9.4)
Diet low in vegetables	14.5 (6.8 to 21.3)	6.8 (0.9 to 13.7)	0.5 (0.0 to 2.3)	-53.0 (-88.6 to -30.1)	-92.0 (-99.9 to -80.9)	17.5 (9.9 to 23.9)	9.7 (2.5 to 16.7)	1.2 (0.0 to 4.2)	-44.4 (-77.4 to -23.9)	-87.9 (-99.2 to -72.3)
Diet low in legumes	54.8 (54.8 to 54.8)	54.8 (54.8 to 54.8)	54.8 (54.8 to 54.8)	0.0 (0.0 to 0.0)	0.0 (0.0 to 0.0)	54.8 (54.8 to 54.8)	54.8 (54.8 to 54.8)	54.8 (54.8 to 54.8)	0.0 (0.0 to 0.0)	0.0 (0.0 to 0.0)
Diet low in whole grains	52.7 (52.3 to 53.0)	52.7 (52.3 to 53.1)	52.8 (52.4 to 53.1)	0.1 (-0.3 to 0.5)	0.1 (-0.3 to 0.6)	52.6 (52.2 to 52.9)	52.7 (52.3 to 53.1)	52.8 (52.5 to 53.2)	0.2 (-0.4 to 0.8)	0.3 (-0.3 to 0.8)
Diet low in nuts and seeds	50.9 (49.9 to 51.7)	50.1 (48.9 to 51.1)	47.3 (45.1 to 49.0)	-1.5 (-3.1 to -0.1)	-5.8 (-8.3 to -3.8)	51.1 (50.1 to 51.9)	50.4 (49.3 to 51.4)	47.7 (45.7 to 49.3)	-1.3 (-2.6 to -0.0)	-5.5 (-7.9 to -3.5)
Diet low in milk	40.5 (37.4 to 43.2)	45.6 (43.6 to 47.3)	45.7 (43.6 to 47.4)	12.6 (9.1 to 17.4)	0.2 (-1.0 to 1.4)	41.3 (38.4 to 43.8)	46.1 (44.2 to 47.7)	46.1 (44.1 to 47.8)	11.7 (8.4 to 16.0)	0.0 (-1.2 to 1.1)
Diet high in red meat	5.1 (1.6 to 8.7)	4.1 (1.1 to 7.4)	11.7 (6.3 to 16.8)	-19.8 (-60.3 to 30.4)	188.3 (81.7 to 682.8)	0.4 (0.0 to 1.5)	0.2 (0.0 to 0.9)	2.2 (0.2 to 5.4)	-51.7 (-99.8 to 559.4)	1131.7 (308.6 to 818069.8)
Diet high in processed meat	0.8 (0.0 to 2.6)	0.6 (0.0 to 2.3)	1.3 (0.1 to 3.4)	-21.0 (-82.6 to 15.7)	107.3 (45.1 to 4895.1)	0.5 (0.0 to 2.0)	0.4 (0.0 to 1.7)	0.8 (0.0 to 2.5)	-20.2 (-90.6 to 6.6)	107.3 (45.0 to 49939.0)
Diet high in sugar-sweetened beverages	2.6 (1.2 to 4.7)	2.4 (1.0 to 4.6)	4.1 (1.7 to 7.8)	-5.0 (-50.0 to 82.9)	66.4 (5.1 to 158.1)	1.9 (0.8 to 3.4)	1.9 (0.8 to 3.5)	3.0 (1.4 to 5.6)	-2.9 (-48.0 to 88.8)	59.9 (6.2 to 154.2)
Diet low in fibre	36.3 (32.0 to 39.6)	32.5 (27.6 to 36.4)	25.7 (19.6 to 31.0)	-10.3 (-15.7 to -5.8)	-20.9 (-30.6 to -13.6)	40.6 (37.4 to 43.3)	37.4 (33.4 to 40.5)	31.7 (26.3 to 35.9)	-7.9 (-12.3 to -4.3)	-15.3 (-22.5 to -10.1)
Diet low in calcium	32.0 (27.4 to 35.7)	32.6 (28.1 to 36.1)	27.3 (21.7 to 31.8)	1.9 (0.1 to 4.2)	-16.3 (-23.4 to -11.7)	34.9 (30.8 to 38.1)	35.3 (31.3 to 38.5)	30.6 (25.6 to 34.5)	1.2 (-0.4 to 3.1)	-13.5 (-18.7 to -9.9)
Diet low in seafood omega-3 fatty acids	46.2 (44.5 to 47.8)	47.0 (45.4 to 48.5)	42.9 (40.5 to 45.1)	1.7 (1.2 to 2.3)	-8.6 (-10.9 to -6.8)	47.4 (45.9 to 48.8)	48.1 (46.7 to 49.4)	44.6 (42.5 to 46.5)	1.4 (1.0 to 1.9)	-7.3 (-9.1 to -5.8)
Diet low in polyunsaturated fatty acids	48.0 (46.3 to 49.3)	46.5 (44.8 to 47.9)	44.2 (41.9 to 46.1)	-3.2 (-5.1 to 0.1)	-4.9 (-7.5 to -2.3)	48.2 (46.4 to 49.6)	46.8 (45.1 to 48.1)	44.5 (42.4 to 46.3)	-3.0 (-5.2 to 0.6)	-4.7 (-6.5 to -3.1)
Diet high in trans fatty acids	0.5 (0.0 to 1.9)	0.4 (0.0 to 1.8)	0.4 (0.0 to 1.8)	-6.6 (-43.2 to 5.8)	-5.0 (-46.5 to 5.9)	0.6 (0.0 to 2.4)	0.6 (0.0 to 2.3)	0.6 (0.0 to 2.2)	-7.2 (-36.0 to 7.5)	-5.1 (-35.5 to 8.2)
Diet high in sodium	12.7 (5.6 to 19.9)	7.5 (0.9 to 15.0)	8.6 (1.3 to 16.5)	-40.8 (-85.7 to -17.1)	13.6 (-14.2 to 82.3)	12.3 (5.1 to 19.2)	6.8 (0.8 to 13.8)	5.8 (0.4 to 12.9)	-45.2 (-86.5 to -23.5)	-14.5 (-57.6 to 0.2)
Intimate partner violence	-	-	-	-	-	4.7 (3.6 to 5.8)	4.0 (3.1 to 4.9)	3.6 (2.8 to 4.4)	-16.7 (-21.8 to -12.0)	-8.4 (-13.3 to -2.7)
Childhood sexual abuse	1.6 (1.3 to 2.0)	1.6 (1.3 to 2.0)	1.6 (1.3 to 2.0)	0.0 (0.0 to 0.0)	0.0 (0.0 to 0.0)	2.2 (1.7 to 2.7)	2.2 (1.7 to 2.7)	2.2 (1.7 to 2.7)	0.0 (0.0 to 0.0)	0.0 (0.0 to 0.0)

Supplementary Results Table 1. Uzbekistan age-standardised Summary Exposure Values (SEVs) for Level 4 risk factors and percentage change, by sex, 1990, 2007, and 2017.Data in parentheses are 95% uncertainty intervals.

	Male					Female				
	**1990**	**2007**	**2017**	**Percentage change 1990-2007**	**Percentage change 2007-2017**	**1990**	**2007**	**2017**	**Percentage change 1990-2007**	**Percentage change 2007-2017**
Bullying victimization	2.5 (1.0 to 5.1)	2.6 (1.1 to 5.5)	2.6 (1.0 to 5.3)	5.6 (1.2 to 11.7)	-1.5 (-4.1 to 1.4)	1.4 (0.6 to 3.1)	1.5 (0.6 to 3.2)	1.5 (0.6 to 3.2)	6.9 (1.9 to 13.5)	-1.4 (-4.1 to 1.7)
Low physical activity	0.3 (0.2 to 0.6)	0.3 (0.2 to 0.6)	0.3 (0.2 to 0.6)	1.1 (-1.3 to 3.4)	2.4 (0.5 to 4.6)	0.3 (0.1 to 0.5)	0.3 (0.1 to 0.5)	0.3 (0.1 to 0.5)	0.8 (-1.0 to 2.9)	2.1 (0.2 to 4.3)
High fasting plasma glucose	4.0 (2.9 to 5.9)	4.5 (3.3 to 6.7)	5.0 (3.5 to 7.3)	12.9 (-6.2 to 37.4)	9.2 (-12.0 to 33.0)	3.2 (2.2 to 4.8)	4.3 (3.1 to 6.2)	4.3 (3.1 to 6.2)	32.6 (8.0 to 71.2)	0.3 (-18.0 to 22.1)
High LDL cholesterol	10.8 (9.3 to 12.5)	11.0 (9.5 to 12.6)	11.1 (9.6 to 12.8)	1.6 (-2.6 to 6.0)	0.9 (-2.4 to 4.3)	12.3 (10.9 to 14.0)	13.0 (11.5 to 14.7)	13.5 (12.0 to 15.3)	5.7 (1.7 to 10.3)	4.0 (0.5 to 7.6)
High systolic blood pressure	5.5 (4.8 to 6.3)	6.7 (5.9 to 7.5)	8.6 (7.7 to 9.5)	21.4 (11.7 to 31.7)	28.3 (20.6 to 36.7)	5.3 (4.6 to 6.0)	6.5 (5.8 to 7.2)	8.0 (7.2 to 8.8)	22.3 (11.0 to 35.6)	22.5 (13.6 to 32.0)
High body-mass index	9.8 (6.8 to 14.8)	10.7 (7.6 to 15.6)	14.3 (10.8 to 19.7)	9.4 (-5.6 to 27.6)	33.6 (19.2 to 51.3)	10.7 (7.5 to 15.2)	12.3 (9.0 to 16.8)	16.9 (13.0 to 22.7)	14.2 (-1.6 to 33.9)	37.6 (23.5 to 56.5)
Low bone mineral density	3.9 (3.1 to 4.8)	3.8 (3.1 to 4.6)	3.8 (3.0 to 4.6)	-1.3 (-16.0 to 15.3)	-2.3 (-17.4 to 14.1)	5.4 (4.5 to 6.5)	5.4 (4.5 to 6.3)	5.3 (4.4 to 6.4)	-0.1 (-14.3 to 16.1)	-1.3 (-14.5 to 13.8)
Impaired kidney function	2.6 (1.2 to 6.5)	2.8 (1.3 to 6.8)	2.9 (1.4 to 7.0)	8.9 (3.1 to 15.1)	3.6 (0.3 to 8.2)	3.1 (1.5 to 7.2)	3.4 (1.7 to 7.6)	3.5 (1.7 to 7.7)	9.0 (3.5 to 14.9)	4.0 (0.3 to 8.8)

Supplementary Results Table 1. Central Europe age-standardised Summary Exposure Values (SEVs) for Level 4 risk factors and percentage change, by sex, 1990, 2007, and 2017.Data in parentheses are 95% uncertainty intervals.

	Male					Female				
	**1990**	**2007**	**2017**	**Percentage change 1990-2007**	**Percentage change 2007-2017**	**1990**	**2007**	**2017**	**Percentage change 1990-2007**	**Percentage change 2007-2017**
Unsafe water source	16.1 (13.9 to 18.3)	7.2 (6.1 to 8.4)	5.3 (4.5 to 6.3)	-55.5 (-63.0 to -46.8)	-25.5 (-35.3 to -14.5)	16.0 (13.8 to 18.3)	7.1 (6.1 to 8.4)	5.3 (4.5 to 6.3)	-55.3 (-62.9 to -46.6)	-25.6 (-35.4 to -14.7)
Unsafe sanitation	19.9 (15.2 to 25.9)	8.7 (6.3 to 12.3)	5.5 (3.8 to 8.2)	-56.2 (-64.0 to -45.7)	-36.6 (-48.2 to -21.8)	19.8 (15.1 to 25.8)	8.7 (6.3 to 12.3)	5.5 (3.8 to 8.1)	-56.2 (-64.0 to -45.5)	-36.6 (-48.3 to -21.8)
No access to handwashing facility	6.1 (5.5 to 6.7)	4.1 (3.7 to 4.5)	3.4 (3.1 to 3.7)	-32.8 (-39.2 to -25.9)	-16.2 (-22.5 to -9.2)	6.0 (5.5 to 6.6)	4.1 (3.7 to 4.4)	3.4 (3.1 to 3.7)	-32.7 (-39.0 to -25.7)	-16.1 (-22.5 to -9.2)
Ambient particulate matter pollution	35.1 (26.5 to 46.0)	38.6 (29.4 to 50.5)	35.4 (26.3 to 48.4)	9.8 (-9.4 to 32.0)	-8.2 (-23.0 to 8.3)	31.8 (23.2 to 42.1)	36.2 (27.2 to 47.9)	33.5 (24.4 to 46.3)	13.8 (-6.6 to 40.6)	-7.4 (-23.4 to 10.3)
Household air pollution from solid fuels	20.5 (15.5 to 27.9)	10.2 (7.2 to 15.4)	7.5 (5.1 to 12.2)	-50.4 (-60.9 to -36.9)	-26.2 (-40.5 to -8.7)	22.2 (16.9 to 29.7)	11.6 (8.3 to 17.2)	8.7 (6.0 to 13.8)	-47.6 (-58.2 to -34.8)	-24.9 (-39.3 to -8.1)
Ambient ozone pollution	43.5 (16.1 to 54.8)	43.5 (16.0 to 54.8)	44.3 (16.6 to 54.8)	-0.2 (-0.6 to 0.0)	2.0 (0.0 to 4.1)	43.6 (16.1 to 54.8)	43.5 (16.1 to 54.8)	44.3 (16.6 to 54.8)	-0.2 (-0.6 to -0.0)	2.0 (0.0 to 4.0)
Residential radon	33.5 (19.8 to 47.4)	32.8 (19.5 to 46.4)	32.5 (19.4 to 45.9)	-1.9 (-3.4 to -0.4)	-1.0 (-2.3 to 0.3)	33.3 (19.7 to 47.1)	32.7 (19.4 to 46.3)	32.3 (19.2 to 45.7)	-1.8 (-3.1 to -0.5)	-1.1 (-2.3 to 0.1)
Lead exposure	5.7 (2.1 to 9.6)	5.4 (2.1 to 9.2)	4.2 (1.5 to 7.7)	-4.8 (-8.4 to 4.0)	-22.9 (-30.8 to -16.5)	2.5 (0.3 to 6.0)	2.4 (0.3 to 5.8)	1.9 (0.2 to 5.0)	-1.7 (-5.8 to 23.1)	-21.4 (-31.0 to -14.7)
Occupational exposure to asbestos	1.1 (0.9 to 1.2)	1.7 (1.6 to 1.8)	1.9 (1.8 to 2.1)	59.2 (35.9 to 84.5)	14.8 (6.9 to 24.5)	0.5 (0.4 to 0.6)	0.6 (0.6 to 0.6)	0.6 (0.6 to 0.7)	31.9 (6.5 to 55.7)	4.1 (-5.8 to 13.9)
Occupational exposure to arsenic	0.3 (0.0 to 0.8)	0.3 (0.0 to 0.7)	0.3 (0.0 to 0.7)	-	-	0.4 (0.0 to 0.9)	0.3 (0.0 to 0.7)	0.3 (0.0 to 0.8)	-	-
Occupational exposure to benzene	0.4 (0.0 to 1.2)	0.4 (0.0 to 1.2)	0.5 (0.0 to 1.3)	5.0 (2.8 to 7.8)	6.8 (4.8 to 9.4)	0.5 (0.0 to 1.5)	0.6 (0.0 to 1.8)	0.7 (0.0 to 2.0)	17.1 (13.7 to 21.6)	11.8 (9.3 to 15.3)
Occupational exposure to beryllium	0.0 (0.0 to 0.0)	0.0 (0.0 to 0.0)	0.0 (0.0 to 0.0)	-5.9 (-7.3 to -4.4)	4.8 (3.8 to 5.9)	0.0 (0.0 to 0.0)	0.0 (0.0 to 0.0)	0.0 (0.0 to 0.0)	-4.0 (-5.9 to -2.1)	6.5 (5.1 to 7.9)
Occupational exposure to cadmium	0.0 (0.0 to 0.0)	0.0 (0.0 to 0.0)	0.0 (0.0 to 0.0)	-7.2 (-10.3 to -4.2)	4.9 (2.2 to 8.1)	0.0 (0.0 to 0.0)	0.0 (0.0 to 0.0)	0.0 (0.0 to 0.0)	-11.9 (-15.7 to -7.8)	2.8 (-0.8 to 6.6)
Occupational exposure to chromium	0.1 (0.1 to 0.1)	0.1 (0.1 to 0.1)	0.1 (0.1 to 0.1)	-4.8 (-7.8 to -1.9)	5.7 (3.1 to 8.7)	0.1 (0.1 to 0.1)	0.1 (0.1 to 0.1)	0.1 (0.1 to 0.1)	-7.7 (-11.6 to -4.0)	4.7 (1.5 to 7.9)
Occupational exposure to diesel engine exhaust	0.4 (0.4 to 0.4)	0.4 (0.4 to 0.4)	0.4 (0.4 to 0.4)	-2.5 (-5.2 to 0.4)	6.3 (4.1 to 8.4)	0.3 (0.3 to 0.3)	0.3 (0.3 to 0.3)	0.4 (0.4 to 0.4)	1.9 (-1.0 to 5.2)	10.2 (7.6 to 13.1)
Occupational exposure to formaldehyde	0.2 (0.2 to 0.2)	0.2 (0.2 to 0.2)	0.2 (0.2 to 0.2)	-7.3 (-10.6 to -3.7)	4.1 (1.1 to 7.7)	0.2 (0.2 to 0.2)	0.2 (0.2 to 0.2)	0.2 (0.2 to 0.2)	-8.7 (-13.0 to -4.4)	3.3 (-0.3 to 7.1)
Occupational exposure to nickel	0.3 (0.0 to 1.5)	0.3 (0.0 to 1.3)	0.3 (0.0 to 1.4)	-	-	0.3 (0.0 to 1.4)	0.3 (0.0 to 1.1)	0.3 (0.0 to 1.1)	-	-
Occupational exposure to polycyclic aromatic hydrocarbons	0.2 (0.2 to 0.2)	0.2 (0.2 to 0.2)	0.2 (0.2 to 0.2)	-4.4 (-7.0 to -1.7)	5.6 (3.4 to 8.2)	0.2 (0.2 to 0.2)	0.2 (0.2 to 0.2)	0.2 (0.2 to 0.2)	-5.7 (-9.2 to -2.1)	5.2 (2.3 to 8.2)
Occupational exposure to silica	4.1 (0.6 to 13.4)	3.7 (0.5 to 11.8)	3.8 (0.6 to 12.4)	-11.3 (-14.6 to -8.0)	4.7 (2.1 to 7.8)	2.9 (0.4 to 9.2)	2.3 (0.3 to 7.3)	2.3 (0.3 to 7.2)	-20.5 (-24.4 to -17.1)	-0.7 (-4.4 to 3.8)
Occupational exposure to sulphuric acid	0.5 (0.1 to 1.6)	0.5 (0.1 to 1.4)	0.5 (0.1 to 1.5)	-8.0 (-10.6 to -5.2)	2.0 (-0.2 to 4.6)	0.5 (0.1 to 1.6)	0.4 (0.1 to 1.4)	0.4 (0.1 to 1.4)	-14.4 (-17.6 to -10.8)	0.2 (-3.1 to 3.7)
Occupational exposure to trichloroethylene	0.0 (0.0 to 0.0)	0.0 (0.0 to 0.0)	0.0 (0.0 to 0.0)	-2.2 (-4.6 to 0.2)	6.0 (4.1 to 8.1)	0.1 (0.0 to 0.1)	0.0 (0.0 to 0.0)	0.1 (0.1 to 0.1)	-4.3 (-7.4 to -1.1)	6.5 (4.1 to 9.0)
Occupational asthmagens	16.6 (14.2 to 19.6)	14.8 (12.6 to 17.6)	14.7 (12.5 to 17.4)	-10.7 (-14.1 to -7.1)	-1.1 (-4.1 to 1.8)	9.5 (7.8 to 11.5)	8.8 (7.2 to 10.6)	8.6 (7.2 to 10.2)	-7.6 (-12.3 to -2.6)	-1.4 (-6.1 to 3.2)
Occupational particulate matter, gases, and fumes	6.8 (4.9 to 9.8)	6.3 (4.5 to 9.0)	5.9 (4.3 to 8.5)	-7.6 (-10.4 to -5.6)	-5.8 (-7.4 to -4.5)	4.2 (3.0 to 6.1)	3.8 (2.7 to 5.4)	3.4 (2.5 to 4.9)	-9.5 (-12.4 to -6.9)	-9.6 (-11.5 to -7.9)
Occupational noise	6.2 (5.9 to 6.6)	5.8 (5.5 to 6.3)	5.5 (5.3 to 6.0)	-6.2 (-7.3 to -5.2)	-4.8 (-5.3 to -4.2)	3.9 (3.7 to 4.3)	3.8 (3.6 to 4.2)	3.6 (3.5 to 4.0)	-3.6 (-5.2 to -1.9)	-4.0 (-4.7 to -3.2)
Occupational ergonomic factors	12.4 (11.4 to 13.7)	10.6 (9.7 to 11.8)	9.9 (8.9 to 11.1)	-14.3 (-18.0 to -10.5)	-7.1 (-10.4 to -3.4)	9.3 (8.5 to 10.3)	8.0 (7.4 to 8.8)	7.3 (6.5 to 8.3)	-13.9 (-19.0 to -8.7)	-9.3 (-14.4 to -4.0)
Non-exclusive breastfeeding	0.5 (0.3 to 0.6)	0.5 (0.3 to 0.6)	0.5 (0.3 to 0.6)	1.0 (-3.5 to 6.2)	-1.5 (-4.1 to 1.3)	0.5 (0.3 to 0.6)	0.5 (0.3 to 0.6)	0.5 (0.3 to 0.6)	0.8 (-3.6 to 6.0)	-1.6 (-4.2 to 1.3)
Discontinued breastfeeding	1.8 (1.8 to 1.9)	2.1 (2.0 to 2.1)	2.1 (2.1 to 2.1)	12.4 (9.5 to 15.3)	1.4 (-0.5 to 3.2)	1.8 (1.8 to 1.9)	2.1 (2.0 to 2.1)	2.1 (2.1 to 2.1)	12.3 (9.4 to 15.2)	1.4 (-0.5 to 3.2)
Child underweight	0.2 (0.1 to 0.3)	0.1 (0.1 to 0.2)	0.1 (0.1 to 0.2)	-35.1 (-43.9 to -29.3)	-13.0 (-17.5 to -9.4)	0.2 (0.1 to 0.3)	0.1 (0.1 to 0.2)	0.1 (0.1 to 0.1)	-39.9 (-48.8 to -33.8)	-13.5 (-18.0 to -9.8)
Child wasting	0.2 (0.2 to 0.3)	0.2 (0.1 to 0.2)	0.2 (0.1 to 0.2)	-10.3 (-22.3 to 2.3)	-11.5 (-20.2 to -3.3)	0.2 (0.1 to 0.3)	0.2 (0.1 to 0.2)	0.1 (0.1 to 0.2)	-24.1 (-33.6 to -14.6)	-9.3 (-16.4 to -2.1)
Child stunting	0.9 (0.6 to 1.2)	0.6 (0.4 to 0.9)	0.5 (0.3 to 0.7)	-28.6 (-43.5 to -13.5)	-15.9 (-28.2 to -4.0)	0.9 (0.6 to 1.1)	0.6 (0.4 to 0.8)	0.5 (0.3 to 0.7)	-30.1 (-43.8 to -14.6)	-16.7 (-28.4 to -4.1)
Short gestation for birth weight	0.0 (0.0 to 0.0)	0.0 (0.0 to 0.0)	0.0 (0.0 to 0.0)	13.9 (1.5 to 25.2)	6.3 (-3.8 to 17.4)	0.0 (0.0 to 0.0)	0.0 (0.0 to 0.0)	0.0 (0.0 to 0.0)	8.0 (-1.4 to 22.0)	5.0 (-4.9 to 15.5)
Low birth weight for gestation	0.0 (0.0 to 0.0)	0.0 (0.0 to 0.0)	0.0 (0.0 to 0.0)	6.5 (-1.7 to 15.8)	-0.1 (-7.6 to 7.2)	0.0 (0.0 to 0.0)	0.0 (0.0 to 0.0)	0.0 (0.0 to 0.0)	5.6 (-1.7 to 13.1)	0.2 (-7.6 to 7.6)
Iron deficiency	-	-	-	-	-	7.2 (5.2 to 9.4)	6.0 (4.3 to 8.1)	5.2 (3.7 to 7.1)	-16.1 (-22.2 to -9.6)	-13.3 (-19.2 to -7.3)
Vitamin A deficiency	2.9 (2.4 to 3.5)	2.3 (2.0 to 2.7)	2.1 (1.8 to 2.5)	-19.4 (-24.9 to -13.0)	-8.4 (-13.3 to -3.4)	2.9 (2.4 to 3.5)	2.4 (2.0 to 2.8)	2.2 (1.9 to 2.6)	-17.9 (-23.3 to -11.9)	-7.7 (-12.1 to -3.5)
Zinc deficiency	0.5 (0.1 to 1.1)	0.4 (0.1 to 1.0)	0.5 (0.1 to 1.0)	-13.4 (-38.4 to 21.5)	5.6 (-23.2 to 57.8)	0.5 (0.2 to 1.0)	0.4 (0.1 to 1.0)	0.5 (0.1 to 1.0)	-13.8 (-39.2 to 20.2)	4.1 (-23.6 to 59.3)
Smoking	14.5 (13.0 to 16.1)	12.7 (11.4 to 14.0)	11.2 (10.0 to 12.5)	-12.7 (-15.6 to -9.5)	-11.8 (-14.1 to -9.5)	7.3 (6.3 to 8.4)	6.7 (5.8 to 7.6)	6.3 (5.5 to 7.2)	-8.8 (-14.3 to -3.4)	-5.6 (-9.0 to -1.8)
Chewing tobacco	0.1 (0.1 to 0.1)	0.1 (0.1 to 0.1)	0.1 (0.1 to 0.1)	1.3 (-10.0 to 15.2)	-5.9 (-14.5 to 3.1)	0.1 (0.0 to 0.1)	0.1 (0.0 to 0.1)	0.1 (0.0 to 0.1)	-0.2 (-13.2 to 15.0)	0.2 (-10.5 to 11.1)
Second-hand smoke	45.1 (43.8 to 46.2)	42.7 (41.8 to 43.6)	38.3 (36.9 to 39.7)	-5.2 (-8.1 to -2.0)	-10.3 (-13.0 to -7.7)	53.2 (52.2 to 54.3)	48.1 (47.2 to 48.9)	42.1 (40.9 to 43.4)	-9.7 (-11.8 to -7.6)	-12.3 (-14.5 to -10.2)
Alcohol use	29.4 (23.0 to 36.3)	29.7 (22.1 to 38.4)	31.6 (22.6 to 42.3)	1.2 (-6.6 to 8.5)	6.3 (-4.6 to 19.1)	11.6 (7.0 to 18.7)	11.3 (6.7 to 18.6)	12.4 (7.4 to 20.1)	-2.5 (-10.2 to 4.2)	10.2 (-1.8 to 27.1)
Drug use	0.4 (0.3 to 0.6)	0.5 (0.4 to 0.7)	0.5 (0.4 to 0.7)	10.9 (2.8 to 20.7)	4.6 (-1.6 to 11.1)	0.2 (0.1 to 0.3)	0.2 (0.2 to 0.3)	0.2 (0.2 to 0.4)	8.7 (-0.2 to 20.3)	3.6 (-3.2 to 10.8)
Diet low in fruits	40.7 (35.5 to 43.6)	38.5 (33.2 to 41.6)	35.8 (30.3 to 39.3)	-5.4 (-7.4 to -3.9)	-6.9 (-9.6 to -4.7)	38.8 (33.4 to 41.9)	36.3 (30.8 to 39.7)	33.4 (27.9 to 37.3)	-6.3 (-8.6 to -4.5)	-8.1 (-11.4 to -5.6)
Diet low in vegetables	22.0 (15.8 to 27.3)	16.9 (11.6 to 22.1)	14.5 (9.3 to 19.5)	-23.4 (-29.3 to -18.1)	-13.9 (-22.7 to -7.8)	24.5 (18.7 to 29.5)	18.7 (13.3 to 23.9)	16.2 (11.0 to 21.2)	-23.8 (-30.6 to -17.8)	-13.5 (-20.5 to -8.3)
Diet low in legumes	37.1 (34.1 to 39.7)	37.1 (34.1 to 39.6)	36.8 (33.9 to 39.4)	-0.2 (-0.9 to 0.5)	-0.6 (-1.5 to 0.4)	40.0 (37.5 to 42.2)	40.1 (37.5 to 42.2)	40.0 (37.5 to 42.2)	0.0 (-0.6 to 0.7)	-0.2 (-1.0 to 0.7)
Diet low in whole grains	45.9 (44.5 to 47.1)	42.5 (40.6 to 44.2)	41.2 (39.0 to 43.0)	-7.5 (-9.1 to -6.2)	-3.1 (-4.2 to -2.2)	46.4 (45.1 to 47.5)	43.3 (41.5 to 44.8)	42.0 (39.9 to 43.8)	-6.8 (-8.1 to -5.6)	-2.9 (-3.9 to -2.0)
Diet low in nuts and seeds	50.9 (50.1 to 51.6)	50.1 (49.1 to 50.9)	48.0 (46.5 to 49.2)	-1.6 (-2.3 to -1.1)	-4.3 (-5.6 to -3.3)	51.2 (50.5 to 51.9)	50.4 (49.4 to 51.2)	48.4 (47.0 to 49.6)	-1.6 (-2.2 to -1.1)	-4.0 (-5.3 to -3.0)
Diet low in milk	34.5 (30.4 to 38.0)	37.3 (33.8 to 40.2)	36.5 (32.8 to 39.6)	8.0 (5.7 to 11.3)	-2.2 (-3.6 to -1.1)	35.5 (31.6 to 38.8)	38.1 (34.6 to 40.9)	37.2 (33.5 to 40.1)	7.1 (5.0 to 10.1)	-2.4 (-3.7 to -1.3)
Diet high in red meat	30.4 (27.8 to 32.8)	25.6 (22.8 to 28.1)	32.2 (29.1 to 35.2)	-16.0 (-21.4 to -10.8)	26.2 (17.4 to 36.7)	13.6 (10.1 to 16.4)	9.2 (6.0 to 12.1)	12.8 (9.0 to 16.3)	-32.5 (-46.6 to -21.6)	40.3 (21.2 to 74.3)
Diet high in processed meat	3.2 (1.3 to 5.5)	4.0 (1.8 to 6.5)	5.2 (2.8 to 7.8)	23.7 (11.6 to 51.9)	30.9 (18.5 to 64.2)	2.0 (0.5 to 4.1)	2.5 (0.7 to 4.8)	3.4 (1.3 to 5.9)	24.9 (12.6 to 64.7)	34.3 (18.6 to 89.2)
Diet high in sugar-sweetened beverages	8.9 (4.6 to 11.3)	10.6 (5.4 to 12.3)	14.6 (7.4 to 17.0)	18.3 (-2.6 to 41.6)	38.0 (22.5 to 55.0)	6.2 (3.2 to 8.1)	7.2 (3.8 to 8.6)	9.8 (5.1 to 11.7)	16.3 (-5.5 to 42.5)	36.2 (17.9 to 56.7)
Diet low in fibre	26.4 (20.9 to 30.9)	27.0 (21.6 to 31.4)	26.4 (21.0 to 31.0)	2.2 (0.3 to 5.0)	-2.0 (-4.9 to 0.4)	31.8 (27.1 to 35.6)	32.3 (27.7 to 36.1)	31.7 (27.0 to 35.7)	1.6 (-0.0 to 3.8)	-1.7 (-4.1 to 0.4)
Diet low in calcium	24.3 (18.2 to 29.1)	23.6 (17.8 to 28.3)	21.6 (15.7 to 26.5)	-2.8 (-4.4 to -1.1)	-8.6 (-12.5 to -6.2)	27.8 (22.4 to 32.1)	27.1 (21.6 to 31.4)	25.0 (19.4 to 29.6)	-2.6 (-4.2 to -1.4)	-7.5 (-10.8 to -5.3)
Diet low in seafood omega-3 fatty acids	39.6 (36.7 to 42.3)	38.9 (35.9 to 41.8)	36.9 (33.4 to 40.0)	-1.7 (-2.5 to -1.1)	-5.3 (-6.9 to -4.0)	41.7 (39.1 to 44.1)	41.1 (38.5 to 43.6)	39.3 (36.3 to 42.1)	-1.3 (-2.0 to -0.9)	-4.3 (-5.7 to -3.2)
Diet low in polyunsaturated fatty acids	39.9 (37.1 to 42.5)	36.5 (33.1 to 39.3)	35.5 (32.0 to 38.4)	-8.7 (-12.1 to -5.6)	-2.7 (-4.2 to -1.4)	40.4 (37.7 to 42.9)	37.0 (33.8 to 39.8)	36.0 (32.6 to 39.0)	-8.4 (-11.6 to -5.6)	-2.6 (-4.0 to -1.3)
Diet high in trans fatty acids	2.9 (0.9 to 5.7)	1.8 (0.3 to 4.3)	1.3 (0.1 to 3.6)	-36.8 (-66.6 to -23.2)	-26.4 (-54.1 to -15.8)	4.0 (1.6 to 7.1)	2.5 (0.6 to 5.3)	1.8 (0.3 to 4.4)	-37.4 (-63.2 to -23.9)	-26.1 (-52.0 to -15.6)
Diet high in sodium	20.3 (14.1 to 25.8)	11.9 (4.2 to 18.7)	12.9 (4.9 to 19.6)	-41.5 (-70.5 to -26.4)	8.6 (1.6 to 26.7)	15.3 (9.5 to 21.0)	6.5 (0.8 to 13.1)	5.2 (0.2 to 11.9)	-57.7 (-92.2 to -37.0)	-19.2 (-71.8 to -9.1)
Intimate partner violence	-	-	-	-	-	2.9 (2.5 to 3.4)	2.6 (2.1 to 3.0)	2.3 (1.9 to 2.7)	-13.2 (-15.2 to -11.4)	-8.8 (-10.9 to -6.9)
Childhood sexual abuse	8.6 (7.0 to 10.6)	8.6 (7.1 to 10.5)	8.6 (7.1 to 10.6)	-0.2 (-1.3 to 1.0)	0.2 (-0.5 to 0.9)	4.3 (3.3 to 5.6)	4.2 (3.4 to 5.2)	4.3 (3.3 to 5.5)	-2.5 (-7.5 to 3.8)	2.0 (-4.0 to 7.4)

Supplementary Results Table 1. Central Europe age-standardised Summary Exposure Values (SEVs) for Level 4 risk factors and percentage change, by sex, 1990, 2007, and 2017.Data in parentheses are 95% uncertainty intervals.

	Male					Female				
	**1990**	**2007**	**2017**	**Percentage change 1990-2007**	**Percentage change 2007-2017**	**1990**	**2007**	**2017**	**Percentage change 1990-2007**	**Percentage change 2007-2017**
Bullying victimization	4.9<br>(2.1 to 9.8)	5.6<br>(2.6 to 10.7)	5.8<br>(2.8 to 11.1)	14.8<br>(-0.8 to 39.9)	3.6<br>(-0.9 to 9.8)	3.6<br>(1.6 to 7.0)	4.1<br>(2.0 to 7.7)	4.2<br>(2.0 to 8.0)	13.3<br>(-3.3 to 38.3)	2.5<br>(-1.4 to 6.7)
Low physical activity	0.3<br>(0.2 to 0.6)	0.3<br>(0.2 to 0.6)	0.3<br>(0.2 to 0.6)	1.1<br>(0.2 to 2.1)	1.5<br>(0.6 to 2.6)	0.3<br>(0.2 to 0.6)	0.3<br>(0.2 to 0.7)	0.4<br>(0.2 to 0.7)	1.4<br>(0.4 to 2.4)	0.9<br>(0.0 to 1.9)
High fasting plasma glucose	2.3<br>(1.7 to 3.1)	2.9<br>(2.1 to 3.9)	3.2<br>(2.4 to 4.4)	26.7<br>(8.9 to 49.6)	11.4<br>(-5.6 to 32.1)	1.9<br>(1.4 to 2.6)	2.1<br>(1.5 to 2.9)	2.4<br>(1.7 to 3.3)	8.9<br>(-4.6 to 26.1)	14.6<br>(-3.1 to 38.2)
High LDL cholesterol	14.7<br>(13.1 to 16.4)	14.0<br>(12.4 to 15.7)	13.8<br>(12.2 to 15.5)	-4.5<br>(-6.1 to -2.8)	-1.3<br>(-2.7 to 0.0)	14.8<br>(13.3 to 16.5)	14.1<br>(12.6 to 15.8)	13.7<br>(12.2 to 15.4)	-4.8<br>(-6.5 to -3.1)	-3.1<br>(-4.4 to -1.9)
High systolic blood pressure	10.9<br>(10.1 to 11.7)	11.0<br>(10.2 to 11.8)	10.5<br>(9.7 to 11.4)	0.8<br>(-1.7 to 3.3)	-4.4<br>(-6.5 to -2.3)	10.6<br>(9.9 to 11.3)	9.8<br>(9.1 to 10.5)	8.8<br>(8.2 to 9.5)	-7.4<br>(-10.4 to -4.7)	-9.5<br>(-12.0 to -6.9)
High body-mass index	9.9<br>(7.1 to 14.1)	13.5<br>(10.3 to 18.2)	15.2<br>(11.7 to 20.1)	36.3<br>(26.5 to 48.7)	12.5<br>(7.9 to 17.6)	10.5<br>(7.8 to 14.2)	13.7<br>(10.6 to 18.1)	15.1<br>(11.9 to 19.6)	30.0<br>(21.5 to 40.9)	10.7<br>(5.9 to 15.5)
Low bone mineral density	3.7<br>(3.0 to 4.4)	3.6<br>(2.9 to 4.3)	3.5<br>(2.8 to 4.2)	-3.2<br>(-10.3 to 3.5)	-2.7<br>(-9.1 to 4.2)	6.3<br>(5.4 to 7.2)	6.1<br>(5.3 to 7.0)	6.0<br>(5.1 to 6.9)	-3.0<br>(-8.1 to 2.6)	-2.2<br>(-7.7 to 3.9)
Impaired kidney function	2.1<br>(0.9 to 5.7)	2.1<br>(0.9 to 5.7)	2.0<br>(0.9 to 5.6)	-2.3<br>(-4.7 to -0.2)	-1.0<br>(-4.3 to 1.9)	2.8<br>(1.3 to 6.7)	2.8<br>(1.3 to 6.7)	2.8<br>(1.3 to 6.7)	-0.3<br>(-3.1 to 1.7)	0.3<br>(-2.2 to 3.3)

Supplementary Results Table 1. Albania age-standardised Summary Exposure Values (SEVs) for Level 4 risk factors and percentage change, by sex, 1990, 2007, and 2017.Data in parentheses are 95% uncertainty intervals.

	Male					Female				
	1990	2007	2017	Percentage change 1990-2007	Percentage change 2007-2017	1990	2007	2017	Percentage change 1990-2007	Percentage change 2007-2017
Unsafe water source	31.7 (24.5 to 40.3)	22.6 (19.5 to 25.7)	18.3 (13.2 to 23.8)	-28.9 (-44.5 to -7.7)	-19.0 (-38.5 to 1.5)	31.7 (24.5 to 40.3)	22.6 (19.5 to 25.7)	18.3 (13.2 to 23.8)	-28.9 (-44.5 to -7.7)	-19.0 (-38.5 to 1.5)
Unsafe sanitation	35.0 (24.3 to 47.6)	18.7 (14.2 to 25.1)	13.7 (9.0 to 20.4)	-46.5 (-59.7 to -27.8)	-26.7 (-47.8 to 0.3)	35.0 (24.3 to 47.6)	18.7 (14.2 to 25.1)	13.7 (9.0 to 20.4)	-46.5 (-59.7 to -27.8)	-26.7 (-47.8 to 0.3)
No access to handwashing facility	9.9 (7.9 to 12.4)	7.2 (5.8 to 9.0)	5.8 (4.5 to 7.1)	-27.2 (-42.3 to -9.3)	-20.0 (-33.1 to -4.7)	9.9 (7.9 to 12.4)	7.2 (5.8 to 9.0)	5.8 (4.5 to 7.1)	-27.2 (-42.3 to -9.3)	-20.0 (-33.1 to -4.7)
Ambient particulate matter pollution	19.7 (11.7 to 33.2)	26.0 (17.4 to 39.7)	26.1 (16.4 to 41.2)	31.5 (-18.5 to 107.3)	0.6 (-37.5 to 60.9)	16.3 (9.3 to 28.8)	22.1 (14.4 to 34.6)	22.6 (13.7 to 37.0)	35.9 (-18.4 to 120.7)	2.1 (-39.4 to 69.6)
Household air pollution from solid fuels	50.6 (38.8 to 66.6)	30.5 (21.0 to 43.0)	23.0 (14.7 to 34.4)	-39.7 (-56.2 to -21.6)	-24.6 (-48.7 to 7.2)	51.7 (39.6 to 67.2)	32.4 (22.9 to 44.9)	24.9 (16.9 to 35.9)	-37.3 (-52.1 to -21.4)	-23.2 (-44.9 to 4.5)
Ambient ozone pollution	47.4 (18.6 to 54.8)	47.3 (18.4 to 54.8)	47.8 (18.9 to 54.8)	-0.4 (-1.7 to 0.0)	1.1 (0.0 to 4.1)	47.4 (18.6 to 54.8)	47.3 (18.4 to 54.8)	47.8 (18.9 to 54.8)	-0.4 (-1.7 to 0.0)	1.1 (0.0 to 4.1)
Residential radon	43.0 (12.6 to 71.2)	43.0 (12.6 to 71.2)	43.0 (12.6 to 71.2)	0.0 (0.0 to 0.0)	0.0 (0.0 to 0.0)	43.0 (12.6 to 71.2)	43.0 (12.6 to 71.2)	43.0 (12.6 to 71.2)	0.0 (0.0 to 0.0)	0.0 (0.0 to 0.0)
Lead exposure	9.4 (4.8 to 13.7)	9.1 (5.0 to 13.4)	7.6 (3.8 to 11.6)	-3.1 (-9.0 to 5.6)	-17.2 (-24.2 to -11.6)	4.5 (1.1 to 8.5)	4.4 (1.3 to 8.2)	3.7 (1.0 to 7.2)	-1.4 (-8.0 to 21.1)	-16.4 (-24.8 to -10.7)
Occupational exposure to asbestos	0.4 (0.2 to 0.7)	0.6 (0.3 to 1.1)	0.6 (0.3 to 1.3)	29.2 (-17.4 to 88.1)	11.3 (-19.5 to 62.0)	0.2 (0.1 to 0.3)	0.3 (0.2 to 0.6)	0.3 (0.1 to 0.5)	52.8 (-1.5 to 137.1)	-16.0 (-45.4 to 26.2)
Occupational exposure to arsenic	0.2 (0.0 to 0.5)	0.2 (0.0 to 0.4)	0.2 (0.0 to 0.4)	-	-	0.2 (0.0 to 0.4)	0.2 (0.0 to 0.5)	0.2 (0.0 to 0.5)	-	-
Occupational exposure to benzene	0.4 (0.0 to 1.0)	0.4 (0.0 to 1.2)	0.4 (0.0 to 1.2)	13.3 (6.4 to 23.8)	1.6 (-3.7 to 9.3)	0.3 (0.0 to 0.9)	0.4 (0.0 to 1.1)	0.4 (0.0 to 1.2)	24.8 (12.0 to 44.1)	11.7 (3.3 to 24.6)
Occupational exposure to beryllium	0.0 (0.0 to 0.0)	0.0 (0.0 to 0.0)	0.0 (0.0 to 0.0)	1.4 (-3.9 to 6.5)	-2.7 (-6.6 to 1.2)	0.0 (0.0 to 0.0)	0.0 (0.0 to 0.0)	0.0 (0.0 to 0.0)	9.5 (0.5 to 20.9)	2.1 (-3.7 to 8.6)
Occupational exposure to cadmium	0.0 (0.0 to 0.0)	0.0 (0.0 to 0.0)	0.0 (0.0 to 0.0)	0.4 (-6.6 to 8.0)	-0.6 (-5.9 to 5.5)	0.0 (0.0 to 0.0)	0.0 (0.0 to 0.0)	0.0 (0.0 to 0.0)	8.5 (-3.7 to 21.4)	5.9 (-2.5 to 15.5)
Occupational exposure to chromium	0.0 (0.0 to 0.1)	0.1 (0.1 to 0.1)	0.1 (0.1 to 0.1)	7.2 (-0.4 to 15.6)	1.8 (-3.8 to 7.7)	0.0 (0.0 to 0.1)	0.1 (0.0 to 0.1)	0.1 (0.1 to 0.1)	12.6 (-0.3 to 26.3)	9.2 (0.6 to 19.1)
Occupational exposure to diesel engine exhaust	0.4 (0.3 to 0.4)	0.4 (0.4 to 0.5)	0.4 (0.4 to 0.5)	22.8 (13.1 to 33.5)	1.9 (-3.8 to 7.8)	0.2 (0.2 to 0.2)	0.2 (0.2 to 0.2)	0.2 (0.2 to 0.3)	16.8 (5.2 to 30.3)	8.5 (0.5 to 16.9)
Occupational exposure to formaldehyde	0.1 (0.1 to 0.1)	0.1 (0.1 to 0.1)	0.1 (0.1 to 0.1)	2.8 (-5.6 to 12.3)	1.0 (-5.1 to 7.8)	0.1 (0.1 to 0.1)	0.1 (0.1 to 0.1)	0.1 (0.1 to 0.1)	11.6 (-3.0 to 27.0)	10.1 (0.2 to 21.4)
Occupational exposure to nickel	0.2 (0.0 to 1.1)	0.2 (0.0 to 1.1)	0.2 (0.0 to 1.1)	-	-	0.2 (0.0 to 0.8)	0.2 (0.0 to 0.8)	0.2 (0.0 to 0.8)	-	-
Occupational exposure to polycyclic aromatic hydrocarbons	0.1 (0.1 to 0.1)	0.1 (0.1 to 0.1)	0.1 (0.1 to 0.1)	9.2 (2.2 to 17.2)	1.2 (-3.8 to 6.5)	0.1 (0.1 to 0.1)	0.1 (0.1 to 0.1)	0.1 (0.1 to 0.1)	13.5 (0.9 to 27.0)	9.1 (0.8 to 18.5)
Occupational exposure to silica	3.4 (0.5 to 11.2)	3.9 (0.5 to 12.2)	3.8 (0.5 to 12.2)	12.8 (2.3 to 27.6)	-2.4 (-10.6 to 7.3)	1.8 (0.3 to 5.5)	1.8 (0.2 to 5.6)	1.8 (0.2 to 5.5)	-0.6 (-10.6 to 12.2)	0.4 (-7.3 to 9.5)
Occupational exposure to sulphuric acid	0.4 (0.1 to 1.2)	0.4 (0.1 to 1.1)	0.4 (0.1 to 1.1)	0.0 (-6.8 to 7.4)	-2.9 (-8.6 to 3.1)	0.3 (0.1 to 1.0)	0.3 (0.1 to 1.1)	0.3 (0.1 to 1.1)	7.1 (-3.2 to 19.2)	2.3 (-5.2 to 10.5)
Occupational exposure to trichloroethylene	0.0 (0.0 to 0.0)	0.0 (0.0 to 0.0)	0.0 (0.0 to 0.0)	11.0 (4.1 to 18.8)	2.2 (-2.8 to 7.6)	0.0 (0.0 to 0.0)	0.0 (0.0 to 0.0)	0.0 (0.0 to 0.0)	14.5 (3.1 to 28.1)	8.6 (0.8 to 17.3)
Occupational asthmagens	15.5 (12.0 to 20.2)	14.9 (11.9 to 18.6)	14.2 (11.4 to 17.8)	-4.0 (-15.0 to 9.1)	-4.6 (-12.6 to 3.9)	10.1 (7.2 to 14.0)	10.6 (7.9 to 14.1)	10.1 (7.5 to 13.6)	4.4 (-8.5 to 20.8)	-4.7 (-15.6 to 6.9)
Occupational particulate matter, gases, and fumes	6.3 (4.5 to 8.8)	6.0 (4.3 to 8.4)	5.6 (4.0 to 7.9)	-5.0 (-8.8 to -1.5)	-6.3 (-8.8 to -3.9)	3.6 (2.5 to 5.2)	3.6 (2.6 to 5.1)	3.6 (2.6 to 5.2)	0.6 (-5.3 to 7.4)	-0.1 (-4.0 to 4.4)
Occupational noise	6.1 (5.6 to 6.7)	5.9 (5.5 to 6.4)	5.6 (5.3 to 6.1)	-3.3 (-6.0 to -0.3)	-4.8 (-6.6 to -2.8)	3.4 (3.0 to 3.9)	3.5 (3.2 to 3.9)	3.6 (3.3 to 3.9)	2.8 (-2.4 to 9.1)	1.8 (-1.6 to 5.9)
Occupational ergonomic factors	20.0 (17.5 to 22.4)	18.0 (16.6 to 19.4)	16.0 (14.3 to 17.8)	-10.0 (-20.4 to 2.0)	-10.7 (-19.5 to -1.2)	15.8 (13.6 to 17.9)	16.0 (15.0 to 17.0)	14.5 (12.7 to 16.1)	1.1 (-10.6 to 16.2)	-9.2 (-20.0 to 2.6)
Non-exclusive breastfeeding	0.5 (0.3 to 0.8)	0.5 (0.3 to 0.7)	0.5 (0.3 to 0.7)	-11.5 (-17.2 to -4.2)	6.0 (-0.7 to 12.8)	0.5 (0.3 to 0.8)	0.5 (0.3 to 0.7)	0.5 (0.3 to 0.7)	-11.6 (-17.3 to -4.3)	6.0 (-0.7 to 12.8)
Discontinued breastfeeding	1.3 (1.2 to 1.4)	1.3 (1.3 to 1.4)	1.5 (1.4 to 1.6)	2.3 (-6.5 to 12.1)	13.0 (4.9 to 21.8)	1.3 (1.2 to 1.4)	1.3 (1.3 to 1.4)	1.5 (1.4 to 1.6)	2.2 (-6.6 to 11.9)	13.0 (4.9 to 21.9)
Child underweight	0.7 (0.5 to 0.9)	0.4 (0.2 to 0.5)	0.3 (0.2 to 0.4)	-48.0 (-62.7 to -33.1)	-22.9 (-37.4 to -9.4)	0.6 (0.4 to 0.8)	0.3 (0.2 to 0.4)	0.2 (0.1 to 0.3)	-50.5 (-64.7 to -33.7)	-25.5 (-40.3 to -9.5)
Child wasting	0.6 (0.4 to 0.8)	0.4 (0.3 to 0.6)	0.4 (0.3 to 0.6)	-22.6 (-46.0 to 11.1)	-7.2 (-29.6 to 18.7)	0.5 (0.3 to 0.8)	0.4 (0.3 to 0.5)	0.3 (0.2 to 0.5)	-30.9 (-53.9 to 0.7)	-15.2 (-36.1 to 11.2)
Child stunting	3.1 (2.1 to 3.8)	1.7 (1.1 to 2.2)	1.4 (0.8 to 2.0)	-43.0 (-57.7 to -22.5)	-21.7 (-41.9 to 2.4)	2.8 (1.8 to 3.7)	1.6 (1.1 to 2.0)	1.4 (0.8 to 2.0)	-41.2 (-58.1 to -17.3)	-16.1 (-40.1 to 11.1)
Short gestation for birth weight	0.0 (0.0 to 0.0)	0.0 (0.0 to 0.0)	0.0 (0.0 to 0.0)	14.4 (-5.9 to 45.5)	4.4 (-20.3 to 32.1)	0.0 (0.0 to 0.0)	0.0 (0.0 to 0.0)	0.0 (0.0 to 0.0)	-3.2 (-27.6 to 37.1)	7.6 (-17.0 to 33.6)
Low birth weight for gestation	0.0 (0.0 to 0.0)	0.0 (0.0 to 0.0)	0.0 (0.0 to 0.0)	6.5 (-7.6 to 24.2)	4.5 (-12.9 to 26.2)	0.0 (0.0 to 0.0)	0.0 (0.0 to 0.0)	0.0 (0.0 to 0.0)	5.5 (-9.8 to 26.9)	6.2 (-10.1 to 24.6)
Iron deficiency	-	-	-	-	-	9.2 (6.7 to 12.3)	7.9 (5.7 to 10.5)	6.8 (4.8 to 9.3)	-14.9 (-25.6 to -2.3)	-12.9 (-23.2 to -1.5)
Vitamin A deficiency	3.4 (2.8 to 4.1)	2.8 (2.3 to 3.4)	2.5 (2.1 to 3.1)	-18.7 (-28.8 to -6.4)	-8.5 (-17.8 to 1.9)	3.4 (2.9 to 4.1)	2.9 (2.4 to 3.4)	2.6 (2.2 to 3.1)	-17.0 (-27.5 to -6.8)	-7.8 (-17.3 to 2.8)
Zinc deficiency	0.6 (0.2 to 1.4)	0.4 (0.1 to 0.9)	0.4 (0.1 to 1.2)	-31.8 (-45.5 to -9.9)	2.9 (-58.2 to 105.8)	0.6 (0.2 to 1.4)	0.4 (0.1 to 0.9)	0.4 (0.1 to 1.1)	-32.0 (-46.8 to -10.7)	1.7 (-59.5 to 106.1)
Smoking	12.6 (10.2 to 15.3)	12.9 (11.3 to 14.6)	12.8 (11.4 to 14.3)	2.3 (-10.2 to 17.2)	-0.6 (-7.2 to 6.4)	1.7 (1.2 to 2.3)	1.5 (1.2 to 1.8)	2.1 (1.8 to 2.6)	-13.0 (-31.6 to 11.3)	44.9 (24.1 to 69.2)
Chewing tobacco	0.8 (0.6 to 1.0)	0.8 (0.6 to 1.0)	0.8 (0.6 to 1.0)	1.4 (-25.4 to 41.2)	-1.5 (-22.9 to 27.7)	0.1 (0.0 to 0.1)	0.1 (0.0 to 0.1)	0.1 (0.0 to 0.1)	0.2 (-30.0 to 40.9)	0.1 (-22.0 to 30.6)
Second-hand smoke	35.4 (32.8 to 37.7)	32.9 (32.0 to 33.7)	32.9 (30.6 to 35.1)	-7.2 (-13.4 to 0.4)	0.0 (-6.1 to 6.1)	55.0 (52.8 to 57.4)	50.1 (49.1 to 51.1)	51.0 (48.9 to 53.2)	-8.9 (-13.1 to -4.4)	1.9 (-1.6 to 5.5)
Alcohol use	9.2 (5.5 to 14.8)	16.9 (11.0 to 23.2)	19.4 (11.5 to 27.8)	83.1 (34.3 to 161.4)	14.7 (-15.2 to 53.4)	3.5 (2.0 to 5.9)	6.2 (4.0 to 9.5)	6.9 (4.0 to 10.6)	77.2 (30.7 to 150.8)	12.0 (-15.7 to 52.7)
Drug use	0.4 (0.3 to 0.5)	0.4 (0.3 to 0.5)	0.4 (0.3 to 0.5)	2.8 (-3.4 to 9.0)	6.1 (-0.2 to 13.1)	0.2 (0.1 to 0.2)	0.2 (0.1 to 0.2)	0.2 (0.1 to 0.2)	1.4 (-3.2 to 6.2)	3.8 (-0.9 to 9.0)
Diet low in fruits	43.6 (38.5 to 46.2)	28.9 (22.9 to 33.5)	21.0 (14.0 to 27.2)	-33.6 (-42.9 to -26.7)	-27.4 (-41.8 to -16.6)	42.1 (37.0 to 44.9)	25.1 (19.0 to 30.2)	17.1 (10.2 to 23.4)	-40.3 (-51.3 to -31.8)	-32.1 (-50.3 to -20.0)
Diet low in vegetables	21.2 (14.3 to 26.9)	3.4 (0.2 to 9.3)	1.2 (0.0 to 4.2)	-83.8 (-99.0 to -64.0)	-65.8 (-95.3 to -14.2)	23.7 (17.1 to 29.0)	5.7 (0.5 to 12.5)	2.2 (0.1 to 6.6)	-75.9 (-97.1 to -55.2)	-61.3 (-91.1 to -25.3)
Diet low in legumes	32.3 (28.4 to 35.6)	28.7 (24.5 to 32.4)	29.4 (25.1 to 33.2)	-11.0 (-14.9 to -8.0)	2.5 (-1.7 to 6.8)	35.8 (32.5 to 38.6)	32.6 (28.8 to 35.9)	33.2 (29.4 to 36.5)	-8.8 (-11.8 to -6.4)	1.8 (-1.2 to 5.6)
Diet low in whole grains	52.1 (51.7 to 52.5)	52.2 (51.8 to 52.6)	52.0 (51.5 to 52.4)	0.2 (-0.0 to 0.4)	-0.4 (-0.7 to -0.2)	52.0 (51.6 to 52.4)	52.2 (51.8 to 52.6)	52.2 (51.7 to 52.5)	0.4 (0.2 to 0.6)	-0.1 (-0.4 to 0.1)
Diet low in nuts and seeds	50.1 (48.9 to 51.1)	46.0 (43.8 to 47.8)	41.8 (38.0 to 44.5)	-8.2 (-11.2 to -5.8)	-9.1 (-13.7 to -5.5)	50.4 (49.2 to 51.3)	46.5 (44.3 to 48.3)	42.5 (39.0 to 45.1)	-7.7 (-10.9 to -5.3)	-8.7 (-13.0 to -5.4)
Diet low in milk	35.6 (31.5 to 39.1)	28.3 (22.8 to 32.8)	24.8 (18.2 to 30.1)	-20.4 (-29.1 to -14.3)	-12.5 (-21.5 to -6.2)	36.6 (32.5 to 39.8)	29.7 (24.5 to 33.9)	26.3 (20.1 to 31.2)	-18.6 (-26.2 to -12.9)	-11.7 (-19.7 to -6.4)
Diet high in red meat	0.2 (0.0 to 1.0)	5.7 (2.0 to 9.6)	12.0 (6.2 to 17.7)	2475.7 (659.3 to 2021318.3)	112.2 (40.6 to 322.7)	0.0 (0.0 to 0.0)	0.3 (0.0 to 1.3)	2.2 (0.2 to 5.4)	22592.7 (4909.8 to [illegible]	666.8 (151.4 to 513746.0)
Diet high in processed meat	5.3 (2.4 to 8.2)	8.2 (5.3 to 10.9)	8.9 (5.6 to 12.0)	53.5 (22.6 to 133.3)	9.0 (-6.0 to 28.0)	3.3 (0.9 to 5.9)	5.2 (2.4 to 7.9)	5.8 (2.9 to 8.7)	58.4 (23.1 to 206.8)	12.6 (-2.6 to 42.6)
Diet high in sugar-sweetened beverages	4.1 (1.8 to 7.5)	5.8 (2.6 to 10.8)	8.8 (3.9 to 15.4)	41.4 (-20.4 to 166.1)	50.5 (-2.4 to 132.4)	3.0 (1.4 to 5.6)	4.2 (1.8 to 7.5)	6.2 (2.7 to 12.0)	38.6 (-23.3 to 155.6)	48.6 (-6.0 to 135.0)
Diet low in fibre	31.3 (26.3 to 35.4)	29.4 (24.1 to 33.8)	25.0 (18.7 to 30.3)	-6.1 (-11.3 to -2.2)	-15.2 (-24.3 to -9.0)	36.3 (32.2 to 39.5)	34.7 (30.1 to 38.2)	30.8 (25.7 to 35.3)	-4.5 (-8.4 to -1.4)	-11.0 (-17.6 to -6.0)
Diet low in calcium	26.8 (21.0 to 31.4)	11.0 (5.1 to 16.8)	8.4 (3.8 to 13.3)	-59.0 (-76.2 to -45.8)	-23.0 (-31.6 to -12.8)	30.1 (25.0 to 34.2)	14.7 (7.8 to 20.8)	11.1 (5.4 to 16.8)	-51.2 (-69.2 to -38.5)	-24.6 (-35.7 to -17.3)
Diet low in seafood omega-3 fatty acids	49.0 (47.8 to 50.2)	41.4 (38.8 to 43.9)	38.1 (34.8 to 41.0)	-15.4 (-18.9 to -12.5)	-8.1 (-10.8 to -6.0)	49.8 (48.8 to 50.8)	43.3 (41.1 to 45.5)	40.4 (37.6 to 43.0)	-12.9 (-15.8 to -10.4)	-6.8 (-9.1 to -5.0)
Diet low in polyunsaturated fatty acids	43.7 (41.3 to 46.0)	45.5 (43.7 to 47.0)	44.8 (42.8 to 46.6)	4.1 (-0.4 to 7.8)	-1.6 (-3.7 to 0.1)	44.0 (41.6 to 46.1)	45.8 (44.1 to 47.3)	45.2 (42.7 to 47.1)	4.0 (-0.5 to 8.6)	-1.4 (-5.9 to 1.9)
Diet high in trans fatty acids	2.4 (0.3 to 5.3)	1.8 (0.2 to 4.4)	1.4 (0.1 to 3.8)	-24.9 (-58.8 to -7.8)	-22.3 (-59.4 to -6.4)	3.3 (0.8 to 6.6)	2.4 (0.4 to 5.4)	1.9 (0.2 to 4.5)	-25.4 (-55.9 to -7.2)	-22.2 (-56.2 to -5.0)
Diet high in sodium	20.0 (12.1 to 26.7)	10.2 (2.4 to 17.6)	11.4 (3.1 to 19.2)	-49.1 (-82.5 to -27.6)	12.2 (-7.5 to 66.6)	16.7 (9.7 to 23.2)	6.2 (0.5 to 13.3)	5.1 (0.1 to 12.2)	-62.6 (-95.2 to -39.2)	-18.1 (-77.7 to -2.9)
Intimate partner violence	-	-	-	-	-	2.4 (2.0 to 2.8)	2.0 (1.7 to 2.3)	1.9 (1.6 to 2.2)	-17.3 (-22.5 to -12.2)	-5.1 (-11.0 to 2.1)
Childhood sexual abuse	8.7 (7.1 to 10.8)	8.7 (7.1 to 10.8)	8.7 (7.1 to 10.8)	0.0 (0.0 to 0.0)	0.0 (0.0 to 0.0)	4.9 (3.6 to 6.5)	4.9 (3.6 to 6.5)	4.9 (3.6 to 6.5)	0.0 (0.0 to 0.0)	0.0 (0.0 to 0.0)

Supplementary Results Table 1. Albania age-standardised Summary Exposure Values (SEVs) for Level 4 risk factors and percentage change, by sex, 1990, 2007, and 2017.Data in parentheses are 95% uncertainty intervals.

	Male					Female				
	1990	2007	2017	Percentage change 1990-2007	Percentage change 2007-2017	1990	2007	2017	Percentage change 1990-2007	Percentage change 2007-2017
Bullying victimization	4.4<br>(1.9 to 8.9)	4.7<br>(2.0 to 9.5)	4.6<br>(2.0 to 9.6)	5.9<br>(1.3 to 12.3)	-0.2<br>(-2.8 to 2.4)	3.3<br>(1.4 to 6.5)	3.6<br>(1.5 to 7.0)	3.6<br>(1.5 to 6.9)	7.6<br>(1.4 to 15.8)	-0.2<br>(-3.2 to 2.6)
Low physical activity	0.3<br>(0.2 to 0.6)	0.3<br>(0.2 to 0.6)	0.3<br>(0.2 to 0.6)	1.4<br>(-0.9 to 3.7)	1.2<br>(-0.8 to 3.4)	0.3<br>(0.1 to 0.6)	0.3<br>(0.1 to 0.6)	0.3<br>(0.1 to 0.6)	1.6<br>(-0.6 to 4.1)	1.4<br>(-0.8 to 3.9)
High fasting plasma glucose	1.4<br>(0.8 to 2.3)	2.0<br>(1.3 to 3.0)	2.0<br>(1.2 to 3.3)	37.2<br>(-5.1 to 139.2)	4.6<br>(-30.8 to 49.3)	0.9<br>(0.6 to 1.5)	1.1<br>(0.7 to 1.7)	1.2<br>(0.7 to 2.0)	12.6<br>(-30.5 to 84.4)	15.2<br>(-27.0 to 82.8)
High LDL cholesterol	13.1<br>(11.5 to 14.8)	12.1<br>(10.5 to 13.9)	12.1<br>(10.5 to 13.8)	-7.6<br>(-11.8 to -3.0)	-0.4<br>(-3.7 to 3.3)	12.5<br>(10.9 to 14.1)	12.0<br>(10.6 to 13.7)	12.1<br>(10.7 to 13.8)	-3.3<br>(-7.2 to 1.0)	0.7<br>(-2.5 to 4.0)
High systolic blood pressure	10.8<br>(9.9 to 11.7)	11.5<br>(10.5 to 12.6)	11.6<br>(10.6 to 12.7)	6.6<br>(-0.3 to 14.3)	0.6<br>(-4.7 to 5.9)	10.5<br>(9.4 to 11.7)	10.8<br>(9.8 to 11.7)	10.9<br>(9.9 to 12.0)	2.1<br>(-6.7 to 12.3)	1.2<br>(-5.6 to 8.0)
High body-mass index	9.7<br>(6.6 to 14.6)	12.7<br>(9.1 to 18.2)	13.5<br>(9.8 to 19.0)	30.7<br>(12.6 to 54.5)	6.5<br>(-4.6 to 17.3)	9.1<br>(6.2 to 13.3)	12.7<br>(9.3 to 17.8)	13.4<br>(9.7 to 18.9)	38.9<br>(18.6 to 66.5)	5.9<br>(-6.1 to 18.5)
Low bone mineral density	3.8<br>(3.1 to 4.7)	3.6<br>(3.0 to 4.3)	3.5<br>(2.8 to 4.4)	-5.2<br>(-18.8 to 10.5)	-1.7<br>(-16.7 to 17.2)	6.2<br>(5.1 to 7.4)	5.9<br>(5.0 to 7.0)	5.9<br>(4.9 to 7.0)	-4.4<br>(-15.9 to 9.0)	-0.5<br>(-12.9 to 13.6)
Impaired kidney function	1.9<br>(0.9 to 5.4)	1.9<br>(0.9 to 5.3)	1.9<br>(0.9 to 5.3)	-0.1<br>(-4.0 to 3.8)	-0.2<br>(-4.8 to 4.0)	2.6<br>(1.2 to 6.5)	2.6<br>(1.2 to 6.4)	2.6<br>(1.2 to 6.5)	1.7<br>(-2.4 to 6.4)	-0.2<br>(-4.6 to 4.5)

Supplementary Results Table 1. Bosnia and Herzegovina age-standardised Summary Exposure Values (SEVs) for Level 4 risk factors and percentage change, by sex, 1990, 2007, and 2017.Data in parentheses are 95% uncertainty intervals.										
	**Male**					**Female**				
	**1990**	**2007**	**2017**	**Percentage change 1990-2007**	**Percentage change 2007-2017**	**1990**	**2007**	**2017**	**Percentage change 1990-2007**	**Percentage change 2007-2017**
Unsafe water source	22.7 (16.8 to 29.6)	10.0 (7.9 to 12.4)	7.2 (5.1 to 9.9)	-55.9 (-68.1 to -37.4)	-27.8 (-48.4 to -2.6)	22.7 (16.8 to 29.6)	10.0 (7.9 to 12.4)	7.2 (5.1 to 9.9)	-55.9 (-68.1 to -37.4)	-27.8 (-48.4 to -2.6)
Unsafe sanitation	35.8 (25.7 to 49.5)	10.5 (7.6 to 14.5)	6.2 (3.8 to 9.7)	-70.8 (-78.9 to -59.0)	-41.2 (-59.9 to -15.9)	35.8 (25.7 to 49.5)	10.5 (7.6 to 14.5)	6.2 (3.8 to 9.7)	-70.8 (-78.9 to -59.0)	-41.2 (-59.9 to -15.9)
No access to handwashing facility	8.4 (6.8 to 10.3)	4.7 (3.7 to 5.8)	4.0 (3.2 to 5.1)	-44.4 (-57.2 to -29.9)	-13.7 (-27.6 to 4.5)	8.4 (6.8 to 10.3)	4.7 (3.7 to 5.8)	4.0 (3.2 to 5.1)	-44.4 (-57.2 to -29.9)	-13.7 (-27.6 to 4.5)
Ambient particulate matter pollution	21.7 (10.9 to 38.3)	37.0 (23.4 to 53.8)	35.6 (19.6 to 52.2)	70.2 (-11.9 to 254.2)	-3.6 (-43.3 to 57.8)	17.9 (8.8 to 32.2)	32.5 (19.6 to 48.5)	31.7 (16.7 to 47.7)	81.9 (-10.2 to 296.6)	-2.4 (-45.4 to 66.5)
Household air pollution from solid fuels	56.8 (41.9 to 73.4)	25.8 (17.0 to 38.0)	20.6 (12.0 to 32.8)	-54.6 (-70.4 to -35.2)	-20.2 (-49.5 to 20.1)	58.1 (44.0 to 74.1)	28.3 (19.3 to 40.6)	22.8 (14.0 to 34.6)	-51.3 (-66.5 to -34.0)	-19.3 (-46.8 to 14.5)
Ambient ozone pollution	45.3 (16.9 to 54.8)	45.0 (16.7 to 54.8)	45.5 (17.1 to 54.8)	-0.6 (-1.5 to 0.0)	1.2 (0.0 to 2.9)	45.3 (16.9 to 54.8)	45.0 (16.7 to 54.8)	45.5 (17.1 to 54.8)	-0.6 (-1.5 to 0.0)	1.2 (0.0 to 2.9)
Residential radon	38.2 (7.2 to 71.2)	38.2 (7.2 to 71.2)	38.2 (7.2 to 71.2)	0.0 (0.0 to 0.0)	0.0 (0.0 to 0.0)	38.2 (7.2 to 71.2)	38.2 (7.2 to 71.2)	38.2 (7.2 to 71.2)	0.0 (0.0 to 0.0)	0.0 (0.0 to 0.0)
Lead exposure	11.4 (6.5 to 15.7)	10.1 (5.6 to 14.3)	7.9 (4.0 to 11.9)	-10.7 (-17.2 to -5.4)	-22.5 (-29.7 to -16.5)	5.7 (1.8 to 9.9)	5.3 (1.8 to 9.3)	4.2 (1.3 to 7.9)	-8.0 (-15.0 to 2.4)	-21.1 (-30.2 to -14.4)
Occupational exposure to asbestos	1.2 (0.9 to 1.7)	1.3 (1.1 to 1.5)	0.9 (0.8 to 1.1)	7.5 (-20.5 to 46.4)	-29.5 (-42.5 to -13.5)	0.2 (0.1 to 0.3)	0.2 (0.2 to 0.3)	0.1 (0.1 to 0.2)	-3.5 (-34.8 to 43.4)	-28.8 (-46.1 to -8.0)
Occupational exposure to arsenic	0.2 (0.0 to 0.6)	0.2 (0.0 to 0.5)	0.2 (0.0 to 0.5)	-	-	0.2 (0.0 to 0.5)	0.2 (0.0 to 0.4)	0.2 (0.0 to 0.4)	-	-
Occupational exposure to benzene	0.3 (0.0 to 0.9)	0.3 (0.0 to 1.0)	0.3 (0.0 to 1.0)	2.6 (-4.6 to 13.8)	1.6 (-5.2 to 11.1)	0.2 (0.0 to 0.7)	0.3 (0.0 to 0.9)	0.4 (0.0 to 1.1)	31.5 (14.1 to 60.9)	20.4 (10.2 to 37.7)
Occupational exposure to beryllium	0.0 (0.0 to 0.0)	0.0 (0.0 to 0.0)	0.0 (0.0 to 0.0)	-15.6 (-20.8 to -9.7)	0.3 (-5.1 to 6.2)	0.0 (0.0 to 0.0)	0.0 (0.0 to 0.0)	0.0 (0.0 to 0.0)	-2.1 (-13.5 to 11.3)	12.2 (3.8 to 22.0)
Occupational exposure to cadmium	0.0 (0.0 to 0.0)	0.0 (0.0 to 0.0)	0.0 (0.0 to 0.0)	-10.9 (-20.3 to -0.5)	1.9 (-6.4 to 10.8)	0.0 (0.0 to 0.0)	0.0 (0.0 to 0.0)	0.0 (0.0 to 0.0)	-3.8 (-17.7 to 14.4)	8.5 (-3.1 to 22.0)
Occupational exposure to chromium	0.1 (0.1 to 0.1)	0.1 (0.1 to 0.1)	0.1 (0.1 to 0.1)	-6.6 (-16.1 to 3.6)	2.6 (-5.3 to 11.1)	0.0 (0.0 to 0.1)	0.0 (0.0 to 0.1)	0.1 (0.0 to 0.1)	3.2 (-11.4 to 22.6)	10.5 (-0.5 to 23.6)
Occupational exposure to diesel engine exhaust	0.4 (0.3 to 0.4)	0.3 (0.3 to 0.4)	0.3 (0.3 to 0.4)	-11.3 (-20.3 to -1.9)	-0.4 (-7.2 to 6.9)	0.2 (0.1 to 0.2)	0.2 (0.2 to 0.2)	0.2 (0.2 to 0.2)	12.5 (-2.2 to 32.0)	13.4 (2.5 to 25.3)
Occupational exposure to formaldehyde	0.1 (0.1 to 0.2)	0.1 (0.1 to 0.1)	0.1 (0.1 to 0.1)	-8.3 (-19.2 to 3.4)	1.1 (-7.6 to 10.7)	0.1 (0.1 to 0.1)	0.1 (0.1 to 0.1)	0.1 (0.1 to 0.1)	1.8 (-14.1 to 22.3)	9.4 (-2.3 to 23.5)
Occupational exposure to nickel	0.3 (0.0 to 1.4)	0.2 (0.0 to 1.1)	0.2 (0.0 to 1.1)	-	-	0.2 (0.0 to 0.7)	0.1 (0.0 to 0.7)	0.2 (0.0 to 0.7)	-	-
Occupational exposure to polycyclic aromatic hydrocarbons	0.1 (0.1 to 0.1)	0.1 (0.1 to 0.1)	0.1 (0.1 to 0.1)	-6.8 (-15.3 to 2.5)	1.6 (-5.6 to 9.4)	0.1 (0.1 to 0.1)	0.1 (0.1 to 0.1)	0.1 (0.1 to 0.1)	4.1 (-10.2 to 22.8)	11.3 (0.7 to 23.8)
Occupational exposure to silica	4.1 (0.6 to 13.2)	3.2 (0.4 to 10.2)	3.0 (0.4 to 9.7)	-21.5 (-31.3 to -10.2)	-5.1 (-13.0 to 4.0)	1.7 (0.2 to 5.7)	1.3 (0.2 to 4.2)	1.4 (0.2 to 4.3)	-22.0 (-33.0 to -6.1)	1.2 (-9.9 to 16.1)
Occupational exposure to sulphuric acid	0.4 (0.1 to 1.3)	0.3 (0.1 to 1.1)	0.4 (0.1 to 1.1)	-10.8 (-19.2 to -1.2)	0.2 (-7.7 to 8.8)	0.3 (0.1 to 0.8)	0.2 (0.1 to 0.8)	0.3 (0.1 to 0.8)	-7.9 (-20.7 to 8.6)	6.5 (-4.7 to 19.7)
Occupational exposure to trichloroethylene	0.0 (0.0 to 0.0)	0.0 (0.0 to 0.0)	0.0 (0.0 to 0.0)	-3.4 (-11.4 to 6.0)	3.8 (-3.0 to 11.4)	0.0 (0.0 to 0.0)	0.0 (0.0 to 0.0)	0.0 (0.0 to 0.0)	8.3 (-5.7 to 27.4)	12.1 (2.1 to 24.8)
Occupational asthmagens	13.6 (11.1 to 16.5)	10.6 (9.0 to 12.7)	10.6 (8.9 to 12.7)	-21.5 (-31.3 to -11.2)	-0.0 (-8.2 to 9.6)	5.5 (4.0 to 7.5)	5.1 (4.1 to 6.3)	5.4 (4.3 to 6.9)	-7.8 (-24.9 to 15.2)	7.0 (-5.6 to 23.5)
Occupational particulate matter, gases, and fumes	6.2 (4.3 to 8.7)	5.5 (3.9 to 7.8)	4.9 (3.4 to 7.0)	-10.9 (-15.0 to -6.9)	-11.2 (-14.4 to -8.0)	2.6 (1.8 to 3.9)	2.4 (1.7 to 3.5)	2.1 (1.5 to 3.1)	-9.5 (-16.6 to -1.9)	-10.2 (-14.7 to -5.4)
Occupational noise	5.8 (5.3 to 6.4)	5.3 (4.8 to 5.8)	4.7 (4.4 to 5.2)	-9.7 (-12.5 to -6.5)	-10.4 (-12.2 to -8.2)	2.4 (2.0 to 2.9)	2.3 (2.0 to 2.7)	2.2 (2.0 to 2.5)	-5.9 (-11.5 to 1.0)	-5.6 (-9.5 to -1.0)
Occupational ergonomic factors	11.3 (9.6 to 13.4)	7.8 (6.8 to 9.0)	7.7 (6.5 to 9.0)	-31.5 (-42.4 to -20.5)	-1.0 (-10.5 to 11.0)	6.4 (4.9 to 8.1)	5.0 (4.3 to 5.8)	5.1 (4.0 to 6.3)	-22.0 (-37.9 to 2.3)	1.2 (-15.1 to 22.1)
Non-exclusive breastfeeding	0.6 (0.4 to 0.8)	0.6 (0.5 to 0.8)	0.6 (0.4 to 0.8)	0.7 (-6.0 to 8.6)	-2.3 (-8.0 to 3.9)	0.6 (0.4 to 0.8)	0.6 (0.5 to 0.8)	0.6 (0.4 to 0.8)	0.7 (-6.0 to 8.6)	-2.3 (-8.0 to 3.9)
Discontinued breastfeeding	2.0 (1.9 to 2.1)	2.2 (2.2 to 2.3)	2.2 (2.1 to 2.3)	12.1 (6.6 to 18.2)	-2.2 (-5.8 to 1.3)	2.0 (1.9 to 2.1)	2.2 (2.2 to 2.3)	2.2 (2.1 to 2.3)	12.2 (6.7 to 18.2)	-2.0 (-5.6 to 1.5)
Child underweight	0.2 (0.1 to 0.3)	0.1 (0.1 to 0.2)	0.1 (0.1 to 0.2)	-49.0 (-62.4 to -35.2)	-11.9 (-20.0 to -3.3)	0.2 (0.1 to 0.2)	0.1 (0.0 to 0.1)	0.1 (0.0 to 0.1)	-45.1 (-55.9 to -34.5)	-13.2 (-21.6 to -4.7)
Child wasting	0.3 (0.2 to 0.4)	0.2 (0.1 to 0.2)	0.1 (0.1 to 0.2)	-39.6 (-59.0 to -11.6)	-28.1 (-41.6 to -13.3)	0.2 (0.1 to 0.3)	0.1 (0.1 to 0.2)	0.1 (0.1 to 0.2)	-38.0 (-56.5 to -5.1)	-17.2 (-29.2 to -2.8)
Child stunting	1.1 (0.6 to 1.6)	0.6 (0.4 to 0.9)	0.5 (0.3 to 0.8)	-42.2 (-65.1 to -5.0)	-19.0 (-43.8 to 14.2)	1.0 (0.6 to 1.6)	0.6 (0.4 to 0.9)	0.5 (0.3 to 0.8)	-41.3 (-63.2 to -6.3)	-17.1 (-42.8 to 14.3)
Short gestation for birth weight	0.0 (0.0 to 0.0)	0.0 (0.0 to 0.0)	0.0 (0.0 to 0.0)	-6.7 (-31.2 to 26.7)	10.8 (-14.3 to 44.9)	0.0 (0.0 to 0.0)	0.0 (0.0 to 0.0)	0.0 (0.0 to 0.0)	-19.0 (-37.0 to 5.7)	14.6 (-11.0 to 44.7)
Low birth weight for gestation	0.0 (0.0 to 0.0)	0.0 (0.0 to 0.0)	0.0 (0.0 to 0.0)	21.9 (1.4 to 50.4)	-4.8 (-23.2 to 14.4)	0.0 (0.0 to 0.0)	0.0 (0.0 to 0.0)	0.0 (0.0 to 0.0)	24.8 (5.1 to 47.5)	-7.1 (-21.9 to 10.0)
Iron deficiency	-	-	-	-	-	9.2 (6.9 to 11.9)	6.9 (5.0 to 9.5)	6.1 (4.2 to 8.5)	-24.6 (-35.1 to -12.5)	-11.9 (-23.5 to 0.6)
Vitamin A deficiency	3.4 (2.8 to 4.0)	2.5 (2.1 to 3.1)	2.4 (2.0 to 2.8)	-24.7 (-35.7 to -13.4)	-7.0 (-16.3 to 2.3)	3.4 (2.8 to 4.0)	2.6 (2.2 to 3.1)	2.5 (2.1 to 2.9)	-22.4 (-33.2 to -10.8)	-6.5 (-15.1 to 2.6)
Zinc deficiency	0.5 (0.1 to 1.0)	0.4 (0.1 to 1.1)	0.5 (0.1 to 1.2)	-10.3 (-58.9 to 93.6)	20.4 (-59.3 to 269.8)	0.5 (0.1 to 1.0)	0.4 (0.1 to 1.0)	0.5 (0.1 to 1.2)	-10.3 (-57.9 to 94.7)	21.1 (-55.1 to 257.5)
Smoking	11.4 (9.8 to 13.2)	11.9 (10.4 to 13.4)	13.4 (11.9 to 15.0)	4.6 (-3.7 to 14.9)	12.8 (6.5 to 19.6)	5.5 (4.3 to 6.9)	5.5 (4.7 to 6.5)	6.9 (6.0 to 8.0)	-0.1 (-15.9 to 20.3)	25.4 (14.3 to 37.3)
Chewing tobacco	0.1 (0.1 to 0.2)	0.1 (0.1 to 0.2)	0.1 (0.1 to 0.2)	0.1 (-32.0 to 46.1)	-3.8 (-27.2 to 25.1)	0.1 (0.0 to 0.1)	0.1 (0.0 to 0.1)	0.1 (0.0 to 0.1)	-0.6 (-29.9 to 41.8)	0.8 (-21.5 to 27.3)
Second-hand smoke	40.6 (38.3 to 42.9)	47.5 (46.3 to 48.8)	45.8 (43.9 to 47.7)	17.2 (9.7 to 25.1)	-3.7 (-7.7 to 0.5)	49.0 (46.8 to 51.2)	58.7 (57.5 to 59.9)	56.2 (54.3 to 57.9)	19.8 (13.8 to 26.3)	-4.2 (-7.4 to -1.1)
Alcohol use	24.6 (19.7 to 28.9)	25.3 (19.8 to 30.1)	27.1 (20.8 to 33.7)	3.0 (-4.2 to 11.0)	7.1 (-4.9 to 21.2)	9.9 (7.2 to 12.8)	9.2 (6.6 to 11.8)	10.4 (7.0 to 13.9)	-6.9 (-23.6 to 12.7)	13.0 (-11.8 to 41.1)
Drug use	0.3 (0.2 to 0.3)	0.3 (0.3 to 0.4)	0.3 (0.3 to 0.4)	25.9 (14.4 to 39.6)	1.2 (-6.0 to 9.4)	0.1 (0.1 to 0.1)	0.1 (0.1 to 0.1)	0.1 (0.1 to 0.1)	6.6 (0.3 to 14.3)	5.5 (-0.6 to 11.3)
Diet low in fruits	48.5 (44.0 to 50.4)	36.8 (31.2 to 40.4)	32.7 (26.8 to 37.2)	-24.2 (-30.4 to -19.2)	-11.0 (-18.3 to -5.6)	47.6 (42.9 to 49.7)	34.2 (28.6 to 38.3)	29.6 (23.6 to 34.5)	-28.1 (-35.3 to -22.3)	-13.5 (-21.9 to -7.2)
Diet low in vegetables	17.0 (9.3 to 23.3)	5.4 (0.5 to 11.9)	5.3 (0.7 to 11.7)	-68.0 (-94.6 to -46.0)	-2.5 (-42.2 to 166.0)	19.8 (12.7 to 25.8)	8.1 (1.6 to 14.8)	7.7 (1.6 to 14.7)	-59.3 (-88.6 to -39.8)	-5.1 (-41.3 to 80.5)
Diet low in legumes	23.5 (18.2 to 28.0)	18.1 (12.4 to 23.1)	19.1 (13.1 to 24.3)	-23.1 (-35.1 to -15.1)	5.4 (-5.0 to 19.4)	28.2 (23.6 to 32.1)	23.5 (18.3 to 27.9)	24.1 (18.9 to 28.7)	-16.8 (-24.7 to -11.2)	2.9 (-4.3 to 11.6)
Diet low in whole grains	40.7 (38.4 to 42.7)	35.6 (32.5 to 38.3)	35.0 (31.7 to 37.9)	-12.3 (-15.9 to -9.3)	-1.7 (-4.9 to 1.3)	41.6 (39.4 to 43.4)	36.8 (33.8 to 39.4)	36.2 (33.0 to 39.0)	-11.5 (-15.0 to -8.6)	-1.6 (-4.6 to 1.1)
Diet low in nuts and seeds	53.2 (52.8 to 53.5)	49.9 (48.5 to 50.9)	48.5 (46.7 to 50.0)	-6.2 (-8.2 to -4.7)	-2.8 (-4.7 to -1.2)	53.3 (52.9 to 53.6)	50.1 (48.9 to 51.1)	48.8 (47.0 to 50.2)	-5.9 (-7.7 to -4.4)	-2.6 (-4.4 to -1.2)
Diet low in milk	42.6 (39.9 to 44.9)	38.6 (35.3 to 41.5)	38.6 (35.1 to 41.6)	-9.3 (-12.8 to -6.5)	-0.0 (-2.4 to 2.3)	43.3 (40.9 to 45.5)	39.3 (36.1 to 42.0)	39.3 (35.9 to 42.1)	-9.2 (-12.7 to -6.6)	-0.2 (-2.4 to 2.0)
Diet high in red meat	0.0 (0.0 to 0.1)	0.0 (0.0 to 0.3)	0.6 (0.0 to 2.1)	413.0 (-61.8 to [illegible]	1327.7 (297.5 to [illegible]	0.0 (0.0 to 0.0)	0.0 (0.0 to 0.0)	0.0 (0.0 to 0.2)	302.3 (-72.4 to [illegible]	55100.4 (-68.3 to [illegible]
Diet high in processed meat	0.7 (0.0 to 2.5)	2.5 (0.4 to 5.2)	3.4 (1.0 to 6.1)	236.0 (93.5 to 6667.9)	36.7 (12.4 to 153.2)	0.4 (0.0 to 1.8)	1.5 (0.1 to 3.8)	2.1 (0.3 to 4.6)	251.5 (101.3 to 21990.8)	39.9 (14.9 to 276.1)
Diet high in sugar-sweetened beverages	1.8 (0.8 to 3.2)	6.4 (2.9 to 11.5)	8.4 (3.8 to 15.0)	261.1 (94.9 to 602.3)	31.6 (-13.4 to 97.8)	1.4 (0.6 to 2.7)	4.6 (2.1 to 8.7)	6.0 (2.7 to 11.2)	221.1 (71.7 to 522.1)	30.9 (-14.5 to 100.2)
Diet low in fibre	13.4 (7.4 to 19.3)	11.2 (5.7 to 16.8)	12.3 (6.4 to 18.4)	-16.4 (-31.7 to -3.7)	10.0 (-5.9 to 35.4)	19.6 (13.0 to 25.5)	17.1 (10.8 to 22.9)	18.1 (11.7 to 24.0)	-13.1 (-25.5 to -2.9)	6.1 (-4.4 to 21.0)
Diet low in calcium	36.0 (32.2 to 39.1)	22.7 (16.4 to 27.8)	20.3 (13.4 to 25.9)	-36.9 (-49.3 to -28.7)	-10.9 (-19.1 to -6.1)	38.6 (35.3 to 41.3)	26.4 (20.6 to 30.9)	24.0 (17.8 to 29.0)	-31.6 (-41.6 to -24.7)	-8.9 (-15.7 to -5.2)
Diet low in seafood omega-3 fatty acids	48.9 (47.6 to 50.0)	45.6 (43.8 to 47.4)	43.1 (40.8 to 45.3)	-6.6 (-8.2 to -5.2)	-5.5 (-7.1 to -4.2)	49.7 (48.7 to 50.7)	46.9 (45.3 to 48.5)	44.8 (42.7 to 46.7)	-5.6 (-6.9 to -4.4)	-4.6 (-6.0 to -3.4)
Diet low in polyunsaturated fatty acids	46.6 (44.6 to 48.2)	43.0 (40.8 to 44.9)	42.4 (40.0 to 44.4)	-7.6 (-10.6 to -3.6)	-1.5 (-3.0 to -0.0)	46.9 (45.1 to 48.5)	43.4 (41.2 to 45.2)	42.8 (40.3 to 44.8)	-7.4 (-10.2 to -3.8)	-1.5 (-3.2 to 0.0)
Diet high in trans fatty acids	0.4 (0.0 to 1.8)	0.4 (0.0 to 1.7)	0.4 (0.0 to 1.7)	-7.7 (-56.3 to 9.2)	-5.1 (-51.1 to 11.8)	0.6 (0.0 to 2.2)	0.5 (0.0 to 2.2)	0.5 (0.0 to 2.1)	-8.4 (-45.3 to 7.9)	-5.0 (-43.5 to 12.9)
Diet high in sodium	16.9 (9.5 to 23.6)	10.5 (2.5 to 18.0)	11.3 (3.1 to 18.9)	-38.1 (-75.9 to -16.5)	8.0 (-11.4 to 55.6)	15.5 (8.3 to 22.0)	6.3 (0.6 to 13.4)	5.1 (0.1 to 11.9)	-59.3 (-94.2 to -34.8)	-19.7 (-79.5 to -5.0)
Intimate partner violence	-	-	-	-	-	3.0 (2.3 to 3.7)	2.9 (2.2 to 3.6)	2.7 (2.1 to 3.4)	-2.4 (-8.0 to 4.0)	-5.2 (-11.0 to 0.3)
Childhood sexual abuse	8.7 (7.1 to 10.8)	8.7 (7.1 to 10.8)	8.7 (7.1 to 10.8)	0.0 (0.0 to 0.0)	0.0 (0.0 to 0.0)	4.9 (3.6 to 6.5)	4.9 (3.6 to 6.5)	4.9 (3.6 to 6.5)	0.0 (0.0 to 0.0)	0.0 (0.0 to 0.0)

Supplementary Results Table 1. Bosnia and Herzegovina age-standardised Summary Exposure Values (SEVs) for Level 4 risk factors and percentage change, by sex, 1990, 2007, and 2017.Data in parentheses are 95% uncertainty intervals.

	Male					Female				
	**1990**	**2007**	**2017**	**Percentage change 1990-2007**	**Percentage change 2007-2017**	**1990**	**2007**	**2017**	**Percentage change 1990-2007**	**Percentage change 2007-2017**
Bullying victimization	4.4 (1.8 to 9.5)	4.8 (2.0 to 9.9)	4.7 (1.9 to 10.0)	7.8 (3.1 to 13.9)	-0.6 (-3.0 to 1.7)	3.4 (1.4 to 6.9)	3.7 (1.5 to 7.5)	3.7 (1.5 to 7.5)	8.0 (3.4 to 14.7)	-0.8 (-3.1 to 1.6)
Low physical activity	0.4 (0.2 to 0.6)	0.4 (0.2 to 0.7)	0.4 (0.2 to 0.7)	3.2 (1.1 to 5.5)	0.6 (-1.2 to 2.3)	0.3 (0.1 to 0.5)	0.3 (0.1 to 0.5)	0.3 (0.1 to 0.5)	1.6 (-0.3 to 3.7)	1.0 (-0.6 to 2.9)
High fasting plasma glucose	2.8 (1.8 to 4.3)	4.2 (3.0 to 6.2)	4.7 (3.3 to 7.2)	51.8 (16.8 to 108.4)	10.8 (-10.7 to 37.1)	2.2 (1.4 to 3.4)	3.2 (2.2 to 4.8)	3.5 (2.4 to 5.2)	46.2 (9.0 to 116.6)	9.9 (-11.1 to 36.4)
High LDL cholesterol	12.7 (11.1 to 14.5)	12.7 (11.0 to 14.5)	12.8 (11.3 to 14.7)	-0.1 (-4.4 to 4.9)	1.4 (-2.2 to 4.9)	12.0 (10.5 to 13.7)	12.6 (11.1 to 14.3)	12.8 (11.3 to 14.5)	5.0 (1.0 to 9.4)	1.6 (-1.6 to 4.9)
High systolic blood pressure	9.5 (8.6 to 10.5)	9.8 (8.8 to 10.7)	9.8 (8.8 to 10.8)	3.0 (-4.4 to 10.9)	0.2 (-5.1 to 6.2)	11.2 (10.1 to 12.2)	11.6 (10.6 to 12.6)	10.9 (9.8 to 11.9)	3.9 (-5.5 to 13.9)	-6.2 (-12.9 to 0.4)
High body-mass index	6.4 (4.3 to 9.6)	11.8 (8.6 to 16.6)	12.6 (9.3 to 18.2)	83.1 (56.0 to 126.2)	7.2 (-2.2 to 17.6)	6.7 (4.5 to 9.9)	12.6 (9.7 to 17.3)	13.6 (10.2 to 18.6)	87.8 (59.1 to 131.2)	8.1 (-1.9 to 19.1)
Low bone mineral density	3.8 (3.1 to 4.7)	3.7 (3.0 to 4.4)	3.6 (2.9 to 4.4)	-3.4 (-18.0 to 12.6)	-2.0 (-17.8 to 14.2)	6.2 (5.2 to 7.4)	6.0 (5.1 to 7.1)	6.0 (5.0 to 7.1)	-3.5 (-15.1 to 9.1)	-1.2 (-15.1 to 13.4)
Impaired kidney function	2.0 (0.9 to 5.7)	2.2 (1.0 to 5.8)	2.2 (1.0 to 5.9)	6.1 (0.9 to 11.6)	1.6 (-2.5 to 6.4)	2.8 (1.3 to 6.6)	3.0 (1.4 to 6.9)	3.0 (1.4 to 7.0)	7.0 (0.8 to 13.6)	1.8 (-2.0 to 6.4)

Supplementary Results Table 1. Bulgaria age-standardised Summary Exposure Values (SEVs) for Level 4 risk factors and percentage change, by sex, 1990, 2007, and 2017.Data in parentheses are 95% uncertainty intervals.

	Male					Female				
	1990	2007	2017	Percentage change 1990-2007	Percentage change 2007-2017	1990	2007	2017	Percentage change 1990-2007	Percentage change 2007-2017
Unsafe water source	13.8 (9.7 to 18.6)	7.7 (5.1 to 10.9)	6.0 (4.1 to 8.7)	-44.0 (-63.6 to -14.0)	-21.8 (-44.4 to 10.8)	13.8 (9.7 to 18.6)	7.7 (5.1 to 10.9)	6.0 (4.1 to 8.7)	-44.0 (-63.6 to -14.0)	-21.8 (-44.4 to 10.8)
Unsafe sanitation	2.9 (1.6 to 4.6)	1.9 (1.1 to 3.1)	1.3 (0.7 to 2.3)	-34.2 (-63.0 to 21.9)	-28.4 (-54.0 to 13.8)	2.9 (1.6 to 4.6)	1.9 (1.1 to 3.1)	1.3 (0.7 to 2.3)	-34.2 (-63.0 to 21.9)	-28.4 (-54.0 to 13.8)
No access to handwashing facility	4.9 (3.9 to 6.0)	3.5 (2.8 to 4.4)	3.2 (2.5 to 4.1)	-27.4 (-44.4 to -7.0)	-8.7 (-24.1 to 9.9)	4.9 (3.9 to 6.0)	3.5 (2.8 to 4.4)	3.2 (2.5 to 4.1)	-27.4 (-44.4 to -7.0)	-8.7 (-24.1 to 9.9)
Ambient particulate matter pollution	36.9 (22.3 to 52.6)	37.8 (26.6 to 53.1)	35.1 (22.2 to 50.4)	2.5 (-29.3 to 59.0)	-7.3 (-39.9 to 32.9)	33.0 (19.2 to 48.9)	34.9 (23.6 to 50.4)	32.7 (20.0 to 48.0)	5.7 (-30.0 to 72.8)	-6.3 (-41.4 to 38.8)
Household air pollution from solid fuels	18.0 (10.7 to 27.3)	10.7 (5.5 to 18.5)	8.4 (3.9 to 15.9)	-40.5 (-68.0 to 1.6)	-22.1 (-57.9 to 41.6)	20.2 (12.4 to 29.2)	12.5 (6.6 to 20.0)	9.9 (4.9 to 18.0)	-37.9 (-65.3 to 1.1)	-20.9 (-56.0 to 35.3)
Ambient ozone pollution	43.1 (15.6 to 54.8)	42.6 (15.2 to 54.8)	43.0 (15.4 to 54.8)	-1.3 (-2.7 to 0.0)	0.9 (0.0 to 2.1)	43.1 (15.6 to 54.8)	42.6 (15.2 to 54.8)	43.0 (15.4 to 54.8)	-1.3 (-2.7 to 0.0)	0.9 (0.0 to 2.1)
Residential radon	31.8 (8.6 to 69.9)	31.8 (8.6 to 69.9)	31.8 (8.6 to 69.9)	0.0 (0.0 to 0.0)	0.0 (0.0 to 0.0)	31.8 (8.6 to 69.9)	31.8 (8.6 to 69.9)	31.8 (8.6 to 69.9)	0.0 (0.0 to 0.0)	0.0 (0.0 to 0.0)
Lead exposure	5.2 (1.5 to 9.3)	4.6 (1.4 to 8.5)	3.6 (1.0 to 7.1)	-10.4 (-17.4 to -1.1)	-23.1 (-33.0 to -16.0)	2.8 (0.3 to 6.5)	2.5 (0.3 to 6.0)	2.0 (0.2 to 5.0)	-7.9 (-14.5 to 13.6)	-22.5 (-35.1 to -15.4)
Occupational exposure to asbestos	0.3 (0.2 to 0.4)	0.3 (0.3 to 0.4)	0.3 (0.2 to 0.4)	23.9 (-5.6 to 80.8)	-10.8 (-26.0 to 5.6)	0.1 (0.1 to 0.2)	0.2 (0.1 to 0.2)	0.2 (0.2 to 0.2)	38.5 (-4.0 to 103.9)	9.2 (-10.6 to 32.3)
Occupational exposure to arsenic	0.3 (0.0 to 0.6)	0.3 (0.0 to 0.6)	0.3 (0.0 to 0.6)	-	-	0.3 (0.0 to 0.8)	0.4 (0.0 to 0.9)	0.4 (0.0 to 0.9)	-	-
Occupational exposure to benzene	0.3 (0.0 to 1.0)	0.4 (0.0 to 1.3)	0.5 (0.0 to 1.3)	28.0 (19.7 to 41.8)	4.6 (0.1 to 11.2)	0.5 (0.0 to 1.3)	0.7 (0.0 to 1.9)	0.7 (0.0 to 2.1)	44.8 (34.1 to 61.7)	8.9 (4.4 to 15.8)
Occupational exposure to beryllium	0.0 (0.0 to 0.0)	0.0 (0.0 to 0.0)	0.0 (0.0 to 0.0)	12.4 (6.7 to 18.5)	3.0 (0.1 to 5.8)	0.0 (0.0 to 0.0)	0.0 (0.0 to 0.0)	0.0 (0.0 to 0.0)	24.8 (16.8 to 33.8)	4.7 (2.0 to 7.6)
Occupational exposure to cadmium	0.0 (0.0 to 0.0)	0.0 (0.0 to 0.0)	0.0 (0.0 to 0.0)	1.1 (-6.4 to 9.3)	2.5 (-3.7 to 8.9)	0.0 (0.0 to 0.0)	0.0 (0.0 to 0.0)	0.0 (0.0 to 0.0)	16.8 (6.4 to 28.9)	-0.7 (-7.6 to 6.1)
Occupational exposure to chromium	0.1 (0.1 to 0.1)	0.1 (0.1 to 0.1)	0.1 (0.1 to 0.1)	5.7 (-1.8 to 13.6)	3.3 (-2.2 to 8.7)	0.1 (0.1 to 0.1)	0.1 (0.1 to 0.1)	0.1 (0.1 to 0.1)	22.4 (12.0 to 34.6)	1.0 (-4.5 to 6.6)
Occupational exposure to diesel engine exhaust	0.3 (0.3 to 0.4)	0.4 (0.4 to 0.4)	0.4 (0.4 to 0.5)	28.9 (20.6 to 38.6)	4.1 (-0.3 to 8.8)	0.3 (0.3 to 0.3)	0.4 (0.4 to 0.4)	0.5 (0.4 to 0.5)	44.2 (33.1 to 56.9)	8.4 (3.2 to 14.4)
Occupational exposure to formaldehyde	0.2 (0.1 to 0.2)	0.2 (0.2 to 0.2)	0.2 (0.2 to 0.2)	-0.4 (-8.2 to 8.0)	1.7 (-4.8 to 8.2)	0.2 (0.2 to 0.2)	0.2 (0.2 to 0.2)	0.2 (0.2 to 0.2)	18.8 (8.1 to 31.9)	-0.7 (-7.2 to 5.8)
Occupational exposure to nickel	0.3 (0.0 to 1.2)	0.3 (0.0 to 1.2)	0.3 (0.0 to 1.2)	-	-	0.3 (0.0 to 1.2)	0.3 (0.0 to 1.4)	0.3 (0.0 to 1.3)	-	-
Occupational exposure to polycyclic aromatic hydrocarbons	0.1 (0.1 to 0.2)	0.2 (0.2 to 0.2)	0.2 (0.2 to 0.2)	8.1 (0.8 to 16.0)	3.0 (-1.9 to 7.9)	0.2 (0.2 to 0.2)	0.2 (0.2 to 0.2)	0.2 (0.2 to 0.2)	23.6 (13.4 to 35.9)	1.5 (-3.7 to 6.7)
Occupational exposure to silica	3.2 (0.5 to 10.4)	3.5 (0.5 to 11.3)	3.5 (0.5 to 11.4)	8.4 (-0.1 to 20.2)	1.1 (-5.0 to 8.6)	2.6 (0.4 to 8.1)	2.7 (0.4 to 8.5)	2.6 (0.4 to 8.2)	6.3 (-3.1 to 18.7)	-3.8 (-11.4 to 4.4)
Occupational exposure to sulphuric acid	0.4 (0.1 to 1.3)	0.4 (0.1 to 1.4)	0.4 (0.1 to 1.4)	5.9 (-1.4 to 14.0)	-1.1 (-7.0 to 4.9)	0.4 (0.1 to 1.4)	0.5 (0.1 to 1.6)	0.5 (0.1 to 1.6)	15.4 (4.7 to 27.0)	-2.6 (-9.2 to 4.0)
Occupational exposure to trichloroethylene	0.0 (0.0 to 0.0)	0.0 (0.0 to 0.0)	0.0 (0.0 to 0.0)	12.7 (5.9 to 20.0)	4.1 (-0.0 to 8.5)	0.0 (0.0 to 0.0)	0.1 (0.1 to 0.1)	0.1 (0.1 to 0.1)	28.9 (19.1 to 40.4)	3.6 (-0.7 to 8.1)
Occupational asthmagens	12.6 (10.6 to 14.9)	12.8 (11.0 to 15.2)	12.6 (10.8 to 14.9)	1.7 (-6.7 to 10.5)	-1.7 (-6.8 to 3.6)	7.2 (5.9 to 8.8)	8.6 (7.2 to 10.3)	8.4 (7.0 to 10.1)	19.5 (6.9 to 34.4)	-2.5 (-9.0 to 3.8)
Occupational particulate matter, gases, and fumes	5.6 (4.0 to 8.1)	5.2 (3.7 to 7.5)	5.0 (3.5 to 7.2)	-7.0 (-10.7 to -3.1)	-3.5 (-5.6 to -1.2)	3.6 (2.5 to 5.3)	3.5 (2.5 to 5.1)	3.3 (2.4 to 4.8)	-3.1 (-8.6 to 3.3)	-4.2 (-7.8 to -0.8)
Occupational noise	5.0 (4.6 to 5.5)	4.8 (4.5 to 5.2)	4.7 (4.5 to 5.1)	-4.6 (-7.6 to -1.3)	-1.4 (-3.2 to 0.6)	3.3 (2.9 to 3.8)	3.4 (3.2 to 3.8)	3.4 (3.2 to 3.8)	2.6 (-2.5 to 8.8)	1.0 (-1.5 to 3.4)
Occupational ergonomic factors	7.5 (6.3 to 8.8)	7.8 (6.8 to 9.0)	7.3 (6.2 to 8.5)	4.6 (-7.6 to 15.5)	-7.4 (-12.9 to -1.4)	5.6 (4.6 to 6.8)	6.1 (5.2 to 7.0)	5.6 (4.8 to 6.7)	7.6 (-8.4 to 23.9)	-6.9 (-13.8 to 0.6)
Non-exclusive breastfeeding	0.4 (0.3 to 0.6)	0.5 (0.3 to 0.6)	0.5 (0.3 to 0.6)	7.5 (-1.9 to 17.2)	-1.6 (-7.7 to 5.4)	0.4 (0.3 to 0.6)	0.5 (0.3 to 0.6)	0.5 (0.3 to 0.6)	7.3 (-2.1 to 17.0)	-1.6 (-7.7 to 5.4)
Discontinued breastfeeding	1.9 (1.8 to 2.0)	2.2 (2.1 to 2.2)	2.1 (2.0 to 2.2)	10.6 (4.3 to 17.8)	-0.8 (-5.4 to 4.0)	1.9 (1.8 to 2.0)	2.2 (2.1 to 2.3)	2.1 (2.0 to 2.2)	11.0 (4.6 to 18.2)	-1.1 (-5.7 to 3.7)
Child underweight	0.2 (0.1 to 0.3)	0.2 (0.1 to 0.3)	0.1 (0.1 to 0.2)	-19.4 (-37.2 to 2.2)	-15.5 (-29.0 to -3.7)	0.2 (0.1 to 0.3)	0.2 (0.1 to 0.2)	0.1 (0.1 to 0.2)	-26.2 (-44.2 to -4.5)	-16.0 (-28.6 to -5.0)
Child wasting	0.2 (0.1 to 0.3)	0.2 (0.1 to 0.3)	0.2 (0.1 to 0.2)	-11.4 (-39.6 to 29.0)	-8.7 (-29.5 to 15.2)	0.2 (0.1 to 0.3)	0.2 (0.1 to 0.2)	0.1 (0.1 to 0.2)	-14.6 (-42.6 to 23.6)	-9.0 (-27.4 to 13.7)
Child stunting	0.9 (0.4 to 1.4)	0.7 (0.4 to 1.0)	0.6 (0.3 to 1.0)	-21.5 (-52.5 to 32.0)	-13.6 (-40.1 to 20.7)	0.8 (0.4 to 1.3)	0.6 (0.3 to 1.0)	0.5 (0.3 to 1.0)	-22.2 (-56.0 to 36.7)	-15.0 (-42.7 to 22.3)
Short gestation for birth weight	0.0 (0.0 to 0.0)	0.0 (0.0 to 0.0)	0.0 (0.0 to 0.0)	5.9 (-20.0 to 29.2)	7.1 (-15.8 to 49.3)	0.0 (0.0 to 0.0)	0.0 (0.0 to 0.0)	0.0 (0.0 to 0.0)	2.7 (-26.2 to 33.4)	6.7 (-18.6 to 42.4)
Low birth weight for gestation	0.0 (0.0 to 0.0)	0.0 (0.0 to 0.0)	0.0 (0.0 to 0.0)	0.7 (-14.9 to 20.2)	-1.5 (-22.4 to 16.2)	0.0 (0.0 to 0.0)	0.0 (0.0 to 0.0)	0.0 (0.0 to 0.0)	0.3 (-16.2 to 31.3)	-1.9 (-22.3 to 19.9)
Iron deficiency	-	-	-	-	-	6.5 (4.5 to 8.8)	5.7 (3.9 to 7.9)	4.9 (3.3 to 6.8)	-11.6 (-26.8 to 5.2)	-14.9 (-28.0 to -0.4)
Vitamin A deficiency	2.9 (2.4 to 3.6)	2.4 (2.0 to 2.9)	2.3 (1.9 to 2.7)	-16.9 (-26.4 to -7.1)	-7.4 (-16.1 to 3.4)	2.9 (2.4 to 3.5)	2.5 (2.1 to 3.0)	2.3 (1.9 to 2.8)	-13.7 (-22.4 to -2.5)	-8.5 (-16.8 to 0.3)
Zinc deficiency	0.4 (0.1 to 0.9)	0.6 (0.1 to 1.3)	0.5 (0.1 to 1.2)	30.8 (0.6 to 146.1)	-8.3 (-25.8 to 13.8)	0.4 (0.1 to 0.9)	0.6 (0.2 to 1.3)	0.5 (0.1 to 1.2)	30.5 (0.2 to 144.2)	-8.9 (-25.6 to 12.7)
Smoking	17.1 (15.1 to 19.2)	15.7 (14.0 to 17.6)	14.5 (13.0 to 16.2)	-7.7 (-15.1 to 0.7)	-7.6 (-13.1 to -2.0)	9.0 (7.2 to 11.0)	8.3 (6.9 to 9.9)	7.9 (6.8 to 9.2)	-6.9 (-21.0 to 9.9)	-4.8 (-13.7 to 6.2)
Chewing tobacco	0.1 (0.1 to 0.2)	0.1 (0.1 to 0.2)	0.1 (0.1 to 0.2)	0.8 (-31.4 to 48.5)	-4.2 (-26.5 to 25.6)	0.1 (0.0 to 0.1)	0.1 (0.0 to 0.1)	0.1 (0.0 to 0.1)	0.4 (-28.6 to 41.5)	0.6 (-24.5 to 30.7)
Second-hand smoke	47.5 (45.8 to 49.4)	47.1 (45.8 to 48.3)	39.7 (37.2 to 42.4)	-0.9 (-5.5 to 3.9)	-15.8 (-20.8 to -10.5)	54.5 (52.7 to 56.5)	53.0 (51.6 to 54.2)	44.8 (42.4 to 47.2)	-2.8 (-7.1 to 1.3)	-15.4 (-19.8 to -11.1)
Alcohol use	31.5 (22.2 to 41.1)	25.3 (16.6 to 36.1)	28.8 (17.6 to 42.3)	-19.8 (-36.5 to -4.5)	13.9 (-8.5 to 43.3)	12.4 (7.3 to 20.4)	10.2 (5.5 to 18.2)	11.4 (5.8 to 19.8)	-18.1 (-37.4 to -2.0)	11.9 (-9.0 to 42.8)
Drug use	0.5 (0.4 to 0.7)	0.5 (0.4 to 0.7)	0.5 (0.4 to 0.8)	5.3 (-4.1 to 16.2)	6.7 (-1.5 to 15.1)	0.2 (0.2 to 0.4)	0.2 (0.2 to 0.4)	0.2 (0.2 to 0.4)	5.8 (-2.7 to 16.2)	1.0 (-6.5 to 8.9)
Diet low in fruits	37.3 (31.9 to 40.7)	43.8 (38.9 to 46.4)	42.1 (36.7 to 45.1)	17.6 (12.7 to 25.3)	-4.1 (-7.3 to -1.6)	34.8 (29.0 to 38.7)	42.4 (37.3 to 45.2)	40.3 (35.0 to 43.6)	21.7 (15.1 to 31.1)	-4.8 (-8.3 to -1.9)
Diet low in vegetables	16.7 (9.0 to 23.1)	6.0 (0.9 to 12.5)	3.8 (0.4 to 9.1)	-63.9 (-91.2 to -42.1)	-37.9 (-75.8 to 2.7)	19.3 (12.0 to 25.4)	7.6 (1.4 to 14.5)	4.9 (0.6 to 11.4)	-60.8 (-88.7 to -40.2)	-35.0 (-72.9 to -6.7)
Diet low in legumes	28.6 (24.3 to 32.4)	37.7 (34.8 to 40.3)	38.2 (35.1 to 40.9)	31.7 (23.5 to 44.5)	1.1 (-1.7 to 4.1)	32.6 (28.7 to 35.9)	41.0 (38.5 to 43.1)	41.7 (38.9 to 43.8)	25.9 (19.4 to 35.1)	1.5 (-0.8 to 3.8)
Diet low in whole grains	50.4 (49.7 to 51.0)	46.3 (44.8 to 47.5)	44.3 (42.6 to 45.9)	-8.2 (-9.9 to -6.8)	-4.1 (-5.7 to -2.7)	50.6 (49.9 to 51.2)	46.8 (45.4 to 47.9)	45.0 (43.3 to 46.5)	-7.5 (-9.0 to -6.2)	-3.8 (-5.3 to -2.5)
Diet low in nuts and seeds	44.6 (42.0 to 46.7)	47.2 (45.2 to 48.8)	44.4 (41.6 to 46.7)	5.8 (2.8 to 9.8)	-5.9 (-9.3 to -3.4)	45.2 (42.7 to 47.2)	47.6 (45.7 to 49.1)	44.8 (42.1 to 47.0)	5.2 (2.4 to 8.8)	-5.7 (-8.9 to -3.2)
Diet low in milk	35.6 (31.6 to 39.0)	42.5 (39.8 to 44.8)	42.9 (40.3 to 45.1)	19.5 (13.9 to 27.9)	1.0 (-0.5 to 2.6)	36.5 (32.6 to 39.9)	43.1 (40.6 to 45.2)	43.4 (40.9 to 45.5)	17.9 (12.6 to 25.6)	0.7 (-0.7 to 2.2)
Diet high in red meat	28.7 (24.1 to 32.6)	30.3 (26.5 to 33.7)	40.5 (34.5 to 45.5)	5.7 (-7.1 to 21.1)	33.6 (16.3 to 54.9)	11.1 (6.6 to 15.4)	10.0 (5.8 to 13.7)	16.0 (10.0 to 21.2)	-10.4 (-34.9 to 18.2)	60.2 (21.5 to 138.2)
Diet high in processed meat	6.2 (3.3 to 9.2)	6.2 (3.2 to 9.0)	7.7 (4.6 to 10.7)	-1.2 (-22.0 to 25.8)	24.4 (6.9 to 57.6)	4.1 (1.4 to 7.0)	4.1 (1.4 to 6.9)	5.2 (2.3 to 8.1)	-0.3 (-26.9 to 35.7)	26.9 (7.3 to 80.5)
Diet high in sugar-sweetened beverages	10.5 (4.8 to 16.7)	9.3 (4.8 to 11.3)	13.6 (6.8 to 17.7)	-10.9 (-41.4 to 43.7)	45.1 (12.3 to 91.1)	7.2 (3.7 to 11.7)	6.4 (3.3 to 8.3)	9.2 (4.6 to 12.5)	-10.3 (-43.9 to 45.0)	42.8 (7.7 to 92.3)
Diet low in fibre	32.2 (27.2 to 36.1)	36.6 (32.5 to 39.9)	34.3 (29.5 to 38.3)	13.8 (8.8 to 21.7)	-6.2 (-11.0 to -2.5)	37.0 (33.0 to 40.2)	40.8 (37.6 to 43.4)	38.9 (35.0 to 41.8)	10.2 (6.3 to 16.1)	-4.7 (-8.5 to -1.6)
Diet low in calcium	24.9 (18.9 to 29.7)	28.4 (23.2 to 32.7)	25.1 (19.0 to 30.0)	14.1 (9.1 to 23.8)	-11.7 (-18.5 to -7.5)	28.4 (23.1 to 32.6)	31.6 (26.9 to 35.4)	28.5 (23.2 to 32.9)	11.3 (7.5 to 17.4)	-9.8 (-14.9 to -6.2)
Diet low in seafood omega-3 fatty acids	38.2 (35.0 to 41.2)	40.9 (38.2 to 43.5)	38.3 (35.2 to 41.3)	7.2 (5.4 to 9.6)	-6.5 (-8.9 to -4.7)	40.5 (37.7 to 43.1)	42.9 (40.6 to 45.1)	40.6 (37.9 to 43.2)	6.0 (4.6 to 7.9)	-5.5 (-7.4 to -4.0)
Diet low in polyunsaturated fatty acids	34.8 (30.8 to 38.7)	24.1 (18.6 to 28.8)	19.7 (13.3 to 25.1)	-30.9 (-41.8 to -22.3)	-18.2 (-30.3 to -10.1)	35.4 (31.5 to 38.7)	24.9 (19.5 to 29.4)	20.6 (14.3 to 26.0)	-29.6 (-39.9 to -21.3)	-17.1 (-29.2 to -9.6)
Diet high in trans fatty acids	1.3 (0.0 to 3.6)	1.2 (0.0 to 3.5)	1.2 (0.0 to 3.5)	-3.5 (-30.3 to 25.5)	-2.8 (-27.7 to 22.1)	1.7 (0.2 to 4.4)	1.6 (0.1 to 4.3)	1.6 (0.1 to 4.3)	-5.5 (-32.5 to 22.3)	-1.2 (-22.4 to 25.4)
Diet high in sodium	24.1 (15.8 to 30.7)	11.7 (3.1 to 19.2)	12.8 (4.0 to 20.5)	-51.6 (-81.2 to -32.0)	9.3 (-8.2 to 56.1)	17.9 (10.7 to 24.4)	6.6 (0.6 to 13.7)	5.4 (0.2 to 12.3)	-63.1 (-94.6 to -40.2)	-19.1 (-74.2 to -4.5)
Intimate partner violence	-	-	-	-	-	4.0 (3.4 to 4.7)	3.6 (3.0 to 4.2)	3.3 (2.8 to 3.9)	-10.3 (-14.3 to -5.5)	-7.4 (-12.1 to -2.5)
Childhood sexual abuse	8.7 (7.1 to 10.8)	8.7 (7.1 to 10.8)	8.7 (7.1 to 10.8)	0.0 (0.0 to 0.0)	0.0 (0.0 to 0.0)	4.9 (3.6 to 6.5)	4.9 (3.6 to 6.5)	4.9 (3.6 to 6.5)	0.0 (0.0 to 0.0)	0.0 (0.0 to 0.0)

Supplementary Results Table 1. Bulgaria age-standardised Summary Exposure Values (SEVs) for Level 4 risk factors and percentage change, by sex, 1990, 2007, and 2017.Data in parentheses are 95% uncertainty intervals.										
	Male					Female				
	**1990**	**2007**	**2017**	**Percentage change 1990-2007**	**Percentage change 2007-2017**	**1990**	**2007**	**2017**	**Percentage change 1990-2007**	**Percentage change 2007-2017**
Bullying victimization	11.1<br>(4.8 to 21.5)	11.1<br>(5.0 to 22.1)	10.9<br>(4.9 to 21.4)	0.6<br>(-19.5 to 20.5)	-1.7<br>(-8.1 to 9.1)	6.4<br>(2.7 to 12.5)	6.6<br>(3.0 to 13.2)	6.5<br>(2.9 to 12.7)	3.9<br>(-14.3 to 26.7)	-2.2<br>(-8.8 to 5.3)
Low physical activity	0.3<br>(0.1 to 0.5)	0.3<br>(0.1 to 0.5)	0.3<br>(0.1 to 0.5)	-2.3<br>(-6.2 to 0.1)	0.8<br>(-2.0 to 4.1)	0.3<br>(0.2 to 0.6)	0.3<br>(0.2 to 0.6)	0.3<br>(0.2 to 0.6)	-1.2<br>(-3.2 to 0.7)	0.2<br>(-1.5 to 2.5)
High fasting plasma glucose	1.6<br>(1.0 to 2.6)	2.0<br>(1.2 to 3.2)	1.9<br>(1.1 to 3.1)	28.1<br>(-20.4 to 122.2)	-4.5<br>(-43.8 to 51.8)	1.4<br>(0.8 to 2.1)	1.5<br>(0.9 to 2.4)	1.6<br>(1.0 to 2.4)	11.9<br>(-29.1 to 87.8)	3.9<br>(-35.4 to 56.8)
High LDL cholesterol	13.4<br>(11.8 to 15.2)	13.8<br>(12.2 to 15.7)	14.0<br>(12.4 to 15.8)	3.0<br>(-1.5 to 7.6)	1.4<br>(-2.2 to 4.7)	13.5<br>(12.0 to 15.3)	13.8<br>(12.3 to 15.6)	14.1<br>(12.6 to 15.9)	2.3<br>(-2.0 to 6.7)	2.1<br>(-1.0 to 5.7)
High systolic blood pressure	10.4<br>(9.4 to 11.4)	10.5<br>(9.5 to 11.6)	10.4<br>(9.4 to 11.4)	1.1<br>(-6.2 to 8.7)	-0.7<br>(-5.5 to 5.3)	9.8<br>(8.8 to 10.7)	9.9<br>(9.0 to 10.9)	9.3<br>(8.4 to 10.3)	1.6<br>(-7.0 to 10.7)	-6.2<br>(-12.7 to 1.2)
High body-mass index	12.4<br>(9.0 to 17.7)	14.7<br>(11.2 to 20.4)	16.9<br>(12.8 to 22.7)	18.9<br>(6.0 to 34.5)	14.9<br>(5.4 to 26.2)	12.8<br>(9.5 to 17.5)	15.3<br>(11.7 to 20.5)	16.8<br>(12.5 to 22.5)	19.3<br>(5.8 to 37.6)	9.7<br>(-0.6 to 20.2)
Low bone mineral density	3.7<br>(3.0 to 4.5)	3.7<br>(3.0 to 4.4)	3.5<br>(2.8 to 4.4)	-0.7<br>(-15.0 to 17.3)	-4.0<br>(-19.2 to 13.5)	6.6<br>(5.9 to 7.3)	6.2<br>(5.6 to 6.8)	5.8<br>(5.2 to 6.5)	-5.9<br>(-14.1 to 3.3)	-6.1<br>(-14.2 to 3.1)
Impaired kidney function	2.1<br>(1.0 to 5.8)	2.1<br>(0.9 to 5.8)	2.1<br>(1.0 to 5.7)	-0.9<br>(-5.8 to 4.3)	-0.1<br>(-4.7 to 4.5)	2.8<br>(1.3 to 6.8)	2.9<br>(1.3 to 6.8)	2.9<br>(1.4 to 6.8)	2.1<br>(-1.9 to 7.3)	0.1<br>(-4.6 to 5.2)

Supplementary Results Table 1. Croatia age-standardised Summary Exposure Values (SEVs) for Level 4 risk factors and percentage change, by sex, 1990, 2007, and 2017.Data in parentheses are 95% uncertainty intervals.										
	Male					Female				
	1990	2007	2017	Percentage change 1990-2007	Percentage change 2007-2017	1990	2007	2017	Percentage change 1990-2007	Percentage change 2007-2017
Unsafe water source	9.0 (6.2 to 12.5)	5.6 (3.7 to 8.1)	4.6 (2.9 to 6.7)	-38.5 (-61.0 to -4.1)	-17.6 (-44.1 to 15.1)	9.0 (6.2 to 12.5)	5.6 (3.7 to 8.1)	4.6 (2.9 to 6.7)	-38.5 (-61.0 to -4.1)	-17.6 (-44.1 to 15.1)
Unsafe sanitation	6.7 (3.9 to 11.0)	3.3 (1.9 to 5.9)	2.4 (1.4 to 4.2)	-50.2 (-72.0 to -12.3)	-27.4 (-53.0 to 17.2)	6.7 (3.9 to 11.0)	3.3 (1.9 to 5.9)	2.4 (1.4 to 4.2)	-50.2 (-72.0 to -12.3)	-27.4 (-53.0 to 17.2)
No access to handwashing facility	3.8 (3.0 to 4.7)	3.1 (2.5 to 3.9)	3.0 (2.4 to 3.7)	-17.7 (-35.9 to 7.3)	-4.5 (-20.2 to 14.3)	3.8 (3.0 to 4.7)	3.1 (2.5 to 3.9)	3.0 (2.4 to 3.7)	-17.7 (-35.9 to 7.3)	-4.5 (-20.2 to 14.3)
Ambient particulate matter pollution	35.2 (22.4 to 56.3)	39.4 (26.6 to 57.3)	35.4 (22.8 to 54.4)	11.8 (-29.4 to 75.4)	-10.1 (-42.7 to 36.6)	32.1 (19.2 to 52.7)	37.2 (24.4 to 55.3)	33.7 (20.7 to 52.3)	15.9 (-28.9 to 92.4)	-9.5 (-44.9 to 43.7)
Household air pollution from solid fuels	12.9 (6.9 to 22.2)	7.0 (3.5 to 14.0)	5.5 (2.5 to 11.7)	-45.5 (-72.8 to 2.7)	-21.2 (-60.9 to 42.4)	14.8 (8.2 to 24.0)	8.5 (4.3 to 15.4)	6.8 (3.1 to 13.6)	-42.6 (-70.8 to 0.8)	-20.1 (-58.9 to 38.1)
Ambient ozone pollution	47.3 (18.4 to 54.8)	47.5 (18.6 to 54.8)	48.0 (19.1 to 54.8)	0.5 (0.0 to 1.5)	1.1 (0.0 to 3.2)	47.3 (18.4 to 54.8)	47.5 (18.6 to 54.8)	48.0 (19.1 to 54.8)	0.5 (0.0 to 1.5)	1.1 (0.0 to 3.2)
Residential radon	34.0 (11.8 to 68.9)	34.0 (11.8 to 68.9)	34.0 (11.8 to 68.9)	0.0 (0.0 to 0.0)	0.0 (0.0 to 0.0)	34.0 (11.8 to 68.9)	34.0 (11.8 to 68.9)	34.0 (11.8 to 68.9)	0.0 (0.0 to 0.0)	0.0 (0.0 to 0.0)
Lead exposure	2.9 (0.4 to 6.6)	2.8 (0.4 to 6.3)	2.2 (0.3 to 5.4)	-3.3 (-9.3 to 27.5)	-21.9 (-33.2 to -15.0)	2.0 (0.1 to 5.2)	1.9 (0.1 to 5.0)	1.5 (0.1 to 4.2)	-4.0 (-11.5 to 32.3)	-23.7 (-37.7 to -15.8)
Occupational exposure to asbestos	1.9 (1.6 to 2.2)	6.7 (6.2 to 7.2)	6.5 (5.9 to 7.3)	251.4 (195.1 to 333.0)	-3.1 (-14.7 to 9.3)	0.6 (0.5 to 0.7)	1.2 (1.1 to 1.3)	0.8 (0.7 to 1.0)	90.9 (56.2 to 128.5)	-29.2 (-38.8 to -18.8)
Occupational exposure to arsenic	0.3 (0.0 to 0.6)	0.3 (0.0 to 0.6)	0.3 (0.0 to 0.6)	-	-	0.3 (0.0 to 0.6)	0.3 (0.0 to 0.7)	0.3 (0.0 to 0.6)	-	-
Occupational exposure to benzene	0.4 (0.0 to 1.1)	0.4 (0.0 to 1.2)	0.4 (0.0 to 1.2)	9.0 (1.9 to 18.9)	-1.0 (-5.3 to 5.8)	0.5 (0.0 to 1.3)	0.6 (0.0 to 1.7)	0.6 (0.0 to 1.9)	29.0 (17.8 to 46.5)	7.7 (2.9 to 14.5)
Occupational exposure to beryllium	0.0 (0.0 to 0.0)	0.0 (0.0 to 0.0)	0.0 (0.0 to 0.0)	8.9 (3.0 to 15.4)	-2.1 (-5.2 to 0.8)	0.0 (0.0 to 0.0)	0.0 (0.0 to 0.0)	0.0 (0.0 to 0.0)	17.9 (9.5 to 27.9)	2.0 (-1.4 to 6.0)
Occupational exposure to cadmium	0.0 (0.0 to 0.0)	0.0 (0.0 to 0.0)	0.0 (0.0 to 0.0)	6.8 (-0.8 to 16.2)	-0.4 (-5.4 to 4.7)	0.0 (0.0 to 0.0)	0.0 (0.0 to 0.0)	0.0 (0.0 to 0.0)	8.2 (-2.8 to 19.8)	-1.0 (-6.7 to 5.1)
Occupational exposure to chromium	0.1 (0.1 to 0.1)	0.1 (0.1 to 0.1)	0.1 (0.1 to 0.1)	8.2 (0.7 to 17.1)	-0.0 (-4.6 to 4.7)	0.1 (0.1 to 0.1)	0.1 (0.1 to 0.1)	0.1 (0.1 to 0.1)	12.0 (1.2 to 23.9)	1.0 (-4.2 to 6.5)
Occupational exposure to diesel engine exhaust	0.4 (0.3 to 0.4)	0.4 (0.4 to 0.4)	0.4 (0.4 to 0.4)	15.7 (7.9 to 23.7)	-2.7 (-6.7 to 1.4)	0.3 (0.3 to 0.3)	0.4 (0.4 to 0.4)	0.4 (0.4 to 0.4)	26.9 (15.4 to 40.1)	5.2 (0.1 to 10.8)
Occupational exposure to formaldehyde	0.2 (0.1 to 0.2)	0.2 (0.2 to 0.2)	0.2 (0.1 to 0.2)	4.8 (-3.1 to 14.3)	-0.9 (-6.0 to 4.4)	0.1 (0.1 to 0.2)	0.2 (0.2 to 0.2)	0.2 (0.1 to 0.2)	8.9 (-2.4 to 20.9)	0.1 (-5.8 to 6.3)
Occupational exposure to nickel	0.2 (0.0 to 1.1)	0.3 (0.0 to 1.2)	0.3 (0.0 to 1.2)	-	-	0.2 (0.0 to 1.0)	0.2 (0.0 to 1.0)	0.2 (0.0 to 1.0)	-	-
Occupational exposure to polycyclic aromatic hydrocarbons	0.1 (0.1 to 0.2)	0.2 (0.1 to 0.2)	0.2 (0.1 to 0.2)	8.5 (1.3 to 17.0)	-0.8 (-4.8 to 3.4)	0.1 (0.1 to 0.2)	0.2 (0.2 to 0.2)	0.2 (0.2 to 0.2)	13.4 (2.6 to 25.0)	1.7 (-3.4 to 6.9)
Occupational exposure to silica	3.0 (0.4 to 9.5)	3.4 (0.5 to 11.0)	3.3 (0.5 to 10.6)	14.0 (5.4 to 25.6)	-4.4 (-9.9 to 1.1)	1.9 (0.3 to 6.1)	2.0 (0.3 to 6.3)	1.9 (0.3 to 6.0)	2.6 (-8.1 to 16.3)	-4.8 (-11.6 to 2.8)
Occupational exposure to sulphuric acid	0.4 (0.1 to 1.3)	0.4 (0.1 to 1.3)	0.4 (0.1 to 1.3)	3.0 (-5.0 to 12.0)	-4.8 (-9.5 to 0.2)	0.4 (0.1 to 1.2)	0.4 (0.1 to 1.2)	0.4 (0.1 to 1.2)	6.2 (-4.5 to 17.7)	-3.8 (-9.4 to 2.5)
Occupational exposure to trichloroethylene	0.0 (0.0 to 0.0)	0.0 (0.0 to 0.0)	0.0 (0.0 to 0.0)	9.5 (2.4 to 17.6)	-0.2 (-4.0 to 4.0)	0.0 (0.0 to 0.0)	0.0 (0.0 to 0.0)	0.0 (0.0 to 0.1)	16.7 (6.1 to 28.5)	2.5 (-2.0 to 7.5)
Occupational asthmagens	13.6 (11.2 to 16.8)	13.6 (11.6 to 16.4)	12.9 (10.9 to 15.3)	-0.1 (-8.8 to 9.3)	-5.6 (-10.9 to -0.3)	7.4 (5.8 to 9.6)	8.5 (7.0 to 10.3)	7.9 (6.5 to 9.7)	14.2 (-0.7 to 32.9)	-6.2 (-14.5 to 2.7)
Occupational particulate matter, gases, and fumes	5.4 (3.8 to 7.8)	5.3 (3.8 to 7.6)	5.1 (3.6 to 7.4)	-2.3 (-6.1 to 2.3)	-3.0 (-5.6 to -0.5)	3.0 (2.1 to 4.5)	3.0 (2.1 to 4.3)	2.8 (2.0 to 4.1)	-1.8 (-8.0 to 5.2)	-4.2 (-7.5 to -0.7)
Occupational noise	5.0 (4.5 to 5.4)	4.9 (4.6 to 5.3)	4.8 (4.5 to 5.2)	-1.3 (-4.4 to 2.6)	-2.1 (-3.8 to -0.0)	3.0 (2.7 to 3.4)	3.1 (2.9 to 3.4)	3.1 (3.0 to 3.4)	2.9 (-2.4 to 9.0)	1.1 (-1.6 to 3.9)
Occupational ergonomic factors	9.6 (8.2 to 11.1)	9.2 (8.2 to 10.4)	8.0 (6.9 to 9.2)	-4.1 (-15.7 to 9.5)	-13.6 (-21.1 to -5.5)	7.1 (5.7 to 8.7)	7.7 (6.9 to 8.6)	6.6 (5.6 to 7.8)	7.8 (-12.6 to 33.8)	-14.1 (-26.1 to -1.2)
Non-exclusive breastfeeding	0.4 (0.3 to 0.6)	0.5 (0.3 to 0.6)	0.5 (0.3 to 0.6)	7.0 (-2.3 to 16.3)	-1.4 (-7.6 to 6.3)	0.4 (0.3 to 0.6)	0.5 (0.3 to 0.6)	0.5 (0.3 to 0.6)	7.0 (-2.2 to 16.3)	-1.4 (-7.6 to 6.3)
Discontinued breastfeeding	2.2 (2.1 to 2.2)	2.3 (2.2 to 2.4)	2.3 (2.2 to 2.3)	6.4 (1.4 to 11.8)	-1.4 (-5.1 to 2.3)	2.2 (2.1 to 2.2)	2.3 (2.2 to 2.4)	2.3 (2.2 to 2.3)	6.4 (1.5 to 11.8)	-1.4 (-5.1 to 2.3)
Child underweight	0.1 (0.1 to 0.2)	0.1 (0.1 to 0.2)	0.1 (0.1 to 0.2)	-13.7 (-25.6 to -1.2)	-7.4 (-15.7 to 1.4)	0.1 (0.1 to 0.2)	0.1 (0.1 to 0.1)	0.1 (0.1 to 0.1)	-19.3 (-31.7 to -7.1)	-8.2 (-16.3 to 0.5)
Child wasting	0.2 (0.1 to 0.3)	0.2 (0.1 to 0.2)	0.1 (0.1 to 0.2)	-10.1 (-35.4 to 30.5)	-7.0 (-25.0 to 14.8)	0.2 (0.1 to 0.2)	0.1 (0.1 to 0.2)	0.1 (0.1 to 0.2)	-12.0 (-33.6 to 16.5)	-4.9 (-21.5 to 15.4)
Child stunting	0.7 (0.3 to 1.2)	0.6 (0.3 to 1.0)	0.5 (0.2 to 0.9)	-16.4 (-50.1 to 38.1)	-11.8 (-38.4 to 25.9)	0.6 (0.3 to 1.1)	0.5 (0.2 to 0.8)	0.5 (0.2 to 0.8)	-16.7 (-51.3 to 36.2)	-10.9 (-40.7 to 30.5)
Short gestation for birth weight	0.0 (0.0 to 0.0)	0.0 (0.0 to 0.0)	0.0 (0.0 to 0.0)	10.2 (-12.5 to 38.4)	2.3 (-18.7 to 25.4)	0.0 (0.0 to 0.0)	0.0 (0.0 to 0.0)	0.0 (0.0 to 0.0)	8.5 (-16.1 to 42.5)	6.1 (-17.6 to 37.1)
Low birth weight for gestation	0.0 (0.0 to 0.0)	0.0 (0.0 to 0.0)	0.0 (0.0 to 0.0)	4.9 (-11.2 to 26.5)	1.7 (-14.0 to 21.3)	0.0 (0.0 to 0.0)	0.0 (0.0 to 0.0)	0.0 (0.0 to 0.0)	1.2 (-17.8 to 21.8)	-0.1 (-17.7 to 21.4)
Iron deficiency	-	-	-	-	-	4.9 (3.3 to 7.0)	4.5 (3.0 to 6.5)	4.0 (2.6 to 5.8)	-7.2 (-26.0 to 16.9)	-11.4 (-26.2 to 4.9)
Vitamin A deficiency	2.8 (2.2 to 3.4)	2.3 (1.9 to 2.8)	2.2 (1.8 to 2.6)	-16.5 (-26.6 to -6.1)	-7.3 (-17.8 to 2.7)	2.8 (2.3 to 3.4)	2.4 (2.0 to 2.8)	2.2 (1.8 to 2.7)	-15.1 (-23.6 to -5.7)	-5.2 (-14.0 to 4.3)
Zinc deficiency	1.1 (0.3 to 2.2)	0.7 (0.2 to 1.5)	0.6 (0.2 to 1.4)	-36.4 (-57.5 to -6.9)	-12.7 (-30.9 to 10.4)	1.1 (0.3 to 2.1)	0.7 (0.2 to 1.5)	0.6 (0.2 to 1.3)	-35.8 (-57.1 to -7.8)	-13.3 (-30.9 to 13.3)
Smoking	15.3 (13.3 to 17.3)	12.5 (11.1 to 14.1)	11.0 (9.7 to 12.3)	-17.8 (-25.0 to -10.3)	-12.5 (-17.7 to -7.0)	9.2 (7.4 to 11.3)	7.6 (6.3 to 8.9)	6.9 (5.9 to 8.1)	-17.9 (-29.5 to -4.3)	-8.5 (-17.1 to 0.6)
Chewing tobacco	0.1 (0.1 to 0.2)	0.1 (0.1 to 0.2)	0.1 (0.1 to 0.2)	0.3 (-30.0 to 41.5)	-4.2 (-26.1 to 25.4)	0.1 (0.0 to 0.1)	0.1 (0.0 to 0.1)	0.1 (0.0 to 0.1)	0.5 (-27.8 to 40.4)	-0.3 (-23.3 to 30.1)
Second-hand smoke	59.7 (55.5 to 63.4)	45.7 (43.9 to 47.4)	43.9 (41.1 to 46.7)	-23.4 (-29.5 to -15.7)	-4.0 (-8.2 to 0.5)	69.6 (66.1 to 73.1)	49.5 (47.5 to 51.2)	45.7 (43.1 to 48.3)	-28.9 (-34.1 to -23.7)	-7.7 (-11.1 to -4.4)
Alcohol use	29.8 (19.2 to 39.9)	29.4 (19.7 to 39.7)	28.4 (16.6 to 42.0)	-1.3 (-19.6 to 23.5)	-3.3 (-27.4 to 21.7)	9.6 (5.3 to 16.4)	9.1 (5.1 to 15.7)	9.1 (4.4 to 16.2)	-5.0 (-23.1 to 15.7)	-0.8 (-26.0 to 24.3)
Drug use	0.4 (0.3 to 0.5)	0.5 (0.5 to 0.6)	0.5 (0.4 to 0.6)	32.0 (17.2 to 52.3)	-8.1 (-17.7 to 2.8)	0.2 (0.1 to 0.2)	0.2 (0.1 to 0.2)	0.2 (0.1 to 0.2)	6.2 (-1.2 to 14.2)	0.8 (-5.5 to 8.7)
Diet low in fruits	35.4 (29.9 to 39.5)	31.9 (25.9 to 36.3)	29.2 (22.8 to 34.2)	-10.0 (-17.2 to -4.5)	-8.4 (-16.8 to -2.4)	32.8 (26.7 to 37.2)	28.7 (22.7 to 33.4)	25.7 (19.3 to 31.2)	-12.6 (-21.0 to -6.3)	-10.5 (-20.3 to -2.9)
Diet low in vegetables	31.7 (26.7 to 35.9)	30.8 (25.8 to 35.0)	29.7 (23.8 to 35.0)	-2.8 (-9.6 to 4.1)	-3.5 (-12.4 to 3.5)	33.6 (28.8 to 37.6)	32.8 (28.3 to 36.8)	31.7 (26.4 to 36.3)	-2.3 (-9.3 to 4.1)	-3.4 (-10.2 to 2.6)
Diet low in legumes	36.1 (32.9 to 39.1)	43.0 (40.9 to 44.9)	45.7 (43.8 to 47.3)	19.1 (14.1 to 25.7)	6.1 (4.1 to 8.5)	39.1 (36.2 to 41.5)	45.0 (43.2 to 46.6)	47.2 (45.6 to 48.5)	15.2 (11.4 to 19.9)	4.9 (3.3 to 6.9)
Diet low in whole grains	43.6 (41.6 to 45.1)	41.9 (39.8 to 43.8)	41.8 (39.6 to 43.7)	-3.7 (-5.6 to -2.0)	-0.3 (-2.1 to 1.5)	44.3 (42.5 to 45.8)	42.8 (40.8 to 44.5)	42.6 (40.5 to 44.4)	-3.4 (-5.0 to -1.9)	-0.4 (-2.0 to 1.3)
Diet low in nuts and seeds	48.5 (46.8 to 50.0)	45.7 (43.3 to 47.7)	43.9 (40.8 to 46.5)	-5.9 (-9.3 to -3.3)	-3.9 (-7.2 to -1.1)	48.9 (47.1 to 50.2)	46.1 (43.9 to 47.9)	44.4 (41.7 to 46.6)	-5.6 (-8.6 to -3.0)	-3.8 (-6.8 to -1.2)
Diet low in milk	36.3 (32.3 to 39.5)	35.2 (31.3 to 38.6)	34.9 (30.6 to 38.4)	-2.8 (-6.3 to 0.3)	-0.9 (-4.2 to 2.1)	37.1 (33.5 to 40.3)	36.0 (32.2 to 39.2)	35.6 (31.6 to 39.0)	-3.1 (-6.1 to -0.3)	-1.0 (-3.9 to 1.7)
Diet high in red meat	5.7 (1.9 to 10.0)	16.3 (11.3 to 20.7)	26.6 (19.5 to 33.8)	183.4 (78.5 to 600.1)	63.9 (30.9 to 118.3)	0.7 (0.0 to 2.4)	3.3 (0.6 to 6.6)	8.5 (3.3 to 14.2)	377.5 (60.2 to 40999.7)	160.0 (54.8 to 860.0)
Diet high in processed meat	0.2 (0.0 to 1.2)	0.2 (0.0 to 1.2)	0.5 (0.0 to 2.0)	-5.2 (-100.0 to 102.5)	131.0 (58.6 to [illegible]	0.1 (0.0 to 0.9)	0.1 (0.0 to 0.9)	0.3 (0.0 to 1.5)	-2.5 (-100.0 to 125.2)	120.8 (-49.2 to [illegible]
Diet high in sugar-sweetened beverages	12.0 (5.6 to 20.2)	13.6 (6.2 to 21.6)	15.3 (7.2 to 24.2)	13.2 (-34.9 to 94.5)	12.9 (-23.3 to 69.7)	8.2 (3.8 to 14.2)	9.2 (4.4 to 15.6)	10.5 (4.8 to 17.6)	13.2 (-37.2 to 98.4)	13.7 (-23.2 to 71.7)
Diet low in fibre	36.3 (32.1 to 39.7)	35.8 (31.8 to 39.2)	36.3 (32.0 to 39.7)	-1.3 (-5.1 to 2.6)	1.2 (-2.6 to 5.4)	40.6 (37.2 to 43.3)	40.2 (36.8 to 42.9)	40.4 (36.8 to 43.2)	-0.9 (-4.4 to 2.2)	0.6 (-2.4 to 3.6)
Diet low in calcium	28.2 (22.9 to 32.5)	22.7 (16.2 to 27.8)	20.9 (14.2 to 26.4)	-19.6 (-29.3 to -13.7)	-7.8 (-14.8 to -3.5)	31.3 (26.5 to 35.1)	26.3 (20.5 to 30.8)	24.7 (18.5 to 29.5)	-16.0 (-23.0 to -11.4)	-6.2 (-11.5 to -2.7)
Diet low in seafood omega-3 fatty acids	45.9 (44.2 to 47.6)	46.0 (44.3 to 47.7)	45.2 (43.3 to 47.0)	0.2 (-0.0 to 0.5)	-1.7 (-2.4 to -1.1)	47.2 (45.6 to 48.7)	47.3 (45.7 to 48.7)	46.6 (45.0 to 48.2)	0.2 (-0.0 to 0.5)	-1.4 (-2.0 to -0.9)
Diet low in polyunsaturated fatty acids	32.9 (28.7 to 36.6)	21.4 (15.4 to 26.4)	19.3 (12.9 to 24.7)	-35.1 (-48.3 to -26.0)	-9.4 (-20.5 to -1.8)	33.5 (29.4 to 37.5)	22.3 (16.3 to 27.2)	20.2 (14.0 to 25.6)	-33.6 (-46.5 to -23.9)	-9.3 (-18.8 to -2.7)
Diet high in trans fatty acids	2.5 (0.4 to 5.4)	1.4 (0.1 to 3.8)	1.0 (0.0 to 3.1)	-43.7 (-80.3 to -25.7)	-30.9 (-76.8 to -14.9)	3.4 (0.8 to 6.7)	1.9 (0.2 to 4.6)	1.3 (0.1 to 3.7)	-43.4 (-76.7 to -25.6)	-30.7 (-71.5 to -14.8)
Diet high in sodium	25.5 (17.7 to 32.3)	12.9 (4.4 to 20.3)	13.2 (4.7 to 20.7)	-49.6 (-77.0 to -30.6)	2.6 (-17.5 to 31.3)	18.6 (11.7 to 25.2)	6.9 (0.7 to 14.1)	5.4 (0.2 to 12.1)	-63.1 (-94.5 to -41.2)	-21.6 (-79.3 to -6.8)
Intimate partner violence	-	-	-	-	-	1.9 (1.6 to 2.2)	1.6 (1.4 to 1.9)	1.5 (1.3 to 1.8)	-12.6 (-16.5 to -8.4)	-6.1 (-10.6 to -1.4)
Childhood sexual abuse	8.7 (7.1 to 10.8)	8.7 (7.1 to 10.8)	8.7 (7.1 to 10.8)	0.0 (0.0 to 0.0)	0.0 (0.0 to 0.0)	4.9 (3.6 to 6.5)	4.9 (3.6 to 6.5)	4.9 (3.6 to 6.5)	0.0 (0.0 to 0.0)	0.0 (0.0 to 0.0)

Supplementary Results Table 1. Croatia age-standardised Summary Exposure Values (SEVs) for Level 4 risk factors and percentage change, by sex, 1990, 2007, and 2017.Data in parentheses are 95% uncertainty intervals.

	Male					Female				
	**1990**	**2007**	**2017**	**Percentage change 1990-2007**	**Percentage change 2007-2017**	**1990**	**2007**	**2017**	**Percentage change 1990-2007**	**Percentage change 2007-2017**
Bullying victimization	4.7 (2.0 to 9.6)	5.1 (2.4 to 9.6)	5.2 (2.4 to 9.9)	9.3 (-17.8 to 49.7)	1.0 (-4.5 to 7.3)	3.9 (1.6 to 8.0)	4.0 (1.9 to 7.6)	4.2 (1.9 to 8.0)	2.7 (-21.4 to 40.7)	4.2 (-2.3 to 10.2)
Low physical activity	0.3 (0.2 to 0.5)	0.3 (0.2 to 0.5)	0.3 (0.2 to 0.6)	2.1 (-0.4 to 4.6)	0.5 (-1.4 to 2.4)	0.3 (0.1 to 0.5)	0.3 (0.1 to 0.5)	0.3 (0.1 to 0.5)	2.5 (0.4 to 5.0)	0.4 (-1.3 to 2.4)
High fasting plasma glucose	2.7 (1.7 to 4.2)	3.4 (2.3 to 5.0)	3.5 (2.3 to 5.1)	24.7 (-7.7 to 74.9)	2.2 (-22.8 to 33.6)	2.0 (1.3 to 3.1)	2.3 (1.5 to 3.6)	2.6 (1.6 to 3.9)	16.0 (-15.9 to 62.7)	11.0 (-20.3 to 60.9)
High LDL cholesterol	13.6 (12.0 to 15.4)	13.4 (11.7 to 15.2)	13.5 (11.7 to 15.3)	-2.1 (-6.4 to 2.7)	1.0 (-2.6 to 4.5)	13.7 (12.1 to 15.3)	13.5 (12.0 to 15.3)	13.7 (12.1 to 15.5)	-0.8 (-4.9 to 3.6)	1.2 (-2.3 to 4.6)
High systolic blood pressure	11.4 (10.4 to 12.4)	11.3 (10.3 to 12.3)	10.7 (9.7 to 11.7)	-1.0 (-7.6 to 5.9)	-4.9 (-9.9 to 0.8)	10.3 (9.3 to 11.2)	9.7 (8.8 to 10.7)	8.9 (7.9 to 9.9)	-5.1 (-13.3 to 4.4)	-8.7 (-15.3 to -2.1)
High body-mass index	11.4 (8.1 to 16.6)	16.0 (12.0 to 22.3)	16.5 (12.4 to 22.8)	40.0 (23.5 to 62.6)	3.1 (-5.2 to 11.7)	10.0 (7.0 to 13.8)	14.0 (10.4 to 18.6)	15.0 (11.4 to 20.1)	40.4 (22.4 to 66.3)	7.2 (-3.2 to 19.5)
Low bone mineral density	3.7 (3.0 to 4.6)	3.5 (2.9 to 4.3)	3.5 (2.8 to 4.3)	-5.2 (-19.3 to 10.2)	-1.3 (-17.0 to 15.3)	6.1 (5.1 to 7.3)	5.8 (4.9 to 6.9)	5.8 (4.7 to 6.9)	-5.2 (-17.4 to 8.6)	-0.7 (-13.9 to 14.0)
Impaired kidney function	2.1 (0.9 to 5.7)	2.1 (0.9 to 5.8)	2.1 (0.9 to 5.7)	-0.6 (-5.1 to 4.5)	-0.1 (-5.5 to 5.3)	2.8 (1.3 to 6.7)	2.8 (1.3 to 6.7)	2.8 (1.3 to 6.7)	1.9 (-2.4 to 7.6)	0.4 (-3.3 to 4.6)

Supplementary Results Table 1. Czech Republic age-standardised Summary Exposure Values (SEVs) for Level 4 risk factors and percentage change, by sex, 1990, 2007, and 2017.Data in parentheses are 95% uncertainty intervals.

	Male					Female				
	1990	2007	2017	Percentage change 1990-2007	Percentage change 2007-2017	1990	2007	2017	Percentage change 1990-2007	Percentage change 2007-2017
Unsafe water source	6.9 (4.7 to 10.0)	2.9 (1.9 to 4.3)	2.7 (1.8 to 4.0)	-57.8 (-73.6 to -32.4)	-6.7 (-35.3 to 34.2)	6.9 (4.7 to 10.0)	2.9 (1.9 to 4.3)	2.7 (1.8 to 4.0)	-57.8 (-73.6 to -32.4)	-6.7 (-35.3 to 34.2)
Unsafe sanitation	2.6 (1.5 to 4.4)	0.8 (0.4 to 1.3)	0.7 (0.4 to 1.2)	-71.2 (-84.6 to -47.4)	-11.2 (-44.6 to 42.2)	2.6 (1.5 to 4.4)	0.8 (0.4 to 1.3)	0.7 (0.4 to 1.2)	-71.2 (-84.6 to -47.4)	-11.2 (-44.6 to 42.2)
No access to handwashing facility	3.5 (2.8 to 4.4)	2.7 (2.1 to 3.3)	2.7 (2.1 to 3.4)	-24.2 (-41.7 to -2.7)	-0.1 (-17.0 to 21.0)	3.5 (2.8 to 4.4)	2.7 (2.1 to 3.3)	2.7 (2.1 to 3.4)	-24.2 (-41.7 to -2.7)	-0.1 (-17.0 to 21.0)
Ambient particulate matter pollution	44.6 (30.1 to 64.5)	40.9 (29.0 to 57.4)	35.6 (23.7 to 55.5)	-8.3 (-37.9 to 28.0)	-13.0 (-37.5 to 27.7)	43.5 (29.2 to 63.5)	40.3 (28.6 to 56.7)	35.1 (23.5 to 54.9)	-7.3 (-38.1 to 31.2)	-13.0 (-38.4 to 28.7)
Household air pollution from solid fuels	2.7 (1.2 to 7.3)	1.4 (0.6 to 3.6)	1.2 (0.4 to 4.1)	-48.1 (-77.7 to 6.8)	-13.6 (-59.3 to 88.0)	3.4 (1.5 to 9.2)	1.9 (0.7 to 7.6)	1.6 (0.6 to 7.5)	-45.3 (-77.3 to 8.0)	-12.7 (-58.7 to 84.3)
Ambient ozone pollution	46.2 (17.5 to 54.8)	46.3 (17.6 to 54.8)	47.0 (18.2 to 54.8)	0.4 (0.0 to 0.9)	1.4 (0.0 to 3.6)	46.2 (17.5 to 54.8)	46.3 (17.6 to 54.8)	47.0 (18.2 to 54.8)	0.4 (0.0 to 0.9)	1.4 (0.0 to 3.6)
Residential radon	49.8 (16.4 to 71.2)	49.8 (16.4 to 71.2)	49.8 (16.4 to 71.2)	0.0 (0.0 to 0.0)	0.0 (0.0 to 0.0)	49.8 (16.4 to 71.2)	49.8 (16.4 to 71.2)	49.8 (16.4 to 71.2)	0.0 (0.0 to 0.0)	0.0 (0.0 to 0.0)
Lead exposure	3.4 (0.6 to 7.1)	3.0 (0.5 to 6.5)	2.3 (0.3 to 5.4)	-10.8 (-17.5 to 0.8)	-23.4 (-37.1 to -15.9)	1.8 (0.1 to 5.0)	1.6 (0.1 to 4.6)	1.2 (0.1 to 3.8)	-10.8 (-18.4 to 5.1)	-23.3 (-41.6 to -15.6)
Occupational exposure to asbestos	1.7 (1.4 to 2.2)	1.5 (1.4 to 1.7)	1.5 (1.3 to 1.8)	-12.2 (-31.9 to 12.5)	-1.0 (-13.4 to 14.1)	0.8 (0.6 to 1.0)	0.7 (0.7 to 0.8)	0.5 (0.5 to 0.6)	-7.1 (-30.6 to 18.9)	-27.2 (-37.0 to -16.6)
Occupational exposure to arsenic	0.4 (0.0 to 1.0)	0.4 (0.0 to 0.9)	0.4 (0.0 to 1.0)	-	-	0.4 (0.0 to 1.1)	0.4 (0.0 to 0.9)	0.4 (0.0 to 1.0)	-	-
Occupational exposure to benzene	0.5 (0.0 to 1.5)	0.5 (0.0 to 1.5)	0.5 (0.0 to 1.5)	-0.2 (-4.1 to 5.0)	4.6 (0.8 to 9.9)	0.7 (0.0 to 2.0)	0.7 (0.0 to 2.1)	0.8 (0.0 to 2.3)	6.0 (0.8 to 14.6)	7.9 (3.1 to 14.2)
Occupational exposure to beryllium	0.0 (0.0 to 0.0)	0.0 (0.0 to 0.0)	0.0 (0.0 to 0.0)	-5.6 (-7.5 to -3.6)	4.2 (2.4 to 6.2)	0.0 (0.0 to 0.0)	0.0 (0.0 to 0.0)	0.0 (0.0 to 0.0)	-4.0 (-6.9 to -1.2)	5.7 (3.2 to 8.2)
Occupational exposure to cadmium	0.0 (0.0 to 0.0)	0.0 (0.0 to 0.0)	0.0 (0.0 to 0.0)	-4.2 (-10.6 to 3.2)	4.4 (-1.6 to 11.0)	0.1 (0.0 to 0.1)	0.0 (0.0 to 0.0)	0.0 (0.0 to 0.1)	-9.0 (-16.8 to 0.0)	2.7 (-5.7 to 11.8)
Occupational exposure to chromium	0.1 (0.1 to 0.1)	0.1 (0.1 to 0.1)	0.1 (0.1 to 0.1)	-3.1 (-8.8 to 3.5)	4.5 (-0.8 to 10.3)	0.1 (0.1 to 0.1)	0.1 (0.1 to 0.1)	0.1 (0.1 to 0.1)	-7.0 (-14.1 to 0.5)	3.4 (-3.5 to 10.8)
Occupational exposure to diesel engine exhaust	0.5 (0.5 to 0.5)	0.5 (0.5 to 0.5)	0.5 (0.5 to 0.5)	-8.1 (-13.4 to -2.4)	2.6 (-2.1 to 7.1)	0.4 (0.4 to 0.5)	0.4 (0.4 to 0.4)	0.4 (0.4 to 0.5)	-5.4 (-11.7 to 1.9)	5.4 (-0.2 to 11.9)
Occupational exposure to formaldehyde	0.2 (0.2 to 0.3)	0.2 (0.2 to 0.2)	0.2 (0.2 to 0.3)	-5.5 (-12.1 to 2.3)	3.2 (-3.0 to 10.0)	0.2 (0.2 to 0.3)	0.2 (0.2 to 0.2)	0.2 (0.2 to 0.2)	-8.1 (-16.1 to 0.9)	2.5 (-5.3 to 11.0)
Occupational exposure to nickel	0.4 (0.0 to 1.9)	0.4 (0.0 to 1.7)	0.4 (0.0 to 1.8)	-	-	0.4 (0.0 to 1.6)	0.3 (0.0 to 1.5)	0.3 (0.0 to 1.5)	-	-
Occupational exposure to polycyclic aromatic hydrocarbons	0.2 (0.2 to 0.2)	0.2 (0.2 to 0.2)	0.2 (0.2 to 0.2)	-4.1 (-8.9 to 1.4)	4.3 (-0.4 to 9.3)	0.2 (0.2 to 0.3)	0.2 (0.2 to 0.2)	0.2 (0.2 to 0.2)	-5.9 (-12.3 to 1.0)	3.9 (-2.1 to 10.4)
Occupational exposure to silica	5.3 (0.8 to 16.9)	4.5 (0.7 to 14.7)	4.6 (0.7 to 14.9)	-14.9 (-20.2 to -9.3)	2.1 (-2.8 to 7.9)	3.3 (0.5 to 10.5)	2.8 (0.4 to 9.0)	2.8 (0.4 to 8.8)	-15.1 (-23.4 to -5.8)	0.9 (-8.8 to 11.7)
Occupational exposure to sulphuric acid	0.6 (0.1 to 1.9)	0.6 (0.1 to 1.7)	0.6 (0.1 to 1.8)	-7.7 (-13.2 to -1.6)	2.2 (-3.3 to 8.4)	0.6 (0.1 to 1.9)	0.5 (0.1 to 1.6)	0.5 (0.1 to 1.7)	-12.8 (-20.3 to -4.7)	1.2 (-7.4 to 10.3)
Occupational exposure to trichloroethylene	0.1 (0.1 to 0.1)	0.1 (0.1 to 0.1)	0.1 (0.1 to 0.1)	-1.3 (-5.4 to 3.4)	4.4 (0.6 to 8.9)	0.1 (0.1 to 0.1)	0.1 (0.1 to 0.1)	0.1 (0.1 to 0.1)	-5.8 (-11.0 to -0.3)	4.2 (-0.5 to 9.6)
Occupational asthmagens	18.2 (15.7 to 21.3)	16.0 (13.7 to 18.8)	16.3 (13.9 to 19.4)	-12.2 (-17.8 to -6.4)	1.8 (-3.8 to 8.4)	10.1 (8.2 to 12.2)	8.9 (7.4 to 10.6)	9.7 (7.9 to 11.6)	-11.7 (-22.6 to 0.7)	8.8 (-2.7 to 21.7)
Occupational particulate matter, gases, and fumes	8.2 (5.9 to 11.6)	7.5 (5.3 to 10.6)	6.9 (5.0 to 9.9)	-9.0 (-14.4 to -5.1)	-7.2 (-10.2 to -4.6)	4.5 (3.2 to 6.5)	4.0 (2.9 to 5.8)	3.7 (2.6 to 5.4)	-10.8 (-17.0 to -4.6)	-9.3 (-12.9 to -6.0)
Occupational noise	7.4 (6.9 to 7.9)	6.8 (6.4 to 7.3)	6.4 (6.0 to 6.9)	-7.7 (-9.7 to -5.7)	-6.4 (-7.4 to -5.5)	4.5 (4.1 to 4.9)	4.2 (3.9 to 4.6)	4.0 (3.8 to 4.4)	-5.7 (-8.9 to -2.3)	-5.2 (-6.6 to -3.6)
Occupational ergonomic factors	8.9 (7.6 to 10.5)	7.6 (6.5 to 9.0)	7.6 (6.4 to 9.1)	-14.7 (-18.5 to -11.1)	0.6 (-3.2 to 4.3)	6.1 (5.2 to 7.2)	5.1 (4.3 to 6.1)	5.3 (4.4 to 6.3)	-16.1 (-22.2 to -9.6)	2.6 (-4.2 to 9.6)
Non-exclusive breastfeeding	0.6 (0.5 to 0.6)	0.6 (0.5 to 0.7)	0.6 (0.5 to 0.6)	1.9 (-7.2 to 12.5)	-2.7 (-10.2 to 5.6)	0.6 (0.5 to 0.6)	0.6 (0.5 to 0.7)	0.6 (0.5 to 0.6)	1.9 (-7.2 to 12.5)	-2.7 (-10.2 to 5.6)
Discontinued breastfeeding	2.1 (2.0 to 2.2)	2.3 (2.3 to 2.4)	2.3 (2.2 to 2.4)	11.3 (6.1 to 17.0)	-1.8 (-5.5 to 2.3)	2.1 (2.0 to 2.2)	2.3 (2.3 to 2.4)	2.3 (2.2 to 2.4)	11.3 (6.1 to 17.0)	-1.8 (-5.5 to 2.3)
Child underweight	0.1 (0.1 to 0.2)	0.1 (0.1 to 0.1)	0.1 (0.1 to 0.1)	-14.4 (-24.2 to -4.6)	-3.2 (-10.8 to 4.5)	0.1 (0.1 to 0.2)	0.1 (0.0 to 0.1)	0.1 (0.0 to 0.1)	-22.3 (-32.7 to -12.3)	-3.2 (-10.3 to 4.6)
Child wasting	0.2 (0.1 to 0.3)	0.2 (0.1 to 0.3)	0.2 (0.1 to 0.3)	-6.6 (-28.6 to 24.6)	-3.6 (-21.1 to 18.9)	0.2 (0.1 to 0.2)	0.1 (0.1 to 0.2)	0.1 (0.1 to 0.2)	-23.5 (-40.5 to -3.1)	-1.1 (-16.3 to 16.4)
Child stunting	0.3 (0.2 to 0.5)	0.3 (0.1 to 0.5)	0.3 (0.1 to 0.5)	-15.8 (-45.2 to 33.0)	2.4 (-21.9 to 38.0)	0.3 (0.1 to 0.5)	0.2 (0.1 to 0.4)	0.2 (0.1 to 0.4)	-22.1 (-49.4 to 16.2)	1.9 (-21.7 to 32.6)
Short gestation for birth weight	0.0 (0.0 to 0.0)	0.0 (0.0 to 0.0)	0.0 (0.0 to 0.0)	27.0 (-2.7 to 59.9)	9.6 (-7.5 to 30.2)	0.0 (0.0 to 0.0)	0.0 (0.0 to 0.0)	0.0 (0.0 to 0.0)	20.6 (-3.4 to 50.9)	6.6 (-17.8 to 29.0)
Low birth weight for gestation	0.0 (0.0 to 0.0)	0.0 (0.0 to 0.0)	0.0 (0.0 to 0.0)	-1.2 (-16.9 to 19.7)	-5.1 (-18.8 to 7.6)	0.0 (0.0 to 0.0)	0.0 (0.0 to 0.0)	0.0 (0.0 to 0.0)	-2.5 (-17.9 to 15.3)	-4.2 (-19.1 to 17.3)
Iron deficiency	-	-	-	-	-	5.2 (3.6 to 7.2)	4.1 (2.8 to 5.9)	3.7 (2.4 to 5.4)	-20.0 (-36.2 to -1.8)	-10.3 (-24.7 to 6.2)
Vitamin A deficiency	2.7 (2.2 to 3.3)	2.2 (1.8 to 2.6)	2.1 (1.7 to 2.5)	-18.3 (-28.1 to -6.8)	-5.0 (-15.7 to 7.4)	2.7 (2.2 to 3.3)	2.2 (1.9 to 2.7)	2.1 (1.7 to 2.5)	-16.9 (-26.8 to -7.1)	-4.9 (-14.7 to 5.6)
Zinc deficiency	0.4 (0.1 to 0.9)	0.4 (0.1 to 0.9)	0.4 (0.1 to 0.9)	10.5 (-9.6 to 156.8)	0.2 (-50.3 to 71.4)	0.4 (0.1 to 0.9)	0.4 (0.1 to 0.9)	0.4 (0.1 to 0.9)	10.0 (-8.9 to 163.7)	0.6 (-52.6 to 32.7)
Smoking	13.7 (12.0 to 15.6)	11.8 (10.4 to 13.3)	10.9 (9.6 to 12.2)	-13.3 (-20.8 to -5.5)	-8.0 (-13.6 to -1.6)	7.4 (6.0 to 9.0)	6.6 (5.6 to 7.7)	5.9 (5.0 to 6.8)	-11.2 (-23.6 to 6.0)	-11.0 (-18.2 to -3.4)
Chewing tobacco	0.1 (0.1 to 0.2)	0.1 (0.1 to 0.2)	0.1 (0.1 to 0.2)	-1.0 (-29.7 to 44.2)	-4.3 (-28.5 to 27.3)	0.1 (0.0 to 0.1)	0.1 (0.0 to 0.1)	0.1 (0.0 to 0.1)	-0.3 (-28.4 to 40.0)	0.1 (-22.5 to 29.9)
Second-hand smoke	45.0 (41.9 to 47.8)	38.8 (36.9 to 40.5)	39.0 (35.9 to 42.1)	-13.7 (-20.7 to -6.0)	0.5 (-5.9 to 6.9)	51.1 (48.6 to 53.5)	42.1 (40.5 to 43.6)	42.2 (39.6 to 44.7)	-17.7 (-22.6 to -12.4)	0.3 (-4.0 to 4.6)
Alcohol use	28.2 (19.2 to 38.0)	32.8 (23.2 to 43.8)	32.2 (21.1 to 45.4)	16.3 (2.1 to 37.2)	-2.0 (-20.3 to 18.3)	13.4 (7.8 to 21.5)	15.2 (9.3 to 23.4)	15.6 (9.0 to 24.8)	13.7 (1.0 to 33.4)	2.8 (-18.8 to 26.5)
Drug use	0.5 (0.3 to 0.8)	0.5 (0.3 to 0.9)	0.7 (0.4 to 1.3)	13.6 (-8.0 to 41.8)	38.9 (25.9 to 49.2)	0.2 (0.1 to 0.4)	0.3 (0.2 to 0.5)	0.4 (0.2 to 0.7)	18.4 (-10.1 to 55.2)	45.5 (34.3 to 54.2)
Diet low in fruits	37.7 (32.3 to 41.1)	37.0 (31.3 to 40.5)	35.8 (30.1 to 39.9)	-1.8 (-6.4 to 2.6)	-3.1 (-7.9 to 1.0)	35.2 (29.4 to 38.9)	34.7 (29.0 to 38.7)	33.3 (27.5 to 37.8)	-1.3 (-6.3 to 3.6)	-4.1 (-9.9 to 1.0)
Diet low in vegetables	36.0 (32.0 to 39.4)	34.8 (30.8 to 38.3)	33.6 (28.9 to 37.6)	-3.1 (-7.4 to 0.8)	-3.6 (-9.3 to 1.7)	37.6 (34.0 to 40.7)	36.5 (32.8 to 39.8)	35.3 (30.5 to 39.1)	-2.8 (-6.9 to 1.3)	-3.4 (-8.9 to 1.1)
Diet low in legumes	42.7 (40.5 to 44.5)	37.1 (33.9 to 39.7)	36.4 (33.0 to 39.3)	-13.1 (-16.5 to -10.4)	-1.8 (-4.9 to 1.0)	44.7 (42.8 to 46.3)	39.9 (37.4 to 42.2)	39.3 (36.4 to 41.8)	-10.7 (-13.2 to -8.5)	-1.5 (-4.0 to 0.8)
Diet low in whole grains	31.2 (27.4 to 34.4)	31.2 (27.3 to 34.4)	32.0 (28.1 to 35.3)	-0.1 (-3.9 to 4.2)	2.7 (-1.1 to 7.1)	32.6 (29.0 to 35.7)	32.5 (28.9 to 35.5)	33.4 (29.7 to 36.6)	-0.3 (-3.6 to 3.3)	2.7 (-0.8 to 6.6)
Diet low in nuts and seeds	50.8 (49.8 to 51.7)	49.4 (48.1 to 50.6)	48.0 (46.1 to 49.7)	-2.7 (-4.5 to -1.2)	-3.0 (-5.0 to -1.2)	51.1 (50.1 to 51.8)	49.8 (48.5 to 50.9)	48.4 (46.5 to 49.9)	-2.5 (-4.2 to -1.1)	-2.8 (-4.7 to -1.2)
Diet low in milk	34.0 (29.6 to 37.6)	34.8 (30.6 to 38.2)	35.4 (31.3 to 38.7)	2.2 (-1.5 to 6.5)	1.8 (-1.2 to 4.9)	35.2 (31.1 to 38.8)	35.5 (31.6 to 38.9)	36.0 (32.1 to 39.4)	1.1 (-2.2 to 5.3)	1.3 (-1.3 to 4.2)
Diet high in red meat	45.6 (40.4 to 49.3)	30.2 (26.7 to 33.3)	32.8 (25.6 to 38.6)	-33.7 (-41.6 to -25.3)	8.5 (-10.2 to 28.0)	22.0 (15.7 to 27.4)	12.6 (7.6 to 16.3)	14.2 (8.0 to 19.8)	-42.8 (-60.0 to -26.5)	12.9 (-16.2 to 50.5)
Diet high in processed meat	5.5 (2.5 to 8.3)	6.3 (3.2 to 9.4)	7.6 (4.1 to 10.7)	14.2 (-10.9 to 54.6)	21.4 (4.5 to 54.0)	3.6 (1.1 to 6.3)	4.1 (1.3 to 7.0)	5.1 (2.0 to 8.1)	16.1 (-13.8 to 68.9)	23.6 (5.2 to 74.9)
Diet high in sugar-sweetened beverages	14.9 (7.2 to 23.9)	17.5 (8.3 to 26.8)	20.3 (9.7 to 30.5)	17.5 (-29.3 to 96.7)	16.3 (-16.3 to 64.6)	9.8 (4.4 to 17.4)	11.7 (5.3 to 20.2)	13.7 (6.6 to 22.1)	19.2 (-30.6 to 111.6)	17.6 (-19.1 to 78.6)
Diet low in fibre	32.3 (27.3 to 36.2)	33.9 (29.4 to 37.5)	33.8 (28.7 to 37.7)	5.1 (0.8 to 11.3)	-0.3 (-4.7 to 4.0)	37.1 (33.1 to 40.3)	38.5 (34.9 to 41.4)	38.3 (34.4 to 41.5)	3.8 (0.4 to 8.2)	-0.5 (-4.4 to 2.8)
Diet low in calcium	21.5 (14.8 to 26.8)	22.0 (15.5 to 27.2)	21.2 (14.5 to 26.8)	2.4 (-1.5 to 7.6)	-3.6 (-9.0 to 0.5)	25.3 (19.4 to 30.0)	25.7 (19.9 to 30.3)	25.0 (19.0 to 29.8)	1.7 (-1.6 to 5.6)	-2.8 (-6.7 to 0.6)
Diet low in seafood omega-3 fatty acids	38.3 (35.1 to 41.2)	35.8 (32.2 to 39.2)	36.5 (33.0 to 39.8)	-6.4 (-8.6 to -4.8)	2.0 (0.5 to 3.9)	40.6 (37.8 to 43.1)	38.4 (35.3 to 41.3)	39.0 (35.9 to 41.9)	-5.4 (-7.1 to -4.0)	1.7 (0.5 to 3.1)
Diet low in polyunsaturated fatty acids	43.0 (40.4 to 45.3)	38.5 (35.5 to 41.2)	38.2 (35.0 to 40.9)	-10.4 (-15.4 to -5.4)	-0.8 (-4.5 to 3.0)	43.3 (40.9 to 45.6)	39.0 (35.9 to 41.8)	38.7 (35.6 to 41.4)	-9.9 (-14.1 to -5.0)	-1.0 (-4.8 to 2.8)
Diet high in trans fatty acids	4.6 (1.5 to 8.2)	2.4 (0.4 to 5.3)	1.5 (0.1 to 4.0)	-47.4 (-78.4 to -29.3)	-36.5 (-76.7 to -19.9)	6.3 (2.7 to 10.3)	3.3 (0.8 to 6.5)	2.1 (0.2 to 5.0)	-47.6 (-73.7 to -30.0)	-35.5 (-70.9 to -17.9)
Diet high in sodium	27.1 (19.2 to 33.6)	13.7 (4.6 to 21.3)	14.3 (5.3 to 21.8)	-49.5 (-78.3 to -31.7)	4.9 (-13.1 to 38.4)	19.4 (12.1 to 26.2)	7.1 (0.8 to 14.2)	5.7 (0.2 to 12.8)	-63.3 (-93.7 to -42.2)	-19.9 (-77.7 to -5.0)
Intimate partner violence	-	-	-	-	-	2.7 (2.2 to 3.1)	2.4 (2.0 to 2.8)	2.3 (1.9 to 2.6)	-10.1 (-14.4 to -5.9)	-5.7 (-10.3 to -1.2)
Childhood sexual abuse	5.9 (4.8 to 7.3)	5.8 (5.0 to 6.7)	5.9 (4.8 to 7.3)	-2.0 (-12.7 to 9.4)	2.1 (-8.6 to 14.5)	6.8 (5.4 to 8.4)	6.4 (5.7 to 7.1)	6.8 (5.4 to 8.4)	-6.5 (-20.4 to 12.4)	6.9 (-11.0 to 25.7)

Supplementary Results Table 1. Czech Republic age-standardised Summary Exposure Values (SEVs) for Level 4 risk factors and percentage change, by sex, 1990, 2007, and 2017.Data in parentheses are 95% uncertainty intervals.

	Male					Female				
	**1990**	**2007**	**2017**	**Percentage change 1990-2007**	**Percentage change 2007-2017**	**1990**	**2007**	**2017**	**Percentage change 1990-2007**	**Percentage change 2007-2017**
Bullying victimization	2.6 (1.1 to 5.4)	2.9 (1.4 to 5.5)	2.9 (1.3 to 5.8)	10.7 (-8.7 to 32.8)	1.6 (-24.6 to 35.4)	2.4 (1.0 to 4.9)	2.5 (1.2 to 5.0)	2.6 (1.2 to 5.1)	6.1 (-22.9 to 49.4)	4.2 (-2.8 to 11.5)
Low physical activity	0.3 (0.2 to 0.6)	0.3 (0.2 to 0.6)	0.3 (0.2 to 0.6)	1.0 (-0.8 to 2.7)	0.5 (-1.1 to 2.3)	0.3 (0.2 to 0.6)	0.3 (0.2 to 0.6)	0.3 (0.2 to 0.6)	0.7 (-1.3 to 2.9)	0.6 (-1.4 to 2.7)
High fasting plasma glucose	2.8 (1.8 to 4.2)	2.9 (2.0 to 4.2)	3.7 (2.4 to 5.5)	3.3 (-25.7 to 55.7)	26.2 (-12.0 to 81.2)	1.9 (1.1 to 2.9)	1.9 (1.2 to 2.8)	2.9 (1.9 to 4.3)	0.8 (-30.2 to 54.6)	53.2 (7.1 to 142.2)
High LDL cholesterol	17.6 (15.9 to 19.5)	14.0 (12.4 to 15.8)	14.0 (12.4 to 15.8)	-20.0 (-23.8 to -16.1)	-0.4 (-3.9 to 3.2)	17.5 (15.8 to 19.3)	14.5 (12.9 to 16.3)	14.4 (12.8 to 16.1)	-16.8 (-20.6 to -12.7)	-1.0 (-4.0 to 2.3)
High systolic blood pressure	10.6 (9.8 to 11.5)	9.8 (9.0 to 10.7)	10.0 (9.1 to 11.0)	-7.6 (-12.8 to -2.5)	1.8 (-3.6 to 7.5)	9.6 (8.7 to 10.4)	8.0 (7.2 to 8.8)	7.9 (7.0 to 8.7)	-16.7 (-23.2 to -9.7)	-0.8 (-7.9 to 6.7)
High body-mass index	11.1 (7.9 to 15.9)	14.7 (10.8 to 20.1)	15.9 (11.8 to 21.4)	32.3 (17.3 to 50.1)	8.1 (-0.7 to 18.4)	11.0 (7.9 to 15.2)	13.8 (10.2 to 18.3)	14.6 (10.6 to 19.4)	24.7 (8.4 to 45.6)	5.8 (-4.0 to 16.4)
Low bone mineral density	3.6 (2.9 to 4.4)	3.6 (2.9 to 4.2)	3.5 (2.8 to 4.4)	-2.1 (-16.8 to 17.1)	-1.2 (-17.3 to 16.6)	5.9 (5.0 to 7.1)	5.8 (4.9 to 6.9)	5.8 (4.8 to 6.9)	-2.3 (-15.2 to 12.2)	-0.5 (-13.5 to 14.5)
Impaired kidney function	2.2 (1.0 to 5.8)	2.1 (0.9 to 5.8)	2.1 (0.9 to 5.8)	-3.9 (-8.5 to -0.1)	0.7 (-4.0 to 5.8)	2.9 (1.4 to 6.9)	2.9 (1.4 to 6.9)	2.9 (1.4 to 6.9)	-0.6 (-5.4 to 4.4)	0.8 (-3.0 to 5.1)

Supplementary Results Table 1. Hungary age-standardised Summary Exposure Values (SEVs) for Level 4 risk factors and percentage change, by sex, 1990, 2007, and 2017.Data in parentheses are 95% uncertainty intervals.

	Male					Female				
	1990	2007	2017	Percentage change 1990-2007	Percentage change 2007-2017	1990	2007	2017	Percentage change 1990-2007	Percentage change 2007-2017
Unsafe water source	10.2 (7.6 to 13.0)	3.7 (2.5 to 5.3)	3.3 (2.2 to 4.9)	-63.2 (-75.5 to -44.9)	-11.1 (-38.0 to 27.2)	10.2 (7.6 to 13.0)	3.7 (2.5 to 5.3)	3.3 (2.2 to 4.9)	-63.2 (-75.5 to -44.9)	-11.1 (-38.0 to 27.2)
Unsafe sanitation	23.5 (14.9 to 34.0)	7.6 (4.2 to 12.3)	5.7 (3.1 to 9.6)	-67.8 (-80.5 to -49.6)	-24.9 (-53.0 to 17.6)	23.5 (14.9 to 34.0)	7.6 (4.2 to 12.3)	5.7 (3.1 to 9.6)	-67.8 (-80.5 to -49.6)	-24.9 (-53.0 to 17.6)
No access to handwashing facility	3.9 (3.1 to 4.9)	2.8 (2.2 to 3.5)	2.7 (2.2 to 3.5)	-28.7 (-43.4 to -9.2)	-1.3 (-16.8 to 18.5)	3.9 (3.1 to 4.9)	2.8 (2.2 to 3.5)	2.7 (2.2 to 3.5)	-28.7 (-43.4 to -9.2)	-1.3 (-16.8 to 18.5)
Ambient particulate matter pollution	30.7 (20.9 to 42.9)	34.6 (25.1 to 47.0)	31.3 (22.2 to 44.1)	12.8 (-17.3 to 57.7)	-9.6 (-31.8 to 16.7)	27.0 (17.8 to 38.8)	32.0 (22.3 to 44.3)	29.0 (19.9 to 41.3)	18.5 (-16.7 to 72.7)	-9.2 (-34.0 to 21.8)
Household air pollution from solid fuels	20.5 (12.7 to 31.1)	9.9 (4.8 to 17.4)	8.3 (3.9 to 15.3)	-51.5 (-73.1 to -15.5)	-16.9 (-55.3 to 43.3)	22.6 (14.2 to 33.3)	11.7 (5.8 to 19.7)	9.8 (4.6 to 17.2)	-48.2 (-70.5 to -14.6)	-16.1 (-52.7 to 39.0)
Ambient ozone pollution	47.1 (18.3 to 54.8)	47.3 (18.4 to 54.8)	48.1 (19.2 to 54.8)	0.5 (0.0 to 1.6)	1.7 (0.0 to 4.8)	47.1 (18.3 to 54.8)	47.3 (18.4 to 54.8)	48.1 (19.2 to 54.8)	0.5 (0.0 to 1.6)	1.7 (0.0 to 4.8)
Residential radon	38.5 (9.7 to 71.2)	38.5 (9.7 to 71.2)	38.5 (9.7 to 71.2)	0.0 (0.0 to 0.0)	0.0 (0.0 to 0.0)	38.5 (9.7 to 71.2)	38.5 (9.7 to 71.2)	38.5 (9.7 to 71.2)	0.0 (0.0 to 0.0)	0.0 (0.0 to 0.0)
Lead exposure	4.6 (1.0 to 8.8)	4.3 (1.1 to 8.3)	3.4 (0.8 to 6.9)	-5.5 (-11.4 to 13.1)	-22.0 (-29.9 to -15.6)	2.5 (0.2 to 6.1)	2.4 (0.2 to 5.8)	1.9 (0.2 to 4.9)	-4.6 (-11.4 to 27.5)	-21.8 (-31.4 to -15.3)
Occupational exposure to asbestos	1.1 (0.9 to 1.3)	1.2 (1.1 to 1.4)	1.2 (1.1 to 1.4)	16.5 (-3.3 to 39.6)	-3.9 (-16.2 to 11.4)	0.7 (0.5 to 0.8)	0.5 (0.4 to 0.5)	0.5 (0.4 to 0.5)	-26.8 (-44.1 to -8.4)	-5.5 (-18.4 to 10.9)
Occupational exposure to arsenic	0.3 (0.0 to 0.7)	0.3 (0.0 to 0.7)	0.3 (0.0 to 0.8)	-	-	0.4 (0.0 to 0.9)	0.3 (0.0 to 0.8)	0.3 (0.0 to 0.8)	-	-
Occupational exposure to benzene	0.4 (0.0 to 1.1)	0.5 (0.0 to 1.3)	0.5 (0.0 to 1.4)	14.8 (9.6 to 22.4)	5.7 (1.4 to 12.7)	0.5 (0.0 to 1.6)	0.7 (0.0 to 2.0)	0.8 (0.0 to 2.2)	25.8 (18.8 to 37.0)	10.4 (5.3 to 17.5)
Occupational exposure to beryllium	0.0 (0.0 to 0.0)	0.0 (0.0 to 0.0)	0.0 (0.0 to 0.0)	1.8 (-1.1 to 4.9)	7.0 (4.5 to 9.7)	0.0 (0.0 to 0.0)	0.0 (0.0 to 0.0)	0.0 (0.0 to 0.0)	1.6 (-2.2 to 5.5)	9.6 (6.4 to 12.8)
Occupational exposure to cadmium	0.0 (0.0 to 0.0)	0.0 (0.0 to 0.0)	0.0 (0.0 to 0.0)	-0.3 (-7.7 to 8.1)	4.2 (-1.4 to 10.7)	0.0 (0.0 to 0.0)	0.0 (0.0 to 0.0)	0.0 (0.0 to 0.0)	-9.2 (-18.6 to 1.2)	5.5 (-1.8 to 14.4)
Occupational exposure to chromium	0.1 (0.1 to 0.1)	0.1 (0.1 to 0.1)	0.1 (0.1 to 0.1)	3.4 (-3.4 to 11.3)	4.0 (-1.0 to 9.4)	0.1 (0.1 to 0.1)	0.1 (0.1 to 0.1)	0.1 (0.1 to 0.1)	-4.6 (-13.0 to 4.7)	6.6 (0.3 to 13.6)
Occupational exposure to diesel engine exhaust	0.3 (0.3 to 0.4)	0.4 (0.4 to 0.4)	0.4 (0.4 to 0.4)	13.8 (7.3 to 20.7)	8.5 (4.0 to 13.1)	0.3 (0.3 to 0.4)	0.4 (0.3 to 0.4)	0.4 (0.4 to 0.4)	6.0 (-1.4 to 13.8)	13.0 (6.4 to 19.7)
Occupational exposure to formaldehyde	0.2 (0.2 to 0.2)	0.2 (0.2 to 0.2)	0.2 (0.2 to 0.2)	-0.3 (-8.1 to 8.8)	2.6 (-3.1 to 9.0)	0.2 (0.2 to 0.2)	0.2 (0.2 to 0.2)	0.2 (0.2 to 0.2)	-6.0 (-15.2 to 4.6)	4.9 (-2.2 to 13.1)
Occupational exposure to nickel	0.3 (0.0 to 1.3)	0.3 (0.0 to 1.2)	0.3 (0.0 to 1.3)	-	-	0.3 (0.0 to 1.3)	0.3 (0.0 to 1.1)	0.3 (0.0 to 1.1)	-	-
Occupational exposure to polycyclic aromatic hydrocarbons	0.2 (0.2 to 0.2)	0.2 (0.2 to 0.2)	0.2 (0.2 to 0.2)	3.3 (-2.9 to 10.6)	5.3 (0.8 to 10.1)	0.2 (0.2 to 0.2)	0.2 (0.2 to 0.2)	0.2 (0.2 to 0.2)	-1.8 (-9.3 to 6.7)	6.9 (1.1 to 13.1)
Occupational exposure to silica	2.9 (0.4 to 9.2)	2.8 (0.4 to 8.9)	3.3 (0.5 to 10.6)	-4.8 (-11.6 to 3.8)	18.8 (12.8 to 26.9)	2.7 (0.4 to 8.3)	2.2 (0.3 to 6.7)	2.3 (0.3 to 7.2)	-18.5 (-28.8 to -6.9)	4.8 (-4.0 to 16.5)
Occupational exposure to sulphuric acid	0.5 (0.1 to 1.6)	0.5 (0.1 to 1.5)	0.5 (0.1 to 1.5)	-4.7 (-10.9 to 2.5)	0.9 (-4.3 to 6.2)	0.5 (0.1 to 1.6)	0.4 (0.1 to 1.4)	0.4 (0.1 to 1.4)	-13.6 (-22.2 to -3.8)	3.3 (-4.1 to 12.2)
Occupational exposure to trichloroethylene	0.0 (0.0 to 0.0)	0.0 (0.0 to 0.0)	0.0 (0.0 to 0.1)	7.2 (2.4 to 13.1)	3.1 (-0.7 to 7.1)	0.1 (0.0 to 0.1)	0.1 (0.0 to 0.1)	0.1 (0.1 to 0.1)	-1.2 (-8.1 to 5.7)	8.6 (3.6 to 14.0)
Occupational asthmagens	14.2 (12.1 to 17.1)	14.4 (12.2 to 17.2)	14.3 (12.2 to 17.2)	0.8 (-6.0 to 8.6)	-0.3 (-4.9 to 4.4)	8.0 (6.6 to 9.5)	7.5 (6.3 to 8.9)	7.9 (6.6 to 9.5)	-6.2 (-13.9 to 1.9)	5.8 (-0.5 to 12.8)
Occupational particulate matter, gases, and fumes	6.4 (4.6 to 9.2)	5.9 (4.2 to 8.4)	5.5 (3.9 to 7.9)	-8.4 (-12.1 to -4.7)	-5.8 (-8.4 to -3.4)	4.0 (2.8 to 6.0)	3.5 (2.5 to 5.1)	3.1 (2.2 to 4.5)	-13.7 (-19.0 to -8.1)	-10.0 (-13.8 to -6.2)
Occupational noise	5.6 (5.2 to 6.1)	5.2 (4.9 to 5.6)	5.0 (4.7 to 5.4)	-6.6 (-8.8 to -4.1)	-3.7 (-4.9 to -2.5)	3.8 (3.4 to 4.2)	3.6 (3.3 to 3.9)	3.4 (3.3 to 3.8)	-5.2 (-8.7 to -1.3)	-3.1 (-5.0 to -1.1)
Occupational ergonomic factors	8.7 (7.5 to 10.1)	7.7 (6.6 to 9.0)	8.0 (6.9 to 9.3)	-12.1 (-17.9 to -5.7)	4.2 (-1.6 to 10.8)	5.2 (4.3 to 6.2)	4.6 (3.9 to 5.5)	5.3 (4.4 to 6.3)	-11.3 (-19.3 to -3.3)	14.0 (7.2 to 22.6)
Non-exclusive breastfeeding	0.4 (0.3 to 0.6)	0.5 (0.3 to 0.6)	0.5 (0.3 to 0.6)	3.8 (-5.0 to 13.4)	-1.0 (-7.1 to 5.7)	0.4 (0.3 to 0.6)	0.5 (0.3 to 0.6)	0.5 (0.3 to 0.6)	3.8 (-5.0 to 13.5)	-1.0 (-7.1 to 5.7)
Discontinued breastfeeding	2.2 (2.1 to 2.3)	2.3 (2.3 to 2.4)	2.3 (2.3 to 2.4)	6.0 (1.5 to 11.0)	0.4 (-3.1 to 4.1)	2.2 (2.1 to 2.3)	2.3 (2.3 to 2.4)	2.3 (2.3 to 2.4)	6.1 (1.5 to 11.0)	0.4 (-3.0 to 4.2)
Child underweight	0.2 (0.1 to 0.2)	0.1 (0.1 to 0.2)	0.1 (0.1 to 0.2)	-27.0 (-38.4 to -16.1)	-7.4 (-15.5 to 1.4)	0.2 (0.1 to 0.2)	0.1 (0.1 to 0.1)	0.1 (0.1 to 0.1)	-33.1 (-45.1 to -21.6)	-8.4 (-17.1 to 0.7)
Child wasting	0.2 (0.1 to 0.3)	0.2 (0.1 to 0.2)	0.1 (0.1 to 0.2)	-21.1 (-44.3 to 10.0)	-7.8 (-26.2 to 13.8)	0.2 (0.1 to 0.3)	0.1 (0.1 to 0.2)	0.1 (0.1 to 0.2)	-21.6 (-43.7 to 6.6)	-5.8 (-21.9 to 13.4)
Child stunting	0.8 (0.4 to 1.4)	0.6 (0.3 to 1.0)	0.5 (0.3 to 0.9)	-27.5 (-57.5 to 22.3)	-10.1 (-39.3 to 28.2)	0.7 (0.4 to 1.3)	0.5 (0.3 to 0.9)	0.5 (0.2 to 0.8)	-29.7 (-60.2 to 19.8)	-11.3 (-39.5 to 29.5)
Short gestation for birth weight	0.0 (0.0 to 0.0)	0.0 (0.0 to 0.0)	0.0 (0.0 to 0.0)	14.2 (-17.2 to 59.2)	4.0 (-17.6 to 38.3)	0.0 (0.0 to 0.0)	0.0 (0.0 to 0.0)	0.0 (0.0 to 0.0)	10.3 (-13.0 to 46.9)	5.1 (-17.7 to 53.2)
Low birth weight for gestation	0.0 (0.0 to 0.0)	0.0 (0.0 to 0.0)	0.0 (0.0 to 0.0)	8.6 (-13.2 to 34.4)	1.0 (-23.1 to 26.7)	0.0 (0.0 to 0.0)	0.0 (0.0 to 0.0)	0.0 (0.0 to 0.0)	6.8 (-13.0 to 28.6)	-0.3 (-29.7 to 23.7)
Iron deficiency	-	-	-	-	-	6.0 (4.1 to 8.0)	4.8 (3.2 to 6.6)	4.4 (2.9 to 6.2)	-19.9 (-34.7 to -3.7)	-9.3 (-23.4 to 7.2)
Vitamin A deficiency	2.8 (2.3 to 3.4)	2.2 (1.9 to 2.7)	2.1 (1.7 to 2.5)	-19.8 (-28.0 to -10.0)	-5.3 (-16.5 to 6.0)	2.8 (2.3 to 3.3)	2.3 (2.0 to 2.8)	2.2 (1.8 to 2.6)	-15.4 (-24.3 to -4.4)	-6.4 (-15.9 to 4.2)
Zinc deficiency	0.4 (0.1 to 0.9)	0.6 (0.2 to 1.3)	0.6 (0.2 to 1.3)	34.2 (2.1 to 185.9)	2.7 (-15.8 to 35.9)	0.4 (0.1 to 0.9)	0.6 (0.2 to 1.3)	0.6 (0.2 to 1.4)	32.5 (1.7 to 166.2)	1.6 (-16.8 to 30.6)
Smoking	14.6 (12.9 to 16.3)	12.5 (11.2 to 13.9)	10.9 (9.6 to 12.2)	-14.2 (-20.5 to -6.9)	-12.9 (-18.3 to -7.2)	9.0 (7.4 to 10.7)	8.1 (7.0 to 9.3)	7.1 (6.1 to 8.1)	-9.6 (-21.0 to 5.7)	-12.8 (-20.1 to -4.7)
Chewing tobacco	0.1 (0.1 to 0.2)	0.1 (0.1 to 0.2)	0.1 (0.1 to 0.2)	-0.1 (-32.8 to 46.9)	-5.0 (-28.9 to 25.9)	0.1 (0.0 to 0.1)	0.1 (0.0 to 0.1)	0.1 (0.0 to 0.1)	-1.3 (-32.5 to 39.0)	-0.3 (-21.0 to 29.7)
Second-hand smoke	42.5 (40.2 to 44.8)	39.3 (37.1 to 41.5)	36.4 (33.5 to 39.3)	-7.5 (-15.8 to 0.5)	-7.5 (-12.4 to -2.3)	47.7 (45.7 to 49.8)	40.7 (38.9 to 42.7)	38.3 (36.0 to 40.7)	-14.6 (-20.8 to -8.1)	-5.9 (-9.7 to -2.0)
Alcohol use	41.2 (32.6 to 49.3)	36.5 (27.3 to 46.7)	33.4 (22.2 to 46.7)	-11.5 (-22.9 to 0.6)	-8.5 (-25.9 to 7.1)	14.7 (8.6 to 23.0)	13.3 (8.0 to 21.1)	13.3 (7.3 to 21.7)	-9.3 (-22.4 to 4.8)	-0.5 (-21.7 to 24.3)
Drug use	0.5 (0.4 to 0.8)	0.6 (0.4 to 0.9)	0.6 (0.4 to 0.9)	7.4 (-4.3 to 22.2)	2.5 (-10.1 to 15.2)	0.3 (0.2 to 0.5)	0.3 (0.2 to 0.5)	0.3 (0.2 to 0.5)	12.9 (-2.3 to 33.5)	-5.3 (-19.6 to 10.8)
Diet low in fruits	38.4 (33.1 to 41.7)	37.5 (32.3 to 41.0)	36.4 (30.8 to 40.4)	-2.4 (-6.0 to 1.2)	-3.0 (-7.9 to 1.0)	36.3 (30.6 to 39.9)	35.3 (29.7 to 39.0)	34.0 (28.4 to 38.1)	-2.8 (-7.3 to 1.4)	-3.7 (-9.3 to 1.1)
Diet low in vegetables	28.8 (23.6 to 33.1)	20.0 (13.1 to 25.7)	14.9 (7.1 to 21.8)	-30.7 (-44.8 to -21.4)	-25.4 (-49.3 to -11.1)	30.9 (26.1 to 34.9)	21.8 (15.2 to 27.2)	16.7 (9.1 to 23.3)	-29.4 (-42.2 to -20.8)	-23.2 (-43.0 to -10.7)
Diet low in legumes	38.0 (35.0 to 40.5)	33.3 (29.6 to 36.4)	32.3 (28.4 to 35.9)	-12.5 (-16.4 to -9.3)	-2.8 (-6.9 to 0.6)	40.6 (38.0 to 42.8)	37.1 (34.0 to 39.8)	37.0 (33.8 to 39.9)	-8.6 (-11.7 to -6.2)	-0.3 (-3.3 to 2.8)
Diet low in whole grains	42.3 (40.3 to 44.2)	43.9 (42.1 to 45.4)	44.2 (42.4 to 45.7)	3.7 (2.1 to 5.8)	0.6 (-0.9 to 2.0)	43.2 (41.2 to 44.8)	44.6 (42.8 to 46.0)	44.8 (43.1 to 46.3)	3.3 (1.7 to 5.0)	0.5 (-0.8 to 1.8)
Diet low in nuts and seeds	51.9 (51.2 to 52.5)	53.0 (52.5 to 53.4)	52.7 (52.0 to 53.2)	2.1 (1.3 to 3.0)	-0.7 (-1.2 to -0.2)	52.1 (51.4 to 52.7)	53.1 (52.6 to 53.5)	52.7 (52.2 to 53.2)	1.9 (1.2 to 2.8)	-0.6 (-1.2 to -0.2)
Diet low in milk	34.9 (30.9 to 38.3)	36.3 (32.6 to 39.5)	35.4 (31.4 to 38.9)	4.0 (1.1 to 7.7)	-2.6 (-5.6 to -0.2)	35.9 (31.9 to 39.1)	36.9 (33.2 to 40.1)	35.9 (31.8 to 39.2)	2.9 (0.0 to 6.6)	-2.8 (-6.2 to -0.2)
Diet high in red meat	51.5 (49.6 to 53.2)	28.3 (23.8 to 32.0)	29.3 (22.8 to 34.9)	-45.1 (-52.5 to -38.2)	3.8 (-15.3 to 24.7)	30.0 (23.7 to 34.9)	10.2 (5.9 to 14.4)	10.8 (5.6 to 15.8)	-65.9 (-79.0 to -52.6)	5.2 (-26.9 to 47.4)
Diet high in processed meat	4.5 (1.7 to 7.4)	5.3 (2.5 to 8.1)	6.4 (3.2 to 9.4)	19.6 (-4.6 to 78.8)	19.4 (2.7 to 53.0)	2.8 (0.5 to 5.5)	3.5 (1.0 to 6.3)	4.2 (1.6 to 7.1)	24.0 (-3.1 to 134.8)	21.1 (2.3 to 82.2)
Diet high in sugar-sweetened beverages	10.1 (4.9 to 16.3)	11.2 (5.8 to 13.3)	13.2 (6.6 to 17.0)	10.1 (-31.5 to 79.3)	18.5 (-8.3 to 51.1)	7.0 (3.7 to 11.3)	7.6 (3.8 to 9.6)	9.0 (4.5 to 11.9)	9.1 (-32.8 to 72.5)	18.3 (-11.9 to 57.7)
Diet low in fibre	29.4 (24.0 to 33.7)	33.8 (29.2 to 37.5)	34.7 (30.1 to 38.3)	15.1 (9.4 to 24.1)	2.5 (-1.3 to 7.2)	34.5 (30.1 to 38.1)	38.4 (34.7 to 41.5)	39.0 (35.2 to 42.1)	11.4 (6.8 to 17.6)	1.6 (-1.7 to 5.1)
Diet low in calcium	26.9 (21.2 to 31.4)	30.3 (25.3 to 34.2)	29.9 (24.8 to 34.1)	12.6 (8.1 to 20.9)	-1.2 (-4.1 to 1.4)	30.2 (25.3 to 34.2)	33.3 (28.9 to 36.8)	32.9 (28.4 to 36.6)	10.1 (6.8 to 15.4)	-1.1 (-3.7 to 1.3)
Diet low in seafood omega-3 fatty acids	33.5 (29.7 to 37.1)	35.4 (31.7 to 38.9)	36.0 (32.3 to 39.2)	5.5 (3.3 to 8.4)	1.6 (-0.7 to 4.2)	36.4 (33.0 to 39.6)	38.1 (34.9 to 41.0)	38.6 (35.4 to 41.6)	4.5 (2.8 to 6.6)	1.4 (-0.4 to 3.3)
Diet low in polyunsaturated fatty acids	38.6 (35.5 to 41.8)	27.4 (22.3 to 31.6)	28.6 (23.6 to 32.9)	-29.2 (-38.3 to -22.0)	4.6 (-0.7 to 10.9)	39.0 (35.9 to 41.8)	28.1 (23.2 to 32.3)	29.3 (24.6 to 33.5)	-28.0 (-36.6 to -21.7)	4.3 (-0.3 to 9.8)
Diet high in trans fatty acids	5.8 (2.5 to 9.8)	3.0 (0.7 to 6.3)	2.0 (0.2 to 4.7)	-48.0 (-76.1 to -29.9)	-34.6 (-69.1 to -17.8)	7.9 (4.0 to 12.0)	4.2 (1.3 to 7.7)	2.7 (0.5 to 5.8)	-47.3 (-71.2 to -29.5)	-34.2 (-65.2 to -18.6)
Diet high in sodium	34.2 (27.6 to 39.7)	19.2 (11.1 to 26.2)	15.3 (6.8 to 22.3)	-43.9 (-63.1 to -29.0)	-20.3 (-44.4 to -7.2)	26.2 (19.7 to 32.2)	8.6 (1.6 to 15.9)	5.8 (0.2 to 12.7)	-67.0 (-92.5 to -48.1)	-33.1 (-89.7 to -15.7)
Intimate partner violence	-	-	-	-	-	4.0 (3.4 to 4.7)	3.4 (2.9 to 3.9)	3.2 (2.7 to 3.6)	-15.0 (-19.4 to -11.0)	-6.6 (-10.8 to -1.9)
Childhood sexual abuse	8.7 (7.1 to 10.8)	8.7 (7.1 to 10.8)	8.7 (7.1 to 10.8)	0.0 (0.0 to 0.0)	0.0 (0.0 to 0.0)	4.9 (3.6 to 6.5)	4.9 (3.6 to 6.5)	4.9 (3.6 to 6.5)	0.0 (0.0 to 0.0)	0.0 (0.0 to 0.0)

Supplementary Results Table 1. Hungary age-standardised Summary Exposure Values (SEVs) for Level 4 risk factors and percentage change, by sex, 1990, 2007, and 2017.Data in parentheses are 95% uncertainty intervals.

	Male					Female				
	**1990**	**2007**	**2017**	**Percentage change 1990-2007**	**Percentage change 2007-2017**	**1990**	**2007**	**2017**	**Percentage change 1990-2007**	**Percentage change 2007-2017**
Bullying victimization	2.5 (1.1 to 5.0)	3.1 (1.5 to 6.1)	3.5 (1.6 to 7.0)	20.3 (3.0 to 42.4)	12.9 (1.9 to 26.6)	2.3 (1.0 to 4.7)	2.9 (1.4 to 5.7)	3.1 (1.5 to 6.1)	26.1 (4.1 to 56.0)	8.3 (-0.2 to 18.2)
Low physical activity	0.3 (0.1 to 0.5)	0.3 (0.1 to 0.5)	0.3 (0.1 to 0.5)	-1.0 (-4.4 to 1.5)	0.4 (-2.3 to 3.5)	0.3 (0.1 to 0.6)	0.3 (0.1 to 0.6)	0.3 (0.1 to 0.6)	-0.6 (-2.6 to 1.2)	0.0 (-1.8 to 1.9)
High fasting plasma glucose	2.8 (1.7 to 4.3)	3.4 (2.3 to 4.8)	3.3 (2.0 to 4.9)	20.1 (-24.1 to 106.4)	-1.3 (-38.9 to 41.3)	2.7 (1.7 to 4.0)	2.7 (1.7 to 3.9)	2.6 (1.6 to 3.9)	0.4 (-32.5 to 49.6)	-3.1 (-36.5 to 44.6)
High LDL cholesterol	17.8 (16.0 to 19.7)	14.1 (12.5 to 15.8)	13.3 (11.6 to 15.0)	-20.5 (-24.6 to -16.5)	-6.0 (-9.5 to -2.8)	17.7 (16.0 to 19.5)	14.3 (12.7 to 16.1)	12.8 (11.2 to 14.5)	-18.9 (-22.8 to -15.0)	-10.8 (-13.9 to -7.6)
High systolic blood pressure	13.3 (12.4 to 14.2)	13.0 (12.0 to 14.1)	12.7 (11.7 to 13.7)	-2.1 (-7.5 to 3.7)	-2.5 (-7.5 to 2.6)	11.4 (10.6 to 12.3)	11.3 (10.3 to 12.4)	11.1 (10.1 to 12.2)	-0.7 (-8.1 to 7.5)	-1.8 (-8.2 to 5.4)
High body-mass index	12.6 (9.4 to 17.3)	15.9 (12.3 to 21.0)	16.5 (12.6 to 22.3)	26.1 (12.2 to 42.0)	3.5 (-4.3 to 12.4)	13.2 (10.0 to 17.4)	16.1 (12.6 to 21.0)	16.4 (12.9 to 21.5)	21.7 (8.2 to 38.2)	2.2 (-6.9 to 12.7)
Low bone mineral density	3.7 (2.4 to 5.2)	3.7 (2.2 to 5.2)	3.5 (2.0 to 5.3)	-0.9 (-25.0 to 31.4)	-3.8 (-25.7 to 20.7)	6.1 (5.1 to 7.3)	6.1 (5.2 to 7.2)	6.0 (5.0 to 7.1)	-0.4 (-13.4 to 15.7)	-1.5 (-14.9 to 12.9)
Impaired kidney function	2.2 (1.0 to 5.9)	2.2 (1.0 to 5.9)	2.2 (1.0 to 5.8)	-0.4 (-4.9 to 4.4)	-0.8 (-4.7 to 3.2)	2.9 (1.4 to 6.9)	3.0 (1.4 to 7.0)	3.0 (1.4 to 6.9)	2.1 (-1.4 to 6.4)	-0.4 (-3.9 to 3.2)

Supplementary Results Table 1. Macedonia age-standardised Summary Exposure Values (SEVs) for Level 4 risk factors and percentage change, by sex, 1990, 2007, and 2017.Data in parentheses are 95% uncertainty intervals.

	Male					Female				
	1990	2007	2017	Percentage change 1990-2007	Percentage change 2007-2017	1990	2007	2017	Percentage change 1990-2007	Percentage change 2007-2017
Unsafe water source	12.9 (8.9 to 18.3)	7.1 (5.1 to 9.5)	5.9 (4.2 to 8.2)	-45.1 (-64.0 to -16.1)	-15.9 (-40.1 to 20.0)	12.9 (8.9 to 18.3)	7.1 (5.1 to 9.5)	5.9 (4.2 to 8.2)	-45.1 (-64.0 to -16.1)	-15.9 (-40.1 to 20.0)
Unsafe sanitation	18.8 (11.6 to 27.6)	9.4 (5.7 to 14.4)	6.9 (4.0 to 11.1)	-50.1 (-69.9 to -16.9)	-26.1 (-52.6 to 13.5)	18.8 (11.6 to 27.6)	9.4 (5.7 to 14.4)	6.9 (4.0 to 11.1)	-50.1 (-69.9 to -16.9)	-26.1 (-52.6 to 13.5)
No access to handwashing facility	5.0 (4.0 to 6.3)	3.4 (2.7 to 4.3)	3.3 (2.6 to 4.1)	-31.8 (-47.4 to -12.6)	-5.0 (-21.9 to 15.3)	5.0 (4.0 to 6.3)	3.4 (2.7 to 4.3)	3.3 (2.6 to 4.1)	-31.8 (-47.4 to -12.6)	-5.0 (-21.9 to 15.3)
Ambient particulate matter pollution	39.0 (24.8 to 58.9)	42.9 (30.6 to 59.0)	42.2 (28.3 to 60.4)	10.0 (-28.5 to 73.8)	-1.7 (-31.8 to 35.7)	34.1 (20.8 to 53.1)	38.8 (26.4 to 55.1)	38.8 (25.3 to 57.3)	13.6 (-29.3 to 85.4)	0.2 (-33.6 to 42.7)
Household air pollution from solid fuels	30.1 (19.0 to 44.0)	18.2 (11.2 to 27.3)	12.9 (6.8 to 21.7)	-39.6 (-62.5 to -2.0)	-29.0 (-57.0 to 11.1)	32.8 (21.8 to 46.2)	20.6 (13.2 to 30.2)	14.9 (8.2 to 23.9)	-37.1 (-59.4 to -3.4)	-27.5 (-54.5 to 9.7)
Ambient ozone pollution	46.1 (17.4 to 54.8)	45.5 (17.1 to 54.8)	45.9 (17.4 to 54.8)	-1.3 (-3.5 to 0.0)	0.9 (0.0 to 2.8)	46.1 (17.4 to 54.8)	45.5 (17.1 to 54.8)	45.9 (17.4 to 54.8)	-1.3 (-3.5 to 0.0)	0.9 (0.0 to 2.8)
Residential radon	42.6 (12.9 to 71.2)	42.6 (12.9 to 71.2)	42.6 (12.9 to 71.2)	0.0 (0.0 to 0.0)	0.0 (0.0 to 0.0)	42.6 (12.9 to 71.2)	42.6 (12.9 to 71.2)	42.6 (12.9 to 71.2)	0.0 (0.0 to 0.0)	0.0 (0.0 to 0.0)
Lead exposure	4.9 (1.4 to 9.0)	4.9 (1.6 to 8.9)	4.0 (1.2 to 7.6)	-0.1 (-6.6 to 21.4)	-18.6 (-27.2 to -12.5)	2.6 (0.2 to 6.2)	2.6 (0.3 to 6.1)	2.2 (0.3 to 5.4)	2.4 (-5.2 to 44.8)	-17.0 (-27.7 to -11.2)
Occupational exposure to asbestos	0.3 (0.2 to 0.4)	0.3 (0.3 to 0.4)	0.2 (0.1 to 0.2)	4.2 (-27.2 to 54.3)	-42.3 (-58.0 to -23.3)	0.1 (0.1 to 0.2)	0.1 (0.1 to 0.1)	0.1 (0.1 to 0.1)	-28.8 (-48.2 to 14.0)	-21.6 (-47.1 to 14.9)
Occupational exposure to arsenic	0.2 (0.0 to 0.5)	0.2 (0.0 to 0.5)	0.2 (0.0 to 0.6)	-	-	0.2 (0.0 to 0.6)	0.2 (0.0 to 0.6)	0.3 (0.0 to 0.6)	-	-
Occupational exposure to benzene	0.3 (0.0 to 0.9)	0.4 (0.0 to 1.0)	0.4 (0.0 to 1.2)	16.2 (6.6 to 30.8)	15.3 (10.3 to 22.1)	0.3 (0.0 to 0.9)	0.4 (0.0 to 1.1)	0.5 (0.0 to 1.3)	22.0 (8.2 to 48.9)	23.3 (16.6 to 33.5)
Occupational exposure to beryllium	0.0 (0.0 to 0.0)	0.0 (0.0 to 0.0)	0.0 (0.0 to 0.0)	4.6 (-2.5 to 13.0)	14.9 (11.3 to 18.4)	0.0 (0.0 to 0.0)	0.0 (0.0 to 0.0)	0.0 (0.0 to 0.0)	2.9 (-7.0 to 15.1)	19.7 (14.6 to 25.3)
Occupational exposure to cadmium	0.0 (0.0 to 0.0)	0.0 (0.0 to 0.0)	0.0 (0.0 to 0.0)	1.8 (-7.2 to 12.3)	17.1 (11.7 to 23.2)	0.0 (0.0 to 0.0)	0.0 (0.0 to 0.0)	0.0 (0.0 to 0.0)	0.0 (-13.0 to 17.3)	15.1 (8.6 to 22.7)
Occupational exposure to chromium	0.1 (0.0 to 0.1)	0.1 (0.1 to 0.1)	0.1 (0.1 to 0.1)	5.3 (-3.7 to 15.7)	18.0 (12.9 to 23.6)	0.1 (0.1 to 0.1)	0.1 (0.1 to 0.1)	0.1 (0.1 to 0.1)	3.2 (-9.9 to 20.8)	16.8 (10.6 to 24.1)
Occupational exposure to diesel engine exhaust	0.3 (0.3 to 0.3)	0.3 (0.3 to 0.3)	0.4 (0.4 to 0.4)	13.1 (3.4 to 24.3)	17.0 (12.4 to 22.2)	0.2 (0.2 to 0.2)	0.2 (0.2 to 0.2)	0.2 (0.2 to 0.3)	7.4 (-5.0 to 21.2)	27.7 (21.0 to 35.6)
Occupational exposure to formaldehyde	0.1 (0.1 to 0.1)	0.1 (0.1 to 0.1)	0.1 (0.1 to 0.1)	1.7 (-7.7 to 13.2)	16.7 (11.0 to 23.4)	0.1 (0.1 to 0.1)	0.1 (0.1 to 0.1)	0.1 (0.1 to 0.2)	2.5 (-11.2 to 21.1)	14.8 (8.3 to 22.4)
Occupational exposure to nickel	0.2 (0.0 to 1.0)	0.2 (0.0 to 0.9)	0.2 (0.0 to 1.0)	-	-	0.2 (0.0 to 0.9)	0.2 (0.0 to 0.9)	0.2 (0.0 to 1.0)	-	-
Occupational exposure to polycyclic aromatic hydrocarbons	0.1 (0.1 to 0.1)	0.1 (0.1 to 0.1)	0.1 (0.1 to 0.1)	6.4 (-2.5 to 16.8)	16.6 (12.0 to 21.6)	0.1 (0.1 to 0.1)	0.1 (0.1 to 0.1)	0.1 (0.1 to 0.2)	4.9 (-8.0 to 22.1)	16.9 (11.0 to 23.8)
Occupational exposure to silica	2.6 (0.4 to 8.3)	2.6 (0.4 to 8.3)	3.0 (0.4 to 9.6)	1.1 (-8.3 to 13.5)	13.8 (7.3 to 21.9)	1.8 (0.3 to 5.7)	1.7 (0.2 to 5.3)	1.9 (0.3 to 6.1)	-6.0 (-17.6 to 10.5)	11.6 (5.3 to 20.6)
Occupational exposure to sulphuric acid	0.4 (0.1 to 1.1)	0.4 (0.1 to 1.2)	0.4 (0.1 to 1.3)	3.7 (-5.8 to 14.4)	8.9 (3.0 to 15.3)	0.3 (0.1 to 1.0)	0.3 (0.1 to 1.0)	0.4 (0.1 to 1.1)	-2.1 (-14.1 to 13.2)	12.7 (6.4 to 20.3)
Occupational exposure to trichloroethylene	0.0 (0.0 to 0.0)	0.0 (0.0 to 0.0)	0.0 (0.0 to 0.0)	8.4 (0.1 to 18.2)	18.2 (13.7 to 22.9)	0.0 (0.0 to 0.0)	0.0 (0.0 to 0.0)	0.0 (0.0 to 0.0)	4.4 (-7.9 to 20.9)	19.9 (14.0 to 26.9)
Occupational asthmagens	9.2 (7.7 to 11.1)	9.3 (8.0 to 10.9)	10.6 (9.1 to 12.6)	0.9 (-9.5 to 12.4)	14.3 (8.3 to 20.5)	4.5 (3.5 to 5.7)	4.4 (3.7 to 5.3)	5.2 (4.3 to 6.2)	-1.3 (-16.4 to 19.3)	16.9 (8.1 to 26.8)
Occupational particulate matter, gases, and fumes	4.5 (3.2 to 6.6)	4.2 (3.0 to 6.1)	4.1 (3.0 to 6.0)	-7.3 (-12.2 to -2.8)	-1.2 (-3.8 to 1.8)	2.6 (1.8 to 3.9)	2.5 (1.7 to 3.6)	2.4 (1.7 to 3.6)	-5.6 (-12.0 to 2.6)	-1.2 (-5.3 to 3.3)
Occupational noise	4.2 (3.8 to 4.8)	4.0 (3.7 to 4.4)	4.0 (3.8 to 4.4)	-5.6 (-9.4 to -1.5)	-0.1 (-2.4 to 2.5)	2.4 (2.1 to 2.9)	2.4 (2.1 to 2.7)	2.4 (2.2 to 2.7)	-2.1 (-7.5 to 4.9)	2.3 (-1.3 to 6.4)
Occupational ergonomic factors	6.9 (5.8 to 8.1)	6.3 (5.4 to 7.3)	6.7 (5.7 to 7.8)	-7.6 (-19.0 to 5.5)	4.9 (-2.5 to 12.5)	4.2 (3.3 to 5.2)	3.9 (3.3 to 4.5)	4.1 (3.4 to 4.9)	-8.2 (-24.5 to 14.1)	5.4 (-4.8 to 16.3)
Non-exclusive breastfeeding	0.6 (0.4 to 0.8)	0.6 (0.4 to 0.8)	0.6 (0.4 to 0.8)	-1.5 (-8.0 to 5.5)	0.4 (-5.5 to 7.6)	0.6 (0.4 to 0.8)	0.6 (0.4 to 0.8)	0.6 (0.4 to 0.8)	-1.5 (-8.0 to 5.5)	0.4 (-5.5 to 7.6)
Discontinued breastfeeding	1.6 (1.5 to 1.7)	1.7 (1.7 to 1.8)	1.8 (1.7 to 1.9)	7.6 (0.2 to 16.0)	3.9 (-1.8 to 10.1)	1.6 (1.5 to 1.7)	1.7 (1.7 to 1.8)	1.8 (1.7 to 1.9)	7.6 (0.2 to 16.1)	3.9 (-1.8 to 10.1)
Child underweight	0.1 (0.1 to 0.1)	0.1 (0.1 to 0.2)	0.1 (0.1 to 0.1)	5.5 (-6.3 to 18.5)	-7.3 (-15.3 to 1.6)	0.1 (0.1 to 0.2)	0.1 (0.1 to 0.1)	0.1 (0.0 to 0.1)	-21.8 (-34.0 to -9.9)	-10.0 (-17.9 to -2.1)
Child wasting	0.1 (0.1 to 0.2)	0.1 (0.1 to 0.2)	0.1 (0.1 to 0.2)	8.2 (-18.4 to 45.5)	-2.3 (-22.3 to 19.5)	0.1 (0.1 to 0.2)	0.1 (0.1 to 0.2)	0.1 (0.1 to 0.2)	-0.9 (-25.1 to 32.2)	-7.2 (-23.3 to 10.8)
Child stunting	0.7 (0.3 to 1.1)	0.6 (0.4 to 0.8)	0.5 (0.2 to 0.7)	-8.9 (-42.2 to 54.3)	-23.2 (-47.6 to 7.2)	0.6 (0.3 to 1.0)	0.5 (0.2 to 0.7)	0.4 (0.2 to 0.6)	-24.4 (-54.7 to 21.0)	-19.2 (-43.0 to 14.4)
Short gestation for birth weight	0.0 (0.0 to 0.0)	0.0 (0.0 to 0.0)	0.0 (0.0 to 0.0)	18.8 (-8.7 to 55.1)	-0.5 (-21.4 to 20.2)	0.0 (0.0 to 0.0)	0.0 (0.0 to 0.0)	0.0 (0.0 to 0.0)	14.6 (-14.1 to 46.2)	8.3 (-18.4 to 39.2)
Low birth weight for gestation	0.0 (0.0 to 0.0)	0.0 (0.0 to 0.0)	0.0 (0.0 to 0.0)	5.5 (-15.6 to 28.7)	3.4 (-11.1 to 23.1)	0.0 (0.0 to 0.0)	0.0 (0.0 to 0.0)	0.0 (0.0 to 0.0)	9.6 (-7.6 to 30.0)	-1.7 (-18.8 to 21.5)
Iron deficiency	-	-	-	-	-	6.1 (4.4 to 8.3)	5.8 (4.1 to 8.0)	5.3 (3.7 to 7.4)	-4.7 (-20.3 to 13.6)	-8.2 (-20.1 to 6.4)
Vitamin A deficiency	2.9 (2.4 to 3.5)	2.3 (1.9 to 2.8)	2.1 (1.8 to 2.6)	-20.1 (-29.2 to -10.0)	-8.7 (-17.5 to 1.4)	2.9 (2.4 to 3.4)	2.4 (2.0 to 2.8)	2.2 (1.8 to 2.6)	-16.5 (-25.2 to -6.4)	-8.9 (-19.6 to 1.6)
Zinc deficiency	0.7 (0.2 to 1.5)	0.6 (0.2 to 1.3)	0.5 (0.1 to 1.2)	-11.3 (-28.7 to 13.4)	-13.1 (-30.0 to 6.7)	0.7 (0.2 to 1.6)	0.6 (0.2 to 1.3)	0.5 (0.1 to 1.1)	-12.3 (-31.8 to 11.9)	-13.1 (-28.3 to 7.1)
Smoking	13.6 (11.0 to 16.2)	13.4 (11.4 to 15.3)	13.1 (11.5 to 14.8)	-1.6 (-13.1 to 12.1)	-2.0 (-9.1 to 6.1)	6.6 (5.0 to 8.5)	6.4 (5.0 to 8.0)	6.5 (5.5 to 7.7)	-2.2 (-20.5 to 19.7)	1.1 (-11.4 to 16.3)
Chewing tobacco	0.1 (0.1 to 0.2)	0.1 (0.1 to 0.2)	0.1 (0.1 to 0.2)	0.9 (-29.8 to 47.0)	-4.4 (-28.4 to 24.9)	0.1 (0.0 to 0.1)	0.1 (0.0 to 0.1)	0.1 (0.0 to 0.1)	-0.3 (-28.5 to 39.7)	0.8 (-21.9 to 29.6)
Second-hand smoke	48.2 (45.3 to 51.0)	48.7 (47.3 to 50.0)	49.5 (47.5 to 51.5)	1.0 (-4.6 to 7.6)	1.6 (-2.6 to 6.3)	58.4 (55.8 to 60.7)	58.7 (57.4 to 60.0)	57.8 (55.9 to 59.6)	0.6 (-3.3 to 4.8)	-1.5 (-4.3 to 1.3)
Alcohol use	12.1 (6.8 to 19.9)	10.2 (5.5 to 18.0)	10.9 (5.4 to 18.8)	-15.8 (-40.9 to 8.5)	7.1 (-19.7 to 36.4)	5.3 (2.5 to 10.0)	4.1 (1.9 to 8.0)	4.3 (1.7 to 8.3)	-21.9 (-45.9 to 8.0)	4.0 (-23.5 to 34.9)
Drug use	0.4 (0.3 to 0.5)	0.4 (0.3 to 0.5)	0.4 (0.3 to 0.6)	6.5 (0.8 to 12.9)	3.9 (-3.1 to 11.3)	0.2 (0.1 to 0.2)	0.2 (0.1 to 0.2)	0.2 (0.1 to 0.2)	3.3 (-1.7 to 9.0)	3.3 (-1.8 to 9.1)
Diet low in fruits	34.6 (28.9 to 38.7)	30.3 (24.4 to 34.8)	27.1 (21.1 to 32.6)	-12.3 (-19.9 to -5.9)	-10.5 (-19.8 to -3.7)	31.9 (26.1 to 36.4)	26.9 (20.6 to 32.0)	23.8 (16.9 to 29.8)	-15.8 (-25.6 to -8.6)	-11.6 (-24.3 to -2.8)
Diet low in vegetables	7.4 (1.3 to 14.4)	9.5 (2.5 to 16.4)	6.2 (1.0 to 13.1)	28.8 (-4.1 to 147.0)	-35.0 (-70.8 to -10.1)	10.2 (3.0 to 17.3)	12.5 (4.9 to 19.1)	8.8 (2.2 to 16.1)	22.4 (-4.4 to 108.3)	-29.5 (-63.8 to -9.0)
Diet low in legumes	24.5 (19.3 to 28.8)	13.3 (7.0 to 18.9)	14.3 (7.8 to 20.4)	-45.6 (-64.8 to -33.4)	7.0 (-9.3 to 31.8)	28.9 (24.4 to 32.7)	19.2 (13.3 to 24.2)	20.0 (13.8 to 25.2)	-33.7 (-46.5 to -25.2)	4.2 (-6.7 to 16.9)
Diet low in whole grains	49.9 (49.1 to 50.6)	49.7 (48.9 to 50.4)	49.5 (48.6 to 50.3)	-0.4 (-0.9 to 0.0)	-0.3 (-0.8 to 0.2)	50.1 (49.3 to 50.8)	50.0 (49.2 to 50.6)	49.9 (49.0 to 50.6)	-0.3 (-0.8 to 0.1)	-0.2 (-0.7 to 0.3)
Diet low in nuts and seeds	47.7 (45.8 to 49.2)	45.7 (43.4 to 47.5)	42.1 (38.7 to 44.8)	-4.1 (-7.3 to -1.5)	-7.9 (-12.2 to -4.5)	48.0 (46.3 to 49.6)	46.2 (44.1 to 48.0)	42.8 (39.6 to 45.4)	-3.8 (-6.7 to -1.3)	-7.5 (-11.7 to -4.3)
Diet low in milk	42.9 (40.2 to 45.1)	42.0 (39.2 to 44.2)	40.3 (37.2 to 42.9)	-2.2 (-4.2 to -0.4)	-4.0 (-6.4 to -2.1)	43.5 (41.0 to 45.5)	42.6 (40.0 to 44.7)	40.9 (37.9 to 43.4)	-2.1 (-4.0 to -0.5)	-3.9 (-6.2 to -2.0)
Diet high in red meat	4.0 (0.9 to 7.7)	2.0 (0.2 to 4.8)	4.0 (0.9 to 7.9)	-50.3 (-88.8 to -3.4)	99.4 (9.7 to 760.0)	0.3 (0.0 to 1.4)	0.1 (0.0 to 0.5)	0.4 (0.0 to 1.9)	-77.0 (-100.0 to 374.9)	505.5 (-33.0 to [illegible]
Diet high in processed meat	3.1 (0.9 to 5.8)	3.0 (0.7 to 5.6)	4.0 (1.4 to 6.8)	-4.4 (-39.5 to 33.0)	35.7 (12.2 to 125.5)	1.9 (0.3 to 4.3)	1.8 (0.2 to 4.1)	2.6 (0.5 to 5.0)	-5.5 (-45.2 to 33.9)	39.8 (14.5 to 209.0)
Diet high in sugar-sweetened beverages	7.9 (3.6 to 14.5)	7.6 (3.5 to 13.4)	10.2 (4.7 to 17.5)	-4.4 (-47.6 to 68.2)	34.4 (-10.3 to 102.5)	5.5 (2.6 to 10.3)	5.5 (2.4 to 10.3)	7.1 (3.0 to 13.4)	-1.7 (-47.0 to 84.8)	30.1 (-14.4 to 103.5)
Diet low in fibre	27.4 (21.6 to 32.1)	26.3 (20.5 to 31.1)	24.8 (18.5 to 30.0)	-3.9 (-10.2 to 1.4)	-5.8 (-13.2 to 0.5)	32.8 (27.9 to 36.7)	31.9 (26.9 to 35.8)	30.5 (25.1 to 35.0)	-2.7 (-6.9 to 2.0)	-4.4 (-10.5 to 0.1)
Diet low in calcium	33.0 (28.6 to 36.5)	27.9 (22.3 to 32.3)	22.4 (15.7 to 27.7)	-15.5 (-22.2 to -11.0)	-19.7 (-30.1 to -13.3)	35.6 (31.6 to 38.8)	31.2 (26.3 to 35.0)	26.2 (20.3 to 30.9)	-12.5 (-17.5 to -9.0)	-16.1 (-23.6 to -11.1)
Diet low in seafood omega-3 fatty acids	42.9 (40.7 to 45.1)	42.2 (39.7 to 44.6)	40.7 (37.9 to 43.3)	-1.7 (-2.4 to -1.1)	-3.6 (-4.9 to -2.4)	44.6 (42.6 to 46.5)	44.0 (41.9 to 46.0)	42.7 (40.3 to 44.9)	-1.4 (-2.0 to -0.9)	-2.9 (-4.0 to -1.9)
Diet low in polyunsaturated fatty acids	43.5 (41.0 to 45.9)	33.0 (29.0 to 36.4)	31.9 (27.6 to 35.6)	-24.1 (-30.8 to -18.1)	-3.5 (-7.6 to 0.5)	43.9 (41.5 to 46.2)	33.7 (29.8 to 37.1)	32.5 (28.0 to 36.1)	-23.4 (-30.0 to -17.7)	-3.5 (-7.6 to 0.1)
Diet high in trans fatty acids	1.0 (0.0 to 3.1)	1.0 (0.0 to 3.1)	1.0 (0.0 to 3.2)	1.5 (-19.4 to 36.5)	-0.6 (-21.4 to 23.5)	1.4 (0.1 to 3.8)	1.4 (0.1 to 3.9)	1.4 (0.1 to 3.8)	0.9 (-24.6 to 39.2)	-0.8 (-24.4 to 22.8)
Diet high in sodium	23.3 (15.3 to 30.2)	10.9 (2.8 to 18.6)	11.8 (3.4 to 19.5)	-53.0 (-82.9 to -33.6)	7.7 (-10.1 to 56.0)	17.9 (10.5 to 24.6)	6.4 (0.5 to 13.6)	5.2 (0.1 to 12.0)	-63.9 (-95.5 to -41.6)	-19.8 (-78.8 to -4.9)
Intimate partner violence	-	-	-	-	-	2.9 (2.2 to 3.6)	2.7 (2.1 to 3.4)	2.6 (2.0 to 3.2)	-7.7 (-13.5 to -1.4)	-4.2 (-10.5 to 1.8)
Childhood sexual abuse	11.4 (9.3 to 14.0)	11.5 (9.4 to 14.0)	11.5 (9.4 to 14.0)	1.0 (-5.8 to 9.4)	0.0 (0.0 to 0.0)	7.6 (6.1 to 9.4)	7.6 (6.1 to 9.4)	7.6 (6.1 to 9.4)	-0.6 (-8.5 to 7.5)	0.0 (0.0 to 0.0)

Supplementary Results Table 1. Macedonia age-standardised Summary Exposure Values (SEVs) for Level 4 risk factors and percentage change, by sex, 1990, 2007, and 2017.Data in parentheses are 95% uncertainty intervals.

	Male					Female				
	**1990**	**2007**	**2017**	**Percentage change 1990-2007**	**Percentage change 2007-2017**	**1990**	**2007**	**2017**	**Percentage change 1990-2007**	**Percentage change 2007-2017**
Bullying victimization	4.6 (1.9 to 9.1)	4.9 (2.3 to 9.4)	4.6 (2.1 to 8.7)	7.9 (-16.1 to 44.6)	-6.7 (-11.7 to -1.5)	3.1 (1.3 to 6.4)	3.3 (1.5 to 6.4)	3.0 (1.4 to 5.7)	7.7 (-24.8 to 60.9)	-9.4 (-15.8 to -3.0)
Low physical activity	0.3 (0.2 to 0.6)	0.3 (0.2 to 0.6)	0.3 (0.2 to 0.6)	1.6 (-0.2 to 3.9)	1.1 (-0.8 to 3.3)	0.3 (0.1 to 0.6)	0.3 (0.2 to 0.6)	0.3 (0.2 to 0.6)	1.3 (-0.8 to 3.8)	0.9 (-1.3 to 3.2)
High fasting plasma glucose	3.0 (1.8 to 4.5)	4.2 (3.0 to 6.1)	4.3 (3.0 to 6.3)	41.7 (6.1 to 104.7)	0.7 (-22.3 to 24.6)	2.2 (1.4 to 3.5)	3.0 (2.0 to 4.6)	2.9 (1.9 to 4.6)	32.6 (-5.6 to 94.9)	-1.6 (-33.2 to 40.4)
High LDL cholesterol	13.1 (11.5 to 14.8)	12.9 (11.3 to 14.6)	12.9 (11.3 to 14.7)	-1.2 (-5.6 to 3.0)	0.1 (-3.4 to 3.7)	13.1 (11.4 to 14.7)	13.0 (11.5 to 14.8)	13.2 (11.6 to 14.8)	-0.3 (-4.4 to 4.0)	1.1 (-2.4 to 4.5)
High systolic blood pressure	10.2 (9.2 to 11.2)	10.0 (9.1 to 11.1)	9.7 (8.8 to 10.8)	-1.6 (-8.5 to 5.9)	-3.1 (-8.4 to 2.2)	10.2 (9.2 to 11.2)	9.7 (8.7 to 10.7)	8.9 (7.9 to 9.9)	-4.3 (-12.8 to 4.6)	-8.5 (-14.8 to -1.4)
High body-mass index	8.8 (5.9 to 12.7)	12.4 (9.0 to 17.5)	13.7 (10.1 to 19.3)	40.4 (22.5 to 64.4)	10.4 (0.9 to 21.9)	8.8 (6.1 to 12.4)	12.3 (9.2 to 16.9)	14.1 (10.4 to 19.4)	40.7 (22.9 to 65.8)	14.7 (4.3 to 26.9)
Low bone mineral density	3.7 (3.0 to 4.6)	3.6 (2.9 to 4.4)	3.5 (2.8 to 4.3)	-3.2 (-16.8 to 13.4)	-1.7 (-17.2 to 14.6)	6.1 (5.0 to 7.3)	5.9 (5.0 to 7.0)	5.8 (4.8 to 6.9)	-2.7 (-14.1 to 12.1)	-1.6 (-13.7 to 12.7)
Impaired kidney function	2.2 (1.0 to 5.7)	2.2 (1.0 to 5.9)	2.2 (1.0 to 5.9)	1.2 (-3.7 to 6.0)	0.5 (-4.2 to 5.3)	2.9 (1.4 to 6.8)	3.0 (1.4 to 6.9)	3.0 (1.4 to 6.9)	3.8 (-0.1 to 9.0)	0.5 (-3.4 to 4.8)

Supplementary Results Table 1. Montenegro age-standardised Summary Exposure Values (SEVs) for Level 4 risk factors and percentage change, by sex, 1990, 2007, and 2017.Data in parentheses are 95% uncertainty intervals.

	Male					Female				
	1990	2007	2017	Percentage change 1990-2007	Percentage change 2007-2017	1990	2007	2017	Percentage change 1990-2007	Percentage change 2007-2017
Unsafe water source	18.2 (12.5 to 25.9)	16.5 (12.4 to 22.0)	13.0 (9.2 to 18.2)	-9.6 (-37.9 to 30.9)	-20.9 (-42.2 to 7.6)	18.2 (12.5 to 25.9)	16.5 (12.4 to 22.0)	13.0 (9.2 to 18.2)	-9.6 (-37.9 to 30.9)	-20.9 (-42.2 to 7.6)
Unsafe sanitation	8.4 (4.8 to 14.4)	5.7 (3.1 to 9.7)	3.5 (1.9 to 6.1)	-32.6 (-61.4 to 21.0)	-38.5 (-60.4 to -3.4)	8.4 (4.8 to 14.4)	5.7 (3.1 to 9.7)	3.5 (1.9 to 6.1)	-32.6 (-61.4 to 21.0)	-38.5 (-60.4 to -3.4)
No access to handwashing facility	4.6 (3.6 to 5.8)	4.3 (3.4 to 5.4)	3.8 (3.0 to 4.7)	-6.3 (-26.5 to 22.8)	-13.2 (-26.9 to 3.5)	4.6 (3.6 to 5.8)	4.3 (3.4 to 5.4)	3.8 (3.0 to 4.7)	-6.3 (-26.5 to 22.8)	-13.2 (-26.9 to 3.5)
Ambient particulate matter pollution	34.6 (21.1 to 52.1)	35.9 (23.4 to 57.0)	32.4 (20.6 to 49.2)	3.7 (-32.1 to 59.9)	-9.7 (-41.2 to 30.2)	30.4 (18.0 to 47.3)	32.1 (20.1 to 52.6)	28.9 (17.6 to 46.1)	5.5 (-34.0 to 72.8)	-9.8 (-43.9 to 34.9)
Household air pollution from solid fuels	25.1 (15.9 to 37.1)	18.6 (11.7 to 27.8)	17.2 (10.4 to 26.8)	-26.1 (-55.5 to 14.5)	-7.3 (-40.6 to 50.2)	27.5 (18.1 to 39.5)	20.8 (13.2 to 30.2)	19.3 (12.2 to 28.9)	-24.3 (-52.3 to 12.0)	-7.2 (-37.8 to 43.1)
Ambient ozone pollution	45.2 (16.8 to 54.8)	45.3 (16.9 to 54.8)	45.6 (17.0 to 54.8)	0.3 (-0.2 to 1.3)	0.7 (0.0 to 2.4)	45.2 (16.8 to 54.8)	45.3 (16.9 to 54.8)	45.6 (17.0 to 54.8)	0.3 (-0.2 to 1.3)	0.7 (0.0 to 2.4)
Residential radon	35.6 (11.9 to 70.7)	35.6 (11.9 to 70.7)	35.6 (11.9 to 70.7)	0.0 (0.0 to 0.0)	0.0 (0.0 to 0.0)	35.6 (11.9 to 70.7)	35.6 (11.9 to 70.7)	35.6 (11.9 to 70.7)	0.0 (0.0 to 0.0)	0.0 (0.0 to 0.0)
Lead exposure	2.4 (0.2 to 5.9)	2.6 (0.3 to 6.0)	2.1 (0.2 to 5.3)	5.6 (-2.8 to 56.5)	-18.4 (-29.2 to -12.0)	1.6 (0.1 to 4.7)	1.7 (0.1 to 4.9)	1.4 (0.1 to 4.3)	6.7 (-1.3 to 69.0)	-17.6 (-30.5 to -11.6)
Occupational exposure to asbestos	0.4 (0.3 to 0.5)	0.7 (0.6 to 0.8)	0.7 (0.5 to 0.8)	64.4 (28.6 to 111.2)	-7.9 (-26.5 to 14.2)	0.4 (0.3 to 0.5)	0.5 (0.5 to 0.6)	0.5 (0.4 to 0.6)	28.9 (3.4 to 64.6)	-15.2 (-34.0 to 6.8)
Occupational exposure to arsenic	0.2 (0.0 to 0.5)	0.2 (0.0 to 0.4)	0.2 (0.0 to 0.4)	-	-	0.2 (0.0 to 0.5)	0.2 (0.0 to 0.4)	0.2 (0.0 to 0.4)	-	-
Occupational exposure to benzene	0.4 (0.0 to 1.2)	0.4 (0.0 to 1.1)	0.4 (0.0 to 1.1)	-5.1 (-13.0 to 5.6)	-1.2 (-6.4 to 7.3)	0.5 (0.0 to 1.3)	0.5 (0.0 to 1.3)	0.5 (0.0 to 1.5)	2.6 (-8.8 to 20.2)	17.0 (9.2 to 28.1)
Occupational exposure to beryllium	0.0 (0.0 to 0.0)	0.0 (0.0 to 0.0)	0.0 (0.0 to 0.0)	-9.1 (-14.8 to -3.1)	2.6 (-2.4 to 7.9)	0.0 (0.0 to 0.0)	0.0 (0.0 to 0.0)	0.0 (0.0 to 0.0)	-7.5 (-16.1 to 2.6)	19.0 (12.1 to 26.2)
Occupational exposure to cadmium	0.0 (0.0 to 0.0)	0.0 (0.0 to 0.0)	0.0 (0.0 to 0.0)	-15.0 (-23.0 to -5.8)	1.1 (-6.6 to 8.7)	0.0 (0.0 to 0.0)	0.0 (0.0 to 0.0)	0.0 (0.0 to 0.0)	-17.3 (-27.8 to -5.4)	14.7 (6.1 to 24.3)
Occupational exposure to chromium	0.1 (0.1 to 0.1)	0.1 (0.1 to 0.1)	0.1 (0.1 to 0.1)	-12.1 (-19.6 to -3.4)	2.1 (-4.7 to 9.0)	0.1 (0.1 to 0.1)	0.1 (0.0 to 0.1)	0.1 (0.1 to 0.1)	-12.9 (-22.9 to -1.6)	16.4 (8.4 to 25.1)
Occupational exposure to diesel engine exhaust	0.4 (0.3 to 0.4)	0.4 (0.3 to 0.4)	0.4 (0.4 to 0.4)	-0.2 (-8.1 to 8.5)	5.5 (-0.5 to 11.7)	0.3 (0.3 to 0.3)	0.3 (0.3 to 0.3)	0.4 (0.4 to 0.4)	0.6 (-10.5 to 14.3)	23.6 (14.0 to 32.9)
Occupational exposure to formaldehyde	0.1 (0.1 to 0.1)	0.1 (0.1 to 0.1)	0.1 (0.1 to 0.1)	-16.2 (-24.5 to -7.1)	-1.2 (-9.4 to 6.6)	0.1 (0.1 to 0.1)	0.1 (0.1 to 0.1)	0.1 (0.1 to 0.1)	-15.6 (-26.4 to -3.3)	12.8 (4.3 to 22.1)
Occupational exposure to nickel	0.2 (0.0 to 1.0)	0.2 (0.0 to 0.8)	0.2 (0.0 to 0.8)	-	-	0.2 (0.0 to 0.7)	0.1 (0.0 to 0.6)	0.1 (0.0 to 0.6)	-	-
Occupational exposure to polycyclic aromatic hydrocarbons	0.1 (0.1 to 0.1)	0.1 (0.1 to 0.1)	0.1 (0.1 to 0.1)	-11.4 (-18.6 to -3.6)	0.0 (-6.5 to 6.5)	0.1 (0.1 to 0.1)	0.1 (0.1 to 0.1)	0.1 (0.1 to 0.1)	-11.3 (-21.0 to -0.2)	15.1 (7.6 to 23.2)
Occupational exposure to silica	2.7 (0.4 to 8.5)	2.4 (0.3 to 7.5)	2.3 (0.3 to 7.4)	-11.2 (-19.9 to -0.4)	-3.6 (-11.4 to 5.5)	1.5 (0.2 to 4.7)	1.2 (0.2 to 3.8)	1.3 (0.2 to 4.1)	-19.1 (-29.8 to -5.9)	5.2 (-3.4 to 16.8)
Occupational exposure to sulphuric acid	0.4 (0.1 to 1.2)	0.3 (0.1 to 1.0)	0.3 (0.1 to 1.0)	-12.9 (-20.6 to -4.1)	-5.6 (-12.7 to 1.6)	0.3 (0.1 to 0.9)	0.2 (0.0 to 0.7)	0.3 (0.1 to 0.8)	-19.6 (-29.4 to -8.4)	11.3 (2.6 to 21.2)
Occupational exposure to trichloroethylene	0.0 (0.0 to 0.0)	0.0 (0.0 to 0.0)	0.0 (0.0 to 0.0)	-8.2 (-14.9 to -0.6)	5.5 (-0.7 to 11.9)	0.0 (0.0 to 0.0)	0.0 (0.0 to 0.0)	0.0 (0.0 to 0.0)	-8.4 (-17.9 to 3.1)	20.7 (12.7 to 29.1)
Occupational asthmagens	13.3 (11.0 to 16.2)	11.7 (10.1 to 14.0)	10.9 (9.2 to 13.2)	-11.9 (-20.7 to -2.0)	-6.9 (-13.7 to 0.1)	6.3 (4.9 to 8.0)	5.7 (4.8 to 6.8)	6.5 (5.4 to 7.9)	-10.3 (-23.7 to 4.1)	14.0 (3.8 to 25.3)
Occupational particulate matter, gases, and fumes	4.8 (3.4 to 7.0)	4.3 (3.0 to 6.3)	3.9 (2.8 to 5.8)	-10.9 (-15.6 to -6.0)	-8.5 (-11.6 to -5.4)	2.2 (1.5 to 3.4)	1.9 (1.3 to 2.8)	1.7 (1.2 to 2.5)	-15.4 (-21.8 to -8.2)	-9.1 (-13.3 to -4.6)
Occupational noise	4.7 (4.2 to 5.2)	4.4 (4.0 to 4.8)	4.0 (3.8 to 4.4)	-6.9 (-10.1 to -3.3)	-7.3 (-9.4 to -5.0)	2.5 (2.2 to 3.0)	2.3 (2.1 to 2.7)	2.2 (2.0 to 2.5)	-8.4 (-13.0 to -2.9)	-3.7 (-7.3 to 0.3)
Occupational ergonomic factors	9.0 (7.6 to 10.7)	7.0 (6.1 to 8.1)	6.7 (5.7 to 7.9)	-21.7 (-32.6 to -9.0)	-4.3 (-13.2 to 6.3)	5.4 (4.2 to 6.8)	4.4 (3.7 to 5.2)	4.8 (4.0 to 5.8)	-18.5 (-35.7 to 2.3)	10.3 (-3.3 to 27.9)
Non-exclusive breastfeeding	0.6 (0.5 to 0.8)	0.6 (0.5 to 0.8)	0.6 (0.5 to 0.8)	0.4 (-5.4 to 6.4)	-1.4 (-6.6 to 4.6)	0.6 (0.5 to 0.8)	0.6 (0.5 to 0.8)	0.6 (0.5 to 0.8)	0.4 (-5.4 to 6.4)	-1.4 (-6.6 to 4.7)
Discontinued breastfeeding	2.0 (1.9 to 2.1)	2.1 (2.0 to 2.2)	2.2 (2.1 to 2.2)	4.2 (-0.9 to 10.1)	3.3 (-1.2 to 7.9)	2.0 (1.9 to 2.1)	2.1 (2.0 to 2.2)	2.2 (2.1 to 2.2)	4.2 (-0.9 to 10.0)	3.6 (-1.0 to 8.2)
Child underweight	0.1 (0.1 to 0.2)	0.1 (0.1 to 0.2)	0.1 (0.1 to 0.2)	-8.9 (-20.6 to 4.8)	-14.5 (-23.4 to -6.3)	0.1 (0.1 to 0.2)	0.1 (0.1 to 0.1)	0.1 (0.0 to 0.1)	-15.4 (-26.7 to -4.1)	-15.3 (-22.9 to -8.3)
Child wasting	0.2 (0.1 to 0.3)	0.2 (0.1 to 0.3)	0.1 (0.1 to 0.2)	-16.3 (-42.6 to 19.5)	-16.7 (-34.0 to 3.1)	0.2 (0.1 to 0.3)	0.2 (0.1 to 0.2)	0.1 (0.1 to 0.2)	-13.4 (-39.3 to 22.8)	-12.1 (-28.5 to 6.9)
Child stunting	0.7 (0.4 to 1.1)	0.6 (0.4 to 0.9)	0.6 (0.3 to 0.9)	-10.4 (-44.3 to 46.0)	-3.4 (-32.0 to 35.6)	0.6 (0.3 to 1.0)	0.6 (0.3 to 0.8)	0.5 (0.3 to 0.9)	-12.1 (-45.5 to 47.9)	-2.0 (-34.3 to 42.4)
Short gestation for birth weight	0.0 (0.0 to 0.0)	0.0 (0.0 to 0.0)	0.0 (0.0 to 0.0)	7.6 (-12.0 to 40.1)	8.4 (-15.5 to 30.6)	0.0 (0.0 to 0.0)	0.0 (0.0 to 0.0)	0.0 (0.0 to 0.0)	10.6 (-24.8 to 52.3)	9.9 (-19.0 to 45.5)
Low birth weight for gestation	0.0 (0.0 to 0.0)	0.0 (0.0 to 0.0)	0.0 (0.0 to 0.0)	5.0 (-11.7 to 22.3)	3.0 (-12.1 to 26.8)	0.0 (0.0 to 0.0)	0.0 (0.0 to 0.0)	0.0 (0.0 to 0.0)	12.8 (-8.1 to 38.3)	4.9 (-11.8 to 25.8)
Iron deficiency	-	-	-	-	-	5.5 (3.8 to 7.8)	5.7 (4.0 to 7.9)	5.0 (3.3 to 6.9)	3.8 (-13.9 to 24.6)	-12.3 (-25.7 to 2.0)
Vitamin A deficiency	2.7 (2.2 to 3.3)	2.4 (2.0 to 2.9)	2.2 (1.8 to 2.6)	-11.8 (-21.9 to -2.6)	-10.1 (-19.5 to 0.7)	2.7 (2.2 to 3.3)	2.5 (2.1 to 2.9)	2.3 (1.9 to 2.7)	-9.3 (-18.0 to 1.5)	-7.7 (-16.9 to 1.7)
Zinc deficiency	0.5 (0.1 to 1.0)	0.5 (0.1 to 1.1)	0.5 (0.1 to 1.0)	5.7 (-10.1 to 84.4)	-5.9 (-50.2 to 13.9)	0.5 (0.1 to 1.0)	0.5 (0.1 to 1.1)	0.5 (0.1 to 1.0)	6.8 (-9.4 to 93.9)	-6.6 (-49.7 to 19.9)
Smoking	14.2 (12.3 to 16.1)	14.6 (13.0 to 16.3)	14.9 (13.3 to 16.5)	2.8 (-5.7 to 11.9)	2.1 (-3.7 to 8.0)	8.4 (6.7 to 10.2)	9.1 (7.6 to 10.7)	9.8 (8.4 to 11.4)	9.0 (-7.3 to 28.3)	8.1 (-2.1 to 20.1)
Chewing tobacco	0.1 (0.1 to 0.2)	0.1 (0.1 to 0.2)	0.1 (0.1 to 0.2)	-0.9 (-32.1 to 45.6)	-3.4 (-25.5 to 26.8)	0.1 (0.0 to 0.1)	0.1 (0.0 to 0.1)	0.1 (0.0 to 0.1)	-0.9 (-27.7 to 39.9)	-0.1 (-21.9 to 28.3)
Second-hand smoke	42.4 (39.7 to 44.9)	53.0 (51.4 to 54.4)	51.7 (49.9 to 53.6)	25.0 (17.1 to 33.0)	-2.4 (-5.7 to 1.4)	43.1 (41.0 to 45.3)	56.9 (55.4 to 58.1)	55.2 (53.5 to 56.6)	32.0 (25.6 to 38.6)	-3.0 (-5.7 to -0.2)
Alcohol use	34.7 (22.5 to 44.7)	27.1 (18.2 to 37.5)	32.1 (20.1 to 44.5)	-21.8 (-43.6 to 4.6)	18.5 (-7.6 to 54.3)	15.4 (8.2 to 25.1)	10.8 (6.2 to 17.8)	12.6 (7.2 to 20.1)	-29.5 (-52.9 to 1.1)	16.2 (-7.1 to 55.2)
Drug use	0.5 (0.5 to 0.7)	0.6 (0.5 to 0.7)	0.5 (0.4 to 0.6)	1.4 (-6.3 to 9.9)	-8.1 (-16.8 to -0.2)	0.2 (0.1 to 0.3)	0.2 (0.2 to 0.3)	0.2 (0.1 to 0.3)	4.9 (-0.5 to 11.9)	-2.6 (-8.2 to 3.0)
Diet low in fruits	22.3 (15.0 to 28.6)	19.6 (12.6 to 25.8)	15.6 (8.6 to 22.4)	-12.0 (-28.5 to 3.8)	-20.3 (-39.4 to -5.1)	18.5 (11.5 to 24.9)	15.6 (8.5 to 22.2)	11.6 (5.0 to 18.4)	-15.8 (-38.0 to 2.6)	-25.9 (-51.3 to -6.9)
Diet low in vegetables	7.6 (1.6 to 14.7)	3.2 (0.1 to 8.8)	0.9 (0.0 to 3.3)	-57.9 (-95.3 to -24.2)	-72.6 (-97.8 to -22.0)	10.1 (3.1 to 17.3)	5.4 (0.5 to 12.3)	1.8 (0.1 to 5.9)	-46.5 (-89.0 to -16.8)	-67.0 (-94.7 to -25.8)
Diet low in legumes	31.7 (27.5 to 35.3)	28.0 (23.5 to 31.7)	29.0 (24.4 to 32.7)	-11.6 (-17.4 to -7.2)	3.6 (-0.8 to 8.8)	35.2 (31.6 to 38.3)	32.0 (28.1 to 35.2)	32.8 (28.8 to 36.2)	-9.1 (-13.2 to -5.6)	2.5 (-0.9 to 6.2)
Diet low in whole grains	47.6 (46.4 to 48.6)	48.2 (47.1 to 49.2)	47.6 (46.3 to 48.7)	1.3 (0.6 to 2.1)	-1.3 (-2.2 to -0.5)	48.0 (46.9 to 49.0)	48.6 (47.6 to 49.4)	48.0 (46.8 to 49.0)	1.2 (0.5 to 2.0)	-1.1 (-1.9 to -0.4)
Diet low in nuts and seeds	49.6 (48.1 to 50.9)	49.5 (48.1 to 50.7)	46.5 (44.3 to 48.4)	-0.2 (-2.0 to 1.6)	-6.0 (-8.7 to -3.8)	49.9 (48.5 to 51.1)	49.8 (48.5 to 50.9)	47.1 (44.9 to 48.8)	-0.2 (-1.8 to 1.6)	-5.6 (-8.2 to -3.6)
Diet low in milk	21.8 (14.9 to 27.5)	20.3 (13.3 to 26.1)	19.2 (12.1 to 25.3)	-6.9 (-18.4 to 3.9)	-5.4 (-17.4 to 4.2)	23.3 (16.7 to 28.8)	22.0 (15.5 to 27.6)	20.8 (13.9 to 26.7)	-5.2 (-15.4 to 4.5)	-5.4 (-17.0 to 2.7)
Diet high in red meat	27.0 (19.5 to 34.1)	28.7 (23.7 to 33.0)	38.9 (31.1 to 45.0)	6.0 (-14.0 to 37.6)	35.6 (13.2 to 62.3)	10.6 (5.2 to 15.8)	10.7 (5.7 to 15.0)	17.4 (10.3 to 23.0)	0.6 (-37.3 to 55.5)	63.0 (17.7 to 145.4)
Diet high in processed meat	2.8 (0.6 to 5.5)	2.4 (0.4 to 4.9)	3.4 (1.0 to 6.2)	-14.4 (-55.8 to 8.6)	45.6 (17.5 to 210.8)	1.7 (0.2 to 4.1)	1.5 (0.1 to 3.7)	2.2 (0.3 to 4.6)	-13.2 (-68.2 to 11.8)	47.0 (17.1 to 352.6)
Diet high in sugar-sweetened beverages	9.5 (4.2 to 16.1)	8.9 (4.0 to 15.5)	11.6 (5.4 to 19.8)	-5.8 (-46.1 to 64.4)	30.9 (-13.7 to 95.6)	6.4 (3.0 to 12.2)	6.2 (2.6 to 11.6)	8.0 (3.4 to 14.3)	-3.7 (-47.0 to 75.3)	29.5 (-17.0 to 99.7)
Diet low in fibre	28.6 (22.7 to 33.3)	29.7 (24.0 to 34.3)	28.4 (22.5 to 33.6)	4.0 (-3.8 to 13.6)	-4.5 (-11.8 to 1.6)	33.9 (28.9 to 37.9)	34.8 (30.1 to 38.7)	33.6 (28.2 to 38.0)	2.8 (-2.8 to 9.8)	-3.4 (-9.2 to 1.7)
Diet low in calcium	26.9 (21.2 to 31.5)	27.1 (21.5 to 31.7)	23.9 (17.6 to 29.1)	0.7 (-3.5 to 5.6)	-11.6 (-19.3 to -6.8)	30.2 (25.1 to 34.2)	30.4 (25.4 to 34.4)	27.5 (21.8 to 32.1)	0.6 (-3.1 to 4.3)	-9.5 (-15.1 to -5.7)
Diet low in seafood omega-3 fatty acids	41.1 (38.4 to 43.6)	41.8 (39.2 to 44.2)	39.4 (36.4 to 42.1)	1.6 (0.8 to 2.5)	-5.6 (-7.7 to -4.1)	43.1 (40.8 to 45.2)	43.6 (41.4 to 45.7)	41.6 (38.9 to 44.1)	1.3 (0.6 to 2.2)	-4.7 (-6.5 to -3.3)
Diet low in polyunsaturated fatty acids	41.0 (38.0 to 43.8)	39.1 (36.0 to 41.8)	37.1 (33.4 to 40.2)	-4.5 (-9.5 to 1.1)	-5.2 (-10.3 to -1.1)	41.4 (38.5 to 44.2)	39.6 (36.6 to 42.1)	37.6 (34.2 to 40.6)	-4.4 (-9.2 to 0.2)	-4.9 (-8.3 to -2.2)
Diet high in trans fatty acids	2.3 (0.3 to 5.2)	1.8 (0.2 to 4.3)	1.4 (0.1 to 3.7)	-24.9 (-58.5 to -6.9)	-22.2 (-58.5 to -7.2)	3.3 (0.8 to 6.5)	2.4 (0.4 to 5.4)	1.9 (0.2 to 4.5)	-25.5 (-57.2 to -7.1)	-23.0 (-56.3 to -8.6)
Diet high in sodium	24.3 (16.1 to 31.4)	11.4 (3.2 to 18.7)	12.2 (3.7 to 19.7)	-53.0 (-81.9 to -33.8)	6.8 (-11.7 to 48.9)	18.1 (11.1 to 24.6)	6.6 (0.6 to 13.5)	5.2 (0.2 to 12.2)	-63.9 (-95.2 to -41.9)	-20.3 (-78.8 to -5.9)
Intimate partner violence	-	-	-	-	-	3.1 (2.4 to 3.9)	2.9 (2.3 to 3.6)	2.8 (2.1 to 3.5)	-5.6 (-11.8 to 1.0)	-4.6 (-10.6 to 1.9)
Childhood sexual abuse	8.7 (7.1 to 10.8)	8.7 (7.1 to 10.8)	8.7 (7.1 to 10.8)	0.0 (0.0 to 0.0)	0.0 (0.0 to 0.0)	4.9 (3.6 to 6.5)	4.9 (3.6 to 6.5)	4.9 (3.6 to 6.5)	0.0 (0.0 to 0.0)	0.0 (0.0 to 0.0)

Supplementary Results Table 1. Montenegro age-standardised Summary Exposure Values (SEVs) for Level 4 risk factors and percentage change, by sex, 1990, 2007, and 2017.Data in parentheses are 95% uncertainty intervals.

	Male					Female				
	**1990**	**2007**	**2017**	**Percentage change 1990-2007**	**Percentage change 2007-2017**	**1990**	**2007**	**2017**	**Percentage change 1990-2007**	**Percentage change 2007-2017**
Bullying victimization	4.7 (1.9 to 9.8)	4.8 (2.0 to 10.0)	4.8 (2.0 to 10.0)	2.1 (-0.4 to 5.2)	-0.1 (-1.7 to 1.4)	3.7 (1.5 to 7.7)	3.7 (1.6 to 7.8)	3.7 (1.6 to 7.8)	2.1 (-24.0 to 36.7)	-0.1 (-1.4 to 1.2)
Low physical activity	0.3 (0.2 to 0.6)	0.3 (0.2 to 0.6)	0.3 (0.2 to 0.6)	0.2 (-1.7 to 2.0)	1.0 (-1.0 to 3.1)	0.3 (0.2 to 0.6)	0.3 (0.2 to 0.6)	0.3 (0.2 to 0.6)	0.2 (-2.0 to 2.5)	0.9 (-1.3 to 3.3)
High fasting plasma glucose	3.4 (2.2 to 5.2)	4.1 (2.8 to 5.9)	4.1 (2.8 to 6.2)	18.2 (-7.6 to 65.8)	1.1 (-20.4 to 29.1)	2.7 (1.8 to 4.2)	3.3 (2.3 to 4.8)	3.4 (2.3 to 5.1)	19.6 (-7.1 to 64.5)	3.4 (-18.6 to 27.9)
High LDL cholesterol	13.9 (12.2 to 15.7)	13.1 (11.5 to 14.9)	13.0 (11.5 to 14.7)	-5.6 (-10.0 to -1.4)	-0.8 (-4.2 to 2.6)	13.9 (12.4 to 15.6)	13.2 (11.7 to 14.9)	13.2 (11.7 to 14.9)	-5.3 (-9.3 to -1.2)	0.4 (-3.1 to 3.8)
High systolic blood pressure	10.8 (9.8 to 11.8)	10.4 (9.4 to 11.4)	10.1 (9.1 to 11.1)	-3.5 (-10.8 to 4.2)	-3.1 (-8.3 to 2.4)	10.3 (9.3 to 11.2)	9.8 (8.8 to 10.8)	8.8 (8.0 to 9.9)	-5.2 (-13.2 to 3.8)	-9.5 (-16.4 to -2.8)
High body-mass index	12.6 (9.0 to 17.7)	14.1 (10.6 to 19.6)	17.5 (13.7 to 23.1)	12.5 (0.2 to 28.4)	23.8 (12.5 to 36.5)	11.8 (8.7 to 16.3)	13.0 (10.0 to 17.4)	15.9 (12.4 to 20.8)	10.5 (-1.8 to 26.3)	21.8 (10.1 to 35.0)
Low bone mineral density	3.7 (2.9 to 4.5)	3.7 (3.0 to 4.4)	3.6 (2.9 to 4.4)	-0.7 (-15.8 to 15.4)	-1.6 (-15.9 to 16.3)	6.1 (5.0 to 7.2)	6.0 (5.1 to 7.0)	5.9 (4.9 to 7.0)	-0.7 (-12.6 to 14.8)	-1.3 (-13.7 to 12.2)
Impaired kidney function	2.2 (1.0 to 5.9)	2.2 (1.0 to 5.8)	2.2 (1.0 to 5.9)	-1.4 (-5.2 to 2.5)	-0.9 (-5.2 to 3.4)	2.9 (1.4 to 6.8)	2.9 (1.4 to 6.9)	2.9 (1.4 to 6.9)	1.9 (-2.0 to 6.2)	-0.4 (-5.0 to 4.0)

Supplementary Results Table 1. Poland age-standardised Summary Exposure Values (SEVs) for Level 4 risk factors and percentage change, by sex, 1990, 2007, and 2017.Data in parentheses are 95% uncertainty intervals.

	Male					Female				
	1990	2007	2017	Percentage change 1990-2007	Percentage change 2007-2017	1990	2007	2017	Percentage change 1990-2007	Percentage change 2007-2017
Unsafe water source	13.2 (9.0 to 18.8)	5.4 (3.6 to 7.9)	4.0 (2.6 to 5.7)	-58.9 (-74.4 to -32.8)	-25.6 (-49.0 to 8.5)	13.2 (9.0 to 18.8)	5.4 (3.6 to 7.9)	4.0 (2.6 to 5.7)	-58.9 (-74.4 to -32.8)	-25.6 (-49.0 to 8.5)
Unsafe sanitation	14.2 (8.2 to 22.2)	3.6 (1.9 to 5.9)	2.2 (1.2 to 3.7)	-74.8 (-85.7 to -57.0)	-38.0 (-60.5 to 1.0)	14.2 (8.2 to 22.2)	3.6 (1.9 to 5.9)	2.2 (1.2 to 3.7)	-74.8 (-85.7 to -57.0)	-38.0 (-60.5 to 1.0)
No access to handwashing facility	4.8 (3.7 to 6.0)	3.1 (2.5 to 3.9)	2.9 (2.3 to 3.6)	-34.9 (-48.8 to -16.8)	-7.8 (-23.5 to 11.0)	4.8 (3.7 to 6.0)	3.1 (2.5 to 3.9)	2.9 (2.3 to 3.6)	-34.9 (-48.8 to -16.8)	-7.8 (-23.5 to 11.0)
Ambient particulate matter pollution	41.0 (24.7 to 60.4)	44.7 (30.2 to 62.5)	39.8 (24.3 to 59.1)	9.0 (-28.1 to 77.6)	-11.0 (-42.2 to 31.8)	37.4 (21.2 to 56.6)	42.8 (28.2 to 60.3)	38.4 (22.8 to 57.7)	14.6 (-27.6 to 96.2)	-10.3 (-43.1 to 36.6)
Household air pollution from solid fuels	14.7 (7.7 to 24.0)	5.6 (2.6 to 11.1)	4.0 (1.7 to 9.1)	-62.2 (-82.8 to -27.2)	-27.6 (-65.8 to 51.5)	16.7 (9.5 to 26.1)	6.8 (3.2 to 12.7)	5.0 (2.2 to 10.4)	-59.4 (-80.7 to -25.9)	-26.1 (-63.9 to 49.2)
Ambient ozone pollution	44.1 (16.1 to 54.8)	43.9 (16.0 to 54.8)	44.9 (16.6 to 54.8)	-0.4 (-0.8 to 0.0)	2.3 (0.0 to 4.8)	44.1 (16.1 to 54.8)	43.9 (16.0 to 54.8)	44.9 (16.6 to 54.8)	-0.4 (-0.8 to 0.0)	2.3 (0.0 to 4.8)
Residential radon	7.1 (0.5 to 19.8)	7.1 (0.5 to 19.8)	7.1 (0.5 to 19.8)	0.0 (0.0 to 0.0)	0.0 (0.0 to 0.0)	7.1 (0.5 to 19.8)	7.1 (0.5 to 19.8)	7.1 (0.5 to 19.8)	0.0 (0.0 to 0.0)	0.0 (0.0 to 0.0)
Lead exposure	6.8 (2.9 to 10.9)	6.6 (3.1 to 10.5)	5.0 (2.1 to 8.5)	-3.0 (-9.8 to 10.0)	-25.2 (-34.1 to -18.5)	2.2 (0.1 to 5.7)	2.3 (0.2 to 5.8)	1.8 (0.1 to 4.9)	6.4 (-2.7 to 67.0)	-21.9 (-34.5 to -14.8)
Occupational exposure to asbestos	1.1 (0.9 to 1.4)	2.0 (1.9 to 2.2)	2.9 (2.6 to 3.2)	81.2 (43.2 to 124.1)	44.2 (26.4 to 64.5)	0.4 (0.3 to 0.5)	0.7 (0.7 to 0.8)	0.9 (0.8 to 1.1)	99.8 (44.2 to 163.5)	27.3 (9.2 to 47.7)
Occupational exposure to arsenic	0.3 (0.0 to 0.7)	0.3 (0.0 to 0.7)	0.3 (0.0 to 0.8)	-	-	0.3 (0.0 to 0.8)	0.3 (0.0 to 0.7)	0.3 (0.0 to 0.7)	-	-
Occupational exposure to benzene	0.4 (0.0 to 1.2)	0.4 (0.0 to 1.2)	0.5 (0.0 to 1.4)	5.1 (0.5 to 12.2)	13.4 (8.4 to 19.8)	0.5 (0.0 to 1.6)	0.6 (0.0 to 1.8)	0.8 (0.0 to 2.2)	17.3 (10.3 to 28.5)	17.9 (12.5 to 26.0)
Occupational exposure to beryllium	0.0 (0.0 to 0.0)	0.0 (0.0 to 0.0)	0.0 (0.0 to 0.0)	-8.1 (-10.8 to -5.3)	9.9 (7.3 to 12.5)	0.0 (0.0 to 0.0)	0.0 (0.0 to 0.0)	0.0 (0.0 to 0.0)	-7.2 (-10.3 to -3.8)	12.5 (9.2 to 16.0)
Occupational exposure to cadmium	0.0 (0.0 to 0.0)	0.0 (0.0 to 0.0)	0.0 (0.0 to 0.0)	-5.7 (-12.5 to 1.9)	10.0 (3.0 to 18.0)	0.0 (0.0 to 0.0)	0.0 (0.0 to 0.0)	0.0 (0.0 to 0.0)	-13.6 (-21.3 to -4.9)	9.4 (0.5 to 18.6)
Occupational exposure to chromium	0.1 (0.1 to 0.1)	0.1 (0.1 to 0.1)	0.1 (0.1 to 0.1)	-3.2 (-9.5 to 4.5)	10.9 (4.6 to 18.0)	0.1 (0.1 to 0.1)	0.1 (0.1 to 0.1)	0.1 (0.1 to 0.1)	-8.5 (-16.1 to -0.3)	11.3 (3.7 to 19.7)
Occupational exposure to diesel engine exhaust	0.4 (0.4 to 0.5)	0.4 (0.4 to 0.4)	0.4 (0.4 to 0.5)	-6.2 (-12.0 to 0.3)	10.8 (5.5 to 16.3)	0.3 (0.3 to 0.4)	0.3 (0.3 to 0.3)	0.4 (0.4 to 0.4)	-2.2 (-9.1 to 5.6)	14.8 (8.7 to 21.9)
Occupational exposure to formaldehyde	0.2 (0.2 to 0.2)	0.2 (0.2 to 0.2)	0.2 (0.2 to 0.2)	-4.8 (-12.3 to 4.0)	9.5 (2.3 to 18.1)	0.2 (0.2 to 0.2)	0.2 (0.2 to 0.2)	0.2 (0.2 to 0.2)	-9.1 (-17.8 to 0.8)	10.4 (1.7 to 20.2)
Occupational exposure to nickel	0.4 (0.0 to 1.6)	0.3 (0.0 to 1.4)	0.3 (0.0 to 1.4)	-	-	0.3 (0.0 to 1.3)	0.2 (0.0 to 1.0)	0.2 (0.0 to 1.1)	-	-
Occupational exposure to polycyclic aromatic hydrocarbons	0.2 (0.2 to 0.2)	0.2 (0.2 to 0.2)	0.2 (0.2 to 0.2)	-2.7 (-8.5 to 4.0)	10.9 (5.5 to 16.9)	0.2 (0.2 to 0.2)	0.2 (0.2 to 0.2)	0.2 (0.2 to 0.2)	-6.1 (-13.1 to 1.9)	12.0 (5.1 to 19.7)
Occupational exposure to silica	4.3 (0.6 to 13.6)	3.8 (0.6 to 12.4)	4.2 (0.6 to 13.1)	-11.1 (-18.0 to -3.0)	8.5 (2.0 to 16.4)	2.7 (0.4 to 8.5)	2.0 (0.3 to 6.4)	2.1 (0.3 to 6.6)	-25.0 (-32.2 to -17.5)	4.6 (-4.9 to 16.6)
Occupational exposure to sulphuric acid	0.5 (0.1 to 1.5)	0.4 (0.1 to 1.4)	0.5 (0.1 to 1.5)	-8.4 (-14.3 to -1.9)	6.9 (1.0 to 13.9)	0.5 (0.1 to 1.5)	0.4 (0.1 to 1.2)	0.4 (0.1 to 1.3)	-18.6 (-25.2 to -11.4)	6.2 (-2.3 to 15.3)
Occupational exposure to trichloroethylene	0.0 (0.0 to 0.0)	0.0 (0.0 to 0.0)	0.0 (0.0 to 0.0)	-2.0 (-6.9 to 3.8)	11.3 (6.5 to 16.3)	0.0 (0.0 to 0.1)	0.0 (0.0 to 0.0)	0.1 (0.0 to 0.1)	-5.7 (-12.0 to 0.7)	12.6 (7.0 to 19.1)
Occupational asthmagens	16.4 (13.8 to 19.6)	15.0 (12.6 to 17.9)	15.2 (12.9 to 18.1)	-8.9 (-15.6 to -1.5)	1.6 (-4.6 to 7.9)	8.7 (7.2 to 10.8)	8.7 (7.2 to 10.7)	8.9 (7.3 to 10.6)	0.1 (-8.4 to 9.7)	1.4 (-6.9 to 10.3)
Occupational particulate matter, gases, and fumes	6.8 (4.9 to 9.9)	6.2 (4.5 to 9.0)	6.0 (4.3 to 8.6)	-9.0 (-13.0 to -5.5)	-4.3 (-6.1 to -2.5)	4.1 (2.9 to 5.9)	3.5 (2.5 to 5.1)	3.2 (2.3 to 4.6)	-14.2 (-19.0 to -9.2)	-10.0 (-12.6 to -7.3)
Occupational noise	6.3 (5.9 to 6.9)	5.8 (5.5 to 6.3)	5.6 (5.3 to 6.1)	-7.8 (-9.9 to -5.3)	-3.6 (-4.8 to -2.3)	4.0 (3.7 to 4.4)	3.7 (3.5 to 4.1)	3.6 (3.4 to 3.9)	-6.9 (-10.1 to -3.0)	-3.5 (-5.1 to -1.8)
Occupational ergonomic factors	12.5 (11.3 to 14.0)	11.0 (10.1 to 12.2)	10.3 (9.1 to 11.7)	-12.2 (-19.0 to -5.3)	-6.6 (-13.5 to 1.1)	8.8 (7.7 to 10.0)	8.0 (7.3 to 8.9)	7.4 (6.4 to 8.6)	-8.5 (-18.0 to 2.0)	-7.6 (-17.5 to 2.9)
Non-exclusive breastfeeding	0.4 (0.3 to 0.5)	0.4 (0.3 to 0.5)	0.4 (0.3 to 0.5)	-0.3 (-8.6 to 10.0)	-1.4 (-7.5 to 5.8)	0.4 (0.3 to 0.5)	0.4 (0.3 to 0.5)	0.4 (0.3 to 0.5)	-0.3 (-8.6 to 10.0)	-1.4 (-7.5 to 5.8)
Discontinued breastfeeding	1.8 (1.7 to 1.9)	2.1 (2.0 to 2.2)	2.1 (2.0 to 2.2)	17.5 (9.8 to 25.3)	0.3 (-4.0 to 4.9)	1.8 (1.7 to 1.9)	2.1 (2.0 to 2.2)	2.1 (2.0 to 2.2)	17.5 (9.8 to 25.4)	0.3 (-4.0 to 4.9)
Child underweight	0.2 (0.1 to 0.2)	0.1 (0.1 to 0.2)	0.1 (0.1 to 0.2)	-27.1 (-38.5 to -15.5)	-10.2 (-18.2 to -1.9)	0.1 (0.1 to 0.2)	0.1 (0.1 to 0.1)	0.1 (0.0 to 0.1)	-33.5 (-45.6 to -22.0)	-11.1 (-19.0 to -3.3)
Child wasting	0.2 (0.1 to 0.3)	0.2 (0.1 to 0.2)	0.1 (0.1 to 0.2)	-21.6 (-45.5 to 8.4)	-9.1 (-28.4 to 10.8)	0.2 (0.1 to 0.3)	0.1 (0.1 to 0.2)	0.1 (0.1 to 0.2)	-22.4 (-44.0 to 4.6)	-7.8 (-24.4 to 10.8)
Child stunting	0.8 (0.4 to 1.4)	0.6 (0.3 to 1.0)	0.5 (0.2 to 0.9)	-29.3 (-59.6 to 19.5)	-14.6 (-41.7 to 19.1)	0.8 (0.4 to 1.4)	0.5 (0.2 to 0.9)	0.4 (0.2 to 0.8)	-32.4 (-61.8 to 14.0)	-15.1 (-42.4 to 23.2)
Short gestation for birth weight	0.0 (0.0 to 0.0)	0.0 (0.0 to 0.0)	0.0 (0.0 to 0.0)	18.6 (-6.1 to 46.3)	5.3 (-13.1 to 30.3)	0.0 (0.0 to 0.0)	0.0 (0.0 to 0.0)	0.0 (0.0 to 0.0)	7.8 (-11.7 to 39.6)	3.9 (-15.3 to 26.5)
Low birth weight for gestation	0.0 (0.0 to 0.0)	0.0 (0.0 to 0.0)	0.0 (0.0 to 0.0)	9.4 (-6.5 to 29.0)	-0.4 (-15.7 to 15.6)	0.0 (0.0 to 0.0)	0.0 (0.0 to 0.0)	0.0 (0.0 to 0.0)	8.9 (-9.0 to 27.9)	0.9 (-14.3 to 17.2)
Iron deficiency	-	-	-	-	-	6.6 (4.5 to 9.0)	4.9 (3.3 to 7.0)	4.1 (2.7 to 5.9)	-24.9 (-37.7 to -10.0)	-16.8 (-29.1 to -2.0)
Vitamin A deficiency	2.8 (2.3 to 3.5)	2.2 (1.8 to 2.6)	2.0 (1.7 to 2.4)	-21.9 (-31.3 to -10.2)	-8.3 (-21.7 to 4.4)	2.9 (2.4 to 3.5)	2.3 (1.9 to 2.7)	2.1 (1.7 to 2.6)	-21.1 (-30.6 to -11.1)	-6.7 (-16.2 to 4.3)
Zinc deficiency	0.5 (0.1 to 1.3)	0.3 (0.1 to 0.8)	0.4 (0.1 to 1.1)	-37.4 (-75.6 to 124.9)	20.0 (-59.7 to 289.3)	0.6 (0.1 to 1.2)	0.3 (0.1 to 0.7)	0.4 (0.1 to 1.0)	-38.6 (-76.1 to 106.7)	16.2 (-59.0 to 295.7)
Smoking	15.9 (13.9 to 17.8)	12.2 (10.8 to 13.7)	9.7 (8.6 to 11.1)	-22.9 (-28.5 to -16.9)	-20.6 (-24.9 to -16.0)	7.9 (6.6 to 9.3)	7.1 (6.1 to 8.2)	6.6 (5.7 to 7.5)	-9.9 (-20.2 to 1.6)	-7.6 (-15.3 to 1.1)
Chewing tobacco	0.1 (0.0 to 0.1)	0.1 (0.0 to 0.1)	0.1 (0.0 to 0.1)	-0.5 (-27.5 to 36.1)	-0.0 (-20.0 to 25.9)	0.1 (0.0 to 0.1)	0.1 (0.0 to 0.1)	0.1 (0.0 to 0.1)	0.1 (-27.9 to 44.0)	-0.0 (-22.7 to 27.4)
Second-hand smoke	46.4 (43.8 to 48.9)	43.0 (41.1 to 45.0)	36.1 (33.3 to 39.0)	-7.4 (-13.7 to -0.2)	-16.1 (-21.2 to -11.0)	54.9 (52.9 to 56.8)	46.4 (44.7 to 48.2)	37.5 (35.1 to 40.0)	-15.4 (-19.6 to -11.1)	-19.2 (-23.1 to -15.1)
Alcohol use	21.9 (14.1 to 31.7)	26.4 (17.4 to 37.4)	29.2 (18.6 to 42.3)	20.6 (5.6 to 40.5)	11.0 (-10.2 to 40.2)	10.1 (5.4 to 18.4)	11.5 (6.5 to 19.7)	12.9 (7.3 to 22.1)	13.4 (1.3 to 34.1)	12.9 (-10.8 to 44.9)
Drug use	0.6 (0.4 to 0.8)	0.6 (0.4 to 0.9)	0.6 (0.4 to 0.9)	8.4 (-1.8 to 21.1)	0.1 (-11.5 to 11.0)	0.2 (0.2 to 0.4)	0.3 (0.2 to 0.4)	0.3 (0.2 to 0.4)	7.8 (-3.3 to 21.0)	-1.6 (-13.5 to 10.6)
Diet low in fruits	45.5 (40.6 to 47.8)	42.2 (37.1 to 44.9)	39.4 (34.0 to 42.9)	-7.4 (-10.3 to -4.9)	-6.5 (-10.5 to -3.0)	44.1 (39.0 to 46.6)	40.4 (35.2 to 43.4)	37.4 (32.0 to 41.2)	-8.4 (-12.0 to -5.6)	-7.2 (-11.7 to -3.7)
Diet low in vegetables	20.7 (13.9 to 26.3)	17.8 (10.6 to 23.8)	14.8 (6.9 to 21.8)	-13.8 (-28.7 to -4.7)	-16.8 (-39.9 to -1.3)	23.3 (16.8 to 28.6)	19.8 (12.9 to 25.5)	16.8 (9.2 to 23.5)	-15.2 (-27.2 to -7.1)	-15.0 (-34.4 to -2.4)
Diet low in legumes	43.1 (41.0 to 44.9)	43.3 (41.3 to 45.1)	43.0 (40.7 to 45.0)	0.6 (-0.2 to 1.5)	-0.6 (-2.4 to 0.9)	45.0 (43.3 to 46.6)	45.3 (43.5 to 46.8)	45.2 (43.3 to 46.8)	0.5 (-0.2 to 1.4)	-0.1 (-1.5 to 1.2)
Diet low in whole grains	51.6 (51.0 to 52.0)	44.3 (42.6 to 45.8)	41.5 (39.2 to 43.5)	-14.1 (-16.6 to -11.9)	-6.3 (-8.4 to -4.5)	51.6 (51.1 to 52.0)	44.9 (43.3 to 46.3)	42.4 (40.2 to 44.2)	-12.9 (-15.3 to -10.9)	-5.7 (-7.8 to -4.0)
Diet low in nuts and seeds	53.8 (53.6 to 54.0)	51.9 (51.1 to 52.5)	49.6 (48.1 to 50.9)	-3.6 (-4.7 to -2.6)	-4.4 (-6.2 to -3.0)	53.9 (53.6 to 54.1)	52.1 (51.4 to 52.6)	50.0 (48.5 to 51.1)	-3.3 (-4.4 to -2.5)	-4.1 (-5.9 to -2.7)
Diet low in milk	30.9 (26.1 to 35.0)	37.0 (33.4 to 40.0)	35.7 (31.6 to 39.0)	19.4 (13.1 to 29.2)	-3.5 (-6.7 to -0.9)	32.2 (27.4 to 36.1)	37.6 (34.0 to 40.6)	36.2 (32.2 to 39.5)	16.8 (11.6 to 25.1)	-3.7 (-6.4 to -1.4)
Diet high in red meat	39.8 (35.0 to 43.9)	32.7 (28.8 to 36.2)	38.8 (32.6 to 44.2)	-17.7 (-26.9 to -6.2)	18.6 (3.7 to 36.1)	17.9 (12.7 to 22.3)	12.9 (8.1 to 16.7)	16.6 (10.4 to 22.0)	-28.0 (-45.1 to -8.8)	28.9 (0.7 to 75.0)
Diet high in processed meat	1.7 (0.2 to 4.1)	2.8 (0.6 to 5.4)	3.8 (1.2 to 6.6)	58.5 (18.2 to 319.2)	37.2 (13.2 to 154.1)	1.0 (0.0 to 3.0)	1.6 (0.1 to 3.9)	2.3 (0.4 to 4.9)	60.0 (22.1 to 491.7)	42.9 (16.8 to 268.3)
Diet high in sugar-sweetened beverages	7.3 (3.4 to 12.0)	10.2 (5.3 to 12.4)	15.6 (7.9 to 19.8)	40.9 (-15.7 to 119.7)	51.9 (16.6 to 95.2)	5.2 (2.5 to 9.1)	7.1 (3.5 to 8.8)	10.4 (5.4 to 13.6)	34.4 (-18.6 to 120.9)	47.1 (11.5 to 97.7)
Diet low in fibre	20.3 (14.0 to 25.7)	22.0 (15.8 to 27.4)	21.9 (15.5 to 27.4)	8.2 (2.2 to 17.8)	-0.5 (-8.4 to 7.8)	26.4 (20.7 to 31.2)	27.9 (22.3 to 32.4)	27.7 (21.8 to 32.6)	5.6 (1.1 to 12.4)	-0.5 (-6.6 to 5.4)
Diet low in calcium	21.0 (14.3 to 26.4)	26.2 (20.3 to 30.8)	23.8 (17.3 to 28.8)	24.5 (15.2 to 43.6)	-9.2 (-16.0 to -5.2)	24.9 (18.8 to 29.6)	29.4 (24.3 to 33.5)	27.3 (21.5 to 31.8)	18.3 (11.8 to 30.6)	-7.4 (-12.6 to -4.1)
Diet low in seafood omega-3 fatty acids	37.3 (33.9 to 40.3)	36.6 (33.1 to 39.8)	32.7 (28.6 to 36.5)	-1.7 (-3.3 to -0.4)	-10.7 (-14.5 to -7.6)	39.7 (36.8 to 42.4)	39.1 (36.0 to 42.0)	35.8 (32.1 to 39.2)	-1.5 (-2.9 to -0.3)	-8.5 (-11.7 to -5.9)
Diet low in polyunsaturated fatty acids	43.4 (40.9 to 45.7)	41.5 (38.9 to 43.7)	40.5 (37.8 to 42.9)	-4.4 (-8.1 to -0.2)	-2.3 (-4.9 to -0.2)	43.7 (41.4 to 45.8)	41.9 (39.5 to 44.0)	41.0 (38.3 to 43.3)	-4.2 (-7.8 to 0.8)	-2.2 (-4.5 to -0.5)
Diet high in trans fatty acids	1.7 (0.1 to 4.4)	1.0 (0.0 to 3.2)	0.7 (0.0 to 2.6)	-38.7 (-80.9 to -21.5)	-29.9 (-80.7 to -14.9)	2.4 (0.3 to 5.1)	1.4 (0.1 to 3.9)	1.0 (0.0 to 3.1)	-39.1 (-76.4 to -21.3)	-29.0 (-73.4 to -14.1)
Diet high in sodium	8.0 (1.0 to 15.2)	9.6 (1.7 to 17.2)	12.1 (3.4 to 19.4)	20.2 (-10.4 to 152.2)	26.8 (5.5 to 142.3)	6.2 (0.6 to 13.1)	5.4 (0.3 to 12.1)	4.9 (0.1 to 11.5)	-11.7 (-68.9 to 8.4)	-10.4 (-69.2 to 5.6)
Intimate partner violence	-	-	-	-	-	1.9 (1.6 to 2.2)	1.6 (1.3 to 1.9)	1.4 (1.2 to 1.7)	-16.4 (-20.3 to -12.5)	-10.5 (-15.2 to -5.5)
Childhood sexual abuse	8.7 (7.1 to 10.8)	8.7 (7.1 to 10.8)	8.7 (7.1 to 10.8)	0.0 (0.0 to 0.0)	0.0 (0.0 to 0.0)	3.1 (2.4 to 3.8)	2.9 (2.5 to 3.4)	3.1 (2.4 to 3.8)	-4.5 (-18.0 to 10.0)	4.8 (-9.1 to 21.9)

Supplementary Results Table 1. Poland age-standardised Summary Exposure Values (SEVs) for Level 4 risk factors and percentage change, by sex, 1990, 2007, and 2017.Data in parentheses are 95% uncertainty intervals.

	Male					Female				
	**1990**	**2007**	**2017**	**Percentage change 1990-2007**	**Percentage change 2007-2017**	**1990**	**2007**	**2017**	**Percentage change 1990-2007**	**Percentage change 2007-2017**
Bullying victimization	3.6 (1.5 to 7.1)	5.1 (2.4 to 9.9)	5.7 (2.7 to 11.3)	41.6 (17.2 to 109.0)	10.8 (0.9 to 28.7)	2.6 (1.1 to 5.5)	3.4 (1.6 to 6.7)	3.6 (1.7 to 7.2)	30.6 (-9.0 to 97.3)	5.8 (-1.8 to 16.7)
Low physical activity	0.3 (0.2 to 0.6)	0.3 (0.2 to 0.6)	0.4 (0.2 to 0.6)	0.6 (-1.1 to 2.4)	1.9 (0.2 to 4.2)	0.4 (0.2 to 0.7)	0.4 (0.2 to 0.7)	0.4 (0.2 to 0.7)	0.9 (-0.8 to 2.9)	1.5 (-0.3 to 3.4)
High fasting plasma glucose	2.5 (1.6 to 3.9)	3.3 (2.2 to 4.8)	4.0 (2.7 to 6.0)	30.7 (-4.9 to 92.8)	20.8 (-9.3 to 67.1)	2.2 (1.4 to 3.3)	2.2 (1.5 to 3.4)	2.7 (1.8 to 4.1)	1.8 (-25.1 to 42.4)	20.7 (-7.4 to 64.4)
High LDL cholesterol	13.6 (12.0 to 15.3)	13.8 (12.2 to 15.6)	13.3 (11.6 to 15.1)	1.6 (-2.3 to 5.8)	-3.5 (-6.6 to -0.4)	13.8 (12.2 to 15.5)	13.6 (12.1 to 15.3)	12.5 (11.1 to 14.2)	-1.4 (-5.2 to 2.3)	-7.9 (-10.7 to -5.0)
High systolic blood pressure	9.6 (8.8 to 10.5)	10.4 (9.5 to 11.4)	9.6 (8.7 to 10.6)	8.5 (2.0 to 15.3)	-8.0 (-13.1 to -2.9)	10.1 (9.2 to 11.0)	8.3 (7.6 to 9.2)	6.9 (6.2 to 7.7)	-17.1 (-23.1 to -10.6)	-17.2 (-23.5 to -11.0)
High body-mass index	9.3 (6.5 to 13.7)	12.9 (9.7 to 17.4)	14.6 (11.0 to 19.4)	38.3 (20.8 to 61.3)	13.1 (3.6 to 23.1)	10.5 (7.5 to 14.6)	13.5 (10.4 to 18.0)	15.1 (11.6 to 20.2)	29.5 (13.3 to 49.4)	11.9 (2.0 to 23.3)
Low bone mineral density	3.7 (2.9 to 4.5)	3.6 (2.9 to 4.3)	3.4 (2.8 to 4.3)	-3.8 (-20.1 to 14.9)	-3.5 (-17.2 to 13.5)	6.7 (5.6 to 8.1)	6.6 (5.6 to 7.7)	6.4 (5.3 to 7.6)	-2.8 (-15.2 to 12.5)	-2.2 (-15.6 to 11.4)
Impaired kidney function	2.1 (0.9 to 5.7)	2.0 (0.9 to 5.6)	2.0 (0.9 to 5.6)	-2.5 (-8.5 to 4.0)	-3.2 (-10.2 to 2.5)	2.7 (1.3 to 6.6)	2.7 (1.2 to 6.5)	2.7 (1.2 to 6.6)	-2.9 (-9.8 to 2.2)	0.4 (-5.5 to 6.2)

Supplementary Results Table 1. Romania age-standardised Summary Exposure Values (SEVs) for Level 4 risk factors and percentage change, by sex, 1990, 2007, and 2017.Data in parentheses are 95% uncertainty intervals.										
	Male					Female				
	1990	2007	2017	Percentage change 1990-2007	Percentage change 2007-2017	1990	2007	2017	Percentage change 1990-2007	Percentage change 2007-2017
Unsafe water source	30.8 (25.7 to 36.0)	11.7 (8.0 to 15.8)	8.5 (5.7 to 12.1)	-62.0 (-73.7 to -47.3)	-27.8 (-47.6 to -0.5)	30.8 (25.7 to 36.0)	11.7 (8.0 to 15.8)	8.5 (5.7 to 12.1)	-62.0 (-73.7 to -47.3)	-27.8 (-47.6 to -0.5)
Unsafe sanitation	44.1 (32.0 to 60.4)	26.3 (17.4 to 39.7)	16.8 (9.7 to 27.2)	-40.3 (-58.7 to -17.7)	-36.1 (-54.9 to -10.5)	44.1 (32.0 to 60.4)	26.3 (17.4 to 39.7)	16.8 (9.7 to 27.2)	-40.3 (-58.7 to -17.7)	-36.1 (-54.9 to -10.5)
No access to handwashing facility	10.6 (8.5 to 12.9)	7.1 (5.6 to 8.7)	5.2 (4.1 to 6.3)	-32.8 (-46.6 to -15.5)	-27.3 (-39.5 to -13.4)	10.6 (8.5 to 12.9)	7.1 (5.6 to 8.7)	5.2 (4.1 to 6.3)	-32.8 (-46.6 to -15.5)	-27.3 (-39.5 to -13.4)
Ambient particulate matter pollution	26.1 (16.4 to 37.8)	29.8 (20.3 to 42.2)	28.7 (18.6 to 41.6)	14.1 (-23.2 to 83.8)	-3.5 (-36.7 to 35.3)	22.4 (13.5 to 33.6)	26.8 (17.5 to 39.3)	26.4 (16.6 to 39.0)	19.7 (-23.0 to 99.4)	-1.4 (-37.9 to 41.1)
Household air pollution from solid fuels	26.7 (17.7 to 38.9)	12.9 (7.7 to 21.1)	8.9 (4.5 to 15.9)	-51.5 (-70.4 to -26.2)	-31.6 (-61.2 to 9.6)	28.6 (20.0 to 40.7)	14.8 (9.2 to 22.5)	10.4 (5.4 to 17.3)	-48.2 (-66.9 to -25.1)	-29.6 (-58.5 to 7.7)
Ambient ozone pollution	36.6 (12.3 to 54.8)	36.4 (12.2 to 54.8)	37.2 (12.6 to 54.8)	-0.5 (-1.2 to 0.0)	2.2 (0.0 to 3.6)	36.6 (12.3 to 54.8)	36.4 (12.2 to 54.8)	37.2 (12.6 to 54.8)	-0.5 (-1.2 to 0.0)	2.2 (0.0 to 3.6)
Residential radon	50.5 (20.5 to 71.2)	50.5 (20.5 to 71.2)	50.5 (20.5 to 71.2)	0.0 (0.0 to 0.0)	0.0 (0.0 to 0.0)	50.5 (20.5 to 71.2)	50.5 (20.5 to 71.2)	50.5 (20.5 to 71.2)	0.0 (0.0 to 0.0)	0.0 (0.0 to 0.0)
Lead exposure	6.1 (2.1 to 10.3)	5.7 (2.1 to 9.6)	4.4 (1.5 to 8.1)	-7.8 (-14.8 to 0.7)	-21.5 (-29.8 to -15.0)	2.9 (0.3 to 6.6)	2.7 (0.4 to 6.3)	2.2 (0.3 to 5.4)	-4.5 (-11.6 to 24.4)	-20.3 (-30.9 to -13.6)
Occupational exposure to asbestos	0.8 (0.6 to 0.9)	1.0 (0.9 to 1.1)	1.0 (0.9 to 1.2)	21.4 (1.7 to 52.7)	5.8 (-8.5 to 20.8)	0.5 (0.4 to 0.6)	0.6 (0.5 to 0.6)	0.6 (0.5 to 0.7)	15.5 (-9.9 to 43.3)	1.5 (-13.9 to 18.6)
Occupational exposure to arsenic	0.4 (0.0 to 1.0)	0.3 (0.0 to 0.8)	0.3 (0.0 to 0.8)	-	-	0.5 (0.0 to 1.3)	0.4 (0.0 to 0.9)	0.4 (0.0 to 0.8)	-	-
Occupational exposure to benzene	0.5 (0.0 to 1.3)	0.4 (0.0 to 1.2)	0.4 (0.0 to 1.2)	-7.0 (-12.3 to 0.3)	1.6 (-2.3 to 8.0)	0.6 (0.0 to 1.6)	0.6 (0.0 to 1.6)	0.6 (0.0 to 1.7)	2.1 (-5.2 to 13.3)	2.6 (-3.7 to 12.2)
Occupational exposure to beryllium	0.0 (0.0 to 0.0)	0.0 (0.0 to 0.0)	0.0 (0.0 to 0.0)	-16.1 (-19.9 to -12.2)	0.5 (-2.1 to 3.1)	0.0 (0.0 to 0.0)	0.0 (0.0 to 0.0)	0.0 (0.0 to 0.0)	-16.8 (-21.9 to -11.5)	-2.7 (-6.4 to 1.2)
Occupational exposure to cadmium	0.0 (0.0 to 0.1)	0.0 (0.0 to 0.0)	0.0 (0.0 to 0.0)	-23.1 (-29.4 to -15.8)	0.4 (-6.1 to 7.7)	0.1 (0.1 to 0.1)	0.0 (0.0 to 0.0)	0.0 (0.0 to 0.0)	-25.7 (-33.8 to -16.0)	-5.3 (-13.3 to 3.3)
Occupational exposure to chromium	0.1 (0.1 to 0.1)	0.1 (0.1 to 0.1)	0.1 (0.1 to 0.1)	-21.8 (-28.0 to -14.7)	1.5 (-4.8 to 8.5)	0.1 (0.1 to 0.1)	0.1 (0.1 to 0.1)	0.1 (0.1 to 0.1)	-23.2 (-31.3 to -13.3)	-3.5 (-11.4 to 5.0)
Occupational exposure to diesel engine exhaust	0.5 (0.4 to 0.5)	0.4 (0.4 to 0.4)	0.4 (0.4 to 0.4)	-16.3 (-22.1 to -9.7)	5.0 (-0.5 to 10.5)	0.4 (0.3 to 0.4)	0.3 (0.3 to 0.3)	0.3 (0.3 to 0.4)	-12.0 (-19.2 to -4.6)	4.2 (-2.7 to 10.4)
Occupational exposure to formaldehyde	0.2 (0.2 to 0.3)	0.2 (0.2 to 0.2)	0.2 (0.2 to 0.2)	-25.2 (-32.0 to -17.5)	-0.4 (-7.6 to 7.8)	0.3 (0.2 to 0.3)	0.2 (0.2 to 0.2)	0.2 (0.2 to 0.2)	-24.9 (-33.6 to -14.2)	-4.9 (-13.9 to 4.3)
Occupational exposure to nickel	0.4 (0.0 to 1.9)	0.3 (0.0 to 1.4)	0.3 (0.0 to 1.4)	-	-	0.5 (0.0 to 2.1)	0.3 (0.0 to 1.5)	0.3 (0.0 to 1.3)	-	-
Occupational exposure to polycyclic aromatic hydrocarbons	0.2 (0.2 to 0.2)	0.2 (0.2 to 0.2)	0.2 (0.2 to 0.2)	-20.8 (-26.6 to -14.1)	1.4 (-4.3 to 7.6)	0.3 (0.2 to 0.3)	0.2 (0.2 to 0.2)	0.2 (0.2 to 0.2)	-21.2 (-29.1 to -11.9)	-3.4 (-10.8 to 4.5)
Occupational exposure to silica	5.1 (0.7 to 16.4)	3.7 (0.5 to 11.9)	3.8 (0.5 to 11.9)	-26.2 (-32.6 to -19.6)	1.3 (-5.3 to 8.4)	4.2 (0.6 to 13.8)	2.9 (0.4 to 9.0)	2.7 (0.4 to 8.2)	-31.3 (-38.9 to -23.0)	-8.1 (-16.2 to 1.1)
Occupational exposure to sulphuric acid	0.6 (0.1 to 2.0)	0.5 (0.1 to 1.6)	0.5 (0.1 to 1.6)	-19.6 (-25.4 to -13.1)	-0.9 (-6.1 to 5.3)	0.7 (0.2 to 2.2)	0.5 (0.1 to 1.7)	0.5 (0.1 to 1.6)	-24.0 (-31.6 to -15.7)	-7.2 (-14.3 to 0.3)
Occupational exposure to trichloroethylene	0.1 (0.1 to 0.1)	0.0 (0.0 to 0.0)	0.0 (0.0 to 0.0)	-18.0 (-23.6 to -12.0)	2.6 (-2.3 to 7.9)	0.1 (0.1 to 0.1)	0.1 (0.0 to 0.1)	0.1 (0.0 to 0.1)	-19.7 (-26.6 to -11.1)	-1.3 (-7.5 to 6.0)
Occupational asthmagens	22.0 (18.5 to 26.4)	16.9 (14.1 to 20.3)	15.8 (13.1 to 19.1)	-23.4 (-29.5 to -16.6)	-6.4 (-13.3 to 0.6)	14.9 (11.8 to 18.7)	11.2 (9.0 to 14.1)	9.9 (7.8 to 12.5)	-24.8 (-32.7 to -15.3)	-12.2 (-21.9 to -1.8)
Occupational particulate matter, gases, and fumes	8.1 (5.9 to 11.3)	7.5 (5.4 to 10.5)	6.8 (5.0 to 9.7)	-7.8 (-11.7 to -4.1)	-8.2 (-10.5 to -5.9)	5.8 (4.1 to 8.3)	5.4 (4.0 to 7.7)	4.8 (3.5 to 7.0)	-5.9 (-10.6 to -1.1)	-11.0 (-14.1 to -8.3)
Occupational noise	7.1 (6.7 to 7.7)	6.7 (6.3 to 7.2)	6.2 (5.9 to 6.7)	-6.4 (-8.8 to -3.8)	-7.0 (-8.3 to -5.6)	5.1 (4.6 to 5.6)	5.0 (4.7 to 5.4)	4.6 (4.3 to 5.1)	-1.6 (-5.4 to 2.5)	-7.2 (-8.9 to -5.5)
Occupational ergonomic factors	18.6 (16.7 to 20.7)	15.1 (14.0 to 16.3)	13.3 (11.9 to 14.9)	-19.2 (-27.3 to -9.9)	-11.5 (-19.0 to -2.7)	17.4 (15.3 to 19.4)	13.0 (11.9 to 14.1)	10.7 (9.2 to 12.2)	-25.4 (-34.4 to -14.5)	-18.0 (-28.7 to -6.3)
Non-exclusive breastfeeding	0.5 (0.3 to 0.7)	0.5 (0.4 to 0.7)	0.5 (0.3 to 0.7)	1.7 (-6.6 to 10.8)	-1.4 (-8.2 to 5.9)	0.5 (0.3 to 0.7)	0.5 (0.3 to 0.7)	0.5 (0.3 to 0.7)	1.7 (-6.5 to 10.8)	-1.3 (-8.2 to 5.9)
Discontinued breastfeeding	1.7 (1.6 to 1.8)	1.8 (1.7 to 1.9)	1.9 (1.8 to 2.0)	8.5 (1.8 to 15.6)	6.3 (1.4 to 11.5)	1.7 (1.6 to 1.8)	1.8 (1.7 to 1.9)	1.9 (1.8 to 2.0)	8.6 (1.8 to 15.7)	6.4 (1.4 to 11.6)
Child underweight	0.3 (0.2 to 0.3)	0.1 (0.1 to 0.2)	0.1 (0.1 to 0.2)	-44.7 (-55.2 to -35.3)	-15.3 (-22.6 to -7.7)	0.3 (0.2 to 0.3)	0.1 (0.1 to 0.2)	0.1 (0.1 to 0.1)	-51.8 (-61.9 to -42.9)	-16.8 (-25.7 to -9.2)
Child wasting	0.2 (0.1 to 0.2)	0.3 (0.2 to 0.4)	0.2 (0.2 to 0.4)	47.8 (20.2 to 85.7)	-16.3 (-35.1 to 6.1)	0.2 (0.1 to 0.2)	0.1 (0.1 to 0.2)	0.1 (0.1 to 0.2)	-24.5 (-36.3 to -10.8)	-10.1 (-22.7 to 5.5)
Child stunting	0.9 (0.6 to 1.3)	0.8 (0.5 to 1.1)	0.7 (0.4 to 1.1)	-12.7 (-38.7 to 24.6)	-16.7 (-42.4 to 12.1)	1.0 (0.6 to 1.3)	0.8 (0.5 to 1.1)	0.7 (0.4 to 1.1)	-15.0 (-41.2 to 19.8)	-17.5 (-42.8 to 9.7)
Short gestation for birth weight	0.0 (0.0 to 0.0)	0.0 (0.0 to 0.0)	0.0 (0.0 to 0.0)	4.0 (-18.0 to 35.4)	9.1 (-9.0 to 49.9)	0.0 (0.0 to 0.0)	0.0 (0.0 to 0.0)	0.0 (0.0 to 0.0)	3.7 (-16.7 to 27.1)	4.1 (-15.7 to 25.1)
Low birth weight for gestation	0.0 (0.0 to 0.0)	0.0 (0.0 to 0.0)	0.0 (0.0 to 0.0)	2.2 (-14.9 to 22.9)	1.1 (-19.0 to 16.2)	0.0 (0.0 to 0.0)	0.0 (0.0 to 0.0)	0.0 (0.0 to 0.0)	0.8 (-13.9 to 22.1)	1.9 (-14.1 to 18.2)
Iron deficiency	-	-	-	-	-	10.4 (7.6 to 13.7)	10.0 (7.3 to 13.3)	9.0 (6.5 to 12.3)	-4.1 (-16.4 to 11.2)	-9.6 (-20.2 to 0.5)
Vitamin A deficiency	2.9 (2.3 to 3.5)	2.3 (1.9 to 2.8)	2.2 (1.8 to 2.6)	-19.7 (-29.6 to -7.6)	-7.1 (-17.1 to 3.8)	2.9 (2.4 to 3.5)	2.4 (2.0 to 2.9)	2.2 (1.9 to 2.7)	-18.2 (-27.5 to -8.6)	-6.9 (-15.5 to 2.7)
Zinc deficiency	0.5 (0.1 to 1.0)	0.4 (0.1 to 0.8)	0.5 (0.1 to 1.2)	-21.7 (-63.9 to 7.0)	26.3 (-56.1 to 309.2)	0.5 (0.1 to 1.0)	0.4 (0.1 to 0.8)	0.4 (0.1 to 1.1)	-21.3 (-62.6 to 6.1)	25.2 (-57.5 to 319.7)
Smoking	13.7 (11.7 to 15.8)	12.9 (11.3 to 14.7)	11.4 (9.9 to 13.0)	-5.3 (-13.3 to 3.6)	-11.6 (-17.1 to -5.7)	5.3 (4.0 to 6.8)	4.9 (3.9 to 5.9)	4.7 (3.9 to 5.6)	-7.9 (-25.3 to 13.4)	-3.8 (-14.9 to 7.6)
Chewing tobacco	0.1 (0.1 to 0.2)	0.1 (0.1 to 0.2)	0.1 (0.1 to 0.2)	-0.4 (-32.8 to 43.2)	-5.1 (-27.8 to 26.7)	0.1 (0.0 to 0.1)	0.1 (0.0 to 0.1)	0.1 (0.0 to 0.1)	-0.2 (-29.8 to 38.5)	0.7 (-22.3 to 30.7)
Second-hand smoke	41.3 (38.1 to 44.4)	41.4 (38.5 to 44.7)	37.5 (34.3 to 40.4)	0.4 (-7.2 to 9.3)	-9.5 (-15.5 to -3.6)	50.7 (48.1 to 53.4)	50.6 (48.0 to 53.2)	43.4 (40.9 to 46.1)	-0.2 (-5.7 to 5.4)	-14.1 (-17.7 to -10.3)
Alcohol use	42.5 (35.0 to 49.4)	40.4 (32.3 to 48.3)	43.1 (32.3 to 53.8)	-5.0 (-15.0 to 4.0)	6.8 (-10.1 to 23.8)	14.0 (8.7 to 21.5)	11.5 (6.9 to 18.3)	12.8 (7.2 to 20.8)	-17.9 (-29.4 to -6.3)	11.2 (-12.2 to 42.6)
Drug use	0.3 (0.2 to 0.4)	0.3 (0.3 to 0.4)	0.3 (0.3 to 0.4)	4.0 (-3.0 to 11.0)	2.8 (-3.5 to 9.5)	0.2 (0.1 to 0.3)	0.2 (0.1 to 0.3)	0.2 (0.1 to 0.3)	1.3 (-4.4 to 7.1)	-1.1 (-6.7 to 4.8)
Diet low in fruits	41.5 (36.4 to 44.3)	38.9 (33.6 to 42.2)	35.2 (29.6 to 39.2)	-6.2 (-9.8 to -3.3)	-9.6 (-15.1 to -5.2)	39.6 (34.3 to 42.7)	36.8 (31.4 to 40.3)	32.5 (26.7 to 36.8)	-7.1 (-11.3 to -4.1)	-11.9 (-18.4 to -6.6)
Diet low in vegetables	13.2 (5.5 to 20.1)	2.5 (0.1 to 7.1)	1.1 (0.0 to 4.0)	-81.3 (-97.7 to -62.8)	-57.2 (-92.6 to -8.5)	16.0 (8.5 to 22.3)	3.7 (0.3 to 9.4)	1.6 (0.1 to 5.1)	-77.0 (-96.8 to -56.1)	-56.6 (-89.0 to -16.4)
Diet low in legumes	40.7 (38.2 to 42.9)	43.2 (41.1 to 45.0)	42.0 (39.5 to 44.2)	6.1 (4.1 to 8.5)	-2.7 (-4.9 to -0.7)	43.0 (40.8 to 44.9)	45.3 (43.5 to 46.8)	44.5 (42.5 to 46.3)	5.4 (3.8 to 7.4)	-1.8 (-3.6 to -0.1)
Diet low in whole grains	45.0 (43.4 to 46.4)	41.3 (39.0 to 43.2)	39.0 (36.3 to 41.3)	-8.3 (-10.8 to -6.3)	-5.5 (-8.0 to -3.1)	45.6 (44.1 to 46.9)	42.1 (40.2 to 43.9)	40.0 (37.4 to 42.1)	-7.7 (-9.7 to -5.9)	-5.1 (-7.5 to -3.1)
Diet low in nuts and seeds	51.1 (50.1 to 51.9)	50.8 (49.7 to 51.6)	48.1 (46.3 to 49.7)	-0.7 (-1.9 to 0.5)	-5.2 (-7.5 to -3.4)	51.3 (50.4 to 52.1)	51.0 (50.0 to 51.8)	48.5 (46.7 to 50.0)	-0.6 (-1.8 to 0.5)	-4.9 (-7.0 to -3.1)
Diet low in milk	35.5 (31.4 to 39.0)	37.1 (33.3 to 40.3)	36.7 (32.9 to 40.1)	4.6 (0.9 to 9.5)	-1.2 (-3.9 to 1.6)	36.5 (32.5 to 39.7)	38.2 (34.8 to 41.1)	37.6 (33.9 to 40.7)	4.6 (1.0 to 8.9)	-1.3 (-4.1 to 1.1)
Diet high in red meat	21.1 (16.4 to 24.9)	23.3 (19.1 to 26.5)	36.0 (30.4 to 40.9)	10.3 (-4.1 to 27.6)	54.6 (31.6 to 85.0)	6.7 (2.8 to 10.5)	7.1 (3.2 to 10.6)	13.5 (8.1 to 18.4)	5.9 (-27.3 to 68.7)	92.0 (40.6 to 225.2)
Diet high in processed meat	4.2 (1.5 to 7.1)	4.9 (2.0 to 7.9)	7.0 (3.8 to 10.2)	16.8 (-7.5 to 67.4)	43.1 (19.0 to 110.5)	2.7 (0.5 to 5.3)	3.2 (0.8 to 5.9)	4.7 (2.0 to 7.6)	18.1 (-10.5 to 103.6)	49.2 (21.1 to 169.3)
Diet high in sugar-sweetened beverages	9.5 (4.7 to 15.1)	9.6 (4.9 to 11.7)	14.7 (7.3 to 19.0)	1.1 (-36.3 to 59.6)	53.0 (16.8 to 98.0)	6.5 (3.1 to 11.1)	6.6 (3.4 to 8.5)	9.9 (4.9 to 13.1)	1.1 (-38.4 to 67.8)	50.5 (11.4 to 103.5)
Diet low in fibre	26.7 (20.9 to 31.4)	22.1 (15.9 to 27.4)	20.6 (14.1 to 26.3)	-17.3 (-26.0 to -11.3)	-6.6 (-16.6 to 1.3)	32.1 (27.3 to 36.1)	28.1 (22.5 to 32.6)	26.5 (20.3 to 31.7)	-12.7 (-19.3 to -7.7)	-5.5 (-13.4 to -0.1)
Diet low in calcium	22.9 (16.6 to 28.0)	12.8 (6.4 to 18.7)	9.3 (4.2 to 15.0)	-44.3 (-61.3 to -32.7)	-26.9 (-41.0 to -18.1)	26.5 (20.9 to 31.1)	16.6 (9.6 to 22.5)	12.6 (6.0 to 18.8)	-37.3 (-54.1 to -27.1)	-24.5 (-40.3 to -15.1)
Diet low in seafood omega-3 fatty acids	42.9 (40.6 to 45.1)	42.0 (39.5 to 44.3)	41.2 (38.5 to 43.7)	-2.1 (-3.0 to -1.4)	-2.0 (-3.3 to -1.0)	44.6 (42.6 to 46.5)	43.8 (41.7 to 45.9)	43.1 (40.7 to 45.3)	-1.7 (-2.4 to -1.2)	-1.7 (-2.8 to -0.8)
Diet low in polyunsaturated fatty acids	37.7 (34.3 to 40.9)	39.3 (36.3 to 41.9)	36.8 (33.3 to 39.7)	4.1 (-1.7 to 9.6)	-6.3 (-9.5 to -3.8)	38.2 (35.0 to 41.4)	39.8 (36.9 to 42.3)	37.3 (33.7 to 40.3)	4.0 (-1.0 to 8.7)	-6.2 (-10.0 to -2.3)
Diet high in trans fatty acids	2.4 (0.4 to 5.3)	1.8 (0.2 to 4.4)	1.4 (0.1 to 3.7)	-25.3 (-61.3 to -7.8)	-22.5 (-59.3 to -6.3)	3.2 (0.8 to 6.4)	2.4 (0.4 to 5.5)	1.9 (0.2 to 4.7)	-24.6 (-55.3 to -6.4)	-22.4 (-56.6 to -6.8)
Diet high in sodium	23.7 (15.7 to 30.4)	11.8 (3.4 to 19.3)	12.9 (4.4 to 20.2)	-50.3 (-81.2 to -30.6)	9.5 (-8.2 to 54.7)	17.9 (11.1 to 24.0)	6.6 (0.6 to 13.6)	5.3 (0.2 to 12.0)	-63.1 (-94.8 to -40.7)	-19.8 (-77.4 to -5.5)
Intimate partner violence	-	-	-	-	-	4.0 (3.4 to 4.7)	3.5 (3.0 to 4.1)	3.2 (2.7 to 3.7)	-13.4 (-18.0 to -8.9)	-9.5 (-14.1 to -4.8)
Childhood sexual abuse	8.7 (7.1 to 10.8)	8.7 (7.1 to 10.8)	8.7 (7.1 to 10.8)	0.0 (0.0 to 0.0)	0.0 (0.0 to 0.0)	4.9 (3.6 to 6.5)	4.9 (3.6 to 6.5)	4.9 (3.6 to 6.5)	0.0 (0.0 to 0.0)	0.0 (0.0 to 0.0)

Supplementary Results Table 1. Romania age-standardised Summary Exposure Values (SEVs) for Level 4 risk factors and percentage change, by sex, 1990, 2007, and 2017.Data in parentheses are 95% uncertainty intervals.										
	**Male**					**Female**				
	**1990**	**2007**	**2017**	**Percentage change 1990-2007**	**Percentage change 2007-2017**	**1990**	**2007**	**2017**	**Percentage change 1990-2007**	**Percentage change 2007-2017**
Bullying victimization	7.7 (3.2 to 15.7)	9.0 (4.2 to 17.1)	9.0 (4.2 to 17.1)	16.8 (-6.5 to 56.3)	0.7 (-5.6 to 7.9)	5.7 (2.4 to 11.5)	6.7 (3.2 to 12.8)	6.8 (3.2 to 13.0)	18.5 (-6.7 to 62.6)	0.6 (-6.8 to 7.3)
Low physical activity	0.3 (0.1 to 0.5)	0.3 (0.2 to 0.5)	0.3 (0.2 to 0.6)	3.1 (0.0 to 6.0)	1.2 (-1.1 to 3.5)	0.4 (0.2 to 0.7)	0.4 (0.2 to 0.7)	0.4 (0.2 to 0.7)	3.8 (1.4 to 6.6)	0.4 (-1.5 to 2.5)
High fasting plasma glucose	1.2 (0.8 to 1.8)	1.5 (1.0 to 2.4)	1.7 (1.0 to 2.6)	26.4 (-15.8 to 105.2)	7.2 (-35.3 to 77.9)	1.0 (0.6 to 1.5)	1.1 (0.7 to 1.7)	1.3 (0.8 to 2.0)	15.7 (-22.6 to 69.6)	15.5 (-24.4 to 75.7)
High LDL cholesterol	14.2 (12.6 to 16.1)	14.0 (12.3 to 15.8)	14.0 (12.3 to 15.9)	-1.9 (-6.1 to 2.5)	0.5 (-3.2 to 4.2)	14.8 (13.2 to 16.6)	14.4 (12.9 to 16.2)	14.6 (13.0 to 16.4)	-2.7 (-6.6 to 1.6)	1.1 (-2.3 to 4.7)
High systolic blood pressure	12.1 (11.0 to 13.2)	12.2 (11.3 to 13.2)	11.5 (10.7 to 12.6)	1.3 (-4.8 to 7.7)	-5.8 (-10.4 to -0.9)	11.7 (10.7 to 12.7)	11.7 (10.8 to 12.6)	10.4 (9.5 to 11.4)	-0.2 (-7.5 to 8.2)	-10.6 (-16.1 to -4.6)
High body-mass index	8.8 (6.1 to 13.0)	12.8 (9.7 to 17.4)	15.4 (12.1 to 20.3)	45.2 (25.4 to 72.1)	20.2 (10.1 to 32.9)	9.8 (7.1 to 13.5)	13.0 (10.3 to 16.9)	15.2 (12.2 to 19.5)	32.7 (15.1 to 57.6)	16.9 (6.9 to 29.4)
Low bone mineral density	3.6 (2.9 to 4.4)	3.5 (2.8 to 4.2)	3.4 (2.7 to 4.2)	-4.4 (-18.7 to 13.8)	-1.9 (-16.9 to 18.1)	6.1 (5.1 to 7.2)	5.9 (5.0 to 6.9)	5.7 (4.8 to 6.8)	-4.0 (-16.2 to 10.0)	-2.6 (-15.2 to 11.6)
Impaired kidney function	2.0 (0.9 to 5.5)	1.9 (0.8 to 5.3)	1.9 (0.8 to 5.3)	-5.1 (-10.7 to -0.6)	0.3 (-4.9 to 6.4)	2.7 (1.3 to 6.5)	2.7 (1.2 to 6.6)	2.7 (1.3 to 6.6)	-1.5 (-7.2 to 2.9)	0.2 (-4.8 to 5.7)

Supplementary Results Table 1. Serbia age-standardised Summary Exposure Values (SEVs) for Level 4 risk factors and percentage change, by sex, 1990, 2007, and 2017.Data in parentheses are 95% uncertainty intervals.

	Male					Female				
	**1990**	**2007**	**2017**	**Percentage change 1990-2007**	**Percentage change 2007-2017**	**1990**	**2007**	**2017**	**Percentage change 1990-2007**	**Percentage change 2007-2017**
Unsafe water source	11.1 (6.4 to 15.6)	8.2 (4.7 to 10.7)	5.3 (2.7 to 7.3)	-25.4 (-44.9 to 2.2)	-35.6 (-51.9 to -17.2)	11.1 (6.4 to 15.6)	8.2 (4.7 to 10.7)	5.3 (2.7 to 7.3)	-25.4 (-44.9 to 2.2)	-35.6 (-51.9 to -17.2)
Unsafe sanitation	16.8 (10.2 to 26.2)	8.2 (5.3 to 12.3)	5.2 (3.0 to 8.3)	-51.4 (-68.3 to -22.5)	-36.5 (-56.5 to -7.3)	16.8 (10.2 to 26.2)	8.2 (5.3 to 12.3)	5.2 (3.0 to 8.3)	-51.4 (-68.3 to -22.5)	-36.5 (-56.5 to -7.3)
No access to handwashing facility	6.6 (5.3 to 8.2)	4.5 (3.6 to 5.6)	3.3 (2.6 to 4.1)	-32.8 (-46.7 to -14.4)	-26.0 (-37.8 to -12.1)	6.6 (5.3 to 8.2)	4.5 (3.6 to 5.6)	3.3 (2.6 to 4.1)	-32.8 (-46.7 to -14.4)	-26.0 (-37.8 to -12.1)
Ambient particulate matter pollution	31.5 (19.5 to 47.2)	36.2 (25.0 to 52.9)	34.9 (22.5 to 51.9)	14.7 (-24.0 to 75.5)	-3.4 (-39.3 to 43.8)	27.1 (15.9 to 42.5)	31.9 (21.6 to 48.0)	31.2 (19.4 to 48.3)	17.9 (-24.9 to 87.9)	-2.3 (-41.1 to 51.9)
Household air pollution from solid fuels	34.0 (23.4 to 47.7)	23.4 (15.5 to 33.2)	18.6 (10.8 to 28.1)	-31.2 (-54.5 to -1.6)	-20.6 (-50.4 to 21.5)	36.4 (26.4 to 49.9)	25.9 (18.0 to 35.6)	20.8 (12.7 to 30.0)	-28.8 (-50.2 to -2.4)	-19.6 (-46.6 to 17.7)
Ambient ozone pollution	43.7 (15.9 to 54.8)	43.1 (15.5 to 54.8)	43.7 (15.9 to 54.8)	-1.4 (-2.9 to 0.0)	1.3 (0.0 to 2.9)	43.7 (15.9 to 54.8)	43.1 (15.5 to 54.8)	43.7 (15.9 to 54.8)	-1.4 (-2.9 to 0.0)	1.3 (0.0 to 2.9)
Residential radon	60.9 (30.3 to 71.2)	60.9 (30.3 to 71.2)	60.9 (30.3 to 71.2)	0.0 (0.0 to 0.0)	0.0 (0.0 to 0.0)	60.9 (30.3 to 71.2)	60.9 (30.3 to 71.2)	60.9 (30.3 to 71.2)	0.0 (0.0 to 0.0)	0.0 (0.0 to 0.0)
Lead exposure	5.4 (1.6 to 9.5)	5.5 (1.9 to 9.5)	4.6 (1.5 to 8.3)	3.1 (-3.4 to 24.4)	-17.8 (-25.3 to -12.1)	2.1 (0.1 to 5.5)	2.2 (0.2 to 5.5)	1.8 (0.2 to 4.9)	4.8 (-2.4 to 50.8)	-16.8 (-27.4 to -10.9)
Occupational exposure to asbestos	1.0 (0.8 to 1.3)	1.6 (1.4 to 1.7)	1.4 (1.1 to 1.6)	54.6 (16.4 to 107.0)	-13.1 (-27.7 to 6.1)	0.4 (0.3 to 0.5)	0.5 (0.5 to 0.6)	0.5 (0.4 to 0.6)	35.5 (6.1 to 84.5)	-1.9 (-19.0 to 18.5)
Occupational exposure to arsenic	0.3 (0.0 to 0.6)	0.3 (0.0 to 0.6)	0.3 (0.0 to 0.6)	-	-	0.3 (0.0 to 0.6)	0.2 (0.0 to 0.6)	0.3 (0.0 to 0.6)	-	-
Occupational exposure to benzene	0.4 (0.0 to 1.1)	0.4 (0.0 to 1.2)	0.4 (0.0 to 1.2)	5.1 (-2.1 to 14.5)	2.0 (-2.4 to 9.4)	0.4 (0.0 to 1.2)	0.5 (0.0 to 1.4)	0.6 (0.0 to 1.6)	23.8 (10.3 to 44.0)	12.2 (5.4 to 22.1)
Occupational exposure to beryllium	0.0 (0.0 to 0.0)	0.0 (0.0 to 0.0)	0.0 (0.0 to 0.0)	-2.2 (-7.5 to 3.5)	-0.8 (-4.4 to 2.9)	0.0 (0.0 to 0.0)	0.0 (0.0 to 0.0)	0.0 (0.0 to 0.0)	3.0 (-5.3 to 14.2)	6.0 (1.3 to 11.3)
Occupational exposure to cadmium	0.0 (0.0 to 0.0)	0.0 (0.0 to 0.0)	0.0 (0.0 to 0.0)	-1.0 (-9.9 to 9.8)	-0.5 (-7.8 to 6.7)	0.0 (0.0 to 0.0)	0.0 (0.0 to 0.0)	0.0 (0.0 to 0.0)	-1.8 (-13.9 to 13.3)	3.1 (-6.6 to 13.6)
Occupational exposure to chromium	0.1 (0.1 to 0.1)	0.1 (0.1 to 0.1)	0.1 (0.1 to 0.1)	0.8 (-8.0 to 11.0)	0.6 (-6.3 to 7.4)	0.1 (0.1 to 0.1)	0.1 (0.1 to 0.1)	0.1 (0.1 to 0.1)	2.0 (-10.1 to 17.6)	5.1 (-3.8 to 14.6)
Occupational exposure to diesel engine exhaust	0.3 (0.3 to 0.4)	0.3 (0.3 to 0.4)	0.3 (0.3 to 0.4)	-0.2 (-7.5 to 8.7)	1.3 (-4.4 to 7.0)	0.3 (0.2 to 0.3)	0.3 (0.3 to 0.3)	0.3 (0.3 to 0.3)	5.7 (-5.4 to 19.4)	9.4 (1.8 to 17.5)
Occupational exposure to formaldehyde	0.2 (0.1 to 0.2)	0.2 (0.1 to 0.2)	0.2 (0.1 to 0.2)	-1.4 (-11.3 to 10.7)	-0.8 (-8.8 to 7.1)	0.1 (0.1 to 0.2)	0.1 (0.1 to 0.1)	0.1 (0.1 to 0.2)	1.6 (-11.6 to 18.1)	4.1 (-5.7 to 14.5)
Occupational exposure to nickel	0.3 (0.0 to 1.2)	0.3 (0.0 to 1.2)	0.3 (0.0 to 1.1)	-	-	0.2 (0.0 to 1.0)	0.2 (0.0 to 0.9)	0.2 (0.0 to 0.9)	-	-
Occupational exposure to polycyclic aromatic hydrocarbons	0.1 (0.1 to 0.2)	0.1 (0.1 to 0.2)	0.1 (0.1 to 0.2)	1.0 (-7.2 to 10.6)	0.2 (-6.1 to 6.5)	0.1 (0.1 to 0.2)	0.1 (0.1 to 0.2)	0.1 (0.1 to 0.2)	4.4 (-7.8 to 19.6)	5.8 (-2.6 to 14.2)
Occupational exposure to silica	3.3 (0.5 to 10.3)	3.1 (0.4 to 9.9)	3.0 (0.4 to 9.5)	-6.2 (-15.6 to 3.9)	-3.4 (-10.1 to 4.6)	2.0 (0.3 to 6.4)	1.8 (0.3 to 5.9)	1.8 (0.2 to 5.8)	-9.2 (-20.5 to 5.4)	-0.2 (-9.7 to 11.0)
Occupational exposure to sulphuric acid	0.4 (0.1 to 1.3)	0.4 (0.1 to 1.4)	0.4 (0.1 to 1.3)	-0.9 (-8.7 to 8.9)	-3.9 (-9.9 to 2.7)	0.4 (0.1 to 1.1)	0.3 (0.1 to 1.1)	0.4 (0.1 to 1.1)	-4.6 (-15.8 to 9.1)	0.6 (-7.8 to 10.1)
Occupational exposure to trichloroethylene	0.0 (0.0 to 0.0)	0.0 (0.0 to 0.0)	0.0 (0.0 to 0.0)	1.9 (-5.3 to 10.7)	1.6 (-3.9 to 7.1)	0.0 (0.0 to 0.0)	0.0 (0.0 to 0.0)	0.0 (0.0 to 0.0)	3.4 (-7.2 to 17.6)	6.5 (-0.4 to 14.2)
Occupational asthmagens	15.2 (12.4 to 18.5)	14.1 (11.8 to 16.9)	13.6 (11.4 to 16.5)	-7.3 (-17.1 to 4.6)	-3.3 (-10.0 to 4.1)	7.7 (5.7 to 10.2)	7.5 (6.0 to 9.3)	7.6 (6.1 to 9.4)	-3.0 (-19.0 to 18.3)	1.6 (-8.7 to 12.6)
Occupational particulate matter, gases, and fumes	5.8 (4.1 to 8.3)	5.5 (3.9 to 7.8)	5.1 (3.7 to 7.4)	-5.0 (-9.1 to -1.1)	-6.8 (-9.7 to -4.1)	3.1 (2.1 to 4.6)	2.9 (2.1 to 4.3)	2.7 (1.9 to 4.0)	-5.6 (-11.8 to 1.4)	-7.2 (-11.6 to -3.3)
Occupational noise	5.4 (4.9 to 6.0)	5.2 (4.8 to 5.7)	4.9 (4.6 to 5.3)	-4.0 (-6.9 to -0.9)	-5.7 (-7.7 to -3.9)	3.0 (2.6 to 3.5)	3.0 (2.7 to 3.3)	2.9 (2.7 to 3.2)	-1.3 (-6.6 to 4.9)	-2.2 (-5.3 to 1.5)
Occupational ergonomic factors	12.9 (10.8 to 15.1)	11.3 (10.4 to 12.3)	10.4 (9.2 to 11.7)	-12.4 (-24.3 to 2.0)	-7.7 (-16.3 to 1.5)	8.5 (6.6 to 10.7)	7.8 (7.1 to 8.6)	7.1 (6.1 to 8.2)	-8.1 (-25.2 to 15.8)	-9.1 (-20.7 to 4.6)
Non-exclusive breastfeeding	0.6 (0.4 to 0.8)	0.6 (0.4 to 0.8)	0.6 (0.4 to 0.8)	0.4 (-5.0 to 6.3)	-0.9 (-6.0 to 5.0)	0.6 (0.4 to 0.8)	0.6 (0.4 to 0.8)	0.6 (0.4 to 0.8)	0.5 (-5.0 to 6.3)	-0.9 (-6.0 to 5.0)
Discontinued breastfeeding	2.0 (1.9 to 2.1)	2.1 (2.0 to 2.1)	2.1 (2.1 to 2.2)	2.2 (-2.2 to 7.1)	3.1 (-0.6 to 7.3)	2.0 (1.9 to 2.1)	2.1 (2.0 to 2.1)	2.1 (2.1 to 2.2)	2.2 (-2.2 to 7.1)	3.1 (-0.6 to 7.3)
Child underweight	0.2 (0.1 to 0.2)	0.1 (0.1 to 0.2)	0.1 (0.1 to 0.2)	-17.3 (-32.9 to -4.4)	-13.1 (-21.2 to -4.5)	0.2 (0.1 to 0.2)	0.1 (0.1 to 0.2)	0.1 (0.1 to 0.2)	-19.6 (-32.4 to -6.2)	-8.6 (-17.8 to 0.5)
Child wasting	0.2 (0.1 to 0.3)	0.2 (0.1 to 0.2)	0.2 (0.1 to 0.2)	-21.1 (-45.0 to 12.3)	-6.2 (-25.3 to 19.2)	0.2 (0.1 to 0.4)	0.2 (0.1 to 0.3)	0.2 (0.1 to 0.2)	-20.3 (-45.7 to 16.5)	-13.4 (-34.3 to 15.4)
Child stunting	0.8 (0.4 to 1.3)	0.6 (0.4 to 0.8)	0.5 (0.3 to 0.8)	-17.6 (-47.2 to 29.8)	-15.0 (-37.7 to 13.9)	0.7 (0.4 to 1.2)	0.6 (0.4 to 0.8)	0.5 (0.3 to 0.7)	-20.6 (-49.4 to 23.5)	-22.8 (-43.9 to 5.0)
Short gestation for birth weight	0.0 (0.0 to 0.0)	0.0 (0.0 to 0.0)	0.0 (0.0 to 0.0)	24.3 (-0.3 to 52.9)	9.6 (-9.6 to 36.9)	0.0 (0.0 to 0.0)	0.0 (0.0 to 0.0)	0.0 (0.0 to 0.0)	11.2 (-12.8 to 38.5)	8.7 (-12.3 to 35.2)
Low birth weight for gestation	0.0 (0.0 to 0.0)	0.0 (0.0 to 0.0)	0.0 (0.0 to 0.0)	7.3 (-5.9 to 23.4)	-0.5 (-16.2 to 15.0)	0.0 (0.0 to 0.0)	0.0 (0.0 to 0.0)	0.0 (0.0 to 0.0)	7.5 (-7.9 to 26.4)	-1.4 (-17.1 to 16.7)
Iron deficiency	-	-	-	-	-	6.7 (4.8 to 9.1)	6.1 (4.3 to 8.3)	5.3 (3.6 to 7.3)	-8.7 (-24.3 to 8.9)	-13.5 (-25.9 to 0.3)
Vitamin A deficiency	3.0 (2.5 to 3.6)	2.8 (2.3 to 3.1)	2.3 (1.9 to 2.8)	-7.5 (-17.9 to 5.5)	-16.5 (-27.5 to -4.9)	3.1 (2.5 to 3.6)	2.8 (2.4 to 3.2)	2.4 (2.0 to 2.9)	-8.3 (-17.6 to 2.8)	-14.7 (-23.5 to -4.1)
Zinc deficiency	0.5 (0.1 to 1.2)	0.6 (0.2 to 1.2)	0.5 (0.1 to 1.1)	5.0 (-14.0 to 32.2)	-11.5 (-27.3 to 8.2)	0.5 (0.2 to 1.2)	0.6 (0.2 to 1.2)	0.5 (0.1 to 1.1)	4.4 (-14.8 to 32.5)	-11.4 (-26.7 to 8.3)
Smoking	11.2 (9.4 to 13.0)	12.7 (11.1 to 14.4)	13.6 (11.9 to 15.4)	13.9 (3.3 to 26.3)	6.6 (-0.5 to 14.8)	6.5 (5.0 to 8.1)	7.3 (6.1 to 8.7)	7.7 (6.5 to 9.1)	12.4 (-3.6 to 33.2)	5.8 (-3.0 to 15.3)
Chewing tobacco	0.1 (0.1 to 0.2)	0.1 (0.1 to 0.2)	0.1 (0.1 to 0.2)	0.9 (-32.4 to 45.8)	-4.6 (-28.3 to 24.3)	0.1 (0.0 to 0.1)	0.1 (0.0 to 0.1)	0.1 (0.0 to 0.1)	-0.8 (-31.3 to 41.2)	-0.0 (-23.2 to 30.2)
Second-hand smoke	46.1 (43.4 to 48.8)	50.3 (49.0 to 51.5)	43.4 (41.4 to 45.3)	9.2 (2.7 to 16.3)	-13.7 (-18.8 to -8.7)	50.1 (47.8 to 52.4)	56.6 (55.1 to 58.0)	48.6 (46.5 to 50.6)	13.0 (7.5 to 18.8)	-14.2 (-18.5 to -9.9)
Alcohol use	26.8 (16.5 to 38.2)	26.5 (17.5 to 35.7)	33.8 (22.4 to 46.9)	-0.9 (-29.4 to 40.2)	27.6 (2.1 to 68.4)	11.2 (6.0 to 18.6)	10.7 (6.0 to 17.7)	13.8 (7.8 to 22.3)	-4.2 (-31.6 to 31.4)	29.1 (1.6 to 77.0)
Drug use	0.3 (0.2 to 0.4)	0.3 (0.3 to 0.4)	0.3 (0.3 to 0.4)	13.1 (4.4 to 22.6)	-3.9 (-12.2 to 4.8)	0.1 (0.1 to 0.1)	0.1 (0.1 to 0.1)	0.1 (0.1 to 0.1)	2.8 (-3.4 to 8.7)	3.3 (-2.5 to 9.5)
Diet low in fruits	32.7 (27.2 to 37.3)	33.2 (27.6 to 37.5)	30.7 (24.8 to 35.6)	1.6 (-5.5 to 10.0)	-7.5 (-15.0 to -1.6)	29.7 (23.2 to 34.9)	30.3 (24.3 to 34.8)	27.4 (21.1 to 32.7)	2.0 (-6.5 to 12.2)	-9.3 (-18.9 to -2.0)
Diet low in vegetables	22.6 (15.4 to 28.7)	24.4 (18.2 to 29.4)	20.6 (13.4 to 26.6)	7.7 (-3.9 to 27.6)	-15.4 (-31.0 to -5.3)	25.2 (18.2 to 30.8)	26.9 (21.2 to 31.6)	23.3 (16.3 to 28.8)	6.7 (-3.5 to 22.3)	-13.4 (-26.3 to -4.3)
Diet low in legumes	20.9 (15.2 to 25.9)	10.9 (4.6 to 16.6)	9.0 (3.0 to 15.0)	-47.7 (-71.2 to -32.4)	-17.6 (-43.9 to -0.3)	25.8 (20.6 to 30.3)	16.9 (10.8 to 22.2)	15.1 (8.6 to 20.8)	-34.4 (-49.4 to -24.3)	-10.8 (-27.4 to 0.7)
Diet low in whole grains	45.7 (44.1 to 47.0)	45.5 (44.0 to 46.9)	45.3 (43.7 to 46.7)	-0.3 (-1.4 to 0.8)	-0.5 (-1.6 to 0.7)	46.3 (44.9 to 47.5)	46.1 (44.7 to 47.4)	45.9 (44.4 to 47.3)	-0.3 (-1.3 to 0.8)	-0.5 (-1.6 to 0.6)
Diet low in nuts and seeds	47.4 (45.3 to 49.2)	47.9 (46.1 to 49.4)	45.8 (43.1 to 47.9)	1.1 (-1.6 to 4.1)	-4.5 (-7.3 to -2.3)	47.8 (45.9 to 49.5)	48.3 (46.5 to 49.8)	46.2 (43.9 to 48.1)	1.0 (-1.5 to 3.6)	-4.3 (-7.0 to -2.2)
Diet low in milk	37.9 (34.2 to 41.0)	40.8 (37.9 to 43.2)	40.2 (37.1 to 42.8)	7.7 (4.0 to 12.3)	-1.6 (-3.8 to 0.3)	38.8 (35.4 to 41.8)	41.5 (38.8 to 43.8)	40.8 (37.8 to 43.3)	7.0 (3.9 to 11.3)	-1.8 (-3.7 to -0.1)
Diet high in red meat	13.6 (7.8 to 18.9)	11.9 (7.5 to 15.7)	17.5 (11.2 to 22.6)	-12.6 (-36.0 to 21.9)	47.2 (12.7 to 101.7)	3.4 (0.5 to 7.2)	2.0 (0.2 to 4.9)	5.0 (1.3 to 9.1)	-40.6 (-89.5 to 49.8)	143.3 (27.9 to 1561.7)
Diet high in processed meat	1.1 (0.0 to 3.1)	0.9 (0.0 to 2.8)	1.6 (0.1 to 3.9)	-16.1 (-79.6 to 9.1)	78.9 (34.1 to 1240.6)	0.7 (0.0 to 2.4)	0.6 (0.0 to 2.2)	1.0 (0.0 to 3.0)	-13.0 (-83.0 to 15.2)	77.5 (34.4 to 2154.2)
Diet high in sugar-sweetened beverages	8.1 (3.5 to 14.6)	8.1 (3.7 to 14.8)	10.4 (4.5 to 17.8)	-0.4 (-44.4 to 75.3)	28.9 (-14.6 to 95.2)	5.7 (2.5 to 10.8)	5.6 (2.4 to 10.7)	7.2 (3.2 to 13.2)	-3.0 (-49.3 to 78.0)	28.7 (-15.3 to 105.7)
Diet low in fibre	29.0 (23.3 to 33.5)	29.7 (24.4 to 34.0)	28.6 (23.0 to 33.4)	2.3 (-2.8 to 9.1)	-3.4 (-9.6 to 1.4)	34.3 (29.4 to 38.0)	34.8 (30.6 to 38.3)	33.9 (29.1 to 37.6)	1.6 (-2.9 to 7.1)	-2.7 (-7.3 to 1.4)
Diet low in calcium	26.5 (20.7 to 31.0)	27.6 (22.1 to 32.0)	26.6 (20.9 to 31.2)	4.4 (0.8 to 9.4)	-3.6 (-7.8 to -0.5)	29.8 (24.7 to 34.0)	30.9 (26.0 to 34.8)	29.9 (24.9 to 33.9)	3.5 (0.7 to 7.8)	-3.2 (-6.5 to -0.8)
Diet low in seafood omega-3 fatty acids	40.3 (37.5 to 43.0)	40.1 (37.3 to 42.8)	37.7 (34.5 to 40.8)	-0.5 (-1.3 to 0.3)	-5.9 (-7.9 to -4.4)	42.4 (40.0 to 44.7)	42.2 (39.7 to 44.5)	40.1 (37.2 to 42.7)	-0.4 (-1.1 to 0.2)	-5.0 (-6.6 to -3.6)
Diet low in polyunsaturated fatty acids	32.8 (28.6 to 36.8)	35.1 (31.4 to 38.2)	37.0 (33.6 to 40.0)	6.8 (-0.3 to 12.5)	5.6 (1.6 to 11.2)	33.4 (29.2 to 37.2)	35.6 (32.1 to 38.7)	37.5 (34.1 to 40.6)	6.6 (-0.2 to 11.9)	5.3 (0.3 to 10.9)
Diet high in trans fatty acids	1.4 (0.1 to 3.8)	1.3 (0.1 to 3.6)	1.3 (0.1 to 3.5)	-5.2 (-31.0 to 21.3)	-2.4 (-27.9 to 21.9)	1.9 (0.2 to 4.6)	1.8 (0.2 to 4.6)	1.8 (0.2 to 4.5)	-6.0 (-31.9 to 19.3)	-1.7 (-23.4 to 22.9)
Diet high in sodium	23.8 (15.7 to 30.6)	11.1 (2.8 to 18.8)	11.9 (3.6 to 19.8)	-53.4 (-83.6 to -33.1)	7.6 (-11.3 to 47.2)	18.0 (10.8 to 24.6)	6.5 (0.6 to 13.3)	5.2 (0.1 to 11.9)	-64.1 (-95.2 to -42.4)	-18.9 (-78.5 to -3.8)
Intimate partner violence	-	-	-	-	-	3.2 (2.5 to 3.9)	2.9 (2.2 to 3.6)	2.8 (2.2 to 3.5)	-8.2 (-13.4 to -2.2)	-4.3 (-10.1 to 1.9)
Childhood sexual abuse	9.8 (8.0 to 11.8)	9.9 (8.2 to 11.9)	9.9 (8.2 to 11.9)	0.8 (-6.4 to 9.6)	0.0 (0.0 to 0.0)	2.5 (2.0 to 3.1)	2.5 (2.1 to 3.0)	2.7 (2.2 to 3.4)	1.0 (-8.8 to 12.3)	7.9 (-3.2 to 21.9)

Supplementary Results Table 1. Serbia age-standardised Summary Exposure Values (SEVs) for Level 4 risk factors and percentage change, by sex, 1990, 2007, and 2017.Data in parentheses are 95% uncertainty intervals.

	Male					Female				
	**1990**	**2007**	**2017**	**Percentage change 1990-2007**	**Percentage change 2007-2017**	**1990**	**2007**	**2017**	**Percentage change 1990-2007**	**Percentage change 2007-2017**
Bullying victimization	4.9<br>(2.0 to 10.5)	4.9<br>(2.0 to 10.4)	4.9<br>(2.0 to 10.4)	-0.4<br>(-2.6 to 2.0)	-0.5<br>(-1.9 to 0.9)	3.8<br>(1.6 to 8.2)	3.8<br>(1.6 to 8.1)	3.8<br>(1.6 to 8.1)	-0.9<br>(-2.9 to 1.2)	-0.3<br>(-1.5 to 1.1)
Low physical activity	0.3<br>(0.2 to 0.6)	0.3<br>(0.2 to 0.6)	0.3<br>(0.2 to 0.6)	0.7<br>(-1.2 to 2.6)	1.3<br>(-0.5 to 3.2)	0.3<br>(0.2 to 0.6)	0.3<br>(0.2 to 0.6)	0.3<br>(0.2 to 0.6)	0.7<br>(-1.6 to 3.1)	1.1<br>(-0.9 to 3.4)
High fasting plasma glucose	3.4<br>(2.3 to 5.3)	4.3<br>(3.0 to 6.2)	4.2<br>(2.9 to 6.2)	24.0<br>(-4.1 to 74.8)	-1.0<br>(-23.2 to 26.8)	2.4<br>(1.5 to 3.7)	3.1<br>(2.2 to 4.6)	3.3<br>(2.2 to 4.8)	28.4<br>(-7.4 to 103.8)	5.5<br>(-18.1 to 38.3)
High LDL cholesterol	16.7<br>(14.9 to 18.4)	16.5<br>(14.7 to 18.3)	16.7<br>(15.1 to 18.6)	-1.4<br>(-5.7 to 3.1)	1.4<br>(-2.0 to 5.1)	16.9<br>(15.3 to 18.8)	16.7<br>(15.0 to 18.4)	17.0<br>(15.4 to 18.8)	-1.7<br>(-6.0 to 2.9)	2.2<br>(-1.2 to 5.9)
High systolic blood pressure	10.8<br>(9.9 to 11.7)	10.1<br>(9.1 to 11.0)	10.0<br>(9.2 to 10.9)	-6.8<br>(-12.3 to -1.1)	-0.4<br>(-6.1 to 5.9)	10.8<br>(9.9 to 11.8)	10.0<br>(9.0 to 11.0)	10.0<br>(9.0 to 11.0)	-7.6<br>(-15.2 to 1.2)	0.0<br>(-7.2 to 7.4)
High body-mass index	9.2<br>(6.4 to 13.7)	12.2<br>(8.8 to 17.1)	13.7<br>(10.1 to 18.5)	33.5<br>(17.2 to 55.2)	12.1<br>(3.2 to 22.1)	9.4<br>(6.4 to 13.2)	12.8<br>(9.5 to 17.6)	14.4<br>(10.7 to 19.0)	35.9<br>(19.8 to 58.7)	12.0<br>(1.5 to 24.3)
Low bone mineral density	3.6<br>(2.9 to 4.5)	3.6<br>(2.9 to 4.3)	3.5<br>(2.8 to 4.3)	-1.4<br>(-15.5 to 16.0)	-2.9<br>(-18.5 to 14.6)	6.0<br>(4.9 to 7.1)	5.9<br>(5.0 to 6.9)	5.8<br>(4.8 to 6.9)	-0.9<br>(-15.0 to 14.0)	-2.6<br>(-15.6 to 11.8)
Impaired kidney function	2.3<br>(1.0 to 6.1)	2.3<br>(1.0 to 6.0)	2.2<br>(1.0 to 5.9)	-2.7<br>(-7.2 to 1.2)	-1.7<br>(-6.6 to 2.9)	3.0<br>(1.4 to 6.9)	3.0<br>(1.4 to 7.0)	3.0<br>(1.5 to 7.1)	1.7<br>(-2.3 to 6.2)	0.9<br>(-2.4 to 4.6)

Supplementary Results Table 1. Slovakia age-standardised Summary Exposure Values (SEVs) for Level 4 risk factors and percentage change, by sex, 1990, 2007, and 2017.Data in parentheses are 95% uncertainty intervals.										
	Male					Female				
	1990	2007	2017	Percentage change 1990-2007	Percentage change 2007-2017	1990	2007	2017	Percentage change 1990-2007	Percentage change 2007-2017
Unsafe water source	8.6 (5.7 to 12.2)	3.5 (2.4 to 5.0)	3.1 (2.0 to 4.5)	-59.2 (-74.6 to -33.9)	-12.8 (-38.5 to 22.5)	8.6 (5.7 to 12.2)	3.5 (2.4 to 5.0)	3.1 (2.0 to 4.5)	-59.2 (-74.6 to -33.9)	-12.8 (-38.5 to 22.5)
Unsafe sanitation	8.2 (4.8 to 13.8)	2.3 (1.3 to 3.8)	1.7 (0.9 to 2.8)	-72.0 (-84.2 to -50.2)	-26.3 (-52.3 to 15.4)	8.2 (4.8 to 13.8)	2.3 (1.3 to 3.8)	1.7 (0.9 to 2.8)	-72.0 (-84.2 to -50.2)	-26.3 (-52.3 to 15.4)
No access to handwashing facility	3.9 (3.1 to 4.9)	2.8 (2.2 to 3.5)	2.7 (2.1 to 3.4)	-28.7 (-45.2 to -9.1)	-2.4 (-17.4 to 15.7)	3.9 (3.1 to 4.9)	2.8 (2.2 to 3.5)	2.7 (2.1 to 3.4)	-28.7 (-45.2 to -9.1)	-2.4 (-17.4 to 15.7)
Ambient particulate matter pollution	44.5 (31.8 to 61.8)	43.0 (31.0 to 58.8)	37.7 (26.6 to 53.7)	-3.4 (-25.1 to 21.9)	-12.3 (-32.2 to 8.7)	43.0 (30.2 to 60.4)	42.2 (30.4 to 57.9)	37.0 (25.8 to 52.9)	-1.9 (-24.4 to 26.3)	-12.2 (-32.6 to 10.0)
Household air pollution from solid fuels	4.1 (1.9 to 9.3)	2.1 (0.9 to 5.4)	1.7 (0.7 to 4.3)	-49.2 (-76.8 to 1.3)	-16.5 (-59.6 to 74.5)	5.1 (2.3 to 10.9)	2.7 (1.0 to 8.5)	2.3 (0.9 to 8.3)	-46.8 (-76.0 to 1.3)	-15.0 (-58.9 to 71.8)
Ambient ozone pollution	47.7 (18.8 to 54.8)	48.0 (19.0 to 54.8)	48.5 (19.5 to 54.8)	0.5 (0.0 to 1.5)	1.0 (0.0 to 3.1)	47.7 (18.8 to 54.8)	48.0 (19.0 to 54.8)	48.5 (19.5 to 54.8)	0.5 (0.0 to 1.5)	1.0 (0.0 to 3.1)
Residential radon	42.4 (12.2 to 71.2)	42.4 (12.2 to 71.2)	42.4 (12.2 to 71.2)	0.0 (0.0 to 0.0)	0.0 (0.0 to 0.0)	42.4 (12.2 to 71.2)	42.4 (12.2 to 71.2)	42.4 (12.2 to 71.2)	0.0 (0.0 to 0.0)	0.0 (0.0 to 0.0)
Lead exposure	3.7 (0.7 to 7.6)	3.5 (0.7 to 7.2)	2.7 (0.5 to 6.0)	-5.1 (-11.8 to 15.8)	-22.9 (-33.9 to -15.9)	2.0 (0.1 to 5.3)	1.9 (0.1 to 5.0)	1.5 (0.1 to 4.3)	-4.2 (-10.9 to 28.8)	-22.4 (-36.7 to -15.2)
Occupational exposure to asbestos	1.2 (0.9 to 1.5)	1.1 (1.0 to 1.3)	1.0 (0.8 to 1.1)	-7.4 (-30.4 to 21.3)	-12.5 (-28.5 to 6.3)	0.5 (0.4 to 0.7)	0.4 (0.3 to 0.4)	0.4 (0.3 to 0.4)	-31.6 (-47.6 to -7.8)	-3.2 (-23.6 to 20.7)
Occupational exposure to arsenic	0.3 (0.0 to 0.8)	0.3 (0.0 to 0.8)	0.3 (0.0 to 0.8)	-	-	0.4 (0.0 to 0.9)	0.3 (0.0 to 0.7)	0.3 (0.0 to 0.8)	-	-
Occupational exposure to benzene	0.4 (0.0 to 1.3)	0.5 (0.0 to 1.3)	0.5 (0.0 to 1.4)	2.7 (-1.7 to 8.5)	4.8 (1.0 to 10.7)	0.6 (0.0 to 1.8)	0.7 (0.0 to 2.0)	0.8 (0.0 to 2.2)	12.3 (5.9 to 22.4)	8.0 (2.8 to 14.8)
Occupational exposure to beryllium	0.0 (0.0 to 0.0)	0.0 (0.0 to 0.0)	0.0 (0.0 to 0.0)	-1.9 (-4.4 to 0.8)	4.3 (2.0 to 7.0)	0.0 (0.0 to 0.0)	0.0 (0.0 to 0.0)	0.0 (0.0 to 0.0)	0.0 (-3.2 to 3.4)	5.8 (3.2 to 8.5)
Occupational exposure to cadmium	0.0 (0.0 to 0.0)	0.0 (0.0 to 0.0)	0.0 (0.0 to 0.0)	-0.2 (-7.0 to 7.6)	4.1 (-2.2 to 12.0)	0.0 (0.0 to 0.0)	0.0 (0.0 to 0.0)	0.0 (0.0 to 0.0)	-11.4 (-19.5 to -2.5)	1.7 (-6.3 to 10.9)
Occupational exposure to chromium	0.1 (0.1 to 0.1)	0.1 (0.1 to 0.1)	0.1 (0.1 to 0.1)	1.4 (-5.0 to 8.5)	4.5 (-1.2 to 11.3)	0.1 (0.1 to 0.1)	0.1 (0.1 to 0.1)	0.1 (0.1 to 0.1)	-7.8 (-15.0 to -0.2)	2.9 (-3.3 to 10.4)
Occupational exposure to diesel engine exhaust	0.4 (0.4 to 0.4)	0.4 (0.4 to 0.4)	0.4 (0.4 to 0.5)	-0.0 (-5.8 to 6.1)	4.6 (-0.4 to 9.7)	0.4 (0.3 to 0.4)	0.4 (0.4 to 0.4)	0.4 (0.4 to 0.4)	4.9 (-2.2 to 12.7)	7.3 (0.8 to 13.9)
Occupational exposure to formaldehyde	0.2 (0.2 to 0.2)	0.2 (0.2 to 0.2)	0.2 (0.2 to 0.2)	-1.2 (-8.6 to 7.1)	3.0 (-3.6 to 11.1)	0.2 (0.2 to 0.2)	0.2 (0.2 to 0.2)	0.2 (0.2 to 0.2)	-10.2 (-18.1 to -1.7)	1.7 (-5.8 to 10.2)
Occupational exposure to nickel	0.4 (0.0 to 1.6)	0.3 (0.0 to 1.5)	0.3 (0.0 to 1.5)	-	-	0.3 (0.0 to 1.3)	0.3 (0.0 to 1.1)	0.2 (0.0 to 1.1)	-	-
Occupational exposure to polycyclic aromatic hydrocarbons	0.2 (0.2 to 0.2)	0.2 (0.2 to 0.2)	0.2 (0.2 to 0.2)	0.4 (-5.1 to 6.8)	4.2 (-0.7 to 10.3)	0.2 (0.2 to 0.2)	0.2 (0.2 to 0.2)	0.2 (0.2 to 0.2)	-6.1 (-12.6 to 0.8)	3.5 (-2.1 to 10.0)
Occupational exposure to silica	4.3 (0.6 to 14.2)	4.1 (0.6 to 13.2)	4.2 (0.6 to 13.7)	-6.6 (-12.8 to 0.6)	3.1 (-1.9 to 9.8)	2.7 (0.4 to 8.3)	2.2 (0.3 to 6.7)	2.2 (0.3 to 6.7)	-18.1 (-26.4 to -9.1)	-0.3 (-10.1 to 11.2)
Occupational exposure to sulphuric acid	0.5 (0.1 to 1.7)	0.5 (0.1 to 1.6)	0.5 (0.1 to 1.6)	-5.5 (-11.3 to 1.3)	0.9 (-4.7 to 7.8)	0.5 (0.1 to 1.5)	0.4 (0.1 to 1.3)	0.4 (0.1 to 1.3)	-15.0 (-22.5 to -6.9)	-0.2 (-8.4 to 9.1)
Occupational exposure to trichloroethylene	0.0 (0.0 to 0.0)	0.0 (0.0 to 0.0)	0.0 (0.0 to 0.1)	3.2 (-1.8 to 9.0)	4.8 (0.7 to 10.1)	0.1 (0.1 to 0.1)	0.1 (0.0 to 0.1)	0.1 (0.1 to 0.1)	-3.4 (-8.7 to 2.6)	4.4 (-0.5 to 9.9)
Occupational asthmagens	14.7 (12.6 to 17.4)	13.5 (11.5 to 16.0)	13.8 (11.8 to 16.4)	-8.3 (-14.3 to -1.9)	2.3 (-3.3 to 8.5)	7.8 (6.5 to 9.4)	7.3 (6.1 to 8.6)	7.3 (6.2 to 8.9)	-6.9 (-14.5 to 0.9)	1.2 (-5.5 to 8.4)
Occupational particulate matter, gases, and fumes	6.9 (4.9 to 10.0)	6.4 (4.6 to 9.2)	6.1 (4.4 to 8.8)	-6.9 (-10.9 to -3.5)	-5.2 (-7.3 to -3.1)	3.8 (2.6 to 5.6)	3.4 (2.3 to 4.9)	3.0 (2.1 to 4.3)	-10.5 (-16.4 to -4.6)	-11.7 (-15.8 to -8.0)
Occupational noise	6.3 (5.9 to 6.7)	5.9 (5.6 to 6.3)	5.6 (5.3 to 6.0)	-6.1 (-8.2 to -4.0)	-4.7 (-5.7 to -3.7)	3.8 (3.5 to 4.2)	3.7 (3.5 to 4.0)	3.5 (3.3 to 3.9)	-3.6 (-7.3 to 0.3)	-5.4 (-7.0 to -3.8)
Occupational ergonomic factors	8.0 (6.8 to 9.5)	6.9 (5.8 to 8.3)	7.1 (5.9 to 8.4)	-13.6 (-18.0 to -9.4)	2.0 (-1.9 to 6.0)	5.1 (4.3 to 6.2)	4.7 (3.9 to 5.6)	4.8 (4.0 to 5.8)	-8.8 (-16.2 to -1.8)	3.4 (-2.7 to 9.9)
Non-exclusive breastfeeding	0.4 (0.3 to 0.5)	0.4 (0.3 to 0.5)	0.3 (0.3 to 0.5)	-0.1 (-8.0 to 9.2)	-0.7 (-5.8 to 5.7)	0.4 (0.3 to 0.5)	0.4 (0.3 to 0.5)	0.3 (0.3 to 0.5)	-0.1 (-8.0 to 9.2)	-0.7 (-5.8 to 5.7)
Discontinued breastfeeding	1.7 (1.6 to 1.8)	1.9 (1.8 to 2.0)	1.9 (1.8 to 2.0)	11.5 (4.8 to 18.3)	-1.1 (-5.3 to 3.5)	1.7 (1.6 to 1.8)	1.9 (1.8 to 2.0)	1.9 (1.8 to 2.0)	11.5 (4.8 to 18.4)	-1.1 (-5.3 to 3.5)
Child underweight	0.1 (0.1 to 0.2)	0.1 (0.1 to 0.2)	0.1 (0.1 to 0.2)	-21.6 (-33.2 to -9.9)	-7.2 (-15.0 to 0.5)	0.1 (0.1 to 0.2)	0.1 (0.1 to 0.1)	0.1 (0.0 to 0.1)	-28.0 (-39.3 to -16.2)	-8.1 (-16.0 to 0.9)
Child wasting	0.2 (0.1 to 0.3)	0.2 (0.1 to 0.2)	0.1 (0.1 to 0.2)	-16.1 (-41.5 to 18.3)	-6.1 (-25.8 to 13.2)	0.2 (0.1 to 0.2)	0.1 (0.1 to 0.2)	0.1 (0.1 to 0.2)	-18.4 (-40.8 to 11.3)	-5.3 (-21.3 to 14.4)
Child stunting	0.7 (0.3 to 1.2)	0.6 (0.3 to 0.9)	0.5 (0.2 to 0.8)	-25.4 (-54.3 to 27.3)	-12.0 (-38.9 to 26.5)	0.7 (0.3 to 1.2)	0.5 (0.2 to 0.8)	0.4 (0.2 to 0.8)	-29.0 (-59.6 to 20.2)	-9.1 (-39.3 to 28.7)
Short gestation for birth weight	0.0 (0.0 to 0.0)	0.0 (0.0 to 0.0)	0.0 (0.0 to 0.0)	11.0 (-9.7 to 44.2)	2.7 (-19.1 to 33.2)	0.0 (0.0 to 0.0)	0.0 (0.0 to 0.0)	0.0 (0.0 to 0.0)	6.6 (-13.9 to 31.5)	3.0 (-18.2 to 28.4)
Low birth weight for gestation	0.0 (0.0 to 0.0)	0.0 (0.0 to 0.0)	0.0 (0.0 to 0.0)	2.9 (-11.8 to 20.4)	-0.3 (-19.5 to 17.5)	0.0 (0.0 to 0.0)	0.0 (0.0 to 0.0)	0.0 (0.0 to 0.0)	1.8 (-11.6 to 19.0)	-0.7 (-16.5 to 18.2)
Iron deficiency	-	-	-	-	-	6.1 (4.3 to 8.4)	4.9 (3.3 to 6.9)	4.3 (2.9 to 6.1)	-19.9 (-35.4 to -2.5)	-11.3 (-25.1 to 4.8)
Vitamin A deficiency	2.8 (2.2 to 3.4)	2.2 (1.8 to 2.7)	2.1 (1.7 to 2.5)	-19.8 (-29.1 to -10.0)	-6.0 (-17.7 to 4.6)	2.8 (2.3 to 3.4)	2.3 (1.9 to 2.8)	2.2 (1.9 to 2.6)	-18.4 (-27.9 to -8.4)	-4.5 (-13.5 to 6.2)
Zinc deficiency	0.5 (0.1 to 1.2)	0.7 (0.2 to 1.5)	0.6 (0.2 to 1.4)	33.5 (4.9 to 85.2)	-9.0 (-28.3 to 19.9)	0.5 (0.2 to 1.1)	0.7 (0.2 to 1.5)	0.6 (0.2 to 1.4)	33.7 (6.7 to 76.3)	-9.3 (-28.3 to 20.4)
Smoking	16.0 (14.2 to 17.9)	12.8 (11.3 to 14.4)	10.3 (9.1 to 11.7)	-20.1 (-25.7 to -13.9)	-19.2 (-24.0 to -13.9)	7.5 (6.0 to 9.2)	6.0 (5.1 to 7.2)	5.0 (4.3 to 5.9)	-19.8 (-33.0 to -3.8)	-16.3 (-26.4 to -4.7)
Chewing tobacco	0.1 (0.1 to 0.2)	0.1 (0.1 to 0.2)	0.1 (0.1 to 0.2)	0.2 (-30.0 to 45.2)	-4.6 (-26.7 to 29.2)	0.1 (0.0 to 0.1)	0.1 (0.0 to 0.1)	0.1 (0.0 to 0.1)	0.7 (-28.9 to 41.9)	-0.2 (-21.9 to 29.4)
Second-hand smoke	46.6 (43.3 to 49.9)	37.4 (35.6 to 39.1)	37.1 (34.3 to 39.7)	-19.6 (-26.0 to -12.2)	-0.9 (-6.6 to 4.9)	58.8 (55.8 to 61.7)	41.6 (40.1 to 43.0)	40.6 (38.3 to 42.9)	-29.3 (-33.9 to -24.5)	-2.2 (-6.6 to 1.8)
Alcohol use	32.4 (22.9 to 42.8)	30.8 (21.1 to 41.8)	31.4 (19.5 to 44.8)	-5.0 (-19.9 to 9.4)	2.2 (-19.7 to 25.1)	11.9 (6.5 to 21.0)	11.3 (6.0 to 20.4)	12.1 (6.2 to 21.4)	-5.2 (-19.5 to 6.7)	6.8 (-14.6 to 33.0)
Drug use	0.4 (0.3 to 0.6)	0.5 (0.4 to 0.7)	0.4 (0.3 to 0.7)	7.1 (-6.5 to 22.3)	-6.3 (-19.1 to 8.2)	0.2 (0.2 to 0.4)	0.2 (0.2 to 0.4)	0.2 (0.2 to 0.4)	5.9 (-8.9 to 23.5)	-4.2 (-18.2 to 11.6)
Diet low in fruits	40.3 (35.0 to 43.6)	39.5 (34.2 to 42.6)	36.5 (30.9 to 40.2)	-2.0 (-5.3 to 1.7)	-7.6 (-12.5 to -3.9)	38.3 (32.8 to 41.8)	37.4 (31.8 to 40.9)	34.0 (28.3 to 38.2)	-2.3 (-6.4 to 1.7)	-9.2 (-14.9 to -4.9)
Diet low in vegetables	32.7 (27.8 to 36.8)	29.2 (24.2 to 33.7)	24.2 (17.5 to 29.6)	-10.6 (-17.8 to -5.1)	-17.3 (-29.2 to -8.8)	34.5 (30.0 to 38.4)	30.9 (25.8 to 34.9)	26.0 (19.7 to 31.3)	-10.6 (-17.4 to -5.0)	-15.9 (-26.1 to -8.5)
Diet low in legumes	30.5 (26.3 to 34.1)	41.3 (38.9 to 43.5)	42.3 (39.9 to 44.3)	35.6 (26.2 to 49.2)	2.4 (0.3 to 4.8)	34.3 (30.7 to 37.5)	44.1 (42.0 to 45.7)	44.9 (42.9 to 46.7)	28.5 (21.2 to 38.2)	2.0 (0.3 to 3.8)
Diet low in whole grains	42.5 (40.4 to 44.2)	43.4 (41.5 to 45.0)	43.7 (41.8 to 45.3)	2.2 (0.7 to 4.1)	0.6 (-1.0 to 2.2)	43.3 (41.3 to 44.9)	44.1 (42.4 to 45.6)	44.4 (42.6 to 45.9)	2.0 (0.4 to 3.7)	0.6 (-0.8 to 1.9)
Diet low in nuts and seeds	49.7 (48.4 to 50.8)	49.0 (47.5 to 50.2)	46.3 (44.0 to 48.2)	-1.5 (-3.3 to 0.2)	-5.4 (-8.2 to -3.2)	50.0 (48.8 to 51.1)	49.3 (47.8 to 50.4)	46.8 (44.5 to 48.6)	-1.5 (-3.2 to 0.3)	-5.1 (-7.6 to -3.1)
Diet low in milk	39.1 (35.6 to 42.0)	41.1 (38.3 to 43.6)	39.1 (35.7 to 41.9)	5.2 (2.7 to 8.5)	-5.1 (-7.7 to -3.0)	39.9 (36.7 to 42.7)	41.7 (38.9 to 44.0)	39.6 (36.2 to 42.3)	4.4 (2.1 to 7.3)	-5.0 (-7.6 to -3.0)
Diet high in red meat	37.1 (31.5 to 42.4)	24.8 (20.9 to 28.3)	31.2 (24.6 to 36.7)	-33.2 (-44.1 to -21.5)	25.7 (5.9 to 49.5)	15.8 (9.3 to 21.4)	8.2 (4.2 to 12.1)	11.3 (5.7 to 16.3)	-47.9 (-69.9 to -24.1)	37.1 (-2.9 to 117.0)
Diet high in processed meat	4.6 (1.9 to 7.5)	5.4 (2.5 to 8.3)	7.7 (4.6 to 10.8)	18.3 (-4.0 to 70.0)	42.2 (19.4 to 104.2)	2.9 (0.7 to 5.6)	3.5 (1.0 to 6.2)	5.2 (2.3 to 8.1)	19.3 (-7.9 to 91.4)	48.3 (20.8 to 148.9)
Diet high in sugar-sweetened beverages	8.1 (3.8 to 12.7)	8.8 (4.5 to 10.8)	12.3 (6.3 to 16.1)	9.2 (-31.3 to 72.6)	39.9 (6.9 to 81.0)	5.6 (2.5 to 9.4)	6.1 (3.0 to 7.7)	8.4 (4.2 to 11.4)	8.8 (-33.1 to 84.1)	37.9 (3.0 to 86.0)
Diet low in fibre	31.7 (26.7 to 35.9)	38.3 (34.6 to 41.2)	37.9 (33.8 to 41.2)	20.9 (13.9 to 32.1)	-0.9 (-4.1 to 2.3)	36.7 (32.6 to 40.0)	42.3 (39.4 to 44.5)	42.0 (38.9 to 44.6)	15.1 (10.1 to 22.6)	-0.7 (-3.1 to 1.9)
Diet low in calcium	31.1 (26.2 to 35.0)	33.6 (29.3 to 37.0)	31.1 (26.2 to 35.1)	8.1 (4.9 to 13.1)	-7.4 (-11.5 to -4.7)	34.1 (29.9 to 37.5)	36.2 (32.3 to 39.2)	34.0 (29.7 to 37.5)	6.1 (3.6 to 9.7)	-6.1 (-9.3 to -3.8)
Diet low in seafood omega-3 fatty acids	40.3 (37.6 to 42.9)	39.8 (36.9 to 42.5)	39.0 (36.0 to 41.9)	-1.2 (-2.3 to -0.3)	-2.1 (-4.0 to -0.6)	42.4 (39.9 to 44.7)	41.9 (39.4 to 44.3)	41.2 (38.5 to 43.7)	-1.0 (-1.8 to -0.2)	-1.8 (-3.2 to -0.6)
Diet low in polyunsaturated fatty acids	41.8 (39.1 to 44.3)	31.5 (27.2 to 35.3)	28.9 (24.0 to 33.2)	-24.4 (-31.6 to -18.0)	-8.2 (-14.2 to -0.4)	42.1 (39.5 to 44.6)	32.2 (27.9 to 35.9)	29.6 (24.6 to 33.8)	-23.6 (-30.8 to -17.7)	-8.0 (-14.3 to 1.6)
Diet high in trans fatty acids	11.8 (7.3 to 16.6)	5.7 (2.4 to 9.5)	3.4 (0.9 to 6.7)	-51.9 (-71.8 to -35.7)	-39.4 (-66.5 to -23.1)	16.1 (11.1 to 21.9)	7.6 (3.7 to 12.1)	4.7 (1.7 to 8.4)	-52.7 (-70.7 to -36.8)	-38.0 (-60.3 to -21.6)
Diet high in sodium	25.0 (16.9 to 31.6)	12.9 (4.5 to 20.4)	13.9 (5.6 to 21.2)	-48.3 (-76.0 to -29.9)	7.7 (-7.8 to 47.0)	18.4 (11.6 to 24.9)	6.9 (0.8 to 14.2)	5.5 (0.3 to 12.6)	-62.4 (-93.6 to -40.1)	-20.1 (-74.9 to -6.0)
Intimate partner violence	-	-	-	-	-	3.8 (3.2 to 4.5)	3.5 (3.0 to 4.0)	3.3 (2.8 to 3.8)	-9.1 (-13.7 to -4.9)	-6.9 (-11.1 to -2.1)
Childhood sexual abuse	8.7 (7.1 to 10.8)	8.7 (7.1 to 10.8)	8.7 (7.1 to 10.8)	0.0 (0.0 to 0.0)	0.0 (0.0 to 0.0)	4.9 (3.6 to 6.5)	4.9 (3.6 to 6.5)	4.9 (3.6 to 6.5)	0.0 (0.0 to 0.0)	0.0 (0.0 to 0.0)

Supplementary Results Table 1. Slovakia age-standardised Summary Exposure Values (SEVs) for Level 4 risk factors and percentage change, by sex, 1990, 2007, and 2017.Data in parentheses are 95% uncertainty intervals.										
	Male					Female				
	1990	2007	2017	Percentage change 1990-2007	Percentage change 2007-2017	1990	2007	2017	Percentage change 1990-2007	Percentage change 2007-2017
Bullying victimization	2.4 (1.0 to 4.8)	3.1 (1.4 to 6.1)	3.9 (1.6 to 8.0)	31.2 (11.8 to 98.0)	24.1 (6.2 to 54.2)	1.9 (0.8 to 4.0)	2.5 (1.1 to 5.0)	3.1 (1.3 to 6.4)	30.0 (10.9 to 98.9)	23.3 (8.3 to 47.1)
Low physical activity	0.3 (0.2 to 0.6)	0.3 (0.2 to 0.6)	0.3 (0.2 to 0.6)	-1.0 (-2.7 to 0.6)	1.6 (0.0 to 3.5)	0.3 (0.1 to 0.6)	0.3 (0.2 to 0.6)	0.3 (0.1 to 0.6)	0.4 (-1.4 to 2.6)	0.1 (-1.7 to 2.1)
High fasting plasma glucose	2.0 (1.1 to 3.2)	2.2 (1.3 to 3.4)	1.9 (1.1 to 3.1)	10.8 (-33.7 to 85.0)	-12.1 (-48.6 to 37.4)	1.5 (0.8 to 2.5)	1.5 (0.9 to 2.3)	1.5 (0.9 to 2.5)	0.1 (-36.3 to 65.5)	3.5 (-36.3 to 63.4)
High LDL cholesterol	14.3 (12.6 to 16.1)	14.2 (12.5 to 15.9)	14.1 (12.5 to 15.9)	-0.7 (-5.2 to 3.8)	-0.3 (-3.7 to 3.0)	14.1 (12.5 to 15.8)	14.3 (12.7 to 16.0)	14.3 (12.6 to 16.1)	1.1 (-3.1 to 5.6)	0.1 (-2.9 to 3.5)
High systolic blood pressure	11.5 (10.5 to 12.5)	11.3 (10.3 to 12.4)	10.8 (9.8 to 11.9)	-1.5 (-8.2 to 5.2)	-4.0 (-9.5 to 1.3)	10.4 (9.4 to 11.4)	10.2 (9.2 to 11.3)	9.2 (8.3 to 10.2)	-1.6 (-9.6 to 7.5)	-9.3 (-16.0 to -2.6)
High body-mass index	9.6 (6.8 to 13.7)	13.4 (9.8 to 18.6)	16.3 (12.2 to 22.3)	40.0 (25.0 to 60.9)	22.0 (11.7 to 34.0)	10.2 (7.5 to 13.9)	12.7 (9.7 to 16.8)	14.5 (11.2 to 19.0)	23.9 (9.1 to 42.5)	14.6 (4.3 to 27.2)
Low bone mineral density	3.8 (2.3 to 5.4)	3.6 (2.1 to 5.3)	3.5 (2.0 to 5.1)	-6.0 (-27.1 to 20.4)	-1.3 (-23.0 to 25.8)	4.9 (4.1 to 5.9)	4.7 (3.9 to 5.6)	4.6 (3.8 to 5.6)	-4.3 (-18.4 to 10.4)	-1.6 (-16.2 to 15.0)
Impaired kidney function	2.1 (0.9 to 5.8)	2.1 (0.9 to 5.6)	2.0 (0.9 to 5.6)	-3.4 (-8.1 to 0.4)	-0.9 (-5.6 to 3.5)	2.8 (1.3 to 6.7)	2.8 (1.3 to 6.6)	2.8 (1.3 to 6.7)	-0.8 (-4.7 to 3.5)	-0.1 (-4.4 to 3.7)

Supplementary Results Table 1. Slovenia age-standardised Summary Exposure Values (SEVs) for Level 4 risk factors and percentage change, by sex, 1990, 2007, and 2017.Data in parentheses are 95% uncertainty intervals.										
	Male					Female				
	1990	2007	2017	Percentage change 1990-2007	Percentage change 2007-2017	1990	2007	2017	Percentage change 1990-2007	Percentage change 2007-2017
Unsafe water source	5.3 (3.6 to 7.7)	2.6 (1.8 to 3.7)	2.4 (1.7 to 3.4)	-50.1 (-68.6 to -23.9)	-7.5 (-35.5 to 32.5)	5.3 (3.6 to 7.7)	2.6 (1.8 to 3.7)	2.4 (1.7 to 3.4)	-50.1 (-68.6 to -23.9)	-7.5 (-35.5 to 32.5)
Unsafe sanitation	8.0 (4.8 to 13.0)	3.0 (1.7 to 5.0)	2.4 (1.3 to 3.9)	-62.4 (-78.5 to -33.8)	-21.6 (-51.3 to 21.7)	8.0 (4.8 to 13.0)	3.0 (1.7 to 5.0)	2.4 (1.3 to 3.9)	-62.4 (-78.5 to -33.8)	-21.6 (-51.3 to 21.7)
No access to handwashing facility	3.8 (3.0 to 4.8)	3.0 (2.4 to 3.8)	2.8 (2.2 to 3.5)	-21.0 (-38.8 to 1.7)	-7.4 (-22.0 to 10.6)	3.8 (3.0 to 4.8)	3.0 (2.4 to 3.8)	2.8 (2.2 to 3.5)	-21.0 (-38.8 to 1.7)	-7.4 (-22.0 to 10.6)
Ambient particulate matter pollution	37.0 (26.1 to 51.4)	38.0 (27.6 to 52.0)	34.5 (23.9 to 48.1)	2.7 (-20.7 to 35.2)	-9.2 (-30.8 to 12.4)	34.2 (23.2 to 48.5)	36.5 (26.1 to 50.7)	33.5 (22.6 to 47.4)	6.9 (-20.0 to 47.2)	-8.4 (-31.9 to 15.9)
Household air pollution from solid fuels	10.6 (5.4 to 17.9)	4.4 (2.0 to 9.7)	3.0 (1.3 to 8.3)	-58.5 (-80.0 to -22.0)	-30.5 (-66.3 to 43.6)	12.4 (6.7 to 20.1)	5.5 (2.5 to 11.4)	3.9 (1.6 to 9.4)	-55.8 (-78.3 to -20.0)	-28.7 (-64.9 to 39.7)
Ambient ozone pollution	48.4 (19.5 to 54.8)	48.6 (19.7 to 54.8)	48.9 (20.1 to 54.8)	0.3 (0.0 to 1.4)	0.7 (0.0 to 2.3)	48.4 (19.5 to 54.8)	48.6 (19.7 to 54.8)	48.9 (20.1 to 54.8)	0.3 (0.0 to 1.4)	0.7 (0.0 to 2.3)
Residential radon	38.6 (10.1 to 71.2)	38.6 (10.1 to 71.2)	38.6 (10.1 to 71.2)	0.0 (0.0 to 0.0)	0.0 (0.0 to 0.0)	38.6 (10.1 to 71.2)	38.6 (10.1 to 71.2)	38.6 (10.1 to 71.2)	0.0 (0.0 to 0.0)	0.0 (0.0 to 0.0)
Lead exposure	3.2 (0.5 to 7.0)	3.0 (0.5 to 6.5)	2.3 (0.4 to 5.3)	-6.5 (-13.5 to 14.7)	-25.2 (-37.3 to -17.7)	1.7 (0.1 to 5.0)	1.7 (0.1 to 4.8)	1.3 (0.1 to 4.0)	-4.0 (-10.7 to 30.3)	-24.8 (-40.8 to -17.2)
Occupational exposure to asbestos	3.2 (2.6 to 3.8)	5.9 (5.5 to 6.4)	5.0 (4.4 to 5.7)	87.7 (54.7 to 130.3)	-15.1 (-27.1 to -1.3)	1.2 (0.9 to 1.5)	1.7 (1.6 to 1.9)	1.4 (1.2 to 1.7)	47.7 (13.3 to 89.8)	-16.0 (-29.7 to -1.8)
Occupational exposure to arsenic	0.4 (0.0 to 0.9)	0.4 (0.0 to 0.9)	0.4 (0.0 to 0.9)	-	-	0.4 (0.0 to 1.0)	0.4 (0.0 to 1.0)	0.4 (0.0 to 0.9)	-	-
Occupational exposure to benzene	0.4 (0.0 to 1.2)	0.5 (0.0 to 1.4)	0.5 (0.0 to 1.4)	15.1 (9.5 to 22.7)	0.1 (-3.7 to 5.5)	0.6 (0.0 to 1.8)	0.8 (0.0 to 2.2)	0.8 (0.0 to 2.3)	25.4 (17.1 to 39.6)	1.8 (-3.4 to 9.6)
Occupational exposure to beryllium	0.0 (0.0 to 0.0)	0.0 (0.0 to 0.0)	0.0 (0.0 to 0.0)	7.5 (3.3 to 11.9)	-0.6 (-3.0 to 1.8)	0.0 (0.0 to 0.0)	0.0 (0.0 to 0.0)	0.0 (0.0 to 0.0)	9.3 (4.2 to 14.7)	-1.0 (-3.8 to 2.0)
Occupational exposure to cadmium	0.0 (0.0 to 0.0)	0.0 (0.0 to 0.0)	0.0 (0.0 to 0.0)	6.9 (-1.4 to 15.2)	-1.2 (-5.7 to 3.3)	0.0 (0.0 to 0.1)	0.0 (0.0 to 0.0)	0.0 (0.0 to 0.0)	-1.3 (-10.9 to 10.1)	-8.2 (-14.2 to -1.6)
Occupational exposure to chromium	0.1 (0.1 to 0.1)	0.1 (0.1 to 0.1)	0.1 (0.1 to 0.1)	8.3 (0.9 to 15.8)	-1.1 (-5.2 to 3.1)	0.1 (0.1 to 0.1)	0.1 (0.1 to 0.1)	0.1 (0.1 to 0.1)	2.4 (-6.7 to 12.4)	-6.6 (-11.7 to -1.0)
Occupational exposure to diesel engine exhaust	0.4 (0.4 to 0.4)	0.4 (0.4 to 0.4)	0.4 (0.4 to 0.4)	9.1 (2.4 to 16.6)	-1.9 (-5.7 to 2.2)	0.3 (0.3 to 0.4)	0.4 (0.4 to 0.4)	0.4 (0.4 to 0.4)	15.7 (7.0 to 25.7)	4.0 (-2.5 to 10.1)
Occupational exposure to formaldehyde	0.2 (0.2 to 0.2)	0.2 (0.2 to 0.2)	0.2 (0.2 to 0.2)	5.8 (-2.6 to 14.7)	-2.4 (-6.9 to 2.2)	0.2 (0.2 to 0.3)	0.2 (0.2 to 0.2)	0.2 (0.2 to 0.2)	0.3 (-9.5 to 11.5)	-8.5 (-13.9 to -2.4)
Occupational exposure to nickel	0.3 (0.0 to 1.5)	0.4 (0.0 to 1.6)	0.4 (0.0 to 1.5)	-	-	0.3 (0.0 to 1.6)	0.3 (0.0 to 1.4)	0.3 (0.0 to 1.3)	-	-
Occupational exposure to polycyclic aromatic hydrocarbons	0.2 (0.2 to 0.2)	0.2 (0.2 to 0.2)	0.2 (0.2 to 0.2)	8.6 (1.8 to 15.7)	-0.9 (-4.7 to 2.8)	0.2 (0.2 to 0.2)	0.2 (0.2 to 0.2)	0.2 (0.2 to 0.2)	4.5 (-4.1 to 13.6)	-5.8 (-10.3 to -0.9)
Occupational exposure to silica	3.7 (0.5 to 11.7)	3.8 (0.5 to 12.7)	3.8 (0.5 to 12.4)	4.0 (-3.3 to 13.0)	-1.3 (-6.5 to 4.4)	3.0 (0.4 to 9.9)	2.7 (0.4 to 8.5)	2.5 (0.4 to 7.8)	-8.0 (-17.4 to 5.0)	-9.5 (-16.6 to -1.5)
Occupational exposure to sulphuric acid	0.5 (0.1 to 1.6)	0.5 (0.1 to 1.7)	0.5 (0.1 to 1.7)	3.0 (-4.1 to 10.5)	-2.7 (-7.3 to 1.9)	0.6 (0.1 to 1.8)	0.5 (0.1 to 1.6)	0.5 (0.1 to 1.5)	-4.4 (-13.4 to 6.3)	-8.9 (-15.2 to -2.0)
Occupational exposure to trichloroethylene	0.0 (0.0 to 0.1)	0.1 (0.1 to 0.1)	0.1 (0.1 to 0.1)	9.2 (3.0 to 15.6)	-1.3 (-4.8 to 2.2)	0.1 (0.1 to 0.1)	0.1 (0.1 to 0.1)	0.1 (0.1 to 0.1)	6.0 (-1.3 to 14.3)	-3.5 (-7.8 to 1.1)
Occupational asthmagens	16.7 (14.2 to 19.9)	18.2 (15.4 to 21.6)	17.2 (14.5 to 20.6)	9.0 (0.5 to 18.7)	-5.3 (-12.6 to 2.4)	9.6 (7.8 to 11.7)	11.0 (9.0 to 13.5)	9.4 (7.6 to 11.5)	14.9 (0.5 to 31.7)	-15.2 (-24.7 to -3.8)
Occupational particulate matter, gases, and fumes	6.7 (4.8 to 9.6)	6.5 (4.6 to 9.4)	6.3 (4.5 to 9.0)	-2.5 (-6.1 to 1.1)	-3.0 (-5.2 to -0.9)	4.4 (3.1 to 6.5)	4.2 (3.0 to 6.0)	3.8 (2.7 to 5.6)	-5.1 (-10.1 to 0.8)	-8.2 (-11.8 to -4.8)
Occupational noise	5.8 (5.3 to 6.3)	5.7 (5.3 to 6.1)	5.6 (5.3 to 6.0)	-2.0 (-4.6 to 0.9)	-1.9 (-3.3 to -0.4)	4.2 (3.8 to 4.6)	4.2 (3.9 to 4.6)	4.1 (3.9 to 4.5)	0.5 (-3.2 to 4.8)	-2.2 (-4.1 to -0.3)
Occupational ergonomic factors	9.5 (8.3 to 10.9)	8.8 (7.7 to 10.0)	7.9 (6.8 to 9.1)	-8.3 (-15.9 to 0.2)	-10.0 (-16.7 to -3.2)	7.5 (6.3 to 8.7)	7.0 (6.0 to 8.0)	6.1 (5.1 to 7.3)	-7.0 (-19.0 to 7.3)	-12.2 (-20.5 to -3.0)
Non-exclusive breastfeeding	0.4 (0.3 to 0.6)	0.5 (0.3 to 0.6)	0.5 (0.3 to 0.6)	7.4 (-1.5 to 17.4)	-2.7 (-8.9 to 3.6)	0.4 (0.3 to 0.6)	0.5 (0.3 to 0.6)	0.5 (0.3 to 0.6)	7.5 (-1.5 to 17.4)	-2.7 (-8.9 to 3.6)
Discontinued breastfeeding	1.8 (1.7 to 1.9)	2.0 (1.9 to 2.1)	1.9 (1.9 to 2.0)	11.9 (5.6 to 18.7)	-3.1 (-7.0 to 0.9)	1.8 (1.7 to 1.9)	2.0 (1.9 to 2.1)	1.9 (1.9 to 2.0)	11.9 (5.6 to 18.7)	-3.1 (-7.0 to 0.9)
Child underweight	0.1 (0.1 to 0.2)	0.1 (0.1 to 0.2)	0.1 (0.1 to 0.1)	-17.3 (-28.6 to -5.8)	-5.8 (-14.0 to 2.4)	0.1 (0.1 to 0.2)	0.1 (0.1 to 0.1)	0.1 (0.0 to 0.1)	-23.1 (-35.0 to -11.2)	-6.0 (-13.9 to 2.7)
Child wasting	0.2 (0.1 to 0.3)	0.2 (0.1 to 0.2)	0.1 (0.1 to 0.2)	-12.9 (-38.9 to 20.5)	-6.4 (-24.3 to 17.2)	0.2 (0.1 to 0.2)	0.1 (0.1 to 0.2)	0.1 (0.1 to 0.2)	-14.5 (-34.6 to 13.4)	-4.1 (-20.9 to 15.0)
Child stunting	0.7 (0.3 to 1.1)	0.5 (0.2 to 0.9)	0.5 (0.2 to 0.8)	-19.5 (-51.9 to 36.3)	-9.3 (-38.9 to 29.4)	0.6 (0.3 to 1.0)	0.5 (0.2 to 0.8)	0.4 (0.2 to 0.8)	-22.6 (-57.5 to 36.6)	-8.9 (-37.4 to 32.4)
Short gestation for birth weight	0.0 (0.0 to 0.0)	0.0 (0.0 to 0.0)	0.0 (0.0 to 0.0)	9.7 (-12.7 to 38.7)	3.6 (-13.9 to 28.6)	0.0 (0.0 to 0.0)	0.0 (0.0 to 0.0)	0.0 (0.0 to 0.0)	5.0 (-15.1 to 36.8)	3.9 (-15.2 to 34.4)
Low birth weight for gestation	0.0 (0.0 to 0.0)	0.0 (0.0 to 0.0)	0.0 (0.0 to 0.0)	2.4 (-14.7 to 20.2)	2.6 (-15.2 to 19.6)	0.0 (0.0 to 0.0)	0.0 (0.0 to 0.0)	0.0 (0.0 to 0.0)	2.2 (-15.7 to 20.8)	1.6 (-18.1 to 20.3)
Iron deficiency	-	-	-	-	-	5.0 (3.4 to 7.1)	4.5 (3.1 to 6.4)	4.2 (2.7 to 6.0)	-10.3 (-27.4 to 10.7)	-6.8 (-22.0 to 10.9)
Vitamin A deficiency	2.6 (2.1 to 3.1)	2.1 (1.8 to 2.5)	2.0 (1.6 to 2.4)	-18.6 (-29.0 to -8.0)	-6.0 (-17.4 to 5.9)	2.6 (2.2 to 3.1)	2.2 (1.8 to 2.6)	2.1 (1.7 to 2.5)	-16.0 (-26.4 to -6.1)	-5.5 (-14.8 to 4.9)
Zinc deficiency	0.4 (0.1 to 0.8)	0.6 (0.1 to 1.2)	0.5 (0.1 to 1.2)	60.5 (-47.9 to 304.6)	-6.5 (-70.4 to 195.4)	0.4 (0.1 to 0.8)	0.6 (0.1 to 1.2)	0.5 (0.1 to 1.2)	56.7 (-48.1 to 312.4)	-6.5 (-71.9 to 166.5)
Smoking	14.0 (12.4 to 15.8)	12.0 (10.7 to 13.4)	11.1 (10.0 to 12.4)	-13.8 (-21.9 to -5.0)	-7.5 (-14.3 to -0.1)	8.2 (6.6 to 10.0)	7.1 (6.0 to 8.4)	7.1 (6.1 to 8.2)	-12.8 (-25.9 to 4.9)	-0.4 (-11.7 to 12.7)
Chewing tobacco	0.1 (0.1 to 0.2)	0.1 (0.1 to 0.2)	0.1 (0.1 to 0.2)	-0.4 (-32.7 to 45.3)	-3.7 (-26.6 to 28.0)	0.1 (0.0 to 0.1)	0.1 (0.0 to 0.1)	0.1 (0.0 to 0.1)	0.4 (-27.2 to 41.9)	-0.5 (-22.4 to 28.5)
Second-hand smoke	46.9 (43.7 to 49.9)	38.6 (36.6 to 40.6)	36.6 (33.7 to 39.3)	-17.7 (-24.2 to -10.4)	-5.3 (-11.0 to 0.1)	55.2 (52.7 to 57.8)	39.5 (37.9 to 41.0)	37.8 (35.5 to 40.2)	-28.5 (-32.6 to -24.0)	-4.3 (-8.5 to -0.3)
Alcohol use	17.6 (9.8 to 28.1)	13.2 (6.2 to 22.9)	13.3 (3.3 to 26.1)	-25.1 (-54.6 to 1.7)	0.7 (-65.6 to 74.4)	8.7 (4.4 to 16.2)	6.3 (2.7 to 12.6)	5.8 (1.3 to 12.8)	-27.5 (-56.0 to 0.2)	-7.9 (-69.8 to 68.8)
Drug use	0.5 (0.4 to 0.8)	0.7 (0.5 to 0.8)	0.7 (0.6 to 0.9)	21.0 (11.5 to 33.1)	11.0 (1.9 to 21.4)	0.2 (0.2 to 0.3)	0.2 (0.2 to 0.3)	0.2 (0.2 to 0.3)	5.3 (-0.7 to 12.2)	5.1 (-1.1 to 12.1)
Diet low in fruits	31.9 (26.3 to 36.6)	23.6 (17.2 to 29.2)	23.3 (15.9 to 29.4)	-25.9 (-39.5 to -15.8)	-1.6 (-12.8 to 9.3)	29.2 (23.1 to 34.2)	19.8 (13.0 to 25.6)	19.7 (12.8 to 26.2)	-32.3 (-46.9 to -21.0)	-0.4 (-15.1 to 15.4)
Diet low in vegetables	37.5 (33.9 to 40.7)	33.4 (29.1 to 37.2)	31.8 (26.5 to 36.0)	-11.0 (-15.8 to -7.1)	-4.9 (-11.5 to 0.9)	39.0 (35.5 to 42.0)	35.4 (31.6 to 38.8)	33.7 (28.7 to 37.9)	-9.1 (-13.5 to -5.6)	-4.9 (-10.7 to -0.1)
Diet low in legumes	40.7 (38.0 to 42.9)	38.0 (35.0 to 40.5)	38.2 (35.0 to 41.0)	-6.7 (-9.5 to -4.4)	0.7 (-2.2 to 3.6)	43.0 (40.8 to 44.9)	40.7 (38.2 to 42.9)	40.9 (38.1 to 43.2)	-5.3 (-7.5 to -3.3)	0.4 (-2.1 to 2.9)
Diet low in whole grains	38.8 (36.1 to 41.0)	38.1 (35.4 to 40.5)	37.8 (34.8 to 40.3)	-1.7 (-4.0 to 0.6)	-0.8 (-3.2 to 1.7)	39.8 (37.3 to 42.0)	39.1 (36.6 to 41.4)	38.9 (36.1 to 41.1)	-1.6 (-3.7 to 0.6)	-0.7 (-3.1 to 1.5)
Diet low in nuts and seeds	47.4 (45.4 to 49.0)	44.6 (42.1 to 46.7)	43.0 (39.7 to 45.6)	-5.7 (-9.3 to -2.8)	-3.7 (-7.3 to -0.9)	47.8 (45.8 to 49.4)	45.2 (42.6 to 47.2)	43.6 (40.6 to 46.2)	-5.3 (-8.7 to -2.3)	-3.6 (-7.0 to -0.8)
Diet low in milk	32.0 (27.3 to 35.9)	32.1 (27.5 to 35.9)	32.1 (27.3 to 36.1)	0.3 (-4.3 to 5.2)	-0.1 (-4.0 to 3.8)	33.1 (28.6 to 36.8)	33.0 (28.4 to 36.7)	32.9 (28.3 to 36.8)	-0.5 (-4.3 to 3.9)	-0.3 (-3.7 to 2.9)
Diet high in red meat	39.6 (34.2 to 44.4)	38.7 (34.7 to 42.7)	36.7 (29.7 to 43.2)	-2.2 (-15.0 to 14.3)	-5.1 (-19.4 to 10.9)	18.7 (11.8 to 24.1)	17.4 (12.1 to 21.4)	17.2 (10.6 to 22.7)	-6.8 (-29.1 to 22.5)	-1.6 (-25.9 to 25.9)
Diet high in processed meat	6.1 (3.2 to 9.1)	7.4 (4.6 to 10.1)	8.1 (5.0 to 11.0)	21.1 (-1.5 to 61.1)	9.2 (-4.1 to 29.9)	4.0 (1.4 to 6.8)	4.9 (2.1 to 7.5)	5.4 (2.5 to 8.1)	22.5 (-0.9 to 79.8)	10.7 (-4.2 to 38.1)
Diet high in sugar-sweetened beverages	14.6 (6.9 to 23.0)	18.1 (8.9 to 27.9)	19.9 (10.2 to 29.7)	24.0 (-24.4 to 103.7)	10.2 (-21.6 to 56.1)	9.9 (4.6 to 17.6)	12.2 (5.6 to 20.0)	13.6 (6.0 to 22.0)	23.1 (-27.7 to 115.5)	10.9 (-24.7 to 65.2)
Diet low in fibre	30.6 (25.3 to 34.8)	27.4 (21.7 to 31.9)	27.8 (21.8 to 32.5)	-10.3 (-16.8 to -5.8)	1.5 (-4.3 to 7.6)	35.5 (31.2 to 38.9)	32.9 (28.0 to 36.6)	33.1 (28.3 to 37.1)	-7.5 (-12.6 to -3.7)	0.9 (-3.9 to 5.1)
Diet low in calcium	22.2 (15.6 to 27.4)	16.4 (9.6 to 22.2)	17.0 (10.1 to 22.9)	-26.0 (-40.2 to -17.8)	3.7 (-2.3 to 12.6)	25.8 (20.0 to 30.5)	20.4 (13.7 to 25.7)	20.8 (14.1 to 26.3)	-21.0 (-32.5 to -14.4)	1.9 (-3.1 to 7.3)
Diet low in seafood omega-3 fatty acids	34.9 (31.2 to 38.4)	32.7 (28.7 to 36.4)	32.3 (28.0 to 36.2)	-6.4 (-9.0 to -4.3)	-1.2 (-4.1 to 1.2)	37.6 (34.3 to 40.6)	35.7 (32.0 to 39.0)	35.4 (31.7 to 38.9)	-5.2 (-7.3 to -3.5)	-0.8 (-2.8 to 1.2)
Diet low in polyunsaturated fatty acids	39.2 (36.0 to 42.1)	27.1 (22.1 to 31.4)	25.0 (19.4 to 29.8)	-30.9 (-40.1 to -24.0)	-7.8 (-15.4 to -2.6)	39.6 (36.6 to 42.5)	27.9 (22.9 to 31.9)	25.8 (20.3 to 30.4)	-29.7 (-38.8 to -22.5)	-7.4 (-13.9 to -0.1)
Diet high in trans fatty acids	6.4 (2.9 to 10.3)	5.9 (2.4 to 9.7)	5.7 (2.3 to 9.5)	-8.3 (-31.8 to 18.0)	-3.3 (-23.3 to 19.8)	8.7 (4.7 to 13.0)	8.0 (4.2 to 12.4)	7.7 (3.9 to 12.0)	-8.2 (-29.2 to 17.2)	-3.1 (-24.3 to 21.4)
Diet high in sodium	26.5 (18.6 to 33.0)	13.9 (5.1 to 21.6)	14.2 (5.4 to 21.5)	-47.6 (-75.3 to -29.0)	1.8 (-18.0 to 35.0)	18.9 (11.5 to 25.4)	7.2 (0.8 to 14.4)	5.6 (0.2 to 12.5)	-62.1 (-93.5 to -40.3)	-21.7 (-77.8 to -6.7)
Intimate partner violence	-	-	-	-	-	1.6 (1.3 to 1.9)	1.4 (1.1 to 1.6)	1.3 (1.1 to 1.5)	-13.9 (-18.1 to -9.7)	-8.1 (-12.8 to -3.3)
Childhood sexual abuse	8.7 (7.1 to 10.8)	8.7 (7.1 to 10.8)	8.7 (7.1 to 10.8)	0.0 (0.0 to 0.0)	0.0 (0.0 to 0.0)	4.9 (3.6 to 6.5)	4.9 (3.6 to 6.5)	4.9 (3.6 to 6.5)	0.0 (0.0 to 0.0)	0.0 (0.0 to 0.0)

Supplementary Results Table 1. Slovenia age-standardised Summary Exposure Values (SEVs) for Level 4 risk factors and percentage change, by sex, 1990, 2007, and 2017.Data in parentheses are 95% uncertainty intervals.										
	**Male**					**Female**				
	**1990**	**2007**	**2017**	**Percentage change 1990-2007**	**Percentage change 2007-2017**	**1990**	**2007**	**2017**	**Percentage change 1990-2007**	**Percentage change 2007-2017**
Bullying victimization	4.5 (1.9 to 9.5)	4.6 (2.2 to 9.2)	4.7 (2.2 to 9.2)	2.5 (-21.8 to 44.2)	0.9 (-4.8 to 6.8)	3.6 (1.5 to 7.8)	3.8 (1.8 to 7.7)	3.9 (1.8 to 7.8)	5.4 (-22.9 to 47.8)	1.8 (-5.1 to 7.6)
Low physical activity	0.3 (0.2 to 0.6)	0.3 (0.2 to 0.6)	0.4 (0.2 to 0.6)	1.7 (-0.2 to 3.9)	1.0 (-0.9 to 3.0)	0.3 (0.1 to 0.6)	0.3 (0.2 to 0.6)	0.3 (0.2 to 0.6)	1.5 (-0.1 to 3.6)	0.4 (-1.3 to 2.3)
High fasting plasma glucose	2.1 (1.2 to 3.3)	2.9 (1.9 to 4.3)	2.4 (1.4 to 3.8)	41.7 (-3.8 to 136.5)	-18.0 (-49.4 to 15.5)	1.9 (1.1 to 2.9)	2.0 (1.3 to 3.1)	1.9 (1.1 to 2.9)	8.3 (-27.8 to 64.0)	-7.2 (-39.8 to 41.5)
High LDL cholesterol	14.6 (13.0 to 16.3)	14.2 (12.5 to 15.9)	14.2 (12.5 to 16.0)	-2.8 (-7.3 to 1.5)	-0.0 (-3.4 to 3.4)	14.5 (12.8 to 16.2)	14.4 (12.7 to 16.2)	14.4 (12.7 to 16.2)	-0.7 (-4.7 to 3.5)	-0.0 (-3.7 to 3.5)
High systolic blood pressure	11.5 (10.5 to 12.6)	11.2 (10.2 to 12.2)	10.8 (9.8 to 11.8)	-2.8 (-9.6 to 4.0)	-4.1 (-8.8 to 1.1)	10.3 (9.3 to 11.2)	9.9 (8.9 to 11.0)	8.9 (8.0 to 9.9)	-3.8 (-12.1 to 4.9)	-9.6 (-15.7 to -2.6)
High body-mass index	11.7 (8.6 to 16.9)	17.6 (13.5 to 23.6)	19.1 (14.5 to 25.0)	50.6 (30.8 to 76.0)	8.4 (0.2 to 17.5)	10.9 (8.0 to 14.8)	15.4 (12.0 to 20.5)	16.9 (13.1 to 22.3)	42.1 (26.4 to 63.2)	9.4 (0.3 to 20.3)
Low bone mineral density	3.8 (3.0 to 4.6)	3.5 (2.8 to 4.3)	3.5 (2.7 to 4.3)	-5.8 (-19.7 to 11.6)	-2.0 (-17.5 to 16.6)	6.1 (5.1 to 7.2)	5.9 (4.9 to 6.9)	5.8 (4.8 to 6.8)	-4.2 (-15.7 to 9.5)	-1.2 (-15.4 to 11.9)
Impaired kidney function	2.1 (0.9 to 5.8)	2.0 (0.9 to 5.7)	2.0 (0.9 to 5.6)	-1.7 (-6.0 to 2.4)	-1.0 (-5.7 to 3.9)	2.8 (1.3 to 6.7)	2.8 (1.3 to 6.7)	2.8 (1.3 to 6.7)	1.1 (-2.7 to 5.3)	-0.8 (-5.6 to 4.1)

Supplementary Results Table 1. Eastern Europe age-standardised Summary Exposure Values (SEVs) for Level 4 risk factors and percentage change, by sex, 1990, 2007, and 2017.Data in parentheses are 95% uncertainty intervals.

	Male					Female				
	**1990**	**2007**	**2017**	**Percentage change 1990-2007**	**Percentage change 2007-2017**	**1990**	**2007**	**2017**	**Percentage change 1990-2007**	**Percentage change 2007-2017**
Unsafe water source	16.3 (13.1 to 20.2)	11.0 (9.1 to 13.3)	9.3 (7.1 to 11.8)	-32.3 (-47.9 to -14.0)	-16.2 (-31.3 to 1.4)	16.3 (13.1 to 20.2)	11.1 (9.1 to 13.4)	9.2 (7.1 to 11.7)	-32.2 (-47.8 to -13.7)	-16.5 (-31.6 to 1.0)
Unsafe sanitation	41.4 (32.0 to 54.0)	29.5 (23.6 to 37.6)	23.0 (17.4 to 30.4)	-28.6 (-41.8 to -11.7)	-22.1 (-37.0 to -3.0)	41.3 (32.0 to 53.9)	29.6 (23.6 to 37.7)	23.0 (17.4 to 30.4)	-28.3 (-41.5 to -11.3)	-22.5 (-37.4 to -3.5)
No access to handwashing facility	11.2 (9.7 to 12.9)	8.5 (7.4 to 9.9)	7.5 (6.4 to 8.7)	-23.5 (-34.8 to -10.7)	-12.5 (-22.6 to -1.4)	11.2 (9.7 to 12.9)	8.5 (7.4 to 9.9)	7.5 (6.4 to 8.7)	-23.5 (-34.9 to -10.7)	-12.6 (-22.6 to -1.4)
Ambient particulate matter pollution	40.6 (30.1 to 55.0)	39.5 (29.3 to 54.2)	34.8 (25.5 to 49.0)	-2.7 (-18.7 to 16.4)	-12.1 (-27.8 to 5.5)	39.9 (29.5 to 54.4)	39.0 (28.9 to 53.7)	34.5 (25.2 to 48.5)	-2.1 (-18.7 to 18.0)	-11.7 (-27.5 to 6.3)
Household air pollution from solid fuels	2.9 (1.7 to 5.0)	1.6 (0.9 to 3.2)	1.0 (0.5 to 2.4)	-45.0 (-67.0 to -12.9)	-39.2 (-63.6 to -7.1)	3.4 (2.0 to 6.0)	2.0 (1.1 to 4.3)	1.3 (0.6 to 3.4)	-41.8 (-65.4 to -10.5)	-36.2 (-62.4 to -5.0)
Ambient ozone pollution	35.9 (12.3 to 53.6)	36.4 (12.5 to 54.0)	38.0 (13.2 to 54.4)	1.2 (0.6 to 1.9)	4.5 (0.8 to 6.9)	35.9 (12.3 to 53.7)	36.3 (12.4 to 54.0)	38.0 (13.2 to 54.4)	1.2 (0.5 to 2.0)	4.5 (0.8 to 6.9)
Residential radon	38.1 (21.7 to 56.7)	38.2 (22.0 to 56.7)	38.2 (22.0 to 56.8)	0.4 (-1.7 to 2.9)	0.1 (-0.7 to 1.2)	38.1 (21.8 to 56.7)	38.2 (21.9 to 56.7)	38.3 (22.0 to 56.6)	0.4 (-1.5 to 2.5)	0.1 (-1.0 to 1.4)
Lead exposure	2.2 (0.2 to 5.5)	2.3 (0.3 to 5.5)	1.9 (0.2 to 4.9)	6.6 (-0.1 to 53.2)	-16.1 (-24.4 to -10.7)	1.2 (0.0 to 4.1)	1.3 (0.1 to 4.2)	1.1 (0.0 to 3.7)	6.3 (-0.0 to 68.0)	-15.8 (-24.3 to -10.6)
Occupational exposure to asbestos	1.0 (0.8 to 1.2)	1.5 (1.4 to 1.6)	1.3 (1.2 to 1.4)	52.9 (16.0 to 97.1)	-14.8 (-21.6 to -3.9)	0.8 (0.6 to 1.0)	0.9 (0.9 to 1.0)	0.9 (0.8 to 0.9)	16.1 (-9.9 to 48.8)	-10.1 (-18.3 to -0.0)
Occupational exposure to arsenic	0.3 (0.0 to 0.6)	0.2 (0.0 to 0.6)	0.2 (0.0 to 0.5)	-	-	0.3 (0.0 to 0.7)	0.3 (0.0 to 0.7)	0.3 (0.0 to 0.7)	-	-
Occupational exposure to benzene	0.4 (0.0 to 1.2)	0.4 (0.0 to 1.3)	0.5 (0.0 to 1.3)	4.7 (-0.6 to 11.5)	2.4 (-1.5 to 7.5)	0.7 (0.0 to 2.0)	0.8 (0.0 to 2.3)	0.8 (0.0 to 2.4)	14.0 (7.4 to 23.9)	8.4 (4.0 to 14.4)
Occupational exposure to beryllium	0.0 (0.0 to 0.0)	0.0 (0.0 to 0.0)	0.0 (0.0 to 0.0)	-6.4 (-9.5 to -3.0)	-2.7 (-4.8 to -0.6)	0.0 (0.0 to 0.0)	0.0 (0.0 to 0.0)	0.0 (0.0 to 0.0)	3.1 (-1.4 to 7.9)	3.1 (0.3 to 5.9)
Occupational exposure to cadmium	0.0 (0.0 to 0.0)	0.0 (0.0 to 0.0)	0.0 (0.0 to 0.0)	-4.8 (-11.1 to 1.7)	-3.6 (-8.3 to 1.5)	0.0 (0.0 to 0.0)	0.0 (0.0 to 0.0)	0.0 (0.0 to 0.0)	-1.7 (-9.8 to 7.2)	-0.9 (-6.5 to 5.0)
Occupational exposure to chromium	0.1 (0.1 to 0.1)	0.1 (0.1 to 0.1)	0.1 (0.1 to 0.1)	-3.0 (-8.8 to 2.9)	-2.5 (-6.8 to 2.1)	0.1 (0.1 to 0.1)	0.1 (0.1 to 0.1)	0.1 (0.1 to 0.1)	1.1 (-6.0 to 9.0)	0.8 (-3.8 to 6.1)
Occupational exposure to diesel engine exhaust	0.4 (0.4 to 0.4)	0.4 (0.4 to 0.4)	0.4 (0.4 to 0.4)	-3.6 (-8.6 to 1.9)	-0.2 (-3.6 to 3.8)	0.4 (0.4 to 0.4)	0.4 (0.4 to 0.4)	0.4 (0.4 to 0.5)	6.4 (-0.3 to 13.8)	4.5 (-0.3 to 9.0)
Occupational exposure to formaldehyde	0.2 (0.1 to 0.2)	0.2 (0.1 to 0.2)	0.2 (0.1 to 0.2)	0.2 (-6.9 to 7.6)	-1.3 (-6.7 to 4.4)	0.2 (0.2 to 0.2)	0.2 (0.2 to 0.2)	0.2 (0.2 to 0.2)	2.0 (-6.3 to 11.1)	0.9 (-4.4 to 6.8)
Occupational exposure to nickel	0.3 (0.0 to 1.3)	0.3 (0.0 to 1.2)	0.2 (0.0 to 1.1)	-	-	0.3 (0.0 to 1.2)	0.3 (0.0 to 1.2)	0.2 (0.0 to 1.1)	-	-
Occupational exposure to polycyclic aromatic hydrocarbons	0.1 (0.1 to 0.2)	0.1 (0.1 to 0.1)	0.1 (0.1 to 0.1)	-3.2 (-8.2 to 2.2)	-2.4 (-6.3 to 1.5)	0.2 (0.2 to 0.2)	0.2 (0.2 to 0.2)	0.2 (0.2 to 0.2)	2.2 (-4.3 to 9.2)	1.6 (-2.5 to 6.1)
Occupational exposure to silica	3.6 (0.5 to 11.6)	3.3 (0.5 to 10.6)	3.2 (0.5 to 10.1)	-9.2 (-15.9 to -1.8)	-3.2 (-8.2 to 2.6)	2.6 (0.4 to 8.9)	2.4 (0.4 to 7.9)	2.3 (0.3 to 7.2)	-8.3 (-15.9 to 0.6)	-5.0 (-11.2 to 2.0)
Occupational exposure to sulphuric acid	0.5 (0.1 to 1.4)	0.4 (0.1 to 1.4)	0.4 (0.1 to 1.3)	-4.9 (-10.6 to 1.5)	-4.5 (-9.2 to 0.7)	0.5 (0.1 to 1.5)	0.5 (0.1 to 1.5)	0.5 (0.1 to 1.4)	-1.9 (-9.8 to 6.1)	-2.1 (-7.8 to 3.9)
Occupational exposure to trichloroethylene	0.0 (0.0 to 0.0)	0.0 (0.0 to 0.0)	0.0 (0.0 to 0.0)	-1.9 (-6.5 to 3.0)	-1.5 (-4.7 to 1.9)	0.1 (0.0 to 0.1)	0.1 (0.1 to 0.1)	0.1 (0.1 to 0.1)	3.6 (-2.1 to 10.3)	2.5 (-1.5 to 6.7)
Occupational asthmagens	12.3 (10.6 to 14.3)	12.8 (11.1 to 14.9)	12.9 (11.1 to 15.1)	4.1 (-2.7 to 10.5)	0.7 (-4.2 to 5.4)	7.8 (6.4 to 9.4)	7.8 (6.6 to 9.2)	8.1 (6.8 to 9.8)	0.3 (-7.8 to 9.4)	4.5 (-2.1 to 12.3)
Occupational particulate matter, gases, and fumes	6.0 (4.3 to 8.6)	5.7 (4.1 to 8.2)	5.7 (4.1 to 8.1)	-3.7 (-6.4 to -0.7)	-1.3 (-3.0 to 0.7)	3.5 (2.5 to 5.0)	3.3 (2.4 to 4.7)	3.2 (2.3 to 4.6)	-4.7 (-8.9 to 0.0)	-4.4 (-7.3 to -1.4)
Occupational noise	5.8 (5.4 to 6.3)	5.6 (5.3 to 6.1)	5.5 (5.3 to 6.0)	-3.1 (-5.1 to -0.9)	-0.6 (-1.9 to 0.6)	3.9 (3.6 to 4.3)	3.9 (3.6 to 4.3)	3.9 (3.7 to 4.3)	-0.3 (-3.2 to 2.6)	0.5 (-1.1 to 2.2)
Occupational ergonomic factors	8.5 (7.3 to 9.9)	8.1 (7.0 to 9.5)	7.9 (6.7 to 9.3)	-4.4 (-9.4 to 1.2)	-3.3 (-7.2 to 0.3)	6.7 (5.8 to 7.8)	6.3 (5.4 to 7.4)	6.3 (5.3 to 7.6)	-6.1 (-15.1 to 3.1)	0.8 (-5.3 to 7.6)
Non-exclusive breastfeeding	0.5 (0.4 to 0.7)	0.6 (0.4 to 0.7)	0.5 (0.4 to 0.7)	3.4 (-2.8 to 9.8)	-3.8 (-8.5 to 1.5)	0.5 (0.4 to 0.7)	0.6 (0.4 to 0.7)	0.5 (0.4 to 0.7)	3.5 (-2.6 to 10.0)	-3.8 (-8.5 to 1.5)
Discontinued breastfeeding	2.0 (2.0 to 2.1)	2.2 (2.2 to 2.3)	2.2 (2.1 to 2.3)	9.8 (5.3 to 14.1)	-0.2 (-3.2 to 3.1)	2.0 (2.0 to 2.1)	2.2 (2.2 to 2.3)	2.2 (2.1 to 2.3)	9.8 (5.4 to 14.2)	-0.2 (-3.2 to 3.1)
Child underweight	0.2 (0.1 to 0.2)	0.1 (0.1 to 0.2)	0.1 (0.1 to 0.1)	-33.3 (-42.5 to -25.8)	-12.0 (-17.2 to -7.2)	0.2 (0.1 to 0.2)	0.1 (0.1 to 0.2)	0.1 (0.1 to 0.1)	-33.5 (-43.5 to -23.6)	-13.2 (-19.6 to -7.5)
Child wasting	0.2 (0.2 to 0.3)	0.2 (0.2 to 0.3)	0.2 (0.1 to 0.3)	-11.2 (-29.5 to 10.1)	-12.7 (-25.0 to 4.1)	0.2 (0.2 to 0.3)	0.2 (0.2 to 0.3)	0.2 (0.1 to 0.3)	-3.9 (-21.7 to 19.7)	-16.2 (-28.9 to -1.3)
Child stunting	1.3 (0.9 to 1.6)	0.9 (0.6 to 1.1)	0.8 (0.5 to 1.1)	-33.1 (-49.2 to -15.2)	-8.6 (-27.2 to 15.5)	1.3 (0.9 to 1.6)	0.7 (0.5 to 1.0)	0.6 (0.4 to 0.9)	-43.1 (-57.4 to -26.6)	-15.7 (-34.7 to 7.9)
Short gestation for birth weight	0.0 (0.0 to 0.0)	0.0 (0.0 to 0.0)	0.0 (0.0 to 0.0)	12.0 (2.9 to 21.6)	3.5 (-3.1 to 9.3)	0.0 (0.0 to 0.0)	0.0 (0.0 to 0.0)	0.0 (0.0 to 0.0)	7.6 (-1.9 to 17.1)	1.7 (-3.9 to 7.6)
Low birth weight for gestation	0.0 (0.0 to 0.0)	0.0 (0.0 to 0.0)	0.0 (0.0 to 0.0)	5.2 (-1.8 to 12.3)	3.2 (-2.2 to 9.7)	0.0 (0.0 to 0.0)	0.0 (0.0 to 0.0)	0.0 (0.0 to 0.0)	3.5 (-2.8 to 11.1)	3.0 (-1.8 to 9.1)
Iron deficiency	-	-	-	-	-	5.9 (4.2 to 7.9)	5.3 (3.7 to 7.2)	4.7 (3.2 to 6.5)	-9.0 (-20.4 to 2.5)	-11.6 (-21.1 to -1.8)
Vitamin A deficiency	0.5 (0.4 to 0.6)	0.4 (0.3 to 0.5)	0.4 (0.3 to 0.5)	-13.6 (-17.7 to -9.6)	-0.4 (-5.5 to 3.8)	0.3 (0.2 to 0.4)	0.2 (0.2 to 0.3)	0.2 (0.2 to 0.3)	-19.7 (-25.3 to -12.5)	3.3 (-2.0 to 8.4)
Zinc deficiency	0.4 (0.1 to 0.9)	0.5 (0.1 to 1.0)	0.4 (0.1 to 0.8)	3.4 (-7.6 to 51.9)	-15.4 (-43.9 to 9.1)	0.4 (0.1 to 0.9)	0.5 (0.1 to 1.0)	0.4 (0.1 to 0.8)	3.6 (-8.2 to 50.5)	-16.0 (-43.4 to 7.9)
Smoking	17.4 (15.5 to 19.6)	16.4 (14.6 to 18.3)	16.1 (14.4 to 18.1)	-6.2 (-9.9 to -2.5)	-1.4 (-4.3 to 1.8)	2.8 (2.2 to 3.5)	2.6 (2.1 to 3.1)	3.4 (2.8 to 4.1)	-8.4 (-20.5 to 6.2)	32.9 (26.2 to 39.9)
Chewing tobacco	0.1 (0.1 to 0.1)	0.1 (0.1 to 0.1)	0.1 (0.1 to 0.1)	1.0 (-18.0 to 27.5)	-0.4 (-16.3 to 18.4)	0.1 (0.0 to 0.1)	0.1 (0.0 to 0.1)	0.1 (0.0 to 0.1)	-0.1 (-21.0 to 28.7)	0.2 (-16.9 to 20.8)
Second-hand smoke	34.6 (33.0 to 36.1)	35.2 (34.2 to 36.1)	34.0 (32.3 to 35.9)	1.7 (-3.6 to 7.4)	-3.1 (-7.2 to 0.7)	59.5 (57.8 to 61.1)	55.9 (54.8 to 56.9)	51.3 (49.6 to 53.1)	-6.1 (-8.9 to -3.0)	-8.2 (-10.6 to -5.6)
Alcohol use	22.6 (15.3 to 30.7)	32.6 (24.5 to 41.3)	34.6 (24.7 to 45.5)	44.2 (21.9 to 75.0)	6.1 (-7.9 to 20.4)	9.9 (5.8 to 16.2)	14.4 (9.3 to 21.3)	16.2 (10.0 to 23.9)	45.1 (22.7 to 78.4)	12.4 (-4.2 to 34.0)
Drug use	0.7 (0.6 to 1.0)	0.9 (0.7 to 1.2)	0.8 (0.6 to 1.1)	28.0 (24.6 to 31.7)	-13.5 (-17.0 to -9.9)	0.3 (0.2 to 0.4)	0.3 (0.2 to 0.4)	0.3 (0.2 to 0.4)	13.2 (8.7 to 18.0)	-8.7 (-13.2 to -4.6)
Diet low in fruits	44.7 (39.7 to 47.1)	42.0 (36.9 to 44.8)	38.5 (33.1 to 42.0)	-5.9 (-8.6 to -3.7)	-8.3 (-12.0 to -5.4)	43.3 (38.2 to 45.9)	40.1 (34.8 to 43.1)	36.2 (30.6 to 39.8)	-7.2 (-10.5 to -4.7)	-9.8 (-13.9 to -6.3)
Diet low in vegetables	29.6 (24.6 to 33.8)	22.2 (16.3 to 27.4)	21.0 (14.9 to 26.5)	-25.1 (-35.3 to -18.1)	-5.5 (-13.1 to 1.0)	32.0 (27.6 to 36.0)	24.9 (19.2 to 29.8)	22.9 (17.0 to 28.1)	-22.1 (-30.8 to -16.1)	-8.2 (-15.9 to -2.3)
Diet low in legumes	42.1 (39.7 to 44.0)	44.1 (42.2 to 45.8)	43.3 (41.3 to 45.2)	4.9 (3.4 to 6.7)	-1.8 (-3.3 to -0.4)	44.3 (42.3 to 45.9)	46.1 (44.5 to 47.5)	45.5 (43.7 to 47.0)	4.0 (2.7 to 5.5)	-1.2 (-2.5 to -0.1)
Diet low in whole grains	49.9 (49.1 to 50.6)	47.3 (46.1 to 48.4)	45.4 (43.8 to 46.7)	-5.2 (-6.3 to -4.3)	-4.1 (-5.3 to -3.0)	50.0 (49.3 to 50.7)	47.7 (46.5 to 48.7)	45.9 (44.4 to 47.2)	-4.7 (-5.8 to -3.8)	-3.7 (-4.8 to -2.7)
Diet low in nuts and seeds	52.1 (51.5 to 52.6)	51.5 (50.7 to 52.1)	50.0 (48.9 to 51.0)	-1.2 (-1.8 to -0.6)	-2.8 (-3.9 to -2.1)	52.2 (51.6 to 52.7)	51.6 (50.9 to 52.2)	50.2 (49.1 to 51.1)	-1.1 (-1.7 to -0.6)	-2.7 (-3.7 to -1.9)
Diet low in milk	38.7 (35.4 to 41.4)	40.1 (37.1 to 42.6)	38.7 (35.4 to 41.5)	3.6 (1.7 to 6.2)	-3.5 (-5.6 to -1.8)	39.5 (36.3 to 42.2)	40.7 (37.9 to 43.2)	39.2 (35.9 to 42.0)	3.1 (1.3 to 5.4)	-3.7 (-5.7 to -2.0)
Diet high in red meat	23.7 (20.3 to 26.8)	12.6 (9.6 to 15.5)	18.1 (13.7 to 21.7)	-47.0 (-55.8 to -38.9)	44.3 (23.6 to 71.9)	9.1 (5.9 to 12.4)	3.6 (2.0 to 5.5)	6.1 (3.5 to 9.0)	-60.3 (-72.7 to -49.4)	69.3 (27.6 to 142.7)
Diet high in processed meat	12.6 (9.2 to 15.8)	9.3 (6.4 to 12.1)	9.6 (6.5 to 12.5)	-26.4 (-39.9 to -12.4)	2.9 (-8.6 to 16.0)	8.6 (5.6 to 11.4)	6.1 (3.6 to 8.8)	6.5 (3.9 to 9.4)	-28.6 (-45.0 to -14.8)	5.9 (-6.8 to 22.8)
Diet high in sugar-sweetened beverages	4.8 (2.2 to 7.3)	3.6 (1.8 to 4.8)	5.2 (2.6 to 7.2)	-23.5 (-47.8 to 11.8)	43.5 (9.8 to 88.1)	3.1 (1.5 to 4.8)	2.5 (1.3 to 3.5)	3.5 (1.8 to 4.9)	-19.9 (-47.8 to 21.9)	40.3 (3.4 to 91.0)
Diet low in fibre	26.0 (20.4 to 30.7)	27.9 (22.5 to 32.4)	26.3 (20.6 to 31.0)	7.2 (3.7 to 12.6)	-5.7 (-11.1 to -1.6)	31.6 (26.8 to 35.5)	33.3 (28.7 to 36.9)	31.8 (26.8 to 35.7)	5.3 (2.5 to 9.5)	-4.5 (-8.6 to -1.5)
Diet low in calcium	29.3 (24.1 to 33.4)	25.5 (19.7 to 30.1)	22.8 (16.4 to 27.8)	-12.9 (-19.1 to -9.1)	-10.8 (-17.2 to -7.2)	32.3 (27.7 to 35.9)	28.9 (23.7 to 33.1)	26.3 (20.5 to 30.8)	-10.3 (-14.8 to -7.4)	-9.1 (-14.1 to -6.0)
Diet low in seafood omega-3 fatty acids	36.4 (33.1 to 39.5)	36.7 (33.4 to 39.8)	32.8 (28.8 to 36.4)	0.7 (0.1 to 1.5)	-10.6 (-13.7 to -8.3)	38.9 (35.9 to 41.6)	39.2 (36.2 to 41.9)	35.7 (32.3 to 39.0)	0.7 (0.2 to 1.3)	-8.8 (-11.3 to -6.8)
Diet low in polyunsaturated fatty acids	41.3 (38.6 to 43.7)	36.8 (33.5 to 39.7)	34.5 (30.6 to 37.8)	-10.8 (-14.6 to -7.7)	-6.2 (-9.3 to -3.8)	41.6 (39.0 to 44.0)	37.3 (34.0 to 40.1)	35.0 (31.4 to 38.3)	-10.3 (-13.9 to -7.4)	-6.1 (-9.3 to -3.8)
Diet high in trans fatty acids	1.2 (0.2 to 3.2)	1.1 (0.1 to 3.1)	1.1 (0.1 to 3.1)	-6.7 (-30.1 to 8.0)	-2.5 (-19.7 to 12.0)	1.6 (0.3 to 4.0)	1.5 (0.3 to 3.8)	1.5 (0.2 to 3.7)	-6.2 (-26.1 to 9.0)	-3.1 (-19.6 to 11.9)
Diet high in sodium	7.5 (1.5 to 14.3)	6.8 (1.0 to 13.7)	7.4 (1.3 to 14.2)	-9.4 (-45.4 to 6.6)	8.2 (-6.8 to 52.6)	4.7 (0.3 to 11.2)	4.2 (0.2 to 10.5)	4.2 (0.1 to 10.6)	-11.1 (-57.9 to 2.3)	-0.2 (-32.4 to 30.3)
Intimate partner violence	-	-	-	-	-	4.6 (3.9 to 5.3)	4.6 (3.9 to 5.3)	4.6 (3.8 to 5.3)	-0.4 (-3.7 to 3.1)	-0.7 (-3.9 to 2.9)
Childhood sexual abuse	4.0 (3.3 to 5.0)	4.0 (3.3 to 5.0)	4.0 (3.3 to 5.0)	0.0 (-0.0 to 0.1)	0.0 (-0.0 to 0.1)	12.1 (9.6 to 15.0)	12.1 (9.5 to 14.9)	12.1 (9.5 to 14.9)	-0.4 (-4.5 to 4.2)	0.0 (-0.0 to 0.1)

Supplementary Results Table 1. Eastern Europe age-standardised Summary Exposure Values (SEVs) for Level 4 risk factors and percentage change, by sex, 1990, 2007, and 2017.Data in parentheses are 95% uncertainty intervals.

	Male					Female				
	**1990**	**2007**	**2017**	**Percentage change 1990-2007**	**Percentage change 2007-2017**	**1990**	**2007**	**2017**	**Percentage change 1990-2007**	**Percentage change 2007-2017**
Bullying victimization	5.4 (2.3 to 11.1)	6.5 (2.8 to 13.5)	6.5 (2.8 to 13.1)	20.6 (12.2 to 32.5)	-0.4 (-5.9 to 4.8)	5.2 (2.3 to 10.7)	6.2 (2.7 to 12.4)	6.2 (2.7 to 12.6)	19.1 (10.8 to 29.0)	1.0 (-3.3 to 6.3)
Low physical activity	0.6 (0.2 to 1.1)	0.5 (0.2 to 1.1)	0.5 (0.2 to 1.1)	-5.0 (-7.5 to -1.7)	1.6 (0.5 to 2.5)	0.5 (0.2 to 1.0)	0.4 (0.2 to 0.9)	0.5 (0.2 to 0.9)	-5.2 (-7.9 to -1.9)	1.5 (0.5 to 2.6)
High fasting plasma glucose	2.4 (1.7 to 3.4)	2.6 (1.8 to 3.6)	2.9 (2.0 to 4.1)	8.0 (-15.6 to 41.3)	9.9 (-9.9 to 36.8)	1.8 (1.3 to 2.5)	2.0 (1.4 to 2.8)	2.2 (1.6 to 3.1)	8.6 (-12.5 to 37.7)	13.8 (-8.5 to 41.8)
High LDL cholesterol	14.5 (12.9 to 16.2)	14.0 (12.4 to 15.8)	13.9 (12.3 to 15.6)	-3.2 (-6.3 to 0.0)	-0.8 (-3.6 to 1.9)	15.7 (14.2 to 17.4)	15.4 (13.9 to 17.3)	15.6 (14.1 to 17.5)	-2.0 (-4.7 to 1.1)	1.2 (-1.5 to 3.9)
High systolic blood pressure	9.8 (9.0 to 10.6)	10.1 (9.3 to 10.9)	10.0 (9.2 to 10.8)	3.0 (0.9 to 5.2)	-1.1 (-2.7 to 0.5)	10.2 (9.5 to 10.9)	9.5 (8.8 to 10.1)	9.1 (8.4 to 9.7)	-7.0 (-9.4 to -4.7)	-4.6 (-6.5 to -2.5)
High body-mass index	9.1 (7.0 to 12.6)	11.2 (8.6 to 15.6)	13.2 (10.3 to 17.9)	22.9 (10.9 to 36.3)	18.1 (9.4 to 27.0)	11.1 (8.5 to 14.8)	12.3 (9.4 to 16.1)	14.0 (10.9 to 18.3)	10.6 (0.6 to 21.7)	13.8 (6.6 to 21.6)
Low bone mineral density	4.1 (3.4 to 4.8)	4.1 (3.4 to 4.8)	4.1 (3.4 to 4.8)	0.1 (-3.6 to 4.2)	-1.4 (-5.4 to 2.7)	5.2 (4.5 to 6.0)	5.1 (4.4 to 5.9)	5.0 (4.3 to 5.9)	-2.1 (-6.0 to 1.8)	-0.8 (-4.9 to 3.6)
Impaired kidney function	3.2 (1.5 to 7.5)	3.2 (1.5 to 7.2)	3.2 (1.5 to 7.3)	-1.8 (-4.4 to 0.6)	1.6 (-0.9 to 4.9)	3.8 (1.9 to 8.2)	3.8 (1.8 to 8.1)	3.9 (2.0 to 8.3)	-0.8 (-3.2 to 1.3)	3.6 (0.8 to 7.8)

Supplementary Results Table 1. Belarus age-standardised Summary Exposure Values (SEVs) for Level 4 risk factors and percentage change, by sex, 1990, 2007, and 2017.Data in parentheses are 95% uncertainty intervals.

	Male					Female				
	1990	2007	2017	Percentage change 1990-2007	Percentage change 2007-2017	1990	2007	2017	Percentage change 1990-2007	Percentage change 2007-2017
Unsafe water source	20.7 (15.8 to 26.2)	8.9 (7.0 to 11.1)	5.7 (4.0 to 7.8)	-56.8 (-68.5 to -41.4)	-36.4 (-53.8 to -12.5)	20.7 (15.8 to 26.2)	8.9 (7.0 to 11.1)	5.7 (4.0 to 7.8)	-56.8 (-68.5 to -41.4)	-36.4 (-53.8 to -12.5)
Unsafe sanitation	40.2 (29.8 to 54.3)	23.1 (17.9 to 29.4)	16.4 (11.8 to 22.7)	-42.7 (-55.7 to -22.0)	-29.0 (-48.2 to -5.1)	40.2 (29.8 to 54.3)	23.1 (17.9 to 29.4)	16.4 (11.8 to 22.7)	-42.7 (-55.7 to -22.0)	-29.0 (-48.2 to -5.1)
No access to handwashing facility	14.1 (11.6 to 17.1)	7.4 (5.8 to 9.2)	6.0 (4.7 to 7.4)	-47.8 (-58.8 to -34.9)	-19.1 (-32.4 to -1.5)	14.1 (11.6 to 17.1)	7.4 (5.8 to 9.2)	6.0 (4.7 to 7.4)	-47.8 (-58.8 to -34.9)	-19.1 (-32.4 to -1.5)
Ambient particulate matter pollution	43.6 (28.6 to 63.2)	44.5 (31.0 to 64.7)	39.6 (26.3 to 58.8)	1.8 (-28.4 to 48.2)	-10.9 (-38.7 to 26.1)	42.0 (26.9 to 61.2)	43.9 (30.6 to 64.3)	39.4 (26.0 to 58.5)	4.7 (-27.3 to 56.4)	-10.3 (-38.5 to 26.6)
Household air pollution from solid fuels	4.4 (2.1 to 9.8)	1.2 (0.5 to 2.9)	0.5 (0.2 to 1.3)	-71.8 (-87.5 to -40.9)	-56.8 (-80.0 to -21.3)	5.5 (2.6 to 11.7)	1.7 (0.6 to 7.7)	0.8 (0.2 to 4.0)	-69.2 (-86.8 to -30.0)	-54.1 (-79.7 to -11.9)
Ambient ozone pollution	32.9 (11.0 to 54.8)	32.6 (11.0 to 54.8)	34.3 (11.5 to 54.8)	-0.9 (-2.1 to 0.1)	5.1 (0.0 to 7.9)	32.9 (11.0 to 54.8)	32.6 (11.0 to 54.8)	34.3 (11.5 to 54.8)	-0.9 (-2.1 to 0.1)	5.1 (0.0 to 7.9)
Residential radon	24.4 (4.5 to 63.8)	24.4 (4.5 to 63.8)	24.4 (4.5 to 63.8)	0.0 (0.0 to 0.0)	0.0 (0.0 to 0.0)	24.4 (4.5 to 63.8)	24.4 (4.5 to 63.8)	24.4 (4.5 to 63.8)	0.0 (0.0 to 0.0)	0.0 (0.0 to 0.0)
Lead exposure	3.9 (0.7 to 7.9)	4.2 (1.0 to 8.2)	3.5 (0.8 to 7.2)	8.4 (0.2 to 51.2)	-16.9 (-25.6 to -10.9)	2.1 (0.1 to 5.6)	2.3 (0.2 to 5.9)	1.9 (0.1 to 5.2)	9.6 (0.9 to 75.6)	-16.4 (-25.7 to -10.2)
Occupational exposure to asbestos	0.5 (0.4 to 0.7)	1.0 (0.8 to 1.1)	0.8 (0.6 to 0.9)	84.0 (26.4 to 160.9)	-20.0 (-33.8 to -1.0)	0.5 (0.3 to 0.6)	0.9 (0.8 to 1.0)	0.7 (0.5 to 0.8)	96.2 (48.4 to 171.1)	-26.7 (-41.9 to -8.4)
Occupational exposure to arsenic	0.3 (0.0 to 0.8)	0.3 (0.0 to 0.7)	0.3 (0.0 to 0.7)	-	-	0.4 (0.0 to 1.0)	0.4 (0.0 to 1.0)	0.4 (0.0 to 1.0)	-	-
Occupational exposure to benzene	0.4 (0.0 to 1.2)	0.5 (0.0 to 1.3)	0.5 (0.0 to 1.3)	9.9 (3.0 to 18.8)	1.9 (-3.0 to 9.3)	0.6 (0.0 to 1.8)	0.8 (0.0 to 2.1)	0.9 (0.1 to 2.5)	24.6 (14.1 to 40.9)	14.8 (8.3 to 23.4)
Occupational exposure to beryllium	0.0 (0.0 to 0.0)	0.0 (0.0 to 0.0)	0.0 (0.0 to 0.0)	-11.7 (-16.1 to -7.1)	-1.6 (-5.4 to 2.5)	0.0 (0.0 to 0.0)	0.0 (0.0 to 0.0)	0.0 (0.0 to 0.0)	3.2 (-3.5 to 10.6)	8.1 (3.3 to 13.0)
Occupational exposure to cadmium	0.0 (0.0 to 0.0)	0.0 (0.0 to 0.0)	0.0 (0.0 to 0.0)	-8.3 (-15.5 to 0.3)	-3.5 (-10.7 to 3.9)	0.0 (0.0 to 0.1)	0.0 (0.0 to 0.1)	0.0 (0.0 to 0.1)	-1.3 (-11.0 to 11.8)	2.5 (-7.4 to 13.5)
Occupational exposure to chromium	0.1 (0.1 to 0.1)	0.1 (0.1 to 0.1)	0.1 (0.1 to 0.1)	-5.3 (-12.7 to 3.0)	-2.4 (-9.0 to 4.4)	0.1 (0.1 to 0.1)	0.1 (0.1 to 0.1)	0.1 (0.1 to 0.1)	1.8 (-7.9 to 14.1)	4.6 (-4.0 to 14.0)
Occupational exposure to diesel engine exhaust	0.4 (0.3 to 0.4)	0.3 (0.3 to 0.3)	0.3 (0.3 to 0.4)	-11.6 (-17.8 to -4.3)	6.9 (1.2 to 13.4)	0.4 (0.3 to 0.4)	0.4 (0.4 to 0.4)	0.4 (0.4 to 0.5)	5.1 (-4.1 to 16.2)	15.6 (7.5 to 24.0)
Occupational exposure to formaldehyde	0.2 (0.2 to 0.2)	0.2 (0.2 to 0.2)	0.2 (0.2 to 0.2)	-1.8 (-10.4 to 8.1)	-1.7 (-9.1 to 6.3)	0.2 (0.2 to 0.3)	0.2 (0.2 to 0.3)	0.2 (0.2 to 0.3)	3.5 (-7.1 to 17.6)	4.0 (-5.5 to 14.5)
Occupational exposure to nickel	0.3 (0.0 to 1.4)	0.3 (0.0 to 1.2)	0.3 (0.0 to 1.1)	-	-	0.4 (0.0 to 1.6)	0.3 (0.0 to 1.6)	0.3 (0.0 to 1.6)	-	-
Occupational exposure to polycyclic aromatic hydrocarbons	0.2 (0.2 to 0.2)	0.2 (0.2 to 0.2)	0.2 (0.1 to 0.2)	-6.2 (-13.2 to 1.7)	-1.5 (-7.5 to 5.0)	0.2 (0.2 to 0.2)	0.2 (0.2 to 0.2)	0.2 (0.2 to 0.3)	4.6 (-5.0 to 16.6)	5.5 (-2.3 to 13.7)
Occupational exposure to silica	3.6 (0.5 to 12.0)	2.7 (0.4 to 8.7)	2.8 (0.4 to 9.0)	-25.4 (-31.6 to -19.1)	4.7 (-2.2 to 13.1)	3.3 (0.5 to 10.5)	3.1 (0.4 to 10.1)	3.1 (0.4 to 10.0)	-7.6 (-16.7 to 4.9)	0.0 (-9.9 to 12.7)
Occupational exposure to sulphuric acid	0.5 (0.1 to 1.5)	0.4 (0.1 to 1.3)	0.4 (0.1 to 1.3)	-11.2 (-17.3 to -3.5)	-0.6 (-7.5 to 6.3)	0.6 (0.1 to 1.9)	0.6 (0.1 to 1.9)	0.6 (0.1 to 1.9)	4.1 (-5.8 to 17.1)	3.5 (-5.9 to 13.2)
Occupational exposure to trichloroethylene	0.0 (0.0 to 0.0)	0.0 (0.0 to 0.0)	0.0 (0.0 to 0.0)	-3.6 (-9.8 to 3.7)	-1.1 (-6.2 to 4.1)	0.1 (0.1 to 0.1)	0.1 (0.1 to 0.1)	0.1 (0.1 to 0.1)	2.8 (-5.9 to 13.5)	7.7 (0.8 to 15.5)
Occupational asthmagens	14.4 (12.0 to 17.1)	14.3 (12.2 to 16.8)	14.2 (11.9 to 16.9)	-0.6 (-9.3 to 9.0)	-0.5 (-6.9 to 6.4)	9.7 (7.7 to 12.1)	10.5 (8.8 to 12.7)	11.0 (8.8 to 13.6)	7.5 (-6.6 to 24.1)	4.7 (-7.6 to 21.9)
Occupational particulate matter, gases, and fumes	7.0 (5.0 to 9.9)	6.6 (4.7 to 9.4)	6.2 (4.5 to 9.0)	-6.3 (-9.9 to -2.8)	-4.9 (-7.4 to -2.1)	4.6 (3.2 to 6.5)	4.4 (3.1 to 6.3)	4.3 (3.1 to 6.2)	-3.8 (-9.4 to 1.9)	-2.0 (-5.7 to 2.5)
Occupational noise	6.2 (5.7 to 6.8)	5.9 (5.4 to 6.3)	5.6 (5.3 to 6.1)	-5.6 (-8.2 to -2.9)	-3.9 (-5.6 to -2.0)	4.4 (3.9 to 4.9)	4.4 (4.0 to 4.8)	4.5 (4.2 to 4.9)	-0.1 (-4.3 to 4.6)	2.5 (-0.4 to 5.7)
Occupational ergonomic factors	9.7 (8.2 to 11.5)	8.3 (7.1 to 9.8)	8.1 (6.8 to 9.6)	-14.5 (-23.9 to -5.4)	-2.9 (-9.3 to 3.7)	7.6 (6.0 to 9.6)	6.7 (5.7 to 8.0)	6.9 (5.7 to 8.4)	-11.7 (-28.1 to 6.8)	3.0 (-8.7 to 17.0)
Non-exclusive breastfeeding	0.6 (0.5 to 0.8)	0.7 (0.5 to 0.8)	0.6 (0.5 to 0.8)	1.5 (-4.5 to 8.6)	-4.9 (-10.3 to 0.7)	0.6 (0.5 to 0.8)	0.7 (0.5 to 0.8)	0.6 (0.5 to 0.8)	1.7 (-4.4 to 8.8)	-4.9 (-10.3 to 0.7)
Discontinued breastfeeding	2.2 (2.1 to 2.2)	2.4 (2.4 to 2.5)	2.3 (2.3 to 2.4)	11.2 (6.7 to 16.1)	-3.1 (-6.5 to 0.3)	2.2 (2.1 to 2.2)	2.4 (2.4 to 2.5)	2.3 (2.3 to 2.4)	11.1 (6.6 to 16.0)	-3.0 (-6.5 to 0.3)
Child underweight	0.1 (0.1 to 0.2)	0.1 (0.0 to 0.1)	0.1 (0.0 to 0.1)	-31.3 (-40.7 to -22.3)	-6.0 (-12.3 to 0.9)	0.1 (0.1 to 0.2)	0.1 (0.1 to 0.1)	0.1 (0.0 to 0.1)	-31.5 (-43.1 to -20.9)	-9.8 (-17.7 to -2.3)
Child wasting	0.2 (0.1 to 0.3)	0.1 (0.1 to 0.2)	0.1 (0.1 to 0.2)	-27.1 (-48.0 to -3.3)	-5.5 (-21.4 to 13.0)	0.1 (0.1 to 0.2)	0.1 (0.1 to 0.2)	0.1 (0.1 to 0.2)	-21.2 (-40.7 to 1.8)	-7.0 (-20.6 to 8.0)
Child stunting	0.6 (0.3 to 0.9)	0.3 (0.2 to 0.5)	0.3 (0.2 to 0.5)	-38.2 (-62.2 to -4.5)	-3.9 (-25.9 to 28.7)	0.5 (0.2 to 0.9)	0.3 (0.2 to 0.5)	0.3 (0.1 to 0.5)	-41.3 (-66.1 to -2.5)	-4.8 (-29.3 to 29.4)
Short gestation for birth weight	0.0 (0.0 to 0.0)	0.0 (0.0 to 0.0)	0.0 (0.0 to 0.0)	11.3 (-15.9 to 51.8)	3.9 (-16.4 to 35.1)	0.0 (0.0 to 0.0)	0.0 (0.0 to 0.0)	0.0 (0.0 to 0.0)	9.3 (-20.7 to 45.6)	-0.2 (-24.9 to 23.2)
Low birth weight for gestation	0.0 (0.0 to 0.0)	0.0 (0.0 to 0.0)	0.0 (0.0 to 0.0)	8.9 (-6.1 to 31.0)	3.7 (-13.3 to 21.3)	0.0 (0.0 to 0.0)	0.0 (0.0 to 0.0)	0.0 (0.0 to 0.0)	3.3 (-15.6 to 23.2)	6.0 (-8.3 to 27.2)
Iron deficiency	-	-	-	-	-	6.5 (4.7 to 8.9)	5.9 (4.2 to 8.3)	4.9 (3.4 to 6.8)	-9.0 (-24.3 to 8.0)	-17.2 (-28.7 to -3.9)
Vitamin A deficiency	0.4 (0.3 to 0.6)	0.3 (0.2 to 0.4)	0.4 (0.3 to 0.5)	-22.9 (-38.7 to 3.8)	10.2 (-11.2 to 41.0)	0.3 (0.2 to 0.4)	0.2 (0.1 to 0.3)	0.2 (0.1 to 0.2)	-26.4 (-43.8 to -5.3)	-6.0 (-23.5 to 15.7)
Zinc deficiency	0.6 (0.2 to 1.1)	0.6 (0.1 to 1.3)	0.6 (0.2 to 1.2)	-2.9 (-67.4 to 82.7)	10.6 (-45.4 to 237.3)	0.6 (0.2 to 1.1)	0.6 (0.1 to 1.3)	0.6 (0.2 to 1.2)	-3.2 (-65.4 to 77.9)	11.0 (-45.3 to 241.9)
Smoking	17.3 (15.3 to 19.5)	16.1 (14.4 to 18.0)	15.7 (13.9 to 17.6)	-6.6 (-11.5 to -1.1)	-2.6 (-7.2 to 2.5)	3.9 (2.9 to 5.1)	3.4 (2.7 to 4.3)	4.0 (3.3 to 4.8)	-11.5 (-26.7 to 9.4)	16.6 (3.4 to 31.6)
Chewing tobacco	0.1 (0.1 to 0.1)	0.1 (0.1 to 0.1)	0.1 (0.1 to 0.1)	-0.1 (-26.6 to 35.6)	0.1 (-21.8 to 27.8)	0.1 (0.0 to 0.1)	0.1 (0.0 to 0.1)	0.1 (0.0 to 0.1)	-0.3 (-28.6 to 44.0)	0.2 (-21.8 to 32.8)
Second-hand smoke	33.7 (31.3 to 35.9)	33.7 (32.5 to 34.8)	34.1 (32.2 to 36.1)	-0.0 (-6.9 to 7.7)	1.4 (-4.0 to 6.8)	49.2 (47.1 to 51.3)	46.0 (44.9 to 47.1)	45.5 (43.8 to 47.1)	-6.5 (-10.5 to -2.1)	-1.1 (-4.7 to 2.5)
Alcohol use	32.2 (22.3 to 42.4)	36.6 (27.3 to 46.2)	42.2 (30.4 to 54.6)	13.9 (-0.5 to 34.9)	15.3 (-3.4 to 38.9)	13.0 (7.6 to 20.9)	15.0 (9.2 to 23.3)	18.9 (11.1 to 29.5)	14.7 (-0.0 to 40.0)	26.1 (-1.4 to 64.9)
Drug use	0.6 (0.5 to 0.9)	0.7 (0.6 to 1.0)	0.7 (0.6 to 1.0)	11.9 (3.9 to 22.4)	-1.2 (-9.5 to 7.3)	0.2 (0.2 to 0.4)	0.3 (0.2 to 0.4)	0.3 (0.2 to 0.4)	3.7 (-2.0 to 10.1)	4.7 (-1.4 to 11.2)
Diet low in fruits	44.6 (39.8 to 47.1)	40.7 (35.4 to 43.7)	36.1 (30.5 to 40.2)	-8.8 (-12.3 to -5.7)	-11.2 (-16.8 to -6.9)	43.3 (38.3 to 46.0)	38.6 (33.3 to 41.9)	33.5 (27.8 to 37.8)	-10.8 (-15.0 to -7.3)	-13.3 (-19.9 to -8.3)
Diet low in vegetables	31.4 (26.5 to 35.6)	18.3 (11.1 to 24.1)	9.6 (2.7 to 16.6)	-41.8 (-58.5 to -30.6)	-47.4 (-77.2 to -27.7)	33.3 (28.7 to 37.1)	21.0 (14.5 to 26.6)	12.4 (4.7 to 19.5)	-36.8 (-50.9 to -27.1)	-41.1 (-70.1 to -23.7)
Diet low in legumes	54.7 (54.6 to 54.8)	53.7 (53.4 to 53.9)	51.8 (51.2 to 52.4)	-1.9 (-2.3 to -1.5)	-3.5 (-4.5 to -2.7)	54.8 (54.7 to 54.8)	54.2 (54.0 to 54.3)	52.7 (52.2 to 53.1)	-1.1 (-1.4 to -0.8)	-2.7 (-3.5 to -2.1)
Diet low in whole grains	52.4 (51.9 to 52.7)	51.6 (51.1 to 52.1)	50.2 (49.5 to 50.9)	-1.4 (-1.7 to -1.1)	-2.7 (-3.3 to -2.2)	52.3 (51.9 to 52.7)	51.7 (51.2 to 52.1)	50.5 (49.8 to 51.1)	-1.3 (-1.6 to -1.0)	-2.2 (-2.8 to -1.7)
Diet low in nuts and seeds	51.5 (50.7 to 52.2)	49.6 (48.2 to 50.7)	46.0 (43.6 to 48.0)	-3.8 (-5.5 to -2.2)	-7.3 (-10.4 to -4.9)	51.7 (50.9 to 52.4)	49.9 (48.6 to 51.0)	46.5 (44.2 to 48.5)	-3.5 (-5.2 to -2.0)	-6.8 (-9.8 to -4.7)
Diet low in milk	31.3 (26.5 to 35.5)	39.3 (36.1 to 42.0)	38.7 (35.2 to 41.5)	25.4 (17.5 to 37.8)	-1.6 (-4.2 to 0.6)	32.6 (27.9 to 36.6)	40.0 (36.9 to 42.6)	39.2 (35.8 to 42.0)	22.6 (15.3 to 34.3)	-2.0 (-4.3 to 0.0)
Diet high in red meat	39.2 (34.1 to 43.2)	29.2 (25.8 to 32.2)	37.5 (30.7 to 43.3)	-25.5 (-34.7 to -15.7)	28.5 (10.3 to 49.3)	17.8 (12.8 to 22.3)	11.6 (7.3 to 15.4)	17.0 (10.5 to 22.8)	-34.7 (-52.7 to -16.8)	45.8 (11.2 to 102.1)
Diet high in processed meat	7.3 (4.0 to 10.5)	6.0 (2.8 to 9.0)	7.3 (4.0 to 10.6)	-18.2 (-41.2 to 0.0)	21.9 (3.3 to 59.1)	4.9 (2.2 to 7.7)	3.9 (1.3 to 6.7)	4.9 (2.0 to 7.8)	-20.2 (-51.7 to 0.2)	25.8 (8.1 to 82.2)
Diet high in sugar-sweetened beverages	3.0 (1.2 to 5.3)	3.2 (1.3 to 5.7)	5.2 (2.2 to 9.3)	6.7 (-41.6 to 90.8)	63.2 (8.3 to 147.8)	2.0 (0.9 to 3.9)	2.1 (0.9 to 4.0)	3.4 (1.5 to 6.3)	5.4 (-44.1 to 95.1)	60.6 (1.8 to 151.8)
Diet low in fibre	13.1 (7.2 to 18.9)	19.8 (13.5 to 25.4)	18.1 (11.6 to 24.1)	50.9 (30.3 to 97.4)	-8.5 (-19.5 to 0.3)	19.3 (12.8 to 25.0)	25.8 (19.8 to 30.7)	24.3 (17.8 to 29.5)	34.0 (19.7 to 62.5)	-6.0 (-14.5 to 0.7)
Diet low in calcium	19.2 (12.4 to 24.8)	22.9 (16.6 to 28.0)	19.4 (12.6 to 25.1)	19.4 (11.4 to 36.4)	-15.3 (-25.5 to -9.3)	23.1 (16.8 to 28.2)	26.5 (20.9 to 31.1)	23.2 (16.6 to 28.3)	14.8 (9.0 to 26.1)	-12.7 (-21.4 to -7.7)
Diet low in seafood omega-3 fatty acids	34.5 (30.7 to 38.0)	38.0 (34.8 to 41.0)	32.1 (27.9 to 35.9)	10.2 (7.6 to 13.7)	-15.6 (-20.5 to -11.9)	37.3 (34.0 to 40.4)	40.3 (37.6 to 42.9)	35.2 (31.5 to 38.6)	8.0 (6.0 to 10.6)	-12.6 (-16.3 to -9.7)
Diet low in polyunsaturated fatty acids	47.2 (45.3 to 48.8)	35.9 (32.2 to 38.9)	32.9 (28.6 to 36.5)	-24.0 (-29.9 to -18.7)	-8.1 (-13.4 to -4.0)	47.4 (45.5 to 49.0)	36.4 (33.0 to 39.5)	33.5 (29.4 to 36.9)	-23.2 (-28.9 to -18.3)	-7.9 (-12.4 to -4.4)
Diet high in trans fatty acids	0.6 (0.0 to 2.3)	0.5 (0.0 to 2.1)	0.5 (0.0 to 2.1)	-15.4 (-64.8 to -1.3)	-3.6 (-37.8 to 14.6)	0.9 (0.0 to 2.8)	0.7 (0.0 to 2.6)	0.7 (0.0 to 2.5)	-14.9 (-56.7 to 2.9)	-3.9 (-31.9 to 18.2)
Diet high in sodium	7.2 (1.0 to 14.1)	6.4 (0.5 to 13.5)	7.4 (0.7 to 14.7)	-10.5 (-64.9 to 20.8)	14.2 (-7.9 to 106.8)	5.2 (0.3 to 11.9)	4.4 (0.2 to 11.1)	4.3 (0.1 to 11.0)	-16.2 (-71.4 to 5.7)	-0.9 (-61.5 to 30.4)
Intimate partner violence	-	-	-	-	-	3.7 (3.0 to 4.4)	3.6 (3.0 to 4.3)	3.4 (2.7 to 4.1)	-2.2 (-7.7 to 3.7)	-6.4 (-12.3 to -0.1)
Childhood sexual abuse	3.9 (3.2 to 4.8)	3.9 (3.2 to 4.8)	3.9 (3.2 to 4.8)	0.0 (0.0 to 0.0)	0.0 (0.0 to 0.0)	11.4 (9.1 to 13.9)	11.4 (9.1 to 13.9)	11.4 (9.1 to 13.9)	0.0 (0.0 to 0.0)	0.0 (0.0 to 0.0)

Supplementary Results Table 1. Belarus age-standardised Summary Exposure Values (SEVs) for Level 4 risk factors and percentage change, by sex, 1990, 2007, and 2017.Data in parentheses are 95% uncertainty intervals.

	Male					Female				
	**1990**	**2007**	**2017**	**Percentage change 1990-2007**	**Percentage change 2007-2017**	**1990**	**2007**	**2017**	**Percentage change 1990-2007**	**Percentage change 2007-2017**
Bullying victimization	8.5 (3.6 to 17.5)	9.0 (3.7 to 18.7)	8.7 (3.5 to 17.7)	5.8 (1.8 to 10.8)	-4.2 (-7.5 to -1.0)	7.7 (3.2 to 16.0)	8.2 (3.4 to 16.8)	7.9 (3.2 to 15.9)	6.6 (2.4 to 12.4)	-4.3 (-7.5 to -1.1)
Low physical activity	0.3 (0.1 to 0.5)	0.3 (0.1 to 0.5)	0.3 (0.2 to 0.5)	0.6 (-2.0 to 3.8)	1.4 (-1.1 to 4.4)	0.3 (0.1 to 0.5)	0.3 (0.1 to 0.5)	0.3 (0.1 to 0.5)	0.5 (-2.1 to 2.9)	1.3 (-1.1 to 3.9)
High fasting plasma glucose	1.7 (1.0 to 2.8)	1.7 (1.0 to 2.5)	1.6 (1.0 to 2.4)	-2.8 (-39.5 to 56.6)	-4.3 (-35.8 to 32.2)	1.5 (0.8 to 2.3)	1.3 (0.8 to 1.9)	1.3 (0.9 to 2.0)	-11.4 (-46.2 to 36.7)	2.6 (-31.7 to 49.0)
High LDL cholesterol	10.2 (8.8 to 11.9)	10.1 (8.7 to 11.8)	10.3 (8.8 to 11.9)	-1.5 (-5.5 to 2.9)	1.9 (-1.4 to 5.3)	11.7 (10.2 to 13.3)	11.9 (10.5 to 13.6)	12.4 (10.9 to 14.1)	2.3 (-1.9 to 6.7)	4.2 (0.8 to 7.8)
High systolic blood pressure	11.2 (10.3 to 12.3)	11.9 (11.0 to 13.0)	12.4 (11.5 to 13.5)	6.3 (-0.5 to 13.6)	4.1 (-0.8 to 9.6)	10.6 (9.5 to 11.7)	11.0 (10.1 to 12.1)	11.2 (10.2 to 12.2)	4.3 (-4.8 to 14.7)	1.1 (-6.3 to 8.7)
High body-mass index	7.9 (5.5 to 11.7)	10.0 (7.3 to 14.5)	13.1 (9.8 to 18.4)	26.3 (11.2 to 48.8)	31.0 (18.6 to 44.8)	9.8 (7.0 to 13.6)	11.0 (8.1 to 15.2)	13.5 (10.1 to 18.3)	12.1 (-1.0 to 29.0)	22.8 (11.5 to 36.3)
Low bone mineral density	4.1 (3.3 to 5.0)	4.1 (3.4 to 4.9)	4.1 (3.3 to 5.0)	-0.1 (-14.7 to 17.1)	-0.9 (-16.6 to 16.1)	5.1 (4.2 to 6.2)	5.1 (4.3 to 6.0)	5.1 (4.2 to 6.1)	-0.5 (-14.4 to 15.4)	-0.4 (-13.3 to 13.6)
Impaired kidney function	3.1 (1.4 to 7.2)	3.0 (1.4 to 7.1)	3.0 (1.4 to 7.1)	-2.2 (-6.4 to 1.7)	0.0 (-5.0 to 4.7)	3.6 (1.8 to 7.9)	3.6 (1.7 to 7.8)	3.6 (1.7 to 7.8)	-1.7 (-5.8 to 2.0)	0.7 (-3.5 to 5.4)

Supplementary Results Table 1. Estonia age-standardised Summary Exposure Values (SEVs) for Level 4 risk factors and percentage change, by sex, 1990, 2007, and 2017.Data in parentheses are 95% uncertainty intervals.

	Male					Female				
	**1990**	**2007**	**2017**	**Percentage change 1990-2007**	**Percentage change 2007-2017**	**1990**	**2007**	**2017**	**Percentage change 1990-2007**	**Percentage change 2007-2017**
Unsafe water source	11.7 (8.0 to 16.1)	5.8 (3.9 to 8.2)	4.6 (2.9 to 6.6)	-50.1 (-67.9 to -23.4)	-22.0 (-44.6 to 14.3)	11.7 (8.0 to 16.1)	5.8 (3.9 to 8.2)	4.6 (2.9 to 6.6)	-50.1 (-67.9 to -23.4)	-22.0 (-44.6 to 14.3)
Unsafe sanitation	22.5 (14.4 to 33.5)	9.8 (5.7 to 15.4)	6.9 (4.0 to 10.7)	-56.5 (-74.5 to -26.4)	-29.8 (-53.7 to 6.8)	22.5 (14.4 to 33.5)	9.8 (5.7 to 15.4)	6.9 (4.0 to 10.7)	-56.5 (-74.5 to -26.4)	-29.8 (-53.7 to 6.8)
No access to handwashing facility	8.8 (6.9 to 10.9)	6.1 (4.9 to 7.5)	5.4 (4.3 to 6.8)	-31.3 (-45.9 to -12.8)	-10.6 (-23.5 to 6.2)	8.8 (6.9 to 10.9)	6.1 (4.9 to 7.5)	5.4 (4.3 to 6.8)	-31.3 (-45.9 to -12.8)	-10.6 (-23.5 to 6.2)
Ambient particulate matter pollution	16.8 (10.8 to 25.9)	18.8 (12.4 to 28.3)	15.3 (7.5 to 25.3)	12.0 (-29.5 to 74.9)	-18.5 (-57.1 to 34.7)	14.3 (8.8 to 22.8)	16.9 (10.9 to 26.5)	14.0 (6.8 to 23.5)	17.5 (-28.7 to 89.8)	-16.8 (-55.9 to 44.4)
Household air pollution from solid fuels	16.1 (9.3 to 25.7)	8.9 (4.4 to 15.9)	5.7 (2.3 to 12.0)	-44.6 (-70.3 to -7.5)	-35.6 (-70.2 to 15.4)	17.4 (10.5 to 26.6)	10.3 (5.3 to 17.1)	6.8 (2.7 to 13.4)	-40.9 (-66.1 to -6.2)	-34.0 (-67.8 to 11.6)
Ambient ozone pollution	26.7 (8.9 to 49.4)	28.6 (9.5 to 52.3)	30.6 (10.2 to 54.8)	7.1 (5.1 to 10.2)	6.9 (4.8 to 9.8)	26.7 (8.9 to 49.4)	28.6 (9.5 to 52.3)	30.6 (10.2 to 54.8)	7.1 (5.1 to 10.2)	6.9 (4.8 to 9.8)
Residential radon	45.2 (16.5 to 71.2)	45.2 (16.5 to 71.2)	45.2 (16.5 to 71.2)	0.0 (0.0 to 0.0)	0.0 (0.0 to 0.0)	45.2 (16.5 to 71.2)	45.2 (16.5 to 71.2)	45.2 (16.5 to 71.2)	0.0 (0.0 to 0.0)	0.0 (0.0 to 0.0)
Lead exposure	1.4 (0.0 to 4.5)	1.7 (0.1 to 5.0)	1.5 (0.1 to 4.5)	25.6 (10.7 to 158.3)	-14.3 (-24.8 to -8.5)	0.9 (0.0 to 3.5)	1.1 (0.0 to 3.9)	1.0 (0.0 to 3.5)	21.2 (8.8 to 164.4)	-14.8 (-28.3 to -8.9)
Occupational exposure to asbestos	0.7 (0.6 to 0.8)	1.3 (1.2 to 1.5)	1.1 (0.8 to 1.3)	96.2 (65.7 to 141.9)	-20.2 (-38.1 to 6.2)	0.3 (0.3 to 0.4)	0.6 (0.5 to 0.7)	0.6 (0.4 to 0.7)	80.5 (37.7 to 125.6)	-3.6 (-26.4 to 21.2)
Occupational exposure to arsenic	0.3 (0.0 to 0.8)	0.3 (0.0 to 0.6)	0.3 (0.0 to 0.6)	-	-	0.5 (0.0 to 1.1)	0.4 (0.0 to 0.9)	0.3 (0.0 to 0.8)	-	-
Occupational exposure to benzene	0.5 (0.0 to 1.4)	0.4 (0.0 to 1.3)	0.5 (0.0 to 1.3)	-8.7 (-12.7 to -4.5)	2.5 (-1.4 to 8.9)	0.8 (0.0 to 2.4)	0.9 (0.1 to 2.5)	0.9 (0.1 to 2.6)	5.2 (0.4 to 12.0)	6.0 (1.6 to 12.8)
Occupational exposure to beryllium	0.0 (0.0 to 0.0)	0.0 (0.0 to 0.0)	0.0 (0.0 to 0.0)	-21.4 (-23.6 to -19.1)	-2.7 (-5.6 to 0.1)	0.0 (0.0 to 0.0)	0.0 (0.0 to 0.0)	0.0 (0.0 to 0.0)	-10.3 (-13.0 to -7.3)	1.6 (-1.5 to 4.6)
Occupational exposure to cadmium	0.0 (0.0 to 0.0)	0.0 (0.0 to 0.0)	0.0 (0.0 to 0.0)	-21.0 (-24.7 to -16.8)	-2.8 (-7.8 to 2.9)	0.1 (0.1 to 0.1)	0.0 (0.0 to 0.0)	0.0 (0.0 to 0.0)	-18.6 (-22.8 to -14.2)	-4.1 (-10.1 to 2.7)
Occupational exposure to chromium	0.1 (0.1 to 0.1)	0.1 (0.1 to 0.1)	0.1 (0.1 to 0.1)	-18.4 (-22.0 to -14.4)	-2.2 (-6.8 to 3.0)	0.1 (0.1 to 0.1)	0.1 (0.1 to 0.1)	0.1 (0.1 to 0.1)	-14.3 (-18.2 to -10.2)	-2.5 (-7.5 to 3.1)
Occupational exposure to diesel engine exhaust	0.5 (0.5 to 0.5)	0.4 (0.4 to 0.4)	0.4 (0.4 to 0.4)	-12.9 (-17.0 to -8.7)	-3.0 (-7.1 to 1.5)	0.5 (0.4 to 0.5)	0.5 (0.5 to 0.5)	0.5 (0.5 to 0.5)	-0.2 (-5.3 to 4.8)	3.1 (-1.9 to 8.2)
Occupational exposure to formaldehyde	0.2 (0.2 to 0.2)	0.2 (0.2 to 0.2)	0.2 (0.2 to 0.2)	-16.0 (-20.3 to -11.1)	-0.3 (-5.9 to 6.0)	0.3 (0.2 to 0.3)	0.2 (0.2 to 0.2)	0.2 (0.2 to 0.2)	-14.6 (-18.9 to -10.1)	-2.7 (-8.2 to 3.8)
Occupational exposure to nickel	0.4 (0.0 to 1.6)	0.3 (0.0 to 1.2)	0.3 (0.0 to 1.1)	-	-	0.4 (0.0 to 1.7)	0.3 (0.0 to 1.3)	0.3 (0.0 to 1.2)	-	-
Occupational exposure to polycyclic aromatic hydrocarbons	0.2 (0.2 to 0.2)	0.2 (0.1 to 0.2)	0.1 (0.1 to 0.2)	-18.2 (-21.4 to -14.5)	-2.3 (-6.4 to 2.3)	0.3 (0.3 to 0.3)	0.2 (0.2 to 0.2)	0.2 (0.2 to 0.2)	-13.5 (-17.1 to -9.6)	-1.7 (-6.1 to 3.2)
Occupational exposure to silica	4.4 (0.6 to 14.1)	3.7 (0.5 to 11.7)	3.5 (0.5 to 11.1)	-16.7 (-22.3 to -10.9)	-3.8 (-9.0 to 2.6)	3.6 (0.5 to 11.2)	2.6 (0.4 to 8.3)	2.4 (0.4 to 7.5)	-26.6 (-31.7 to -22.1)	-6.5 (-13.6 to 2.5)
Occupational exposure to sulphuric acid	0.6 (0.1 to 1.8)	0.4 (0.1 to 1.3)	0.4 (0.1 to 1.3)	-26.5 (-30.7 to -22.1)	-6.3 (-11.1 to -0.7)	0.7 (0.1 to 2.1)	0.5 (0.1 to 1.6)	0.5 (0.1 to 1.5)	-23.5 (-27.7 to -19.0)	-5.6 (-11.5 to 1.4)
Occupational exposure to trichloroethylene	0.0 (0.0 to 0.1)	0.0 (0.0 to 0.0)	0.0 (0.0 to 0.0)	-17.3 (-20.5 to -13.8)	-2.2 (-6.0 to 1.9)	0.1 (0.1 to 0.1)	0.1 (0.1 to 0.1)	0.1 (0.1 to 0.1)	-9.5 (-13.1 to -5.6)	-0.5 (-4.6 to 3.8)
Occupational asthmagens	15.3 (13.2 to 17.9)	13.2 (11.3 to 15.5)	13.4 (11.5 to 15.9)	-13.5 (-18.6 to -8.1)	1.6 (-3.8 to 7.4)	10.5 (8.8 to 12.7)	9.1 (7.7 to 10.8)	9.5 (8.0 to 11.2)	-13.7 (-21.5 to -6.0)	4.0 (-2.7 to 11.7)
Occupational particulate matter, gases, and fumes	7.9 (5.7 to 11.1)	7.0 (5.1 to 10.0)	6.5 (4.7 to 9.2)	-10.9 (-15.2 to -7.5)	-8.0 (-10.5 to -5.8)	4.8 (3.5 to 7.0)	4.1 (3.0 to 5.9)	3.6 (2.6 to 5.2)	-14.5 (-19.3 to -9.7)	-11.7 (-14.4 to -9.1)
Occupational noise	7.1 (6.7 to 7.6)	6.5 (6.2 to 7.0)	6.1 (5.8 to 6.5)	-9.0 (-11.0 to -7.2)	-6.6 (-7.6 to -5.5)	4.9 (4.5 to 5.3)	4.5 (4.3 to 4.9)	4.3 (4.0 to 4.7)	-8.0 (-10.6 to -5.2)	-5.9 (-7.2 to -4.5)
Occupational ergonomic factors	10.3 (8.9 to 11.9)	7.8 (6.6 to 9.1)	7.7 (6.5 to 9.1)	-24.3 (-28.8 to -19.9)	-1.3 (-6.4 to 4.3)	7.9 (7.0 to 9.0)	6.0 (5.1 to 7.1)	6.0 (5.0 to 7.1)	-23.9 (-30.6 to -17.5)	-0.5 (-7.5 to 7.7)
Non-exclusive breastfeeding	0.4 (0.3 to 0.6)	0.5 (0.3 to 0.6)	0.4 (0.3 to 0.6)	3.7 (-4.4 to 12.4)	-3.3 (-10.1 to 3.8)	0.4 (0.3 to 0.6)	0.5 (0.3 to 0.6)	0.4 (0.3 to 0.6)	3.7 (-4.4 to 12.4)	-3.2 (-10.1 to 3.8)
Discontinued breastfeeding	2.0 (1.9 to 2.1)	2.3 (2.2 to 2.4)	2.2 (2.1 to 2.3)	11.9 (6.0 to 18.7)	-3.0 (-6.9 to 1.2)	2.0 (1.9 to 2.1)	2.3 (2.2 to 2.4)	2.2 (2.1 to 2.3)	11.6 (5.6 to 18.3)	-2.9 (-6.8 to 1.4)
Child underweight	0.1 (0.1 to 0.2)	0.1 (0.1 to 0.1)	0.1 (0.1 to 0.1)	-29.9 (-40.5 to -19.3)	-6.5 (-14.4 to 1.4)	0.1 (0.1 to 0.2)	0.1 (0.1 to 0.1)	0.1 (0.0 to 0.1)	-31.1 (-41.8 to -19.9)	-8.5 (-16.5 to -0.4)
Child wasting	0.2 (0.1 to 0.3)	0.1 (0.1 to 0.2)	0.1 (0.1 to 0.2)	-25.6 (-47.9 to 5.3)	-7.4 (-24.2 to 11.6)	0.2 (0.1 to 0.3)	0.1 (0.1 to 0.2)	0.1 (0.1 to 0.2)	-19.5 (-41.5 to 6.3)	-8.8 (-23.8 to 8.3)
Child stunting	0.8 (0.4 to 1.4)	0.6 (0.3 to 1.0)	0.5 (0.2 to 0.9)	-29.5 (-58.6 to 16.5)	-13.1 (-40.0 to 24.1)	0.7 (0.3 to 1.3)	0.5 (0.2 to 0.9)	0.4 (0.2 to 0.8)	-33.5 (-62.5 to 10.3)	-10.5 (-39.1 to 28.5)
Short gestation for birth weight	0.0 (0.0 to 0.0)	0.0 (0.0 to 0.0)	0.0 (0.0 to 0.0)	17.6 (-10.2 to 55.8)	4.8 (-17.8 to 34.6)	0.0 (0.0 to 0.0)	0.0 (0.0 to 0.0)	0.0 (0.0 to 0.0)	12.7 (-15.2 to 44.8)	4.7 (-18.0 to 39.2)
Low birth weight for gestation	0.0 (0.0 to 0.0)	0.0 (0.0 to 0.0)	0.0 (0.0 to 0.0)	10.2 (-6.8 to 34.3)	3.7 (-15.5 to 25.1)	0.0 (0.0 to 0.0)	0.0 (0.0 to 0.0)	0.0 (0.0 to 0.0)	5.5 (-11.9 to 29.8)	1.8 (-20.5 to 25.0)
Iron deficiency	-	-	-	-	-	5.4 (3.8 to 7.5)	4.6 (3.2 to 6.6)	4.0 (2.7 to 5.7)	-13.4 (-29.2 to 5.1)	-14.3 (-27.8 to 0.3)
Vitamin A deficiency	0.3 (0.2 to 0.4)	0.3 (0.2 to 0.4)	0.3 (0.2 to 0.3)	-13.0 (-27.9 to 6.0)	-8.6 (-25.4 to 9.0)	0.2 (0.1 to 0.3)	0.2 (0.1 to 0.2)	0.1 (0.1 to 0.2)	-15.9 (-40.4 to 22.3)	-10.2 (-33.8 to 28.0)
Zinc deficiency	0.4 (0.1 to 0.8)	0.4 (0.1 to 0.9)	0.6 (0.2 to 1.3)	10.1 (-59.2 to 231.5)	63.1 (-17.6 to 261.0)	0.4 (0.1 to 0.9)	0.4 (0.1 to 0.9)	0.6 (0.2 to 1.3)	10.7 (-60.5 to 231.4)	63.0 (-17.3 to 262.7)
Smoking	15.1 (13.2 to 17.2)	12.2 (10.8 to 13.8)	12.3 (10.8 to 13.8)	-19.1 (-25.0 to -12.2)	0.4 (-4.7 to 6.2)	5.8 (4.5 to 7.1)	4.5 (3.7 to 5.4)	5.1 (4.2 to 6.1)	-21.7 (-33.6 to -7.7)	13.5 (5.8 to 22.4)
Chewing tobacco	0.1 (0.1 to 0.1)	0.1 (0.1 to 0.1)	0.1 (0.1 to 0.1)	0.2 (-29.0 to 39.7)	0.5 (-20.5 to 28.4)	0.1 (0.0 to 0.1)	0.1 (0.0 to 0.1)	0.1 (0.0 to 0.1)	0.0 (-26.8 to 41.4)	0.3 (-22.6 to 27.9)
Second-hand smoke	37.0 (34.3 to 39.6)	35.1 (33.4 to 36.7)	34.4 (31.4 to 37.3)	-5.3 (-12.8 to 3.5)	-2.0 (-8.7 to 5.3)	49.8 (47.4 to 52.0)	43.2 (41.7 to 44.5)	40.1 (37.5 to 42.5)	-13.3 (-18.0 to -8.1)	-7.1 (-12.2 to -2.4)
Alcohol use	14.8 (8.6 to 24.0)	34.5 (25.0 to 44.2)	39.2 (25.8 to 53.0)	133.3 (53.7 to 259.3)	13.6 (-11.9 to 40.2)	6.4 (3.0 to 12.3)	13.6 (8.0 to 22.1)	15.9 (8.6 to 25.5)	113.5 (48.1 to 254.6)	17.4 (-6.5 to 51.7)
Drug use	0.8 (0.6 to 1.1)	1.1 (0.9 to 1.6)	1.2 (0.9 to 1.6)	48.1 (33.4 to 67.6)	4.2 (-5.1 to 14.9)	0.3 (0.2 to 0.4)	0.3 (0.3 to 0.5)	0.3 (0.3 to 0.5)	17.1 (8.0 to 27.7)	4.4 (-3.2 to 12.2)
Diet low in fruits	40.7 (35.3 to 44.0)	36.2 (30.7 to 39.9)	34.6 (28.9 to 38.8)	-11.1 (-17.0 to -6.7)	-4.3 (-9.8 to 0.3)	38.6 (33.2 to 42.2)	33.4 (27.5 to 37.5)	31.8 (25.9 to 36.5)	-13.7 (-20.5 to -8.5)	-4.6 (-10.9 to 0.7)
Diet low in vegetables	38.1 (34.7 to 41.2)	29.1 (23.9 to 33.5)	21.6 (14.4 to 27.7)	-23.6 (-31.8 to -17.5)	-25.7 (-41.2 to -15.6)	39.5 (36.3 to 42.4)	31.2 (26.4 to 35.3)	24.3 (17.8 to 30.0)	-21.0 (-28.4 to -15.4)	-22.3 (-34.6 to -13.6)
Diet low in legumes	48.7 (47.4 to 49.7)	36.8 (33.6 to 39.7)	33.4 (29.1 to 37.0)	-24.4 (-29.6 to -19.7)	-9.3 (-14.9 to -5.0)	49.8 (48.7 to 50.7)	40.0 (37.3 to 42.4)	37.1 (33.7 to 40.1)	-19.7 (-24.1 to -15.9)	-7.1 (-11.4 to -3.7)
Diet low in whole grains	49.0 (48.1 to 49.8)	42.5 (40.5 to 44.3)	39.6 (37.0 to 41.9)	-13.3 (-16.0 to -11.0)	-6.9 (-9.6 to -4.6)	49.3 (48.4 to 50.0)	43.3 (41.4 to 44.9)	40.6 (38.1 to 42.7)	-12.1 (-14.6 to -10.0)	-6.3 (-8.6 to -4.3)
Diet low in nuts and seeds	50.3 (49.0 to 51.3)	48.2 (46.5 to 49.7)	45.3 (42.6 to 47.5)	-4.1 (-6.4 to -2.1)	-6.1 (-9.2 to -3.6)	50.6 (49.3 to 51.5)	48.6 (46.9 to 50.0)	45.8 (43.0 to 48.0)	-3.9 (-6.1 to -2.0)	-5.8 (-9.0 to -3.2)
Diet low in milk	25.4 (19.4 to 30.6)	33.1 (28.6 to 36.9)	33.4 (28.8 to 37.3)	30.0 (18.4 to 49.9)	0.9 (-2.8 to 4.4)	27.1 (21.6 to 31.9)	34.1 (30.0 to 37.5)	34.2 (29.9 to 37.8)	25.6 (15.8 to 41.5)	0.3 (-2.8 to 3.8)
Diet high in red meat	26.1 (21.3 to 30.5)	18.6 (14.4 to 21.9)	20.9 (14.5 to 26.2)	-28.9 (-41.4 to -16.0)	12.3 (-9.5 to 37.0)	9.5 (4.8 to 14.1)	5.5 (2.0 to 9.0)	7.0 (2.8 to 11.6)	-42.7 (-71.4 to -13.7)	27.4 (-17.9 to 142.2)
Diet high in processed meat	10.4 (7.0 to 13.6)	12.9 (9.7 to 15.6)	14.1 (10.3 to 17.2)	24.5 (4.4 to 56.3)	9.1 (-3.7 to 24.4)	7.1 (3.9 to 10.1)	9.0 (6.1 to 11.7)	9.9 (6.7 to 13.0)	27.3 (5.9 to 66.8)	10.6 (-2.1 to 29.8)
Diet high in sugar-sweetened beverages	3.9 (1.7 to 7.2)	5.6 (2.3 to 10.6)	7.4 (3.3 to 13.5)	44.1 (-20.9 to 163.4)	31.7 (-14.2 to 100.5)	2.6 (1.1 to 4.6)	3.6 (1.5 to 6.7)	4.7 (2.0 to 8.6)	41.3 (-20.6 to 165.0)	28.9 (-19.4 to 104.9)
Diet low in fibre	27.3 (21.4 to 32.0)	23.6 (17.5 to 28.8)	21.2 (14.6 to 26.8)	-13.5 (-22.5 to -7.5)	-10.1 (-21.0 to -2.6)	32.7 (27.7 to 36.7)	29.4 (24.1 to 33.8)	27.3 (21.3 to 32.3)	-10.1 (-16.6 to -5.4)	-7.2 (-14.6 to -1.6)
Diet low in calcium	14.3 (7.7 to 20.3)	13.7 (7.1 to 19.7)	11.9 (6.0 to 17.8)	-4.3 (-13.4 to 2.0)	-13.1 (-22.6 to -5.7)	18.3 (11.4 to 24.1)	17.6 (10.6 to 23.4)	15.4 (8.6 to 21.5)	-3.7 (-11.2 to 2.0)	-12.4 (-22.6 to -6.5)
Diet low in seafood omega-3 fatty acids	39.1 (36.0 to 41.9)	37.6 (34.3 to 40.6)	35.3 (31.5 to 38.8)	-3.8 (-5.3 to -2.6)	-6.2 (-8.9 to -4.1)	41.3 (38.7 to 43.7)	40.0 (37.0 to 42.6)	37.9 (34.6 to 41.0)	-3.2 (-4.5 to -2.2)	-5.0 (-7.1 to -3.4)
Diet low in polyunsaturated fatty acids	42.2 (39.6 to 44.7)	36.3 (32.8 to 39.2)	40.2 (37.3 to 42.6)	-14.0 (-19.6 to -9.3)	10.7 (7.4 to 15.2)	42.6 (39.9 to 44.9)	36.8 (33.5 to 39.8)	40.6 (37.8 to 43.1)	-13.5 (-18.4 to -9.2)	10.3 (7.0 to 14.8)
Diet high in trans fatty acids	4.5 (1.5 to 8.2)	4.4 (1.4 to 8.0)	4.3 (1.5 to 8.0)	-3.8 (-29.3 to 27.2)	-0.3 (-21.5 to 27.4)	6.2 (2.6 to 10.3)	5.9 (2.5 to 9.9)	5.9 (2.6 to 9.8)	-4.2 (-27.1 to 23.0)	-0.4 (-22.4 to 24.9)
Diet high in sodium	7.9 (1.3 to 15.0)	7.5 (0.9 to 14.7)	8.0 (1.2 to 15.4)	-5.0 (-48.1 to 33.5)	6.4 (-23.7 to 66.5)	5.5 (0.4 to 12.3)	4.7 (0.2 to 11.3)	4.5 (0.1 to 11.3)	-14.8 (-71.2 to 6.4)	-4.1 (-60.7 to 21.9)
Intimate partner violence	-	-	-	-	-	2.4 (2.0 to 2.8)	2.1 (1.8 to 2.5)	1.9 (1.6 to 2.2)	-11.3 (-15.4 to -6.9)	-9.8 (-14.1 to -5.0)
Childhood sexual abuse	3.9 (3.2 to 4.8)	3.9 (3.2 to 4.8)	3.9 (3.2 to 4.8)	0.0 (0.0 to 0.0)	0.0 (0.0 to 0.0)	11.4 (9.1 to 13.9)	11.4 (9.1 to 13.9)	11.4 (9.1 to 13.9)	0.0 (0.0 to 0.0)	0.0 (0.0 to 0.0)

Supplementary Results Table 1. Estonia age-standardised Summary Exposure Values (SEVs) for Level 4 risk factors and percentage change, by sex, 1990, 2007, and 2017.Data in parentheses are 95% uncertainty intervals.

	Male					Female				
	**1990**	**2007**	**2017**	**Percentage change 1990-2007**	**Percentage change 2007-2017**	**1990**	**2007**	**2017**	**Percentage change 1990-2007**	**Percentage change 2007-2017**
Bullying victimization	5.9 (2.4 to 12.6)	8.7 (4.2 to 17.7)	9.5 (4.7 to 18.9)	47.7 (21.9 to 124.6)	8.9 (-1.1 to 28.3)	5.1 (2.1 to 10.6)	7.1 (3.4 to 14.1)	7.7 (3.8 to 15.2)	39.7 (14.7 to 111.7)	8.5 (-0.2 to 24.8)
Low physical activity	0.3 (0.1 to 0.5)	0.3 (0.1 to 0.5)	0.3 (0.1 to 0.5)	1.9 (-0.2 to 4.0)	2.4 (0.4 to 5.5)	0.3 (0.1 to 0.4)	0.3 (0.1 to 0.5)	0.3 (0.1 to 0.5)	3.0 (0.9 to 5.2)	1.6 (-0.1 to 3.8)
High fasting plasma glucose	3.0 (1.9 to 4.7)	4.0 (2.8 to 5.9)	3.7 (2.4 to 5.5)	31.5 (2.7 to 85.1)	-6.7 (-31.5 to 20.1)	2.3 (1.4 to 3.5)	2.8 (1.8 to 4.3)	2.8 (1.8 to 4.5)	25.4 (-9.2 to 76.7)	-0.2 (-29.5 to 40.1)
High LDL cholesterol	16.6 (14.7 to 18.4)	16.6 (14.9 to 18.4)	16.1 (14.4 to 17.9)	0.1 (-4.7 to 5.2)	-3.0 (-6.5 to 0.7)	16.3 (14.6 to 18.1)	17.0 (15.3 to 18.8)	17.0 (15.3 to 18.8)	4.0 (-0.7 to 9.0)	0.2 (-3.1 to 3.9)
High systolic blood pressure	12.0 (11.1 to 12.9)	11.6 (10.6 to 12.6)	11.9 (10.9 to 13.0)	-3.5 (-9.2 to 2.8)	3.3 (-2.3 to 9.0)	10.3 (9.4 to 11.3)	9.3 (8.4 to 10.2)	9.4 (8.5 to 10.3)	-10.1 (-18.0 to -1.5)	1.0 (-6.2 to 8.9)
High body-mass index	9.5 (6.8 to 13.6)	14.4 (11.1 to 19.1)	17.3 (13.6 to 22.4)	52.0 (32.3 to 77.5)	20.0 (9.9 to 31.2)	11.2 (8.1 to 15.5)	13.9 (10.6 to 18.2)	16.6 (12.8 to 21.5)	23.5 (7.7 to 43.3)	19.8 (8.9 to 33.1)
Low bone mineral density	4.1 (3.3 to 5.0)	4.1 (3.3 to 4.9)	4.0 (3.2 to 4.9)	-0.6 (-14.9 to 16.9)	-1.4 (-16.9 to 15.5)	5.1 (4.2 to 6.1)	5.1 (4.2 to 6.0)	5.0 (4.1 to 6.0)	-0.7 (-12.8 to 13.9)	-0.9 (-13.8 to 14.2)
Impaired kidney function	3.2 (1.5 to 7.4)	3.3 (1.6 to 7.5)	3.3 (1.5 to 7.5)	3.3 (0.2 to 7.8)	-1.1 (-4.9 to 2.3)	3.7 (1.8 to 8.1)	3.9 (1.9 to 8.4)	3.9 (1.9 to 8.3)	4.0 (0.8 to 8.6)	0.2 (-3.3 to 3.7)

Supplementary Results Table 1. Latvia age-standardised Summary Exposure Values (SEVs) for Level 4 risk factors and percentage change, by sex, 1990, 2007, and 2017.Data in parentheses are 95% uncertainty intervals.

	Male					Female				
	1990	2007	2017	Percentage change 1990-2007	Percentage change 2007-2017	1990	2007	2017	Percentage change 1990-2007	Percentage change 2007-2017
Unsafe water source	13.0 (9.3 to 17.2)	7.3 (4.9 to 10.1)	5.8 (3.8 to 8.2)	-44.1 (-63.0 to -10.5)	-19.8 (-44.8 to 9.8)	13.0 (9.3 to 17.2)	7.3 (4.9 to 10.1)	5.8 (3.8 to 8.2)	-44.1 (-63.0 to -10.5)	-19.8 (-44.8 to 9.8)
Unsafe sanitation	29.2 (19.1 to 42.1)	15.6 (9.8 to 23.0)	11.4 (6.8 to 17.2)	-46.6 (-66.0 to -15.0)	-26.7 (-51.7 to 7.7)	29.2 (19.1 to 42.1)	15.6 (9.8 to 23.0)	11.4 (6.8 to 17.2)	-46.6 (-66.0 to -15.0)	-26.7 (-51.7 to 7.7)
No access to handwashing facility	9.5 (7.6 to 11.7)	6.6 (5.2 to 8.2)	5.9 (4.7 to 7.4)	-30.5 (-45.2 to -12.5)	-10.3 (-25.4 to 7.7)	9.5 (7.6 to 11.7)	6.6 (5.2 to 8.2)	5.9 (4.7 to 7.4)	-30.5 (-45.2 to -12.5)	-10.3 (-25.4 to 7.7)
Ambient particulate matter pollution	33.4 (21.3 to 48.7)	33.6 (22.7 to 48.0)	29.4 (18.5 to 44.4)	0.6 (-34.0 to 52.3)	-12.4 (-39.3 to 18.1)	30.8 (18.8 to 46.3)	31.8 (20.9 to 46.3)	28.1 (17.0 to 43.2)	3.4 (-34.8 to 62.3)	-11.5 (-40.5 to 22.7)
Household air pollution from solid fuels	9.5 (4.9 to 16.8)	5.5 (2.6 to 10.9)	3.9 (1.5 to 9.3)	-41.4 (-70.3 to 14.4)	-30.2 (-66.1 to 26.6)	11.1 (5.9 to 19.0)	6.8 (3.2 to 12.7)	4.8 (2.0 to 10.7)	-38.9 (-68.2 to 13.6)	-28.6 (-64.6 to 23.6)
Ambient ozone pollution	24.7 (8.3 to 46.1)	25.3 (8.5 to 47.3)	27.0 (9.0 to 49.7)	2.5 (1.4 to 4.2)	6.7 (5.0 to 9.6)	24.7 (8.3 to 46.1)	25.3 (8.5 to 47.3)	27.0 (9.0 to 49.7)	2.5 (1.4 to 4.2)	6.7 (5.0 to 9.6)
Residential radon	37.4 (8.4 to 71.2)	37.4 (8.4 to 71.2)	37.4 (8.4 to 71.2)	0.0 (0.0 to 0.0)	0.0 (0.0 to 0.0)	37.4 (8.4 to 71.2)	37.4 (8.4 to 71.2)	37.4 (8.4 to 71.2)	0.0 (0.0 to 0.0)	0.0 (0.0 to 0.0)
Lead exposure	2.1 (0.1 to 5.6)	2.3 (0.2 to 5.8)	1.9 (0.2 to 5.2)	10.2 (1.5 to 72.6)	-16.9 (-26.5 to -10.8)	1.3 (0.0 to 4.3)	1.4 (0.0 to 4.4)	1.2 (0.0 to 3.9)	10.6 (1.8 to 90.0)	-16.4 (-27.7 to -10.4)
Occupational exposure to asbestos	1.0 (0.8 to 1.2)	1.7 (1.5 to 1.9)	1.4 (1.1 to 1.7)	67.2 (35.0 to 119.2)	-18.2 (-34.6 to 4.9)	0.5 (0.4 to 0.6)	0.7 (0.6 to 0.8)	0.6 (0.5 to 0.7)	40.9 (10.6 to 80.2)	-16.1 (-35.6 to 8.6)
Occupational exposure to arsenic	0.3 (0.0 to 0.6)	0.2 (0.0 to 0.5)	0.2 (0.0 to 0.5)	-	-	0.3 (0.0 to 0.7)	0.3 (0.0 to 0.7)	0.3 (0.0 to 0.7)	-	-
Occupational exposure to benzene	0.4 (0.0 to 1.2)	0.4 (0.0 to 1.3)	0.4 (0.0 to 1.2)	4.5 (-2.1 to 13.2)	-3.0 (-7.0 to 2.6)	0.7 (0.0 to 1.8)	0.8 (0.0 to 2.4)	0.9 (0.1 to 2.5)	25.3 (17.3 to 37.4)	6.0 (1.8 to 12.1)
Occupational exposure to beryllium	0.0 (0.0 to 0.0)	0.0 (0.0 to 0.0)	0.0 (0.0 to 0.0)	-5.9 (-10.2 to -0.7)	-6.1 (-9.1 to -3.0)	0.0 (0.0 to 0.0)	0.0 (0.0 to 0.0)	0.0 (0.0 to 0.0)	9.8 (4.0 to 16.1)	1.7 (-1.1 to 4.3)
Occupational exposure to cadmium	0.0 (0.0 to 0.0)	0.0 (0.0 to 0.0)	0.0 (0.0 to 0.0)	-10.9 (-17.5 to -3.5)	-5.3 (-10.4 to 0.3)	0.0 (0.0 to 0.0)	0.0 (0.0 to 0.0)	0.0 (0.0 to 0.0)	1.1 (-7.7 to 10.5)	-1.8 (-7.1 to 3.7)
Occupational exposure to chromium	0.1 (0.1 to 0.1)	0.1 (0.1 to 0.1)	0.1 (0.1 to 0.1)	-8.1 (-14.7 to -1.0)	-4.4 (-9.2 to 0.7)	0.1 (0.1 to 0.1)	0.1 (0.1 to 0.1)	0.1 (0.1 to 0.1)	6.2 (-1.9 to 15.1)	-0.4 (-4.8 to 4.3)
Occupational exposure to diesel engine exhaust	0.4 (0.4 to 0.4)	0.4 (0.4 to 0.4)	0.4 (0.4 to 0.4)	6.3 (-0.1 to 14.2)	-3.7 (-7.7 to 0.5)	0.4 (0.3 to 0.4)	0.4 (0.4 to 0.5)	0.5 (0.4 to 0.5)	21.7 (12.3 to 31.7)	2.8 (-1.8 to 7.7)
Occupational exposure to formaldehyde	0.2 (0.1 to 0.2)	0.1 (0.1 to 0.1)	0.1 (0.1 to 0.1)	-6.9 (-14.3 to 1.6)	-2.9 (-8.4 to 3.1)	0.2 (0.2 to 0.2)	0.2 (0.2 to 0.2)	0.2 (0.2 to 0.2)	5.9 (-2.5 to 15.7)	-0.0 (-5.2 to 5.5)
Occupational exposure to nickel	0.3 (0.0 to 1.1)	0.2 (0.0 to 1.0)	0.2 (0.0 to 1.0)	-	-	0.3 (0.0 to 1.1)	0.2 (0.0 to 1.1)	0.2 (0.0 to 1.0)	-	-
Occupational exposure to polycyclic aromatic hydrocarbons	0.1 (0.1 to 0.2)	0.1 (0.1 to 0.1)	0.1 (0.1 to 0.1)	-6.5 (-12.5 to 0.2)	-5.4 (-9.7 to -1.0)	0.2 (0.2 to 0.2)	0.2 (0.2 to 0.2)	0.2 (0.2 to 0.2)	7.7 (-0.1 to 16.2)	0.2 (-4.1 to 4.6)
Occupational exposure to silica	3.2 (0.5 to 10.0)	3.3 (0.5 to 10.5)	3.0 (0.4 to 9.8)	3.0 (-5.6 to 14.3)	-7.0 (-13.3 to -0.7)	2.3 (0.3 to 6.9)	2.1 (0.3 to 6.6)	2.1 (0.3 to 6.3)	-5.3 (-14.1 to 5.7)	-3.7 (-10.5 to 3.5)
Occupational exposure to sulphuric acid	0.4 (0.1 to 1.4)	0.4 (0.1 to 1.2)	0.3 (0.1 to 1.1)	-12.8 (-19.5 to -5.4)	-9.7 (-14.6 to -4.5)	0.5 (0.1 to 1.5)	0.4 (0.1 to 1.4)	0.4 (0.1 to 1.3)	-3.3 (-11.8 to 5.4)	-4.8 (-10.2 to 0.9)
Occupational exposure to trichloroethylene	0.0 (0.0 to 0.0)	0.0 (0.0 to 0.0)	0.0 (0.0 to 0.0)	-5.8 (-11.6 to 0.5)	-3.6 (-7.8 to 0.9)	0.0 (0.0 to 0.1)	0.1 (0.1 to 0.1)	0.1 (0.1 to 0.1)	11.0 (3.5 to 19.8)	0.8 (-2.9 to 4.6)
Occupational asthmagens	13.2 (11.0 to 15.8)	12.2 (10.4 to 14.6)	12.1 (10.3 to 14.4)	-7.3 (-15.5 to 1.6)	-1.0 (-6.7 to 5.0)	8.3 (6.8 to 10.2)	8.6 (7.3 to 10.2)	8.8 (7.5 to 10.6)	4.0 (-8.0 to 17.1)	2.5 (-3.5 to 8.8)
Occupational particulate matter, gases, and fumes	6.0 (4.2 to 8.7)	5.8 (4.2 to 8.4)	5.6 (4.0 to 8.1)	-2.7 (-6.3 to 1.2)	-4.0 (-6.4 to -1.8)	3.5 (2.4 to 5.1)	3.3 (2.4 to 4.8)	3.1 (2.2 to 4.5)	-5.1 (-10.3 to 1.2)	-7.3 (-10.3 to -4.0)
Occupational noise	5.6 (5.2 to 6.1)	5.5 (5.2 to 5.9)	5.4 (5.1 to 5.8)	-1.4 (-4.3 to 1.9)	-2.6 (-4.2 to -0.8)	3.8 (3.4 to 4.2)	3.8 (3.6 to 4.2)	3.8 (3.6 to 4.1)	0.4 (-3.6 to 4.9)	-1.2 (-3.2 to 0.8)
Occupational ergonomic factors	10.5 (9.1 to 12.0)	8.8 (7.6 to 10.2)	8.2 (7.0 to 9.6)	-16.3 (-25.8 to -6.9)	-6.7 (-12.6 to 0.1)	7.4 (6.2 to 8.9)	6.6 (5.7 to 7.6)	6.4 (5.4 to 7.6)	-11.6 (-25.0 to 2.7)	-2.8 (-10.3 to 5.7)
Non-exclusive breastfeeding	0.4 (0.3 to 0.6)	0.5 (0.3 to 0.6)	0.4 (0.3 to 0.6)	2.7 (-5.8 to 13.0)	-2.7 (-9.2 to 4.2)	0.4 (0.3 to 0.6)	0.5 (0.3 to 0.6)	0.4 (0.3 to 0.6)	2.6 (-5.8 to 12.9)	-2.7 (-9.2 to 4.2)
Discontinued breastfeeding	1.7 (1.6 to 1.8)	1.9 (1.8 to 2.0)	2.0 (1.9 to 2.1)	13.3 (6.4 to 20.5)	2.0 (-2.6 to 6.9)	1.7 (1.6 to 1.8)	1.9 (1.9 to 2.0)	2.0 (1.9 to 2.1)	14.3 (7.4 to 21.5)	1.2 (-3.4 to 6.1)
Child underweight	0.1 (0.1 to 0.2)	0.1 (0.1 to 0.1)	0.1 (0.1 to 0.1)	-31.6 (-41.9 to -21.2)	-6.0 (-13.6 to 2.6)	0.1 (0.1 to 0.2)	0.1 (0.1 to 0.2)	0.1 (0.1 to 0.1)	-33.0 (-44.6 to -21.4)	-8.2 (-16.9 to 0.5)
Child wasting	0.2 (0.1 to 0.3)	0.2 (0.1 to 0.2)	0.1 (0.1 to 0.2)	-28.0 (-50.2 to 1.1)	-7.2 (-24.9 to 12.8)	0.2 (0.1 to 0.3)	0.1 (0.1 to 0.2)	0.1 (0.1 to 0.2)	-20.2 (-39.9 to 5.9)	-8.8 (-24.3 to 8.7)
Child stunting	0.8 (0.4 to 1.4)	0.6 (0.3 to 1.1)	0.6 (0.3 to 1.0)	-26.7 (-55.9 to 18.8)	-11.0 (-39.9 to 26.6)	0.8 (0.4 to 1.4)	0.5 (0.2 to 0.9)	0.5 (0.2 to 0.8)	-32.8 (-62.5 to 13.9)	-10.1 (-38.0 to 31.5)
Short gestation for birth weight	0.0 (0.0 to 0.0)	0.0 (0.0 to 0.0)	0.0 (0.0 to 0.0)	7.5 (-14.3 to 43.1)	5.9 (-17.9 to 26.9)	0.0 (0.0 to 0.0)	0.0 (0.0 to 0.0)	0.0 (0.0 to 0.0)	6.8 (-16.2 to 38.4)	5.0 (-14.7 to 33.6)
Low birth weight for gestation	0.0 (0.0 to 0.0)	0.0 (0.0 to 0.0)	0.0 (0.0 to 0.0)	6.1 (-11.3 to 24.4)	2.1 (-11.3 to 21.5)	0.0 (0.0 to 0.0)	0.0 (0.0 to 0.0)	0.0 (0.0 to 0.0)	2.6 (-15.1 to 23.1)	3.4 (-14.2 to 22.9)
Iron deficiency	-	-	-	-	-	5.5 (3.8 to 7.7)	4.8 (3.2 to 6.7)	4.1 (2.7 to 5.8)	-13.4 (-28.6 to 3.9)	-14.2 (-27.1 to 0.8)
Vitamin A deficiency	0.3 (0.2 to 0.4)	0.3 (0.2 to 0.4)	0.3 (0.2 to 0.3)	-14.6 (-36.0 to 11.0)	-4.0 (-16.6 to 14.7)	0.2 (0.1 to 0.2)	0.1 (0.1 to 0.2)	0.2 (0.1 to 0.2)	-17.3 (-35.7 to -0.1)	13.6 (-13.4 to 44.7)
Zinc deficiency	0.4 (0.1 to 0.8)	0.4 (0.1 to 0.9)	0.4 (0.1 to 0.8)	18.8 (-8.1 to 159.0)	-17.5 (-60.0 to 16.5)	0.4 (0.1 to 0.8)	0.4 (0.1 to 0.9)	0.4 (0.1 to 0.8)	18.5 (-7.1 to 161.9)	-19.1 (-59.8 to 13.5)
Smoking	12.5 (10.7 to 14.4)	12.5 (11.0 to 14.1)	12.8 (11.3 to 14.5)	0.1 (-6.4 to 8.3)	2.7 (-2.0 to 8.0)	3.6 (2.7 to 4.6)	3.6 (3.0 to 4.3)	4.1 (3.4 to 5.0)	0.7 (-16.4 to 23.2)	14.3 (4.2 to 25.8)
Chewing tobacco	0.1 (0.1 to 0.1)	0.1 (0.1 to 0.1)	0.1 (0.1 to 0.1)	0.1 (-29.2 to 37.4)	-0.5 (-21.1 to 26.0)	0.1 (0.0 to 0.1)	0.1 (0.0 to 0.1)	0.1 (0.0 to 0.1)	0.1 (-28.4 to 40.6)	0.5 (-22.2 to 29.0)
Second-hand smoke	34.8 (32.3 to 37.4)	36.5 (34.7 to 38.6)	34.1 (31.5 to 36.4)	4.9 (-3.7 to 13.9)	-6.7 (-12.5 to -1.0)	49.8 (47.5 to 52.2)	52.1 (50.4 to 53.9)	48.6 (46.1 to 50.9)	4.6 (-1.0 to 10.5)	-6.8 (-10.5 to -3.0)
Alcohol use	22.6 (14.2 to 32.5)	31.6 (22.2 to 42.0)	35.8 (23.1 to 49.6)	39.7 (12.4 to 83.1)	13.2 (-10.8 to 42.5)	9.6 (5.0 to 17.4)	12.3 (7.0 to 20.6)	13.9 (7.6 to 22.6)	27.2 (6.6 to 66.4)	13.6 (-8.4 to 45.1)
Drug use	0.7 (0.6 to 1.0)	0.9 (0.7 to 1.2)	1.0 (0.8 to 1.3)	20.5 (8.9 to 34.4)	9.3 (0.9 to 19.5)	0.4 (0.3 to 0.6)	0.4 (0.3 to 0.6)	0.4 (0.3 to 0.6)	11.6 (-1.0 to 29.4)	5.7 (-2.1 to 14.6)
Diet low in fruits	42.3 (37.0 to 45.3)	41.7 (36.5 to 44.7)	40.7 (35.2 to 44.1)	-1.3 (-4.9 to 2.3)	-2.5 (-6.2 to 0.5)	40.3 (34.9 to 43.6)	39.7 (34.4 to 42.9)	38.8 (33.4 to 42.4)	-1.5 (-5.1 to 2.4)	-2.3 (-6.3 to 1.1)
Diet low in vegetables	29.8 (24.5 to 34.3)	24.5 (18.6 to 29.5)	19.8 (12.3 to 26.0)	-17.7 (-27.0 to -11.0)	-19.3 (-36.3 to -8.9)	31.7 (26.6 to 35.8)	26.9 (21.4 to 31.7)	22.4 (15.5 to 28.0)	-15.2 (-23.2 to -8.9)	-16.7 (-30.1 to -7.9)
Diet low in legumes	54.7 (54.6 to 54.8)	52.5 (52.1 to 52.9)	52.5 (52.0 to 53.0)	-4.0 (-4.8 to -3.3)	0.0 (-0.5 to 0.6)	54.8 (54.8 to 54.8)	53.2 (52.8 to 53.5)	53.1 (52.7 to 53.5)	-3.0 (-3.6 to -2.4)	-0.1 (-0.5 to 0.4)
Diet low in whole grains	52.0 (51.5 to 52.4)	47.5 (46.3 to 48.5)	44.7 (42.9 to 46.2)	-8.6 (-10.2 to -7.3)	-6.0 (-7.8 to -4.5)	51.8 (51.4 to 52.3)	47.9 (46.8 to 48.9)	45.2 (43.6 to 46.7)	-7.6 (-9.0 to -6.3)	-5.6 (-7.3 to -4.3)
Diet low in nuts and seeds	51.6 (50.7 to 52.3)	49.3 (47.8 to 50.5)	46.2 (43.6 to 48.2)	-4.5 (-6.6 to -2.9)	-6.3 (-9.2 to -3.9)	51.8 (50.9 to 52.5)	49.6 (48.2 to 50.8)	46.7 (44.3 to 48.6)	-4.2 (-6.1 to -2.6)	-5.9 (-8.9 to -3.7)
Diet low in milk	28.0 (22.5 to 32.7)	34.6 (30.3 to 38.0)	34.4 (29.9 to 38.1)	23.7 (15.0 to 37.7)	-0.4 (-3.8 to 2.6)	29.2 (23.9 to 33.6)	35.5 (31.5 to 38.8)	35.2 (30.9 to 38.7)	21.2 (13.7 to 34.7)	-0.9 (-4.0 to 1.8)
Diet high in red meat	28.6 (23.1 to 33.3)	16.2 (11.4 to 20.2)	24.9 (17.9 to 31.2)	-43.2 (-56.3 to -28.6)	53.6 (23.0 to 99.2)	10.7 (5.5 to 15.4)	3.9 (0.8 to 7.5)	8.5 (3.5 to 13.6)	-63.6 (-89.6 to -38.8)	117.2 (35.7 to 546.5)
Diet high in processed meat	14.4 (10.3 to 18.0)	15.2 (11.9 to 17.9)	16.2 (12.0 to 19.7)	5.3 (-11.1 to 26.5)	6.9 (-5.8 to 21.2)	10.0 (6.6 to 13.2)	10.5 (7.2 to 13.3)	11.3 (8.0 to 14.6)	4.9 (-12.3 to 26.4)	8.4 (-4.5 to 24.2)
Diet high in sugar-sweetened beverages	4.4 (2.0 to 7.9)	4.7 (2.0 to 8.8)	6.4 (2.8 to 11.5)	6.6 (-42.8 to 90.4)	37.3 (-10.1 to 113.8)	2.8 (1.3 to 5.1)	3.0 (1.3 to 5.3)	4.0 (1.7 to 7.8)	6.2 (-41.1 to 92.1)	34.9 (-14.7 to 110.7)
Diet low in fibre	29.2 (23.9 to 33.5)	31.0 (25.9 to 35.1)	29.4 (23.9 to 34.1)	6.1 (1.7 to 12.1)	-5.0 (-10.2 to -0.6)	34.4 (29.6 to 38.1)	36.0 (31.9 to 39.3)	34.6 (29.8 to 38.5)	4.5 (0.8 to 9.5)	-3.7 (-8.2 to 0.1)
Diet low in calcium	15.5 (8.8 to 21.4)	17.7 (10.7 to 23.5)	16.8 (9.8 to 22.7)	14.2 (7.1 to 27.7)	-5.3 (-13.3 to 0.0)	19.4 (12.5 to 25.0)	21.7 (15.1 to 26.9)	20.6 (13.8 to 26.1)	11.6 (5.5 to 23.4)	-4.8 (-11.5 to -0.5)
Diet low in seafood omega-3 fatty acids	32.3 (28.2 to 36.0)	28.4 (23.8 to 32.7)	22.8 (17.8 to 27.6)	-12.0 (-16.4 to -8.8)	-19.7 (-26.4 to -14.7)	35.4 (31.8 to 38.8)	32.2 (27.9 to 36.0)	26.6 (21.9 to 31.2)	-9.2 (-12.7 to -6.7)	-17.1 (-22.5 to -12.7)
Diet low in polyunsaturated fatty acids	41.8 (39.0 to 44.4)	22.3 (16.3 to 27.4)	28.1 (23.0 to 32.4)	-46.6 (-58.6 to -36.7)	25.7 (14.8 to 46.6)	42.3 (39.5 to 44.9)	23.2 (17.4 to 28.1)	28.8 (23.5 to 33.2)	-45.1 (-56.9 to -35.7)	23.9 (14.1 to 40.8)
Diet high in trans fatty acids	14.9 (10.0 to 20.2)	14.0 (9.3 to 19.0)	13.8 (9.0 to 19.3)	-6.0 (-29.1 to 22.8)	-1.3 (-20.8 to 22.2)	20.3 (14.7 to 27.9)	19.1 (13.5 to 26.6)	18.7 (13.1 to 25.2)	-6.0 (-28.7 to 23.7)	-1.9 (-22.1 to 21.2)
Diet high in sodium	8.0 (1.3 to 15.1)	7.1 (0.7 to 14.3)	7.8 (1.0 to 15.4)	-10.8 (-60.2 to 15.7)	8.6 (-15.2 to 82.8)	5.5 (0.4 to 12.1)	4.6 (0.2 to 11.1)	4.5 (0.1 to 11.0)	-17.0 (-75.6 to 4.8)	-3.0 (-60.7 to 21.1)
Intimate partner violence	-	-	-	-	-	3.8 (3.2 to 4.4)	3.5 (3.0 to 4.1)	3.2 (2.8 to 3.7)	-7.6 (-11.3 to -3.2)	-8.6 (-13.0 to -4.1)
Childhood sexual abuse	3.9 (3.2 to 4.8)	3.9 (3.2 to 4.8)	3.9 (3.2 to 4.8)	0.0 (0.0 to 0.0)	0.0 (0.0 to 0.0)	11.4 (9.1 to 13.9)	11.4 (9.1 to 13.9)	11.4 (9.1 to 13.9)	0.0 (0.0 to 0.0)	0.0 (0.0 to 0.0)

Supplementary Results Table 1. Latvia age-standardised Summary Exposure Values (SEVs) for Level 4 risk factors and percentage change, by sex, 1990, 2007, and 2017.Data in parentheses are 95% uncertainty intervals.										
	**Male**					**Female**				
	**1990**	**2007**	**2017**	**Percentage change 1990-2007**	**Percentage change 2007-2017**	**1990**	**2007**	**2017**	**Percentage change 1990-2007**	**Percentage change 2007-2017**
Bullying victimization	6.8 (2.9 to 13.9)	9.3 (4.3 to 18.1)	9.9 (4.7 to 19.0)	38.4 (15.9 to 103.9)	5.9 (-2.5 to 15.9)	6.1 (2.5 to 12.7)	7.8 (3.7 to 15.1)	8.3 (3.9 to 16.1)	28.5 (-11.5 to 99.4)	6.4 (-2.2 to 17.8)
Low physical activity	0.3 (0.1 to 0.5)	0.3 (0.2 to 0.5)	0.3 (0.2 to 0.5)	1.2 (-1.1 to 3.6)	1.6 (-0.5 to 4.4)	0.3 (0.2 to 0.6)	0.3 (0.2 to 0.6)	0.3 (0.2 to 0.6)	2.9 (0.5 to 5.5)	-0.4 (-2.4 to 1.7)
High fasting plasma glucose	1.7 (1.0 to 2.6)	2.6 (1.6 to 4.0)	2.7 (1.6 to 4.3)	56.1 (2.4 to 165.3)	4.9 (-32.9 to 57.6)	1.5 (0.8 to 2.4)	1.9 (1.2 to 2.9)	2.2 (1.3 to 3.4)	32.5 (-15.2 to 123.5)	15.8 (-26.3 to 76.9)
High LDL cholesterol	15.2 (13.6 to 17.1)	13.6 (11.9 to 15.3)	12.7 (11.1 to 14.4)	-11.0 (-15.2 to -7.0)	-6.5 (-9.8 to -3.1)	16.6 (14.9 to 18.4)	15.1 (13.5 to 16.9)	14.6 (13.0 to 16.4)	-8.8 (-13.0 to -4.8)	-3.3 (-6.5 to -0.1)
High systolic blood pressure	10.0 (9.0 to 11.0)	10.7 (9.7 to 11.7)	11.4 (10.3 to 12.4)	7.3 (-0.3 to 15.1)	6.3 (0.2 to 12.5)	9.9 (8.8 to 10.9)	9.9 (8.9 to 10.9)	10.3 (9.3 to 11.4)	0.3 (-8.9 to 10.5)	4.3 (-2.9 to 12.5)
High body-mass index	9.4 (6.8 to 13.7)	12.3 (9.3 to 17.2)	14.2 (10.9 to 19.2)	30.8 (15.8 to 49.8)	15.5 (5.3 to 26.5)	10.9 (7.8 to 14.8)	12.4 (9.1 to 16.5)	14.1 (10.6 to 18.7)	13.5 (0.2 to 29.6)	14.0 (3.6 to 25.7)
Low bone mineral density	4.0 (3.2 to 4.8)	4.0 (3.3 to 4.8)	4.0 (3.2 to 4.8)	0.7 (-14.6 to 19.6)	-1.6 (-16.2 to 15.0)	5.0 (4.2 to 6.0)	5.0 (4.2 to 6.0)	5.0 (4.1 to 5.9)	0.4 (-13.1 to 16.5)	-0.7 (-14.4 to 16.1)
Impaired kidney function	3.1 (1.4 to 7.2)	3.2 (1.5 to 7.4)	3.2 (1.5 to 7.5)	1.5 (-2.3 to 6.2)	2.1 (-1.6 to 6.0)	3.6 (1.7 to 8.0)	3.7 (1.8 to 8.1)	3.8 (1.9 to 8.2)	2.8 (-1.6 to 8.0)	2.9 (-0.1 to 7.1)

Supplementary Results Table 1. Lithuania age-standardised Summary Exposure Values (SEVs) for Level 4 risk factors and percentage change, by sex, 1990, 2007, and 2017.Data in parentheses are 95% uncertainty intervals.

	Male					Female				
	**1990**	**2007**	**2017**	**Percentage change 1990-2007**	**Percentage change 2007-2017**	**1990**	**2007**	**2017**	**Percentage change 1990-2007**	**Percentage change 2007-2017**
Unsafe water source	13.8 (9.5 to 18.8)	7.3 (4.7 to 10.4)	5.7 (3.7 to 8.2)	-46.8 (-64.4 to -19.9)	-22.5 (-45.5 to 11.0)	13.8 (9.5 to 18.8)	7.3 (4.7 to 10.4)	5.7 (3.7 to 8.2)	-46.8 (-64.4 to -19.9)	-22.5 (-45.5 to 11.0)
Unsafe sanitation	29.9 (19.9 to 42.4)	16.5 (10.1 to 25.0)	12.0 (7.3 to 18.9)	-44.8 (-65.0 to -14.9)	-27.3 (-52.3 to 9.1)	29.9 (19.9 to 42.4)	16.5 (10.1 to 25.0)	12.0 (7.3 to 18.9)	-44.8 (-65.0 to -14.9)	-27.3 (-52.3 to 9.1)
No access to handwashing facility	9.7 (7.8 to 12.0)	6.7 (5.4 to 8.3)	5.8 (4.6 to 7.2)	-31.2 (-46.1 to -11.6)	-13.5 (-26.4 to 4.1)	9.7 (7.8 to 12.0)	6.7 (5.4 to 8.3)	5.8 (4.6 to 7.2)	-31.2 (-46.1 to -11.6)	-13.5 (-26.4 to 4.1)
Ambient particulate matter pollution	36.1 (25.5 to 51.9)	33.7 (23.6 to 49.2)	29.1 (19.2 to 42.6)	-6.6 (-27.9 to 16.5)	-13.5 (-35.2 to 13.8)	35.5 (24.9 to 51.1)	33.3 (23.2 to 48.8)	28.8 (19.0 to 42.1)	-6.2 (-28.2 to 18.5)	-13.5 (-35.2 to 14.2)
Household air pollution from solid fuels	1.4 (0.6 to 3.8)	1.1 (0.4 to 2.7)	0.9 (0.3 to 2.3)	-23.2 (-63.1 to 51.5)	-17.3 (-58.6 to 61.7)	1.9 (0.8 to 7.7)	1.5 (0.5 to 7.4)	1.3 (0.4 to 7.3)	-21.8 (-62.3 to 47.7)	-14.5 (-57.3 to 59.3)
Ambient ozone pollution	32.8 (11.0 to 54.8)	32.6 (11.0 to 54.8)	34.2 (11.5 to 54.8)	-0.5 (-1.2 to 0.0)	4.8 (0.0 to 6.8)	32.8 (11.0 to 54.8)	32.6 (11.0 to 54.8)	34.2 (11.5 to 54.8)	-0.5 (-1.2 to 0.0)	4.8 (0.0 to 6.8)
Residential radon	29.9 (6.7 to 70.7)	29.9 (6.7 to 70.7)	29.9 (6.7 to 70.7)	0.0 (0.0 to 0.0)	0.0 (0.0 to 0.0)	29.9 (6.7 to 70.7)	29.9 (6.7 to 70.7)	29.9 (6.7 to 70.7)	0.0 (0.0 to 0.0)	0.0 (0.0 to 0.0)
Lead exposure	1.7 (0.1 to 5.0)	1.9 (0.1 to 5.2)	1.6 (0.1 to 4.6)	12.2 (2.4 to 91.8)	-17.6 (-29.2 to -11.1)	1.1 (0.0 to 4.0)	1.3 (0.0 to 4.2)	1.0 (0.0 to 3.7)	12.6 (3.2 to 105.8)	-17.4 (-30.5 to -10.8)
Occupational exposure to asbestos	1.3 (1.1 to 1.6)	1.5 (1.3 to 1.7)	0.7 (0.6 to 0.9)	17.6 (-6.6 to 51.2)	-51.7 (-59.1 to -41.6)	1.0 (0.8 to 1.2)	0.9 (0.8 to 1.0)	0.7 (0.6 to 0.8)	-5.1 (-23.6 to 18.6)	-26.5 (-38.6 to -12.3)
Occupational exposure to arsenic	0.2 (0.0 to 0.6)	0.2 (0.0 to 0.5)	0.2 (0.0 to 0.5)	-	-	0.3 (0.0 to 0.8)	0.3 (0.0 to 0.8)	0.3 (0.0 to 0.8)	-	-
Occupational exposure to benzene	0.4 (0.0 to 1.2)	0.4 (0.0 to 1.2)	0.4 (0.0 to 1.2)	1.5 (-4.2 to 10.1)	1.5 (-2.1 to 6.7)	0.7 (0.0 to 1.9)	0.8 (0.0 to 2.3)	0.9 (0.1 to 2.5)	19.4 (12.1 to 31.9)	8.9 (5.0 to 14.7)
Occupational exposure to beryllium	0.0 (0.0 to 0.0)	0.0 (0.0 to 0.0)	0.0 (0.0 to 0.0)	-8.1 (-12.1 to -3.8)	-3.5 (-5.9 to -0.8)	0.0 (0.0 to 0.0)	0.0 (0.0 to 0.0)	0.0 (0.0 to 0.0)	3.9 (-1.3 to 9.4)	4.6 (2.1 to 7.0)
Occupational exposure to cadmium	0.0 (0.0 to 0.0)	0.0 (0.0 to 0.0)	0.0 (0.0 to 0.0)	-8.4 (-14.7 to -1.8)	-3.5 (-8.0 to 1.3)	0.0 (0.0 to 0.0)	0.0 (0.0 to 0.0)	0.0 (0.0 to 0.0)	-1.6 (-10.8 to 7.4)	0.7 (-4.3 to 5.3)
Occupational exposure to chromium	0.1 (0.1 to 0.1)	0.1 (0.1 to 0.1)	0.1 (0.1 to 0.1)	-6.1 (-12.2 to 0.5)	-2.1 (-6.4 to 2.3)	0.1 (0.1 to 0.1)	0.1 (0.1 to 0.1)	0.1 (0.1 to 0.1)	2.4 (-6.4 to 11.4)	2.5 (-1.7 to 6.5)
Occupational exposure to diesel engine exhaust	0.4 (0.3 to 0.4)	0.4 (0.4 to 0.4)	0.4 (0.4 to 0.4)	1.3 (-5.0 to 8.2)	0.2 (-3.7 to 4.3)	0.4 (0.3 to 0.4)	0.4 (0.4 to 0.4)	0.4 (0.4 to 0.5)	11.5 (4.1 to 19.8)	9.1 (4.4 to 14.0)
Occupational exposure to formaldehyde	0.1 (0.1 to 0.2)	0.1 (0.1 to 0.1)	0.1 (0.1 to 0.1)	-3.6 (-10.6 to 3.9)	-0.6 (-5.5 to 4.5)	0.2 (0.2 to 0.2)	0.2 (0.2 to 0.2)	0.2 (0.2 to 0.2)	3.1 (-6.5 to 13.0)	2.4 (-2.3 to 6.8)
Occupational exposure to nickel	0.2 (0.0 to 1.1)	0.2 (0.0 to 1.0)	0.2 (0.0 to 1.0)	-	-	0.3 (0.0 to 1.2)	0.3 (0.0 to 1.2)	0.3 (0.0 to 1.1)	-	-
Occupational exposure to polycyclic aromatic hydrocarbons	0.1 (0.1 to 0.1)	0.1 (0.1 to 0.1)	0.1 (0.1 to 0.1)	-5.7 (-11.5 to 0.3)	-3.0 (-6.5 to 0.9)	0.2 (0.2 to 0.2)	0.2 (0.2 to 0.2)	0.2 (0.2 to 0.2)	3.8 (-4.5 to 11.9)	2.8 (-1.1 to 6.4)
Occupational exposure to silica	3.1 (0.5 to 10.2)	3.1 (0.4 to 10.1)	3.0 (0.4 to 9.6)	-0.3 (-9.0 to 10.4)	-4.6 (-10.1 to 2.3)	2.5 (0.4 to 8.2)	2.3 (0.3 to 7.3)	2.3 (0.3 to 7.1)	-7.6 (-16.6 to 2.5)	-2.0 (-7.5 to 4.3)
Occupational exposure to sulphuric acid	0.4 (0.1 to 1.4)	0.4 (0.1 to 1.2)	0.3 (0.1 to 1.0)	-14.7 (-20.8 to -7.4)	-9.4 (-13.9 to -4.5)	0.5 (0.1 to 1.6)	0.5 (0.1 to 1.4)	0.4 (0.1 to 1.4)	-6.8 (-15.1 to 1.8)	-2.8 (-8.0 to 2.2)
Occupational exposure to trichloroethylene	0.0 (0.0 to 0.0)	0.0 (0.0 to 0.0)	0.0 (0.0 to 0.0)	-5.3 (-10.5 to 0.5)	-0.9 (-4.6 to 3.0)	0.1 (0.0 to 0.1)	0.1 (0.1 to 0.1)	0.1 (0.1 to 0.1)	5.1 (-1.8 to 13.1)	4.7 (1.0 to 8.3)
Occupational asthmagens	13.2 (10.8 to 16.3)	12.1 (10.2 to 14.5)	11.9 (10.1 to 14.2)	-8.3 (-17.2 to 1.7)	-1.3 (-7.1 to 4.8)	9.2 (7.4 to 11.6)	8.8 (7.3 to 10.5)	9.1 (7.6 to 10.9)	-4.5 (-17.6 to 9.2)	3.7 (-3.1 to 10.4)
Occupational particulate matter, gases, and fumes	5.7 (4.1 to 8.2)	5.5 (4.0 to 8.0)	5.3 (3.8 to 7.6)	-3.2 (-7.1 to 0.8)	-4.1 (-6.3 to -1.9)	3.8 (2.7 to 5.5)	3.6 (2.6 to 5.2)	3.3 (2.4 to 4.8)	-5.6 (-11.1 to 0.1)	-7.4 (-10.5 to -4.5)
Occupational noise	5.4 (4.9 to 5.9)	5.3 (4.9 to 5.7)	5.1 (4.8 to 5.5)	-1.8 (-4.5 to 1.4)	-3.0 (-4.6 to -1.3)	4.1 (3.7 to 4.5)	4.1 (3.8 to 4.4)	4.0 (3.8 to 4.4)	-0.2 (-4.0 to 4.0)	-2.0 (-3.9 to -0.2)
Occupational ergonomic factors	12.1 (10.5 to 14.0)	9.8 (8.7 to 11.1)	8.7 (7.5 to 10.1)	-19.0 (-28.3 to -8.0)	-11.0 (-17.9 to -3.8)	9.2 (7.5 to 11.0)	7.4 (6.5 to 8.5)	6.9 (5.9 to 8.1)	-19.4 (-32.9 to -2.1)	-6.8 (-15.1 to 3.2)
Non-exclusive breastfeeding	0.4 (0.3 to 0.6)	0.4 (0.3 to 0.6)	0.4 (0.3 to 0.6)	0.1 (-8.8 to 10.8)	-2.8 (-10.3 to 5.0)	0.4 (0.3 to 0.6)	0.4 (0.3 to 0.6)	0.4 (0.3 to 0.6)	-0.0 (-8.9 to 10.7)	-2.7 (-10.3 to 5.0)
Discontinued breastfeeding	2.3 (2.2 to 2.4)	2.5 (2.4 to 2.5)	2.5 (2.4 to 2.5)	5.6 (1.7 to 9.6)	1.4 (-1.3 to 4.3)	2.3 (2.2 to 2.4)	2.5 (2.4 to 2.5)	2.5 (2.4 to 2.5)	6.0 (2.2 to 10.1)	1.0 (-1.7 to 4.0)
Child underweight	0.1 (0.1 to 0.2)	0.1 (0.1 to 0.1)	0.1 (0.1 to 0.1)	-31.4 (-43.2 to -20.8)	-6.2 (-14.0 to 1.6)	0.1 (0.1 to 0.2)	0.1 (0.1 to 0.1)	0.1 (0.1 to 0.1)	-32.8 (-44.9 to -21.1)	-8.6 (-16.9 to -0.3)
Child wasting	0.2 (0.1 to 0.3)	0.1 (0.1 to 0.2)	0.1 (0.1 to 0.2)	-26.9 (-48.6 to 0.6)	-7.3 (-23.5 to 12.4)	0.2 (0.1 to 0.3)	0.1 (0.1 to 0.2)	0.1 (0.1 to 0.2)	-20.2 (-43.2 to 7.5)	-9.4 (-24.1 to 8.4)
Child stunting	0.8 (0.4 to 1.4)	0.6 (0.3 to 1.0)	0.5 (0.2 to 0.9)	-28.8 (-58.0 to 13.1)	-11.5 (-39.8 to 23.2)	0.7 (0.3 to 1.3)	0.5 (0.2 to 0.9)	0.5 (0.2 to 0.8)	-32.2 (-62.4 to 16.8)	-10.5 (-38.5 to 30.1)
Short gestation for birth weight	0.0 (0.0 to 0.0)	0.0 (0.0 to 0.0)	0.0 (0.0 to 0.0)	11.1 (-14.8 to 46.5)	-1.9 (-24.5 to 19.2)	0.0 (0.0 to 0.0)	0.0 (0.0 to 0.0)	0.0 (0.0 to 0.0)	9.0 (-12.0 to 40.9)	-1.9 (-24.2 to 26.5)
Low birth weight for gestation	0.0 (0.0 to 0.0)	0.0 (0.0 to 0.0)	0.0 (0.0 to 0.0)	5.6 (-13.8 to 32.2)	3.5 (-14.1 to 23.4)	0.0 (0.0 to 0.0)	0.0 (0.0 to 0.0)	0.0 (0.0 to 0.0)	3.0 (-13.4 to 23.8)	3.8 (-14.3 to 24.8)
Iron deficiency	-	-	-	-	-	5.3 (3.7 to 7.3)	4.7 (3.1 to 6.6)	4.0 (2.7 to 5.7)	-11.9 (-27.1 to 7.3)	-14.8 (-28.7 to 1.5)
Vitamin A deficiency	0.4 (0.2 to 0.5)	0.3 (0.2 to 0.3)	0.3 (0.2 to 0.4)	-27.3 (-42.9 to -7.7)	4.8 (-11.6 to 28.0)	0.2 (0.1 to 0.2)	0.1 (0.1 to 0.2)	0.1 (0.1 to 0.2)	-19.4 (-39.3 to 10.7)	-2.2 (-28.9 to 21.9)
Zinc deficiency	0.5 (0.1 to 1.2)	0.4 (0.1 to 0.8)	0.5 (0.1 to 1.2)	-27.9 (-71.5 to 128.0)	42.9 (-50.6 to 267.0)	0.5 (0.1 to 1.2)	0.4 (0.1 to 0.9)	0.5 (0.1 to 1.2)	-26.5 (-71.6 to 131.0)	40.4 (-48.2 to 257.1)
Smoking	14.9 (13.2 to 16.8)	12.0 (10.6 to 13.5)	10.8 (9.5 to 12.2)	-19.4 (-25.5 to -13.0)	-10.0 (-15.6 to -4.2)	4.5 (3.6 to 5.8)	3.4 (2.8 to 4.1)	3.8 (3.1 to 4.6)	-25.2 (-37.1 to -9.2)	12.8 (1.5 to 25.0)
Chewing tobacco	0.1 (0.1 to 0.1)	0.1 (0.1 to 0.1)	0.1 (0.1 to 0.1)	0.3 (-27.5 to 36.9)	-0.7 (-22.2 to 24.5)	0.1 (0.0 to 0.1)	0.1 (0.0 to 0.1)	0.1 (0.0 to 0.1)	-0.5 (-29.8 to 43.2)	0.3 (-22.1 to 27.0)
Second-hand smoke	37.6 (33.9 to 41.4)	30.8 (27.9 to 33.5)	29.4 (26.7 to 32.3)	-18.2 (-27.4 to -8.1)	-4.3 (-10.8 to 2.4)	54.6 (50.6 to 58.4)	41.0 (38.5 to 43.8)	38.4 (35.8 to 41.2)	-24.8 (-30.8 to -18.2)	-6.4 (-10.9 to -2.0)
Alcohol use	25.8 (17.1 to 35.6)	37.2 (27.9 to 46.2)	42.1 (30.7 to 53.9)	44.5 (18.0 to 82.9)	13.1 (-2.7 to 35.8)	10.5 (5.7 to 18.7)	13.8 (8.2 to 22.2)	16.8 (10.0 to 26.2)	31.5 (4.6 to 78.0)	21.9 (0.8 to 54.9)
Drug use	0.5 (0.3 to 0.6)	0.5 (0.4 to 0.6)	0.6 (0.5 to 0.8)	5.0 (-4.1 to 14.8)	23.0 (12.2 to 39.4)	0.2 (0.1 to 0.2)	0.2 (0.1 to 0.2)	0.2 (0.1 to 0.2)	-8.1 (-17.1 to -0.2)	16.4 (4.7 to 31.9)
Diet low in fruits	40.1 (34.6 to 43.3)	38.2 (32.7 to 41.7)	36.0 (30.2 to 40.3)	-4.8 (-9.7 to -0.0)	-5.6 (-10.2 to -1.3)	37.4 (32.0 to 41.1)	35.5 (29.7 to 39.5)	33.1 (27.0 to 37.8)	-5.1 (-11.1 to 1.0)	-6.7 (-13.2 to -1.7)
Diet low in vegetables	32.4 (27.8 to 36.4)	25.9 (20.1 to 30.7)	20.3 (12.5 to 26.4)	-20.1 (-29.1 to -13.6)	-21.4 (-39.5 to -10.7)	34.1 (29.6 to 37.8)	28.2 (22.8 to 32.7)	22.8 (16.1 to 28.7)	-17.4 (-25.7 to -11.1)	-18.9 (-32.0 to -9.2)
Diet low in legumes	39.3 (36.3 to 41.8)	29.7 (25.3 to 33.4)	29.5 (24.7 to 33.6)	-24.5 (-31.9 to -18.8)	-0.5 (-6.6 to 5.1)	41.7 (39.2 to 43.8)	33.6 (29.8 to 36.8)	33.5 (29.5 to 37.0)	-19.5 (-25.1 to -15.2)	-0.1 (-4.4 to 4.1)
Diet low in whole grains	51.6 (51.0 to 52.0)	44.5 (42.8 to 46.0)	40.7 (38.3 to 42.8)	-13.6 (-16.2 to -11.5)	-8.5 (-11.1 to -6.3)	51.5 (51.0 to 52.0)	45.2 (43.6 to 46.6)	41.6 (39.4 to 43.6)	-12.3 (-14.6 to -10.3)	-7.9 (-10.2 to -5.9)
Diet low in nuts and seeds	51.7 (50.8 to 52.4)	50.9 (49.8 to 51.7)	48.9 (47.2 to 50.2)	-1.6 (-2.9 to -0.5)	-3.9 (-5.9 to -2.4)	51.8 (51.0 to 52.5)	51.1 (50.1 to 51.9)	49.2 (47.6 to 50.5)	-1.5 (-2.6 to -0.4)	-3.7 (-5.5 to -2.3)
Diet low in milk	34.7 (30.4 to 38.3)	34.3 (30.1 to 37.9)	31.9 (26.8 to 36.0)	-1.0 (-4.9 to 3.1)	-7.1 (-11.9 to -3.7)	35.6 (31.6 to 39.0)	35.4 (31.4 to 38.8)	32.8 (28.1 to 36.7)	-0.7 (-4.1 to 3.2)	-7.2 (-12.1 to -3.6)
Diet high in red meat	29.0 (24.5 to 33.4)	26.0 (21.7 to 29.6)	34.4 (27.3 to 40.6)	-10.2 (-23.6 to 5.2)	31.9 (10.2 to 55.6)	11.4 (6.4 to 16.4)	9.3 (5.0 to 13.4)	14.6 (8.7 to 20.4)	-18.5 (-44.7 to 15.7)	57.2 (12.2 to 136.5)
Diet high in processed meat	14.9 (10.7 to 18.5)	16.6 (13.3 to 19.4)	18.7 (14.2 to 22.3)	11.5 (-5.9 to 34.1)	12.6 (-0.5 to 29.1)	10.4 (6.9 to 13.5)	11.6 (8.3 to 14.3)	13.3 (9.3 to 16.8)	12.0 (-5.8 to 36.8)	14.5 (1.2 to 30.8)
Diet high in sugar-sweetened beverages	4.2 (1.9 to 7.5)	4.9 (2.4 to 8.8)	7.4 (3.6 to 13.1)	16.4 (-36.3 to 121.7)	51.3 (-2.3 to 129.2)	2.7 (1.2 to 5.2)	3.1 (1.3 to 5.9)	4.6 (2.0 to 8.7)	14.3 (-37.8 to 125.0)	46.3 (-5.0 to 135.7)
Diet low in fibre	16.9 (10.6 to 22.8)	23.3 (17.1 to 28.5)	24.1 (18.0 to 29.6)	37.9 (21.7 to 71.0)	3.4 (-4.3 to 12.6)	23.2 (16.8 to 28.5)	29.0 (23.5 to 33.4)	29.6 (23.9 to 34.4)	24.8 (13.4 to 45.2)	2.0 (-3.7 to 8.3)
Diet low in calcium	26.0 (20.2 to 30.7)	20.0 (13.2 to 25.5)	16.4 (9.3 to 22.4)	-23.1 (-35.4 to -15.8)	-17.9 (-31.4 to -10.7)	29.3 (24.2 to 33.5)	23.9 (17.7 to 28.8)	20.3 (13.4 to 25.8)	-18.5 (-27.9 to -12.8)	-15.0 (-25.3 to -9.2)
Diet low in seafood omega-3 fatty acids	34.5 (30.7 to 38.0)	34.4 (30.5 to 37.9)	30.1 (25.7 to 34.0)	-0.3 (-1.7 to 0.9)	-12.4 (-16.5 to -9.2)	37.3 (33.9 to 40.4)	37.1 (33.8 to 40.3)	33.5 (29.5 to 37.2)	-0.4 (-1.4 to 0.6)	-9.7 (-13.0 to -7.3)
Diet low in polyunsaturated fatty acids	45.3 (42.9 to 47.5)	29.8 (25.0 to 34.8)	33.1 (28.9 to 36.7)	-34.3 (-43.2 to -24.9)	11.1 (-1.4 to 22.4)	45.7 (43.1 to 47.8)	30.2 (25.6 to 34.2)	33.8 (29.7 to 37.3)	-33.9 (-42.1 to -26.6)	11.8 (6.6 to 19.3)
Diet high in trans fatty acids	3.3 (0.8 to 6.6)	3.0 (0.7 to 6.2)	3.0 (0.7 to 6.2)	-6.7 (-36.0 to 23.7)	-0.6 (-23.6 to 29.8)	4.5 (1.6 to 8.0)	4.2 (1.3 to 7.6)	4.1 (1.2 to 7.6)	-6.7 (-33.2 to 21.8)	-1.1 (-23.3 to 26.8)
Diet high in sodium	7.9 (1.3 to 14.8)	7.2 (0.8 to 14.7)	8.0 (1.1 to 15.4)	-8.2 (-57.9 to 19.2)	10.4 (-12.5 to 89.4)	5.5 (0.4 to 12.3)	4.6 (0.2 to 11.4)	4.5 (0.1 to 11.2)	-16.9 (-71.2 to 2.9)	-2.8 (-59.0 to 26.8)
Intimate partner violence	-	-	-	-	-	2.8 (2.4 to 3.3)	2.7 (2.3 to 3.1)	2.4 (2.0 to 2.8)	-5.1 (-9.8 to -0.1)	-10.3 (-14.4 to -5.9)
Childhood sexual abuse	3.9 (3.2 to 4.8)	3.9 (3.2 to 4.8)	3.9 (3.2 to 4.8)	0.0 (0.0 to 0.0)	0.0 (0.0 to 0.0)	11.4 (9.1 to 13.9)	11.4 (9.1 to 13.9)	11.4 (9.1 to 13.9)	0.0 (0.0 to 0.0)	0.0 (0.0 to 0.0)

Supplementary Results Table 1. Lithuania age-standardised Summary Exposure Values (SEVs) for Level 4 risk factors and percentage change, by sex, 1990, 2007, and 2017.Data in parentheses are 95% uncertainty intervals.										
	**Male**					**Female**				
	**1990**	**2007**	**2017**	**Percentage change 1990-2007**	**Percentage change 2007-2017**	**1990**	**2007**	**2017**	**Percentage change 1990-2007**	**Percentage change 2007-2017**
Bullying victimization	13.0 (6.2 to 25.1)	14.2 (7.0 to 26.2)	14.3 (7.0 to 26.4)	9.9 (-0.6 to 26.4)	0.4 (-7.6 to 8.0)	12.7 (6.0 to 25.0)	13.5 (6.6 to 25.3)	13.1 (6.3 to 24.8)	6.3 (-5.5 to 19.2)	-3.1 (-11.3 to 3.4)
Low physical activity	0.3 (0.2 to 0.6)	0.4 (0.2 to 0.7)	0.4 (0.2 to 0.7)	3.2 (1.1 to 5.6)	0.4 (-1.5 to 2.3)	0.3 (0.2 to 0.6)	0.4 (0.2 to 0.7)	0.4 (0.2 to 0.7)	4.0 (1.3 to 6.8)	-0.9 (-2.9 to 1.1)
High fasting plasma glucose	2.5 (1.5 to 3.8)	2.6 (1.6 to 3.9)	2.5 (1.5 to 3.8)	3.0 (-33.4 to 54.8)	-3.6 (-36.7 to 40.4)	1.7 (0.9 to 2.6)	1.5 (0.9 to 2.2)	1.5 (0.9 to 2.3)	-11.5 (-47.8 to 45.2)	2.0 (-32.8 to 55.3)
High LDL cholesterol	18.6 (16.7 to 20.4)	14.2 (12.5 to 16.0)	13.4 (11.8 to 15.2)	-23.7 (-27.5 to -19.9)	-5.7 (-8.9 to -2.4)	20.7 (19.0 to 22.5)	13.9 (12.3 to 15.5)	13.9 (12.3 to 15.5)	-33.1 (-36.9 to -29.6)	0.2 (-2.9 to 3.5)
High systolic blood pressure	12.5 (11.5 to 13.4)	13.2 (12.1 to 14.2)	13.6 (12.4 to 14.7)	5.4 (-0.6 to 11.9)	3.0 (-1.9 to 8.8)	12.3 (11.4 to 13.4)	11.5 (10.5 to 12.5)	11.8 (10.7 to 12.8)	-6.7 (-13.3 to 0.1)	2.3 (-4.2 to 9.0)
High body-mass index	7.7 (5.2 to 11.0)	11.9 (8.7 to 16.9)	13.7 (10.0 to 19.0)	54.8 (36.2 to 82.5)	15.1 (6.1 to 26.2)	9.3 (6.6 to 13.0)	11.7 (8.6 to 15.8)	12.6 (9.1 to 17.2)	25.5 (11.4 to 44.5)	7.3 (-2.3 to 18.4)
Low bone mineral density	4.1 (3.3 to 4.9)	3.9 (3.2 to 4.7)	3.9 (3.1 to 4.8)	-3.2 (-17.6 to 13.8)	-0.9 (-16.6 to 15.0)	4.7 (4.2 to 5.3)	4.5 (4.0 to 5.0)	4.5 (3.7 to 5.3)	-4.4 (-13.0 to 5.2)	-1.0 (-17.4 to 17.4)
Impaired kidney function	3.1 (1.4 to 7.2)	3.1 (1.4 to 7.1)	3.1 (1.4 to 7.2)	0.7 (-3.1 to 5.0)	-0.7 (-5.0 to 3.6)	3.6 (1.7 to 7.9)	3.6 (1.8 to 7.9)	3.7 (1.8 to 8.0)	1.2 (-2.5 to 5.4)	0.2 (-4.1 to 5.2)

Supplementary Results Table 1. Moldova age-standardised Summary Exposure Values (SEVs) for Level 4 risk factors and percentage change, by sex, 1990, 2007, and 2017.Data in parentheses are 95% uncertainty intervals.

	Male					Female				
	**1990**	**2007**	**2017**	**Percentage change 1990-2007**	**Percentage change 2007-2017**	**1990**	**2007**	**2017**	**Percentage change 1990-2007**	**Percentage change 2007-2017**
Unsafe water source	35.7 (29.7 to 41.8)	29.5 (25.8 to 33.2)	22.3 (17.9 to 26.6)	-17.2 (-30.3 to 0.5)	-24.5 (-37.6 to -10.2)	35.7 (29.7 to 41.8)	29.5 (25.8 to 33.2)	22.3 (17.9 to 26.6)	-17.2 (-30.3 to 0.5)	-24.5 (-37.6 to -10.2)
Unsafe sanitation	65.9 (55.3 to 78.7)	65.1 (58.7 to 72.9)	55.9 (48.1 to 64.0)	-1.1 (-13.4 to 16.2)	-14.2 (-26.4 to -1.5)	65.9 (55.3 to 78.7)	65.1 (58.7 to 72.9)	55.9 (48.1 to 64.0)	-1.1 (-13.4 to 16.2)	-14.2 (-26.4 to -1.5)
No access to handwashing facility	27.6 (23.2 to 32.3)	22.6 (19.0 to 26.4)	15.6 (12.8 to 18.7)	-18.2 (-31.4 to -2.7)	-31.0 (-41.2 to -19.1)	27.6 (23.2 to 32.3)	22.6 (19.0 to 26.4)	15.6 (12.8 to 18.7)	-18.2 (-31.4 to -2.7)	-31.0 (-41.2 to -19.1)
Ambient particulate matter pollution	25.6 (15.6 to 40.3)	29.9 (20.5 to 41.9)	31.8 (18.8 to 49.2)	16.7 (-27.6 to 94.8)	6.7 (-34.7 to 70.3)	21.7 (12.9 to 34.8)	26.4 (17.0 to 38.6)	29.7 (17.0 to 46.3)	21.8 (-27.6 to 110.0)	12.4 (-34.5 to 87.0)
Household air pollution from solid fuels	31.7 (22.1 to 44.1)	15.9 (10.3 to 24.0)	7.0 (3.5 to 13.0)	-49.7 (-66.8 to -27.5)	-56.3 (-75.1 to -32.4)	33.4 (24.0 to 45.9)	17.8 (12.1 to 25.5)	8.4 (4.4 to 14.9)	-46.6 (-62.7 to -26.6)	-52.9 (-72.7 to -30.8)
Ambient ozone pollution	38.2 (12.9 to 54.8)	38.4 (13.1 to 54.8)	39.6 (13.6 to 54.8)	0.5 (-0.6 to 2.0)	3.1 (0.0 to 5.6)	38.2 (12.9 to 54.8)	38.4 (13.1 to 54.8)	39.6 (13.6 to 54.8)	0.5 (-0.6 to 2.0)	3.1 (0.0 to 5.6)
Residential radon	32.7 (4.9 to 71.2)	32.7 (4.9 to 71.2)	32.7 (4.9 to 71.2)	0.0 (0.0 to 0.0)	0.0 (0.0 to 0.0)	32.7 (4.9 to 71.2)	32.7 (4.9 to 71.2)	32.7 (4.9 to 71.2)	0.0 (0.0 to 0.0)	0.0 (0.0 to 0.0)
Lead exposure	5.0 (1.2 to 9.2)	5.7 (1.9 to 9.8)	5.0 (1.6 to 9.0)	13.6 (4.5 to 53.2)	-11.8 (-18.7 to -7.1)	2.8 (0.3 to 6.6)	3.2 (0.5 to 7.1)	2.9 (0.4 to 6.5)	14.5 (4.0 to 85.2)	-10.7 (-17.2 to -6.2)
Occupational exposure to asbestos	0.5 (0.3 to 0.6)	0.4 (0.3 to 0.5)	0.3 (0.3 to 0.4)	-13.8 (-36.9 to 24.0)	-17.4 (-30.2 to -1.0)	0.3 (0.2 to 0.4)	0.4 (0.3 to 0.4)	0.3 (0.3 to 0.4)	30.2 (-1.7 to 82.0)	-7.1 (-23.7 to 17.1)
Occupational exposure to arsenic	0.2 (0.0 to 0.4)	0.1 (0.0 to 0.3)	0.1 (0.0 to 0.3)	-	-	0.3 (0.0 to 0.6)	0.2 (0.0 to 0.5)	0.2 (0.0 to 0.5)	-	-
Occupational exposure to benzene	0.3 (0.0 to 0.8)	0.3 (0.0 to 0.8)	0.3 (0.0 to 0.8)	-5.1 (-12.2 to 5.8)	1.4 (-5.0 to 11.6)	0.5 (0.0 to 1.4)	0.5 (0.0 to 1.4)	0.6 (0.0 to 1.6)	4.6 (-5.4 to 22.2)	12.5 (4.5 to 24.2)
Occupational exposure to beryllium	0.0 (0.0 to 0.0)	0.0 (0.0 to 0.0)	0.0 (0.0 to 0.0)	-18.2 (-24.1 to -11.6)	-6.0 (-11.1 to -0.3)	0.0 (0.0 to 0.0)	0.0 (0.0 to 0.0)	0.0 (0.0 to 0.0)	-11.5 (-18.8 to -2.6)	0.7 (-4.7 to 6.9)
Occupational exposure to cadmium	0.0 (0.0 to 0.0)	0.0 (0.0 to 0.0)	0.0 (0.0 to 0.0)	-26.4 (-33.2 to -18.9)	-3.9 (-10.8 to 4.4)	0.0 (0.0 to 0.0)	0.0 (0.0 to 0.0)	0.0 (0.0 to 0.0)	-21.8 (-30.6 to -11.7)	2.7 (-5.7 to 12.0)
Occupational exposure to chromium	0.0 (0.0 to 0.1)	0.0 (0.0 to 0.0)	0.0 (0.0 to 0.0)	-23.7 (-30.8 to -16.0)	-1.4 (-8.4 to 6.7)	0.1 (0.1 to 0.1)	0.1 (0.1 to 0.1)	0.1 (0.1 to 0.1)	-18.2 (-27.4 to -7.9)	6.4 (-1.8 to 15.7)
Occupational exposure to diesel engine exhaust	0.3 (0.2 to 0.3)	0.2 (0.2 to 0.2)	0.2 (0.2 to 0.3)	-6.7 (-14.6 to 2.3)	1.9 (-4.8 to 8.7)	0.3 (0.2 to 0.3)	0.3 (0.2 to 0.3)	0.3 (0.3 to 0.3)	-4.5 (-13.8 to 6.4)	8.0 (0.4 to 16.6)
Occupational exposure to formaldehyde	0.1 (0.1 to 0.1)	0.1 (0.1 to 0.1)	0.1 (0.1 to 0.1)	-24.2 (-31.9 to -16.1)	0.3 (-8.0 to 9.9)	0.1 (0.1 to 0.2)	0.1 (0.1 to 0.1)	0.1 (0.1 to 0.1)	-18.7 (-28.5 to -7.5)	7.8 (-1.6 to 17.9)
Occupational exposure to nickel	0.2 (0.0 to 0.9)	0.1 (0.0 to 0.6)	0.1 (0.0 to 0.6)	-	-	0.2 (0.0 to 1.0)	0.2 (0.0 to 0.7)	0.2 (0.0 to 0.7)	-	-
Occupational exposure to polycyclic aromatic hydrocarbons	0.1 (0.1 to 0.1)	0.1 (0.1 to 0.1)	0.1 (0.1 to 0.1)	-20.6 (-27.7 to -12.9)	-1.6 (-8.1 to 6.2)	0.1 (0.1 to 0.2)	0.1 (0.1 to 0.1)	0.1 (0.1 to 0.1)	-15.7 (-24.9 to -4.9)	6.5 (-1.3 to 15.5)
Occupational exposure to silica	2.4 (0.3 to 7.8)	2.0 (0.3 to 6.2)	2.0 (0.3 to 6.2)	-16.2 (-24.8 to -6.3)	-1.8 (-9.7 to 7.9)	2.1 (0.3 to 6.7)	1.6 (0.2 to 4.8)	1.5 (0.2 to 4.6)	-25.9 (-34.4 to -15.5)	-5.1 (-13.5 to 5.5)
Occupational exposure to sulphuric acid	0.3 (0.1 to 1.0)	0.3 (0.1 to 0.8)	0.2 (0.1 to 0.8)	-17.4 (-24.7 to -9.1)	-6.1 (-13.1 to 1.7)	0.4 (0.1 to 1.2)	0.3 (0.1 to 0.9)	0.3 (0.1 to 1.0)	-20.0 (-28.3 to -10.3)	-2.6 (-9.9 to 6.4)
Occupational exposure to trichloroethylene	0.0 (0.0 to 0.0)	0.0 (0.0 to 0.0)	0.0 (0.0 to 0.0)	-19.5 (-26.4 to -11.8)	-0.9 (-6.9 to 6.3)	0.0 (0.0 to 0.0)	0.0 (0.0 to 0.0)	0.0 (0.0 to 0.0)	-13.9 (-22.7 to -3.4)	6.8 (-0.3 to 15.1)
Occupational asthmagens	11.2 (8.9 to 14.0)	8.5 (6.9 to 10.4)	7.6 (6.2 to 9.3)	-24.7 (-34.9 to -12.3)	-10.0 (-19.8 to 0.2)	9.1 (6.8 to 12.1)	6.7 (5.2 to 8.5)	5.8 (4.6 to 7.2)	-26.7 (-39.5 to -9.2)	-13.9 (-26.7 to 1.1)
Occupational particulate matter, gases, and fumes	4.9 (3.5 to 7.0)	4.5 (3.2 to 6.4)	4.1 (2.9 to 5.9)	-9.6 (-14.4 to -5.0)	-9.0 (-12.3 to -5.6)	3.5 (2.5 to 5.1)	3.2 (2.3 to 4.7)	2.9 (2.0 to 4.2)	-8.4 (-13.8 to -2.2)	-11.4 (-15.5 to -7.3)
Occupational noise	4.6 (4.1 to 5.2)	4.3 (3.9 to 4.7)	4.0 (3.7 to 4.4)	-6.9 (-10.5 to -2.7)	-7.0 (-9.5 to -4.5)	3.5 (3.0 to 4.0)	3.4 (3.1 to 3.8)	3.2 (3.0 to 3.6)	-3.0 (-7.7 to 2.8)	-6.2 (-9.1 to -3.0)
Occupational ergonomic factors	12.9 (10.8 to 15.0)	9.6 (8.4 to 10.8)	7.7 (6.6 to 9.0)	-25.1 (-36.7 to -12.1)	-20.3 (-29.6 to -10.2)	11.9 (9.5 to 14.2)	8.9 (7.7 to 10.1)	6.7 (5.5 to 8.2)	-25.8 (-40.2 to -5.9)	-24.4 (-35.8 to -10.5)
Non-exclusive breastfeeding	0.5 (0.4 to 0.7)	0.5 (0.4 to 0.7)	0.5 (0.4 to 0.7)	0.9 (-6.9 to 9.3)	-1.0 (-8.5 to 7.1)	0.5 (0.4 to 0.7)	0.5 (0.4 to 0.7)	0.5 (0.4 to 0.7)	0.9 (-6.9 to 9.3)	-1.0 (-8.5 to 7.1)
Discontinued breastfeeding	1.7 (1.6 to 1.8)	1.8 (1.8 to 1.9)	1.8 (1.8 to 1.9)	8.4 (2.1 to 14.9)	0.2 (-4.2 to 5.4)	1.7 (1.6 to 1.8)	1.9 (1.8 to 1.9)	1.9 (1.8 to 1.9)	8.5 (2.3 to 15.0)	-0.2 (-4.5 to 5.0)
Child underweight	0.2 (0.1 to 0.3)	0.1 (0.1 to 0.2)	0.1 (0.1 to 0.2)	-31.1 (-46.4 to -16.5)	-12.5 (-22.1 to -3.1)	0.3 (0.2 to 0.4)	0.2 (0.1 to 0.2)	0.1 (0.1 to 0.2)	-39.6 (-55.9 to -21.4)	-20.4 (-31.5 to -9.6)
Child wasting	0.3 (0.2 to 0.4)	0.2 (0.1 to 0.3)	0.1 (0.1 to 0.2)	-29.8 (-52.8 to 3.6)	-22.3 (-36.6 to -5.2)	0.2 (0.1 to 0.3)	0.2 (0.1 to 0.2)	0.1 (0.1 to 0.2)	-21.9 (-47.9 to 11.1)	-25.5 (-39.0 to -10.7)
Child stunting	0.8 (0.4 to 1.4)	0.6 (0.3 to 1.0)	0.5 (0.2 to 0.8)	-25.5 (-53.5 to 24.1)	-22.5 (-47.3 to 6.6)	0.9 (0.4 to 1.4)	0.6 (0.4 to 0.9)	0.5 (0.3 to 0.8)	-26.1 (-54.3 to 19.9)	-18.6 (-42.6 to 10.9)
Short gestation for birth weight	0.0 (0.0 to 0.0)	0.0 (0.0 to 0.0)	0.0 (0.0 to 0.0)	7.0 (-18.1 to 38.3)	4.1 (-21.8 to 26.5)	0.0 (0.0 to 0.0)	0.0 (0.0 to 0.0)	0.0 (0.0 to 0.0)	7.0 (-16.2 to 38.9)	3.9 (-19.4 to 29.6)
Low birth weight for gestation	0.0 (0.0 to 0.0)	0.0 (0.0 to 0.0)	0.0 (0.0 to 0.0)	3.1 (-12.0 to 23.8)	2.2 (-13.7 to 23.0)	0.0 (0.0 to 0.0)	0.0 (0.0 to 0.0)	0.0 (0.0 to 0.0)	4.6 (-10.4 to 21.7)	2.7 (-11.1 to 21.6)
Iron deficiency	-	-	-	-	-	9.3 (6.9 to 12.3)	9.2 (6.9 to 12.0)	7.5 (5.4 to 10.1)	-0.8 (-12.3 to 13.2)	-18.3 (-26.7 to -9.4)
Vitamin A deficiency	0.5 (0.4 to 0.6)	0.5 (0.3 to 0.6)	0.5 (0.4 to 0.6)	-4.8 (-19.1 to 13.1)	2.5 (-17.3 to 24.6)	0.3 (0.2 to 0.4)	0.3 (0.2 to 0.4)	0.3 (0.2 to 0.4)	-2.1 (-29.0 to 35.1)	-10.8 (-29.0 to 12.4)
Zinc deficiency	0.5 (0.1 to 1.1)	0.6 (0.2 to 1.2)	0.5 (0.1 to 1.1)	12.2 (-2.9 to 39.0)	-11.5 (-22.4 to 10.6)	0.5 (0.1 to 1.1)	0.6 (0.2 to 1.2)	0.5 (0.1 to 1.1)	12.3 (-0.9 to 32.4)	-11.9 (-23.8 to -0.6)
Smoking	14.3 (12.5 to 16.3)	13.3 (11.6 to 15.1)	14.2 (12.7 to 15.9)	-7.1 (-15.9 to 2.7)	7.1 (-1.3 to 15.0)	1.3 (0.9 to 1.8)	1.3 (1.0 to 1.6)	1.5 (1.2 to 1.9)	-3.5 (-28.4 to 28.3)	20.5 (0.1 to 44.7)
Chewing tobacco	0.1 (0.1 to 0.1)	0.1 (0.1 to 0.1)	0.1 (0.1 to 0.1)	0.5 (-27.4 to 38.4)	-0.4 (-22.4 to 27.3)	0.1 (0.0 to 0.1)	0.1 (0.0 to 0.1)	0.1 (0.0 to 0.1)	-0.6 (-27.7 to 38.4)	-0.2 (-23.3 to 27.3)
Second-hand smoke	30.7 (28.4 to 32.9)	29.5 (28.4 to 30.6)	27.0 (25.3 to 28.8)	-3.9 (-11.4 to 4.4)	-8.5 (-14.0 to -2.5)	50.8 (48.5 to 53.1)	46.3 (45.2 to 47.3)	41.4 (39.7 to 43.1)	-9.0 (-13.4 to -4.3)	-10.6 (-14.1 to -7.0)
Alcohol use	37.6 (24.7 to 47.8)	30.3 (18.9 to 42.4)	31.5 (16.8 to 46.7)	-19.3 (-42.3 to 2.4)	3.8 (-23.3 to 32.9)	16.3 (8.8 to 26.0)	12.4 (7.0 to 20.2)	12.3 (6.2 to 20.4)	-24.3 (-46.2 to 1.8)	-0.6 (-22.8 to 25.3)
Drug use	0.6 (0.4 to 0.9)	0.6 (0.5 to 0.9)	0.7 (0.5 to 0.9)	2.5 (-3.1 to 8.7)	10.7 (3.7 to 18.6)	0.2 (0.2 to 0.3)	0.2 (0.2 to 0.4)	0.3 (0.2 to 0.4)	1.0 (-4.5 to 6.8)	6.8 (-0.1 to 13.8)
Diet low in fruits	36.5 (30.8 to 40.3)	43.8 (38.8 to 46.3)	43.2 (38.0 to 46.1)	19.9 (13.0 to 29.8)	-1.4 (-4.2 to 1.0)	34.1 (28.2 to 38.2)	42.4 (37.3 to 45.2)	41.8 (36.6 to 44.9)	24.4 (15.9 to 36.3)	-1.4 (-4.6 to 1.1)
Diet low in vegetables	24.2 (18.0 to 29.4)	26.8 (21.0 to 31.5)	22.0 (14.8 to 27.9)	11.0 (1.8 to 24.6)	-18.2 (-32.6 to -8.4)	26.4 (20.5 to 31.3)	29.1 (23.9 to 33.5)	24.3 (17.5 to 30.0)	10.3 (1.5 to 22.4)	-16.5 (-29.2 to -8.1)
Diet low in legumes	53.0 (52.6 to 53.3)	51.5 (50.8 to 52.1)	48.7 (47.4 to 49.9)	-2.8 (-3.7 to -2.1)	-5.4 (-7.0 to -4.1)	53.6 (53.3 to 53.9)	52.3 (51.8 to 52.8)	50.2 (49.1 to 51.0)	-2.3 (-3.0 to -1.7)	-4.2 (-5.5 to -3.1)
Diet low in whole grains	42.6 (40.6 to 44.3)	41.0 (38.7 to 43.0)	39.6 (36.9 to 41.8)	-3.7 (-5.7 to -1.9)	-3.5 (-5.9 to -1.5)	43.4 (41.5 to 45.0)	41.9 (39.7 to 43.6)	40.5 (38.1 to 42.5)	-3.5 (-5.4 to -1.9)	-3.3 (-5.4 to -1.4)
Diet low in nuts and seeds	47.8 (45.9 to 49.3)	51.4 (50.4 to 52.1)	49.6 (48.0 to 50.8)	7.4 (5.0 to 10.7)	-3.4 (-5.1 to -2.1)	48.2 (46.3 to 49.7)	51.6 (50.7 to 52.3)	49.9 (48.4 to 51.0)	7.0 (4.5 to 10.1)	-3.2 (-4.7 to -1.9)
Diet low in milk	38.1 (34.6 to 41.0)	38.8 (35.5 to 41.6)	36.9 (33.0 to 40.1)	2.0 (-0.5 to 5.0)	-5.0 (-8.1 to -2.5)	38.8 (35.6 to 41.6)	39.7 (36.6 to 42.3)	37.8 (34.1 to 40.8)	2.2 (-0.1 to 5.1)	-4.9 (-7.8 to -2.4)
Diet high in red meat	4.5 (1.3 to 8.4)	0.3 (0.0 to 1.1)	1.9 (0.1 to 4.8)	-94.2 (-100.0 to -83.0)	642.0 (177.7 to 252202.7)	0.3 (0.0 to 1.4)	0.0 (0.0 to 0.0)	0.1 (0.0 to 0.7)	-99.4 (-100.0 to -95.9)	4729.4 (-38.0 to [illegible]
Diet high in processed meat	5.3 (2.3 to 8.3)	2.2 (0.2 to 4.8)	2.6 (0.4 to 5.3)	-59.1 (-91.5 to -38.1)	20.6 (-0.6 to 144.5)	3.3 (0.9 to 6.1)	1.2 (0.0 to 3.4)	1.5 (0.1 to 3.9)	-62.8 (-97.8 to -39.8)	24.2 (0.2 to 374.9)
Diet high in sugar-sweetened beverages	1.9 (0.9 to 3.4)	1.0 (0.4 to 1.9)	1.6 (0.7 to 2.9)	-48.9 (-73.1 to -2.8)	59.9 (3.2 to 157.1)	1.3 (0.6 to 2.5)	0.7 (0.3 to 1.4)	1.1 (0.4 to 2.0)	-46.0 (-72.0 to -2.4)	51.2 (-5.5 to 146.6)
Diet low in fibre	22.4 (16.1 to 27.8)	26.2 (20.5 to 31.0)	26.1 (20.1 to 31.1)	17.0 (8.2 to 32.4)	-0.5 (-7.1 to 6.3)	28.3 (22.7 to 32.9)	31.7 (26.7 to 35.7)	31.5 (26.3 to 35.8)	12.3 (5.8 to 21.8)	-0.8 (-6.0 to 4.2)
Diet low in calcium	30.8 (25.8 to 34.6)	31.7 (27.1 to 35.4)	28.2 (22.7 to 32.5)	3.1 (0.8 to 6.1)	-11.1 (-16.9 to -7.6)	33.7 (29.4 to 37.1)	34.6 (30.6 to 37.9)	31.4 (26.6 to 35.2)	2.8 (0.9 to 5.4)	-9.4 (-13.7 to -6.3)
Diet low in seafood omega-3 fatty acids	45.5 (43.7 to 47.3)	45.3 (43.4 to 47.1)	42.1 (39.4 to 44.4)	-0.6 (-1.3 to 0.1)	-7.1 (-9.4 to -5.3)	46.9 (45.3 to 48.4)	46.6 (45.0 to 48.2)	43.8 (41.6 to 45.9)	-0.5 (-1.1 to 0.0)	-6.0 (-7.7 to -4.5)
Diet low in polyunsaturated fatty acids	41.5 (38.8 to 44.1)	39.1 (36.1 to 41.6)	33.6 (29.5 to 37.2)	-5.9 (-10.3 to -1.7)	-14.0 (-19.1 to -10.1)	41.9 (39.2 to 44.4)	39.6 (36.6 to 42.0)	34.2 (30.4 to 37.5)	-5.6 (-9.6 to -1.4)	-13.6 (-18.4 to -9.4)
Diet high in trans fatty acids	2.2 (0.3 to 5.0)	1.9 (0.2 to 4.5)	1.7 (0.1 to 4.3)	-13.8 (-46.3 to 7.7)	-7.8 (-35.9 to 13.0)	3.0 (0.6 to 6.3)	2.6 (0.4 to 5.5)	2.4 (0.3 to 5.2)	-14.2 (-43.6 to 8.1)	-8.0 (-31.1 to 13.1)
Diet high in sodium	6.5 (0.7 to 13.6)	4.7 (0.1 to 11.3)	5.3 (0.2 to 12.3)	-28.5 (-85.8 to -7.5)	13.2 (-18.8 to 124.6)	5.0 (0.3 to 11.5)	3.8 (0.1 to 10.1)	3.8 (0.0 to 10.2)	-23.3 (-81.9 to -3.4)	-1.5 (-65.9 to 24.5)
Intimate partner violence	-	-	-	-	-	5.2 (4.4 to 6.0)	5.0 (4.4 to 5.6)	4.5 (3.8 to 5.1)	-3.2 (-10.6 to 5.6)	-11.0 (-18.7 to -3.4)
Childhood sexual abuse	3.9 (3.2 to 4.8)	3.9 (3.2 to 4.8)	3.9 (3.2 to 4.8)	0.0 (0.0 to 0.0)	0.0 (0.0 to 0.0)	11.4 (9.1 to 13.9)	11.4 (9.1 to 13.9)	11.4 (9.1 to 13.9)	0.0 (0.0 to 0.0)	0.0 (0.0 to 0.0)

Supplementary Results Table 1. Moldova age-standardised Summary Exposure Values (SEVs) for Level 4 risk factors and percentage change, by sex, 1990, 2007, and 2017.Data in parentheses are 95% uncertainty intervals.										
	**Male**					**Female**				
	**1990**	**2007**	**2017**	**Percentage change 1990-2007**	**Percentage change 2007-2017**	**1990**	**2007**	**2017**	**Percentage change 1990-2007**	**Percentage change 2007-2017**
Bullying victimization	8.0 (3.2 to 16.8)	8.5 (3.5 to 18.1)	8.2 (3.4 to 17.4)	6.9 (1.3 to 14.6)	-3.8 (-7.4 to -0.7)	7.4 (2.8 to 16.2)	7.8 (3.0 to 16.8)	7.5 (2.9 to 16.2)	5.8 (0.4 to 12.5)	-4.0 (-7.6 to -0.6)
Low physical activity	0.3 (0.1 to 0.5)	0.3 (0.1 to 0.5)	0.3 (0.1 to 0.5)	0.6 (-1.9 to 3.3)	1.6 (-0.8 to 5.0)	0.3 (0.1 to 0.5)	0.3 (0.1 to 0.5)	0.3 (0.1 to 0.5)	0.5 (-1.9 to 3.1)	1.4 (-0.8 to 4.1)
High fasting plasma glucose	2.7 (1.6 to 4.3)	3.1 (2.1 to 4.7)	2.8 (1.7 to 4.3)	12.8 (-18.6 to 71.4)	-10.6 (-40.0 to 22.8)	2.7 (1.7 to 4.1)	2.5 (1.7 to 3.7)	2.5 (1.6 to 3.7)	-6.9 (-35.2 to 28.3)	-1.3 (-30.2 to 36.3)
High LDL cholesterol	10.0 (8.5 to 11.6)	9.5 (8.1 to 11.1)	9.7 (8.3 to 11.3)	-4.3 (-8.3 to -0.1)	1.8 (-1.5 to 5.3)	10.9 (9.5 to 12.5)	10.5 (9.2 to 12.1)	11.0 (9.6 to 12.6)	-3.3 (-7.1 to 0.9)	4.2 (0.9 to 7.7)
High systolic blood pressure	10.0 (9.0 to 11.0)	10.5 (9.5 to 11.4)	11.7 (10.7 to 12.7)	5.1 (-2.1 to 13.0)	11.2 (5.4 to 17.4)	10.4 (9.4 to 11.4)	11.3 (10.3 to 12.2)	12.2 (11.2 to 13.3)	8.4 (-0.6 to 18.0)	8.5 (1.0 to 16.2)
High body-mass index	5.5 (3.5 to 8.1)	6.5 (4.4 to 9.5)	10.4 (7.4 to 14.3)	19.9 (4.4 to 38.7)	58.7 (40.0 to 85.0)	7.2 (4.9 to 10.3)	8.3 (5.7 to 11.5)	12.1 (8.9 to 16.0)	14.9 (1.9 to 31.5)	45.2 (31.1 to 66.1)
Low bone mineral density	4.1 (3.3 to 4.9)	4.2 (3.4 to 5.0)	4.0 (3.2 to 5.0)	1.7 (-13.3 to 19.9)	-2.6 (-17.3 to 14.6)	5.1 (4.2 to 6.2)	5.2 (4.3 to 6.1)	5.1 (4.2 to 6.1)	1.1 (-11.7 to 16.5)	-1.3 (-15.8 to 13.9)
Impaired kidney function	3.2 (1.5 to 7.4)	3.2 (1.5 to 7.4)	3.2 (1.4 to 7.4)	1.1 (-3.5 to 6.7)	-1.7 (-7.1 to 2.5)	3.7 (1.7 to 8.1)	3.9 (1.9 to 8.2)	3.8 (1.8 to 8.2)	4.5 (-0.4 to 13.4)	-2.5 (-8.9 to 1.9)

Supplementary Results Table 1. Russian Federation age-standardised Summary Exposure Values (SEVs) for Level 4 risk factors and percentage change, by sex, 1990, 2007, and 2017.Data in parentheses are 95% uncertainty intervals.										
	Male					Female				
	1990	2007	2017	Percentage change 1990-2007	Percentage change 2007-2017	1990	2007	2017	Percentage change 1990-2007	Percentage change 2007-2017
Unsafe water source	15.5 (11.1 to 20.7)	9.8 (7.3 to 12.9)	8.0 (5.5 to 11.2)	-36.7 (-57.3 to -9.4)	-18.7 (-39.3 to 7.8)	15.5 (11.1 to 20.7)	9.9 (7.4 to 12.9)	8.0 (5.5 to 11.2)	-36.4 (-57.1 to -8.9)	-19.3 (-39.8 to 6.9)
Unsafe sanitation	43.9 (32.3 to 57.4)	28.7 (22.3 to 36.5)	20.8 (14.2 to 29.8)	-34.7 (-50.1 to -12.7)	-27.5 (-47.4 to -1.2)	43.8 (32.3 to 57.2)	28.8 (22.4 to 36.6)	20.7 (14.1 to 29.7)	-34.3 (-49.8 to -12.1)	-28.1 (-47.8 to -2.0)
No access to handwashing facility	10.3 (8.2 to 12.5)	7.7 (6.1 to 9.5)	6.7 (5.3 to 8.3)	-25.1 (-41.6 to -5.8)	-13.1 (-27.3 to 3.6)	10.3 (8.2 to 12.5)	7.7 (6.1 to 9.5)	6.7 (5.3 to 8.3)	-25.0 (-41.5 to -5.8)	-13.3 (-27.5 to 3.4)
Ambient particulate matter pollution	40.1 (30.1 to 54.2)	38.9 (29.0 to 52.6)	33.6 (25.1 to 46.3)	-3.0 (-14.7 to 9.8)	-13.6 (-24.2 to -2.8)	40.0 (30.1 to 54.2)	38.8 (29.0 to 52.4)	33.6 (25.1 to 46.3)	-3.1 (-14.9 to 9.4)	-13.4 (-24.2 to -2.6)
Household air pollution from solid fuels	0.2 (0.1 to 0.5)	0.2 (0.1 to 0.5)	0.2 (0.1 to 0.3)	11.9 (-32.5 to 105.9)	-33.6 (-62.7 to -1.4)	0.3 (0.1 to 1.3)	0.4 (0.1 to 1.5)	0.3 (0.1 to 1.1)	11.9 (-32.1 to 108.0)	-31.2 (-62.3 to -1.2)
Ambient ozone pollution	35.2 (12.1 to 53.3)	35.7 (12.3 to 53.7)	37.3 (12.9 to 54.3)	1.3 (0.6 to 2.2)	4.6 (1.0 to 7.0)	35.2 (12.1 to 53.3)	35.7 (12.3 to 53.7)	37.3 (12.9 to 54.3)	1.4 (0.6 to 2.3)	4.7 (1.0 to 7.0)
Residential radon	40.6 (24.0 to 57.0)	40.7 (23.7 to 57.2)	40.6 (23.7 to 57.2)	0.0 (-2.1 to 2.1)	-0.0 (-0.9 to 0.8)	40.6 (23.9 to 57.1)	40.7 (23.7 to 57.1)	40.7 (23.7 to 57.3)	0.0 (-1.6 to 1.5)	0.0 (-1.4 to 1.4)
Lead exposure	2.1 (0.1 to 5.5)	2.3 (0.2 to 5.6)	1.9 (0.2 to 5.0)	8.3 (-0.2 to 77.9)	-15.3 (-24.5 to -9.8)	1.2 (0.0 to 4.1)	1.3 (0.0 to 4.2)	1.1 (0.0 to 3.8)	7.8 (0.1 to 89.1)	-15.3 (-25.2 to -9.8)
Occupational exposure to asbestos	1.0 (0.8 to 1.4)	1.5 (1.5 to 1.6)	1.3 (1.2 to 1.4)	48.1 (12.5 to 92.8)	-16.6 (-21.5 to -11.3)	0.9 (0.7 to 1.2)	1.0 (1.0 to 1.1)	0.9 (0.8 to 0.9)	6.9 (-18.4 to 38.5)	-13.1 (-19.0 to -7.4)
Occupational exposure to arsenic	0.3 (0.0 to 0.6)	0.2 (0.0 to 0.6)	0.2 (0.0 to 0.6)	-	-	0.3 (0.0 to 0.7)	0.3 (0.0 to 0.7)	0.3 (0.0 to 0.7)	-	-
Occupational exposure to benzene	0.4 (0.0 to 1.3)	0.5 (0.0 to 1.3)	0.5 (0.0 to 1.4)	7.1 (0.0 to 16.6)	3.6 (-1.6 to 11.1)	0.7 (0.0 to 2.0)	0.8 (0.0 to 2.4)	0.9 (0.1 to 2.5)	18.6 (9.1 to 33.3)	10.0 (4.3 to 18.5)
Occupational exposure to beryllium	0.0 (0.0 to 0.0)	0.0 (0.0 to 0.0)	0.0 (0.0 to 0.0)	-4.1 (-8.6 to 0.9)	-0.9 (-3.7 to 2.0)	0.0 (0.0 to 0.0)	0.0 (0.0 to 0.0)	0.0 (0.0 to 0.0)	5.8 (-0.7 to 12.5)	4.9 (1.1 to 8.8)
Occupational exposure to cadmium	0.0 (0.0 to 0.0)	0.0 (0.0 to 0.0)	0.0 (0.0 to 0.0)	-6.2 (-14.8 to 3.1)	-2.2 (-8.8 to 4.8)	0.0 (0.0 to 0.0)	0.0 (0.0 to 0.0)	0.0 (0.0 to 0.0)	-2.6 (-14.1 to 10.1)	0.7 (-7.1 to 9.4)
Occupational exposure to chromium	0.1 (0.1 to 0.1)	0.1 (0.1 to 0.1)	0.1 (0.1 to 0.1)	-4.2 (-12.3 to 4.6)	-1.2 (-7.0 to 4.8)	0.1 (0.1 to 0.1)	0.1 (0.1 to 0.1)	0.1 (0.1 to 0.1)	0.8 (-9.6 to 11.3)	2.4 (-4.0 to 9.8)
Occupational exposure to diesel engine exhaust	0.4 (0.4 to 0.5)	0.4 (0.4 to 0.4)	0.4 (0.4 to 0.4)	-0.3 (-7.2 to 7.5)	1.3 (-3.3 to 6.3)	0.4 (0.4 to 0.4)	0.4 (0.4 to 0.5)	0.5 (0.4 to 0.5)	9.2 (0.1 to 20.2)	5.7 (-0.8 to 11.9)
Occupational exposure to formaldehyde	0.2 (0.1 to 0.2)	0.2 (0.1 to 0.2)	0.2 (0.1 to 0.2)	-1.9 (-11.6 to 8.5)	-0.1 (-7.3 to 7.7)	0.2 (0.2 to 0.2)	0.2 (0.2 to 0.2)	0.2 (0.2 to 0.2)	1.5 (-9.9 to 14.5)	2.5 (-4.9 to 11.3)
Occupational exposure to nickel	0.3 (0.0 to 1.3)	0.3 (0.0 to 1.2)	0.2 (0.0 to 1.1)	-	-	0.3 (0.0 to 1.2)	0.3 (0.0 to 1.1)	0.3 (0.0 to 1.1)	-	-
Occupational exposure to polycyclic aromatic hydrocarbons	0.2 (0.1 to 0.2)	0.1 (0.1 to 0.2)	0.1 (0.1 to 0.2)	-3.7 (-10.6 to 4.2)	-1.1 (-6.1 to 4.1)	0.2 (0.2 to 0.2)	0.2 (0.2 to 0.2)	0.2 (0.2 to 0.2)	2.8 (-6.9 to 12.6)	3.3 (-2.5 to 9.9)
Occupational exposure to silica	3.7 (0.5 to 11.8)	3.3 (0.5 to 10.7)	3.3 (0.5 to 10.6)	-8.4 (-17.4 to 1.6)	-1.6 (-8.5 to 6.3)	2.7 (0.3 to 9.3)	2.4 (0.4 to 8.0)	2.4 (0.3 to 7.6)	-8.8 (-19.7 to 4.6)	-3.4 (-11.9 to 5.8)
Occupational exposure to sulphuric acid	0.5 (0.1 to 1.5)	0.4 (0.1 to 1.4)	0.4 (0.1 to 1.4)	-3.3 (-11.2 to 5.3)	-2.9 (-9.2 to 4.0)	0.5 (0.1 to 1.6)	0.5 (0.1 to 1.4)	0.5 (0.1 to 1.5)	-1.5 (-12.9 to 11.3)	-0.3 (-8.3 to 8.4)
Occupational exposure to trichloroethylene	0.0 (0.0 to 0.0)	0.0 (0.0 to 0.0)	0.0 (0.0 to 0.0)	-1.6 (-8.0 to 5.4)	-0.2 (-4.7 to 4.5)	0.1 (0.0 to 0.1)	0.1 (0.1 to 0.1)	0.1 (0.1 to 0.1)	4.1 (-4.0 to 13.4)	4.0 (-1.4 to 10.1)
Occupational asthmagens	12.7 (10.9 to 14.9)	13.4 (11.6 to 15.6)	13.6 (11.7 to 16.0)	4.8 (-4.4 to 14.4)	1.7 (-4.7 to 7.8)	7.9 (6.5 to 9.7)	8.1 (6.7 to 9.6)	8.5 (7.0 to 10.3)	1.6 (-9.2 to 13.9)	5.9 (-3.2 to 16.7)
Occupational particulate matter, gases, and fumes	5.8 (4.2 to 8.4)	5.6 (4.0 to 8.1)	5.6 (4.0 to 8.0)	-3.1 (-6.9 to 1.1)	0.4 (-2.1 to 3.2)	3.3 (2.4 to 4.9)	3.2 (2.2 to 4.6)	3.1 (2.2 to 4.5)	-4.9 (-11.5 to 1.8)	-3.5 (-7.4 to 0.4)
Occupational noise	5.6 (5.1 to 6.2)	5.5 (5.1 to 6.0)	5.5 (5.2 to 6.0)	-2.2 (-5.1 to 1.0)	1.1 (-0.7 to 3.2)	3.8 (3.4 to 4.2)	3.8 (3.5 to 4.2)	3.9 (3.6 to 4.2)	0.5 (-3.7 to 5.1)	2.2 (-0.2 to 4.6)
Occupational ergonomic factors	8.2 (7.0 to 9.6)	8.3 (7.1 to 9.7)	8.1 (6.9 to 9.6)	1.2 (-6.0 to 9.3)	-2.0 (-7.2 to 3.2)	6.3 (5.3 to 7.5)	6.4 (5.5 to 7.5)	6.7 (5.6 to 8.0)	1.5 (-11.4 to 15.1)	4.1 (-4.1 to 13.5)
Non-exclusive breastfeeding	0.5 (0.4 to 0.7)	0.6 (0.4 to 0.7)	0.5 (0.4 to 0.7)	4.3 (-4.3 to 13.8)	-3.7 (-10.3 to 3.7)	0.5 (0.4 to 0.7)	0.6 (0.4 to 0.7)	0.5 (0.4 to 0.7)	4.5 (-4.2 to 13.9)	-3.7 (-10.3 to 3.7)
Discontinued breastfeeding	2.0 (1.9 to 2.1)	2.2 (2.1 to 2.3)	2.3 (2.2 to 2.4)	9.4 (3.3 to 15.4)	1.5 (-2.6 to 6.0)	2.0 (1.9 to 2.1)	2.2 (2.1 to 2.3)	2.3 (2.2 to 2.4)	9.5 (3.4 to 15.6)	1.5 (-2.6 to 6.0)
Child underweight	0.2 (0.1 to 0.2)	0.1 (0.1 to 0.2)	0.1 (0.1 to 0.1)	-37.8 (-48.6 to -28.2)	-12.8 (-19.5 to -6.6)	0.2 (0.1 to 0.3)	0.1 (0.1 to 0.2)	0.1 (0.1 to 0.1)	-37.6 (-49.2 to -25.3)	-13.9 (-21.6 to -5.8)
Child wasting	0.2 (0.2 to 0.3)	0.2 (0.1 to 0.3)	0.2 (0.1 to 0.3)	-4.7 (-25.7 to 22.3)	-10.7 (-26.3 to 10.7)	0.2 (0.2 to 0.3)	0.2 (0.1 to 0.3)	0.2 (0.1 to 0.3)	3.4 (-21.1 to 34.1)	-16.0 (-31.5 to 2.1)
Child stunting	1.4 (1.0 to 1.8)	0.9 (0.6 to 1.2)	0.8 (0.5 to 1.3)	-38.4 (-57.1 to -16.7)	-6.5 (-29.9 to 25.0)	1.3 (0.9 to 1.7)	0.6 (0.4 to 0.9)	0.6 (0.3 to 0.9)	-52.0 (-68.3 to -32.5)	-12.9 (-38.2 to 18.7)
Short gestation for birth weight	0.0 (0.0 to 0.0)	0.0 (0.0 to 0.0)	0.0 (0.0 to 0.0)	10.8 (1.6 to 20.9)	3.9 (-2.4 to 10.0)	0.0 (0.0 to 0.0)	0.0 (0.0 to 0.0)	0.0 (0.0 to 0.0)	6.7 (-2.1 to 16.2)	2.5 (-3.0 to 8.4)
Low birth weight for gestation	0.0 (0.0 to 0.0)	0.0 (0.0 to 0.0)	0.0 (0.0 to 0.0)	4.8 (-2.6 to 13.1)	3.3 (-2.1 to 9.2)	0.0 (0.0 to 0.0)	0.0 (0.0 to 0.0)	0.0 (0.0 to 0.0)	3.0 (-5.0 to 11.7)	2.6 (-2.1 to 8.7)
Iron deficiency	-	-	-	-	-	6.0 (4.3 to 8.1)	5.5 (3.8 to 7.5)	4.9 (3.3 to 6.8)	-7.8 (-23.4 to 9.0)	-11.9 (-23.7 to 1.3)
Vitamin A deficiency	0.5 (0.4 to 0.6)	0.4 (0.3 to 0.5)	0.4 (0.3 to 0.5)	-14.8 (-18.8 to -11.5)	1.8 (-4.9 to 7.3)	0.3 (0.2 to 0.4)	0.2 (0.2 to 0.3)	0.2 (0.2 to 0.3)	-24.5 (-29.7 to -16.4)	6.6 (1.6 to 11.7)
Zinc deficiency	0.4 (0.1 to 0.9)	0.4 (0.1 to 1.0)	0.4 (0.1 to 0.8)	3.0 (-7.2 to 96.0)	-21.3 (-61.5 to 6.5)	0.4 (0.1 to 0.9)	0.5 (0.1 to 1.0)	0.4 (0.1 to 0.8)	3.1 (-7.1 to 101.9)	-21.9 (-61.1 to 5.8)
Smoking	17.4 (15.4 to 19.5)	16.8 (14.9 to 18.9)	16.6 (14.8 to 18.7)	-3.2 (-8.1 to 2.1)	-1.1 (-4.9 to 2.9)	2.5 (1.9 to 3.3)	2.5 (2.1 to 3.0)	3.5 (2.9 to 4.2)	-0.7 (-19.4 to 25.1)	40.6 (31.5 to 50.3)
Chewing tobacco	0.1 (0.1 to 0.1)	0.1 (0.1 to 0.1)	0.1 (0.1 to 0.1)	0.6 (-25.8 to 36.3)	-0.4 (-21.2 to 25.9)	0.1 (0.0 to 0.1)	0.1 (0.0 to 0.1)	0.1 (0.0 to 0.1)	-0.1 (-28.9 to 42.7)	0.0 (-21.7 to 30.2)
Second-hand smoke	33.1 (30.9 to 35.2)	35.5 (34.1 to 36.9)	34.3 (31.9 to 36.9)	7.4 (-0.4 to 16.0)	-3.5 (-8.9 to 2.1)	58.3 (56.1 to 60.5)	57.1 (55.6 to 58.6)	51.8 (49.5 to 54.4)	-2.1 (-6.1 to 2.4)	-9.3 (-12.4 to -5.8)
Alcohol use	18.8 (11.9 to 27.5)	29.9 (21.4 to 38.8)	31.9 (21.0 to 43.9)	58.6 (24.9 to 108.9)	6.9 (-13.5 to 27.1)	7.8 (4.2 to 14.4)	12.0 (7.1 to 19.2)	13.5 (7.5 to 21.4)	53.9 (21.9 to 109.5)	12.5 (-10.4 to 42.8)
Drug use	0.8 (0.6 to 1.0)	1.0 (0.8 to 1.4)	0.8 (0.6 to 1.1)	33.7 (30.3 to 37.5)	-20.7 (-24.4 to -16.9)	0.3 (0.2 to 0.4)	0.3 (0.3 to 0.4)	0.3 (0.2 to 0.4)	16.8 (11.1 to 22.4)	-14.1 (-19.5 to -9.5)
Diet low in fruits	44.8 (39.8 to 47.3)	41.1 (35.8 to 44.1)	37.1 (31.3 to 40.9)	-8.3 (-11.8 to -5.0)	-9.8 (-14.8 to -5.8)	43.4 (38.3 to 46.1)	39.2 (33.6 to 42.4)	34.6 (28.6 to 38.7)	-9.9 (-14.3 to -6.3)	-11.7 (-17.4 to -7.0)
Diet low in vegetables	29.8 (24.8 to 34.1)	23.9 (18.5 to 28.8)	24.6 (18.7 to 29.9)	-19.8 (-28.6 to -13.2)	2.9 (-5.8 to 12.9)	32.4 (27.8 to 36.3)	26.8 (21.4 to 31.5)	26.4 (20.6 to 31.5)	-17.2 (-24.7 to -11.4)	-1.5 (-9.7 to 6.4)
Diet low in legumes	42.1 (39.8 to 44.1)	44.2 (42.3 to 45.8)	42.8 (40.5 to 44.8)	4.9 (3.1 to 7.1)	-3.2 (-5.2 to -1.3)	44.4 (42.3 to 46.1)	46.1 (44.5 to 47.5)	45.0 (43.1 to 46.7)	3.7 (2.2 to 5.7)	-2.4 (-4.2 to -0.8)
Diet low in whole grains	49.0 (48.1 to 49.8)	46.3 (44.9 to 47.6)	44.6 (42.9 to 46.2)	-5.5 (-6.8 to -4.3)	-3.6 (-5.1 to -2.3)	49.3 (48.4 to 50.1)	46.8 (45.5 to 48.0)	45.2 (43.6 to 46.7)	-5.0 (-6.3 to -4.0)	-3.3 (-4.7 to -2.1)
Diet low in nuts and seeds	52.9 (52.4 to 53.3)	51.7 (51.0 to 52.4)	50.2 (49.1 to 51.2)	-2.2 (-3.0 to -1.5)	-3.0 (-4.2 to -2.1)	52.9 (52.5 to 53.3)	51.9 (51.1 to 52.5)	50.4 (49.2 to 51.3)	-2.0 (-2.8 to -1.4)	-2.8 (-4.0 to -2.0)
Diet low in milk	40.5 (37.4 to 43.0)	39.7 (36.6 to 42.3)	37.5 (34.0 to 40.7)	-2.0 (-4.8 to 0.6)	-5.4 (-8.6 to -3.0)	41.2 (38.1 to 43.6)	40.3 (37.4 to 42.9)	38.1 (34.4 to 41.1)	-2.1 (-4.6 to 0.3)	-5.5 (-8.7 to -3.1)
Diet high in red meat	24.9 (21.1 to 28.4)	13.9 (10.7 to 17.0)	20.1 (15.1 to 24.4)	-44.1 (-54.2 to -34.1)	44.7 (19.8 to 77.4)	10.1 (6.4 to 13.7)	4.1 (2.2 to 6.3)	7.0 (3.7 to 10.6)	-59.5 (-74.5 to -44.4)	70.7 (20.3 to 164.9)
Diet high in processed meat	16.3 (12.1 to 20.2)	11.6 (8.3 to 14.7)	11.7 (8.1 to 15.0)	-28.5 (-43.0 to -13.1)	0.5 (-12.5 to 15.2)	11.1 (7.4 to 14.2)	7.7 (4.7 to 10.6)	8.0 (4.9 to 11.2)	-30.9 (-47.9 to -15.8)	3.9 (-10.1 to 22.0)
Diet high in sugar-sweetened beverages	5.7 (2.6 to 9.1)	4.3 (2.1 to 5.9)	6.2 (3.0 to 8.8)	-24.6 (-51.7 to 20.7)	43.8 (4.0 to 97.9)	3.7 (1.8 to 6.3)	3.0 (1.5 to 4.3)	4.1 (2.1 to 6.0)	-20.7 (-52.6 to 32.3)	40.0 (-3.6 to 101.1)
Diet low in fibre	27.7 (22.2 to 32.2)	29.2 (24.0 to 33.5)	27.2 (21.6 to 32.0)	5.5 (0.8 to 11.8)	-6.9 (-13.4 to -1.4)	33.1 (28.6 to 36.9)	34.4 (30.1 to 37.9)	32.6 (27.6 to 36.5)	4.0 (0.4 to 8.7)	-5.4 (-10.7 to -1.6)
Diet low in calcium	31.3 (26.5 to 35.1)	25.7 (19.9 to 30.3)	22.3 (15.8 to 27.5)	-17.9 (-25.5 to -12.7)	-13.3 (-21.4 to -8.5)	34.1 (29.8 to 37.5)	29.1 (24.0 to 33.2)	25.8 (20.0 to 30.4)	-14.6 (-20.2 to -10.5)	-11.2 (-17.4 to -7.3)
Diet low in seafood omega-3 fatty acids	34.4 (30.8 to 37.8)	35.1 (31.6 to 38.5)	31.2 (27.1 to 35.0)	2.1 (1.1 to 3.3)	-11.3 (-15.0 to -8.6)	37.1 (33.9 to 40.1)	37.8 (34.6 to 40.8)	34.3 (30.6 to 37.8)	1.9 (1.0 to 2.9)	-9.3 (-12.2 to -6.9)
Diet low in polyunsaturated fatty acids	39.7 (36.8 to 42.5)	36.5 (33.1 to 39.5)	33.6 (29.5 to 37.1)	-8.2 (-11.9 to -5.0)	-7.9 (-12.1 to -4.6)	40.1 (37.2 to 42.7)	37.0 (33.6 to 39.9)	34.1 (30.1 to 37.5)	-7.8 (-11.5 to -4.7)	-7.8 (-12.1 to -4.7)
Diet high in trans fatty acids	1.1 (0.0 to 3.2)	1.0 (0.0 to 3.1)	1.0 (0.0 to 3.2)	-2.9 (-31.5 to 25.8)	-1.0 (-25.6 to 24.3)	1.5 (0.1 to 4.0)	1.5 (0.1 to 3.9)	1.4 (0.1 to 3.8)	-2.6 (-31.8 to 27.2)	-1.3 (-22.6 to 26.7)
Diet high in sodium	7.6 (1.3 to 14.7)	7.2 (1.1 to 14.3)	7.8 (1.4 to 14.7)	-5.3 (-42.4 to 27.9)	8.5 (-12.0 to 62.7)	4.4 (0.2 to 10.8)	4.2 (0.1 to 10.5)	4.3 (0.1 to 10.7)	-5.9 (-53.1 to 46.3)	2.1 (-30.3 to 70.8)
Intimate partner violence	-	-	-	-	-	4.4 (3.7 to 5.1)	4.5 (3.8 to 5.2)	4.3 (3.6 to 4.9)	1.4 (-3.2 to 6.2)	-4.9 (-9.3 to -0.2)
Childhood sexual abuse	4.1 (3.3 to 5.0)	4.1 (3.3 to 5.0)	4.1 (3.3 to 5.0)	0.0 (-0.0 to 0.0)	0.0 (-0.0 to 0.0)	12.2 (9.6 to 15.1)	12.1 (9.6 to 15.0)	12.1 (9.6 to 15.0)	-0.7 (-6.6 to 6.3)	0.0 (-0.0 to 0.0)

Supplementary Results Table 1. Russian Federation age-standardised Summary Exposure Values (SEVs) for Level 4 risk factors and percentage change, by sex, 1990, 2007, and 2017.Data in parentheses are 95% uncertainty intervals.

	Male					Female				
	**1990**	**2007**	**2017**	**Percentage change 1990-2007**	**Percentage change 2007-2017**	**1990**	**2007**	**2017**	**Percentage change 1990-2007**	**Percentage change 2007-2017**
Bullying victimization	4.3 (1.8 to 8.6)	5.7 (2.4 to 11.6)	5.8 (2.5 to 11.9)	32.7 (17.2 to 51.5)	3.4 (-3.4 to 11.2)	4.0 (1.7 to 8.2)	5.2 (2.2 to 10.7)	5.5 (2.4 to 11.3)	28.8 (16.3 to 44.0)	6.5 (-0.4 to 14.6)
Low physical activity	0.6 (0.2 to 1.2)	0.6 (0.2 to 1.2)	0.6 (0.2 to 1.2)	-5.6 (-8.2 to -2.1)	1.4 (0.4 to 2.5)	0.5 (0.2 to 1.0)	0.5 (0.2 to 0.9)	0.5 (0.2 to 1.0)	-5.8 (-8.7 to -2.2)	1.5 (0.3 to 2.7)
High fasting plasma glucose	2.6 (1.8 to 3.8)	2.7 (1.8 to 3.8)	3.1 (2.1 to 4.6)	4.2 (-23.7 to 44.5)	14.7 (-10.2 to 49.9)	1.9 (1.3 to 2.7)	2.1 (1.4 to 3.0)	2.5 (1.7 to 3.6)	12.4 (-15.7 to 50.1)	18.2 (-8.8 to 53.8)
High LDL cholesterol	14.6 (12.9 to 16.3)	14.7 (13.0 to 16.5)	14.8 (13.1 to 16.6)	0.6 (-3.8 to 5.2)	0.8 (-2.9 to 4.5)	15.9 (14.3 to 17.7)	16.1 (14.6 to 18.1)	16.5 (14.9 to 18.5)	1.9 (-2.1 to 6.2)	2.4 (-1.1 to 5.9)
High systolic blood pressure	9.1 (8.3 to 9.9)	9.1 (8.4 to 9.9)	8.9 (8.2 to 9.6)	0.4 (-0.7 to 1.6)	-2.6 (-3.7 to -1.7)	10.1 (9.4 to 10.7)	8.9 (8.3 to 9.5)	8.2 (7.7 to 8.9)	-11.8 (-13.3 to -10.4)	-7.4 (-8.8 to -6.2)
High body-mass index	9.6 (7.4 to 13.2)	12.0 (9.2 to 16.8)	14.0 (10.8 to 19.0)	24.9 (10.0 to 42.1)	17.2 (6.6 to 28.2)	11.6 (8.9 to 15.5)	13.0 (9.9 to 17.1)	14.7 (11.4 to 19.5)	11.5 (-2.0 to 25.4)	13.5 (4.3 to 22.8)
Low bone mineral density	4.1 (3.5 to 4.8)	4.1 (3.5 to 4.8)	4.1 (3.4 to 4.8)	0.6 (-1.8 to 3.2)	-1.3 (-3.9 to 1.7)	4.8 (4.1 to 5.6)	4.7 (4.1 to 5.5)	4.7 (4.0 to 5.5)	-1.2 (-3.4 to 1.2)	-0.9 (-3.5 to 1.6)
Impaired kidney function	3.3 (1.6 to 7.5)	3.2 (1.5 to 7.3)	3.3 (1.6 to 7.3)	-1.7 (-5.0 to 1.3)	2.2 (-1.2 to 6.3)	3.9 (1.9 to 8.2)	3.8 (1.9 to 8.2)	4.0 (2.0 to 8.4)	-0.9 (-3.9 to 2.1)	4.8 (1.1 to 10.0)

Supplementary Results Table 1. Ukraine age-standardised Summary Exposure Values (SEVs) for Level 4 risk factors and percentage change, by sex, 1990, 2007, and 2017.Data in parentheses are 95% uncertainty intervals.

	Male					Female				
	1990	2007	2017	Percentage change 1990-2007	Percentage change 2007-2017	1990	2007	2017	Percentage change 1990-2007	Percentage change 2007-2017
Unsafe water source	16.5 (12.0 to 21.4)	14.2 (11.4 to 17.2)	13.7 (10.2 to 17.2)	-13.9 (-36.9 to 20.1)	-4.0 (-26.7 to 22.3)	16.5 (12.0 to 21.4)	14.2 (11.4 to 17.2)	13.7 (10.2 to 17.2)	-13.9 (-37.0 to 20.0)	-4.0 (-26.7 to 22.3)
Unsafe sanitation	34.2 (22.7 to 50.0)	32.5 (23.9 to 45.0)	30.7 (21.7 to 43.2)	-4.9 (-27.8 to 31.3)	-5.8 (-26.4 to 19.3)	34.2 (22.7 to 50.0)	32.5 (23.9 to 45.0)	30.7 (21.7 to 43.2)	-4.9 (-27.7 to 31.3)	-5.8 (-26.4 to 19.2)
No access to handwashing facility	12.0 (9.6 to 14.5)	10.5 (8.4 to 12.9)	10.0 (8.1 to 12.2)	-12.4 (-31.2 to 11.4)	-4.9 (-19.2 to 11.9)	12.0 (9.6 to 14.5)	10.5 (8.4 to 12.9)	10.0 (8.1 to 12.2)	-12.4 (-31.2 to 11.4)	-4.8 (-19.1 to 12.0)
Ambient particulate matter pollution	44.3 (22.3 to 74.9)	42.6 (25.2 to 66.9)	39.0 (20.5 to 66.0)	-3.8 (-49.1 to 101.1)	-8.5 (-54.9 to 75.7)	42.0 (20.4 to 72.5)	41.1 (23.9 to 65.6)	37.9 (19.6 to 65.3)	-2.3 (-50.3 to 115.5)	-7.7 (-56.5 to 80.5)
Household air pollution from solid fuels	7.0 (3.2 to 13.4)	4.2 (1.8 to 9.3)	2.9 (1.2 to 8.0)	-40.5 (-74.0 to 32.7)	-30.4 (-68.9 to 40.6)	8.4 (3.8 to 15.3)	5.2 (2.3 to 10.9)	3.7 (1.4 to 9.1)	-38.0 (-72.4 to 27.2)	-28.6 (-67.8 to 39.6)
Ambient ozone pollution	39.5 (13.7 to 54.8)	40.0 (14.0 to 54.8)	41.6 (14.8 to 54.8)	1.3 (0.0 to 2.4)	4.2 (0.0 to 7.2)	39.5 (13.7 to 54.8)	40.0 (14.0 to 54.8)	41.6 (14.8 to 54.8)	1.3 (0.0 to 2.4)	4.2 (0.0 to 7.2)
Residential radon	34.4 (6.9 to 69.6)	34.4 (7.1 to 69.5)	34.4 (7.1 to 69.5)	0.0 (-0.4 to 2.1)	0.0 (-0.1 to 0.3)	34.4 (6.9 to 69.6)	34.4 (7.1 to 69.4)	34.4 (7.1 to 69.5)	0.0 (-0.4 to 2.2)	0.0 (-0.2 to 0.0)
Lead exposure	1.8 (0.2 to 4.8)	1.8 (0.2 to 4.7)	1.5 (0.2 to 4.1)	-1.6 (-8.5 to 26.4)	-19.4 (-31.5 to -13.5)	1.0 (0.0 to 3.4)	1.0 (0.0 to 3.3)	0.8 (0.0 to 2.8)	-3.8 (-10.4 to 26.0)	-19.4 (-32.9 to -13.1)
Occupational exposure to asbestos	1.0 (0.8 to 1.2)	1.6 (1.1 to 1.8)	1.5 (1.3 to 1.7)	64.1 (19.8 to 117.4)	-5.5 (-22.1 to 33.0)	0.6 (0.5 to 0.8)	0.8 (0.6 to 1.0)	0.9 (0.8 to 1.0)	40.1 (10.2 to 87.6)	5.9 (-16.1 to 51.2)
Occupational exposure to arsenic	0.2 (0.0 to 0.5)	0.2 (0.0 to 0.5)	0.2 (0.0 to 0.5)	-	-	0.3 (0.0 to 0.7)	0.3 (0.0 to 0.7)	0.3 (0.0 to 0.6)	-	-
Occupational exposure to benzene	0.4 (0.0 to 1.2)	0.4 (0.0 to 1.2)	0.4 (0.0 to 1.1)	-3.1 (-9.8 to 6.3)	-2.2 (-6.3 to 4.8)	0.7 (0.0 to 2.0)	0.7 (0.0 to 2.0)	0.7 (0.0 to 2.0)	-1.9 (-10.0 to 12.6)	0.4 (-5.2 to 8.6)
Occupational exposure to beryllium	0.0 (0.0 to 0.0)	0.0 (0.0 to 0.0)	0.0 (0.0 to 0.0)	-10.3 (-14.9 to -5.7)	-8.6 (-11.8 to -5.0)	0.0 (0.0 to 0.0)	0.0 (0.0 to 0.0)	0.0 (0.0 to 0.0)	-3.2 (-9.0 to 3.5)	-4.3 (-7.6 to -0.6)
Occupational exposure to cadmium	0.0 (0.0 to 0.0)	0.0 (0.0 to 0.0)	0.0 (0.0 to 0.0)	3.6 (-4.9 to 13.0)	-8.6 (-14.9 to -1.4)	0.0 (0.0 to 0.0)	0.0 (0.0 to 0.0)	0.0 (0.0 to 0.0)	2.9 (-7.7 to 15.5)	-7.9 (-15.0 to -0.4)
Occupational exposure to chromium	0.1 (0.1 to 0.1)	0.1 (0.1 to 0.1)	0.1 (0.1 to 0.1)	4.0 (-4.5 to 13.3)	-7.4 (-13.3 to -1.1)	0.1 (0.1 to 0.1)	0.1 (0.1 to 0.1)	0.1 (0.1 to 0.1)	3.1 (-6.7 to 14.3)	-6.3 (-12.9 to 0.2)
Occupational exposure to diesel engine exhaust	0.4 (0.4 to 0.5)	0.4 (0.4 to 0.4)	0.4 (0.4 to 0.4)	-11.5 (-17.9 to -4.5)	-6.7 (-11.7 to -1.2)	0.4 (0.4 to 0.4)	0.4 (0.4 to 0.4)	0.4 (0.4 to 0.4)	-1.8 (-10.4 to 7.6)	-2.6 (-7.9 to 3.2)
Occupational exposure to formaldehyde	0.1 (0.1 to 0.1)	0.1 (0.1 to 0.1)	0.1 (0.1 to 0.1)	12.1 (1.1 to 23.7)	-6.0 (-13.1 to 2.2)	0.2 (0.1 to 0.2)	0.2 (0.2 to 0.2)	0.2 (0.1 to 0.2)	5.0 (-6.6 to 18.3)	-6.5 (-14.0 to 1.4)
Occupational exposure to nickel	0.3 (0.0 to 1.2)	0.3 (0.0 to 1.2)	0.2 (0.0 to 1.1)	-	-	0.2 (0.0 to 1.1)	0.2 (0.0 to 1.1)	0.2 (0.0 to 1.0)	-	-
Occupational exposure to polycyclic aromatic hydrocarbons	0.1 (0.1 to 0.1)	0.1 (0.1 to 0.1)	0.1 (0.1 to 0.1)	1.5 (-6.0 to 9.5)	-7.5 (-12.7 to -2.0)	0.2 (0.2 to 0.2)	0.2 (0.2 to 0.2)	0.2 (0.2 to 0.2)	1.1 (-8.0 to 11.3)	-6.0 (-11.7 to 0.1)
Occupational exposure to silica	3.7 (0.5 to 11.8)	3.4 (0.5 to 10.7)	3.1 (0.5 to 10.2)	-8.0 (-16.9 to 2.8)	-9.4 (-16.2 to -1.7)	2.4 (0.3 to 7.8)	2.3 (0.3 to 7.2)	2.0 (0.3 to 6.5)	-5.5 (-16.1 to 7.5)	-11.9 (-19.8 to -3.2)
Occupational exposure to sulphuric acid	0.4 (0.1 to 1.4)	0.4 (0.1 to 1.3)	0.4 (0.1 to 1.2)	-5.5 (-13.7 to 3.8)	-10.9 (-17.0 to -4.6)	0.5 (0.1 to 1.5)	0.5 (0.1 to 1.5)	0.4 (0.1 to 1.3)	-2.1 (-12.1 to 9.4)	-9.6 (-16.0 to -2.4)
Occupational exposure to trichloroethylene	0.0 (0.0 to 0.0)	0.0 (0.0 to 0.0)	0.0 (0.0 to 0.0)	-0.5 (-7.1 to 6.6)	-6.6 (-11.0 to -1.6)	0.0 (0.0 to 0.1)	0.0 (0.0 to 0.1)	0.0 (0.0 to 0.0)	2.9 (-5.5 to 12.9)	-4.4 (-9.6 to 1.1)
Occupational asthmagens	10.4 (9.0 to 12.1)	11.1 (9.7 to 12.9)	10.9 (9.4 to 12.7)	6.4 (-3.6 to 18.0)	-1.9 (-8.5 to 5.6)	6.6 (5.3 to 8.0)	6.3 (5.4 to 7.5)	6.4 (5.4 to 7.5)	-4.0 (-17.0 to 10.8)	0.7 (-6.2 to 7.8)
Occupational particulate matter, gases, and fumes	6.2 (4.5 to 8.9)	6.0 (4.3 to 8.6)	5.8 (4.1 to 8.3)	-3.5 (-7.4 to 0.1)	-4.0 (-6.3 to -1.6)	3.6 (2.6 to 5.3)	3.5 (2.5 to 5.1)	3.3 (2.4 to 4.8)	-2.8 (-8.5 to 3.5)	-6.0 (-9.4 to -2.2)
Occupational noise	6.2 (5.7 to 6.8)	6.0 (5.6 to 6.5)	5.7 (5.4 to 6.2)	-4.0 (-6.8 to -0.9)	-4.1 (-5.9 to -2.2)	4.2 (3.8 to 4.7)	4.1 (3.8 to 4.5)	4.0 (3.7 to 4.3)	-1.4 (-5.2 to 3.1)	-3.5 (-5.6 to -1.1)
Occupational ergonomic factors	8.4 (7.1 to 9.9)	7.3 (6.2 to 8.6)	6.9 (5.8 to 8.2)	-13.1 (-19.9 to -6.6)	-5.6 (-9.7 to -1.5)	7.0 (5.8 to 8.5)	5.6 (4.7 to 6.7)	5.2 (4.3 to 6.2)	-20.3 (-32.3 to -8.4)	-7.7 (-12.9 to -2.1)
Non-exclusive breastfeeding	0.6 (0.4 to 0.8)	0.6 (0.4 to 0.8)	0.5 (0.4 to 0.8)	0.6 (-4.7 to 5.9)	-4.5 (-9.3 to 0.4)	0.6 (0.4 to 0.8)	0.6 (0.4 to 0.8)	0.5 (0.4 to 0.8)	0.7 (-4.6 to 6.0)	-4.5 (-9.3 to 0.4)
Discontinued breastfeeding	1.9 (1.9 to 2.0)	2.1 (2.1 to 2.2)	2.0 (1.9 to 2.1)	10.7 (5.3 to 16.5)	-8.0 (-11.9 to -3.4)	1.9 (1.9 to 2.0)	2.1 (2.1 to 2.2)	2.0 (1.9 to 2.1)	10.7 (5.2 to 16.5)	-7.9 (-11.8 to -3.3)
Child underweight	0.2 (0.1 to 0.3)	0.1 (0.1 to 0.2)	0.1 (0.1 to 0.2)	-19.0 (-35.9 to -2.3)	-8.3 (-17.7 to 1.8)	0.2 (0.1 to 0.2)	0.1 (0.1 to 0.2)	0.1 (0.1 to 0.2)	-17.1 (-33.4 to -2.1)	-9.9 (-19.5 to 0.2)
Child wasting	0.4 (0.2 to 0.5)	0.3 (0.2 to 0.4)	0.3 (0.1 to 0.4)	-16.7 (-43.8 to 24.4)	-16.2 (-36.2 to 11.9)	0.3 (0.2 to 0.4)	0.2 (0.2 to 0.4)	0.2 (0.1 to 0.3)	-15.5 (-44.4 to 30.7)	-18.2 (-37.9 to 7.6)
Child stunting	1.2 (0.7 to 1.8)	1.1 (0.6 to 1.5)	0.9 (0.5 to 1.4)	-14.7 (-47.3 to 39.1)	-14.7 (-40.0 to 16.8)	1.5 (0.9 to 2.2)	1.2 (0.8 to 1.7)	1.0 (0.5 to 1.6)	-18.2 (-50.7 to 37.6)	-18.8 (-42.7 to 7.9)
Short gestation for birth weight	0.0 (0.0 to 0.0)	0.0 (0.0 to 0.0)	0.0 (0.0 to 0.0)	16.3 (-5.5 to 52.2)	-0.1 (-18.5 to 23.3)	0.0 (0.0 to 0.0)	0.0 (0.0 to 0.0)	0.0 (0.0 to 0.0)	8.5 (-14.6 to 43.1)	-3.0 (-23.3 to 18.4)
Low birth weight for gestation	0.0 (0.0 to 0.0)	0.0 (0.0 to 0.0)	0.0 (0.0 to 0.0)	6.1 (-9.8 to 25.1)	3.8 (-11.5 to 19.6)	0.0 (0.0 to 0.0)	0.0 (0.0 to 0.0)	0.0 (0.0 to 0.0)	5.5 (-10.4 to 28.9)	4.4 (-11.3 to 22.6)
Iron deficiency	-	-	-	-	-	5.1 (3.5 to 6.9)	4.3 (2.9 to 5.9)	4.0 (2.7 to 5.6)	-15.8 (-30.1 to 1.9)	-7.0 (-19.4 to 7.1)
Vitamin A deficiency	0.5 (0.4 to 0.7)	0.5 (0.4 to 0.6)	0.4 (0.3 to 0.6)	-8.3 (-22.3 to 7.3)	-8.9 (-22.3 to 7.1)	0.3 (0.2 to 0.4)	0.3 (0.2 to 0.4)	0.3 (0.2 to 0.4)	-4.2 (-18.0 to 10.5)	-2.3 (-21.5 to 17.4)
Zinc deficiency	0.5 (0.1 to 0.9)	0.5 (0.1 to 1.1)	0.5 (0.1 to 1.0)	8.4 (-4.2 to 37.7)	-5.2 (-15.3 to 15.9)	0.5 (0.1 to 0.9)	0.5 (0.1 to 1.1)	0.5 (0.1 to 1.0)	8.7 (-4.0 to 39.1)	-5.3 (-15.8 to 12.3)
Smoking	18.4 (16.3 to 20.8)	16.0 (14.0 to 17.9)	15.5 (13.7 to 17.3)	-13.2 (-17.5 to -8.7)	-3.2 (-7.6 to 1.3)	3.2 (2.3 to 4.2)	2.5 (2.0 to 3.1)	2.9 (2.4 to 3.5)	-22.6 (-37.7 to -4.3)	18.2 (5.2 to 32.6)
Chewing tobacco	0.1 (0.0 to 0.1)	0.1 (0.0 to 0.1)	0.1 (0.0 to 0.1)	-0.2 (-26.8 to 39.4)	-1.9 (-21.7 to 23.8)	0.1 (0.0 to 0.1)	0.1 (0.0 to 0.1)	0.1 (0.0 to 0.1)	-0.0 (-30.8 to 39.0)	0.7 (-21.1 to 31.2)
Second-hand smoke	39.0 (36.2 to 41.8)	35.0 (34.0 to 36.0)	33.8 (32.0 to 35.8)	-10.3 (-16.6 to -2.9)	-3.4 (-8.4 to 2.0)	66.8 (63.9 to 69.6)	56.5 (55.0 to 57.8)	52.7 (50.6 to 54.7)	-15.4 (-19.1 to -11.8)	-6.7 (-9.8 to -3.4)
Alcohol use	30.5 (19.7 to 40.9)	40.2 (30.7 to 49.4)	41.4 (30.3 to 53.4)	32.0 (11.8 to 65.5)	2.9 (-12.7 to 20.1)	14.9 (8.6 to 23.1)	22.2 (14.0 to 31.1)	24.8 (14.3 to 36.4)	48.4 (19.0 to 88.8)	11.9 (-14.2 to 44.4)
Drug use	0.6 (0.4 to 0.8)	0.6 (0.5 to 0.9)	0.7 (0.6 to 1.0)	8.4 (1.9 to 15.9)	15.1 (8.4 to 23.1)	0.2 (0.2 to 0.3)	0.2 (0.2 to 0.3)	0.3 (0.2 to 0.4)	1.5 (-4.0 to 6.6)	8.5 (3.0 to 15.3)
Diet low in fruits	45.4 (40.4 to 47.7)	45.2 (40.5 to 47.5)	43.4 (38.5 to 46.2)	-0.3 (-2.4 to 1.9)	-4.0 (-7.0 to -1.8)	44.1 (39.1 to 46.7)	43.7 (38.6 to 46.2)	41.7 (36.6 to 44.7)	-1.1 (-3.6 to 1.3)	-4.6 (-7.9 to -1.9)
Diet low in vegetables	28.8 (23.1 to 33.3)	16.8 (9.7 to 22.8)	11.6 (4.4 to 18.8)	-41.5 (-59.8 to -30.2)	-30.8 (-57.7 to -14.5)	30.8 (25.7 to 35.1)	19.2 (12.1 to 25.0)	13.7 (6.0 to 20.5)	-37.7 (-53.5 to -27.1)	-28.3 (-53.9 to -13.5)
Diet low in legumes	37.8 (34.8 to 40.5)	42.2 (39.9 to 44.2)	43.6 (41.4 to 45.5)	11.6 (8.2 to 16.0)	3.4 (1.3 to 5.6)	40.7 (38.0 to 42.9)	44.5 (42.6 to 46.2)	45.8 (43.9 to 47.4)	9.5 (6.7 to 13.2)	2.9 (1.1 to 4.7)
Diet low in whole grains	52.2 (51.8 to 52.6)	50.1 (49.3 to 50.8)	47.6 (46.3 to 48.6)	-4.1 (-4.8 to -3.4)	-5.1 (-6.4 to -4.1)	52.1 (51.6 to 52.5)	50.3 (49.5 to 50.9)	48.0 (46.8 to 49.0)	-3.5 (-4.2 to -2.9)	-4.5 (-5.7 to -3.6)
Diet low in nuts and seeds	50.5 (49.3 to 51.4)	51.3 (50.3 to 52.1)	50.6 (49.4 to 51.6)	1.7 (0.4 to 3.2)	-1.3 (-2.5 to -0.4)	50.7 (49.6 to 51.6)	51.5 (50.7 to 52.2)	50.9 (49.8 to 51.8)	1.6 (0.4 to 3.0)	-1.2 (-2.2 to -0.3)
Diet low in milk	36.4 (32.4 to 39.7)	42.3 (39.7 to 44.6)	43.2 (40.7 to 45.3)	16.2 (11.1 to 23.5)	2.1 (0.4 to 3.9)	37.4 (33.7 to 40.5)	43.0 (40.4 to 45.1)	43.7 (41.3 to 45.8)	15.0 (10.3 to 21.3)	1.8 (0.4 to 3.6)
Diet high in red meat	18.1 (13.1 to 22.2)	4.7 (1.7 to 8.0)	7.7 (3.3 to 12.3)	-73.7 (-88.6 to -59.4)	61.1 (8.7 to 211.8)	4.9 (1.5 to 8.7)	0.3 (0.0 to 1.1)	0.9 (0.0 to 2.7)	-94.0 (-99.8 to -83.5)	192.7 (-24.9 to 3331.3)
Diet high in processed meat	3.8 (1.3 to 6.6)	2.6 (0.5 to 5.1)	2.8 (0.6 to 5.4)	-33.4 (-71.1 to -12.6)	10.0 (-10.2 to 61.7)	2.4 (0.5 to 4.9)	1.6 (0.1 to 3.8)	1.7 (0.2 to 4.0)	-35.7 (-82.3 to -15.7)	11.4 (-11.4 to 113.4)
Diet high in sugar-sweetened beverages	2.8 (1.3 to 5.0)	1.8 (0.9 to 3.1)	2.3 (1.2 to 3.7)	-35.2 (-62.5 to 10.3)	27.6 (-14.7 to 91.7)	1.7 (0.8 to 3.2)	1.2 (0.6 to 2.1)	1.5 (0.7 to 2.6)	-32.7 (-62.9 to 15.7)	28.5 (-15.7 to 103.0)
Diet low in fibre	24.6 (18.6 to 29.6)	26.0 (20.2 to 30.7)	25.2 (19.1 to 30.3)	5.6 (0.0 to 13.4)	-2.8 (-9.9 to 3.6)	30.3 (25.0 to 34.6)	31.6 (26.6 to 35.6)	30.9 (25.7 to 35.3)	4.2 (-0.5 to 10.4)	-2.1 (-7.1 to 2.3)
Diet low in calcium	27.0 (21.3 to 31.5)	26.0 (20.2 to 30.6)	25.5 (19.5 to 30.2)	-3.4 (-7.1 to -0.6)	-2.0 (-5.7 to 1.0)	30.2 (25.3 to 34.2)	29.5 (24.3 to 33.5)	28.9 (23.5 to 33.1)	-2.5 (-5.6 to 0.2)	-2.0 (-5.1 to 0.6)
Diet low in seafood omega-3 fatty acids	41.9 (39.4 to 44.3)	40.9 (38.1 to 43.4)	37.9 (34.6 to 40.9)	-2.5 (-3.7 to -1.4)	-7.3 (-10.1 to -5.2)	43.7 (41.5 to 45.8)	42.9 (40.6 to 45.1)	40.2 (37.3 to 42.9)	-1.9 (-2.9 to -1.1)	-6.4 (-8.5 to -4.5)
Diet low in polyunsaturated fatty acids	44.0 (41.7 to 46.1)	39.0 (36.0 to 41.6)	38.0 (34.8 to 40.9)	-11.4 (-15.4 to -7.2)	-2.4 (-5.6 to 0.5)	44.4 (42.0 to 46.4)	39.5 (36.6 to 41.9)	38.5 (35.3 to 41.4)	-11.1 (-14.8 to -7.0)	-2.4 (-4.9 to 0.3)
Diet high in trans fatty acids	0.5 (0.0 to 2.1)	0.5 (0.0 to 2.0)	0.5 (0.0 to 1.9)	-9.3 (-48.4 to 6.8)	-2.8 (-33.6 to 18.5)	0.7 (0.0 to 2.5)	0.7 (0.0 to 2.4)	0.6 (0.0 to 2.4)	-8.6 (-44.0 to 12.9)	-3.2 (-33.3 to 17.6)
Diet high in sodium	7.3 (1.1 to 14.2)	5.8 (0.4 to 12.8)	6.1 (0.5 to 13.2)	-20.3 (-73.7 to 2.4)	4.2 (-34.3 to 82.7)	5.3 (0.4 to 11.9)	4.3 (0.1 to 10.6)	4.0 (0.1 to 10.5)	-19.9 (-75.7 to -1.3)	-5.4 (-63.6 to 17.9)
Intimate partner violence	-	-	-	-	-	5.5 (4.6 to 6.3)	5.3 (4.4 to 6.1)	5.9 (5.0 to 6.8)	-3.5 (-9.1 to 2.1)	12.6 (6.8 to 18.4)
Childhood sexual abuse	4.1 (3.3 to 5.0)	4.1 (3.3 to 5.0)	4.1 (3.3 to 5.0)	0.0 (-0.0 to 0.0)	0.0 (-0.0 to 0.0)	12.1 (9.6 to 15.0)	12.1 (9.6 to 15.0)	12.1 (9.6 to 15.0)	-0.0 (-0.0 to 0.0)	0.0 (-0.0 to 0.0)

Supplementary Results Table 1. Ukraine age-standardised Summary Exposure Values (SEVs) for Level 4 risk factors and percentage change, by sex, 1990, 2007, and 2017.Data in parentheses are 95% uncertainty intervals.

	Male					Female				
	**1990**	**2007**	**2017**	**Percentage change 1990-2007**	**Percentage change 2007-2017**	**1990**	**2007**	**2017**	**Percentage change 1990-2007**	**Percentage change 2007-2017**
Bullying victimization	7.1 (3.0 to 14.9)	7.6 (3.2 to 15.9)	7.2 (3.0 to 14.9)	6.5 (-6.0 to 19.8)	-5.4 (-12.6 to 1.1)	7.2 (3.0 to 15.0)	7.9 (3.3 to 16.4)	7.5 (3.1 to 15.5)	10.9 (-3.1 to 25.7)	-5.5 (-11.0 to 0.7)
Low physical activity	0.6 (0.2 to 1.2)	0.6 (0.2 to 1.1)	0.6 (0.2 to 1.1)	-5.7 (-8.4 to -2.1)	1.1 (0.2 to 2.1)	0.5 (0.2 to 1.0)	0.4 (0.2 to 0.9)	0.4 (0.2 to 0.9)	-6.2 (-9.3 to -2.4)	1.0 (-0.0 to 2.1)
High fasting plasma glucose	2.0 (1.1 to 3.3)	2.4 (1.5 to 3.6)	2.4 (1.4 to 3.6)	19.7 (-18.2 to 93.4)	-0.2 (-33.4 to 46.8)	1.6 (1.0 to 2.7)	1.6 (1.0 to 2.5)	1.6 (1.0 to 2.5)	-0.7 (-35.6 to 58.4)	-0.6 (-33.3 to 49.9)
High LDL cholesterol	14.9 (13.3 to 16.7)	13.1 (11.5 to 14.8)	12.2 (10.6 to 13.9)	-12.3 (-16.3 to -8.3)	-7.2 (-10.6 to -3.8)	16.1 (14.5 to 17.9)	14.3 (12.8 to 16.2)	13.8 (12.3 to 15.6)	-10.9 (-14.9 to -7.1)	-3.9 (-6.9 to -0.6)
High systolic blood pressure	11.2 (10.1 to 12.3)	12.2 (11.2 to 13.3)	12.4 (11.3 to 13.5)	9.7 (2.6 to 17.6)	1.2 (-4.4 to 6.8)	10.3 (9.3 to 11.4)	10.6 (9.7 to 11.6)	10.6 (9.6 to 11.7)	3.1 (-5.6 to 12.0)	0.4 (-6.4 to 7.8)
High body-mass index	8.4 (6.0 to 11.7)	9.3 (6.9 to 12.9)	10.5 (7.7 to 14.9)	11.3 (-2.2 to 27.3)	12.3 (2.3 to 23.7)	10.2 (7.7 to 13.9)	10.7 (8.0 to 14.0)	11.6 (8.7 to 15.1)	4.2 (-9.3 to 17.8)	8.6 (-1.0 to 20.0)
Low bone mineral density	4.1 (3.4 to 5.0)	4.1 (3.4 to 4.9)	4.0 (3.3 to 4.9)	-1.0 (-13.4 to 15.1)	-1.8 (-15.9 to 12.6)	6.3 (5.3 to 7.4)	6.1 (5.2 to 7.2)	6.1 (5.1 to 7.2)	-2.5 (-13.6 to 9.8)	0.1 (-12.1 to 14.9)
Impaired kidney function	3.1 (1.4 to 7.2)	3.0 (1.4 to 7.1)	3.0 (1.4 to 7.0)	-2.9 (-7.1 to 0.6)	-0.0 (-4.1 to 4.0)	3.8 (1.9 to 8.2)	3.8 (1.8 to 8.2)	3.8 (1.9 to 8.2)	-1.4 (-5.1 to 2.0)	1.2 (-2.5 to 5.0)

Supplementary Results Table 1. High-income age-standardised Summary Exposure Values (SEVs) for Level 4 risk factors and percentage change, by sex, 1990, 2007, and 2017.Data in parentheses are 95% uncertainty intervals.										
	Male					Female				
	1990	2007	2017	Percentage change 1990-2007	Percentage change 2007-2017	1990	2007	2017	Percentage change 1990-2007	Percentage change 2007-2017
Unsafe water source	3.2 (2.9 to 3.6)	1.3 (1.1 to 1.6)	1.1 (0.9 to 1.3)	-58.7 (-65.0 to -51.2)	-18.9 (-30.1 to -6.5)	3.3 (2.9 to 3.7)	1.3 (1.1 to 1.6)	1.1 (0.9 to 1.3)	-58.8 (-65.0 to -51.2)	-19.1 (-30.5 to -6.5)
Unsafe sanitation	6.7 (5.9 to 7.8)	3.0 (2.3 to 3.9)	2.1 (1.6 to 2.8)	-55.1 (-64.7 to -43.0)	-29.7 (-43.7 to -12.1)	6.8 (5.9 to 7.9)	3.0 (2.3 to 3.9)	2.1 (1.6 to 2.9)	-55.2 (-64.8 to -43.1)	-29.7 (-43.7 to -12.2)
No access to handwashing facility	5.2 (4.9 to 5.6)	4.2 (3.9 to 4.6)	3.9 (3.7 to 4.3)	-19.3 (-25.4 to -12.7)	-6.8 (-12.3 to -1.1)	5.3 (4.9 to 5.7)	4.2 (3.9 to 4.6)	3.9 (3.7 to 4.3)	-19.5 (-25.6 to -12.9)	-6.9 (-12.4 to -1.2)
Ambient particulate matter pollution	31.4 (23.5 to 42.9)	31.2 (22.8 to 43.8)	26.3 (19.3 to 37.2)	-0.5 (-7.3 to 7.0)	-16.0 (-24.3 to -9.2)	30.8 (22.9 to 42.1)	31.0 (22.7 to 43.4)	26.0 (19.2 to 37.0)	0.6 (-6.5 to 8.4)	-15.9 (-24.4 to -9.1)
Household air pollution from solid fuels	1.9 (1.2 to 3.9)	0.7 (0.4 to 1.6)	0.5 (0.2 to 1.2)	-63.4 (-72.4 to -55.1)	-34.3 (-51.9 to -17.1)	2.4 (1.5 to 6.2)	1.0 (0.5 to 3.5)	0.7 (0.3 to 3.0)	-59.9 (-70.8 to -44.8)	-31.0 (-50.6 to -15.3)
Ambient ozone pollution	41.5 (16.5 to 51.3)	41.7 (16.6 to 51.6)	42.9 (17.4 to 52.7)	0.5 (0.3 to 0.7)	2.9 (1.8 to 5.2)	41.5 (16.5 to 51.3)	41.6 (16.6 to 51.5)	42.8 (17.4 to 52.7)	0.2 (-0.0 to 0.5)	2.9 (1.8 to 5.2)
Residential radon	19.3 (10.5 to 30.0)	18.9 (10.4 to 29.7)	18.7 (10.3 to 29.4)	-1.7 (-3.3 to 0.0)	-1.1 (-2.2 to -0.1)	19.3 (10.5 to 30.0)	18.9 (10.4 to 29.6)	18.7 (10.3 to 29.4)	-1.9 (-3.6 to -0.0)	-1.3 (-2.3 to -0.3)
Lead exposure	7.1 (3.4 to 10.9)	5.1 (2.2 to 8.6)	3.6 (1.4 to 6.6)	-27.6 (-35.5 to -20.6)	-30.1 (-38.6 to -22.9)	4.4 (1.6 to 8.0)	3.4 (1.1 to 6.7)	2.4 (0.7 to 5.2)	-22.7 (-29.9 to -16.3)	-28.9 (-38.3 to -21.6)
Occupational exposure to asbestos	6.7 (5.8 to 7.3)	5.8 (5.8 to 5.9)	5.4 (5.2 to 5.7)	-12.5 (-19.7 to 0.9)	-6.7 (-10.3 to -2.7)	1.3 (1.1 to 1.6)	1.3 (1.3 to 1.3)	1.2 (1.2 to 1.3)	-3.0 (-16.7 to 14.3)	-4.4 (-10.3 to 0.7)
Occupational exposure to arsenic	0.4 (0.0 to 0.9)	0.4 (0.0 to 0.9)	0.4 (0.0 to 0.9)	-5.9 (-8.5 to 9.8)	0.0 (-2.4 to 17.2)	0.3 (0.0 to 0.8)	0.3 (0.0 to 0.7)	0.3 (0.0 to 0.7)	-2.3 (-5.9 to 21.9)	-0.0 (-2.9 to 21.4)
Occupational exposure to benzene	0.6 (0.1 to 1.6)	0.6 (0.1 to 1.7)	0.6 (0.1 to 1.7)	4.6 (3.1 to 15.7)	1.5 (0.1 to 9.4)	0.7 (0.1 to 2.0)	0.9 (0.1 to 2.4)	0.9 (0.1 to 2.6)	25.0 (22.9 to 39.4)	7.6 (6.1 to 17.2)
Occupational exposure to beryllium	0.0 (0.0 to 0.0)	0.0 (0.0 to 0.0)	0.0 (0.0 to 0.0)	2.4 (1.5 to 3.2)	4.3 (3.4 to 5.1)	0.0 (0.0 to 0.0)	0.0 (0.0 to 0.0)	0.0 (0.0 to 0.0)	14.0 (12.5 to 15.5)	7.5 (6.3 to 8.7)
Occupational exposure to cadmium	0.1 (0.1 to 0.1)	0.1 (0.0 to 0.1)	0.1 (0.1 to 0.1)	-2.9 (-5.1 to -0.7)	2.8 (0.7 to 5.1)	0.0 (0.0 to 0.0)	0.0 (0.0 to 0.0)	0.0 (0.0 to 0.0)	2.5 (-0.7 to 5.8)	3.6 (1.0 to 6.4)
Occupational exposure to chromium	0.1 (0.1 to 0.1)	0.1 (0.1 to 0.1)	0.1 (0.1 to 0.1)	-0.9 (-2.8 to 1.0)	3.3 (1.4 to 5.2)	0.1 (0.1 to 0.1)	0.1 (0.1 to 0.1)	0.1 (0.1 to 0.1)	6.8 (4.0 to 9.6)	4.8 (2.6 to 7.1)
Occupational exposure to diesel engine exhaust	0.6 (0.6 to 0.6)	0.7 (0.6 to 0.7)	0.7 (0.7 to 0.7)	9.2 (7.3 to 11.2)	6.3 (4.7 to 7.9)	0.5 (0.4 to 0.5)	0.5 (0.5 to 0.5)	0.6 (0.6 to 0.6)	17.1 (14.0 to 20.0)	6.9 (4.5 to 9.2)
Occupational exposure to formaldehyde	0.3 (0.3 to 0.3)	0.3 (0.2 to 0.3)	0.3 (0.2 to 0.3)	-2.7 (-5.1 to -0.4)	2.3 (0.1 to 4.8)	0.2 (0.2 to 0.2)	0.2 (0.2 to 0.2)	0.2 (0.2 to 0.2)	6.6 (3.5 to 9.9)	5.1 (2.6 to 7.8)
Occupational exposure to nickel	0.4 (0.0 to 1.6)	0.3 (0.0 to 1.4)	0.3 (0.0 to 1.4)	-6.9 (-9.2 to 28.1)	0.0 (-2.1 to 24.1)	0.3 (0.0 to 1.2)	0.3 (0.0 to 1.1)	0.2 (0.0 to 1.1)	-5.8 (-9.5 to 29.4)	-0.8 (-3.6 to 26.2)
Occupational exposure to polycyclic aromatic hydrocarbons	0.2 (0.2 to 0.2)	0.2 (0.2 to 0.2)	0.3 (0.2 to 0.3)	0.6 (-1.3 to 2.4)	3.4 (1.8 to 5.1)	0.2 (0.2 to 0.2)	0.2 (0.2 to 0.2)	0.3 (0.2 to 0.3)	10.1 (7.6 to 12.7)	5.6 (3.6 to 7.6)
Occupational exposure to silica	3.8 (0.6 to 12.0)	3.6 (0.6 to 11.6)	3.7 (0.6 to 11.5)	-3.5 (-5.8 to 2.1)	0.7 (-1.3 to 4.9)	2.3 (0.4 to 7.2)	2.1 (0.3 to 6.6)	2.1 (0.3 to 6.4)	-7.5 (-11.4 to -2.7)	-1.3 (-4.5 to 2.9)
Occupational exposure to sulphuric acid	0.6 (0.1 to 1.8)	0.6 (0.1 to 1.7)	0.6 (0.1 to 1.7)	-2.3 (-4.8 to 0.5)	-0.9 (-3.2 to 2.4)	0.5 (0.1 to 1.4)	0.5 (0.1 to 1.5)	0.5 (0.1 to 1.5)	5.1 (1.1 to 9.9)	-1.4 (-5.2 to 2.9)
Occupational exposure to trichloroethylene	0.1 (0.1 to 0.1)	0.1 (0.1 to 0.1)	0.1 (0.1 to 0.1)	1.5 (0.1 to 2.9)	4.0 (2.7 to 5.4)	0.1 (0.1 to 0.1)	0.1 (0.1 to 0.1)	0.1 (0.1 to 0.1)	10.0 (7.8 to 12.5)	5.7 (3.9 to 7.7)
Occupational asthmagens	16.8 (14.6 to 19.8)	15.4 (13.3 to 18.1)	15.2 (13.0 to 17.8)	-8.4 (-11.2 to -5.7)	-1.4 (-4.0 to 1.3)	7.7 (6.5 to 9.0)	8.0 (6.8 to 9.4)	8.0 (6.9 to 9.4)	4.0 (0.8 to 7.2)	0.8 (-1.8 to 3.5)
Occupational particulate matter, gases, and fumes	6.6 (4.8 to 9.4)	6.2 (4.5 to 8.8)	5.9 (4.3 to 8.4)	-6.5 (-8.2 to -5.0)	-4.6 (-5.6 to -3.5)	3.3 (2.4 to 4.8)	3.1 (2.3 to 4.5)	3.0 (2.2 to 4.3)	-4.0 (-6.9 to -1.6)	-4.6 (-6.4 to -2.7)
Occupational noise	6.1 (5.8 to 6.5)	5.8 (5.6 to 6.3)	5.7 (5.4 to 6.1)	-4.0 (-4.6 to -3.4)	-2.8 (-3.2 to -2.4)	3.6 (3.4 to 3.9)	3.8 (3.6 to 4.0)	3.9 (3.7 to 4.1)	5.0 (4.1 to 5.9)	2.6 (2.1 to 3.1)
Occupational ergonomic factors	9.0 (7.9 to 10.4)	8.1 (7.0 to 9.4)	7.9 (6.8 to 9.2)	-10.5 (-12.7 to -8.5)	-2.6 (-4.5 to -0.7)	5.0 (4.3 to 5.8)	5.2 (4.4 to 6.2)	5.4 (4.6 to 6.5)	5.2 (2.0 to 8.4)	3.6 (0.8 to 6.4)
Non-exclusive breastfeeding	0.5 (0.4 to 0.6)	0.5 (0.4 to 0.6)	0.5 (0.4 to 0.6)	-0.4 (-3.8 to 3.3)	-3.4 (-6.2 to -0.8)	0.5 (0.4 to 0.6)	0.5 (0.4 to 0.6)	0.5 (0.4 to 0.6)	-0.5 (-3.9 to 3.2)	-3.4 (-6.2 to -0.8)
Discontinued breastfeeding	2.0 (2.0 to 2.0)	2.0 (2.0 to 2.1)	2.0 (2.0 to 2.1)	1.0 (-1.2 to 3.2)	0.9 (-1.0 to 2.8)	2.0 (2.0 to 2.0)	2.0 (2.0 to 2.1)	2.0 (2.0 to 2.1)	1.0 (-1.1 to 3.3)	0.8 (-1.1 to 2.7)
Child underweight	0.1 (0.1 to 0.2)	0.1 (0.1 to 0.2)	0.1 (0.1 to 0.1)	-17.8 (-23.2 to -13.7)	-8.8 (-11.5 to -6.4)	0.1 (0.1 to 0.2)	0.1 (0.1 to 0.1)	0.1 (0.1 to 0.1)	-20.2 (-26.2 to -16.0)	-6.4 (-9.0 to -4.2)
Child wasting	0.1 (0.1 to 0.1)	0.1 (0.1 to 0.1)	0.1 (0.1 to 0.1)	-7.6 (-12.6 to -2.6)	-4.9 (-8.4 to -1.5)	0.1 (0.1 to 0.1)	0.1 (0.1 to 0.1)	0.1 (0.1 to 0.1)	-6.4 (-11.4 to -1.4)	-5.4 (-9.0 to -1.6)
Child stunting	0.3 (0.2 to 0.4)	0.3 (0.2 to 0.4)	0.3 (0.1 to 0.4)	-16.1 (-26.9 to -5.7)	-7.3 (-14.7 to 1.3)	0.3 (0.2 to 0.5)	0.3 (0.2 to 0.4)	0.3 (0.1 to 0.4)	-20.2 (-29.3 to -11.6)	-4.5 (-11.1 to 2.8)
Short gestation for birth weight	0.0 (0.0 to 0.0)	0.0 (0.0 to 0.0)	0.0 (0.0 to 0.0)	7.8 (3.3 to 14.4)	2.5 (-1.8 to 6.6)	0.0 (0.0 to 0.0)	0.0 (0.0 to 0.0)	0.0 (0.0 to 0.0)	7.7 (2.4 to 14.1)	2.3 (-2.4 to 6.6)
Low birth weight for gestation	0.0 (0.0 to 0.0)	0.0 (0.0 to 0.0)	0.0 (0.0 to 0.0)	5.0 (-1.1 to 11.2)	0.6 (-4.3 to 8.8)	0.0 (0.0 to 0.0)	0.0 (0.0 to 0.0)	0.0 (0.0 to 0.0)	3.3 (-2.6 to 10.0)	0.3 (-4.5 to 5.7)
Iron deficiency	-	-	-	-	-	6.4 (4.7 to 8.6)	5.6 (4.0 to 7.5)	5.1 (3.7 to 6.9)	-13.5 (-19.2 to -7.8)	-7.7 (-12.9 to -2.2)
Vitamin A deficiency	1.0 (0.8 to 1.2)	0.9 (0.7 to 1.0)	0.9 (0.6 to 1.1)	-13.0 (-16.8 to -9.8)	-0.6 (-5.7 to 3.0)	1.0 (0.8 to 1.2)	0.9 (0.7 to 1.1)	0.9 (0.7 to 1.1)	-8.0 (-12.1 to -4.6)	-1.6 (-5.0 to 1.2)
Zinc deficiency	0.5 (0.2 to 1.0)	0.4 (0.1 to 0.7)	0.4 (0.1 to 0.7)	-19.2 (-31.4 to 6.2)	2.4 (-13.6 to 21.6)	0.5 (0.2 to 1.0)	0.4 (0.1 to 0.7)	0.4 (0.1 to 0.8)	-20.7 (-32.1 to 7.2)	3.7 (-14.7 to 24.6)
Smoking	14.4 (13.0 to 15.9)	9.9 (8.8 to 11.1)	8.2 (7.3 to 9.2)	-31.3 (-33.5 to -29.1)	-17.3 (-19.1 to -15.4)	7.6 (6.8 to 8.6)	5.4 (4.7 to 6.2)	4.7 (4.1 to 5.4)	-28.8 (-32.6 to -25.2)	-13.5 (-15.7 to -11.2)
Chewing tobacco	0.9 (0.9 to 1.0)	0.8 (0.7 to 1.0)	0.8 (0.7 to 1.0)	-10.3 (-21.5 to 3.8)	-1.3 (-15.2 to 15.1)	0.1 (0.1 to 0.1)	0.1 (0.1 to 0.1)	0.1 (0.1 to 0.1)	-5.9 (-17.7 to 8.5)	-2.5 (-11.8 to 8.8)
Second-hand smoke	43.5 (41.8 to 45.3)	32.7 (31.9 to 33.4)	31.6 (30.7 to 32.5)	-24.9 (-29.0 to -21.2)	-3.2 (-5.6 to -0.8)	48.8 (47.1 to 50.7)	35.1 (34.4 to 35.8)	33.2 (32.5 to 33.8)	-28.1 (-31.6 to -24.8)	-5.6 (-7.4 to -3.8)
Alcohol use	26.1 (19.3 to 33.6)	24.3 (16.9 to 32.8)	25.2 (17.2 to 34.6)	-6.8 (-15.3 to 0.7)	3.6 (-5.5 to 14.0)	14.5 (9.2 to 21.9)	13.6 (8.5 to 20.9)	14.1 (8.7 to 21.7)	-6.6 (-13.8 to -0.4)	4.0 (-5.7 to 15.5)
Drug use	1.0 (0.7 to 1.5)	1.2 (0.9 to 1.7)	1.4 (1.1 to 2.0)	23.2 (15.3 to 33.5)	18.9 (14.3 to 24.7)	0.6 (0.5 to 0.9)	0.8 (0.7 to 1.1)	1.0 (0.8 to 1.3)	29.2 (21.1 to 39.7)	17.1 (12.1 to 22.4)
Diet low in fruits	30.9 (25.1 to 35.0)	29.4 (23.5 to 33.6)	29.6 (23.8 to 33.8)	-5.1 (-7.8 to -3.2)	0.7 (-1.3 to 2.8)	27.7 (22.0 to 32.2)	26.0 (20.1 to 30.7)	26.2 (20.4 to 31.0)	-6.1 (-9.8 to -3.8)	0.6 (-2.0 to 3.6)
Diet low in vegetables	22.1 (16.9 to 27.0)	22.4 (17.0 to 27.3)	23.3 (18.0 to 28.0)	1.3 (-1.0 to 3.6)	3.7 (1.0 to 7.5)	24.5 (19.3 to 29.2)	24.5 (19.2 to 29.2)	25.3 (20.0 to 29.9)	-0.3 (-2.3 to 1.8)	3.3 (0.7 to 7.0)
Diet low in legumes	27.0 (23.5 to 30.3)	26.4 (22.7 to 29.9)	27.5 (23.8 to 30.9)	-2.2 (-4.6 to -0.2)	4.1 (1.7 to 7.0)	30.7 (27.2 to 33.7)	30.3 (26.9 to 33.5)	31.5 (28.1 to 34.7)	-1.1 (-2.7 to 0.5)	3.9 (2.0 to 6.1)
Diet low in whole grains	39.0 (36.5 to 41.1)	40.5 (38.3 to 42.5)	40.6 (38.3 to 42.5)	3.9 (2.8 to 5.3)	0.2 (-0.6 to 1.0)	39.9 (37.6 to 41.9)	41.4 (39.3 to 43.2)	41.5 (39.4 to 43.3)	3.8 (2.8 to 5.1)	0.3 (-0.4 to 1.1)
Diet low in nuts and seeds	46.1 (44.2 to 47.6)	41.4 (38.8 to 43.5)	40.6 (37.7 to 43.0)	-10.2 (-12.8 to -8.2)	-1.7 (-3.6 to -0.3)	46.6 (44.9 to 48.0)	42.1 (39.6 to 44.1)	41.4 (38.6 to 43.6)	-9.7 (-12.2 to -7.8)	-1.7 (-3.4 to -0.4)
Diet low in milk	32.2 (27.7 to 36.0)	30.5 (25.8 to 34.5)	30.5 (25.7 to 34.5)	-5.1 (-7.7 to -3.3)	-0.0 (-1.4 to 1.5)	33.3 (29.0 to 36.8)	31.1 (26.4 to 35.0)	31.0 (26.3 to 34.9)	-6.4 (-9.3 to -4.5)	-0.4 (-1.9 to 1.1)
Diet high in red meat	34.4 (33.1 to 36.1)	33.7 (32.1 to 35.5)	33.7 (31.7 to 35.9)	-2.3 (-5.1 to 0.4)	0.2 (-3.6 to 4.1)	18.2 (15.9 to 20.5)	15.9 (13.3 to 18.4)	16.1 (13.2 to 18.8)	-12.7 (-19.1 to -6.8)	1.5 (-5.8 to 9.3)
Diet high in processed meat	19.0 (16.4 to 21.2)	22.1 (20.0 to 24.1)	21.2 (18.4 to 23.5)	16.7 (10.2 to 25.0)	-4.1 (-8.9 to 0.3)	12.5 (9.7 to 14.9)	15.1 (12.6 to 17.4)	14.7 (11.9 to 17.2)	20.7 (11.7 to 33.5)	-2.6 (-8.1 to 2.8)
Diet high in sugar-sweetened beverages	16.2 (8.4 to 18.7)	17.3 (8.9 to 18.8)	16.8 (8.6 to 18.8)	6.4 (-3.9 to 19.6)	-2.7 (-10.0 to 4.6)	12.7 (6.4 to 14.9)	12.6 (6.5 to 13.9)	12.2 (6.2 to 13.8)	-0.6 (-13.0 to 15.3)	-3.5 (-12.1 to 5.7)
Diet low in fibre	30.3 (25.3 to 34.3)	28.4 (23.3 to 32.7)	29.2 (24.1 to 33.5)	-6.1 (-8.9 to -4.0)	2.8 (0.8 to 5.0)	34.6 (30.4 to 38.0)	32.7 (28.2 to 36.4)	33.5 (29.0 to 37.0)	-5.4 (-7.9 to -3.6)	2.3 (0.8 to 4.3)
Diet low in calcium	22.5 (16.6 to 27.4)	21.4 (15.5 to 26.4)	21.4 (15.5 to 26.4)	-5.0 (-7.7 to -3.2)	-0.0 (-1.8 to 1.8)	26.0 (20.4 to 30.5)	24.8 (19.0 to 29.5)	24.8 (19.0 to 29.5)	-4.8 (-7.6 to -2.9)	0.1 (-1.2 to 1.6)
Diet low in seafood omega-3 fatty acids	29.3 (26.0 to 32.4)	26.6 (23.3 to 29.9)	26.6 (23.1 to 29.9)	-9.1 (-10.9 to -7.5)	-0.3 (-1.6 to 0.7)	31.8 (28.8 to 34.6)	29.5 (26.3 to 32.5)	29.4 (26.0 to 32.5)	-7.2 (-8.8 to -5.9)	-0.3 (-1.3 to 0.5)
Diet low in polyunsaturated fatty acids	24.4 (20.0 to 28.6)	20.5 (16.0 to 24.8)	19.4 (15.1 to 23.6)	-15.8 (-21.7 to -11.1)	-5.3 (-7.5 to -3.1)	25.0 (20.7 to 29.1)	21.2 (16.6 to 25.5)	20.1 (15.9 to 24.2)	-15.3 (-21.0 to -11.0)	-5.2 (-7.3 to -2.8)
Diet high in trans fatty acids	7.0 (4.5 to 10.3)	4.4 (2.2 to 7.2)	3.4 (1.4 to 6.1)	-38.0 (-51.3 to -28.5)	-22.7 (-37.4 to -14.9)	9.7 (6.7 to 14.1)	5.8 (3.4 to 8.9)	4.5 (2.3 to 7.5)	-40.2 (-50.7 to -31.1)	-22.3 (-33.7 to -15.5)
Diet high in sodium	9.9 (4.1 to 16.3)	10.4 (3.7 to 16.8)	10.3 (3.5 to 16.8)	4.9 (-12.6 to 15.4)	-0.8 (-12.2 to 5.6)	9.2 (4.2 to 15.5)	8.6 (2.8 to 15.1)	8.0 (2.3 to 14.5)	-5.8 (-35.3 to 3.4)	-7.2 (-23.4 to -1.5)
Intimate partner violence	-	-	-	-	-	2.2 (1.9 to 2.6)	2.0 (1.6 to 2.3)	1.9 (1.6 to 2.3)	-11.9 (-12.9 to -11.0)	-0.8 (-1.8 to 0.3)
Childhood sexual abuse	4.8 (4.0 to 5.9)	4.8 (3.9 to 5.8)	4.8 (3.9 to 5.8)	-1.3 (-3.1 to 0.7)	-0.2 (-1.8 to 1.5)	9.5 (7.8 to 11.6)	9.2 (7.7 to 11.0)	9.4 (7.7 to 11.3)	-3.4 (-6.2 to -0.7)	1.6 (-0.3 to 3.7)

Supplementary Results Table 1. High-income age-standardised Summary Exposure Values (SEVs) for Level 4 risk factors and percentage change, by sex, 1990, 2007, and 2017.Data in parentheses are 95% uncertainty intervals.										
	**Male**					**Female**				
	**1990**	**2007**	**2017**	**Percentage change 1990-2007**	**Percentage change 2007-2017**	**1990**	**2007**	**2017**	**Percentage change 1990-2007**	**Percentage change 2007-2017**
Bullying victimization	5.7 (2.5 to 11.8)	6.9 (3.2 to 13.8)	7.1 (3.3 to 14.0)	22.1 (13.0 to 34.2)	2.4 (-0.4 to 5.2)	4.5 (2.0 to 9.0)	5.6 (2.6 to 11.2)	5.8 (2.7 to 11.4)	26.6 (17.2 to 40.0)	3.6 (0.6 to 7.3)
Low physical activity	0.5 (0.2 to 0.9)	0.5 (0.2 to 0.9)	0.4 (0.2 to 0.9)	0.9 (0.2 to 1.6)	-1.6 (-2.3 to -0.8)	0.5 (0.2 to 0.9)	0.5 (0.2 to 0.9)	0.5 (0.2 to 0.9)	0.3 (-0.3 to 1.0)	0.2 (-0.2 to 0.5)
High fasting plasma glucose	1.9 (1.5 to 2.5)	2.5 (1.9 to 3.4)	2.9 (2.2 to 3.9)	30.3 (19.5 to 44.3)	16.5 (6.5 to 29.9)	1.7 (1.3 to 2.2)	2.1 (1.6 to 2.8)	2.3 (1.7 to 3.0)	25.4 (15.9 to 38.2)	7.4 (-3.8 to 21.7)
High LDL cholesterol	16.7 (15.1 to 18.5)	13.1 (11.5 to 14.8)	12.7 (11.2 to 14.4)	-21.9 (-24.4 to -19.6)	-3.0 (-4.0 to -2.0)	16.4 (14.9 to 18.1)	13.1 (11.6 to 14.8)	12.9 (11.4 to 14.5)	-20.1 (-22.4 to -18.1)	-1.6 (-2.7 to -0.6)
High systolic blood pressure	8.8 (8.1 to 9.5)	7.1 (6.4 to 7.8)	6.9 (6.2 to 7.6)	-19.6 (-21.6 to -17.8)	-2.2 (-3.8 to -0.5)	7.8 (7.2 to 8.4)	5.5 (5.1 to 6.0)	5.3 (4.8 to 5.8)	-28.9 (-31.0 to -26.8)	-4.5 (-6.8 to -2.2)
High body-mass index	10.8 (8.0 to 14.9)	16.1 (12.8 to 21.1)	17.1 (13.7 to 22.1)	49.6 (40.0 to 62.8)	6.2 (3.2 to 9.4)	10.6 (8.0 to 14.3)	15.5 (12.3 to 20.0)	16.6 (13.2 to 21.1)	45.8 (37.3 to 55.8)	6.7 (3.2 to 10.3)
Low bone mineral density	4.7 (4.1 to 5.4)	4.5 (3.9 to 5.2)	4.6 (3.9 to 5.3)	-4.5 (-6.8 to -2.2)	1.2 (-1.4 to 3.6)	5.9 (5.3 to 6.6)	5.8 (5.1 to 6.5)	5.9 (5.1 to 6.8)	-2.3 (-4.4 to -0.2)	2.0 (-1.2 to 5.2)
Impaired kidney function	2.1 (1.0 to 5.3)	2.0 (0.9 to 5.2)	2.0 (0.9 to 5.2)	-2.2 (-4.3 to -0.5)	-0.9 (-3.7 to 2.1)	2.6 (1.2 to 6.3)	2.6 (1.2 to 6.2)	2.6 (1.2 to 6.2)	-1.8 (-3.9 to -0.4)	0.5 (-1.8 to 3.0)

Supplementary Results Table 1. Australasia age-standardised Summary Exposure Values (SEVs) for Level 4 risk factors and percentage change, by sex, 1990, 2007, and 2017.Data in parentheses are 95% uncertainty intervals.										
	Male					Female				
	1990	2007	2017	Percentage change 1990-2007	Percentage change 2007-2017	1990	2007	2017	Percentage change 1990-2007	Percentage change 2007-2017
Unsafe water source	2.5 (1.7 to 3.4)	1.9 (1.3 to 2.6)	1.8 (1.3 to 2.5)	-23.9 (-48.1 to 10.9)	-2.9 (-29.5 to 29.6)	2.5 (1.7 to 3.4)	1.9 (1.3 to 2.6)	1.8 (1.3 to 2.5)	-23.7 (-48.0 to 11.2)	-2.9 (-29.4 to 29.4)
Unsafe sanitation	4.4 (2.9 to 6.4)	2.0 (1.2 to 2.9)	1.5 (1.0 to 2.3)	-55.2 (-73.4 to -27.8)	-24.1 (-49.0 to 9.3)	4.4 (2.9 to 6.4)	2.0 (1.3 to 2.9)	1.5 (1.0 to 2.3)	-55.0 (-73.2 to -27.9)	-24.1 (-48.9 to 9.2)
No access to handwashing facility	6.8 (5.6 to 8.2)	6.3 (5.2 to 7.6)	5.8 (4.7 to 7.0)	-6.5 (-23.6 to 12.5)	-8.5 (-21.2 to 6.3)	6.8 (5.6 to 8.2)	6.3 (5.2 to 7.6)	5.8 (4.7 to 7.0)	-6.5 (-23.6 to 12.4)	-8.5 (-21.2 to 6.2)
Ambient particulate matter pollution	23.5 (8.9 to 42.8)	24.1 (11.3 to 44.2)	19.1 (5.0 to 40.2)	2.5 (-55.7 to 166.1)	-20.7 (-71.6 to 16.3)	23.3 (8.9 to 42.4)	24.0 (11.3 to 44.0)	19.0 (5.0 to 40.0)	3.1 (-55.4 to 167.2)	-20.6 (-71.6 to 16.4)
Household air pollution from solid fuels	0.7 (0.3 to 2.1)	0.3 (0.1 to 1.0)	0.2 (0.1 to 0.8)	-55.5 (-81.4 to -8.0)	-25.4 (-71.4 to 34.4)	1.0 (0.3 to 6.3)	0.5 (0.1 to 3.0)	0.4 (0.1 to 2.2)	-51.3 (-81.3 to -4.7)	-24.8 (-71.7 to 34.6)
Ambient ozone pollution	3.4 (0.7 to 8.4)	4.8 (1.0 to 12.1)	11.3 (3.4 to 24.5)	43.4 (32.2 to 48.2)	135.1 (84.4 to 311.3)	3.4 (0.7 to 8.4)	4.8 (1.0 to 12.0)	11.3 (3.4 to 24.4)	43.0 (31.7 to 47.7)	135.5 (84.7 to 311.7)
Residential radon	4.1 (0.4 to 11.7)	4.1 (0.4 to 11.5)	3.9 (0.3 to 11.4)	-1.0 (-3.4 to 3.8)	-3.1 (-7.2 to 0.3)	4.1 (0.4 to 11.7)	4.1 (0.4 to 11.7)	4.0 (0.4 to 11.4)	-0.2 (-2.5 to 8.8)	-3.0 (-7.0 to 0.3)
Lead exposure	10.7 (6.4 to 14.8)	8.3 (4.7 to 12.2)	5.8 (2.9 to 9.3)	-21.9 (-28.1 to -16.2)	-30.7 (-37.6 to -23.7)	6.8 (3.0 to 10.8)	5.4 (2.2 to 9.0)	3.7 (1.3 to 6.8)	-21.4 (-27.6 to -15.5)	-31.6 (-40.9 to -23.6)
Occupational exposure to asbestos	13.1 (11.0 to 14.4)	11.1 (10.7 to 11.4)	9.9 (9.2 to 10.6)	-15.7 (-23.5 to 0.3)	-11.0 (-17.8 to -3.9)	1.8 (1.3 to 2.1)	2.7 (2.6 to 2.9)	2.6 (2.2 to 3.0)	52.7 (25.7 to 105.7)	-4.2 (-18.9 to 10.9)
Occupational exposure to arsenic	0.4 (0.0 to 0.8)	0.4 (0.0 to 0.9)	0.4 (0.0 to 0.9)	-	-	0.3 (0.0 to 0.7)	0.3 (0.0 to 0.8)	0.3 (0.0 to 0.8)	-	-
Occupational exposure to benzene	0.5 (0.0 to 1.5)	0.6 (0.0 to 1.6)	0.6 (0.0 to 1.7)	9.6 (6.8 to 13.9)	3.0 (0.4 to 6.8)	0.6 (0.0 to 1.8)	0.8 (0.0 to 2.3)	0.9 (0.1 to 2.5)	30.8 (25.8 to 38.0)	7.7 (3.9 to 12.8)
Occupational exposure to beryllium	0.0 (0.0 to 0.0)	0.0 (0.0 to 0.0)	0.0 (0.0 to 0.0)	6.2 (4.7 to 7.8)	2.6 (0.9 to 4.3)	0.0 (0.0 to 0.0)	0.0 (0.0 to 0.0)	0.0 (0.0 to 0.0)	22.6 (20.3 to 24.8)	6.0 (3.6 to 8.3)
Occupational exposure to cadmium	0.0 (0.0 to 0.0)	0.0 (0.0 to 0.0)	0.0 (0.0 to 0.0)	3.1 (-3.8 to 9.7)	3.0 (-3.2 to 9.0)	0.0 (0.0 to 0.0)	0.0 (0.0 to 0.0)	0.0 (0.0 to 0.0)	14.9 (7.1 to 23.1)	4.7 (-1.8 to 11.3)
Occupational exposure to chromium	0.1 (0.1 to 0.1)	0.1 (0.1 to 0.1)	0.1 (0.1 to 0.1)	4.4 (-1.7 to 10.2)	3.3 (-2.0 to 8.3)	0.1 (0.1 to 0.1)	0.1 (0.1 to 0.1)	0.1 (0.1 to 0.1)	17.5 (10.8 to 24.4)	5.3 (-0.4 to 10.9)
Occupational exposure to diesel engine exhaust	0.5 (0.5 to 0.5)	0.6 (0.5 to 0.6)	0.6 (0.6 to 0.6)	8.3 (4.2 to 12.2)	3.0 (-0.4 to 6.6)	0.4 (0.4 to 0.5)	0.5 (0.5 to 0.6)	0.6 (0.5 to 0.6)	22.1 (16.9 to 27.9)	5.3 (0.7 to 9.8)
Occupational exposure to formaldehyde	0.2 (0.2 to 0.2)	0.2 (0.2 to 0.2)	0.2 (0.2 to 0.2)	2.0 (-5.6 to 9.3)	2.5 (-4.0 to 9.2)	0.2 (0.1 to 0.2)	0.2 (0.2 to 0.2)	0.2 (0.2 to 0.2)	15.5 (7.4 to 23.7)	4.8 (-1.7 to 11.8)
Occupational exposure to nickel	0.3 (0.0 to 1.5)	0.3 (0.0 to 1.6)	0.4 (0.0 to 1.6)	-	-	0.2 (0.0 to 1.0)	0.3 (0.0 to 1.2)	0.3 (0.0 to 1.2)	-	-
Occupational exposure to polycyclic aromatic hydrocarbons	0.2 (0.2 to 0.2)	0.2 (0.2 to 0.2)	0.2 (0.2 to 0.2)	4.5 (-1.1 to 9.8)	3.3 (-1.4 to 8.2)	0.2 (0.2 to 0.2)	0.2 (0.2 to 0.2)	0.2 (0.2 to 0.2)	19.4 (13.3 to 25.7)	5.8 (0.8 to 11.1)
Occupational exposure to silica	3.8 (0.5 to 12.0)	3.9 (0.6 to 12.8)	4.0 (0.6 to 12.7)	3.9 (-2.3 to 11.5)	2.5 (-2.8 to 8.6)	2.1 (0.3 to 6.9)	2.4 (0.3 to 7.3)	2.4 (0.3 to 7.5)	9.8 (1.3 to 20.5)	2.5 (-4.4 to 10.7)
Occupational exposure to sulphuric acid	0.5 (0.1 to 1.7)	0.5 (0.1 to 1.7)	0.6 (0.1 to 1.7)	-2.0 (-8.3 to 4.1)	2.2 (-3.2 to 8.2)	0.4 (0.1 to 1.1)	0.4 (0.1 to 1.3)	0.4 (0.1 to 1.4)	11.9 (3.7 to 20.5)	4.0 (-3.0 to 10.9)
Occupational exposure to trichloroethylene	0.1 (0.1 to 0.1)	0.1 (0.1 to 0.1)	0.1 (0.1 to 0.1)	6.0 (2.2 to 9.7)	3.3 (-0.0 to 6.8)	0.1 (0.1 to 0.1)	0.1 (0.1 to 0.1)	0.1 (0.1 to 0.1)	19.4 (14.8 to 24.3)	5.4 (1.5 to 9.5)
Occupational asthmagens	16.1 (13.8 to 18.8)	15.4 (13.1 to 18.1)	15.2 (13.0 to 18.1)	-4.4 (-12.3 to 4.3)	-1.1 (-8.0 to 6.6)	8.1 (6.8 to 9.5)	8.8 (7.4 to 10.3)	8.9 (7.6 to 10.6)	9.3 (2.1 to 17.1)	1.5 (-3.9 to 7.0)
Occupational particulate matter, gases, and fumes	6.8 (4.9 to 9.8)	6.3 (4.5 to 9.0)	6.0 (4.3 to 8.7)	-8.5 (-11.0 to -6.2)	-3.5 (-5.2 to -1.8)	3.0 (2.1 to 4.4)	3.0 (2.1 to 4.3)	3.0 (2.1 to 4.3)	-0.1 (-4.0 to 3.8)	-0.3 (-3.7 to 2.7)
Occupational noise	6.3 (6.0 to 6.7)	5.9 (5.6 to 6.3)	5.7 (5.4 to 6.2)	-6.6 (-7.8 to -5.4)	-2.7 (-3.5 to -1.7)	3.3 (3.1 to 3.5)	3.5 (3.3 to 3.7)	3.7 (3.5 to 3.9)	6.4 (4.5 to 8.4)	4.3 (3.1 to 5.6)
Occupational ergonomic factors	9.0 (7.9 to 10.4)	9.0 (7.9 to 10.4)	8.7 (7.6 to 10.0)	0.6 (-5.8 to 7.4)	-3.6 (-9.1 to 2.7)	5.3 (4.5 to 6.2)	5.5 (4.7 to 6.5)	5.5 (4.6 to 6.6)	3.6 (-6.5 to 14.3)	-0.1 (-7.5 to 7.8)
Non-exclusive breastfeeding	0.5 (0.4 to 0.6)	0.5 (0.4 to 0.6)	0.5 (0.4 to 0.6)	1.9 (-5.8 to 11.2)	-1.4 (-8.3 to 5.8)	0.5 (0.4 to 0.6)	0.5 (0.4 to 0.6)	0.5 (0.4 to 0.6)	2.0 (-5.7 to 11.3)	-1.4 (-8.3 to 5.8)
Discontinued breastfeeding	1.8 (1.7 to 1.9)	1.9 (1.8 to 2.0)	1.9 (1.8 to 2.0)	5.7 (-0.1 to 12.1)	-1.7 (-5.9 to 2.8)	1.8 (1.7 to 1.9)	1.9 (1.8 to 2.0)	1.9 (1.8 to 2.0)	5.6 (-0.2 to 12.0)	-1.6 (-5.8 to 2.9)
Child underweight	0.1 (0.1 to 0.2)	0.1 (0.1 to 0.2)	0.1 (0.1 to 0.1)	-9.4 (-19.1 to 1.1)	-6.0 (-13.0 to 1.0)	0.1 (0.1 to 0.2)	0.1 (0.1 to 0.1)	0.1 (0.1 to 0.1)	-11.1 (-21.2 to 0.7)	-4.9 (-12.7 to 2.6)
Child wasting	0.1 (0.1 to 0.1)	0.1 (0.1 to 0.1)	0.1 (0.1 to 0.1)	-7.0 (-22.9 to 10.1)	-1.2 (-11.7 to 9.9)	0.1 (0.1 to 0.1)	0.1 (0.1 to 0.1)	0.1 (0.1 to 0.1)	-6.4 (-20.4 to 9.5)	-1.3 (-11.2 to 10.4)
Child stunting	0.4 (0.2 to 0.6)	0.3 (0.2 to 0.5)	0.3 (0.1 to 0.5)	-14.0 (-43.2 to 28.3)	-4.6 (-25.3 to 24.1)	0.4 (0.2 to 0.7)	0.4 (0.2 to 0.6)	0.3 (0.2 to 0.6)	-17.0 (-46.3 to 25.6)	-5.1 (-27.5 to 24.0)
Short gestation for birth weight	0.0 (0.0 to 0.0)	0.0 (0.0 to 0.0)	0.0 (0.0 to 0.0)	6.1 (-9.7 to 29.9)	2.3 (-12.9 to 25.1)	0.0 (0.0 to 0.0)	0.0 (0.0 to 0.0)	0.0 (0.0 to 0.0)	7.0 (-9.6 to 27.0)	2.6 (-14.3 to 25.6)
Low birth weight for gestation	0.0 (0.0 to 0.0)	0.0 (0.0 to 0.0)	0.0 (0.0 to 0.0)	0.8 (-14.4 to 16.9)	1.3 (-14.7 to 17.1)	0.0 (0.0 to 0.0)	0.0 (0.0 to 0.0)	0.0 (0.0 to 0.0)	-0.4 (-14.6 to 15.1)	-0.3 (-17.6 to 16.9)
Iron deficiency	-	-	-	-	-	4.4 (2.9 to 6.3)	3.6 (2.4 to 5.1)	3.2 (2.1 to 4.6)	-18.6 (-34.9 to 2.0)	-10.7 (-26.1 to 7.7)
Vitamin A deficiency	0.2 (0.1 to 0.3)	0.2 (0.1 to 0.2)	0.2 (0.1 to 0.2)	-11.0 (-22.5 to 8.6)	-11.3 (-24.3 to 3.7)	0.2 (0.1 to 0.2)	0.2 (0.1 to 0.2)	0.1 (0.1 to 0.2)	-16.5 (-27.1 to -3.5)	-13.1 (-31.0 to 2.9)
Zinc deficiency	0.6 (0.1 to 1.1)	0.5 (0.1 to 0.9)	0.5 (0.1 to 0.9)	-20.4 (-76.6 to 74.9)	1.4 (-73.8 to 344.8)	0.6 (0.2 to 1.1)	0.5 (0.1 to 0.9)	0.5 (0.1 to 1.0)	-12.5 (-73.8 to 73.3)	1.1 (-71.1 to 260.8)
Smoking	11.9 (10.6 to 13.4)	7.9 (6.9 to 8.9)	6.8 (5.9 to 7.8)	-33.7 (-37.4 to -29.9)	-13.3 (-17.4 to -9.4)	8.3 (7.2 to 9.5)	5.9 (5.1 to 6.8)	5.2 (4.4 to 6.1)	-28.6 (-33.6 to -23.5)	-12.3 (-17.2 to -7.1)
Chewing tobacco	1.4 (1.1 to 1.7)	0.9 (0.7 to 1.2)	0.7 (0.6 to 0.9)	-32.5 (-49.0 to -13.3)	-18.7 (-32.2 to -0.7)	0.1 (0.1 to 0.1)	0.1 (0.1 to 0.1)	0.1 (0.1 to 0.1)	-3.8 (-27.8 to 29.7)	-2.8 (-21.3 to 19.1)
Second-hand smoke	37.2 (34.8 to 39.8)	33.7 (32.2 to 35.3)	32.5 (29.9 to 35.0)	-9.3 (-16.2 to -1.8)	-3.6 (-8.9 to 2.2)	37.0 (35.2 to 39.0)	32.8 (31.6 to 34.1)	31.5 (29.5 to 33.5)	-11.3 (-16.4 to -5.5)	-4.0 (-8.2 to 0.4)
Alcohol use	33.5 (24.2 to 43.4)	29.2 (19.9 to 39.8)	31.3 (20.7 to 43.6)	-12.8 (-25.6 to -2.0)	7.1 (-12.8 to 33.4)	13.2 (7.9 to 21.5)	11.9 (6.8 to 20.5)	12.3 (6.7 to 21.1)	-10.0 (-24.0 to 0.8)	3.4 (-12.2 to 23.8)
Drug use	1.4 (1.0 to 2.3)	1.5 (1.0 to 2.3)	1.8 (1.2 to 2.8)	4.6 (-3.3 to 14.3)	20.7 (10.5 to 30.4)	0.7 (0.4 to 1.1)	0.7 (0.5 to 1.1)	0.9 (0.6 to 1.4)	3.5 (-4.2 to 13.4)	23.2 (13.8 to 33.0)
Diet low in fruits	31.8 (26.1 to 35.9)	29.9 (24.1 to 34.1)	30.0 (24.1 to 34.6)	-5.9 (-10.0 to -2.6)	0.3 (-4.8 to 5.6)	28.7 (22.7 to 33.2)	26.8 (20.8 to 31.5)	26.8 (20.9 to 31.8)	-6.5 (-11.5 to -2.8)	-0.0 (-5.9 to 6.1)
Diet low in vegetables	29.7 (24.8 to 33.9)	26.6 (20.9 to 31.2)	27.3 (21.5 to 32.1)	-10.5 (-16.3 to -6.9)	2.6 (-4.0 to 9.0)	31.8 (27.3 to 35.8)	28.7 (23.6 to 33.1)	29.4 (24.0 to 33.7)	-9.8 (-14.6 to -6.4)	2.3 (-3.4 to 8.6)
Diet low in legumes	28.0 (23.5 to 31.8)	36.3 (33.1 to 39.0)	34.8 (31.2 to 37.9)	29.7 (21.8 to 42.1)	-4.1 (-7.4 to -1.4)	32.2 (28.4 to 35.5)	39.6 (36.9 to 41.9)	38.5 (35.4 to 41.1)	22.9 (17.2 to 31.2)	-2.7 (-5.1 to -0.2)
Diet low in whole grains	34.9 (31.7 to 37.7)	38.0 (35.2 to 40.4)	38.1 (35.3 to 40.4)	8.8 (5.8 to 12.9)	0.3 (-1.9 to 2.7)	36.1 (33.1 to 38.7)	39.0 (36.4 to 41.2)	39.1 (36.6 to 41.4)	8.0 (5.4 to 11.5)	0.3 (-1.6 to 2.3)
Diet low in nuts and seeds	47.1 (45.2 to 48.7)	38.0 (33.9 to 41.5)	34.3 (28.9 to 38.6)	-19.3 (-26.0 to -14.4)	-9.7 (-16.8 to -4.4)	47.6 (45.8 to 49.1)	39.0 (35.3 to 42.1)	35.5 (30.8 to 39.6)	-18.0 (-23.6 to -13.4)	-8.9 (-15.4 to -4.0)
Diet low in milk	29.5 (24.4 to 33.9)	23.3 (17.0 to 28.5)	21.1 (14.2 to 26.6)	-21.2 (-31.5 to -14.4)	-9.3 (-19.8 to -3.4)	30.6 (25.6 to 34.7)	23.8 (17.7 to 28.9)	21.6 (15.0 to 27.2)	-22.2 (-32.9 to -15.7)	-9.3 (-18.5 to -3.6)
Diet high in red meat	52.3 (51.6 to 53.4)	51.5 (50.9 to 52.6)	51.3 (49.6 to 53.0)	-1.4 (-3.2 to 0.4)	-0.5 (-3.6 to 2.0)	33.2 (30.0 to 36.2)	28.6 (25.5 to 31.6)	30.0 (24.7 to 34.2)	-13.8 (-21.3 to -5.9)	4.9 (-9.2 to 19.2)
Diet high in processed meat	5.9 (2.9 to 8.8)	7.5 (4.2 to 10.4)	8.1 (4.8 to 11.0)	26.8 (4.6 to 67.7)	8.8 (-3.6 to 26.7)	3.8 (1.4 to 6.5)	5.0 (2.2 to 7.8)	5.5 (2.7 to 8.3)	30.8 (8.1 to 90.2)	9.1 (-5.5 to 34.3)
Diet high in sugar-sweetened beverages	10.6 (5.3 to 16.0)	11.7 (6.0 to 13.6)	11.8 (6.0 to 15.0)	9.7 (-26.6 to 66.1)	0.9 (-18.4 to 28.3)	7.2 (3.8 to 11.6)	7.9 (4.0 to 9.6)	8.1 (4.3 to 10.7)	9.7 (-28.4 to 68.6)	3.0 (-20.5 to 31.8)
Diet low in fibre	35.0 (30.7 to 38.4)	35.5 (31.3 to 38.8)	34.8 (30.3 to 38.4)	1.5 (-1.1 to 4.5)	-2.1 (-5.8 to 1.1)	39.5 (36.1 to 42.2)	39.9 (36.7 to 42.5)	39.4 (35.9 to 42.3)	1.2 (-0.8 to 3.3)	-1.2 (-4.0 to 1.2)
Diet low in calcium	22.0 (15.5 to 27.1)	24.1 (18.0 to 29.0)	22.2 (15.8 to 27.4)	9.5 (5.5 to 17.3)	-7.8 (-14.0 to -3.8)	25.7 (19.8 to 30.4)	27.7 (22.2 to 32.0)	26.0 (20.2 to 30.7)	7.6 (4.4 to 13.0)	-6.1 (-10.6 to -3.1)
Diet low in seafood omega-3 fatty acids	30.7 (26.5 to 34.7)	26.9 (22.3 to 31.3)	25.2 (20.5 to 29.7)	-12.4 (-16.3 to -9.3)	-6.5 (-10.3 to -3.6)	34.1 (30.2 to 37.6)	30.7 (26.4 to 34.7)	29.0 (24.6 to 33.2)	-10.0 (-13.1 to -7.5)	-5.3 (-8.3 to -3.0)
Diet low in polyunsaturated fatty acids	39.5 (36.6 to 42.3)	39.4 (36.4 to 42.0)	38.1 (34.8 to 40.9)	-0.4 (-4.8 to 4.9)	-3.2 (-6.5 to 0.7)	40.0 (37.0 to 42.7)	39.8 (36.9 to 42.5)	38.6 (35.6 to 41.2)	-0.4 (-5.1 to 3.8)	-3.2 (-6.7 to 0.4)
Diet high in trans fatty acids	6.3 (2.8 to 10.0)	3.6 (1.0 to 6.9)	2.5 (0.4 to 5.5)	-42.4 (-67.9 to -27.6)	-29.5 (-61.4 to -15.3)	8.5 (4.8 to 12.6)	4.9 (1.9 to 8.6)	3.5 (0.9 to 6.8)	-42.2 (-63.7 to -26.6)	-29.0 (-53.7 to -14.5)
Diet high in sodium	5.5 (0.3 to 12.4)	5.7 (0.6 to 12.4)	5.6 (0.4 to 12.2)	4.0 (-28.4 to 157.6)	-1.7 (-40.7 to 37.0)	3.1 (0.1 to 8.9)	3.7 (0.3 to 9.6)	3.6 (0.2 to 9.6)	20.0 (0.2 to 321.1)	-3.9 (-53.3 to 11.7)
Intimate partner violence	-	-	-	-	-	1.9 (1.6 to 2.1)	1.7 (1.5 to 2.0)	1.6 (1.3 to 1.8)	-7.0 (-11.4 to -3.1)	-7.9 (-12.0 to -3.8)
Childhood sexual abuse	4.7 (4.3 to 5.2)	4.6 (4.0 to 5.2)	4.7 (4.0 to 5.5)	-3.2 (-8.2 to 1.8)	3.2 (-4.4 to 10.3)	9.8 (8.3 to 11.4)	10.4 (9.1 to 11.6)	10.7 (8.9 to 12.8)	5.3 (-0.7 to 11.3)	3.5 (-6.2 to 13.5)

Supplementary Results Table 1. Australasia age-standardised Summary Exposure Values (SEVs) for Level 4 risk factors and percentage change, by sex, 1990, 2007, and 2017.Data in parentheses are 95% uncertainty intervals.

	Male					Female				
	**1990**	**2007**	**2017**	**Percentage change 1990-2007**	**Percentage change 2007-2017**	**1990**	**2007**	**2017**	**Percentage change 1990-2007**	**Percentage change 2007-2017**
Bullying victimization	4.8<br>(2.1 to 9.7)	4.7<br>(2.2 to 9.2)	5.6<br>(2.5 to 10.7)	-2.3<br>(-14.2 to 13.1)	17.8<br>(10.6 to 27.7)	4.3<br>(1.8 to 8.6)	4.2<br>(1.9 to 8.2)	5.1<br>(2.3 to 9.6)	-2.4<br>(-12.8 to 15.6)	19.7<br>(12.0 to 29.8)
Low physical activity	0.5<br>(0.2 to 1.0)	0.5<br>(0.2 to 1.1)	0.5<br>(0.2 to 1.1)	2.9<br>(1.2 to 4.6)	0.1<br>(-1.1 to 1.4)	0.5<br>(0.2 to 1.0)	0.5<br>(0.2 to 1.1)	0.5<br>(0.2 to 1.1)	2.6<br>(0.8 to 4.3)	-0.2<br>(-1.4 to 1.0)
High fasting plasma glucose	1.8<br>(1.0 to 2.9)	1.8<br>(1.1 to 2.7)	1.9<br>(1.2 to 3.0)	-4.4<br>(-37.2 to 48.9)	8.1<br>(-29.4 to 67.4)	1.4<br>(0.8 to 2.1)	1.2<br>(0.8 to 1.8)	1.3<br>(0.8 to 2.0)	-12.7<br>(-45.6 to 33.8)	7.2<br>(-23.1 to 55.5)
High LDL cholesterol	16.3<br>(14.7 to 18.1)	15.2<br>(13.5 to 17.0)	15.3<br>(13.7 to 17.2)	-7.0<br>(-10.3 to -3.5)	1.0<br>(-2.1 to 3.7)	16.4<br>(14.8 to 18.1)	15.2<br>(13.6 to 16.9)	15.6<br>(13.9 to 17.4)	-7.4<br>(-10.4 to -4.3)	3.0<br>(0.2 to 6.0)
High systolic blood pressure	8.6<br>(7.8 to 9.4)	7.3<br>(6.6 to 8.1)	7.1<br>(6.4 to 7.9)	-14.9<br>(-20.0 to -10.1)	-2.8<br>(-7.8 to 2.6)	7.1<br>(6.4 to 7.7)	5.8<br>(5.2 to 6.5)	5.8<br>(5.1 to 6.4)	-17.6<br>(-23.8 to -10.9)	-1.4<br>(-8.7 to 5.6)
High body-mass index	11.2<br>(8.2 to 15.7)	16.8<br>(13.0 to 22.0)	18.4<br>(14.4 to 23.8)	49.7<br>(34.2 to 72.2)	9.8<br>(3.4 to 16.6)	11.8<br>(8.6 to 16.0)	17.5<br>(13.9 to 22.1)	18.8<br>(15.1 to 23.7)	47.8<br>(30.9 to 69.3)	7.7<br>(0.8 to 15.1)
Low bone mineral density	5.2<br>(4.4 to 6.0)	5.1<br>(4.6 to 5.7)	4.9<br>(4.0 to 5.8)	-1.2<br>(-12.7 to 11.2)	-5.1<br>(-17.2 to 9.3)	5.7<br>(5.3 to 6.1)	5.6<br>(5.0 to 6.2)	5.5<br>(4.6 to 6.5)	-1.1<br>(-10.0 to 8.2)	-2.2<br>(-13.5 to 10.7)
Impaired kidney function	2.0<br>(0.9 to 5.5)	2.0<br>(0.9 to 5.4)	2.0<br>(0.9 to 5.3)	-2.7<br>(-7.3 to 0.8)	0.7<br>(-4.2 to 6.1)	2.7<br>(1.2 to 6.5)	2.7<br>(1.2 to 6.5)	2.7<br>(1.3 to 6.5)	-0.7<br>(-5.1 to 3.2)	1.0<br>(-3.2 to 6.6)

Supplementary Results Table 1. Australia age-standardised Summary Exposure Values (SEVs) for Level 4 risk factors and percentage change, by sex, 1990, 2007, and 2017.Data in parentheses are 95% uncertainty intervals.

	Male					Female				
	1990	2007	2017	Percentage change 1990-2007	Percentage change 2007-2017	1990	2007	2017	Percentage change 1990-2007	Percentage change 2007-2017
Unsafe water source	2.4 (1.6 to 3.4)	1.8 (1.2 to 2.6)	1.7 (1.2 to 2.6)	-25.4 (-53.1 to 17.8)	-2.2 (-33.5 to 38.6)	2.4 (1.6 to 3.4)	1.8 (1.2 to 2.6)	1.7 (1.2 to 2.6)	-25.4 (-53.1 to 17.8)	-2.2 (-33.5 to 38.6)
Unsafe sanitation	4.2 (2.6 to 6.6)	1.8 (1.1 to 3.0)	1.4 (0.8 to 2.3)	-56.6 (-76.5 to -22.1)	-23.5 (-53.1 to 19.3)	4.2 (2.6 to 6.6)	1.8 (1.1 to 3.0)	1.4 (0.8 to 2.3)	-56.6 (-76.5 to -22.1)	-23.5 (-53.1 to 19.3)
No access to handwashing facility	6.8 (5.4 to 8.5)	6.3 (5.0 to 7.8)	5.8 (4.6 to 7.2)	-7.4 (-27.4 to 14.6)	-8.5 (-23.4 to 9.7)	6.8 (5.4 to 8.5)	6.3 (5.0 to 7.8)	5.8 (4.6 to 7.2)	-7.4 (-27.4 to 14.6)	-8.5 (-23.4 to 9.7)
Ambient particulate matter pollution	24.7 (7.4 to 46.8)	25.4 (10.5 to 48.9)	20.2 (3.8 to 43.6)	2.9 (-62.0 to 204.7)	-20.5 (-75.6 to 21.9)	24.4 (7.4 to 46.6)	25.3 (10.4 to 48.9)	20.1 (3.8 to 43.5)	3.5 (-61.9 to 209.6)	-20.4 (-75.5 to 22.0)
Household air pollution from solid fuels	0.7 (0.2 to 2.3)	0.3 (0.1 to 0.9)	0.2 (0.0 to 0.8)	-58.3 (-85.6 to -3.1)	-24.5 (-74.7 to 56.4)	1.1 (0.3 to 7.1)	0.5 (0.1 to 3.2)	0.4 (0.1 to 2.2)	-54.3 (-85.4 to -0.9)	-24.0 (-75.2 to 57.1)
Ambient ozone pollution	4.0 (0.8 to 9.7)	5.6 (1.1 to 14.0)	12.4 (3.8 to 26.3)	42.4 (31.7 to 47.3)	120.5 (72.9 to 290.9)	4.0 (0.8 to 9.7)	5.6 (1.1 to 14.0)	12.4 (3.8 to 26.3)	42.4 (31.7 to 47.3)	120.5 (72.9 to 290.9)
Residential radon	2.2 (0.0 to 10.2)	2.2 (0.0 to 10.2)	2.2 (0.0 to 10.2)	0.0 (0.0 to 0.0)	0.0 (0.0 to 0.0)	2.2 (0.0 to 10.2)	2.2 (0.0 to 10.2)	2.2 (0.0 to 10.2)	0.0 (0.0 to 0.0)	0.0 (0.0 to 0.0)
Lead exposure	11.7 (7.2 to 15.8)	8.9 (5.2 to 12.9)	6.1 (3.2 to 9.6)	-23.5 (-29.7 to -17.7)	-31.4 (-38.0 to -24.5)	7.5 (3.3 to 11.6)	5.7 (2.5 to 9.4)	3.9 (1.4 to 7.1)	-23.1 (-29.3 to -17.1)	-32.4 (-41.6 to -24.5)
Occupational exposure to asbestos	13.5 (11.4 to 14.8)	11.2 (10.8 to 11.7)	10.1 (9.3 to 11.0)	-16.5 (-24.7 to -1.3)	-10.0 (-17.8 to -1.7)	1.9 (1.4 to 2.3)	2.9 (2.7 to 3.1)	2.8 (2.4 to 3.3)	56.2 (28.1 to 109.6)	-4.4 (-20.5 to 11.8)
Occupational exposure to arsenic	0.4 (0.0 to 0.8)	0.4 (0.0 to 0.9)	0.4 (0.0 to 0.9)	-	-	0.3 (0.0 to 0.6)	0.3 (0.0 to 0.8)	0.3 (0.0 to 0.8)	-	-
Occupational exposure to benzene	0.5 (0.0 to 1.5)	0.6 (0.0 to 1.6)	0.6 (0.0 to 1.7)	7.9 (4.5 to 13.1)	2.8 (-0.3 to 7.4)	0.6 (0.0 to 1.8)	0.8 (0.0 to 2.2)	0.8 (0.0 to 2.4)	28.2 (22.3 to 37.1)	8.2 (3.5 to 14.2)
Occupational exposure to beryllium	0.0 (0.0 to 0.0)	0.0 (0.0 to 0.0)	0.0 (0.0 to 0.0)	5.7 (3.9 to 7.5)	2.4 (0.5 to 4.3)	0.0 (0.0 to 0.0)	0.0 (0.0 to 0.0)	0.0 (0.0 to 0.0)	23.7 (21.0 to 26.5)	5.8 (3.0 to 8.5)
Occupational exposure to cadmium	0.0 (0.0 to 0.0)	0.0 (0.0 to 0.0)	0.0 (0.0 to 0.0)	3.9 (-4.2 to 11.9)	3.2 (-3.9 to 10.2)	0.0 (0.0 to 0.0)	0.0 (0.0 to 0.0)	0.0 (0.0 to 0.0)	17.7 (8.1 to 27.6)	4.5 (-3.1 to 12.3)
Occupational exposure to chromium	0.1 (0.1 to 0.1)	0.1 (0.1 to 0.1)	0.1 (0.1 to 0.1)	4.7 (-2.4 to 11.6)	3.4 (-2.8 to 9.5)	0.1 (0.1 to 0.1)	0.1 (0.1 to 0.1)	0.1 (0.1 to 0.1)	19.4 (11.3 to 27.8)	5.2 (-1.1 to 11.8)
Occupational exposure to diesel engine exhaust	0.5 (0.5 to 0.5)	0.6 (0.5 to 0.6)	0.6 (0.5 to 0.6)	5.6 (0.9 to 10.2)	2.7 (-1.3 to 6.8)	0.4 (0.4 to 0.5)	0.5 (0.5 to 0.6)	0.6 (0.5 to 0.6)	22.9 (16.7 to 29.9)	4.9 (-0.2 to 10.3)
Occupational exposure to formaldehyde	0.2 (0.2 to 0.2)	0.2 (0.2 to 0.2)	0.2 (0.2 to 0.2)	2.6 (-6.5 to 11.3)	2.7 (-5.0 to 10.6)	0.2 (0.1 to 0.2)	0.2 (0.2 to 0.2)	0.2 (0.2 to 0.2)	17.2 (7.2 to 27.3)	4.8 (-2.9 to 13.0)
Occupational exposure to nickel	0.3 (0.0 to 1.5)	0.3 (0.0 to 1.6)	0.4 (0.0 to 1.6)	-	-	0.2 (0.0 to 1.0)	0.3 (0.0 to 1.2)	0.3 (0.0 to 1.2)	-	-
Occupational exposure to polycyclic aromatic hydrocarbons	0.2 (0.2 to 0.2)	0.2 (0.2 to 0.2)	0.2 (0.2 to 0.2)	4.4 (-2.2 to 10.7)	3.4 (-2.3 to 9.2)	0.2 (0.2 to 0.2)	0.2 (0.2 to 0.2)	0.2 (0.2 to 0.2)	20.5 (13.1 to 28.0)	5.7 (-0.2 to 12.0)
Occupational exposure to silica	3.8 (0.5 to 12.1)	3.8 (0.5 to 12.5)	3.9 (0.6 to 12.4)	1.0 (-6.1 to 10.0)	2.5 (-3.8 to 9.8)	2.1 (0.3 to 6.8)	2.4 (0.3 to 7.3)	2.4 (0.3 to 7.7)	12.4 (2.1 to 24.9)	1.9 (-6.4 to 11.6)
Occupational exposure to sulphuric acid	0.6 (0.1 to 1.8)	0.5 (0.1 to 1.7)	0.6 (0.1 to 1.7)	-1.6 (-8.7 to 5.6)	2.4 (-4.0 to 9.4)	0.4 (0.1 to 1.1)	0.4 (0.1 to 1.4)	0.4 (0.1 to 1.4)	14.8 (5.1 to 25.5)	3.9 (-4.2 to 12.0)
Occupational exposure to trichloroethylene	0.1 (0.1 to 0.1)	0.1 (0.1 to 0.1)	0.1 (0.1 to 0.1)	6.1 (1.5 to 10.6)	3.3 (-0.8 to 7.3)	0.1 (0.0 to 0.1)	0.1 (0.1 to 0.1)	0.1 (0.1 to 0.1)	21.2 (15.6 to 27.6)	5.2 (0.6 to 10.0)
Occupational asthmagens	15.9 (13.6 to 18.7)	15.2 (12.9 to 17.9)	14.9 (12.7 to 17.8)	-4.5 (-14.0 to 5.9)	-1.7 (-9.4 to 6.8)	7.9 (6.7 to 9.3)	8.8 (7.4 to 10.4)	8.8 (7.4 to 10.5)	11.2 (2.3 to 20.7)	0.5 (-5.7 to 7.3)
Occupational particulate matter, gases, and fumes	6.8 (4.9 to 9.8)	6.2 (4.5 to 8.9)	6.0 (4.3 to 8.7)	-8.8 (-11.5 to -6.2)	-3.3 (-5.2 to -1.3)	2.9 (2.0 to 4.2)	2.9 (2.1 to 4.2)	3.0 (2.1 to 4.3)	2.2 (-2.2 to 6.8)	0.9 (-2.9 to 4.3)
Occupational noise	6.3 (6.0 to 6.7)	5.8 (5.6 to 6.3)	5.7 (5.4 to 6.1)	-7.0 (-8.4 to -5.7)	-2.8 (-3.8 to -1.7)	3.2 (3.0 to 3.4)	3.4 (3.3 to 3.7)	3.6 (3.4 to 3.8)	7.9 (5.4 to 10.4)	4.9 (3.5 to 6.5)
Occupational ergonomic factors	8.4 (7.3 to 9.9)	8.6 (7.5 to 10.0)	8.4 (7.2 to 9.7)	2.1 (-6.0 to 10.5)	-2.4 (-9.2 to 5.0)	5.1 (4.3 to 6.1)	5.3 (4.6 to 6.3)	5.4 (4.5 to 6.6)	4.6 (-7.7 to 18.0)	1.0 (-7.6 to 10.9)
Non-exclusive breastfeeding	0.5 (0.4 to 0.6)	0.5 (0.4 to 0.6)	0.5 (0.4 to 0.6)	2.2 (-6.6 to 13.0)	-1.5 (-9.8 to 6.7)	0.5 (0.4 to 0.6)	0.5 (0.4 to 0.6)	0.5 (0.4 to 0.6)	2.3 (-6.6 to 13.0)	-1.5 (-9.8 to 6.7)
Discontinued breastfeeding	1.8 (1.7 to 1.9)	2.0 (1.9 to 2.1)	1.9 (1.8 to 2.0)	7.3 (0.4 to 15.1)	-1.6 (-6.4 to 3.3)	1.8 (1.7 to 1.9)	2.0 (1.9 to 2.1)	1.9 (1.8 to 2.0)	7.3 (0.4 to 15.0)	-1.5 (-6.3 to 3.4)
Child underweight	0.1 (0.1 to 0.2)	0.1 (0.1 to 0.2)	0.1 (0.1 to 0.1)	-9.6 (-20.4 to 3.3)	-5.8 (-14.0 to 2.8)	0.1 (0.1 to 0.2)	0.1 (0.1 to 0.1)	0.1 (0.1 to 0.1)	-11.4 (-23.3 to 2.3)	-4.8 (-13.6 to 4.4)
Child wasting	0.1 (0.1 to 0.1)	0.1 (0.1 to 0.1)	0.1 (0.1 to 0.1)	-7.2 (-25.3 to 12.8)	-1.2 (-13.9 to 11.8)	0.1 (0.1 to 0.1)	0.1 (0.1 to 0.1)	0.1 (0.1 to 0.1)	-6.4 (-23.3 to 13.5)	-1.2 (-12.9 to 13.0)
Child stunting	0.4 (0.2 to 0.7)	0.3 (0.1 to 0.6)	0.3 (0.1 to 0.5)	-14.4 (-47.5 to 38.3)	-4.3 (-28.0 to 30.1)	0.4 (0.2 to 0.8)	0.4 (0.2 to 0.6)	0.3 (0.1 to 0.6)	-17.3 (-49.3 to 32.6)	-5.1 (-30.5 to 28.9)
Short gestation for birth weight	0.0 (0.0 to 0.0)	0.0 (0.0 to 0.0)	0.0 (0.0 to 0.0)	6.4 (-11.9 to 36.4)	2.3 (-16.4 to 29.3)	0.0 (0.0 to 0.0)	0.0 (0.0 to 0.0)	0.0 (0.0 to 0.0)	8.2 (-13.1 to 31.8)	2.7 (-17.8 to 28.2)
Low birth weight for gestation	0.0 (0.0 to 0.0)	0.0 (0.0 to 0.0)	0.0 (0.0 to 0.0)	-0.5 (-18.2 to 20.7)	1.4 (-17.4 to 22.1)	0.0 (0.0 to 0.0)	0.0 (0.0 to 0.0)	0.0 (0.0 to 0.0)	-2.1 (-17.8 to 16.2)	-0.5 (-19.6 to 19.6)
Iron deficiency	-	-	-	-	-	4.5 (2.9 to 6.5)	3.6 (2.4 to 5.2)	3.2 (2.1 to 4.6)	-19.7 (-38.5 to 3.9)	-11.0 (-28.7 to 10.0)
Vitamin A deficiency	0.2 (0.1 to 0.2)	0.2 (0.1 to 0.2)	0.1 (0.1 to 0.2)	-6.8 (-22.1 to 21.5)	-14.3 (-30.6 to 3.4)	0.2 (0.1 to 0.2)	0.1 (0.1 to 0.2)	0.1 (0.1 to 0.2)	-21.8 (-33.4 to -6.7)	-14.0 (-36.9 to 6.0)
Zinc deficiency	0.6 (0.1 to 1.2)	0.5 (0.1 to 0.9)	0.5 (0.1 to 1.0)	-20.9 (-82.8 to 105.0)	1.2 (-81.2 to 475.3)	0.6 (0.2 to 1.2)	0.6 (0.1 to 1.0)	0.6 (0.1 to 1.0)	-11.7 (-80.1 to 77.3)	0.9 (-77.5 to 402.2)
Smoking	12.1 (10.7 to 13.6)	8.1 (7.1 to 9.2)	7.1 (6.1 to 8.0)	-32.7 (-37.0 to -28.4)	-13.2 (-17.7 to -8.8)	8.3 (7.1 to 9.5)	6.0 (5.2 to 7.0)	5.4 (4.5 to 6.3)	-27.2 (-32.7 to -21.1)	-10.9 (-16.4 to -5.0)
Chewing tobacco	1.4 (1.0 to 1.8)	0.9 (0.7 to 1.2)	0.8 (0.6 to 1.0)	-32.5 (-51.9 to -9.9)	-18.6 (-34.0 to 2.7)	0.1 (0.1 to 0.1)	0.1 (0.1 to 0.1)	0.1 (0.1 to 0.1)	-3.8 (-31.0 to 36.7)	-2.8 (-23.6 to 22.3)
Second-hand smoke	36.0 (33.2 to 39.1)	33.2 (31.4 to 34.9)	32.0 (29.0 to 34.8)	-8.0 (-16.7 to 1.0)	-3.6 (-10.0 to 3.1)	36.4 (34.0 to 38.7)	32.5 (31.1 to 34.1)	31.2 (28.9 to 33.4)	-10.5 (-17.0 to -3.4)	-4.0 (-9.0 to 1.1)
Alcohol use	32.9 (23.2 to 43.1)	28.5 (19.0 to 39.2)	30.8 (19.4 to 44.0)	-13.2 (-27.4 to -1.3)	8.1 (-15.6 to 39.7)	14.2 (8.5 to 22.9)	12.8 (7.3 to 21.8)	13.1 (7.1 to 22.3)	-10.4 (-25.7 to 2.0)	3.1 (-14.2 to 26.2)
Drug use	1.5 (1.0 to 2.4)	1.6 (1.1 to 2.3)	1.9 (1.3 to 3.1)	2.4 (-6.7 to 13.0)	23.9 (12.0 to 34.9)	0.7 (0.5 to 1.2)	0.7 (0.5 to 1.2)	0.9 (0.6 to 1.5)	3.8 (-4.8 to 15.1)	24.8 (14.3 to 35.5)
Diet low in fruits	32.6 (26.9 to 36.7)	30.4 (24.7 to 34.6)	30.4 (24.4 to 35.0)	-6.7 (-11.2 to -3.1)	0.1 (-5.8 to 5.8)	29.6 (23.6 to 34.0)	27.4 (21.5 to 32.1)	27.3 (21.4 to 32.3)	-7.4 (-12.7 to -3.2)	-0.5 (-6.8 to 5.9)
Diet low in vegetables	31.5 (26.9 to 35.5)	27.8 (22.4 to 32.3)	28.0 (22.2 to 32.7)	-11.8 (-17.5 to -8.0)	0.9 (-6.1 to 7.7)	33.5 (29.3 to 37.2)	29.9 (25.1 to 34.0)	30.1 (24.7 to 34.4)	-10.9 (-15.9 to -7.5)	0.8 (-5.1 to 7.4)
Diet low in legumes	27.9 (23.4 to 31.8)	38.5 (35.6 to 41.0)	36.1 (32.6 to 39.1)	38.3 (28.4 to 54.1)	-6.2 (-9.8 to -3.1)	32.1 (28.3 to 35.5)	41.5 (39.1 to 43.7)	39.7 (36.8 to 42.2)	29.3 (22.1 to 39.6)	-4.4 (-7.2 to -1.6)
Diet low in whole grains	34.8 (31.5 to 37.7)	37.6 (34.8 to 40.1)	37.7 (34.8 to 40.2)	8.1 (4.6 to 12.4)	0.2 (-2.4 to 3.0)	36.1 (33.0 to 38.7)	38.7 (36.0 to 41.0)	38.8 (36.1 to 41.1)	7.4 (4.5 to 11.0)	0.2 (-2.1 to 2.6)
Diet low in nuts and seeds	46.5 (44.4 to 48.2)	36.7 (32.1 to 40.5)	32.9 (27.0 to 37.6)	-21.1 (-28.6 to -15.4)	-10.3 (-18.5 to -4.0)	47.0 (45.0 to 48.6)	37.7 (33.5 to 41.2)	34.1 (28.8 to 38.6)	-19.8 (-26.3 to -14.5)	-9.5 (-17.0 to -3.5)
Diet low in milk	29.3 (24.1 to 33.8)	23.0 (16.6 to 28.3)	20.3 (13.1 to 25.9)	-21.5 (-32.3 to -14.4)	-11.6 (-24.8 to -4.7)	30.5 (25.4 to 34.6)	23.6 (17.3 to 28.7)	20.8 (14.0 to 26.5)	-22.7 (-34.2 to -15.7)	-11.6 (-23.1 to -4.6)
Diet high in red meat	52.5 (51.9 to 53.6)	52.0 (51.4 to 53.1)	51.7 (49.8 to 53.4)	-1.0 (-2.9 to 1.0)	-0.6 (-4.2 to 2.3)	33.1 (29.5 to 36.3)	29.0 (25.6 to 32.0)	30.4 (24.8 to 35.3)	-12.6 (-21.1 to -4.2)	5.0 (-10.8 to 21.6)
Diet high in processed meat	5.9 (2.9 to 9.0)	7.5 (4.1 to 10.7)	8.1 (4.7 to 11.2)	27.3 (2.1 to 79.3)	8.1 (-6.5 to 28.2)	3.9 (1.3 to 6.6)	5.1 (2.2 to 8.0)	5.5 (2.7 to 8.5)	31.7 (5.3 to 99.6)	8.3 (-7.8 to 37.0)
Diet high in sugar-sweetened beverages	11.4 (5.6 to 17.9)	12.5 (6.4 to 14.7)	12.4 (6.3 to 16.1)	9.3 (-29.5 to 73.9)	-1.0 (-21.5 to 26.7)	7.7 (4.0 to 12.8)	8.5 (4.2 to 10.4)	8.6 (4.5 to 11.5)	9.3 (-31.1 to 78.8)	1.3 (-24.0 to 33.0)
Diet low in fibre	35.8 (31.6 to 39.1)	36.5 (32.5 to 39.7)	35.5 (31.1 to 39.0)	2.1 (-0.8 to 5.5)	-3.0 (-6.9 to 0.4)	40.2 (36.8 to 42.8)	40.9 (37.7 to 43.4)	40.1 (36.6 to 42.9)	1.7 (-0.5 to 4.3)	-1.9 (-4.9 to 0.8)
Diet low in calcium	21.3 (14.7 to 26.5)	22.0 (15.5 to 27.2)	20.1 (13.2 to 25.6)	3.2 (-0.2 to 8.3)	-8.7 (-16.7 to -3.7)	25.1 (19.2 to 29.8)	25.7 (19.8 to 30.3)	24.0 (17.7 to 29.0)	2.6 (0.1 to 6.5)	-6.7 (-12.5 to -3.1)
Diet low in seafood omega-3 fatty acids	30.1 (25.7 to 34.1)	26.3 (21.6 to 30.7)	24.5 (19.7 to 29.1)	-12.7 (-16.9 to -9.5)	-6.6 (-11.0 to -3.2)	33.5 (29.6 to 37.1)	30.0 (25.6 to 34.1)	28.4 (23.9 to 32.6)	-10.4 (-13.7 to -7.6)	-5.5 (-9.0 to -2.9)
Diet low in polyunsaturated fatty acids	40.8 (38.0 to 43.5)	40.7 (37.9 to 43.2)	39.2 (36.0 to 41.8)	-0.4 (-5.2 to 5.4)	-3.6 (-7.1 to 0.8)	41.3 (38.5 to 44.0)	41.1 (38.4 to 43.7)	39.7 (36.8 to 42.2)	-0.3 (-5.4 to 4.6)	-3.6 (-7.6 to 0.7)
Diet high in trans fatty acids	6.4 (2.9 to 10.2)	3.7 (1.0 to 7.1)	2.6 (0.4 to 5.6)	-42.0 (-67.8 to -26.7)	-29.4 (-60.9 to -14.3)	8.7 (4.8 to 12.7)	5.1 (2.0 to 8.8)	3.6 (0.9 to 6.9)	-41.7 (-63.2 to -24.7)	-28.9 (-54.9 to -13.5)
Diet high in sodium	4.9 (0.1 to 11.8)	4.8 (0.1 to 11.5)	4.8 (0.1 to 11.5)	-2.8 (-77.8 to 236.1)	1.6 (-57.2 to 251.0)	2.4 (0.0 to 7.9)	2.6 (0.0 to 8.3)	2.7 (0.0 to 8.5)	10.1 (-55.5 to 705.6)	4.0 (-56.5 to 265.7)
Intimate partner violence	-	-	-	-	-	1.4 (1.2 to 1.6)	1.3 (1.1 to 1.5)	1.2 (1.0 to 1.4)	-8.0 (-13.4 to -2.4)	-5.4 (-10.9 to 0.5)
Childhood sexual abuse	3.2 (3.1 to 3.2)	3.1 (2.8 to 3.4)	3.4 (2.8 to 3.9)	-3.8 (-11.5 to 5.2)	10.1 (-3.2 to 22.6)	8.6 (7.4 to 9.8)	8.7 (7.9 to 9.5)	9.3 (7.7 to 11.0)	1.5 (-6.3 to 10.1)	6.9 (-6.8 to 21.3)

Supplementary Results Table 1. Australia age-standardised Summary Exposure Values (SEVs) for Level 4 risk factors and percentage change, by sex, 1990, 2007, and 2017.Data in parentheses are 95% uncertainty intervals.

	Male					Female				
	**1990**	**2007**	**2017**	**Percentage change 1990-2007**	**Percentage change 2007-2017**	**1990**	**2007**	**2017**	**Percentage change 1990-2007**	**Percentage change 2007-2017**
Bullying victimization	4.7<br>(2.0 to 9.5)	4.5<br>(2.1 to 9.0)	5.5<br>(2.5 to 10.9)	-4.2<br>(-18.5 to 14.6)	22.4<br>(13.3 to 35.1)	4.2<br>(1.8 to 8.6)	4.0<br>(1.8 to 7.9)	5.0<br>(2.3 to 9.6)	-4.9<br>(-17.8 to 16.2)	25.0<br>(15.2 to 38.3)
Low physical activity	0.5<br>(0.2 to 0.9)	0.5<br>(0.2 to 1.0)	0.5<br>(0.2 to 1.0)	3.7<br>(1.5 to 6.0)	1.4<br>(-0.1 to 3.1)	0.5<br>(0.2 to 1.0)	0.5<br>(0.2 to 1.0)	0.5<br>(0.2 to 1.0)	3.1<br>(0.9 to 5.2)	0.7<br>(-0.7 to 2.2)
High fasting plasma glucose	1.9<br>(1.0 to 3.2)	1.9<br>(1.2 to 2.9)	1.9<br>(1.2 to 3.1)	-3.5<br>(-38.7 to 60.4)	3.5<br>(-37.0 to 64.1)	1.4<br>(0.8 to 2.3)	1.2<br>(0.8 to 1.9)	1.3<br>(0.8 to 2.0)	-13.2<br>(-49.1 to 43.0)	3.4<br>(-29.3 to 57.9)
High LDL cholesterol	16.4<br>(14.7 to 18.1)	15.4<br>(13.7 to 17.2)	15.6<br>(13.9 to 17.5)	-5.7<br>(-9.5 to -1.6)	0.8<br>(-2.6 to 4.1)	16.3<br>(14.7 to 18.1)	15.2<br>(13.6 to 17.0)	15.7<br>(14.0 to 17.5)	-6.7<br>(-10.2 to -3.2)	3.1<br>(-0.1 to 6.7)
High systolic blood pressure	8.6<br>(7.7 to 9.4)	7.2<br>(6.4 to 8.0)	7.0<br>(6.2 to 7.8)	-16.5<br>(-22.2 to -10.9)	-2.9<br>(-8.6 to 3.3)	7.1<br>(6.4 to 7.8)	5.9<br>(5.2 to 6.7)	5.9<br>(5.2 to 6.6)	-16.6<br>(-23.8 to -8.6)	-0.9<br>(-8.8 to 7.2)
High body-mass index	11.1<br>(8.0 to 15.5)	16.6<br>(12.9 to 21.8)	18.2<br>(14.1 to 23.7)	50.4<br>(32.1 to 75.4)	9.2<br>(1.6 to 17.6)	11.7<br>(8.3 to 16.0)	17.4<br>(13.8 to 21.9)	18.6<br>(14.7 to 23.1)	48.8<br>(28.6 to 75.4)	6.9<br>(-1.0 to 15.2)
Low bone mineral density	5.2<br>(4.4 to 6.1)	5.2<br>(4.7 to 5.7)	4.9<br>(4.0 to 5.8)	-0.6<br>(-13.5 to 14.4)	-5.6<br>(-19.7 to 11.7)	5.7<br>(5.3 to 6.1)	5.6<br>(5.0 to 6.3)	5.5<br>(4.6 to 6.6)	-0.6<br>(-10.5 to 10.6)	-2.3<br>(-15.6 to 13.0)
Impaired kidney function	2.0<br>(0.9 to 5.5)	2.0<br>(0.9 to 5.5)	2.0<br>(0.9 to 5.4)	-2.5<br>(-7.5 to 1.4)	0.6<br>(-4.6 to 6.6)	2.7<br>(1.2 to 6.5)	2.7<br>(1.2 to 6.6)	2.7<br>(1.3 to 6.5)	-1.1<br>(-6.6 to 3.3)	1.1<br>(-3.7 to 7.6)

Supplementary Results Table 1. New Zealand age-standardised Summary Exposure Values (SEVs) for Level 4 risk factors and percentage change, by sex, 1990, 2007, and 2017.Data in parentheses are 95% uncertainty intervals.										
	Male					Female				
	1990	2007	2017	Percentage change 1990-2007	Percentage change 2007-2017	1990	2007	2017	Percentage change 1990-2007	Percentage change 2007-2017
Unsafe water source	2.9 (1.9 to 4.2)	2.4 (1.5 to 3.4)	2.3 (1.5 to 3.2)	-17.9 (-50.4 to 33.6)	-3.5 (-32.5 to 41.3)	2.9 (1.9 to 4.2)	2.4 (1.5 to 3.5)	2.3 (1.5 to 3.3)	-17.3 (-50.2 to 34.3)	-3.5 (-32.6 to 41.6)
Unsafe sanitation	5.2 (3.1 to 8.3)	2.6 (1.5 to 4.2)	2.0 (1.1 to 3.2)	-49.6 (-72.3 to -11.7)	-24.4 (-52.9 to 20.9)	5.2 (3.1 to 8.3)	2.7 (1.6 to 4.2)	2.0 (1.1 to 3.2)	-49.0 (-72.0 to -10.7)	-24.4 (-53.2 to 21.0)
No access to handwashing facility	6.6 (5.2 to 8.2)	6.5 (5.1 to 8.0)	5.9 (4.7 to 7.4)	-2.3 (-23.8 to 26.2)	-8.4 (-22.9 to 9.1)	6.6 (5.2 to 8.2)	6.5 (5.1 to 8.0)	5.9 (4.7 to 7.4)	-2.2 (-23.6 to 26.4)	-8.4 (-22.9 to 9.1)
Ambient particulate matter pollution	17.8 (6.8 to 32.4)	17.7 (8.3 to 29.9)	13.3 (2.8 to 27.5)	-0.3 (-56.0 to 156.8)	-24.8 (-80.8 to 55.0)	17.6 (6.8 to 32.3)	17.6 (8.2 to 29.7)	13.3 (2.8 to 27.3)	0.0 (-55.9 to 158.1)	-24.7 (-81.0 to 55.6)
Household air pollution from solid fuels	0.6 (0.2 to 1.8)	0.4 (0.1 to 1.3)	0.3 (0.0 to 1.1)	-39.2 (-73.7 to 21.0)	-28.2 (-78.7 to 27.5)	0.9 (0.3 to 3.2)	0.6 (0.2 to 2.8)	0.4 (0.1 to 2.3)	-32.6 (-71.1 to 28.5)	-27.0 (-78.8 to 27.5)
Ambient ozone pollution	0.3 (0.0 to 1.7)	0.5 (0.0 to 2.8)	5.1 (1.0 to 13.1)	69.8 (-84.0 to 243.0)	837.0 (320.7 to 923772.5)	0.3 (0.0 to 1.7)	0.5 (0.0 to 2.8)	5.1 (1.0 to 13.1)	69.8 (-83.9 to 242.8)	837.0 (320.7 to 922032.4)
Residential radon	13.7 (1.3 to 41.6)	13.7 (1.3 to 41.0)	13.7 (1.3 to 40.8)	0.1 (-1.5 to 8.4)	0.0 (-0.6 to 3.2)	13.7 (1.3 to 41.5)	13.7 (1.3 to 40.7)	13.7 (1.4 to 40.4)	0.1 (-2.3 to 13.3)	0.0 (-0.8 to 3.8)
Lead exposure	5.5 (2.4 to 9.4)	5.2 (2.1 to 9.1)	4.0 (1.4 to 7.5)	-6.0 (-13.9 to -0.8)	-23.8 (-32.4 to -17.0)	3.5 (1.1 to 7.0)	3.3 (1.0 to 6.8)	2.5 (0.7 to 5.6)	-4.5 (-13.9 to 1.3)	-24.6 (-33.5 to -17.4)
Occupational exposure to asbestos	11.5 (8.9 to 12.8)	10.3 (9.8 to 10.8)	8.5 (7.8 to 9.3)	-11.0 (-21.2 to 15.8)	-16.9 (-24.8 to -9.4)	1.3 (0.9 to 1.6)	1.6 (1.5 to 1.8)	1.6 (1.4 to 1.8)	23.6 (-1.3 to 78.5)	-3.5 (-16.8 to 9.9)
Occupational exposure to arsenic	0.4 (0.0 to 0.9)	0.4 (0.0 to 0.8)	0.4 (0.0 to 0.9)	-	-	0.3 (0.0 to 0.7)	0.3 (0.0 to 0.7)	0.3 (0.0 to 0.8)	-	-
Occupational exposure to benzene	0.5 (0.0 to 1.6)	0.6 (0.0 to 1.8)	0.7 (0.0 to 1.9)	17.9 (14.4 to 22.5)	5.5 (2.2 to 10.5)	0.7 (0.0 to 2.0)	1.0 (0.1 to 2.8)	1.1 (0.1 to 3.0)	42.3 (36.9 to 51.6)	8.1 (3.9 to 14.8)
Occupational exposure to beryllium	0.0 (0.0 to 0.0)	0.0 (0.0 to 0.0)	0.0 (0.0 to 0.0)	8.8 (7.1 to 10.6)	3.9 (1.9 to 5.7)	0.0 (0.0 to 0.0)	0.0 (0.0 to 0.0)	0.0 (0.0 to 0.0)	17.2 (15.2 to 19.3)	7.1 (4.7 to 9.4)
Occupational exposure to cadmium	0.0 (0.0 to 0.0)	0.0 (0.0 to 0.0)	0.0 (0.0 to 0.0)	-0.8 (-4.5 to 2.8)	1.8 (-3.2 to 7.5)	0.0 (0.0 to 0.0)	0.0 (0.0 to 0.0)	0.0 (0.0 to 0.0)	2.2 (-2.2 to 7.3)	5.4 (-0.2 to 11.5)
Occupational exposure to chromium	0.1 (0.1 to 0.1)	0.1 (0.1 to 0.1)	0.1 (0.1 to 0.1)	2.5 (-0.8 to 5.7)	2.5 (-1.9 to 7.2)	0.1 (0.1 to 0.1)	0.1 (0.1 to 0.1)	0.1 (0.1 to 0.1)	8.6 (4.8 to 13.0)	6.1 (1.5 to 10.9)
Occupational exposure to diesel engine exhaust	0.5 (0.5 to 0.5)	0.6 (0.6 to 0.6)	0.6 (0.6 to 0.6)	23.3 (19.3 to 27.0)	5.3 (1.6 to 9.2)	0.5 (0.4 to 0.5)	0.5 (0.5 to 0.6)	0.6 (0.5 to 0.6)	18.0 (12.5 to 23.6)	7.3 (1.6 to 13.6)
Occupational exposure to formaldehyde	0.2 (0.2 to 0.2)	0.2 (0.2 to 0.2)	0.2 (0.2 to 0.2)	-1.1 (-5.0 to 2.7)	1.3 (-3.7 to 7.1)	0.2 (0.2 to 0.2)	0.2 (0.2 to 0.2)	0.2 (0.2 to 0.2)	8.1 (3.6 to 13.3)	5.5 (0.5 to 11.0)
Occupational exposure to nickel	0.3 (0.0 to 1.5)	0.4 (0.0 to 1.6)	0.4 (0.0 to 1.6)	-	-	0.3 (0.0 to 1.1)	0.2 (0.0 to 1.1)	0.3 (0.0 to 1.1)	-	-
Occupational exposure to polycyclic aromatic hydrocarbons	0.2 (0.2 to 0.2)	0.2 (0.2 to 0.2)	0.2 (0.2 to 0.2)	5.2 (2.0 to 8.2)	3.0 (-1.0 to 7.2)	0.2 (0.2 to 0.2)	0.2 (0.2 to 0.2)	0.2 (0.2 to 0.2)	14.4 (10.6 to 18.3)	6.6 (2.7 to 10.8)
Occupational exposure to silica	3.7 (0.5 to 11.9)	4.5 (0.7 to 14.7)	4.6 (0.7 to 15.1)	19.2 (12.9 to 27.6)	3.9 (-1.9 to 11.4)	2.3 (0.3 to 7.0)	2.2 (0.3 to 7.1)	2.3 (0.3 to 7.2)	-2.8 (-8.1 to 3.5)	5.4 (-1.9 to 14.3)
Occupational exposure to sulphuric acid	0.5 (0.1 to 1.6)	0.5 (0.1 to 1.5)	0.5 (0.1 to 1.6)	-4.4 (-8.0 to -0.8)	0.7 (-4.1 to 6.0)	0.4 (0.1 to 1.3)	0.4 (0.1 to 1.3)	0.4 (0.1 to 1.3)	-1.4 (-6.0 to 4.0)	4.3 (-2.0 to 11.4)
Occupational exposure to trichloroethylene	0.1 (0.1 to 0.1)	0.1 (0.1 to 0.1)	0.1 (0.1 to 0.1)	5.8 (3.3 to 8.7)	3.2 (-0.1 to 6.8)	0.1 (0.1 to 0.1)	0.1 (0.1 to 0.1)	0.1 (0.1 to 0.1)	10.9 (7.6 to 14.6)	6.4 (2.1 to 11.0)
Occupational asthmagens	17.2 (14.5 to 20.4)	16.5 (14.0 to 19.6)	17.0 (14.0 to 20.5)	-3.7 (-10.6 to 3.2)	2.7 (-7.8 to 14.0)	8.8 (7.4 to 10.4)	8.9 (7.5 to 10.4)	9.5 (7.9 to 11.3)	0.8 (-5.5 to 7.7)	6.5 (-2.9 to 18.4)
Occupational particulate matter, gases, and fumes	6.9 (5.0 to 9.8)	6.4 (4.6 to 9.2)	6.1 (4.4 to 8.8)	-6.8 (-9.9 to -3.8)	-4.5 (-6.2 to -2.8)	3.5 (2.5 to 5.2)	3.2 (2.3 to 4.6)	3.0 (2.1 to 4.3)	-9.4 (-14.4 to -4.9)	-6.7 (-9.2 to -3.9)
Occupational noise	6.3 (6.0 to 6.8)	6.1 (5.8 to 6.5)	6.0 (5.7 to 6.4)	-4.2 (-5.8 to -2.4)	-1.7 (-2.6 to -0.8)	3.9 (3.6 to 4.2)	3.9 (3.7 to 4.2)	4.0 (3.8 to 4.3)	0.7 (-2.0 to 3.2)	1.8 (0.6 to 3.0)
Occupational ergonomic factors	11.7 (10.4 to 13.1)	11.2 (10.0 to 12.5)	10.4 (9.0 to 12.2)	-4.2 (-11.4 to 3.1)	-7.1 (-15.8 to 2.9)	6.1 (5.3 to 7.1)	6.1 (5.3 to 7.1)	5.8 (4.8 to 7.0)	-0.3 (-8.6 to 8.1)	-4.6 (-15.3 to 8.5)
Non-exclusive breastfeeding	0.5 (0.4 to 0.6)	0.5 (0.4 to 0.6)	0.5 (0.4 to 0.6)	0.2 (-8.8 to 11.8)	-1.4 (-10.2 to 8.2)	0.5 (0.4 to 0.6)	0.5 (0.4 to 0.6)	0.5 (0.4 to 0.6)	0.4 (-8.6 to 12.1)	-1.4 (-10.2 to 8.2)
Discontinued breastfeeding	1.8 (1.7 to 1.9)	1.7 (1.6 to 1.8)	1.7 (1.6 to 1.8)	-2.3 (-9.4 to 5.6)	-3.8 (-9.0 to 1.7)	1.8 (1.7 to 1.9)	1.7 (1.6 to 1.8)	1.7 (1.6 to 1.8)	-2.6 (-9.6 to 5.3)	-3.6 (-8.8 to 1.9)
Child underweight	0.1 (0.1 to 0.2)	0.1 (0.1 to 0.2)	0.1 (0.1 to 0.2)	-8.4 (-20.0 to 4.6)	-6.2 (-14.8 to 2.1)	0.1 (0.1 to 0.2)	0.1 (0.1 to 0.2)	0.1 (0.1 to 0.2)	-10.0 (-23.4 to 4.0)	-5.3 (-14.9 to 3.8)
Child wasting	0.1 (0.1 to 0.1)	0.1 (0.1 to 0.1)	0.1 (0.1 to 0.1)	-6.0 (-23.5 to 14.5)	-1.3 (-13.3 to 12.9)	0.1 (0.1 to 0.1)	0.1 (0.1 to 0.1)	0.1 (0.1 to 0.1)	-6.4 (-23.7 to 14.3)	-1.8 (-14.3 to 12.9)
Child stunting	0.4 (0.2 to 0.7)	0.3 (0.2 to 0.6)	0.3 (0.1 to 0.6)	-12.3 (-46.1 to 41.4)	-5.3 (-32.0 to 28.8)	0.5 (0.2 to 0.8)	0.4 (0.2 to 0.7)	0.4 (0.2 to 0.7)	-15.6 (-48.4 to 36.0)	-4.4 (-30.7 to 31.8)
Short gestation for birth weight	0.0 (0.0 to 0.0)	0.0 (0.0 to 0.0)	0.0 (0.0 to 0.0)	4.5 (-14.8 to 25.9)	1.6 (-17.6 to 25.0)	0.0 (0.0 to 0.0)	0.0 (0.0 to 0.0)	0.0 (0.0 to 0.0)	2.2 (-16.4 to 22.8)	1.9 (-14.9 to 21.1)
Low birth weight for gestation	0.0 (0.0 to 0.0)	0.0 (0.0 to 0.0)	0.0 (0.0 to 0.0)	6.9 (-7.0 to 26.8)	0.7 (-15.4 to 19.6)	0.0 (0.0 to 0.0)	0.0 (0.0 to 0.0)	0.0 (0.0 to 0.0)	8.2 (-7.1 to 27.8)	-0.4 (-15.0 to 17.3)
Iron deficiency	-	-	-	-	-	4.0 (2.8 to 5.7)	3.6 (2.3 to 5.1)	3.2 (2.1 to 4.7)	-12.3 (-31.3 to 11.7)	-9.4 (-26.2 to 10.0)
Vitamin A deficiency	0.3 (0.2 to 0.4)	0.2 (0.2 to 0.3)	0.2 (0.2 to 0.3)	-22.7 (-34.3 to -11.8)	2.7 (-13.7 to 20.5)	0.2 (0.1 to 0.3)	0.2 (0.2 to 0.3)	0.2 (0.1 to 0.3)	5.4 (-8.3 to 22.5)	-7.1 (-19.8 to 5.0)
Zinc deficiency	0.4 (0.1 to 1.1)	0.4 (0.1 to 0.8)	0.4 (0.1 to 0.8)	-17.4 (-72.9 to 167.7)	-0.0 (-60.1 to 254.5)	0.4 (0.1 to 1.1)	0.3 (0.1 to 0.8)	0.3 (0.1 to 0.8)	-17.5 (-72.2 to 169.7)	-3.0 (-61.8 to 185.6)
Smoking	10.8 (9.4 to 12.4)	6.6 (5.7 to 7.6)	5.6 (4.7 to 6.5)	-39.1 (-44.1 to -33.6)	-15.6 (-19.6 to -11.1)	8.5 (7.1 to 10.0)	5.5 (4.6 to 6.5)	4.3 (3.6 to 5.1)	-35.3 (-41.7 to -28.0)	-21.7 (-26.0 to -17.2)
Chewing tobacco	1.4 (1.0 to 1.8)	0.9 (0.7 to 1.2)	0.7 (0.6 to 1.0)	-32.6 (-50.4 to -9.8)	-19.0 (-35.4 to 1.8)	0.1 (0.1 to 0.1)	0.1 (0.1 to 0.1)	0.1 (0.1 to 0.1)	-3.9 (-30.8 to 33.2)	-3.3 (-25.2 to 23.9)
Second-hand smoke	42.9 (40.1 to 46.2)	36.6 (34.5 to 38.8)	35.7 (32.6 to 39.0)	-14.7 (-22.3 to -6.7)	-2.5 (-8.2 to 3.7)	40.1 (38.0 to 42.4)	34.1 (32.5 to 35.8)	33.0 (30.6 to 35.4)	-14.9 (-21.4 to -8.5)	-3.4 (-8.2 to 1.4)
Alcohol use	36.9 (28.1 to 45.5)	33.0 (23.6 to 42.5)	34.2 (21.9 to 45.6)	-10.5 (-22.7 to -0.6)	3.5 (-18.0 to 27.3)	8.3 (4.3 to 15.7)	7.8 (4.0 to 15.0)	7.9 (3.8 to 14.9)	-5.8 (-17.0 to 5.6)	1.3 (-19.5 to 24.7)
Drug use	0.9 (0.6 to 1.5)	1.1 (0.7 to 1.8)	0.9 (0.6 to 1.4)	21.5 (12.6 to 30.5)	-11.2 (-20.4 to 0.7)	0.5 (0.3 to 0.8)	0.5 (0.3 to 0.8)	0.5 (0.3 to 0.7)	1.0 (-4.6 to 7.1)	2.1 (-5.1 to 11.4)
Diet low in fruits	27.7 (21.9 to 32.7)	27.5 (21.4 to 32.3)	27.8 (21.5 to 32.9)	-0.9 (-7.3 to 5.5)	1.0 (-6.6 to 8.8)	24.2 (17.8 to 29.5)	23.8 (17.5 to 29.1)	24.3 (17.8 to 29.7)	-1.6 (-8.6 to 5.8)	1.9 (-7.9 to 11.7)
Diet low in vegetables	20.7 (13.7 to 26.6)	20.3 (13.3 to 26.1)	22.9 (16.0 to 28.7)	-1.6 (-13.4 to 11.5)	12.9 (0.6 to 33.9)	23.3 (16.9 to 28.9)	22.9 (16.3 to 28.2)	25.2 (18.4 to 30.7)	-1.9 (-12.4 to 9.3)	10.4 (0.2 to 25.2)
Diet low in legumes	28.6 (24.0 to 32.5)	24.5 (19.2 to 28.9)	27.0 (21.7 to 31.4)	-14.1 (-22.4 to -7.6)	10.0 (2.5 to 19.6)	32.4 (28.5 to 35.9)	29.5 (25.0 to 33.3)	31.7 (27.4 to 35.4)	-9.1 (-15.3 to -4.0)	7.5 (2.2 to 14.8)
Diet low in whole grains	35.2 (32.0 to 37.9)	39.7 (37.2 to 41.9)	40.2 (37.7 to 42.5)	12.8 (8.8 to 18.3)	1.4 (-1.0 to 3.7)	36.4 (33.3 to 39.0)	40.5 (38.1 to 42.6)	41.1 (38.8 to 43.0)	11.3 (7.9 to 15.9)	1.3 (-0.5 to 3.3)
Diet low in nuts and seeds	50.4 (49.2 to 51.4)	45.1 (42.5 to 47.1)	42.5 (38.9 to 45.2)	-10.5 (-14.6 to -7.5)	-5.8 (-10.0 to -2.5)	50.6 (49.6 to 51.5)	45.7 (43.4 to 47.6)	43.2 (40.1 to 45.8)	-9.7 (-12.9 to -7.1)	-5.4 (-9.2 to -2.5)
Diet low in milk	30.5 (25.4 to 34.9)	24.6 (18.5 to 29.9)	25.5 (19.3 to 30.7)	-19.3 (-29.5 to -11.9)	3.3 (-3.0 to 10.8)	31.5 (26.6 to 35.6)	25.2 (19.0 to 30.1)	25.9 (19.7 to 30.9)	-20.0 (-30.7 to -13.0)	2.9 (-3.8 to 10.2)
Diet high in red meat	51.2 (47.6 to 53.2)	49.2 (45.5 to 51.6)	49.0 (45.3 to 51.8)	-3.9 (-9.7 to 1.7)	-0.5 (-7.2 to 5.6)	33.3 (27.3 to 38.3)	26.8 (22.0 to 30.6)	27.7 (20.9 to 33.4)	-19.7 (-32.2 to -5.8)	3.5 (-13.3 to 23.0)
Diet high in processed meat	5.7 (2.8 to 8.7)	7.1 (3.9 to 10.1)	7.9 (4.6 to 11.2)	24.0 (-2.5 to 69.6)	12.0 (-2.7 to 34.9)	3.7 (1.3 to 6.5)	4.7 (1.8 to 7.6)	5.3 (2.4 to 8.3)	26.5 (0.4 to 84.6)	13.3 (-4.2 to 48.7)
Diet high in sugar-sweetened beverages	6.6 (2.9 to 11.1)	7.3 (3.8 to 9.3)	8.3 (4.4 to 11.2)	10.9 (-31.1 to 79.7)	13.1 (-14.2 to 49.7)	4.5 (2.1 to 7.6)	5.0 (2.6 to 6.7)	5.7 (2.7 to 8.2)	12.5 (-30.3 to 85.3)	13.1 (-18.6 to 58.2)
Diet low in fibre	30.8 (25.7 to 34.9)	30.1 (24.8 to 34.4)	30.8 (25.4 to 35.2)	-2.1 (-7.1 to 2.4)	2.4 (-2.8 to 8.6)	35.8 (31.6 to 39.3)	35.1 (30.9 to 38.7)	35.7 (31.0 to 39.4)	-2.1 (-5.9 to 1.6)	1.7 (-2.5 to 6.1)
Diet low in calcium	25.5 (19.7 to 30.3)	35.1 (31.1 to 38.4)	34.5 (30.3 to 37.9)	37.6 (25.7 to 59.1)	-1.8 (-4.1 to 0.4)	29.1 (23.7 to 33.3)	37.6 (34.0 to 40.5)	37.0 (33.2 to 40.1)	29.5 (21.0 to 44.5)	-1.6 (-3.7 to 0.3)
Diet low in seafood omega-3 fatty acids	34.1 (30.3 to 37.7)	30.5 (26.1 to 34.7)	29.1 (24.5 to 33.3)	-10.6 (-14.3 to -7.7)	-4.6 (-7.8 to -2.2)	37.0 (33.6 to 40.1)	33.9 (30.0 to 37.6)	32.7 (28.5 to 36.4)	-8.2 (-11.2 to -6.1)	-3.6 (-6.0 to -1.6)
Diet low in polyunsaturated fatty acids	32.8 (28.6 to 36.5)	32.5 (28.3 to 36.0)	31.9 (27.6 to 35.9)	-0.7 (-7.5 to 4.5)	-1.8 (-6.5 to 5.5)	33.4 (29.3 to 37.5)	33.2 (29.2 to 36.5)	32.5 (28.3 to 36.3)	-0.7 (-7.6 to 4.7)	-2.1 (-7.5 to 4.9)
Diet high in trans fatty acids	5.4 (2.2 to 9.2)	3.0 (0.7 to 6.1)	2.0 (0.2 to 4.8)	-45.0 (-72.1 to -28.7)	-31.6 (-66.7 to -16.4)	7.4 (3.7 to 11.6)	4.1 (1.2 to 7.5)	2.8 (0.6 to 6.0)	-45.1 (-69.4 to -29.0)	-30.8 (-59.4 to -15.3)
Diet high in sodium	8.4 (1.2 to 15.7)	10.6 (2.4 to 18.3)	10.0 (2.0 to 17.5)	26.6 (-3.3 to 204.7)	-6.2 (-38.6 to 21.6)	6.7 (0.5 to 14.1)	9.4 (1.5 to 17.0)	8.3 (1.0 to 15.7)	38.7 (8.5 to 344.9)	-11.6 (-53.0 to 4.2)
Intimate partner violence	-	-	-	-	-	4.0 (3.3 to 4.6)	3.8 (3.2 to 4.4)	3.5 (3.0 to 4.1)	-5.6 (-11.2 to 0.2)	-6.0 (-11.7 to 0.2)
Childhood sexual abuse	12.5 (10.2 to 15.5)	12.4 (10.2 to 15.1)	12.3 (10.1 to 15.1)	-0.9 (-6.0 to 4.8)	-0.2 (-5.3 to 5.3)	16.2 (12.9 to 19.7)	18.6 (14.8 to 22.7)	18.5 (14.8 to 22.5)	14.9 (8.0 to 22.4)	-0.7 (-7.7 to 5.3)

Supplementary Results Table 1. New Zealand age-standardised Summary Exposure Values (SEVs) for Level 4 risk factors and percentage change, by sex, 1990, 2007, and 2017.Data in parentheses are 95% uncertainty intervals.

	Male					Female				
	1990	2007	2017	Percentage change 1990-2007	Percentage change 2007-2017	1990	2007	2017	Percentage change 1990-2007	Percentage change 2007-2017
Bullying victimization	5.3 (2.2 to 10.7)	5.6 (2.3 to 11.2)	5.6 (2.4 to 11.3)	6.0 (-2.8 to 15.6)	0.5 (-3.9 to 5.1)	5.0 (2.0 to 10.0)	5.4 (2.2 to 10.8)	5.5 (2.3 to 11.3)	7.2 (-1.1 to 17.4)	2.4 (-2.5 to 6.9)
Low physical activity	0.7 (0.2 to 1.5)	0.7 (0.2 to 1.5)	0.7 (0.2 to 1.5)	0.5 (-0.1 to 1.1)	-2.5 (-3.8 to -0.6)	0.6 (0.2 to 1.3)	0.6 (0.2 to 1.4)	0.6 (0.2 to 1.3)	0.5 (-0.0 to 1.1)	-2.7 (-4.2 to -0.5)
High fasting plasma glucose	1.4 (0.8 to 2.3)	1.2 (0.8 to 1.8)	1.7 (1.0 to 2.7)	-15.3 (-49.3 to 32.2)	49.4 (2.9 to 139.0)	1.2 (0.8 to 1.8)	1.1 (0.7 to 1.5)	1.4 (0.9 to 2.0)	-11.6 (-41.5 to 24.6)	30.9 (-0.0 to 93.5)
High LDL cholesterol	16.2 (14.4 to 18.1)	13.9 (12.3 to 15.8)	14.1 (12.4 to 15.9)	-14.0 (-17.8 to -10.1)	1.0 (-2.4 to 4.6)	16.7 (15.0 to 18.5)	14.9 (13.3 to 16.7)	15.2 (13.6 to 17.0)	-10.8 (-14.7 to -7.0)	2.0 (-1.1 to 5.7)
High systolic blood pressure	8.6 (7.8 to 9.4)	8.0 (7.2 to 8.9)	7.9 (7.1 to 8.8)	-6.3 (-12.1 to -0.7)	-2.1 (-7.8 to 3.8)	6.9 (6.2 to 7.7)	5.4 (4.8 to 6.0)	5.1 (4.5 to 5.8)	-22.7 (-28.9 to -16.0)	-4.2 (-11.3 to 3.8)
High body-mass index	12.0 (8.7 to 17.1)	17.5 (13.6 to 23.1)	19.8 (15.5 to 25.8)	45.8 (28.1 to 67.3)	13.1 (4.9 to 23.0)	12.6 (9.5 to 16.9)	18.0 (14.4 to 23.6)	20.1 (16.0 to 25.9)	42.0 (23.2 to 65.7)	11.8 (1.6 to 22.8)
Low bone mineral density	5.1 (4.3 to 6.1)	4.9 (4.1 to 5.7)	4.8 (4.0 to 5.7)	-4.4 (-15.6 to 9.4)	-2.2 (-14.6 to 10.4)	5.9 (4.9 to 7.0)	5.6 (4.8 to 6.6)	5.5 (4.6 to 6.6)	-3.8 (-15.0 to 10.9)	-1.5 (-14.1 to 11.1)
Impaired kidney function	1.9 (0.9 to 5.2)	1.8 (0.8 to 5.0)	1.8 (0.8 to 4.9)	-4.0 (-8.8 to 0.2)	0.5 (-4.4 to 6.1)	2.7 (1.2 to 6.5)	2.7 (1.3 to 6.5)	2.7 (1.3 to 6.6)	1.3 (-2.8 to 5.9)	0.7 (-3.5 to 5.6)

Supplementary Results Table 1. High-income Asia Pacific age-standardised Summary Exposure Values (SEVs) for Level 4 risk factors and percentage change, by sex, 1990, 2007, and 2017.Data in parentheses are 95% uncertainty intervals.

	Male					Female				
	**1990**	**2007**	**2017**	**Percentage change 1990-2007**	**Percentage change 2007-2017**	**1990**	**2007**	**2017**	**Percentage change 1990-2007**	**Percentage change 2007-2017**
Unsafe water source	2.6 (1.9 to 3.4)	1.8 (1.3 to 2.4)	1.8 (1.3 to 2.5)	-31.6 (-52.8 to -3.2)	1.9 (-22.8 to 38.8)	2.6 (1.9 to 3.4)	1.8 (1.3 to 2.4)	1.8 (1.3 to 2.5)	-31.6 (-52.8 to -3.1)	1.9 (-23.1 to 38.8)
Unsafe sanitation	4.9 (3.5 to 6.8)	1.8 (1.3 to 2.7)	1.5 (1.0 to 2.2)	-63.0 (-75.6 to -44.1)	-18.6 (-42.8 to 17.1)	4.9 (3.5 to 6.8)	1.8 (1.3 to 2.7)	1.5 (1.0 to 2.2)	-63.0 (-75.6 to -43.8)	-18.6 (-42.9 to 16.9)
No access to handwashing facility	6.9 (5.9 to 8.1)	6.3 (5.4 to 7.4)	5.8 (5.0 to 6.8)	-8.8 (-23.6 to 9.3)	-7.8 (-19.0 to 4.2)	6.9 (5.9 to 8.1)	6.3 (5.4 to 7.4)	5.8 (5.0 to 6.8)	-8.8 (-23.5 to 9.2)	-7.9 (-19.0 to 4.2)
Ambient particulate matter pollution	38.5 (27.9 to 53.5)	39.8 (29.1 to 55.6)	35.3 (25.4 to 49.3)	3.3 (-4.1 to 12.0)	-11.3 (-20.0 to -3.1)	38.1 (27.6 to 53.2)	39.6 (29.0 to 55.5)	35.2 (25.3 to 49.1)	4.0 (-3.4 to 12.8)	-11.3 (-19.9 to -3.1)
Household air pollution from solid fuels	0.8 (0.4 to 2.0)	0.1 (0.0 to 0.2)	0.1 (0.0 to 0.2)	-84.8 (-93.1 to -82.2)	-20.4 (-51.6 to -0.1)	1.2 (0.5 to 5.4)	0.2 (0.1 to 0.8)	0.2 (0.0 to 0.6)	-81.7 (-92.9 to -80.3)	-18.5 (-51.5 to -0.3)
Ambient ozone pollution	49.7 (23.2 to 54.2)	49.7 (23.2 to 54.4)	50.5 (24.2 to 54.7)	0.1 (-0.2 to 0.5)	1.6 (0.6 to 4.2)	49.7 (23.1 to 54.3)	49.7 (23.2 to 54.4)	50.5 (24.1 to 54.7)	-0.0 (-0.4 to 0.4)	1.6 (0.5 to 4.4)
Residential radon	16.8 (8.2 to 28.6)	17.0 (8.3 to 29.2)	17.3 (8.3 to 30.0)	1.2 (-2.4 to 4.7)	1.7 (-2.2 to 5.3)	16.8 (8.2 to 28.7)	17.0 (8.3 to 29.3)	17.2 (8.2 to 29.6)	1.0 (-2.6 to 4.4)	0.9 (-2.4 to 3.7)
Lead exposure	4.2 (1.2 to 7.9)	3.0 (0.8 to 6.2)	2.2 (0.5 to 5.0)	-28.1 (-34.5 to -21.5)	-26.4 (-34.4 to -19.7)	2.5 (0.4 to 5.8)	1.8 (0.3 to 4.5)	1.3 (0.2 to 3.6)	-29.6 (-36.6 to -22.9)	-27.7 (-38.3 to -20.2)
Occupational exposure to asbestos	2.2 (2.0 to 2.4)	2.9 (2.8 to 3.0)	2.8 (2.6 to 3.0)	30.4 (20.9 to 47.6)	-5.2 (-11.3 to 3.1)	0.6 (0.5 to 0.7)	0.6 (0.5 to 0.6)	0.5 (0.5 to 0.6)	-4.5 (-16.9 to 16.8)	-6.7 (-14.6 to 2.6)
Occupational exposure to arsenic	0.5 (0.0 to 1.1)	0.4 (0.0 to 1.0)	0.4 (0.0 to 1.0)	-	-	0.4 (0.0 to 1.0)	0.4 (0.0 to 0.9)	0.4 (0.0 to 0.9)	-	-
Occupational exposure to benzene	0.6 (0.0 to 1.8)	0.6 (0.0 to 1.8)	0.7 (0.0 to 1.9)	4.0 (1.6 to 7.9)	3.0 (0.3 to 6.8)	0.6 (0.0 to 1.6)	0.7 (0.0 to 2.1)	0.8 (0.0 to 2.3)	23.2 (18.9 to 29.6)	13.1 (8.8 to 19.8)
Occupational exposure to beryllium	0.0 (0.0 to 0.0)	0.0 (0.0 to 0.0)	0.0 (0.0 to 0.0)	-4.7 (-6.0 to -3.4)	1.5 (-0.0 to 3.0)	0.0 (0.0 to 0.0)	0.0 (0.0 to 0.0)	0.0 (0.0 to 0.0)	1.4 (-0.7 to 3.5)	7.2 (4.9 to 9.7)
Occupational exposure to cadmium	0.1 (0.0 to 0.1)	0.0 (0.0 to 0.1)	0.0 (0.0 to 0.1)	-6.8 (-12.2 to -1.4)	0.6 (-4.2 to 5.7)	0.0 (0.0 to 0.1)	0.0 (0.0 to 0.0)	0.0 (0.0 to 0.0)	-10.9 (-17.5 to -3.6)	3.0 (-3.5 to 9.6)
Occupational exposure to chromium	0.1 (0.1 to 0.1)	0.1 (0.1 to 0.1)	0.1 (0.1 to 0.1)	-4.9 (-9.7 to -0.1)	1.1 (-2.9 to 5.5)	0.1 (0.1 to 0.1)	0.1 (0.1 to 0.1)	0.1 (0.1 to 0.1)	-5.6 (-11.6 to 1.0)	4.7 (-0.7 to 10.1)
Occupational exposure to diesel engine exhaust	0.6 (0.6 to 0.6)	0.6 (0.6 to 0.6)	0.6 (0.6 to 0.6)	-0.1 (-3.2 to 3.5)	2.6 (-0.5 to 5.8)	0.5 (0.4 to 0.5)	0.5 (0.5 to 0.5)	0.6 (0.5 to 0.6)	14.6 (9.0 to 20.6)	8.5 (3.6 to 13.5)
Occupational exposure to formaldehyde	0.3 (0.2 to 0.3)	0.2 (0.2 to 0.2)	0.2 (0.2 to 0.2)	-6.2 (-11.6 to -0.6)	0.3 (-4.8 to 5.5)	0.2 (0.2 to 0.2)	0.2 (0.2 to 0.2)	0.2 (0.2 to 0.2)	-8.4 (-15.2 to -0.7)	4.1 (-2.1 to 10.4)
Occupational exposure to nickel	0.4 (0.0 to 1.9)	0.4 (0.0 to 1.7)	0.4 (0.0 to 1.7)	-	-	0.4 (0.0 to 1.6)	0.3 (0.0 to 1.3)	0.3 (0.0 to 1.3)	-	-
Occupational exposure to polycyclic aromatic hydrocarbons	0.2 (0.2 to 0.3)	0.2 (0.2 to 0.2)	0.2 (0.2 to 0.2)	-3.9 (-8.3 to 0.6)	1.3 (-2.5 to 5.4)	0.2 (0.2 to 0.2)	0.2 (0.2 to 0.2)	0.2 (0.2 to 0.2)	-3.5 (-9.3 to 2.7)	5.7 (0.9 to 10.6)
Occupational exposure to silica	4.7 (0.7 to 15.1)	4.4 (0.7 to 14.2)	4.5 (0.7 to 14.3)	-5.2 (-9.8 to 0.0)	0.6 (-3.5 to 5.6)	3.2 (0.5 to 10.0)	2.6 (0.4 to 8.1)	2.6 (0.4 to 8.0)	-16.8 (-24.0 to -9.3)	-0.2 (-7.0 to 7.9)
Occupational exposure to sulphuric acid	0.6 (0.1 to 1.9)	0.6 (0.1 to 1.8)	0.6 (0.1 to 1.8)	-7.3 (-12.2 to -2.2)	0.5 (-3.9 to 5.3)	0.5 (0.1 to 1.7)	0.5 (0.1 to 1.4)	0.5 (0.1 to 1.4)	-12.7 (-19.4 to -5.7)	1.9 (-4.6 to 9.0)
Occupational exposure to trichloroethylene	0.1 (0.1 to 0.1)	0.1 (0.1 to 0.1)	0.1 (0.1 to 0.1)	-3.7 (-6.9 to -0.4)	1.6 (-1.1 to 4.5)	0.1 (0.1 to 0.1)	0.1 (0.1 to 0.1)	0.1 (0.1 to 0.1)	0.5 (-3.6 to 4.7)	6.1 (2.2 to 10.3)
Occupational asthmagens	18.4 (15.7 to 21.9)	17.6 (15.1 to 21.0)	17.8 (15.0 to 21.2)	-4.1 (-12.4 to 5.7)	1.1 (-6.6 to 9.0)	8.4 (7.1 to 10.0)	8.4 (7.1 to 9.9)	8.8 (7.4 to 10.3)	0.2 (-6.7 to 6.9)	3.9 (-2.0 to 10.4)
Occupational particulate matter, gases, and fumes	6.4 (4.6 to 9.2)	6.0 (4.3 to 8.6)	5.7 (4.1 to 8.2)	-5.9 (-8.5 to -3.2)	-4.6 (-6.3 to -3.0)	3.7 (2.7 to 5.4)	3.5 (2.5 to 5.0)	3.2 (2.3 to 4.7)	-7.3 (-11.9 to -3.2)	-6.4 (-9.0 to -4.0)
Occupational noise	5.9 (5.6 to 6.4)	5.7 (5.3 to 6.1)	5.5 (5.2 to 5.9)	-4.5 (-5.8 to -3.2)	-3.3 (-4.0 to -2.5)	3.5 (3.3 to 3.9)	3.5 (3.3 to 3.8)	3.5 (3.3 to 3.8)	-1.1 (-3.2 to 1.1)	0.2 (-0.9 to 1.5)
Occupational ergonomic factors	10.6 (9.2 to 12.2)	8.9 (7.8 to 10.3)	8.7 (7.5 to 10.1)	-15.6 (-20.1 to -10.9)	-2.6 (-7.5 to 2.5)	6.2 (5.4 to 7.1)	5.8 (5.1 to 6.8)	6.2 (5.3 to 7.4)	-5.5 (-12.7 to 1.3)	6.4 (-1.4 to 15.3)
Non-exclusive breastfeeding	0.4 (0.3 to 0.6)	0.4 (0.3 to 0.6)	0.4 (0.3 to 0.5)	0.2 (-7.0 to 8.1)	-2.0 (-7.7 to 4.0)	0.4 (0.3 to 0.6)	0.4 (0.3 to 0.6)	0.4 (0.3 to 0.5)	0.1 (-7.1 to 8.1)	-2.0 (-7.7 to 3.9)
Discontinued breastfeeding	1.8 (1.7 to 1.9)	1.8 (1.8 to 1.9)	1.8 (1.7 to 1.9)	1.8 (-2.7 to 6.7)	-1.4 (-5.0 to 2.4)	1.8 (1.7 to 1.9)	1.8 (1.8 to 1.9)	1.8 (1.7 to 1.9)	2.3 (-2.2 to 7.3)	-1.7 (-5.3 to 2.1)
Child underweight	0.2 (0.1 to 0.3)	0.2 (0.1 to 0.2)	0.2 (0.1 to 0.2)	-11.9 (-16.7 to -8.0)	-6.3 (-9.1 to -3.9)	0.2 (0.1 to 0.3)	0.2 (0.1 to 0.2)	0.2 (0.1 to 0.2)	-15.2 (-20.4 to -10.8)	-7.2 (-10.3 to -4.4)
Child wasting	0.2 (0.1 to 0.2)	0.2 (0.1 to 0.2)	0.1 (0.1 to 0.2)	-4.6 (-10.0 to 0.9)	-2.2 (-6.0 to 1.9)	0.2 (0.1 to 0.2)	0.2 (0.1 to 0.2)	0.2 (0.1 to 0.2)	-8.6 (-14.9 to -2.2)	-4.0 (-8.6 to 1.0)
Child stunting	0.5 (0.3 to 0.6)	0.4 (0.3 to 0.6)	0.4 (0.2 to 0.5)	-8.2 (-16.6 to -0.4)	-4.0 (-10.4 to 2.1)	0.5 (0.3 to 0.7)	0.5 (0.3 to 0.6)	0.4 (0.3 to 0.6)	-13.5 (-24.2 to -3.8)	-4.2 (-11.2 to 2.9)
Short gestation for birth weight	0.0 (0.0 to 0.0)	0.0 (0.0 to 0.0)	0.0 (0.0 to 0.0)	8.7 (-1.5 to 19.1)	2.2 (-4.3 to 12.2)	0.0 (0.0 to 0.0)	0.0 (0.0 to 0.0)	0.0 (0.0 to 0.0)	13.5 (4.0 to 26.4)	0.7 (-8.1 to 8.6)
Low birth weight for gestation	0.0 (0.0 to 0.0)	0.0 (0.0 to 0.0)	0.0 (0.0 to 0.0)	4.6 (-4.0 to 15.2)	2.3 (-4.5 to 8.6)	0.0 (0.0 to 0.0)	0.0 (0.0 to 0.0)	0.0 (0.0 to 0.0)	-2.4 (-12.4 to 8.4)	3.5 (-4.7 to 12.6)
Iron deficiency	-	-	-	-	-	8.5 (6.3 to 11.1)	7.3 (5.3 to 9.7)	6.6 (4.8 to 8.9)	-14.2 (-24.7 to -3.4)	-8.9 (-17.5 to 0.3)
Vitamin A deficiency	1.0 (0.7 to 1.3)	0.7 (0.5 to 0.9)	0.6 (0.4 to 0.8)	-27.9 (-33.4 to -17.7)	-14.6 (-20.0 to -10.6)	0.7 (0.5 to 0.9)	0.7 (0.5 to 0.9)	0.6 (0.4 to 0.9)	-0.9 (-8.8 to 5.4)	-11.2 (-17.8 to -4.9)
Zinc deficiency	0.5 (0.1 to 1.0)	0.5 (0.2 to 0.9)	0.4 (0.1 to 0.8)	-2.9 (-15.2 to 14.7)	-8.7 (-25.1 to 4.5)	0.5 (0.2 to 1.0)	0.4 (0.1 to 0.8)	0.4 (0.1 to 0.8)	-17.7 (-26.4 to -4.9)	-1.0 (-18.8 to 13.3)
Smoking	17.2 (15.3 to 19.3)	12.9 (11.5 to 14.5)	10.6 (9.5 to 12.0)	-25.1 (-28.1 to -22.2)	-17.4 (-20.0 to -14.5)	3.4 (2.9 to 4.0)	2.8 (2.4 to 3.3)	2.6 (2.2 to 3.0)	-18.5 (-26.1 to -10.3)	-8.1 (-14.2 to -1.6)
Chewing tobacco	1.4 (1.1 to 1.7)	0.9 (0.7 to 1.1)	0.8 (0.6 to 0.9)	-32.9 (-47.5 to -15.1)	-18.9 (-31.6 to -3.6)	0.1 (0.1 to 0.1)	0.1 (0.1 to 0.1)	0.1 (0.1 to 0.1)	-4.0 (-25.4 to 23.5)	-2.5 (-17.6 to 19.2)
Second-hand smoke	38.6 (36.5 to 40.6)	35.2 (32.9 to 37.4)	34.2 (31.9 to 36.4)	-8.7 (-14.3 to -2.6)	-2.9 (-7.8 to 2.1)	61.2 (59.3 to 63.2)	49.5 (47.5 to 51.4)	47.0 (44.8 to 49.0)	-19.2 (-22.2 to -16.3)	-4.9 (-8.1 to -1.8)
Alcohol use	14.6 (8.8 to 23.7)	15.4 (9.1 to 24.6)	18.0 (9.9 to 28.4)	4.9 (-4.6 to 17.0)	16.9 (-3.0 to 40.2)	11.4 (6.6 to 19.4)	11.8 (6.9 to 19.8)	13.7 (7.6 to 22.1)	3.2 (-5.9 to 14.9)	15.6 (-3.7 to 39.6)
Drug use	0.5 (0.4 to 0.7)	0.5 (0.4 to 0.8)	0.6 (0.4 to 0.8)	1.9 (-0.3 to 4.5)	9.5 (5.5 to 14.3)	0.3 (0.2 to 0.4)	0.3 (0.2 to 0.4)	0.3 (0.3 to 0.4)	3.1 (0.7 to 5.6)	7.6 (4.3 to 11.5)
Diet low in fruits	40.5 (35.2 to 43.4)	35.5 (30.0 to 39.1)	35.3 (29.7 to 38.9)	-12.2 (-16.5 to -8.8)	-0.8 (-3.6 to 1.9)	37.4 (31.8 to 40.8)	30.9 (25.2 to 35.0)	31.5 (25.7 to 35.8)	-17.4 (-23.1 to -12.8)	2.0 (-1.9 to 6.2)
Diet low in vegetables	15.4 (10.0 to 20.8)	12.2 (6.0 to 18.2)	15.0 (8.5 to 21.1)	-21.0 (-43.0 to -8.2)	23.4 (7.6 to 55.8)	17.5 (11.7 to 23.0)	14.6 (8.2 to 20.5)	17.2 (10.9 to 23.1)	-16.2 (-33.6 to -5.6)	17.8 (4.9 to 42.9)
Diet low in legumes	4.4 (1.5 to 8.2)	8.3 (4.0 to 12.6)	11.3 (6.6 to 16.2)	86.4 (46.1 to 192.4)	37.2 (19.7 to 76.3)	8.6 (3.9 to 13.7)	11.8 (7.2 to 16.4)	15.2 (10.2 to 20.1)	36.3 (15.9 to 89.8)	29.3 (16.9 to 51.0)
Diet low in whole grains	28.4 (24.4 to 31.9)	33.6 (30.3 to 36.5)	34.0 (30.6 to 36.9)	18.2 (13.0 to 26.1)	1.2 (-1.4 to 3.9)	29.9 (26.0 to 33.3)	35.0 (31.8 to 37.7)	35.4 (32.2 to 38.1)	16.9 (12.1 to 23.7)	1.2 (-0.7 to 3.4)
Diet low in nuts and seeds	50.1 (49.0 to 51.0)	48.2 (46.6 to 49.4)	47.1 (45.3 to 48.7)	-3.8 (-5.4 to -2.5)	-2.1 (-3.6 to -0.9)	50.3 (49.2 to 51.1)	48.3 (46.8 to 49.5)	47.3 (45.6 to 48.8)	-4.0 (-5.6 to -2.8)	-2.0 (-3.5 to -0.8)
Diet low in milk	47.4 (45.9 to 48.7)	47.0 (45.4 to 48.4)	47.1 (45.4 to 48.5)	-0.9 (-1.6 to -0.3)	0.2 (-0.4 to 0.8)	47.4 (45.9 to 48.8)	46.5 (44.8 to 48.0)	46.7 (45.0 to 48.2)	-2.0 (-2.9 to -1.2)	0.5 (-0.2 to 1.2)
Diet high in red meat	3.8 (1.9 to 6.1)	13.7 (11.0 to 16.4)	16.5 (12.9 to 19.8)	257.8 (158.3 to 515.2)	20.6 (5.2 to 39.6)	0.5 (0.1 to 1.2)	3.5 (1.9 to 5.4)	4.6 (2.3 to 7.0)	598.5 (333.5 to 2066.4)	30.3 (-4.0 to 81.7)
Diet high in processed meat	9.5 (6.6 to 12.2)	15.1 (12.7 to 17.4)	16.4 (13.5 to 19.1)	58.4 (38.0 to 93.1)	9.1 (0.3 to 19.8)	7.8 (5.2 to 10.3)	10.9 (8.5 to 13.3)	11.6 (8.9 to 14.0)	40.2 (22.5 to 71.9)	5.8 (-3.0 to 16.8)
Diet high in sugar-sweetened beverages	6.1 (3.0 to 9.0)	7.8 (4.0 to 9.4)	9.3 (4.7 to 11.4)	28.3 (-10.3 to 89.2)	18.4 (-3.3 to 42.8)	4.2 (2.0 to 6.5)	5.2 (2.7 to 6.5)	6.4 (3.2 to 8.1)	22.1 (-16.0 to 87.5)	23.3 (-2.1 to 54.8)
Diet low in fibre	24.9 (19.2 to 29.8)	27.1 (22.0 to 31.6)	28.5 (23.4 to 33.0)	9.0 (1.9 to 18.2)	5.0 (0.6 to 9.9)	26.9 (21.4 to 31.6)	29.2 (24.3 to 33.3)	30.8 (26.0 to 34.9)	8.4 (2.2 to 16.5)	5.5 (1.8 to 10.4)
Diet low in calcium	35.0 (31.0 to 38.2)	33.6 (29.3 to 37.0)	33.7 (29.6 to 37.1)	-4.2 (-6.3 to -2.7)	0.5 (-0.9 to 2.0)	37.3 (33.7 to 40.1)	35.5 (31.6 to 38.7)	36.1 (32.3 to 39.2)	-4.7 (-6.7 to -3.1)	1.6 (0.4 to 3.2)
Diet low in seafood omega-3 fatty acids	13.5 (12.1 to 14.7)	11.9 (11.1 to 12.9)	12.7 (11.5 to 13.9)	-11.5 (-15.1 to -5.1)	6.4 (3.8 to 8.6)	14.3 (12.8 to 15.5)	12.8 (11.9 to 13.7)	13.3 (12.3 to 14.5)	-10.5 (-14.1 to -4.4)	4.6 (2.4 to 6.5)
Diet low in polyunsaturated fatty acids	30.4 (25.9 to 34.3)	23.0 (17.8 to 27.7)	22.0 (17.0 to 26.6)	-24.2 (-32.2 to -18.0)	-4.5 (-8.0 to -0.8)	31.0 (26.6 to 34.8)	23.7 (18.4 to 28.3)	22.9 (17.9 to 27.5)	-23.5 (-31.6 to -17.5)	-3.5 (-7.2 to 0.4)
Diet high in trans fatty acids	1.7 (0.2 to 4.1)	1.5 (0.2 to 3.9)	1.4 (0.2 to 3.7)	-9.7 (-26.2 to -5.4)	-4.8 (-17.5 to -1.5)	2.3 (0.5 to 5.0)	2.1 (0.4 to 4.7)	1.9 (0.3 to 4.6)	-10.1 (-23.9 to -5.8)	-6.1 (-17.2 to -2.5)
Diet high in sodium	24.7 (17.2 to 30.8)	17.3 (9.5 to 23.3)	15.2 (7.4 to 21.5)	-30.1 (-49.0 to -18.7)	-11.9 (-26.8 to -4.2)	27.4 (20.6 to 33.2)	17.0 (9.5 to 23.0)	13.8 (6.2 to 20.3)	-38.0 (-55.8 to -26.6)	-18.6 (-36.1 to -10.1)
Intimate partner violence	-	-	-	-	-	2.4 (2.0 to 2.7)	2.1 (1.8 to 2.5)	2.0 (1.7 to 2.4)	-9.1 (-10.6 to -7.7)	-5.5 (-7.0 to -3.9)
Childhood sexual abuse	5.2 (4.3 to 6.4)	5.2 (4.3 to 6.4)	5.2 (4.2 to 6.4)	-0.2 (-0.6 to 0.2)	-0.2 (-0.5 to 0.1)	7.2 (5.9 to 8.8)	7.2 (5.9 to 8.7)	7.3 (5.9 to 9.0)	-0.2 (-0.7 to 0.3)	1.7 (-3.0 to 6.7)

Supplementary Results Table 1. High-income Asia Pacific age-standardised Summary Exposure Values (SEVs) for Level 4 risk factors and percentage change, by sex, 1990, 2007, and 2017.Data in parentheses are 95% uncertainty intervals.										
	**Male**					**Female**				
	**1990**	**2007**	**2017**	**Percentage change 1990-2007**	**Percentage change 2007-2017**	**1990**	**2007**	**2017**	**Percentage change 1990-2007**	**Percentage change 2007-2017**
Bullying victimization	3.9 (1.7 to 7.6)	4.0 (1.7 to 8.0)	4.0 (1.7 to 8.1)	3.9 (-0.8 to 8.2)	1.1 (-1.9 to 3.9)	2.3 (1.0 to 4.8)	2.4 (1.0 to 5.0)	2.4 (1.0 to 4.9)	3.9 (-0.6 to 8.3)	0.6 (-2.7 to 3.6)
Low physical activity	0.5 (0.2 to 0.9)	0.5 (0.2 to 0.9)	0.4 (0.2 to 0.8)	-1.8 (-2.8 to -0.9)	-8.4 (-11.4 to -4.5)	0.5 (0.2 to 1.0)	0.5 (0.2 to 0.9)	0.5 (0.2 to 0.9)	-3.6 (-5.3 to -1.3)	1.4 (0.6 to 2.3)
High fasting plasma glucose	1.7 (1.2 to 2.3)	1.7 (1.2 to 2.3)	1.9 (1.4 to 2.6)	1.8 (-14.0 to 20.2)	13.0 (-3.9 to 34.0)	1.4 (1.0 to 1.9)	1.4 (1.0 to 1.9)	1.6 (1.1 to 2.2)	0.1 (-16.7 to 23.0)	11.6 (-6.9 to 34.5)
High LDL cholesterol	11.5 (10.0 to 13.1)	12.2 (10.7 to 13.9)	12.3 (10.8 to 14.0)	6.6 (3.3 to 10.0)	0.2 (-2.1 to 2.6)	12.2 (10.8 to 13.8)	12.7 (11.2 to 14.3)	12.6 (11.2 to 14.3)	3.8 (1.0 to 6.7)	-0.2 (-2.4 to 2.2)
High systolic blood pressure	9.9 (9.2 to 10.7)	7.3 (6.6 to 7.9)	6.9 (6.2 to 7.6)	-26.7 (-29.3 to -24.1)	-5.2 (-9.0 to -1.5)	8.7 (8.1 to 9.2)	5.5 (5.0 to 6.0)	4.9 (4.4 to 5.5)	-36.3 (-38.9 to -33.8)	-10.5 (-15.5 to -5.1)
High body-mass index	6.0 (4.0 to 9.2)	10.5 (7.8 to 15.6)	10.8 (8.0 to 15.7)	75.7 (52.0 to 107.7)	2.6 (-5.1 to 10.0)	5.8 (3.7 to 8.9)	8.2 (5.6 to 12.2)	8.2 (5.5 to 12.1)	42.1 (25.7 to 65.5)	0.7 (-7.2 to 9.8)
Low bone mineral density	5.2 (4.4 to 6.1)	5.1 (4.5 to 5.8)	5.1 (4.4 to 5.8)	-2.0 (-5.7 to 2.3)	-0.5 (-3.7 to 2.7)	7.0 (6.1 to 8.0)	6.8 (6.1 to 7.7)	6.9 (6.1 to 7.7)	-3.0 (-6.4 to 0.8)	0.8 (-2.0 to 3.5)
Impaired kidney function	2.6 (1.2 to 6.5)	2.5 (1.1 to 6.4)	2.4 (1.1 to 6.1)	-2.7 (-4.8 to -0.7)	-5.5 (-10.2 to -1.8)	3.2 (1.5 to 7.4)	3.2 (1.5 to 7.4)	3.0 (1.4 to 7.2)	1.4 (-0.5 to 3.4)	-5.8 (-9.7 to -2.0)

Supplementary Results Table 1. Brunei age-standardised Summary Exposure Values (SEVs) for Level 4 risk factors and percentage change, by sex, 1990, 2007, and 2017.Data in parentheses are 95% uncertainty intervals.

	Male					Female				
	1990	2007	2017	Percentage change 1990-2007	Percentage change 2007-2017	1990	2007	2017	Percentage change 1990-2007	Percentage change 2007-2017
Unsafe water source	3.5 (2.3 to 5.1)	2.1 (1.3 to 3.0)	2.0 (1.3 to 2.8)	-41.4 (-63.7 to -7.3)	-4.7 (-34.6 to 39.8)	3.5 (2.3 to 5.1)	2.1 (1.3 to 3.0)	2.0 (1.3 to 2.8)	-41.4 (-63.7 to -7.3)	-4.7 (-34.6 to 39.8)
Unsafe sanitation	7.2 (4.3 to 11.2)	2.3 (1.3 to 3.6)	1.7 (1.0 to 2.7)	-68.7 (-82.2 to -46.3)	-25.7 (-53.5 to 15.8)	7.2 (4.3 to 11.2)	2.3 (1.3 to 3.6)	1.7 (1.0 to 2.7)	-68.7 (-82.2 to -46.3)	-25.7 (-53.5 to 15.8)
No access to handwashing facility	7.1 (5.6 to 8.8)	6.3 (4.9 to 7.9)	5.8 (4.6 to 7.2)	-10.6 (-29.5 to 14.0)	-8.6 (-24.0 to 8.5)	7.1 (5.6 to 8.8)	6.3 (4.9 to 7.9)	5.8 (4.6 to 7.2)	-10.6 (-29.5 to 14.0)	-8.6 (-24.0 to 8.5)
Ambient particulate matter pollution	14.9 (6.0 to 25.3)	19.2 (8.1 to 32.9)	13.7 (3.3 to 28.6)	28.4 (-29.8 to 131.3)	-28.6 (-73.5 to 16.3)	13.5 (5.4 to 23.6)	19.0 (8.1 to 32.8)	13.6 (3.3 to 28.5)	40.8 (-23.3 to 152.7)	-28.4 (-73.4 to 17.0)
Household air pollution from solid fuels	6.0 (2.5 to 13.0)	0.4 (0.1 to 1.4)	0.2 (0.0 to 0.6)	-93.2 (-97.4 to -84.7)	-56.0 (-87.8 to -11.5)	7.0 (3.0 to 13.8)	0.6 (0.2 to 3.9)	0.3 (0.0 to 1.2)	-91.1 (-97.1 to -61.5)	-54.6 (-88.6 to -8.4)
Ambient ozone pollution	11.1 (3.0 to 25.1)	11.2 (2.8 to 23.9)	11.9 (3.0 to 26.7)	0.4 (-35.0 to 70.3)	6.4 (-39.4 to 89.4)	11.1 (3.0 to 25.1)	11.2 (2.8 to 23.9)	11.9 (3.0 to 26.7)	0.4 (-35.0 to 70.3)	6.4 (-39.4 to 89.4)
Residential radon	5.9 (0.1 to 23.7)	5.9 (0.1 to 23.7)	5.9 (0.1 to 23.7)	0.0 (0.0 to 0.0)	0.0 (0.0 to 0.0)	5.9 (0.1 to 23.7)	5.9 (0.1 to 23.7)	5.9 (0.1 to 23.7)	0.0 (0.0 to 0.0)	0.0 (0.0 to 0.0)
Lead exposure	6.7 (2.7 to 10.9)	4.9 (1.7 to 8.5)	3.4 (1.1 to 6.7)	-27.2 (-36.3 to -19.8)	-29.2 (-39.9 to -21.5)	3.5 (0.7 to 7.4)	2.6 (0.5 to 5.8)	1.8 (0.2 to 4.6)	-27.8 (-39.3 to -19.8)	-30.3 (-45.7 to -21.6)
Occupational exposure to asbestos	1.6 (1.0 to 2.5)	1.6 (1.3 to 1.8)	1.8 (1.5 to 2.1)	-3.9 (-35.1 to 48.6)	16.0 (-6.9 to 43.0)	1.6 (0.9 to 2.2)	1.1 (1.0 to 1.3)	1.3 (1.1 to 1.5)	-30.2 (-50.0 to 13.0)	16.8 (-3.2 to 41.5)
Occupational exposure to arsenic	0.3 (0.0 to 0.8)	0.3 (0.0 to 0.7)	0.3 (0.0 to 0.6)	-	-	0.3 (0.0 to 0.7)	0.3 (0.0 to 0.7)	0.3 (0.0 to 0.7)	-	-
Occupational exposure to benzene	0.8 (0.0 to 2.3)	0.9 (0.0 to 2.6)	0.9 (0.0 to 2.6)	5.8 (-2.4 to 17.1)	2.6 (-4.1 to 11.9)	0.8 (0.0 to 2.3)	1.0 (0.1 to 3.0)	1.1 (0.1 to 3.3)	30.0 (19.7 to 46.0)	10.6 (4.5 to 20.7)
Occupational exposure to beryllium	0.0 (0.0 to 0.0)	0.0 (0.0 to 0.0)	0.0 (0.0 to 0.0)	-7.9 (-12.0 to -3.7)	-1.9 (-5.3 to 1.9)	0.0 (0.0 to 0.0)	0.0 (0.0 to 0.0)	0.0 (0.0 to 0.0)	11.4 (3.6 to 20.1)	5.3 (0.1 to 11.0)
Occupational exposure to cadmium	0.0 (0.0 to 0.0)	0.0 (0.0 to 0.0)	0.0 (0.0 to 0.0)	-8.5 (-15.8 to -0.1)	-5.2 (-11.1 to 1.3)	0.0 (0.0 to 0.0)	0.0 (0.0 to 0.0)	0.0 (0.0 to 0.0)	2.8 (-8.0 to 15.9)	0.3 (-7.6 to 8.7)
Occupational exposure to chromium	0.1 (0.1 to 0.1)	0.1 (0.1 to 0.1)	0.1 (0.1 to 0.1)	-6.0 (-12.5 to 1.1)	-3.2 (-8.3 to 2.5)	0.1 (0.1 to 0.1)	0.1 (0.1 to 0.1)	0.1 (0.1 to 0.1)	7.0 (-2.8 to 18.2)	2.4 (-4.5 to 9.9)
Occupational exposure to diesel engine exhaust	0.7 (0.7 to 0.8)	0.6 (0.6 to 0.7)	0.6 (0.6 to 0.7)	-12.9 (-19.8 to -6.0)	-0.4 (-6.5 to 5.8)	0.4 (0.4 to 0.5)	0.4 (0.4 to 0.5)	0.5 (0.4 to 0.5)	1.7 (-8.0 to 12.6)	3.7 (-3.5 to 11.9)
Occupational exposure to formaldehyde	0.2 (0.2 to 0.2)	0.2 (0.2 to 0.2)	0.2 (0.2 to 0.2)	-5.3 (-13.2 to 3.4)	-4.1 (-9.9 to 1.9)	0.2 (0.2 to 0.2)	0.2 (0.2 to 0.2)	0.2 (0.2 to 0.2)	9.0 (-1.7 to 21.4)	2.7 (-4.4 to 10.9)
Occupational exposure to nickel	0.4 (0.0 to 1.7)	0.3 (0.0 to 1.4)	0.3 (0.0 to 1.3)	-	-	0.2 (0.0 to 1.1)	0.2 (0.0 to 1.0)	0.2 (0.0 to 1.0)	-	-
Occupational exposure to polycyclic aromatic hydrocarbons	0.2 (0.2 to 0.2)	0.2 (0.2 to 0.2)	0.2 (0.2 to 0.2)	-4.6 (-10.8 to 1.5)	-2.3 (-7.0 to 2.7)	0.2 (0.2 to 0.2)	0.2 (0.2 to 0.2)	0.2 (0.2 to 0.2)	11.9 (2.2 to 22.5)	4.7 (-1.6 to 11.5)
Occupational exposure to silica	5.5 (0.8 to 17.5)	4.5 (0.6 to 14.5)	4.2 (0.6 to 14.1)	-18.5 (-28.9 to -7.2)	-5.8 (-14.8 to 3.9)	2.4 (0.3 to 7.5)	2.2 (0.3 to 6.7)	2.2 (0.3 to 7.0)	-8.5 (-20.9 to 6.4)	0.2 (-9.6 to 11.9)
Occupational exposure to sulphuric acid	0.5 (0.1 to 1.6)	0.5 (0.1 to 1.5)	0.4 (0.1 to 1.4)	-9.9 (-18.5 to -0.9)	-5.5 (-12.6 to 2.6)	0.4 (0.1 to 1.2)	0.4 (0.1 to 1.2)	0.4 (0.1 to 1.2)	-1.2 (-12.1 to 12.8)	-0.7 (-9.4 to 8.5)
Occupational exposure to trichloroethylene	0.1 (0.1 to 0.1)	0.1 (0.1 to 0.1)	0.1 (0.0 to 0.1)	-6.2 (-11.9 to -0.0)	-1.8 (-6.3 to 3.4)	0.1 (0.0 to 0.1)	0.1 (0.0 to 0.1)	0.1 (0.1 to 0.1)	5.8 (-3.1 to 15.9)	2.3 (-4.2 to 8.9)
Occupational asthmagens	14.9 (12.9 to 17.2)	15.6 (13.3 to 18.2)	16.0 (13.7 to 18.8)	4.6 (-5.7 to 15.3)	2.4 (-4.3 to 9.7)	7.5 (6.4 to 9.0)	9.7 (8.2 to 11.4)	10.4 (8.7 to 12.4)	28.5 (14.2 to 43.4)	7.9 (-0.3 to 17.3)
Occupational particulate matter, gases, and fumes	4.7 (3.3 to 6.8)	4.3 (3.1 to 6.2)	4.0 (2.9 to 5.8)	-8.4 (-13.3 to -3.6)	-6.6 (-10.0 to -3.2)	1.8 (1.2 to 2.6)	1.7 (1.2 to 2.5)	1.7 (1.2 to 2.5)	-1.1 (-9.0 to 9.0)	-0.7 (-5.2 to 4.9)
Occupational noise	5.5 (5.0 to 6.1)	5.3 (4.9 to 5.8)	5.1 (4.7 to 5.6)	-4.3 (-7.8 to -0.7)	-3.1 (-5.2 to -0.6)	2.3 (2.1 to 2.6)	2.7 (2.4 to 3.0)	2.9 (2.7 to 3.3)	14.5 (9.3 to 20.6)	9.8 (6.9 to 13.0)
Occupational ergonomic factors	8.8 (7.4 to 10.3)	8.4 (7.1 to 10.0)	8.1 (6.9 to 9.6)	-4.3 (-10.8 to 2.8)	-3.5 (-8.9 to 2.2)	3.7 (3.1 to 4.5)	4.5 (3.7 to 5.4)	4.5 (3.6 to 5.4)	19.6 (7.7 to 33.9)	-0.1 (-9.0 to 9.3)
Non-exclusive breastfeeding	0.4 (0.3 to 0.5)	0.4 (0.3 to 0.5)	0.4 (0.3 to 0.5)	-1.6 (-9.9 to 8.4)	-1.2 (-8.0 to 6.0)	0.4 (0.3 to 0.5)	0.4 (0.3 to 0.5)	0.4 (0.3 to 0.5)	-1.6 (-9.9 to 8.4)	-1.2 (-8.0 to 6.0)
Discontinued breastfeeding	1.7 (1.6 to 1.8)	1.7 (1.6 to 1.8)	1.7 (1.6 to 1.8)	-2.3 (-8.8 to 4.7)	3.1 (-2.2 to 8.8)	1.7 (1.6 to 1.8)	1.7 (1.6 to 1.8)	1.7 (1.6 to 1.8)	-2.3 (-8.8 to 4.7)	3.1 (-2.2 to 8.8)
Child underweight	0.4 (0.3 to 0.6)	0.4 (0.2 to 0.5)	0.4 (0.2 to 0.5)	-4.9 (-26.7 to 21.3)	-7.8 (-22.0 to 9.8)	0.4 (0.3 to 0.6)	0.4 (0.2 to 0.6)	0.4 (0.2 to 0.5)	-10.9 (-32.6 to 17.0)	-9.6 (-26.4 to 8.3)
Child wasting	0.1 (0.1 to 0.2)	0.1 (0.1 to 0.2)	0.1 (0.1 to 0.2)	-5.4 (-25.6 to 20.8)	-1.8 (-17.3 to 17.4)	0.2 (0.1 to 0.4)	0.3 (0.2 to 0.4)	0.2 (0.1 to 0.4)	6.0 (-28.7 to 60.7)	-6.1 (-32.1 to 26.0)
Child stunting	0.7 (0.3 to 1.1)	0.7 (0.4 to 1.1)	0.7 (0.3 to 1.1)	1.0 (-36.2 to 63.1)	-4.1 (-33.0 to 34.8)	0.8 (0.4 to 1.3)	0.8 (0.4 to 1.1)	0.7 (0.4 to 1.1)	-6.2 (-42.3 to 54.9)	-4.7 (-35.3 to 35.2)
Short gestation for birth weight	0.0 (0.0 to 0.0)	0.0 (0.0 to 0.0)	0.0 (0.0 to 0.0)	4.8 (-17.0 to 28.8)	1.7 (-17.9 to 23.0)	0.0 (0.0 to 0.0)	0.0 (0.0 to 0.0)	0.0 (0.0 to 0.0)	2.5 (-15.2 to 24.7)	0.9 (-16.2 to 25.5)
Low birth weight for gestation	0.0 (0.0 to 0.0)	0.0 (0.0 to 0.0)	0.0 (0.0 to 0.0)	2.5 (-11.8 to 20.8)	0.5 (-12.0 to 19.1)	0.0 (0.0 to 0.0)	0.0 (0.0 to 0.0)	0.0 (0.0 to 0.0)	2.5 (-13.2 to 19.6)	1.6 (-15.5 to 17.2)
Iron deficiency	-	-	-	-	-	7.6 (5.5 to 10.2)	6.2 (4.2 to 8.3)	5.4 (3.7 to 7.5)	-19.4 (-33.4 to -3.0)	-12.1 (-26.1 to 1.7)
Vitamin A deficiency	1.0 (0.6 to 1.4)	0.7 (0.5 to 0.9)	0.5 (0.4 to 0.7)	-29.0 (-42.3 to -13.2)	-21.9 (-36.6 to -4.4)	0.7 (0.5 to 0.9)	0.7 (0.4 to 0.9)	0.6 (0.4 to 0.8)	-1.1 (-23.4 to 27.1)	-14.6 (-31.0 to 6.1)
Zinc deficiency	0.3 (0.1 to 0.7)	0.3 (0.1 to 0.7)	0.3 (0.1 to 0.7)	0.3 (-60.3 to 175.9)	-0.4 (-60.8 to 179.3)	0.3 (0.1 to 0.7)	0.3 (0.1 to 0.7)	0.3 (0.1 to 0.7)	-1.2 (-61.6 to 185.4)	-0.7 (-62.4 to 161.6)
Smoking	12.8 (10.9 to 14.8)	10.4 (8.6 to 12.1)	9.5 (7.8 to 11.3)	-18.6 (-28.2 to -7.8)	-9.0 (-17.2 to -0.1)	2.7 (2.1 to 3.5)	2.3 (1.7 to 2.8)	2.1 (1.6 to 2.6)	-17.6 (-34.0 to 0.6)	-6.6 (-19.2 to 8.8)
Chewing tobacco	1.4 (1.0 to 1.8)	0.9 (0.7 to 1.2)	0.8 (0.6 to 1.0)	-32.4 (-49.3 to -9.5)	-18.4 (-34.5 to 2.1)	0.1 (0.1 to 0.1)	0.1 (0.1 to 0.1)	0.1 (0.1 to 0.1)	-4.3 (-31.8 to 31.0)	-3.8 (-25.4 to 22.4)
Second-hand smoke	33.8 (30.8 to 37.2)	29.8 (26.9 to 32.6)	27.9 (25.3 to 30.8)	-12.0 (-20.3 to -2.6)	-6.3 (-13.4 to 1.2)	49.0 (46.5 to 51.7)	37.8 (35.3 to 40.3)	34.4 (32.0 to 37.0)	-22.9 (-27.8 to -17.4)	-8.8 (-13.9 to -3.3)
Alcohol use	7.0 (3.2 to 13.9)	2.6 (0.7 to 6.6)	3.3 (1.0 to 8.0)	-63.0 (-83.9 to -39.2)	28.4 (-2.7 to 86.5)	4.9 (2.1 to 10.1)	1.7 (0.4 to 4.4)	2.0 (0.6 to 5.1)	-65.6 (-85.8 to -44.3)	21.2 (-7.7 to 79.7)
Drug use	0.6 (0.4 to 0.9)	0.6 (0.4 to 0.8)	0.6 (0.4 to 0.8)	-8.0 (-12.5 to -2.7)	1.9 (-4.2 to 8.4)	0.3 (0.2 to 0.4)	0.3 (0.2 to 0.4)	0.3 (0.3 to 0.4)	-1.1 (-6.3 to 4.6)	3.9 (-1.2 to 9.7)
Diet low in fruits	34.3 (28.8 to 38.2)	30.9 (25.1 to 35.3)	32.1 (25.9 to 36.5)	-10.1 (-16.3 to -5.1)	4.0 (-1.7 to 10.7)	31.7 (26.3 to 35.9)	27.8 (22.1 to 32.4)	29.3 (23.2 to 34.0)	-12.6 (-19.9 to -7.4)	5.5 (-1.9 to 13.3)
Diet low in vegetables	35.3 (31.3 to 39.0)	34.0 (29.6 to 37.9)	36.9 (32.1 to 40.5)	-3.6 (-9.2 to 1.5)	8.2 (2.9 to 15.4)	37.0 (33.0 to 40.3)	35.7 (31.6 to 39.1)	38.3 (34.1 to 41.6)	-3.3 (-8.4 to 1.3)	7.2 (2.2 to 13.2)
Diet low in legumes	25.5 (20.4 to 29.7)	32.8 (28.9 to 36.0)	31.0 (26.6 to 34.8)	28.6 (19.9 to 43.6)	-5.5 (-10.8 to -1.2)	30.0 (25.7 to 33.7)	36.1 (32.7 to 38.9)	34.7 (31.0 to 38.0)	20.5 (14.3 to 29.3)	-3.8 (-8.0 to -0.3)
Diet low in whole grains	33.7 (30.2 to 36.6)	35.9 (32.8 to 38.6)	36.4 (33.3 to 39.1)	6.6 (3.1 to 10.5)	1.3 (-1.6 to 4.3)	35.0 (31.8 to 37.8)	37.1 (34.2 to 39.7)	37.6 (34.6 to 40.1)	6.0 (3.0 to 9.7)	1.2 (-1.3 to 3.9)
Diet low in nuts and seeds	45.0 (42.2 to 47.1)	44.5 (41.7 to 46.6)	45.2 (42.3 to 47.3)	-1.1 (-4.5 to 2.6)	1.5 (-1.4 to 4.4)	45.5 (42.8 to 47.5)	45.1 (42.5 to 47.2)	45.7 (43.2 to 47.7)	-0.9 (-4.6 to 2.8)	1.4 (-1.1 to 4.1)
Diet low in milk	47.2 (45.5 to 48.6)	45.1 (43.2 to 46.9)	45.1 (43.0 to 47.0)	-4.4 (-6.0 to -2.9)	-0.2 (-1.5 to 1.1)	47.6 (46.1 to 48.9)	45.7 (43.7 to 47.3)	45.6 (43.6 to 47.4)	-4.1 (-5.7 to -2.7)	-0.1 (-1.3 to 1.0)
Diet high in red meat	7.7 (3.6 to 11.8)	0.8 (0.0 to 2.7)	1.0 (0.0 to 3.0)	-89.0 (-99.7 to -73.3)	13.5 (-88.4 to 1102.4)	0.8 (0.0 to 2.5)	0.0 (0.0 to 0.2)	0.0 (0.0 to 0.4)	-97.8 (-100.0 to -86.7)	141.7 (-100.0 to [illegible]
Diet high in processed meat	13.7 (9.2 to 17.5)	11.0 (7.2 to 14.4)	10.2 (6.6 to 13.4)	-19.6 (-36.3 to -1.4)	-7.5 (-20.6 to 7.4)	10.0 (6.5 to 13.4)	7.6 (4.3 to 10.8)	6.9 (3.8 to 10.1)	-24.2 (-43.2 to -6.2)	-8.8 (-25.4 to 6.0)
Diet high in sugar-sweetened beverages	16.3 (7.6 to 25.7)	16.1 (8.2 to 25.2)	15.8 (8.1 to 24.4)	-1.3 (-38.8 to 63.4)	-2.1 (-33.8 to 43.5)	10.0 (4.4 to 17.0)	10.2 (4.7 to 17.3)	10.1 (4.3 to 17.3)	2.3 (-42.6 to 82.7)	-1.2 (-35.5 to 49.2)
Diet low in fibre	32.4 (27.6 to 36.4)	37.1 (33.0 to 40.3)	40.0 (36.3 to 43.0)	14.3 (8.7 to 22.6)	7.8 (4.0 to 13.1)	37.3 (33.4 to 40.5)	41.2 (38.1 to 43.7)	43.6 (40.7 to 45.9)	10.3 (6.1 to 15.8)	5.7 (2.7 to 9.4)
Diet low in calcium	34.8 (30.7 to 38.1)	30.2 (25.2 to 34.1)	30.9 (26.0 to 34.9)	-13.2 (-18.5 to -9.7)	2.5 (0.2 to 5.7)	37.3 (33.8 to 40.1)	33.3 (28.8 to 36.8)	33.9 (29.5 to 37.4)	-10.7 (-14.9 to -7.9)	2.0 (-0.2 to 4.6)
Diet low in seafood omega-3 fatty acids	32.8 (28.8 to 36.5)	24.1 (19.2 to 28.7)	24.4 (19.5 to 28.8)	-26.6 (-33.9 to -21.0)	1.2 (-3.7 to 6.4)	35.8 (32.2 to 39.1)	27.9 (23.4 to 32.3)	28.2 (23.5 to 32.4)	-22.0 (-28.3 to -17.2)	1.0 (-3.0 to 5.1)
Diet low in polyunsaturated fatty acids	48.4 (46.7 to 49.8)	46.3 (44.6 to 47.7)	47.3 (45.8 to 48.6)	-4.4 (-6.6 to -0.6)	2.2 (1.1 to 3.4)	48.6 (46.9 to 50.1)	46.6 (44.8 to 48.1)	47.6 (46.0 to 49.0)	-4.3 (-7.3 to -1.2)	2.2 (-0.5 to 5.5)
Diet high in trans fatty acids	1.7 (0.1 to 4.4)	1.5 (0.1 to 4.0)	1.3 (0.1 to 3.7)	-16.2 (-41.6 to -9.2)	-9.7 (-31.5 to -4.4)	2.4 (0.4 to 5.4)	2.0 (0.2 to 4.8)	1.8 (0.2 to 4.5)	-16.3 (-37.1 to -8.9)	-9.5 (-27.3 to -3.9)
Diet high in sodium	27.0 (18.1 to 34.1)	22.6 (13.3 to 29.6)	21.0 (11.4 to 28.5)	-16.4 (-36.4 to 0.3)	-7.1 (-23.2 to 7.6)	26.2 (17.8 to 33.2)	21.3 (12.6 to 28.4)	19.1 (10.5 to 26.1)	-18.8 (-38.4 to -4.5)	-10.2 (-25.4 to 0.8)
Intimate partner violence	-	-	-	-	-	2.6 (2.1 to 3.0)	2.2 (1.8 to 2.6)	2.2 (1.8 to 2.5)	-13.0 (-17.1 to -9.4)	-3.2 (-7.6 to 1.5)
Childhood sexual abuse	5.0 (4.1 to 6.2)	5.0 (4.1 to 6.2)	5.0 (4.1 to 6.2)	0.0 (0.0 to 0.0)	0.0 (0.0 to 0.0)	7.1 (5.7 to 8.6)	7.1 (5.7 to 8.6)	7.1 (5.7 to 8.6)	0.0 (0.0 to 0.0)	0.0 (0.0 to 0.0)

Supplementary Results Table 1. Brunei age-standardised Summary Exposure Values (SEVs) for Level 4 risk factors and percentage change, by sex, 1990, 2007, and 2017.Data in parentheses are 95% uncertainty intervals.										
	Male					Female				
	1990	2007	2017	Percentage change 1990-2007	Percentage change 2007-2017	1990	2007	2017	Percentage change 1990-2007	Percentage change 2007-2017
Bullying victimization	5.9 (2.5 to 12.5)	6.7 (2.9 to 14.1)	6.9 (3.0 to 14.5)	14.5 (-1.0 to 26.7)	3.1 (-7.5 to 9.1)	4.0 (1.7 to 8.4)	4.6 (2.0 to 9.8)	4.8 (2.1 to 9.7)	15.4 (3.8 to 29.4)	3.0 (-3.8 to 9.9)
Low physical activity	0.5 (0.2 to 0.9)	0.5 (0.2 to 0.9)	0.4 (0.2 to 0.8)	-1.6 (-3.7 to 0.4)	-7.8 (-11.3 to -3.9)	0.5 (0.2 to 1.0)	0.5 (0.2 to 0.9)	0.5 (0.2 to 1.0)	-2.4 (-4.4 to -0.4)	2.5 (0.6 to 4.7)
High fasting plasma glucose	4.6 (3.0 to 6.8)	4.9 (3.4 to 7.3)	4.8 (3.2 to 7.1)	7.1 (-22.9 to 57.6)	-2.1 (-29.8 to 31.8)	4.8 (3.5 to 6.9)	4.7 (3.5 to 6.9)	4.5 (3.2 to 6.6)	-2.6 (-20.5 to 20.5)	-4.2 (-21.4 to 14.6)
High LDL cholesterol	12.4 (10.9 to 14.1)	13.2 (11.6 to 15.0)	13.7 (12.1 to 15.4)	6.2 (1.4 to 11.4)	3.5 (-0.2 to 7.0)	12.6 (11.1 to 14.2)	13.7 (12.1 to 15.4)	14.2 (12.6 to 16.0)	8.2 (3.9 to 12.6)	4.3 (0.7 to 8.0)
High systolic blood pressure	8.4 (7.5 to 9.3)	7.8 (6.9 to 8.7)	8.1 (7.3 to 9.0)	-7.6 (-14.5 to 0.1)	4.4 (-1.7 to 11.0)	6.9 (6.1 to 7.7)	5.9 (5.2 to 6.6)	5.9 (5.2 to 6.6)	-15.2 (-23.1 to -6.3)	-0.2 (-8.7 to 8.4)
High body-mass index	5.8 (3.7 to 9.7)	11.1 (7.8 to 16.4)	12.4 (9.0 to 17.9)	92.5 (60.2 to 144.0)	11.9 (1.8 to 25.1)	5.4 (3.3 to 8.3)	9.9 (7.1 to 14.2)	11.2 (8.0 to 16.2)	83.3 (48.8 to 137.0)	13.5 (0.1 to 29.2)
Low bone mineral density	5.3 (4.4 to 6.3)	5.2 (4.3 to 6.0)	5.1 (4.2 to 6.1)	-3.1 (-15.4 to 10.8)	-0.9 (-14.5 to 13.2)	7.2 (6.0 to 8.4)	7.0 (6.0 to 8.1)	6.9 (5.7 to 8.2)	-3.0 (-14.7 to 9.4)	-0.7 (-12.9 to 12.3)
Impaired kidney function	3.3 (1.6 to 7.3)	3.1 (1.4 to 7.1)	3.0 (1.4 to 7.0)	-6.6 (-11.2 to -2.2)	-2.2 (-6.6 to 1.8)	4.1 (1.9 to 8.6)	3.9 (1.8 to 8.5)	3.8 (1.7 to 8.4)	-3.3 (-7.6 to 0.0)	-3.1 (-7.5 to 0.4)

Supplementary Results Table 1. Japan age-standardised Summary Exposure Values (SEVs) for Level 4 risk factors and percentage change, by sex, 1990, 2007, and 2017.Data in parentheses are 95% uncertainty intervals.

	Male					Female				
	**1990**	**2007**	**2017**	**Percentage change 1990-2007**	**Percentage change 2007-2017**	**1990**	**2007**	**2017**	**Percentage change 1990-2007**	**Percentage change 2007-2017**
Unsafe water source	2.1 (1.4 to 3.1)	1.7 (1.1 to 2.5)	1.8 (1.2 to 2.7)	-17.2 (-48.8 to 32.4)	6.0 (-27.8 to 57.7)	2.1 (1.4 to 3.1)	1.7 (1.1 to 2.5)	1.8 (1.2 to 2.7)	-17.4 (-48.8 to 32.0)	5.8 (-27.8 to 57.3)
Unsafe sanitation	3.5 (2.1 to 5.6)	1.8 (1.1 to 2.9)	1.5 (0.9 to 2.5)	-50.2 (-72.0 to -10.4)	-14.3 (-45.8 to 39.6)	3.6 (2.1 to 5.6)	1.8 (1.1 to 2.9)	1.5 (0.9 to 2.5)	-50.3 (-72.1 to -10.3)	-14.5 (-45.9 to 39.4)
No access to handwashing facility	6.7 (5.3 to 8.1)	6.3 (5.0 to 7.8)	5.8 (4.7 to 7.2)	-5.5 (-25.6 to 21.4)	-7.2 (-21.9 to 9.5)	6.7 (5.3 to 8.1)	6.3 (5.0 to 7.8)	5.8 (4.7 to 7.2)	-5.6 (-25.7 to 21.5)	-7.3 (-21.9 to 9.5)
Ambient particulate matter pollution	33.5 (24.2 to 47.3)	33.8 (24.6 to 47.6)	29.2 (20.9 to 42.1)	0.6 (-4.3 to 5.9)	-13.5 (-19.1 to -8.6)	33.3 (23.9 to 47.0)	33.7 (24.5 to 47.6)	29.2 (20.9 to 42.0)	1.2 (-3.6 to 6.7)	-13.4 (-18.9 to -8.6)
Household air pollution from solid fuels	0.5 (0.2 to 1.4)	0.1 (0.0 to 0.3)	0.1 (0.0 to 0.2)	-75.1 (-89.2 to -66.0)	-15.4 (-50.8 to 15.0)	0.8 (0.3 to 4.2)	0.2 (0.1 to 0.9)	0.2 (0.0 to 0.7)	-71.9 (-89.1 to -64.1)	-14.0 (-50.5 to 15.1)
Ambient ozone pollution	50.6 (24.2 to 54.8)	50.8 (24.6 to 54.8)	51.3 (25.7 to 54.8)	0.3 (-0.1 to 1.5)	0.9 (0.0 to 4.5)	50.6 (24.1 to 54.8)	50.7 (24.4 to 54.8)	51.2 (25.6 to 54.8)	0.3 (-0.1 to 1.6)	1.0 (0.0 to 4.7)
Residential radon	14.4 (6.8 to 24.4)	14.3 (6.8 to 24.4)	14.3 (6.7 to 24.6)	-0.3 (-1.8 to 1.2)	-0.4 (-2.2 to 1.5)	14.4 (6.9 to 24.3)	14.3 (6.8 to 24.3)	14.3 (6.7 to 24.6)	-0.4 (-2.3 to 1.5)	-0.5 (-2.7 to 2.0)
Lead exposure	3.2 (0.6 to 6.8)	2.0 (0.3 to 5.0)	1.4 (0.2 to 3.9)	-36.0 (-47.9 to -26.8)	-30.4 (-44.6 to -22.2)	2.1 (0.3 to 5.1)	1.3 (0.2 to 3.6)	0.9 (0.1 to 2.7)	-39.3 (-49.9 to -29.7)	-31.6 (-43.8 to -23.0)
Occupational exposure to asbestos	2.4 (2.1 to 2.6)	3.3 (3.2 to 3.4)	3.2 (3.0 to 3.5)	37.4 (28.3 to 54.2)	-1.4 (-7.6 to 6.5)	0.6 (0.5 to 0.7)	0.6 (0.6 to 0.7)	0.6 (0.6 to 0.7)	-2.0 (-14.0 to 18.7)	-4.1 (-11.6 to 4.9)
Occupational exposure to arsenic	0.5 (0.0 to 1.1)	0.4 (0.0 to 1.0)	0.4 (0.0 to 1.0)	-	-	0.4 (0.0 to 1.0)	0.4 (0.0 to 0.9)	0.4 (0.0 to 0.9)	-	-
Occupational exposure to benzene	0.6 (0.0 to 1.8)	0.7 (0.0 to 1.9)	0.7 (0.0 to 1.9)	2.5 (-0.4 to 7.1)	3.5 (-0.1 to 8.7)	0.6 (0.0 to 1.7)	0.7 (0.0 to 2.1)	0.9 (0.1 to 2.4)	21.0 (15.5 to 28.9)	13.9 (8.1 to 22.5)
Occupational exposure to beryllium	0.0 (0.0 to 0.0)	0.0 (0.0 to 0.0)	0.0 (0.0 to 0.0)	-5.2 (-6.8 to -3.7)	2.0 (0.0 to 3.7)	0.0 (0.0 to 0.0)	0.0 (0.0 to 0.0)	0.0 (0.0 to 0.0)	1.9 (-0.6 to 4.4)	8.8 (5.8 to 11.9)
Occupational exposure to cadmium	0.1 (0.0 to 0.1)	0.0 (0.0 to 0.1)	0.0 (0.0 to 0.1)	-7.2 (-14.1 to -0.1)	0.9 (-5.3 to 7.9)	0.0 (0.0 to 0.1)	0.0 (0.0 to 0.0)	0.0 (0.0 to 0.0)	-9.4 (-17.9 to -0.2)	4.5 (-3.8 to 13.0)
Occupational exposure to chromium	0.1 (0.1 to 0.1)	0.1 (0.1 to 0.1)	0.1 (0.1 to 0.1)	-5.6 (-11.6 to 0.6)	1.4 (-4.0 to 7.1)	0.1 (0.1 to 0.1)	0.1 (0.1 to 0.1)	0.1 (0.1 to 0.1)	-4.6 (-12.3 to 3.5)	6.3 (-0.5 to 13.3)
Occupational exposure to diesel engine exhaust	0.6 (0.6 to 0.6)	0.6 (0.6 to 0.6)	0.6 (0.6 to 0.6)	-2.0 (-6.1 to 2.5)	3.0 (-1.0 to 7.3)	0.5 (0.4 to 0.5)	0.5 (0.5 to 0.5)	0.6 (0.5 to 0.6)	12.3 (5.2 to 19.6)	10.8 (4.2 to 17.5)
Occupational exposure to formaldehyde	0.3 (0.2 to 0.3)	0.2 (0.2 to 0.3)	0.2 (0.2 to 0.3)	-6.7 (-14.0 to 1.1)	0.5 (-6.1 to 7.5)	0.2 (0.2 to 0.2)	0.2 (0.2 to 0.2)	0.2 (0.2 to 0.2)	-6.8 (-16.0 to 3.0)	5.3 (-3.1 to 13.5)
Occupational exposure to nickel	0.4 (0.0 to 1.9)	0.4 (0.0 to 1.8)	0.4 (0.0 to 1.7)	-	-	0.4 (0.0 to 1.6)	0.3 (0.0 to 1.3)	0.3 (0.0 to 1.4)	-	-
Occupational exposure to polycyclic aromatic hydrocarbons	0.2 (0.2 to 0.3)	0.2 (0.2 to 0.2)	0.2 (0.2 to 0.3)	-4.5 (-10.1 to 1.4)	1.8 (-3.1 to 6.7)	0.2 (0.2 to 0.2)	0.2 (0.2 to 0.2)	0.2 (0.2 to 0.2)	-2.4 (-9.8 to 5.3)	7.2 (0.8 to 13.6)
Occupational exposure to silica	4.7 (0.7 to 15.2)	4.4 (0.7 to 14.1)	4.5 (0.6 to 14.3)	-6.2 (-12.1 to 0.2)	1.4 (-3.9 to 7.8)	3.2 (0.5 to 10.2)	2.8 (0.4 to 8.5)	2.8 (0.4 to 8.8)	-14.2 (-23.0 to -3.9)	1.6 (-7.2 to 12.4)
Occupational exposure to sulphuric acid	0.6 (0.1 to 1.9)	0.6 (0.1 to 1.8)	0.6 (0.1 to 1.8)	-7.3 (-13.6 to -0.6)	1.3 (-5.0 to 7.6)	0.5 (0.1 to 1.7)	0.5 (0.1 to 1.5)	0.5 (0.1 to 1.5)	-10.8 (-19.4 to -1.7)	3.8 (-4.7 to 13.0)
Occupational exposure to trichloroethylene	0.1 (0.1 to 0.1)	0.1 (0.1 to 0.1)	0.1 (0.1 to 0.1)	-4.6 (-8.6 to -0.6)	1.9 (-1.8 to 5.7)	0.1 (0.1 to 0.1)	0.1 (0.1 to 0.1)	0.1 (0.1 to 0.1)	0.5 (-4.5 to 6.1)	7.9 (3.0 to 13.6)
Occupational asthmagens	19.2 (16.2 to 23.1)	18.3 (15.4 to 22.1)	18.3 (15.3 to 22.0)	-4.4 (-15.2 to 9.4)	-0.2 (-10.7 to 10.8)	8.5 (7.1 to 9.9)	8.4 (7.1 to 10.0)	8.9 (7.3 to 10.6)	-0.3 (-8.9 to 8.1)	5.2 (-2.5 to 14.2)
Occupational particulate matter, gases, and fumes	6.4 (4.6 to 9.2)	6.1 (4.4 to 8.8)	5.9 (4.2 to 8.4)	-4.4 (-7.3 to -1.3)	-3.7 (-5.7 to -1.8)	3.7 (2.7 to 5.3)	3.5 (2.5 to 5.1)	3.3 (2.4 to 4.9)	-5.7 (-10.9 to -0.7)	-4.8 (-8.3 to -1.6)
Occupational noise	5.9 (5.6 to 6.4)	5.7 (5.4 to 6.2)	5.6 (5.3 to 6.1)	-3.1 (-4.7 to -1.4)	-2.3 (-3.2 to -1.3)	3.5 (3.3 to 3.9)	3.5 (3.3 to 3.9)	3.6 (3.4 to 3.9)	0.4 (-2.3 to 3.2)	1.8 (0.3 to 3.4)
Occupational ergonomic factors	10.2 (8.7 to 12.0)	9.0 (7.8 to 10.6)	8.9 (7.5 to 10.5)	-11.6 (-17.7 to -4.7)	-1.3 (-7.8 to 5.7)	5.5 (4.7 to 6.5)	5.6 (4.8 to 6.5)	6.3 (5.3 to 7.6)	1.6 (-9.1 to 12.9)	12.8 (1.7 to 26.0)
Non-exclusive breastfeeding	0.4 (0.3 to 0.6)	0.4 (0.3 to 0.6)	0.4 (0.3 to 0.6)	-1.0 (-11.0 to 9.4)	-1.7 (-9.6 to 7.0)	0.4 (0.3 to 0.6)	0.4 (0.3 to 0.6)	0.4 (0.3 to 0.6)	-1.0 (-11.0 to 9.4)	-1.7 (-9.6 to 7.0)
Discontinued breastfeeding	1.7 (1.6 to 1.8)	1.7 (1.6 to 1.8)	1.7 (1.6 to 1.8)	1.9 (-4.3 to 9.1)	-2.5 (-7.6 to 3.1)	1.7 (1.6 to 1.8)	1.7 (1.6 to 1.8)	1.7 (1.6 to 1.8)	1.9 (-4.3 to 9.1)	-2.5 (-7.6 to 3.1)
Child underweight	0.2 (0.2 to 0.3)	0.2 (0.1 to 0.3)	0.2 (0.1 to 0.3)	-14.2 (-19.6 to -9.8)	-5.0 (-8.0 to -2.2)	0.3 (0.2 to 0.3)	0.2 (0.1 to 0.3)	0.2 (0.1 to 0.3)	-16.1 (-21.9 to -11.6)	-5.7 (-8.9 to -2.5)
Child wasting	0.2 (0.1 to 0.2)	0.2 (0.1 to 0.2)	0.2 (0.1 to 0.2)	-7.7 (-12.9 to -2.6)	-2.2 (-5.9 to 1.9)	0.2 (0.1 to 0.2)	0.2 (0.1 to 0.2)	0.2 (0.1 to 0.2)	-9.1 (-14.6 to -3.8)	-2.8 (-6.7 to 1.7)
Child stunting	0.6 (0.4 to 0.8)	0.5 (0.3 to 0.7)	0.5 (0.3 to 0.7)	-11.8 (-20.5 to -3.7)	-3.2 (-9.6 to 3.5)	0.6 (0.4 to 0.8)	0.5 (0.3 to 0.7)	0.5 (0.3 to 0.7)	-13.3 (-22.2 to -5.2)	-3.7 (-10.4 to 2.5)
Short gestation for birth weight	0.0 (0.0 to 0.0)	0.0 (0.0 to 0.0)	0.0 (0.0 to 0.0)	9.1 (0.3 to 20.5)	2.2 (-4.1 to 11.2)	0.0 (0.0 to 0.0)	0.0 (0.0 to 0.0)	0.0 (0.0 to 0.0)	14.2 (3.4 to 28.6)	0.8 (-7.0 to 9.8)
Low birth weight for gestation	0.0 (0.0 to 0.0)	0.0 (0.0 to 0.0)	0.0 (0.0 to 0.0)	4.0 (-5.0 to 14.1)	2.2 (-4.7 to 8.4)	0.0 (0.0 to 0.0)	0.0 (0.0 to 0.0)	0.0 (0.0 to 0.0)	-4.3 (-17.4 to 8.6)	3.6 (-3.2 to 11.5)
Iron deficiency	-	-	-	-	-	7.8 (5.6 to 10.7)	7.9 (5.7 to 10.6)	7.2 (5.1 to 9.9)	0.1 (-15.3 to 19.5)	-8.6 (-20.1 to 3.1)
Vitamin A deficiency	0.9 (0.6 to 1.2)	0.8 (0.5 to 1.0)	0.6 (0.4 to 0.8)	-15.5 (-18.1 to -11.9)	-18.2 (-20.8 to -15.4)	0.7 (0.5 to 0.9)	0.8 (0.5 to 1.0)	0.7 (0.4 to 0.8)	5.5 (-0.2 to 10.0)	-14.4 (-17.3 to -11.3)
Zinc deficiency	0.5 (0.1 to 1.0)	0.5 (0.2 to 0.9)	0.4 (0.1 to 0.8)	-0.1 (-17.2 to 23.0)	-9.6 (-23.4 to 7.7)	0.5 (0.1 to 1.1)	0.4 (0.1 to 0.8)	0.4 (0.1 to 0.8)	-21.1 (-32.8 to -4.6)	1.0 (-15.1 to 20.4)
Smoking	16.9 (14.9 to 19.0)	11.9 (10.5 to 13.5)	10.1 (8.9 to 11.5)	-29.6 (-32.8 to -26.2)	-15.2 (-19.2 to -11.2)	3.8 (3.2 to 4.5)	3.2 (2.7 to 3.7)	3.1 (2.6 to 3.6)	-16.5 (-25.2 to -6.9)	-3.2 (-10.1 to 4.5)
Chewing tobacco	1.4 (1.0 to 1.8)	0.9 (0.7 to 1.2)	0.7 (0.6 to 1.0)	-33.1 (-50.7 to -9.5)	-18.8 (-34.7 to 1.3)	0.1 (0.1 to 0.1)	0.1 (0.1 to 0.1)	0.1 (0.1 to 0.1)	-4.1 (-30.4 to 32.8)	-2.6 (-23.1 to 26.3)
Second-hand smoke	37.7 (34.7 to 40.5)	36.7 (33.7 to 39.7)	36.5 (33.2 to 39.8)	-2.8 (-10.5 to 6.4)	-0.6 (-7.1 to 6.3)	57.3 (54.8 to 59.9)	49.8 (47.1 to 52.2)	49.5 (46.4 to 52.5)	-13.2 (-17.8 to -8.6)	-0.6 (-5.2 to 3.7)
Alcohol use	14.7 (8.8 to 23.7)	14.9 (8.7 to 24.1)	17.2 (9.4 to 28.3)	1.4 (-12.2 to 14.9)	15.1 (-7.0 to 42.7)	11.8 (6.8 to 19.8)	11.9 (6.9 to 20.1)	13.7 (7.4 to 22.6)	1.2 (-12.5 to 14.9)	15.1 (-7.5 to 42.2)
Drug use	0.5 (0.4 to 0.7)	0.5 (0.4 to 0.7)	0.6 (0.4 to 0.8)	0.5 (-1.9 to 2.8)	11.5 (7.3 to 17.4)	0.3 (0.2 to 0.4)	0.3 (0.2 to 0.4)	0.3 (0.3 to 0.4)	2.3 (0.1 to 4.6)	9.0 (5.2 to 13.3)
Diet low in fruits	40.5 (35.2 to 43.5)	38.8 (33.4 to 42.0)	40.2 (34.7 to 43.4)	-4.1 (-7.4 to -1.1)	3.6 (0.4 to 6.8)	37.4 (32.0 to 41.0)	34.6 (28.9 to 38.4)	37.0 (31.4 to 40.8)	-7.4 (-11.9 to -3.4)	6.8 (2.9 to 11.8)
Diet low in vegetables	18.9 (12.6 to 24.6)	13.3 (7.3 to 19.1)	15.3 (9.0 to 21.3)	-29.9 (-46.6 to -18.4)	15.5 (-2.2 to 41.3)	21.2 (14.9 to 26.7)	15.3 (9.2 to 21.1)	17.1 (10.9 to 23.0)	-27.7 (-42.9 to -16.2)	11.7 (-4.3 to 34.5)
Diet low in legumes	4.3 (1.5 to 7.9)	5.8 (2.3 to 9.9)	7.8 (3.5 to 12.7)	33.1 (10.5 to 86.7)	35.0 (7.6 to 88.8)	8.3 (3.9 to 13.2)	8.2 (4.0 to 12.8)	10.7 (5.6 to 16.1)	-0.9 (-16.3 to 18.9)	30.4 (8.9 to 67.8)
Diet low in whole grains	30.2 (26.3 to 33.6)	35.0 (31.8 to 37.8)	36.4 (33.2 to 39.1)	15.9 (10.7 to 23.3)	3.8 (0.7 to 7.2)	31.6 (27.9 to 34.8)	36.3 (33.1 to 38.8)	37.5 (34.5 to 40.0)	14.6 (9.7 to 20.9)	3.5 (0.8 to 6.4)
Diet low in nuts and seeds	50.4 (49.4 to 51.3)	49.1 (47.7 to 50.2)	48.9 (47.3 to 50.2)	-2.7 (-4.2 to -1.4)	-0.4 (-1.9 to 0.8)	50.6 (49.6 to 51.4)	49.1 (47.7 to 50.2)	48.8 (47.2 to 50.1)	-3.0 (-4.5 to -1.7)	-0.5 (-2.0 to 0.8)
Diet low in milk	46.0 (44.1 to 47.6)	46.3 (44.6 to 47.8)	46.8 (45.0 to 48.3)	0.7 (-0.2 to 1.8)	1.0 (0.2 to 1.9)	46.0 (44.1 to 47.5)	45.8 (43.9 to 47.3)	46.4 (44.6 to 48.0)	-0.4 (-1.4 to 0.5)	1.4 (0.5 to 2.5)
Diet high in red meat	4.2 (2.3 to 6.4)	8.2 (5.4 to 11.1)	9.5 (5.6 to 13.0)	94.2 (57.0 to 168.6)	15.7 (-12.7 to 52.4)	0.6 (0.1 to 1.4)	1.7 (0.6 to 3.1)	2.2 (0.7 to 4.2)	170.7 (80.9 to 579.8)	30.7 (-26.0 to 132.7)
Diet high in processed meat	12.0 (8.6 to 15.0)	20.0 (17.5 to 22.3)	23.3 (19.9 to 26.3)	66.6 (43.8 to 104.5)	16.6 (5.9 to 29.3)	10.1 (7.0 to 12.8)	14.9 (12.2 to 17.3)	16.6 (13.4 to 19.4)	47.8 (28.3 to 81.0)	11.3 (1.3 to 23.5)
Diet high in sugar-sweetened beverages	7.7 (3.7 to 11.7)	8.9 (4.5 to 10.9)	9.4 (4.8 to 12.3)	15.5 (-23.8 to 82.9)	6.0 (-18.8 to 35.3)	5.4 (2.6 to 8.8)	6.1 (3.2 to 7.9)	6.5 (3.2 to 9.0)	12.8 (-26.1 to 84.7)	7.5 (-22.2 to 47.1)
Diet low in fibre	21.8 (15.5 to 27.3)	22.9 (17.0 to 28.0)	23.9 (18.1 to 29.3)	5.1 (-4.9 to 17.5)	4.5 (-2.6 to 13.2)	23.0 (16.8 to 28.4)	24.0 (18.2 to 29.0)	25.4 (19.5 to 30.6)	4.5 (-4.5 to 15.5)	5.8 (-0.6 to 14.3)
Diet low in calcium	34.2 (30.0 to 37.6)	34.0 (29.8 to 37.4)	34.9 (30.9 to 38.1)	-0.5 (-2.1 to 1.1)	2.4 (0.5 to 4.6)	36.5 (32.7 to 39.4)	35.4 (31.4 to 38.6)	36.6 (32.9 to 39.6)	-2.9 (-4.8 to -1.3)	3.5 (1.8 to 5.9)
Diet low in seafood omega-3 fatty acids	3.0 (1.4 to 4.2)	0.2 (0.0 to 0.9)	0.3 (0.0 to 1.0)	-92.1 (-99.7 to -73.3)	6.6 (-35.7 to 182.9)	3.5 (1.8 to 4.8)	0.3 (0.0 to 1.2)	0.4 (0.0 to 1.2)	-90.2 (-99.4 to -70.1)	7.1 (-29.4 to 131.4)
Diet low in polyunsaturated fatty acids	30.0 (25.4 to 33.9)	27.1 (22.2 to 31.3)	27.8 (22.8 to 32.0)	-9.5 (-15.1 to -5.6)	2.3 (-1.1 to 6.3)	30.7 (26.2 to 34.4)	27.9 (22.9 to 32.0)	28.5 (23.6 to 32.6)	-9.2 (-14.7 to -5.3)	2.2 (-1.1 to 6.5)
Diet high in trans fatty acids	1.1 (0.0 to 3.3)	0.9 (0.0 to 3.0)	0.9 (0.0 to 2.9)	-12.5 (-36.8 to -7.6)	-8.4 (-32.8 to -4.8)	1.5 (0.1 to 4.0)	1.3 (0.1 to 3.7)	1.2 (0.0 to 3.5)	-12.5 (-33.9 to -7.7)	-8.4 (-28.1 to -4.8)
Diet high in sodium	27.6 (20.1 to 33.5)	18.0 (10.5 to 23.9)	15.4 (7.7 to 21.7)	-35.0 (-52.3 to -23.2)	-14.3 (-31.6 to -4.7)	31.1 (24.3 to 36.6)	18.7 (11.4 to 24.5)	15.5 (7.8 to 21.8)	-39.7 (-56.5 to -28.5)	-17.5 (-34.8 to -8.8)
Intimate partner violence	-	-	-	-	-	2.3 (1.9 to 2.6)	2.1 (1.8 to 2.4)	2.0 (1.7 to 2.3)	-7.9 (-8.9 to -7.0)	-4.0 (-5.0 to -3.0)
Childhood sexual abuse	5.3 (4.3 to 6.5)	5.3 (4.3 to 6.5)	5.3 (4.3 to 6.5)	-0.0 (-0.0 to 0.0)	0.0 (-0.0 to 0.0)	7.3 (5.9 to 8.9)	7.3 (5.9 to 8.9)	7.5 (6.0 to 9.2)	-0.0 (-0.0 to 0.0)	2.8 (-4.0 to 10.6)

Supplementary Results Table 1. Japan age-standardised Summary Exposure Values (SEVs) for Level 4 risk factors and percentage change, by sex, 1990, 2007, and 2017.Data in parentheses are 95% uncertainty intervals.

	Male					Female				
	**1990**	**2007**	**2017**	**Percentage change 1990-2007**	**Percentage change 2007-2017**	**1990**	**2007**	**2017**	**Percentage change 1990-2007**	**Percentage change 2007-2017**
Bullying victimization	3.3<br>(1.4 to 6.6)	3.3<br>(1.4 to 6.7)	3.5<br>(1.5 to 7.1)	1.4<br>(-6.0 to 8.8)	3.9<br>(-1.0 to 8.1)	1.9<br>(0.8 to 3.8)	1.9<br>(0.8 to 3.8)	2.0<br>(0.8 to 4.0)	-0.5<br>(-7.8 to 7.7)	3.0<br>(-2.5 to 7.9)
Low physical activity	0.5<br>(0.2 to 0.9)	0.5<br>(0.2 to 0.9)	0.4<br>(0.2 to 0.8)	-1.8<br>(-2.7 to -0.9)	-8.8<br>(-11.7 to -4.8)	0.5<br>(0.2 to 1.0)	0.5<br>(0.2 to 0.9)	0.5<br>(0.2 to 0.9)	-3.8<br>(-5.7 to -1.4)	0.8<br>(0.1 to 1.6)
High fasting plasma glucose	1.4<br>(1.0 to 2.0)	1.4<br>(1.0 to 2.0)	1.6<br>(1.1 to 2.2)	-0.4<br>(-22.4 to 23.9)	11.9<br>(-6.7 to 40.7)	1.2<br>(0.8 to 1.7)	1.2<br>(0.8 to 1.8)	1.4<br>(0.9 to 2.0)	4.1<br>(-20.7 to 35.1)	10.0<br>(-11.2 to 40.3)
High LDL cholesterol	12.1<br>(10.5 to 13.8)	12.6<br>(11.0 to 14.4)	12.6<br>(11.1 to 14.3)	4.3<br>(0.2 to 8.4)	0.0<br>(-3.0 to 3.2)	12.9<br>(11.5 to 14.6)	13.0<br>(11.5 to 14.7)	13.1<br>(11.6 to 14.8)	0.8<br>(-2.6 to 4.2)	0.2<br>(-2.7 to 3.3)
High systolic blood pressure	10.8<br>(10.1 to 11.7)	8.1<br>(7.4 to 8.8)	8.0<br>(7.2 to 8.8)	-25.3<br>(-28.1 to -22.3)	-1.2<br>(-6.0 to 3.9)	9.2<br>(8.6 to 9.9)	5.8<br>(5.3 to 6.4)	5.4<br>(4.8 to 6.1)	-36.9<br>(-39.6 to -34.3)	-6.7<br>(-13.0 to 0.1)
High body-mass index	6.4<br>(4.4 to 9.6)	9.3<br>(6.8 to 13.8)	9.7<br>(7.0 to 14.3)	45.0<br>(25.1 to 70.4)	4.4<br>(-6.1 to 15.3)	5.8<br>(3.8 to 8.8)	7.5<br>(5.0 to 11.0)	7.5<br>(4.9 to 11.2)	29.5<br>(11.7 to 53.9)	0.6<br>(-10.4 to 13.6)
Low bone mineral density	5.3<br>(4.5 to 6.1)	5.2<br>(4.4 to 6.0)	5.2<br>(4.4 to 6.0)	-1.8<br>(-4.6 to 1.1)	0.3<br>(-2.8 to 3.3)	7.1<br>(6.2 to 8.1)	6.9<br>(6.0 to 7.9)	7.0<br>(6.0 to 8.0)	-2.6<br>(-4.9 to -0.3)	1.4<br>(-1.5 to 4.2)
Impaired kidney function	2.6<br>(1.2 to 6.6)	2.6<br>(1.2 to 6.5)	2.4<br>(1.1 to 6.2)	-2.6<br>(-4.7 to -0.5)	-5.2<br>(-9.1 to -1.7)	3.2<br>(1.5 to 7.4)	3.3<br>(1.5 to 7.6)	3.1<br>(1.4 to 7.2)	2.6<br>(0.7 to 4.6)	-6.1<br>(-9.0 to -2.3)

Supplementary Results Table 1. Aichi age-standardised Summary Exposure Values (SEVs) for Level 4 risk factors and percentage change, by sex, 1990, 2007, and 2017.Data in parentheses are 95% uncertainty intervals.										
	Male					Female				
	1990	2007	2017	Percentage change 1990-2007	Percentage change 2007-2017	1990	2007	2017	Percentage change 1990-2007	Percentage change 2007-2017
Unsafe water source	1.9 (1.1 to 3.1)	1.6 (0.9 to 2.6)	1.7 (0.9 to 2.8)	-16.8 (-55.9 to 60.6)	5.3 (-36.3 to 74.2)	1.9 (1.1 to 3.1)	1.6 (0.9 to 2.6)	1.7 (0.9 to 2.8)	-16.8 (-55.9 to 60.6)	5.3 (-36.3 to 74.2)
Unsafe sanitation	3.0 (1.5 to 5.4)	1.5 (0.8 to 2.9)	1.3 (0.6 to 2.5)	-49.5 (-78.4 to 17.2)	-15.7 (-55.0 to 65.6)	3.0 (1.5 to 5.4)	1.5 (0.8 to 2.9)	1.3 (0.6 to 2.5)	-49.5 (-78.4 to 17.2)	-15.7 (-55.0 to 65.6)
No access to handwashing facility	6.6 (4.8 to 8.9)	6.2 (4.4 to 8.3)	5.8 (4.1 to 7.7)	-6.0 (-32.4 to 29.9)	-7.3 (-27.8 to 19.3)	6.6 (4.8 to 8.9)	6.2 (4.4 to 8.3)	5.8 (4.1 to 7.7)	-6.0 (-32.4 to 29.9)	-7.3 (-27.8 to 19.3)
Ambient particulate matter pollution	35.2 (25.0 to 49.9)	35.0 (24.4 to 50.5)	30.0 (20.9 to 43.4)	-0.6 (-19.0 to 22.4)	-14.2 (-28.7 to 2.1)	35.0 (24.9 to 49.8)	34.9 (24.4 to 50.5)	30.0 (20.9 to 43.4)	-0.3 (-18.6 to 22.7)	-14.1 (-28.7 to 2.1)
Household air pollution from solid fuels	0.4 (0.1 to 1.1)	0.1 (0.0 to 0.2)	0.1 (0.0 to 0.2)	-73.6 (-92.9 to -50.8)	-9.5 (-68.2 to 91.4)	0.6 (0.1 to 3.0)	0.2 (0.0 to 0.5)	0.2 (0.0 to 0.6)	-70.1 (-92.9 to -48.5)	-10.3 (-68.2 to 92.8)
Ambient ozone pollution	52.1 (26.4 to 54.8)	52.1 (26.3 to 54.8)	52.2 (26.8 to 54.8)	-0.0 (-0.7 to 0.2)	0.2 (0.0 to 1.9)	52.1 (26.4 to 54.8)	52.1 (26.3 to 54.8)	52.2 (26.8 to 54.8)	-0.0 (-0.7 to 0.2)	0.2 (0.0 to 1.9)
Residential radon	13.5 (0.8 to 44.1)	13.5 (0.8 to 44.1)	13.5 (0.8 to 44.1)	0.0 (0.0 to 0.0)	0.0 (0.0 to 0.0)	13.5 (0.8 to 44.1)	13.5 (0.8 to 44.1)	13.5 (0.8 to 44.1)	0.0 (0.0 to 0.0)	0.0 (0.0 to 0.0)
Lead exposure	3.8 (0.8 to 7.6)	2.3 (0.4 to 5.3)	1.5 (0.2 to 3.9)	-39.9 (-54.2 to -29.6)	-33.5 (-48.5 to -25.3)	2.0 (0.2 to 5.1)	1.1 (0.1 to 3.2)	0.7 (0.0 to 2.3)	-47.0 (-65.2 to -36.3)	-38.1 (-60.1 to -28.2)
Occupational exposure to asbestos	2.1 (1.8 to 2.3)	3.0 (2.8 to 3.3)	3.1 (2.6 to 3.6)	48.6 (29.8 to 74.8)	1.1 (-16.0 to 21.7)	0.5 (0.4 to 0.6)	0.5 (0.4 to 0.5)	0.5 (0.4 to 0.6)	-9.1 (-24.9 to 19.6)	-6.1 (-24.8 to 16.7)
Occupational exposure to arsenic	0.5 (0.0 to 1.1)	0.4 (0.0 to 1.0)	0.4 (0.0 to 1.0)	-	-	0.4 (0.0 to 1.1)	0.4 (0.0 to 0.9)	0.4 (0.0 to 0.9)	-	-
Occupational exposure to benzene	0.6 (0.0 to 1.9)	0.7 (0.0 to 1.9)	0.7 (0.0 to 2.0)	2.7 (-4.5 to 12.8)	3.7 (-2.2 to 11.7)	0.6 (0.0 to 1.9)	0.8 (0.0 to 2.2)	0.9 (0.1 to 2.4)	20.5 (8.0 to 42.7)	13.6 (4.2 to 27.4)
Occupational exposure to beryllium	0.0 (0.0 to 0.0)	0.0 (0.0 to 0.0)	0.0 (0.0 to 0.0)	-5.4 (-9.9 to -0.3)	1.9 (-2.0 to 5.7)	0.0 (0.0 to 0.0)	0.0 (0.0 to 0.0)	0.0 (0.0 to 0.0)	1.2 (-6.6 to 10.0)	8.4 (2.3 to 14.9)
Occupational exposure to cadmium	0.1 (0.0 to 0.1)	0.0 (0.0 to 0.1)	0.1 (0.0 to 0.1)	-7.8 (-17.7 to 4.2)	0.7 (-8.7 to 10.3)	0.1 (0.0 to 0.1)	0.0 (0.0 to 0.1)	0.0 (0.0 to 0.1)	-10.6 (-24.0 to 5.1)	3.8 (-8.5 to 17.1)
Occupational exposure to chromium	0.1 (0.1 to 0.1)	0.1 (0.1 to 0.1)	0.1 (0.1 to 0.1)	-6.2 (-15.1 to 4.3)	1.2 (-7.2 to 9.5)	0.1 (0.1 to 0.1)	0.1 (0.1 to 0.1)	0.1 (0.1 to 0.1)	-5.9 (-18.5 to 8.3)	5.6 (-5.0 to 17.0)
Occupational exposure to diesel engine exhaust	0.6 (0.5 to 0.6)	0.6 (0.5 to 0.6)	0.6 (0.6 to 0.6)	-2.6 (-10.1 to 5.8)	2.9 (-3.8 to 10.1)	0.5 (0.4 to 0.5)	0.5 (0.5 to 0.6)	0.6 (0.5 to 0.7)	11.3 (-1.8 to 25.7)	10.4 (0.1 to 22.2)
Occupational exposure to formaldehyde	0.3 (0.2 to 0.3)	0.2 (0.2 to 0.3)	0.2 (0.2 to 0.3)	-7.3 (-17.9 to 5.5)	0.3 (-9.3 to 10.0)	0.2 (0.2 to 0.3)	0.2 (0.2 to 0.2)	0.2 (0.2 to 0.3)	-8.1 (-22.7 to 8.5)	4.6 (-7.3 to 17.9)
Occupational exposure to nickel	0.4 (0.0 to 2.0)	0.4 (0.0 to 1.8)	0.4 (0.0 to 1.8)	-	-	0.4 (0.0 to 1.6)	0.3 (0.0 to 1.4)	0.3 (0.0 to 1.4)	-	-
Occupational exposure to polycyclic aromatic hydrocarbons	0.3 (0.2 to 0.3)	0.2 (0.2 to 0.3)	0.2 (0.2 to 0.3)	-5.1 (-13.8 to 4.4)	1.6 (-6.0 to 9.3)	0.2 (0.2 to 0.3)	0.2 (0.2 to 0.3)	0.2 (0.2 to 0.3)	-3.7 (-16.7 to 9.9)	6.5 (-3.3 to 17.4)
Occupational exposure to silica	4.9 (0.7 to 15.9)	4.5 (0.6 to 14.8)	4.6 (0.6 to 14.7)	-7.1 (-17.3 to 5.1)	1.1 (-7.5 to 12.2)	3.3 (0.5 to 11.2)	2.8 (0.4 to 9.2)	2.8 (0.4 to 8.7)	-15.8 (-28.9 to 0.9)	0.6 (-12.7 to 17.4)
Occupational exposure to sulphuric acid	0.6 (0.1 to 2.0)	0.6 (0.1 to 1.9)	0.6 (0.1 to 1.8)	-7.6 (-17.0 to 3.8)	0.9 (-8.3 to 10.5)	0.5 (0.1 to 1.8)	0.5 (0.1 to 1.6)	0.5 (0.1 to 1.6)	-12.0 (-25.6 to 3.7)	3.3 (-9.7 to 18.1)
Occupational exposure to trichloroethylene	0.1 (0.1 to 0.1)	0.1 (0.1 to 0.1)	0.1 (0.1 to 0.1)	-5.1 (-11.7 to 2.6)	1.7 (-4.6 to 8.1)	0.1 (0.1 to 0.1)	0.1 (0.1 to 0.1)	0.1 (0.1 to 0.1)	-0.7 (-11.1 to 10.7)	7.3 (-0.9 to 16.8)
Occupational asthmagens	19.7 (15.6 to 24.5)	18.6 (14.8 to 23.6)	18.5 (14.7 to 23.4)	-5.8 (-22.8 to 15.6)	-0.5 (-15.3 to 17.6)	8.7 (7.1 to 10.9)	8.6 (7.0 to 10.6)	9.0 (7.3 to 11.0)	-1.1 (-16.8 to 15.7)	4.9 (-6.8 to 17.0)
Occupational particulate matter, gases, and fumes	6.4 (4.6 to 9.2)	6.1 (4.3 to 8.8)	5.9 (4.2 to 8.5)	-4.6 (-9.3 to 0.3)	-3.7 (-6.9 to -0.5)	3.8 (2.6 to 5.7)	3.5 (2.5 to 5.3)	3.4 (2.4 to 5.0)	-6.2 (-14.1 to 3.5)	-5.1 (-10.2 to 0.9)
Occupational noise	6.0 (5.5 to 6.5)	5.8 (5.3 to 6.3)	5.6 (5.2 to 6.1)	-3.2 (-6.1 to -0.0)	-2.3 (-4.4 to -0.3)	3.6 (3.2 to 4.2)	3.6 (3.2 to 4.1)	3.7 (3.3 to 4.1)	0.0 (-5.6 to 6.9)	1.5 (-2.0 to 5.6)
Occupational ergonomic factors	9.8 (8.1 to 11.9)	8.7 (7.2 to 10.4)	8.6 (7.1 to 10.4)	-11.6 (-21.8 to 0.1)	-1.3 (-11.3 to 9.6)	5.3 (4.2 to 6.7)	5.4 (4.2 to 6.9)	6.1 (4.7 to 7.8)	2.3 (-17.7 to 26.8)	13.0 (-5.4 to 34.0)
Non-exclusive breastfeeding	0.4 (0.3 to 0.6)	0.4 (0.3 to 0.6)	0.4 (0.3 to 0.6)	-1.0 (-13.3 to 13.1)	-1.7 (-11.8 to 10.6)	0.4 (0.3 to 0.6)	0.4 (0.3 to 0.6)	0.4 (0.3 to 0.6)	-0.8 (-13.2 to 13.2)	-1.6 (-11.7 to 10.7)
Discontinued breastfeeding	1.7 (1.6 to 1.9)	1.8 (1.6 to 1.9)	1.7 (1.6 to 1.9)	2.2 (-6.5 to 11.8)	-3.1 (-10.0 to 4.4)	1.7 (1.6 to 1.9)	1.8 (1.6 to 1.9)	1.7 (1.6 to 1.9)	2.2 (-6.5 to 11.8)	-2.7 (-9.7 to 4.7)
Child underweight	0.2 (0.1 to 0.3)	0.2 (0.1 to 0.3)	0.2 (0.1 to 0.3)	-12.3 (-29.3 to 6.8)	-4.6 (-16.0 to 8.5)	0.2 (0.1 to 0.3)	0.2 (0.1 to 0.3)	0.2 (0.1 to 0.3)	-13.9 (-32.2 to 7.7)	-5.0 (-17.7 to 9.2)
Child wasting	0.2 (0.1 to 0.3)	0.2 (0.1 to 0.2)	0.2 (0.1 to 0.2)	-8.8 (-30.8 to 18.3)	-1.6 (-18.4 to 17.7)	0.2 (0.1 to 0.3)	0.2 (0.1 to 0.2)	0.2 (0.1 to 0.2)	-9.8 (-32.1 to 17.6)	-1.9 (-19.2 to 17.9)
Child stunting	0.5 (0.3 to 0.9)	0.5 (0.2 to 0.7)	0.4 (0.2 to 0.7)	-15.9 (-47.5 to 25.9)	-1.9 (-29.0 to 36.6)	0.6 (0.3 to 0.9)	0.5 (0.2 to 0.7)	0.5 (0.2 to 0.7)	-17.5 (-48.4 to 28.4)	-2.3 (-31.6 to 38.8)
Short gestation for birth weight	0.0 (0.0 to 0.0)	0.0 (0.0 to 0.0)	0.0 (0.0 to 0.0)	8.6 (-11.1 to 36.7)	2.7 (-16.4 to 26.2)	0.0 (0.0 to 0.0)	0.0 (0.0 to 0.0)	0.0 (0.0 to 0.0)	15.1 (-20.7 to 105.3)	1.5 (-20.7 to 32.7)
Low birth weight for gestation	0.0 (0.0 to 0.0)	0.0 (0.0 to 0.0)	0.0 (0.0 to 0.0)	2.9 (-13.6 to 25.7)	1.2 (-15.2 to 19.2)	0.0 (0.0 to 0.0)	0.0 (0.0 to 0.0)	0.0 (0.0 to 0.0)	-7.9 (-63.5 to 35.6)	2.0 (-17.9 to 26.3)
Iron deficiency	-	-	-	-	-	7.6 (5.1 to 10.6)	7.6 (5.2 to 10.7)	7.0 (4.7 to 9.9)	0.2 (-21.5 to 29.1)	-8.4 (-26.0 to 10.3)
Vitamin A deficiency	0.9 (0.6 to 1.2)	0.7 (0.5 to 1.0)	0.6 (0.4 to 0.8)	-18.9 (-30.0 to -1.2)	-24.8 (-42.2 to -5.8)	0.7 (0.5 to 1.0)	0.7 (0.5 to 1.0)	0.6 (0.4 to 0.9)	-0.5 (-19.8 to 21.7)	-13.4 (-36.0 to 20.5)
Zinc deficiency	0.5 (0.1 to 1.0)	0.6 (0.1 to 1.2)	0.5 (0.1 to 1.2)	28.2 (-55.4 to 207.8)	-13.8 (-68.4 to 97.3)	0.5 (0.1 to 1.0)	0.4 (0.1 to 1.1)	0.4 (0.1 to 1.0)	-11.8 (-64.3 to 73.9)	-13.6 (-63.4 to 169.0)
Smoking	16.8 (13.4 to 20.2)	11.8 (9.5 to 14.4)	10.0 (7.9 to 12.1)	-29.3 (-38.1 to -19.3)	-15.2 (-23.4 to -6.0)	3.8 (2.7 to 5.1)	3.2 (2.3 to 4.3)	3.0 (2.2 to 4.2)	-16.1 (-36.6 to 9.6)	-3.9 (-19.4 to 14.6)
Chewing tobacco	1.4 (0.9 to 2.0)	0.9 (0.6 to 1.4)	0.8 (0.5 to 1.1)	-32.9 (-57.0 to 1.7)	-18.8 (-41.0 to 9.8)	0.1 (0.1 to 0.1)	0.1 (0.1 to 0.1)	0.1 (0.1 to 0.1)	-3.5 (-41.0 to 61.5)	-1.9 (-31.2 to 41.5)
Second-hand smoke	37.8 (34.0 to 41.7)	37.4 (33.3 to 42.0)	36.8 (32.6 to 41.2)	-1.1 (-12.3 to 11.0)	-1.7 (-10.3 to 7.6)	57.3 (53.7 to 60.6)	50.9 (47.2 to 54.3)	50.1 (46.2 to 53.9)	-11.2 (-17.6 to -5.3)	-1.6 (-7.2 to 4.3)
Alcohol use	14.7 (7.5 to 24.9)	14.9 (7.6 to 25.4)	16.8 (8.4 to 29.5)	1.0 (-32.8 to 47.0)	13.1 (-18.8 to 63.4)	11.7 (5.8 to 20.5)	11.8 (5.8 to 20.7)	13.4 (6.3 to 23.3)	0.5 (-34.3 to 46.0)	13.4 (-18.9 to 64.1)
Drug use	0.5 (0.4 to 0.7)	0.5 (0.4 to 0.7)	0.6 (0.4 to 0.8)	0.1 (-5.9 to 6.2)	11.5 (4.5 to 20.3)	0.3 (0.2 to 0.4)	0.3 (0.2 to 0.4)	0.3 (0.3 to 0.4)	2.4 (-3.3 to 8.1)	8.6 (1.9 to 16.3)
Diet low in fruits	39.4 (33.8 to 43.4)	37.8 (32.0 to 41.9)	39.4 (33.6 to 43.4)	-4.0 (-10.8 to 2.6)	4.3 (-0.7 to 10.7)	36.3 (30.4 to 40.9)	33.6 (27.8 to 38.5)	36.2 (30.3 to 41.1)	-7.5 (-16.3 to 1.7)	7.8 (1.2 to 16.4)
Diet low in vegetables	17.1 (8.8 to 25.0)	12.1 (4.9 to 19.3)	14.5 (6.4 to 22.2)	-29.4 (-62.9 to 0.5)	20.6 (-11.1 to 92.8)	19.3 (10.9 to 26.7)	14.1 (5.9 to 21.8)	16.2 (8.1 to 23.4)	-27.1 (-57.5 to 0.2)	15.2 (-13.1 to 72.5)
Diet low in legumes	4.4 (0.7 to 9.5)	6.6 (1.8 to 12.2)	9.0 (3.1 to 15.9)	49.7 (-22.7 to 336.4)	36.6 (-10.2 to 192.2)	8.7 (2.9 to 15.3)	9.4 (3.4 to 15.6)	12.3 (5.6 to 19.1)	9.0 (-32.4 to 97.0)	30.2 (-3.5 to 117.3)
Diet low in whole grains	29.4 (24.3 to 33.7)	34.4 (30.4 to 37.8)	35.8 (31.9 to 39.1)	17.1 (7.4 to 30.0)	4.0 (-1.3 to 9.7)	30.9 (25.6 to 35.1)	35.6 (31.6 to 38.9)	36.9 (33.2 to 40.1)	15.3 (6.8 to 26.8)	3.6 (-0.8 to 9.1)
Diet low in nuts and seeds	49.8 (48.2 to 51.1)	48.3 (46.2 to 50.0)	48.2 (45.9 to 49.9)	-3.0 (-6.1 to -0.2)	-0.2 (-2.7 to 2.0)	50.0 (48.3 to 51.3)	48.4 (46.3 to 50.0)	48.2 (46.0 to 50.0)	-3.2 (-6.3 to -0.7)	-0.3 (-3.1 to 2.0)
Diet low in milk	45.6 (43.5 to 47.4)	45.9 (43.8 to 47.7)	46.4 (44.3 to 48.2)	0.7 (-1.1 to 2.8)	1.0 (-0.4 to 2.7)	45.6 (43.4 to 47.4)	45.4 (43.1 to 47.2)	46.0 (43.7 to 47.9)	-0.4 (-2.4 to 1.7)	1.5 (-0.2 to 3.1)
Diet high in red meat	5.0 (1.1 to 10.2)	9.5 (3.5 to 16.2)	10.8 (3.9 to 18.5)	89.9 (-2.8 to 473.0)	13.8 (-35.1 to 95.7)	0.8 (0.0 to 2.9)	2.1 (0.1 to 5.6)	2.7 (0.2 to 7.0)	179.5 (-68.2 to 102438.9)	27.2 (-74.6 to 679.7)
Diet high in processed meat	13.6 (9.1 to 17.9)	22.4 (16.7 to 27.3)	26.0 (19.3 to 31.7)	65.3 (31.7 to 120.9)	16.2 (-0.3 to 36.1)	11.6 (7.5 to 15.3)	17.1 (12.6 to 21.2)	19.0 (14.1 to 23.7)	47.9 (17.7 to 97.0)	10.9 (-5.7 to 29.2)
Diet high in sugar-sweetened beverages	8.7 (3.8 to 16.5)	10.2 (4.4 to 17.4)	10.7 (4.7 to 18.7)	16.7 (-43.2 to 144.8)	5.0 (-36.1 to 68.1)	6.1 (2.3 to 12.1)	6.8 (3.2 to 12.8)	7.2 (3.2 to 13.1)	10.4 (-46.8 to 150.0)	6.7 (-36.9 to 76.0)
Diet low in fibre	21.2 (13.7 to 27.7)	22.7 (15.6 to 28.9)	24.1 (16.8 to 30.4)	7.0 (-10.3 to 33.6)	5.9 (-5.3 to 21.0)	22.4 (15.1 to 29.0)	24.0 (17.1 to 30.1)	25.6 (18.7 to 32.0)	6.8 (-9.9 to 28.8)	7.0 (-4.3 to 21.7)
Diet low in calcium	32.9 (28.0 to 36.7)	32.8 (28.0 to 36.7)	33.7 (29.2 to 37.4)	-0.2 (-4.7 to 4.2)	2.9 (-0.4 to 6.7)	35.2 (30.9 to 38.7)	34.3 (29.7 to 38.0)	35.7 (31.6 to 39.2)	-2.6 (-6.7 to 1.1)	4.1 (1.1 to 8.3)
Diet low in seafood omega-3 fatty acids	2.6 (0.8 to 4.0)	0.1 (0.0 to 0.7)	0.1 (0.0 to 0.7)	-95.5 (-100.0 to -78.3)	13.0 (-97.4 to [illegible]	2.9 (1.0 to 4.7)	0.2 (0.0 to 0.8)	0.2 (0.0 to 0.9)	-94.8 (-100.0 to -79.9)	17.9 (-98.3 to [illegible]
Diet low in polyunsaturated fatty acids	28.8 (23.0 to 34.2)	25.9 (19.6 to 31.3)	26.5 (20.1 to 31.7)	-10.2 (-23.4 to 0.2)	2.4 (-6.1 to 12.8)	29.4 (23.7 to 34.4)	26.6 (20.4 to 31.7)	27.3 (21.5 to 32.4)	-9.7 (-20.9 to 0.0)	2.8 (-6.5 to 13.5)
Diet high in trans fatty acids	1.1 (0.0 to 3.3)	0.9 (0.0 to 3.0)	0.9 (0.0 to 2.9)	-12.7 (-39.3 to -6.4)	-8.4 (-34.3 to -3.7)	1.5 (0.1 to 4.0)	1.3 (0.1 to 3.7)	1.2 (0.0 to 3.5)	-12.7 (-35.0 to -6.6)	-8.4 (-28.1 to -3.4)
Diet high in sodium	28.9 (20.1 to 37.2)	18.7 (9.8 to 25.5)	16.0 (7.3 to 23.2)	-35.6 (-58.3 to -17.9)	-14.0 (-37.1 to 1.9)	32.1 (23.8 to 39.0)	19.4 (11.0 to 26.1)	16.1 (7.5 to 23.1)	-39.7 (-59.6 to -24.9)	-16.9 (-38.4 to -3.9)
Intimate partner violence	-	-	-	-	-	2.2 (1.8 to 2.6)	2.0 (1.7 to 2.3)	2.0 (1.6 to 2.3)	-7.3 (-11.4 to -2.2)	-3.8 (-7.8 to 0.3)
Childhood sexual abuse	5.3 (4.3 to 6.5)	5.3 (4.3 to 6.5)	5.3 (4.3 to 6.5)	0.0 (0.0 to 0.0)	0.0 (0.0 to 0.0)	7.3 (5.9 to 8.9)	7.3 (5.9 to 8.9)	7.5 (6.0 to 9.2)	0.0 (0.0 to 0.0)	2.8 (-4.0 to 10.6)

Supplementary Results Table 1. Aichi age-standardised Summary Exposure Values (SEVs) for Level 4 risk factors and percentage change, by sex, 1990, 2007, and 2017.Data in parentheses are 95% uncertainty intervals.

	Male					Female				
	**1990**	**2007**	**2017**	**Percentage change 1990-2007**	**Percentage change 2007-2017**	**1990**	**2007**	**2017**	**Percentage change 1990-2007**	**Percentage change 2007-2017**
Bullying victimization	3.2 (1.4 to 6.8)	3.3 (1.4 to 6.9)	3.4 (1.4 to 7.2)	0.8 (-7.2 to 8.8)	5.0 (-0.4 to 9.9)	1.9 (0.8 to 3.9)	1.9 (0.8 to 4.0)	2.0 (0.8 to 4.1)	-0.5 (-8.3 to 8.0)	4.0 (-1.6 to 9.1)
Low physical activity	0.5 (0.2 to 0.9)	0.5 (0.2 to 0.9)	0.4 (0.2 to 0.8)	-1.7 (-3.7 to 0.1)	-8.9 (-12.7 to -4.7)	0.5 (0.2 to 1.0)	0.5 (0.2 to 0.9)	0.5 (0.2 to 0.9)	-3.7 (-5.8 to -1.2)	0.9 (-0.8 to 2.4)
High fasting plasma glucose	1.4 (0.9 to 2.2)	1.4 (0.9 to 2.2)	1.6 (1.0 to 2.6)	1.7 (-34.8 to 64.2)	12.9 (-24.6 to 80.4)	1.2 (0.7 to 2.0)	1.3 (0.8 to 2.2)	1.4 (0.8 to 2.4)	7.9 (-32.5 to 81.2)	10.1 (-30.7 to 87.7)
High LDL cholesterol	12.1 (10.5 to 13.9)	12.7 (11.0 to 14.4)	12.7 (11.0 to 14.5)	4.6 (-1.3 to 11.0)	-0.1 (-4.6 to 4.6)	13.0 (11.5 to 14.8)	13.1 (11.6 to 14.9)	13.2 (11.6 to 14.9)	0.7 (-4.8 to 6.1)	0.2 (-3.9 to 4.6)
High systolic blood pressure	11.4 (10.4 to 12.4)	8.5 (7.6 to 9.4)	8.3 (7.4 to 9.3)	-25.8 (-31.2 to -20.0)	-1.6 (-8.9 to 5.8)	9.7 (8.8 to 10.7)	6.1 (5.4 to 6.9)	5.7 (4.9 to 6.4)	-37.1 (-43.6 to -30.6)	-7.3 (-16.8 to 3.1)
High body-mass index	7.5 (5.0 to 11.2)	11.0 (7.9 to 15.7)	11.4 (8.2 to 16.6)	47.1 (19.7 to 84.1)	3.7 (-10.8 to 19.6)	6.2 (3.9 to 9.6)	8.1 (5.4 to 12.5)	8.1 (5.2 to 12.7)	29.7 (2.1 to 68.1)	0.4 (-15.8 to 21.3)
Low bone mineral density	5.8 (4.8 to 6.9)	5.8 (4.9 to 6.8)	5.7 (4.7 to 6.8)	0.0 (-12.0 to 14.9)	-1.5 (-13.5 to 13.4)	6.9 (5.8 to 8.0)	6.9 (5.9 to 8.0)	6.9 (5.8 to 8.1)	0.5 (-11.1 to 14.8)	-0.0 (-12.4 to 13.1)
Impaired kidney function	2.6 (1.2 to 6.5)	2.5 (1.2 to 6.4)	2.4 (1.1 to 6.1)	-3.6 (-8.1 to 0.2)	-6.5 (-12.2 to -1.9)	3.2 (1.5 to 7.4)	3.3 (1.5 to 7.6)	3.1 (1.4 to 7.2)	2.7 (-1.7 to 7.9)	-7.2 (-12.0 to -2.4)

Supplementary Results Table 1. Akita age-standardised Summary Exposure Values (SEVs) for Level 4 risk factors and percentage change, by sex, 1990, 2007, and 2017.Data in parentheses are 95% uncertainty intervals.										
	Male					Female				
	**1990**	**2007**	**2017**	**Percentage change 1990-2007**	**Percentage change 2007-2017**	**1990**	**2007**	**2017**	**Percentage change 1990-2007**	**Percentage change 2007-2017**
Unsafe water source	2.6 (1.4 to 4.4)	2.2 (1.2 to 3.6)	2.3 (1.2 to 3.8)	-16.6 (-56.6 to 78.4)	6.0 (-38.0 to 77.1)	2.6 (1.4 to 4.4)	2.2 (1.2 to 3.6)	2.3 (1.2 to 3.8)	-16.6 (-56.6 to 78.4)	6.0 (-38.0 to 77.1)
Unsafe sanitation	4.7 (2.3 to 8.5)	2.4 (1.1 to 4.6)	2.0 (0.9 to 3.9)	-49.3 (-78.6 to 17.7)	-15.7 (-56.8 to 64.8)	4.7 (2.3 to 8.5)	2.4 (1.1 to 4.6)	2.0 (0.9 to 3.9)	-49.3 (-78.6 to 17.7)	-15.7 (-56.8 to 64.8)
No access to handwashing facility	6.9 (5.0 to 9.3)	6.5 (4.6 to 8.8)	6.0 (4.4 to 8.1)	-5.8 (-32.4 to 31.9)	-7.6 (-27.2 to 18.1)	6.9 (5.0 to 9.3)	6.5 (4.6 to 8.8)	6.0 (4.4 to 8.1)	-5.8 (-32.4 to 31.9)	-7.6 (-27.2 to 18.1)
Ambient particulate matter pollution	24.7 (12.4 to 42.3)	25.3 (15.4 to 40.6)	21.0 (7.6 to 36.8)	2.4 (-41.2 to 100.7)	-17.1 (-67.2 to 43.7)	24.3 (12.1 to 41.7)	25.3 (15.3 to 40.5)	20.9 (7.6 to 36.7)	3.8 (-40.7 to 103.7)	-17.0 (-67.2 to 44.0)
Household air pollution from solid fuels	1.1 (0.4 to 3.5)	0.3 (0.1 to 0.7)	0.2 (0.0 to 0.6)	-76.7 (-92.3 to -46.1)	-17.8 (-70.3 to 79.0)	1.5 (0.4 to 7.4)	0.4 (0.1 to 3.0)	0.3 (0.1 to 2.8)	-72.5 (-92.2 to -31.3)	-17.8 (-70.0 to 75.5)
Ambient ozone pollution	47.9 (19.0 to 54.8)	47.9 (18.9 to 54.8)	48.3 (19.3 to 54.8)	-0.1 (-0.7 to 0.3)	0.8 (0.0 to 2.7)	47.9 (19.0 to 54.8)	47.9 (18.9 to 54.8)	48.3 (19.3 to 54.8)	-0.1 (-0.7 to 0.3)	0.8 (0.0 to 2.7)
Residential radon	17.6 (1.5 to 51.6)	17.6 (1.5 to 51.6)	17.6 (1.5 to 51.6)	0.0 (0.0 to 0.0)	0.0 (0.0 to 0.0)	17.6 (1.5 to 51.6)	17.6 (1.5 to 51.6)	17.6 (1.5 to 51.6)	0.0 (0.0 to 0.0)	0.0 (0.0 to 0.0)
Lead exposure	3.0 (0.4 to 6.6)	2.1 (0.3 to 5.1)	1.6 (0.2 to 4.1)	-29.8 (-42.7 to -21.3)	-25.6 (-40.7 to -17.7)	1.6 (0.1 to 4.5)	1.1 (0.1 to 3.4)	0.8 (0.0 to 2.8)	-33.6 (-49.7 to -24.5)	-26.3 (-47.3 to -17.0)
Occupational exposure to asbestos	1.4 (1.2 to 1.5)	1.9 (1.8 to 2.1)	1.8 (1.5 to 2.2)	43.8 (25.3 to 70.2)	-5.6 (-22.8 to 16.2)	0.3 (0.3 to 0.4)	0.4 (0.4 to 0.4)	0.4 (0.3 to 0.5)	13.5 (-5.7 to 45.4)	2.5 (-19.5 to 27.0)
Occupational exposure to arsenic	0.4 (0.0 to 1.0)	0.4 (0.0 to 0.9)	0.4 (0.0 to 1.0)	-	-	0.4 (0.0 to 0.9)	0.3 (0.0 to 0.8)	0.4 (0.0 to 0.9)	-	-
Occupational exposure to benzene	0.6 (0.0 to 1.7)	0.6 (0.0 to 1.8)	0.7 (0.0 to 1.8)	2.6 (-4.0 to 11.6)	2.8 (-2.8 to 11.9)	0.6 (0.0 to 1.5)	0.7 (0.0 to 2.0)	0.8 (0.0 to 2.3)	22.4 (8.4 to 41.2)	14.7 (4.2 to 30.5)
Occupational exposure to beryllium	0.0 (0.0 to 0.0)	0.0 (0.0 to 0.0)	0.0 (0.0 to 0.0)	-5.1 (-10.1 to -0.0)	1.8 (-2.2 to 5.9)	0.0 (0.0 to 0.0)	0.0 (0.0 to 0.0)	0.0 (0.0 to 0.0)	2.5 (-7.1 to 12.7)	9.5 (2.8 to 17.3)
Occupational exposure to cadmium	0.1 (0.0 to 0.1)	0.0 (0.0 to 0.1)	0.0 (0.0 to 0.1)	-6.4 (-17.2 to 5.8)	0.6 (-9.1 to 10.7)	0.0 (0.0 to 0.1)	0.0 (0.0 to 0.0)	0.0 (0.0 to 0.1)	-7.5 (-22.6 to 11.6)	5.3 (-7.1 to 19.8)
Occupational exposure to chromium	0.1 (0.1 to 0.1)	0.1 (0.1 to 0.1)	0.1 (0.1 to 0.1)	-4.6 (-14.4 to 6.9)	1.2 (-7.3 to 10.0)	0.1 (0.1 to 0.1)	0.1 (0.1 to 0.1)	0.1 (0.1 to 0.1)	-2.9 (-17.0 to 14.2)	7.1 (-3.8 to 19.4)
Occupational exposure to diesel engine exhaust	0.6 (0.5 to 0.6)	0.6 (0.5 to 0.6)	0.6 (0.5 to 0.6)	-1.0 (-9.2 to 7.6)	3.2 (-3.5 to 10.1)	0.4 (0.4 to 0.5)	0.5 (0.4 to 0.5)	0.5 (0.5 to 0.6)	12.9 (-0.5 to 28.6)	12.0 (1.5 to 23.8)
Occupational exposure to formaldehyde	0.2 (0.2 to 0.3)	0.2 (0.2 to 0.3)	0.2 (0.2 to 0.3)	-5.6 (-17.5 to 8.0)	0.1 (-10.1 to 10.7)	0.2 (0.2 to 0.2)	0.2 (0.2 to 0.2)	0.2 (0.2 to 0.2)	-4.7 (-20.7 to 14.4)	6.0 (-6.6 to 20.8)
Occupational exposure to nickel	0.4 (0.0 to 1.8)	0.4 (0.0 to 1.6)	0.4 (0.0 to 1.6)	-	-	0.3 (0.0 to 1.4)	0.3 (0.0 to 1.3)	0.3 (0.0 to 1.3)	-	-
Occupational exposure to polycyclic aromatic hydrocarbons	0.2 (0.2 to 0.3)	0.2 (0.2 to 0.2)	0.2 (0.2 to 0.3)	-3.7 (-13.1 to 7.2)	1.4 (-6.2 to 9.8)	0.2 (0.2 to 0.2)	0.2 (0.2 to 0.2)	0.2 (0.2 to 0.2)	-0.8 (-15.0 to 15.4)	7.9 (-2.4 to 19.8)
Occupational exposure to silica	4.4 (0.6 to 14.3)	4.2 (0.6 to 13.3)	4.2 (0.6 to 14.1)	-5.5 (-16.1 to 7.1)	1.0 (-7.6 to 12.0)	3.0 (0.4 to 9.4)	2.6 (0.4 to 8.6)	2.7 (0.4 to 8.8)	-12.1 (-27.1 to 8.2)	2.8 (-10.9 to 20.8)
Occupational exposure to sulphuric acid	0.6 (0.1 to 1.9)	0.6 (0.1 to 1.8)	0.6 (0.1 to 1.8)	-6.5 (-17.1 to 5.7)	0.9 (-8.1 to 10.4)	0.5 (0.1 to 1.5)	0.4 (0.1 to 1.4)	0.5 (0.1 to 1.4)	-9.0 (-24.4 to 9.1)	4.7 (-7.9 to 19.4)
Occupational exposure to trichloroethylene	0.1 (0.1 to 0.1)	0.1 (0.1 to 0.1)	0.1 (0.1 to 0.1)	-3.6 (-11.1 to 4.6)	1.8 (-4.2 to 8.2)	0.1 (0.1 to 0.1)	0.1 (0.1 to 0.1)	0.1 (0.1 to 0.1)	1.7 (-9.9 to 15.4)	8.8 (0.1 to 19.5)
Occupational asthmagens	17.3 (13.9 to 21.4)	17.0 (13.9 to 21.2)	17.0 (13.6 to 21.4)	-1.3 (-18.2 to 20.9)	-0.2 (-14.8 to 15.5)	7.9 (6.2 to 10.1)	8.0 (6.2 to 10.0)	8.5 (6.7 to 10.6)	0.8 (-16.1 to 20.1)	5.9 (-7.7 to 21.5)
Occupational particulate matter, gases, and fumes	6.4 (4.5 to 9.2)	6.1 (4.3 to 8.7)	5.9 (4.2 to 8.3)	-4.6 (-9.7 to 0.3)	-3.8 (-7.2 to -0.5)	3.6 (2.4 to 5.2)	3.4 (2.3 to 5.0)	3.2 (2.3 to 4.8)	-5.3 (-13.8 to 4.0)	-4.4 (-10.3 to 2.1)
Occupational noise	6.0 (5.5 to 6.5)	5.8 (5.4 to 6.3)	5.6 (5.2 to 6.1)	-3.5 (-6.5 to -0.1)	-2.6 (-4.7 to -0.5)	3.4 (2.9 to 3.9)	3.4 (3.0 to 3.9)	3.4 (3.0 to 4.0)	0.3 (-5.8 to 7.4)	1.7 (-2.3 to 6.3)
Occupational ergonomic factors	11.3 (9.3 to 13.7)	10.1 (8.4 to 12.1)	10.0 (8.2 to 12.3)	-10.6 (-23.5 to 5.1)	-0.3 (-12.7 to 14.1)	6.0 (4.6 to 7.8)	6.1 (4.7 to 7.9)	6.9 (5.4 to 9.2)	1.6 (-21.8 to 32.1)	13.4 (-7.9 to 40.1)
Non-exclusive breastfeeding	0.4 (0.3 to 0.6)	0.4 (0.3 to 0.6)	0.4 (0.3 to 0.6)	-1.3 (-15.5 to 12.6)	-1.1 (-11.7 to 9.7)	0.4 (0.3 to 0.6)	0.4 (0.3 to 0.6)	0.4 (0.3 to 0.6)	-1.0 (-15.3 to 13.0)	-1.4 (-11.9 to 9.4)
Discontinued breastfeeding	1.6 (1.5 to 1.8)	1.7 (1.5 to 1.8)	1.6 (1.5 to 1.8)	0.9 (-8.0 to 11.7)	-1.5 (-8.9 to 6.5)	1.6 (1.5 to 1.8)	1.7 (1.5 to 1.8)	1.6 (1.5 to 1.8)	2.2 (-6.8 to 13.1)	-2.3 (-9.6 to 5.6)
Child underweight	0.3 (0.2 to 0.4)	0.2 (0.1 to 0.3)	0.2 (0.1 to 0.3)	-15.0 (-34.1 to 8.0)	-5.9 (-21.7 to 10.8)	0.3 (0.2 to 0.4)	0.2 (0.1 to 0.3)	0.2 (0.1 to 0.3)	-17.1 (-39.7 to 9.2)	-7.0 (-23.7 to 11.2)
Child wasting	0.2 (0.1 to 0.3)	0.2 (0.1 to 0.3)	0.2 (0.1 to 0.3)	-7.2 (-34.1 to 33.7)	-2.6 (-22.4 to 19.8)	0.2 (0.1 to 0.3)	0.2 (0.1 to 0.3)	0.2 (0.1 to 0.3)	-8.1 (-35.7 to 28.5)	-3.0 (-24.0 to 24.4)
Child stunting	0.6 (0.3 to 1.0)	0.6 (0.3 to 1.0)	0.5 (0.3 to 1.0)	-10.2 (-45.5 to 45.5)	-2.1 (-31.4 to 35.9)	0.6 (0.3 to 1.1)	0.6 (0.3 to 1.0)	0.5 (0.3 to 0.9)	-12.1 (-45.6 to 49.9)	-3.6 (-34.7 to 35.9)
Short gestation for birth weight	0.0 (0.0 to 0.0)	0.0 (0.0 to 0.0)	0.0 (0.0 to 0.0)	10.1 (-17.6 to 44.0)	3.6 (-12.6 to 28.6)	0.0 (0.0 to 0.0)	0.0 (0.0 to 0.0)	0.0 (0.0 to 0.0)	13.4 (-13.8 to 46.1)	-1.6 (-24.1 to 24.4)
Low birth weight for gestation	0.0 (0.0 to 0.0)	0.0 (0.0 to 0.0)	0.0 (0.0 to 0.0)	4.2 (-15.9 to 34.6)	2.4 (-13.7 to 17.5)	0.0 (0.0 to 0.0)	0.0 (0.0 to 0.0)	0.0 (0.0 to 0.0)	-1.3 (-20.9 to 24.4)	6.4 (-10.0 to 31.1)
Iron deficiency	-	-	-	-	-	8.4 (5.8 to 11.6)	8.4 (5.8 to 11.6)	7.7 (5.3 to 10.7)	-0.0 (-21.3 to 26.2)	-8.2 (-23.9 to 8.8)
Vitamin A deficiency	1.1 (0.6 to 1.4)	0.9 (0.6 to 1.1)	0.8 (0.5 to 1.0)	-20.2 (-35.4 to 2.0)	-12.6 (-26.8 to 2.6)	0.8 (0.5 to 1.1)	0.9 (0.5 to 1.2)	0.7 (0.4 to 0.9)	9.1 (-7.7 to 30.8)	-20.0 (-35.3 to 1.9)
Zinc deficiency	0.5 (0.2 to 1.1)	0.4 (0.1 to 1.1)	0.4 (0.1 to 0.9)	-15.6 (-63.8 to 72.4)	-11.9 (-66.9 to 167.3)	0.5 (0.2 to 1.1)	0.3 (0.1 to 0.8)	0.4 (0.1 to 0.8)	-31.6 (-64.1 to 8.8)	8.4 (-59.9 to 170.2)
Smoking	18.1 (14.8 to 21.7)	12.8 (10.3 to 15.7)	10.8 (8.7 to 13.2)	-29.3 (-38.3 to -18.2)	-15.4 (-23.7 to -6.5)	4.1 (2.9 to 5.6)	3.4 (2.4 to 4.6)	3.4 (2.4 to 4.6)	-16.5 (-36.8 to 11.6)	-1.7 (-18.6 to 18.6)
Chewing tobacco	1.4 (0.9 to 2.0)	0.9 (0.6 to 1.4)	0.8 (0.5 to 1.1)	-33.0 (-56.1 to 6.4)	-18.8 (-43.2 to 16.6)	0.1 (0.1 to 0.2)	0.1 (0.1 to 0.1)	0.1 (0.1 to 0.1)	-4.6 (-41.0 to 57.9)	-1.9 (-30.9 to 43.0)
Second-hand smoke	37.8 (33.6 to 42.0)	35.3 (31.1 to 39.4)	35.4 (31.0 to 40.0)	-6.5 (-17.4 to 5.8)	0.3 (-9.4 to 10.6)	57.2 (53.7 to 60.8)	47.3 (43.3 to 50.9)	47.2 (42.9 to 51.2)	-17.4 (-23.6 to -11.5)	-0.2 (-6.8 to 6.3)
Alcohol use	14.4 (7.2 to 25.0)	14.6 (7.4 to 25.1)	17.2 (8.3 to 30.0)	1.5 (-34.1 to 50.2)	18.2 (-18.6 to 65.0)	11.3 (5.5 to 20.1)	11.4 (5.6 to 20.2)	13.1 (6.2 to 23.8)	0.8 (-33.2 to 50.0)	14.8 (-20.3 to 61.1)
Drug use	0.5 (0.4 to 0.7)	0.5 (0.4 to 0.7)	0.6 (0.4 to 0.8)	0.2 (-6.2 to 6.3)	11.5 (3.7 to 20.8)	0.3 (0.2 to 0.4)	0.3 (0.2 to 0.4)	0.3 (0.3 to 0.4)	1.2 (-4.7 to 7.3)	9.7 (2.4 to 18.2)
Diet low in fruits	39.5 (33.6 to 43.4)	39.1 (33.6 to 43.2)	40.7 (35.0 to 44.3)	-0.8 (-7.4 to 6.1)	3.8 (-0.9 to 9.7)	36.2 (30.1 to 40.9)	34.9 (28.9 to 39.6)	37.4 (31.4 to 41.8)	-3.6 (-12.1 to 5.4)	7.2 (0.4 to 15.3)
Diet low in vegetables	19.3 (10.7 to 26.7)	14.0 (6.2 to 21.4)	16.1 (7.9 to 24.0)	-27.5 (-56.1 to -2.2)	14.9 (-16.7 to 67.8)	20.5 (11.8 to 27.6)	15.6 (7.0 to 23.3)	17.6 (9.3 to 25.4)	-24.1 (-51.9 to 3.2)	12.9 (-14.8 to 57.9)
Diet low in legumes	4.4 (0.8 to 9.7)	5.3 (1.1 to 11.3)	7.0 (1.8 to 13.4)	20.9 (-48.9 to 229.0)	32.1 (-20.1 to 208.7)	8.2 (2.6 to 14.8)	7.9 (2.4 to 14.2)	10.2 (3.8 to 17.2)	-4.0 (-48.5 to 79.7)	29.3 (-13.3 to 131.7)
Diet low in whole grains	31.0 (25.9 to 35.3)	35.7 (31.7 to 39.1)	37.0 (33.3 to 40.2)	15.3 (6.4 to 28.6)	3.7 (-1.1 to 9.5)	32.4 (27.9 to 36.2)	36.9 (33.2 to 40.2)	38.2 (34.5 to 41.1)	14.0 (6.5 to 23.9)	3.4 (-0.8 to 8.2)
Diet low in nuts and seeds	50.9 (49.7 to 51.9)	49.7 (47.8 to 51.0)	49.5 (47.5 to 50.9)	-2.5 (-5.0 to -0.5)	-0.4 (-2.6 to 1.5)	51.0 (49.7 to 52.0)	49.6 (47.8 to 50.8)	49.2 (47.3 to 50.8)	-2.9 (-5.4 to -1.0)	-0.7 (-2.9 to 1.3)
Diet low in milk	45.8 (43.8 to 47.6)	46.5 (44.6 to 48.2)	47.1 (45.2 to 48.7)	1.5 (-0.5 to 3.6)	1.2 (-0.3 to 2.6)	45.6 (43.3 to 47.4)	46.0 (44.0 to 47.8)	46.8 (44.8 to 48.5)	0.9 (-1.3 to 3.1)	1.7 (0.2 to 3.4)
Diet high in red meat	3.5 (0.3 to 8.8)	7.0 (2.0 to 13.5)	8.2 (2.6 to 14.7)	98.5 (-18.5 to 1109.2)	16.1 (-43.3 to 145.9)	0.5 (0.0 to 2.4)	1.4 (0.0 to 4.3)	1.9 (0.0 to 5.7)	180.5 (-76.6 to 7794576.8)	33.8 (-87.0 to 2575.5)
Diet high in processed meat	10.7 (6.5 to 14.8)	17.6 (12.6 to 22.7)	20.4 (14.6 to 26.0)	65.3 (29.5 to 136.0)	15.5 (-2.1 to 39.6)	8.9 (5.2 to 12.7)	13.0 (9.0 to 16.7)	14.2 (9.7 to 18.3)	46.5 (14.2 to 103.3)	9.6 (-8.2 to 32.3)
Diet high in sugar-sweetened beverages	6.7 (2.7 to 13.6)	7.7 (3.4 to 14.2)	8.2 (3.5 to 14.8)	15.2 (-46.9 to 145.5)	5.8 (-39.1 to 81.5)	4.8 (1.7 to 10.7)	5.4 (2.4 to 10.7)	5.8 (2.5 to 11.8)	12.6 (-50.2 to 160.6)	6.1 (-39.5 to 83.5)
Diet low in fibre	22.2 (14.9 to 29.0)	22.8 (15.8 to 29.0)	23.5 (16.3 to 29.9)	2.9 (-14.1 to 24.6)	3.2 (-10.2 to 18.7)	23.3 (15.8 to 29.9)	23.8 (16.9 to 29.8)	25.0 (17.6 to 31.3)	2.3 (-13.0 to 23.5)	4.8 (-7.1 to 19.9)
Diet low in calcium	34.8 (30.3 to 38.4)	34.9 (30.6 to 38.4)	35.8 (31.5 to 39.2)	0.2 (-3.4 to 3.9)	2.7 (-0.2 to 6.0)	36.9 (32.8 to 40.0)	36.2 (32.2 to 39.6)	37.6 (33.8 to 40.8)	-1.7 (-5.2 to 1.7)	3.8 (1.2 to 7.3)
Diet low in seafood omega-3 fatty acids	3.4 (1.8 to 4.8)	0.4 (0.0 to 1.3)	0.4 (0.0 to 1.4)	-88.8 (-99.9 to -61.6)	5.8 (-81.7 to 601.5)	3.9 (2.2 to 5.2)	0.6 (0.0 to 1.7)	0.6 (0.0 to 1.7)	-85.7 (-99.8 to -56.4)	6.9 (-71.3 to 452.6)
Diet low in polyunsaturated fatty acids	31.1 (25.6 to 35.9)	28.4 (22.8 to 33.2)	29.1 (23.2 to 33.7)	-8.7 (-20.7 to 1.0)	2.3 (-5.8 to 11.4)	31.7 (26.5 to 36.6)	29.1 (23.7 to 33.8)	29.8 (24.5 to 34.7)	-8.3 (-18.3 to 1.0)	2.6 (-5.4 to 13.8)
Diet high in trans fatty acids	1.1 (0.0 to 3.3)	0.9 (0.0 to 3.0)	0.9 (0.0 to 2.9)	-12.5 (-39.3 to -6.6)	-8.1 (-31.8 to -3.3)	1.5 (0.1 to 4.0)	1.3 (0.1 to 3.7)	1.2 (0.0 to 3.5)	-12.6 (-36.4 to -6.4)	-8.5 (-29.0 to -3.6)
Diet high in sodium	30.7 (21.9 to 38.5)	19.5 (11.0 to 26.7)	16.7 (8.1 to 23.8)	-36.3 (-57.0 to -18.4)	-14.3 (-34.4 to 1.6)	36.0 (28.2 to 42.6)	21.1 (13.0 to 27.9)	17.3 (8.8 to 24.2)	-41.6 (-60.1 to -27.3)	-17.9 (-39.4 to -5.7)
Intimate partner violence	-	-	-	-	-	2.3 (1.9 to 2.6)	2.1 (1.8 to 2.4)	2.0 (1.7 to 2.3)	-7.5 (-11.8 to -3.2)	-4.2 (-8.8 to 0.5)
Childhood sexual abuse	5.3 (4.3 to 6.5)	5.3 (4.3 to 6.5)	5.3 (4.3 to 6.5)	0.0 (0.0 to 0.0)	0.0 (0.0 to 0.0)	7.3 (5.9 to 8.9)	7.3 (5.9 to 8.9)	7.5 (6.0 to 9.2)	0.0 (0.0 to 0.0)	2.8 (-4.0 to 10.6)

Supplementary Results Table 1. Akita age-standardised Summary Exposure Values (SEVs) for Level 4 risk factors and percentage change, by sex, 1990, 2007, and 2017.Data in parentheses are 95% uncertainty intervals.

	Male					Female				
	**1990**	**2007**	**2017**	**Percentage change 1990-2007**	**Percentage change 2007-2017**	**1990**	**2007**	**2017**	**Percentage change 1990-2007**	**Percentage change 2007-2017**
Bullying victimization	3.3 (1.4 to 6.7)	3.4 (1.5 to 7.1)	3.5 (1.5 to 7.2)	3.4 (-4.3 to 11.4)	2.9 (-2.4 to 7.6)	1.9 (0.8 to 3.8)	2.0 (0.8 to 3.8)	2.0 (0.9 to 4.0)	1.7 (-5.9 to 9.5)	2.1 (-3.4 to 6.9)
Low physical activity	0.5 (0.2 to 0.9)	0.5 (0.2 to 0.9)	0.4 (0.2 to 0.8)	-1.8 (-3.9 to 0.1)	-8.8 (-12.5 to -4.5)	0.5 (0.2 to 1.0)	0.5 (0.2 to 0.9)	0.5 (0.2 to 0.9)	-3.9 (-6.1 to -1.3)	0.7 (-0.8 to 2.2)
High fasting plasma glucose	1.4 (0.9 to 2.3)	1.4 (0.8 to 2.1)	1.5 (0.9 to 2.5)	-4.4 (-39.6 to 47.0)	12.7 (-23.8 to 71.3)	1.1 (0.7 to 1.9)	1.2 (0.7 to 2.0)	1.3 (0.8 to 2.3)	4.0 (-34.8 to 73.1)	11.6 (-27.6 to 84.0)
High LDL cholesterol	11.9 (10.3 to 13.7)	12.4 (10.8 to 14.2)	12.4 (10.8 to 14.2)	4.3 (-1.6 to 10.7)	0.0 (-4.7 to 5.3)	12.7 (11.2 to 14.4)	12.8 (11.2 to 14.5)	12.8 (11.2 to 14.6)	0.8 (-4.5 to 6.5)	0.3 (-4.1 to 4.7)
High systolic blood pressure	11.1 (10.1 to 12.2)	8.2 (7.3 to 9.1)	8.0 (7.1 to 9.1)	-26.4 (-32.6 to -20.1)	-1.8 (-8.9 to 6.8)	9.6 (8.6 to 10.6)	6.0 (5.2 to 6.7)	5.6 (4.8 to 6.4)	-37.3 (-44.1 to -30.3)	-7.1 (-16.8 to 4.0)
High body-mass index	4.7 (2.9 to 7.4)	6.5 (4.2 to 10.4)	6.6 (4.2 to 10.7)	39.0 (10.5 to 78.8)	1.6 (-14.5 to 18.5)	5.1 (3.0 to 8.3)	6.4 (4.0 to 10.2)	6.4 (3.8 to 10.4)	27.4 (-0.3 to 69.0)	-0.3 (-17.5 to 20.9)
Low bone mineral density	5.3 (4.4 to 6.2)	5.2 (4.4 to 6.0)	5.2 (4.2 to 6.2)	-2.3 (-15.4 to 13.0)	0.5 (-13.0 to 14.6)	6.3 (5.2 to 7.5)	6.0 (5.1 to 7.0)	6.1 (5.1 to 7.2)	-4.3 (-16.3 to 10.5)	0.9 (-12.4 to 15.3)
Impaired kidney function	2.6 (1.2 to 6.4)	2.5 (1.2 to 6.4)	2.4 (1.1 to 6.2)	-2.2 (-6.9 to 2.2)	-6.6 (-11.7 to -2.0)	3.2 (1.5 to 7.4)	3.3 (1.5 to 7.6)	3.1 (1.4 to 7.2)	3.8 (-0.1 to 9.0)	-6.9 (-12.1 to -2.0)

Supplementary Results Table 1. Aomori age-standardised Summary Exposure Values (SEVs) for Level 4 risk factors and percentage change, by sex, 1990, 2007, and 2017.Data in parentheses are 95% uncertainty intervals.

	Male					Female				
	**1990**	**2007**	**2017**	**Percentage change 1990-2007**	**Percentage change 2007-2017**	**1990**	**2007**	**2017**	**Percentage change 1990-2007**	**Percentage change 2007-2017**
Unsafe water source	2.7 (1.5 to 4.4)	2.2 (1.2 to 3.6)	2.4 (1.2 to 4.1)	-18.6 (-58.4 to 63.5)	7.0 (-36.1 to 84.0)	2.7 (1.5 to 4.4)	2.2 (1.2 to 3.6)	2.4 (1.2 to 4.1)	-18.6 (-58.4 to 63.5)	7.0 (-36.1 to 84.0)
Unsafe sanitation	5.0 (2.5 to 9.2)	2.5 (1.2 to 4.6)	2.1 (1.0 to 4.1)	-50.1 (-78.2 to 12.3)	-14.7 (-56.7 to 67.8)	5.0 (2.5 to 9.2)	2.5 (1.2 to 4.6)	2.1 (1.0 to 4.1)	-50.1 (-78.2 to 12.3)	-14.7 (-56.7 to 67.8)
No access to handwashing facility	6.9 (4.8 to 9.4)	6.6 (4.7 to 8.9)	6.1 (4.4 to 8.2)	-5.1 (-33.8 to 33.7)	-7.6 (-28.0 to 19.8)	6.9 (4.8 to 9.4)	6.6 (4.7 to 8.9)	6.1 (4.4 to 8.2)	-5.1 (-33.8 to 33.7)	-7.6 (-28.0 to 19.8)
Ambient particulate matter pollution	23.9 (11.0 to 40.6)	24.9 (14.2 to 39.7)	20.3 (7.8 to 35.6)	4.1 (-40.7 to 133.4)	-18.5 (-69.6 to 45.1)	23.5 (10.3 to 40.1)	24.8 (14.2 to 39.6)	20.2 (7.8 to 35.6)	5.6 (-40.4 to 143.1)	-18.5 (-69.6 to 45.1)
Household air pollution from solid fuels	1.2 (0.4 to 3.9)	0.3 (0.1 to 0.9)	0.2 (0.0 to 0.6)	-77.0 (-92.7 to -47.3)	-20.0 (-72.0 to 71.2)	1.6 (0.5 to 7.4)	0.4 (0.1 to 2.4)	0.4 (0.1 to 2.1)	-73.2 (-92.3 to -23.7)	-19.8 (-72.6 to 70.7)
Ambient ozone pollution	46.2 (17.5 to 54.8)	45.7 (17.2 to 54.8)	46.2 (17.5 to 54.8)	-0.9 (-2.8 to 0.0)	1.1 (0.0 to 3.4)	46.2 (17.5 to 54.8)	45.7 (17.2 to 54.8)	46.2 (17.5 to 54.8)	-0.9 (-2.8 to 0.0)	1.1 (0.0 to 3.4)
Residential radon	18.2 (1.6 to 55.7)	18.2 (1.6 to 55.7)	18.2 (1.6 to 55.7)	0.0 (0.0 to 0.0)	0.0 (0.0 to 0.0)	18.2 (1.6 to 55.7)	18.2 (1.6 to 55.7)	18.2 (1.6 to 55.7)	0.0 (0.0 to 0.0)	0.0 (0.0 to 0.0)
Lead exposure	3.1 (0.5 to 6.9)	2.2 (0.3 to 5.3)	1.6 (0.2 to 4.3)	-30.2 (-43.2 to -21.6)	-25.9 (-40.4 to -18.3)	1.7 (0.1 to 4.6)	1.1 (0.1 to 3.5)	0.8 (0.0 to 2.8)	-33.8 (-49.4 to -24.9)	-26.8 (-47.6 to -18.4)
Occupational exposure to asbestos	1.3 (1.1 to 1.5)	1.9 (1.7 to 2.1)	1.9 (1.5 to 2.3)	45.7 (26.4 to 72.9)	-1.1 (-19.1 to 23.4)	0.3 (0.3 to 0.4)	0.4 (0.3 to 0.4)	0.4 (0.3 to 0.5)	6.6 (-11.1 to 34.1)	1.1 (-19.2 to 25.3)
Occupational exposure to arsenic	0.4 (0.0 to 1.0)	0.4 (0.0 to 1.0)	0.4 (0.0 to 1.0)	-	-	0.4 (0.0 to 0.9)	0.4 (0.0 to 0.8)	0.4 (0.0 to 0.9)	-	-
Occupational exposure to benzene	0.6 (0.0 to 1.8)	0.6 (0.0 to 1.8)	0.7 (0.0 to 1.9)	2.5 (-4.6 to 12.4)	3.6 (-2.3 to 11.6)	0.6 (0.0 to 1.7)	0.7 (0.0 to 1.9)	0.8 (0.0 to 2.2)	21.8 (8.0 to 46.8)	14.7 (4.4 to 31.1)
Occupational exposure to beryllium	0.0 (0.0 to 0.0)	0.0 (0.0 to 0.0)	0.0 (0.0 to 0.0)	-5.0 (-10.4 to 0.3)	2.0 (-1.9 to 6.1)	0.0 (0.0 to 0.0)	0.0 (0.0 to 0.0)	0.0 (0.0 to 0.0)	2.6 (-7.3 to 12.8)	9.5 (2.4 to 16.7)
Occupational exposure to cadmium	0.1 (0.0 to 0.1)	0.0 (0.0 to 0.1)	0.0 (0.0 to 0.1)	-6.0 (-17.9 to 7.6)	1.1 (-8.4 to 11.6)	0.0 (0.0 to 0.1)	0.0 (0.0 to 0.0)	0.0 (0.0 to 0.1)	-7.6 (-23.6 to 10.9)	5.4 (-7.6 to 20.3)
Occupational exposure to chromium	0.1 (0.1 to 0.1)	0.1 (0.1 to 0.1)	0.1 (0.1 to 0.1)	-4.2 (-14.8 to 7.8)	1.7 (-6.7 to 10.7)	0.1 (0.1 to 0.1)	0.1 (0.1 to 0.1)	0.1 (0.1 to 0.1)	-2.9 (-17.4 to 14.3)	7.2 (-4.2 to 19.5)
Occupational exposure to diesel engine exhaust	0.6 (0.5 to 0.6)	0.6 (0.5 to 0.6)	0.6 (0.5 to 0.6)	-1.2 (-8.8 to 7.7)	3.2 (-3.2 to 10.1)	0.4 (0.4 to 0.5)	0.5 (0.4 to 0.5)	0.5 (0.5 to 0.6)	13.2 (-0.1 to 28.9)	11.9 (1.7 to 24.6)
Occupational exposure to formaldehyde	0.2 (0.2 to 0.3)	0.2 (0.2 to 0.3)	0.2 (0.2 to 0.3)	-5.3 (-17.6 to 9.0)	0.8 (-9.3 to 12.0)	0.2 (0.2 to 0.2)	0.2 (0.2 to 0.2)	0.2 (0.2 to 0.2)	-4.9 (-21.2 to 14.5)	6.2 (-7.2 to 21.4)
Occupational exposure to nickel	0.4 (0.0 to 1.9)	0.4 (0.0 to 1.7)	0.4 (0.0 to 1.7)	-	-	0.3 (0.0 to 1.5)	0.3 (0.0 to 1.3)	0.3 (0.0 to 1.3)	-	-
Occupational exposure to polycyclic aromatic hydrocarbons	0.2 (0.2 to 0.3)	0.2 (0.2 to 0.2)	0.2 (0.2 to 0.3)	-3.4 (-13.3 to 7.9)	2.0 (-5.9 to 10.3)	0.2 (0.2 to 0.2)	0.2 (0.2 to 0.2)	0.2 (0.2 to 0.3)	-0.9 (-14.6 to 16.0)	8.0 (-2.9 to 19.7)
Occupational exposure to silica	4.5 (0.7 to 14.4)	4.3 (0.7 to 13.8)	4.3 (0.6 to 13.9)	-5.9 (-17.0 to 6.4)	1.3 (-7.3 to 12.7)	3.1 (0.5 to 9.7)	2.7 (0.4 to 8.4)	2.7 (0.4 to 8.5)	-12.6 (-28.0 to 7.2)	2.4 (-10.9 to 20.0)
Occupational exposure to sulphuric acid	0.6 (0.1 to 1.9)	0.6 (0.1 to 1.8)	0.6 (0.1 to 1.8)	-6.2 (-17.2 to 7.0)	1.3 (-7.5 to 11.6)	0.5 (0.1 to 1.6)	0.5 (0.1 to 1.4)	0.5 (0.1 to 1.4)	-9.3 (-25.6 to 9.0)	4.5 (-8.7 to 20.0)
Occupational exposure to trichloroethylene	0.1 (0.1 to 0.1)	0.1 (0.1 to 0.1)	0.1 (0.1 to 0.1)	-3.2 (-11.1 to 5.3)	2.1 (-3.7 to 8.7)	0.1 (0.1 to 0.1)	0.1 (0.1 to 0.1)	0.1 (0.1 to 0.1)	1.9 (-10.5 to 17.0)	8.8 (-0.3 to 19.2)
Occupational asthmagens	17.4 (14.2 to 22.1)	17.1 (13.6 to 21.6)	17.2 (13.6 to 22.0)	-1.9 (-20.4 to 20.3)	0.6 (-14.2 to 17.6)	8.0 (6.4 to 10.1)	8.0 (6.4 to 9.9)	8.5 (6.8 to 10.5)	0.3 (-15.1 to 18.5)	5.4 (-7.9 to 19.8)
Occupational particulate matter, gases, and fumes	6.4 (4.6 to 9.2)	6.1 (4.4 to 8.7)	5.9 (4.2 to 8.4)	-4.4 (-9.6 to 0.9)	-3.6 (-7.0 to -0.3)	3.6 (2.4 to 5.3)	3.4 (2.3 to 5.1)	3.3 (2.3 to 4.8)	-5.1 (-13.7 to 5.0)	-4.3 (-10.3 to 2.3)
Occupational noise	6.0 (5.5 to 6.6)	5.8 (5.4 to 6.3)	5.6 (5.2 to 6.1)	-3.5 (-6.8 to 0.0)	-2.6 (-4.8 to -0.5)	3.4 (2.9 to 3.9)	3.4 (3.0 to 3.9)	3.5 (3.1 to 3.9)	0.5 (-5.9 to 7.9)	1.8 (-2.3 to 6.5)
Occupational ergonomic factors	11.2 (9.2 to 13.5)	10.0 (8.3 to 12.1)	9.9 (8.0 to 12.2)	-10.7 (-23.7 to 5.0)	-0.9 (-12.4 to 12.3)	6.0 (4.6 to 7.8)	6.1 (4.6 to 7.6)	6.8 (5.2 to 8.7)	0.6 (-21.8 to 28.0)	12.0 (-8.5 to 36.3)
Non-exclusive breastfeeding	0.4 (0.3 to 0.6)	0.4 (0.3 to 0.6)	0.4 (0.3 to 0.6)	-1.6 (-14.0 to 13.6)	-1.8 (-11.9 to 9.9)	0.4 (0.3 to 0.6)	0.4 (0.3 to 0.6)	0.4 (0.3 to 0.6)	-1.5 (-13.9 to 13.7)	-1.3 (-11.5 to 10.4)
Discontinued breastfeeding	1.6 (1.5 to 1.8)	1.6 (1.5 to 1.8)	1.6 (1.5 to 1.8)	0.0 (-9.8 to 10.6)	0.5 (-6.9 to 9.1)	1.6 (1.5 to 1.8)	1.6 (1.5 to 1.8)	1.6 (1.5 to 1.8)	2.1 (-8.0 to 12.9)	-1.6 (-8.8 to 6.8)
Child underweight	0.3 (0.2 to 0.4)	0.2 (0.1 to 0.3)	0.2 (0.1 to 0.3)	-15.9 (-35.1 to 5.5)	-5.2 (-20.9 to 12.6)	0.3 (0.2 to 0.4)	0.2 (0.1 to 0.3)	0.2 (0.1 to 0.3)	-17.5 (-38.4 to 7.1)	-6.7 (-23.0 to 13.8)
Child wasting	0.2 (0.1 to 0.3)	0.2 (0.1 to 0.3)	0.2 (0.1 to 0.3)	-6.1 (-32.7 to 31.4)	-2.0 (-22.7 to 22.7)	0.2 (0.1 to 0.3)	0.2 (0.1 to 0.3)	0.2 (0.1 to 0.3)	-7.9 (-34.6 to 28.2)	-2.7 (-23.3 to 24.2)
Child stunting	0.7 (0.3 to 1.1)	0.6 (0.3 to 1.0)	0.6 (0.3 to 1.0)	-8.8 (-44.3 to 49.7)	-4.3 (-32.6 to 35.2)	0.7 (0.3 to 1.2)	0.6 (0.3 to 1.1)	0.6 (0.3 to 1.0)	-11.8 (-47.7 to 44.4)	-4.1 (-34.2 to 35.7)
Short gestation for birth weight	0.0 (0.0 to 0.0)	0.0 (0.0 to 0.0)	0.0 (0.0 to 0.0)	10.6 (-8.6 to 40.2)	3.9 (-14.3 to 34.0)	0.0 (0.0 to 0.0)	0.0 (0.0 to 0.0)	0.0 (0.0 to 0.0)	14.2 (-18.8 to 94.8)	2.3 (-21.6 to 39.3)
Low birth weight for gestation	0.0 (0.0 to 0.0)	0.0 (0.0 to 0.0)	0.0 (0.0 to 0.0)	3.8 (-14.5 to 24.5)	1.5 (-16.7 to 20.2)	0.0 (0.0 to 0.0)	0.0 (0.0 to 0.0)	0.0 (0.0 to 0.0)	-6.7 (-62.4 to 30.3)	2.9 (-20.0 to 24.9)
Iron deficiency	-	-	-	-	-	8.5 (5.9 to 11.7)	8.5 (6.0 to 11.4)	7.8 (5.3 to 10.7)	-0.5 (-21.0 to 24.9)	-8.3 (-24.1 to 8.4)
Vitamin A deficiency	1.1 (0.7 to 1.5)	0.9 (0.6 to 1.2)	0.8 (0.5 to 1.0)	-18.7 (-31.0 to -1.1)	-12.8 (-28.9 to 7.3)	0.9 (0.6 to 1.2)	0.8 (0.5 to 1.1)	0.7 (0.4 to 1.0)	-8.0 (-27.2 to 27.2)	-7.4 (-27.2 to 8.7)
Zinc deficiency	0.5 (0.1 to 1.0)	0.4 (0.1 to 1.1)	0.4 (0.1 to 0.9)	-18.0 (-66.8 to 72.0)	-10.7 (-69.2 to 159.5)	0.5 (0.2 to 1.1)	0.4 (0.1 to 0.8)	0.4 (0.1 to 0.9)	-30.0 (-63.8 to 8.6)	8.0 (-60.0 to 170.7)
Smoking	18.1 (14.7 to 21.5)	12.8 (10.2 to 15.6)	10.9 (8.6 to 13.4)	-29.2 (-38.5 to -18.7)	-15.3 (-23.3 to -6.3)	4.1 (2.9 to 5.5)	3.5 (2.5 to 4.6)	3.4 (2.3 to 4.6)	-15.9 (-35.3 to 10.3)	-2.3 (-19.3 to 17.6)
Chewing tobacco	1.4 (0.9 to 2.0)	0.9 (0.6 to 1.4)	0.8 (0.5 to 1.2)	-33.1 (-57.9 to 6.5)	-18.9 (-40.4 to 9.7)	0.1 (0.1 to 0.1)	0.1 (0.1 to 0.1)	0.1 (0.1 to 0.1)	-4.1 (-40.3 to 57.2)	-2.0 (-32.9 to 44.0)
Second-hand smoke	36.9 (32.8 to 40.7)	36.1 (31.6 to 40.5)	37.0 (32.4 to 41.8)	-2.2 (-13.4 to 9.8)	2.7 (-7.0 to 13.1)	55.6 (51.9 to 59.3)	48.5 (44.6 to 52.3)	49.5 (45.0 to 54.1)	-12.7 (-19.0 to -5.6)	2.1 (-5.0 to 8.7)
Alcohol use	14.8 (7.6 to 25.4)	14.6 (7.1 to 24.8)	17.1 (8.0 to 29.0)	-1.1 (-34.0 to 47.7)	17.0 (-19.3 to 65.8)	11.5 (5.8 to 20.3)	11.4 (5.4 to 20.6)	13.4 (6.2 to 22.9)	-0.9 (-33.6 to 47.8)	18.5 (-18.2 to 70.8)
Drug use	0.5 (0.4 to 0.7)	0.5 (0.4 to 0.7)	0.6 (0.4 to 0.8)	0.1 (-5.7 to 6.4)	11.8 (4.4 to 20.3)	0.3 (0.2 to 0.4)	0.3 (0.2 to 0.4)	0.3 (0.3 to 0.4)	2.2 (-3.4 to 8.6)	9.2 (2.1 to 16.9)
Diet low in fruits	41.8 (35.7 to 45.5)	40.0 (34.2 to 43.9)	41.2 (35.5 to 45.1)	-4.3 (-10.0 to 1.0)	3.2 (-1.3 to 8.3)	38.7 (33.0 to 42.9)	36.0 (30.2 to 40.5)	38.1 (32.3 to 42.5)	-6.8 (-14.8 to 0.5)	5.8 (-0.2 to 13.3)
Diet low in vegetables	20.7 (11.9 to 28.3)	14.9 (6.7 to 22.9)	17.0 (8.5 to 25.0)	-28.0 (-54.7 to -3.6)	14.0 (-16.2 to 61.9)	23.0 (14.5 to 30.3)	16.7 (8.1 to 24.4)	18.4 (9.7 to 26.4)	-27.5 (-53.3 to -5.0)	10.7 (-12.6 to 50.6)
Diet low in legumes	4.2 (0.7 to 9.6)	5.2 (0.9 to 10.6)	6.9 (1.9 to 13.3)	23.3 (-51.9 to 270.0)	33.3 (-23.9 to 228.1)	8.2 (2.7 to 14.6)	7.7 (2.4 to 14.1)	10.0 (3.6 to 16.8)	-5.7 (-47.6 to 62.4)	29.5 (-13.1 to 137.1)
Diet low in whole grains	31.0 (26.2 to 35.1)	35.7 (31.3 to 39.1)	37.0 (33.0 to 40.3)	15.0 (6.8 to 26.7)	3.7 (-1.0 to 9.5)	32.4 (28.0 to 36.2)	36.9 (33.0 to 40.0)	38.2 (34.5 to 41.1)	13.9 (6.3 to 24.4)	3.4 (-1.0 to 8.2)
Diet low in nuts and seeds	51.1 (49.9 to 52.0)	49.8 (48.1 to 51.1)	49.5 (47.6 to 51.0)	-2.5 (-4.9 to -0.6)	-0.5 (-2.5 to 1.3)	51.1 (49.9 to 52.0)	49.7 (48.0 to 51.0)	49.4 (47.3 to 50.9)	-2.8 (-5.1 to -0.9)	-0.6 (-2.6 to 1.2)
Diet low in milk	46.5 (44.5 to 48.2)	46.8 (44.9 to 48.4)	47.3 (45.5 to 48.8)	0.7 (-1.1 to 2.5)	1.0 (-0.4 to 2.6)	46.5 (44.5 to 48.1)	46.2 (44.1 to 47.9)	46.8 (44.7 to 48.5)	-0.5 (-2.5 to 1.4)	1.3 (-0.2 to 2.9)
Diet high in red meat	3.6 (0.4 to 8.4)	7.1 (2.2 to 13.4)	8.2 (2.4 to 15.2)	97.8 (-19.6 to 868.9)	15.4 (-40.3 to 131.8)	0.5 (0.0 to 2.3)	1.3 (0.0 to 4.3)	1.7 (0.0 to 5.0)	175.5 (-80.1 to [illegible]	32.6 (-90.8 to 3479.4)
Diet high in processed meat	11.0 (6.9 to 15.1)	18.7 (13.6 to 23.5)	22.0 (16.3 to 27.2)	69.5 (33.2 to 130.0)	17.4 (-0.9 to 40.6)	8.9 (5.3 to 12.9)	13.3 (9.0 to 17.1)	14.7 (10.2 to 19.1)	48.3 (17.8 to 106.2)	10.8 (-7.0 to 33.9)
Diet high in sugar-sweetened beverages	6.5 (2.6 to 12.7)	7.5 (3.2 to 13.6)	8.1 (3.4 to 15.4)	15.1 (-45.6 to 148.7)	7.3 (-39.8 to 73.5)	4.8 (1.8 to 10.8)	5.3 (2.2 to 9.9)	5.8 (2.3 to 11.4)	11.4 (-49.3 to 159.6)	9.3 (-39.0 to 92.0)
Diet low in fibre	15.2 (8.1 to 21.7)	18.5 (11.2 to 25.2)	21.0 (13.6 to 27.8)	22.4 (-3.0 to 65.5)	13.0 (-3.4 to 35.0)	16.5 (9.4 to 23.5)	19.9 (12.6 to 26.2)	22.5 (15.3 to 28.9)	20.3 (-3.6 to 64.5)	13.2 (-0.3 to 35.2)
Diet low in calcium	35.6 (31.3 to 39.0)	35.3 (30.9 to 38.7)	36.0 (32.0 to 39.4)	-0.9 (-4.5 to 2.7)	2.2 (-0.9 to 5.4)	37.8 (34.0 to 40.8)	36.6 (32.6 to 39.8)	37.8 (33.9 to 40.8)	-3.0 (-6.6 to 0.3)	3.1 (0.6 to 6.5)
Diet low in seafood omega-3 fatty acids	3.4 (1.7 to 4.7)	0.4 (0.0 to 1.4)	0.4 (0.0 to 1.5)	-88.5 (-99.9 to -61.0)	5.5 (-82.1 to 689.3)	3.9 (2.1 to 5.2)	0.5 (0.0 to 1.6)	0.5 (0.0 to 1.7)	-86.9 (-99.8 to -59.1)	5.5 (-72.2 to 708.6)
Diet low in polyunsaturated fatty acids	31.2 (26.0 to 36.1)	28.5 (22.6 to 33.2)	29.1 (23.1 to 33.8)	-8.7 (-20.7 to 1.1)	2.3 (-5.2 to 10.7)	31.9 (26.6 to 36.5)	29.2 (23.5 to 34.0)	29.8 (24.5 to 34.3)	-8.4 (-19.4 to 1.2)	2.2 (-4.7 to 10.3)
Diet high in trans fatty acids	1.1 (0.0 to 3.3)	0.9 (0.0 to 3.0)	0.9 (0.0 to 2.9)	-12.7 (-37.4 to -6.8)	-8.4 (-34.1 to -3.8)	1.5 (0.1 to 4.0)	1.3 (0.1 to 3.7)	1.2 (0.0 to 3.5)	-12.5 (-35.8 to -6.4)	-8.2 (-27.8 to -3.2)
Diet high in sodium	27.8 (18.5 to 35.8)	18.4 (9.6 to 25.7)	15.9 (7.1 to 23.4)	-33.9 (-57.0 to -15.7)	-13.3 (-36.5 to 3.4)	31.9 (23.3 to 39.1)	19.4 (10.9 to 26.4)	16.2 (7.7 to 23.3)	-39.1 (-58.5 to -24.5)	-16.4 (-38.3 to -3.2)
Intimate partner violence	-	-	-	-	-	2.3 (2.0 to 2.7)	2.2 (1.8 to 2.5)	2.1 (1.7 to 2.4)	-7.0 (-11.0 to -2.8)	-4.2 (-8.7 to 0.3)
Childhood sexual abuse	5.3 (4.3 to 6.5)	5.3 (4.3 to 6.5)	5.3 (4.3 to 6.5)	0.0 (0.0 to 0.0)	0.0 (0.0 to 0.0)	7.3 (5.9 to 8.9)	7.3 (5.9 to 8.9)	7.5 (6.0 to 9.2)	0.0 (0.0 to 0.0)	2.8 (-4.0 to 10.6)

Supplementary Results Table 1. Aomori age-standardised Summary Exposure Values (SEVs) for Level 4 risk factors and percentage change, by sex, 1990, 2007, and 2017.Data in parentheses are 95% uncertainty intervals.

	Male					Female				
	**1990**	**2007**	**2017**	**Percentage change 1990-2007**	**Percentage change 2007-2017**	**1990**	**2007**	**2017**	**Percentage change 1990-2007**	**Percentage change 2007-2017**
Bullying victimization	3.3 (1.3 to 6.6)	3.4 (1.4 to 6.8)	3.5 (1.4 to 7.2)	2.6 (-4.9 to 10.6)	3.4 (-2.0 to 8.0)	1.9 (0.7 to 3.9)	1.9 (0.8 to 3.9)	2.0 (0.8 to 4.0)	0.9 (-6.6 to 9.1)	2.3 (-3.1 to 7.1)
Low physical activity	0.5 (0.2 to 0.9)	0.5 (0.2 to 0.9)	0.4 (0.2 to 0.8)	-1.8 (-3.6 to -0.0)	-8.8 (-12.5 to -4.7)	0.5 (0.2 to 1.0)	0.5 (0.2 to 0.9)	0.5 (0.2 to 0.9)	-3.9 (-6.2 to -1.5)	0.7 (-0.7 to 2.3)
High fasting plasma glucose	1.5 (0.9 to 2.5)	1.4 (0.9 to 2.3)	1.6 (0.9 to 2.5)	-7.2 (-45.1 to 53.0)	11.2 (-24.7 to 77.2)	1.2 (0.7 to 1.9)	1.2 (0.7 to 2.0)	1.3 (0.8 to 2.3)	2.1 (-36.8 to 65.9)	10.6 (-27.0 to 74.0)
High LDL cholesterol	11.9 (10.3 to 13.7)	12.4 (10.8 to 14.3)	12.4 (10.8 to 14.2)	4.3 (-1.7 to 10.7)	0.1 (-4.5 to 4.8)	12.6 (11.0 to 14.3)	12.8 (11.2 to 14.5)	12.8 (11.2 to 14.5)	0.9 (-4.2 to 6.4)	0.2 (-4.2 to 4.9)
High systolic blood pressure	11.2 (10.1 to 12.2)	8.2 (7.4 to 9.2)	8.1 (7.2 to 9.1)	-26.1 (-31.7 to -19.7)	-1.9 (-9.3 to 5.5)	9.6 (8.7 to 10.6)	6.0 (5.3 to 6.8)	5.6 (4.8 to 6.4)	-37.3 (-44.0 to -30.2)	-7.5 (-16.9 to 2.0)
High body-mass index	4.7 (2.8 to 7.7)	6.6 (4.3 to 10.7)	6.8 (4.4 to 10.8)	39.7 (11.8 to 81.0)	2.5 (-13.1 to 20.8)	5.0 (3.0 to 7.9)	6.4 (3.9 to 10.1)	6.4 (4.0 to 10.2)	27.8 (1.4 to 71.8)	0.6 (-17.5 to 22.7)
Low bone mineral density	5.3 (4.3 to 6.3)	5.2 (4.4 to 6.1)	5.2 (4.4 to 6.1)	-2.5 (-14.1 to 13.0)	0.5 (-12.1 to 15.5)	7.2 (6.1 to 8.5)	7.0 (6.0 to 8.1)	7.1 (6.0 to 8.4)	-3.6 (-15.1 to 9.2)	1.8 (-11.1 to 15.6)
Impaired kidney function	2.7 (1.2 to 6.6)	2.6 (1.2 to 6.5)	2.4 (1.1 to 6.2)	-3.1 (-7.3 to 1.1)	-7.3 (-12.6 to -2.2)	3.3 (1.5 to 7.6)	3.4 (1.6 to 7.7)	3.1 (1.4 to 7.3)	3.0 (-0.5 to 7.5)	-7.5 (-12.6 to -2.5)

Supplementary Results Table 1. Chiba age-standardised Summary Exposure Values (SEVs) for Level 4 risk factors and percentage change, by sex, 1990, 2007, and 2017.Data in parentheses are 95% uncertainty intervals.	Male					Female				
	1990	2007	2017	Percentage change 1990-2007	Percentage change 2007-2017	1990	2007	2017	Percentage change 1990-2007	Percentage change 2007-2017
Unsafe water source	2.0 (1.1 to 3.4)	1.7 (1.0 to 2.9)	1.8 (1.0 to 3.1)	-14.8 (-54.5 to 68.2)	7.4 (-36.2 to 79.2)	2.0 (1.1 to 3.4)	1.7 (1.0 to 2.9)	1.8 (1.0 to 3.1)	-14.8 (-54.5 to 68.2)	7.4 (-36.2 to 79.2)
Unsafe sanitation	3.3 (1.7 to 6.1)	1.7 (0.8 to 3.1)	1.5 (0.7 to 2.9)	-47.9 (-77.3 to 16.4)	-12.5 (-54.3 to 68.4)	3.3 (1.7 to 6.1)	1.7 (0.8 to 3.1)	1.5 (0.7 to 2.9)	-47.9 (-77.3 to 16.4)	-12.5 (-54.3 to 68.4)
No access to handwashing facility	6.7 (4.8 to 8.8)	6.3 (4.5 to 8.5)	5.9 (4.3 to 7.9)	-5.3 (-32.7 to 32.7)	-6.8 (-26.8 to 19.3)	6.7 (4.8 to 8.8)	6.3 (4.5 to 8.5)	5.9 (4.3 to 7.9)	-5.3 (-32.7 to 32.7)	-6.8 (-26.8 to 19.3)
Ambient particulate matter pollution	32.7 (22.6 to 48.1)	32.6 (23.1 to 49.1)	28.0 (18.6 to 43.3)	-0.2 (-23.8 to 29.0)	-14.1 (-33.0 to 6.9)	32.5 (22.4 to 48.1)	32.6 (23.0 to 49.0)	28.0 (18.6 to 43.3)	0.2 (-23.5 to 29.8)	-14.1 (-33.0 to 6.9)
Household air pollution from solid fuels	0.4 (0.1 to 1.1)	0.1 (0.0 to 0.3)	0.1 (0.0 to 0.2)	-72.3 (-92.2 to -47.6)	-12.6 (-67.9 to 85.6)	0.6 (0.2 to 3.3)	0.2 (0.0 to 0.5)	0.2 (0.0 to 0.6)	-69.6 (-92.1 to -41.5)	-10.0 (-67.6 to 85.5)
Ambient ozone pollution	50.9 (23.3 to 54.8)	50.9 (23.2 to 54.8)	51.2 (23.9 to 54.8)	-0.1 (-1.0 to 0.0)	0.6 (0.0 to 3.5)	50.9 (23.3 to 54.8)	50.9 (23.2 to 54.8)	51.2 (23.9 to 54.8)	-0.1 (-1.0 to 0.0)	0.6 (0.0 to 3.5)
Residential radon	12.6 (0.7 to 40.1)	12.6 (0.7 to 40.1)	12.6 (0.7 to 40.1)	0.0 (0.0 to 0.0)	0.0 (0.0 to 0.0)	12.6 (0.7 to 40.1)	12.6 (0.7 to 40.1)	12.6 (0.7 to 40.1)	0.0 (0.0 to 0.0)	0.0 (0.0 to 0.0)
Lead exposure	3.2 (0.5 to 6.9)	2.0 (0.3 to 5.0)	1.4 (0.2 to 3.8)	-37.2 (-51.9 to -27.3)	-31.1 (-47.0 to -22.8)	1.7 (0.1 to 4.6)	1.0 (0.1 to 3.1)	0.6 (0.0 to 2.3)	-43.8 (-62.7 to -33.5)	-35.6 (-58.3 to -25.2)
Occupational exposure to asbestos	1.7 (1.5 to 1.9)	2.5 (2.3 to 2.7)	2.6 (2.2 to 3.1)	42.8 (24.4 to 66.0)	6.3 (-12.3 to 27.8)	0.6 (0.5 to 0.7)	0.5 (0.5 to 0.6)	0.5 (0.4 to 0.7)	-6.4 (-22.0 to 19.5)	-0.0 (-20.2 to 23.9)
Occupational exposure to arsenic	0.5 (0.0 to 1.1)	0.4 (0.0 to 1.0)	0.4 (0.0 to 1.0)	-	-	0.4 (0.0 to 1.0)	0.4 (0.0 to 0.9)	0.4 (0.0 to 0.9)	-	-
Occupational exposure to benzene	0.6 (0.0 to 1.8)	0.7 (0.0 to 1.9)	0.7 (0.0 to 1.9)	2.2 (-4.2 to 12.1)	3.2 (-2.3 to 11.6)	0.6 (0.0 to 1.8)	0.8 (0.0 to 2.2)	0.9 (0.1 to 2.5)	20.0 (7.9 to 38.2)	13.8 (4.8 to 27.5)
Occupational exposure to beryllium	0.0 (0.0 to 0.0)	0.0 (0.0 to 0.0)	0.0 (0.0 to 0.0)	-5.4 (-10.0 to -0.0)	1.9 (-1.7 to 5.8)	0.0 (0.0 to 0.0)	0.0 (0.0 to 0.0)	0.0 (0.0 to 0.0)	1.0 (-7.3 to 10.1)	8.6 (2.4 to 14.9)
Occupational exposure to cadmium	0.1 (0.0 to 0.1)	0.0 (0.0 to 0.1)	0.1 (0.0 to 0.1)	-7.8 (-19.3 to 5.2)	0.9 (-7.9 to 11.2)	0.0 (0.0 to 0.1)	0.0 (0.0 to 0.1)	0.0 (0.0 to 0.1)	-10.2 (-24.1 to 6.3)	4.6 (-8.1 to 19.0)
Occupational exposure to chromium	0.1 (0.1 to 0.1)	0.1 (0.1 to 0.1)	0.1 (0.1 to 0.1)	-6.2 (-16.8 to 5.1)	1.4 (-6.6 to 10.4)	0.1 (0.1 to 0.1)	0.1 (0.1 to 0.1)	0.1 (0.1 to 0.1)	-5.5 (-18.4 to 9.5)	6.3 (-4.6 to 17.9)
Occupational exposure to diesel engine exhaust	0.6 (0.6 to 0.6)	0.6 (0.5 to 0.6)	0.6 (0.5 to 0.6)	-2.6 (-10.5 to 5.8)	2.9 (-3.5 to 9.8)	0.5 (0.4 to 0.5)	0.5 (0.5 to 0.6)	0.6 (0.5 to 0.6)	10.7 (-2.0 to 25.4)	10.3 (0.8 to 21.7)
Occupational exposure to formaldehyde	0.3 (0.2 to 0.3)	0.2 (0.2 to 0.3)	0.2 (0.2 to 0.3)	-7.3 (-19.4 to 6.5)	0.5 (-8.8 to 11.1)	0.2 (0.2 to 0.3)	0.2 (0.2 to 0.2)	0.2 (0.2 to 0.2)	-7.5 (-22.4 to 10.0)	5.4 (-7.1 to 18.9)
Occupational exposure to nickel	0.4 (0.0 to 1.9)	0.4 (0.0 to 1.7)	0.4 (0.0 to 1.7)	-	-	0.4 (0.0 to 1.6)	0.3 (0.0 to 1.4)	0.3 (0.0 to 1.4)	-	-
Occupational exposure to polycyclic aromatic hydrocarbons	0.2 (0.2 to 0.3)	0.2 (0.2 to 0.3)	0.2 (0.2 to 0.3)	-5.1 (-14.8 to 5.4)	1.7 (-5.7 to 9.7)	0.2 (0.2 to 0.3)	0.2 (0.2 to 0.2)	0.2 (0.2 to 0.3)	-3.4 (-16.2 to 11.4)	7.1 (-2.7 to 18.3)
Occupational exposure to silica	4.8 (0.7 to 15.2)	4.5 (0.7 to 14.8)	4.5 (0.6 to 14.8)	-7.1 (-16.9 to 4.8)	1.2 (-7.6 to 11.7)	3.3 (0.5 to 10.2)	2.8 (0.4 to 8.7)	2.8 (0.4 to 9.0)	-15.0 (-28.7 to 2.7)	1.7 (-11.7 to 18.5)
Occupational exposure to sulphuric acid	0.6 (0.1 to 2.0)	0.6 (0.1 to 1.8)	0.6 (0.1 to 1.8)	-7.7 (-18.5 to 4.2)	1.3 (-7.4 to 11.5)	0.5 (0.1 to 1.7)	0.5 (0.1 to 1.5)	0.5 (0.1 to 1.5)	-11.6 (-25.5 to 5.3)	3.9 (-9.3 to 18.7)
Occupational exposure to trichloroethylene	0.1 (0.1 to 0.1)	0.1 (0.1 to 0.1)	0.1 (0.1 to 0.1)	-5.1 (-12.3 to 3.2)	1.8 (-4.1 to 8.4)	0.1 (0.1 to 0.1)	0.1 (0.1 to 0.1)	0.1 (0.1 to 0.1)	-0.6 (-10.9 to 11.8)	7.7 (-0.9 to 17.4)
Occupational asthmagens	20.0 (15.7 to 25.0)	18.7 (14.9 to 23.7)	18.5 (14.7 to 23.4)	-6.8 (-23.8 to 15.6)	-0.9 (-15.5 to 15.9)	8.6 (6.9 to 10.6)	8.4 (6.7 to 10.5)	8.9 (7.1 to 11.2)	-1.2 (-17.3 to 16.4)	5.2 (-7.2 to 18.7)
Occupational particulate matter, gases, and fumes	6.4 (4.5 to 9.1)	6.1 (4.4 to 8.8)	5.9 (4.1 to 8.5)	-4.5 (-9.7 to 0.8)	-3.7 (-7.1 to -0.6)	3.7 (2.6 to 5.6)	3.5 (2.5 to 5.3)	3.3 (2.3 to 5.0)	-5.8 (-14.4 to 3.8)	-4.8 (-10.4 to 1.1)
Occupational noise	6.0 (5.5 to 6.6)	5.8 (5.3 to 6.3)	5.6 (5.2 to 6.2)	-3.1 (-6.4 to 0.2)	-2.3 (-4.3 to -0.2)	3.6 (3.1 to 4.1)	3.6 (3.2 to 4.0)	3.6 (3.2 to 4.1)	0.3 (-5.6 to 6.7)	1.6 (-2.1 to 6.0)
Occupational ergonomic factors	9.9 (8.2 to 11.9)	8.9 (7.3 to 10.7)	8.7 (7.2 to 10.6)	-10.9 (-21.7 to 1.5)	-1.3 (-11.6 to 9.7)	5.3 (4.2 to 6.7)	5.5 (4.2 to 6.9)	6.2 (4.8 to 7.9)	2.2 (-18.1 to 24.9)	13.6 (-5.5 to 35.0)
Non-exclusive breastfeeding	0.4 (0.3 to 0.6)	0.4 (0.3 to 0.6)	0.4 (0.3 to 0.6)	-0.9 (-13.7 to 13.3)	-1.6 (-11.7 to 9.9)	0.4 (0.3 to 0.6)	0.4 (0.3 to 0.6)	0.4 (0.3 to 0.6)	-0.6 (-13.5 to 13.6)	-1.7 (-11.8 to 9.8)
Discontinued breastfeeding	1.7 (1.6 to 1.9)	1.8 (1.6 to 1.9)	1.7 (1.6 to 1.8)	1.5 (-7.0 to 11.1)	-3.4 (-10.5 to 4.2)	1.7 (1.6 to 1.9)	1.8 (1.6 to 1.9)	1.7 (1.6 to 1.8)	1.5 (-7.1 to 11.1)	-3.2 (-10.4 to 4.4)
Child underweight	0.2 (0.1 to 0.3)	0.2 (0.1 to 0.3)	0.2 (0.1 to 0.3)	-12.0 (-29.4 to 7.1)	-3.7 (-15.3 to 8.9)	0.2 (0.1 to 0.3)	0.2 (0.1 to 0.3)	0.2 (0.1 to 0.3)	-14.2 (-33.3 to 6.9)	-4.4 (-17.4 to 9.5)
Child wasting	0.1 (0.1 to 0.2)	0.1 (0.1 to 0.2)	0.1 (0.1 to 0.2)	-5.3 (-26.5 to 20.2)	-1.6 (-17.8 to 17.5)	0.1 (0.1 to 0.2)	0.1 (0.1 to 0.2)	0.1 (0.1 to 0.2)	-6.6 (-27.6 to 21.7)	-1.9 (-19.4 to 17.3)
Child stunting	0.5 (0.3 to 0.9)	0.5 (0.3 to 0.7)	0.5 (0.2 to 0.7)	-12.4 (-45.1 to 35.9)	-2.3 (-29.0 to 33.4)	0.6 (0.3 to 0.9)	0.5 (0.3 to 0.7)	0.5 (0.2 to 0.7)	-14.9 (-48.7 to 33.4)	-2.2 (-34.3 to 38.5)
Short gestation for birth weight	0.0 (0.0 to 0.0)	0.0 (0.0 to 0.0)	0.0 (0.0 to 0.0)	6.4 (-13.9 to 32.7)	3.7 (-13.4 to 34.3)	0.0 (0.0 to 0.0)	0.0 (0.0 to 0.0)	0.0 (0.0 to 0.0)	14.7 (-18.6 to 95.4)	-0.6 (-20.8 to 35.9)
Low birth weight for gestation	0.0 (0.0 to 0.0)	0.0 (0.0 to 0.0)	0.0 (0.0 to 0.0)	3.9 (-12.9 to 25.4)	1.3 (-19.7 to 17.2)	0.0 (0.0 to 0.0)	0.0 (0.0 to 0.0)	0.0 (0.0 to 0.0)	-6.3 (-63.2 to 28.9)	4.4 (-18.5 to 25.6)
Iron deficiency	-	-	-	-	-	7.8 (5.4 to 10.7)	7.9 (5.5 to 10.7)	7.2 (5.0 to 10.2)	0.8 (-20.5 to 27.7)	-8.0 (-23.9 to 10.6)
Vitamin A deficiency	0.9 (0.6 to 1.2)	0.8 (0.5 to 1.0)	0.6 (0.4 to 0.9)	-17.3 (-31.9 to 4.6)	-18.6 (-35.9 to -0.6)	0.6 (0.4 to 0.9)	0.7 (0.5 to 1.0)	0.6 (0.4 to 0.9)	16.2 (-5.6 to 37.5)	-14.7 (-31.1 to 5.9)
Zinc deficiency	0.5 (0.2 to 1.1)	0.4 (0.1 to 1.1)	0.4 (0.1 to 0.8)	-21.1 (-64.4 to 67.7)	-9.2 (-67.1 to 165.1)	0.5 (0.2 to 1.1)	0.4 (0.1 to 0.8)	0.4 (0.1 to 0.8)	-31.1 (-64.3 to 4.5)	8.4 (-51.9 to 176.3)
Smoking	15.3 (11.8 to 18.9)	11.3 (8.7 to 13.8)	9.7 (7.7 to 11.9)	-26.2 (-35.7 to -14.4)	-13.7 (-21.9 to -4.1)	3.4 (2.4 to 4.6)	3.0 (2.1 to 4.1)	2.9 (2.1 to 3.9)	-12.3 (-32.5 to 15.3)	-2.2 (-18.2 to 18.3)
Chewing tobacco	1.4 (0.9 to 2.0)	0.9 (0.6 to 1.3)	0.7 (0.5 to 1.1)	-33.5 (-57.1 to 4.1)	-18.2 (-42.3 to 12.1)	0.1 (0.1 to 0.2)	0.1 (0.1 to 0.1)	0.1 (0.1 to 0.1)	-3.8 (-42.0 to 58.9)	-2.8 (-32.7 to 43.2)
Second-hand smoke	38.3 (34.2 to 42.1)	37.4 (33.1 to 41.5)	37.3 (32.8 to 41.7)	-2.3 (-13.4 to 10.6)	-0.4 (-8.7 to 9.8)	58.9 (55.4 to 62.1)	51.7 (47.8 to 55.4)	51.3 (47.0 to 55.2)	-12.2 (-18.1 to -6.0)	-0.8 (-6.8 to 5.1)
Alcohol use	14.6 (7.2 to 24.8)	14.8 (7.6 to 25.4)	17.0 (8.4 to 30.0)	0.9 (-34.9 to 48.9)	15.5 (-18.0 to 61.9)	11.5 (5.6 to 20.2)	11.6 (5.8 to 20.8)	13.5 (6.5 to 24.5)	0.6 (-34.1 to 50.0)	15.8 (-18.3 to 64.6)
Drug use	0.5 (0.4 to 0.7)	0.5 (0.4 to 0.7)	0.6 (0.4 to 0.8)	0.5 (-4.9 to 6.6)	11.8 (5.0 to 20.4)	0.3 (0.2 to 0.4)	0.3 (0.2 to 0.4)	0.3 (0.3 to 0.4)	2.2 (-3.0 to 8.1)	9.3 (2.3 to 17.6)
Diet low in fruits	41.5 (35.7 to 45.2)	39.8 (33.7 to 43.5)	40.9 (35.1 to 44.7)	-4.2 (-9.9 to 1.4)	2.9 (-1.7 to 8.2)	38.6 (32.8 to 42.7)	36.0 (30.1 to 40.4)	38.0 (32.2 to 42.5)	-6.7 (-14.1 to -0.4)	5.4 (-0.2 to 12.5)
Diet low in vegetables	20.9 (12.9 to 28.2)	14.8 (6.7 to 22.5)	16.8 (8.3 to 24.4)	-29.0 (-57.7 to -6.0)	13.3 (-13.7 to 56.9)	23.2 (14.7 to 29.9)	16.9 (8.3 to 24.6)	18.7 (10.0 to 26.0)	-26.8 (-51.9 to -5.5)	10.2 (-14.5 to 51.5)
Diet low in legumes	4.4 (0.6 to 10.0)	5.6 (1.3 to 11.6)	7.5 (2.1 to 14.1)	29.8 (-38.2 to 301.8)	33.4 (-16.9 to 180.1)	8.2 (2.4 to 14.8)	8.0 (2.4 to 14.4)	10.3 (3.9 to 17.3)	-2.5 (-47.2 to 73.4)	29.3 (-13.3 to 126.4)
Diet low in whole grains	31.2 (26.8 to 35.0)	35.8 (32.1 to 39.0)	37.1 (33.4 to 40.2)	14.9 (7.0 to 25.9)	3.6 (-0.6 to 8.5)	32.4 (27.9 to 36.3)	36.9 (33.3 to 40.0)	38.1 (34.5 to 41.1)	13.9 (6.0 to 23.9)	3.3 (-0.9 to 8.0)
Diet low in nuts and seeds	51.2 (50.0 to 52.1)	49.9 (48.4 to 51.1)	49.7 (47.9 to 51.1)	-2.4 (-4.5 to -0.7)	-0.5 (-2.4 to 1.2)	51.3 (50.2 to 52.2)	50.0 (48.4 to 51.2)	49.7 (48.1 to 51.1)	-2.6 (-4.6 to -0.8)	-0.5 (-2.4 to 1.2)
Diet low in milk	46.5 (44.5 to 48.2)	46.8 (44.9 to 48.3)	47.2 (45.4 to 48.7)	0.6 (-1.1 to 2.5)	1.0 (-0.3 to 2.4)	46.5 (44.5 to 48.2)	46.3 (44.3 to 48.0)	46.9 (45.0 to 48.6)	-0.4 (-2.3 to 1.5)	1.3 (-0.1 to 2.9)
Diet high in red meat	3.2 (0.2 to 7.7)	6.6 (1.8 to 12.3)	7.7 (2.3 to 14.3)	108.6 (-17.3 to 1507.1)	16.4 (-39.2 to 141.9)	0.4 (0.0 to 2.0)	1.2 (0.0 to 4.2)	1.6 (0.0 to 5.1)	173.3 (-84.8 to 19239823.8)	36.1 (-92.3 to 3705.2)
Diet high in processed meat	10.8 (6.9 to 14.7)	18.4 (13.5 to 23.0)	21.5 (15.9 to 26.6)	69.7 (33.8 to 138.2)	16.8 (-1.4 to 39.9)	9.3 (5.7 to 13.0)	14.1 (9.9 to 17.9)	16.0 (11.3 to 20.1)	51.9 (18.7 to 108.7)	12.9 (-3.5 to 34.2)
Diet high in sugar-sweetened beverages	6.4 (2.6 to 12.6)	7.3 (3.2 to 13.1)	7.8 (3.4 to 14.3)	15.2 (-43.4 to 161.1)	6.5 (-36.1 to 75.0)	4.5 (1.7 to 9.3)	5.1 (2.2 to 9.3)	5.5 (2.3 to 10.8)	12.5 (-49.0 to 154.1)	7.4 (-38.7 to 91.2)
Diet low in fibre	22.6 (15.4 to 29.1)	23.3 (16.2 to 29.3)	24.0 (16.8 to 30.3)	3.1 (-12.8 to 24.0)	3.2 (-8.8 to 17.0)	23.7 (16.5 to 30.1)	24.4 (17.3 to 30.4)	25.6 (18.6 to 31.7)	3.1 (-11.3 to 22.7)	4.9 (-6.8 to 19.0)
Diet low in calcium	35.7 (31.5 to 39.1)	35.3 (31.0 to 38.8)	36.0 (31.7 to 39.4)	-1.1 (-5.0 to 2.6)	1.9 (-0.8 to 5.0)	37.8 (34.0 to 40.8)	36.6 (32.8 to 39.8)	37.8 (34.1 to 40.8)	-3.1 (-6.5 to -0.1)	3.1 (0.4 to 6.1)
Diet low in seafood omega-3 fatty acids	3.4 (1.7 to 4.8)	0.3 (0.0 to 1.3)	0.4 (0.0 to 1.3)	-90.0 (-99.9 to -63.2)	5.4 (-85.2 to 742.1)	4.0 (2.3 to 5.3)	0.5 (0.0 to 1.6)	0.5 (0.0 to 1.7)	-87.5 (-99.9 to -58.1)	3.3 (-79.9 to 554.9)
Diet low in polyunsaturated fatty acids	31.2 (25.9 to 36.0)	28.7 (22.9 to 33.6)	29.3 (23.4 to 34.3)	-8.2 (-18.4 to 1.1)	2.2 (-4.7 to 10.1)	32.0 (26.5 to 36.6)	29.3 (23.6 to 34.0)	29.9 (24.3 to 34.6)	-8.2 (-18.9 to 0.2)	2.0 (-4.8 to 10.0)
Diet high in trans fatty acids	1.1 (0.0 to 3.3)	1.0 (0.0 to 3.1)	0.9 (0.0 to 2.9)	-12.5 (-38.1 to -6.4)	-8.7 (-32.6 to -4.0)	1.5 (0.1 to 4.0)	1.3 (0.1 to 3.7)	1.2 (0.0 to 3.5)	-12.3 (-35.8 to -6.3)	-8.4 (-28.2 to -3.3)
Diet high in sodium	27.8 (18.4 to 35.9)	18.4 (9.4 to 25.7)	16.0 (7.1 to 23.1)	-33.9 (-57.3 to -14.9)	-13.2 (-36.8 to 3.1)	32.3 (23.9 to 39.9)	19.9 (11.6 to 26.8)	16.6 (8.1 to 23.6)	-38.4 (-57.9 to -24.2)	-16.3 (-36.3 to -2.9)
Intimate partner violence	-	-	-	-	-	2.3 (1.9 to 2.6)	2.1 (1.8 to 2.4)	2.0 (1.7 to 2.3)	-7.2 (-11.1 to -2.8)	-3.7 (-8.0 to 0.5)
Childhood sexual abuse	5.3 (4.3 to 6.5)	5.3 (4.3 to 6.5)	5.3 (4.3 to 6.5)	0.0 (0.0 to 0.0)	0.0 (0.0 to 0.0)	7.3 (5.9 to 8.9)	7.3 (5.9 to 8.9)	7.5 (6.0 to 9.2)	0.0 (0.0 to 0.0)	2.8 (-4.0 to 10.6)

Supplementary Results Table 1. Chiba age-standardised Summary Exposure Values (SEVs) for Level 4 risk factors and percentage change, by sex, 1990, 2007, and 2017.Data in parentheses are 95% uncertainty intervals.

	Male					Female				
	**1990**	**2007**	**2017**	**Percentage change 1990-2007**	**Percentage change 2007-2017**	**1990**	**2007**	**2017**	**Percentage change 1990-2007**	**Percentage change 2007-2017**
Bullying victimization	3.3 (1.4 to 6.7)	3.3 (1.4 to 6.7)	3.5 (1.4 to 7.1)	-0.5 (-8.4 to 7.5)	4.7 (-0.6 to 9.7)	1.9 (0.8 to 4.0)	1.9 (0.8 to 3.9)	2.0 (0.8 to 4.0)	-2.1 (-9.9 to 6.5)	3.8 (-1.9 to 8.6)
Low physical activity	0.5 (0.2 to 0.9)	0.5 (0.2 to 0.9)	0.4 (0.2 to 0.8)	-1.8 (-3.7 to -0.1)	-8.8 (-12.5 to -4.6)	0.5 (0.2 to 1.0)	0.5 (0.2 to 0.9)	0.5 (0.2 to 0.9)	-3.9 (-6.2 to -1.2)	0.8 (-0.6 to 2.3)
High fasting plasma glucose	1.4 (0.8 to 2.2)	1.3 (0.8 to 2.1)	1.4 (0.9 to 2.4)	-4.3 (-42.2 to 57.3)	10.2 (-27.2 to 72.9)	1.2 (0.7 to 1.9)	1.2 (0.7 to 2.0)	1.3 (0.7 to 2.4)	4.8 (-34.9 to 72.0)	9.3 (-30.8 to 83.7)
High LDL cholesterol	12.2 (10.6 to 14.0)	12.7 (11.0 to 14.5)	12.7 (11.1 to 14.6)	4.3 (-1.7 to 10.2)	-0.1 (-4.6 to 4.6)	13.0 (11.4 to 14.7)	13.1 (11.5 to 14.8)	13.1 (11.5 to 14.9)	0.5 (-4.7 to 5.8)	0.1 (-4.4 to 4.6)
High systolic blood pressure	11.4 (10.3 to 12.4)	8.4 (7.5 to 9.3)	8.3 (7.3 to 9.2)	-26.1 (-32.1 to -19.9)	-1.7 (-9.1 to 6.2)	9.7 (8.6 to 10.7)	6.1 (5.3 to 6.8)	5.6 (4.9 to 6.4)	-37.1 (-43.7 to -30.4)	-7.4 (-17.5 to 3.4)
High body-mass index	6.5 (4.3 to 10.3)	9.4 (6.5 to 14.2)	9.6 (6.6 to 14.7)	43.9 (16.3 to 84.0)	3.0 (-11.4 to 20.5)	5.6 (3.5 to 8.8)	7.3 (4.6 to 11.6)	7.2 (4.5 to 11.3)	29.3 (2.3 to 69.4)	-0.8 (-20.5 to 20.4)
Low bone mineral density	5.3 (4.4 to 6.3)	5.2 (4.3 to 6.1)	5.2 (4.3 to 6.2)	-2.5 (-15.1 to 11.1)	0.3 (-13.4 to 14.8)	7.2 (6.1 to 8.5)	7.0 (6.0 to 8.2)	7.1 (6.0 to 8.3)	-3.4 (-14.3 to 11.1)	1.5 (-10.7 to 14.6)
Impaired kidney function	2.6 (1.2 to 6.5)	2.6 (1.1 to 6.5)	2.4 (1.1 to 6.1)	-3.1 (-7.6 to 1.4)	-6.8 (-12.4 to -1.9)	3.2 (1.5 to 7.5)	3.3 (1.5 to 7.7)	3.1 (1.4 to 7.2)	3.3 (-0.5 to 8.9)	-7.4 (-12.4 to -2.6)

Supplementary Results Table 1. Ehime age-standardised Summary Exposure Values (SEVs) for Level 4 risk factors and percentage change, by sex, 1990, 2007, and 2017.Data in parentheses are 95% uncertainty intervals.

	Male					Female				
	1990	2007	2017	Percentage change 1990-2007	Percentage change 2007-2017	1990	2007	2017	Percentage change 1990-2007	Percentage change 2007-2017
Unsafe water source	2.4 (1.4 to 4.0)	2.0 (1.1 to 3.3)	2.1 (1.1 to 3.6)	-17.6 (-57.6 to 66.2)	5.1 (-38.4 to 84.2)	2.4 (1.4 to 4.0)	2.0 (1.1 to 3.3)	2.1 (1.1 to 3.6)	-17.6 (-57.6 to 66.2)	5.1 (-38.4 to 84.2)
Unsafe sanitation	4.4 (2.1 to 7.8)	2.2 (1.0 to 4.1)	1.9 (0.9 to 3.6)	-49.9 (-78.5 to 11.5)	-13.8 (-56.2 to 80.2)	4.4 (2.1 to 7.8)	2.2 (1.0 to 4.1)	1.9 (0.9 to 3.6)	-49.9 (-78.5 to 11.5)	-13.8 (-56.2 to 80.2)
No access to handwashing facility	6.8 (4.9 to 8.9)	6.4 (4.7 to 8.7)	6.0 (4.4 to 8.0)	-5.4 (-32.3 to 33.3)	-6.9 (-26.5 to 19.2)	6.8 (4.9 to 8.9)	6.4 (4.7 to 8.7)	6.0 (4.4 to 8.0)	-5.4 (-32.3 to 33.3)	-6.9 (-26.5 to 19.2)
Ambient particulate matter pollution	30.6 (19.2 to 46.8)	31.0 (20.2 to 46.7)	26.4 (15.4 to 40.6)	1.5 (-27.9 to 45.1)	-15.0 (-44.4 to 19.6)	30.3 (19.0 to 46.3)	31.0 (20.2 to 46.6)	26.3 (15.4 to 40.5)	2.1 (-27.6 to 46.3)	-15.0 (-44.4 to 19.6)
Household air pollution from solid fuels	0.7 (0.2 to 1.9)	0.2 (0.0 to 0.4)	0.1 (0.0 to 0.3)	-75.0 (-92.9 to -48.7)	-17.5 (-65.1 to 81.5)	1.0 (0.3 to 7.0)	0.3 (0.0 to 1.2)	0.2 (0.0 to 1.0)	-72.3 (-92.7 to -41.1)	-14.8 (-64.3 to 85.1)
Ambient ozone pollution	51.0 (23.5 to 54.8)	51.1 (23.4 to 54.8)	51.8 (25.4 to 54.8)	0.0 (-0.6 to 0.8)	1.4 (0.0 to 8.5)	51.0 (23.5 to 54.8)	51.1 (23.4 to 54.8)	51.8 (25.4 to 54.8)	0.0 (-0.6 to 0.8)	1.4 (0.0 to 8.5)
Residential radon	13.2 (0.9 to 41.5)	13.2 (0.9 to 41.5)	13.2 (0.9 to 41.5)	0.0 (0.0 to 0.0)	0.0 (0.0 to 0.0)	13.2 (0.9 to 41.5)	13.2 (0.9 to 41.5)	13.2 (0.9 to 41.5)	0.0 (0.0 to 0.0)	0.0 (0.0 to 0.0)
Lead exposure	3.4 (0.6 to 7.1)	2.2 (0.3 to 5.3)	1.6 (0.2 to 4.2)	-33.3 (-47.6 to -24.4)	-28.5 (-43.4 to -20.3)	1.7 (0.1 to 4.7)	1.1 (0.1 to 3.3)	0.7 (0.0 to 2.6)	-38.7 (-56.1 to -28.7)	-30.7 (-52.8 to -21.2)
Occupational exposure to asbestos	2.2 (1.9 to 2.4)	3.1 (2.8 to 3.4)	3.1 (2.5 to 3.8)	43.2 (24.2 to 66.7)	1.7 (-18.7 to 26.3)	0.6 (0.5 to 0.7)	0.6 (0.6 to 0.7)	0.6 (0.5 to 0.7)	-0.3 (-15.9 to 24.1)	-7.0 (-26.3 to 15.2)
Occupational exposure to arsenic	0.5 (0.0 to 1.0)	0.4 (0.0 to 1.0)	0.4 (0.0 to 1.0)	-	-	0.4 (0.0 to 1.0)	0.4 (0.0 to 0.9)	0.4 (0.0 to 0.9)	-	-
Occupational exposure to benzene	0.6 (0.0 to 1.8)	0.6 (0.0 to 1.9)	0.7 (0.0 to 2.0)	2.4 (-4.5 to 13.0)	3.5 (-2.2 to 11.1)	0.6 (0.0 to 1.7)	0.7 (0.0 to 2.1)	0.8 (0.0 to 2.3)	21.6 (7.0 to 40.1)	14.2 (4.2 to 29.4)
Occupational exposure to beryllium	0.0 (0.0 to 0.0)	0.0 (0.0 to 0.0)	0.0 (0.0 to 0.0)	-5.4 (-10.3 to -0.1)	1.9 (-1.9 to 5.9)	0.0 (0.0 to 0.0)	0.0 (0.0 to 0.0)	0.0 (0.0 to 0.0)	1.9 (-7.7 to 12.8)	9.2 (2.3 to 16.4)
Occupational exposure to cadmium	0.1 (0.0 to 0.1)	0.0 (0.0 to 0.1)	0.0 (0.0 to 0.1)	-7.1 (-17.6 to 5.3)	0.8 (-8.7 to 11.3)	0.0 (0.0 to 0.1)	0.0 (0.0 to 0.1)	0.0 (0.0 to 0.1)	-8.9 (-22.8 to 8.3)	5.2 (-6.6 to 18.9)
Occupational exposure to chromium	0.1 (0.1 to 0.1)	0.1 (0.1 to 0.1)	0.1 (0.1 to 0.1)	-5.4 (-15.1 to 5.9)	1.3 (-7.0 to 10.4)	0.1 (0.1 to 0.1)	0.1 (0.1 to 0.1)	0.1 (0.1 to 0.1)	-4.2 (-17.5 to 12.6)	6.9 (-3.8 to 18.8)
Occupational exposure to diesel engine exhaust	0.6 (0.5 to 0.6)	0.6 (0.5 to 0.6)	0.6 (0.5 to 0.6)	-2.2 (-9.7 to 6.3)	3.0 (-3.2 to 10.3)	0.4 (0.4 to 0.5)	0.5 (0.4 to 0.6)	0.6 (0.5 to 0.6)	12.0 (-1.4 to 27.6)	11.5 (0.9 to 23.2)
Occupational exposure to formaldehyde	0.3 (0.2 to 0.3)	0.2 (0.2 to 0.3)	0.2 (0.2 to 0.3)	-6.5 (-17.7 to 6.8)	0.4 (-9.7 to 11.3)	0.2 (0.2 to 0.3)	0.2 (0.2 to 0.2)	0.2 (0.2 to 0.2)	-6.3 (-21.0 to 12.6)	5.9 (-6.0 to 19.5)
Occupational exposure to nickel	0.4 (0.0 to 1.9)	0.4 (0.0 to 1.7)	0.4 (0.0 to 1.7)	-	-	0.3 (0.0 to 1.5)	0.3 (0.0 to 1.2)	0.3 (0.0 to 1.4)	-	-
Occupational exposure to polycyclic aromatic hydrocarbons	0.2 (0.2 to 0.3)	0.2 (0.2 to 0.3)	0.2 (0.2 to 0.3)	-4.5 (-13.7 to 6.0)	1.7 (-5.9 to 9.8)	0.2 (0.2 to 0.3)	0.2 (0.2 to 0.2)	0.2 (0.2 to 0.3)	-2.2 (-15.5 to 14.3)	7.7 (-2.5 to 18.9)
Occupational exposure to silica	4.7 (0.7 to 15.3)	4.4 (0.6 to 14.3)	4.4 (0.6 to 14.6)	-7.1 (-17.1 to 4.7)	1.1 (-7.9 to 11.7)	3.2 (0.4 to 10.5)	2.7 (0.4 to 8.5)	2.8 (0.4 to 8.6)	-14.0 (-28.6 to 4.6)	2.2 (-10.5 to 19.7)
Occupational exposure to sulphuric acid	0.6 (0.1 to 1.9)	0.6 (0.1 to 1.8)	0.6 (0.1 to 1.8)	-7.2 (-17.2 to 5.0)	1.0 (-7.6 to 11.2)	0.5 (0.1 to 1.6)	0.5 (0.1 to 1.4)	0.5 (0.1 to 1.5)	-10.6 (-24.3 to 6.9)	4.6 (-7.4 to 19.8)
Occupational exposure to trichloroethylene	0.1 (0.1 to 0.1)	0.1 (0.1 to 0.1)	0.1 (0.1 to 0.1)	-4.2 (-11.3 to 4.5)	1.8 (-4.0 to 8.1)	0.1 (0.1 to 0.1)	0.1 (0.1 to 0.1)	0.1 (0.1 to 0.1)	0.7 (-10.7 to 14.8)	8.5 (-0.6 to 18.3)
Occupational asthmagens	18.8 (14.9 to 23.8)	18.0 (14.3 to 23.1)	17.9 (14.2 to 22.9)	-3.9 (-21.8 to 18.9)	-0.6 (-15.2 to 17.6)	8.3 (6.6 to 10.3)	8.2 (6.6 to 10.2)	8.7 (6.9 to 10.8)	-0.5 (-16.4 to 17.2)	5.4 (-7.3 to 20.3)
Occupational particulate matter, gases, and fumes	6.4 (4.6 to 9.3)	6.1 (4.4 to 8.8)	5.9 (4.2 to 8.6)	-4.5 (-9.7 to 1.1)	-3.8 (-7.2 to -0.3)	3.7 (2.5 to 5.4)	3.5 (2.4 to 5.0)	3.3 (2.3 to 4.9)	-5.8 (-14.4 to 3.5)	-4.7 (-10.3 to 1.0)
Occupational noise	6.0 (5.5 to 6.6)	5.8 (5.4 to 6.3)	5.6 (5.2 to 6.2)	-3.4 (-6.7 to -0.3)	-2.6 (-4.8 to -0.6)	3.5 (3.0 to 4.0)	3.5 (3.1 to 4.0)	3.5 (3.1 to 4.0)	0.1 (-6.2 to 6.9)	1.6 (-2.4 to 5.9)
Occupational ergonomic factors	10.6 (8.8 to 12.7)	9.5 (7.8 to 11.3)	9.3 (7.7 to 11.3)	-11.0 (-23.4 to 2.9)	-1.2 (-12.6 to 11.1)	5.7 (4.4 to 7.4)	5.8 (4.5 to 7.3)	6.5 (5.0 to 8.5)	1.1 (-21.5 to 29.5)	12.1 (-7.7 to 36.4)
Non-exclusive breastfeeding	0.4 (0.3 to 0.6)	0.4 (0.3 to 0.6)	0.4 (0.3 to 0.6)	-0.5 (-13.1 to 13.8)	-2.1 (-12.7 to 9.3)	0.4 (0.3 to 0.6)	0.4 (0.3 to 0.6)	0.4 (0.3 to 0.6)	-1.1 (-13.6 to 13.2)	-1.2 (-11.8 to 10.3)
Discontinued breastfeeding	1.7 (1.5 to 1.8)	1.7 (1.5 to 1.8)	1.7 (1.5 to 1.8)	1.0 (-8.8 to 12.4)	-1.5 (-9.0 to 6.7)	1.7 (1.5 to 1.8)	1.7 (1.6 to 1.9)	1.6 (1.5 to 1.8)	3.2 (-6.8 to 14.8)	-3.9 (-11.2 to 4.2)
Child underweight	0.3 (0.2 to 0.4)	0.2 (0.1 to 0.3)	0.2 (0.1 to 0.3)	-15.0 (-34.2 to 8.6)	-4.9 (-19.5 to 11.6)	0.3 (0.2 to 0.4)	0.2 (0.1 to 0.3)	0.2 (0.1 to 0.3)	-18.0 (-37.9 to 8.9)	-5.8 (-22.5 to 12.5)
Child wasting	0.2 (0.1 to 0.3)	0.2 (0.1 to 0.3)	0.2 (0.1 to 0.3)	-7.1 (-33.7 to 25.7)	-1.7 (-21.7 to 21.6)	0.2 (0.1 to 0.3)	0.2 (0.1 to 0.3)	0.2 (0.1 to 0.3)	-7.1 (-35.2 to 30.5)	-2.7 (-21.8 to 24.7)
Child stunting	0.6 (0.3 to 1.1)	0.6 (0.3 to 1.0)	0.6 (0.3 to 1.0)	-9.5 (-47.2 to 54.1)	-4.0 (-33.8 to 36.5)	0.7 (0.3 to 1.2)	0.6 (0.3 to 1.0)	0.6 (0.3 to 1.0)	-10.8 (-47.6 to 47.2)	-2.8 (-33.9 to 38.5)
Short gestation for birth weight	0.0 (0.0 to 0.0)	0.0 (0.0 to 0.0)	0.0 (0.0 to 0.0)	10.0 (-13.1 to 35.6)	2.8 (-19.8 to 23.7)	0.0 (0.0 to 0.0)	0.0 (0.0 to 0.0)	0.0 (0.0 to 0.0)	12.0 (-21.8 to 54.0)	-0.3 (-22.4 to 31.2)
Low birth weight for gestation	0.0 (0.0 to 0.0)	0.0 (0.0 to 0.0)	0.0 (0.0 to 0.0)	4.7 (-12.9 to 32.6)	1.0 (-17.3 to 24.9)	0.0 (0.0 to 0.0)	0.0 (0.0 to 0.0)	0.0 (0.0 to 0.0)	0.1 (-21.6 to 35.8)	3.8 (-19.6 to 30.0)
Iron deficiency	-	-	-	-	-	8.2 (5.7 to 11.2)	8.3 (5.7 to 11.4)	7.6 (5.2 to 10.8)	0.3 (-20.0 to 29.0)	-8.4 (-24.5 to 10.0)
Vitamin A deficiency	1.1 (0.6 to 1.5)	1.0 (0.6 to 1.4)	0.7 (0.5 to 0.9)	-10.4 (-25.1 to 7.8)	-31.8 (-48.7 to -8.4)	0.7 (0.5 to 1.0)	0.9 (0.6 to 1.1)	0.7 (0.4 to 1.0)	16.5 (-5.4 to 42.6)	-16.2 (-36.7 to 1.3)
Zinc deficiency	0.5 (0.2 to 1.1)	0.4 (0.1 to 1.1)	0.4 (0.1 to 1.0)	-13.6 (-64.2 to 71.9)	-12.4 (-66.1 to 169.1)	0.5 (0.1 to 1.1)	0.3 (0.1 to 0.8)	0.4 (0.1 to 0.8)	-32.2 (-64.2 to 7.7)	7.2 (-59.3 to 164.5)
Smoking	17.9 (14.5 to 21.2)	12.5 (10.0 to 15.3)	10.5 (8.3 to 12.9)	-30.2 (-39.1 to -19.2)	-15.8 (-24.2 to -6.4)	4.0 (2.9 to 5.4)	3.3 (2.4 to 4.6)	3.2 (2.3 to 4.5)	-17.4 (-36.6 to 8.8)	-2.9 (-19.8 to 17.3)
Chewing tobacco	1.4 (0.9 to 2.0)	0.9 (0.6 to 1.3)	0.7 (0.5 to 1.1)	-33.4 (-56.9 to 3.5)	-18.7 (-42.3 to 16.4)	0.1 (0.1 to 0.2)	0.1 (0.1 to 0.1)	0.1 (0.1 to 0.1)	-4.4 (-41.0 to 57.6)	-2.7 (-34.7 to 41.9)
Second-hand smoke	37.4 (33.3 to 41.7)	35.8 (31.8 to 40.2)	35.7 (31.1 to 40.4)	-4.3 (-15.4 to 7.8)	-0.3 (-9.0 to 9.0)	56.4 (52.8 to 59.8)	47.9 (44.2 to 51.4)	47.6 (43.7 to 51.6)	-15.1 (-21.2 to -8.7)	-0.6 (-6.8 to 6.3)
Alcohol use	15.3 (7.9 to 26.2)	15.3 (7.9 to 25.5)	16.8 (7.9 to 29.2)	0.1 (-32.1 to 46.5)	10.2 (-21.2 to 52.7)	11.9 (6.1 to 20.8)	12.0 (6.1 to 20.2)	14.3 (6.6 to 24.6)	0.8 (-31.7 to 46.5)	19.0 (-14.1 to 66.9)
Drug use	0.5 (0.4 to 0.7)	0.5 (0.4 to 0.7)	0.6 (0.4 to 0.8)	0.6 (-5.1 to 6.8)	11.7 (4.7 to 21.3)	0.3 (0.2 to 0.4)	0.3 (0.2 to 0.4)	0.3 (0.3 to 0.4)	2.3 (-3.4 to 8.7)	8.7 (2.1 to 17.6)
Diet low in fruits	41.1 (35.5 to 45.0)	39.6 (33.9 to 43.5)	40.7 (34.9 to 44.5)	-3.8 (-10.0 to 1.9)	2.9 (-2.2 to 8.2)	38.3 (32.6 to 42.7)	35.7 (29.9 to 40.4)	37.9 (31.8 to 42.4)	-6.7 (-15.1 to 0.3)	6.0 (-0.4 to 13.8)
Diet low in vegetables	20.5 (11.9 to 28.0)	14.8 (6.6 to 23.0)	17.1 (8.4 to 24.8)	-27.6 (-56.8 to -2.7)	15.1 (-12.7 to 69.4)	22.5 (14.1 to 30.0)	16.8 (8.6 to 25.0)	18.6 (10.1 to 26.5)	-25.5 (-52.2 to -3.2)	11.0 (-13.0 to 50.5)
Diet low in legumes	4.3 (0.8 to 9.6)	5.4 (0.9 to 10.8)	7.2 (1.8 to 13.6)	24.2 (-53.0 to 215.9)	33.1 (-24.1 to 235.1)	8.2 (2.5 to 15.3)	7.8 (2.4 to 14.5)	10.2 (3.8 to 17.2)	-4.3 (-50.8 to 83.2)	30.1 (-10.9 to 127.3)
Diet low in whole grains	30.8 (25.7 to 34.9)	35.5 (31.6 to 38.9)	36.9 (32.9 to 40.1)	15.2 (7.0 to 28.0)	3.7 (-0.9 to 9.2)	32.2 (27.5 to 36.1)	36.7 (32.8 to 40.0)	37.9 (34.1 to 41.1)	14.0 (6.4 to 24.1)	3.3 (-1.1 to 8.3)
Diet low in nuts and seeds	50.9 (49.7 to 51.8)	49.6 (47.7 to 50.9)	49.4 (47.3 to 50.9)	-2.7 (-5.3 to -0.7)	-0.4 (-2.6 to 1.5)	51.0 (49.9 to 51.9)	49.6 (47.9 to 50.9)	49.3 (47.4 to 50.9)	-2.9 (-5.1 to -0.9)	-0.5 (-2.6 to 1.5)
Diet low in milk	46.3 (44.3 to 48.1)	46.7 (44.8 to 48.3)	47.1 (45.3 to 48.7)	0.7 (-1.2 to 2.8)	1.0 (-0.4 to 2.6)	46.3 (44.3 to 48.1)	46.1 (44.0 to 47.8)	46.7 (44.6 to 48.4)	-0.5 (-2.4 to 1.4)	1.3 (-0.1 to 3.0)
Diet high in red meat	3.7 (0.4 to 8.7)	7.4 (2.2 to 13.7)	8.4 (2.6 to 14.9)	98.9 (-13.9 to 975.7)	14.4 (-39.3 to 125.5)	0.5 (0.0 to 2.6)	1.4 (0.0 to 4.5)	1.8 (0.0 to 5.4)	156.3 (-88.7 to 2268460.1)	28.5 (-88.7 to 2817.9)
Diet high in processed meat	11.1 (6.8 to 15.4)	18.5 (13.1 to 23.4)	21.4 (15.7 to 26.9)	66.7 (31.5 to 131.6)	15.7 (-2.7 to 39.7)	9.2 (5.5 to 12.8)	13.6 (9.4 to 17.8)	15.1 (10.6 to 19.5)	47.3 (14.9 to 99.2)	10.8 (-6.7 to 31.9)
Diet high in sugar-sweetened beverages	6.9 (2.5 to 13.5)	7.9 (3.3 to 14.4)	8.3 (3.7 to 15.2)	14.3 (-47.0 to 157.9)	5.8 (-36.7 to 85.0)	4.9 (1.8 to 10.8)	5.5 (2.5 to 10.2)	5.9 (2.5 to 11.7)	10.6 (-49.4 to 164.2)	7.6 (-39.0 to 90.2)
Diet low in fibre	22.3 (14.9 to 28.8)	23.4 (16.4 to 29.6)	24.4 (17.1 to 30.4)	4.7 (-12.4 to 29.5)	4.3 (-9.2 to 19.2)	23.6 (16.2 to 29.9)	24.6 (17.5 to 30.6)	26.0 (18.8 to 32.3)	4.2 (-12.3 to 27.6)	5.8 (-4.6 to 19.4)
Diet low in calcium	35.2 (30.9 to 38.9)	34.9 (30.6 to 38.5)	35.7 (31.4 to 39.2)	-0.8 (-4.9 to 2.9)	2.2 (-0.9 to 5.6)	37.4 (33.5 to 40.5)	36.2 (32.2 to 39.6)	37.4 (33.6 to 40.6)	-3.0 (-6.6 to 0.4)	3.2 (0.7 to 6.5)
Diet low in seafood omega-3 fatty acids	3.3 (1.4 to 4.7)	0.3 (0.0 to 1.1)	0.3 (0.0 to 1.1)	-91.9 (-100.0 to -69.0)	5.6 (-93.1 to 1424.2)	3.8 (2.0 to 5.3)	0.4 (0.0 to 1.5)	0.5 (0.0 to 1.5)	-88.7 (-99.9 to -61.4)	6.0 (-80.0 to 619.5)
Diet low in polyunsaturated fatty acids	30.8 (25.3 to 35.6)	28.1 (22.3 to 32.9)	28.8 (22.8 to 33.6)	-8.8 (-20.7 to 0.1)	2.5 (-5.8 to 11.2)	31.5 (26.1 to 36.2)	28.8 (23.2 to 33.6)	29.6 (24.0 to 34.3)	-8.4 (-19.0 to 0.8)	2.5 (-6.0 to 15.0)
Diet high in trans fatty acids	1.1 (0.0 to 3.3)	0.9 (0.0 to 3.0)	0.9 (0.0 to 2.9)	-12.4 (-39.0 to -6.4)	-8.4 (-32.6 to -3.7)	1.5 (0.1 to 4.0)	1.3 (0.1 to 3.7)	1.2 (0.0 to 3.5)	-12.7 (-34.7 to -6.9)	-8.4 (-27.8 to -3.3)
Diet high in sodium	27.4 (18.0 to 35.5)	17.7 (8.8 to 25.1)	15.1 (6.5 to 22.4)	-35.6 (-57.3 to -17.9)	-14.6 (-40.1 to 3.4)	31.5 (22.9 to 38.7)	18.9 (10.4 to 26.0)	15.7 (7.0 to 22.9)	-39.9 (-60.4 to -25.0)	-17.0 (-40.3 to -4.7)
Intimate partner violence	-	-	-	-	-	2.4 (2.0 to 2.8)	2.2 (1.8 to 2.5)	2.1 (1.7 to 2.4)	-8.8 (-12.7 to -4.0)	-3.3 (-7.9 to 1.0)
Childhood sexual abuse	5.3 (4.3 to 6.5)	5.3 (4.3 to 6.5)	5.3 (4.3 to 6.5)	0.0 (0.0 to 0.0)	0.0 (0.0 to 0.0)	7.3 (5.9 to 8.9)	7.3 (5.9 to 8.9)	7.5 (6.0 to 9.2)	0.0 (0.0 to 0.0)	2.8 (-4.0 to 10.6)

Supplementary Results Table 1. Ehime age-standardised Summary Exposure Values (SEVs) for Level 4 risk factors and percentage change, by sex, 1990, 2007, and 2017.Data in parentheses are 95% uncertainty intervals.

	Male					Female				
	**1990**	**2007**	**2017**	**Percentage change 1990-2007**	**Percentage change 2007-2017**	**1990**	**2007**	**2017**	**Percentage change 1990-2007**	**Percentage change 2007-2017**
Bullying victimization	3.3 (1.4 to 7.1)	3.4 (1.5 to 7.1)	3.5 (1.5 to 7.3)	2.1 (-5.9 to 9.9)	3.2 (-2.2 to 8.0)	1.9 (0.8 to 4.1)	1.9 (0.8 to 4.0)	2.0 (0.8 to 4.2)	0.4 (-7.3 to 8.5)	2.6 (-3.0 to 7.6)
Low physical activity	0.5 (0.2 to 0.9)	0.5 (0.2 to 0.9)	0.4 (0.2 to 0.8)	-1.8 (-3.7 to -0.1)	-8.8 (-12.3 to -4.5)	0.5 (0.2 to 1.0)	0.5 (0.2 to 0.9)	0.5 (0.2 to 0.9)	-3.9 (-6.1 to -1.3)	0.8 (-0.8 to 2.2)
High fasting plasma glucose	1.4 (0.9 to 2.2)	1.4 (0.9 to 2.1)	1.5 (0.9 to 2.5)	-2.3 (-37.2 to 56.2)	11.3 (-25.1 to 73.6)	1.2 (0.7 to 2.0)	1.2 (0.7 to 2.0)	1.3 (0.8 to 2.3)	3.5 (-37.7 to 68.6)	9.8 (-32.1 to 88.6)
High LDL cholesterol	12.0 (10.3 to 13.8)	12.5 (10.9 to 14.4)	12.5 (10.9 to 14.4)	4.5 (-1.5 to 10.8)	0.1 (-4.6 to 4.8)	12.7 (11.2 to 14.5)	12.9 (11.3 to 14.6)	12.9 (11.3 to 14.6)	0.9 (-4.5 to 6.4)	0.2 (-4.0 to 4.8)
High systolic blood pressure	11.2 (10.2 to 12.2)	8.3 (7.4 to 9.2)	8.1 (7.2 to 9.2)	-26.2 (-32.0 to -19.9)	-1.6 (-9.4 to 7.0)	9.6 (8.6 to 10.6)	6.0 (5.3 to 6.8)	5.6 (4.8 to 6.4)	-37.1 (-43.9 to -30.5)	-7.0 (-16.6 to 3.5)
High body-mass index	5.4 (3.4 to 8.6)	7.6 (5.1 to 12.4)	7.9 (5.2 to 12.8)	40.9 (12.5 to 80.7)	3.7 (-12.4 to 22.4)	5.3 (3.2 to 8.3)	6.9 (4.3 to 10.9)	6.9 (4.2 to 11.2)	29.6 (1.2 to 74.4)	0.3 (-18.7 to 21.3)
Low bone mineral density	5.3 (4.3 to 6.4)	5.2 (4.3 to 6.1)	5.2 (4.3 to 6.2)	-2.3 (-14.9 to 12.7)	0.5 (-12.5 to 15.7)	7.2 (6.0 to 8.5)	6.9 (5.9 to 8.2)	7.1 (6.0 to 8.3)	-3.6 (-14.8 to 10.4)	2.2 (-10.9 to 16.3)
Impaired kidney function	2.6 (1.2 to 6.5)	2.6 (1.2 to 6.4)	2.4 (1.1 to 6.2)	-2.7 (-7.1 to 1.8)	-6.9 (-12.2 to -2.2)	3.2 (1.5 to 7.5)	3.3 (1.5 to 7.7)	3.1 (1.4 to 7.3)	3.1 (-0.5 to 7.9)	-7.3 (-12.2 to -2.3)

Supplementary Results Table 1. Fukui age-standardised Summary Exposure Values (SEVs) for Level 4 risk factors and percentage change, by sex, 1990, 2007, and 2017.Data in parentheses are 95% uncertainty intervals.

	Male					Female				
	**1990**	**2007**	**2017**	**Percentage change 1990-2007**	**Percentage change 2007-2017**	**1990**	**2007**	**2017**	**Percentage change 1990-2007**	**Percentage change 2007-2017**
Unsafe water source	2.3 (1.3 to 3.9)	1.9 (1.0 to 3.1)	2.0 (1.0 to 3.2)	-20.1 (-58.0 to 72.2)	4.7 (-39.1 to 80.2)	2.3 (1.3 to 3.9)	1.9 (1.0 to 3.1)	2.0 (1.0 to 3.2)	-20.1 (-58.0 to 72.2)	4.7 (-39.1 to 80.2)
Unsafe sanitation	4.0 (1.9 to 7.3)	1.9 (0.9 to 3.7)	1.6 (0.7 to 3.0)	-52.2 (-79.6 to 8.5)	-15.6 (-55.0 to 63.4)	4.0 (1.9 to 7.3)	1.9 (0.9 to 3.7)	1.6 (0.7 to 3.0)	-52.2 (-79.6 to 8.5)	-15.6 (-55.0 to 63.4)
No access to handwashing facility	6.7 (4.9 to 9.1)	6.3 (4.6 to 8.7)	5.9 (4.3 to 7.9)	-6.1 (-33.6 to 33.2)	-7.5 (-27.5 to 18.5)	6.7 (4.9 to 9.1)	6.3 (4.6 to 8.7)	5.9 (4.3 to 7.9)	-6.1 (-33.6 to 33.2)	-7.5 (-27.5 to 18.5)
Ambient particulate matter pollution	32.0 (20.1 to 47.6)	32.3 (22.2 to 46.6)	27.4 (16.7 to 43.6)	1.1 (-27.2 to 44.3)	-15.3 (-42.8 to 17.9)	31.7 (20.0 to 47.2)	32.3 (22.1 to 46.5)	27.3 (16.7 to 43.6)	1.8 (-27.0 to 46.3)	-15.3 (-42.9 to 18.0)
Household air pollution from solid fuels	0.8 (0.2 to 2.2)	0.2 (0.0 to 0.4)	0.1 (0.0 to 0.4)	-77.0 (-94.3 to -48.8)	-15.3 (-66.8 to 73.9)	1.1 (0.3 to 6.4)	0.3 (0.0 to 1.3)	0.2 (0.0 to 1.1)	-73.2 (-94.2 to -43.6)	-14.6 (-66.9 to 75.2)
Ambient ozone pollution	51.0 (23.5 to 54.8)	51.4 (24.3 to 54.8)	51.8 (25.5 to 54.8)	0.6 (0.0 to 3.8)	0.9 (0.0 to 5.6)	51.0 (23.5 to 54.8)	51.4 (24.3 to 54.8)	51.8 (25.5 to 54.8)	0.6 (0.0 to 3.8)	0.9 (0.0 to 5.6)
Residential radon	14.6 (0.9 to 45.5)	14.6 (0.9 to 45.5)	14.6 (0.9 to 45.5)	0.0 (0.0 to 0.0)	0.0 (0.0 to 0.0)	14.6 (0.9 to 45.5)	14.6 (0.9 to 45.5)	14.6 (0.9 to 45.5)	0.0 (0.0 to 0.0)	0.0 (0.0 to 0.0)
Lead exposure	2.9 (0.4 to 6.5)	2.0 (0.2 to 4.9)	1.4 (0.1 to 4.0)	-32.4 (-46.7 to -23.5)	-27.6 (-43.8 to -19.5)	1.6 (0.1 to 4.5)	1.0 (0.0 to 3.2)	0.7 (0.0 to 2.6)	-37.0 (-54.3 to -27.3)	-29.5 (-52.5 to -20.6)
Occupational exposure to asbestos	1.6 (1.4 to 1.9)	2.2 (2.0 to 2.4)	2.3 (1.9 to 2.8)	35.8 (17.1 to 60.9)	2.6 (-14.9 to 23.2)	0.5 (0.4 to 0.6)	0.5 (0.4 to 0.5)	0.4 (0.4 to 0.5)	-4.8 (-21.9 to 22.2)	-6.0 (-23.6 to 14.1)
Occupational exposure to arsenic	0.4 (0.0 to 1.1)	0.4 (0.0 to 1.0)	0.4 (0.0 to 1.0)	-	-	0.4 (0.0 to 1.0)	0.4 (0.0 to 0.9)	0.4 (0.0 to 0.9)	-	-
Occupational exposure to benzene	0.6 (0.0 to 1.8)	0.6 (0.0 to 1.8)	0.7 (0.0 to 1.9)	2.6 (-4.9 to 12.9)	3.5 (-2.0 to 11.1)	0.6 (0.0 to 1.7)	0.7 (0.0 to 2.0)	0.8 (0.1 to 2.3)	22.5 (8.4 to 42.3)	14.6 (5.1 to 30.7)
Occupational exposure to beryllium	0.0 (0.0 to 0.0)	0.0 (0.0 to 0.0)	0.0 (0.0 to 0.0)	-5.3 (-10.4 to -0.1)	2.0 (-2.0 to 6.3)	0.0 (0.0 to 0.0)	0.0 (0.0 to 0.0)	0.0 (0.0 to 0.0)	2.9 (-5.7 to 14.1)	9.2 (2.8 to 16.6)
Occupational exposure to cadmium	0.1 (0.0 to 0.1)	0.0 (0.0 to 0.1)	0.0 (0.0 to 0.1)	-7.0 (-17.6 to 5.9)	0.8 (-8.7 to 11.3)	0.0 (0.0 to 0.1)	0.0 (0.0 to 0.0)	0.0 (0.0 to 0.1)	-7.8 (-21.9 to 10.2)	4.8 (-7.0 to 19.4)
Occupational exposure to chromium	0.1 (0.1 to 0.1)	0.1 (0.1 to 0.1)	0.1 (0.1 to 0.1)	-5.2 (-15.0 to 6.3)	1.4 (-7.1 to 10.7)	0.1 (0.1 to 0.1)	0.1 (0.1 to 0.1)	0.1 (0.1 to 0.1)	-3.0 (-16.5 to 13.8)	6.7 (-3.6 to 19.1)
Occupational exposure to diesel engine exhaust	0.6 (0.5 to 0.6)	0.6 (0.5 to 0.6)	0.6 (0.6 to 0.7)	-1.3 (-8.9 to 7.2)	3.5 (-3.3 to 10.7)	0.4 (0.4 to 0.5)	0.5 (0.4 to 0.6)	0.6 (0.5 to 0.6)	13.7 (-0.0 to 28.4)	11.5 (1.3 to 22.3)
Occupational exposure to formaldehyde	0.2 (0.2 to 0.3)	0.2 (0.2 to 0.3)	0.2 (0.2 to 0.3)	-6.3 (-17.7 to 7.6)	0.4 (-9.5 to 11.8)	0.2 (0.2 to 0.2)	0.2 (0.2 to 0.2)	0.2 (0.2 to 0.2)	-5.1 (-19.9 to 14.3)	5.7 (-6.3 to 20.5)
Occupational exposure to nickel	0.4 (0.0 to 1.9)	0.4 (0.0 to 1.7)	0.4 (0.0 to 1.7)	-	-	0.3 (0.0 to 1.5)	0.3 (0.0 to 1.3)	0.3 (0.0 to 1.4)	-	-
Occupational exposure to polycyclic aromatic hydrocarbons	0.2 (0.2 to 0.3)	0.2 (0.2 to 0.3)	0.2 (0.2 to 0.3)	-4.2 (-13.5 to 6.6)	1.7 (-6.0 to 10.1)	0.2 (0.2 to 0.2)	0.2 (0.2 to 0.2)	0.2 (0.2 to 0.3)	-1.0 (-13.7 to 15.6)	7.6 (-2.1 to 19.7)
Occupational exposure to silica	4.5 (0.7 to 14.9)	4.3 (0.6 to 14.0)	4.3 (0.6 to 14.4)	-5.8 (-15.9 to 6.2)	1.6 (-7.7 to 13.2)	3.1 (0.5 to 10.2)	2.7 (0.4 to 8.4)	2.8 (0.4 to 8.7)	-12.8 (-27.1 to 7.3)	2.0 (-11.1 to 19.8)
Occupational exposure to sulphuric acid	0.6 (0.1 to 1.9)	0.6 (0.1 to 1.8)	0.6 (0.1 to 1.8)	-7.1 (-17.1 to 5.3)	1.1 (-8.0 to 11.1)	0.5 (0.1 to 1.7)	0.5 (0.1 to 1.5)	0.5 (0.1 to 1.6)	-9.5 (-23.6 to 8.4)	4.2 (-8.0 to 19.2)
Occupational exposure to trichloroethylene	0.1 (0.1 to 0.1)	0.1 (0.1 to 0.1)	0.1 (0.1 to 0.1)	-4.2 (-11.2 to 4.2)	2.0 (-4.2 to 8.6)	0.1 (0.1 to 0.1)	0.1 (0.1 to 0.1)	0.1 (0.1 to 0.1)	2.0 (-9.0 to 16.0)	8.3 (0.5 to 19.1)
Occupational asthmagens	18.1 (14.4 to 23.3)	17.6 (13.9 to 22.1)	17.6 (13.9 to 22.1)	-2.6 (-21.5 to 19.7)	-0.3 (-14.9 to 17.1)	8.1 (6.5 to 10.1)	8.2 (6.5 to 10.1)	8.6 (6.9 to 10.6)	0.9 (-14.5 to 19.1)	5.3 (-7.4 to 19.5)
Occupational particulate matter, gases, and fumes	6.4 (4.5 to 9.0)	6.1 (4.3 to 8.8)	5.9 (4.2 to 8.4)	-4.6 (-9.4 to 0.3)	-3.8 (-7.1 to -0.2)	3.6 (2.5 to 5.3)	3.4 (2.4 to 5.1)	3.3 (2.3 to 4.9)	-5.4 (-13.4 to 4.1)	-4.4 (-10.1 to 1.7)
Occupational noise	5.9 (5.5 to 6.5)	5.7 (5.3 to 6.2)	5.6 (5.2 to 6.1)	-3.5 (-6.6 to -0.3)	-2.5 (-4.5 to -0.3)	3.4 (3.0 to 4.0)	3.4 (3.0 to 3.9)	3.5 (3.1 to 3.9)	0.4 (-5.7 to 7.4)	1.9 (-2.1 to 6.0)
Occupational ergonomic factors	10.7 (8.8 to 12.7)	9.5 (7.8 to 11.4)	9.4 (7.7 to 11.4)	-11.6 (-23.2 to 2.5)	-1.2 (-12.9 to 11.5)	5.8 (4.4 to 7.4)	5.9 (4.5 to 7.5)	6.6 (5.0 to 8.5)	1.6 (-22.1 to 31.2)	12.4 (-8.9 to 37.0)
Non-exclusive breastfeeding	0.4 (0.3 to 0.6)	0.4 (0.3 to 0.6)	0.4 (0.3 to 0.6)	-1.1 (-13.9 to 12.6)	-1.5 (-12.4 to 9.9)	0.4 (0.3 to 0.6)	0.4 (0.3 to 0.6)	0.4 (0.3 to 0.6)	-1.7 (-14.3 to 12.0)	-1.5 (-12.4 to 10.0)
Discontinued breastfeeding	1.7 (1.6 to 1.8)	1.7 (1.6 to 1.9)	1.7 (1.5 to 1.8)	2.3 (-6.5 to 12.2)	-2.9 (-9.9 to 5.0)	1.7 (1.6 to 1.8)	1.7 (1.6 to 1.9)	1.7 (1.6 to 1.8)	2.0 (-6.7 to 11.9)	-2.0 (-9.0 to 5.9)
Child underweight	0.3 (0.2 to 0.4)	0.2 (0.1 to 0.3)	0.2 (0.1 to 0.3)	-16.2 (-34.8 to 6.3)	-5.5 (-19.6 to 10.9)	0.3 (0.2 to 0.4)	0.2 (0.1 to 0.3)	0.2 (0.1 to 0.3)	-18.5 (-38.9 to 6.2)	-6.3 (-22.5 to 12.6)
Child wasting	0.2 (0.1 to 0.3)	0.2 (0.1 to 0.3)	0.2 (0.1 to 0.3)	-7.3 (-33.6 to 29.3)	-2.2 (-21.5 to 21.7)	0.2 (0.1 to 0.3)	0.2 (0.1 to 0.3)	0.2 (0.1 to 0.3)	-8.7 (-36.2 to 28.1)	-3.6 (-24.7 to 21.7)
Child stunting	0.6 (0.3 to 1.0)	0.5 (0.3 to 1.0)	0.5 (0.3 to 0.9)	-11.1 (-45.3 to 48.9)	-4.0 (-33.0 to 39.2)	0.6 (0.3 to 1.1)	0.5 (0.3 to 1.0)	0.5 (0.2 to 0.9)	-15.2 (-51.2 to 49.9)	-3.9 (-33.5 to 38.5)
Short gestation for birth weight	0.0 (0.0 to 0.0)	0.0 (0.0 to 0.0)	0.0 (0.0 to 0.0)	10.5 (-6.9 to 35.5)	2.9 (-14.4 to 29.0)	0.0 (0.0 to 0.0)	0.0 (0.0 to 0.0)	0.0 (0.0 to 0.0)	14.1 (-19.1 to 77.8)	-1.8 (-20.4 to 26.6)
Low birth weight for gestation	0.0 (0.0 to 0.0)	0.0 (0.0 to 0.0)	0.0 (0.0 to 0.0)	4.0 (-11.3 to 23.5)	1.7 (-16.5 to 19.6)	0.0 (0.0 to 0.0)	0.0 (0.0 to 0.0)	0.0 (0.0 to 0.0)	-4.0 (-57.2 to 32.9)	6.0 (-14.3 to 25.8)
Iron deficiency	-	-	-	-	-	8.1 (5.4 to 11.2)	8.0 (5.6 to 11.1)	7.4 (4.9 to 10.4)	-0.4 (-22.3 to 29.0)	-8.2 (-24.0 to 9.3)
Vitamin A deficiency	0.9 (0.6 to 1.3)	0.8 (0.5 to 1.1)	0.7 (0.4 to 1.0)	-12.0 (-31.4 to 26.2)	-19.5 (-34.2 to -3.5)	0.8 (0.5 to 1.1)	0.7 (0.5 to 1.0)	0.7 (0.4 to 0.9)	-6.4 (-22.0 to 14.6)	-12.5 (-36.8 to 10.2)
Zinc deficiency	0.5 (0.1 to 1.0)	0.5 (0.1 to 1.2)	0.4 (0.1 to 1.2)	12.3 (-58.6 to 78.6)	-16.5 (-69.0 to 111.8)	0.5 (0.1 to 1.0)	0.4 (0.1 to 0.9)	0.3 (0.1 to 0.8)	-24.9 (-64.9 to 71.6)	-7.1 (-64.0 to 168.2)
Smoking	18.0 (14.5 to 21.2)	12.5 (9.9 to 15.3)	10.5 (8.4 to 12.8)	-30.5 (-39.7 to -20.3)	-15.9 (-24.5 to -7.8)	4.0 (2.9 to 5.4)	3.3 (2.4 to 4.5)	3.2 (2.3 to 4.3)	-17.6 (-38.1 to 9.9)	-2.9 (-18.9 to 16.1)
Chewing tobacco	1.4 (0.9 to 2.0)	0.9 (0.6 to 1.4)	0.7 (0.5 to 1.1)	-33.2 (-56.7 to 7.2)	-19.1 (-41.6 to 15.5)	0.1 (0.1 to 0.2)	0.1 (0.1 to 0.1)	0.1 (0.1 to 0.1)	-3.4 (-43.0 to 58.3)	-2.4 (-33.2 to 40.3)
Second-hand smoke	37.4 (33.1 to 41.4)	35.8 (31.9 to 40.1)	35.7 (31.4 to 40.4)	-4.2 (-15.6 to 7.3)	-0.1 (-9.5 to 10.0)	56.4 (52.8 to 60.1)	48.1 (44.4 to 52.0)	47.8 (43.6 to 52.1)	-14.8 (-20.6 to -8.4)	-0.6 (-6.8 to 6.1)
Alcohol use	14.6 (7.1 to 25.2)	14.6 (7.1 to 24.9)	16.4 (7.9 to 28.2)	0.0 (-33.6 to 47.1)	12.0 (-20.0 to 58.5)	11.5 (5.6 to 20.4)	11.5 (5.5 to 20.4)	13.0 (6.1 to 23.1)	-0.1 (-33.5 to 46.4)	13.4 (-18.7 to 60.2)
Drug use	0.5 (0.4 to 0.7)	0.5 (0.4 to 0.7)	0.6 (0.4 to 0.8)	0.2 (-5.7 to 6.1)	11.4 (4.4 to 19.8)	0.3 (0.2 to 0.4)	0.3 (0.2 to 0.4)	0.3 (0.3 to 0.4)	2.0 (-3.6 to 8.6)	8.4 (1.7 to 15.7)
Diet low in fruits	40.4 (34.7 to 44.3)	38.7 (32.9 to 42.9)	40.2 (34.6 to 44.1)	-4.2 (-10.5 to 2.2)	3.7 (-1.4 to 9.6)	37.3 (31.3 to 41.8)	34.5 (27.9 to 39.4)	36.9 (30.7 to 41.7)	-7.6 (-16.4 to 0.8)	7.1 (0.7 to 16.2)
Diet low in vegetables	18.3 (9.5 to 26.2)	12.4 (4.7 to 20.5)	14.5 (6.2 to 22.7)	-32.1 (-63.8 to -1.1)	16.8 (-16.8 to 82.6)	20.5 (11.9 to 28.0)	14.6 (6.7 to 22.6)	16.4 (8.0 to 24.8)	-28.9 (-57.2 to -1.9)	12.7 (-17.7 to 67.3)
Diet low in legumes	4.5 (0.8 to 10.1)	5.9 (1.0 to 11.8)	8.0 (2.0 to 14.7)	32.7 (-45.4 to 239.3)	34.3 (-19.8 to 192.4)	8.4 (3.0 to 15.2)	8.6 (3.2 to 15.1)	11.2 (4.4 to 18.5)	1.8 (-39.0 to 85.7)	30.0 (-11.7 to 115.0)
Diet low in whole grains	30.0 (24.9 to 34.3)	34.8 (30.5 to 38.5)	36.2 (32.2 to 39.8)	16.3 (7.0 to 30.0)	3.9 (-1.2 to 9.8)	31.5 (26.7 to 35.4)	36.1 (32.1 to 39.4)	37.4 (33.5 to 40.7)	14.7 (6.2 to 25.0)	3.5 (-1.4 to 8.8)
Diet low in nuts and seeds	50.3 (48.9 to 51.5)	48.9 (46.9 to 50.5)	48.7 (46.5 to 50.4)	-2.9 (-5.7 to -0.5)	-0.4 (-2.8 to 1.8)	50.4 (49.0 to 51.5)	48.7 (46.8 to 50.3)	48.4 (46.0 to 50.2)	-3.3 (-6.1 to -0.9)	-0.6 (-3.1 to 1.6)
Diet low in milk	46.0 (43.9 to 47.8)	46.5 (44.5 to 48.2)	47.0 (45.0 to 48.7)	0.9 (-0.9 to 3.0)	1.2 (-0.3 to 2.7)	46.0 (43.8 to 47.7)	45.8 (43.6 to 47.6)	46.5 (44.4 to 48.2)	-0.4 (-2.4 to 1.8)	1.5 (-0.0 to 3.0)
Diet high in red meat	4.4 (0.7 to 9.7)	8.6 (2.9 to 15.9)	9.8 (3.7 to 17.5)	95.3 (-12.3 to 601.1)	14.2 (-36.3 to 114.2)	0.7 (0.0 to 2.6)	1.9 (0.0 to 5.5)	2.4 (0.1 to 6.6)	179.5 (-68.5 to 373252.2)	26.6 (-83.2 to 2359.2)
Diet high in processed meat	12.2 (7.9 to 16.6)	20.2 (14.7 to 25.4)	23.4 (17.1 to 29.5)	65.2 (28.7 to 125.6)	15.9 (-1.3 to 37.9)	10.3 (6.3 to 14.0)	15.2 (10.8 to 19.1)	16.8 (11.9 to 21.1)	47.6 (17.2 to 102.3)	10.4 (-6.2 to 33.7)
Diet high in sugar-sweetened beverages	8.1 (3.2 to 15.1)	9.2 (4.1 to 16.0)	9.6 (4.4 to 17.3)	14.1 (-44.3 to 143.0)	3.9 (-36.4 to 67.8)	5.7 (2.1 to 12.3)	6.4 (2.7 to 12.3)	6.7 (2.6 to 12.7)	12.4 (-50.5 to 162.3)	5.2 (-40.6 to 85.4)
Diet low in fibre	21.6 (13.9 to 28.3)	22.6 (15.2 to 29.1)	23.6 (16.2 to 30.0)	4.8 (-12.9 to 28.0)	4.5 (-8.3 to 19.6)	22.7 (15.5 to 29.2)	23.7 (16.7 to 29.7)	25.1 (18.2 to 31.3)	4.3 (-13.2 to 27.0)	6.0 (-5.4 to 19.0)
Diet low in calcium	33.9 (29.4 to 37.6)	33.8 (29.1 to 37.5)	34.6 (30.2 to 38.3)	-0.4 (-4.8 to 3.8)	2.5 (-0.5 to 6.0)	36.3 (32.3 to 39.7)	35.3 (30.9 to 38.8)	36.5 (32.5 to 40.0)	-2.8 (-6.5 to 0.9)	3.6 (0.8 to 7.3)
Diet low in seafood omega-3 fatty acids	2.9 (1.2 to 4.3)	0.2 (0.0 to 0.9)	0.2 (0.0 to 1.0)	-92.2 (-100.0 to -68.5)	6.9 (-92.5 to 3897.7)	3.5 (1.5 to 5.0)	0.3 (0.0 to 1.2)	0.3 (0.0 to 1.3)	-90.9 (-100.0 to -68.2)	7.8 (-85.7 to 2914.0)
Diet low in polyunsaturated fatty acids	29.7 (24.2 to 34.8)	26.9 (20.7 to 32.2)	27.7 (21.2 to 32.7)	-9.3 (-22.2 to 0.8)	2.7 (-6.4 to 13.7)	30.4 (24.6 to 35.1)	27.6 (21.2 to 32.7)	28.3 (22.5 to 33.4)	-9.1 (-20.3 to 0.2)	2.6 (-5.8 to 13.6)
Diet high in trans fatty acids	1.1 (0.0 to 3.3)	0.9 (0.0 to 3.0)	0.9 (0.0 to 2.9)	-12.5 (-38.2 to -6.6)	-8.4 (-31.8 to -3.8)	1.5 (0.1 to 4.0)	1.3 (0.1 to 3.7)	1.2 (0.0 to 3.5)	-12.5 (-34.7 to -6.6)	-8.4 (-28.3 to -3.8)
Diet high in sodium	28.8 (19.4 to 36.5)	18.9 (10.6 to 26.1)	16.3 (7.5 to 23.3)	-34.2 (-55.6 to -15.6)	-13.9 (-39.3 to 2.5)	32.3 (23.8 to 39.5)	19.7 (11.2 to 26.7)	16.4 (7.6 to 23.8)	-39.1 (-58.6 to -25.0)	-16.6 (-37.9 to -3.7)
Intimate partner violence	-	-	-	-	-	2.2 (1.8 to 2.5)	2.0 (1.6 to 2.3)	1.9 (1.6 to 2.2)	-8.8 (-12.9 to -4.5)	-3.3 (-7.5 to 1.1)
Childhood sexual abuse	5.3 (4.3 to 6.5)	5.3 (4.3 to 6.5)	5.3 (4.3 to 6.5)	0.0 (0.0 to 0.0)	0.0 (0.0 to 0.0)	7.3 (5.9 to 8.9)	7.3 (5.9 to 8.9)	7.5 (6.0 to 9.2)	0.0 (0.0 to 0.0)	2.8 (-4.0 to 10.6)

Supplementary Results Table 1. Fukui age-standardised Summary Exposure Values (SEVs) for Level 4 risk factors and percentage change, by sex, 1990, 2007, and 2017.Data in parentheses are 95% uncertainty intervals.										
	**Male**					**Female**				
	**1990**	**2007**	**2017**	**Percentage change 1990-2007**	**Percentage change 2007-2017**	**1990**	**2007**	**2017**	**Percentage change 1990-2007**	**Percentage change 2007-2017**
Bullying victimization	3.3 (1.4 to 6.9)	3.4 (1.4 to 6.9)	3.5 (1.4 to 7.2)	2.3 (-5.6 to 10.1)	3.4 (-1.8 to 8.1)	1.9 (0.8 to 4.0)	1.9 (0.8 to 4.0)	2.0 (0.8 to 4.0)	0.8 (-7.0 to 8.6)	2.6 (-3.1 to 7.6)
Low physical activity	0.5 (0.2 to 0.9)	0.5 (0.2 to 0.9)	0.4 (0.2 to 0.8)	-1.8 (-3.7 to 0.1)	-8.8 (-12.3 to -4.4)	0.5 (0.2 to 1.0)	0.5 (0.2 to 0.9)	0.5 (0.2 to 0.9)	-3.9 (-5.9 to -1.4)	0.7 (-0.9 to 2.2)
High fasting plasma glucose	1.4 (0.9 to 2.3)	1.4 (0.9 to 2.2)	1.6 (0.9 to 2.7)	-0.3 (-39.6 to 55.2)	15.3 (-22.0 to 88.1)	1.2 (0.7 to 1.9)	1.2 (0.7 to 2.1)	1.3 (0.8 to 2.3)	2.2 (-39.3 to 69.0)	9.6 (-30.7 to 81.9)
High LDL cholesterol	12.0 (10.3 to 13.8)	12.5 (10.9 to 14.3)	12.5 (10.9 to 14.3)	4.6 (-1.0 to 11.3)	0.1 (-4.5 to 5.1)	12.8 (11.3 to 14.6)	12.9 (11.4 to 14.6)	13.0 (11.4 to 14.8)	1.0 (-4.4 to 6.8)	0.3 (-4.0 to 4.8)
High systolic blood pressure	11.2 (10.1 to 12.3)	8.3 (7.4 to 9.2)	8.1 (7.2 to 9.2)	-26.1 (-32.4 to -19.7)	-1.5 (-9.3 to 6.1)	9.6 (8.6 to 10.6)	6.0 (5.2 to 6.8)	5.6 (4.8 to 6.4)	-37.2 (-43.9 to -30.0)	-7.1 (-16.5 to 2.8)
High body-mass index	5.4 (3.4 to 8.6)	7.6 (5.1 to 12.0)	7.9 (5.1 to 12.5)	42.7 (17.3 to 81.6)	3.1 (-13.5 to 21.4)	5.5 (3.3 to 8.9)	7.1 (4.4 to 10.8)	7.2 (4.4 to 11.1)	29.1 (-1.0 to 71.8)	1.1 (-17.4 to 23.7)
Low bone mineral density	5.3 (4.4 to 6.3)	5.2 (4.3 to 6.1)	5.2 (4.2 to 6.2)	-2.3 (-14.4 to 12.9)	0.3 (-12.1 to 15.8)	7.2 (6.0 to 8.6)	7.0 (6.0 to 8.1)	7.1 (5.9 to 8.3)	-3.4 (-15.2 to 9.3)	1.8 (-10.9 to 15.7)
Impaired kidney function	2.6 (1.2 to 6.5)	2.5 (1.1 to 6.3)	2.4 (1.1 to 6.1)	-3.2 (-7.8 to 0.8)	-6.2 (-11.7 to -1.8)	3.2 (1.5 to 7.4)	3.3 (1.5 to 7.6)	3.1 (1.4 to 7.3)	2.8 (-0.5 to 7.4)	-7.1 (-11.9 to -2.3)

Supplementary Results Table 1. Fukuoka age-standardised Summary Exposure Values (SEVs) for Level 4 risk factors and percentage change, by sex, 1990, 2007, and 2017.Data in parentheses are 95% uncertainty intervals.

	Male					Female				
	1990	2007	2017	Percentage change 1990-2007	Percentage change 2007-2017	1990	2007	2017	Percentage change 1990-2007	Percentage change 2007-2017
Unsafe water source	2.1 (1.1 to 3.5)	1.8 (1.0 to 3.0)	1.9 (1.1 to 3.2)	-15.4 (-59.1 to 70.5)	7.4 (-37.4 to 84.1)	2.1 (1.1 to 3.5)	1.8 (1.0 to 3.0)	1.9 (1.1 to 3.2)	-15.4 (-59.1 to 70.5)	7.4 (-37.4 to 84.1)
Unsafe sanitation	3.5 (1.7 to 6.3)	1.8 (0.8 to 3.2)	1.6 (0.7 to 3.0)	-48.1 (-77.3 to 17.5)	-12.4 (-55.8 to 61.8)	3.5 (1.7 to 6.3)	1.8 (0.8 to 3.2)	1.6 (0.7 to 3.0)	-48.1 (-77.3 to 17.5)	-12.4 (-55.8 to 61.8)
No access to handwashing facility	6.7 (4.8 to 9.0)	6.4 (4.6 to 8.6)	5.9 (4.3 to 7.8)	-4.7 (-33.5 to 32.8)	-7.6 (-28.6 to 15.8)	6.7 (4.8 to 9.0)	6.4 (4.6 to 8.6)	5.9 (4.3 to 7.8)	-4.7 (-33.5 to 32.8)	-7.6 (-28.6 to 15.8)
Ambient particulate matter pollution	37.0 (25.3 to 53.2)	37.3 (25.9 to 52.0)	32.7 (22.0 to 46.5)	0.7 (-19.6 to 30.6)	-12.3 (-29.2 to 4.8)	36.9 (25.1 to 53.1)	37.3 (25.9 to 52.0)	32.7 (22.0 to 46.5)	1.0 (-19.4 to 31.2)	-12.3 (-29.3 to 4.8)
Household air pollution from solid fuels	0.4 (0.1 to 0.9)	0.1 (0.0 to 0.2)	0.1 (0.0 to 0.2)	-72.2 (-92.3 to -45.2)	-13.2 (-66.3 to 79.7)	0.6 (0.2 to 3.4)	0.2 (0.0 to 0.7)	0.2 (0.0 to 0.5)	-69.1 (-92.3 to -39.8)	-9.4 (-66.2 to 83.9)
Ambient ozone pollution	49.1 (20.3 to 54.8)	49.7 (21.2 to 54.8)	51.3 (24.1 to 54.8)	1.3 (0.0 to 5.1)	3.1 (0.0 to 14.5)	49.1 (20.3 to 54.8)	49.7 (21.2 to 54.8)	51.3 (24.1 to 54.8)	1.3 (0.0 to 5.1)	3.1 (0.0 to 14.5)
Residential radon	12.5 (0.8 to 39.6)	12.5 (0.8 to 39.6)	12.5 (0.8 to 39.6)	0.0 (0.0 to 0.0)	0.0 (0.0 to 0.0)	12.5 (0.8 to 39.6)	12.5 (0.8 to 39.6)	12.5 (0.8 to 39.6)	0.0 (0.0 to 0.0)	0.0 (0.0 to 0.0)
Lead exposure	3.4 (0.7 to 7.2)	2.1 (0.3 to 5.1)	1.5 (0.2 to 3.9)	-37.3 (-51.4 to -27.7)	-31.2 (-46.4 to -23.0)	1.8 (0.2 to 4.8)	1.0 (0.1 to 3.2)	0.7 (0.0 to 2.4)	-44.2 (-62.4 to -33.9)	-35.3 (-57.4 to -25.4)
Occupational exposure to asbestos	2.7 (2.3 to 3.0)	3.6 (3.4 to 3.9)	3.4 (2.8 to 4.1)	34.7 (17.4 to 62.5)	-7.1 (-23.6 to 14.7)	0.7 (0.6 to 0.8)	0.7 (0.6 to 0.8)	0.6 (0.5 to 0.8)	-0.2 (-16.3 to 26.2)	-10.3 (-29.4 to 11.0)
Occupational exposure to arsenic	0.5 (0.0 to 1.1)	0.4 (0.0 to 1.0)	0.4 (0.0 to 1.1)	-	-	0.4 (0.0 to 1.0)	0.4 (0.0 to 0.9)	0.4 (0.0 to 0.9)	-	-
Occupational exposure to benzene	0.6 (0.0 to 1.8)	0.7 (0.0 to 1.9)	0.7 (0.0 to 2.0)	2.3 (-3.8 to 12.7)	3.2 (-2.5 to 11.5)	0.6 (0.0 to 1.8)	0.7 (0.0 to 2.1)	0.8 (0.0 to 2.5)	20.0 (7.9 to 38.9)	13.3 (4.5 to 26.6)
Occupational exposure to beryllium	0.0 (0.0 to 0.0)	0.0 (0.0 to 0.0)	0.0 (0.0 to 0.0)	-5.3 (-10.2 to -0.3)	1.9 (-2.0 to 5.7)	0.0 (0.0 to 0.0)	0.0 (0.0 to 0.0)	0.0 (0.0 to 0.0)	1.3 (-6.8 to 9.9)	8.4 (2.0 to 15.3)
Occupational exposure to cadmium	0.1 (0.0 to 0.1)	0.0 (0.0 to 0.1)	0.1 (0.0 to 0.1)	-7.5 (-18.3 to 4.0)	0.9 (-8.8 to 11.1)	0.0 (0.0 to 0.1)	0.0 (0.0 to 0.1)	0.0 (0.0 to 0.1)	-9.6 (-22.4 to 6.7)	4.1 (-8.6 to 18.8)
Occupational exposure to chromium	0.1 (0.1 to 0.1)	0.1 (0.1 to 0.1)	0.1 (0.1 to 0.1)	-5.9 (-15.3 to 4.3)	1.4 (-7.2 to 10.2)	0.1 (0.1 to 0.1)	0.1 (0.1 to 0.1)	0.1 (0.1 to 0.1)	-5.1 (-17.0 to 9.5)	5.8 (-5.0 to 18.3)
Occupational exposure to diesel engine exhaust	0.6 (0.6 to 0.6)	0.6 (0.5 to 0.6)	0.6 (0.6 to 0.6)	-2.7 (-10.9 to 5.8)	2.8 (-3.7 to 9.7)	0.5 (0.4 to 0.5)	0.5 (0.5 to 0.6)	0.6 (0.5 to 0.6)	10.6 (-2.7 to 26.2)	10.3 (0.0 to 22.3)
Occupational exposure to formaldehyde	0.3 (0.2 to 0.3)	0.2 (0.2 to 0.3)	0.2 (0.2 to 0.3)	-6.9 (-18.3 to 5.2)	0.5 (-9.8 to 11.1)	0.2 (0.2 to 0.3)	0.2 (0.2 to 0.2)	0.2 (0.2 to 0.2)	-7.0 (-20.5 to 10.1)	4.9 (-7.8 to 19.2)
Occupational exposure to nickel	0.4 (0.0 to 2.0)	0.4 (0.0 to 1.8)	0.4 (0.0 to 1.7)	-	-	0.4 (0.0 to 1.6)	0.3 (0.0 to 1.3)	0.3 (0.0 to 1.4)	-	-
Occupational exposure to polycyclic aromatic hydrocarbons	0.2 (0.2 to 0.3)	0.2 (0.2 to 0.3)	0.2 (0.2 to 0.3)	-4.9 (-13.8 to 4.5)	1.7 (-6.0 to 9.6)	0.2 (0.2 to 0.3)	0.2 (0.2 to 0.2)	0.2 (0.2 to 0.3)	-2.9 (-14.8 to 10.9)	6.7 (-3.5 to 18.1)
Occupational exposure to silica	4.8 (0.7 to 15.3)	4.5 (0.6 to 14.4)	4.6 (0.7 to 14.6)	-7.2 (-17.4 to 4.8)	1.2 (-7.4 to 11.3)	3.3 (0.5 to 10.5)	2.8 (0.4 to 8.8)	2.8 (0.4 to 9.1)	-14.8 (-28.3 to 2.2)	1.3 (-11.9 to 20.2)
Occupational exposure to sulphuric acid	0.6 (0.1 to 2.0)	0.6 (0.1 to 1.8)	0.6 (0.1 to 1.9)	-7.5 (-17.9 to 3.4)	1.2 (-8.6 to 11.2)	0.5 (0.1 to 1.7)	0.5 (0.1 to 1.5)	0.5 (0.1 to 1.5)	-11.1 (-23.8 to 5.7)	3.7 (-9.7 to 19.3)
Occupational exposure to trichloroethylene	0.1 (0.1 to 0.1)	0.1 (0.1 to 0.1)	0.1 (0.1 to 0.1)	-4.8 (-12.0 to 2.5)	1.8 (-4.4 to 8.4)	0.1 (0.1 to 0.1)	0.1 (0.1 to 0.1)	0.1 (0.1 to 0.1)	-0.2 (-10.3 to 11.1)	7.4 (-1.0 to 17.0)
Occupational asthmagens	19.7 (15.6 to 24.4)	18.6 (14.9 to 23.2)	18.5 (14.7 to 23.3)	-5.6 (-22.5 to 16.5)	-0.8 (-15.5 to 16.7)	8.5 (6.9 to 10.6)	8.5 (6.8 to 10.3)	8.9 (7.2 to 10.7)	-0.8 (-14.9 to 15.5)	4.7 (-8.6 to 18.3)
Occupational particulate matter, gases, and fumes	6.4 (4.6 to 9.1)	6.1 (4.4 to 8.8)	5.9 (4.2 to 8.5)	-4.6 (-9.7 to 0.5)	-3.7 (-7.1 to -0.5)	3.7 (2.6 to 5.7)	3.5 (2.4 to 5.3)	3.3 (2.3 to 5.0)	-5.7 (-13.7 to 3.1)	-4.8 (-10.0 to 1.1)
Occupational noise	6.0 (5.5 to 6.6)	5.8 (5.4 to 6.3)	5.7 (5.3 to 6.2)	-3.2 (-6.3 to 0.1)	-2.4 (-4.4 to -0.4)	3.6 (3.1 to 4.1)	3.6 (3.2 to 4.1)	3.6 (3.3 to 4.1)	0.2 (-5.5 to 6.7)	1.6 (-2.1 to 5.6)
Occupational ergonomic factors	10.0 (8.3 to 12.1)	8.9 (7.4 to 10.7)	8.8 (7.3 to 10.5)	-11.2 (-21.6 to 1.1)	-1.0 (-11.3 to 10.1)	5.4 (4.1 to 6.8)	5.5 (4.4 to 6.9)	6.2 (4.8 to 7.9)	2.2 (-18.1 to 28.9)	13.4 (-5.3 to 36.7)
Non-exclusive breastfeeding	0.4 (0.3 to 0.6)	0.4 (0.3 to 0.6)	0.4 (0.3 to 0.6)	-1.0 (-14.2 to 13.3)	-1.7 (-12.0 to 9.8)	0.4 (0.3 to 0.6)	0.4 (0.3 to 0.6)	0.4 (0.3 to 0.6)	-1.0 (-14.2 to 13.3)	-1.7 (-12.0 to 9.8)
Discontinued breastfeeding	1.7 (1.6 to 1.8)	1.7 (1.6 to 1.9)	1.7 (1.6 to 1.8)	1.9 (-7.0 to 12.9)	-2.8 (-9.7 to 5.0)	1.7 (1.6 to 1.8)	1.7 (1.6 to 1.9)	1.7 (1.6 to 1.8)	1.9 (-7.0 to 12.9)	-2.8 (-9.7 to 5.0)
Child underweight	0.3 (0.2 to 0.4)	0.2 (0.1 to 0.3)	0.2 (0.1 to 0.3)	-12.9 (-30.6 to 6.9)	-4.5 (-17.6 to 11.4)	0.3 (0.2 to 0.4)	0.2 (0.1 to 0.3)	0.2 (0.1 to 0.3)	-15.0 (-34.8 to 7.5)	-5.2 (-19.7 to 12.0)
Child wasting	0.2 (0.1 to 0.3)	0.2 (0.1 to 0.3)	0.2 (0.1 to 0.3)	-6.5 (-30.8 to 26.6)	-1.8 (-20.1 to 20.3)	0.2 (0.1 to 0.3)	0.2 (0.1 to 0.3)	0.2 (0.1 to 0.3)	-8.1 (-33.5 to 25.8)	-2.1 (-21.1 to 21.5)
Child stunting	0.7 (0.4 to 1.2)	0.7 (0.4 to 0.9)	0.7 (0.4 to 1.0)	-5.4 (-36.9 to 43.9)	-3.1 (-31.3 to 34.7)	0.7 (0.4 to 1.2)	0.7 (0.4 to 1.0)	0.7 (0.4 to 1.0)	-6.1 (-37.7 to 44.8)	-3.9 (-32.2 to 33.3)
Short gestation for birth weight	0.0 (0.0 to 0.0)	0.0 (0.0 to 0.0)	0.0 (0.0 to 0.0)	7.8 (-19.0 to 32.3)	2.7 (-14.1 to 25.9)	0.0 (0.0 to 0.0)	0.0 (0.0 to 0.0)	0.0 (0.0 to 0.0)	13.8 (-19.8 to 54.5)	-0.4 (-19.6 to 39.5)
Low birth weight for gestation	0.0 (0.0 to 0.0)	0.0 (0.0 to 0.0)	0.0 (0.0 to 0.0)	5.3 (-11.3 to 33.0)	1.5 (-18.5 to 17.4)	0.0 (0.0 to 0.0)	0.0 (0.0 to 0.0)	0.0 (0.0 to 0.0)	-1.1 (-31.8 to 32.2)	4.0 (-20.4 to 23.6)
Iron deficiency	-	-	-	-	-	8.0 (5.4 to 11.2)	8.1 (5.7 to 11.0)	7.4 (4.9 to 10.3)	1.2 (-20.3 to 31.3)	-8.3 (-24.9 to 9.5)
Vitamin A deficiency	0.9 (0.6 to 1.2)	0.8 (0.5 to 1.1)	0.6 (0.4 to 0.9)	-7.0 (-20.6 to 9.2)	-20.9 (-40.7 to -5.2)	0.8 (0.5 to 1.1)	0.8 (0.5 to 1.1)	0.6 (0.4 to 0.8)	-4.7 (-22.0 to 18.1)	-22.3 (-35.8 to -0.8)
Zinc deficiency	0.5 (0.2 to 1.1)	0.5 (0.1 to 1.1)	0.4 (0.1 to 1.0)	-8.0 (-64.4 to 74.6)	-15.1 (-70.2 to 157.6)	0.5 (0.2 to 1.1)	0.3 (0.1 to 0.8)	0.3 (0.1 to 0.8)	-32.5 (-65.5 to 42.6)	2.7 (-61.3 to 176.1)
Smoking	17.2 (13.9 to 20.5)	12.1 (9.6 to 14.8)	10.2 (8.1 to 12.5)	-29.7 (-38.8 to -19.5)	-15.3 (-23.8 to -6.4)	3.9 (2.8 to 5.3)	3.2 (2.3 to 4.4)	3.1 (2.2 to 4.3)	-16.3 (-36.1 to 10.2)	-2.8 (-18.6 to 16.9)
Chewing tobacco	1.4 (0.9 to 2.0)	0.9 (0.6 to 1.4)	0.7 (0.5 to 1.1)	-32.5 (-56.6 to 6.6)	-19.6 (-43.5 to 10.7)	0.1 (0.1 to 0.2)	0.1 (0.1 to 0.1)	0.1 (0.1 to 0.1)	-3.9 (-40.2 to 55.3)	-2.9 (-34.3 to 41.3)
Second-hand smoke	37.7 (33.5 to 41.6)	36.5 (32.2 to 40.9)	35.9 (31.5 to 40.4)	-3.1 (-14.1 to 9.4)	-1.8 (-10.3 to 8.0)	56.9 (53.3 to 60.6)	49.3 (45.3 to 53.0)	48.5 (44.1 to 52.4)	-13.4 (-19.6 to -6.9)	-1.7 (-7.5 to 5.0)
Alcohol use	14.8 (7.5 to 25.5)	14.7 (7.1 to 25.4)	16.7 (7.8 to 29.1)	-0.1 (-32.4 to 48.7)	13.3 (-18.0 to 59.2)	11.8 (5.8 to 21.0)	11.8 (5.5 to 21.2)	13.6 (6.1 to 23.8)	0.6 (-32.7 to 51.2)	14.5 (-18.1 to 63.2)
Drug use	0.5 (0.4 to 0.7)	0.5 (0.4 to 0.7)	0.6 (0.4 to 0.8)	1.0 (-4.4 to 7.3)	11.1 (3.8 to 19.8)	0.3 (0.2 to 0.4)	0.3 (0.2 to 0.4)	0.3 (0.3 to 0.4)	2.4 (-3.2 to 8.5)	9.0 (2.0 to 16.2)
Diet low in fruits	41.1 (35.6 to 44.9)	39.8 (34.2 to 43.7)	41.3 (35.7 to 45.0)	-3.1 (-9.2 to 2.9)	3.7 (-0.8 to 8.6)	38.3 (32.3 to 42.4)	35.5 (29.2 to 40.3)	37.8 (31.7 to 42.3)	-7.2 (-15.5 to 0.2)	6.5 (0.7 to 14.8)
Diet low in vegetables	20.0 (11.5 to 27.5)	14.5 (6.0 to 22.3)	16.8 (8.2 to 24.7)	-27.4 (-55.6 to -2.6)	15.6 (-12.5 to 72.7)	22.2 (13.8 to 29.7)	16.5 (8.2 to 24.2)	18.4 (10.1 to 25.9)	-25.6 (-51.7 to -3.3)	11.6 (-12.3 to 50.3)
Diet low in legumes	4.2 (0.7 to 9.5)	5.5 (1.1 to 11.4)	7.3 (2.0 to 13.5)	29.0 (-47.1 to 256.8)	33.7 (-20.0 to 192.5)	8.2 (2.5 to 14.8)	7.9 (2.4 to 14.6)	10.2 (3.9 to 17.4)	-4.0 (-47.8 to 83.5)	28.9 (-13.4 to 135.4)
Diet low in whole grains	30.6 (25.6 to 34.7)	35.3 (31.4 to 38.6)	36.6 (33.0 to 39.8)	15.5 (7.3 to 28.2)	3.9 (-1.2 to 9.6)	31.9 (27.5 to 35.9)	36.5 (32.6 to 39.8)	37.8 (34.0 to 40.9)	14.3 (6.2 to 24.8)	3.6 (-0.5 to 8.8)
Diet low in nuts and seeds	50.8 (49.6 to 51.8)	49.5 (47.7 to 50.8)	49.3 (47.3 to 50.7)	-2.6 (-4.9 to -0.6)	-0.4 (-2.5 to 1.4)	50.9 (49.6 to 51.9)	49.4 (47.6 to 50.8)	49.1 (47.1 to 50.7)	-2.9 (-5.5 to -0.9)	-0.6 (-2.7 to 1.3)
Diet low in milk	46.2 (44.1 to 47.9)	46.5 (44.5 to 48.1)	46.9 (45.0 to 48.5)	0.6 (-1.3 to 2.7)	1.0 (-0.4 to 2.5)	46.3 (44.2 to 48.0)	46.2 (44.1 to 47.9)	46.8 (44.8 to 48.4)	-0.2 (-2.2 to 1.9)	1.4 (-0.1 to 3.0)
Diet high in red meat	3.8 (0.5 to 8.3)	7.7 (2.4 to 14.3)	8.9 (2.9 to 16.4)	102.6 (-12.5 to 795.2)	15.3 (-37.7 to 142.5)	0.5 (0.0 to 2.3)	1.4 (0.0 to 4.6)	1.9 (0.0 to 5.6)	178.9 (-74.2 to 727318.2)	32.8 (-84.6 to 1956.9)
Diet high in processed meat	11.2 (7.0 to 15.3)	18.4 (13.3 to 23.2)	21.1 (15.5 to 26.6)	64.6 (28.5 to 127.7)	14.6 (-3.8 to 36.4)	9.5 (5.9 to 12.9)	13.9 (9.5 to 17.8)	15.3 (10.7 to 19.4)	46.9 (14.8 to 105.2)	9.9 (-6.9 to 33.0)
Diet high in sugar-sweetened beverages	7.2 (2.7 to 14.1)	8.3 (3.4 to 14.5)	8.7 (3.8 to 16.2)	15.6 (-45.5 to 155.3)	5.6 (-35.5 to 76.2)	5.1 (2.0 to 11.0)	5.6 (2.6 to 10.6)	5.9 (2.5 to 11.5)	10.5 (-51.2 to 152.3)	6.2 (-40.0 to 90.2)
Diet low in fibre	22.4 (15.0 to 29.0)	23.8 (16.9 to 30.1)	25.0 (17.9 to 30.9)	5.9 (-11.0 to 30.9)	5.1 (-6.3 to 19.1)	23.4 (16.2 to 30.0)	24.5 (17.5 to 30.7)	26.0 (19.1 to 32.1)	4.8 (-10.7 to 26.5)	6.1 (-4.6 to 20.6)
Diet low in calcium	34.9 (30.6 to 38.4)	34.8 (30.5 to 38.3)	35.7 (31.4 to 39.1)	-0.4 (-4.1 to 3.4)	2.6 (-0.3 to 6.0)	37.1 (33.2 to 40.3)	36.2 (32.2 to 39.6)	37.4 (33.6 to 40.7)	-2.6 (-6.4 to 1.0)	3.5 (0.8 to 6.4)
Diet low in seafood omega-3 fatty acids	3.2 (1.3 to 4.7)	0.2 (0.0 to 1.0)	0.2 (0.0 to 1.0)	-93.3 (-100.0 to -71.3)	9.9 (-92.5 to 2410.3)	3.7 (1.7 to 5.2)	0.3 (0.0 to 1.3)	0.4 (0.0 to 1.5)	-90.7 (-100.0 to -67.5)	8.8 (-83.9 to 1623.8)
Diet low in polyunsaturated fatty acids	30.5 (25.1 to 35.4)	27.7 (21.9 to 32.7)	28.4 (22.5 to 33.5)	-9.1 (-20.0 to 0.7)	2.6 (-7.0 to 15.1)	31.2 (26.0 to 35.7)	28.4 (22.8 to 33.2)	29.1 (23.5 to 33.8)	-8.7 (-20.2 to -0.0)	2.2 (-5.1 to 10.3)
Diet high in trans fatty acids	1.1 (0.0 to 3.3)	1.0 (0.0 to 3.0)	0.9 (0.0 to 2.9)	-12.4 (-38.4 to -6.5)	-8.4 (-33.3 to -3.4)	1.5 (0.1 to 4.0)	1.3 (0.1 to 3.7)	1.2 (0.0 to 3.5)	-12.7 (-34.2 to -6.6)	-8.3 (-28.0 to -3.6)
Diet high in sodium	27.1 (18.1 to 34.8)	17.6 (8.8 to 24.9)	15.0 (6.2 to 22.2)	-35.2 (-58.7 to -17.2)	-14.7 (-39.8 to 2.8)	31.2 (22.6 to 37.9)	18.7 (10.1 to 25.7)	15.5 (6.4 to 22.6)	-39.9 (-60.3 to -25.0)	-17.0 (-42.6 to -3.3)
Intimate partner violence	-	-	-	-	-	2.3 (2.0 to 2.7)	2.1 (1.8 to 2.5)	2.1 (1.7 to 2.4)	-8.2 (-12.5 to -4.3)	-4.1 (-7.9 to 0.4)
Childhood sexual abuse	5.3 (4.3 to 6.5)	5.3 (4.3 to 6.5)	5.3 (4.3 to 6.5)	0.0 (0.0 to 0.0)	0.0 (0.0 to 0.0)	7.3 (5.9 to 8.9)	7.3 (5.9 to 8.9)	7.5 (6.0 to 9.2)	0.0 (0.0 to 0.0)	2.8 (-4.0 to 10.6)

Supplementary Results Table 1. Fukuoka age-standardised Summary Exposure Values (SEVs) for Level 4 risk factors and percentage change, by sex, 1990, 2007, and 2017.Data in parentheses are 95% uncertainty intervals.

	Male					Female				
	**1990**	**2007**	**2017**	**Percentage change 1990-2007**	**Percentage change 2007-2017**	**1990**	**2007**	**2017**	**Percentage change 1990-2007**	**Percentage change 2007-2017**
Bullying victimization	3.2<br>(1.4 to 6.7)	3.3<br>(1.4 to 6.8)	3.4<br>(1.5 to 7.2)	1.6<br>(-6.3 to 9.4)	3.4<br>(-2.0 to 8.1)	1.9<br>(0.8 to 3.9)	1.9<br>(0.8 to 3.8)	1.9<br>(0.8 to 3.9)	-0.3<br>(-7.9 to 7.5)	2.4<br>(-3.6 to 7.2)
Low physical activity	0.5<br>(0.2 to 0.9)	0.5<br>(0.2 to 0.9)	0.4<br>(0.2 to 0.8)	-1.8<br>(-3.6 to 0.1)	-8.8<br>(-12.3 to -4.4)	0.5<br>(0.2 to 1.0)	0.5<br>(0.2 to 0.9)	0.5<br>(0.2 to 0.9)	-3.8<br>(-6.0 to -1.3)	0.8<br>(-0.8 to 2.3)
High fasting plasma glucose	1.5<br>(0.9 to 2.4)	1.6<br>(0.9 to 2.4)	1.7<br>(0.9 to 2.8)	4.0<br>(-32.0 to 68.1)	7.8<br>(-35.5 to 70.1)	1.2<br>(0.7 to 2.0)	0.9<br>(0.5 to 1.5)	1.0<br>(0.6 to 1.7)	-28.9<br>(-53.9 to 7.8)	15.9<br>(-17.3 to 68.9)
High LDL cholesterol	11.9<br>(10.3 to 13.8)	12.3<br>(10.7 to 14.2)	12.3<br>(10.7 to 14.1)	3.7<br>(-1.8 to 9.8)	0.1<br>(-4.3 to 4.8)	13.0<br>(11.4 to 14.7)	13.2<br>(11.6 to 15.0)	13.2<br>(11.6 to 15.0)	2.0<br>(-3.6 to 7.6)	0.1<br>(-4.3 to 4.6)
High systolic blood pressure	9.2<br>(8.3 to 10.1)	7.2<br>(6.3 to 8.0)	7.3<br>(6.4 to 8.3)	-21.7<br>(-27.8 to -15.2)	1.8<br>(-6.0 to 10.1)	7.9<br>(7.0 to 8.7)	4.7<br>(4.1 to 5.4)	4.7<br>(4.0 to 5.4)	-40.4<br>(-46.3 to -34.3)	-1.0<br>(-10.7 to 10.1)
High body-mass index	6.5<br>(4.2 to 10.2)	9.4<br>(6.5 to 14.1)	9.7<br>(6.7 to 15.0)	44.7<br>(18.1 to 82.4)	3.7<br>(-10.7 to 20.3)	5.7<br>(3.5 to 8.6)	7.3<br>(4.8 to 11.3)	7.3<br>(4.6 to 11.5)	28.7<br>(-0.1 to 67.6)	-0.1<br>(-17.4 to 21.0)
Low bone mineral density	5.3<br>(4.4 to 6.3)	5.1<br>(4.3 to 6.1)	5.2<br>(4.3 to 6.1)	-2.1<br>(-14.5 to 12.2)	0.0<br>(-13.6 to 15.1)	7.2<br>(6.1 to 8.5)	7.0<br>(5.9 to 8.2)	7.1<br>(5.9 to 8.3)	-3.4<br>(-14.7 to 10.2)	1.5<br>(-10.1 to 15.7)
Impaired kidney function	3.6<br>(1.7 to 7.9)	3.5<br>(1.7 to 7.7)	3.6<br>(1.7 to 7.9)	-2.5<br>(-6.6 to 1.4)	1.7<br>(-3.1 to 6.9)	4.1<br>(1.9 to 8.7)	4.3<br>(1.9 to 8.9)	4.1<br>(1.9 to 8.8)	2.7<br>(-1.1 to 7.7)	-2.6<br>(-7.1 to 1.1)

Supplementary Results Table 1. Fukushima age-standardised Summary Exposure Values (SEVs) for Level 4 risk factors and percentage change, by sex, 1990, 2007, and 2017.Data in parentheses are 95% uncertainty intervals.

	Male					Female				
	1990	2007	2017	Percentage change 1990-2007	Percentage change 2007-2017	1990	2007	2017	Percentage change 1990-2007	Percentage change 2007-2017
Unsafe water source	2.6 (1.4 to 4.3)	2.1 (1.2 to 3.5)	2.3 (1.3 to 3.8)	-16.4 (-58.5 to 62.5)	5.4 (-37.7 to 79.1)	2.6 (1.4 to 4.3)	2.1 (1.2 to 3.5)	2.3 (1.3 to 3.8)	-16.4 (-58.5 to 62.5)	5.4 (-37.7 to 79.1)
Unsafe sanitation	4.7 (2.3 to 8.6)	2.4 (1.1 to 4.3)	2.0 (0.9 to 3.8)	-49.6 (-77.4 to 14.8)	-15.1 (-53.5 to 68.3)	4.7 (2.3 to 8.6)	2.4 (1.1 to 4.3)	2.0 (0.9 to 3.8)	-49.6 (-77.4 to 14.8)	-15.1 (-53.5 to 68.3)
No access to handwashing facility	6.8 (4.9 to 9.1)	6.5 (4.7 to 8.7)	6.0 (4.3 to 8.2)	-5.3 (-33.3 to 35.3)	-7.1 (-27.5 to 19.0)	6.8 (4.9 to 9.1)	6.5 (4.7 to 8.7)	6.0 (4.3 to 8.2)	-5.3 (-33.3 to 35.3)	-7.1 (-27.5 to 19.0)
Ambient particulate matter pollution	27.9 (18.7 to 45.0)	28.4 (19.0 to 43.4)	23.6 (14.8 to 37.2)	2.0 (-22.0 to 37.9)	-17.0 (-40.9 to 8.5)	27.5 (18.5 to 44.5)	28.4 (18.9 to 43.2)	23.5 (14.7 to 37.1)	3.1 (-21.2 to 39.8)	-17.0 (-41.0 to 8.6)
Household air pollution from solid fuels	1.0 (0.3 to 3.1)	0.2 (0.1 to 0.6)	0.2 (0.0 to 0.5)	-77.6 (-92.8 to -50.7)	-13.8 (-63.7 to 81.2)	1.4 (0.4 to 7.4)	0.4 (0.1 to 1.8)	0.3 (0.1 to 1.3)	-74.1 (-92.6 to -44.6)	-13.9 (-63.1 to 80.6)
Ambient ozone pollution	49.8 (21.3 to 54.8)	49.7 (21.1 to 54.8)	50.1 (21.8 to 54.8)	-0.2 (-0.9 to 0.0)	0.7 (0.0 to 3.0)	49.8 (21.3 to 54.8)	49.7 (21.1 to 54.8)	50.1 (21.8 to 54.8)	-0.2 (-0.9 to 0.0)	0.7 (0.0 to 3.0)
Residential radon	17.5 (1.6 to 57.2)	17.5 (1.6 to 57.2)	17.5 (1.6 to 57.2)	0.0 (0.0 to 0.0)	0.0 (0.0 to 0.0)	17.5 (1.6 to 57.2)	17.5 (1.6 to 57.2)	17.5 (1.6 to 57.2)	0.0 (0.0 to 0.0)	0.0 (0.0 to 0.0)
Lead exposure	2.8 (0.4 to 6.3)	2.0 (0.2 to 4.9)	1.5 (0.1 to 4.1)	-29.3 (-42.2 to -20.7)	-25.4 (-41.0 to -17.4)	1.5 (0.1 to 4.3)	1.0 (0.0 to 3.3)	0.7 (0.0 to 2.7)	-33.0 (-49.2 to -23.8)	-26.2 (-47.9 to -17.6)
Occupational exposure to asbestos	1.9 (1.7 to 2.2)	2.7 (2.5 to 2.9)	2.6 (2.1 to 3.1)	40.2 (21.9 to 65.1)	-4.4 (-22.0 to 15.7)	0.5 (0.4 to 0.6)	0.5 (0.4 to 0.5)	0.5 (0.4 to 0.6)	2.7 (-14.5 to 29.9)	3.7 (-18.6 to 30.3)
Occupational exposure to arsenic	0.4 (0.0 to 1.0)	0.4 (0.0 to 1.0)	0.4 (0.0 to 1.0)	-	-	0.4 (0.0 to 0.9)	0.3 (0.0 to 0.8)	0.4 (0.0 to 0.9)	-	-
Occupational exposure to benzene	0.6 (0.0 to 1.8)	0.6 (0.0 to 1.8)	0.7 (0.0 to 1.9)	3.3 (-3.6 to 13.1)	3.5 (-2.4 to 11.1)	0.6 (0.0 to 1.6)	0.7 (0.0 to 1.8)	0.8 (0.0 to 2.2)	23.1 (8.9 to 46.4)	14.9 (5.6 to 31.1)
Occupational exposure to beryllium	0.0 (0.0 to 0.0)	0.0 (0.0 to 0.0)	0.0 (0.0 to 0.0)	-4.8 (-9.5 to 0.1)	2.0 (-1.7 to 6.4)	0.0 (0.0 to 0.0)	0.0 (0.0 to 0.0)	0.0 (0.0 to 0.0)	2.9 (-7.2 to 14.4)	9.7 (2.8 to 16.9)
Occupational exposure to cadmium	0.1 (0.0 to 0.1)	0.0 (0.0 to 0.1)	0.0 (0.0 to 0.1)	-5.9 (-17.8 to 6.4)	1.1 (-8.6 to 11.7)	0.0 (0.0 to 0.1)	0.0 (0.0 to 0.0)	0.0 (0.0 to 0.1)	-7.0 (-22.9 to 10.0)	5.6 (-7.3 to 20.3)
Occupational exposure to chromium	0.1 (0.1 to 0.1)	0.1 (0.1 to 0.1)	0.1 (0.1 to 0.1)	-4.1 (-15.0 to 7.2)	1.7 (-7.0 to 11.0)	0.1 (0.1 to 0.1)	0.1 (0.1 to 0.1)	0.1 (0.1 to 0.1)	-2.3 (-17.2 to 13.9)	7.4 (-3.7 to 20.2)
Occupational exposure to diesel engine exhaust	0.6 (0.5 to 0.6)	0.6 (0.5 to 0.6)	0.6 (0.5 to 0.6)	-0.4 (-8.1 to 8.0)	3.5 (-3.1 to 11.0)	0.4 (0.4 to 0.5)	0.5 (0.4 to 0.5)	0.5 (0.5 to 0.6)	13.4 (-0.6 to 30.3)	12.3 (1.1 to 23.5)
Occupational exposure to formaldehyde	0.2 (0.2 to 0.3)	0.2 (0.2 to 0.3)	0.2 (0.2 to 0.3)	-5.1 (-18.1 to 8.5)	0.7 (-9.2 to 11.9)	0.2 (0.2 to 0.2)	0.2 (0.2 to 0.2)	0.2 (0.2 to 0.2)	-4.2 (-21.2 to 14.5)	6.3 (-6.3 to 21.4)
Occupational exposure to nickel	0.4 (0.0 to 1.8)	0.4 (0.0 to 1.7)	0.4 (0.0 to 1.8)	-	-	0.3 (0.0 to 1.5)	0.3 (0.0 to 1.3)	0.3 (0.0 to 1.3)	-	-
Occupational exposure to polycyclic aromatic hydrocarbons	0.2 (0.2 to 0.3)	0.2 (0.2 to 0.2)	0.2 (0.2 to 0.3)	-3.1 (-13.4 to 7.5)	2.0 (-5.8 to 10.3)	0.2 (0.2 to 0.2)	0.2 (0.2 to 0.2)	0.2 (0.2 to 0.3)	-0.2 (-14.5 to 16.0)	8.2 (-2.3 to 20.2)
Occupational exposure to silica	4.4 (0.6 to 13.9)	4.2 (0.6 to 13.0)	4.3 (0.6 to 13.6)	-4.7 (-14.7 to 6.7)	1.8 (-7.4 to 13.1)	3.0 (0.4 to 9.6)	2.6 (0.4 to 8.3)	2.7 (0.4 to 8.7)	-11.8 (-27.2 to 7.8)	2.6 (-10.5 to 20.9)
Occupational exposure to sulphuric acid	0.6 (0.1 to 1.9)	0.6 (0.1 to 1.8)	0.6 (0.1 to 1.8)	-6.0 (-17.0 to 5.3)	1.4 (-7.7 to 10.9)	0.5 (0.1 to 1.6)	0.4 (0.1 to 1.4)	0.5 (0.1 to 1.5)	-8.5 (-24.4 to 8.3)	4.9 (-8.2 to 19.8)
Occupational exposure to trichloroethylene	0.1 (0.1 to 0.1)	0.1 (0.1 to 0.1)	0.1 (0.1 to 0.1)	-3.2 (-11.1 to 5.0)	2.2 (-3.6 to 8.8)	0.1 (0.1 to 0.1)	0.1 (0.1 to 0.1)	0.1 (0.1 to 0.1)	2.3 (-10.3 to 16.3)	9.1 (0.4 to 19.4)
Occupational asthmagens	17.3 (13.9 to 21.8)	17.2 (13.8 to 21.7)	17.3 (13.7 to 21.9)	-0.4 (-18.5 to 22.5)	0.4 (-13.7 to 17.0)	7.9 (6.3 to 9.9)	8.0 (6.4 to 10.0)	8.5 (6.7 to 10.6)	1.0 (-15.1 to 20.4)	5.7 (-7.6 to 20.8)
Occupational particulate matter, gases, and fumes	6.4 (4.5 to 9.0)	6.1 (4.3 to 8.6)	5.9 (4.1 to 8.4)	-4.4 (-9.5 to 0.8)	-3.7 (-7.2 to -0.4)	3.5 (2.4 to 5.3)	3.4 (2.3 to 5.0)	3.2 (2.2 to 4.8)	-5.2 (-14.4 to 5.0)	-4.3 (-10.0 to 2.5)
Occupational noise	5.9 (5.4 to 6.5)	5.7 (5.3 to 6.3)	5.6 (5.2 to 6.1)	-3.3 (-6.5 to 0.2)	-2.4 (-4.5 to -0.4)	3.4 (2.9 to 3.9)	3.4 (3.0 to 3.9)	3.4 (3.0 to 3.9)	0.4 (-6.3 to 7.8)	1.9 (-2.2 to 6.4)
Occupational ergonomic factors	11.3 (9.4 to 13.6)	10.0 (8.2 to 12.0)	9.9 (8.0 to 12.0)	-11.6 (-25.0 to 3.6)	-1.2 (-13.1 to 11.7)	6.0 (4.6 to 7.9)	6.0 (4.7 to 7.8)	6.8 (5.2 to 8.7)	0.4 (-22.1 to 28.8)	11.9 (-8.0 to 36.0)
Non-exclusive breastfeeding	0.4 (0.3 to 0.6)	0.4 (0.3 to 0.6)	0.4 (0.3 to 0.6)	-1.8 (-14.5 to 13.1)	-1.4 (-11.5 to 10.3)	0.4 (0.3 to 0.6)	0.4 (0.3 to 0.6)	0.4 (0.3 to 0.6)	-1.6 (-14.3 to 13.4)	-1.1 (-11.2 to 10.6)
Discontinued breastfeeding	1.7 (1.5 to 1.8)	1.7 (1.5 to 1.8)	1.7 (1.5 to 1.8)	0.5 (-9.4 to 10.7)	-0.7 (-8.6 to 7.3)	1.7 (1.5 to 1.8)	1.7 (1.5 to 1.8)	1.6 (1.5 to 1.8)	0.7 (-9.2 to 10.9)	-1.6 (-9.4 to 6.4)
Child underweight	0.2 (0.1 to 0.3)	0.2 (0.1 to 0.2)	0.2 (0.1 to 0.2)	-13.5 (-29.9 to 3.6)	-4.2 (-14.1 to 7.3)	0.2 (0.1 to 0.3)	0.2 (0.1 to 0.2)	0.2 (0.1 to 0.2)	-15.0 (-31.9 to 4.6)	-4.6 (-17.1 to 8.0)
Child wasting	0.2 (0.1 to 0.3)	0.2 (0.1 to 0.2)	0.2 (0.1 to 0.2)	-8.2 (-34.1 to 23.4)	-2.8 (-20.9 to 18.1)	0.2 (0.1 to 0.3)	0.2 (0.1 to 0.2)	0.2 (0.1 to 0.2)	-9.8 (-35.5 to 22.3)	-2.1 (-21.0 to 21.7)
Child stunting	0.5 (0.3 to 0.9)	0.4 (0.2 to 0.7)	0.4 (0.2 to 0.7)	-16.2 (-46.4 to 26.8)	-1.2 (-29.0 to 33.7)	0.5 (0.3 to 0.9)	0.4 (0.2 to 0.7)	0.4 (0.2 to 0.7)	-18.4 (-48.0 to 29.5)	-2.3 (-31.0 to 35.7)
Short gestation for birth weight	0.0 (0.0 to 0.0)	0.0 (0.0 to 0.0)	0.0 (0.0 to 0.0)	8.9 (-11.0 to 35.3)	2.3 (-16.1 to 29.3)	0.0 (0.0 to 0.0)	0.0 (0.0 to 0.0)	0.0 (0.0 to 0.0)	16.0 (-11.9 to 57.8)	0.1 (-18.8 to 27.4)
Low birth weight for gestation	0.0 (0.0 to 0.0)	0.0 (0.0 to 0.0)	0.0 (0.0 to 0.0)	5.2 (-13.7 to 28.3)	1.7 (-15.9 to 20.9)	0.0 (0.0 to 0.0)	0.0 (0.0 to 0.0)	0.0 (0.0 to 0.0)	-3.1 (-23.5 to 25.1)	3.9 (-14.4 to 24.4)
Iron deficiency	-	-	-	-	-	8.4 (5.7 to 11.9)	8.4 (5.8 to 11.6)	7.7 (5.3 to 10.9)	0.1 (-21.6 to 29.4)	-8.5 (-23.8 to 10.1)
Vitamin A deficiency	1.1 (0.6 to 1.4)	0.9 (0.6 to 1.1)	0.7 (0.4 to 0.9)	-19.7 (-35.0 to 3.0)	-20.4 (-36.9 to -3.5)	0.8 (0.5 to 1.0)	0.8 (0.5 to 1.0)	0.6 (0.4 to 0.9)	0.5 (-15.0 to 17.8)	-18.6 (-33.0 to -0.0)
Zinc deficiency	0.5 (0.1 to 1.0)	0.5 (0.1 to 1.1)	0.4 (0.1 to 1.0)	-7.7 (-65.1 to 71.6)	-14.0 (-66.7 to 179.4)	0.5 (0.2 to 1.1)	0.3 (0.1 to 0.8)	0.3 (0.1 to 0.8)	-31.7 (-65.4 to 57.2)	2.3 (-62.0 to 175.4)
Smoking	18.0 (14.7 to 21.4)	12.6 (10.0 to 15.4)	10.7 (8.5 to 13.0)	-29.9 (-39.3 to -19.0)	-15.4 (-23.3 to -6.1)	4.1 (2.9 to 5.5)	3.4 (2.4 to 4.5)	3.3 (2.3 to 4.5)	-17.3 (-36.8 to 7.4)	-2.4 (-18.6 to 17.6)
Chewing tobacco	1.4 (0.9 to 2.0)	0.9 (0.6 to 1.4)	0.8 (0.5 to 1.1)	-32.9 (-57.1 to 6.5)	-18.8 (-42.4 to 15.2)	0.1 (0.1 to 0.1)	0.1 (0.1 to 0.1)	0.1 (0.1 to 0.1)	-4.1 (-40.8 to 56.0)	-3.1 (-32.5 to 43.2)
Second-hand smoke	37.0 (32.8 to 41.1)	36.2 (32.1 to 40.5)	35.9 (31.8 to 40.5)	-2.1 (-13.3 to 11.8)	-0.8 (-9.8 to 8.9)	56.2 (52.6 to 59.7)	48.8 (45.1 to 52.5)	48.3 (44.1 to 52.3)	-13.1 (-19.7 to -6.0)	-1.1 (-6.7 to 5.5)
Alcohol use	14.2 (7.0 to 25.3)	14.2 (7.1 to 25.6)	16.7 (7.9 to 30.5)	-0.4 (-37.4 to 45.3)	18.1 (-20.3 to 71.6)	11.5 (5.4 to 20.7)	11.4 (5.4 to 20.8)	12.8 (5.9 to 23.4)	-1.1 (-36.8 to 42.1)	11.9 (-22.6 to 60.6)
Drug use	0.5 (0.4 to 0.7)	0.5 (0.4 to 0.7)	0.6 (0.4 to 0.8)	0.3 (-5.7 to 6.4)	12.2 (5.5 to 21.1)	0.3 (0.2 to 0.4)	0.3 (0.2 to 0.4)	0.3 (0.3 to 0.4)	2.0 (-3.5 to 7.9)	9.7 (3.1 to 18.3)
Diet low in fruits	41.2 (35.6 to 44.8)	39.3 (33.6 to 43.3)	40.6 (35.0 to 44.5)	-4.4 (-10.6 to 1.9)	3.3 (-1.5 to 8.5)	38.3 (32.3 to 42.9)	35.6 (29.4 to 40.2)	37.9 (31.6 to 42.2)	-7.2 (-15.1 to 1.0)	6.6 (0.3 to 14.3)
Diet low in vegetables	19.7 (11.4 to 27.0)	13.8 (5.9 to 21.5)	15.8 (7.5 to 23.9)	-30.0 (-58.7 to -5.3)	14.7 (-14.6 to 65.1)	22.1 (13.5 to 29.3)	16.1 (8.0 to 23.9)	17.9 (9.4 to 25.5)	-27.2 (-54.0 to -5.2)	11.2 (-14.7 to 50.5)
Diet low in legumes	4.4 (0.7 to 9.8)	5.6 (1.0 to 11.6)	7.4 (2.0 to 14.2)	26.5 (-49.4 to 262.1)	32.7 (-19.1 to 217.2)	8.4 (2.7 to 15.2)	8.3 (2.5 to 15.1)	10.7 (3.9 to 17.8)	-1.9 (-47.2 to 67.1)	28.7 (-10.1 to 134.4)
Diet low in whole grains	30.5 (25.6 to 34.8)	35.3 (31.4 to 38.8)	36.7 (32.9 to 40.1)	15.7 (7.2 to 28.5)	3.9 (-1.2 to 9.5)	32.0 (27.1 to 36.0)	36.5 (32.7 to 39.8)	37.7 (34.1 to 40.9)	14.2 (6.0 to 25.5)	3.4 (-1.0 to 8.5)
Diet low in nuts and seeds	50.9 (49.5 to 51.9)	49.7 (48.0 to 51.0)	49.5 (47.5 to 50.9)	-2.4 (-4.8 to -0.4)	-0.3 (-2.4 to 1.6)	51.2 (49.8 to 52.1)	50.1 (48.4 to 51.4)	50.0 (48.2 to 51.3)	-2.1 (-4.4 to -0.3)	-0.1 (-2.1 to 1.8)
Diet low in milk	46.3 (44.3 to 48.1)	46.8 (44.8 to 48.4)	47.3 (45.5 to 48.9)	1.0 (-0.7 to 3.1)	1.2 (-0.2 to 2.5)	46.3 (44.3 to 48.0)	46.1 (44.1 to 47.7)	46.7 (44.8 to 48.3)	-0.4 (-2.3 to 1.5)	1.4 (-0.1 to 3.0)
Diet high in red meat	3.8 (0.4 to 8.7)	7.5 (2.0 to 13.4)	8.5 (2.5 to 15.2)	96.8 (-22.0 to 1103.9)	14.3 (-37.5 to 122.4)	0.5 (0.0 to 2.6)	1.5 (0.0 to 4.4)	2.0 (0.0 to 5.6)	173.1 (-75.8 to 3065852.3)	38.0 (-81.2 to 2537.5)
Diet high in processed meat	11.3 (7.0 to 15.1)	18.7 (13.8 to 23.2)	21.6 (16.0 to 27.1)	65.5 (28.9 to 130.9)	15.7 (-2.2 to 38.4)	9.5 (5.7 to 13.2)	13.9 (9.9 to 17.8)	15.3 (10.9 to 19.5)	47.1 (14.8 to 104.5)	10.3 (-6.4 to 31.4)
Diet high in sugar-sweetened beverages	7.2 (2.8 to 13.7)	8.2 (3.4 to 15.4)	8.5 (3.6 to 15.8)	14.1 (-48.5 to 161.5)	4.1 (-37.3 to 70.2)	5.1 (1.9 to 11.0)	5.8 (2.7 to 11.3)	6.2 (2.6 to 11.6)	14.7 (-45.1 to 155.8)	6.4 (-43.0 to 92.4)
Diet low in fibre	22.0 (14.6 to 28.7)	22.8 (15.5 to 29.1)	23.6 (16.5 to 30.1)	3.3 (-14.4 to 25.4)	3.8 (-8.8 to 19.3)	23.4 (15.5 to 30.1)	24.4 (17.3 to 30.6)	25.8 (18.7 to 31.9)	4.3 (-11.2 to 27.5)	5.6 (-6.6 to 20.5)
Diet low in calcium	35.0 (30.6 to 38.4)	34.7 (30.3 to 38.2)	35.6 (31.3 to 39.2)	-0.6 (-4.7 to 3.5)	2.4 (-0.7 to 5.7)	37.1 (33.3 to 40.2)	36.1 (31.9 to 39.5)	37.3 (33.4 to 40.5)	-2.8 (-6.5 to 0.6)	3.5 (0.9 to 6.9)
Diet low in seafood omega-3 fatty acids	3.2 (1.6 to 4.5)	0.3 (0.0 to 1.2)	0.4 (0.0 to 1.3)	-89.7 (-99.9 to -61.6)	6.6 (-84.5 to 762.6)	3.7 (1.9 to 5.1)	0.4 (0.0 to 1.6)	0.5 (0.0 to 1.5)	-88.4 (-99.9 to -60.5)	6.4 (-79.5 to 708.4)
Diet low in polyunsaturated fatty acids	30.6 (25.0 to 35.4)	27.7 (21.5 to 32.7)	28.3 (22.4 to 33.3)	-9.4 (-20.8 to 0.0)	2.3 (-6.1 to 12.3)	31.1 (25.7 to 35.6)	28.5 (22.4 to 33.3)	29.2 (23.2 to 34.0)	-8.4 (-19.8 to 1.0)	2.5 (-5.2 to 11.4)
Diet high in trans fatty acids	1.1 (0.0 to 3.3)	1.0 (0.0 to 3.0)	0.9 (0.0 to 2.9)	-12.5 (-37.5 to -6.5)	-8.3 (-32.6 to -3.4)	1.5 (0.1 to 4.0)	1.3 (0.1 to 3.7)	1.2 (0.0 to 3.5)	-12.5 (-34.9 to -6.5)	-8.4 (-28.5 to -3.3)
Diet high in sodium	28.5 (18.9 to 36.3)	18.9 (9.8 to 26.4)	16.4 (7.0 to 24.0)	-33.7 (-57.1 to -16.0)	-13.1 (-36.3 to 3.3)	32.3 (23.9 to 39.6)	19.8 (11.8 to 26.5)	16.7 (8.3 to 24.1)	-38.5 (-56.2 to -23.4)	-16.1 (-37.3 to -2.1)
Intimate partner violence	-	-	-	-	-	2.3 (1.9 to 2.6)	2.1 (1.7 to 2.4)	2.0 (1.7 to 2.3)	-9.4 (-13.4 to -4.8)	-4.1 (-8.5 to 0.3)
Childhood sexual abuse	5.3 (4.3 to 6.5)	5.3 (4.3 to 6.5)	5.3 (4.3 to 6.5)	0.0 (0.0 to 0.0)	0.0 (0.0 to 0.0)	7.3 (5.9 to 8.9)	7.3 (5.9 to 8.9)	7.5 (6.0 to 9.2)	0.0 (0.0 to 0.0)	2.8 (-4.0 to 10.6)

Supplementary Results Table 1. Fukushima age-standardised Summary Exposure Values (SEVs) for Level 4 risk factors and percentage change, by sex, 1990, 2007, and 2017.Data in parentheses are 95% uncertainty intervals.										
	**Male**					**Female**				
	**1990**	**2007**	**2017**	**Percentage change 1990-2007**	**Percentage change 2007-2017**	**1990**	**2007**	**2017**	**Percentage change 1990-2007**	**Percentage change 2007-2017**
Bullying victimization	3.3 (1.4 to 7.1)	3.4 (1.4 to 7.3)	3.5 (1.5 to 7.5)	3.2 (-4.5 to 11.0)	3.2 (-2.1 to 7.8)	1.9 (0.8 to 4.0)	2.0 (0.8 to 4.1)	2.0 (0.8 to 4.2)	1.4 (-6.2 to 9.6)	2.3 (-3.3 to 7.0)
Low physical activity	0.5 (0.2 to 0.9)	0.5 (0.2 to 0.9)	0.4 (0.2 to 0.8)	-1.7 (-3.8 to 0.3)	-8.8 (-12.2 to -4.7)	0.5 (0.2 to 1.0)	0.5 (0.2 to 0.9)	0.5 (0.2 to 0.9)	-3.9 (-6.2 to -1.3)	0.7 (-0.8 to 2.1)
High fasting plasma glucose	1.4 (0.9 to 2.2)	1.4 (0.9 to 2.1)	1.5 (0.9 to 2.5)	-1.0 (-39.6 to 53.0)	11.5 (-23.2 to 72.9)	1.2 (0.7 to 1.9)	1.2 (0.7 to 2.1)	1.3 (0.8 to 2.3)	4.6 (-35.3 to 74.3)	10.7 (-29.3 to 84.1)
High LDL cholesterol	11.9 (10.3 to 13.7)	12.4 (10.8 to 14.3)	12.5 (10.9 to 14.3)	4.1 (-1.9 to 10.0)	0.2 (-4.3 to 5.2)	12.7 (11.2 to 14.5)	12.8 (11.2 to 14.5)	12.8 (11.3 to 14.6)	0.6 (-4.6 to 6.0)	0.4 (-4.0 to 4.8)
High systolic blood pressure	11.1 (10.1 to 12.2)	8.2 (7.3 to 9.1)	8.1 (7.1 to 9.1)	-26.3 (-32.7 to -19.5)	-1.7 (-9.7 to 6.7)	9.6 (8.6 to 10.6)	6.0 (5.3 to 6.8)	5.6 (4.8 to 6.4)	-37.6 (-43.7 to -30.4)	-7.2 (-16.4 to 3.5)
High body-mass index	4.8 (3.0 to 7.9)	7.0 (4.6 to 11.5)	7.1 (4.6 to 11.6)	43.8 (16.1 to 85.5)	2.8 (-13.6 to 20.3)	5.2 (3.1 to 8.1)	6.7 (4.1 to 10.2)	6.7 (4.0 to 11.0)	29.0 (1.8 to 69.2)	0.6 (-17.7 to 23.9)
Low bone mineral density	5.3 (4.3 to 6.2)	5.2 (4.4 to 6.1)	5.2 (4.3 to 6.1)	-2.0 (-14.2 to 12.1)	0.4 (-13.6 to 14.8)	6.7 (5.9 to 7.5)	6.6 (5.9 to 7.5)	6.8 (5.7 to 8.0)	-1.0 (-10.3 to 10.4)	2.4 (-11.2 to 17.6)
Impaired kidney function	2.6 (1.2 to 6.5)	2.6 (1.2 to 6.4)	2.4 (1.1 to 6.1)	-2.3 (-6.7 to 2.5)	-6.8 (-12.0 to -1.9)	3.2 (1.5 to 7.4)	3.3 (1.5 to 7.7)	3.1 (1.4 to 7.3)	3.5 (-0.8 to 8.7)	-7.2 (-12.1 to -2.3)

Supplementary Results Table 1. Gifu age-standardised Summary Exposure Values (SEVs) for Level 4 risk factors and percentage change, by sex, 1990, 2007, and 2017.Data in parentheses are 95% uncertainty intervals.										
	Male					Female				
	1990	2007	2017	Percentage change 1990-2007	Percentage change 2007-2017	1990	2007	2017	Percentage change 1990-2007	Percentage change 2007-2017
Unsafe water source	2.3 (1.3 to 4.0)	1.9 (1.0 to 3.0)	2.0 (1.1 to 3.2)	-19.7 (-59.7 to 64.8)	7.0 (-36.0 to 83.2)	2.3 (1.3 to 4.0)	1.9 (1.0 to 3.0)	2.0 (1.1 to 3.2)	-19.7 (-59.7 to 64.8)	7.0 (-36.0 to 83.2)
Unsafe sanitation	3.9 (2.0 to 7.2)	1.9 (0.9 to 3.5)	1.7 (0.8 to 3.2)	-50.5 (-78.7 to 11.7)	-14.3 (-56.9 to 64.4)	3.9 (2.0 to 7.2)	1.9 (0.9 to 3.5)	1.7 (0.8 to 3.2)	-50.5 (-78.7 to 11.7)	-14.3 (-56.9 to 64.4)
No access to handwashing facility	6.8 (4.9 to 9.1)	6.4 (4.6 to 8.7)	5.9 (4.3 to 7.9)	-5.6 (-34.5 to 33.1)	-7.3 (-28.4 to 19.4)	6.8 (4.9 to 9.1)	6.4 (4.6 to 8.7)	5.9 (4.3 to 7.9)	-5.6 (-34.5 to 33.1)	-7.3 (-28.4 to 19.4)
Ambient particulate matter pollution	32.8 (22.7 to 46.9)	32.9 (23.4 to 47.1)	28.0 (19.0 to 41.4)	0.4 (-19.9 to 27.0)	-14.7 (-36.9 to 8.0)	32.5 (22.6 to 46.6)	32.8 (23.4 to 47.1)	28.0 (18.9 to 41.3)	0.9 (-19.6 to 28.2)	-14.7 (-36.9 to 8.1)
Household air pollution from solid fuels	0.6 (0.2 to 1.5)	0.1 (0.0 to 0.3)	0.1 (0.0 to 0.3)	-75.1 (-92.8 to -46.4)	-14.3 (-66.3 to 86.1)	0.8 (0.2 to 5.9)	0.2 (0.0 to 0.9)	0.2 (0.0 to 0.8)	-72.0 (-92.7 to -44.6)	-11.5 (-66.1 to 87.8)
Ambient ozone pollution	52.0 (26.4 to 54.8)	51.9 (25.7 to 54.8)	52.0 (26.0 to 54.8)	-0.3 (-2.1 to 0.0)	0.2 (0.0 to 1.5)	52.0 (26.4 to 54.8)	51.9 (25.7 to 54.8)	52.0 (26.0 to 54.8)	-0.3 (-2.1 to 0.0)	0.2 (0.0 to 1.5)
Residential radon	13.9 (1.0 to 42.9)	13.9 (1.0 to 42.9)	13.9 (1.0 to 42.9)	0.0 (0.0 to 0.0)	0.0 (0.0 to 0.0)	13.9 (1.0 to 42.9)	13.9 (1.0 to 42.9)	13.9 (1.0 to 42.9)	0.0 (0.0 to 0.0)	0.0 (0.0 to 0.0)
Lead exposure	3.6 (0.8 to 7.3)	2.3 (0.4 to 5.4)	1.6 (0.2 to 4.2)	-36.1 (-49.4 to -26.1)	-30.5 (-44.9 to -21.9)	1.9 (0.2 to 5.0)	1.1 (0.1 to 3.3)	0.7 (0.0 to 2.5)	-42.4 (-60.4 to -32.4)	-34.0 (-55.8 to -24.4)
Occupational exposure to asbestos	1.8 (1.6 to 2.0)	2.6 (2.3 to 2.8)	2.7 (2.3 to 3.3)	40.5 (22.0 to 64.3)	5.9 (-13.4 to 28.2)	0.6 (0.5 to 0.7)	0.6 (0.5 to 0.6)	0.6 (0.5 to 0.7)	-5.0 (-21.9 to 24.5)	-0.5 (-19.0 to 21.2)
Occupational exposure to arsenic	0.5 (0.0 to 1.1)	0.4 (0.0 to 1.0)	0.4 (0.0 to 1.0)	-	-	0.4 (0.0 to 1.0)	0.4 (0.0 to 0.9)	0.4 (0.0 to 0.9)	-	-
Occupational exposure to benzene	0.6 (0.0 to 1.8)	0.7 (0.0 to 1.8)	0.7 (0.0 to 1.9)	2.2 (-4.6 to 11.7)	3.5 (-3.0 to 11.9)	0.6 (0.0 to 1.7)	0.7 (0.0 to 2.0)	0.8 (0.0 to 2.4)	21.4 (7.4 to 41.4)	13.8 (4.4 to 28.0)
Occupational exposure to beryllium	0.0 (0.0 to 0.0)	0.0 (0.0 to 0.0)	0.0 (0.0 to 0.0)	-5.3 (-10.1 to -0.6)	1.9 (-2.1 to 6.2)	0.0 (0.0 to 0.0)	0.0 (0.0 to 0.0)	0.0 (0.0 to 0.0)	1.8 (-6.8 to 11.4)	8.9 (2.2 to 15.9)
Occupational exposure to cadmium	0.1 (0.0 to 0.1)	0.0 (0.0 to 0.1)	0.0 (0.0 to 0.1)	-7.3 (-18.6 to 5.0)	0.7 (-9.0 to 11.7)	0.0 (0.0 to 0.1)	0.0 (0.0 to 0.1)	0.0 (0.0 to 0.1)	-9.4 (-23.5 to 7.9)	4.5 (-8.7 to 19.5)
Occupational exposure to chromium	0.1 (0.1 to 0.1)	0.1 (0.1 to 0.1)	0.1 (0.1 to 0.1)	-5.7 (-15.9 to 5.1)	1.2 (-7.3 to 10.7)	0.1 (0.1 to 0.1)	0.1 (0.1 to 0.1)	0.1 (0.1 to 0.1)	-4.7 (-18.0 to 11.4)	6.3 (-5.2 to 18.7)
Occupational exposure to diesel engine exhaust	0.6 (0.5 to 0.6)	0.6 (0.5 to 0.6)	0.6 (0.6 to 0.6)	-1.9 (-9.3 to 6.6)	3.1 (-3.7 to 10.6)	0.5 (0.4 to 0.5)	0.5 (0.5 to 0.6)	0.6 (0.5 to 0.6)	12.3 (-0.7 to 28.2)	11.1 (0.4 to 23.3)
Occupational exposure to formaldehyde	0.3 (0.2 to 0.3)	0.2 (0.2 to 0.3)	0.2 (0.2 to 0.3)	-6.8 (-18.7 to 6.4)	0.3 (-9.6 to 11.7)	0.2 (0.2 to 0.3)	0.2 (0.2 to 0.2)	0.2 (0.2 to 0.2)	-6.8 (-21.3 to 10.9)	5.2 (-8.0 to 19.3)
Occupational exposure to nickel	0.4 (0.0 to 1.9)	0.4 (0.0 to 1.7)	0.4 (0.0 to 1.7)	-	-	0.4 (0.0 to 1.6)	0.3 (0.0 to 1.3)	0.3 (0.0 to 1.3)	-	-
Occupational exposure to polycyclic aromatic hydrocarbons	0.2 (0.2 to 0.3)	0.2 (0.2 to 0.3)	0.2 (0.2 to 0.3)	-4.7 (-14.2 to 5.9)	1.6 (-6.4 to 10.1)	0.2 (0.2 to 0.3)	0.2 (0.2 to 0.2)	0.2 (0.2 to 0.3)	-2.6 (-15.4 to 12.8)	7.1 (-3.8 to 18.2)
Occupational exposure to silica	4.7 (0.7 to 15.8)	4.4 (0.7 to 14.5)	4.5 (0.7 to 14.7)	-5.5 (-15.5 to 6.1)	1.6 (-7.3 to 11.8)	3.2 (0.5 to 10.2)	2.8 (0.4 to 8.7)	2.8 (0.4 to 8.7)	-13.7 (-28.5 to 6.1)	1.7 (-12.3 to 19.0)
Occupational exposure to sulphuric acid	0.6 (0.1 to 2.0)	0.6 (0.1 to 1.8)	0.6 (0.1 to 1.8)	-7.5 (-17.6 to 4.8)	0.9 (-8.4 to 11.2)	0.5 (0.1 to 1.6)	0.5 (0.1 to 1.5)	0.5 (0.1 to 1.6)	-10.6 (-25.4 to 6.9)	3.8 (-9.8 to 19.7)
Occupational exposure to trichloroethylene	0.1 (0.1 to 0.1)	0.1 (0.1 to 0.1)	0.1 (0.1 to 0.1)	-4.9 (-12.3 to 3.0)	1.7 (-4.6 to 8.6)	0.1 (0.1 to 0.1)	0.1 (0.1 to 0.1)	0.1 (0.1 to 0.1)	0.3 (-10.8 to 13.3)	7.9 (-1.2 to 18.0)
Occupational asthmagens	19.0 (15.0 to 24.0)	18.3 (14.5 to 23.1)	18.3 (14.4 to 23.2)	-3.5 (-22.2 to 20.4)	-0.1 (-14.9 to 17.5)	8.3 (6.7 to 10.3)	8.3 (6.8 to 10.5)	8.8 (7.1 to 10.9)	-0.3 (-15.6 to 17.5)	5.5 (-7.5 to 19.6)
Occupational particulate matter, gases, and fumes	6.4 (4.5 to 9.1)	6.1 (4.3 to 8.8)	5.9 (4.2 to 8.5)	-4.3 (-9.2 to 0.9)	-3.6 (-6.9 to 0.0)	3.7 (2.6 to 5.5)	3.5 (2.4 to 5.1)	3.3 (2.3 to 4.9)	-5.6 (-13.8 to 4.0)	-4.8 (-10.1 to 1.8)
Occupational noise	5.9 (5.4 to 6.4)	5.7 (5.3 to 6.2)	5.6 (5.2 to 6.1)	-3.0 (-6.1 to 0.5)	-2.2 (-4.1 to 0.0)	3.5 (3.0 to 4.1)	3.5 (3.1 to 4.0)	3.6 (3.2 to 4.1)	0.4 (-5.3 to 7.6)	1.7 (-2.2 to 6.3)
Occupational ergonomic factors	10.3 (8.5 to 12.2)	9.1 (7.5 to 10.9)	9.0 (7.3 to 10.8)	-11.1 (-22.2 to 2.3)	-1.5 (-11.6 to 9.8)	5.5 (4.3 to 7.0)	5.6 (4.4 to 6.9)	6.3 (4.9 to 8.0)	1.3 (-21.5 to 27.6)	12.9 (-8.0 to 37.5)
Non-exclusive breastfeeding	0.4 (0.3 to 0.6)	0.4 (0.3 to 0.6)	0.4 (0.3 to 0.6)	-1.3 (-14.1 to 13.8)	-1.6 (-11.7 to 9.9)	0.4 (0.3 to 0.6)	0.4 (0.3 to 0.6)	0.4 (0.3 to 0.6)	-1.6 (-14.3 to 13.5)	-1.2 (-11.4 to 10.4)
Discontinued breastfeeding	1.7 (1.6 to 1.8)	1.7 (1.6 to 1.8)	1.7 (1.6 to 1.8)	0.9 (-7.9 to 11.5)	-1.3 (-8.5 to 6.6)	1.7 (1.6 to 1.8)	1.7 (1.6 to 1.9)	1.7 (1.5 to 1.8)	1.6 (-7.2 to 12.3)	-3.1 (-10.2 to 4.8)
Child underweight	0.3 (0.2 to 0.4)	0.2 (0.1 to 0.3)	0.2 (0.1 to 0.3)	-14.8 (-34.5 to 11.6)	-5.2 (-21.0 to 11.2)	0.3 (0.2 to 0.4)	0.2 (0.1 to 0.3)	0.2 (0.1 to 0.3)	-16.8 (-38.0 to 8.8)	-6.2 (-23.1 to 14.6)
Child wasting	0.2 (0.1 to 0.3)	0.2 (0.1 to 0.3)	0.2 (0.1 to 0.3)	-7.0 (-34.4 to 29.8)	-2.5 (-21.6 to 22.1)	0.2 (0.1 to 0.3)	0.2 (0.1 to 0.3)	0.2 (0.1 to 0.3)	-8.5 (-37.3 to 30.2)	-3.5 (-24.5 to 21.4)
Child stunting	0.6 (0.3 to 1.0)	0.6 (0.3 to 0.9)	0.5 (0.3 to 0.9)	-9.5 (-44.8 to 49.5)	-3.7 (-33.4 to 33.0)	0.6 (0.3 to 1.1)	0.6 (0.3 to 1.0)	0.5 (0.3 to 0.9)	-11.5 (-46.9 to 52.6)	-3.0 (-33.1 to 40.6)
Short gestation for birth weight	0.0 (0.0 to 0.0)	0.0 (0.0 to 0.0)	0.0 (0.0 to 0.0)	9.1 (-10.8 to 42.8)	0.7 (-14.7 to 19.8)	0.0 (0.0 to 0.0)	0.0 (0.0 to 0.0)	0.0 (0.0 to 0.0)	13.7 (-19.6 to 119.8)	1.3 (-21.2 to 35.1)
Low birth weight for gestation	0.0 (0.0 to 0.0)	0.0 (0.0 to 0.0)	0.0 (0.0 to 0.0)	2.7 (-16.9 to 26.1)	4.0 (-10.9 to 21.2)	0.0 (0.0 to 0.0)	0.0 (0.0 to 0.0)	0.0 (0.0 to 0.0)	-7.2 (-65.5 to 33.0)	3.0 (-18.5 to 24.5)
Iron deficiency	-	-	-	-	-	8.0 (5.4 to 11.1)	8.0 (5.6 to 10.9)	7.4 (5.1 to 10.4)	0.0 (-22.0 to 29.7)	-7.4 (-23.5 to 11.0)
Vitamin A deficiency	0.9 (0.6 to 1.3)	0.8 (0.5 to 1.0)	0.7 (0.5 to 0.9)	-19.9 (-33.3 to -1.4)	-11.3 (-27.8 to 6.5)	0.7 (0.5 to 1.0)	0.8 (0.5 to 1.0)	0.7 (0.5 to 1.0)	8.1 (-12.0 to 31.2)	-6.1 (-23.3 to 13.4)
Zinc deficiency	0.5 (0.2 to 1.0)	0.4 (0.1 to 1.1)	0.4 (0.1 to 1.0)	-11.1 (-64.5 to 74.5)	-11.9 (-67.1 to 177.6)	0.5 (0.2 to 1.1)	0.3 (0.1 to 0.8)	0.4 (0.1 to 0.9)	-31.9 (-65.0 to 9.0)	7.9 (-60.4 to 184.5)
Smoking	17.5 (14.2 to 21.0)	12.3 (9.7 to 15.2)	10.5 (8.3 to 12.7)	-29.7 (-38.7 to -19.7)	-15.2 (-23.4 to -5.7)	4.0 (2.8 to 5.4)	3.3 (2.3 to 4.5)	3.2 (2.3 to 4.4)	-16.4 (-36.3 to 8.3)	-2.8 (-19.2 to 17.4)
Chewing tobacco	1.4 (0.9 to 2.0)	0.9 (0.6 to 1.4)	0.8 (0.5 to 1.1)	-32.6 (-56.9 to 5.8)	-19.2 (-41.5 to 13.9)	0.1 (0.1 to 0.1)	0.1 (0.1 to 0.1)	0.1 (0.1 to 0.1)	-3.8 (-41.0 to 63.5)	-2.8 (-32.8 to 43.1)
Second-hand smoke	36.9 (32.9 to 40.9)	36.8 (32.3 to 41.2)	36.8 (32.3 to 41.3)	-0.3 (-12.3 to 12.6)	0.1 (-8.7 to 9.8)	55.8 (52.4 to 59.3)	49.6 (45.6 to 53.5)	49.4 (45.1 to 53.8)	-11.2 (-17.6 to -4.2)	-0.3 (-7.0 to 6.1)
Alcohol use	14.3 (7.2 to 24.7)	14.2 (7.2 to 24.4)	16.0 (7.7 to 27.0)	-0.6 (-35.4 to 46.3)	12.7 (-21.4 to 62.8)	11.5 (5.7 to 21.0)	11.4 (5.6 to 20.1)	13.0 (6.1 to 23.0)	-0.7 (-34.6 to 46.3)	13.7 (-21.9 to 64.6)
Drug use	0.5 (0.4 to 0.7)	0.5 (0.4 to 0.7)	0.6 (0.4 to 0.8)	0.2 (-5.5 to 5.8)	11.4 (4.5 to 19.2)	0.3 (0.2 to 0.4)	0.3 (0.2 to 0.4)	0.3 (0.3 to 0.4)	1.7 (-3.9 to 8.9)	9.2 (2.4 to 17.4)
Diet low in fruits	41.3 (35.4 to 45.0)	39.6 (33.9 to 43.4)	41.1 (35.4 to 44.8)	-3.9 (-9.8 to 1.7)	3.6 (-0.9 to 8.4)	38.4 (32.4 to 42.7)	35.9 (29.6 to 40.5)	38.2 (32.6 to 42.4)	-6.4 (-13.9 to 1.4)	6.4 (0.1 to 14.2)
Diet low in vegetables	20.2 (11.4 to 28.1)	14.5 (6.2 to 22.3)	16.6 (8.2 to 24.9)	-28.4 (-56.4 to -3.2)	15.2 (-13.1 to 70.1)	22.4 (13.7 to 29.4)	16.4 (8.0 to 24.2)	18.3 (10.0 to 26.0)	-26.6 (-53.1 to -5.0)	11.4 (-13.1 to 60.1)
Diet low in legumes	4.4 (0.7 to 10.2)	6.1 (1.4 to 12.5)	8.2 (2.5 to 15.4)	36.5 (-43.1 to 302.5)	35.0 (-14.8 to 190.4)	8.3 (2.8 to 15.0)	8.3 (2.7 to 14.7)	10.8 (3.9 to 17.8)	-0.5 (-47.5 to 77.8)	30.4 (-9.3 to 115.3)
Diet low in whole grains	30.7 (25.9 to 34.7)	35.4 (31.5 to 38.7)	36.7 (32.8 to 40.0)	15.5 (6.8 to 27.3)	3.8 (-1.3 to 9.1)	32.0 (27.3 to 36.0)	36.6 (32.9 to 39.9)	37.9 (34.5 to 40.8)	14.4 (6.5 to 26.1)	3.5 (-0.7 to 8.2)
Diet low in nuts and seeds	50.9 (49.5 to 51.9)	49.6 (47.8 to 50.9)	49.4 (47.4 to 50.9)	-2.5 (-5.0 to -0.5)	-0.4 (-2.5 to 1.5)	51.0 (49.7 to 51.9)	49.5 (47.7 to 50.9)	49.2 (47.2 to 50.7)	-2.9 (-5.4 to -0.9)	-0.6 (-2.7 to 1.5)
Diet low in milk	46.3 (44.2 to 48.0)	46.6 (44.6 to 48.2)	47.0 (45.2 to 48.6)	0.7 (-1.2 to 2.6)	1.0 (-0.5 to 2.4)	46.3 (44.4 to 48.0)	46.0 (44.0 to 47.7)	46.6 (44.5 to 48.3)	-0.6 (-2.6 to 1.5)	1.3 (-0.2 to 2.9)
Diet high in red meat	3.9 (0.4 to 8.9)	7.5 (2.3 to 13.4)	8.6 (2.9 to 15.8)	94.3 (-17.2 to 957.3)	15.2 (-40.0 to 138.7)	0.5 (0.0 to 2.4)	1.5 (0.0 to 4.4)	2.0 (0.1 to 5.7)	180.4 (-84.2 to 2154668.9)	35.1 (-80.8 to 3149.1)
Diet high in processed meat	11.6 (7.2 to 15.9)	19.8 (14.5 to 24.9)	23.3 (17.4 to 29.1)	70.7 (33.9 to 134.8)	17.6 (-0.6 to 41.1)	9.7 (5.8 to 13.2)	14.7 (10.5 to 18.4)	16.4 (11.9 to 20.8)	50.7 (18.0 to 105.5)	12.0 (-6.4 to 34.2)
Diet high in sugar-sweetened beverages	7.0 (2.7 to 14.1)	8.0 (3.6 to 14.5)	8.4 (3.6 to 15.7)	13.3 (-44.0 to 154.8)	5.2 (-39.6 to 70.7)	5.0 (1.8 to 10.6)	5.6 (2.6 to 10.7)	5.9 (2.4 to 11.6)	11.7 (-50.7 to 154.4)	6.4 (-38.9 to 85.5)
Diet low in fibre	22.4 (14.8 to 28.9)	23.5 (16.4 to 29.8)	24.6 (17.6 to 31.0)	5.3 (-11.4 to 29.7)	4.4 (-7.8 to 20.6)	23.4 (16.2 to 29.8)	24.5 (17.4 to 30.5)	25.9 (18.9 to 31.9)	4.6 (-11.3 to 26.8)	5.7 (-5.3 to 19.3)
Diet low in calcium	35.1 (30.7 to 38.6)	34.8 (30.3 to 38.4)	35.6 (31.3 to 39.1)	-0.7 (-4.5 to 3.1)	2.3 (-0.7 to 5.8)	37.3 (33.5 to 40.4)	36.2 (32.2 to 39.5)	37.3 (33.5 to 40.5)	-3.0 (-6.8 to 0.5)	3.2 (0.6 to 6.6)
Diet low in seafood omega-3 fatty acids	3.2 (1.3 to 4.7)	0.2 (0.0 to 1.0)	0.2 (0.0 to 1.0)	-93.2 (-100.0 to -72.5)	9.4 (-92.7 to 2931.6)	3.7 (1.6 to 5.3)	0.3 (0.0 to 1.2)	0.4 (0.0 to 1.4)	-91.3 (-100.0 to -72.0)	12.9 (-86.0 to 3299.7)
Diet low in polyunsaturated fatty acids	30.7 (25.3 to 35.7)	28.0 (22.0 to 33.2)	28.7 (22.8 to 33.8)	-8.9 (-21.2 to 1.2)	2.5 (-5.7 to 14.0)	31.3 (26.1 to 36.0)	28.7 (22.9 to 33.6)	29.3 (23.7 to 34.1)	-8.6 (-19.0 to 1.3)	2.2 (-7.2 to 12.6)
Diet high in trans fatty acids	1.1 (0.0 to 3.3)	1.0 (0.0 to 3.0)	0.9 (0.0 to 2.9)	-12.6 (-38.1 to -6.7)	-8.5 (-32.6 to -3.9)	1.5 (0.1 to 4.0)	1.3 (0.1 to 3.7)	1.2 (0.0 to 3.5)	-12.4 (-34.8 to -6.3)	-8.5 (-28.2 to -3.8)
Diet high in sodium	27.6 (18.3 to 35.9)	17.9 (9.3 to 25.2)	15.3 (6.3 to 22.9)	-35.1 (-58.1 to -17.5)	-14.3 (-40.4 to 3.0)	31.9 (23.5 to 39.1)	19.3 (10.7 to 26.1)	16.0 (6.9 to 23.1)	-39.4 (-59.3 to -24.1)	-17.2 (-39.7 to -4.7)
Intimate partner violence	-	-	-	-	-	2.2 (1.9 to 2.6)	2.1 (1.7 to 2.4)	2.0 (1.7 to 2.3)	-7.8 (-11.9 to -3.9)	-3.4 (-8.1 to 1.2)
Childhood sexual abuse	5.3 (4.3 to 6.5)	5.3 (4.3 to 6.5)	5.3 (4.3 to 6.5)	0.0 (0.0 to 0.0)	0.0 (0.0 to 0.0)	7.3 (5.9 to 8.9)	7.3 (5.9 to 8.9)	7.5 (6.0 to 9.2)	0.0 (0.0 to 0.0)	2.8 (-4.0 to 10.6)

Supplementary Results Table 1. Gifu age-standardised Summary Exposure Values (SEVs) for Level 4 risk factors and percentage change, by sex, 1990, 2007, and 2017.Data in parentheses are 95% uncertainty intervals.										
	**Male**					**Female**				
	**1990**	**2007**	**2017**	**Percentage change 1990-2007**	**Percentage change 2007-2017**	**1990**	**2007**	**2017**	**Percentage change 1990-2007**	**Percentage change 2007-2017**
Bullying victimization	3.3 (1.4 to 6.9)	3.4 (1.4 to 7.1)	3.5 (1.5 to 7.5)	1.2 (-6.9 to 9.2)	4.3 (-0.9 to 9.3)	1.9 (0.8 to 3.9)	1.9 (0.8 to 4.0)	2.0 (0.8 to 4.1)	0.4 (-7.7 to 8.2)	3.5 (-2.1 to 8.4)
Low physical activity	0.5 (0.2 to 0.9)	0.5 (0.2 to 0.9)	0.4 (0.2 to 0.8)	-1.9 (-3.9 to 0.0)	-8.8 (-12.4 to -4.5)	0.5 (0.2 to 1.0)	0.5 (0.2 to 0.9)	0.5 (0.2 to 0.9)	-3.9 (-6.1 to -1.4)	0.7 (-0.9 to 2.3)
High fasting plasma glucose	1.3 (0.8 to 2.1)	1.3 (0.8 to 2.1)	1.5 (0.9 to 2.4)	-0.1 (-37.2 to 59.7)	13.1 (-24.6 to 84.7)	1.2 (0.7 to 1.9)	1.2 (0.7 to 2.1)	1.4 (0.8 to 2.4)	5.4 (-35.5 to 75.4)	10.4 (-27.2 to 79.1)
High LDL cholesterol	12.1 (10.4 to 13.8)	12.6 (11.0 to 14.3)	12.6 (11.0 to 14.3)	4.3 (-1.5 to 10.7)	0.1 (-4.4 to 4.8)	12.8 (11.3 to 14.5)	13.0 (11.4 to 14.7)	13.0 (11.4 to 14.8)	0.9 (-4.5 to 6.5)	0.2 (-4.4 to 4.9)
High systolic blood pressure	11.2 (10.2 to 12.3)	8.3 (7.5 to 9.2)	8.2 (7.2 to 9.2)	-25.8 (-31.8 to -19.6)	-1.4 (-8.9 to 7.1)	9.6 (8.6 to 10.5)	6.0 (5.3 to 6.8)	5.6 (4.8 to 6.5)	-36.8 (-43.9 to -29.7)	-7.2 (-17.2 to 3.6)
High body-mass index	5.9 (3.8 to 9.2)	8.6 (5.8 to 13.4)	8.9 (6.0 to 13.9)	44.6 (15.4 to 82.4)	3.4 (-10.8 to 21.2)	5.4 (3.4 to 8.4)	7.0 (4.6 to 11.0)	7.1 (4.4 to 11.2)	29.9 (1.0 to 70.5)	0.6 (-19.4 to 25.7)
Low bone mineral density	5.3 (4.4 to 6.3)	5.2 (4.3 to 6.1)	5.2 (4.2 to 6.1)	-2.4 (-14.5 to 10.7)	0.4 (-11.9 to 13.9)	7.2 (6.0 to 8.7)	7.0 (5.9 to 8.1)	7.1 (5.9 to 8.4)	-3.4 (-15.2 to 11.4)	1.7 (-10.3 to 16.0)
Impaired kidney function	2.6 (1.2 to 6.5)	2.5 (1.1 to 6.4)	2.4 (1.1 to 6.1)	-3.0 (-7.7 to 1.5)	-6.4 (-11.4 to -1.9)	3.2 (1.4 to 7.5)	3.3 (1.5 to 7.6)	3.1 (1.4 to 7.3)	3.1 (-0.6 to 7.7)	-7.0 (-11.9 to -2.3)

Supplementary Results Table 1. Gunma age-standardised Summary Exposure Values (SEVs) for Level 4 risk factors and percentage change, by sex, 1990, 2007, and 2017.Data in parentheses are 95% uncertainty intervals.

	Male					Female				
	**1990**	**2007**	**2017**	**Percentage change 1990-2007**	**Percentage change 2007-2017**	**1990**	**2007**	**2017**	**Percentage change 1990-2007**	**Percentage change 2007-2017**
Unsafe water source	2.2 (1.2 to 3.7)	1.9 (1.0 to 3.1)	2.0 (1.1 to 3.4)	-17.3 (-57.5 to 69.7)	6.0 (-38.7 to 76.7)	2.2 (1.2 to 3.7)	1.9 (1.0 to 3.1)	2.0 (1.1 to 3.4)	-17.3 (-57.5 to 69.7)	6.0 (-38.7 to 76.7)
Unsafe sanitation	3.9 (1.8 to 6.8)	1.9 (0.9 to 3.6)	1.7 (0.8 to 3.2)	-50.3 (-79.6 to 12.0)	-14.0 (-56.7 to 65.1)	3.9 (1.8 to 6.8)	1.9 (0.9 to 3.6)	1.7 (0.8 to 3.2)	-50.3 (-79.6 to 12.0)	-14.0 (-56.7 to 65.1)
No access to handwashing facility	6.7 (4.8 to 9.1)	6.4 (4.6 to 8.6)	5.9 (4.2 to 7.9)	-5.2 (-33.2 to 34.2)	-7.6 (-27.2 to 17.9)	6.7 (4.8 to 9.1)	6.4 (4.6 to 8.6)	5.9 (4.2 to 7.9)	-5.2 (-33.2 to 34.2)	-7.6 (-27.2 to 17.9)
Ambient particulate matter pollution	33.2 (21.8 to 48.9)	32.9 (22.6 to 48.2)	28.0 (18.1 to 42.2)	-0.9 (-24.3 to 36.3)	-15.1 (-39.8 to 10.1)	33.0 (21.5 to 48.8)	32.9 (22.6 to 48.1)	27.9 (18.1 to 42.2)	-0.4 (-24.0 to 37.5)	-15.1 (-39.9 to 10.1)
Household air pollution from solid fuels	0.6 (0.2 to 1.6)	0.1 (0.0 to 0.4)	0.1 (0.0 to 0.2)	-75.2 (-93.1 to -45.0)	-14.0 (-64.7 to 77.2)	0.8 (0.2 to 5.3)	0.2 (0.0 to 0.9)	0.2 (0.0 to 1.0)	-71.6 (-93.0 to -41.6)	-10.9 (-64.3 to 78.8)
Ambient ozone pollution	52.9 (30.1 to 54.8)	53.1 (30.8 to 54.8)	53.2 (32.1 to 54.8)	0.2 (0.0 to 2.4)	0.4 (0.0 to 4.5)	52.9 (30.1 to 54.8)	53.1 (30.8 to 54.8)	53.2 (32.1 to 54.8)	0.2 (0.0 to 2.4)	0.4 (0.0 to 4.5)
Residential radon	16.6 (1.2 to 53.5)	16.6 (1.2 to 53.5)	16.6 (1.2 to 53.5)	0.0 (0.0 to 0.0)	0.0 (0.0 to 0.0)	16.6 (1.2 to 53.5)	16.6 (1.2 to 53.5)	16.6 (1.2 to 53.5)	0.0 (0.0 to 0.0)	0.0 (0.0 to 0.0)
Lead exposure	2.9 (0.4 to 6.5)	1.9 (0.2 to 4.8)	1.4 (0.1 to 3.9)	-33.7 (-47.6 to -24.5)	-28.6 (-44.5 to -20.3)	1.5 (0.1 to 4.4)	0.9 (0.0 to 3.1)	0.6 (0.0 to 2.4)	-39.7 (-58.0 to -29.3)	-31.6 (-54.6 to -22.0)
Occupational exposure to asbestos	1.5 (1.3 to 1.7)	2.0 (1.8 to 2.2)	2.0 (1.7 to 2.5)	35.2 (16.9 to 58.5)	2.7 (-16.9 to 26.2)	0.5 (0.4 to 0.6)	0.5 (0.4 to 0.5)	0.5 (0.4 to 0.6)	-7.4 (-24.0 to 19.8)	-0.9 (-20.2 to 20.8)
Occupational exposure to arsenic	0.5 (0.0 to 1.1)	0.4 (0.0 to 1.0)	0.4 (0.0 to 1.0)	-	-	0.4 (0.0 to 1.0)	0.4 (0.0 to 0.9)	0.4 (0.0 to 0.9)	-	-
Occupational exposure to benzene	0.6 (0.0 to 1.8)	0.7 (0.0 to 1.9)	0.7 (0.0 to 2.0)	2.2 (-4.3 to 12.2)	3.3 (-2.0 to 11.3)	0.6 (0.0 to 1.7)	0.7 (0.0 to 2.0)	0.8 (0.0 to 2.3)	21.4 (7.2 to 40.7)	14.2 (4.5 to 29.2)
Occupational exposure to beryllium	0.0 (0.0 to 0.0)	0.0 (0.0 to 0.0)	0.0 (0.0 to 0.0)	-5.1 (-9.9 to -0.0)	1.9 (-1.9 to 6.1)	0.0 (0.0 to 0.0)	0.0 (0.0 to 0.0)	0.0 (0.0 to 0.0)	2.0 (-7.6 to 12.1)	8.9 (2.7 to 16.2)
Occupational exposure to cadmium	0.1 (0.0 to 0.1)	0.0 (0.0 to 0.1)	0.0 (0.0 to 0.1)	-7.3 (-18.6 to 5.0)	0.7 (-8.5 to 11.3)	0.0 (0.0 to 0.1)	0.0 (0.0 to 0.1)	0.0 (0.0 to 0.1)	-8.9 (-23.5 to 9.1)	4.7 (-7.7 to 18.4)
Occupational exposure to chromium	0.1 (0.1 to 0.1)	0.1 (0.1 to 0.1)	0.1 (0.1 to 0.1)	-5.6 (-15.8 to 5.3)	1.2 (-6.9 to 10.5)	0.1 (0.1 to 0.1)	0.1 (0.1 to 0.1)	0.1 (0.1 to 0.1)	-4.3 (-18.1 to 12.4)	6.4 (-3.9 to 18.5)
Occupational exposure to diesel engine exhaust	0.6 (0.5 to 0.6)	0.6 (0.5 to 0.6)	0.6 (0.6 to 0.7)	-1.3 (-9.0 to 7.5)	3.2 (-2.9 to 10.2)	0.5 (0.4 to 0.5)	0.5 (0.4 to 0.6)	0.6 (0.5 to 0.6)	12.4 (-2.2 to 28.4)	11.1 (0.4 to 23.2)
Occupational exposure to formaldehyde	0.3 (0.2 to 0.3)	0.2 (0.2 to 0.3)	0.2 (0.2 to 0.3)	-6.8 (-18.6 to 6.1)	0.3 (-9.5 to 11.3)	0.2 (0.2 to 0.3)	0.2 (0.2 to 0.2)	0.2 (0.2 to 0.2)	-6.3 (-21.8 to 12.5)	5.5 (-6.7 to 19.7)
Occupational exposure to nickel	0.4 (0.0 to 1.9)	0.4 (0.0 to 1.7)	0.4 (0.0 to 1.7)	-	-	0.3 (0.0 to 1.6)	0.3 (0.0 to 1.3)	0.3 (0.0 to 1.3)	-	-
Occupational exposure to polycyclic aromatic hydrocarbons	0.2 (0.2 to 0.3)	0.2 (0.2 to 0.3)	0.2 (0.2 to 0.3)	-4.6 (-14.1 to 5.8)	1.6 (-5.8 to 10.0)	0.2 (0.2 to 0.3)	0.2 (0.2 to 0.2)	0.2 (0.2 to 0.3)	-2.1 (-15.7 to 13.4)	7.3 (-2.6 to 18.7)
Occupational exposure to silica	4.6 (0.7 to 14.9)	4.4 (0.6 to 14.4)	4.5 (0.6 to 14.5)	-5.3 (-15.8 to 6.9)	1.3 (-6.9 to 11.9)	3.2 (0.5 to 10.2)	2.7 (0.4 to 8.5)	2.8 (0.4 to 9.3)	-13.1 (-27.5 to 6.2)	1.7 (-11.2 to 19.0)
Occupational exposure to sulphuric acid	0.6 (0.1 to 2.0)	0.6 (0.1 to 1.8)	0.6 (0.1 to 1.8)	-7.2 (-17.9 to 4.9)	1.2 (-8.0 to 11.5)	0.5 (0.1 to 1.6)	0.5 (0.1 to 1.5)	0.5 (0.1 to 1.5)	-10.8 (-24.9 to 7.4)	3.8 (-9.0 to 18.9)
Occupational exposure to trichloroethylene	0.1 (0.1 to 0.1)	0.1 (0.1 to 0.1)	0.1 (0.1 to 0.1)	-4.7 (-12.3 to 3.6)	1.7 (-4.2 to 8.7)	0.1 (0.1 to 0.1)	0.1 (0.1 to 0.1)	0.1 (0.1 to 0.1)	0.6 (-11.4 to 14.8)	8.0 (-0.3 to 17.9)
Occupational asthmagens	18.8 (14.9 to 23.7)	18.1 (14.4 to 22.4)	18.1 (14.2 to 22.5)	-3.7 (-22.5 to 19.3)	-0.2 (-15.4 to 18.3)	8.2 (6.6 to 10.3)	8.2 (6.6 to 10.1)	8.7 (7.0 to 10.8)	0.0 (-16.6 to 18.4)	5.5 (-7.9 to 19.9)
Occupational particulate matter, gases, and fumes	6.3 (4.6 to 9.2)	6.1 (4.4 to 8.7)	5.9 (4.2 to 8.5)	-4.1 (-8.9 to 1.3)	-3.5 (-6.8 to 0.0)	3.6 (2.5 to 5.5)	3.4 (2.4 to 5.2)	3.3 (2.3 to 4.9)	-5.4 (-14.0 to 4.6)	-4.6 (-10.3 to 1.7)
Occupational noise	5.9 (5.4 to 6.4)	5.7 (5.3 to 6.3)	5.6 (5.2 to 6.1)	-2.7 (-5.5 to 0.3)	-2.1 (-4.1 to -0.0)	3.5 (3.0 to 4.0)	3.5 (3.1 to 4.0)	3.6 (3.1 to 4.0)	0.6 (-5.7 to 7.6)	1.9 (-2.4 to 6.2)
Occupational ergonomic factors	10.3 (8.5 to 12.5)	9.2 (7.7 to 11.2)	9.1 (7.5 to 11.0)	-11.2 (-23.0 to 2.7)	-1.0 (-11.4 to 10.7)	5.6 (4.3 to 7.1)	5.6 (4.4 to 7.1)	6.4 (4.9 to 8.4)	1.5 (-21.0 to 28.9)	13.5 (-6.3 to 36.7)
Non-exclusive breastfeeding	0.4 (0.3 to 0.6)	0.4 (0.3 to 0.6)	0.4 (0.3 to 0.6)	-1.8 (-14.6 to 11.9)	-1.3 (-12.7 to 10.3)	0.4 (0.3 to 0.6)	0.4 (0.3 to 0.6)	0.4 (0.3 to 0.6)	-1.1 (-14.1 to 12.6)	-1.3 (-12.8 to 10.3)
Discontinued breastfeeding	1.7 (1.6 to 1.8)	1.7 (1.6 to 1.8)	1.7 (1.5 to 1.8)	0.6 (-8.6 to 11.8)	-1.3 (-9.1 to 6.7)	1.7 (1.6 to 1.8)	1.7 (1.6 to 1.9)	1.7 (1.6 to 1.8)	0.7 (-8.5 to 11.9)	-1.9 (-9.6 to 6.0)
Child underweight	0.2 (0.1 to 0.3)	0.2 (0.1 to 0.3)	0.2 (0.1 to 0.3)	-12.8 (-29.5 to 6.1)	-4.7 (-17.6 to 8.4)	0.2 (0.1 to 0.4)	0.2 (0.1 to 0.3)	0.2 (0.1 to 0.3)	-14.9 (-34.1 to 8.2)	-5.4 (-18.9 to 10.2)
Child wasting	0.2 (0.1 to 0.2)	0.2 (0.1 to 0.2)	0.2 (0.1 to 0.2)	-7.4 (-29.3 to 19.0)	-1.6 (-17.9 to 18.5)	0.2 (0.1 to 0.3)	0.2 (0.1 to 0.2)	0.2 (0.1 to 0.2)	-8.6 (-32.1 to 17.8)	-2.5 (-18.9 to 16.5)
Child stunting	0.7 (0.4 to 1.1)	0.6 (0.4 to 0.9)	0.6 (0.3 to 0.9)	-7.4 (-41.9 to 46.3)	-4.2 (-31.3 to 32.9)	0.7 (0.4 to 1.2)	0.7 (0.4 to 1.0)	0.6 (0.3 to 1.0)	-8.7 (-41.7 to 45.8)	-4.3 (-34.4 to 34.4)
Short gestation for birth weight	0.0 (0.0 to 0.0)	0.0 (0.0 to 0.0)	0.0 (0.0 to 0.0)	7.2 (-24.2 to 42.2)	3.0 (-12.7 to 27.5)	0.0 (0.0 to 0.0)	0.0 (0.0 to 0.0)	0.0 (0.0 to 0.0)	10.2 (-23.9 to 40.9)	2.3 (-15.0 to 35.6)
Low birth weight for gestation	0.0 (0.0 to 0.0)	0.0 (0.0 to 0.0)	0.0 (0.0 to 0.0)	5.3 (-14.3 to 40.6)	2.6 (-14.2 to 19.9)	0.0 (0.0 to 0.0)	0.0 (0.0 to 0.0)	0.0 (0.0 to 0.0)	3.4 (-15.9 to 37.7)	1.7 (-20.5 to 18.9)
Iron deficiency	-	-	-	-	-	8.2 (5.6 to 11.4)	8.1 (5.7 to 11.2)	7.4 (4.9 to 10.5)	-0.5 (-22.7 to 28.2)	-8.5 (-24.5 to 8.7)
Vitamin A deficiency	0.9 (0.5 to 1.2)	0.9 (0.5 to 1.3)	0.7 (0.4 to 0.9)	4.1 (-18.6 to 38.0)	-26.1 (-40.4 to -4.6)	0.8 (0.5 to 1.1)	0.8 (0.5 to 1.0)	0.7 (0.4 to 0.9)	-5.8 (-21.3 to 13.8)	-13.8 (-28.4 to 6.9)
Zinc deficiency	0.5 (0.1 to 1.0)	0.5 (0.1 to 1.1)	0.4 (0.1 to 1.0)	-3.4 (-63.5 to 77.0)	-14.4 (-68.5 to 155.0)	0.5 (0.1 to 1.1)	0.3 (0.1 to 0.8)	0.4 (0.1 to 0.8)	-30.3 (-64.9 to 71.0)	1.4 (-62.8 to 180.7)
Smoking	17.4 (13.6 to 21.0)	12.2 (9.7 to 14.8)	10.4 (8.2 to 12.7)	-29.5 (-38.3 to -18.4)	-15.2 (-23.4 to -5.7)	3.9 (2.7 to 5.3)	3.3 (2.3 to 4.5)	3.2 (2.2 to 4.4)	-16.3 (-36.9 to 11.0)	-2.8 (-19.7 to 18.2)
Chewing tobacco	1.4 (0.9 to 1.9)	0.9 (0.6 to 1.4)	0.8 (0.5 to 1.1)	-32.3 (-56.2 to 4.4)	-19.0 (-42.8 to 12.4)	0.1 (0.1 to 0.2)	0.1 (0.1 to 0.1)	0.1 (0.1 to 0.1)	-5.0 (-42.4 to 55.2)	-2.4 (-33.2 to 44.9)
Second-hand smoke	37.9 (33.5 to 42.1)	36.4 (32.1 to 40.4)	35.9 (31.5 to 40.5)	-3.9 (-15.2 to 8.9)	-1.5 (-10.5 to 8.3)	57.5 (53.8 to 61.1)	49.5 (45.8 to 53.0)	48.6 (44.6 to 52.6)	-13.9 (-20.2 to -7.0)	-1.9 (-7.5 to 4.0)
Alcohol use	14.8 (7.2 to 25.9)	14.9 (7.4 to 25.7)	16.6 (8.1 to 28.1)	0.8 (-30.9 to 48.8)	10.8 (-23.0 to 55.0)	12.0 (5.8 to 21.5)	11.9 (5.9 to 21.4)	13.0 (6.3 to 22.6)	-0.5 (-32.1 to 46.8)	9.0 (-23.8 to 57.4)
Drug use	0.5 (0.4 to 0.7)	0.5 (0.4 to 0.7)	0.6 (0.4 to 0.8)	0.6 (-5.1 to 6.6)	11.4 (4.4 to 19.8)	0.3 (0.2 to 0.4)	0.3 (0.2 to 0.4)	0.3 (0.3 to 0.4)	2.2 (-3.5 to 8.9)	9.0 (3.1 to 16.2)
Diet low in fruits	40.8 (35.2 to 44.7)	39.2 (33.5 to 43.1)	40.7 (35.1 to 44.5)	-4.1 (-10.0 to 2.5)	3.7 (-1.5 to 9.0)	38.0 (32.1 to 42.3)	35.2 (29.4 to 39.8)	37.5 (32.1 to 42.0)	-7.5 (-16.1 to 0.0)	6.7 (0.5 to 14.5)
Diet low in vegetables	19.3 (10.5 to 26.7)	13.3 (5.0 to 21.1)	15.3 (6.8 to 23.5)	-30.9 (-62.4 to -5.8)	15.1 (-15.1 to 74.5)	21.5 (12.9 to 28.9)	15.3 (7.2 to 23.5)	17.1 (8.7 to 25.2)	-28.6 (-53.5 to -5.9)	11.3 (-15.4 to 52.2)
Diet low in legumes	4.2 (0.7 to 9.4)	5.2 (1.0 to 11.0)	6.9 (1.8 to 13.3)	24.1 (-50.4 to 253.6)	32.2 (-20.3 to 198.3)	8.2 (2.6 to 14.7)	7.7 (2.1 to 13.6)	9.8 (3.5 to 16.7)	-6.6 (-51.3 to 76.0)	28.0 (-17.6 to 127.4)
Diet low in whole grains	30.4 (25.7 to 34.6)	35.1 (31.1 to 38.7)	36.5 (32.5 to 39.9)	15.7 (6.9 to 27.4)	3.9 (-1.0 to 9.0)	31.7 (27.0 to 35.8)	36.4 (32.6 to 39.7)	37.7 (34.0 to 40.8)	14.7 (6.1 to 26.1)	3.5 (-0.9 to 8.4)
Diet low in nuts and seeds	50.6 (49.2 to 51.6)	49.2 (47.4 to 50.6)	49.0 (46.9 to 50.5)	-2.8 (-5.3 to -0.7)	-0.4 (-2.8 to 1.6)	50.8 (49.4 to 51.9)	49.5 (47.6 to 50.9)	49.3 (47.1 to 50.8)	-2.7 (-5.3 to -0.4)	-0.3 (-2.5 to 1.8)
Diet low in milk	46.1 (44.0 to 47.9)	46.4 (44.3 to 48.0)	46.8 (44.8 to 48.4)	0.6 (-1.4 to 2.6)	0.9 (-0.5 to 2.5)	46.1 (44.0 to 47.9)	45.8 (43.7 to 47.6)	46.4 (44.3 to 48.1)	-0.6 (-2.7 to 1.4)	1.3 (-0.2 to 3.0)
Diet high in red meat	4.2 (0.6 to 9.1)	8.1 (2.4 to 14.4)	9.2 (2.8 to 16.6)	93.7 (-18.1 to 666.3)	14.2 (-40.9 to 132.8)	0.6 (0.0 to 2.5)	1.6 (0.0 to 4.7)	2.1 (0.0 to 5.9)	183.7 (-84.1 to 1260994.9)	27.7 (-84.3 to 2238.1)
Diet high in processed meat	11.8 (7.4 to 15.9)	19.6 (14.5 to 24.4)	22.7 (16.7 to 28.4)	66.5 (29.1 to 132.2)	15.6 (-1.7 to 37.4)	10.0 (6.2 to 13.8)	14.8 (10.5 to 18.8)	16.5 (11.9 to 21.0)	48.6 (19.9 to 100.4)	11.1 (-5.1 to 33.1)
Diet high in sugar-sweetened beverages	7.4 (2.8 to 15.2)	8.6 (3.7 to 15.2)	9.0 (3.8 to 16.7)	15.2 (-45.2 to 155.0)	5.4 (-38.2 to 73.1)	5.3 (2.1 to 11.8)	6.0 (2.4 to 11.4)	6.4 (2.5 to 12.5)	12.0 (-47.3 to 149.5)	7.4 (-39.1 to 88.7)
Diet low in fibre	21.9 (14.9 to 28.4)	22.8 (15.7 to 29.0)	23.8 (16.4 to 30.0)	4.2 (-14.1 to 27.5)	4.2 (-8.5 to 19.0)	23.1 (15.7 to 29.6)	24.0 (17.1 to 30.2)	25.3 (18.4 to 31.4)	3.8 (-11.3 to 23.4)	5.3 (-5.6 to 19.4)
Diet low in calcium	34.5 (29.9 to 38.2)	34.3 (29.7 to 37.9)	35.1 (30.7 to 38.7)	-0.6 (-4.6 to 3.1)	2.4 (-0.4 to 5.8)	36.8 (32.8 to 40.1)	35.7 (31.3 to 39.1)	36.9 (32.7 to 40.2)	-3.0 (-6.7 to 0.5)	3.4 (0.6 to 6.8)
Diet low in seafood omega-3 fatty acids	3.1 (1.3 to 4.5)	0.2 (0.0 to 1.0)	0.2 (0.0 to 1.0)	-93.0 (-100.0 to -71.2)	5.5 (-95.4 to 6125.6)	3.6 (1.8 to 5.1)	0.3 (0.0 to 1.3)	0.4 (0.0 to 1.3)	-91.1 (-100.0 to -67.5)	10.4 (-90.4 to 2316.6)
Diet low in polyunsaturated fatty acids	30.1 (24.6 to 34.7)	27.4 (21.3 to 32.2)	28.1 (22.0 to 32.9)	-8.8 (-20.9 to 0.7)	2.4 (-5.4 to 10.8)	30.9 (25.3 to 35.7)	28.1 (22.2 to 33.1)	28.9 (23.2 to 33.9)	-9.0 (-21.0 to 0.6)	2.9 (-5.4 to 16.2)
Diet high in trans fatty acids	1.1 (0.0 to 3.3)	1.0 (0.0 to 3.0)	0.9 (0.0 to 2.9)	-12.5 (-38.1 to -6.3)	-8.2 (-33.5 to -3.2)	1.5 (0.1 to 4.0)	1.3 (0.1 to 3.7)	1.2 (0.0 to 3.5)	-12.7 (-34.7 to -6.5)	-8.4 (-28.9 to -3.5)
Diet high in sodium	28.4 (19.3 to 36.6)	18.6 (10.2 to 25.6)	16.0 (7.2 to 23.5)	-34.3 (-56.6 to -16.3)	-14.0 (-38.0 to 1.9)	32.1 (23.6 to 39.1)	19.5 (11.0 to 26.7)	16.3 (7.5 to 23.7)	-39.3 (-59.5 to -23.7)	-16.4 (-38.9 to -2.7)
Intimate partner violence	-	-	-	-	-	2.3 (1.9 to 2.7)	2.1 (1.8 to 2.4)	2.0 (1.7 to 2.4)	-8.5 (-12.5 to -3.8)	-3.4 (-7.4 to 1.5)
Childhood sexual abuse	5.3 (4.3 to 6.5)	5.3 (4.3 to 6.5)	5.3 (4.3 to 6.5)	0.0 (0.0 to 0.0)	0.0 (0.0 to 0.0)	7.3 (5.9 to 8.9)	7.3 (5.9 to 8.9)	7.5 (6.0 to 9.2)	0.0 (0.0 to 0.0)	2.8 (-4.0 to 10.6)

Supplementary Results Table 1. Gunma age-standardised Summary Exposure Values (SEVs) for Level 4 risk factors and percentage change, by sex, 1990, 2007, and 2017.Data in parentheses are 95% uncertainty intervals.										
	**Male**					**Female**				
	**1990**	**2007**	**2017**	**Percentage change 1990-2007**	**Percentage change 2007-2017**	**1990**	**2007**	**2017**	**Percentage change 1990-2007**	**Percentage change 2007-2017**
Bullying victimization	3.3 (1.4 to 7.1)	3.4 (1.4 to 7.2)	3.5 (1.4 to 7.6)	1.6 (-6.1 to 9.8)	4.3 (-0.9 to 9.1)	1.9 (0.8 to 3.8)	1.9 (0.8 to 3.9)	2.0 (0.8 to 4.1)	-0.2 (-7.9 to 7.9)	3.3 (-2.4 to 8.1)
Low physical activity	0.5 (0.2 to 0.9)	0.5 (0.2 to 0.9)	0.4 (0.2 to 0.8)	-1.8 (-3.8 to 0.0)	-8.8 (-12.3 to -4.5)	0.5 (0.2 to 1.0)	0.5 (0.2 to 0.9)	0.5 (0.2 to 0.9)	-3.9 (-6.1 to -1.3)	0.7 (-0.8 to 2.2)
High fasting plasma glucose	1.4 (0.9 to 2.2)	1.4 (0.9 to 2.4)	1.6 (1.0 to 2.6)	3.9 (-36.1 to 59.9)	12.0 (-25.1 to 77.3)	1.2 (0.7 to 1.9)	1.2 (0.7 to 2.1)	1.3 (0.8 to 2.4)	4.3 (-34.5 to 73.3)	9.0 (-29.7 to 77.6)
High LDL cholesterol	12.0 (10.4 to 13.7)	12.6 (10.9 to 14.3)	12.6 (10.9 to 14.4)	4.4 (-1.5 to 10.4)	0.2 (-4.5 to 4.6)	12.9 (11.2 to 14.6)	13.0 (11.5 to 14.7)	13.0 (11.4 to 14.8)	0.8 (-4.6 to 6.5)	0.2 (-4.4 to 5.0)
High systolic blood pressure	11.3 (10.2 to 12.3)	8.3 (7.4 to 9.2)	8.2 (7.2 to 9.2)	-26.2 (-32.3 to -19.9)	-1.7 (-8.8 to 6.5)	9.6 (8.6 to 10.7)	6.0 (5.2 to 6.8)	5.6 (4.8 to 6.4)	-37.4 (-44.0 to -30.8)	-7.3 (-17.3 to 3.0)
High body-mass index	5.9 (3.8 to 9.2)	8.4 (5.7 to 13.2)	8.7 (5.9 to 13.7)	42.6 (14.5 to 82.9)	3.2 (-11.9 to 20.5)	5.6 (3.4 to 8.7)	7.1 (4.6 to 11.5)	7.2 (4.5 to 11.4)	28.6 (1.3 to 66.2)	0.6 (-18.0 to 23.5)
Low bone mineral density	5.3 (4.4 to 6.3)	5.2 (4.3 to 6.0)	5.2 (4.3 to 6.2)	-2.2 (-15.4 to 12.5)	0.2 (-13.3 to 14.4)	7.2 (6.0 to 8.6)	7.0 (5.9 to 8.2)	7.1 (6.0 to 8.3)	-3.1 (-15.5 to 10.2)	1.5 (-11.1 to 13.9)
Impaired kidney function	2.6 (1.2 to 6.5)	2.5 (1.1 to 6.4)	2.4 (1.1 to 6.1)	-2.1 (-7.0 to 2.7)	-6.2 (-11.9 to -1.8)	3.2 (1.5 to 7.4)	3.3 (1.5 to 7.6)	3.1 (1.4 to 7.2)	3.3 (-0.7 to 7.7)	-6.6 (-11.2 to -2.0)

Supplementary Results Table 1. Hiroshima age-standardised Summary Exposure Values (SEVs) for Level 4 risk factors and percentage change, by sex, 1990, 2007, and 2017.Data in parentheses are 95% uncertainty intervals.										
	Male					Female				
	1990	2007	2017	Percentage change 1990-2007	Percentage change 2007-2017	1990	2007	2017	Percentage change 1990-2007	Percentage change 2007-2017
Unsafe water source	2.1 (1.1 to 3.6)	1.7 (0.9 to 2.9)	1.8 (1.0 to 3.0)	-15.8 (-57.0 to 63.9)	5.9 (-37.2 to 81.2)	2.1 (1.1 to 3.6)	1.7 (0.9 to 2.9)	1.8 (1.0 to 3.0)	-15.8 (-57.0 to 63.9)	5.9 (-37.2 to 81.2)
Unsafe sanitation	3.4 (1.6 to 6.4)	1.7 (0.8 to 3.2)	1.5 (0.7 to 2.8)	-50.0 (-77.6 to 15.3)	-13.8 (-56.2 to 71.2)	3.4 (1.6 to 6.4)	1.7 (0.8 to 3.2)	1.5 (0.7 to 2.8)	-50.0 (-77.6 to 15.3)	-13.8 (-56.2 to 71.2)
No access to handwashing facility	6.7 (4.8 to 8.9)	6.3 (4.6 to 8.5)	5.8 (4.2 to 7.9)	-5.6 (-32.4 to 30.5)	-7.3 (-27.7 to 18.2)	6.7 (4.8 to 8.9)	6.3 (4.6 to 8.5)	5.8 (4.2 to 7.9)	-5.6 (-32.4 to 30.5)	-7.3 (-27.7 to 18.2)
Ambient particulate matter pollution	30.6 (19.7 to 46.8)	30.7 (20.4 to 46.3)	26.5 (16.0 to 41.3)	0.2 (-25.8 to 39.2)	-13.8 (-39.3 to 16.0)	30.4 (19.6 to 46.4)	30.6 (20.4 to 46.2)	26.4 (16.0 to 41.3)	0.7 (-25.6 to 40.6)	-13.8 (-39.3 to 16.0)
Household air pollution from solid fuels	0.5 (0.2 to 1.4)	0.1 (0.0 to 0.3)	0.1 (0.0 to 0.3)	-74.0 (-93.0 to -46.3)	-12.9 (-66.3 to 81.6)	0.8 (0.2 to 4.3)	0.2 (0.0 to 1.0)	0.2 (0.0 to 0.8)	-69.8 (-92.9 to -38.9)	-14.0 (-66.5 to 81.3)
Ambient ozone pollution	51.4 (24.6 to 54.8)	51.8 (25.4 to 54.8)	52.3 (27.0 to 54.8)	0.7 (0.0 to 4.2)	1.0 (0.0 to 7.1)	51.4 (24.6 to 54.8)	51.8 (25.4 to 54.8)	52.3 (27.0 to 54.8)	0.7 (0.0 to 4.2)	1.0 (0.0 to 7.1)
Residential radon	13.9 (0.9 to 45.4)	13.9 (0.9 to 45.4)	13.9 (0.9 to 45.4)	0.0 (0.0 to 0.0)	0.0 (0.0 to 0.0)	13.9 (0.9 to 45.4)	13.9 (0.9 to 45.4)	13.9 (0.9 to 45.4)	0.0 (0.0 to 0.0)	0.0 (0.0 to 0.0)
Lead exposure	2.9 (0.4 to 6.5)	1.9 (0.2 to 4.8)	1.3 (0.1 to 3.8)	-34.8 (-49.3 to -25.3)	-29.2 (-45.2 to -21.1)	1.5 (0.1 to 4.4)	0.9 (0.0 to 3.1)	0.6 (0.0 to 2.4)	-40.5 (-58.8 to -30.8)	-32.1 (-55.1 to -22.4)
Occupational exposure to asbestos	3.9 (3.4 to 4.4)	5.5 (5.1 to 5.9)	4.9 (4.1 to 5.8)	39.5 (22.6 to 61.4)	-10.0 (-24.6 to 7.8)	0.8 (0.6 to 1.0)	0.7 (0.7 to 0.8)	0.7 (0.6 to 0.9)	-8.0 (-24.0 to 18.0)	-0.5 (-21.9 to 22.7)
Occupational exposure to arsenic	0.5 (0.0 to 1.1)	0.4 (0.0 to 1.0)	0.4 (0.0 to 1.0)	-	-	0.4 (0.0 to 1.0)	0.4 (0.0 to 0.9)	0.4 (0.0 to 0.9)	-	-
Occupational exposure to benzene	0.6 (0.0 to 1.8)	0.7 (0.0 to 1.9)	0.7 (0.0 to 1.9)	2.9 (-3.9 to 12.0)	3.3 (-2.1 to 11.7)	0.6 (0.0 to 1.8)	0.7 (0.0 to 2.1)	0.8 (0.1 to 2.4)	21.2 (9.1 to 40.7)	13.9 (4.0 to 28.6)
Occupational exposure to beryllium	0.0 (0.0 to 0.0)	0.0 (0.0 to 0.0)	0.0 (0.0 to 0.0)	-5.0 (-9.8 to 0.0)	2.1 (-2.1 to 6.0)	0.0 (0.0 to 0.0)	0.0 (0.0 to 0.0)	0.0 (0.0 to 0.0)	1.8 (-6.7 to 11.0)	8.9 (2.5 to 16.1)
Occupational exposure to cadmium	0.1 (0.0 to 0.1)	0.0 (0.0 to 0.1)	0.0 (0.0 to 0.1)	-7.0 (-18.3 to 5.6)	1.1 (-8.5 to 11.2)	0.0 (0.0 to 0.1)	0.0 (0.0 to 0.1)	0.0 (0.0 to 0.1)	-9.4 (-24.0 to 7.2)	4.6 (-7.3 to 18.7)
Occupational exposure to chromium	0.1 (0.1 to 0.1)	0.1 (0.1 to 0.1)	0.1 (0.1 to 0.1)	-5.4 (-15.6 to 5.8)	1.7 (-6.7 to 10.2)	0.1 (0.1 to 0.1)	0.1 (0.1 to 0.1)	0.1 (0.1 to 0.1)	-4.6 (-18.1 to 11.1)	6.4 (-4.1 to 18.6)
Occupational exposure to diesel engine exhaust	0.6 (0.5 to 0.6)	0.6 (0.5 to 0.6)	0.6 (0.6 to 0.7)	-1.9 (-9.5 to 5.9)	3.2 (-3.6 to 10.4)	0.5 (0.4 to 0.5)	0.5 (0.5 to 0.6)	0.6 (0.5 to 0.6)	12.2 (-1.2 to 27.3)	11.0 (0.8 to 23.4)
Occupational exposure to formaldehyde	0.3 (0.2 to 0.3)	0.2 (0.2 to 0.3)	0.2 (0.2 to 0.3)	-6.4 (-18.7 to 7.5)	0.7 (-9.0 to 11.1)	0.2 (0.2 to 0.3)	0.2 (0.2 to 0.2)	0.2 (0.2 to 0.2)	-6.8 (-22.3 to 10.9)	5.4 (-6.4 to 20.0)
Occupational exposure to nickel	0.4 (0.0 to 1.9)	0.4 (0.0 to 1.7)	0.4 (0.0 to 1.7)	-	-	0.3 (0.0 to 1.6)	0.3 (0.0 to 1.3)	0.3 (0.0 to 1.3)	-	-
Occupational exposure to polycyclic aromatic hydrocarbons	0.2 (0.2 to 0.3)	0.2 (0.2 to 0.3)	0.2 (0.2 to 0.3)	-4.3 (-13.9 to 6.2)	2.0 (-5.7 to 10.1)	0.2 (0.2 to 0.3)	0.2 (0.2 to 0.2)	0.2 (0.2 to 0.3)	-2.5 (-15.9 to 12.4)	7.2 (-2.4 to 18.7)
Occupational exposure to silica	4.7 (0.7 to 15.3)	4.4 (0.6 to 14.2)	4.4 (0.6 to 14.8)	-6.1 (-16.4 to 5.7)	1.4 (-7.1 to 12.4)	3.2 (0.5 to 10.2)	2.7 (0.4 to 8.7)	2.8 (0.4 to 8.8)	-14.2 (-28.9 to 4.0)	1.5 (-11.0 to 19.0)
Occupational exposure to sulphuric acid	0.6 (0.1 to 1.9)	0.6 (0.1 to 1.8)	0.6 (0.1 to 1.8)	-7.1 (-17.6 to 5.6)	1.4 (-7.6 to 11.8)	0.5 (0.1 to 1.7)	0.5 (0.1 to 1.5)	0.5 (0.1 to 1.5)	-10.7 (-25.1 to 6.0)	3.6 (-8.5 to 18.9)
Occupational exposure to trichloroethylene	0.1 (0.1 to 0.1)	0.1 (0.1 to 0.1)	0.1 (0.1 to 0.1)	-4.4 (-11.6 to 4.4)	2.1 (-3.7 to 8.7)	0.1 (0.1 to 0.1)	0.1 (0.1 to 0.1)	0.1 (0.1 to 0.1)	0.5 (-10.4 to 12.9)	8.0 (-1.1 to 18.2)
Occupational asthmagens	18.9 (14.9 to 23.8)	18.2 (14.4 to 23.0)	18.2 (14.4 to 23.4)	-3.6 (-22.6 to 19.9)	0.1 (-15.1 to 18.1)	8.4 (6.7 to 10.4)	8.3 (6.6 to 10.3)	8.8 (7.1 to 10.9)	-0.6 (-15.6 to 17.4)	5.3 (-8.5 to 20.1)
Occupational particulate matter, gases, and fumes	6.4 (4.5 to 9.1)	6.1 (4.3 to 8.6)	5.9 (4.2 to 8.4)	-4.4 (-9.5 to 0.7)	-3.5 (-7.0 to 0.1)	3.7 (2.5 to 5.4)	3.4 (2.4 to 5.1)	3.3 (2.3 to 4.8)	-6.0 (-14.7 to 3.6)	-4.8 (-10.4 to 1.3)
Occupational noise	5.9 (5.4 to 6.5)	5.7 (5.3 to 6.3)	5.6 (5.2 to 6.1)	-3.1 (-6.1 to 0.1)	-2.2 (-4.4 to -0.0)	3.5 (3.0 to 4.0)	3.5 (3.1 to 4.0)	3.6 (3.2 to 4.0)	0.2 (-5.8 to 6.9)	1.6 (-2.2 to 5.7)
Occupational ergonomic factors	10.3 (8.5 to 12.4)	9.1 (7.5 to 10.9)	9.0 (7.4 to 10.9)	-11.4 (-22.7 to 0.7)	-1.4 (-12.5 to 10.0)	5.6 (4.3 to 7.1)	5.6 (4.4 to 7.1)	6.4 (4.9 to 8.2)	1.5 (-19.4 to 26.2)	12.9 (-6.2 to 36.9)
Non-exclusive breastfeeding	0.4 (0.3 to 0.6)	0.4 (0.3 to 0.6)	0.4 (0.3 to 0.6)	-1.0 (-14.0 to 13.3)	-1.5 (-11.8 to 9.4)	0.4 (0.3 to 0.6)	0.4 (0.3 to 0.6)	0.4 (0.3 to 0.6)	-1.0 (-14.0 to 13.3)	-1.5 (-11.8 to 9.4)
Discontinued breastfeeding	1.7 (1.6 to 1.9)	1.7 (1.6 to 1.9)	1.7 (1.6 to 1.8)	1.8 (-7.3 to 13.1)	-2.5 (-9.5 to 5.0)	1.7 (1.6 to 1.9)	1.7 (1.6 to 1.9)	1.7 (1.6 to 1.8)	1.8 (-7.3 to 13.1)	-2.5 (-9.5 to 5.0)
Child underweight	0.2 (0.1 to 0.3)	0.2 (0.1 to 0.3)	0.2 (0.1 to 0.2)	-15.2 (-33.6 to 4.2)	-5.1 (-17.5 to 10.6)	0.2 (0.1 to 0.3)	0.2 (0.1 to 0.3)	0.2 (0.1 to 0.2)	-17.9 (-36.9 to 3.1)	-5.5 (-19.5 to 10.5)
Child wasting	0.2 (0.1 to 0.3)	0.2 (0.1 to 0.2)	0.2 (0.1 to 0.2)	-8.7 (-33.7 to 23.2)	-2.2 (-20.6 to 19.8)	0.2 (0.1 to 0.3)	0.2 (0.1 to 0.2)	0.2 (0.1 to 0.2)	-9.7 (-34.9 to 22.3)	-2.2 (-22.4 to 20.7)
Child stunting	0.5 (0.3 to 0.9)	0.4 (0.2 to 0.7)	0.4 (0.2 to 0.7)	-14.9 (-46.4 to 33.7)	-2.2 (-31.2 to 33.9)	0.5 (0.3 to 0.9)	0.5 (0.2 to 0.7)	0.4 (0.2 to 0.7)	-16.2 (-47.7 to 35.4)	-2.3 (-31.2 to 34.3)
Short gestation for birth weight	0.0 (0.0 to 0.0)	0.0 (0.0 to 0.0)	0.0 (0.0 to 0.0)	7.9 (-14.4 to 34.7)	0.3 (-17.3 to 23.1)	0.0 (0.0 to 0.0)	0.0 (0.0 to 0.0)	0.0 (0.0 to 0.0)	15.1 (-16.2 to 116.7)	0.9 (-20.6 to 39.1)
Low birth weight for gestation	0.0 (0.0 to 0.0)	0.0 (0.0 to 0.0)	0.0 (0.0 to 0.0)	4.8 (-14.2 to 28.7)	3.1 (-13.8 to 21.1)	0.0 (0.0 to 0.0)	0.0 (0.0 to 0.0)	0.0 (0.0 to 0.0)	-4.7 (-61.7 to 32.9)	2.6 (-23.2 to 26.1)
Iron deficiency	-	-	-	-	-	7.8 (5.3 to 10.8)	7.8 (5.4 to 10.7)	7.2 (4.8 to 9.9)	0.3 (-22.2 to 28.7)	-8.2 (-23.8 to 10.8)
Vitamin A deficiency	0.9 (0.6 to 1.2)	0.8 (0.5 to 1.1)	0.6 (0.4 to 0.8)	-9.1 (-22.2 to 5.3)	-27.1 (-42.2 to -11.6)	0.7 (0.5 to 0.9)	0.7 (0.4 to 1.0)	0.7 (0.4 to 0.9)	8.3 (-11.2 to 48.1)	-10.2 (-27.7 to 10.8)
Zinc deficiency	0.5 (0.1 to 1.0)	0.5 (0.1 to 1.2)	0.4 (0.1 to 1.1)	6.3 (-61.1 to 77.0)	-16.0 (-65.7 to 140.1)	0.5 (0.2 to 1.0)	0.3 (0.1 to 0.8)	0.3 (0.1 to 0.8)	-29.1 (-65.7 to 67.4)	-4.3 (-63.2 to 160.0)
Smoking	16.9 (13.3 to 20.4)	11.9 (9.4 to 14.5)	10.1 (8.1 to 12.3)	-29.5 (-39.2 to -18.9)	-15.3 (-23.7 to -5.5)	3.8 (2.7 to 5.1)	3.2 (2.2 to 4.3)	3.1 (2.2 to 4.2)	-16.5 (-37.4 to 9.5)	-2.9 (-19.7 to 16.9)
Chewing tobacco	1.4 (0.9 to 2.0)	0.9 (0.6 to 1.4)	0.8 (0.5 to 1.1)	-32.7 (-56.3 to 5.3)	-19.0 (-42.5 to 11.3)	0.1 (0.1 to 0.1)	0.1 (0.1 to 0.1)	0.1 (0.1 to 0.1)	-3.5 (-39.4 to 63.5)	-3.2 (-31.8 to 40.8)
Second-hand smoke	37.4 (33.4 to 41.1)	36.7 (32.3 to 40.8)	35.4 (31.0 to 40.0)	-1.9 (-13.3 to 10.1)	-3.5 (-11.8 to 5.8)	56.5 (52.7 to 60.2)	49.4 (45.3 to 53.3)	47.6 (43.5 to 51.6)	-12.6 (-19.2 to -5.9)	-3.5 (-9.5 to 2.6)
Alcohol use	15.1 (7.9 to 25.8)	15.1 (7.8 to 25.4)	17.1 (7.9 to 30.0)	0.1 (-33.3 to 47.0)	13.3 (-18.8 to 59.2)	11.8 (6.0 to 20.8)	11.7 (5.8 to 20.2)	13.6 (6.3 to 24.4)	-0.8 (-35.4 to 48.0)	16.5 (-18.2 to 69.2)
Drug use	0.5 (0.4 to 0.7)	0.5 (0.4 to 0.7)	0.6 (0.4 to 0.8)	0.3 (-5.1 to 5.9)	11.7 (4.8 to 20.4)	0.3 (0.2 to 0.4)	0.3 (0.2 to 0.4)	0.3 (0.3 to 0.4)	2.7 (-3.6 to 8.8)	9.2 (2.3 to 17.1)
Diet low in fruits	40.5 (34.6 to 44.2)	38.7 (32.4 to 42.7)	40.0 (34.0 to 43.9)	-4.4 (-10.9 to 1.8)	3.4 (-1.5 to 9.1)	37.4 (31.5 to 41.7)	34.7 (28.4 to 39.5)	37.0 (30.9 to 41.6)	-7.3 (-16.6 to 0.7)	6.6 (-0.5 to 15.4)
Diet low in vegetables	18.8 (10.4 to 26.5)	13.2 (5.4 to 20.9)	15.4 (7.1 to 23.1)	-29.9 (-59.0 to -0.2)	16.6 (-16.7 to 75.0)	21.0 (12.5 to 28.3)	15.2 (6.9 to 23.2)	17.0 (8.3 to 25.0)	-27.8 (-55.9 to -3.2)	12.3 (-14.7 to 59.4)
Diet low in legumes	4.4 (0.8 to 9.7)	6.1 (1.3 to 12.6)	8.3 (2.6 to 15.4)	39.4 (-43.9 to 285.8)	35.7 (-15.5 to 202.4)	8.3 (2.8 to 14.4)	8.1 (2.5 to 14.7)	10.6 (4.3 to 18.0)	-1.8 (-46.8 to 91.5)	30.3 (-11.3 to 137.3)
Diet low in whole grains	30.2 (25.0 to 34.4)	35.0 (30.8 to 38.5)	36.3 (32.3 to 39.6)	16.0 (7.2 to 29.0)	3.9 (-1.6 to 10.0)	31.6 (27.0 to 35.8)	36.2 (31.9 to 39.5)	37.5 (33.6 to 40.5)	14.6 (6.6 to 26.4)	3.5 (-0.8 to 8.4)
Diet low in nuts and seeds	50.4 (49.0 to 51.5)	48.8 (46.9 to 50.4)	48.6 (46.4 to 50.3)	-3.0 (-5.7 to -0.8)	-0.5 (-3.0 to 1.7)	50.7 (49.3 to 51.7)	49.2 (47.4 to 50.7)	49.0 (46.9 to 50.7)	-2.9 (-5.7 to -0.5)	-0.4 (-2.7 to 1.7)
Diet low in milk	46.0 (43.8 to 47.8)	46.3 (44.2 to 48.0)	46.8 (44.7 to 48.5)	0.7 (-1.3 to 2.9)	1.0 (-0.4 to 2.7)	46.0 (43.9 to 47.8)	45.7 (43.6 to 47.5)	46.3 (44.2 to 48.1)	-0.6 (-2.6 to 1.5)	1.4 (-0.3 to 3.0)
Diet high in red meat	4.4 (0.7 to 9.3)	8.4 (2.5 to 15.3)	9.6 (3.3 to 17.2)	90.5 (-18.2 to 588.1)	13.7 (-35.3 to 115.9)	0.6 (0.0 to 2.7)	1.8 (0.0 to 5.4)	2.3 (0.1 to 6.3)	183.3 (-74.2 to 289705.8)	24.9 (-79.9 to 1357.1)
Diet high in processed meat	12.1 (7.9 to 16.1)	20.0 (14.8 to 25.3)	23.2 (17.1 to 28.6)	66.1 (30.5 to 130.4)	16.0 (-2.6 to 37.0)	10.2 (6.4 to 13.9)	14.9 (10.8 to 19.1)	16.5 (11.8 to 20.8)	47.0 (15.8 to 104.3)	10.6 (-6.4 to 31.2)
Diet high in sugar-sweetened beverages	7.7 (3.2 to 15.2)	9.0 (3.9 to 15.6)	9.5 (4.0 to 17.6)	17.6 (-42.4 to 146.3)	5.5 (-37.4 to 76.5)	5.4 (2.2 to 11.7)	6.1 (2.9 to 11.2)	6.6 (2.7 to 12.7)	12.6 (-47.4 to 168.6)	7.1 (-38.4 to 89.6)
Diet low in fibre	21.6 (14.1 to 28.3)	22.7 (15.5 to 29.1)	23.8 (16.5 to 30.0)	5.3 (-13.6 to 27.4)	4.7 (-7.3 to 20.3)	22.9 (15.7 to 29.5)	24.0 (16.8 to 29.7)	25.4 (18.3 to 31.3)	4.4 (-12.2 to 24.4)	5.9 (-5.5 to 21.0)
Diet low in calcium	34.1 (29.5 to 37.8)	33.9 (29.3 to 37.6)	34.8 (30.3 to 38.4)	-0.6 (-4.7 to 3.5)	2.5 (-0.7 to 6.3)	36.4 (32.4 to 39.7)	35.2 (30.9 to 38.7)	36.4 (32.4 to 39.7)	-3.2 (-7.0 to 0.2)	3.4 (0.7 to 6.9)
Diet low in seafood omega-3 fatty acids	2.9 (1.1 to 4.4)	0.2 (0.0 to 0.9)	0.2 (0.0 to 0.9)	-93.9 (-100.0 to -74.8)	10.4 (-97.0 to 20166.6)	3.4 (1.4 to 5.1)	0.2 (0.0 to 1.1)	0.3 (0.0 to 1.1)	-93.0 (-100.0 to -76.0)	10.5 (-94.7 to 3845.4)
Diet low in polyunsaturated fatty acids	29.8 (24.2 to 34.7)	27.1 (20.9 to 32.2)	27.7 (21.4 to 32.9)	-9.3 (-21.4 to 0.7)	2.5 (-6.1 to 13.1)	30.5 (24.9 to 35.3)	27.7 (21.4 to 32.9)	28.4 (22.4 to 33.4)	-9.3 (-21.4 to -0.3)	2.5 (-4.6 to 11.2)
Diet high in trans fatty acids	1.1 (0.0 to 3.3)	1.0 (0.0 to 3.0)	0.9 (0.0 to 2.9)	-12.4 (-39.1 to -6.3)	-8.5 (-33.0 to -3.8)	1.5 (0.1 to 4.0)	1.3 (0.1 to 3.7)	1.2 (0.0 to 3.5)	-12.6 (-33.9 to -6.7)	-8.5 (-29.0 to -3.6)
Diet high in sodium	28.4 (19.1 to 36.2)	18.3 (9.7 to 25.4)	15.7 (7.1 to 23.0)	-35.3 (-58.9 to -17.3)	-14.4 (-38.0 to 2.1)	31.6 (22.9 to 38.6)	18.8 (10.2 to 25.5)	15.5 (6.8 to 22.9)	-40.4 (-61.6 to -25.1)	-17.4 (-40.6 to -4.7)
Intimate partner violence	-	-	-	-	-	2.3 (1.9 to 2.7)	2.1 (1.8 to 2.5)	2.0 (1.7 to 2.4)	-8.5 (-12.4 to -4.3)	-4.2 (-8.5 to 0.0)
Childhood sexual abuse	5.3 (4.3 to 6.5)	5.3 (4.3 to 6.5)	5.3 (4.3 to 6.5)	0.0 (0.0 to 0.0)	0.0 (0.0 to 0.0)	7.3 (5.9 to 8.9)	7.3 (5.9 to 8.9)	7.5 (6.0 to 9.2)	0.0 (0.0 to 0.0)	2.8 (-4.0 to 10.6)

Supplementary Results Table 1. Hiroshima age-standardised Summary Exposure Values (SEVs) for Level 4 risk factors and percentage change, by sex, 1990, 2007, and 2017.Data in parentheses are 95% uncertainty intervals.

	Male					Female				
	**1990**	**2007**	**2017**	**Percentage change 1990-2007**	**Percentage change 2007-2017**	**1990**	**2007**	**2017**	**Percentage change 1990-2007**	**Percentage change 2007-2017**
Bullying victimization	3.3<br>(1.4 to 7.1)	3.3<br>(1.4 to 7.2)	3.5<br>(1.4 to 7.5)	0.8<br>(-7.4 to 8.6)	3.9<br>(-1.2 to 9.1)	1.9<br>(0.8 to 3.9)	1.9<br>(0.8 to 4.0)	2.0<br>(0.8 to 4.2)	-0.9<br>(-8.6 to 7.2)	3.0<br>(-2.5 to 8.0)
Low physical activity	0.5<br>(0.2 to 0.9)	0.5<br>(0.2 to 0.9)	0.4<br>(0.2 to 0.8)	-1.8<br>(-3.8 to 0.1)	-8.8<br>(-12.5 to -4.6)	0.5<br>(0.2 to 1.0)	0.5<br>(0.2 to 0.9)	0.5<br>(0.2 to 0.9)	-3.8<br>(-5.9 to -1.3)	0.7<br>(-0.8 to 2.3)
High fasting plasma glucose	1.4<br>(0.9 to 2.3)	1.4<br>(0.9 to 2.2)	1.6<br>(0.9 to 2.6)	-3.9<br>(-41.7 to 57.3)	13.1<br>(-24.4 to 76.3)	1.2<br>(0.7 to 2.0)	1.2<br>(0.7 to 2.1)	1.4<br>(0.8 to 2.4)	2.1<br>(-37.6 to 73.5)	10.8<br>(-29.1 to 83.6)
High LDL cholesterol	12.1<br>(10.5 to 13.9)	12.6<br>(11.0 to 14.4)	12.6<br>(11.0 to 14.4)	4.2<br>(-1.6 to 10.1)	0.1<br>(-4.4 to 4.6)	13.0<br>(11.3 to 14.7)	13.1<br>(11.5 to 14.8)	13.1<br>(11.4 to 14.9)	0.7<br>(-4.4 to 6.4)	0.1<br>(-4.6 to 4.7)
High systolic blood pressure	11.3<br>(10.2 to 12.3)	8.4<br>(7.4 to 9.3)	8.2<br>(7.3 to 9.2)	-26.0<br>(-32.2 to -19.6)	-1.5<br>(-9.0 to 6.7)	9.7<br>(8.7 to 10.6)	6.0<br>(5.3 to 6.8)	5.6<br>(4.8 to 6.5)	-37.5<br>(-44.2 to -29.7)	-6.9<br>(-16.7 to 3.6)
High body-mass index	6.0<br>(3.8 to 9.3)	8.6<br>(5.7 to 13.1)	8.8<br>(5.9 to 13.3)	42.8<br>(14.8 to 80.4)	3.3<br>(-12.8 to 22.4)	5.8<br>(3.5 to 9.6)	7.5<br>(4.6 to 11.9)	7.5<br>(4.6 to 11.6)	29.3<br>(0.9 to 71.9)	0.1<br>(-18.4 to 22.0)
Low bone mineral density	4.8<br>(4.0 to 5.8)	5.0<br>(4.2 to 5.9)	5.1<br>(4.2 to 6.1)	3.9<br>(-9.1 to 21.2)	1.7<br>(-11.4 to 16.1)	6.2<br>(5.2 to 7.4)	6.3<br>(5.4 to 7.4)	6.1<br>(5.0 to 7.2)	2.1<br>(-10.8 to 17.5)	-4.3<br>(-17.0 to 10.1)
Impaired kidney function	2.6<br>(1.2 to 6.5)	2.5<br>(1.1 to 6.4)	2.4<br>(1.1 to 6.1)	-3.7<br>(-8.9 to 0.8)	-6.7<br>(-12.1 to -2.0)	3.3<br>(1.5 to 7.6)	3.3<br>(1.5 to 7.7)	3.1<br>(1.4 to 7.2)	2.1<br>(-1.7 to 6.7)	-7.2<br>(-12.3 to -2.4)

Supplementary Results Table 1. Hokkaido age-standardised Summary Exposure Values (SEVs) for Level 4 risk factors and percentage change, by sex, 1990, 2007, and 2017.Data in parentheses are 95% uncertainty intervals.

	Male					Female				
	**1990**	**2007**	**2017**	**Percentage change 1990-2007**	**Percentage change 2007-2017**	**1990**	**2007**	**2017**	**Percentage change 1990-2007**	**Percentage change 2007-2017**
Unsafe water source	2.3 (1.3 to 4.0)	2.0 (1.1 to 3.3)	2.1 (1.1 to 3.6)	-16.5 (-57.5 to 71.6)	7.2 (-35.5 to 85.5)	2.3 (1.3 to 4.0)	2.0 (1.1 to 3.3)	2.1 (1.1 to 3.6)	-16.5 (-57.5 to 71.6)	7.2 (-35.5 to 85.5)
Unsafe sanitation	4.1 (1.9 to 7.4)	2.1 (1.0 to 3.8)	1.8 (0.8 to 3.3)	-48.9 (-77.2 to 16.9)	-13.1 (-53.6 to 67.8)	4.1 (1.9 to 7.4)	2.1 (1.0 to 3.8)	1.8 (0.8 to 3.3)	-48.9 (-77.2 to 16.9)	-13.1 (-53.6 to 67.8)
No access to handwashing facility	6.8 (5.0 to 9.1)	6.4 (4.6 to 8.6)	6.0 (4.2 to 8.0)	-4.9 (-31.8 to 30.4)	-7.4 (-26.9 to 20.1)	6.8 (5.0 to 9.1)	6.4 (4.6 to 8.6)	6.0 (4.2 to 8.0)	-4.9 (-31.8 to 30.4)	-7.4 (-26.9 to 20.1)
Ambient particulate matter pollution	24.3 (12.1 to 39.3)	24.3 (14.3 to 39.1)	20.7 (9.2 to 35.5)	0.2 (-38.5 to 93.7)	-15.1 (-56.2 to 37.5)	24.0 (11.8 to 39.0)	24.3 (14.2 to 38.9)	20.6 (9.2 to 35.4)	1.2 (-37.9 to 96.0)	-15.1 (-56.2 to 37.5)
Household air pollution from solid fuels	0.8 (0.2 to 2.4)	0.2 (0.0 to 0.6)	0.2 (0.0 to 0.5)	-74.4 (-92.0 to -43.0)	-20.4 (-69.8 to 68.6)	1.2 (0.3 to 7.1)	0.3 (0.1 to 1.9)	0.3 (0.0 to 1.2)	-70.6 (-91.9 to -37.3)	-17.8 (-69.8 to 69.4)
Ambient ozone pollution	40.9 (14.4 to 54.8)	40.0 (14.0 to 54.8)	40.9 (14.3 to 54.8)	-2.1 (-4.0 to 0.0)	2.3 (0.0 to 4.2)	40.9 (14.4 to 54.8)	40.0 (14.0 to 54.8)	40.9 (14.3 to 54.8)	-2.1 (-4.0 to 0.0)	2.3 (0.0 to 4.2)
Residential radon	22.0 (2.3 to 66.9)	22.0 (2.3 to 66.9)	22.0 (2.3 to 66.9)	0.0 (0.0 to 0.0)	0.0 (0.0 to 0.0)	22.0 (2.3 to 66.9)	22.0 (2.3 to 66.9)	22.0 (2.3 to 66.9)	0.0 (0.0 to 0.0)	0.0 (0.0 to 0.0)
Lead exposure	2.9 (0.5 to 6.6)	2.0 (0.3 to 5.0)	1.4 (0.2 to 4.0)	-32.5 (-45.8 to -23.7)	-27.4 (-42.7 to -19.6)	1.6 (0.1 to 4.4)	1.0 (0.1 to 3.2)	0.7 (0.0 to 2.6)	-37.5 (-55.2 to -28.0)	-29.6 (-51.5 to -20.0)
Occupational exposure to asbestos	2.4 (2.1 to 2.7)	3.3 (3.0 to 3.5)	3.3 (2.8 to 4.0)	35.4 (17.8 to 59.3)	2.1 (-15.8 to 23.8)	0.7 (0.5 to 0.8)	0.6 (0.6 to 0.7)	0.7 (0.5 to 0.8)	-4.0 (-20.5 to 25.8)	0.3 (-20.4 to 23.7)
Occupational exposure to arsenic	0.4 (0.0 to 1.1)	0.4 (0.0 to 1.0)	0.4 (0.0 to 1.0)	-	-	0.4 (0.0 to 1.0)	0.4 (0.0 to 0.9)	0.4 (0.0 to 0.9)	-	-
Occupational exposure to benzene	0.6 (0.0 to 1.8)	0.6 (0.0 to 1.9)	0.7 (0.0 to 2.0)	2.6 (-4.3 to 12.4)	3.2 (-2.2 to 10.8)	0.6 (0.0 to 1.7)	0.7 (0.0 to 2.0)	0.8 (0.0 to 2.3)	20.9 (8.4 to 41.7)	14.3 (4.7 to 29.2)
Occupational exposure to beryllium	0.0 (0.0 to 0.0)	0.0 (0.0 to 0.0)	0.0 (0.0 to 0.0)	-5.1 (-10.0 to -0.3)	2.0 (-2.1 to 5.9)	0.0 (0.0 to 0.0)	0.0 (0.0 to 0.0)	0.0 (0.0 to 0.0)	1.8 (-7.4 to 11.1)	9.1 (2.2 to 16.4)
Occupational exposure to cadmium	0.1 (0.0 to 0.1)	0.0 (0.0 to 0.1)	0.0 (0.0 to 0.1)	-6.7 (-17.4 to 4.8)	1.1 (-8.8 to 11.4)	0.0 (0.0 to 0.1)	0.0 (0.0 to 0.0)	0.0 (0.0 to 0.1)	-9.0 (-23.5 to 7.5)	4.9 (-7.9 to 18.7)
Occupational exposure to chromium	0.1 (0.1 to 0.1)	0.1 (0.1 to 0.1)	0.1 (0.1 to 0.1)	-5.0 (-14.9 to 5.4)	1.6 (-7.2 to 10.6)	0.1 (0.1 to 0.1)	0.1 (0.1 to 0.1)	0.1 (0.1 to 0.1)	-4.3 (-17.9 to 11.4)	6.7 (-4.5 to 18.5)
Occupational exposure to diesel engine exhaust	0.6 (0.5 to 0.6)	0.6 (0.5 to 0.6)	0.6 (0.6 to 0.6)	-1.9 (-9.2 to 6.3)	3.1 (-3.1 to 10.5)	0.4 (0.4 to 0.5)	0.5 (0.4 to 0.6)	0.6 (0.5 to 0.6)	12.5 (-0.8 to 27.3)	11.0 (0.3 to 22.6)
Occupational exposure to formaldehyde	0.3 (0.2 to 0.3)	0.2 (0.2 to 0.3)	0.2 (0.2 to 0.3)	-6.0 (-17.6 to 6.5)	0.7 (-9.5 to 11.3)	0.2 (0.2 to 0.2)	0.2 (0.2 to 0.2)	0.2 (0.2 to 0.2)	-6.3 (-21.6 to 11.3)	5.8 (-6.9 to 19.5)
Occupational exposure to nickel	0.4 (0.0 to 1.9)	0.4 (0.0 to 1.7)	0.4 (0.0 to 1.8)	-	-	0.3 (0.0 to 1.5)	0.3 (0.0 to 1.4)	0.3 (0.0 to 1.4)	-	-
Occupational exposure to polycyclic aromatic hydrocarbons	0.2 (0.2 to 0.3)	0.2 (0.2 to 0.3)	0.2 (0.2 to 0.3)	-4.0 (-13.1 to 5.9)	2.0 (-6.1 to 9.9)	0.2 (0.2 to 0.2)	0.2 (0.2 to 0.2)	0.2 (0.2 to 0.3)	-2.2 (-15.6 to 12.8)	7.6 (-2.9 to 18.6)
Occupational exposure to silica	4.6 (0.7 to 14.3)	4.3 (0.6 to 14.1)	4.4 (0.6 to 14.1)	-6.1 (-16.2 to 4.8)	1.7 (-7.3 to 12.2)	3.1 (0.5 to 10.0)	2.7 (0.4 to 9.0)	2.8 (0.4 to 9.0)	-13.6 (-28.4 to 3.8)	2.0 (-11.6 to 19.1)
Occupational exposure to sulphuric acid	0.6 (0.1 to 1.9)	0.6 (0.1 to 1.8)	0.6 (0.1 to 1.9)	-6.6 (-17.1 to 4.9)	1.6 (-8.2 to 11.5)	0.5 (0.1 to 1.6)	0.5 (0.1 to 1.5)	0.5 (0.1 to 1.5)	-10.3 (-24.6 to 6.1)	3.9 (-8.7 to 18.8)
Occupational exposure to trichloroethylene	0.1 (0.1 to 0.1)	0.1 (0.1 to 0.1)	0.1 (0.1 to 0.1)	-4.1 (-11.2 to 4.1)	2.0 (-4.7 to 8.4)	0.1 (0.1 to 0.1)	0.1 (0.1 to 0.1)	0.1 (0.1 to 0.1)	0.7 (-10.6 to 13.6)	8.2 (-0.8 to 18.1)
Occupational asthmagens	18.2 (14.5 to 22.7)	17.6 (14.2 to 22.1)	17.6 (13.9 to 22.5)	-3.2 (-20.9 to 18.2)	0.0 (-15.7 to 17.5)	8.1 (6.5 to 10.0)	8.2 (6.6 to 10.1)	8.6 (6.9 to 10.5)	0.3 (-15.0 to 17.7)	5.2 (-7.0 to 18.7)
Occupational particulate matter, gases, and fumes	6.4 (4.6 to 9.1)	6.1 (4.4 to 8.8)	5.9 (4.2 to 8.5)	-4.4 (-9.2 to 0.4)	-3.6 (-6.8 to -0.2)	3.6 (2.5 to 5.4)	3.4 (2.4 to 5.0)	3.3 (2.3 to 4.9)	-5.7 (-13.7 to 3.7)	-4.6 (-10.4 to 1.6)
Occupational noise	5.9 (5.5 to 6.5)	5.8 (5.4 to 6.2)	5.6 (5.2 to 6.1)	-3.1 (-6.1 to -0.1)	-2.3 (-4.3 to -0.3)	3.5 (3.0 to 4.0)	3.5 (3.1 to 3.9)	3.5 (3.1 to 4.0)	0.1 (-5.4 to 6.8)	1.6 (-2.2 to 6.0)
Occupational ergonomic factors	10.6 (8.7 to 12.8)	9.5 (7.8 to 11.5)	9.4 (7.6 to 11.4)	-10.9 (-22.3 to 2.8)	-1.0 (-11.2 to 10.7)	5.7 (4.4 to 7.3)	5.8 (4.6 to 7.4)	6.5 (5.0 to 8.5)	1.8 (-19.5 to 29.9)	12.2 (-8.0 to 34.1)
Non-exclusive breastfeeding	0.4 (0.3 to 0.6)	0.4 (0.3 to 0.6)	0.4 (0.3 to 0.6)	-1.6 (-13.2 to 12.0)	-1.0 (-10.4 to 10.3)	0.4 (0.3 to 0.6)	0.4 (0.3 to 0.6)	0.4 (0.3 to 0.6)	-0.4 (-12.2 to 13.4)	-2.1 (-11.4 to 9.1)
Discontinued breastfeeding	1.7 (1.5 to 1.8)	1.7 (1.6 to 1.8)	1.7 (1.5 to 1.8)	2.6 (-6.8 to 13.2)	-3.0 (-10.2 to 5.8)	1.7 (1.5 to 1.8)	1.7 (1.6 to 1.8)	1.7 (1.5 to 1.8)	1.0 (-8.2 to 11.4)	-2.0 (-9.2 to 7.0)
Child underweight	0.3 (0.2 to 0.4)	0.2 (0.2 to 0.3)	0.2 (0.1 to 0.3)	-13.3 (-31.9 to 9.9)	-5.2 (-20.1 to 12.7)	0.3 (0.2 to 0.4)	0.3 (0.2 to 0.4)	0.2 (0.1 to 0.3)	-15.0 (-35.1 to 9.0)	-6.4 (-22.4 to 13.9)
Child wasting	0.2 (0.1 to 0.3)	0.2 (0.1 to 0.2)	0.2 (0.1 to 0.2)	-8.3 (-33.7 to 21.9)	-1.6 (-18.6 to 22.4)	0.2 (0.1 to 0.3)	0.2 (0.1 to 0.2)	0.2 (0.1 to 0.2)	-9.1 (-33.8 to 22.0)	-1.8 (-20.5 to 22.7)
Child stunting	0.6 (0.3 to 1.0)	0.5 (0.3 to 0.7)	0.5 (0.3 to 0.7)	-14.0 (-45.7 to 33.7)	-1.9 (-29.9 to 34.6)	0.6 (0.3 to 1.1)	0.5 (0.3 to 0.7)	0.5 (0.3 to 0.7)	-16.2 (-46.1 to 29.6)	-3.5 (-31.4 to 38.1)
Short gestation for birth weight	0.0 (0.0 to 0.0)	0.0 (0.0 to 0.0)	0.0 (0.0 to 0.0)	9.4 (-12.2 to 34.0)	4.1 (-14.4 to 30.9)	0.0 (0.0 to 0.0)	0.0 (0.0 to 0.0)	0.0 (0.0 to 0.0)	11.9 (-17.5 to 64.6)	0.4 (-22.1 to 32.6)
Low birth weight for gestation	0.0 (0.0 to 0.0)	0.0 (0.0 to 0.0)	0.0 (0.0 to 0.0)	3.6 (-11.8 to 24.3)	0.7 (-15.9 to 20.4)	0.0 (0.0 to 0.0)	0.0 (0.0 to 0.0)	0.0 (0.0 to 0.0)	-1.8 (-24.4 to 32.6)	3.9 (-18.2 to 25.7)
Iron deficiency	-	-	-	-	-	8.2 (5.6 to 11.1)	8.2 (5.7 to 11.3)	7.5 (5.2 to 10.6)	0.6 (-21.2 to 28.6)	-8.4 (-23.9 to 9.3)
Vitamin A deficiency	1.1 (0.7 to 1.6)	0.8 (0.5 to 1.0)	0.7 (0.5 to 1.0)	-30.9 (-46.2 to -17.4)	-9.0 (-26.9 to 14.8)	0.8 (0.5 to 1.0)	0.9 (0.6 to 1.2)	0.7 (0.5 to 0.9)	15.6 (-7.0 to 44.8)	-24.5 (-40.5 to -7.3)
Zinc deficiency	0.5 (0.1 to 1.1)	0.4 (0.1 to 1.1)	0.4 (0.1 to 0.8)	-17.3 (-64.2 to 72.6)	-11.7 (-68.3 to 152.0)	0.5 (0.1 to 1.1)	0.3 (0.1 to 0.8)	0.4 (0.1 to 0.8)	-31.5 (-64.0 to 12.3)	7.8 (-61.2 to 166.7)
Smoking	17.4 (14.0 to 20.7)	12.3 (10.0 to 14.9)	10.5 (8.4 to 12.7)	-28.9 (-37.3 to -18.3)	-14.8 (-23.2 to -4.6)	3.9 (2.7 to 5.2)	3.3 (2.4 to 4.5)	3.2 (2.3 to 4.4)	-15.5 (-33.7 to 9.5)	-1.9 (-17.1 to 15.8)
Chewing tobacco	1.4 (0.9 to 2.1)	0.9 (0.6 to 1.3)	0.7 (0.5 to 1.1)	-33.7 (-58.7 to 1.2)	-18.2 (-40.5 to 16.8)	0.1 (0.1 to 0.2)	0.1 (0.1 to 0.1)	0.1 (0.1 to 0.1)	-3.8 (-42.3 to 59.4)	-2.2 (-31.9 to 42.3)
Second-hand smoke	38.1 (34.0 to 42.1)	36.3 (32.3 to 40.4)	35.8 (31.5 to 40.0)	-4.8 (-15.5 to 7.8)	-1.5 (-10.0 to 7.7)	57.8 (54.3 to 61.4)	49.1 (45.3 to 52.6)	48.3 (44.1 to 52.2)	-15.1 (-21.3 to -8.9)	-1.6 (-7.6 to 4.5)
Alcohol use	14.3 (7.0 to 24.3)	14.5 (7.4 to 24.9)	17.2 (8.0 to 29.8)	1.0 (-31.9 to 47.4)	18.5 (-16.1 to 67.7)	11.4 (5.3 to 19.9)	11.4 (5.8 to 20.7)	13.7 (6.4 to 23.9)	0.8 (-31.9 to 47.1)	19.7 (-16.8 to 72.5)
Drug use	0.5 (0.4 to 0.7)	0.5 (0.4 to 0.7)	0.6 (0.4 to 0.8)	0.5 (-5.9 to 6.1)	11.8 (4.7 to 20.6)	0.3 (0.2 to 0.4)	0.3 (0.2 to 0.4)	0.3 (0.3 to 0.4)	2.5 (-3.1 to 9.1)	8.9 (1.5 to 16.9)
Diet low in fruits	41.5 (36.0 to 44.9)	39.7 (34.0 to 43.3)	41.0 (35.0 to 44.7)	-4.4 (-10.4 to 1.1)	3.2 (-1.4 to 8.0)	38.4 (32.4 to 42.5)	35.8 (29.8 to 40.2)	37.9 (31.7 to 42.3)	-6.8 (-14.3 to 0.4)	5.9 (-0.1 to 13.5)
Diet low in vegetables	20.7 (11.9 to 28.5)	15.2 (7.4 to 22.8)	17.4 (9.0 to 24.8)	-26.5 (-53.1 to -2.8)	14.4 (-12.2 to 65.2)	22.9 (14.5 to 29.9)	16.9 (8.9 to 24.8)	18.7 (10.8 to 26.5)	-26.1 (-49.5 to -4.4)	10.6 (-11.9 to 50.7)
Diet low in legumes	4.4 (0.7 to 9.8)	5.9 (1.2 to 11.6)	8.0 (2.3 to 14.7)	36.0 (-37.4 to 337.5)	35.4 (-15.8 to 186.9)	8.2 (2.6 to 14.8)	8.0 (2.5 to 14.3)	10.4 (3.7 to 17.1)	-2.1 (-45.7 to 78.1)	29.8 (-10.1 to 115.0)
Diet low in whole grains	30.9 (26.2 to 34.9)	35.5 (31.8 to 39.0)	36.9 (33.1 to 40.1)	15.1 (6.6 to 26.6)	3.7 (-1.2 to 9.2)	32.2 (27.5 to 36.0)	36.8 (33.2 to 40.0)	38.1 (34.5 to 41.0)	14.2 (6.7 to 25.7)	3.4 (-0.7 to 8.0)
Diet low in nuts and seeds	51.1 (49.9 to 52.0)	50.0 (48.4 to 51.3)	49.9 (48.2 to 51.3)	-2.2 (-4.5 to -0.4)	-0.3 (-2.0 to 1.5)	51.3 (50.0 to 52.2)	50.0 (48.3 to 51.3)	49.9 (48.0 to 51.3)	-2.4 (-4.6 to -0.6)	-0.3 (-2.3 to 1.5)
Diet low in milk	46.3 (44.4 to 48.0)	46.6 (44.8 to 48.2)	47.1 (45.2 to 48.6)	0.6 (-1.2 to 2.6)	1.0 (-0.4 to 2.4)	46.3 (44.2 to 48.1)	46.1 (44.0 to 47.8)	46.7 (44.5 to 48.3)	-0.6 (-2.5 to 1.3)	1.3 (-0.2 to 3.0)
Diet high in red meat	3.6 (0.4 to 8.4)	7.2 (1.9 to 13.8)	8.3 (2.7 to 15.3)	101.5 (-15.1 to 849.1)	15.1 (-39.1 to 133.4)	0.4 (0.0 to 2.1)	1.3 (0.0 to 4.1)	1.7 (0.0 to 4.9)	193.0 (-80.5 to 4997482.4)	33.8 (-87.4 to 5305.0)
Diet high in processed meat	10.7 (6.8 to 14.9)	17.6 (12.9 to 22.5)	20.3 (14.6 to 25.5)	64.2 (27.2 to 124.6)	15.1 (-3.2 to 37.6)	8.9 (5.3 to 12.4)	13.0 (8.7 to 16.8)	14.3 (9.8 to 18.3)	46.4 (15.0 to 105.5)	9.8 (-5.8 to 30.5)
Diet high in sugar-sweetened beverages	6.7 (2.6 to 13.4)	7.8 (3.5 to 14.2)	8.2 (3.7 to 14.6)	16.8 (-45.1 to 165.0)	5.3 (-40.3 to 75.1)	4.7 (1.9 to 9.8)	5.3 (2.5 to 9.8)	5.8 (2.4 to 11.1)	13.4 (-45.0 to 154.7)	8.5 (-37.8 to 88.3)
Diet low in fibre	22.6 (15.2 to 29.0)	23.7 (16.7 to 29.9)	24.8 (17.8 to 31.0)	5.1 (-11.0 to 28.9)	4.3 (-7.3 to 18.0)	23.8 (16.4 to 30.0)	24.7 (18.0 to 30.6)	26.1 (19.2 to 32.0)	4.1 (-11.4 to 24.9)	5.5 (-5.2 to 17.9)
Diet low in calcium	35.4 (31.1 to 38.8)	35.2 (30.8 to 38.6)	36.0 (31.9 to 39.4)	-0.5 (-4.2 to 3.4)	2.4 (-0.4 to 5.9)	37.5 (33.8 to 40.6)	36.4 (32.4 to 39.6)	37.6 (33.7 to 40.7)	-2.9 (-6.3 to 0.4)	3.2 (0.6 to 6.2)
Diet low in seafood omega-3 fatty acids	3.3 (1.5 to 4.8)	0.3 (0.0 to 1.2)	0.3 (0.0 to 1.2)	-91.6 (-100.0 to -67.3)	5.6 (-86.4 to 2102.5)	3.9 (2.0 to 5.3)	0.4 (0.0 to 1.5)	0.4 (0.0 to 1.6)	-89.2 (-99.9 to -64.4)	7.9 (-80.9 to 1055.4)
Diet low in polyunsaturated fatty acids	31.0 (25.6 to 35.8)	28.3 (22.3 to 33.3)	28.9 (23.2 to 33.7)	-8.8 (-20.6 to 0.4)	2.1 (-5.3 to 10.5)	31.6 (26.2 to 36.2)	28.9 (23.1 to 33.8)	29.6 (23.7 to 34.1)	-8.5 (-19.3 to 0.4)	2.2 (-4.6 to 10.8)
Diet high in trans fatty acids	1.1 (0.0 to 3.3)	0.9 (0.0 to 3.0)	0.9 (0.0 to 2.9)	-12.4 (-39.2 to -6.5)	-8.4 (-32.8 to -3.9)	1.5 (0.1 to 4.0)	1.3 (0.1 to 3.7)	1.2 (0.0 to 3.5)	-12.4 (-33.5 to -6.5)	-8.1 (-28.3 to -3.4)
Diet high in sodium	27.5 (17.4 to 35.8)	17.5 (8.5 to 24.6)	14.9 (5.9 to 22.3)	-36.5 (-60.2 to -18.9)	-14.4 (-40.5 to 2.5)	32.0 (23.4 to 39.1)	19.4 (10.8 to 26.3)	16.3 (7.4 to 23.6)	-39.6 (-60.0 to -25.1)	-16.0 (-38.1 to -3.1)
Intimate partner violence	-	-	-	-	-	2.3 (1.9 to 2.7)	2.1 (1.8 to 2.5)	2.0 (1.7 to 2.4)	-7.1 (-10.9 to -2.7)	-3.8 (-8.3 to 0.1)
Childhood sexual abuse	5.3 (4.3 to 6.5)	5.3 (4.3 to 6.5)	5.3 (4.3 to 6.5)	0.0 (0.0 to 0.0)	0.0 (0.0 to 0.0)	7.3 (5.9 to 8.9)	7.3 (5.9 to 8.9)	7.5 (6.0 to 9.2)	0.0 (0.0 to 0.0)	2.8 (-4.0 to 10.6)

Supplementary Results Table 1. Hokkaido age-standardised Summary Exposure Values (SEVs) for Level 4 risk factors and percentage change, by sex, 1990, 2007, and 2017.Data in parentheses are 95% uncertainty intervals.										
	**Male**					**Female**				
	**1990**	**2007**	**2017**	**Percentage change 1990-2007**	**Percentage change 2007-2017**	**1990**	**2007**	**2017**	**Percentage change 1990-2007**	**Percentage change 2007-2017**
Bullying victimization	3.3 (1.3 to 6.8)	3.3 (1.3 to 7.0)	3.5 (1.4 to 7.4)	1.8 (-6.0 to 9.9)	3.3 (-1.8 to 8.1)	1.9 (0.7 to 4.0)	1.9 (0.8 to 4.0)	2.0 (0.8 to 4.1)	0.1 (-7.6 to 8.1)	2.5 (-3.0 to 7.2)
Low physical activity	0.5 (0.2 to 0.9)	0.5 (0.2 to 0.9)	0.4 (0.2 to 0.8)	-1.8 (-3.8 to 0.0)	-8.8 (-12.3 to -4.5)	0.5 (0.2 to 1.0)	0.5 (0.2 to 0.9)	0.5 (0.2 to 0.9)	-3.9 (-6.2 to -1.4)	0.7 (-0.8 to 2.3)
High fasting plasma glucose	1.3 (0.8 to 2.1)	1.3 (0.8 to 2.1)	1.5 (0.9 to 2.4)	2.6 (-36.8 to 53.6)	9.7 (-22.8 to 74.0)	1.2 (0.7 to 1.9)	1.2 (0.7 to 2.0)	1.3 (0.7 to 2.3)	1.7 (-36.4 to 69.4)	10.0 (-26.9 to 77.1)
High LDL cholesterol	11.8 (10.3 to 13.6)	12.2 (10.6 to 13.9)	12.2 (10.6 to 14.0)	2.7 (-3.1 to 8.9)	0.1 (-4.5 to 4.8)	12.5 (10.9 to 14.2)	12.3 (10.8 to 14.0)	12.4 (10.8 to 14.1)	-1.2 (-6.6 to 4.2)	0.5 (-3.9 to 5.3)
High systolic blood pressure	10.3 (9.3 to 11.2)	6.6 (5.9 to 7.5)	6.8 (6.0 to 7.7)	-35.2 (-40.7 to -30.2)	2.7 (-4.6 to 10.4)	8.6 (7.7 to 9.5)	4.6 (4.1 to 5.3)	4.5 (3.9 to 5.2)	-45.9 (-52.0 to -40.2)	-3.0 (-13.4 to 8.5)
High body-mass index	5.3 (3.3 to 8.4)	7.4 (5.0 to 11.8)	7.7 (5.0 to 12.4)	40.4 (13.5 to 80.5)	3.0 (-13.1 to 20.9)	5.3 (3.2 to 8.2)	6.7 (4.3 to 10.6)	6.7 (4.1 to 10.7)	27.2 (-0.7 to 65.8)	0.5 (-17.7 to 22.6)
Low bone mineral density	5.3 (4.3 to 6.2)	5.2 (4.4 to 6.1)	5.2 (4.2 to 6.2)	-1.8 (-14.1 to 14.5)	0.1 (-12.5 to 14.9)	6.6 (5.7 to 7.5)	6.5 (5.7 to 7.4)	6.7 (5.6 to 7.9)	-1.0 (-11.1 to 9.9)	2.7 (-9.8 to 18.8)
Impaired kidney function	2.6 (1.2 to 6.5)	2.5 (1.1 to 6.4)	2.4 (1.1 to 6.1)	-3.4 (-8.2 to 1.2)	-6.7 (-12.5 to -1.9)	3.2 (1.5 to 7.4)	3.3 (1.5 to 7.6)	3.1 (1.4 to 7.2)	2.0 (-2.4 to 6.7)	-7.0 (-12.2 to -2.2)

Supplementary Results Table 1. Hyogo age-standardised Summary Exposure Values (SEVs) for Level 4 risk factors and percentage change, by sex, 1990, 2007, and 2017.Data in parentheses are 95% uncertainty intervals.	Male					Female				
	1990	2007	2017	Percentage change 1990-2007	Percentage change 2007-2017	1990	2007	2017	Percentage change 1990-2007	Percentage change 2007-2017
Unsafe water source	2.1 (1.2 to 3.5)	1.7 (1.0 to 3.0)	1.9 (1.0 to 3.1)	-16.5 (-57.8 to 70.5)	8.7 (-37.7 to 88.0)	2.1 (1.2 to 3.5)	1.7 (1.0 to 3.0)	1.9 (1.0 to 3.1)	-16.5 (-57.8 to 70.5)	8.7 (-37.7 to 88.0)
Unsafe sanitation	3.5 (1.7 to 6.6)	1.7 (0.8 to 3.3)	1.5 (0.7 to 2.8)	-50.9 (-79.0 to 14.1)	-13.0 (-55.0 to 70.9)	3.5 (1.7 to 6.6)	1.7 (0.8 to 3.3)	1.5 (0.7 to 2.8)	-50.9 (-79.0 to 14.1)	-13.0 (-55.0 to 70.9)
No access to handwashing facility	6.7 (4.9 to 8.9)	6.3 (4.5 to 8.5)	5.9 (4.2 to 7.8)	-6.6 (-33.6 to 30.9)	-6.7 (-26.7 to 17.9)	6.7 (4.9 to 8.9)	6.3 (4.5 to 8.5)	5.9 (4.2 to 7.8)	-6.6 (-33.6 to 30.9)	-6.7 (-26.7 to 17.9)
Ambient particulate matter pollution	33.7 (23.1 to 48.6)	33.9 (23.7 to 49.5)	29.1 (19.8 to 43.2)	0.5 (-21.0 to 29.1)	-14.2 (-33.2 to 6.5)	33.6 (23.0 to 48.3)	33.9 (23.7 to 49.4)	29.1 (19.8 to 43.2)	0.9 (-20.9 to 30.0)	-14.2 (-33.2 to 6.5)
Household air pollution from solid fuels	0.4 (0.1 to 1.3)	0.1 (0.0 to 0.2)	0.1 (0.0 to 0.2)	-74.3 (-92.8 to -47.9)	-9.7 (-67.0 to 87.9)	0.7 (0.2 to 4.7)	0.2 (0.0 to 0.7)	0.2 (0.0 to 0.6)	-70.9 (-92.7 to -42.6)	-8.7 (-66.7 to 90.4)
Ambient ozone pollution	51.6 (24.9 to 54.8)	51.9 (25.7 to 54.8)	52.3 (27.1 to 54.8)	0.6 (0.0 to 4.0)	0.8 (0.0 to 6.3)	51.6 (24.9 to 54.8)	51.9 (25.7 to 54.8)	52.3 (27.1 to 54.8)	0.6 (0.0 to 4.0)	0.8 (0.0 to 6.3)
Residential radon	13.5 (0.8 to 45.2)	13.5 (0.8 to 45.2)	13.5 (0.8 to 45.2)	0.0 (0.0 to 0.0)	0.0 (0.0 to 0.0)	13.5 (0.8 to 45.2)	13.5 (0.8 to 45.2)	13.5 (0.8 to 45.2)	0.0 (0.0 to 0.0)	0.0 (0.0 to 0.0)
Lead exposure	3.2 (0.6 to 6.9)	2.0 (0.3 to 4.9)	1.4 (0.2 to 3.8)	-36.0 (-50.7 to -26.8)	-30.5 (-46.3 to -22.0)	1.7 (0.1 to 4.6)	1.0 (0.1 to 3.2)	0.7 (0.0 to 2.4)	-42.2 (-60.0 to -32.3)	-33.0 (-56.2 to -23.9)
Occupational exposure to asbestos	4.8 (4.1 to 5.4)	6.8 (6.4 to 7.2)	6.6 (5.6 to 7.7)	41.1 (23.9 to 66.1)	-2.6 (-18.0 to 13.9)	1.3 (1.0 to 1.5)	1.3 (1.2 to 1.4)	1.2 (1.0 to 1.5)	2.4 (-14.9 to 31.4)	-5.1 (-23.1 to 15.7)
Occupational exposure to arsenic	0.5 (0.0 to 1.1)	0.4 (0.0 to 1.0)	0.4 (0.0 to 1.0)	-	-	0.4 (0.0 to 1.0)	0.4 (0.0 to 0.9)	0.4 (0.0 to 0.9)	-	-
Occupational exposure to benzene	0.6 (0.0 to 1.8)	0.7 (0.0 to 1.9)	0.7 (0.0 to 1.9)	2.5 (-4.1 to 13.9)	3.7 (-2.0 to 11.1)	0.6 (0.0 to 1.8)	0.7 (0.0 to 2.2)	0.8 (0.1 to 2.4)	20.7 (9.1 to 40.3)	13.9 (4.0 to 28.7)
Occupational exposure to beryllium	0.0 (0.0 to 0.0)	0.0 (0.0 to 0.0)	0.0 (0.0 to 0.0)	-5.3 (-10.2 to 0.0)	2.0 (-1.7 to 6.1)	0.0 (0.0 to 0.0)	0.0 (0.0 to 0.0)	0.0 (0.0 to 0.0)	2.0 (-6.0 to 11.3)	8.6 (2.6 to 15.2)
Occupational exposure to cadmium	0.1 (0.0 to 0.1)	0.0 (0.0 to 0.1)	0.0 (0.0 to 0.1)	-7.5 (-17.8 to 4.5)	1.0 (-8.6 to 10.9)	0.0 (0.0 to 0.1)	0.0 (0.0 to 0.1)	0.0 (0.0 to 0.1)	-9.3 (-22.5 to 7.6)	4.4 (-8.4 to 17.9)
Occupational exposure to chromium	0.1 (0.1 to 0.1)	0.1 (0.1 to 0.1)	0.1 (0.1 to 0.1)	-5.8 (-15.1 to 4.6)	1.5 (-7.0 to 10.1)	0.1 (0.1 to 0.1)	0.1 (0.1 to 0.1)	0.1 (0.1 to 0.1)	-4.6 (-16.6 to 10.8)	6.1 (-4.4 to 17.5)
Occupational exposure to diesel engine exhaust	0.6 (0.6 to 0.6)	0.6 (0.5 to 0.6)	0.6 (0.6 to 0.6)	-2.2 (-9.8 to 6.2)	3.1 (-3.0 to 9.5)	0.5 (0.4 to 0.5)	0.5 (0.5 to 0.6)	0.6 (0.5 to 0.6)	12.1 (-0.9 to 27.1)	10.5 (0.4 to 21.6)
Occupational exposure to formaldehyde	0.3 (0.2 to 0.3)	0.2 (0.2 to 0.3)	0.2 (0.2 to 0.3)	-7.0 (-17.7 to 5.9)	0.6 (-9.3 to 10.9)	0.2 (0.2 to 0.3)	0.2 (0.2 to 0.2)	0.2 (0.2 to 0.2)	-6.7 (-20.7 to 11.2)	5.2 (-7.3 to 18.9)
Occupational exposure to nickel	0.4 (0.0 to 2.0)	0.4 (0.0 to 1.8)	0.4 (0.0 to 1.7)	-	-	0.4 (0.0 to 1.6)	0.3 (0.0 to 1.3)	0.3 (0.0 to 1.4)	-	-
Occupational exposure to polycyclic aromatic hydrocarbons	0.2 (0.2 to 0.3)	0.2 (0.2 to 0.3)	0.2 (0.2 to 0.3)	-4.8 (-13.3 to 5.1)	1.9 (-5.7 to 9.6)	0.2 (0.2 to 0.3)	0.2 (0.2 to 0.2)	0.2 (0.2 to 0.3)	-2.4 (-14.3 to 12.3)	7.0 (-2.7 to 17.7)
Occupational exposure to silica	4.7 (0.7 to 15.2)	4.4 (0.6 to 13.8)	4.5 (0.7 to 14.2)	-6.7 (-15.9 to 5.8)	1.4 (-6.9 to 11.9)	3.2 (0.4 to 10.3)	2.8 (0.4 to 8.6)	2.8 (0.4 to 8.7)	-14.7 (-27.5 to 3.6)	1.5 (-11.4 to 18.4)
Occupational exposure to sulphuric acid	0.6 (0.1 to 1.9)	0.6 (0.1 to 1.8)	0.6 (0.1 to 1.8)	-7.5 (-17.4 to 4.7)	1.3 (-8.0 to 11.4)	0.5 (0.1 to 1.7)	0.5 (0.1 to 1.5)	0.5 (0.1 to 1.5)	-10.6 (-24.2 to 5.7)	3.3 (-9.2 to 18.2)
Occupational exposure to trichloroethylene	0.1 (0.1 to 0.1)	0.1 (0.1 to 0.1)	0.1 (0.1 to 0.1)	-4.7 (-11.4 to 3.3)	1.9 (-3.9 to 8.4)	0.1 (0.1 to 0.1)	0.1 (0.1 to 0.1)	0.1 (0.1 to 0.1)	0.5 (-9.4 to 12.6)	7.7 (-0.2 to 17.3)
Occupational asthmagens	19.5 (15.5 to 24.1)	18.5 (14.7 to 23.2)	18.4 (14.5 to 23.2)	-4.9 (-23.0 to 18.7)	-0.7 (-15.7 to 15.3)	8.4 (6.8 to 10.5)	8.4 (6.9 to 10.3)	8.8 (7.1 to 10.9)	-0.4 (-14.3 to 16.8)	5.0 (-7.7 to 18.3)
Occupational particulate matter, gases, and fumes	6.4 (4.5 to 9.3)	6.1 (4.3 to 8.8)	5.9 (4.1 to 8.4)	-4.6 (-9.4 to 0.7)	-3.7 (-7.2 to -0.2)	3.7 (2.5 to 5.5)	3.5 (2.4 to 5.1)	3.3 (2.3 to 4.8)	-5.9 (-14.4 to 2.9)	-4.9 (-10.4 to 1.0)
Occupational noise	5.9 (5.4 to 6.5)	5.8 (5.3 to 6.3)	5.6 (5.2 to 6.1)	-3.2 (-6.1 to 0.1)	-2.4 (-4.4 to -0.2)	3.5 (3.1 to 4.0)	3.5 (3.2 to 4.0)	3.6 (3.2 to 4.1)	0.2 (-5.4 to 6.3)	1.6 (-1.9 to 5.4)
Occupational ergonomic factors	10.2 (8.5 to 12.2)	9.0 (7.5 to 10.7)	8.9 (7.4 to 10.7)	-11.7 (-23.6 to 1.5)	-1.1 (-11.1 to 10.8)	5.5 (4.3 to 7.1)	5.6 (4.4 to 7.0)	6.3 (4.8 to 8.0)	1.3 (-19.4 to 28.4)	12.8 (-6.6 to 34.4)
Non-exclusive breastfeeding	0.4 (0.3 to 0.6)	0.4 (0.3 to 0.6)	0.4 (0.3 to 0.6)	-0.8 (-13.8 to 13.4)	-1.7 (-11.7 to 9.9)	0.4 (0.3 to 0.6)	0.4 (0.3 to 0.6)	0.4 (0.3 to 0.6)	-1.5 (-14.4 to 12.6)	-1.6 (-11.5 to 10.1)
Discontinued breastfeeding	1.7 (1.6 to 1.8)	1.7 (1.6 to 1.9)	1.7 (1.6 to 1.8)	1.4 (-7.7 to 12.3)	-3.0 (-9.9 to 4.4)	1.7 (1.6 to 1.8)	1.7 (1.6 to 1.9)	1.7 (1.6 to 1.8)	2.4 (-6.8 to 13.3)	-2.5 (-9.4 to 5.0)
Child underweight	0.2 (0.1 to 0.3)	0.2 (0.1 to 0.3)	0.2 (0.1 to 0.3)	-14.1 (-32.1 to 4.9)	-4.1 (-17.2 to 9.1)	0.2 (0.1 to 0.3)	0.2 (0.1 to 0.3)	0.2 (0.1 to 0.3)	-16.2 (-34.0 to 6.3)	-4.5 (-18.3 to 11.1)
Child wasting	0.2 (0.1 to 0.3)	0.2 (0.1 to 0.3)	0.2 (0.1 to 0.3)	-7.3 (-32.7 to 26.3)	-2.0 (-20.4 to 23.2)	0.2 (0.1 to 0.3)	0.2 (0.1 to 0.3)	0.2 (0.1 to 0.3)	-9.7 (-35.9 to 23.2)	-2.7 (-22.3 to 22.2)
Child stunting	0.6 (0.3 to 0.9)	0.5 (0.3 to 0.7)	0.5 (0.2 to 0.7)	-14.4 (-45.5 to 33.5)	-3.0 (-31.8 to 35.3)	0.6 (0.3 to 1.0)	0.5 (0.3 to 0.7)	0.5 (0.3 to 0.7)	-17.7 (-46.2 to 28.7)	-2.8 (-31.6 to 43.6)
Short gestation for birth weight	0.0 (0.0 to 0.0)	0.0 (0.0 to 0.0)	0.0 (0.0 to 0.0)	10.3 (-11.7 to 37.2)	0.2 (-18.8 to 23.0)	0.0 (0.0 to 0.0)	0.0 (0.0 to 0.0)	0.0 (0.0 to 0.0)	16.8 (-16.8 to 104.2)	0.5 (-19.8 to 35.9)
Low birth weight for gestation	0.0 (0.0 to 0.0)	0.0 (0.0 to 0.0)	0.0 (0.0 to 0.0)	4.8 (-14.3 to 28.3)	3.1 (-13.0 to 22.6)	0.0 (0.0 to 0.0)	0.0 (0.0 to 0.0)	0.0 (0.0 to 0.0)	-6.5 (-62.2 to 28.4)	3.7 (-18.7 to 22.6)
Iron deficiency	-	-	-	-	-	7.9 (5.3 to 10.9)	7.9 (5.5 to 10.9)	7.2 (4.8 to 10.1)	-0.2 (-22.3 to 29.7)	-8.5 (-24.8 to 9.8)
Vitamin A deficiency	0.9 (0.6 to 1.3)	0.8 (0.5 to 1.0)	0.7 (0.4 to 1.0)	-18.0 (-30.2 to -2.5)	-8.3 (-24.9 to 11.7)	0.8 (0.5 to 1.0)	0.7 (0.4 to 1.0)	0.8 (0.4 to 1.1)	-5.1 (-22.8 to 17.1)	6.3 (-15.7 to 30.3)
Zinc deficiency	0.5 (0.1 to 1.0)	0.4 (0.1 to 1.1)	0.4 (0.1 to 0.9)	-13.5 (-65.9 to 74.1)	-12.7 (-66.8 to 175.0)	0.5 (0.2 to 1.1)	0.4 (0.1 to 0.8)	0.4 (0.1 to 0.8)	-30.4 (-64.1 to 51.1)	5.8 (-60.8 to 174.3)
Smoking	16.7 (13.4 to 20.4)	11.8 (9.2 to 14.4)	10.0 (7.9 to 12.3)	-29.6 (-39.2 to -19.5)	-15.1 (-23.5 to -5.3)	3.8 (2.7 to 5.1)	3.1 (2.2 to 4.2)	3.1 (2.1 to 4.1)	-16.6 (-36.0 to 9.0)	-2.8 (-19.6 to 17.6)
Chewing tobacco	1.4 (0.9 to 1.9)	0.9 (0.6 to 1.4)	0.7 (0.5 to 1.1)	-32.5 (-56.3 to 4.0)	-18.9 (-42.0 to 12.0)	0.1 (0.1 to 0.1)	0.1 (0.1 to 0.1)	0.1 (0.1 to 0.1)	-4.3 (-41.4 to 52.7)	-1.9 (-31.0 to 41.7)
Second-hand smoke	37.5 (33.6 to 41.7)	36.7 (32.6 to 41.0)	36.9 (32.3 to 41.4)	-2.1 (-13.3 to 10.2)	0.6 (-9.2 to 10.2)	57.0 (53.8 to 60.3)	49.7 (46.2 to 53.4)	49.9 (45.6 to 53.9)	-12.8 (-19.1 to -6.7)	0.4 (-5.6 to 6.6)
Alcohol use	14.5 (7.2 to 25.0)	14.7 (7.6 to 24.5)	16.7 (8.1 to 27.9)	1.4 (-31.0 to 56.2)	13.5 (-18.3 to 59.2)	11.7 (5.7 to 20.9)	11.9 (6.0 to 20.4)	13.6 (6.4 to 23.3)	1.5 (-30.8 to 53.3)	14.6 (-17.7 to 61.2)
Drug use	0.5 (0.4 to 0.7)	0.5 (0.4 to 0.7)	0.6 (0.4 to 0.8)	1.0 (-5.4 to 7.7)	11.5 (4.1 to 20.0)	0.3 (0.2 to 0.4)	0.3 (0.2 to 0.4)	0.3 (0.3 to 0.4)	2.7 (-2.7 to 8.7)	9.4 (2.3 to 18.2)
Diet low in fruits	41.4 (35.6 to 45.2)	39.7 (34.0 to 43.7)	41.0 (35.5 to 44.7)	-4.1 (-10.1 to 1.5)	3.2 (-1.8 to 8.5)	38.4 (32.4 to 42.6)	35.9 (29.6 to 40.3)	38.0 (31.8 to 42.4)	-6.6 (-14.1 to 1.0)	5.8 (-0.5 to 13.1)
Diet low in vegetables	20.6 (11.9 to 27.9)	14.6 (6.3 to 22.5)	16.7 (8.1 to 24.6)	-29.0 (-57.4 to -4.6)	14.5 (-12.9 to 65.3)	22.8 (14.6 to 30.3)	16.6 (8.4 to 24.5)	18.4 (10.1 to 26.0)	-27.0 (-52.1 to -5.1)	11.0 (-12.8 to 49.2)
Diet low in legumes	4.3 (0.7 to 9.2)	5.3 (0.9 to 11.1)	7.1 (1.8 to 13.6)	23.6 (-51.6 to 243.0)	32.9 (-19.5 to 211.7)	8.1 (2.6 to 14.9)	7.7 (2.3 to 13.9)	9.9 (3.7 to 17.0)	-5.3 (-51.8 to 77.9)	29.1 (-12.6 to 128.4)
Diet low in whole grains	30.9 (26.1 to 35.2)	35.6 (31.5 to 39.1)	36.9 (33.1 to 40.2)	15.2 (6.6 to 26.5)	3.7 (-1.1 to 9.2)	32.3 (27.9 to 36.2)	36.8 (33.0 to 39.9)	38.0 (34.5 to 41.0)	14.0 (6.2 to 24.1)	3.4 (-0.6 to 8.2)
Diet low in nuts and seeds	51.0 (49.8 to 52.0)	49.7 (48.1 to 51.0)	49.5 (47.6 to 51.0)	-2.5 (-4.7 to -0.6)	-0.4 (-2.5 to 1.4)	51.1 (49.9 to 52.0)	49.8 (48.2 to 51.1)	49.5 (47.6 to 50.9)	-2.7 (-5.0 to -0.7)	-0.5 (-2.5 to 1.3)
Diet low in milk	46.4 (44.3 to 48.1)	46.7 (44.9 to 48.4)	47.2 (45.3 to 48.7)	0.8 (-1.0 to 2.8)	1.0 (-0.3 to 2.4)	46.4 (44.5 to 48.1)	46.2 (44.2 to 48.0)	46.9 (44.9 to 48.5)	-0.4 (-2.2 to 1.5)	1.4 (0.0 to 2.9)
Diet high in red meat	3.5 (0.5 to 8.2)	7.0 (2.4 to 13.3)	8.1 (2.8 to 14.5)	98.7 (-11.3 to 1056.3)	15.5 (-37.0 to 128.7)	0.5 (0.0 to 2.2)	1.3 (0.0 to 4.1)	1.8 (0.0 to 5.3)	162.4 (-80.0 to 3773980.0)	31.8 (-82.1 to 3393.6)
Diet high in processed meat	11.0 (6.9 to 14.9)	18.4 (13.6 to 23.1)	21.3 (15.7 to 26.7)	66.4 (31.6 to 133.1)	16.2 (-2.3 to 36.9)	9.2 (5.5 to 12.8)	13.7 (9.3 to 17.4)	15.2 (10.5 to 19.4)	48.1 (15.9 to 104.2)	10.9 (-6.2 to 31.7)
Diet high in sugar-sweetened beverages	6.7 (2.7 to 13.6)	7.7 (3.4 to 13.8)	8.1 (3.5 to 15.1)	14.5 (-43.2 to 155.1)	5.9 (-37.4 to 74.1)	4.9 (1.9 to 10.9)	5.4 (2.4 to 10.3)	5.7 (2.4 to 11.2)	11.0 (-47.5 to 143.1)	5.9 (-39.1 to 83.6)
Diet low in fibre	22.5 (15.2 to 29.2)	23.7 (16.8 to 29.7)	24.7 (17.8 to 30.8)	5.1 (-11.2 to 28.2)	4.4 (-7.7 to 18.5)	23.6 (16.4 to 29.9)	24.5 (17.5 to 30.5)	25.7 (18.9 to 32.2)	3.7 (-11.0 to 24.0)	5.3 (-5.0 to 18.1)
Diet low in calcium	35.3 (31.1 to 38.7)	35.0 (30.6 to 38.5)	35.8 (31.7 to 39.2)	-0.8 (-4.6 to 2.9)	2.2 (-0.5 to 5.8)	37.4 (33.6 to 40.5)	36.2 (32.1 to 39.6)	37.3 (33.3 to 40.5)	-3.2 (-6.7 to -0.0)	3.0 (0.6 to 6.1)
Diet low in seafood omega-3 fatty acids	3.3 (1.5 to 4.8)	0.3 (0.0 to 1.2)	0.3 (0.0 to 1.2)	-91.0 (-100.0 to -64.0)	5.4 (-83.7 to 1740.0)	3.9 (2.0 to 5.3)	0.4 (0.0 to 1.4)	0.5 (0.0 to 1.5)	-89.0 (-99.9 to -64.8)	6.7 (-77.3 to 733.8)
Diet low in polyunsaturated fatty acids	30.9 (25.5 to 35.8)	28.2 (22.3 to 33.1)	28.8 (22.9 to 33.6)	-8.7 (-19.7 to 1.0)	2.1 (-5.6 to 10.5)	31.5 (26.5 to 36.5)	28.9 (23.5 to 33.7)	29.5 (24.1 to 34.1)	-8.5 (-18.7 to 0.2)	2.2 (-5.1 to 10.2)
Diet high in trans fatty acids	1.1 (0.0 to 3.3)	1.0 (0.0 to 3.0)	0.9 (0.0 to 2.9)	-12.5 (-38.6 to -6.4)	-8.3 (-33.2 to -3.7)	1.5 (0.1 to 4.0)	1.3 (0.1 to 3.7)	1.2 (0.0 to 3.5)	-12.6 (-34.8 to -6.6)	-8.3 (-28.1 to -3.5)
Diet high in sodium	27.5 (17.9 to 35.3)	17.7 (9.2 to 24.9)	15.2 (6.5 to 22.7)	-35.6 (-58.2 to -17.0)	-14.2 (-39.1 to 3.1)	31.7 (23.0 to 39.4)	19.1 (10.8 to 25.9)	15.9 (7.4 to 23.1)	-39.6 (-58.8 to -25.0)	-16.7 (-39.6 to -2.8)
Intimate partner violence	-	-	-	-	-	2.3 (1.9 to 2.7)	2.1 (1.8 to 2.5)	2.1 (1.7 to 2.4)	-7.3 (-11.3 to -2.7)	-4.3 (-8.6 to -0.1)
Childhood sexual abuse	5.3 (4.3 to 6.5)	5.3 (4.3 to 6.5)	5.3 (4.3 to 6.5)	0.0 (0.0 to 0.0)	0.0 (0.0 to 0.0)	7.3 (5.9 to 8.9)	7.3 (5.9 to 8.9)	7.5 (6.0 to 9.2)	0.0 (0.0 to 0.0)	2.8 (-4.0 to 10.6)

Supplementary Results Table 1. Hyogo age-standardised Summary Exposure Values (SEVs) for Level 4 risk factors and percentage change, by sex, 1990, 2007, and 2017.Data in parentheses are 95% uncertainty intervals.										
	**Male**					**Female**				
	**1990**	**2007**	**2017**	**Percentage change 1990-2007**	**Percentage change 2007-2017**	**1990**	**2007**	**2017**	**Percentage change 1990-2007**	**Percentage change 2007-2017**
Bullying victimization	3.3<br>(1.3 to 7.1)	3.3<br>(1.4 to 7.3)	3.5<br>(1.4 to 7.4)	0.9<br>(-7.1 to 8.9)	4.4<br>(-1.0 to 9.2)	1.9<br>(0.8 to 4.1)	1.9<br>(0.8 to 4.0)	2.0<br>(0.8 to 4.2)	-0.7<br>(-8.5 to 7.8)	3.7<br>(-1.9 to 8.7)
Low physical activity	0.5<br>(0.2 to 0.9)	0.5<br>(0.2 to 0.9)	0.4<br>(0.2 to 0.8)	-1.8<br>(-3.8 to 0.1)	-8.8<br>(-12.3 to -4.6)	0.5<br>(0.2 to 1.0)	0.5<br>(0.2 to 0.9)	0.5<br>(0.2 to 0.9)	-3.9<br>(-6.0 to -1.4)	0.8<br>(-0.7 to 2.4)
High fasting plasma glucose	1.4<br>(0.9 to 2.3)	1.4<br>(0.8 to 2.2)	1.5<br>(0.9 to 2.5)	-3.8<br>(-42.7 to 58.9)	8.7<br>(-26.8 to 72.9)	1.2<br>(0.7 to 2.0)	1.2<br>(0.7 to 2.1)	1.3<br>(0.8 to 2.4)	3.4<br>(-37.5 to 72.4)	9.4<br>(-31.7 to 81.6)
High LDL cholesterol	12.1<br>(10.6 to 14.0)	12.7<br>(11.1 to 14.4)	12.7<br>(11.1 to 14.5)	4.4<br>(-1.7 to 10.3)	-0.0<br>(-4.6 to 4.9)	13.0<br>(11.4 to 14.7)	13.1<br>(11.5 to 14.8)	13.1<br>(11.4 to 15.0)	0.8<br>(-4.4 to 6.5)	0.0<br>(-4.4 to 5.0)
High systolic blood pressure	11.3<br>(10.3 to 12.3)	8.4<br>(7.5 to 9.3)	8.2<br>(7.3 to 9.3)	-26.0<br>(-32.2 to -19.3)	-1.7<br>(-9.2 to 5.7)	9.7<br>(8.7 to 10.8)	6.1<br>(5.3 to 6.9)	5.6<br>(4.9 to 6.5)	-37.5<br>(-43.9 to -30.5)	-7.2<br>(-17.1 to 3.3)
High body-mass index	6.0<br>(3.8 to 9.6)	8.7<br>(6.0 to 13.7)	9.0<br>(6.1 to 13.7)	44.6<br>(15.1 to 87.2)	3.0<br>(-12.2 to 20.4)	5.6<br>(3.4 to 8.8)	7.2<br>(4.6 to 11.3)	7.2<br>(4.5 to 11.2)	30.1<br>(2.7 to 72.2)	-0.2<br>(-17.9 to 20.4)
Low bone mineral density	5.3<br>(4.4 to 6.2)	5.2<br>(4.3 to 6.1)	5.2<br>(4.2 to 6.2)	-2.1<br>(-14.6 to 11.9)	-0.1<br>(-13.1 to 14.4)	7.2<br>(6.0 to 8.5)	7.0<br>(6.0 to 8.2)	7.1<br>(5.9 to 8.4)	-3.1<br>(-14.0 to 10.4)	1.7<br>(-11.0 to 15.1)
Impaired kidney function	2.6<br>(1.2 to 6.5)	2.6<br>(1.2 to 6.4)	2.4<br>(1.1 to 6.1)	-3.5<br>(-8.3 to 0.7)	-6.8<br>(-12.4 to -1.9)	3.3<br>(1.5 to 7.6)	3.4<br>(1.6 to 7.7)	3.2<br>(1.5 to 7.4)	2.9<br>(-0.9 to 7.4)	-6.8<br>(-11.6 to -2.2)

Supplementary Results Table 1. Ibaraki age-standardised Summary Exposure Values (SEVs) for Level 4 risk factors and percentage change, by sex, 1990, 2007, and 2017.Data in parentheses are 95% uncertainty intervals.										
	Male					Female				
	**1990**	**2007**	**2017**	**Percentage change 1990-2007**	**Percentage change 2007-2017**	**1990**	**2007**	**2017**	**Percentage change 1990-2007**	**Percentage change 2007-2017**
Unsafe water source	2.3 (1.2 to 3.9)	1.9 (1.0 to 3.0)	2.0 (1.1 to 3.4)	-17.3 (-58.5 to 61.3)	5.4 (-36.5 to 77.5)	2.3 (1.2 to 3.9)	1.9 (1.0 to 3.0)	2.0 (1.1 to 3.4)	-17.3 (-58.5 to 61.3)	5.4 (-36.5 to 77.5)
Unsafe sanitation	3.8 (1.8 to 7.2)	1.9 (0.9 to 3.7)	1.7 (0.7 to 3.4)	-49.8 (-78.1 to 18.3)	-13.8 (-54.7 to 68.0)	3.8 (1.8 to 7.2)	1.9 (0.9 to 3.7)	1.7 (0.7 to 3.4)	-49.8 (-78.1 to 18.3)	-13.8 (-54.7 to 68.0)
No access to handwashing facility	6.7 (4.8 to 9.0)	6.4 (4.6 to 8.8)	5.9 (4.2 to 8.1)	-5.2 (-34.0 to 36.4)	-7.5 (-28.3 to 19.1)	6.7 (4.8 to 9.0)	6.4 (4.6 to 8.8)	5.9 (4.2 to 8.1)	-5.2 (-34.0 to 36.4)	-7.5 (-28.3 to 19.1)
Ambient particulate matter pollution	31.2 (20.8 to 46.7)	31.3 (21.8 to 45.6)	26.7 (17.7 to 40.4)	0.2 (-18.6 to 25.8)	-14.8 (-33.5 to 4.6)	31.0 (20.7 to 46.5)	31.3 (21.7 to 45.6)	26.6 (17.7 to 40.4)	0.8 (-18.4 to 27.2)	-14.8 (-33.6 to 4.6)
Household air pollution from solid fuels	0.6 (0.2 to 1.6)	0.1 (0.0 to 0.3)	0.1 (0.0 to 0.2)	-75.7 (-92.9 to -51.4)	-14.5 (-68.9 to 83.5)	0.9 (0.2 to 4.7)	0.2 (0.0 to 1.2)	0.2 (0.0 to 0.9)	-72.0 (-92.8 to -45.7)	-15.8 (-68.0 to 81.3)
Ambient ozone pollution	51.5 (24.5 to 54.8)	51.7 (25.1 to 54.8)	52.1 (26.4 to 54.8)	0.4 (0.0 to 2.5)	0.9 (0.0 to 6.0)	51.5 (24.5 to 54.8)	51.7 (25.1 to 54.8)	52.1 (26.4 to 54.8)	0.4 (0.0 to 2.5)	0.9 (0.0 to 6.0)
Residential radon	14.0 (1.0 to 43.3)	14.0 (1.0 to 43.3)	14.0 (1.0 to 43.3)	0.0 (0.0 to 0.0)	0.0 (0.0 to 0.0)	14.0 (1.0 to 43.3)	14.0 (1.0 to 43.3)	14.0 (1.0 to 43.3)	0.0 (0.0 to 0.0)	0.0 (0.0 to 0.0)
Lead exposure	2.8 (0.4 to 6.4)	1.9 (0.2 to 4.8)	1.3 (0.1 to 3.8)	-33.9 (-48.0 to -24.4)	-28.5 (-44.6 to -20.4)	1.5 (0.1 to 4.4)	0.9 (0.0 to 3.0)	0.6 (0.0 to 2.4)	-40.3 (-58.8 to -30.5)	-31.9 (-55.3 to -22.3)
Occupational exposure to asbestos	1.7 (1.5 to 2.0)	2.4 (2.1 to 2.6)	2.4 (2.0 to 2.8)	34.7 (17.1 to 57.5)	0.3 (-17.7 to 21.2)	0.5 (0.4 to 0.6)	0.4 (0.4 to 0.5)	0.4 (0.3 to 0.5)	-7.8 (-25.1 to 19.7)	-0.0 (-21.0 to 24.6)
Occupational exposure to arsenic	0.5 (0.0 to 1.1)	0.4 (0.0 to 1.0)	0.4 (0.0 to 1.0)	-	-	0.4 (0.0 to 1.0)	0.4 (0.0 to 0.9)	0.4 (0.0 to 0.9)	-	-
Occupational exposure to benzene	0.6 (0.0 to 1.9)	0.7 (0.0 to 1.8)	0.7 (0.0 to 1.9)	2.5 (-4.1 to 12.4)	3.6 (-2.4 to 11.0)	0.6 (0.0 to 1.8)	0.7 (0.0 to 2.1)	0.8 (0.1 to 2.5)	22.3 (8.4 to 40.9)	13.7 (4.3 to 29.0)
Occupational exposure to beryllium	0.0 (0.0 to 0.0)	0.0 (0.0 to 0.0)	0.0 (0.0 to 0.0)	-5.1 (-10.5 to -0.2)	2.0 (-2.3 to 6.0)	0.0 (0.0 to 0.0)	0.0 (0.0 to 0.0)	0.0 (0.0 to 0.0)	2.0 (-6.3 to 11.9)	8.8 (2.3 to 15.5)
Occupational exposure to cadmium	0.1 (0.0 to 0.1)	0.0 (0.0 to 0.1)	0.0 (0.0 to 0.1)	-7.1 (-19.4 to 5.7)	1.1 (-9.6 to 10.9)	0.0 (0.0 to 0.1)	0.0 (0.0 to 0.1)	0.0 (0.0 to 0.1)	-9.4 (-22.7 to 8.0)	4.8 (-7.4 to 19.1)
Occupational exposure to chromium	0.1 (0.1 to 0.1)	0.1 (0.1 to 0.1)	0.1 (0.1 to 0.1)	-5.5 (-16.3 to 5.8)	1.6 (-7.8 to 10.3)	0.1 (0.1 to 0.1)	0.1 (0.1 to 0.1)	0.1 (0.1 to 0.1)	-4.6 (-17.3 to 11.7)	6.5 (-4.1 to 18.6)
Occupational exposure to diesel engine exhaust	0.6 (0.5 to 0.6)	0.6 (0.5 to 0.6)	0.6 (0.6 to 0.6)	-2.0 (-9.9 to 7.3)	3.1 (-3.3 to 9.4)	0.5 (0.4 to 0.5)	0.5 (0.5 to 0.6)	0.6 (0.5 to 0.6)	12.1 (-1.4 to 27.4)	10.7 (-1.0 to 22.4)
Occupational exposure to formaldehyde	0.3 (0.2 to 0.3)	0.2 (0.2 to 0.3)	0.2 (0.2 to 0.3)	-6.6 (-19.4 to 7.2)	0.7 (-10.3 to 11.4)	0.2 (0.2 to 0.3)	0.2 (0.2 to 0.2)	0.2 (0.2 to 0.2)	-6.7 (-20.8 to 11.3)	5.7 (-6.2 to 20.1)
Occupational exposure to nickel	0.4 (0.0 to 1.9)	0.4 (0.0 to 1.8)	0.4 (0.0 to 1.8)	-	-	0.4 (0.0 to 1.6)	0.3 (0.0 to 1.3)	0.3 (0.0 to 1.3)	-	-
Occupational exposure to polycyclic aromatic hydrocarbons	0.2 (0.2 to 0.3)	0.2 (0.2 to 0.3)	0.2 (0.2 to 0.3)	-4.5 (-14.6 to 6.0)	1.9 (-6.8 to 9.7)	0.2 (0.2 to 0.3)	0.2 (0.2 to 0.2)	0.2 (0.2 to 0.3)	-2.4 (-14.5 to 13.0)	7.4 (-2.6 to 18.7)
Occupational exposure to silica	4.7 (0.7 to 15.2)	4.4 (0.7 to 14.4)	4.5 (0.7 to 14.8)	-6.2 (-16.6 to 5.9)	1.3 (-7.2 to 11.3)	3.2 (0.5 to 10.3)	2.8 (0.4 to 8.9)	2.8 (0.4 to 8.8)	-14.2 (-27.6 to 3.8)	2.0 (-10.6 to 18.9)
Occupational exposure to sulphuric acid	0.6 (0.1 to 1.9)	0.6 (0.1 to 1.7)	0.6 (0.1 to 1.8)	-7.1 (-18.3 to 5.0)	1.4 (-8.7 to 11.0)	0.5 (0.1 to 1.6)	0.5 (0.1 to 1.5)	0.5 (0.1 to 1.5)	-11.0 (-24.8 to 6.8)	4.1 (-8.3 to 18.3)
Occupational exposure to trichloroethylene	0.1 (0.1 to 0.1)	0.1 (0.1 to 0.1)	0.1 (0.1 to 0.1)	-4.5 (-12.2 to 3.7)	2.0 (-4.4 to 8.5)	0.1 (0.1 to 0.1)	0.1 (0.1 to 0.1)	0.1 (0.1 to 0.1)	0.4 (-9.8 to 13.5)	8.0 (-0.5 to 17.8)
Occupational asthmagens	19.0 (15.1 to 23.9)	18.1 (14.4 to 23.2)	18.1 (14.4 to 22.9)	-4.5 (-22.9 to 17.8)	-0.2 (-15.3 to 17.9)	8.4 (6.7 to 10.4)	8.3 (6.7 to 10.3)	8.8 (7.0 to 10.9)	-0.5 (-15.1 to 16.8)	5.1 (-7.7 to 19.3)
Occupational particulate matter, gases, and fumes	6.4 (4.5 to 9.2)	6.1 (4.3 to 8.9)	5.9 (4.2 to 8.5)	-4.3 (-9.4 to 0.7)	-3.6 (-7.1 to -0.3)	3.7 (2.5 to 5.5)	3.5 (2.4 to 5.3)	3.3 (2.3 to 5.1)	-5.3 (-13.6 to 3.9)	-4.6 (-10.4 to 1.7)
Occupational noise	5.9 (5.5 to 6.5)	5.8 (5.3 to 6.3)	5.6 (5.2 to 6.2)	-3.0 (-6.2 to 0.1)	-2.3 (-4.4 to -0.3)	3.5 (3.0 to 4.0)	3.5 (3.1 to 4.0)	3.6 (3.2 to 4.0)	0.8 (-5.4 to 7.8)	1.9 (-1.8 to 6.6)
Occupational ergonomic factors	10.3 (8.6 to 12.5)	9.1 (7.5 to 11.0)	9.0 (7.4 to 11.0)	-11.5 (-22.6 to 1.9)	-1.2 (-11.3 to 10.0)	5.6 (4.3 to 7.2)	5.6 (4.4 to 7.1)	6.3 (4.9 to 8.2)	0.8 (-20.7 to 24.4)	12.3 (-6.7 to 33.3)
Non-exclusive breastfeeding	0.4 (0.3 to 0.6)	0.4 (0.3 to 0.6)	0.4 (0.3 to 0.6)	-1.3 (-13.8 to 12.4)	-1.3 (-11.5 to 10.9)	0.4 (0.3 to 0.6)	0.4 (0.3 to 0.6)	0.4 (0.3 to 0.6)	-1.2 (-13.8 to 12.5)	-1.4 (-11.7 to 10.7)
Discontinued breastfeeding	1.7 (1.6 to 1.8)	1.7 (1.6 to 1.9)	1.7 (1.6 to 1.8)	0.9 (-8.1 to 11.2)	-1.7 (-8.6 to 6.5)	1.7 (1.6 to 1.8)	1.7 (1.6 to 1.9)	1.7 (1.5 to 1.8)	1.3 (-7.7 to 11.6)	-2.4 (-9.2 to 5.8)
Child underweight	0.2 (0.1 to 0.3)	0.2 (0.1 to 0.3)	0.2 (0.1 to 0.3)	-15.8 (-34.7 to 5.2)	-4.9 (-19.6 to 10.3)	0.2 (0.1 to 0.4)	0.2 (0.1 to 0.3)	0.2 (0.1 to 0.3)	-17.5 (-36.8 to 7.7)	-5.4 (-20.4 to 11.7)
Child wasting	0.2 (0.1 to 0.2)	0.2 (0.1 to 0.2)	0.2 (0.1 to 0.2)	-7.0 (-31.1 to 24.7)	-2.2 (-19.2 to 18.4)	0.2 (0.1 to 0.2)	0.2 (0.1 to 0.2)	0.1 (0.1 to 0.2)	-8.3 (-30.8 to 23.3)	-2.3 (-20.4 to 20.3)
Child stunting	0.6 (0.3 to 1.0)	0.5 (0.3 to 0.8)	0.5 (0.3 to 0.8)	-12.8 (-43.5 to 33.4)	-2.4 (-32.5 to 36.2)	0.6 (0.3 to 1.1)	0.5 (0.3 to 0.8)	0.5 (0.3 to 0.8)	-16.0 (-46.9 to 35.8)	-3.1 (-30.4 to 38.2)
Short gestation for birth weight	0.0 (0.0 to 0.0)	0.0 (0.0 to 0.0)	0.0 (0.0 to 0.0)	9.4 (-12.5 to 46.3)	2.3 (-15.1 to 36.2)	0.0 (0.0 to 0.0)	0.0 (0.0 to 0.0)	0.0 (0.0 to 0.0)	17.9 (-14.7 to 90.3)	-2.5 (-22.4 to 35.0)
Low birth weight for gestation	0.0 (0.0 to 0.0)	0.0 (0.0 to 0.0)	0.0 (0.0 to 0.0)	4.1 (-17.7 to 30.1)	1.9 (-20.3 to 17.7)	0.0 (0.0 to 0.0)	0.0 (0.0 to 0.0)	0.0 (0.0 to 0.0)	-10.7 (-62.1 to 24.5)	6.1 (-18.0 to 28.2)
Iron deficiency	-	-	-	-	-	8.0 (5.4 to 11.3)	8.0 (5.6 to 11.0)	7.4 (4.8 to 10.4)	0.0 (-22.6 to 28.7)	-8.5 (-24.5 to 9.3)
Vitamin A deficiency	1.0 (0.6 to 1.3)	0.8 (0.5 to 1.0)	0.7 (0.4 to 0.9)	-17.7 (-30.0 to -0.6)	-15.8 (-31.0 to 3.0)	0.8 (0.4 to 1.1)	0.8 (0.5 to 1.0)	0.7 (0.5 to 1.0)	0.0 (-14.2 to 16.2)	-5.8 (-24.8 to 14.7)
Zinc deficiency	0.5 (0.1 to 1.0)	0.5 (0.1 to 1.1)	0.4 (0.1 to 1.1)	1.8 (-62.1 to 76.9)	-15.2 (-70.1 to 136.2)	0.5 (0.2 to 1.1)	0.4 (0.1 to 0.8)	0.3 (0.1 to 0.8)	-28.7 (-65.8 to 67.9)	-3.6 (-64.6 to 165.8)
Smoking	17.1 (13.6 to 20.6)	12.1 (9.7 to 14.7)	10.3 (8.2 to 12.7)	-29.3 (-39.4 to -18.4)	-14.9 (-23.0 to -5.3)	3.9 (2.8 to 5.2)	3.2 (2.3 to 4.4)	3.1 (2.2 to 4.3)	-16.1 (-35.9 to 9.3)	-2.8 (-19.6 to 16.8)
Chewing tobacco	1.4 (0.9 to 2.0)	0.9 (0.6 to 1.4)	0.8 (0.5 to 1.1)	-32.9 (-56.2 to 7.5)	-18.4 (-41.5 to 14.2)	0.1 (0.1 to 0.1)	0.1 (0.1 to 0.1)	0.1 (0.1 to 0.1)	-3.9 (-39.2 to 60.7)	-3.3 (-32.0 to 40.7)
Second-hand smoke	37.8 (33.9 to 41.6)	36.7 (32.6 to 41.1)	37.0 (32.8 to 41.7)	-2.8 (-12.8 to 10.3)	0.7 (-7.8 to 10.0)	57.4 (53.8 to 60.8)	49.8 (46.2 to 53.6)	50.1 (45.6 to 54.4)	-13.2 (-19.2 to -6.7)	0.6 (-5.4 to 7.1)
Alcohol use	14.5 (7.1 to 24.6)	14.5 (7.1 to 24.9)	17.0 (8.1 to 29.7)	0.2 (-33.0 to 50.4)	16.9 (-19.8 to 64.9)	11.5 (5.6 to 20.1)	11.4 (5.4 to 20.3)	13.2 (6.2 to 23.8)	-0.9 (-33.8 to 46.7)	15.7 (-18.6 to 65.5)
Drug use	0.5 (0.4 to 0.7)	0.5 (0.4 to 0.7)	0.6 (0.4 to 0.8)	0.3 (-5.9 to 6.5)	11.3 (4.5 to 20.5)	0.3 (0.2 to 0.4)	0.3 (0.2 to 0.4)	0.3 (0.3 to 0.4)	2.2 (-3.9 to 8.7)	9.1 (2.4 to 17.7)
Diet low in fruits	40.6 (35.1 to 44.3)	39.1 (33.0 to 43.0)	40.5 (34.7 to 44.3)	-3.9 (-9.8 to 2.3)	3.8 (-0.7 to 9.4)	37.7 (32.1 to 42.3)	34.8 (28.5 to 39.5)	37.2 (31.3 to 41.8)	-7.7 (-16.6 to 0.3)	6.9 (0.7 to 14.9)
Diet low in vegetables	19.3 (11.2 to 26.5)	13.6 (5.7 to 21.3)	15.8 (7.7 to 23.5)	-29.7 (-58.9 to -2.5)	16.2 (-13.0 to 69.4)	21.4 (12.7 to 29.1)	15.5 (7.4 to 23.3)	17.4 (8.8 to 25.0)	-27.5 (-55.1 to -3.9)	12.2 (-12.9 to 56.9)
Diet low in legumes	4.4 (0.7 to 9.8)	5.8 (1.1 to 11.9)	7.7 (2.1 to 14.7)	32.2 (-45.0 to 256.3)	33.8 (-19.5 to 194.7)	8.4 (2.7 to 15.2)	8.5 (2.5 to 14.7)	11.2 (4.5 to 18.3)	1.4 (-44.9 to 85.9)	31.0 (-6.8 to 131.4)
Diet low in whole grains	30.2 (25.1 to 34.6)	35.0 (30.7 to 38.5)	36.4 (32.4 to 39.9)	15.7 (6.8 to 27.1)	4.0 (-1.1 to 9.4)	31.7 (27.0 to 35.5)	36.3 (32.5 to 39.6)	37.6 (33.9 to 40.7)	14.7 (6.6 to 26.1)	3.5 (-1.0 to 8.1)
Diet low in nuts and seeds	50.6 (49.2 to 51.7)	49.3 (47.3 to 50.8)	49.2 (47.2 to 50.8)	-2.5 (-5.4 to -0.3)	-0.3 (-2.4 to 1.9)	50.6 (49.2 to 51.7)	49.1 (47.3 to 50.6)	48.9 (46.7 to 50.5)	-3.0 (-5.6 to -0.7)	-0.5 (-3.0 to 1.6)
Diet low in milk	46.2 (44.1 to 48.0)	46.6 (44.7 to 48.2)	47.2 (45.2 to 48.7)	0.9 (-0.8 to 3.2)	1.2 (-0.2 to 2.9)	46.1 (44.0 to 47.9)	45.9 (43.6 to 47.7)	46.5 (44.5 to 48.3)	-0.5 (-2.7 to 1.4)	1.4 (-0.0 to 3.1)
Diet high in red meat	4.2 (0.6 to 9.1)	8.2 (2.7 to 15.5)	9.3 (2.7 to 16.7)	95.0 (-14.0 to 720.8)	14.5 (-36.8 to 112.0)	0.6 (0.0 to 2.7)	1.7 (0.0 to 5.1)	2.2 (0.0 to 6.2)	162.5 (-77.8 to 688773.5)	27.3 (-82.2 to 2154.2)
Diet high in processed meat	12.0 (7.7 to 16.2)	19.9 (14.6 to 24.9)	23.1 (17.1 to 28.9)	66.1 (29.2 to 127.4)	16.2 (-1.5 to 37.9)	9.8 (5.8 to 13.5)	14.3 (10.1 to 18.2)	15.8 (11.0 to 20.0)	45.9 (13.0 to 104.3)	10.0 (-8.3 to 29.7)
Diet high in sugar-sweetened beverages	7.6 (2.9 to 14.1)	8.6 (3.5 to 15.7)	9.1 (3.8 to 16.1)	13.8 (-44.1 to 135.4)	5.5 (-37.1 to 72.2)	5.4 (2.1 to 11.3)	6.0 (2.7 to 10.7)	6.4 (2.7 to 12.4)	10.7 (-51.7 to 138.5)	8.0 (-35.7 to 87.5)
Diet low in fibre	21.9 (14.3 to 28.4)	23.1 (16.1 to 29.3)	24.1 (17.2 to 30.6)	5.4 (-12.1 to 31.1)	4.6 (-7.4 to 19.4)	23.1 (15.6 to 29.6)	24.2 (17.2 to 30.3)	25.6 (18.7 to 31.6)	4.7 (-10.8 to 27.2)	6.0 (-5.4 to 19.9)
Diet low in calcium	34.5 (30.0 to 38.1)	34.4 (30.1 to 38.0)	35.4 (31.3 to 38.9)	-0.1 (-3.9 to 3.8)	2.7 (-0.4 to 6.4)	36.7 (32.8 to 39.9)	35.7 (31.5 to 39.1)	37.0 (33.1 to 40.2)	-2.6 (-6.3 to 0.9)	3.5 (0.8 to 7.2)
Diet low in seafood omega-3 fatty acids	3.0 (1.1 to 4.5)	0.2 (0.0 to 0.9)	0.2 (0.0 to 1.0)	-93.7 (-100.0 to -72.1)	13.2 (-94.7 to 4312.3)	3.5 (1.6 to 5.0)	0.3 (0.0 to 1.3)	0.3 (0.0 to 1.3)	-91.7 (-100.0 to -69.0)	10.2 (-93.4 to 1687.1)
Diet low in polyunsaturated fatty acids	30.1 (24.6 to 34.7)	27.3 (21.4 to 32.5)	27.9 (22.0 to 33.0)	-9.3 (-22.3 to 0.9)	2.2 (-5.6 to 10.7)	30.7 (25.0 to 35.5)	28.0 (21.9 to 33.0)	28.6 (23.0 to 33.5)	-8.9 (-19.4 to 0.8)	2.4 (-5.2 to 11.2)
Diet high in trans fatty acids	1.1 (0.0 to 3.3)	0.9 (0.0 to 3.0)	0.9 (0.0 to 2.9)	-12.5 (-39.5 to -6.6)	-8.4 (-32.0 to -3.7)	1.5 (0.1 to 4.0)	1.3 (0.1 to 3.7)	1.2 (0.0 to 3.5)	-12.5 (-35.9 to -6.6)	-8.3 (-27.6 to -3.1)
Diet high in sodium	28.3 (19.1 to 36.5)	18.3 (9.8 to 25.6)	15.8 (7.2 to 23.3)	-35.2 (-58.0 to -16.8)	-13.6 (-36.5 to 2.9)	32.2 (24.2 to 39.9)	19.7 (11.3 to 26.4)	16.5 (7.4 to 23.7)	-38.9 (-58.0 to -24.3)	-16.4 (-38.4 to -3.6)
Intimate partner violence	-	-	-	-	-	2.3 (1.9 to 2.6)	2.1 (1.7 to 2.4)	2.0 (1.7 to 2.4)	-8.3 (-12.2 to -4.1)	-3.3 (-7.8 to 1.2)
Childhood sexual abuse	5.3 (4.3 to 6.5)	5.3 (4.3 to 6.5)	5.3 (4.3 to 6.5)	0.0 (0.0 to 0.0)	0.0 (0.0 to 0.0)	7.3 (5.9 to 8.9)	7.3 (5.9 to 8.9)	7.5 (6.0 to 9.2)	0.0 (0.0 to 0.0)	2.8 (-4.0 to 10.6)

Supplementary Results Table 1. Ibaraki age-standardised Summary Exposure Values (SEVs) for Level 4 risk factors and percentage change, by sex, 1990, 2007, and 2017.Data in parentheses are 95% uncertainty intervals.										
	**Male**					**Female**				
	**1990**	**2007**	**2017**	**Percentage change 1990-2007**	**Percentage change 2007-2017**	**1990**	**2007**	**2017**	**Percentage change 1990-2007**	**Percentage change 2007-2017**
Bullying victimization	3.3<br>(1.3 to 6.5)	3.3<br>(1.4 to 6.7)	3.5<br>(1.4 to 7.0)	1.2<br>(-6.9 to 9.6)	3.8<br>(-1.5 to 8.5)	1.9<br>(0.8 to 3.9)	1.9<br>(0.7 to 3.8)	2.0<br>(0.8 to 4.2)	-0.7<br>(-8.5 to 6.7)	3.3<br>(-24.2 to 39.9)
Low physical activity	0.5<br>(0.2 to 0.9)	0.5<br>(0.2 to 0.9)	0.4<br>(0.2 to 0.8)	-1.8<br>(-3.8 to 0.1)	-8.8<br>(-12.4 to -4.5)	0.5<br>(0.2 to 1.0)	0.5<br>(0.2 to 0.9)	0.5<br>(0.2 to 0.9)	-3.9<br>(-6.1 to -1.3)	0.8<br>(-0.8 to 2.3)
High fasting plasma glucose	1.5<br>(0.9 to 2.4)	1.4<br>(0.9 to 2.2)	1.6<br>(1.0 to 2.6)	-7.3<br>(-44.0 to 44.5)	13.8<br>(-22.8 to 84.5)	1.2<br>(0.7 to 1.9)	1.2<br>(0.7 to 2.1)	1.4<br>(0.8 to 2.3)	2.8<br>(-39.5 to 75.8)	10.0<br>(-30.0 to 78.6)
High LDL cholesterol	12.0<br>(10.4 to 13.8)	12.6<br>(10.9 to 14.3)	12.6<br>(11.0 to 14.3)	4.3<br>(-1.4 to 11.1)	0.1<br>(-4.7 to 4.9)	12.9<br>(11.2 to 14.5)	13.0<br>(11.4 to 14.7)	13.0<br>(11.4 to 14.8)	0.9<br>(-4.3 to 6.3)	0.0<br>(-4.6 to 5.0)
High systolic blood pressure	11.3<br>(10.3 to 12.3)	8.3<br>(7.5 to 9.3)	8.2<br>(7.3 to 9.2)	-26.1<br>(-32.0 to -19.9)	-1.5<br>(-9.2 to 6.3)	9.6<br>(8.7 to 10.7)	6.0<br>(5.3 to 6.8)	5.6<br>(4.8 to 6.4)	-37.6<br>(-43.9 to -30.5)	-7.1<br>(-17.1 to 3.8)
High body-mass index	6.0<br>(3.7 to 9.3)	8.7<br>(5.9 to 13.6)	9.1<br>(6.1 to 14.2)	45.2<br>(18.8 to 84.3)	3.8<br>(-13.1 to 22.3)	5.6<br>(3.5 to 8.8)	7.3<br>(4.7 to 11.3)	7.3<br>(4.5 to 11.5)	29.5<br>(0.9 to 72.6)	0.5<br>(-17.7 to 22.6)
Low bone mineral density	5.3<br>(4.4 to 6.3)	5.2<br>(4.4 to 6.1)	5.2<br>(4.2 to 6.2)	-2.0<br>(-14.5 to 12.5)	0.4<br>(-13.6 to 14.7)	7.2<br>(6.0 to 8.5)	7.0<br>(6.0 to 8.0)	7.1<br>(5.9 to 8.4)	-3.3<br>(-14.4 to 8.9)	1.3<br>(-11.2 to 14.6)
Impaired kidney function	2.6<br>(1.1 to 6.4)	2.5<br>(1.1 to 6.3)	2.3<br>(1.0 to 6.1)	-2.7<br>(-7.2 to 2.4)	-6.1<br>(-11.7 to -1.5)	3.3<br>(1.5 to 7.5)	3.4<br>(1.6 to 7.8)	3.2<br>(1.5 to 7.4)	3.7<br>(-0.4 to 8.5)	-6.6<br>(-12.1 to -1.7)

Supplementary Results Table 1. Ishikawa age-standardised Summary Exposure Values (SEVs) for Level 4 risk factors and percentage change, by sex, 1990, 2007, and 2017.Data in parentheses are 95% uncertainty intervals.										
	Male					Female				
	**1990**	**2007**	**2017**	**Percentage change 1990-2007**	**Percentage change 2007-2017**	**1990**	**2007**	**2017**	**Percentage change 1990-2007**	**Percentage change 2007-2017**
Unsafe water source	2.2 (1.2 to 3.6)	1.8 (1.0 to 3.0)	1.9 (1.0 to 3.2)	-19.0 (-58.7 to 61.0)	5.1 (-38.4 to 77.1)	2.2 (1.2 to 3.6)	1.8 (1.0 to 3.0)	1.9 (1.0 to 3.2)	-19.0 (-58.7 to 61.0)	5.1 (-38.4 to 77.1)
Unsafe sanitation	3.8 (1.9 to 7.0)	1.8 (0.8 to 3.4)	1.6 (0.7 to 3.1)	-52.6 (-79.3 to 5.9)	-13.0 (-55.6 to 67.9)	3.8 (1.9 to 7.0)	1.8 (0.8 to 3.4)	1.6 (0.7 to 3.1)	-52.6 (-79.3 to 5.9)	-13.0 (-55.6 to 67.9)
No access to handwashing facility	6.7 (4.8 to 9.1)	6.3 (4.5 to 8.7)	5.9 (4.2 to 8.0)	-5.4 (-31.8 to 33.0)	-7.6 (-27.7 to 17.8)	6.7 (4.8 to 9.1)	6.3 (4.5 to 8.7)	5.9 (4.2 to 8.0)	-5.4 (-31.8 to 33.0)	-7.6 (-27.7 to 17.8)
Ambient particulate matter pollution	32.2 (19.2 to 50.0)	32.5 (21.5 to 48.3)	27.2 (14.8 to 43.5)	1.0 (-32.3 to 58.8)	-16.4 (-50.8 to 24.9)	32.0 (18.8 to 49.8)	32.5 (21.4 to 48.3)	27.2 (14.8 to 43.5)	1.6 (-31.9 to 60.6)	-16.4 (-50.8 to 25.0)
Household air pollution from solid fuels	0.6 (0.2 to 1.7)	0.1 (0.0 to 0.3)	0.1 (0.0 to 0.3)	-76.5 (-93.3 to -49.8)	-14.9 (-66.6 to 84.9)	0.8 (0.2 to 5.2)	0.2 (0.0 to 1.0)	0.2 (0.0 to 0.8)	-72.9 (-93.2 to -45.7)	-13.2 (-66.3 to 87.1)
Ambient ozone pollution	51.3 (24.0 to 54.8)	51.3 (24.1 to 54.8)	51.6 (25.2 to 54.8)	-0.0 (-0.7 to 0.4)	0.6 (0.0 to 4.0)	51.3 (24.0 to 54.8)	51.3 (24.1 to 54.8)	51.6 (25.2 to 54.8)	-0.0 (-0.7 to 0.4)	0.6 (0.0 to 4.0)
Residential radon	15.2 (1.3 to 47.5)	15.2 (1.3 to 47.5)	15.2 (1.3 to 47.5)	0.0 (0.0 to 0.0)	0.0 (0.0 to 0.0)	15.2 (1.3 to 47.5)	15.2 (1.3 to 47.5)	15.2 (1.3 to 47.5)	0.0 (0.0 to 0.0)	0.0 (0.0 to 0.0)
Lead exposure	3.6 (0.7 to 7.3)	2.3 (0.4 to 5.3)	1.6 (0.2 to 4.1)	-36.1 (-49.9 to -26.7)	-30.5 (-45.5 to -22.3)	1.9 (0.2 to 4.9)	1.1 (0.1 to 3.3)	0.7 (0.0 to 2.5)	-41.9 (-59.4 to -31.8)	-33.5 (-55.6 to -23.7)
Occupational exposure to asbestos	2.2 (1.9 to 2.4)	2.9 (2.6 to 3.1)	2.9 (2.4 to 3.5)	33.0 (15.8 to 58.0)	0.2 (-18.1 to 22.3)	0.5 (0.4 to 0.6)	0.5 (0.5 to 0.6)	0.5 (0.4 to 0.6)	-3.8 (-20.6 to 23.5)	-1.9 (-21.4 to 20.4)
Occupational exposure to arsenic	0.5 (0.0 to 1.1)	0.4 (0.0 to 1.0)	0.4 (0.0 to 1.0)	-	-	0.4 (0.0 to 1.0)	0.4 (0.0 to 0.9)	0.4 (0.0 to 0.9)	-	-
Occupational exposure to benzene	0.6 (0.0 to 1.8)	0.6 (0.0 to 1.8)	0.7 (0.0 to 1.9)	2.0 (-4.7 to 12.9)	3.4 (-2.3 to 12.0)	0.6 (0.0 to 1.7)	0.7 (0.0 to 2.1)	0.8 (0.1 to 2.4)	22.2 (9.0 to 44.0)	13.9 (4.7 to 28.4)
Occupational exposure to beryllium	0.0 (0.0 to 0.0)	0.0 (0.0 to 0.0)	0.0 (0.0 to 0.0)	-5.4 (-10.2 to 0.0)	2.0 (-2.2 to 6.0)	0.0 (0.0 to 0.0)	0.0 (0.0 to 0.0)	0.0 (0.0 to 0.0)	2.4 (-6.5 to 12.8)	8.7 (2.0 to 15.7)
Occupational exposure to cadmium	0.1 (0.0 to 0.1)	0.0 (0.0 to 0.1)	0.0 (0.0 to 0.1)	-7.3 (-18.0 to 5.4)	0.7 (-8.9 to 11.0)	0.0 (0.0 to 0.1)	0.0 (0.0 to 0.1)	0.0 (0.0 to 0.1)	-8.7 (-23.2 to 8.8)	4.4 (-7.0 to 18.2)
Occupational exposure to chromium	0.1 (0.1 to 0.1)	0.1 (0.1 to 0.1)	0.1 (0.1 to 0.1)	-5.6 (-15.2 to 5.6)	1.3 (-7.4 to 10.2)	0.1 (0.1 to 0.1)	0.1 (0.1 to 0.1)	0.1 (0.1 to 0.1)	-3.9 (-17.4 to 12.3)	6.2 (-4.0 to 17.7)
Occupational exposure to diesel engine exhaust	0.6 (0.5 to 0.6)	0.6 (0.5 to 0.6)	0.6 (0.6 to 0.6)	-2.3 (-10.6 to 7.0)	3.3 (-2.9 to 9.9)	0.5 (0.4 to 0.5)	0.5 (0.5 to 0.6)	0.6 (0.5 to 0.6)	12.9 (-0.8 to 28.7)	10.7 (0.3 to 22.0)
Occupational exposure to formaldehyde	0.3 (0.2 to 0.3)	0.2 (0.2 to 0.3)	0.2 (0.2 to 0.3)	-6.7 (-18.2 to 6.5)	0.3 (-9.5 to 11.2)	0.2 (0.2 to 0.3)	0.2 (0.2 to 0.2)	0.2 (0.2 to 0.2)	-6.1 (-21.6 to 12.5)	5.2 (-6.7 to 19.0)
Occupational exposure to nickel	0.4 (0.0 to 2.0)	0.4 (0.0 to 1.8)	0.4 (0.0 to 1.8)	-	-	0.4 (0.0 to 1.5)	0.3 (0.0 to 1.3)	0.3 (0.0 to 1.3)	-	-
Occupational exposure to polycyclic aromatic hydrocarbons	0.2 (0.2 to 0.3)	0.2 (0.2 to 0.3)	0.2 (0.2 to 0.3)	-4.6 (-13.8 to 5.7)	1.6 (-6.3 to 9.9)	0.2 (0.2 to 0.3)	0.2 (0.2 to 0.2)	0.2 (0.2 to 0.3)	-1.8 (-15.2 to 13.5)	7.1 (-2.8 to 18.1)
Occupational exposure to silica	4.7 (0.7 to 15.9)	4.4 (0.6 to 14.1)	4.5 (0.6 to 14.2)	-6.9 (-17.5 to 5.6)	1.2 (-8.2 to 12.5)	3.2 (0.4 to 10.3)	2.8 (0.4 to 8.6)	2.8 (0.4 to 8.9)	-13.7 (-29.4 to 4.7)	1.2 (-11.1 to 19.7)
Occupational exposure to sulphuric acid	0.6 (0.1 to 1.9)	0.6 (0.1 to 1.8)	0.6 (0.1 to 1.8)	-7.3 (-17.4 to 5.0)	1.0 (-8.0 to 10.9)	0.5 (0.1 to 1.6)	0.5 (0.1 to 1.5)	0.5 (0.1 to 1.5)	-10.1 (-24.7 to 7.2)	3.7 (-8.1 to 18.1)
Occupational exposure to trichloroethylene	0.1 (0.1 to 0.1)	0.1 (0.1 to 0.1)	0.1 (0.1 to 0.1)	-4.5 (-11.6 to 3.4)	1.9 (-4.2 to 8.6)	0.1 (0.1 to 0.1)	0.1 (0.1 to 0.1)	0.1 (0.1 to 0.1)	1.2 (-9.4 to 14.6)	7.8 (-0.6 to 17.7)
Occupational asthmagens	18.9 (15.2 to 23.7)	18.1 (14.3 to 22.9)	18.0 (14.1 to 22.9)	-4.4 (-23.4 to 18.4)	-0.5 (-15.7 to 17.7)	8.3 (6.8 to 10.3)	8.4 (6.7 to 10.3)	8.8 (6.9 to 10.8)	0.2 (-15.0 to 18.6)	5.1 (-7.3 to 20.0)
Occupational particulate matter, gases, and fumes	6.4 (4.6 to 9.3)	6.1 (4.3 to 8.9)	5.9 (4.2 to 8.5)	-4.8 (-9.5 to 0.1)	-3.9 (-7.2 to -0.3)	3.7 (2.5 to 5.5)	3.5 (2.4 to 5.3)	3.3 (2.3 to 5.0)	-5.6 (-14.1 to 4.8)	-4.7 (-10.2 to 1.4)
Occupational noise	6.0 (5.5 to 6.6)	5.8 (5.3 to 6.3)	5.6 (5.2 to 6.1)	-3.7 (-6.9 to -0.5)	-2.6 (-4.7 to -0.3)	3.5 (3.0 to 4.1)	3.5 (3.1 to 4.0)	3.6 (3.2 to 4.1)	0.5 (-5.6 to 7.9)	1.7 (-2.1 to 6.1)
Occupational ergonomic factors	10.3 (8.6 to 12.5)	9.1 (7.5 to 10.9)	9.0 (7.4 to 10.9)	-12.0 (-23.8 to 1.8)	-0.9 (-11.9 to 11.0)	5.5 (4.3 to 7.0)	5.6 (4.4 to 6.9)	6.4 (4.9 to 8.1)	1.6 (-20.6 to 30.4)	13.2 (-6.7 to 36.0)
Non-exclusive breastfeeding	0.4 (0.3 to 0.6)	0.4 (0.3 to 0.6)	0.4 (0.3 to 0.6)	-1.2 (-14.1 to 13.1)	-1.7 (-12.9 to 9.7)	0.4 (0.3 to 0.6)	0.4 (0.3 to 0.6)	0.4 (0.3 to 0.6)	-0.8 (-13.8 to 13.5)	-1.7 (-12.9 to 9.7)
Discontinued breastfeeding	1.7 (1.6 to 1.8)	1.7 (1.6 to 1.9)	1.7 (1.6 to 1.8)	2.9 (-6.8 to 13.5)	-1.9 (-9.3 to 5.9)	1.7 (1.6 to 1.9)	1.7 (1.6 to 1.9)	1.7 (1.6 to 1.8)	1.1 (-8.4 to 11.5)	-2.8 (-10.1 to 5.0)
Child underweight	0.3 (0.2 to 0.4)	0.2 (0.1 to 0.3)	0.2 (0.1 to 0.3)	-15.1 (-34.2 to 8.1)	-4.9 (-20.4 to 12.2)	0.3 (0.2 to 0.4)	0.2 (0.1 to 0.3)	0.2 (0.1 to 0.3)	-18.0 (-37.6 to 8.2)	-5.9 (-21.8 to 13.6)
Child wasting	0.2 (0.1 to 0.3)	0.2 (0.1 to 0.3)	0.2 (0.1 to 0.3)	-6.8 (-34.3 to 33.0)	-2.2 (-22.3 to 21.7)	0.2 (0.1 to 0.3)	0.2 (0.1 to 0.3)	0.2 (0.1 to 0.3)	-8.6 (-36.5 to 31.2)	-2.6 (-23.6 to 25.0)
Child stunting	0.6 (0.3 to 1.1)	0.6 (0.3 to 1.0)	0.5 (0.3 to 0.9)	-11.5 (-46.3 to 46.9)	-3.8 (-33.5 to 38.4)	0.7 (0.3 to 1.1)	0.6 (0.3 to 1.0)	0.6 (0.3 to 1.0)	-12.8 (-48.9 to 51.5)	-4.2 (-33.2 to 32.4)
Short gestation for birth weight	0.0 (0.0 to 0.0)	0.0 (0.0 to 0.0)	0.0 (0.0 to 0.0)	9.0 (-13.8 to 37.5)	2.1 (-16.8 to 41.8)	0.0 (0.0 to 0.0)	0.0 (0.0 to 0.0)	0.0 (0.0 to 0.0)	11.5 (-17.4 to 49.0)	-0.7 (-24.9 to 34.4)
Low birth weight for gestation	0.0 (0.0 to 0.0)	0.0 (0.0 to 0.0)	0.0 (0.0 to 0.0)	3.6 (-14.6 to 25.9)	2.8 (-20.9 to 22.2)	0.0 (0.0 to 0.0)	0.0 (0.0 to 0.0)	0.0 (0.0 to 0.0)	0.9 (-20.4 to 26.4)	5.3 (-18.5 to 31.3)
Iron deficiency	-	-	-	-	-	8.0 (5.5 to 11.0)	8.0 (5.6 to 10.8)	7.3 (5.1 to 10.5)	-0.8 (-23.3 to 27.3)	-8.1 (-23.9 to 10.7)
Vitamin A deficiency	0.9 (0.6 to 1.2)	0.8 (0.5 to 1.0)	0.6 (0.4 to 0.8)	-14.3 (-26.7 to 1.1)	-22.9 (-34.7 to -8.3)	0.8 (0.5 to 1.0)	0.8 (0.5 to 1.1)	0.7 (0.4 to 1.0)	6.3 (-11.8 to 24.4)	-16.7 (-32.8 to 2.2)
Zinc deficiency	0.5 (0.1 to 1.0)	0.5 (0.1 to 1.2)	0.4 (0.1 to 1.1)	4.9 (-61.8 to 74.5)	-15.9 (-68.1 to 147.6)	0.5 (0.1 to 1.0)	0.4 (0.1 to 0.8)	0.3 (0.1 to 0.8)	-28.6 (-65.0 to 73.9)	-2.4 (-64.0 to 180.4)
Smoking	17.6 (14.2 to 21.4)	12.4 (10.0 to 15.0)	10.4 (8.3 to 12.7)	-29.8 (-39.0 to -19.0)	-15.7 (-23.9 to -5.9)	4.0 (2.8 to 5.3)	3.3 (2.3 to 4.5)	3.2 (2.3 to 4.3)	-16.7 (-36.5 to 7.4)	-3.4 (-20.6 to 16.4)
Chewing tobacco	1.4 (0.9 to 2.0)	0.9 (0.6 to 1.4)	0.8 (0.5 to 1.1)	-32.8 (-56.4 to 6.2)	-18.8 (-42.8 to 11.2)	0.1 (0.1 to 0.2)	0.1 (0.1 to 0.1)	0.1 (0.1 to 0.1)	-4.6 (-41.5 to 58.8)	-2.4 (-32.1 to 41.9)
Second-hand smoke	37.9 (33.9 to 42.0)	36.0 (32.0 to 40.3)	36.4 (31.8 to 41.1)	-4.9 (-16.2 to 7.3)	1.1 (-9.3 to 11.1)	56.9 (53.1 to 60.5)	48.7 (44.8 to 52.2)	49.1 (45.2 to 52.8)	-14.5 (-20.7 to -8.1)	0.9 (-5.4 to 7.3)
Alcohol use	15.3 (7.8 to 26.1)	15.3 (7.8 to 26.3)	16.7 (7.7 to 29.2)	0.3 (-31.3 to 47.3)	9.1 (-22.3 to 54.9)	12.0 (6.0 to 20.8)	12.0 (6.0 to 20.6)	13.4 (6.3 to 24.3)	0.3 (-31.6 to 48.2)	11.6 (-21.7 to 60.8)
Drug use	0.5 (0.4 to 0.7)	0.5 (0.4 to 0.7)	0.6 (0.4 to 0.8)	0.5 (-5.7 to 6.2)	11.5 (4.8 to 20.3)	0.3 (0.2 to 0.4)	0.3 (0.2 to 0.4)	0.3 (0.3 to 0.4)	2.3 (-3.6 to 8.7)	8.9 (1.9 to 17.0)
Diet low in fruits	40.5 (34.8 to 44.3)	38.7 (33.2 to 42.7)	40.1 (34.5 to 44.0)	-4.2 (-10.6 to 3.1)	3.4 (-1.8 to 8.9)	37.0 (30.7 to 41.8)	34.4 (27.9 to 39.2)	36.8 (30.6 to 41.2)	-7.2 (-15.7 to 2.2)	7.0 (0.2 to 15.8)
Diet low in vegetables	17.9 (9.5 to 25.4)	12.8 (4.5 to 21.0)	15.0 (6.6 to 23.1)	-28.6 (-61.5 to 0.0)	17.5 (-13.8 to 89.6)	20.4 (11.7 to 27.8)	15.0 (6.8 to 22.7)	17.1 (8.5 to 24.8)	-26.7 (-55.1 to -0.3)	14.4 (-12.1 to 69.8)
Diet low in legumes	4.4 (0.7 to 9.8)	5.5 (1.1 to 11.6)	7.3 (2.1 to 13.7)	25.9 (-44.4 to 252.5)	31.4 (-22.0 to 180.4)	8.1 (2.6 to 14.9)	7.6 (2.1 to 13.9)	9.7 (3.5 to 16.6)	-6.3 (-51.6 to 71.7)	27.6 (-18.1 to 127.3)
Diet low in whole grains	30.2 (24.9 to 34.6)	35.0 (30.9 to 38.7)	36.4 (32.4 to 39.8)	16.0 (6.8 to 29.7)	3.9 (-1.2 to 9.6)	31.6 (26.7 to 35.6)	36.2 (32.3 to 39.5)	37.5 (33.7 to 40.7)	14.6 (6.1 to 26.6)	3.6 (-0.9 to 9.1)
Diet low in nuts and seeds	52.3 (51.4 to 53.0)	50.5 (49.0 to 51.7)	49.9 (48.1 to 51.3)	-3.4 (-5.5 to -1.6)	-1.2 (-3.2 to 0.5)	52.3 (51.4 to 53.0)	50.6 (49.1 to 51.8)	50.1 (48.2 to 51.5)	-3.3 (-5.6 to -1.5)	-1.0 (-3.1 to 0.7)
Diet low in milk	45.8 (43.6 to 47.7)	46.1 (44.1 to 47.9)	46.6 (44.6 to 48.3)	0.7 (-1.2 to 3.0)	1.0 (-0.6 to 2.7)	45.0 (42.7 to 47.0)	45.4 (43.1 to 47.2)	46.2 (44.1 to 48.0)	0.8 (-1.4 to 3.1)	1.8 (0.2 to 3.7)
Diet high in red meat	4.2 (0.6 to 9.8)	8.2 (2.6 to 14.7)	9.4 (3.4 to 16.7)	93.5 (-11.3 to 689.5)	14.3 (-38.7 to 120.8)	0.6 (0.0 to 2.8)	1.7 (0.0 to 5.0)	2.2 (0.0 to 6.4)	172.9 (-73.1 to 308438.0)	26.3 (-85.7 to 1910.7)
Diet high in processed meat	12.0 (7.7 to 16.1)	19.9 (14.4 to 25.3)	23.1 (16.6 to 29.0)	66.0 (29.1 to 130.0)	16.0 (-0.9 to 38.8)	10.0 (6.2 to 13.6)	14.6 (10.4 to 18.6)	16.1 (11.6 to 20.2)	46.6 (14.0 to 100.4)	10.2 (-7.4 to 31.4)
Diet high in sugar-sweetened beverages	7.8 (3.1 to 15.6)	8.8 (3.8 to 16.2)	9.2 (3.8 to 16.2)	12.5 (-46.2 to 142.8)	4.3 (-37.7 to 83.5)	5.4 (2.0 to 12.0)	6.1 (2.6 to 11.6)	6.5 (2.6 to 12.8)	12.7 (-50.5 to 149.5)	7.2 (-38.4 to 87.3)
Diet low in fibre	21.7 (14.7 to 28.6)	22.7 (15.4 to 29.1)	23.7 (16.5 to 30.2)	4.5 (-13.4 to 30.0)	4.4 (-9.1 to 20.2)	22.9 (15.6 to 29.4)	23.7 (16.8 to 29.7)	25.0 (17.9 to 31.4)	3.6 (-12.7 to 25.1)	5.6 (-5.8 to 19.7)
Diet low in calcium	34.5 (30.1 to 38.0)	34.0 (29.4 to 37.6)	34.7 (30.3 to 38.3)	-1.4 (-5.7 to 2.4)	2.2 (-1.0 to 6.0)	36.3 (32.4 to 39.7)	35.2 (31.0 to 38.7)	36.4 (32.5 to 39.7)	-3.2 (-7.0 to 0.2)	3.4 (0.6 to 6.8)
Diet low in seafood omega-3 fatty acids	3.1 (1.4 to 4.5)	0.3 (0.0 to 1.0)	0.3 (0.0 to 1.1)	-91.7 (-100.0 to -67.7)	5.8 (-89.5 to 3350.9)	3.5 (1.7 to 5.0)	0.3 (0.0 to 1.4)	0.4 (0.0 to 1.3)	-90.4 (-99.9 to -65.3)	6.3 (-77.7 to 827.1)
Diet low in polyunsaturated fatty acids	30.0 (24.5 to 35.2)	27.3 (21.6 to 32.4)	28.0 (21.8 to 33.0)	-9.1 (-20.9 to 0.7)	2.6 (-6.0 to 12.3)	30.7 (25.2 to 35.8)	27.9 (22.4 to 32.9)	28.6 (22.8 to 33.3)	-8.8 (-20.0 to 0.8)	2.3 (-5.2 to 10.4)
Diet high in trans fatty acids	1.1 (0.0 to 3.3)	0.9 (0.0 to 3.0)	0.9 (0.0 to 2.9)	-12.5 (-38.2 to -6.4)	-8.4 (-33.7 to -3.5)	1.5 (0.1 to 4.0)	1.3 (0.1 to 3.7)	1.2 (0.0 to 3.5)	-12.4 (-34.4 to -6.5)	-8.5 (-28.8 to -3.6)
Diet high in sodium	25.8 (16.4 to 34.1)	18.5 (10.1 to 25.4)	16.4 (7.8 to 23.9)	-28.3 (-49.8 to -9.1)	-11.4 (-33.9 to 6.2)	29.1 (20.6 to 36.1)	19.3 (10.3 to 26.2)	16.4 (7.3 to 23.8)	-33.7 (-54.8 to -18.8)	-14.9 (-37.2 to -1.6)
Intimate partner violence	-	-	-	-	-	2.2 (1.8 to 2.5)	2.0 (1.7 to 2.3)	1.9 (1.6 to 2.2)	-8.0 (-11.9 to -3.8)	-4.5 (-8.4 to 0.3)
Childhood sexual abuse	5.3 (4.3 to 6.5)	5.3 (4.3 to 6.5)	5.3 (4.3 to 6.5)	0.0 (0.0 to 0.0)	0.0 (0.0 to 0.0)	7.3 (5.9 to 8.9)	7.3 (5.9 to 8.9)	7.5 (6.0 to 9.2)	0.0 (0.0 to 0.0)	2.8 (-4.0 to 10.6)

Supplementary Results Table 1. Ishikawa age-standardised Summary Exposure Values (SEVs) for Level 4 risk factors and percentage change, by sex, 1990, 2007, and 2017.Data in parentheses are 95% uncertainty intervals.

	**Male**					**Female**				
	**1990**	**2007**	**2017**	**Percentage change 1990-2007**	**Percentage change 2007-2017**	**1990**	**2007**	**2017**	**Percentage change 1990-2007**	**Percentage change 2007-2017**
Bullying victimization	3.3 (1.3 to 6.7)	3.3 (1.3 to 6.8)	3.4 (1.4 to 7.0)	-0.0 (-7.7 to 8.0)	4.1 (-1.3 to 9.2)	1.9 (0.7 to 3.9)	1.9 (0.8 to 3.9)	2.0 (0.8 to 4.1)	-0.7 (-8.5 to 7.4)	5.1 (-22.5 to 42.3)
Low physical activity	0.5 (0.2 to 0.9)	0.5 (0.2 to 0.9)	0.4 (0.2 to 0.8)	-1.8 (-3.8 to -0.0)	-8.8 (-12.1 to -4.6)	0.5 (0.2 to 1.0)	0.5 (0.2 to 0.9)	0.5 (0.2 to 0.9)	-3.8 (-5.9 to -1.3)	0.7 (-0.9 to 2.3)
High fasting plasma glucose	1.4 (0.9 to 2.2)	1.4 (0.9 to 2.2)	1.6 (1.0 to 2.7)	-0.0 (-36.6 to 59.5)	12.4 (-24.8 to 74.2)	1.2 (0.7 to 1.9)	1.3 (0.7 to 2.1)	1.4 (0.8 to 2.4)	4.5 (-35.8 to 75.8)	10.2 (-30.8 to 92.7)
High LDL cholesterol	12.0 (10.4 to 13.9)	12.6 (11.0 to 14.4)	12.6 (11.0 to 14.5)	4.6 (-1.6 to 10.7)	0.1 (-4.3 to 4.8)	12.9 (11.3 to 14.6)	13.0 (11.5 to 14.7)	13.0 (11.5 to 14.8)	1.1 (-4.0 to 6.8)	0.2 (-4.5 to 5.0)
High systolic blood pressure	11.2 (10.2 to 12.3)	8.4 (7.5 to 9.3)	8.2 (7.2 to 9.2)	-25.6 (-31.6 to -19.7)	-1.6 (-9.2 to 6.2)	9.6 (8.6 to 10.5)	6.0 (5.3 to 6.8)	5.6 (4.8 to 6.5)	-37.0 (-43.9 to -29.7)	-6.9 (-17.1 to 4.2)
High body-mass index	5.9 (3.7 to 9.1)	8.6 (5.8 to 13.5)	8.8 (6.0 to 14.1)	45.6 (17.6 to 86.7)	2.7 (-13.4 to 19.4)	5.6 (3.5 to 8.7)	7.2 (4.6 to 11.2)	7.3 (4.5 to 11.6)	29.0 (-1.3 to 70.3)	0.4 (-19.6 to 22.6)
Low bone mineral density	5.3 (4.3 to 6.3)	5.2 (4.3 to 6.0)	5.2 (4.3 to 6.2)	-2.2 (-15.9 to 11.7)	0.5 (-13.3 to 16.4)	7.2 (6.0 to 8.4)	7.0 (5.9 to 8.1)	7.1 (5.9 to 8.3)	-3.1 (-13.9 to 10.1)	1.4 (-11.0 to 15.5)
Impaired kidney function	2.9 (1.4 to 6.8)	2.8 (1.3 to 6.6)	2.7 (1.3 to 6.5)	-3.3 (-8.2 to 0.7)	-2.4 (-7.9 to 3.1)	3.4 (1.6 to 7.5)	3.4 (1.6 to 7.8)	3.3 (1.5 to 7.4)	2.3 (-2.0 to 6.9)	-5.1 (-9.9 to -0.7)

Supplementary Results Table 1. Iwate age-standardised Summary Exposure Values (SEVs) for Level 4 risk factors and percentage change, by sex, 1990, 2007, and 2017.Data in parentheses are 95% uncertainty intervals.

	Male					Female				
	1990	2007	2017	Percentage change 1990-2007	Percentage change 2007-2017	1990	2007	2017	Percentage change 1990-2007	Percentage change 2007-2017
Unsafe water source	2.8 (1.5 to 4.8)	2.3 (1.2 to 3.8)	2.4 (1.3 to 4.0)	-18.7 (-60.5 to 66.1)	4.4 (-41.1 to 79.4)	2.8 (1.5 to 4.8)	2.3 (1.2 to 3.8)	2.4 (1.3 to 4.0)	-18.7 (-60.5 to 66.1)	4.4 (-41.1 to 79.4)
Unsafe sanitation	5.1 (2.3 to 9.1)	2.5 (1.2 to 4.9)	2.1 (1.0 to 3.9)	-50.5 (-78.8 to 16.1)	-16.4 (-55.3 to 58.9)	5.1 (2.3 to 9.1)	2.5 (1.2 to 4.9)	2.1 (1.0 to 3.9)	-50.5 (-78.8 to 16.1)	-16.4 (-55.3 to 58.9)
No access to handwashing facility	7.0 (5.1 to 9.2)	6.6 (4.9 to 8.8)	6.1 (4.4 to 8.4)	-6.4 (-34.4 to 31.9)	-7.4 (-27.2 to 19.1)	7.0 (5.1 to 9.2)	6.6 (4.9 to 8.8)	6.1 (4.4 to 8.4)	-6.4 (-34.4 to 31.9)	-7.4 (-27.2 to 19.1)
Ambient particulate matter pollution	23.8 (13.9 to 38.3)	24.7 (15.4 to 38.9)	20.2 (8.7 to 33.9)	3.5 (-30.8 to 68.2)	-18.1 (-61.7 to 31.2)	23.2 (13.3 to 37.7)	24.6 (15.3 to 38.8)	20.1 (8.6 to 33.8)	5.8 (-29.8 to 71.6)	-18.1 (-61.8 to 32.1)
Household air pollution from solid fuels	1.8 (0.6 to 6.0)	0.4 (0.1 to 1.0)	0.3 (0.1 to 1.0)	-79.0 (-93.2 to -53.1)	-20.6 (-67.1 to 67.8)	2.4 (0.7 to 8.2)	0.6 (0.1 to 4.3)	0.5 (0.1 to 2.7)	-75.5 (-92.9 to -33.5)	-20.4 (-67.4 to 68.0)
Ambient ozone pollution	45.8 (17.2 to 54.8)	45.9 (17.3 to 54.8)	46.5 (17.8 to 54.8)	0.3 (0.0 to 1.1)	1.1 (0.0 to 3.0)	45.8 (17.2 to 54.8)	45.9 (17.3 to 54.8)	46.5 (17.8 to 54.8)	0.3 (0.0 to 1.1)	1.1 (0.0 to 3.0)
Residential radon	18.9 (2.1 to 57.9)	18.9 (2.1 to 57.9)	18.9 (2.1 to 57.9)	0.0 (0.0 to 0.0)	0.0 (0.0 to 0.0)	18.9 (2.1 to 57.9)	18.9 (2.1 to 57.9)	18.9 (2.1 to 57.9)	0.0 (0.0 to 0.0)	0.0 (0.0 to 0.0)
Lead exposure	2.4 (0.2 to 5.9)	1.8 (0.2 to 4.7)	1.4 (0.1 to 4.0)	-26.8 (-39.3 to -19.0)	-23.4 (-39.1 to -16.0)	1.4 (0.1 to 4.2)	1.0 (0.0 to 3.4)	0.8 (0.0 to 2.9)	-28.1 (-41.8 to -20.7)	-22.7 (-42.9 to -14.3)
Occupational exposure to asbestos	1.4 (1.2 to 1.6)	2.1 (1.9 to 2.3)	1.9 (1.6 to 2.4)	45.6 (25.0 to 72.4)	-6.8 (-24.5 to 15.5)	0.4 (0.3 to 0.5)	0.4 (0.4 to 0.5)	0.4 (0.3 to 0.5)	-5.2 (-22.2 to 21.9)	4.5 (-17.7 to 31.6)
Occupational exposure to arsenic	0.4 (0.0 to 1.0)	0.4 (0.0 to 0.9)	0.4 (0.0 to 0.9)	-	-	0.4 (0.0 to 0.8)	0.3 (0.0 to 0.8)	0.3 (0.0 to 0.8)	-	-
Occupational exposure to benzene	0.6 (0.0 to 1.8)	0.6 (0.0 to 1.8)	0.6 (0.0 to 1.8)	3.7 (-3.3 to 13.5)	3.7 (-2.3 to 11.3)	0.5 (0.0 to 1.5)	0.6 (0.0 to 1.8)	0.7 (0.0 to 2.0)	24.5 (9.5 to 46.4)	15.7 (5.8 to 31.2)
Occupational exposure to beryllium	0.0 (0.0 to 0.0)	0.0 (0.0 to 0.0)	0.0 (0.0 to 0.0)	-4.7 (-9.5 to 0.4)	1.9 (-1.9 to 5.8)	0.0 (0.0 to 0.0)	0.0 (0.0 to 0.0)	0.0 (0.0 to 0.0)	3.9 (-6.5 to 14.9)	10.3 (3.3 to 18.5)
Occupational exposure to cadmium	0.0 (0.0 to 0.1)	0.0 (0.0 to 0.1)	0.0 (0.0 to 0.1)	-4.9 (-16.1 to 7.9)	0.8 (-8.5 to 11.4)	0.0 (0.0 to 0.0)	0.0 (0.0 to 0.0)	0.0 (0.0 to 0.0)	-5.3 (-20.9 to 12.9)	6.5 (-6.7 to 22.0)
Occupational exposure to chromium	0.1 (0.1 to 0.1)	0.1 (0.1 to 0.1)	0.1 (0.1 to 0.1)	-2.7 (-13.5 to 9.5)	1.5 (-6.8 to 10.6)	0.1 (0.1 to 0.1)	0.1 (0.1 to 0.1)	0.1 (0.1 to 0.1)	-0.4 (-15.7 to 16.6)	8.4 (-3.3 to 22.0)
Occupational exposure to diesel engine exhaust	0.5 (0.5 to 0.6)	0.6 (0.5 to 0.6)	0.6 (0.5 to 0.6)	1.2 (-6.8 to 10.9)	3.6 (-3.4 to 10.7)	0.4 (0.3 to 0.4)	0.4 (0.4 to 0.5)	0.5 (0.4 to 0.6)	15.8 (0.7 to 34.1)	13.3 (2.3 to 25.0)
Occupational exposure to formaldehyde	0.2 (0.2 to 0.3)	0.2 (0.2 to 0.2)	0.2 (0.2 to 0.2)	-3.7 (-16.0 to 11.1)	0.5 (-9.7 to 11.9)	0.2 (0.2 to 0.2)	0.2 (0.2 to 0.2)	0.2 (0.2 to 0.2)	-2.3 (-19.4 to 17.4)	7.3 (-6.3 to 23.3)
Occupational exposure to nickel	0.4 (0.0 to 1.7)	0.4 (0.0 to 1.6)	0.4 (0.0 to 1.6)	-	-	0.3 (0.0 to 1.4)	0.3 (0.0 to 1.2)	0.3 (0.0 to 1.3)	-	-
Occupational exposure to polycyclic aromatic hydrocarbons	0.2 (0.2 to 0.2)	0.2 (0.2 to 0.2)	0.2 (0.2 to 0.2)	-1.8 (-11.7 to 9.6)	1.8 (-5.7 to 10.6)	0.2 (0.2 to 0.2)	0.2 (0.2 to 0.2)	0.2 (0.2 to 0.2)	1.6 (-13.5 to 18.2)	9.2 (-2.1 to 21.6)
Occupational exposure to silica	4.1 (0.6 to 13.1)	3.9 (0.6 to 12.5)	4.0 (0.6 to 12.7)	-3.2 (-13.5 to 10.2)	1.5 (-7.2 to 12.2)	2.8 (0.4 to 8.7)	2.5 (0.4 to 7.7)	2.6 (0.3 to 8.1)	-9.7 (-24.4 to 9.5)	3.4 (-10.5 to 22.0)
Occupational exposure to sulphuric acid	0.6 (0.1 to 1.8)	0.5 (0.1 to 1.7)	0.5 (0.1 to 1.7)	-5.4 (-15.2 to 6.5)	1.0 (-7.8 to 10.8)	0.5 (0.1 to 1.5)	0.4 (0.1 to 1.3)	0.5 (0.1 to 1.4)	-7.3 (-22.6 to 9.9)	5.6 (-7.5 to 21.5)
Occupational exposure to trichloroethylene	0.1 (0.1 to 0.1)	0.1 (0.1 to 0.1)	0.1 (0.1 to 0.1)	-2.1 (-9.8 to 6.5)	2.1 (-3.7 to 8.5)	0.1 (0.0 to 0.1)	0.1 (0.0 to 0.1)	0.1 (0.1 to 0.1)	4.2 (-9.0 to 19.0)	10.0 (0.5 to 21.5)
Occupational asthmagens	15.8 (12.8 to 19.9)	16.2 (13.1 to 20.3)	16.3 (13.0 to 20.5)	2.1 (-16.3 to 24.6)	0.6 (-14.4 to 18.0)	7.7 (6.0 to 9.9)	7.8 (6.2 to 9.8)	8.3 (6.6 to 10.6)	2.1 (-18.1 to 24.3)	6.3 (-8.0 to 23.3)
Occupational particulate matter, gases, and fumes	6.3 (4.5 to 9.1)	6.0 (4.3 to 8.6)	5.8 (4.1 to 8.3)	-4.6 (-9.7 to 0.4)	-3.9 (-7.0 to -0.8)	3.4 (2.4 to 5.3)	3.3 (2.3 to 5.0)	3.1 (2.2 to 4.7)	-4.9 (-13.0 to 5.7)	-3.9 (-9.6 to 2.4)
Occupational noise	5.9 (5.4 to 6.5)	5.7 (5.2 to 6.2)	5.5 (5.1 to 6.1)	-3.6 (-6.7 to -0.5)	-2.7 (-4.7 to -0.5)	3.2 (2.7 to 3.8)	3.2 (2.8 to 3.7)	3.3 (2.9 to 3.7)	0.5 (-6.0 to 8.5)	2.0 (-2.6 to 6.7)
Occupational ergonomic factors	12.5 (10.3 to 15.1)	11.1 (9.1 to 13.7)	11.0 (8.9 to 13.4)	-11.4 (-25.7 to 6.5)	-1.0 (-13.9 to 14.2)	6.7 (5.1 to 8.7)	6.6 (5.0 to 8.5)	7.4 (5.6 to 9.7)	-0.4 (-26.8 to 30.9)	11.5 (-11.2 to 36.9)
Non-exclusive breastfeeding	0.4 (0.3 to 0.6)	0.4 (0.3 to 0.6)	0.4 (0.3 to 0.6)	-0.7 (-13.9 to 14.0)	-2.0 (-12.3 to 9.1)	0.4 (0.3 to 0.6)	0.4 (0.3 to 0.6)	0.4 (0.3 to 0.6)	-2.2 (-15.2 to 12.2)	-0.5 (-11.0 to 10.8)
Discontinued breastfeeding	1.7 (1.5 to 1.8)	1.6 (1.5 to 1.8)	1.6 (1.5 to 1.8)	-0.8 (-10.0 to 9.8)	0.3 (-7.3 to 9.3)	1.6 (1.5 to 1.8)	1.7 (1.5 to 1.8)	1.6 (1.5 to 1.8)	2.0 (-7.5 to 12.9)	-2.3 (-9.6 to 6.6)
Child underweight	0.3 (0.2 to 0.4)	0.2 (0.1 to 0.3)	0.2 (0.1 to 0.3)	-16.1 (-35.2 to 6.0)	-5.8 (-21.9 to 13.0)	0.3 (0.2 to 0.4)	0.2 (0.1 to 0.4)	0.2 (0.1 to 0.3)	-17.6 (-37.4 to 7.8)	-6.8 (-22.7 to 12.4)
Child wasting	0.2 (0.1 to 0.3)	0.2 (0.1 to 0.3)	0.2 (0.1 to 0.3)	-6.4 (-32.7 to 34.3)	-2.6 (-23.2 to 23.7)	0.2 (0.1 to 0.3)	0.2 (0.1 to 0.3)	0.2 (0.1 to 0.3)	-8.9 (-36.5 to 31.1)	-2.8 (-24.9 to 24.2)
Child stunting	0.7 (0.3 to 1.2)	0.6 (0.3 to 1.1)	0.6 (0.3 to 1.0)	-8.8 (-42.0 to 45.0)	-5.3 (-33.8 to 34.1)	0.7 (0.3 to 1.3)	0.6 (0.3 to 1.1)	0.6 (0.3 to 1.0)	-10.6 (-47.4 to 50.1)	-5.1 (-33.7 to 34.7)
Short gestation for birth weight	0.0 (0.0 to 0.0)	0.0 (0.0 to 0.0)	0.0 (0.0 to 0.0)	8.0 (-18.8 to 40.4)	1.5 (-24.5 to 41.1)	0.0 (0.0 to 0.0)	0.0 (0.0 to 0.0)	0.0 (0.0 to 0.0)	14.0 (-20.2 to 89.6)	-1.4 (-20.9 to 34.7)
Low birth weight for gestation	0.0 (0.0 to 0.0)	0.0 (0.0 to 0.0)	0.0 (0.0 to 0.0)	3.3 (-17.3 to 27.2)	2.9 (-21.8 to 31.5)	0.0 (0.0 to 0.0)	0.0 (0.0 to 0.0)	0.0 (0.0 to 0.0)	-5.6 (-58.2 to 33.0)	5.0 (-17.0 to 27.1)
Iron deficiency	-	-	-	-	-	8.5 (5.9 to 11.6)	8.5 (5.9 to 11.7)	7.8 (5.3 to 10.9)	-0.4 (-21.7 to 27.3)	-8.1 (-23.7 to 9.1)
Vitamin A deficiency	1.1 (0.7 to 1.5)	0.8 (0.5 to 1.1)	0.8 (0.5 to 1.0)	-22.9 (-38.1 to -0.1)	-7.2 (-20.3 to 7.1)	0.9 (0.5 to 1.2)	0.9 (0.5 to 1.1)	0.8 (0.5 to 1.0)	-0.2 (-19.5 to 18.2)	-10.1 (-23.6 to 4.9)
Zinc deficiency	0.5 (0.2 to 1.1)	0.4 (0.1 to 1.1)	0.4 (0.1 to 0.9)	-16.3 (-64.1 to 72.3)	-10.1 (-66.1 to 160.4)	0.5 (0.1 to 1.1)	0.4 (0.1 to 0.8)	0.4 (0.1 to 0.8)	-31.4 (-63.6 to 7.0)	10.4 (-60.0 to 169.4)
Smoking	18.3 (14.9 to 21.7)	12.9 (10.3 to 15.5)	10.9 (8.7 to 13.1)	-29.6 (-38.4 to -19.4)	-15.4 (-23.2 to -6.9)	4.2 (3.0 to 5.5)	3.5 (2.5 to 4.7)	3.4 (2.4 to 4.7)	-16.8 (-35.9 to 7.6)	-2.2 (-18.1 to 19.7)
Chewing tobacco	1.4 (0.9 to 2.0)	0.9 (0.6 to 1.4)	0.8 (0.5 to 1.1)	-32.9 (-58.1 to 4.6)	-18.9 (-41.3 to 14.6)	0.1 (0.1 to 0.1)	0.1 (0.1 to 0.2)	0.1 (0.1 to 0.1)	-4.0 (-42.7 to 60.1)	-2.7 (-33.2 to 41.6)
Second-hand smoke	37.3 (33.0 to 41.2)	36.2 (32.2 to 40.5)	35.3 (31.2 to 39.7)	-2.8 (-14.3 to 10.1)	-2.7 (-11.3 to 6.9)	56.4 (52.9 to 60.3)	48.7 (45.1 to 52.6)	47.3 (43.0 to 51.1)	-13.6 (-19.4 to -7.1)	-2.9 (-9.0 to 3.3)
Alcohol use	14.8 (7.4 to 25.8)	14.9 (7.4 to 25.7)	17.0 (8.1 to 30.0)	0.2 (-35.5 to 49.5)	14.7 (-20.7 to 59.1)	11.4 (5.5 to 20.6)	11.4 (5.6 to 19.7)	13.0 (6.4 to 23.2)	-0.1 (-35.8 to 52.4)	14.8 (-20.1 to 63.2)
Drug use	0.5 (0.4 to 0.7)	0.5 (0.4 to 0.7)	0.6 (0.4 to 0.8)	-0.2 (-6.0 to 5.8)	11.9 (5.2 to 20.7)	0.3 (0.2 to 0.4)	0.3 (0.2 to 0.4)	0.3 (0.3 to 0.4)	1.5 (-4.0 to 7.5)	9.4 (2.6 to 17.3)
Diet low in fruits	39.2 (33.2 to 43.2)	39.2 (33.1 to 43.1)	40.8 (35.0 to 44.7)	-0.2 (-6.8 to 7.2)	4.1 (-0.7 to 9.5)	35.9 (29.9 to 40.8)	35.2 (29.1 to 40.0)	37.8 (32.0 to 42.3)	-2.0 (-11.0 to 7.2)	7.2 (0.9 to 15.6)
Diet low in vegetables	18.4 (10.1 to 26.0)	13.5 (5.6 to 21.3)	15.7 (7.4 to 24.1)	-26.4 (-55.7 to 1.8)	16.5 (-14.4 to 76.5)	20.8 (12.4 to 28.5)	15.8 (7.7 to 23.7)	17.8 (9.6 to 25.5)	-24.0 (-50.1 to -1.3)	12.8 (-13.0 to 54.7)
Diet low in legumes	4.2 (0.6 to 9.3)	4.9 (0.9 to 10.0)	6.2 (1.4 to 12.3)	16.5 (-52.6 to 225.8)	26.5 (-34.5 to 212.1)	8.0 (2.4 to 14.2)	7.1 (1.8 to 13.2)	9.1 (2.9 to 15.8)	-11.0 (-56.4 to 65.1)	27.2 (-16.3 to 126.4)
Diet low in whole grains	31.0 (25.9 to 35.1)	35.7 (31.7 to 39.2)	37.0 (32.9 to 40.3)	15.0 (6.1 to 26.9)	3.7 (-1.3 to 8.8)	32.3 (27.7 to 36.4)	36.8 (33.1 to 40.2)	38.1 (34.3 to 41.0)	14.0 (5.9 to 25.4)	3.4 (-0.9 to 8.0)
Diet low in nuts and seeds	50.6 (49.1 to 51.6)	49.4 (47.6 to 50.7)	49.2 (47.2 to 50.7)	-2.3 (-4.6 to -0.3)	-0.4 (-2.4 to 1.6)	50.4 (49.0 to 51.5)	49.4 (47.5 to 50.8)	49.2 (47.1 to 50.8)	-2.1 (-4.6 to 0.1)	-0.3 (-2.4 to 1.7)
Diet low in milk	45.2 (42.8 to 47.1)	46.3 (44.3 to 48.0)	47.0 (45.1 to 48.6)	2.6 (0.5 to 5.0)	1.4 (-0.0 to 3.0)	45.0 (42.8 to 47.0)	45.6 (43.5 to 47.5)	46.4 (44.3 to 48.2)	1.3 (-1.0 to 3.8)	1.8 (0.1 to 3.6)
Diet high in red meat	3.5 (0.4 to 8.3)	7.1 (1.9 to 13.6)	8.2 (2.3 to 15.4)	103.0 (-22.1 to 967.0)	15.0 (-38.6 to 140.4)	0.5 (0.0 to 2.1)	1.4 (0.0 to 4.4)	1.8 (0.0 to 5.7)	189.3 (-81.7 to 8745508.2)	35.2 (-89.3 to 3085.1)
Diet high in processed meat	10.5 (6.4 to 14.6)	17.2 (12.4 to 22.1)	19.8 (14.5 to 25.0)	64.5 (27.9 to 130.2)	15.2 (-2.7 to 37.2)	8.7 (5.0 to 12.5)	12.6 (8.5 to 16.3)	13.7 (9.4 to 17.7)	44.3 (12.4 to 104.9)	8.8 (-8.5 to 31.0)
Diet high in sugar-sweetened beverages	6.6 (2.6 to 13.2)	7.6 (3.4 to 14.3)	8.0 (3.3 to 14.8)	15.0 (-49.1 to 140.2)	4.8 (-37.7 to 84.8)	4.7 (1.7 to 10.3)	5.4 (2.4 to 11.0)	5.7 (2.3 to 11.2)	15.2 (-50.4 to 192.6)	5.5 (-41.8 to 84.2)
Diet low in fibre	22.2 (14.7 to 29.0)	22.9 (15.9 to 29.2)	23.7 (16.3 to 30.2)	3.0 (-14.4 to 26.3)	3.5 (-10.1 to 17.9)	23.5 (16.4 to 30.2)	24.2 (17.0 to 30.4)	25.4 (18.2 to 31.6)	3.1 (-13.8 to 25.3)	4.9 (-7.0 to 19.1)
Diet low in calcium	34.0 (29.4 to 37.7)	34.5 (29.9 to 38.0)	35.4 (31.0 to 39.0)	1.4 (-2.3 to 5.9)	2.8 (-0.2 to 6.4)	36.2 (32.3 to 39.6)	35.8 (31.6 to 39.2)	37.1 (33.1 to 40.3)	-1.3 (-5.3 to 2.1)	3.7 (1.0 to 7.2)
Diet low in seafood omega-3 fatty acids	3.4 (1.8 to 4.7)	0.4 (0.0 to 1.3)	0.4 (0.0 to 1.4)	-88.2 (-99.9 to -60.2)	5.0 (-78.1 to 600.3)	3.9 (2.3 to 5.3)	0.5 (0.0 to 1.7)	0.5 (0.0 to 1.7)	-87.1 (-99.8 to -58.9)	6.8 (-73.3 to 517.4)
Diet low in polyunsaturated fatty acids	31.2 (25.6 to 36.1)	28.5 (22.9 to 33.3)	29.1 (23.4 to 34.0)	-8.8 (-20.1 to 0.8)	2.3 (-4.4 to 10.6)	31.8 (26.7 to 36.4)	29.2 (23.7 to 34.2)	29.8 (24.2 to 34.8)	-8.3 (-19.1 to 0.6)	2.3 (-5.2 to 10.5)
Diet high in trans fatty acids	1.1 (0.0 to 3.3)	0.9 (0.0 to 3.1)	0.9 (0.0 to 2.9)	-12.5 (-38.7 to -6.5)	-8.2 (-32.0 to -3.3)	1.5 (0.1 to 4.0)	1.3 (0.1 to 3.7)	1.2 (0.0 to 3.5)	-12.4 (-36.4 to -6.4)	-8.5 (-28.1 to -3.9)
Diet high in sodium	30.8 (20.9 to 38.2)	20.0 (10.9 to 27.4)	17.4 (8.1 to 24.6)	-34.9 (-57.1 to -17.4)	-13.3 (-36.8 to 3.4)	34.6 (27.0 to 41.4)	21.5 (13.6 to 28.0)	18.2 (9.8 to 25.1)	-38.0 (-56.5 to -24.1)	-15.2 (-34.6 to -2.0)
Intimate partner violence	-	-	-	-	-	2.3 (1.9 to 2.6)	2.1 (1.8 to 2.4)	2.0 (1.7 to 2.3)	-7.9 (-12.3 to -3.5)	-4.1 (-8.1 to 0.3)
Childhood sexual abuse	5.3 (4.3 to 6.5)	5.3 (4.3 to 6.5)	5.3 (4.3 to 6.5)	0.0 (0.0 to 0.0)	0.0 (0.0 to 0.0)	7.3 (5.9 to 8.9)	7.3 (5.9 to 8.9)	7.5 (6.0 to 9.2)	0.0 (0.0 to 0.0)	2.8 (-4.0 to 10.6)

Supplementary Results Table 1. Iwate age-standardised Summary Exposure Values (SEVs) for Level 4 risk factors and percentage change, by sex, 1990, 2007, and 2017.Data in parentheses are 95% uncertainty intervals.										
	**Male**					**Female**				
	**1990**	**2007**	**2017**	**Percentage change 1990-2007**	**Percentage change 2007-2017**	**1990**	**2007**	**2017**	**Percentage change 1990-2007**	**Percentage change 2007-2017**
Bullying victimization	3.3 (1.4 to 6.8)	3.4 (1.3 to 7.1)	3.5 (1.4 to 7.4)	2.9 (-4.7 to 10.7)	3.0 (-2.0 to 7.6)	1.9 (0.8 to 4.0)	2.0 (0.8 to 4.0)	2.0 (0.8 to 4.2)	0.8 (-7.0 to 8.6)	2.5 (-24.0 to 38.2)
Low physical activity	0.5 (0.2 to 0.9)	0.5 (0.2 to 0.9)	0.4 (0.2 to 0.8)	-1.7 (-3.7 to 0.1)	-8.7 (-12.1 to -4.5)	0.5 (0.2 to 1.0)	0.5 (0.2 to 0.9)	0.5 (0.2 to 0.9)	-3.9 (-6.2 to -1.5)	0.7 (-0.9 to 2.3)
High fasting plasma glucose	1.4 (0.9 to 2.3)	1.6 (0.9 to 2.5)	1.7 (1.0 to 2.8)	10.2 (-30.7 to 87.1)	5.8 (-32.4 to 75.5)	1.2 (0.7 to 2.0)	1.4 (0.8 to 2.3)	1.5 (0.8 to 2.6)	16.5 (-31.6 to 102.5)	5.8 (-37.1 to 74.4)
High LDL cholesterol	11.4 (9.8 to 13.2)	11.7 (10.1 to 13.5)	11.8 (10.3 to 13.5)	2.6 (-2.9 to 8.9)	1.0 (-3.4 to 5.7)	11.9 (10.4 to 13.6)	11.9 (10.3 to 13.6)	12.0 (10.5 to 13.8)	-0.5 (-5.7 to 5.0)	1.4 (-3.1 to 5.6)
High systolic blood pressure	8.8 (7.9 to 9.8)	5.8 (5.0 to 6.5)	6.2 (5.4 to 7.0)	-34.5 (-40.3 to -28.3)	6.7 (-2.0 to 15.6)	7.0 (6.2 to 7.8)	3.8 (3.3 to 4.3)	3.9 (3.3 to 4.6)	-46.2 (-52.5 to -39.6)	4.2 (-6.7 to 17.2)
High body-mass index	4.3 (2.5 to 6.9)	5.9 (3.6 to 9.5)	6.1 (3.7 to 9.7)	39.1 (10.8 to 81.2)	2.8 (-12.6 to 20.8)	4.9 (2.9 to 7.8)	6.3 (3.8 to 10.2)	6.3 (3.8 to 10.2)	27.1 (-0.5 to 71.4)	0.7 (-18.4 to 22.9)
Low bone mineral density	5.3 (4.4 to 6.4)	5.2 (4.4 to 6.1)	5.2 (4.3 to 6.1)	-2.1 (-15.4 to 12.0)	-0.0 (-12.9 to 14.1)	6.6 (5.7 to 7.5)	6.5 (5.7 to 7.4)	6.6 (5.5 to 7.9)	-1.2 (-11.7 to 11.4)	2.1 (-12.7 to 17.0)
Impaired kidney function	2.5 (1.1 to 6.3)	2.4 (1.1 to 6.1)	2.3 (1.0 to 5.9)	-4.2 (-9.2 to 0.7)	-5.0 (-10.5 to -0.7)	3.0 (1.4 to 7.2)	3.1 (1.4 to 7.2)	2.9 (1.3 to 6.9)	1.5 (-2.1 to 5.8)	-5.0 (-9.8 to -1.0)

Supplementary Results Table 1. Kagawa age-standardised Summary Exposure Values (SEVs) for Level 4 risk factors and percentage change, by sex, 1990, 2007, and 2017.Data in parentheses are 95% uncertainty intervals.

	Male					Female				
	1990	2007	2017	Percentage change 1990-2007	Percentage change 2007-2017	1990	2007	2017	Percentage change 1990-2007	Percentage change 2007-2017
Unsafe water source	2.3 (1.2 to 3.9)	1.9 (1.0 to 3.1)	2.0 (1.1 to 3.4)	-15.9 (-57.2 to 58.2)	5.2 (-39.6 to 84.3)	2.3 (1.2 to 3.9)	1.9 (1.0 to 3.1)	2.0 (1.1 to 3.4)	-15.9 (-57.2 to 58.2)	5.2 (-39.6 to 84.3)
Unsafe sanitation	3.9 (1.9 to 7.1)	1.9 (0.9 to 3.8)	1.7 (0.8 to 3.3)	-50.7 (-79.5 to 9.8)	-13.5 (-56.3 to 65.6)	3.9 (1.9 to 7.1)	1.9 (0.9 to 3.8)	1.7 (0.8 to 3.3)	-50.7 (-79.5 to 9.8)	-13.5 (-56.3 to 65.6)
No access to handwashing facility	6.8 (4.9 to 9.0)	6.3 (4.5 to 8.6)	5.8 (4.3 to 7.7)	-6.8 (-33.9 to 31.9)	-7.5 (-28.1 to 18.9)	6.8 (4.9 to 9.0)	6.3 (4.5 to 8.6)	5.8 (4.3 to 7.7)	-6.8 (-33.9 to 31.9)	-7.5 (-28.1 to 18.9)
Ambient particulate matter pollution	31.6 (20.4 to 47.3)	31.8 (21.4 to 47.1)	27.2 (17.0 to 41.6)	0.7 (-23.9 to 35.0)	-14.5 (-38.1 to 12.9)	31.4 (20.3 to 47.1)	31.8 (21.4 to 47.0)	27.2 (17.0 to 41.6)	1.2 (-23.7 to 36.2)	-14.5 (-38.2 to 12.9)
Household air pollution from solid fuels	0.6 (0.2 to 1.6)	0.1 (0.0 to 0.3)	0.1 (0.0 to 0.2)	-75.1 (-93.1 to -45.8)	-13.4 (-66.3 to 75.7)	0.8 (0.2 to 5.5)	0.2 (0.0 to 1.0)	0.2 (0.0 to 0.9)	-71.3 (-93.0 to -39.0)	-11.7 (-65.6 to 76.3)
Ambient ozone pollution	49.9 (21.5 to 54.8)	51.3 (24.2 to 54.8)	52.2 (27.0 to 54.8)	2.8 (0.0 to 13.5)	1.9 (0.0 to 12.7)	49.9 (21.5 to 54.8)	51.3 (24.2 to 54.8)	52.2 (27.0 to 54.8)	2.8 (0.0 to 13.5)	1.9 (0.0 to 12.7)
Residential radon	13.0 (0.9 to 44.6)	13.0 (0.9 to 44.6)	13.0 (0.9 to 44.6)	0.0 (0.0 to 0.0)	0.0 (0.0 to 0.0)	13.0 (0.9 to 44.6)	13.0 (0.9 to 44.6)	13.0 (0.9 to 44.6)	0.0 (0.0 to 0.0)	0.0 (0.0 to 0.0)
Lead exposure	3.3 (0.6 to 7.0)	2.2 (0.3 to 5.2)	1.5 (0.2 to 4.1)	-34.8 (-49.1 to -25.1)	-29.4 (-45.2 to -21.3)	1.7 (0.1 to 4.7)	1.0 (0.1 to 3.3)	0.7 (0.0 to 2.5)	-40.6 (-58.3 to -30.4)	-32.4 (-54.5 to -22.7)
Occupational exposure to asbestos	2.6 (2.2 to 2.9)	3.4 (3.1 to 3.7)	3.4 (2.8 to 4.1)	33.0 (15.2 to 57.7)	-0.7 (-17.9 to 20.1)	0.6 (0.5 to 0.7)	0.6 (0.5 to 0.7)	0.6 (0.5 to 0.7)	0.6 (-15.9 to 25.2)	-5.4 (-24.5 to 18.2)
Occupational exposure to arsenic	0.5 (0.0 to 1.1)	0.4 (0.0 to 1.0)	0.4 (0.0 to 1.0)	-	-	0.4 (0.0 to 1.0)	0.4 (0.0 to 0.9)	0.4 (0.0 to 0.9)	-	-
Occupational exposure to benzene	0.6 (0.0 to 1.8)	0.7 (0.0 to 1.8)	0.7 (0.0 to 2.0)	2.6 (-4.5 to 13.2)	3.5 (-2.1 to 11.4)	0.6 (0.0 to 1.7)	0.7 (0.0 to 2.0)	0.8 (0.0 to 2.4)	21.2 (7.8 to 41.2)	14.5 (4.7 to 29.9)
Occupational exposure to beryllium	0.0 (0.0 to 0.0)	0.0 (0.0 to 0.0)	0.0 (0.0 to 0.0)	-5.2 (-10.3 to 0.3)	1.9 (-2.0 to 5.9)	0.0 (0.0 to 0.0)	0.0 (0.0 to 0.0)	0.0 (0.0 to 0.0)	1.9 (-7.0 to 10.9)	9.1 (2.4 to 16.2)
Occupational exposure to cadmium	0.1 (0.0 to 0.1)	0.0 (0.0 to 0.1)	0.0 (0.0 to 0.1)	-6.7 (-18.6 to 5.9)	0.8 (-8.7 to 11.2)	0.0 (0.0 to 0.1)	0.0 (0.0 to 0.1)	0.0 (0.0 to 0.1)	-9.2 (-23.6 to 9.2)	4.9 (-7.0 to 19.6)
Occupational exposure to chromium	0.1 (0.1 to 0.1)	0.1 (0.1 to 0.1)	0.1 (0.1 to 0.1)	-5.1 (-15.9 to 6.3)	1.3 (-7.1 to 10.0)	0.1 (0.1 to 0.1)	0.1 (0.1 to 0.1)	0.1 (0.1 to 0.1)	-4.4 (-17.9 to 12.4)	6.6 (-3.8 to 18.9)
Occupational exposure to diesel engine exhaust	0.6 (0.6 to 0.6)	0.6 (0.5 to 0.6)	0.6 (0.6 to 0.6)	-2.4 (-10.6 to 6.1)	3.0 (-3.5 to 9.9)	0.5 (0.4 to 0.5)	0.5 (0.4 to 0.6)	0.6 (0.5 to 0.6)	12.0 (-1.4 to 26.5)	11.0 (1.2 to 22.6)
Occupational exposure to formaldehyde	0.3 (0.2 to 0.3)	0.2 (0.2 to 0.3)	0.2 (0.2 to 0.3)	-6.1 (-18.9 to 7.7)	0.4 (-9.6 to 11.0)	0.2 (0.2 to 0.3)	0.2 (0.2 to 0.2)	0.2 (0.2 to 0.2)	-6.5 (-21.5 to 13.2)	5.7 (-6.0 to 20.2)
Occupational exposure to nickel	0.4 (0.0 to 1.9)	0.4 (0.0 to 1.7)	0.4 (0.0 to 1.7)	-	-	0.4 (0.0 to 1.6)	0.3 (0.0 to 1.3)	0.3 (0.0 to 1.3)	-	-
Occupational exposure to polycyclic aromatic hydrocarbons	0.2 (0.2 to 0.3)	0.2 (0.2 to 0.3)	0.2 (0.2 to 0.3)	-4.2 (-13.6 to 6.5)	1.7 (-5.9 to 9.8)	0.2 (0.2 to 0.3)	0.2 (0.2 to 0.2)	0.2 (0.2 to 0.3)	-2.3 (-15.3 to 13.9)	7.5 (-2.0 to 18.8)
Occupational exposure to silica	4.7 (0.7 to 15.2)	4.4 (0.6 to 14.4)	4.5 (0.7 to 14.4)	-6.7 (-16.1 to 5.0)	1.4 (-7.3 to 12.3)	3.2 (0.5 to 10.4)	2.8 (0.4 to 8.9)	2.8 (0.4 to 8.7)	-14.0 (-28.7 to 4.8)	1.6 (-10.4 to 18.9)
Occupational exposure to sulphuric acid	0.6 (0.1 to 2.0)	0.6 (0.1 to 1.8)	0.6 (0.1 to 1.8)	-6.9 (-17.6 to 5.8)	1.2 (-8.1 to 11.1)	0.5 (0.1 to 1.7)	0.5 (0.1 to 1.5)	0.5 (0.1 to 1.5)	-10.8 (-25.4 to 7.7)	3.9 (-8.1 to 19.4)
Occupational exposure to trichloroethylene	0.1 (0.1 to 0.1)	0.1 (0.1 to 0.1)	0.1 (0.1 to 0.1)	-4.2 (-11.5 to 4.1)	1.8 (-4.4 to 8.0)	0.1 (0.1 to 0.1)	0.1 (0.1 to 0.1)	0.1 (0.1 to 0.1)	0.6 (-10.0 to 13.9)	8.2 (-0.3 to 18.4)
Occupational asthmagens	19.0 (15.2 to 23.8)	18.3 (14.5 to 23.1)	18.2 (14.3 to 22.8)	-3.8 (-22.5 to 20.9)	-0.3 (-15.2 to 16.9)	8.3 (6.6 to 10.5)	8.3 (6.6 to 10.2)	8.7 (6.9 to 10.9)	-0.6 (-15.7 to 17.8)	5.4 (-7.5 to 19.6)
Occupational particulate matter, gases, and fumes	6.4 (4.6 to 9.1)	6.1 (4.4 to 8.8)	5.9 (4.2 to 8.6)	-4.5 (-9.2 to 1.0)	-3.7 (-6.9 to -0.3)	3.7 (2.5 to 5.6)	3.5 (2.4 to 5.2)	3.3 (2.3 to 4.9)	-5.8 (-13.9 to 4.0)	-4.8 (-10.6 to 1.2)
Occupational noise	6.0 (5.5 to 6.6)	5.8 (5.4 to 6.3)	5.6 (5.2 to 6.2)	-3.4 (-6.5 to -0.3)	-2.5 (-4.5 to -0.4)	3.5 (3.0 to 4.1)	3.5 (3.1 to 4.0)	3.6 (3.2 to 4.1)	0.2 (-5.3 to 6.8)	1.7 (-2.2 to 6.0)
Occupational ergonomic factors	10.4 (8.7 to 12.5)	9.2 (7.6 to 11.2)	9.1 (7.4 to 11.1)	-11.2 (-22.3 to 1.8)	-1.2 (-12.5 to 10.8)	5.6 (4.3 to 7.2)	5.7 (4.4 to 7.2)	6.4 (4.8 to 8.1)	1.4 (-21.6 to 28.2)	12.3 (-6.5 to 33.2)
Non-exclusive breastfeeding	0.4 (0.3 to 0.6)	0.4 (0.3 to 0.6)	0.4 (0.3 to 0.6)	-1.0 (-14.7 to 13.6)	-1.6 (-12.4 to 10.7)	0.4 (0.3 to 0.6)	0.4 (0.3 to 0.6)	0.4 (0.3 to 0.6)	-1.3 (-15.0 to 13.3)	-1.6 (-12.4 to 10.7)
Discontinued breastfeeding	1.7 (1.5 to 1.8)	1.7 (1.6 to 1.9)	1.7 (1.5 to 1.8)	2.7 (-6.8 to 13.9)	-1.7 (-9.0 to 6.1)	1.7 (1.6 to 1.8)	1.7 (1.6 to 1.9)	1.7 (1.5 to 1.8)	2.4 (-7.1 to 13.5)	-3.4 (-10.5 to 4.4)
Child underweight	0.3 (0.2 to 0.4)	0.2 (0.1 to 0.3)	0.2 (0.1 to 0.3)	-15.2 (-33.1 to 7.0)	-5.3 (-19.6 to 11.1)	0.3 (0.2 to 0.4)	0.2 (0.1 to 0.3)	0.2 (0.1 to 0.3)	-16.6 (-38.4 to 9.5)	-6.5 (-23.0 to 13.4)
Child wasting	0.2 (0.1 to 0.3)	0.2 (0.1 to 0.3)	0.2 (0.1 to 0.3)	-7.1 (-33.5 to 28.2)	-2.2 (-21.9 to 20.1)	0.2 (0.1 to 0.3)	0.2 (0.1 to 0.3)	0.2 (0.1 to 0.3)	-7.3 (-33.7 to 29.4)	-2.8 (-23.7 to 24.2)
Child stunting	0.6 (0.3 to 1.1)	0.6 (0.3 to 1.0)	0.6 (0.3 to 1.0)	-8.6 (-46.6 to 54.3)	-3.0 (-33.3 to 39.1)	0.7 (0.3 to 1.1)	0.6 (0.3 to 1.0)	0.6 (0.3 to 1.0)	-11.4 (-48.4 to 54.8)	-3.9 (-33.9 to 37.4)
Short gestation for birth weight	0.0 (0.0 to 0.0)	0.0 (0.0 to 0.0)	0.0 (0.0 to 0.0)	10.3 (-11.9 to 37.3)	1.8 (-11.7 to 22.4)	0.0 (0.0 to 0.0)	0.0 (0.0 to 0.0)	0.0 (0.0 to 0.0)	12.8 (-19.8 to 96.1)	1.9 (-19.4 to 30.8)
Low birth weight for gestation	0.0 (0.0 to 0.0)	0.0 (0.0 to 0.0)	0.0 (0.0 to 0.0)	4.3 (-12.4 to 32.5)	1.7 (-12.8 to 17.1)	0.0 (0.0 to 0.0)	0.0 (0.0 to 0.0)	0.0 (0.0 to 0.0)	-4.9 (-63.8 to 31.7)	2.6 (-15.9 to 24.4)
Iron deficiency	-	-	-	-	-	8.0 (5.5 to 11.0)	8.0 (5.6 to 11.1)	7.3 (4.9 to 10.3)	-0.4 (-21.7 to 28.3)	-8.3 (-23.7 to 9.3)
Vitamin A deficiency	0.9 (0.6 to 1.2)	0.8 (0.5 to 1.1)	0.6 (0.4 to 0.9)	-10.7 (-26.9 to 7.1)	-22.4 (-37.7 to -3.8)	0.7 (0.4 to 1.0)	0.8 (0.5 to 1.0)	0.7 (0.4 to 0.9)	12.7 (-2.5 to 29.1)	-14.7 (-27.5 to 0.6)
Zinc deficiency	0.5 (0.1 to 1.0)	0.5 (0.1 to 1.2)	0.4 (0.1 to 1.1)	-2.2 (-63.2 to 75.3)	-14.7 (-68.1 to 155.8)	0.5 (0.1 to 1.0)	0.3 (0.1 to 0.8)	0.4 (0.1 to 0.8)	-30.0 (-64.9 to 66.5)	1.1 (-64.3 to 164.8)
Smoking	17.4 (13.9 to 20.8)	12.2 (9.8 to 14.7)	10.3 (8.2 to 12.5)	-30.0 (-39.3 to -19.2)	-15.7 (-23.8 to -6.3)	3.9 (2.7 to 5.2)	3.3 (2.3 to 4.5)	3.2 (2.2 to 4.3)	-16.2 (-36.2 to 11.0)	-3.0 (-20.0 to 17.4)
Chewing tobacco	1.4 (0.9 to 2.0)	0.9 (0.6 to 1.4)	0.8 (0.5 to 1.1)	-33.1 (-56.8 to 6.6)	-18.9 (-41.7 to 12.6)	0.1 (0.1 to 0.1)	0.1 (0.1 to 0.1)	0.1 (0.1 to 0.1)	-4.2 (-42.8 to 58.3)	-2.0 (-32.5 to 41.7)
Second-hand smoke	36.7 (32.6 to 40.8)	36.3 (32.2 to 40.5)	35.7 (31.3 to 40.2)	-1.2 (-12.7 to 11.5)	-1.4 (-10.6 to 8.1)	55.4 (51.4 to 58.8)	48.6 (44.7 to 52.3)	47.7 (43.5 to 51.8)	-12.2 (-18.9 to -5.3)	-1.9 (-7.6 to 4.4)
Alcohol use	14.9 (7.3 to 25.6)	14.8 (7.3 to 25.3)	17.1 (8.0 to 29.7)	-0.6 (-34.4 to 50.8)	15.5 (-18.6 to 62.7)	11.8 (5.6 to 20.7)	11.6 (5.6 to 20.3)	13.2 (6.0 to 22.9)	-1.3 (-35.0 to 50.3)	13.7 (-21.7 to 66.8)
Drug use	0.5 (0.4 to 0.7)	0.5 (0.4 to 0.7)	0.6 (0.4 to 0.8)	0.9 (-5.3 to 7.1)	11.0 (3.8 to 19.8)	0.3 (0.2 to 0.4)	0.3 (0.2 to 0.4)	0.3 (0.3 to 0.4)	2.2 (-3.4 to 8.5)	8.8 (2.0 to 16.1)
Diet low in fruits	40.7 (34.7 to 44.4)	39.1 (33.1 to 43.2)	40.4 (34.6 to 44.4)	-4.0 (-10.4 to 2.3)	3.4 (-1.8 to 9.1)	37.7 (31.7 to 42.0)	35.0 (28.7 to 39.7)	37.2 (31.5 to 41.8)	-7.0 (-15.6 to 1.4)	6.2 (-0.6 to 14.6)
Diet low in vegetables	19.4 (11.2 to 27.0)	13.3 (5.6 to 21.2)	15.3 (7.0 to 23.6)	-31.1 (-60.4 to -6.7)	14.3 (-16.6 to 71.4)	21.5 (13.2 to 28.8)	15.6 (7.6 to 24.0)	17.5 (9.2 to 25.1)	-27.6 (-53.4 to -3.9)	12.4 (-14.7 to 56.3)
Diet low in legumes	4.4 (0.8 to 9.9)	5.6 (1.1 to 11.7)	7.5 (1.8 to 14.3)	26.4 (-44.4 to 219.0)	33.5 (-22.3 to 197.4)	8.3 (2.5 to 14.9)	8.1 (2.3 to 14.6)	10.6 (4.1 to 17.7)	-2.3 (-50.1 to 86.8)	31.0 (-12.1 to 132.7)
Diet low in whole grains	30.4 (25.7 to 34.4)	35.1 (31.1 to 38.6)	36.5 (32.7 to 39.8)	15.6 (6.9 to 27.4)	3.9 (-1.0 to 9.7)	31.8 (27.0 to 36.0)	36.4 (32.5 to 39.8)	37.7 (33.9 to 40.9)	14.5 (6.6 to 25.4)	3.4 (-1.1 to 8.1)
Diet low in nuts and seeds	50.7 (49.3 to 51.8)	49.4 (47.6 to 50.8)	49.2 (47.1 to 50.7)	-2.6 (-5.1 to -0.2)	-0.4 (-2.6 to 1.5)	50.8 (49.5 to 51.9)	49.4 (47.6 to 50.8)	49.3 (47.3 to 50.7)	-2.7 (-5.3 to -0.6)	-0.3 (-2.4 to 1.6)
Diet low in milk	46.1 (43.9 to 47.8)	46.4 (44.4 to 48.1)	46.9 (44.9 to 48.5)	0.7 (-1.3 to 2.7)	1.0 (-0.6 to 2.6)	46.1 (43.9 to 47.9)	45.8 (43.6 to 47.7)	46.4 (44.3 to 48.2)	-0.6 (-2.7 to 1.5)	1.3 (-0.3 to 3.1)
Diet high in red meat	4.2 (0.5 to 9.8)	8.1 (2.8 to 15.0)	9.2 (3.1 to 16.8)	93.2 (-14.1 to 803.3)	14.0 (-39.0 to 110.3)	0.6 (0.0 to 2.7)	1.7 (0.0 to 5.1)	2.2 (0.1 to 6.1)	165.4 (-79.9 to 785432.6)	29.6 (-81.0 to 3134.3)
Diet high in processed meat	11.5 (7.6 to 15.5)	19.0 (13.7 to 24.1)	21.9 (15.3 to 27.4)	65.2 (27.8 to 125.7)	14.9 (-3.2 to 38.3)	9.7 (6.0 to 13.3)	14.2 (10.0 to 18.2)	15.6 (11.2 to 19.9)	46.7 (13.4 to 101.7)	9.7 (-7.3 to 30.3)
Diet high in sugar-sweetened beverages	7.5 (3.1 to 14.8)	8.7 (3.9 to 15.6)	9.2 (3.8 to 17.0)	15.8 (-43.6 to 153.7)	6.4 (-39.1 to 81.7)	5.2 (1.9 to 11.4)	6.0 (2.5 to 11.3)	6.3 (2.5 to 11.9)	14.5 (-47.9 to 163.6)	5.6 (-41.7 to 88.3)
Diet low in fibre	21.9 (14.4 to 28.2)	22.8 (16.1 to 29.2)	23.8 (17.2 to 30.4)	4.4 (-12.0 to 27.6)	4.1 (-8.1 to 18.2)	23.1 (16.0 to 29.8)	24.2 (17.3 to 30.3)	25.5 (19.1 to 31.9)	4.4 (-12.8 to 25.2)	5.6 (-6.1 to 20.4)
Diet low in calcium	34.5 (30.0 to 38.0)	34.2 (29.8 to 37.8)	35.0 (30.7 to 38.5)	-0.9 (-4.8 to 3.3)	2.3 (-0.7 to 5.8)	36.8 (32.7 to 40.0)	35.6 (31.5 to 39.0)	36.8 (32.8 to 40.1)	-3.1 (-6.7 to 0.0)	3.3 (0.5 to 6.9)
Diet low in seafood omega-3 fatty acids	3.1 (1.2 to 4.6)	0.2 (0.0 to 1.0)	0.2 (0.0 to 1.0)	-93.3 (-100.0 to -71.1)	9.5 (-94.3 to 6296.5)	3.6 (1.7 to 5.1)	0.3 (0.0 to 1.2)	0.3 (0.0 to 1.3)	-91.5 (-100.0 to -71.7)	12.1 (-89.6 to 1574.1)
Diet low in polyunsaturated fatty acids	30.3 (25.1 to 35.1)	27.5 (21.4 to 32.4)	28.1 (22.0 to 33.1)	-9.2 (-20.8 to 0.7)	2.2 (-6.5 to 11.4)	31.0 (25.7 to 35.8)	28.3 (22.5 to 33.1)	28.8 (23.1 to 33.7)	-8.9 (-20.1 to 0.1)	2.1 (-5.6 to 10.8)
Diet high in trans fatty acids	1.1 (0.0 to 3.3)	0.9 (0.0 to 3.0)	0.9 (0.0 to 2.8)	-12.4 (-38.5 to -6.5)	-8.3 (-33.0 to -3.4)	1.5 (0.1 to 4.0)	1.3 (0.1 to 3.7)	1.2 (0.0 to 3.5)	-12.5 (-34.7 to -6.1)	-8.5 (-28.4 to -3.8)
Diet high in sodium	27.7 (17.7 to 36.0)	17.9 (8.9 to 25.2)	15.3 (6.5 to 22.7)	-35.5 (-58.8 to -17.2)	-14.3 (-40.2 to 4.5)	31.4 (23.3 to 38.5)	18.6 (10.0 to 25.5)	15.3 (6.6 to 22.6)	-40.8 (-60.9 to -25.9)	-17.7 (-42.3 to -3.9)
Intimate partner violence	-	-	-	-	-	2.3 (1.9 to 2.7)	2.1 (1.8 to 2.5)	2.0 (1.7 to 2.4)	-8.3 (-12.1 to -4.2)	-3.5 (-7.5 to 1.1)
Childhood sexual abuse	5.3 (4.3 to 6.5)	5.3 (4.3 to 6.5)	5.3 (4.3 to 6.5)	0.0 (0.0 to 0.0)	0.0 (0.0 to 0.0)	7.3 (5.9 to 8.9)	7.3 (5.9 to 8.9)	7.5 (6.0 to 9.2)	0.0 (0.0 to 0.0)	2.8 (-4.0 to 10.6)

Supplementary Results Table 1. Kagawa age-standardised Summary Exposure Values (SEVs) for Level 4 risk factors and percentage change, by sex, 1990, 2007, and 2017.Data in parentheses are 95% uncertainty intervals.										
	**Male**					**Female**				
	**1990**	**2007**	**2017**	**Percentage change 1990-2007**	**Percentage change 2007-2017**	**1990**	**2007**	**2017**	**Percentage change 1990-2007**	**Percentage change 2007-2017**
Bullying victimization	3.3 (1.4 to 6.7)	3.4 (1.4 to 7.0)	3.5 (1.4 to 7.3)	0.6 (-7.3 to 8.7)	4.4 (-0.8 to 9.5)	1.9 (0.8 to 3.9)	1.9 (0.8 to 3.8)	2.0 (0.8 to 4.1)	-0.2 (-7.9 to 7.7)	3.9 (-1.9 to 8.9)
Low physical activity	0.5 (0.2 to 0.9)	0.5 (0.2 to 0.9)	0.4 (0.2 to 0.8)	-1.8 (-3.7 to 0.1)	-8.8 (-12.1 to -4.7)	0.5 (0.2 to 1.0)	0.5 (0.2 to 0.9)	0.5 (0.2 to 0.9)	-3.8 (-5.9 to -1.3)	0.7 (-0.8 to 2.2)
High fasting plasma glucose	1.4 (0.9 to 2.3)	1.4 (0.9 to 2.2)	1.6 (0.9 to 2.6)	-0.5 (-38.7 to 61.1)	12.8 (-24.6 to 78.0)	1.2 (0.7 to 1.9)	1.2 (0.7 to 2.1)	1.4 (0.8 to 2.4)	5.6 (-34.1 to 72.2)	10.6 (-31.5 to 87.7)
High LDL cholesterol	12.0 (10.4 to 13.8)	12.6 (10.9 to 14.4)	12.6 (10.9 to 14.4)	4.4 (-1.3 to 10.8)	0.1 (-4.3 to 4.9)	12.9 (11.3 to 14.6)	13.0 (11.4 to 14.7)	13.0 (11.4 to 14.7)	0.8 (-4.4 to 6.3)	0.2 (-4.5 to 5.2)
High systolic blood pressure	11.2 (10.2 to 12.3)	8.3 (7.4 to 9.2)	8.2 (7.2 to 9.2)	-25.8 (-31.6 to -19.3)	-1.6 (-8.9 to 6.3)	9.6 (8.6 to 10.6)	6.0 (5.3 to 6.8)	5.6 (4.9 to 6.4)	-37.2 (-44.1 to -29.7)	-7.1 (-16.2 to 3.6)
High body-mass index	5.8 (3.7 to 9.4)	8.4 (5.7 to 13.4)	8.8 (5.9 to 13.9)	44.2 (17.0 to 83.8)	4.0 (-12.3 to 21.0)	5.6 (3.4 to 8.9)	7.3 (4.7 to 11.3)	7.3 (4.5 to 11.3)	29.0 (1.8 to 72.0)	0.3 (-17.3 to 22.8)
Low bone mineral density	5.3 (4.4 to 6.3)	5.2 (4.4 to 6.1)	5.1 (4.3 to 6.1)	-2.3 (-15.2 to 12.8)	-0.3 (-12.4 to 12.8)	6.7 (5.9 to 7.5)	6.6 (5.9 to 7.5)	6.7 (5.7 to 7.9)	-0.9 (-10.1 to 9.6)	1.8 (-11.4 to 16.1)
Impaired kidney function	2.6 (1.2 to 6.5)	2.6 (1.2 to 6.5)	2.4 (1.1 to 6.1)	-2.4 (-6.6 to 2.0)	-6.7 (-12.3 to -1.9)	3.2 (1.5 to 7.4)	3.4 (1.5 to 7.7)	3.1 (1.4 to 7.3)	3.6 (-0.2 to 8.7)	-7.2 (-12.0 to -2.4)

Supplementary Results Table 1. Kagoshima age-standardised Summary Exposure Values (SEVs) for Level 4 risk factors and percentage change, by sex, 1990, 2007, and 2017.Data in parentheses are 95% uncertainty intervals.

	Male					Female				
	1990	2007	2017	Percentage change 1990-2007	Percentage change 2007-2017	1990	2007	2017	Percentage change 1990-2007	Percentage change 2007-2017
Unsafe water source	2.6 (1.4 to 4.3)	2.2 (1.2 to 3.6)	2.3 (1.3 to 3.7)	-16.0 (-59.1 to 72.5)	4.0 (-36.7 to 87.7)	2.6 (1.4 to 4.3)	2.2 (1.2 to 3.6)	2.3 (1.3 to 3.7)	-16.0 (-59.1 to 72.5)	4.0 (-36.7 to 87.7)
Unsafe sanitation	4.6 (2.2 to 8.6)	2.4 (1.1 to 4.7)	2.1 (0.9 to 3.9)	-47.5 (-77.5 to 22.5)	-15.2 (-57.1 to 63.4)	4.6 (2.2 to 8.6)	2.4 (1.1 to 4.7)	2.1 (0.9 to 3.9)	-47.5 (-77.5 to 22.5)	-15.2 (-57.1 to 63.4)
No access to handwashing facility	6.9 (5.0 to 9.2)	6.5 (4.7 to 8.8)	6.0 (4.4 to 8.1)	-5.8 (-33.2 to 33.2)	-7.1 (-28.7 to 19.3)	6.9 (5.0 to 9.2)	6.5 (4.7 to 8.8)	6.0 (4.4 to 8.1)	-5.8 (-33.2 to 33.2)	-7.1 (-28.7 to 19.3)
Ambient particulate matter pollution	36.6 (24.2 to 52.9)	37.6 (26.8 to 54.0)	33.0 (22.2 to 49.2)	2.7 (-20.9 to 39.7)	-12.4 (-35.4 to 16.1)	36.3 (23.8 to 52.5)	37.6 (26.7 to 53.8)	32.9 (22.1 to 49.1)	3.5 (-20.5 to 40.9)	-12.4 (-35.4 to 16.2)
Household air pollution from solid fuels	0.9 (0.3 to 2.4)	0.2 (0.1 to 0.5)	0.2 (0.0 to 0.4)	-76.9 (-92.8 to -51.5)	-14.6 (-67.1 to 74.7)	1.3 (0.4 to 6.9)	0.3 (0.1 to 1.6)	0.3 (0.0 to 1.2)	-74.2 (-92.8 to -46.2)	-13.6 (-66.7 to 74.5)
Ambient ozone pollution	46.8 (18.0 to 54.8)	47.3 (18.4 to 54.8)	48.9 (20.1 to 54.8)	1.1 (0.0 to 3.5)	3.5 (0.0 to 10.8)	46.8 (18.0 to 54.8)	47.3 (18.4 to 54.8)	48.9 (20.1 to 54.8)	1.1 (0.0 to 3.5)	3.5 (0.0 to 10.8)
Residential radon	11.5 (0.6 to 37.8)	11.5 (0.6 to 37.8)	11.5 (0.6 to 37.8)	0.0 (0.0 to 0.0)	0.0 (0.0 to 0.0)	11.5 (0.6 to 37.8)	11.5 (0.6 to 37.8)	11.5 (0.6 to 37.8)	0.0 (0.0 to 0.0)	0.0 (0.0 to 0.0)
Lead exposure	2.7 (0.3 to 6.2)	1.9 (0.2 to 4.8)	1.4 (0.1 to 4.0)	-28.8 (-41.3 to -20.5)	-24.5 (-40.0 to -17.0)	1.5 (0.1 to 4.3)	1.0 (0.0 to 3.3)	0.8 (0.0 to 2.7)	-31.8 (-47.9 to -23.4)	-25.2 (-46.3 to -16.6)
Occupational exposure to asbestos	1.9 (1.7 to 2.2)	2.6 (2.4 to 2.9)	2.7 (2.2 to 3.2)	36.6 (17.0 to 62.1)	0.9 (-17.3 to 23.0)	0.6 (0.5 to 0.7)	0.6 (0.5 to 0.6)	0.5 (0.4 to 0.7)	-3.0 (-20.0 to 26.4)	-5.8 (-25.0 to 17.4)
Occupational exposure to arsenic	0.4 (0.0 to 1.0)	0.4 (0.0 to 1.0)	0.4 (0.0 to 1.0)	-	-	0.4 (0.0 to 0.9)	0.3 (0.0 to 0.8)	0.4 (0.0 to 0.9)	-	-
Occupational exposure to benzene	0.6 (0.0 to 1.8)	0.6 (0.0 to 1.8)	0.7 (0.0 to 1.9)	3.1 (-4.3 to 13.6)	3.6 (-1.7 to 11.5)	0.6 (0.0 to 1.5)	0.7 (0.0 to 1.9)	0.8 (0.0 to 2.2)	22.1 (9.7 to 45.2)	15.0 (5.3 to 32.0)
Occupational exposure to beryllium	0.0 (0.0 to 0.0)	0.0 (0.0 to 0.0)	0.0 (0.0 to 0.0)	-4.9 (-10.0 to 0.4)	1.9 (-2.0 to 6.1)	0.0 (0.0 to 0.0)	0.0 (0.0 to 0.0)	0.0 (0.0 to 0.0)	2.9 (-6.2 to 12.9)	9.8 (2.6 to 16.6)
Occupational exposure to cadmium	0.1 (0.0 to 0.1)	0.0 (0.0 to 0.1)	0.0 (0.0 to 0.1)	-6.0 (-16.5 to 6.2)	0.9 (-8.5 to 11.6)	0.0 (0.0 to 0.1)	0.0 (0.0 to 0.0)	0.0 (0.0 to 0.1)	-7.1 (-22.0 to 11.0)	5.8 (-7.1 to 20.9)
Occupational exposure to chromium	0.1 (0.1 to 0.1)	0.1 (0.1 to 0.1)	0.1 (0.1 to 0.1)	-4.2 (-13.7 to 7.1)	1.5 (-6.7 to 11.1)	0.1 (0.1 to 0.1)	0.1 (0.1 to 0.1)	0.1 (0.1 to 0.1)	-2.4 (-16.7 to 14.4)	7.6 (-3.6 to 20.9)
Occupational exposure to diesel engine exhaust	0.6 (0.5 to 0.6)	0.6 (0.5 to 0.6)	0.6 (0.5 to 0.6)	-0.8 (-9.4 to 8.3)	3.4 (-3.3 to 10.7)	0.4 (0.4 to 0.5)	0.5 (0.4 to 0.5)	0.5 (0.5 to 0.6)	13.6 (-0.9 to 29.8)	12.2 (1.3 to 23.8)
Occupational exposure to formaldehyde	0.2 (0.2 to 0.3)	0.2 (0.2 to 0.3)	0.2 (0.2 to 0.3)	-5.3 (-16.8 to 8.1)	0.5 (-9.4 to 12.2)	0.2 (0.2 to 0.2)	0.2 (0.2 to 0.2)	0.2 (0.2 to 0.2)	-4.4 (-20.1 to 14.7)	6.6 (-6.5 to 22.2)
Occupational exposure to nickel	0.4 (0.0 to 1.8)	0.4 (0.0 to 1.6)	0.4 (0.0 to 1.6)	-	-	0.3 (0.0 to 1.5)	0.3 (0.0 to 1.2)	0.3 (0.0 to 1.4)	-	-
Occupational exposure to polycyclic aromatic hydrocarbons	0.2 (0.2 to 0.3)	0.2 (0.2 to 0.2)	0.2 (0.2 to 0.3)	-3.3 (-12.2 to 7.2)	1.8 (-5.9 to 10.7)	0.2 (0.2 to 0.2)	0.2 (0.2 to 0.2)	0.2 (0.2 to 0.2)	-0.3 (-14.1 to 15.6)	8.4 (-2.4 to 20.9)
Occupational exposure to silica	4.4 (0.6 to 14.3)	4.2 (0.6 to 13.7)	4.2 (0.6 to 13.6)	-5.3 (-15.6 to 7.7)	1.3 (-7.6 to 12.0)	3.0 (0.4 to 9.6)	2.6 (0.4 to 8.3)	2.7 (0.4 to 8.7)	-11.7 (-26.1 to 7.5)	2.7 (-10.8 to 21.4)
Occupational exposure to sulphuric acid	0.6 (0.1 to 1.9)	0.6 (0.1 to 1.8)	0.6 (0.1 to 1.8)	-6.3 (-16.5 to 5.9)	1.2 (-8.0 to 11.3)	0.5 (0.1 to 1.5)	0.4 (0.1 to 1.5)	0.5 (0.1 to 1.5)	-8.5 (-23.7 to 8.5)	4.9 (-7.9 to 20.4)
Occupational exposure to trichloroethylene	0.1 (0.1 to 0.1)	0.1 (0.1 to 0.1)	0.1 (0.1 to 0.1)	-3.2 (-10.6 to 5.2)	2.1 (-3.7 to 8.3)	0.1 (0.1 to 0.1)	0.1 (0.1 to 0.1)	0.1 (0.1 to 0.1)	2.4 (-9.7 to 17.0)	9.2 (0.4 to 20.5)
Occupational asthmagens	17.3 (13.9 to 21.8)	17.1 (13.8 to 21.6)	17.2 (13.6 to 21.9)	-1.3 (-19.3 to 20.3)	0.2 (-13.9 to 17.4)	8.0 (6.3 to 9.9)	8.1 (6.5 to 10.2)	8.5 (6.7 to 10.7)	1.2 (-14.7 to 20.1)	5.6 (-7.2 to 20.9)
Occupational particulate matter, gases, and fumes	6.4 (4.5 to 9.3)	6.1 (4.3 to 8.9)	5.9 (4.1 to 8.5)	-4.4 (-9.3 to 0.7)	-3.7 (-7.0 to -0.4)	3.5 (2.5 to 5.4)	3.3 (2.3 to 5.0)	3.2 (2.2 to 4.9)	-5.0 (-13.5 to 4.4)	-4.2 (-9.9 to 2.4)
Occupational noise	5.9 (5.5 to 6.5)	5.7 (5.3 to 6.3)	5.6 (5.2 to 6.1)	-3.4 (-6.4 to -0.2)	-2.6 (-4.6 to -0.4)	3.3 (2.9 to 3.9)	3.4 (2.9 to 3.8)	3.4 (3.0 to 3.9)	0.4 (-5.4 to 7.3)	1.8 (-2.3 to 6.5)
Occupational ergonomic factors	11.4 (9.4 to 13.7)	10.1 (8.4 to 12.2)	10.0 (8.2 to 12.0)	-11.1 (-23.2 to 3.8)	-1.2 (-12.3 to 12.5)	6.1 (4.7 to 7.9)	6.2 (4.8 to 8.0)	6.9 (5.3 to 9.1)	1.4 (-22.4 to 29.7)	11.3 (-8.8 to 36.0)
Non-exclusive breastfeeding	0.4 (0.3 to 0.6)	0.4 (0.3 to 0.6)	0.4 (0.3 to 0.6)	-1.0 (-13.9 to 13.5)	-1.6 (-12.4 to 9.6)	0.4 (0.3 to 0.6)	0.4 (0.3 to 0.6)	0.4 (0.3 to 0.6)	-0.8 (-13.7 to 13.7)	-2.1 (-12.8 to 9.1)
Discontinued breastfeeding	1.6 (1.5 to 1.8)	1.7 (1.6 to 1.8)	1.7 (1.5 to 1.8)	4.1 (-5.1 to 15.3)	-3.4 (-10.7 to 4.4)	1.7 (1.5 to 1.8)	1.7 (1.6 to 1.8)	1.7 (1.5 to 1.8)	2.4 (-6.7 to 13.4)	-2.3 (-9.7 to 5.6)
Child underweight	0.3 (0.2 to 0.4)	0.2 (0.1 to 0.3)	0.2 (0.1 to 0.3)	-15.0 (-33.7 to 7.8)	-6.4 (-20.6 to 11.6)	0.3 (0.2 to 0.4)	0.2 (0.1 to 0.3)	0.2 (0.1 to 0.3)	-16.1 (-36.8 to 12.0)	-7.1 (-23.2 to 10.8)
Child wasting	0.2 (0.1 to 0.3)	0.2 (0.1 to 0.3)	0.2 (0.1 to 0.3)	-7.4 (-34.6 to 29.5)	-2.3 (-22.4 to 23.1)	0.2 (0.1 to 0.3)	0.2 (0.1 to 0.3)	0.2 (0.1 to 0.3)	-7.8 (-36.9 to 33.0)	-3.5 (-23.9 to 21.1)
Child stunting	0.6 (0.3 to 1.1)	0.6 (0.3 to 1.0)	0.5 (0.3 to 1.0)	-10.5 (-48.3 to 50.1)	-4.5 (-33.1 to 36.9)	0.6 (0.3 to 1.1)	0.6 (0.3 to 1.0)	0.5 (0.3 to 1.0)	-12.0 (-47.0 to 46.9)	-3.8 (-35.1 to 38.7)
Short gestation for birth weight	0.0 (0.0 to 0.0)	0.0 (0.0 to 0.0)	0.0 (0.0 to 0.0)	9.7 (-14.4 to 32.9)	0.4 (-17.5 to 27.6)	0.0 (0.0 to 0.0)	0.0 (0.0 to 0.0)	0.0 (0.0 to 0.0)	12.5 (-19.2 to 51.7)	0.4 (-20.2 to 39.7)
Low birth weight for gestation	0.0 (0.0 to 0.0)	0.0 (0.0 to 0.0)	0.0 (0.0 to 0.0)	3.7 (-12.3 to 27.6)	3.5 (-15.8 to 22.7)	0.0 (0.0 to 0.0)	0.0 (0.0 to 0.0)	0.0 (0.0 to 0.0)	-3.0 (-42.2 to 28.8)	3.5 (-20.6 to 24.2)
Iron deficiency	-	-	-	-	-	8.4 (5.7 to 11.3)	8.4 (5.9 to 11.5)	7.7 (5.2 to 11.0)	0.5 (-21.3 to 29.6)	-8.0 (-24.9 to 10.0)
Vitamin A deficiency	1.0 (0.7 to 1.4)	0.8 (0.5 to 1.1)	0.7 (0.4 to 1.0)	-21.5 (-35.1 to -5.6)	-11.7 (-25.9 to 4.7)	0.8 (0.6 to 1.0)	0.8 (0.5 to 1.1)	0.7 (0.4 to 1.0)	4.3 (-14.8 to 26.5)	-16.9 (-35.4 to 0.2)
Zinc deficiency	0.5 (0.2 to 1.1)	0.4 (0.1 to 1.0)	0.4 (0.1 to 0.8)	-20.5 (-65.1 to 68.3)	-10.4 (-67.5 to 177.5)	0.5 (0.2 to 1.1)	0.4 (0.1 to 0.8)	0.4 (0.1 to 0.9)	-31.3 (-64.7 to 4.7)	7.1 (-53.4 to 157.4)
Smoking	18.4 (14.8 to 21.8)	12.9 (10.3 to 15.5)	10.8 (8.6 to 13.1)	-30.2 (-39.2 to -20.3)	-15.9 (-24.5 to -6.9)	4.2 (3.0 to 5.7)	3.4 (2.5 to 4.6)	3.4 (2.4 to 4.5)	-17.7 (-37.4 to 7.6)	-2.5 (-19.6 to 16.4)
Chewing tobacco	1.4 (0.9 to 2.1)	0.9 (0.6 to 1.4)	0.8 (0.5 to 1.1)	-32.8 (-57.6 to 2.8)	-18.4 (-41.9 to 15.7)	0.1 (0.1 to 0.1)	0.1 (0.1 to 0.1)	0.1 (0.1 to 0.1)	-4.0 (-41.6 to 65.2)	-2.4 (-33.0 to 46.4)
Second-hand smoke	37.3 (33.2 to 41.2)	35.5 (30.9 to 39.8)	35.8 (31.4 to 40.4)	-5.0 (-16.2 to 7.4)	0.8 (-8.5 to 10.6)	56.3 (52.6 to 59.9)	47.6 (43.7 to 51.4)	48.0 (43.9 to 51.9)	-15.5 (-22.1 to -9.1)	0.8 (-5.5 to 7.2)
Alcohol use	14.5 (7.2 to 25.6)	14.6 (7.6 to 24.9)	16.9 (8.2 to 29.9)	0.4 (-31.5 to 48.8)	15.6 (-20.0 to 60.2)	11.7 (5.6 to 21.1)	11.7 (6.0 to 20.7)	13.1 (6.5 to 23.4)	-0.5 (-31.7 to 47.6)	12.5 (-20.3 to 59.7)
Drug use	0.5 (0.4 to 0.7)	0.5 (0.4 to 0.7)	0.6 (0.4 to 0.8)	0.5 (-5.5 to 6.7)	11.3 (3.9 to 19.8)	0.3 (0.2 to 0.4)	0.3 (0.2 to 0.4)	0.3 (0.3 to 0.4)	2.3 (-3.8 to 9.2)	9.0 (2.2 to 16.2)
Diet low in fruits	41.9 (36.2 to 45.4)	40.1 (34.5 to 43.9)	41.3 (36.0 to 45.0)	-4.2 (-9.8 to 1.0)	3.0 (-1.5 to 7.5)	38.8 (32.8 to 43.2)	36.2 (30.1 to 41.0)	38.3 (32.5 to 42.7)	-6.7 (-14.5 to 0.5)	5.6 (-0.5 to 13.1)
Diet low in vegetables	21.3 (13.1 to 28.7)	15.6 (7.5 to 23.4)	17.6 (9.2 to 25.7)	-27.0 (-53.7 to -3.8)	12.9 (-13.7 to 60.6)	23.3 (15.0 to 30.4)	17.2 (8.8 to 25.0)	18.9 (10.2 to 26.8)	-26.2 (-51.4 to -4.8)	9.7 (-13.9 to 49.1)
Diet low in legumes	4.3 (0.7 to 9.4)	5.4 (1.1 to 10.8)	7.0 (1.9 to 13.6)	24.7 (-50.1 to 317.6)	30.2 (-26.3 to 182.7)	8.1 (2.5 to 14.7)	7.7 (2.0 to 13.9)	9.8 (3.4 to 16.9)	-5.8 (-52.8 to 82.8)	28.2 (-14.0 to 141.1)
Diet low in whole grains	31.1 (26.2 to 35.2)	35.8 (32.0 to 39.0)	37.1 (33.2 to 40.2)	15.0 (5.7 to 27.4)	3.6 (-1.0 to 9.0)	32.4 (27.6 to 36.3)	37.0 (33.1 to 40.2)	38.2 (34.5 to 41.1)	13.9 (6.2 to 25.4)	3.3 (-1.3 to 8.2)
Diet low in nuts and seeds	51.1 (49.9 to 52.0)	49.9 (48.2 to 51.1)	49.6 (47.8 to 51.0)	-2.5 (-4.6 to -0.6)	-0.5 (-2.3 to 1.2)	51.4 (50.3 to 52.2)	50.1 (48.5 to 51.3)	49.9 (48.1 to 51.2)	-2.5 (-4.7 to -0.7)	-0.4 (-2.3 to 1.1)
Diet low in milk	46.6 (44.6 to 48.3)	47.0 (45.2 to 48.6)	47.5 (45.7 to 49.0)	0.9 (-0.9 to 2.9)	1.0 (-0.3 to 2.3)	46.6 (44.6 to 48.3)	46.4 (44.3 to 48.1)	47.1 (45.0 to 48.7)	-0.3 (-2.2 to 1.5)	1.4 (0.0 to 2.9)
Diet high in red meat	3.4 (0.3 to 8.0)	6.9 (2.0 to 13.3)	7.9 (2.5 to 14.6)	102.6 (-14.6 to 1124.7)	15.4 (-40.5 to 146.7)	0.4 (0.0 to 2.2)	1.2 (0.0 to 4.4)	1.6 (0.0 to 5.4)	176.7 (-87.2 to [illegible])	31.1 (-87.6 to 5972.7)
Diet high in processed meat	10.2 (6.3 to 14.1)	16.9 (12.1 to 21.7)	19.4 (13.7 to 24.6)	65.7 (27.8 to 133.5)	14.5 (-3.4 to 37.5)	8.4 (4.6 to 11.9)	12.1 (8.3 to 15.9)	13.2 (9.0 to 17.1)	44.7 (13.2 to 108.1)	8.9 (-8.6 to 30.7)
Diet high in sugar-sweetened beverages	6.4 (2.6 to 13.4)	7.3 (3.2 to 13.0)	7.7 (3.2 to 13.9)	14.0 (-45.2 to 143.0)	6.1 (-41.9 to 87.8)	4.6 (1.8 to 10.3)	5.3 (2.2 to 10.5)	5.6 (2.1 to 11.0)	14.4 (-46.3 to 161.6)	6.0 (-38.8 to 80.6)
Diet low in fibre	22.7 (15.6 to 29.0)	23.6 (16.4 to 29.6)	24.6 (17.5 to 30.8)	4.0 (-11.8 to 28.1)	4.0 (-8.5 to 18.0)	23.7 (16.7 to 30.0)	24.3 (17.3 to 30.6)	25.4 (18.6 to 31.8)	2.4 (-15.2 to 22.1)	4.5 (-6.7 to 18.2)
Diet low in calcium	35.7 (31.5 to 39.1)	35.3 (31.2 to 38.7)	36.0 (31.9 to 39.3)	-1.1 (-4.8 to 2.6)	1.9 (-1.1 to 5.1)	37.8 (34.0 to 40.9)	36.6 (32.6 to 39.8)	37.6 (33.8 to 40.7)	-3.3 (-6.9 to -0.2)	2.8 (0.3 to 6.0)
Diet low in seafood omega-3 fatty acids	3.5 (2.0 to 4.8)	0.4 (0.0 to 1.3)	0.4 (0.0 to 1.4)	-89.0 (-99.9 to -60.9)	4.3 (-86.1 to 681.9)	4.0 (2.1 to 5.4)	0.5 (0.0 to 1.6)	0.6 (0.0 to 1.7)	-86.7 (-99.8 to -59.0)	5.6 (-72.4 to 473.9)
Diet low in polyunsaturated fatty acids	31.3 (25.8 to 36.3)	28.6 (22.8 to 33.6)	29.3 (23.5 to 34.1)	-8.5 (-18.9 to 0.5)	2.3 (-5.5 to 11.0)	32.0 (26.9 to 36.5)	29.3 (23.9 to 34.4)	30.0 (24.7 to 34.8)	-8.3 (-18.1 to 0.7)	2.2 (-4.4 to 10.3)
Diet high in trans fatty acids	1.1 (0.0 to 3.3)	1.0 (0.0 to 3.0)	0.9 (0.0 to 2.9)	-12.5 (-39.4 to -6.5)	-8.4 (-33.5 to -3.8)	1.5 (0.1 to 4.0)	1.3 (0.1 to 3.7)	1.2 (0.0 to 3.5)	-12.7 (-35.6 to -6.5)	-8.2 (-28.0 to -3.5)
Diet high in sodium	27.4 (17.5 to 35.6)	17.8 (9.6 to 25.1)	15.4 (6.9 to 22.9)	-34.9 (-57.9 to -16.0)	-13.8 (-37.1 to 3.9)	31.8 (23.5 to 39.0)	19.4 (10.8 to 26.4)	16.3 (7.5 to 23.8)	-39.0 (-59.3 to -24.3)	-16.2 (-37.8 to -3.3)
Intimate partner violence	-	-	-	-	-	2.3 (2.0 to 2.7)	2.1 (1.8 to 2.5)	2.1 (1.7 to 2.4)	-8.4 (-12.5 to -4.1)	-3.6 (-8.0 to 0.8)
Childhood sexual abuse	5.3 (4.3 to 6.5)	5.3 (4.3 to 6.5)	5.3 (4.3 to 6.5)	0.0 (0.0 to 0.0)	0.0 (0.0 to 0.0)	7.3 (5.9 to 8.9)	7.3 (5.9 to 8.9)	7.5 (6.0 to 9.2)	0.0 (0.0 to 0.0)	2.8 (-4.0 to 10.6)

Supplementary Results Table 1. Kagoshima age-standardised Summary Exposure Values (SEVs) for Level 4 risk factors and percentage change, by sex, 1990, 2007, and 2017.Data in parentheses are 95% uncertainty intervals.										
	**Male**					**Female**				
	**1990**	**2007**	**2017**	**Percentage change 1990-2007**	**Percentage change 2007-2017**	**1990**	**2007**	**2017**	**Percentage change 1990-2007**	**Percentage change 2007-2017**
Bullying victimization	3.3 (1.3 to 6.7)	3.4 (1.4 to 7.2)	3.5 (1.4 to 7.2)	3.4 (-4.4 to 11.5)	2.0 (-3.3 to 6.5)	1.9 (0.8 to 3.9)	1.9 (0.8 to 4.0)	2.0 (0.8 to 4.1)	1.0 (-6.4 to 8.9)	1.3 (-4.5 to 6.4)
Low physical activity	0.5 (0.2 to 0.9)	0.5 (0.2 to 0.9)	0.4 (0.2 to 0.8)	-1.9 (-3.9 to -0.0)	-8.7 (-12.1 to -4.5)	0.5 (0.2 to 1.0)	0.5 (0.2 to 0.9)	0.5 (0.2 to 0.9)	-3.9 (-6.1 to -1.4)	0.7 (-0.8 to 2.4)
High fasting plasma glucose	1.4 (0.9 to 2.2)	1.3 (0.8 to 2.1)	1.5 (0.9 to 2.4)	-2.9 (-38.3 to 55.3)	11.2 (-24.2 to 78.7)	1.1 (0.7 to 1.7)	0.9 (0.6 to 1.5)	0.9 (0.6 to 1.6)	-14.9 (-43.9 to 33.5)	1.4 (-26.7 to 51.4)
High LDL cholesterol	11.9 (10.3 to 13.7)	12.4 (10.9 to 14.2)	12.5 (10.8 to 14.2)	4.2 (-1.7 to 10.7)	0.1 (-4.3 to 4.2)	12.7 (11.1 to 14.5)	12.8 (11.1 to 14.6)	12.8 (11.2 to 14.6)	0.6 (-4.8 to 6.4)	0.3 (-4.5 to 5.1)
High systolic blood pressure	11.2 (10.1 to 12.3)	8.2 (7.3 to 9.2)	8.1 (7.1 to 9.1)	-26.4 (-32.3 to -20.2)	-1.7 (-9.3 to 6.7)	9.6 (8.6 to 10.7)	6.0 (5.3 to 6.7)	5.6 (4.8 to 6.4)	-37.3 (-43.6 to -29.5)	-7.0 (-16.2 to 3.2)
High body-mass index	4.6 (2.7 to 7.4)	6.5 (4.1 to 10.7)	6.7 (4.3 to 11.4)	41.2 (12.7 to 84.3)	3.5 (-12.6 to 20.4)	5.0 (2.9 to 8.0)	6.4 (3.9 to 10.5)	6.5 (3.9 to 10.4)	28.9 (1.2 to 67.1)	1.1 (-16.4 to 25.9)
Low bone mineral density	5.3 (4.4 to 6.3)	5.2 (4.3 to 6.1)	5.2 (4.3 to 6.1)	-2.2 (-15.6 to 12.2)	0.7 (-13.2 to 15.1)	7.2 (6.1 to 8.6)	7.0 (6.0 to 8.2)	7.1 (6.0 to 8.4)	-3.8 (-14.9 to 9.0)	2.1 (-9.8 to 16.3)
Impaired kidney function	2.6 (1.2 to 6.5)	2.5 (1.1 to 6.3)	2.4 (1.1 to 6.2)	-3.9 (-8.6 to 0.4)	-4.5 (-9.9 to -0.4)	3.2 (1.5 to 7.5)	3.3 (1.5 to 7.6)	3.1 (1.4 to 7.4)	1.8 (-2.1 to 6.2)	-5.2 (-10.5 to -1.0)

Supplementary Results Table 1. Kagawa age-standardised Summary Exposure Values (SEVs) for Level 4 risk factors and percentage change, by sex, 1990, 2007, and 2017.Data in parentheses are 95% uncertainty intervals.										
	Male					Female				
	1990	2007	2017	Percentage change 1990-2007	Percentage change 2007-2017	1990	2007	2017	Percentage change 1990-2007	Percentage change 2007-2017
Unsafe water source	1.8 (1.0 to 3.0)	1.5 (0.9 to 2.5)	1.7 (0.9 to 2.8)	-15.6 (-54.9 to 69.7)	8.1 (-37.5 to 81.8)	1.8 (1.0 to 3.0)	1.5 (0.9 to 2.5)	1.7 (0.9 to 2.8)	-15.6 (-54.9 to 69.7)	8.1 (-37.5 to 81.8)
Unsafe sanitation	2.9 (1.4 to 5.1)	1.5 (0.7 to 2.8)	1.3 (0.6 to 2.5)	-48.7 (-77.9 to 23.3)	-11.8 (-54.7 to 73.7)	2.9 (1.4 to 5.1)	1.5 (0.7 to 2.8)	1.3 (0.6 to 2.5)	-48.7 (-77.9 to 23.3)	-11.8 (-54.7 to 73.7)
No access to handwashing facility	6.5 (4.7 to 8.7)	6.1 (4.5 to 8.3)	5.7 (4.2 to 7.7)	-5.7 (-31.5 to 34.3)	-6.6 (-27.5 to 18.1)	6.5 (4.7 to 8.7)	6.1 (4.5 to 8.3)	5.7 (4.2 to 7.7)	-5.7 (-31.5 to 34.3)	-6.6 (-27.5 to 18.1)
Ambient particulate matter pollution	36.9 (25.9 to 52.5)	36.7 (26.6 to 51.2)	32.0 (21.9 to 46.5)	-0.7 (-22.4 to 29.9)	-12.8 (-29.0 to 2.1)	36.8 (25.8 to 52.4)	36.6 (26.5 to 51.1)	31.9 (21.9 to 46.5)	-0.5 (-22.2 to 30.7)	-12.8 (-29.0 to 2.1)
Household air pollution from solid fuels	0.3 (0.1 to 0.8)	0.1 (0.0 to 0.2)	0.1 (0.0 to 0.1)	-70.5 (-92.6 to -44.2)	-8.9 (-65.6 to 91.6)	0.5 (0.1 to 2.3)	0.1 (0.0 to 0.6)	0.1 (0.0 to 0.4)	-68.2 (-92.5 to -40.1)	-8.8 (-65.2 to 93.9)
Ambient ozone pollution	52.4 (27.4 to 54.8)	52.2 (26.8 to 54.8)	52.2 (26.8 to 54.8)	-0.4 (-3.1 to 0.0)	0.0 (-0.4 to 0.7)	52.4 (27.4 to 54.8)	52.2 (26.8 to 54.8)	52.2 (26.8 to 54.8)	-0.4 (-3.1 to 0.0)	0.0 (-0.4 to 0.7)
Residential radon	13.6 (0.7 to 46.4)	13.6 (0.7 to 46.4)	13.6 (0.7 to 46.4)	0.0 (0.0 to 0.0)	0.0 (0.0 to 0.0)	13.6 (0.7 to 46.4)	13.6 (0.7 to 46.4)	13.6 (0.7 to 46.4)	0.0 (0.0 to 0.0)	0.0 (0.0 to 0.0)
Lead exposure	3.5 (0.7 to 7.3)	2.1 (0.3 to 5.0)	1.4 (0.2 to 3.8)	-40.0 (-54.7 to -30.0)	-33.4 (-49.1 to -25.0)	1.9 (0.2 to 4.9)	1.0 (0.1 to 3.1)	0.6 (0.0 to 2.2)	-47.2 (-65.0 to -36.8)	-38.1 (-59.7 to -28.3)
Occupational exposure to asbestos	3.1 (2.7 to 3.4)	4.0 (3.8 to 4.3)	3.7 (3.1 to 4.5)	31.9 (15.4 to 53.7)	-7.8 (-23.2 to 11.5)	0.7 (0.6 to 0.9)	0.7 (0.6 to 0.8)	0.7 (0.5 to 0.8)	-4.4 (-20.2 to 22.4)	-4.3 (-22.7 to 19.2)
Occupational exposure to arsenic	0.5 (0.0 to 1.1)	0.4 (0.0 to 1.0)	0.4 (0.0 to 1.0)	-	-	0.4 (0.0 to 1.0)	0.4 (0.0 to 0.9)	0.4 (0.0 to 0.9)	-	-
Occupational exposure to benzene	0.6 (0.0 to 1.8)	0.7 (0.0 to 1.9)	0.7 (0.0 to 1.9)	2.5 (-3.9 to 12.8)	3.4 (-1.9 to 13.7)	0.7 (0.0 to 1.9)	0.8 (0.0 to 2.3)	0.9 (0.1 to 2.5)	19.4 (7.7 to 40.6)	13.3 (4.5 to 28.0)
Occupational exposure to beryllium	0.0 (0.0 to 0.0)	0.0 (0.0 to 0.0)	0.0 (0.0 to 0.0)	-5.2 (-9.9 to -0.4)	2.0 (-1.9 to 5.8)	0.0 (0.0 to 0.0)	0.0 (0.0 to 0.0)	0.0 (0.0 to 0.0)	0.9 (-7.2 to 10.0)	8.0 (2.1 to 15.3)
Occupational exposure to cadmium	0.1 (0.0 to 0.1)	0.1 (0.0 to 0.1)	0.1 (0.0 to 0.1)	-7.8 (-18.8 to 4.2)	0.9 (-8.5 to 11.1)	0.1 (0.0 to 0.1)	0.0 (0.0 to 0.1)	0.0 (0.0 to 0.1)	-10.7 (-24.9 to 6.3)	3.8 (-8.5 to 18.5)
Occupational exposure to chromium	0.1 (0.1 to 0.1)	0.1 (0.1 to 0.1)	0.1 (0.1 to 0.1)	-6.2 (-16.2 to 4.0)	1.4 (-6.9 to 10.1)	0.1 (0.1 to 0.1)	0.1 (0.1 to 0.1)	0.1 (0.1 to 0.1)	-5.9 (-19.0 to 8.8)	5.5 (-5.0 to 17.7)
Occupational exposure to diesel engine exhaust	0.6 (0.6 to 0.6)	0.6 (0.5 to 0.6)	0.6 (0.6 to 0.6)	-2.6 (-10.0 to 5.9)	2.9 (-3.7 to 9.4)	0.5 (0.4 to 0.5)	0.5 (0.5 to 0.6)	0.6 (0.5 to 0.7)	10.8 (-2.4 to 24.5)	9.8 (-0.4 to 21.5)
Occupational exposure to formaldehyde	0.3 (0.2 to 0.3)	0.2 (0.2 to 0.3)	0.2 (0.2 to 0.3)	-7.3 (-19.3 to 5.0)	0.5 (-9.2 to 11.2)	0.2 (0.2 to 0.3)	0.2 (0.2 to 0.2)	0.2 (0.2 to 0.3)	-8.0 (-22.6 to 9.5)	4.6 (-7.7 to 18.8)
Occupational exposure to nickel	0.4 (0.0 to 1.9)	0.4 (0.0 to 1.7)	0.4 (0.0 to 1.8)	-	-	0.4 (0.0 to 1.6)	0.3 (0.0 to 1.4)	0.3 (0.0 to 1.4)	-	-
Occupational exposure to polycyclic aromatic hydrocarbons	0.3 (0.2 to 0.3)	0.2 (0.2 to 0.3)	0.2 (0.2 to 0.3)	-5.1 (-14.7 to 4.3)	1.7 (-5.8 to 9.7)	0.2 (0.2 to 0.3)	0.2 (0.2 to 0.3)	0.2 (0.2 to 0.3)	-3.8 (-16.3 to 10.3)	6.4 (-3.6 to 17.9)
Occupational exposure to silica	4.9 (0.7 to 15.7)	4.5 (0.7 to 14.5)	4.6 (0.6 to 15.0)	-7.0 (-16.8 to 4.1)	1.4 (-6.7 to 10.4)	3.3 (0.5 to 10.3)	2.8 (0.4 to 8.7)	2.8 (0.4 to 8.7)	-15.6 (-29.9 to 3.3)	1.0 (-12.7 to 18.0)
Occupational exposure to sulphuric acid	0.6 (0.1 to 2.0)	0.6 (0.1 to 1.9)	0.6 (0.1 to 1.8)	-7.8 (-18.5 to 3.8)	1.2 (-7.9 to 11.4)	0.5 (0.1 to 1.7)	0.5 (0.1 to 1.5)	0.5 (0.1 to 1.6)	-11.9 (-26.2 to 5.3)	3.1 (-9.5 to 18.6)
Occupational exposure to trichloroethylene	0.1 (0.1 to 0.1)	0.1 (0.1 to 0.1)	0.1 (0.1 to 0.1)	-5.1 (-12.7 to 2.5)	1.8 (-4.3 to 8.1)	0.1 (0.1 to 0.1)	0.1 (0.1 to 0.1)	0.1 (0.1 to 0.1)	-0.9 (-11.5 to 11.1)	7.0 (-1.2 to 16.9)
Occupational asthmagens	20.4 (16.1 to 25.4)	19.1 (15.3 to 23.9)	19.0 (14.9 to 23.9)	-6.2 (-23.0 to 15.4)	-0.8 (-16.7 to 18.0)	8.8 (7.1 to 11.0)	8.6 (6.9 to 10.7)	9.1 (7.2 to 11.1)	-1.5 (-17.2 to 17.7)	5.0 (-7.7 to 19.3)
Occupational particulate matter, gases, and fumes	6.4 (4.5 to 9.2)	6.1 (4.4 to 8.9)	5.9 (4.2 to 8.5)	-4.5 (-9.6 to 0.6)	-3.6 (-7.0 to -0.1)	3.8 (2.6 to 5.6)	3.5 (2.5 to 5.4)	3.3 (2.4 to 5.0)	-6.2 (-14.3 to 3.3)	-5.1 (-10.8 to 0.8)
Occupational noise	5.9 (5.4 to 6.5)	5.8 (5.3 to 6.3)	5.6 (5.2 to 6.2)	-3.0 (-6.0 to 0.2)	-2.2 (-4.1 to -0.2)	3.6 (3.1 to 4.2)	3.6 (3.2 to 4.1)	3.7 (3.3 to 4.2)	-0.1 (-5.7 to 6.7)	1.5 (-2.1 to 5.4)
Occupational ergonomic factors	9.7 (8.0 to 11.7)	8.6 (7.2 to 10.4)	8.5 (7.0 to 10.4)	-11.1 (-21.8 to 1.3)	-0.7 (-10.1 to 10.5)	5.2 (4.0 to 6.6)	5.3 (4.2 to 6.7)	6.1 (4.7 to 7.8)	2.4 (-17.2 to 26.2)	13.9 (-5.1 to 35.3)
Non-exclusive breastfeeding	0.4 (0.3 to 0.6)	0.4 (0.3 to 0.6)	0.4 (0.3 to 0.6)	-1.1 (-13.8 to 13.1)	-1.5 (-11.8 to 10.1)	0.4 (0.3 to 0.6)	0.4 (0.3 to 0.6)	0.4 (0.3 to 0.6)	-1.1 (-13.8 to 13.1)	-1.6 (-11.9 to 10.1)
Discontinued breastfeeding	1.8 (1.6 to 1.9)	1.8 (1.7 to 1.9)	1.7 (1.6 to 1.9)	0.0 (-8.1 to 9.8)	-3.0 (-9.5 to 4.9)	1.8 (1.6 to 1.9)	1.8 (1.7 to 1.9)	1.7 (1.6 to 1.9)	-0.2 (-8.3 to 9.6)	-3.2 (-9.7 to 4.7)
Child underweight	0.2 (0.2 to 0.4)	0.2 (0.1 to 0.3)	0.2 (0.1 to 0.3)	-13.5 (-33.0 to 9.2)	-4.5 (-19.6 to 12.5)	0.3 (0.2 to 0.4)	0.2 (0.1 to 0.3)	0.2 (0.1 to 0.3)	-14.4 (-34.6 to 9.1)	-4.7 (-20.0 to 11.4)
Child wasting	0.2 (0.1 to 0.3)	0.2 (0.1 to 0.2)	0.2 (0.1 to 0.2)	-7.9 (-31.3 to 20.4)	-1.6 (-18.4 to 20.9)	0.2 (0.1 to 0.3)	0.2 (0.1 to 0.2)	0.2 (0.1 to 0.2)	-8.2 (-31.1 to 19.5)	-2.1 (-20.0 to 20.8)
Child stunting	0.6 (0.3 to 1.0)	0.5 (0.3 to 0.7)	0.5 (0.3 to 0.7)	-13.0 (-44.3 to 37.3)	-2.5 (-32.1 to 33.7)	0.6 (0.3 to 1.0)	0.5 (0.3 to 0.7)	0.5 (0.3 to 0.7)	-13.6 (-46.7 to 37.5)	-2.2 (-30.7 to 40.2)
Short gestation for birth weight	0.0 (0.0 to 0.0)	0.0 (0.0 to 0.0)	0.0 (0.0 to 0.0)	7.9 (-10.5 to 36.1)	3.6 (-11.1 to 33.6)	0.0 (0.0 to 0.0)	0.0 (0.0 to 0.0)	0.0 (0.0 to 0.0)	16.1 (-6.2 to 88.8)	0.5 (-24.6 to 38.7)
Low birth weight for gestation	0.0 (0.0 to 0.0)	0.0 (0.0 to 0.0)	0.0 (0.0 to 0.0)	5.0 (-14.1 to 31.7)	0.8 (-21.8 to 16.4)	0.0 (0.0 to 0.0)	0.0 (0.0 to 0.0)	0.0 (0.0 to 0.0)	-6.0 (-59.5 to 21.0)	4.8 (-20.6 to 33.4)
Iron deficiency	-	-	-	-	-	7.5 (5.1 to 10.4)	7.6 (5.3 to 10.6)	7.0 (4.8 to 9.8)	1.0 (-21.5 to 29.9)	-8.2 (-23.9 to 10.6)
Vitamin A deficiency	0.9 (0.6 to 1.3)	0.8 (0.5 to 1.1)	0.6 (0.4 to 0.9)	-7.7 (-21.2 to 9.8)	-26.7 (-38.0 to -13.6)	0.6 (0.4 to 0.8)	0.8 (0.5 to 1.0)	0.6 (0.4 to 0.8)	24.1 (-6.1 to 50.6)	-27.2 (-42.7 to -8.9)
Zinc deficiency	0.5 (0.1 to 1.0)	0.4 (0.1 to 1.1)	0.4 (0.1 to 0.9)	-10.0 (-64.8 to 72.2)	-14.0 (-68.6 to 159.9)	0.5 (0.1 to 1.1)	0.3 (0.1 to 0.9)	0.4 (0.1 to 0.8)	-31.0 (-63.7 to 40.8)	5.3 (-60.8 to 168.6)
Smoking	15.0 (11.6 to 18.4)	10.9 (8.6 to 13.6)	9.4 (7.5 to 11.7)	-27.3 (-37.3 to -16.2)	-13.8 (-22.1 to -4.6)	3.4 (2.3 to 4.7)	2.9 (2.1 to 3.8)	2.8 (2.0 to 3.8)	-13.6 (-34.5 to 13.2)	-2.6 (-18.1 to 17.1)
Chewing tobacco	1.4 (0.9 to 2.0)	0.9 (0.6 to 1.3)	0.7 (0.5 to 1.1)	-33.2 (-55.5 to 2.6)	-18.9 (-42.0 to 13.7)	0.1 (0.1 to 0.2)	0.1 (0.1 to 0.1)	0.1 (0.1 to 0.1)	-4.2 (-39.5 to 55.1)	-2.4 (-31.2 to 41.4)
Second-hand smoke	37.9 (34.2 to 41.7)	38.2 (34.1 to 42.5)	37.8 (33.5 to 42.1)	0.8 (-9.7 to 14.7)	-1.0 (-9.7 to 7.7)	58.7 (55.3 to 62.1)	52.8 (49.0 to 56.3)	52.2 (48.3 to 56.3)	-10.1 (-16.6 to -4.0)	-1.2 (-6.6 to 4.6)
Alcohol use	14.6 (7.6 to 24.8)	15.1 (7.6 to 25.2)	17.3 (8.2 to 30.3)	3.1 (-29.1 to 50.0)	14.6 (-16.8 to 57.9)	11.6 (5.8 to 20.6)	12.1 (5.9 to 21.2)	14.0 (6.6 to 25.0)	3.5 (-28.9 to 50.6)	16.4 (-17.2 to 60.4)
Drug use	0.5 (0.4 to 0.7)	0.5 (0.4 to 0.7)	0.6 (0.4 to 0.8)	0.1 (-5.7 to 5.6)	11.7 (4.8 to 20.9)	0.3 (0.2 to 0.4)	0.3 (0.2 to 0.4)	0.3 (0.3 to 0.4)	1.5 (-4.3 to 8.0)	9.0 (2.5 to 17.1)
Diet low in fruits	41.4 (36.1 to 45.0)	39.7 (34.2 to 43.6)	41.0 (35.6 to 44.6)	-4.2 (-9.7 to 0.9)	3.4 (-1.2 to 8.1)	38.6 (33.0 to 42.9)	35.8 (29.6 to 40.4)	38.0 (32.0 to 42.4)	-7.3 (-14.7 to -0.3)	6.4 (0.3 to 14.2)
Diet low in vegetables	20.3 (11.8 to 27.7)	14.3 (6.2 to 22.3)	16.2 (7.6 to 24.3)	-29.5 (-58.3 to -4.6)	13.6 (-15.7 to 64.7)	22.6 (14.8 to 29.6)	16.6 (8.3 to 24.4)	18.4 (10.0 to 25.8)	-26.6 (-52.6 to -5.1)	11.1 (-13.9 to 50.2)
Diet low in legumes	4.4 (0.8 to 9.8)	6.3 (1.6 to 12.6)	8.6 (2.8 to 15.8)	43.0 (-27.1 to 295.6)	36.7 (-14.0 to 182.2)	8.5 (2.8 to 15.4)	8.8 (2.8 to 15.5)	11.6 (5.0 to 18.4)	4.4 (-38.9 to 97.6)	31.5 (-6.8 to 119.5)
Diet low in whole grains	30.7 (26.0 to 34.9)	35.4 (31.7 to 38.9)	36.8 (33.1 to 40.2)	15.4 (7.0 to 27.1)	3.9 (-0.7 to 9.0)	32.1 (27.7 to 36.1)	36.7 (33.1 to 39.9)	37.9 (34.3 to 41.0)	14.1 (6.7 to 25.6)	3.4 (-1.1 to 8.2)
Diet low in nuts and seeds	51.0 (49.7 to 51.9)	49.7 (48.2 to 51.0)	49.5 (47.7 to 50.9)	-2.5 (-4.5 to -0.5)	-0.4 (-2.5 to 1.4)	51.1 (49.9 to 52.0)	49.8 (48.2 to 51.0)	49.6 (47.8 to 51.0)	-2.6 (-4.8 to -0.8)	-0.4 (-2.2 to 1.5)
Diet low in milk	46.3 (44.1 to 48.0)	46.5 (44.5 to 48.2)	47.0 (45.0 to 48.6)	0.5 (-1.2 to 2.4)	1.0 (-0.3 to 2.5)	46.3 (44.3 to 48.0)	46.0 (43.9 to 47.7)	46.6 (44.5 to 48.3)	-0.7 (-2.6 to 1.3)	1.3 (-0.1 to 2.8)
Diet high in red meat	3.5 (0.4 to 7.9)	7.1 (1.9 to 13.0)	8.4 (2.4 to 15.3)	104.5 (-14.4 to 909.3)	17.9 (-35.8 to 129.7)	0.5 (0.0 to 2.2)	1.3 (0.0 to 4.2)	1.7 (0.0 to 4.9)	183.7 (-75.4 to 3419971.5)	28.8 (-86.4 to 2817.4)
Diet high in processed meat	11.6 (7.6 to 15.5)	19.9 (15.0 to 24.1)	23.4 (17.7 to 28.6)	71.0 (33.5 to 130.2)	18.1 (1.1 to 38.7)	9.7 (6.0 to 13.2)	14.9 (10.6 to 18.6)	16.8 (12.0 to 20.8)	52.6 (20.2 to 110.8)	13.4 (-1.3 to 33.6)
Diet high in sugar-sweetened beverages	6.7 (2.8 to 13.4)	7.7 (3.5 to 13.3)	8.1 (3.6 to 14.3)	14.2 (-45.0 to 134.8)	5.4 (-34.7 to 74.8)	4.9 (1.8 to 10.4)	5.3 (2.4 to 9.6)	5.7 (2.5 to 11.0)	9.1 (-48.6 to 143.3)	6.7 (-36.3 to 78.3)
Diet low in fibre	22.4 (14.8 to 29.0)	23.5 (16.4 to 29.5)	24.6 (17.3 to 30.8)	5.2 (-11.6 to 28.4)	4.4 (-6.2 to 18.3)	23.6 (16.5 to 30.1)	24.7 (17.6 to 30.6)	26.2 (19.5 to 32.0)	4.4 (-11.2 to 25.3)	6.0 (-4.9 to 20.1)
Diet low in calcium	35.2 (30.9 to 38.7)	35.0 (30.7 to 38.5)	35.9 (31.7 to 39.2)	-0.4 (-4.1 to 3.3)	2.4 (-0.3 to 5.6)	37.3 (33.4 to 40.4)	36.2 (32.1 to 39.6)	37.4 (33.5 to 40.6)	-2.9 (-6.3 to 0.1)	3.3 (1.0 to 6.6)
Diet low in seafood omega-3 fatty acids	3.3 (1.5 to 4.7)	0.2 (0.0 to 1.0)	0.3 (0.0 to 1.2)	-92.4 (-100.0 to -69.7)	6.8 (-89.2 to 2235.3)	3.8 (1.8 to 5.3)	0.3 (0.0 to 1.4)	0.4 (0.0 to 1.5)	-91.0 (-100.0 to -68.7)	10.7 (-87.4 to 1412.4)
Diet low in polyunsaturated fatty acids	30.8 (25.2 to 35.6)	28.0 (22.4 to 33.2)	28.8 (23.0 to 33.7)	-9.0 (-20.6 to 0.7)	2.5 (-4.9 to 12.3)	31.4 (26.3 to 35.8)	28.8 (23.0 to 33.8)	29.5 (23.7 to 34.4)	-8.5 (-19.4 to 0.3)	2.6 (-4.8 to 12.5)
Diet high in trans fatty acids	1.1 (0.0 to 3.3)	1.0 (0.0 to 3.0)	0.9 (0.0 to 2.9)	-12.4 (-37.7 to -6.5)	-8.5 (-32.6 to -3.3)	1.5 (0.1 to 4.0)	1.3 (0.1 to 3.7)	1.2 (0.0 to 3.5)	-12.6 (-33.4 to -6.5)	-8.3 (-28.3 to -3.4)
Diet high in sodium	27.9 (17.9 to 36.1)	18.1 (9.5 to 25.2)	15.6 (6.8 to 22.9)	-35.0 (-57.6 to -16.5)	-13.8 (-37.6 to 2.8)	31.7 (23.3 to 38.9)	19.0 (10.7 to 25.8)	15.8 (7.1 to 23.0)	-40.1 (-59.2 to -25.8)	-17.1 (-38.6 to -4.3)
Intimate partner violence	-	-	-	-	-	2.3 (1.9 to 2.7)	2.1 (1.8 to 2.5)	2.1 (1.7 to 2.4)	-6.8 (-11.0 to -2.5)	-3.8 (-8.4 to 0.9)
Childhood sexual abuse	5.3 (4.3 to 6.5)	5.3 (4.3 to 6.5)	5.3 (4.3 to 6.5)	0.0 (0.0 to 0.0)	0.0 (0.0 to 0.0)	7.3 (5.9 to 8.9)	7.3 (5.9 to 8.9)	7.5 (6.0 to 9.2)	0.0 (0.0 to 0.0)	2.8 (-4.0 to 10.6)

Supplementary Results Table 1. Kanagawa age-standardised Summary Exposure Values (SEVs) for Level 4 risk factors and percentage change, by sex, 1990, 2007, and 2017.Data in parentheses are 95% uncertainty intervals.

	Male					Female				
	**1990**	**2007**	**2017**	**Percentage change 1990-2007**	**Percentage change 2007-2017**	**1990**	**2007**	**2017**	**Percentage change 1990-2007**	**Percentage change 2007-2017**
Bullying victimization	3.2 (1.4 to 6.6)	3.3 (1.4 to 6.7)	3.4 (1.4 to 7.1)	0.9 (-7.0 to 9.0)	4.7 (-0.6 to 9.9)	1.9 (0.8 to 3.8)	1.9 (0.8 to 3.7)	2.0 (0.8 to 3.9)	-1.6 (-9.3 to 6.5)	4.0 (-1.5 to 9.2)
Low physical activity	0.5 (0.2 to 0.9)	0.5 (0.2 to 0.9)	0.4 (0.2 to 0.8)	-1.8 (-3.6 to 0.3)	-8.8 (-12.2 to -4.7)	0.5 (0.2 to 1.0)	0.5 (0.2 to 1.0)	0.5 (0.2 to 0.9)	-3.8 (-6.0 to -1.3)	0.8 (-0.7 to 2.3)
High fasting plasma glucose	1.3 (0.8 to 2.2)	1.3 (0.8 to 2.0)	1.5 (0.9 to 2.4)	-1.4 (-36.3 to 51.8)	12.7 (-23.5 to 81.8)	1.2 (0.7 to 1.9)	1.3 (0.7 to 2.1)	1.4 (0.8 to 2.4)	6.7 (-34.9 to 74.6)	10.2 (-29.1 to 83.6)
High LDL cholesterol	12.3 (10.7 to 14.0)	12.8 (11.2 to 14.6)	12.8 (11.2 to 14.6)	4.2 (-1.7 to 10.5)	-0.1 (-4.5 to 4.6)	13.2 (11.6 to 14.9)	13.2 (11.7 to 15.0)	13.2 (11.7 to 15.0)	0.6 (-4.8 to 6.3)	0.0 (-3.9 to 4.4)
High systolic blood pressure	11.5 (10.5 to 12.5)	8.5 (7.6 to 9.4)	8.3 (7.4 to 9.3)	-25.9 (-31.3 to -19.5)	-1.6 (-8.7 to 5.8)	9.7 (8.8 to 10.7)	6.1 (5.4 to 6.9)	5.7 (4.9 to 6.5)	-37.3 (-43.4 to -30.3)	-7.3 (-16.1 to 2.7)
High body-mass index	7.4 (4.9 to 11.3)	10.7 (7.7 to 16.1)	11.0 (7.8 to 16.3)	43.9 (15.1 to 82.6)	2.7 (-11.5 to 17.5)	6.0 (3.8 to 9.2)	7.8 (5.1 to 12.0)	7.7 (4.8 to 12.0)	29.1 (1.3 to 68.5)	-0.8 (-18.0 to 19.6)
Low bone mineral density	5.3 (4.4 to 6.3)	5.2 (4.3 to 6.1)	5.2 (4.3 to 6.1)	-2.4 (-14.7 to 13.1)	0.4 (-13.0 to 16.1)	7.2 (6.1 to 8.6)	7.0 (6.0 to 8.1)	7.1 (6.0 to 8.4)	-3.5 (-14.5 to 9.6)	2.0 (-10.4 to 15.0)
Impaired kidney function	2.6 (1.2 to 6.5)	2.5 (1.1 to 6.3)	2.3 (1.1 to 6.1)	-3.1 (-7.4 to 1.5)	-6.6 (-12.1 to -1.9)	3.2 (1.5 to 7.4)	3.3 (1.5 to 7.6)	3.1 (1.4 to 7.2)	3.0 (-1.2 to 7.9)	-6.9 (-11.2 to -2.4)

Supplementary Results Table 1. Kochi age-standardised Summary Exposure Values (SEVs) for Level 4 risk factors and percentage change, by sex, 1990, 2007, and 2017.Data in parentheses are 95% uncertainty intervals.

	Male					Female				
	1990	2007	2017	Percentage change 1990-2007	Percentage change 2007-2017	1990	2007	2017	Percentage change 1990-2007	Percentage change 2007-2017
Unsafe water source	2.8 (1.5 to 4.6)	2.2 (1.2 to 3.8)	2.4 (1.3 to 3.9)	-20.1 (-60.1 to 66.2)	7.2 (-39.1 to 85.4)	2.8 (1.5 to 4.6)	2.2 (1.2 to 3.8)	2.4 (1.3 to 3.9)	-20.1 (-60.1 to 66.2)	7.2 (-39.1 to 85.4)
Unsafe sanitation	5.1 (2.5 to 9.5)	2.4 (1.1 to 4.6)	2.1 (1.0 to 4.0)	-52.3 (-80.1 to 11.1)	-13.5 (-55.3 to 68.7)	5.1 (2.5 to 9.5)	2.4 (1.1 to 4.6)	2.1 (1.0 to 4.0)	-52.3 (-80.1 to 11.1)	-13.5 (-55.3 to 68.7)
No access to handwashing facility	7.0 (5.0 to 9.4)	6.5 (4.8 to 8.9)	6.0 (4.5 to 8.1)	-6.4 (-34.4 to 33.6)	-7.7 (-29.1 to 18.9)	7.0 (5.0 to 9.4)	6.5 (4.8 to 8.9)	6.0 (4.5 to 8.1)	-6.4 (-34.4 to 33.6)	-7.7 (-29.1 to 18.9)
Ambient particulate matter pollution	31.2 (21.2 to 46.5)	31.8 (22.3 to 46.7)	27.0 (17.1 to 41.6)	1.7 (-23.6 to 38.8)	-15.1 (-41.0 to 13.3)	30.9 (20.8 to 46.0)	31.7 (22.3 to 46.6)	26.9 (17.1 to 41.5)	2.7 (-23.3 to 41.6)	-15.1 (-41.1 to 13.4)
Household air pollution from solid fuels	1.0 (0.3 to 2.9)	0.2 (0.1 to 0.5)	0.2 (0.0 to 0.4)	-78.2 (-93.9 to -51.8)	-14.8 (-63.4 to 78.2)	1.4 (0.4 to 7.2)	0.3 (0.1 to 1.7)	0.3 (0.1 to 1.5)	-75.0 (-93.8 to -47.9)	-12.6 (-62.8 to 79.0)
Ambient ozone pollution	48.2 (19.2 to 54.8)	50.1 (21.9 to 54.8)	51.8 (25.5 to 54.8)	4.1 (0.0 to 15.3)	3.3 (0.0 to 17.6)	48.2 (19.2 to 54.8)	50.1 (21.9 to 54.8)	51.8 (25.5 to 54.8)	4.1 (0.0 to 15.3)	3.3 (0.0 to 17.6)
Residential radon	14.1 (1.0 to 44.5)	14.1 (1.0 to 44.5)	14.1 (1.0 to 44.5)	0.0 (0.0 to 0.0)	0.0 (0.0 to 0.0)	14.1 (1.0 to 44.5)	14.1 (1.0 to 44.5)	14.1 (1.0 to 44.5)	0.0 (0.0 to 0.0)	0.0 (0.0 to 0.0)
Lead exposure	2.9 (0.4 to 6.6)	2.1 (0.3 to 5.1)	1.6 (0.2 to 4.2)	-29.2 (-41.2 to -21.0)	-25.1 (-40.0 to -17.2)	1.6 (0.1 to 4.5)	1.1 (0.1 to 3.4)	0.8 (0.0 to 2.9)	-32.1 (-47.9 to -23.4)	-25.7 (-46.8 to -16.8)
Occupational exposure to asbestos	1.6 (1.4 to 1.9)	2.4 (2.2 to 2.6)	2.1 (1.7 to 2.6)	46.1 (26.6 to 74.2)	-12.4 (-29.1 to 8.1)	0.5 (0.4 to 0.6)	0.5 (0.5 to 0.6)	0.5 (0.4 to 0.6)	-2.4 (-20.7 to 29.1)	-9.9 (-28.3 to 12.8)
Occupational exposure to arsenic	0.4 (0.0 to 1.0)	0.4 (0.0 to 0.9)	0.4 (0.0 to 1.0)	-	-	0.4 (0.0 to 0.9)	0.3 (0.0 to 0.8)	0.4 (0.0 to 0.9)	-	-
Occupational exposure to benzene	0.6 (0.0 to 1.7)	0.6 (0.0 to 1.7)	0.7 (0.0 to 1.8)	3.2 (-4.4 to 13.7)	3.5 (-2.5 to 11.9)	0.5 (0.0 to 1.5)	0.7 (0.0 to 1.9)	0.8 (0.0 to 2.2)	24.5 (9.8 to 47.6)	14.7 (4.6 to 30.3)
Occupational exposure to beryllium	0.0 (0.0 to 0.0)	0.0 (0.0 to 0.0)	0.0 (0.0 to 0.0)	-5.0 (-10.6 to 0.4)	1.9 (-2.0 to 6.3)	0.0 (0.0 to 0.0)	0.0 (0.0 to 0.0)	0.0 (0.0 to 0.0)	3.8 (-6.6 to 16.2)	9.6 (2.5 to 17.7)
Occupational exposure to cadmium	0.1 (0.0 to 0.1)	0.0 (0.0 to 0.1)	0.0 (0.0 to 0.1)	-6.0 (-17.7 to 6.7)	1.0 (-9.4 to 11.5)	0.0 (0.0 to 0.1)	0.0 (0.0 to 0.0)	0.0 (0.0 to 0.1)	-6.4 (-22.3 to 13.1)	5.7 (-7.1 to 20.9)
Occupational exposure to chromium	0.1 (0.1 to 0.1)	0.1 (0.1 to 0.1)	0.1 (0.1 to 0.1)	-4.0 (-14.8 to 7.6)	1.6 (-7.5 to 10.6)	0.1 (0.1 to 0.1)	0.1 (0.1 to 0.1)	0.1 (0.1 to 0.1)	-1.5 (-16.4 to 16.9)	7.4 (-4.0 to 21.0)
Occupational exposure to diesel engine exhaust	0.6 (0.5 to 0.6)	0.6 (0.5 to 0.6)	0.6 (0.5 to 0.6)	-0.3 (-8.3 to 8.5)	3.3 (-3.6 to 10.3)	0.4 (0.4 to 0.5)	0.5 (0.4 to 0.5)	0.5 (0.5 to 0.6)	14.8 (0.4 to 31.6)	11.9 (0.9 to 23.4)
Occupational exposure to formaldehyde	0.2 (0.2 to 0.3)	0.2 (0.2 to 0.3)	0.2 (0.2 to 0.3)	-5.1 (-17.8 to 9.0)	0.7 (-10.6 to 11.7)	0.2 (0.2 to 0.2)	0.2 (0.2 to 0.2)	0.2 (0.2 to 0.2)	-3.5 (-20.4 to 17.2)	6.5 (-6.7 to 21.7)
Occupational exposure to nickel	0.4 (0.0 to 1.9)	0.4 (0.0 to 1.7)	0.4 (0.0 to 1.7)	-	-	0.3 (0.0 to 1.4)	0.3 (0.0 to 1.2)	0.3 (0.0 to 1.3)	-	-
Occupational exposure to polycyclic aromatic hydrocarbons	0.2 (0.2 to 0.3)	0.2 (0.2 to 0.2)	0.2 (0.2 to 0.3)	-3.1 (-13.2 to 8.3)	1.9 (-6.3 to 10.3)	0.2 (0.2 to 0.2)	0.2 (0.2 to 0.2)	0.2 (0.2 to 0.2)	0.6 (-13.8 to 18.4)	8.3 (-2.7 to 20.7)
Occupational exposure to silica	4.4 (0.7 to 13.9)	4.2 (0.6 to 13.2)	4.2 (0.6 to 13.4)	-5.5 (-16.8 to 7.0)	1.5 (-7.0 to 12.6)	2.9 (0.4 to 9.6)	2.6 (0.4 to 8.3)	2.7 (0.4 to 9.2)	-11.2 (-27.3 to 8.9)	3.1 (-10.7 to 22.0)
Occupational exposure to sulphuric acid	0.6 (0.1 to 1.8)	0.6 (0.1 to 1.8)	0.6 (0.1 to 1.8)	-6.3 (-17.1 to 5.2)	1.3 (-8.0 to 11.2)	0.5 (0.1 to 1.5)	0.4 (0.1 to 1.4)	0.5 (0.1 to 1.5)	-8.3 (-24.1 to 11.3)	4.7 (-8.3 to 20.9)
Occupational exposure to trichloroethylene	0.1 (0.1 to 0.1)	0.1 (0.1 to 0.1)	0.1 (0.1 to 0.1)	-2.9 (-11.0 to 5.6)	2.1 (-4.2 to 8.9)	0.1 (0.0 to 0.1)	0.1 (0.1 to 0.1)	0.1 (0.1 to 0.1)	3.3 (-9.4 to 18.6)	9.0 (-0.3 to 19.9)
Occupational asthmagens	17.1 (13.8 to 21.6)	17.0 (13.7 to 21.3)	17.1 (13.6 to 21.4)	-0.6 (-19.3 to 22.2)	0.3 (-14.4 to 17.9)	7.9 (6.2 to 10.0)	8.0 (6.3 to 10.0)	8.4 (6.7 to 10.6)	0.8 (-16.8 to 19.9)	5.7 (-9.1 to 21.8)
Occupational particulate matter, gases, and fumes	6.4 (4.6 to 9.1)	6.1 (4.4 to 8.7)	5.9 (4.2 to 8.5)	-4.7 (-9.9 to 0.5)	-3.9 (-7.5 to -0.6)	3.5 (2.4 to 5.4)	3.3 (2.3 to 5.0)	3.2 (2.2 to 4.8)	-5.1 (-13.3 to 4.7)	-4.3 (-10.5 to 2.4)
Occupational noise	6.0 (5.5 to 6.6)	5.8 (5.3 to 6.3)	5.6 (5.2 to 6.1)	-3.7 (-6.7 to -0.3)	-2.7 (-4.7 to -0.4)	3.3 (2.8 to 3.9)	3.3 (2.9 to 3.8)	3.4 (3.0 to 3.9)	0.4 (-6.3 to 7.9)	1.9 (-2.6 to 6.8)
Occupational ergonomic factors	11.6 (9.5 to 13.9)	10.2 (8.4 to 12.3)	10.1 (8.2 to 12.4)	-12.0 (-25.6 to 3.3)	-1.0 (-13.3 to 13.2)	6.2 (4.8 to 8.1)	6.2 (4.8 to 7.9)	6.9 (5.3 to 9.0)	-0.3 (-24.0 to 28.7)	11.7 (-9.8 to 36.3)
Non-exclusive breastfeeding	0.4 (0.3 to 0.6)	0.4 (0.3 to 0.6)	0.4 (0.3 to 0.6)	-1.3 (-14.1 to 13.6)	-1.6 (-12.3 to 10.7)	0.4 (0.3 to 0.6)	0.4 (0.3 to 0.6)	0.4 (0.3 to 0.6)	-1.8 (-14.5 to 13.0)	-1.1 (-11.9 to 11.2)
Discontinued breastfeeding	1.6 (1.5 to 1.8)	1.7 (1.5 to 1.8)	1.6 (1.5 to 1.8)	2.5 (-7.5 to 13.9)	-1.1 (-8.7 to 7.2)	1.6 (1.5 to 1.8)	1.7 (1.5 to 1.8)	1.6 (1.5 to 1.8)	3.5 (-6.6 to 15.0)	-2.7 (-10.1 to 5.5)
Child underweight	0.3 (0.2 to 0.4)	0.2 (0.1 to 0.3)	0.2 (0.1 to 0.3)	-17.2 (-36.7 to 7.2)	-5.1 (-20.4 to 12.3)	0.3 (0.2 to 0.4)	0.2 (0.1 to 0.3)	0.2 (0.1 to 0.3)	-19.4 (-40.5 to 7.8)	-6.6 (-23.3 to 12.5)
Child wasting	0.2 (0.1 to 0.3)	0.2 (0.1 to 0.3)	0.2 (0.1 to 0.3)	-7.4 (-35.6 to 27.9)	-2.2 (-22.9 to 21.9)	0.2 (0.1 to 0.3)	0.2 (0.1 to 0.3)	0.2 (0.1 to 0.3)	-9.1 (-36.5 to 31.2)	-3.2 (-23.6 to 20.7)
Child stunting	0.7 (0.3 to 1.1)	0.6 (0.3 to 1.1)	0.6 (0.3 to 1.0)	-9.5 (-45.1 to 53.5)	-2.7 (-32.1 to 40.6)	0.7 (0.3 to 1.2)	0.6 (0.3 to 1.1)	0.6 (0.3 to 1.1)	-12.5 (-48.2 to 50.2)	-4.2 (-33.5 to 36.3)
Short gestation for birth weight	0.0 (0.0 to 0.0)	0.0 (0.0 to 0.0)	0.0 (0.0 to 0.0)	8.3 (-10.3 to 35.7)	4.2 (-13.4 to 30.7)	0.0 (0.0 to 0.0)	0.0 (0.0 to 0.0)	0.0 (0.0 to 0.0)	12.9 (-24.8 to 45.3)	0.6 (-20.9 to 35.2)
Low birth weight for gestation	0.0 (0.0 to 0.0)	0.0 (0.0 to 0.0)	0.0 (0.0 to 0.0)	5.2 (-12.9 to 25.9)	1.2 (-16.6 to 19.1)	0.0 (0.0 to 0.0)	0.0 (0.0 to 0.0)	0.0 (0.0 to 0.0)	-0.7 (-18.3 to 37.7)	4.1 (-18.0 to 26.2)
Iron deficiency	-	-	-	-	-	8.5 (5.9 to 11.6)	8.4 (5.9 to 11.7)	7.7 (5.2 to 10.9)	-1.1 (-21.3 to 26.8)	-8.2 (-24.4 to 10.4)
Vitamin A deficiency	1.0 (0.6 to 1.4)	0.9 (0.6 to 1.2)	0.7 (0.5 to 0.9)	-10.6 (-24.0 to 4.8)	-21.5 (-37.1 to -2.9)	0.8 (0.5 to 1.1)	0.9 (0.6 to 1.1)	0.7 (0.5 to 1.0)	6.4 (-9.0 to 30.3)	-16.8 (-31.8 to 5.7)
Zinc deficiency	0.5 (0.2 to 1.1)	0.4 (0.1 to 0.9)	0.4 (0.1 to 0.8)	-29.5 (-66.1 to 69.8)	-2.0 (-66.1 to 181.8)	0.5 (0.2 to 1.1)	0.4 (0.1 to 0.8)	0.4 (0.1 to 0.9)	-27.2 (-62.9 to 3.5)	8.4 (-46.5 to 168.9)
Smoking	18.4 (15.0 to 21.7)	12.9 (10.3 to 15.6)	10.9 (8.6 to 13.3)	-30.0 (-39.0 to -20.2)	-15.5 (-23.7 to -6.8)	4.2 (2.9 to 5.7)	3.5 (2.4 to 4.7)	3.4 (2.4 to 4.6)	-17.5 (-37.8 to 8.0)	-2.6 (-19.0 to 16.4)
Chewing tobacco	1.4 (0.9 to 2.0)	0.9 (0.6 to 1.4)	0.8 (0.5 to 1.1)	-32.7 (-56.0 to 7.5)	-18.3 (-40.4 to 14.3)	0.1 (0.1 to 0.2)	0.1 (0.1 to 0.1)	0.1 (0.1 to 0.1)	-5.0 (-44.1 to 57.8)	-2.3 (-33.3 to 39.7)
Second-hand smoke	37.6 (33.5 to 42.0)	35.6 (31.4 to 39.9)	35.8 (31.5 to 40.4)	-5.5 (-17.0 to 6.9)	0.5 (-8.6 to 11.4)	56.9 (53.4 to 60.4)	47.8 (44.4 to 51.3)	47.9 (44.0 to 52.0)	-16.0 (-21.9 to -9.5)	0.1 (-6.5 to 7.0)
Alcohol use	15.5 (7.8 to 27.3)	15.2 (7.5 to 26.2)	16.6 (7.9 to 29.2)	-2.0 (-35.2 to 42.1)	9.5 (-23.3 to 52.8)	11.9 (5.8 to 21.1)	11.6 (5.6 to 20.6)	12.7 (5.8 to 22.6)	-2.3 (-33.8 to 43.6)	8.8 (-24.2 to 57.7)
Drug use	0.5 (0.4 to 0.7)	0.5 (0.4 to 0.7)	0.6 (0.4 to 0.8)	1.2 (-4.5 to 7.0)	11.3 (4.1 to 20.2)	0.3 (0.2 to 0.4)	0.3 (0.2 to 0.4)	0.3 (0.3 to 0.4)	2.5 (-3.2 to 9.1)	8.9 (1.7 to 16.6)
Diet low in fruits	42.3 (36.7 to 45.7)	40.6 (35.1 to 44.4)	41.7 (35.8 to 45.3)	-4.0 (-9.5 to 1.3)	2.8 (-1.6 to 7.4)	39.3 (33.6 to 43.3)	36.6 (30.5 to 41.2)	38.4 (32.7 to 42.5)	-6.8 (-15.0 to -0.0)	5.0 (-0.7 to 12.9)
Diet low in vegetables	22.0 (13.7 to 29.1)	15.9 (7.8 to 23.5)	17.9 (9.4 to 25.5)	-27.8 (-54.8 to -6.3)	12.4 (-12.8 to 59.5)	24.2 (16.2 to 31.3)	18.1 (9.5 to 26.4)	19.8 (10.8 to 27.5)	-25.2 (-50.4 to -3.9)	9.1 (-12.7 to 43.0)
Diet low in legumes	4.6 (0.8 to 10.1)	5.6 (1.1 to 11.4)	7.4 (1.8 to 14.0)	21.9 (-51.6 to 215.4)	32.6 (-21.7 to 190.8)	8.2 (2.6 to 14.8)	7.9 (2.2 to 14.5)	10.3 (3.8 to 17.8)	-3.9 (-51.1 to 68.4)	30.8 (-11.3 to 138.1)
Diet low in whole grains	31.5 (26.8 to 35.5)	36.1 (32.3 to 39.4)	37.4 (33.8 to 40.6)	14.6 (6.4 to 26.0)	3.6 (-1.2 to 9.1)	32.9 (28.2 to 36.8)	37.3 (33.3 to 40.4)	38.5 (34.9 to 41.5)	13.5 (5.5 to 23.6)	3.2 (-1.0 to 8.0)
Diet low in nuts and seeds	51.7 (50.6 to 52.5)	50.7 (49.4 to 51.8)	50.7 (49.2 to 51.8)	-1.8 (-3.7 to -0.1)	-0.2 (-1.6 to 1.3)	51.7 (50.6 to 52.5)	50.5 (49.1 to 51.6)	50.4 (48.8 to 51.6)	-2.2 (-4.0 to -0.5)	-0.3 (-2.0 to 1.1)
Diet low in milk	46.7 (44.8 to 48.4)	47.1 (45.3 to 48.6)	47.6 (45.8 to 49.1)	0.8 (-1.0 to 2.7)	1.0 (-0.3 to 2.5)	46.8 (44.9 to 48.4)	46.6 (44.6 to 48.3)	47.2 (45.3 to 48.8)	-0.4 (-2.4 to 1.4)	1.3 (-0.0 to 2.9)
Diet high in red meat	3.1 (0.2 to 7.9)	6.4 (1.6 to 13.4)	7.4 (1.9 to 14.4)	106.1 (-18.9 to 1909.8)	15.2 (-46.5 to 158.0)	0.4 (0.0 to 1.9)	1.1 (0.0 to 4.1)	1.5 (0.0 to 4.9)	195.8 (-94.3 to [illegible]	32.5 (-96.8 to 11877.6)
Diet high in processed meat	9.7 (6.1 to 13.6)	16.2 (11.3 to 20.9)	18.7 (13.3 to 24.3)	66.3 (26.2 to 127.9)	15.5 (-2.9 to 39.5)	7.9 (4.4 to 11.4)	11.4 (7.4 to 15.2)	12.4 (8.3 to 16.1)	44.9 (12.2 to 111.5)	8.0 (-9.9 to 30.4)
Diet high in sugar-sweetened beverages	6.1 (2.3 to 12.7)	6.8 (2.8 to 12.9)	7.2 (3.0 to 14.0)	11.5 (-49.1 to 153.4)	5.5 (-39.8 to 78.2)	4.3 (1.6 to 9.4)	4.8 (2.1 to 9.5)	5.1 (2.1 to 10.1)	10.6 (-52.5 to 144.6)	6.5 (-38.8 to 78.5)
Diet low in fibre	23.3 (16.2 to 29.5)	24.4 (17.3 to 30.5)	25.3 (17.9 to 31.6)	4.6 (-10.9 to 26.5)	3.7 (-7.8 to 17.3)	24.2 (17.1 to 30.7)	25.1 (18.2 to 31.2)	26.3 (19.3 to 32.4)	3.6 (-12.0 to 23.1)	4.9 (-5.5 to 16.6)
Diet low in calcium	36.3 (32.2 to 39.6)	35.9 (31.6 to 39.3)	36.6 (32.4 to 39.9)	-1.1 (-4.7 to 2.7)	1.8 (-0.8 to 4.9)	38.4 (34.8 to 41.3)	37.1 (33.3 to 40.3)	38.1 (34.4 to 41.2)	-3.3 (-6.9 to -0.3)	2.7 (0.3 to 5.8)
Diet low in seafood omega-3 fatty acids	3.6 (2.1 to 5.0)	0.4 (0.0 to 1.4)	0.5 (0.0 to 1.5)	-87.7 (-99.9 to -59.0)	5.1 (-73.1 to 487.7)	4.1 (2.4 to 5.6)	0.6 (0.0 to 1.8)	0.6 (0.0 to 1.8)	-85.8 (-99.7 to -59.3)	5.2 (-71.2 to 436.2)
Diet low in polyunsaturated fatty acids	31.8 (26.4 to 36.6)	29.2 (23.1 to 34.1)	29.8 (23.9 to 34.5)	-8.2 (-19.4 to 1.5)	2.1 (-4.5 to 11.3)	32.4 (27.2 to 36.9)	29.9 (24.7 to 34.4)	30.6 (25.0 to 35.1)	-7.8 (-18.5 to 0.8)	2.3 (-6.4 to 12.7)
Diet high in trans fatty acids	1.1 (0.0 to 3.3)	0.9 (0.0 to 3.0)	0.9 (0.0 to 2.9)	-12.8 (-39.1 to -6.8)	-8.0 (-31.6 to -3.3)	1.5 (0.1 to 4.0)	1.3 (0.1 to 3.7)	1.2 (0.0 to 3.5)	-12.7 (-35.4 to -6.4)	-8.5 (-28.5 to -3.4)
Diet high in sodium	26.2 (16.4 to 34.6)	16.6 (7.6 to 23.8)	14.1 (5.2 to 21.6)	-36.8 (-61.2 to -17.1)	-15.0 (-42.4 to 2.8)	30.9 (22.7 to 38.6)	18.4 (10.1 to 25.6)	15.2 (6.7 to 22.9)	-40.4 (-59.7 to -25.1)	-17.2 (-42.0 to -2.3)
Intimate partner violence	-	-	-	-	-	2.4 (2.0 to 2.8)	2.2 (1.9 to 2.6)	2.1 (1.8 to 2.5)	-8.1 (-11.8 to -4.0)	-4.0 (-8.5 to 0.5)
Childhood sexual abuse	5.3 (4.3 to 6.5)	5.3 (4.3 to 6.5)	5.3 (4.3 to 6.5)	0.0 (0.0 to 0.0)	0.0 (0.0 to 0.0)	7.3 (5.9 to 8.9)	7.3 (5.9 to 8.9)	7.5 (6.0 to 9.2)	0.0 (0.0 to 0.0)	2.8 (-4.0 to 10.6)

Supplementary Results Table 1. Kochi age-standardised Summary Exposure Values (SEVs) for Level 4 risk factors and percentage change, by sex, 1990, 2007, and 2017.Data in parentheses are 95% uncertainty intervals.										
	**Male**					**Female**				
	**1990**	**2007**	**2017**	**Percentage change 1990-2007**	**Percentage change 2007-2017**	**1990**	**2007**	**2017**	**Percentage change 1990-2007**	**Percentage change 2007-2017**
Bullying victimization	3.3 (1.4 to 6.9)	3.4 (1.4 to 7.0)	3.5 (1.4 to 7.2)	1.2 (-6.6 to 9.1)	3.4 (-1.7 to 8.3)	1.9 (0.8 to 3.9)	1.9 (0.8 to 4.0)	2.0 (0.8 to 4.1)	-0.3 (-7.8 to 7.5)	2.4 (-3.1 to 7.4)
Low physical activity	0.5 (0.2 to 0.9)	0.5 (0.2 to 0.9)	0.4 (0.2 to 0.8)	-1.7 (-3.8 to 0.1)	-8.8 (-12.3 to -4.4)	0.5 (0.2 to 1.0)	0.5 (0.2 to 0.9)	0.5 (0.2 to 0.9)	-3.9 (-6.1 to -1.3)	0.7 (-1.0 to 2.2)
High fasting plasma glucose	1.4 (0.9 to 2.3)	1.3 (0.8 to 2.0)	1.5 (0.9 to 2.4)	-8.6 (-42.7 to 38.3)	10.6 (-24.4 to 75.9)	1.2 (0.7 to 1.9)	1.2 (0.7 to 2.0)	1.3 (0.7 to 2.2)	3.3 (-37.9 to 77.0)	8.9 (-31.0 to 71.4)
High LDL cholesterol	11.9 (10.3 to 13.7)	12.5 (10.9 to 14.3)	12.5 (10.9 to 14.3)	4.5 (-1.8 to 11.0)	-0.0 (-4.4 to 4.9)	12.6 (11.1 to 14.4)	12.8 (11.2 to 14.5)	12.8 (11.2 to 14.6)	1.1 (-4.0 to 6.7)	0.0 (-4.2 to 4.6)
High systolic blood pressure	11.1 (10.0 to 12.1)	8.2 (7.3 to 9.1)	8.1 (7.1 to 9.0)	-25.8 (-31.8 to -19.1)	-1.8 (-9.5 to 5.5)	9.5 (8.5 to 10.5)	6.0 (5.3 to 6.8)	5.6 (4.8 to 6.4)	-36.9 (-44.0 to -29.7)	-7.3 (-17.1 to 3.2)
High body-mass index	4.5 (2.7 to 7.2)	6.3 (3.9 to 10.6)	6.5 (4.0 to 10.7)	41.6 (13.2 to 82.0)	2.7 (-14.1 to 21.9)	4.9 (2.9 to 7.7)	6.3 (3.8 to 10.3)	6.3 (3.8 to 10.4)	29.2 (2.0 to 74.6)	0.5 (-17.4 to 23.0)
Low bone mineral density	5.3 (4.4 to 6.3)	5.2 (4.3 to 6.1)	5.2 (4.3 to 6.3)	-2.6 (-15.0 to 12.5)	0.5 (-12.6 to 14.6)	7.3 (6.1 to 8.6)	7.0 (6.0 to 8.2)	7.1 (6.0 to 8.3)	-3.6 (-15.1 to 10.1)	1.3 (-10.2 to 15.4)
Impaired kidney function	2.6 (1.2 to 6.5)	2.6 (1.2 to 6.5)	2.4 (1.1 to 6.1)	-3.2 (-7.7 to 1.4)	-7.3 (-12.9 to -2.2)	3.2 (1.5 to 7.5)	3.3 (1.5 to 7.6)	3.1 (1.4 to 7.3)	2.8 (-1.4 to 7.5)	-7.3 (-12.2 to -2.4)

Supplementary Results Table 1. Kumamoto age-standardised Summary Exposure Values (SEVs) for Level 4 risk factors and percentage change, by sex, 1990, 2007, and 2017.Data in parentheses are 95% uncertainty intervals.										
	Male					Female				
	1990	2007	2017	Percentage change 1990-2007	Percentage change 2007-2017	1990	2007	2017	Percentage change 1990-2007	Percentage change 2007-2017
Unsafe water source	2.5 (1.4 to 4.2)	2.1 (1.1 to 3.6)	2.3 (1.2 to 3.8)	-15.9 (-57.2 to 71.1)	5.5 (-39.8 to 85.7)	2.5 (1.4 to 4.2)	2.1 (1.1 to 3.6)	2.3 (1.2 to 3.8)	-15.9 (-57.2 to 71.1)	5.5 (-39.8 to 85.7)
Unsafe sanitation	4.6 (2.2 to 8.6)	2.3 (1.1 to 4.4)	2.0 (0.9 to 3.7)	-49.0 (-77.9 to 17.6)	-15.0 (-55.9 to 69.0)	4.6 (2.2 to 8.6)	2.3 (1.1 to 4.4)	2.0 (0.9 to 3.7)	-49.0 (-77.9 to 17.6)	-15.0 (-55.9 to 69.0)
No access to handwashing facility	6.9 (4.8 to 9.3)	6.5 (4.7 to 8.8)	6.0 (4.4 to 8.2)	-5.3 (-33.8 to 34.4)	-7.1 (-28.4 to 21.3)	6.9 (4.8 to 9.3)	6.5 (4.7 to 8.8)	6.0 (4.4 to 8.2)	-5.3 (-33.8 to 34.4)	-7.1 (-28.4 to 21.3)
Ambient particulate matter pollution	37.3 (25.1 to 54.0)	37.5 (26.5 to 54.5)	33.1 (22.1 to 50.0)	0.8 (-24.6 to 34.7)	-11.8 (-35.8 to 16.4)	37.0 (24.8 to 53.7)	37.5 (26.5 to 54.3)	33.1 (22.1 to 50.0)	1.3 (-24.2 to 35.9)	-11.8 (-35.8 to 16.5)
Household air pollution from solid fuels	0.6 (0.2 to 1.7)	0.2 (0.0 to 0.4)	0.1 (0.0 to 0.3)	-75.3 (-92.1 to -48.3)	-11.9 (-65.7 to 70.9)	0.9 (0.3 to 5.4)	0.3 (0.1 to 1.1)	0.2 (0.0 to 1.2)	-72.1 (-92.1 to -44.9)	-11.8 (-66.0 to 72.5)
Ambient ozone pollution	47.9 (18.9 to 54.8)	49.2 (20.4 to 54.8)	50.5 (22.3 to 54.8)	2.7 (0.0 to 8.9)	2.7 (0.0 to 10.7)	47.9 (18.9 to 54.8)	49.2 (20.4 to 54.8)	50.5 (22.3 to 54.8)	2.7 (0.0 to 8.9)	2.7 (0.0 to 10.7)
Residential radon	13.8 (0.9 to 46.2)	13.8 (0.9 to 46.2)	13.8 (0.9 to 46.2)	0.0 (0.0 to 0.0)	0.0 (0.0 to 0.0)	13.8 (0.9 to 46.2)	13.8 (0.9 to 46.2)	13.8 (0.9 to 46.2)	0.0 (0.0 to 0.0)	0.0 (0.0 to 0.0)
Lead exposure	3.3 (0.6 to 7.1)	2.2 (0.3 to 5.4)	1.6 (0.2 to 4.3)	-32.2 (-46.1 to -23.5)	-27.3 (-42.5 to -19.5)	1.7 (0.1 to 4.7)	1.1 (0.1 to 3.4)	0.8 (0.0 to 2.7)	-37.5 (-54.5 to -27.6)	-29.7 (-51.5 to -20.4)
Occupational exposure to asbestos	1.8 (1.5 to 2.0)	2.2 (2.1 to 2.5)	2.3 (1.8 to 2.8)	25.7 (8.3 to 52.9)	1.5 (-19.1 to 24.8)	0.5 (0.3 to 0.5)	0.5 (0.4 to 0.5)	0.4 (0.3 to 0.5)	-0.6 (-19.0 to 31.2)	-9.4 (-27.9 to 13.2)
Occupational exposure to arsenic	0.5 (0.0 to 1.1)	0.4 (0.0 to 1.0)	0.4 (0.0 to 1.0)	-	-	0.4 (0.0 to 1.0)	0.4 (0.0 to 0.8)	0.4 (0.0 to 0.9)	-	-
Occupational exposure to benzene	0.6 (0.0 to 1.8)	0.6 (0.0 to 1.9)	0.7 (0.0 to 2.0)	2.6 (-4.1 to 13.4)	3.3 (-2.2 to 11.3)	0.6 (0.0 to 1.6)	0.7 (0.0 to 2.0)	0.8 (0.0 to 2.3)	22.1 (8.4 to 42.3)	15.1 (5.5 to 29.4)
Occupational exposure to beryllium	0.0 (0.0 to 0.0)	0.0 (0.0 to 0.0)	0.0 (0.0 to 0.0)	-5.1 (-10.1 to 0.1)	1.9 (-2.1 to 6.0)	0.0 (0.0 to 0.0)	0.0 (0.0 to 0.0)	0.0 (0.0 to 0.0)	2.2 (-6.8 to 12.5)	9.6 (2.7 to 17.2)
Occupational exposure to cadmium	0.1 (0.0 to 0.1)	0.0 (0.0 to 0.1)	0.0 (0.0 to 0.1)	-6.5 (-18.2 to 6.2)	0.7 (-8.9 to 11.0)	0.0 (0.0 to 0.1)	0.0 (0.0 to 0.0)	0.0 (0.0 to 0.1)	-8.3 (-22.9 to 9.6)	5.6 (-6.8 to 20.6)
Occupational exposure to chromium	0.1 (0.1 to 0.1)	0.1 (0.1 to 0.1)	0.1 (0.1 to 0.1)	-4.8 (-15.4 to 6.4)	1.3 (-7.0 to 10.2)	0.1 (0.1 to 0.1)	0.1 (0.1 to 0.1)	0.1 (0.1 to 0.1)	-3.7 (-17.6 to 13.0)	7.3 (-3.6 to 20.5)
Occupational exposure to diesel engine exhaust	0.6 (0.5 to 0.6)	0.6 (0.5 to 0.6)	0.6 (0.6 to 0.7)	-2.0 (-10.1 to 6.9)	3.3 (-2.8 to 10.2)	0.4 (0.4 to 0.5)	0.5 (0.4 to 0.6)	0.5 (0.5 to 0.6)	12.1 (-2.4 to 27.4)	11.8 (0.2 to 23.1)
Occupational exposure to formaldehyde	0.3 (0.2 to 0.3)	0.2 (0.2 to 0.3)	0.2 (0.2 to 0.3)	-5.9 (-18.3 to 7.7)	0.3 (-9.4 to 11.0)	0.2 (0.2 to 0.2)	0.2 (0.2 to 0.2)	0.2 (0.2 to 0.2)	-5.7 (-21.7 to 14.0)	6.4 (-6.1 to 21.2)
Occupational exposure to nickel	0.4 (0.0 to 2.0)	0.4 (0.0 to 1.8)	0.4 (0.0 to 1.8)	-	-	0.3 (0.0 to 1.5)	0.3 (0.0 to 1.4)	0.3 (0.0 to 1.3)	-	-
Occupational exposure to polycyclic aromatic hydrocarbons	0.2 (0.2 to 0.3)	0.2 (0.2 to 0.3)	0.2 (0.2 to 0.3)	-3.9 (-13.8 to 7.0)	1.7 (-5.7 to 9.8)	0.2 (0.2 to 0.2)	0.2 (0.2 to 0.2)	0.2 (0.2 to 0.3)	-1.6 (-15.2 to 15.0)	8.2 (-1.9 to 20.5)
Occupational exposure to silica	4.7 (0.6 to 14.8)	4.4 (0.6 to 14.2)	4.4 (0.6 to 14.3)	-6.7 (-17.0 to 4.8)	1.2 (-7.6 to 11.5)	3.1 (0.4 to 10.1)	2.7 (0.4 to 8.7)	2.8 (0.4 to 9.1)	-12.9 (-27.7 to 5.5)	2.6 (-10.6 to 19.9)
Occupational exposure to sulphuric acid	0.6 (0.1 to 1.9)	0.6 (0.1 to 1.9)	0.6 (0.1 to 1.8)	-6.5 (-17.5 to 5.3)	0.9 (-7.7 to 10.4)	0.5 (0.1 to 1.6)	0.5 (0.1 to 1.4)	0.5 (0.1 to 1.5)	-9.6 (-24.6 to 7.9)	4.6 (-7.7 to 20.4)
Occupational exposure to trichloroethylene	0.1 (0.1 to 0.1)	0.1 (0.1 to 0.1)	0.1 (0.1 to 0.1)	-3.8 (-11.3 to 4.7)	1.9 (-4.0 to 8.3)	0.1 (0.1 to 0.1)	0.1 (0.1 to 0.1)	0.1 (0.1 to 0.1)	1.0 (-9.8 to 14.6)	8.9 (-0.0 to 19.2)
Occupational asthmagens	18.7 (14.8 to 23.6)	18.0 (14.3 to 22.8)	17.9 (14.1 to 22.6)	-3.6 (-21.6 to 20.4)	-0.6 (-15.8 to 17.3)	8.1 (6.6 to 10.1)	8.1 (6.6 to 10.1)	8.6 (6.9 to 10.9)	0.1 (-15.4 to 18.0)	5.7 (-6.5 to 20.2)
Occupational particulate matter, gases, and fumes	6.4 (4.6 to 9.2)	6.1 (4.4 to 8.9)	5.9 (4.2 to 8.6)	-4.5 (-9.3 to 0.2)	-3.7 (-6.8 to -0.1)	3.6 (2.5 to 5.3)	3.4 (2.3 to 5.1)	3.3 (2.3 to 5.0)	-5.3 (-13.2 to 4.1)	-4.4 (-10.0 to 2.2)
Occupational noise	6.0 (5.5 to 6.5)	5.8 (5.4 to 6.3)	5.6 (5.2 to 6.2)	-3.4 (-6.6 to -0.4)	-2.5 (-4.5 to -0.4)	3.4 (2.9 to 4.0)	3.4 (3.0 to 4.0)	3.5 (3.1 to 4.0)	0.4 (-5.5 to 8.0)	1.8 (-2.3 to 6.4)
Occupational ergonomic factors	10.7 (8.8 to 12.9)	9.6 (7.9 to 11.6)	9.4 (7.7 to 11.3)	-11.0 (-23.2 to 2.3)	-1.5 (-12.9 to 10.7)	5.7 (4.4 to 7.2)	5.8 (4.5 to 7.4)	6.5 (5.0 to 8.4)	1.7 (-20.7 to 29.3)	12.4 (-7.2 to 35.5)
Non-exclusive breastfeeding	0.4 (0.3 to 0.6)	0.4 (0.3 to 0.6)	0.4 (0.3 to 0.6)	-0.6 (-13.2 to 14.1)	-2.1 (-12.4 to 9.8)	0.4 (0.3 to 0.6)	0.4 (0.3 to 0.6)	0.4 (0.3 to 0.6)	-1.3 (-13.9 to 13.3)	-1.6 (-11.9 to 10.3)
Discontinued breastfeeding	1.7 (1.5 to 1.8)	1.7 (1.6 to 1.8)	1.6 (1.5 to 1.8)	1.5 (-7.9 to 11.8)	-2.7 (-10.0 to 5.8)	1.6 (1.5 to 1.8)	1.7 (1.6 to 1.9)	1.7 (1.5 to 1.8)	4.3 (-5.4 to 14.9)	-3.4 (-10.7 to 4.9)
Child underweight	0.3 (0.2 to 0.4)	0.2 (0.1 to 0.3)	0.2 (0.1 to 0.3)	-14.8 (-34.4 to 7.6)	-6.2 (-20.9 to 11.4)	0.3 (0.2 to 0.4)	0.2 (0.1 to 0.3)	0.2 (0.1 to 0.3)	-17.4 (-38.6 to 9.0)	-6.7 (-24.1 to 11.5)
Child wasting	0.2 (0.1 to 0.3)	0.2 (0.1 to 0.3)	0.2 (0.1 to 0.3)	-7.5 (-35.3 to 30.3)	-2.1 (-21.6 to 21.5)	0.2 (0.1 to 0.3)	0.2 (0.1 to 0.3)	0.2 (0.1 to 0.3)	-7.8 (-35.5 to 28.3)	-3.1 (-24.2 to 20.9)
Child stunting	0.6 (0.3 to 1.1)	0.6 (0.3 to 1.0)	0.6 (0.3 to 0.9)	-10.4 (-44.9 to 49.0)	-3.3 (-32.4 to 34.9)	0.7 (0.3 to 1.1)	0.6 (0.3 to 1.0)	0.6 (0.3 to 1.0)	-10.8 (-47.1 to 48.8)	-3.8 (-35.6 to 41.8)
Short gestation for birth weight	0.0 (0.0 to 0.0)	0.0 (0.0 to 0.0)	0.0 (0.0 to 0.0)	11.4 (-12.9 to 41.1)	1.6 (-15.9 to 25.9)	0.0 (0.0 to 0.0)	0.0 (0.0 to 0.0)	0.0 (0.0 to 0.0)	11.6 (-21.8 to 63.8)	0.7 (-21.1 to 37.5)
Low birth weight for gestation	0.0 (0.0 to 0.0)	0.0 (0.0 to 0.0)	0.0 (0.0 to 0.0)	2.8 (-18.3 to 25.3)	2.8 (-15.1 to 22.8)	0.0 (0.0 to 0.0)	0.0 (0.0 to 0.0)	0.0 (0.0 to 0.0)	-4.2 (-56.4 to 33.1)	4.3 (-17.2 to 26.1)
Iron deficiency	-	-	-	-	-	8.4 (5.8 to 11.4)	8.4 (5.8 to 11.5)	7.7 (5.2 to 10.7)	0.6 (-20.0 to 26.0)	-8.5 (-24.3 to 7.6)
Vitamin A deficiency	0.9 (0.5 to 1.2)	0.9 (0.6 to 1.2)	0.8 (0.5 to 1.1)	0.2 (-19.3 to 23.8)	-4.1 (-25.4 to 21.0)	0.7 (0.5 to 1.0)	0.7 (0.5 to 1.0)	0.7 (0.5 to 1.0)	6.4 (-14.1 to 38.5)	-0.3 (-18.3 to 18.4)
Zinc deficiency	0.5 (0.1 to 1.1)	0.4 (0.1 to 1.0)	0.4 (0.1 to 0.8)	-23.6 (-65.3 to 69.2)	-7.7 (-64.9 to 174.0)	0.5 (0.2 to 1.1)	0.4 (0.1 to 0.8)	0.4 (0.1 to 0.8)	-29.3 (-63.2 to 6.8)	5.6 (-56.0 to 158.2)
Smoking	18.2 (14.7 to 21.5)	12.7 (10.2 to 15.4)	10.7 (8.5 to 13.0)	-30.1 (-39.0 to -20.3)	-15.8 (-24.2 to -6.0)	4.1 (3.0 to 5.4)	3.4 (2.4 to 4.5)	3.3 (2.4 to 4.5)	-17.3 (-35.5 to 7.2)	-2.9 (-19.5 to 16.9)
Chewing tobacco	1.4 (0.9 to 2.0)	0.9 (0.6 to 1.4)	0.8 (0.5 to 1.1)	-32.9 (-55.8 to 3.7)	-18.8 (-42.3 to 12.1)	0.1 (0.1 to 0.2)	0.1 (0.1 to 0.1)	0.1 (0.1 to 0.1)	-4.8 (-41.1 to 52.7)	-2.0 (-32.1 to 44.5)
Second-hand smoke	37.6 (33.5 to 41.6)	35.9 (31.5 to 40.3)	35.7 (31.2 to 40.4)	-4.6 (-15.2 to 7.3)	-0.5 (-9.5 to 9.6)	56.9 (53.2 to 60.5)	48.1 (44.6 to 52.0)	47.7 (43.6 to 51.6)	-15.4 (-21.8 to -8.8)	-0.8 (-6.7 to 5.7)
Alcohol use	14.2 (7.3 to 24.9)	14.1 (7.1 to 25.1)	16.2 (7.5 to 28.0)	-0.5 (-33.8 to 50.2)	14.6 (-19.3 to 63.5)	11.7 (5.9 to 20.5)	11.7 (5.7 to 21.4)	13.1 (6.0 to 23.2)	-0.4 (-35.1 to 51.2)	12.1 (-20.1 to 58.0)
Drug use	0.5 (0.4 to 0.7)	0.5 (0.4 to 0.7)	0.6 (0.4 to 0.8)	0.7 (-5.1 to 6.7)	11.7 (4.6 to 20.2)	0.3 (0.2 to 0.4)	0.3 (0.2 to 0.4)	0.3 (0.3 to 0.4)	2.2 (-4.0 to 7.8)	9.2 (2.8 to 17.1)
Diet low in fruits	42.1 (36.3 to 45.6)	40.3 (34.7 to 44.1)	41.5 (36.1 to 45.1)	-4.2 (-9.8 to 1.5)	2.9 (-2.0 to 7.7)	39.4 (33.0 to 43.6)	36.6 (30.2 to 41.0)	38.9 (33.0 to 43.1)	-6.9 (-14.1 to 0.3)	6.2 (0.5 to 13.2)
Diet low in vegetables	21.6 (12.5 to 28.8)	15.9 (7.8 to 23.2)	17.9 (9.9 to 25.7)	-26.5 (-51.9 to -1.9)	12.9 (-12.0 to 55.1)	23.7 (14.9 to 30.7)	17.9 (9.7 to 25.5)	19.7 (11.3 to 27.1)	-24.5 (-49.3 to -3.6)	9.9 (-12.6 to 47.2)
Diet low in legumes	4.5 (0.7 to 10.0)	5.8 (1.1 to 11.3)	7.7 (2.1 to 14.0)	29.3 (-42.9 to 300.8)	33.3 (-19.7 to 202.8)	8.1 (2.5 to 15.1)	7.7 (2.1 to 14.5)	9.9 (3.5 to 17.4)	-4.7 (-48.6 to 81.4)	29.3 (-16.1 to 142.8)
Diet low in whole grains	31.2 (26.5 to 35.2)	35.8 (31.8 to 39.1)	37.1 (33.3 to 40.3)	14.7 (6.7 to 26.2)	3.6 (-1.0 to 8.8)	32.6 (28.1 to 36.6)	37.0 (33.0 to 40.2)	38.3 (34.5 to 41.3)	13.7 (6.1 to 24.4)	3.3 (-1.0 to 8.3)
Diet low in nuts and seeds	51.0 (49.7 to 51.9)	49.5 (47.7 to 50.8)	49.1 (47.2 to 50.7)	-2.9 (-5.2 to -1.1)	-0.7 (-2.9 to 1.1)	51.2 (50.1 to 52.1)	49.8 (48.2 to 51.0)	49.5 (47.8 to 51.0)	-2.8 (-4.8 to -1.0)	-0.6 (-2.8 to 1.1)
Diet low in milk	46.6 (44.5 to 48.2)	46.9 (45.1 to 48.5)	47.4 (45.6 to 48.9)	0.8 (-1.0 to 2.8)	1.0 (-0.3 to 2.5)	46.6 (44.7 to 48.3)	46.4 (44.3 to 48.1)	47.1 (45.1 to 48.6)	-0.3 (-2.2 to 1.7)	1.4 (-0.2 to 3.0)
Diet high in red meat	3.3 (0.3 to 8.0)	6.6 (1.7 to 13.1)	7.7 (2.1 to 15.4)	98.2 (-15.6 to 996.7)	16.0 (-43.0 to 149.6)	0.4 (0.0 to 2.1)	1.2 (0.0 to 4.0)	1.6 (0.0 to 5.0)	186.7 (-81.7 to [illegible]	27.7 (-90.9 to 4151.2)
Diet high in processed meat	10.3 (6.3 to 14.1)	17.2 (12.2 to 22.1)	19.9 (14.5 to 25.3)	66.1 (29.1 to 130.6)	15.7 (-3.3 to 41.4)	8.5 (5.0 to 12.0)	12.5 (8.4 to 16.4)	13.7 (9.4 to 17.6)	46.5 (14.9 to 101.2)	9.5 (-7.8 to 29.5)
Diet high in sugar-sweetened beverages	6.4 (2.5 to 13.6)	7.2 (3.2 to 12.9)	7.6 (3.2 to 13.6)	12.0 (-48.8 to 144.2)	5.3 (-37.8 to 78.2)	4.6 (1.8 to 9.8)	5.2 (2.2 to 9.8)	5.5 (2.2 to 10.9)	13.4 (-45.2 to 161.7)	6.2 (-41.9 to 87.3)
Diet low in fibre	22.9 (15.4 to 29.3)	24.0 (16.6 to 30.0)	25.0 (17.9 to 31.2)	4.5 (-12.2 to 26.6)	4.3 (-7.8 to 19.1)	24.1 (16.9 to 30.6)	25.1 (17.9 to 31.2)	26.5 (19.6 to 32.6)	4.3 (-10.6 to 24.0)	5.4 (-5.0 to 19.7)
Diet low in calcium	35.9 (31.6 to 39.3)	35.8 (31.6 to 39.1)	36.6 (32.6 to 39.9)	-0.4 (-4.0 to 3.4)	2.3 (-0.5 to 5.6)	38.1 (34.4 to 41.0)	37.0 (33.1 to 40.1)	38.2 (34.6 to 41.2)	-2.7 (-6.0 to 0.3)	3.2 (0.6 to 6.2)
Diet low in seafood omega-3 fatty acids	3.5 (1.8 to 4.9)	0.4 (0.0 to 1.3)	0.4 (0.0 to 1.4)	-89.1 (-99.9 to -62.7)	2.8 (-75.5 to 1052.0)	4.0 (2.2 to 5.5)	0.5 (0.0 to 1.6)	0.5 (0.0 to 1.7)	-87.4 (-99.8 to -62.6)	7.5 (-76.3 to 541.9)
Diet low in polyunsaturated fatty acids	31.5 (26.1 to 36.2)	28.8 (23.2 to 33.7)	29.4 (23.8 to 34.2)	-8.5 (-18.8 to 0.4)	2.1 (-5.5 to 10.3)	32.0 (26.7 to 36.6)	29.4 (23.5 to 34.1)	30.1 (24.1 to 34.7)	-8.0 (-19.0 to 0.9)	2.3 (-4.7 to 10.0)
Diet high in trans fatty acids	1.1 (0.0 to 3.3)	1.0 (0.0 to 3.0)	0.9 (0.0 to 2.9)	-12.4 (-39.0 to -6.5)	-8.4 (-32.2 to -3.7)	1.5 (0.1 to 4.0)	1.3 (0.1 to 3.7)	1.2 (0.0 to 3.5)	-12.7 (-36.0 to -6.6)	-8.3 (-29.1 to -3.7)
Diet high in sodium	26.9 (17.2 to 35.0)	17.3 (8.3 to 24.5)	14.9 (6.3 to 22.0)	-35.6 (-59.4 to -17.4)	-14.1 (-38.7 to 3.3)	31.4 (22.7 to 38.5)	19.0 (10.3 to 26.0)	15.7 (6.9 to 22.9)	-39.7 (-60.9 to -24.1)	-17.3 (-39.8 to -3.9)
Intimate partner violence	-	-	-	-	-	2.3 (2.0 to 2.7)	2.1 (1.8 to 2.5)	2.0 (1.7 to 2.4)	-8.8 (-12.7 to -4.8)	-3.7 (-8.0 to 0.4)
Childhood sexual abuse	5.3 (4.3 to 6.5)	5.3 (4.3 to 6.5)	5.3 (4.3 to 6.5)	0.0 (0.0 to 0.0)	0.0 (0.0 to 0.0)	7.3 (5.9 to 8.9)	7.3 (5.9 to 8.9)	7.5 (6.0 to 9.2)	0.0 (0.0 to 0.0)	2.8 (-4.0 to 10.6)

Supplementary Results Table 1. Kumamoto age-standardised Summary Exposure Values (SEVs) for Level 4 risk factors and percentage change, by sex, 1990, 2007, and 2017.Data in parentheses are 95% uncertainty intervals.

	**Male**					**Female**				
	**1990**	**2007**	**2017**	**Percentage change 1990-2007**	**Percentage change 2007-2017**	**1990**	**2007**	**2017**	**Percentage change 1990-2007**	**Percentage change 2007-2017**
Bullying victimization	3.3 (1.3 to 6.9)	3.4 (1.4 to 7.2)	3.5 (1.4 to 7.3)	3.1 (-4.6 to 11.3)	2.9 (-2.5 to 7.6)	1.9 (0.8 to 4.0)	1.9 (0.8 to 4.1)	2.0 (0.8 to 4.2)	1.4 (-6.8 to 9.5)	1.4 (-4.2 to 6.0)
Low physical activity	0.5 (0.2 to 0.9)	0.5 (0.2 to 0.9)	0.4 (0.2 to 0.8)	-1.8 (-3.7 to 0.1)	-8.8 (-12.3 to -4.5)	0.5 (0.2 to 1.0)	0.5 (0.2 to 0.9)	0.5 (0.2 to 0.9)	-3.9 (-6.1 to -1.2)	0.7 (-0.7 to 2.2)
High fasting plasma glucose	1.4 (0.9 to 2.1)	1.3 (0.8 to 2.0)	1.5 (0.9 to 2.4)	-4.5 (-38.5 to 48.8)	12.5 (-24.2 to 79.8)	1.1 (0.7 to 1.8)	1.2 (0.7 to 2.1)	1.3 (0.8 to 2.4)	5.2 (-33.2 to 70.2)	10.1 (-30.7 to 84.6)
High LDL cholesterol	12.0 (10.4 to 13.7)	12.5 (10.9 to 14.3)	12.5 (10.8 to 14.3)	4.3 (-1.6 to 10.7)	0.1 (-4.6 to 4.8)	12.7 (11.2 to 14.4)	12.8 (11.3 to 14.6)	12.8 (11.3 to 14.5)	0.7 (-4.8 to 6.4)	0.3 (-4.2 to 5.0)
High systolic blood pressure	11.1 (10.1 to 12.2)	8.2 (7.4 to 9.1)	8.1 (7.2 to 9.1)	-26.2 (-32.2 to -19.5)	-1.8 (-8.9 to 6.0)	9.6 (8.6 to 10.5)	6.0 (5.3 to 6.8)	5.6 (4.8 to 6.4)	-37.2 (-43.7 to -29.9)	-7.2 (-17.2 to 3.4)
High body-mass index	5.1 (3.2 to 8.1)	7.4 (4.8 to 12.2)	7.6 (5.0 to 11.9)	44.0 (16.3 to 86.2)	3.0 (-14.0 to 21.6)	5.1 (3.1 to 8.0)	6.6 (4.1 to 10.0)	6.6 (4.1 to 10.2)	29.5 (1.9 to 72.4)	0.5 (-18.5 to 23.2)
Low bone mineral density	5.3 (4.4 to 6.3)	5.2 (4.3 to 6.1)	5.2 (4.3 to 6.2)	-2.6 (-16.4 to 12.5)	0.6 (-13.5 to 16.4)	7.2 (6.0 to 8.5)	7.0 (6.0 to 8.2)	7.1 (5.9 to 8.3)	-3.4 (-15.3 to 10.6)	1.6 (-11.5 to 14.4)
Impaired kidney function	2.6 (1.2 to 6.5)	2.5 (1.1 to 6.4)	2.4 (1.1 to 6.1)	-2.9 (-7.2 to 1.9)	-6.8 (-12.2 to -2.1)	3.2 (1.5 to 7.4)	3.3 (1.5 to 7.6)	3.1 (1.4 to 7.2)	3.0 (-0.7 to 7.5)	-7.2 (-12.1 to -2.3)

Supplementary Results Table 1. Kyoto age-standardised Summary Exposure Values (SEVs) for Level 4 risk factors and percentage change, by sex, 1990, 2007, and 2017.Data in parentheses are 95% uncertainty intervals.

	Male					Female				
	1990	2007	2017	Percentage change 1990-2007	Percentage change 2007-2017	1990	2007	2017	Percentage change 1990-2007	Percentage change 2007-2017
Unsafe water source	1.9 (1.1 to 3.2)	1.6 (0.9 to 2.7)	1.7 (1.0 to 2.8)	-16.9 (-55.3 to 65.3)	7.1 (-37.8 to 77.4)	1.9 (1.1 to 3.2)	1.6 (0.9 to 2.7)	1.7 (1.0 to 2.8)	-16.9 (-55.3 to 65.3)	7.1 (-37.8 to 77.4)
Unsafe sanitation	3.0 (1.4 to 5.5)	1.5 (0.7 to 2.8)	1.3 (0.6 to 2.7)	-50.4 (-79.5 to 16.1)	-11.3 (-53.6 to 68.9)	3.0 (1.4 to 5.5)	1.5 (0.7 to 2.8)	1.3 (0.6 to 2.7)	-50.4 (-79.5 to 16.1)	-11.3 (-53.6 to 68.9)
No access to handwashing facility	6.6 (4.7 to 8.8)	6.2 (4.5 to 8.5)	5.8 (4.1 to 7.8)	-5.5 (-32.8 to 32.4)	-6.9 (-28.8 to 20.8)	6.6 (4.7 to 8.8)	6.2 (4.5 to 8.5)	5.8 (4.1 to 7.8)	-5.5 (-32.8 to 32.4)	-6.9 (-28.8 to 20.8)
Ambient particulate matter pollution	33.3 (23.4 to 47.5)	33.0 (23.3 to 47.9)	28.3 (19.1 to 42.8)	-0.9 (-20.2 to 26.8)	-14.4 (-33.5 to 6.5)	33.2 (23.2 to 47.4)	33.0 (23.3 to 47.9)	28.2 (19.1 to 42.8)	-0.5 (-20.0 to 27.3)	-14.4 (-33.5 to 6.5)
Household air pollution from solid fuels	0.4 (0.1 to 1.1)	0.1 (0.0 to 0.2)	0.1 (0.0 to 0.2)	-73.1 (-92.8 to -47.1)	-11.5 (-65.8 to 86.8)	0.6 (0.2 to 3.1)	0.2 (0.0 to 0.7)	0.2 (0.0 to 0.6)	-69.1 (-92.7 to -45.1)	-7.8 (-65.5 to 89.5)
Ambient ozone pollution	52.4 (27.4 to 54.8)	52.3 (27.1 to 54.8)	52.5 (27.7 to 54.8)	-0.2 (-1.8 to 0.0)	0.3 (0.0 to 2.8)	52.4 (27.4 to 54.8)	52.3 (27.1 to 54.8)	52.5 (27.7 to 54.8)	-0.2 (-1.8 to 0.0)	0.3 (0.0 to 2.8)
Residential radon	13.7 (0.9 to 42.7)	13.7 (0.9 to 42.7)	13.7 (0.9 to 42.7)	0.0 (0.0 to 0.0)	0.0 (0.0 to 0.0)	13.7 (0.9 to 42.7)	13.7 (0.9 to 42.7)	13.7 (0.9 to 42.7)	0.0 (0.0 to 0.0)	0.0 (0.0 to 0.0)
Lead exposure	2.8 (0.4 to 6.4)	1.8 (0.2 to 4.7)	1.2 (0.1 to 3.6)	-36.0 (-51.3 to -26.6)	-30.5 (-47.0 to -22.1)	9.9 (5.4 to 14.1)	6.7 (3.3 to 10.5)	4.9 (2.1 to 8.2)	-32.4 (-41.1 to -24.4)	-27.5 (-35.3 to -20.8)
Occupational exposure to asbestos	2.1 (1.8 to 2.3)	2.8 (2.5 to 3.0)	2.5 (2.1 to 3.0)	31.6 (13.8 to 54.2)	-7.5 (-23.7 to 11.2)	0.6 (0.5 to 0.7)	0.5 (0.5 to 0.6)	0.5 (0.4 to 0.6)	-9.5 (-25.8 to 18.0)	-9.8 (-29.0 to 15.5)
Occupational exposure to arsenic	0.5 (0.0 to 1.1)	0.4 (0.0 to 1.0)	0.4 (0.0 to 1.0)	-	-	0.4 (0.0 to 1.0)	0.4 (0.0 to 0.9)	0.4 (0.0 to 0.9)	-	-
Occupational exposure to benzene	0.6 (0.0 to 1.8)	0.7 (0.0 to 1.9)	0.7 (0.0 to 1.9)	2.3 (-4.4 to 12.0)	3.5 (-2.3 to 11.7)	0.6 (0.0 to 1.9)	0.8 (0.0 to 2.1)	0.9 (0.1 to 2.4)	20.3 (7.5 to 40.6)	13.4 (3.7 to 27.5)
Occupational exposure to beryllium	0.0 (0.0 to 0.0)	0.0 (0.0 to 0.0)	0.0 (0.0 to 0.0)	-5.4 (-10.3 to -0.5)	2.0 (-1.7 to 6.2)	0.0 (0.0 to 0.0)	0.0 (0.0 to 0.0)	0.0 (0.0 to 0.0)	1.2 (-7.2 to 10.6)	8.4 (2.3 to 14.9)
Occupational exposure to cadmium	0.1 (0.0 to 0.1)	0.0 (0.0 to 0.1)	0.0 (0.0 to 0.1)	-7.9 (-18.3 to 4.1)	0.8 (-8.8 to 11.3)	0.0 (0.0 to 0.1)	0.0 (0.0 to 0.1)	0.0 (0.0 to 0.1)	-10.6 (-24.1 to 6.6)	4.3 (-7.7 to 18.2)
Occupational exposure to chromium	0.1 (0.1 to 0.1)	0.1 (0.1 to 0.1)	0.1 (0.1 to 0.1)	-6.2 (-15.6 to 4.7)	1.3 (-7.0 to 10.5)	0.1 (0.1 to 0.1)	0.1 (0.1 to 0.1)	0.1 (0.1 to 0.1)	-5.8 (-18.8 to 9.9)	6.0 (-4.3 to 17.7)
Occupational exposure to diesel engine exhaust	0.6 (0.6 to 0.6)	0.6 (0.5 to 0.6)	0.6 (0.6 to 0.7)	-1.9 (-9.5 to 6.4)	3.0 (-3.6 to 9.9)	0.5 (0.4 to 0.5)	0.5 (0.5 to 0.6)	0.6 (0.5 to 0.7)	11.9 (-1.0 to 26.8)	10.1 (-0.0 to 20.5)
Occupational exposure to formaldehyde	0.3 (0.2 to 0.3)	0.2 (0.2 to 0.3)	0.2 (0.2 to 0.3)	-7.3 (-18.2 to 5.5)	0.4 (-9.6 to 11.3)	0.2 (0.2 to 0.3)	0.2 (0.2 to 0.2)	0.2 (0.2 to 0.2)	-8.0 (-21.9 to 10.2)	5.1 (-6.7 to 19.3)
Occupational exposure to nickel	0.4 (0.0 to 1.9)	0.4 (0.0 to 1.7)	0.4 (0.0 to 1.7)	-	-	0.4 (0.0 to 1.6)	0.3 (0.0 to 1.3)	0.3 (0.0 to 1.3)	-	-
Occupational exposure to polycyclic aromatic hydrocarbons	0.2 (0.2 to 0.3)	0.2 (0.2 to 0.3)	0.2 (0.2 to 0.3)	-5.1 (-14.0 to 5.4)	1.7 (-6.0 to 9.9)	0.2 (0.2 to 0.3)	0.2 (0.2 to 0.2)	0.2 (0.2 to 0.3)	-3.6 (-16.1 to 11.6)	6.9 (-2.8 to 18.3)
Occupational exposure to silica	4.7 (0.7 to 15.3)	4.4 (0.6 to 14.0)	4.5 (0.6 to 14.3)	-6.0 (-16.5 to 7.1)	1.5 (-7.3 to 12.9)	3.2 (0.5 to 10.4)	2.7 (0.4 to 8.5)	2.8 (0.4 to 8.4)	-15.1 (-29.4 to 4.2)	1.7 (-11.4 to 19.3)
Occupational exposure to sulphuric acid	0.6 (0.1 to 2.0)	0.6 (0.1 to 1.8)	0.6 (0.1 to 1.8)	-8.0 (-18.0 to 4.1)	1.1 (-8.3 to 11.4)	0.5 (0.1 to 1.6)	0.5 (0.1 to 1.5)	0.5 (0.1 to 1.5)	-11.9 (-26.0 to 5.4)	3.2 (-9.0 to 18.6)
Occupational exposure to trichloroethylene	0.1 (0.1 to 0.1)	0.1 (0.1 to 0.1)	0.1 (0.1 to 0.1)	-5.1 (-12.3 to 3.1)	1.8 (-4.4 to 8.7)	0.1 (0.1 to 0.1)	0.1 (0.1 to 0.1)	0.1 (0.1 to 0.1)	-0.5 (-11.3 to 12.1)	7.4 (-0.6 to 17.0)
Occupational asthmagens	19.4 (15.2 to 24.2)	18.5 (14.8 to 23.9)	18.4 (14.4 to 23.2)	-4.7 (-21.4 to 19.5)	-0.5 (-15.9 to 17.4)	8.5 (6.9 to 10.4)	8.5 (6.9 to 10.3)	8.9 (7.2 to 11.2)	-0.6 (-16.5 to 17.7)	5.0 (-7.1 to 19.5)
Occupational particulate matter, gases, and fumes	6.3 (4.5 to 9.3)	6.0 (4.3 to 8.9)	5.8 (4.2 to 8.4)	-4.3 (-9.5 to 1.0)	-3.6 (-7.1 to -0.1)	3.7 (2.5 to 5.5)	3.5 (2.4 to 5.1)	3.3 (2.3 to 4.9)	-6.3 (-14.5 to 3.3)	-5.0 (-10.6 to 1.0)
Occupational noise	5.8 (5.4 to 6.4)	5.7 (5.3 to 6.2)	5.6 (5.2 to 6.1)	-2.8 (-6.0 to 0.5)	-2.1 (-4.3 to 0.1)	3.6 (3.1 to 4.1)	3.6 (3.2 to 4.0)	3.6 (3.2 to 4.0)	-0.0 (-5.8 to 7.0)	1.6 (-2.3 to 5.4)
Occupational ergonomic factors	10.0 (8.2 to 12.1)	8.9 (7.3 to 10.9)	8.8 (7.1 to 10.6)	-11.2 (-22.6 to 2.8)	-1.2 (-11.3 to 9.8)	5.4 (4.2 to 6.7)	5.5 (4.4 to 7.0)	6.3 (4.9 to 8.2)	2.2 (-17.0 to 26.7)	13.6 (-5.2 to 36.4)
Non-exclusive breastfeeding	0.4 (0.3 to 0.6)	0.4 (0.3 to 0.6)	0.4 (0.3 to 0.6)	-0.8 (-13.7 to 13.8)	-1.5 (-11.8 to 10.3)	0.4 (0.3 to 0.6)	0.4 (0.3 to 0.6)	0.4 (0.3 to 0.6)	-1.0 (-13.8 to 13.6)	-1.6 (-11.9 to 10.2)
Discontinued breastfeeding	1.7 (1.6 to 1.9)	1.8 (1.6 to 1.9)	1.7 (1.6 to 1.9)	2.5 (-7.0 to 13.4)	-3.2 (-10.5 to 4.8)	1.7 (1.6 to 1.8)	1.8 (1.6 to 1.9)	1.7 (1.6 to 1.8)	2.2 (-7.2 to 13.1)	-3.0 (-10.2 to 5.1)
Child underweight	0.2 (0.1 to 0.3)	0.2 (0.1 to 0.3)	0.2 (0.1 to 0.3)	-12.9 (-32.0 to 7.8)	-4.0 (-17.4 to 10.0)	0.2 (0.1 to 0.4)	0.2 (0.1 to 0.3)	0.2 (0.1 to 0.3)	-14.4 (-34.2 to 9.7)	-4.9 (-18.6 to 10.3)
Child wasting	0.2 (0.1 to 0.3)	0.2 (0.1 to 0.3)	0.2 (0.1 to 0.3)	-6.5 (-32.2 to 28.5)	-1.6 (-21.2 to 24.0)	0.2 (0.1 to 0.3)	0.2 (0.1 to 0.3)	0.2 (0.1 to 0.3)	-8.2 (-35.6 to 29.8)	-2.3 (-22.2 to 23.7)
Child stunting	0.6 (0.3 to 1.1)	0.6 (0.3 to 0.9)	0.6 (0.3 to 0.9)	-8.8 (-43.6 to 44.8)	-3.1 (-31.2 to 34.7)	0.6 (0.3 to 1.1)	0.6 (0.3 to 0.9)	0.5 (0.3 to 0.9)	-10.7 (-44.6 to 46.7)	-3.3 (-31.7 to 37.2)
Short gestation for birth weight	0.0 (0.0 to 0.0)	0.0 (0.0 to 0.0)	0.0 (0.0 to 0.0)	11.6 (-9.5 to 41.8)	0.4 (-15.5 to 20.5)	0.0 (0.0 to 0.0)	0.0 (0.0 to 0.0)	0.0 (0.0 to 0.0)	11.4 (-21.9 to 47.6)	-1.4 (-24.2 to 23.6)
Low birth weight for gestation	0.0 (0.0 to 0.0)	0.0 (0.0 to 0.0)	0.0 (0.0 to 0.0)	3.6 (-14.4 to 25.9)	3.4 (-11.8 to 19.6)	0.0 (0.0 to 0.0)	0.0 (0.0 to 0.0)	0.0 (0.0 to 0.0)	-2.1 (-52.2 to 34.1)	4.9 (-10.4 to 30.3)
Iron deficiency	-	-	-	-	-	7.7 (5.2 to 10.8)	7.7 (5.3 to 10.7)	7.1 (4.7 to 10.2)	-0.3 (-22.2 to 29.3)	-7.8 (-24.6 to 10.0)
Vitamin A deficiency	0.8 (0.6 to 1.2)	0.7 (0.5 to 1.0)	0.5 (0.4 to 0.7)	-15.0 (-33.8 to 5.3)	-24.0 (-39.3 to 4.5)	0.7 (0.5 to 1.0)	0.7 (0.5 to 0.9)	0.7 (0.4 to 1.0)	1.2 (-14.7 to 24.8)	0.8 (-13.9 to 18.7)
Zinc deficiency	0.5 (0.1 to 1.0)	0.5 (0.1 to 1.2)	0.4 (0.1 to 1.1)	1.5 (-62.7 to 76.2)	-15.0 (-69.0 to 147.4)	0.5 (0.1 to 1.0)	0.4 (0.1 to 0.8)	0.3 (0.1 to 0.7)	-28.4 (-64.3 to 63.1)	-2.0 (-63.5 to 176.3)
Smoking	16.8 (13.5 to 20.2)	11.8 (9.4 to 14.4)	10.0 (8.0 to 12.4)	-29.8 (-38.6 to -19.1)	-15.2 (-23.8 to -6.2)	3.8 (2.7 to 5.1)	3.1 (2.2 to 4.3)	3.0 (2.1 to 4.2)	-17.0 (-36.5 to 7.6)	-3.4 (-18.9 to 15.8)
Chewing tobacco	1.4 (0.9 to 2.0)	0.9 (0.6 to 1.4)	0.8 (0.5 to 1.1)	-32.7 (-56.0 to 0.5)	-19.1 (-41.4 to 14.3)	0.1 (0.1 to 0.1)	0.1 (0.1 to 0.1)	0.1 (0.1 to 0.1)	-3.9 (-41.5 to 58.3)	-2.6 (-32.5 to 44.3)
Second-hand smoke	37.3 (33.2 to 41.2)	36.7 (32.4 to 40.9)	36.9 (32.4 to 41.2)	-1.5 (-13.3 to 11.6)	0.6 (-8.7 to 10.1)	56.4 (52.8 to 59.9)	49.5 (45.7 to 53.2)	49.7 (45.5 to 53.8)	-12.2 (-19.0 to -5.7)	0.4 (-5.4 to 6.5)
Alcohol use	15.0 (7.5 to 25.5)	15.3 (7.8 to 26.2)	17.6 (8.2 to 30.4)	2.0 (-31.9 to 49.3)	15.2 (-19.2 to 64.0)	11.7 (5.7 to 20.8)	12.0 (6.0 to 21.1)	14.0 (6.5 to 24.4)	2.0 (-32.1 to 49.9)	17.4 (-16.0 to 72.1)
Drug use	0.5 (0.4 to 0.7)	0.5 (0.4 to 0.7)	0.6 (0.4 to 0.8)	0.6 (-4.7 to 6.2)	11.6 (4.3 to 20.3)	0.3 (0.2 to 0.4)	0.3 (0.2 to 0.4)	0.3 (0.3 to 0.4)	2.5 (-3.4 to 8.0)	9.2 (2.1 to 16.9)
Diet low in fruits	40.7 (35.1 to 44.6)	39.3 (33.5 to 43.3)	40.9 (34.9 to 44.5)	-3.3 (-9.4 to 2.6)	4.0 (-0.6 to 9.2)	38.0 (31.7 to 42.3)	35.2 (29.0 to 39.8)	37.6 (32.0 to 41.9)	-7.3 (-14.8 to 0.8)	6.9 (0.4 to 15.9)
Diet low in vegetables	19.3 (10.7 to 27.0)	13.6 (5.6 to 21.7)	15.9 (7.7 to 24.1)	-29.3 (-59.0 to -3.6)	16.6 (-14.6 to 76.7)	21.4 (13.1 to 28.5)	15.5 (7.6 to 23.8)	17.4 (9.3 to 25.2)	-27.5 (-53.6 to -5.3)	12.5 (-14.1 to 59.7)
Diet low in legumes	4.5 (0.8 to 9.9)	6.7 (1.5 to 12.9)	9.1 (3.0 to 16.2)	47.7 (-27.0 to 355.3)	36.4 (-10.2 to 169.4)	8.4 (2.8 to 15.1)	8.6 (2.8 to 15.4)	11.3 (4.5 to 18.4)	2.2 (-41.6 to 88.1)	31.0 (-8.5 to 130.4)
Diet low in whole grains	30.3 (25.1 to 34.4)	35.1 (31.1 to 38.7)	36.5 (32.5 to 39.6)	16.1 (7.1 to 28.8)	3.9 (-1.3 to 9.5)	31.8 (27.2 to 35.7)	36.4 (32.6 to 39.6)	37.7 (34.1 to 40.7)	14.5 (6.7 to 25.2)	3.5 (-0.9 to 8.6)
Diet low in nuts and seeds	50.6 (49.2 to 51.6)	49.1 (47.3 to 50.6)	48.9 (46.9 to 50.5)	-2.8 (-5.4 to -0.6)	-0.4 (-2.6 to 1.5)	50.9 (49.6 to 51.9)	49.5 (47.8 to 50.9)	49.4 (47.4 to 50.9)	-2.6 (-5.2 to -0.4)	-0.3 (-2.4 to 1.7)
Diet low in milk	46.0 (44.0 to 47.8)	46.3 (44.3 to 48.0)	46.7 (44.8 to 48.3)	0.6 (-1.3 to 2.7)	0.9 (-0.5 to 2.5)	46.0 (43.9 to 47.8)	45.7 (43.6 to 47.5)	46.3 (44.3 to 48.1)	-0.6 (-2.6 to 1.4)	1.3 (-0.2 to 3.1)
Diet high in red meat	4.2 (0.6 to 9.3)	8.0 (2.5 to 14.9)	9.0 (2.6 to 16.4)	90.0 (-14.1 to 706.8)	13.3 (-39.4 to 127.7)	0.6 (0.0 to 2.4)	1.6 (0.0 to 5.1)	2.0 (0.0 to 5.8)	186.6 (-78.3 to 319498.5)	24.0 (-85.4 to 2777.8)
Diet high in processed meat	11.5 (7.3 to 15.6)	18.9 (14.0 to 23.6)	21.7 (16.3 to 27.0)	64.8 (30.5 to 128.9)	14.7 (-4.0 to 38.3)	9.8 (5.9 to 13.6)	14.3 (9.9 to 18.2)	15.8 (11.5 to 20.1)	46.3 (14.5 to 102.0)	10.1 (-6.1 to 32.3)
Diet high in sugar-sweetened beverages	7.4 (2.9 to 15.0)	8.7 (3.9 to 15.3)	9.1 (4.0 to 16.9)	17.1 (-44.3 to 154.4)	4.2 (-39.0 to 76.9)	5.3 (2.0 to 11.6)	5.9 (2.5 to 11.4)	6.4 (2.6 to 12.7)	11.3 (-48.5 to 163.3)	7.9 (-38.3 to 87.4)
Diet low in fibre	22.0 (14.5 to 28.7)	23.3 (16.4 to 29.6)	24.5 (17.2 to 31.0)	5.8 (-12.0 to 30.6)	5.0 (-7.6 to 19.7)	23.2 (15.8 to 29.7)	24.3 (17.2 to 30.4)	25.7 (18.8 to 31.6)	4.9 (-11.3 to 25.8)	5.9 (-5.2 to 20.6)
Diet low in calcium	34.5 (30.0 to 38.0)	34.3 (29.7 to 37.9)	35.2 (30.9 to 38.7)	-0.5 (-4.2 to 3.5)	2.6 (-0.6 to 6.0)	36.7 (32.6 to 40.0)	35.6 (31.4 to 39.0)	36.8 (32.7 to 40.1)	-3.0 (-6.9 to 0.6)	3.4 (0.6 to 7.0)
Diet low in seafood omega-3 fatty acids	3.1 (1.4 to 4.4)	0.2 (0.0 to 1.0)	0.3 (0.0 to 1.1)	-92.3 (-100.0 to -68.5)	7.3 (-92.6 to 2578.2)	3.7 (2.0 to 5.0)	0.5 (0.0 to 1.5)	0.5 (0.0 to 1.6)	-87.7 (-99.9 to -58.8)	4.9 (-77.6 to 745.7)
Diet low in polyunsaturated fatty acids	30.2 (25.0 to 35.0)	27.4 (21.6 to 32.3)	28.0 (22.0 to 32.9)	-9.2 (-20.1 to 0.4)	2.3 (-5.7 to 11.0)	30.8 (25.4 to 35.7)	28.1 (22.2 to 33.0)	28.7 (23.0 to 33.5)	-8.8 (-19.8 to 1.8)	2.2 (-5.4 to 10.9)
Diet high in trans fatty acids	1.1 (0.0 to 3.3)	1.0 (0.0 to 3.0)	0.9 (0.0 to 2.9)	-12.4 (-38.5 to -6.3)	-8.4 (-33.5 to -3.5)	1.5 (0.1 to 4.0)	1.3 (0.1 to 3.7)	1.2 (0.0 to 3.5)	-12.5 (-34.1 to -6.4)	-8.4 (-27.2 to -3.6)
Diet high in sodium	27.8 (18.2 to 35.9)	17.8 (9.0 to 25.1)	15.2 (6.4 to 22.7)	-36.2 (-59.5 to -18.0)	-14.5 (-39.0 to 2.2)	31.8 (23.4 to 38.7)	19.2 (10.9 to 25.8)	16.0 (7.2 to 23.1)	-39.6 (-58.6 to -25.4)	-16.6 (-38.2 to -3.7)
Intimate partner violence	-	-	-	-	-	2.3 (1.9 to 2.6)	2.1 (1.7 to 2.4)	2.0 (1.7 to 2.4)	-8.0 (-11.7 to -3.5)	-3.6 (-7.9 to 0.4)
Childhood sexual abuse	5.3 (4.3 to 6.5)	5.3 (4.3 to 6.5)	5.3 (4.3 to 6.5)	0.0 (0.0 to 0.0)	0.0 (0.0 to 0.0)	7.3 (5.9 to 8.9)	7.3 (5.9 to 8.9)	7.5 (6.0 to 9.2)	0.0 (0.0 to 0.0)	2.8 (-4.0 to 10.6)

Supplementary Results Table 1. Kyoto age-standardised Summary Exposure Values (SEVs) for Level 4 risk factors and percentage change, by sex, 1990, 2007, and 2017.Data in parentheses are 95% uncertainty intervals.

	Male					Female				
	**1990**	**2007**	**2017**	**Percentage change 1990-2007**	**Percentage change 2007-2017**	**1990**	**2007**	**2017**	**Percentage change 1990-2007**	**Percentage change 2007-2017**
Bullying victimization	3.2 (1.3 to 6.5)	3.3 (1.3 to 6.7)	3.4 (1.3 to 6.9)	1.0 (-6.9 to 8.8)	4.1 (-1.3 to 9.0)	1.9 (0.8 to 3.9)	1.9 (0.8 to 3.8)	1.9 (0.8 to 4.2)	-1.2 (-8.3 to 6.9)	4.5 (-21.6 to 37.9)
Low physical activity	0.5 (0.2 to 0.9)	0.5 (0.2 to 0.9)	0.4 (0.2 to 0.8)	-1.9 (-3.8 to 0.0)	-8.8 (-12.3 to -4.6)	0.5 (0.2 to 1.0)	0.5 (0.2 to 0.9)	0.5 (0.2 to 0.9)	-3.8 (-6.1 to -1.4)	0.8 (-0.8 to 2.3)
High fasting plasma glucose	1.4 (0.9 to 2.2)	1.4 (0.8 to 2.1)	1.5 (0.9 to 2.5)	-1.5 (-36.6 to 55.4)	12.8 (-23.4 to 79.4)	1.2 (0.7 to 1.9)	1.2 (0.7 to 2.1)	1.4 (0.8 to 2.3)	5.3 (-35.7 to 69.6)	10.7 (-30.2 to 86.4)
High LDL cholesterol	12.2 (10.6 to 14.0)	12.7 (11.1 to 14.5)	12.7 (11.1 to 14.5)	4.3 (-1.1 to 10.7)	-0.0 (-4.6 to 4.9)	13.1 (11.5 to 14.8)	13.2 (11.6 to 14.9)	13.2 (11.6 to 15.0)	0.7 (-4.5 to 6.7)	-0.0 (-4.7 to 4.6)
High systolic blood pressure	11.4 (10.4 to 12.4)	8.4 (7.6 to 9.3)	8.3 (7.3 to 9.3)	-26.1 (-32.1 to -20.3)	-1.9 (-9.2 to 6.1)	9.7 (8.8 to 10.8)	6.1 (5.4 to 6.9)	5.7 (4.9 to 6.5)	-37.3 (-43.9 to -30.4)	-7.3 (-18.1 to 2.9)
High body-mass index	6.4 (4.1 to 9.6)	9.2 (6.3 to 13.9)	9.4 (6.5 to 14.2)	42.9 (14.4 to 82.8)	3.0 (-13.3 to 19.3)	5.9 (3.6 to 9.1)	7.6 (4.8 to 11.8)	7.6 (4.7 to 12.2)	28.0 (1.6 to 70.2)	-0.2 (-18.3 to 21.8)
Low bone mineral density	5.3 (4.4 to 6.2)	5.1 (4.3 to 6.0)	5.2 (4.3 to 6.2)	-2.3 (-15.0 to 12.4)	0.1 (-13.1 to 14.9)	7.2 (6.1 to 8.5)	7.0 (5.9 to 8.2)	7.1 (6.0 to 8.3)	-3.1 (-15.1 to 11.1)	1.7 (-10.0 to 14.8)
Impaired kidney function	2.6 (1.2 to 6.5)	2.5 (1.1 to 6.4)	2.4 (1.0 to 6.1)	-3.9 (-8.5 to 0.3)	-6.9 (-12.1 to -2.3)	3.3 (1.5 to 7.4)	3.3 (1.5 to 7.7)	3.1 (1.4 to 7.2)	1.7 (-2.3 to 6.2)	-7.5 (-12.2 to -2.5)

Supplementary Results Table 1. Mie age-standardised Summary Exposure Values (SEVs) for Level 4 risk factors and percentage change, by sex, 1990, 2007, and 2017.Data in parentheses are 95% uncertainty intervals.

	Male					Female				
	1990	2007	2017	Percentage change 1990-2007	Percentage change 2007-2017	1990	2007	2017	Percentage change 1990-2007	Percentage change 2007-2017
Unsafe water source	2.3 (1.3 to 3.7)	1.9 (1.0 to 3.0)	2.0 (1.1 to 3.3)	-18.5 (-57.1 to 58.8)	5.2 (-38.1 to 84.5)	2.3 (1.3 to 3.7)	1.9 (1.0 to 3.0)	2.0 (1.1 to 3.3)	-18.5 (-57.1 to 58.8)	5.2 (-38.1 to 84.5)
Unsafe sanitation	4.0 (1.9 to 7.3)	1.9 (0.9 to 3.6)	1.6 (0.8 to 3.1)	-51.9 (-80.0 to 15.2)	-15.0 (-55.7 to 71.7)	4.0 (1.9 to 7.3)	1.9 (0.9 to 3.6)	1.6 (0.8 to 3.1)	-51.9 (-80.0 to 15.2)	-15.0 (-55.7 to 71.7)
No access to handwashing facility	6.7 (4.9 to 9.1)	6.4 (4.6 to 8.5)	5.9 (4.2 to 8.0)	-5.5 (-31.3 to 33.5)	-7.4 (-28.9 to 17.2)	6.7 (4.9 to 9.1)	6.4 (4.6 to 8.5)	5.9 (4.2 to 8.0)	-5.5 (-31.3 to 33.5)	-7.4 (-28.9 to 17.2)
Ambient particulate matter pollution	31.8 (21.9 to 47.2)	31.7 (22.3 to 45.7)	27.0 (17.6 to 41.3)	-0.4 (-17.8 to 24.4)	-14.8 (-32.9 to 1.7)	31.6 (21.6 to 46.9)	31.7 (22.3 to 45.7)	27.0 (17.6 to 41.2)	0.2 (-17.4 to 25.0)	-14.8 (-32.9 to 1.7)
Household air pollution from solid fuels	0.6 (0.2 to 1.7)	0.2 (0.0 to 0.3)	0.1 (0.0 to 0.3)	-75.7 (-93.3 to -45.5)	-16.9 (-65.5 to 86.4)	0.9 (0.3 to 5.6)	0.2 (0.0 to 1.1)	0.2 (0.0 to 0.7)	-73.0 (-93.2 to -43.9)	-15.4 (-65.2 to 86.4)
Ambient ozone pollution	52.3 (27.3 to 54.8)	52.1 (26.3 to 54.8)	52.4 (27.5 to 54.8)	-0.4 (-3.5 to 0.0)	0.6 (0.0 to 4.7)	52.3 (27.3 to 54.8)	52.1 (26.3 to 54.8)	52.4 (27.5 to 54.8)	-0.4 (-3.5 to 0.0)	0.6 (0.0 to 4.7)
Residential radon	12.9 (0.8 to 40.5)	12.9 (0.8 to 40.5)	12.9 (0.8 to 40.5)	0.0 (0.0 to 0.0)	0.0 (0.0 to 0.0)	12.9 (0.8 to 40.5)	12.9 (0.8 to 40.5)	12.9 (0.8 to 40.5)	0.0 (0.0 to 0.0)	0.0 (0.0 to 0.0)
Lead exposure	3.4 (0.7 to 7.2)	2.2 (0.3 to 5.2)	1.5 (0.2 to 4.0)	-35.3 (-49.2 to -26.0)	-30.2 (-45.3 to -21.7)	1.8 (0.1 to 4.8)	1.0 (0.1 to 3.3)	0.7 (0.0 to 2.5)	-41.4 (-58.7 to -31.2)	-33.1 (-55.1 to -23.4)
Occupational exposure to asbestos	1.6 (1.4 to 1.8)	2.1 (1.9 to 2.3)	2.2 (1.8 to 2.7)	33.3 (13.5 to 59.4)	4.6 (-14.8 to 29.5)	0.6 (0.4 to 0.7)	0.5 (0.5 to 0.6)	0.5 (0.4 to 0.6)	-6.2 (-22.7 to 19.9)	-4.3 (-23.9 to 18.6)
Occupational exposure to arsenic	0.5 (0.0 to 1.1)	0.4 (0.0 to 1.0)	0.4 (0.0 to 1.0)	-	-	0.4 (0.0 to 1.0)	0.4 (0.0 to 0.9)	0.4 (0.0 to 0.9)	-	-
Occupational exposure to benzene	0.6 (0.0 to 1.9)	0.7 (0.0 to 1.8)	0.7 (0.0 to 1.9)	2.2 (-4.3 to 12.5)	3.2 (-2.4 to 11.5)	0.6 (0.0 to 1.7)	0.7 (0.0 to 2.1)	0.8 (0.1 to 2.4)	22.4 (9.1 to 41.2)	14.8 (4.0 to 31.0)
Occupational exposure to beryllium	0.0 (0.0 to 0.0)	0.0 (0.0 to 0.0)	0.0 (0.0 to 0.0)	-5.3 (-10.0 to -0.4)	1.9 (-2.0 to 5.7)	0.0 (0.0 to 0.0)	0.0 (0.0 to 0.0)	0.0 (0.0 to 0.0)	2.3 (-6.8 to 12.7)	9.0 (2.7 to 16.5)
Occupational exposure to cadmium	0.1 (0.0 to 0.1)	0.0 (0.0 to 0.1)	0.0 (0.0 to 0.1)	-7.2 (-17.9 to 4.7)	0.8 (-8.8 to 10.8)	0.0 (0.0 to 0.1)	0.0 (0.0 to 0.1)	0.0 (0.0 to 0.1)	-9.0 (-23.1 to 8.4)	4.8 (-8.3 to 18.2)
Occupational exposure to chromium	0.1 (0.1 to 0.1)	0.1 (0.1 to 0.1)	0.1 (0.1 to 0.1)	-5.6 (-15.3 to 5.1)	1.3 (-6.9 to 9.7)	0.1 (0.1 to 0.1)	0.1 (0.1 to 0.1)	0.1 (0.1 to 0.1)	-4.2 (-17.5 to 11.8)	6.6 (-4.6 to 18.1)
Occupational exposure to diesel engine exhaust	0.6 (0.5 to 0.6)	0.6 (0.5 to 0.6)	0.6 (0.6 to 0.7)	-2.3 (-10.7 to 7.0)	3.2 (-3.3 to 10.0)	0.5 (0.4 to 0.5)	0.5 (0.4 to 0.6)	0.6 (0.5 to 0.6)	12.7 (-1.8 to 28.4)	11.1 (0.5 to 22.8)
Occupational exposure to formaldehyde	0.3 (0.2 to 0.3)	0.2 (0.2 to 0.3)	0.2 (0.2 to 0.3)	-6.6 (-17.9 to 6.3)	0.4 (-9.6 to 11.0)	0.2 (0.2 to 0.3)	0.2 (0.2 to 0.2)	0.2 (0.2 to 0.2)	-6.3 (-21.1 to 11.9)	5.6 (-6.7 to 18.9)
Occupational exposure to nickel	0.4 (0.0 to 1.9)	0.4 (0.0 to 1.7)	0.4 (0.0 to 1.8)	-	-	0.4 (0.0 to 1.6)	0.3 (0.0 to 1.3)	0.3 (0.0 to 1.3)	-	-
Occupational exposure to polycyclic aromatic hydrocarbons	0.2 (0.2 to 0.3)	0.2 (0.2 to 0.3)	0.2 (0.2 to 0.3)	-4.6 (-13.8 to 5.4)	1.7 (-6.0 to 9.4)	0.2 (0.2 to 0.3)	0.2 (0.2 to 0.2)	0.2 (0.2 to 0.3)	-2.1 (-14.9 to 13.5)	7.5 (-2.8 to 18.2)
Occupational exposure to silica	4.8 (0.7 to 15.2)	4.4 (0.6 to 13.9)	4.5 (0.6 to 14.2)	-7.1 (-17.3 to 5.2)	1.4 (-7.2 to 11.5)	3.2 (0.5 to 10.6)	2.8 (0.4 to 8.5)	2.8 (0.4 to 8.8)	-14.1 (-28.1 to 5.6)	1.4 (-11.3 to 18.9)
Occupational exposure to sulphuric acid	0.6 (0.1 to 1.9)	0.6 (0.1 to 1.9)	0.6 (0.1 to 1.9)	-7.2 (-17.2 to 4.5)	1.1 (-8.0 to 11.0)	0.5 (0.1 to 1.6)	0.5 (0.1 to 1.5)	0.5 (0.1 to 1.5)	-10.4 (-24.3 to 6.7)	4.2 (-9.5 to 18.1)
Occupational exposure to trichloroethylene	0.1 (0.1 to 0.1)	0.1 (0.1 to 0.1)	0.1 (0.1 to 0.1)	-4.4 (-11.6 to 2.9)	1.8 (-4.2 to 8.1)	0.1 (0.1 to 0.1)	0.1 (0.1 to 0.1)	0.1 (0.1 to 0.1)	0.9 (-10.7 to 14.1)	8.2 (-0.7 to 17.4)
Occupational asthmagens	19.1 (14.9 to 24.3)	18.2 (14.5 to 23.0)	18.1 (14.5 to 22.8)	-4.6 (-22.2 to 18.2)	-0.6 (-16.0 to 15.9)	8.3 (6.7 to 10.3)	8.3 (6.7 to 10.2)	8.8 (6.9 to 11.0)	-0.0 (-16.0 to 17.6)	5.3 (-7.5 to 18.5)
Occupational particulate matter, gases, and fumes	6.4 (4.6 to 9.2)	6.1 (4.3 to 8.9)	5.9 (4.2 to 8.5)	-4.7 (-9.9 to 0.3)	-3.7 (-7.2 to -0.3)	3.7 (2.5 to 5.5)	3.5 (2.4 to 5.2)	3.3 (2.3 to 5.0)	-5.7 (-14.1 to 4.2)	-4.7 (-10.6 to 1.4)
Occupational noise	6.0 (5.5 to 6.6)	5.8 (5.3 to 6.3)	5.6 (5.2 to 6.2)	-3.5 (-6.6 to -0.4)	-2.5 (-4.7 to -0.4)	3.5 (3.0 to 4.1)	3.5 (3.1 to 4.0)	3.6 (3.2 to 4.0)	0.3 (-5.9 to 7.4)	1.7 (-2.3 to 6.0)
Occupational ergonomic factors	10.4 (8.6 to 12.3)	9.2 (7.5 to 11.2)	9.1 (7.4 to 11.0)	-11.6 (-23.4 to 1.5)	-1.4 (-11.3 to 10.8)	5.6 (4.3 to 7.1)	5.6 (4.4 to 7.1)	6.3 (4.9 to 8.2)	1.1 (-19.9 to 28.0)	12.6 (-6.0 to 34.8)
Non-exclusive breastfeeding	0.4 (0.3 to 0.6)	0.4 (0.3 to 0.6)	0.4 (0.3 to 0.6)	-1.6 (-14.8 to 13.7)	-1.5 (-12.0 to 9.9)	0.4 (0.3 to 0.6)	0.4 (0.3 to 0.6)	0.4 (0.3 to 0.6)	-1.1 (-14.4 to 14.2)	-1.3 (-11.8 to 10.2)
Discontinued breastfeeding	1.7 (1.6 to 1.8)	1.7 (1.6 to 1.8)	1.7 (1.6 to 1.8)	0.7 (-8.4 to 10.7)	-1.3 (-9.0 to 6.6)	1.7 (1.6 to 1.8)	1.7 (1.6 to 1.9)	1.7 (1.6 to 1.8)	1.5 (-7.7 to 11.6)	-2.9 (-10.5 to 4.9)
Child underweight	0.3 (0.2 to 0.4)	0.2 (0.1 to 0.3)	0.2 (0.1 to 0.3)	-15.6 (-35.2 to 7.9)	-6.1 (-21.2 to 9.9)	0.3 (0.2 to 0.4)	0.2 (0.1 to 0.3)	0.2 (0.1 to 0.3)	-17.6 (-37.5 to 5.4)	-6.2 (-22.2 to 11.6)
Child wasting	0.2 (0.1 to 0.3)	0.2 (0.1 to 0.3)	0.2 (0.1 to 0.3)	-6.6 (-34.9 to 35.5)	-2.5 (-23.0 to 21.8)	0.2 (0.1 to 0.3)	0.2 (0.1 to 0.3)	0.2 (0.1 to 0.3)	-8.7 (-35.6 to 31.4)	-3.5 (-23.4 to 20.6)
Child stunting	0.6 (0.3 to 1.1)	0.5 (0.3 to 0.9)	0.5 (0.2 to 0.9)	-11.2 (-47.8 to 47.5)	-3.4 (-33.3 to 40.4)	0.6 (0.3 to 1.1)	0.6 (0.3 to 1.0)	0.5 (0.2 to 0.9)	-12.2 (-48.4 to 45.9)	-4.0 (-31.8 to 35.6)
Short gestation for birth weight	0.0 (0.0 to 0.0)	0.0 (0.0 to 0.0)	0.0 (0.0 to 0.0)	11.5 (-11.1 to 39.5)	2.0 (-22.7 to 40.7)	0.0 (0.0 to 0.0)	0.0 (0.0 to 0.0)	0.0 (0.0 to 0.0)	11.2 (-22.8 to 66.9)	-0.9 (-20.7 to 17.8)
Low birth weight for gestation	0.0 (0.0 to 0.0)	0.0 (0.0 to 0.0)	0.0 (0.0 to 0.0)	4.0 (-12.7 to 26.6)	2.2 (-23.3 to 26.3)	0.0 (0.0 to 0.0)	0.0 (0.0 to 0.0)	0.0 (0.0 to 0.0)	-1.8 (-54.0 to 35.7)	4.2 (-10.4 to 25.9)
Iron deficiency	-	-	-	-	-	8.0 (5.4 to 11.1)	8.0 (5.6 to 11.0)	7.3 (4.9 to 10.4)	-0.4 (-21.1 to 27.3)	-8.5 (-23.8 to 8.3)
Vitamin A deficiency	0.9 (0.6 to 1.4)	0.9 (0.6 to 1.3)	0.7 (0.4 to 0.9)	-0.4 (-26.4 to 33.8)	-29.0 (-44.6 to -8.5)	0.8 (0.5 to 1.0)	0.9 (0.5 to 1.1)	0.7 (0.4 to 0.9)	11.2 (-5.4 to 31.3)	-23.9 (-40.8 to -6.6)
Zinc deficiency	0.5 (0.1 to 1.0)	0.5 (0.1 to 1.2)	0.4 (0.1 to 1.1)	8.9 (-61.0 to 76.0)	-16.0 (-68.3 to 124.9)	0.5 (0.2 to 1.0)	0.4 (0.1 to 0.9)	0.3 (0.1 to 0.8)	-25.9 (-66.2 to 70.4)	-5.4 (-64.3 to 164.4)
Smoking	17.9 (14.5 to 21.4)	12.5 (9.9 to 15.1)	10.5 (8.3 to 12.7)	-30.3 (-39.2 to -19.6)	-16.0 (-24.2 to -6.0)	4.0 (2.8 to 5.3)	3.3 (2.4 to 4.6)	3.2 (2.3 to 4.4)	-16.9 (-35.2 to 9.9)	-3.4 (-20.4 to 15.8)
Chewing tobacco	1.4 (0.9 to 2.1)	0.9 (0.6 to 1.4)	0.8 (0.5 to 1.1)	-33.1 (-57.8 to 4.1)	-19.2 (-41.5 to 14.0)	0.1 (0.1 to 0.2)	0.1 (0.1 to 0.1)	0.1 (0.1 to 0.1)	-4.1 (-39.6 to 59.1)	-3.0 (-33.3 to 45.0)
Second-hand smoke	36.9 (33.0 to 40.8)	36.6 (32.3 to 40.8)	35.0 (30.9 to 39.4)	-1.0 (-12.6 to 12.6)	-4.2 (-13.0 to 4.8)	55.7 (52.0 to 59.3)	49.3 (45.5 to 53.2)	47.2 (43.2 to 51.0)	-11.6 (-18.4 to -5.1)	-4.3 (-10.0 to 1.5)
Alcohol use	14.7 (7.7 to 24.9)	14.7 (7.4 to 25.3)	16.9 (8.1 to 30.3)	-0.1 (-32.6 to 46.7)	14.9 (-19.6 to 63.5)	11.9 (6.1 to 21.1)	11.8 (5.8 to 21.0)	13.2 (6.2 to 23.4)	-1.2 (-33.9 to 45.7)	11.6 (-20.2 to 59.5)
Drug use	0.5 (0.4 to 0.7)	0.5 (0.4 to 0.7)	0.6 (0.4 to 0.8)	0.4 (-5.2 to 6.4)	11.5 (4.6 to 20.4)	0.3 (0.2 to 0.4)	0.3 (0.2 to 0.4)	0.3 (0.3 to 0.4)	2.5 (-3.1 to 8.6)	8.7 (2.0 to 16.4)
Diet low in fruits	40.0 (34.2 to 43.9)	38.4 (32.4 to 42.3)	39.7 (33.9 to 43.5)	-4.0 (-10.8 to 2.1)	3.3 (-2.1 to 9.6)	36.7 (30.7 to 41.2)	34.1 (28.2 to 39.2)	36.3 (30.4 to 41.0)	-7.2 (-15.4 to 1.1)	6.6 (-0.5 to 15.3)
Diet low in vegetables	18.4 (10.1 to 25.7)	12.6 (5.1 to 20.2)	14.9 (6.5 to 22.8)	-31.2 (-62.7 to -4.2)	17.4 (-15.5 to 81.4)	20.6 (12.0 to 28.3)	14.8 (7.1 to 22.8)	16.6 (8.5 to 24.1)	-28.1 (-56.0 to -1.7)	12.5 (-14.2 to 59.6)
Diet low in legumes	4.3 (0.6 to 10.0)	5.3 (1.1 to 11.5)	7.1 (1.9 to 13.6)	23.7 (-44.1 to 305.4)	32.4 (-18.0 to 198.4)	8.1 (2.4 to 15.1)	7.5 (2.3 to 13.8)	9.7 (3.7 to 16.8)	-6.7 (-53.2 to 76.9)	28.8 (-17.7 to 133.1)
Diet low in whole grains	29.9 (24.6 to 34.3)	34.8 (30.7 to 38.4)	36.2 (32.3 to 39.6)	16.5 (7.1 to 29.1)	4.0 (-1.0 to 9.9)	31.4 (26.8 to 35.6)	36.1 (32.2 to 39.3)	37.4 (33.6 to 40.7)	14.9 (6.8 to 26.0)	3.7 (-0.9 to 8.4)
Diet low in nuts and seeds	50.4 (48.8 to 51.6)	49.1 (47.1 to 50.6)	49.0 (46.8 to 50.6)	-2.5 (-5.0 to -0.1)	-0.2 (-2.5 to 2.1)	50.5 (49.0 to 51.7)	49.1 (47.0 to 50.6)	48.9 (46.7 to 50.5)	-2.9 (-5.6 to -0.6)	-0.4 (-2.5 to 1.8)
Diet low in milk	45.9 (43.7 to 47.7)	46.3 (44.2 to 48.1)	46.8 (44.8 to 48.6)	0.9 (-1.2 to 3.0)	1.1 (-0.3 to 2.6)	45.9 (43.7 to 47.7)	45.7 (43.5 to 47.6)	46.4 (44.4 to 48.1)	-0.3 (-2.4 to 1.6)	1.5 (-0.1 to 3.3)
Diet high in red meat	4.5 (0.8 to 9.6)	8.6 (2.9 to 15.4)	9.7 (3.3 to 16.9)	92.7 (-12.9 to 597.1)	12.8 (-37.1 to 110.8)	0.7 (0.0 to 2.8)	1.8 (0.0 to 5.1)	2.4 (0.1 to 6.6)	161.3 (-70.6 to 299520.6)	30.9 (-76.9 to 1704.0)
Diet high in processed meat	12.3 (8.0 to 16.7)	20.3 (14.9 to 25.9)	23.4 (16.4 to 29.3)	65.0 (29.8 to 126.3)	15.4 (-3.5 to 37.6)	10.4 (6.5 to 14.1)	15.0 (10.7 to 19.3)	16.4 (11.6 to 20.6)	44.9 (12.8 to 100.2)	9.5 (-7.9 to 30.9)
Diet high in sugar-sweetened beverages	8.0 (3.2 to 15.7)	9.4 (4.0 to 16.5)	9.9 (4.2 to 17.7)	18.0 (-45.3 to 170.6)	5.4 (-36.9 to 76.5)	5.7 (2.1 to 12.6)	6.6 (2.7 to 12.7)	6.9 (3.0 to 13.3)	15.0 (-47.0 to 157.5)	5.6 (-39.0 to 89.7)
Diet low in fibre	21.4 (13.7 to 27.9)	22.5 (15.3 to 28.3)	23.5 (16.2 to 29.6)	5.0 (-12.4 to 29.3)	4.5 (-8.4 to 18.9)	22.7 (15.4 to 29.6)	23.8 (16.8 to 30.1)	25.2 (18.1 to 31.7)	4.7 (-11.8 to 25.2)	6.1 (-5.2 to 21.0)
Diet low in calcium	33.9 (29.3 to 37.7)	33.7 (29.3 to 37.4)	34.6 (30.1 to 38.2)	-0.5 (-5.1 to 3.8)	2.5 (-0.9 to 6.4)	36.2 (32.0 to 39.5)	35.1 (30.9 to 38.6)	36.4 (32.3 to 39.7)	-2.9 (-6.7 to 0.7)	3.6 (0.7 to 7.2)
Diet low in seafood omega-3 fatty acids	2.9 (1.1 to 4.3)	0.2 (0.0 to 0.9)	0.2 (0.0 to 0.9)	-93.5 (-100.0 to -72.6)	10.8 (-98.1 to 11393.3)	3.4 (1.4 to 4.9)	0.3 (0.0 to 1.1)	0.3 (0.0 to 1.2)	-91.9 (-100.0 to -70.6)	10.9 (-92.6 to 2716.1)
Diet low in polyunsaturated fatty acids	29.6 (23.7 to 34.7)	26.7 (20.7 to 31.9)	27.4 (21.4 to 32.4)	-9.7 (-22.8 to 0.5)	2.4 (-6.0 to 12.8)	30.2 (24.5 to 35.3)	27.4 (21.2 to 32.6)	28.1 (22.1 to 33.2)	-9.4 (-21.1 to 1.0)	2.6 (-4.9 to 11.1)
Diet high in trans fatty acids	1.1 (0.0 to 3.3)	0.9 (0.0 to 3.0)	0.9 (0.0 to 2.9)	-12.5 (-38.4 to -6.3)	-8.3 (-32.5 to -3.4)	1.5 (0.1 to 4.0)	1.3 (0.1 to 3.7)	1.2 (0.0 to 3.5)	-12.8 (-35.2 to -6.5)	-8.1 (-28.5 to -3.0)
Diet high in sodium	28.5 (19.1 to 36.8)	18.5 (10.0 to 25.9)	15.9 (7.0 to 23.2)	-35.0 (-56.7 to -16.2)	-14.1 (-38.6 to 2.8)	32.3 (24.0 to 39.3)	19.5 (11.4 to 26.2)	16.2 (7.6 to 23.4)	-39.5 (-59.6 to -24.8)	-16.9 (-39.4 to -3.7)
Intimate partner violence	-	-	-	-	-	2.3 (1.9 to 2.7)	2.1 (1.7 to 2.4)	2.0 (1.7 to 2.4)	-8.3 (-12.0 to -4.2)	-3.1 (-7.4 to 1.2)
Childhood sexual abuse	5.3 (4.3 to 6.5)	5.3 (4.3 to 6.5)	5.3 (4.3 to 6.5)	0.0 (0.0 to 0.0)	0.0 (0.0 to 0.0)	7.3 (5.9 to 8.9)	7.3 (5.9 to 8.9)	7.5 (6.0 to 9.2)	0.0 (0.0 to 0.0)	2.8 (-4.0 to 10.6)

Supplementary Results Table 1. Mie age-standardised Summary Exposure Values (SEVs) for Level 4 risk factors and percentage change, by sex, 1990, 2007, and 2017.Data in parentheses are 95% uncertainty intervals.

	Male					Female				
	1990	2007	2017	Percentage change 1990-2007	Percentage change 2007-2017	1990	2007	2017	Percentage change 1990-2007	Percentage change 2007-2017
Bullying victimization	3.3<br>(1.4 to 6.6)	3.4<br>(1.4 to 6.8)	3.5<br>(1.5 to 7.1)	1.8<br>(-5.9 to 9.9)	4.0<br>(-1.2 to 8.7)	1.9<br>(0.8 to 3.7)	1.9<br>(0.8 to 3.8)	2.0<br>(0.8 to 4.0)	0.4<br>(-6.9 to 8.7)	3.0<br>(-2.7 to 7.8)
Low physical activity	0.5<br>(0.2 to 0.9)	0.5<br>(0.2 to 0.9)	0.4<br>(0.2 to 0.8)	-1.8<br>(-3.7 to -0.0)	-8.8<br>(-12.2 to -4.4)	0.5<br>(0.2 to 1.0)	0.5<br>(0.2 to 0.9)	0.5<br>(0.2 to 0.9)	-3.8<br>(-6.0 to -1.3)	0.7<br>(-0.8 to 2.3)
High fasting plasma glucose	1.5<br>(0.9 to 2.3)	1.4<br>(0.9 to 2.3)	1.6<br>(1.0 to 2.6)	-3.8<br>(-41.0 to 54.7)	12.5<br>(-25.6 to 78.4)	1.2<br>(0.7 to 2.1)	1.3<br>(0.7 to 2.2)	1.4<br>(0.8 to 2.4)	1.4<br>(-37.3 to 71.7)	10.4<br>(-27.6 to 88.3)
High LDL cholesterol	12.0<br>(10.4 to 13.8)	12.6<br>(10.9 to 14.2)	12.6<br>(11.0 to 14.3)	4.5<br>(-1.4 to 11.2)	0.1<br>(-4.3 to 4.9)	12.8<br>(11.3 to 14.6)	13.0<br>(11.4 to 14.7)	13.0<br>(11.4 to 14.9)	1.0<br>(-4.7 to 6.7)	0.3<br>(-4.0 to 5.0)
High systolic blood pressure	11.2<br>(10.2 to 12.2)	8.3<br>(7.5 to 9.2)	8.2<br>(7.2 to 9.2)	-25.8<br>(-32.1 to -19.2)	-1.5<br>(-8.7 to 6.8)	9.6<br>(8.7 to 10.5)	6.0<br>(5.3 to 6.8)	5.6<br>(4.8 to 6.4)	-37.2<br>(-43.8 to -30.4)	-7.4<br>(-16.7 to 3.7)
High body-mass index	5.9<br>(3.8 to 9.1)	8.6<br>(5.8 to 13.3)	8.9<br>(6.0 to 13.4)	45.5<br>(15.1 to 86.7)	3.1<br>(-12.5 to 20.2)	5.6<br>(3.5 to 8.7)	7.3<br>(4.7 to 11.1)	7.3<br>(4.5 to 11.4)	29.9<br>(1.3 to 72.3)	0.5<br>(-18.4 to 22.5)
Low bone mineral density	5.3<br>(4.3 to 6.3)	5.2<br>(4.3 to 6.1)	5.2<br>(4.2 to 6.1)	-2.2<br>(-14.6 to 12.5)	0.5<br>(-12.3 to 14.7)	7.2<br>(6.1 to 8.4)	7.0<br>(6.0 to 8.0)	7.1<br>(5.9 to 8.3)	-3.3<br>(-16.0 to 10.5)	1.6<br>(-10.2 to 14.9)
Impaired kidney function	2.7<br>(1.2 to 6.5)	2.6<br>(1.2 to 6.4)	2.4<br>(1.1 to 6.2)	-3.3<br>(-7.7 to 0.8)	-6.7<br>(-12.3 to -1.9)	3.3<br>(1.5 to 7.4)	3.4<br>(1.5 to 7.7)	3.1<br>(1.4 to 7.3)	2.6<br>(-1.4 to 7.0)	-7.2<br>(-12.1 to -2.4)

Supplementary Results Table 1. Miyagi age-standardised Summary Exposure Values (SEVs) for Level 4 risk factors and percentage change, by sex, 1990, 2007, and 2017.Data in parentheses are 95% uncertainty intervals.

	Male					Female				
	1990	2007	2017	Percentage change 1990-2007	Percentage change 2007-2017	1990	2007	2017	Percentage change 1990-2007	Percentage change 2007-2017
Unsafe water source	2.3 (1.3 to 3.8)	1.9 (1.0 to 3.1)	2.0 (1.1 to 3.3)	-16.0 (-58.4 to 60.3)	5.0 (-37.5 to 80.8)	2.3 (1.3 to 3.8)	1.9 (1.0 to 3.1)	2.0 (1.1 to 3.3)	-16.0 (-58.4 to 60.3)	5.0 (-37.5 to 80.8)
Unsafe sanitation	3.8 (1.9 to 6.5)	1.9 (0.9 to 3.6)	1.7 (0.8 to 3.2)	-49.9 (-78.1 to 14.2)	-13.0 (-55.4 to 68.0)	3.8 (1.9 to 6.5)	1.9 (0.9 to 3.6)	1.7 (0.8 to 3.2)	-49.9 (-78.1 to 14.2)	-13.0 (-55.4 to 68.0)
No access to handwashing facility	6.7 (4.9 to 9.0)	6.4 (4.7 to 8.6)	5.9 (4.3 to 8.0)	-5.6 (-32.3 to 31.1)	-6.8 (-27.7 to 19.2)	6.7 (4.9 to 9.0)	6.4 (4.7 to 8.6)	5.9 (4.3 to 8.0)	-5.6 (-32.3 to 31.1)	-6.8 (-27.7 to 19.2)
Ambient particulate matter pollution	27.0 (18.0 to 40.3)	27.1 (18.4 to 41.3)	22.7 (12.9 to 36.3)	0.3 (-25.0 to 41.1)	-16.1 (-46.1 to 12.1)	26.7 (17.8 to 39.9)	27.0 (18.3 to 41.2)	22.7 (12.9 to 36.3)	1.1 (-24.5 to 42.7)	-16.1 (-46.2 to 12.2)
Household air pollution from solid fuels	0.7 (0.2 to 2.2)	0.2 (0.0 to 0.4)	0.1 (0.0 to 0.3)	-75.7 (-92.8 to -46.8)	-17.4 (-68.5 to 76.2)	1.0 (0.3 to 6.4)	0.3 (0.0 to 1.5)	0.2 (0.0 to 1.1)	-71.7 (-92.7 to -40.8)	-15.0 (-68.5 to 77.9)
Ambient ozone pollution	47.4 (18.5 to 54.8)	47.5 (18.7 to 54.8)	48.1 (19.2 to 54.8)	0.3 (0.0 to 1.2)	1.3 (0.0 to 3.9)	47.4 (18.5 to 54.8)	47.5 (18.7 to 54.8)	48.1 (19.2 to 54.8)	0.3 (0.0 to 1.2)	1.3 (0.0 to 3.9)
Residential radon	17.0 (1.4 to 48.8)	17.0 (1.4 to 48.8)	17.0 (1.4 to 48.8)	0.0 (0.0 to 0.0)	0.0 (0.0 to 0.0)	17.0 (1.4 to 48.8)	17.0 (1.4 to 48.8)	17.0 (1.4 to 48.8)	0.0 (0.0 to 0.0)	0.0 (0.0 to 0.0)
Lead exposure	3.1 (0.5 to 6.6)	2.0 (0.3 to 5.0)	1.4 (0.1 to 4.0)	-33.8 (-47.4 to -24.5)	-28.4 (-44.2 to -20.4)	3.2 (0.5 to 7.0)	2.2 (0.3 to 5.3)	1.6 (0.2 to 4.2)	-32.0 (-45.5 to -23.2)	-27.4 (-42.6 to -19.5)
Occupational exposure to asbestos	2.1 (1.8 to 2.4)	2.8 (2.6 to 3.1)	2.7 (2.2 to 3.3)	32.8 (14.8 to 56.1)	-3.3 (-21.6 to 21.3)	0.5 (0.4 to 0.6)	0.5 (0.5 to 0.6)	0.5 (0.4 to 0.6)	-3.4 (-19.9 to 22.6)	-5.7 (-25.2 to 16.0)
Occupational exposure to arsenic	0.5 (0.0 to 1.1)	0.4 (0.0 to 1.0)	0.4 (0.0 to 1.0)	-	-	0.4 (0.0 to 1.0)	0.4 (0.0 to 0.9)	0.4 (0.0 to 0.9)	-	-
Occupational exposure to benzene	0.6 (0.0 to 1.8)	0.6 (0.0 to 1.8)	0.7 (0.0 to 1.9)	2.8 (-4.0 to 14.1)	3.8 (-2.5 to 11.4)	0.6 (0.0 to 1.7)	0.7 (0.0 to 2.0)	0.8 (0.1 to 2.4)	21.8 (8.4 to 44.2)	14.3 (4.1 to 31.3)
Occupational exposure to beryllium	0.0 (0.0 to 0.0)	0.0 (0.0 to 0.0)	0.0 (0.0 to 0.0)	-5.2 (-10.3 to 0.1)	2.0 (-2.0 to 6.1)	0.0 (0.0 to 0.0)	0.0 (0.0 to 0.0)	0.0 (0.0 to 0.0)	2.4 (-6.2 to 12.6)	9.0 (2.5 to 16.2)
Occupational exposure to cadmium	0.1 (0.0 to 0.1)	0.0 (0.0 to 0.1)	0.0 (0.0 to 0.1)	-7.0 (-18.2 to 5.2)	1.1 (-8.1 to 12.0)	0.0 (0.0 to 0.1)	0.0 (0.0 to 0.1)	0.0 (0.0 to 0.1)	-8.4 (-23.6 to 10.3)	4.9 (-7.5 to 18.7)
Occupational exposure to chromium	0.1 (0.1 to 0.1)	0.1 (0.1 to 0.1)	0.1 (0.1 to 0.1)	-5.4 (-15.4 to 5.5)	1.6 (-6.5 to 11.0)	0.1 (0.1 to 0.1)	0.1 (0.1 to 0.1)	0.1 (0.1 to 0.1)	-3.7 (-17.9 to 13.6)	6.7 (-3.9 to 18.5)
Occupational exposure to diesel engine exhaust	0.6 (0.5 to 0.6)	0.6 (0.5 to 0.6)	0.6 (0.6 to 0.7)	-1.9 (-9.6 to 6.6)	3.1 (-3.5 to 10.3)	0.5 (0.4 to 0.5)	0.5 (0.4 to 0.6)	0.6 (0.5 to 0.6)	12.7 (-0.5 to 29.1)	11.0 (0.1 to 23.2)
Occupational exposure to formaldehyde	0.3 (0.2 to 0.3)	0.2 (0.2 to 0.3)	0.2 (0.2 to 0.3)	-6.4 (-18.1 to 6.6)	0.7 (-9.2 to 12.3)	0.2 (0.2 to 0.3)	0.2 (0.2 to 0.2)	0.2 (0.2 to 0.2)	-5.7 (-21.6 to 14.0)	5.7 (-6.4 to 19.6)
Occupational exposure to nickel	0.4 (0.0 to 1.9)	0.4 (0.0 to 1.7)	0.4 (0.0 to 1.8)	-	-	0.3 (0.0 to 1.6)	0.3 (0.0 to 1.3)	0.3 (0.0 to 1.3)	-	-
Occupational exposure to polycyclic aromatic hydrocarbons	0.2 (0.2 to 0.3)	0.2 (0.2 to 0.3)	0.2 (0.2 to 0.3)	-4.3 (-13.7 to 5.9)	1.9 (-5.5 to 10.6)	0.2 (0.2 to 0.3)	0.2 (0.2 to 0.2)	0.2 (0.2 to 0.3)	-1.6 (-15.5 to 15.1)	7.5 (-2.7 to 18.6)
Occupational exposure to silica	4.7 (0.7 to 14.9)	4.4 (0.6 to 13.9)	4.4 (0.7 to 14.3)	-6.1 (-16.2 to 6.7)	1.3 (-7.7 to 12.9)	3.2 (0.4 to 10.5)	2.7 (0.4 to 8.7)	2.8 (0.4 to 8.9)	-13.2 (-28.2 to 5.6)	1.8 (-11.1 to 19.1)
Occupational exposure to sulphuric acid	0.6 (0.1 to 2.0)	0.6 (0.1 to 1.9)	0.6 (0.1 to 1.9)	-7.1 (-17.6 to 4.2)	1.4 (-8.0 to 12.2)	0.5 (0.1 to 1.6)	0.5 (0.1 to 1.4)	0.5 (0.1 to 1.4)	-9.8 (-25.2 to 8.5)	4.3 (-8.8 to 18.3)
Occupational exposure to trichloroethylene	0.1 (0.1 to 0.1)	0.1 (0.1 to 0.1)	0.1 (0.1 to 0.1)	-4.4 (-11.9 to 3.5)	2.0 (-4.0 to 8.8)	0.1 (0.1 to 0.1)	0.1 (0.1 to 0.1)	0.1 (0.1 to 0.1)	1.2 (-10.2 to 16.1)	8.2 (-0.4 to 17.8)
Occupational asthmagens	18.6 (14.8 to 23.3)	18.0 (14.4 to 22.4)	18.0 (14.2 to 22.8)	-3.5 (-21.8 to 20.2)	0.1 (-15.1 to 18.5)	8.2 (6.5 to 10.3)	8.3 (6.6 to 10.3)	8.7 (7.0 to 10.8)	0.7 (-14.8 to 19.6)	5.4 (-7.1 to 19.4)
Occupational particulate matter, gases, and fumes	6.4 (4.5 to 9.1)	6.1 (4.4 to 8.8)	5.9 (4.2 to 8.5)	-4.4 (-9.6 to 0.9)	-3.7 (-7.1 to -0.2)	3.7 (2.5 to 5.4)	3.5 (2.4 to 5.2)	3.3 (2.3 to 5.0)	-5.5 (-14.2 to 3.9)	-4.5 (-10.3 to 1.6)
Occupational noise	6.0 (5.5 to 6.6)	5.8 (5.3 to 6.3)	5.6 (5.2 to 6.1)	-3.2 (-6.4 to 0.1)	-2.4 (-4.5 to -0.3)	3.5 (3.0 to 4.1)	3.5 (3.1 to 4.0)	3.6 (3.2 to 4.1)	0.5 (-5.6 to 7.4)	1.8 (-2.2 to 6.2)
Occupational ergonomic factors	10.5 (8.6 to 12.6)	9.3 (7.6 to 11.3)	9.2 (7.6 to 11.1)	-11.6 (-22.8 to 1.4)	-1.4 (-12.6 to 10.8)	5.6 (4.3 to 7.1)	5.7 (4.4 to 7.2)	6.4 (5.0 to 8.4)	1.4 (-20.5 to 28.5)	12.7 (-8.0 to 37.9)
Non-exclusive breastfeeding	0.4 (0.3 to 0.6)	0.4 (0.3 to 0.6)	0.4 (0.3 to 0.6)	-0.4 (-13.0 to 14.6)	-1.8 (-11.9 to 9.3)	0.4 (0.3 to 0.6)	0.4 (0.3 to 0.6)	0.4 (0.3 to 0.6)	-1.4 (-13.8 to 13.6)	-1.1 (-11.2 to 10.1)
Discontinued breastfeeding	1.7 (1.6 to 1.8)	1.7 (1.6 to 1.9)	1.7 (1.6 to 1.8)	1.9 (-7.2 to 12.9)	-1.4 (-8.9 to 6.6)	1.7 (1.6 to 1.8)	1.7 (1.6 to 1.9)	1.7 (1.6 to 1.8)	2.1 (-7.1 to 13.1)	-2.1 (-9.5 to 5.8)
Child underweight	0.3 (0.2 to 0.4)	0.2 (0.1 to 0.3)	0.2 (0.1 to 0.3)	-14.6 (-33.5 to 8.3)	-5.6 (-20.9 to 10.5)	0.3 (0.2 to 0.4)	0.2 (0.1 to 0.3)	0.2 (0.1 to 0.3)	-16.5 (-36.8 to 10.0)	-6.0 (-22.7 to 12.7)
Child wasting	0.2 (0.1 to 0.3)	0.2 (0.1 to 0.3)	0.2 (0.1 to 0.3)	-6.5 (-33.8 to 31.4)	-2.1 (-20.9 to 22.4)	0.2 (0.1 to 0.3)	0.2 (0.1 to 0.3)	0.2 (0.1 to 0.3)	-7.2 (-33.9 to 27.8)	-3.3 (-22.8 to 19.3)
Child stunting	0.6 (0.3 to 1.1)	0.6 (0.3 to 0.9)	0.5 (0.3 to 0.9)	-10.7 (-45.7 to 50.9)	-2.7 (-28.8 to 37.5)	0.6 (0.3 to 1.1)	0.6 (0.3 to 1.0)	0.6 (0.3 to 1.0)	-9.4 (-47.0 to 52.5)	-3.0 (-31.6 to 37.0)
Short gestation for birth weight	0.0 (0.0 to 0.0)	0.0 (0.0 to 0.0)	0.0 (0.0 to 0.0)	9.0 (-13.4 to 41.8)	4.1 (-18.0 to 39.9)	0.0 (0.0 to 0.0)	0.0 (0.0 to 0.0)	0.0 (0.0 to 0.0)	12.1 (-22.3 to 49.7)	2.1 (-21.7 to 39.4)
Low birth weight for gestation	0.0 (0.0 to 0.0)	0.0 (0.0 to 0.0)	0.0 (0.0 to 0.0)	4.6 (-13.2 to 26.9)	0.8 (-22.2 to 22.2)	0.0 (0.0 to 0.0)	0.0 (0.0 to 0.0)	0.0 (0.0 to 0.0)	-0.7 (-22.0 to 33.9)	2.2 (-21.3 to 28.7)
Iron deficiency	-	-	-	-	-	7.8 (5.4 to 10.7)	7.8 (5.3 to 10.4)	7.1 (4.8 to 10.0)	-1.1 (-22.8 to 29.2)	-8.5 (-24.8 to 9.8)
Vitamin A deficiency	1.0 (0.6 to 1.4)	0.8 (0.5 to 1.1)	0.6 (0.4 to 0.9)	-17.6 (-35.5 to 4.2)	-21.8 (-34.9 to -5.1)	0.7 (0.4 to 0.9)	0.8 (0.5 to 1.0)	0.7 (0.4 to 0.9)	11.8 (-3.6 to 34.3)	-11.1 (-25.3 to 8.3)
Zinc deficiency	0.5 (0.1 to 1.0)	0.4 (0.1 to 1.1)	0.4 (0.1 to 0.9)	-11.6 (-62.4 to 71.3)	-13.3 (-68.4 to 146.3)	0.5 (0.2 to 1.1)	0.3 (0.1 to 0.8)	0.4 (0.1 to 0.8)	-31.8 (-64.5 to 8.2)	6.5 (-59.4 to 172.0)
Smoking	17.1 (13.4 to 20.5)	12.1 (9.5 to 14.8)	10.3 (8.2 to 12.7)	-29.2 (-38.0 to -17.0)	-15.1 (-23.4 to -4.9)	3.9 (2.8 to 5.3)	3.2 (2.3 to 4.3)	3.2 (2.2 to 4.3)	-16.6 (-36.8 to 10.8)	-2.3 (-18.2 to 18.6)
Chewing tobacco	1.4 (0.9 to 2.0)	0.9 (0.6 to 1.4)	0.8 (0.5 to 1.1)	-33.1 (-56.3 to 1.2)	-18.8 (-42.7 to 11.7)	0.1 (0.1 to 0.2)	0.1 (0.1 to 0.1)	0.1 (0.1 to 0.1)	-3.9 (-41.8 to 60.0)	-2.3 (-34.4 to 46.4)
Second-hand smoke	37.3 (33.3 to 41.3)	37.1 (32.8 to 41.6)	36.8 (32.5 to 41.4)	-0.5 (-11.8 to 13.1)	-0.9 (-9.1 to 8.8)	56.6 (52.9 to 60.2)	50.4 (46.8 to 54.1)	50.1 (46.2 to 54.2)	-10.8 (-17.2 to -3.7)	-0.7 (-6.6 to 5.3)
Alcohol use	14.4 (7.3 to 25.1)	14.3 (7.1 to 24.8)	16.0 (7.6 to 28.8)	-0.6 (-34.5 to 42.6)	12.0 (-22.8 to 58.7)	11.4 (5.7 to 20.5)	11.5 (5.4 to 20.4)	13.1 (6.1 to 23.3)	0.5 (-34.1 to 45.4)	14.2 (-20.3 to 65.7)
Drug use	0.5 (0.4 to 0.7)	0.5 (0.4 to 0.7)	0.6 (0.4 to 0.8)	0.4 (-5.4 to 6.9)	11.7 (4.2 to 20.2)	0.3 (0.2 to 0.4)	0.3 (0.2 to 0.4)	0.3 (0.3 to 0.4)	2.0 (-3.7 to 8.6)	9.1 (2.5 to 17.9)
Diet low in fruits	41.3 (35.7 to 44.9)	39.5 (34.0 to 43.3)	40.8 (35.2 to 44.5)	-4.4 (-10.4 to 0.9)	3.3 (-1.7 to 8.3)	38.1 (32.3 to 42.5)	35.5 (29.5 to 40.1)	37.6 (31.6 to 41.9)	-6.9 (-14.4 to 0.4)	5.9 (-0.5 to 14.3)
Diet low in vegetables	20.0 (11.6 to 27.8)	14.1 (5.9 to 21.7)	16.3 (7.7 to 24.0)	-29.2 (-58.2 to -6.2)	15.3 (-15.4 to 70.2)	22.3 (13.8 to 29.6)	16.3 (8.4 to 24.1)	18.1 (9.7 to 26.0)	-26.8 (-53.8 to -2.4)	11.2 (-13.6 to 52.6)
Diet low in legumes	4.3 (0.7 to 9.8)	5.4 (1.0 to 11.1)	7.1 (2.0 to 13.7)	25.2 (-45.9 to 245.1)	31.9 (-21.2 to 204.2)	8.3 (2.6 to 14.8)	7.9 (2.3 to 14.7)	10.2 (3.7 to 17.3)	-4.4 (-49.5 to 74.4)	28.7 (-11.8 to 136.9)
Diet low in whole grains	30.6 (25.8 to 34.9)	35.4 (31.2 to 38.8)	36.8 (33.0 to 40.0)	15.8 (6.7 to 28.8)	3.7 (-0.6 to 9.1)	32.0 (27.4 to 36.0)	36.6 (33.1 to 39.8)	37.9 (34.4 to 41.0)	14.2 (6.7 to 24.1)	3.5 (-1.1 to 8.3)
Diet low in nuts and seeds	51.0 (49.6 to 52.0)	49.8 (48.0 to 51.1)	49.7 (47.9 to 51.1)	-2.3 (-4.7 to -0.3)	-0.2 (-2.1 to 1.7)	51.2 (49.8 to 52.2)	50.1 (48.4 to 51.4)	50.1 (48.2 to 51.4)	-2.2 (-4.4 to -0.2)	-0.1 (-1.9 to 1.6)
Diet low in milk	46.3 (44.2 to 48.0)	46.6 (44.6 to 48.3)	47.1 (45.2 to 48.7)	0.7 (-1.1 to 2.7)	1.0 (-0.3 to 2.5)	46.3 (44.4 to 48.0)	46.1 (44.0 to 47.9)	46.8 (44.7 to 48.4)	-0.4 (-2.4 to 1.6)	1.4 (-0.0 to 2.9)
Diet high in red meat	3.7 (0.4 to 8.6)	7.5 (2.0 to 14.4)	8.6 (2.6 to 15.9)	101.8 (-19.7 to 758.3)	14.8 (-36.4 to 121.8)	0.6 (0.0 to 2.5)	1.5 (0.0 to 4.6)	2.0 (0.0 to 6.2)	170.7 (-77.2 to 1106264.6)	33.2 (-84.0 to 2649.3)
Diet high in processed meat	11.2 (7.2 to 15.5)	18.8 (13.6 to 23.4)	21.9 (16.4 to 27.1)	67.4 (30.7 to 133.0)	16.5 (-2.3 to 38.2)	9.6 (6.0 to 13.1)	14.2 (9.9 to 17.9)	15.7 (11.1 to 19.8)	48.0 (16.4 to 99.8)	10.7 (-7.3 to 32.6)
Diet high in sugar-sweetened beverages	7.2 (2.9 to 13.7)	8.2 (3.7 to 14.8)	8.6 (3.8 to 16.0)	14.9 (-45.8 to 161.2)	4.2 (-37.0 to 74.8)	5.0 (1.9 to 11.1)	5.6 (2.4 to 10.6)	5.9 (2.3 to 11.5)	13.0 (-47.8 to 161.6)	5.0 (-40.9 to 87.8)
Diet low in fibre	22.9 (15.5 to 29.4)	23.9 (16.7 to 30.0)	24.6 (17.6 to 30.9)	4.3 (-12.1 to 28.4)	3.1 (-8.2 to 17.9)	23.9 (16.2 to 30.4)	24.9 (17.7 to 31.0)	26.2 (19.0 to 32.1)	4.3 (-10.6 to 24.3)	5.0 (-5.9 to 19.1)
Diet low in calcium	33.5 (29.0 to 37.2)	33.8 (29.1 to 37.4)	35.0 (30.5 to 38.6)	0.9 (-3.5 to 5.0)	3.4 (0.2 to 7.2)	35.4 (31.1 to 38.8)	35.0 (30.7 to 38.5)	36.7 (32.6 to 40.0)	-1.1 (-5.1 to 2.6)	4.7 (1.8 to 8.5)
Diet low in seafood omega-3 fatty acids	1.8 (0.3 to 3.6)	0.2 (0.0 to 0.8)	0.3 (0.0 to 1.1)	-91.5 (-100.0 to -74.4)	66.1 (-79.9 to [illegible]	2.6 (0.7 to 4.5)	0.3 (0.0 to 1.2)	0.4 (0.0 to 1.4)	-89.5 (-100.0 to -70.5)	41.6 (-85.0 to [illegible]
Diet low in polyunsaturated fatty acids	30.2 (24.4 to 35.0)	27.1 (21.1 to 32.1)	28.0 (21.8 to 32.9)	-10.0 (-22.1 to 1.1)	3.2 (-5.3 to 13.7)	31.0 (25.6 to 35.6)	27.9 (22.0 to 32.8)	28.7 (22.8 to 33.5)	-10.0 (-21.8 to -0.4)	3.0 (-4.5 to 12.3)
Diet high in trans fatty acids	1.1 (0.0 to 3.3)	0.9 (0.0 to 3.0)	0.9 (0.0 to 2.9)	-12.5 (-37.5 to -6.6)	-8.2 (-32.8 to -3.7)	1.5 (0.1 to 4.0)	1.3 (0.1 to 3.7)	1.2 (0.0 to 3.5)	-12.5 (-36.5 to -6.6)	-8.4 (-28.4 to -3.5)
Diet high in sodium	28.2 (18.5 to 36.2)	18.5 (9.5 to 25.6)	16.0 (7.2 to 23.4)	-34.4 (-56.4 to -16.5)	-13.6 (-35.3 to 4.8)	32.3 (23.7 to 39.4)	19.8 (11.4 to 27.0)	16.7 (8.0 to 24.0)	-38.6 (-58.2 to -23.5)	-16.0 (-36.7 to -2.2)
Intimate partner violence	-	-	-	-	-	2.3 (1.9 to 2.7)	2.1 (1.8 to 2.4)	2.0 (1.7 to 2.3)	-7.5 (-11.7 to -3.0)	-4.0 (-8.1 to 0.4)
Childhood sexual abuse	5.3 (4.3 to 6.5)	5.3 (4.3 to 6.5)	5.3 (4.3 to 6.5)	0.0 (0.0 to 0.0)	0.0 (0.0 to 0.0)	7.3 (5.9 to 8.9)	7.3 (5.9 to 8.9)	7.5 (6.0 to 9.2)	0.0 (0.0 to 0.0)	2.8 (-4.0 to 10.6)

Supplementary Results Table 1. Miyagi age-standardised Summary Exposure Values (SEVs) for Level 4 risk factors and percentage change, by sex, 1990, 2007, and 2017.Data in parentheses are 95% uncertainty intervals.

	Male					Female				
	**1990**	**2007**	**2017**	**Percentage change 1990-2007**	**Percentage change 2007-2017**	**1990**	**2007**	**2017**	**Percentage change 1990-2007**	**Percentage change 2007-2017**
Bullying victimization	3.2 (1.3 to 6.9)	3.3 (1.4 to 7.1)	3.4 (1.4 to 7.3)	2.5 (-5.4 to 10.2)	3.8 (-1.6 to 8.5)	1.9 (0.8 to 3.9)	1.9 (0.8 to 4.0)	1.9 (0.8 to 4.1)	0.2 (-7.4 to 8.1)	2.6 (-3.0 to 7.5)
Low physical activity	0.5 (0.2 to 0.9)	0.5 (0.2 to 0.9)	0.4 (0.2 to 0.8)	-1.8 (-3.7 to 0.1)	-8.8 (-12.4 to -4.4)	0.5 (0.2 to 1.0)	0.5 (0.2 to 0.9)	0.5 (0.2 to 0.9)	-3.8 (-6.1 to -1.3)	0.8 (-0.7 to 2.3)
High fasting plasma glucose	1.4 (0.8 to 2.2)	1.4 (0.9 to 2.1)	1.5 (0.9 to 2.5)	-1.7 (-36.9 to 54.7)	11.8 (-25.2 to 75.2)	1.2 (0.7 to 1.9)	1.3 (0.7 to 2.2)	1.5 (0.8 to 2.6)	9.3 (-34.5 to 86.5)	9.0 (-32.7 to 84.4)
High LDL cholesterol	12.1 (10.5 to 13.8)	12.6 (11.0 to 14.4)	12.6 (11.0 to 14.5)	4.3 (-1.4 to 10.8)	0.0 (-4.4 to 4.6)	12.9 (11.4 to 14.7)	13.1 (11.5 to 14.8)	13.1 (11.4 to 14.8)	1.0 (-4.9 to 6.7)	0.2 (-4.5 to 4.8)
High systolic blood pressure	10.8 (9.8 to 11.8)	7.6 (6.8 to 8.5)	7.6 (6.6 to 8.5)	-29.4 (-35.6 to -22.7)	-1.0 (-8.3 to 6.9)	9.4 (8.4 to 10.4)	5.9 (5.2 to 6.7)	5.5 (4.7 to 6.3)	-37.2 (-43.8 to -29.9)	-6.5 (-16.5 to 4.5)
High body-mass index	5.6 (3.5 to 9.1)	8.0 (5.3 to 13.0)	8.2 (5.5 to 13.0)	41.7 (12.4 to 81.6)	2.9 (-12.5 to 20.5)	5.6 (3.4 to 8.8)	7.0 (4.5 to 10.9)	7.0 (4.4 to 10.6)	25.4 (0.2 to 64.8)	-0.4 (-17.8 to 18.6)
Low bone mineral density	5.2 (4.4 to 6.2)	5.2 (4.3 to 6.0)	5.2 (4.2 to 6.1)	-1.7 (-14.5 to 13.8)	0.1 (-13.4 to 15.9)	7.2 (6.1 to 8.6)	6.9 (5.9 to 8.1)	7.1 (5.9 to 8.3)	-3.4 (-14.6 to 9.8)	1.7 (-10.1 to 14.5)
Impaired kidney function	2.6 (1.2 to 6.5)	2.5 (1.1 to 6.4)	2.4 (1.1 to 6.1)	-1.8 (-6.3 to 3.4)	-6.6 (-12.4 to -1.8)	3.2 (1.4 to 7.4)	3.3 (1.5 to 7.6)	3.1 (1.4 to 7.2)	4.1 (-0.1 to 9.2)	-7.0 (-12.3 to -2.4)

Supplementary Results Table 1. Miyazaki age-standardised Summary Exposure Values (SEVs) for Level 4 risk factors and percentage change, by sex, 1990, 2007, and 2017.Data in parentheses are 95% uncertainty intervals.

	Male					Female				
	**1990**	**2007**	**2017**	**Percentage change 1990-2007**	**Percentage change 2007-2017**	**1990**	**2007**	**2017**	**Percentage change 1990-2007**	**Percentage change 2007-2017**
Unsafe water source	2.7 (1.4 to 4.5)	2.3 (1.2 to 3.8)	2.4 (1.4 to 3.9)	-14.2 (-55.7 to 71.5)	5.8 (-39.6 to 82.2)	2.7 (1.4 to 4.5)	2.3 (1.2 to 3.8)	2.4 (1.4 to 3.9)	-14.2 (-55.7 to 71.5)	5.8 (-39.6 to 82.2)
Unsafe sanitation	4.9 (2.3 to 8.7)	2.6 (1.2 to 4.7)	2.2 (1.0 to 4.2)	-47.9 (-78.6 to 19.6)	-15.7 (-56.5 to 60.8)	4.9 (2.3 to 8.7)	2.6 (1.2 to 4.7)	2.2 (1.0 to 4.2)	-47.9 (-78.6 to 19.6)	-15.7 (-56.5 to 60.8)
No access to handwashing facility	6.9 (4.9 to 9.3)	6.5 (4.7 to 8.9)	6.0 (4.3 to 8.2)	-5.5 (-33.4 to 32.5)	-7.7 (-28.7 to 17.4)	6.9 (4.9 to 9.3)	6.5 (4.7 to 8.9)	6.0 (4.3 to 8.2)	-5.5 (-33.4 to 32.5)	-7.7 (-28.7 to 17.4)
Ambient particulate matter pollution	34.6 (23.0 to 50.5)	35.6 (24.9 to 52.1)	31.0 (20.2 to 46.6)	3.0 (-22.3 to 37.8)	-13.1 (-34.8 to 14.5)	34.3 (22.8 to 50.3)	35.6 (24.8 to 52.0)	30.9 (20.1 to 46.6)	3.8 (-21.9 to 39.4)	-13.1 (-34.9 to 14.5)
Household air pollution from solid fuels	0.8 (0.3 to 2.4)	0.2 (0.0 to 0.4)	0.2 (0.0 to 0.4)	-76.5 (-92.5 to -50.3)	-16.1 (-66.4 to 75.5)	1.1 (0.3 to 6.4)	0.3 (0.1 to 1.3)	0.3 (0.0 to 1.1)	-73.5 (-92.4 to -47.7)	-14.2 (-66.1 to 78.8)
Ambient ozone pollution	46.7 (17.8 to 54.8)	47.4 (18.5 to 54.8)	48.8 (20.0 to 54.8)	1.7 (0.0 to 5.0)	2.8 (0.0 to 8.9)	46.7 (17.8 to 54.8)	47.4 (18.5 to 54.8)	48.8 (20.0 to 54.8)	1.7 (0.0 to 5.0)	2.8 (0.0 to 8.9)
Residential radon	12.2 (0.7 to 40.5)	12.2 (0.7 to 40.5)	12.2 (0.7 to 40.5)	0.0 (0.0 to 0.0)	0.0 (0.0 to 0.0)	12.2 (0.7 to 40.5)	12.2 (0.7 to 40.5)	12.2 (0.7 to 40.5)	0.0 (0.0 to 0.0)	0.0 (0.0 to 0.0)
Lead exposure	3.5 (0.6 to 7.2)	2.4 (0.4 to 5.5)	1.7 (0.2 to 4.4)	-31.6 (-44.8 to -22.9)	-26.9 (-41.3 to -19.1)	1.8 (0.1 to 4.9)	1.2 (0.1 to 3.5)	0.8 (0.0 to 2.8)	-36.2 (-52.6 to -27.1)	-28.8 (-50.2 to -19.3)
Occupational exposure to asbestos	1.8 (1.5 to 2.0)	2.3 (2.1 to 2.6)	2.4 (1.9 to 2.9)	31.1 (12.7 to 59.4)	0.5 (-19.3 to 23.4)	0.5 (0.4 to 0.6)	0.5 (0.4 to 0.5)	0.4 (0.3 to 0.6)	-4.7 (-21.6 to 25.7)	-6.2 (-26.0 to 19.8)
Occupational exposure to arsenic	0.4 (0.0 to 1.1)	0.4 (0.0 to 1.0)	0.4 (0.0 to 1.0)	-	-	0.4 (0.0 to 1.0)	0.4 (0.0 to 0.8)	0.4 (0.0 to 0.9)	-	-
Occupational exposure to benzene	0.6 (0.0 to 1.8)	0.6 (0.0 to 1.8)	0.7 (0.0 to 1.9)	2.5 (-4.3 to 13.2)	3.6 (-1.7 to 11.0)	0.6 (0.0 to 1.6)	0.7 (0.0 to 1.9)	0.8 (0.1 to 2.2)	21.9 (7.8 to 42.6)	15.1 (4.8 to 31.1)
Occupational exposure to beryllium	0.0 (0.0 to 0.0)	0.0 (0.0 to 0.0)	0.0 (0.0 to 0.0)	-5.1 (-9.9 to 0.5)	1.9 (-2.1 to 6.0)	0.0 (0.0 to 0.0)	0.0 (0.0 to 0.0)	0.0 (0.0 to 0.0)	1.9 (-7.3 to 11.9)	9.9 (2.9 to 17.6)
Occupational exposure to cadmium	0.1 (0.0 to 0.1)	0.0 (0.0 to 0.1)	0.0 (0.0 to 0.1)	-6.3 (-17.4 to 5.7)	1.0 (-8.7 to 11.5)	0.0 (0.0 to 0.1)	0.0 (0.0 to 0.0)	0.0 (0.0 to 0.1)	-8.4 (-23.3 to 10.4)	6.1 (-6.9 to 19.9)
Occupational exposure to chromium	0.1 (0.1 to 0.1)	0.1 (0.1 to 0.1)	0.1 (0.1 to 0.1)	-4.6 (-14.9 to 6.0)	1.5 (-6.8 to 10.6)	0.1 (0.1 to 0.1)	0.1 (0.1 to 0.1)	0.1 (0.1 to 0.1)	-3.9 (-18.1 to 13.4)	7.9 (-3.3 to 19.9)
Occupational exposure to diesel engine exhaust	0.6 (0.5 to 0.6)	0.6 (0.5 to 0.6)	0.6 (0.6 to 0.6)	-2.0 (-10.2 to 6.8)	3.3 (-3.3 to 10.1)	0.4 (0.4 to 0.5)	0.5 (0.4 to 0.5)	0.5 (0.5 to 0.6)	11.9 (-1.8 to 27.8)	12.1 (0.5 to 24.6)
Occupational exposure to formaldehyde	0.3 (0.2 to 0.3)	0.2 (0.2 to 0.3)	0.2 (0.2 to 0.3)	-5.6 (-17.5 to 7.1)	0.6 (-9.3 to 11.4)	0.2 (0.2 to 0.2)	0.2 (0.2 to 0.2)	0.2 (0.2 to 0.2)	-5.8 (-21.7 to 13.8)	6.9 (-5.9 to 21.3)
Occupational exposure to nickel	0.4 (0.0 to 1.9)	0.4 (0.0 to 1.8)	0.4 (0.0 to 1.8)	-	-	0.3 (0.0 to 1.5)	0.3 (0.0 to 1.3)	0.3 (0.0 to 1.3)	-	-
Occupational exposure to polycyclic aromatic hydrocarbons	0.2 (0.2 to 0.3)	0.2 (0.2 to 0.3)	0.2 (0.2 to 0.3)	-3.7 (-13.3 to 6.5)	1.9 (-5.6 to 10.2)	0.2 (0.2 to 0.2)	0.2 (0.2 to 0.2)	0.2 (0.2 to 0.3)	-1.8 (-15.4 to 15.0)	8.7 (-1.8 to 20.3)
Occupational exposure to silica	4.6 (0.7 to 15.1)	4.3 (0.6 to 14.0)	4.4 (0.6 to 14.4)	-6.3 (-16.6 to 6.9)	1.5 (-8.2 to 13.0)	3.1 (0.4 to 9.7)	2.7 (0.4 to 8.4)	2.8 (0.4 to 8.8)	-13.1 (-27.8 to 5.7)	3.2 (-9.7 to 20.1)
Occupational exposure to sulphuric acid	0.6 (0.1 to 2.0)	0.6 (0.1 to 1.8)	0.6 (0.1 to 1.8)	-6.1 (-17.0 to 5.3)	1.4 (-7.7 to 11.5)	0.5 (0.1 to 1.5)	0.5 (0.1 to 1.4)	0.5 (0.1 to 1.5)	-9.8 (-25.0 to 8.4)	5.3 (-7.8 to 19.6)
Occupational exposure to trichloroethylene	0.1 (0.1 to 0.1)	0.1 (0.1 to 0.1)	0.1 (0.1 to 0.1)	-3.7 (-11.1 to 4.3)	2.1 (-4.0 to 8.7)	0.1 (0.1 to 0.1)	0.1 (0.1 to 0.1)	0.1 (0.1 to 0.1)	0.8 (-11.1 to 15.1)	9.3 (-0.1 to 19.9)
Occupational asthmagens	18.2 (14.6 to 23.1)	17.7 (14.2 to 22.3)	17.7 (14.1 to 22.6)	-2.8 (-21.0 to 18.2)	-0.1 (-15.3 to 17.0)	8.1 (6.4 to 10.0)	8.1 (6.5 to 10.1)	8.5 (6.9 to 10.5)	-0.0 (-15.9 to 18.0)	5.7 (-7.1 to 20.4)
Occupational particulate matter, gases, and fumes	6.4 (4.6 to 9.3)	6.2 (4.4 to 8.8)	5.9 (4.2 to 8.5)	-4.5 (-9.2 to 0.8)	-3.7 (-7.0 to -0.3)	3.6 (2.5 to 5.4)	3.4 (2.4 to 5.1)	3.2 (2.3 to 4.8)	-5.5 (-14.0 to 4.3)	-4.3 (-10.4 to 2.0)
Occupational noise	6.0 (5.5 to 6.6)	5.8 (5.3 to 6.4)	5.7 (5.2 to 6.2)	-3.4 (-6.4 to 0.0)	-2.6 (-4.7 to -0.4)	3.4 (2.9 to 3.9)	3.4 (3.0 to 3.9)	3.5 (3.1 to 3.9)	0.2 (-5.9 to 7.1)	1.8 (-2.0 to 6.1)
Occupational ergonomic factors	11.0 (9.1 to 13.3)	9.8 (8.2 to 11.8)	9.6 (7.9 to 11.7)	-10.7 (-23.4 to 3.8)	-1.7 (-13.0 to 11.3)	5.8 (4.5 to 7.5)	5.9 (4.6 to 7.5)	6.6 (5.1 to 8.4)	1.4 (-21.7 to 31.7)	11.6 (-9.4 to 36.6)
Non-exclusive breastfeeding	0.4 (0.3 to 0.6)	0.4 (0.3 to 0.6)	0.4 (0.3 to 0.6)	-0.8 (-13.4 to 13.8)	-2.0 (-13.2 to 9.3)	0.4 (0.3 to 0.6)	0.4 (0.3 to 0.6)	0.4 (0.3 to 0.6)	-0.8 (-13.4 to 13.8)	-1.4 (-12.7 to 9.9)
Discontinued breastfeeding	1.6 (1.5 to 1.8)	1.7 (1.5 to 1.8)	1.6 (1.5 to 1.8)	3.8 (-6.1 to 15.6)	-2.6 (-10.3 to 5.9)	1.6 (1.5 to 1.8)	1.7 (1.5 to 1.8)	1.6 (1.5 to 1.8)	2.6 (-7.3 to 14.2)	-2.8 (-10.4 to 5.7)
Child underweight	0.3 (0.2 to 0.4)	0.2 (0.1 to 0.4)	0.2 (0.1 to 0.3)	-13.9 (-35.7 to 9.1)	-5.5 (-20.8 to 12.9)	0.3 (0.2 to 0.4)	0.2 (0.1 to 0.4)	0.2 (0.1 to 0.3)	-16.5 (-36.4 to 8.1)	-7.5 (-24.2 to 12.0)
Child wasting	0.2 (0.1 to 0.3)	0.2 (0.1 to 0.3)	0.2 (0.1 to 0.3)	-6.6 (-33.0 to 30.7)	-3.0 (-22.6 to 22.0)	0.2 (0.1 to 0.3)	0.2 (0.1 to 0.3)	0.2 (0.1 to 0.3)	-7.3 (-35.6 to 32.3)	-3.3 (-25.4 to 22.3)
Child stunting	0.6 (0.3 to 1.1)	0.6 (0.3 to 1.0)	0.5 (0.3 to 0.9)	-10.0 (-47.4 to 48.5)	-4.6 (-34.1 to 35.7)	0.6 (0.3 to 1.1)	0.6 (0.3 to 1.0)	0.6 (0.3 to 1.0)	-10.4 (-47.1 to 58.5)	-3.8 (-33.0 to 36.7)
Short gestation for birth weight	0.0 (0.0 to 0.0)	0.0 (0.0 to 0.0)	0.0 (0.0 to 0.0)	11.5 (-8.3 to 41.0)	3.0 (-13.6 to 30.6)	0.0 (0.0 to 0.0)	0.0 (0.0 to 0.0)	0.0 (0.0 to 0.0)	14.4 (-22.3 to 63.6)	1.8 (-18.6 to 37.7)
Low birth weight for gestation	0.0 (0.0 to 0.0)	0.0 (0.0 to 0.0)	0.0 (0.0 to 0.0)	4.1 (-13.0 to 26.1)	1.5 (-15.9 to 21.1)	0.0 (0.0 to 0.0)	0.0 (0.0 to 0.0)	0.0 (0.0 to 0.0)	-3.0 (-49.8 to 31.4)	2.7 (-21.5 to 23.0)
Iron deficiency	-	-	-	-	-	8.4 (5.7 to 11.5)	8.5 (5.9 to 11.7)	7.8 (5.4 to 10.9)	1.1 (-20.6 to 28.4)	-8.2 (-24.0 to 8.7)
Vitamin A deficiency	0.9 (0.6 to 1.3)	0.8 (0.6 to 1.1)	0.7 (0.5 to 0.9)	-9.9 (-23.1 to 7.3)	-15.2 (-29.4 to 0.7)	0.8 (0.5 to 1.0)	0.8 (0.6 to 1.2)	0.7 (0.5 to 1.0)	6.0 (-11.7 to 33.9)	-17.5 (-43.4 to 12.5)
Zinc deficiency	0.5 (0.1 to 1.1)	0.4 (0.1 to 1.1)	0.4 (0.1 to 0.9)	-21.3 (-65.7 to 70.6)	-10.3 (-66.7 to 170.1)	0.5 (0.2 to 1.1)	0.4 (0.1 to 0.8)	0.4 (0.1 to 0.8)	-30.4 (-64.2 to 6.0)	7.8 (-57.1 to 159.8)
Smoking	18.1 (14.6 to 21.4)	12.8 (10.3 to 15.4)	10.8 (8.7 to 13.1)	-29.6 (-38.8 to -19.6)	-15.4 (-23.3 to -6.5)	4.1 (2.9 to 5.4)	3.4 (2.5 to 4.6)	3.4 (2.5 to 4.7)	-16.7 (-36.8 to 10.6)	-2.0 (-19.6 to 18.3)
Chewing tobacco	1.4 (0.9 to 2.0)	0.9 (0.6 to 1.4)	0.8 (0.5 to 1.1)	-33.5 (-59.1 to 4.0)	-18.9 (-42.0 to 14.0)	0.1 (0.1 to 0.2)	0.1 (0.1 to 0.1)	0.1 (0.1 to 0.1)	-3.9 (-39.6 to 57.8)	-2.6 (-31.7 to 41.9)
Second-hand smoke	38.2 (34.0 to 42.2)	35.2 (30.7 to 39.5)	35.7 (31.0 to 40.5)	-7.8 (-18.7 to 4.3)	1.5 (-7.8 to 11.9)	57.6 (53.9 to 61.2)	47.1 (43.4 to 50.8)	47.7 (43.5 to 51.8)	-18.2 (-24.1 to -11.6)	1.2 (-5.4 to 7.7)
Alcohol use	14.5 (7.1 to 24.6)	14.6 (7.2 to 25.3)	16.6 (7.9 to 29.0)	0.8 (-32.2 to 52.7)	13.8 (-18.8 to 63.6)	11.3 (5.5 to 20.3)	11.3 (5.4 to 19.9)	13.1 (6.1 to 23.1)	0.0 (-33.5 to 52.4)	15.7 (-17.8 to 67.4)
Drug use	0.5 (0.4 to 0.7)	0.5 (0.4 to 0.7)	0.6 (0.4 to 0.8)	0.9 (-5.2 to 7.4)	11.3 (3.5 to 20.6)	0.3 (0.2 to 0.4)	0.3 (0.2 to 0.4)	0.3 (0.3 to 0.4)	2.1 (-3.8 to 8.4)	9.3 (2.6 to 16.8)
Diet low in fruits	42.0 (36.2 to 45.7)	40.3 (34.5 to 44.0)	41.6 (35.9 to 45.2)	-4.0 (-9.6 to 1.9)	3.2 (-1.4 to 8.2)	39.0 (33.2 to 43.1)	36.5 (30.5 to 41.0)	38.5 (32.7 to 42.7)	-6.5 (-14.3 to 0.7)	5.5 (-0.4 to 13.0)
Diet low in vegetables	21.6 (13.3 to 29.0)	16.1 (7.8 to 24.4)	18.2 (9.5 to 26.2)	-25.4 (-51.6 to -3.2)	13.3 (-12.8 to 52.6)	23.6 (15.5 to 30.5)	17.7 (9.7 to 25.0)	19.5 (11.7 to 27.4)	-24.8 (-50.0 to -2.4)	10.0 (-12.8 to 44.6)
Diet low in legumes	4.6 (0.8 to 10.4)	6.3 (1.5 to 11.9)	8.4 (2.5 to 15.1)	36.5 (-43.0 to 284.2)	32.8 (-17.0 to 176.4)	8.4 (2.9 to 15.3)	8.5 (2.6 to 14.8)	10.9 (4.2 to 18.1)	0.2 (-45.3 to 93.6)	28.9 (-10.6 to 114.0)
Diet low in whole grains	31.2 (26.4 to 35.2)	35.8 (31.8 to 39.1)	37.2 (33.3 to 40.3)	15.0 (6.7 to 26.0)	3.7 (-1.1 to 8.8)	32.6 (28.2 to 36.6)	37.1 (33.3 to 40.2)	38.3 (34.5 to 41.2)	13.6 (5.8 to 23.5)	3.3 (-1.1 to 8.2)
Diet low in nuts and seeds	51.2 (50.1 to 52.2)	50.1 (48.4 to 51.3)	49.9 (48.1 to 51.2)	-2.3 (-4.5 to -0.5)	-0.4 (-2.3 to 1.2)	51.5 (50.3 to 52.4)	50.4 (48.8 to 51.6)	50.2 (48.6 to 51.5)	-2.2 (-4.2 to -0.6)	-0.3 (-2.0 to 1.3)
Diet low in milk	46.4 (44.4 to 48.2)	46.7 (44.7 to 48.3)	47.1 (45.1 to 48.7)	0.5 (-1.3 to 2.5)	0.9 (-0.6 to 2.4)	46.6 (44.7 to 48.3)	46.4 (44.5 to 48.1)	47.0 (45.1 to 48.7)	-0.4 (-2.4 to 1.5)	1.3 (-0.1 to 2.9)
Diet high in red meat	3.3 (0.4 to 7.8)	6.8 (1.7 to 12.9)	7.9 (2.3 to 14.6)	104.7 (-20.4 to 970.4)	16.3 (-43.4 to 144.2)	0.5 (0.0 to 2.2)	1.3 (0.0 to 4.5)	1.7 (0.0 to 5.1)	193.4 (-71.4 to [illegible]	27.7 (-93.8 to 2765.1)
Diet high in processed meat	10.6 (6.8 to 14.7)	18.0 (13.2 to 22.7)	21.0 (15.6 to 26.5)	69.1 (31.1 to 132.9)	17.0 (-1.0 to 38.7)	9.0 (5.2 to 12.7)	13.5 (9.5 to 17.4)	15.0 (10.7 to 18.9)	50.5 (18.2 to 109.2)	11.1 (-6.1 to 34.2)
Diet high in sugar-sweetened beverages	6.4 (2.5 to 12.8)	7.1 (3.1 to 12.9)	7.6 (3.3 to 14.4)	12.4 (-48.2 to 141.2)	6.1 (-39.9 to 85.1)	4.6 (1.7 to 9.8)	5.1 (2.2 to 9.8)	5.4 (2.3 to 11.0)	11.3 (-48.7 to 149.4)	5.8 (-41.6 to 88.9)
Diet low in fibre	23.1 (16.1 to 29.5)	24.3 (17.7 to 30.2)	25.5 (18.6 to 31.6)	5.6 (-11.4 to 26.4)	4.6 (-6.8 to 18.4)	24.1 (16.6 to 30.7)	25.1 (17.9 to 31.3)	26.5 (19.2 to 32.6)	4.1 (-11.0 to 25.5)	5.5 (-5.5 to 19.4)
Diet low in calcium	35.8 (31.7 to 39.2)	35.5 (31.2 to 38.9)	36.2 (32.2 to 39.4)	-1.0 (-4.7 to 2.7)	2.0 (-1.0 to 5.6)	38.0 (34.3 to 41.0)	36.8 (32.8 to 40.0)	37.9 (34.2 to 41.0)	-3.1 (-6.7 to -0.0)	3.0 (0.5 to 6.2)
Diet low in seafood omega-3 fatty acids	3.4 (1.7 to 4.8)	0.3 (0.0 to 1.2)	0.3 (0.0 to 1.2)	-91.0 (-100.0 to -68.2)	8.0 (-86.4 to 881.2)	4.0 (2.0 to 5.4)	0.5 (0.0 to 1.6)	0.5 (0.0 to 1.7)	-88.4 (-99.9 to -65.7)	9.3 (-73.4 to 918.0)
Diet low in polyunsaturated fatty acids	31.4 (26.2 to 36.0)	28.8 (23.0 to 33.5)	29.4 (23.7 to 33.9)	-8.1 (-19.1 to 0.8)	2.2 (-5.0 to 10.2)	32.1 (26.7 to 36.6)	29.4 (23.8 to 34.3)	30.2 (24.8 to 35.4)	-8.1 (-18.3 to 0.6)	2.5 (-5.6 to 13.9)
Diet high in trans fatty acids	1.1 (0.0 to 3.3)	1.0 (0.0 to 3.0)	0.9 (0.0 to 2.9)	-12.4 (-38.7 to -6.6)	-8.3 (-31.9 to -3.6)	1.5 (0.1 to 4.0)	1.3 (0.1 to 3.7)	1.2 (0.0 to 3.5)	-12.2 (-34.8 to -6.0)	-8.6 (-28.9 to -3.4)
Diet high in sodium	27.1 (17.5 to 35.1)	17.6 (8.7 to 25.1)	15.0 (6.0 to 22.5)	-35.2 (-59.5 to -16.4)	-14.6 (-39.5 to 2.6)	31.9 (23.5 to 39.1)	19.5 (11.1 to 26.3)	16.3 (7.6 to 23.5)	-38.7 (-58.4 to -24.1)	-16.3 (-38.7 to -2.3)
Intimate partner violence	-	-	-	-	-	2.3 (1.9 to 2.7)	2.1 (1.8 to 2.4)	2.0 (1.7 to 2.4)	-9.4 (-13.5 to -5.2)	-3.5 (-7.8 to 1.1)
Childhood sexual abuse	5.3 (4.3 to 6.5)	5.3 (4.3 to 6.5)	5.3 (4.3 to 6.5)	0.0 (0.0 to 0.0)	0.0 (0.0 to 0.0)	7.3 (5.9 to 8.9)	7.3 (5.9 to 8.9)	7.5 (6.0 to 9.2)	0.0 (0.0 to 0.0)	2.8 (-4.0 to 10.6)

Supplementary Results Table 1. Miyazaki age-standardised Summary Exposure Values (SEVs) for Level 4 risk factors and percentage change, by sex, 1990, 2007, and 2017.Data in parentheses are 95% uncertainty intervals.

	Male					Female				
	**1990**	**2007**	**2017**	**Percentage change 1990-2007**	**Percentage change 2007-2017**	**1990**	**2007**	**2017**	**Percentage change 1990-2007**	**Percentage change 2007-2017**
Bullying victimization	3.3 (1.4 to 6.8)	3.4 (1.4 to 7.1)	3.5 (1.4 to 7.3)	2.1 (-6.0 to 10.3)	2.7 (-2.7 to 7.6)	1.9 (0.8 to 3.9)	2.0 (0.8 to 4.0)	2.0 (0.8 to 4.0)	0.3 (-7.4 to 8.3)	1.4 (-24.7 to 36.1)
Low physical activity	0.5 (0.2 to 0.9)	0.5 (0.2 to 0.9)	0.4 (0.2 to 0.8)	-1.8 (-3.8 to 0.1)	-8.8 (-12.3 to -4.6)	0.5 (0.2 to 1.0)	0.5 (0.2 to 0.9)	0.5 (0.2 to 0.9)	-3.9 (-6.1 to -1.3)	0.7 (-0.9 to 2.1)
High fasting plasma glucose	1.3 (0.8 to 2.0)	1.3 (0.8 to 2.1)	1.5 (0.9 to 2.4)	-1.3 (-36.5 to 56.3)	13.0 (-22.3 to 82.7)	1.1 (0.7 to 1.9)	1.2 (0.7 to 2.0)	1.3 (0.8 to 2.3)	4.6 (-33.3 to 77.5)	9.8 (-31.4 to 84.5)
High LDL cholesterol	11.9 (10.3 to 13.7)	12.4 (10.8 to 14.1)	12.4 (10.9 to 14.3)	4.1 (-1.8 to 10.3)	0.1 (-4.5 to 4.9)	12.7 (11.1 to 14.4)	12.7 (11.2 to 14.5)	12.7 (11.1 to 14.5)	0.4 (-5.1 to 6.0)	0.3 (-4.3 to 5.3)
High systolic blood pressure	11.1 (10.2 to 12.2)	8.2 (7.3 to 9.1)	8.0 (7.0 to 9.0)	-26.3 (-32.1 to -19.6)	-1.7 (-9.2 to 6.0)	9.6 (8.6 to 10.5)	6.0 (5.2 to 6.7)	5.5 (4.8 to 6.4)	-37.4 (-44.2 to -29.9)	-7.3 (-17.3 to 3.3)
High body-mass index	4.9 (3.0 to 7.9)	7.0 (4.6 to 11.5)	7.2 (4.7 to 11.7)	41.9 (12.3 to 85.5)	3.5 (-12.6 to 21.1)	5.0 (3.1 to 8.0)	6.4 (3.9 to 10.3)	6.5 (3.9 to 10.3)	28.2 (-0.4 to 74.2)	0.7 (-18.5 to 22.9)
Low bone mineral density	5.3 (4.3 to 6.4)	5.2 (4.3 to 6.1)	5.2 (4.3 to 6.1)	-2.5 (-16.0 to 12.6)	0.3 (-13.2 to 14.8)	7.2 (6.0 to 8.6)	7.0 (5.9 to 8.2)	7.1 (6.0 to 8.2)	-3.3 (-14.0 to 11.4)	1.6 (-10.8 to 15.1)
Impaired kidney function	2.6 (1.2 to 6.4)	2.5 (1.1 to 6.3)	2.4 (1.1 to 6.1)	-2.5 (-7.0 to 2.0)	-6.6 (-11.8 to -2.0)	3.2 (1.5 to 7.5)	3.3 (1.5 to 7.6)	3.1 (1.4 to 7.3)	3.4 (-0.4 to 8.4)	-7.2 (-12.0 to -2.4)

Supplementary Results Table 1. Nagano age-standardised Summary Exposure Values (SEVs) for Level 4 risk factors and percentage change, by sex, 1990, 2007, and 2017.Data in parentheses are 95% uncertainty intervals.

	Male					Female				
	1990	2007	2017	Percentage change 1990-2007	Percentage change 2007-2017	1990	2007	2017	Percentage change 1990-2007	Percentage change 2007-2017
Unsafe water source	2.2 (1.2 to 3.5)	1.8 (1.0 to 3.1)	2.0 (1.1 to 3.2)	-16.7 (-56.6 to 67.9)	7.8 (-36.2 to 84.8)	2.2 (1.2 to 3.5)	1.8 (1.0 to 3.1)	2.0 (1.1 to 3.2)	-16.7 (-56.6 to 67.9)	7.8 (-36.2 to 84.8)
Unsafe sanitation	3.7 (1.7 to 6.6)	1.9 (0.9 to 3.5)	1.6 (0.8 to 3.1)	-48.0 (-77.4 to 13.7)	-14.0 (-56.2 to 70.5)	3.7 (1.7 to 6.6)	1.9 (0.9 to 3.5)	1.6 (0.8 to 3.1)	-48.0 (-77.4 to 13.7)	-14.0 (-56.2 to 70.5)
No access to handwashing facility	6.7 (4.9 to 9.1)	6.3 (4.5 to 8.6)	5.9 (4.3 to 7.9)	-5.4 (-30.7 to 34.8)	-6.8 (-27.0 to 22.2)	6.7 (4.9 to 9.1)	6.3 (4.5 to 8.6)	5.9 (4.3 to 7.9)	-5.4 (-30.7 to 34.8)	-6.8 (-27.0 to 22.2)
Ambient particulate matter pollution	30.9 (19.6 to 47.9)	30.8 (20.2 to 46.3)	26.3 (15.8 to 41.3)	-0.2 (-27.8 to 39.2)	-14.6 (-41.4 to 19.0)	30.7 (19.4 to 47.7)	30.8 (20.1 to 46.3)	26.3 (15.7 to 41.2)	0.4 (-27.3 to 41.0)	-14.6 (-41.5 to 19.2)
Household air pollution from solid fuels	0.7 (0.2 to 1.8)	0.2 (0.0 to 0.4)	0.1 (0.0 to 0.3)	-74.6 (-92.5 to -44.9)	-15.1 (-67.8 to 81.1)	0.9 (0.3 to 4.2)	0.3 (0.0 to 1.3)	0.2 (0.0 to 1.2)	-70.9 (-92.5 to -40.9)	-13.1 (-67.2 to 83.8)
Ambient ozone pollution	52.1 (26.3 to 54.8)	52.0 (26.2 to 54.8)	52.2 (26.8 to 54.8)	-0.0 (-0.3 to 0.2)	0.3 (0.0 to 2.6)	52.1 (26.3 to 54.8)	52.0 (26.2 to 54.8)	52.2 (26.8 to 54.8)	-0.0 (-0.3 to 0.2)	0.3 (0.0 to 2.6)
Residential radon	19.1 (2.1 to 63.4)	19.1 (2.1 to 63.4)	19.1 (2.1 to 63.4)	0.0 (0.0 to 0.0)	0.0 (0.0 to 0.0)	19.1 (2.1 to 63.4)	19.1 (2.1 to 63.4)	19.1 (2.1 to 63.4)	0.0 (0.0 to 0.0)	0.0 (0.0 to 0.0)
Lead exposure	2.0 (0.1 to 5.2)	1.4 (0.1 to 4.1)	1.1 (0.1 to 3.4)	-28.3 (-42.5 to -20.3)	-24.6 (-41.2 to -16.5)	1.2 (0.0 to 3.8)	0.8 (0.0 to 2.8)	0.6 (0.0 to 2.3)	-33.8 (-51.1 to -24.8)	-26.5 (-49.4 to -18.0)
Occupational exposure to asbestos	1.3 (1.1 to 1.5)	1.8 (1.7 to 2.0)	1.9 (1.5 to 2.3)	40.9 (21.4 to 66.5)	2.7 (-17.7 to 27.9)	0.5 (0.4 to 0.6)	0.5 (0.4 to 0.5)	0.4 (0.3 to 0.5)	-4.7 (-21.6 to 23.5)	-10.9 (-30.1 to 11.9)
Occupational exposure to arsenic	0.4 (0.0 to 1.1)	0.4 (0.0 to 1.0)	0.4 (0.0 to 1.0)	-	-	0.4 (0.0 to 1.0)	0.4 (0.0 to 0.9)	0.4 (0.0 to 0.9)	-	-
Occupational exposure to benzene	0.6 (0.0 to 1.8)	0.6 (0.0 to 1.8)	0.7 (0.0 to 1.9)	2.3 (-4.1 to 13.1)	3.2 (-2.6 to 11.7)	0.6 (0.0 to 1.6)	0.7 (0.0 to 2.0)	0.8 (0.1 to 2.3)	21.3 (8.8 to 41.0)	14.0 (4.6 to 28.0)
Occupational exposure to beryllium	0.0 (0.0 to 0.0)	0.0 (0.0 to 0.0)	0.0 (0.0 to 0.0)	-4.9 (-9.7 to 0.2)	1.9 (-1.9 to 6.0)	0.0 (0.0 to 0.0)	0.0 (0.0 to 0.0)	0.0 (0.0 to 0.0)	2.1 (-7.1 to 11.3)	9.0 (2.6 to 16.1)
Occupational exposure to cadmium	0.1 (0.0 to 0.1)	0.0 (0.0 to 0.1)	0.0 (0.0 to 0.1)	-6.5 (-17.9 to 6.9)	0.9 (-8.8 to 11.5)	0.0 (0.0 to 0.1)	0.0 (0.0 to 0.0)	0.0 (0.0 to 0.1)	-8.5 (-23.3 to 9.1)	4.9 (-7.4 to 20.1)
Occupational exposure to chromium	0.1 (0.1 to 0.1)	0.1 (0.1 to 0.1)	0.1 (0.1 to 0.1)	-4.8 (-15.0 to 7.0)	1.4 (-7.0 to 10.8)	0.1 (0.1 to 0.1)	0.1 (0.1 to 0.1)	0.1 (0.1 to 0.1)	-3.8 (-17.9 to 12.5)	6.6 (-4.0 to 19.5)
Occupational exposure to diesel engine exhaust	0.6 (0.5 to 0.6)	0.6 (0.5 to 0.6)	0.6 (0.6 to 0.6)	-1.0 (-8.8 to 7.8)	3.2 (-3.5 to 10.1)	0.4 (0.4 to 0.5)	0.5 (0.4 to 0.6)	0.6 (0.5 to 0.6)	12.7 (-0.1 to 28.5)	11.1 (0.4 to 21.8)
Occupational exposure to formaldehyde	0.2 (0.2 to 0.3)	0.2 (0.2 to 0.3)	0.2 (0.2 to 0.3)	-5.9 (-18.2 to 8.6)	0.5 (-9.7 to 11.7)	0.2 (0.2 to 0.2)	0.2 (0.2 to 0.2)	0.2 (0.2 to 0.2)	-5.9 (-22.0 to 12.8)	5.7 (-6.9 to 20.7)
Occupational exposure to nickel	0.4 (0.0 to 1.8)	0.4 (0.0 to 1.6)	0.4 (0.0 to 1.6)	-	-	0.3 (0.0 to 1.5)	0.3 (0.0 to 1.2)	0.3 (0.0 to 1.3)	-	-
Occupational exposure to polycyclic aromatic hydrocarbons	0.2 (0.2 to 0.3)	0.2 (0.2 to 0.3)	0.2 (0.2 to 0.3)	-3.8 (-13.6 to 7.5)	1.8 (-6.1 to 10.1)	0.2 (0.2 to 0.2)	0.2 (0.2 to 0.2)	0.2 (0.2 to 0.3)	-1.7 (-15.2 to 13.8)	7.5 (-2.8 to 19.4)
Occupational exposure to silica	4.5 (0.7 to 14.4)	4.3 (0.6 to 13.4)	4.3 (0.6 to 14.0)	-4.6 (-14.9 to 9.2)	1.7 (-6.9 to 12.3)	3.1 (0.4 to 10.3)	2.7 (0.4 to 8.5)	2.7 (0.4 to 8.7)	-12.6 (-27.0 to 6.8)	2.2 (-10.8 to 20.2)
Occupational exposure to sulphuric acid	0.6 (0.1 to 1.9)	0.6 (0.1 to 1.8)	0.6 (0.1 to 1.8)	-6.6 (-17.8 to 5.5)	1.3 (-8.0 to 11.8)	0.5 (0.1 to 1.6)	0.5 (0.1 to 1.5)	0.5 (0.1 to 1.5)	-10.1 (-24.9 to 7.5)	4.2 (-8.1 to 20.2)
Occupational exposure to trichloroethylene	0.1 (0.1 to 0.1)	0.1 (0.1 to 0.1)	0.1 (0.1 to 0.1)	-4.1 (-11.4 to 4.8)	1.8 (-4.0 to 8.7)	0.1 (0.1 to 0.1)	0.1 (0.1 to 0.1)	0.1 (0.1 to 0.1)	0.9 (-10.2 to 14.6)	8.2 (-0.3 to 18.5)
Occupational asthmagens	18.1 (14.2 to 22.8)	17.8 (14.3 to 22.4)	17.8 (14.0 to 23.1)	-1.6 (-20.7 to 23.3)	0.4 (-14.1 to 19.2)	8.1 (6.5 to 10.2)	8.2 (6.5 to 10.1)	8.6 (6.8 to 10.8)	0.6 (-15.4 to 19.3)	5.5 (-7.2 to 19.9)
Occupational particulate matter, gases, and fumes	6.2 (4.5 to 9.0)	6.0 (4.3 to 8.7)	5.8 (4.2 to 8.4)	-3.6 (-8.7 to 1.4)	-3.3 (-6.5 to 0.2)	3.6 (2.4 to 5.4)	3.4 (2.3 to 5.2)	3.2 (2.3 to 5.0)	-4.6 (-13.4 to 4.7)	-4.2 (-10.3 to 2.2)
Occupational noise	5.8 (5.3 to 6.3)	5.6 (5.2 to 6.2)	5.5 (5.1 to 6.1)	-2.3 (-5.6 to 1.0)	-1.9 (-3.9 to 0.2)	3.4 (2.9 to 3.9)	3.4 (3.0 to 3.9)	3.5 (3.1 to 3.9)	1.4 (-5.0 to 8.8)	2.2 (-1.7 to 6.6)
Occupational ergonomic factors	10.7 (8.8 to 12.9)	9.5 (7.9 to 11.5)	9.4 (7.6 to 11.4)	-11.4 (-23.0 to 2.2)	-0.9 (-12.2 to 11.7)	5.8 (4.4 to 7.6)	5.8 (4.5 to 7.4)	6.6 (5.1 to 8.6)	1.1 (-22.1 to 27.0)	13.0 (-6.2 to 36.2)
Non-exclusive breastfeeding	0.4 (0.3 to 0.6)	0.4 (0.3 to 0.6)	0.4 (0.3 to 0.6)	-1.6 (-14.4 to 12.0)	-1.4 (-11.4 to 10.7)	0.4 (0.3 to 0.6)	0.4 (0.3 to 0.6)	0.4 (0.3 to 0.6)	-2.0 (-14.7 to 11.6)	-1.2 (-11.2 to 10.9)
Discontinued breastfeeding	1.7 (1.6 to 1.9)	1.7 (1.6 to 1.9)	1.7 (1.5 to 1.8)	-0.5 (-10.4 to 9.8)	-2.2 (-9.4 to 5.6)	1.7 (1.6 to 1.9)	1.7 (1.6 to 1.9)	1.7 (1.6 to 1.8)	-0.8 (-10.7 to 9.5)	-1.1 (-8.4 to 6.9)
Child underweight	0.3 (0.2 to 0.4)	0.2 (0.1 to 0.3)	0.2 (0.1 to 0.3)	-13.5 (-32.7 to 7.8)	-4.6 (-18.7 to 13.1)	0.3 (0.2 to 0.4)	0.2 (0.1 to 0.3)	0.2 (0.1 to 0.3)	-16.4 (-38.1 to 8.7)	-6.0 (-21.7 to 12.2)
Child wasting	0.2 (0.1 to 0.3)	0.2 (0.1 to 0.3)	0.2 (0.1 to 0.3)	-6.1 (-33.7 to 31.4)	-2.0 (-22.6 to 23.0)	0.2 (0.1 to 0.3)	0.2 (0.1 to 0.3)	0.2 (0.1 to 0.3)	-7.5 (-35.8 to 33.2)	-2.1 (-22.8 to 26.1)
Child stunting	0.6 (0.3 to 1.1)	0.6 (0.3 to 0.9)	0.5 (0.3 to 0.9)	-10.7 (-46.0 to 49.1)	-4.2 (-31.9 to 35.9)	0.6 (0.3 to 1.1)	0.6 (0.3 to 1.0)	0.5 (0.3 to 0.9)	-11.9 (-46.7 to 44.4)	-5.2 (-36.5 to 29.0)
Short gestation for birth weight	0.0 (0.0 to 0.0)	0.0 (0.0 to 0.0)	0.0 (0.0 to 0.0)	12.5 (-7.2 to 47.8)	0.6 (-18.4 to 25.8)	0.0 (0.0 to 0.0)	0.0 (0.0 to 0.0)	0.0 (0.0 to 0.0)	11.6 (-19.8 to 40.2)	3.5 (-19.9 to 40.7)
Low birth weight for gestation	0.0 (0.0 to 0.0)	0.0 (0.0 to 0.0)	0.0 (0.0 to 0.0)	2.2 (-15.8 to 25.6)	3.1 (-14.1 to 22.8)	0.0 (0.0 to 0.0)	0.0 (0.0 to 0.0)	0.0 (0.0 to 0.0)	2.0 (-20.1 to 34.2)	0.8 (-22.5 to 23.4)
Iron deficiency	-	-	-	-	-	8.0 (5.5 to 10.9)	8.0 (5.7 to 11.0)	7.4 (5.0 to 10.3)	0.1 (-21.3 to 29.4)	-8.1 (-24.2 to 10.2)
Vitamin A deficiency	1.0 (0.5 to 1.4)	0.8 (0.6 to 1.2)	0.7 (0.5 to 0.9)	-15.2 (-31.3 to 8.2)	-19.3 (-33.2 to 0.6)	0.7 (0.4 to 1.0)	0.8 (0.5 to 1.1)	0.6 (0.4 to 0.9)	19.0 (-3.9 to 46.9)	-21.4 (-36.4 to -1.8)
Zinc deficiency	0.5 (0.1 to 1.0)	0.5 (0.1 to 1.2)	0.4 (0.1 to 1.1)	0.3 (-63.2 to 74.2)	-13.8 (-68.6 to 181.5)	0.5 (0.1 to 1.1)	0.3 (0.1 to 0.8)	0.4 (0.1 to 0.8)	-30.8 (-65.1 to 61.0)	4.0 (-61.5 to 186.6)
Smoking	17.8 (14.4 to 21.0)	12.4 (9.8 to 15.0)	10.4 (8.3 to 12.6)	-30.4 (-39.3 to -19.7)	-16.1 (-24.6 to -6.1)	4.0 (2.8 to 5.4)	3.3 (2.4 to 4.5)	3.2 (2.2 to 4.3)	-17.7 (-37.3 to 9.0)	-3.8 (-20.0 to 17.1)
Chewing tobacco	1.4 (0.9 to 2.0)	0.9 (0.6 to 1.4)	0.8 (0.5 to 1.1)	-33.1 (-56.6 to 1.6)	-18.7 (-40.7 to 13.4)	0.1 (0.1 to 0.2)	0.1 (0.1 to 0.1)	0.1 (0.1 to 0.1)	-3.2 (-40.3 to 68.8)	-2.7 (-31.7 to 39.2)
Second-hand smoke	37.5 (33.5 to 41.5)	35.9 (32.0 to 40.4)	34.8 (30.5 to 39.4)	-4.2 (-15.6 to 8.3)	-3.3 (-12.1 to 5.9)	56.8 (53.0 to 60.5)	48.4 (44.7 to 51.9)	46.6 (42.7 to 50.5)	-14.8 (-21.2 to -8.5)	-3.6 (-9.5 to 2.5)
Alcohol use	14.9 (7.7 to 25.3)	14.8 (7.8 to 25.5)	16.7 (8.1 to 28.9)	-0.6 (-32.3 to 49.4)	12.7 (-21.7 to 62.2)	11.8 (6.1 to 20.9)	11.7 (5.9 to 21.0)	13.1 (6.2 to 22.8)	-1.3 (-33.7 to 48.7)	12.5 (-22.1 to 61.4)
Drug use	0.5 (0.4 to 0.7)	0.5 (0.4 to 0.7)	0.6 (0.4 to 0.8)	0.0 (-5.9 to 6.4)	11.7 (4.6 to 20.3)	0.3 (0.2 to 0.4)	0.3 (0.2 to 0.4)	0.3 (0.3 to 0.4)	2.3 (-3.5 to 8.6)	9.0 (2.4 to 16.0)
Diet low in fruits	38.7 (32.8 to 42.8)	38.2 (32.6 to 42.3)	39.8 (34.1 to 43.9)	-1.3 (-8.1 to 5.6)	4.2 (-1.1 to 10.3)	35.1 (28.9 to 40.2)	33.9 (27.5 to 38.8)	36.6 (30.5 to 41.1)	-3.4 (-12.2 to 5.9)	7.7 (0.7 to 15.8)
Diet low in vegetables	17.1 (8.8 to 24.7)	11.9 (4.4 to 19.7)	14.0 (6.1 to 21.4)	-30.0 (-62.7 to -1.3)	17.3 (-15.9 to 81.7)	19.1 (10.9 to 26.5)	13.7 (5.8 to 21.3)	15.6 (7.4 to 23.4)	-28.3 (-58.6 to 1.3)	13.6 (-17.1 to 65.9)
Diet low in legumes	4.5 (0.8 to 9.9)	6.1 (1.2 to 12.3)	8.2 (2.3 to 14.8)	36.3 (-41.0 to 265.1)	34.3 (-18.1 to 185.0)	8.3 (2.8 to 14.5)	8.2 (2.6 to 14.4)	10.6 (4.3 to 17.4)	-2.2 (-46.8 to 79.1)	30.2 (-10.3 to 136.5)
Diet low in whole grains	30.4 (25.2 to 34.8)	35.3 (31.2 to 38.7)	36.6 (32.6 to 39.8)	16.1 (7.1 to 28.8)	3.9 (-1.1 to 9.4)	31.8 (27.4 to 35.8)	36.4 (32.5 to 39.7)	37.7 (33.9 to 40.8)	14.5 (5.9 to 25.5)	3.5 (-0.9 to 8.6)
Diet low in nuts and seeds	49.8 (48.2 to 51.1)	48.5 (46.3 to 50.0)	48.3 (46.2 to 50.0)	-2.6 (-5.6 to -0.1)	-0.4 (-2.8 to 1.7)	50.0 (48.4 to 51.2)	48.4 (46.3 to 50.0)	48.1 (45.8 to 49.9)	-3.2 (-5.8 to -0.8)	-0.6 (-3.3 to 1.7)
Diet low in milk	44.7 (42.3 to 46.7)	45.9 (43.9 to 47.7)	46.6 (44.7 to 48.2)	2.7 (0.3 to 5.4)	1.5 (-0.1 to 2.9)	44.7 (42.4 to 46.8)	45.3 (43.0 to 47.2)	46.1 (43.9 to 47.9)	1.3 (-1.1 to 3.8)	1.8 (0.2 to 3.8)
Diet high in red meat	4.0 (0.6 to 8.9)	7.9 (2.4 to 15.0)	9.1 (2.7 to 16.6)	98.1 (-15.2 to 754.0)	14.7 (-36.9 to 117.8)	0.6 (0.0 to 2.6)	1.6 (0.0 to 5.0)	2.0 (0.0 to 6.1)	179.1 (-77.0 to 529712.0)	31.3 (-91.5 to 3112.1)
Diet high in processed meat	11.6 (7.6 to 15.7)	19.3 (14.3 to 24.1)	22.3 (16.6 to 28.2)	66.1 (28.3 to 128.4)	15.7 (-1.4 to 36.8)	9.7 (6.0 to 13.6)	14.3 (10.2 to 18.5)	15.8 (11.5 to 20.1)	47.3 (16.7 to 104.2)	10.5 (-6.4 to 33.1)
Diet high in sugar-sweetened beverages	7.4 (3.0 to 14.6)	8.4 (3.7 to 15.2)	8.8 (3.6 to 16.0)	12.4 (-46.0 to 142.8)	5.4 (-37.4 to 79.1)	5.2 (2.0 to 11.8)	5.8 (2.5 to 11.2)	6.3 (2.5 to 12.4)	12.3 (-48.3 to 163.0)	7.2 (-38.6 to 82.5)
Diet low in fibre	21.5 (14.2 to 27.9)	22.0 (14.9 to 28.6)	22.7 (15.4 to 29.2)	2.5 (-15.4 to 24.9)	3.0 (-10.5 to 18.3)	22.6 (15.2 to 29.3)	23.0 (16.0 to 29.3)	24.0 (16.8 to 30.4)	1.7 (-14.5 to 23.8)	4.3 (-7.2 to 18.9)
Diet low in calcium	33.1 (28.4 to 36.9)	33.5 (28.8 to 37.2)	34.6 (30.1 to 38.2)	1.2 (-3.0 to 5.9)	3.1 (-0.2 to 6.8)	35.5 (31.3 to 39.1)	34.8 (30.7 to 38.5)	36.1 (31.9 to 39.6)	-2.0 (-5.9 to 1.8)	3.7 (0.8 to 7.3)
Diet low in seafood omega-3 fatty acids	3.2 (1.4 to 4.5)	0.3 (0.0 to 1.1)	0.3 (0.0 to 1.2)	-91.2 (-100.0 to -67.6)	6.4 (-87.7 to 1079.0)	3.7 (2.0 to 5.0)	0.4 (0.0 to 1.6)	0.5 (0.0 to 1.5)	-88.1 (-99.9 to -59.6)	6.0 (-82.1 to 761.7)
Diet low in polyunsaturated fatty acids	30.4 (25.1 to 35.1)	27.6 (21.6 to 32.6)	28.3 (22.1 to 33.3)	-9.2 (-21.4 to 0.0)	2.6 (-5.5 to 11.8)	31.1 (25.9 to 35.9)	28.3 (22.4 to 33.2)	29.0 (23.2 to 33.8)	-8.9 (-19.9 to 0.6)	2.4 (-5.2 to 11.4)
Diet high in trans fatty acids	1.1 (0.0 to 3.3)	1.0 (0.0 to 3.0)	0.9 (0.0 to 2.9)	-12.5 (-38.4 to -6.3)	-8.4 (-32.6 to -3.8)	1.5 (0.1 to 4.0)	1.3 (0.1 to 3.7)	1.2 (0.0 to 3.5)	-12.5 (-35.9 to -6.1)	-8.5 (-28.1 to -3.9)
Diet high in sodium	32.1 (23.2 to 39.8)	20.3 (12.0 to 27.2)	17.4 (8.7 to 24.2)	-36.5 (-56.8 to -19.7)	-14.5 (-35.8 to 1.5)	35.5 (27.7 to 42.6)	21.6 (13.6 to 28.5)	18.0 (9.6 to 25.1)	-39.3 (-57.5 to -25.4)	-16.5 (-35.2 to -4.2)
Intimate partner violence	-	-	-	-	-	2.2 (1.9 to 2.5)	2.0 (1.7 to 2.3)	1.9 (1.6 to 2.2)	-9.0 (-12.9 to -5.1)	-3.9 (-8.5 to 0.7)
Childhood sexual abuse	5.3 (4.3 to 6.5)	5.3 (4.3 to 6.5)	5.3 (4.3 to 6.5)	0.0 (0.0 to 0.0)	0.0 (0.0 to 0.0)	7.3 (5.9 to 8.9)	7.3 (5.9 to 8.9)	7.5 (6.0 to 9.2)	0.0 (0.0 to 0.0)	2.8 (-4.0 to 10.6)

Supplementary Results Table 1. Nagano age-standardised Summary Exposure Values (SEVs) for Level 4 risk factors and percentage change, by sex, 1990, 2007, and 2017.Data in parentheses are 95% uncertainty intervals.

	Male					Female				
	**1990**	**2007**	**2017**	**Percentage change 1990-2007**	**Percentage change 2007-2017**	**1990**	**2007**	**2017**	**Percentage change 1990-2007**	**Percentage change 2007-2017**
Bullying victimization	3.3<br>(1.4 to 6.8)	3.4<br>(1.4 to 6.9)	3.5<br>(1.5 to 7.3)	1.7<br>(-6.2 to 9.9)	4.3<br>(-1.0 to 9.4)	1.9<br>(0.8 to 3.9)	1.9<br>(0.8 to 4.0)	2.0<br>(0.8 to 4.3)	0.5<br>(-7.0 to 8.7)	3.2<br>(-21.3 to 37.8)
Low physical activity	0.5<br>(0.2 to 0.9)	0.5<br>(0.2 to 0.9)	0.4<br>(0.2 to 0.8)	-1.7<br>(-3.7 to 0.2)	-8.8<br>(-12.2 to -4.5)	0.5<br>(0.2 to 1.0)	0.5<br>(0.2 to 0.9)	0.5<br>(0.2 to 0.9)	-3.8<br>(-5.9 to -1.3)	0.7<br>(-0.9 to 2.2)
High fasting plasma glucose	1.3<br>(0.8 to 2.1)	1.4<br>(0.9 to 2.1)	1.5<br>(0.9 to 2.6)	2.2<br>(-33.9 to 56.4)	12.6<br>(-24.6 to 77.6)	1.2<br>(0.7 to 1.9)	1.2<br>(0.7 to 2.1)	1.3<br>(0.8 to 2.4)	4.5<br>(-35.8 to 67.4)	10.7<br>(-28.7 to 84.0)
High LDL cholesterol	12.1<br>(10.5 to 13.8)	12.5<br>(10.9 to 14.3)	12.5<br>(10.9 to 14.4)	4.0<br>(-1.7 to 10.6)	-0.0<br>(-4.3 to 4.9)	12.9<br>(11.3 to 14.6)	13.0<br>(11.4 to 14.7)	13.0<br>(11.4 to 14.8)	0.5<br>(-4.8 to 6.0)	0.0<br>(-4.6 to 4.8)
High systolic blood pressure	11.2<br>(10.2 to 12.3)	8.3<br>(7.4 to 9.2)	8.1<br>(7.2 to 9.2)	-26.3<br>(-32.2 to -20.0)	-1.6<br>(-9.1 to 6.6)	9.6<br>(8.6 to 10.7)	6.0<br>(5.2 to 6.8)	5.6<br>(4.8 to 6.4)	-37.6<br>(-44.1 to -30.1)	-7.2<br>(-17.4 to 3.3)
High body-mass index	5.3<br>(3.4 to 8.6)	7.6<br>(5.1 to 12.2)	7.8<br>(5.2 to 12.6)	43.1<br>(15.9 to 83.1)	2.7<br>(-12.5 to 20.3)	5.5<br>(3.3 to 8.6)	7.0<br>(4.3 to 10.7)	7.1<br>(4.3 to 11.0)	29.0<br>(-0.6 to 73.0)	0.3<br>(-18.1 to 23.9)
Low bone mineral density	5.3<br>(4.4 to 6.3)	5.1<br>(4.3 to 6.1)	5.2<br>(4.2 to 6.1)	-2.8<br>(-15.5 to 10.9)	0.7<br>(-13.3 to 15.3)	7.2<br>(6.1 to 8.5)	7.0<br>(6.0 to 8.2)	7.1<br>(5.9 to 8.3)	-3.0<br>(-14.0 to 10.6)	1.6<br>(-10.2 to 15.3)
Impaired kidney function	2.6<br>(1.1 to 6.5)	2.5<br>(1.1 to 6.3)	2.4<br>(1.0 to 6.1)	-2.1<br>(-6.4 to 2.3)	-6.1<br>(-11.5 to -1.7)	3.2<br>(1.4 to 7.4)	3.3<br>(1.5 to 7.7)	3.1<br>(1.4 to 7.2)	3.6<br>(-0.4 to 8.4)	-6.5<br>(-11.6 to -1.9)

Supplementary Results Table 1. Nagasaki age-standardised Summary Exposure Values (SEVs) for Level 4 risk factors and percentage change, by sex, 1990, 2007, and 2017.Data in parentheses are 95% uncertainty intervals.										
	Male					Female				
	1990	2007	2017	Percentage change 1990-2007	Percentage change 2007-2017	1990	2007	2017	Percentage change 1990-2007	Percentage change 2007-2017
Unsafe water source	2.7 (1.5 to 4.4)	2.2 (1.2 to 3.6)	2.3 (1.3 to 3.9)	-16.2 (-55.3 to 66.1)	5.4 (-40.7 to 81.5)	2.7 (1.5 to 4.4)	2.2 (1.2 to 3.6)	2.3 (1.3 to 3.9)	-16.2 (-55.3 to 66.1)	5.4 (-40.7 to 81.5)
Unsafe sanitation	4.8 (2.3 to 8.8)	2.5 (1.1 to 4.5)	2.1 (1.0 to 4.1)	-48.9 (-78.6 to 19.2)	-14.0 (-54.5 to 67.2)	4.8 (2.3 to 8.8)	2.5 (1.1 to 4.5)	2.1 (1.0 to 4.1)	-48.9 (-78.6 to 19.2)	-14.0 (-54.5 to 67.2)
No access to handwashing facility	6.9 (5.0 to 9.1)	6.5 (4.7 to 8.9)	6.0 (4.3 to 8.2)	-5.9 (-33.3 to 29.6)	-7.6 (-29.8 to 18.3)	6.9 (5.0 to 9.1)	6.5 (4.7 to 8.9)	6.0 (4.3 to 8.2)	-5.9 (-33.3 to 29.6)	-7.6 (-29.8 to 18.3)
Ambient particulate matter pollution	39.4 (26.2 to 58.5)	40.0 (27.2 to 57.4)	36.0 (23.7 to 54.8)	1.5 (-22.1 to 34.2)	-9.8 (-32.0 to 15.9)	39.1 (25.9 to 58.3)	39.9 (27.1 to 57.3)	36.0 (23.7 to 54.8)	2.2 (-21.8 to 35.2)	-9.8 (-32.0 to 16.0)
Household air pollution from solid fuels	0.7 (0.2 to 2.0)	0.2 (0.0 to 0.4)	0.1 (0.0 to 0.3)	-77.3 (-93.0 to -48.9)	-13.9 (-65.6 to 87.6)	1.1 (0.3 to 7.1)	0.3 (0.1 to 1.3)	0.2 (0.0 to 1.2)	-73.6 (-92.9 to -43.2)	-13.9 (-65.3 to 91.0)
Ambient ozone pollution	50.4 (22.2 to 54.8)	49.6 (20.9 to 54.8)	50.8 (23.0 to 54.8)	-1.8 (-7.0 to 0.0)	2.4 (0.0 to 10.4)	50.4 (22.2 to 54.8)	49.6 (20.9 to 54.8)	50.8 (23.0 to 54.8)	-1.8 (-7.0 to 0.0)	2.4 (0.0 to 10.4)
Residential radon	12.4 (0.7 to 42.5)	12.4 (0.7 to 42.5)	12.4 (0.7 to 42.5)	0.0 (0.0 to 0.0)	0.0 (0.0 to 0.0)	12.4 (0.7 to 42.5)	12.4 (0.7 to 42.5)	12.4 (0.7 to 42.5)	0.0 (0.0 to 0.0)	0.0 (0.0 to 0.0)
Lead exposure	3.0 (0.4 to 6.6)	2.1 (0.3 to 5.1)	1.5 (0.2 to 4.2)	-30.0 (-42.4 to -21.3)	-25.6 (-40.2 to -17.5)	1.6 (0.1 to 4.5)	1.0 (0.1 to 3.4)	0.8 (0.0 to 2.8)	-34.3 (-50.5 to -24.9)	-26.8 (-48.4 to -18.1)
Occupational exposure to asbestos	4.1 (3.5 to 4.6)	5.5 (5.1 to 6.0)	5.3 (4.4 to 6.6)	33.6 (16.5 to 58.9)	-2.4 (-19.9 to 21.7)	1.0 (0.8 to 1.1)	0.9 (0.9 to 1.0)	1.0 (0.8 to 1.2)	-2.2 (-18.4 to 26.9)	2.0 (-18.1 to 26.0)
Occupational exposure to arsenic	0.4 (0.0 to 1.0)	0.4 (0.0 to 1.0)	0.4 (0.0 to 1.0)	-	-	0.4 (0.0 to 0.9)	0.4 (0.0 to 0.8)	0.4 (0.0 to 0.9)	-	-
Occupational exposure to benzene	0.6 (0.0 to 1.8)	0.6 (0.0 to 1.8)	0.7 (0.0 to 1.9)	2.7 (-3.8 to 12.4)	3.6 (-2.0 to 12.1)	0.6 (0.0 to 1.6)	0.7 (0.0 to 1.9)	0.8 (0.0 to 2.3)	22.3 (8.6 to 45.4)	14.9 (5.1 to 31.1)
Occupational exposure to beryllium	0.0 (0.0 to 0.0)	0.0 (0.0 to 0.0)	0.0 (0.0 to 0.0)	-5.1 (-9.9 to -0.4)	1.9 (-1.7 to 6.2)	0.0 (0.0 to 0.0)	0.0 (0.0 to 0.0)	0.0 (0.0 to 0.0)	2.3 (-7.0 to 13.0)	9.5 (2.8 to 17.7)
Occupational exposure to cadmium	0.1 (0.0 to 0.1)	0.0 (0.0 to 0.1)	0.0 (0.0 to 0.1)	-6.2 (-16.7 to 5.9)	1.1 (-8.5 to 12.2)	0.0 (0.0 to 0.1)	0.0 (0.0 to 0.0)	0.0 (0.0 to 0.1)	-7.9 (-22.9 to 8.9)	5.4 (-7.3 to 20.7)
Occupational exposure to chromium	0.1 (0.1 to 0.1)	0.1 (0.1 to 0.1)	0.1 (0.1 to 0.1)	-4.5 (-14.1 to 6.6)	1.6 (-6.8 to 11.2)	0.1 (0.1 to 0.1)	0.1 (0.1 to 0.1)	0.1 (0.1 to 0.1)	-3.3 (-17.7 to 13.4)	7.2 (-3.9 to 20.2)
Occupational exposure to diesel engine exhaust	0.6 (0.5 to 0.6)	0.6 (0.5 to 0.6)	0.6 (0.5 to 0.6)	-2.1 (-9.6 to 6.7)	3.2 (-3.6 to 10.4)	0.4 (0.4 to 0.5)	0.5 (0.4 to 0.5)	0.5 (0.5 to 0.6)	12.2 (-1.2 to 27.8)	11.8 (1.1 to 23.9)
Occupational exposure to formaldehyde	0.2 (0.2 to 0.3)	0.2 (0.2 to 0.3)	0.2 (0.2 to 0.3)	-5.5 (-17.0 to 8.0)	0.7 (-9.4 to 12.6)	0.2 (0.2 to 0.2)	0.2 (0.2 to 0.2)	0.2 (0.2 to 0.2)	-5.2 (-21.3 to 13.4)	6.2 (-6.2 to 21.1)
Occupational exposure to nickel	0.4 (0.0 to 1.9)	0.4 (0.0 to 1.7)	0.4 (0.0 to 1.7)	-	-	0.3 (0.0 to 1.5)	0.3 (0.0 to 1.3)	0.3 (0.0 to 1.4)	-	-
Occupational exposure to polycyclic aromatic hydrocarbons	0.2 (0.2 to 0.3)	0.2 (0.2 to 0.3)	0.2 (0.2 to 0.3)	-3.7 (-12.5 to 6.9)	1.9 (-5.7 to 10.9)	0.2 (0.2 to 0.2)	0.2 (0.2 to 0.2)	0.2 (0.2 to 0.3)	-1.2 (-15.3 to 15.0)	8.1 (-2.3 to 20.4)
Occupational exposure to silica	4.6 (0.7 to 14.8)	4.3 (0.6 to 14.2)	4.4 (0.6 to 13.9)	-6.5 (-16.5 to 5.0)	1.2 (-7.6 to 12.6)	3.1 (0.5 to 10.2)	2.7 (0.4 to 8.5)	2.7 (0.4 to 8.6)	-13.0 (-27.6 to 6.3)	2.3 (-10.8 to 20.3)
Occupational exposure to sulphuric acid	0.6 (0.1 to 1.8)	0.6 (0.1 to 1.7)	0.6 (0.1 to 1.7)	-6.3 (-16.3 to 5.1)	1.4 (-7.6 to 12.0)	0.5 (0.1 to 1.5)	0.5 (0.1 to 1.4)	0.5 (0.1 to 1.5)	-9.4 (-24.1 to 7.4)	4.3 (-8.5 to 20.1)
Occupational exposure to trichloroethylene	0.1 (0.1 to 0.1)	0.1 (0.1 to 0.1)	0.1 (0.1 to 0.1)	-3.6 (-10.6 to 4.4)	2.1 (-4.2 to 8.8)	0.1 (0.1 to 0.1)	0.1 (0.1 to 0.1)	0.1 (0.1 to 0.1)	1.4 (-10.7 to 17.0)	8.8 (0.1 to 19.5)
Occupational asthmagens	18.0 (14.4 to 22.6)	17.6 (14.0 to 22.1)	17.6 (13.9 to 22.6)	-2.4 (-19.8 to 19.6)	0.3 (-15.8 to 19.1)	8.1 (6.5 to 10.0)	8.1 (6.5 to 10.1)	8.6 (6.9 to 10.6)	0.1 (-15.6 to 17.8)	5.3 (-7.7 to 20.1)
Occupational particulate matter, gases, and fumes	6.4 (4.6 to 9.2)	6.1 (4.4 to 8.8)	5.9 (4.2 to 8.5)	-4.3 (-9.1 to 0.5)	-3.6 (-6.9 to -0.2)	3.6 (2.4 to 5.3)	3.4 (2.4 to 5.1)	3.3 (2.3 to 4.9)	-5.1 (-13.7 to 4.2)	-4.4 (-10.1 to 1.6)
Occupational noise	6.0 (5.5 to 6.6)	5.8 (5.4 to 6.3)	5.6 (5.2 to 6.2)	-3.3 (-6.3 to -0.1)	-2.5 (-4.7 to -0.3)	3.4 (2.9 to 4.0)	3.4 (3.0 to 3.9)	3.5 (3.1 to 4.0)	0.5 (-5.9 to 7.3)	1.8 (-2.2 to 6.8)
Occupational ergonomic factors	11.0 (9.0 to 13.2)	9.8 (8.2 to 11.8)	9.7 (7.9 to 11.7)	-10.7 (-23.7 to 2.9)	-1.1 (-12.1 to 11.5)	5.9 (4.5 to 7.6)	6.0 (4.6 to 7.7)	6.7 (5.1 to 8.7)	1.6 (-22.7 to 29.0)	12.0 (-7.9 to 36.0)
Non-exclusive breastfeeding	0.4 (0.3 to 0.6)	0.4 (0.3 to 0.6)	0.4 (0.3 to 0.6)	-1.4 (-15.1 to 14.5)	-1.7 (-12.0 to 9.9)	0.4 (0.3 to 0.6)	0.4 (0.3 to 0.6)	0.4 (0.3 to 0.6)	-1.2 (-14.8 to 14.8)	-1.8 (-12.1 to 9.7)
Discontinued breastfeeding	1.6 (1.5 to 1.8)	1.7 (1.5 to 1.8)	1.6 (1.5 to 1.8)	3.1 (-6.3 to 14.4)	-3.4 (-10.7 to 4.9)	1.7 (1.5 to 1.8)	1.7 (1.5 to 1.8)	1.6 (1.5 to 1.8)	1.6 (-7.7 to 12.8)	-2.0 (-9.4 to 6.3)
Child underweight	0.3 (0.2 to 0.4)	0.2 (0.1 to 0.4)	0.2 (0.1 to 0.3)	-15.0 (-34.3 to 8.9)	-5.2 (-20.4 to 12.3)	0.3 (0.2 to 0.4)	0.2 (0.1 to 0.4)	0.2 (0.1 to 0.3)	-17.5 (-36.5 to 6.3)	-6.6 (-23.7 to 12.7)
Child wasting	0.2 (0.1 to 0.3)	0.2 (0.1 to 0.2)	0.2 (0.1 to 0.2)	-7.7 (-32.9 to 23.3)	-1.3 (-19.5 to 21.7)	0.2 (0.1 to 0.3)	0.2 (0.1 to 0.2)	0.2 (0.1 to 0.2)	-9.3 (-35.3 to 22.7)	-2.9 (-21.9 to 19.5)
Child stunting	0.7 (0.4 to 1.2)	0.7 (0.4 to 1.0)	0.6 (0.3 to 1.0)	-7.0 (-39.5 to 45.0)	-3.6 (-31.4 to 33.2)	0.7 (0.4 to 1.3)	0.7 (0.4 to 1.0)	0.6 (0.4 to 1.0)	-8.5 (-41.3 to 42.4)	-4.6 (-33.1 to 35.4)
Short gestation for birth weight	0.0 (0.0 to 0.0)	0.0 (0.0 to 0.0)	0.0 (0.0 to 0.0)	6.6 (-11.6 to 31.9)	4.9 (-13.2 to 46.5)	0.0 (0.0 to 0.0)	0.0 (0.0 to 0.0)	0.0 (0.0 to 0.0)	12.6 (-24.5 to 50.9)	-0.0 (-19.9 to 28.1)
Low birth weight for gestation	0.0 (0.0 to 0.0)	0.0 (0.0 to 0.0)	0.0 (0.0 to 0.0)	3.9 (-11.3 to 23.5)	1.6 (-23.3 to 17.2)	0.0 (0.0 to 0.0)	0.0 (0.0 to 0.0)	0.0 (0.0 to 0.0)	-1.5 (-20.0 to 36.5)	4.4 (-15.6 to 24.9)
Iron deficiency	-	-	-	-	-	8.6 (5.9 to 11.8)	8.6 (6.0 to 11.9)	7.9 (5.4 to 11.1)	0.1 (-20.4 to 28.5)	-8.6 (-24.4 to 8.8)
Vitamin A deficiency	1.1 (0.6 to 1.5)	0.9 (0.6 to 1.2)	0.8 (0.5 to 1.0)	-17.4 (-33.3 to 1.7)	-14.8 (-27.2 to -0.0)	0.8 (0.5 to 1.1)	0.9 (0.6 to 1.2)	0.8 (0.5 to 1.0)	14.6 (-7.8 to 53.4)	-11.1 (-26.0 to 4.8)
Zinc deficiency	0.5 (0.2 to 1.1)	0.4 (0.1 to 1.0)	0.4 (0.1 to 0.8)	-22.1 (-66.2 to 71.7)	-9.9 (-66.7 to 166.4)	0.5 (0.2 to 1.1)	0.4 (0.1 to 0.8)	0.4 (0.1 to 0.9)	-29.3 (-63.0 to 8.7)	8.0 (-56.4 to 161.1)
Smoking	18.3 (14.8 to 21.6)	12.8 (10.2 to 15.4)	10.8 (8.7 to 13.2)	-29.9 (-38.9 to -19.2)	-15.5 (-24.0 to -6.3)	4.2 (3.0 to 5.6)	3.5 (2.5 to 4.7)	3.4 (2.4 to 4.6)	-16.8 (-36.2 to 9.2)	-2.8 (-18.9 to 18.6)
Chewing tobacco	1.4 (0.9 to 2.0)	0.9 (0.6 to 1.4)	0.8 (0.5 to 1.1)	-32.9 (-56.7 to 5.0)	-18.7 (-42.6 to 15.0)	0.1 (0.1 to 0.1)	0.1 (0.1 to 0.1)	0.1 (0.1 to 0.1)	-4.4 (-42.0 to 58.4)	-2.1 (-32.8 to 44.0)
Second-hand smoke	36.6 (32.7 to 40.4)	36.0 (31.9 to 40.0)	37.2 (32.6 to 42.0)	-1.5 (-12.1 to 11.6)	3.4 (-5.7 to 14.5)	55.2 (51.7 to 58.7)	48.4 (44.5 to 52.0)	49.7 (45.2 to 53.8)	-12.4 (-19.3 to -5.5)	2.7 (-4.3 to 9.5)
Alcohol use	14.1 (7.1 to 24.4)	14.1 (7.1 to 24.4)	16.8 (8.2 to 28.4)	0.0 (-35.7 to 45.9)	18.4 (-18.3 to 72.9)	11.8 (5.8 to 21.2)	11.9 (5.8 to 21.4)	13.7 (6.3 to 23.9)	0.8 (-32.9 to 49.3)	14.6 (-21.4 to 68.5)
Drug use	0.5 (0.4 to 0.7)	0.5 (0.4 to 0.7)	0.6 (0.4 to 0.8)	0.6 (-5.7 to 6.9)	11.0 (3.7 to 19.7)	0.3 (0.2 to 0.4)	0.3 (0.2 to 0.4)	0.3 (0.3 to 0.4)	2.3 (-3.5 to 8.3)	9.4 (2.6 to 17.7)
Diet low in fruits	42.1 (36.4 to 45.7)	40.5 (34.9 to 44.3)	41.9 (36.6 to 45.5)	-3.6 (-9.4 to 2.1)	3.3 (-1.1 to 8.0)	39.5 (33.8 to 43.7)	36.8 (30.8 to 41.3)	39.0 (33.1 to 43.2)	-6.9 (-14.3 to -0.0)	5.9 (0.1 to 13.3)
Diet low in vegetables	21.7 (13.2 to 29.0)	16.0 (7.8 to 24.0)	18.1 (9.5 to 26.0)	-26.0 (-54.2 to -3.7)	13.0 (-13.1 to 57.1)	24.0 (15.8 to 31.2)	18.3 (9.9 to 26.0)	20.2 (11.6 to 27.5)	-23.6 (-46.5 to -2.4)	10.4 (-11.2 to 47.6)
Diet low in legumes	4.5 (0.8 to 10.0)	5.7 (1.0 to 11.5)	7.5 (2.0 to 14.1)	26.1 (-46.8 to 242.2)	32.9 (-23.9 to 208.8)	8.6 (2.8 to 15.7)	8.9 (2.8 to 15.7)	11.6 (4.9 to 18.9)	3.7 (-43.3 to 85.5)	31.0 (-6.5 to 115.1)
Diet low in whole grains	31.2 (26.5 to 35.4)	36.0 (31.8 to 39.3)	37.3 (33.6 to 40.5)	15.1 (6.8 to 26.6)	3.7 (-0.9 to 9.2)	32.6 (28.0 to 36.4)	37.0 (33.2 to 40.3)	38.3 (34.7 to 41.3)	13.7 (6.1 to 24.0)	3.4 (-1.3 to 8.3)
Diet low in nuts and seeds	51.5 (50.5 to 52.4)	50.7 (49.2 to 51.7)	50.6 (49.0 to 51.8)	-1.7 (-3.6 to 0.1)	-0.1 (-1.7 to 1.5)	51.6 (50.6 to 52.5)	50.6 (49.1 to 51.7)	50.5 (48.9 to 51.7)	-2.0 (-4.1 to -0.3)	-0.1 (-1.6 to 1.4)
Diet low in milk	46.5 (44.4 to 48.2)	46.7 (44.7 to 48.3)	47.1 (45.2 to 48.7)	0.4 (-1.4 to 2.4)	0.8 (-0.6 to 2.3)	46.6 (44.6 to 48.2)	46.3 (44.1 to 48.0)	46.9 (44.8 to 48.5)	-0.6 (-2.5 to 1.4)	1.3 (-0.2 to 2.8)
Diet high in red meat	3.3 (0.3 to 8.0)	6.7 (1.7 to 13.2)	7.7 (2.3 to 14.9)	104.6 (-20.3 to 1196.0)	14.7 (-43.6 to 145.3)	0.4 (0.0 to 2.2)	1.2 (0.0 to 3.9)	1.6 (0.0 to 4.9)	176.2 (-82.5 to 85829385.3)	33.0 (-93.8 to 3442.8)
Diet high in processed meat	10.5 (6.7 to 14.3)	17.7 (12.9 to 22.3)	20.6 (14.9 to 26.2)	68.9 (30.3 to 131.1)	16.5 (-1.6 to 40.9)	8.6 (5.2 to 12.1)	12.7 (8.6 to 16.3)	14.0 (9.9 to 18.1)	48.1 (15.6 to 107.2)	10.5 (-7.4 to 32.5)
Diet high in sugar-sweetened beverages	6.4 (2.5 to 12.9)	7.2 (3.1 to 13.5)	7.6 (3.4 to 14.1)	11.7 (-46.7 to 141.3)	6.1 (-37.9 to 88.4)	4.5 (1.7 to 10.0)	5.0 (2.2 to 8.8)	5.5 (2.3 to 11.4)	11.8 (-49.0 to 171.7)	8.8 (-40.1 to 92.0)
Diet low in fibre	23.1 (15.6 to 29.4)	24.2 (17.4 to 30.4)	25.3 (18.4 to 31.5)	5.1 (-11.7 to 26.9)	4.3 (-7.1 to 18.7)	24.4 (17.1 to 30.7)	25.6 (18.4 to 31.6)	27.2 (20.1 to 33.2)	4.9 (-10.4 to 23.3)	6.1 (-4.1 to 19.5)
Diet low in calcium	35.9 (31.8 to 39.4)	35.6 (31.4 to 39.0)	36.3 (32.3 to 39.6)	-1.0 (-4.5 to 2.7)	1.9 (-0.9 to 5.4)	38.1 (34.4 to 41.2)	37.1 (33.1 to 40.3)	38.3 (34.5 to 41.3)	-2.7 (-6.2 to 0.2)	3.1 (0.6 to 6.4)
Diet low in seafood omega-3 fatty acids	3.5 (2.0 to 4.9)	0.5 (0.0 to 1.4)	0.5 (0.0 to 1.5)	-87.1 (-99.9 to -58.8)	3.5 (-78.7 to 502.3)	4.0 (2.2 to 5.4)	0.5 (0.0 to 1.5)	0.5 (0.0 to 1.7)	-88.7 (-99.9 to -64.0)	8.0 (-77.4 to 1073.1)
Diet low in polyunsaturated fatty acids	31.5 (26.4 to 36.3)	29.0 (23.5 to 33.9)	29.5 (23.7 to 34.4)	-8.0 (-18.3 to 1.0)	1.9 (-5.7 to 10.0)	32.2 (26.9 to 36.8)	29.6 (24.0 to 34.2)	30.2 (24.5 to 34.8)	-8.0 (-18.3 to 0.9)	2.0 (-5.8 to 10.8)
Diet high in trans fatty acids	1.1 (0.0 to 3.3)	0.9 (0.0 to 3.1)	0.9 (0.0 to 2.9)	-12.7 (-39.4 to -6.8)	-8.2 (-32.4 to -3.4)	1.5 (0.1 to 4.0)	1.3 (0.1 to 3.7)	1.2 (0.0 to 3.5)	-12.7 (-35.6 to -6.7)	-8.0 (-27.8 to -2.9)
Diet high in sodium	27.2 (17.5 to 35.4)	17.6 (8.8 to 25.1)	15.2 (6.3 to 22.6)	-35.3 (-57.4 to -15.7)	-13.7 (-36.5 to 2.9)	31.1 (23.1 to 38.6)	18.6 (10.2 to 25.4)	15.4 (6.3 to 22.6)	-40.1 (-60.9 to -25.0)	-17.4 (-42.1 to -3.8)
Intimate partner violence	-	-	-	-	-	2.3 (2.0 to 2.7)	2.1 (1.8 to 2.5)	2.0 (1.7 to 2.4)	-8.7 (-12.6 to -4.4)	-3.9 (-7.9 to 0.5)
Childhood sexual abuse	5.3 (4.3 to 6.5)	5.3 (4.3 to 6.5)	5.3 (4.3 to 6.5)	0.0 (0.0 to 0.0)	0.0 (0.0 to 0.0)	7.3 (5.9 to 8.9)	7.3 (5.9 to 8.9)	7.5 (6.0 to 9.2)	0.0 (0.0 to 0.0)	2.8 (-4.0 to 10.6)

Supplementary Results Table 1. Nagasaki age-standardised Summary Exposure Values (SEVs) for Level 4 risk factors and percentage change, by sex, 1990, 2007, and 2017.Data in parentheses are 95% uncertainty intervals.

	Male					Female				
	**1990**	**2007**	**2017**	**Percentage change 1990-2007**	**Percentage change 2007-2017**	**1990**	**2007**	**2017**	**Percentage change 1990-2007**	**Percentage change 2007-2017**
Bullying victimization	3.3 (1.4 to 7.1)	3.4 (1.4 to 7.3)	3.5 (1.4 to 7.6)	3.1 (-4.7 to 11.3)	2.3 (-2.9 to 6.8)	1.9 (0.8 to 4.2)	1.9 (0.8 to 4.3)	2.0 (0.8 to 4.1)	0.6 (-6.9 to 8.6)	1.8 (-24.1 to 34.3)
Low physical activity	0.5 (0.2 to 0.9)	0.5 (0.2 to 0.9)	0.4 (0.2 to 0.8)	-1.8 (-3.8 to 0.0)	-8.7 (-12.0 to -4.5)	0.5 (0.2 to 1.0)	0.5 (0.2 to 0.9)	0.5 (0.2 to 0.9)	-3.9 (-6.1 to -1.4)	0.8 (-0.8 to 2.2)
High fasting plasma glucose	1.2 (0.8 to 1.9)	1.4 (0.9 to 2.3)	1.6 (0.9 to 2.7)	13.1 (-27.6 to 86.8)	12.0 (-31.6 to 79.0)	1.1 (0.7 to 1.8)	1.1 (0.7 to 2.0)	1.3 (0.7 to 2.3)	5.8 (-37.6 to 75.1)	11.6 (-31.1 to 87.5)
High LDL cholesterol	12.0 (10.3 to 13.8)	12.5 (10.9 to 14.2)	12.5 (10.8 to 14.2)	4.3 (-2.0 to 10.5)	-0.0 (-4.6 to 4.5)	12.7 (11.1 to 14.4)	12.8 (11.2 to 14.5)	12.8 (11.3 to 14.5)	0.7 (-4.5 to 6.8)	0.2 (-4.4 to 5.1)
High systolic blood pressure	11.1 (10.2 to 12.2)	8.2 (7.4 to 9.1)	8.1 (7.1 to 9.0)	-26.3 (-32.3 to -19.8)	-1.9 (-10.2 to 5.8)	9.6 (8.6 to 10.6)	6.0 (5.3 to 6.8)	5.6 (4.7 to 6.4)	-37.2 (-44.1 to -29.7)	-7.6 (-17.2 to 3.1)
High body-mass index	4.8 (2.9 to 7.7)	6.8 (4.4 to 11.0)	7.0 (4.6 to 11.5)	41.0 (14.6 to 81.4)	3.3 (-12.3 to 21.1)	5.0 (2.9 to 8.0)	6.4 (3.9 to 10.2)	6.4 (3.8 to 10.3)	28.7 (-0.1 to 68.7)	0.8 (-16.6 to 24.2)
Low bone mineral density	4.8 (4.0 to 5.8)	5.0 (4.2 to 5.8)	5.1 (4.3 to 6.1)	3.9 (-10.5 to 18.5)	1.5 (-12.2 to 15.9)	6.2 (5.1 to 7.4)	6.4 (5.4 to 7.4)	6.1 (5.0 to 7.2)	2.6 (-10.6 to 18.0)	-4.8 (-16.2 to 8.3)
Impaired kidney function	2.6 (1.2 to 6.5)	2.5 (1.1 to 6.4)	2.4 (1.1 to 6.1)	-2.9 (-7.7 to 1.2)	-6.7 (-11.9 to -2.0)	3.2 (1.5 to 7.4)	3.3 (1.5 to 7.6)	3.1 (1.4 to 7.2)	2.6 (-1.2 to 7.2)	-6.9 (-11.9 to -1.9)

Supplementary Results Table 1. Nara age-standardised Summary Exposure Values (SEVs) for Level 4 risk factors and percentage change, by sex, 1990, 2007, and 2017.Data in parentheses are 95% uncertainty intervals.										
	**Male**					**Female**				
	**1990**	**2007**	**2017**	**Percentage change 1990-2007**	**Percentage change 2007-2017**	**1990**	**2007**	**2017**	**Percentage change 1990-2007**	**Percentage change 2007-2017**
Unsafe water source	2.2 (1.2 to 3.9)	1.9 (1.0 to 3.0)	2.0 (1.1 to 3.4)	-16.9 (-56.9 to 65.0)	8.0 (-36.2 to 84.8)	2.2 (1.2 to 3.9)	1.9 (1.0 to 3.0)	2.0 (1.1 to 3.4)	-16.9 (-56.9 to 65.0)	8.0 (-36.2 to 84.8)
Unsafe sanitation	3.8 (1.9 to 6.5)	1.9 (0.9 to 3.6)	1.7 (0.8 to 3.2)	-49.7 (-77.6 to 13.9)	-10.2 (-54.7 to 80.8)	3.8 (1.9 to 6.5)	1.9 (0.9 to 3.6)	1.7 (0.8 to 3.2)	-49.7 (-77.6 to 13.9)	-10.2 (-54.7 to 80.8)
No access to handwashing facility	6.8 (4.9 to 9.0)	6.3 (4.6 to 8.4)	5.9 (4.2 to 8.0)	-6.2 (-31.4 to 32.9)	-6.6 (-27.0 to 19.6)	6.8 (4.9 to 9.0)	6.3 (4.6 to 8.4)	5.9 (4.2 to 8.0)	-6.2 (-31.4 to 32.9)	-6.6 (-27.0 to 19.6)
Ambient particulate matter pollution	33.2 (22.4 to 49.9)	33.3 (22.8 to 49.2)	28.2 (18.8 to 42.4)	0.0 (-19.6 to 25.2)	-15.2 (-33.7 to 3.8)	33.1 (22.2 to 49.7)	33.2 (22.7 to 49.2)	28.2 (18.8 to 42.4)	0.4 (-19.4 to 25.7)	-15.2 (-33.8 to 3.8)
Household air pollution from solid fuels	0.4 (0.1 to 1.2)	0.1 (0.0 to 0.2)	0.1 (0.0 to 0.2)	-73.7 (-92.5 to -46.9)	-12.4 (-65.7 to 90.9)	0.7 (0.2 to 3.4)	0.2 (0.0 to 0.8)	0.2 (0.0 to 0.6)	-70.0 (-92.5 to -44.2)	-11.5 (-65.6 to 90.4)
Ambient ozone pollution	52.1 (26.5 to 54.8)	52.3 (27.3 to 54.8)	52.6 (28.4 to 54.8)	0.4 (0.0 to 3.7)	0.6 (0.0 to 5.1)	52.1 (26.5 to 54.8)	52.3 (27.3 to 54.8)	52.6 (28.4 to 54.8)	0.4 (0.0 to 3.7)	0.6 (0.0 to 5.1)
Residential radon	13.7 (1.1 to 43.5)	13.7 (1.1 to 43.5)	13.7 (1.1 to 43.5)	0.0 (0.0 to 0.0)	0.0 (0.0 to 0.0)	13.7 (1.1 to 43.5)	13.7 (1.1 to 43.5)	13.7 (1.1 to 43.5)	0.0 (0.0 to 0.0)	0.0 (0.0 to 0.0)
Lead exposure	3.2 (0.6 to 6.9)	2.1 (0.3 to 5.1)	1.5 (0.2 to 4.1)	-34.7 (-48.9 to -25.3)	-29.1 (-44.4 to -20.9)	1.7 (0.1 to 4.5)	1.0 (0.1 to 3.1)	0.7 (0.0 to 2.4)	-40.5 (-57.6 to -30.5)	-31.9 (-53.5 to -21.8)
Occupational exposure to asbestos	3.2 (2.7 to 3.7)	3.9 (3.6 to 4.3)	3.9 (3.2 to 4.6)	22.1 (3.8 to 48.5)	-1.7 (-18.5 to 18.7)	1.3 (1.0 to 1.5)	1.1 (1.0 to 1.2)	1.1 (0.9 to 1.3)	-11.9 (-27.4 to 14.7)	-2.9 (-21.6 to 17.8)
Occupational exposure to arsenic	0.5 (0.0 to 1.1)	0.4 (0.0 to 1.0)	0.4 (0.0 to 1.0)	-	-	0.4 (0.0 to 1.0)	0.4 (0.0 to 0.9)	0.4 (0.0 to 0.9)	-	-
Occupational exposure to benzene	0.6 (0.0 to 1.8)	0.7 (0.0 to 1.8)	0.7 (0.0 to 1.9)	2.2 (-4.7 to 12.1)	3.3 (-2.9 to 11.3)	0.6 (0.0 to 1.7)	0.7 (0.0 to 2.1)	0.8 (0.1 to 2.4)	21.5 (8.2 to 44.9)	13.9 (3.5 to 28.4)
Occupational exposure to beryllium	0.0 (0.0 to 0.0)	0.0 (0.0 to 0.0)	0.0 (0.0 to 0.0)	-5.0 (-10.1 to 0.1)	1.9 (-2.0 to 5.9)	0.0 (0.0 to 0.0)	0.0 (0.0 to 0.0)	0.0 (0.0 to 0.0)	2.1 (-7.1 to 11.9)	8.6 (2.0 to 15.7)
Occupational exposure to cadmium	0.1 (0.0 to 0.1)	0.0 (0.0 to 0.1)	0.0 (0.0 to 0.1)	-7.0 (-18.0 to 6.3)	0.9 (-8.7 to 11.5)	0.0 (0.0 to 0.1)	0.0 (0.0 to 0.1)	0.0 (0.0 to 0.1)	-8.8 (-23.6 to 8.2)	4.4 (-8.6 to 17.6)
Occupational exposure to chromium	0.1 (0.1 to 0.1)	0.1 (0.1 to 0.1)	0.1 (0.1 to 0.1)	-5.4 (-15.2 to 6.5)	1.4 (-6.9 to 10.7)	0.1 (0.1 to 0.1)	0.1 (0.1 to 0.1)	0.1 (0.1 to 0.1)	-4.1 (-17.9 to 11.7)	6.1 (-5.3 to 17.5)
Occupational exposure to diesel engine exhaust	0.6 (0.5 to 0.6)	0.6 (0.5 to 0.6)	0.6 (0.5 to 0.6)	-1.4 (-10.0 to 7.0)	3.2 (-3.8 to 9.8)	0.5 (0.4 to 0.5)	0.5 (0.4 to 0.6)	0.6 (0.5 to 0.6)	12.3 (-1.9 to 27.9)	10.5 (0.1 to 22.3)
Occupational exposure to formaldehyde	0.3 (0.2 to 0.3)	0.2 (0.2 to 0.3)	0.2 (0.2 to 0.3)	-6.5 (-17.7 to 7.8)	0.4 (-9.3 to 11.2)	0.2 (0.2 to 0.3)	0.2 (0.2 to 0.2)	0.2 (0.2 to 0.2)	-6.1 (-21.3 to 12.6)	5.2 (-7.7 to 18.2)
Occupational exposure to nickel	0.4 (0.0 to 2.0)	0.4 (0.0 to 1.8)	0.4 (0.0 to 1.7)	-	-	0.3 (0.0 to 1.5)	0.3 (0.0 to 1.3)	0.3 (0.0 to 1.3)	-	-
Occupational exposure to polycyclic aromatic hydrocarbons	0.2 (0.2 to 0.3)	0.2 (0.2 to 0.3)	0.2 (0.2 to 0.3)	-4.4 (-13.8 to 6.8)	1.7 (-5.9 to 10.1)	0.2 (0.2 to 0.3)	0.2 (0.2 to 0.2)	0.2 (0.2 to 0.3)	-2.0 (-15.1 to 13.8)	7.0 (-3.4 to 17.6)
Occupational exposure to silica	4.6 (0.6 to 15.1)	4.4 (0.6 to 14.0)	4.4 (0.7 to 14.3)	-5.4 (-15.2 to 7.4)	1.5 (-7.4 to 12.2)	3.1 (0.5 to 10.7)	2.7 (0.4 to 8.5)	2.8 (0.4 to 9.0)	-13.0 (-27.9 to 5.6)	1.4 (-12.0 to 19.4)
Occupational exposure to sulphuric acid	0.6 (0.1 to 1.9)	0.6 (0.1 to 1.8)	0.6 (0.1 to 1.9)	-6.8 (-17.7 to 5.1)	1.0 (-7.5 to 11.7)	0.5 (0.1 to 1.6)	0.5 (0.1 to 1.5)	0.5 (0.1 to 1.5)	-10.2 (-25.5 to 6.8)	3.5 (-9.9 to 17.5)
Occupational exposure to trichloroethylene	0.1 (0.1 to 0.1)	0.1 (0.1 to 0.1)	0.1 (0.1 to 0.1)	-4.6 (-11.6 to 4.0)	1.8 (-4.0 to 8.6)	0.1 (0.1 to 0.1)	0.1 (0.1 to 0.1)	0.1 (0.1 to 0.1)	0.7 (-10.2 to 13.3)	7.6 (-1.7 to 17.2)
Occupational asthmagens	19.3 (15.1 to 24.3)	18.5 (14.6 to 23.1)	18.5 (14.7 to 23.3)	-3.7 (-20.9 to 20.1)	-0.5 (-15.9 to 17.9)	8.3 (6.6 to 10.3)	8.3 (6.7 to 10.2)	8.7 (7.0 to 10.9)	0.4 (-16.0 to 18.0)	4.8 (-7.8 to 20.0)
Occupational particulate matter, gases, and fumes	6.4 (4.5 to 9.2)	6.1 (4.3 to 8.8)	5.9 (4.2 to 8.5)	-4.1 (-9.3 to 0.8)	-3.4 (-7.1 to 0.0)	3.6 (2.5 to 5.5)	3.4 (2.4 to 5.2)	3.3 (2.2 to 5.0)	-5.7 (-14.1 to 4.2)	-4.7 (-10.5 to 1.3)
Occupational noise	5.9 (5.4 to 6.4)	5.7 (5.3 to 6.2)	5.6 (5.2 to 6.1)	-2.8 (-6.0 to 0.5)	-2.0 (-4.1 to -0.0)	3.5 (3.0 to 4.0)	3.5 (3.1 to 4.0)	3.6 (3.2 to 4.0)	0.4 (-5.9 to 8.0)	1.8 (-2.0 to 6.2)
Occupational ergonomic factors	10.3 (8.6 to 12.2)	9.1 (7.6 to 11.1)	9.0 (7.5 to 11.0)	-11.3 (-22.9 to 1.5)	-0.8 (-11.0 to 10.5)	5.5 (4.2 to 7.1)	5.6 (4.4 to 7.2)	6.4 (4.9 to 8.4)	1.9 (-21.3 to 29.4)	13.5 (-7.4 to 36.5)
Non-exclusive breastfeeding	0.4 (0.3 to 0.6)	0.4 (0.3 to 0.6)	0.4 (0.3 to 0.6)	-1.1 (-14.2 to 13.4)	-1.7 (-12.1 to 10.6)	0.4 (0.3 to 0.6)	0.4 (0.3 to 0.6)	0.4 (0.3 to 0.6)	-1.9 (-15.0 to 12.5)	-1.5 (-11.9 to 10.9)
Discontinued breastfeeding	1.7 (1.6 to 1.9)	1.7 (1.6 to 1.8)	1.7 (1.5 to 1.8)	-1.0 (-10.2 to 9.3)	-2.4 (-9.4 to 5.9)	1.7 (1.6 to 1.8)	1.7 (1.6 to 1.9)	1.7 (1.5 to 1.8)	1.3 (-8.2 to 11.8)	-3.1 (-10.0 to 5.2)
Child underweight	0.3 (0.2 to 0.4)	0.2 (0.1 to 0.3)	0.2 (0.1 to 0.3)	-14.5 (-32.6 to 10.3)	-4.7 (-19.5 to 12.0)	0.3 (0.2 to 0.4)	0.2 (0.1 to 0.3)	0.2 (0.1 to 0.3)	-16.8 (-37.2 to 8.1)	-5.1 (-21.9 to 12.1)
Child wasting	0.2 (0.1 to 0.3)	0.2 (0.1 to 0.3)	0.2 (0.1 to 0.3)	-6.9 (-34.4 to 28.7)	-1.7 (-21.6 to 24.5)	0.2 (0.1 to 0.3)	0.2 (0.1 to 0.3)	0.2 (0.1 to 0.3)	-7.8 (-36.4 to 34.6)	-2.9 (-23.3 to 23.3)
Child stunting	0.6 (0.3 to 1.0)	0.5 (0.3 to 0.9)	0.5 (0.3 to 0.9)	-9.6 (-43.4 to 44.4)	-3.2 (-30.0 to 34.4)	0.6 (0.3 to 1.0)	0.5 (0.3 to 0.9)	0.5 (0.2 to 0.9)	-10.5 (-45.8 to 51.6)	-4.3 (-32.3 to 32.1)
Short gestation for birth weight	0.0 (0.0 to 0.0)	0.0 (0.0 to 0.0)	0.0 (0.0 to 0.0)	9.5 (-12.2 to 45.6)	1.2 (-24.6 to 36.8)	0.0 (0.0 to 0.0)	0.0 (0.0 to 0.0)	0.0 (0.0 to 0.0)	13.5 (-15.6 to 56.5)	1.9 (-22.9 to 29.4)
Low birth weight for gestation	0.0 (0.0 to 0.0)	0.0 (0.0 to 0.0)	0.0 (0.0 to 0.0)	4.2 (-16.7 to 26.2)	3.5 (-17.3 to 28.8)	0.0 (0.0 to 0.0)	0.0 (0.0 to 0.0)	0.0 (0.0 to 0.0)	-2.0 (-34.1 to 27.9)	2.5 (-17.6 to 29.2)
Iron deficiency	-	-	-	-	-	8.0 (5.5 to 11.0)	8.0 (5.5 to 11.1)	7.4 (5.0 to 10.4)	-0.3 (-21.5 to 28.3)	-7.8 (-23.5 to 10.4)
Vitamin A deficiency	1.0 (0.6 to 1.3)	0.8 (0.6 to 1.1)	0.8 (0.4 to 1.0)	-18.6 (-32.4 to -2.2)	-5.4 (-26.3 to 16.0)	0.8 (0.5 to 1.2)	0.8 (0.5 to 1.0)	0.7 (0.4 to 1.0)	-7.7 (-29.5 to 10.1)	-8.8 (-28.0 to 15.5)
Zinc deficiency	0.5 (0.2 to 1.2)	0.4 (0.1 to 0.9)	0.4 (0.1 to 0.8)	-31.0 (-64.0 to 60.4)	5.0 (-63.7 to 178.9)	0.5 (0.2 to 1.2)	0.4 (0.1 to 0.9)	0.4 (0.1 to 0.9)	-23.9 (-60.6 to 6.1)	6.4 (-10.0 to 162.9)
Smoking	16.2 (12.8 to 19.9)	11.7 (9.2 to 14.4)	10.0 (8.0 to 12.3)	-27.9 (-37.2 to -15.5)	-14.3 (-22.8 to -4.0)	3.7 (2.6 to 5.1)	3.1 (2.2 to 4.2)	3.1 (2.2 to 4.2)	-15.0 (-36.2 to 11.9)	-2.0 (-18.2 to 17.5)
Chewing tobacco	1.4 (0.9 to 2.1)	0.9 (0.6 to 1.4)	0.8 (0.5 to 1.1)	-33.1 (-56.6 to 4.0)	-18.9 (-43.1 to 10.7)	0.1 (0.1 to 0.2)	0.1 (0.1 to 0.1)	0.1 (0.1 to 0.1)	-4.9 (-44.7 to 59.6)	-3.1 (-34.5 to 44.2)
Second-hand smoke	38.0 (33.9 to 42.1)	36.7 (32.7 to 41.0)	36.7 (32.2 to 41.4)	-3.3 (-13.8 to 8.8)	-0.1 (-10.0 to 9.8)	57.9 (54.3 to 61.5)	50.0 (46.0 to 53.7)	49.6 (45.3 to 53.8)	-13.6 (-19.9 to -7.9)	-0.7 (-6.8 to 5.9)
Alcohol use	14.6 (7.4 to 24.9)	14.7 (7.5 to 25.5)	16.9 (8.0 to 29.1)	0.9 (-36.0 to 53.5)	15.2 (-19.7 to 61.2)	11.9 (5.8 to 20.9)	11.8 (5.9 to 21.5)	13.0 (6.0 to 22.9)	-0.7 (-36.8 to 50.5)	10.1 (-23.0 to 57.7)
Drug use	0.5 (0.4 to 0.7)	0.5 (0.4 to 0.7)	0.6 (0.4 to 0.8)	0.4 (-5.8 to 6.5)	11.8 (4.2 to 20.3)	0.3 (0.2 to 0.4)	0.3 (0.2 to 0.4)	0.3 (0.3 to 0.4)	2.2 (-3.5 to 8.7)	9.1 (1.7 to 18.1)
Diet low in fruits	42.8 (37.5 to 46.0)	41.2 (35.9 to 44.9)	42.2 (36.7 to 45.8)	-3.7 (-8.8 to 1.5)	2.5 (-2.1 to 7.1)	40.2 (34.5 to 44.2)	37.7 (31.7 to 42.0)	39.5 (33.9 to 43.7)	-6.3 (-13.1 to 0.1)	5.0 (-0.3 to 11.6)
Diet low in vegetables	23.5 (15.4 to 30.6)	17.7 (9.0 to 25.3)	19.8 (10.7 to 27.4)	-24.4 (-50.6 to -1.5)	11.4 (-13.7 to 48.9)	25.6 (17.4 to 32.6)	20.1 (11.6 to 27.8)	21.9 (13.8 to 29.0)	-21.8 (-44.2 to -2.4)	9.3 (-10.7 to 40.6)
Diet low in legumes	4.6 (0.8 to 9.9)	6.0 (1.3 to 11.9)	8.0 (2.3 to 14.4)	30.5 (-40.6 to 302.9)	33.4 (-17.0 to 183.9)	8.2 (2.7 to 15.0)	8.1 (2.4 to 14.8)	10.7 (4.3 to 18.1)	-1.0 (-48.0 to 80.1)	31.5 (-8.6 to 128.4)
Diet low in whole grains	31.9 (27.3 to 35.7)	36.4 (32.8 to 39.5)	37.7 (34.1 to 40.9)	14.2 (5.9 to 25.1)	3.4 (-1.4 to 8.4)	33.3 (28.7 to 37.2)	37.7 (34.1 to 40.9)	38.9 (35.6 to 41.7)	13.4 (5.9 to 23.7)	3.3 (-0.9 to 8.3)
Diet low in nuts and seeds	51.8 (50.8 to 52.5)	50.8 (49.5 to 51.8)	50.7 (49.2 to 51.7)	-1.9 (-3.7 to -0.4)	-0.3 (-1.8 to 0.9)	51.9 (51.0 to 52.7)	50.9 (49.5 to 51.9)	50.7 (49.2 to 51.8)	-2.1 (-3.8 to -0.5)	-0.3 (-1.7 to 1.0)
Diet low in milk	46.9 (45.0 to 48.5)	47.1 (45.4 to 48.7)	47.5 (45.7 to 49.1)	0.5 (-1.4 to 2.4)	0.9 (-0.4 to 2.2)	47.0 (45.1 to 48.6)	46.8 (44.8 to 48.4)	47.4 (45.5 to 48.9)	-0.4 (-2.2 to 1.4)	1.2 (-0.2 to 2.7)
Diet high in red meat	2.7 (0.2 to 7.1)	5.7 (1.3 to 12.1)	6.7 (1.7 to 13.1)	109.1 (-16.5 to 1409.5)	17.8 (-42.8 to 170.3)	0.3 (0.0 to 1.8)	1.0 (0.0 to 3.6)	1.3 (0.0 to 4.6)	201.6 (-88.8 to [illegible]	33.3 (-95.5 to 20360.3)
Diet high in processed meat	9.5 (5.8 to 13.2)	16.2 (11.3 to 20.7)	19.0 (13.4 to 24.2)	70.0 (30.2 to 143.3)	17.7 (-0.5 to 40.5)	7.8 (4.3 to 11.2)	11.7 (7.8 to 15.4)	13.0 (8.7 to 17.2)	49.7 (16.2 to 122.1)	11.4 (-7.3 to 34.8)
Diet high in sugar-sweetened beverages	5.5 (2.2 to 11.3)	6.2 (2.7 to 11.8)	6.5 (2.7 to 11.9)	13.2 (-47.3 to 158.0)	5.3 (-36.0 to 77.0)	4.0 (1.5 to 9.3)	4.5 (1.9 to 9.3)	4.8 (2.0 to 9.4)	13.0 (-45.0 to 153.0)	6.2 (-40.8 to 91.6)
Diet low in fibre	23.7 (16.5 to 30.0)	24.6 (17.6 to 30.4)	25.5 (18.4 to 31.7)	3.7 (-13.2 to 25.7)	3.6 (-8.3 to 18.4)	24.9 (17.8 to 31.4)	26.0 (18.8 to 32.0)	27.3 (20.5 to 33.3)	4.5 (-9.9 to 25.4)	5.0 (-5.5 to 18.2)
Diet low in calcium	37.1 (33.2 to 40.3)	36.6 (32.7 to 39.9)	37.2 (33.4 to 40.4)	-1.2 (-4.7 to 2.3)	1.6 (-1.0 to 4.6)	39.1 (35.6 to 42.0)	37.9 (34.3 to 41.0)	38.9 (35.4 to 41.9)	-3.1 (-6.7 to -0.0)	2.7 (0.2 to 5.5)
Diet low in seafood omega-3 fatty acids	3.8 (2.3 to 5.2)	0.5 (0.0 to 1.6)	0.5 (0.0 to 1.6)	-86.5 (-99.8 to -58.2)	3.1 (-78.2 to 668.1)	4.4 (2.9 to 5.7)	0.7 (0.0 to 2.0)	0.8 (0.0 to 2.1)	-83.1 (-99.7 to -52.7)	4.5 (-62.6 to 344.7)
Diet low in polyunsaturated fatty acids	32.6 (27.7 to 37.1)	29.9 (24.6 to 34.5)	30.6 (25.0 to 35.4)	-8.1 (-18.6 to 0.3)	2.1 (-5.0 to 9.8)	33.2 (28.3 to 37.6)	30.7 (25.1 to 35.3)	31.2 (25.9 to 35.7)	-7.6 (-17.5 to 0.4)	1.9 (-4.3 to 9.1)
Diet high in trans fatty acids	1.1 (0.0 to 3.3)	1.0 (0.0 to 3.0)	0.9 (0.0 to 2.9)	-12.4 (-38.8 to -6.4)	-8.6 (-33.9 to -4.1)	1.5 (0.1 to 4.0)	1.3 (0.1 to 3.7)	1.2 (0.0 to 3.5)	-12.5 (-34.3 to -6.1)	-8.2 (-27.3 to -3.4)
Diet high in sodium	26.2 (16.8 to 34.1)	16.9 (8.2 to 23.9)	14.5 (5.9 to 22.0)	-35.4 (-58.7 to -16.4)	-14.2 (-39.1 to 2.9)	31.2 (22.4 to 38.4)	18.7 (9.8 to 25.3)	15.5 (6.5 to 22.6)	-40.1 (-60.8 to -24.9)	-16.9 (-40.3 to -2.5)
Intimate partner violence	-	-	-	-	-	2.3 (2.0 to 2.7)	2.2 (1.8 to 2.5)	2.1 (1.7 to 2.4)	-8.2 (-12.2 to -3.5)	-4.0 (-8.1 to 0.1)
Childhood sexual abuse	5.3 (4.3 to 6.5)	5.3 (4.3 to 6.5)	5.3 (4.3 to 6.5)	0.0 (0.0 to 0.0)	0.0 (0.0 to 0.0)	7.3 (5.9 to 8.9)	7.3 (5.9 to 8.9)	7.5 (6.0 to 9.2)	0.0 (0.0 to 0.0)	2.8 (-4.0 to 10.6)

Supplementary Results Table 1. Nara age-standardised Summary Exposure Values (SEVs) for Level 4 risk factors and percentage change, by sex, 1990, 2007, and 2017.Data in parentheses are 95% uncertainty intervals.										
	**Male**					**Female**				
	**1990**	**2007**	**2017**	**Percentage change 1990-2007**	**Percentage change 2007-2017**	**1990**	**2007**	**2017**	**Percentage change 1990-2007**	**Percentage change 2007-2017**
Bullying victimization	3.3 (1.4 to 7.2)	3.3 (1.4 to 7.1)	3.4 (1.5 to 7.4)	0.9 (-7.1 to 8.9)	4.1 (-1.1 to 9.2)	1.9 (0.8 to 4.0)	1.9 (0.8 to 4.0)	1.9 (0.8 to 4.1)	-0.4 (-8.5 to 7.6)	2.9 (-2.7 to 8.0)
Low physical activity	0.5 (0.2 to 0.9)	0.5 (0.2 to 0.9)	0.4 (0.2 to 0.8)	-1.8 (-3.8 to -0.0)	-8.8 (-12.3 to -4.5)	0.5 (0.2 to 1.0)	0.5 (0.2 to 0.9)	0.5 (0.2 to 0.9)	-3.9 (-6.1 to -1.4)	0.8 (-0.7 to 2.3)
High fasting plasma glucose	1.4 (0.9 to 2.3)	1.3 (0.8 to 1.9)	1.4 (0.9 to 2.4)	-10.6 (-45.2 to 41.2)	12.9 (-21.5 to 77.2)	1.2 (0.7 to 1.8)	1.2 (0.7 to 2.0)	1.3 (0.8 to 2.3)	2.2 (-38.9 to 72.4)	9.0 (-34.0 to 76.6)
High LDL cholesterol	12.1 (10.6 to 13.9)	12.7 (11.0 to 14.5)	12.7 (11.0 to 14.5)	4.5 (-1.5 to 10.9)	-0.1 (-4.8 to 4.9)	12.9 (11.3 to 14.6)	13.0 (11.5 to 14.8)	13.0 (11.5 to 14.8)	0.8 (-4.6 to 6.7)	-0.0 (-4.3 to 4.6)
High systolic blood pressure	11.3 (10.2 to 12.3)	8.3 (7.5 to 9.3)	8.2 (7.2 to 9.2)	-26.0 (-31.8 to -19.8)	-1.9 (-9.5 to 6.0)	9.7 (8.7 to 10.7)	6.1 (5.4 to 6.9)	5.6 (4.9 to 6.5)	-37.1 (-43.6 to -30.0)	-7.3 (-17.3 to 2.9)
High body-mass index	5.5 (3.4 to 9.0)	7.8 (5.2 to 12.3)	8.0 (5.4 to 12.5)	43.0 (15.8 to 86.6)	2.5 (-12.5 to 19.7)	5.2 (3.1 to 8.2)	6.6 (4.2 to 10.5)	6.6 (4.1 to 10.8)	28.4 (1.8 to 72.6)	-0.3 (-18.5 to 23.1)
Low bone mineral density	5.1 (4.3 to 6.1)	5.3 (4.5 to 6.1)	5.3 (4.5 to 6.3)	3.9 (-10.4 to 19.0)	0.9 (-12.1 to 15.4)	7.3 (6.0 to 8.6)	7.0 (6.1 to 8.2)	7.1 (6.0 to 8.4)	-3.4 (-14.1 to 10.2)	1.5 (-11.0 to 15.1)
Impaired kidney function	2.6 (1.2 to 6.5)	2.5 (1.1 to 6.4)	2.4 (1.1 to 6.1)	-3.7 (-8.4 to 0.6)	-6.3 (-11.6 to -2.0)	3.2 (1.5 to 7.4)	3.3 (1.5 to 7.6)	3.1 (1.4 to 7.2)	2.2 (-1.7 to 6.7)	-7.1 (-11.9 to -2.5)

Supplementary Results Table 1. Niigata age-standardised Summary Exposure Values (SEVs) for Level 4 risk factors and percentage change, by sex, 1990, 2007, and 2017.Data in parentheses are 95% uncertainty intervals.

	Male					Female				
	**1990**	**2007**	**2017**	**Percentage change 1990-2007**	**Percentage change 2007-2017**	**1990**	**2007**	**2017**	**Percentage change 1990-2007**	**Percentage change 2007-2017**
Unsafe water source	2.5 (1.3 to 4.1)	2.0 (1.1 to 3.2)	2.1 (1.1 to 3.5)	-19.8 (-60.4 to 54.4)	5.1 (-37.4 to 82.2)	2.5 (1.3 to 4.1)	2.0 (1.1 to 3.2)	2.1 (1.1 to 3.5)	-19.8 (-60.4 to 54.4)	5.1 (-37.4 to 82.2)
Unsafe sanitation	4.4 (2.0 to 8.0)	2.1 (1.0 to 3.9)	1.8 (0.8 to 3.4)	-51.9 (-79.4 to 1.9)	-15.2 (-57.0 to 63.5)	4.4 (2.0 to 8.0)	2.1 (1.0 to 3.9)	1.8 (0.8 to 3.4)	-51.9 (-79.4 to 1.9)	-15.2 (-57.0 to 63.5)
No access to handwashing facility	6.8 (4.9 to 9.0)	6.4 (4.7 to 8.6)	6.0 (4.3 to 7.9)	-5.5 (-32.6 to 35.5)	-7.3 (-27.3 to 17.4)	6.8 (4.9 to 9.0)	6.4 (4.7 to 8.6)	6.0 (4.3 to 7.9)	-5.5 (-32.6 to 35.5)	-7.3 (-27.3 to 17.4)
Ambient particulate matter pollution	30.1 (20.0 to 44.1)	30.2 (20.8 to 44.2)	25.4 (16.0 to 40.8)	0.3 (-24.6 to 33.8)	-15.7 (-40.1 to 12.0)	29.8 (19.6 to 43.6)	30.1 (20.8 to 44.1)	25.4 (16.0 to 40.7)	1.1 (-24.3 to 35.3)	-15.7 (-40.1 to 12.0)
Household air pollution from solid fuels	0.8 (0.3 to 2.4)	0.2 (0.0 to 0.4)	0.1 (0.0 to 0.4)	-77.0 (-93.1 to -49.5)	-18.4 (-65.3 to 76.8)	1.1 (0.3 to 7.0)	0.3 (0.1 to 1.2)	0.2 (0.0 to 0.9)	-73.1 (-93.0 to -44.0)	-17.6 (-65.2 to 77.7)
Ambient ozone pollution	50.8 (22.9 to 54.8)	51.0 (23.3 to 54.8)	51.4 (24.2 to 54.8)	0.4 (0.0 to 2.2)	0.8 (0.0 to 4.1)	50.8 (22.9 to 54.8)	51.0 (23.3 to 54.8)	51.4 (24.2 to 54.8)	0.4 (0.0 to 2.2)	0.8 (0.0 to 4.1)
Residential radon	15.8 (1.3 to 52.8)	15.8 (1.3 to 52.8)	15.8 (1.3 to 52.8)	0.0 (0.0 to 0.0)	0.0 (0.0 to 0.0)	15.8 (1.3 to 52.8)	15.8 (1.3 to 52.8)	15.8 (1.3 to 52.8)	0.0 (0.0 to 0.0)	0.0 (0.0 to 0.0)
Lead exposure	3.1 (0.5 to 6.9)	2.1 (0.3 to 5.2)	1.5 (0.2 to 4.1)	-32.7 (-46.3 to -24.0)	-28.0 (-43.4 to -20.1)	1.7 (0.1 to 4.6)	1.0 (0.1 to 3.3)	0.7 (0.0 to 2.6)	-37.9 (-55.2 to -27.7)	-30.5 (-52.4 to -20.6)
Occupational exposure to asbestos	1.8 (1.6 to 2.1)	2.7 (2.4 to 2.9)	2.5 (2.1 to 3.1)	45.2 (27.4 to 71.1)	-4.1 (-23.3 to 17.8)	0.5 (0.4 to 0.5)	0.4 (0.4 to 0.5)	0.4 (0.3 to 0.5)	-7.0 (-23.5 to 21.1)	-0.3 (-20.0 to 22.7)
Occupational exposure to arsenic	0.5 (0.0 to 1.1)	0.4 (0.0 to 1.0)	0.4 (0.0 to 1.0)	-	-	0.4 (0.0 to 1.0)	0.4 (0.0 to 0.9)	0.4 (0.0 to 0.9)	-	-
Occupational exposure to benzene	0.6 (0.0 to 1.8)	0.6 (0.0 to 1.8)	0.7 (0.0 to 1.9)	2.3 (-4.2 to 12.6)	3.8 (-2.4 to 11.5)	0.6 (0.0 to 1.6)	0.7 (0.0 to 2.0)	0.8 (0.0 to 2.3)	22.1 (9.6 to 41.6)	14.2 (4.3 to 30.8)
Occupational exposure to beryllium	0.0 (0.0 to 0.0)	0.0 (0.0 to 0.0)	0.0 (0.0 to 0.0)	-5.2 (-9.8 to -0.4)	2.1 (-1.8 to 5.8)	0.0 (0.0 to 0.0)	0.0 (0.0 to 0.0)	0.0 (0.0 to 0.0)	2.5 (-6.6 to 12.6)	9.3 (2.9 to 16.7)
Occupational exposure to cadmium	0.1 (0.0 to 0.1)	0.0 (0.0 to 0.1)	0.0 (0.0 to 0.1)	-6.7 (-16.9 to 5.3)	1.1 (-8.5 to 11.8)	0.0 (0.0 to 0.1)	0.0 (0.0 to 0.0)	0.0 (0.0 to 0.1)	-8.2 (-22.5 to 9.4)	5.3 (-6.8 to 19.3)
Occupational exposure to chromium	0.1 (0.1 to 0.1)	0.1 (0.1 to 0.1)	0.1 (0.1 to 0.1)	-5.0 (-14.3 to 5.7)	1.6 (-6.7 to 10.8)	0.1 (0.1 to 0.1)	0.1 (0.1 to 0.1)	0.1 (0.1 to 0.1)	-3.5 (-17.5 to 12.9)	7.1 (-3.3 to 19.2)
Occupational exposure to diesel engine exhaust	0.6 (0.5 to 0.6)	0.6 (0.5 to 0.6)	0.6 (0.6 to 0.6)	-1.8 (-9.6 to 7.1)	3.3 (-2.9 to 10.2)	0.4 (0.4 to 0.5)	0.5 (0.4 to 0.6)	0.6 (0.5 to 0.6)	12.9 (-1.1 to 27.5)	11.6 (1.2 to 23.1)
Occupational exposure to formaldehyde	0.3 (0.2 to 0.3)	0.2 (0.2 to 0.3)	0.2 (0.2 to 0.3)	-6.1 (-17.1 to 7.2)	0.7 (-9.2 to 11.2)	0.2 (0.2 to 0.2)	0.2 (0.2 to 0.2)	0.2 (0.2 to 0.2)	-5.6 (-20.7 to 13.3)	6.1 (-5.9 to 19.9)
Occupational exposure to nickel	0.4 (0.0 to 1.9)	0.4 (0.0 to 1.7)	0.4 (0.0 to 1.7)	-	-	0.3 (0.0 to 1.5)	0.3 (0.0 to 1.3)	0.3 (0.0 to 1.3)	-	-
Occupational exposure to polycyclic aromatic hydrocarbons	0.2 (0.2 to 0.3)	0.2 (0.2 to 0.3)	0.2 (0.2 to 0.3)	-4.1 (-12.9 to 6.1)	2.0 (-5.7 to 10.1)	0.2 (0.2 to 0.2)	0.2 (0.2 to 0.2)	0.2 (0.2 to 0.3)	-1.4 (-14.9 to 14.3)	7.9 (-1.8 to 19.3)
Occupational exposure to silica	4.7 (0.7 to 15.2)	4.3 (0.6 to 14.1)	4.4 (0.6 to 14.3)	-6.5 (-16.8 to 5.9)	1.6 (-7.1 to 12.4)	3.1 (0.4 to 10.3)	2.7 (0.4 to 8.7)	2.8 (0.4 to 9.0)	-13.4 (-27.8 to 5.0)	2.2 (-10.7 to 19.6)
Occupational exposure to sulphuric acid	0.6 (0.1 to 2.0)	0.6 (0.1 to 1.8)	0.6 (0.1 to 1.8)	-6.8 (-16.2 to 4.8)	1.5 (-7.5 to 11.4)	0.5 (0.1 to 1.6)	0.5 (0.1 to 1.5)	0.5 (0.1 to 1.6)	-9.9 (-24.6 to 7.6)	4.6 (-8.0 to 19.3)
Occupational exposure to trichloroethylene	0.1 (0.1 to 0.1)	0.1 (0.1 to 0.1)	0.1 (0.1 to 0.1)	-4.0 (-10.9 to 4.1)	2.1 (-3.9 to 9.1)	0.1 (0.1 to 0.1)	0.1 (0.1 to 0.1)	0.1 (0.1 to 0.1)	1.4 (-10.0 to 15.1)	8.6 (0.5 to 18.6)
Occupational asthmagens	18.4 (14.7 to 23.1)	17.8 (14.3 to 22.3)	17.8 (14.1 to 22.6)	-3.4 (-21.1 to 18.2)	0.1 (-15.3 to 17.7)	8.2 (6.5 to 10.1)	8.2 (6.6 to 10.2)	8.7 (7.0 to 10.8)	0.4 (-15.1 to 17.4)	5.5 (-7.3 to 19.0)
Occupational particulate matter, gases, and fumes	6.4 (4.5 to 9.1)	6.1 (4.4 to 8.7)	5.9 (4.2 to 8.4)	-4.4 (-9.3 to 0.7)	-3.7 (-7.0 to -0.5)	3.6 (2.5 to 5.5)	3.4 (2.4 to 5.2)	3.3 (2.3 to 4.9)	-5.6 (-14.3 to 3.9)	-4.5 (-10.2 to 1.4)
Occupational noise	6.0 (5.5 to 6.6)	5.8 (5.3 to 6.3)	5.6 (5.2 to 6.2)	-3.4 (-6.3 to -0.1)	-2.5 (-4.6 to -0.5)	3.5 (3.0 to 4.0)	3.5 (3.0 to 3.9)	3.5 (3.2 to 4.0)	0.3 (-5.6 to 6.9)	1.8 (-2.3 to 6.0)
Occupational ergonomic factors	10.7 (8.9 to 12.8)	9.4 (7.8 to 11.3)	9.3 (7.7 to 11.3)	-11.8 (-23.6 to 2.2)	-1.5 (-11.6 to 10.9)	5.7 (4.4 to 7.3)	5.8 (4.5 to 7.3)	6.5 (4.9 to 8.5)	0.9 (-21.8 to 28.6)	12.4 (-7.8 to 38.1)
Non-exclusive breastfeeding	0.4 (0.3 to 0.6)	0.4 (0.3 to 0.6)	0.4 (0.3 to 0.6)	-1.4 (-14.7 to 13.0)	-1.7 (-12.7 to 10.4)	0.4 (0.3 to 0.6)	0.4 (0.3 to 0.6)	0.4 (0.3 to 0.6)	-1.5 (-14.8 to 12.9)	-1.1 (-12.2 to 11.0)
Discontinued breastfeeding	1.7 (1.5 to 1.8)	1.7 (1.6 to 1.8)	1.7 (1.6 to 1.8)	1.7 (-8.1 to 11.8)	-1.3 (-8.7 to 6.0)	1.7 (1.5 to 1.8)	1.7 (1.6 to 1.8)	1.7 (1.5 to 1.8)	1.7 (-8.1 to 11.9)	-1.9 (-9.2 to 5.4)
Child underweight	0.3 (0.2 to 0.4)	0.3 (0.2 to 0.4)	0.3 (0.2 to 0.4)	-14.5 (-33.1 to 6.9)	-6.1 (-20.5 to 9.8)	0.3 (0.2 to 0.5)	0.3 (0.2 to 0.4)	0.3 (0.2 to 0.4)	-15.8 (-35.6 to 7.0)	-7.2 (-22.9 to 10.7)
Child wasting	0.2 (0.1 to 0.3)	0.2 (0.1 to 0.3)	0.2 (0.1 to 0.3)	-7.1 (-35.6 to 29.9)	-2.5 (-22.0 to 21.9)	0.2 (0.1 to 0.4)	0.2 (0.1 to 0.3)	0.2 (0.1 to 0.3)	-9.4 (-37.8 to 30.6)	-2.6 (-22.7 to 22.5)
Child stunting	0.7 (0.4 to 1.1)	0.6 (0.3 to 0.9)	0.6 (0.3 to 0.9)	-10.0 (-44.6 to 40.7)	-4.0 (-31.9 to 31.1)	0.7 (0.4 to 1.2)	0.6 (0.4 to 0.9)	0.6 (0.3 to 0.9)	-12.7 (-47.0 to 38.6)	-3.6 (-31.9 to 39.6)
Short gestation for birth weight	0.0 (0.0 to 0.0)	0.0 (0.0 to 0.0)	0.0 (0.0 to 0.0)	9.2 (-17.0 to 33.3)	2.6 (-15.0 to 29.3)	0.0 (0.0 to 0.0)	0.0 (0.0 to 0.0)	0.0 (0.0 to 0.0)	14.2 (-11.4 to 61.3)	3.0 (-20.0 to 40.3)
Low birth weight for gestation	0.0 (0.0 to 0.0)	0.0 (0.0 to 0.0)	0.0 (0.0 to 0.0)	2.4 (-12.3 to 27.6)	2.0 (-16.0 to 18.8)	0.0 (0.0 to 0.0)	0.0 (0.0 to 0.0)	0.0 (0.0 to 0.0)	-4.0 (-52.9 to 28.7)	1.7 (-21.5 to 22.9)
Iron deficiency	-	-	-	-	-	8.4 (5.9 to 11.6)	8.3 (5.8 to 11.2)	7.6 (5.1 to 10.4)	-1.0 (-22.0 to 25.7)	-8.6 (-24.8 to 10.8)
Vitamin A deficiency	1.0 (0.6 to 1.4)	0.9 (0.6 to 1.2)	0.6 (0.4 to 0.8)	-9.2 (-25.8 to 19.9)	-27.8 (-37.7 to -16.9)	0.8 (0.5 to 1.1)	0.8 (0.5 to 1.1)	0.7 (0.5 to 0.9)	0.5 (-23.4 to 45.3)	-15.5 (-32.3 to 0.0)
Zinc deficiency	0.5 (0.2 to 1.1)	0.5 (0.1 to 1.1)	0.4 (0.1 to 1.0)	-3.0 (-63.0 to 73.7)	-16.3 (-69.1 to 146.1)	0.5 (0.1 to 1.1)	0.3 (0.1 to 0.8)	0.3 (0.1 to 0.8)	-30.0 (-64.1 to 66.5)	-0.2 (-60.1 to 163.3)
Smoking	18.1 (14.8 to 21.5)	12.7 (10.2 to 15.4)	10.7 (8.6 to 13.0)	-29.9 (-38.7 to -20.9)	-15.5 (-23.9 to -6.1)	4.1 (2.9 to 5.5)	3.4 (2.4 to 4.7)	3.3 (2.4 to 4.6)	-17.0 (-35.2 to 7.4)	-2.8 (-19.1 to 17.2)
Chewing tobacco	1.4 (0.9 to 2.0)	0.9 (0.6 to 1.4)	0.7 (0.5 to 1.1)	-33.5 (-57.3 to 3.2)	-18.7 (-41.6 to 14.0)	0.1 (0.1 to 0.1)	0.1 (0.1 to 0.1)	0.1 (0.1 to 0.1)	-3.8 (-39.3 to 60.5)	-2.3 (-31.6 to 42.3)
Second-hand smoke	37.2 (32.8 to 41.1)	35.9 (31.4 to 40.1)	35.5 (31.0 to 39.9)	-3.5 (-14.1 to 9.3)	-1.0 (-10.1 to 9.1)	56.4 (52.9 to 60.1)	48.4 (44.6 to 52.2)	47.8 (44.0 to 51.6)	-14.3 (-20.4 to -7.8)	-1.2 (-7.4 to 5.0)
Alcohol use	14.5 (7.6 to 24.2)	14.7 (7.2 to 24.5)	17.6 (7.7 to 30.2)	1.0 (-36.2 to 53.6)	20.2 (-17.8 to 72.5)	11.4 (5.8 to 19.7)	11.4 (5.4 to 19.8)	13.4 (5.7 to 23.7)	0.1 (-37.6 to 54.3)	17.1 (-19.0 to 66.9)
Drug use	0.5 (0.4 to 0.7)	0.5 (0.4 to 0.7)	0.6 (0.4 to 0.8)	0.4 (-5.4 to 6.6)	11.5 (4.0 to 19.6)	0.3 (0.2 to 0.4)	0.3 (0.2 to 0.4)	0.3 (0.3 to 0.4)	1.3 (-4.1 to 7.1)	9.5 (1.7 to 18.5)
Diet low in fruits	40.5 (34.9 to 44.3)	38.7 (32.9 to 42.6)	39.9 (34.1 to 43.9)	-4.6 (-11.2 to 2.1)	3.2 (-1.9 to 9.3)	37.5 (32.1 to 41.8)	34.8 (29.1 to 39.6)	36.9 (31.1 to 41.5)	-7.2 (-15.7 to 0.9)	6.0 (-0.5 to 14.0)
Diet low in vegetables	19.2 (10.1 to 26.6)	13.0 (5.5 to 20.5)	15.1 (6.9 to 23.0)	-31.9 (-59.1 to -5.4)	15.6 (-15.7 to 71.1)	21.4 (12.6 to 29.4)	15.1 (6.7 to 23.3)	16.8 (8.1 to 24.8)	-29.5 (-58.9 to -6.8)	10.9 (-16.2 to 57.3)
Diet low in legumes	4.3 (0.7 to 9.5)	5.6 (1.1 to 11.3)	7.6 (1.9 to 14.2)	29.9 (-44.6 to 288.9)	34.6 (-22.4 to 218.9)	8.3 (2.6 to 14.9)	8.1 (2.4 to 14.6)	10.6 (3.7 to 17.6)	-2.7 (-50.3 to 86.1)	30.5 (-10.4 to 136.6)
Diet low in whole grains	30.4 (25.4 to 34.7)	35.2 (31.0 to 38.7)	36.6 (32.9 to 39.9)	15.8 (6.3 to 28.2)	3.9 (-1.1 to 9.5)	31.8 (27.0 to 35.9)	36.4 (32.7 to 39.9)	37.7 (33.9 to 40.8)	14.4 (6.4 to 26.0)	3.5 (-1.0 to 8.5)
Diet low in nuts and seeds	50.7 (49.3 to 51.8)	49.6 (47.8 to 50.9)	49.5 (47.5 to 50.9)	-2.3 (-4.8 to -0.2)	-0.2 (-2.2 to 1.6)	50.9 (49.5 to 51.9)	49.5 (47.7 to 50.9)	49.3 (47.3 to 50.8)	-2.7 (-5.1 to -0.7)	-0.4 (-2.5 to 1.6)
Diet low in milk	46.2 (44.1 to 48.0)	46.6 (44.8 to 48.3)	47.1 (45.3 to 48.7)	0.9 (-0.9 to 3.1)	1.1 (-0.2 to 2.5)	46.2 (44.1 to 47.9)	46.0 (43.8 to 47.7)	46.6 (44.5 to 48.3)	-0.4 (-2.5 to 1.5)	1.4 (-0.1 to 3.2)
Diet high in red meat	4.0 (0.5 to 9.1)	7.9 (2.4 to 14.1)	9.0 (3.2 to 16.0)	96.3 (-13.1 to 837.8)	14.4 (-35.1 to 110.1)	0.6 (0.0 to 2.6)	1.7 (0.0 to 5.0)	2.1 (0.0 to 6.1)	179.9 (-84.3 to 634219.3)	25.0 (-87.3 to 2107.2)
Diet high in processed meat	11.4 (7.3 to 15.6)	18.9 (13.5 to 23.7)	21.7 (15.7 to 27.1)	64.9 (26.8 to 128.3)	14.8 (-1.9 to 36.9)	9.4 (5.5 to 13.1)	13.6 (9.3 to 17.3)	14.8 (10.7 to 18.8)	44.3 (12.1 to 104.2)	8.9 (-8.3 to 31.1)
Diet high in sugar-sweetened beverages	7.3 (3.1 to 14.6)	8.4 (4.0 to 14.8)	8.9 (3.9 to 16.0)	15.3 (-45.1 to 155.3)	5.4 (-37.7 to 71.1)	5.3 (2.0 to 11.1)	5.8 (2.5 to 10.8)	6.2 (2.7 to 11.7)	10.5 (-49.8 to 143.9)	6.1 (-40.8 to 90.7)
Diet low in fibre	21.6 (14.2 to 28.2)	22.3 (15.1 to 28.6)	23.1 (15.9 to 29.5)	3.0 (-14.4 to 25.3)	3.5 (-9.3 to 19.7)	22.8 (15.3 to 29.2)	23.4 (15.9 to 29.8)	24.6 (17.5 to 30.9)	2.8 (-12.3 to 22.3)	5.1 (-6.7 to 20.9)
Diet low in calcium	34.6 (30.2 to 38.1)	34.5 (30.1 to 38.0)	35.4 (31.0 to 38.9)	-0.4 (-4.3 to 3.8)	2.5 (-0.7 to 6.1)	36.8 (32.9 to 40.0)	35.8 (31.6 to 39.1)	37.0 (33.1 to 40.2)	-2.9 (-6.5 to 0.7)	3.4 (0.5 to 6.7)
Diet low in seafood omega-3 fatty acids	3.1 (1.4 to 4.5)	0.2 (0.0 to 1.0)	0.3 (0.0 to 1.1)	-92.2 (-100.0 to -68.7)	5.6 (-92.8 to 1685.1)	3.6 (1.9 to 5.0)	0.4 (0.0 to 1.4)	0.4 (0.0 to 1.4)	-89.6 (-100.0 to -64.5)	8.2 (-86.1 to 1464.9)
Diet low in polyunsaturated fatty acids	30.3 (24.6 to 35.2)	27.4 (21.5 to 32.5)	28.2 (22.1 to 33.0)	-9.3 (-21.9 to 1.3)	2.7 (-5.7 to 12.5)	30.9 (26.0 to 35.7)	28.1 (22.4 to 33.1)	28.8 (23.0 to 33.5)	-9.0 (-21.1 to 0.7)	2.3 (-5.0 to 11.0)
Diet high in trans fatty acids	1.1 (0.0 to 3.3)	0.9 (0.0 to 3.0)	0.9 (0.0 to 2.9)	-12.6 (-38.7 to -6.6)	-8.3 (-32.9 to -3.3)	1.5 (0.1 to 4.0)	1.3 (0.1 to 3.7)	1.2 (0.0 to 3.5)	-12.6 (-36.6 to -6.6)	-8.5 (-28.2 to -3.6)
Diet high in sodium	28.7 (19.3 to 36.9)	18.6 (10.1 to 25.6)	16.0 (7.2 to 23.3)	-35.3 (-56.6 to -17.4)	-14.1 (-35.6 to 2.1)	32.7 (24.2 to 39.5)	19.9 (11.6 to 26.6)	16.6 (7.6 to 23.6)	-39.0 (-58.0 to -23.0)	-16.7 (-37.2 to -3.5)
Intimate partner violence	-	-	-	-	-	2.2 (1.8 to 2.6)	2.0 (1.7 to 2.4)	1.9 (1.6 to 2.3)	-8.1 (-12.4 to -4.0)	-3.7 (-7.5 to 0.5)
Childhood sexual abuse	5.3 (4.3 to 6.5)	5.3 (4.3 to 6.5)	5.3 (4.3 to 6.5)	0.0 (0.0 to 0.0)	0.0 (0.0 to 0.0)	7.3 (5.9 to 8.9)	7.3 (5.9 to 8.9)	7.5 (6.0 to 9.2)	0.0 (0.0 to 0.0)	2.8 (-4.0 to 10.6)

Supplementary Results Table 1. Niigata age-standardised Summary Exposure Values (SEVs) for Level 4 risk factors and percentage change, by sex, 1990, 2007, and 2017.Data in parentheses are 95% uncertainty intervals.

	Male					Female				
	**1990**	**2007**	**2017**	**Percentage change 1990-2007**	**Percentage change 2007-2017**	**1990**	**2007**	**2017**	**Percentage change 1990-2007**	**Percentage change 2007-2017**
Bullying victimization	3.3 (1.4 to 6.9)	3.4 (1.4 to 6.9)	3.5 (1.4 to 7.1)	2.2 (-5.7 to 9.9)	3.2 (-2.0 to 8.3)	1.9 (0.8 to 3.9)	1.9 (0.8 to 4.0)	2.0 (0.8 to 4.1)	0.2 (-7.5 to 8.3)	2.5 (-22.9 to 38.6)
Low physical activity	0.5 (0.2 to 0.9)	0.5 (0.2 to 0.9)	0.4 (0.2 to 0.8)	-1.8 (-3.7 to 0.0)	-8.8 (-12.3 to -4.6)	0.5 (0.2 to 1.0)	0.5 (0.2 to 0.9)	0.5 (0.2 to 0.9)	-3.9 (-6.2 to -1.3)	0.7 (-0.8 to 2.3)
High fasting plasma glucose	1.4 (0.8 to 2.2)	1.4 (0.9 to 2.2)	1.6 (0.9 to 2.5)	1.7 (-36.6 to 52.8)	12.6 (-26.9 to 78.9)	1.2 (0.7 to 1.9)	1.2 (0.7 to 2.1)	1.3 (0.7 to 2.3)	5.3 (-35.9 to 73.0)	10.1 (-32.6 to 76.9)
High LDL cholesterol	12.0 (10.3 to 13.8)	12.5 (10.9 to 14.3)	12.5 (10.9 to 14.3)	4.6 (-1.2 to 10.7)	0.0 (-4.6 to 4.7)	12.7 (11.1 to 14.5)	12.9 (11.3 to 14.6)	12.9 (11.3 to 14.7)	1.2 (-4.2 to 7.1)	0.3 (-4.4 to 4.8)
High systolic blood pressure	9.8 (8.8 to 10.8)	5.8 (5.0 to 6.6)	5.1 (4.3 to 5.9)	-41.0 (-46.5 to -35.2)	-12.3 (-20.0 to -3.6)	8.5 (7.7 to 9.4)	4.6 (3.9 to 5.2)	3.9 (3.3 to 4.6)	-46.4 (-52.1 to -40.3)	-14.3 (-24.7 to -2.6)
High body-mass index	5.4 (3.3 to 8.6)	7.7 (5.1 to 12.3)	8.0 (5.2 to 12.4)	43.6 (14.3 to 87.7)	3.7 (-11.8 to 22.0)	5.3 (3.2 to 8.4)	6.9 (4.3 to 10.7)	7.0 (4.3 to 10.9)	29.5 (2.1 to 72.6)	0.8 (-17.4 to 21.6)
Low bone mineral density	5.3 (4.4 to 6.3)	5.1 (4.3 to 6.0)	5.2 (4.3 to 6.2)	-2.6 (-14.8 to 11.4)	0.6 (-12.9 to 16.1)	6.6 (5.7 to 7.5)	6.5 (5.6 to 7.3)	6.6 (5.5 to 7.8)	-1.0 (-11.9 to 10.0)	1.9 (-11.1 to 15.5)
Impaired kidney function	2.5 (1.1 to 6.4)	2.5 (1.1 to 6.4)	2.3 (1.0 to 6.1)	-1.5 (-5.3 to 3.2)	-6.1 (-11.4 to -1.5)	3.1 (1.4 to 7.3)	3.3 (1.5 to 7.5)	3.0 (1.4 to 7.2)	4.8 (0.7 to 10.0)	-6.5 (-11.5 to -2.0)

Supplementary Results Table 1. Oita age-standardised Summary Exposure Values (SEVs) for Level 4 risk factors and percentage change, by sex, 1990, 2007, and 2017.Data in parentheses are 95% uncertainty intervals.

	Male					Female				
	1990	2007	2017	Percentage change 1990-2007	Percentage change 2007-2017	1990	2007	2017	Percentage change 1990-2007	Percentage change 2007-2017
Unsafe water source	2.3 (1.3 to 3.8)	1.9 (1.0 to 3.2)	2.0 (1.1 to 3.4)	-16.8 (-56.9 to 60.7)	5.9 (-39.4 to 83.3)	2.3 (1.3 to 3.8)	1.9 (1.0 to 3.2)	2.0 (1.1 to 3.4)	-16.8 (-56.9 to 60.7)	5.9 (-39.4 to 83.3)
Unsafe sanitation	3.9 (1.9 to 7.1)	2.0 (0.9 to 3.8)	1.7 (0.8 to 3.3)	-49.2 (-77.4 to 8.3)	-13.6 (-55.8 to 66.4)	3.9 (1.9 to 7.1)	2.0 (0.9 to 3.8)	1.7 (0.8 to 3.3)	-49.2 (-77.4 to 8.3)	-13.6 (-55.8 to 66.4)
No access to handwashing facility	6.7 (4.9 to 9.0)	6.4 (4.6 to 8.7)	5.9 (4.3 to 8.0)	-5.5 (-33.4 to 36.8)	-7.0 (-27.5 to 20.0)	6.7 (4.9 to 9.0)	6.4 (4.6 to 8.7)	5.9 (4.3 to 8.0)	-5.5 (-33.4 to 36.8)	-7.0 (-27.5 to 20.0)
Ambient particulate matter pollution	32.3 (21.3 to 49.1)	32.8 (22.0 to 48.7)	28.2 (17.7 to 42.6)	1.5 (-24.5 to 40.0)	-14.1 (-42.0 to 15.5)	32.1 (21.0 to 48.9)	32.7 (22.0 to 48.6)	28.1 (17.7 to 42.5)	2.2 (-24.4 to 41.4)	-14.1 (-42.0 to 15.6)
Household air pollution from solid fuels	0.7 (0.2 to 2.3)	0.2 (0.0 to 0.4)	0.2 (0.0 to 0.4)	-74.8 (-92.7 to -46.3)	-13.3 (-63.9 to 89.8)	1.0 (0.3 to 5.3)	0.3 (0.1 to 1.5)	0.3 (0.0 to 1.4)	-70.8 (-92.6 to -43.0)	-11.9 (-63.7 to 93.0)
Ambient ozone pollution	50.5 (22.4 to 54.8)	49.6 (21.0 to 54.8)	50.7 (22.9 to 54.8)	-1.8 (-7.0 to 0.0)	2.4 (0.0 to 10.2)	50.5 (22.4 to 54.8)	49.6 (21.0 to 54.8)	50.7 (22.9 to 54.8)	-1.8 (-7.0 to 0.0)	2.4 (0.0 to 10.2)
Residential radon	13.3 (0.9 to 43.9)	13.3 (0.9 to 43.9)	13.3 (0.9 to 43.9)	0.0 (0.0 to 0.0)	0.0 (0.0 to 0.0)	13.3 (0.9 to 43.9)	13.3 (0.9 to 43.9)	13.3 (0.9 to 43.9)	0.0 (0.0 to 0.0)	0.0 (0.0 to 0.0)
Lead exposure	2.6 (0.3 to 6.1)	1.8 (0.2 to 4.7)	1.3 (0.1 to 3.8)	-30.5 (-44.2 to -21.9)	-25.9 (-41.7 to -18.0)	1.4 (0.1 to 4.2)	0.9 (0.0 to 3.1)	0.7 (0.0 to 2.6)	-34.2 (-51.3 to -25.3)	-27.4 (-49.5 to -18.1)
Occupational exposure to asbestos	2.1 (1.8 to 2.3)	2.6 (2.4 to 2.9)	2.4 (1.9 to 2.9)	28.2 (9.8 to 53.6)	-9.9 (-26.7 to 9.8)	0.5 (0.4 to 0.6)	0.5 (0.4 to 0.5)	0.5 (0.4 to 0.6)	-6.6 (-23.7 to 22.5)	-6.2 (-25.7 to 15.5)
Occupational exposure to arsenic	0.4 (0.0 to 1.0)	0.4 (0.0 to 1.0)	0.4 (0.0 to 1.0)	-	-	0.4 (0.0 to 0.9)	0.4 (0.0 to 0.9)	0.4 (0.0 to 0.9)	-	-
Occupational exposure to benzene	0.6 (0.0 to 1.7)	0.6 (0.0 to 1.8)	0.7 (0.0 to 1.8)	3.0 (-3.6 to 12.0)	3.6 (-2.1 to 12.0)	0.6 (0.0 to 1.6)	0.7 (0.0 to 1.9)	0.8 (0.0 to 2.3)	22.4 (7.9 to 41.4)	14.6 (4.2 to 30.9)
Occupational exposure to beryllium	0.0 (0.0 to 0.0)	0.0 (0.0 to 0.0)	0.0 (0.0 to 0.0)	-4.9 (-9.9 to 0.1)	2.0 (-1.8 to 6.2)	0.0 (0.0 to 0.0)	0.0 (0.0 to 0.0)	0.0 (0.0 to 0.0)	2.6 (-6.6 to 12.5)	9.3 (2.2 to 16.5)
Occupational exposure to cadmium	0.1 (0.0 to 0.1)	0.0 (0.0 to 0.1)	0.0 (0.0 to 0.1)	-6.5 (-17.5 to 6.2)	0.9 (-8.6 to 11.4)	0.0 (0.0 to 0.1)	0.0 (0.0 to 0.0)	0.0 (0.0 to 0.1)	-7.6 (-22.2 to 10.4)	5.2 (-8.4 to 19.9)
Occupational exposure to chromium	0.1 (0.1 to 0.1)	0.1 (0.1 to 0.1)	0.1 (0.1 to 0.1)	-4.7 (-14.5 to 6.7)	1.5 (-6.6 to 10.4)	0.1 (0.1 to 0.1)	0.1 (0.1 to 0.1)	0.1 (0.1 to 0.1)	-2.9 (-16.3 to 14.0)	7.0 (-5.1 to 19.5)
Occupational exposure to diesel engine exhaust	0.6 (0.5 to 0.6)	0.6 (0.5 to 0.6)	0.6 (0.6 to 0.6)	-0.9 (-9.2 to 7.9)	3.4 (-2.8 to 10.4)	0.4 (0.4 to 0.5)	0.5 (0.4 to 0.6)	0.5 (0.5 to 0.6)	13.0 (-1.4 to 29.3)	11.6 (0.5 to 22.8)
Occupational exposure to formaldehyde	0.2 (0.2 to 0.3)	0.2 (0.2 to 0.3)	0.2 (0.2 to 0.3)	-5.9 (-17.4 to 8.5)	0.5 (-9.4 to 11.4)	0.2 (0.2 to 0.2)	0.2 (0.2 to 0.2)	0.2 (0.2 to 0.2)	-4.9 (-20.4 to 13.9)	6.0 (-7.9 to 20.1)
Occupational exposure to nickel	0.4 (0.0 to 1.8)	0.4 (0.0 to 1.7)	0.4 (0.0 to 1.7)	-	-	0.3 (0.0 to 1.5)	0.3 (0.0 to 1.3)	0.3 (0.0 to 1.3)	-	-
Occupational exposure to polycyclic aromatic hydrocarbons	0.2 (0.2 to 0.3)	0.2 (0.2 to 0.2)	0.2 (0.2 to 0.3)	-3.7 (-13.0 to 7.3)	1.8 (-5.7 to 10.0)	0.2 (0.2 to 0.2)	0.2 (0.2 to 0.2)	0.2 (0.2 to 0.3)	-0.8 (-14.1 to 15.1)	7.8 (-3.5 to 19.2)
Occupational exposure to silica	4.5 (0.7 to 14.2)	4.2 (0.6 to 13.7)	4.3 (0.6 to 13.8)	-5.4 (-15.8 to 8.5)	1.4 (-7.4 to 13.4)	3.0 (0.4 to 9.8)	2.7 (0.4 to 8.5)	2.7 (0.4 to 8.7)	-12.4 (-26.9 to 6.6)	2.5 (-12.1 to 19.6)
Occupational exposure to sulphuric acid	0.6 (0.1 to 1.8)	0.6 (0.1 to 1.8)	0.6 (0.1 to 1.8)	-6.7 (-17.3 to 5.5)	0.9 (-7.6 to 11.0)	0.5 (0.1 to 1.6)	0.5 (0.1 to 1.4)	0.5 (0.1 to 1.5)	-9.3 (-23.7 to 9.1)	4.5 (-9.3 to 20.1)
Occupational exposure to trichloroethylene	0.1 (0.1 to 0.1)	0.1 (0.1 to 0.1)	0.1 (0.1 to 0.1)	-3.8 (-11.0 to 4.5)	2.0 (-3.7 to 8.5)	0.1 (0.1 to 0.1)	0.1 (0.1 to 0.1)	0.1 (0.1 to 0.1)	1.8 (-9.3 to 16.1)	8.6 (-1.0 to 18.5)
Occupational asthmagens	17.8 (14.3 to 22.5)	17.4 (14.0 to 22.3)	17.4 (14.0 to 22.0)	-2.2 (-20.4 to 19.5)	0.0 (-14.3 to 17.8)	8.1 (6.4 to 10.2)	8.1 (6.5 to 10.1)	8.6 (6.9 to 10.7)	0.8 (-15.5 to 18.0)	5.4 (-7.0 to 21.5)
Occupational particulate matter, gases, and fumes	6.3 (4.5 to 9.2)	6.1 (4.2 to 8.8)	5.8 (4.1 to 8.4)	-4.6 (-10.0 to 0.8)	-3.7 (-7.1 to -0.1)	3.6 (2.5 to 5.3)	3.4 (2.4 to 5.0)	3.2 (2.2 to 4.8)	-5.5 (-13.8 to 4.4)	-4.4 (-9.8 to 1.9)
Occupational noise	5.9 (5.4 to 6.4)	5.7 (5.3 to 6.2)	5.6 (5.2 to 6.1)	-3.3 (-6.5 to 0.1)	-2.4 (-4.5 to -0.2)	3.4 (2.9 to 4.0)	3.4 (3.0 to 3.9)	3.5 (3.1 to 3.9)	0.3 (-6.2 to 7.5)	1.8 (-2.1 to 6.2)
Occupational ergonomic factors	11.0 (9.0 to 13.2)	9.7 (8.1 to 11.7)	9.6 (7.8 to 11.6)	-11.2 (-23.8 to 3.3)	-1.3 (-12.8 to 12.0)	5.9 (4.6 to 7.6)	6.0 (4.7 to 7.6)	6.7 (5.2 to 8.5)	0.8 (-22.8 to 29.9)	12.4 (-8.8 to 36.5)
Non-exclusive breastfeeding	0.4 (0.3 to 0.6)	0.4 (0.3 to 0.6)	0.4 (0.3 to 0.6)	-0.4 (-14.3 to 13.8)	-2.3 (-12.4 to 9.9)	0.4 (0.3 to 0.6)	0.4 (0.3 to 0.6)	0.4 (0.3 to 0.6)	-1.2 (-15.0 to 12.9)	-1.3 (-11.5 to 11.0)
Discontinued breastfeeding	1.7 (1.5 to 1.8)	1.7 (1.6 to 1.9)	1.7 (1.5 to 1.8)	2.1 (-7.3 to 13.1)	-2.7 (-10.1 to 5.3)	1.7 (1.5 to 1.8)	1.7 (1.6 to 1.9)	1.7 (1.6 to 1.8)	4.2 (-5.4 to 15.5)	-3.6 (-10.9 to 4.3)
Child underweight	0.3 (0.2 to 0.4)	0.2 (0.1 to 0.3)	0.2 (0.1 to 0.3)	-14.2 (-32.0 to 9.9)	-5.3 (-19.8 to 11.9)	0.3 (0.2 to 0.4)	0.2 (0.1 to 0.3)	0.2 (0.1 to 0.3)	-16.2 (-36.4 to 10.6)	-6.0 (-24.4 to 14.2)
Child wasting	0.2 (0.1 to 0.3)	0.2 (0.1 to 0.3)	0.2 (0.1 to 0.3)	-6.0 (-33.9 to 32.6)	-2.4 (-22.5 to 22.2)	0.2 (0.1 to 0.3)	0.2 (0.1 to 0.3)	0.2 (0.1 to 0.3)	-8.6 (-37.8 to 32.7)	-2.8 (-24.4 to 24.4)
Child stunting	0.6 (0.3 to 1.1)	0.6 (0.3 to 1.0)	0.6 (0.3 to 0.9)	-9.8 (-45.0 to 46.7)	-2.7 (-31.8 to 39.9)	0.7 (0.3 to 1.1)	0.6 (0.3 to 1.0)	0.6 (0.3 to 1.0)	-11.4 (-47.2 to 52.7)	-3.4 (-32.3 to 37.4)
Short gestation for birth weight	0.0 (0.0 to 0.0)	0.0 (0.0 to 0.0)	0.0 (0.0 to 0.0)	11.2 (-10.1 to 41.4)	1.9 (-18.9 to 31.0)	0.0 (0.0 to 0.0)	0.0 (0.0 to 0.0)	0.0 (0.0 to 0.0)	15.1 (-20.3 to 96.8)	0.3 (-24.3 to 32.1)
Low birth weight for gestation	0.0 (0.0 to 0.0)	0.0 (0.0 to 0.0)	0.0 (0.0 to 0.0)	1.8 (-15.1 to 21.7)	2.9 (-16.7 to 25.4)	0.0 (0.0 to 0.0)	0.0 (0.0 to 0.0)	0.0 (0.0 to 0.0)	-6.6 (-60.8 to 31.8)	4.0 (-15.6 to 28.4)
Iron deficiency	-	-	-	-	-	8.1 (5.6 to 11.3)	8.1 (5.7 to 11.1)	7.4 (4.9 to 10.4)	0.1 (-20.1 to 29.9)	-8.3 (-24.5 to 9.9)
Vitamin A deficiency	0.9 (0.6 to 1.2)	0.8 (0.5 to 1.0)	0.7 (0.5 to 0.9)	-17.3 (-32.3 to 7.5)	-7.9 (-24.0 to 9.6)	0.7 (0.5 to 1.0)	0.8 (0.5 to 1.1)	0.7 (0.4 to 0.9)	13.2 (-7.0 to 36.6)	-20.2 (-32.6 to -5.1)
Zinc deficiency	0.5 (0.1 to 1.1)	0.5 (0.1 to 1.1)	0.4 (0.1 to 1.0)	-6.6 (-64.2 to 71.4)	-16.3 (-68.4 to 151.3)	0.5 (0.2 to 1.1)	0.3 (0.1 to 0.7)	0.3 (0.1 to 0.8)	-32.0 (-64.2 to 56.2)	1.4 (-61.7 to 162.6)
Smoking	17.8 (14.3 to 21.2)	12.5 (10.0 to 15.2)	10.5 (8.4 to 12.9)	-29.9 (-39.2 to -19.4)	-15.7 (-24.2 to -5.4)	4.0 (2.8 to 5.4)	3.4 (2.4 to 4.6)	3.3 (2.4 to 4.5)	-16.1 (-36.7 to 11.0)	-2.6 (-19.0 to 17.3)
Chewing tobacco	1.4 (0.9 to 2.0)	0.9 (0.6 to 1.4)	0.8 (0.5 to 1.1)	-32.5 (-56.8 to 4.6)	-18.9 (-42.4 to 13.7)	0.1 (0.1 to 0.1)	0.1 (0.1 to 0.1)	0.1 (0.1 to 0.1)	-3.8 (-40.6 to 56.5)	-2.9 (-33.4 to 39.7)
Second-hand smoke	37.8 (33.6 to 42.1)	35.3 (31.1 to 39.8)	34.7 (30.4 to 39.4)	-6.6 (-17.7 to 5.5)	-1.9 (-10.9 to 7.7)	56.9 (53.2 to 60.5)	47.3 (43.5 to 50.7)	46.2 (42.4 to 50.0)	-16.9 (-23.0 to -10.7)	-2.3 (-8.3 to 4.0)
Alcohol use	14.5 (7.5 to 24.7)	14.4 (7.3 to 24.6)	16.6 (7.8 to 28.8)	-0.6 (-33.2 to 47.2)	15.1 (-18.6 to 68.2)	11.8 (6.1 to 20.5)	11.7 (5.8 to 20.3)	13.4 (5.9 to 23.9)	-0.4 (-34.7 to 48.5)	14.5 (-19.4 to 66.9)
Drug use	0.5 (0.4 to 0.7)	0.5 (0.4 to 0.7)	0.6 (0.4 to 0.8)	0.6 (-5.1 to 6.8)	11.7 (4.4 to 21.3)	0.3 (0.2 to 0.4)	0.3 (0.2 to 0.4)	0.3 (0.3 to 0.4)	1.8 (-4.4 to 8.3)	9.4 (1.8 to 17.8)
Diet low in fruits	41.0 (35.4 to 44.8)	39.4 (33.6 to 43.5)	40.9 (35.0 to 44.7)	-3.9 (-9.6 to 2.4)	3.7 (-1.4 to 9.1)	38.2 (32.4 to 42.6)	35.4 (29.4 to 40.1)	37.7 (31.6 to 42.3)	-7.4 (-15.7 to 1.0)	6.6 (-0.2 to 14.5)
Diet low in vegetables	19.7 (10.8 to 27.2)	14.2 (6.4 to 21.8)	16.4 (8.0 to 24.2)	-27.8 (-58.0 to 0.2)	15.1 (-15.8 to 69.7)	22.0 (13.3 to 29.1)	16.1 (7.7 to 23.9)	18.0 (9.5 to 25.6)	-26.6 (-53.7 to -3.8)	11.2 (-12.0 to 52.4)
Diet low in legumes	4.3 (0.7 to 10.1)	5.7 (1.1 to 11.7)	7.6 (2.0 to 14.2)	32.2 (-42.1 to 285.8)	33.0 (-18.4 to 219.5)	8.3 (2.6 to 15.0)	8.5 (2.7 to 15.3)	11.1 (4.3 to 18.4)	2.2 (-40.7 to 86.0)	31.0 (-7.4 to 128.6)
Diet low in whole grains	30.4 (25.7 to 34.7)	35.2 (31.1 to 38.6)	36.6 (32.8 to 40.1)	15.7 (7.1 to 28.0)	4.0 (-1.0 to 10.2)	31.9 (27.0 to 35.8)	36.5 (32.4 to 39.6)	37.8 (34.1 to 40.8)	14.6 (6.1 to 25.3)	3.5 (-0.8 to 8.7)
Diet low in nuts and seeds	50.7 (49.4 to 51.8)	49.3 (47.5 to 50.8)	49.1 (47.2 to 50.7)	-2.7 (-5.2 to -0.6)	-0.4 (-2.6 to 1.5)	50.9 (49.5 to 51.9)	49.4 (47.6 to 50.8)	49.2 (47.2 to 50.9)	-2.8 (-5.3 to -0.6)	-0.5 (-2.5 to 1.5)
Diet low in milk	46.2 (44.1 to 48.0)	46.6 (44.6 to 48.3)	47.1 (45.2 to 48.7)	0.8 (-1.1 to 3.0)	1.1 (-0.4 to 2.6)	46.2 (44.2 to 48.0)	46.0 (44.0 to 47.8)	46.7 (44.7 to 48.3)	-0.4 (-2.5 to 1.7)	1.3 (-0.2 to 3.0)
Diet high in red meat	4.0 (0.6 to 9.1)	7.9 (2.4 to 14.4)	9.0 (3.1 to 16.5)	97.8 (-18.6 to 805.6)	14.3 (-39.4 to 137.6)	0.6 (0.0 to 3.0)	1.6 (0.0 to 5.4)	2.1 (0.0 to 6.0)	167.5 (-73.7 to 1397234.5)	28.4 (-84.2 to 1955.8)
Diet high in processed meat	11.4 (7.0 to 15.6)	18.9 (13.3 to 24.0)	21.8 (15.7 to 27.4)	66.1 (27.2 to 131.7)	15.5 (-1.4 to 37.8)	9.3 (5.5 to 12.9)	13.5 (9.5 to 17.3)	14.7 (10.6 to 18.7)	45.4 (14.1 to 102.9)	9.1 (-8.7 to 31.1)
Diet high in sugar-sweetened beverages	7.4 (2.9 to 15.1)	8.3 (3.8 to 14.6)	8.7 (3.8 to 15.4)	11.9 (-42.7 to 156.8)	5.2 (-37.0 to 76.1)	5.1 (2.0 to 10.7)	5.8 (2.3 to 11.1)	6.2 (2.4 to 12.1)	13.3 (-48.3 to 153.5)	6.8 (-38.9 to 84.1)
Diet low in fibre	22.1 (14.8 to 28.7)	23.3 (16.2 to 29.5)	24.3 (17.1 to 30.8)	5.1 (-12.3 to 27.5)	4.6 (-7.8 to 20.0)	23.3 (16.0 to 29.9)	24.4 (17.1 to 30.5)	25.9 (18.8 to 31.8)	4.7 (-11.6 to 26.4)	5.8 (-5.0 to 18.9)
Diet low in calcium	34.9 (30.5 to 38.4)	34.7 (30.2 to 38.1)	35.5 (31.2 to 38.9)	-0.5 (-4.6 to 3.5)	2.4 (-0.5 to 5.9)	37.0 (33.1 to 40.2)	36.0 (31.7 to 39.4)	37.2 (33.3 to 40.4)	-2.7 (-6.5 to 0.7)	3.4 (0.7 to 6.9)
Diet low in seafood omega-3 fatty acids	3.2 (1.3 to 4.7)	0.2 (0.0 to 1.1)	0.3 (0.0 to 1.1)	-92.5 (-100.0 to -68.8)	8.8 (-94.5 to 2189.8)	3.7 (1.7 to 5.2)	0.4 (0.0 to 1.4)	0.4 (0.0 to 1.4)	-90.4 (-100.0 to -69.3)	9.5 (-87.1 to 1442.6)
Diet low in polyunsaturated fatty acids	30.4 (25.2 to 35.1)	27.7 (21.5 to 32.6)	28.4 (22.0 to 33.3)	-9.0 (-19.7 to 0.9)	2.4 (-6.0 to 11.7)	31.1 (25.6 to 35.8)	28.4 (22.7 to 33.4)	29.1 (23.5 to 33.9)	-8.8 (-19.7 to 0.7)	2.4 (-5.4 to 11.8)
Diet high in trans fatty acids	1.1 (0.0 to 3.3)	0.9 (0.0 to 3.0)	0.9 (0.0 to 2.9)	-12.7 (-38.8 to -6.9)	-8.2 (-31.6 to -3.4)	1.5 (0.1 to 4.0)	1.3 (0.1 to 3.7)	1.2 (0.0 to 3.5)	-12.5 (-34.4 to -6.6)	-8.4 (-28.1 to -3.3)
Diet high in sodium	28.0 (18.1 to 36.5)	18.2 (9.4 to 25.7)	15.6 (6.6 to 23.1)	-35.0 (-58.2 to -17.1)	-14.4 (-37.2 to 1.6)	31.6 (22.9 to 38.6)	18.9 (10.6 to 25.8)	15.6 (6.9 to 22.9)	-40.1 (-60.3 to -24.4)	-17.5 (-40.7 to -3.6)
Intimate partner violence	-	-	-	-	-	2.3 (2.0 to 2.7)	2.1 (1.8 to 2.5)	2.0 (1.7 to 2.4)	-9.1 (-13.3 to -4.8)	-4.0 (-8.8 to 0.3)
Childhood sexual abuse	5.3 (4.3 to 6.5)	5.3 (4.3 to 6.5)	5.3 (4.3 to 6.5)	0.0 (0.0 to 0.0)	0.0 (0.0 to 0.0)	7.3 (5.9 to 8.9)	7.3 (5.9 to 8.9)	7.5 (6.0 to 9.2)	0.0 (0.0 to 0.0)	2.8 (-4.0 to 10.6)

Supplementary Results Table 1. Oita age-standardised Summary Exposure Values (SEVs) for Level 4 risk factors and percentage change, by sex, 1990, 2007, and 2017.Data in parentheses are 95% uncertainty intervals.

	Male					Female				
	**1990**	**2007**	**2017**	**Percentage change 1990-2007**	**Percentage change 2007-2017**	**1990**	**2007**	**2017**	**Percentage change 1990-2007**	**Percentage change 2007-2017**
Bullying victimization	3.3 (1.4 to 6.9)	3.4 (1.4 to 7.2)	3.5 (1.5 to 7.3)	1.7 (-6.0 to 9.2)	2.4 (-2.6 to 7.1)	1.9 (0.8 to 4.0)	1.9 (0.8 to 4.0)	2.0 (0.8 to 4.1)	0.5 (-7.6 to 8.6)	1.8 (-3.6 to 6.9)
Low physical activity	0.5 (0.2 to 0.9)	0.5 (0.2 to 0.9)	0.4 (0.2 to 0.8)	-1.7 (-3.6 to 0.1)	-8.8 (-12.3 to -4.7)	0.5 (0.2 to 1.0)	0.5 (0.2 to 0.9)	0.5 (0.2 to 0.9)	-3.9 (-6.0 to -1.3)	0.7 (-0.9 to 2.3)
High fasting plasma glucose	1.4 (0.9 to 2.2)	1.4 (0.9 to 2.1)	1.5 (0.9 to 2.5)	-1.9 (-38.8 to 50.0)	10.7 (-26.8 to 75.8)	1.1 (0.7 to 1.9)	1.2 (0.7 to 2.0)	1.3 (0.7 to 2.3)	5.2 (-33.6 to 64.2)	9.7 (-28.6 to 77.9)
High LDL cholesterol	12.0 (10.4 to 13.8)	12.5 (10.9 to 14.3)	12.5 (10.9 to 14.4)	4.3 (-1.6 to 10.9)	0.1 (-4.1 to 4.8)	12.8 (11.2 to 14.6)	12.9 (11.4 to 14.6)	12.9 (11.3 to 14.8)	0.8 (-4.5 to 5.9)	0.2 (-4.2 to 5.0)
High systolic blood pressure	11.2 (10.1 to 12.3)	8.3 (7.4 to 9.2)	8.1 (7.2 to 9.2)	-26.1 (-32.2 to -20.1)	-1.6 (-8.8 to 6.8)	9.6 (8.6 to 10.6)	6.0 (5.3 to 6.9)	5.6 (4.8 to 6.4)	-37.3 (-43.8 to -30.0)	-7.3 (-17.3 to 3.0)
High body-mass index	5.1 (3.1 to 8.0)	7.2 (4.7 to 11.8)	7.4 (4.7 to 11.9)	42.2 (12.1 to 82.7)	2.4 (-14.0 to 19.9)	5.4 (3.2 to 8.5)	6.9 (4.3 to 10.7)	6.9 (4.4 to 10.9)	27.9 (-0.7 to 69.8)	0.2 (-16.7 to 20.7)
Low bone mineral density	5.3 (4.4 to 6.2)	5.2 (4.3 to 6.1)	5.2 (4.2 to 6.2)	-2.3 (-14.5 to 11.3)	0.2 (-13.2 to 14.7)	6.6 (5.7 to 7.6)	6.5 (5.7 to 7.4)	6.7 (5.6 to 8.0)	-1.3 (-11.6 to 9.7)	2.6 (-10.7 to 17.5)
Impaired kidney function	2.6 (1.2 to 6.5)	2.5 (1.2 to 6.4)	2.4 (1.1 to 6.1)	-3.0 (-7.6 to 1.5)	-6.9 (-12.7 to -2.0)	3.2 (1.5 to 7.4)	3.3 (1.5 to 7.6)	3.1 (1.4 to 7.3)	2.9 (-1.1 to 7.6)	-7.2 (-12.2 to -2.4)

Supplementary Results Table 1. Okayama age-standardised Summary Exposure Values (SEVs) for Level 4 risk factors and percentage change, by sex, 1990, 2007, and 2017.Data in parentheses are 95% uncertainty intervals.

	Male					Female				
	**1990**	**2007**	**2017**	**Percentage change 1990-2007**	**Percentage change 2007-2017**	**1990**	**2007**	**2017**	**Percentage change 1990-2007**	**Percentage change 2007-2017**
Unsafe water source	2.2 (1.2 to 3.7)	1.8 (1.0 to 3.1)	1.9 (1.0 to 3.2)	-18.9 (-60.6 to 62.9)	5.2 (-42.1 to 79.3)	2.2 (1.2 to 3.7)	1.8 (1.0 to 3.1)	1.9 (1.0 to 3.2)	-18.9 (-60.6 to 62.9)	5.2 (-42.1 to 79.3)
Unsafe sanitation	3.8 (1.8 to 6.8)	1.8 (0.8 to 3.3)	1.6 (0.7 to 2.9)	-52.0 (-79.6 to 8.4)	-14.8 (-54.5 to 67.2)	3.8 (1.8 to 6.8)	1.8 (0.8 to 3.3)	1.6 (0.7 to 2.9)	-52.0 (-79.6 to 8.4)	-14.8 (-54.5 to 67.2)
No access to handwashing facility	6.7 (4.9 to 9.1)	6.4 (4.5 to 8.6)	5.9 (4.2 to 7.9)	-5.2 (-31.8 to 32.2)	-7.6 (-27.7 to 17.9)	6.7 (4.9 to 9.1)	6.4 (4.5 to 8.6)	5.9 (4.2 to 7.9)	-5.2 (-31.8 to 32.2)	-7.6 (-27.7 to 17.9)
Ambient particulate matter pollution	32.1 (21.3 to 47.3)	32.3 (21.8 to 47.7)	28.1 (17.2 to 44.0)	0.8 (-23.9 to 33.2)	-13.1 (-37.3 to 14.3)	31.9 (21.2 to 47.1)	32.3 (21.8 to 47.6)	28.1 (17.2 to 43.9)	1.3 (-23.7 to 34.4)	-13.1 (-37.4 to 14.4)
Household air pollution from solid fuels	0.5 (0.2 to 1.4)	0.1 (0.0 to 0.3)	0.1 (0.0 to 0.2)	-73.7 (-92.3 to -43.4)	-14.7 (-67.3 to 98.7)	0.8 (0.2 to 4.1)	0.2 (0.0 to 0.7)	0.2 (0.0 to 0.8)	-70.8 (-92.1 to -38.9)	-12.2 (-67.4 to 95.6)
Ambient ozone pollution	51.3 (24.0 to 54.8)	51.7 (25.2 to 54.8)	52.2 (26.9 to 54.8)	0.9 (0.0 to 5.6)	1.0 (0.0 to 7.2)	51.3 (24.0 to 54.8)	51.7 (25.2 to 54.8)	52.2 (26.9 to 54.8)	0.9 (0.0 to 5.6)	1.0 (0.0 to 7.2)
Residential radon	14.3 (1.0 to 46.3)	14.3 (1.0 to 46.3)	14.3 (1.0 to 46.3)	0.0 (0.0 to 0.0)	0.0 (0.0 to 0.0)	14.3 (1.0 to 46.3)	14.3 (1.0 to 46.3)	14.3 (1.0 to 46.3)	0.0 (0.0 to 0.0)	0.0 (0.0 to 0.0)
Lead exposure	3.1 (0.5 to 6.7)	2.1 (0.3 to 5.0)	1.4 (0.2 to 3.9)	-34.7 (-49.2 to -25.1)	-29.4 (-45.4 to -21.0)	1.7 (0.1 to 4.6)	1.0 (0.1 to 3.2)	0.7 (0.0 to 2.4)	-40.6 (-59.3 to -30.4)	-32.4 (-55.0 to -22.5)
Occupational exposure to asbestos	3.2 (2.7 to 3.5)	4.0 (3.7 to 4.4)	3.9 (3.2 to 4.7)	27.3 (10.2 to 50.8)	-2.8 (-19.6 to 16.5)	0.6 (0.5 to 0.7)	0.5 (0.5 to 0.6)	0.6 (0.4 to 0.7)	-6.1 (-23.3 to 21.3)	0.5 (-18.5 to 22.7)
Occupational exposure to arsenic	0.5 (0.0 to 1.1)	0.4 (0.0 to 1.0)	0.4 (0.0 to 1.0)	-	-	0.4 (0.0 to 1.0)	0.4 (0.0 to 0.8)	0.4 (0.0 to 0.9)	-	-
Occupational exposure to benzene	0.6 (0.0 to 1.8)	0.7 (0.0 to 1.9)	0.7 (0.0 to 1.9)	2.5 (-4.4 to 13.6)	3.5 (-2.3 to 10.9)	0.6 (0.0 to 1.7)	0.7 (0.0 to 2.1)	0.8 (0.0 to 2.4)	21.4 (7.9 to 40.2)	14.0 (4.6 to 29.3)
Occupational exposure to beryllium	0.0 (0.0 to 0.0)	0.0 (0.0 to 0.0)	0.0 (0.0 to 0.0)	-5.2 (-9.8 to -0.3)	2.0 (-1.8 to 5.7)	0.0 (0.0 to 0.0)	0.0 (0.0 to 0.0)	0.0 (0.0 to 0.0)	2.2 (-6.7 to 11.8)	8.8 (2.6 to 15.4)
Occupational exposure to cadmium	0.1 (0.0 to 0.1)	0.0 (0.0 to 0.1)	0.0 (0.0 to 0.1)	-7.1 (-18.4 to 4.0)	0.9 (-8.5 to 10.7)	0.0 (0.0 to 0.1)	0.0 (0.0 to 0.1)	0.0 (0.0 to 0.1)	-8.6 (-23.7 to 8.6)	4.4 (-8.0 to 20.3)
Occupational exposure to chromium	0.1 (0.1 to 0.1)	0.1 (0.1 to 0.1)	0.1 (0.1 to 0.1)	-5.4 (-15.3 to 4.4)	1.4 (-6.8 to 10.0)	0.1 (0.1 to 0.1)	0.1 (0.1 to 0.1)	0.1 (0.1 to 0.1)	-3.9 (-18.4 to 12.0)	6.2 (-4.5 to 19.4)
Occupational exposure to diesel engine exhaust	0.6 (0.5 to 0.6)	0.6 (0.5 to 0.6)	0.6 (0.6 to 0.7)	-2.0 (-9.9 to 6.8)	3.3 (-2.9 to 10.2)	0.5 (0.4 to 0.5)	0.5 (0.5 to 0.6)	0.6 (0.5 to 0.6)	12.2 (-1.7 to 27.1)	11.0 (0.2 to 22.8)
Occupational exposure to formaldehyde	0.3 (0.2 to 0.3)	0.2 (0.2 to 0.3)	0.2 (0.2 to 0.3)	-6.6 (-18.7 to 5.4)	0.5 (-9.5 to 10.9)	0.2 (0.2 to 0.3)	0.2 (0.2 to 0.2)	0.2 (0.2 to 0.2)	-6.0 (-21.9 to 11.8)	5.2 (-7.2 to 20.5)
Occupational exposure to nickel	0.4 (0.0 to 2.0)	0.4 (0.0 to 1.8)	0.4 (0.0 to 1.8)	-	-	0.3 (0.0 to 1.6)	0.3 (0.0 to 1.3)	0.3 (0.0 to 1.3)	-	-
Occupational exposure to polycyclic aromatic hydrocarbons	0.2 (0.2 to 0.3)	0.2 (0.2 to 0.3)	0.2 (0.2 to 0.3)	-4.4 (-13.7 to 5.3)	1.7 (-5.9 to 9.5)	0.2 (0.2 to 0.3)	0.2 (0.2 to 0.2)	0.2 (0.2 to 0.3)	-1.9 (-15.7 to 13.3)	7.1 (-3.3 to 18.9)
Occupational exposure to silica	4.7 (0.7 to 14.8)	4.4 (0.6 to 14.2)	4.5 (0.6 to 14.4)	-6.5 (-16.6 to 5.2)	1.4 (-7.6 to 12.8)	3.2 (0.4 to 10.7)	2.7 (0.4 to 8.8)	2.8 (0.4 to 8.9)	-13.5 (-28.6 to 4.8)	1.6 (-11.8 to 20.4)
Occupational exposure to sulphuric acid	0.6 (0.1 to 2.0)	0.6 (0.1 to 1.8)	0.6 (0.1 to 1.8)	-7.4 (-17.6 to 3.2)	1.3 (-7.9 to 10.8)	0.5 (0.1 to 1.7)	0.5 (0.1 to 1.4)	0.5 (0.1 to 1.5)	-10.4 (-25.5 to 7.5)	3.8 (-9.0 to 20.3)
Occupational exposure to trichloroethylene	0.1 (0.1 to 0.1)	0.1 (0.1 to 0.1)	0.1 (0.1 to 0.1)	-4.4 (-11.7 to 3.7)	2.0 (-3.7 to 7.9)	0.1 (0.1 to 0.1)	0.1 (0.1 to 0.1)	0.1 (0.1 to 0.1)	0.9 (-10.6 to 14.5)	7.9 (-0.7 to 18.1)
Occupational asthmagens	19.0 (14.7 to 23.9)	18.1 (14.5 to 23.0)	18.1 (14.2 to 23.1)	-4.3 (-21.9 to 18.9)	-0.4 (-14.9 to 16.9)	8.3 (6.8 to 10.3)	8.3 (6.7 to 10.3)	8.8 (7.0 to 10.9)	0.2 (-15.9 to 18.7)	5.0 (-7.1 to 18.6)
Occupational particulate matter, gases, and fumes	6.4 (4.5 to 9.2)	6.1 (4.3 to 8.9)	5.9 (4.2 to 8.3)	-4.4 (-9.6 to 0.5)	-3.7 (-7.1 to -0.4)	3.6 (2.5 to 5.4)	3.4 (2.4 to 5.0)	3.3 (2.3 to 4.8)	-5.5 (-14.2 to 4.8)	-4.6 (-10.4 to 1.7)
Occupational noise	6.0 (5.4 to 6.5)	5.8 (5.3 to 6.3)	5.6 (5.2 to 6.1)	-3.2 (-6.3 to 0.1)	-2.4 (-4.4 to -0.4)	3.5 (3.0 to 4.0)	3.5 (3.1 to 4.0)	3.6 (3.2 to 4.0)	0.5 (-5.8 to 7.9)	1.8 (-2.2 to 5.8)
Occupational ergonomic factors	10.3 (8.5 to 12.5)	9.2 (7.5 to 11.0)	9.1 (7.4 to 11.0)	-11.4 (-23.9 to 1.7)	-1.1 (-11.9 to 10.7)	5.5 (4.2 to 7.2)	5.7 (4.3 to 7.3)	6.4 (4.9 to 8.3)	2.5 (-19.4 to 31.4)	12.6 (-6.8 to 35.1)
Non-exclusive breastfeeding	0.4 (0.3 to 0.6)	0.4 (0.3 to 0.6)	0.4 (0.3 to 0.6)	-1.3 (-15.1 to 14.3)	-1.4 (-12.3 to 9.7)	0.4 (0.3 to 0.6)	0.4 (0.3 to 0.6)	0.4 (0.3 to 0.6)	-0.8 (-14.7 to 14.8)	-1.2 (-12.1 to 9.9)
Discontinued breastfeeding	1.7 (1.5 to 1.8)	1.7 (1.6 to 1.9)	1.7 (1.6 to 1.8)	3.8 (-5.9 to 14.7)	-3.1 (-10.2 to 4.7)	1.7 (1.6 to 1.8)	1.7 (1.6 to 1.9)	1.7 (1.6 to 1.8)	1.0 (-8.5 to 11.6)	-0.3 (-7.6 to 7.7)
Child underweight	0.2 (0.1 to 0.3)	0.2 (0.1 to 0.2)	0.1 (0.1 to 0.2)	-11.3 (-23.7 to 3.3)	-4.0 (-13.1 to 6.4)	0.2 (0.1 to 0.3)	0.2 (0.1 to 0.2)	0.2 (0.1 to 0.2)	-12.2 (-27.7 to 5.1)	-4.6 (-16.4 to 8.8)
Child wasting	0.1 (0.1 to 0.2)	0.1 (0.1 to 0.2)	0.1 (0.1 to 0.2)	-4.6 (-26.3 to 20.8)	-2.1 (-17.2 to 15.3)	0.1 (0.1 to 0.2)	0.1 (0.1 to 0.2)	0.1 (0.1 to 0.2)	-5.0 (-24.0 to 20.7)	-2.4 (-17.5 to 16.5)
Child stunting	0.5 (0.2 to 0.9)	0.5 (0.2 to 0.7)	0.4 (0.2 to 0.7)	-10.7 (-45.3 to 40.2)	-3.5 (-30.4 to 32.2)	0.6 (0.3 to 0.9)	0.5 (0.2 to 0.8)	0.5 (0.2 to 0.8)	-12.6 (-45.0 to 34.8)	-4.3 (-30.5 to 37.8)
Short gestation for birth weight	0.0 (0.0 to 0.0)	0.0 (0.0 to 0.0)	0.0 (0.0 to 0.0)	9.8 (-10.5 to 43.6)	2.5 (-15.5 to 29.2)	0.0 (0.0 to 0.0)	0.0 (0.0 to 0.0)	0.0 (0.0 to 0.0)	13.2 (-17.9 to 40.3)	0.1 (-21.3 to 35.4)
Low birth weight for gestation	0.0 (0.0 to 0.0)	0.0 (0.0 to 0.0)	0.0 (0.0 to 0.0)	3.3 (-15.1 to 27.3)	1.9 (-16.0 to 20.9)	0.0 (0.0 to 0.0)	0.0 (0.0 to 0.0)	0.0 (0.0 to 0.0)	0.9 (-14.5 to 28.9)	3.8 (-18.7 to 25.0)
Iron deficiency	-	-	-	-	-	8.0 (5.5 to 11.0)	8.0 (5.6 to 11.0)	7.3 (5.0 to 10.4)	-0.5 (-20.8 to 28.9)	-8.6 (-24.6 to 10.8)
Vitamin A deficiency	0.9 (0.6 to 1.2)	0.8 (0.6 to 1.1)	0.7 (0.4 to 0.9)	-7.6 (-19.7 to 7.1)	-20.3 (-33.1 to -4.5)	0.8 (0.5 to 1.1)	0.8 (0.5 to 1.1)	0.7 (0.4 to 1.0)	1.5 (-12.5 to 19.2)	-6.1 (-28.5 to 14.6)
Zinc deficiency	0.5 (0.1 to 1.0)	0.5 (0.1 to 1.1)	0.4 (0.1 to 1.1)	-0.6 (-63.4 to 76.9)	-14.2 (-69.1 to 147.4)	0.5 (0.1 to 1.1)	0.3 (0.1 to 0.9)	0.3 (0.1 to 0.7)	-30.3 (-64.8 to 67.1)	0.8 (-62.4 to 175.2)
Smoking	17.4 (14.0 to 20.8)	12.2 (9.7 to 15.0)	10.3 (8.2 to 12.7)	-30.0 (-38.7 to -18.9)	-15.4 (-24.0 to -6.2)	3.9 (2.8 to 5.4)	3.3 (2.3 to 4.4)	3.2 (2.2 to 4.3)	-17.0 (-36.3 to 7.9)	-2.8 (-19.4 to 18.3)
Chewing tobacco	1.4 (0.9 to 2.1)	0.9 (0.6 to 1.4)	0.7 (0.5 to 1.2)	-33.8 (-56.8 to 3.5)	-19.2 (-43.0 to 13.0)	0.1 (0.1 to 0.2)	0.1 (0.1 to 0.1)	0.1 (0.1 to 0.1)	-3.4 (-40.5 to 58.2)	-3.1 (-32.6 to 41.3)
Second-hand smoke	37.2 (33.3 to 41.2)	36.1 (32.1 to 40.3)	36.2 (31.8 to 40.7)	-3.1 (-14.7 to 8.9)	0.3 (-9.3 to 10.9)	56.0 (52.5 to 59.7)	48.4 (44.3 to 52.3)	48.5 (44.4 to 52.8)	-13.6 (-20.6 to -6.9)	0.2 (-6.4 to 6.6)
Alcohol use	14.8 (7.6 to 24.9)	14.7 (7.4 to 24.8)	16.8 (7.9 to 28.8)	-0.1 (-30.1 to 51.4)	13.7 (-22.0 to 60.8)	11.9 (5.9 to 20.5)	11.7 (5.8 to 20.1)	12.9 (6.0 to 22.7)	-1.5 (-32.2 to 52.2)	10.1 (-23.8 to 56.9)
Drug use	0.5 (0.4 to 0.7)	0.5 (0.4 to 0.7)	0.6 (0.4 to 0.8)	0.2 (-5.8 to 6.3)	11.8 (4.7 to 21.1)	0.3 (0.2 to 0.4)	0.3 (0.2 to 0.4)	0.3 (0.3 to 0.4)	1.7 (-4.0 to 7.3)	8.9 (2.3 to 17.4)
Diet low in fruits	40.6 (35.1 to 44.4)	39.0 (32.9 to 43.0)	40.3 (34.6 to 44.2)	-3.9 (-9.6 to 2.4)	3.2 (-1.7 to 8.9)	37.6 (31.5 to 42.1)	35.0 (28.8 to 39.8)	37.2 (31.1 to 41.7)	-7.1 (-15.7 to 0.7)	6.4 (0.1 to 14.8)
Diet low in vegetables	19.3 (10.5 to 26.9)	13.7 (5.8 to 21.7)	15.9 (7.4 to 23.7)	-28.9 (-57.4 to -1.7)	15.4 (-14.6 to 71.9)	21.5 (13.1 to 28.8)	15.5 (7.7 to 23.5)	17.4 (9.2 to 24.8)	-27.7 (-53.6 to -3.6)	11.8 (-14.1 to 56.8)
Diet low in legumes	4.5 (0.7 to 10.0)	5.8 (1.1 to 11.8)	7.9 (1.9 to 14.6)	30.3 (-47.0 to 270.3)	35.2 (-19.2 to 215.5)	8.4 (2.5 to 15.8)	8.4 (2.7 to 15.0)	11.0 (4.8 to 18.3)	0.5 (-45.8 to 79.3)	31.0 (-9.7 to 136.1)
Diet low in whole grains	30.3 (25.3 to 34.5)	35.1 (31.2 to 38.6)	36.5 (32.5 to 39.7)	16.0 (6.9 to 28.0)	3.9 (-1.0 to 10.1)	31.8 (27.1 to 35.7)	36.4 (32.6 to 39.7)	37.7 (33.9 to 40.9)	14.4 (6.5 to 25.9)	3.5 (-0.9 to 8.4)
Diet low in nuts and seeds	50.5 (49.1 to 51.7)	49.1 (47.2 to 50.6)	48.9 (46.8 to 50.5)	-2.9 (-5.5 to -0.5)	-0.5 (-3.0 to 1.6)	50.8 (49.5 to 51.8)	49.4 (47.5 to 50.9)	49.2 (47.2 to 50.8)	-2.8 (-5.4 to -0.5)	-0.4 (-2.4 to 1.6)
Diet low in milk	46.1 (43.9 to 47.9)	46.5 (44.5 to 48.2)	47.0 (45.1 to 48.6)	0.8 (-1.0 to 2.8)	1.1 (-0.3 to 2.5)	46.1 (44.0 to 47.9)	45.8 (43.6 to 47.6)	46.4 (44.4 to 48.1)	-0.6 (-2.7 to 1.4)	1.3 (-0.3 to 3.1)
Diet high in red meat	4.1 (0.6 to 9.1)	8.0 (2.6 to 15.3)	9.2 (3.2 to 16.8)	95.1 (-15.0 to 775.9)	14.1 (-35.9 to 120.3)	0.6 (0.0 to 2.5)	1.7 (0.0 to 5.1)	2.1 (0.0 to 6.3)	170.5 (-77.0 to 396459.7)	25.8 (-81.9 to 2109.2)
Diet high in processed meat	11.6 (7.4 to 15.6)	19.2 (14.0 to 23.9)	22.1 (15.9 to 27.6)	66.1 (29.3 to 128.1)	15.0 (-2.7 to 37.5)	9.7 (5.9 to 13.6)	14.2 (10.0 to 18.2)	15.6 (11.0 to 19.7)	46.0 (14.4 to 98.1)	9.4 (-6.9 to 31.0)
Diet high in sugar-sweetened beverages	7.4 (3.2 to 14.7)	8.5 (3.9 to 14.8)	9.1 (4.0 to 16.6)	16.1 (-43.3 to 149.2)	6.1 (-38.4 to 78.7)	5.3 (2.1 to 11.4)	5.9 (2.7 to 11.3)	6.3 (2.7 to 12.3)	11.1 (-48.2 to 144.0)	7.2 (-39.4 to 85.2)
Diet low in fibre	21.8 (14.8 to 28.0)	22.7 (15.3 to 28.8)	23.5 (16.3 to 29.8)	4.0 (-12.7 to 26.0)	3.7 (-9.1 to 18.5)	23.1 (15.9 to 29.7)	24.1 (17.3 to 30.0)	25.5 (18.0 to 31.7)	4.1 (-12.7 to 25.8)	5.8 (-5.6 to 20.0)
Diet low in calcium	34.5 (30.0 to 38.2)	34.3 (30.0 to 37.9)	35.1 (30.9 to 38.6)	-0.6 (-4.7 to 3.4)	2.4 (-0.7 to 5.8)	36.7 (32.8 to 39.9)	35.6 (31.4 to 39.0)	36.8 (32.9 to 40.1)	-3.1 (-7.0 to 0.4)	3.4 (0.8 to 6.7)
Diet low in seafood omega-3 fatty acids	3.1 (1.3 to 4.5)	0.3 (0.0 to 1.1)	0.3 (0.0 to 1.1)	-91.7 (-100.0 to -67.9)	6.4 (-94.2 to 2371.7)	3.6 (1.8 to 5.0)	0.4 (0.0 to 1.4)	0.4 (0.0 to 1.4)	-89.8 (-100.0 to -64.8)	9.8 (-81.7 to 1911.2)
Diet low in polyunsaturated fatty acids	30.2 (24.8 to 35.2)	27.3 (21.3 to 32.3)	28.1 (22.0 to 33.0)	-9.4 (-21.3 to 0.7)	2.7 (-5.7 to 13.0)	30.8 (25.4 to 35.7)	28.1 (22.3 to 33.1)	28.8 (23.2 to 33.7)	-8.7 (-20.5 to 0.9)	2.5 (-4.9 to 11.1)
Diet high in trans fatty acids	1.1 (0.0 to 3.3)	0.9 (0.0 to 3.0)	0.9 (0.0 to 2.9)	-12.8 (-38.7 to -6.6)	-8.2 (-33.5 to -3.3)	1.5 (0.1 to 4.0)	1.3 (0.1 to 3.7)	1.2 (0.0 to 3.5)	-12.9 (-36.1 to -6.4)	-8.6 (-30.0 to -3.4)
Diet high in sodium	28.0 (18.2 to 36.2)	18.1 (9.0 to 25.2)	15.5 (6.7 to 23.0)	-35.3 (-57.6 to -16.5)	-14.2 (-39.7 to 5.0)	31.8 (23.0 to 39.1)	19.1 (10.8 to 25.9)	15.8 (7.3 to 23.0)	-39.9 (-58.4 to -25.5)	-17.4 (-39.4 to -4.6)
Intimate partner violence	-	-	-	-	-	2.3 (1.9 to 2.7)	2.1 (1.7 to 2.4)	2.0 (1.7 to 2.3)	-9.2 (-13.2 to -5.0)	-3.5 (-8.0 to 1.0)
Childhood sexual abuse	5.3 (4.3 to 6.5)	5.3 (4.3 to 6.5)	5.3 (4.3 to 6.5)	0.0 (0.0 to 0.0)	0.0 (0.0 to 0.0)	7.3 (5.9 to 8.9)	7.3 (5.9 to 8.9)	7.5 (6.0 to 9.2)	0.0 (0.0 to 0.0)	2.8 (-4.0 to 10.6)

Supplementary Results Table 1. Okayama age-standardised Summary Exposure Values (SEVs) for Level 4 risk factors and percentage change, by sex, 1990, 2007, and 2017.Data in parentheses are 95% uncertainty intervals.

	Male					Female				
	**1990**	**2007**	**2017**	**Percentage change 1990-2007**	**Percentage change 2007-2017**	**1990**	**2007**	**2017**	**Percentage change 1990-2007**	**Percentage change 2007-2017**
Bullying victimization	3.3 (1.4 to 6.7)	3.3 (1.4 to 6.6)	3.5 (1.4 to 7.1)	0.4 (-7.5 to 8.6)	4.1 (-1.4 to 9.0)	1.9 (0.8 to 3.7)	1.9 (0.8 to 3.7)	2.0 (0.8 to 3.9)	-0.6 (-8.6 to 7.7)	3.3 (-2.1 to 8.1)
Low physical activity	0.5 (0.2 to 0.9)	0.5 (0.2 to 0.9)	0.4 (0.2 to 0.8)	-1.8 (-3.9 to 0.1)	-8.7 (-12.3 to -4.5)	0.5 (0.2 to 1.0)	0.5 (0.2 to 0.9)	0.5 (0.2 to 0.9)	-3.8 (-6.0 to -1.3)	0.7 (-0.9 to 2.2)
High fasting plasma glucose	1.4 (0.8 to 2.2)	1.4 (0.9 to 2.2)	1.6 (0.9 to 2.6)	-0.1 (-35.7 to 63.4)	12.8 (-23.3 to 72.1)	1.2 (0.7 to 2.0)	1.2 (0.7 to 2.1)	1.4 (0.8 to 2.3)	5.4 (-35.1 to 75.2)	10.2 (-30.9 to 80.3)
High LDL cholesterol	12.1 (10.4 to 13.9)	12.6 (10.9 to 14.4)	12.6 (10.9 to 14.5)	4.5 (-1.4 to 10.8)	0.1 (-4.1 to 5.0)	12.9 (11.3 to 14.5)	13.0 (11.5 to 14.8)	13.0 (11.5 to 14.8)	1.0 (-4.5 to 6.8)	0.1 (-4.1 to 4.8)
High systolic blood pressure	11.2 (10.2 to 12.3)	8.3 (7.4 to 9.3)	8.2 (7.3 to 9.2)	-25.9 (-32.1 to -19.6)	-1.4 (-8.5 to 6.5)	9.6 (8.7 to 10.6)	6.0 (5.3 to 6.8)	5.6 (4.8 to 6.4)	-37.1 (-44.0 to -29.8)	-7.1 (-16.3 to 2.6)
High body-mass index	5.9 (3.9 to 9.0)	8.5 (5.8 to 13.2)	8.8 (5.9 to 13.5)	43.5 (16.4 to 81.4)	3.5 (-12.2 to 19.8)	5.7 (3.5 to 8.9)	7.3 (4.6 to 11.6)	7.3 (4.5 to 11.7)	29.5 (1.6 to 72.9)	0.2 (-18.2 to 22.9)
Low bone mineral density	5.3 (4.4 to 6.2)	5.2 (4.3 to 6.0)	5.2 (4.2 to 6.1)	-2.0 (-14.0 to 11.7)	0.1 (-12.2 to 12.7)	7.2 (6.0 to 8.5)	7.0 (5.9 to 8.2)	7.1 (5.9 to 8.4)	-3.6 (-15.2 to 9.3)	2.1 (-11.0 to 16.7)
Impaired kidney function	2.6 (1.2 to 6.5)	2.5 (1.1 to 6.4)	2.4 (1.1 to 6.1)	-2.8 (-7.1 to 1.4)	-6.5 (-11.9 to -1.9)	3.2 (1.5 to 7.4)	3.3 (1.5 to 7.6)	3.1 (1.4 to 7.2)	3.4 (-0.4 to 8.1)	-7.1 (-12.0 to -2.2)

Supplementary Results Table 1. Okinawa age-standardised Summary Exposure Values (SEVs) for Level 4 risk factors and percentage change, by sex, 1990, 2007, and 2017.Data in parentheses are 95% uncertainty intervals.										
	Male					Female				
	1990	2007	2017	Percentage change 1990-2007	Percentage change 2007-2017	1990	2007	2017	Percentage change 1990-2007	Percentage change 2007-2017
Unsafe water source	2.8 (1.6 to 4.8)	2.4 (1.3 to 4.1)	2.5 (1.4 to 4.2)	-17.1 (-58.1 to 68.6)	4.5 (-37.0 to 85.0)	2.8 (1.6 to 4.8)	2.4 (1.3 to 4.1)	2.5 (1.4 to 4.2)	-17.1 (-58.1 to 68.6)	4.5 (-37.0 to 85.0)
Unsafe sanitation	5.4 (2.6 to 9.7)	2.7 (1.3 to 5.0)	2.3 (1.1 to 4.4)	-49.9 (-77.8 to 10.8)	-14.9 (-55.4 to 65.7)	5.4 (2.6 to 9.7)	2.7 (1.3 to 5.0)	2.3 (1.1 to 4.4)	-49.9 (-77.8 to 10.8)	-14.9 (-55.4 to 65.7)
No access to handwashing facility	7.0 (5.1 to 9.5)	6.5 (4.8 to 8.8)	6.1 (4.4 to 8.1)	-5.9 (-32.6 to 35.1)	-7.4 (-28.0 to 16.3)	7.0 (5.1 to 9.5)	6.5 (4.8 to 8.8)	6.1 (4.4 to 8.1)	-5.9 (-32.6 to 35.1)	-7.4 (-28.0 to 16.3)
Ambient particulate matter pollution	37.9 (24.9 to 56.9)	38.9 (25.5 to 58.7)	33.0 (20.8 to 51.2)	2.7 (-29.0 to 47.9)	-15.2 (-20.2 to -11.3)	37.6 (24.6 to 56.7)	38.8 (25.5 to 58.7)	32.9 (20.8 to 51.2)	3.3 (-28.7 to 49.8)	-15.2 (-20.2 to -11.3)
Household air pollution from solid fuels	0.7 (0.2 to 1.9)	0.2 (0.0 to 0.4)	0.1 (0.0 to 0.3)	-76.7 (-92.9 to -49.1)	-13.1 (-62.2 to 78.3)	1.0 (0.3 to 5.9)	0.3 (0.0 to 1.2)	0.2 (0.0 to 1.2)	-73.2 (-92.9 to -44.4)	-10.8 (-61.4 to 79.9)
Ambient ozone pollution	41.1 (14.4 to 54.8)	41.4 (14.6 to 54.8)	42.1 (14.9 to 54.8)	0.7 (-3.2 to 5.4)	1.6 (-1.8 to 7.1)	41.1 (14.4 to 54.8)	41.4 (14.6 to 54.8)	42.1 (14.9 to 54.8)	0.7 (-3.2 to 5.4)	1.6 (-1.8 to 7.1)
Residential radon	8.5 (0.2 to 28.0)	8.5 (0.2 to 28.0)	8.5 (0.2 to 28.0)	0.0 (0.0 to 0.0)	0.0 (0.0 to 0.0)	8.5 (0.2 to 28.0)	8.5 (0.2 to 28.0)	8.5 (0.2 to 28.0)	0.0 (0.0 to 0.0)	0.0 (0.0 to 0.0)
Lead exposure	3.9 (0.9 to 7.9)	2.6 (0.5 to 5.9)	1.9 (0.3 to 4.6)	-32.8 (-45.5 to -24.3)	-28.0 (-42.0 to -20.3)	2.0 (0.2 to 5.2)	1.2 (0.1 to 3.7)	0.9 (0.0 to 2.9)	-38.3 (-54.6 to -28.7)	-30.6 (-51.5 to -21.1)
Occupational exposure to asbestos	1.7 (1.5 to 1.9)	2.6 (2.4 to 2.9)	2.5 (2.1 to 3.1)	51.6 (33.3 to 75.1)	-3.5 (-21.2 to 17.3)	0.5 (0.4 to 0.5)	0.6 (0.6 to 0.7)	0.7 (0.5 to 0.8)	29.8 (11.5 to 53.6)	8.2 (-14.5 to 34.3)
Occupational exposure to arsenic	0.5 (0.0 to 1.1)	0.4 (0.0 to 1.0)	0.4 (0.0 to 1.0)	-	-	0.4 (0.0 to 0.9)	0.4 (0.0 to 0.9)	0.4 (0.0 to 0.9)	-	-
Occupational exposure to benzene	0.6 (0.0 to 1.8)	0.6 (0.0 to 1.8)	0.7 (0.0 to 1.9)	2.2 (-4.7 to 13.2)	3.4 (-2.3 to 11.3)	0.6 (0.0 to 1.6)	0.7 (0.0 to 2.0)	0.8 (0.0 to 2.3)	22.8 (8.5 to 42.1)	14.8 (5.1 to 30.5)
Occupational exposure to beryllium	0.0 (0.0 to 0.0)	0.0 (0.0 to 0.0)	0.0 (0.0 to 0.0)	-5.5 (-10.2 to -0.3)	2.0 (-1.8 to 6.4)	0.0 (0.0 to 0.0)	0.0 (0.0 to 0.0)	0.0 (0.0 to 0.0)	2.6 (-7.0 to 13.2)	9.7 (3.3 to 17.1)
Occupational exposure to cadmium	0.1 (0.0 to 0.1)	0.0 (0.0 to 0.1)	0.0 (0.0 to 0.1)	-6.4 (-17.2 to 6.2)	1.0 (-9.0 to 12.2)	0.0 (0.0 to 0.1)	0.0 (0.0 to 0.0)	0.0 (0.0 to 0.1)	-7.5 (-21.8 to 10.6)	5.5 (-7.6 to 19.7)
Occupational exposure to chromium	0.1 (0.1 to 0.1)	0.1 (0.1 to 0.1)	0.1 (0.1 to 0.1)	-4.8 (-14.7 to 6.5)	1.6 (-7.4 to 11.6)	0.1 (0.1 to 0.1)	0.1 (0.1 to 0.1)	0.1 (0.1 to 0.1)	-2.9 (-16.7 to 13.9)	7.3 (-4.1 to 19.8)
Occupational exposure to diesel engine exhaust	0.6 (0.6 to 0.6)	0.6 (0.5 to 0.6)	0.6 (0.5 to 0.6)	-3.1 (-11.3 to 5.9)	3.2 (-3.2 to 9.7)	0.4 (0.4 to 0.5)	0.5 (0.4 to 0.5)	0.5 (0.5 to 0.6)	11.9 (-2.6 to 28.2)	12.0 (0.6 to 24.5)
Occupational exposure to formaldehyde	0.3 (0.2 to 0.3)	0.2 (0.2 to 0.3)	0.2 (0.2 to 0.3)	-5.7 (-17.3 to 8.0)	0.6 (-9.9 to 12.0)	0.2 (0.2 to 0.2)	0.2 (0.2 to 0.2)	0.2 (0.2 to 0.2)	-4.7 (-19.6 to 14.0)	6.2 (-6.6 to 20.8)
Occupational exposure to nickel	0.4 (0.0 to 2.1)	0.4 (0.0 to 1.8)	0.4 (0.0 to 1.7)	-	-	0.3 (0.0 to 1.6)	0.3 (0.0 to 1.3)	0.3 (0.0 to 1.4)	-	-
Occupational exposure to polycyclic aromatic hydrocarbons	0.2 (0.2 to 0.3)	0.2 (0.2 to 0.3)	0.2 (0.2 to 0.3)	-4.1 (-13.3 to 6.7)	1.9 (-6.5 to 10.8)	0.2 (0.2 to 0.2)	0.2 (0.2 to 0.2)	0.2 (0.2 to 0.3)	-0.9 (-14.0 to 14.8)	8.2 (-2.3 to 19.8)
Occupational exposure to silica	4.8 (0.7 to 15.5)	4.4 (0.6 to 14.6)	4.5 (0.6 to 14.1)	-8.3 (-17.8 to 3.4)	1.1 (-7.8 to 11.9)	3.2 (0.4 to 10.2)	2.7 (0.4 to 8.9)	2.8 (0.4 to 9.0)	-12.8 (-26.8 to 6.5)	2.4 (-11.8 to 20.1)
Occupational exposure to sulphuric acid	0.6 (0.1 to 2.0)	0.6 (0.1 to 1.9)	0.6 (0.1 to 1.8)	-6.3 (-16.3 to 5.8)	1.8 (-7.8 to 12.2)	0.5 (0.1 to 1.7)	0.5 (0.1 to 1.5)	0.5 (0.1 to 1.6)	-8.9 (-23.8 to 8.3)	4.7 (-8.4 to 20.4)
Occupational exposure to trichloroethylene	0.1 (0.1 to 0.1)	0.1 (0.1 to 0.1)	0.1 (0.1 to 0.1)	-3.8 (-11.3 to 4.4)	2.1 (-4.4 to 9.1)	0.1 (0.1 to 0.1)	0.1 (0.1 to 0.1)	0.1 (0.1 to 0.1)	1.7 (-10.2 to 16.1)	9.0 (0.3 to 19.5)
Occupational asthmagens	18.8 (15.1 to 23.8)	17.9 (14.4 to 22.6)	17.8 (14.3 to 22.5)	-4.8 (-22.8 to 17.4)	-0.7 (-16.4 to 17.4)	8.2 (6.5 to 10.2)	8.2 (6.5 to 10.1)	8.6 (7.0 to 10.9)	-0.2 (-15.8 to 18.7)	5.4 (-7.4 to 21.0)
Occupational particulate matter, gases, and fumes	6.5 (4.6 to 9.4)	6.2 (4.4 to 9.0)	6.0 (4.3 to 8.5)	-4.6 (-9.6 to 0.3)	-3.8 (-7.2 to -0.5)	3.6 (2.5 to 5.4)	3.4 (2.4 to 5.1)	3.3 (2.3 to 4.9)	-4.9 (-13.0 to 4.6)	-4.2 (-9.8 to 1.8)
Occupational noise	6.1 (5.6 to 6.8)	5.9 (5.4 to 6.5)	5.7 (5.3 to 6.3)	-3.8 (-6.9 to -0.5)	-2.9 (-5.0 to -0.7)	3.4 (2.9 to 4.0)	3.5 (3.0 to 3.9)	3.5 (3.1 to 4.0)	0.7 (-5.4 to 7.4)	1.9 (-2.3 to 6.2)
Occupational ergonomic factors	10.8 (8.9 to 12.9)	9.6 (8.0 to 11.6)	9.5 (7.7 to 11.4)	-11.2 (-22.8 to 2.2)	-1.3 (-12.3 to 11.6)	5.8 (4.4 to 7.4)	5.8 (4.5 to 7.3)	6.5 (5.0 to 8.4)	0.8 (-21.8 to 31.0)	11.6 (-8.8 to 34.8)
Non-exclusive breastfeeding	0.4 (0.3 to 0.6)	0.4 (0.3 to 0.6)	0.4 (0.3 to 0.6)	-1.4 (-14.9 to 13.7)	-1.6 (-11.7 to 9.7)	0.4 (0.3 to 0.6)	0.4 (0.3 to 0.6)	0.4 (0.3 to 0.6)	-0.7 (-14.2 to 14.5)	-2.1 (-12.2 to 9.2)
Discontinued breastfeeding	1.6 (1.5 to 1.8)	1.7 (1.5 to 1.8)	1.6 (1.5 to 1.8)	3.9 (-5.6 to 15.4)	-2.5 (-10.3 to 5.6)	1.6 (1.5 to 1.8)	1.7 (1.5 to 1.8)	1.7 (1.5 to 1.8)	2.9 (-6.4 to 14.3)	-1.6 (-9.4 to 6.6)
Child underweight	0.3 (0.2 to 0.4)	0.2 (0.1 to 0.4)	0.2 (0.1 to 0.3)	-15.9 (-36.1 to 8.7)	-5.8 (-21.3 to 12.6)	0.3 (0.2 to 0.4)	0.3 (0.2 to 0.4)	0.2 (0.1 to 0.3)	-18.1 (-39.2 to 6.7)	-7.2 (-24.6 to 12.6)
Child wasting	0.2 (0.1 to 0.3)	0.2 (0.1 to 0.3)	0.2 (0.1 to 0.3)	-8.3 (-36.9 to 31.2)	-2.6 (-24.7 to 23.3)	0.2 (0.1 to 0.4)	0.2 (0.1 to 0.3)	0.2 (0.1 to 0.3)	-10.0 (-36.4 to 31.4)	-2.9 (-24.8 to 24.0)
Child stunting	0.7 (0.3 to 1.1)	0.6 (0.3 to 1.0)	0.6 (0.3 to 1.0)	-10.2 (-45.1 to 49.8)	-3.8 (-33.4 to 36.7)	0.7 (0.3 to 1.2)	0.6 (0.3 to 1.1)	0.6 (0.3 to 1.0)	-11.3 (-48.2 to 51.2)	-5.0 (-34.6 to 34.2)
Short gestation for birth weight	0.0 (0.0 to 0.0)	0.0 (0.0 to 0.0)	0.0 (0.0 to 0.0)	11.5 (-8.7 to 43.2)	2.6 (-16.1 to 34.3)	0.0 (0.0 to 0.0)	0.0 (0.0 to 0.0)	0.0 (0.0 to 0.0)	17.8 (-7.1 to 118.5)	0.3 (-20.6 to 33.9)
Low birth weight for gestation	0.0 (0.0 to 0.0)	0.0 (0.0 to 0.0)	0.0 (0.0 to 0.0)	4.7 (-18.5 to 25.5)	0.7 (-18.8 to 20.0)	0.0 (0.0 to 0.0)	0.0 (0.0 to 0.0)	0.0 (0.0 to 0.0)	-7.6 (-64.9 to 21.8)	3.8 (-17.6 to 26.5)
Iron deficiency	-	-	-	-	-	8.6 (5.9 to 11.6)	8.7 (6.2 to 11.8)	8.0 (5.5 to 11.2)	0.5 (-20.5 to 30.6)	-8.2 (-24.5 to 9.0)
Vitamin A deficiency	1.1 (0.7 to 1.5)	0.9 (0.6 to 1.2)	0.8 (0.5 to 1.0)	-19.1 (-36.9 to -0.1)	-16.1 (-30.8 to 11.6)	0.9 (0.6 to 1.2)	0.9 (0.6 to 1.2)	0.8 (0.5 to 1.2)	4.4 (-10.3 to 22.1)	-9.0 (-29.3 to 10.8)
Zinc deficiency	0.5 (0.2 to 1.1)	0.4 (0.1 to 0.9)	0.4 (0.1 to 0.8)	-28.9 (-65.2 to 71.6)	-0.5 (-64.0 to 174.3)	0.5 (0.1 to 1.1)	0.4 (0.1 to 0.9)	0.4 (0.1 to 0.9)	-24.8 (-61.0 to 124.4)	5.2 (-44.6 to 158.5)
Smoking	17.3 (13.9 to 20.8)	12.3 (9.9 to 15.0)	10.5 (8.4 to 12.9)	-28.8 (-38.5 to -18.8)	-14.9 (-23.2 to -6.0)	3.9 (2.8 to 5.3)	3.3 (2.4 to 4.5)	3.2 (2.3 to 4.4)	-15.2 (-34.3 to 10.2)	-3.1 (-19.3 to 16.2)
Chewing tobacco	1.4 (0.9 to 2.0)	0.9 (0.6 to 1.4)	0.8 (0.5 to 1.1)	-32.7 (-56.9 to 3.9)	-18.6 (-42.2 to 13.1)	0.1 (0.1 to 0.1)	0.1 (0.1 to 0.1)	0.1 (0.1 to 0.1)	-4.4 (-40.8 to 55.8)	-2.4 (-32.3 to 40.1)
Second-hand smoke	38.7 (34.3 to 43.0)	37.5 (33.4 to 41.7)	37.1 (32.9 to 41.3)	-3.1 (-13.8 to 8.9)	-0.9 (-9.4 to 8.2)	59.0 (55.1 to 62.4)	51.6 (48.0 to 55.4)	51.3 (47.4 to 55.3)	-12.5 (-18.4 to -6.1)	-0.6 (-6.0 to 5.3)
Alcohol use	13.6 (6.8 to 23.1)	13.5 (6.9 to 23.9)	15.4 (7.4 to 27.5)	-0.5 (-36.4 to 48.3)	14.1 (-20.6 to 62.8)	11.0 (5.4 to 19.3)	10.9 (5.4 to 19.5)	11.9 (5.5 to 21.1)	-0.8 (-36.7 to 50.0)	9.6 (-23.0 to 55.5)
Drug use	0.5 (0.4 to 0.7)	0.5 (0.4 to 0.7)	0.6 (0.4 to 0.8)	0.8 (-5.5 to 6.6)	11.5 (4.5 to 21.0)	0.3 (0.2 to 0.4)	0.3 (0.2 to 0.4)	0.3 (0.3 to 0.4)	2.2 (-3.7 to 8.5)	9.6 (3.1 to 17.8)
Diet low in fruits	42.7 (37.0 to 46.0)	41.6 (36.0 to 45.2)	43.0 (37.3 to 46.4)	-2.5 (-7.5 to 2.3)	3.4 (-0.1 to 7.6)	40.1 (34.5 to 43.9)	38.3 (32.3 to 42.5)	40.4 (34.9 to 44.4)	-4.6 (-11.5 to 1.7)	5.4 (0.3 to 12.4)
Diet low in vegetables	23.1 (14.4 to 30.1)	17.4 (9.1 to 25.0)	19.6 (10.9 to 27.4)	-24.4 (-50.2 to -2.4)	12.5 (-11.7 to 52.2)	25.5 (16.8 to 32.3)	19.8 (11.2 to 27.2)	21.6 (13.1 to 28.8)	-22.4 (-46.2 to -2.1)	9.1 (-11.0 to 42.0)
Diet low in legumes	4.5 (0.7 to 10.1)	5.6 (1.1 to 11.3)	7.4 (2.2 to 13.8)	22.7 (-46.0 to 267.8)	32.2 (-21.7 to 183.6)	8.2 (2.6 to 14.9)	7.5 (2.0 to 13.5)	9.5 (3.2 to 16.2)	-8.4 (-53.6 to 57.7)	27.3 (-18.2 to 134.1)
Diet low in whole grains	31.8 (27.1 to 35.7)	36.4 (32.8 to 39.7)	37.7 (34.1 to 41.0)	14.3 (6.0 to 25.9)	3.6 (-1.0 to 8.6)	33.2 (28.7 to 36.9)	37.5 (33.8 to 40.7)	38.7 (35.3 to 41.7)	13.2 (6.1 to 22.6)	3.2 (-0.8 to 7.9)
Diet low in nuts and seeds	51.8 (50.8 to 52.6)	50.9 (49.6 to 51.9)	50.8 (49.4 to 51.9)	-1.7 (-3.5 to -0.2)	-0.2 (-1.6 to 1.1)	52.0 (51.1 to 52.8)	51.2 (50.0 to 52.2)	51.2 (49.9 to 52.2)	-1.5 (-3.2 to -0.2)	-0.1 (-1.4 to 1.2)
Diet low in milk	46.8 (44.8 to 48.5)	47.0 (45.2 to 48.6)	47.4 (45.7 to 49.0)	0.5 (-1.2 to 2.3)	0.9 (-0.4 to 2.3)	46.9 (45.0 to 48.5)	46.7 (44.7 to 48.3)	47.3 (45.4 to 48.8)	-0.4 (-2.3 to 1.4)	1.3 (-0.1 to 2.8)
Diet high in red meat	2.8 (0.2 to 7.1)	5.8 (1.4 to 12.2)	6.7 (1.9 to 13.3)	105.9 (-27.4 to 2050.6)	16.6 (-43.4 to 192.1)	0.3 (0.0 to 1.6)	1.0 (0.0 to 3.7)	1.4 (0.0 to 4.9)	206.9 (-82.5 to [illegible]	42.3 (-95.3 to 8172.4)
Diet high in processed meat	10.4 (6.5 to 14.2)	18.6 (13.9 to 23.3)	22.4 (16.7 to 27.6)	78.2 (39.1 to 146.7)	20.6 (1.6 to 45.1)	8.8 (5.1 to 12.3)	13.7 (9.5 to 17.4)	15.7 (11.0 to 20.1)	55.5 (23.0 to 118.0)	14.7 (-4.3 to 37.9)
Diet high in sugar-sweetened beverages	5.7 (2.2 to 11.8)	6.5 (2.6 to 12.3)	6.8 (3.1 to 13.0)	12.8 (-48.5 to 153.2)	5.0 (-37.2 to 72.8)	4.1 (1.5 to 9.3)	4.6 (2.0 to 9.1)	5.0 (2.0 to 10.1)	12.8 (-48.9 to 156.0)	7.0 (-38.4 to 96.5)
Diet low in fibre	24.0 (16.6 to 30.4)	25.4 (18.2 to 31.5)	26.6 (19.3 to 32.9)	5.7 (-9.6 to 26.7)	4.8 (-5.8 to 17.7)	25.3 (18.2 to 31.5)	26.8 (19.9 to 32.7)	28.3 (21.4 to 34.0)	5.8 (-9.5 to 24.9)	5.8 (-3.6 to 19.6)
Diet low in calcium	36.9 (33.0 to 40.1)	36.8 (32.8 to 40.0)	37.6 (33.7 to 40.7)	-0.4 (-3.6 to 2.9)	2.2 (-0.6 to 5.0)	39.0 (35.5 to 41.9)	38.2 (34.4 to 41.2)	39.3 (35.9 to 42.2)	-2.2 (-5.5 to 0.9)	3.1 (0.7 to 5.9)
Diet low in seafood omega-3 fatty acids	3.7 (1.9 to 5.2)	0.4 (0.0 to 1.3)	0.4 (0.0 to 1.4)	-90.4 (-99.9 to -68.6)	5.2 (-86.2 to 831.3)	4.3 (2.5 to 5.8)	0.5 (0.0 to 1.6)	0.6 (0.0 to 1.7)	-87.9 (-99.8 to -64.8)	10.2 (-69.5 to 504.9)
Diet low in polyunsaturated fatty acids	32.3 (27.2 to 36.7)	29.7 (24.1 to 34.5)	30.3 (24.8 to 35.1)	-7.9 (-18.1 to 1.2)	2.1 (-5.5 to 11.6)	32.9 (28.0 to 37.5)	30.5 (25.1 to 35.2)	31.1 (25.7 to 35.5)	-7.4 (-16.9 to 1.2)	2.0 (-4.5 to 8.7)
Diet high in trans fatty acids	1.1 (0.0 to 3.3)	0.9 (0.0 to 3.0)	0.9 (0.0 to 2.9)	-12.5 (-38.4 to -6.8)	-8.3 (-32.2 to -3.7)	1.5 (0.1 to 4.0)	1.3 (0.1 to 3.7)	1.2 (0.1 to 3.5)	-12.3 (-34.0 to -6.3)	-8.5 (-29.0 to -3.8)
Diet high in sodium	19.0 (9.7 to 26.7)	15.0 (6.0 to 22.4)	13.1 (4.4 to 20.7)	-20.7 (-49.7 to 2.7)	-12.8 (-39.1 to 6.6)	20.3 (11.1 to 28.1)	15.8 (7.2 to 22.8)	13.4 (4.7 to 20.8)	-22.4 (-48.3 to -4.0)	-15.0 (-42.1 to -0.0)
Intimate partner violence	-	-	-	-	-	2.6 (2.2 to 3.0)	2.4 (2.0 to 2.7)	2.3 (1.9 to 2.6)	-7.9 (-11.9 to -3.7)	-4.2 (-8.1 to 0.2)
Childhood sexual abuse	5.3 (4.3 to 6.5)	5.3 (4.3 to 6.5)	5.3 (4.3 to 6.5)	0.0 (0.0 to 0.0)	0.0 (0.0 to 0.0)	7.3 (5.9 to 8.9)	7.3 (5.9 to 8.9)	7.5 (6.0 to 9.2)	0.0 (0.0 to 0.0)	2.8 (-4.0 to 10.6)

Supplementary Results Table 1. Okinawa age-standardised Summary Exposure Values (SEVs) for Level 4 risk factors and percentage change, by sex, 1990, 2007, and 2017.Data in parentheses are 95% uncertainty intervals.

	Male					Female				
	**1990**	**2007**	**2017**	**Percentage change 1990-2007**	**Percentage change 2007-2017**	**1990**	**2007**	**2017**	**Percentage change 1990-2007**	**Percentage change 2007-2017**
Bullying victimization	3.3 (1.3 to 6.9)	3.4 (1.4 to 7.3)	3.5 (1.4 to 7.6)	3.2 (-4.7 to 10.9)	2.4 (-2.7 to 7.2)	1.9 (0.8 to 4.0)	2.0 (0.8 to 4.1)	2.0 (0.8 to 4.2)	1.1 (-6.5 to 9.1)	1.3 (-4.4 to 6.1)
Low physical activity	0.5 (0.2 to 0.9)	0.5 (0.2 to 0.9)	0.4 (0.2 to 0.8)	-1.9 (-3.8 to 0.1)	-8.7 (-12.2 to -4.6)	0.5 (0.2 to 1.0)	0.5 (0.2 to 0.9)	0.5 (0.2 to 0.9)	-3.9 (-6.2 to -1.3)	0.7 (-0.7 to 2.3)
High fasting plasma glucose	1.3 (0.8 to 2.1)	1.2 (0.8 to 1.9)	1.4 (0.9 to 2.2)	-1.0 (-39.7 to 51.0)	11.6 (-20.6 to 76.5)	0.8 (0.5 to 1.4)	1.0 (0.6 to 1.7)	1.2 (0.7 to 2.1)	23.9 (-15.3 to 86.2)	14.8 (-21.6 to 74.2)
High LDL cholesterol	11.9 (10.2 to 13.7)	12.4 (10.9 to 14.3)	12.5 (10.8 to 14.3)	4.5 (-1.1 to 10.4)	0.1 (-4.1 to 5.0)	12.6 (11.0 to 14.3)	12.7 (11.1 to 14.4)	12.7 (11.2 to 14.5)	0.9 (-4.6 to 6.8)	0.3 (-4.0 to 4.9)
High systolic blood pressure	8.3 (7.4 to 9.2)	7.4 (6.5 to 8.2)	7.5 (6.5 to 8.4)	-10.8 (-17.8 to -3.5)	1.1 (-6.5 to 9.7)	7.6 (6.8 to 8.4)	5.9 (5.1 to 6.7)	5.6 (4.8 to 6.4)	-22.1 (-29.5 to -13.8)	-5.0 (-15.5 to 5.5)
High body-mass index	5.1 (3.1 to 8.5)	7.3 (4.8 to 11.5)	7.5 (4.9 to 12.0)	43.6 (15.0 to 84.0)	3.7 (-13.0 to 21.7)	4.9 (3.0 to 8.1)	6.3 (4.0 to 9.9)	6.3 (3.9 to 10.2)	28.0 (-1.8 to 70.9)	0.7 (-17.1 to 23.1)
Low bone mineral density	5.3 (4.4 to 6.3)	5.2 (4.4 to 6.1)	5.2 (4.3 to 6.2)	-2.1 (-14.9 to 12.6)	0.6 (-12.7 to 14.6)	6.6 (5.7 to 7.5)	6.5 (5.7 to 7.4)	6.7 (5.5 to 7.9)	-0.8 (-11.0 to 11.3)	1.8 (-11.1 to 15.4)
Impaired kidney function	2.7 (1.2 to 6.5)	2.6 (1.2 to 6.6)	2.5 (1.2 to 6.3)	-1.0 (-5.6 to 3.5)	-4.3 (-9.2 to 0.1)	3.3 (1.5 to 7.5)	3.5 (1.6 to 7.9)	3.3 (1.6 to 7.5)	5.3 (1.1 to 10.2)	-5.2 (-10.3 to -0.9)

Supplementary Results Table 1. Osaka age-standardised Summary Exposure Values (SEVs) for Level 4 risk factors and percentage change, by sex, 1990, 2007, and 2017.Data in parentheses are 95% uncertainty intervals.										
	Male					Female				
	1990	2007	2017	Percentage change 1990-2007	Percentage change 2007-2017	1990	2007	2017	Percentage change 1990-2007	Percentage change 2007-2017
Unsafe water source	1.9 (1.0 to 3.1)	1.6 (0.9 to 2.6)	1.7 (0.9 to 2.8)	-16.0 (-55.1 to 63.3)	7.4 (-35.6 to 83.9)	1.9 (1.0 to 3.1)	1.6 (0.9 to 2.6)	1.7 (0.9 to 2.8)	-16.0 (-55.1 to 63.3)	7.4 (-35.6 to 83.9)
Unsafe sanitation	3.0 (1.4 to 5.3)	1.5 (0.7 to 2.8)	1.3 (0.6 to 2.6)	-49.3 (-77.6 to 16.1)	-12.8 (-54.5 to 73.7)	3.0 (1.4 to 5.3)	1.5 (0.7 to 2.8)	1.3 (0.6 to 2.6)	-49.3 (-77.6 to 16.1)	-12.8 (-54.5 to 73.7)
No access to handwashing facility	6.5 (4.8 to 8.8)	6.2 (4.5 to 8.4)	5.8 (4.2 to 7.8)	-4.2 (-32.1 to 34.9)	-6.8 (-25.9 to 19.4)	6.5 (4.8 to 8.8)	6.2 (4.5 to 8.4)	5.8 (4.2 to 7.8)	-4.2 (-32.1 to 34.9)	-6.8 (-25.9 to 19.4)
Ambient particulate matter pollution	36.7 (25.6 to 52.0)	36.8 (25.7 to 52.6)	31.7 (21.5 to 47.5)	0.1 (-18.7 to 21.2)	-13.9 (-29.1 to 3.1)	36.6 (25.5 to 52.0)	36.8 (25.7 to 52.6)	31.7 (21.5 to 47.4)	0.4 (-18.7 to 21.5)	-13.9 (-29.1 to 3.1)
Household air pollution from solid fuels	0.3 (0.1 to 0.8)	0.1 (0.0 to 0.2)	0.1 (0.0 to 0.1)	-70.9 (-92.9 to -45.8)	-9.5 (-65.7 to 95.0)	0.5 (0.1 to 2.8)	0.2 (0.0 to 0.5)	0.2 (0.0 to 0.5)	-67.9 (-93.0 to -42.1)	-9.0 (-65.8 to 96.0)
Ambient ozone pollution	51.9 (26.0 to 54.8)	52.2 (26.8 to 54.8)	52.6 (28.2 to 54.8)	0.6 (0.0 to 4.5)	0.6 (0.0 to 5.6)	51.9 (26.0 to 54.8)	52.2 (26.8 to 54.8)	52.6 (28.2 to 54.8)	0.6 (0.0 to 4.5)	0.6 (0.0 to 5.6)
Residential radon	12.3 (0.8 to 42.7)	12.3 (0.8 to 42.7)	12.3 (0.8 to 42.7)	0.0 (0.0 to 0.0)	0.0 (0.0 to 0.0)	12.3 (0.8 to 42.7)	12.3 (0.8 to 42.7)	12.3 (0.8 to 42.7)	0.0 (0.0 to 0.0)	0.0 (0.0 to 0.0)
Lead exposure	3.7 (0.8 to 7.4)	2.2 (0.4 to 5.2)	1.5 (0.2 to 3.9)	-39.8 (-54.7 to -29.6)	-33.2 (-48.7 to -24.8)	2.5 (0.3 to 6.0)	1.5 (0.1 to 4.2)	1.0 (0.1 to 3.1)	-40.6 (-58.8 to -29.7)	-33.8 (-52.6 to -26.1)
Occupational exposure to asbestos	3.8 (3.2 to 4.2)	4.8 (4.5 to 5.2)	4.8 (4.0 to 5.6)	28.2 (11.7 to 54.7)	-1.1 (-17.7 to 17.6)	1.0 (0.8 to 1.2)	1.1 (1.0 to 1.2)	1.0 (0.8 to 1.2)	1.8 (-15.6 to 31.0)	-8.9 (-26.9 to 12.3)
Occupational exposure to arsenic	0.5 (0.0 to 1.1)	0.4 (0.0 to 1.0)	0.4 (0.0 to 1.0)	-	-	0.4 (0.0 to 1.0)	0.4 (0.0 to 0.9)	0.4 (0.0 to 0.9)	-	-
Occupational exposure to benzene	0.6 (0.0 to 1.8)	0.7 (0.0 to 1.9)	0.7 (0.0 to 2.0)	2.6 (-3.9 to 13.8)	3.5 (-2.3 to 11.7)	0.6 (0.0 to 1.8)	0.8 (0.0 to 2.1)	0.9 (0.1 to 2.4)	19.1 (7.5 to 37.8)	13.3 (4.0 to 26.1)
Occupational exposure to beryllium	0.0 (0.0 to 0.0)	0.0 (0.0 to 0.0)	0.0 (0.0 to 0.0)	-5.5 (-9.9 to -0.7)	1.9 (-1.6 to 5.7)	0.0 (0.0 to 0.0)	0.0 (0.0 to 0.0)	0.0 (0.0 to 0.0)	0.6 (-7.4 to 9.0)	8.2 (2.3 to 14.8)
Occupational exposure to cadmium	0.1 (0.0 to 0.1)	0.0 (0.0 to 0.1)	0.1 (0.0 to 0.1)	-8.0 (-18.5 to 3.2)	0.9 (-8.2 to 10.9)	0.1 (0.0 to 0.1)	0.0 (0.0 to 0.1)	0.0 (0.0 to 0.1)	-11.0 (-23.6 to 4.4)	3.9 (-7.6 to 16.6)
Occupational exposure to chromium	0.1 (0.1 to 0.1)	0.1 (0.1 to 0.1)	0.1 (0.1 to 0.1)	-6.5 (-15.8 to 3.6)	1.4 (-6.4 to 10.1)	0.1 (0.1 to 0.1)	0.1 (0.1 to 0.1)	0.1 (0.1 to 0.1)	-6.3 (-18.4 to 7.2)	5.6 (-4.4 to 16.4)
Occupational exposure to diesel engine exhaust	0.6 (0.6 to 0.6)	0.6 (0.5 to 0.6)	0.6 (0.6 to 0.6)	-3.0 (-10.6 to 5.1)	2.8 (-3.4 to 9.6)	0.5 (0.4 to 0.5)	0.5 (0.5 to 0.6)	0.6 (0.5 to 0.7)	10.3 (-2.5 to 24.3)	10.0 (-0.3 to 20.5)
Occupational exposure to formaldehyde	0.3 (0.2 to 0.3)	0.2 (0.2 to 0.3)	0.2 (0.2 to 0.3)	-7.5 (-18.7 to 4.2)	0.5 (-8.8 to 10.9)	0.2 (0.2 to 0.3)	0.2 (0.2 to 0.2)	0.2 (0.2 to 0.2)	-8.3 (-21.8 to 7.7)	4.6 (-6.9 to 16.9)
Occupational exposure to nickel	0.4 (0.0 to 2.0)	0.4 (0.0 to 1.8)	0.4 (0.0 to 1.8)	-	-	0.4 (0.0 to 1.6)	0.3 (0.0 to 1.4)	0.3 (0.0 to 1.4)	-	-
Occupational exposure to polycyclic aromatic hydrocarbons	0.3 (0.2 to 0.3)	0.2 (0.2 to 0.3)	0.2 (0.2 to 0.3)	-5.4 (-14.2 to 3.9)	1.7 (-5.4 to 9.5)	0.2 (0.2 to 0.3)	0.2 (0.2 to 0.3)	0.2 (0.2 to 0.3)	-4.2 (-16.1 to 9.0)	6.5 (-3.0 to 16.4)
Occupational exposure to silica	4.9 (0.7 to 15.7)	4.5 (0.6 to 14.8)	4.6 (0.6 to 14.5)	-7.4 (-16.6 to 3.6)	1.1 (-6.8 to 11.1)	3.3 (0.5 to 11.3)	2.8 (0.4 to 9.2)	2.8 (0.4 to 9.1)	-15.9 (-28.9 to -0.1)	1.0 (-11.5 to 17.1)
Occupational exposure to sulphuric acid	0.6 (0.1 to 2.1)	0.6 (0.1 to 1.9)	0.6 (0.1 to 1.9)	-8.1 (-17.7 to 2.8)	1.4 (-7.0 to 11.0)	0.5 (0.1 to 1.7)	0.5 (0.1 to 1.5)	0.5 (0.1 to 1.6)	-12.2 (-25.2 to 3.0)	3.1 (-9.0 to 17.1)
Occupational exposure to trichloroethylene	0.1 (0.1 to 0.1)	0.1 (0.1 to 0.1)	0.1 (0.1 to 0.1)	-5.4 (-12.1 to 1.7)	1.8 (-3.8 to 8.3)	0.1 (0.1 to 0.1)	0.1 (0.1 to 0.1)	0.1 (0.1 to 0.1)	-1.3 (-11.2 to 9.8)	7.2 (-0.9 to 16.4)
Occupational asthmagens	20.1 (16.1 to 25.2)	18.8 (15.1 to 23.9)	18.8 (14.9 to 23.7)	-6.2 (-23.0 to 14.2)	-0.3 (-15.9 to 17.1)	8.7 (7.0 to 10.8)	8.6 (6.9 to 10.7)	9.0 (7.2 to 11.1)	-1.6 (-18.3 to 14.5)	4.6 (-9.1 to 18.0)
Occupational particulate matter, gases, and fumes	6.4 (4.5 to 9.2)	6.1 (4.4 to 9.0)	5.9 (4.2 to 8.5)	-4.5 (-9.5 to 0.9)	-3.7 (-6.8 to -0.3)	3.8 (2.6 to 5.7)	3.6 (2.5 to 5.4)	3.4 (2.4 to 5.1)	-6.3 (-14.0 to 2.1)	-5.2 (-10.4 to 0.1)
Occupational noise	5.9 (5.5 to 6.5)	5.8 (5.3 to 6.3)	5.6 (5.2 to 6.2)	-3.0 (-6.2 to 0.1)	-2.3 (-4.2 to -0.2)	3.6 (3.2 to 4.2)	3.6 (3.3 to 4.1)	3.7 (3.3 to 4.1)	-0.2 (-5.6 to 5.7)	1.3 (-2.0 to 4.9)
Occupational ergonomic factors	9.7 (8.1 to 11.7)	8.6 (7.2 to 10.4)	8.6 (7.1 to 10.3)	-11.3 (-21.6 to 0.1)	-1.0 (-10.3 to 9.0)	5.2 (4.1 to 6.6)	5.3 (4.2 to 6.7)	6.1 (4.6 to 7.8)	2.5 (-17.5 to 27.6)	13.5 (-5.1 to 34.7)
Non-exclusive breastfeeding	0.4 (0.3 to 0.6)	0.4 (0.3 to 0.6)	0.4 (0.3 to 0.6)	-1.2 (-13.8 to 12.3)	-1.7 (-12.0 to 9.5)	0.4 (0.3 to 0.6)	0.4 (0.3 to 0.6)	0.4 (0.3 to 0.6)	-1.1 (-13.7 to 12.4)	-1.8 (-12.1 to 9.3)
Discontinued breastfeeding	1.7 (1.6 to 1.9)	1.8 (1.6 to 1.9)	1.7 (1.6 to 1.8)	1.0 (-7.8 to 11.0)	-2.5 (-9.2 to 5.2)	1.7 (1.6 to 1.9)	1.8 (1.6 to 1.9)	1.7 (1.6 to 1.8)	1.1 (-7.8 to 11.1)	-2.8 (-9.5 to 4.8)
Child underweight	0.2 (0.1 to 0.3)	0.2 (0.1 to 0.3)	0.2 (0.1 to 0.3)	-12.6 (-31.2 to 7.9)	-4.3 (-17.8 to 11.9)	0.2 (0.1 to 0.4)	0.2 (0.1 to 0.3)	0.2 (0.1 to 0.3)	-14.4 (-33.4 to 10.4)	-4.8 (-19.2 to 12.0)
Child wasting	0.2 (0.1 to 0.3)	0.2 (0.1 to 0.2)	0.2 (0.1 to 0.2)	-7.5 (-32.1 to 20.5)	-1.3 (-19.0 to 19.1)	0.2 (0.1 to 0.3)	0.2 (0.1 to 0.2)	0.2 (0.1 to 0.2)	-9.5 (-35.2 to 24.8)	-1.9 (-20.2 to 19.3)
Child stunting	0.6 (0.3 to 0.9)	0.5 (0.3 to 0.7)	0.5 (0.3 to 0.8)	-12.8 (-44.2 to 34.8)	-2.2 (-31.0 to 39.1)	0.6 (0.3 to 1.0)	0.5 (0.3 to 0.7)	0.5 (0.3 to 0.7)	-11.3 (-44.8 to 41.7)	-3.1 (-30.5 to 35.7)
Short gestation for birth weight	0.0 (0.0 to 0.0)	0.0 (0.0 to 0.0)	0.0 (0.0 to 0.0)	10.0 (-11.3 to 33.6)	2.4 (-13.9 to 33.5)	0.0 (0.0 to 0.0)	0.0 (0.0 to 0.0)	0.0 (0.0 to 0.0)	17.9 (-17.8 to 128.7)	1.5 (-15.4 to 37.2)
Low birth weight for gestation	0.0 (0.0 to 0.0)	0.0 (0.0 to 0.0)	0.0 (0.0 to 0.0)	3.3 (-12.8 to 23.0)	2.5 (-17.5 to 22.2)	0.0 (0.0 to 0.0)	0.0 (0.0 to 0.0)	0.0 (0.0 to 0.0)	-10.3 (-65.7 to 29.9)	3.1 (-18.8 to 22.5)
Iron deficiency	-	-	-	-	-	7.6 (5.1 to 10.6)	7.7 (5.4 to 10.6)	7.0 (4.7 to 10.0)	1.1 (-20.4 to 29.5)	-8.3 (-25.0 to 8.8)
Vitamin A deficiency	0.9 (0.5 to 1.1)	0.7 (0.5 to 0.9)	0.6 (0.4 to 0.8)	-17.7 (-32.2 to 1.1)	-8.3 (-27.1 to 13.4)	0.7 (0.5 to 0.9)	0.7 (0.5 to 1.0)	0.7 (0.4 to 0.9)	12.5 (-9.1 to 36.5)	-10.4 (-23.2 to 4.3)
Zinc deficiency	0.5 (0.1 to 1.0)	0.5 (0.1 to 1.2)	0.5 (0.1 to 1.2)	16.6 (-57.7 to 173.4)	-13.7 (-69.3 to 99.6)	0.5 (0.1 to 1.0)	0.4 (0.1 to 0.9)	0.3 (0.1 to 0.8)	-20.2 (-65.2 to 72.5)	-9.3 (-64.4 to 187.6)
Smoking	16.5 (13.3 to 20.1)	11.8 (9.3 to 14.3)	10.0 (7.9 to 12.2)	-28.9 (-37.7 to -19.2)	-15.1 (-23.3 to -5.9)	3.7 (2.6 to 5.0)	3.1 (2.3 to 4.3)	3.0 (2.2 to 4.1)	-15.6 (-34.9 to 10.9)	-3.5 (-19.1 to 13.2)
Chewing tobacco	1.4 (0.9 to 2.0)	0.9 (0.6 to 1.4)	0.7 (0.5 to 1.1)	-33.3 (-56.0 to 1.8)	-18.9 (-41.1 to 13.6)	0.1 (0.1 to 0.1)	0.1 (0.1 to 0.1)	0.1 (0.1 to 0.1)	-4.5 (-38.2 to 49.9)	-2.3 (-33.1 to 41.1)
Second-hand smoke	37.5 (33.4 to 41.5)	37.1 (33.1 to 41.4)	36.3 (31.9 to 40.6)	-0.9 (-12.2 to 11.3)	-2.1 (-11.4 to 7.6)	57.1 (53.7 to 60.3)	50.3 (46.4 to 53.9)	49.1 (45.3 to 53.0)	-11.8 (-17.8 to -5.5)	-2.4 (-8.2 to 3.7)
Alcohol use	14.7 (7.7 to 25.4)	15.0 (7.8 to 25.0)	17.4 (9.3 to 29.5)	2.1 (-29.3 to 45.5)	16.3 (-14.7 to 58.5)	11.9 (6.2 to 21.0)	12.2 (6.3 to 21.3)	14.2 (7.2 to 24.1)	2.9 (-28.8 to 50.2)	16.5 (-16.0 to 63.4)
Drug use	0.5 (0.4 to 0.7)	0.5 (0.4 to 0.7)	0.6 (0.4 to 0.8)	0.8 (-4.5 to 7.1)	11.5 (4.4 to 20.9)	0.3 (0.2 to 0.4)	0.3 (0.2 to 0.4)	0.3 (0.3 to 0.4)	3.7 (-2.2 to 10.5)	8.3 (1.5 to 16.0)
Diet low in fruits	40.1 (34.4 to 44.0)	38.4 (32.5 to 42.3)	39.9 (34.2 to 43.8)	-4.2 (-10.8 to 2.1)	3.9 (-0.9 to 9.1)	35.9 (29.7 to 40.8)	32.3 (26.3 to 37.0)	35.6 (29.6 to 40.4)	-10.1 (-18.7 to -1.7)	10.3 (3.1 to 19.6)
Diet low in vegetables	18.3 (9.8 to 25.8)	12.9 (5.2 to 20.7)	15.3 (7.1 to 23.5)	-29.1 (-62.0 to -2.2)	18.5 (-13.4 to 82.5)	20.5 (11.9 to 27.6)	15.1 (6.6 to 22.6)	17.2 (9.1 to 24.5)	-26.4 (-54.8 to -0.7)	14.0 (-12.5 to 65.5)
Diet low in legumes	4.3 (0.7 to 9.7)	5.7 (1.1 to 11.3)	7.7 (1.9 to 14.1)	31.9 (-44.2 to 287.0)	34.3 (-20.9 to 218.0)	8.3 (2.7 to 15.1)	8.6 (3.1 to 15.5)	11.3 (5.0 to 18.6)	3.4 (-42.8 to 95.7)	31.5 (-6.5 to 120.9)
Diet low in whole grains	29.7 (24.7 to 34.0)	34.7 (30.6 to 38.2)	36.1 (32.2 to 39.5)	16.7 (7.8 to 30.0)	4.0 (-1.1 to 9.8)	31.1 (26.4 to 35.0)	35.9 (32.1 to 39.0)	37.2 (33.4 to 40.2)	15.3 (7.1 to 26.7)	3.6 (-0.6 to 8.5)
Diet low in nuts and seeds	50.2 (48.7 to 51.4)	48.8 (46.7 to 50.3)	48.6 (46.4 to 50.3)	-2.9 (-5.7 to -0.5)	-0.4 (-3.0 to 1.8)	50.3 (48.8 to 51.4)	48.8 (47.0 to 50.3)	48.9 (46.8 to 50.5)	-2.9 (-5.5 to -0.4)	0.1 (-2.1 to 2.1)
Diet low in milk	45.8 (43.8 to 47.7)	46.2 (44.1 to 48.0)	46.7 (44.7 to 48.4)	0.8 (-1.1 to 2.8)	1.1 (-0.3 to 2.6)	45.4 (43.2 to 47.3)	45.1 (42.7 to 47.0)	46.0 (43.8 to 47.8)	-0.8 (-3.1 to 1.2)	2.1 (0.6 to 4.0)
Diet high in red meat	4.5 (0.7 to 9.3)	8.7 (3.0 to 15.6)	10.0 (3.6 to 17.4)	92.1 (0.3 to 675.8)	15.1 (-34.0 to 111.9)	0.6 (0.0 to 2.7)	1.7 (0.0 to 5.1)	2.3 (0.1 to 6.1)	171.8 (-70.4 to 203811.1)	31.9 (-77.1 to 1228.4)
Diet high in processed meat	12.4 (8.3 to 16.3)	20.3 (14.9 to 25.3)	23.2 (17.4 to 29.3)	63.8 (28.0 to 118.1)	14.8 (-2.5 to 35.5)	10.5 (6.6 to 14.0)	15.4 (10.7 to 19.2)	16.9 (12.0 to 21.3)	46.6 (16.5 to 98.1)	9.7 (-6.5 to 29.5)
Diet high in sugar-sweetened beverages	8.1 (3.2 to 15.2)	9.3 (4.0 to 15.7)	9.8 (4.3 to 17.5)	13.9 (-40.3 to 142.6)	5.6 (-35.7 to 69.8)	5.5 (2.3 to 11.6)	6.4 (3.0 to 11.6)	6.8 (3.0 to 12.2)	14.6 (-45.6 to 125.7)	6.7 (-35.9 to 79.9)
Diet low in fibre	21.8 (14.5 to 28.3)	23.4 (16.8 to 29.8)	24.8 (17.9 to 31.2)	7.3 (-9.3 to 30.2)	6.0 (-5.7 to 20.9)	22.9 (15.8 to 29.2)	24.1 (17.1 to 30.1)	25.7 (18.9 to 31.8)	5.0 (-9.7 to 25.5)	6.8 (-4.6 to 21.4)
Diet low in calcium	33.6 (29.2 to 37.4)	33.6 (29.3 to 37.3)	34.5 (30.1 to 38.1)	-0.2 (-4.0 to 3.5)	2.8 (-0.2 to 6.4)	35.5 (31.3 to 38.9)	34.3 (29.9 to 37.9)	35.9 (31.6 to 39.2)	-3.5 (-7.8 to -0.1)	4.7 (1.7 to 8.3)
Diet low in seafood omega-3 fatty acids	2.7 (0.9 to 4.2)	0.1 (0.0 to 0.7)	0.2 (0.0 to 0.8)	-95.1 (-100.0 to -77.8)	12.7 (-96.8 to 10622.4)	3.3 (1.3 to 4.8)	0.2 (0.0 to 1.0)	0.2 (0.0 to 1.0)	-93.4 (-100.0 to -74.6)	10.1 (-97.8 to 6666.9)
Diet low in polyunsaturated fatty acids	29.4 (23.5 to 34.6)	26.4 (20.4 to 31.6)	27.1 (21.1 to 32.2)	-10.1 (-22.6 to -0.4)	2.5 (-5.5 to 11.2)	29.9 (24.7 to 34.9)	27.1 (21.0 to 32.1)	27.8 (21.5 to 32.8)	-9.5 (-21.2 to -0.3)	2.7 (-5.6 to 12.7)
Diet high in trans fatty acids	1.1 (0.0 to 3.3)	1.0 (0.0 to 3.0)	0.9 (0.0 to 2.9)	-12.3 (-37.9 to -6.4)	-8.3 (-33.2 to -3.8)	1.5 (0.1 to 4.0)	1.3 (0.1 to 3.7)	1.2 (0.1 to 3.5)	-12.4 (-36.2 to -6.1)	-8.5 (-29.2 to -3.6)
Diet high in sodium	21.7 (13.0 to 28.9)	13.3 (4.8 to 20.7)	10.4 (2.5 to 17.6)	-38.6 (-68.7 to -19.5)	-22.0 (-54.7 to -5.8)	21.3 (13.1 to 28.7)	10.8 (3.6 to 17.6)	7.2 (1.4 to 14.1)	-49.3 (-75.1 to -31.7)	-33.0 (-67.4 to -17.1)
Intimate partner violence	-	-	-	-	-	2.3 (2.0 to 2.7)	2.2 (1.8 to 2.5)	2.1 (1.8 to 2.4)	-7.6 (-11.8 to -3.1)	-4.2 (-8.4 to -0.0)
Childhood sexual abuse	5.3 (4.3 to 6.5)	5.3 (4.3 to 6.5)	5.3 (4.3 to 6.5)	0.0 (0.0 to 0.0)	0.0 (0.0 to 0.0)	7.3 (5.9 to 8.9)	7.3 (5.9 to 8.9)	7.5 (6.0 to 9.2)	0.0 (0.0 to 0.0)	2.8 (-4.0 to 10.6)

Supplementary Results Table 1. Osaka age-standardised Summary Exposure Values (SEVs) for Level 4 risk factors and percentage change, by sex, 1990, 2007, and 2017.Data in parentheses are 95% uncertainty intervals.

	Male					Female				
	**1990**	**2007**	**2017**	**Percentage change 1990-2007**	**Percentage change 2007-2017**	**1990**	**2007**	**2017**	**Percentage change 1990-2007**	**Percentage change 2007-2017**
Bullying victimization	3.3 (1.4 to 6.8)	3.3 (1.4 to 6.9)	3.5 (1.5 to 7.3)	0.6 (-7.7 to 8.8)	5.3 (0.1 to 10.2)	1.9 (0.8 to 3.9)	1.9 (0.8 to 3.8)	2.0 (0.8 to 4.0)	-1.3 (-9.6 to 7.7)	4.3 (-1.2 to 9.5)
Low physical activity	0.5 (0.2 to 0.9)	0.5 (0.2 to 0.9)	0.4 (0.2 to 0.8)	-1.7 (-3.6 to 0.2)	-8.9 (-12.4 to -4.6)	0.5 (0.2 to 1.0)	0.5 (0.2 to 0.9)	0.5 (0.2 to 0.9)	-3.7 (-5.8 to -1.2)	0.9 (-0.9 to 2.4)
High fasting plasma glucose	1.5 (0.9 to 2.4)	1.4 (0.9 to 2.3)	1.6 (1.0 to 2.6)	-3.6 (-41.3 to 61.1)	12.7 (-25.1 to 83.2)	1.2 (0.8 to 1.9)	1.3 (0.8 to 2.1)	1.4 (0.8 to 2.4)	3.7 (-36.2 to 72.0)	9.2 (-29.8 to 77.7)
High LDL cholesterol	12.2 (10.6 to 14.0)	12.7 (11.1 to 14.5)	12.7 (11.0 to 14.5)	4.3 (-1.5 to 10.8)	-0.1 (-4.5 to 4.8)	13.1 (11.6 to 14.9)	13.2 (11.7 to 14.9)	13.2 (11.6 to 15.0)	0.5 (-4.5 to 5.8)	0.1 (-4.6 to 4.3)
High systolic blood pressure	8.6 (7.7 to 9.5)	7.7 (6.9 to 8.5)	7.6 (6.8 to 8.5)	-10.8 (-17.3 to -3.7)	-0.3 (-7.4 to 7.6)	7.8 (7.0 to 8.6)	5.7 (5.0 to 6.5)	5.4 (4.6 to 6.1)	-26.5 (-33.5 to -18.9)	-6.5 (-16.2 to 4.2)
High body-mass index	7.7 (5.3 to 11.3)	11.1 (8.1 to 16.5)	11.5 (8.2 to 16.6)	44.7 (16.4 to 83.6)	3.0 (-11.3 to 19.3)	6.2 (4.0 to 9.8)	8.0 (5.3 to 12.0)	8.0 (5.2 to 11.9)	28.8 (1.8 to 69.1)	0.1 (-16.7 to 20.7)
Low bone mineral density	5.3 (4.3 to 6.3)	5.1 (4.3 to 6.1)	5.1 (4.3 to 6.1)	-2.3 (-16.2 to 13.2)	-0.2 (-13.7 to 15.1)	7.2 (6.0 to 8.5)	6.9 (5.9 to 8.1)	7.0 (5.9 to 8.3)	-3.7 (-14.8 to 8.4)	1.4 (-11.4 to 16.8)
Impaired kidney function	2.6 (1.2 to 6.4)	2.5 (1.1 to 6.4)	2.4 (1.1 to 6.1)	-3.2 (-7.4 to 0.7)	-6.3 (-11.7 to -1.9)	3.2 (1.5 to 7.4)	3.3 (1.5 to 7.5)	3.1 (1.4 to 7.2)	2.4 (-1.9 to 7.3)	-7.3 (-12.1 to -2.5)

Supplementary Results Table 1. Saga age-standardised Summary Exposure Values (SEVs) for Level 4 risk factors and percentage change, by sex, 1990, 2007, and 2017.Data in parentheses are 95% uncertainty intervals.

	Male					Female				
	**1990**	**2007**	**2017**	**Percentage change 1990-2007**	**Percentage change 2007-2017**	**1990**	**2007**	**2017**	**Percentage change 1990-2007**	**Percentage change 2007-2017**
Unsafe water source	2.5 (1.4 to 4.1)	2.1 (1.2 to 3.5)	2.2 (1.2 to 3.7)	-15.3 (-55.1 to 63.8)	5.9 (-40.2 to 78.6)	2.5 (1.4 to 4.1)	2.1 (1.2 to 3.5)	2.2 (1.2 to 3.7)	-15.3 (-55.1 to 63.8)	5.9 (-40.2 to 78.6)
Unsafe sanitation	4.5 (2.2 to 8.5)	2.3 (1.0 to 4.3)	1.9 (0.9 to 3.6)	-49.2 (-79.2 to 17.8)	-15.3 (-56.1 to 72.4)	4.5 (2.2 to 8.5)	2.3 (1.0 to 4.3)	1.9 (0.9 to 3.6)	-49.2 (-79.2 to 17.8)	-15.3 (-56.1 to 72.4)
No access to handwashing facility	6.9 (4.9 to 9.3)	6.5 (4.7 to 8.9)	6.0 (4.3 to 8.0)	-5.8 (-33.7 to 33.0)	-7.6 (-29.1 to 20.2)	6.9 (4.9 to 9.3)	6.5 (4.7 to 8.9)	6.0 (4.3 to 8.0)	-5.8 (-33.7 to 33.0)	-7.6 (-29.1 to 20.2)
Ambient particulate matter pollution	37.9 (26.4 to 55.0)	38.3 (26.7 to 55.1)	33.6 (22.8 to 50.1)	1.0 (-18.3 to 25.2)	-12.2 (-31.5 to 8.2)	37.7 (26.1 to 54.7)	38.3 (26.6 to 55.0)	33.6 (22.7 to 50.1)	1.5 (-17.8 to 26.7)	-12.2 (-31.5 to 8.3)
Household air pollution from solid fuels	0.6 (0.2 to 1.7)	0.2 (0.0 to 0.4)	0.1 (0.0 to 0.3)	-75.0 (-92.6 to -41.5)	-13.7 (-65.4 to 88.1)	0.9 (0.3 to 5.2)	0.2 (0.0 to 1.0)	0.2 (0.0 to 0.8)	-71.9 (-92.4 to -38.8)	-11.0 (-65.1 to 88.6)
Ambient ozone pollution	51.3 (24.1 to 54.8)	50.0 (21.6 to 54.8)	51.0 (23.4 to 54.8)	-2.6 (-11.6 to 0.0)	2.1 (0.0 to 9.7)	51.3 (24.1 to 54.8)	50.0 (21.6 to 54.8)	51.0 (23.4 to 54.8)	-2.6 (-11.6 to 0.0)	2.1 (0.0 to 9.7)
Residential radon	13.0 (0.7 to 41.5)	13.0 (0.7 to 41.5)	13.0 (0.7 to 41.5)	0.0 (0.0 to 0.0)	0.0 (0.0 to 0.0)	13.0 (0.7 to 41.5)	13.0 (0.7 to 41.5)	13.0 (0.7 to 41.5)	0.0 (0.0 to 0.0)	0.0 (0.0 to 0.0)
Lead exposure	3.0 (0.5 to 6.7)	2.1 (0.3 to 5.1)	1.5 (0.2 to 4.1)	-32.0 (-45.7 to -23.2)	-27.3 (-42.7 to -19.3)	1.6 (0.1 to 4.5)	1.0 (0.1 to 3.2)	0.7 (0.0 to 2.6)	-37.7 (-55.0 to -28.0)	-29.8 (-52.3 to -20.5)
Occupational exposure to asbestos	2.2 (1.9 to 2.5)	3.0 (2.7 to 3.2)	2.8 (2.3 to 3.4)	33.4 (15.3 to 59.0)	-7.0 (-24.9 to 16.0)	0.9 (0.7 to 1.0)	0.8 (0.8 to 0.9)	0.8 (0.7 to 1.0)	-1.1 (-17.0 to 23.7)	-2.9 (-21.1 to 19.2)
Occupational exposure to arsenic	0.5 (0.0 to 1.1)	0.4 (0.0 to 1.0)	0.4 (0.0 to 1.0)	-	-	0.4 (0.0 to 1.0)	0.4 (0.0 to 0.9)	0.4 (0.0 to 0.9)	-	-
Occupational exposure to benzene	0.6 (0.0 to 1.8)	0.7 (0.0 to 1.9)	0.7 (0.0 to 2.0)	2.3 (-4.1 to 12.3)	3.5 (-2.5 to 11.8)	0.6 (0.0 to 1.7)	0.7 (0.0 to 2.1)	0.8 (0.0 to 2.3)	20.8 (7.6 to 42.8)	15.0 (5.2 to 30.0)
Occupational exposure to beryllium	0.0 (0.0 to 0.0)	0.0 (0.0 to 0.0)	0.0 (0.0 to 0.0)	-5.2 (-10.0 to -0.2)	1.9 (-2.0 to 5.9)	0.0 (0.0 to 0.0)	0.0 (0.0 to 0.0)	0.0 (0.0 to 0.0)	1.7 (-7.4 to 12.0)	9.4 (2.9 to 17.1)
Occupational exposure to cadmium	0.1 (0.0 to 0.1)	0.0 (0.0 to 0.1)	0.0 (0.0 to 0.1)	-6.7 (-18.0 to 5.9)	1.0 (-9.0 to 11.1)	0.0 (0.0 to 0.1)	0.0 (0.0 to 0.0)	0.0 (0.0 to 0.1)	-8.8 (-23.2 to 9.8)	5.4 (-7.1 to 21.2)
Occupational exposure to chromium	0.1 (0.1 to 0.1)	0.1 (0.1 to 0.1)	0.1 (0.1 to 0.1)	-5.1 (-15.4 to 6.2)	1.5 (-7.1 to 10.4)	0.1 (0.1 to 0.1)	0.1 (0.1 to 0.1)	0.1 (0.1 to 0.1)	-4.2 (-17.8 to 12.9)	7.1 (-3.8 to 20.4)
Occupational exposure to diesel engine exhaust	0.6 (0.5 to 0.6)	0.6 (0.5 to 0.6)	0.6 (0.6 to 0.7)	-2.2 (-9.9 to 5.7)	3.0 (-3.7 to 10.4)	0.4 (0.4 to 0.5)	0.5 (0.4 to 0.6)	0.6 (0.5 to 0.6)	11.7 (-1.9 to 26.8)	11.5 (1.0 to 23.0)
Occupational exposure to formaldehyde	0.3 (0.2 to 0.3)	0.2 (0.2 to 0.3)	0.2 (0.2 to 0.3)	-6.2 (-18.5 to 7.6)	0.6 (-9.8 to 11.1)	0.2 (0.2 to 0.3)	0.2 (0.2 to 0.2)	0.2 (0.2 to 0.2)	-6.2 (-21.0 to 13.1)	6.2 (-6.4 to 21.5)
Occupational exposure to nickel	0.4 (0.0 to 1.9)	0.4 (0.0 to 1.7)	0.4 (0.0 to 1.7)	-	-	0.3 (0.0 to 1.5)	0.3 (0.0 to 1.3)	0.3 (0.0 to 1.4)	-	-
Occupational exposure to polycyclic aromatic hydrocarbons	0.2 (0.2 to 0.3)	0.2 (0.2 to 0.3)	0.2 (0.2 to 0.3)	-4.2 (-13.7 to 6.3)	1.8 (-6.0 to 9.9)	0.2 (0.2 to 0.3)	0.2 (0.2 to 0.2)	0.2 (0.2 to 0.3)	-2.1 (-15.2 to 14.4)	8.0 (-2.2 to 19.9)
Occupational exposure to silica	4.7 (0.7 to 15.1)	4.4 (0.6 to 14.3)	4.5 (0.6 to 14.4)	-6.9 (-16.4 to 5.1)	1.1 (-7.6 to 11.6)	3.2 (0.5 to 10.5)	2.7 (0.4 to 8.8)	2.8 (0.4 to 8.9)	-13.5 (-28.3 to 4.9)	2.4 (-11.2 to 21.5)
Occupational exposure to sulphuric acid	0.6 (0.1 to 1.9)	0.6 (0.1 to 1.8)	0.6 (0.1 to 1.8)	-6.9 (-17.2 to 5.2)	1.1 (-7.9 to 10.9)	0.5 (0.1 to 1.7)	0.5 (0.1 to 1.5)	0.5 (0.1 to 1.5)	-10.2 (-25.0 to 7.5)	4.6 (-8.2 to 20.6)
Occupational exposure to trichloroethylene	0.1 (0.1 to 0.1)	0.1 (0.1 to 0.1)	0.1 (0.1 to 0.1)	-4.1 (-11.2 to 4.2)	2.0 (-4.5 to 8.4)	0.1 (0.1 to 0.1)	0.1 (0.1 to 0.1)	0.1 (0.1 to 0.1)	0.6 (-10.6 to 14.8)	8.7 (-0.2 to 19.0)
Occupational asthmagens	18.9 (15.0 to 24.0)	18.0 (14.3 to 23.0)	17.9 (14.0 to 22.4)	-4.6 (-22.9 to 18.0)	-0.4 (-15.4 to 18.2)	8.2 (6.6 to 10.2)	8.2 (6.6 to 10.1)	8.7 (6.9 to 10.8)	-0.6 (-16.5 to 17.1)	5.8 (-6.6 to 20.5)
Occupational particulate matter, gases, and fumes	6.4 (4.6 to 9.3)	6.1 (4.4 to 8.8)	5.9 (4.3 to 8.5)	-4.4 (-9.2 to 0.8)	-3.7 (-6.9 to -0.3)	3.6 (2.5 to 5.6)	3.4 (2.4 to 5.3)	3.3 (2.3 to 5.0)	-5.3 (-13.5 to 3.6)	-4.5 (-10.0 to 1.8)
Occupational noise	6.0 (5.5 to 6.6)	5.8 (5.4 to 6.3)	5.7 (5.3 to 6.2)	-3.2 (-6.1 to -0.0)	-2.5 (-4.5 to -0.5)	3.5 (3.0 to 4.0)	3.5 (3.0 to 4.0)	3.5 (3.1 to 4.0)	0.4 (-5.4 to 7.1)	1.7 (-2.0 to 6.1)
Occupational ergonomic factors	10.6 (8.7 to 12.9)	9.5 (7.8 to 11.4)	9.3 (7.7 to 11.5)	-10.8 (-22.7 to 2.5)	-1.5 (-12.2 to 11.4)	5.7 (4.4 to 7.3)	5.8 (4.5 to 7.3)	6.5 (4.9 to 8.4)	1.4 (-20.3 to 29.2)	12.8 (-7.7 to 38.8)
Non-exclusive breastfeeding	0.4 (0.3 to 0.6)	0.4 (0.3 to 0.6)	0.4 (0.3 to 0.6)	-2.2 (-15.9 to 12.9)	-1.0 (-12.1 to 11.4)	0.4 (0.3 to 0.6)	0.4 (0.3 to 0.6)	0.4 (0.3 to 0.6)	0.2 (-13.8 to 15.7)	-2.3 (-13.3 to 9.9)
Discontinued breastfeeding	1.6 (1.5 to 1.8)	1.7 (1.6 to 1.8)	1.7 (1.5 to 1.8)	3.1 (-7.2 to 14.2)	-2.6 (-10.1 to 6.1)	1.7 (1.5 to 1.8)	1.7 (1.5 to 1.8)	1.7 (1.5 to 1.8)	0.5 (-9.6 to 11.3)	-0.4 (-8.1 to 8.5)
Child underweight	0.3 (0.2 to 0.4)	0.2 (0.1 to 0.3)	0.2 (0.1 to 0.3)	-14.4 (-34.6 to 10.5)	-5.8 (-22.7 to 12.2)	0.3 (0.2 to 0.4)	0.2 (0.1 to 0.3)	0.2 (0.1 to 0.3)	-17.3 (-38.5 to 9.1)	-6.5 (-23.3 to 13.5)
Child wasting	0.2 (0.1 to 0.3)	0.2 (0.1 to 0.3)	0.2 (0.1 to 0.3)	-5.9 (-33.4 to 30.0)	-2.2 (-20.9 to 22.2)	0.2 (0.1 to 0.3)	0.2 (0.1 to 0.3)	0.2 (0.1 to 0.3)	-8.5 (-36.4 to 33.6)	-2.4 (-22.7 to 23.1)
Child stunting	0.7 (0.3 to 1.1)	0.6 (0.3 to 1.0)	0.6 (0.3 to 1.0)	-9.6 (-45.9 to 51.4)	-3.7 (-33.0 to 36.8)	0.7 (0.3 to 1.2)	0.6 (0.3 to 1.1)	0.6 (0.3 to 1.0)	-10.1 (-47.8 to 48.4)	-4.1 (-33.1 to 40.1)
Short gestation for birth weight	0.0 (0.0 to 0.0)	0.0 (0.0 to 0.0)	0.0 (0.0 to 0.0)	8.5 (-13.6 to 45.1)	2.0 (-19.0 to 42.9)	0.0 (0.0 to 0.0)	0.0 (0.0 to 0.0)	0.0 (0.0 to 0.0)	14.3 (-24.2 to 110.2)	0.3 (-19.8 to 34.9)
Low birth weight for gestation	0.0 (0.0 to 0.0)	0.0 (0.0 to 0.0)	0.0 (0.0 to 0.0)	5.5 (-16.3 to 30.6)	2.1 (-21.1 to 22.6)	0.0 (0.0 to 0.0)	0.0 (0.0 to 0.0)	0.0 (0.0 to 0.0)	-6.5 (-63.7 to 39.7)	4.2 (-18.2 to 24.9)
Iron deficiency	-	-	-	-	-	8.3 (5.7 to 11.7)	8.3 (5.8 to 11.5)	7.7 (5.3 to 10.8)	0.1 (-21.0 to 29.3)	-8.3 (-24.3 to 9.2)
Vitamin A deficiency	1.1 (0.7 to 1.5)	0.8 (0.6 to 1.1)	0.7 (0.4 to 0.9)	-21.0 (-33.5 to -6.4)	-18.7 (-34.6 to -3.2)	0.8 (0.6 to 1.2)	0.8 (0.5 to 1.1)	0.7 (0.5 to 1.0)	-2.3 (-28.8 to 36.8)	-9.7 (-24.5 to 7.9)
Zinc deficiency	0.5 (0.2 to 1.1)	0.4 (0.1 to 1.1)	0.4 (0.1 to 0.9)	-15.1 (-65.2 to 70.4)	-12.4 (-65.9 to 166.9)	0.5 (0.2 to 1.1)	0.4 (0.1 to 0.8)	0.4 (0.1 to 0.8)	-30.9 (-62.9 to 6.1)	6.1 (-59.5 to 169.3)
Smoking	18.3 (15.0 to 21.6)	12.7 (10.3 to 15.3)	10.7 (8.5 to 13.0)	-30.6 (-39.4 to -20.9)	-15.7 (-24.2 to -5.9)	4.1 (2.9 to 5.5)	3.4 (2.4 to 4.6)	3.3 (2.3 to 4.4)	-17.3 (-36.9 to 8.8)	-2.6 (-19.1 to 16.7)
Chewing tobacco	1.4 (0.9 to 2.1)	0.9 (0.6 to 1.3)	0.8 (0.5 to 1.1)	-33.3 (-56.9 to 3.4)	-18.9 (-40.6 to 12.0)	0.1 (0.1 to 0.2)	0.1 (0.1 to 0.1)	0.1 (0.1 to 0.1)	-4.1 (-41.2 to 57.5)	-2.2 (-33.7 to 43.3)
Second-hand smoke	36.9 (33.0 to 40.8)	35.9 (31.8 to 40.0)	36.4 (31.9 to 41.5)	-2.7 (-14.4 to 10.1)	1.4 (-7.6 to 12.0)	55.6 (51.9 to 59.2)	48.2 (44.5 to 51.8)	48.8 (44.4 to 52.9)	-13.2 (-19.8 to -6.9)	1.2 (-5.3 to 7.3)
Alcohol use	14.1 (7.1 to 23.7)	14.0 (6.8 to 24.2)	15.4 (7.1 to 26.9)	-1.2 (-34.2 to 47.8)	10.3 (-22.4 to 58.2)	11.6 (5.6 to 20.3)	11.5 (5.3 to 20.5)	12.8 (5.7 to 22.7)	-1.0 (-33.7 to 43.3)	10.7 (-22.6 to 61.5)
Drug use	0.5 (0.4 to 0.7)	0.5 (0.4 to 0.7)	0.6 (0.4 to 0.8)	0.6 (-5.1 to 6.5)	11.8 (4.4 to 20.3)	0.3 (0.2 to 0.4)	0.3 (0.2 to 0.4)	0.3 (0.3 to 0.4)	1.7 (-3.8 to 7.9)	9.0 (2.2 to 15.8)
Diet low in fruits	41.7 (35.9 to 45.4)	40.0 (34.5 to 43.9)	41.4 (35.6 to 44.9)	-3.9 (-9.7 to 1.5)	3.3 (-1.4 to 8.0)	38.8 (32.9 to 43.2)	36.5 (30.4 to 40.9)	38.7 (32.7 to 42.9)	-6.1 (-13.6 to 1.2)	6.2 (0.5 to 13.8)
Diet low in vegetables	20.8 (12.1 to 28.1)	15.3 (7.0 to 23.2)	17.5 (9.3 to 25.4)	-26.6 (-53.7 to -1.9)	14.4 (-11.4 to 67.0)	23.1 (14.7 to 30.2)	17.5 (9.4 to 25.3)	19.3 (11.0 to 27.2)	-24.3 (-46.9 to -1.6)	10.6 (-13.6 to 48.6)
Diet low in legumes	4.6 (0.8 to 10.0)	6.2 (1.4 to 12.3)	8.4 (2.6 to 15.0)	35.0 (-38.8 to 316.7)	34.8 (-18.2 to 191.6)	8.5 (2.7 to 14.7)	8.6 (2.9 to 15.6)	11.2 (4.5 to 18.3)	1.8 (-41.9 to 95.2)	30.1 (-12.3 to 123.9)
Diet low in whole grains	30.9 (25.7 to 34.9)	35.6 (31.8 to 39.0)	36.9 (33.3 to 40.1)	15.2 (6.5 to 27.5)	3.7 (-1.0 to 9.5)	32.4 (27.7 to 36.5)	36.8 (33.0 to 40.2)	38.1 (34.5 to 41.2)	13.8 (6.0 to 24.7)	3.4 (-1.0 to 8.4)
Diet low in nuts and seeds	50.9 (49.7 to 51.9)	49.5 (47.7 to 50.9)	49.3 (47.3 to 50.7)	-2.7 (-5.3 to -0.7)	-0.5 (-2.7 to 1.4)	51.1 (50.0 to 52.1)	49.7 (48.0 to 51.1)	49.4 (47.5 to 50.9)	-2.8 (-5.3 to -1.0)	-0.5 (-2.6 to 1.3)
Diet low in milk	46.5 (44.5 to 48.2)	46.8 (45.1 to 48.4)	47.3 (45.6 to 48.8)	0.8 (-1.0 to 2.8)	1.0 (-0.3 to 2.5)	46.5 (44.5 to 48.1)	46.3 (44.1 to 48.0)	46.9 (44.9 to 48.6)	-0.4 (-2.4 to 1.4)	1.4 (-0.1 to 3.1)
Diet high in red meat	3.7 (0.5 to 8.9)	7.0 (1.9 to 13.3)	8.3 (2.5 to 15.6)	91.2 (-23.6 to 930.4)	18.4 (-40.7 to 139.7)	0.5 (0.0 to 2.3)	1.4 (0.0 to 4.3)	1.8 (0.0 to 5.2)	171.8 (-81.5 to 2982329.0)	32.2 (-89.5 to 3919.5)
Diet high in processed meat	10.3 (6.3 to 14.1)	16.9 (11.9 to 21.5)	19.4 (13.6 to 24.8)	63.7 (27.4 to 128.5)	14.7 (-3.5 to 40.4)	8.5 (4.8 to 12.1)	12.2 (8.0 to 16.0)	13.1 (8.9 to 17.1)	43.8 (12.2 to 101.2)	7.5 (-9.4 to 29.0)
Diet high in sugar-sweetened beverages	6.5 (2.4 to 12.7)	7.6 (3.2 to 13.9)	8.1 (3.3 to 15.3)	16.7 (-49.0 to 157.8)	6.3 (-40.2 to 78.0)	4.8 (1.8 to 10.4)	5.4 (2.4 to 10.4)	5.8 (2.4 to 11.4)	11.5 (-47.8 to 167.0)	8.2 (-38.0 to 95.3)
Diet low in fibre	22.7 (14.8 to 28.8)	24.0 (16.9 to 30.0)	25.1 (18.1 to 31.4)	5.5 (-10.7 to 30.6)	4.9 (-6.8 to 19.5)	24.1 (16.8 to 30.3)	25.2 (18.2 to 31.3)	26.7 (19.9 to 32.5)	4.7 (-10.6 to 26.2)	5.9 (-4.6 to 19.8)
Diet low in calcium	35.5 (31.1 to 38.9)	35.3 (31.0 to 38.8)	36.1 (31.9 to 39.4)	-0.6 (-4.3 to 3.1)	2.3 (-0.5 to 5.5)	37.7 (33.9 to 40.9)	36.7 (32.7 to 40.0)	37.9 (34.0 to 40.9)	-2.7 (-6.4 to 0.7)	3.3 (0.8 to 6.4)
Diet low in seafood omega-3 fatty acids	3.3 (1.5 to 4.8)	0.3 (0.0 to 1.1)	0.3 (0.0 to 1.2)	-92.0 (-100.0 to -71.1)	6.8 (-93.9 to 1192.2)	3.9 (1.9 to 5.4)	0.4 (0.0 to 1.4)	0.4 (0.0 to 1.5)	-90.4 (-99.9 to -68.3)	9.0 (-85.8 to 1728.8)
Diet low in polyunsaturated fatty acids	31.1 (25.6 to 35.9)	28.3 (22.5 to 33.3)	29.1 (23.1 to 33.9)	-8.7 (-18.8 to 1.2)	2.5 (-5.1 to 11.2)	31.6 (26.1 to 36.1)	29.0 (23.2 to 33.8)	29.7 (24.0 to 34.7)	-8.3 (-19.3 to 0.8)	2.4 (-5.6 to 12.5)
Diet high in trans fatty acids	1.1 (0.0 to 3.3)	0.9 (0.0 to 3.0)	0.9 (0.0 to 2.9)	-12.6 (-38.6 to -6.6)	-8.1 (-32.0 to -3.4)	1.5 (0.1 to 4.0)	1.3 (0.1 to 3.7)	1.2 (0.0 to 3.5)	-12.5 (-34.6 to -6.4)	-8.4 (-28.2 to -3.1)
Diet high in sodium	27.1 (18.0 to 35.3)	17.4 (8.3 to 24.6)	14.8 (6.0 to 22.3)	-35.9 (-59.3 to -17.9)	-14.9 (-40.2 to 1.8)	31.0 (22.7 to 38.4)	18.4 (9.7 to 25.3)	15.1 (6.2 to 22.3)	-40.7 (-62.8 to -25.8)	-17.6 (-42.3 to -4.9)
Intimate partner violence	-	-	-	-	-	2.3 (1.9 to 2.7)	2.1 (1.8 to 2.4)	2.0 (1.7 to 2.3)	-9.7 (-13.6 to -5.6)	-3.6 (-7.6 to 0.7)
Childhood sexual abuse	5.3 (4.3 to 6.5)	5.3 (4.3 to 6.5)	5.3 (4.3 to 6.5)	0.0 (0.0 to 0.0)	0.0 (0.0 to 0.0)	7.3 (5.9 to 8.9)	7.3 (5.9 to 8.9)	7.5 (6.0 to 9.2)	0.0 (0.0 to 0.0)	2.8 (-4.0 to 10.6)

Supplementary Results Table 1. Saga age-standardised Summary Exposure Values (SEVs) for Level 4 risk factors and percentage change, by sex, 1990, 2007, and 2017.Data in parentheses are 95% uncertainty intervals.										
	**Male**					**Female**				
	**1990**	**2007**	**2017**	**Percentage change 1990-2007**	**Percentage change 2007-2017**	**1990**	**2007**	**2017**	**Percentage change 1990-2007**	**Percentage change 2007-2017**
Bullying victimization	3.3 (1.4 to 6.8)	3.4 (1.5 to 7.1)	3.5 (1.5 to 7.2)	2.9 (-5.1 to 11.1)	2.8 (-2.6 to 7.5)	1.9 (0.8 to 4.0)	1.9 (0.8 to 4.1)	2.0 (0.8 to 4.2)	1.1 (-6.2 to 9.4)	2.2 (-3.6 to 7.0)
Low physical activity	0.5 (0.2 to 0.9)	0.5 (0.2 to 0.9)	0.4 (0.2 to 0.8)	-1.8 (-3.6 to 0.1)	-8.9 (-12.3 to -4.5)	0.5 (0.2 to 1.0)	0.5 (0.2 to 0.9)	0.5 (0.2 to 0.9)	-3.9 (-6.0 to -1.4)	0.7 (-0.8 to 2.3)
High fasting plasma glucose	1.4 (0.8 to 2.2)	1.3 (0.8 to 2.1)	1.5 (0.9 to 2.5)	-3.0 (-40.0 to 57.4)	13.0 (-20.8 to 80.2)	1.2 (0.7 to 1.9)	1.2 (0.7 to 2.1)	1.3 (0.8 to 2.3)	4.4 (-37.9 to 78.8)	9.6 (-29.2 to 85.4)
High LDL cholesterol	12.0 (10.3 to 13.8)	12.5 (10.9 to 14.3)	12.5 (10.9 to 14.3)	4.3 (-1.9 to 10.8)	0.1 (-4.5 to 5.0)	12.7 (11.2 to 14.4)	12.8 (11.3 to 14.6)	12.9 (11.4 to 14.6)	0.7 (-5.1 to 6.1)	0.3 (-4.2 to 5.1)
High systolic blood pressure	11.2 (10.2 to 12.2)	8.3 (7.4 to 9.2)	8.1 (7.1 to 9.1)	-26.2 (-32.2 to -20.1)	-1.7 (-9.1 to 6.6)	9.6 (8.6 to 10.6)	6.0 (5.2 to 6.8)	5.6 (4.8 to 6.5)	-37.4 (-44.6 to -30.6)	-7.3 (-17.9 to 4.1)
High body-mass index	5.3 (3.4 to 8.6)	7.7 (5.1 to 12.3)	8.0 (5.2 to 12.5)	43.9 (15.5 to 83.6)	3.6 (-13.1 to 21.4)	5.2 (3.2 to 8.2)	6.7 (4.3 to 10.7)	6.8 (4.1 to 11.0)	29.4 (0.3 to 69.8)	0.9 (-18.5 to 25.9)
Low bone mineral density	5.3 (4.3 to 6.3)	5.1 (4.3 to 6.0)	5.2 (4.2 to 6.2)	-2.8 (-14.4 to 11.0)	0.6 (-13.2 to 15.8)	7.2 (6.0 to 8.5)	7.0 (5.9 to 8.2)	7.1 (5.9 to 8.3)	-3.3 (-14.6 to 10.1)	1.0 (-11.0 to 15.2)
Impaired kidney function	2.6 (1.2 to 6.5)	2.5 (1.1 to 6.3)	2.4 (1.1 to 6.1)	-2.8 (-7.3 to 1.6)	-6.5 (-11.9 to -1.8)	3.2 (1.4 to 7.4)	3.3 (1.5 to 7.7)	3.1 (1.4 to 7.3)	3.0 (-1.0 to 8.0)	-6.8 (-12.0 to -2.0)

Supplementary Results Table 1. Saitama age-standardised Summary Exposure Values (SEVs) for Level 4 risk factors and percentage change, by sex, 1990, 2007, and 2017.Data in parentheses are 95% uncertainty intervals.

	Male					Female				
	1990	2007	2017	Percentage change 1990-2007	Percentage change 2007-2017	1990	2007	2017	Percentage change 1990-2007	Percentage change 2007-2017
Unsafe water source	2.2 (1.2 to 3.5)	1.8 (1.0 to 3.0)	1.9 (1.1 to 3.2)	-15.5 (-57.1 to 71.4)	6.7 (-36.4 to 83.8)	2.2 (1.2 to 3.5)	1.8 (1.0 to 3.0)	1.9 (1.1 to 3.2)	-15.5 (-57.1 to 71.4)	6.7 (-36.4 to 83.8)
Unsafe sanitation	3.6 (1.7 to 6.7)	1.8 (0.9 to 3.6)	1.6 (0.8 to 3.0)	-49.3 (-78.4 to 15.4)	-12.0 (-54.0 to 66.5)	3.6 (1.7 to 6.7)	1.8 (0.9 to 3.6)	1.6 (0.8 to 3.0)	-49.3 (-78.4 to 15.4)	-12.0 (-54.0 to 66.5)
No access to handwashing facility	6.8 (4.9 to 9.1)	6.4 (4.7 to 8.5)	5.9 (4.3 to 8.0)	-5.6 (-31.6 to 34.7)	-7.4 (-27.4 to 16.2)	6.8 (4.9 to 9.1)	6.4 (4.7 to 8.5)	5.9 (4.3 to 8.0)	-5.6 (-31.6 to 34.7)	-7.4 (-27.4 to 16.2)
Ambient particulate matter pollution	36.2 (25.1 to 54.2)	36.0 (25.7 to 51.7)	31.2 (21.3 to 46.8)	-0.5 (-21.7 to 23.9)	-13.4 (-30.3 to 5.3)	36.0 (25.0 to 53.9)	36.0 (25.7 to 51.6)	31.2 (21.3 to 46.8)	-0.1 (-21.6 to 24.5)	-13.3 (-30.3 to 5.3)
Household air pollution from solid fuels	0.4 (0.1 to 1.1)	0.1 (0.0 to 0.2)	0.1 (0.0 to 0.2)	-72.0 (-92.7 to -48.0)	-14.4 (-65.9 to 77.1)	0.6 (0.1 to 3.4)	0.2 (0.0 to 0.6)	0.2 (0.0 to 0.5)	-69.9 (-92.6 to -45.8)	-11.2 (-65.8 to 78.9)
Ambient ozone pollution	52.8 (29.7 to 54.8)	53.0 (30.2 to 54.8)	53.2 (31.6 to 54.8)	0.2 (0.0 to 2.3)	0.4 (0.0 to 5.0)	52.8 (29.7 to 54.8)	53.0 (30.2 to 54.8)	53.2 (31.6 to 54.8)	0.2 (0.0 to 2.3)	0.4 (0.0 to 5.0)
Residential radon	14.3 (1.0 to 45.2)	14.3 (1.0 to 45.2)	14.3 (1.0 to 45.2)	0.0 (0.0 to 0.0)	0.0 (0.0 to 0.0)	14.3 (1.0 to 45.2)	14.3 (1.0 to 45.2)	14.3 (1.0 to 45.2)	0.0 (0.0 to 0.0)	0.0 (0.0 to 0.0)
Lead exposure	4.0 (1.0 to 8.0)	2.4 (0.5 to 5.6)	1.6 (0.3 to 4.2)	-39.1 (-52.9 to -29.2)	-32.7 (-47.3 to -24.5)	2.1 (0.3 to 5.1)	1.1 (0.1 to 3.3)	0.7 (0.0 to 2.4)	-46.2 (-63.7 to -35.8)	-37.0 (-58.1 to -27.3)
Occupational exposure to asbestos	2.4 (2.0 to 2.7)	3.2 (2.9 to 3.4)	3.2 (2.7 to 3.8)	32.8 (15.1 to 57.2)	1.1 (-15.5 to 22.0)	0.7 (0.5 to 0.8)	0.6 (0.6 to 0.7)	0.6 (0.5 to 0.7)	-2.7 (-20.0 to 23.6)	-4.7 (-23.5 to 18.0)
Occupational exposure to arsenic	0.5 (0.0 to 1.2)	0.4 (0.0 to 1.0)	0.4 (0.0 to 1.0)	-	-	0.4 (0.0 to 1.0)	0.4 (0.0 to 0.9)	0.4 (0.0 to 0.9)	-	-
Occupational exposure to benzene	0.7 (0.0 to 1.9)	0.7 (0.0 to 1.9)	0.7 (0.0 to 2.0)	1.6 (-4.3 to 11.7)	3.1 (-1.9 to 11.3)	0.6 (0.0 to 1.8)	0.8 (0.0 to 2.2)	0.9 (0.1 to 2.4)	19.7 (8.0 to 39.7)	13.5 (3.7 to 28.0)
Occupational exposure to beryllium	0.0 (0.0 to 0.0)	0.0 (0.0 to 0.0)	0.0 (0.0 to 0.0)	-5.3 (-9.8 to -0.3)	2.0 (-1.8 to 6.0)	0.0 (0.0 to 0.0)	0.0 (0.0 to 0.0)	0.0 (0.0 to 0.0)	1.4 (-7.7 to 10.5)	8.3 (2.8 to 14.7)
Occupational exposure to cadmium	0.1 (0.0 to 0.1)	0.1 (0.0 to 0.1)	0.1 (0.0 to 0.1)	-7.7 (-18.2 to 3.8)	0.8 (-8.5 to 10.9)	0.0 (0.0 to 0.1)	0.0 (0.0 to 0.1)	0.0 (0.0 to 0.1)	-10.2 (-23.7 to 6.2)	4.1 (-7.5 to 17.1)
Occupational exposure to chromium	0.1 (0.1 to 0.1)	0.1 (0.1 to 0.1)	0.1 (0.1 to 0.1)	-6.2 (-15.5 to 4.3)	1.3 (-6.8 to 9.9)	0.1 (0.1 to 0.1)	0.1 (0.1 to 0.1)	0.1 (0.1 to 0.1)	-5.5 (-17.8 to 9.7)	5.8 (-4.0 to 17.1)
Occupational exposure to diesel engine exhaust	0.6 (0.5 to 0.6)	0.6 (0.5 to 0.6)	0.6 (0.5 to 0.6)	-2.2 (-10.0 to 6.1)	3.0 (-3.3 to 10.0)	0.5 (0.4 to 0.5)	0.5 (0.5 to 0.6)	0.6 (0.5 to 0.6)	11.4 (-2.0 to 25.8)	10.0 (0.5 to 20.3)
Occupational exposure to formaldehyde	0.3 (0.2 to 0.3)	0.2 (0.2 to 0.3)	0.2 (0.2 to 0.3)	-7.2 (-18.2 to 5.0)	0.4 (-9.1 to 10.7)	0.2 (0.2 to 0.3)	0.2 (0.2 to 0.2)	0.2 (0.2 to 0.3)	-7.5 (-21.1 to 9.4)	5.0 (-6.7 to 18.1)
Occupational exposure to nickel	0.4 (0.0 to 2.0)	0.4 (0.0 to 1.7)	0.4 (0.0 to 1.8)	-	-	0.4 (0.0 to 1.6)	0.3 (0.0 to 1.3)	0.3 (0.0 to 1.4)	-	-
Occupational exposure to polycyclic aromatic hydrocarbons	0.3 (0.2 to 0.3)	0.2 (0.2 to 0.3)	0.2 (0.2 to 0.3)	-5.1 (-14.0 to 4.4)	1.7 (-5.9 to 9.6)	0.2 (0.2 to 0.3)	0.2 (0.2 to 0.3)	0.2 (0.2 to 0.3)	-3.3 (-15.1 to 11.2)	6.8 (-2.5 to 17.3)
Occupational exposure to silica	4.8 (0.7 to 15.4)	4.5 (0.7 to 14.4)	4.6 (0.7 to 14.9)	-6.3 (-16.2 to 5.1)	1.5 (-7.1 to 12.8)	3.3 (0.4 to 10.9)	2.8 (0.4 to 9.2)	2.8 (0.4 to 9.6)	-14.6 (-29.1 to 3.6)	1.2 (-10.7 to 17.4)
Occupational exposure to sulphuric acid	0.6 (0.1 to 2.0)	0.6 (0.1 to 1.8)	0.6 (0.1 to 1.9)	-7.6 (-17.9 to 3.9)	1.4 (-7.8 to 10.9)	0.5 (0.1 to 1.7)	0.5 (0.1 to 1.5)	0.5 (0.1 to 1.5)	-11.4 (-25.5 to 4.7)	3.6 (-8.5 to 17.1)
Occupational exposure to trichloroethylene	0.1 (0.1 to 0.1)	0.1 (0.1 to 0.1)	0.1 (0.1 to 0.1)	-5.2 (-12.1 to 2.9)	1.8 (-3.9 to 8.0)	0.1 (0.1 to 0.1)	0.1 (0.1 to 0.1)	0.1 (0.1 to 0.1)	-0.5 (-11.1 to 11.8)	7.3 (-0.5 to 16.4)
Occupational asthmagens	20.0 (16.0 to 25.1)	18.9 (15.0 to 23.7)	18.8 (14.8 to 23.6)	-5.4 (-22.8 to 16.8)	-0.8 (-15.9 to 16.8)	8.6 (6.9 to 10.7)	8.5 (6.8 to 10.5)	8.9 (7.1 to 11.0)	-0.8 (-18.2 to 17.0)	4.7 (-7.4 to 18.1)
Occupational particulate matter, gases, and fumes	6.4 (4.5 to 9.3)	6.1 (4.3 to 8.9)	5.9 (4.2 to 8.5)	-4.2 (-9.2 to 1.1)	-3.5 (-6.8 to -0.1)	3.7 (2.6 to 5.6)	3.5 (2.5 to 5.2)	3.4 (2.3 to 5.0)	-5.8 (-14.4 to 3.9)	-4.8 (-10.8 to 1.1)
Occupational noise	5.9 (5.5 to 6.5)	5.8 (5.3 to 6.3)	5.6 (5.2 to 6.1)	-2.8 (-5.9 to 0.5)	-2.1 (-4.1 to 0.1)	3.6 (3.1 to 4.1)	3.6 (3.2 to 4.0)	3.7 (3.3 to 4.1)	0.4 (-5.6 to 7.2)	1.7 (-2.1 to 5.6)
Occupational ergonomic factors	9.9 (8.3 to 12.0)	8.8 (7.3 to 10.6)	8.7 (7.1 to 10.6)	-11.2 (-21.5 to -0.1)	-0.8 (-11.0 to 9.8)	5.3 (4.1 to 6.8)	5.4 (4.3 to 6.9)	6.2 (4.8 to 7.9)	2.3 (-19.8 to 25.7)	13.2 (-5.2 to 34.4)
Non-exclusive breastfeeding	0.4 (0.3 to 0.6)	0.4 (0.3 to 0.6)	0.4 (0.3 to 0.6)	-1.2 (-13.3 to 12.7)	-1.6 (-12.4 to 9.5)	0.4 (0.3 to 0.6)	0.4 (0.3 to 0.6)	0.4 (0.3 to 0.6)	-1.3 (-13.4 to 12.5)	-1.5 (-12.3 to 9.6)
Discontinued breastfeeding	1.7 (1.6 to 1.9)	1.7 (1.6 to 1.9)	1.7 (1.6 to 1.8)	-1.0 (-9.8 to 9.1)	-3.1 (-9.9 to 4.3)	1.8 (1.6 to 1.9)	1.7 (1.6 to 1.9)	1.7 (1.6 to 1.8)	-1.0 (-9.8 to 9.1)	-3.1 (-9.9 to 4.3)
Child underweight	0.2 (0.1 to 0.3)	0.2 (0.1 to 0.3)	0.2 (0.1 to 0.3)	-12.0 (-29.1 to 7.3)	-4.2 (-15.9 to 7.9)	0.2 (0.1 to 0.4)	0.2 (0.1 to 0.3)	0.2 (0.1 to 0.3)	-15.0 (-35.2 to 5.0)	-5.0 (-17.6 to 10.1)
Child wasting	0.2 (0.1 to 0.3)	0.2 (0.1 to 0.2)	0.2 (0.1 to 0.2)	-7.9 (-31.3 to 21.2)	-1.4 (-18.1 to 19.4)	0.2 (0.1 to 0.3)	0.2 (0.1 to 0.2)	0.2 (0.1 to 0.2)	-10.7 (-35.1 to 19.2)	-2.4 (-19.6 to 19.9)
Child stunting	0.6 (0.3 to 1.0)	0.5 (0.3 to 0.8)	0.5 (0.3 to 0.8)	-12.4 (-41.9 to 36.0)	-1.8 (-28.2 to 36.7)	0.6 (0.3 to 1.1)	0.5 (0.3 to 0.8)	0.5 (0.3 to 0.8)	-13.8 (-45.2 to 32.8)	-2.9 (-29.9 to 34.1)
Short gestation for birth weight	0.0 (0.0 to 0.0)	0.0 (0.0 to 0.0)	0.0 (0.0 to 0.0)	9.9 (-10.7 to 40.0)	1.3 (-17.6 to 27.1)	0.0 (0.0 to 0.0)	0.0 (0.0 to 0.0)	0.0 (0.0 to 0.0)	14.2 (-17.1 to 50.7)	-0.2 (-17.3 to 31.8)
Low birth weight for gestation	0.0 (0.0 to 0.0)	0.0 (0.0 to 0.0)	0.0 (0.0 to 0.0)	3.9 (-14.7 to 23.6)	3.0 (-16.5 to 23.8)	0.0 (0.0 to 0.0)	0.0 (0.0 to 0.0)	0.0 (0.0 to 0.0)	-2.6 (-35.5 to 27.6)	3.8 (-15.7 to 21.3)
Iron deficiency	-	-	-	-	-	8.0 (5.5 to 11.0)	8.1 (5.6 to 11.1)	7.4 (5.0 to 10.3)	0.4 (-21.2 to 28.0)	-8.5 (-24.5 to 10.0)
Vitamin A deficiency	0.9 (0.6 to 1.2)	0.8 (0.6 to 1.1)	0.7 (0.4 to 0.9)	-13.2 (-29.8 to 3.1)	-18.7 (-35.5 to -0.6)	0.8 (0.4 to 1.1)	0.8 (0.5 to 1.1)	0.6 (0.4 to 0.8)	3.9 (-14.1 to 36.5)	-22.9 (-40.3 to -4.6)
Zinc deficiency	0.5 (0.2 to 1.1)	0.4 (0.1 to 1.0)	0.4 (0.1 to 0.8)	-27.7 (-66.3 to 69.6)	-3.0 (-64.1 to 180.1)	0.5 (0.2 to 1.2)	0.4 (0.1 to 0.9)	0.4 (0.1 to 0.9)	-28.4 (-61.5 to 1.6)	8.3 (-48.4 to 150.0)
Smoking	15.3 (11.8 to 18.9)	11.3 (8.9 to 13.9)	9.8 (7.8 to 12.0)	-26.2 (-35.6 to -14.3)	-13.4 (-21.4 to -3.5)	3.4 (2.4 to 4.6)	3.0 (2.1 to 4.1)	3.0 (2.1 to 4.0)	-11.3 (-32.1 to 15.4)	-2.4 (-18.1 to 15.9)
Chewing tobacco	1.4 (0.9 to 2.0)	0.9 (0.6 to 1.3)	0.7 (0.5 to 1.1)	-33.8 (-56.3 to -0.2)	-18.8 (-41.5 to 11.1)	0.1 (0.1 to 0.1)	0.1 (0.1 to 0.1)	0.1 (0.1 to 0.1)	-3.4 (-40.2 to 54.2)	-2.6 (-30.1 to 37.7)
Second-hand smoke	39.7 (35.7 to 43.7)	37.4 (33.5 to 41.6)	37.4 (33.1 to 41.9)	-5.8 (-16.0 to 5.4)	-0.0 (-8.9 to 9.4)	61.4 (57.9 to 65.1)	51.8 (48.1 to 55.2)	51.6 (47.5 to 55.4)	-15.7 (-21.5 to -9.9)	-0.3 (-6.0 to 5.3)
Alcohol use	14.1 (7.5 to 23.9)	14.2 (7.4 to 24.3)	16.8 (8.2 to 28.6)	1.0 (-31.0 to 42.8)	18.0 (-14.0 to 64.3)	11.3 (5.8 to 19.9)	11.5 (5.7 to 20.3)	13.2 (6.1 to 22.9)	1.4 (-30.7 to 43.2)	15.5 (-16.2 to 66.2)
Drug use	0.5 (0.4 to 0.7)	0.5 (0.4 to 0.7)	0.6 (0.4 to 0.8)	0.3 (-5.8 to 6.9)	11.7 (4.7 to 20.3)	0.3 (0.2 to 0.4)	0.3 (0.2 to 0.4)	0.3 (0.3 to 0.4)	2.2 (-3.4 to 8.9)	8.9 (2.0 to 17.4)
Diet low in fruits	42.4 (36.8 to 45.9)	40.8 (35.3 to 44.3)	42.2 (36.8 to 45.6)	-3.8 (-9.3 to 1.2)	3.3 (-0.8 to 8.0)	39.9 (34.3 to 43.7)	37.1 (31.3 to 41.2)	39.2 (33.4 to 43.1)	-7.0 (-14.1 to -0.3)	5.7 (-0.1 to 12.2)
Diet low in vegetables	22.2 (13.5 to 29.4)	16.0 (7.9 to 23.6)	17.8 (8.9 to 25.4)	-28.0 (-53.6 to -5.9)	11.6 (-16.2 to 56.3)	24.4 (16.6 to 30.9)	18.1 (9.8 to 25.2)	19.7 (11.4 to 27.0)	-25.7 (-49.1 to -5.8)	9.0 (-13.5 to 39.2)
Diet low in legumes	4.6 (0.7 to 10.2)	6.0 (1.3 to 11.9)	8.1 (2.2 to 15.0)	31.8 (-36.8 to 258.0)	33.9 (-17.4 to 181.2)	8.5 (2.5 to 15.2)	8.7 (3.1 to 15.1)	11.5 (5.1 to 18.3)	3.2 (-41.0 to 104.4)	31.5 (-5.4 to 125.2)
Diet low in whole grains	31.6 (26.6 to 35.5)	36.1 (32.3 to 39.4)	37.4 (33.9 to 40.6)	14.4 (6.3 to 25.8)	3.6 (-0.9 to 8.8)	32.8 (28.7 to 36.5)	37.3 (33.7 to 40.4)	38.6 (35.2 to 41.4)	13.7 (6.4 to 23.4)	3.3 (-0.8 to 7.7)
Diet low in nuts and seeds	51.5 (50.5 to 52.3)	50.5 (49.1 to 51.6)	50.3 (48.8 to 51.5)	-2.1 (-4.0 to -0.5)	-0.4 (-2.1 to 1.2)	51.6 (50.7 to 52.4)	50.4 (49.0 to 51.5)	50.2 (48.5 to 51.4)	-2.3 (-4.2 to -0.8)	-0.4 (-2.1 to 1.2)
Diet low in milk	46.7 (44.6 to 48.4)	46.9 (45.1 to 48.5)	47.4 (45.5 to 48.9)	0.5 (-1.2 to 2.3)	0.9 (-0.4 to 2.3)	46.8 (44.9 to 48.4)	46.6 (44.6 to 48.3)	47.2 (45.3 to 48.8)	-0.4 (-2.3 to 1.4)	1.3 (-0.2 to 2.8)
Diet high in red meat	2.9 (0.3 to 6.9)	6.0 (1.3 to 11.7)	7.1 (2.0 to 13.2)	106.2 (-22.6 to 1165.6)	18.7 (-40.5 to 179.0)	0.3 (0.0 to 1.8)	1.0 (0.0 to 3.4)	1.4 (0.0 to 4.4)	201.2 (-89.6 to [illegible]	39.8 (-95.9 to 10014.4)
Diet high in processed meat	10.4 (6.4 to 14.2)	17.8 (12.9 to 22.2)	21.0 (15.6 to 26.4)	71.6 (34.1 to 137.9)	18.1 (-0.1 to 39.1)	8.7 (5.2 to 12.4)	13.2 (9.2 to 16.9)	14.9 (10.4 to 19.0)	52.1 (21.9 to 110.6)	12.6 (-3.7 to 33.6)
Diet high in sugar-sweetened beverages	5.9 (2.2 to 12.8)	6.7 (3.0 to 12.1)	7.0 (3.1 to 13.4)	13.6 (-49.8 to 151.0)	5.1 (-35.5 to 73.9)	4.2 (1.6 to 9.1)	4.7 (2.1 to 8.8)	5.0 (2.0 to 9.8)	12.2 (-46.5 to 163.9)	5.8 (-37.1 to 87.7)
Diet low in fibre	23.0 (15.7 to 29.5)	23.9 (17.0 to 30.2)	24.8 (17.9 to 31.3)	4.1 (-12.1 to 27.5)	3.7 (-8.1 to 18.3)	24.3 (17.3 to 30.6)	25.1 (18.3 to 30.9)	26.3 (19.4 to 32.3)	3.2 (-11.9 to 20.7)	4.6 (-5.8 to 17.0)
Diet low in calcium	36.4 (32.3 to 39.8)	35.9 (31.8 to 39.2)	36.6 (32.5 to 39.8)	-1.3 (-4.8 to 2.0)	1.9 (-0.8 to 5.0)	38.5 (34.9 to 41.4)	37.4 (33.5 to 40.4)	38.5 (34.8 to 41.4)	-2.9 (-6.0 to 0.0)	3.0 (0.7 to 5.8)
Diet low in seafood omega-3 fatty acids	3.6 (2.1 to 5.0)	0.5 (0.0 to 1.4)	0.5 (0.0 to 1.6)	-87.5 (-99.9 to -59.4)	4.8 (-73.9 to 714.4)	4.2 (2.5 to 5.4)	0.7 (0.0 to 1.9)	0.7 (0.0 to 1.9)	-84.0 (-99.7 to -54.9)	4.1 (-63.7 to 337.3)
Diet low in polyunsaturated fatty acids	31.8 (26.4 to 36.6)	29.2 (23.7 to 34.1)	29.9 (24.4 to 34.6)	-8.2 (-18.9 to 0.4)	2.3 (-4.8 to 11.7)	32.5 (27.6 to 37.1)	29.8 (24.3 to 34.6)	30.5 (24.9 to 35.0)	-8.1 (-17.2 to 0.0)	2.3 (-4.1 to 9.1)
Diet high in trans fatty acids	1.1 (0.0 to 3.3)	0.9 (0.0 to 3.0)	0.9 (0.0 to 2.9)	-12.7 (-37.1 to -7.1)	-8.4 (-34.1 to -3.3)	1.5 (0.1 to 4.0)	1.3 (0.1 to 3.7)	1.2 (0.0 to 3.5)	-12.7 (-35.7 to -6.6)	-8.4 (-27.8 to -3.4)
Diet high in sodium	27.5 (17.7 to 35.6)	18.2 (9.7 to 25.3)	15.9 (7.1 to 23.2)	-33.8 (-56.1 to -15.1)	-12.5 (-35.6 to 2.6)	32.0 (23.7 to 39.3)	19.8 (11.3 to 26.4)	16.7 (8.2 to 24.0)	-38.2 (-57.3 to -23.5)	-15.6 (-35.4 to -2.6)
Intimate partner violence	-	-	-	-	-	2.3 (1.9 to 2.6)	2.1 (1.8 to 2.4)	2.0 (1.7 to 2.4)	-7.0 (-11.1 to -2.4)	-3.5 (-7.7 to 0.9)
Childhood sexual abuse	5.3 (4.3 to 6.5)	5.3 (4.3 to 6.5)	5.3 (4.3 to 6.5)	0.0 (0.0 to 0.0)	0.0 (0.0 to 0.0)	7.3 (5.9 to 8.9)	7.3 (5.9 to 8.9)	7.5 (6.0 to 9.2)	0.0 (0.0 to 0.0)	2.8 (-4.0 to 10.6)

Supplementary Results Table 1. Saitama age-standardised Summary Exposure Values (SEVs) for Level 4 risk factors and percentage change, by sex, 1990, 2007, and 2017.Data in parentheses are 95% uncertainty intervals.										
	**Male**					**Female**				
	**1990**	**2007**	**2017**	**Percentage change 1990-2007**	**Percentage change 2007-2017**	**1990**	**2007**	**2017**	**Percentage change 1990-2007**	**Percentage change 2007-2017**
Bullying victimization	3.3 (1.4 to 6.8)	3.3 (1.4 to 6.8)	3.4 (1.4 to 7.2)	-0.3 (-8.3 to 8.4)	4.2 (-1.0 to 9.1)	1.9 (0.8 to 4.0)	1.9 (0.8 to 3.9)	2.0 (0.8 to 4.0)	-2.0 (-9.8 to 6.7)	3.4 (-2.0 to 8.4)
Low physical activity	0.5 (0.2 to 0.9)	0.5 (0.2 to 0.9)	0.4 (0.2 to 0.8)	-1.8 (-3.7 to 0.1)	-8.9 (-12.5 to -4.7)	0.5 (0.2 to 1.0)	0.5 (0.2 to 0.9)	0.5 (0.2 to 0.9)	-3.8 (-6.0 to -1.2)	0.7 (-0.9 to 2.2)
High fasting plasma glucose	1.4 (0.9 to 2.1)	1.3 (0.8 to 2.0)	1.4 (0.9 to 2.4)	-7.1 (-42.1 to 44.7)	12.4 (-20.3 to 77.4)	1.2 (0.7 to 1.9)	1.2 (0.7 to 2.1)	1.3 (0.8 to 2.2)	5.4 (-35.1 to 74.6)	7.4 (-30.4 to 74.3)
High LDL cholesterol	12.2 (10.6 to 14.0)	12.7 (11.1 to 14.5)	12.7 (11.1 to 14.5)	4.4 (-1.9 to 10.4)	-0.2 (-4.8 to 4.6)	12.9 (11.4 to 14.7)	13.0 (11.4 to 14.8)	13.0 (11.5 to 14.8)	0.8 (-4.4 to 6.1)	0.1 (-4.4 to 4.7)
High systolic blood pressure	11.4 (10.4 to 12.4)	8.4 (7.5 to 9.3)	8.2 (7.3 to 9.2)	-26.0 (-32.0 to -19.8)	-1.8 (-9.0 to 6.1)	9.7 (8.7 to 10.7)	6.1 (5.3 to 6.9)	5.6 (4.8 to 6.4)	-37.3 (-43.8 to -29.9)	-7.4 (-16.5 to 3.1)
High body-mass index	6.7 (4.4 to 10.3)	9.7 (6.9 to 14.1)	10.0 (7.1 to 14.4)	46.2 (16.1 to 85.4)	2.9 (-11.9 to 18.1)	5.5 (3.4 to 8.4)	7.1 (4.6 to 10.5)	7.0 (4.3 to 10.7)	29.5 (0.4 to 70.5)	-0.6 (-18.8 to 20.5)
Low bone mineral density	5.3 (4.4 to 6.3)	5.2 (4.4 to 6.2)	5.2 (4.3 to 6.2)	-2.0 (-13.6 to 13.2)	-0.1 (-12.8 to 13.2)	7.3 (6.1 to 8.6)	7.0 (6.0 to 8.1)	7.1 (5.9 to 8.5)	-3.3 (-14.3 to 10.6)	1.4 (-10.8 to 14.3)
Impaired kidney function	2.6 (1.2 to 6.4)	2.5 (1.1 to 6.4)	2.4 (1.1 to 6.1)	-2.9 (-7.3 to 1.5)	-6.1 (-11.5 to -1.5)	3.2 (1.5 to 7.5)	3.3 (1.5 to 7.7)	3.1 (1.4 to 7.2)	2.8 (-1.3 to 7.4)	-7.0 (-11.9 to -2.1)

Supplementary Results Table 1. Shiga age-standardised Summary Exposure Values (SEVs) for Level 4 risk factors and percentage change, by sex, 1990, 2007, and 2017.Data in parentheses are 95% uncertainty intervals.

	Male					Female				
	1990	2007	2017	Percentage change 1990-2007	Percentage change 2007-2017	1990	2007	2017	Percentage change 1990-2007	Percentage change 2007-2017
Unsafe water source	2.0 (1.1 to 3.4)	1.6 (0.9 to 2.7)	1.7 (1.0 to 2.9)	-19.6 (-59.7 to 59.4)	6.5 (-36.8 to 80.8)	2.0 (1.1 to 3.4)	1.6 (0.9 to 2.7)	1.7 (1.0 to 2.9)	-19.6 (-59.7 to 59.4)	6.5 (-36.8 to 80.8)
Unsafe sanitation	3.3 (1.5 to 5.8)	1.6 (0.7 to 3.0)	1.3 (0.6 to 2.6)	-52.4 (-79.8 to 6.4)	-13.0 (-55.3 to 72.3)	3.3 (1.5 to 5.8)	1.6 (0.7 to 3.0)	1.3 (0.6 to 2.6)	-52.4 (-79.8 to 6.4)	-13.0 (-55.3 to 72.3)
No access to handwashing facility	6.6 (4.8 to 8.8)	6.2 (4.5 to 8.4)	5.8 (4.3 to 7.8)	-5.6 (-31.0 to 32.9)	-7.1 (-28.0 to 19.2)	6.6 (4.8 to 8.8)	6.2 (4.5 to 8.4)	5.8 (4.3 to 7.8)	-5.6 (-31.0 to 32.9)	-7.1 (-28.0 to 19.2)
Ambient particulate matter pollution	32.4 (22.3 to 46.9)	32.8 (23.0 to 47.7)	27.8 (18.7 to 43.5)	1.2 (-18.2 to 25.7)	-15.2 (-34.1 to 4.3)	32.2 (22.1 to 46.8)	32.7 (23.0 to 47.7)	27.8 (18.7 to 43.4)	1.7 (-18.0 to 26.5)	-15.2 (-34.1 to 4.4)
Household air pollution from solid fuels	0.5 (0.2 to 1.4)	0.1 (0.0 to 0.3)	0.1 (0.0 to 0.2)	-75.3 (-93.2 to -49.0)	-16.7 (-68.8 to 73.3)	0.8 (0.2 to 5.8)	0.2 (0.0 to 0.8)	0.2 (0.0 to 0.7)	-72.7 (-93.1 to -47.3)	-15.2 (-68.5 to 73.5)
Ambient ozone pollution	52.4 (27.4 to 54.8)	52.3 (27.0 to 54.8)	52.4 (27.5 to 54.8)	-0.2 (-1.7 to 0.0)	0.3 (0.0 to 2.4)	52.4 (27.4 to 54.8)	52.3 (27.0 to 54.8)	52.4 (27.5 to 54.8)	-0.2 (-1.7 to 0.0)	0.3 (0.0 to 2.4)
Residential radon	14.3 (1.0 to 49.6)	14.3 (1.0 to 49.6)	14.3 (1.0 to 49.6)	0.0 (0.0 to 0.0)	0.0 (0.0 to 0.0)	14.3 (1.0 to 49.6)	14.3 (1.0 to 49.6)	14.3 (1.0 to 49.6)	0.0 (0.0 to 0.0)	0.0 (0.0 to 0.0)
Lead exposure	2.6 (0.3 to 6.1)	1.7 (0.2 to 4.6)	1.2 (0.1 to 3.6)	-33.8 (-48.4 to -24.8)	-28.7 (-45.5 to -20.6)	1.4 (0.1 to 4.2)	0.8 (0.0 to 3.0)	0.6 (0.0 to 2.3)	-39.1 (-58.4 to -29.3)	-31.4 (-54.8 to -21.8)
Occupational exposure to asbestos	2.5 (2.1 to 2.8)	3.1 (2.8 to 3.4)	2.9 (2.4 to 3.5)	23.5 (7.2 to 48.1)	-5.9 (-22.2 to 13.9)	0.8 (0.6 to 0.9)	0.7 (0.6 to 0.7)	0.6 (0.5 to 0.7)	-13.5 (-30.4 to 14.6)	-7.6 (-26.1 to 14.7)
Occupational exposure to arsenic	0.5 (0.0 to 1.1)	0.4 (0.0 to 1.0)	0.4 (0.0 to 1.0)	-	-	0.4 (0.0 to 1.0)	0.4 (0.0 to 0.9)	0.4 (0.0 to 0.9)	-	-
Occupational exposure to benzene	0.6 (0.0 to 1.8)	0.6 (0.0 to 1.8)	0.7 (0.0 to 1.9)	2.2 (-5.2 to 12.1)	3.7 (-2.5 to 12.3)	0.6 (0.0 to 1.8)	0.7 (0.0 to 2.1)	0.8 (0.0 to 2.4)	22.4 (9.5 to 42.0)	14.6 (4.4 to 29.6)
Occupational exposure to beryllium	0.0 (0.0 to 0.0)	0.0 (0.0 to 0.0)	0.0 (0.0 to 0.0)	-5.8 (-10.4 to -0.6)	2.3 (-1.7 to 6.3)	0.0 (0.0 to 0.0)	0.0 (0.0 to 0.0)	0.0 (0.0 to 0.0)	2.7 (-6.5 to 12.8)	9.0 (2.9 to 16.1)
Occupational exposure to cadmium	0.1 (0.0 to 0.1)	0.0 (0.0 to 0.1)	0.0 (0.0 to 0.1)	-8.0 (-19.1 to 5.1)	1.4 (-8.4 to 11.9)	0.0 (0.0 to 0.1)	0.0 (0.0 to 0.1)	0.0 (0.0 to 0.1)	-9.0 (-24.5 to 8.9)	4.8 (-8.0 to 19.1)
Occupational exposure to chromium	0.1 (0.1 to 0.1)	0.1 (0.1 to 0.1)	0.1 (0.1 to 0.1)	-6.3 (-16.4 to 5.3)	1.9 (-6.6 to 10.8)	0.1 (0.1 to 0.1)	0.1 (0.1 to 0.1)	0.1 (0.1 to 0.1)	-4.1 (-18.6 to 12.3)	6.6 (-4.4 to 18.5)
Occupational exposure to diesel engine exhaust	0.6 (0.5 to 0.6)	0.6 (0.5 to 0.6)	0.6 (0.6 to 0.7)	-1.6 (-9.8 to 6.6)	3.2 (-3.1 to 9.6)	0.5 (0.4 to 0.5)	0.5 (0.5 to 0.6)	0.6 (0.5 to 0.7)	14.1 (0.8 to 30.4)	11.0 (-0.0 to 22.7)
Occupational exposure to formaldehyde	0.3 (0.2 to 0.3)	0.2 (0.2 to 0.3)	0.2 (0.2 to 0.3)	-7.1 (-19.1 to 7.2)	1.0 (-9.0 to 12.0)	0.2 (0.2 to 0.3)	0.2 (0.2 to 0.2)	0.2 (0.2 to 0.2)	-6.4 (-22.2 to 12.4)	5.7 (-7.3 to 19.2)
Occupational exposure to nickel	0.4 (0.0 to 1.9)	0.4 (0.0 to 1.7)	0.4 (0.0 to 1.7)	-	-	0.3 (0.0 to 1.5)	0.3 (0.0 to 1.3)	0.3 (0.0 to 1.3)	-	-
Occupational exposure to polycyclic aromatic hydrocarbons	0.2 (0.2 to 0.3)	0.2 (0.2 to 0.3)	0.2 (0.2 to 0.3)	-5.2 (-14.4 to 5.6)	2.2 (-5.8 to 10.3)	0.2 (0.2 to 0.3)	0.2 (0.2 to 0.2)	0.2 (0.2 to 0.3)	-1.9 (-15.9 to 13.7)	7.5 (-2.8 to 18.1)
Occupational exposure to silica	4.5 (0.7 to 14.5)	4.3 (0.6 to 14.1)	4.4 (0.6 to 14.5)	-5.3 (-15.8 to 7.1)	2.0 (-6.8 to 13.0)	3.1 (0.5 to 10.2)	2.7 (0.4 to 8.7)	2.8 (0.4 to 8.8)	-13.6 (-28.9 to 5.8)	1.6 (-12.0 to 18.9)
Occupational exposure to sulphuric acid	0.6 (0.1 to 1.9)	0.6 (0.1 to 1.8)	0.6 (0.1 to 1.8)	-8.3 (-18.6 to 3.8)	1.9 (-7.8 to 12.1)	0.5 (0.1 to 1.7)	0.5 (0.1 to 1.5)	0.5 (0.1 to 1.5)	-10.2 (-25.8 to 7.5)	3.8 (-9.2 to 19.1)
Occupational exposure to trichloroethylene	0.1 (0.1 to 0.1)	0.1 (0.1 to 0.1)	0.1 (0.1 to 0.1)	-5.3 (-12.2 to 2.9)	2.1 (-3.9 to 8.7)	0.1 (0.1 to 0.1)	0.1 (0.1 to 0.1)	0.1 (0.1 to 0.1)	1.3 (-10.3 to 14.4)	8.2 (-0.7 to 18.5)
Occupational asthmagens	18.5 (14.6 to 23.5)	18.0 (14.3 to 22.8)	18.1 (14.4 to 23.0)	-2.5 (-21.6 to 23.3)	0.4 (-16.2 to 19.0)	8.2 (6.6 to 10.1)	8.3 (6.7 to 10.1)	8.8 (7.0 to 10.8)	0.9 (-15.7 to 19.2)	5.6 (-7.3 to 21.2)
Occupational particulate matter, gases, and fumes	6.3 (4.5 to 9.1)	6.0 (4.3 to 8.6)	5.8 (4.2 to 8.5)	-4.3 (-9.4 to 0.8)	-3.5 (-6.9 to -0.3)	3.6 (2.6 to 5.5)	3.4 (2.4 to 5.1)	3.3 (2.3 to 4.8)	-5.7 (-14.1 to 3.6)	-4.7 (-10.1 to 1.6)
Occupational noise	5.8 (5.3 to 6.4)	5.6 (5.2 to 6.2)	5.5 (5.1 to 6.0)	-2.9 (-6.0 to 0.5)	-2.0 (-4.2 to 0.0)	3.5 (3.0 to 4.0)	3.5 (3.1 to 3.9)	3.6 (3.2 to 4.0)	0.5 (-5.5 to 7.4)	2.0 (-1.6 to 6.4)
Occupational ergonomic factors	10.4 (8.6 to 12.7)	9.1 (7.5 to 11.0)	9.0 (7.4 to 10.9)	-12.3 (-23.7 to 2.5)	-1.6 (-12.1 to 10.6)	5.6 (4.4 to 7.2)	5.6 (4.5 to 7.1)	6.4 (4.9 to 8.0)	0.8 (-20.3 to 27.1)	13.0 (-6.7 to 34.7)
Non-exclusive breastfeeding	0.4 (0.3 to 0.6)	0.4 (0.3 to 0.6)	0.4 (0.3 to 0.6)	-1.7 (-14.0 to 12.7)	-1.2 (-11.8 to 10.4)	0.4 (0.3 to 0.6)	0.4 (0.3 to 0.6)	0.4 (0.3 to 0.6)	-0.7 (-13.1 to 13.8)	-2.0 (-12.5 to 9.5)
Discontinued breastfeeding	1.7 (1.6 to 1.9)	1.8 (1.6 to 1.9)	1.7 (1.6 to 1.9)	2.7 (-6.6 to 13.4)	-2.8 (-9.7 to 5.0)	1.7 (1.6 to 1.9)	1.8 (1.6 to 1.9)	1.7 (1.6 to 1.9)	1.3 (-7.8 to 11.9)	-1.7 (-8.7 to 6.3)
Child underweight	0.2 (0.1 to 0.3)	0.2 (0.1 to 0.3)	0.2 (0.1 to 0.2)	-13.1 (-31.5 to 5.2)	-4.1 (-15.1 to 6.9)	0.2 (0.1 to 0.3)	0.2 (0.1 to 0.3)	0.2 (0.1 to 0.2)	-14.8 (-34.2 to 4.4)	-5.2 (-17.4 to 7.6)
Child wasting	0.2 (0.1 to 0.3)	0.2 (0.1 to 0.2)	0.2 (0.1 to 0.2)	-7.9 (-32.4 to 21.2)	-1.2 (-20.7 to 21.8)	0.2 (0.1 to 0.3)	0.2 (0.1 to 0.2)	0.2 (0.1 to 0.2)	-10.0 (-33.6 to 22.5)	-2.4 (-20.2 to 21.4)
Child stunting	0.6 (0.3 to 1.0)	0.6 (0.3 to 0.9)	0.5 (0.3 to 0.9)	-9.4 (-42.4 to 42.3)	-3.4 (-32.3 to 32.3)	0.6 (0.3 to 1.1)	0.6 (0.3 to 0.9)	0.6 (0.3 to 0.9)	-9.9 (-43.5 to 48.8)	-3.6 (-31.2 to 36.1)
Short gestation for birth weight	0.0 (0.0 to 0.0)	0.0 (0.0 to 0.0)	0.0 (0.0 to 0.0)	10.6 (-9.7 to 43.3)	2.1 (-19.9 to 33.2)	0.0 (0.0 to 0.0)	0.0 (0.0 to 0.0)	0.0 (0.0 to 0.0)	12.7 (-16.2 to 104.9)	-0.1 (-21.3 to 41.6)
Low birth weight for gestation	0.0 (0.0 to 0.0)	0.0 (0.0 to 0.0)	0.0 (0.0 to 0.0)	3.8 (-16.4 to 26.1)	2.9 (-17.7 to 25.2)	0.0 (0.0 to 0.0)	0.0 (0.0 to 0.0)	0.0 (0.0 to 0.0)	-7.3 (-62.4 to 28.2)	5.0 (-21.0 to 27.1)
Iron deficiency	-	-	-	-	-	7.9 (5.4 to 10.9)	7.8 (5.5 to 10.8)	7.1 (4.8 to 9.9)	-1.3 (-22.8 to 26.3)	-8.4 (-24.3 to 9.3)
Vitamin A deficiency	0.9 (0.6 to 1.3)	0.7 (0.5 to 1.0)	0.6 (0.4 to 0.9)	-19.8 (-35.2 to -0.8)	-12.5 (-31.1 to 7.3)	0.7 (0.5 to 0.9)	0.7 (0.4 to 1.0)	0.7 (0.4 to 0.9)	9.1 (-12.1 to 30.3)	-7.2 (-22.9 to 11.6)
Zinc deficiency	0.5 (0.1 to 1.0)	0.6 (0.1 to 1.2)	0.5 (0.1 to 1.2)	19.0 (-59.7 to 188.7)	-13.5 (-64.3 to 133.7)	0.5 (0.2 to 1.0)	0.4 (0.1 to 1.0)	0.3 (0.1 to 0.8)	-22.5 (-65.2 to 72.5)	-7.3 (-65.1 to 178.6)
Smoking	17.0 (13.4 to 20.3)	11.9 (9.6 to 14.6)	10.1 (7.9 to 12.4)	-29.7 (-39.1 to -18.9)	-15.5 (-23.9 to -6.2)	3.8 (2.7 to 5.2)	3.2 (2.3 to 4.4)	3.1 (2.2 to 4.2)	-16.2 (-35.2 to 9.1)	-3.4 (-20.1 to 15.5)
Chewing tobacco	1.4 (1.0 to 2.0)	0.9 (0.6 to 1.3)	0.8 (0.5 to 1.1)	-33.1 (-57.7 to 3.6)	-18.4 (-41.9 to 12.7)	0.1 (0.1 to 0.2)	0.1 (0.1 to 0.1)	0.1 (0.1 to 0.1)	-4.4 (-42.6 to 59.3)	-2.0 (-34.2 to 41.9)
Second-hand smoke	38.4 (34.5 to 42.9)	36.5 (32.3 to 40.8)	36.9 (32.5 to 41.4)	-5.2 (-16.2 to 7.1)	1.3 (-7.7 to 11.3)	58.0 (54.0 to 61.8)	49.3 (45.7 to 53.1)	50.2 (46.1 to 54.3)	-15.0 (-21.0 to -9.0)	1.8 (-4.0 to 8.3)
Alcohol use	14.8 (7.6 to 25.6)	15.0 (8.0 to 25.3)	16.9 (8.1 to 29.8)	1.3 (-30.3 to 51.5)	12.7 (-21.2 to 59.9)	11.5 (5.8 to 20.7)	11.5 (5.9 to 20.6)	13.0 (6.1 to 23.2)	0.1 (-33.0 to 48.7)	12.3 (-21.9 to 61.1)
Drug use	0.5 (0.4 to 0.7)	0.5 (0.4 to 0.7)	0.6 (0.4 to 0.8)	0.3 (-5.7 to 6.2)	11.5 (4.6 to 20.0)	0.3 (0.2 to 0.4)	0.3 (0.2 to 0.4)	0.3 (0.3 to 0.4)	1.9 (-3.8 to 8.5)	8.6 (2.7 to 16.4)
Diet low in fruits	40.0 (34.6 to 44.0)	38.1 (32.1 to 42.3)	39.6 (33.4 to 43.6)	-4.7 (-11.5 to 2.0)	3.8 (-1.3 to 9.7)	37.0 (31.1 to 41.5)	34.1 (27.6 to 38.8)	36.6 (30.3 to 41.1)	-7.9 (-16.4 to 0.5)	7.6 (0.7 to 16.3)
Diet low in vegetables	18.0 (9.8 to 25.8)	12.6 (5.0 to 20.5)	14.8 (6.4 to 22.9)	-29.9 (-63.5 to -2.0)	17.6 (-17.4 to 80.6)	20.3 (11.5 to 28.3)	15.0 (6.9 to 23.0)	17.0 (8.5 to 24.7)	-26.3 (-54.6 to 1.6)	13.8 (-12.2 to 60.8)
Diet low in legumes	4.5 (0.9 to 9.8)	6.4 (1.6 to 12.5)	8.8 (2.9 to 15.7)	43.7 (-30.3 to 304.1)	36.4 (-14.7 to 165.1)	8.7 (2.6 to 15.5)	9.5 (3.4 to 16.0)	12.4 (5.6 to 19.6)	9.0 (-32.5 to 108.4)	30.3 (-4.3 to 106.7)
Diet low in whole grains	29.7 (24.8 to 34.0)	34.7 (30.6 to 38.2)	36.1 (32.0 to 39.4)	16.7 (7.2 to 30.2)	4.0 (-1.8 to 9.4)	31.2 (26.3 to 35.3)	35.9 (32.0 to 39.3)	37.2 (33.4 to 40.3)	15.2 (6.4 to 27.3)	3.6 (-0.8 to 8.7)
Diet low in nuts and seeds	50.1 (48.5 to 51.3)	48.5 (46.5 to 50.1)	48.4 (46.1 to 50.1)	-3.1 (-5.9 to -0.5)	-0.4 (-2.8 to 2.0)	50.2 (48.6 to 51.4)	48.5 (46.6 to 50.1)	48.2 (46.0 to 50.0)	-3.4 (-6.2 to -0.9)	-0.5 (-3.0 to 1.8)
Diet low in milk	45.8 (43.5 to 47.7)	46.2 (44.2 to 47.9)	46.7 (44.9 to 48.3)	0.8 (-1.2 to 3.0)	1.1 (-0.3 to 2.7)	45.8 (43.8 to 47.7)	45.7 (43.5 to 47.5)	46.4 (44.3 to 48.1)	-0.3 (-2.5 to 1.7)	1.5 (-0.1 to 3.3)
Diet high in red meat	4.9 (1.0 to 10.6)	9.0 (3.2 to 16.5)	10.2 (3.6 to 18.2)	85.1 (-17.5 to 546.8)	13.8 (-35.1 to 106.9)	0.7 (0.0 to 3.0)	1.9 (0.1 to 5.7)	2.5 (0.1 to 6.9)	160.2 (-62.1 to 126816.8)	29.5 (-77.3 to 936.2)
Diet high in processed meat	12.8 (8.2 to 17.0)	21.1 (15.5 to 26.2)	24.4 (18.0 to 30.3)	65.2 (32.2 to 125.6)	15.8 (-2.4 to 38.7)	10.7 (6.9 to 14.5)	15.6 (11.0 to 19.8)	17.2 (12.2 to 21.7)	45.5 (14.3 to 100.4)	10.4 (-6.0 to 31.6)
Diet high in sugar-sweetened beverages	8.3 (3.3 to 15.7)	9.7 (4.1 to 17.3)	10.1 (4.4 to 18.2)	16.2 (-42.8 to 147.9)	4.1 (-35.9 to 78.1)	5.8 (2.1 to 12.1)	6.6 (2.8 to 12.7)	7.0 (2.8 to 13.1)	14.3 (-47.1 to 157.9)	6.2 (-40.5 to 87.4)
Diet low in fibre	21.5 (14.2 to 28.4)	22.7 (15.5 to 29.3)	23.9 (16.7 to 30.5)	5.6 (-12.4 to 29.2)	5.3 (-6.3 to 21.5)	22.8 (15.7 to 29.5)	24.2 (17.3 to 30.4)	25.9 (19.0 to 32.1)	6.2 (-9.7 to 30.0)	6.7 (-4.2 to 21.0)
Diet low in calcium	33.6 (29.0 to 37.4)	33.4 (28.8 to 37.2)	34.3 (30.0 to 38.0)	-0.4 (-4.4 to 3.9)	2.7 (-0.8 to 6.4)	35.9 (31.8 to 39.2)	35.0 (30.6 to 38.4)	36.3 (32.2 to 39.6)	-2.6 (-6.6 to 0.9)	3.8 (0.9 to 7.5)
Diet low in seafood omega-3 fatty acids	2.8 (1.1 to 4.3)	0.2 (0.0 to 0.8)	0.2 (0.0 to 1.0)	-94.0 (-100.0 to -74.0)	11.0 (-97.3 to 7181.4)	3.3 (1.4 to 4.9)	0.2 (0.0 to 1.0)	0.3 (0.0 to 1.2)	-92.9 (-100.0 to -74.6)	14.9 (-92.6 to 12463.3)
Diet low in polyunsaturated fatty acids	29.3 (23.3 to 34.5)	26.3 (19.5 to 31.7)	27.1 (20.6 to 32.3)	-9.9 (-22.6 to 0.8)	2.7 (-6.1 to 13.4)	30.0 (24.6 to 34.8)	27.2 (21.2 to 32.3)	27.9 (21.6 to 33.5)	-9.3 (-20.9 to 0.1)	2.7 (-5.2 to 13.6)
Diet high in trans fatty acids	1.1 (0.0 to 3.3)	0.9 (0.0 to 3.0)	0.9 (0.0 to 2.9)	-12.6 (-39.4 to -6.9)	-8.3 (-32.5 to -3.3)	1.5 (0.1 to 4.0)	1.3 (0.1 to 3.7)	1.2 (0.0 to 3.5)	-12.5 (-36.4 to -5.9)	-8.5 (-29.1 to -3.6)
Diet high in sodium	28.9 (19.2 to 37.0)	18.9 (10.0 to 25.9)	16.3 (7.5 to 23.8)	-34.7 (-58.3 to -15.6)	-13.7 (-37.2 to 3.1)	32.2 (24.0 to 39.2)	19.4 (11.0 to 26.1)	16.0 (6.9 to 23.0)	-39.7 (-59.8 to -25.1)	-17.5 (-40.2 to -4.5)
Intimate partner violence	-	-	-	-	-	2.2 (1.9 to 2.6)	2.0 (1.7 to 2.3)	1.9 (1.6 to 2.2)	-9.3 (-13.5 to -4.9)	-4.4 (-8.3 to 0.0)
Childhood sexual abuse	5.3 (4.3 to 6.5)	5.3 (4.3 to 6.5)	5.3 (4.3 to 6.5)	0.0 (0.0 to 0.0)	0.0 (0.0 to 0.0)	7.3 (5.9 to 8.9)	7.3 (5.9 to 8.9)	7.5 (6.0 to 9.2)	0.0 (0.0 to 0.0)	2.8 (-4.0 to 10.6)

Supplementary Results Table 1. Shiga age-standardised Summary Exposure Values (SEVs) for Level 4 risk factors and percentage change, by sex, 1990, 2007, and 2017.Data in parentheses are 95% uncertainty intervals.										
	**Male**					**Female**				
	**1990**	**2007**	**2017**	**Percentage change 1990-2007**	**Percentage change 2007-2017**	**1990**	**2007**	**2017**	**Percentage change 1990-2007**	**Percentage change 2007-2017**
Bullying victimization	3.3 (1.3 to 7.0)	3.3 (1.3 to 7.0)	3.5 (1.4 to 7.3)	1.0 (-6.9 to 8.8)	3.9 (-1.2 to 8.9)	1.9 (0.8 to 4.0)	1.9 (0.8 to 4.0)	2.0 (0.8 to 4.1)	-0.3 (-8.0 to 7.6)	3.3 (-2.1 to 8.4)
Low physical activity	0.5 (0.2 to 0.9)	0.5 (0.2 to 0.9)	0.4 (0.2 to 0.8)	-1.8 (-3.6 to 0.1)	-8.8 (-12.4 to -4.4)	0.5 (0.2 to 1.0)	0.5 (0.2 to 0.9)	0.5 (0.2 to 0.9)	-3.8 (-6.2 to -1.2)	0.8 (-0.6 to 2.2)
High fasting plasma glucose	1.4 (0.9 to 2.2)	1.4 (0.9 to 2.2)	1.6 (0.9 to 2.7)	2.7 (-36.8 to 71.9)	13.5 (-26.2 to 79.7)	1.2 (0.7 to 2.0)	1.3 (0.7 to 2.0)	1.4 (0.8 to 2.4)	3.0 (-38.7 to 73.3)	10.0 (-29.3 to 79.2)
High LDL cholesterol	12.1 (10.5 to 13.9)	12.6 (11.0 to 14.5)	12.7 (11.0 to 14.4)	4.5 (-1.2 to 10.7)	0.1 (-4.2 to 4.9)	13.0 (11.5 to 14.7)	13.1 (11.5 to 14.9)	13.1 (11.5 to 14.9)	1.1 (-4.3 to 6.7)	0.1 (-4.3 to 4.5)
High systolic blood pressure	11.3 (10.3 to 12.4)	8.4 (7.5 to 9.3)	8.3 (7.3 to 9.2)	-26.0 (-32.5 to -19.3)	-1.5 (-9.0 to 6.5)	9.7 (8.6 to 10.7)	6.1 (5.3 to 6.8)	5.6 (4.8 to 6.5)	-37.5 (-44.5 to -29.8)	-7.1 (-16.5 to 4.0)
High body-mass index	5.9 (3.8 to 9.4)	8.6 (5.9 to 13.3)	9.0 (6.1 to 13.9)	45.9 (17.7 to 87.9)	5.1 (-11.1 to 22.5)	5.8 (3.6 to 9.2)	7.6 (4.8 to 12.2)	7.6 (4.8 to 12.0)	30.3 (2.4 to 75.6)	0.2 (-17.9 to 21.9)
Low bone mineral density	5.3 (4.4 to 6.3)	5.2 (4.3 to 6.1)	5.2 (4.3 to 6.2)	-2.3 (-14.6 to 11.5)	0.6 (-11.1 to 16.0)	7.2 (6.1 to 8.5)	7.0 (5.9 to 8.1)	7.1 (6.0 to 8.3)	-3.3 (-14.1 to 10.0)	1.6 (-9.4 to 13.9)
Impaired kidney function	2.6 (1.2 to 6.5)	2.5 (1.1 to 6.4)	2.4 (1.0 to 6.1)	-3.6 (-8.0 to 0.8)	-6.5 (-11.9 to -2.0)	3.2 (1.5 to 7.4)	3.3 (1.5 to 7.6)	3.1 (1.4 to 7.3)	2.3 (-1.5 to 6.8)	-6.8 (-11.5 to -2.1)

Supplementary Results Table 1. Shimane age-standardised Summary Exposure Values (SEVs) for Level 4 risk factors and percentage change, by sex, 1990, 2007, and 2017.Data in parentheses are 95% uncertainty intervals.										
	**Male**					**Female**				
	**1990**	**2007**	**2017**	**Percentage change 1990-2007**	**Percentage change 2007-2017**	**1990**	**2007**	**2017**	**Percentage change 1990-2007**	**Percentage change 2007-2017**
Unsafe water source	2.7 (1.5 to 4.5)	2.2 (1.1 to 3.7)	2.3 (1.2 to 3.9)	-19.2 (-59.9 to 71.2)	5.6 (-40.1 to 88.5)	2.7 (1.5 to 4.5)	2.2 (1.1 to 3.7)	2.3 (1.2 to 3.9)	-19.2 (-59.9 to 71.2)	5.6 (-40.1 to 88.5)
Unsafe sanitation	5.0 (2.4 to 8.8)	2.4 (1.1 to 4.5)	2.0 (0.9 to 3.9)	-52.8 (-80.4 to 10.0)	-16.0 (-57.7 to 70.6)	5.0 (2.4 to 8.8)	2.4 (1.1 to 4.5)	2.0 (0.9 to 3.9)	-52.8 (-80.4 to 10.0)	-16.0 (-57.7 to 70.6)
No access to handwashing facility	6.9 (5.1 to 9.2)	6.4 (4.7 to 8.8)	6.0 (4.4 to 8.1)	-6.2 (-33.1 to 34.2)	-6.9 (-26.6 to 19.3)	6.9 (5.1 to 9.2)	6.4 (4.7 to 8.8)	6.0 (4.4 to 8.1)	-6.2 (-33.1 to 34.2)	-6.9 (-26.6 to 19.3)
Ambient particulate matter pollution	30.7 (18.6 to 46.8)	31.3 (21.1 to 46.8)	26.8 (15.6 to 41.0)	2.0 (-30.2 to 57.6)	-14.6 (-46.7 to 22.7)	30.3 (18.3 to 46.4)	31.3 (21.1 to 46.7)	26.7 (15.6 to 41.0)	3.0 (-30.0 to 59.4)	-14.6 (-46.7 to 22.7)
Household air pollution from solid fuels	1.0 (0.3 to 2.7)	0.2 (0.1 to 0.5)	0.2 (0.0 to 0.4)	-77.7 (-93.0 to -48.9)	-17.6 (-64.5 to 87.8)	1.3 (0.4 to 7.2)	0.3 (0.1 to 1.7)	0.3 (0.1 to 1.5)	-74.4 (-92.9 to -41.0)	-14.8 (-65.4 to 89.4)
Ambient ozone pollution	51.5 (24.6 to 54.8)	51.5 (24.5 to 54.8)	51.7 (25.1 to 54.8)	-0.0 (-0.6 to 0.4)	0.4 (0.0 to 2.6)	51.5 (24.6 to 54.8)	51.5 (24.5 to 54.8)	51.7 (25.1 to 54.8)	-0.0 (-0.6 to 0.4)	0.4 (0.0 to 2.6)
Residential radon	14.6 (1.3 to 48.9)	14.6 (1.3 to 48.9)	14.6 (1.3 to 48.9)	0.0 (0.0 to 0.0)	0.0 (0.0 to 0.0)	14.6 (1.3 to 48.9)	14.6 (1.3 to 48.9)	14.6 (1.3 to 48.9)	0.0 (0.0 to 0.0)	0.0 (0.0 to 0.0)
Lead exposure	2.9 (0.4 to 6.5)	2.0 (0.3 to 5.1)	1.5 (0.2 to 4.2)	-29.3 (-42.1 to -20.9)	-25.4 (-40.3 to -17.6)	1.6 (0.1 to 4.5)	1.1 (0.1 to 3.4)	0.8 (0.0 to 2.8)	-32.5 (-48.5 to -23.8)	-26.1 (-47.6 to -17.4)
Occupational exposure to asbestos	1.6 (1.4 to 1.8)	2.3 (2.1 to 2.6)	2.1 (1.8 to 2.6)	48.4 (29.3 to 75.1)	-8.5 (-25.0 to 10.1)	0.3 (0.2 to 0.4)	0.3 (0.3 to 0.3)	0.3 (0.2 to 0.4)	0.4 (-18.7 to 32.8)	1.1 (-20.1 to 27.4)
Occupational exposure to arsenic	0.4 (0.0 to 1.0)	0.4 (0.0 to 1.0)	0.4 (0.0 to 1.0)	-	-	0.4 (0.0 to 0.9)	0.3 (0.0 to 0.8)	0.4 (0.0 to 0.9)	-	-
Occupational exposure to benzene	0.6 (0.0 to 1.7)	0.6 (0.0 to 1.8)	0.7 (0.0 to 1.8)	2.7 (-4.3 to 12.6)	3.5 (-1.9 to 12.2)	0.5 (0.0 to 1.5)	0.7 (0.0 to 2.0)	0.8 (0.0 to 2.3)	24.2 (9.9 to 49.3)	15.1 (5.1 to 34.5)
Occupational exposure to beryllium	0.0 (0.0 to 0.0)	0.0 (0.0 to 0.0)	0.0 (0.0 to 0.0)	-5.0 (-10.1 to -0.1)	2.0 (-1.8 to 5.8)	0.0 (0.0 to 0.0)	0.0 (0.0 to 0.0)	0.0 (0.0 to 0.0)	3.7 (-5.5 to 15.0)	9.7 (3.1 to 18.1)
Occupational exposure to cadmium	0.1 (0.0 to 0.1)	0.0 (0.0 to 0.1)	0.0 (0.0 to 0.1)	-6.1 (-17.3 to 6.0)	1.1 (-8.6 to 11.4)	0.0 (0.0 to 0.1)	0.0 (0.0 to 0.0)	0.0 (0.0 to 0.1)	-6.5 (-21.2 to 11.8)	5.7 (-7.7 to 20.1)
Occupational exposure to chromium	0.1 (0.1 to 0.1)	0.1 (0.1 to 0.1)	0.1 (0.1 to 0.1)	-4.2 (-14.7 to 6.6)	1.7 (-7.0 to 10.8)	0.1 (0.1 to 0.1)	0.1 (0.1 to 0.1)	0.1 (0.1 to 0.1)	-1.6 (-15.8 to 15.7)	7.5 (-4.2 to 20.2)
Occupational exposure to diesel engine exhaust	0.6 (0.5 to 0.6)	0.6 (0.5 to 0.6)	0.6 (0.5 to 0.6)	-0.6 (-9.0 to 7.9)	3.5 (-3.2 to 10.4)	0.4 (0.4 to 0.5)	0.5 (0.4 to 0.5)	0.5 (0.5 to 0.6)	14.8 (0.4 to 31.1)	12.1 (0.9 to 23.7)
Occupational exposure to formaldehyde	0.2 (0.2 to 0.3)	0.2 (0.2 to 0.3)	0.2 (0.2 to 0.3)	-5.4 (-17.8 to 8.1)	0.8 (-9.4 to 11.8)	0.2 (0.2 to 0.2)	0.2 (0.2 to 0.2)	0.2 (0.2 to 0.2)	-3.7 (-19.1 to 16.6)	6.6 (-6.6 to 21.5)
Occupational exposure to nickel	0.4 (0.0 to 1.9)	0.4 (0.0 to 1.6)	0.4 (0.0 to 1.6)	-	-	0.3 (0.0 to 1.4)	0.3 (0.0 to 1.3)	0.3 (0.0 to 1.3)	-	-
Occupational exposure to polycyclic aromatic hydrocarbons	0.2 (0.2 to 0.3)	0.2 (0.2 to 0.2)	0.2 (0.2 to 0.3)	-3.4 (-13.2 to 6.8)	2.0 (-5.9 to 10.3)	0.2 (0.2 to 0.2)	0.2 (0.2 to 0.2)	0.2 (0.2 to 0.2)	0.4 (-13.3 to 17.9)	8.3 (-2.2 to 20.8)
Occupational exposure to silica	4.4 (0.7 to 14.2)	4.2 (0.6 to 13.3)	4.2 (0.6 to 13.4)	-5.8 (-16.4 to 6.9)	1.4 (-7.2 to 12.7)	3.0 (0.4 to 9.6)	2.6 (0.4 to 8.5)	2.7 (0.4 to 8.8)	-11.5 (-25.4 to 7.3)	2.5 (-11.0 to 20.5)
Occupational exposure to sulphuric acid	0.6 (0.1 to 1.9)	0.6 (0.1 to 1.7)	0.6 (0.1 to 1.7)	-6.5 (-17.0 to 5.4)	1.4 (-8.0 to 11.7)	0.5 (0.1 to 1.6)	0.4 (0.1 to 1.4)	0.5 (0.1 to 1.5)	-8.5 (-23.1 to 9.8)	5.0 (-8.6 to 20.6)
Occupational exposure to trichloroethylene	0.1 (0.1 to 0.1)	0.1 (0.1 to 0.1)	0.1 (0.1 to 0.1)	-3.2 (-11.0 to 5.0)	2.2 (-4.1 to 8.5)	0.1 (0.0 to 0.1)	0.1 (0.1 to 0.1)	0.1 (0.1 to 0.1)	3.3 (-8.7 to 17.6)	9.1 (-0.2 to 20.2)
Occupational asthmagens	17.4 (14.1 to 21.8)	17.2 (13.8 to 21.9)	17.3 (13.6 to 22.0)	-1.1 (-19.3 to 21.0)	0.2 (-15.2 to 18.5)	7.9 (6.2 to 9.9)	8.0 (6.3 to 9.9)	8.5 (6.8 to 10.8)	1.3 (-15.3 to 22.0)	5.8 (-7.9 to 22.2)
Occupational particulate matter, gases, and fumes	6.4 (4.5 to 9.2)	6.1 (4.3 to 8.7)	5.9 (4.1 to 8.4)	-4.7 (-9.6 to 0.3)	-3.8 (-7.1 to -0.6)	3.5 (2.4 to 5.5)	3.4 (2.3 to 5.2)	3.2 (2.2 to 4.9)	-5.4 (-13.5 to 4.4)	-4.3 (-9.9 to 2.3)
Occupational noise	6.0 (5.5 to 6.5)	5.8 (5.3 to 6.3)	5.6 (5.2 to 6.1)	-3.6 (-6.7 to -0.5)	-2.6 (-4.7 to -0.5)	3.4 (2.8 to 3.9)	3.4 (2.9 to 3.9)	3.4 (3.0 to 3.9)	0.3 (-6.0 to 7.8)	1.9 (-2.0 to 6.8)
Occupational ergonomic factors	11.4 (9.4 to 13.8)	10.1 (8.4 to 12.3)	10.0 (8.1 to 12.1)	-11.4 (-24.2 to 3.7)	-1.4 (-13.5 to 13.5)	6.1 (4.6 to 7.9)	6.1 (4.7 to 7.8)	6.9 (5.2 to 8.9)	0.8 (-22.4 to 31.6)	12.2 (-8.9 to 37.0)
Non-exclusive breastfeeding	0.4 (0.3 to 0.6)	0.4 (0.3 to 0.6)	0.4 (0.3 to 0.6)	-1.0 (-13.5 to 15.0)	-1.9 (-12.4 to 10.0)	0.4 (0.3 to 0.6)	0.4 (0.3 to 0.6)	0.4 (0.3 to 0.6)	-1.2 (-13.8 to 14.7)	-1.8 (-12.3 to 10.1)
Discontinued breastfeeding	1.6 (1.5 to 1.8)	1.7 (1.5 to 1.8)	1.6 (1.5 to 1.8)	2.4 (-7.0 to 13.4)	-3.0 (-10.7 to 5.2)	1.6 (1.5 to 1.8)	1.7 (1.6 to 1.8)	1.7 (1.5 to 1.8)	3.0 (-6.5 to 14.1)	-1.6 (-9.3 to 6.6)
Child underweight	0.3 (0.2 to 0.4)	0.2 (0.1 to 0.3)	0.2 (0.1 to 0.3)	-17.0 (-36.5 to 6.6)	-6.0 (-22.3 to 12.1)	0.3 (0.2 to 0.4)	0.2 (0.1 to 0.4)	0.2 (0.1 to 0.3)	-18.8 (-39.8 to 6.7)	-7.3 (-24.0 to 12.8)
Child wasting	0.2 (0.1 to 0.3)	0.2 (0.1 to 0.3)	0.2 (0.1 to 0.3)	-7.6 (-34.5 to 29.6)	-1.8 (-22.3 to 23.0)	0.2 (0.1 to 0.3)	0.2 (0.1 to 0.3)	0.2 (0.1 to 0.3)	-9.9 (-35.7 to 26.4)	-3.2 (-25.3 to 21.5)
Child stunting	0.7 (0.4 to 1.2)	0.6 (0.3 to 1.1)	0.6 (0.3 to 1.0)	-11.4 (-45.8 to 48.4)	-4.2 (-33.4 to 34.4)	0.7 (0.3 to 1.2)	0.6 (0.3 to 1.1)	0.6 (0.3 to 1.1)	-11.3 (-47.3 to 47.5)	-3.7 (-35.1 to 36.7)
Short gestation for birth weight	0.0 (0.0 to 0.0)	0.0 (0.0 to 0.0)	0.0 (0.0 to 0.0)	9.8 (-10.5 to 32.0)	3.9 (-20.9 to 44.5)	0.0 (0.0 to 0.0)	0.0 (0.0 to 0.0)	0.0 (0.0 to 0.0)	15.9 (-18.1 to 109.4)	0.6 (-21.7 to 42.8)
Low birth weight for gestation	0.0 (0.0 to 0.0)	0.0 (0.0 to 0.0)	0.0 (0.0 to 0.0)	4.3 (-10.6 to 24.8)	1.2 (-23.8 to 25.0)	0.0 (0.0 to 0.0)	0.0 (0.0 to 0.0)	0.0 (0.0 to 0.0)	-7.0 (-61.2 to 26.4)	3.6 (-21.4 to 26.0)
Iron deficiency	-	-	-	-	-	8.5 (5.8 to 11.8)	8.4 (5.8 to 11.5)	7.7 (5.3 to 10.7)	-0.8 (-22.8 to 28.4)	-8.4 (-24.0 to 9.4)
Vitamin A deficiency	1.0 (0.6 to 1.4)	0.9 (0.6 to 1.2)	0.7 (0.5 to 1.0)	-11.8 (-27.1 to 7.3)	-18.0 (-30.0 to -6.6)	0.7 (0.5 to 1.0)	0.8 (0.5 to 1.1)	0.7 (0.5 to 1.0)	14.2 (-8.8 to 43.9)	-14.0 (-28.5 to 4.1)
Zinc deficiency	0.5 (0.2 to 1.0)	0.4 (0.1 to 1.1)	0.4 (0.1 to 0.9)	-14.3 (-64.7 to 72.0)	-13.3 (-69.3 to 153.4)	0.5 (0.2 to 1.1)	0.4 (0.1 to 0.8)	0.4 (0.1 to 0.9)	-30.7 (-64.3 to 9.9)	6.3 (-59.6 to 155.5)
Smoking	18.5 (15.2 to 22.1)	12.9 (10.5 to 15.5)	10.8 (8.6 to 13.2)	-30.6 (-39.1 to -21.3)	-16.2 (-24.3 to -7.2)	4.2 (3.0 to 5.6)	3.4 (2.5 to 4.6)	3.4 (2.4 to 4.6)	-17.7 (-37.4 to 8.0)	-2.6 (-18.7 to 15.8)
Chewing tobacco	1.4 (0.9 to 2.0)	0.9 (0.6 to 1.4)	0.8 (0.5 to 1.1)	-32.4 (-58.5 to 8.4)	-18.6 (-42.7 to 14.6)	0.1 (0.1 to 0.1)	0.1 (0.1 to 0.1)	0.1 (0.1 to 0.1)	-4.3 (-42.0 to 57.6)	-1.8 (-32.8 to 42.0)
Second-hand smoke	37.0 (32.9 to 40.9)	35.1 (31.0 to 39.6)	35.4 (31.0 to 39.9)	-5.1 (-16.2 to 7.7)	0.6 (-8.4 to 11.0)	55.8 (52.1 to 59.3)	47.0 (43.3 to 50.6)	47.4 (43.4 to 51.3)	-15.8 (-22.2 to -9.3)	0.9 (-5.3 to 7.4)
Alcohol use	14.4 (7.4 to 24.8)	14.3 (7.1 to 24.8)	16.5 (8.1 to 28.8)	-0.3 (-34.9 to 53.9)	15.0 (-19.3 to 69.2)	12.0 (6.1 to 20.8)	11.9 (5.8 to 20.9)	13.1 (6.4 to 23.7)	-1.0 (-34.7 to 53.7)	10.2 (-21.3 to 60.4)
Drug use	0.5 (0.4 to 0.7)	0.5 (0.4 to 0.7)	0.6 (0.4 to 0.8)	0.3 (-5.5 to 6.5)	11.6 (3.5 to 19.9)	0.3 (0.2 to 0.4)	0.3 (0.2 to 0.4)	0.3 (0.3 to 0.4)	2.6 (-2.9 to 8.7)	8.3 (0.7 to 17.1)
Diet low in fruits	41.5 (36.1 to 45.2)	39.8 (34.2 to 43.8)	41.0 (35.3 to 44.7)	-4.2 (-10.5 to 1.6)	3.0 (-1.8 to 8.1)	38.6 (33.0 to 42.8)	36.0 (29.6 to 40.5)	38.1 (32.1 to 42.3)	-6.9 (-14.6 to 0.4)	5.9 (0.2 to 13.4)
Diet low in vegetables	20.2 (11.7 to 27.5)	13.9 (5.8 to 21.9)	15.6 (7.4 to 23.3)	-31.3 (-60.6 to -5.5)	12.7 (-14.4 to 64.9)	22.6 (14.2 to 29.5)	16.1 (7.9 to 23.8)	17.6 (9.3 to 25.3)	-28.7 (-55.0 to -6.3)	9.6 (-16.8 to 49.4)
Diet low in legumes	4.3 (0.6 to 9.5)	5.4 (0.9 to 10.6)	7.1 (1.8 to 13.4)	24.9 (-52.9 to 285.3)	32.4 (-22.5 to 216.2)	8.2 (2.7 to 14.9)	7.8 (2.4 to 14.3)	10.2 (3.4 to 17.4)	-4.8 (-50.9 to 70.9)	30.0 (-10.6 to 135.6)
Diet low in whole grains	30.9 (26.0 to 35.2)	35.5 (31.5 to 39.1)	36.9 (32.7 to 40.2)	15.1 (6.0 to 26.9)	3.8 (-1.1 to 9.0)	32.3 (27.7 to 36.2)	36.8 (33.2 to 40.1)	38.0 (34.6 to 41.2)	14.2 (6.3 to 24.4)	3.3 (-1.1 to 8.0)
Diet low in nuts and seeds	51.1 (49.9 to 52.1)	50.0 (48.4 to 51.2)	49.8 (48.1 to 51.2)	-2.3 (-4.6 to -0.3)	-0.3 (-2.2 to 1.5)	51.3 (50.1 to 52.2)	50.0 (48.6 to 51.3)	49.8 (48.2 to 51.2)	-2.5 (-4.4 to -0.5)	-0.3 (-2.4 to 1.4)
Diet low in milk	46.4 (44.3 to 48.1)	46.7 (44.8 to 48.3)	47.2 (45.3 to 48.7)	0.7 (-1.0 to 2.7)	1.0 (-0.4 to 2.5)	46.4 (44.3 to 48.1)	46.1 (44.0 to 47.8)	46.7 (44.7 to 48.4)	-0.6 (-2.6 to 1.4)	1.3 (-0.2 to 2.9)
Diet high in red meat	3.6 (0.3 to 8.5)	7.4 (2.2 to 13.8)	8.4 (2.7 to 15.4)	105.8 (-19.8 to 1117.6)	14.9 (-40.5 to 132.8)	0.5 (0.0 to 2.4)	1.4 (0.0 to 4.4)	1.9 (0.0 to 5.6)	182.8 (-74.8 to 1333302.1)	31.0 (-89.3 to 4106.6)
Diet high in processed meat	10.7 (6.6 to 15.0)	17.8 (12.6 to 22.4)	20.6 (14.9 to 26.1)	65.7 (29.7 to 134.2)	15.6 (-3.0 to 37.6)	8.7 (5.0 to 12.2)	12.5 (8.5 to 16.4)	13.5 (9.2 to 17.6)	44.3 (10.8 to 103.4)	7.9 (-8.6 to 30.4)
Diet high in sugar-sweetened beverages	6.8 (2.6 to 14.3)	7.8 (3.4 to 14.1)	8.1 (3.3 to 15.2)	14.2 (-45.6 to 142.0)	3.9 (-40.5 to 78.1)	4.8 (1.9 to 10.5)	5.4 (2.3 to 10.0)	5.7 (2.4 to 10.8)	11.5 (-49.5 to 150.3)	4.9 (-39.7 to 88.2)
Diet low in fibre	22.1 (14.8 to 28.5)	22.9 (15.8 to 29.1)	23.8 (16.6 to 30.0)	3.6 (-12.0 to 27.9)	3.6 (-9.7 to 18.9)	23.3 (16.3 to 29.9)	23.9 (16.9 to 30.1)	25.1 (18.1 to 31.3)	2.7 (-12.6 to 23.8)	4.7 (-7.6 to 19.0)
Diet low in calcium	35.3 (31.1 to 38.8)	35.0 (30.7 to 38.5)	35.7 (31.4 to 39.1)	-1.0 (-4.8 to 2.7)	2.0 (-0.9 to 5.3)	37.5 (33.7 to 40.6)	36.2 (32.1 to 39.5)	37.2 (33.4 to 40.4)	-3.6 (-7.3 to -0.3)	3.0 (0.4 to 6.5)
Diet low in seafood omega-3 fatty acids	3.4 (1.7 to 4.8)	0.3 (0.0 to 1.2)	0.3 (0.0 to 1.2)	-90.5 (-99.9 to -64.3)	3.0 (-91.2 to 969.6)	3.8 (1.8 to 5.3)	0.4 (0.0 to 1.4)	0.4 (0.0 to 1.5)	-90.5 (-100.0 to -68.3)	12.2 (-78.9 to 1558.7)
Diet low in polyunsaturated fatty acids	31.0 (25.7 to 35.6)	28.3 (22.6 to 33.2)	29.0 (23.3 to 33.7)	-8.6 (-19.7 to 0.7)	2.4 (-5.2 to 10.8)	31.6 (26.3 to 36.3)	29.0 (23.5 to 33.7)	29.6 (24.0 to 34.2)	-8.4 (-19.4 to 0.7)	2.1 (-4.9 to 10.6)
Diet high in trans fatty acids	1.1 (0.0 to 3.3)	0.9 (0.0 to 3.0)	0.9 (0.0 to 2.9)	-12.6 (-39.8 to -6.8)	-8.3 (-31.8 to -3.6)	1.5 (0.1 to 4.0)	1.3 (0.1 to 3.7)	1.2 (0.0 to 3.5)	-12.5 (-35.3 to -5.9)	-8.5 (-29.1 to -3.5)
Diet high in sodium	23.4 (13.7 to 31.4)	17.2 (8.1 to 24.3)	15.1 (5.9 to 22.4)	-26.6 (-52.0 to -6.3)	-12.3 (-37.4 to 6.0)	24.2 (14.8 to 31.7)	17.9 (9.4 to 25.0)	15.5 (6.7 to 22.7)	-26.2 (-47.4 to -10.8)	-13.3 (-36.4 to 1.0)
Intimate partner violence	-	-	-	-	-	2.3 (2.0 to 2.7)	2.1 (1.8 to 2.5)	2.0 (1.7 to 2.3)	-10.0 (-14.4 to -5.8)	-3.6 (-7.9 to 0.7)
Childhood sexual abuse	5.3 (4.3 to 6.5)	5.3 (4.3 to 6.5)	5.3 (4.3 to 6.5)	0.0 (0.0 to 0.0)	0.0 (0.0 to 0.0)	7.3 (5.9 to 8.9)	7.3 (5.9 to 8.9)	7.5 (6.0 to 9.2)	0.0 (0.0 to 0.0)	2.8 (-4.0 to 10.6)

Supplementary Results Table 1. Shimane age-standardised Summary Exposure Values (SEVs) for Level 4 risk factors and percentage change, by sex, 1990, 2007, and 2017.Data in parentheses are 95% uncertainty intervals.

	Male					Female				
	1990	2007	2017	Percentage change 1990-2007	Percentage change 2007-2017	1990	2007	2017	Percentage change 1990-2007	Percentage change 2007-2017
Bullying victimization	3.3 (1.4 to 6.6)	3.4 (1.4 to 6.9)	3.5 (1.4 to 7.2)	2.2 (-5.5 to 10.4)	2.5 (-2.6 to 7.4)	1.9 (0.8 to 4.0)	2.0 (0.8 to 4.0)	2.0 (0.8 to 4.1)	0.7 (-6.9 to 8.8)	1.7 (-3.8 to 6.4)
Low physical activity	0.5 (0.2 to 0.9)	0.5 (0.2 to 0.9)	0.4 (0.2 to 0.8)	-1.8 (-3.8 to -0.1)	-8.7 (-12.2 to -4.6)	0.5 (0.2 to 1.0)	0.5 (0.2 to 0.9)	0.5 (0.2 to 0.9)	-3.9 (-6.3 to -1.3)	0.8 (-0.8 to 2.4)
High fasting plasma glucose	1.3 (0.8 to 2.1)	1.3 (0.9 to 2.1)	1.5 (0.9 to 2.5)	0.2 (-34.5 to 54.5)	10.7 (-22.5 to 64.6)	1.1 (0.7 to 1.8)	1.2 (0.7 to 2.0)	1.3 (0.8 to 2.3)	6.9 (-34.0 to 71.9)	10.8 (-30.4 to 76.7)
High LDL cholesterol	11.9 (10.3 to 13.7)	12.4 (10.8 to 14.3)	12.4 (10.8 to 14.2)	4.6 (-1.5 to 10.8)	0.0 (-4.5 to 4.7)	12.6 (11.1 to 14.4)	12.8 (11.3 to 14.5)	12.8 (11.2 to 14.6)	1.1 (-4.2 to 6.7)	0.3 (-4.1 to 4.8)
High systolic blood pressure	11.1 (10.1 to 12.2)	8.2 (7.3 to 9.1)	8.1 (7.1 to 9.1)	-25.9 (-31.5 to -19.5)	-1.7 (-9.7 to 6.4)	9.5 (8.5 to 10.5)	6.0 (5.2 to 6.7)	5.6 (4.8 to 6.4)	-37.3 (-43.9 to -30.2)	-7.0 (-16.5 to 3.1)
High body-mass index	4.7 (2.8 to 7.3)	6.6 (4.3 to 11.1)	6.8 (4.4 to 10.8)	41.6 (13.9 to 82.8)	2.9 (-12.5 to 20.1)	5.1 (3.0 to 8.0)	6.5 (4.0 to 10.7)	6.6 (4.1 to 10.8)	28.4 (-1.6 to 72.4)	0.7 (-18.1 to 22.5)
Low bone mineral density	5.3 (4.4 to 6.3)	5.2 (4.4 to 6.0)	5.2 (4.3 to 6.2)	-2.5 (-14.0 to 12.3)	0.1 (-13.0 to 14.0)	7.3 (6.1 to 8.6)	7.0 (6.0 to 8.2)	7.1 (5.9 to 8.4)	-3.9 (-15.2 to 8.3)	1.7 (-11.2 to 14.2)
Impaired kidney function	2.6 (1.1 to 6.4)	2.5 (1.1 to 6.3)	2.4 (1.1 to 6.1)	-2.4 (-7.1 to 1.9)	-6.4 (-11.7 to -1.9)	3.2 (1.4 to 7.4)	3.3 (1.5 to 7.6)	3.1 (1.4 to 7.2)	3.7 (0.3 to 8.5)	-6.8 (-11.5 to -2.3)

Supplementary Results Table 1. Shizuoka age-standardised Summary Exposure Values (SEVs) for Level 4 risk factors and percentage change, by sex, 1990, 2007, and 2017.Data in parentheses are 95% uncertainty intervals.										
	Male					Female				
	1990	2007	2017	Percentage change 1990-2007	Percentage change 2007-2017	1990	2007	2017	Percentage change 1990-2007	Percentage change 2007-2017
Unsafe water source	2.1 (1.1 to 3.5)	1.8 (0.9 to 2.9)	1.9 (1.0 to 3.1)	-15.7 (-56.7 to 71.1)	7.0 (-38.3 to 81.3)	2.1 (1.1 to 3.5)	1.8 (0.9 to 2.9)	1.9 (1.0 to 3.1)	-15.7 (-56.7 to 71.1)	7.0 (-38.3 to 81.3)
Unsafe sanitation	3.5 (1.7 to 6.3)	1.8 (0.8 to 3.4)	1.5 (0.7 to 3.0)	-48.2 (-78.5 to 22.3)	-15.1 (-55.2 to 67.9)	3.5 (1.7 to 6.3)	1.8 (0.8 to 3.4)	1.5 (0.7 to 3.0)	-48.2 (-78.5 to 22.3)	-15.1 (-55.2 to 67.9)
No access to handwashing facility	6.7 (4.8 to 9.0)	6.3 (4.5 to 8.5)	5.9 (4.2 to 8.1)	-5.8 (-33.3 to 33.7)	-7.2 (-28.4 to 21.3)	6.7 (4.8 to 9.0)	6.3 (4.5 to 8.5)	5.9 (4.2 to 8.1)	-5.8 (-33.3 to 33.7)	-7.2 (-28.4 to 21.3)
Ambient particulate matter pollution	32.0 (21.9 to 46.5)	31.7 (22.2 to 46.2)	27.3 (17.9 to 40.1)	-0.9 (-20.1 to 23.9)	-14.0 (-29.3 to 2.9)	31.8 (21.7 to 46.0)	31.7 (22.2 to 46.1)	27.3 (17.8 to 40.0)	-0.3 (-19.7 to 24.3)	-14.0 (-29.3 to 2.9)
Household air pollution from solid fuels	0.6 (0.2 to 1.5)	0.1 (0.0 to 0.3)	0.1 (0.0 to 0.3)	-75.1 (-93.0 to -49.3)	-15.5 (-66.8 to 72.4)	0.8 (0.2 to 5.9)	0.2 (0.0 to 1.0)	0.2 (0.0 to 0.8)	-71.6 (-92.9 to -41.9)	-13.7 (-66.2 to 72.9)
Ambient ozone pollution	52.0 (26.3 to 54.8)	52.1 (26.6 to 54.8)	52.4 (27.4 to 54.8)	0.2 (0.0 to 1.7)	0.4 (0.0 to 3.4)	52.0 (26.3 to 54.8)	52.1 (26.6 to 54.8)	52.4 (27.4 to 54.8)	0.2 (0.0 to 1.7)	0.4 (0.0 to 3.4)
Residential radon	14.9 (1.1 to 43.0)	14.9 (1.1 to 43.0)	14.9 (1.1 to 43.0)	0.0 (0.0 to 0.0)	0.0 (0.0 to 0.0)	14.9 (1.1 to 43.0)	14.9 (1.1 to 43.0)	14.9 (1.1 to 43.0)	0.0 (0.0 to 0.0)	0.0 (0.0 to 0.0)
Lead exposure	2.7 (0.4 to 6.1)	1.6 (0.2 to 4.4)	1.1 (0.1 to 3.4)	-38.3 (-56.3 to -27.9)	-31.5 (-50.2 to -22.7)	1.7 (0.1 to 4.7)	1.0 (0.1 to 3.3)	0.7 (0.0 to 2.5)	-39.7 (-59.5 to -28.8)	-31.8 (-54.5 to -23.8)
Occupational exposure to asbestos	2.0 (1.7 to 2.2)	2.6 (2.4 to 2.8)	2.8 (2.4 to 3.4)	31.6 (13.3 to 56.3)	9.4 (-8.4 to 31.1)	0.6 (0.5 to 0.7)	0.6 (0.5 to 0.6)	0.5 (0.4 to 0.6)	-2.8 (-19.4 to 23.0)	-6.1 (-24.3 to 18.2)
Occupational exposure to arsenic	0.5 (0.0 to 1.1)	0.4 (0.0 to 1.0)	0.4 (0.0 to 1.0)	-	-	0.4 (0.0 to 1.0)	0.4 (0.0 to 0.9)	0.4 (0.0 to 0.9)	-	-
Occupational exposure to benzene	0.6 (0.0 to 1.8)	0.7 (0.0 to 1.9)	0.7 (0.0 to 1.9)	2.6 (-4.2 to 12.0)	3.6 (-2.0 to 11.4)	0.6 (0.0 to 1.8)	0.7 (0.0 to 2.1)	0.9 (0.0 to 2.4)	20.8 (7.4 to 39.2)	14.1 (4.5 to 29.1)
Occupational exposure to beryllium	0.0 (0.0 to 0.0)	0.0 (0.0 to 0.0)	0.0 (0.0 to 0.0)	-5.2 (-9.9 to 0.1)	1.8 (-2.0 to 5.5)	0.0 (0.0 to 0.0)	0.0 (0.0 to 0.0)	0.0 (0.0 to 0.0)	1.2 (-7.4 to 10.5)	8.8 (2.6 to 16.1)
Occupational exposure to cadmium	0.1 (0.0 to 0.1)	0.0 (0.0 to 0.1)	0.0 (0.0 to 0.1)	-7.2 (-17.2 to 5.4)	0.6 (-8.8 to 10.0)	0.0 (0.0 to 0.1)	0.0 (0.0 to 0.1)	0.0 (0.0 to 0.1)	-10.1 (-24.8 to 6.0)	4.7 (-8.1 to 19.3)
Occupational exposure to chromium	0.1 (0.1 to 0.1)	0.1 (0.1 to 0.1)	0.1 (0.1 to 0.1)	-5.6 (-14.7 to 6.0)	1.2 (-6.9 to 9.2)	0.1 (0.1 to 0.1)	0.1 (0.1 to 0.1)	0.1 (0.1 to 0.1)	-5.4 (-18.8 to 8.8)	6.4 (-4.3 to 18.6)
Occupational exposure to diesel engine exhaust	0.6 (0.6 to 0.6)	0.6 (0.5 to 0.6)	0.6 (0.6 to 0.6)	-2.2 (-9.8 to 6.6)	2.9 (-3.7 to 9.6)	0.5 (0.4 to 0.5)	0.5 (0.5 to 0.6)	0.6 (0.5 to 0.6)	11.3 (-1.8 to 26.2)	10.8 (0.2 to 22.7)
Occupational exposure to formaldehyde	0.3 (0.2 to 0.3)	0.2 (0.2 to 0.3)	0.2 (0.2 to 0.3)	-6.7 (-17.1 to 7.1)	0.3 (-9.7 to 10.4)	0.2 (0.2 to 0.3)	0.2 (0.2 to 0.2)	0.2 (0.2 to 0.2)	-7.5 (-22.5 to 9.2)	5.4 (-7.0 to 19.8)
Occupational exposure to nickel	0.4 (0.0 to 1.9)	0.4 (0.0 to 1.8)	0.4 (0.0 to 1.7)	-	-	0.4 (0.0 to 1.6)	0.3 (0.0 to 1.3)	0.3 (0.0 to 1.4)	-	-
Occupational exposure to polycyclic aromatic hydrocarbons	0.2 (0.2 to 0.3)	0.2 (0.2 to 0.3)	0.2 (0.2 to 0.3)	-4.6 (-13.2 to 6.2)	1.5 (-6.0 to 9.0)	0.2 (0.2 to 0.3)	0.2 (0.2 to 0.2)	0.2 (0.2 to 0.3)	-3.3 (-16.2 to 10.3)	7.2 (-2.7 to 18.8)
Occupational exposure to silica	4.7 (0.7 to 15.0)	4.5 (0.7 to 14.2)	4.5 (0.6 to 14.3)	-6.2 (-15.9 to 7.9)	1.1 (-7.5 to 11.1)	3.3 (0.5 to 10.4)	2.8 (0.4 to 9.0)	2.8 (0.4 to 9.0)	-14.7 (-29.0 to 3.1)	1.6 (-11.6 to 20.7)
Occupational exposure to sulphuric acid	0.6 (0.1 to 2.0)	0.6 (0.1 to 1.8)	0.6 (0.1 to 1.8)	-7.1 (-17.2 to 4.8)	1.0 (-8.0 to 10.2)	0.5 (0.1 to 1.7)	0.5 (0.1 to 1.5)	0.5 (0.1 to 1.5)	-11.7 (-26.0 to 4.5)	4.0 (-9.2 to 19.3)
Occupational exposure to trichloroethylene	0.1 (0.1 to 0.1)	0.1 (0.1 to 0.1)	0.1 (0.1 to 0.1)	-4.6 (-11.6 to 3.6)	1.7 (-4.3 to 7.5)	0.1 (0.1 to 0.1)	0.1 (0.1 to 0.1)	0.1 (0.1 to 0.1)	-0.4 (-11.4 to 12.3)	8.0 (-0.1 to 18.4)
Occupational asthmagens	19.3 (15.2 to 24.4)	18.3 (14.7 to 23.0)	18.2 (14.5 to 23.4)	-4.8 (-22.0 to 17.7)	-0.5 (-16.4 to 17.1)	8.4 (6.8 to 10.4)	8.4 (6.8 to 10.3)	8.8 (7.1 to 10.9)	-0.9 (-15.6 to 15.3)	5.4 (-6.7 to 18.9)
Occupational particulate matter, gases, and fumes	6.4 (4.5 to 9.3)	6.1 (4.4 to 9.0)	5.9 (4.2 to 8.6)	-4.5 (-9.9 to 0.9)	-3.7 (-7.2 to -0.2)	3.7 (2.6 to 5.5)	3.5 (2.4 to 5.3)	3.3 (2.3 to 4.9)	-6.1 (-14.4 to 3.5)	-4.9 (-10.5 to 1.1)
Occupational noise	6.0 (5.5 to 6.5)	5.8 (5.3 to 6.3)	5.6 (5.2 to 6.1)	-3.2 (-6.3 to 0.2)	-2.4 (-4.4 to -0.2)	3.6 (3.1 to 4.1)	3.6 (3.2 to 4.0)	3.6 (3.3 to 4.1)	0.0 (-5.9 to 7.6)	1.5 (-2.3 to 5.6)
Occupational ergonomic factors	10.2 (8.3 to 12.2)	9.0 (7.4 to 10.8)	8.9 (7.3 to 10.7)	-11.2 (-22.6 to 1.8)	-1.6 (-11.3 to 10.0)	5.5 (4.2 to 7.0)	5.6 (4.4 to 7.0)	6.3 (4.9 to 8.1)	1.6 (-19.7 to 27.4)	12.6 (-6.2 to 34.7)
Non-exclusive breastfeeding	0.4 (0.3 to 0.6)	0.4 (0.3 to 0.6)	0.4 (0.3 to 0.6)	-0.7 (-13.3 to 13.7)	-1.7 (-12.0 to 10.0)	0.4 (0.3 to 0.6)	0.4 (0.3 to 0.6)	0.4 (0.3 to 0.6)	-1.1 (-13.7 to 13.2)	-1.7 (-12.0 to 10.1)
Discontinued breastfeeding	1.7 (1.6 to 1.9)	1.7 (1.6 to 1.9)	1.7 (1.6 to 1.8)	1.7 (-7.7 to 11.9)	-2.5 (-9.7 to 5.1)	1.7 (1.6 to 1.8)	1.7 (1.6 to 1.9)	1.7 (1.6 to 1.8)	2.4 (-7.0 to 12.7)	-3.3 (-10.4 to 4.3)
Child underweight	0.3 (0.2 to 0.4)	0.2 (0.2 to 0.4)	0.2 (0.1 to 0.3)	-11.9 (-30.6 to 9.4)	-5.5 (-19.6 to 10.7)	0.3 (0.2 to 0.4)	0.3 (0.2 to 0.4)	0.2 (0.1 to 0.3)	-13.2 (-31.9 to 11.7)	-6.1 (-21.3 to 12.5)
Child wasting	0.2 (0.1 to 0.3)	0.2 (0.1 to 0.3)	0.2 (0.1 to 0.3)	-8.0 (-33.0 to 24.4)	-2.5 (-21.4 to 20.3)	0.2 (0.1 to 0.3)	0.2 (0.1 to 0.3)	0.2 (0.1 to 0.3)	-9.1 (-35.6 to 24.9)	-2.4 (-21.6 to 22.6)
Child stunting	0.7 (0.3 to 1.1)	0.6 (0.3 to 1.0)	0.6 (0.3 to 1.0)	-8.2 (-42.5 to 42.2)	-3.0 (-32.9 to 37.5)	0.7 (0.3 to 1.2)	0.6 (0.3 to 1.0)	0.6 (0.3 to 0.9)	-10.3 (-44.0 to 48.0)	-3.6 (-34.9 to 40.5)
Short gestation for birth weight	0.0 (0.0 to 0.0)	0.0 (0.0 to 0.0)	0.0 (0.0 to 0.0)	8.8 (-12.6 to 35.0)	1.9 (-17.6 to 24.6)	0.0 (0.0 to 0.0)	0.0 (0.0 to 0.0)	0.0 (0.0 to 0.0)	12.5 (-15.7 to 76.1)	2.0 (-20.2 to 37.6)
Low birth weight for gestation	0.0 (0.0 to 0.0)	0.0 (0.0 to 0.0)	0.0 (0.0 to 0.0)	3.5 (-14.1 to 25.2)	2.4 (-13.6 to 20.5)	0.0 (0.0 to 0.0)	0.0 (0.0 to 0.0)	0.0 (0.0 to 0.0)	-2.7 (-57.1 to 26.2)	3.2 (-18.2 to 25.2)
Iron deficiency	-	-	-	-	-	8.0 (5.4 to 11.1)	8.0 (5.6 to 11.0)	7.3 (4.9 to 10.4)	0.4 (-21.7 to 29.4)	-8.9 (-25.1 to 9.7)
Vitamin A deficiency	0.8 (0.6 to 1.1)	0.8 (0.5 to 1.0)	0.7 (0.4 to 0.9)	-5.0 (-21.9 to 12.3)	-16.4 (-31.3 to 1.5)	0.7 (0.5 to 1.0)	0.7 (0.5 to 1.0)	0.7 (0.4 to 0.9)	1.4 (-18.5 to 22.5)	-12.0 (-31.9 to 7.7)
Zinc deficiency	0.5 (0.1 to 1.0)	0.5 (0.1 to 1.2)	0.5 (0.1 to 1.2)	15.2 (-59.6 to 182.0)	-14.4 (-63.3 to 118.4)	0.5 (0.1 to 1.0)	0.4 (0.1 to 1.0)	0.3 (0.1 to 0.8)	-22.4 (-64.5 to 72.2)	-7.8 (-64.6 to 165.8)
Smoking	17.3 (14.0 to 20.8)	12.2 (9.7 to 14.8)	10.3 (8.2 to 12.6)	-29.7 (-39.3 to -19.2)	-15.2 (-23.4 to -6.0)	3.9 (2.7 to 5.2)	3.2 (2.3 to 4.3)	3.1 (2.2 to 4.3)	-16.0 (-35.3 to 10.2)	-3.1 (-19.1 to 17.8)
Chewing tobacco	1.4 (0.9 to 2.0)	0.9 (0.6 to 1.4)	0.8 (0.5 to 1.1)	-32.7 (-57.4 to 5.9)	-18.7 (-41.9 to 13.0)	0.1 (0.1 to 0.1)	0.1 (0.1 to 0.1)	0.1 (0.1 to 0.1)	-4.2 (-42.5 to 54.7)	-1.5 (-29.4 to 38.0)
Second-hand smoke	37.4 (33.5 to 41.3)	36.5 (32.5 to 40.7)	36.3 (31.6 to 40.8)	-2.3 (-13.4 to 10.1)	-0.6 (-9.9 to 9.4)	56.6 (53.0 to 60.2)	49.4 (45.6 to 52.8)	49.0 (44.7 to 52.9)	-12.7 (-19.0 to -6.4)	-0.8 (-6.9 to 5.3)
Alcohol use	14.3 (7.3 to 24.3)	14.6 (7.4 to 25.2)	17.4 (8.3 to 29.5)	2.1 (-32.7 to 50.7)	19.2 (-16.7 to 67.2)	11.6 (5.8 to 20.4)	11.8 (5.8 to 21.0)	13.6 (6.3 to 24.0)	1.4 (-33.4 to 51.2)	15.9 (-18.3 to 69.2)
Drug use	0.5 (0.4 to 0.7)	0.5 (0.4 to 0.7)	0.6 (0.4 to 0.8)	0.4 (-5.4 to 6.5)	11.9 (5.1 to 20.6)	0.3 (0.2 to 0.4)	0.3 (0.2 to 0.4)	0.3 (0.3 to 0.4)	2.1 (-4.1 to 8.4)	9.0 (2.0 to 16.8)
Diet low in fruits	39.9 (34.3 to 43.8)	38.1 (32.1 to 42.1)	39.6 (33.7 to 43.5)	-4.6 (-11.1 to 2.0)	3.7 (-1.8 to 9.5)	36.7 (30.4 to 41.4)	34.0 (27.7 to 39.0)	36.3 (30.3 to 41.1)	-7.4 (-16.3 to 1.0)	7.0 (0.1 to 15.3)
Diet low in vegetables	18.0 (9.5 to 25.6)	12.3 (4.6 to 20.4)	14.5 (6.4 to 22.3)	-31.4 (-62.0 to -4.5)	17.7 (-15.6 to 87.9)	20.0 (11.4 to 27.6)	14.2 (6.5 to 21.7)	16.2 (8.3 to 23.7)	-29.0 (-56.5 to -4.5)	13.5 (-14.2 to 64.7)
Diet low in legumes	4.4 (0.7 to 9.7)	6.2 (1.3 to 12.2)	8.3 (2.5 to 14.6)	39.6 (-34.3 to 315.7)	34.4 (-20.2 to 193.5)	8.3 (2.4 to 15.1)	8.4 (2.5 to 14.8)	11.0 (4.4 to 18.2)	0.9 (-46.9 to 81.6)	30.9 (-8.8 to 141.2)
Diet low in whole grains	29.7 (24.4 to 33.9)	34.7 (30.5 to 38.2)	36.1 (31.9 to 39.5)	16.6 (7.1 to 31.6)	4.0 (-1.2 to 9.5)	31.2 (26.2 to 35.1)	35.9 (31.9 to 39.3)	37.2 (33.3 to 40.6)	15.2 (6.3 to 26.9)	3.7 (-0.7 to 8.7)
Diet low in nuts and seeds	50.1 (48.7 to 51.3)	48.6 (46.5 to 50.2)	48.5 (46.3 to 50.3)	-3.0 (-5.8 to -0.6)	-0.4 (-2.9 to 1.9)	50.2 (48.7 to 51.3)	48.4 (46.4 to 50.1)	48.1 (45.7 to 49.9)	-3.5 (-6.5 to -1.0)	-0.6 (-3.1 to 1.7)
Diet low in milk	45.8 (43.6 to 47.6)	46.1 (44.1 to 47.8)	46.6 (44.6 to 48.2)	0.7 (-1.2 to 2.8)	1.0 (-0.5 to 2.6)	45.8 (43.6 to 47.7)	45.7 (43.5 to 47.6)	46.4 (44.2 to 48.2)	-0.3 (-2.3 to 1.7)	1.5 (-0.0 to 3.3)
Diet high in red meat	4.8 (0.9 to 10.2)	8.9 (3.3 to 15.9)	10.1 (3.7 to 17.9)	84.2 (-11.0 to 573.8)	13.6 (-34.9 to 103.0)	0.8 (0.0 to 2.9)	2.0 (0.0 to 5.8)	2.5 (0.1 to 6.7)	160.2 (-66.1 to 165621.5)	27.7 (-81.2 to 1060.2)
Diet high in processed meat	12.8 (8.5 to 17.0)	21.2 (15.9 to 26.4)	24.6 (19.1 to 30.5)	66.0 (29.6 to 121.3)	16.3 (-2.3 to 38.6)	10.7 (6.9 to 14.6)	15.6 (11.2 to 19.8)	17.2 (12.6 to 21.7)	45.5 (14.7 to 96.6)	10.4 (-5.2 to 30.3)
Diet high in sugar-sweetened beverages	8.2 (3.5 to 15.7)	9.4 (4.3 to 16.3)	9.9 (4.1 to 17.4)	14.9 (-43.7 to 148.3)	5.1 (-36.9 to 74.1)	5.8 (2.3 to 12.6)	6.5 (2.8 to 12.5)	6.9 (3.0 to 13.0)	12.6 (-48.7 to 151.5)	5.8 (-38.0 to 83.9)
Diet low in fibre	21.3 (13.9 to 28.1)	22.5 (15.2 to 28.6)	23.7 (16.2 to 30.1)	5.6 (-12.5 to 28.9)	5.1 (-7.1 to 20.7)	22.5 (15.2 to 29.2)	23.5 (16.5 to 29.7)	24.9 (17.8 to 31.4)	4.5 (-12.9 to 28.1)	6.0 (-5.8 to 21.1)
Diet low in calcium	33.6 (29.1 to 37.3)	33.5 (28.8 to 37.2)	34.4 (29.9 to 37.9)	-0.4 (-4.6 to 3.6)	2.7 (-0.4 to 6.3)	35.9 (31.8 to 39.3)	35.0 (30.7 to 38.5)	36.4 (32.1 to 39.7)	-2.4 (-6.2 to 1.2)	3.9 (1.0 to 7.6)
Diet low in seafood omega-3 fatty acids	2.7 (1.0 to 4.3)	0.1 (0.0 to 0.8)	0.2 (0.0 to 0.8)	-95.1 (-100.0 to -77.7)	14.6 (-100.0 to 28901.2)	3.3 (1.3 to 4.8)	0.2 (0.0 to 1.1)	0.3 (0.0 to 1.1)	-92.6 (-100.0 to -72.0)	10.8 (-89.1 to 4698.2)
Diet low in polyunsaturated fatty acids	29.2 (23.7 to 34.2)	26.5 (20.3 to 31.8)	27.2 (20.7 to 32.4)	-9.5 (-22.4 to 0.6)	2.7 (-5.7 to 12.4)	30.0 (24.8 to 35.0)	27.2 (21.1 to 32.5)	28.0 (21.8 to 33.3)	-9.5 (-22.4 to 1.2)	2.9 (-6.0 to 15.9)
Diet high in trans fatty acids	1.1 (0.0 to 3.3)	0.9 (0.0 to 3.1)	0.9 (0.0 to 2.9)	-12.7 (-38.4 to -6.6)	-8.3 (-33.0 to -3.4)	1.5 (0.1 to 4.0)	1.3 (0.1 to 3.7)	1.2 (0.0 to 3.5)	-12.5 (-34.5 to -6.6)	-8.4 (-29.5 to -3.5)
Diet high in sodium	28.8 (19.2 to 36.9)	18.8 (10.0 to 26.0)	16.3 (7.4 to 23.6)	-34.6 (-57.0 to -18.0)	-13.4 (-35.1 to 2.9)	32.5 (24.6 to 39.6)	19.8 (11.3 to 26.4)	16.6 (7.8 to 23.5)	-39.1 (-58.6 to -24.2)	-16.3 (-38.6 to -3.2)
Intimate partner violence	-	-	-	-	-	2.2 (1.9 to 2.6)	2.1 (1.7 to 2.4)	2.0 (1.7 to 2.3)	-8.0 (-12.3 to -3.7)	-3.6 (-8.7 to 1.5)
Childhood sexual abuse	5.3 (4.3 to 6.5)	5.3 (4.3 to 6.5)	5.3 (4.3 to 6.5)	0.0 (0.0 to 0.0)	0.0 (0.0 to 0.0)	7.3 (5.9 to 8.9)	7.3 (5.9 to 8.9)	7.5 (6.0 to 9.2)	0.0 (0.0 to 0.0)	2.8 (-4.0 to 10.6)

Supplementary Results Table 1. Shizuoka age-standardised Summary Exposure Values (SEVs) for Level 4 risk factors and percentage change, by sex, 1990, 2007, and 2017.Data in parentheses are 95% uncertainty intervals.

	Male					Female				
	**1990**	**2007**	**2017**	**Percentage change 1990-2007**	**Percentage change 2007-2017**	**1990**	**2007**	**2017**	**Percentage change 1990-2007**	**Percentage change 2007-2017**
Bullying victimization	3.8 (1.6 to 7.9)	3.8 (1.7 to 7.6)	3.9 (1.7 to 7.7)	0.1 (-13.6 to 20.3)	2.0 (-7.8 to 13.0)	2.6 (1.0 to 5.3)	2.5 (1.1 to 5.0)	2.5 (1.1 to 4.9)	-2.4 (-17.4 to 18.2)	0.3 (-11.2 to 12.0)
Low physical activity	0.5 (0.2 to 0.9)	0.5 (0.2 to 0.9)	0.4 (0.2 to 0.8)	-1.7 (-3.5 to 0.1)	-8.8 (-12.2 to -4.7)	0.5 (0.2 to 1.0)	0.5 (0.2 to 0.9)	0.5 (0.2 to 0.9)	-3.8 (-6.1 to -1.3)	0.8 (-0.8 to 2.5)
High fasting plasma glucose	1.5 (0.9 to 2.3)	1.5 (0.9 to 2.4)	1.6 (1.0 to 2.7)	0.5 (-36.5 to 62.9)	13.1 (-27.0 to 82.7)	1.2 (0.7 to 1.9)	1.3 (0.8 to 2.1)	1.4 (0.8 to 2.4)	6.2 (-34.9 to 81.9)	9.8 (-30.8 to 78.5)
High LDL cholesterol	12.1 (10.4 to 14.0)	12.6 (11.0 to 14.4)	12.6 (11.0 to 14.4)	4.4 (-1.8 to 10.7)	0.0 (-4.4 to 4.8)	12.9 (11.4 to 14.6)	13.0 (11.5 to 14.7)	13.0 (11.5 to 14.8)	0.5 (-4.9 to 5.8)	0.4 (-4.0 to 5.0)
High systolic blood pressure	11.3 (10.2 to 12.3)	8.4 (7.5 to 9.3)	8.2 (7.2 to 9.3)	-25.9 (-31.5 to -19.8)	-1.5 (-8.9 to 6.2)	9.6 (8.6 to 10.6)	6.0 (5.3 to 6.8)	5.6 (4.8 to 6.4)	-37.2 (-44.0 to -30.2)	-7.2 (-16.7 to 3.0)
High body-mass index	6.3 (4.1 to 9.7)	9.0 (6.1 to 13.7)	9.4 (6.4 to 14.2)	43.4 (15.0 to 83.8)	4.2 (-11.0 to 21.4)	5.8 (3.6 to 9.4)	7.4 (4.9 to 11.5)	7.5 (4.7 to 11.5)	28.3 (1.0 to 70.9)	0.6 (-16.8 to 21.1)
Low bone mineral density	5.3 (4.4 to 6.3)	5.1 (4.3 to 6.0)	5.2 (4.3 to 6.1)	-2.4 (-13.8 to 10.9)	0.2 (-12.4 to 14.5)	7.2 (6.0 to 8.5)	7.0 (6.0 to 8.1)	7.1 (5.9 to 8.3)	-3.6 (-14.5 to 9.9)	1.8 (-11.0 to 15.8)
Impaired kidney function	2.6 (1.2 to 6.5)	2.6 (1.2 to 6.5)	2.4 (1.1 to 6.2)	-2.6 (-7.4 to 2.5)	-6.4 (-11.7 to -1.7)	3.3 (1.5 to 7.5)	3.4 (1.5 to 7.7)	3.1 (1.4 to 7.2)	3.1 (-0.9 to 7.7)	-6.9 (-12.0 to -2.0)

Supplementary Results Table 1. Tochigi age-standardised Summary Exposure Values (SEVs) for Level 4 risk factors and percentage change, by sex, 1990, 2007, and 2017.Data in parentheses are 95% uncertainty intervals.										
	Male					Female				
	1990	2007	2017	Percentage change 1990-2007	Percentage change 2007-2017	1990	2007	2017	Percentage change 1990-2007	Percentage change 2007-2017
Unsafe water source	2.3 (1.3 to 3.8)	1.8 (1.0 to 3.1)	1.9 (1.1 to 3.2)	-18.8 (-58.8 to 61.5)	5.8 (-38.9 to 84.3)	2.3 (1.3 to 3.8)	1.8 (1.0 to 3.1)	1.9 (1.1 to 3.2)	-18.8 (-58.8 to 61.5)	5.8 (-38.9 to 84.3)
Unsafe sanitation	3.9 (1.9 to 7.4)	1.9 (0.9 to 3.6)	1.6 (0.8 to 3.0)	-51.0 (-79.4 to 7.0)	-15.6 (-55.9 to 70.7)	3.9 (1.9 to 7.4)	1.9 (0.9 to 3.6)	1.6 (0.8 to 3.0)	-51.0 (-79.4 to 7.0)	-15.6 (-55.9 to 70.7)
No access to handwashing facility	6.7 (4.9 to 9.1)	6.3 (4.6 to 8.5)	5.8 (4.3 to 7.8)	-6.0 (-32.5 to 33.7)	-7.7 (-29.7 to 18.3)	6.7 (4.9 to 9.1)	6.3 (4.6 to 8.5)	5.8 (4.3 to 7.8)	-6.0 (-32.5 to 33.7)	-7.7 (-29.7 to 18.3)
Ambient particulate matter pollution	31.8 (22.0 to 45.7)	31.6 (21.6 to 45.7)	26.9 (17.4 to 41.1)	-0.5 (-22.1 to 26.5)	-14.8 (-33.8 to 7.5)	31.5 (21.7 to 45.4)	31.5 (21.5 to 45.7)	26.9 (17.3 to 41.0)	0.1 (-21.8 to 27.8)	-14.8 (-33.8 to 7.6)
Household air pollution from solid fuels	0.7 (0.2 to 1.9)	0.2 (0.0 to 0.4)	0.1 (0.0 to 0.3)	-76.1 (-92.8 to -47.7)	-15.5 (-66.8 to 80.7)	1.0 (0.3 to 5.9)	0.3 (0.0 to 1.1)	0.2 (0.0 to 1.0)	-72.9 (-92.7 to -40.6)	-12.0 (-66.5 to 82.2)
Ambient ozone pollution	52.6 (28.1 to 54.8)	52.6 (28.2 to 54.8)	52.7 (28.9 to 54.8)	0.0 (0.0 to 0.6)	0.3 (0.0 to 2.8)	52.6 (28.1 to 54.8)	52.6 (28.2 to 54.8)	52.7 (28.9 to 54.8)	0.0 (0.0 to 0.6)	0.3 (0.0 to 2.8)
Residential radon	15.2 (1.1 to 49.7)	15.2 (1.1 to 49.7)	15.2 (1.1 to 49.7)	0.0 (0.0 to 0.0)	0.0 (0.0 to 0.0)	15.2 (1.1 to 49.7)	15.2 (1.1 to 49.7)	15.2 (1.1 to 49.7)	0.0 (0.0 to 0.0)	0.0 (0.0 to 0.0)
Lead exposure	2.5 (0.3 to 6.0)	1.7 (0.2 to 4.6)	1.3 (0.1 to 3.7)	-32.0 (-46.5 to -23.3)	-27.2 (-43.7 to -19.3)	1.4 (0.1 to 4.2)	0.9 (0.0 to 3.0)	0.6 (0.0 to 2.4)	-37.5 (-55.6 to -27.9)	-29.9 (-52.5 to -20.8)
Occupational exposure to asbestos	1.5 (1.3 to 1.7)	1.9 (1.8 to 2.1)	1.8 (1.5 to 2.2)	28.9 (11.8 to 55.4)	-4.5 (-21.9 to 16.2)	0.4 (0.3 to 0.5)	0.4 (0.4 to 0.4)	0.4 (0.3 to 0.5)	-7.9 (-24.7 to 19.4)	-0.7 (-22.2 to 25.2)
Occupational exposure to arsenic	0.5 (0.0 to 1.0)	0.4 (0.0 to 1.0)	0.4 (0.0 to 1.0)	-	-	0.4 (0.0 to 1.0)	0.4 (0.0 to 0.9)	0.4 (0.0 to 0.9)	-	-
Occupational exposure to benzene	0.6 (0.0 to 1.8)	0.7 (0.0 to 1.9)	0.7 (0.0 to 1.9)	2.6 (-4.4 to 12.6)	3.6 (-2.0 to 12.0)	0.6 (0.0 to 1.7)	0.7 (0.0 to 2.1)	0.8 (0.0 to 2.5)	21.6 (8.6 to 42.5)	15.0 (4.7 to 28.1)
Occupational exposure to beryllium	0.0 (0.0 to 0.0)	0.0 (0.0 to 0.0)	0.0 (0.0 to 0.0)	-5.1 (-10.2 to -0.2)	2.0 (-1.8 to 6.0)	0.0 (0.0 to 0.0)	0.0 (0.0 to 0.0)	0.0 (0.0 to 0.0)	2.2 (-7.1 to 12.2)	9.2 (2.8 to 16.5)
Occupational exposure to cadmium	0.1 (0.0 to 0.1)	0.0 (0.0 to 0.1)	0.0 (0.0 to 0.1)	-7.0 (-17.9 to 6.0)	0.8 (-8.4 to 10.9)	0.0 (0.0 to 0.1)	0.0 (0.0 to 0.1)	0.0 (0.0 to 0.1)	-8.8 (-22.9 to 9.1)	4.8 (-8.1 to 18.4)
Occupational exposure to chromium	0.1 (0.1 to 0.1)	0.1 (0.1 to 0.1)	0.1 (0.1 to 0.1)	-5.3 (-15.0 to 6.3)	1.4 (-7.0 to 10.0)	0.1 (0.1 to 0.1)	0.1 (0.1 to 0.1)	0.1 (0.1 to 0.1)	-4.1 (-17.0 to 12.5)	6.6 (-4.5 to 18.4)
Occupational exposure to diesel engine exhaust	0.6 (0.5 to 0.6)	0.6 (0.5 to 0.6)	0.6 (0.6 to 0.7)	-1.0 (-9.0 to 8.2)	3.3 (-3.1 to 10.4)	0.4 (0.4 to 0.5)	0.5 (0.4 to 0.6)	0.6 (0.5 to 0.6)	13.1 (-0.6 to 28.9)	11.5 (0.7 to 23.3)
Occupational exposure to formaldehyde	0.3 (0.2 to 0.3)	0.2 (0.2 to 0.3)	0.2 (0.2 to 0.3)	-6.5 (-18.1 to 7.3)	0.4 (-9.2 to 11.2)	0.2 (0.2 to 0.3)	0.2 (0.2 to 0.2)	0.2 (0.2 to 0.2)	-6.2 (-21.0 to 12.8)	5.6 (-7.1 to 19.6)
Occupational exposure to nickel	0.4 (0.0 to 1.9)	0.4 (0.0 to 1.8)	0.4 (0.0 to 1.7)	-	-	0.3 (0.0 to 1.6)	0.3 (0.0 to 1.3)	0.3 (0.0 to 1.4)	-	-
Occupational exposure to polycyclic aromatic hydrocarbons	0.2 (0.2 to 0.3)	0.2 (0.2 to 0.3)	0.2 (0.2 to 0.3)	-4.3 (-13.4 to 6.5)	1.7 (-6.1 to 9.9)	0.2 (0.2 to 0.3)	0.2 (0.2 to 0.2)	0.2 (0.2 to 0.3)	-2.0 (-14.9 to 14.1)	7.5 (-3.1 to 18.8)
Occupational exposure to silica	4.6 (0.7 to 14.8)	4.4 (0.6 to 13.8)	4.4 (0.6 to 13.7)	-4.8 (-15.6 to 8.3)	1.5 (-6.6 to 11.9)	3.1 (0.5 to 9.9)	2.7 (0.4 to 8.4)	2.8 (0.4 to 8.6)	-13.1 (-26.8 to 5.9)	2.0 (-11.5 to 18.7)
Occupational exposure to sulphuric acid	0.6 (0.1 to 1.9)	0.6 (0.1 to 1.8)	0.6 (0.1 to 1.9)	-7.1 (-17.3 to 4.8)	1.1 (-7.8 to 11.2)	0.5 (0.1 to 1.7)	0.5 (0.1 to 1.5)	0.5 (0.1 to 1.5)	-10.3 (-24.3 to 7.2)	3.8 (-9.1 to 17.9)
Occupational exposure to trichloroethylene	0.1 (0.1 to 0.1)	0.1 (0.1 to 0.1)	0.1 (0.1 to 0.1)	-4.4 (-11.6 to 3.7)	1.9 (-4.0 to 8.2)	0.1 (0.1 to 0.1)	0.1 (0.1 to 0.1)	0.1 (0.1 to 0.1)	0.9 (-10.2 to 13.7)	8.3 (-0.2 to 18.1)
Occupational asthmagens	18.4 (14.4 to 23.2)	17.9 (14.3 to 22.3)	17.9 (14.1 to 22.6)	-2.6 (-21.8 to 22.7)	0.0 (-15.3 to 16.9)	8.2 (6.5 to 10.3)	8.2 (6.5 to 10.1)	8.7 (7.0 to 10.7)	0.2 (-14.6 to 18.1)	5.6 (-6.8 to 20.3)
Occupational particulate matter, gases, and fumes	6.3 (4.5 to 9.2)	6.1 (4.3 to 8.7)	5.9 (4.2 to 8.5)	-4.1 (-9.1 to 1.0)	-3.4 (-7.0 to -0.0)	3.6 (2.5 to 5.4)	3.4 (2.4 to 5.1)	3.3 (2.3 to 4.9)	-5.2 (-13.6 to 4.6)	-4.5 (-10.0 to 1.3)
Occupational noise	5.8 (5.4 to 6.4)	5.7 (5.2 to 6.2)	5.6 (5.1 to 6.1)	-2.7 (-5.8 to 0.6)	-2.1 (-4.2 to 0.3)	3.4 (3.0 to 4.0)	3.5 (3.1 to 4.0)	3.5 (3.2 to 4.0)	0.9 (-5.0 to 7.9)	2.0 (-2.0 to 6.2)
Occupational ergonomic factors	10.5 (8.7 to 12.6)	9.3 (7.6 to 11.2)	9.2 (7.5 to 11.2)	-11.7 (-23.3 to 0.8)	-1.2 (-12.4 to 10.4)	5.7 (4.3 to 7.1)	5.7 (4.4 to 7.1)	6.4 (4.9 to 8.2)	0.5 (-20.7 to 29.9)	12.7 (-6.9 to 36.6)
Non-exclusive breastfeeding	0.4 (0.3 to 0.6)	0.4 (0.3 to 0.6)	0.4 (0.3 to 0.6)	-1.4 (-13.6 to 13.5)	-1.2 (-11.8 to 10.3)	0.4 (0.3 to 0.6)	0.4 (0.3 to 0.6)	0.4 (0.3 to 0.6)	-0.5 (-12.8 to 14.6)	-1.6 (-12.1 to 9.9)
Discontinued breastfeeding	1.7 (1.5 to 1.8)	1.7 (1.6 to 1.9)	1.7 (1.6 to 1.8)	3.0 (-6.6 to 14.0)	-2.8 (-10.1 to 4.9)	1.7 (1.5 to 1.8)	1.7 (1.6 to 1.9)	1.7 (1.6 to 1.8)	2.4 (-7.2 to 13.3)	-1.4 (-8.8 to 6.4)
Child underweight	0.2 (0.1 to 0.4)	0.2 (0.1 to 0.3)	0.2 (0.1 to 0.3)	-14.4 (-32.3 to 7.2)	-5.1 (-18.1 to 9.5)	0.3 (0.2 to 0.4)	0.2 (0.1 to 0.3)	0.2 (0.1 to 0.3)	-16.7 (-36.6 to 8.2)	-5.4 (-20.9 to 10.0)
Child wasting	0.2 (0.1 to 0.3)	0.2 (0.1 to 0.2)	0.2 (0.1 to 0.2)	-8.4 (-32.6 to 23.2)	-2.1 (-20.5 to 21.1)	0.2 (0.1 to 0.3)	0.2 (0.1 to 0.2)	0.2 (0.1 to 0.2)	-8.9 (-32.3 to 24.8)	-2.3 (-21.4 to 20.8)
Child stunting	0.6 (0.3 to 1.0)	0.6 (0.3 to 0.8)	0.5 (0.3 to 0.8)	-9.6 (-40.0 to 38.1)	-3.2 (-30.0 to 34.0)	0.7 (0.3 to 1.1)	0.6 (0.3 to 0.9)	0.6 (0.3 to 0.9)	-11.7 (-42.3 to 42.9)	-4.1 (-31.1 to 33.5)
Short gestation for birth weight	0.0 (0.0 to 0.0)	0.0 (0.0 to 0.0)	0.0 (0.0 to 0.0)	10.7 (-11.8 to 41.7)	3.8 (-16.3 to 49.0)	0.0 (0.0 to 0.0)	0.0 (0.0 to 0.0)	0.0 (0.0 to 0.0)	14.0 (-17.7 to 100.0)	0.6 (-22.0 to 35.8)
Low birth weight for gestation	0.0 (0.0 to 0.0)	0.0 (0.0 to 0.0)	0.0 (0.0 to 0.0)	3.8 (-14.7 to 25.2)	1.5 (-23.6 to 19.2)	0.0 (0.0 to 0.0)	0.0 (0.0 to 0.0)	0.0 (0.0 to 0.0)	-4.0 (-60.8 to 30.4)	4.6 (-17.9 to 24.8)
Iron deficiency	-	-	-	-	-	8.1 (5.5 to 11.3)	8.1 (5.6 to 11.1)	7.4 (5.0 to 10.4)	0.1 (-19.9 to 27.4)	-8.8 (-24.4 to 10.1)
Vitamin A deficiency	1.0 (0.7 to 1.3)	0.8 (0.5 to 1.0)	0.6 (0.4 to 0.9)	-23.4 (-33.1 to -11.9)	-17.0 (-31.3 to -0.7)	0.8 (0.4 to 1.1)	0.7 (0.5 to 1.0)	0.6 (0.4 to 0.8)	-8.6 (-23.6 to 28.6)	-18.1 (-34.7 to 2.6)
Zinc deficiency	0.5 (0.1 to 1.0)	0.5 (0.1 to 1.2)	0.4 (0.1 to 1.1)	9.8 (-59.5 to 78.7)	-15.1 (-71.0 to 126.9)	0.5 (0.2 to 1.0)	0.4 (0.1 to 0.8)	0.3 (0.1 to 0.8)	-26.1 (-64.7 to 71.5)	-5.5 (-64.9 to 160.2)
Smoking	17.3 (13.7 to 20.9)	12.2 (9.6 to 15.2)	10.3 (8.2 to 12.9)	-29.5 (-39.0 to -18.8)	-15.2 (-23.4 to -5.7)	3.9 (2.7 to 5.5)	3.3 (2.3 to 4.5)	3.2 (2.2 to 4.5)	-16.7 (-36.6 to 9.1)	-3.2 (-18.7 to 15.6)
Chewing tobacco	1.4 (0.9 to 2.0)	0.9 (0.6 to 1.3)	0.7 (0.5 to 1.1)	-33.2 (-57.1 to 2.9)	-19.1 (-42.0 to 12.8)	0.1 (0.1 to 0.1)	0.1 (0.1 to 0.1)	0.1 (0.1 to 0.1)	-3.5 (-40.2 to 59.4)	-3.1 (-33.1 to 36.9)
Second-hand smoke	37.9 (33.7 to 41.8)	36.8 (32.7 to 41.1)	36.6 (32.2 to 41.5)	-3.0 (-14.0 to 8.9)	-0.5 (-9.7 to 9.2)	57.7 (53.9 to 61.4)	50.0 (46.4 to 53.4)	49.9 (45.9 to 53.9)	-13.4 (-19.7 to -7.2)	-0.1 (-5.5 to 5.8)
Alcohol use	14.6 (7.4 to 25.2)	14.6 (7.6 to 24.8)	16.7 (8.1 to 29.8)	0.0 (-33.4 to 48.2)	14.1 (-18.3 to 58.0)	11.8 (5.8 to 20.9)	11.7 (5.9 to 20.7)	13.1 (6.3 to 23.5)	-0.6 (-35.6 to 49.3)	12.3 (-19.7 to 58.8)
Drug use	0.5 (0.4 to 0.7)	0.5 (0.4 to 0.7)	0.6 (0.4 to 0.8)	0.7 (-5.4 to 6.8)	11.5 (4.1 to 20.4)	0.3 (0.2 to 0.4)	0.3 (0.2 to 0.4)	0.3 (0.3 to 0.4)	1.5 (-4.4 to 8.1)	9.5 (2.3 to 17.6)
Diet low in fruits	40.5 (34.7 to 44.3)	38.8 (33.1 to 42.6)	40.3 (34.7 to 43.9)	-4.1 (-10.7 to 2.5)	3.9 (-1.0 to 9.6)	37.4 (31.7 to 41.7)	34.7 (28.5 to 39.4)	37.2 (31.1 to 41.8)	-7.2 (-15.5 to 1.2)	7.2 (0.5 to 16.4)
Diet low in vegetables	18.6 (10.2 to 26.1)	12.8 (4.9 to 20.4)	14.9 (6.8 to 23.3)	-31.1 (-61.8 to -3.6)	16.2 (-16.2 to 74.2)	20.8 (12.3 to 28.0)	14.8 (6.9 to 22.3)	16.6 (8.7 to 24.1)	-28.9 (-55.1 to -4.8)	12.4 (-15.3 to 58.8)
Diet low in legumes	4.4 (0.8 to 9.8)	6.0 (1.3 to 12.1)	7.9 (2.4 to 14.6)	34.2 (-36.1 to 314.5)	33.0 (-19.8 to 171.6)	8.3 (2.6 to 15.1)	8.1 (2.5 to 14.5)	10.5 (4.0 to 17.3)	-2.4 (-49.4 to 86.2)	30.5 (-8.3 to 132.7)
Diet low in whole grains	30.0 (25.4 to 34.1)	35.0 (31.0 to 38.5)	36.3 (32.5 to 39.7)	16.4 (7.3 to 29.1)	3.9 (-0.9 to 10.1)	31.4 (26.6 to 35.5)	36.1 (32.3 to 39.6)	37.4 (33.6 to 40.7)	14.9 (6.5 to 26.0)	3.7 (-0.8 to 8.9)
Diet low in nuts and seeds	50.5 (49.0 to 51.7)	49.3 (47.3 to 50.8)	49.2 (47.2 to 50.8)	-2.4 (-5.1 to -0.2)	-0.1 (-2.3 to 2.0)	50.6 (49.2 to 51.7)	49.1 (47.2 to 50.7)	49.0 (46.9 to 50.6)	-2.9 (-5.5 to -0.4)	-0.3 (-2.6 to 1.8)
Diet low in milk	46.0 (43.8 to 47.8)	46.4 (44.4 to 48.1)	46.9 (44.9 to 48.6)	0.9 (-1.2 to 3.0)	1.1 (-0.3 to 2.6)	46.0 (43.9 to 47.8)	45.9 (43.7 to 47.7)	46.6 (44.5 to 48.3)	-0.2 (-2.2 to 1.9)	1.5 (0.1 to 3.1)
Diet high in red meat	4.4 (0.8 to 9.1)	8.5 (2.7 to 15.6)	9.7 (3.4 to 17.2)	93.5 (-13.4 to 630.9)	13.5 (-35.8 to 117.6)	0.7 (0.0 to 2.7)	1.8 (0.0 to 5.3)	2.3 (0.1 to 6.6)	166.6 (-73.1 to 343140.6)	28.0 (-86.0 to 1727.9)
Diet high in processed meat	12.3 (7.9 to 16.8)	20.5 (15.3 to 25.4)	24.0 (17.8 to 30.2)	66.8 (31.0 to 128.8)	16.9 (-1.8 to 38.3)	10.1 (6.2 to 13.7)	14.6 (10.4 to 18.7)	16.0 (11.4 to 20.4)	44.9 (14.2 to 102.1)	9.6 (-7.1 to 30.3)
Diet high in sugar-sweetened beverages	7.9 (3.0 to 15.7)	8.9 (4.0 to 15.7)	9.4 (4.1 to 17.0)	12.8 (-46.8 to 151.6)	5.7 (-35.2 to 74.0)	5.4 (2.1 to 11.2)	6.1 (2.7 to 11.4)	6.6 (2.9 to 12.9)	13.7 (-47.5 to 148.0)	7.3 (-39.8 to 87.6)
Diet low in fibre	21.5 (13.9 to 28.4)	22.7 (15.4 to 29.0)	23.7 (16.6 to 30.2)	5.5 (-12.1 to 30.5)	4.5 (-8.3 to 21.2)	22.9 (15.3 to 29.8)	23.9 (16.8 to 30.0)	25.3 (18.2 to 31.2)	4.5 (-10.7 to 26.5)	5.9 (-5.7 to 21.2)
Diet low in calcium	34.1 (29.5 to 37.8)	34.1 (29.7 to 37.8)	35.1 (30.8 to 38.7)	0.0 (-3.9 to 4.2)	2.9 (-0.2 to 6.6)	36.4 (32.3 to 39.8)	35.6 (31.4 to 39.2)	37.0 (32.9 to 40.3)	-2.3 (-6.4 to 1.2)	3.8 (1.0 to 7.4)
Diet low in seafood omega-3 fatty acids	3.0 (1.2 to 4.3)	0.2 (0.0 to 1.1)	0.2 (0.0 to 1.1)	-92.0 (-100.0 to -66.8)	4.4 (-92.5 to 2377.4)	3.5 (1.5 to 5.0)	0.3 (0.0 to 1.2)	0.3 (0.0 to 1.3)	-91.5 (-100.0 to -69.0)	10.3 (-87.9 to 2012.9)
Diet low in polyunsaturated fatty acids	29.7 (24.0 to 34.4)	27.0 (20.8 to 32.2)	27.7 (21.1 to 33.0)	-9.3 (-21.6 to 0.2)	2.6 (-6.6 to 12.2)	30.5 (24.9 to 35.1)	27.7 (22.1 to 32.9)	28.4 (22.9 to 33.4)	-9.1 (-19.5 to 0.7)	2.6 (-5.0 to 12.1)
Diet high in trans fatty acids	1.1 (0.0 to 3.3)	1.0 (0.0 to 3.0)	0.9 (0.0 to 2.9)	-12.6 (-38.6 to -6.5)	-8.4 (-32.2 to -3.9)	1.5 (0.1 to 4.0)	1.3 (0.1 to 3.7)	1.2 (0.0 to 3.5)	-12.5 (-34.9 to -6.5)	-8.2 (-28.8 to -3.4)
Diet high in sodium	21.3 (12.4 to 28.9)	13.1 (4.7 to 20.4)	9.9 (2.3 to 17.2)	-38.4 (-67.2 to -19.5)	-24.8 (-58.1 to -8.3)	24.6 (16.2 to 31.6)	14.1 (5.8 to 21.3)	10.4 (2.5 to 17.8)	-42.6 (-69.3 to -26.2)	-26.2 (-60.5 to -11.9)
Intimate partner violence	-	-	-	-	-	2.3 (1.9 to 2.7)	2.1 (1.8 to 2.4)	2.0 (1.7 to 2.3)	-8.8 (-13.0 to -4.6)	-3.9 (-8.7 to 0.8)
Childhood sexual abuse	5.3 (4.3 to 6.5)	5.3 (4.3 to 6.5)	5.3 (4.3 to 6.5)	0.0 (0.0 to 0.0)	0.0 (0.0 to 0.0)	7.3 (5.9 to 8.9)	7.3 (5.9 to 8.9)	7.5 (6.0 to 9.2)	0.0 (0.0 to 0.0)	2.8 (-4.0 to 10.6)

Supplementary Results Table 1. Tochigi age-standardised Summary Exposure Values (SEVs) for Level 4 risk factors and percentage change, by sex, 1990, 2007, and 2017.Data in parentheses are 95% uncertainty intervals.

	Male					Female				
	**1990**	**2007**	**2017**	**Percentage change 1990-2007**	**Percentage change 2007-2017**	**1990**	**2007**	**2017**	**Percentage change 1990-2007**	**Percentage change 2007-2017**
Bullying victimization	3.3<br>(1.3 to 6.8)	3.4<br>(1.4 to 6.9)	3.5<br>(1.4 to 7.1)	1.4<br>(-6.6 to 9.1)	4.1<br>(-1.3 to 9.0)	1.9<br>(0.8 to 4.0)	1.9<br>(0.8 to 4.1)	2.0<br>(0.8 to 4.2)	-0.1<br>(-8.2 to 7.8)	3.1<br>(-2.4 to 7.8)
Low physical activity	0.5<br>(0.2 to 0.9)	0.5<br>(0.2 to 0.9)	0.4<br>(0.2 to 0.8)	-1.8<br>(-3.9 to 0.2)	-8.8<br>(-12.4 to -4.4)	0.5<br>(0.2 to 1.0)	0.5<br>(0.2 to 0.9)	0.5<br>(0.2 to 0.9)	-3.9<br>(-6.1 to -1.3)	0.8<br>(-0.8 to 2.3)
High fasting plasma glucose	1.4<br>(0.9 to 2.2)	1.4<br>(0.9 to 2.2)	1.6<br>(0.9 to 2.7)	-0.2<br>(-36.6 to 60.3)	12.3<br>(-24.7 to 79.1)	1.2<br>(0.7 to 2.0)	1.2<br>(0.7 to 2.1)	1.4<br>(0.8 to 2.4)	3.9<br>(-36.5 to 69.7)	9.5<br>(-29.6 to 76.7)
High LDL cholesterol	12.0<br>(10.5 to 13.8)	12.6<br>(11.0 to 14.4)	12.6<br>(11.0 to 14.4)	4.5<br>(-1.6 to 10.8)	0.1<br>(-4.8 to 4.7)	12.8<br>(11.3 to 14.6)	13.0<br>(11.4 to 14.8)	13.0<br>(11.4 to 14.8)	1.0<br>(-4.3 to 6.5)	0.2<br>(-4.2 to 4.6)
High systolic blood pressure	7.8<br>(6.9 to 8.6)	7.0<br>(6.2 to 7.8)	7.0<br>(6.2 to 7.9)	-10.4<br>(-17.6 to -2.9)	0.8<br>(-6.8 to 9.1)	5.8<br>(5.1 to 6.5)	4.8<br>(4.1 to 5.5)	4.6<br>(3.9 to 5.3)	-16.7<br>(-26.3 to -6.6)	-4.8<br>(-15.1 to 7.1)
High body-mass index	5.6<br>(3.7 to 8.5)	8.1<br>(5.4 to 12.6)	8.4<br>(5.7 to 13.1)	44.1<br>(15.7 to 79.6)	3.6<br>(-12.1 to 22.3)	5.6<br>(3.4 to 8.8)	7.2<br>(4.6 to 11.4)	7.3<br>(4.5 to 11.5)	29.3<br>(1.4 to 69.8)	0.4<br>(-18.3 to 21.0)
Low bone mineral density	5.3<br>(4.4 to 6.4)	5.2<br>(4.4 to 6.1)	5.2<br>(4.2 to 6.2)	-2.1<br>(-17.0 to 12.3)	0.0<br>(-13.3 to 14.4)	7.2<br>(6.0 to 8.5)	7.0<br>(5.9 to 8.1)	7.1<br>(6.0 to 8.4)	-3.3<br>(-14.3 to 9.6)	1.7<br>(-10.6 to 15.5)
Impaired kidney function	2.5<br>(1.1 to 6.3)	2.5<br>(1.1 to 6.2)	2.3<br>(1.0 to 6.0)	-0.9<br>(-5.2 to 3.5)	-6.4<br>(-11.7 to -1.6)	3.1<br>(1.4 to 7.2)	3.2<br>(1.5 to 7.6)	3.0<br>(1.3 to 7.1)	4.7<br>(0.7 to 9.9)	-6.8<br>(-11.8 to -2.0)

Supplementary Results Table 1. Tokushima age-standardised Summary Exposure Values (SEVs) for Level 4 risk factors and percentage change, by sex, 1990, 2007, and 2017.Data in parentheses are 95% uncertainty intervals.

	Male					Female				
	1990	2007	2017	Percentage change 1990-2007	Percentage change 2007-2017	1990	2007	2017	Percentage change 1990-2007	Percentage change 2007-2017
Unsafe water source	2.4 (1.4 to 4.0)	1.9 (1.1 to 3.2)	2.1 (1.1 to 3.5)	-21.0 (-59.4 to 54.5)	6.6 (-36.7 to 74.8)	2.4 (1.4 to 4.0)	1.9 (1.1 to 3.2)	2.1 (1.1 to 3.5)	-21.0 (-59.4 to 54.5)	6.6 (-36.7 to 74.8)
Unsafe sanitation	4.4 (2.1 to 7.9)	2.0 (1.0 to 3.8)	1.8 (0.8 to 3.4)	-53.5 (-79.7 to 3.7)	-13.4 (-55.4 to 63.8)	4.4 (2.1 to 7.9)	2.0 (1.0 to 3.8)	1.8 (0.8 to 3.4)	-53.5 (-79.7 to 3.7)	-13.4 (-55.4 to 63.8)
No access to handwashing facility	6.8 (5.0 to 9.2)	6.4 (4.7 to 8.5)	5.9 (4.3 to 7.8)	-6.1 (-33.7 to 32.5)	-6.7 (-27.7 to 19.3)	6.8 (5.0 to 9.2)	6.4 (4.7 to 8.5)	5.9 (4.3 to 7.8)	-6.1 (-33.7 to 32.5)	-6.7 (-27.7 to 19.3)
Ambient particulate matter pollution	31.5 (21.6 to 46.9)	31.9 (22.2 to 46.8)	26.8 (17.1 to 41.5)	1.1 (-20.8 to 29.7)	-15.9 (-36.1 to 8.0)	31.3 (21.4 to 46.6)	31.8 (22.2 to 46.7)	26.7 (17.1 to 41.5)	1.7 (-20.5 to 30.6)	-15.9 (-36.1 to 8.1)
Household air pollution from solid fuels	0.7 (0.2 to 1.8)	0.2 (0.0 to 0.4)	0.1 (0.0 to 0.3)	-76.3 (-92.8 to -52.5)	-17.2 (-67.5 to 83.1)	0.9 (0.3 to 5.4)	0.3 (0.0 to 1.1)	0.2 (0.0 to 0.9)	-72.2 (-92.9 to -47.8)	-18.9 (-67.3 to 84.9)
Ambient ozone pollution	51.5 (24.6 to 54.8)	51.4 (24.2 to 54.8)	51.6 (24.9 to 54.8)	-0.2 (-1.8 to 0.0)	0.4 (0.0 to 3.1)	51.5 (24.6 to 54.8)	51.4 (24.2 to 54.8)	51.6 (24.9 to 54.8)	-0.2 (-1.8 to 0.0)	0.4 (0.0 to 3.1)
Residential radon	12.6 (0.8 to 39.9)	12.6 (0.8 to 39.9)	12.6 (0.8 to 39.9)	0.0 (0.0 to 0.0)	0.0 (0.0 to 0.0)	12.6 (0.8 to 39.9)	12.6 (0.8 to 39.9)	12.6 (0.8 to 39.9)	0.0 (0.0 to 0.0)	0.0 (0.0 to 0.0)
Lead exposure	3.3 (0.6 to 7.0)	2.2 (0.3 to 5.3)	1.6 (0.2 to 4.2)	-33.7 (-47.1 to -24.7)	-28.5 (-43.3 to -20.5)	1.8 (0.1 to 4.7)	1.1 (0.1 to 3.3)	0.7 (0.0 to 2.6)	-39.0 (-56.8 to -29.3)	-31.3 (-53.3 to -21.8)
Occupational exposure to asbestos	2.2 (1.8 to 2.5)	3.0 (2.7 to 3.2)	2.9 (2.4 to 3.5)	36.2 (17.7 to 63.5)	-1.0 (-19.5 to 18.8)	0.4 (0.3 to 0.5)	0.4 (0.4 to 0.5)	0.5 (0.4 to 0.5)	-3.7 (-22.4 to 27.8)	5.1 (-15.7 to 30.4)
Occupational exposure to arsenic	0.5 (0.0 to 1.1)	0.4 (0.0 to 1.0)	0.4 (0.0 to 1.0)	-	-	0.4 (0.0 to 1.0)	0.4 (0.0 to 0.9)	0.4 (0.0 to 0.9)	-	-
Occupational exposure to benzene	0.6 (0.0 to 1.8)	0.6 (0.0 to 1.9)	0.7 (0.0 to 1.9)	2.4 (-4.4 to 13.2)	3.7 (-2.2 to 11.5)	0.6 (0.0 to 1.6)	0.7 (0.0 to 2.1)	0.8 (0.0 to 2.4)	22.1 (8.6 to 44.9)	14.5 (4.1 to 31.4)
Occupational exposure to beryllium	0.0 (0.0 to 0.0)	0.0 (0.0 to 0.0)	0.0 (0.0 to 0.0)	-5.3 (-10.3 to -0.6)	2.1 (-1.7 to 6.1)	0.0 (0.0 to 0.0)	0.0 (0.0 to 0.0)	0.0 (0.0 to 0.0)	2.6 (-6.9 to 13.2)	9.2 (2.6 to 16.4)
Occupational exposure to cadmium	0.1 (0.0 to 0.1)	0.0 (0.0 to 0.1)	0.0 (0.0 to 0.1)	-6.8 (-17.7 to 5.2)	1.2 (-8.1 to 11.6)	0.0 (0.0 to 0.1)	0.0 (0.0 to 0.0)	0.0 (0.0 to 0.1)	-8.6 (-22.4 to 9.8)	5.0 (-7.7 to 19.3)
Occupational exposure to chromium	0.1 (0.1 to 0.1)	0.1 (0.1 to 0.1)	0.1 (0.1 to 0.1)	-5.0 (-15.2 to 6.2)	1.7 (-6.7 to 10.7)	0.1 (0.1 to 0.1)	0.1 (0.1 to 0.1)	0.1 (0.1 to 0.1)	-3.8 (-17.2 to 13.2)	6.8 (-4.5 to 18.2)
Occupational exposure to diesel engine exhaust	0.6 (0.5 to 0.6)	0.6 (0.5 to 0.6)	0.6 (0.6 to 0.7)	-2.0 (-9.8 to 6.4)	3.1 (-3.1 to 9.9)	0.4 (0.4 to 0.5)	0.5 (0.4 to 0.6)	0.6 (0.5 to 0.6)	13.1 (-0.2 to 28.7)	11.4 (0.9 to 23.3)
Occupational exposure to formaldehyde	0.3 (0.2 to 0.3)	0.2 (0.2 to 0.3)	0.2 (0.2 to 0.3)	-6.1 (-18.0 to 7.1)	0.8 (-8.9 to 11.7)	0.2 (0.2 to 0.2)	0.2 (0.2 to 0.2)	0.2 (0.2 to 0.2)	-5.9 (-20.6 to 13.9)	5.8 (-7.0 to 20.0)
Occupational exposure to nickel	0.4 (0.0 to 1.9)	0.4 (0.0 to 1.8)	0.4 (0.0 to 1.8)	-	-	0.3 (0.0 to 1.5)	0.3 (0.0 to 1.3)	0.3 (0.0 to 1.4)	-	-
Occupational exposure to polycyclic aromatic hydrocarbons	0.2 (0.2 to 0.3)	0.2 (0.2 to 0.3)	0.2 (0.2 to 0.3)	-4.2 (-13.8 to 6.1)	2.0 (-5.7 to 10.1)	0.2 (0.2 to 0.2)	0.2 (0.2 to 0.2)	0.2 (0.2 to 0.3)	-1.7 (-14.8 to 15.7)	7.6 (-3.0 to 18.8)
Occupational exposure to silica	4.7 (0.7 to 15.1)	4.4 (0.7 to 13.9)	4.4 (0.6 to 14.7)	-6.9 (-16.5 to 4.8)	1.5 (-7.5 to 12.0)	3.1 (0.5 to 9.9)	2.7 (0.4 to 8.7)	2.8 (0.4 to 9.0)	-13.6 (-28.2 to 4.7)	2.3 (-11.8 to 18.8)
Occupational exposure to sulphuric acid	0.6 (0.1 to 1.9)	0.6 (0.1 to 1.8)	0.6 (0.1 to 1.8)	-6.9 (-17.6 to 4.8)	1.5 (-7.4 to 11.3)	0.5 (0.1 to 1.6)	0.5 (0.1 to 1.4)	0.5 (0.1 to 1.5)	-10.2 (-24.7 to 8.2)	4.3 (-8.8 to 19.3)
Occupational exposure to trichloroethylene	0.1 (0.1 to 0.1)	0.1 (0.1 to 0.1)	0.1 (0.1 to 0.1)	-4.0 (-11.1 to 4.1)	2.1 (-3.8 to 8.4)	0.1 (0.1 to 0.1)	0.1 (0.1 to 0.1)	0.1 (0.1 to 0.1)	1.3 (-9.7 to 15.1)	8.3 (-0.5 to 18.1)
Occupational asthmagens	18.6 (14.9 to 23.5)	18.0 (14.4 to 22.7)	18.0 (14.3 to 22.6)	-3.2 (-21.8 to 20.2)	-0.3 (-16.3 to 17.8)	8.2 (6.6 to 10.3)	8.2 (6.6 to 10.1)	8.7 (6.9 to 10.7)	-0.3 (-15.5 to 17.7)	5.6 (-7.5 to 19.6)
Occupational particulate matter, gases, and fumes	6.4 (4.6 to 9.3)	6.1 (4.4 to 8.9)	5.9 (4.2 to 8.6)	-4.6 (-10.0 to 0.4)	-3.7 (-7.4 to -0.4)	3.6 (2.5 to 5.4)	3.4 (2.4 to 5.2)	3.3 (2.3 to 4.9)	-5.7 (-14.0 to 4.8)	-4.7 (-10.5 to 1.9)
Occupational noise	6.0 (5.5 to 6.6)	5.8 (5.3 to 6.3)	5.6 (5.2 to 6.2)	-3.5 (-6.8 to -0.3)	-2.6 (-4.7 to -0.5)	3.5 (3.0 to 4.0)	3.5 (3.1 to 4.0)	3.5 (3.1 to 4.0)	0.1 (-6.0 to 7.2)	1.7 (-2.2 to 6.2)
Occupational ergonomic factors	10.6 (8.8 to 12.7)	9.3 (7.9 to 11.3)	9.2 (7.5 to 11.1)	-12.3 (-23.7 to 1.3)	-1.1 (-12.1 to 12.0)	5.7 (4.4 to 7.1)	5.7 (4.5 to 7.3)	6.5 (5.0 to 8.4)	0.7 (-21.6 to 28.3)	12.9 (-6.3 to 35.9)
Non-exclusive breastfeeding	0.4 (0.3 to 0.6)	0.4 (0.3 to 0.6)	0.4 (0.3 to 0.6)	-1.2 (-14.7 to 13.1)	-1.4 (-11.5 to 10.3)	0.4 (0.3 to 0.6)	0.4 (0.3 to 0.6)	0.4 (0.3 to 0.6)	-1.3 (-14.8 to 13.0)	-1.7 (-11.7 to 10.0)
Discontinued breastfeeding	1.7 (1.5 to 1.8)	1.7 (1.6 to 1.8)	1.7 (1.5 to 1.8)	2.3 (-7.4 to 12.4)	-1.9 (-9.2 to 6.2)	1.7 (1.5 to 1.8)	1.7 (1.6 to 1.8)	1.7 (1.5 to 1.8)	3.0 (-6.8 to 13.2)	-2.1 (-9.4 to 6.0)
Child underweight	0.3 (0.2 to 0.4)	0.2 (0.1 to 0.3)	0.2 (0.1 to 0.3)	-16.3 (-36.3 to 4.9)	-4.9 (-20.1 to 11.0)	0.3 (0.2 to 0.4)	0.2 (0.1 to 0.3)	0.2 (0.1 to 0.3)	-18.6 (-38.9 to 4.2)	-5.9 (-22.4 to 12.9)
Child wasting	0.2 (0.1 to 0.3)	0.2 (0.1 to 0.3)	0.2 (0.1 to 0.3)	-7.3 (-34.2 to 30.7)	-2.2 (-23.2 to 23.1)	0.2 (0.1 to 0.3)	0.2 (0.1 to 0.3)	0.2 (0.1 to 0.3)	-8.7 (-38.6 to 27.3)	-2.9 (-23.5 to 22.4)
Child stunting	0.7 (0.3 to 1.1)	0.6 (0.3 to 1.1)	0.6 (0.3 to 1.0)	-8.5 (-45.3 to 55.1)	-3.5 (-30.8 to 36.8)	0.7 (0.3 to 1.1)	0.6 (0.3 to 1.0)	0.6 (0.3 to 1.0)	-11.4 (-47.2 to 47.8)	-3.9 (-34.8 to 37.6)
Short gestation for birth weight	0.0 (0.0 to 0.0)	0.0 (0.0 to 0.0)	0.0 (0.0 to 0.0)	9.6 (-11.9 to 42.4)	1.8 (-17.4 to 48.2)	0.0 (0.0 to 0.0)	0.0 (0.0 to 0.0)	0.0 (0.0 to 0.0)	12.2 (-14.7 to 80.5)	1.2 (-20.2 to 34.7)
Low birth weight for gestation	0.0 (0.0 to 0.0)	0.0 (0.0 to 0.0)	0.0 (0.0 to 0.0)	2.7 (-18.5 to 24.2)	1.9 (-25.6 to 23.8)	0.0 (0.0 to 0.0)	0.0 (0.0 to 0.0)	0.0 (0.0 to 0.0)	-2.9 (-58.9 to 25.4)	3.0 (-18.2 to 24.1)
Iron deficiency	-	-	-	-	-	8.3 (5.6 to 11.5)	8.2 (5.7 to 11.1)	7.5 (5.1 to 10.5)	-1.6 (-22.0 to 27.0)	-8.0 (-24.8 to 10.8)
Vitamin A deficiency	1.0 (0.6 to 1.4)	0.8 (0.5 to 1.0)	0.7 (0.5 to 1.0)	-22.0 (-39.5 to 5.6)	-6.6 (-22.4 to 13.6)	0.8 (0.5 to 1.1)	0.8 (0.5 to 1.1)	0.6 (0.4 to 0.8)	0.9 (-17.3 to 24.1)	-27.6 (-45.1 to 3.1)
Zinc deficiency	0.5 (0.1 to 1.1)	0.5 (0.1 to 1.1)	0.4 (0.1 to 1.0)	-6.7 (-63.3 to 74.2)	-13.3 (-67.2 to 153.6)	0.5 (0.2 to 1.1)	0.3 (0.1 to 0.8)	0.4 (0.1 to 0.8)	-31.3 (-64.7 to 50.3)	2.5 (-62.5 to 169.5)
Smoking	17.9 (14.3 to 21.5)	12.5 (10.0 to 15.2)	10.5 (8.4 to 12.9)	-30.3 (-39.6 to -19.1)	-15.8 (-23.8 to -5.8)	4.1 (2.8 to 5.4)	3.4 (2.4 to 4.6)	3.3 (2.2 to 4.4)	-17.5 (-36.4 to 10.2)	-3.0 (-20.0 to 17.6)
Chewing tobacco	1.4 (0.9 to 2.1)	0.9 (0.6 to 1.3)	0.8 (0.5 to 1.1)	-33.7 (-57.4 to 3.4)	-18.7 (-41.4 to 13.2)	0.1 (0.1 to 0.1)	0.1 (0.1 to 0.1)	0.1 (0.1 to 0.1)	-4.6 (-41.4 to 56.8)	-1.6 (-34.2 to 45.5)
Second-hand smoke	38.2 (33.7 to 41.9)	35.4 (31.1 to 39.6)	34.5 (30.1 to 38.8)	-7.3 (-18.2 to 5.6)	-2.5 (-11.2 to 6.7)	57.7 (54.1 to 61.5)	47.4 (43.7 to 51.0)	46.0 (42.1 to 49.5)	-17.9 (-24.0 to -11.5)	-3.1 (-9.1 to 3.0)
Alcohol use	14.3 (7.1 to 24.0)	14.4 (7.3 to 24.3)	16.9 (8.0 to 29.3)	0.6 (-33.2 to 45.2)	17.3 (-17.8 to 66.2)	11.8 (5.8 to 20.7)	11.6 (5.8 to 20.5)	12.3 (5.9 to 21.5)	-1.8 (-36.2 to 43.8)	6.5 (-23.2 to 46.9)
Drug use	0.5 (0.4 to 0.7)	0.5 (0.4 to 0.7)	0.6 (0.4 to 0.8)	0.5 (-5.1 to 6.1)	11.6 (3.8 to 20.3)	0.3 (0.2 to 0.4)	0.3 (0.2 to 0.4)	0.3 (0.3 to 0.4)	1.9 (-3.3 to 7.8)	8.8 (2.5 to 17.0)
Diet low in fruits	41.1 (35.7 to 44.8)	39.4 (33.7 to 43.3)	40.6 (34.7 to 44.4)	-4.2 (-10.4 to 1.8)	3.2 (-1.4 to 8.5)	38.0 (32.3 to 42.4)	35.4 (29.2 to 40.3)	37.5 (31.4 to 42.0)	-6.8 (-15.0 to 0.3)	5.8 (-0.4 to 13.5)
Diet low in vegetables	20.1 (11.9 to 27.7)	14.6 (6.7 to 22.7)	16.8 (8.4 to 24.7)	-27.4 (-56.7 to -3.6)	15.2 (-13.7 to 63.0)	22.1 (13.2 to 29.0)	16.3 (7.5 to 23.8)	18.3 (9.3 to 26.1)	-26.0 (-53.2 to -3.4)	12.2 (-12.9 to 57.6)
Diet low in legumes	4.2 (0.7 to 9.6)	5.1 (0.8 to 10.3)	6.6 (1.6 to 13.0)	20.1 (-53.7 to 274.4)	30.0 (-27.7 to 224.1)	8.3 (2.8 to 14.6)	8.1 (2.4 to 14.6)	10.4 (3.7 to 17.4)	-2.6 (-48.2 to 72.8)	28.7 (-13.6 to 125.8)
Diet low in whole grains	30.6 (25.9 to 34.7)	35.3 (31.5 to 38.9)	36.7 (32.8 to 40.3)	15.5 (6.7 to 27.6)	3.8 (-1.3 to 9.4)	32.0 (27.2 to 36.0)	36.6 (32.7 to 39.8)	37.9 (34.2 to 40.9)	14.3 (6.2 to 25.0)	3.5 (-0.7 to 8.3)
Diet low in nuts and seeds	50.9 (49.6 to 51.9)	49.7 (47.9 to 51.0)	49.5 (47.6 to 51.0)	-2.4 (-4.8 to -0.5)	-0.3 (-2.5 to 1.7)	51.0 (49.7 to 51.9)	49.6 (47.9 to 51.0)	49.4 (47.5 to 50.9)	-2.7 (-5.1 to -0.6)	-0.4 (-2.4 to 1.5)
Diet low in milk	46.2 (44.1 to 48.0)	46.6 (44.6 to 48.2)	47.1 (45.1 to 48.6)	0.8 (-1.2 to 2.9)	1.0 (-0.4 to 2.5)	46.2 (44.2 to 48.0)	46.0 (43.9 to 47.7)	46.6 (44.6 to 48.3)	-0.5 (-2.5 to 1.6)	1.3 (-0.2 to 2.9)
Diet high in red meat	3.8 (0.5 to 9.0)	7.7 (2.5 to 14.5)	8.9 (3.1 to 15.9)	100.2 (-14.3 to 831.1)	15.6 (-35.9 to 118.1)	0.6 (0.0 to 2.6)	1.6 (0.0 to 5.2)	2.1 (0.0 to 6.4)	177.0 (-76.5 to 867526.0)	28.0 (-84.0 to 2206.1)
Diet high in processed meat	11.1 (7.1 to 15.0)	18.3 (13.3 to 22.8)	21.0 (15.6 to 26.2)	64.7 (30.1 to 125.1)	14.9 (-4.4 to 37.7)	9.3 (5.6 to 13.0)	13.5 (9.4 to 17.5)	14.7 (10.2 to 18.7)	45.4 (13.6 to 101.1)	8.8 (-8.3 to 30.6)
Diet high in sugar-sweetened beverages	7.0 (2.7 to 14.4)	8.2 (3.4 to 15.1)	8.6 (3.8 to 15.4)	16.6 (-43.0 to 155.6)	4.8 (-38.4 to 73.2)	5.1 (2.0 to 11.1)	5.7 (2.6 to 11.0)	6.1 (2.4 to 12.0)	12.7 (-50.0 to 154.9)	6.3 (-38.4 to 87.7)
Diet low in fibre	22.4 (15.1 to 29.0)	23.6 (16.9 to 29.5)	24.7 (17.7 to 30.8)	5.2 (-11.9 to 29.3)	4.8 (-8.0 to 19.4)	23.5 (16.1 to 29.9)	24.5 (17.2 to 30.9)	25.9 (18.5 to 32.2)	4.3 (-12.9 to 26.4)	5.7 (-5.7 to 20.1)
Diet low in calcium	34.9 (30.6 to 38.4)	34.6 (30.4 to 38.2)	35.4 (31.1 to 38.9)	-0.8 (-4.6 to 3.2)	2.3 (-1.0 to 5.8)	37.1 (33.2 to 40.3)	35.9 (31.6 to 39.3)	37.0 (33.0 to 40.2)	-3.3 (-7.5 to -0.1)	3.2 (0.3 to 6.7)
Diet low in seafood omega-3 fatty acids	3.2 (1.4 to 4.6)	0.3 (0.0 to 1.1)	0.3 (0.0 to 1.2)	-91.9 (-100.0 to -68.0)	8.5 (-91.4 to 1550.6)	3.7 (1.8 to 5.2)	0.4 (0.0 to 1.4)	0.4 (0.0 to 1.4)	-89.1 (-100.0 to -67.1)	7.9 (-75.5 to 1262.2)
Diet low in polyunsaturated fatty acids	30.6 (25.1 to 35.5)	27.8 (22.0 to 32.8)	28.5 (22.5 to 33.7)	-9.0 (-21.4 to 0.9)	2.4 (-5.7 to 11.8)	31.2 (26.0 to 36.1)	28.5 (22.4 to 33.5)	29.2 (23.2 to 33.9)	-8.6 (-20.3 to 1.2)	2.2 (-5.0 to 10.6)
Diet high in trans fatty acids	1.1 (0.0 to 3.3)	1.0 (0.0 to 3.0)	0.9 (0.0 to 2.9)	-12.3 (-37.9 to -6.5)	-8.6 (-33.6 to -3.8)	1.5 (0.1 to 4.0)	1.3 (0.1 to 3.7)	1.2 (0.0 to 3.5)	-12.4 (-35.0 to -6.4)	-8.2 (-27.8 to -3.5)
Diet high in sodium	27.7 (17.3 to 35.8)	17.8 (8.6 to 24.7)	15.2 (6.1 to 22.7)	-35.7 (-60.6 to -18.3)	-14.5 (-40.4 to 3.1)	31.5 (22.7 to 39.1)	18.9 (10.6 to 25.8)	15.6 (7.1 to 22.9)	-40.0 (-60.4 to -25.2)	-17.4 (-41.1 to -3.7)
Intimate partner violence	-	-	-	-	-	2.3 (2.0 to 2.7)	2.2 (1.8 to 2.5)	2.1 (1.8 to 2.4)	-7.9 (-12.3 to -3.3)	-3.0 (-7.1 to 1.3)
Childhood sexual abuse	5.3 (4.3 to 6.5)	5.3 (4.3 to 6.5)	5.3 (4.3 to 6.5)	0.0 (0.0 to 0.0)	0.0 (0.0 to 0.0)	7.3 (5.9 to 8.9)	7.3 (5.9 to 8.9)	7.5 (6.0 to 9.2)	0.0 (0.0 to 0.0)	2.8 (-4.0 to 10.6)

Supplementary Results Table 1. Tokushima age-standardised Summary Exposure Values (SEVs) for Level 4 risk factors and percentage change, by sex, 1990, 2007, and 2017.Data in parentheses are 95% uncertainty intervals.										
	**Male**					**Female**				
	**1990**	**2007**	**2017**	**Percentage change 1990-2007**	**Percentage change 2007-2017**	**1990**	**2007**	**2017**	**Percentage change 1990-2007**	**Percentage change 2007-2017**
Bullying victimization	3.3 (1.4 to 6.6)	3.4 (1.4 to 6.9)	3.5 (1.5 to 7.2)	2.1 (-5.6 to 9.6)	2.8 (-2.4 to 7.5)	1.9 (0.8 to 3.9)	1.9 (0.8 to 4.0)	2.0 (0.8 to 4.1)	0.6 (-6.9 to 8.7)	3.1 (-2.2 to 8.1)
Low physical activity	0.5 (0.2 to 0.9)	0.5 (0.2 to 0.9)	0.4 (0.2 to 0.8)	-1.8 (-3.6 to -0.0)	-8.8 (-12.3 to -4.5)	0.5 (0.2 to 1.0)	0.5 (0.2 to 0.9)	0.5 (0.2 to 0.9)	-3.8 (-6.0 to -1.3)	0.8 (-0.7 to 2.3)
High fasting plasma glucose	1.5 (0.9 to 2.4)	1.4 (0.9 to 2.2)	1.6 (0.9 to 2.5)	-6.7 (-41.9 to 56.7)	11.0 (-28.4 to 77.4)	1.2 (0.7 to 2.0)	1.3 (0.7 to 2.0)	1.4 (0.8 to 2.4)	1.5 (-38.6 to 69.4)	10.0 (-32.9 to 82.8)
High LDL cholesterol	12.0 (10.3 to 13.8)	12.6 (11.0 to 14.4)	12.5 (11.0 to 14.3)	4.8 (-1.4 to 11.4)	-0.1 (-4.7 to 4.7)	12.8 (11.2 to 14.5)	12.9 (11.3 to 14.6)	12.9 (11.3 to 14.8)	1.3 (-3.7 to 6.8)	0.2 (-4.3 to 4.8)
High systolic blood pressure	11.2 (10.2 to 12.3)	8.3 (7.4 to 9.2)	8.2 (7.2 to 9.2)	-25.7 (-31.7 to -19.5)	-1.5 (-9.0 to 6.4)	9.6 (8.6 to 10.6)	6.1 (5.3 to 6.8)	5.6 (4.8 to 6.5)	-36.8 (-43.4 to -30.0)	-6.9 (-16.6 to 3.3)
High body-mass index	5.4 (3.3 to 8.5)	7.8 (5.2 to 12.3)	8.1 (5.4 to 12.7)	44.4 (17.8 to 87.2)	3.4 (-13.3 to 21.5)	5.4 (3.3 to 8.2)	6.9 (4.4 to 10.8)	7.0 (4.3 to 11.2)	29.2 (1.4 to 73.6)	0.5 (-18.0 to 21.4)
Low bone mineral density	5.3 (4.4 to 6.3)	5.2 (4.3 to 6.1)	5.2 (4.3 to 6.2)	-2.4 (-16.4 to 11.8)	0.6 (-12.4 to 14.5)	7.2 (6.1 to 8.5)	6.9 (5.9 to 8.1)	7.1 (5.9 to 8.4)	-3.5 (-15.8 to 9.5)	2.3 (-10.4 to 16.9)
Impaired kidney function	2.7 (1.2 to 6.6)	2.6 (1.2 to 6.5)	2.4 (1.1 to 6.1)	-3.1 (-7.7 to 1.5)	-7.0 (-12.3 to -2.3)	3.3 (1.5 to 7.5)	3.4 (1.6 to 7.8)	3.2 (1.4 to 7.3)	2.6 (-1.2 to 7.2)	-7.1 (-12.0 to -2.4)

Supplementary Results Table 1. Tokyo age-standardised Summary Exposure Values (SEVs) for Level 4 risk factors and percentage change, by sex, 1990, 2007, and 2017.Data in parentheses are 95% uncertainty intervals.

	Male					Female				
	**1990**	**2007**	**2017**	**Percentage change 1990-2007**	**Percentage change 2007-2017**	**1990**	**2007**	**2017**	**Percentage change 1990-2007**	**Percentage change 2007-2017**
Unsafe water source	1.3 (0.7 to 2.1)	1.1 (0.6 to 1.7)	1.2 (0.7 to 2.0)	-17.0 (-55.4 to 53.4)	12.0 (-34.4 to 86.7)	1.3 (0.7 to 2.1)	1.1 (0.6 to 1.7)	1.2 (0.7 to 2.0)	-17.0 (-55.4 to 53.4)	12.0 (-34.4 to 86.7)
Unsafe sanitation	1.7 (0.8 to 3.2)	0.8 (0.4 to 1.7)	0.8 (0.4 to 1.6)	-50.8 (-79.7 to 15.5)	-6.9 (-51.7 to 80.3)	1.7 (0.8 to 3.2)	0.8 (0.4 to 1.7)	0.8 (0.4 to 1.6)	-50.8 (-79.7 to 15.5)	-6.9 (-51.7 to 80.3)
No access to handwashing facility	6.2 (4.6 to 8.2)	5.9 (4.3 to 7.9)	5.5 (4.1 to 7.3)	-5.0 (-30.8 to 32.3)	-7.0 (-26.2 to 19.0)	6.2 (4.6 to 8.2)	5.9 (4.3 to 7.9)	5.5 (4.1 to 7.3)	-5.0 (-30.8 to 32.3)	-7.0 (-26.2 to 19.0)
Ambient particulate matter pollution	39.6 (27.8 to 56.1)	39.4 (28.4 to 56.2)	34.4 (23.9 to 50.0)	-0.5 (-20.2 to 25.1)	-12.7 (-29.8 to 4.7)	39.6 (27.7 to 55.8)	39.4 (28.4 to 56.2)	34.4 (23.9 to 50.0)	-0.4 (-20.1 to 25.3)	-12.7 (-29.8 to 4.8)
Household air pollution from solid fuels	0.2 (0.1 to 0.5)	0.1 (0.0 to 0.1)	0.1 (0.0 to 0.1)	-66.5 (-93.3 to -39.4)	-9.1 (-65.6 to 123.4)	0.4 (0.1 to 1.7)	0.1 (0.0 to 0.3)	0.1 (0.0 to 0.3)	-65.5 (-93.3 to -34.6)	-5.5 (-64.9 to 124.9)
Ambient ozone pollution	52.3 (27.1 to 54.8)	52.3 (26.9 to 54.8)	52.4 (27.7 to 54.8)	-0.0 (-0.8 to 0.2)	0.4 (0.0 to 3.2)	52.3 (27.1 to 54.8)	52.3 (26.9 to 54.8)	52.4 (27.7 to 54.8)	-0.0 (-0.8 to 0.2)	0.4 (0.0 to 3.2)
Residential radon	13.5 (0.7 to 46.2)	13.5 (0.7 to 46.2)	13.5 (0.7 to 46.2)	0.0 (0.0 to 0.0)	0.0 (0.0 to 0.0)	13.5 (0.7 to 46.2)	13.5 (0.7 to 46.2)	13.5 (0.7 to 46.2)	0.0 (0.0 to 0.0)	0.0 (0.0 to 0.0)
Lead exposure	3.4 (0.7 to 7.0)	2.0 (0.3 to 4.7)	1.3 (0.2 to 3.5)	-41.8 (-56.7 to -31.9)	-34.7 (-51.4 to -26.3)	3.2 (0.6 to 6.8)	2.0 (0.3 to 4.7)	1.3 (0.2 to 3.5)	-39.0 (-53.2 to -29.6)	-32.2 (-47.9 to -24.5)
Occupational exposure to asbestos	2.0 (1.7 to 2.3)	2.9 (2.7 to 3.1)	2.9 (2.5 to 3.5)	45.6 (26.1 to 70.0)	0.3 (-17.1 to 20.4)	0.6 (0.5 to 0.8)	0.6 (0.6 to 0.7)	0.5 (0.4 to 0.7)	-4.0 (-20.5 to 20.8)	-11.9 (-28.7 to 7.5)
Occupational exposure to arsenic	0.5 (0.0 to 1.1)	0.4 (0.0 to 1.0)	0.4 (0.0 to 1.0)	-	-	0.4 (0.0 to 1.1)	0.4 (0.0 to 0.9)	0.4 (0.0 to 1.0)	-	-
Occupational exposure to benzene	0.6 (0.0 to 1.8)	0.7 (0.0 to 1.9)	0.7 (0.0 to 2.0)	2.3 (-3.9 to 12.6)	3.6 (-2.1 to 11.8)	0.7 (0.0 to 1.9)	0.8 (0.0 to 2.3)	0.9 (0.1 to 2.6)	18.3 (7.2 to 37.0)	11.6 (3.2 to 23.8)
Occupational exposure to beryllium	0.0 (0.0 to 0.0)	0.0 (0.0 to 0.0)	0.0 (0.0 to 0.0)	-5.5 (-10.1 to -1.0)	2.1 (-1.5 to 5.8)	0.0 (0.0 to 0.0)	0.0 (0.0 to 0.0)	0.0 (0.0 to 0.0)	0.5 (-6.4 to 8.3)	7.1 (1.7 to 12.5)
Occupational exposure to cadmium	0.1 (0.0 to 0.1)	0.0 (0.0 to 0.1)	0.0 (0.0 to 0.1)	-8.8 (-19.0 to 2.8)	1.0 (-7.7 to 10.4)	0.1 (0.0 to 0.1)	0.0 (0.0 to 0.1)	0.0 (0.0 to 0.1)	-12.1 (-25.1 to 3.1)	3.1 (-7.9 to 15.7)
Occupational exposure to chromium	0.1 (0.1 to 0.1)	0.1 (0.1 to 0.1)	0.1 (0.1 to 0.1)	-7.1 (-16.4 to 3.1)	1.5 (-6.1 to 9.5)	0.1 (0.1 to 0.1)	0.1 (0.1 to 0.1)	0.1 (0.1 to 0.1)	-7.1 (-18.9 to 6.7)	4.7 (-4.5 to 15.0)
Occupational exposure to diesel engine exhaust	0.6 (0.6 to 0.6)	0.6 (0.5 to 0.6)	0.6 (0.6 to 0.6)	-2.3 (-9.7 to 5.5)	2.7 (-3.4 to 9.3)	0.5 (0.5 to 0.6)	0.6 (0.5 to 0.6)	0.6 (0.6 to 0.7)	11.4 (-0.3 to 26.2)	8.6 (-1.1 to 18.4)
Occupational exposure to formaldehyde	0.3 (0.2 to 0.3)	0.2 (0.2 to 0.3)	0.2 (0.2 to 0.3)	-8.4 (-19.1 to 4.2)	0.6 (-8.4 to 10.3)	0.2 (0.2 to 0.3)	0.2 (0.2 to 0.2)	0.2 (0.2 to 0.3)	-9.6 (-22.9 to 6.7)	3.8 (-7.1 to 16.1)
Occupational exposure to nickel	0.4 (0.0 to 1.9)	0.4 (0.0 to 1.7)	0.4 (0.0 to 1.6)	-	-	0.4 (0.0 to 1.6)	0.3 (0.0 to 1.3)	0.3 (0.0 to 1.3)	-	-
Occupational exposure to polycyclic aromatic hydrocarbons	0.2 (0.2 to 0.3)	0.2 (0.2 to 0.3)	0.2 (0.2 to 0.3)	-5.9 (-14.7 to 3.8)	1.8 (-5.0 to 9.1)	0.2 (0.2 to 0.3)	0.2 (0.2 to 0.3)	0.2 (0.2 to 0.3)	-5.0 (-16.3 to 8.5)	5.6 (-3.0 to 14.9)
Occupational exposure to silica	4.8 (0.7 to 15.1)	4.5 (0.7 to 14.3)	4.5 (0.6 to 14.7)	-6.5 (-16.1 to 4.2)	1.3 (-6.9 to 11.7)	3.4 (0.5 to 10.7)	2.8 (0.4 to 8.8)	2.8 (0.4 to 8.8)	-17.1 (-30.5 to 0.4)	0.4 (-12.0 to 16.5)
Occupational exposure to sulphuric acid	0.6 (0.1 to 2.0)	0.6 (0.1 to 1.9)	0.6 (0.1 to 1.8)	-8.6 (-18.4 to 2.1)	1.4 (-7.1 to 10.5)	0.6 (0.1 to 1.8)	0.5 (0.1 to 1.6)	0.5 (0.1 to 1.6)	-13.5 (-26.5 to 2.3)	2.8 (-9.3 to 16.3)
Occupational exposure to trichloroethylene	0.1 (0.1 to 0.1)	0.1 (0.1 to 0.1)	0.1 (0.1 to 0.1)	-6.0 (-12.8 to 1.3)	1.8 (-3.6 to 7.7)	0.1 (0.1 to 0.1)	0.1 (0.1 to 0.1)	0.1 (0.1 to 0.1)	-1.5 (-10.8 to 9.1)	6.1 (-1.3 to 14.9)
Occupational asthmagens	20.3 (16.1 to 25.4)	19.0 (15.1 to 24.1)	18.9 (15.0 to 24.0)	-6.0 (-22.5 to 14.4)	-0.5 (-15.7 to 17.4)	9.1 (7.4 to 11.5)	9.1 (7.4 to 11.2)	9.4 (7.5 to 11.8)	-0.9 (-17.6 to 18.0)	4.2 (-9.9 to 20.9)
Occupational particulate matter, gases, and fumes	6.2 (4.5 to 9.1)	6.0 (4.3 to 8.6)	5.8 (4.1 to 8.5)	-4.3 (-9.1 to 0.9)	-3.5 (-6.7 to -0.4)	3.8 (2.6 to 5.6)	3.5 (2.5 to 5.2)	3.3 (2.4 to 5.0)	-6.9 (-14.7 to 2.2)	-5.4 (-10.6 to 0.2)
Occupational noise	5.8 (5.3 to 6.4)	5.6 (5.2 to 6.2)	5.5 (5.1 to 6.1)	-2.4 (-5.3 to 0.6)	-1.8 (-3.8 to 0.1)	3.7 (3.2 to 4.2)	3.7 (3.3 to 4.1)	3.8 (3.4 to 4.2)	-0.3 (-5.5 to 5.4)	1.3 (-2.0 to 4.7)
Occupational ergonomic factors	9.2 (7.7 to 11.1)	8.1 (6.7 to 9.8)	8.1 (6.7 to 9.7)	-11.9 (-21.6 to -1.0)	-0.5 (-9.4 to 10.3)	5.1 (4.1 to 6.4)	5.3 (4.2 to 6.6)	6.0 (4.7 to 7.8)	3.3 (-13.7 to 27.1)	14.9 (-1.9 to 35.5)
Non-exclusive breastfeeding	0.4 (0.3 to 0.6)	0.4 (0.3 to 0.6)	0.4 (0.3 to 0.6)	0.0 (-12.7 to 14.0)	-2.0 (-12.5 to 9.3)	0.4 (0.3 to 0.6)	0.4 (0.3 to 0.6)	0.4 (0.3 to 0.6)	-0.1 (-12.8 to 13.8)	-2.0 (-12.6 to 9.3)
Discontinued breastfeeding	1.8 (1.7 to 1.9)	1.9 (1.8 to 2.0)	1.8 (1.7 to 1.9)	4.0 (-4.4 to 13.2)	-4.2 (-10.5 to 2.1)	1.8 (1.7 to 1.9)	1.9 (1.8 to 2.0)	1.8 (1.7 to 1.9)	4.1 (-4.4 to 13.3)	-3.9 (-10.2 to 2.4)
Child underweight	0.2 (0.1 to 0.3)	0.2 (0.1 to 0.3)	0.2 (0.1 to 0.3)	-12.0 (-30.0 to 6.8)	-2.7 (-14.9 to 11.6)	0.2 (0.1 to 0.3)	0.2 (0.1 to 0.3)	0.2 (0.1 to 0.3)	-13.1 (-31.7 to 6.4)	-2.5 (-15.7 to 14.0)
Child wasting	0.2 (0.1 to 0.2)	0.1 (0.1 to 0.2)	0.1 (0.1 to 0.2)	-7.6 (-27.7 to 19.1)	-0.8 (-17.5 to 19.3)	0.2 (0.1 to 0.2)	0.1 (0.1 to 0.2)	0.1 (0.1 to 0.2)	-8.4 (-29.0 to 15.7)	-1.0 (-18.7 to 18.7)
Child stunting	0.5 (0.3 to 0.8)	0.4 (0.2 to 0.6)	0.4 (0.2 to 0.7)	-12.3 (-42.6 to 30.8)	-1.3 (-27.6 to 33.2)	0.5 (0.2 to 0.9)	0.4 (0.2 to 0.7)	0.4 (0.2 to 0.7)	-13.8 (-46.4 to 30.4)	-0.8 (-29.8 to 39.0)
Short gestation for birth weight	0.0 (0.0 to 0.0)	0.0 (0.0 to 0.0)	0.0 (0.0 to 0.0)	7.0 (-23.5 to 38.7)	1.3 (-18.6 to 21.8)	0.0 (0.0 to 0.0)	0.0 (0.0 to 0.0)	0.0 (0.0 to 0.0)	13.3 (-10.2 to 56.7)	2.1 (-20.4 to 40.5)
Low birth weight for gestation	0.0 (0.0 to 0.0)	0.0 (0.0 to 0.0)	0.0 (0.0 to 0.0)	4.4 (-14.5 to 38.4)	3.5 (-12.4 to 22.3)	0.0 (0.0 to 0.0)	0.0 (0.0 to 0.0)	0.0 (0.0 to 0.0)	-2.2 (-24.0 to 25.4)	3.4 (-21.2 to 24.1)
Iron deficiency	-	-	-	-	-	6.6 (4.4 to 9.3)	6.7 (4.5 to 9.4)	6.2 (4.2 to 8.9)	1.0 (-22.6 to 31.4)	-7.6 (-24.6 to 10.9)
Vitamin A deficiency	0.7 (0.5 to 0.9)	0.6 (0.4 to 0.9)	0.5 (0.3 to 0.8)	-10.6 (-26.9 to 12.2)	-15.8 (-29.6 to -0.3)	0.5 (0.4 to 0.8)	0.6 (0.4 to 0.8)	0.6 (0.4 to 0.8)	11.9 (-18.4 to 40.0)	-9.4 (-31.4 to 10.6)
Zinc deficiency	0.4 (0.1 to 0.8)	0.6 (0.2 to 1.2)	0.7 (0.2 to 1.2)	76.3 (-1.6 to 332.8)	2.5 (-44.0 to 75.0)	0.4 (0.1 to 0.9)	0.7 (0.2 to 1.2)	0.6 (0.1 to 1.2)	66.8 (-13.0 to 306.5)	-7.8 (-46.7 to 85.0)
Smoking	15.9 (12.7 to 19.1)	11.1 (8.7 to 13.6)	9.4 (7.4 to 11.7)	-30.2 (-38.9 to -20.8)	-15.5 (-23.2 to -7.0)	3.5 (2.5 to 4.8)	2.9 (2.1 to 3.9)	2.8 (2.0 to 3.8)	-17.1 (-35.6 to 5.1)	-4.2 (-20.3 to 14.8)
Chewing tobacco	1.4 (0.9 to 2.0)	0.9 (0.6 to 1.3)	0.7 (0.5 to 1.1)	-33.1 (-56.6 to 1.4)	-19.0 (-41.7 to 10.0)	0.1 (0.1 to 0.1)	0.1 (0.1 to 0.1)	0.1 (0.1 to 0.1)	-4.3 (-41.0 to 55.0)	-3.1 (-30.1 to 37.3)
Second-hand smoke	38.0 (34.0 to 42.0)	36.5 (32.6 to 40.5)	36.8 (32.5 to 41.5)	-3.8 (-14.5 to 9.2)	0.7 (-8.3 to 9.5)	57.6 (54.2 to 61.2)	49.5 (46.0 to 53.2)	50.3 (46.1 to 54.2)	-13.9 (-20.3 to -7.7)	1.5 (-4.8 to 7.0)
Alcohol use	16.1 (8.8 to 26.6)	17.0 (8.8 to 28.2)	19.5 (9.8 to 33.6)	5.7 (-26.3 to 48.0)	14.4 (-14.4 to 56.9)	13.0 (7.0 to 21.9)	13.9 (6.9 to 23.8)	16.2 (7.9 to 28.2)	6.5 (-26.4 to 49.6)	16.9 (-13.2 to 62.9)
Drug use	0.5 (0.4 to 0.7)	0.5 (0.4 to 0.7)	0.6 (0.4 to 0.8)	0.9 (-5.0 to 6.3)	11.1 (3.7 to 19.5)	0.3 (0.2 to 0.4)	0.3 (0.2 to 0.4)	0.3 (0.3 to 0.4)	2.6 (-2.6 to 8.4)	9.0 (1.7 to 17.5)
Diet low in fruits	36.3 (30.3 to 40.7)	34.4 (28.2 to 38.9)	36.3 (30.6 to 40.8)	-5.4 (-13.5 to 2.5)	5.5 (-0.3 to 12.5)	32.4 (26.0 to 37.7)	29.2 (22.7 to 34.6)	32.3 (25.9 to 37.5)	-9.7 (-20.4 to 1.1)	10.4 (1.7 to 21.5)
Diet low in vegetables	11.8 (4.4 to 19.6)	6.9 (1.2 to 13.8)	9.0 (2.3 to 16.6)	-41.4 (-80.2 to -7.4)	30.2 (-19.1 to 193.9)	13.9 (5.4 to 21.8)	8.6 (2.2 to 15.6)	10.5 (3.5 to 18.0)	-38.2 (-74.7 to -5.0)	22.9 (-18.7 to 134.8)
Diet low in legumes	3.8 (0.6 to 8.5)	5.2 (1.0 to 10.5)	7.2 (1.9 to 13.5)	35.4 (-40.3 to 324.1)	38.9 (-13.5 to 238.7)	8.1 (2.5 to 15.0)	7.7 (2.3 to 13.7)	10.0 (3.7 to 16.8)	-5.1 (-48.5 to 71.2)	30.4 (-10.9 to 127.9)
Diet low in whole grains	27.3 (21.8 to 32.0)	32.7 (28.4 to 36.5)	34.2 (30.0 to 37.8)	19.5 (8.9 to 35.4)	4.6 (-0.9 to 11.4)	28.9 (23.9 to 33.2)	34.0 (29.8 to 37.5)	35.5 (31.5 to 38.7)	17.7 (8.2 to 31.6)	4.4 (-0.5 to 10.0)
Diet low in nuts and seeds	47.4 (45.1 to 49.3)	45.6 (42.9 to 47.8)	45.6 (42.7 to 48.1)	-3.8 (-7.6 to -0.4)	-0.0 (-3.4 to 3.3)	47.5 (45.2 to 49.4)	45.1 (42.0 to 47.5)	44.9 (41.6 to 47.5)	-5.1 (-9.2 to -1.2)	-0.4 (-4.1 to 3.0)
Diet low in milk	44.5 (42.0 to 46.5)	44.8 (42.5 to 46.8)	45.4 (43.1 to 47.2)	0.8 (-1.3 to 2.8)	1.3 (-0.3 to 2.9)	44.4 (41.9 to 46.5)	44.1 (41.8 to 46.3)	44.9 (42.6 to 46.9)	-0.5 (-2.8 to 1.8)	1.8 (0.1 to 3.8)
Diet high in red meat	7.3 (2.3 to 13.7)	12.9 (5.8 to 20.9)	14.6 (6.2 to 22.7)	76.3 (2.9 to 283.0)	13.0 (-26.8 to 73.6)	1.4 (0.0 to 4.0)	3.2 (0.5 to 7.1)	4.0 (0.6 to 8.9)	137.0 (-31.4 to 9946.2)	22.7 (-55.0 to 271.2)
Diet high in processed meat	16.6 (11.6 to 21.4)	26.6 (20.2 to 31.5)	30.4 (23.2 to 36.1)	60.1 (28.1 to 104.2)	14.6 (-1.0 to 33.1)	14.3 (10.0 to 18.5)	20.3 (15.3 to 24.6)	22.1 (16.8 to 26.9)	41.7 (13.3 to 81.6)	8.9 (-5.8 to 25.8)
Diet high in sugar-sweetened beverages	12.1 (5.2 to 21.0)	13.9 (6.8 to 21.0)	14.4 (6.8 to 22.4)	15.0 (-35.9 to 113.8)	4.1 (-30.8 to 55.1)	8.1 (3.2 to 15.4)	9.1 (4.3 to 15.1)	9.7 (4.5 to 16.9)	12.1 (-42.0 to 125.6)	6.0 (-33.8 to 65.1)
Diet low in fibre	18.9 (11.8 to 25.7)	20.0 (13.2 to 26.3)	21.1 (14.0 to 27.5)	6.1 (-14.1 to 32.4)	5.5 (-7.4 to 22.4)	20.0 (12.7 to 26.4)	21.2 (14.4 to 27.6)	22.8 (16.3 to 29.2)	6.0 (-11.5 to 32.1)	7.5 (-4.8 to 23.8)
Diet low in calcium	29.4 (23.9 to 33.8)	29.6 (24.2 to 33.8)	30.8 (25.8 to 34.9)	0.8 (-4.1 to 6.0)	4.0 (0.6 to 8.8)	32.1 (27.1 to 36.0)	31.2 (25.9 to 35.3)	32.8 (28.0 to 36.6)	-2.8 (-7.3 to 1.3)	5.1 (1.7 to 9.8)
Diet low in seafood omega-3 fatty acids	1.7 (0.3 to 3.1)	0.0 (0.0 to 0.3)	0.0 (0.0 to 0.4)	-97.9 (-100.0 to -89.3)	22.0 (-100.0 to [illegible]	2.0 (0.4 to 3.7)	0.1 (0.0 to 0.4)	0.1 (0.0 to 0.4)	-97.2 (-100.0 to -86.8)	19.8 (-100.0 to [illegible]
Diet low in polyunsaturated fatty acids	25.7 (19.3 to 31.0)	22.5 (15.8 to 28.2)	23.2 (16.5 to 28.8)	-12.3 (-26.2 to -0.5)	3.3 (-6.5 to 15.1)	26.4 (20.3 to 31.5)	23.3 (16.5 to 28.8)	24.0 (17.2 to 29.3)	-11.7 (-26.4 to -0.6)	3.0 (-7.5 to 14.3)
Diet high in trans fatty acids	1.1 (0.0 to 3.3)	1.0 (0.0 to 3.0)	0.9 (0.0 to 2.8)	-12.2 (-37.8 to -6.4)	-8.4 (-32.7 to -3.8)	1.5 (0.1 to 4.0)	1.3 (0.1 to 3.7)	1.2 (0.0 to 3.5)	-12.5 (-35.5 to -6.4)	-8.4 (-27.6 to -3.5)
Diet high in sodium	31.1 (22.3 to 38.6)	20.2 (11.6 to 27.0)	17.3 (8.5 to 24.4)	-34.9 (-55.0 to -17.8)	-14.2 (-36.0 to 2.1)	33.3 (25.6 to 40.6)	20.1 (11.9 to 26.6)	16.7 (8.0 to 23.7)	-39.5 (-58.7 to -25.5)	-17.2 (-37.9 to -5.4)
Intimate partner violence	-	-	-	-	-	2.1 (1.8 to 2.5)	1.9 (1.6 to 2.2)	1.9 (1.6 to 2.2)	-7.7 (-12.2 to -3.2)	-4.0 (-8.2 to 0.6)
Childhood sexual abuse	5.3 (4.3 to 6.5)	5.3 (4.3 to 6.5)	5.3 (4.3 to 6.5)	0.0 (0.0 to 0.0)	0.0 (0.0 to 0.0)	7.3 (5.9 to 8.9)	7.3 (5.9 to 8.9)	7.5 (6.0 to 9.2)	0.0 (0.0 to 0.0)	2.8 (-4.0 to 10.6)

Supplementary Results Table 1. Tokyo age-standardised Summary Exposure Values (SEVs) for Level 4 risk factors and percentage change, by sex, 1990, 2007, and 2017.Data in parentheses are 95% uncertainty intervals.

	**Male**					**Female**				
	**1990**	**2007**	**2017**	**Percentage change 1990-2007**	**Percentage change 2007-2017**	**1990**	**2007**	**2017**	**Percentage change 1990-2007**	**Percentage change 2007-2017**
Bullying victimization	3.2 (1.3 to 6.5)	3.2 (1.4 to 6.6)	3.4 (1.4 to 6.9)	2.5 (-5.2 to 10.7)	3.9 (-1.4 to 8.9)	1.9 (0.7 to 4.0)	1.9 (0.8 to 3.8)	1.9 (0.8 to 3.9)	0.2 (-7.2 to 8.2)	3.0 (-2.8 to 7.9)
Low physical activity	0.5 (0.2 to 0.9)	0.5 (0.2 to 0.9)	0.4 (0.2 to 0.8)	-1.7 (-3.6 to 0.4)	-8.9 (-12.4 to -4.5)	0.5 (0.2 to 1.0)	0.5 (0.2 to 0.9)	0.5 (0.2 to 1.0)	-3.6 (-6.0 to -1.1)	0.9 (-0.7 to 2.5)
High fasting plasma glucose	1.5 (0.9 to 2.5)	1.7 (1.0 to 2.6)	1.9 (1.1 to 3.1)	8.6 (-32.4 to 78.9)	12.5 (-28.6 to 77.9)	1.2 (0.7 to 2.0)	1.4 (0.8 to 2.2)	1.5 (0.9 to 2.6)	11.2 (-34.2 to 86.5)	9.8 (-30.9 to 84.4)
High LDL cholesterol	12.4 (10.7 to 14.2)	12.9 (11.3 to 14.7)	12.8 (11.3 to 14.7)	4.1 (-1.4 to 10.1)	-0.3 (-4.6 to 4.2)	13.5 (11.9 to 15.3)	13.6 (12.0 to 15.4)	13.5 (12.0 to 15.3)	0.6 (-4.3 to 6.0)	-0.3 (-4.3 to 4.0)
High systolic blood pressure	11.7 (10.6 to 12.6)	8.7 (7.8 to 9.6)	8.5 (7.6 to 9.5)	-25.4 (-31.0 to -19.3)	-1.9 (-9.2 to 5.7)	9.8 (8.9 to 10.9)	6.1 (5.4 to 6.9)	5.7 (4.9 to 6.4)	-37.4 (-43.6 to -30.9)	-7.5 (-16.1 to 1.8)
High body-mass index	9.7 (6.9 to 13.8)	13.8 (10.4 to 19.3)	14.1 (10.6 to 19.1)	43.1 (15.9 to 78.1)	2.3 (-12.0 to 17.1)	7.7 (5.2 to 11.0)	10.0 (7.0 to 14.7)	9.9 (6.6 to 14.6)	29.7 (3.0 to 69.5)	-1.4 (-18.0 to 16.3)
Low bone mineral density	5.2 (4.3 to 6.2)	5.1 (4.3 to 6.0)	5.2 (4.2 to 6.2)	-2.5 (-14.5 to 11.4)	1.1 (-12.0 to 16.1)	7.1 (6.1 to 8.4)	6.9 (5.9 to 8.1)	7.0 (5.9 to 8.4)	-3.3 (-14.2 to 10.2)	1.6 (-10.7 to 16.1)
Impaired kidney function	2.6 (1.2 to 6.5)	2.6 (1.2 to 6.5)	2.6 (1.2 to 6.5)	-0.5 (-5.0 to 4.0)	-0.2 (-5.3 to 4.6)	2.9 (1.4 to 6.8)	2.9 (1.4 to 6.8)	2.9 (1.4 to 6.8)	-0.2 (-5.5 to 4.5)	0.1 (-4.2 to 4.7)

Supplementary Results Table 1. Tottori age-standardised Summary Exposure Values (SEVs) for Level 4 risk factors and percentage change, by sex, 1990, 2007, and 2017.Data in parentheses are 95% uncertainty intervals.

	Male					Female				
	**1990**	**2007**	**2017**	**Percentage change 1990-2007**	**Percentage change 2007-2017**	**1990**	**2007**	**2017**	**Percentage change 1990-2007**	**Percentage change 2007-2017**
Unsafe water source	2.6 (1.4 to 4.3)	2.1 (1.1 to 3.6)	2.2 (1.2 to 3.8)	-18.0 (-58.6 to 66.0)	5.6 (-40.2 to 87.4)	2.6 (1.4 to 4.3)	2.1 (1.1 to 3.6)	2.2 (1.2 to 3.8)	-18.0 (-58.6 to 66.0)	5.6 (-40.2 to 87.4)
Unsafe sanitation	4.6 (2.2 to 8.1)	2.3 (1.1 to 4.2)	1.9 (0.9 to 3.7)	-50.3 (-79.4 to 11.3)	-14.4 (-57.0 to 69.7)	4.6 (2.2 to 8.1)	2.3 (1.1 to 4.2)	1.9 (0.9 to 3.7)	-50.3 (-79.4 to 11.3)	-14.4 (-57.0 to 69.7)
No access to handwashing facility	6.9 (4.9 to 9.2)	6.5 (4.7 to 8.8)	6.0 (4.3 to 8.0)	-5.5 (-32.1 to 33.4)	-7.6 (-29.6 to 18.1)	6.9 (4.9 to 9.2)	6.5 (4.7 to 8.8)	6.0 (4.3 to 8.0)	-5.5 (-32.1 to 33.4)	-7.6 (-29.6 to 18.1)
Ambient particulate matter pollution	31.7 (19.9 to 47.0)	32.3 (21.7 to 47.4)	27.8 (16.7 to 43.1)	2.0 (-25.2 to 42.2)	-13.9 (-42.0 to 18.4)	31.4 (19.7 to 46.6)	32.3 (21.7 to 47.3)	27.8 (16.6 to 43.0)	2.7 (-24.9 to 43.6)	-13.9 (-42.1 to 18.5)
Household air pollution from solid fuels	0.7 (0.2 to 1.8)	0.2 (0.0 to 0.4)	0.2 (0.0 to 0.4)	-75.4 (-92.7 to -47.1)	-12.8 (-67.1 to 76.4)	1.0 (0.3 to 7.1)	0.3 (0.1 to 1.3)	0.3 (0.0 to 1.2)	-71.8 (-92.7 to -42.9)	-11.5 (-66.7 to 77.9)
Ambient ozone pollution	51.6 (24.8 to 54.8)	51.5 (24.7 to 54.8)	51.6 (24.9 to 54.8)	-0.2 (-1.7 to 0.0)	0.1 (0.0 to 1.4)	51.6 (24.8 to 54.8)	51.5 (24.7 to 54.8)	51.6 (24.9 to 54.8)	-0.2 (-1.7 to 0.0)	0.1 (0.0 to 1.4)
Residential radon	15.7 (1.1 to 49.6)	15.7 (1.1 to 49.6)	15.7 (1.1 to 49.6)	0.0 (0.0 to 0.0)	0.0 (0.0 to 0.0)	15.7 (1.1 to 49.6)	15.7 (1.1 to 49.6)	15.7 (1.1 to 49.6)	0.0 (0.0 to 0.0)	0.0 (0.0 to 0.0)
Lead exposure	3.2 (0.5 to 6.9)	2.2 (0.3 to 5.3)	1.6 (0.2 to 4.2)	-31.7 (-44.8 to -23.2)	-27.4 (-42.3 to -19.3)	1.7 (0.1 to 4.7)	1.1 (0.1 to 3.4)	0.8 (0.0 to 2.7)	-36.4 (-53.4 to -26.9)	-29.1 (-51.1 to -19.6)
Occupational exposure to asbestos	1.6 (1.4 to 1.8)	2.3 (2.1 to 2.5)	2.3 (1.9 to 2.7)	40.2 (21.9 to 65.7)	-1.9 (-19.2 to 18.2)	0.7 (0.5 to 0.8)	0.7 (0.6 to 0.8)	0.6 (0.5 to 0.7)	4.7 (-13.6 to 34.2)	-10.4 (-29.0 to 11.8)
Occupational exposure to arsenic	0.4 (0.0 to 1.1)	0.4 (0.0 to 1.0)	0.4 (0.0 to 1.0)	-	-	0.4 (0.0 to 0.9)	0.4 (0.0 to 0.8)	0.4 (0.0 to 0.9)	-	-
Occupational exposure to benzene	0.6 (0.0 to 1.8)	0.6 (0.0 to 1.8)	0.7 (0.0 to 1.9)	2.7 (-4.2 to 13.3)	3.5 (-2.0 to 13.0)	0.6 (0.0 to 1.6)	0.7 (0.0 to 2.0)	0.8 (0.0 to 2.3)	22.4 (8.0 to 44.8)	14.8 (4.7 to 29.8)
Occupational exposure to beryllium	0.0 (0.0 to 0.0)	0.0 (0.0 to 0.0)	0.0 (0.0 to 0.0)	-5.0 (-10.1 to 0.3)	2.0 (-1.6 to 6.1)	0.0 (0.0 to 0.0)	0.0 (0.0 to 0.0)	0.0 (0.0 to 0.0)	2.3 (-6.5 to 11.9)	9.5 (3.1 to 16.6)
Occupational exposure to cadmium	0.1 (0.0 to 0.1)	0.0 (0.0 to 0.1)	0.0 (0.0 to 0.1)	-6.3 (-17.7 to 6.8)	1.2 (-8.3 to 11.9)	0.0 (0.0 to 0.1)	0.0 (0.0 to 0.0)	0.0 (0.0 to 0.1)	-8.0 (-23.1 to 8.5)	5.5 (-6.7 to 19.3)
Occupational exposure to chromium	0.1 (0.1 to 0.1)	0.1 (0.1 to 0.1)	0.1 (0.1 to 0.1)	-4.6 (-14.9 to 7.2)	1.7 (-6.5 to 10.9)	0.1 (0.1 to 0.1)	0.1 (0.1 to 0.1)	0.1 (0.1 to 0.1)	-3.3 (-17.6 to 11.6)	7.3 (-3.5 to 19.2)
Occupational exposure to diesel engine exhaust	0.6 (0.5 to 0.6)	0.6 (0.5 to 0.6)	0.6 (0.5 to 0.6)	-1.6 (-9.7 to 6.7)	3.3 (-3.2 to 10.3)	0.4 (0.4 to 0.5)	0.5 (0.4 to 0.6)	0.5 (0.5 to 0.6)	12.2 (-0.2 to 27.4)	11.6 (1.3 to 24.5)
Occupational exposure to formaldehyde	0.2 (0.2 to 0.3)	0.2 (0.2 to 0.3)	0.2 (0.2 to 0.3)	-5.7 (-17.7 to 8.4)	0.8 (-8.9 to 11.9)	0.2 (0.2 to 0.2)	0.2 (0.2 to 0.2)	0.2 (0.2 to 0.2)	-5.3 (-20.9 to 12.5)	6.3 (-5.7 to 20.6)
Occupational exposure to nickel	0.4 (0.0 to 1.8)	0.4 (0.0 to 1.6)	0.4 (0.0 to 1.7)	-	-	0.3 (0.0 to 1.5)	0.3 (0.0 to 1.3)	0.3 (0.0 to 1.3)	-	-
Occupational exposure to polycyclic aromatic hydrocarbons	0.2 (0.2 to 0.3)	0.2 (0.2 to 0.3)	0.2 (0.2 to 0.3)	-3.7 (-13.3 to 7.6)	2.0 (-5.2 to 10.3)	0.2 (0.2 to 0.2)	0.2 (0.2 to 0.2)	0.2 (0.2 to 0.3)	-1.2 (-14.9 to 13.5)	8.1 (-2.0 to 19.4)
Occupational exposure to silica	4.6 (0.7 to 15.1)	4.3 (0.6 to 13.8)	4.4 (0.6 to 14.2)	-6.5 (-17.1 to 6.1)	1.6 (-7.5 to 12.7)	3.1 (0.4 to 10.3)	2.7 (0.4 to 8.7)	2.8 (0.4 to 9.0)	-12.8 (-26.8 to 5.7)	2.4 (-10.1 to 20.3)
Occupational exposure to sulphuric acid	0.6 (0.1 to 2.0)	0.6 (0.1 to 1.8)	0.6 (0.1 to 1.8)	-6.1 (-16.8 to 6.2)	1.6 (-7.7 to 11.6)	0.5 (0.1 to 1.6)	0.5 (0.1 to 1.4)	0.5 (0.1 to 1.5)	-9.6 (-24.3 to 7.2)	4.9 (-7.9 to 19.7)
Occupational exposure to trichloroethylene	0.1 (0.1 to 0.1)	0.1 (0.1 to 0.1)	0.1 (0.1 to 0.1)	-3.6 (-10.9 to 4.6)	2.2 (-3.7 to 8.4)	0.1 (0.1 to 0.1)	0.1 (0.1 to 0.1)	0.1 (0.1 to 0.1)	1.4 (-10.7 to 13.7)	8.8 (0.2 to 18.9)
Occupational asthmagens	18.3 (14.4 to 22.8)	17.7 (13.9 to 22.2)	17.6 (13.9 to 22.5)	-3.3 (-21.3 to 19.0)	-0.3 (-15.2 to 17.8)	8.2 (6.5 to 10.2)	8.2 (6.5 to 10.0)	8.6 (6.8 to 10.6)	-0.1 (-15.6 to 18.2)	5.4 (-7.3 to 19.8)
Occupational particulate matter, gases, and fumes	6.4 (4.5 to 9.3)	6.1 (4.4 to 8.8)	5.9 (4.2 to 8.4)	-4.6 (-9.5 to 0.4)	-3.7 (-7.0 to -0.4)	3.6 (2.5 to 5.3)	3.4 (2.4 to 5.1)	3.3 (2.3 to 4.9)	-5.5 (-13.4 to 4.1)	-4.5 (-10.0 to 1.5)
Occupational noise	6.0 (5.5 to 6.6)	5.8 (5.4 to 6.3)	5.6 (5.2 to 6.2)	-3.4 (-6.6 to -0.0)	-2.5 (-4.5 to -0.3)	3.4 (3.0 to 4.0)	3.5 (3.0 to 3.9)	3.5 (3.1 to 4.0)	0.2 (-5.6 to 7.2)	1.7 (-2.2 to 5.8)
Occupational ergonomic factors	10.9 (9.0 to 13.4)	9.7 (8.0 to 11.7)	9.5 (7.8 to 11.6)	-11.1 (-23.4 to 4.3)	-1.5 (-13.0 to 11.5)	5.8 (4.5 to 7.5)	5.9 (4.5 to 7.5)	6.6 (5.1 to 8.6)	1.1 (-21.5 to 30.4)	11.8 (-8.9 to 35.6)
Non-exclusive breastfeeding	0.4 (0.3 to 0.6)	0.4 (0.3 to 0.6)	0.4 (0.3 to 0.6)	-1.6 (-14.6 to 14.5)	-0.9 (-11.4 to 11.1)	0.4 (0.3 to 0.6)	0.4 (0.3 to 0.6)	0.4 (0.3 to 0.6)	-1.2 (-14.3 to 14.9)	-1.6 (-12.1 to 10.3)
Discontinued breastfeeding	1.7 (1.5 to 1.8)	1.7 (1.6 to 1.8)	1.7 (1.5 to 1.8)	2.1 (-8.1 to 12.5)	-1.1 (-8.3 to 7.2)	1.7 (1.5 to 1.8)	1.7 (1.6 to 1.8)	1.7 (1.5 to 1.8)	1.5 (-8.7 to 11.8)	-2.0 (-9.1 to 6.2)
Child underweight	0.3 (0.2 to 0.4)	0.2 (0.1 to 0.3)	0.2 (0.1 to 0.3)	-15.7 (-34.9 to 7.9)	-5.3 (-20.0 to 12.1)	0.3 (0.2 to 0.4)	0.2 (0.1 to 0.4)	0.2 (0.1 to 0.3)	-17.4 (-37.3 to 9.3)	-6.7 (-23.7 to 13.6)
Child wasting	0.2 (0.1 to 0.3)	0.2 (0.1 to 0.3)	0.2 (0.1 to 0.3)	-8.1 (-34.9 to 31.3)	-2.5 (-22.7 to 21.8)	0.2 (0.1 to 0.3)	0.2 (0.1 to 0.3)	0.2 (0.1 to 0.3)	-8.6 (-33.9 to 30.3)	-3.3 (-23.9 to 24.0)
Child stunting	0.7 (0.3 to 1.1)	0.6 (0.3 to 1.1)	0.6 (0.3 to 1.1)	-8.8 (-45.1 to 52.4)	-3.5 (-32.4 to 39.9)	0.7 (0.4 to 1.2)	0.6 (0.3 to 1.1)	0.6 (0.3 to 1.0)	-11.1 (-47.3 to 51.7)	-4.2 (-36.9 to 33.9)
Short gestation for birth weight	0.0 (0.0 to 0.0)	0.0 (0.0 to 0.0)	0.0 (0.0 to 0.0)	7.0 (-17.7 to 35.2)	1.8 (-16.7 to 27.5)	0.0 (0.0 to 0.0)	0.0 (0.0 to 0.0)	0.0 (0.0 to 0.0)	13.3 (-22.1 to 74.1)	-0.2 (-21.2 to 34.8)
Low birth weight for gestation	0.0 (0.0 to 0.0)	0.0 (0.0 to 0.0)	0.0 (0.0 to 0.0)	4.6 (-15.0 to 30.3)	2.0 (-16.8 to 20.9)	0.0 (0.0 to 0.0)	0.0 (0.0 to 0.0)	0.0 (0.0 to 0.0)	-5.0 (-58.5 to 33.6)	4.0 (-17.3 to 25.2)
Iron deficiency	-	-	-	-	-	8.4 (5.7 to 11.7)	8.3 (5.8 to 11.4)	7.6 (5.2 to 10.8)	-0.3 (-20.7 to 29.4)	-8.4 (-24.0 to 9.7)
Vitamin A deficiency	1.1 (0.7 to 1.5)	0.9 (0.5 to 1.1)	0.7 (0.5 to 0.9)	-19.6 (-34.8 to 0.9)	-20.5 (-33.9 to -4.4)	0.8 (0.5 to 1.1)	0.9 (0.6 to 1.2)	0.8 (0.4 to 1.1)	12.6 (-8.2 to 39.8)	-12.7 (-32.6 to 25.6)
Zinc deficiency	0.5 (0.2 to 1.1)	0.4 (0.1 to 1.0)	0.4 (0.1 to 0.9)	-19.0 (-64.8 to 71.2)	-11.1 (-66.2 to 163.3)	0.5 (0.2 to 1.1)	0.4 (0.1 to 0.8)	0.4 (0.1 to 0.8)	-31.5 (-64.8 to 7.6)	8.7 (-56.1 to 182.1)
Smoking	17.9 (14.6 to 21.2)	12.5 (10.0 to 15.1)	10.6 (8.5 to 12.8)	-30.2 (-39.2 to -20.5)	-15.6 (-23.9 to -6.3)	4.1 (2.9 to 5.5)	3.4 (2.4 to 4.6)	3.3 (2.3 to 4.5)	-17.0 (-37.3 to 8.1)	-2.3 (-18.9 to 18.6)
Chewing tobacco	1.4 (0.9 to 2.0)	0.9 (0.6 to 1.4)	0.8 (0.5 to 1.1)	-33.1 (-55.8 to 4.8)	-18.7 (-41.7 to 11.3)	0.1 (0.1 to 0.2)	0.1 (0.1 to 0.1)	0.1 (0.1 to 0.1)	-4.8 (-41.2 to 55.5)	-2.2 (-31.7 to 44.4)
Second-hand smoke	36.9 (32.6 to 41.2)	36.2 (31.8 to 40.6)	36.1 (31.5 to 40.5)	-2.0 (-13.1 to 11.1)	-0.1 (-9.1 to 9.8)	55.8 (52.4 to 59.4)	48.7 (45.1 to 52.4)	48.6 (44.7 to 52.5)	-12.8 (-18.9 to -6.1)	-0.2 (-6.3 to 5.8)
Alcohol use	14.6 (7.5 to 24.8)	14.4 (7.3 to 24.6)	16.2 (7.6 to 27.7)	-0.9 (-36.8 to 50.7)	12.0 (-20.7 to 64.0)	11.6 (5.8 to 20.5)	11.3 (5.5 to 20.0)	12.5 (6.0 to 22.3)	-1.8 (-37.0 to 52.2)	10.5 (-21.4 to 62.4)
Drug use	0.5 (0.4 to 0.7)	0.5 (0.4 to 0.7)	0.6 (0.4 to 0.8)	0.1 (-6.3 to 6.2)	11.7 (3.8 to 20.6)	0.3 (0.2 to 0.4)	0.3 (0.2 to 0.4)	0.3 (0.3 to 0.4)	1.8 (-4.0 to 8.8)	9.3 (2.4 to 16.6)
Diet low in fruits	41.8 (36.5 to 45.4)	40.1 (34.4 to 43.8)	41.3 (35.6 to 44.9)	-4.2 (-10.3 to 1.5)	3.0 (-1.5 to 7.9)	38.7 (32.5 to 42.8)	36.2 (29.9 to 40.6)	38.3 (32.2 to 42.8)	-6.6 (-14.1 to 0.9)	5.8 (-0.8 to 13.4)
Diet low in vegetables	21.1 (12.6 to 28.3)	15.1 (7.1 to 22.6)	17.2 (8.9 to 24.5)	-28.2 (-55.0 to -3.2)	13.4 (-16.0 to 59.8)	23.0 (14.6 to 30.4)	17.0 (8.9 to 24.7)	18.8 (10.4 to 26.3)	-26.0 (-50.8 to -3.6)	10.3 (-13.2 to 49.4)
Diet low in legumes	4.5 (0.8 to 10.1)	5.9 (1.3 to 11.9)	7.9 (2.3 to 14.6)	30.3 (-39.5 to 240.6)	34.7 (-17.6 to 173.6)	8.4 (2.6 to 15.0)	8.3 (2.4 to 14.8)	10.8 (3.9 to 17.6)	-1.2 (-48.7 to 80.5)	30.5 (-11.2 to 136.9)
Diet low in whole grains	31.0 (26.4 to 35.3)	35.7 (31.6 to 39.2)	37.1 (33.2 to 40.4)	15.1 (6.8 to 27.2)	3.7 (-0.9 to 9.0)	32.4 (27.6 to 36.4)	36.9 (33.0 to 40.3)	38.1 (34.3 to 41.2)	14.0 (5.9 to 25.4)	3.4 (-0.7 to 8.1)
Diet low in nuts and seeds	51.0 (49.7 to 52.0)	49.6 (47.7 to 51.0)	49.3 (47.2 to 50.9)	-2.7 (-5.2 to -0.8)	-0.6 (-2.7 to 1.4)	51.2 (50.0 to 52.1)	49.8 (48.2 to 51.0)	49.5 (47.7 to 51.0)	-2.8 (-5.1 to -0.8)	-0.6 (-2.5 to 1.3)
Diet low in milk	46.4 (44.4 to 48.1)	46.7 (44.7 to 48.3)	47.1 (45.2 to 48.7)	0.6 (-1.3 to 2.5)	0.9 (-0.4 to 2.4)	46.5 (44.5 to 48.1)	46.2 (44.2 to 47.9)	46.8 (44.8 to 48.4)	-0.5 (-2.7 to 1.4)	1.3 (-0.1 to 3.0)
Diet high in red meat	3.5 (0.4 to 8.1)	7.0 (1.9 to 13.3)	8.2 (2.2 to 15.7)	99.0 (-17.4 to 1102.3)	16.5 (-41.6 to 141.0)	0.5 (0.0 to 2.4)	1.3 (0.0 to 4.4)	1.8 (0.0 to 5.7)	177.8 (-83.9 to 2907131.0)	31.5 (-92.7 to 5352.6)
Diet high in processed meat	10.6 (6.6 to 14.5)	17.8 (12.2 to 22.2)	20.5 (14.9 to 25.7)	67.1 (28.4 to 133.8)	15.6 (-3.0 to 37.6)	8.9 (5.1 to 12.4)	13.1 (9.0 to 17.0)	14.4 (10.0 to 18.5)	47.3 (15.6 to 105.2)	10.3 (-6.5 to 35.3)
Diet high in sugar-sweetened beverages	6.6 (2.7 to 13.3)	7.6 (3.1 to 14.5)	8.0 (3.3 to 15.2)	15.8 (-47.4 to 170.6)	5.1 (-39.3 to 83.3)	4.6 (1.7 to 10.3)	5.2 (2.3 to 10.1)	5.6 (2.2 to 11.3)	11.9 (-52.0 to 155.7)	9.2 (-37.6 to 96.5)
Diet low in fibre	22.8 (15.5 to 29.2)	23.9 (16.4 to 30.0)	24.9 (17.6 to 30.9)	4.6 (-11.1 to 27.1)	4.1 (-7.8 to 17.7)	23.7 (16.3 to 30.0)	24.5 (17.6 to 30.5)	25.9 (19.2 to 31.9)	3.5 (-11.8 to 23.4)	5.5 (-5.6 to 19.6)
Diet low in calcium	35.6 (31.3 to 39.0)	35.3 (30.9 to 38.8)	36.0 (31.9 to 39.5)	-0.9 (-4.6 to 2.7)	2.1 (-0.7 to 5.6)	37.8 (33.8 to 40.9)	36.6 (32.5 to 39.9)	37.7 (33.8 to 40.9)	-3.1 (-6.6 to 0.2)	3.1 (0.4 to 6.4)
Diet low in seafood omega-3 fatty acids	3.4 (1.6 to 4.8)	0.3 (0.0 to 1.2)	0.3 (0.0 to 1.3)	-91.1 (-100.0 to -66.0)	3.7 (-89.5 to 1482.2)	3.9 (2.0 to 5.4)	0.4 (0.0 to 1.4)	0.5 (0.0 to 1.5)	-89.0 (-99.9 to -67.3)	8.0 (-80.3 to 757.7)
Diet low in polyunsaturated fatty acids	31.3 (25.9 to 36.2)	28.6 (22.9 to 33.5)	29.2 (23.3 to 33.9)	-8.7 (-20.2 to 0.2)	2.1 (-5.3 to 10.6)	31.8 (26.6 to 36.4)	29.1 (23.4 to 33.9)	29.8 (24.3 to 34.3)	-8.3 (-18.2 to 1.4)	2.3 (-5.5 to 11.4)
Diet high in trans fatty acids	1.1 (0.0 to 3.3)	0.9 (0.0 to 3.0)	0.9 (0.0 to 2.9)	-12.6 (-37.7 to -6.6)	-8.3 (-32.5 to -3.5)	1.5 (0.1 to 4.0)	1.3 (0.1 to 3.7)	1.2 (0.0 to 3.5)	-12.6 (-35.3 to -6.3)	-8.2 (-27.4 to -3.0)
Diet high in sodium	27.3 (17.5 to 35.3)	17.6 (8.6 to 24.9)	15.0 (6.1 to 22.4)	-35.6 (-59.4 to -16.3)	-14.5 (-40.0 to 2.3)	31.5 (23.3 to 39.0)	18.9 (10.5 to 26.1)	15.7 (6.9 to 23.0)	-40.0 (-61.4 to -24.3)	-16.8 (-38.5 to -2.5)
Intimate partner violence	-	-	-	-	-	2.3 (2.0 to 2.7)	2.1 (1.8 to 2.5)	2.0 (1.7 to 2.4)	-9.2 (-13.6 to -4.8)	-4.1 (-8.3 to 0.2)
Childhood sexual abuse	5.3 (4.3 to 6.5)	5.3 (4.3 to 6.5)	5.3 (4.3 to 6.5)	0.0 (0.0 to 0.0)	0.0 (0.0 to 0.0)	7.3 (5.9 to 8.9)	7.3 (5.9 to 8.9)	7.5 (6.0 to 9.2)	0.0 (0.0 to 0.0)	2.8 (-4.0 to 10.6)

Supplementary Results Table 1. Tottori age-standardised Summary Exposure Values (SEVs) for Level 4 risk factors and percentage change, by sex, 1990, 2007, and 2017.Data in parentheses are 95% uncertainty intervals.										
	**Male**					**Female**				
	**1990**	**2007**	**2017**	**Percentage change 1990-2007**	**Percentage change 2007-2017**	**1990**	**2007**	**2017**	**Percentage change 1990-2007**	**Percentage change 2007-2017**
Bullying victimization	3.3 (1.4 to 6.7)	3.4 (1.4 to 7.0)	3.5 (1.4 to 7.3)	2.9 (-5.0 to 10.7)	2.1 (-3.3 to 6.9)	1.9 (0.8 to 4.1)	2.0 (0.8 to 4.0)	2.0 (0.8 to 4.0)	1.3 (-6.3 to 9.1)	1.8 (-3.7 to 6.7)
Low physical activity	0.5 (0.2 to 0.9)	0.5 (0.2 to 0.9)	0.4 (0.2 to 0.8)	-1.8 (-3.7 to 0.1)	-8.8 (-12.5 to -4.6)	0.5 (0.2 to 1.0)	0.5 (0.2 to 0.9)	0.5 (0.2 to 1.0)	-3.9 (-6.2 to -1.3)	0.8 (-0.8 to 2.2)
High fasting plasma glucose	1.5 (0.9 to 2.5)	1.4 (0.9 to 2.1)	1.6 (0.9 to 2.5)	-9.0 (-45.8 to 48.4)	10.9 (-25.3 to 73.0)	1.1 (0.7 to 1.8)	1.1 (0.6 to 1.7)	1.2 (0.7 to 2.0)	-4.8 (-41.2 to 52.5)	13.3 (-23.2 to 71.3)
High LDL cholesterol	12.0 (10.4 to 13.7)	12.5 (10.9 to 14.3)	12.5 (10.8 to 14.3)	4.4 (-1.4 to 10.5)	0.1 (-4.7 to 5.1)	12.7 (11.2 to 14.5)	12.8 (11.3 to 14.6)	12.8 (11.3 to 14.7)	0.9 (-4.3 to 6.3)	0.2 (-4.3 to 4.6)
High systolic blood pressure	11.1 (10.1 to 12.2)	8.2 (7.4 to 9.1)	8.1 (7.1 to 9.1)	-25.9 (-32.2 to -19.9)	-1.5 (-8.6 to 6.0)	9.6 (8.6 to 10.6)	6.0 (5.3 to 6.8)	5.6 (4.8 to 6.3)	-37.4 (-44.1 to -29.9)	-7.3 (-16.9 to 4.1)
High body-mass index	5.1 (3.2 to 8.0)	7.2 (4.7 to 11.5)	7.5 (4.9 to 11.9)	42.4 (11.9 to 85.5)	3.7 (-12.2 to 22.8)	5.2 (3.1 to 8.2)	6.7 (4.0 to 10.8)	6.7 (4.0 to 10.9)	29.1 (2.6 to 70.6)	0.2 (-18.0 to 22.7)
Low bone mineral density	5.3 (4.4 to 6.3)	5.2 (4.4 to 6.1)	5.2 (4.3 to 6.2)	-2.1 (-14.9 to 12.9)	0.1 (-13.0 to 15.1)	7.2 (6.1 to 8.5)	7.0 (5.9 to 8.1)	7.1 (5.9 to 8.5)	-3.6 (-14.5 to 8.8)	2.0 (-9.5 to 15.1)
Impaired kidney function	2.6 (1.2 to 6.5)	2.6 (1.2 to 6.5)	2.4 (1.1 to 6.1)	-2.3 (-6.6 to 2.2)	-6.7 (-11.9 to -2.2)	3.2 (1.5 to 7.5)	3.3 (1.5 to 7.7)	3.1 (1.4 to 7.2)	3.6 (-0.2 to 8.1)	-7.0 (-11.8 to -2.2)

Supplementary Results Table 1. Toyama age-standardised Summary Exposure Values (SEVs) for Level 4 risk factors and percentage change, by sex, 1990, 2007, and 2017.Data in parentheses are 95% uncertainty intervals.	Male					Female				
	1990	2007	2017	Percentage change 1990-2007	Percentage change 2007-2017	1990	2007	2017	Percentage change 1990-2007	Percentage change 2007-2017
Unsafe water source	2.2 (1.2 to 3.6)	1.8 (0.9 to 3.0)	1.8 (1.0 to 3.2)	-19.8 (-59.9 to 65.8)	4.6 (-38.7 to 79.0)	2.2 (1.2 to 3.6)	1.8 (0.9 to 3.0)	1.8 (1.0 to 3.2)	-19.8 (-59.9 to 65.8)	4.6 (-38.7 to 79.0)
Unsafe sanitation	3.8 (1.8 to 6.7)	1.8 (0.8 to 3.4)	1.5 (0.7 to 2.9)	-53.1 (-80.1 to 5.2)	-15.6 (-57.0 to 69.4)	3.8 (1.8 to 6.7)	1.8 (0.8 to 3.4)	1.5 (0.7 to 2.9)	-53.1 (-80.1 to 5.2)	-15.6 (-57.0 to 69.4)
No access to handwashing facility	6.7 (4.9 to 8.9)	6.3 (4.5 to 8.7)	5.9 (4.2 to 8.0)	-5.2 (-32.1 to 32.0)	-7.5 (-27.5 to 18.0)	6.7 (4.9 to 8.9)	6.3 (4.5 to 8.7)	5.9 (4.2 to 8.0)	-5.2 (-32.1 to 32.0)	-7.5 (-27.5 to 18.0)
Ambient particulate matter pollution	32.2 (19.8 to 49.1)	32.1 (21.2 to 48.7)	27.2 (15.4 to 44.2)	-0.1 (-31.5 to 47.1)	-15.5 (-47.5 to 23.5)	32.0 (19.5 to 49.0)	32.1 (21.2 to 48.7)	27.1 (15.4 to 44.2)	0.4 (-31.4 to 49.2)	-15.5 (-47.5 to 23.7)
Household air pollution from solid fuels	0.6 (0.2 to 1.9)	0.1 (0.0 to 0.4)	0.1 (0.0 to 0.3)	-75.6 (-93.1 to -47.8)	-17.0 (-67.4 to 76.4)	0.9 (0.2 to 6.7)	0.2 (0.0 to 1.0)	0.2 (0.0 to 0.8)	-73.2 (-93.0 to -45.2)	-12.1 (-67.3 to 81.3)
Ambient ozone pollution	51.4 (24.3 to 54.8)	51.2 (23.9 to 54.8)	51.7 (25.2 to 54.8)	-0.4 (-2.4 to 0.0)	0.9 (0.0 to 5.3)	51.4 (24.3 to 54.8)	51.2 (23.9 to 54.8)	51.7 (25.2 to 54.8)	-0.4 (-2.4 to 0.0)	0.9 (0.0 to 5.3)
Residential radon	16.2 (1.2 to 54.1)	16.2 (1.2 to 54.1)	16.2 (1.2 to 54.1)	0.0 (0.0 to 0.0)	0.0 (0.0 to 0.0)	16.2 (1.2 to 54.1)	16.2 (1.2 to 54.1)	16.2 (1.2 to 54.1)	0.0 (0.0 to 0.0)	0.0 (0.0 to 0.0)
Lead exposure	3.2 (0.5 to 7.0)	2.1 (0.3 to 5.1)	1.5 (0.2 to 4.0)	-35.0 (-49.0 to -25.2)	-29.7 (-45.6 to -21.4)	1.7 (0.1 to 4.8)	1.0 (0.1 to 3.3)	0.7 (0.0 to 2.5)	-40.6 (-58.5 to -30.7)	-32.5 (-54.8 to -23.0)
Occupational exposure to asbestos	3.2 (2.7 to 3.6)	4.2 (3.8 to 4.5)	4.1 (3.5 to 5.0)	31.1 (13.2 to 54.3)	-0.1 (-18.3 to 21.8)	0.7 (0.6 to 0.9)	0.7 (0.7 to 0.8)	0.7 (0.6 to 0.9)	-0.6 (-17.2 to 24.7)	-3.9 (-23.8 to 19.2)
Occupational exposure to arsenic	0.5 (0.0 to 1.1)	0.4 (0.0 to 1.0)	0.4 (0.0 to 1.0)	-	-	0.4 (0.0 to 1.0)	0.4 (0.0 to 0.9)	0.4 (0.0 to 0.9)	-	-
Occupational exposure to benzene	0.6 (0.0 to 1.8)	0.6 (0.0 to 1.9)	0.7 (0.0 to 2.0)	2.6 (-4.3 to 13.2)	3.5 (-2.5 to 11.5)	0.6 (0.0 to 1.7)	0.7 (0.0 to 2.0)	0.8 (0.1 to 2.3)	22.6 (8.3 to 42.2)	14.1 (5.0 to 28.5)
Occupational exposure to beryllium	0.0 (0.0 to 0.0)	0.0 (0.0 to 0.0)	0.0 (0.0 to 0.0)	-5.2 (-10.3 to -0.2)	1.9 (-2.1 to 5.8)	0.0 (0.0 to 0.0)	0.0 (0.0 to 0.0)	0.0 (0.0 to 0.0)	2.4 (-6.8 to 12.3)	9.0 (2.6 to 16.5)
Occupational exposure to cadmium	0.1 (0.0 to 0.1)	0.0 (0.0 to 0.1)	0.0 (0.0 to 0.1)	-7.4 (-18.0 to 5.4)	0.9 (-8.2 to 11.3)	0.0 (0.0 to 0.1)	0.0 (0.0 to 0.1)	0.0 (0.0 to 0.1)	-9.0 (-22.7 to 8.7)	4.6 (-7.2 to 19.0)
Occupational exposure to chromium	0.1 (0.1 to 0.1)	0.1 (0.1 to 0.1)	0.1 (0.1 to 0.1)	-5.6 (-15.2 to 5.9)	1.4 (-6.7 to 10.4)	0.1 (0.1 to 0.1)	0.1 (0.1 to 0.1)	0.1 (0.1 to 0.1)	-4.2 (-16.7 to 12.0)	6.4 (-4.0 to 18.2)
Occupational exposure to diesel engine exhaust	0.6 (0.5 to 0.6)	0.6 (0.5 to 0.6)	0.6 (0.6 to 0.7)	-1.6 (-9.4 to 6.9)	3.0 (-3.4 to 10.2)	0.5 (0.4 to 0.5)	0.5 (0.5 to 0.6)	0.6 (0.5 to 0.6)	13.2 (-0.4 to 29.6)	11.1 (0.6 to 23.0)
Occupational exposure to formaldehyde	0.3 (0.2 to 0.3)	0.2 (0.2 to 0.3)	0.2 (0.2 to 0.3)	-6.8 (-18.1 to 6.7)	0.5 (-8.9 to 11.6)	0.2 (0.2 to 0.3)	0.2 (0.2 to 0.2)	0.2 (0.2 to 0.2)	-6.4 (-20.5 to 12.2)	5.4 (-6.8 to 19.6)
Occupational exposure to nickel	0.4 (0.0 to 1.9)	0.4 (0.0 to 1.8)	0.4 (0.0 to 1.7)	-	-	0.4 (0.0 to 1.6)	0.3 (0.0 to 1.3)	0.3 (0.0 to 1.3)	-	-
Occupational exposure to polycyclic aromatic hydrocarbons	0.2 (0.2 to 0.3)	0.2 (0.2 to 0.3)	0.2 (0.2 to 0.3)	-4.6 (-13.6 to 6.0)	1.8 (-5.5 to 10.3)	0.2 (0.2 to 0.3)	0.2 (0.2 to 0.2)	0.2 (0.2 to 0.3)	-2.0 (-14.3 to 13.5)	7.2 (-2.6 to 18.5)
Occupational exposure to silica	4.7 (0.7 to 14.8)	4.4 (0.6 to 13.5)	4.4 (0.6 to 14.4)	-6.5 (-16.4 to 6.1)	1.4 (-7.2 to 13.0)	3.2 (0.4 to 10.4)	2.7 (0.4 to 8.7)	2.8 (0.4 to 9.2)	-13.9 (-28.3 to 3.5)	1.6 (-11.1 to 20.7)
Occupational exposure to sulphuric acid	0.6 (0.1 to 1.9)	0.6 (0.1 to 1.8)	0.6 (0.1 to 1.8)	-7.3 (-17.6 to 5.2)	1.2 (-7.7 to 10.9)	0.5 (0.1 to 1.6)	0.5 (0.1 to 1.5)	0.5 (0.1 to 1.5)	-10.4 (-24.2 to 7.2)	3.9 (-8.8 to 19.3)
Occupational exposure to trichloroethylene	0.1 (0.1 to 0.1)	0.1 (0.1 to 0.1)	0.1 (0.1 to 0.1)	-4.4 (-11.5 to 4.0)	1.8 (-3.9 to 8.7)	0.1 (0.1 to 0.1)	0.1 (0.1 to 0.1)	0.1 (0.1 to 0.1)	1.0 (-9.8 to 15.2)	8.0 (-0.5 to 18.1)
Occupational asthmagens	18.8 (14.9 to 23.8)	18.1 (14.2 to 22.7)	18.1 (14.3 to 23.1)	-3.9 (-22.0 to 17.7)	0.1 (-15.8 to 18.9)	8.3 (6.6 to 10.4)	8.3 (6.6 to 10.4)	8.8 (7.0 to 11.0)	0.2 (-15.2 to 18.8)	5.4 (-7.4 to 18.5)
Occupational particulate matter, gases, and fumes	6.4 (4.5 to 9.2)	6.1 (4.3 to 8.9)	5.9 (4.2 to 8.5)	-4.7 (-9.7 to 0.4)	-3.8 (-7.1 to -0.5)	3.7 (2.5 to 5.4)	3.5 (2.4 to 5.0)	3.3 (2.3 to 4.8)	-5.8 (-13.8 to 3.2)	-4.8 (-10.3 to 1.4)
Occupational noise	6.0 (5.5 to 6.5)	5.7 (5.3 to 6.3)	5.6 (5.2 to 6.2)	-3.5 (-6.5 to -0.3)	-2.5 (-4.7 to -0.4)	3.5 (3.0 to 4.0)	3.5 (3.1 to 4.0)	3.6 (3.2 to 4.0)	0.3 (-5.7 to 6.9)	1.7 (-2.2 to 5.9)
Occupational ergonomic factors	10.4 (8.5 to 12.6)	9.2 (7.6 to 11.0)	9.0 (7.4 to 11.0)	-12.2 (-23.6 to 1.6)	-1.5 (-12.1 to 9.7)	5.6 (4.3 to 7.3)	5.7 (4.5 to 7.2)	6.4 (4.9 to 8.3)	1.9 (-19.4 to 30.3)	12.7 (-7.9 to 36.3)
Non-exclusive breastfeeding	0.4 (0.3 to 0.6)	0.4 (0.3 to 0.6)	0.4 (0.3 to 0.6)	-1.4 (-14.4 to 14.2)	-1.0 (-11.5 to 10.4)	0.4 (0.3 to 0.6)	0.4 (0.3 to 0.6)	0.4 (0.3 to 0.6)	-1.2 (-14.2 to 14.4)	-1.6 (-12.1 to 9.7)
Discontinued breastfeeding	1.7 (1.6 to 1.8)	1.7 (1.6 to 1.9)	1.7 (1.6 to 1.8)	2.1 (-8.0 to 12.7)	-2.3 (-9.7 to 5.9)	1.7 (1.5 to 1.8)	1.7 (1.6 to 1.8)	1.7 (1.6 to 1.8)	2.1 (-8.0 to 12.8)	-0.7 (-8.3 to 7.6)
Child underweight	0.3 (0.2 to 0.4)	0.2 (0.1 to 0.3)	0.2 (0.1 to 0.3)	-16.0 (-35.3 to 7.5)	-5.5 (-20.3 to 10.4)	0.3 (0.2 to 0.4)	0.2 (0.1 to 0.3)	0.2 (0.1 to 0.3)	-18.0 (-37.3 to 5.7)	-6.8 (-23.1 to 11.0)
Child wasting	0.2 (0.1 to 0.3)	0.2 (0.1 to 0.3)	0.2 (0.1 to 0.3)	-8.2 (-35.3 to 27.3)	-2.8 (-23.7 to 21.9)	0.2 (0.1 to 0.3)	0.2 (0.1 to 0.3)	0.2 (0.1 to 0.3)	-9.1 (-35.4 to 32.0)	-3.5 (-23.2 to 21.5)
Child stunting	0.6 (0.3 to 1.1)	0.6 (0.3 to 1.0)	0.5 (0.3 to 1.0)	-10.9 (-44.8 to 49.9)	-3.7 (-33.3 to 36.4)	0.7 (0.3 to 1.2)	0.6 (0.3 to 1.0)	0.6 (0.3 to 1.0)	-13.5 (-47.4 to 46.6)	-4.4 (-33.6 to 38.8)
Short gestation for birth weight	0.0 (0.0 to 0.0)	0.0 (0.0 to 0.0)	0.0 (0.0 to 0.0)	10.2 (-17.0 to 41.9)	4.1 (-18.6 to 41.6)	0.0 (0.0 to 0.0)	0.0 (0.0 to 0.0)	0.0 (0.0 to 0.0)	16.3 (-16.1 to 48.2)	-2.1 (-22.8 to 32.4)
Low birth weight for gestation	0.0 (0.0 to 0.0)	0.0 (0.0 to 0.0)	0.0 (0.0 to 0.0)	5.2 (-14.6 to 34.5)	1.5 (-19.6 to 20.5)	0.0 (0.0 to 0.0)	0.0 (0.0 to 0.0)	0.0 (0.0 to 0.0)	-1.2 (-20.2 to 27.3)	5.8 (-15.7 to 28.4)
Iron deficiency	-	-	-	-	-	8.0 (5.3 to 10.9)	7.9 (5.4 to 10.8)	7.2 (4.8 to 10.2)	-1.0 (-24.0 to 27.4)	-8.8 (-24.2 to 9.4)
Vitamin A deficiency	0.9 (0.6 to 1.3)	0.8 (0.5 to 1.0)	0.7 (0.4 to 1.0)	-18.3 (-31.0 to 2.6)	-8.0 (-23.3 to 7.9)	0.7 (0.5 to 1.0)	0.8 (0.5 to 1.2)	0.8 (0.5 to 1.1)	12.5 (-13.9 to 40.3)	-6.1 (-24.7 to 14.2)
Zinc deficiency	0.5 (0.1 to 1.0)	0.5 (0.1 to 1.2)	0.5 (0.1 to 1.2)	11.3 (-62.0 to 114.4)	-14.9 (-68.3 to 128.4)	0.5 (0.1 to 1.0)	0.4 (0.1 to 0.9)	0.3 (0.1 to 0.8)	-24.8 (-65.5 to 71.0)	-5.3 (-62.8 to 185.1)
Smoking	17.7 (14.1 to 21.2)	12.4 (9.9 to 15.3)	10.4 (8.3 to 12.9)	-30.0 (-38.9 to -19.2)	-16.0 (-23.6 to -7.4)	4.0 (2.8 to 5.3)	3.3 (2.4 to 4.4)	3.2 (2.3 to 4.3)	-17.2 (-36.4 to 10.2)	-3.3 (-18.7 to 17.2)
Chewing tobacco	1.4 (0.9 to 2.0)	0.9 (0.6 to 1.4)	0.8 (0.5 to 1.1)	-32.8 (-57.6 to 4.7)	-19.0 (-41.8 to 14.1)	0.1 (0.1 to 0.1)	0.1 (0.1 to 0.1)	0.1 (0.1 to 0.1)	-3.6 (-39.5 to 59.3)	-3.1 (-32.9 to 38.8)
Second-hand smoke	37.8 (34.0 to 41.5)	35.6 (31.5 to 39.6)	35.5 (31.0 to 39.8)	-5.9 (-16.5 to 5.9)	-0.4 (-9.2 to 9.5)	56.9 (53.1 to 60.6)	47.8 (44.2 to 51.4)	47.5 (43.6 to 51.4)	-15.9 (-21.9 to -9.7)	-0.7 (-6.8 to 5.8)
Alcohol use	15.7 (8.0 to 26.3)	15.8 (7.9 to 28.0)	17.9 (8.7 to 31.8)	0.8 (-33.1 to 46.4)	13.2 (-18.0 to 54.1)	11.9 (5.8 to 20.7)	11.9 (5.9 to 21.3)	13.8 (6.8 to 25.1)	-0.1 (-34.8 to 45.7)	15.4 (-17.5 to 58.5)
Drug use	0.5 (0.4 to 0.7)	0.5 (0.4 to 0.7)	0.6 (0.4 to 0.8)	0.5 (-5.3 to 6.6)	11.5 (4.8 to 19.8)	0.3 (0.2 to 0.4)	0.3 (0.2 to 0.4)	0.3 (0.3 to 0.4)	3.4 (-2.8 to 10.0)	8.7 (2.3 to 16.3)
Diet low in fruits	40.2 (34.2 to 44.2)	38.3 (32.2 to 42.5)	39.7 (33.9 to 43.8)	-4.6 (-11.4 to 2.0)	3.6 (-1.7 to 9.7)	36.9 (31.2 to 41.6)	34.1 (27.9 to 39.0)	36.5 (30.1 to 41.0)	-7.5 (-16.1 to 1.0)	6.9 (0.1 to 16.0)
Diet low in vegetables	18.2 (9.3 to 25.9)	12.8 (5.3 to 20.7)	15.0 (6.6 to 23.0)	-29.9 (-60.8 to -1.8)	17.0 (-15.9 to 83.6)	20.5 (12.0 to 28.2)	14.6 (6.2 to 23.0)	16.5 (7.8 to 25.0)	-28.8 (-56.8 to -4.0)	13.2 (-15.3 to 66.2)
Diet low in legumes	4.4 (0.6 to 9.9)	5.5 (1.1 to 11.3)	7.2 (1.8 to 13.8)	24.3 (-49.9 to 303.9)	32.5 (-25.3 to 213.0)	8.0 (2.5 to 14.6)	7.5 (2.3 to 13.9)	9.7 (3.5 to 16.5)	-6.0 (-51.1 to 69.1)	28.7 (-14.0 to 138.8)
Diet low in whole grains	29.9 (25.1 to 34.2)	34.8 (30.7 to 38.3)	36.2 (32.3 to 39.7)	16.2 (6.6 to 28.8)	4.0 (-1.0 to 10.0)	31.4 (26.6 to 35.3)	36.1 (32.0 to 39.4)	37.4 (33.4 to 40.5)	14.9 (7.1 to 27.1)	3.6 (-1.4 to 8.9)
Diet low in nuts and seeds	50.4 (49.0 to 51.6)	49.0 (47.0 to 50.5)	48.9 (46.7 to 50.5)	-2.7 (-5.5 to -0.3)	-0.3 (-2.7 to 1.9)	50.4 (48.9 to 51.5)	48.6 (46.6 to 50.2)	48.3 (46.0 to 50.1)	-3.4 (-6.2 to -1.1)	-0.6 (-3.3 to 1.5)
Diet low in milk	45.9 (43.7 to 47.7)	46.3 (44.2 to 48.0)	46.8 (44.8 to 48.4)	0.8 (-1.1 to 2.9)	1.1 (-0.4 to 2.9)	45.9 (43.8 to 47.6)	45.7 (43.6 to 47.4)	46.4 (44.3 to 48.1)	-0.4 (-2.5 to 1.8)	1.5 (-0.1 to 3.3)
Diet high in red meat	4.5 (0.8 to 10.2)	8.7 (3.2 to 15.0)	9.9 (3.4 to 17.6)	91.9 (-13.8 to 599.8)	13.5 (-35.1 to 100.3)	0.7 (0.0 to 3.0)	1.9 (0.0 to 5.7)	2.5 (0.1 to 6.8)	164.4 (-69.7 to 381715.3)	29.1 (-79.4 to 1283.7)
Diet high in processed meat	12.5 (8.2 to 17.0)	20.8 (15.7 to 26.2)	24.2 (18.1 to 30.2)	66.8 (28.5 to 129.5)	16.2 (-1.4 to 38.8)	10.4 (6.4 to 14.3)	15.1 (11.0 to 19.0)	16.6 (11.9 to 20.9)	45.5 (11.9 to 98.2)	10.4 (-5.9 to 30.5)
Diet high in sugar-sweetened beverages	8.0 (3.3 to 15.8)	9.4 (4.2 to 16.2)	9.8 (4.2 to 17.4)	17.1 (-45.3 to 150.6)	4.4 (-40.3 to 73.8)	5.7 (2.2 to 11.9)	6.5 (2.7 to 11.9)	6.8 (2.7 to 12.9)	12.9 (-44.8 to 154.7)	6.2 (-41.4 to 87.0)
Diet low in fibre	21.3 (13.9 to 28.3)	22.3 (15.3 to 28.5)	23.3 (16.1 to 29.9)	4.5 (-12.8 to 30.5)	4.4 (-7.4 to 19.9)	22.5 (15.1 to 29.2)	23.5 (16.1 to 29.8)	24.8 (17.7 to 31.0)	4.3 (-12.0 to 24.4)	5.6 (-5.8 to 20.1)
Diet low in calcium	33.8 (29.3 to 37.6)	33.6 (29.2 to 37.4)	34.5 (29.9 to 38.1)	-0.7 (-4.8 to 3.4)	2.5 (-0.5 to 6.2)	36.1 (32.0 to 39.4)	35.0 (30.7 to 38.5)	36.2 (32.1 to 39.6)	-3.1 (-7.5 to 0.7)	3.5 (0.5 to 7.2)
Diet low in seafood omega-3 fatty acids	2.9 (1.2 to 4.3)	0.2 (0.0 to 1.0)	0.2 (0.0 to 1.1)	-92.5 (-100.0 to -68.0)	6.4 (-94.7 to 5268.2)	3.5 (1.5 to 4.9)	0.3 (0.0 to 1.3)	0.4 (0.0 to 1.3)	-90.5 (-100.0 to -65.6)	8.1 (-86.4 to 1701.4)
Diet low in polyunsaturated fatty acids	29.5 (23.9 to 35.0)	26.7 (20.3 to 31.9)	27.4 (21.0 to 32.7)	-9.5 (-21.6 to 0.2)	2.3 (-7.6 to 11.7)	30.3 (24.6 to 35.1)	27.5 (21.6 to 32.8)	28.2 (22.4 to 33.1)	-9.0 (-20.6 to 1.0)	2.4 (-6.3 to 11.8)
Diet high in trans fatty acids	1.1 (0.0 to 3.3)	1.0 (0.0 to 3.0)	0.9 (0.0 to 2.8)	-12.3 (-39.0 to -6.4)	-8.4 (-33.2 to -3.9)	1.5 (0.1 to 4.0)	1.3 (0.1 to 3.7)	1.2 (0.0 to 3.5)	-12.3 (-33.9 to -6.1)	-8.4 (-28.6 to -3.3)
Diet high in sodium	24.5 (15.7 to 32.2)	16.3 (7.6 to 23.2)	13.8 (5.0 to 20.9)	-33.5 (-57.7 to -15.7)	-15.2 (-41.6 to 1.3)	26.3 (18.0 to 33.4)	15.6 (7.6 to 22.6)	12.3 (4.0 to 19.8)	-40.6 (-64.2 to -24.2)	-21.2 (-52.3 to -6.3)
Intimate partner violence	-	-	-	-	-	2.2 (1.8 to 2.5)	2.0 (1.7 to 2.3)	1.9 (1.6 to 2.2)	-8.8 (-12.9 to -3.8)	-3.5 (-8.1 to 1.3)
Childhood sexual abuse	5.3 (4.3 to 6.5)	5.3 (4.3 to 6.5)	5.3 (4.3 to 6.5)	0.0 (0.0 to 0.0)	0.0 (0.0 to 0.0)	7.3 (5.9 to 8.9)	7.3 (5.9 to 8.9)	7.5 (6.0 to 9.2)	0.0 (0.0 to 0.0)	2.8 (-4.0 to 10.6)

Supplementary Results Table 1. Toyama age-standardised Summary Exposure Values (SEVs) for Level 4 risk factors and percentage change, by sex, 1990, 2007, and 2017.Data in parentheses are 95% uncertainty intervals.

	Male					Female				
	**1990**	**2007**	**2017**	**Percentage change 1990-2007**	**Percentage change 2007-2017**	**1990**	**2007**	**2017**	**Percentage change 1990-2007**	**Percentage change 2007-2017**
Bullying victimization	3.3 (1.4 to 6.9)	3.3 (1.4 to 7.2)	3.5 (1.4 to 7.6)	0.5 (-7.6 to 8.7)	5.0 (-0.4 to 10.0)	1.9 (0.8 to 4.0)	1.9 (0.8 to 4.1)	2.0 (0.8 to 4.2)	-0.2 (-7.8 to 8.0)	4.4 (-21.9 to 37.5)
Low physical activity	0.5 (0.2 to 0.9)	0.5 (0.2 to 0.9)	0.4 (0.2 to 0.8)	-1.8 (-3.8 to 0.0)	-8.8 (-12.4 to -4.6)	0.5 (0.2 to 1.0)	0.5 (0.2 to 0.9)	0.5 (0.2 to 0.9)	-3.9 (-6.0 to -1.3)	0.8 (-0.9 to 2.2)
High fasting plasma glucose	1.5 (0.9 to 2.4)	1.5 (0.9 to 2.4)	1.6 (1.0 to 2.7)	-1.4 (-40.7 to 67.5)	12.4 (-27.0 to 74.4)	1.2 (0.7 to 1.9)	1.2 (0.7 to 2.0)	1.4 (0.8 to 2.3)	2.9 (-39.2 to 72.1)	11.0 (-26.4 to 79.6)
High LDL cholesterol	12.0 (10.5 to 13.8)	12.6 (10.9 to 14.4)	12.6 (11.0 to 14.4)	4.6 (-1.3 to 10.9)	0.1 (-4.4 to 5.2)	12.9 (11.3 to 14.6)	13.0 (11.4 to 14.8)	13.1 (11.5 to 14.9)	1.1 (-4.5 to 7.5)	0.3 (-3.9 to 4.9)
High systolic blood pressure	9.2 (8.3 to 10.1)	7.8 (7.0 to 8.6)	7.8 (6.9 to 8.7)	-15.2 (-22.7 to -8.3)	-0.4 (-7.7 to 8.0)	7.6 (6.8 to 8.4)	5.6 (4.9 to 6.4)	5.3 (4.5 to 6.1)	-25.7 (-33.7 to -17.0)	-6.0 (-17.1 to 4.6)
High body-mass index	5.8 (3.7 to 9.0)	8.4 (5.6 to 13.1)	8.7 (5.8 to 13.8)	44.4 (15.9 to 85.7)	3.6 (-12.1 to 21.0)	5.6 (3.4 to 8.8)	7.3 (4.6 to 11.1)	7.4 (4.6 to 11.5)	29.5 (-0.6 to 71.7)	1.0 (-17.3 to 21.7)
Low bone mineral density	5.3 (4.4 to 6.3)	5.1 (4.3 to 6.0)	5.1 (4.2 to 6.1)	-2.6 (-15.0 to 11.0)	0.3 (-13.4 to 15.3)	7.2 (6.1 to 8.5)	7.0 (6.0 to 8.1)	7.1 (6.0 to 8.4)	-3.4 (-14.8 to 10.3)	2.1 (-9.7 to 15.6)
Impaired kidney function	2.6 (1.1 to 6.5)	2.5 (1.1 to 6.4)	2.3 (1.0 to 6.1)	-2.0 (-6.3 to 3.1)	-6.3 (-11.8 to -2.0)	3.2 (1.4 to 7.3)	3.3 (1.5 to 7.6)	3.1 (1.4 to 7.2)	3.7 (-0.0 to 8.3)	-6.9 (-11.9 to -2.0)

Supplementary Results Table 1. Wakayama age-standardised Summary Exposure Values (SEVs) for Level 4 risk factors and percentage change, by sex, 1990, 2007, and 2017.Data in parentheses are 95% uncertainty intervals.

	Male					Female				
	1990	2007	2017	Percentage change 1990-2007	Percentage change 2007-2017	1990	2007	2017	Percentage change 1990-2007	Percentage change 2007-2017
Unsafe water source	2.5 (1.3 to 4.1)	2.0 (1.1 to 3.3)	2.1 (1.2 to 3.5)	-18.8 (-57.6 to 61.7)	5.6 (-39.1 to 81.0)	2.5 (1.3 to 4.1)	2.0 (1.1 to 3.3)	2.1 (1.2 to 3.5)	-18.8 (-57.6 to 61.7)	5.6 (-39.1 to 81.0)
Unsafe sanitation	4.4 (2.1 to 8.2)	2.2 (1.0 to 4.3)	1.8 (0.9 to 3.6)	-50.6 (-78.5 to 9.6)	-14.3 (-55.3 to 63.9)	4.4 (2.1 to 8.2)	2.2 (1.0 to 4.3)	1.8 (0.9 to 3.6)	-50.6 (-78.5 to 9.6)	-14.3 (-55.3 to 63.9)
No access to handwashing facility	6.8 (4.9 to 9.1)	6.4 (4.6 to 8.6)	6.0 (4.3 to 8.1)	-5.7 (-33.6 to 34.0)	-7.1 (-27.5 to 20.0)	6.8 (4.9 to 9.1)	6.4 (4.6 to 8.6)	6.0 (4.3 to 8.1)	-5.7 (-33.6 to 34.0)	-7.1 (-27.5 to 20.0)
Ambient particulate matter pollution	31.4 (21.6 to 46.3)	31.9 (22.4 to 46.4)	26.8 (17.6 to 41.7)	1.7 (-18.6 to 25.2)	-15.9 (-35.6 to 4.7)	31.1 (21.3 to 46.1)	31.8 (22.3 to 46.3)	26.8 (17.6 to 41.7)	2.5 (-18.2 to 26.4)	-15.8 (-35.6 to 4.7)
Household air pollution from solid fuels	0.8 (0.3 to 2.4)	0.2 (0.0 to 0.5)	0.2 (0.0 to 0.4)	-76.0 (-92.5 to -48.9)	-17.2 (-66.0 to 74.9)	1.1 (0.3 to 7.3)	0.3 (0.1 to 1.3)	0.3 (0.0 to 1.3)	-72.5 (-92.5 to -40.6)	-13.6 (-64.9 to 76.1)
Ambient ozone pollution	51.2 (23.8 to 54.8)	51.8 (25.2 to 54.8)	52.3 (27.1 to 54.8)	1.1 (0.0 to 6.9)	1.0 (0.0 to 7.7)	51.2 (23.8 to 54.8)	51.8 (25.2 to 54.8)	52.3 (27.1 to 54.8)	1.1 (0.0 to 6.9)	1.0 (0.0 to 7.7)
Residential radon	13.0 (0.7 to 41.7)	13.0 (0.7 to 41.7)	13.0 (0.7 to 41.7)	0.0 (0.0 to 0.0)	0.0 (0.0 to 0.0)	13.0 (0.7 to 41.7)	13.0 (0.7 to 41.7)	13.0 (0.7 to 41.7)	0.0 (0.0 to 0.0)	0.0 (0.0 to 0.0)
Lead exposure	2.9 (0.4 to 6.5)	2.0 (0.2 to 5.0)	1.5 (0.1 to 4.1)	-31.2 (-44.4 to -22.1)	-26.4 (-42.3 to -18.5)	1.6 (0.1 to 4.4)	1.0 (0.1 to 3.3)	0.7 (0.0 to 2.7)	-35.4 (-52.3 to -25.8)	-27.6 (-49.9 to -18.4)
Occupational exposure to asbestos	2.1 (1.8 to 2.4)	2.8 (2.6 to 3.1)	2.8 (2.3 to 3.4)	33.8 (15.1 to 60.4)	-1.4 (-18.8 to 19.7)	0.5 (0.4 to 0.6)	0.5 (0.4 to 0.5)	0.5 (0.4 to 0.6)	-6.7 (-24.8 to 22.5)	-0.8 (-20.3 to 23.0)
Occupational exposure to arsenic	0.4 (0.0 to 1.1)	0.4 (0.0 to 1.0)	0.4 (0.0 to 1.0)	-	-	0.4 (0.0 to 0.9)	0.4 (0.0 to 0.9)	0.4 (0.0 to 0.9)	-	-
Occupational exposure to benzene	0.6 (0.0 to 1.8)	0.6 (0.0 to 1.8)	0.7 (0.0 to 1.9)	2.5 (-4.8 to 12.7)	3.4 (-2.1 to 11.9)	0.6 (0.0 to 1.6)	0.7 (0.0 to 2.0)	0.8 (0.0 to 2.2)	22.8 (9.3 to 44.1)	14.4 (4.8 to 30.4)
Occupational exposure to beryllium	0.0 (0.0 to 0.0)	0.0 (0.0 to 0.0)	0.0 (0.0 to 0.0)	-5.3 (-10.5 to -0.1)	1.9 (-1.7 to 6.1)	0.0 (0.0 to 0.0)	0.0 (0.0 to 0.0)	0.0 (0.0 to 0.0)	3.1 (-6.6 to 13.7)	9.1 (2.5 to 16.7)
Occupational exposure to cadmium	0.1 (0.0 to 0.1)	0.0 (0.0 to 0.1)	0.0 (0.0 to 0.1)	-6.7 (-18.6 to 6.2)	1.0 (-8.3 to 11.6)	0.0 (0.0 to 0.1)	0.0 (0.0 to 0.0)	0.0 (0.0 to 0.1)	-7.5 (-22.0 to 11.5)	4.9 (-8.0 to 20.7)
Occupational exposure to chromium	0.1 (0.1 to 0.1)	0.1 (0.1 to 0.1)	0.1 (0.1 to 0.1)	-4.9 (-15.4 to 6.7)	1.5 (-6.5 to 10.6)	0.1 (0.1 to 0.1)	0.1 (0.1 to 0.1)	0.1 (0.1 to 0.1)	-2.7 (-16.7 to 14.8)	6.7 (-4.4 to 19.5)
Occupational exposure to diesel engine exhaust	0.6 (0.5 to 0.6)	0.6 (0.5 to 0.6)	0.6 (0.5 to 0.6)	-1.5 (-9.3 to 7.5)	3.2 (-3.0 to 10.0)	0.4 (0.4 to 0.5)	0.5 (0.4 to 0.6)	0.5 (0.5 to 0.6)	13.7 (0.1 to 30.2)	11.3 (0.1 to 22.6)
Occupational exposure to formaldehyde	0.2 (0.2 to 0.3)	0.2 (0.2 to 0.3)	0.2 (0.2 to 0.3)	-6.0 (-18.4 to 7.5)	0.6 (-9.1 to 11.4)	0.2 (0.2 to 0.2)	0.2 (0.2 to 0.2)	0.2 (0.2 to 0.2)	-4.9 (-20.3 to 15.2)	5.6 (-7.5 to 20.8)
Occupational exposure to nickel	0.4 (0.0 to 1.8)	0.4 (0.0 to 1.6)	0.4 (0.0 to 1.6)	-	-	0.3 (0.0 to 1.5)	0.3 (0.0 to 1.3)	0.3 (0.0 to 1.3)	-	-
Occupational exposure to polycyclic aromatic hydrocarbons	0.2 (0.2 to 0.3)	0.2 (0.2 to 0.3)	0.2 (0.2 to 0.3)	-4.0 (-13.8 to 6.9)	1.8 (-5.4 to 9.9)	0.2 (0.2 to 0.2)	0.2 (0.2 to 0.2)	0.2 (0.2 to 0.3)	-0.6 (-14.0 to 16.4)	7.5 (-2.7 to 19.7)
Occupational exposure to silica	4.6 (0.6 to 14.6)	4.3 (0.6 to 13.5)	4.3 (0.6 to 13.6)	-6.6 (-16.9 to 6.4)	1.3 (-7.6 to 12.8)	3.1 (0.5 to 10.1)	2.7 (0.4 to 8.4)	2.7 (0.4 to 8.6)	-12.5 (-27.4 to 7.4)	1.9 (-11.7 to 21.1)
Occupational exposure to sulphuric acid	0.6 (0.1 to 1.9)	0.6 (0.1 to 1.8)	0.6 (0.1 to 1.8)	-6.6 (-17.3 to 5.4)	1.2 (-7.6 to 10.8)	0.5 (0.1 to 1.6)	0.5 (0.1 to 1.4)	0.5 (0.1 to 1.5)	-9.2 (-23.7 to 9.6)	4.2 (-9.2 to 20.8)
Occupational exposure to trichloroethylene	0.1 (0.1 to 0.1)	0.1 (0.1 to 0.1)	0.1 (0.1 to 0.1)	-3.9 (-11.4 to 4.8)	2.0 (-3.8 to 8.5)	0.1 (0.1 to 0.1)	0.1 (0.1 to 0.1)	0.1 (0.1 to 0.1)	2.2 (-9.8 to 16.8)	8.3 (-0.8 to 18.9)
Occupational asthmagens	18.0 (14.2 to 22.7)	17.5 (13.9 to 22.2)	17.6 (13.7 to 22.4)	-2.5 (-20.7 to 20.0)	0.3 (-15.0 to 18.8)	8.1 (6.4 to 10.0)	8.1 (6.5 to 10.1)	8.6 (6.9 to 10.7)	0.5 (-15.8 to 19.6)	5.3 (-7.6 to 19.1)
Occupational particulate matter, gases, and fumes	6.4 (4.6 to 9.2)	6.1 (4.3 to 8.8)	5.9 (4.2 to 8.5)	-4.7 (-9.6 to 0.4)	-3.8 (-7.3 to -0.5)	3.6 (2.4 to 5.3)	3.4 (2.4 to 5.0)	3.2 (2.3 to 4.8)	-5.2 (-13.9 to 5.0)	-4.5 (-10.2 to 2.1)
Occupational noise	6.0 (5.5 to 6.6)	5.8 (5.3 to 6.3)	5.6 (5.2 to 6.1)	-3.6 (-6.7 to -0.2)	-2.6 (-4.7 to -0.5)	3.4 (2.9 to 3.9)	3.4 (3.0 to 3.9)	3.5 (3.1 to 3.9)	0.5 (-6.1 to 7.8)	1.7 (-2.4 to 6.3)
Occupational ergonomic factors	11.0 (9.0 to 13.3)	9.7 (8.0 to 11.6)	9.6 (7.8 to 11.7)	-11.3 (-23.2 to 3.5)	-1.0 (-12.8 to 12.1)	5.9 (4.5 to 7.6)	5.9 (4.6 to 7.5)	6.7 (5.2 to 8.7)	0.5 (-22.1 to 28.4)	12.8 (-7.5 to 36.9)
Non-exclusive breastfeeding	0.4 (0.3 to 0.6)	0.4 (0.3 to 0.6)	0.4 (0.3 to 0.6)	-0.9 (-14.1 to 13.6)	-2.2 (-12.4 to 9.6)	0.4 (0.3 to 0.6)	0.4 (0.3 to 0.6)	0.4 (0.3 to 0.6)	-1.6 (-14.7 to 12.8)	-1.2 (-11.5 to 10.6)
Discontinued breastfeeding	1.6 (1.5 to 1.8)	1.7 (1.5 to 1.8)	1.6 (1.5 to 1.8)	1.6 (-7.8 to 12.3)	-1.6 (-9.0 to 6.3)	1.7 (1.5 to 1.8)	1.7 (1.6 to 1.8)	1.7 (1.5 to 1.8)	0.8 (-8.5 to 11.4)	-2.6 (-9.9 to 5.3)
Child underweight	0.3 (0.2 to 0.4)	0.2 (0.1 to 0.3)	0.2 (0.1 to 0.3)	-15.8 (-34.3 to 6.8)	-5.6 (-21.1 to 9.7)	0.3 (0.2 to 0.4)	0.2 (0.1 to 0.3)	0.2 (0.1 to 0.3)	-17.1 (-36.9 to 8.7)	-5.8 (-21.0 to 12.6)
Child wasting	0.2 (0.1 to 0.3)	0.2 (0.1 to 0.3)	0.2 (0.1 to 0.3)	-6.8 (-35.0 to 32.2)	-2.2 (-22.5 to 22.8)	0.2 (0.1 to 0.3)	0.2 (0.1 to 0.3)	0.2 (0.1 to 0.3)	-7.7 (-35.4 to 33.3)	-2.7 (-22.8 to 23.1)
Child stunting	0.7 (0.3 to 1.1)	0.6 (0.3 to 1.0)	0.6 (0.3 to 1.0)	-9.0 (-47.1 to 48.4)	-3.6 (-32.1 to 35.5)	0.7 (0.3 to 1.2)	0.6 (0.3 to 1.1)	0.6 (0.3 to 1.0)	-12.3 (-48.8 to 49.3)	-6.0 (-34.6 to 37.5)
Short gestation for birth weight	0.0 (0.0 to 0.0)	0.0 (0.0 to 0.0)	0.0 (0.0 to 0.0)	8.1 (-13.2 to 37.3)	2.7 (-13.7 to 29.1)	0.0 (0.0 to 0.0)	0.0 (0.0 to 0.0)	0.0 (0.0 to 0.0)	14.0 (-18.7 to 56.2)	0.9 (-20.1 to 35.2)
Low birth weight for gestation	0.0 (0.0 to 0.0)	0.0 (0.0 to 0.0)	0.0 (0.0 to 0.0)	5.0 (-12.6 to 26.4)	1.9 (-15.8 to 19.5)	0.0 (0.0 to 0.0)	0.0 (0.0 to 0.0)	0.0 (0.0 to 0.0)	-3.2 (-21.5 to 30.2)	3.5 (-18.2 to 24.8)
Iron deficiency	-	-	-	-	-	8.3 (5.7 to 11.4)	8.2 (5.8 to 11.3)	7.6 (5.1 to 10.6)	-0.6 (-21.7 to 26.6)	-8.1 (-23.7 to 10.2)
Vitamin A deficiency	0.9 (0.6 to 1.2)	0.8 (0.5 to 1.0)	0.7 (0.5 to 1.0)	-15.6 (-32.2 to 6.6)	-7.4 (-26.5 to 10.8)	0.8 (0.5 to 1.1)	0.8 (0.5 to 1.1)	0.7 (0.5 to 0.9)	0.1 (-15.6 to 18.2)	-16.0 (-28.6 to -0.3)
Zinc deficiency	0.5 (0.1 to 1.0)	0.4 (0.1 to 1.1)	0.4 (0.1 to 0.9)	-11.7 (-63.9 to 73.8)	-14.0 (-69.0 to 176.8)	0.5 (0.2 to 1.1)	0.3 (0.1 to 0.8)	0.4 (0.1 to 0.8)	-31.4 (-64.5 to 8.6)	4.8 (-59.8 to 169.0)
Smoking	18.0 (14.5 to 21.4)	12.6 (10.1 to 15.4)	10.6 (8.5 to 13.2)	-30.1 (-39.3 to -19.9)	-15.7 (-24.4 to -5.9)	4.1 (2.9 to 5.4)	3.4 (2.4 to 4.5)	3.3 (2.3 to 4.4)	-17.7 (-37.2 to 8.5)	-2.9 (-20.3 to 15.8)
Chewing tobacco	1.4 (0.9 to 2.1)	0.9 (0.6 to 1.5)	0.8 (0.5 to 1.2)	-32.9 (-57.6 to 5.6)	-18.8 (-43.6 to 14.5)	0.1 (0.1 to 0.1)	0.1 (0.1 to 0.1)	0.1 (0.1 to 0.1)	-3.8 (-41.4 to 65.7)	-2.5 (-32.9 to 44.5)
Second-hand smoke	36.9 (32.9 to 40.9)	35.6 (31.2 to 39.9)	35.5 (31.1 to 39.9)	-3.5 (-14.7 to 8.5)	-0.4 (-9.3 to 10.0)	55.7 (52.1 to 59.3)	47.6 (44.0 to 51.4)	47.2 (43.0 to 50.9)	-14.5 (-21.3 to -7.7)	-0.8 (-7.3 to 6.1)
Alcohol use	14.9 (7.5 to 25.7)	15.1 (7.5 to 25.2)	17.5 (8.1 to 30.4)	1.1 (-32.4 to 47.6)	16.0 (-18.3 to 59.5)	11.7 (5.8 to 20.7)	11.8 (5.7 to 20.7)	13.1 (5.8 to 23.7)	0.4 (-32.1 to 45.3)	11.4 (-20.7 to 56.1)
Drug use	0.5 (0.4 to 0.7)	0.5 (0.4 to 0.7)	0.6 (0.4 to 0.8)	0.8 (-4.6 to 7.0)	11.4 (4.1 to 19.8)	0.3 (0.2 to 0.4)	0.3 (0.2 to 0.4)	0.3 (0.3 to 0.4)	2.6 (-3.2 to 8.9)	8.9 (2.1 to 16.2)
Diet low in fruits	41.0 (35.5 to 44.6)	39.4 (33.4 to 43.4)	40.6 (34.9 to 44.4)	-3.9 (-9.9 to 1.8)	3.1 (-1.7 to 8.7)	38.2 (32.0 to 42.5)	35.6 (29.3 to 40.2)	37.7 (31.8 to 42.2)	-6.8 (-14.6 to 1.3)	6.0 (-0.4 to 13.1)
Diet low in vegetables	20.2 (11.8 to 27.6)	14.5 (5.9 to 22.4)	16.7 (8.1 to 24.5)	-27.9 (-58.1 to -2.8)	15.0 (-12.2 to 68.8)	22.4 (14.1 to 29.5)	16.8 (8.2 to 24.2)	18.7 (10.5 to 26.3)	-24.9 (-50.8 to -2.3)	10.9 (-11.9 to 51.9)
Diet low in legumes	4.7 (0.8 to 10.6)	6.7 (1.6 to 13.3)	9.0 (3.0 to 16.1)	42.0 (-35.2 to 311.5)	35.5 (-11.8 to 195.9)	8.6 (2.7 to 15.4)	9.0 (2.9 to 15.6)	11.7 (4.9 to 18.8)	4.7 (-39.9 to 101.4)	30.4 (-9.5 to 136.8)
Diet low in whole grains	30.7 (25.5 to 35.1)	35.4 (31.4 to 38.9)	36.8 (33.0 to 40.1)	15.5 (7.0 to 28.2)	3.9 (-0.9 to 9.5)	32.1 (27.2 to 36.2)	36.6 (32.8 to 39.9)	37.9 (34.1 to 41.0)	14.2 (6.1 to 25.1)	3.5 (-0.7 to 8.9)
Diet low in nuts and seeds	51.2 (50.0 to 52.2)	50.4 (48.9 to 51.6)	50.5 (48.9 to 51.7)	-1.6 (-3.6 to 0.3)	0.2 (-1.5 to 1.8)	51.2 (50.0 to 52.2)	50.2 (48.5 to 51.5)	50.1 (48.4 to 51.5)	-2.1 (-4.4 to -0.1)	-0.1 (-2.0 to 1.5)
Diet low in milk	46.3 (44.1 to 48.0)	46.7 (44.6 to 48.3)	47.1 (45.2 to 48.7)	0.7 (-1.1 to 2.8)	1.0 (-0.4 to 2.5)	46.3 (44.3 to 48.0)	46.1 (43.9 to 47.9)	46.7 (44.8 to 48.4)	-0.4 (-2.4 to 1.5)	1.4 (-0.0 to 3.1)
Diet high in red meat	3.8 (0.5 to 9.1)	7.6 (2.2 to 14.5)	8.6 (2.7 to 16.2)	97.9 (-14.5 to 755.4)	13.7 (-43.3 to 128.2)	0.5 (0.0 to 2.4)	1.5 (0.0 to 4.9)	2.0 (0.0 to 5.7)	177.8 (-77.9 to 3333790.7)	30.0 (-84.3 to 2270.8)
Diet high in processed meat	11.1 (7.0 to 15.2)	18.5 (13.4 to 23.5)	21.4 (15.9 to 27.0)	66.1 (27.4 to 129.9)	16.0 (-3.1 to 37.6)	9.1 (5.4 to 12.8)	13.2 (9.0 to 17.0)	14.3 (9.9 to 18.3)	44.7 (10.9 to 98.7)	8.9 (-8.7 to 29.8)
Diet high in sugar-sweetened beverages	7.1 (2.7 to 14.5)	8.2 (3.5 to 14.6)	8.7 (3.7 to 15.8)	15.1 (-46.5 to 157.8)	6.3 (-39.5 to 79.9)	4.9 (1.9 to 10.6)	5.7 (2.4 to 10.7)	6.0 (2.4 to 12.1)	14.9 (-47.8 to 163.6)	6.5 (-39.2 to 89.8)
Diet low in fibre	22.6 (14.9 to 29.1)	24.0 (17.1 to 30.2)	25.1 (18.2 to 31.1)	6.2 (-10.3 to 27.9)	4.8 (-7.1 to 19.0)	23.8 (16.6 to 30.3)	25.2 (17.9 to 31.4)	26.8 (19.7 to 32.9)	5.7 (-10.5 to 28.2)	6.4 (-4.1 to 20.5)
Diet low in calcium	35.1 (30.7 to 38.7)	35.0 (30.8 to 38.5)	35.9 (31.8 to 39.3)	-0.3 (-4.1 to 3.7)	2.5 (-0.5 to 5.9)	37.2 (33.4 to 40.5)	36.2 (32.2 to 39.5)	37.4 (33.6 to 40.6)	-2.8 (-6.5 to 0.5)	3.3 (0.8 to 6.6)
Diet low in seafood omega-3 fatty acids	3.2 (1.3 to 4.6)	0.2 (0.0 to 1.1)	0.3 (0.0 to 1.1)	-92.2 (-100.0 to -69.4)	8.8 (-89.9 to 1387.2)	3.8 (1.8 to 5.3)	0.4 (0.0 to 1.3)	0.4 (0.0 to 1.4)	-90.4 (-100.0 to -68.1)	8.9 (-85.8 to 2136.9)
Diet low in polyunsaturated fatty acids	30.7 (25.1 to 35.3)	28.0 (22.1 to 33.1)	28.7 (22.6 to 33.6)	-8.7 (-20.2 to 0.9)	2.3 (-6.8 to 12.3)	31.4 (25.8 to 36.3)	28.7 (22.8 to 33.6)	29.3 (23.6 to 34.1)	-8.6 (-20.8 to 0.7)	2.2 (-4.9 to 11.2)
Diet high in trans fatty acids	1.1 (0.0 to 3.3)	1.0 (0.0 to 3.0)	0.9 (0.0 to 2.9)	-12.5 (-38.0 to -6.2)	-8.3 (-33.7 to -3.7)	1.5 (0.1 to 4.0)	1.3 (0.1 to 3.7)	1.2 (0.0 to 3.5)	-12.7 (-35.5 to -6.5)	-8.2 (-28.6 to -3.2)
Diet high in sodium	27.4 (16.9 to 36.3)	17.5 (8.3 to 24.9)	14.9 (6.0 to 22.1)	-36.2 (-60.5 to -17.4)	-14.4 (-39.5 to 4.9)	31.5 (23.0 to 39.1)	18.9 (10.4 to 25.7)	15.6 (6.7 to 22.9)	-40.2 (-60.8 to -24.8)	-17.5 (-41.8 to -4.1)
Intimate partner violence	-	-	-	-	-	2.4 (2.0 to 2.8)	2.2 (1.9 to 2.6)	2.1 (1.8 to 2.5)	-8.7 (-12.9 to -4.0)	-3.9 (-8.2 to 0.4)
Childhood sexual abuse	5.3 (4.3 to 6.5)	5.3 (4.3 to 6.5)	5.3 (4.3 to 6.5)	0.0 (0.0 to 0.0)	0.0 (0.0 to 0.0)	7.3 (5.9 to 8.9)	7.3 (5.9 to 8.9)	7.5 (6.0 to 9.2)	0.0 (0.0 to 0.0)	2.8 (-4.0 to 10.6)

Supplementary Results Table 1. Wakayama age-standardised Summary Exposure Values (SEVs) for Level 4 risk factors and percentage change, by sex, 1990, 2007, and 2017.Data in parentheses are 95% uncertainty intervals.										
	**Male**					**Female**				
	**1990**	**2007**	**2017**	**Percentage change 1990-2007**	**Percentage change 2007-2017**	**1990**	**2007**	**2017**	**Percentage change 1990-2007**	**Percentage change 2007-2017**
Bullying victimization	3.3 (1.4 to 6.6)	3.4 (1.4 to 6.8)	3.5 (1.4 to 7.2)	2.0 (-5.8 to 10.0)	3.3 (-2.0 to 8.1)	1.9 (0.8 to 3.8)	1.9 (0.8 to 3.8)	2.0 (0.8 to 4.1)	0.7 (-7.4 to 8.9)	2.9 (-22.9 to 36.7)
Low physical activity	0.5 (0.2 to 0.9)	0.5 (0.2 to 0.9)	0.4 (0.2 to 0.8)	-1.8 (-3.6 to -0.1)	-8.8 (-11.9 to -4.6)	0.5 (0.2 to 1.0)	0.5 (0.2 to 0.9)	0.5 (0.2 to 0.9)	-3.9 (-6.1 to -1.4)	0.7 (-0.7 to 2.3)
High fasting plasma glucose	1.4 (0.9 to 2.2)	1.4 (0.9 to 2.1)	1.5 (0.9 to 2.4)	-4.1 (-40.3 to 52.1)	12.5 (-22.9 to 79.9)	1.2 (0.7 to 1.8)	1.2 (0.7 to 2.1)	1.3 (0.8 to 2.3)	3.0 (-36.3 to 71.7)	9.8 (-26.2 to 79.9)
High LDL cholesterol	12.0 (10.3 to 13.8)	12.5 (10.9 to 14.2)	12.5 (10.8 to 14.3)	4.5 (-1.8 to 10.8)	0.0 (-4.8 to 4.9)	12.7 (11.2 to 14.5)	12.9 (11.4 to 14.7)	12.9 (11.4 to 14.6)	1.0 (-4.5 to 6.7)	0.2 (-4.3 to 5.1)
High systolic blood pressure	11.2 (10.2 to 12.2)	8.2 (7.4 to 9.1)	8.1 (7.2 to 9.2)	-26.1 (-32.2 to -19.0)	-1.6 (-9.3 to 6.5)	9.6 (8.6 to 10.6)	6.0 (5.3 to 6.8)	5.6 (4.8 to 6.4)	-37.0 (-43.4 to -29.8)	-7.6 (-16.5 to 2.9)
High body-mass index	5.0 (3.1 to 8.0)	7.1 (4.6 to 11.3)	7.3 (4.6 to 11.9)	41.5 (12.8 to 81.4)	2.8 (-13.3 to 20.2)	5.2 (3.1 to 8.5)	6.7 (4.2 to 10.8)	6.8 (4.1 to 10.9)	28.4 (-0.1 to 69.7)	0.1 (-18.2 to 21.5)
Low bone mineral density	5.5 (4.8 to 6.3)	5.6 (4.6 to 6.5)	5.6 (4.6 to 6.6)	0.9 (-11.9 to 15.2)	0.1 (-13.1 to 13.5)	8.4 (7.1 to 9.6)	8.4 (7.1 to 9.9)	8.4 (7.0 to 10.0)	0.3 (-11.2 to 14.6)	-0.2 (-12.0 to 12.5)
Impaired kidney function	2.6 (1.2 to 6.5)	2.6 (1.2 to 6.4)	2.4 (1.1 to 6.1)	-3.0 (-7.4 to 1.2)	-6.7 (-12.2 to -2.1)	3.2 (1.5 to 7.5)	3.3 (1.5 to 7.7)	3.1 (1.4 to 7.3)	2.9 (-1.3 to 7.5)	-7.0 (-11.9 to -2.3)

Supplementary Results Table 1. Yamagata age-standardised Summary Exposure Values (SEVs) for Level 4 risk factors and percentage change, by sex, 1990, 2007, and 2017.Data in parentheses are 95% uncertainty intervals.

	Male					Female				
	**1990**	**2007**	**2017**	**Percentage change 1990-2007**	**Percentage change 2007-2017**	**1990**	**2007**	**2017**	**Percentage change 1990-2007**	**Percentage change 2007-2017**
Unsafe water source	2.6 (1.4 to 4.4)	2.2 (1.2 to 3.6)	2.3 (1.3 to 3.7)	-16.8 (-57.0 to 71.5)	4.7 (-39.9 to 76.4)	2.6 (1.4 to 4.4)	2.2 (1.2 to 3.6)	2.3 (1.3 to 3.7)	-16.8 (-57.0 to 71.5)	4.7 (-39.9 to 76.4)
Unsafe sanitation	4.8 (2.4 to 8.6)	2.4 (1.1 to 4.4)	2.0 (0.9 to 4.0)	-50.3 (-78.1 to 5.8)	-15.5 (-56.3 to 65.7)	4.8 (2.4 to 8.6)	2.4 (1.1 to 4.4)	2.0 (0.9 to 4.0)	-50.3 (-78.1 to 5.8)	-15.5 (-56.3 to 65.7)
No access to handwashing facility	6.9 (4.9 to 9.3)	6.5 (4.6 to 8.7)	6.0 (4.3 to 8.2)	-6.1 (-34.8 to 32.2)	-6.8 (-27.7 to 21.2)	6.9 (4.9 to 9.3)	6.5 (4.6 to 8.7)	6.0 (4.3 to 8.2)	-6.1 (-34.8 to 32.2)	-6.8 (-27.7 to 21.2)
Ambient particulate matter pollution	26.3 (16.2 to 40.3)	26.7 (17.6 to 40.7)	22.5 (12.6 to 36.2)	1.3 (-27.9 to 47.5)	-15.8 (-49.2 to 23.3)	25.9 (15.8 to 39.6)	26.6 (17.5 to 40.7)	22.4 (12.6 to 36.1)	2.5 (-27.4 to 50.1)	-15.8 (-49.3 to 23.4)
Household air pollution from solid fuels	1.1 (0.3 to 3.4)	0.2 (0.1 to 0.7)	0.2 (0.0 to 0.5)	-76.5 (-92.4 to -44.1)	-19.4 (-68.2 to 79.7)	1.5 (0.4 to 7.5)	0.4 (0.1 to 2.1)	0.3 (0.1 to 1.4)	-72.9 (-92.3 to -33.8)	-18.3 (-68.4 to 77.6)
Ambient ozone pollution	48.9 (20.2 to 54.8)	48.8 (19.9 to 54.8)	49.2 (20.5 to 54.8)	-0.3 (-1.2 to 0.0)	0.8 (0.0 to 2.9)	48.9 (20.2 to 54.8)	48.8 (19.9 to 54.8)	49.2 (20.5 to 54.8)	-0.3 (-1.2 to 0.0)	0.8 (0.0 to 2.9)
Residential radon	17.6 (1.7 to 55.7)	17.6 (1.7 to 55.7)	17.6 (1.7 to 55.7)	0.0 (0.0 to 0.0)	0.0 (0.0 to 0.0)	17.6 (1.7 to 55.7)	17.6 (1.7 to 55.7)	17.6 (1.7 to 55.7)	0.0 (0.0 to 0.0)	0.0 (0.0 to 0.0)
Lead exposure	2.8 (0.4 to 6.5)	2.0 (0.2 to 5.1)	1.5 (0.1 to 4.1)	-29.2 (-42.0 to -21.0)	-25.4 (-40.4 to -17.9)	1.5 (0.1 to 4.4)	1.0 (0.1 to 3.3)	0.8 (0.0 to 2.8)	-32.7 (-48.8 to -24.1)	-26.2 (-47.6 to -17.5)
Occupational exposure to asbestos	1.3 (1.1 to 1.4)	1.8 (1.6 to 1.9)	1.8 (1.4 to 2.2)	39.3 (20.2 to 68.1)	0.2 (-19.7 to 23.5)	0.4 (0.3 to 0.5)	0.4 (0.3 to 0.4)	0.4 (0.3 to 0.5)	-7.3 (-24.5 to 21.1)	-0.3 (-21.0 to 24.9)
Occupational exposure to arsenic	0.4 (0.0 to 1.0)	0.4 (0.0 to 1.0)	0.4 (0.0 to 1.0)	-	-	0.4 (0.0 to 0.9)	0.3 (0.0 to 0.8)	0.4 (0.0 to 0.9)	-	-
Occupational exposure to benzene	0.6 (0.0 to 1.7)	0.6 (0.0 to 1.8)	0.7 (0.0 to 1.8)	2.2 (-4.3 to 13.0)	3.5 (-2.2 to 11.3)	0.6 (0.0 to 1.6)	0.7 (0.0 to 2.0)	0.8 (0.0 to 2.2)	22.7 (9.4 to 41.5)	15.2 (4.7 to 33.9)
Occupational exposure to beryllium	0.0 (0.0 to 0.0)	0.0 (0.0 to 0.0)	0.0 (0.0 to 0.0)	-5.1 (-10.0 to 0.2)	2.0 (-1.9 to 6.2)	0.0 (0.0 to 0.0)	0.0 (0.0 to 0.0)	0.0 (0.0 to 0.0)	2.9 (-6.9 to 14.0)	9.8 (2.7 to 17.9)
Occupational exposure to cadmium	0.1 (0.0 to 0.1)	0.0 (0.0 to 0.1)	0.0 (0.0 to 0.1)	-6.2 (-17.2 to 7.3)	0.8 (-8.2 to 11.3)	0.0 (0.0 to 0.1)	0.0 (0.0 to 0.0)	0.0 (0.0 to 0.1)	-7.0 (-22.6 to 10.2)	5.6 (-7.2 to 22.5)
Occupational exposure to chromium	0.1 (0.1 to 0.1)	0.1 (0.1 to 0.1)	0.1 (0.1 to 0.1)	-4.3 (-14.2 to 8.0)	1.5 (-6.6 to 10.5)	0.1 (0.1 to 0.1)	0.1 (0.1 to 0.1)	0.1 (0.1 to 0.1)	-2.3 (-17.0 to 14.5)	7.4 (-4.0 to 21.2)
Occupational exposure to diesel engine exhaust	0.6 (0.5 to 0.6)	0.6 (0.5 to 0.6)	0.6 (0.5 to 0.6)	-0.8 (-8.9 to 8.1)	3.6 (-2.8 to 10.4)	0.4 (0.4 to 0.5)	0.5 (0.4 to 0.5)	0.5 (0.5 to 0.6)	13.9 (-0.4 to 30.5)	12.5 (1.4 to 24.1)
Occupational exposure to formaldehyde	0.2 (0.2 to 0.3)	0.2 (0.2 to 0.3)	0.2 (0.2 to 0.3)	-5.4 (-17.4 to 9.3)	0.5 (-9.0 to 11.7)	0.2 (0.2 to 0.2)	0.2 (0.2 to 0.2)	0.2 (0.2 to 0.2)	-4.3 (-20.4 to 14.2)	6.4 (-6.7 to 22.6)
Occupational exposure to nickel	0.4 (0.0 to 1.8)	0.4 (0.0 to 1.6)	0.4 (0.0 to 1.6)	-	-	0.3 (0.0 to 1.4)	0.3 (0.0 to 1.2)	0.3 (0.0 to 1.3)	-	-
Occupational exposure to polycyclic aromatic hydrocarbons	0.2 (0.2 to 0.3)	0.2 (0.2 to 0.2)	0.2 (0.2 to 0.3)	-3.4 (-13.1 to 8.2)	1.8 (-5.7 to 10.1)	0.2 (0.2 to 0.2)	0.2 (0.2 to 0.2)	0.2 (0.2 to 0.2)	-0.3 (-14.3 to 16.0)	8.3 (-2.6 to 21.1)
Occupational exposure to silica	4.4 (0.7 to 13.9)	4.2 (0.6 to 13.1)	4.2 (0.6 to 13.2)	-5.1 (-15.4 to 7.7)	1.5 (-7.4 to 13.4)	3.0 (0.4 to 9.7)	2.6 (0.4 to 8.3)	2.7 (0.4 to 8.6)	-11.5 (-26.9 to 6.1)	2.4 (-10.8 to 22.7)
Occupational exposure to sulphuric acid	0.6 (0.1 to 1.9)	0.6 (0.1 to 1.7)	0.6 (0.1 to 1.8)	-6.2 (-16.5 to 6.4)	1.0 (-7.4 to 11.7)	0.5 (0.1 to 1.6)	0.4 (0.1 to 1.4)	0.5 (0.1 to 1.4)	-8.6 (-24.2 to 8.2)	4.9 (-8.5 to 22.1)
Occupational exposure to trichloroethylene	0.1 (0.1 to 0.1)	0.1 (0.1 to 0.1)	0.1 (0.1 to 0.1)	-3.5 (-10.6 to 5.1)	2.1 (-3.9 to 9.0)	0.1 (0.1 to 0.1)	0.1 (0.1 to 0.1)	0.1 (0.1 to 0.1)	2.4 (-10.3 to 16.8)	9.2 (-0.2 to 20.3)
Occupational asthmagens	17.3 (13.8 to 21.8)	17.1 (13.7 to 21.6)	17.2 (13.8 to 21.9)	-0.8 (-18.9 to 22.7)	0.4 (-14.7 to 17.4)	7.9 (6.4 to 9.9)	8.0 (6.4 to 9.9)	8.5 (6.8 to 10.7)	1.2 (-14.7 to 22.0)	5.6 (-7.9 to 21.8)
Occupational particulate matter, gases, and fumes	6.4 (4.5 to 9.2)	6.1 (4.3 to 8.6)	5.9 (4.2 to 8.4)	-4.4 (-9.5 to 0.7)	-3.8 (-7.2 to -0.4)	3.5 (2.4 to 5.3)	3.4 (2.3 to 5.2)	3.2 (2.2 to 4.9)	-5.2 (-14.2 to 4.5)	-4.2 (-9.7 to 1.9)
Occupational noise	5.9 (5.5 to 6.4)	5.7 (5.3 to 6.2)	5.6 (5.2 to 6.0)	-3.3 (-6.5 to 0.1)	-2.5 (-4.6 to -0.3)	3.4 (2.9 to 3.9)	3.4 (2.9 to 3.8)	3.4 (3.0 to 3.9)	0.3 (-6.3 to 8.1)	1.9 (-2.7 to 6.8)
Occupational ergonomic factors	11.4 (9.4 to 13.7)	10.0 (8.3 to 12.0)	9.9 (8.0 to 12.0)	-11.5 (-24.8 to 3.0)	-1.1 (-12.5 to 12.6)	6.1 (4.7 to 7.9)	6.1 (4.7 to 7.7)	6.9 (5.2 to 8.8)	0.9 (-21.7 to 29.3)	11.6 (-8.2 to 36.6)
Non-exclusive breastfeeding	0.4 (0.3 to 0.6)	0.4 (0.3 to 0.6)	0.4 (0.3 to 0.6)	-1.1 (-14.5 to 13.7)	-1.5 (-11.0 to 10.1)	0.4 (0.3 to 0.6)	0.4 (0.3 to 0.6)	0.4 (0.3 to 0.6)	-2.0 (-15.3 to 12.7)	-1.0 (-10.6 to 10.7)
Discontinued breastfeeding	1.7 (1.5 to 1.8)	1.7 (1.5 to 1.8)	1.7 (1.5 to 1.8)	0.7 (-8.8 to 11.2)	-0.8 (-8.3 to 8.0)	1.7 (1.5 to 1.8)	1.7 (1.5 to 1.8)	1.7 (1.5 to 1.8)	0.4 (-9.0 to 11.0)	-0.7 (-8.2 to 8.0)
Child underweight	0.3 (0.2 to 0.4)	0.2 (0.1 to 0.3)	0.2 (0.1 to 0.3)	-15.6 (-36.0 to 8.6)	-5.8 (-21.0 to 11.5)	0.3 (0.2 to 0.4)	0.2 (0.1 to 0.4)	0.2 (0.1 to 0.3)	-18.0 (-39.0 to 7.6)	-6.8 (-22.6 to 11.5)
Child wasting	0.2 (0.1 to 0.3)	0.2 (0.1 to 0.3)	0.2 (0.1 to 0.3)	-7.2 (-33.5 to 30.9)	-2.3 (-22.0 to 22.3)	0.2 (0.1 to 0.3)	0.2 (0.1 to 0.3)	0.2 (0.1 to 0.3)	-8.0 (-35.8 to 31.6)	-4.1 (-24.1 to 22.1)
Child stunting	0.7 (0.3 to 1.1)	0.6 (0.3 to 1.0)	0.6 (0.3 to 1.0)	-10.5 (-45.4 to 49.1)	-3.4 (-33.1 to 34.8)	0.7 (0.3 to 1.2)	0.6 (0.3 to 1.1)	0.6 (0.3 to 1.1)	-11.7 (-47.0 to 47.8)	-4.9 (-33.1 to 37.2)
Short gestation for birth weight	0.0 (0.0 to 0.0)	0.0 (0.0 to 0.0)	0.0 (0.0 to 0.0)	7.8 (-15.5 to 39.4)	2.2 (-17.8 to 25.0)	0.0 (0.0 to 0.0)	0.0 (0.0 to 0.0)	0.0 (0.0 to 0.0)	14.1 (-24.8 to 84.8)	2.3 (-15.2 to 33.1)
Low birth weight for gestation	0.0 (0.0 to 0.0)	0.0 (0.0 to 0.0)	0.0 (0.0 to 0.0)	3.5 (-17.2 to 28.6)	1.7 (-14.3 to 23.4)	0.0 (0.0 to 0.0)	0.0 (0.0 to 0.0)	0.0 (0.0 to 0.0)	-6.8 (-60.6 to 36.8)	1.8 (-18.6 to 19.7)
Iron deficiency	-	-	-	-	-	8.4 (5.8 to 11.7)	8.4 (5.9 to 11.7)	7.7 (5.2 to 10.6)	-0.3 (-21.4 to 25.7)	-8.7 (-24.6 to 8.7)
Vitamin A deficiency	1.0 (0.6 to 1.4)	0.8 (0.6 to 1.0)	0.7 (0.5 to 0.9)	-24.6 (-39.2 to -2.9)	-10.5 (-27.7 to 9.8)	0.8 (0.5 to 1.2)	0.8 (0.5 to 1.1)	0.7 (0.5 to 1.0)	-2.6 (-22.0 to 19.0)	-12.6 (-32.1 to 9.5)
Zinc deficiency	0.5 (0.1 to 1.1)	0.4 (0.1 to 1.1)	0.4 (0.1 to 0.9)	-18.5 (-63.7 to 71.9)	-8.6 (-64.6 to 173.3)	0.5 (0.2 to 1.1)	0.4 (0.1 to 0.8)	0.4 (0.1 to 0.8)	-30.3 (-65.1 to 11.4)	9.1 (-58.3 to 182.8)
Smoking	18.2 (14.8 to 21.5)	12.8 (10.3 to 15.4)	10.9 (8.7 to 13.1)	-29.6 (-38.6 to -19.4)	-15.3 (-23.5 to -5.9)	4.2 (3.0 to 5.6)	3.5 (2.4 to 4.7)	3.4 (2.4 to 4.6)	-17.0 (-37.1 to 8.1)	-2.0 (-19.1 to 18.0)
Chewing tobacco	1.4 (0.9 to 2.1)	0.9 (0.6 to 1.4)	0.8 (0.5 to 1.1)	-33.7 (-57.3 to 5.4)	-18.6 (-41.2 to 15.0)	0.1 (0.1 to 0.1)	0.1 (0.1 to 0.1)	0.1 (0.1 to 0.1)	-3.7 (-40.8 to 57.5)	-2.8 (-33.6 to 41.7)
Second-hand smoke	38.0 (33.6 to 42.2)	35.3 (30.9 to 39.6)	35.7 (31.1 to 40.3)	-7.1 (-18.1 to 4.9)	1.1 (-8.2 to 11.1)	57.7 (54.1 to 61.5)	47.5 (43.9 to 51.1)	47.8 (43.6 to 51.7)	-17.6 (-23.5 to -11.2)	0.7 (-5.8 to 7.1)
Alcohol use	14.6 (7.6 to 25.4)	14.8 (7.8 to 25.3)	17.1 (8.4 to 29.6)	1.0 (-31.3 to 46.3)	15.7 (-19.3 to 63.4)	11.8 (6.0 to 20.9)	11.5 (6.1 to 20.7)	12.6 (6.2 to 21.9)	-2.0 (-34.6 to 42.0)	9.6 (-25.4 to 55.7)
Drug use	0.5 (0.4 to 0.7)	0.5 (0.4 to 0.7)	0.6 (0.4 to 0.8)	0.3 (-5.2 to 6.6)	11.5 (4.8 to 19.9)	0.3 (0.2 to 0.4)	0.3 (0.2 to 0.4)	0.3 (0.3 to 0.4)	2.0 (-4.2 to 8.1)	9.1 (2.5 to 16.2)
Diet low in fruits	41.2 (35.4 to 45.0)	39.5 (33.6 to 43.3)	40.6 (35.1 to 44.4)	-4.3 (-10.3 to 1.6)	2.8 (-2.0 to 8.5)	38.5 (32.6 to 42.8)	35.6 (29.6 to 40.4)	37.6 (32.1 to 42.1)	-7.4 (-15.3 to 0.3)	5.5 (-1.0 to 13.5)
Diet low in vegetables	20.5 (11.6 to 28.0)	14.3 (6.3 to 21.6)	16.1 (7.5 to 23.5)	-30.5 (-58.7 to -7.1)	13.2 (-15.6 to 65.0)	22.8 (14.4 to 30.3)	16.3 (7.9 to 24.2)	18.0 (9.5 to 25.7)	-28.3 (-55.7 to -5.5)	10.4 (-14.4 to 58.8)
Diet low in legumes	4.4 (0.7 to 10.0)	5.6 (1.0 to 11.4)	7.5 (1.9 to 14.5)	27.5 (-49.9 to 275.1)	34.2 (-22.0 to 210.6)	8.3 (2.6 to 14.9)	7.8 (2.4 to 14.7)	10.1 (3.3 to 17.4)	-5.5 (-53.2 to 73.6)	29.2 (-16.3 to 129.8)
Diet low in whole grains	30.9 (25.8 to 34.9)	35.6 (31.5 to 39.1)	36.9 (33.0 to 40.1)	15.1 (6.5 to 26.3)	3.8 (-1.3 to 9.5)	32.4 (27.8 to 36.3)	36.9 (32.9 to 40.1)	38.1 (34.4 to 41.2)	13.8 (6.0 to 24.7)	3.3 (-0.9 to 8.2)
Diet low in nuts and seeds	51.0 (49.7 to 52.0)	49.7 (48.0 to 51.0)	49.4 (47.5 to 51.0)	-2.6 (-4.8 to -0.8)	-0.5 (-2.5 to 1.2)	51.2 (50.1 to 52.1)	49.9 (48.2 to 51.2)	49.7 (47.9 to 51.1)	-2.6 (-4.9 to -0.8)	-0.4 (-2.2 to 1.2)
Diet low in milk	46.4 (44.4 to 48.2)	46.6 (44.7 to 48.3)	47.0 (45.2 to 48.6)	0.5 (-1.5 to 2.5)	0.9 (-0.5 to 2.4)	46.4 (44.3 to 48.1)	46.2 (44.0 to 48.0)	46.8 (44.8 to 48.5)	-0.5 (-2.5 to 1.5)	1.3 (-0.3 to 3.1)
Diet high in red meat	3.5 (0.4 to 8.4)	7.1 (1.9 to 13.2)	8.2 (2.4 to 15.5)	101.0 (-25.3 to 1131.8)	15.7 (-36.9 to 132.4)	0.5 (0.0 to 2.2)	1.4 (0.0 to 4.1)	1.8 (0.0 to 5.2)	178.9 (-86.0 to 2047205.6)	32.3 (-87.0 to 2387.7)
Diet high in processed meat	10.8 (6.5 to 14.7)	18.2 (13.1 to 23.0)	21.2 (15.5 to 26.6)	68.3 (31.1 to 134.6)	16.2 (-2.4 to 40.1)	8.9 (5.2 to 12.4)	13.2 (9.2 to 17.0)	14.6 (10.3 to 18.5)	47.4 (14.2 to 104.2)	10.5 (-5.7 to 34.7)
Diet high in sugar-sweetened beverages	6.7 (2.6 to 13.3)	7.8 (3.5 to 14.1)	8.2 (3.5 to 15.4)	15.4 (-44.9 to 156.6)	6.3 (-38.4 to 82.2)	4.8 (1.8 to 10.7)	5.5 (2.3 to 10.3)	5.9 (2.3 to 11.4)	14.3 (-48.1 to 160.0)	7.4 (-40.0 to 85.4)
Diet low in fibre	23.6 (16.0 to 30.4)	24.5 (17.4 to 30.6)	24.6 (17.1 to 30.7)	3.9 (-13.0 to 25.7)	0.3 (-11.5 to 14.2)	24.6 (17.0 to 31.3)	25.3 (18.1 to 31.5)	25.8 (18.5 to 32.0)	2.8 (-12.6 to 24.9)	2.1 (-8.5 to 15.3)
Diet low in calcium	35.5 (31.4 to 39.0)	35.2 (30.9 to 38.7)	35.7 (31.5 to 39.3)	-1.0 (-4.8 to 2.8)	1.6 (-1.4 to 4.7)	37.6 (33.8 to 40.8)	36.4 (32.5 to 39.8)	37.5 (33.6 to 40.6)	-3.2 (-6.7 to 0.0)	2.9 (0.2 to 6.1)
Diet low in seafood omega-3 fatty acids	3.4 (1.7 to 4.7)	0.4 (0.0 to 1.3)	0.4 (0.0 to 1.4)	-88.8 (-99.9 to -61.8)	5.9 (-81.1 to 570.2)	3.9 (2.3 to 5.3)	0.6 (0.0 to 1.7)	0.6 (0.0 to 1.6)	-85.8 (-99.7 to -57.2)	4.8 (-74.6 to 507.3)
Diet low in polyunsaturated fatty acids	30.4 (25.0 to 35.2)	26.9 (21.0 to 32.1)	27.9 (22.1 to 33.1)	-11.7 (-24.4 to -1.0)	4.0 (-4.4 to 13.8)	30.7 (25.3 to 35.4)	26.7 (20.8 to 31.8)	28.0 (22.2 to 33.2)	-13.1 (-26.0 to -3.7)	4.7 (-3.0 to 15.6)
Diet high in trans fatty acids	1.1 (0.0 to 3.3)	0.9 (0.0 to 3.0)	0.9 (0.0 to 2.8)	-12.5 (-38.0 to -6.9)	-8.2 (-33.7 to -2.8)	1.5 (0.1 to 4.0)	1.3 (0.1 to 3.7)	1.2 (0.0 to 3.5)	-12.5 (-36.8 to -6.2)	-8.5 (-28.7 to -3.6)
Diet high in sodium	28.4 (18.6 to 36.3)	19.4 (10.4 to 26.5)	16.9 (7.9 to 24.4)	-31.7 (-54.1 to -14.1)	-12.6 (-34.8 to 6.6)	33.2 (25.6 to 40.1)	21.3 (13.3 to 28.1)	18.0 (9.4 to 25.3)	-35.8 (-53.1 to -22.4)	-15.5 (-34.4 to -2.8)
Intimate partner violence	-	-	-	-	-	2.3 (1.9 to 2.6)	2.1 (1.7 to 2.4)	2.0 (1.7 to 2.4)	-8.4 (-12.5 to -4.5)	-2.4 (-6.9 to 2.6)
Childhood sexual abuse	5.3 (4.3 to 6.5)	5.3 (4.3 to 6.5)	5.3 (4.3 to 6.5)	0.0 (0.0 to 0.0)	0.0 (0.0 to 0.0)	7.3 (5.9 to 8.9)	7.3 (5.9 to 8.9)	7.5 (6.0 to 9.2)	0.0 (0.0 to 0.0)	2.8 (-4.0 to 10.6)

Supplementary Results Table 1. Yamagata age-standardised Summary Exposure Values (SEVs) for Level 4 risk factors and percentage change, by sex, 1990, 2007, and 2017.Data in parentheses are 95% uncertainty intervals.

	Male					Female				
	**1990**	**2007**	**2017**	**Percentage change 1990-2007**	**Percentage change 2007-2017**	**1990**	**2007**	**2017**	**Percentage change 1990-2007**	**Percentage change 2007-2017**
Bullying victimization	3.3 (1.3 to 7.0)	3.4 (1.4 to 7.3)	3.5 (1.4 to 7.6)	3.7 (-4.4 to 11.9)	3.4 (-1.9 to 8.2)	1.9 (0.8 to 4.1)	2.0 (0.8 to 4.2)	2.0 (0.8 to 4.3)	2.0 (-5.7 to 10.2)	2.0 (-3.4 to 7.0)
Low physical activity	0.5 (0.2 to 0.9)	0.5 (0.2 to 0.9)	0.4 (0.2 to 0.8)	-1.8 (-3.7 to 0.1)	-8.8 (-12.2 to -4.5)	0.5 (0.2 to 1.0)	0.5 (0.2 to 0.9)	0.5 (0.2 to 0.9)	-3.9 (-6.2 to -1.4)	0.8 (-0.9 to 2.4)
High fasting plasma glucose	1.2 (0.8 to 1.8)	1.2 (0.8 to 1.9)	1.4 (0.8 to 2.3)	3.3 (-29.6 to 45.7)	12.5 (-19.9 to 77.1)	1.2 (0.7 to 1.9)	1.3 (0.8 to 2.1)	1.4 (0.8 to 2.3)	10.9 (-31.8 to 93.9)	8.0 (-30.5 to 72.2)
High LDL cholesterol	11.3 (9.7 to 13.1)	11.8 (10.3 to 13.6)	11.9 (10.3 to 13.5)	4.3 (-1.5 to 10.1)	0.5 (-4.1 to 5.4)	12.7 (11.1 to 14.4)	12.8 (11.3 to 14.5)	12.8 (11.3 to 14.6)	0.9 (-4.1 to 6.3)	0.3 (-4.1 to 4.9)
High systolic blood pressure	10.9 (9.9 to 11.9)	8.1 (7.2 to 9.0)	8.1 (7.1 to 9.0)	-25.6 (-31.6 to -19.3)	-0.4 (-8.6 to 7.1)	9.3 (8.3 to 10.3)	5.8 (5.1 to 6.5)	5.4 (4.7 to 6.3)	-37.6 (-44.0 to -30.8)	-5.8 (-16.1 to 4.6)
High body-mass index	4.7 (2.8 to 7.6)	6.6 (4.2 to 11.3)	6.8 (4.4 to 11.4)	41.3 (12.4 to 79.7)	3.1 (-13.3 to 21.7)	5.1 (3.0 to 8.0)	6.5 (3.9 to 10.8)	6.5 (3.9 to 10.5)	28.1 (0.6 to 70.6)	0.1 (-17.5 to 22.3)
Low bone mineral density	5.3 (4.4 to 6.3)	5.2 (4.4 to 6.0)	5.2 (4.2 to 6.2)	-2.3 (-14.0 to 13.1)	0.2 (-13.3 to 15.3)	7.3 (6.0 to 8.5)	7.0 (6.0 to 8.1)	7.1 (6.0 to 8.4)	-3.8 (-15.1 to 10.4)	1.7 (-10.6 to 14.8)
Impaired kidney function	2.1 (0.9 to 5.5)	2.0 (0.9 to 5.4)	2.0 (0.9 to 5.4)	-0.6 (-4.7 to 5.3)	-1.2 (-7.8 to 4.3)	2.4 (1.1 to 6.1)	2.5 (1.1 to 6.3)	2.4 (1.1 to 6.1)	3.0 (-1.3 to 7.4)	-3.4 (-8.4 to 0.5)

Supplementary Results Table 1. Yamaguchi age-standardised Summary Exposure Values (SEVs) for Level 4 risk factors and percentage change, by sex, 1990, 2007, and 2017.Data in parentheses are 95% uncertainty intervals.										
	Male					Female				
	1990	2007	2017	Percentage change 1990-2007	Percentage change 2007-2017	1990	2007	2017	Percentage change 1990-2007	Percentage change 2007-2017
Unsafe water source	2.2 (1.2 to 4.0)	1.8 (1.0 to 3.2)	2.0 (1.1 to 3.4)	-16.6 (-57.8 to 67.2)	7.1 (-39.1 to 87.4)	2.2 (1.2 to 4.0)	1.8 (1.0 to 3.2)	2.0 (1.1 to 3.4)	-16.6 (-57.8 to 67.2)	7.1 (-39.1 to 87.4)
Unsafe sanitation	3.7 (1.8 to 6.7)	1.9 (1.0 to 3.6)	1.7 (0.7 to 3.3)	-47.6 (-77.1 to 23.1)	-13.6 (-58.0 to 70.1)	3.7 (1.8 to 6.7)	1.9 (1.0 to 3.6)	1.7 (0.7 to 3.3)	-47.6 (-77.1 to 23.1)	-13.6 (-58.0 to 70.1)
No access to handwashing facility	6.7 (4.8 to 9.1)	6.4 (4.6 to 8.9)	5.9 (4.2 to 8.0)	-5.5 (-32.5 to 35.3)	-7.4 (-28.4 to 17.6)	6.7 (4.8 to 9.1)	6.4 (4.6 to 8.9)	5.9 (4.2 to 8.0)	-5.5 (-32.5 to 35.3)	-7.4 (-28.4 to 17.6)
Ambient particulate matter pollution	30.3 (19.9 to 46.2)	30.7 (20.7 to 45.7)	26.4 (16.6 to 40.3)	1.2 (-22.2 to 36.9)	-13.9 (-36.5 to 11.7)	30.0 (19.6 to 45.9)	30.6 (20.6 to 45.6)	26.3 (16.6 to 40.3)	1.9 (-21.8 to 38.4)	-13.9 (-36.6 to 11.7)
Household air pollution from solid fuels	0.7 (0.2 to 2.0)	0.2 (0.0 to 0.5)	0.2 (0.0 to 0.4)	-74.5 (-92.0 to -45.7)	-14.4 (-65.2 to 87.4)	1.0 (0.3 to 6.6)	0.3 (0.1 to 1.4)	0.3 (0.0 to 1.3)	-71.1 (-91.7 to -40.0)	-11.4 (-64.7 to 88.5)
Ambient ozone pollution	50.7 (22.8 to 54.8)	50.7 (22.8 to 54.8)	51.3 (24.1 to 54.8)	-0.0 (-0.6 to 0.3)	1.2 (0.0 to 6.5)	50.7 (22.8 to 54.8)	50.7 (22.8 to 54.8)	51.3 (24.1 to 54.8)	-0.0 (-0.6 to 0.3)	1.2 (0.0 to 6.5)
Residential radon	13.9 (0.9 to 47.3)	13.9 (0.9 to 47.3)	13.9 (0.9 to 47.3)	0.0 (0.0 to 0.0)	0.0 (0.0 to 0.0)	13.9 (0.9 to 47.3)	13.9 (0.9 to 47.3)	13.9 (0.9 to 47.3)	0.0 (0.0 to 0.0)	0.0 (0.0 to 0.0)
Lead exposure	2.6 (0.3 to 6.1)	1.8 (0.2 to 4.7)	1.3 (0.1 to 3.8)	-31.2 (-44.4 to -22.3)	-26.1 (-41.9 to -18.3)	1.4 (0.1 to 4.2)	0.9 (0.0 to 3.1)	0.7 (0.0 to 2.5)	-35.5 (-53.1 to -26.5)	-27.9 (-50.8 to -19.0)
Occupational exposure to asbestos	3.1 (2.7 to 3.4)	4.3 (4.0 to 4.7)	4.1 (3.4 to 5.1)	40.1 (22.4 to 63.2)	-4.5 (-22.7 to 17.5)	0.6 (0.5 to 0.6)	0.6 (0.5 to 0.6)	0.6 (0.4 to 0.7)	5.6 (-10.8 to 31.7)	-6.0 (-26.4 to 21.3)
Occupational exposure to arsenic	0.4 (0.0 to 1.1)	0.4 (0.0 to 1.0)	0.4 (0.0 to 1.0)	-	-	0.4 (0.0 to 1.0)	0.4 (0.0 to 0.9)	0.4 (0.0 to 0.9)	-	-
Occupational exposure to benzene	0.6 (0.0 to 1.8)	0.6 (0.0 to 1.9)	0.7 (0.0 to 1.9)	2.8 (-4.1 to 12.4)	3.5 (-2.6 to 11.8)	0.6 (0.0 to 1.7)	0.7 (0.0 to 2.0)	0.8 (0.0 to 2.3)	21.2 (7.4 to 45.6)	14.6 (4.3 to 28.4)
Occupational exposure to beryllium	0.0 (0.0 to 0.0)	0.0 (0.0 to 0.0)	0.0 (0.0 to 0.0)	-4.9 (-10.0 to 0.4)	1.9 (-2.1 to 6.0)	0.0 (0.0 to 0.0)	0.0 (0.0 to 0.0)	0.0 (0.0 to 0.0)	1.9 (-7.1 to 12.3)	9.3 (2.6 to 16.7)
Occupational exposure to cadmium	0.1 (0.0 to 0.1)	0.0 (0.0 to 0.1)	0.0 (0.0 to 0.1)	-6.5 (-17.1 to 5.6)	0.8 (-8.5 to 10.9)	0.0 (0.0 to 0.1)	0.0 (0.0 to 0.0)	0.0 (0.0 to 0.1)	-8.7 (-22.7 to 9.9)	5.3 (-7.5 to 19.9)
Occupational exposure to chromium	0.1 (0.1 to 0.1)	0.1 (0.1 to 0.1)	0.1 (0.1 to 0.1)	-4.7 (-14.5 to 6.4)	1.4 (-7.0 to 10.2)	0.1 (0.1 to 0.1)	0.1 (0.1 to 0.1)	0.1 (0.1 to 0.1)	-4.0 (-17.1 to 13.7)	7.0 (-3.9 to 19.1)
Occupational exposure to diesel engine exhaust	0.6 (0.5 to 0.6)	0.6 (0.5 to 0.6)	0.6 (0.6 to 0.7)	-1.2 (-8.7 to 6.5)	3.1 (-3.2 to 9.9)	0.4 (0.4 to 0.5)	0.5 (0.4 to 0.6)	0.6 (0.5 to 0.6)	12.4 (-1.3 to 29.0)	11.3 (0.3 to 22.9)
Occupational exposure to formaldehyde	0.2 (0.2 to 0.3)	0.2 (0.2 to 0.3)	0.2 (0.2 to 0.3)	-5.8 (-17.2 to 7.6)	0.5 (-9.5 to 11.2)	0.2 (0.2 to 0.2)	0.2 (0.2 to 0.2)	0.2 (0.2 to 0.2)	-6.0 (-20.9 to 13.5)	6.1 (-6.5 to 20.3)
Occupational exposure to nickel	0.4 (0.0 to 1.9)	0.4 (0.0 to 1.7)	0.4 (0.0 to 1.7)	-	-	0.3 (0.0 to 1.5)	0.3 (0.0 to 1.3)	0.3 (0.0 to 1.3)	-	-
Occupational exposure to polycyclic aromatic hydrocarbons	0.2 (0.2 to 0.3)	0.2 (0.2 to 0.2)	0.2 (0.2 to 0.3)	-3.7 (-12.9 to 6.9)	1.8 (-5.8 to 10.0)	0.2 (0.2 to 0.2)	0.2 (0.2 to 0.2)	0.2 (0.2 to 0.3)	-1.9 (-15.0 to 14.8)	7.9 (-2.3 to 18.6)
Occupational exposure to silica	4.5 (0.6 to 14.2)	4.2 (0.6 to 13.8)	4.3 (0.6 to 13.9)	-5.5 (-14.9 to 7.2)	1.4 (-7.6 to 12.2)	3.1 (0.5 to 9.6)	2.7 (0.4 to 8.7)	2.7 (0.4 to 8.7)	-13.2 (-27.5 to 6.9)	2.3 (-11.3 to 20.9)
Occupational exposure to sulphuric acid	0.6 (0.1 to 1.9)	0.6 (0.1 to 1.8)	0.6 (0.1 to 1.8)	-6.5 (-17.1 to 5.6)	0.9 (-8.1 to 11.0)	0.5 (0.1 to 1.6)	0.5 (0.1 to 1.4)	0.5 (0.1 to 1.4)	-10.1 (-24.0 to 8.4)	4.5 (-8.7 to 19.9)
Occupational exposure to trichloroethylene	0.1 (0.1 to 0.1)	0.1 (0.1 to 0.1)	0.1 (0.1 to 0.1)	-3.8 (-11.3 to 4.4)	1.9 (-4.2 to 8.4)	0.1 (0.1 to 0.1)	0.1 (0.1 to 0.1)	0.1 (0.1 to 0.1)	0.9 (-10.2 to 14.5)	8.5 (-0.3 to 18.2)
Occupational asthmagens	18.0 (14.3 to 23.0)	17.6 (14.0 to 22.5)	17.6 (13.8 to 22.6)	-2.3 (-20.8 to 22.4)	0.0 (-15.0 to 17.3)	8.1 (6.5 to 10.2)	8.1 (6.5 to 10.0)	8.6 (6.9 to 10.7)	0.2 (-15.7 to 19.5)	5.5 (-6.8 to 19.5)
Occupational particulate matter, gases, and fumes	6.3 (4.5 to 9.1)	6.1 (4.3 to 8.7)	5.8 (4.1 to 8.5)	-4.4 (-9.3 to 0.7)	-3.7 (-7.0 to -0.4)	3.6 (2.5 to 5.4)	3.4 (2.3 to 5.2)	3.2 (2.2 to 5.0)	-5.7 (-14.0 to 3.6)	-4.5 (-10.0 to 2.0)
Occupational noise	5.9 (5.4 to 6.4)	5.7 (5.3 to 6.2)	5.6 (5.1 to 6.1)	-3.2 (-6.4 to 0.2)	-2.4 (-4.4 to -0.3)	3.4 (3.0 to 4.0)	3.4 (3.0 to 3.9)	3.5 (3.1 to 3.9)	0.1 (-6.0 to 7.4)	1.7 (-2.4 to 6.4)
Occupational ergonomic factors	10.8 (8.9 to 13.0)	9.6 (7.9 to 11.7)	9.5 (7.7 to 11.5)	-11.1 (-22.4 to 3.1)	-1.1 (-12.7 to 11.1)	5.8 (4.4 to 7.4)	5.9 (4.6 to 7.4)	6.6 (5.0 to 8.5)	1.1 (-19.9 to 29.2)	12.7 (-8.6 to 37.1)
Non-exclusive breastfeeding	0.4 (0.3 to 0.6)	0.4 (0.3 to 0.6)	0.4 (0.3 to 0.6)	-0.8 (-12.9 to 13.6)	-2.0 (-13.1 to 10.1)	0.4 (0.3 to 0.6)	0.4 (0.3 to 0.6)	0.4 (0.3 to 0.6)	-0.6 (-12.8 to 13.8)	-1.7 (-12.8 to 10.4)
Discontinued breastfeeding	1.7 (1.5 to 1.8)	1.7 (1.6 to 1.9)	1.7 (1.5 to 1.8)	3.1 (-6.3 to 13.7)	-2.3 (-9.6 to 6.1)	1.7 (1.5 to 1.8)	1.7 (1.6 to 1.9)	1.7 (1.5 to 1.8)	3.2 (-6.1 to 13.8)	-3.2 (-10.4 to 5.1)
Child underweight	0.3 (0.2 to 0.4)	0.2 (0.1 to 0.3)	0.2 (0.1 to 0.3)	-13.0 (-32.8 to 11.9)	-4.8 (-20.0 to 11.7)	0.3 (0.2 to 0.4)	0.2 (0.1 to 0.3)	0.2 (0.1 to 0.3)	-16.3 (-37.2 to 10.6)	-5.7 (-23.0 to 13.8)
Child wasting	0.2 (0.1 to 0.3)	0.2 (0.1 to 0.3)	0.2 (0.1 to 0.3)	-6.2 (-34.1 to 33.2)	-1.3 (-21.7 to 22.8)	0.2 (0.1 to 0.3)	0.2 (0.1 to 0.3)	0.2 (0.1 to 0.3)	-7.7 (-36.1 to 30.1)	-2.5 (-22.4 to 22.3)
Child stunting	0.7 (0.3 to 1.1)	0.6 (0.3 to 1.0)	0.6 (0.3 to 1.0)	-9.5 (-45.5 to 49.0)	-3.3 (-31.9 to 31.9)	0.7 (0.3 to 1.2)	0.6 (0.3 to 1.1)	0.6 (0.3 to 1.0)	-10.5 (-44.8 to 55.3)	-5.7 (-34.6 to 31.7)
Short gestation for birth weight	0.0 (0.0 to 0.0)	0.0 (0.0 to 0.0)	0.0 (0.0 to 0.0)	9.9 (-7.8 to 30.6)	2.8 (-17.6 to 44.9)	0.0 (0.0 to 0.0)	0.0 (0.0 to 0.0)	0.0 (0.0 to 0.0)	12.0 (-18.9 to 71.5)	2.0 (-18.9 to 30.9)
Low birth weight for gestation	0.0 (0.0 to 0.0)	0.0 (0.0 to 0.0)	0.0 (0.0 to 0.0)	5.3 (-6.2 to 25.4)	1.6 (-21.6 to 21.6)	0.0 (0.0 to 0.0)	0.0 (0.0 to 0.0)	0.0 (0.0 to 0.0)	-1.6 (-28.0 to 32.1)	2.9 (-16.5 to 24.9)
Iron deficiency	-	-	-	-	-	8.0 (5.6 to 11.1)	8.1 (5.6 to 11.0)	7.4 (5.1 to 10.4)	0.7 (-21.7 to 27.8)	-7.9 (-25.4 to 10.3)
Vitamin A deficiency	1.0 (0.6 to 1.4)	0.8 (0.5 to 1.0)	0.6 (0.4 to 0.9)	-26.8 (-41.6 to -10.0)	-14.3 (-31.5 to 7.5)	0.7 (0.4 to 0.9)	0.7 (0.5 to 0.9)	0.6 (0.4 to 0.8)	5.2 (-9.8 to 26.5)	-12.3 (-27.6 to 7.3)
Zinc deficiency	0.5 (0.1 to 1.0)	0.5 (0.1 to 1.1)	0.4 (0.1 to 1.1)	5.1 (-63.1 to 74.9)	-16.7 (-67.1 to 127.8)	0.5 (0.2 to 1.0)	0.4 (0.1 to 0.9)	0.3 (0.1 to 0.8)	-28.8 (-64.5 to 68.4)	-2.6 (-64.6 to 179.8)
Smoking	17.7 (14.6 to 21.0)	12.5 (10.1 to 14.8)	10.5 (8.5 to 12.6)	-29.7 (-38.4 to -19.4)	-15.6 (-24.0 to -6.2)	4.0 (2.8 to 5.3)	3.3 (2.3 to 4.5)	3.2 (2.3 to 4.5)	-16.3 (-36.6 to 11.8)	-2.3 (-19.6 to 19.8)
Chewing tobacco	1.4 (0.9 to 2.0)	0.9 (0.6 to 1.4)	0.8 (0.5 to 1.1)	-32.6 (-56.2 to 5.8)	-18.9 (-42.8 to 12.9)	0.1 (0.1 to 0.2)	0.1 (0.1 to 0.1)	0.1 (0.1 to 0.1)	-4.3 (-42.5 to 62.5)	-3.2 (-32.8 to 42.8)
Second-hand smoke	37.0 (33.2 to 40.9)	35.5 (31.3 to 39.7)	35.7 (31.1 to 40.1)	-4.3 (-15.8 to 7.6)	0.6 (-8.5 to 10.2)	55.5 (51.8 to 59.2)	47.1 (43.5 to 50.6)	47.2 (43.0 to 51.0)	-15.1 (-21.3 to -8.1)	0.2 (-6.1 to 6.6)
Alcohol use	14.9 (7.3 to 25.3)	15.0 (7.5 to 26.9)	17.7 (8.5 to 30.6)	0.9 (-31.9 to 47.5)	17.6 (-17.5 to 68.2)	12.2 (6.0 to 21.7)	12.2 (5.9 to 22.3)	13.5 (6.4 to 23.7)	-0.6 (-32.3 to 47.0)	10.7 (-21.4 to 57.3)
Drug use	0.5 (0.4 to 0.7)	0.5 (0.4 to 0.7)	0.6 (0.4 to 0.8)	0.5 (-5.4 to 6.6)	11.4 (3.9 to 19.8)	0.3 (0.2 to 0.4)	0.3 (0.2 to 0.4)	0.3 (0.3 to 0.4)	1.5 (-4.4 to 7.7)	9.2 (2.6 to 17.4)
Diet low in fruits	40.4 (34.6 to 44.2)	38.7 (32.7 to 42.8)	40.1 (34.6 to 44.2)	-4.4 (-10.9 to 2.0)	3.8 (-1.1 to 9.4)	37.4 (31.8 to 41.9)	34.6 (28.6 to 39.7)	37.1 (31.2 to 41.8)	-7.3 (-15.6 to 0.9)	7.2 (0.1 to 16.3)
Diet low in vegetables	18.6 (10.4 to 26.2)	12.6 (4.5 to 20.3)	14.7 (6.3 to 22.6)	-32.3 (-66.4 to -6.5)	16.6 (-16.0 to 85.8)	21.0 (12.0 to 28.4)	14.9 (6.8 to 23.0)	16.8 (8.5 to 24.7)	-28.9 (-56.3 to -4.8)	12.3 (-16.8 to 63.7)
Diet low in legumes	4.2 (0.6 to 9.6)	5.4 (1.0 to 11.1)	7.2 (1.8 to 13.8)	28.0 (-47.2 to 286.7)	32.8 (-25.8 to 204.8)	8.1 (2.5 to 14.9)	7.5 (1.7 to 13.9)	9.7 (3.2 to 17.0)	-6.7 (-57.2 to 72.5)	28.6 (-15.8 to 167.3)
Diet low in whole grains	30.1 (25.1 to 34.3)	35.0 (30.8 to 38.6)	36.3 (32.5 to 39.7)	16.2 (7.2 to 29.2)	3.9 (-1.0 to 9.8)	31.5 (26.6 to 35.8)	36.2 (32.3 to 39.5)	37.5 (33.7 to 40.6)	14.7 (6.4 to 26.2)	3.6 (-1.2 to 8.8)
Diet low in nuts and seeds	50.3 (48.9 to 51.5)	48.8 (46.9 to 50.3)	48.6 (46.5 to 50.3)	-3.0 (-5.7 to -0.6)	-0.5 (-2.8 to 1.7)	50.5 (49.1 to 51.6)	48.9 (46.8 to 50.4)	48.6 (46.4 to 50.4)	-3.2 (-6.1 to -0.7)	-0.6 (-3.2 to 1.7)
Diet low in milk	45.9 (43.8 to 47.8)	46.2 (44.2 to 47.9)	46.6 (44.7 to 48.3)	0.6 (-1.4 to 2.7)	0.9 (-0.6 to 2.5)	46.0 (43.8 to 47.8)	45.8 (43.5 to 47.6)	46.4 (44.2 to 48.2)	-0.5 (-2.6 to 1.5)	1.4 (-0.2 to 3.2)
Diet high in red meat	4.5 (0.7 to 9.9)	8.6 (2.6 to 16.2)	9.7 (3.1 to 17.9)	91.1 (-14.5 to 616.6)	12.9 (-35.2 to 116.5)	0.7 (0.0 to 2.8)	1.8 (0.0 to 5.1)	2.4 (0.1 to 6.4)	175.4 (-65.6 to 297119.2)	31.7 (-79.4 to 1418.5)
Diet high in processed meat	12.4 (8.2 to 16.9)	20.7 (15.5 to 25.5)	24.0 (17.8 to 29.7)	66.8 (30.9 to 124.8)	16.1 (-1.1 to 39.7)	10.4 (6.6 to 14.0)	15.3 (11.0 to 19.5)	17.0 (12.0 to 21.4)	47.4 (15.0 to 96.8)	10.9 (-5.5 to 30.8)
Diet high in sugar-sweetened beverages	7.9 (3.1 to 15.7)	9.0 (4.2 to 16.2)	9.4 (4.0 to 17.0)	12.9 (-48.0 to 144.9)	4.9 (-37.7 to 76.2)	5.6 (2.1 to 12.3)	6.3 (2.7 to 12.2)	6.6 (2.7 to 12.7)	12.5 (-49.8 to 174.9)	5.4 (-37.9 to 88.0)
Diet low in fibre	21.6 (14.0 to 27.9)	22.7 (15.4 to 29.1)	23.6 (16.6 to 30.3)	5.2 (-13.6 to 30.2)	4.1 (-8.6 to 18.6)	22.8 (15.4 to 29.5)	23.7 (16.4 to 29.7)	25.1 (18.0 to 31.0)	4.0 (-13.6 to 27.7)	6.0 (-5.6 to 20.7)
Diet low in calcium	34.0 (29.5 to 37.7)	33.7 (29.1 to 37.4)	34.4 (29.8 to 38.0)	-1.0 (-5.5 to 3.2)	2.2 (-1.1 to 5.7)	36.4 (32.4 to 39.6)	35.2 (31.0 to 38.7)	36.5 (32.4 to 39.9)	-3.1 (-7.1 to 0.4)	3.5 (0.6 to 7.0)
Diet low in seafood omega-3 fatty acids	2.9 (1.1 to 4.4)	0.2 (0.0 to 0.9)	0.2 (0.0 to 1.0)	-93.7 (-100.0 to -72.3)	9.2 (-95.7 to 3449.9)	3.4 (1.5 to 5.1)	0.2 (0.0 to 1.1)	0.3 (0.0 to 1.2)	-92.7 (-100.0 to -73.2)	13.5 (-94.2 to 5674.0)
Diet low in polyunsaturated fatty acids	29.8 (24.5 to 34.7)	27.0 (21.1 to 32.0)	27.7 (21.7 to 32.6)	-9.4 (-21.1 to 1.7)	2.3 (-7.2 to 12.3)	30.5 (25.1 to 35.2)	27.8 (21.9 to 32.8)	28.5 (22.6 to 33.8)	-9.0 (-21.0 to 0.7)	2.8 (-5.7 to 15.3)
Diet high in trans fatty acids	1.1 (0.0 to 3.3)	1.0 (0.0 to 3.0)	0.9 (0.0 to 2.9)	-12.4 (-37.5 to -6.9)	-8.4 (-33.8 to -3.7)	1.5 (0.1 to 3.9)	1.3 (0.1 to 3.7)	1.2 (0.0 to 3.5)	-12.6 (-36.0 to -6.0)	-8.5 (-28.5 to -3.2)
Diet high in sodium	28.5 (19.5 to 36.9)	18.6 (9.8 to 25.8)	16.0 (7.0 to 23.4)	-34.7 (-57.7 to -17.9)	-13.8 (-36.2 to 3.3)	31.8 (22.9 to 39.1)	19.1 (10.7 to 26.0)	15.8 (7.1 to 23.3)	-40.0 (-60.4 to -23.9)	-17.4 (-39.8 to -4.1)
Intimate partner violence	-	-	-	-	-	2.4 (2.0 to 2.7)	2.2 (1.8 to 2.5)	2.1 (1.8 to 2.4)	-8.2 (-12.3 to -3.9)	-4.1 (-8.1 to 0.2)
Childhood sexual abuse	5.3 (4.3 to 6.5)	5.3 (4.3 to 6.5)	5.3 (4.3 to 6.5)	0.0 (0.0 to 0.0)	0.0 (0.0 to 0.0)	7.3 (5.9 to 8.9)	7.3 (5.9 to 8.9)	7.5 (6.0 to 9.2)	0.0 (0.0 to 0.0)	2.8 (-4.0 to 10.6)

Supplementary Results Table 1. Yamaguchi age-standardised Summary Exposure Values (SEVs) for Level 4 risk factors and percentage change, by sex, 1990, 2007, and 2017.Data in parentheses are 95% uncertainty intervals.										
	**Male**					**Female**				
	**1990**	**2007**	**2017**	**Percentage change 1990-2007**	**Percentage change 2007-2017**	**1990**	**2007**	**2017**	**Percentage change 1990-2007**	**Percentage change 2007-2017**
Bullying victimization	3.3 (1.4 to 6.8)	3.4 (1.4 to 6.9)	3.5 (1.5 to 7.2)	0.8 (-7.4 to 8.6)	3.5 (-1.7 to 8.5)	1.9 (0.8 to 3.9)	1.9 (0.8 to 3.9)	2.0 (0.8 to 4.0)	-0.4 (-7.8 to 7.9)	3.0 (-2.8 to 7.9)
Low physical activity	0.5 (0.2 to 0.9)	0.5 (0.2 to 0.9)	0.4 (0.2 to 0.8)	-1.8 (-3.6 to 0.2)	-8.8 (-12.3 to -4.5)	0.5 (0.2 to 1.0)	0.5 (0.2 to 0.9)	0.5 (0.2 to 0.9)	-3.8 (-6.1 to -1.2)	0.9 (-0.6 to 2.5)
High fasting plasma glucose	1.4 (0.9 to 2.2)	1.4 (0.9 to 2.2)	1.6 (0.9 to 2.6)	-1.8 (-36.0 to 56.5)	12.8 (-22.6 to 72.6)	1.2 (0.7 to 2.0)	1.3 (0.7 to 2.1)	1.4 (0.8 to 2.6)	8.0 (-34.1 to 83.0)	10.5 (-30.3 to 78.7)
High LDL cholesterol	12.1 (10.5 to 13.9)	12.6 (11.0 to 14.4)	12.6 (10.9 to 14.4)	4.3 (-1.4 to 11.0)	-0.1 (-4.7 to 4.7)	12.9 (11.3 to 14.7)	13.0 (11.4 to 14.7)	13.0 (11.4 to 14.8)	0.8 (-4.6 to 6.6)	0.1 (-4.1 to 4.8)
High systolic blood pressure	11.3 (10.3 to 12.4)	8.4 (7.5 to 9.4)	8.3 (7.2 to 9.2)	-25.8 (-31.6 to -19.5)	-1.8 (-9.2 to 6.2)	9.6 (8.6 to 10.6)	6.0 (5.3 to 6.8)	5.6 (4.8 to 6.5)	-37.4 (-43.8 to -30.5)	-7.3 (-17.0 to 3.5)
High body-mass index	5.3 (3.3 to 8.5)	7.3 (4.8 to 11.3)	7.5 (4.8 to 12.2)	36.5 (9.8 to 72.5)	2.9 (-13.5 to 20.1)	5.5 (3.3 to 8.8)	6.4 (4.0 to 9.8)	6.4 (3.9 to 10.2)	16.4 (-8.4 to 52.2)	-0.7 (-17.5 to 21.1)
Low bone mineral density	5.2 (4.3 to 6.2)	5.1 (4.3 to 6.1)	5.1 (4.2 to 6.1)	-2.2 (-14.7 to 12.9)	0.3 (-13.2 to 15.3)	7.1 (6.0 to 8.4)	6.9 (5.9 to 8.0)	7.0 (5.9 to 8.2)	-3.6 (-14.2 to 9.0)	1.6 (-11.5 to 13.4)
Impaired kidney function	2.6 (1.2 to 6.5)	2.5 (1.2 to 6.3)	2.4 (1.1 to 6.1)	-2.9 (-7.2 to 1.5)	-6.8 (-11.8 to -2.0)	3.2 (1.5 to 7.4)	3.3 (1.5 to 7.6)	3.1 (1.4 to 7.3)	3.2 (-0.7 to 8.2)	-7.2 (-12.4 to -2.2)

Supplementary Results Table 1. Yamanashi age-standardised Summary Exposure Values (SEVs) for Level 4 risk factors and percentage change, by sex, 1990, 2007, and 2017.Data in parentheses are 95% uncertainty intervals.

	Male					Female				
	**1990**	**2007**	**2017**	**Percentage change 1990-2007**	**Percentage change 2007-2017**	**1990**	**2007**	**2017**	**Percentage change 1990-2007**	**Percentage change 2007-2017**
Unsafe water source	2.2 (1.2 to 3.6)	1.8 (1.0 to 3.1)	1.9 (1.0 to 3.2)	-17.2 (-57.2 to 65.4)	7.5 (-37.1 to 87.5)	2.2 (1.2 to 3.6)	1.8 (1.0 to 3.1)	1.9 (1.0 to 3.2)	-17.2 (-57.2 to 65.4)	7.5 (-37.1 to 87.5)
Unsafe sanitation	3.7 (1.8 to 6.9)	1.8 (0.9 to 3.5)	1.6 (0.8 to 3.0)	-50.9 (-78.7 to 7.7)	-13.1 (-54.6 to 74.2)	3.7 (1.8 to 6.9)	1.8 (0.9 to 3.5)	1.6 (0.8 to 3.0)	-50.9 (-78.7 to 7.7)	-13.1 (-54.6 to 74.2)
No access to handwashing facility	6.7 (4.9 to 9.1)	6.3 (4.6 to 8.4)	5.9 (4.2 to 8.0)	-5.6 (-33.1 to 29.8)	-7.0 (-28.4 to 19.6)	6.7 (4.9 to 9.1)	6.3 (4.6 to 8.4)	5.9 (4.2 to 8.0)	-5.6 (-33.1 to 29.8)	-7.0 (-28.4 to 19.6)
Ambient particulate matter pollution	31.4 (21.2 to 46.5)	31.5 (22.3 to 46.2)	26.6 (17.1 to 40.4)	0.2 (-21.1 to 29.5)	-15.5 (-36.3 to 8.3)	31.2 (21.0 to 46.1)	31.4 (22.3 to 46.1)	26.6 (17.1 to 40.3)	0.8 (-20.8 to 30.3)	-15.4 (-36.2 to 8.4)
Household air pollution from solid fuels	0.7 (0.2 to 1.9)	0.2 (0.0 to 0.4)	0.1 (0.0 to 0.3)	-75.4 (-93.4 to -46.9)	-17.0 (-66.7 to 80.1)	1.0 (0.3 to 6.6)	0.3 (0.0 to 1.5)	0.2 (0.0 to 1.0)	-72.7 (-93.3 to -44.8)	-12.5 (-66.5 to 82.1)
Ambient ozone pollution	52.6 (28.3 to 54.8)	52.5 (28.2 to 54.8)	52.7 (29.0 to 54.8)	-0.1 (-1.3 to 0.0)	0.4 (0.0 to 3.3)	52.6 (28.3 to 54.8)	52.5 (28.2 to 54.8)	52.7 (29.0 to 54.8)	-0.1 (-1.3 to 0.0)	0.4 (0.0 to 3.3)
Residential radon	17.8 (1.7 to 60.2)	17.8 (1.7 to 60.2)	17.8 (1.7 to 60.2)	0.0 (0.0 to 0.0)	0.0 (0.0 to 0.0)	17.8 (1.7 to 60.2)	17.8 (1.7 to 60.2)	17.8 (1.7 to 60.2)	0.0 (0.0 to 0.0)	0.0 (0.0 to 0.0)
Lead exposure	2.5 (0.3 to 5.9)	1.7 (0.2 to 4.5)	1.2 (0.1 to 3.7)	-31.3 (-45.3 to -22.6)	-26.5 (-42.8 to -18.5)	1.4 (0.1 to 4.1)	0.9 (0.0 to 3.0)	0.6 (0.0 to 2.4)	-35.6 (-53.0 to -26.2)	-28.2 (-50.7 to -19.0)
Occupational exposure to asbestos	1.5 (1.3 to 1.7)	2.0 (1.9 to 2.3)	2.0 (1.7 to 2.5)	37.7 (18.5 to 65.5)	-0.7 (-19.9 to 20.3)	0.5 (0.4 to 0.6)	0.5 (0.5 to 0.6)	0.5 (0.4 to 0.6)	-1.8 (-19.7 to 29.3)	-3.9 (-23.6 to 17.2)
Occupational exposure to arsenic	0.4 (0.0 to 1.1)	0.4 (0.0 to 1.0)	0.4 (0.0 to 1.0)	-	-	0.4 (0.0 to 1.0)	0.4 (0.0 to 0.8)	0.4 (0.0 to 0.9)	-	-
Occupational exposure to benzene	0.6 (0.0 to 1.8)	0.6 (0.0 to 1.9)	0.7 (0.0 to 1.9)	2.4 (-4.4 to 12.5)	3.5 (-2.6 to 12.1)	0.6 (0.0 to 1.8)	0.7 (0.0 to 2.1)	0.8 (0.0 to 2.3)	22.3 (8.8 to 42.3)	14.3 (4.6 to 29.1)
Occupational exposure to beryllium	0.0 (0.0 to 0.0)	0.0 (0.0 to 0.0)	0.0 (0.0 to 0.0)	-5.1 (-10.6 to 0.1)	1.9 (-1.9 to 5.9)	0.0 (0.0 to 0.0)	0.0 (0.0 to 0.0)	0.0 (0.0 to 0.0)	2.4 (-6.4 to 12.5)	9.0 (2.6 to 16.3)
Occupational exposure to cadmium	0.1 (0.0 to 0.1)	0.0 (0.0 to 0.1)	0.0 (0.0 to 0.1)	-6.9 (-18.4 to 5.3)	0.7 (-8.8 to 11.7)	0.0 (0.0 to 0.1)	0.0 (0.0 to 0.0)	0.0 (0.0 to 0.1)	-8.5 (-23.3 to 10.1)	4.7 (-7.7 to 19.8)
Occupational exposure to chromium	0.1 (0.1 to 0.1)	0.1 (0.1 to 0.1)	0.1 (0.1 to 0.1)	-5.1 (-15.6 to 5.7)	1.3 (-6.9 to 10.7)	0.1 (0.1 to 0.1)	0.1 (0.1 to 0.1)	0.1 (0.1 to 0.1)	-3.7 (-17.7 to 13.7)	6.5 (-4.5 to 19.8)
Occupational exposure to diesel engine exhaust	0.6 (0.5 to 0.6)	0.6 (0.5 to 0.6)	0.6 (0.6 to 0.6)	-0.8 (-8.3 to 8.1)	3.3 (-3.4 to 10.2)	0.4 (0.4 to 0.5)	0.5 (0.4 to 0.6)	0.6 (0.5 to 0.6)	13.4 (-0.9 to 28.6)	11.2 (1.5 to 22.6)
Occupational exposure to formaldehyde	0.2 (0.2 to 0.3)	0.2 (0.2 to 0.3)	0.2 (0.2 to 0.3)	-6.3 (-18.3 to 6.7)	0.4 (-9.5 to 11.7)	0.2 (0.2 to 0.2)	0.2 (0.2 to 0.2)	0.2 (0.2 to 0.2)	-5.8 (-21.4 to 13.5)	5.6 (-6.6 to 20.6)
Occupational exposure to nickel	0.4 (0.0 to 1.9)	0.4 (0.0 to 1.7)	0.4 (0.0 to 1.6)	-	-	0.3 (0.0 to 1.6)	0.3 (0.0 to 1.3)	0.3 (0.0 to 1.3)	-	-
Occupational exposure to polycyclic aromatic hydrocarbons	0.2 (0.2 to 0.3)	0.2 (0.2 to 0.3)	0.2 (0.2 to 0.3)	-4.1 (-14.0 to 6.2)	1.7 (-6.1 to 10.1)	0.2 (0.2 to 0.2)	0.2 (0.2 to 0.2)	0.2 (0.2 to 0.3)	-1.5 (-15.2 to 15.0)	7.4 (-2.5 to 19.6)
Occupational exposure to silica	4.5 (0.6 to 13.9)	4.3 (0.6 to 13.5)	4.3 (0.6 to 13.9)	-4.4 (-15.4 to 7.9)	1.8 (-7.2 to 12.8)	3.1 (0.4 to 10.3)	2.7 (0.4 to 8.5)	2.7 (0.4 to 8.7)	-12.9 (-28.0 to 7.3)	1.9 (-12.0 to 20.2)
Occupational exposure to sulphuric acid	0.6 (0.1 to 1.9)	0.6 (0.1 to 1.8)	0.6 (0.1 to 1.8)	-6.9 (-17.9 to 4.8)	1.1 (-7.7 to 11.5)	0.5 (0.1 to 1.7)	0.5 (0.1 to 1.4)	0.5 (0.1 to 1.5)	-10.3 (-24.7 to 8.3)	4.2 (-8.5 to 20.1)
Occupational exposure to trichloroethylene	0.1 (0.1 to 0.1)	0.1 (0.1 to 0.1)	0.1 (0.1 to 0.1)	-4.3 (-11.7 to 3.7)	1.8 (-4.1 to 8.5)	0.1 (0.1 to 0.1)	0.1 (0.1 to 0.1)	0.1 (0.1 to 0.1)	1.2 (-10.2 to 15.2)	8.1 (-0.4 to 18.1)
Occupational asthmagens	17.9 (14.1 to 22.8)	17.6 (14.0 to 22.5)	17.5 (13.9 to 22.1)	-1.7 (-20.7 to 21.7)	-0.1 (-15.8 to 17.6)	8.1 (6.4 to 10.0)	8.2 (6.5 to 10.0)	8.7 (6.9 to 10.6)	1.0 (-14.8 to 18.7)	5.4 (-7.9 to 20.4)
Occupational particulate matter, gases, and fumes	6.3 (4.5 to 9.1)	6.0 (4.3 to 8.7)	5.8 (4.1 to 8.3)	-4.2 (-9.5 to 1.2)	-3.6 (-7.2 to 0.1)	3.6 (2.5 to 5.5)	3.4 (2.4 to 5.2)	3.2 (2.3 to 4.9)	-5.5 (-14.2 to 4.3)	-4.6 (-10.2 to 1.3)
Occupational noise	5.8 (5.3 to 6.4)	5.6 (5.2 to 6.2)	5.5 (5.1 to 6.1)	-2.8 (-6.0 to 0.4)	-2.1 (-4.4 to 0.3)	3.4 (3.0 to 3.9)	3.4 (3.0 to 3.9)	3.5 (3.1 to 4.0)	0.4 (-5.2 to 7.0)	1.9 (-2.2 to 5.9)
Occupational ergonomic factors	10.7 (8.9 to 12.8)	9.5 (7.9 to 11.3)	9.4 (7.7 to 11.3)	-11.6 (-23.3 to 2.8)	-1.5 (-13.2 to 11.3)	5.8 (4.4 to 7.3)	5.9 (4.6 to 7.4)	6.6 (5.1 to 8.5)	1.7 (-21.6 to 28.3)	12.3 (-7.6 to 36.5)
Non-exclusive breastfeeding	0.4 (0.3 to 0.6)	0.4 (0.3 to 0.6)	0.4 (0.3 to 0.6)	-1.4 (-14.3 to 13.1)	-1.7 (-12.8 to 9.8)	0.4 (0.3 to 0.6)	0.4 (0.3 to 0.6)	0.4 (0.3 to 0.6)	-1.7 (-14.6 to 12.8)	-1.2 (-12.2 to 10.4)
Discontinued breastfeeding	1.7 (1.6 to 1.9)	1.7 (1.5 to 1.8)	1.7 (1.5 to 1.8)	-2.0 (-11.1 to 8.2)	-0.7 (-7.7 to 7.6)	1.7 (1.6 to 1.9)	1.7 (1.6 to 1.8)	1.7 (1.6 to 1.8)	-0.5 (-9.8 to 9.7)	-2.0 (-8.9 to 6.2)
Child underweight	0.3 (0.2 to 0.4)	0.2 (0.1 to 0.3)	0.2 (0.1 to 0.3)	-15.2 (-33.9 to 8.0)	-5.0 (-19.6 to 12.2)	0.3 (0.2 to 0.4)	0.2 (0.1 to 0.3)	0.2 (0.1 to 0.3)	-17.4 (-38.2 to 7.4)	-6.4 (-23.8 to 12.8)
Child wasting	0.2 (0.1 to 0.3)	0.2 (0.1 to 0.3)	0.2 (0.1 to 0.3)	-6.9 (-33.9 to 30.0)	-1.7 (-22.5 to 20.8)	0.2 (0.1 to 0.3)	0.2 (0.1 to 0.3)	0.2 (0.1 to 0.3)	-9.0 (-34.8 to 29.4)	-2.9 (-23.2 to 22.1)
Child stunting	0.6 (0.3 to 1.1)	0.6 (0.3 to 1.0)	0.6 (0.3 to 1.0)	-9.9 (-44.2 to 44.4)	-3.9 (-31.3 to 35.6)	0.7 (0.3 to 1.1)	0.6 (0.3 to 1.0)	0.6 (0.3 to 1.0)	-12.2 (-47.9 to 46.2)	-4.5 (-33.6 to 34.1)
Short gestation for birth weight	0.0 (0.0 to 0.0)	0.0 (0.0 to 0.0)	0.0 (0.0 to 0.0)	9.4 (-13.4 to 35.9)	2.2 (-16.2 to 39.8)	0.0 (0.0 to 0.0)	0.0 (0.0 to 0.0)	0.0 (0.0 to 0.0)	10.9 (-21.2 to 39.9)	2.4 (-18.3 to 39.8)
Low birth weight for gestation	0.0 (0.0 to 0.0)	0.0 (0.0 to 0.0)	0.0 (0.0 to 0.0)	4.2 (-13.1 to 27.3)	2.1 (-22.0 to 22.9)	0.0 (0.0 to 0.0)	0.0 (0.0 to 0.0)	0.0 (0.0 to 0.0)	-1.1 (-15.3 to 32.3)	2.6 (-21.3 to 24.6)
Iron deficiency	-	-	-	-	-	8.1 (5.5 to 11.2)	8.0 (5.6 to 11.1)	7.3 (4.9 to 10.5)	-0.4 (-21.4 to 27.9)	-8.4 (-24.9 to 9.5)
Vitamin A deficiency	1.0 (0.6 to 1.4)	0.9 (0.5 to 1.1)	0.6 (0.4 to 0.9)	-14.7 (-32.9 to 2.1)	-24.9 (-37.9 to -9.0)	0.7 (0.4 to 0.9)	0.7 (0.5 to 1.0)	0.7 (0.5 to 0.9)	8.4 (-9.1 to 30.7)	-5.6 (-21.6 to 12.7)
Zinc deficiency	0.5 (0.1 to 1.0)	0.5 (0.1 to 1.2)	0.4 (0.1 to 1.1)	-4.5 (-63.1 to 76.4)	-13.2 (-66.9 to 147.3)	0.5 (0.1 to 1.0)	0.3 (0.1 to 0.8)	0.4 (0.1 to 0.8)	-30.2 (-63.7 to 67.1)	4.0 (-61.1 to 171.3)
Smoking	17.8 (14.5 to 21.3)	12.3 (9.7 to 15.0)	10.4 (8.2 to 12.8)	-30.8 (-39.1 to -21.4)	-15.7 (-24.3 to -6.5)	4.0 (2.9 to 5.4)	3.3 (2.3 to 4.4)	3.2 (2.2 to 4.4)	-17.9 (-37.2 to 6.5)	-3.4 (-20.1 to 17.0)
Chewing tobacco	1.4 (0.9 to 2.1)	0.9 (0.6 to 1.4)	0.8 (0.5 to 1.1)	-33.1 (-56.7 to 7.0)	-18.8 (-43.0 to 13.8)	0.1 (0.1 to 0.2)	0.1 (0.1 to 0.1)	0.1 (0.1 to 0.1)	-3.7 (-38.7 to 55.0)	-3.1 (-35.4 to 42.4)
Second-hand smoke	36.8 (32.7 to 41.0)	36.7 (32.3 to 40.9)	35.4 (31.0 to 39.8)	-0.3 (-12.0 to 12.1)	-3.6 (-12.2 to 6.1)	56.0 (52.5 to 59.4)	49.6 (45.5 to 53.4)	47.7 (43.3 to 51.7)	-11.4 (-18.1 to -4.5)	-3.8 (-10.0 to 2.2)
Alcohol use	14.8 (7.4 to 25.6)	14.7 (7.6 to 25.7)	16.7 (8.4 to 29.8)	-0.3 (-34.2 to 51.3)	13.4 (-19.1 to 62.5)	11.8 (5.7 to 20.8)	11.8 (5.8 to 20.9)	13.4 (6.6 to 23.9)	-0.2 (-35.2 to 50.5)	14.2 (-19.4 to 64.3)
Drug use	0.5 (0.4 to 0.7)	0.5 (0.4 to 0.7)	0.6 (0.4 to 0.8)	1.0 (-4.8 to 6.7)	11.1 (4.3 to 19.4)	0.3 (0.2 to 0.4)	0.3 (0.2 to 0.4)	0.3 (0.3 to 0.4)	1.8 (-4.2 to 8.3)	8.7 (2.2 to 16.4)
Diet low in fruits	40.9 (35.3 to 44.9)	39.3 (33.4 to 43.2)	40.7 (35.3 to 44.5)	-4.1 (-10.5 to 1.9)	3.7 (-0.9 to 8.9)	37.9 (31.8 to 42.3)	35.2 (29.0 to 39.7)	37.5 (31.8 to 42.0)	-7.2 (-15.6 to 0.5)	6.5 (-0.3 to 15.4)
Diet low in vegetables	19.4 (10.3 to 27.1)	13.4 (5.1 to 21.4)	15.3 (7.0 to 23.5)	-31.0 (-61.7 to -3.5)	14.7 (-17.3 to 78.8)	21.6 (13.3 to 29.1)	15.4 (7.3 to 23.3)	17.2 (8.6 to 25.2)	-28.7 (-55.7 to -4.6)	11.2 (-15.4 to 56.5)
Diet low in legumes	4.5 (0.8 to 9.7)	6.0 (1.2 to 11.8)	8.1 (2.3 to 14.8)	33.9 (-40.9 to 289.4)	34.9 (-15.5 to 214.1)	8.2 (2.6 to 15.4)	8.1 (2.8 to 14.7)	10.5 (4.0 to 17.4)	-1.2 (-45.4 to 76.8)	29.6 (-12.7 to 124.1)
Diet low in whole grains	30.4 (25.4 to 34.6)	35.2 (31.2 to 38.7)	36.6 (32.7 to 40.1)	15.8 (7.5 to 29.1)	3.9 (-1.0 to 9.2)	32.0 (27.2 to 36.2)	36.5 (32.5 to 39.8)	37.8 (34.1 to 40.9)	14.2 (6.0 to 24.7)	3.5 (-0.7 to 8.5)
Diet low in nuts and seeds	50.8 (49.3 to 51.8)	49.5 (47.7 to 50.9)	49.4 (47.3 to 50.9)	-2.5 (-5.1 to -0.4)	-0.3 (-2.4 to 1.5)	51.0 (49.7 to 52.0)	49.6 (47.9 to 51.0)	49.5 (47.6 to 51.0)	-2.6 (-4.9 to -0.5)	-0.3 (-2.2 to 1.6)
Diet low in milk	46.2 (44.2 to 48.0)	46.6 (44.6 to 48.3)	47.2 (45.2 to 48.7)	0.8 (-1.2 to 3.0)	1.1 (-0.3 to 2.6)	46.3 (44.3 to 48.0)	46.2 (44.1 to 47.9)	46.9 (44.9 to 48.6)	-0.2 (-2.1 to 1.8)	1.5 (0.0 to 3.3)
Diet high in red meat	4.1 (0.6 to 9.3)	7.9 (2.2 to 15.0)	9.1 (2.9 to 16.9)	95.3 (-16.9 to 702.1)	14.2 (-36.5 to 125.7)	0.6 (0.0 to 2.3)	1.7 (0.0 to 5.3)	2.1 (0.0 to 6.0)	196.3 (-72.8 to 443729.0)	26.7 (-87.4 to 2089.1)
Diet high in processed meat	11.6 (7.4 to 15.5)	19.1 (14.0 to 23.7)	22.1 (16.3 to 27.5)	64.9 (26.8 to 129.0)	16.0 (-2.2 to 38.6)	9.8 (5.9 to 13.4)	14.6 (10.2 to 18.2)	16.2 (11.7 to 20.4)	48.7 (15.9 to 109.3)	10.8 (-6.7 to 30.8)
Diet high in sugar-sweetened beverages	7.2 (3.0 to 14.2)	8.3 (3.7 to 14.7)	8.9 (3.8 to 16.0)	15.1 (-44.6 to 150.8)	7.6 (-36.9 to 73.3)	5.3 (2.0 to 11.5)	5.9 (2.5 to 11.5)	6.3 (2.6 to 12.3)	10.9 (-51.2 to 160.3)	6.2 (-40.4 to 87.8)
Diet low in fibre	21.8 (14.4 to 28.4)	22.7 (15.6 to 29.2)	23.5 (16.4 to 29.8)	4.3 (-14.8 to 28.9)	3.5 (-8.5 to 18.0)	23.1 (15.6 to 29.8)	23.9 (16.9 to 29.7)	25.2 (18.1 to 31.3)	3.7 (-12.2 to 25.2)	5.4 (-6.8 to 20.1)
Diet low in calcium	34.8 (30.3 to 38.3)	34.7 (30.3 to 38.2)	35.6 (31.6 to 38.9)	-0.2 (-4.1 to 3.7)	2.6 (-0.6 to 6.2)	37.0 (33.2 to 40.1)	36.0 (31.9 to 39.4)	37.3 (33.2 to 40.5)	-2.7 (-6.5 to 0.6)	3.6 (0.9 to 6.7)
Diet low in seafood omega-3 fatty acids	3.1 (1.2 to 4.6)	0.2 (0.0 to 1.0)	0.3 (0.0 to 1.1)	-92.7 (-100.0 to -71.2)	9.8 (-95.8 to 2893.0)	3.7 (1.8 to 5.1)	0.4 (0.0 to 1.3)	0.4 (0.0 to 1.4)	-89.2 (-99.9 to -65.6)	7.2 (-85.7 to 866.8)
Diet low in polyunsaturated fatty acids	30.3 (24.7 to 35.3)	27.6 (21.4 to 32.6)	28.4 (22.4 to 33.7)	-8.9 (-22.1 to 1.2)	2.9 (-6.3 to 16.1)	31.1 (25.4 to 36.1)	28.3 (22.5 to 33.4)	29.0 (23.2 to 34.1)	-8.7 (-19.5 to 1.3)	2.5 (-5.3 to 12.0)
Diet high in trans fatty acids	1.1 (0.0 to 3.3)	0.9 (0.0 to 3.0)	0.9 (0.0 to 2.9)	-12.6 (-39.5 to -6.7)	-8.3 (-33.9 to -3.6)	1.5 (0.1 to 4.0)	1.3 (0.1 to 3.7)	1.2 (0.0 to 3.5)	-12.6 (-34.8 to -6.5)	-8.3 (-27.5 to -3.4)
Diet high in sodium	28.7 (19.2 to 36.8)	19.0 (10.2 to 26.2)	16.5 (7.4 to 23.6)	-33.8 (-55.7 to -14.9)	-13.3 (-37.3 to 3.1)	32.4 (23.6 to 40.0)	20.0 (11.9 to 27.1)	16.9 (8.1 to 24.3)	-38.2 (-57.5 to -24.1)	-15.6 (-37.2 to -1.6)
Intimate partner violence	-	-	-	-	-	2.3 (1.9 to 2.7)	2.1 (1.8 to 2.4)	2.0 (1.7 to 2.3)	-9.6 (-14.0 to -4.9)	-3.7 (-8.8 to 1.0)
Childhood sexual abuse	5.3 (4.3 to 6.5)	5.3 (4.3 to 6.5)	5.3 (4.3 to 6.5)	0.0 (0.0 to 0.0)	0.0 (0.0 to 0.0)	7.3 (5.9 to 8.9)	7.3 (5.9 to 8.9)	7.5 (6.0 to 9.2)	0.0 (0.0 to 0.0)	2.8 (-4.0 to 10.6)

Supplementary Results Table 1. Yamanashi age-standardised Summary Exposure Values (SEVs) for Level 4 risk factors and percentage change, by sex, 1990, 2007, and 2017.Data in parentheses are 95% uncertainty intervals.										
	**Male**					**Female**				
	**1990**	**2007**	**2017**	**Percentage change 1990-2007**	**Percentage change 2007-2017**	**1990**	**2007**	**2017**	**Percentage change 1990-2007**	**Percentage change 2007-2017**
Bullying victimization	3.3 (1.4 to 7.3)	3.4 (1.4 to 7.5)	3.5 (1.4 to 7.5)	2.4 (-5.2 to 10.6)	3.9 (-1.4 to 8.7)	1.9 (0.8 to 4.1)	1.9 (0.8 to 4.3)	2.0 (0.8 to 4.4)	1.1 (-6.4 to 9.1)	1.8 (-4.0 to 6.6)
Low physical activity	0.5 (0.2 to 0.9)	0.5 (0.2 to 0.9)	0.4 (0.2 to 0.8)	-1.8 (-3.6 to 0.1)	-8.8 (-12.2 to -4.6)	0.5 (0.2 to 1.0)	0.5 (0.2 to 0.9)	0.5 (0.2 to 0.9)	-3.9 (-6.0 to -1.3)	0.8 (-0.7 to 2.2)
High fasting plasma glucose	1.4 (0.9 to 2.3)	1.4 (0.9 to 2.1)	1.6 (0.9 to 2.6)	-2.4 (-40.4 to 60.5)	12.9 (-27.8 to 75.8)	1.2 (0.7 to 1.8)	1.2 (0.7 to 2.1)	1.4 (0.8 to 2.4)	5.3 (-34.2 to 69.1)	11.3 (-29.5 to 98.9)
High LDL cholesterol	12.1 (10.5 to 13.8)	12.6 (10.9 to 14.3)	12.6 (11.0 to 14.4)	4.4 (-1.5 to 10.1)	0.0 (-4.7 to 4.9)	12.9 (11.3 to 14.6)	13.0 (11.4 to 14.8)	13.0 (11.4 to 14.8)	0.9 (-4.5 to 6.6)	0.2 (-3.8 to 4.8)
High systolic blood pressure	11.2 (10.2 to 12.3)	8.3 (7.4 to 9.3)	8.2 (7.2 to 9.1)	-25.8 (-31.7 to -19.5)	-1.7 (-9.0 to 6.3)	9.6 (8.6 to 10.6)	6.0 (5.3 to 6.8)	5.6 (4.8 to 6.5)	-37.1 (-44.2 to -29.6)	-7.3 (-17.0 to 3.1)
High body-mass index	5.2 (3.2 to 8.2)	7.5 (5.0 to 11.9)	7.7 (5.0 to 12.8)	42.6 (15.3 to 82.3)	3.8 (-12.1 to 22.4)	5.5 (3.3 to 8.8)	7.0 (4.4 to 10.7)	7.0 (4.3 to 11.0)	28.3 (0.9 to 71.2)	0.3 (-17.7 to 21.6)
Low bone mineral density	5.3 (4.4 to 6.2)	5.2 (4.3 to 6.1)	5.2 (4.2 to 6.1)	-1.8 (-14.0 to 12.5)	-0.3 (-13.2 to 14.1)	7.2 (6.1 to 8.5)	7.0 (5.9 to 8.1)	7.1 (6.0 to 8.4)	-3.3 (-15.5 to 10.5)	1.7 (-11.4 to 15.3)
Impaired kidney function	2.6 (1.2 to 6.5)	2.5 (1.2 to 6.4)	2.4 (1.1 to 6.1)	-2.7 (-7.2 to 1.9)	-6.3 (-11.3 to -1.8)	3.2 (1.5 to 7.4)	3.3 (1.5 to 7.6)	3.1 (1.4 to 7.2)	3.2 (-0.2 to 7.5)	-6.5 (-11.4 to -1.7)

Supplementary Results Table 1. South Korea age-standardised Summary Exposure Values (SEVs) for Level 4 risk factors and percentage change, by sex, 1990, 2007, and 2017.Data in parentheses are 95% uncertainty intervals.										
	Male					Female				
	1990	2007	2017	Percentage change 1990-2007	Percentage change 2007-2017	1990	2007	2017	Percentage change 1990-2007	Percentage change 2007-2017
Unsafe water source	3.9 (2.5 to 5.7)	1.9 (1.2 to 2.9)	1.8 (1.2 to 2.7)	-51.8 (-71.3 to -20.1)	-5.8 (-33.8 to 36.7)	3.9 (2.5 to 5.7)	1.9 (1.2 to 2.9)	1.8 (1.2 to 2.7)	-51.8 (-71.3 to -20.1)	-5.8 (-33.8 to 36.7)
Unsafe sanitation	8.5 (5.0 to 13.1)	2.0 (1.2 to 3.2)	1.4 (0.8 to 2.3)	-76.9 (-87.3 to -58.2)	-26.4 (-54.8 to 19.8)	8.5 (5.0 to 13.1)	2.0 (1.2 to 3.2)	1.4 (0.8 to 2.3)	-76.9 (-87.3 to -58.2)	-26.4 (-54.8 to 19.8)
No access to handwashing facility	7.6 (6.1 to 9.5)	6.4 (5.0 to 7.9)	5.8 (4.6 to 7.2)	-16.2 (-35.3 to 7.1)	-9.1 (-23.9 to 8.5)	7.6 (6.1 to 9.5)	6.4 (5.0 to 7.9)	5.8 (4.6 to 7.2)	-16.2 (-35.3 to 7.1)	-9.1 (-23.9 to 8.5)
Ambient particulate matter pollution	51.5 (35.9 to 72.7)	53.7 (38.1 to 74.0)	48.1 (33.0 to 67.2)	4.4 (-13.8 to 27.1)	-10.4 (-27.5 to 9.9)	50.8 (35.4 to 71.8)	53.7 (38.1 to 73.9)	48.1 (33.0 to 67.1)	5.6 (-12.8 to 29.5)	-10.4 (-27.5 to 9.9)
Household air pollution from solid fuels	1.5 (0.7 to 4.0)	0.1 (0.0 to 0.1)	0.1 (0.0 to 0.1)	-93.9 (-98.3 to -91.9)	-27.6 (-75.4 to 21.0)	2.0 (0.9 to 7.9)	0.2 (0.0 to 0.4)	0.1 (0.0 to 0.3)	-92.2 (-98.2 to -90.5)	-25.7 (-75.6 to 21.6)
Ambient ozone pollution	49.9 (21.4 to 54.8)	49.9 (21.4 to 54.8)	50.4 (22.3 to 54.8)	0.0 (0.0 to 0.2)	1.1 (0.0 to 4.3)	49.9 (21.4 to 54.8)	49.9 (21.4 to 54.8)	50.4 (22.3 to 54.8)	0.0 (0.0 to 0.2)	1.1 (0.0 to 4.3)
Residential radon	25.0 (7.9 to 52.6)	25.0 (7.9 to 52.6)	25.0 (7.9 to 52.6)	0.0 (0.0 to 0.0)	0.0 (0.0 to 0.0)	25.0 (7.9 to 52.6)	25.0 (7.9 to 52.6)	25.0 (7.9 to 52.6)	0.0 (0.0 to 0.0)	0.0 (0.0 to 0.0)
Lead exposure	8.0 (3.9 to 12.3)	6.0 (2.7 to 9.8)	4.2 (1.7 to 7.6)	-24.9 (-33.3 to -17.9)	-29.7 (-38.7 to -22.0)	4.2 (1.1 to 8.1)	3.3 (0.8 to 6.8)	2.3 (0.5 to 5.3)	-20.9 (-29.0 to -14.3)	-29.4 (-41.4 to -21.2)
Occupational exposure to asbestos	0.9 (0.7 to 1.1)	1.0 (0.9 to 1.1)	0.9 (0.8 to 1.1)	5.3 (-13.3 to 32.4)	-3.5 (-18.8 to 18.6)	0.3 (0.2 to 0.3)	0.3 (0.2 to 0.3)	0.3 (0.2 to 0.3)	3.0 (-22.9 to 45.7)	1.7 (-16.5 to 27.4)
Occupational exposure to arsenic	0.4 (0.0 to 1.0)	0.4 (0.0 to 1.0)	0.4 (0.0 to 1.0)	-	-	0.4 (0.0 to 0.9)	0.3 (0.0 to 0.8)	0.3 (0.0 to 0.8)	-	-
Occupational exposure to benzene	0.6 (0.0 to 1.6)	0.6 (0.0 to 1.7)	0.6 (0.0 to 1.7)	8.8 (4.4 to 14.4)	2.7 (-1.0 to 8.2)	0.5 (0.0 to 1.4)	0.7 (0.0 to 1.9)	0.7 (0.0 to 2.1)	31.3 (24.4 to 40.3)	11.9 (5.7 to 21.5)
Occupational exposure to beryllium	0.0 (0.0 to 0.0)	0.0 (0.0 to 0.0)	0.0 (0.0 to 0.0)	-2.9 (-5.2 to -0.3)	1.3 (-1.6 to 4.4)	0.0 (0.0 to 0.0)	0.0 (0.0 to 0.0)	0.0 (0.0 to 0.0)	0.6 (-2.6 to 4.4)	4.6 (0.4 to 8.8)
Occupational exposure to cadmium	0.0 (0.0 to 0.1)	0.0 (0.0 to 0.0)	0.0 (0.0 to 0.1)	-4.3 (-10.2 to 2.5)	0.8 (-6.0 to 8.3)	0.0 (0.0 to 0.1)	0.0 (0.0 to 0.0)	0.0 (0.0 to 0.0)	-14.4 (-21.3 to -6.2)	0.7 (-7.2 to 9.0)
Occupational exposure to chromium	0.1 (0.1 to 0.1)	0.1 (0.1 to 0.1)	0.1 (0.1 to 0.1)	-1.4 (-7.0 to 4.8)	1.3 (-4.8 to 7.8)	0.1 (0.1 to 0.1)	0.1 (0.1 to 0.1)	0.1 (0.1 to 0.1)	-7.0 (-14.2 to 1.0)	2.3 (-4.9 to 9.4)
Occupational exposure to diesel engine exhaust	0.5 (0.5 to 0.6)	0.6 (0.6 to 0.6)	0.6 (0.6 to 0.6)	6.2 (1.3 to 11.4)	1.7 (-3.1 to 6.3)	0.4 (0.4 to 0.4)	0.5 (0.5 to 0.5)	0.5 (0.5 to 0.6)	24.2 (15.5 to 34.4)	4.3 (-3.2 to 12.8)
Occupational exposure to formaldehyde	0.2 (0.2 to 0.3)	0.2 (0.2 to 0.2)	0.2 (0.2 to 0.2)	-3.1 (-9.7 to 4.4)	0.5 (-6.6 to 8.2)	0.2 (0.2 to 0.2)	0.2 (0.2 to 0.2)	0.2 (0.2 to 0.2)	-11.1 (-19.0 to -1.5)	2.1 (-6.0 to 10.2)
Occupational exposure to nickel	0.4 (0.0 to 1.8)	0.4 (0.0 to 1.7)	0.4 (0.0 to 1.7)	-	-	0.3 (0.0 to 1.5)	0.3 (0.0 to 1.1)	0.3 (0.0 to 1.1)	-	-
Occupational exposure to polycyclic aromatic hydrocarbons	0.2 (0.2 to 0.2)	0.2 (0.2 to 0.2)	0.2 (0.2 to 0.2)	-0.4 (-5.7 to 5.4)	1.3 (-4.4 to 7.3)	0.2 (0.2 to 0.2)	0.2 (0.2 to 0.2)	0.2 (0.2 to 0.2)	-5.1 (-12.0 to 2.9)	3.6 (-3.0 to 10.0)
Occupational exposure to silica	4.6 (0.7 to 14.6)	4.5 (0.7 to 14.3)	4.5 (0.7 to 14.4)	-1.5 (-7.5 to 4.8)	-0.7 (-7.1 to 6.3)	3.0 (0.4 to 9.6)	2.3 (0.3 to 7.0)	2.2 (0.3 to 7.0)	-22.7 (-30.1 to -14.2)	-2.3 (-11.6 to 8.7)
Occupational exposure to sulphuric acid	0.6 (0.1 to 1.9)	0.5 (0.1 to 1.7)	0.5 (0.1 to 1.7)	-6.3 (-11.7 to -0.3)	0.0 (-6.4 to 7.2)	0.5 (0.1 to 1.6)	0.4 (0.1 to 1.3)	0.4 (0.1 to 1.3)	-17.5 (-24.2 to -10.0)	-1.4 (-9.5 to 7.4)
Occupational exposure to trichloroethylene	0.1 (0.1 to 0.1)	0.1 (0.1 to 0.1)	0.1 (0.1 to 0.1)	0.3 (-3.8 to 5.0)	1.7 (-2.6 to 6.3)	0.1 (0.1 to 0.1)	0.1 (0.1 to 0.1)	0.1 (0.1 to 0.1)	1.7 (-4.0 to 8.6)	3.0 (-2.6 to 9.3)
Occupational asthmagens	16.5 (14.1 to 19.6)	16.1 (13.7 to 19.0)	16.9 (14.0 to 20.3)	-2.1 (-10.1 to 6.3)	4.9 (-5.8 to 17.2)	8.5 (7.1 to 10.2)	8.6 (7.3 to 10.0)	8.6 (7.2 to 10.2)	0.5 (-8.4 to 9.8)	0.2 (-7.7 to 8.1)
Occupational particulate matter, gases, and fumes	6.6 (4.8 to 9.4)	6.0 (4.3 to 8.5)	5.6 (4.0 to 8.0)	-9.6 (-13.3 to -6.5)	-6.3 (-8.4 to -4.1)	3.9 (2.9 to 5.7)	3.5 (2.5 to 5.0)	3.1 (2.3 to 4.5)	-11.3 (-16.1 to -6.6)	-10.1 (-12.8 to -7.5)
Occupational noise	6.0 (5.7 to 6.5)	5.5 (5.2 to 6.0)	5.3 (5.0 to 5.7)	-8.2 (-9.9 to -6.7)	-5.0 (-6.0 to -3.9)	3.5 (3.2 to 3.9)	3.3 (3.1 to 3.7)	3.2 (3.1 to 3.6)	-4.7 (-7.5 to -1.5)	-3.0 (-4.4 to -1.3)
Occupational ergonomic factors	12.0 (10.7 to 13.6)	9.1 (8.0 to 10.4)	8.7 (7.4 to 10.1)	-24.4 (-30.4 to -18.7)	-4.8 (-11.7 to 2.5)	8.5 (7.6 to 9.5)	6.7 (5.9 to 7.7)	6.3 (5.4 to 7.5)	-20.6 (-27.7 to -13.0)	-5.5 (-14.6 to 4.5)
Non-exclusive breastfeeding	0.4 (0.3 to 0.5)	0.4 (0.3 to 0.5)	0.4 (0.3 to 0.5)	-1.4 (-10.0 to 8.8)	-1.8 (-8.7 to 5.5)	0.4 (0.3 to 0.5)	0.4 (0.3 to 0.5)	0.4 (0.3 to 0.5)	-1.4 (-10.0 to 8.8)	-1.8 (-8.7 to 5.5)
Discontinued breastfeeding	2.0 (1.9 to 2.1)	2.1 (2.0 to 2.2)	2.1 (2.0 to 2.1)	4.8 (-1.2 to 11.9)	-0.4 (-4.8 to 4.2)	2.0 (1.9 to 2.1)	2.1 (2.0 to 2.2)	2.1 (2.0 to 2.1)	6.0 (0.0 to 13.2)	-1.5 (-5.9 to 3.1)
Child underweight	0.1 (0.1 to 0.2)	0.1 (0.0 to 0.1)	0.1 (0.0 to 0.1)	-19.0 (-29.4 to -8.2)	-3.9 (-11.3 to 2.9)	0.1 (0.1 to 0.2)	0.1 (0.0 to 0.1)	0.1 (0.0 to 0.1)	-22.9 (-34.8 to -9.9)	-5.0 (-13.4 to 4.9)
Child wasting	0.1 (0.1 to 0.1)	0.1 (0.1 to 0.1)	0.1 (0.1 to 0.1)	-4.2 (-21.6 to 18.7)	3.7 (-9.6 to 21.1)	0.1 (0.1 to 0.2)	0.1 (0.1 to 0.2)	0.1 (0.1 to 0.1)	-13.8 (-31.2 to 9.7)	-3.7 (-17.5 to 13.5)
Child stunting	0.2 (0.1 to 0.4)	0.2 (0.1 to 0.3)	0.2 (0.1 to 0.3)	-10.0 (-37.8 to 21.2)	3.5 (-17.1 to 34.6)	0.4 (0.2 to 0.6)	0.3 (0.2 to 0.5)	0.3 (0.1 to 0.5)	-21.2 (-48.7 to 15.8)	0.5 (-23.3 to 35.4)
Short gestation for birth weight	0.0 (0.0 to 0.0)	0.0 (0.0 to 0.0)	0.0 (0.0 to 0.0)	6.0 (-14.6 to 32.9)	3.0 (-14.5 to 22.5)	0.0 (0.0 to 0.0)	0.0 (0.0 to 0.0)	0.0 (0.0 to 0.0)	6.4 (-12.4 to 27.1)	3.1 (-15.3 to 32.4)
Low birth weight for gestation	0.0 (0.0 to 0.0)	0.0 (0.0 to 0.0)	0.0 (0.0 to 0.0)	6.2 (-9.5 to 26.2)	1.7 (-13.1 to 18.0)	0.0 (0.0 to 0.0)	0.0 (0.0 to 0.0)	0.0 (0.0 to 0.0)	3.8 (-10.0 to 22.0)	1.4 (-18.7 to 17.5)
Iron deficiency	-	-	-	-	-	9.9 (7.6 to 12.6)	6.1 (4.4 to 8.3)	5.6 (3.9 to 7.6)	-38.1 (-48.2 to -27.3)	-9.6 (-22.9 to 4.9)
Vitamin A deficiency	1.2 (0.8 to 1.5)	0.6 (0.4 to 0.8)	0.6 (0.4 to 0.8)	-49.1 (-58.6 to -25.3)	-0.4 (-18.3 to 16.9)	0.7 (0.5 to 1.0)	0.6 (0.4 to 0.8)	0.6 (0.4 to 0.9)	-14.1 (-26.3 to 0.7)	-0.7 (-26.5 to 23.7)
Zinc deficiency	0.5 (0.2 to 1.1)	0.5 (0.2 to 1.0)	0.4 (0.1 to 0.9)	-6.8 (-15.5 to 1.9)	-10.7 (-58.0 to -0.9)	0.5 (0.2 to 1.1)	0.5 (0.1 to 0.9)	0.4 (0.1 to 0.9)	-8.5 (-16.4 to -1.2)	-10.2 (-56.4 to -0.7)
Smoking	18.6 (16.5 to 20.9)	16.1 (14.4 to 17.9)	12.3 (11.0 to 13.7)	-13.8 (-19.2 to -8.1)	-23.4 (-27.8 to -19.5)	2.1 (1.6 to 2.6)	1.9 (1.5 to 2.3)	1.6 (1.3 to 1.9)	-7.2 (-25.1 to 16.3)	-18.9 (-30.5 to -5.6)
Chewing tobacco	1.4 (1.0 to 1.8)	0.9 (0.7 to 1.3)	0.8 (0.6 to 1.0)	-32.3 (-49.9 to -6.9)	-19.1 (-36.3 to 2.1)	0.1 (0.1 to 0.1)	0.1 (0.1 to 0.1)	0.1 (0.1 to 0.1)	-3.9 (-29.8 to 33.6)	-2.3 (-24.5 to 24.6)
Second-hand smoke	40.4 (37.6 to 43.4)	32.8 (30.0 to 35.5)	30.5 (27.7 to 33.3)	-18.9 (-25.6 to -11.6)	-7.1 (-12.9 to -0.8)	73.3 (70.8 to 76.1)	51.1 (48.6 to 53.6)	44.2 (41.4 to 47.0)	-30.3 (-33.8 to -26.7)	-13.5 (-17.0 to -10.0)
Alcohol use	15.2 (8.9 to 24.4)	17.1 (10.0 to 27.0)	20.3 (11.0 to 32.2)	12.4 (-1.9 to 37.6)	19.3 (-8.8 to 53.3)	11.3 (6.4 to 19.3)	12.3 (7.1 to 20.1)	14.4 (7.7 to 23.0)	9.4 (-4.6 to 35.1)	16.9 (-10.4 to 53.8)
Drug use	0.5 (0.4 to 0.8)	0.6 (0.4 to 0.8)	0.6 (0.4 to 0.8)	3.4 (-1.5 to 9.9)	5.8 (-0.5 to 12.3)	0.3 (0.2 to 0.4)	0.3 (0.3 to 0.4)	0.3 (0.3 to 0.4)	4.4 (-1.0 to 10.2)	5.5 (-0.4 to 11.8)
Diet low in fruits	41.6 (36.4 to 44.4)	28.9 (23.2 to 33.4)	26.3 (20.2 to 31.3)	-30.6 (-39.2 to -23.9)	-9.0 (-17.3 to -2.4)	38.5 (33.1 to 41.9)	23.2 (16.9 to 28.4)	21.0 (14.5 to 27.0)	-39.8 (-51.3 to -30.9)	-9.3 (-21.6 to 1.2)
Diet low in vegetables	2.4 (0.1 to 7.5)	8.8 (1.7 to 15.9)	14.2 (6.0 to 21.4)	263.4 (100.5 to 2533.5)	60.7 (21.4 to 354.3)	4.4 (0.3 to 10.7)	12.9 (5.2 to 19.5)	17.5 (9.7 to 24.1)	189.8 (75.1 to 1828.1)	35.7 (12.1 to 118.0)
Diet low in legumes	3.4 (0.4 to 8.4)	14.2 (7.8 to 19.8)	18.3 (12.2 to 23.6)	316.1 (128.0 to 2226.6)	28.6 (10.7 to 73.5)	8.6 (2.7 to 14.6)	20.3 (14.7 to 25.4)	24.2 (18.7 to 28.9)	135.4 (67.4 to 473.0)	19.0 (8.4 to 36.1)
Diet low in whole grains	22.0 (16.8 to 26.5)	29.6 (25.6 to 33.1)	28.9 (24.5 to 32.6)	34.9 (22.4 to 55.6)	-2.6 (-7.6 to 2.2)	24.0 (18.8 to 28.3)	31.8 (28.0 to 34.9)	30.9 (26.7 to 34.3)	32.4 (21.6 to 50.5)	-2.9 (-7.0 to 1.2)
Diet low in nuts and seeds	48.9 (47.3 to 50.1)	46.5 (44.5 to 48.2)	44.6 (41.9 to 46.9)	-4.9 (-7.3 to -2.6)	-4.2 (-7.3 to -1.7)	49.3 (47.8 to 50.5)	46.8 (45.0 to 48.5)	45.0 (42.4 to 47.2)	-4.9 (-7.3 to -2.8)	-3.8 (-6.8 to -1.4)
Diet low in milk	52.5 (52.0 to 52.9)	49.2 (48.0 to 50.2)	48.0 (46.5 to 49.3)	-6.3 (-7.9 to -5.1)	-2.3 (-3.3 to -1.4)	52.5 (51.9 to 52.9)	48.7 (47.4 to 49.8)	47.7 (46.2 to 49.0)	-7.2 (-8.9 to -5.8)	-2.0 (-3.0 to -1.1)
Diet high in red meat	2.2 (0.2 to 5.0)	26.3 (22.4 to 29.6)	30.1 (23.9 to 35.3)	1117.5 (461.8 to 11547.3)	14.4 (-2.8 to 34.1)	0.1 (0.0 to 0.6)	7.4 (4.3 to 10.7)	9.1 (4.7 to 13.6)	8352.4 (1629.2 to [illegible]	23.1 (-17.9 to 84.6)
Diet high in processed meat	2.4 (0.5 to 4.9)	4.0 (1.5 to 6.7)	3.7 (1.2 to 6.3)	66.0 (27.6 to 282.3)	-7.8 (-31.4 to 10.1)	1.5 (0.1 to 3.6)	2.2 (0.4 to 4.6)	1.9 (0.2 to 4.3)	48.3 (12.2 to 320.0)	-14.0 (-54.1 to 1.9)
Diet high in sugar-sweetened beverages	1.7 (0.8 to 2.7)	5.2 (2.7 to 6.2)	8.8 (4.4 to 11.4)	212.4 (102.2 to 396.4)	69.3 (31.4 to 119.6)	1.0 (0.5 to 1.6)	2.9 (1.5 to 3.5)	5.8 (3.0 to 7.8)	192.7 (95.0 to 380.8)	103.2 (54.8 to 170.2)
Diet low in fibre	35.0 (30.7 to 38.5)	38.7 (35.1 to 41.5)	38.5 (34.8 to 41.5)	10.4 (6.2 to 16.5)	-0.4 (-3.4 to 3.1)	39.6 (36.0 to 42.4)	42.7 (40.0 to 44.8)	42.6 (39.6 to 45.0)	7.9 (4.5 to 12.6)	-0.2 (-3.0 to 2.4)
Diet low in calcium	38.0 (34.6 to 40.7)	33.5 (29.3 to 37.0)	32.5 (28.0 to 36.2)	-11.7 (-15.9 to -8.7)	-2.9 (-5.7 to -0.8)	40.3 (37.4 to 42.7)	37.0 (33.4 to 39.9)	36.2 (32.4 to 39.3)	-8.1 (-11.1 to -6.0)	-2.2 (-4.2 to -0.6)
Diet low in seafood omega-3 fatty acids	46.9 (45.3 to 48.5)	42.0 (39.5 to 44.3)	38.8 (35.7 to 41.7)	-10.5 (-13.0 to -8.5)	-7.6 (-9.8 to -5.8)	48.1 (46.7 to 49.4)	43.8 (41.6 to 45.8)	41.0 (38.4 to 43.6)	-8.9 (-10.9 to -7.1)	-6.3 (-8.1 to -4.9)
Diet low in polyunsaturated fatty acids	31.0 (26.5 to 35.2)	10.8 (4.7 to 16.8)	8.4 (3.1 to 14.3)	-65.1 (-82.4 to -50.7)	-22.4 (-43.6 to -7.1)	31.7 (27.2 to 35.7)	11.8 (5.6 to 17.7)	9.3 (3.6 to 15.2)	-62.8 (-79.8 to -48.9)	-21.0 (-42.5 to -6.7)
Diet high in trans fatty acids	3.8 (1.1 to 7.1)	3.1 (0.7 to 6.3)	2.8 (0.5 to 5.8)	-17.5 (-36.3 to -9.8)	-11.7 (-27.8 to -5.4)	5.2 (2.0 to 8.8)	4.3 (1.4 to 7.7)	3.8 (1.1 to 7.1)	-17.8 (-33.6 to -10.7)	-11.3 (-24.8 to -5.5)
Diet high in sodium	15.1 (6.0 to 22.9)	15.0 (6.2 to 22.5)	14.3 (5.5 to 21.5)	-0.7 (-23.0 to 30.9)	-4.8 (-25.2 to 14.0)	16.0 (7.5 to 23.3)	12.4 (4.2 to 19.3)	10.0 (2.5 to 17.1)	-22.4 (-51.6 to -7.0)	-19.2 (-48.9 to -7.2)
Intimate partner violence	-	-	-	-	-	2.6 (2.1 to 3.0)	2.3 (1.9 to 2.7)	2.1 (1.8 to 2.5)	-9.8 (-13.9 to -5.8)	-7.9 (-11.9 to -3.5)
Childhood sexual abuse	5.0 (4.1 to 6.2)	5.0 (4.1 to 6.2)	5.0 (4.1 to 6.2)	0.0 (0.0 to 0.0)	0.0 (0.0 to 0.0)	7.1 (5.7 to 8.6)	7.1 (5.7 to 8.6)	7.1 (5.7 to 8.6)	0.0 (0.0 to 0.0)	0.0 (0.0 to 0.0)

Supplementary Results Table 1. South Korea age-standardised Summary Exposure Values (SEVs) for Level 4 risk factors and percentage change, by sex, 1990, 2007, and 2017.Data in parentheses are 95% uncertainty intervals.

	Male					Female				
	**1990**	**2007**	**2017**	**Percentage change 1990-2007**	**Percentage change 2007-2017**	**1990**	**2007**	**2017**	**Percentage change 1990-2007**	**Percentage change 2007-2017**
Bullying victimization	4.9 (2.1 to 10.3)	5.1 (2.1 to 10.7)	5.1 (2.1 to 10.7)	3.9 (1.0 to 6.4)	-0.5 (-2.7 to 1.5)	3.1 (1.3 to 6.3)	3.2 (1.4 to 6.7)	3.2 (1.4 to 6.7)	6.0 (3.1 to 9.0)	-0.4 (-2.4 to 1.5)
Low physical activity	0.5 (0.2 to 0.9)	0.5 (0.2 to 0.9)	0.4 (0.2 to 0.8)	-1.8 (-3.9 to 0.4)	-7.8 (-11.2 to -3.9)	0.5 (0.2 to 1.0)	0.5 (0.2 to 0.9)	0.5 (0.2 to 0.9)	-2.7 (-4.6 to -0.7)	2.5 (0.6 to 4.5)
High fasting plasma glucose	3.5 (2.4 to 5.1)	3.4 (2.4 to 4.9)	3.5 (2.4 to 5.1)	-3.6 (-22.4 to 25.0)	3.2 (-17.3 to 26.2)	2.6 (1.6 to 3.8)	2.5 (1.7 to 3.5)	2.7 (1.8 to 3.9)	-4.2 (-28.8 to 40.5)	7.9 (-19.7 to 39.7)
High LDL cholesterol	8.8 (7.4 to 10.3)	11.0 (9.4 to 12.6)	11.3 (9.8 to 13.0)	24.6 (19.6 to 30.9)	3.0 (-0.0 to 6.1)	9.1 (7.8 to 10.7)	11.5 (10.0 to 13.2)	11.6 (10.1 to 13.2)	25.9 (20.7 to 32.3)	0.5 (-2.6 to 3.8)
High systolic blood pressure	6.1 (5.4 to 6.9)	4.5 (3.9 to 5.1)	4.1 (3.5 to 4.7)	-26.5 (-31.3 to -21.5)	-8.9 (-14.6 to -3.4)	6.2 (5.5 to 6.8)	4.4 (4.0 to 5.0)	3.6 (3.1 to 4.1)	-27.9 (-33.3 to -22.5)	-18.3 (-24.6 to -12.0)
High body-mass index	5.2 (3.2 to 8.8)	13.2 (9.6 to 19.1)	13.3 (9.7 to 18.9)	152.9 (103.3 to 232.8)	0.9 (-8.0 to 9.9)	5.8 (3.5 to 9.1)	9.6 (6.4 to 14.8)	9.6 (6.3 to 14.9)	65.3 (36.2 to 111.8)	-0.2 (-11.8 to 13.3)
Low bone mineral density	4.8 (4.0 to 5.8)	4.9 (4.3 to 5.3)	4.8 (4.2 to 5.3)	0.3 (-14.3 to 17.7)	-1.0 (-9.5 to 8.3)	6.7 (5.6 to 7.9)	6.6 (6.0 to 7.2)	6.5 (5.9 to 7.2)	-1.0 (-14.9 to 15.3)	-1.1 (-8.5 to 6.0)
Impaired kidney function	2.4 (1.1 to 6.1)	2.3 (1.0 to 6.0)	2.2 (1.0 to 5.9)	-3.3 (-10.2 to 2.6)	-3.7 (-14.1 to 3.0)	2.9 (1.3 to 6.9)	2.9 (1.3 to 7.0)	2.7 (1.2 to 6.8)	-1.4 (-7.9 to 3.9)	-4.1 (-13.2 to 1.0)

Supplementary Results Table 1. Singapore age-standardised Summary Exposure Values (SEVs) for Level 4 risk factors and percentage change, by sex, 1990, 2007, and 2017.Data in parentheses are 95% uncertainty intervals.

	Male					Female				
	**1990**	**2007**	**2017**	**Percentage change 1990-2007**	**Percentage change 2007-2017**	**1990**	**2007**	**2017**	**Percentage change 1990-2007**	**Percentage change 2007-2017**
Unsafe water source	3.4 (2.2 to 4.9)	1.9 (1.2 to 2.7)	1.7 (1.1 to 2.5)	-44.6 (-66.0 to -11.4)	-6.6 (-34.4 to 38.4)	3.4 (2.2 to 4.9)	1.9 (1.2 to 2.7)	1.7 (1.1 to 2.5)	-44.6 (-66.0 to -11.4)	-6.6 (-34.4 to 38.4)
Unsafe sanitation	6.9 (4.2 to 10.7)	2.0 (1.2 to 3.1)	1.4 (0.8 to 2.3)	-71.3 (-84.0 to -49.3)	-28.1 (-56.2 to 16.4)	6.9 (4.2 to 10.7)	2.0 (1.2 to 3.1)	1.4 (0.8 to 2.3)	-71.3 (-84.0 to -49.3)	-28.1 (-56.2 to 16.4)
No access to handwashing facility	7.4 (5.9 to 9.1)	6.4 (5.1 to 7.9)	5.8 (4.6 to 7.1)	-13.6 (-31.9 to 10.3)	-9.1 (-23.0 to 9.3)	7.4 (5.9 to 9.1)	6.4 (5.1 to 7.9)	5.8 (4.6 to 7.1)	-13.6 (-31.9 to 10.3)	-9.1 (-23.0 to 9.3)
Ambient particulate matter pollution	38.9 (24.0 to 57.1)	42.8 (31.0 to 60.0)	40.6 (27.3 to 57.5)	10.1 (-19.7 to 60.3)	-5.2 (-27.5 to 20.7)	38.1 (23.3 to 56.1)	42.7 (31.0 to 59.9)	40.5 (27.3 to 57.5)	12.3 (-18.7 to 66.0)	-5.1 (-27.4 to 20.8)
Household air pollution from solid fuels	2.1 (0.9 to 5.9)	0.2 (0.1 to 0.3)	0.1 (0.0 to 0.2)	-91.2 (-96.9 to -83.1)	-40.2 (-77.5 to 10.8)	2.8 (1.1 to 8.6)	0.3 (0.1 to 1.1)	0.2 (0.0 to 0.7)	-89.4 (-96.8 to -78.2)	-38.3 (-77.4 to 11.3)
Ambient ozone pollution	11.6 (2.7 to 25.0)	22.7 (7.7 to 42.8)	39.8 (13.5 to 54.8)	95.8 (43.1 to 325.1)	75.5 (28.1 to 134.3)	11.6 (2.7 to 25.0)	22.7 (7.7 to 42.8)	39.8 (13.5 to 54.8)	95.8 (43.1 to 325.1)	75.5 (28.1 to 134.3)
Residential radon	5.6 (0.1 to 23.9)	5.6 (0.1 to 23.9)	5.6 (0.1 to 23.9)	0.0 (0.0 to 0.0)	0.0 (0.0 to 0.0)	5.6 (0.1 to 23.9)	5.6 (0.1 to 23.9)	5.6 (0.1 to 23.9)	0.0 (0.0 to 0.0)	0.0 (0.0 to 0.0)
Lead exposure	8.7 (4.4 to 12.9)	6.6 (3.1 to 10.6)	4.8 (2.0 to 8.3)	-24.2 (-32.2 to -17.3)	-27.6 (-36.4 to -20.3)	5.0 (1.4 to 9.0)	3.9 (1.1 to 7.5)	2.8 (0.7 to 6.0)	-22.4 (-30.8 to -15.7)	-27.0 (-38.8 to -19.1)
Occupational exposure to asbestos	4.2 (2.8 to 5.4)	3.1 (2.7 to 3.6)	2.3 (2.0 to 2.6)	-24.6 (-40.4 to 5.0)	-26.6 (-39.3 to -7.4)	0.9 (0.5 to 1.3)	0.7 (0.6 to 0.8)	0.3 (0.3 to 0.4)	-23.4 (-44.7 to 26.1)	-49.3 (-58.8 to -35.4)
Occupational exposure to arsenic	0.4 (0.0 to 0.9)	0.4 (0.0 to 0.8)	0.4 (0.0 to 0.9)	-	-	0.4 (0.0 to 1.0)	0.3 (0.0 to 0.8)	0.4 (0.0 to 0.9)	-	-
Occupational exposure to benzene	0.6 (0.0 to 1.8)	0.7 (0.0 to 1.9)	0.7 (0.0 to 2.0)	10.0 (2.3 to 22.2)	5.4 (-0.8 to 13.6)	0.6 (0.0 to 1.7)	0.8 (0.0 to 2.2)	0.9 (0.1 to 2.5)	24.1 (13.3 to 40.3)	13.4 (6.3 to 23.6)
Occupational exposure to beryllium	0.0 (0.0 to 0.0)	0.0 (0.0 to 0.0)	0.0 (0.0 to 0.0)	2.9 (-2.9 to 8.5)	4.5 (1.0 to 8.1)	0.0 (0.0 to 0.0)	0.0 (0.0 to 0.0)	0.0 (0.0 to 0.0)	6.0 (-2.5 to 14.8)	10.0 (4.3 to 16.1)
Occupational exposure to cadmium	0.0 (0.0 to 0.0)	0.0 (0.0 to 0.0)	0.0 (0.0 to 0.0)	-7.5 (-14.1 to -0.3)	3.0 (-3.5 to 10.2)	0.0 (0.0 to 0.1)	0.0 (0.0 to 0.0)	0.0 (0.0 to 0.0)	-15.7 (-24.6 to -6.2)	4.9 (-4.4 to 15.1)
Occupational exposure to chromium	0.1 (0.1 to 0.1)	0.1 (0.1 to 0.1)	0.1 (0.1 to 0.1)	-5.3 (-11.6 to 1.5)	3.4 (-2.3 to 9.3)	0.1 (0.1 to 0.1)	0.1 (0.1 to 0.1)	0.1 (0.1 to 0.1)	-11.3 (-20.1 to -2.2)	6.1 (-2.3 to 14.8)
Occupational exposure to diesel engine exhaust	0.6 (0.6 to 0.6)	0.6 (0.6 to 0.7)	0.7 (0.6 to 0.7)	4.7 (-2.6 to 12.3)	3.8 (-1.5 to 8.9)	0.4 (0.4 to 0.5)	0.5 (0.4 to 0.5)	0.5 (0.5 to 0.6)	13.5 (1.6 to 25.7)	9.8 (2.0 to 18.3)
Occupational exposure to formaldehyde	0.2 (0.2 to 0.2)	0.2 (0.2 to 0.2)	0.2 (0.2 to 0.2)	-8.2 (-14.5 to -1.0)	2.4 (-4.2 to 9.7)	0.2 (0.2 to 0.2)	0.2 (0.2 to 0.2)	0.2 (0.2 to 0.2)	-14.6 (-23.7 to -5.0)	5.0 (-4.0 to 14.6)
Occupational exposure to nickel	0.4 (0.0 to 1.6)	0.3 (0.0 to 1.5)	0.3 (0.0 to 1.5)	-	-	0.4 (0.0 to 1.6)	0.3 (0.0 to 1.3)	0.3 (0.0 to 1.2)	-	-
Occupational exposure to polycyclic aromatic hydrocarbons	0.2 (0.2 to 0.2)	0.2 (0.2 to 0.2)	0.2 (0.2 to 0.2)	-3.8 (-9.6 to 2.7)	3.3 (-2.0 to 9.1)	0.2 (0.2 to 0.2)	0.2 (0.2 to 0.2)	0.2 (0.2 to 0.2)	-8.3 (-17.0 to 0.7)	7.1 (-0.7 to 14.8)
Occupational exposure to silica	4.1 (0.6 to 13.1)	3.9 (0.6 to 12.3)	3.9 (0.6 to 12.5)	-5.8 (-14.0 to 4.9)	0.3 (-6.5 to 8.8)	3.0 (0.4 to 9.7)	2.4 (0.3 to 7.7)	2.5 (0.3 to 8.1)	-18.6 (-28.4 to -7.6)	2.7 (-7.7 to 15.0)
Occupational exposure to sulphuric acid	0.5 (0.1 to 1.7)	0.5 (0.1 to 1.6)	0.5 (0.1 to 1.6)	-6.6 (-13.3 to 0.6)	2.3 (-4.6 to 9.6)	0.5 (0.1 to 1.7)	0.4 (0.1 to 1.4)	0.5 (0.1 to 1.4)	-17.1 (-26.1 to -7.3)	3.5 (-6.2 to 14.3)
Occupational exposure to trichloroethylene	0.1 (0.1 to 0.1)	0.1 (0.1 to 0.1)	0.1 (0.1 to 0.1)	-1.7 (-7.9 to 4.8)	4.1 (-0.7 to 8.9)	0.1 (0.1 to 0.1)	0.1 (0.1 to 0.1)	0.1 (0.1 to 0.1)	-4.2 (-12.9 to 4.7)	7.5 (0.4 to 15.4)
Occupational asthmagens	15.2 (13.0 to 17.8)	18.0 (14.7 to 21.7)	19.9 (16.1 to 24.2)	18.5 (5.7 to 32.8)	10.7 (-0.1 to 23.6)	7.7 (6.5 to 9.0)	8.3 (6.9 to 9.9)	9.1 (7.5 to 11.2)	8.8 (-2.4 to 20.9)	9.3 (-1.5 to 24.7)
Occupational particulate matter, gases, and fumes	5.3 (3.7 to 7.6)	5.0 (3.6 to 7.2)	4.9 (3.5 to 7.1)	-4.6 (-8.4 to -0.2)	-1.6 (-4.1 to 0.8)	3.4 (2.4 to 4.8)	3.0 (2.1 to 4.3)	2.9 (2.1 to 4.1)	-11.2 (-17.2 to -3.7)	-4.0 (-8.5 to 0.8)
Occupational noise	4.9 (4.5 to 5.4)	4.9 (4.5 to 5.3)	4.9 (4.6 to 5.4)	-1.3 (-4.3 to 2.0)	1.3 (-0.6 to 3.1)	3.1 (2.8 to 3.5)	3.0 (2.8 to 3.5)	3.2 (3.0 to 3.6)	-1.8 (-6.2 to 3.4)	4.8 (1.8 to 8.1)
Occupational ergonomic factors	6.7 (5.6 to 7.9)	6.4 (5.3 to 7.7)	6.5 (5.4 to 7.8)	-4.0 (-10.4 to 2.3)	0.9 (-4.2 to 6.2)	3.9 (3.1 to 4.8)	4.0 (3.3 to 4.9)	4.3 (3.5 to 5.3)	3.1 (-8.4 to 16.3)	8.4 (-2.4 to 19.4)
Non-exclusive breastfeeding	0.7 (0.6 to 0.8)	0.6 (0.5 to 0.7)	0.6 (0.5 to 0.7)	-9.0 (-15.6 to -2.5)	-6.7 (-13.6 to -0.1)	0.7 (0.6 to 0.8)	0.6 (0.5 to 0.7)	0.6 (0.5 to 0.7)	-9.1 (-15.8 to -2.7)	-6.5 (-13.5 to 0.0)
Discontinued breastfeeding	1.7 (1.6 to 1.8)	1.8 (1.7 to 1.9)	1.7 (1.6 to 1.8)	4.9 (-2.6 to 13.1)	-2.0 (-6.9 to 3.7)	1.7 (1.6 to 1.8)	1.8 (1.7 to 1.9)	1.7 (1.6 to 1.8)	4.7 (-2.7 to 12.9)	-2.0 (-7.0 to 3.7)
Child underweight	0.2 (0.1 to 0.3)	0.1 (0.1 to 0.2)	0.1 (0.1 to 0.2)	-20.7 (-33.6 to -7.0)	-6.7 (-15.4 to 3.2)	0.3 (0.2 to 0.4)	0.2 (0.1 to 0.3)	0.2 (0.1 to 0.3)	-36.3 (-52.3 to -16.3)	-12.7 (-25.4 to 0.9)
Child wasting	0.2 (0.1 to 0.3)	0.2 (0.1 to 0.3)	0.2 (0.1 to 0.2)	-18.3 (-43.6 to 19.7)	-9.5 (-25.2 to 9.5)	0.3 (0.2 to 0.4)	0.2 (0.1 to 0.3)	0.2 (0.1 to 0.3)	-23.9 (-50.1 to 19.1)	-13.8 (-31.2 to 10.0)
Child stunting	0.3 (0.1 to 0.4)	0.2 (0.1 to 0.3)	0.2 (0.1 to 0.3)	-15.3 (-42.3 to 23.1)	-0.2 (-19.2 to 25.5)	0.4 (0.2 to 0.6)	0.3 (0.2 to 0.5)	0.3 (0.1 to 0.5)	-21.2 (-51.6 to 25.6)	-2.9 (-24.7 to 25.5)
Short gestation for birth weight	0.0 (0.0 to 0.0)	0.0 (0.0 to 0.0)	0.0 (0.0 to 0.0)	11.1 (-10.6 to 49.1)	4.2 (-13.6 to 35.1)	0.0 (0.0 to 0.0)	0.0 (0.0 to 0.0)	0.0 (0.0 to 0.0)	16.6 (-13.2 to 42.6)	3.4 (-17.5 to 39.7)
Low birth weight for gestation	0.0 (0.0 to 0.0)	0.0 (0.0 to 0.0)	0.0 (0.0 to 0.0)	7.9 (-13.5 to 30.7)	-0.2 (-19.0 to 15.0)	0.0 (0.0 to 0.0)	0.0 (0.0 to 0.0)	0.0 (0.0 to 0.0)	1.6 (-15.0 to 35.0)	-0.9 (-23.2 to 19.5)
Iron deficiency	-	-	-	-	-	8.2 (6.0 to 11.0)	6.4 (4.3 to 8.7)	5.6 (3.8 to 8.0)	-22.7 (-36.7 to -7.1)	-12.1 (-25.0 to 3.1)
Vitamin A deficiency	1.0 (0.7 to 1.4)	0.7 (0.4 to 0.9)	0.5 (0.3 to 0.7)	-32.5 (-46.2 to -13.9)	-28.2 (-39.6 to -16.6)	0.8 (0.5 to 1.1)	0.6 (0.4 to 0.8)	0.6 (0.4 to 0.7)	-18.4 (-33.4 to 2.5)	-10.5 (-29.6 to 16.8)
Zinc deficiency	0.4 (0.1 to 0.9)	0.3 (0.1 to 0.8)	0.5 (0.1 to 1.2)	-25.0 (-57.5 to 26.8)	48.1 (-54.2 to 263.1)	0.4 (0.1 to 0.9)	0.3 (0.1 to 0.8)	0.5 (0.1 to 1.2)	-25.1 (-56.9 to 25.8)	52.3 (-48.1 to 259.8)
Smoking	7.8 (6.1 to 9.4)	6.1 (5.0 to 7.3)	5.5 (4.6 to 6.4)	-21.5 (-32.3 to -8.6)	-9.7 (-18.2 to 0.7)	2.3 (1.6 to 3.2)	1.9 (1.4 to 2.5)	1.8 (1.5 to 2.3)	-16.4 (-36.7 to 9.7)	-5.2 (-19.7 to 12.7)
Chewing tobacco	1.4 (1.0 to 1.8)	0.9 (0.7 to 1.2)	0.7 (0.6 to 1.0)	-32.3 (-49.8 to -7.5)	-19.1 (-35.1 to -1.0)	0.1 (0.1 to 0.1)	0.1 (0.1 to 0.1)	0.1 (0.1 to 0.1)	-3.1 (-30.5 to 32.8)	-2.6 (-23.9 to 23.2)
Second-hand smoke	30.1 (26.9 to 33.2)	26.2 (23.6 to 28.9)	25.8 (23.1 to 28.6)	-12.7 (-22.1 to -2.0)	-1.5 (-9.1 to 6.4)	34.8 (32.0 to 37.5)	29.4 (27.2 to 31.6)	27.9 (25.7 to 30.2)	-15.5 (-22.1 to -7.7)	-5.0 (-10.5 to 0.8)
Alcohol use	9.9 (5.0 to 18.7)	11.4 (5.9 to 20.6)	13.2 (6.5 to 23.4)	15.1 (-6.0 to 43.6)	15.6 (-6.0 to 42.0)	6.2 (3.2 to 12.1)	7.1 (3.5 to 12.8)	8.1 (3.9 to 14.9)	14.2 (-8.3 to 42.9)	14.0 (-6.4 to 38.9)
Drug use	0.5 (0.4 to 0.8)	0.5 (0.4 to 0.8)	0.6 (0.4 to 0.8)	2.0 (-3.3 to 7.8)	5.9 (-0.2 to 12.2)	0.3 (0.2 to 0.4)	0.3 (0.3 to 0.4)	0.3 (0.3 to 0.4)	2.4 (-2.2 to 7.9)	4.4 (-1.1 to 10.1)
Diet low in fruits	36.7 (30.8 to 40.7)	28.1 (21.8 to 33.0)	26.3 (19.6 to 31.8)	-23.5 (-33.4 to -16.0)	-6.5 (-16.6 to 1.3)	34.1 (28.4 to 38.3)	24.4 (18.1 to 29.8)	23.0 (16.3 to 29.0)	-28.5 (-40.7 to -19.5)	-5.6 (-17.0 to 4.4)
Diet low in vegetables	24.4 (17.5 to 30.3)	19.9 (12.6 to 25.6)	17.8 (9.9 to 24.4)	-18.6 (-35.0 to -7.1)	-10.5 (-29.6 to 4.0)	26.6 (20.2 to 31.9)	21.5 (14.8 to 27.1)	19.4 (11.5 to 25.8)	-19.2 (-34.6 to -8.0)	-9.7 (-27.7 to 3.5)
Diet low in legumes	22.2 (16.4 to 27.1)	23.9 (18.8 to 28.6)	23.7 (18.0 to 28.4)	7.5 (-1.3 to 19.8)	-0.8 (-9.1 to 7.7)	27.1 (22.2 to 31.5)	29.4 (24.8 to 33.4)	29.3 (24.5 to 33.4)	8.2 (1.2 to 18.3)	-0.4 (-6.4 to 5.9)
Diet low in whole grains	30.7 (26.8 to 34.0)	32.0 (28.2 to 35.2)	31.9 (27.9 to 35.1)	4.4 (0.2 to 9.5)	-0.5 (-4.5 to 3.4)	32.1 (28.4 to 35.2)	33.4 (29.8 to 36.4)	33.3 (29.6 to 36.4)	4.0 (0.1 to 8.5)	-0.4 (-3.9 to 3.4)
Diet low in nuts and seeds	49.1 (47.3 to 50.6)	41.2 (37.2 to 44.4)	36.7 (31.2 to 41.0)	-16.1 (-22.5 to -11.3)	-11.0 (-18.0 to -5.9)	49.4 (47.8 to 50.7)	42.0 (38.2 to 45.0)	37.6 (32.3 to 41.7)	-15.1 (-21.2 to -10.6)	-10.4 (-16.9 to -5.3)
Diet low in milk	47.7 (46.0 to 49.0)	46.6 (44.8 to 48.0)	45.9 (43.9 to 47.6)	-2.3 (-3.8 to -0.9)	-1.5 (-2.7 to -0.4)	48.0 (46.5 to 49.4)	46.8 (45.0 to 48.3)	46.1 (44.2 to 47.8)	-2.5 (-3.8 to -1.1)	-1.5 (-2.8 to -0.4)
Diet high in red meat	8.1 (3.3 to 12.8)	21.1 (16.2 to 24.8)	24.7 (18.6 to 30.1)	159.1 (74.0 to 447.7)	17.3 (-3.8 to 43.5)	1.2 (0.0 to 3.6)	5.4 (1.8 to 9.4)	7.7 (3.1 to 12.4)	338.2 (84.7 to 18825.7)	43.0 (-9.2 to 181.0)
Diet high in processed meat	0.8 (0.0 to 2.6)	1.5 (0.1 to 3.9)	1.9 (0.2 to 4.3)	88.9 (34.3 to 1669.2)	22.8 (1.2 to 177.2)	0.5 (0.0 to 1.9)	0.9 (0.0 to 2.8)	1.1 (0.0 to 3.2)	92.2 (38.2 to 2853.8)	23.4 (1.9 to 291.5)
Diet high in sugar-sweetened beverages	4.5 (2.3 to 7.8)	7.5 (3.9 to 9.4)	9.6 (4.7 to 13.1)	66.1 (-0.6 to 177.0)	27.8 (-3.3 to 73.5)	3.2 (1.6 to 5.3)	5.2 (2.7 to 6.9)	6.6 (3.2 to 9.2)	61.9 (-1.1 to 165.3)	27.1 (-5.7 to 71.5)
Diet low in fibre	35.2 (30.3 to 38.8)	34.1 (29.3 to 38.0)	33.6 (28.5 to 37.8)	-3.0 (-8.4 to 2.5)	-1.6 (-7.0 to 3.2)	39.7 (35.9 to 42.6)	38.7 (34.9 to 41.9)	38.2 (34.0 to 41.6)	-2.4 (-7.0 to 2.4)	-1.3 (-5.4 to 2.5)
Diet low in calcium	33.3 (28.8 to 36.9)	27.0 (21.3 to 31.5)	24.6 (18.5 to 29.6)	-19.0 (-27.0 to -13.8)	-9.0 (-14.9 to -4.9)	36.0 (32.1 to 39.1)	30.3 (25.3 to 34.2)	28.0 (22.4 to 32.4)	-15.9 (-21.9 to -11.6)	-7.5 (-12.8 to -4.3)
Diet low in seafood omega-3 fatty acids	40.4 (37.6 to 43.0)	31.0 (26.6 to 35.0)	26.6 (22.0 to 31.0)	-23.3 (-29.2 to -18.5)	-14.0 (-18.7 to -10.2)	42.4 (40.0 to 44.7)	34.3 (30.4 to 37.8)	30.5 (26.2 to 34.4)	-19.2 (-23.9 to -15.3)	-11.2 (-15.3 to -8.1)
Diet low in polyunsaturated fatty acids	38.0 (34.6 to 41.1)	29.9 (25.3 to 33.7)	27.7 (22.7 to 32.0)	-21.4 (-28.8 to -15.5)	-7.4 (-12.8 to -3.7)	38.5 (35.3 to 41.4)	30.6 (26.0 to 34.4)	28.4 (23.6 to 32.5)	-20.6 (-27.8 to -14.5)	-6.9 (-12.2 to -2.4)
Diet high in trans fatty acids	0.9 (0.0 to 2.9)	0.8 (0.0 to 2.7)	0.7 (0.0 to 2.6)	-10.7 (-37.7 to -5.6)	-4.4 (-23.1 to -0.5)	1.2 (0.0 to 3.5)	1.1 (0.0 to 3.3)	1.0 (0.0 to 3.2)	-11.1 (-33.7 to -5.8)	-4.1 (-18.1 to 0.3)
Diet high in sodium	21.9 (12.5 to 29.1)	21.3 (12.1 to 28.6)	20.9 (11.7 to 28.6)	-2.7 (-23.2 to 22.5)	-1.8 (-15.4 to 13.4)	24.2 (15.9 to 31.3)	20.7 (12.4 to 27.5)	19.0 (10.7 to 25.7)	-14.5 (-31.9 to -0.1)	-8.2 (-22.3 to 2.5)
Intimate partner violence	-	-	-	-	-	2.5 (2.1 to 3.0)	1.9 (1.6 to 2.3)	1.8 (1.5 to 2.1)	-23.7 (-27.0 to -19.9)	-6.9 (-10.9 to -2.3)
Childhood sexual abuse	5.0 (4.1 to 6.2)	5.0 (4.1 to 6.2)	5.0 (4.1 to 6.2)	0.0 (0.0 to 0.0)	0.0 (0.0 to 0.0)	7.1 (5.7 to 8.6)	7.1 (5.7 to 8.6)	7.1 (5.7 to 8.6)	0.0 (0.0 to 0.0)	0.0 (0.0 to 0.0)

Supplementary Results Table 1. Singapore age-standardised Summary Exposure Values (SEVs) for Level 4 risk factors and percentage change, by sex, 1990, 2007, and 2017.Data in parentheses are 95% uncertainty intervals.

	Male					Female				
	**1990**	**2007**	**2017**	**Percentage change 1990-2007**	**Percentage change 2007-2017**	**1990**	**2007**	**2017**	**Percentage change 1990-2007**	**Percentage change 2007-2017**
Bullying victimization	5.0 (2.1 to 10.7)	5.0 (2.2 to 11.0)	5.1 (2.2 to 11.0)	0.7 (-2.1 to 3.8)	0.7 (-1.7 to 3.0)	3.2 (1.4 to 6.9)	3.3 (1.4 to 6.9)	3.2 (1.4 to 6.8)	1.1 (-1.2 to 3.5)	-2.1 (-4.7 to 0.6)
Low physical activity	0.5 (0.2 to 0.9)	0.5 (0.2 to 0.9)	0.4 (0.2 to 0.8)	-1.8 (-4.0 to 0.1)	-7.2 (-10.2 to -3.6)	0.5 (0.2 to 0.9)	0.5 (0.2 to 0.9)	0.5 (0.2 to 1.0)	-1.1 (-2.8 to 0.6)	4.2 (1.7 to 6.5)
High fasting plasma glucose	3.2 (2.1 to 4.7)	3.4 (2.2 to 4.8)	2.1 (1.3 to 3.2)	4.9 (-26.6 to 58.3)	-36.3 (-61.5 to -8.9)	3.0 (2.0 to 4.4)	2.7 (1.8 to 4.0)	1.6 (1.0 to 2.4)	-11.2 (-41.1 to 34.0)	-41.0 (-63.6 to -10.6)
High LDL cholesterol	16.7 (15.0 to 18.5)	14.8 (13.2 to 16.5)	14.3 (12.6 to 16.1)	-11.1 (-15.2 to -7.0)	-3.5 (-6.8 to -0.2)	16.1 (14.4 to 17.8)	14.3 (12.8 to 16.1)	13.6 (12.1 to 15.4)	-10.9 (-14.5 to -7.2)	-5.0 (-8.0 to -2.0)
High systolic blood pressure	6.5 (5.7 to 7.2)	5.4 (4.7 to 6.1)	5.1 (4.5 to 5.9)	-17.2 (-23.4 to -10.1)	-4.1 (-10.4 to 2.9)	6.0 (5.3 to 6.6)	3.9 (3.4 to 4.5)	3.6 (3.1 to 4.2)	-33.9 (-40.7 to -26.9)	-7.9 (-16.2 to 0.9)
High body-mass index	4.9 (3.0 to 7.8)	10.2 (7.2 to 15.2)	12.0 (8.7 to 16.9)	107.5 (68.3 to 163.7)	17.1 (5.8 to 31.3)	5.0 (3.0 to 7.9)	9.0 (6.2 to 12.8)	10.6 (7.6 to 14.6)	80.2 (45.2 to 139.7)	16.9 (3.0 to 33.6)
Low bone mineral density	5.7 (5.1 to 6.3)	5.5 (4.9 to 6.1)	5.5 (4.5 to 6.5)	-3.6 (-11.6 to 5.9)	-0.1 (-13.9 to 14.9)	7.5 (6.3 to 8.9)	7.0 (6.2 to 7.8)	7.1 (6.0 to 8.4)	-7.3 (-19.7 to 7.0)	2.8 (-11.9 to 19.2)
Impaired kidney function	2.9 (1.4 to 6.9)	2.8 (1.3 to 6.8)	2.7 (1.2 to 6.6)	-4.4 (-10.3 to 1.4)	-4.7 (-11.5 to 0.2)	3.8 (1.7 to 8.3)	3.7 (1.6 to 8.1)	3.5 (1.6 to 8.0)	-3.8 (-9.1 to 0.4)	-3.0 (-7.9 to 1.3)

Supplementary Results Table 1. High-income North America age-standardised Summary Exposure Values (SEVs) for Level 4 risk factors and percentage change, by sex, 1990, 2007, and 2017.Data in parentheses are 95% uncertainty intervals.

	Male					Female				
	1990	2007	2017	Percentage change 1990-2007	Percentage change 2007-2017	1990	2007	2017	Percentage change 1990-2007	Percentage change 2007-2017
Unsafe water source	1.5 (1.0 to 2.1)	0.5 (0.4 to 0.7)	0.3 (0.2 to 0.4)	-65.9 (-77.6 to -47.5)	-36.3 (-52.4 to -12.3)	1.5 (1.0 to 2.1)	0.5 (0.4 to 0.7)	0.3 (0.2 to 0.4)	-65.9 (-77.6 to -47.4)	-36.3 (-52.4 to -12.3)
Unsafe sanitation	6.9 (4.9 to 9.4)	4.2 (2.5 to 6.8)	2.9 (1.7 to 4.8)	-38.7 (-63.7 to 0.1)	-31.2 (-55.8 to 6.0)	6.9 (4.9 to 9.4)	4.2 (2.5 to 6.8)	2.9 (1.7 to 4.8)	-38.8 (-63.6 to 0.0)	-31.1 (-55.8 to 6.1)
No access to handwashing facility	3.4 (2.8 to 4.2)	3.1 (2.6 to 3.9)	3.0 (2.5 to 3.8)	-8.7 (-26.5 to 13.7)	-3.5 (-18.6 to 13.8)	3.4 (2.8 to 4.2)	3.1 (2.6 to 3.9)	3.0 (2.5 to 3.8)	-8.7 (-26.5 to 13.7)	-3.5 (-18.5 to 13.8)
Ambient particulate matter pollution	24.3 (17.1 to 35.0)	24.3 (17.5 to 36.6)	18.4 (12.0 to 28.0)	-0.0 (-8.5 to 11.9)	-24.6 (-38.7 to -14.5)	24.3 (17.1 to 34.9)	24.3 (17.5 to 36.5)	18.3 (12.0 to 27.9)	0.1 (-8.3 to 12.0)	-24.6 (-38.7 to -14.6)
Household air pollution from solid fuels	0.3 (0.1 to 0.8)	0.2 (0.1 to 0.5)	0.2 (0.0 to 0.5)	-36.0 (-65.0 to -1.0)	-14.9 (-51.5 to 16.3)	0.5 (0.1 to 2.6)	0.3 (0.1 to 1.8)	0.3 (0.1 to 1.3)	-32.5 (-64.9 to -1.1)	-14.0 (-50.9 to 17.1)
Ambient ozone pollution	45.2 (17.7 to 54.5)	45.4 (17.8 to 54.5)	46.4 (18.8 to 54.6)	0.3 (-0.1 to 0.6)	2.4 (0.1 to 6.3)	45.3 (17.8 to 54.5)	45.4 (17.8 to 54.5)	46.5 (18.8 to 54.6)	0.2 (-0.1 to 0.5)	2.4 (0.1 to 6.4)
Residential radon	13.3 (6.4 to 23.4)	13.1 (6.2 to 23.2)	13.0 (6.2 to 22.9)	-1.4 (-4.2 to 1.8)	-0.8 (-2.2 to 0.7)	13.4 (6.4 to 23.5)	13.1 (6.2 to 23.2)	13.0 (6.2 to 22.9)	-1.6 (-4.7 to 1.6)	-0.9 (-2.5 to 0.8)
Lead exposure	8.7 (4.4 to 12.8)	5.6 (2.4 to 9.3)	3.8 (1.4 to 6.8)	-35.8 (-46.8 to -26.8)	-32.9 (-41.2 to -26.5)	5.5 (1.9 to 9.5)	3.6 (1.0 to 7.1)	2.4 (0.6 to 5.2)	-34.4 (-49.0 to -24.3)	-32.2 (-42.4 to -25.7)
Occupational exposure to asbestos	6.4 (5.6 to 6.9)	5.2 (5.1 to 5.3)	4.6 (4.4 to 4.8)	-17.8 (-23.8 to -6.5)	-12.1 (-16.4 to -7.9)	0.9 (0.8 to 1.0)	0.9 (0.8 to 0.9)	0.9 (0.8 to 0.9)	1.3 (-7.3 to 8.5)	-0.2 (-6.8 to 5.6)
Occupational exposure to arsenic	0.4 (0.0 to 0.9)	0.4 (0.0 to 0.9)	0.4 (0.0 to 0.9)	-	-	0.3 (0.0 to 0.8)	0.3 (0.0 to 0.8)	0.3 (0.0 to 0.8)	-	-
Occupational exposure to benzene	0.6 (0.0 to 1.7)	0.6 (0.0 to 1.8)	0.6 (0.0 to 1.7)	1.4 (-2.5 to 6.2)	-2.5 (-5.9 to 2.2)	0.8 (0.1 to 2.4)	0.9 (0.1 to 2.7)	1.0 (0.1 to 2.8)	13.1 (8.5 to 20.1)	0.6 (-3.3 to 6.1)
Occupational exposure to beryllium	0.0 (0.0 to 0.0)	0.0 (0.0 to 0.0)	0.0 (0.0 to 0.0)	0.6 (-0.9 to 2.0)	0.6 (-1.3 to 2.6)	0.0 (0.0 to 0.0)	0.0 (0.0 to 0.0)	0.0 (0.0 to 0.0)	6.7 (4.7 to 8.7)	-0.2 (-2.5 to 2.2)
Occupational exposure to cadmium	0.0 (0.0 to 0.0)	0.0 (0.0 to 0.0)	0.0 (0.0 to 0.0)	-0.0 (-6.0 to 6.2)	0.7 (-5.1 to 6.4)	0.0 (0.0 to 0.0)	0.0 (0.0 to 0.0)	0.0 (0.0 to 0.0)	3.5 (-3.7 to 11.6)	-1.7 (-8.0 to 5.6)
Occupational exposure to chromium	0.1 (0.1 to 0.1)	0.1 (0.1 to 0.1)	0.1 (0.1 to 0.1)	2.2 (-2.8 to 7.4)	0.6 (-4.1 to 5.2)	0.1 (0.1 to 0.1)	0.1 (0.1 to 0.1)	0.1 (0.1 to 0.1)	5.8 (0.1 to 12.1)	-1.7 (-6.7 to 4.0)
Occupational exposure to diesel engine exhaust	0.5 (0.5 to 0.5)	0.6 (0.5 to 0.6)	0.6 (0.5 to 0.6)	9.1 (4.8 to 13.5)	1.7 (-2.0 to 5.8)	0.5 (0.4 to 0.5)	0.5 (0.5 to 0.5)	0.5 (0.5 to 0.5)	9.2 (2.9 to 15.9)	-3.5 (-8.5 to 2.2)
Occupational exposure to formaldehyde	0.2 (0.2 to 0.2)	0.2 (0.2 to 0.2)	0.2 (0.2 to 0.2)	-2.0 (-8.0 to 4.4)	-0.9 (-6.8 to 5.0)	0.2 (0.2 to 0.2)	0.2 (0.2 to 0.2)	0.2 (0.2 to 0.2)	4.1 (-2.4 to 11.7)	-1.7 (-7.2 to 4.8)
Occupational exposure to nickel	0.3 (0.0 to 1.4)	0.3 (0.0 to 1.3)	0.3 (0.0 to 1.3)	-	-	0.3 (0.0 to 1.2)	0.3 (0.0 to 1.2)	0.3 (0.0 to 1.2)	-	-
Occupational exposure to polycyclic aromatic hydrocarbons	0.2 (0.2 to 0.2)	0.2 (0.2 to 0.2)	0.2 (0.2 to 0.2)	2.2 (-2.4 to 7.1)	0.0 (-4.4 to 4.4)	0.2 (0.2 to 0.2)	0.2 (0.2 to 0.2)	0.2 (0.2 to 0.2)	8.6 (3.6 to 14.1)	-1.4 (-5.7 to 3.2)
Occupational exposure to silica	2.9 (0.4 to 9.4)	2.7 (0.4 to 8.4)	2.7 (0.4 to 8.4)	-8.7 (-14.7 to -1.7)	0.6 (-5.8 to 7.5)	2.2 (0.3 to 7.0)	2.1 (0.3 to 6.6)	2.1 (0.3 to 6.5)	-4.6 (-13.3 to 7.0)	-2.6 (-11.1 to 8.2)
Occupational exposure to sulphuric acid	0.6 (0.1 to 1.8)	0.6 (0.1 to 2.0)	0.6 (0.1 to 2.0)	9.6 (2.6 to 16.8)	-1.4 (-6.9 to 4.8)	0.6 (0.1 to 1.7)	0.7 (0.1 to 2.1)	0.6 (0.1 to 2.0)	20.0 (9.7 to 31.0)	-4.5 (-12.1 to 3.5)
Occupational exposure to trichloroethylene	0.1 (0.1 to 0.1)	0.1 (0.1 to 0.1)	0.1 (0.1 to 0.1)	5.3 (2.1 to 8.8)	1.2 (-1.9 to 4.1)	0.1 (0.1 to 0.1)	0.1 (0.1 to 0.1)	0.1 (0.1 to 0.1)	7.1 (2.5 to 12.1)	-2.1 (-6.1 to 2.7)
Occupational asthmagens	16.3 (14.1 to 19.1)	15.1 (13.0 to 17.7)	14.7 (12.6 to 17.3)	-7.3 (-12.9 to -1.8)	-2.4 (-7.3 to 2.6)	7.8 (6.6 to 9.3)	8.0 (6.8 to 9.4)	7.6 (6.4 to 9.0)	2.3 (-4.2 to 9.6)	-4.9 (-11.2 to 1.8)
Occupational particulate matter, gases, and fumes	6.7 (4.9 to 9.6)	6.4 (4.6 to 9.1)	6.1 (4.4 to 8.7)	-5.5 (-7.9 to -3.2)	-4.5 (-6.6 to -2.5)	3.7 (2.7 to 5.3)	3.6 (2.6 to 5.2)	3.4 (2.5 to 4.9)	-2.7 (-6.5 to 1.4)	-4.3 (-7.4 to -1.3)
Occupational noise	6.1 (5.7 to 6.5)	5.9 (5.6 to 6.3)	5.7 (5.4 to 6.1)	-3.1 (-4.3 to -1.9)	-3.7 (-4.5 to -2.8)	4.2 (4.0 to 4.5)	4.4 (4.2 to 4.7)	4.4 (4.2 to 4.7)	4.6 (3.1 to 6.4)	-0.1 (-1.0 to 0.8)
Occupational ergonomic factors	8.7 (7.4 to 10.4)	7.9 (6.7 to 9.4)	7.5 (6.4 to 9.0)	-9.1 (-13.7 to -4.7)	-4.6 (-8.0 to -0.9)	4.9 (4.1 to 5.9)	5.0 (4.2 to 6.1)	4.9 (4.1 to 6.0)	2.7 (-3.1 to 8.6)	-1.5 (-7.4 to 4.6)
Non-exclusive breastfeeding	0.5 (0.5 to 0.6)	0.5 (0.5 to 0.6)	0.5 (0.4 to 0.6)	-0.7 (-7.4 to 6.6)	-6.3 (-12.1 to -0.8)	0.5 (0.5 to 0.6)	0.5 (0.5 to 0.6)	0.5 (0.4 to 0.6)	-0.7 (-7.4 to 6.6)	-6.3 (-12.1 to -0.8)
Discontinued breastfeeding	2.2 (2.1 to 2.3)	2.1 (2.0 to 2.2)	2.2 (2.1 to 2.2)	-2.6 (-7.1 to 2.3)	2.1 (-2.0 to 6.4)	2.2 (2.1 to 2.3)	2.1 (2.0 to 2.2)	2.2 (2.1 to 2.2)	-2.6 (-7.1 to 2.3)	2.1 (-2.0 to 6.4)
Child underweight	0.1 (0.1 to 0.1)	0.1 (0.1 to 0.1)	0.1 (0.0 to 0.1)	-5.1 (-14.2 to 3.9)	-13.4 (-18.9 to -7.6)	0.1 (0.0 to 0.1)	0.1 (0.0 to 0.1)	0.1 (0.0 to 0.1)	-3.1 (-12.7 to 7.5)	-4.8 (-10.8 to 1.7)
Child wasting	0.1 (0.0 to 0.1)	0.1 (0.0 to 0.1)	0.1 (0.0 to 0.1)	-10.3 (-21.3 to 2.6)	-3.2 (-11.2 to 5.0)	0.1 (0.0 to 0.1)	0.1 (0.0 to 0.1)	0.1 (0.0 to 0.1)	5.2 (-8.7 to 21.5)	-5.1 (-13.6 to 4.2)
Child stunting	0.3 (0.1 to 0.4)	0.2 (0.1 to 0.4)	0.2 (0.1 to 0.3)	-9.5 (-37.1 to 27.2)	-11.5 (-29.2 to 8.6)	0.2 (0.1 to 0.3)	0.2 (0.1 to 0.3)	0.2 (0.1 to 0.3)	-10.7 (-33.1 to 19.3)	-3.9 (-20.0 to 17.2)
Short gestation for birth weight	0.0 (0.0 to 0.0)	0.0 (0.0 to 0.0)	0.0 (0.0 to 0.0)	6.3 (-2.4 to 17.8)	2.0 (-5.7 to 8.8)	0.0 (0.0 to 0.0)	0.0 (0.0 to 0.0)	0.0 (0.0 to 0.0)	6.5 (-3.9 to 17.6)	2.5 (-6.3 to 11.2)
Low birth weight for gestation	0.0 (0.0 to 0.0)	0.0 (0.0 to 0.0)	0.0 (0.0 to 0.0)	2.5 (-7.7 to 14.2)	-1.0 (-8.2 to 5.9)	0.0 (0.0 to 0.0)	0.0 (0.0 to 0.0)	0.0 (0.0 to 0.0)	0.9 (-8.0 to 11.7)	-1.3 (-9.2 to 6.9)
Iron deficiency	-	-	-	-	-	4.3 (3.0 to 5.9)	3.9 (2.7 to 5.4)	3.9 (2.7 to 5.5)	-8.0 (-21.0 to 8.0)	0.7 (-13.5 to 16.3)
Vitamin A deficiency	0.6 (0.4 to 0.7)	0.6 (0.4 to 0.7)	0.6 (0.4 to 0.8)	-0.2 (-5.9 to 5.4)	5.8 (-1.0 to 11.8)	0.7 (0.5 to 0.8)	0.7 (0.5 to 0.8)	0.7 (0.5 to 0.9)	-1.2 (-5.8 to 3.7)	4.4 (-1.3 to 8.3)
Zinc deficiency	0.5 (0.1 to 0.9)	0.3 (0.1 to 0.5)	0.3 (0.1 to 0.5)	-47.1 (-66.8 to 10.1)	0.5 (-38.6 to 74.3)	0.5 (0.1 to 0.9)	0.3 (0.1 to 0.5)	0.3 (0.1 to 0.5)	-46.8 (-65.9 to 7.8)	1.8 (-39.9 to 67.0)
Smoking	13.9 (12.4 to 15.6)	7.8 (6.8 to 8.8)	6.2 (5.3 to 7.2)	-44.2 (-49.0 to -39.9)	-20.8 (-25.9 to -15.4)	10.1 (8.9 to 11.6)	5.7 (5.0 to 6.6)	4.4 (3.7 to 5.1)	-43.4 (-49.4 to -37.2)	-24.0 (-28.7 to -19.2)
Chewing tobacco	1.9 (1.8 to 2.0)	1.9 (1.6 to 2.3)	1.9 (1.5 to 2.5)	-0.0 (-16.5 to 21.6)	2.0 (-16.6 to 22.5)	0.1 (0.1 to 0.1)	0.1 (0.1 to 0.1)	0.1 (0.1 to 0.1)	-7.5 (-31.2 to 24.1)	-4.7 (-23.3 to 19.5)
Second-hand smoke	43.7 (40.9 to 46.8)	26.5 (25.4 to 27.7)	26.8 (25.1 to 28.8)	-39.2 (-45.0 to -33.4)	1.0 (-5.8 to 7.8)	41.4 (38.9 to 44.0)	25.5 (24.6 to 26.3)	25.2 (24.1 to 26.3)	-38.5 (-44.0 to -32.9)	-0.8 (-6.2 to 4.6)
Alcohol use	20.8 (14.0 to 29.2)	22.5 (15.3 to 31.2)	24.3 (15.6 to 34.9)	8.2 (-4.3 to 23.3)	8.0 (-12.3 to 32.1)	12.7 (7.9 to 19.7)	13.4 (8.4 to 20.2)	14.0 (8.3 to 21.7)	5.2 (-5.3 to 18.6)	4.9 (-13.9 to 29.3)
Drug use	1.7 (1.2 to 2.5)	2.1 (1.6 to 2.9)	2.7 (2.1 to 3.5)	26.2 (15.0 to 41.9)	27.1 (20.3 to 35.3)	1.3 (1.0 to 1.8)	1.7 (1.3 to 2.2)	2.0 (1.6 to 2.6)	31.1 (21.4 to 43.9)	18.5 (12.3 to 25.4)
Diet low in fruits	26.2 (20.2 to 31.0)	26.7 (20.9 to 31.5)	28.3 (22.1 to 33.0)	2.0 (-2.4 to 7.3)	5.8 (-0.1 to 12.5)	22.9 (16.8 to 28.2)	24.1 (18.1 to 29.2)	25.1 (19.2 to 30.5)	4.9 (-0.4 to 12.7)	4.4 (-2.6 to 12.9)
Diet low in vegetables	22.6 (16.7 to 27.8)	24.3 (19.2 to 28.9)	24.2 (19.3 to 28.6)	7.6 (2.9 to 16.1)	-0.7 (-5.6 to 5.6)	25.5 (20.0 to 30.3)	25.9 (20.7 to 30.4)	25.9 (20.8 to 30.3)	1.4 (-1.4 to 5.3)	0.0 (-4.6 to 5.6)
Diet low in legumes	23.2 (17.9 to 27.7)	22.0 (17.2 to 26.5)	23.1 (18.2 to 27.5)	-4.9 (-10.5 to 1.3)	4.7 (-2.6 to 15.0)	28.0 (23.3 to 31.8)	27.3 (22.8 to 31.2)	28.1 (23.8 to 32.0)	-2.6 (-7.4 to 2.4)	3.2 (-2.0 to 9.4)
Diet low in whole grains	38.1 (35.3 to 40.5)	39.2 (36.6 to 41.4)	38.8 (36.0 to 41.1)	2.9 (0.7 to 5.4)	-1.1 (-3.4 to 0.8)	39.0 (36.4 to 41.3)	40.1 (37.7 to 42.2)	39.8 (37.3 to 41.9)	2.7 (0.7 to 5.3)	-0.9 (-2.8 to 1.1)
Diet low in nuts and seeds	45.7 (43.5 to 47.4)	37.2 (33.9 to 40.1)	37.4 (33.5 to 40.7)	-18.5 (-23.3 to -14.5)	0.4 (-4.3 to 4.7)	46.4 (44.5 to 48.1)	38.3 (35.0 to 41.0)	38.3 (34.6 to 41.6)	-17.6 (-22.4 to -13.9)	0.2 (-4.1 to 4.3)
Diet low in milk	27.8 (22.4 to 32.4)	26.5 (20.9 to 31.2)	26.9 (21.0 to 31.7)	-4.7 (-9.7 to -0.8)	1.4 (-3.0 to 6.4)	29.5 (24.3 to 33.7)	27.5 (22.0 to 32.0)	27.7 (22.2 to 32.2)	-6.6 (-11.8 to -2.9)	0.6 (-3.9 to 5.2)
Diet high in red meat	43.8 (41.7 to 45.8)	38.6 (36.1 to 40.8)	36.8 (33.4 to 40.1)	-11.9 (-16.5 to -7.0)	-4.5 (-12.0 to 2.9)	25.0 (21.7 to 28.2)	20.1 (16.8 to 23.1)	18.9 (14.1 to 22.7)	-19.4 (-27.7 to -10.6)	-6.2 (-21.8 to 9.9)
Diet high in processed meat	40.3 (35.8 to 43.7)	41.5 (38.9 to 43.8)	35.8 (31.1 to 39.3)	3.2 (-5.2 to 12.8)	-13.7 (-20.4 to -7.2)	22.5 (18.4 to 26.0)	26.6 (23.5 to 29.6)	24.0 (20.0 to 27.5)	18.2 (4.3 to 35.8)	-9.8 (-18.6 to -1.4)
Diet high in sugar-sweetened beverages	31.0 (15.8 to 36.5)	29.7 (15.3 to 32.5)	25.3 (13.1 to 29.6)	-4.3 (-16.8 to 14.4)	-14.6 (-25.5 to -3.5)	25.6 (12.8 to 31.7)	22.8 (11.7 to 25.3)	19.1 (9.6 to 22.7)	-11.0 (-25.9 to 11.3)	-16.0 (-28.8 to -1.4)
Diet low in fibre	33.0 (28.3 to 36.7)	25.4 (19.9 to 30.0)	26.7 (20.9 to 31.4)	-23.0 (-31.1 to -17.2)	5.1 (-0.6 to 10.9)	37.7 (33.8 to 40.7)	30.1 (25.0 to 34.2)	31.1 (26.0 to 35.2)	-20.2 (-26.9 to -15.1)	3.4 (-0.9 to 8.4)
Diet low in calcium	17.1 (10.5 to 22.9)	16.0 (9.8 to 21.6)	16.6 (10.4 to 22.3)	-6.6 (-13.1 to -0.9)	3.7 (-2.2 to 11.3)	21.1 (14.6 to 26.4)	19.8 (13.2 to 25.2)	20.4 (13.8 to 25.8)	-6.4 (-13.6 to -1.5)	3.1 (-1.2 to 9.3)
Diet low in seafood omega-3 fatty acids	27.8 (23.4 to 32.0)	23.6 (19.1 to 28.0)	24.8 (20.3 to 29.3)	-15.0 (-19.2 to -11.8)	5.1 (2.0 to 9.0)	31.4 (27.2 to 35.2)	27.8 (23.3 to 32.0)	28.6 (24.3 to 32.8)	-11.4 (-14.8 to -8.8)	3.0 (0.7 to 6.0)
Diet low in polyunsaturated fatty acids	11.2 (6.8 to 16.1)	8.6 (5.0 to 13.0)	7.4 (4.5 to 11.4)	-23.5 (-36.0 to -13.1)	-14.0 (-24.9 to -1.0)	11.9 (7.2 to 16.8)	9.1 (5.3 to 13.8)	7.8 (4.7 to 11.9)	-22.9 (-35.6 to -13.2)	-14.9 (-24.3 to -2.8)
Diet high in trans fatty acids	17.2 (13.0 to 22.5)	10.2 (6.4 to 14.4)	7.5 (3.9 to 11.4)	-40.7 (-51.2 to -33.2)	-26.6 (-38.9 to -19.5)	24.1 (19.0 to 32.1)	13.6 (9.6 to 18.1)	10.1 (6.2 to 14.1)	-43.4 (-51.0 to -35.7)	-26.1 (-35.3 to -20.1)
Diet high in sodium	7.2 (1.1 to 14.2)	12.0 (4.4 to 18.7)	12.5 (4.7 to 19.4)	66.8 (27.0 to 391.0)	4.1 (-10.2 to 29.5)	6.0 (0.5 to 12.8)	10.2 (2.8 to 17.2)	10.5 (3.0 to 17.5)	69.7 (30.2 to 553.7)	3.3 (-11.7 to 31.7)
Intimate partner violence	-	-	-	-	-	1.2 (1.0 to 1.5)	0.9 (0.8 to 1.1)	1.1 (0.9 to 1.3)	-24.0 (-26.0 to -21.7)	15.3 (12.2 to 18.0)
Childhood sexual abuse	5.1 (4.2 to 6.3)	4.8 (4.0 to 5.8)	4.8 (4.0 to 5.8)	-6.1 (-10.5 to -0.7)	0.4 (-3.9 to 4.7)	12.9 (10.4 to 15.8)	11.8 (9.9 to 14.1)	11.9 (9.9 to 14.1)	-8.4 (-13.5 to -3.1)	0.5 (-3.4 to 5.2)

Supplementary Results Table 1. High-income North America age-standardised Summary Exposure Values (SEVs) for Level 4 risk factors and percentage change, by sex, 1990, 2007, and 2017.Data in parentheses are 95% uncertainty intervals.										
	**Male**					**Female**				
	**1990**	**2007**	**2017**	**Percentage change 1990-2007**	**Percentage change 2007-2017**	**1990**	**2007**	**2017**	**Percentage change 1990-2007**	**Percentage change 2007-2017**
Bullying victimization	6.4 (2.8 to 13.2)	8.8 (3.9 to 17.6)	8.8 (3.9 to 17.6)	36.2 (24.4 to 51.6)	0.2 (-4.5 to 5.3)	5.1 (2.2 to 10.1)	7.4 (3.3 to 14.8)	7.5 (3.3 to 15.1)	45.3 (32.4 to 65.0)	1.5 (-3.8 to 7.9)
Low physical activity	0.4 (0.2 to 0.8)	0.4 (0.2 to 0.8)	0.4 (0.2 to 0.8)	0.2 (-1.1 to 1.4)	0.1 (-0.4 to 0.6)	0.4 (0.2 to 0.9)	0.4 (0.2 to 0.9)	0.4 (0.2 to 0.9)	-0.0 (-1.5 to 1.4)	-0.1 (-0.5 to 0.4)
High fasting plasma glucose	1.5 (1.1 to 1.9)	2.6 (1.9 to 3.5)	3.2 (2.4 to 4.3)	76.4 (55.4 to 109.3)	24.1 (6.2 to 47.8)	1.6 (1.2 to 2.1)	2.7 (2.0 to 3.7)	2.6 (1.9 to 3.4)	70.3 (49.6 to 103.8)	-5.3 (-20.1 to 14.7)
High LDL cholesterol	20.4 (18.7 to 22.4)	11.7 (10.1 to 13.4)	10.4 (8.9 to 12.1)	-42.9 (-46.9 to -39.0)	-10.7 (-13.6 to -7.9)	19.2 (17.6 to 21.1)	11.6 (10.1 to 13.2)	10.8 (9.4 to 12.5)	-39.7 (-43.4 to -36.0)	-6.7 (-9.3 to -4.0)
High systolic blood pressure	6.5 (5.7 to 7.2)	5.0 (4.3 to 5.6)	5.1 (4.5 to 5.8)	-23.2 (-28.2 to -17.9)	2.7 (-3.1 to 8.1)	6.1 (5.5 to 6.7)	4.5 (4.0 to 5.0)	4.4 (3.9 to 5.0)	-25.9 (-31.1 to -20.4)	-2.4 (-8.8 to 4.0)
High body-mass index	14.3 (10.7 to 19.3)	20.9 (16.7 to 26.5)	22.0 (17.6 to 27.3)	46.0 (33.6 to 62.3)	5.3 (-0.3 to 11.7)	15.7 (12.3 to 20.1)	21.9 (18.0 to 27.1)	23.1 (18.7 to 28.2)	39.3 (30.4 to 51.0)	5.3 (-0.2 to 11.7)
Low bone mineral density	5.0 (4.3 to 5.8)	4.6 (3.9 to 5.3)	4.8 (4.0 to 5.6)	-9.5 (-12.3 to -6.5)	4.9 (1.2 to 8.3)	5.8 (5.0 to 6.6)	5.6 (4.9 to 6.4)	6.0 (5.2 to 6.9)	-2.6 (-5.3 to 0.1)	7.1 (3.9 to 10.1)
Impaired kidney function	2.2 (1.1 to 5.3)	2.2 (1.0 to 5.4)	2.2 (1.1 to 5.5)	-1.0 (-4.2 to 2.1)	2.2 (-2.1 to 6.9)	2.9 (1.4 to 6.7)	2.8 (1.3 to 6.6)	2.9 (1.4 to 6.8)	-2.3 (-5.7 to 0.6)	3.7 (0.3 to 7.7)

Supplementary Results Table 1. Canada age-standardised Summary Exposure Values (SEVs) for Level 4 risk factors and percentage change, by sex, 1990, 2007, and 2017.Data in parentheses are 95% uncertainty intervals.

	Male					Female				
	**1990**	**2007**	**2017**	**Percentage change 1990-2007**	**Percentage change 2007-2017**	**1990**	**2007**	**2017**	**Percentage change 1990-2007**	**Percentage change 2007-2017**
Unsafe water source	1.3 (0.9 to 1.9)	0.4 (0.3 to 0.6)	0.3 (0.2 to 0.4)	-68.1 (-80.0 to -47.9)	-22.6 (-44.3 to 6.6)	1.3 (0.9 to 1.9)	0.4 (0.3 to 0.6)	0.3 (0.2 to 0.4)	-68.1 (-80.0 to -47.9)	-22.6 (-44.3 to 6.6)
Unsafe sanitation	5.5 (3.3 to 8.5)	2.4 (1.4 to 3.8)	1.9 (1.1 to 3.2)	-55.7 (-76.0 to -19.9)	-21.4 (-51.8 to 29.5)	5.5 (3.3 to 8.5)	2.4 (1.4 to 3.8)	1.9 (1.1 to 3.2)	-55.7 (-76.0 to -19.9)	-21.4 (-51.8 to 29.5)
No access to handwashing facility	3.3 (2.6 to 4.1)	3.0 (2.4 to 3.8)	3.0 (2.4 to 3.7)	-7.6 (-28.4 to 21.7)	-1.1 (-16.9 to 17.6)	3.3 (2.6 to 4.1)	3.0 (2.4 to 3.8)	3.0 (2.4 to 3.7)	-7.6 (-28.4 to 21.7)	-1.1 (-16.9 to 17.6)
Ambient particulate matter pollution	21.0 (8.1 to 34.3)	22.1 (11.8 to 34.7)	15.4 (3.6 to 28.8)	5.6 (-38.1 to 142.4)	-30.5 (-82.4 to 35.1)	20.9 (8.0 to 34.3)	22.1 (11.8 to 34.6)	15.4 (3.6 to 28.7)	5.7 (-38.1 to 143.0)	-30.5 (-82.5 to 35.2)
Household air pollution from solid fuels	0.2 (0.1 to 0.6)	0.1 (0.0 to 0.2)	0.1 (0.0 to 0.2)	-46.4 (-81.2 to 5.1)	-13.8 (-78.9 to 68.1)	0.3 (0.1 to 1.5)	0.2 (0.0 to 0.6)	0.2 (0.0 to 0.4)	-41.2 (-81.3 to 6.5)	-17.9 (-79.8 to 67.5)
Ambient ozone pollution	35.3 (11.8 to 54.8)	35.8 (12.0 to 54.8)	37.0 (12.5 to 54.8)	1.5 (0.0 to 2.2)	3.4 (0.0 to 5.0)	35.3 (11.8 to 54.8)	35.8 (12.0 to 54.8)	37.0 (12.5 to 54.8)	1.5 (0.0 to 2.2)	3.4 (0.0 to 5.0)
Residential radon	9.5 (0.5 to 29.8)	9.5 (0.5 to 29.8)	9.5 (0.5 to 29.8)	0.0 (0.0 to 0.0)	0.0 (0.0 to 0.0)	9.5 (0.5 to 29.8)	9.5 (0.5 to 29.8)	9.5 (0.5 to 29.8)	0.0 (0.0 to 0.0)	0.0 (0.0 to 0.0)
Lead exposure	4.1 (0.9 to 8.1)	2.9 (0.5 to 6.5)	2.1 (0.3 to 5.1)	-27.9 (-42.5 to -18.5)	-27.1 (-39.9 to -20.0)	2.6 (0.3 to 6.2)	1.8 (0.2 to 4.9)	1.3 (0.1 to 3.9)	-30.1 (-49.8 to -19.7)	-27.8 (-45.1 to -20.8)
Occupational exposure to asbestos	9.5 (7.9 to 10.6)	7.9 (7.7 to 8.2)	7.0 (6.4 to 7.6)	-16.2 (-26.0 to 1.1)	-11.5 (-19.4 to -3.8)	0.8 (0.7 to 1.0)	1.0 (0.9 to 1.1)	1.0 (0.9 to 1.1)	18.1 (-3.0 to 44.7)	-3.7 (-15.4 to 9.1)
Occupational exposure to arsenic	0.4 (0.0 to 0.8)	0.4 (0.0 to 0.9)	0.4 (0.0 to 0.9)	-	-	0.3 (0.0 to 0.8)	0.3 (0.0 to 0.8)	0.3 (0.0 to 0.8)	-	-
Occupational exposure to benzene	0.5 (0.0 to 1.5)	0.5 (0.0 to 1.5)	0.6 (0.0 to 1.6)	5.3 (0.7 to 11.1)	3.3 (-0.3 to 8.6)	0.7 (0.0 to 2.1)	0.9 (0.1 to 2.6)	1.0 (0.1 to 2.7)	25.2 (18.2 to 36.5)	6.3 (1.3 to 13.4)
Occupational exposure to beryllium	0.0 (0.0 to 0.0)	0.0 (0.0 to 0.0)	0.0 (0.0 to 0.0)	4.7 (2.9 to 6.5)	2.7 (0.8 to 4.8)	0.0 (0.0 to 0.0)	0.0 (0.0 to 0.0)	0.0 (0.0 to 0.0)	16.7 (14.3 to 19.0)	3.0 (1.0 to 5.2)
Occupational exposure to cadmium	0.0 (0.0 to 0.0)	0.0 (0.0 to 0.0)	0.0 (0.0 to 0.0)	4.2 (-3.8 to 12.7)	2.0 (-4.3 to 8.8)	0.0 (0.0 to 0.0)	0.0 (0.0 to 0.0)	0.0 (0.0 to 0.0)	10.9 (0.4 to 22.4)	0.5 (-7.0 to 8.6)
Occupational exposure to chromium	0.1 (0.1 to 0.1)	0.1 (0.1 to 0.1)	0.1 (0.1 to 0.1)	4.4 (-2.2 to 11.3)	2.1 (-3.2 to 7.8)	0.1 (0.1 to 0.1)	0.1 (0.1 to 0.1)	0.1 (0.1 to 0.1)	12.4 (4.0 to 21.8)	1.3 (-5.1 to 7.8)
Occupational exposure to diesel engine exhaust	0.6 (0.5 to 0.6)	0.6 (0.6 to 0.6)	0.6 (0.6 to 0.6)	3.0 (-2.7 to 8.4)	1.9 (-2.1 to 6.4)	0.5 (0.5 to 0.5)	0.6 (0.5 to 0.6)	0.6 (0.5 to 0.6)	10.1 (2.0 to 18.3)	1.2 (-4.8 to 7.9)
Occupational exposure to formaldehyde	0.2 (0.2 to 0.2)	0.2 (0.2 to 0.2)	0.2 (0.2 to 0.2)	1.9 (-6.6 to 10.9)	1.0 (-5.8 to 8.4)	0.2 (0.2 to 0.2)	0.2 (0.2 to 0.2)	0.2 (0.2 to 0.2)	12.7 (2.3 to 23.9)	1.3 (-5.9 to 9.2)
Occupational exposure to nickel	0.3 (0.0 to 1.4)	0.3 (0.0 to 1.4)	0.3 (0.0 to 1.4)	-	-	0.2 (0.0 to 1.1)	0.3 (0.0 to 1.2)	0.3 (0.0 to 1.2)	-	-
Occupational exposure to polycyclic aromatic hydrocarbons	0.2 (0.2 to 0.2)	0.2 (0.2 to 0.2)	0.2 (0.2 to 0.2)	4.4 (-2.1 to 10.8)	2.3 (-2.9 to 7.9)	0.2 (0.2 to 0.2)	0.2 (0.2 to 0.2)	0.2 (0.2 to 0.2)	12.8 (5.7 to 20.3)	1.5 (-3.8 to 7.1)
Occupational exposure to silica	3.0 (0.4 to 9.7)	3.0 (0.4 to 9.4)	3.1 (0.4 to 10.1)	0.4 (-7.9 to 11.0)	3.1 (-4.0 to 11.1)	2.1 (0.3 to 6.5)	2.2 (0.3 to 7.0)	2.2 (0.3 to 6.7)	4.7 (-7.9 to 21.4)	-1.4 (-11.3 to 10.7)
Occupational exposure to sulphuric acid	0.6 (0.1 to 1.9)	0.6 (0.1 to 1.9)	0.6 (0.1 to 1.9)	1.7 (-5.9 to 8.8)	-0.0 (-5.8 to 6.1)	0.7 (0.1 to 2.1)	0.7 (0.1 to 2.1)	0.6 (0.1 to 2.0)	-4.4 (-15.1 to 7.2)	-5.6 (-13.3 to 3.2)
Occupational exposure to trichloroethylene	0.1 (0.1 to 0.1)	0.1 (0.1 to 0.1)	0.1 (0.1 to 0.1)	4.9 (1.1 to 9.0)	2.0 (-1.4 to 5.4)	0.1 (0.1 to 0.1)	0.1 (0.1 to 0.1)	0.1 (0.1 to 0.1)	12.6 (6.6 to 18.9)	1.5 (-3.1 to 6.4)
Occupational asthmagens	17.4 (14.9 to 20.4)	16.7 (14.2 to 20.0)	17.0 (14.2 to 20.5)	-3.7 (-12.3 to 5.9)	1.5 (-6.7 to 10.8)	8.1 (6.9 to 9.5)	9.0 (7.6 to 10.7)	9.3 (7.8 to 11.1)	11.5 (2.6 to 21.4)	3.4 (-4.3 to 13.8)
Occupational particulate matter, gases, and fumes	6.9 (4.9 to 9.8)	6.5 (4.7 to 9.3)	6.3 (4.5 to 9.2)	-5.2 (-7.8 to -2.3)	-2.9 (-4.9 to -1.1)	3.8 (2.7 to 5.6)	3.8 (2.7 to 5.5)	3.6 (2.6 to 5.3)	-1.5 (-6.4 to 4.4)	-3.0 (-6.3 to 0.2)
Occupational noise	6.1 (5.8 to 6.6)	5.8 (5.5 to 6.3)	5.7 (5.4 to 6.1)	-5.3 (-6.9 to -3.6)	-2.5 (-3.6 to -1.5)	4.1 (3.9 to 4.4)	4.3 (4.1 to 4.6)	4.4 (4.2 to 4.7)	4.9 (2.8 to 7.1)	2.3 (1.1 to 3.6)
Occupational ergonomic factors	9.0 (7.9 to 10.3)	8.2 (7.0 to 9.6)	7.7 (6.6 to 9.0)	-8.8 (-12.4 to -5.1)	-5.7 (-10.3 to -1.0)	4.7 (4.0 to 5.6)	5.0 (4.3 to 6.1)	5.0 (4.2 to 6.1)	7.7 (3.0 to 12.4)	-0.9 (-6.5 to 5.1)
Non-exclusive breastfeeding	0.6 (0.5 to 0.7)	0.6 (0.5 to 0.7)	0.6 (0.5 to 0.7)	-2.8 (-10.7 to 5.7)	-0.9 (-8.0 to 7.1)	0.6 (0.5 to 0.7)	0.6 (0.5 to 0.7)	0.6 (0.5 to 0.7)	-2.7 (-10.6 to 5.8)	-0.9 (-8.0 to 7.1)
Discontinued breastfeeding	2.1 (2.0 to 2.2)	2.0 (1.9 to 2.1)	2.1 (2.0 to 2.2)	-1.0 (-7.2 to 5.0)	1.5 (-3.3 to 6.9)	2.1 (2.0 to 2.2)	2.0 (1.9 to 2.1)	2.1 (2.0 to 2.2)	-1.0 (-7.2 to 5.0)	1.4 (-3.4 to 6.8)
Child underweight	0.1 (0.1 to 0.2)	0.1 (0.1 to 0.1)	0.1 (0.1 to 0.1)	-8.0 (-19.0 to 3.5)	-9.4 (-17.3 to -1.4)	0.1 (0.1 to 0.1)	0.1 (0.1 to 0.1)	0.1 (0.0 to 0.1)	-7.1 (-18.6 to 5.7)	-2.8 (-10.6 to 6.1)
Child wasting	0.1 (0.1 to 0.1)	0.1 (0.1 to 0.1)	0.1 (0.1 to 0.1)	-11.2 (-27.5 to 7.5)	-1.3 (-12.6 to 11.7)	0.1 (0.1 to 0.1)	0.1 (0.1 to 0.1)	0.1 (0.1 to 0.1)	-2.0 (-19.1 to 20.1)	-2.2 (-14.5 to 12.4)
Child stunting	0.3 (0.1 to 0.5)	0.2 (0.1 to 0.4)	0.2 (0.1 to 0.4)	-17.0 (-45.7 to 26.0)	-3.3 (-28.3 to 29.8)	0.3 (0.1 to 0.5)	0.2 (0.1 to 0.4)	0.2 (0.1 to 0.4)	-16.6 (-46.2 to 27.3)	-1.2 (-26.6 to 27.4)
Short gestation for birth weight	0.0 (0.0 to 0.0)	0.0 (0.0 to 0.0)	0.0 (0.0 to 0.0)	2.1 (-19.2 to 23.9)	-0.4 (-18.6 to 21.7)	0.0 (0.0 to 0.0)	0.0 (0.0 to 0.0)	0.0 (0.0 to 0.0)	3.8 (-14.8 to 28.7)	0.8 (-22.4 to 25.6)
Low birth weight for gestation	0.0 (0.0 to 0.0)	0.0 (0.0 to 0.0)	0.0 (0.0 to 0.0)	5.7 (-9.2 to 28.3)	2.9 (-14.1 to 20.9)	0.0 (0.0 to 0.0)	0.0 (0.0 to 0.0)	0.0 (0.0 to 0.0)	2.7 (-13.4 to 22.0)	1.1 (-16.6 to 24.1)
Iron deficiency	-	-	-	-	-	4.6 (3.1 to 6.6)	3.9 (2.6 to 5.7)	3.7 (2.4 to 5.4)	-14.1 (-33.5 to 11.0)	-6.2 (-21.9 to 13.5)
Vitamin A deficiency	1.7 (1.4 to 2.1)	1.6 (1.3 to 2.0)	1.4 (1.0 to 1.8)	-5.6 (-18.1 to 6.9)	-16.1 (-29.7 to 1.2)	1.6 (1.3 to 2.1)	1.5 (1.2 to 1.9)	1.4 (1.0 to 1.8)	-6.8 (-20.6 to 12.0)	-8.0 (-23.6 to 9.3)
Zinc deficiency	0.4 (0.1 to 0.9)	0.7 (0.2 to 1.3)	0.6 (0.2 to 1.2)	84.3 (-7.6 to 347.6)	-3.5 (-47.6 to 84.0)	0.4 (0.1 to 0.9)	0.7 (0.2 to 1.3)	0.6 (0.2 to 1.2)	81.8 (-7.4 to 344.6)	-2.5 (-48.1 to 86.6)
Smoking	15.3 (13.8 to 16.9)	9.4 (8.4 to 10.6)	7.7 (6.8 to 8.8)	-38.1 (-43.0 to -32.9)	-18.4 (-24.1 to -12.4)	11.6 (10.1 to 13.1)	7.5 (6.5 to 8.6)	6.1 (5.2 to 7.0)	-35.4 (-41.6 to -28.7)	-19.0 (-25.5 to -11.1)
Chewing tobacco	0.8 (0.6 to 1.0)	0.9 (0.7 to 1.1)	0.9 (0.7 to 1.2)	8.7 (-18.3 to 40.8)	0.2 (-18.6 to 22.1)	0.1 (0.0 to 0.1)	0.1 (0.0 to 0.1)	0.1 (0.0 to 0.1)	1.2 (-27.2 to 40.4)	-0.2 (-20.0 to 25.5)
Second-hand smoke	38.1 (34.8 to 41.3)	30.6 (27.7 to 33.8)	30.2 (27.3 to 33.4)	-19.5 (-27.3 to -10.8)	-1.4 (-8.6 to 6.4)	36.3 (33.9 to 38.8)	28.6 (26.4 to 30.8)	27.8 (25.4 to 30.3)	-21.2 (-27.3 to -14.6)	-2.7 (-8.9 to 3.7)
Alcohol use	20.0 (12.7 to 29.9)	20.8 (13.1 to 30.4)	22.8 (13.7 to 34.2)	4.0 (-7.2 to 18.2)	9.9 (-11.3 to 40.8)	14.1 (8.4 to 22.5)	14.6 (8.7 to 23.2)	15.7 (9.1 to 24.8)	3.3 (-7.3 to 15.0)	7.6 (-11.3 to 35.6)
Drug use	1.2 (0.9 to 1.6)	1.4 (1.1 to 1.9)	1.6 (1.3 to 2.1)	17.8 (7.5 to 30.4)	20.1 (7.3 to 35.6)	0.7 (0.6 to 1.0)	0.8 (0.6 to 1.0)	1.0 (0.8 to 1.3)	10.7 (-1.2 to 23.5)	18.9 (8.0 to 32.2)
Diet low in fruits	26.2 (20.2 to 31.1)	23.2 (16.6 to 28.6)	22.8 (15.9 to 28.5)	-11.7 (-21.1 to -4.9)	-1.5 (-12.2 to 8.5)	22.4 (16.1 to 27.9)	19.3 (12.8 to 25.1)	19.1 (11.7 to 25.4)	-13.8 (-25.3 to -5.0)	-1.2 (-17.5 to 13.6)
Diet low in vegetables	23.4 (17.2 to 28.6)	22.0 (15.6 to 27.5)	23.0 (16.7 to 28.5)	-5.8 (-12.8 to -0.2)	4.7 (-3.9 to 16.1)	25.8 (20.0 to 30.7)	24.5 (18.7 to 29.6)	25.5 (19.5 to 30.5)	-4.8 (-10.8 to 0.2)	3.8 (-3.1 to 12.1)
Diet low in legumes	16.0 (9.8 to 21.3)	3.7 (0.4 to 8.7)	1.9 (0.1 to 5.5)	-77.1 (-96.2 to -59.1)	-48.1 (-78.5 to -20.9)	21.5 (16.1 to 26.2)	9.0 (3.0 to 14.7)	6.0 (1.3 to 11.7)	-58.3 (-82.0 to -42.2)	-33.1 (-63.9 to -14.8)
Diet low in whole grains	38.3 (35.7 to 40.7)	37.9 (35.2 to 40.3)	38.0 (35.1 to 40.4)	-1.1 (-3.3 to 1.2)	0.2 (-2.3 to 2.5)	39.4 (36.8 to 41.5)	39.0 (36.3 to 41.2)	39.0 (36.3 to 41.4)	-1.0 (-3.3 to 1.2)	0.2 (-1.9 to 2.6)
Diet low in nuts and seeds	47.3 (45.3 to 48.8)	44.3 (41.8 to 46.5)	43.1 (40.0 to 45.7)	-6.2 (-9.4 to -3.5)	-2.7 (-5.7 to -0.1)	47.7 (45.8 to 49.2)	44.9 (42.4 to 46.9)	43.7 (40.6 to 46.1)	-5.8 (-9.0 to -3.2)	-2.6 (-5.8 to 0.0)
Diet low in milk	31.3 (26.4 to 35.3)	30.7 (25.8 to 34.7)	30.4 (25.4 to 34.6)	-2.2 (-5.9 to 1.3)	-0.7 (-4.7 to 3.4)	32.5 (27.9 to 36.3)	31.4 (26.8 to 35.4)	31.1 (26.3 to 35.3)	-3.3 (-7.0 to -0.1)	-1.0 (-4.9 to 2.7)
Diet high in red meat	44.8 (41.9 to 47.7)	36.9 (33.6 to 40.2)	35.4 (29.3 to 40.8)	-17.6 (-24.5 to -10.4)	-4.1 (-17.5 to 9.9)	21.7 (17.6 to 25.1)	16.6 (12.3 to 20.2)	16.0 (10.3 to 21.2)	-23.7 (-36.6 to -11.9)	-3.5 (-24.7 to 21.0)
Diet high in processed meat	26.7 (20.0 to 32.4)	25.3 (19.0 to 30.6)	24.2 (18.6 to 29.3)	-5.1 (-21.6 to 13.6)	-4.4 (-16.7 to 7.4)	15.9 (11.6 to 20.0)	16.4 (11.8 to 20.4)	16.1 (11.6 to 20.2)	3.2 (-13.8 to 24.8)	-2.0 (-14.6 to 12.3)
Diet high in sugar-sweetened beverages	24.1 (11.4 to 35.0)	26.8 (13.2 to 36.9)	26.7 (13.2 to 37.1)	11.1 (-22.6 to 67.3)	-0.4 (-22.6 to 29.9)	17.8 (8.5 to 27.3)	19.7 (8.8 to 29.5)	19.1 (8.5 to 29.4)	10.7 (-30.5 to 77.5)	-3.1 (-31.6 to 34.4)
Diet low in fibre	31.9 (26.9 to 35.9)	26.7 (21.0 to 31.4)	25.9 (19.9 to 30.8)	-16.2 (-23.4 to -11.2)	-3.2 (-9.7 to 2.5)	36.7 (32.7 to 39.9)	32.2 (27.3 to 36.1)	31.5 (26.1 to 35.6)	-12.4 (-18.3 to -8.4)	-2.2 (-6.8 to 2.0)
Diet low in calcium	23.8 (17.5 to 28.7)	24.8 (18.8 to 29.6)	24.6 (18.6 to 29.5)	4.1 (1.3 to 8.8)	-0.9 (-5.0 to 2.5)	27.4 (21.9 to 31.8)	28.2 (22.9 to 32.5)	28.0 (22.6 to 32.5)	2.9 (0.6 to 6.1)	-0.6 (-3.4 to 2.1)
Diet low in seafood omega-3 fatty acids	24.3 (19.5 to 28.7)	18.9 (14.2 to 23.5)	18.7 (14.2 to 23.4)	-22.2 (-28.5 to -17.0)	-1.1 (-5.9 to 4.7)	28.1 (23.6 to 32.4)	23.0 (18.3 to 27.7)	22.8 (18.0 to 27.5)	-18.0 (-23.4 to -13.9)	-0.9 (-5.2 to 3.2)
Diet low in polyunsaturated fatty acids	39.2 (36.0 to 41.9)	38.5 (35.4 to 41.1)	37.8 (34.4 to 40.6)	-1.8 (-6.8 to 2.7)	-1.7 (-4.8 to 1.6)	39.6 (36.5 to 42.4)	38.9 (35.9 to 41.5)	38.3 (35.2 to 41.1)	-1.8 (-6.6 to 2.1)	-1.6 (-4.0 to 0.4)
Diet high in trans fatty acids	19.5 (14.5 to 25.8)	10.5 (6.4 to 15.0)	7.4 (3.6 to 11.3)	-46.1 (-60.1 to -33.8)	-29.8 (-46.6 to -18.4)	27.4 (21.2 to 37.0)	14.1 (9.4 to 18.6)	9.9 (5.8 to 14.0)	-48.7 (-59.7 to -37.7)	-29.7 (-42.8 to -19.1)
Diet high in sodium	7.8 (0.6 to 15.2)	9.0 (1.4 to 16.5)	9.3 (1.5 to 16.8)	15.4 (-12.6 to 166.3)	3.1 (-27.7 to 54.6)	6.9 (0.7 to 14.0)	6.5 (0.7 to 13.5)	5.7 (0.6 to 12.4)	-5.8 (-42.6 to 41.1)	-12.6 (-40.4 to 3.1)
Intimate partner violence	-	-	-	-	-	2.2 (1.9 to 2.6)	2.0 (1.7 to 2.3)	2.0 (1.7 to 2.4)	-8.3 (-12.9 to -3.5)	-0.1 (-5.4 to 5.5)
Childhood sexual abuse	2.9 (2.5 to 3.5)	3.8 (3.5 to 4.0)	3.8 (3.6 to 4.1)	27.4 (10.7 to 45.9)	1.6 (-3.0 to 7.0)	7.6 (6.7 to 8.7)	9.8 (9.4 to 10.3)	10.0 (9.6 to 10.4)	29.1 (13.3 to 43.4)	1.9 (-2.3 to 5.9)

Supplementary Results Table 1. Canada age-standardised Summary Exposure Values (SEVs) for Level 4 risk factors and percentage change, by sex, 1990, 2007, and 2017.Data in parentheses are 95% uncertainty intervals.

	Male					Female				
	**1990**	**2007**	**2017**	**Percentage change 1990-2007**	**Percentage change 2007-2017**	**1990**	**2007**	**2017**	**Percentage change 1990-2007**	**Percentage change 2007-2017**
Bullying victimization	5.6 (2.6 to 10.9)	7.4 (3.6 to 14.1)	8.0 (3.8 to 15.6)	32.1 (13.6 to 51.8)	8.6 (0.6 to 20.4)	4.4 (2.0 to 8.6)	6.1 (2.9 to 11.6)	6.9 (3.3 to 13.1)	38.7 (1.0 to 93.6)	13.6 (-16.1 to 52.1)
Low physical activity	0.4 (0.2 to 0.7)	0.4 (0.2 to 0.7)	0.4 (0.2 to 0.7)	0.5 (-1.2 to 2.3)	0.7 (-0.9 to 2.5)	0.4 (0.2 to 0.8)	0.4 (0.2 to 0.8)	0.4 (0.2 to 0.8)	0.2 (-1.9 to 2.3)	2.0 (-0.1 to 4.3)
High fasting plasma glucose	3.1 (2.1 to 4.6)	3.2 (2.3 to 4.7)	3.1 (2.1 to 4.7)	3.7 (-16.7 to 30.2)	-2.4 (-23.6 to 19.4)	2.7 (1.8 to 4.1)	2.0 (1.2 to 3.1)	1.6 (0.8 to 2.7)	-26.1 (-53.5 to 2.5)	-18.7 (-56.5 to 29.2)
High LDL cholesterol	14.3 (12.6 to 16.1)	12.5 (10.9 to 14.3)	12.3 (10.7 to 14.1)	-12.3 (-16.4 to -8.1)	-1.6 (-4.8 to 1.4)	13.5 (12.1 to 15.2)	13.1 (11.6 to 14.9)	13.0 (11.5 to 14.7)	-3.3 (-7.0 to 0.4)	-0.4 (-3.5 to 3.0)
High systolic blood pressure	7.3 (6.5 to 8.1)	6.4 (5.7 to 7.3)	6.6 (5.8 to 7.4)	-11.2 (-18.1 to -3.6)	1.9 (-4.5 to 9.0)	5.7 (5.0 to 6.3)	5.1 (4.5 to 5.7)	5.2 (4.5 to 5.8)	-10.6 (-19.3 to -1.1)	1.5 (-6.6 to 10.5)
High body-mass index	13.1 (9.5 to 18.2)	18.0 (13.9 to 23.2)	18.9 (14.8 to 24.0)	37.5 (20.3 to 57.5)	5.0 (-2.9 to 12.9)	13.2 (9.8 to 17.6)	17.6 (14.2 to 22.1)	18.4 (14.6 to 23.6)	34.0 (18.6 to 54.1)	4.3 (-3.9 to 13.4)
Low bone mineral density	4.6 (4.1 to 5.0)	4.3 (3.9 to 4.7)	3.9 (3.1 to 4.8)	-7.4 (-13.7 to 1.4)	-7.3 (-23.4 to 8.8)	5.8 (5.3 to 6.5)	5.3 (4.7 to 5.8)	5.4 (4.4 to 6.4)	-9.7 (-17.6 to -1.0)	2.3 (-12.9 to 17.5)
Impaired kidney function	1.7 (0.8 to 4.5)	1.7 (0.8 to 4.4)	1.7 (0.8 to 4.6)	-2.6 (-9.1 to 2.7)	4.2 (-1.6 to 12.4)	2.2 (1.0 to 5.8)	2.2 (1.0 to 6.0)	2.4 (1.1 to 6.1)	0.8 (-4.8 to 6.2)	7.3 (1.8 to 14.3)

Supplementary Results Table 1. Greenland age-standardised Summary Exposure Values (SEVs) for Level 4 risk factors and percentage change, by sex, 1990, 2007, and 2017.Data in parentheses are 95% uncertainty intervals.

	Male					Female				
	**1990**	**2007**	**2017**	**Percentage change 1990-2007**	**Percentage change 2007-2017**	**1990**	**2007**	**2017**	**Percentage change 1990-2007**	**Percentage change 2007-2017**
Unsafe water source	3.2 (2.1 to 4.6)	1.0 (0.7 to 1.5)	0.7 (0.4 to 1.0)	-69.4 (-80.9 to -49.0)	-33.2 (-52.8 to -4.0)	3.2 (2.1 to 4.6)	1.0 (0.7 to 1.5)	0.7 (0.4 to 1.0)	-69.4 (-80.9 to -49.0)	-33.2 (-52.8 to -4.0)
Unsafe sanitation	17.7 (11.2 to 26.2)	8.9 (5.3 to 13.5)	6.3 (3.6 to 10.1)	-49.6 (-69.9 to -15.6)	-29.4 (-54.5 to 7.9)	17.7 (11.2 to 26.2)	8.9 (5.3 to 13.5)	6.3 (3.6 to 10.1)	-49.6 (-69.9 to -15.6)	-29.4 (-54.5 to 7.9)
No access to handwashing facility	3.8 (3.0 to 4.8)	3.3 (2.6 to 4.2)	3.2 (2.5 to 4.0)	-12.1 (-32.7 to 12.5)	-3.4 (-19.0 to 14.6)	3.8 (3.0 to 4.8)	3.3 (2.6 to 4.2)	3.2 (2.5 to 4.0)	-12.1 (-32.7 to 12.5)	-3.4 (-19.0 to 14.6)
Ambient particulate matter pollution	32.9 (14.9 to 56.4)	32.2 (16.5 to 52.7)	27.6 (8.2 to 50.8)	-2.3 (-50.4 to 118.8)	-14.2 (-75.4 to 75.9)	32.3 (14.2 to 56.0)	31.9 (16.1 to 52.4)	27.4 (8.1 to 50.6)	-1.2 (-51.1 to 126.4)	-14.1 (-75.3 to 78.3)
Household air pollution from solid fuels	1.7 (0.7 to 4.6)	0.9 (0.3 to 2.8)	0.6 (0.2 to 1.9)	-49.5 (-78.3 to 10.6)	-27.8 (-68.1 to 40.0)	2.3 (0.9 to 8.1)	1.2 (0.4 to 7.4)	0.9 (0.3 to 7.1)	-46.8 (-78.0 to 12.7)	-25.4 (-68.6 to 41.7)
Ambient ozone pollution	32.0 (10.7 to 54.8)	30.6 (10.5 to 54.8)	31.5 (10.8 to 54.8)	-4.6 (-15.6 to 7.0)	3.0 (-7.9 to 15.9)	32.0 (10.7 to 54.8)	30.6 (10.5 to 54.8)	31.5 (10.8 to 54.8)	-4.6 (-15.6 to 7.0)	3.0 (-7.9 to 15.9)
Residential radon	32.6 (4.7 to 71.2)	32.6 (4.7 to 71.2)	32.6 (4.7 to 71.2)	0.0 (0.0 to 0.0)	0.0 (0.0 to 0.0)	32.6 (4.7 to 71.2)	32.6 (4.7 to 71.2)	32.6 (4.7 to 71.2)	0.0 (0.0 to 0.0)	0.0 (0.0 to 0.0)
Lead exposure	8.5 (4.2 to 12.7)	7.8 (4.0 to 11.8)	6.0 (2.8 to 9.7)	-8.4 (-14.3 to -1.8)	-23.9 (-32.3 to -17.3)	4.4 (1.1 to 8.3)	4.3 (1.2 to 8.0)	3.3 (0.9 to 6.8)	-3.5 (-9.8 to 19.9)	-22.7 (-33.1 to -15.5)
Occupational exposure to asbestos	4.8 (3.7 to 5.8)	7.1 (6.5 to 7.7)	6.8 (6.1 to 7.5)	48.8 (20.9 to 94.0)	-3.8 (-15.1 to 7.0)	5.4 (3.2 to 7.5)	3.9 (2.9 to 4.9)	2.0 (1.7 to 2.4)	-27.2 (-42.2 to 0.8)	-49.2 (-61.2 to -32.1)
Occupational exposure to arsenic	0.3 (0.0 to 0.8)	0.2 (0.0 to 0.6)	0.2 (0.0 to 0.5)	-	-	0.3 (0.0 to 0.7)	0.2 (0.0 to 0.6)	0.2 (0.0 to 0.6)	-	-
Occupational exposure to benzene	0.5 (0.0 to 1.5)	0.6 (0.0 to 1.6)	0.6 (0.0 to 1.7)	9.8 (2.4 to 21.0)	8.2 (0.6 to 18.5)	0.6 (0.0 to 1.9)	1.0 (0.1 to 2.7)	1.1 (0.1 to 3.2)	48.7 (36.7 to 72.7)	14.1 (7.6 to 24.0)
Occupational exposure to beryllium	0.0 (0.0 to 0.0)	0.0 (0.0 to 0.0)	0.0 (0.0 to 0.0)	-7.3 (-11.5 to -2.3)	-1.4 (-5.9 to 3.4)	0.0 (0.0 to 0.0)	0.0 (0.0 to 0.0)	0.0 (0.0 to 0.0)	9.2 (1.1 to 19.1)	3.3 (-1.7 to 8.8)
Occupational exposure to cadmium	0.0 (0.0 to 0.0)	0.0 (0.0 to 0.0)	0.0 (0.0 to 0.0)	-22.4 (-29.0 to -14.8)	-6.0 (-12.4 to 1.2)	0.0 (0.0 to 0.0)	0.0 (0.0 to 0.0)	0.0 (0.0 to 0.0)	-14.2 (-23.8 to -3.2)	-2.5 (-8.8 to 4.1)
Occupational exposure to chromium	0.1 (0.1 to 0.1)	0.1 (0.1 to 0.1)	0.1 (0.1 to 0.1)	-17.7 (-23.9 to -10.7)	-3.9 (-9.8 to 2.3)	0.1 (0.1 to 0.1)	0.1 (0.1 to 0.1)	0.1 (0.1 to 0.1)	-4.5 (-14.0 to 6.4)	0.1 (-5.6 to 6.0)
Occupational exposure to diesel engine exhaust	0.6 (0.5 to 0.6)	0.6 (0.6 to 0.6)	0.6 (0.5 to 0.6)	1.5 (-5.1 to 9.3)	-2.1 (-8.1 to 4.3)	0.5 (0.4 to 0.6)	0.5 (0.5 to 0.6)	0.5 (0.5 to 0.6)	7.4 (-3.1 to 20.9)	-4.1 (-11.4 to 3.7)
Occupational exposure to formaldehyde	0.2 (0.2 to 0.2)	0.1 (0.1 to 0.2)	0.1 (0.1 to 0.1)	-23.0 (-29.9 to -15.1)	-4.4 (-10.8 to 3.1)	0.2 (0.1 to 0.2)	0.2 (0.2 to 0.2)	0.2 (0.2 to 0.2)	-5.3 (-15.4 to 6.5)	2.6 (-3.6 to 9.4)
Occupational exposure to nickel	0.3 (0.0 to 1.4)	0.2 (0.0 to 1.1)	0.2 (0.0 to 1.0)	-	-	0.2 (0.0 to 1.1)	0.2 (0.0 to 0.8)	0.2 (0.0 to 0.8)	-	-
Occupational exposure to polycyclic aromatic hydrocarbons	0.2 (0.2 to 0.2)	0.2 (0.2 to 0.2)	0.2 (0.2 to 0.2)	-14.2 (-20.1 to -7.4)	-1.9 (-7.2 to 4.1)	0.2 (0.2 to 0.2)	0.2 (0.2 to 0.2)	0.2 (0.2 to 0.2)	3.0 (-6.7 to 14.3)	3.7 (-1.8 to 9.6)
Occupational exposure to silica	3.7 (0.5 to 12.1)	3.4 (0.5 to 11.1)	3.3 (0.5 to 10.6)	-9.0 (-18.6 to 2.9)	-3.0 (-12.0 to 8.3)	2.3 (0.3 to 7.2)	1.7 (0.2 to 5.1)	1.5 (0.2 to 4.7)	-26.8 (-36.3 to -15.0)	-9.2 (-17.2 to -0.6)
Occupational exposure to sulphuric acid	0.6 (0.1 to 1.7)	0.4 (0.1 to 1.4)	0.4 (0.1 to 1.3)	-21.0 (-27.6 to -13.1)	-8.0 (-15.5 to -0.5)	0.4 (0.1 to 1.4)	0.4 (0.1 to 1.1)	0.3 (0.1 to 1.0)	-20.9 (-29.8 to -9.9)	-8.1 (-14.7 to -1.0)
Occupational exposure to trichloroethylene	0.1 (0.1 to 0.1)	0.0 (0.0 to 0.1)	0.0 (0.0 to 0.1)	-11.8 (-17.2 to -5.5)	-3.7 (-9.1 to 1.9)	0.1 (0.1 to 0.1)	0.1 (0.1 to 0.1)	0.1 (0.1 to 0.1)	0.1 (-8.6 to 10.9)	-1.6 (-7.3 to 4.9)
Occupational asthmagens	15.2 (12.9 to 18.0)	13.1 (11.2 to 15.6)	13.2 (11.2 to 15.5)	-13.7 (-21.3 to -4.9)	0.5 (-6.4 to 8.0)	8.7 (7.1 to 10.8)	13.9 (11.0 to 17.7)	17.3 (13.8 to 21.6)	60.1 (34.3 to 88.3)	23.9 (5.4 to 46.5)
Occupational particulate matter, gases, and fumes	6.9 (5.0 to 9.8)	6.1 (4.4 to 8.6)	5.3 (3.8 to 7.6)	-11.4 (-16.2 to -7.5)	-13.1 (-16.3 to -10.1)	3.4 (2.4 to 5.1)	2.9 (2.0 to 4.4)	2.4 (1.7 to 3.6)	-14.7 (-21.0 to -8.0)	-17.4 (-22.0 to -13.1)
Occupational noise	6.2 (5.7 to 6.8)	5.8 (5.3 to 6.3)	5.3 (5.0 to 5.8)	-7.6 (-10.2 to -4.9)	-7.5 (-9.2 to -5.7)	3.6 (3.1 to 4.1)	3.5 (3.2 to 4.0)	3.6 (3.3 to 3.9)	-1.7 (-6.1 to 3.6)	0.2 (-2.9 to 3.8)
Occupational ergonomic factors	9.2 (7.8 to 10.8)	7.7 (6.5 to 9.1)	7.3 (6.2 to 8.5)	-15.8 (-24.7 to -6.2)	-6.0 (-13.0 to 1.2)	4.5 (3.6 to 5.7)	4.7 (3.8 to 5.9)	4.8 (3.8 to 5.9)	4.9 (-11.9 to 24.1)	1.1 (-9.5 to 12.9)
Non-exclusive breastfeeding	0.4 (0.3 to 0.5)	0.4 (0.3 to 0.5)	0.4 (0.3 to 0.5)	0.5 (-7.5 to 10.9)	0.6 (-6.1 to 8.1)	0.4 (0.3 to 0.5)	0.4 (0.3 to 0.5)	0.4 (0.3 to 0.5)	3.8 (-4.3 to 14.6)	-1.6 (-8.2 to 5.7)
Discontinued breastfeeding	1.8 (1.7 to 1.9)	1.7 (1.6 to 1.8)	1.8 (1.7 to 1.9)	-6.5 (-13.0 to 0.4)	4.6 (-1.3 to 11.0)	1.9 (1.8 to 2.0)	1.7 (1.6 to 1.8)	1.8 (1.7 to 1.9)	-9.3 (-15.5 to -2.5)	3.6 (-2.4 to 9.9)
Child underweight	0.1 (0.1 to 0.2)	0.1 (0.1 to 0.2)	0.1 (0.1 to 0.2)	-9.6 (-21.9 to 3.8)	-14.0 (-22.6 to -5.2)	0.1 (0.1 to 0.2)	0.1 (0.1 to 0.2)	0.1 (0.1 to 0.2)	-8.4 (-20.7 to 5.6)	-7.3 (-16.7 to 3.8)
Child wasting	0.1 (0.1 to 0.2)	0.1 (0.1 to 0.1)	0.1 (0.1 to 0.1)	-12.7 (-28.8 to 8.0)	-3.1 (-15.9 to 11.8)	0.1 (0.1 to 0.2)	0.1 (0.1 to 0.1)	0.1 (0.1 to 0.1)	-1.9 (-21.0 to 22.5)	-3.8 (-16.7 to 10.6)
Child stunting	0.4 (0.2 to 0.6)	0.3 (0.1 to 0.5)	0.3 (0.1 to 0.5)	-18.0 (-50.8 to 30.5)	-6.7 (-31.5 to 26.7)	0.4 (0.2 to 0.7)	0.3 (0.1 to 0.5)	0.3 (0.1 to 0.5)	-18.1 (-49.0 to 26.1)	-4.7 (-31.9 to 32.5)
Short gestation for birth weight	0.0 (0.0 to 0.0)	0.0 (0.0 to 0.0)	0.0 (0.0 to 0.0)	20.9 (-6.2 to 48.5)	1.7 (-21.3 to 31.1)	0.0 (0.0 to 0.0)	0.0 (0.0 to 0.0)	0.0 (0.0 to 0.0)	14.5 (-11.5 to 51.5)	4.0 (-14.5 to 35.8)
Low birth weight for gestation	0.0 (0.0 to 0.0)	0.0 (0.0 to 0.0)	0.0 (0.0 to 0.0)	11.5 (-5.8 to 31.6)	3.7 (-14.1 to 26.1)	0.0 (0.0 to 0.0)	0.0 (0.0 to 0.0)	0.0 (0.0 to 0.0)	8.2 (-10.4 to 29.6)	2.9 (-15.2 to 18.6)
Iron deficiency	-	-	-	-	-	6.5 (4.5 to 9.1)	6.1 (4.1 to 8.5)	5.4 (3.7 to 7.6)	-7.2 (-24.1 to 11.3)	-10.7 (-24.2 to 5.6)
Vitamin A deficiency	1.4 (0.9 to 1.9)	1.4 (0.8 to 1.9)	1.3 (0.7 to 1.8)	5.4 (-30.7 to 42.3)	-12.2 (-29.4 to 8.7)	1.5 (1.0 to 2.0)	1.5 (1.0 to 1.9)	1.1 (0.7 to 1.6)	1.1 (-13.6 to 23.5)	-25.1 (-40.8 to -5.8)
Zinc deficiency	0.5 (0.1 to 1.1)	0.4 (0.0 to 1.0)	0.3 (0.0 to 1.0)	-28.1 (-84.9 to 323.4)	-4.1 (-82.6 to 528.3)	0.5 (0.1 to 1.1)	0.4 (0.0 to 1.0)	0.3 (0.0 to 1.0)	-26.9 (-85.4 to 280.6)	-10.8 (-81.9 to 509.5)
Smoking	14.1 (11.1 to 17.0)	11.2 (9.0 to 13.5)	10.2 (8.2 to 12.3)	-20.8 (-30.4 to -8.6)	-8.7 (-16.5 to -0.1)	11.1 (8.4 to 14.2)	9.7 (7.6 to 12.2)	9.1 (7.2 to 11.2)	-12.8 (-26.4 to 2.5)	-5.8 (-15.3 to 6.4)
Chewing tobacco	1.9 (1.5 to 2.5)	2.0 (1.5 to 2.5)	2.0 (1.5 to 2.5)	0.9 (-26.1 to 36.9)	-0.6 (-20.6 to 21.8)	0.1 (0.1 to 0.1)	0.1 (0.1 to 0.1)	0.1 (0.1 to 0.1)	-1.9 (-30.6 to 34.3)	0.4 (-20.5 to 28.0)
Second-hand smoke	73.2 (68.7 to 77.3)	53.5 (50.3 to 56.8)	50.8 (47.3 to 54.2)	-26.8 (-32.2 to -20.3)	-5.0 (-9.5 to -0.4)	75.4 (71.6 to 78.7)	53.3 (50.7 to 56.1)	50.2 (47.6 to 52.9)	-29.4 (-33.9 to -24.5)	-5.8 (-9.1 to -2.3)
Alcohol use	14.1 (7.1 to 23.4)	14.4 (7.4 to 24.5)	15.4 (8.2 to 26.0)	1.9 (-32.1 to 51.6)	6.5 (-21.3 to 42.7)	10.3 (4.7 to 18.3)	10.4 (5.0 to 18.9)	10.8 (5.2 to 19.4)	1.5 (-30.2 to 48.3)	3.4 (-22.7 to 35.0)
Drug use	1.1 (0.8 to 1.6)	1.1 (0.8 to 1.6)	1.3 (0.9 to 1.8)	1.6 (-5.5 to 8.5)	12.8 (5.2 to 22.7)	0.8 (0.6 to 1.0)	0.8 (0.6 to 1.0)	0.9 (0.7 to 1.2)	3.3 (-5.3 to 13.6)	16.1 (4.2 to 29.2)
Diet low in fruits	24.0 (16.5 to 30.7)	24.1 (16.5 to 30.5)	22.5 (14.9 to 29.2)	0.5 (-17.2 to 19.2)	-6.9 (-19.3 to 4.8)	20.4 (12.6 to 27.6)	20.5 (12.7 to 27.5)	19.0 (11.0 to 26.3)	0.4 (-21.4 to 27.4)	-7.3 (-25.2 to 10.9)
Diet low in vegetables	20.7 (12.5 to 27.8)	21.4 (13.5 to 28.6)	20.0 (11.6 to 27.5)	3.4 (-18.9 to 35.4)	-6.9 (-24.5 to 10.5)	23.2 (14.8 to 30.3)	23.3 (14.4 to 30.2)	21.9 (13.5 to 29.3)	0.6 (-20.4 to 24.8)	-5.9 (-22.2 to 8.8)
Diet low in legumes	26.1 (20.0 to 31.5)	29.9 (24.4 to 34.5)	29.7 (24.2 to 34.3)	14.7 (2.1 to 34.9)	-0.7 (-8.2 to 6.8)	31.2 (26.0 to 35.8)	34.6 (30.1 to 38.6)	34.4 (29.8 to 38.4)	11.1 (1.9 to 22.6)	-0.6 (-5.8 to 5.4)
Diet low in whole grains	38.5 (35.1 to 41.3)	38.7 (35.4 to 41.5)	38.2 (34.8 to 41.2)	0.6 (-3.8 to 5.5)	-1.2 (-4.4 to 1.8)	39.6 (36.5 to 42.1)	39.7 (36.6 to 42.3)	39.3 (35.9 to 42.0)	0.4 (-3.5 to 4.4)	-1.1 (-4.2 to 1.8)
Diet low in nuts and seeds	42.6 (38.6 to 45.6)	37.5 (32.7 to 41.6)	35.1 (29.5 to 39.5)	-11.9 (-19.9 to -5.4)	-6.3 (-13.2 to -0.6)	43.5 (40.2 to 46.2)	38.7 (33.9 to 42.6)	36.5 (31.0 to 40.7)	-11.0 (-18.5 to -4.9)	-5.9 (-12.1 to -0.1)
Diet low in milk	29.4 (23.7 to 34.3)	30.4 (24.8 to 35.1)	29.6 (23.6 to 34.4)	3.6 (-4.0 to 13.0)	-2.7 (-8.5 to 2.5)	30.4 (25.0 to 35.0)	31.1 (25.7 to 35.8)	30.2 (24.5 to 35.0)	2.2 (-4.7 to 9.4)	-2.8 (-8.0 to 2.1)
Diet high in red meat	43.0 (33.5 to 50.0)	41.1 (30.5 to 49.1)	42.2 (30.9 to 49.2)	-4.3 (-22.6 to 15.0)	2.6 (-10.9 to 20.3)	25.3 (15.4 to 34.7)	22.5 (12.1 to 32.1)	23.5 (12.7 to 32.8)	-11.1 (-39.1 to 26.7)	4.8 (-20.5 to 40.0)
Diet high in processed meat	33.3 (26.2 to 39.8)	30.4 (23.6 to 36.4)	30.9 (23.9 to 36.7)	-8.8 (-23.6 to 8.1)	1.6 (-9.3 to 14.3)	20.2 (14.9 to 24.7)	20.0 (15.1 to 24.8)	20.8 (15.8 to 25.9)	-0.8 (-16.4 to 18.1)	4.2 (-7.9 to 18.0)
Diet high in sugar-sweetened beverages	30.5 (15.8 to 40.9)	32.0 (16.3 to 41.4)	33.3 (16.4 to 43.3)	5.0 (-22.6 to 43.4)	4.0 (-17.2 to 33.1)	22.9 (10.9 to 33.7)	24.0 (11.4 to 33.9)	25.2 (12.1 to 35.2)	4.7 (-29.1 to 61.5)	5.1 (-20.7 to 46.6)
Diet low in fibre	29.9 (23.8 to 35.0)	26.7 (20.1 to 32.3)	25.9 (19.2 to 31.6)	-10.6 (-22.8 to -0.8)	-3.0 (-11.9 to 5.7)	34.9 (29.3 to 39.4)	32.0 (26.2 to 36.9)	31.2 (25.5 to 36.1)	-8.4 (-17.5 to -0.8)	-2.4 (-8.7 to 4.1)
Diet low in calcium	17.3 (10.0 to 23.4)	16.7 (9.7 to 23.0)	15.8 (9.3 to 22.0)	-3.4 (-15.8 to 9.1)	-5.3 (-13.6 to 2.8)	21.3 (14.5 to 27.0)	20.7 (13.7 to 26.5)	19.6 (12.7 to 25.5)	-3.1 (-12.7 to 5.5)	-5.0 (-12.6 to 1.2)
Diet low in seafood omega-3 fatty acids	22.2 (15.9 to 28.2)	19.5 (13.0 to 25.5)	17.5 (11.2 to 23.7)	-12.4 (-28.6 to 3.6)	-10.0 (-22.1 to 4.0)	26.2 (20.1 to 31.7)	23.7 (16.9 to 29.7)	21.5 (14.9 to 27.6)	-9.6 (-23.5 to 2.9)	-9.1 (-20.6 to 1.4)
Diet low in polyunsaturated fatty acids	23.9 (17.0 to 29.8)	21.2 (14.1 to 27.3)	19.6 (12.5 to 26.2)	-11.2 (-29.1 to 2.8)	-7.6 (-20.8 to 4.8)	24.6 (17.8 to 30.4)	21.7 (14.4 to 27.8)	20.1 (12.9 to 26.5)	-11.5 (-26.9 to 2.7)	-7.4 (-20.1 to 3.3)
Diet high in trans fatty acids	9.5 (5.5 to 13.8)	5.9 (2.6 to 9.8)	4.3 (1.4 to 7.8)	-38.6 (-58.1 to -25.8)	-26.2 (-46.8 to -14.7)	12.8 (8.4 to 17.1)	7.9 (4.2 to 11.9)	5.9 (2.5 to 9.6)	-38.1 (-53.6 to -26.0)	-25.7 (-43.0 to -14.2)
Diet high in sodium	8.1 (1.1 to 15.5)	11.6 (3.2 to 19.0)	12.2 (3.7 to 19.7)	42.5 (8.5 to 320.9)	5.7 (-14.9 to 44.9)	6.3 (0.4 to 13.6)	9.6 (1.7 to 17.0)	9.4 (1.5 to 16.7)	52.1 (15.6 to 503.1)	-2.3 (-31.5 to 23.5)
Intimate partner violence	-	-	-	-	-	2.5 (1.9 to 3.2)	2.3 (1.7 to 3.0)	2.2 (1.6 to 2.8)	-5.6 (-10.2 to -0.8)	-5.6 (-10.0 to -0.6)
Childhood sexual abuse	4.4 (3.6 to 5.4)	4.4 (3.6 to 5.4)	4.4 (3.6 to 5.4)	0.0 (0.0 to 0.0)	0.0 (0.0 to 0.0)	11.1 (9.0 to 13.5)	11.1 (9.0 to 13.5)	11.1 (9.0 to 13.5)	0.0 (0.0 to 0.0)	0.0 (0.0 to 0.0)

Supplementary Results Table 1. Greenland age-standardised Summary Exposure Values (SEVs) for Level 4 risk factors and percentage change, by sex, 1990, 2007, and 2017.Data in parentheses are 95% uncertainty intervals.										
	**Male**					**Female**				
	**1990**	**2007**	**2017**	**Percentage change 1990-2007**	**Percentage change 2007-2017**	**1990**	**2007**	**2017**	**Percentage change 1990-2007**	**Percentage change 2007-2017**
Bullying victimization	7.1 (3.3 to 14.6)	9.6 (4.5 to 19.7)	10.4 (4.9 to 21.8)	33.8 (14.8 to 58.1)	9.1 (0.9 to 19.0)	6.5 (3.0 to 12.9)	9.4 (4.5 to 19.6)	9.8 (4.6 to 21.0)	46.0 (22.5 to 84.4)	4.5 (-5.8 to 13.5)
Low physical activity	0.4 (0.2 to 0.7)	0.4 (0.2 to 0.7)	0.4 (0.2 to 0.7)	1.1 (-1.0 to 3.2)	1.0 (-0.8 to 3.1)	0.4 (0.2 to 0.8)	0.4 (0.2 to 0.8)	0.4 (0.2 to 0.8)	0.8 (-1.1 to 3.0)	1.2 (-0.7 to 3.1)
High fasting plasma glucose	3.3 (2.4 to 5.0)	2.8 (1.9 to 4.2)	2.5 (1.7 to 3.9)	-16.1 (-33.8 to 0.7)	-9.1 (-32.1 to 11.4)	2.8 (1.9 to 4.3)	2.5 (1.8 to 3.9)	2.1 (1.3 to 3.4)	-10.6 (-26.3 to 6.1)	-14.9 (-41.2 to 5.1)
High LDL cholesterol	12.5 (10.9 to 14.2)	11.5 (10.0 to 13.2)	11.2 (9.6 to 12.9)	-7.9 (-11.8 to -4.2)	-2.3 (-5.6 to 1.1)	12.1 (10.6 to 13.8)	11.5 (10.0 to 13.1)	11.4 (10.0 to 13.0)	-5.1 (-9.2 to -1.1)	-0.3 (-3.3 to 3.1)
High systolic blood pressure	6.0 (5.2 to 6.7)	5.0 (4.3 to 5.7)	4.9 (4.2 to 5.7)	-15.9 (-23.5 to -8.1)	-1.6 (-8.1 to 5.5)	5.8 (5.1 to 6.5)	4.9 (4.3 to 5.6)	4.7 (4.0 to 5.3)	-14.4 (-23.6 to -4.8)	-5.3 (-13.2 to 3.5)
High body-mass index	8.5 (5.7 to 13.0)	11.8 (8.4 to 16.6)	13.2 (9.5 to 18.2)	38.7 (19.9 to 62.4)	11.9 (2.5 to 23.1)	10.8 (7.6 to 15.1)	14.9 (11.3 to 20.3)	16.6 (12.5 to 22.3)	38.3 (19.7 to 63.2)	11.7 (1.5 to 23.7)
Low bone mineral density	4.6 (3.7 to 5.5)	4.5 (3.8 to 5.4)	4.5 (3.6 to 5.4)	-0.9 (-14.6 to 15.6)	-1.5 (-14.6 to 14.6)	6.0 (4.9 to 7.1)	5.9 (5.0 to 6.9)	5.9 (4.8 to 7.1)	-1.1 (-13.5 to 13.8)	-0.3 (-12.4 to 13.5)
Impaired kidney function	2.0 (0.9 to 5.1)	1.8 (0.9 to 4.6)	1.9 (0.9 to 4.8)	-6.0 (-10.7 to -1.4)	1.2 (-3.9 to 6.5)	2.4 (1.1 to 6.2)	2.4 (1.1 to 6.1)	2.4 (1.1 to 6.2)	-2.6 (-6.5 to 1.6)	1.8 (-2.4 to 6.9)

Supplementary Results Table 1. United States age-standardised Summary Exposure Values (SEVs) for Level 4 risk factors and percentage change, by sex, 1990, 2007, and 2017.Data in parentheses are 95% uncertainty intervals.

	Male					Female				
	1990	2007	2017	Percentage change 1990-2007	Percentage change 2007-2017	1990	2007	2017	Percentage change 1990-2007	Percentage change 2007-2017
Unsafe water source	1.5 (1.0 to 2.2)	0.5 (0.4 to 0.7)	0.3 (0.2 to 0.4)	-65.7 (-78.4 to -44.7)	-37.5 (-54.8 to -11.5)	1.5 (1.0 to 2.2)	0.5 (0.4 to 0.7)	0.3 (0.2 to 0.4)	-65.7 (-78.4 to -44.6)	-37.5 (-54.7 to -11.5)
Unsafe sanitation	7.0 (4.9 to 9.8)	4.4 (2.5 to 7.1)	3.0 (1.6 to 5.0)	-37.4 (-64.5 to 5.1)	-31.7 (-57.5 to 7.9)	7.0 (4.9 to 9.8)	4.4 (2.5 to 7.1)	3.0 (1.6 to 5.0)	-37.4 (-64.6 to 4.9)	-31.7 (-57.5 to 8.0)
No access to handwashing facility	3.5 (2.8 to 4.3)	3.2 (2.5 to 4.0)	3.0 (2.4 to 3.8)	-8.9 (-27.9 to 16.0)	-3.8 (-20.2 to 15.6)	3.5 (2.8 to 4.3)	3.2 (2.5 to 4.0)	3.0 (2.4 to 3.8)	-8.9 (-27.9 to 15.9)	-3.8 (-20.2 to 15.6)
Ambient particulate matter pollution	24.7 (17.5 to 35.4)	24.6 (17.5 to 37.0)	18.7 (12.3 to 28.9)	-0.6 (-8.6 to 10.8)	-24.0 (-38.6 to -14.3)	24.6 (17.4 to 35.5)	24.5 (17.5 to 36.8)	18.7 (12.2 to 28.9)	-0.4 (-8.5 to 11.1)	-24.0 (-38.5 to -14.3)
Household air pollution from solid fuels	0.3 (0.1 to 0.8)	0.2 (0.1 to 0.5)	0.2 (0.0 to 0.5)	-35.2 (-65.4 to 2.0)	-14.9 (-51.7 to 20.5)	0.5 (0.1 to 2.7)	0.3 (0.1 to 2.0)	0.3 (0.1 to 1.4)	-31.7 (-65.3 to 1.7)	-13.6 (-51.7 to 20.0)
Ambient ozone pollution	46.3 (18.4 to 54.5)	46.4 (18.4 to 54.5)	47.5 (19.5 to 54.5)	0.2 (-0.1 to 0.5)	2.3 (0.1 to 6.5)	46.4 (18.4 to 54.5)	46.5 (18.4 to 54.5)	47.6 (19.5 to 54.6)	0.2 (-0.1 to 0.5)	2.3 (0.1 to 6.5)
Residential radon	13.7 (6.6 to 23.8)	13.5 (6.5 to 23.6)	13.4 (6.4 to 23.5)	-1.5 (-4.5 to 1.9)	-0.9 (-2.4 to 0.8)	13.8 (6.6 to 23.8)	13.5 (6.6 to 23.6)	13.4 (6.4 to 23.4)	-1.7 (-4.8 to 1.8)	-1.0 (-2.7 to 0.9)
Lead exposure	9.3 (4.8 to 13.4)	5.9 (2.6 to 9.6)	4.0 (1.5 to 7.0)	-35.9 (-46.6 to -27.1)	-33.1 (-41.1 to -26.9)	5.8 (2.0 to 9.9)	3.8 (1.1 to 7.4)	2.6 (0.6 to 5.4)	-34.3 (-48.6 to -24.2)	-32.3 (-42.3 to -26.0)
Occupational exposure to asbestos	6.0 (5.3 to 6.5)	4.9 (4.8 to 5.0)	4.3 (4.1 to 4.5)	-18.8 (-24.7 to -8.0)	-12.4 (-16.8 to -7.8)	0.9 (0.8 to 0.9)	0.9 (0.8 to 0.9)	0.9 (0.8 to 0.9)	-0.5 (-8.5 to 6.0)	0.4 (-6.4 to 6.4)
Occupational exposure to arsenic	0.4 (0.0 to 0.9)	0.4 (0.0 to 0.9)	0.4 (0.0 to 0.9)	-	-	0.3 (0.0 to 0.8)	0.3 (0.0 to 0.8)	0.3 (0.0 to 0.8)	-	-
Occupational exposure to benzene	0.6 (0.0 to 1.8)	0.6 (0.0 to 1.8)	0.6 (0.0 to 1.7)	1.0 (-3.3 to 6.3)	-3.0 (-6.8 to 2.1)	0.9 (0.1 to 2.4)	1.0 (0.1 to 2.7)	1.0 (0.1 to 2.8)	11.9 (6.8 to 19.8)	0.0 (-4.2 to 6.0)
Occupational exposure to beryllium	0.0 (0.0 to 0.0)	0.0 (0.0 to 0.0)	0.0 (0.0 to 0.0)	0.1 (-1.4 to 1.7)	0.4 (-1.7 to 2.5)	0.0 (0.0 to 0.0)	0.0 (0.0 to 0.0)	0.0 (0.0 to 0.0)	5.6 (3.5 to 7.8)	-0.6 (-3.0 to 2.1)
Occupational exposure to cadmium	0.0 (0.0 to 0.0)	0.0 (0.0 to 0.0)	0.0 (0.0 to 0.0)	-0.5 (-6.9 to 6.4)	0.5 (-5.9 to 6.9)	0.0 (0.0 to 0.0)	0.0 (0.0 to 0.0)	0.0 (0.0 to 0.0)	2.7 (-4.7 to 11.5)	-2.0 (-8.9 to 6.1)
Occupational exposure to chromium	0.1 (0.1 to 0.1)	0.1 (0.1 to 0.1)	0.1 (0.1 to 0.1)	1.9 (-3.4 to 7.8)	0.5 (-4.7 to 5.6)	0.1 (0.1 to 0.1)	0.1 (0.1 to 0.1)	0.1 (0.1 to 0.1)	5.1 (-1.0 to 12.0)	-2.1 (-7.7 to 4.3)
Occupational exposure to diesel engine exhaust	0.5 (0.5 to 0.5)	0.6 (0.5 to 0.6)	0.6 (0.5 to 0.6)	9.9 (5.0 to 14.9)	1.7 (-2.4 to 6.4)	0.5 (0.4 to 0.5)	0.5 (0.5 to 0.5)	0.5 (0.5 to 0.5)	9.0 (2.2 to 16.7)	-4.1 (-9.8 to 2.2)
Occupational exposure to formaldehyde	0.2 (0.2 to 0.2)	0.2 (0.2 to 0.2)	0.2 (0.2 to 0.2)	-2.4 (-9.0 to 4.7)	-1.1 (-7.6 to 5.3)	0.2 (0.2 to 0.2)	0.2 (0.2 to 0.2)	0.2 (0.2 to 0.2)	3.3 (-3.8 to 11.4)	-2.0 (-8.1 to 5.3)
Occupational exposure to nickel	0.3 (0.0 to 1.4)	0.3 (0.0 to 1.3)	0.3 (0.0 to 1.3)	-	-	0.3 (0.0 to 1.2)	0.3 (0.0 to 1.2)	0.3 (0.0 to 1.2)	-	-
Occupational exposure to polycyclic aromatic hydrocarbons	0.2 (0.2 to 0.2)	0.2 (0.2 to 0.2)	0.2 (0.2 to 0.2)	2.0 (-3.1 to 7.4)	-0.2 (-5.2 to 4.7)	0.2 (0.2 to 0.2)	0.2 (0.2 to 0.2)	0.2 (0.2 to 0.2)	8.1 (2.7 to 14.1)	-1.7 (-6.4 to 3.3)
Occupational exposure to silica	2.9 (0.4 to 9.3)	2.6 (0.4 to 8.3)	2.7 (0.4 to 8.4)	-9.7 (-16.4 to -2.0)	0.3 (-6.9 to 8.2)	2.2 (0.3 to 7.0)	2.1 (0.3 to 6.6)	2.1 (0.3 to 6.5)	-5.5 (-14.5 to 6.4)	-2.8 (-12.5 to 9.5)
Occupational exposure to sulphuric acid	0.6 (0.1 to 1.8)	0.7 (0.1 to 2.1)	0.6 (0.1 to 2.0)	10.6 (2.9 to 18.5)	-1.5 (-7.7 to 5.1)	0.5 (0.1 to 1.7)	0.7 (0.1 to 2.1)	0.6 (0.1 to 2.0)	23.4 (11.4 to 36.0)	-4.4 (-12.8 to 4.7)
Occupational exposure to trichloroethylene	0.1 (0.1 to 0.1)	0.1 (0.1 to 0.1)	0.1 (0.1 to 0.1)	5.4 (1.8 to 9.2)	1.1 (-2.2 to 4.3)	0.1 (0.1 to 0.1)	0.1 (0.1 to 0.1)	0.1 (0.1 to 0.1)	6.4 (1.5 to 11.7)	-2.5 (-7.0 to 2.6)
Occupational asthmagens	16.2 (13.9 to 19.1)	14.9 (12.9 to 17.5)	14.5 (12.3 to 17.1)	-7.7 (-13.7 to -1.7)	-2.9 (-8.3 to 2.5)	7.8 (6.6 to 9.3)	7.9 (6.7 to 9.3)	7.4 (6.3 to 8.8)	1.3 (-5.9 to 9.5)	-6.0 (-12.9 to 1.5)
Occupational particulate matter, gases, and fumes	6.7 (4.9 to 9.6)	6.3 (4.6 to 9.0)	6.1 (4.4 to 8.6)	-5.5 (-8.2 to -3.0)	-4.6 (-6.9 to -2.5)	3.7 (2.7 to 5.3)	3.6 (2.6 to 5.2)	3.4 (2.5 to 4.9)	-2.9 (-6.9 to 1.6)	-4.4 (-7.7 to -1.1)
Occupational noise	6.1 (5.7 to 6.5)	5.9 (5.6 to 6.3)	5.7 (5.3 to 6.1)	-2.8 (-4.2 to -1.5)	-3.8 (-4.6 to -2.8)	4.2 (4.0 to 4.5)	4.4 (4.2 to 4.7)	4.4 (4.2 to 4.7)	4.6 (3.0 to 6.6)	-0.3 (-1.3 to 0.7)
Occupational ergonomic factors	8.7 (7.4 to 10.4)	7.9 (6.7 to 9.4)	7.5 (6.3 to 9.0)	-9.1 (-14.1 to -4.3)	-4.5 (-8.3 to -0.5)	4.9 (4.1 to 5.9)	5.0 (4.2 to 6.1)	4.9 (4.1 to 6.0)	2.3 (-3.9 to 8.8)	-1.6 (-8.0 to 5.4)
Non-exclusive breastfeeding	0.5 (0.5 to 0.6)	0.5 (0.5 to 0.6)	0.5 (0.4 to 0.6)	-0.4 (-7.5 to 7.7)	-7.1 (-13.3 to -0.9)	0.5 (0.5 to 0.6)	0.5 (0.5 to 0.6)	0.5 (0.4 to 0.6)	-0.4 (-7.5 to 7.7)	-7.1 (-13.3 to -0.9)
Discontinued breastfeeding	2.2 (2.1 to 2.3)	2.1 (2.0 to 2.2)	2.2 (2.1 to 2.3)	-2.8 (-7.7 to 2.4)	2.2 (-2.2 to 7.0)	2.2 (2.1 to 2.3)	2.1 (2.0 to 2.2)	2.2 (2.1 to 2.3)	-2.8 (-7.7 to 2.4)	2.2 (-2.2 to 7.0)
Child underweight	0.1 (0.1 to 0.1)	0.1 (0.1 to 0.1)	0.1 (0.0 to 0.1)	-4.7 (-14.9 to 5.4)	-13.9 (-19.9 to -7.7)	0.1 (0.0 to 0.1)	0.1 (0.0 to 0.1)	0.1 (0.0 to 0.1)	-2.4 (-13.6 to 9.7)	-5.2 (-11.9 to 1.8)
Child wasting	0.1 (0.0 to 0.1)	0.1 (0.0 to 0.1)	0.1 (0.0 to 0.1)	-9.8 (-21.9 to 4.2)	-3.8 (-12.5 to 5.4)	0.1 (0.0 to 0.1)	0.1 (0.0 to 0.1)	0.1 (0.0 to 0.1)	6.4 (-9.3 to 25.1)	-5.8 (-15.0 to 4.7)
Child stunting	0.3 (0.1 to 0.4)	0.2 (0.1 to 0.4)	0.2 (0.1 to 0.3)	-8.7 (-38.3 to 31.0)	-12.3 (-31.2 to 10.0)	0.2 (0.1 to 0.3)	0.2 (0.1 to 0.3)	0.2 (0.1 to 0.3)	-9.6 (-34.8 to 26.6)	-4.5 (-22.1 to 17.4)
Short gestation for birth weight	0.0 (0.0 to 0.0)	0.0 (0.0 to 0.0)	0.0 (0.0 to 0.0)	6.6 (-2.9 to 20.5)	2.3 (-5.8 to 8.9)	0.0 (0.0 to 0.0)	0.0 (0.0 to 0.0)	0.0 (0.0 to 0.0)	6.6 (-4.5 to 18.4)	2.9 (-7.0 to 12.2)
Low birth weight for gestation	0.0 (0.0 to 0.0)	0.0 (0.0 to 0.0)	0.0 (0.0 to 0.0)	2.1 (-9.2 to 14.2)	-1.1 (-8.3 to 6.1)	0.0 (0.0 to 0.0)	0.0 (0.0 to 0.0)	0.0 (0.0 to 0.0)	0.7 (-8.6 to 12.1)	-1.5 (-9.5 to 8.1)
Iron deficiency	-	-	-	-	-	4.2 (2.9 to 5.9)	3.9 (2.7 to 5.4)	4.0 (2.8 to 5.6)	-7.2 (-21.3 to 11.0)	1.4 (-13.6 to 18.6)
Vitamin A deficiency	0.5 (0.3 to 0.6)	0.5 (0.3 to 0.6)	0.5 (0.4 to 0.7)	4.5 (-1.2 to 9.6)	9.9 (4.5 to 14.3)	0.6 (0.4 to 0.7)	0.6 (0.4 to 0.7)	0.6 (0.4 to 0.8)	2.3 (-2.1 to 6.5)	5.3 (1.1 to 8.9)
Zinc deficiency	0.5 (0.2 to 0.9)	0.2 (0.1 to 0.4)	0.2 (0.1 to 0.4)	-55.4 (-76.0 to 5.0)	-1.0 (-46.5 to 94.1)	0.5 (0.2 to 0.9)	0.2 (0.1 to 0.4)	0.2 (0.1 to 0.4)	-55.2 (-73.2 to 4.2)	0.3 (-46.4 to 89.1)
Smoking	13.8 (12.2 to 15.5)	7.6 (6.6 to 8.6)	6.0 (5.1 to 7.0)	-45.0 (-50.0 to -40.3)	-21.2 (-26.7 to -15.2)	10.0 (8.6 to 11.5)	5.5 (4.8 to 6.4)	4.2 (3.5 to 4.9)	-44.5 (-51.1 to -37.6)	-24.9 (-30.3 to -19.5)
Chewing tobacco	2.0 (1.9 to 2.2)	2.0 (1.7 to 2.4)	2.1 (1.6 to 2.6)	-0.4 (-17.8 to 21.9)	2.2 (-17.5 to 24.0)	0.1 (0.1 to 0.2)	0.1 (0.1 to 0.1)	0.1 (0.1 to 0.1)	-7.6 (-33.0 to 25.6)	-4.9 (-25.1 to 21.1)
Second-hand smoke	44.3 (41.2 to 47.7)	26.1 (24.9 to 27.3)	26.4 (24.5 to 28.6)	-41.0 (-47.2 to -34.6)	1.2 (-6.1 to 9.0)	41.9 (39.1 to 44.9)	25.1 (24.1 to 26.0)	25.0 (23.7 to 26.2)	-40.1 (-46.1 to -34.1)	-0.6 (-6.4 to 5.5)
Alcohol use	20.9 (14.1 to 29.2)	22.7 (15.5 to 31.2)	24.4 (15.5 to 35.4)	8.6 (-5.0 to 24.9)	7.8 (-13.8 to 34.6)	12.6 (7.8 to 19.4)	13.3 (8.3 to 20.1)	13.9 (8.1 to 21.7)	5.4 (-6.3 to 20.1)	4.6 (-16.1 to 31.6)
Drug use	1.7 (1.2 to 2.6)	2.2 (1.7 to 3.0)	2.8 (2.2 to 3.7)	26.6 (14.7 to 43.3)	27.7 (20.7 to 36.2)	1.3 (1.0 to 1.9)	1.8 (1.4 to 2.3)	2.1 (1.7 to 2.7)	32.2 (21.8 to 46.2)	18.5 (11.9 to 25.7)
Diet low in fruits	26.2 (20.2 to 31.0)	27.1 (21.3 to 31.8)	28.9 (22.8 to 33.7)	3.6 (-1.3 to 9.5)	6.6 (0.2 to 14.2)	23.0 (16.9 to 28.2)	24.6 (18.6 to 29.6)	25.8 (19.8 to 31.2)	7.0 (1.3 to 16.0)	5.0 (-2.5 to 14.3)
Diet low in vegetables	22.5 (16.6 to 27.7)	24.6 (19.6 to 29.0)	24.3 (19.4 to 28.7)	9.2 (3.9 to 18.8)	-1.2 (-6.5 to 5.3)	25.5 (20.0 to 30.2)	26.0 (21.0 to 30.5)	25.9 (20.9 to 30.3)	2.1 (-1.1 to 6.5)	-0.4 (-5.4 to 5.8)
Diet low in legumes	24.0 (18.8 to 28.5)	24.1 (19.0 to 28.5)	25.5 (20.3 to 30.1)	0.6 (-5.3 to 7.4)	5.8 (-1.7 to 15.7)	28.7 (24.0 to 32.5)	29.3 (25.0 to 33.1)	30.7 (26.3 to 34.4)	2.3 (-2.6 to 8.2)	4.6 (-0.8 to 10.5)
Diet low in whole grains	38.1 (35.2 to 40.5)	39.3 (36.7 to 41.6)	38.8 (36.0 to 41.2)	3.3 (1.0 to 6.1)	-1.2 (-3.8 to 0.8)	39.0 (36.4 to 41.3)	40.3 (37.8 to 42.3)	39.9 (37.3 to 42.0)	3.2 (0.9 to 6.0)	-1.0 (-3.1 to 1.2)
Diet low in nuts and seeds	45.5 (43.2 to 47.3)	36.4 (33.0 to 39.4)	36.7 (32.6 to 40.2)	-20.0 (-25.2 to -15.5)	0.8 (-4.5 to 5.4)	46.3 (44.3 to 48.0)	37.5 (34.0 to 40.4)	37.7 (33.8 to 41.2)	-19.0 (-24.3 to -14.9)	0.6 (-4.1 to 5.2)
Diet low in milk	27.4 (21.9 to 32.0)	26.0 (20.3 to 30.8)	26.5 (20.5 to 31.4)	-5.0 (-10.6 to -0.4)	1.6 (-3.3 to 7.3)	29.1 (24.0 to 33.4)	27.1 (21.4 to 31.6)	27.3 (21.7 to 31.9)	-7.1 (-13.0 to -3.0)	0.7 (-4.3 to 6.0)
Diet high in red meat	43.7 (41.5 to 45.8)	38.8 (36.2 to 41.1)	37.0 (33.4 to 40.5)	-11.2 (-16.2 to -5.9)	-4.5 (-12.9 to 3.5)	25.3 (22.0 to 28.7)	20.5 (17.0 to 23.6)	19.2 (14.4 to 23.3)	-18.9 (-27.7 to -9.3)	-6.4 (-22.4 to 10.7)
Diet high in processed meat	41.8 (37.0 to 45.7)	43.4 (40.4 to 45.7)	37.2 (32.2 to 40.9)	3.8 (-5.0 to 13.9)	-14.2 (-21.2 to -7.5)	23.3 (19.0 to 27.0)	27.8 (24.5 to 30.8)	24.9 (20.4 to 28.6)	19.5 (5.1 to 38.2)	-10.3 (-19.6 to -1.4)
Diet high in sugar-sweetened beverages	31.8 (16.1 to 37.7)	30.0 (15.5 to 32.9)	25.2 (13.0 to 29.6)	-5.5 (-18.7 to 14.5)	-16.1 (-27.9 to -3.3)	26.5 (13.2 to 32.7)	23.2 (11.9 to 25.8)	19.2 (9.6 to 23.1)	-12.6 (-27.8 to 10.7)	-17.3 (-31.2 to -0.9)
Diet low in fibre	33.1 (28.4 to 36.9)	25.2 (19.7 to 29.9)	26.8 (21.0 to 31.5)	-23.8 (-32.2 to -17.7)	6.1 (-0.1 to 12.8)	37.8 (33.9 to 40.8)	29.8 (24.6 to 34.0)	31.1 (25.9 to 35.3)	-21.0 (-28.2 to -15.7)	4.1 (-0.8 to 9.7)
Diet low in calcium	16.4 (9.7 to 22.2)	15.0 (8.8 to 20.7)	15.7 (9.4 to 21.5)	-8.3 (-16.5 to -1.9)	4.4 (-2.7 to 14.1)	20.5 (13.8 to 25.9)	18.8 (12.1 to 24.4)	19.5 (12.7 to 25.1)	-8.0 (-16.8 to -2.3)	3.6 (-1.5 to 11.2)
Diet low in seafood omega-3 fatty acids	28.2 (23.8 to 32.4)	24.2 (19.6 to 28.6)	25.6 (21.0 to 30.0)	-14.3 (-18.4 to -11.2)	5.7 (2.4 to 9.9)	31.7 (27.6 to 35.5)	28.3 (23.9 to 32.5)	29.3 (25.0 to 33.4)	-10.7 (-14.1 to -8.2)	3.4 (1.0 to 6.6)
Diet low in polyunsaturated fatty acids	8.1 (3.5 to 13.3)	5.2 (1.5 to 9.8)	3.9 (1.1 to 8.1)	-35.7 (-63.2 to -18.4)	-25.6 (-46.9 to -3.1)	8.8 (3.9 to 14.0)	5.8 (1.8 to 10.6)	4.3 (1.2 to 8.5)	-34.8 (-61.3 to -19.7)	-25.8 (-46.6 to -5.7)
Diet high in trans fatty acids	17.0 (12.8 to 22.0)	10.2 (6.3 to 14.4)	7.5 (4.0 to 11.4)	-40.1 (-50.8 to -32.6)	-26.2 (-37.9 to -19.3)	23.7 (18.7 to 31.7)	13.6 (9.6 to 18.0)	10.1 (6.2 to 14.2)	-42.7 (-50.3 to -35.3)	-25.7 (-34.8 to -19.6)
Diet high in sodium	7.1 (1.0 to 14.1)	12.4 (4.8 to 19.0)	12.9 (5.0 to 19.7)	73.0 (28.7 to 456.3)	4.2 (-11.1 to 30.5)	5.9 (0.5 to 12.8)	10.6 (2.9 to 17.7)	11.1 (3.3 to 18.1)	79.1 (33.9 to 680.2)	4.5 (-11.1 to 34.0)
Intimate partner violence	-	-	-	-	-	1.1 (0.9 to 1.3)	0.8 (0.7 to 1.0)	1.0 (0.8 to 1.2)	-27.3 (-29.5 to -25.0)	19.4 (16.1 to 22.8)
Childhood sexual abuse	5.4 (4.4 to 6.6)	4.9 (4.0 to 6.0)	4.9 (4.1 to 6.0)	-8.2 (-12.8 to -3.0)	0.4 (-4.1 to 5.0)	13.5 (10.8 to 16.6)	12.1 (9.9 to 14.5)	12.1 (9.9 to 14.6)	-10.7 (-15.7 to -5.5)	0.4 (-4.0 to 5.5)

Supplementary Results Table 1. United States age-standardised Summary Exposure Values (SEVs) for Level 4 risk factors and percentage change, by sex, 1990, 2007, and 2017.Data in parentheses are 95% uncertainty intervals.

	Male					Female				
	**1990**	**2007**	**2017**	**Percentage change 1990-2007**	**Percentage change 2007-2017**	**1990**	**2007**	**2017**	**Percentage change 1990-2007**	**Percentage change 2007-2017**
Bullying victimization	6.5 (2.8 to 13.5)	8.9 (3.9 to 18.0)	8.9 (3.9 to 18.0)	36.4 (24.1 to 53.1)	-0.4 (-5.3 to 5.0)	5.1 (2.2 to 10.3)	7.5 (3.4 to 15.2)	7.5 (3.3 to 15.2)	45.7 (32.1 to 67.7)	0.5 (-4.7 to 6.9)
Low physical activity	0.4 (0.2 to 0.8)	0.4 (0.2 to 0.8)	0.4 (0.2 to 0.8)	0.2 (-1.3 to 1.5)	0.1 (-0.5 to 0.5)	0.4 (0.2 to 0.9)	0.4 (0.2 to 0.9)	0.4 (0.2 to 0.9)	-0.0 (-1.6 to 1.6)	-0.3 (-0.8 to 0.2)
High fasting plasma glucose	1.3 (0.9 to 1.7)	2.5 (1.8 to 3.3)	3.2 (2.4 to 4.2)	92.9 (66.1 to 139.6)	28.2 (7.6 to 56.3)	1.5 (1.1 to 2.1)	2.8 (2.1 to 3.8)	2.7 (2.0 to 3.6)	86.9 (60.9 to 137.2)	-4.1 (-19.5 to 17.1)
High LDL cholesterol	21.1 (19.3 to 23.1)	11.6 (10.0 to 13.3)	10.2 (8.7 to 11.9)	-45.3 (-49.4 to -41.2)	-11.9 (-15.0 to -8.7)	19.9 (18.2 to 21.8)	11.4 (9.9 to 13.0)	10.6 (9.1 to 12.2)	-42.4 (-46.3 to -38.6)	-7.5 (-10.4 to -4.5)
High systolic blood pressure	6.4 (5.6 to 7.2)	4.8 (4.2 to 5.5)	4.9 (4.3 to 5.6)	-24.9 (-30.2 to -19.0)	2.7 (-3.7 to 9.1)	6.1 (5.5 to 6.8)	4.5 (4.0 to 5.0)	4.3 (3.8 to 4.9)	-27.4 (-33.0 to -21.4)	-3.0 (-9.9 to 4.2)
High body-mass index	14.5 (10.8 to 19.5)	21.2 (16.9 to 26.8)	22.4 (17.8 to 27.8)	46.8 (33.3 to 63.9)	5.3 (-0.5 to 12.1)	16.0 (12.5 to 20.5)	22.4 (18.3 to 27.6)	23.6 (19.1 to 28.8)	39.6 (29.9 to 51.3)	5.4 (-0.6 to 12.1)
Low bone mineral density	5.1 (4.3 to 5.9)	4.6 (3.9 to 5.4)	4.9 (4.2 to 5.7)	-9.7 (-12.5 to -6.6)	6.2 (2.9 to 9.7)	5.8 (5.0 to 6.7)	5.7 (4.9 to 6.6)	6.1 (5.2 to 7.0)	-1.8 (-4.6 to 1.2)	7.7 (4.7 to 10.7)
Impaired kidney function	2.2 (1.1 to 5.5)	2.2 (1.1 to 5.5)	2.3 (1.1 to 5.6)	-0.6 (-4.1 to 2.6)	2.1 (-2.4 to 7.0)	2.9 (1.4 to 6.7)	2.9 (1.4 to 6.7)	3.0 (1.4 to 6.9)	-2.3 (-5.9 to 0.8)	3.4 (-0.2 to 7.7)

Supplementary Results Table 1. Alabama age-standardised Summary Exposure Values (SEVs) for Level 4 risk factors and percentage change, by sex, 1990, 2007, and 2017.Data in parentheses are 95% uncertainty intervals.										
	Male					Female				
	1990	2007	2017	Percentage change 1990-2007	Percentage change 2007-2017	1990	2007	2017	Percentage change 1990-2007	Percentage change 2007-2017
Unsafe water source	1.9 (1.0 to 3.3)	0.6 (0.4 to 1.0)	0.4 (0.2 to 0.6)	-67.2 (-82.9 to -35.4)	-40.2 (-63.4 to -0.5)	1.9 (1.0 to 3.3)	0.6 (0.4 to 1.0)	0.4 (0.2 to 0.6)	-67.2 (-82.9 to -35.4)	-40.2 (-63.4 to -0.5)
Unsafe sanitation	9.4 (5.0 to 16.0)	5.7 (2.7 to 10.7)	3.9 (1.7 to 7.3)	-38.8 (-71.7 to 24.2)	-32.4 (-64.5 to 28.9)	9.4 (5.0 to 16.0)	5.7 (2.7 to 10.7)	3.9 (1.7 to 7.3)	-38.8 (-71.7 to 24.2)	-32.4 (-64.5 to 28.9)
No access to handwashing facility	3.5 (2.6 to 4.8)	3.2 (2.2 to 4.5)	3.1 (2.2 to 4.2)	-9.6 (-35.4 to 33.2)	-3.9 (-26.6 to 24.6)	3.5 (2.6 to 4.8)	3.2 (2.2 to 4.5)	3.1 (2.2 to 4.2)	-9.6 (-35.4 to 33.2)	-3.9 (-26.6 to 24.6)
Ambient particulate matter pollution	28.0 (19.0 to 40.6)	27.6 (18.6 to 41.6)	21.2 (12.9 to 32.6)	-1.4 (-24.2 to 29.3)	-22.9 (-48.4 to 1.2)	27.8 (18.9 to 40.5)	27.5 (18.5 to 41.4)	21.2 (12.9 to 32.5)	-1.1 (-24.2 to 30.0)	-23.0 (-48.5 to 1.2)
Household air pollution from solid fuels	0.5 (0.1 to 1.4)	0.3 (0.1 to 0.7)	0.2 (0.1 to 0.8)	-42.2 (-78.6 to 37.6)	-11.7 (-63.0 to 73.3)	0.7 (0.2 to 4.7)	0.4 (0.1 to 3.2)	0.4 (0.1 to 2.7)	-36.9 (-77.8 to 38.0)	-11.1 (-63.2 to 75.4)
Ambient ozone pollution	46.5 (17.8 to 54.8)	46.9 (18.1 to 54.8)	48.5 (19.6 to 54.8)	0.9 (0.0 to 2.4)	3.4 (0.0 to 9.9)	46.5 (17.8 to 54.8)	46.9 (18.1 to 54.8)	48.5 (19.6 to 54.8)	0.9 (0.0 to 2.4)	3.4 (0.0 to 9.9)
Residential radon	11.0 (0.5 to 38.2)	11.0 (0.5 to 38.2)	11.0 (0.5 to 38.2)	0.0 (0.0 to 0.0)	0.0 (0.0 to 0.0)	11.0 (0.5 to 38.2)	11.0 (0.5 to 38.2)	11.0 (0.5 to 38.2)	0.0 (0.0 to 0.0)	0.0 (0.0 to 0.0)
Lead exposure	7.8 (3.2 to 12.1)	5.3 (1.9 to 9.1)	3.6 (1.2 to 6.8)	-32.4 (-43.5 to -23.6)	-30.8 (-40.4 to -24.3)	4.3 (0.9 to 8.4)	3.1 (0.6 to 6.6)	2.2 (0.3 to 5.1)	-29.3 (-43.1 to -19.7)	-29.1 (-41.0 to -22.1)
Occupational exposure to asbestos	5.2 (4.6 to 5.9)	4.4 (4.1 to 4.7)	4.1 (3.4 to 4.8)	-16.1 (-26.4 to -2.5)	-7.2 (-22.9 to 10.2)	0.6 (0.5 to 0.6)	0.6 (0.6 to 0.7)	0.7 (0.5 to 0.8)	13.3 (-2.1 to 32.1)	5.4 (-15.3 to 28.5)
Occupational exposure to arsenic	0.4 (0.0 to 0.9)	0.4 (0.0 to 0.9)	0.4 (0.0 to 0.9)	-	-	0.3 (0.0 to 0.8)	0.4 (0.0 to 0.8)	0.3 (0.0 to 0.8)	-	-
Occupational exposure to benzene	0.6 (0.0 to 1.6)	0.6 (0.0 to 1.6)	0.6 (0.0 to 1.6)	1.0 (-6.1 to 12.4)	-2.2 (-8.4 to 5.4)	0.8 (0.0 to 2.2)	0.9 (0.1 to 2.5)	0.9 (0.1 to 2.5)	11.8 (0.2 to 29.4)	1.1 (-6.8 to 12.3)
Occupational exposure to beryllium	0.0 (0.0 to 0.0)	0.0 (0.0 to 0.0)	0.0 (0.0 to 0.0)	0.2 (-5.3 to 5.9)	-0.0 (-4.4 to 4.4)	0.0 (0.0 to 0.0)	0.0 (0.0 to 0.0)	0.0 (0.0 to 0.0)	6.6 (-2.1 to 16.1)	-0.6 (-6.8 to 5.6)
Occupational exposure to cadmium	0.0 (0.0 to 0.1)	0.0 (0.0 to 0.1)	0.0 (0.0 to 0.1)	1.1 (-9.7 to 13.7)	-1.0 (-9.6 to 8.8)	0.0 (0.0 to 0.0)	0.0 (0.0 to 0.0)	0.0 (0.0 to 0.0)	4.0 (-10.4 to 21.7)	-2.6 (-14.4 to 9.7)
Occupational exposure to chromium	0.1 (0.1 to 0.1)	0.1 (0.1 to 0.1)	0.1 (0.1 to 0.1)	3.3 (-6.7 to 14.3)	-0.7 (-8.2 to 7.4)	0.1 (0.1 to 0.1)	0.1 (0.1 to 0.1)	0.1 (0.1 to 0.1)	6.3 (-6.4 to 21.4)	-2.6 (-12.6 to 7.5)
Occupational exposure to diesel engine exhaust	0.5 (0.5 to 0.6)	0.6 (0.5 to 0.6)	0.6 (0.5 to 0.6)	9.2 (-0.1 to 19.0)	1.9 (-5.2 to 9.7)	0.4 (0.4 to 0.5)	0.5 (0.4 to 0.5)	0.5 (0.4 to 0.5)	12.6 (-1.1 to 27.7)	-4.6 (-13.4 to 4.8)
Occupational exposure to formaldehyde	0.2 (0.2 to 0.3)	0.2 (0.2 to 0.3)	0.2 (0.2 to 0.3)	-0.8 (-12.4 to 11.9)	-2.5 (-11.4 to 7.7)	0.2 (0.2 to 0.2)	0.2 (0.2 to 0.2)	0.2 (0.2 to 0.2)	4.0 (-9.9 to 21.0)	-2.4 (-13.9 to 8.8)
Occupational exposure to nickel	0.3 (0.0 to 1.5)	0.3 (0.0 to 1.5)	0.3 (0.0 to 1.5)	-	-	0.3 (0.0 to 1.2)	0.3 (0.0 to 1.2)	0.3 (0.0 to 1.2)	-	-
Occupational exposure to polycyclic aromatic hydrocarbons	0.2 (0.2 to 0.2)	0.2 (0.2 to 0.2)	0.2 (0.2 to 0.2)	3.1 (-6.7 to 13.7)	-1.2 (-8.2 to 6.7)	0.2 (0.2 to 0.2)	0.2 (0.2 to 0.3)	0.2 (0.2 to 0.3)	9.0 (-2.5 to 23.1)	-2.1 (-11.1 to 6.8)
Occupational exposure to silica	3.3 (0.5 to 10.5)	3.0 (0.4 to 9.6)	3.0 (0.4 to 9.7)	-8.7 (-18.9 to 3.9)	-1.2 (-10.3 to 10.4)	2.3 (0.3 to 7.3)	2.2 (0.3 to 7.1)	2.2 (0.3 to 7.2)	-3.3 (-18.8 to 19.2)	-3.4 (-17.8 to 13.2)
Occupational exposure to sulphuric acid	0.6 (0.1 to 1.9)	0.7 (0.1 to 2.1)	0.6 (0.1 to 2.1)	11.2 (-0.3 to 24.5)	-2.4 (-10.3 to 7.3)	0.5 (0.1 to 1.6)	0.7 (0.1 to 2.1)	0.6 (0.1 to 2.0)	27.3 (8.0 to 50.8)	-5.6 (-17.8 to 7.7)
Occupational exposure to trichloroethylene	0.1 (0.1 to 0.1)	0.1 (0.1 to 0.1)	0.1 (0.1 to 0.1)	6.2 (-1.7 to 13.8)	0.3 (-4.9 to 6.1)	0.1 (0.1 to 0.1)	0.1 (0.1 to 0.1)	0.1 (0.1 to 0.1)	8.3 (-3.3 to 21.0)	-3.0 (-11.0 to 6.1)
Occupational asthmagens	16.9 (14.2 to 20.1)	15.9 (13.5 to 19.0)	15.3 (12.7 to 18.5)	-6.1 (-16.0 to 5.1)	-3.8 (-11.7 to 5.5)	7.6 (6.1 to 9.2)	7.7 (6.3 to 9.4)	7.2 (5.8 to 8.9)	1.8 (-11.1 to 17.5)	-6.3 (-16.3 to 5.5)
Occupational particulate matter, gases, and fumes	7.1 (5.1 to 10.1)	6.8 (5.0 to 9.7)	6.5 (4.7 to 9.4)	-3.7 (-8.3 to 0.6)	-4.5 (-8.5 to -0.8)	3.6 (2.5 to 5.4)	3.6 (2.5 to 5.5)	3.5 (2.5 to 5.2)	0.3 (-7.5 to 9.1)	-3.7 (-9.2 to 2.3)
Occupational noise	6.1 (5.6 to 6.7)	6.0 (5.5 to 6.6)	5.8 (5.3 to 6.4)	-2.1 (-5.2 to 1.3)	-3.6 (-5.9 to -1.1)	4.0 (3.4 to 4.6)	4.2 (3.7 to 4.8)	4.2 (3.7 to 4.7)	6.3 (0.1 to 13.2)	0.1 (-3.3 to 4.0)
Occupational ergonomic factors	9.3 (7.7 to 11.3)	8.2 (6.9 to 9.8)	7.8 (6.4 to 9.5)	-11.6 (-21.4 to -0.5)	-4.7 (-12.3 to 2.7)	5.0 (4.0 to 6.3)	5.0 (4.0 to 6.3)	4.9 (3.9 to 6.2)	0.0 (-14.9 to 17.4)	-2.2 (-13.5 to 10.2)
Non-exclusive breastfeeding	0.5 (0.5 to 0.6)	0.5 (0.5 to 0.6)	0.5 (0.4 to 0.6)	-0.7 (-12.0 to 12.5)	-6.9 (-16.0 to 2.8)	0.5 (0.5 to 0.6)	0.5 (0.5 to 0.6)	0.5 (0.4 to 0.6)	0.1 (-11.3 to 13.4)	-7.6 (-16.6 to 2.0)
Discontinued breastfeeding	2.1 (2.0 to 2.3)	2.1 (2.0 to 2.2)	2.1 (2.0 to 2.2)	-1.2 (-8.9 to 7.1)	1.1 (-4.8 to 8.2)	2.1 (2.0 to 2.3)	2.1 (2.0 to 2.2)	2.1 (2.0 to 2.3)	-1.3 (-8.9 to 6.9)	2.0 (-4.0 to 9.1)
Child underweight	0.1 (0.1 to 0.2)	0.1 (0.1 to 0.1)	0.1 (0.0 to 0.1)	-5.8 (-20.0 to 10.2)	-14.5 (-24.1 to -5.7)	0.1 (0.0 to 0.1)	0.1 (0.0 to 0.1)	0.1 (0.0 to 0.1)	-4.3 (-21.2 to 13.3)	-5.3 (-16.1 to 6.2)
Child wasting	0.1 (0.0 to 0.1)	0.1 (0.0 to 0.1)	0.1 (0.0 to 0.1)	-10.6 (-30.9 to 10.6)	-3.8 (-17.6 to 9.8)	0.1 (0.0 to 0.1)	0.1 (0.0 to 0.1)	0.1 (0.0 to 0.1)	6.1 (-17.1 to 39.2)	-6.6 (-21.0 to 9.6)
Child stunting	0.3 (0.1 to 0.5)	0.3 (0.1 to 0.5)	0.2 (0.1 to 0.4)	-9.2 (-50.1 to 64.4)	-12.0 (-41.5 to 29.1)	0.2 (0.1 to 0.4)	0.2 (0.1 to 0.4)	0.2 (0.1 to 0.4)	-10.2 (-47.9 to 51.5)	-4.6 (-31.5 to 32.9)
Short gestation for birth weight	0.0 (0.0 to 0.0)	0.0 (0.0 to 0.0)	0.0 (0.0 to 0.0)	9.5 (-12.9 to 40.7)	-1.4 (-25.6 to 27.9)	0.0 (0.0 to 0.0)	0.0 (0.0 to 0.0)	0.0 (0.0 to 0.0)	7.6 (-29.3 to 44.6)	2.3 (-26.1 to 41.9)
Low birth weight for gestation	0.0 (0.0 to 0.0)	0.0 (0.0 to 0.0)	0.0 (0.0 to 0.0)	0.5 (-20.1 to 26.3)	0.5 (-19.1 to 27.8)	0.0 (0.0 to 0.0)	0.0 (0.0 to 0.0)	0.0 (0.0 to 0.0)	-1.3 (-23.3 to 50.6)	-1.9 (-29.0 to 25.5)
Iron deficiency	-	-	-	-	-	4.7 (3.0 to 6.8)	4.4 (2.8 to 6.2)	4.3 (2.7 to 6.3)	-7.8 (-31.7 to 24.5)	-0.8 (-22.5 to 27.8)
Vitamin A deficiency	0.5 (0.3 to 0.7)	0.5 (0.4 to 0.7)	0.6 (0.4 to 0.8)	5.7 (-14.1 to 28.2)	7.0 (-18.2 to 31.5)	0.6 (0.4 to 0.8)	0.7 (0.4 to 0.9)	0.8 (0.4 to 1.3)	11.5 (-7.6 to 37.1)	18.0 (-15.1 to 66.8)
Zinc deficiency	0.5 (0.1 to 1.2)	0.2 (0.0 to 1.0)	0.2 (0.0 to 1.0)	-56.4 (-87.9 to 319.7)	12.5 (-87.9 to 840.4)	0.5 (0.1 to 1.1)	0.2 (0.0 to 1.0)	0.2 (0.0 to 1.1)	-55.6 (-87.4 to 246.6)	13.0 (-87.1 to 846.6)
Smoking	15.4 (12.6 to 18.1)	8.4 (6.8 to 10.1)	6.8 (5.3 to 8.5)	-45.2 (-52.9 to -36.3)	-19.4 (-28.8 to -10.0)	10.1 (7.9 to 12.5)	5.5 (4.4 to 6.8)	4.4 (3.4 to 5.5)	-45.4 (-55.6 to -32.4)	-20.2 (-29.3 to -9.7)
Chewing tobacco	5.2 (4.6 to 5.9)	4.2 (3.2 to 5.5)	4.1 (2.9 to 5.8)	-18.1 (-39.9 to 9.1)	-2.6 (-26.2 to 24.9)	0.3 (0.2 to 0.4)	0.2 (0.1 to 0.3)	0.2 (0.1 to 0.2)	-35.7 (-60.4 to 5.4)	-13.6 (-40.0 to 21.9)
Second-hand smoke	41.8 (38.3 to 45.7)	27.9 (26.3 to 29.4)	28.2 (25.3 to 31.1)	-33.2 (-41.1 to -24.7)	1.2 (-8.9 to 11.6)	41.0 (38.1 to 44.1)	27.6 (26.3 to 28.7)	27.1 (25.2 to 29.1)	-32.6 (-39.7 to -25.4)	-1.9 (-9.1 to 5.7)
Alcohol use	20.9 (11.5 to 31.8)	22.3 (12.1 to 33.3)	23.7 (12.3 to 36.7)	7.0 (-34.4 to 69.9)	6.3 (-28.5 to 56.8)	11.2 (5.9 to 18.5)	11.9 (6.3 to 19.5)	12.2 (6.0 to 21.5)	6.2 (-33.4 to 64.6)	2.9 (-28.9 to 54.7)
Drug use	1.6 (1.2 to 2.4)	2.3 (1.8 to 2.9)	3.1 (2.5 to 4.0)	41.5 (21.9 to 69.1)	36.1 (23.8 to 51.5)	1.2 (0.9 to 1.7)	1.6 (1.2 to 2.1)	2.1 (1.7 to 2.8)	34.2 (20.6 to 52.8)	35.2 (22.8 to 50.3)
Diet low in fruits	29.4 (23.1 to 34.5)	32.1 (26.3 to 36.5)	34.7 (28.7 to 39.3)	9.4 (-0.3 to 21.8)	8.2 (1.5 to 16.2)	25.7 (19.4 to 31.3)	29.0 (23.0 to 33.8)	31.2 (25.0 to 36.5)	12.8 (0.6 to 33.0)	7.8 (-1.2 to 18.2)
Diet low in vegetables	29.3 (22.3 to 35.2)	38.5 (33.6 to 42.4)	41.1 (36.8 to 44.6)	31.4 (16.8 to 58.3)	6.6 (0.8 to 13.7)	31.4 (24.6 to 36.7)	38.3 (33.2 to 42.4)	41.1 (36.9 to 44.7)	22.1 (10.0 to 41.2)	7.2 (1.6 to 14.7)
Diet low in legumes	24.2 (17.2 to 30.1)	24.3 (17.7 to 30.2)	25.7 (18.9 to 31.3)	0.5 (-15.5 to 20.2)	5.8 (-6.9 to 20.7)	28.7 (22.7 to 33.7)	29.4 (24.0 to 34.4)	30.7 (25.0 to 35.4)	2.4 (-9.1 to 16.9)	4.4 (-4.6 to 14.6)
Diet low in whole grains	38.7 (35.1 to 41.6)	39.9 (36.7 to 42.5)	39.5 (36.1 to 42.3)	3.0 (-1.5 to 8.7)	-1.0 (-4.6 to 2.6)	39.6 (36.2 to 42.3)	40.8 (37.6 to 43.2)	40.5 (37.3 to 43.1)	2.9 (-1.1 to 8.1)	-0.7 (-4.2 to 2.8)
Diet low in nuts and seeds	47.3 (44.7 to 49.4)	38.9 (34.5 to 42.5)	39.7 (34.9 to 43.4)	-17.9 (-25.2 to -12.7)	2.0 (-4.7 to 8.8)	48.1 (45.8 to 49.9)	40.0 (35.8 to 43.5)	40.6 (36.0 to 44.2)	-16.7 (-23.5 to -11.5)	1.4 (-4.4 to 7.4)
Diet low in milk	28.9 (22.8 to 33.8)	27.4 (20.9 to 32.4)	28.0 (21.7 to 33.1)	-5.3 (-14.4 to 3.5)	2.2 (-5.8 to 11.8)	30.7 (24.9 to 35.4)	28.5 (22.5 to 33.7)	28.9 (22.9 to 33.9)	-7.1 (-16.8 to 1.1)	1.1 (-6.3 to 9.0)
Diet high in red meat	39.7 (29.9 to 47.9)	33.7 (22.3 to 43.1)	31.8 (20.1 to 41.3)	-15.1 (-35.1 to 8.1)	-5.7 (-26.0 to 16.9)	20.9 (11.3 to 30.3)	15.0 (7.4 to 23.5)	14.0 (6.2 to 22.8)	-28.2 (-57.5 to 12.1)	-6.5 (-42.6 to 40.4)
Diet high in processed meat	36.1 (28.1 to 43.8)	33.1 (25.2 to 40.1)	26.0 (19.6 to 32.4)	-8.3 (-24.8 to 12.4)	-21.3 (-33.6 to -7.1)	19.0 (13.5 to 23.9)	18.6 (13.6 to 23.4)	16.0 (11.1 to 20.8)	-2.5 (-24.1 to 22.5)	-13.7 (-28.4 to 1.8)
Diet high in sugar-sweetened beverages	28.5 (13.8 to 39.4)	26.8 (13.3 to 37.2)	21.8 (10.6 to 32.6)	-6.0 (-38.0 to 42.4)	-18.5 (-44.0 to 13.3)	23.6 (11.2 to 35.0)	20.6 (9.6 to 30.8)	16.5 (7.1 to 26.6)	-12.6 (-48.1 to 46.8)	-19.8 (-49.0 to 21.3)
Diet low in fibre	34.0 (28.2 to 38.7)	26.0 (19.1 to 32.1)	27.7 (20.8 to 33.6)	-23.5 (-36.0 to -13.8)	6.5 (-4.5 to 20.7)	38.6 (33.9 to 42.4)	30.7 (24.3 to 35.9)	32.0 (25.8 to 37.3)	-20.5 (-31.0 to -12.6)	4.2 (-4.3 to 14.1)
Diet low in calcium	18.8 (11.3 to 24.8)	16.9 (9.9 to 23.3)	17.9 (10.8 to 24.3)	-9.7 (-23.3 to 1.7)	5.7 (-3.4 to 19.3)	22.8 (15.8 to 28.4)	20.8 (14.0 to 26.6)	21.8 (14.8 to 27.6)	-8.6 (-20.0 to 0.7)	4.6 (-3.0 to 14.8)
Diet low in seafood omega-3 fatty acids	31.2 (25.8 to 35.8)	26.9 (21.0 to 32.6)	28.6 (22.6 to 33.9)	-13.7 (-25.0 to -4.2)	6.3 (-2.4 to 16.7)	34.5 (29.5 to 38.7)	31.1 (25.3 to 35.8)	32.2 (26.9 to 36.8)	-9.8 (-18.4 to -2.4)	3.6 (-2.7 to 11.4)
Diet low in polyunsaturated fatty acids	9.8 (3.6 to 16.8)	6.4 (1.5 to 13.0)	5.1 (0.9 to 11.0)	-34.6 (-75.0 to -1.6)	-20.8 (-60.0 to 49.7)	10.6 (4.0 to 17.7)	7.2 (1.9 to 13.9)	5.5 (1.2 to 11.8)	-32.4 (-71.8 to 1.6)	-23.1 (-59.2 to 13.8)
Diet high in trans fatty acids	16.7 (12.1 to 21.9)	9.8 (5.8 to 14.2)	7.2 (3.4 to 11.2)	-41.3 (-56.3 to -29.1)	-27.2 (-43.0 to -15.2)	23.8 (18.1 to 31.6)	13.3 (9.0 to 17.9)	9.7 (5.9 to 14.0)	-44.2 (-55.8 to -31.9)	-26.6 (-39.9 to -16.1)
Diet high in sodium	6.8 (0.5 to 14.5)	12.1 (3.6 to 19.6)	12.6 (3.6 to 20.1)	76.2 (20.2 to 745.9)	4.6 (-23.4 to 49.7)	6.0 (0.3 to 13.0)	10.8 (2.4 to 18.3)	11.2 (2.7 to 18.7)	80.7 (26.5 to 967.5)	4.0 (-24.6 to 52.0)
Intimate partner violence	-	-	-	-	-	1.5 (1.2 to 1.7)	1.0 (0.9 to 1.2)	1.3 (1.1 to 1.5)	-29.5 (-33.7 to -25.5)	25.3 (19.6 to 32.2)
Childhood sexual abuse	5.6 (4.6 to 6.9)	5.1 (4.1 to 6.2)	5.1 (4.1 to 6.2)	-9.8 (-15.1 to -4.3)	0.4 (-4.7 to 6.0)	13.8 (11.0 to 16.9)	12.1 (9.8 to 14.7)	12.2 (9.9 to 14.8)	-11.9 (-17.2 to -6.0)	0.4 (-4.8 to 6.5)

Supplementary Results Table 1. Alabama age-standardised Summary Exposure Values (SEVs) for Level 4 risk factors and percentage change, by sex, 1990, 2007, and 2017.Data in parentheses are 95% uncertainty intervals.										
	**Male**					**Female**				
	**1990**	**2007**	**2017**	**Percentage change 1990-2007**	**Percentage change 2007-2017**	**1990**	**2007**	**2017**	**Percentage change 1990-2007**	**Percentage change 2007-2017**
Bullying victimization	17.9 (8.0 to 31.5)	19.5 (9.3 to 34.3)	19.8 (9.2 to 34.6)	9.0 (-6.6 to 34.1)	1.3 (-6.8 to 10.9)	18.0 (8.0 to 32.5)	18.9 (9.0 to 33.4)	19.1 (8.9 to 33.7)	5.0 (-11.0 to 37.2)	1.0 (-8.5 to 10.7)
Low physical activity	0.4 (0.2 to 0.8)	0.4 (0.2 to 0.8)	0.4 (0.2 to 0.8)	-0.6 (-3.1 to 1.7)	0.4 (-1.2 to 2.2)	0.5 (0.2 to 0.9)	0.5 (0.2 to 0.9)	0.5 (0.2 to 0.9)	-0.4 (-3.3 to 2.9)	-0.3 (-2.1 to 1.6)
High fasting plasma glucose	1.3 (0.8 to 2.3)	3.2 (2.1 to 4.7)	3.8 (2.4 to 5.8)	140.6 (41.4 to 318.8)	17.7 (-21.3 to 75.7)	1.6 (1.0 to 2.8)	3.4 (2.0 to 5.4)	3.2 (1.7 to 5.0)	109.5 (22.6 to 273.2)	-8.3 (-49.4 to 61.5)
High LDL cholesterol	21.1 (19.1 to 23.3)	11.5 (9.9 to 13.2)	10.1 (8.6 to 11.9)	-45.5 (-50.5 to -40.8)	-11.9 (-16.0 to -7.5)	19.5 (17.7 to 21.5)	11.5 (10.0 to 13.2)	10.6 (9.2 to 12.3)	-41.2 (-45.8 to -36.6)	-7.3 (-11.6 to -3.3)
High systolic blood pressure	6.8 (5.9 to 7.8)	5.3 (4.6 to 6.0)	5.3 (4.6 to 6.2)	-23.0 (-31.2 to -14.9)	1.8 (-6.6 to 10.7)	6.8 (5.9 to 7.7)	4.8 (4.3 to 5.4)	4.5 (3.9 to 5.2)	-28.8 (-36.7 to -19.9)	-6.2 (-15.5 to 4.4)
High body-mass index	12.9 (9.1 to 18.1)	20.8 (16.1 to 26.8)	20.9 (16.0 to 27.1)	61.2 (39.9 to 88.6)	0.4 (-7.5 to 8.7)	15.0 (11.3 to 19.9)	23.3 (18.6 to 29.3)	24.3 (19.3 to 30.4)	55.1 (36.0 to 78.5)	4.6 (-3.9 to 14.3)
Low bone mineral density	5.4 (4.4 to 6.3)	5.8 (4.8 to 6.8)	6.2 (5.2 to 7.4)	7.5 (-7.6 to 25.2)	8.1 (-5.8 to 24.0)	5.7 (4.7 to 6.9)	5.6 (4.7 to 6.6)	6.2 (5.1 to 7.3)	-2.4 (-14.9 to 13.4)	9.9 (-4.6 to 25.9)
Impaired kidney function	2.5 (1.2 to 6.1)	2.5 (1.2 to 6.3)	2.6 (1.3 to 6.3)	2.4 (-3.2 to 8.5)	1.4 (-4.2 to 7.3)	3.2 (1.6 to 7.3)	3.2 (1.6 to 7.3)	3.3 (1.6 to 7.5)	0.7 (-4.3 to 5.9)	3.3 (-1.7 to 9.2)

Supplementary Results Table 1. Alaska age-standardised Summary Exposure Values (SEVs) for Level 4 risk factors and percentage change, by sex, 1990, 2007, and 2017.Data in parentheses are 95% uncertainty intervals.

	Male					Female				
	1990	2007	2017	Percentage change 1990-2007	Percentage change 2007-2017	1990	2007	2017	Percentage change 1990-2007	Percentage change 2007-2017
Unsafe water source	1.7 (1.0 to 2.9)	0.6 (0.3 to 0.9)	0.3 (0.2 to 0.5)	-68.2 (-83.6 to -38.7)	-40.7 (-64.4 to -3.6)	1.7 (1.0 to 2.9)	0.6 (0.3 to 0.9)	0.3 (0.2 to 0.5)	-68.2 (-83.6 to -38.7)	-40.7 (-64.4 to -3.6)
Unsafe sanitation	8.5 (4.3 to 14.6)	4.8 (2.3 to 9.2)	3.0 (1.4 to 5.9)	-43.2 (-73.5 to 28.4)	-37.5 (-68.0 to 13.7)	8.5 (4.3 to 14.6)	4.8 (2.3 to 9.2)	3.0 (1.4 to 5.9)	-43.2 (-73.5 to 28.4)	-37.5 (-68.0 to 13.7)
No access to handwashing facility	3.4 (2.5 to 4.7)	3.2 (2.3 to 4.3)	3.0 (2.2 to 4.1)	-8.1 (-35.4 to 30.7)	-4.1 (-25.4 to 24.5)	3.4 (2.5 to 4.7)	3.2 (2.3 to 4.3)	3.0 (2.2 to 4.1)	-8.1 (-35.4 to 30.7)	-4.1 (-25.4 to 24.5)
Ambient particulate matter pollution	15.8 (0.3 to 35.0)	18.0 (1.7 to 35.3)	13.4 (0.1 to 30.0)	13.5 (-84.1 to 5986.9)	-25.6 (-99.0 to 380.2)	15.7 (0.3 to 34.8)	17.9 (1.7 to 35.2)	13.3 (0.1 to 29.9)	13.9 (-84.0 to 5494.6)	-25.5 (-98.9 to 380.3)
Household air pollution from solid fuels	0.4 (0.0 to 1.8)	0.2 (0.0 to 0.8)	0.2 (0.0 to 0.6)	-37.7 (-86.6 to 853.8)	-23.5 (-97.2 to 138.6)	0.6 (0.0 to 4.3)	0.4 (0.0 to 2.1)	0.3 (0.0 to 1.6)	-34.0 (-87.7 to 836.7)	-23.5 (-96.7 to 138.8)
Ambient ozone pollution	16.2 (5.3 to 32.3)	18.3 (6.1 to 35.9)	20.9 (7.0 to 40.1)	13.5 (7.5 to 24.9)	14.0 (7.7 to 25.2)	16.2 (5.3 to 32.3)	18.3 (6.1 to 35.9)	20.9 (7.0 to 40.1)	13.5 (7.5 to 24.9)	14.0 (7.7 to 25.2)
Residential radon	26.7 (3.2 to 71.2)	26.7 (3.2 to 71.2)	26.7 (3.2 to 71.2)	0.0 (0.0 to 0.0)	0.0 (0.0 to 0.0)	26.7 (3.2 to 71.2)	26.7 (3.2 to 71.2)	26.7 (3.2 to 71.2)	0.0 (0.0 to 0.0)	0.0 (0.0 to 0.0)
Lead exposure	6.7 (2.6 to 11.1)	4.6 (1.5 to 8.4)	3.2 (0.9 to 6.2)	-32.2 (-44.8 to -22.7)	-31.0 (-41.7 to -24.0)	3.9 (0.7 to 7.8)	2.7 (0.4 to 6.2)	1.9 (0.3 to 4.7)	-29.2 (-44.3 to -19.7)	-29.5 (-42.4 to -22.4)
Occupational exposure to asbestos	8.1 (7.1 to 9.1)	6.6 (6.1 to 7.1)	6.1 (5.5 to 6.9)	-18.6 (-28.6 to -4.4)	-6.9 (-18.3 to 5.9)	1.0 (0.8 to 1.1)	0.9 (0.8 to 1.0)	1.0 (0.8 to 1.1)	-3.3 (-17.0 to 11.7)	3.9 (-11.0 to 22.5)
Occupational exposure to arsenic	0.2 (0.0 to 0.6)	0.3 (0.0 to 0.6)	0.3 (0.0 to 0.6)	-	-	0.2 (0.0 to 0.6)	0.3 (0.0 to 0.6)	0.3 (0.0 to 0.6)	-	-
Occupational exposure to benzene	0.5 (0.0 to 1.5)	0.6 (0.0 to 1.5)	0.5 (0.0 to 1.5)	5.2 (-4.1 to 18.8)	-2.4 (-9.1 to 8.0)	0.7 (0.0 to 2.1)	0.9 (0.1 to 2.5)	0.9 (0.1 to 2.5)	18.7 (6.4 to 37.9)	0.6 (-7.4 to 15.3)
Occupational exposure to beryllium	0.0 (0.0 to 0.0)	0.0 (0.0 to 0.0)	0.0 (0.0 to 0.0)	0.6 (-5.5 to 6.6)	0.4 (-3.9 to 5.3)	0.0 (0.0 to 0.0)	0.0 (0.0 to 0.0)	0.0 (0.0 to 0.0)	7.2 (-1.0 to 16.2)	0.4 (-5.5 to 7.1)
Occupational exposure to cadmium	0.0 (0.0 to 0.0)	0.0 (0.0 to 0.0)	0.0 (0.0 to 0.0)	7.2 (-4.3 to 19.0)	2.6 (-5.7 to 12.0)	0.0 (0.0 to 0.0)	0.0 (0.0 to 0.0)	0.0 (0.0 to 0.0)	9.5 (-2.7 to 24.0)	-0.1 (-9.2 to 10.6)
Occupational exposure to chromium	0.1 (0.1 to 0.1)	0.1 (0.1 to 0.1)	0.1 (0.1 to 0.1)	11.7 (0.3 to 23.0)	2.7 (-4.7 to 11.0)	0.1 (0.1 to 0.1)	0.1 (0.1 to 0.1)	0.1 (0.1 to 0.1)	13.1 (1.5 to 26.2)	-0.7 (-8.8 to 8.6)
Occupational exposure to diesel engine exhaust	0.5 (0.4 to 0.5)	0.6 (0.5 to 0.6)	0.6 (0.5 to 0.6)	13.8 (2.3 to 25.2)	1.5 (-5.5 to 9.1)	0.4 (0.4 to 0.5)	0.5 (0.4 to 0.5)	0.5 (0.4 to 0.5)	10.5 (-3.0 to 25.8)	-5.0 (-13.3 to 4.9)
Occupational exposure to formaldehyde	0.1 (0.1 to 0.1)	0.1 (0.1 to 0.2)	0.1 (0.1 to 0.2)	8.4 (-5.0 to 22.2)	1.5 (-8.0 to 11.9)	0.1 (0.1 to 0.2)	0.2 (0.1 to 0.2)	0.2 (0.1 to 0.2)	13.2 (0.4 to 27.8)	0.1 (-8.9 to 10.4)
Occupational exposure to nickel	0.2 (0.0 to 1.0)	0.2 (0.0 to 1.0)	0.2 (0.0 to 1.0)	-	-	0.2 (0.0 to 0.9)	0.2 (0.0 to 0.9)	0.2 (0.0 to 0.9)	-	-
Occupational exposure to polycyclic aromatic hydrocarbons	0.1 (0.1 to 0.2)	0.2 (0.1 to 0.2)	0.2 (0.1 to 0.2)	11.5 (0.9 to 23.3)	1.4 (-6.1 to 9.6)	0.2 (0.1 to 0.2)	0.2 (0.2 to 0.2)	0.2 (0.2 to 0.2)	17.2 (6.2 to 29.9)	-0.5 (-7.8 to 8.0)
Occupational exposure to silica	2.4 (0.3 to 7.4)	2.2 (0.3 to 7.3)	2.1 (0.3 to 6.9)	-7.3 (-22.1 to 9.1)	-3.2 (-13.9 to 9.9)	1.7 (0.2 to 5.5)	1.7 (0.2 to 5.4)	1.6 (0.2 to 5.2)	-3.4 (-17.4 to 15.5)	-2.5 (-14.0 to 12.7)
Occupational exposure to sulphuric acid	0.4 (0.1 to 1.3)	0.5 (0.1 to 1.6)	0.5 (0.1 to 1.6)	23.5 (7.0 to 42.5)	-0.5 (-10.7 to 10.6)	0.4 (0.1 to 1.4)	0.6 (0.1 to 1.9)	0.6 (0.1 to 1.8)	35.3 (13.9 to 60.8)	-3.9 (-18.1 to 10.7)
Occupational exposure to trichloroethylene	0.0 (0.0 to 0.0)	0.1 (0.0 to 0.1)	0.1 (0.0 to 0.1)	13.5 (4.3 to 23.2)	3.1 (-2.9 to 10.1)	0.0 (0.0 to 0.1)	0.1 (0.0 to 0.1)	0.1 (0.0 to 0.1)	11.9 (0.9 to 24.1)	-1.7 (-8.2 to 6.5)
Occupational asthmagens	12.2 (10.3 to 14.5)	12.0 (10.2 to 14.2)	11.6 (9.7 to 13.9)	-1.7 (-12.9 to 10.3)	-3.5 (-11.8 to 4.8)	6.5 (5.3 to 7.8)	6.8 (5.6 to 8.4)	6.4 (5.2 to 7.9)	5.9 (-8.4 to 22.9)	-6.1 (-17.1 to 6.5)
Occupational particulate matter, gases, and fumes	4.8 (3.4 to 7.0)	4.8 (3.5 to 7.0)	4.7 (3.4 to 6.9)	0.3 (-4.7 to 5.7)	-1.8 (-5.8 to 2.3)	2.8 (1.9 to 4.2)	2.8 (2.0 to 4.1)	2.7 (1.9 to 4.0)	1.4 (-6.0 to 10.0)	-2.3 (-7.5 to 3.6)
Occupational noise	4.9 (4.4 to 5.4)	5.0 (4.6 to 5.4)	4.9 (4.5 to 5.3)	1.9 (-1.9 to 6.1)	-1.8 (-4.4 to 1.2)	3.5 (3.1 to 4.0)	3.8 (3.4 to 4.2)	3.8 (3.4 to 4.3)	8.0 (2.2 to 15.0)	1.4 (-2.0 to 5.3)
Occupational ergonomic factors	9.1 (7.5 to 11.0)	8.1 (6.7 to 9.7)	7.7 (6.4 to 9.3)	-11.3 (-22.0 to -0.3)	-5.2 (-12.1 to 3.0)	5.0 (4.0 to 6.3)	5.0 (4.0 to 6.3)	5.0 (3.9 to 6.4)	1.1 (-13.9 to 18.3)	-1.5 (-12.9 to 11.3)
Non-exclusive breastfeeding	0.5 (0.5 to 0.6)	0.5 (0.5 to 0.6)	0.5 (0.4 to 0.6)	0.1 (-10.5 to 13.7)	-7.5 (-16.5 to 2.4)	0.5 (0.5 to 0.6)	0.5 (0.5 to 0.6)	0.5 (0.4 to 0.6)	0.1 (-10.4 to 13.8)	-7.2 (-16.3 to 2.7)
Discontinued breastfeeding	2.1 (2.0 to 2.2)	2.1 (2.0 to 2.2)	2.1 (2.0 to 2.3)	0.5 (-7.4 to 8.6)	2.5 (-3.5 to 9.2)	2.1 (2.0 to 2.2)	2.1 (2.0 to 2.2)	2.2 (2.0 to 2.3)	-0.6 (-8.3 to 7.5)	3.5 (-2.7 to 10.2)
Child underweight	0.1 (0.1 to 0.2)	0.1 (0.1 to 0.1)	0.1 (0.0 to 0.1)	-7.0 (-21.6 to 9.3)	-14.9 (-23.6 to -6.0)	0.1 (0.0 to 0.1)	0.1 (0.0 to 0.1)	0.1 (0.0 to 0.1)	-4.7 (-20.2 to 13.5)	-5.9 (-16.0 to 5.8)
Child wasting	0.1 (0.0 to 0.1)	0.1 (0.0 to 0.1)	0.1 (0.0 to 0.1)	-10.7 (-31.9 to 11.2)	-4.4 (-18.7 to 10.6)	0.1 (0.0 to 0.1)	0.1 (0.0 to 0.1)	0.1 (0.0 to 0.1)	5.6 (-17.3 to 36.2)	-6.3 (-20.2 to 10.4)
Child stunting	0.3 (0.1 to 0.5)	0.3 (0.1 to 0.4)	0.2 (0.1 to 0.4)	-10.7 (-50.3 to 61.8)	-13.1 (-45.0 to 26.0)	0.2 (0.1 to 0.4)	0.2 (0.1 to 0.3)	0.2 (0.1 to 0.3)	-10.9 (-47.6 to 45.4)	-5.1 (-30.7 to 32.2)
Short gestation for birth weight	0.0 (0.0 to 0.0)	0.0 (0.0 to 0.0)	0.0 (0.0 to 0.0)	7.8 (-16.7 to 35.1)	3.3 (-18.6 to 29.1)	0.0 (0.0 to 0.0)	0.0 (0.0 to 0.0)	0.0 (0.0 to 0.0)	12.5 (-11.0 to 44.5)	3.1 (-18.7 to 29.7)
Low birth weight for gestation	0.0 (0.0 to 0.0)	0.0 (0.0 to 0.0)	0.0 (0.0 to 0.0)	3.1 (-15.0 to 29.2)	-1.9 (-21.4 to 18.9)	0.0 (0.0 to 0.0)	0.0 (0.0 to 0.0)	0.0 (0.0 to 0.0)	-2.6 (-20.4 to 22.8)	-3.6 (-20.5 to 18.5)
Iron deficiency	-	-	-	-	-	4.4 (2.8 to 6.4)	4.0 (2.5 to 5.7)	3.9 (2.3 to 5.7)	-10.2 (-32.9 to 24.6)	-1.6 (-26.3 to 26.0)
Vitamin A deficiency	0.5 (0.3 to 0.7)	0.5 (0.3 to 0.6)	0.6 (0.3 to 0.8)	-9.2 (-24.0 to 17.1)	16.5 (-1.3 to 42.0)	0.6 (0.4 to 0.8)	0.6 (0.4 to 0.8)	0.7 (0.4 to 0.9)	2.0 (-18.2 to 30.3)	11.9 (-14.2 to 36.3)
Zinc deficiency	0.5 (0.1 to 1.1)	0.2 (0.0 to 1.1)	0.2 (0.0 to 1.0)	-54.8 (-88.3 to 530.2)	-0.3 (-88.8 to 781.0)	0.5 (0.1 to 1.2)	0.2 (0.0 to 1.0)	0.2 (0.0 to 1.0)	-53.7 (-88.5 to 396.5)	-2.3 (-89.2 to 946.3)
Smoking	12.9 (10.1 to 15.9)	7.2 (5.6 to 8.9)	5.7 (4.4 to 7.2)	-44.5 (-52.6 to -34.2)	-20.7 (-30.2 to -10.6)	9.1 (6.8 to 11.8)	5.6 (4.3 to 7.0)	4.2 (3.2 to 5.4)	-38.6 (-50.0 to -23.8)	-24.3 (-32.9 to -14.5)
Chewing tobacco	1.9 (1.6 to 2.3)	2.3 (1.7 to 3.1)	2.3 (1.5 to 3.6)	19.8 (-17.6 to 74.4)	0.7 (-27.8 to 39.2)	0.4 (0.3 to 0.6)	0.4 (0.2 to 0.6)	0.3 (0.2 to 0.5)	-5.5 (-43.6 to 54.2)	-22.3 (-47.2 to 13.5)
Second-hand smoke	46.3 (42.6 to 50.0)	27.3 (25.6 to 28.9)	28.3 (25.6 to 31.3)	-41.0 (-48.0 to -33.5)	3.8 (-6.1 to 15.6)	45.9 (42.7 to 49.4)	26.7 (25.3 to 28.0)	26.9 (25.0 to 29.0)	-41.8 (-47.8 to -35.2)	0.8 (-7.7 to 10.2)
Alcohol use	19.7 (10.3 to 31.8)	21.2 (11.3 to 34.6)	22.9 (11.6 to 38.0)	7.4 (-31.7 to 72.9)	8.1 (-27.0 to 59.2)	12.6 (6.3 to 21.6)	13.1 (6.8 to 23.0)	13.6 (6.5 to 24.0)	3.8 (-33.9 to 62.1)	3.6 (-27.8 to 49.4)
Drug use	1.7 (1.2 to 2.5)	2.1 (1.6 to 2.9)	2.9 (2.2 to 4.0)	21.3 (9.3 to 37.0)	43.1 (28.5 to 62.2)	1.2 (0.9 to 1.7)	1.6 (1.3 to 2.1)	2.0 (1.6 to 2.6)	35.1 (20.8 to 52.6)	26.8 (15.3 to 39.9)
Diet low in fruits	25.4 (19.0 to 30.8)	28.2 (22.3 to 33.2)	29.2 (22.6 to 34.3)	11.1 (0.9 to 27.1)	3.7 (-5.3 to 14.2)	20.5 (13.1 to 26.8)	25.5 (19.1 to 30.8)	25.2 (18.3 to 30.8)	24.5 (7.4 to 58.9)	-1.0 (-13.3 to 11.2)
Diet low in vegetables	18.6 (10.2 to 25.8)	28.1 (21.4 to 33.9)	28.2 (21.4 to 34.0)	51.0 (21.6 to 127.8)	0.5 (-10.0 to 13.2)	20.0 (11.4 to 27.2)	28.0 (21.0 to 34.0)	29.4 (22.5 to 35.2)	40.4 (13.5 to 105.9)	5.1 (-5.9 to 18.3)
Diet low in legumes	23.8 (16.5 to 30.1)	24.1 (17.3 to 29.8)	25.5 (18.7 to 31.3)	1.6 (-15.2 to 23.6)	5.9 (-7.2 to 22.0)	28.7 (22.7 to 33.9)	29.3 (23.4 to 34.5)	30.6 (25.2 to 35.4)	2.0 (-9.9 to 15.8)	4.3 (-4.8 to 15.7)
Diet low in whole grains	37.4 (33.8 to 40.6)	39.1 (35.8 to 42.0)	38.4 (34.8 to 41.3)	4.7 (-0.7 to 10.9)	-1.9 (-5.8 to 2.1)	38.3 (34.7 to 41.4)	40.0 (37.0 to 42.8)	39.3 (36.0 to 42.2)	4.5 (-0.7 to 10.5)	-1.6 (-5.6 to 2.2)
Diet low in nuts and seeds	43.2 (39.5 to 46.4)	35.4 (30.2 to 39.4)	34.4 (28.3 to 39.2)	-18.1 (-26.7 to -10.9)	-2.9 (-11.5 to 5.3)	44.3 (40.5 to 47.1)	36.4 (31.4 to 40.7)	35.4 (29.2 to 40.3)	-17.7 (-26.4 to -10.4)	-2.9 (-12.4 to 4.9)
Diet low in milk	25.7 (18.6 to 31.2)	25.4 (18.8 to 31.1)	25.2 (18.0 to 30.9)	-1.0 (-12.1 to 12.4)	-0.8 (-10.4 to 9.0)	27.5 (21.0 to 32.9)	26.5 (20.2 to 32.0)	26.1 (19.7 to 31.6)	-3.4 (-14.2 to 7.3)	-1.7 (-10.3 to 7.8)
Diet high in red meat	47.2 (42.0 to 51.1)	40.8 (29.9 to 47.8)	40.0 (29.0 to 48.1)	-13.6 (-30.1 to 2.2)	-2.0 (-18.4 to 14.9)	26.6 (20.3 to 32.1)	22.4 (12.7 to 30.8)	22.3 (11.9 to 32.4)	-15.8 (-44.9 to 19.6)	-0.0 (-29.9 to 34.0)
Diet high in processed meat	36.4 (29.6 to 41.8)	44.8 (38.0 to 49.7)	41.4 (32.5 to 47.8)	23.0 (3.2 to 45.6)	-7.6 (-19.1 to 3.0)	17.6 (13.2 to 21.3)	26.3 (20.5 to 31.4)	26.6 (19.9 to 33.1)	48.9 (19.8 to 89.0)	1.2 (-14.0 to 17.4)
Diet high in sugar-sweetened beverages	34.9 (17.8 to 44.6)	31.2 (15.6 to 41.3)	27.6 (13.4 to 37.9)	-10.7 (-36.8 to 25.5)	-11.4 (-36.2 to 17.3)	29.5 (14.5 to 40.0)	24.2 (11.8 to 35.1)	21.3 (10.2 to 32.1)	-18.2 (-47.1 to 23.8)	-11.9 (-40.3 to 23.3)
Diet low in fibre	32.4 (26.3 to 37.4)	25.9 (19.1 to 32.0)	26.6 (19.5 to 33.0)	-20.2 (-33.8 to -10.0)	3.0 (-8.4 to 15.1)	36.9 (31.8 to 41.1)	30.0 (23.5 to 35.4)	30.7 (24.2 to 35.8)	-18.5 (-29.8 to -10.1)	2.2 (-6.4 to 11.9)
Diet low in calcium	15.9 (8.2 to 22.5)	17.7 (10.3 to 24.1)	16.3 (8.5 to 23.1)	11.0 (-2.7 to 38.7)	-7.9 (-23.1 to 1.5)	20.7 (13.2 to 26.6)	22.7 (15.9 to 28.3)	21.5 (14.2 to 27.4)	9.6 (-1.0 to 28.9)	-5.2 (-15.0 to 1.6)
Diet low in seafood omega-3 fatty acids	24.8 (18.7 to 30.4)	22.9 (16.8 to 28.7)	23.2 (16.7 to 29.2)	-7.7 (-22.8 to 5.3)	1.3 (-9.8 to 14.3)	28.8 (23.0 to 34.1)	27.4 (21.1 to 32.8)	27.2 (21.1 to 32.5)	-5.1 (-15.2 to 5.4)	-0.6 (-8.6 to 8.9)
Diet low in polyunsaturated fatty acids	6.3 (1.4 to 12.8)	4.6 (0.7 to 10.3)	3.0 (0.3 to 7.6)	-26.8 (-77.5 to 46.9)	-34.5 (-77.6 to 40.7)	8.1 (2.3 to 14.9)	9.5 (3.5 to 16.5)	6.5 (1.8 to 13.0)	17.7 (-26.7 to 120.8)	-31.9 (-61.2 to -4.6)
Diet high in trans fatty acids	16.8 (12.0 to 22.5)	9.9 (5.9 to 14.3)	7.1 (3.4 to 11.1)	-41.3 (-55.2 to -29.0)	-27.8 (-44.2 to -16.0)	23.8 (18.1 to 31.8)	13.3 (9.1 to 17.9)	9.7 (5.7 to 14.0)	-44.2 (-55.9 to -32.1)	-26.8 (-39.9 to -16.4)
Diet high in sodium	7.7 (0.7 to 15.3)	12.9 (4.1 to 20.4)	13.7 (4.4 to 21.8)	67.2 (18.5 to 715.2)	5.9 (-16.5 to 51.2)	6.2 (0.4 to 13.6)	11.0 (2.7 to 18.3)	11.6 (3.2 to 19.2)	77.5 (25.5 to 1043.6)	5.2 (-19.0 to 50.7)
Intimate partner violence	-	-	-	-	-	1.4 (1.1 to 1.6)	1.0 (0.8 to 1.1)	1.2 (1.0 to 1.4)	-29.1 (-33.0 to -24.6)	23.8 (17.8 to 30.8)
Childhood sexual abuse	5.6 (4.6 to 6.9)	5.1 (4.1 to 6.2)	5.1 (4.1 to 6.2)	-9.8 (-15.1 to -4.3)	0.4 (-4.7 to 6.0)	13.8 (11.0 to 16.9)	12.1 (9.8 to 14.7)	12.2 (9.9 to 14.8)	-11.9 (-17.2 to -6.0)	0.4 (-4.8 to 6.5)

Supplementary Results Table 1. Alaska age-standardised Summary Exposure Values (SEVs) for Level 4 risk factors and percentage change, by sex, 1990, 2007, and 2017.Data in parentheses are 95% uncertainty intervals.										
	Male					Female				
	**1990**	**2007**	**2017**	**Percentage change 1990-2007**	**Percentage change 2007-2017**	**1990**	**2007**	**2017**	**Percentage change 1990-2007**	**Percentage change 2007-2017**
Bullying victimization	6.1 (2.6 to 12.8)	9.1 (3.7 to 18.4)	8.6 (3.6 to 18.5)	49.9 (33.3 to 70.7)	-4.8 (-12.9 to 3.2)	4.6 (1.9 to 9.7)	7.8 (3.2 to 16.3)	7.5 (3.2 to 15.9)	67.3 (47.9 to 93.5)	-3.0 (-11.1 to 6.6)
Low physical activity	0.4 (0.2 to 0.7)	0.4 (0.2 to 0.7)	0.4 (0.2 to 0.7)	0.0 (-1.8 to 1.9)	-0.3 (-1.8 to 1.1)	0.4 (0.2 to 0.8)	0.4 (0.2 to 0.8)	0.4 (0.2 to 0.8)	-1.0 (-3.8 to 1.6)	-1.6 (-3.3 to 0.1)
High fasting plasma glucose	1.3 (0.8 to 2.1)	2.0 (1.2 to 3.2)	2.6 (1.4 to 4.2)	59.0 (-6.9 to 159.4)	27.5 (-26.2 to 106.1)	1.8 (1.0 to 2.9)	2.9 (1.6 to 4.5)	2.4 (1.3 to 4.0)	62.8 (-9.5 to 210.4)	-15.6 (-52.5 to 51.3)
High LDL cholesterol	21.3 (19.2 to 23.4)	11.5 (9.9 to 13.3)	10.2 (8.7 to 11.9)	-45.8 (-50.5 to -40.6)	-11.6 (-15.9 to -7.5)	20.4 (18.5 to 22.5)	11.7 (10.2 to 13.4)	10.7 (9.2 to 12.4)	-42.8 (-47.6 to -38.1)	-8.1 (-12.2 to -3.9)
High systolic blood pressure	6.3 (5.5 to 7.1)	4.7 (4.0 to 5.4)	4.9 (4.1 to 5.6)	-25.1 (-32.8 to -16.8)	3.2 (-6.3 to 12.5)	6.2 (5.4 to 7.0)	4.4 (3.9 to 5.0)	4.2 (3.6 to 4.9)	-28.8 (-37.1 to -19.0)	-4.7 (-14.7 to 5.8)
High body-mass index	13.9 (9.8 to 19.1)	19.9 (15.3 to 25.7)	20.9 (16.1 to 26.8)	43.0 (27.1 to 64.7)	5.2 (-3.2 to 15.0)	18.5 (14.4 to 24.0)	23.2 (18.5 to 29.4)	24.2 (19.0 to 30.0)	25.0 (12.0 to 42.0)	4.2 (-4.5 to 15.1)
Low bone mineral density	5.2 (4.3 to 6.1)	4.5 (3.7 to 5.3)	4.7 (3.8 to 5.6)	-14.1 (-25.5 to 0.0)	5.5 (-8.2 to 21.2)	5.8 (4.8 to 6.9)	5.7 (4.8 to 6.7)	6.2 (5.1 to 7.5)	-1.5 (-13.0 to 13.5)	9.5 (-4.6 to 25.3)
Impaired kidney function	2.3 (1.1 to 5.7)	2.3 (1.1 to 5.8)	2.3 (1.1 to 5.8)	0.4 (-5.0 to 6.5)	1.8 (-3.9 to 7.9)	3.0 (1.4 to 6.9)	2.9 (1.4 to 6.9)	3.0 (1.4 to 7.0)	-1.6 (-6.6 to 3.1)	4.1 (-0.6 to 9.7)

Supplementary Results Table 1. Arizona age-standardised Summary Exposure Values (SEVs) for Level 4 risk factors and percentage change, by sex, 1990, 2007, and 2017.Data in parentheses are 95% uncertainty intervals.										
	Male					Female				
	1990	2007	2017	Percentage change 1990-2007	Percentage change 2007-2017	1990	2007	2017	Percentage change 1990-2007	Percentage change 2007-2017
Unsafe water source	1.8 (1.0 to 3.0)	0.6 (0.3 to 1.0)	0.4 (0.2 to 0.6)	-66.2 (-82.7 to -30.9)	-42.1 (-63.1 to -6.6)	1.8 (1.0 to 3.0)	0.6 (0.3 to 1.0)	0.4 (0.2 to 0.6)	-66.2 (-82.7 to -30.9)	-42.1 (-63.1 to -6.6)
Unsafe sanitation	8.9 (4.7 to 14.8)	5.7 (2.7 to 10.4)	3.6 (1.6 to 6.9)	-35.5 (-69.5 to 33.6)	-37.6 (-66.4 to 20.5)	8.9 (4.7 to 14.8)	5.7 (2.7 to 10.4)	3.6 (1.6 to 6.9)	-35.5 (-69.5 to 33.6)	-37.6 (-66.4 to 20.5)
No access to handwashing facility	3.5 (2.5 to 4.8)	3.2 (2.3 to 4.4)	3.1 (2.2 to 4.3)	-8.4 (-35.4 to 30.8)	-3.9 (-27.5 to 24.4)	3.5 (2.5 to 4.8)	3.2 (2.3 to 4.4)	3.1 (2.2 to 4.3)	-8.4 (-35.4 to 30.8)	-3.9 (-27.5 to 24.4)
Ambient particulate matter pollution	19.6 (4.9 to 33.4)	20.5 (7.2 to 35.0)	16.3 (1.7 to 29.7)	4.6 (-54.1 to 261.4)	-20.4 (-88.9 to 53.5)	19.5 (4.8 to 33.2)	20.4 (7.2 to 34.9)	16.3 (1.7 to 29.6)	4.8 (-54.1 to 263.0)	-20.3 (-88.8 to 53.5)
Household air pollution from solid fuels	0.3 (0.1 to 0.9)	0.2 (0.0 to 0.6)	0.2 (0.0 to 0.5)	-32.7 (-75.2 to 107.3)	-23.4 (-83.5 to 67.8)	0.5 (0.1 to 3.4)	0.4 (0.1 to 1.9)	0.3 (0.0 to 1.5)	-28.4 (-75.2 to 101.4)	-20.7 (-85.2 to 66.0)
Ambient ozone pollution	50.5 (22.5 to 54.8)	50.4 (22.3 to 54.8)	51.3 (24.1 to 54.8)	-0.2 (-1.1 to 0.0)	1.7 (0.0 to 8.5)	50.5 (22.5 to 54.8)	50.4 (22.3 to 54.8)	51.3 (24.1 to 54.8)	-0.2 (-1.1 to 0.0)	1.7 (0.0 to 8.5)
Residential radon	8.5 (0.2 to 29.0)	8.5 (0.2 to 29.0)	8.5 (0.2 to 29.0)	0.0 (0.0 to 0.0)	0.0 (0.0 to 0.0)	8.5 (0.2 to 29.0)	8.5 (0.2 to 29.0)	8.5 (0.2 to 29.0)	0.0 (0.0 to 0.0)	0.0 (0.0 to 0.0)
Lead exposure	10.6 (5.7 to 14.9)	6.8 (3.2 to 10.7)	4.6 (1.9 to 7.7)	-35.3 (-46.5 to -26.3)	-33.1 (-41.4 to -27.0)	6.5 (2.4 to 10.7)	4.3 (1.2 to 8.0)	2.9 (0.7 to 5.9)	-33.9 (-49.3 to -23.0)	-32.5 (-43.7 to -26.0)
Occupational exposure to asbestos	5.0 (4.3 to 5.7)	4.2 (3.9 to 4.6)	4.1 (3.4 to 4.8)	-15.8 (-26.9 to 0.6)	-3.9 (-19.3 to 15.1)	0.8 (0.7 to 0.9)	0.8 (0.7 to 0.9)	0.8 (0.7 to 1.0)	0.2 (-13.6 to 14.3)	5.7 (-14.6 to 29.9)
Occupational exposure to arsenic	0.3 (0.0 to 0.7)	0.3 (0.0 to 0.8)	0.3 (0.0 to 0.8)	-	-	0.3 (0.0 to 0.7)	0.3 (0.0 to 0.8)	0.3 (0.0 to 0.8)	-	-
Occupational exposure to benzene	0.6 (0.0 to 1.7)	0.6 (0.0 to 1.8)	0.6 (0.0 to 1.7)	1.8 (-6.2 to 12.8)	-3.3 (-9.4 to 5.7)	0.8 (0.0 to 2.4)	0.9 (0.1 to 2.7)	0.9 (0.1 to 2.7)	12.3 (0.9 to 30.4)	0.9 (-7.0 to 12.5)
Occupational exposure to beryllium	0.0 (0.0 to 0.0)	0.0 (0.0 to 0.0)	0.0 (0.0 to 0.0)	1.0 (-4.3 to 6.7)	0.5 (-4.0 to 5.2)	0.0 (0.0 to 0.0)	0.0 (0.0 to 0.0)	0.0 (0.0 to 0.0)	6.1 (-2.3 to 15.3)	0.5 (-5.6 to 6.9)
Occupational exposure to cadmium	0.0 (0.0 to 0.0)	0.0 (0.0 to 0.0)	0.0 (0.0 to 0.0)	4.8 (-6.3 to 17.0)	1.8 (-7.7 to 11.7)	0.0 (0.0 to 0.0)	0.0 (0.0 to 0.0)	0.0 (0.0 to 0.0)	5.1 (-8.5 to 21.2)	-0.3 (-10.4 to 11.4)
Occupational exposure to chromium	0.1 (0.1 to 0.1)	0.1 (0.1 to 0.1)	0.1 (0.1 to 0.1)	6.8 (-3.2 to 17.2)	1.4 (-6.6 to 9.8)	0.1 (0.1 to 0.1)	0.1 (0.1 to 0.1)	0.1 (0.1 to 0.1)	7.2 (-4.5 to 21.8)	-0.7 (-9.4 to 9.2)
Occupational exposure to diesel engine exhaust	0.5 (0.5 to 0.6)	0.6 (0.5 to 0.6)	0.6 (0.5 to 0.6)	7.5 (-0.5 to 16.4)	0.5 (-6.4 to 7.7)	0.5 (0.4 to 0.5)	0.5 (0.5 to 0.6)	0.5 (0.4 to 0.6)	8.8 (-3.8 to 23.5)	-4.1 (-13.4 to 5.8)
Occupational exposure to formaldehyde	0.2 (0.2 to 0.2)	0.2 (0.2 to 0.2)	0.2 (0.2 to 0.2)	3.2 (-7.9 to 16.0)	0.2 (-9.4 to 10.4)	0.2 (0.2 to 0.2)	0.2 (0.2 to 0.2)	0.2 (0.2 to 0.2)	5.7 (-7.6 to 22.1)	-0.3 (-10.2 to 11.3)
Occupational exposure to nickel	0.3 (0.0 to 1.2)	0.3 (0.0 to 1.2)	0.3 (0.0 to 1.2)	-	-	0.2 (0.0 to 1.1)	0.2 (0.0 to 1.1)	0.2 (0.0 to 1.1)	-	-
Occupational exposure to polycyclic aromatic hydrocarbons	0.2 (0.2 to 0.2)	0.2 (0.2 to 0.2)	0.2 (0.2 to 0.2)	6.5 (-2.6 to 16.2)	0.3 (-7.2 to 8.1)	0.2 (0.2 to 0.2)	0.2 (0.2 to 0.2)	0.2 (0.2 to 0.2)	10.3 (-0.7 to 23.7)	-0.6 (-8.4 to 8.3)
Occupational exposure to silica	2.8 (0.4 to 8.8)	2.5 (0.3 to 7.8)	2.5 (0.4 to 8.0)	-9.9 (-21.1 to 2.9)	1.3 (-9.4 to 13.2)	2.1 (0.3 to 6.2)	2.0 (0.3 to 6.1)	1.9 (0.3 to 6.6)	-4.3 (-19.4 to 17.7)	-0.9 (-14.0 to 16.3)
Occupational exposure to sulphuric acid	0.6 (0.1 to 1.8)	0.7 (0.1 to 2.1)	0.6 (0.1 to 2.0)	16.7 (2.3 to 31.4)	-2.6 (-12.0 to 7.8)	0.5 (0.1 to 1.7)	0.7 (0.1 to 2.2)	0.6 (0.1 to 2.1)	26.9 (6.7 to 50.9)	-4.2 (-17.0 to 9.4)
Occupational exposure to trichloroethylene	0.1 (0.1 to 0.1)	0.1 (0.1 to 0.1)	0.1 (0.1 to 0.1)	8.6 (0.4 to 17.1)	1.4 (-4.4 to 7.5)	0.1 (0.0 to 0.1)	0.1 (0.1 to 0.1)	0.1 (0.1 to 0.1)	7.9 (-2.4 to 21.4)	-1.5 (-9.3 to 8.0)
Occupational asthmagens	14.9 (12.7 to 17.9)	14.2 (12.1 to 16.8)	13.8 (11.5 to 16.6)	-5.1 (-14.6 to 4.7)	-2.8 (-10.8 to 5.6)	7.3 (6.0 to 8.9)	7.5 (6.1 to 9.1)	7.1 (5.7 to 8.7)	1.7 (-12.2 to 17.5)	-5.2 (-16.5 to 6.9)
Occupational particulate matter, gases, and fumes	6.3 (4.5 to 9.0)	6.0 (4.3 to 8.7)	5.8 (4.2 to 8.4)	-3.3 (-8.1 to 1.3)	-3.7 (-7.5 to -0.1)	3.5 (2.4 to 5.2)	3.4 (2.4 to 5.0)	3.3 (2.3 to 4.8)	-2.0 (-8.8 to 5.8)	-3.7 (-9.2 to 2.0)
Occupational noise	5.9 (5.4 to 6.5)	5.8 (5.4 to 6.3)	5.6 (5.2 to 6.1)	-2.0 (-5.3 to 1.5)	-3.5 (-5.9 to -1.2)	4.1 (3.5 to 4.6)	4.2 (3.8 to 4.7)	4.2 (3.8 to 4.8)	4.5 (-0.6 to 10.5)	0.0 (-3.2 to 3.7)
Occupational ergonomic factors	8.7 (7.3 to 10.6)	8.0 (6.6 to 9.6)	7.6 (6.3 to 9.2)	-8.8 (-17.9 to 1.4)	-4.9 (-12.1 to 2.3)	4.8 (3.8 to 6.1)	4.9 (3.9 to 6.1)	4.9 (3.8 to 6.2)	1.9 (-12.7 to 17.7)	-0.9 (-11.8 to 11.3)
Non-exclusive breastfeeding	0.5 (0.5 to 0.6)	0.5 (0.5 to 0.6)	0.5 (0.4 to 0.6)	-0.6 (-10.9 to 11.9)	-6.8 (-16.1 to 2.2)	0.5 (0.5 to 0.6)	0.5 (0.5 to 0.6)	0.5 (0.4 to 0.6)	-0.9 (-11.2 to 11.5)	-6.7 (-16.0 to 2.3)
Discontinued breastfeeding	2.2 (2.0 to 2.3)	2.1 (2.0 to 2.3)	2.1 (2.0 to 2.2)	-2.1 (-9.6 to 5.3)	0.2 (-6.2 to 6.6)	2.2 (2.0 to 2.3)	2.1 (2.0 to 2.3)	2.2 (2.0 to 2.3)	-1.7 (-9.2 to 5.8)	3.1 (-3.5 to 9.8)
Child underweight	0.1 (0.1 to 0.2)	0.1 (0.1 to 0.1)	0.1 (0.0 to 0.1)	-5.1 (-19.8 to 11.5)	-15.5 (-24.7 to -6.1)	0.1 (0.0 to 0.1)	0.1 (0.0 to 0.1)	0.1 (0.0 to 0.1)	-2.8 (-18.2 to 15.1)	-6.9 (-16.9 to 4.0)
Child wasting	0.1 (0.0 to 0.1)	0.1 (0.0 to 0.1)	0.1 (0.0 to 0.1)	-10.0 (-28.4 to 11.9)	-4.1 (-18.1 to 10.9)	0.1 (0.0 to 0.1)	0.1 (0.0 to 0.1)	0.1 (0.0 to 0.1)	6.4 (-16.9 to 34.3)	-6.5 (-21.0 to 8.4)
Child stunting	0.3 (0.1 to 0.6)	0.3 (0.1 to 0.5)	0.2 (0.1 to 0.4)	-9.1 (-51.4 to 69.7)	-14.2 (-41.2 to 23.1)	0.2 (0.1 to 0.4)	0.2 (0.1 to 0.3)	0.2 (0.1 to 0.3)	-10.0 (-46.2 to 47.8)	-5.6 (-31.9 to 30.9)
Short gestation for birth weight	0.0 (0.0 to 0.0)	0.0 (0.0 to 0.0)	0.0 (0.0 to 0.0)	7.1 (-19.0 to 34.3)	1.9 (-22.6 to 30.2)	0.0 (0.0 to 0.0)	0.0 (0.0 to 0.0)	0.0 (0.0 to 0.0)	3.2 (-23.0 to 32.3)	3.5 (-15.8 to 34.6)
Low birth weight for gestation	0.0 (0.0 to 0.0)	0.0 (0.0 to 0.0)	0.0 (0.0 to 0.0)	1.8 (-15.7 to 30.3)	0.3 (-17.8 to 24.7)	0.0 (0.0 to 0.0)	0.0 (0.0 to 0.0)	0.0 (0.0 to 0.0)	3.2 (-15.6 to 33.8)	-1.4 (-21.0 to 16.6)
Iron deficiency	-	-	-	-	-	4.8 (3.1 to 6.9)	4.3 (2.8 to 6.1)	4.3 (2.7 to 6.4)	-10.9 (-33.2 to 18.7)	0.9 (-20.5 to 25.2)
Vitamin A deficiency	0.5 (0.3 to 0.7)	0.6 (0.4 to 0.8)	0.5 (0.4 to 0.7)	8.1 (-8.5 to 43.0)	-8.0 (-32.0 to 15.3)	0.6 (0.4 to 0.8)	0.6 (0.4 to 0.8)	0.6 (0.4 to 0.9)	1.7 (-16.0 to 22.6)	6.8 (-12.8 to 29.4)
Zinc deficiency	0.5 (0.1 to 1.1)	0.2 (0.0 to 1.0)	0.2 (0.0 to 1.0)	-54.5 (-88.8 to 291.3)	3.3 (-88.2 to 809.7)	0.5 (0.1 to 1.2)	0.2 (0.0 to 1.0)	0.2 (0.0 to 1.0)	-52.9 (-87.3 to 294.7)	-2.7 (-88.1 to 824.6)
Smoking	12.4 (10.0 to 14.9)	7.2 (5.8 to 8.7)	5.9 (4.7 to 7.2)	-41.9 (-50.4 to -31.5)	-18.4 (-27.4 to -7.5)	8.8 (6.8 to 10.9)	5.1 (4.0 to 6.2)	4.0 (3.1 to 4.9)	-42.0 (-53.0 to -28.8)	-22.1 (-30.3 to -12.4)
Chewing tobacco	1.2 (0.9 to 1.5)	1.2 (0.9 to 1.7)	1.3 (0.8 to 2.0)	3.4 (-30.5 to 55.2)	3.4 (-27.0 to 50.2)	0.1 (0.1 to 0.1)	0.1 (0.0 to 0.1)	0.1 (0.0 to 0.1)	-6.2 (-44.4 to 55.1)	3.2 (-30.8 to 51.8)
Second-hand smoke	43.0 (39.7 to 46.7)	24.4 (22.9 to 26.0)	26.3 (23.7 to 29.2)	-43.2 (-50.1 to -36.0)	7.5 (-4.5 to 20.3)	40.4 (37.4 to 43.4)	23.8 (22.6 to 25.0)	25.0 (23.1 to 27.1)	-41.2 (-47.2 to -34.3)	5.1 (-4.3 to 15.2)
Alcohol use	20.4 (11.0 to 32.9)	22.2 (11.7 to 35.1)	23.9 (12.0 to 38.6)	8.7 (-28.8 to 77.8)	7.9 (-25.9 to 53.7)	12.5 (6.4 to 21.2)	13.0 (6.7 to 22.3)	13.4 (6.6 to 23.3)	4.1 (-31.0 to 62.4)	3.3 (-28.1 to 49.7)
Drug use	2.1 (1.6 to 3.0)	2.8 (2.3 to 3.7)	3.3 (2.6 to 4.4)	33.4 (19.0 to 53.3)	17.6 (6.7 to 28.0)	1.7 (1.2 to 2.4)	2.2 (1.7 to 3.0)	2.6 (2.0 to 3.6)	32.8 (18.9 to 51.0)	17.7 (7.5 to 28.7)
Diet low in fruits	26.7 (20.3 to 31.9)	26.0 (19.7 to 31.1)	28.0 (21.7 to 33.6)	-2.8 (-12.6 to 7.9)	7.9 (-2.2 to 21.5)	22.9 (16.4 to 28.6)	22.9 (16.3 to 28.5)	25.2 (17.8 to 31.5)	-0.0 (-13.0 to 14.4)	10.1 (-2.9 to 27.5)
Diet low in vegetables	13.5 (5.9 to 21.5)	8.4 (4.6 to 12.2)	8.9 (4.8 to 12.6)	-38.0 (-56.6 to -0.6)	6.5 (-19.7 to 47.1)	18.9 (10.8 to 25.9)	19.3 (13.5 to 24.3)	21.7 (16.1 to 26.5)	2.4 (-17.9 to 38.3)	12.4 (-1.5 to 35.1)
Diet low in legumes	24.0 (17.1 to 30.0)	24.2 (17.1 to 30.3)	25.7 (19.3 to 31.4)	1.0 (-14.7 to 21.3)	6.0 (-6.8 to 23.4)	28.7 (22.7 to 33.8)	29.4 (23.3 to 34.6)	30.7 (24.8 to 35.6)	2.5 (-8.7 to 16.5)	4.4 (-5.3 to 15.3)
Diet low in whole grains	38.3 (34.9 to 41.4)	39.7 (36.3 to 42.6)	39.5 (35.9 to 42.3)	3.6 (-1.3 to 9.3)	-0.6 (-4.2 to 3.1)	39.3 (35.9 to 42.1)	40.6 (37.4 to 43.0)	40.4 (37.3 to 43.0)	3.3 (-1.1 to 8.8)	-0.5 (-3.9 to 2.8)
Diet low in nuts and seeds	46.2 (43.2 to 48.6)	37.9 (33.5 to 41.6)	39.2 (34.1 to 43.2)	-18.0 (-26.1 to -11.9)	3.5 (-3.1 to 10.5)	47.0 (44.4 to 49.1)	38.9 (34.2 to 42.6)	40.2 (35.5 to 43.9)	-17.2 (-24.9 to -11.4)	3.2 (-2.8 to 10.0)
Diet low in milk	28.0 (21.9 to 33.2)	27.0 (20.9 to 32.2)	27.8 (21.5 to 33.0)	-3.9 (-14.4 to 5.1)	3.3 (-4.7 to 12.4)	29.7 (24.0 to 34.7)	27.9 (22.0 to 33.1)	28.6 (22.8 to 33.8)	-6.1 (-16.1 to 2.7)	2.6 (-5.2 to 10.6)
Diet high in red meat	43.2 (37.3 to 48.0)	35.9 (25.5 to 44.1)	34.7 (24.0 to 43.6)	-16.9 (-37.1 to 3.1)	-3.2 (-22.1 to 17.4)	21.8 (16.1 to 26.7)	20.2 (11.5 to 28.6)	19.3 (10.2 to 28.6)	-7.4 (-40.3 to 36.2)	-4.1 (-34.9 to 32.1)
Diet high in processed meat	41.0 (34.4 to 45.9)	47.7 (41.2 to 51.9)	43.5 (35.2 to 49.4)	16.3 (0.9 to 34.0)	-8.8 (-20.5 to 0.2)	19.6 (15.0 to 23.3)	31.7 (24.7 to 37.2)	30.0 (22.1 to 36.7)	61.3 (29.0 to 106.1)	-5.3 (-18.9 to 10.1)
Diet high in sugar-sweetened beverages	30.6 (15.2 to 41.7)	28.1 (14.5 to 39.1)	22.4 (11.0 to 32.7)	-8.2 (-37.7 to 35.4)	-20.4 (-44.8 to 9.6)	25.2 (12.2 to 36.8)	21.4 (9.5 to 31.9)	17.0 (6.9 to 27.6)	-15.2 (-49.4 to 33.2)	-20.6 (-48.9 to 16.1)
Diet low in fibre	33.5 (27.8 to 38.3)	25.7 (18.8 to 31.6)	27.6 (20.8 to 33.4)	-23.1 (-35.8 to -13.7)	7.1 (-3.3 to 19.5)	38.1 (33.0 to 41.9)	30.3 (24.1 to 35.5)	31.8 (25.6 to 36.9)	-20.4 (-30.6 to -11.7)	5.2 (-3.1 to 15.8)
Diet low in calcium	17.3 (10.2 to 23.4)	16.1 (9.3 to 22.2)	17.5 (10.4 to 23.7)	-6.8 (-21.2 to 6.7)	8.5 (-1.9 to 23.5)	21.3 (14.0 to 27.0)	20.0 (12.8 to 25.8)	21.4 (14.2 to 27.2)	-6.1 (-18.3 to 3.7)	7.1 (-0.4 to 19.1)
Diet low in seafood omega-3 fatty acids	29.4 (23.7 to 34.4)	25.8 (19.9 to 31.2)	28.2 (22.0 to 33.6)	-12.0 (-23.7 to -1.7)	9.2 (0.9 to 21.4)	32.8 (27.5 to 37.6)	30.1 (24.4 to 35.3)	31.9 (26.6 to 36.6)	-8.5 (-18.2 to 0.4)	6.0 (-0.6 to 14.4)
Diet low in polyunsaturated fatty acids	8.6 (2.9 to 15.5)	5.8 (1.2 to 12.1)	4.8 (0.8 to 10.7)	-32.2 (-74.0 to 11.9)	-18.0 (-56.4 to 42.3)	9.4 (3.3 to 16.1)	6.5 (1.4 to 13.1)	5.3 (1.1 to 11.3)	-31.3 (-72.7 to 9.6)	-18.3 (-58.1 to 37.9)
Diet high in trans fatty acids	16.8 (12.0 to 21.9)	9.9 (5.9 to 14.1)	7.1 (3.5 to 11.1)	-41.2 (-55.3 to -28.7)	-27.5 (-44.0 to -16.4)	23.7 (18.1 to 31.9)	13.3 (9.0 to 18.0)	9.7 (5.8 to 13.9)	-44.0 (-55.2 to -32.8)	-26.8 (-40.2 to -16.2)
Diet high in sodium	7.1 (0.7 to 14.6)	12.3 (3.5 to 19.9)	12.7 (3.7 to 20.5)	72.6 (19.7 to 672.0)	3.0 (-23.9 to 46.1)	6.0 (0.3 to 13.1)	10.9 (2.6 to 18.7)	11.3 (2.8 to 18.9)	80.2 (28.8 to 1004.5)	3.8 (-20.8 to 49.9)
Intimate partner violence	-	-	-	-	-	0.8 (0.6 to 1.0)	0.8 (0.6 to 0.9)	0.7 (0.6 to 1.0)	-3.2 (-13.4 to 11.2)	-1.3 (-12.3 to 10.6)
Childhood sexual abuse	5.6 (4.6 to 6.9)	5.1 (4.1 to 6.2)	5.1 (4.1 to 6.2)	-9.8 (-15.1 to -4.3)	0.4 (-4.7 to 6.0)	13.8 (11.0 to 16.9)	12.1 (9.8 to 14.7)	12.2 (9.9 to 14.8)	-11.9 (-17.2 to -6.0)	0.4 (-4.8 to 6.5)

Supplementary Results Table 1. Arizona age-standardised Summary Exposure Values (SEVs) for Level 4 risk factors and percentage change, by sex, 1990, 2007, and 2017.Data in parentheses are 95% uncertainty intervals.

	Male					Female				
	**1990**	**2007**	**2017**	**Percentage change 1990-2007**	**Percentage change 2007-2017**	**1990**	**2007**	**2017**	**Percentage change 1990-2007**	**Percentage change 2007-2017**
Bullying victimization	6.0 (2.5 to 12.8)	8.8 (3.7 to 18.1)	8.6 (3.6 to 18.4)	46.4 (31.0 to 67.1)	-2.5 (-10.7 to 5.7)	4.6 (1.9 to 9.3)	7.5 (3.2 to 15.8)	7.5 (3.1 to 16.0)	64.1 (44.8 to 89.9)	-0.5 (-9.0 to 8.9)
Low physical activity	0.4 (0.2 to 0.8)	0.4 (0.2 to 0.8)	0.4 (0.2 to 0.8)	-0.3 (-2.5 to 1.7)	-0.1 (-1.7 to 1.5)	0.4 (0.2 to 0.9)	0.5 (0.2 to 0.9)	0.5 (0.2 to 0.9)	0.1 (-1.9 to 2.3)	0.3 (-1.2 to 1.7)
High fasting plasma glucose	1.2 (0.8 to 1.9)	2.3 (1.4 to 3.5)	3.2 (2.0 to 4.8)	95.9 (19.3 to 222.9)	37.7 (-7.3 to 119.8)	1.4 (0.8 to 2.5)	2.3 (1.2 to 3.8)	2.3 (1.2 to 3.9)	62.5 (-4.4 to 195.4)	-0.4 (-42.4 to 78.5)
High LDL cholesterol	20.7 (18.7 to 22.9)	11.5 (9.9 to 13.2)	10.2 (8.7 to 11.9)	-44.5 (-49.4 to -39.6)	-11.3 (-15.7 to -7.1)	19.5 (17.6 to 21.4)	11.2 (9.7 to 12.8)	10.5 (9.1 to 12.1)	-42.5 (-47.2 to -38.2)	-6.4 (-10.4 to -2.1)
High systolic blood pressure	6.2 (5.4 to 7.1)	4.6 (3.9 to 5.3)	4.8 (4.0 to 5.5)	-26.1 (-34.0 to -17.5)	3.8 (-5.2 to 13.0)	5.8 (5.1 to 6.7)	4.2 (3.7 to 4.7)	4.1 (3.5 to 4.7)	-28.7 (-36.9 to -19.0)	-1.8 (-11.5 to 10.2)
High body-mass index	13.6 (10.0 to 18.2)	21.2 (16.6 to 26.9)	22.3 (17.4 to 28.0)	55.7 (35.2 to 80.3)	5.1 (-2.9 to 14.4)	14.5 (11.0 to 19.2)	20.4 (16.5 to 25.8)	21.9 (17.5 to 27.8)	40.6 (23.3 to 62.5)	7.2 (-2.9 to 18.1)
Low bone mineral density	5.2 (4.2 to 6.1)	4.4 (3.7 to 5.3)	4.7 (3.9 to 5.7)	-14.6 (-25.6 to -3.0)	6.0 (-9.5 to 23.2)	5.8 (4.8 to 6.9)	5.7 (4.8 to 6.6)	6.2 (5.1 to 7.3)	-2.2 (-15.2 to 12.4)	9.1 (-4.2 to 24.3)
Impaired kidney function	2.3 (1.1 to 5.8)	2.3 (1.1 to 5.8)	2.4 (1.1 to 5.9)	0.8 (-5.0 to 6.4)	3.1 (-3.1 to 10.6)	2.9 (1.4 to 6.9)	2.9 (1.4 to 6.9)	3.1 (1.5 to 7.2)	-0.5 (-6.3 to 5.2)	4.9 (-0.6 to 11.5)

Supplementary Results Table 1. Arkansas age-standardised Summary Exposure Values (SEVs) for Level 4 risk factors and percentage change, by sex, 1990, 2007, and 2017.Data in parentheses are 95% uncertainty intervals.										
	Male					Female				
	1990	2007	2017	Percentage change 1990-2007	Percentage change 2007-2017	1990	2007	2017	Percentage change 1990-2007	Percentage change 2007-2017
Unsafe water source	2.2 (1.2 to 3.7)	0.7 (0.4 to 1.2)	0.4 (0.2 to 0.6)	-67.8 (-83.2 to -37.0)	-44.7 (-64.8 to -6.6)	2.2 (1.2 to 3.7)	0.7 (0.4 to 1.2)	0.4 (0.2 to 0.6)	-67.8 (-83.2 to -37.0)	-44.7 (-64.8 to -6.6)
Unsafe sanitation	11.5 (6.3 to 18.7)	7.1 (3.2 to 13.0)	4.2 (1.9 to 8.1)	-38.8 (-73.3 to 30.0)	-39.8 (-68.1 to 11.2)	11.5 (6.3 to 18.7)	7.1 (3.2 to 13.0)	4.2 (1.9 to 8.1)	-38.8 (-73.3 to 30.0)	-39.8 (-68.1 to 11.2)
No access to handwashing facility	3.6 (2.6 to 4.9)	3.2 (2.3 to 4.5)	3.1 (2.2 to 4.2)	-10.5 (-35.5 to 29.5)	-4.6 (-27.3 to 23.9)	3.6 (2.6 to 4.9)	3.2 (2.3 to 4.5)	3.1 (2.2 to 4.2)	-10.5 (-35.5 to 29.5)	-4.6 (-27.3 to 23.9)
Ambient particulate matter pollution	27.0 (17.9 to 41.7)	26.6 (18.4 to 39.6)	20.8 (12.5 to 34.2)	-1.3 (-23.6 to 30.4)	-22.1 (-48.3 to 1.2)	26.8 (17.7 to 41.5)	26.5 (18.3 to 39.5)	20.7 (12.5 to 34.0)	-0.9 (-23.4 to 31.6)	-22.1 (-48.3 to 1.3)
Household air pollution from solid fuels	0.6 (0.2 to 1.9)	0.3 (0.1 to 0.9)	0.3 (0.1 to 0.9)	-43.0 (-77.7 to 29.9)	-17.6 (-65.3 to 75.5)	0.9 (0.2 to 4.6)	0.5 (0.1 to 3.6)	0.5 (0.1 to 2.7)	-39.0 (-77.2 to 29.6)	-15.3 (-65.2 to 71.0)
Ambient ozone pollution	47.6 (18.7 to 54.8)	48.0 (19.1 to 54.8)	48.9 (20.1 to 54.8)	0.7 (0.0 to 2.1)	1.9 (0.0 to 6.2)	47.6 (18.7 to 54.8)	48.0 (19.1 to 54.8)	48.9 (20.1 to 54.8)	0.7 (0.0 to 2.1)	1.9 (0.0 to 6.2)
Residential radon	11.5 (0.5 to 37.3)	11.5 (0.5 to 37.3)	11.5 (0.5 to 37.3)	0.0 (0.0 to 0.0)	0.0 (0.0 to 0.0)	11.5 (0.5 to 37.3)	11.5 (0.5 to 37.3)	11.5 (0.5 to 37.3)	0.0 (0.0 to 0.0)	0.0 (0.0 to 0.0)
Lead exposure	7.4 (3.0 to 11.8)	5.2 (1.9 to 9.1)	3.7 (1.2 to 6.9)	-29.8 (-40.3 to -21.2)	-29.5 (-38.9 to -22.9)	4.1 (0.9 to 8.1)	3.0 (0.6 to 6.6)	2.2 (0.4 to 5.2)	-26.4 (-38.6 to -17.5)	-27.6 (-39.6 to -21.0)
Occupational exposure to asbestos	3.7 (3.3 to 4.3)	3.2 (2.9 to 3.5)	3.3 (2.7 to 3.9)	-13.3 (-26.2 to 0.8)	1.4 (-16.8 to 21.7)	0.4 (0.3 to 0.4)	0.4 (0.4 to 0.5)	0.5 (0.4 to 0.6)	9.3 (-5.8 to 26.9)	14.0 (-8.9 to 43.6)
Occupational exposure to arsenic	0.4 (0.0 to 0.9)	0.4 (0.0 to 0.9)	0.4 (0.0 to 0.9)	-	-	0.3 (0.0 to 0.8)	0.3 (0.0 to 0.8)	0.3 (0.0 to 0.8)	-	-
Occupational exposure to benzene	0.6 (0.0 to 1.6)	0.6 (0.0 to 1.6)	0.6 (0.0 to 1.6)	1.2 (-6.3 to 10.8)	-2.5 (-8.3 to 6.3)	0.7 (0.0 to 2.1)	0.8 (0.0 to 2.3)	0.9 (0.1 to 2.5)	12.1 (0.2 to 32.2)	2.6 (-6.7 to 17.0)
Occupational exposure to beryllium	0.0 (0.0 to 0.0)	0.0 (0.0 to 0.0)	0.0 (0.0 to 0.0)	0.7 (-5.3 to 6.6)	-0.1 (-4.9 to 4.6)	0.0 (0.0 to 0.0)	0.0 (0.0 to 0.0)	0.0 (0.0 to 0.0)	6.5 (-2.3 to 16.9)	0.4 (-5.9 to 7.0)
Occupational exposure to cadmium	0.0 (0.0 to 0.0)	0.0 (0.0 to 0.1)	0.0 (0.0 to 0.1)	3.2 (-8.0 to 16.1)	-1.4 (-9.9 to 8.8)	0.0 (0.0 to 0.0)	0.0 (0.0 to 0.0)	0.0 (0.0 to 0.0)	2.5 (-12.6 to 19.8)	-1.8 (-14.1 to 11.8)
Occupational exposure to chromium	0.1 (0.1 to 0.1)	0.1 (0.1 to 0.1)	0.1 (0.1 to 0.1)	5.1 (-5.4 to 15.7)	-0.8 (-8.4 to 7.9)	0.1 (0.1 to 0.1)	0.1 (0.1 to 0.1)	0.1 (0.1 to 0.1)	4.9 (-8.4 to 19.8)	-1.8 (-12.0 to 9.4)
Occupational exposure to diesel engine exhaust	0.5 (0.5 to 0.6)	0.6 (0.5 to 0.6)	0.6 (0.5 to 0.6)	8.8 (-0.2 to 19.7)	2.9 (-3.9 to 9.9)	0.4 (0.4 to 0.5)	0.5 (0.4 to 0.5)	0.4 (0.4 to 0.5)	12.3 (-1.8 to 28.2)	-4.3 (-13.2 to 5.1)
Occupational exposure to formaldehyde	0.2 (0.2 to 0.3)	0.2 (0.2 to 0.3)	0.2 (0.2 to 0.3)	1.5 (-10.0 to 14.4)	-2.9 (-11.9 to 7.5)	0.2 (0.2 to 0.2)	0.2 (0.2 to 0.2)	0.2 (0.2 to 0.2)	2.5 (-12.3 to 19.4)	-1.6 (-12.9 to 11.2)
Occupational exposure to nickel	0.3 (0.0 to 1.5)	0.3 (0.0 to 1.4)	0.3 (0.0 to 1.4)	-	-	0.3 (0.0 to 1.2)	0.3 (0.0 to 1.2)	0.3 (0.0 to 1.2)	-	-
Occupational exposure to polycyclic aromatic hydrocarbons	0.2 (0.2 to 0.2)	0.2 (0.2 to 0.2)	0.2 (0.2 to 0.2)	4.8 (-5.1 to 15.1)	-1.4 (-8.5 to 6.9)	0.2 (0.2 to 0.2)	0.2 (0.2 to 0.2)	0.2 (0.2 to 0.3)	7.6 (-4.8 to 21.5)	-1.1 (-10.2 to 8.9)
Occupational exposure to silica	3.1 (0.4 to 9.9)	3.0 (0.4 to 9.3)	2.9 (0.4 to 9.3)	-5.6 (-16.1 to 7.3)	-1.7 (-11.1 to 10.8)	2.4 (0.3 to 7.5)	2.2 (0.3 to 7.3)	2.2 (0.3 to 7.1)	-5.4 (-21.5 to 15.5)	-2.9 (-17.6 to 15.4)
Occupational exposure to sulphuric acid	0.6 (0.1 to 1.9)	0.7 (0.1 to 2.0)	0.6 (0.1 to 2.0)	11.3 (0.1 to 23.6)	-2.3 (-10.5 to 7.1)	0.5 (0.1 to 1.7)	0.7 (0.1 to 2.1)	0.6 (0.1 to 2.0)	22.7 (5.2 to 43.6)	-4.2 (-15.8 to 8.9)
Occupational exposure to trichloroethylene	0.1 (0.1 to 0.1)	0.1 (0.1 to 0.1)	0.1 (0.1 to 0.1)	7.0 (-0.9 to 15.2)	0.6 (-5.3 to 6.9)	0.1 (0.0 to 0.1)	0.1 (0.1 to 0.1)	0.1 (0.1 to 0.1)	7.4 (-3.8 to 20.9)	-2.3 (-10.5 to 6.6)
Occupational asthmagens	16.7 (14.0 to 20.1)	15.9 (13.3 to 19.0)	15.3 (12.6 to 18.3)	-4.5 (-14.6 to 6.4)	-3.9 (-11.7 to 4.9)	7.5 (6.1 to 9.1)	7.6 (6.2 to 9.2)	7.1 (5.7 to 8.8)	1.1 (-12.6 to 16.4)	-6.0 (-16.4 to 4.9)
Occupational particulate matter, gases, and fumes	7.2 (5.1 to 10.1)	7.0 (5.0 to 10.0)	6.6 (4.8 to 9.6)	-3.2 (-7.9 to 1.1)	-4.5 (-8.7 to -1.1)	3.8 (2.6 to 5.5)	3.7 (2.6 to 5.4)	3.6 (2.5 to 5.2)	-1.0 (-8.3 to 7.2)	-4.3 (-10.2 to 1.7)
Occupational noise	6.3 (5.7 to 6.8)	6.1 (5.6 to 6.7)	5.9 (5.4 to 6.4)	-2.3 (-5.6 to 0.9)	-3.9 (-6.4 to -1.6)	4.0 (3.4 to 4.5)	4.2 (3.7 to 4.7)	4.2 (3.7 to 4.6)	5.3 (-0.1 to 11.8)	-0.2 (-3.8 to 3.8)
Occupational ergonomic factors	9.7 (8.0 to 11.7)	8.4 (7.0 to 10.1)	7.9 (6.6 to 9.5)	-13.1 (-25.0 to -1.8)	-5.5 (-13.0 to 2.6)	5.1 (4.0 to 6.5)	5.0 (4.0 to 6.3)	4.9 (3.9 to 6.2)	-1.3 (-16.9 to 16.7)	-2.0 (-13.0 to 9.9)
Non-exclusive breastfeeding	0.5 (0.5 to 0.6)	0.5 (0.5 to 0.6)	0.5 (0.4 to 0.6)	-0.1 (-11.8 to 13.5)	-7.4 (-16.6 to 2.2)	0.5 (0.5 to 0.6)	0.5 (0.5 to 0.6)	0.5 (0.4 to 0.6)	-0.2 (-12.0 to 13.4)	-7.3 (-16.5 to 2.3)
Discontinued breastfeeding	2.1 (2.0 to 2.2)	2.1 (1.9 to 2.2)	2.1 (2.0 to 2.3)	-1.0 (-8.7 to 7.3)	2.7 (-3.7 to 9.3)	2.1 (2.0 to 2.2)	2.1 (1.9 to 2.2)	2.1 (2.0 to 2.2)	-0.7 (-8.4 to 7.6)	1.5 (-4.9 to 8.0)
Child underweight	0.1 (0.1 to 0.2)	0.1 (0.1 to 0.1)	0.1 (0.0 to 0.1)	-6.2 (-20.6 to 10.1)	-16.2 (-25.4 to -7.4)	0.1 (0.0 to 0.1)	0.1 (0.0 to 0.1)	0.1 (0.0 to 0.1)	-3.9 (-19.2 to 14.4)	-7.0 (-17.5 to 3.8)
Child wasting	0.1 (0.0 to 0.1)	0.1 (0.0 to 0.1)	0.1 (0.0 to 0.1)	-11.1 (-30.1 to 9.2)	-4.4 (-17.4 to 8.4)	0.1 (0.0 to 0.1)	0.1 (0.0 to 0.1)	0.1 (0.0 to 0.1)	6.3 (-16.3 to 34.9)	-7.1 (-20.3 to 9.7)
Child stunting	0.3 (0.1 to 0.6)	0.3 (0.1 to 0.5)	0.2 (0.1 to 0.4)	-9.0 (-51.5 to 68.8)	-14.7 (-44.5 to 28.4)	0.2 (0.1 to 0.4)	0.2 (0.1 to 0.3)	0.2 (0.1 to 0.3)	-13.3 (-51.1 to 44.6)	-5.6 (-32.0 to 35.9)
Short gestation for birth weight	0.0 (0.0 to 0.0)	0.0 (0.0 to 0.0)	0.0 (0.0 to 0.0)	4.7 (-24.2 to 38.6)	1.8 (-21.3 to 40.8)	0.0 (0.0 to 0.0)	0.0 (0.0 to 0.0)	0.0 (0.0 to 0.0)	6.1 (-22.7 to 42.4)	1.7 (-25.4 to 44.5)
Low birth weight for gestation	0.0 (0.0 to 0.0)	0.0 (0.0 to 0.0)	0.0 (0.0 to 0.0)	1.8 (-22.0 to 30.9)	-1.9 (-26.4 to 22.4)	0.0 (0.0 to 0.0)	0.0 (0.0 to 0.0)	0.0 (0.0 to 0.0)	-0.8 (-22.2 to 32.7)	-1.1 (-26.8 to 27.6)
Iron deficiency	-	-	-	-	-	5.0 (3.2 to 7.1)	4.5 (3.0 to 6.5)	4.5 (2.9 to 6.6)	-8.7 (-31.2 to 21.3)	-1.3 (-22.7 to 23.3)
Vitamin A deficiency	0.6 (0.4 to 0.9)	0.6 (0.4 to 0.7)	0.6 (0.4 to 0.8)	-10.4 (-22.5 to 6.2)	2.1 (-16.7 to 24.7)	0.6 (0.4 to 0.8)	0.7 (0.4 to 0.9)	0.6 (0.4 to 0.8)	10.9 (-8.9 to 39.6)	-10.9 (-29.3 to 15.1)
Zinc deficiency	0.5 (0.1 to 1.1)	0.2 (0.0 to 1.0)	0.2 (0.0 to 1.0)	-55.8 (-88.9 to 238.2)	6.2 (-88.2 to 782.5)	0.5 (0.1 to 1.1)	0.2 (0.0 to 1.0)	0.2 (0.0 to 1.0)	-54.9 (-87.1 to 270.2)	5.0 (-87.8 to 809.0)
Smoking	15.8 (12.9 to 18.7)	8.5 (6.8 to 10.5)	6.5 (5.0 to 8.2)	-45.9 (-53.6 to -37.9)	-23.7 (-32.5 to -14.1)	10.1 (7.7 to 12.8)	5.5 (4.3 to 6.9)	4.6 (3.5 to 5.9)	-45.3 (-54.9 to -33.1)	-17.2 (-27.4 to -7.1)
Chewing tobacco	1.6 (1.2 to 2.1)	1.9 (1.3 to 2.7)	2.1 (1.4 to 3.1)	22.6 (-17.5 to 83.3)	9.7 (-21.3 to 52.4)	0.1 (0.1 to 0.1)	0.1 (0.1 to 0.1)	0.1 (0.1 to 0.1)	-6.1 (-42.1 to 54.7)	3.7 (-30.0 to 50.8)
Second-hand smoke	43.7 (40.7 to 46.7)	30.3 (28.8 to 31.7)	28.5 (25.9 to 31.3)	-30.8 (-37.0 to -24.2)	-5.9 (-13.8 to 2.6)	44.3 (41.7 to 47.1)	29.5 (28.5 to 30.4)	27.0 (25.2 to 28.8)	-33.5 (-38.9 to -28.0)	-8.4 (-14.5 to -2.4)
Alcohol use	21.0 (11.7 to 31.4)	22.1 (12.0 to 32.8)	23.2 (12.1 to 35.7)	5.5 (-33.3 to 67.7)	5.0 (-26.5 to 53.6)	10.9 (5.8 to 18.2)	11.7 (6.0 to 19.4)	12.5 (6.2 to 21.6)	7.5 (-30.0 to 63.9)	6.5 (-27.1 to 59.8)
Drug use	2.2 (1.5 to 3.6)	2.6 (1.9 to 3.7)	3.3 (2.4 to 4.6)	16.6 (0.4 to 41.4)	27.0 (16.0 to 39.4)	1.8 (1.2 to 2.7)	2.2 (1.6 to 3.1)	2.6 (1.9 to 3.8)	22.5 (10.3 to 38.8)	20.2 (10.1 to 32.9)
Diet low in fruits	29.4 (23.0 to 34.3)	31.4 (25.6 to 35.8)	31.9 (25.9 to 36.9)	6.8 (-2.2 to 19.1)	1.8 (-6.1 to 9.7)	26.8 (20.3 to 32.2)	28.9 (23.0 to 33.7)	29.2 (22.9 to 34.8)	7.7 (-3.1 to 21.8)	1.3 (-8.6 to 11.3)
Diet low in vegetables	24.8 (16.9 to 31.6)	33.1 (27.4 to 37.9)	33.8 (28.0 to 38.5)	33.6 (14.3 to 74.7)	1.8 (-5.9 to 10.5)	29.2 (22.2 to 34.7)	35.2 (29.6 to 39.7)	36.4 (30.8 to 40.7)	20.6 (6.3 to 42.9)	3.2 (-3.6 to 11.1)
Diet low in legumes	24.2 (17.0 to 30.2)	24.3 (17.4 to 30.2)	25.6 (18.9 to 31.5)	0.4 (-15.5 to 18.3)	5.6 (-7.0 to 24.2)	28.6 (22.8 to 33.8)	29.4 (23.5 to 34.4)	30.7 (25.1 to 35.6)	2.5 (-9.9 to 17.9)	4.6 (-4.8 to 15.8)
Diet low in whole grains	39.0 (35.8 to 41.8)	40.1 (37.1 to 42.8)	39.5 (36.3 to 42.3)	2.8 (-1.8 to 8.2)	-1.5 (-5.5 to 2.2)	39.9 (36.4 to 42.6)	41.0 (37.9 to 43.4)	40.4 (37.4 to 43.2)	2.8 (-1.6 to 7.6)	-1.3 (-4.5 to 2.1)
Diet low in nuts and seeds	47.7 (45.3 to 49.6)	39.6 (35.3 to 43.0)	39.6 (35.1 to 43.4)	-17.0 (-23.7 to -11.7)	-0.1 (-6.6 to 6.0)	48.4 (46.2 to 50.1)	40.7 (36.6 to 43.9)	40.5 (36.2 to 44.2)	-15.9 (-22.4 to -11.0)	-0.5 (-6.5 to 5.3)
Diet low in milk	29.3 (23.5 to 34.3)	27.8 (21.4 to 33.0)	27.9 (21.7 to 33.4)	-5.1 (-15.1 to 3.8)	0.4 (-7.3 to 9.2)	31.0 (25.3 to 35.6)	28.9 (23.0 to 33.6)	28.7 (23.0 to 33.7)	-6.8 (-15.1 to 1.7)	-0.5 (-7.7 to 7.4)
Diet high in red meat	38.7 (27.9 to 46.5)	33.1 (21.4 to 42.4)	32.2 (20.4 to 42.0)	-14.6 (-36.3 to 9.5)	-2.5 (-21.7 to 20.8)	20.4 (10.9 to 30.1)	14.7 (7.1 to 23.5)	14.1 (6.1 to 23.1)	-27.9 (-57.1 to 13.7)	-3.8 (-39.3 to 49.7)
Diet high in processed meat	35.2 (27.2 to 42.2)	32.2 (24.9 to 38.7)	26.2 (19.6 to 32.4)	-8.7 (-26.0 to 12.6)	-18.5 (-31.1 to -5.2)	18.6 (13.2 to 23.7)	18.0 (12.8 to 22.8)	16.1 (11.2 to 21.0)	-3.3 (-24.1 to 22.0)	-10.6 (-25.3 to 5.5)
Diet high in sugar-sweetened beverages	28.0 (13.9 to 39.3)	25.9 (13.0 to 36.6)	22.1 (10.7 to 32.8)	-7.3 (-37.6 to 42.7)	-15.0 (-39.8 to 17.9)	22.9 (10.8 to 34.8)	19.7 (9.6 to 29.7)	16.8 (7.8 to 25.9)	-14.0 (-48.6 to 41.0)	-14.7 (-44.7 to 25.9)
Diet low in fibre	34.2 (28.7 to 38.8)	26.3 (19.7 to 32.0)	27.6 (20.6 to 33.4)	-23.3 (-35.8 to -13.4)	4.9 (-5.3 to 17.8)	38.8 (34.3 to 42.6)	30.9 (24.7 to 36.1)	31.9 (25.9 to 36.9)	-20.4 (-30.5 to -12.4)	3.4 (-4.7 to 12.9)
Diet low in calcium	19.2 (12.1 to 25.3)	17.4 (10.3 to 23.8)	17.7 (10.6 to 23.9)	-9.4 (-24.7 to 2.0)	1.5 (-9.1 to 13.7)	23.4 (16.8 to 28.8)	21.4 (14.4 to 27.1)	21.7 (14.5 to 27.5)	-8.1 (-20.5 to -0.4)	1.0 (-6.7 to 10.2)
Diet low in seafood omega-3 fatty acids	31.9 (27.0 to 36.7)	27.7 (21.9 to 33.0)	28.4 (22.8 to 33.5)	-13.1 (-23.3 to -4.0)	2.5 (-6.1 to 11.9)	35.0 (30.1 to 39.0)	31.7 (26.2 to 36.4)	32.0 (26.6 to 36.7)	-9.5 (-17.9 to -2.2)	1.0 (-5.3 to 8.3)
Diet low in polyunsaturated fatty acids	10.2 (3.8 to 17.3)	6.8 (1.5 to 13.3)	5.0 (0.9 to 10.9)	-32.8 (-72.6 to 4.1)	-27.0 (-66.6 to 22.5)	11.1 (4.8 to 18.0)	7.5 (1.8 to 14.3)	5.4 (1.0 to 11.3)	-31.9 (-70.6 to 3.2)	-27.8 (-64.1 to 12.2)
Diet high in trans fatty acids	16.8 (12.1 to 22.0)	9.9 (5.9 to 14.2)	7.1 (3.5 to 11.1)	-41.0 (-55.5 to -28.3)	-27.8 (-44.2 to -16.0)	23.8 (17.8 to 32.2)	13.3 (9.0 to 18.0)	9.7 (5.8 to 14.1)	-44.0 (-55.4 to -32.6)	-26.9 (-39.4 to -16.0)
Diet high in sodium	6.8 (0.5 to 14.4)	12.0 (3.5 to 20.1)	12.6 (4.1 to 20.5)	75.9 (21.0 to 850.5)	5.6 (-20.0 to 55.3)	5.9 (0.2 to 13.0)	10.7 (2.4 to 18.2)	11.1 (2.7 to 18.7)	81.8 (27.5 to 1194.3)	4.5 (-23.6 to 56.6)
Intimate partner violence	-	-	-	-	-	1.0 (0.8 to 1.3)	1.0 (0.9 to 1.2)	1.0 (0.8 to 1.2)	-2.5 (-12.3 to 8.9)	-0.8 (-8.8 to 8.6)
Childhood sexual abuse	2.9 (2.0 to 4.3)	3.3 (2.5 to 4.4)	3.3 (2.5 to 4.4)	15.6 (-1.5 to 39.3)	-0.5 (-6.1 to 4.7)	12.2 (9.6 to 15.4)	11.6 (9.9 to 13.6)	11.6 (9.8 to 13.8)	-5.1 (-17.8 to 8.7)	-0.1 (-4.9 to 4.8)

Supplementary Results Table 1. Arkansas age-standardised Summary Exposure Values (SEVs) for Level 4 risk factors and percentage change, by sex, 1990, 2007, and 2017.Data in parentheses are 95% uncertainty intervals.

	Male					Female				
	**1990**	**2007**	**2017**	**Percentage change 1990-2007**	**Percentage change 2007-2017**	**1990**	**2007**	**2017**	**Percentage change 1990-2007**	**Percentage change 2007-2017**
Bullying victimization	6.1<br>(2.5 to 12.6)	8.8<br>(3.6 to 18.1)	8.6<br>(3.6 to 17.9)	44.5<br>(29.0 to 65.2)	-2.6<br>(-9.6 to 5.1)	4.6<br>(1.9 to 9.5)	7.5<br>(3.1 to 15.3)	7.4<br>(3.0 to 15.4)	62.1<br>(43.8 to 87.6)	-0.6<br>(-8.3 to 8.7)
Low physical activity	0.4<br>(0.2 to 0.7)	0.4<br>(0.2 to 0.7)	0.4<br>(0.2 to 0.7)	0.4<br>(-2.1 to 3.0)	0.1<br>(-1.8 to 1.8)	0.5<br>(0.2 to 0.9)	0.5<br>(0.2 to 0.9)	0.5<br>(0.2 to 0.9)	-0.4<br>(-2.9 to 2.4)	-0.3<br>(-2.1 to 1.3)
High fasting plasma glucose	1.2<br>(0.7 to 1.9)	2.3<br>(1.4 to 3.4)	3.2<br>(2.0 to 4.9)	96.0<br>(13.4 to 226.9)	41.9<br>(-9.1 to 135.8)	1.5<br>(0.9 to 2.5)	2.7<br>(1.5 to 4.5)	2.5<br>(1.3 to 4.3)	78.6<br>(1.5 to 214.5)	-7.9<br>(-49.8 to 62.3)
High LDL cholesterol	20.9<br>(18.9 to 22.9)	11.4<br>(9.9 to 13.2)	10.1<br>(8.7 to 11.8)	-45.3<br>(-50.3 to -40.5)	-11.3<br>(-15.4 to -7.2)	19.7<br>(17.8 to 21.7)	11.4<br>(10.0 to 13.1)	10.7<br>(9.2 to 12.3)	-42.0<br>(-46.7 to -37.3)	-6.8<br>(-11.0 to -2.5)
High systolic blood pressure	6.5<br>(5.7 to 7.4)	4.9<br>(4.2 to 5.6)	5.1<br>(4.3 to 5.8)	-24.2<br>(-32.0 to -16.4)	2.5<br>(-6.1 to 11.6)	6.4<br>(5.5 to 7.3)	4.5<br>(4.0 to 5.1)	4.4<br>(3.7 to 5.0)	-28.8<br>(-37.1 to -20.1)	-4.0<br>(-14.1 to 6.6)
High body-mass index	12.3<br>(8.7 to 17.3)	20.1<br>(15.5 to 26.0)	20.9<br>(16.1 to 27.0)	62.6<br>(39.2 to 92.1)	4.1<br>(-4.9 to 13.2)	15.2<br>(11.4 to 20.1)	22.5<br>(18.1 to 28.5)	23.9<br>(18.8 to 29.8)	48.5<br>(29.6 to 71.0)	6.1<br>(-2.3 to 15.5)
Low bone mineral density	5.2<br>(4.3 to 6.2)	4.4<br>(3.7 to 5.3)	4.7<br>(3.8 to 5.6)	-14.6<br>(-25.6 to -1.6)	5.9<br>(-8.9 to 23.1)	5.8<br>(4.8 to 7.0)	5.7<br>(4.8 to 6.6)	6.2<br>(5.2 to 7.4)	-3.0<br>(-16.2 to 11.4)	9.9<br>(-5.1 to 25.6)
Impaired kidney function	2.3<br>(1.1 to 5.9)	2.4<br>(1.1 to 6.1)	2.5<br>(1.2 to 6.1)	2.1<br>(-4.0 to 8.4)	3.5<br>(-2.5 to 9.7)	3.0<br>(1.4 to 7.0)	3.0<br>(1.5 to 7.0)	3.2<br>(1.6 to 7.2)	0.6<br>(-4.6 to 6.4)	5.3<br>(0.2 to 11.8)

Supplementary Results Table 1. California age-standardised Summary Exposure Values (SEVs) for Level 4 risk factors and percentage change, by sex, 1990, 2007, and 2017.Data in parentheses are 95% uncertainty intervals.

	Male					Female				
	1990	2007	2017	Percentage change 1990-2007	Percentage change 2007-2017	1990	2007	2017	Percentage change 1990-2007	Percentage change 2007-2017
Unsafe water source	1.6 (0.9 to 2.5)	0.5 (0.3 to 0.8)	0.3 (0.2 to 0.5)	-68.8 (-83.4 to -42.6)	-36.2 (-58.6 to -0.6)	1.6 (0.9 to 2.5)	0.5 (0.3 to 0.8)	0.3 (0.2 to 0.5)	-68.8 (-83.4 to -42.6)	-36.2 (-58.6 to -0.6)
Unsafe sanitation	7.3 (3.9 to 11.8)	3.9 (1.9 to 7.2)	2.7 (1.3 to 5.0)	-46.5 (-75.6 to 8.7)	-31.0 (-62.3 to 25.7)	7.3 (3.9 to 11.8)	3.9 (1.9 to 7.2)	2.7 (1.3 to 5.0)	-46.5 (-75.6 to 8.7)	-31.0 (-62.3 to 25.7)
No access to handwashing facility	3.5 (2.5 to 4.7)	3.1 (2.3 to 4.3)	3.0 (2.2 to 4.1)	-9.5 (-34.1 to 26.7)	-3.8 (-24.0 to 21.5)	3.5 (2.5 to 4.7)	3.1 (2.3 to 4.3)	3.0 (2.2 to 4.1)	-9.5 (-34.1 to 26.7)	-3.8 (-24.0 to 21.5)
Ambient particulate matter pollution	26.2 (13.2 to 41.6)	26.5 (15.4 to 41.6)	22.5 (7.8 to 39.4)	1.1 (-36.9 to 79.5)	-15.0 (-63.9 to 47.2)	26.1 (13.1 to 41.5)	26.4 (15.4 to 41.6)	22.4 (7.8 to 39.4)	1.2 (-37.0 to 80.1)	-15.0 (-64.0 to 47.4)
Household air pollution from solid fuels	0.3 (0.1 to 0.8)	0.2 (0.0 to 0.4)	0.2 (0.0 to 0.4)	-28.7 (-74.7 to 71.2)	-12.9 (-69.0 to 88.1)	0.4 (0.1 to 2.3)	0.3 (0.1 to 1.4)	0.3 (0.0 to 1.4)	-26.4 (-74.6 to 70.5)	-10.9 (-68.8 to 90.6)
Ambient ozone pollution	49.2 (20.4 to 54.8)	49.0 (20.1 to 54.8)	50.1 (21.7 to 54.8)	-0.5 (-1.7 to 0.0)	2.2 (0.0 to 8.4)	49.2 (20.4 to 54.8)	49.0 (20.1 to 54.8)	50.1 (21.7 to 54.8)	-0.5 (-1.7 to 0.0)	2.2 (0.0 to 8.4)
Residential radon	11.8 (0.6 to 36.7)	11.8 (0.6 to 36.7)	11.8 (0.6 to 36.7)	0.0 (0.0 to 0.0)	0.0 (0.0 to 0.0)	11.8 (0.6 to 36.7)	11.8 (0.6 to 36.7)	11.8 (0.6 to 36.7)	0.0 (0.0 to 0.0)	0.0 (0.0 to 0.0)
Lead exposure	11.6 (6.8 to 16.0)	7.3 (3.7 to 11.4)	4.8 (2.2 to 8.0)	-37.0 (-47.8 to -28.0)	-34.2 (-41.6 to -28.5)	7.9 (3.5 to 12.1)	5.1 (1.8 to 8.9)	3.4 (1.1 to 6.4)	-35.4 (-50.2 to -25.0)	-33.6 (-43.0 to -28.0)
Occupational exposure to asbestos	5.6 (4.6 to 6.6)	4.6 (4.4 to 4.9)	3.8 (3.3 to 4.4)	-17.6 (-29.1 to 0.6)	-17.2 (-29.2 to -3.6)	0.8 (0.7 to 1.0)	0.9 (0.8 to 0.9)	0.8 (0.7 to 1.0)	4.3 (-12.8 to 24.1)	-6.6 (-23.2 to 11.7)
Occupational exposure to arsenic	0.4 (0.0 to 0.8)	0.4 (0.0 to 0.8)	0.4 (0.0 to 0.8)	-	-	0.3 (0.0 to 0.8)	0.4 (0.0 to 0.8)	0.3 (0.0 to 0.8)	-	-
Occupational exposure to benzene	0.6 (0.0 to 1.9)	0.7 (0.0 to 1.9)	0.6 (0.0 to 1.8)	0.2 (-7.6 to 10.6)	-3.8 (-9.7 to 3.8)	0.9 (0.1 to 2.5)	1.0 (0.1 to 2.8)	1.0 (0.1 to 2.8)	12.9 (3.4 to 29.3)	-0.6 (-7.3 to 9.3)
Occupational exposure to beryllium	0.0 (0.0 to 0.0)	0.0 (0.0 to 0.0)	0.0 (0.0 to 0.0)	0.1 (-4.9 to 5.3)	0.7 (-3.4 to 5.0)	0.0 (0.0 to 0.0)	0.0 (0.0 to 0.0)	0.0 (0.0 to 0.0)	6.7 (-0.2 to 14.9)	-0.5 (-5.6 to 4.6)
Occupational exposure to cadmium	0.0 (0.0 to 0.0)	0.0 (0.0 to 0.0)	0.0 (0.0 to 0.0)	-0.0 (-10.4 to 11.3)	1.8 (-7.5 to 11.2)	0.0 (0.0 to 0.0)	0.0 (0.0 to 0.0)	0.0 (0.0 to 0.0)	4.7 (-9.3 to 19.4)	-1.7 (-12.1 to 10.1)
Occupational exposure to chromium	0.1 (0.1 to 0.1)	0.1 (0.1 to 0.1)	0.1 (0.1 to 0.1)	2.5 (-6.6 to 12.3)	1.4 (-6.4 to 9.4)	0.1 (0.1 to 0.1)	0.1 (0.1 to 0.1)	0.1 (0.1 to 0.1)	6.5 (-5.4 to 18.9)	-1.9 (-10.5 to 7.7)
Occupational exposure to diesel engine exhaust	0.5 (0.5 to 0.5)	0.5 (0.5 to 0.6)	0.6 (0.5 to 0.6)	11.8 (3.2 to 20.8)	1.6 (-4.4 to 8.6)	0.5 (0.4 to 0.5)	0.5 (0.5 to 0.6)	0.5 (0.4 to 0.5)	7.3 (-4.3 to 20.4)	-3.4 (-11.9 to 5.7)
Occupational exposure to formaldehyde	0.2 (0.2 to 0.2)	0.2 (0.2 to 0.2)	0.2 (0.2 to 0.2)	-2.1 (-12.5 to 9.2)	-0.0 (-9.4 to 9.6)	0.2 (0.2 to 0.2)	0.2 (0.2 to 0.2)	0.2 (0.2 to 0.2)	5.3 (-7.7 to 18.8)	-2.0 (-11.7 to 8.8)
Occupational exposure to nickel	0.3 (0.0 to 1.3)	0.3 (0.0 to 1.2)	0.3 (0.0 to 1.3)	-	-	0.3 (0.0 to 1.2)	0.3 (0.0 to 1.2)	0.3 (0.0 to 1.1)	-	-
Occupational exposure to polycyclic aromatic hydrocarbons	0.2 (0.2 to 0.2)	0.2 (0.2 to 0.2)	0.2 (0.2 to 0.2)	2.3 (-6.6 to 11.7)	0.4 (-6.9 to 8.0)	0.2 (0.2 to 0.2)	0.2 (0.2 to 0.3)	0.2 (0.2 to 0.3)	9.6 (-0.8 to 20.7)	-1.7 (-9.3 to 6.4)
Occupational exposure to silica	2.7 (0.4 to 8.7)	2.5 (0.3 to 7.8)	2.5 (0.3 to 8.0)	-8.5 (-19.3 to 4.8)	2.1 (-8.2 to 14.5)	2.2 (0.3 to 7.1)	2.2 (0.3 to 6.7)	2.1 (0.3 to 6.9)	-3.2 (-19.1 to 15.0)	-2.6 (-15.9 to 15.0)
Occupational exposure to sulphuric acid	0.6 (0.1 to 1.9)	0.7 (0.1 to 2.0)	0.6 (0.1 to 2.0)	10.9 (-1.9 to 23.7)	-1.0 (-10.4 to 8.7)	0.5 (0.1 to 1.7)	0.7 (0.1 to 2.0)	0.6 (0.1 to 2.0)	23.7 (6.5 to 43.3)	-3.6 (-15.6 to 9.2)
Occupational exposure to trichloroethylene	0.1 (0.1 to 0.1)	0.1 (0.1 to 0.1)	0.1 (0.1 to 0.1)	6.1 (-1.1 to 14.1)	1.6 (-4.1 to 7.5)	0.1 (0.1 to 0.1)	0.1 (0.1 to 0.1)	0.1 (0.1 to 0.1)	6.9 (-2.8 to 17.8)	-2.1 (-9.0 to 5.8)
Occupational asthmagens	15.5 (13.1 to 18.3)	14.4 (12.2 to 17.0)	14.1 (11.8 to 16.7)	-7.3 (-16.1 to 2.3)	-2.0 (-9.5 to 6.1)	8.0 (6.6 to 9.7)	8.0 (6.6 to 9.8)	7.6 (6.2 to 9.3)	0.1 (-11.7 to 14.3)	-5.5 (-15.2 to 5.9)
Occupational particulate matter, gases, and fumes	6.4 (4.6 to 9.3)	6.1 (4.4 to 8.9)	5.9 (4.2 to 8.4)	-4.8 (-9.4 to -0.6)	-4.0 (-7.4 to -0.5)	3.7 (2.6 to 5.4)	3.6 (2.6 to 5.4)	3.5 (2.5 to 5.1)	-2.4 (-8.4 to 4.3)	-4.1 (-9.4 to 1.0)
Occupational noise	5.9 (5.4 to 6.5)	5.8 (5.4 to 6.3)	5.6 (5.2 to 6.1)	-1.6 (-4.6 to 1.3)	-3.3 (-5.4 to -1.2)	4.3 (3.8 to 4.8)	4.5 (4.1 to 5.0)	4.5 (4.1 to 4.9)	4.6 (0.2 to 9.7)	-0.3 (-3.1 to 2.9)
Occupational ergonomic factors	8.5 (7.1 to 10.2)	7.8 (6.5 to 9.5)	7.5 (6.1 to 9.1)	-8.3 (-16.7 to 0.6)	-4.2 (-10.7 to 2.8)	4.8 (3.8 to 6.0)	5.0 (4.0 to 6.2)	4.9 (3.9 to 6.2)	4.1 (-10.4 to 19.7)	-1.1 (-11.7 to 10.4)
Non-exclusive breastfeeding	0.5 (0.5 to 0.6)	0.5 (0.5 to 0.6)	0.5 (0.4 to 0.6)	-1.0 (-12.2 to 11.5)	-6.7 (-15.3 to 3.0)	0.5 (0.5 to 0.6)	0.5 (0.5 to 0.6)	0.5 (0.4 to 0.6)	-0.8 (-12.0 to 11.7)	-6.7 (-15.3 to 3.0)
Discontinued breastfeeding	2.2 (2.1 to 2.4)	2.1 (2.0 to 2.3)	2.2 (2.1 to 2.3)	-4.6 (-10.9 to 2.7)	2.4 (-3.0 to 8.1)	2.2 (2.1 to 2.4)	2.1 (2.0 to 2.3)	2.2 (2.1 to 2.3)	-4.8 (-11.1 to 2.5)	1.8 (-3.6 to 7.5)
Child underweight	0.1 (0.1 to 0.1)	0.1 (0.1 to 0.1)	0.1 (0.0 to 0.1)	-7.2 (-20.1 to 8.2)	-13.7 (-22.1 to -5.2)	0.1 (0.0 to 0.1)	0.1 (0.0 to 0.1)	0.1 (0.0 to 0.1)	-4.5 (-19.0 to 13.1)	-5.1 (-13.8 to 4.8)
Child wasting	0.1 (0.0 to 0.1)	0.1 (0.0 to 0.1)	0.1 (0.0 to 0.1)	-10.6 (-28.1 to 7.7)	-3.9 (-15.3 to 10.4)	0.1 (0.0 to 0.1)	0.1 (0.0 to 0.1)	0.1 (0.0 to 0.1)	5.3 (-14.5 to 30.3)	-6.0 (-18.0 to 9.6)
Child stunting	0.3 (0.1 to 0.5)	0.2 (0.1 to 0.4)	0.2 (0.1 to 0.4)	-11.6 (-49.4 to 55.1)	-11.7 (-36.8 to 21.6)	0.2 (0.1 to 0.4)	0.2 (0.1 to 0.3)	0.2 (0.1 to 0.3)	-11.7 (-43.8 to 35.9)	-4.0 (-28.5 to 27.5)
Short gestation for birth weight	0.0 (0.0 to 0.0)	0.0 (0.0 to 0.0)	0.0 (0.0 to 0.0)	6.2 (-17.5 to 40.5)	4.5 (-18.6 to 34.5)	0.0 (0.0 to 0.0)	0.0 (0.0 to 0.0)	0.0 (0.0 to 0.0)	6.7 (-17.5 to 37.1)	1.9 (-19.7 to 27.2)
Low birth weight for gestation	0.0 (0.0 to 0.0)	0.0 (0.0 to 0.0)	0.0 (0.0 to 0.0)	2.4 (-17.9 to 30.7)	-1.5 (-21.6 to 22.4)	0.0 (0.0 to 0.0)	0.0 (0.0 to 0.0)	0.0 (0.0 to 0.0)	0.3 (-20.0 to 23.9)	0.0 (-18.4 to 23.3)
Iron deficiency	-	-	-	-	-	4.2 (2.7 to 6.2)	3.8 (2.5 to 5.5)	3.8 (2.4 to 5.7)	-10.2 (-32.7 to 22.4)	0.3 (-22.6 to 27.0)
Vitamin A deficiency	0.5 (0.3 to 0.7)	0.5 (0.3 to 0.7)	0.6 (0.4 to 0.7)	3.7 (-18.3 to 38.5)	11.8 (-6.5 to 35.8)	0.6 (0.4 to 0.8)	0.6 (0.4 to 0.7)	0.6 (0.4 to 0.8)	-6.2 (-24.1 to 18.6)	1.0 (-28.4 to 25.8)
Zinc deficiency	0.5 (0.1 to 1.1)	0.2 (0.0 to 1.1)	0.2 (0.0 to 1.0)	-53.7 (-88.7 to 364.6)	-4.9 (-89.3 to 844.3)	0.5 (0.1 to 1.1)	0.2 (0.0 to 0.9)	0.2 (0.0 to 1.0)	-56.2 (-88.5 to 250.9)	-4.8 (-87.8 to 886.3)
Smoking	12.6 (10.4 to 14.8)	7.0 (5.8 to 8.2)	5.9 (4.7 to 7.2)	-44.6 (-52.8 to -35.5)	-15.2 (-24.5 to -5.0)	9.3 (7.3 to 11.4)	4.8 (3.9 to 5.7)	3.5 (2.7 to 4.3)	-48.6 (-57.7 to -37.7)	-27.2 (-36.0 to -17.6)
Chewing tobacco	1.1 (0.8 to 1.4)	1.4 (1.0 to 1.9)	1.5 (1.0 to 2.2)	29.8 (-8.6 to 87.5)	9.3 (-20.1 to 48.4)	0.1 (0.1 to 0.1)	0.1 (0.1 to 0.1)	0.1 (0.1 to 0.1)	-3.5 (-38.1 to 49.6)	3.6 (-26.4 to 48.3)
Second-hand smoke	40.6 (37.6 to 43.7)	22.5 (21.1 to 23.7)	22.6 (20.2 to 25.3)	-44.7 (-50.4 to -38.5)	0.6 (-9.1 to 11.4)	37.8 (35.3 to 40.6)	21.5 (20.6 to 22.3)	21.2 (19.6 to 22.8)	-43.1 (-48.3 to -37.8)	-1.2 (-9.0 to 6.7)
Alcohol use	21.0 (11.3 to 33.8)	23.1 (12.7 to 35.5)	25.5 (13.2 to 39.8)	10.1 (-27.2 to 71.7)	10.4 (-23.7 to 60.0)	11.0 (5.8 to 18.6)	11.5 (6.0 to 19.0)	11.9 (6.1 to 21.0)	4.2 (-27.9 to 55.7)	3.9 (-25.1 to 45.7)
Drug use	1.7 (1.3 to 2.5)	1.9 (1.4 to 2.6)	2.3 (1.7 to 3.2)	8.5 (1.3 to 17.0)	22.4 (11.2 to 36.1)	1.4 (1.0 to 2.0)	1.7 (1.3 to 2.4)	1.8 (1.3 to 2.5)	19.5 (9.7 to 31.1)	7.3 (-1.6 to 17.0)
Diet low in fruits	24.6 (18.3 to 30.1)	23.3 (16.9 to 28.9)	23.5 (16.6 to 29.4)	-5.2 (-16.1 to 5.5)	0.6 (-13.0 to 15.2)	22.0 (15.4 to 27.6)	21.1 (14.6 to 26.8)	20.4 (13.2 to 26.8)	-4.3 (-17.8 to 8.2)	-3.4 (-19.3 to 12.4)
Diet low in vegetables	12.2 (4.8 to 20.0)	8.3 (2.4 to 15.2)	6.3 (1.8 to 12.4)	-31.6 (-64.1 to -1.5)	-24.1 (-56.9 to 16.9)	17.1 (9.0 to 24.5)	17.6 (9.9 to 24.7)	17.8 (9.9 to 24.7)	2.8 (-18.6 to 40.5)	1.4 (-17.1 to 24.0)
Diet low in legumes	24.1 (17.5 to 29.3)	24.1 (17.8 to 29.7)	25.5 (19.2 to 31.2)	0.3 (-14.5 to 17.4)	5.7 (-6.3 to 21.2)	28.8 (23.0 to 33.7)	29.3 (23.5 to 34.0)	30.7 (25.2 to 35.4)	2.0 (-9.5 to 15.8)	4.7 (-3.2 to 14.6)
Diet low in whole grains	37.7 (34.2 to 40.7)	39.1 (36.1 to 41.8)	38.5 (35.0 to 41.4)	3.6 (-1.1 to 9.1)	-1.5 (-5.3 to 2.1)	38.7 (35.3 to 41.5)	40.0 (37.0 to 42.6)	39.6 (36.4 to 42.2)	3.5 (-0.7 to 8.6)	-1.2 (-4.5 to 2.0)
Diet low in nuts and seeds	44.5 (41.2 to 47.2)	35.4 (30.6 to 39.3)	35.2 (29.7 to 39.7)	-20.6 (-28.7 to -14.3)	-0.4 (-9.5 to 7.6)	45.4 (42.5 to 47.8)	36.5 (31.7 to 40.4)	36.2 (30.7 to 40.7)	-19.8 (-27.4 to -13.2)	-0.6 (-8.6 to 6.0)
Diet low in milk	26.7 (20.4 to 31.8)	25.4 (18.9 to 30.9)	25.7 (19.1 to 31.2)	-4.6 (-14.8 to 5.8)	0.9 (-7.6 to 10.9)	28.3 (22.2 to 33.3)	26.5 (20.3 to 31.7)	26.5 (20.1 to 31.8)	-6.6 (-16.7 to 1.7)	0.0 (-7.5 to 8.4)
Diet high in red meat	44.1 (34.0 to 50.7)	37.9 (27.3 to 46.3)	36.6 (26.2 to 45.7)	-13.9 (-31.0 to 4.4)	-3.6 (-20.2 to 14.7)	25.0 (15.5 to 34.4)	17.9 (9.6 to 26.4)	17.1 (8.8 to 25.7)	-28.3 (-54.0 to 2.9)	-4.5 (-35.7 to 35.8)
Diet high in processed meat	42.0 (33.3 to 48.1)	38.2 (31.0 to 44.4)	31.1 (23.9 to 37.5)	-9.1 (-23.1 to 7.2)	-18.6 (-30.3 to -6.2)	23.2 (16.7 to 28.6)	21.9 (16.1 to 27.0)	19.4 (14.1 to 24.3)	-5.7 (-23.4 to 17.5)	-11.5 (-24.4 to 3.7)
Diet high in sugar-sweetened beverages	33.8 (17.5 to 43.3)	31.9 (15.9 to 41.6)	27.2 (14.0 to 37.6)	-5.7 (-32.1 to 30.7)	-14.8 (-36.9 to 8.3)	28.0 (13.4 to 39.1)	24.4 (12.3 to 34.7)	20.4 (10.3 to 30.2)	-12.8 (-41.9 to 30.6)	-16.4 (-42.3 to 17.1)
Diet low in fibre	32.6 (27.0 to 37.3)	24.8 (18.1 to 30.5)	26.3 (19.7 to 31.7)	-24.0 (-36.3 to -14.6)	5.9 (-4.6 to 19.2)	37.3 (32.5 to 41.3)	29.4 (23.1 to 34.7)	30.6 (24.6 to 35.6)	-21.1 (-32.0 to -13.0)	3.9 (-4.2 to 14.1)
Diet low in calcium	15.1 (8.1 to 21.3)	14.1 (7.8 to 20.2)	14.5 (8.0 to 20.6)	-6.4 (-19.6 to 9.1)	2.7 (-8.0 to 16.1)	19.1 (11.9 to 25.2)	17.8 (10.9 to 23.9)	18.2 (11.2 to 24.3)	-6.8 (-19.4 to 4.0)	2.4 (-5.9 to 12.7)
Diet low in seafood omega-3 fatty acids	26.6 (20.8 to 31.7)	23.0 (16.9 to 28.5)	24.1 (17.9 to 29.5)	-13.4 (-26.5 to -3.0)	4.6 (-5.7 to 15.9)	30.3 (25.1 to 35.2)	27.2 (21.2 to 32.5)	28.0 (22.1 to 33.2)	-10.1 (-20.5 to -1.1)	2.8 (-5.0 to 12.2)
Diet low in polyunsaturated fatty acids	6.9 (1.8 to 13.6)	4.4 (0.6 to 9.9)	3.1 (0.3 to 7.6)	-37.1 (-79.6 to 8.5)	-29.8 (-75.1 to 44.5)	7.8 (2.5 to 14.0)	4.9 (0.7 to 10.8)	3.4 (0.4 to 8.6)	-36.5 (-83.4 to 3.3)	-30.1 (-69.9 to 34.8)
Diet high in trans fatty acids	16.9 (12.2 to 22.5)	9.8 (6.0 to 14.2)	7.1 (3.5 to 11.1)	-41.7 (-56.5 to -28.5)	-27.7 (-44.1 to -16.2)	23.7 (17.9 to 32.1)	13.3 (9.0 to 17.9)	9.7 (5.8 to 14.0)	-43.7 (-55.5 to -31.9)	-27.0 (-40.0 to -16.6)
Diet high in sodium	7.4 (0.7 to 14.8)	12.8 (4.2 to 20.2)	13.3 (4.8 to 21.1)	73.0 (20.0 to 648.5)	4.2 (-18.8 to 45.8)	6.1 (0.3 to 13.1)	10.9 (2.7 to 18.1)	11.4 (2.9 to 18.9)	79.4 (27.6 to 973.8)	4.4 (-18.0 to 46.8)
Intimate partner violence	-	-	-	-	-	1.4 (1.2 to 1.6)	0.9 (0.8 to 1.1)	1.2 (1.0 to 1.3)	-33.0 (-36.5 to -29.3)	23.0 (16.4 to 30.0)
Childhood sexual abuse	5.6 (4.6 to 6.9)	5.1 (4.1 to 6.2)	5.1 (4.1 to 6.2)	-9.8 (-15.1 to -4.3)	0.4 (-4.7 to 6.0)	13.8 (11.0 to 16.9)	12.1 (9.8 to 14.7)	12.2 (9.9 to 14.8)	-11.9 (-17.2 to -6.0)	0.4 (-4.8 to 6.5)

Supplementary Results Table 1. California age-standardised Summary Exposure Values (SEVs) for Level 4 risk factors and percentage change, by sex, 1990, 2007, and 2017.Data in parentheses are 95% uncertainty intervals.										
	**Male**					**Female**				
	**1990**	**2007**	**2017**	**Percentage change 1990-2007**	**Percentage change 2007-2017**	**1990**	**2007**	**2017**	**Percentage change 1990-2007**	**Percentage change 2007-2017**
Bullying victimization	6.0 (2.4 to 12.9)	8.9 (3.7 to 19.4)	8.6 (3.5 to 18.9)	49.3 (33.8 to 70.2)	-3.6 (-11.4 to 3.3)	4.6 (1.9 to 10.0)	7.6 (3.2 to 16.4)	7.5 (3.1 to 16.5)	67.0 (47.9 to 93.4)	-1.8 (-9.8 to 7.1)
Low physical activity	0.4 (0.2 to 0.8)	0.4 (0.2 to 0.8)	0.4 (0.2 to 0.8)	0.7 (-1.3 to 2.7)	0.6 (-0.8 to 2.0)	0.5 (0.2 to 0.9)	0.5 (0.2 to 0.9)	0.5 (0.2 to 0.9)	0.1 (-2.3 to 2.5)	0.5 (-0.9 to 2.0)
High fasting plasma glucose	1.1 (0.7 to 1.7)	2.0 (1.2 to 3.0)	2.9 (1.8 to 4.4)	79.8 (17.6 to 164.8)	46.8 (-7.5 to 143.0)	1.3 (0.8 to 2.1)	2.3 (1.3 to 3.7)	2.0 (1.1 to 3.4)	75.7 (4.7 to 200.4)	-11.7 (-48.9 to 57.1)
High LDL cholesterol	21.2 (19.1 to 23.2)	11.6 (10.0 to 13.4)	10.2 (8.7 to 11.9)	-45.4 (-50.3 to -40.9)	-11.9 (-15.8 to -7.9)	19.4 (17.6 to 21.4)	11.4 (9.9 to 13.0)	10.5 (9.1 to 12.1)	-41.3 (-45.8 to -37.0)	-7.3 (-11.4 to -3.4)
High systolic blood pressure	6.3 (5.4 to 7.1)	4.8 (4.1 to 5.4)	4.9 (4.2 to 5.6)	-24.1 (-31.2 to -16.5)	2.1 (-5.8 to 11.3)	6.2 (5.5 to 7.0)	4.3 (3.8 to 4.8)	3.9 (3.3 to 4.6)	-31.2 (-38.1 to -23.5)	-7.9 (-16.5 to 1.5)
High body-mass index	14.8 (11.1 to 19.8)	21.9 (17.5 to 27.4)	23.0 (18.5 to 28.6)	47.8 (30.1 to 68.6)	5.2 (-2.8 to 14.8)	16.0 (12.5 to 20.4)	22.0 (17.8 to 27.5)	22.8 (18.3 to 28.1)	37.2 (22.1 to 55.1)	3.6 (-4.7 to 12.7)
Low bone mineral density	5.3 (4.4 to 6.5)	5.8 (4.8 to 6.8)	6.3 (5.1 to 7.4)	7.9 (-5.8 to 23.9)	8.4 (-7.0 to 25.0)	5.8 (4.8 to 6.9)	5.6 (4.7 to 6.7)	6.1 (5.1 to 7.3)	-3.0 (-15.7 to 11.5)	9.4 (-4.2 to 24.6)
Impaired kidney function	1.7 (0.9 to 4.3)	1.7 (0.8 to 4.1)	1.8 (0.9 to 4.3)	-2.7 (-10.5 to 5.6)	4.8 (-0.7 to 12.0)	2.7 (1.3 to 6.5)	2.6 (1.3 to 6.3)	2.7 (1.3 to 6.4)	-1.8 (-7.6 to 4.4)	1.5 (-3.3 to 6.6)

Supplementary Results Table 1. Colorado age-standardised Summary Exposure Values (SEVs) for Level 4 risk factors and percentage change, by sex, 1990, 2007, and 2017.Data in parentheses are 95% uncertainty intervals.										
	Male					Female				
	1990	2007	2017	Percentage change 1990-2007	Percentage change 2007-2017	1990	2007	2017	Percentage change 1990-2007	Percentage change 2007-2017
Unsafe water source	1.3 (0.7 to 2.1)	0.5 (0.3 to 0.7)	0.3 (0.2 to 0.5)	-64.7 (-81.5 to -27.8)	-36.4 (-59.5 to 1.8)	1.3 (0.7 to 2.1)	0.5 (0.3 to 0.7)	0.3 (0.2 to 0.5)	-64.7 (-81.5 to -27.8)	-36.4 (-59.5 to 1.8)
Unsafe sanitation	5.9 (3.1 to 10.3)	3.6 (1.6 to 6.7)	2.5 (1.1 to 4.8)	-37.8 (-71.9 to 31.2)	-32.0 (-64.4 to 33.5)	5.9 (3.1 to 10.3)	3.6 (1.6 to 6.7)	2.5 (1.1 to 4.8)	-37.8 (-71.9 to 31.2)	-32.0 (-64.4 to 33.5)
No access to handwashing facility	3.4 (2.5 to 4.7)	3.1 (2.2 to 4.4)	3.0 (2.1 to 4.1)	-8.6 (-36.2 to 27.7)	-4.1 (-26.0 to 23.3)	3.4 (2.5 to 4.7)	3.1 (2.2 to 4.4)	3.0 (2.1 to 4.1)	-8.6 (-36.2 to 27.7)	-4.1 (-26.0 to 23.3)
Ambient particulate matter pollution	17.9 (7.1 to 29.6)	17.4 (8.0 to 29.5)	10.8 (2.1 to 23.3)	-3.1 (-49.6 to 113.0)	-37.9 (-84.0 to 14.4)	17.9 (7.0 to 29.6)	17.3 (8.0 to 29.4)	10.8 (2.1 to 23.3)	-3.0 (-49.6 to 113.0)	-37.8 (-84.0 to 14.4)
Household air pollution from solid fuels	0.2 (0.0 to 0.6)	0.2 (0.0 to 0.5)	0.1 (0.0 to 0.4)	-27.2 (-77.3 to 80.0)	-22.3 (-84.4 to 73.7)	0.4 (0.1 to 2.1)	0.3 (0.0 to 1.6)	0.2 (0.0 to 0.9)	-27.2 (-77.0 to 80.3)	-24.4 (-85.9 to 83.2)
Ambient ozone pollution	50.8 (23.1 to 54.8)	50.6 (22.6 to 54.8)	51.1 (23.6 to 54.8)	-0.4 (-1.9 to 0.0)	0.9 (0.0 to 4.4)	50.8 (23.1 to 54.8)	50.6 (22.6 to 54.8)	51.1 (23.6 to 54.8)	-0.4 (-1.9 to 0.0)	0.9 (0.0 to 4.4)
Residential radon	18.8 (1.5 to 61.0)	18.8 (1.5 to 61.0)	18.8 (1.5 to 61.0)	0.0 (0.0 to 0.0)	0.0 (0.0 to 0.0)	18.8 (1.5 to 61.0)	18.8 (1.5 to 61.0)	18.8 (1.5 to 61.0)	0.0 (0.0 to 0.0)	0.0 (0.0 to 0.0)
Lead exposure	9.1 (4.4 to 13.6)	5.7 (2.3 to 9.5)	3.8 (1.4 to 6.8)	-37.1 (-48.9 to -27.5)	-33.9 (-43.0 to -27.6)	5.8 (1.8 to 10.0)	3.7 (0.9 to 7.4)	2.5 (0.5 to 5.4)	-35.4 (-51.8 to -23.9)	-33.1 (-44.7 to -26.8)
Occupational exposure to asbestos	5.0 (4.3 to 5.7)	4.1 (3.7 to 4.5)	3.6 (3.0 to 4.3)	-18.7 (-30.3 to -0.9)	-11.3 (-27.2 to 7.2)	0.7 (0.6 to 0.8)	0.7 (0.6 to 0.8)	0.7 (0.6 to 0.9)	-2.5 (-15.3 to 11.5)	3.0 (-17.1 to 27.8)
Occupational exposure to arsenic	0.3 (0.0 to 0.8)	0.3 (0.0 to 0.8)	0.3 (0.0 to 0.8)	-	-	0.3 (0.0 to 0.7)	0.3 (0.0 to 0.8)	0.3 (0.0 to 0.8)	-	-
Occupational exposure to benzene	0.6 (0.0 to 1.8)	0.6 (0.0 to 1.8)	0.6 (0.0 to 1.8)	1.5 (-6.6 to 12.9)	-3.8 (-10.2 to 4.7)	0.9 (0.1 to 2.5)	1.0 (0.1 to 2.8)	1.0 (0.1 to 2.8)	11.8 (0.7 to 30.4)	-0.1 (-7.5 to 10.5)
Occupational exposure to beryllium	0.0 (0.0 to 0.0)	0.0 (0.0 to 0.0)	0.0 (0.0 to 0.0)	0.7 (-4.5 to 6.4)	0.4 (-4.1 to 5.1)	0.0 (0.0 to 0.0)	0.0 (0.0 to 0.0)	0.0 (0.0 to 0.0)	5.5 (-2.4 to 14.1)	-0.3 (-5.4 to 5.6)
Occupational exposure to cadmium	0.0 (0.0 to 0.0)	0.0 (0.0 to 0.0)	0.0 (0.0 to 0.0)	3.2 (-8.0 to 16.4)	2.0 (-6.7 to 12.3)	0.0 (0.0 to 0.0)	0.0 (0.0 to 0.0)	0.0 (0.0 to 0.0)	5.5 (-7.0 to 20.3)	-1.0 (-11.1 to 10.6)
Occupational exposure to chromium	0.1 (0.1 to 0.1)	0.1 (0.1 to 0.1)	0.1 (0.1 to 0.1)	5.5 (-4.5 to 17.0)	1.4 (-5.7 to 9.8)	0.1 (0.1 to 0.1)	0.1 (0.1 to 0.1)	0.1 (0.1 to 0.1)	7.4 (-4.4 to 20.8)	-1.5 (-9.5 to 8.1)
Occupational exposure to diesel engine exhaust	0.5 (0.5 to 0.6)	0.6 (0.5 to 0.6)	0.6 (0.5 to 0.6)	8.6 (-0.6 to 18.3)	0.5 (-6.1 to 7.2)	0.5 (0.4 to 0.6)	0.5 (0.5 to 0.6)	0.5 (0.4 to 0.6)	6.4 (-6.1 to 19.9)	-4.7 (-13.2 to 4.9)
Occupational exposure to formaldehyde	0.2 (0.2 to 0.2)	0.2 (0.2 to 0.2)	0.2 (0.2 to 0.2)	1.6 (-9.4 to 15.3)	0.3 (-8.6 to 10.6)	0.2 (0.2 to 0.2)	0.2 (0.2 to 0.2)	0.2 (0.2 to 0.2)	6.5 (-6.6 to 21.2)	-1.1 (-10.0 to 9.9)
Occupational exposure to nickel	0.3 (0.0 to 1.2)	0.3 (0.0 to 1.1)	0.3 (0.0 to 1.2)	-	-	0.2 (0.0 to 1.1)	0.2 (0.0 to 1.1)	0.2 (0.0 to 1.1)	-	-
Occupational exposure to polycyclic aromatic hydrocarbons	0.2 (0.2 to 0.2)	0.2 (0.2 to 0.2)	0.2 (0.2 to 0.2)	5.3 (-3.7 to 15.6)	0.5 (-6.1 to 8.1)	0.2 (0.2 to 0.2)	0.2 (0.2 to 0.2)	0.2 (0.2 to 0.2)	10.4 (-0.5 to 23.0)	-1.2 (-8.3 to 7.1)
Occupational exposure to silica	2.6 (0.4 to 8.4)	2.3 (0.3 to 7.3)	2.4 (0.3 to 7.3)	-10.3 (-21.2 to 3.0)	2.1 (-7.8 to 15.7)	2.1 (0.3 to 6.6)	2.0 (0.3 to 6.3)	2.0 (0.3 to 6.1)	-4.5 (-19.1 to 13.8)	-1.8 (-15.1 to 15.9)
Occupational exposure to sulphuric acid	0.6 (0.1 to 1.7)	0.6 (0.1 to 2.0)	0.6 (0.1 to 2.0)	15.2 (0.8 to 30.1)	-1.4 (-10.8 to 8.6)	0.5 (0.1 to 1.7)	0.7 (0.1 to 2.2)	0.6 (0.1 to 2.1)	25.1 (4.9 to 47.3)	-3.6 (-15.8 to 10.3)
Occupational exposure to trichloroethylene	0.1 (0.1 to 0.1)	0.1 (0.1 to 0.1)	0.1 (0.1 to 0.1)	7.9 (0.3 to 16.4)	1.3 (-4.3 to 7.3)	0.1 (0.1 to 0.1)	0.1 (0.1 to 0.1)	0.1 (0.1 to 0.1)	7.4 (-2.3 to 19.0)	-2.3 (-9.0 to 6.2)
Occupational asthmagens	14.8 (12.5 to 17.6)	14.0 (11.8 to 16.8)	13.6 (11.4 to 16.4)	-5.6 (-15.2 to 4.9)	-2.2 (-10.0 to 6.1)	7.7 (6.3 to 9.3)	7.9 (6.5 to 9.6)	7.4 (6.0 to 9.0)	1.8 (-11.4 to 17.1)	-5.7 (-15.6 to 5.7)
Occupational particulate matter, gases, and fumes	6.2 (4.4 to 8.9)	5.9 (4.2 to 8.5)	5.7 (4.1 to 8.1)	-4.4 (-9.2 to 0.3)	-3.8 (-7.7 to -0.1)	3.5 (2.4 to 5.3)	3.4 (2.4 to 5.0)	3.3 (2.3 to 4.9)	-2.4 (-9.1 to 5.6)	-3.9 (-9.0 to 2.0)
Occupational noise	5.9 (5.4 to 6.5)	5.7 (5.3 to 6.3)	5.5 (5.1 to 6.1)	-2.7 (-6.0 to 0.6)	-3.6 (-5.9 to -1.2)	4.2 (3.7 to 4.9)	4.4 (3.9 to 4.9)	4.4 (3.9 to 4.9)	4.4 (-0.7 to 10.6)	-0.3 (-3.5 to 3.1)
Occupational ergonomic factors	8.4 (6.9 to 10.3)	7.7 (6.4 to 9.5)	7.4 (6.1 to 9.0)	-8.0 (-17.3 to 2.5)	-4.5 (-11.5 to 2.7)	4.8 (3.9 to 6.1)	5.0 (4.0 to 6.3)	5.0 (3.9 to 6.4)	3.2 (-11.7 to 20.9)	-0.7 (-12.4 to 11.9)
Non-exclusive breastfeeding	0.5 (0.5 to 0.6)	0.5 (0.5 to 0.6)	0.5 (0.4 to 0.6)	0.4 (-10.1 to 13.5)	-7.3 (-16.9 to 2.0)	0.5 (0.5 to 0.6)	0.5 (0.5 to 0.6)	0.5 (0.4 to 0.6)	0.4 (-10.2 to 13.5)	-7.2 (-16.8 to 2.1)
Discontinued breastfeeding	2.2 (2.0 to 2.3)	2.1 (2.0 to 2.3)	2.2 (2.1 to 2.3)	-1.1 (-8.4 to 5.9)	3.2 (-2.6 to 9.9)	2.2 (2.0 to 2.3)	2.1 (2.0 to 2.3)	2.2 (2.1 to 2.4)	-1.3 (-8.6 to 5.7)	4.4 (-1.4 to 11.2)
Child underweight	0.1 (0.1 to 0.1)	0.1 (0.1 to 0.1)	0.1 (0.0 to 0.1)	-4.1 (-18.5 to 12.1)	-13.6 (-22.4 to -4.7)	0.1 (0.0 to 0.1)	0.1 (0.0 to 0.1)	0.1 (0.0 to 0.1)	-2.0 (-17.1 to 16.4)	-5.3 (-15.6 to 5.5)
Child wasting	0.1 (0.0 to 0.1)	0.1 (0.0 to 0.1)	0.1 (0.0 to 0.1)	-9.8 (-30.3 to 11.5)	-3.6 (-16.3 to 10.5)	0.1 (0.0 to 0.1)	0.1 (0.0 to 0.1)	0.1 (0.0 to 0.1)	6.2 (-16.4 to 34.6)	-6.0 (-20.6 to 10.5)
Child stunting	0.3 (0.1 to 0.5)	0.2 (0.1 to 0.4)	0.2 (0.1 to 0.4)	-8.1 (-48.9 to 68.7)	-12.1 (-41.9 to 28.8)	0.2 (0.1 to 0.4)	0.2 (0.1 to 0.3)	0.2 (0.1 to 0.3)	-8.9 (-42.5 to 49.5)	-4.5 (-29.9 to 26.9)
Short gestation for birth weight	0.0 (0.0 to 0.0)	0.0 (0.0 to 0.0)	0.0 (0.0 to 0.0)	3.5 (-25.0 to 38.8)	2.9 (-19.5 to 29.1)	0.0 (0.0 to 0.0)	0.0 (0.0 to 0.0)	0.0 (0.0 to 0.0)	2.9 (-29.0 to 41.7)	6.0 (-21.4 to 54.0)
Low birth weight for gestation	0.0 (0.0 to 0.0)	0.0 (0.0 to 0.0)	0.0 (0.0 to 0.0)	3.1 (-20.2 to 39.0)	-0.3 (-23.6 to 23.0)	0.0 (0.0 to 0.0)	0.0 (0.0 to 0.0)	0.0 (0.0 to 0.0)	2.3 (-23.0 to 43.6)	-2.9 (-31.5 to 25.7)
Iron deficiency	-	-	-	-	-	4.1 (2.6 to 6.1)	3.7 (2.4 to 5.5)	3.9 (2.5 to 5.7)	-8.6 (-32.4 to 24.3)	3.5 (-19.7 to 32.0)
Vitamin A deficiency	0.5 (0.3 to 0.7)	0.5 (0.3 to 0.6)	0.6 (0.3 to 0.8)	-4.3 (-19.4 to 17.1)	24.5 (-5.0 to 59.5)	0.5 (0.3 to 0.7)	0.6 (0.4 to 0.8)	0.6 (0.4 to 0.8)	15.9 (-6.2 to 44.0)	4.9 (-11.9 to 25.3)
Zinc deficiency	0.5 (0.1 to 1.1)	0.2 (0.0 to 1.0)	0.2 (0.0 to 0.9)	-56.2 (-88.6 to 221.0)	-3.6 (-88.8 to 870.7)	0.5 (0.1 to 1.1)	0.2 (0.0 to 1.0)	0.2 (0.0 to 1.0)	-53.3 (-88.1 to 276.6)	-0.4 (-88.8 to 889.2)
Smoking	12.9 (10.3 to 15.5)	6.8 (5.3 to 8.4)	5.4 (4.1 to 6.8)	-47.6 (-55.3 to -38.0)	-20.5 (-30.4 to -9.6)	8.8 (6.7 to 11.3)	4.9 (3.8 to 6.2)	3.9 (3.0 to 4.9)	-44.7 (-55.9 to -31.3)	-20.3 (-29.7 to -9.8)
Chewing tobacco	2.2 (1.8 to 2.7)	2.6 (1.9 to 3.5)	2.5 (1.6 to 3.7)	16.6 (-19.8 to 68.1)	-2.3 (-30.3 to 33.2)	0.1 (0.1 to 0.1)	0.1 (0.1 to 0.1)	0.1 (0.1 to 0.1)	-1.3 (-38.8 to 68.2)	-0.7 (-33.5 to 45.4)
Second-hand smoke	43.1 (39.6 to 46.9)	23.7 (22.0 to 25.3)	25.1 (22.5 to 27.9)	-44.9 (-51.9 to -37.6)	5.9 (-5.2 to 18.3)	40.6 (37.7 to 43.9)	22.3 (21.1 to 23.4)	23.3 (21.5 to 25.2)	-45.1 (-51.4 to -38.5)	4.4 (-4.2 to 14.9)
Alcohol use	19.1 (10.2 to 30.3)	20.7 (10.9 to 33.3)	21.9 (11.1 to 35.5)	8.1 (-29.8 to 71.0)	5.9 (-27.1 to 47.1)	14.4 (7.3 to 24.6)	15.3 (7.8 to 26.3)	16.4 (8.1 to 28.8)	6.4 (-26.2 to 61.9)	7.0 (-25.1 to 49.1)
Drug use	1.8 (1.2 to 2.9)	2.0 (1.5 to 3.1)	2.8 (2.0 to 4.0)	14.4 (2.2 to 33.0)	34.3 (21.3 to 51.6)	1.3 (0.9 to 2.0)	1.6 (1.2 to 2.3)	2.0 (1.5 to 2.8)	25.0 (13.2 to 41.9)	23.2 (12.7 to 37.3)
Diet low in fruits	28.3 (22.1 to 33.2)	26.9 (20.7 to 31.8)	27.0 (20.2 to 32.4)	-5.0 (-14.5 to 3.9)	0.3 (-10.3 to 11.1)	23.9 (17.3 to 29.5)	23.3 (17.0 to 28.9)	22.7 (15.6 to 28.7)	-2.5 (-15.3 to 11.3)	-2.7 (-16.7 to 11.3)
Diet low in vegetables	20.1 (11.9 to 27.3)	24.1 (16.8 to 30.0)	24.5 (16.9 to 30.8)	20.0 (-1.1 to 61.6)	1.8 (-12.8 to 20.3)	21.9 (13.2 to 28.9)	26.3 (18.8 to 32.4)	28.0 (20.7 to 34.2)	20.0 (-0.7 to 63.4)	6.5 (-5.2 to 23.4)
Diet low in legumes	24.0 (17.2 to 30.1)	24.1 (16.9 to 30.1)	25.5 (18.8 to 31.2)	0.4 (-16.3 to 21.1)	5.7 (-6.8 to 21.4)	28.6 (22.5 to 33.9)	29.3 (23.5 to 34.2)	30.7 (24.8 to 35.6)	2.7 (-8.7 to 15.8)	4.7 (-4.2 to 16.5)
Diet low in whole grains	38.0 (34.4 to 41.1)	39.2 (35.7 to 42.0)	38.7 (35.1 to 41.6)	3.2 (-1.7 to 8.4)	-1.1 (-5.1 to 2.3)	38.9 (35.4 to 41.7)	40.1 (36.8 to 42.6)	39.7 (36.5 to 42.5)	2.9 (-1.7 to 8.5)	-0.9 (-4.6 to 2.7)
Diet low in nuts and seeds	45.3 (42.2 to 47.8)	35.4 (30.4 to 39.6)	35.8 (30.5 to 40.3)	-21.9 (-31.0 to -14.9)	1.1 (-7.7 to 9.7)	46.1 (43.3 to 48.5)	36.4 (31.6 to 40.4)	36.9 (31.6 to 41.4)	-20.9 (-29.4 to -14.2)	1.1 (-8.0 to 9.7)
Diet low in milk	27.2 (21.0 to 32.5)	25.5 (18.8 to 31.0)	26.0 (18.9 to 31.5)	-6.3 (-17.8 to 3.9)	2.0 (-6.7 to 12.7)	29.0 (23.1 to 34.1)	26.5 (19.7 to 31.8)	26.8 (20.1 to 32.1)	-8.5 (-19.3 to -0.3)	1.1 (-7.8 to 11.7)
Diet high in red meat	49.6 (45.0 to 53.4)	42.6 (32.4 to 49.1)	39.6 (29.1 to 47.1)	-14.2 (-30.5 to -0.4)	-7.1 (-22.1 to 8.7)	31.2 (23.5 to 37.8)	24.4 (14.5 to 33.2)	21.9 (11.6 to 30.9)	-21.7 (-45.7 to 5.8)	-10.3 (-36.5 to 21.1)
Diet high in processed meat	44.3 (37.9 to 49.3)	48.0 (41.5 to 52.3)	40.8 (31.2 to 47.2)	8.4 (-5.3 to 24.1)	-15.0 (-25.9 to -4.8)	22.6 (17.5 to 26.8)	29.7 (23.0 to 35.3)	26.2 (19.4 to 32.4)	31.4 (6.3 to 62.4)	-11.6 (-25.1 to 3.4)
Diet high in sugar-sweetened beverages	32.0 (15.9 to 42.2)	31.1 (15.7 to 41.4)	26.2 (13.5 to 37.1)	-2.7 (-31.4 to 42.0)	-15.7 (-38.4 to 10.6)	26.9 (13.2 to 37.7)	24.1 (11.8 to 34.7)	20.0 (9.6 to 30.0)	-10.4 (-43.0 to 38.5)	-17.0 (-44.8 to 18.6)
Diet low in fibre	33.0 (26.9 to 37.7)	24.9 (17.8 to 31.2)	26.5 (19.5 to 32.6)	-24.4 (-37.7 to -13.8)	6.5 (-4.2 to 20.4)	37.6 (32.8 to 41.7)	29.5 (22.9 to 34.9)	30.8 (24.3 to 36.0)	-21.7 (-33.3 to -12.5)	4.4 (-4.0 to 15.0)
Diet low in calcium	16.1 (9.0 to 22.5)	14.4 (7.9 to 20.5)	15.1 (8.6 to 21.3)	-10.5 (-25.2 to 3.8)	5.1 (-7.2 to 19.7)	20.1 (13.0 to 26.2)	18.1 (11.0 to 24.3)	18.9 (11.6 to 25.0)	-10.1 (-24.2 to 0.3)	4.4 (-4.3 to 17.3)
Diet low in seafood omega-3 fatty acids	27.9 (22.3 to 33.1)	23.3 (17.5 to 29.0)	24.8 (18.8 to 30.4)	-16.4 (-29.0 to -5.6)	6.6 (-3.4 to 19.4)	31.4 (26.5 to 36.2)	27.4 (21.6 to 32.5)	28.6 (22.8 to 33.6)	-12.7 (-23.6 to -3.5)	4.1 (-3.9 to 13.6)
Diet low in polyunsaturated fatty acids	7.8 (2.2 to 14.6)	4.6 (0.7 to 10.5)	3.4 (0.5 to 8.6)	-40.7 (-81.7 to 0.9)	-25.4 (-69.4 to 44.9)	8.6 (2.7 to 15.4)	5.2 (0.9 to 11.0)	3.9 (0.5 to 8.8)	-39.4 (-79.7 to -1.5)	-26.1 (-69.0 to 36.1)
Diet high in trans fatty acids	16.8 (12.1 to 22.1)	9.8 (5.7 to 14.0)	7.1 (3.5 to 10.9)	-41.7 (-55.9 to -29.7)	-27.6 (-43.8 to -15.9)	23.8 (17.8 to 33.0)	13.3 (9.0 to 17.9)	9.7 (5.8 to 14.0)	-44.2 (-55.4 to -31.8)	-26.7 (-39.4 to -16.2)
Diet high in sodium	7.3 (0.7 to 14.7)	12.8 (4.1 to 20.0)	13.3 (4.7 to 21.0)	74.8 (23.0 to 613.2)	3.7 (-23.0 to 47.1)	6.0 (0.3 to 13.4)	10.9 (2.7 to 18.4)	11.4 (3.0 to 19.3)	80.4 (27.2 to 1095.9)	4.8 (-21.1 to 46.4)
Intimate partner violence	-	-	-	-	-	0.5 (0.3 to 0.7)	0.4 (0.3 to 0.6)	0.5 (0.3 to 0.7)	-11.7 (-24.9 to 9.2)	6.1 (-8.5 to 20.5)
Childhood sexual abuse	5.6 (4.6 to 6.9)	5.1 (4.1 to 6.2)	5.1 (4.1 to 6.2)	-9.8 (-15.1 to -4.3)	0.4 (-4.7 to 6.0)	13.8 (11.0 to 16.9)	12.1 (9.8 to 14.7)	12.2 (9.9 to 14.8)	-11.9 (-17.2 to -6.0)	0.4 (-4.8 to 6.5)

Supplementary Results Table 1. Colorado age-standardised Summary Exposure Values (SEVs) for Level 4 risk factors and percentage change, by sex, 1990, 2007, and 2017.Data in parentheses are 95% uncertainty intervals.

	Male					Female				
	**1990**	**2007**	**2017**	**Percentage change 1990-2007**	**Percentage change 2007-2017**	**1990**	**2007**	**2017**	**Percentage change 1990-2007**	**Percentage change 2007-2017**
Bullying victimization	6.0 (2.5 to 12.4)	8.8 (3.6 to 18.0)	8.6 (3.5 to 18.0)	46.6 (31.3 to 67.1)	-2.3 (-10.2 to 6.2)	4.6 (1.9 to 9.5)	7.5 (3.1 to 15.4)	7.5 (3.0 to 15.6)	64.4 (45.0 to 90.5)	-0.2 (-8.3 to 8.9)
Low physical activity	0.4 (0.2 to 0.8)	0.4 (0.2 to 0.8)	0.4 (0.2 to 0.8)	-0.4 (-2.5 to 1.7)	0.4 (-1.1 to 1.8)	0.4 (0.2 to 0.8)	0.4 (0.2 to 0.8)	0.4 (0.2 to 0.8)	-1.1 (-3.3 to 1.4)	-0.1 (-1.5 to 1.3)
High fasting plasma glucose	1.1 (0.7 to 1.8)	1.8 (1.1 to 2.8)	2.3 (1.3 to 3.8)	61.1 (1.0 to 155.4)	29.4 (-20.9 to 124.5)	1.4 (0.8 to 2.3)	1.9 (1.0 to 3.2)	1.7 (0.9 to 3.0)	41.3 (-20.2 to 147.8)	-9.2 (-46.4 to 54.0)
High LDL cholesterol	20.6 (18.5 to 22.7)	11.4 (9.8 to 13.2)	10.1 (8.6 to 11.8)	-44.7 (-49.6 to -39.9)	-11.4 (-15.5 to -7.0)	19.9 (18.1 to 21.9)	11.4 (9.9 to 13.1)	10.5 (9.1 to 12.2)	-42.9 (-47.8 to -38.4)	-7.4 (-11.3 to -3.1)
High systolic blood pressure	6.2 (5.3 to 7.1)	4.4 (3.8 to 5.1)	4.5 (3.8 to 5.3)	-28.3 (-35.9 to -20.2)	2.8 (-7.0 to 12.1)	5.8 (5.0 to 6.6)	4.1 (3.6 to 4.7)	4.1 (3.5 to 4.7)	-28.9 (-37.1 to -18.7)	-1.2 (-11.7 to 10.9)
High body-mass index	14.0 (10.3 to 19.2)	19.2 (14.9 to 24.6)	21.0 (16.3 to 26.8)	37.2 (20.6 to 59.8)	9.4 (0.4 to 19.3)	15.3 (11.6 to 20.0)	20.5 (16.8 to 25.7)	21.7 (17.2 to 26.9)	34.1 (16.0 to 56.0)	5.8 (-3.9 to 17.4)
Low bone mineral density	5.2 (4.3 to 6.2)	4.5 (3.8 to 5.3)	4.8 (3.9 to 5.8)	-14.1 (-25.7 to -0.5)	5.8 (-9.9 to 22.4)	5.9 (4.9 to 7.0)	5.8 (4.9 to 6.8)	6.3 (5.3 to 7.4)	-1.9 (-15.1 to 11.7)	8.6 (-5.8 to 24.2)
Impaired kidney function	2.1 (1.0 to 5.4)	2.1 (1.0 to 5.6)	2.2 (1.1 to 5.6)	-0.3 (-6.1 to 5.4)	2.5 (-3.8 to 9.6)	2.8 (1.3 to 6.7)	2.7 (1.3 to 6.6)	2.8 (1.4 to 6.8)	-2.6 (-8.2 to 2.3)	4.4 (-0.5 to 10.5)

Supplementary Results Table 1. Connecticut age-standardised Summary Exposure Values (SEVs) for Level 4 risk factors and percentage change, by sex, 1990, 2007, and 2017.Data in parentheses are 95% uncertainty intervals.

	Male					Female				
	**1990**	**2007**	**2017**	**Percentage change 1990-2007**	**Percentage change 2007-2017**	**1990**	**2007**	**2017**	**Percentage change 1990-2007**	**Percentage change 2007-2017**
Unsafe water source	1.0 (0.6 to 1.6)	0.4 (0.2 to 0.6)	0.3 (0.2 to 0.4)	-61.8 (-78.9 to -29.7)	-29.2 (-54.0 to 10.2)	1.0 (0.6 to 1.6)	0.4 (0.2 to 0.6)	0.3 (0.2 to 0.4)	-61.8 (-78.9 to -29.7)	-29.2 (-54.0 to 10.2)
Unsafe sanitation	3.8 (1.9 to 6.7)	2.5 (1.1 to 4.9)	2.0 (0.9 to 4.1)	-34.4 (-71.8 to 47.8)	-20.9 (-59.3 to 59.4)	3.8 (1.9 to 6.7)	2.5 (1.1 to 4.9)	2.0 (0.9 to 4.1)	-34.4 (-71.8 to 47.8)	-20.9 (-59.3 to 59.4)
No access to handwashing facility	3.3 (2.4 to 4.7)	3.1 (2.2 to 4.2)	3.0 (2.1 to 4.1)	-7.8 (-34.6 to 30.6)	-3.3 (-26.6 to 25.1)	3.3 (2.4 to 4.7)	3.1 (2.2 to 4.2)	3.0 (2.1 to 4.1)	-7.8 (-34.6 to 30.6)	-3.3 (-26.6 to 25.1)
Ambient particulate matter pollution	22.5 (14.6 to 33.8)	22.4 (15.0 to 33.6)	16.4 (8.5 to 26.7)	-0.3 (-24.2 to 34.8)	-27.1 (-58.9 to -4.1)	22.4 (14.6 to 33.7)	22.4 (15.0 to 33.5)	16.3 (8.5 to 26.7)	-0.2 (-24.2 to 35.0)	-27.1 (-59.0 to -4.1)
Household air pollution from solid fuels	0.2 (0.0 to 0.5)	0.1 (0.0 to 0.3)	0.1 (0.0 to 0.4)	-30.3 (-78.2 to 59.8)	-7.1 (-69.6 to 98.7)	0.3 (0.1 to 1.7)	0.2 (0.0 to 1.3)	0.2 (0.0 to 1.0)	-25.2 (-78.0 to 60.5)	-10.3 (-69.6 to 98.9)
Ambient ozone pollution	47.7 (18.8 to 54.8)	47.6 (18.7 to 54.8)	47.9 (19.0 to 54.8)	-0.2 (-0.8 to 0.0)	0.7 (0.0 to 2.3)	47.7 (18.8 to 54.8)	47.6 (18.7 to 54.8)	47.9 (19.0 to 54.8)	-0.2 (-0.8 to 0.0)	0.7 (0.0 to 2.3)
Residential radon	15.7 (1.1 to 52.2)	15.7 (1.1 to 52.2)	15.7 (1.1 to 52.2)	0.0 (0.0 to 0.0)	0.0 (0.0 to 0.0)	15.7 (1.1 to 52.2)	15.7 (1.1 to 52.2)	15.7 (1.1 to 52.2)	0.0 (0.0 to 0.0)	0.0 (0.0 to 0.0)
Lead exposure	7.4 (3.1 to 11.7)	4.6 (1.5 to 8.2)	3.0 (0.9 to 5.9)	-38.4 (-51.3 to -28.1)	-34.3 (-44.6 to -27.7)	4.8 (1.3 to 8.8)	3.0 (0.6 to 6.5)	2.0 (0.3 to 4.7)	-36.7 (-53.4 to -25.3)	-33.5 (-46.2 to -27.2)
Occupational exposure to asbestos	6.3 (5.3 to 7.1)	5.2 (4.7 to 5.6)	4.5 (3.7 to 5.3)	-17.5 (-29.3 to 0.7)	-13.0 (-27.9 to 4.1)	1.0 (0.9 to 1.1)	1.0 (0.9 to 1.0)	0.9 (0.7 to 1.1)	-5.2 (-18.7 to 10.9)	-5.8 (-24.1 to 15.6)
Occupational exposure to arsenic	0.4 (0.0 to 1.0)	0.4 (0.0 to 0.9)	0.4 (0.0 to 0.9)	-	-	0.4 (0.0 to 0.9)	0.4 (0.0 to 0.8)	0.3 (0.0 to 0.8)	-	-
Occupational exposure to benzene	0.6 (0.0 to 1.8)	0.6 (0.0 to 1.8)	0.6 (0.0 to 1.7)	1.4 (-5.8 to 13.4)	-3.6 (-9.7 to 4.9)	0.9 (0.1 to 2.7)	1.0 (0.1 to 3.0)	1.0 (0.1 to 2.9)	12.4 (2.0 to 27.9)	-1.7 (-8.8 to 8.7)
Occupational exposure to beryllium	0.0 (0.0 to 0.0)	0.0 (0.0 to 0.0)	0.0 (0.0 to 0.0)	-1.1 (-7.0 to 4.7)	0.8 (-3.5 to 5.4)	0.0 (0.0 to 0.0)	0.0 (0.0 to 0.0)	0.0 (0.0 to 0.0)	3.8 (-3.7 to 12.2)	-1.3 (-7.2 to 4.3)
Occupational exposure to cadmium	0.0 (0.0 to 0.1)	0.0 (0.0 to 0.0)	0.0 (0.0 to 0.1)	-7.9 (-18.7 to 3.0)	1.8 (-7.1 to 11.8)	0.0 (0.0 to 0.0)	0.0 (0.0 to 0.0)	0.0 (0.0 to 0.0)	-3.6 (-16.5 to 13.4)	-1.9 (-11.9 to 10.1)
Occupational exposure to chromium	0.1 (0.1 to 0.1)	0.1 (0.1 to 0.1)	0.1 (0.1 to 0.1)	-4.5 (-14.2 to 5.3)	1.4 (-6.3 to 9.8)	0.1 (0.1 to 0.1)	0.1 (0.1 to 0.1)	0.1 (0.1 to 0.1)	-0.1 (-11.2 to 13.8)	-2.2 (-10.6 to 7.4)
Occupational exposure to diesel engine exhaust	0.5 (0.4 to 0.5)	0.5 (0.5 to 0.6)	0.5 (0.5 to 0.6)	14.0 (4.6 to 23.7)	1.5 (-5.3 to 8.9)	0.5 (0.4 to 0.5)	0.5 (0.5 to 0.6)	0.5 (0.4 to 0.6)	8.7 (-2.5 to 22.6)	-3.9 (-12.5 to 5.8)
Occupational exposure to formaldehyde	0.2 (0.2 to 0.3)	0.2 (0.2 to 0.2)	0.2 (0.2 to 0.3)	-10.0 (-21.0 to 1.4)	0.0 (-9.2 to 10.0)	0.2 (0.2 to 0.2)	0.2 (0.2 to 0.2)	0.2 (0.2 to 0.2)	-2.2 (-14.5 to 12.8)	-2.3 (-11.5 to 8.7)
Occupational exposure to nickel	0.3 (0.0 to 1.5)	0.3 (0.0 to 1.3)	0.3 (0.0 to 1.3)	-	-	0.3 (0.0 to 1.3)	0.3 (0.0 to 1.2)	0.3 (0.0 to 1.2)	-	-
Occupational exposure to polycyclic aromatic hydrocarbons	0.2 (0.2 to 0.2)	0.2 (0.2 to 0.2)	0.2 (0.2 to 0.2)	-4.1 (-13.4 to 5.5)	0.7 (-6.7 to 8.5)	0.2 (0.2 to 0.3)	0.2 (0.2 to 0.3)	0.2 (0.2 to 0.3)	3.2 (-7.0 to 16.0)	-2.0 (-9.5 to 6.4)
Occupational exposure to silica	3.0 (0.4 to 9.3)	2.6 (0.4 to 8.1)	2.7 (0.4 to 8.5)	-13.6 (-25.0 to -0.6)	2.5 (-7.3 to 15.4)	2.4 (0.3 to 7.7)	2.1 (0.3 to 6.7)	2.1 (0.3 to 6.5)	-11.5 (-27.1 to 9.4)	-1.9 (-14.9 to 15.0)
Occupational exposure to sulphuric acid	0.6 (0.1 to 2.0)	0.6 (0.1 to 2.0)	0.6 (0.1 to 2.0)	2.9 (-8.5 to 14.5)	0.1 (-8.5 to 10.3)	0.5 (0.1 to 1.8)	0.6 (0.1 to 2.0)	0.6 (0.1 to 1.9)	12.9 (-3.6 to 33.0)	-2.4 (-14.5 to 9.7)
Occupational exposure to trichloroethylene	0.1 (0.1 to 0.1)	0.1 (0.1 to 0.1)	0.1 (0.1 to 0.1)	1.3 (-6.2 to 9.2)	1.4 (-4.3 to 7.6)	0.1 (0.1 to 0.1)	0.1 (0.1 to 0.1)	0.1 (0.1 to 0.1)	2.6 (-7.1 to 14.0)	-2.6 (-9.8 to 5.8)
Occupational asthmagens	17.5 (14.6 to 21.4)	15.1 (12.7 to 18.2)	14.9 (12.2 to 17.9)	-13.7 (-25.2 to -1.4)	-1.6 (-10.2 to 8.8)	8.5 (7.0 to 10.5)	8.5 (6.9 to 10.5)	8.0 (6.4 to 9.9)	-0.3 (-14.3 to 17.1)	-6.3 (-17.2 to 5.2)
Occupational particulate matter, gases, and fumes	7.2 (5.1 to 10.4)	6.5 (4.6 to 9.4)	6.1 (4.3 to 8.8)	-9.5 (-14.2 to -5.3)	-6.0 (-9.7 to -2.4)	4.0 (2.8 to 5.9)	3.7 (2.5 to 5.3)	3.4 (2.4 to 5.1)	-8.7 (-15.2 to -1.7)	-6.4 (-11.8 to -1.0)
Occupational noise	6.2 (5.6 to 6.8)	5.9 (5.4 to 6.5)	5.6 (5.2 to 6.2)	-4.3 (-7.1 to -1.2)	-4.2 (-6.5 to -1.7)	4.5 (3.9 to 5.1)	4.6 (4.1 to 5.1)	4.5 (4.1 to 5.1)	2.9 (-1.8 to 8.5)	-1.1 (-4.3 to 2.3)
Occupational ergonomic factors	8.2 (6.8 to 9.9)	7.6 (6.3 to 9.2)	7.3 (6.0 to 8.9)	-7.5 (-17.0 to 2.8)	-3.9 (-11.1 to 3.9)	4.9 (3.9 to 6.2)	5.1 (4.1 to 6.5)	5.0 (3.9 to 6.4)	3.4 (-12.2 to 23.1)	-1.7 (-13.4 to 11.3)
Non-exclusive breastfeeding	0.5 (0.5 to 0.6)	0.5 (0.5 to 0.6)	0.5 (0.4 to 0.6)	-0.5 (-11.9 to 12.8)	-6.9 (-15.7 to 2.7)	0.5 (0.5 to 0.6)	0.5 (0.5 to 0.6)	0.5 (0.4 to 0.6)	-0.1 (-11.6 to 13.2)	-7.0 (-15.8 to 2.6)
Discontinued breastfeeding	2.3 (2.2 to 2.4)	2.2 (2.1 to 2.3)	2.2 (2.1 to 2.3)	-4.8 (-11.0 to 2.2)	1.5 (-4.1 to 7.7)	2.3 (2.2 to 2.4)	2.2 (2.1 to 2.3)	2.2 (2.1 to 2.4)	-4.8 (-10.9 to 2.2)	2.7 (-2.9 to 9.0)
Child underweight	0.1 (0.0 to 0.1)	0.1 (0.0 to 0.1)	0.1 (0.0 to 0.1)	-3.4 (-17.5 to 11.8)	-11.5 (-19.8 to -3.4)	0.1 (0.0 to 0.1)	0.1 (0.0 to 0.1)	0.1 (0.0 to 0.1)	-0.4 (-17.3 to 66.0)	-3.4 (-14.1 to 8.9)
Child wasting	0.1 (0.0 to 0.1)	0.1 (0.0 to 0.1)	0.1 (0.0 to 0.1)	-8.3 (-27.5 to 11.7)	-3.1 (-14.8 to 11.4)	0.1 (0.0 to 0.1)	0.1 (0.0 to 0.1)	0.1 (0.0 to 0.1)	7.0 (-14.0 to 38.9)	-4.5 (-18.2 to 12.9)
Child stunting	0.2 (0.1 to 0.5)	0.2 (0.1 to 0.4)	0.2 (0.1 to 0.4)	-7.8 (-48.4 to 62.8)	-10.0 (-38.9 to 30.5)	0.2 (0.1 to 0.3)	0.2 (0.1 to 0.3)	0.2 (0.1 to 0.3)	-7.4 (-39.6 to 41.0)	-2.6 (-28.5 to 29.7)
Short gestation for birth weight	0.0 (0.0 to 0.0)	0.0 (0.0 to 0.0)	0.0 (0.0 to 0.0)	7.9 (-16.4 to 31.5)	3.7 (-17.9 to 25.8)	0.0 (0.0 to 0.0)	0.0 (0.0 to 0.0)	0.0 (0.0 to 0.0)	9.0 (-13.1 to 49.2)	2.8 (-24.9 to 33.8)
Low birth weight for gestation	0.0 (0.0 to 0.0)	0.0 (0.0 to 0.0)	0.0 (0.0 to 0.0)	1.8 (-14.4 to 30.8)	-1.5 (-18.4 to 21.3)	0.0 (0.0 to 0.0)	0.0 (0.0 to 0.0)	0.0 (0.0 to 0.0)	-0.8 (-25.5 to 19.9)	-0.4 (-20.8 to 31.8)
Iron deficiency	-	-	-	-	-	3.6 (2.2 to 5.4)	3.4 (2.1 to 5.0)	3.5 (2.2 to 5.3)	-5.3 (-31.0 to 33.4)	4.5 (-21.7 to 38.0)
Vitamin A deficiency	0.4 (0.2 to 0.5)	0.4 (0.3 to 0.6)	0.5 (0.3 to 0.6)	21.7 (-0.0 to 46.9)	12.4 (-8.7 to 34.8)	0.5 (0.3 to 0.7)	0.6 (0.4 to 0.7)	0.5 (0.4 to 0.7)	16.8 (-7.3 to 66.9)	-6.2 (-24.5 to 13.1)
Zinc deficiency	0.5 (0.1 to 1.1)	0.2 (0.0 to 1.1)	0.2 (0.0 to 1.0)	-55.9 (-88.6 to 338.0)	-0.1 (-89.6 to 878.6)	0.5 (0.1 to 1.1)	0.2 (0.0 to 1.1)	0.2 (0.0 to 1.1)	-52.2 (-88.5 to 312.8)	-6.4 (-88.5 to 899.6)
Smoking	13.2 (10.6 to 15.8)	7.1 (5.7 to 8.6)	5.6 (4.4 to 6.9)	-46.6 (-54.3 to -37.4)	-21.3 (-30.5 to -11.1)	9.9 (7.7 to 12.3)	5.7 (4.4 to 7.0)	4.3 (3.3 to 5.4)	-43.0 (-53.4 to -29.3)	-24.2 (-33.2 to -13.8)
Chewing tobacco	0.9 (0.7 to 1.2)	1.3 (0.9 to 1.8)	1.4 (0.9 to 2.1)	42.6 (-4.9 to 111.5)	10.0 (-22.1 to 57.3)	0.1 (0.1 to 0.1)	0.1 (0.1 to 0.1)	0.1 (0.1 to 0.1)	-6.8 (-43.1 to 55.1)	3.1 (-28.4 to 51.5)
Second-hand smoke	45.6 (41.7 to 49.7)	24.8 (23.5 to 26.2)	24.4 (21.8 to 27.3)	-45.6 (-51.7 to -38.8)	-1.7 (-11.1 to 8.7)	41.0 (38.1 to 44.4)	22.4 (21.4 to 23.4)	22.0 (20.3 to 23.8)	-45.4 (-51.2 to -39.4)	-1.9 (-9.4 to 6.1)
Alcohol use	21.8 (11.5 to 34.4)	24.0 (13.3 to 37.1)	25.1 (14.0 to 39.3)	10.0 (-29.8 to 69.5)	4.8 (-27.8 to 50.1)	15.0 (7.7 to 24.7)	15.8 (8.3 to 26.1)	16.3 (8.4 to 27.6)	5.2 (-30.6 to 63.8)	3.2 (-29.9 to 48.2)
Drug use	2.3 (1.6 to 3.6)	3.0 (2.3 to 4.1)	3.7 (2.8 to 5.1)	29.1 (13.0 to 51.1)	23.0 (13.0 to 33.5)	1.6 (1.2 to 2.4)	2.0 (1.6 to 2.9)	2.4 (1.8 to 3.4)	25.7 (12.7 to 42.4)	17.6 (8.4 to 29.2)
Diet low in fruits	21.6 (14.9 to 27.5)	23.6 (17.4 to 29.1)	25.8 (18.9 to 31.5)	9.2 (-4.7 to 32.4)	9.0 (-3.0 to 25.0)	19.7 (12.4 to 26.1)	21.1 (14.5 to 26.9)	21.6 (14.2 to 28.1)	7.4 (-9.9 to 32.2)	2.1 (-15.5 to 20.7)
Diet low in vegetables	16.7 (8.6 to 24.3)	23.1 (15.5 to 29.4)	24.1 (16.5 to 30.4)	38.3 (8.1 to 115.8)	4.5 (-9.7 to 23.3)	21.6 (13.6 to 28.5)	25.7 (18.2 to 31.7)	27.3 (20.0 to 33.1)	19.1 (0.2 to 56.2)	6.2 (-6.4 to 24.4)
Diet low in legumes	23.7 (16.7 to 29.5)	23.9 (16.9 to 30.0)	25.3 (18.5 to 31.3)	0.8 (-17.1 to 21.1)	5.9 (-7.0 to 22.8)	28.6 (22.8 to 33.6)	29.3 (24.1 to 34.4)	30.6 (25.4 to 35.4)	2.6 (-9.4 to 17.9)	4.3 (-4.5 to 15.4)
Diet low in whole grains	37.1 (33.3 to 40.2)	38.3 (34.9 to 41.5)	37.7 (33.8 to 41.0)	3.1 (-2.3 to 9.2)	-1.5 (-5.7 to 2.9)	38.1 (34.5 to 41.0)	39.1 (35.8 to 41.8)	38.7 (35.2 to 41.5)	2.8 (-2.0 to 8.6)	-1.2 (-5.1 to 3.0)
Diet low in nuts and seeds	42.7 (39.0 to 46.1)	31.2 (25.7 to 36.2)	30.9 (24.4 to 36.5)	-26.9 (-37.3 to -18.0)	-0.9 (-13.5 to 10.4)	43.6 (40.1 to 46.6)	32.2 (26.6 to 36.9)	32.0 (26.0 to 37.5)	-26.1 (-36.6 to -17.8)	-0.7 (-11.8 to 10.5)
Diet low in milk	25.3 (18.4 to 31.1)	23.3 (16.3 to 29.3)	23.6 (16.5 to 29.7)	-7.9 (-20.6 to 4.4)	1.5 (-8.5 to 12.6)	27.0 (20.5 to 32.5)	24.2 (17.3 to 30.1)	24.3 (17.7 to 30.1)	-10.5 (-22.9 to 0.4)	0.5 (-9.5 to 13.0)
Diet high in red meat	45.1 (35.6 to 51.2)	40.6 (30.6 to 48.5)	39.1 (28.1 to 47.3)	-10.1 (-27.6 to 8.3)	-3.7 (-19.5 to 14.3)	27.3 (16.2 to 36.6)	21.5 (11.4 to 30.4)	20.4 (10.6 to 30.0)	-21.4 (-49.1 to 13.3)	-4.8 (-34.1 to 34.4)
Diet high in processed meat	44.4 (34.7 to 50.1)	42.7 (35.0 to 48.6)	35.4 (27.4 to 42.7)	-3.9 (-17.4 to 12.8)	-17.0 (-28.5 to -4.7)	25.9 (18.9 to 31.6)	25.6 (18.3 to 30.9)	22.5 (16.4 to 28.3)	-0.9 (-21.1 to 25.0)	-12.2 (-26.3 to 3.2)
Diet high in sugar-sweetened beverages	35.9 (17.9 to 45.4)	35.8 (18.4 to 45.6)	31.0 (15.6 to 41.6)	-0.2 (-25.9 to 38.4)	-13.4 (-35.6 to 10.8)	30.2 (16.0 to 40.8)	28.1 (14.4 to 39.0)	23.9 (12.0 to 34.5)	-6.7 (-36.4 to 35.2)	-15.2 (-39.1 to 12.6)
Diet low in fibre	31.8 (25.8 to 36.8)	23.5 (16.8 to 29.9)	25.0 (18.3 to 31.3)	-26.3 (-40.0 to -14.8)	6.4 (-5.6 to 20.9)	36.6 (31.4 to 40.7)	28.1 (21.4 to 34.2)	29.3 (22.9 to 35.2)	-23.2 (-34.6 to -13.9)	4.2 (-5.4 to 16.6)
Diet low in calcium	13.5 (7.0 to 19.8)	11.9 (6.3 to 17.9)	12.5 (6.7 to 18.5)	-11.7 (-26.1 to 5.1)	4.6 (-8.9 to 21.8)	17.4 (10.2 to 23.7)	15.2 (8.5 to 21.6)	15.8 (9.2 to 21.9)	-12.5 (-28.3 to 0.8)	3.7 (-6.7 to 17.9)
Diet low in seafood omega-3 fatty acids	24.2 (18.0 to 30.1)	19.0 (13.4 to 25.2)	20.3 (14.7 to 26.2)	-21.5 (-35.7 to -7.2)	6.8 (-6.5 to 24.9)	28.0 (22.4 to 33.5)	23.4 (17.4 to 29.3)	24.2 (17.9 to 29.7)	-16.4 (-29.1 to -5.5)	3.5 (-7.5 to 16.0)
Diet low in polyunsaturated fatty acids	5.9 (1.3 to 12.0)	3.1 (0.2 to 8.2)	2.2 (0.1 to 6.0)	-46.9 (-91.1 to 14.7)	-29.9 (-78.7 to 117.3)	6.6 (1.6 to 13.6)	3.5 (0.4 to 8.6)	2.5 (0.2 to 6.4)	-46.5 (-87.8 to 4.5)	-29.5 (-76.1 to 57.7)
Diet high in trans fatty acids	16.8 (12.3 to 21.7)	9.9 (5.8 to 14.1)	7.1 (3.5 to 11.0)	-41.2 (-55.9 to -29.3)	-27.7 (-43.9 to -16.1)	23.7 (17.9 to 31.8)	13.3 (9.0 to 17.6)	9.7 (5.8 to 13.8)	-44.0 (-55.1 to -32.5)	-26.7 (-39.7 to -16.7)
Diet high in sodium	7.8 (0.8 to 15.2)	13.6 (4.7 to 21.3)	14.2 (4.8 to 21.7)	75.4 (20.5 to 594.4)	4.3 (-18.6 to 46.2)	6.3 (0.3 to 13.5)	11.3 (2.8 to 19.1)	11.8 (3.1 to 19.6)	80.4 (27.6 to 919.1)	4.9 (-19.5 to 44.7)
Intimate partner violence	-	-	-	-	-	0.5 (0.3 to 0.7)	0.4 (0.3 to 0.5)	0.5 (0.3 to 0.6)	-14.4 (-26.8 to 5.6)	8.6 (-7.3 to 22.5)
Childhood sexual abuse	5.6 (4.6 to 6.9)	5.1 (4.1 to 6.2)	5.1 (4.1 to 6.2)	-9.8 (-15.1 to -4.3)	0.4 (-4.7 to 6.0)	13.8 (11.0 to 16.9)	12.1 (9.8 to 14.7)	12.2 (9.9 to 14.8)	-11.9 (-17.2 to -6.0)	0.4 (-4.8 to 6.5)

Supplementary Results Table 1. Connecticut age-standardised Summary Exposure Values (SEVs) for Level 4 risk factors and percentage change, by sex, 1990, 2007, and 2017.Data in parentheses are 95% uncertainty intervals.										
	**Male**					**Female**				
	**1990**	**2007**	**2017**	**Percentage change 1990-2007**	**Percentage change 2007-2017**	**1990**	**2007**	**2017**	**Percentage change 1990-2007**	**Percentage change 2007-2017**
Bullying victimization	6.1 (2.6 to 13.0)	9.0 (3.9 to 19.4)	8.7 (3.7 to 19.0)	48.5 (31.2 to 69.2)	-3.2 (-10.8 to 4.6)	4.6 (2.0 to 10.1)	7.7 (3.3 to 16.3)	7.6 (3.3 to 16.1)	66.4 (47.1 to 93.0)	-1.2 (-9.5 to 7.7)
Low physical activity	0.4 (0.2 to 0.8)	0.4 (0.2 to 0.8)	0.4 (0.2 to 0.8)	-0.1 (-2.2 to 2.0)	0.9 (-0.7 to 2.5)	0.4 (0.2 to 0.9)	0.4 (0.2 to 0.9)	0.4 (0.2 to 0.9)	-0.3 (-2.4 to 2.2)	-0.1 (-1.5 to 1.4)
High fasting plasma glucose	1.3 (0.8 to 2.1)	2.4 (1.4 to 3.7)	3.2 (1.9 to 4.7)	87.1 (5.0 to 212.4)	33.9 (-18.6 to 123.2)	1.4 (0.8 to 2.4)	2.4 (1.3 to 3.8)	2.4 (1.3 to 4.1)	63.8 (-5.5 to 184.4)	2.9 (-41.1 to 97.7)
High LDL cholesterol	21.4 (19.3 to 23.5)	11.5 (10.0 to 13.3)	10.2 (8.6 to 11.9)	-46.0 (-50.9 to -40.7)	-12.0 (-16.1 to -7.8)	20.3 (18.5 to 22.4)	11.5 (10.0 to 13.2)	10.6 (9.1 to 12.3)	-43.4 (-48.2 to -38.9)	-8.1 (-12.0 to -4.3)
High systolic blood pressure	6.2 (5.3 to 7.0)	4.5 (3.8 to 5.1)	4.6 (3.9 to 5.4)	-28.1 (-35.0 to -20.4)	2.6 (-6.7 to 12.3)	6.0 (5.2 to 6.7)	4.1 (3.6 to 4.6)	3.9 (3.3 to 4.6)	-31.1 (-38.8 to -21.8)	-3.9 (-13.8 to 6.5)
High body-mass index	15.0 (11.1 to 20.2)	21.5 (16.9 to 27.4)	22.7 (18.0 to 28.5)	43.6 (26.8 to 65.1)	5.6 (-2.9 to 15.0)	15.0 (11.1 to 19.8)	21.6 (17.5 to 27.4)	22.6 (18.1 to 28.6)	43.8 (25.8 to 67.8)	4.9 (-4.9 to 14.7)
Low bone mineral density	5.1 (4.2 to 6.2)	4.4 (3.7 to 5.2)	4.7 (3.8 to 5.6)	-14.1 (-25.6 to 0.2)	5.4 (-10.0 to 21.6)	5.8 (4.8 to 6.9)	5.7 (4.8 to 6.6)	6.1 (5.1 to 7.3)	-1.3 (-14.4 to 12.8)	8.2 (-5.1 to 23.4)
Impaired kidney function	2.3 (1.1 to 5.8)	2.3 (1.1 to 5.8)	2.3 (1.1 to 5.9)	1.2 (-4.9 to 7.6)	2.3 (-3.6 to 8.7)	2.9 (1.4 to 6.9)	2.9 (1.4 to 6.9)	3.0 (1.5 to 7.1)	-0.8 (-6.3 to 4.6)	4.6 (-0.4 to 10.9)

Supplementary Results Table 1. Delaware age-standardised Summary Exposure Values (SEVs) for Level 4 risk factors and percentage change, by sex, 1990, 2007, and 2017.Data in parentheses are 95% uncertainty intervals.										
	Male					Female				
	1990	2007	2017	Percentage change 1990-2007	Percentage change 2007-2017	1990	2007	2017	Percentage change 1990-2007	Percentage change 2007-2017
Unsafe water source	1.3 (0.7 to 2.1)	0.5 (0.3 to 0.8)	0.3 (0.2 to 0.5)	-63.6 (-80.9 to -30.6)	-34.5 (-58.6 to 3.3)	1.3 (0.7 to 2.1)	0.5 (0.3 to 0.8)	0.3 (0.2 to 0.5)	-63.6 (-80.9 to -30.6)	-34.5 (-58.6 to 3.3)
Unsafe sanitation	5.6 (3.0 to 9.8)	3.8 (1.8 to 7.4)	2.7 (1.2 to 5.1)	-32.5 (-70.7 to 50.9)	-28.4 (-64.1 to 43.4)	5.6 (3.0 to 9.8)	3.8 (1.8 to 7.4)	2.7 (1.2 to 5.1)	-32.5 (-70.7 to 50.9)	-28.4 (-64.1 to 43.4)
No access to handwashing facility	3.4 (2.4 to 4.8)	3.1 (2.2 to 4.3)	3.0 (2.2 to 4.2)	-8.7 (-36.0 to 30.2)	-3.4 (-24.7 to 22.0)	3.4 (2.4 to 4.8)	3.1 (2.2 to 4.3)	3.0 (2.2 to 4.2)	-8.7 (-36.0 to 30.2)	-3.4 (-24.7 to 22.0)
Ambient particulate matter pollution	26.5 (18.0 to 39.7)	26.7 (18.5 to 39.3)	20.7 (13.0 to 32.7)	0.4 (-19.4 to 27.8)	-22.3 (-43.2 to -1.9)	26.5 (18.0 to 39.6)	26.6 (18.4 to 39.2)	20.7 (13.0 to 32.7)	0.5 (-19.1 to 28.2)	-22.4 (-43.2 to -1.7)
Household air pollution from solid fuels	0.2 (0.1 to 0.6)	0.2 (0.0 to 0.4)	0.2 (0.0 to 0.4)	-29.7 (-76.6 to 60.6)	-9.6 (-64.9 to 93.9)	0.4 (0.1 to 1.9)	0.3 (0.0 to 1.5)	0.3 (0.0 to 1.0)	-28.2 (-76.3 to 59.8)	-7.3 (-64.6 to 93.4)
Ambient ozone pollution	50.0 (21.6 to 54.8)	50.2 (21.9 to 54.8)	50.6 (22.6 to 54.8)	0.5 (0.0 to 2.2)	0.8 (0.0 to 3.6)	50.0 (21.6 to 54.8)	50.2 (21.9 to 54.8)	50.6 (22.6 to 54.8)	0.5 (0.0 to 2.2)	0.8 (0.0 to 3.6)
Residential radon	14.1 (0.9 to 44.1)	14.1 (0.9 to 44.1)	14.1 (0.9 to 44.1)	0.0 (0.0 to 0.0)	0.0 (0.0 to 0.0)	14.1 (0.9 to 44.1)	14.1 (0.9 to 44.1)	14.1 (0.9 to 44.1)	0.0 (0.0 to 0.0)	0.0 (0.0 to 0.0)
Lead exposure	8.3 (3.8 to 12.6)	5.2 (2.1 to 9.0)	3.5 (1.2 to 6.4)	-36.6 (-48.8 to -26.9)	-33.2 (-42.5 to -27.0)	5.1 (1.5 to 9.4)	3.3 (0.7 to 7.0)	2.3 (0.4 to 5.1)	-35.1 (-51.6 to -23.8)	-32.4 (-45.1 to -26.1)
Occupational exposure to asbestos	8.1 (6.8 to 9.0)	6.3 (5.8 to 6.8)	5.5 (4.7 to 6.3)	-21.9 (-31.8 to -5.4)	-12.9 (-25.3 to 0.2)	1.0 (0.9 to 1.2)	0.9 (0.8 to 1.0)	0.9 (0.8 to 1.1)	-13.2 (-26.2 to 4.4)	0.9 (-14.1 to 18.1)
Occupational exposure to arsenic	0.4 (0.0 to 0.9)	0.4 (0.0 to 0.9)	0.4 (0.0 to 0.9)	-	-	0.3 (0.0 to 0.8)	0.3 (0.0 to 0.8)	0.3 (0.0 to 0.8)	-	-
Occupational exposure to benzene	0.6 (0.0 to 1.8)	0.6 (0.0 to 1.7)	0.6 (0.0 to 1.7)	0.7 (-7.4 to 11.4)	-3.0 (-9.1 to 6.1)	0.9 (0.0 to 2.5)	0.9 (0.1 to 2.8)	0.9 (0.1 to 2.8)	10.4 (-0.2 to 26.6)	-0.3 (-8.1 to 11.3)
Occupational exposure to beryllium	0.0 (0.0 to 0.0)	0.0 (0.0 to 0.0)	0.0 (0.0 to 0.0)	-0.3 (-5.5 to 5.2)	0.5 (-3.8 to 5.4)	0.0 (0.0 to 0.0)	0.0 (0.0 to 0.0)	0.0 (0.0 to 0.0)	4.4 (-3.3 to 12.1)	-1.0 (-6.8 to 5.1)
Occupational exposure to cadmium	0.0 (0.0 to 0.0)	0.0 (0.0 to 0.0)	0.0 (0.0 to 0.0)	-3.3 (-13.5 to 8.5)	1.1 (-7.8 to 11.2)	0.0 (0.0 to 0.0)	0.0 (0.0 to 0.0)	0.0 (0.0 to 0.0)	0.5 (-13.9 to 15.5)	-1.5 (-12.0 to 10.8)
Occupational exposure to chromium	0.1 (0.1 to 0.1)	0.1 (0.1 to 0.1)	0.1 (0.1 to 0.1)	-0.5 (-10.0 to 9.4)	0.9 (-6.5 to 9.2)	0.1 (0.1 to 0.1)	0.1 (0.1 to 0.1)	0.1 (0.1 to 0.1)	3.1 (-8.9 to 15.8)	-1.9 (-10.6 to 7.9)
Occupational exposure to diesel engine exhaust	0.5 (0.5 to 0.5)	0.6 (0.5 to 0.6)	0.6 (0.5 to 0.6)	11.7 (2.6 to 20.8)	1.6 (-5.3 to 9.2)	0.5 (0.4 to 0.5)	0.5 (0.5 to 0.6)	0.5 (0.4 to 0.6)	8.5 (-4.0 to 23.4)	-5.1 (-14.5 to 4.6)
Occupational exposure to formaldehyde	0.2 (0.2 to 0.2)	0.2 (0.2 to 0.2)	0.2 (0.2 to 0.2)	-5.5 (-15.9 to 6.6)	-0.5 (-9.7 to 9.9)	0.2 (0.2 to 0.2)	0.2 (0.2 to 0.2)	0.2 (0.2 to 0.2)	0.8 (-12.4 to 15.4)	-1.4 (-11.4 to 10.2)
Occupational exposure to nickel	0.3 (0.0 to 1.3)	0.3 (0.0 to 1.3)	0.3 (0.0 to 1.3)	-	-	0.3 (0.0 to 1.1)	0.2 (0.0 to 1.1)	0.2 (0.0 to 1.1)	-	-
Occupational exposure to polycyclic aromatic hydrocarbons	0.2 (0.2 to 0.2)	0.2 (0.2 to 0.2)	0.2 (0.2 to 0.2)	-0.2 (-9.4 to 9.3)	0.2 (-6.9 to 8.1)	0.2 (0.2 to 0.2)	0.2 (0.2 to 0.2)	0.2 (0.2 to 0.2)	5.9 (-4.9 to 17.5)	-1.5 (-9.4 to 7.5)
Occupational exposure to silica	2.8 (0.4 to 9.0)	2.5 (0.3 to 7.9)	2.6 (0.4 to 8.0)	-10.7 (-21.3 to 2.6)	1.8 (-8.3 to 14.8)	2.2 (0.3 to 6.9)	2.0 (0.3 to 6.1)	2.0 (0.3 to 6.4)	-7.6 (-23.9 to 12.9)	-1.6 (-15.7 to 16.4)
Occupational exposure to sulphuric acid	0.6 (0.1 to 1.8)	0.7 (0.1 to 2.1)	0.6 (0.1 to 2.0)	9.6 (-2.6 to 23.0)	-1.4 (-10.1 to 7.6)	0.5 (0.1 to 1.7)	0.6 (0.1 to 2.0)	0.6 (0.1 to 2.0)	20.2 (1.3 to 43.3)	-3.5 (-15.6 to 9.5)
Occupational exposure to trichloroethylene	0.1 (0.1 to 0.1)	0.1 (0.1 to 0.1)	0.1 (0.1 to 0.1)	3.9 (-3.6 to 12.1)	1.2 (-4.0 to 7.6)	0.1 (0.1 to 0.1)	0.1 (0.1 to 0.1)	0.1 (0.1 to 0.1)	5.1 (-5.6 to 16.9)	-2.9 (-10.3 to 5.3)
Occupational asthmagens	16.1 (13.4 to 19.5)	14.6 (12.4 to 17.5)	14.4 (11.9 to 17.4)	-9.1 (-19.1 to 1.6)	-1.9 (-10.3 to 7.7)	7.8 (6.3 to 9.3)	7.8 (6.4 to 9.6)	7.4 (5.9 to 9.2)	0.7 (-12.2 to 16.1)	-5.6 (-16.4 to 6.0)
Occupational particulate matter, gases, and fumes	6.8 (4.9 to 10.0)	6.3 (4.6 to 9.2)	6.0 (4.3 to 8.7)	-7.3 (-11.9 to -2.9)	-5.1 (-9.0 to -1.8)	3.7 (2.6 to 5.6)	3.5 (2.5 to 5.1)	3.3 (2.3 to 4.9)	-6.4 (-13.4 to 0.6)	-5.6 (-10.9 to 0.4)
Occupational noise	6.0 (5.5 to 6.6)	5.8 (5.3 to 6.4)	5.5 (5.1 to 6.1)	-3.5 (-6.6 to -0.4)	-3.8 (-6.3 to -1.6)	4.2 (3.8 to 4.8)	4.3 (3.9 to 4.8)	4.3 (3.8 to 4.8)	2.1 (-2.9 to 7.6)	-1.2 (-4.4 to 2.2)
Occupational ergonomic factors	8.5 (7.0 to 10.3)	7.8 (6.5 to 9.5)	7.5 (6.2 to 9.1)	-8.1 (-17.5 to 1.5)	-4.2 (-11.6 to 3.3)	4.9 (3.9 to 6.2)	5.0 (4.0 to 6.3)	4.9 (3.9 to 6.3)	2.8 (-11.3 to 20.7)	-1.7 (-12.8 to 11.9)
Non-exclusive breastfeeding	0.5 (0.5 to 0.6)	0.5 (0.5 to 0.6)	0.5 (0.4 to 0.6)	-0.5 (-12.2 to 13.0)	-7.3 (-16.7 to 2.6)	0.5 (0.5 to 0.6)	0.5 (0.5 to 0.6)	0.5 (0.4 to 0.6)	-0.4 (-12.1 to 13.1)	-6.8 (-16.2 to 3.1)
Discontinued breastfeeding	2.2 (2.1 to 2.4)	2.2 (2.0 to 2.3)	2.2 (2.1 to 2.3)	-3.5 (-10.4 to 3.7)	1.0 (-5.0 to 7.2)	2.2 (2.1 to 2.4)	2.2 (2.1 to 2.3)	2.2 (2.0 to 2.3)	-1.8 (-8.8 to 5.6)	-1.0 (-7.0 to 5.1)
Child underweight	0.1 (0.1 to 0.1)	0.1 (0.1 to 0.1)	0.1 (0.0 to 0.1)	-3.7 (-18.1 to 14.4)	-12.9 (-21.9 to -4.0)	0.1 (0.0 to 0.1)	0.1 (0.0 to 0.1)	0.1 (0.0 to 0.1)	-1.5 (-15.9 to 16.9)	-4.7 (-14.7 to 6.3)
Child wasting	0.1 (0.0 to 0.1)	0.1 (0.0 to 0.1)	0.1 (0.0 to 0.1)	-9.7 (-30.3 to 11.3)	-3.6 (-19.1 to 13.8)	0.1 (0.0 to 0.1)	0.1 (0.0 to 0.1)	0.1 (0.0 to 0.1)	6.6 (-16.2 to 34.1)	-5.3 (-20.5 to 10.5)
Child stunting	0.3 (0.1 to 0.5)	0.2 (0.1 to 0.4)	0.2 (0.1 to 0.4)	-8.7 (-48.8 to 52.8)	-10.6 (-38.2 to 26.3)	0.2 (0.1 to 0.4)	0.2 (0.1 to 0.3)	0.2 (0.1 to 0.3)	-8.5 (-44.1 to 46.8)	-3.5 (-30.2 to 34.3)
Short gestation for birth weight	0.0 (0.0 to 0.0)	0.0 (0.0 to 0.0)	0.0 (0.0 to 0.0)	7.2 (-22.0 to 41.3)	0.6 (-18.7 to 23.2)	0.0 (0.0 to 0.0)	0.0 (0.0 to 0.0)	0.0 (0.0 to 0.0)	15.0 (-8.7 to 55.2)	2.8 (-23.2 to 56.8)
Low birth weight for gestation	0.0 (0.0 to 0.0)	0.0 (0.0 to 0.0)	0.0 (0.0 to 0.0)	1.1 (-19.6 to 38.8)	-0.1 (-18.7 to 20.2)	0.0 (0.0 to 0.0)	0.0 (0.0 to 0.0)	0.0 (0.0 to 0.0)	-5.0 (-27.1 to 20.3)	-2.1 (-31.1 to 25.0)
Iron deficiency	-	-	-	-	-	4.0 (2.5 to 5.8)	3.8 (2.4 to 5.5)	3.9 (2.4 to 5.8)	-5.2 (-30.8 to 31.8)	3.4 (-21.5 to 32.2)
Vitamin A deficiency	0.5 (0.3 to 0.6)	0.5 (0.3 to 0.6)	0.5 (0.3 to 0.6)	-0.4 (-18.7 to 22.9)	2.3 (-21.4 to 36.2)	0.5 (0.4 to 0.7)	0.6 (0.4 to 0.7)	0.6 (0.4 to 0.8)	3.5 (-15.1 to 29.1)	12.0 (-4.8 to 36.2)
Zinc deficiency	0.5 (0.1 to 1.1)	0.2 (0.0 to 1.1)	0.2 (0.0 to 1.0)	-54.4 (-88.1 to 331.4)	-4.9 (-89.9 to 790.9)	0.5 (0.1 to 1.2)	0.2 (0.0 to 1.0)	0.2 (0.0 to 1.0)	-56.4 (-88.4 to 214.5)	1.3 (-87.0 to 884.7)
Smoking	13.8 (11.1 to 16.6)	7.6 (6.0 to 9.3)	5.9 (4.6 to 7.4)	-45.3 (-53.5 to -35.3)	-22.4 (-31.6 to -12.4)	10.0 (7.6 to 12.7)	5.7 (4.4 to 7.2)	4.2 (3.2 to 5.4)	-43.1 (-53.8 to -28.6)	-25.3 (-34.5 to -14.9)
Chewing tobacco	1.1 (0.8 to 1.4)	1.2 (0.9 to 1.7)	1.3 (0.9 to 2.0)	15.7 (-21.9 to 70.0)	7.6 (-23.3 to 51.1)	0.1 (0.1 to 0.1)	0.1 (0.1 to 0.1)	0.1 (0.1 to 0.1)	-3.7 (-42.2 to 66.5)	2.4 (-30.7 to 52.4)
Second-hand smoke	48.1 (44.1 to 53.0)	27.1 (25.5 to 28.7)	27.7 (25.0 to 30.6)	-43.6 (-50.3 to -35.8)	2.3 (-7.8 to 13.1)	46.6 (42.8 to 50.5)	26.1 (24.8 to 27.3)	26.3 (24.4 to 28.3)	-43.9 (-50.6 to -37.1)	0.6 (-7.4 to 8.9)
Alcohol use	21.8 (11.5 to 34.7)	24.2 (13.4 to 38.7)	25.5 (13.7 to 41.6)	11.1 (-28.8 to 74.5)	5.4 (-25.2 to 52.3)	13.0 (6.3 to 21.6)	14.3 (7.6 to 24.1)	15.6 (8.0 to 27.8)	10.2 (-26.8 to 72.3)	9.4 (-22.9 to 60.8)
Drug use	1.9 (1.4 to 2.9)	2.6 (2.0 to 3.4)	3.3 (2.5 to 4.4)	33.7 (18.4 to 54.2)	27.6 (16.2 to 41.6)	1.5 (1.1 to 2.0)	2.2 (1.8 to 2.7)	2.6 (2.1 to 3.3)	45.0 (27.6 to 67.0)	22.1 (9.4 to 34.3)
Diet low in fruits	26.5 (20.2 to 32.0)	29.5 (23.6 to 34.2)	33.2 (27.1 to 38.2)	11.1 (-1.0 to 27.8)	12.6 (4.1 to 24.4)	22.6 (15.9 to 28.6)	26.9 (20.5 to 32.1)	30.3 (23.7 to 35.8)	19.1 (3.8 to 44.0)	12.6 (2.0 to 25.9)
Diet low in vegetables	24.7 (16.7 to 31.4)	35.6 (29.9 to 39.9)	39.2 (34.2 to 43.1)	44.2 (21.5 to 89.7)	10.3 (3.4 to 19.2)	27.7 (20.7 to 33.9)	35.8 (30.3 to 40.4)	39.5 (34.9 to 43.5)	28.9 (12.6 to 55.8)	10.6 (3.8 to 20.2)
Diet low in legumes	23.8 (16.7 to 30.1)	24.0 (17.2 to 30.0)	25.5 (18.8 to 31.1)	0.7 (-15.5 to 21.1)	6.4 (-6.3 to 23.4)	28.7 (22.6 to 34.0)	29.4 (23.7 to 34.3)	30.7 (24.9 to 35.6)	2.1 (-10.3 to 16.5)	4.5 (-5.6 to 15.0)
Diet low in whole grains	37.7 (34.1 to 40.8)	39.0 (35.6 to 42.0)	38.9 (35.2 to 41.8)	3.3 (-1.7 to 9.4)	-0.3 (-3.9 to 3.6)	38.7 (35.2 to 41.8)	39.9 (36.6 to 42.4)	39.8 (36.6 to 42.6)	3.1 (-2.2 to 8.9)	-0.1 (-3.6 to 3.9)
Diet low in nuts and seeds	44.7 (41.2 to 47.5)	34.8 (29.6 to 38.9)	36.8 (31.2 to 41.2)	-22.2 (-30.8 to -15.1)	5.9 (-2.6 to 15.5)	45.6 (42.5 to 48.1)	35.8 (30.9 to 39.9)	37.9 (32.7 to 42.0)	-21.5 (-29.8 to -14.8)	5.8 (-1.6 to 15.5)
Diet low in milk	26.7 (20.2 to 32.1)	25.1 (18.3 to 30.9)	26.4 (19.9 to 32.0)	-6.0 (-18.3 to 5.9)	5.4 (-3.2 to 16.5)	28.5 (22.2 to 33.4)	26.1 (19.6 to 31.5)	27.3 (21.2 to 32.7)	-8.3 (-19.6 to 1.7)	4.4 (-3.9 to 15.6)
Diet high in red meat	43.0 (32.5 to 49.9)	37.7 (26.7 to 45.7)	34.5 (23.7 to 43.4)	-12.4 (-30.1 to 6.8)	-8.4 (-27.2 to 10.7)	24.7 (14.2 to 34.8)	18.6 (10.0 to 27.3)	16.2 (7.9 to 25.2)	-24.8 (-50.2 to 11.2)	-12.8 (-43.7 to 26.2)
Diet high in processed meat	41.4 (32.9 to 47.8)	38.6 (31.2 to 45.2)	29.3 (22.1 to 35.9)	-6.6 (-22.9 to 11.4)	-24.1 (-36.0 to -11.7)	23.1 (16.8 to 29.0)	22.4 (16.2 to 28.0)	18.1 (12.6 to 23.0)	-3.2 (-22.8 to 22.3)	-19.1 (-33.1 to -4.2)
Diet high in sugar-sweetened beverages	32.8 (16.7 to 43.6)	31.8 (16.2 to 42.2)	25.0 (12.8 to 35.8)	-3.2 (-31.2 to 38.4)	-21.3 (-44.2 to 3.6)	27.5 (13.4 to 38.8)	25.1 (12.3 to 35.6)	19.3 (9.1 to 29.6)	-8.7 (-41.0 to 44.2)	-22.9 (-48.7 to 7.4)
Diet low in fibre	32.8 (26.8 to 37.7)	24.6 (17.4 to 30.8)	26.8 (19.4 to 32.8)	-24.9 (-38.5 to -14.6)	8.9 (-2.9 to 23.9)	37.5 (32.6 to 41.4)	29.3 (22.8 to 34.7)	31.1 (25.1 to 36.3)	-21.8 (-33.1 to -13.4)	6.1 (-2.9 to 17.7)
Diet low in calcium	15.4 (8.5 to 21.9)	13.8 (7.6 to 20.2)	15.8 (9.0 to 22.0)	-10.1 (-23.8 to 4.6)	14.1 (1.0 to 34.5)	19.5 (12.4 to 25.5)	17.5 (10.4 to 23.6)	19.6 (12.4 to 25.5)	-9.9 (-24.8 to 1.4)	11.5 (2.1 to 27.4)
Diet low in seafood omega-3 fatty acids	26.9 (20.6 to 32.5)	22.6 (16.0 to 28.7)	25.7 (19.6 to 31.4)	-16.1 (-30.1 to -3.8)	13.9 (2.3 to 31.2)	30.6 (25.2 to 35.6)	26.9 (20.7 to 32.4)	29.5 (23.6 to 34.5)	-12.3 (-24.0 to -3.0)	9.8 (1.2 to 21.4)
Diet low in polyunsaturated fatty acids	7.4 (2.1 to 14.6)	4.4 (0.6 to 9.8)	3.9 (0.6 to 9.1)	-40.0 (-81.7 to 7.4)	-12.1 (-58.0 to 101.7)	7.9 (2.5 to 14.9)	4.9 (0.9 to 10.7)	4.2 (0.7 to 9.5)	-38.6 (-81.1 to 7.0)	-13.5 (-57.7 to 67.1)
Diet high in trans fatty acids	16.8 (12.0 to 22.3)	9.9 (6.0 to 14.1)	7.1 (3.5 to 11.2)	-41.2 (-54.9 to -29.6)	-27.5 (-44.0 to -16.2)	23.8 (17.9 to 32.3)	13.3 (8.8 to 17.9)	9.7 (5.7 to 13.9)	-44.1 (-56.2 to -32.2)	-26.8 (-40.4 to -16.4)
Diet high in sodium	7.4 (0.7 to 15.1)	13.0 (4.5 to 20.3)	13.2 (4.3 to 20.9)	74.9 (19.5 to 686.5)	1.2 (-26.9 to 42.7)	6.2 (0.3 to 13.5)	11.0 (2.8 to 18.6)	11.4 (2.9 to 18.9)	78.8 (25.3 to 983.1)	2.9 (-27.4 to 49.1)
Intimate partner violence	-	-	-	-	-	0.4 (0.2 to 0.7)	0.3 (0.2 to 0.5)	0.4 (0.2 to 0.6)	-24.2 (-36.8 to 4.1)	18.7 (-4.4 to 39.9)
Childhood sexual abuse	5.6 (4.6 to 6.9)	5.1 (4.1 to 6.2)	5.1 (4.1 to 6.2)	-9.8 (-15.1 to -4.3)	0.4 (-4.7 to 6.0)	13.8 (11.0 to 16.9)	12.1 (9.8 to 14.7)	12.2 (9.9 to 14.8)	-11.9 (-17.2 to -6.0)	0.4 (-4.8 to 6.5)

Supplementary Results Table 1. Delaware age-standardised Summary Exposure Values (SEVs) for Level 4 risk factors and percentage change, by sex, 1990, 2007, and 2017.Data in parentheses are 95% uncertainty intervals.

	Male					Female				
	**1990**	**2007**	**2017**	**Percentage change 1990-2007**	**Percentage change 2007-2017**	**1990**	**2007**	**2017**	**Percentage change 1990-2007**	**Percentage change 2007-2017**
Bullying victimization	6.0<br>(2.6 to 13.1)	8.9<br>(3.8 to 18.7)	8.6<br>(3.7 to 18.4)	48.1<br>(31.3 to 68.7)	-3.4<br>(-11.1 to 4.7)	4.6<br>(1.9 to 10.0)	7.5<br>(3.2 to 16.0)	7.4<br>(3.2 to 15.8)	65.4<br>(45.8 to 93.2)	-1.2<br>(-9.2 to 8.1)
Low physical activity	0.4<br>(0.2 to 0.8)	0.4<br>(0.2 to 0.8)	0.4<br>(0.2 to 0.8)	0.7<br>(-1.5 to 2.9)	-1.3<br>(-3.2 to 0.4)	0.5<br>(0.2 to 0.9)	0.5<br>(0.2 to 1.0)	0.5<br>(0.2 to 0.9)	0.2<br>(-2.2 to 3.0)	-1.6<br>(-3.5 to 0.0)
High fasting plasma glucose	1.6<br>(0.9 to 2.7)	3.1<br>(2.0 to 4.6)	3.4<br>(2.2 to 5.1)	97.2<br>(12.9 to 260.1)	9.0<br>(-27.7 to 64.8)	1.9<br>(1.0 to 3.2)	3.1<br>(1.8 to 5.0)	3.2<br>(1.8 to 5.0)	66.5<br>(-1.6 to 196.0)	3.6<br>(-40.3 to 80.5)
High LDL cholesterol	21.3<br>(19.3 to 23.4)	11.5<br>(9.9 to 13.3)	10.2<br>(8.6 to 11.8)	-45.9<br>(-50.7 to -41.0)	-11.9<br>(-16.0 to -7.8)	20.3<br>(18.4 to 22.5)	11.5<br>(10.1 to 13.2)	10.6<br>(9.1 to 12.3)	-43.3<br>(-47.9 to -38.7)	-7.7<br>(-12.0 to -3.5)
High systolic blood pressure	6.6<br>(5.7 to 7.5)	4.8<br>(4.1 to 5.4)	4.7<br>(4.0 to 5.5)	-28.1<br>(-35.2 to -20.9)	-0.5<br>(-8.9 to 8.9)	6.5<br>(5.7 to 7.4)	4.3<br>(3.8 to 4.8)	3.9<br>(3.3 to 4.5)	-34.4<br>(-41.6 to -26.2)	-8.6<br>(-17.6 to 1.1)
High body-mass index	15.5<br>(11.4 to 20.8)	21.4<br>(16.7 to 27.2)	22.4<br>(17.3 to 28.2)	38.2<br>(22.7 to 58.4)	4.8<br>(-4.0 to 13.9)	17.1<br>(13.3 to 22.0)	22.8<br>(18.6 to 28.7)	23.5<br>(18.8 to 29.4)	33.4<br>(17.9 to 51.5)	2.9<br>(-6.3 to 12.4)
Low bone mineral density	5.1<br>(4.2 to 6.2)	4.4<br>(3.7 to 5.3)	4.6<br>(3.8 to 5.6)	-14.4<br>(-25.7 to -0.6)	6.0<br>(-8.1 to 21.7)	5.7<br>(4.7 to 6.8)	5.6<br>(4.8 to 6.6)	6.1<br>(5.1 to 7.4)	-1.7<br>(-14.5 to 13.5)	9.1<br>(-5.2 to 25.6)
Impaired kidney function	2.4<br>(1.2 to 6.0)	2.5<br>(1.2 to 6.2)	2.5<br>(1.2 to 6.2)	0.4<br>(-5.2 to 6.2)	1.3<br>(-4.0 to 7.1)	3.2<br>(1.5 to 7.2)	3.1<br>(1.5 to 7.1)	3.2<br>(1.6 to 7.3)	-1.6<br>(-7.2 to 3.2)	4.3<br>(-0.8 to 10.2)

Supplementary Results Table 1. District of Columbia age-standardised Summary Exposure Values (SEVs) for Level 4 risk factors and percentage change, by sex, 1990, 2007, and 2017.Data in parentheses are 95% uncertainty intervals.										
	**Male**					**Female**				
	**1990**	**2007**	**2017**	**Percentage change 1990-2007**	**Percentage change 2007-2017**	**1990**	**2007**	**2017**	**Percentage change 1990-2007**	**Percentage change 2007-2017**
Unsafe water source	1.3 (0.7 to 2.2)	0.4 (0.3 to 0.6)	0.3 (0.2 to 0.4)	-68.8 (-83.8 to -39.2)	-30.7 (-54.7 to 7.4)	1.3 (0.7 to 2.2)	0.4 (0.3 to 0.6)	0.3 (0.2 to 0.4)	-68.8 (-83.8 to -39.2)	-30.7 (-54.7 to 7.4)
Unsafe sanitation	5.8 (3.0 to 10.2)	2.9 (1.3 to 5.6)	2.3 (1.0 to 4.5)	-50.0 (-77.1 to 5.5)	-20.8 (-58.7 to 50.1)	5.8 (3.0 to 10.2)	2.9 (1.3 to 5.6)	2.3 (1.0 to 4.5)	-50.0 (-77.1 to 5.5)	-20.8 (-58.7 to 50.1)
No access to handwashing facility	3.4 (2.4 to 4.6)	3.1 (2.1 to 4.2)	3.0 (2.1 to 4.1)	-9.3 (-35.7 to 28.0)	-3.5 (-26.5 to 27.8)	3.4 (2.4 to 4.6)	3.1 (2.1 to 4.2)	3.0 (2.1 to 4.1)	-9.3 (-35.7 to 28.0)	-3.5 (-26.5 to 27.8)
Ambient particulate matter pollution	28.3 (19.7 to 43.6)	28.2 (19.4 to 42.7)	21.8 (14.2 to 35.4)	-0.6 (-15.7 to 17.4)	-22.5 (-38.9 to -6.5)	28.3 (19.7 to 43.5)	28.1 (19.3 to 42.7)	21.8 (14.2 to 35.4)	-0.5 (-15.7 to 17.5)	-22.5 (-39.0 to -6.5)
Household air pollution from solid fuels	0.2 (0.0 to 0.4)	0.1 (0.0 to 0.2)	0.1 (0.0 to 0.2)	-37.7 (-81.5 to 32.6)	-7.6 (-65.1 to 91.6)	0.3 (0.0 to 1.4)	0.2 (0.0 to 0.7)	0.2 (0.0 to 0.6)	-36.5 (-81.4 to 32.6)	-5.9 (-64.8 to 92.9)
Ambient ozone pollution	50.4 (22.3 to 54.8)	50.0 (21.5 to 54.8)	50.3 (22.0 to 54.8)	-0.8 (-3.7 to 0.0)	0.6 (0.0 to 3.1)	50.4 (22.3 to 54.8)	50.0 (21.5 to 54.8)	50.3 (22.0 to 54.8)	-0.8 (-3.7 to 0.0)	0.6 (0.0 to 3.1)
Residential radon	13.4 (0.8 to 46.6)	13.4 (0.8 to 46.6)	13.4 (0.8 to 46.6)	0.0 (0.0 to 0.0)	0.0 (0.0 to 0.0)	13.4 (0.8 to 46.6)	13.4 (0.8 to 46.6)	13.4 (0.8 to 46.6)	0.0 (0.0 to 0.0)	0.0 (0.0 to 0.0)
Lead exposure	19.6 (14.2 to 24.3)	11.8 (7.4 to 16.1)	7.6 (4.4 to 10.9)	-39.4 (-48.8 to -31.1)	-35.9 (-41.5 to -31.3)	15.5 (10.3 to 20.2)	9.8 (5.5 to 14.3)	6.4 (3.2 to 9.9)	-36.4 (-48.3 to -26.4)	-34.7 (-41.1 to -30.3)
Occupational exposure to asbestos	3.8 (3.2 to 4.5)	2.8 (2.5 to 3.1)	2.3 (1.9 to 2.7)	-27.7 (-39.2 to -10.4)	-18.0 (-32.9 to 0.2)	0.4 (0.4 to 0.6)	0.4 (0.3 to 0.4)	0.4 (0.3 to 0.4)	-19.9 (-34.9 to -3.8)	-0.7 (-20.1 to 21.0)
Occupational exposure to arsenic	0.2 (0.0 to 0.6)	0.3 (0.0 to 0.6)	0.3 (0.0 to 0.6)	-	-	0.2 (0.0 to 0.6)	0.3 (0.0 to 0.6)	0.3 (0.0 to 0.7)	-	-
Occupational exposure to benzene	0.7 (0.0 to 2.1)	0.8 (0.0 to 2.1)	0.7 (0.0 to 2.1)	4.3 (-5.1 to 19.0)	-4.5 (-11.5 to 5.2)	0.9 (0.1 to 2.6)	1.0 (0.1 to 3.0)	1.0 (0.1 to 2.9)	18.6 (7.2 to 38.1)	-1.9 (-10.0 to 9.6)
Occupational exposure to beryllium	0.0 (0.0 to 0.0)	0.0 (0.0 to 0.0)	0.0 (0.0 to 0.0)	1.3 (-4.2 to 7.5)	1.4 (-2.7 to 6.0)	0.0 (0.0 to 0.0)	0.0 (0.0 to 0.0)	0.0 (0.0 to 0.0)	8.3 (1.1 to 16.9)	-1.1 (-6.4 to 4.8)
Occupational exposure to cadmium	0.0 (0.0 to 0.0)	0.0 (0.0 to 0.0)	0.0 (0.0 to 0.0)	6.7 (-3.9 to 19.8)	4.2 (-4.3 to 13.6)	0.0 (0.0 to 0.0)	0.0 (0.0 to 0.0)	0.0 (0.0 to 0.0)	13.5 (1.2 to 27.3)	-0.8 (-9.1 to 8.8)
Occupational exposure to chromium	0.1 (0.1 to 0.1)	0.1 (0.1 to 0.1)	0.1 (0.1 to 0.1)	9.9 (-0.1 to 21.7)	3.5 (-4.0 to 11.3)	0.1 (0.1 to 0.1)	0.1 (0.1 to 0.1)	0.1 (0.1 to 0.1)	18.0 (6.7 to 31.0)	-0.9 (-8.7 to 7.5)
Occupational exposure to diesel engine exhaust	0.4 (0.4 to 0.4)	0.4 (0.4 to 0.5)	0.5 (0.4 to 0.5)	13.7 (3.4 to 24.8)	4.1 (-3.0 to 12.7)	0.3 (0.3 to 0.4)	0.4 (0.4 to 0.5)	0.4 (0.4 to 0.5)	18.8 (5.8 to 34.5)	-1.5 (-9.9 to 8.2)
Occupational exposure to formaldehyde	0.1 (0.1 to 0.2)	0.2 (0.1 to 0.2)	0.2 (0.1 to 0.2)	6.6 (-4.7 to 20.0)	1.7 (-6.9 to 11.1)	0.1 (0.1 to 0.2)	0.2 (0.2 to 0.2)	0.2 (0.2 to 0.2)	17.7 (5.5 to 31.6)	-1.3 (-9.8 to 7.8)
Occupational exposure to nickel	0.2 (0.0 to 0.8)	0.2 (0.0 to 0.8)	0.2 (0.0 to 0.9)	-	-	0.2 (0.0 to 0.8)	0.2 (0.0 to 0.9)	0.2 (0.0 to 0.9)	-	-
Occupational exposure to polycyclic aromatic hydrocarbons	0.2 (0.1 to 0.2)	0.2 (0.2 to 0.2)	0.2 (0.2 to 0.2)	10.7 (0.5 to 21.5)	1.6 (-5.4 to 8.5)	0.2 (0.2 to 0.2)	0.2 (0.2 to 0.2)	0.2 (0.2 to 0.2)	21.9 (10.7 to 34.3)	-1.4 (-8.9 to 6.0)
Occupational exposure to silica	1.6 (0.2 to 5.1)	1.6 (0.2 to 4.8)	1.7 (0.2 to 5.1)	-2.5 (-15.5 to 13.7)	5.7 (-5.2 to 20.2)	1.4 (0.2 to 4.4)	1.5 (0.2 to 4.9)	1.5 (0.2 to 4.8)	7.5 (-8.8 to 28.4)	-0.7 (-12.7 to 15.4)
Occupational exposure to sulphuric acid	0.4 (0.1 to 1.3)	0.5 (0.1 to 1.8)	0.5 (0.1 to 1.8)	25.8 (7.1 to 46.3)	0.3 (-11.3 to 13.7)	0.4 (0.1 to 1.2)	0.5 (0.1 to 1.8)	0.5 (0.1 to 1.8)	48.7 (25.7 to 75.8)	-3.6 (-16.0 to 10.8)
Occupational exposure to trichloroethylene	0.0 (0.0 to 0.0)	0.0 (0.0 to 0.1)	0.1 (0.0 to 0.1)	10.7 (2.5 to 20.4)	3.8 (-2.1 to 10.3)	0.0 (0.0 to 0.0)	0.1 (0.0 to 0.1)	0.1 (0.0 to 0.1)	16.3 (6.2 to 28.3)	-1.0 (-8.0 to 7.4)
Occupational asthmagens	11.1 (9.3 to 13.5)	10.8 (9.0 to 13.1)	10.8 (8.9 to 13.2)	-2.7 (-13.9 to 10.2)	-0.2 (-8.9 to 9.8)	6.4 (5.2 to 7.9)	7.0 (5.7 to 8.5)	6.6 (5.3 to 8.1)	9.5 (-6.4 to 26.8)	-5.5 (-15.8 to 7.2)
Occupational particulate matter, gases, and fumes	3.7 (2.6 to 5.5)	3.7 (2.6 to 5.4)	3.7 (2.6 to 5.5)	-1.2 (-6.6 to 5.3)	-0.4 (-4.8 to 4.1)	2.1 (1.4 to 3.1)	2.2 (1.6 to 3.3)	2.3 (1.6 to 3.4)	7.3 (-0.5 to 16.4)	1.2 (-4.7 to 7.5)
Occupational noise	4.5 (4.1 to 5.0)	4.7 (4.3 to 5.1)	4.6 (4.3 to 5.1)	3.0 (-0.7 to 7.0)	-0.7 (-3.3 to 2.1)	3.4 (3.0 to 3.8)	3.8 (3.3 to 4.2)	3.9 (3.5 to 4.3)	12.0 (6.4 to 18.4)	3.0 (-0.6 to 6.5)
Occupational ergonomic factors	8.3 (6.9 to 10.1)	7.6 (6.3 to 9.2)	7.3 (6.0 to 8.8)	-8.2 (-17.0 to 0.6)	-4.1 (-10.3 to 2.7)	4.7 (3.7 to 6.0)	4.9 (3.9 to 6.2)	4.9 (3.8 to 6.2)	4.8 (-9.7 to 24.2)	-1.6 (-13.3 to 11.4)
Non-exclusive breastfeeding	0.5 (0.5 to 0.6)	0.5 (0.5 to 0.6)	0.5 (0.4 to 0.6)	0.6 (-10.6 to 14.4)	-7.7 (-16.7 to 2.5)	0.5 (0.5 to 0.6)	0.5 (0.5 to 0.6)	0.5 (0.4 to 0.6)	0.4 (-10.7 to 14.2)	-7.8 (-16.9 to 2.4)
Discontinued breastfeeding	2.3 (2.1 to 2.4)	2.2 (2.1 to 2.4)	2.2 (2.1 to 2.3)	-2.1 (-9.5 to 5.6)	-0.5 (-5.7 to 5.8)	2.3 (2.1 to 2.4)	2.2 (2.1 to 2.3)	2.2 (2.1 to 2.3)	-1.6 (-9.0 to 6.1)	0.2 (-5.0 to 6.6)
Child underweight	0.1 (0.1 to 0.1)	0.1 (0.0 to 0.1)	0.1 (0.0 to 0.1)	-7.9 (-21.6 to 6.0)	-11.7 (-20.3 to -2.9)	0.1 (0.0 to 0.1)	0.1 (0.0 to 0.1)	0.1 (0.0 to 0.1)	-5.3 (-20.0 to 10.5)	-3.1 (-13.4 to 7.9)
Child wasting	0.1 (0.0 to 0.1)	0.1 (0.0 to 0.1)	0.1 (0.0 to 0.1)	-10.5 (-31.3 to 10.5)	-2.9 (-15.7 to 11.4)	0.1 (0.0 to 0.1)	0.1 (0.0 to 0.1)	0.1 (0.0 to 0.1)	4.3 (-19.4 to 31.6)	-4.4 (-18.5 to 13.3)
Child stunting	0.3 (0.1 to 0.5)	0.2 (0.1 to 0.4)	0.2 (0.1 to 0.4)	-12.8 (-55.3 to 51.7)	-9.9 (-40.1 to 29.8)	0.2 (0.1 to 0.4)	0.2 (0.1 to 0.3)	0.2 (0.1 to 0.3)	-11.4 (-46.9 to 37.0)	-2.2 (-27.2 to 32.6)
Short gestation for birth weight	0.0 (0.0 to 0.0)	0.0 (0.0 to 0.0)	0.0 (0.0 to 0.0)	7.1 (-32.5 to 52.2)	5.2 (-21.7 to 40.8)	0.0 (0.0 to 0.0)	0.0 (0.0 to 0.0)	0.0 (0.0 to 0.0)	3.6 (-28.3 to 42.3)	7.6 (-18.1 to 56.1)
Low birth weight for gestation	0.0 (0.0 to 0.0)	0.0 (0.0 to 0.0)	0.0 (0.0 to 0.0)	7.2 (-18.5 to 52.1)	-0.9 (-23.9 to 25.0)	0.0 (0.0 to 0.0)	0.0 (0.0 to 0.0)	0.0 (0.0 to 0.0)	5.6 (-18.7 to 42.5)	-2.8 (-30.8 to 22.8)
Iron deficiency	-	-	-	-	-	3.9 (2.5 to 5.8)	3.7 (2.3 to 5.3)	3.6 (2.2 to 5.3)	-7.0 (-32.2 to 28.1)	-1.2 (-25.7 to 28.8)
Vitamin A deficiency	0.5 (0.3 to 0.7)	0.4 (0.3 to 0.6)	0.5 (0.3 to 0.8)	-10.0 (-25.1 to 11.9)	22.1 (-3.7 to 58.2)	0.6 (0.4 to 0.7)	0.6 (0.4 to 0.7)	0.6 (0.4 to 0.7)	-0.7 (-15.9 to 22.4)	2.3 (-14.1 to 21.1)
Zinc deficiency	0.5 (0.1 to 1.1)	0.2 (0.0 to 1.0)	0.2 (0.0 to 1.0)	-54.9 (-89.8 to 339.2)	-1.6 (-89.5 to 889.5)	0.5 (0.1 to 1.1)	0.2 (0.0 to 1.0)	0.2 (0.0 to 1.0)	-56.3 (-89.6 to 318.0)	-0.9 (-88.1 to 862.1)
Smoking	13.4 (11.2 to 15.7)	7.0 (5.7 to 8.4)	5.7 (4.5 to 7.0)	-47.6 (-55.6 to -38.6)	-18.4 (-27.5 to -8.9)	10.6 (8.5 to 12.9)	5.4 (4.3 to 6.5)	4.1 (3.2 to 5.1)	-49.3 (-58.4 to -38.5)	-23.7 (-32.1 to -14.8)
Chewing tobacco	0.6 (0.4 to 0.7)	0.7 (0.5 to 0.9)	0.7 (0.5 to 1.0)	14.6 (-19.5 to 59.7)	7.9 (-21.3 to 43.6)	0.1 (0.1 to 0.2)	0.1 (0.1 to 0.1)	0.1 (0.1 to 0.1)	-11.8 (-47.6 to 41.4)	-1.5 (-31.6 to 42.6)
Second-hand smoke	40.8 (37.1 to 44.8)	20.4 (18.8 to 22.1)	23.5 (20.9 to 26.6)	-50.0 (-57.1 to -42.7)	15.6 (1.7 to 31.6)	36.2 (33.4 to 39.8)	18.4 (17.3 to 19.6)	21.2 (19.4 to 23.2)	-49.1 (-56.1 to -42.0)	15.2 (3.7 to 28.6)
Alcohol use	21.2 (10.8 to 35.1)	22.5 (12.0 to 34.8)	23.5 (11.6 to 37.2)	6.2 (-31.8 to 69.0)	4.5 (-29.1 to 51.0)	17.2 (8.6 to 28.9)	17.3 (9.1 to 28.5)	17.0 (8.0 to 29.3)	0.7 (-33.9 to 53.5)	-1.9 (-34.0 to 42.3)
Drug use	2.8 (1.8 to 4.6)	3.1 (2.0 to 5.0)	3.6 (2.2 to 5.9)	9.1 (0.5 to 18.6)	16.2 (6.2 to 25.2)	1.8 (1.1 to 2.9)	2.0 (1.4 to 3.3)	2.3 (1.6 to 3.7)	15.5 (6.5 to 27.1)	14.8 (5.3 to 24.0)
Diet low in fruits	14.4 (7.5 to 20.7)	23.1 (16.4 to 28.9)	26.0 (18.3 to 31.9)	60.5 (29.6 to 143.7)	12.6 (0.0 to 31.3)	12.8 (6.2 to 19.2)	19.8 (13.0 to 26.3)	22.9 (15.4 to 29.4)	54.6 (22.9 to 139.7)	15.6 (-1.7 to 41.5)
Diet low in vegetables	21.6 (13.8 to 28.8)	32.5 (26.2 to 37.5)	35.7 (29.8 to 40.3)	50.2 (23.8 to 108.2)	10.0 (2.0 to 21.4)	24.3 (16.7 to 31.1)	32.1 (25.8 to 37.4)	35.5 (29.2 to 40.1)	32.1 (12.6 to 69.7)	10.4 (2.5 to 22.3)
Diet low in legumes	23.8 (16.9 to 29.8)	23.9 (17.1 to 29.6)	25.3 (18.5 to 30.9)	0.4 (-16.7 to 19.9)	5.9 (-6.9 to 22.3)	28.7 (22.7 to 33.7)	29.3 (23.4 to 34.3)	30.7 (24.7 to 35.5)	2.3 (-10.8 to 17.7)	4.7 (-4.6 to 15.4)
Diet low in whole grains	37.3 (33.6 to 40.7)	38.2 (34.7 to 41.3)	37.5 (33.9 to 40.8)	2.2 (-3.0 to 8.0)	-1.8 (-6.1 to 2.2)	38.3 (34.7 to 41.1)	39.1 (35.8 to 41.9)	38.6 (35.3 to 41.6)	2.3 (-3.0 to 7.3)	-1.5 (-5.6 to 2.6)
Diet low in nuts and seeds	43.4 (39.5 to 46.3)	31.1 (25.6 to 36.0)	30.2 (23.3 to 36.0)	-28.3 (-38.4 to -19.8)	-2.9 (-15.1 to 8.3)	44.3 (40.8 to 47.0)	31.9 (26.5 to 36.8)	31.1 (24.5 to 36.9)	-27.9 (-37.6 to -19.6)	-2.5 (-14.8 to 9.8)
Diet low in milk	25.8 (18.8 to 31.3)	23.2 (15.5 to 29.0)	23.3 (15.5 to 29.6)	-10.2 (-23.4 to 1.1)	0.7 (-10.9 to 11.9)	27.6 (21.0 to 32.8)	24.1 (17.0 to 30.0)	24.0 (16.8 to 30.0)	-12.5 (-24.9 to -2.6)	-0.7 (-10.7 to 10.4)
Diet high in red meat	42.4 (35.0 to 48.1)	42.0 (33.7 to 48.9)	41.6 (30.5 to 49.4)	-0.8 (-18.3 to 18.7)	-1.1 (-15.8 to 13.2)	21.4 (14.7 to 27.7)	21.8 (12.8 to 29.7)	22.3 (12.5 to 31.2)	2.1 (-32.2 to 51.7)	2.2 (-24.5 to 37.8)
Diet high in processed meat	44.3 (38.0 to 49.2)	51.2 (46.5 to 54.1)	47.7 (40.1 to 52.6)	15.6 (3.5 to 31.0)	-6.9 (-15.9 to 0.3)	29.7 (23.3 to 35.1)	41.9 (35.1 to 47.4)	37.9 (29.5 to 44.5)	41.0 (16.6 to 72.1)	-9.6 (-21.2 to 1.7)
Diet high in sugar-sweetened beverages	34.8 (17.7 to 45.0)	35.9 (18.9 to 45.7)	31.9 (16.4 to 42.7)	3.4 (-24.9 to 41.2)	-11.4 (-33.3 to 10.4)	29.4 (14.2 to 40.2)	28.7 (14.3 to 39.6)	25.0 (11.3 to 35.4)	-2.3 (-33.2 to 40.7)	-13.0 (-37.7 to 18.2)
Diet low in fibre	32.1 (26.2 to 37.2)	23.4 (16.2 to 29.5)	24.8 (17.5 to 30.9)	-27.2 (-41.7 to -16.8)	5.9 (-6.3 to 19.5)	36.9 (32.0 to 40.9)	28.1 (21.8 to 33.4)	29.2 (22.6 to 34.8)	-23.8 (-35.3 to -15.3)	3.8 (-5.2 to 14.4)
Diet low in calcium	14.2 (7.8 to 20.5)	12.0 (6.4 to 17.7)	12.2 (6.7 to 18.2)	-15.6 (-29.8 to 0.2)	2.3 (-10.7 to 18.4)	18.0 (11.0 to 24.2)	15.1 (8.6 to 21.4)	15.3 (8.9 to 21.6)	-16.4 (-31.4 to -4.4)	1.8 (-8.5 to 14.7)
Diet low in seafood omega-3 fatty acids	25.1 (19.2 to 30.8)	18.7 (12.6 to 24.6)	19.5 (13.1 to 25.7)	-25.5 (-40.9 to -13.1)	4.3 (-10.0 to 20.9)	29.0 (23.4 to 34.1)	23.2 (17.2 to 29.2)	23.6 (17.6 to 29.4)	-19.9 (-33.1 to -8.4)	1.7 (-9.0 to 14.1)
Diet low in polyunsaturated fatty acids	6.4 (1.4 to 12.8)	3.0 (0.3 to 7.8)	2.1 (0.1 to 5.9)	-52.7 (-91.6 to -4.3)	-32.1 (-79.3 to 114.4)	7.2 (1.9 to 13.8)	3.6 (0.4 to 9.2)	2.4 (0.2 to 6.8)	-50.3 (-89.4 to -12.3)	-32.7 (-75.5 to 68.8)
Diet high in trans fatty acids	16.8 (12.2 to 22.2)	9.9 (5.9 to 14.0)	7.2 (3.6 to 11.0)	-41.4 (-56.5 to -29.1)	-27.3 (-43.2 to -16.0)	23.7 (17.9 to 31.6)	13.3 (9.0 to 18.1)	9.7 (5.9 to 14.0)	-43.9 (-54.6 to -31.8)	-26.7 (-40.5 to -16.3)
Diet high in sodium	7.6 (0.9 to 15.0)	13.7 (4.9 to 21.4)	14.4 (5.2 to 22.3)	79.7 (25.4 to 691.3)	5.0 (-19.8 to 49.3)	6.2 (0.3 to 13.5)	11.4 (2.8 to 19.0)	11.9 (3.2 to 20.0)	83.2 (27.8 to 1011.8)	4.5 (-24.1 to 47.8)
Intimate partner violence	-	-	-	-	-	1.6 (1.4 to 1.9)	1.0 (0.9 to 1.2)	1.2 (1.0 to 1.5)	-36.8 (-40.2 to -33.0)	20.0 (13.1 to 26.7)
Childhood sexual abuse	3.7 (2.7 to 5.1)	3.9 (3.1 to 4.8)	3.8 (3.1 to 4.8)	6.4 (-10.3 to 24.8)	-1.0 (-5.6 to 4.4)	11.0 (8.4 to 14.3)	10.5 (8.8 to 12.4)	10.4 (8.7 to 12.4)	-4.9 (-20.9 to 8.9)	-0.6 (-5.2 to 4.7)

Supplementary Results Table 1. District of Columbia age-standardised Summary Exposure Values (SEVs) for Level 4 risk factors and percentage change, by sex, 1990, 2007, and 2017.Data in parentheses are 95% uncertainty intervals.

	Male					Female				
	**1990**	**2007**	**2017**	**Percentage change 1990-2007**	**Percentage change 2007-2017**	**1990**	**2007**	**2017**	**Percentage change 1990-2007**	**Percentage change 2007-2017**
Bullying victimization	5.9 (2.6 to 12.4)	8.6 (3.7 to 17.6)	8.2 (3.5 to 17.0)	44.3 (28.5 to 64.0)	-4.5 (-12.1 to 3.5)	4.5 (1.9 to 9.5)	7.2 (3.2 to 14.9)	7.1 (3.0 to 14.5)	60.6 (42.3 to 86.2)	-2.3 (-10.1 to 6.2)
Low physical activity	0.5 (0.2 to 0.9)	0.5 (0.2 to 0.9)	0.5 (0.2 to 0.9)	-0.5 (-3.2 to 2.1)	0.8 (-0.8 to 2.7)	0.5 (0.2 to 1.0)	0.5 (0.2 to 1.0)	0.5 (0.2 to 1.0)	0.2 (-2.2 to 2.6)	-0.4 (-2.0 to 1.0)
High fasting plasma glucose	1.4 (0.9 to 2.2)	1.8 (1.0 to 2.8)	2.0 (1.1 to 3.3)	31.4 (-25.5 to 116.6)	11.6 (-36.2 to 89.5)	2.0 (1.1 to 3.4)	3.1 (1.6 to 5.0)	2.8 (1.4 to 4.7)	57.9 (-11.7 to 197.2)	-11.1 (-48.8 to 50.0)
High LDL cholesterol	20.6 (18.5 to 22.7)	11.4 (9.7 to 13.1)	10.1 (8.5 to 11.8)	-44.8 (-49.4 to -40.0)	-11.2 (-15.3 to -6.8)	19.2 (17.3 to 21.3)	11.2 (9.6 to 12.9)	10.4 (8.9 to 12.1)	-42.0 (-46.6 to -37.1)	-6.8 (-11.0 to -2.6)
High systolic blood pressure	7.3 (6.4 to 8.2)	5.4 (4.7 to 6.2)	5.6 (4.8 to 6.4)	-25.2 (-32.6 to -17.3)	2.7 (-6.2 to 12.0)	6.6 (5.7 to 7.5)	4.8 (4.2 to 5.5)	4.7 (4.0 to 5.4)	-26.8 (-35.2 to -16.9)	-3.3 (-13.5 to 7.9)
High body-mass index	17.8 (13.7 to 23.2)	23.9 (19.7 to 28.9)	25.9 (21.3 to 30.9)	34.6 (19.1 to 52.4)	8.6 (-0.4 to 18.4)	18.5 (14.7 to 23.2)	25.0 (20.8 to 30.3)	26.6 (21.9 to 31.9)	35.2 (19.4 to 54.1)	6.4 (-3.2 to 16.1)
Low bone mineral density	5.1 (4.1 to 6.0)	4.4 (3.6 to 5.2)	4.6 (3.7 to 5.6)	-13.9 (-24.9 to -1.1)	5.7 (-9.3 to 22.2)	5.7 (4.7 to 6.8)	5.6 (4.8 to 6.6)	6.1 (5.0 to 7.2)	-0.6 (-14.6 to 14.8)	8.3 (-5.4 to 22.8)
Impaired kidney function	2.5 (1.2 to 6.1)	2.3 (1.1 to 6.0)	2.3 (1.1 to 5.7)	-4.9 (-10.5 to -0.1)	-0.9 (-7.0 to 4.8)	3.2 (1.6 to 7.1)	3.0 (1.4 to 6.9)	3.0 (1.5 to 7.0)	-6.6 (-11.9 to -2.0)	1.3 (-3.4 to 6.2)

Supplementary Results Table 1. Florida age-standardised Summary Exposure Values (SEVs) for Level 4 risk factors and percentage change, by sex, 1990, 2007, and 2017.Data in parentheses are 95% uncertainty intervals.										
	Male					Female				
	**1990**	**2007**	**2017**	**Percentage change 1990-2007**	**Percentage change 2007-2017**	**1990**	**2007**	**2017**	**Percentage change 1990-2007**	**Percentage change 2007-2017**
Unsafe water source	1.5 (0.9 to 2.5)	0.5 (0.3 to 0.8)	0.3 (0.2 to 0.5)	-67.0 (-83.0 to -37.5)	-36.5 (-59.0 to 1.3)	1.5 (0.9 to 2.5)	0.5 (0.3 to 0.8)	0.3 (0.2 to 0.5)	-67.0 (-83.0 to -37.5)	-36.5 (-59.0 to 1.3)
Unsafe sanitation	7.3 (3.9 to 12.3)	4.2 (2.0 to 7.9)	3.0 (1.4 to 5.8)	-41.5 (-74.9 to 23.2)	-29.5 (-62.3 to 35.2)	7.3 (3.9 to 12.3)	4.2 (2.0 to 7.9)	3.0 (1.4 to 5.8)	-41.5 (-74.9 to 23.2)	-29.5 (-62.3 to 35.2)
No access to handwashing facility	3.5 (2.6 to 4.8)	3.2 (2.2 to 4.3)	3.1 (2.2 to 4.2)	-8.8 (-34.4 to 27.3)	-3.6 (-25.8 to 23.1)	3.5 (2.6 to 4.8)	3.2 (2.2 to 4.3)	3.1 (2.2 to 4.2)	-8.8 (-34.4 to 27.3)	-3.6 (-25.8 to 23.1)
Ambient particulate matter pollution	21.8 (13.0 to 34.4)	21.2 (13.4 to 34.4)	14.3 (5.7 to 26.1)	-2.8 (-30.0 to 35.4)	-32.4 (-70.0 to 4.6)	21.7 (12.9 to 34.3)	21.1 (13.4 to 34.4)	14.3 (5.7 to 26.0)	-2.6 (-30.0 to 35.8)	-32.4 (-70.0 to 4.7)
Household air pollution from solid fuels	0.3 (0.1 to 0.8)	0.2 (0.0 to 0.5)	0.2 (0.0 to 0.5)	-33.8 (-75.6 to 56.1)	-16.4 (-73.3 to 77.8)	0.4 (0.1 to 2.8)	0.3 (0.1 to 1.7)	0.3 (0.0 to 1.3)	-30.9 (-75.6 to 55.8)	-16.2 (-73.6 to 77.5)
Ambient ozone pollution	37.6 (12.7 to 54.8)	40.0 (13.9 to 54.8)	43.5 (15.7 to 54.8)	6.3 (0.0 to 9.9)	8.7 (0.0 to 15.5)	37.6 (12.7 to 54.8)	40.0 (13.9 to 54.8)	43.5 (15.7 to 54.8)	6.3 (0.0 to 9.9)	8.7 (0.0 to 15.5)
Residential radon	7.5 (0.2 to 28.3)	7.5 (0.2 to 28.3)	7.5 (0.2 to 28.3)	0.0 (0.0 to 0.0)	0.0 (0.0 to 0.0)	7.5 (0.2 to 28.3)	7.5 (0.2 to 28.3)	7.5 (0.2 to 28.3)	0.0 (0.0 to 0.0)	0.0 (0.0 to 0.0)
Lead exposure	11.4 (6.7 to 16.1)	7.1 (3.6 to 11.2)	4.7 (2.1 to 7.9)	-37.5 (-48.2 to -28.2)	-34.2 (-42.0 to -28.2)	7.2 (2.9 to 11.6)	4.6 (1.5 to 8.5)	3.1 (0.8 to 6.1)	-35.8 (-50.4 to -25.1)	-33.4 (-43.8 to -27.4)
Occupational exposure to asbestos	5.2 (4.5 to 6.0)	4.2 (3.9 to 4.4)	3.7 (3.1 to 4.3)	-20.5 (-31.7 to -5.8)	-11.8 (-26.0 to 4.4)	0.7 (0.6 to 0.8)	0.6 (0.6 to 0.7)	0.7 (0.5 to 0.8)	-6.9 (-19.3 to 6.6)	4.9 (-14.1 to 27.5)
Occupational exposure to arsenic	0.3 (0.0 to 0.7)	0.3 (0.0 to 0.8)	0.3 (0.0 to 0.8)	-	-	0.3 (0.0 to 0.7)	0.3 (0.0 to 0.7)	0.3 (0.0 to 0.7)	-	-
Occupational exposure to benzene	0.6 (0.0 to 1.8)	0.6 (0.0 to 1.9)	0.6 (0.0 to 1.8)	0.4 (-7.2 to 12.8)	-3.3 (-9.5 to 5.6)	0.9 (0.0 to 2.5)	1.0 (0.1 to 2.8)	1.0 (0.1 to 2.7)	11.9 (1.8 to 29.5)	-0.3 (-7.6 to 10.0)
Occupational exposure to beryllium	0.0 (0.0 to 0.0)	0.0 (0.0 to 0.0)	0.0 (0.0 to 0.0)	0.8 (-4.4 to 6.2)	0.9 (-3.5 to 5.1)	0.0 (0.0 to 0.0)	0.0 (0.0 to 0.0)	0.0 (0.0 to 0.0)	6.6 (-0.9 to 14.8)	-0.6 (-6.0 to 5.3)
Occupational exposure to cadmium	0.0 (0.0 to 0.0)	0.0 (0.0 to 0.0)	0.0 (0.0 to 0.0)	2.8 (-8.2 to 15.4)	2.8 (-5.9 to 11.7)	0.0 (0.0 to 0.0)	0.0 (0.0 to 0.0)	0.0 (0.0 to 0.0)	5.1 (-7.5 to 20.0)	-0.7 (-10.6 to 10.9)
Occupational exposure to chromium	0.1 (0.1 to 0.1)	0.1 (0.1 to 0.1)	0.1 (0.1 to 0.1)	4.9 (-4.9 to 15.8)	2.1 (-5.1 to 9.3)	0.1 (0.1 to 0.1)	0.1 (0.1 to 0.1)	0.1 (0.1 to 0.1)	7.1 (-4.2 to 20.0)	-1.3 (-9.6 to 8.0)
Occupational exposure to diesel engine exhaust	0.5 (0.5 to 0.6)	0.6 (0.6 to 0.6)	0.6 (0.5 to 0.7)	8.9 (0.6 to 18.2)	0.8 (-5.7 to 7.8)	0.5 (0.4 to 0.6)	0.5 (0.5 to 0.6)	0.5 (0.5 to 0.6)	8.5 (-3.8 to 22.9)	-5.0 (-13.5 to 4.7)
Occupational exposure to formaldehyde	0.2 (0.2 to 0.2)	0.2 (0.2 to 0.2)	0.2 (0.2 to 0.2)	0.9 (-10.8 to 13.5)	1.2 (-7.5 to 10.5)	0.2 (0.2 to 0.2)	0.2 (0.2 to 0.2)	0.2 (0.2 to 0.2)	5.6 (-6.7 to 19.3)	-0.7 (-9.9 to 10.2)
Occupational exposure to nickel	0.3 (0.0 to 1.1)	0.2 (0.0 to 1.1)	0.3 (0.0 to 1.1)	-	-	0.2 (0.0 to 1.0)	0.2 (0.0 to 1.1)	0.2 (0.0 to 1.1)	-	-
Occupational exposure to polycyclic aromatic hydrocarbons	0.2 (0.2 to 0.2)	0.2 (0.2 to 0.2)	0.2 (0.2 to 0.2)	5.0 (-3.9 to 14.5)	1.1 (-5.7 to 8.1)	0.2 (0.2 to 0.2)	0.2 (0.2 to 0.2)	0.2 (0.2 to 0.2)	10.3 (-0.4 to 21.5)	-1.1 (-8.3 to 6.9)
Occupational exposure to silica	2.4 (0.3 to 7.5)	2.2 (0.3 to 7.0)	2.3 (0.3 to 7.2)	-6.9 (-18.1 to 7.5)	3.8 (-6.3 to 16.3)	1.9 (0.3 to 6.2)	1.9 (0.3 to 5.6)	1.8 (0.3 to 5.7)	-3.2 (-18.2 to 16.4)	-0.9 (-13.8 to 16.1)
Occupational exposure to sulphuric acid	0.6 (0.1 to 1.7)	0.6 (0.1 to 2.0)	0.6 (0.1 to 1.9)	14.9 (0.8 to 30.6)	-2.1 (-11.4 to 8.1)	0.5 (0.1 to 1.6)	0.7 (0.1 to 2.0)	0.6 (0.1 to 1.9)	27.5 (6.9 to 50.2)	-4.6 (-16.8 to 9.6)
Occupational exposure to trichloroethylene	0.1 (0.1 to 0.1)	0.1 (0.1 to 0.1)	0.1 (0.1 to 0.1)	6.9 (-0.4 to 15.4)	1.7 (-3.6 to 7.5)	0.1 (0.1 to 0.1)	0.1 (0.1 to 0.1)	0.1 (0.1 to 0.1)	7.6 (-2.2 to 19.7)	-2.3 (-9.5 to 6.2)
Occupational asthmagens	14.2 (11.9 to 16.8)	13.5 (11.3 to 16.1)	13.3 (11.0 to 16.0)	-5.1 (-14.6 to 5.0)	-1.4 (-8.4 to 6.5)	7.6 (6.2 to 9.2)	7.7 (6.3 to 9.4)	7.2 (5.9 to 8.9)	1.4 (-11.5 to 17.0)	-5.4 (-16.2 to 5.4)
Occupational particulate matter, gases, and fumes	6.0 (4.3 to 8.6)	5.8 (4.2 to 8.3)	5.6 (4.1 to 8.0)	-4.4 (-8.9 to 0.2)	-3.6 (-7.0 to -0.1)	3.5 (2.4 to 5.1)	3.4 (2.4 to 4.9)	3.2 (2.3 to 4.7)	-2.2 (-9.1 to 5.2)	-3.9 (-9.4 to 1.4)
Occupational noise	5.7 (5.2 to 6.3)	5.6 (5.2 to 6.2)	5.4 (5.0 to 6.0)	-1.7 (-4.8 to 1.5)	-3.1 (-5.3 to -0.8)	4.1 (3.6 to 4.7)	4.3 (3.9 to 4.8)	4.3 (3.9 to 4.9)	5.0 (-0.2 to 10.7)	-0.1 (-3.3 to 3.0)
Occupational ergonomic factors	8.6 (7.0 to 10.5)	7.8 (6.5 to 9.5)	7.5 (6.2 to 9.0)	-8.4 (-17.9 to 1.1)	-4.2 (-10.7 to 3.3)	4.8 (3.8 to 6.1)	5.0 (4.0 to 6.2)	4.9 (3.9 to 6.2)	3.3 (-10.6 to 22.9)	-1.6 (-12.6 to 11.6)
Non-exclusive breastfeeding	0.5 (0.5 to 0.6)	0.5 (0.5 to 0.6)	0.5 (0.4 to 0.6)	-0.4 (-11.5 to 12.7)	-6.9 (-15.8 to 3.0)	0.5 (0.5 to 0.6)	0.5 (0.5 to 0.6)	0.5 (0.4 to 0.6)	-0.6 (-11.7 to 12.4)	-6.7 (-15.6 to 3.1)
Discontinued breastfeeding	2.2 (2.1 to 2.4)	2.2 (2.0 to 2.3)	2.2 (2.1 to 2.3)	-2.5 (-9.4 to 5.2)	2.0 (-3.5 to 8.7)	2.2 (2.1 to 2.4)	2.2 (2.0 to 2.3)	2.2 (2.1 to 2.3)	-2.0 (-8.9 to 5.7)	0.2 (-5.2 to 6.7)
Child underweight	0.1 (0.1 to 0.1)	0.1 (0.1 to 0.1)	0.1 (0.0 to 0.1)	-5.6 (-19.3 to 9.5)	-13.5 (-23.1 to -4.0)	0.1 (0.0 to 0.1)	0.1 (0.0 to 0.1)	0.1 (0.0 to 0.1)	-3.2 (-17.9 to 14.8)	-4.8 (-15.1 to 6.4)
Child wasting	0.1 (0.0 to 0.1)	0.1 (0.0 to 0.1)	0.1 (0.0 to 0.1)	-9.9 (-27.9 to 9.0)	-3.6 (-16.5 to 10.9)	0.1 (0.0 to 0.1)	0.1 (0.0 to 0.1)	0.1 (0.0 to 0.1)	6.4 (-14.8 to 31.2)	-5.6 (-19.3 to 10.1)
Child stunting	0.3 (0.1 to 0.5)	0.2 (0.1 to 0.4)	0.2 (0.1 to 0.4)	-9.7 (-49.5 to 59.8)	-12.6 (-40.8 to 27.7)	0.2 (0.1 to 0.4)	0.2 (0.1 to 0.3)	0.2 (0.1 to 0.3)	-10.2 (-45.8 to 39.6)	-4.2 (-31.8 to 30.7)
Short gestation for birth weight	0.0 (0.0 to 0.0)	0.0 (0.0 to 0.0)	0.0 (0.0 to 0.0)	7.1 (-18.4 to 38.4)	2.2 (-15.6 to 24.3)	0.0 (0.0 to 0.0)	0.0 (0.0 to 0.0)	0.0 (0.0 to 0.0)	7.7 (-22.8 to 42.2)	3.1 (-18.5 to 47.3)
Low birth weight for gestation	0.0 (0.0 to 0.0)	0.0 (0.0 to 0.0)	0.0 (0.0 to 0.0)	1.0 (-19.9 to 31.2)	-1.5 (-18.2 to 15.6)	0.0 (0.0 to 0.0)	0.0 (0.0 to 0.0)	0.0 (0.0 to 0.0)	-0.8 (-22.6 to 35.8)	-1.7 (-27.4 to 21.0)
Iron deficiency	-	-	-	-	-	4.3 (2.8 to 6.1)	3.9 (2.6 to 5.7)	4.1 (2.6 to 6.1)	-8.4 (-33.6 to 26.3)	4.9 (-17.5 to 32.2)
Vitamin A deficiency	0.4 (0.3 to 0.6)	0.5 (0.3 to 0.6)	0.5 (0.3 to 0.7)	10.0 (-7.7 to 36.8)	13.9 (-10.5 to 41.1)	0.7 (0.4 to 0.9)	0.6 (0.4 to 0.9)	0.6 (0.4 to 0.8)	-6.6 (-23.9 to 9.5)	-4.9 (-20.1 to 13.3)
Zinc deficiency	0.5 (0.1 to 1.2)	0.2 (0.0 to 1.0)	0.2 (0.0 to 1.0)	-55.5 (-88.7 to 324.7)	0.9 (-88.7 to 912.0)	0.5 (0.1 to 1.1)	0.2 (0.0 to 1.0)	0.2 (0.0 to 1.0)	-52.9 (-88.7 to 402.5)	-5.5 (-88.4 to 829.1)
Smoking	13.4 (10.6 to 16.0)	7.8 (6.3 to 9.5)	6.1 (4.7 to 7.6)	-41.3 (-49.8 to -31.2)	-21.8 (-29.9 to -12.0)	10.1 (7.7 to 12.6)	5.9 (4.6 to 7.2)	4.3 (3.4 to 5.5)	-41.7 (-52.0 to -29.8)	-26.1 (-34.2 to -17.5)
Chewing tobacco	1.8 (1.4 to 2.3)	2.0 (1.4 to 2.6)	2.1 (1.5 to 3.1)	9.0 (-22.7 to 51.4)	7.3 (-20.3 to 41.2)	0.1 (0.1 to 0.1)	0.1 (0.1 to 0.1)	0.1 (0.1 to 0.1)	-4.0 (-39.9 to 50.8)	4.7 (-27.0 to 48.0)
Second-hand smoke	44.7 (41.3 to 48.4)	26.4 (24.8 to 27.9)	27.7 (25.0 to 30.8)	-41.0 (-47.7 to -33.5)	5.2 (-5.4 to 17.0)	42.6 (39.6 to 45.8)	25.5 (24.3 to 26.6)	26.4 (24.6 to 28.4)	-40.2 (-46.3 to -33.4)	3.8 (-4.8 to 12.8)
Alcohol use	21.3 (11.7 to 33.2)	23.2 (12.7 to 35.8)	24.7 (12.6 to 38.9)	8.9 (-31.0 to 71.8)	6.8 (-26.4 to 59.0)	13.5 (7.4 to 22.4)	14.0 (7.4 to 23.1)	14.5 (7.0 to 24.8)	3.7 (-33.5 to 61.8)	3.7 (-29.8 to 53.0)
Drug use	1.7 (1.2 to 2.6)	2.4 (1.9 to 3.3)	2.9 (2.2 to 3.9)	41.8 (24.1 to 66.8)	18.3 (8.2 to 31.4)	1.1 (0.9 to 1.6)	1.6 (1.3 to 2.1)	1.9 (1.5 to 2.5)	39.6 (24.9 to 59.3)	17.6 (7.5 to 28.5)
Diet low in fruits	24.4 (17.5 to 30.1)	25.7 (19.4 to 30.8)	28.2 (21.7 to 33.6)	5.3 (-6.7 to 22.8)	9.9 (-1.2 to 23.6)	22.1 (15.2 to 28.1)	23.9 (17.6 to 29.5)	27.0 (20.1 to 32.8)	8.1 (-6.3 to 28.5)	12.8 (1.1 to 29.5)
Diet low in vegetables	21.4 (12.9 to 28.4)	30.4 (24.0 to 35.6)	33.4 (27.8 to 38.3)	42.3 (19.1 to 99.9)	9.7 (0.7 to 23.3)	25.6 (17.5 to 32.0)	31.5 (25.2 to 36.5)	34.7 (28.9 to 39.3)	22.7 (6.7 to 53.1)	10.3 (1.5 to 22.3)
Diet low in legumes	23.9 (16.6 to 29.8)	24.2 (17.3 to 29.6)	25.6 (19.1 to 31.3)	1.2 (-14.2 to 20.5)	5.9 (-6.5 to 21.8)	28.6 (22.5 to 33.9)	29.3 (23.7 to 34.1)	30.7 (25.0 to 35.4)	2.5 (-9.0 to 16.5)	4.6 (-4.3 to 14.8)
Diet low in whole grains	38.0 (34.5 to 40.9)	39.3 (36.1 to 42.1)	39.1 (35.5 to 41.9)	3.6 (-1.2 to 9.1)	-0.7 (-4.6 to 2.9)	39.0 (35.4 to 41.9)	40.3 (37.2 to 42.7)	40.1 (36.9 to 42.7)	3.3 (-1.2 to 8.3)	-0.5 (-3.9 to 3.0)
Diet low in nuts and seeds	45.4 (42.3 to 47.8)	36.4 (31.5 to 40.3)	37.6 (32.1 to 41.7)	-19.7 (-27.7 to -13.7)	3.1 (-4.7 to 10.7)	46.1 (43.0 to 48.3)	37.5 (32.6 to 41.3)	38.6 (33.5 to 42.5)	-18.7 (-26.9 to -12.8)	2.9 (-4.4 to 11.0)
Diet low in milk	27.3 (20.8 to 32.5)	26.0 (19.4 to 31.4)	26.9 (20.3 to 32.4)	-4.5 (-14.4 to 5.3)	3.4 (-4.5 to 13.3)	28.8 (22.5 to 34.0)	27.0 (20.5 to 32.3)	27.6 (21.2 to 32.8)	-6.5 (-16.0 to 2.2)	2.5 (-4.9 to 12.0)
Diet high in red meat	42.7 (32.3 to 49.8)	36.4 (25.1 to 44.8)	33.9 (22.7 to 42.6)	-14.7 (-33.1 to 4.9)	-6.8 (-24.9 to 12.7)	23.8 (13.4 to 32.8)	17.2 (8.8 to 25.7)	15.6 (7.4 to 23.6)	-27.8 (-53.7 to 6.7)	-9.3 (-38.7 to 32.6)
Diet high in processed meat	40.4 (31.5 to 46.8)	36.6 (28.5 to 43.2)	28.5 (21.0 to 35.3)	-9.3 (-24.5 to 8.3)	-22.3 (-34.7 to -9.1)	22.0 (16.1 to 27.6)	20.8 (15.4 to 26.0)	17.5 (12.5 to 22.4)	-5.5 (-26.0 to 19.8)	-15.8 (-28.7 to -0.5)
Diet high in sugar-sweetened beverages	32.1 (15.7 to 42.3)	30.2 (14.7 to 41.2)	24.3 (11.8 to 34.8)	-5.7 (-32.8 to 34.8)	-19.6 (-42.6 to 6.7)	26.7 (13.3 to 37.2)	23.0 (11.3 to 33.4)	18.3 (8.9 to 28.2)	-13.9 (-46.0 to 33.7)	-20.5 (-46.9 to 11.3)
Diet low in fibre	32.9 (27.2 to 37.9)	25.2 (18.0 to 31.4)	27.0 (19.9 to 32.9)	-23.6 (-37.4 to -13.5)	7.4 (-2.5 to 22.2)	37.6 (33.0 to 41.6)	29.8 (23.5 to 35.1)	31.3 (25.1 to 36.5)	-20.7 (-31.9 to -12.4)	5.0 (-2.7 to 14.5)
Diet low in calcium	16.0 (8.9 to 22.1)	14.9 (8.5 to 21.2)	16.2 (9.3 to 22.5)	-6.8 (-21.2 to 7.9)	8.5 (-2.0 to 23.7)	20.1 (12.9 to 26.1)	18.8 (11.7 to 24.8)	20.1 (13.0 to 26.0)	-6.7 (-18.8 to 3.9)	6.9 (-1.7 to 19.6)
Diet low in seafood omega-3 fatty acids	27.9 (21.8 to 32.9)	24.1 (17.7 to 29.9)	26.4 (20.6 to 32.0)	-13.4 (-25.4 to -2.2)	9.3 (-0.7 to 23.0)	31.5 (25.8 to 36.2)	28.4 (22.1 to 33.5)	30.1 (24.8 to 35.1)	-9.9 (-20.0 to -1.5)	6.3 (-0.8 to 16.6)
Diet low in polyunsaturated fatty acids	7.8 (2.2 to 14.4)	5.1 (0.8 to 11.0)	4.0 (0.6 to 9.4)	-35.4 (-79.8 to 12.3)	-20.9 (-64.7 to 61.0)	8.5 (2.6 to 15.2)	5.5 (1.0 to 11.4)	4.4 (0.7 to 9.8)	-34.6 (-77.8 to 9.5)	-20.6 (-60.2 to 46.0)
Diet high in trans fatty acids	16.8 (12.2 to 22.2)	9.9 (5.8 to 14.2)	7.2 (3.5 to 11.2)	-41.3 (-55.9 to -29.5)	-27.4 (-43.8 to -15.8)	23.6 (17.8 to 32.3)	13.3 (9.0 to 17.9)	9.7 (5.6 to 14.0)	-43.9 (-55.4 to -31.8)	-26.9 (-40.3 to -17.1)
Diet high in sodium	7.2 (0.7 to 14.9)	12.6 (4.0 to 20.2)	13.0 (4.2 to 20.5)	74.1 (22.1 to 631.7)	2.7 (-21.2 to 42.4)	6.1 (0.3 to 13.2)	10.9 (2.7 to 18.6)	11.4 (2.7 to 18.9)	79.8 (27.8 to 950.1)	4.2 (-22.7 to 52.7)
Intimate partner violence	-	-	-	-	-	0.5 (0.3 to 0.8)	0.4 (0.3 to 0.5)	0.4 (0.2 to 0.7)	-23.0 (-38.1 to 8.1)	15.7 (-8.8 to 38.5)
Childhood sexual abuse	5.6 (4.6 to 6.9)	5.1 (4.1 to 6.2)	5.1 (4.1 to 6.2)	-9.8 (-15.1 to -4.3)	0.4 (-4.7 to 6.0)	13.8 (11.0 to 16.9)	12.1 (9.8 to 14.7)	12.2 (9.9 to 14.8)	-11.9 (-17.2 to -6.0)	0.4 (-4.8 to 6.5)

Supplementary Results Table 1. Florida age-standardised Summary Exposure Values (SEVs) for Level 4 risk factors and percentage change, by sex, 1990, 2007, and 2017.Data in parentheses are 95% uncertainty intervals.										
	**Male**					**Female**				
	**1990**	**2007**	**2017**	**Percentage change 1990-2007**	**Percentage change 2007-2017**	**1990**	**2007**	**2017**	**Percentage change 1990-2007**	**Percentage change 2007-2017**
Bullying victimization	6.1 (2.6 to 12.3)	8.9 (3.9 to 18.4)	8.6 (3.7 to 18.1)	47.6 (32.0 to 67.2)	-3.6 (-10.9 to 4.2)	4.6 (1.9 to 9.4)	7.6 (3.3 to 16.1)	7.5 (3.2 to 15.7)	65.5 (45.7 to 92.4)	-1.7 (-9.3 to 7.2)
Low physical activity	0.4 (0.2 to 0.8)	0.4 (0.2 to 0.8)	0.4 (0.2 to 0.8)	-0.5 (-2.8 to 1.6)	-0.3 (-1.8 to 1.2)	0.4 (0.2 to 0.9)	0.4 (0.2 to 0.9)	0.4 (0.2 to 0.9)	-0.3 (-2.4 to 1.7)	-0.4 (-1.8 to 0.9)
High fasting plasma glucose	1.2 (0.8 to 1.9)	2.4 (1.5 to 3.7)	3.3 (2.1 to 5.0)	105.7 (28.2 to 237.5)	35.9 (-12.4 to 118.4)	1.4 (0.8 to 2.2)	2.5 (1.4 to 4.0)	2.6 (1.4 to 4.1)	79.1 (10.2 to 205.4)	4.0 (-41.6 to 83.3)
High LDL cholesterol	21.3 (19.3 to 23.4)	11.7 (10.1 to 13.4)	10.3 (8.8 to 11.9)	-45.1 (-50.1 to -40.4)	-12.0 (-16.4 to -8.1)	19.9 (18.1 to 21.8)	11.5 (9.9 to 13.1)	10.7 (9.2 to 12.3)	-42.3 (-47.2 to -37.9)	-6.9 (-11.1 to -2.8)
High systolic blood pressure	6.5 (5.7 to 7.4)	4.9 (4.3 to 5.6)	5.1 (4.3 to 5.8)	-24.6 (-32.1 to -16.2)	3.0 (-5.7 to 12.3)	6.1 (5.4 to 6.9)	4.4 (3.8 to 4.9)	4.3 (3.7 to 4.9)	-28.7 (-37.3 to -19.7)	-2.8 (-13.0 to 7.6)
High body-mass index	14.8 (11.0 to 20.3)	21.5 (16.7 to 27.1)	22.8 (18.1 to 28.5)	45.8 (27.2 to 68.8)	6.1 (-2.5 to 15.6)	16.2 (12.4 to 21.0)	21.8 (17.6 to 27.1)	22.9 (18.2 to 28.1)	34.6 (19.2 to 52.8)	4.7 (-4.3 to 14.7)
Low bone mineral density	5.1 (4.3 to 6.1)	4.4 (3.7 to 5.3)	4.6 (3.8 to 5.6)	-14.2 (-25.6 to 0.1)	5.2 (-8.6 to 21.6)	5.8 (4.8 to 6.9)	5.6 (4.7 to 6.6)	6.1 (5.1 to 7.3)	-2.7 (-15.6 to 11.4)	9.1 (-3.8 to 24.5)
Impaired kidney function	2.3 (1.1 to 5.8)	2.4 (1.1 to 6.1)	2.4 (1.2 to 6.1)	2.3 (-4.1 to 8.4)	2.9 (-2.2 to 8.7)	3.0 (1.4 to 7.0)	3.0 (1.4 to 7.0)	3.1 (1.5 to 7.1)	0.2 (-5.3 to 6.1)	5.0 (-0.1 to 10.8)

Supplementary Results Table 1. Georgia age-standardised Summary Exposure Values (SEVs) for Level 4 risk factors and percentage change, by sex, 1990, 2007, and 2017.Data in parentheses are 95% uncertainty intervals.

	Male					Female				
	**1990**	**2007**	**2017**	**Percentage change 1990-2007**	**Percentage change 2007-2017**	**1990**	**2007**	**2017**	**Percentage change 1990-2007**	**Percentage change 2007-2017**
Unsafe water source	1.7 (1.0 to 2.9)	0.6 (0.3 to 1.0)	0.3 (0.2 to 0.5)	-66.6 (-82.5 to -34.2)	-40.8 (-63.5 to -5.2)	1.7 (1.0 to 2.9)	0.6 (0.3 to 1.0)	0.3 (0.2 to 0.5)	-66.6 (-82.5 to -34.2)	-40.8 (-63.5 to -5.2)
Unsafe sanitation	8.6 (4.5 to 14.7)	5.4 (2.4 to 10.1)	3.4 (1.4 to 6.5)	-37.5 (-73.5 to 36.7)	-35.9 (-66.4 to 21.1)	8.6 (4.5 to 14.7)	5.4 (2.4 to 10.1)	3.4 (1.4 to 6.5)	-37.5 (-73.5 to 36.7)	-35.9 (-66.4 to 21.1)
No access to handwashing facility	3.5 (2.5 to 4.7)	3.2 (2.3 to 4.3)	3.0 (2.2 to 4.1)	-8.8 (-35.8 to 28.3)	-3.9 (-26.2 to 24.6)	3.5 (2.5 to 4.7)	3.2 (2.3 to 4.3)	3.0 (2.2 to 4.1)	-8.8 (-35.8 to 28.3)	-3.9 (-26.2 to 24.6)
Ambient particulate matter pollution	28.2 (19.4 to 41.2)	28.1 (19.5 to 41.6)	22.1 (14.3 to 33.7)	-0.1 (-19.9 to 27.1)	-21.4 (-41.9 to 0.4)	28.0 (19.2 to 41.1)	28.1 (19.5 to 41.6)	22.1 (14.3 to 33.7)	0.1 (-20.0 to 27.4)	-21.4 (-41.9 to 0.5)
Household air pollution from solid fuels	0.3 (0.1 to 0.9)	0.2 (0.1 to 0.5)	0.2 (0.0 to 0.5)	-39.9 (-77.7 to 30.9)	-9.1 (-64.0 to 84.6)	0.5 (0.1 to 2.9)	0.3 (0.1 to 1.8)	0.3 (0.1 to 1.8)	-36.3 (-77.6 to 31.1)	-8.2 (-63.4 to 87.0)
Ambient ozone pollution	47.9 (19.0 to 54.8)	48.0 (19.0 to 54.8)	49.1 (20.4 to 54.8)	0.1 (0.0 to 0.4)	2.4 (0.0 to 7.7)	47.9 (19.0 to 54.8)	48.0 (19.0 to 54.8)	49.1 (20.4 to 54.8)	0.1 (0.0 to 0.4)	2.4 (0.0 to 7.7)
Residential radon	10.8 (0.5 to 38.6)	10.8 (0.5 to 38.6)	10.8 (0.5 to 38.6)	0.0 (0.0 to 0.0)	0.0 (0.0 to 0.0)	10.8 (0.5 to 38.6)	10.8 (0.5 to 38.6)	10.8 (0.5 to 38.6)	0.0 (0.0 to 0.0)	0.0 (0.0 to 0.0)
Lead exposure	9.1 (4.4 to 13.8)	5.9 (2.4 to 10.0)	4.0 (1.5 to 7.3)	-34.9 (-46.2 to -25.8)	-32.3 (-41.0 to -26.0)	5.3 (1.4 to 9.5)	3.5 (0.8 to 7.2)	2.4 (0.5 to 5.3)	-33.1 (-47.6 to -22.8)	-31.5 (-43.1 to -24.8)
Occupational exposure to asbestos	4.3 (3.6 to 4.9)	3.4 (3.1 to 3.7)	2.9 (2.4 to 3.4)	-20.0 (-31.3 to -3.5)	-15.5 (-30.0 to 1.0)	0.5 (0.5 to 0.6)	0.5 (0.5 to 0.6)	0.6 (0.5 to 0.7)	0.0 (-13.7 to 15.3)	5.7 (-17.0 to 29.9)
Occupational exposure to arsenic	0.4 (0.0 to 0.9)	0.4 (0.0 to 0.9)	0.4 (0.0 to 0.9)	-	-	0.3 (0.0 to 0.8)	0.3 (0.0 to 0.8)	0.3 (0.0 to 0.8)	-	-
Occupational exposure to benzene	0.6 (0.0 to 1.6)	0.6 (0.0 to 1.6)	0.6 (0.0 to 1.6)	2.0 (-5.5 to 13.6)	-2.6 (-9.3 to 5.3)	0.8 (0.0 to 2.3)	0.9 (0.1 to 2.5)	0.9 (0.1 to 2.5)	12.2 (0.7 to 28.5)	0.8 (-6.6 to 11.4)
Occupational exposure to beryllium	0.0 (0.0 to 0.0)	0.0 (0.0 to 0.0)	0.0 (0.0 to 0.0)	0.5 (-4.8 to 6.3)	0.4 (-4.0 to 5.2)	0.0 (0.0 to 0.0)	0.0 (0.0 to 0.0)	0.0 (0.0 to 0.0)	6.0 (-2.3 to 14.6)	-0.2 (-6.0 to 5.8)
Occupational exposure to cadmium	0.0 (0.0 to 0.0)	0.0 (0.0 to 0.0)	0.0 (0.0 to 0.0)	0.4 (-10.7 to 12.4)	0.7 (-8.3 to 10.4)	0.0 (0.0 to 0.0)	0.0 (0.0 to 0.0)	0.0 (0.0 to 0.0)	1.3 (-12.5 to 18.2)	-1.6 (-12.5 to 10.4)
Occupational exposure to chromium	0.1 (0.1 to 0.1)	0.1 (0.1 to 0.1)	0.1 (0.1 to 0.1)	2.9 (-7.1 to 13.4)	0.6 (-6.8 to 9.1)	0.1 (0.1 to 0.1)	0.1 (0.1 to 0.1)	0.1 (0.1 to 0.1)	4.2 (-8.6 to 18.9)	-1.9 (-10.9 to 8.3)
Occupational exposure to diesel engine exhaust	0.5 (0.5 to 0.6)	0.6 (0.5 to 0.6)	0.6 (0.5 to 0.6)	10.6 (1.8 to 20.6)	1.2 (-5.4 to 8.6)	0.4 (0.4 to 0.5)	0.5 (0.4 to 0.6)	0.5 (0.4 to 0.5)	13.5 (0.1 to 28.4)	-4.8 (-13.6 to 4.7)
Occupational exposure to formaldehyde	0.2 (0.2 to 0.2)	0.2 (0.2 to 0.2)	0.2 (0.2 to 0.2)	-1.3 (-13.0 to 11.0)	-0.9 (-10.0 to 9.1)	0.2 (0.2 to 0.2)	0.2 (0.2 to 0.2)	0.2 (0.2 to 0.2)	1.5 (-12.4 to 17.4)	-1.5 (-11.9 to 9.6)
Occupational exposure to nickel	0.3 (0.0 to 1.3)	0.3 (0.0 to 1.3)	0.3 (0.0 to 1.3)	-	-	0.3 (0.0 to 1.2)	0.3 (0.0 to 1.2)	0.3 (0.0 to 1.3)	-	-
Occupational exposure to polycyclic aromatic hydrocarbons	0.2 (0.2 to 0.2)	0.2 (0.2 to 0.2)	0.2 (0.2 to 0.2)	3.1 (-6.2 to 13.1)	-0.1 (-7.0 to 7.9)	0.2 (0.2 to 0.2)	0.2 (0.2 to 0.3)	0.2 (0.2 to 0.3)	7.2 (-5.0 to 20.3)	-1.4 (-9.4 to 7.1)
Occupational exposure to silica	2.8 (0.4 to 9.0)	2.6 (0.4 to 8.7)	2.7 (0.4 to 8.4)	-7.7 (-18.4 to 5.2)	1.2 (-8.6 to 13.1)	2.3 (0.3 to 7.5)	2.2 (0.3 to 6.9)	2.1 (0.3 to 7.0)	-6.5 (-21.9 to 15.6)	-2.1 (-16.5 to 14.9)
Occupational exposure to sulphuric acid	0.6 (0.1 to 1.8)	0.6 (0.1 to 2.0)	0.6 (0.1 to 2.0)	11.9 (-0.6 to 25.2)	-1.7 (-9.8 to 7.5)	0.5 (0.1 to 1.8)	0.7 (0.1 to 2.1)	0.6 (0.1 to 2.1)	24.4 (6.6 to 44.8)	-4.6 (-16.4 to 8.6)
Occupational exposure to trichloroethylene	0.1 (0.1 to 0.1)	0.1 (0.1 to 0.1)	0.1 (0.1 to 0.1)	5.9 (-1.3 to 14.2)	0.9 (-4.6 to 7.2)	0.1 (0.1 to 0.1)	0.1 (0.1 to 0.1)	0.1 (0.1 to 0.1)	7.1 (-3.8 to 19.2)	-2.5 (-10.0 to 5.8)
Occupational asthmagens	15.8 (13.2 to 19.0)	14.9 (12.5 to 17.8)	14.4 (11.9 to 17.7)	-5.9 (-16.0 to 5.3)	-2.8 (-11.2 to 5.9)	7.7 (6.3 to 9.4)	7.8 (6.4 to 9.5)	7.4 (5.9 to 9.2)	1.7 (-10.9 to 16.1)	-6.0 (-16.3 to 5.2)
Occupational particulate matter, gases, and fumes	6.7 (4.8 to 9.6)	6.4 (4.5 to 9.3)	6.1 (4.4 to 8.9)	-4.7 (-9.3 to -0.3)	-4.3 (-8.2 to -0.8)	3.8 (2.6 to 5.5)	3.7 (2.6 to 5.5)	3.5 (2.5 to 5.2)	-2.2 (-9.5 to 5.5)	-4.3 (-10.2 to 1.6)
Occupational noise	5.9 (5.4 to 6.4)	5.7 (5.3 to 6.3)	5.5 (5.1 to 6.1)	-2.0 (-5.0 to 1.3)	-3.3 (-5.5 to -1.0)	4.1 (3.5 to 4.6)	4.3 (3.8 to 4.8)	4.3 (3.8 to 4.8)	5.4 (0.0 to 11.6)	-0.0 (-3.4 to 3.6)
Occupational ergonomic factors	8.9 (7.3 to 10.7)	8.0 (6.6 to 9.6)	7.6 (6.3 to 9.1)	-10.3 (-20.1 to -0.1)	-4.7 (-11.6 to 3.1)	4.9 (3.8 to 6.2)	5.0 (3.9 to 6.2)	4.9 (3.8 to 6.2)	0.9 (-13.9 to 18.1)	-1.7 (-13.3 to 9.5)
Non-exclusive breastfeeding	0.5 (0.5 to 0.6)	0.5 (0.5 to 0.6)	0.5 (0.4 to 0.6)	-0.1 (-11.7 to 14.1)	-7.1 (-16.9 to 2.4)	0.5 (0.5 to 0.6)	0.5 (0.5 to 0.6)	0.5 (0.4 to 0.6)	-0.3 (-11.9 to 13.8)	-7.0 (-16.9 to 2.5)
Discontinued breastfeeding	2.2 (2.0 to 2.3)	2.1 (2.0 to 2.3)	2.1 (2.0 to 2.3)	-2.8 (-10.2 to 5.7)	0.8 (-4.8 to 7.6)	2.2 (2.0 to 2.3)	2.1 (2.0 to 2.3)	2.2 (2.0 to 2.3)	-2.8 (-10.2 to 5.7)	2.4 (-3.4 to 9.2)
Child underweight	0.1 (0.1 to 0.1)	0.1 (0.1 to 0.1)	0.1 (0.0 to 0.1)	-5.2 (-18.3 to 10.9)	-14.9 (-24.1 to -6.1)	0.1 (0.0 to 0.1)	0.1 (0.0 to 0.1)	0.1 (0.0 to 0.1)	-2.6 (-17.4 to 14.4)	-6.2 (-16.5 to 4.5)
Child wasting	0.1 (0.0 to 0.1)	0.1 (0.0 to 0.1)	0.1 (0.0 to 0.1)	-10.1 (-27.0 to 9.2)	-3.9 (-17.4 to 11.0)	0.1 (0.0 to 0.1)	0.1 (0.0 to 0.1)	0.1 (0.0 to 0.1)	5.6 (-15.3 to 33.3)	-6.2 (-20.2 to 10.6)
Child stunting	0.3 (0.1 to 0.6)	0.3 (0.1 to 0.5)	0.2 (0.1 to 0.4)	-10.5 (-54.2 to 53.7)	-13.6 (-41.5 to 25.9)	0.2 (0.1 to 0.4)	0.2 (0.1 to 0.3)	0.2 (0.1 to 0.3)	-9.3 (-44.9 to 46.5)	-5.2 (-31.8 to 28.5)
Short gestation for birth weight	0.0 (0.0 to 0.0)	0.0 (0.0 to 0.0)	0.0 (0.0 to 0.0)	8.3 (-15.5 to 52.1)	-0.1 (-23.9 to 34.0)	0.0 (0.0 to 0.0)	0.0 (0.0 to 0.0)	0.0 (0.0 to 0.0)	9.2 (-26.7 to 55.1)	2.2 (-26.8 to 46.8)
Low birth weight for gestation	0.0 (0.0 to 0.0)	0.0 (0.0 to 0.0)	0.0 (0.0 to 0.0)	0.7 (-23.6 to 24.9)	0.1 (-21.0 to 28.3)	0.0 (0.0 to 0.0)	0.0 (0.0 to 0.0)	0.0 (0.0 to 0.0)	-2.6 (-25.9 to 39.3)	-1.2 (-28.9 to 30.1)
Iron deficiency	-	-	-	-	-	4.4 (2.9 to 6.4)	4.2 (2.8 to 6.1)	4.2 (2.7 to 6.3)	-4.9 (-29.0 to 31.2)	-0.6 (-23.0 to 26.6)
Vitamin A deficiency	0.4 (0.3 to 0.6)	0.5 (0.4 to 0.6)	0.6 (0.4 to 0.8)	13.3 (-10.4 to 40.6)	24.7 (-5.7 to 54.8)	0.6 (0.4 to 0.9)	0.6 (0.4 to 0.9)	0.7 (0.4 to 0.9)	0.9 (-21.6 to 31.1)	4.0 (-10.5 to 22.9)
Zinc deficiency	0.5 (0.1 to 1.2)	0.2 (0.0 to 1.1)	0.2 (0.0 to 1.0)	-56.3 (-88.7 to 292.4)	5.6 (-88.8 to 811.0)	0.5 (0.1 to 1.1)	0.2 (0.0 to 1.0)	0.2 (0.0 to 1.0)	-56.9 (-88.5 to 268.0)	10.6 (-87.7 to 850.2)
Smoking	15.0 (12.5 to 17.5)	7.7 (6.2 to 9.4)	6.1 (4.8 to 7.5)	-48.4 (-55.4 to -40.3)	-21.1 (-29.8 to -11.1)	9.4 (7.3 to 11.7)	5.3 (4.2 to 6.5)	4.0 (3.1 to 5.1)	-43.8 (-53.7 to -30.6)	-24.1 (-32.9 to -14.9)
Chewing tobacco	3.7 (3.2 to 4.2)	3.5 (2.7 to 4.5)	3.3 (2.4 to 4.6)	-5.0 (-28.8 to 26.4)	-4.3 (-26.0 to 23.1)	0.5 (0.4 to 0.7)	0.3 (0.2 to 0.4)	0.2 (0.1 to 0.3)	-43.3 (-62.7 to -13.3)	-22.1 (-44.2 to 8.4)
Second-hand smoke	41.2 (37.9 to 45.0)	25.9 (24.4 to 27.4)	26.5 (23.7 to 29.4)	-37.1 (-44.6 to -29.2)	2.0 (-8.7 to 12.7)	40.2 (37.3 to 43.4)	25.1 (23.9 to 26.1)	25.1 (23.1 to 27.0)	-37.6 (-43.9 to -31.0)	-0.2 (-8.4 to 7.8)
Alcohol use	25.1 (14.4 to 36.5)	26.5 (15.0 to 38.8)	27.7 (14.5 to 40.5)	5.7 (-33.3 to 68.0)	4.6 (-28.1 to 52.0)	14.6 (7.9 to 23.7)	15.5 (8.3 to 24.7)	16.2 (8.1 to 26.6)	6.2 (-32.5 to 76.6)	4.5 (-30.0 to 54.6)
Drug use	1.6 (1.1 to 2.5)	2.0 (1.4 to 2.8)	2.6 (1.9 to 3.6)	21.9 (8.3 to 42.5)	32.5 (20.8 to 48.4)	1.4 (1.0 to 2.1)	1.9 (1.5 to 2.5)	2.2 (1.7 to 3.0)	31.1 (16.7 to 48.4)	18.8 (8.8 to 30.1)
Diet low in fruits	25.3 (18.8 to 30.8)	28.6 (22.7 to 33.6)	31.1 (24.5 to 35.9)	13.3 (2.4 to 30.7)	8.6 (-0.7 to 19.5)	21.4 (14.4 to 27.3)	27.0 (20.7 to 32.0)	29.6 (22.9 to 34.7)	25.8 (10.7 to 58.2)	9.7 (-0.5 to 22.9)
Diet low in vegetables	26.0 (18.9 to 32.2)	30.6 (24.6 to 35.8)	32.8 (27.4 to 37.6)	17.7 (2.4 to 42.8)	7.2 (-1.5 to 18.5)	23.0 (15.1 to 29.6)	17.7 (12.0 to 23.0)	18.6 (13.0 to 23.5)	-22.9 (-37.3 to -4.2)	5.2 (-9.1 to 25.6)
Diet low in legumes	24.0 (17.3 to 29.8)	24.1 (17.6 to 29.9)	25.6 (18.5 to 31.4)	0.7 (-15.3 to 19.4)	6.0 (-6.0 to 22.1)	28.6 (22.3 to 33.7)	29.4 (23.7 to 34.5)	30.7 (25.1 to 35.6)	2.8 (-9.4 to 15.9)	4.7 (-4.5 to 16.4)
Diet low in whole grains	38.4 (34.9 to 41.4)	39.5 (36.2 to 42.3)	39.3 (35.8 to 42.3)	3.0 (-1.9 to 8.4)	-0.6 (-4.3 to 2.8)	39.3 (35.9 to 42.2)	40.5 (37.3 to 43.0)	40.3 (37.1 to 42.8)	3.0 (-1.9 to 8.1)	-0.5 (-3.8 to 3.1)
Diet low in nuts and seeds	46.4 (43.6 to 48.6)	37.4 (32.3 to 41.1)	38.9 (33.3 to 42.7)	-19.4 (-27.7 to -13.4)	4.0 (-3.5 to 11.2)	47.1 (44.4 to 49.2)	38.5 (33.9 to 42.3)	39.9 (35.0 to 43.8)	-18.4 (-26.1 to -12.5)	3.7 (-2.7 to 10.6)
Diet low in milk	28.1 (21.8 to 33.4)	26.6 (20.2 to 32.0)	27.5 (21.2 to 33.1)	-5.5 (-15.6 to 3.9)	3.7 (-4.1 to 13.7)	29.8 (24.0 to 34.7)	27.6 (21.3 to 32.9)	28.4 (22.1 to 33.3)	-7.4 (-17.8 to 1.0)	2.8 (-4.3 to 12.0)
Diet high in red meat	40.8 (30.3 to 48.4)	35.0 (24.0 to 44.2)	32.7 (21.5 to 42.6)	-14.1 (-34.4 to 8.5)	-6.8 (-26.3 to 15.4)	22.4 (12.9 to 32.5)	16.3 (8.3 to 25.2)	14.7 (6.5 to 24.5)	-27.1 (-56.9 to 13.4)	-9.7 (-42.9 to 34.4)
Diet high in processed meat	38.2 (29.4 to 45.0)	35.1 (26.3 to 41.4)	26.8 (19.1 to 33.4)	-8.2 (-25.1 to 10.8)	-23.4 (-35.6 to -10.3)	20.6 (15.0 to 26.0)	19.9 (14.9 to 24.6)	16.5 (11.4 to 21.4)	-3.3 (-21.6 to 21.8)	-17.0 (-31.8 to -3.0)
Diet high in sugar-sweetened beverages	30.3 (14.8 to 41.0)	28.6 (14.1 to 38.9)	22.8 (11.0 to 33.7)	-5.6 (-35.2 to 39.2)	-20.5 (-44.6 to 7.7)	25.2 (11.6 to 36.2)	22.1 (10.5 to 32.8)	17.3 (7.4 to 27.7)	-12.3 (-44.4 to 36.0)	-21.9 (-50.2 to 12.1)
Diet low in fibre	33.5 (27.9 to 38.1)	25.5 (18.6 to 31.5)	27.4 (20.3 to 33.4)	-23.8 (-37.2 to -13.4)	7.3 (-3.2 to 20.9)	38.2 (33.6 to 41.8)	30.1 (24.2 to 35.4)	31.8 (26.0 to 36.9)	-21.0 (-30.9 to -12.8)	5.4 (-2.0 to 15.7)
Diet low in calcium	17.4 (10.2 to 23.7)	15.7 (9.1 to 21.9)	17.2 (10.4 to 23.5)	-9.4 (-23.3 to 2.8)	9.3 (-1.3 to 25.2)	21.4 (14.2 to 27.1)	19.5 (12.6 to 25.5)	21.1 (14.2 to 26.9)	-8.6 (-22.3 to 1.5)	7.9 (0.7 to 20.7)
Diet low in seafood omega-3 fatty acids	29.6 (24.0 to 34.6)	25.3 (19.0 to 31.0)	27.8 (21.7 to 33.4)	-14.8 (-27.4 to -3.7)	10.0 (0.9 to 23.8)	33.1 (28.2 to 37.2)	29.4 (23.6 to 34.4)	31.3 (25.9 to 36.0)	-11.0 (-20.6 to -2.6)	6.4 (-0.4 to 15.0)
Diet low in polyunsaturated fatty acids	8.7 (3.1 to 15.8)	5.6 (1.0 to 11.7)	4.7 (0.9 to 10.5)	-35.5 (-76.3 to 2.6)	-16.8 (-57.6 to 45.3)	9.5 (3.3 to 16.4)	6.2 (1.3 to 12.5)	5.1 (0.9 to 10.9)	-35.1 (-75.5 to -1.4)	-18.0 (-56.7 to 40.3)
Diet high in trans fatty acids	16.8 (12.3 to 22.1)	9.8 (5.7 to 14.1)	7.1 (3.4 to 11.0)	-41.6 (-56.5 to -29.1)	-27.4 (-43.7 to -15.3)	23.8 (18.0 to 32.3)	13.3 (8.9 to 17.9)	9.7 (5.9 to 14.0)	-44.2 (-56.8 to -32.0)	-26.7 (-39.1 to -16.6)
Diet high in sodium	7.2 (0.7 to 14.9)	12.5 (3.7 to 20.3)	12.7 (3.8 to 20.5)	74.3 (23.2 to 692.4)	1.9 (-26.5 to 44.1)	6.0 (0.3 to 13.3)	10.8 (2.5 to 18.6)	11.2 (2.7 to 18.8)	80.5 (26.2 to 1020.6)	3.8 (-20.9 to 54.7)
Intimate partner violence	-	-	-	-	-	1.4 (1.2 to 1.7)	1.0 (0.8 to 1.1)	1.2 (1.0 to 1.4)	-31.2 (-34.8 to -27.1)	24.8 (18.4 to 31.4)
Childhood sexual abuse	5.6 (4.6 to 6.9)	5.1 (4.1 to 6.2)	5.1 (4.1 to 6.2)	-9.8 (-15.1 to -4.3)	0.4 (-4.7 to 6.0)	13.8 (11.0 to 16.9)	12.1 (9.8 to 14.7)	12.2 (9.9 to 14.8)	-11.9 (-17.2 to -6.0)	0.4 (-4.8 to 6.5)

Supplementary Results Table 1. Georgia age-standardised Summary Exposure Values (SEVs) for Level 4 risk factors and percentage change, by sex, 1990, 2007, and 2017.Data in parentheses are 95% uncertainty intervals.

	Male					Female				
	**1990**	**2007**	**2017**	**Percentage change 1990-2007**	**Percentage change 2007-2017**	**1990**	**2007**	**2017**	**Percentage change 1990-2007**	**Percentage change 2007-2017**
Bullying victimization	6.1<br>(2.5 to 13.3)	8.9<br>(3.7 to 18.3)	8.7<br>(3.6 to 18.5)	45.6<br>(29.6 to 67.2)	-2.1<br>(-9.5 to 5.7)	4.6<br>(1.9 to 9.6)	7.5<br>(3.2 to 16.0)	7.5<br>(3.2 to 16.0)	63.2<br>(44.8 to 88.1)	-0.1<br>(-7.9 to 9.7)
Low physical activity	0.4<br>(0.2 to 0.8)	0.4<br>(0.2 to 0.8)	0.4<br>(0.2 to 0.7)	0.0<br>(-2.1 to 1.9)	-1.4<br>(-2.9 to 0.1)	0.4<br>(0.2 to 0.8)	0.4<br>(0.2 to 0.8)	0.4<br>(0.2 to 0.8)	-0.5<br>(-2.9 to 1.7)	-2.0<br>(-3.6 to -0.5)
High fasting plasma glucose	1.3<br>(0.8 to 2.2)	3.0<br>(1.9 to 4.5)	3.4<br>(2.2 to 5.1)	127.6<br>(39.2 to 302.9)	14.9<br>(-22.6 to 80.6)	1.5<br>(0.9 to 2.6)	3.1<br>(1.8 to 5.0)	3.3<br>(1.9 to 5.3)	104.3<br>(19.0 to 258.8)	5.9<br>(-35.2 to 85.7)
High LDL cholesterol	21.1<br>(19.2 to 23.3)	11.4<br>(9.9 to 13.2)	10.1<br>(8.7 to 11.8)	-45.8<br>(-50.4 to -41.1)	-11.3<br>(-15.7 to -7.2)	19.6<br>(17.9 to 21.6)	11.3<br>(9.7 to 13.0)	10.5<br>(8.9 to 12.1)	-42.4<br>(-46.9 to -37.7)	-7.3<br>(-11.7 to -3.2)
High systolic blood pressure	6.7<br>(5.9 to 7.6)	5.0<br>(4.4 to 5.7)	5.1<br>(4.3 to 5.8)	-25.4<br>(-32.8 to -17.3)	1.0<br>(-7.9 to 10.1)	6.7<br>(5.8 to 7.5)	4.7<br>(4.1 to 5.2)	4.4<br>(3.8 to 5.1)	-29.4<br>(-37.6 to -20.8)	-5.8<br>(-15.6 to 4.1)
High body-mass index	13.6<br>(9.8 to 19.0)	20.8<br>(16.2 to 26.6)	21.5<br>(16.9 to 26.9)	52.9<br>(34.0 to 78.9)	3.0<br>(-5.0 to 12.7)	15.9<br>(12.1 to 21.0)	22.8<br>(18.2 to 28.4)	24.0<br>(19.2 to 29.7)	43.5<br>(26.5 to 64.4)	5.2<br>(-3.7 to 14.8)
Low bone mineral density	5.1<br>(4.2 to 6.2)	4.4<br>(3.6 to 5.2)	4.7<br>(3.8 to 5.6)	-14.3<br>(-26.0 to -1.5)	6.4<br>(-8.8 to 23.8)	5.7<br>(4.8 to 6.7)	5.6<br>(4.8 to 6.7)	6.2<br>(5.1 to 7.2)	-2.1<br>(-13.9 to 11.5)	9.5<br>(-5.2 to 26.6)
Impaired kidney function	2.4<br>(1.2 to 6.0)	2.5<br>(1.2 to 6.2)	2.5<br>(1.2 to 6.1)	1.5<br>(-4.1 to 7.3)	0.6<br>(-5.3 to 7.2)	3.2<br>(1.6 to 7.2)	3.2<br>(1.5 to 7.2)	3.3<br>(1.6 to 7.4)	-0.7<br>(-5.9 to 4.5)	3.0<br>(-2.6 to 8.9)

Supplementary Results Table 1. Hawaii age-standardised Summary Exposure Values (SEVs) for Level 4 risk factors and percentage change, by sex, 1990, 2007, and 2017.Data in parentheses are 95% uncertainty intervals.

	Male					Female				
	1990	2007	2017	Percentage change 1990-2007	Percentage change 2007-2017	1990	2007	2017	Percentage change 1990-2007	Percentage change 2007-2017
Unsafe water source	1.5 (0.8 to 2.5)	0.5 (0.3 to 0.8)	0.3 (0.2 to 0.5)	-67.4 (-83.0 to -34.6)	-37.3 (-61.2 to -2.5)	1.5 (0.8 to 2.5)	0.5 (0.3 to 0.8)	0.3 (0.2 to 0.5)	-67.4 (-83.0 to -34.6)	-37.3 (-61.2 to -2.5)
Unsafe sanitation	6.7 (3.6 to 11.4)	4.0 (1.8 to 7.5)	2.8 (1.2 to 5.2)	-40.3 (-73.7 to 24.8)	-30.7 (-64.2 to 38.4)	6.7 (3.6 to 11.4)	4.0 (1.8 to 7.5)	2.8 (1.2 to 5.2)	-40.3 (-73.7 to 24.8)	-30.7 (-64.2 to 38.4)
No access to handwashing facility	3.4 (2.5 to 4.6)	3.1 (2.3 to 4.3)	3.0 (2.1 to 4.2)	-8.4 (-36.5 to 30.9)	-3.5 (-24.3 to 24.8)	3.4 (2.5 to 4.6)	3.1 (2.3 to 4.3)	3.0 (2.1 to 4.2)	-8.4 (-36.5 to 30.9)	-3.5 (-24.3 to 24.8)
Ambient particulate matter pollution	9.1 (2.8 to 19.4)	9.2 (2.9 to 23.3)	4.8 (0.6 to 18.7)	2.0 (-61.5 to 246.5)	-48.4 (-91.4 to 82.4)	9.0 (2.8 to 19.4)	9.2 (2.9 to 23.2)	4.8 (0.6 to 18.7)	2.2 (-61.5 to 246.5)	-48.3 (-91.3 to 82.1)
Household air pollution from solid fuels	0.2 (0.0 to 0.8)	0.2 (0.0 to 0.6)	0.1 (0.0 to 0.3)	-28.9 (-80.0 to 137.3)	-32.8 (-93.8 to 80.5)	0.3 (0.0 to 2.3)	0.2 (0.0 to 1.1)	0.1 (0.0 to 0.6)	-28.2 (-80.7 to 131.7)	-39.5 (-94.2 to 77.3)
Ambient ozone pollution	18.2 (6.1 to 35.5)	16.9 (5.5 to 33.6)	15.5 (5.0 to 31.4)	-6.7 (-14.1 to -1.6)	-8.6 (-17.4 to -2.7)	18.2 (6.1 to 35.5)	16.9 (5.5 to 33.6)	15.5 (5.0 to 31.4)	-6.7 (-14.1 to -1.6)	-8.6 (-17.4 to -2.7)
Residential radon	6.7 (0.1 to 26.0)	6.7 (0.1 to 26.0)	6.7 (0.1 to 26.0)	0.0 (0.0 to 0.0)	0.0 (0.0 to 0.0)	6.7 (0.1 to 26.0)	6.7 (0.1 to 26.0)	6.7 (0.1 to 26.0)	0.0 (0.0 to 0.0)	0.0 (0.0 to 0.0)
Lead exposure	7.6 (3.2 to 12.0)	4.9 (1.8 to 8.8)	3.3 (1.1 to 6.5)	-34.8 (-46.7 to -25.1)	-32.4 (-41.7 to -25.8)	4.6 (1.1 to 8.7)	3.1 (0.6 to 6.6)	2.1 (0.4 to 4.9)	-33.3 (-49.3 to -22.6)	-31.4 (-43.9 to -24.9)
Occupational exposure to asbestos	3.8 (3.3 to 4.4)	3.2 (2.9 to 3.6)	2.9 (2.4 to 3.5)	-15.3 (-28.3 to 0.3)	-8.9 (-25.8 to 10.7)	0.3 (0.3 to 0.4)	0.3 (0.3 to 0.4)	0.3 (0.3 to 0.4)	-1.4 (-17.5 to 18.7)	1.7 (-16.4 to 21.1)
Occupational exposure to arsenic	0.3 (0.0 to 0.6)	0.3 (0.0 to 0.6)	0.3 (0.0 to 0.6)	-	-	0.3 (0.0 to 0.7)	0.3 (0.0 to 0.7)	0.3 (0.0 to 0.7)	-	-
Occupational exposure to benzene	0.5 (0.0 to 1.6)	0.6 (0.0 to 1.6)	0.6 (0.0 to 1.6)	5.7 (-4.3 to 21.2)	-2.7 (-10.1 to 6.9)	0.8 (0.0 to 2.3)	0.9 (0.1 to 2.6)	0.9 (0.1 to 2.6)	14.3 (3.6 to 32.6)	0.6 (-7.2 to 12.4)
Occupational exposure to beryllium	0.0 (0.0 to 0.0)	0.0 (0.0 to 0.0)	0.0 (0.0 to 0.0)	0.9 (-4.8 to 6.8)	0.7 (-3.6 to 5.3)	0.0 (0.0 to 0.0)	0.0 (0.0 to 0.0)	0.0 (0.0 to 0.0)	5.8 (-2.2 to 13.8)	-0.6 (-6.2 to 5.9)
Occupational exposure to cadmium	0.0 (0.0 to 0.0)	0.0 (0.0 to 0.0)	0.0 (0.0 to 0.0)	5.3 (-5.7 to 17.7)	2.9 (-5.9 to 12.5)	0.0 (0.0 to 0.0)	0.0 (0.0 to 0.0)	0.0 (0.0 to 0.0)	5.3 (-7.7 to 19.0)	-0.8 (-10.3 to 9.8)
Occupational exposure to chromium	0.1 (0.1 to 0.1)	0.1 (0.1 to 0.1)	0.1 (0.1 to 0.1)	9.4 (-2.1 to 21.7)	3.2 (-5.0 to 11.8)	0.1 (0.1 to 0.1)	0.1 (0.1 to 0.1)	0.1 (0.1 to 0.1)	7.6 (-3.7 to 19.5)	-1.2 (-9.1 to 8.0)
Occupational exposure to diesel engine exhaust	0.4 (0.4 to 0.5)	0.5 (0.5 to 0.5)	0.5 (0.5 to 0.6)	15.1 (4.6 to 26.7)	3.9 (-3.5 to 11.5)	0.5 (0.4 to 0.5)	0.5 (0.5 to 0.6)	0.5 (0.4 to 0.6)	6.7 (-5.8 to 21.1)	-5.5 (-14.2 to 4.4)
Occupational exposure to formaldehyde	0.1 (0.1 to 0.2)	0.2 (0.1 to 0.2)	0.2 (0.1 to 0.2)	5.8 (-7.2 to 19.9)	2.1 (-7.8 to 12.7)	0.2 (0.1 to 0.2)	0.2 (0.2 to 0.2)	0.2 (0.2 to 0.2)	7.0 (-6.1 to 21.0)	-0.1 (-9.4 to 10.2)
Occupational exposure to nickel	0.2 (0.0 to 0.9)	0.2 (0.0 to 0.9)	0.2 (0.0 to 1.0)	-	-	0.2 (0.0 to 0.9)	0.2 (0.0 to 0.9)	0.2 (0.0 to 1.0)	-	-
Occupational exposure to polycyclic aromatic hydrocarbons	0.2 (0.1 to 0.2)	0.2 (0.2 to 0.2)	0.2 (0.2 to 0.2)	10.7 (-0.5 to 22.9)	1.1 (-6.6 to 9.2)	0.2 (0.2 to 0.2)	0.2 (0.2 to 0.2)	0.2 (0.2 to 0.2)	12.3 (1.7 to 23.4)	-1.5 (-8.6 to 6.6)
Occupational exposure to silica	1.8 (0.3 to 5.8)	1.8 (0.3 to 5.8)	1.9 (0.3 to 6.0)	-1.3 (-15.0 to 14.7)	4.3 (-7.1 to 18.3)	1.8 (0.2 to 5.6)	1.8 (0.2 to 5.3)	1.7 (0.2 to 5.3)	-1.6 (-17.2 to 20.9)	-1.3 (-14.0 to 14.8)
Occupational exposure to sulphuric acid	0.5 (0.1 to 1.7)	0.7 (0.1 to 2.1)	0.6 (0.1 to 2.0)	21.4 (2.7 to 43.4)	-5.4 (-16.2 to 6.3)	0.6 (0.1 to 1.9)	0.8 (0.2 to 2.6)	0.7 (0.2 to 2.4)	29.5 (7.2 to 55.7)	-8.6 (-21.8 to 5.4)
Occupational exposure to trichloroethylene	0.0 (0.0 to 0.1)	0.1 (0.0 to 0.1)	0.1 (0.0 to 0.1)	10.5 (0.7 to 20.4)	3.3 (-3.1 to 9.7)	0.1 (0.0 to 0.1)	0.1 (0.1 to 0.1)	0.1 (0.1 to 0.1)	6.7 (-3.4 to 18.2)	-2.4 (-10.0 to 5.9)
Occupational asthmagens	11.2 (9.3 to 13.4)	11.1 (9.2 to 13.3)	11.1 (9.2 to 13.4)	-1.4 (-13.1 to 10.7)	0.3 (-8.5 to 9.4)	7.0 (5.7 to 8.5)	7.2 (6.0 to 8.8)	6.8 (5.5 to 8.3)	3.6 (-9.6 to 18.2)	-5.2 (-15.8 to 6.1)
Occupational particulate matter, gases, and fumes	4.8 (3.5 to 7.0)	4.8 (3.4 to 7.1)	4.7 (3.4 to 6.9)	-1.4 (-6.7 to 4.3)	-1.7 (-6.2 to 2.4)	3.4 (2.4 to 5.0)	3.4 (2.4 to 4.9)	3.2 (2.3 to 4.7)	-1.5 (-8.5 to 5.9)	-4.0 (-9.5 to 1.6)
Occupational noise	4.7 (4.3 to 5.2)	4.8 (4.5 to 5.3)	4.8 (4.4 to 5.2)	2.3 (-1.5 to 6.5)	-1.0 (-3.8 to 2.0)	3.9 (3.4 to 4.5)	4.2 (3.7 to 4.6)	4.2 (3.8 to 4.6)	6.2 (0.7 to 12.0)	0.2 (-2.9 to 3.7)
Occupational ergonomic factors	8.6 (7.2 to 10.3)	7.9 (6.5 to 9.5)	7.5 (6.2 to 9.0)	-8.7 (-18.6 to 1.9)	-4.5 (-11.5 to 2.7)	4.9 (3.9 to 6.1)	5.0 (3.9 to 6.3)	4.9 (3.9 to 6.3)	2.7 (-12.4 to 20.0)	-1.3 (-12.7 to 11.8)
Non-exclusive breastfeeding	0.5 (0.5 to 0.6)	0.5 (0.5 to 0.6)	0.5 (0.4 to 0.6)	-0.7 (-12.7 to 12.9)	-6.9 (-16.7 to 2.9)	0.5 (0.5 to 0.6)	0.5 (0.5 to 0.6)	0.5 (0.4 to 0.6)	-0.3 (-12.4 to 13.3)	-7.2 (-16.9 to 2.5)
Discontinued breastfeeding	2.2 (2.0 to 2.3)	2.2 (2.0 to 2.3)	2.2 (2.0 to 2.3)	-1.0 (-8.2 to 7.2)	0.6 (-5.5 to 6.5)	2.2 (2.1 to 2.3)	2.2 (2.0 to 2.3)	2.2 (2.0 to 2.3)	-1.9 (-9.1 to 6.2)	1.4 (-4.7 to 7.4)
Child underweight	0.1 (0.1 to 0.1)	0.1 (0.1 to 0.1)	0.1 (0.0 to 0.1)	-5.6 (-19.0 to 10.0)	-13.7 (-22.8 to -4.6)	0.1 (0.0 to 0.1)	0.1 (0.0 to 0.1)	0.1 (0.0 to 0.1)	-2.8 (-18.5 to 15.5)	-5.4 (-15.2 to 5.3)
Child wasting	0.1 (0.0 to 0.1)	0.1 (0.0 to 0.1)	0.1 (0.0 to 0.1)	-10.1 (-31.3 to 10.1)	-3.9 (-18.4 to 12.0)	0.1 (0.0 to 0.1)	0.1 (0.0 to 0.1)	0.1 (0.0 to 0.1)	5.9 (-15.6 to 34.6)	-5.3 (-20.2 to 11.1)
Child stunting	0.3 (0.1 to 0.5)	0.2 (0.1 to 0.4)	0.2 (0.1 to 0.4)	-9.1 (-50.5 to 62.8)	-12.1 (-44.2 to 25.4)	0.2 (0.1 to 0.4)	0.2 (0.1 to 0.3)	0.2 (0.1 to 0.3)	-10.2 (-45.5 to 47.6)	-4.3 (-30.1 to 30.5)
Short gestation for birth weight	0.0 (0.0 to 0.0)	0.0 (0.0 to 0.0)	0.0 (0.0 to 0.0)	5.6 (-19.8 to 52.7)	2.6 (-19.6 to 29.2)	0.0 (0.0 to 0.0)	0.0 (0.0 to 0.0)	0.0 (0.0 to 0.0)	2.7 (-22.8 to 41.9)	3.5 (-21.9 to 44.4)
Low birth weight for gestation	0.0 (0.0 to 0.0)	0.0 (0.0 to 0.0)	0.0 (0.0 to 0.0)	1.5 (-25.0 to 32.0)	-1.4 (-20.2 to 21.2)	0.0 (0.0 to 0.0)	0.0 (0.0 to 0.0)	0.0 (0.0 to 0.0)	2.5 (-24.0 to 35.9)	-2.0 (-28.8 to 23.4)
Iron deficiency	-	-	-	-	-	4.2 (2.7 to 6.2)	4.0 (2.6 to 5.9)	4.0 (2.4 to 6.1)	-5.4 (-31.6 to 27.1)	-0.0 (-24.2 to 30.2)
Vitamin A deficiency	0.5 (0.3 to 0.6)	0.5 (0.3 to 0.6)	0.5 (0.4 to 0.7)	2.2 (-13.0 to 21.7)	9.5 (-14.6 to 36.5)	0.5 (0.4 to 0.7)	0.6 (0.4 to 0.7)	0.6 (0.4 to 0.8)	10.0 (-8.2 to 35.4)	1.5 (-15.3 to 25.3)
Zinc deficiency	0.5 (0.1 to 1.2)	0.2 (0.0 to 1.0)	0.2 (0.0 to 1.0)	-55.8 (-88.3 to 285.7)	1.4 (-89.4 to 841.5)	0.5 (0.1 to 1.1)	0.2 (0.0 to 1.0)	0.2 (0.0 to 1.0)	-55.6 (-88.2 to 321.5)	-5.1 (-88.1 to 855.0)
Smoking	13.2 (10.8 to 15.7)	7.4 (5.8 to 9.0)	6.0 (4.6 to 7.5)	-44.3 (-53.1 to -34.8)	-19.1 (-28.3 to -8.9)	8.8 (6.8 to 10.9)	5.0 (3.9 to 6.2)	3.9 (2.9 to 4.9)	-43.5 (-54.7 to -29.1)	-22.5 (-31.8 to -12.0)
Chewing tobacco	0.9 (0.6 to 1.2)	1.2 (0.8 to 1.8)	1.4 (0.9 to 2.1)	40.1 (-6.7 to 110.6)	10.1 (-20.9 to 49.3)	0.1 (0.1 to 0.1)	0.1 (0.1 to 0.1)	0.1 (0.1 to 0.1)	-2.5 (-40.8 to 60.9)	3.2 (-27.8 to 50.8)
Second-hand smoke	44.5 (41.2 to 47.9)	27.1 (25.5 to 28.7)	28.8 (25.8 to 31.9)	-39.1 (-45.3 to -31.8)	6.2 (-4.6 to 17.8)	44.7 (41.4 to 47.8)	27.0 (25.8 to 28.2)	27.8 (25.8 to 29.9)	-39.5 (-45.6 to -33.2)	2.9 (-5.4 to 12.7)
Alcohol use	22.5 (11.8 to 35.0)	24.9 (13.9 to 37.3)	26.3 (14.0 to 40.5)	10.4 (-30.6 to 75.8)	5.7 (-26.2 to 53.1)	10.7 (5.6 to 18.0)	11.5 (5.9 to 18.9)	12.1 (6.0 to 20.3)	7.3 (-31.0 to 70.9)	5.7 (-27.1 to 54.7)
Drug use	1.4 (0.9 to 2.2)	1.6 (1.1 to 2.5)	2.1 (1.5 to 3.0)	16.3 (5.5 to 32.2)	27.4 (16.2 to 42.2)	1.1 (0.8 to 1.6)	1.2 (0.9 to 1.8)	1.4 (1.0 to 2.0)	18.2 (8.3 to 30.9)	10.5 (0.9 to 20.0)
Diet low in fruits	27.0 (20.8 to 32.1)	27.4 (21.3 to 32.5)	30.0 (23.6 to 35.4)	1.6 (-8.6 to 13.4)	9.6 (-0.6 to 22.4)	24.2 (17.7 to 29.8)	24.4 (17.8 to 29.8)	26.6 (19.2 to 32.7)	0.6 (-12.6 to 15.6)	9.0 (-3.6 to 24.9)
Diet low in vegetables	19.7 (11.4 to 26.6)	32.3 (25.8 to 37.3)	36.0 (30.5 to 40.7)	63.7 (32.4 to 141.8)	11.4 (3.0 to 22.8)	21.1 (12.6 to 28.2)	31.7 (24.9 to 36.7)	35.6 (29.9 to 40.1)	50.3 (22.1 to 116.1)	12.5 (4.2 to 25.2)
Diet low in legumes	23.9 (16.7 to 30.0)	24.2 (17.6 to 30.1)	25.5 (19.2 to 31.2)	1.2 (-15.6 to 21.4)	5.7 (-7.4 to 23.8)	28.7 (22.6 to 33.9)	29.3 (23.6 to 34.2)	30.6 (24.8 to 35.4)	2.2 (-10.2 to 16.4)	4.3 (-4.6 to 15.5)
Diet low in whole grains	37.7 (34.2 to 40.8)	39.3 (36.2 to 42.2)	38.8 (35.3 to 41.8)	4.3 (-1.0 to 10.2)	-1.3 (-5.3 to 2.3)	38.7 (35.3 to 41.6)	40.2 (37.3 to 42.9)	39.8 (36.5 to 42.4)	3.9 (-1.0 to 9.2)	-1.1 (-4.4 to 2.3)
Diet low in nuts and seeds	44.7 (41.5 to 47.3)	36.4 (31.5 to 40.4)	36.5 (31.0 to 41.0)	-18.4 (-27.5 to -11.7)	0.2 (-8.4 to 8.6)	45.5 (42.4 to 48.0)	37.4 (32.5 to 41.2)	37.5 (32.1 to 41.7)	-17.8 (-25.8 to -11.8)	0.2 (-7.3 to 7.3)
Diet low in milk	26.7 (20.3 to 32.3)	26.0 (19.6 to 31.6)	26.4 (19.6 to 32.1)	-2.4 (-12.3 to 8.8)	1.3 (-8.4 to 11.0)	28.5 (22.4 to 33.8)	27.0 (20.4 to 32.5)	27.1 (20.8 to 32.4)	-5.2 (-15.6 to 4.6)	0.4 (-8.0 to 9.9)
Diet high in red meat	50.0 (46.8 to 52.7)	43.4 (34.4 to 49.9)	41.0 (30.4 to 48.6)	-13.2 (-26.7 to -0.8)	-5.5 (-20.8 to 8.8)	36.5 (29.8 to 41.9)	27.9 (18.3 to 36.7)	25.1 (14.5 to 34.9)	-23.5 (-45.6 to 0.2)	-10.1 (-33.6 to 15.0)
Diet high in processed meat	34.3 (27.7 to 39.2)	43.8 (36.5 to 49.0)	39.1 (29.7 to 45.9)	27.8 (6.4 to 51.7)	-10.8 (-22.4 to 0.3)	19.9 (15.2 to 23.8)	28.7 (22.2 to 34.3)	27.2 (19.7 to 34.0)	44.3 (17.1 to 80.9)	-5.1 (-20.7 to 11.2)
Diet high in sugar-sweetened beverages	33.3 (16.9 to 43.9)	29.9 (15.1 to 40.3)	25.4 (12.8 to 36.0)	-10.1 (-37.8 to 30.2)	-15.3 (-39.2 to 16.7)	27.6 (13.7 to 39.1)	23.0 (11.3 to 33.2)	19.3 (8.7 to 29.9)	-16.6 (-48.3 to 31.7)	-16.2 (-42.9 to 17.9)
Diet low in fibre	32.6 (26.6 to 37.7)	25.0 (18.2 to 31.2)	26.6 (19.5 to 32.8)	-23.3 (-36.6 to -12.6)	6.1 (-4.8 to 19.1)	37.4 (32.1 to 41.4)	29.7 (23.0 to 35.3)	31.0 (24.3 to 36.4)	-20.4 (-31.1 to -12.1)	4.1 (-4.9 to 13.9)
Diet low in calcium	15.3 (8.3 to 21.6)	14.9 (8.2 to 21.1)	15.5 (8.6 to 21.8)	-2.4 (-17.1 to 16.3)	4.0 (-7.6 to 18.7)	19.3 (11.7 to 25.4)	18.7 (11.2 to 24.8)	19.2 (11.5 to 25.4)	-3.2 (-16.2 to 10.0)	2.9 (-5.9 to 15.3)
Diet low in seafood omega-3 fatty acids	27.0 (21.1 to 32.3)	24.1 (18.1 to 29.9)	25.3 (19.4 to 30.9)	-10.5 (-23.0 to 3.0)	4.7 (-5.2 to 18.2)	30.4 (24.8 to 35.4)	28.3 (22.3 to 33.3)	29.1 (23.3 to 34.0)	-7.0 (-17.2 to 2.6)	2.8 (-4.8 to 11.2)
Diet low in polyunsaturated fatty acids	7.4 (2.0 to 14.3)	5.2 (0.9 to 11.2)	3.7 (0.5 to 8.8)	-30.1 (-75.2 to 23.1)	-27.5 (-70.5 to 43.9)	8.0 (2.3 to 14.6)	5.7 (1.1 to 11.9)	4.2 (0.6 to 9.3)	-28.4 (-74.6 to 22.7)	-26.4 (-67.0 to 41.0)
Diet high in trans fatty acids	16.8 (11.9 to 22.2)	9.9 (5.8 to 14.2)	7.2 (3.5 to 11.2)	-41.2 (-55.3 to -29.7)	-27.3 (-43.5 to -15.7)	23.7 (17.9 to 31.9)	13.3 (9.0 to 18.1)	9.8 (5.8 to 13.9)	-43.7 (-56.2 to -31.9)	-26.8 (-39.6 to -16.8)
Diet high in sodium	5.7 (0.2 to 13.1)	7.5 (0.7 to 14.9)	7.7 (0.8 to 14.8)	31.2 (-18.6 to 617.9)	2.8 (-34.8 to 91.2)	4.1 (0.0 to 10.7)	5.1 (0.1 to 11.9)	5.2 (0.1 to 12.2)	25.6 (-12.6 to 1199.3)	1.4 (-46.3 to 159.4)
Intimate partner violence	-	-	-	-	-	1.1 (0.9 to 1.3)	1.0 (0.9 to 1.2)	1.1 (0.9 to 1.3)	-7.6 (-14.6 to 0.9)	3.5 (-3.8 to 11.7)
Childhood sexual abuse	3.9 (3.0 to 5.2)	4.4 (3.6 to 5.5)	4.4 (3.5 to 5.5)	13.8 (-3.7 to 34.4)	-0.3 (-5.0 to 5.1)	11.2 (8.6 to 14.4)	10.4 (8.9 to 12.4)	10.4 (8.9 to 12.3)	-7.0 (-20.2 to 7.8)	0.0 (-5.0 to 5.4)

Supplementary Results Table 1. Hawaii age-standardised Summary Exposure Values (SEVs) for Level 4 risk factors and percentage change, by sex, 1990, 2007, and 2017.Data in parentheses are 95% uncertainty intervals.										
	**Male**					**Female**				
	**1990**	**2007**	**2017**	**Percentage change 1990-2007**	**Percentage change 2007-2017**	**1990**	**2007**	**2017**	**Percentage change 1990-2007**	**Percentage change 2007-2017**
Bullying victimization	16.9 (7.2 to 30.1)	21.5 (10.2 to 35.2)	21.5 (9.8 to 35.3)	27.5 (10.9 to 53.0)	-0.2 (-7.0 to 5.3)	15.4 (6.3 to 29.3)	19.1 (8.4 to 33.1)	18.7 (8.3 to 33.0)	23.9 (10.1 to 46.1)	-2.1 (-9.1 to 4.6)
Low physical activity	0.4 (0.2 to 0.7)	0.4 (0.2 to 0.7)	0.4 (0.2 to 0.7)	-0.8 (-3.0 to 1.3)	-1.1 (-2.8 to 0.3)	0.4 (0.2 to 0.8)	0.4 (0.2 to 0.8)	0.4 (0.2 to 0.8)	0.2 (-2.2 to 2.5)	-3.1 (-5.0 to -1.3)
High fasting plasma glucose	1.2 (0.7 to 1.9)	2.3 (1.4 to 3.6)	3.0 (1.8 to 4.5)	97.8 (21.1 to 217.3)	29.7 (-18.4 to 107.8)	1.3 (0.8 to 2.3)	2.0 (1.1 to 3.3)	1.9 (1.0 to 3.1)	51.8 (-5.1 to 150.5)	-7.8 (-44.8 to 56.5)
High LDL cholesterol	20.8 (18.9 to 22.9)	11.4 (9.9 to 13.2)	10.1 (8.6 to 11.9)	-45.1 (-49.6 to -40.4)	-11.5 (-15.9 to -7.0)	19.8 (17.9 to 21.8)	11.3 (9.8 to 13.1)	10.5 (8.9 to 12.1)	-42.7 (-47.6 to -38.2)	-7.4 (-11.4 to -3.2)
High systolic blood pressure	7.1 (6.2 to 8.0)	4.8 (4.1 to 5.4)	4.8 (4.1 to 5.5)	-33.0 (-39.5 to -25.6)	0.9 (-7.7 to 9.1)	7.2 (6.3 to 8.0)	4.7 (4.1 to 5.2)	4.4 (3.8 to 5.0)	-34.9 (-42.3 to -27.0)	-5.8 (-15.7 to 3.7)
High body-mass index	14.6 (10.9 to 19.8)	20.3 (15.7 to 26.1)	21.2 (16.4 to 26.7)	38.7 (23.4 to 58.4)	4.6 (-3.9 to 13.7)	14.8 (11.1 to 19.7)	20.8 (17.0 to 26.4)	21.7 (17.5 to 27.3)	40.4 (22.2 to 63.7)	4.1 (-5.7 to 15.0)
Low bone mineral density	5.2 (4.3 to 6.2)	4.5 (3.7 to 5.3)	4.7 (3.9 to 5.6)	-14.6 (-25.8 to -1.4)	5.7 (-10.4 to 22.0)	5.8 (4.9 to 7.0)	5.7 (4.9 to 6.7)	6.3 (5.3 to 7.5)	-1.9 (-13.9 to 14.8)	9.2 (-6.6 to 25.7)
Impaired kidney function	2.3 (1.1 to 5.8)	2.3 (1.1 to 5.9)	2.4 (1.1 to 5.9)	1.3 (-4.2 to 7.2)	1.8 (-4.4 to 8.3)	3.0 (1.4 to 6.9)	2.9 (1.4 to 6.9)	3.1 (1.5 to 7.1)	-1.1 (-6.6 to 4.2)	4.2 (-0.6 to 10.0)

Supplementary Results Table 1. Idaho age-standardised Summary Exposure Values (SEVs) for Level 4 risk factors and percentage change, by sex, 1990, 2007, and 2017.Data in parentheses are 95% uncertainty intervals.										
	Male					Female				
	1990	2007	2017	Percentage change 1990-2007	Percentage change 2007-2017	1990	2007	2017	Percentage change 1990-2007	Percentage change 2007-2017
Unsafe water source	1.7 (1.0 to 3.0)	0.6 (0.3 to 1.0)	0.4 (0.2 to 0.6)	-65.6 (-82.3 to -30.4)	-38.6 (-62.0 to -0.3)	1.7 (1.0 to 3.0)	0.6 (0.3 to 1.0)	0.4 (0.2 to 0.6)	-65.6 (-82.3 to -30.4)	-38.6 (-62.0 to -0.3)
Unsafe sanitation	8.5 (4.6 to 14.0)	5.5 (2.6 to 10.1)	3.7 (1.8 to 7.0)	-35.1 (-70.8 to 34.5)	-32.2 (-63.4 to 26.9)	8.5 (4.6 to 14.0)	5.5 (2.6 to 10.1)	3.7 (1.8 to 7.0)	-35.1 (-70.8 to 34.5)	-32.2 (-63.4 to 26.9)
No access to handwashing facility	3.5 (2.5 to 4.8)	3.2 (2.3 to 4.4)	3.1 (2.2 to 4.2)	-8.9 (-35.5 to 30.8)	-4.2 (-26.3 to 23.8)	3.5 (2.5 to 4.8)	3.2 (2.3 to 4.4)	3.1 (2.2 to 4.2)	-8.9 (-35.5 to 30.8)	-4.2 (-26.3 to 23.8)
Ambient particulate matter pollution	15.3 (3.8 to 29.6)	16.3 (5.9 to 29.8)	10.5 (1.5 to 23.9)	6.7 (-61.3 to 275.8)	-35.3 (-90.6 to 82.7)	15.1 (3.8 to 29.5)	16.2 (5.9 to 29.8)	10.5 (1.5 to 23.8)	7.1 (-61.4 to 277.5)	-35.2 (-90.6 to 83.1)
Household air pollution from solid fuels	0.4 (0.1 to 1.3)	0.3 (0.1 to 0.9)	0.2 (0.0 to 0.7)	-35.5 (-77.9 to 102.1)	-29.4 (-89.4 to 61.4)	0.6 (0.1 to 4.7)	0.4 (0.1 to 2.7)	0.3 (0.0 to 2.1)	-32.8 (-78.4 to 92.9)	-26.7 (-89.6 to 62.5)
Ambient ozone pollution	45.1 (16.8 to 54.8)	44.9 (16.7 to 54.8)	45.4 (16.9 to 54.8)	-0.6 (-1.9 to 0.0)	1.2 (0.0 to 3.3)	45.1 (16.8 to 54.8)	44.9 (16.7 to 54.8)	45.4 (16.9 to 54.8)	-0.6 (-1.9 to 0.0)	1.2 (0.0 to 3.3)
Residential radon	18.6 (1.7 to 56.4)	18.6 (1.7 to 56.4)	18.6 (1.7 to 56.4)	0.0 (0.0 to 0.0)	0.0 (0.0 to 0.0)	18.6 (1.7 to 56.4)	18.6 (1.7 to 56.4)	18.6 (1.7 to 56.4)	0.0 (0.0 to 0.0)	0.0 (0.0 to 0.0)
Lead exposure	6.9 (2.7 to 11.3)	4.7 (1.6 to 8.5)	3.3 (1.0 to 6.4)	-31.9 (-43.3 to -22.8)	-30.6 (-40.7 to -24.0)	3.9 (0.7 to 8.0)	2.8 (0.4 to 6.4)	2.0 (0.3 to 5.0)	-29.1 (-44.0 to -19.9)	-29.2 (-42.3 to -22.2)
Occupational exposure to asbestos	5.5 (4.7 to 6.2)	4.8 (4.4 to 5.3)	4.5 (3.8 to 5.3)	-12.6 (-25.1 to 6.5)	-7.6 (-23.8 to 11.5)	0.9 (0.8 to 1.0)	1.0 (0.9 to 1.1)	1.1 (0.9 to 1.2)	10.4 (-3.4 to 27.3)	7.9 (-8.5 to 25.7)
Occupational exposure to arsenic	0.3 (0.0 to 0.8)	0.3 (0.0 to 0.8)	0.4 (0.0 to 0.8)	-	-	0.3 (0.0 to 0.7)	0.3 (0.0 to 0.8)	0.3 (0.0 to 0.8)	-	-
Occupational exposure to benzene	0.6 (0.0 to 1.6)	0.6 (0.0 to 1.7)	0.6 (0.0 to 1.7)	2.5 (-5.6 to 14.7)	-2.0 (-8.7 to 5.8)	0.8 (0.0 to 2.2)	0.9 (0.1 to 2.5)	0.9 (0.1 to 2.5)	13.2 (1.6 to 33.8)	0.5 (-7.5 to 12.6)
Occupational exposure to beryllium	0.0 (0.0 to 0.0)	0.0 (0.0 to 0.0)	0.0 (0.0 to 0.0)	1.6 (-4.2 to 7.5)	0.5 (-4.0 to 5.4)	0.0 (0.0 to 0.0)	0.0 (0.0 to 0.0)	0.0 (0.0 to 0.0)	7.0 (-1.2 to 16.8)	-0.3 (-6.8 to 6.2)
Occupational exposure to cadmium	0.0 (0.0 to 0.0)	0.0 (0.0 to 0.0)	0.0 (0.0 to 0.0)	7.6 (-4.4 to 20.4)	1.2 (-8.1 to 11.5)	0.0 (0.0 to 0.0)	0.0 (0.0 to 0.0)	0.0 (0.0 to 0.0)	8.5 (-6.0 to 27.3)	-1.4 (-12.7 to 12.1)
Occupational exposure to chromium	0.1 (0.1 to 0.1)	0.1 (0.1 to 0.1)	0.1 (0.1 to 0.1)	9.5 (-1.1 to 20.2)	1.2 (-6.6 to 9.7)	0.1 (0.1 to 0.1)	0.1 (0.1 to 0.1)	0.1 (0.1 to 0.1)	10.3 (-2.3 to 26.5)	-1.6 (-10.7 to 9.5)
Occupational exposure to diesel engine exhaust	0.5 (0.5 to 0.6)	0.6 (0.5 to 0.6)	0.6 (0.5 to 0.6)	9.1 (-0.0 to 19.2)	1.8 (-5.1 to 9.0)	0.5 (0.4 to 0.5)	0.5 (0.4 to 0.6)	0.5 (0.4 to 0.5)	8.7 (-3.9 to 24.1)	-4.3 (-12.9 to 6.0)
Occupational exposure to formaldehyde	0.2 (0.2 to 0.2)	0.2 (0.2 to 0.2)	0.2 (0.2 to 0.2)	6.7 (-6.4 to 19.9)	-0.2 (-9.8 to 10.5)	0.2 (0.2 to 0.2)	0.2 (0.2 to 0.2)	0.2 (0.2 to 0.2)	9.6 (-4.4 to 27.6)	-1.3 (-11.6 to 11.0)
Occupational exposure to nickel	0.3 (0.0 to 1.2)	0.3 (0.0 to 1.2)	0.3 (0.0 to 1.3)	-	-	0.2 (0.0 to 1.0)	0.3 (0.0 to 1.1)	0.2 (0.0 to 1.1)	-	-
Occupational exposure to polycyclic aromatic hydrocarbons	0.2 (0.2 to 0.2)	0.2 (0.2 to 0.2)	0.2 (0.2 to 0.2)	9.1 (-0.9 to 19.1)	0.6 (-6.7 to 8.6)	0.2 (0.2 to 0.2)	0.2 (0.2 to 0.2)	0.2 (0.2 to 0.2)	13.0 (1.3 to 27.7)	-1.2 (-9.5 to 8.2)
Occupational exposure to silica	2.7 (0.4 to 8.3)	2.6 (0.4 to 8.4)	2.6 (0.4 to 8.3)	-3.8 (-15.3 to 10.2)	0.9 (-9.0 to 13.4)	2.1 (0.3 to 6.2)	2.1 (0.3 to 6.5)	2.0 (0.3 to 6.6)	0.1 (-15.9 to 24.6)	-2.0 (-16.0 to 16.5)
Occupational exposure to sulphuric acid	0.5 (0.1 to 1.7)	0.6 (0.1 to 2.0)	0.6 (0.1 to 2.0)	17.2 (4.0 to 32.1)	-0.9 (-9.6 to 9.2)	0.5 (0.1 to 1.6)	0.7 (0.1 to 2.1)	0.7 (0.1 to 2.1)	26.1 (7.2 to 49.6)	-3.6 (-16.5 to 10.1)
Occupational exposure to trichloroethylene	0.1 (0.1 to 0.1)	0.1 (0.1 to 0.1)	0.1 (0.1 to 0.1)	10.0 (2.1 to 18.3)	1.5 (-4.0 to 8.0)	0.1 (0.0 to 0.1)	0.1 (0.1 to 0.1)	0.1 (0.1 to 0.1)	9.7 (-1.0 to 23.8)	-2.2 (-10.3 to 7.2)
Occupational asthmagens	14.5 (12.2 to 17.2)	14.3 (12.1 to 17.0)	14.0 (11.7 to 16.8)	-1.1 (-11.5 to 9.4)	-2.2 (-10.0 to 6.6)	7.1 (5.8 to 8.6)	7.5 (6.1 to 9.2)	7.1 (5.7 to 8.8)	5.3 (-8.1 to 21.6)	-5.6 (-15.7 to 5.5)
Occupational particulate matter, gases, and fumes	6.6 (4.7 to 9.4)	6.4 (4.6 to 9.2)	6.1 (4.5 to 8.8)	-2.6 (-7.6 to 1.9)	-3.6 (-7.3 to -0.1)	3.5 (2.4 to 5.3)	3.5 (2.5 to 5.4)	3.4 (2.4 to 5.1)	0.7 (-7.0 to 9.0)	-3.0 (-8.6 to 3.0)
Occupational noise	6.1 (5.5 to 6.6)	5.9 (5.4 to 6.5)	5.7 (5.2 to 6.3)	-2.2 (-5.3 to 1.0)	-3.4 (-5.8 to -1.0)	3.9 (3.4 to 4.5)	4.2 (3.7 to 4.7)	4.2 (3.8 to 4.7)	6.5 (1.0 to 13.4)	0.4 (-3.1 to 4.5)
Occupational ergonomic factors	9.0 (7.5 to 11.0)	8.1 (6.7 to 9.7)	7.7 (6.3 to 9.3)	-10.9 (-20.9 to -0.4)	-4.6 (-12.1 to 3.9)	5.0 (3.9 to 6.3)	5.0 (3.9 to 6.3)	4.9 (3.9 to 6.2)	0.1 (-14.9 to 17.1)	-2.1 (-14.0 to 10.4)
Non-exclusive breastfeeding	0.5 (0.5 to 0.6)	0.5 (0.5 to 0.6)	0.5 (0.4 to 0.6)	0.1 (-11.2 to 13.6)	-7.8 (-17.1 to 2.1)	0.5 (0.5 to 0.6)	0.5 (0.5 to 0.6)	0.5 (0.4 to 0.6)	-1.0 (-12.2 to 12.3)	-7.5 (-16.9 to 2.4)
Discontinued breastfeeding	2.1 (2.0 to 2.2)	2.1 (2.0 to 2.3)	2.1 (2.0 to 2.3)	1.6 (-6.0 to 10.0)	-0.0 (-6.2 to 6.8)	2.1 (2.0 to 2.2)	2.1 (2.0 to 2.3)	2.2 (2.0 to 2.3)	2.4 (-5.2 to 10.9)	0.6 (-5.6 to 7.4)
Child underweight	0.1 (0.1 to 0.1)	0.1 (0.1 to 0.1)	0.1 (0.0 to 0.1)	-4.8 (-20.0 to 12.1)	-14.6 (-23.5 to -5.2)	0.1 (0.0 to 0.1)	0.1 (0.0 to 0.1)	0.1 (0.0 to 0.1)	-2.3 (-17.8 to 15.4)	-5.5 (-15.4 to 5.1)
Child wasting	0.1 (0.0 to 0.1)	0.1 (0.0 to 0.1)	0.1 (0.0 to 0.1)	-10.2 (-29.3 to 12.1)	-4.2 (-16.7 to 9.3)	0.1 (0.0 to 0.1)	0.1 (0.0 to 0.1)	0.1 (0.0 to 0.1)	6.7 (-16.4 to 36.8)	-6.2 (-20.5 to 11.7)
Child stunting	0.3 (0.1 to 0.5)	0.3 (0.1 to 0.5)	0.2 (0.1 to 0.4)	-6.8 (-53.2 to 81.0)	-12.8 (-43.6 to 30.4)	0.2 (0.1 to 0.4)	0.2 (0.1 to 0.3)	0.2 (0.1 to 0.3)	-10.4 (-48.8 to 48.8)	-4.9 (-30.6 to 35.7)
Short gestation for birth weight	0.0 (0.0 to 0.0)	0.0 (0.0 to 0.0)	0.0 (0.0 to 0.0)	5.8 (-18.0 to 35.8)	4.1 (-17.1 to 38.9)	0.0 (0.0 to 0.0)	0.0 (0.0 to 0.0)	0.0 (0.0 to 0.0)	8.2 (-19.4 to 41.8)	1.3 (-17.0 to 30.2)
Low birth weight for gestation	0.0 (0.0 to 0.0)	0.0 (0.0 to 0.0)	0.0 (0.0 to 0.0)	3.9 (-18.2 to 35.4)	-2.2 (-23.0 to 18.9)	0.0 (0.0 to 0.0)	0.0 (0.0 to 0.0)	0.0 (0.0 to 0.0)	0.6 (-19.1 to 35.0)	0.2 (-19.9 to 18.8)
Iron deficiency	-	-	-	-	-	4.5 (3.0 to 6.5)	4.3 (2.8 to 6.1)	4.3 (2.8 to 6.2)	-6.1 (-32.0 to 30.1)	1.8 (-20.8 to 27.6)
Vitamin A deficiency	0.6 (0.4 to 0.8)	0.5 (0.3 to 0.6)	0.6 (0.4 to 0.8)	-17.2 (-29.7 to -2.9)	25.4 (4.8 to 52.5)	0.6 (0.4 to 0.8)	0.7 (0.4 to 0.9)	0.7 (0.4 to 0.9)	21.1 (1.9 to 44.4)	-4.5 (-24.5 to 16.7)
Zinc deficiency	0.5 (0.1 to 1.2)	0.2 (0.0 to 1.0)	0.2 (0.0 to 0.9)	-56.3 (-88.9 to 306.0)	2.6 (-87.1 to 836.3)	0.5 (0.1 to 1.1)	0.2 (0.0 to 1.0)	0.2 (0.0 to 0.9)	-57.8 (-88.4 to 307.6)	6.8 (-87.9 to 867.9)
Smoking	12.8 (10.6 to 15.2)	7.0 (5.6 to 8.5)	5.6 (4.4 to 7.0)	-45.3 (-53.7 to -36.8)	-20.3 (-29.2 to -9.9)	9.0 (7.1 to 11.2)	5.5 (4.3 to 6.9)	4.1 (3.2 to 5.2)	-38.1 (-49.7 to -24.2)	-25.8 (-33.8 to -16.7)
Chewing tobacco	2.5 (2.1 to 3.0)	3.0 (2.2 to 4.1)	2.9 (1.9 to 4.4)	19.4 (-16.5 to 71.8)	-2.6 (-30.5 to 30.6)	0.1 (0.1 to 0.2)	0.1 (0.1 to 0.2)	0.1 (0.1 to 0.1)	-3.5 (-46.0 to 64.6)	-3.5 (-34.7 to 42.8)
Second-hand smoke	40.6 (37.3 to 43.8)	25.2 (23.7 to 26.7)	26.4 (23.7 to 29.5)	-38.0 (-44.8 to -30.6)	5.0 (-5.8 to 16.6)	37.4 (34.8 to 40.3)	23.7 (22.6 to 24.9)	24.3 (22.5 to 26.3)	-36.6 (-43.4 to -29.8)	2.5 (-5.8 to 11.8)
Alcohol use	18.4 (9.8 to 29.0)	19.6 (11.0 to 29.8)	21.0 (10.8 to 33.1)	6.2 (-31.6 to 68.5)	7.1 (-27.7 to 59.6)	13.4 (6.7 to 22.4)	13.7 (7.3 to 22.8)	14.1 (7.1 to 24.5)	2.8 (-33.7 to 57.9)	2.7 (-30.3 to 49.8)
Drug use	1.3 (0.9 to 2.0)	1.5 (1.1 to 2.2)	2.4 (1.8 to 3.3)	15.8 (4.7 to 31.0)	61.4 (42.8 to 88.8)	1.6 (1.2 to 2.1)	2.3 (1.9 to 3.0)	2.7 (2.2 to 3.5)	48.0 (30.5 to 69.4)	16.0 (3.8 to 28.9)
Diet low in fruits	29.7 (23.8 to 34.4)	30.8 (24.9 to 35.4)	33.4 (27.3 to 38.2)	3.6 (-5.2 to 13.3)	8.5 (0.5 to 18.1)	26.1 (19.8 to 31.4)	28.6 (22.6 to 33.4)	30.5 (24.1 to 36.0)	9.3 (-0.4 to 22.7)	6.9 (-3.3 to 18.2)
Diet low in vegetables	23.7 (15.3 to 30.6)	34.2 (28.6 to 39.1)	38.0 (32.8 to 42.1)	44.4 (21.2 to 93.4)	11.1 (3.7 to 20.7)	24.9 (16.9 to 31.6)	33.6 (27.4 to 38.5)	37.4 (32.3 to 41.5)	34.8 (14.8 to 71.6)	11.5 (3.4 to 22.3)
Diet low in legumes	24.1 (17.1 to 30.1)	24.2 (17.0 to 30.1)	25.6 (18.7 to 31.4)	0.5 (-14.7 to 19.8)	5.8 (-7.4 to 22.0)	28.6 (22.4 to 33.8)	29.3 (23.3 to 34.3)	30.6 (25.0 to 35.7)	2.3 (-9.6 to 16.6)	4.5 (-4.7 to 15.4)
Diet low in whole grains	38.7 (35.4 to 41.7)	39.9 (36.8 to 42.6)	39.5 (36.2 to 42.3)	2.9 (-1.7 to 8.5)	-0.9 (-4.5 to 2.7)	39.7 (36.4 to 42.5)	40.8 (38.0 to 43.3)	40.5 (37.4 to 43.1)	2.8 (-1.5 to 8.4)	-0.8 (-4.2 to 2.6)
Diet low in nuts and seeds	47.3 (44.7 to 49.3)	38.8 (34.4 to 42.5)	39.7 (35.0 to 43.6)	-17.9 (-25.0 to -12.1)	2.3 (-4.4 to 9.4)	48.0 (45.6 to 49.8)	40.0 (35.2 to 43.3)	40.7 (35.7 to 44.2)	-16.7 (-24.1 to -11.5)	1.7 (-4.6 to 7.7)
Diet low in milk	28.9 (22.9 to 34.1)	27.3 (20.9 to 32.4)	28.0 (21.6 to 33.2)	-5.3 (-14.7 to 4.6)	2.3 (-5.6 to 11.7)	30.5 (24.8 to 35.3)	28.3 (21.9 to 33.4)	28.8 (22.5 to 33.8)	-7.2 (-16.4 to 1.2)	1.6 (-6.0 to 10.4)
Diet high in red meat	47.9 (43.4 to 51.4)	43.3 (33.4 to 49.8)	40.3 (29.5 to 48.3)	-9.6 (-25.6 to 4.9)	-7.1 (-21.4 to 8.1)	26.4 (20.2 to 31.6)	25.5 (15.6 to 33.9)	23.4 (13.8 to 32.9)	-3.3 (-32.8 to 33.3)	-8.3 (-34.9 to 20.9)
Diet high in processed meat	43.6 (37.1 to 48.2)	51.0 (46.8 to 53.6)	46.8 (39.4 to 51.7)	17.0 (4.6 to 32.8)	-8.2 (-17.9 to -0.4)	22.2 (17.5 to 26.2)	34.9 (27.9 to 40.5)	32.2 (24.0 to 39.0)	56.8 (25.8 to 96.5)	-7.7 (-21.3 to 6.5)
Diet high in sugar-sweetened beverages	28.8 (14.8 to 38.8)	27.0 (13.1 to 37.7)	22.0 (10.4 to 32.4)	-6.2 (-37.4 to 44.1)	-18.7 (-43.8 to 11.7)	24.0 (11.3 to 35.6)	20.7 (9.8 to 30.3)	16.8 (7.5 to 26.6)	-13.4 (-48.0 to 42.3)	-18.9 (-47.2 to 15.8)
Diet low in fibre	33.9 (28.3 to 38.5)	26.1 (19.1 to 31.9)	27.7 (20.4 to 33.6)	-23.2 (-36.1 to -12.6)	6.2 (-5.5 to 19.2)	38.5 (34.0 to 42.3)	30.7 (24.8 to 35.8)	32.1 (26.2 to 37.1)	-20.4 (-30.7 to -12.0)	4.4 (-3.4 to 14.1)
Diet low in calcium	18.6 (11.2 to 25.1)	16.8 (9.8 to 23.1)	17.8 (10.7 to 24.2)	-9.7 (-23.4 to 1.4)	5.8 (-4.2 to 19.4)	22.7 (15.9 to 28.2)	20.8 (13.6 to 26.6)	21.7 (14.5 to 27.4)	-8.5 (-19.5 to 1.2)	4.5 (-2.8 to 15.4)
Diet low in seafood omega-3 fatty acids	31.0 (25.7 to 35.9)	26.8 (20.9 to 32.1)	28.6 (23.1 to 33.6)	-13.5 (-24.1 to -3.9)	6.4 (-1.9 to 17.9)	34.3 (29.3 to 38.6)	31.0 (25.4 to 35.5)	32.2 (26.7 to 36.7)	-9.8 (-17.8 to -2.1)	3.9 (-2.5 to 11.8)
Diet low in polyunsaturated fatty acids	9.7 (3.4 to 16.6)	6.4 (1.4 to 12.7)	5.0 (0.9 to 10.8)	-34.4 (-74.2 to 1.4)	-21.5 (-62.6 to 27.8)	10.6 (4.0 to 18.1)	7.0 (1.6 to 13.9)	5.6 (1.2 to 11.9)	-33.5 (-70.9 to 4.8)	-19.9 (-58.7 to 42.5)
Diet high in trans fatty acids	16.7 (12.3 to 22.1)	9.8 (5.9 to 14.1)	7.1 (3.6 to 11.1)	-41.4 (-56.2 to -28.6)	-27.1 (-42.9 to -14.8)	23.7 (17.8 to 31.7)	13.3 (9.0 to 18.2)	9.7 (5.7 to 13.9)	-43.8 (-55.4 to -32.3)	-26.9 (-40.5 to -16.2)
Diet high in sodium	6.9 (0.5 to 14.3)	12.2 (3.4 to 19.9)	12.7 (3.7 to 20.3)	76.5 (20.0 to 799.5)	3.5 (-25.3 to 52.3)	6.0 (0.3 to 13.1)	10.8 (2.5 to 18.5)	11.3 (2.8 to 19.0)	80.2 (27.3 to 1023.6)	4.2 (-19.9 to 59.8)
Intimate partner violence	-	-	-	-	-	0.4 (0.2 to 0.7)	0.4 (0.2 to 0.5)	0.4 (0.2 to 0.6)	-19.8 (-34.2 to 8.3)	14.3 (-9.1 to 36.0)
Childhood sexual abuse	5.6 (4.6 to 6.9)	5.1 (4.1 to 6.2)	5.1 (4.1 to 6.2)	-9.8 (-15.1 to -4.3)	0.4 (-4.7 to 6.0)	13.8 (11.0 to 16.9)	12.1 (9.8 to 14.7)	12.2 (9.9 to 14.8)	-11.9 (-17.2 to -6.0)	0.4 (-4.8 to 6.5)

Supplementary Results Table 1. Idaho age-standardised Summary Exposure Values (SEVs) for Level 4 risk factors and percentage change, by sex, 1990, 2007, and 2017.Data in parentheses are 95% uncertainty intervals.

	Male					Female				
	1990	2007	2017	Percentage change 1990-2007	Percentage change 2007-2017	1990	2007	2017	Percentage change 1990-2007	Percentage change 2007-2017
Bullying victimization	6.2<br>(2.6 to 12.9)	8.9<br>(3.8 to 17.9)	8.7<br>(3.7 to 17.6)	43.3<br>(28.5 to 62.3)	-2.1<br>(-9.3 to 5.5)	4.7<br>(2.0 to 9.4)	7.5<br>(3.2 to 15.3)	7.5<br>(3.2 to 15.5)	60.7<br>(42.6 to 85.8)	0.0<br>(-8.0 to 9.1)
Low physical activity	0.4<br>(0.2 to 0.7)	0.4<br>(0.2 to 0.8)	0.4<br>(0.2 to 0.8)	0.6<br>(-1.7 to 2.8)	1.7<br>(0.2 to 3.6)	0.4<br>(0.2 to 0.8)	0.4<br>(0.2 to 0.8)	0.4<br>(0.2 to 0.8)	0.2<br>(-1.7 to 2.5)	0.7<br>(-0.6 to 2.2)
High fasting plasma glucose	1.1<br>(0.7 to 1.8)	2.2<br>(1.3 to 3.4)	3.0<br>(1.9 to 4.6)	91.4<br>(15.7 to 213.7)	37.5<br>(-11.5 to 118.7)	1.4<br>(0.8 to 2.3)	2.6<br>(1.4 to 4.1)	2.3<br>(1.2 to 3.8)	84.9<br>(8.1 to 221.8)	-9.1<br>(-50.8 to 53.3)
High LDL cholesterol	20.9<br>(18.9 to 23.1)	11.4<br>(9.8 to 13.1)	10.1<br>(8.7 to 11.9)	-45.5<br>(-50.2 to -40.5)	-10.8<br>(-15.1 to -6.4)	19.7<br>(17.8 to 21.7)	11.4<br>(9.8 to 13.1)	10.6<br>(9.1 to 12.3)	-42.4<br>(-47.0 to -37.9)	-7.1<br>(-11.2 to -2.8)
High systolic blood pressure	6.1<br>(5.3 to 7.0)	4.6<br>(3.9 to 5.3)	4.7<br>(4.0 to 5.5)	-25.4<br>(-32.6 to -17.3)	3.6<br>(-6.2 to 13.2)	6.0<br>(5.2 to 6.8)	4.3<br>(3.7 to 4.8)	4.1<br>(3.5 to 4.8)	-28.5<br>(-36.6 to -20.4)	-3.0<br>(-12.7 to 7.6)
High body-mass index	12.6<br>(8.9 to 17.5)	19.3<br>(14.7 to 25.2)	20.5<br>(15.8 to 26.5)	53.9<br>(35.8 to 77.7)	6.1<br>(-2.0 to 15.6)	14.9<br>(11.1 to 19.6)	21.6<br>(17.2 to 27.2)	22.8<br>(18.1 to 28.4)	44.9<br>(25.9 to 68.3)	5.7<br>(-3.3 to 15.9)
Low bone mineral density	5.2<br>(4.3 to 6.2)	4.4<br>(3.7 to 5.2)	4.7<br>(3.8 to 5.6)	-14.3<br>(-25.1 to -0.7)	5.9<br>(-9.9 to 23.2)	5.8<br>(4.8 to 6.9)	5.7<br>(4.8 to 6.7)	6.2<br>(5.2 to 7.3)	-1.7<br>(-13.6 to 11.7)	9.1<br>(-5.0 to 22.8)
Impaired kidney function	2.2<br>(1.1 to 5.6)	2.2<br>(1.1 to 5.8)	2.3<br>(1.1 to 5.9)	1.4<br>(-4.3 to 7.3)	2.4<br>(-3.0 to 8.6)	2.9<br>(1.4 to 6.7)	2.9<br>(1.4 to 6.8)	3.0<br>(1.4 to 6.9)	-0.6<br>(-6.3 to 5.1)	4.8<br>(0.5 to 10.1)

Supplementary Results Table 1. Illinois age-standardised Summary Exposure Values (SEVs) for Level 4 risk factors and percentage change, by sex, 1990, 2007, and 2017.Data in parentheses are 95% uncertainty intervals.	Male					Female				
	1990	2007	2017	Percentage change 1990-2007	Percentage change 2007-2017	1990	2007	2017	Percentage change 1990-2007	Percentage change 2007-2017
Unsafe water source	1.4 (0.8 to 2.4)	0.5 (0.3 to 0.8)	0.3 (0.2 to 0.5)	-67.6 (-83.5 to -38.7)	-34.6 (-58.4 to 5.3)	1.4 (0.8 to 2.4)	0.5 (0.3 to 0.8)	0.3 (0.2 to 0.5)	-67.6 (-83.5 to -38.7)	-34.6 (-58.4 to 5.3)
Unsafe sanitation	6.4 (3.3 to 11.1)	3.6 (1.6 to 6.7)	2.6 (1.2 to 5.0)	-43.7 (-74.7 to 20.5)	-28.4 (-62.1 to 36.1)	6.4 (3.3 to 11.1)	3.6 (1.6 to 6.7)	2.6 (1.2 to 5.0)	-43.7 (-74.7 to 20.5)	-28.4 (-62.1 to 36.1)
No access to handwashing facility	3.4 (2.4 to 4.7)	3.1 (2.3 to 4.3)	3.0 (2.2 to 4.1)	-8.1 (-35.0 to 32.5)	-3.5 (-26.0 to 25.6)	3.4 (2.4 to 4.7)	3.1 (2.3 to 4.3)	3.0 (2.2 to 4.1)	-8.1 (-35.0 to 32.5)	-3.5 (-26.0 to 25.6)
Ambient particulate matter pollution	29.0 (19.7 to 42.5)	28.6 (20.3 to 42.4)	22.8 (14.4 to 35.2)	-1.1 (-20.0 to 23.9)	-20.4 (-40.7 to -0.5)	28.9 (19.7 to 42.3)	28.6 (20.3 to 42.3)	22.8 (14.4 to 35.1)	-1.0 (-19.9 to 24.5)	-20.4 (-40.6 to -0.5)
Household air pollution from solid fuels	0.2 (0.1 to 0.5)	0.1 (0.0 to 0.4)	0.1 (0.0 to 0.3)	-34.6 (-78.6 to 53.6)	-9.1 (-63.0 to 98.4)	0.4 (0.1 to 1.7)	0.2 (0.0 to 1.1)	0.2 (0.0 to 1.0)	-31.3 (-78.3 to 51.8)	-7.8 (-62.7 to 97.8)
Ambient ozone pollution	46.9 (18.1 to 54.8)	46.9 (18.1 to 54.8)	47.7 (18.7 to 54.8)	-0.2 (-0.6 to 0.0)	1.8 (0.0 to 4.8)	46.9 (18.1 to 54.8)	46.9 (18.1 to 54.8)	47.7 (18.7 to 54.8)	-0.2 (-0.6 to 0.0)	1.8 (0.0 to 4.8)
Residential radon	17.0 (1.4 to 54.9)	17.0 (1.4 to 54.9)	17.0 (1.4 to 54.9)	0.0 (0.0 to 0.0)	0.0 (0.0 to 0.0)	17.0 (1.4 to 54.9)	17.0 (1.4 to 54.9)	17.0 (1.4 to 54.9)	0.0 (0.0 to 0.0)	0.0 (0.0 to 0.0)
Lead exposure	11.1 (6.2 to 15.5)	7.0 (3.3 to 10.9)	4.6 (2.0 to 7.7)	-37.5 (-48.5 to -28.4)	-34.3 (-41.9 to -28.2)	7.3 (3.0 to 11.5)	4.6 (1.5 to 8.5)	3.1 (0.9 to 6.1)	-36.0 (-50.9 to -24.7)	-33.6 (-43.8 to -27.4)
Occupational exposure to asbestos	7.1 (6.0 to 8.0)	5.6 (5.3 to 5.9)	4.7 (4.0 to 5.5)	-21.0 (-31.2 to -5.1)	-15.7 (-28.9 to -0.5)	1.1 (1.0 to 1.3)	1.1 (1.0 to 1.2)	1.0 (0.8 to 1.2)	-2.6 (-15.4 to 11.4)	-3.3 (-21.1 to 16.3)
Occupational exposure to arsenic	0.4 (0.0 to 1.0)	0.4 (0.0 to 0.9)	0.4 (0.0 to 1.0)	-	-	0.4 (0.0 to 0.9)	0.4 (0.0 to 0.8)	0.3 (0.0 to 0.8)	-	-
Occupational exposure to benzene	0.6 (0.0 to 1.8)	0.6 (0.0 to 1.8)	0.6 (0.0 to 1.7)	0.8 (-6.5 to 12.5)	-3.0 (-9.1 to 4.3)	0.9 (0.1 to 2.5)	1.0 (0.1 to 2.9)	1.0 (0.1 to 2.9)	14.3 (4.0 to 31.3)	-0.7 (-7.9 to 9.6)
Occupational exposure to beryllium	0.0 (0.0 to 0.0)	0.0 (0.0 to 0.0)	0.0 (0.0 to 0.0)	-0.5 (-5.8 to 5.0)	0.4 (-3.8 to 4.8)	0.0 (0.0 to 0.0)	0.0 (0.0 to 0.0)	0.0 (0.0 to 0.0)	5.6 (-2.1 to 14.3)	-1.0 (-6.4 to 4.8)
Occupational exposure to cadmium	0.0 (0.0 to 0.1)	0.0 (0.0 to 0.0)	0.0 (0.0 to 0.1)	-3.6 (-13.9 to 7.9)	0.5 (-8.4 to 9.8)	0.0 (0.0 to 0.0)	0.0 (0.0 to 0.0)	0.0 (0.0 to 0.0)	0.8 (-13.1 to 17.2)	-2.4 (-13.1 to 10.6)
Occupational exposure to chromium	0.1 (0.1 to 0.1)	0.1 (0.1 to 0.1)	0.1 (0.1 to 0.1)	-1.2 (-10.1 to 9.3)	0.4 (-7.0 to 8.3)	0.1 (0.1 to 0.1)	0.1 (0.1 to 0.1)	0.1 (0.1 to 0.1)	3.5 (-8.4 to 16.6)	-2.5 (-11.5 to 7.7)
Occupational exposure to diesel engine exhaust	0.5 (0.5 to 0.5)	0.5 (0.5 to 0.6)	0.6 (0.5 to 0.6)	9.9 (1.1 to 19.1)	1.6 (-4.9 to 7.9)	0.5 (0.4 to 0.5)	0.5 (0.5 to 0.6)	0.5 (0.4 to 0.6)	6.8 (-4.7 to 20.9)	-4.0 (-12.7 to 5.1)
Occupational exposure to formaldehyde	0.2 (0.2 to 0.3)	0.2 (0.2 to 0.3)	0.2 (0.2 to 0.3)	-5.8 (-16.0 to 6.2)	-1.1 (-10.1 to 8.2)	0.2 (0.2 to 0.2)	0.2 (0.2 to 0.2)	0.2 (0.2 to 0.2)	2.2 (-11.2 to 16.5)	-2.6 (-12.4 to 9.3)
Occupational exposure to nickel	0.3 (0.0 to 1.5)	0.3 (0.0 to 1.4)	0.3 (0.0 to 1.4)	-	-	0.3 (0.0 to 1.2)	0.3 (0.0 to 1.2)	0.3 (0.0 to 1.2)	-	-
Occupational exposure to polycyclic aromatic hydrocarbons	0.2 (0.2 to 0.2)	0.2 (0.2 to 0.2)	0.2 (0.2 to 0.2)	-0.8 (-9.6 to 9.3)	-0.2 (-7.3 to 7.0)	0.2 (0.2 to 0.2)	0.2 (0.2 to 0.3)	0.2 (0.2 to 0.3)	6.9 (-4.2 to 18.6)	-2.1 (-9.7 to 7.1)
Occupational exposure to silica	3.1 (0.5 to 9.9)	2.8 (0.4 to 8.6)	2.8 (0.4 to 8.7)	-11.5 (-21.9 to 1.5)	1.0 (-8.8 to 13.0)	2.4 (0.3 to 7.5)	2.2 (0.3 to 7.2)	2.1 (0.3 to 7.0)	-7.3 (-22.8 to 14.8)	-3.1 (-17.3 to 16.2)
Occupational exposure to sulphuric acid	0.6 (0.1 to 2.0)	0.7 (0.1 to 2.1)	0.7 (0.1 to 2.1)	7.7 (-3.8 to 20.3)	-1.3 (-8.9 to 7.6)	0.6 (0.1 to 1.8)	0.7 (0.1 to 2.0)	0.6 (0.1 to 2.1)	17.7 (0.8 to 36.8)	-3.3 (-15.2 to 10.7)
Occupational exposure to trichloroethylene	0.1 (0.1 to 0.1)	0.1 (0.1 to 0.1)	0.1 (0.1 to 0.1)	3.0 (-3.9 to 10.8)	0.9 (-4.8 to 6.6)	0.1 (0.1 to 0.1)	0.1 (0.1 to 0.1)	0.1 (0.1 to 0.1)	4.6 (-5.4 to 16.3)	-2.8 (-10.7 to 5.8)
Occupational asthmagens	17.3 (14.5 to 21.0)	15.4 (13.0 to 18.6)	15.0 (12.5 to 18.2)	-10.8 (-21.6 to 1.9)	-2.6 (-10.8 to 6.7)	8.2 (6.7 to 9.9)	8.3 (6.8 to 10.1)	7.8 (6.2 to 9.7)	1.1 (-12.3 to 16.9)	-6.3 (-16.8 to 5.1)
Occupational particulate matter, gases, and fumes	7.1 (5.1 to 10.0)	6.6 (4.8 to 9.3)	6.2 (4.5 to 9.0)	-7.0 (-11.5 to -2.6)	-5.2 (-8.7 to -1.8)	3.9 (2.8 to 5.8)	3.7 (2.7 to 5.4)	3.5 (2.5 to 5.2)	-4.9 (-11.8 to 2.3)	-5.3 (-10.7 to 0.3)
Occupational noise	6.2 (5.6 to 6.8)	6.0 (5.5 to 6.5)	5.7 (5.3 to 6.3)	-3.4 (-6.5 to -0.2)	-4.0 (-6.2 to -1.7)	4.3 (3.8 to 4.9)	4.5 (4.0 to 5.0)	4.5 (4.0 to 5.0)	4.1 (-0.8 to 9.9)	-0.5 (-3.6 to 2.9)
Occupational ergonomic factors	8.5 (7.1 to 10.4)	7.8 (6.4 to 9.5)	7.4 (6.1 to 9.0)	-8.6 (-17.6 to 2.0)	-4.4 (-10.8 to 3.0)	4.8 (3.8 to 6.2)	5.0 (4.0 to 6.4)	4.9 (3.9 to 6.4)	3.8 (-10.7 to 21.4)	-1.8 (-12.5 to 10.9)
Non-exclusive breastfeeding	0.5 (0.5 to 0.6)	0.5 (0.5 to 0.6)	0.5 (0.4 to 0.6)	-0.5 (-11.5 to 12.0)	-7.2 (-16.3 to 2.4)	0.5 (0.5 to 0.6)	0.5 (0.5 to 0.6)	0.5 (0.4 to 0.6)	-0.5 (-11.4 to 12.0)	-7.0 (-16.2 to 2.6)
Discontinued breastfeeding	2.2 (2.1 to 2.3)	2.1 (2.0 to 2.2)	2.2 (2.1 to 2.3)	-3.3 (-10.0 to 4.4)	3.1 (-2.5 to 9.5)	2.2 (2.1 to 2.3)	2.1 (2.0 to 2.2)	2.2 (2.1 to 2.3)	-3.2 (-9.8 to 4.6)	2.4 (-3.1 to 8.7)
Child underweight	0.1 (0.1 to 0.1)	0.1 (0.1 to 0.1)	0.1 (0.0 to 0.1)	-5.9 (-18.4 to 9.1)	-13.3 (-21.8 to -4.3)	0.1 (0.0 to 0.1)	0.1 (0.0 to 0.1)	0.1 (0.0 to 0.1)	-3.6 (-19.8 to 15.4)	-4.4 (-14.5 to 8.1)
Child wasting	0.1 (0.0 to 0.1)	0.1 (0.0 to 0.1)	0.1 (0.0 to 0.1)	-10.5 (-29.3 to 8.4)	-3.8 (-17.8 to 9.9)	0.1 (0.0 to 0.1)	0.1 (0.0 to 0.1)	0.1 (0.0 to 0.1)	5.4 (-16.2 to 31.8)	-5.4 (-18.9 to 10.0)
Child stunting	0.3 (0.1 to 0.5)	0.2 (0.1 to 0.4)	0.2 (0.1 to 0.4)	-11.4 (-50.2 to 54.4)	-11.5 (-38.4 to 23.5)	0.2 (0.1 to 0.4)	0.2 (0.1 to 0.3)	0.2 (0.1 to 0.3)	-11.0 (-43.0 to 41.1)	-3.9 (-29.7 to 30.8)
Short gestation for birth weight	0.0 (0.0 to 0.0)	0.0 (0.0 to 0.0)	0.0 (0.0 to 0.0)	5.6 (-18.1 to 34.8)	2.6 (-18.4 to 28.9)	0.0 (0.0 to 0.0)	0.0 (0.0 to 0.0)	0.0 (0.0 to 0.0)	6.1 (-29.3 to 45.5)	4.1 (-22.1 to 50.9)
Low birth weight for gestation	0.0 (0.0 to 0.0)	0.0 (0.0 to 0.0)	0.0 (0.0 to 0.0)	3.7 (-18.4 to 31.7)	-1.3 (-20.0 to 21.3)	0.0 (0.0 to 0.0)	0.0 (0.0 to 0.0)	0.0 (0.0 to 0.0)	2.0 (-22.7 to 45.6)	-2.8 (-29.9 to 24.9)
Iron deficiency	-	-	-	-	-	4.0 (2.6 to 5.9)	3.8 (2.4 to 5.4)	3.9 (2.5 to 5.7)	-7.1 (-31.6 to 28.5)	3.3 (-20.6 to 32.2)
Vitamin A deficiency	0.5 (0.3 to 0.7)	0.4 (0.3 to 0.6)	0.5 (0.3 to 0.6)	-7.7 (-24.2 to 19.8)	2.4 (-12.8 to 24.3)	0.5 (0.3 to 0.7)	0.6 (0.4 to 0.8)	0.5 (0.3 to 0.6)	16.3 (-5.3 to 39.2)	-19.9 (-35.7 to -6.4)
Zinc deficiency	0.5 (0.1 to 1.1)	0.2 (0.0 to 1.1)	0.2 (0.0 to 1.0)	-55.0 (-87.8 to 334.0)	-1.8 (-89.6 to 822.5)	0.5 (0.1 to 1.1)	0.2 (0.0 to 1.0)	0.2 (0.0 to 1.0)	-55.4 (-88.4 to 357.7)	4.7 (-88.7 to 907.6)
Smoking	13.9 (11.5 to 16.3)	7.6 (6.2 to 9.3)	6.0 (4.6 to 7.4)	-45.4 (-53.7 to -36.7)	-21.7 (-30.0 to -12.2)	10.5 (8.3 to 13.0)	5.6 (4.5 to 6.9)	4.0 (3.1 to 5.1)	-46.3 (-55.3 to -35.0)	-28.3 (-36.4 to -19.8)
Chewing tobacco	1.2 (0.9 to 1.5)	1.4 (0.9 to 1.9)	1.4 (0.9 to 2.1)	16.6 (-22.5 to 73.4)	4.1 (-25.7 to 43.7)	0.1 (0.1 to 0.1)	0.1 (0.1 to 0.1)	0.1 (0.1 to 0.1)	-10.1 (-43.9 to 46.8)	2.8 (-29.0 to 45.7)
Second-hand smoke	46.2 (42.5 to 50.2)	26.3 (24.7 to 27.9)	27.6 (24.7 to 30.5)	-43.0 (-50.0 to -35.9)	4.8 (-5.5 to 16.2)	43.7 (40.5 to 47.4)	25.5 (24.3 to 26.7)	26.2 (24.3 to 28.2)	-41.7 (-48.0 to -35.3)	2.8 (-5.8 to 11.8)
Alcohol use	21.0 (11.1 to 33.0)	22.8 (12.1 to 35.1)	25.1 (13.2 to 39.4)	8.8 (-29.0 to 69.9)	10.2 (-22.9 to 61.6)	12.8 (6.5 to 20.6)	13.5 (7.0 to 21.8)	14.1 (7.0 to 23.5)	5.5 (-29.8 to 57.3)	4.6 (-27.4 to 53.3)
Drug use	1.7 (1.2 to 2.5)	2.0 (1.5 to 2.8)	2.5 (1.9 to 3.4)	18.6 (7.3 to 33.4)	23.9 (13.0 to 36.9)	1.2 (0.9 to 1.7)	1.5 (1.2 to 2.0)	1.7 (1.3 to 2.3)	23.1 (12.1 to 38.9)	16.3 (6.5 to 26.8)
Diet low in fruits	30.8 (25.0 to 35.2)	32.4 (26.6 to 36.6)	33.8 (27.8 to 38.5)	5.0 (-2.1 to 13.9)	4.6 (-3.0 to 12.1)	27.2 (21.1 to 32.4)	28.8 (22.6 to 33.7)	29.9 (23.2 to 35.2)	5.9 (-2.8 to 17.0)	3.6 (-6.0 to 13.2)
Diet low in vegetables	29.3 (22.1 to 35.0)	37.7 (32.8 to 41.6)	40.2 (36.0 to 43.9)	28.4 (14.2 to 55.4)	6.8 (0.8 to 13.8)	31.4 (25.0 to 36.5)	38.1 (33.3 to 42.1)	41.0 (36.6 to 44.7)	21.4 (9.9 to 40.0)	7.6 (1.9 to 14.9)
Diet low in legumes	23.9 (16.8 to 29.7)	24.2 (17.0 to 29.9)	25.6 (18.5 to 31.2)	0.9 (-15.1 to 20.3)	5.9 (-6.5 to 22.4)	28.6 (22.6 to 33.6)	29.2 (23.1 to 34.1)	30.6 (24.5 to 35.3)	2.1 (-10.4 to 15.5)	4.7 (-4.8 to 15.7)
Diet low in whole grains	37.9 (34.4 to 40.8)	39.1 (35.9 to 42.0)	38.7 (35.1 to 41.7)	3.3 (-1.7 to 8.8)	-1.1 (-5.0 to 2.8)	38.8 (35.4 to 41.6)	40.1 (37.0 to 42.7)	39.7 (36.4 to 42.4)	3.3 (-1.5 to 8.8)	-0.9 (-4.8 to 2.5)
Diet low in nuts and seeds	44.9 (41.7 to 47.5)	35.5 (30.2 to 39.7)	36.1 (30.2 to 40.6)	-20.9 (-30.3 to -14.2)	1.5 (-7.2 to 10.1)	45.8 (42.8 to 48.2)	36.5 (31.0 to 40.6)	37.0 (31.0 to 41.6)	-20.3 (-29.3 to -13.6)	1.3 (-6.3 to 9.5)
Diet low in milk	26.9 (20.3 to 32.3)	25.5 (18.8 to 31.3)	26.1 (19.0 to 32.0)	-5.3 (-16.1 to 4.6)	2.1 (-6.9 to 11.8)	28.6 (22.7 to 33.6)	26.5 (20.0 to 32.0)	26.9 (20.5 to 32.3)	-7.4 (-17.5 to 2.1)	1.4 (-6.4 to 11.1)
Diet high in red meat	49.0 (45.0 to 51.9)	42.8 (33.6 to 49.3)	40.7 (30.0 to 48.7)	-12.7 (-27.4 to 0.6)	-4.9 (-20.2 to 10.0)	25.7 (20.0 to 31.3)	23.6 (13.7 to 32.1)	22.6 (12.9 to 31.8)	-8.1 (-39.0 to 26.4)	-4.4 (-29.4 to 27.9)
Diet high in processed meat	45.5 (39.1 to 49.7)	51.9 (47.9 to 54.1)	48.2 (41.4 to 52.5)	14.2 (3.9 to 26.9)	-7.2 (-15.7 to -0.9)	23.9 (19.0 to 28.1)	36.7 (29.8 to 42.6)	34.4 (27.0 to 40.9)	53.9 (26.8 to 89.3)	-6.4 (-19.1 to 6.6)
Diet high in sugar-sweetened beverages	32.7 (15.8 to 42.7)	31.2 (15.8 to 41.9)	26.0 (13.2 to 36.6)	-4.6 (-32.8 to 33.9)	-16.5 (-39.6 to 8.9)	27.5 (13.0 to 38.7)	24.3 (11.6 to 34.7)	19.9 (8.8 to 30.4)	-11.6 (-44.4 to 40.7)	-17.9 (-44.6 to 16.4)
Diet low in fibre	32.7 (26.6 to 37.6)	24.9 (18.1 to 30.7)	26.5 (19.7 to 32.5)	-24.0 (-37.4 to -14.3)	6.6 (-3.6 to 19.0)	37.6 (32.7 to 41.7)	29.6 (23.4 to 35.0)	30.9 (24.6 to 36.1)	-21.2 (-31.6 to -12.4)	4.5 (-4.5 to 14.1)
Diet low in calcium	15.6 (8.6 to 21.8)	14.4 (8.1 to 20.5)	15.2 (8.6 to 21.6)	-7.9 (-21.1 to 8.1)	5.9 (-6.4 to 20.9)	19.7 (12.3 to 25.7)	18.1 (11.0 to 24.3)	19.0 (11.6 to 25.1)	-8.2 (-21.0 to 3.4)	5.0 (-3.6 to 16.8)
Diet low in seafood omega-3 fatty acids	27.3 (21.4 to 32.7)	23.2 (16.8 to 29.0)	24.8 (18.9 to 30.4)	-15.0 (-27.2 to -3.9)	6.7 (-2.9 to 19.8)	30.9 (25.6 to 35.6)	27.6 (21.9 to 32.6)	28.9 (23.2 to 33.9)	-10.9 (-21.3 to -2.1)	4.7 (-3.4 to 14.4)
Diet low in polyunsaturated fatty acids	7.4 (2.1 to 14.2)	4.6 (0.7 to 10.1)	3.5 (0.4 to 8.6)	-38.5 (-82.2 to 4.8)	-23.9 (-69.6 to 64.8)	8.2 (2.5 to 14.9)	5.2 (1.0 to 11.3)	4.0 (0.6 to 9.2)	-36.1 (-76.4 to 5.7)	-24.4 (-66.9 to 39.2)
Diet high in trans fatty acids	16.8 (12.1 to 21.7)	9.9 (6.0 to 14.1)	7.1 (3.4 to 10.9)	-41.2 (-55.5 to -29.4)	-27.8 (-44.1 to -17.2)	23.7 (18.0 to 32.3)	13.3 (9.1 to 18.0)	9.8 (5.8 to 14.1)	-44.0 (-54.4 to -31.7)	-26.4 (-40.1 to -16.2)
Diet high in sodium	5.6 (0.2 to 12.7)	7.6 (0.7 to 14.8)	7.6 (0.7 to 15.1)	34.8 (-5.7 to 487.3)	0.6 (-42.8 to 93.2)	3.6 (0.0 to 10.1)	4.5 (0.1 to 11.2)	4.4 (0.1 to 11.1)	24.6 (-17.7 to 1418.4)	-2.5 (-57.6 to 144.6)
Intimate partner violence	-	-	-	-	-	1.4 (1.2 to 1.6)	0.9 (0.8 to 1.1)	1.2 (1.0 to 1.4)	-31.7 (-35.5 to -27.9)	24.2 (17.6 to 30.9)
Childhood sexual abuse	5.6 (4.6 to 6.9)	5.1 (4.1 to 6.2)	5.1 (4.1 to 6.2)	-9.8 (-15.1 to -4.3)	0.4 (-4.7 to 6.0)	13.8 (11.0 to 16.9)	12.1 (9.8 to 14.7)	12.2 (9.9 to 14.8)	-11.9 (-17.2 to -6.0)	0.4 (-4.8 to 6.5)

Supplementary Results Table 1. Illinois age-standardised Summary Exposure Values (SEVs) for Level 4 risk factors and percentage change, by sex, 1990, 2007, and 2017.Data in parentheses are 95% uncertainty intervals.										
	**Male**					**Female**				
	**1990**	**2007**	**2017**	**Percentage change 1990-2007**	**Percentage change 2007-2017**	**1990**	**2007**	**2017**	**Percentage change 1990-2007**	**Percentage change 2007-2017**
Bullying victimization	6.1 (2.5 to 12.5)	8.9 (3.7 to 18.0)	8.7 (3.7 to 17.7)	46.8 (30.8 to 67.0)	-2.6 (-10.0 to 4.7)	4.6 (1.9 to 9.5)	7.6 (3.2 to 16.2)	7.5 (3.2 to 15.5)	63.8 (22.2 to 123.5)	-0.4 (-26.1 to 32.0)
Low physical activity	0.4 (0.2 to 0.7)	0.4 (0.2 to 0.7)	0.4 (0.2 to 0.7)	0.7 (-1.3 to 2.7)	1.6 (0.2 to 3.4)	0.4 (0.2 to 0.8)	0.4 (0.2 to 0.8)	0.4 (0.2 to 0.8)	0.4 (-1.8 to 2.5)	2.0 (0.4 to 3.8)
High fasting plasma glucose	1.3 (0.8 to 2.0)	2.3 (1.4 to 3.4)	3.1 (1.9 to 4.7)	78.3 (5.8 to 189.9)	37.4 (-12.7 to 117.6)	1.6 (0.9 to 2.6)	2.8 (1.6 to 4.4)	2.7 (1.4 to 4.4)	82.0 (3.2 to 227.9)	-6.5 (-45.5 to 64.8)
High LDL cholesterol	21.0 (18.8 to 23.1)	11.6 (10.0 to 13.4)	10.3 (8.8 to 12.0)	-44.7 (-49.6 to -40.1)	-11.7 (-16.1 to -7.5)	19.9 (18.0 to 21.9)	11.5 (9.9 to 13.3)	10.6 (9.1 to 12.4)	-42.3 (-47.0 to -38.0)	-7.6 (-11.8 to -3.6)
High systolic blood pressure	6.5 (5.7 to 7.4)	4.8 (4.1 to 5.5)	4.9 (4.2 to 5.6)	-26.4 (-33.9 to -18.4)	1.9 (-6.1 to 10.6)	6.3 (5.6 to 7.1)	4.4 (3.9 to 4.9)	4.2 (3.6 to 4.9)	-30.7 (-38.2 to -22.2)	-4.5 (-14.0 to 5.6)
High body-mass index	15.0 (11.1 to 20.5)	21.6 (16.9 to 27.7)	22.8 (18.0 to 28.6)	43.7 (26.4 to 64.5)	5.4 (-3.5 to 14.4)	17.0 (13.2 to 22.2)	22.8 (18.5 to 28.2)	24.0 (19.3 to 29.4)	34.2 (18.2 to 52.8)	4.9 (-4.0 to 15.4)
Low bone mineral density	5.2 (4.3 to 6.1)	4.4 (3.7 to 5.2)	4.6 (3.8 to 5.6)	-14.8 (-26.0 to -1.4)	5.3 (-9.5 to 22.2)	5.8 (4.8 to 6.9)	5.7 (4.8 to 6.6)	6.2 (5.1 to 7.3)	-2.0 (-14.7 to 11.9)	9.1 (-3.4 to 26.5)
Impaired kidney function	2.3 (1.1 to 5.9)	2.3 (1.1 to 5.9)	2.3 (1.1 to 5.9)	-0.7 (-6.8 to 5.2)	1.0 (-5.3 to 7.5)	3.0 (1.5 to 6.9)	2.9 (1.4 to 6.9)	3.0 (1.5 to 7.1)	-2.1 (-8.2 to 3.3)	3.3 (-2.0 to 9.1)

Supplementary Results Table 1. Indiana age-standardised Summary Exposure Values (SEVs) for Level 4 risk factors and percentage change, by sex, 1990, 2007, and 2017.Data in parentheses are 95% uncertainty intervals.

	Male					Female				
	**1990**	**2007**	**2017**	**Percentage change 1990-2007**	**Percentage change 2007-2017**	**1990**	**2007**	**2017**	**Percentage change 1990-2007**	**Percentage change 2007-2017**
Unsafe water source	1.6 (0.9 to 2.7)	0.6 (0.3 to 0.9)	0.3 (0.2 to 0.6)	-64.0 (-81.2 to -26.3)	-38.6 (-62.2 to 5.2)	1.6 (0.9 to 2.7)	0.6 (0.3 to 0.9)	0.3 (0.2 to 0.6)	-64.0 (-81.2 to -26.3)	-38.6 (-62.2 to 5.2)
Unsafe sanitation	7.5 (4.0 to 12.8)	5.2 (2.3 to 9.8)	3.5 (1.5 to 6.6)	-31.3 (-68.8 to 40.1)	-32.6 (-65.5 to 28.3)	7.5 (4.0 to 12.8)	5.2 (2.3 to 9.8)	3.5 (1.5 to 6.6)	-31.3 (-68.8 to 40.1)	-32.6 (-65.5 to 28.3)
No access to handwashing facility	3.5 (2.5 to 4.6)	3.2 (2.2 to 4.3)	3.0 (2.2 to 4.1)	-9.0 (-35.6 to 28.6)	-3.4 (-25.3 to 24.6)	3.5 (2.5 to 4.6)	3.2 (2.2 to 4.3)	3.0 (2.2 to 4.1)	-9.0 (-35.6 to 28.6)	-3.4 (-25.3 to 24.6)
Ambient particulate matter pollution	29.3 (19.9 to 45.7)	29.3 (20.2 to 45.0)	23.2 (15.1 to 37.4)	-0.2 (-22.9 to 28.4)	-20.6 (-42.1 to 1.0)	29.2 (19.7 to 45.7)	29.2 (20.2 to 45.0)	23.2 (15.0 to 37.3)	-0.0 (-22.9 to 28.7)	-20.6 (-42.0 to 1.1)
Household air pollution from solid fuels	0.3 (0.1 to 0.8)	0.2 (0.1 to 0.5)	0.2 (0.0 to 0.5)	-32.7 (-75.8 to 57.2)	-11.7 (-61.7 to 86.1)	0.5 (0.1 to 3.3)	0.3 (0.1 to 1.7)	0.3 (0.1 to 1.6)	-30.5 (-75.7 to 58.9)	-7.9 (-61.8 to 91.0)
Ambient ozone pollution	49.0 (20.3 to 54.8)	48.8 (19.9 to 54.8)	49.3 (20.5 to 54.8)	-0.5 (-1.8 to 0.0)	0.9 (0.0 to 3.3)	49.0 (20.3 to 54.8)	48.8 (19.9 to 54.8)	49.3 (20.5 to 54.8)	-0.5 (-1.8 to 0.0)	0.9 (0.0 to 3.3)
Residential radon	15.8 (1.2 to 54.9)	15.8 (1.2 to 54.9)	15.8 (1.2 to 54.9)	0.0 (0.0 to 0.0)	0.0 (0.0 to 0.0)	15.8 (1.2 to 54.9)	15.8 (1.2 to 54.9)	15.8 (1.2 to 54.9)	0.0 (0.0 to 0.0)	0.0 (0.0 to 0.0)
Lead exposure	8.1 (3.6 to 12.3)	5.3 (2.0 to 9.2)	3.6 (1.2 to 6.8)	-34.3 (-45.9 to -25.0)	-31.8 (-41.3 to -25.3)	4.8 (1.3 to 8.9)	3.2 (0.7 to 6.7)	2.2 (0.4 to 5.1)	-32.6 (-48.1 to -22.4)	-30.9 (-43.2 to -24.4)
Occupational exposure to asbestos	5.9 (5.2 to 6.7)	5.2 (4.8 to 5.6)	4.8 (4.0 to 5.6)	-13.3 (-24.8 to 2.2)	-7.1 (-23.4 to 10.1)	0.9 (0.8 to 1.0)	0.9 (0.8 to 1.0)	1.0 (0.8 to 1.2)	0.6 (-11.4 to 14.8)	7.7 (-12.2 to 31.7)
Occupational exposure to arsenic	0.5 (0.0 to 1.1)	0.5 (0.0 to 1.1)	0.4 (0.0 to 1.1)	-	-	0.4 (0.0 to 0.9)	0.4 (0.0 to 0.9)	0.4 (0.0 to 0.9)	-	-
Occupational exposure to benzene	0.6 (0.0 to 1.7)	0.6 (0.0 to 1.7)	0.6 (0.0 to 1.7)	0.6 (-6.1 to 11.6)	-2.4 (-8.0 to 5.9)	0.8 (0.0 to 2.3)	0.9 (0.1 to 2.5)	0.9 (0.1 to 2.7)	10.8 (0.4 to 26.4)	0.7 (-6.9 to 11.9)
Occupational exposure to beryllium	0.0 (0.0 to 0.0)	0.0 (0.0 to 0.0)	0.0 (0.0 to 0.0)	-0.4 (-5.5 to 6.0)	-0.1 (-4.8 to 4.3)	0.0 (0.0 to 0.0)	0.0 (0.0 to 0.0)	0.0 (0.0 to 0.0)	4.7 (-3.9 to 13.3)	-0.7 (-6.4 to 5.4)
Occupational exposure to cadmium	0.1 (0.0 to 0.1)	0.1 (0.0 to 0.1)	0.1 (0.0 to 0.1)	-3.0 (-12.2 to 8.5)	-1.8 (-10.4 to 6.5)	0.0 (0.0 to 0.1)	0.0 (0.0 to 0.1)	0.0 (0.0 to 0.1)	2.0 (-11.8 to 19.5)	-3.1 (-14.2 to 8.3)
Occupational exposure to chromium	0.1 (0.1 to 0.1)	0.1 (0.1 to 0.1)	0.1 (0.1 to 0.1)	-0.9 (-9.3 to 9.5)	-1.4 (-8.9 to 5.6)	0.1 (0.1 to 0.1)	0.1 (0.1 to 0.1)	0.1 (0.1 to 0.1)	4.2 (-8.0 to 19.0)	-3.0 (-12.3 to 6.9)
Occupational exposure to diesel engine exhaust	0.5 (0.4 to 0.5)	0.5 (0.5 to 0.6)	0.5 (0.5 to 0.6)	10.3 (0.6 to 20.0)	2.8 (-4.6 to 10.0)	0.5 (0.4 to 0.5)	0.5 (0.4 to 0.6)	0.5 (0.4 to 0.5)	8.8 (-3.8 to 24.0)	-4.1 (-13.2 to 5.5)
Occupational exposure to formaldehyde	0.3 (0.3 to 0.3)	0.3 (0.2 to 0.3)	0.3 (0.2 to 0.3)	-5.1 (-14.4 to 6.2)	-3.3 (-11.9 to 5.2)	0.2 (0.2 to 0.2)	0.2 (0.2 to 0.3)	0.2 (0.2 to 0.3)	2.5 (-11.1 to 18.6)	-3.0 (-13.3 to 8.0)
Occupational exposure to nickel	0.4 (0.0 to 1.8)	0.4 (0.0 to 1.7)	0.4 (0.0 to 1.6)	-	-	0.3 (0.0 to 1.3)	0.3 (0.0 to 1.3)	0.3 (0.0 to 1.3)	-	-
Occupational exposure to polycyclic aromatic hydrocarbons	0.2 (0.2 to 0.3)	0.2 (0.2 to 0.3)	0.2 (0.2 to 0.3)	-0.7 (-8.8 to 9.0)	-1.8 (-9.0 to 4.7)	0.2 (0.2 to 0.3)	0.2 (0.2 to 0.3)	0.2 (0.2 to 0.3)	6.7 (-4.9 to 20.0)	-2.4 (-10.6 to 6.3)
Occupational exposure to silica	3.6 (0.5 to 11.2)	3.2 (0.5 to 10.3)	3.2 (0.5 to 10.2)	-8.6 (-18.2 to 2.9)	-1.9 (-10.8 to 7.6)	2.5 (0.3 to 8.1)	2.4 (0.3 to 7.6)	2.3 (0.3 to 7.3)	-4.7 (-19.8 to 16.8)	-3.7 (-17.7 to 12.4)
Occupational exposure to sulphuric acid	0.7 (0.1 to 2.2)	0.7 (0.2 to 2.3)	0.7 (0.2 to 2.3)	6.0 (-4.1 to 17.8)	-2.5 (-10.5 to 5.5)	0.6 (0.1 to 2.0)	0.7 (0.2 to 2.3)	0.7 (0.1 to 2.2)	18.4 (0.8 to 39.3)	-4.4 (-15.3 to 8.3)
Occupational exposure to trichloroethylene	0.1 (0.1 to 0.1)	0.1 (0.1 to 0.1)	0.1 (0.1 to 0.1)	2.7 (-4.2 to 11.2)	-0.0 (-5.6 to 5.5)	0.1 (0.1 to 0.1)	0.1 (0.1 to 0.1)	0.1 (0.1 to 0.1)	5.6 (-5.0 to 18.2)	-3.1 (-10.7 to 5.3)
Occupational asthmagens	19.6 (16.1 to 24.0)	17.5 (14.7 to 21.2)	16.7 (13.8 to 20.4)	-10.8 (-22.5 to 2.6)	-4.3 (-13.3 to 5.6)	8.1 (6.6 to 10.0)	8.3 (6.8 to 10.2)	7.8 (6.3 to 9.6)	2.4 (-10.7 to 17.8)	-6.2 (-15.9 to 4.5)
Occupational particulate matter, gases, and fumes	7.9 (5.6 to 11.1)	7.5 (5.3 to 10.6)	7.1 (5.1 to 10.0)	-5.3 (-9.6 to -1.1)	-5.4 (-9.1 to -2.1)	4.1 (2.8 to 5.9)	4.0 (2.8 to 5.8)	3.8 (2.7 to 5.5)	-1.4 (-8.8 to 6.4)	-4.4 (-10.1 to 1.4)
Occupational noise	6.5 (6.0 to 7.2)	6.3 (5.8 to 6.9)	6.1 (5.6 to 6.6)	-2.9 (-5.8 to 0.3)	-4.0 (-6.1 to -1.7)	4.3 (3.7 to 4.9)	4.5 (4.0 to 5.0)	4.5 (4.0 to 5.0)	4.9 (-0.5 to 11.2)	-0.5 (-3.9 to 2.9)
Occupational ergonomic factors	8.8 (7.2 to 10.7)	8.0 (6.6 to 9.7)	7.6 (6.3 to 9.3)	-9.2 (-18.8 to 1.2)	-4.4 (-10.9 to 2.7)	4.9 (3.9 to 6.1)	5.0 (4.0 to 6.1)	4.9 (3.9 to 6.2)	1.3 (-14.2 to 19.2)	-1.4 (-12.5 to 10.7)
Non-exclusive breastfeeding	0.5 (0.5 to 0.6)	0.5 (0.5 to 0.6)	0.5 (0.4 to 0.6)	-0.6 (-11.6 to 12.6)	-7.3 (-16.9 to 2.7)	0.5 (0.5 to 0.6)	0.5 (0.5 to 0.6)	0.5 (0.4 to 0.6)	-0.8 (-11.8 to 12.4)	-7.1 (-16.7 to 2.9)
Discontinued breastfeeding	2.2 (2.0 to 2.3)	2.1 (2.0 to 2.2)	2.1 (2.0 to 2.3)	-4.0 (-11.5 to 4.1)	2.8 (-3.3 to 9.8)	2.2 (2.0 to 2.3)	2.1 (2.0 to 2.2)	2.1 (2.0 to 2.3)	-3.3 (-10.9 to 4.8)	2.5 (-3.6 to 9.5)
Child underweight	0.1 (0.1 to 0.1)	0.1 (0.1 to 0.1)	0.1 (0.0 to 0.1)	-3.4 (-18.8 to 13.3)	-14.2 (-23.0 to -5.1)	0.1 (0.0 to 0.1)	0.1 (0.0 to 0.1)	0.1 (0.0 to 0.1)	-1.1 (-17.2 to 17.2)	-5.4 (-14.9 to 6.2)
Child wasting	0.1 (0.0 to 0.1)	0.1 (0.0 to 0.1)	0.1 (0.0 to 0.1)	-9.9 (-29.0 to 12.8)	-4.0 (-17.6 to 10.1)	0.1 (0.0 to 0.1)	0.1 (0.0 to 0.1)	0.1 (0.0 to 0.1)	7.4 (-15.6 to 40.0)	-5.6 (-20.3 to 11.1)
Child stunting	0.3 (0.1 to 0.5)	0.3 (0.1 to 0.4)	0.2 (0.1 to 0.4)	-8.6 (-51.1 to 71.3)	-12.2 (-40.4 to 29.8)	0.2 (0.1 to 0.4)	0.2 (0.1 to 0.3)	0.2 (0.1 to 0.3)	-7.9 (-43.4 to 51.1)	-4.4 (-31.2 to 30.4)
Short gestation for birth weight	0.0 (0.0 to 0.0)	0.0 (0.0 to 0.0)	0.0 (0.0 to 0.0)	6.2 (-10.8 to 32.1)	0.9 (-18.0 to 26.0)	0.0 (0.0 to 0.0)	0.0 (0.0 to 0.0)	0.0 (0.0 to 0.0)	5.7 (-16.9 to 29.3)	5.7 (-18.4 to 54.2)
Low birth weight for gestation	0.0 (0.0 to 0.0)	0.0 (0.0 to 0.0)	0.0 (0.0 to 0.0)	2.9 (-13.4 to 23.7)	-1.0 (-20.3 to 15.6)	0.0 (0.0 to 0.0)	0.0 (0.0 to 0.0)	0.0 (0.0 to 0.0)	1.3 (-15.1 to 28.6)	-3.3 (-30.6 to 21.0)
Iron deficiency	-	-	-	-	-	4.3 (2.8 to 6.4)	4.3 (2.8 to 6.1)	4.3 (2.8 to 6.3)	-1.8 (-25.4 to 34.6)	0.5 (-22.7 to 29.0)
Vitamin A deficiency	0.5 (0.3 to 0.6)	0.5 (0.3 to 0.7)	0.6 (0.4 to 0.8)	11.0 (-10.2 to 43.3)	12.9 (-6.0 to 38.1)	0.6 (0.4 to 0.9)	0.6 (0.4 to 0.8)	0.6 (0.4 to 0.9)	-7.0 (-22.9 to 17.0)	6.8 (-8.3 to 27.5)
Zinc deficiency	0.5 (0.1 to 1.1)	0.2 (0.0 to 1.1)	0.2 (0.0 to 1.0)	-55.4 (-88.0 to 194.6)	3.0 (-88.3 to 839.0)	0.5 (0.1 to 1.1)	0.2 (0.0 to 1.0)	0.2 (0.0 to 0.9)	-55.1 (-88.0 to 204.9)	-1.7 (-88.9 to 714.3)
Smoking	14.3 (11.8 to 16.7)	8.3 (6.7 to 10.1)	6.4 (5.0 to 8.0)	-42.0 (-50.4 to -33.0)	-22.3 (-30.8 to -12.3)	9.6 (7.6 to 11.9)	5.8 (4.5 to 7.2)	4.5 (3.5 to 5.7)	-39.9 (-50.7 to -26.4)	-21.5 (-30.4 to -11.0)
Chewing tobacco	2.6 (2.2 to 3.0)	2.1 (1.6 to 2.8)	2.1 (1.4 to 3.0)	-17.6 (-40.0 to 12.3)	-2.0 (-25.9 to 31.4)	0.1 (0.1 to 0.2)	0.1 (0.1 to 0.1)	0.1 (0.1 to 0.1)	-20.0 (-48.9 to 32.2)	-4.4 (-32.5 to 39.7)
Second-hand smoke	44.9 (41.5 to 48.6)	30.3 (28.6 to 31.7)	29.1 (26.4 to 32.2)	-32.6 (-39.8 to -25.0)	-3.7 (-12.4 to 6.1)	42.4 (39.6 to 45.4)	29.6 (28.4 to 30.7)	27.8 (25.8 to 29.7)	-30.3 (-36.9 to -23.5)	-6.1 (-12.9 to 0.6)
Alcohol use	20.1 (10.8 to 32.0)	21.4 (11.5 to 33.4)	22.9 (11.9 to 36.8)	6.2 (-30.8 to 74.0)	7.3 (-28.7 to 58.2)	13.0 (6.7 to 21.8)	13.4 (7.1 to 22.3)	13.7 (6.9 to 23.7)	3.6 (-31.0 to 56.8)	2.4 (-28.7 to 47.4)
Drug use	1.4 (1.0 to 2.1)	1.9 (1.5 to 2.6)	2.7 (2.1 to 3.6)	37.8 (17.8 to 64.9)	40.1 (26.3 to 58.1)	1.3 (1.0 to 1.7)	1.8 (1.5 to 2.3)	2.3 (1.8 to 2.9)	39.8 (24.8 to 60.1)	25.7 (12.0 to 38.8)
Diet low in fruits	30.0 (24.0 to 34.7)	30.1 (24.3 to 34.8)	32.0 (25.8 to 37.1)	0.4 (-8.2 to 10.4)	6.3 (-2.4 to 15.9)	26.5 (20.3 to 31.7)	27.0 (21.1 to 32.1)	27.9 (21.4 to 33.4)	2.1 (-8.6 to 14.3)	3.0 (-8.3 to 15.1)
Diet low in vegetables	26.0 (18.2 to 32.2)	33.7 (28.1 to 38.7)	36.3 (30.8 to 40.6)	29.4 (11.4 to 64.8)	7.8 (0.1 to 17.5)	27.5 (20.3 to 33.7)	34.1 (27.8 to 38.6)	37.2 (31.9 to 41.4)	23.8 (8.4 to 49.3)	9.0 (1.6 to 19.3)
Diet low in legumes	24.0 (16.7 to 29.9)	24.2 (17.8 to 30.1)	25.6 (19.0 to 31.3)	0.9 (-14.6 to 20.7)	5.7 (-6.6 to 21.8)	28.6 (22.5 to 33.9)	29.3 (23.7 to 34.3)	30.7 (25.1 to 35.9)	2.5 (-9.0 to 16.5)	4.7 (-4.2 to 15.4)
Diet low in whole grains	38.4 (35.0 to 41.4)	39.7 (36.5 to 42.5)	39.2 (35.9 to 42.1)	3.3 (-1.4 to 8.9)	-1.1 (-4.8 to 2.3)	39.4 (36.1 to 42.2)	40.6 (37.5 to 43.0)	40.2 (37.1 to 42.8)	3.1 (-1.3 to 8.3)	-0.9 (-4.1 to 2.9)
Diet low in nuts and seeds	46.4 (43.3 to 48.8)	38.0 (33.3 to 41.9)	38.6 (33.6 to 42.7)	-18.1 (-25.7 to -12.4)	1.5 (-5.5 to 8.3)	47.2 (44.5 to 49.3)	39.1 (34.7 to 42.8)	39.6 (34.6 to 43.4)	-17.2 (-24.5 to -11.1)	1.3 (-6.0 to 8.2)
Diet low in milk	28.1 (21.7 to 33.2)	26.8 (20.4 to 32.1)	27.3 (20.9 to 32.7)	-4.4 (-14.8 to 6.3)	1.8 (-5.7 to 11.4)	29.9 (23.9 to 34.7)	27.9 (21.5 to 33.2)	28.2 (22.1 to 33.4)	-6.5 (-16.4 to 1.8)	0.9 (-6.5 to 9.4)
Diet high in red meat	40.8 (30.1 to 48.9)	34.7 (22.9 to 43.7)	33.2 (21.6 to 41.9)	-15.0 (-35.3 to 6.6)	-4.3 (-23.5 to 20.3)	22.3 (12.4 to 32.2)	16.0 (7.7 to 25.2)	15.0 (6.5 to 24.0)	-28.3 (-56.2 to 11.2)	-6.5 (-42.3 to 41.6)
Diet high in processed meat	38.2 (29.9 to 44.9)	34.5 (27.0 to 40.8)	27.4 (20.0 to 33.8)	-9.7 (-26.3 to 9.0)	-20.7 (-32.9 to -7.2)	20.5 (14.9 to 26.5)	19.4 (14.2 to 24.5)	16.8 (11.4 to 21.8)	-5.5 (-25.4 to 18.8)	-13.4 (-28.3 to 2.6)
Diet high in sugar-sweetened beverages	30.3 (14.6 to 40.9)	28.0 (14.4 to 38.7)	23.0 (11.8 to 33.4)	-7.6 (-37.7 to 34.4)	-17.6 (-42.5 to 12.9)	25.1 (12.2 to 35.7)	21.6 (10.2 to 32.1)	17.7 (8.5 to 28.6)	-13.9 (-47.5 to 35.4)	-18.0 (-46.0 to 20.7)
Diet low in fibre	33.5 (27.6 to 38.2)	25.7 (18.9 to 31.5)	27.3 (20.4 to 33.2)	-23.2 (-35.9 to -12.7)	6.2 (-4.2 to 17.9)	38.1 (33.2 to 42.0)	30.4 (24.3 to 35.7)	31.6 (25.6 to 36.8)	-20.4 (-31.1 to -12.2)	4.1 (-4.2 to 13.8)
Diet low in calcium	17.5 (10.2 to 23.8)	16.2 (9.2 to 22.4)	16.9 (9.6 to 23.3)	-7.3 (-22.1 to 6.3)	4.4 (-7.1 to 17.1)	21.5 (14.0 to 27.1)	20.0 (12.7 to 26.1)	20.8 (13.7 to 26.9)	-6.7 (-19.4 to 3.3)	3.9 (-4.4 to 14.9)
Diet low in seafood omega-3 fatty acids	29.7 (23.7 to 34.5)	25.9 (19.7 to 31.3)	27.4 (21.7 to 32.8)	-12.8 (-24.7 to -2.7)	5.9 (-2.0 to 17.2)	33.1 (28.1 to 37.5)	30.1 (24.2 to 34.8)	31.1 (25.2 to 36.0)	-9.2 (-19.4 to -0.8)	3.4 (-3.5 to 11.1)
Diet low in polyunsaturated fatty acids	8.7 (3.0 to 15.8)	5.9 (1.1 to 12.0)	4.5 (0.6 to 9.9)	-32.2 (-74.9 to 14.1)	-24.0 (-67.9 to 38.4)	9.5 (3.6 to 16.7)	6.4 (1.3 to 12.8)	4.9 (0.9 to 10.9)	-32.7 (-73.5 to 4.2)	-24.4 (-62.4 to 31.8)
Diet high in trans fatty acids	16.9 (12.0 to 22.1)	9.8 (5.8 to 13.9)	7.1 (3.5 to 11.2)	-41.8 (-56.3 to -29.2)	-27.3 (-44.0 to -16.6)	23.7 (18.1 to 32.3)	13.3 (8.9 to 18.2)	9.7 (5.8 to 14.0)	-44.1 (-55.6 to -31.5)	-26.9 (-40.3 to -16.5)
Diet high in sodium	7.1 (0.6 to 14.6)	12.4 (3.7 to 20.2)	12.9 (4.0 to 20.9)	73.7 (19.7 to 637.9)	4.3 (-22.1 to 57.6)	6.0 (0.3 to 13.2)	10.9 (2.7 to 18.4)	11.3 (2.8 to 18.8)	80.1 (26.7 to 1104.6)	3.9 (-21.2 to 55.3)
Intimate partner violence	-	-	-	-	-	1.3 (1.1 to 1.5)	1.0 (0.8 to 1.1)	1.2 (1.0 to 1.4)	-27.6 (-31.4 to -23.0)	26.6 (19.3 to 33.5)
Childhood sexual abuse	5.6 (4.6 to 6.9)	5.1 (4.1 to 6.2)	5.1 (4.1 to 6.2)	-9.8 (-15.1 to -4.3)	0.4 (-4.7 to 6.0)	13.8 (11.0 to 16.9)	12.1 (9.8 to 14.7)	12.2 (9.9 to 14.8)	-11.9 (-17.2 to -6.0)	0.4 (-4.8 to 6.5)

Supplementary Results Table 1. Indiana age-standardised Summary Exposure Values (SEVs) for Level 4 risk factors and percentage change, by sex, 1990, 2007, and 2017.Data in parentheses are 95% uncertainty intervals.										
	**Male**					**Female**				
	**1990**	**2007**	**2017**	**Percentage change 1990-2007**	**Percentage change 2007-2017**	**1990**	**2007**	**2017**	**Percentage change 1990-2007**	**Percentage change 2007-2017**
Bullying victimization	2.8 (1.0 to 6.2)	4.1 (1.5 to 8.8)	4.3 (1.6 to 9.4)	46.1 (27.2 to 77.5)	4.0 (-6.8 to 13.3)	2.9 (1.1 to 6.2)	3.4 (1.3 to 7.6)	3.4 (1.3 to 7.5)	19.5 (5.0 to 39.4)	-0.6 (-10.3 to 8.3)
Low physical activity	0.4 (0.2 to 0.8)	0.4 (0.2 to 0.8)	0.4 (0.2 to 0.7)	0.3 (-1.7 to 2.6)	-1.8 (-3.4 to -0.2)	0.4 (0.2 to 0.8)	0.4 (0.2 to 0.8)	0.4 (0.2 to 0.8)	-0.5 (-3.2 to 1.9)	-2.5 (-4.1 to -0.9)
High fasting plasma glucose	1.4 (0.8 to 2.4)	3.0 (1.9 to 4.5)	3.4 (2.1 to 5.0)	113.6 (22.1 to 302.4)	10.6 (-27.2 to 71.5)	1.7 (1.0 to 2.9)	3.4 (2.0 to 5.5)	3.4 (1.9 to 5.4)	101.2 (17.0 to 283.4)	1.0 (-40.0 to 65.9)
High LDL cholesterol	21.1 (19.2 to 23.2)	11.5 (9.9 to 13.2)	10.2 (8.6 to 11.9)	-45.7 (-50.4 to -40.9)	-11.4 (-15.6 to -7.2)	20.2 (18.4 to 22.3)	11.5 (10.0 to 13.2)	10.6 (9.1 to 12.3)	-43.1 (-48.0 to -38.5)	-7.5 (-11.9 to -3.2)
High systolic blood pressure	6.3 (5.5 to 7.2)	4.7 (4.1 to 5.4)	4.9 (4.1 to 5.6)	-25.3 (-32.7 to -17.6)	2.9 (-5.5 to 12.3)	6.2 (5.4 to 7.1)	4.4 (3.9 to 4.9)	4.2 (3.6 to 4.9)	-29.2 (-36.9 to -20.1)	-4.8 (-14.6 to 5.8)
High body-mass index	14.2 (10.5 to 19.4)	20.6 (16.2 to 26.2)	21.6 (17.0 to 27.2)	44.9 (27.7 to 66.7)	4.5 (-3.3 to 13.1)	16.0 (12.2 to 20.6)	23.2 (18.7 to 29.2)	24.2 (19.4 to 30.4)	44.9 (28.3 to 67.1)	4.6 (-4.5 to 15.5)
Low bone mineral density	5.1 (4.3 to 6.1)	4.4 (3.7 to 5.3)	4.7 (3.8 to 5.7)	-13.8 (-24.6 to -2.0)	5.7 (-8.9 to 22.4)	5.8 (4.8 to 6.9)	5.7 (4.8 to 6.7)	6.2 (5.1 to 7.4)	-1.6 (-13.3 to 13.0)	9.2 (-5.4 to 25.9)
Impaired kidney function	2.4 (1.1 to 5.9)	2.4 (1.2 to 6.1)	2.5 (1.2 to 6.0)	1.5 (-4.3 to 7.4)	2.1 (-3.0 to 8.3)	3.1 (1.5 to 7.1)	3.1 (1.5 to 7.1)	3.2 (1.5 to 7.3)	-0.5 (-5.7 to 4.8)	4.3 (-0.6 to 10.3)

Supplementary Results Table 1. Iowa age-standardised Summary Exposure Values (SEVs) for Level 4 risk factors and percentage change, by sex, 1990, 2007, and 2017.Data in parentheses are 95% uncertainty intervals.										
	**Male**					**Female**				
	**1990**	**2007**	**2017**	**Percentage change 1990-2007**	**Percentage change 2007-2017**	**1990**	**2007**	**2017**	**Percentage change 1990-2007**	**Percentage change 2007-2017**
Unsafe water source	1.4 (0.8 to 2.3)	0.5 (0.3 to 0.8)	0.3 (0.2 to 0.5)	-63.1 (-80.8 to -29.6)	-37.6 (-60.7 to 1.1)	1.4 (0.8 to 2.3)	0.5 (0.3 to 0.8)	0.3 (0.2 to 0.5)	-63.1 (-80.8 to -29.6)	-37.6 (-60.7 to 1.1)
Unsafe sanitation	6.0 (3.1 to 10.3)	4.1 (1.8 to 7.8)	2.8 (1.2 to 5.5)	-32.5 (-71.2 to 44.3)	-31.3 (-66.2 to 34.4)	6.0 (3.1 to 10.3)	4.1 (1.8 to 7.8)	2.8 (1.2 to 5.5)	-32.5 (-71.2 to 44.3)	-31.3 (-66.2 to 34.4)
No access to handwashing facility	3.4 (2.5 to 4.5)	3.2 (2.3 to 4.4)	3.0 (2.2 to 4.2)	-8.0 (-34.7 to 26.8)	-4.0 (-24.8 to 24.9)	3.4 (2.5 to 4.5)	3.2 (2.3 to 4.4)	3.0 (2.2 to 4.2)	-8.0 (-34.7 to 26.8)	-4.0 (-24.8 to 24.9)
Ambient particulate matter pollution	25.2 (16.4 to 39.3)	25.1 (17.0 to 37.9)	19.0 (10.8 to 30.9)	-0.4 (-22.7 to 34.7)	-24.5 (-57.7 to 2.5)	25.1 (16.3 to 39.1)	25.0 (17.0 to 37.8)	18.9 (10.8 to 30.9)	-0.2 (-22.5 to 34.9)	-24.5 (-57.7 to 2.5)
Household air pollution from solid fuels	0.4 (0.1 to 1.0)	0.2 (0.1 to 0.6)	0.2 (0.0 to 0.6)	-39.7 (-78.0 to 38.1)	-17.1 (-71.3 to 72.5)	0.6 (0.1 to 4.0)	0.4 (0.1 to 2.0)	0.3 (0.0 to 1.6)	-37.1 (-77.8 to 37.9)	-15.0 (-70.8 to 73.9)
Ambient ozone pollution	43.0 (15.5 to 54.8)	43.1 (15.5 to 54.8)	44.0 (16.1 to 54.8)	0.3 (0.0 to 0.8)	2.1 (0.0 to 4.3)	43.0 (15.5 to 54.8)	43.1 (15.5 to 54.8)	44.0 (16.1 to 54.8)	0.3 (0.0 to 0.8)	2.1 (0.0 to 4.3)
Residential radon	17.7 (1.7 to 59.1)	17.7 (1.7 to 59.1)	17.7 (1.7 to 59.1)	0.0 (0.0 to 0.0)	0.0 (0.0 to 0.0)	17.7 (1.7 to 59.1)	17.7 (1.7 to 59.1)	17.7 (1.7 to 59.1)	0.0 (0.0 to 0.0)	0.0 (0.0 to 0.0)
Lead exposure	5.2 (1.6 to 9.3)	3.6 (0.9 to 7.1)	2.5 (0.6 to 5.4)	-31.8 (-44.4 to -22.4)	-30.7 (-42.0 to -23.6)	3.0 (0.4 to 6.7)	2.1 (0.2 to 5.3)	1.5 (0.1 to 4.1)	-28.9 (-43.7 to -19.4)	-28.7 (-43.3 to -21.9)
Occupational exposure to asbestos	4.6 (3.9 to 5.2)	3.8 (3.5 to 4.2)	3.7 (3.1 to 4.4)	-16.7 (-29.1 to 0.6)	-3.4 (-20.2 to 16.0)	0.8 (0.7 to 0.8)	0.8 (0.8 to 0.9)	0.9 (0.8 to 1.1)	12.8 (-0.9 to 29.6)	11.9 (-8.5 to 36.8)
Occupational exposure to arsenic	0.4 (0.0 to 0.9)	0.4 (0.0 to 1.0)	0.4 (0.0 to 0.9)	-	-	0.3 (0.0 to 0.8)	0.3 (0.0 to 0.8)	0.3 (0.0 to 0.8)	-	-
Occupational exposure to benzene	0.6 (0.0 to 1.6)	0.6 (0.0 to 1.7)	0.6 (0.0 to 1.7)	2.5 (-5.1 to 14.4)	-2.3 (-8.5 to 5.6)	0.8 (0.0 to 2.4)	0.9 (0.1 to 2.6)	0.9 (0.1 to 2.7)	11.6 (0.9 to 27.7)	0.7 (-7.1 to 12.1)
Occupational exposure to beryllium	0.0 (0.0 to 0.0)	0.0 (0.0 to 0.0)	0.0 (0.0 to 0.0)	0.8 (-4.8 to 6.8)	0.2 (-4.6 to 5.0)	0.0 (0.0 to 0.0)	0.0 (0.0 to 0.0)	0.0 (0.0 to 0.0)	5.8 (-2.2 to 14.6)	-0.3 (-6.8 to 6.2)
Occupational exposure to cadmium	0.0 (0.0 to 0.0)	0.0 (0.0 to 0.1)	0.0 (0.0 to 0.1)	2.9 (-7.8 to 15.0)	-0.6 (-10.2 to 9.1)	0.0 (0.0 to 0.0)	0.0 (0.0 to 0.0)	0.0 (0.0 to 0.0)	5.9 (-8.4 to 22.6)	-2.1 (-13.1 to 11.1)
Occupational exposure to chromium	0.1 (0.1 to 0.1)	0.1 (0.1 to 0.1)	0.1 (0.1 to 0.1)	4.8 (-4.9 to 15.3)	-0.2 (-8.6 to 8.1)	0.1 (0.1 to 0.1)	0.1 (0.1 to 0.1)	0.1 (0.1 to 0.1)	7.9 (-4.9 to 22.2)	-2.2 (-11.1 to 8.4)
Occupational exposure to diesel engine exhaust	0.5 (0.5 to 0.5)	0.5 (0.5 to 0.6)	0.6 (0.5 to 0.6)	8.7 (-0.8 to 19.2)	2.7 (-4.2 to 10.4)	0.5 (0.4 to 0.5)	0.5 (0.4 to 0.6)	0.5 (0.4 to 0.5)	9.2 (-3.2 to 23.7)	-4.0 (-13.1 to 6.2)
Occupational exposure to formaldehyde	0.2 (0.2 to 0.2)	0.2 (0.2 to 0.3)	0.2 (0.2 to 0.3)	1.5 (-9.5 to 14.0)	-2.1 (-11.8 to 7.3)	0.2 (0.2 to 0.2)	0.2 (0.2 to 0.2)	0.2 (0.2 to 0.2)	6.7 (-7.0 to 22.6)	-2.1 (-11.8 to 9.2)
Occupational exposure to nickel	0.3 (0.0 to 1.3)	0.3 (0.0 to 1.4)	0.3 (0.0 to 1.4)	-	-	0.3 (0.0 to 1.1)	0.3 (0.0 to 1.2)	0.3 (0.0 to 1.1)	-	-
Occupational exposure to polycyclic aromatic hydrocarbons	0.2 (0.2 to 0.2)	0.2 (0.2 to 0.2)	0.2 (0.2 to 0.2)	4.9 (-4.4 to 15.2)	-0.7 (-8.6 to 7.4)	0.2 (0.2 to 0.2)	0.2 (0.2 to 0.3)	0.2 (0.2 to 0.3)	10.1 (-1.7 to 23.0)	-1.6 (-9.5 to 7.4)
Occupational exposure to silica	2.9 (0.4 to 9.1)	2.8 (0.4 to 8.8)	2.8 (0.4 to 8.7)	-3.4 (-14.7 to 9.6)	-0.3 (-11.1 to 11.1)	2.2 (0.3 to 6.8)	2.1 (0.3 to 6.9)	2.1 (0.3 to 6.7)	-1.3 (-17.1 to 19.9)	-3.0 (-17.1 to 16.0)
Occupational exposure to sulphuric acid	0.6 (0.1 to 1.8)	0.7 (0.1 to 2.0)	0.7 (0.1 to 2.0)	10.3 (-0.9 to 22.8)	-1.4 (-10.0 to 8.1)	0.5 (0.1 to 1.7)	0.6 (0.1 to 2.1)	0.6 (0.1 to 2.0)	20.7 (2.3 to 42.3)	-3.3 (-15.7 to 10.8)
Occupational exposure to trichloroethylene	0.1 (0.1 to 0.1)	0.1 (0.1 to 0.1)	0.1 (0.1 to 0.1)	6.2 (-1.4 to 14.5)	0.8 (-5.3 to 7.2)	0.1 (0.1 to 0.1)	0.1 (0.1 to 0.1)	0.1 (0.1 to 0.1)	8.3 (-2.8 to 20.1)	-2.5 (-10.3 to 6.7)
Occupational asthmagens	16.2 (13.6 to 19.5)	15.6 (13.1 to 18.6)	15.2 (12.4 to 18.4)	-3.6 (-14.7 to 7.7)	-2.8 (-11.2 to 6.8)	7.5 (6.2 to 9.2)	7.9 (6.5 to 9.6)	7.4 (6.0 to 9.2)	5.0 (-8.5 to 19.8)	-6.0 (-16.4 to 5.6)
Occupational particulate matter, gases, and fumes	7.2 (5.2 to 10.2)	6.9 (5.0 to 9.9)	6.6 (4.7 to 9.3)	-4.4 (-8.9 to -0.1)	-4.7 (-8.7 to -1.4)	3.6 (2.5 to 5.4)	3.6 (2.5 to 5.3)	3.4 (2.4 to 5.2)	-0.5 (-7.5 to 7.8)	-3.7 (-9.8 to 2.0)
Occupational noise	6.2 (5.7 to 6.9)	6.1 (5.6 to 6.7)	5.8 (5.4 to 6.4)	-2.8 (-5.9 to 0.6)	-3.8 (-6.0 to -1.4)	4.1 (3.6 to 4.6)	4.3 (3.9 to 4.8)	4.3 (3.9 to 4.8)	5.4 (0.1 to 11.8)	-0.1 (-3.6 to 3.7)
Occupational ergonomic factors	8.9 (7.4 to 10.9)	8.0 (6.7 to 9.6)	7.6 (6.3 to 9.2)	-10.5 (-19.9 to 1.0)	-4.7 (-12.4 to 3.2)	5.1 (4.0 to 6.4)	5.1 (4.1 to 6.4)	5.0 (3.9 to 6.4)	0.7 (-13.8 to 17.5)	-2.0 (-12.9 to 11.5)
Non-exclusive breastfeeding	0.5 (0.5 to 0.6)	0.5 (0.5 to 0.6)	0.5 (0.4 to 0.6)	-0.3 (-11.7 to 13.8)	-7.3 (-16.0 to 2.4)	0.5 (0.5 to 0.6)	0.5 (0.5 to 0.6)	0.5 (0.4 to 0.6)	0.1 (-11.4 to 14.2)	-7.6 (-16.2 to 2.2)
Discontinued breastfeeding	2.2 (2.0 to 2.3)	2.1 (2.0 to 2.3)	2.2 (2.0 to 2.3)	-0.5 (-7.7 to 7.3)	1.3 (-4.8 to 7.6)	2.2 (2.0 to 2.3)	2.1 (2.0 to 2.3)	2.2 (2.1 to 2.3)	-0.8 (-8.0 to 7.0)	2.3 (-3.9 to 8.6)
Child underweight	0.1 (0.1 to 0.1)	0.1 (0.1 to 0.1)	0.1 (0.0 to 0.1)	-3.1 (-17.6 to 12.3)	-13.6 (-22.6 to -4.7)	0.1 (0.0 to 0.1)	0.1 (0.0 to 0.1)	0.1 (0.0 to 0.1)	-1.2 (-16.4 to 15.1)	-5.2 (-16.5 to 6.4)
Child wasting	0.1 (0.0 to 0.1)	0.1 (0.0 to 0.1)	0.1 (0.0 to 0.1)	-9.6 (-27.4 to 12.2)	-3.9 (-18.0 to 11.4)	0.1 (0.0 to 0.1)	0.1 (0.0 to 0.1)	0.1 (0.0 to 0.1)	7.2 (-14.3 to 35.2)	-5.9 (-21.5 to 10.8)
Child stunting	0.3 (0.1 to 0.5)	0.2 (0.1 to 0.5)	0.2 (0.1 to 0.4)	-6.6 (-49.3 to 69.5)	-12.9 (-41.8 to 30.5)	0.2 (0.1 to 0.4)	0.2 (0.1 to 0.3)	0.2 (0.1 to 0.3)	-9.1 (-44.8 to 45.7)	-4.3 (-30.1 to 30.2)
Short gestation for birth weight	0.0 (0.0 to 0.0)	0.0 (0.0 to 0.0)	0.0 (0.0 to 0.0)	7.2 (-16.9 to 45.6)	3.6 (-18.3 to 25.0)	0.0 (0.0 to 0.0)	0.0 (0.0 to 0.0)	0.0 (0.0 to 0.0)	7.8 (-9.9 to 39.8)	3.4 (-18.7 to 31.5)
Low birth weight for gestation	0.0 (0.0 to 0.0)	0.0 (0.0 to 0.0)	0.0 (0.0 to 0.0)	4.5 (-18.3 to 31.5)	-1.7 (-17.4 to 21.9)	0.0 (0.0 to 0.0)	0.0 (0.0 to 0.0)	0.0 (0.0 to 0.0)	1.2 (-17.7 to 20.2)	-2.4 (-20.3 to 21.4)
Iron deficiency	-	-	-	-	-	4.1 (2.7 to 6.0)	3.8 (2.6 to 5.5)	3.8 (2.4 to 5.7)	-6.5 (-30.9 to 30.4)	-0.2 (-24.6 to 27.3)
Vitamin A deficiency	0.5 (0.3 to 0.7)	0.5 (0.3 to 0.7)	0.5 (0.3 to 0.7)	11.5 (-17.1 to 71.5)	0.3 (-25.5 to 24.5)	0.5 (0.4 to 0.7)	0.6 (0.4 to 0.9)	0.5 (0.4 to 0.7)	17.1 (-18.1 to 62.1)	-15.2 (-30.9 to 2.8)
Zinc deficiency	0.5 (0.1 to 1.2)	0.2 (0.0 to 1.0)	0.2 (0.0 to 1.0)	-55.0 (-88.2 to 291.1)	-3.9 (-87.9 to 764.8)	0.5 (0.1 to 1.2)	0.2 (0.0 to 1.0)	0.2 (0.0 to 1.0)	-58.1 (-87.8 to 330.1)	6.7 (-87.1 to 841.2)
Smoking	13.6 (11.1 to 15.9)	7.8 (6.2 to 9.4)	6.0 (4.7 to 7.4)	-42.8 (-51.0 to -33.4)	-23.3 (-32.0 to -13.1)	10.2 (8.0 to 12.6)	5.6 (4.4 to 6.9)	4.3 (3.2 to 5.4)	-45.6 (-55.9 to -33.0)	-23.4 (-33.0 to -13.4)
Chewing tobacco	2.7 (2.3 to 3.1)	2.6 (1.9 to 3.5)	2.5 (1.7 to 3.6)	-1.8 (-32.6 to 37.5)	-4.4 (-29.9 to 29.5)	0.1 (0.1 to 0.1)	0.1 (0.0 to 0.1)	0.1 (0.0 to 0.1)	-15.7 (-49.1 to 41.2)	-1.0 (-31.7 to 44.2)
Second-hand smoke	45.0 (40.9 to 49.7)	26.2 (24.6 to 27.7)	25.7 (23.2 to 28.7)	-41.8 (-49.6 to -33.9)	-1.9 (-11.1 to 8.3)	41.5 (38.0 to 45.2)	25.2 (24.0 to 26.3)	24.2 (22.4 to 26.0)	-39.2 (-46.1 to -31.6)	-3.8 (-10.9 to 3.1)
Alcohol use	21.0 (10.5 to 33.4)	22.3 (12.2 to 36.0)	23.8 (12.2 to 37.8)	6.3 (-33.0 to 66.7)	6.6 (-26.8 to 54.3)	13.1 (6.6 to 22.3)	14.0 (7.5 to 24.2)	14.9 (7.4 to 25.1)	6.4 (-31.5 to 65.6)	6.4 (-26.1 to 50.5)
Drug use	1.0 (0.7 to 1.5)	1.2 (0.9 to 1.8)	1.9 (1.4 to 2.7)	19.6 (8.4 to 35.1)	63.0 (44.5 to 92.1)	0.8 (0.5 to 1.1)	1.0 (0.8 to 1.5)	1.3 (1.0 to 1.9)	35.1 (22.7 to 50.7)	28.0 (16.9 to 40.8)
Diet low in fruits	30.3 (24.3 to 34.8)	29.4 (23.4 to 34.1)	29.4 (23.0 to 34.8)	-3.0 (-11.4 to 5.1)	0.2 (-9.1 to 9.3)	26.0 (19.4 to 31.4)	26.8 (20.7 to 31.9)	26.3 (19.2 to 31.9)	3.3 (-6.7 to 16.8)	-2.1 (-13.8 to 9.2)
Diet low in vegetables	24.1 (16.0 to 30.7)	29.4 (22.7 to 34.6)	31.4 (24.8 to 36.6)	21.8 (4.4 to 54.5)	6.9 (-2.6 to 18.3)	26.1 (18.7 to 32.5)	32.3 (26.1 to 37.3)	34.8 (29.3 to 39.6)	23.8 (7.5 to 50.7)	7.9 (-0.5 to 18.9)
Diet low in legumes	24.0 (17.4 to 29.7)	24.1 (17.5 to 29.9)	25.6 (18.7 to 31.5)	0.7 (-16.3 to 18.4)	5.8 (-7.1 to 22.4)	28.7 (22.7 to 34.1)	29.4 (23.9 to 34.6)	30.7 (25.2 to 35.5)	2.4 (-9.2 to 18.5)	4.4 (-4.3 to 15.7)
Diet low in whole grains	38.4 (35.0 to 41.4)	39.6 (36.5 to 42.3)	39.0 (35.7 to 41.9)	3.2 (-1.8 to 9.2)	-1.5 (-5.1 to 1.9)	39.3 (36.0 to 42.2)	40.5 (37.4 to 43.1)	40.0 (36.8 to 42.7)	3.1 (-1.5 to 8.9)	-1.2 (-4.8 to 2.4)
Diet low in nuts and seeds	46.3 (43.5 to 48.6)	37.5 (32.6 to 41.4)	37.4 (32.2 to 41.7)	-19.1 (-27.3 to -12.9)	-0.2 (-8.1 to 7.2)	47.0 (44.3 to 49.1)	38.6 (34.1 to 42.4)	38.5 (33.4 to 42.5)	-17.9 (-25.5 to -12.1)	-0.4 (-7.6 to 7.0)
Diet low in milk	28.0 (21.7 to 33.1)	26.6 (20.2 to 32.0)	26.9 (20.2 to 32.4)	-4.9 (-15.9 to 5.5)	0.8 (-7.3 to 10.1)	29.7 (23.6 to 34.4)	27.7 (21.3 to 32.9)	27.7 (21.3 to 32.8)	-6.8 (-17.2 to 1.4)	-0.2 (-8.0 to 8.2)
Diet high in red meat	52.8 (50.1 to 54.6)	48.0 (39.5 to 52.9)	45.0 (34.3 to 51.5)	-9.1 (-21.2 to -0.0)	-6.2 (-19.2 to 5.0)	35.8 (29.2 to 41.3)	31.6 (21.2 to 40.6)	28.7 (17.9 to 38.7)	-11.7 (-35.7 to 16.3)	-9.1 (-30.7 to 16.0)
Diet high in processed meat	49.3 (44.7 to 52.5)	53.2 (50.0 to 54.7)	50.5 (44.5 to 54.0)	8.0 (1.0 to 17.6)	-5.0 (-12.6 to -0.1)	27.9 (22.7 to 32.6)	41.3 (34.3 to 47.1)	38.7 (30.9 to 45.4)	47.7 (24.4 to 77.6)	-6.2 (-17.9 to 5.0)
Diet high in sugar-sweetened beverages	30.6 (14.9 to 41.3)	28.9 (14.5 to 39.9)	24.5 (12.2 to 35.9)	-5.5 (-35.2 to 37.9)	-15.1 (-41.6 to 15.8)	25.2 (11.7 to 36.4)	22.0 (10.8 to 32.6)	18.7 (9.0 to 29.8)	-13.0 (-45.6 to 38.5)	-14.8 (-44.3 to 24.2)
Diet low in fibre	33.5 (27.3 to 38.4)	25.7 (18.2 to 31.5)	27.0 (19.8 to 33.1)	-23.3 (-37.1 to -13.2)	5.3 (-4.8 to 18.0)	38.1 (33.3 to 42.1)	30.1 (23.7 to 35.5)	31.2 (24.6 to 36.4)	-20.8 (-32.2 to -12.4)	3.7 (-5.2 to 13.8)
Diet low in calcium	17.2 (10.2 to 23.7)	15.8 (9.1 to 21.9)	16.1 (9.3 to 22.6)	-8.6 (-23.3 to 5.4)	2.2 (-9.2 to 16.3)	21.4 (14.2 to 27.3)	19.7 (12.6 to 25.9)	20.0 (12.8 to 26.1)	-7.8 (-20.8 to 2.1)	1.6 (-6.4 to 11.7)
Diet low in seafood omega-3 fatty acids	29.6 (23.8 to 34.6)	25.6 (19.6 to 31.0)	26.4 (20.5 to 32.0)	-13.4 (-24.5 to -2.9)	3.2 (-5.9 to 14.4)	32.9 (27.6 to 37.4)	29.7 (23.7 to 34.8)	30.2 (24.7 to 35.3)	-9.9 (-19.3 to -1.8)	1.9 (-5.1 to 10.3)
Diet low in polyunsaturated fatty acids	8.7 (3.0 to 15.7)	5.7 (1.2 to 12.0)	4.1 (0.6 to 9.6)	-34.3 (-75.3 to 6.0)	-28.4 (-71.7 to 29.5)	9.5 (3.3 to 16.5)	6.3 (1.4 to 12.6)	4.5 (0.7 to 10.1)	-33.6 (-73.6 to 5.1)	-28.5 (-68.2 to 20.4)
Diet high in trans fatty acids	16.7 (12.1 to 21.8)	9.8 (5.8 to 13.9)	7.1 (3.5 to 11.0)	-41.3 (-55.9 to -30.0)	-27.3 (-42.8 to -15.9)	23.7 (17.7 to 31.3)	13.3 (8.9 to 17.7)	9.7 (5.7 to 13.9)	-44.1 (-55.2 to -32.3)	-26.7 (-39.7 to -16.5)
Diet high in sodium	7.1 (0.6 to 14.7)	12.5 (4.0 to 20.4)	13.1 (4.1 to 21.0)	75.0 (20.8 to 787.6)	4.9 (-19.8 to 53.4)	6.0 (0.3 to 13.4)	10.8 (2.5 to 18.6)	11.3 (2.9 to 19.2)	80.0 (26.5 to 1168.6)	5.2 (-19.7 to 51.2)
Intimate partner violence	-	-	-	-	-	0.5 (0.3 to 0.7)	0.5 (0.3 to 0.6)	0.5 (0.3 to 0.7)	-13.7 (-25.1 to 4.0)	10.0 (-4.8 to 24.4)
Childhood sexual abuse	4.0 (3.0 to 5.4)	3.9 (3.3 to 4.6)	3.9 (3.3 to 4.6)	-2.0 (-17.1 to 16.6)	-0.4 (-5.6 to 5.1)	11.5 (8.8 to 14.8)	10.7 (9.0 to 12.6)	10.7 (8.9 to 12.6)	-6.5 (-20.1 to 7.2)	-0.1 (-4.5 to 4.4)

Supplementary Results Table 1. Iowa age-standardised Summary Exposure Values (SEVs) for Level 4 risk factors and percentage change, by sex, 1990, 2007, and 2017.Data in parentheses are 95% uncertainty intervals.										
	**Male**					**Female**				
	**1990**	**2007**	**2017**	**Percentage change 1990-2007**	**Percentage change 2007-2017**	**1990**	**2007**	**2017**	**Percentage change 1990-2007**	**Percentage change 2007-2017**
Bullying victimization	6.0 (2.5 to 12.9)	8.8 (3.6 to 18.3)	8.6 (3.6 to 18.0)	46.3 (31.2 to 65.5)	-2.9 (-10.0 to 5.0)	4.6 (1.9 to 9.8)	7.5 (3.1 to 15.5)	7.4 (3.0 to 15.5)	63.8 (45.0 to 88.8)	-0.7 (-8.5 to 8.4)
Low physical activity	0.4 (0.2 to 0.8)	0.4 (0.2 to 0.8)	0.4 (0.2 to 0.8)	0.3 (-2.1 to 2.8)	-0.7 (-2.4 to 0.8)	0.5 (0.2 to 0.9)	0.5 (0.2 to 0.9)	0.4 (0.2 to 0.9)	-0.4 (-3.2 to 3.1)	-1.2 (-3.3 to 0.9)
High fasting plasma glucose	1.1 (0.7 to 1.8)	2.2 (1.3 to 3.4)	3.0 (1.9 to 4.6)	96.1 (23.0 to 207.0)	37.4 (-13.1 to 124.3)	1.4 (0.8 to 2.5)	2.7 (1.4 to 4.2)	2.3 (1.1 to 4.0)	83.8 (3.9 to 237.8)	-13.7 (-53.3 to 57.2)
High LDL cholesterol	21.2 (19.2 to 23.2)	11.5 (9.9 to 13.3)	10.1 (8.6 to 11.8)	-45.8 (-50.6 to -40.7)	-11.8 (-16.2 to -7.5)	20.1 (18.2 to 22.2)	11.5 (10.0 to 13.3)	10.7 (9.2 to 12.3)	-42.6 (-47.4 to -38.1)	-7.6 (-11.6 to -3.3)
High systolic blood pressure	6.3 (5.5 to 7.2)	4.6 (3.9 to 5.3)	4.7 (4.0 to 5.4)	-27.4 (-35.1 to -19.0)	1.9 (-7.7 to 11.4)	6.2 (5.4 to 7.0)	4.3 (3.8 to 4.8)	4.2 (3.6 to 4.8)	-30.2 (-38.4 to -20.7)	-3.1 (-12.9 to 7.6)
High body-mass index	13.6 (9.8 to 18.7)	20.5 (15.9 to 26.2)	21.3 (16.5 to 27.4)	50.5 (32.3 to 73.7)	4.1 (-4.5 to 13.8)	15.7 (12.0 to 20.4)	21.9 (17.4 to 27.4)	23.6 (18.6 to 29.4)	39.0 (21.0 to 60.1)	7.9 (-1.6 to 18.6)
Low bone mineral density	5.1 (4.2 to 6.1)	4.4 (3.6 to 5.2)	4.6 (3.8 to 5.6)	-14.4 (-25.8 to -1.3)	6.4 (-8.3 to 23.0)	5.7 (4.8 to 6.7)	5.6 (4.7 to 6.6)	6.1 (5.1 to 7.2)	-1.3 (-13.5 to 12.2)	9.0 (-4.8 to 24.4)
Impaired kidney function	2.2 (1.0 to 5.6)	2.2 (1.1 to 5.8)	2.3 (1.1 to 5.8)	1.4 (-4.9 to 7.1)	2.9 (-2.6 to 9.4)	2.9 (1.4 to 6.8)	2.8 (1.3 to 6.8)	3.0 (1.4 to 6.9)	-0.6 (-6.0 to 4.5)	4.9 (0.3 to 11.1)

Supplementary Results Table 1. Kansas age-standardised Summary Exposure Values (SEVs) for Level 4 risk factors and percentage change, by sex, 1990, 2007, and 2017.Data in parentheses are 95% uncertainty intervals.										
	Male					Female				
	**1990**	**2007**	**2017**	**Percentage change 1990-2007**	**Percentage change 2007-2017**	**1990**	**2007**	**2017**	**Percentage change 1990-2007**	**Percentage change 2007-2017**
Unsafe water source	1.5 (0.8 to 2.4)	0.5 (0.3 to 0.9)	0.3 (0.2 to 0.5)	-63.4 (-81.4 to -25.5)	-40.0 (-63.3 to -3.7)	1.5 (0.8 to 2.4)	0.5 (0.3 to 0.9)	0.3 (0.2 to 0.5)	-63.4 (-81.4 to -25.5)	-40.0 (-63.3 to -3.7)
Unsafe sanitation	6.6 (3.6 to 11.4)	4.6 (2.0 to 8.6)	3.0 (1.3 to 5.9)	-30.8 (-69.3 to 50.9)	-35.3 (-66.3 to 21.9)	6.6 (3.6 to 11.4)	4.6 (2.0 to 8.6)	3.0 (1.3 to 5.9)	-30.8 (-69.3 to 50.9)	-35.3 (-66.3 to 21.9)
No access to handwashing facility	3.4 (2.5 to 4.8)	3.2 (2.3 to 4.3)	3.0 (2.1 to 4.2)	-8.1 (-35.9 to 30.3)	-4.2 (-25.6 to 23.8)	3.4 (2.5 to 4.8)	3.2 (2.3 to 4.3)	3.0 (2.1 to 4.2)	-8.1 (-35.9 to 30.3)	-4.2 (-25.6 to 23.8)
Ambient particulate matter pollution	24.7 (15.5 to 38.3)	24.0 (16.1 to 37.9)	18.0 (8.7 to 31.0)	-2.6 (-28.7 to 36.4)	-25.0 (-61.1 to 4.7)	24.6 (15.4 to 38.2)	24.0 (16.0 to 37.9)	18.0 (8.7 to 30.9)	-2.4 (-28.7 to 36.5)	-25.0 (-61.2 to 4.9)
Household air pollution from solid fuels	0.3 (0.1 to 0.8)	0.2 (0.0 to 0.5)	0.2 (0.0 to 0.5)	-33.8 (-76.5 to 66.7)	-13.9 (-69.6 to 77.2)	0.4 (0.1 to 2.2)	0.3 (0.1 to 1.7)	0.3 (0.0 to 1.5)	-31.5 (-76.4 to 65.2)	-10.6 (-69.5 to 74.8)
Ambient ozone pollution	48.8 (20.0 to 54.8)	48.9 (20.0 to 54.8)	49.4 (20.7 to 54.8)	0.1 (-0.1 to 0.5)	1.2 (0.0 to 4.2)	48.8 (20.0 to 54.8)	48.9 (20.0 to 54.8)	49.4 (20.7 to 54.8)	0.1 (-0.1 to 0.5)	1.2 (0.0 to 4.2)
Residential radon	13.2 (0.8 to 41.9)	13.2 (0.8 to 41.9)	13.2 (0.8 to 41.9)	0.0 (0.0 to 0.0)	0.0 (0.0 to 0.0)	13.2 (0.8 to 41.9)	13.2 (0.8 to 41.9)	13.2 (0.8 to 41.9)	0.0 (0.0 to 0.0)	0.0 (0.0 to 0.0)
Lead exposure	7.3 (3.0 to 11.7)	4.8 (1.7 to 8.7)	3.3 (1.0 to 6.3)	-34.0 (-46.5 to -24.4)	-31.9 (-42.1 to -25.4)	4.3 (1.0 to 8.4)	2.9 (0.5 to 6.4)	2.0 (0.3 to 4.8)	-32.3 (-48.8 to -21.6)	-31.0 (-44.8 to -24.2)
Occupational exposure to asbestos	4.9 (4.2 to 5.5)	3.8 (3.5 to 4.2)	3.5 (2.9 to 4.1)	-21.9 (-32.7 to -7.7)	-9.5 (-26.2 to 9.6)	0.6 (0.5 to 0.7)	0.7 (0.6 to 0.7)	0.7 (0.6 to 0.9)	14.6 (-3.3 to 34.1)	10.7 (-11.8 to 37.2)
Occupational exposure to arsenic	0.4 (0.0 to 0.9)	0.4 (0.0 to 0.9)	0.4 (0.0 to 0.9)	-	-	0.3 (0.0 to 0.8)	0.3 (0.0 to 0.8)	0.3 (0.0 to 0.8)	-	-
Occupational exposure to benzene	0.6 (0.0 to 1.7)	0.6 (0.0 to 1.7)	0.6 (0.0 to 1.6)	2.5 (-5.4 to 15.2)	-2.5 (-8.7 to 5.5)	0.9 (0.1 to 2.5)	0.9 (0.1 to 2.9)	1.0 (0.1 to 2.8)	10.8 (0.8 to 28.7)	0.9 (-6.4 to 12.5)
Occupational exposure to beryllium	0.0 (0.0 to 0.0)	0.0 (0.0 to 0.0)	0.0 (0.0 to 0.0)	0.8 (-4.8 to 6.9)	0.3 (-4.0 to 5.0)	0.0 (0.0 to 0.0)	0.0 (0.0 to 0.0)	0.0 (0.0 to 0.0)	5.2 (-3.2 to 14.2)	0.3 (-5.6 to 6.2)
Occupational exposure to cadmium	0.0 (0.0 to 0.0)	0.0 (0.0 to 0.0)	0.0 (0.0 to 0.0)	3.2 (-7.8 to 16.3)	0.2 (-8.2 to 10.3)	0.0 (0.0 to 0.0)	0.0 (0.0 to 0.0)	0.0 (0.0 to 0.0)	4.6 (-9.3 to 20.4)	-1.0 (-11.5 to 11.7)
Occupational exposure to chromium	0.1 (0.1 to 0.1)	0.1 (0.1 to 0.1)	0.1 (0.1 to 0.1)	5.0 (-4.8 to 16.7)	0.3 (-7.3 to 8.9)	0.1 (0.1 to 0.1)	0.1 (0.1 to 0.1)	0.1 (0.1 to 0.1)	6.6 (-5.8 to 20.1)	-1.2 (-9.9 to 9.0)
Occupational exposure to diesel engine exhaust	0.5 (0.5 to 0.5)	0.5 (0.5 to 0.6)	0.6 (0.5 to 0.6)	7.4 (-1.8 to 17.5)	2.0 (-5.0 to 9.6)	0.5 (0.4 to 0.5)	0.5 (0.4 to 0.5)	0.5 (0.4 to 0.5)	7.7 (-5.4 to 22.1)	-3.6 (-12.6 to 6.6)
Occupational exposure to formaldehyde	0.2 (0.2 to 0.2)	0.2 (0.2 to 0.2)	0.2 (0.2 to 0.2)	1.6 (-9.9 to 15.0)	-1.3 (-10.3 to 8.9)	0.2 (0.2 to 0.2)	0.2 (0.2 to 0.2)	0.2 (0.2 to 0.2)	5.3 (-7.9 to 20.1)	-1.0 (-10.4 to 10.1)
Occupational exposure to nickel	0.3 (0.0 to 1.4)	0.3 (0.0 to 1.4)	0.3 (0.0 to 1.4)	-	-	0.2 (0.0 to 1.0)	0.3 (0.0 to 1.1)	0.3 (0.0 to 1.1)	-	-
Occupational exposure to polycyclic aromatic hydrocarbons	0.2 (0.2 to 0.2)	0.2 (0.2 to 0.2)	0.2 (0.2 to 0.2)	5.2 (-4.6 to 16.4)	-0.4 (-7.5 to 7.6)	0.2 (0.2 to 0.2)	0.2 (0.2 to 0.2)	0.2 (0.2 to 0.3)	9.3 (-1.9 to 21.5)	-0.8 (-8.5 to 7.9)
Occupational exposure to silica	3.0 (0.4 to 9.1)	2.8 (0.4 to 8.9)	2.8 (0.4 to 8.6)	-6.6 (-17.5 to 7.2)	-0.2 (-9.9 to 11.7)	2.1 (0.3 to 6.7)	2.1 (0.3 to 6.6)	2.0 (0.3 to 6.5)	-3.3 (-19.2 to 19.3)	-2.0 (-15.9 to 15.8)
Occupational exposure to sulphuric acid	0.6 (0.1 to 1.9)	0.7 (0.1 to 2.1)	0.6 (0.1 to 2.0)	14.1 (1.7 to 28.2)	-1.8 (-9.9 to 8.0)	0.5 (0.1 to 1.6)	0.6 (0.1 to 2.0)	0.6 (0.1 to 2.0)	23.6 (4.2 to 45.0)	-3.3 (-16.4 to 10.7)
Occupational exposure to trichloroethylene	0.1 (0.1 to 0.1)	0.1 (0.1 to 0.1)	0.1 (0.1 to 0.1)	6.6 (-0.8 to 15.3)	1.1 (-4.5 to 7.5)	0.1 (0.1 to 0.1)	0.1 (0.1 to 0.1)	0.1 (0.1 to 0.1)	7.0 (-4.3 to 19.6)	-1.7 (-9.4 to 7.2)
Occupational asthmagens	15.7 (13.2 to 18.8)	15.1 (12.7 to 17.9)	14.6 (12.2 to 17.6)	-3.9 (-13.8 to 7.4)	-3.1 (-11.3 to 6.6)	7.5 (6.1 to 9.1)	7.8 (6.4 to 9.5)	7.3 (5.9 to 9.0)	3.3 (-9.9 to 20.3)	-5.8 (-15.7 to 5.6)
Occupational particulate matter, gases, and fumes	6.8 (4.8 to 9.8)	6.5 (4.7 to 9.5)	6.2 (4.5 to 9.1)	-3.9 (-8.6 to 0.6)	-4.3 (-8.3 to -0.8)	3.5 (2.4 to 5.2)	3.4 (2.4 to 5.1)	3.3 (2.3 to 4.9)	-1.8 (-9.0 to 6.3)	-3.7 (-9.2 to 2.1)
Occupational noise	6.1 (5.6 to 6.7)	5.9 (5.5 to 6.5)	5.7 (5.3 to 6.3)	-2.6 (-5.7 to 0.7)	-3.6 (-5.9 to -1.3)	4.1 (3.5 to 4.7)	4.3 (3.8 to 4.8)	4.3 (3.9 to 4.8)	5.2 (-0.4 to 12.0)	-0.0 (-3.4 to 3.6)
Occupational ergonomic factors	8.7 (7.1 to 10.5)	7.9 (6.6 to 9.6)	7.5 (6.2 to 9.2)	-8.8 (-18.9 to 2.1)	-4.9 (-11.6 to 2.6)	4.9 (3.9 to 6.1)	5.0 (4.0 to 6.2)	4.9 (3.9 to 6.2)	1.3 (-13.3 to 20.3)	-1.1 (-12.1 to 13.2)
Non-exclusive breastfeeding	0.5 (0.5 to 0.6)	0.5 (0.5 to 0.6)	0.5 (0.4 to 0.6)	-0.0 (-11.5 to 13.0)	-7.4 (-16.5 to 2.2)	0.5 (0.5 to 0.6)	0.5 (0.5 to 0.6)	0.5 (0.4 to 0.6)	-0.1 (-11.6 to 12.9)	-7.4 (-16.5 to 2.1)
Discontinued breastfeeding	2.2 (2.0 to 2.3)	2.1 (2.0 to 2.2)	2.1 (2.0 to 2.3)	-1.8 (-8.8 to 5.6)	1.4 (-4.4 to 8.1)	2.1 (2.0 to 2.3)	2.1 (2.0 to 2.2)	2.2 (2.0 to 2.3)	-1.2 (-8.3 to 6.2)	2.3 (-3.6 to 9.1)
Child underweight	0.1 (0.1 to 0.1)	0.1 (0.1 to 0.1)	0.1 (0.0 to 0.1)	-3.3 (-18.0 to 12.8)	-14.6 (-22.9 to -5.7)	0.1 (0.0 to 0.1)	0.1 (0.0 to 0.1)	0.1 (0.0 to 0.1)	-1.1 (-16.3 to 16.7)	-6.0 (-16.6 to 5.4)
Child wasting	0.1 (0.0 to 0.1)	0.1 (0.0 to 0.1)	0.1 (0.0 to 0.1)	-9.3 (-30.0 to 11.7)	-4.0 (-18.2 to 10.6)	0.1 (0.0 to 0.1)	0.1 (0.0 to 0.1)	0.1 (0.0 to 0.1)	7.2 (-16.2 to 37.0)	-5.7 (-20.1 to 11.1)
Child stunting	0.3 (0.1 to 0.5)	0.2 (0.1 to 0.5)	0.2 (0.1 to 0.4)	-7.9 (-49.0 to 66.0)	-12.8 (-41.8 to 26.1)	0.2 (0.1 to 0.4)	0.2 (0.1 to 0.3)	0.2 (0.1 to 0.3)	-8.7 (-44.8 to 43.7)	-5.9 (-31.8 to 30.1)
Short gestation for birth weight	0.0 (0.0 to 0.0)	0.0 (0.0 to 0.0)	0.0 (0.0 to 0.0)	5.6 (-14.3 to 33.4)	2.1 (-19.6 to 26.9)	0.0 (0.0 to 0.0)	0.0 (0.0 to 0.0)	0.0 (0.0 to 0.0)	2.5 (-20.9 to 40.0)	5.3 (-14.2 to 54.4)
Low birth weight for gestation	0.0 (0.0 to 0.0)	0.0 (0.0 to 0.0)	0.0 (0.0 to 0.0)	0.9 (-19.2 to 19.7)	-2.2 (-19.9 to 20.5)	0.0 (0.0 to 0.0)	0.0 (0.0 to 0.0)	0.0 (0.0 to 0.0)	2.2 (-23.0 to 30.9)	-3.2 (-32.9 to 15.4)
Iron deficiency	-	-	-	-	-	4.2 (2.7 to 6.1)	4.0 (2.6 to 5.7)	4.0 (2.6 to 5.9)	-6.3 (-31.4 to 30.1)	0.8 (-23.5 to 30.6)
Vitamin A deficiency	0.5 (0.3 to 0.6)	0.5 (0.3 to 0.7)	0.6 (0.3 to 0.8)	3.5 (-13.9 to 27.2)	14.8 (-8.2 to 41.5)	0.5 (0.3 to 0.7)	0.6 (0.4 to 0.7)	0.7 (0.4 to 0.9)	13.8 (-2.9 to 37.0)	17.6 (-7.7 to 38.8)
Zinc deficiency	0.5 (0.1 to 1.2)	0.2 (0.0 to 1.0)	0.2 (0.0 to 1.0)	-55.6 (-88.4 to 258.4)	2.3 (-88.7 to 791.9)	0.5 (0.1 to 1.1)	0.2 (0.0 to 1.0)	0.2 (0.0 to 1.0)	-55.9 (-88.5 to 206.7)	3.4 (-88.7 to 806.4)
Smoking	14.3 (11.7 to 16.9)	7.3 (5.7 to 8.8)	5.7 (4.4 to 7.1)	-49.1 (-56.4 to -41.1)	-21.8 (-30.6 to -12.1)	9.7 (7.4 to 12.4)	5.3 (4.1 to 6.5)	4.2 (3.2 to 5.3)	-46.0 (-56.8 to -31.9)	-20.0 (-29.6 to -8.9)
Chewing tobacco	3.1 (2.5 to 3.6)	3.3 (2.4 to 4.4)	3.2 (2.1 to 4.5)	6.5 (-26.1 to 52.6)	-2.7 (-28.1 to 29.7)	0.1 (0.1 to 0.1)	0.1 (0.0 to 0.1)	0.1 (0.0 to 0.1)	-6.1 (-43.0 to 59.4)	1.4 (-30.3 to 50.8)
Second-hand smoke	41.9 (38.6 to 45.5)	25.8 (24.3 to 27.4)	26.1 (23.4 to 29.0)	-38.4 (-45.2 to -30.9)	1.2 (-9.0 to 12.0)	40.9 (38.2 to 43.9)	24.4 (23.3 to 25.5)	24.5 (22.6 to 26.5)	-40.3 (-46.4 to -34.0)	0.5 (-7.4 to 8.2)
Alcohol use	19.8 (10.7 to 31.4)	21.2 (11.4 to 33.2)	23.1 (11.8 to 37.0)	7.0 (-30.5 to 69.7)	8.9 (-24.2 to 56.8)	13.9 (7.3 to 23.4)	14.2 (7.4 to 24.1)	14.4 (7.0 to 25.2)	2.7 (-32.2 to 54.1)	1.0 (-28.8 to 42.2)
Drug use	1.1 (0.7 to 1.7)	1.3 (1.0 to 1.9)	1.9 (1.5 to 2.6)	25.1 (11.6 to 47.0)	47.3 (30.0 to 70.8)	1.0 (0.8 to 1.4)	1.4 (1.2 to 1.8)	1.7 (1.4 to 2.2)	37.4 (23.0 to 55.7)	21.3 (9.8 to 32.6)
Diet low in fruits	28.7 (22.6 to 33.5)	30.8 (24.9 to 35.2)	31.6 (25.4 to 36.3)	7.3 (-0.9 to 19.1)	2.7 (-4.6 to 11.4)	25.5 (19.2 to 31.1)	28.5 (22.7 to 33.4)	28.9 (22.4 to 34.4)	11.6 (1.3 to 25.5)	1.4 (-7.5 to 11.2)
Diet low in vegetables	20.1 (12.0 to 27.4)	28.8 (22.2 to 34.2)	30.7 (24.4 to 35.8)	43.0 (16.9 to 103.9)	6.5 (-5.0 to 19.1)	22.3 (13.9 to 29.0)	29.7 (23.1 to 35.3)	32.0 (25.5 to 37.6)	33.1 (12.0 to 77.4)	7.8 (-2.0 to 19.8)
Diet low in legumes	24.1 (17.2 to 30.0)	24.1 (17.6 to 29.8)	25.5 (18.9 to 31.4)	0.3 (-16.3 to 21.2)	5.8 (-7.2 to 23.0)	28.7 (22.6 to 33.7)	29.4 (23.8 to 34.2)	30.7 (25.0 to 35.4)	2.4 (-10.2 to 17.0)	4.5 (-4.7 to 15.4)
Diet low in whole grains	38.1 (34.6 to 41.3)	39.5 (36.2 to 42.4)	38.9 (35.3 to 41.9)	3.6 (-1.4 to 9.3)	-1.7 (-5.6 to 2.0)	39.1 (35.7 to 42.0)	40.5 (37.4 to 43.0)	39.9 (36.7 to 42.6)	3.4 (-1.4 to 8.9)	-1.4 (-5.1 to 1.8)
Diet low in nuts and seeds	45.8 (42.5 to 48.1)	37.3 (32.8 to 41.0)	36.8 (31.3 to 41.2)	-18.5 (-25.5 to -12.5)	-1.2 (-10.2 to 6.1)	46.6 (43.7 to 48.9)	38.4 (33.7 to 42.2)	37.9 (32.8 to 42.1)	-17.5 (-25.6 to -11.7)	-1.3 (-8.9 to 6.1)
Diet low in milk	27.6 (21.4 to 32.8)	26.6 (20.1 to 31.7)	26.5 (20.2 to 31.9)	-3.9 (-14.1 to 6.5)	-0.1 (-8.4 to 8.7)	29.3 (23.4 to 34.1)	27.6 (21.5 to 32.9)	27.4 (21.2 to 32.8)	-5.8 (-15.6 to 3.5)	-0.7 (-8.5 to 7.6)
Diet high in red meat	49.8 (44.8 to 53.1)	42.9 (33.8 to 49.8)	40.3 (30.0 to 48.3)	-13.8 (-29.3 to 0.2)	-6.1 (-20.8 to 8.5)	34.5 (27.1 to 41.2)	25.8 (15.9 to 34.4)	23.2 (13.4 to 33.0)	-25.4 (-49.3 to 1.9)	-10.0 (-34.7 to 18.1)
Diet high in processed meat	44.1 (37.5 to 48.9)	47.4 (40.6 to 52.2)	41.5 (32.5 to 48.0)	7.4 (-6.0 to 22.4)	-12.5 (-23.2 to -2.3)	25.5 (20.1 to 30.4)	31.6 (24.5 to 38.1)	28.5 (21.2 to 35.0)	24.0 (0.6 to 54.2)	-9.9 (-23.0 to 3.8)
Diet high in sugar-sweetened beverages	31.3 (15.4 to 41.7)	28.9 (13.9 to 38.8)	25.0 (12.4 to 35.2)	-7.9 (-36.0 to 34.0)	-13.4 (-37.7 to 17.9)	26.2 (12.8 to 37.0)	22.2 (10.9 to 33.6)	19.0 (8.7 to 29.2)	-15.0 (-47.7 to 31.6)	-14.6 (-43.4 to 22.1)
Diet low in fibre	33.1 (27.2 to 37.8)	25.5 (19.2 to 31.3)	26.8 (20.3 to 32.6)	-23.0 (-35.5 to -12.9)	5.3 (-5.5 to 18.4)	37.8 (32.8 to 41.9)	30.1 (23.4 to 35.2)	31.0 (24.6 to 36.3)	-20.5 (-31.1 to -12.1)	3.2 (-5.3 to 13.1)
Diet low in calcium	16.6 (9.5 to 22.9)	15.7 (8.8 to 21.8)	15.8 (9.0 to 22.1)	-5.5 (-19.3 to 10.5)	0.6 (-10.5 to 13.1)	20.7 (13.6 to 26.3)	19.6 (12.4 to 25.4)	19.6 (12.3 to 25.5)	-5.5 (-18.2 to 5.7)	0.3 (-8.5 to 9.6)
Diet low in seafood omega-3 fatty acids	28.8 (23.5 to 34.1)	25.4 (19.5 to 30.9)	25.9 (19.9 to 31.4)	-11.7 (-23.0 to -0.8)	2.0 (-7.5 to 13.0)	32.2 (26.7 to 36.8)	29.5 (23.8 to 34.6)	29.6 (23.6 to 34.6)	-8.6 (-17.9 to 0.2)	0.4 (-7.0 to 8.7)
Diet low in polyunsaturated fatty acids	8.3 (2.4 to 15.1)	5.6 (1.0 to 11.7)	3.9 (0.5 to 9.4)	-32.6 (-76.7 to 9.3)	-29.6 (-70.8 to 32.1)	9.1 (3.2 to 16.1)	6.2 (1.3 to 12.8)	4.3 (0.6 to 9.7)	-31.4 (-73.6 to 13.8)	-30.5 (-67.1 to 17.1)
Diet high in trans fatty acids	16.8 (12.1 to 22.4)	9.9 (5.9 to 14.2)	7.1 (3.5 to 11.0)	-41.4 (-56.9 to -28.9)	-27.6 (-44.0 to -16.4)	23.7 (18.1 to 32.1)	13.3 (9.0 to 18.0)	9.7 (5.7 to 13.9)	-43.9 (-55.0 to -32.6)	-26.8 (-40.9 to -16.3)
Diet high in sodium	7.2 (0.6 to 14.6)	12.5 (3.8 to 20.2)	13.2 (4.5 to 21.3)	73.9 (23.7 to 658.0)	5.4 (-20.8 to 56.5)	6.0 (0.4 to 13.4)	10.9 (2.4 to 18.6)	11.4 (2.8 to 19.0)	80.3 (27.5 to 1000.9)	4.7 (-21.1 to 52.3)
Intimate partner violence	-	-	-	-	-	1.3 (1.1 to 1.5)	0.9 (0.8 to 1.1)	1.1 (1.0 to 1.3)	-27.8 (-31.6 to -23.8)	24.8 (18.2 to 31.6)
Childhood sexual abuse	5.6 (4.6 to 6.9)	5.1 (4.1 to 6.2)	5.1 (4.1 to 6.2)	-9.8 (-15.1 to -4.3)	0.4 (-4.7 to 6.0)	13.8 (11.0 to 16.9)	12.1 (9.8 to 14.7)	12.2 (9.9 to 14.8)	-11.9 (-17.2 to -6.0)	0.4 (-4.8 to 6.5)

Supplementary Results Table 1. Kansas age-standardised Summary Exposure Values (SEVs) for Level 4 risk factors and percentage change, by sex, 1990, 2007, and 2017.Data in parentheses are 95% uncertainty intervals.										
	**Male**					**Female**				
	**1990**	**2007**	**2017**	**Percentage change 1990-2007**	**Percentage change 2007-2017**	**1990**	**2007**	**2017**	**Percentage change 1990-2007**	**Percentage change 2007-2017**
Bullying victimization	6.0 (2.5 to 12.4)	8.9 (3.8 to 18.2)	8.6 (3.6 to 18.0)	47.7 (31.2 to 67.6)	-2.7 (-9.9 to 5.1)	4.6 (1.9 to 9.3)	7.5 (3.3 to 15.4)	7.5 (3.2 to 15.6)	64.9 (45.8 to 89.4)	-0.6 (-8.5 to 8.6)
Low physical activity	0.4 (0.2 to 0.8)	0.4 (0.2 to 0.8)	0.4 (0.2 to 0.8)	0.8 (-1.3 to 3.1)	-0.4 (-1.8 to 0.9)	0.4 (0.2 to 0.8)	0.4 (0.2 to 0.8)	0.4 (0.2 to 0.8)	-0.1 (-2.7 to 2.0)	-0.7 (-2.1 to 0.5)
High fasting plasma glucose	1.2 (0.8 to 2.0)	2.4 (1.4 to 3.6)	3.1 (1.9 to 4.8)	99.4 (24.2 to 231.5)	31.8 (-15.6 to 116.2)	1.5 (0.9 to 2.5)	2.9 (1.5 to 4.6)	2.8 (1.4 to 4.6)	94.7 (12.3 to 254.4)	-2.7 (-45.9 to 70.1)
High LDL cholesterol	21.1 (19.1 to 23.4)	11.4 (9.9 to 13.2)	10.1 (8.6 to 11.8)	-45.9 (-50.5 to -41.1)	-11.4 (-15.7 to -6.8)	20.1 (18.3 to 22.0)	11.5 (9.9 to 13.1)	10.6 (9.1 to 12.3)	-43.0 (-47.9 to -38.4)	-7.3 (-11.4 to -3.3)
High systolic blood pressure	6.1 (5.3 to 7.0)	4.6 (3.9 to 5.3)	4.8 (4.1 to 5.6)	-24.5 (-32.3 to -15.8)	4.7 (-4.5 to 14.7)	5.8 (5.0 to 6.6)	4.3 (3.8 to 4.8)	4.3 (3.7 to 5.0)	-25.9 (-34.8 to -15.7)	-0.3 (-10.5 to 10.6)
High body-mass index	14.0 (10.0 to 19.3)	21.1 (16.5 to 27.1)	22.4 (17.7 to 28.6)	51.3 (32.0 to 78.2)	5.9 (-2.4 to 15.5)	16.5 (12.5 to 21.6)	22.7 (18.7 to 28.9)	24.1 (19.4 to 30.0)	37.9 (20.2 to 59.9)	6.1 (-3.0 to 16.1)
Low bone mineral density	5.1 (4.2 to 6.1)	4.4 (3.6 to 5.2)	4.6 (3.8 to 5.5)	-15.0 (-26.5 to -0.3)	5.8 (-8.2 to 22.8)	5.7 (4.8 to 6.8)	5.6 (4.7 to 6.6)	6.1 (5.1 to 7.2)	-2.6 (-14.5 to 10.8)	9.6 (-4.1 to 25.6)
Impaired kidney function	2.3 (1.1 to 5.8)	2.3 (1.1 to 5.9)	2.4 (1.1 to 5.9)	1.7 (-4.1 to 7.6)	2.2 (-3.4 to 8.4)	3.0 (1.4 to 6.9)	3.0 (1.4 to 6.9)	3.1 (1.5 to 7.3)	-0.4 (-5.8 to 4.9)	5.0 (0.1 to 11.2)

Supplementary Results Table 1. Kentucky age-standardised Summary Exposure Values (SEVs) for Level 4 risk factors and percentage change, by sex, 1990, 2007, and 2017.Data in parentheses are 95% uncertainty intervals.										
	Male					Female				
	1990	2007	2017	Percentage change 1990-2007	Percentage change 2007-2017	1990	2007	2017	Percentage change 1990-2007	Percentage change 2007-2017
Unsafe water source	1.9 (1.0 to 3.2)	0.6 (0.4 to 1.1)	0.4 (0.2 to 0.6)	-66.1 (-83.0 to -32.8)	-40.6 (-65.6 to -2.7)	1.9 (1.0 to 3.2)	0.6 (0.4 to 1.1)	0.4 (0.2 to 0.6)	-66.1 (-83.0 to -32.8)	-40.6 (-65.6 to -2.7)
Unsafe sanitation	9.6 (5.0 to 15.5)	6.1 (2.8 to 11.5)	4.1 (1.8 to 7.5)	-36.1 (-69.9 to 35.4)	-33.4 (-64.2 to 30.2)	9.6 (5.0 to 15.5)	6.1 (2.8 to 11.5)	4.1 (1.8 to 7.5)	-36.1 (-69.9 to 35.4)	-33.4 (-64.2 to 30.2)
No access to handwashing facility	3.6 (2.6 to 4.8)	3.2 (2.3 to 4.4)	3.1 (2.2 to 4.2)	-10.5 (-36.1 to 29.2)	-4.0 (-25.8 to 23.4)	3.6 (2.6 to 4.8)	3.2 (2.3 to 4.4)	3.1 (2.2 to 4.2)	-10.5 (-36.1 to 29.2)	-4.0 (-25.8 to 23.4)
Ambient particulate matter pollution	27.9 (19.1 to 41.5)	27.8 (19.1 to 40.8)	21.5 (13.4 to 33.0)	-0.6 (-21.5 to 24.2)	-22.6 (-47.5 to -1.0)	27.7 (19.0 to 41.3)	27.7 (19.0 to 40.7)	21.4 (13.4 to 32.9)	-0.3 (-21.3 to 25.4)	-22.6 (-47.6 to -0.9)
Household air pollution from solid fuels	0.5 (0.2 to 1.3)	0.3 (0.1 to 0.8)	0.2 (0.1 to 0.8)	-44.8 (-79.7 to 26.9)	-14.5 (-66.0 to 89.9)	0.7 (0.2 to 4.2)	0.4 (0.1 to 2.6)	0.4 (0.1 to 2.5)	-40.7 (-79.4 to 26.8)	-12.7 (-66.2 to 78.5)
Ambient ozone pollution	49.3 (20.5 to 54.8)	49.2 (20.5 to 54.8)	49.7 (21.1 to 54.8)	-0.1 (-0.7 to 0.0)	1.0 (0.0 to 3.5)	49.3 (20.5 to 54.8)	49.2 (20.5 to 54.8)	49.7 (21.1 to 54.8)	-0.1 (-0.7 to 0.0)	1.0 (0.0 to 3.5)
Residential radon	13.8 (0.9 to 46.1)	13.8 (0.9 to 46.1)	13.8 (0.9 to 46.1)	0.0 (0.0 to 0.0)	0.0 (0.0 to 0.0)	13.8 (0.9 to 46.1)	13.8 (0.9 to 46.1)	13.8 (0.9 to 46.1)	0.0 (0.0 to 0.0)	0.0 (0.0 to 0.0)
Lead exposure	7.8 (3.5 to 12.1)	5.3 (2.1 to 9.1)	3.7 (1.3 to 6.8)	-32.1 (-42.8 to -23.2)	-30.4 (-39.7 to -23.6)	4.4 (1.1 to 8.5)	3.1 (0.6 to 6.6)	2.2 (0.4 to 5.1)	-29.1 (-43.5 to -19.7)	-29.1 (-41.5 to -22.1)
Occupational exposure to asbestos	4.7 (4.1 to 5.4)	4.1 (3.7 to 4.4)	3.9 (3.2 to 4.6)	-14.4 (-26.5 to -0.0)	-3.8 (-20.3 to 14.6)	0.7 (0.6 to 0.7)	0.7 (0.6 to 0.8)	0.8 (0.6 to 1.0)	7.1 (-6.5 to 22.6)	12.9 (-9.8 to 39.9)
Occupational exposure to arsenic	0.4 (0.0 to 0.9)	0.4 (0.0 to 1.0)	0.4 (0.0 to 0.9)	-	-	0.3 (0.0 to 0.8)	0.3 (0.0 to 0.8)	0.3 (0.0 to 0.8)	-	-
Occupational exposure to benzene	0.6 (0.0 to 1.6)	0.6 (0.0 to 1.6)	0.6 (0.0 to 1.6)	3.1 (-4.3 to 14.8)	-2.5 (-8.4 to 6.1)	0.8 (0.0 to 2.3)	0.9 (0.0 to 2.5)	0.9 (0.0 to 2.5)	13.0 (1.7 to 30.6)	0.9 (-6.8 to 12.7)
Occupational exposure to beryllium	0.0 (0.0 to 0.0)	0.0 (0.0 to 0.0)	0.0 (0.0 to 0.0)	0.5 (-5.1 to 6.7)	-0.1 (-4.7 to 4.4)	0.0 (0.0 to 0.0)	0.0 (0.0 to 0.0)	0.0 (0.0 to 0.0)	6.1 (-2.7 to 16.4)	-0.2 (-6.2 to 6.4)
Occupational exposure to cadmium	0.0 (0.0 to 0.0)	0.0 (0.0 to 0.1)	0.0 (0.0 to 0.1)	4.8 (-6.6 to 17.7)	-0.9 (-10.0 to 8.2)	0.0 (0.0 to 0.0)	0.0 (0.0 to 0.0)	0.0 (0.0 to 0.0)	3.5 (-11.2 to 23.2)	-2.1 (-13.8 to 10.2)
Occupational exposure to chromium	0.1 (0.1 to 0.1)	0.1 (0.1 to 0.1)	0.1 (0.1 to 0.1)	6.7 (-3.7 to 18.4)	-0.6 (-8.4 to 7.3)	0.1 (0.1 to 0.1)	0.1 (0.1 to 0.1)	0.1 (0.1 to 0.1)	5.9 (-7.3 to 23.3)	-2.1 (-11.7 to 8.5)
Occupational exposure to diesel engine exhaust	0.5 (0.5 to 0.6)	0.6 (0.5 to 0.6)	0.6 (0.5 to 0.6)	5.0 (-4.4 to 13.9)	1.4 (-5.7 to 9.0)	0.4 (0.4 to 0.5)	0.5 (0.4 to 0.5)	0.5 (0.4 to 0.5)	10.7 (-2.7 to 25.9)	-4.0 (-12.9 to 6.3)
Occupational exposure to formaldehyde	0.2 (0.2 to 0.2)	0.2 (0.2 to 0.3)	0.2 (0.2 to 0.3)	3.6 (-8.5 to 17.5)	-2.5 (-11.9 to 7.0)	0.2 (0.2 to 0.2)	0.2 (0.2 to 0.2)	0.2 (0.2 to 0.2)	4.0 (-10.2 to 23.0)	-2.0 (-12.5 to 9.5)
Occupational exposure to nickel	0.4 (0.0 to 1.6)	0.3 (0.0 to 1.5)	0.3 (0.0 to 1.5)	-	-	0.3 (0.0 to 1.2)	0.3 (0.0 to 1.2)	0.3 (0.0 to 1.2)	-	-
Occupational exposure to polycyclic aromatic hydrocarbons	0.2 (0.2 to 0.2)	0.2 (0.2 to 0.2)	0.2 (0.2 to 0.2)	6.2 (-3.5 to 17.3)	-1.2 (-8.8 to 6.4)	0.2 (0.2 to 0.2)	0.2 (0.2 to 0.3)	0.2 (0.2 to 0.3)	8.9 (-3.5 to 24.7)	-1.7 (-9.9 to 7.6)
Occupational exposure to silica	3.5 (0.5 to 11.2)	3.1 (0.4 to 10.0)	3.0 (0.4 to 9.6)	-11.3 (-22.2 to 2.1)	-2.9 (-12.7 to 7.6)	2.3 (0.3 to 7.4)	2.2 (0.3 to 7.0)	2.1 (0.3 to 6.8)	-4.5 (-20.2 to 19.0)	-2.8 (-17.5 to 13.8)
Occupational exposure to sulphuric acid	0.6 (0.1 to 1.8)	0.7 (0.1 to 2.0)	0.6 (0.1 to 2.1)	14.3 (2.3 to 27.6)	-2.0 (-10.3 to 6.6)	0.6 (0.1 to 1.8)	0.7 (0.1 to 2.1)	0.7 (0.1 to 2.2)	24.9 (5.5 to 48.7)	-4.7 (-17.2 to 9.1)
Occupational exposure to trichloroethylene	0.1 (0.1 to 0.1)	0.1 (0.1 to 0.1)	0.1 (0.1 to 0.1)	8.4 (0.7 to 17.1)	0.5 (-5.3 to 6.7)	0.1 (0.0 to 0.1)	0.1 (0.1 to 0.1)	0.1 (0.1 to 0.1)	7.5 (-4.0 to 21.8)	-2.4 (-10.4 to 6.6)
Occupational asthmagens	17.1 (14.4 to 20.2)	16.1 (13.6 to 19.0)	15.4 (12.8 to 18.4)	-5.8 (-15.5 to 4.7)	-4.3 (-12.0 to 4.5)	7.5 (6.1 to 9.1)	7.7 (6.2 to 9.3)	7.2 (5.8 to 8.8)	2.8 (-10.6 to 18.3)	-6.1 (-16.6 to 5.0)
Occupational particulate matter, gases, and fumes	7.1 (5.1 to 10.1)	6.9 (4.9 to 9.8)	6.6 (4.7 to 9.4)	-3.5 (-8.0 to 1.0)	-4.4 (-8.1 to -1.0)	3.7 (2.5 to 5.4)	3.6 (2.6 to 5.4)	3.5 (2.5 to 5.2)	-0.7 (-8.5 to 7.8)	-3.7 (-9.2 to 2.4)
Occupational noise	6.5 (5.9 to 7.0)	6.2 (5.7 to 6.8)	6.0 (5.5 to 6.5)	-3.6 (-6.8 to -0.0)	-4.2 (-6.6 to -1.8)	4.0 (3.4 to 4.5)	4.2 (3.8 to 4.7)	4.2 (3.8 to 4.7)	6.3 (0.1 to 13.0)	0.1 (-3.5 to 3.7)
Occupational ergonomic factors	9.3 (7.6 to 11.3)	8.2 (6.8 to 9.9)	7.8 (6.4 to 9.5)	-11.6 (-20.7 to 0.0)	-4.8 (-12.1 to 2.7)	5.0 (4.0 to 6.4)	5.0 (4.0 to 6.4)	4.9 (3.9 to 6.3)	-0.0 (-16.1 to 18.8)	-1.8 (-13.6 to 11.7)
Non-exclusive breastfeeding	0.5 (0.4 to 0.6)	0.5 (0.5 to 0.6)	0.5 (0.4 to 0.6)	-0.5 (-10.9 to 13.2)	-7.5 (-16.8 to 2.2)	0.5 (0.4 to 0.6)	0.5 (0.5 to 0.6)	0.5 (0.4 to 0.6)	-0.4 (-10.8 to 13.4)	-7.5 (-16.9 to 2.1)
Discontinued breastfeeding	2.1 (2.0 to 2.2)	2.1 (1.9 to 2.2)	2.1 (2.0 to 2.3)	-1.6 (-9.5 to 6.7)	2.4 (-3.5 to 9.6)	2.1 (2.0 to 2.3)	2.1 (1.9 to 2.2)	2.1 (2.0 to 2.2)	-1.8 (-9.6 to 6.5)	1.8 (-4.0 to 9.0)
Child underweight	0.1 (0.1 to 0.2)	0.1 (0.1 to 0.1)	0.1 (0.0 to 0.1)	-5.2 (-19.6 to 11.4)	-14.6 (-23.5 to -5.4)	0.1 (0.0 to 0.1)	0.1 (0.0 to 0.1)	0.1 (0.0 to 0.1)	-2.9 (-18.5 to 14.3)	-5.9 (-15.4 to 5.1)
Child wasting	0.1 (0.0 to 0.1)	0.1 (0.0 to 0.1)	0.1 (0.0 to 0.1)	-10.2 (-31.4 to 12.2)	-4.0 (-17.1 to 9.0)	0.1 (0.0 to 0.1)	0.1 (0.0 to 0.1)	0.1 (0.0 to 0.1)	5.8 (-16.6 to 34.0)	-6.3 (-21.0 to 10.2)
Child stunting	0.3 (0.1 to 0.6)	0.3 (0.1 to 0.5)	0.2 (0.1 to 0.4)	-9.1 (-52.1 to 66.4)	-13.2 (-43.1 to 28.8)	0.2 (0.1 to 0.4)	0.2 (0.1 to 0.3)	0.2 (0.1 to 0.3)	-9.9 (-45.2 to 44.9)	-5.3 (-30.7 to 29.8)
Short gestation for birth weight	0.0 (0.0 to 0.0)	0.0 (0.0 to 0.0)	0.0 (0.0 to 0.0)	4.5 (-20.2 to 32.5)	3.2 (-15.3 to 24.9)	0.0 (0.0 to 0.0)	0.0 (0.0 to 0.0)	0.0 (0.0 to 0.0)	7.7 (-19.7 to 50.2)	3.3 (-26.1 to 53.3)
Low birth weight for gestation	0.0 (0.0 to 0.0)	0.0 (0.0 to 0.0)	0.0 (0.0 to 0.0)	4.5 (-16.1 to 32.1)	-4.3 (-19.1 to 12.6)	0.0 (0.0 to 0.0)	0.0 (0.0 to 0.0)	0.0 (0.0 to 0.0)	0.5 (-26.9 to 32.8)	-4.3 (-35.4 to 27.8)
Iron deficiency	-	-	-	-	-	4.8 (3.1 to 6.9)	4.4 (2.9 to 6.3)	4.4 (2.8 to 6.4)	-8.1 (-30.4 to 21.4)	-1.0 (-23.4 to 24.4)
Vitamin A deficiency	0.5 (0.3 to 0.7)	0.5 (0.4 to 0.7)	0.6 (0.4 to 0.9)	3.7 (-17.3 to 32.4)	18.7 (-5.3 to 48.6)	0.7 (0.4 to 0.9)	0.6 (0.4 to 0.9)	0.8 (0.4 to 1.1)	-3.7 (-22.0 to 24.9)	19.2 (-5.0 to 62.7)
Zinc deficiency	0.5 (0.1 to 1.1)	0.2 (0.0 to 1.0)	0.2 (0.0 to 0.9)	-54.0 (-87.9 to 411.3)	-2.3 (-88.3 to 794.1)	0.5 (0.1 to 1.1)	0.2 (0.0 to 1.0)	0.2 (0.0 to 1.0)	-54.4 (-87.9 to 207.4)	2.2 (-87.1 to 808.7)
Smoking	15.9 (13.1 to 18.6)	9.0 (7.1 to 10.9)	6.8 (5.3 to 8.4)	-43.7 (-51.0 to -35.4)	-24.6 (-33.0 to -15.1)	11.1 (8.8 to 13.7)	6.6 (5.1 to 8.0)	5.1 (3.9 to 6.4)	-40.8 (-50.9 to -28.5)	-22.9 (-31.8 to -12.9)
Chewing tobacco	4.4 (3.8 to 5.0)	4.3 (3.3 to 5.6)	4.4 (3.1 to 6.0)	-0.9 (-26.9 to 33.6)	0.7 (-21.8 to 29.6)	0.2 (0.1 to 0.2)	0.1 (0.1 to 0.2)	0.1 (0.1 to 0.2)	-28.1 (-56.6 to 19.5)	-5.1 (-34.6 to 38.6)
Second-hand smoke	45.6 (42.5 to 49.1)	33.4 (31.9 to 35.1)	32.2 (29.2 to 35.3)	-26.7 (-34.0 to -18.9)	-3.8 (-12.4 to 5.6)	44.7 (41.9 to 47.8)	32.5 (31.3 to 33.6)	30.7 (28.7 to 32.7)	-27.3 (-33.5 to -21.2)	-5.4 (-12.0 to 1.2)
Alcohol use	20.4 (11.9 to 31.2)	22.4 (12.4 to 33.2)	24.1 (13.6 to 36.5)	9.7 (-29.6 to 70.3)	7.4 (-24.6 to 56.2)	10.7 (5.8 to 18.5)	11.4 (6.1 to 18.4)	11.8 (6.1 to 20.3)	7.2 (-31.6 to 69.7)	3.5 (-30.4 to 51.0)
Drug use	1.6 (1.2 to 2.1)	2.9 (2.4 to 3.5)	3.9 (3.2 to 4.8)	83.8 (56.2 to 119.9)	34.4 (21.3 to 49.9)	1.4 (1.1 to 1.8)	2.4 (1.9 to 2.9)	3.2 (2.6 to 3.9)	68.4 (47.4 to 95.2)	35.1 (21.4 to 52.0)
Diet low in fruits	33.4 (27.7 to 37.6)	33.6 (27.9 to 37.8)	34.6 (28.6 to 39.0)	0.4 (-6.4 to 8.2)	3.1 (-3.5 to 10.3)	28.1 (22.1 to 33.2)	31.8 (26.2 to 36.1)	32.4 (26.4 to 37.1)	13.2 (3.6 to 27.0)	1.8 (-6.1 to 9.5)
Diet low in vegetables	29.5 (22.6 to 35.5)	35.8 (30.4 to 40.0)	37.9 (33.0 to 41.9)	21.3 (8.2 to 43.4)	5.9 (-0.6 to 13.1)	31.7 (24.8 to 37.1)	36.4 (31.0 to 40.7)	38.8 (33.8 to 42.8)	14.8 (3.5 to 31.5)	6.4 (0.3 to 14.6)
Diet low in legumes	24.0 (17.1 to 29.8)	24.2 (17.8 to 30.0)	25.6 (19.2 to 31.2)	0.8 (-16.3 to 19.9)	5.9 (-7.3 to 23.2)	28.6 (22.4 to 33.7)	29.3 (23.5 to 34.2)	30.7 (25.1 to 35.7)	2.3 (-9.6 to 15.9)	4.8 (-4.8 to 15.6)
Diet low in whole grains	38.8 (35.3 to 41.6)	40.0 (36.6 to 42.6)	39.5 (36.1 to 42.3)	2.9 (-2.0 to 7.9)	-1.1 (-4.8 to 2.3)	39.7 (36.5 to 42.5)	40.8 (37.7 to 43.3)	40.4 (37.1 to 43.1)	2.7 (-1.8 to 8.1)	-0.9 (-4.3 to 2.4)
Diet low in nuts and seeds	47.4 (45.0 to 49.3)	39.1 (34.9 to 42.5)	39.7 (35.0 to 43.5)	-17.5 (-24.3 to -12.1)	1.5 (-4.9 to 7.4)	48.1 (45.8 to 50.0)	40.1 (35.7 to 43.7)	40.6 (36.0 to 44.2)	-16.5 (-23.6 to -11.2)	1.2 (-5.0 to 6.9)
Diet low in milk	29.0 (23.1 to 34.1)	27.6 (21.1 to 32.6)	28.1 (21.6 to 33.4)	-5.0 (-15.2 to 3.9)	1.8 (-5.7 to 10.1)	30.7 (25.1 to 35.4)	28.6 (22.5 to 33.6)	28.9 (22.7 to 33.8)	-6.8 (-15.7 to 1.6)	0.8 (-6.5 to 8.3)
Diet high in red meat	46.2 (40.2 to 50.7)	38.6 (29.1 to 46.9)	35.8 (24.9 to 45.0)	-16.4 (-34.8 to 1.3)	-7.2 (-24.5 to 12.2)	30.3 (22.5 to 36.6)	21.9 (12.6 to 30.8)	19.2 (9.6 to 28.5)	-27.6 (-53.1 to 2.9)	-12.6 (-40.4 to 19.4)
Diet high in processed meat	51.0 (46.5 to 53.6)	49.8 (45.1 to 53.2)	42.0 (33.6 to 47.8)	-2.4 (-11.0 to 5.7)	-15.6 (-27.0 to -5.1)	32.7 (26.7 to 38.1)	34.2 (27.4 to 40.1)	28.0 (21.1 to 34.3)	4.5 (-14.8 to 28.1)	-17.9 (-30.3 to -5.3)
Diet high in sugar-sweetened beverages	28.4 (14.1 to 39.5)	26.6 (13.1 to 36.4)	21.8 (10.5 to 31.9)	-6.4 (-38.1 to 42.5)	-17.9 (-42.6 to 10.3)	23.5 (10.8 to 35.8)	20.4 (9.6 to 31.5)	16.6 (7.7 to 26.2)	-13.0 (-49.5 to 42.5)	-18.8 (-46.5 to 15.5)
Diet low in fibre	34.0 (28.1 to 38.8)	26.1 (18.9 to 32.3)	27.6 (21.0 to 33.5)	-23.1 (-36.0 to -12.6)	5.8 (-3.9 to 18.5)	38.6 (33.8 to 42.3)	30.8 (24.6 to 35.8)	32.1 (26.1 to 37.1)	-20.3 (-31.0 to -12.6)	4.2 (-3.8 to 14.3)
Diet low in calcium	18.8 (11.6 to 24.7)	17.0 (9.9 to 23.1)	17.9 (10.7 to 24.0)	-9.5 (-22.7 to 2.2)	4.9 (-5.2 to 18.5)	22.9 (16.0 to 28.3)	21.0 (13.9 to 26.9)	21.8 (14.7 to 27.6)	-8.1 (-20.7 to 0.8)	3.8 (-3.3 to 14.2)
Diet low in seafood omega-3 fatty acids	31.3 (26.0 to 36.2)	27.1 (21.0 to 32.4)	28.7 (22.9 to 33.8)	-13.4 (-23.5 to -3.9)	5.7 (-2.5 to 16.0)	34.6 (29.7 to 38.8)	31.3 (25.6 to 36.1)	32.3 (26.8 to 36.9)	-9.6 (-18.9 to -2.3)	3.3 (-2.8 to 11.5)
Diet low in polyunsaturated fatty acids	9.7 (3.6 to 16.5)	6.5 (1.6 to 13.0)	5.0 (1.0 to 10.9)	-33.6 (-72.7 to 1.9)	-22.6 (-63.1 to 33.5)	10.7 (4.0 to 17.7)	7.2 (1.8 to 14.0)	5.6 (1.2 to 11.6)	-32.5 (-72.3 to -0.4)	-22.7 (-60.1 to 29.0)
Diet high in trans fatty acids	16.7 (12.1 to 22.4)	9.9 (5.9 to 14.2)	7.2 (3.5 to 11.2)	-41.0 (-55.6 to -28.4)	-27.5 (-44.0 to -16.5)	23.6 (17.9 to 31.6)	13.3 (9.0 to 17.9)	9.7 (5.7 to 14.0)	-43.7 (-55.4 to -32.5)	-26.9 (-40.2 to -16.4)
Diet high in sodium	6.8 (0.5 to 14.1)	12.1 (3.5 to 19.8)	12.6 (3.9 to 20.4)	77.9 (26.0 to 807.2)	3.9 (-19.7 to 49.4)	5.9 (0.3 to 12.8)	10.8 (2.4 to 18.4)	11.2 (2.7 to 18.7)	82.5 (29.0 to 928.8)	4.0 (-23.1 to 53.0)
Intimate partner violence	-	-	-	-	-	1.3 (1.1 to 1.6)	1.0 (0.8 to 1.1)	1.2 (1.0 to 1.4)	-28.3 (-32.3 to -24.0)	25.8 (19.4 to 32.9)
Childhood sexual abuse	5.6 (4.6 to 6.9)	5.1 (4.1 to 6.2)	5.1 (4.1 to 6.2)	-9.8 (-15.1 to -4.3)	0.4 (-4.7 to 6.0)	13.8 (11.0 to 16.9)	12.1 (9.8 to 14.7)	12.2 (9.9 to 14.8)	-11.9 (-17.2 to -6.0)	0.4 (-4.8 to 6.5)

Supplementary Results Table 1. Kentucky age-standardised Summary Exposure Values (SEVs) for Level 4 risk factors and percentage change, by sex, 1990, 2007, and 2017.Data in parentheses are 95% uncertainty intervals.

	Male					Female				
	1990	2007	2017	Percentage change 1990-2007	Percentage change 2007-2017	1990	2007	2017	Percentage change 1990-2007	Percentage change 2007-2017
Bullying victimization	6.1<br>(2.6 to 12.5)	8.8<br>(3.9 to 17.8)	8.6<br>(3.8 to 17.2)	45.3<br>(29.7 to 65.0)	-2.2<br>(-9.6 to 5.2)	4.6<br>(2.0 to 9.3)	7.5<br>(3.3 to 14.9)	7.5<br>(3.3 to 15.2)	61.9<br>(43.5 to 89.5)	-0.3<br>(-8.0 to 8.6)
Low physical activity	0.4<br>(0.2 to 0.8)	0.4<br>(0.2 to 0.8)	0.4<br>(0.2 to 0.7)	0.5<br>(-1.5 to 2.5)	-2.1<br>(-4.0 to -0.5)	0.4<br>(0.2 to 0.9)	0.4<br>(0.2 to 0.9)	0.4<br>(0.2 to 0.8)	0.6<br>(-2.1 to 3.2)	-3.2<br>(-5.3 to -1.1)
High fasting plasma glucose	1.4<br>(0.8 to 2.4)	3.2<br>(2.1 to 4.8)	3.6<br>(2.3 to 5.5)	133.9<br>(33.6 to 303.1)	10.6<br>(-25.4 to 63.6)	1.7<br>(1.0 to 2.7)	3.4<br>(2.0 to 5.5)	3.5<br>(2.0 to 5.3)	106.8<br>(23.8 to 277.6)	1.6<br>(-40.4 to 74.0)
High LDL cholesterol	21.2<br>(19.2 to 23.3)	11.6<br>(9.9 to 13.3)	10.2<br>(8.7 to 11.8)	-45.4<br>(-50.4 to -40.6)	-11.9<br>(-16.3 to -7.7)	19.9<br>(18.0 to 21.9)	11.5<br>(10.0 to 13.2)	10.5<br>(9.1 to 12.2)	-42.3<br>(-47.0 to -37.7)	-8.2<br>(-12.4 to -3.7)
High systolic blood pressure	6.4<br>(5.5 to 7.3)	5.0<br>(4.2 to 5.8)	5.1<br>(4.4 to 5.9)	-21.9<br>(-30.1 to -13.2)	3.1<br>(-6.5 to 13.3)	6.1<br>(5.2 to 6.8)	4.7<br>(4.1 to 5.4)	4.8<br>(4.0 to 5.5)	-22.2<br>(-31.2 to -11.9)	0.8<br>(-9.2 to 12.6)
High body-mass index	13.5<br>(9.9 to 18.2)	20.9<br>(16.2 to 26.6)	21.1<br>(16.2 to 26.5)	55.0<br>(35.5 to 77.1)	1.0<br>(-7.0 to 10.1)	16.0<br>(12.2 to 21.3)	22.9<br>(18.3 to 29.0)	23.7<br>(18.9 to 29.7)	42.9<br>(25.2 to 64.1)	3.4<br>(-5.7 to 13.2)
Low bone mineral density	5.2<br>(4.2 to 6.1)	4.4<br>(3.6 to 5.2)	4.7<br>(3.7 to 5.7)	-14.7<br>(-25.8 to -1.2)	5.8<br>(-8.3 to 22.0)	5.8<br>(4.8 to 6.9)	5.7<br>(4.8 to 6.6)	6.2<br>(5.2 to 7.4)	-1.8<br>(-14.5 to 12.2)	9.6<br>(-3.9 to 24.7)
Impaired kidney function	2.4<br>(1.2 to 6.0)	2.5<br>(1.2 to 6.2)	2.5<br>(1.2 to 6.2)	2.4<br>(-2.4 to 8.5)	1.6<br>(-4.0 to 8.1)	3.1<br>(1.5 to 7.1)	3.1<br>(1.5 to 7.2)	3.3<br>(1.6 to 7.4)	0.5<br>(-4.7 to 6.1)	3.6<br>(-1.0 to 9.0)

Supplementary Results Table 1. Louisiana age-standardised Summary Exposure Values (SEVs) for Level 4 risk factors and percentage change, by sex, 1990, 2007, and 2017.Data in parentheses are 95% uncertainty intervals.

	Male					Female				
	1990	2007	2017	Percentage change 1990-2007	Percentage change 2007-2017	1990	2007	2017	Percentage change 1990-2007	Percentage change 2007-2017
Unsafe water source	2.0 (1.1 to 3.4)	0.6 (0.4 to 1.0)	0.4 (0.2 to 0.6)	-68.7 (-83.9 to -36.7)	-41.4 (-63.6 to -3.4)	2.0 (1.1 to 3.4)	0.6 (0.4 to 1.0)	0.4 (0.2 to 0.6)	-68.7 (-83.9 to -36.7)	-41.4 (-63.6 to -3.4)
Unsafe sanitation	10.2 (5.5 to 17.9)	6.1 (2.9 to 11.1)	4.0 (1.7 to 7.5)	-40.8 (-73.0 to 29.1)	-34.4 (-65.6 to 20.9)	10.2 (5.5 to 17.9)	6.1 (2.9 to 11.1)	4.0 (1.7 to 7.5)	-40.8 (-73.0 to 29.1)	-34.4 (-65.6 to 20.9)
No access to handwashing facility	3.6 (2.6 to 4.9)	3.2 (2.3 to 4.4)	3.1 (2.2 to 4.1)	-10.3 (-36.6 to 28.3)	-4.3 (-26.9 to 24.1)	3.6 (2.6 to 4.9)	3.2 (2.3 to 4.4)	3.1 (2.2 to 4.1)	-10.3 (-36.6 to 28.3)	-4.3 (-26.9 to 24.1)
Ambient particulate matter pollution	26.2 (17.0 to 40.8)	25.5 (17.0 to 39.0)	19.6 (11.4 to 32.0)	-2.5 (-23.6 to 29.1)	-23.3 (-50.5 to 2.1)	26.0 (16.9 to 40.5)	25.4 (16.9 to 38.9)	19.5 (11.3 to 32.0)	-2.2 (-23.8 to 30.0)	-23.3 (-50.5 to 2.1)
Household air pollution from solid fuels	0.4 (0.1 to 1.2)	0.3 (0.1 to 0.6)	0.2 (0.0 to 0.6)	-40.8 (-79.7 to 35.6)	-15.2 (-66.7 to 66.4)	0.6 (0.2 to 3.8)	0.4 (0.1 to 2.2)	0.3 (0.1 to 2.0)	-37.6 (-79.5 to 36.0)	-13.8 (-66.5 to 66.0)
Ambient ozone pollution	43.4 (15.7 to 54.8)	46.5 (17.8 to 54.8)	49.7 (21.1 to 54.8)	7.1 (0.0 to 15.9)	6.9 (0.0 to 21.5)	43.4 (15.7 to 54.8)	46.5 (17.8 to 54.8)	49.7 (21.1 to 54.8)	7.1 (0.0 to 15.9)	6.9 (0.0 to 21.5)
Residential radon	9.4 (0.3 to 32.2)	9.4 (0.3 to 32.2)	9.4 (0.3 to 32.2)	0.0 (0.0 to 0.0)	0.0 (0.0 to 0.0)	9.4 (0.3 to 32.2)	9.4 (0.3 to 32.2)	9.4 (0.3 to 32.2)	0.0 (0.0 to 0.0)	0.0 (0.0 to 0.0)
Lead exposure	10.2 (5.4 to 14.6)	6.7 (3.1 to 10.6)	4.6 (1.9 to 7.8)	-33.8 (-44.2 to -24.4)	-31.9 (-39.8 to -25.6)	5.9 (1.9 to 10.0)	4.0 (1.1 to 7.7)	2.8 (0.6 to 5.8)	-31.5 (-45.9 to -21.3)	-30.7 (-41.5 to -24.3)
Occupational exposure to asbestos	6.9 (6.1 to 7.8)	5.7 (5.3 to 6.2)	5.7 (4.8 to 6.5)	-17.7 (-27.8 to -4.2)	-0.8 (-16.8 to 16.4)	1.2 (1.1 to 1.4)	1.3 (1.2 to 1.4)	1.3 (1.1 to 1.6)	2.2 (-10.5 to 16.8)	5.1 (-15.5 to 29.0)
Occupational exposure to arsenic	0.3 (0.0 to 0.8)	0.3 (0.0 to 0.8)	0.3 (0.0 to 0.8)	-	-	0.3 (0.0 to 0.6)	0.3 (0.0 to 0.7)	0.3 (0.0 to 0.7)	-	-
Occupational exposure to benzene	0.6 (0.0 to 1.7)	0.6 (0.0 to 1.7)	0.6 (0.0 to 1.6)	1.6 (-6.1 to 13.0)	-2.9 (-9.0 to 5.7)	0.8 (0.0 to 2.3)	0.9 (0.1 to 2.6)	0.9 (0.1 to 2.6)	11.5 (0.7 to 27.2)	0.6 (-7.0 to 11.9)
Occupational exposure to beryllium	0.0 (0.0 to 0.0)	0.0 (0.0 to 0.0)	0.0 (0.0 to 0.0)	-0.6 (-6.0 to 5.0)	-0.1 (-4.3 to 4.5)	0.0 (0.0 to 0.0)	0.0 (0.0 to 0.0)	0.0 (0.0 to 0.0)	6.9 (-1.5 to 16.1)	-0.1 (-5.8 to 6.7)
Occupational exposure to cadmium	0.0 (0.0 to 0.0)	0.0 (0.0 to 0.0)	0.0 (0.0 to 0.0)	2.8 (-8.4 to 16.0)	1.2 (-7.8 to 11.0)	0.0 (0.0 to 0.0)	0.0 (0.0 to 0.0)	0.0 (0.0 to 0.0)	8.0 (-5.7 to 24.3)	-0.5 (-10.9 to 11.8)
Occupational exposure to chromium	0.1 (0.1 to 0.1)	0.1 (0.1 to 0.1)	0.1 (0.1 to 0.1)	4.3 (-5.3 to 15.4)	1.2 (-6.6 to 9.6)	0.1 (0.1 to 0.1)	0.1 (0.1 to 0.1)	0.1 (0.1 to 0.1)	9.1 (-2.6 to 22.9)	-0.9 (-9.5 to 9.0)
Occupational exposure to diesel engine exhaust	0.6 (0.6 to 0.7)	0.6 (0.6 to 0.7)	0.6 (0.6 to 0.7)	0.9 (-8.4 to 9.9)	0.0 (-6.5 to 7.5)	0.5 (0.4 to 0.5)	0.5 (0.4 to 0.6)	0.5 (0.4 to 0.5)	6.1 (-6.4 to 21.8)	-4.8 (-13.5 to 5.3)
Occupational exposure to formaldehyde	0.2 (0.2 to 0.2)	0.2 (0.2 to 0.2)	0.2 (0.2 to 0.2)	1.2 (-10.4 to 15.1)	-0.2 (-9.7 to 10.0)	0.2 (0.1 to 0.2)	0.2 (0.2 to 0.2)	0.2 (0.2 to 0.2)	8.0 (-4.3 to 22.8)	-0.3 (-9.6 to 11.1)
Occupational exposure to nickel	0.3 (0.0 to 1.5)	0.3 (0.0 to 1.3)	0.3 (0.0 to 1.3)	-	-	0.2 (0.0 to 0.9)	0.2 (0.0 to 1.0)	0.2 (0.0 to 1.0)	-	-
Occupational exposure to polycyclic aromatic hydrocarbons	0.2 (0.2 to 0.2)	0.2 (0.2 to 0.2)	0.2 (0.2 to 0.2)	4.2 (-5.2 to 14.5)	-0.1 (-7.3 to 7.6)	0.2 (0.2 to 0.2)	0.2 (0.2 to 0.2)	0.2 (0.2 to 0.2)	12.4 (1.3 to 24.6)	-1.0 (-8.3 to 7.7)
Occupational exposure to silica	3.5 (0.5 to 10.9)	2.9 (0.4 to 9.3)	2.8 (0.4 to 8.9)	-15.7 (-27.4 to -1.4)	-5.1 (-15.1 to 6.0)	1.9 (0.3 to 6.0)	1.8 (0.3 to 6.0)	1.8 (0.3 to 5.9)	-4.0 (-19.9 to 15.3)	-2.1 (-15.6 to 15.0)
Occupational exposure to sulphuric acid	0.5 (0.1 to 1.7)	0.6 (0.1 to 1.9)	0.6 (0.1 to 1.9)	16.7 (4.2 to 31.6)	-2.3 (-10.7 to 6.9)	0.5 (0.1 to 1.5)	0.6 (0.1 to 2.1)	0.6 (0.1 to 2.0)	35.9 (14.2 to 61.4)	-6.1 (-18.5 to 8.7)
Occupational exposure to trichloroethylene	0.1 (0.1 to 0.1)	0.1 (0.1 to 0.1)	0.1 (0.1 to 0.1)	6.0 (-1.7 to 14.5)	2.0 (-3.6 to 8.4)	0.1 (0.0 to 0.1)	0.1 (0.1 to 0.1)	0.1 (0.0 to 0.1)	8.7 (-2.4 to 21.1)	-1.8 (-9.4 to 7.4)
Occupational asthmagens	16.6 (14.2 to 19.5)	15.1 (12.9 to 17.8)	14.4 (12.1 to 17.1)	-9.3 (-18.4 to 0.7)	-4.6 (-12.0 to 3.1)	7.1 (5.9 to 8.6)	7.2 (5.9 to 8.7)	6.8 (5.4 to 8.2)	1.2 (-11.9 to 15.8)	-6.2 (-16.5 to 5.9)
Occupational particulate matter, gases, and fumes	6.8 (4.9 to 9.7)	6.4 (4.6 to 9.2)	6.1 (4.3 to 8.7)	-5.3 (-10.1 to -0.8)	-4.8 (-8.7 to -1.3)	3.1 (2.1 to 4.6)	3.1 (2.2 to 4.5)	3.0 (2.1 to 4.4)	0.4 (-7.0 to 9.6)	-3.1 (-8.9 to 2.9)
Occupational noise	6.4 (5.9 to 7.0)	6.2 (5.7 to 6.8)	5.9 (5.4 to 6.5)	-3.5 (-7.0 to -0.1)	-4.8 (-7.1 to -2.4)	3.9 (3.3 to 4.4)	4.1 (3.7 to 4.6)	4.1 (3.7 to 4.6)	6.4 (0.8 to 12.8)	0.2 (-3.2 to 4.2)
Occupational ergonomic factors	9.1 (7.7 to 11.1)	8.1 (6.7 to 9.8)	7.7 (6.4 to 9.4)	-11.2 (-21.0 to -1.1)	-5.0 (-12.1 to 3.1)	4.9 (3.9 to 6.2)	5.0 (4.0 to 6.2)	4.9 (3.9 to 6.2)	0.9 (-14.4 to 17.4)	-1.8 (-12.0 to 10.4)
Non-exclusive breastfeeding	0.5 (0.4 to 0.6)	0.5 (0.5 to 0.6)	0.5 (0.4 to 0.6)	0.3 (-11.4 to 14.3)	-7.2 (-16.2 to 2.4)	0.5 (0.4 to 0.6)	0.5 (0.5 to 0.6)	0.5 (0.4 to 0.6)	0.4 (-11.3 to 14.4)	-7.6 (-16.6 to 2.0)
Discontinued breastfeeding	2.0 (1.9 to 2.2)	2.0 (1.9 to 2.2)	2.1 (2.0 to 2.3)	-0.6 (-8.0 to 7.8)	4.5 (-1.6 to 11.6)	2.0 (1.9 to 2.2)	2.0 (1.9 to 2.2)	2.1 (2.0 to 2.3)	-1.0 (-8.4 to 7.4)	5.5 (-0.7 to 12.6)
Child underweight	0.1 (0.1 to 0.2)	0.1 (0.1 to 0.1)	0.1 (0.0 to 0.1)	-6.9 (-21.4 to 10.2)	-14.9 (-23.4 to -5.8)	0.1 (0.0 to 0.1)	0.1 (0.0 to 0.1)	0.1 (0.0 to 0.1)	-4.7 (-20.1 to 13.0)	-6.0 (-16.3 to 4.8)
Child wasting	0.1 (0.0 to 0.1)	0.1 (0.0 to 0.1)	0.1 (0.0 to 0.1)	-11.2 (-29.4 to 9.6)	-4.3 (-18.2 to 9.9)	0.1 (0.0 to 0.1)	0.1 (0.0 to 0.1)	0.1 (0.0 to 0.1)	5.9 (-16.1 to 33.7)	-6.3 (-20.1 to 10.1)
Child stunting	0.3 (0.1 to 0.6)	0.3 (0.1 to 0.5)	0.2 (0.1 to 0.4)	-9.5 (-54.1 to 58.3)	-13.6 (-43.0 to 21.7)	0.2 (0.1 to 0.4)	0.2 (0.1 to 0.3)	0.2 (0.1 to 0.3)	-11.8 (-47.5 to 40.7)	-6.3 (-34.2 to 32.5)
Short gestation for birth weight	0.0 (0.0 to 0.0)	0.0 (0.0 to 0.0)	0.0 (0.0 to 0.0)	10.8 (-12.9 to 44.5)	2.0 (-27.6 to 42.1)	0.0 (0.0 to 0.0)	0.0 (0.0 to 0.0)	0.0 (0.0 to 0.0)	7.0 (-25.0 to 52.4)	2.5 (-19.3 to 39.7)
Low birth weight for gestation	0.0 (0.0 to 0.0)	0.0 (0.0 to 0.0)	0.0 (0.0 to 0.0)	-1.8 (-22.1 to 23.7)	-1.8 (-26.3 to 27.5)	0.0 (0.0 to 0.0)	0.0 (0.0 to 0.0)	0.0 (0.0 to 0.0)	-1.1 (-27.9 to 38.3)	-1.7 (-25.3 to 22.6)
Iron deficiency	-	-	-	-	-	4.9 (3.2 to 7.0)	4.4 (2.9 to 6.3)	4.3 (2.8 to 6.4)	-10.6 (-33.5 to 21.1)	-0.9 (-22.4 to 26.1)
Vitamin A deficiency	0.4 (0.3 to 0.6)	0.5 (0.4 to 0.7)	0.6 (0.4 to 0.9)	19.9 (-2.0 to 44.2)	15.4 (-5.3 to 42.4)	0.6 (0.4 to 0.8)	0.6 (0.4 to 0.8)	0.6 (0.4 to 0.9)	7.4 (-16.6 to 29.2)	5.8 (-10.5 to 28.7)
Zinc deficiency	0.5 (0.1 to 1.1)	0.2 (0.0 to 1.0)	0.2 (0.0 to 1.0)	-55.2 (-88.9 to 252.4)	-3.4 (-88.8 to 861.6)	0.5 (0.1 to 1.1)	0.2 (0.0 to 1.0)	0.2 (0.0 to 0.9)	-53.9 (-87.9 to 182.1)	1.6 (-87.0 to 763.9)
Smoking	14.8 (11.9 to 17.3)	7.9 (6.2 to 9.5)	6.4 (4.9 to 8.0)	-46.6 (-54.7 to -37.4)	-19.1 (-28.7 to -8.4)	9.8 (7.4 to 12.2)	5.2 (4.0 to 6.5)	4.2 (3.2 to 5.4)	-46.3 (-57.4 to -32.9)	-19.5 (-28.6 to -9.1)
Chewing tobacco	2.0 (1.7 to 2.4)	2.2 (1.6 to 2.9)	2.1 (1.4 to 3.1)	8.3 (-22.3 to 49.5)	-3.6 (-28.0 to 27.4)	0.1 (0.1 to 0.2)	0.1 (0.1 to 0.2)	0.1 (0.1 to 0.2)	-16.4 (-50.6 to 38.8)	-12.2 (-39.9 to 32.0)
Second-hand smoke	43.8 (40.2 to 48.0)	27.9 (26.3 to 29.4)	26.9 (24.2 to 29.6)	-36.2 (-43.5 to -27.8)	-3.7 (-12.3 to 6.1)	43.6 (40.3 to 47.1)	27.5 (26.3 to 28.7)	26.2 (24.4 to 28.1)	-37.0 (-43.9 to -29.9)	-4.7 (-11.1 to 1.9)
Alcohol use	19.8 (10.5 to 31.3)	21.2 (11.0 to 33.3)	22.6 (11.4 to 36.5)	7.1 (-31.7 to 70.3)	6.5 (-28.3 to 56.8)	11.9 (6.1 to 20.3)	12.6 (6.7 to 20.8)	13.3 (6.7 to 22.7)	5.7 (-29.1 to 61.2)	5.7 (-26.1 to 55.7)
Drug use	2.0 (1.4 to 3.0)	2.8 (2.2 to 3.8)	3.5 (2.7 to 4.7)	44.4 (24.7 to 72.4)	23.0 (13.3 to 34.1)	1.5 (1.1 to 2.2)	2.1 (1.7 to 2.8)	2.6 (2.1 to 3.5)	36.3 (21.0 to 56.8)	25.6 (14.9 to 37.6)
Diet low in fruits	30.4 (24.5 to 35.1)	30.7 (24.7 to 35.4)	31.7 (25.5 to 36.6)	0.8 (-7.5 to 10.5)	3.4 (-4.2 to 12.8)	25.7 (19.4 to 31.2)	29.1 (23.1 to 34.0)	30.0 (23.5 to 35.3)	13.1 (2.1 to 29.6)	3.3 (-6.3 to 13.1)
Diet low in vegetables	28.7 (21.2 to 34.6)	36.1 (30.8 to 40.5)	37.8 (32.9 to 42.0)	26.0 (11.5 to 51.6)	4.7 (-2.1 to 12.6)	31.8 (24.9 to 37.0)	36.5 (31.1 to 40.6)	38.2 (32.9 to 42.3)	14.6 (2.8 to 32.9)	4.7 (-1.8 to 12.5)
Diet low in legumes	24.1 (17.2 to 30.0)	24.2 (17.8 to 30.4)	25.5 (18.8 to 31.1)	0.3 (-15.9 to 20.7)	5.3 (-7.6 to 21.9)	28.7 (22.9 to 34.0)	29.4 (23.6 to 34.5)	30.7 (25.0 to 35.9)	2.5 (-9.6 to 16.2)	4.5 (-4.8 to 14.6)
Diet low in whole grains	38.9 (35.4 to 41.8)	39.9 (36.6 to 42.7)	39.2 (35.7 to 42.2)	2.6 (-2.2 to 7.8)	-1.6 (-5.6 to 1.8)	39.7 (36.2 to 42.5)	40.7 (37.6 to 43.3)	40.1 (36.8 to 42.8)	2.4 (-2.0 to 7.2)	-1.3 (-4.6 to 2.1)
Diet low in nuts and seeds	47.5 (44.9 to 49.4)	38.6 (34.2 to 42.2)	38.3 (33.0 to 42.4)	-18.6 (-26.2 to -12.9)	-0.9 (-8.2 to 5.4)	48.1 (45.8 to 49.9)	39.8 (35.3 to 43.1)	39.3 (34.3 to 43.2)	-17.3 (-24.4 to -11.9)	-1.2 (-8.4 to 5.1)
Diet low in milk	29.0 (23.2 to 34.0)	27.3 (20.9 to 32.4)	27.3 (21.1 to 32.7)	-6.1 (-16.0 to 3.0)	0.1 (-7.7 to 8.2)	30.8 (25.3 to 35.4)	28.4 (22.4 to 33.4)	28.1 (21.9 to 33.2)	-7.8 (-17.5 to -0.1)	-0.9 (-8.3 to 7.0)
Diet high in red meat	49.2 (43.6 to 52.7)	41.9 (32.8 to 48.6)	39.1 (28.3 to 47.3)	-14.9 (-30.2 to -1.4)	-6.7 (-23.5 to 8.3)	38.9 (31.7 to 44.7)	26.2 (16.7 to 34.9)	22.8 (12.9 to 32.8)	-32.6 (-52.1 to -10.8)	-13.3 (-38.8 to 13.0)
Diet high in processed meat	39.9 (33.4 to 45.0)	44.1 (36.9 to 49.5)	37.9 (28.8 to 45.4)	10.5 (-5.5 to 28.5)	-14.0 (-25.3 to -2.5)	26.7 (21.1 to 31.4)	31.0 (24.2 to 37.1)	27.3 (20.4 to 34.0)	16.0 (-5.1 to 44.4)	-11.9 (-25.3 to 2.3)
Diet high in sugar-sweetened beverages	28.1 (14.2 to 39.2)	26.9 (13.4 to 37.4)	23.5 (11.2 to 33.7)	-4.2 (-36.3 to 47.9)	-12.7 (-39.1 to 18.1)	23.3 (11.1 to 34.2)	20.7 (10.3 to 31.1)	17.6 (8.3 to 28.0)	-11.0 (-45.4 to 47.9)	-15.0 (-42.7 to 26.6)
Diet low in fibre	34.1 (28.2 to 38.6)	26.1 (19.3 to 31.8)	27.4 (20.5 to 33.2)	-23.3 (-36.0 to -13.1)	4.8 (-5.4 to 17.4)	38.7 (33.5 to 42.4)	30.6 (24.3 to 35.5)	31.6 (25.6 to 36.6)	-20.8 (-30.7 to -12.8)	3.3 (-4.6 to 12.8)
Diet low in calcium	20.1 (12.9 to 26.1)	17.3 (9.9 to 23.6)	17.1 (9.8 to 23.5)	-14.1 (-29.4 to -3.4)	-1.3 (-11.4 to 10.7)	24.4 (17.9 to 29.7)	21.5 (14.5 to 27.1)	21.2 (14.0 to 27.0)	-12.2 (-24.5 to -3.5)	-1.2 (-8.4 to 7.3)
Diet low in seafood omega-3 fatty acids	31.5 (26.1 to 36.3)	26.8 (20.5 to 32.1)	27.2 (20.9 to 32.6)	-15.1 (-25.8 to -6.3)	1.7 (-7.1 to 11.8)	34.7 (29.9 to 38.9)	30.8 (25.6 to 35.6)	30.9 (25.5 to 35.9)	-11.2 (-19.5 to -4.2)	0.4 (-6.2 to 7.6)
Diet low in polyunsaturated fatty acids	10.0 (3.7 to 16.9)	6.2 (1.4 to 12.1)	4.4 (0.8 to 9.6)	-37.6 (-76.1 to -4.1)	-29.6 (-67.2 to 19.8)	10.8 (4.3 to 18.2)	6.9 (1.7 to 13.5)	4.8 (0.9 to 10.5)	-35.8 (-74.4 to -1.4)	-30.2 (-66.5 to 13.0)
Diet high in trans fatty acids	16.8 (12.1 to 22.2)	9.8 (5.8 to 14.4)	7.1 (3.5 to 11.2)	-41.5 (-56.1 to -28.8)	-27.5 (-42.0 to -16.4)	23.6 (18.2 to 32.0)	13.3 (9.1 to 18.6)	9.7 (5.8 to 13.9)	-43.5 (-54.4 to -32.1)	-27.0 (-40.3 to -16.4)
Diet high in sodium	6.9 (0.6 to 14.4)	12.2 (3.6 to 20.1)	12.9 (4.3 to 20.9)	77.2 (22.4 to 771.6)	5.5 (-20.5 to 62.5)	5.9 (0.3 to 12.8)	10.7 (2.6 to 18.2)	11.3 (2.8 to 18.8)	82.3 (28.2 to 1073.8)	5.3 (-18.3 to 55.1)
Intimate partner violence	-	-	-	-	-	1.0 (0.8 to 1.3)	1.0 (0.8 to 1.1)	1.0 (0.8 to 1.2)	-4.7 (-14.0 to 6.4)	0.5 (-8.0 to 9.4)
Childhood sexual abuse	3.5 (2.6 to 4.9)	3.7 (3.2 to 4.4)	3.7 (3.1 to 4.4)	6.4 (-14.2 to 28.7)	-0.1 (-4.7 to 5.1)	11.0 (8.3 to 14.3)	10.4 (8.8 to 12.2)	10.4 (8.8 to 12.3)	-5.5 (-21.6 to 11.1)	0.2 (-4.8 to 5.6)

Supplementary Results Table 1. Louisiana age-standardised Summary Exposure Values (SEVs) for Level 4 risk factors and percentage change, by sex, 1990, 2007, and 2017.Data in parentheses are 95% uncertainty intervals.										
	**Male**					**Female**				
	**1990**	**2007**	**2017**	**Percentage change 1990-2007**	**Percentage change 2007-2017**	**1990**	**2007**	**2017**	**Percentage change 1990-2007**	**Percentage change 2007-2017**
Bullying victimization	6.1 (2.5 to 13.0)	8.8 (3.6 to 19.3)	8.6 (3.5 to 19.2)	46.0 (30.0 to 66.7)	-3.1 (-10.2 to 4.2)	4.6 (1.8 to 10.2)	7.5 (3.1 to 16.7)	7.4 (3.1 to 16.5)	63.2 (44.3 to 91.2)	-1.0 (-8.7 to 7.9)
Low physical activity	0.4 (0.2 to 0.8)	0.4 (0.2 to 0.8)	0.4 (0.2 to 0.8)	-0.1 (-2.5 to 2.4)	0.9 (-0.6 to 2.7)	0.5 (0.2 to 0.9)	0.5 (0.2 to 0.9)	0.5 (0.2 to 0.9)	0.6 (-1.8 to 3.4)	-0.9 (-2.6 to 0.5)
High fasting plasma glucose	1.7 (0.9 to 2.9)	3.0 (1.9 to 4.4)	3.4 (2.1 to 5.1)	80.9 (5.4 to 247.7)	11.9 (-25.2 to 71.6)	2.0 (1.1 to 3.3)	3.7 (2.1 to 5.7)	3.6 (2.0 to 5.7)	82.3 (2.4 to 235.0)	-3.0 (-42.2 to 62.7)
High LDL cholesterol	20.6 (18.6 to 22.7)	11.5 (9.8 to 13.2)	10.1 (8.6 to 11.8)	-44.4 (-49.4 to -39.6)	-11.5 (-15.8 to -7.0)	19.3 (17.5 to 21.3)	11.3 (9.8 to 13.0)	10.6 (9.0 to 12.2)	-41.4 (-46.2 to -37.0)	-6.7 (-10.8 to -2.3)
High systolic blood pressure	6.7 (5.9 to 7.6)	5.3 (4.6 to 6.0)	5.5 (4.7 to 6.3)	-21.4 (-29.7 to -12.6)	3.1 (-5.9 to 12.6)	6.4 (5.5 to 7.3)	5.0 (4.4 to 5.8)	5.1 (4.3 to 5.8)	-21.0 (-30.2 to -10.1)	0.2 (-9.8 to 10.9)
High body-mass index	13.6 (9.8 to 18.4)	21.2 (16.5 to 27.1)	22.1 (17.1 to 28.4)	56.4 (37.2 to 82.6)	4.0 (-4.6 to 13.6)	16.9 (13.0 to 21.9)	23.1 (18.9 to 28.8)	24.6 (19.7 to 30.7)	36.1 (20.0 to 56.1)	6.8 (-2.1 to 17.5)
Low bone mineral density	4.5 (3.7 to 5.3)	4.2 (3.5 to 4.9)	4.2 (3.4 to 5.0)	-5.3 (-17.1 to 9.2)	0.1 (-13.6 to 16.0)	6.0 (5.0 to 7.1)	5.7 (4.9 to 6.8)	5.9 (5.0 to 6.9)	-3.9 (-17.1 to 11.5)	2.9 (-10.1 to 18.8)
Impaired kidney function	2.5 (1.2 to 6.3)	2.5 (1.2 to 6.4)	2.5 (1.3 to 6.3)	2.0 (-3.2 to 7.9)	-0.8 (-6.9 to 5.7)	3.2 (1.6 to 7.2)	3.2 (1.6 to 7.4)	3.3 (1.6 to 7.4)	-0.1 (-4.9 to 4.8)	1.1 (-4.2 to 5.9)

Supplementary Results Table 1. Maine age-standardised Summary Exposure Values (SEVs) for Level 4 risk factors and percentage change, by sex, 1990, 2007, and 2017.Data in parentheses are 95% uncertainty intervals.										
	Male					Female				
	1990	2007	2017	Percentage change 1990-2007	Percentage change 2007-2017	1990	2007	2017	Percentage change 1990-2007	Percentage change 2007-2017
Unsafe water source	1.4 (0.7 to 2.4)	0.5 (0.3 to 0.8)	0.3 (0.2 to 0.5)	-66.5 (-81.8 to -33.7)	-33.4 (-57.5 to 6.4)	1.4 (0.7 to 2.4)	0.5 (0.3 to 0.8)	0.3 (0.2 to 0.5)	-66.5 (-81.8 to -33.7)	-33.4 (-57.5 to 6.4)
Unsafe sanitation	6.3 (3.5 to 10.8)	3.7 (1.7 to 6.9)	2.8 (1.2 to 5.2)	-40.5 (-74.0 to 24.2)	-26.0 (-61.6 to 47.9)	6.3 (3.5 to 10.8)	3.7 (1.7 to 6.9)	2.8 (1.2 to 5.2)	-40.5 (-74.0 to 24.2)	-26.0 (-61.6 to 47.9)
No access to handwashing facility	3.5 (2.5 to 4.8)	3.1 (2.3 to 4.3)	3.0 (2.2 to 4.2)	-9.1 (-34.5 to 29.5)	-3.0 (-25.6 to 25.3)	3.5 (2.5 to 4.8)	3.1 (2.3 to 4.3)	3.0 (2.2 to 4.2)	-9.1 (-34.5 to 29.5)	-3.0 (-25.6 to 25.3)
Ambient particulate matter pollution	13.1 (3.5 to 25.0)	13.9 (5.4 to 25.6)	7.2 (1.3 to 17.9)	6.4 (-57.6 to 247.0)	-48.1 (-88.9 to 20.6)	12.9 (3.5 to 24.7)	13.8 (5.3 to 25.5)	7.2 (1.4 to 17.8)	7.0 (-57.6 to 249.9)	-48.1 (-88.6 to 20.9)
Household air pollution from solid fuels	0.5 (0.1 to 2.0)	0.3 (0.1 to 1.0)	0.2 (0.0 to 0.9)	-41.7 (-82.3 to 107.3)	-34.3 (-90.4 to 53.7)	0.7 (0.1 to 5.1)	0.5 (0.1 to 3.5)	0.3 (0.0 to 1.9)	-35.2 (-82.1 to 103.8)	-37.0 (-91.7 to 47.6)
Ambient ozone pollution	37.6 (12.7 to 54.8)	37.1 (12.5 to 54.8)	37.9 (12.9 to 54.8)	-1.3 (-2.1 to 0.0)	2.2 (0.0 to 3.5)	37.6 (12.7 to 54.8)	37.1 (12.5 to 54.8)	37.9 (12.9 to 54.8)	-1.3 (-2.1 to 0.0)	2.2 (0.0 to 3.5)
Residential radon	20.5 (2.2 to 65.4)	20.5 (2.2 to 65.4)	20.5 (2.2 to 65.4)	0.0 (0.0 to 0.0)	0.0 (0.0 to 0.0)	20.5 (2.2 to 65.4)	20.5 (2.2 to 65.4)	20.5 (2.2 to 65.4)	0.0 (0.0 to 0.0)	0.0 (0.0 to 0.0)
Lead exposure	4.5 (1.2 to 8.6)	3.1 (0.7 to 6.4)	2.2 (0.4 to 4.9)	-31.6 (-43.9 to -22.9)	-30.0 (-42.2 to -22.9)	2.6 (0.3 to 6.3)	1.9 (0.2 to 5.0)	1.4 (0.1 to 4.0)	-26.8 (-39.4 to -18.3)	-27.0 (-40.5 to -20.3)
Occupational exposure to asbestos	8.6 (7.5 to 9.6)	7.3 (6.7 to 7.8)	6.6 (5.7 to 7.6)	-15.8 (-25.2 to -1.0)	-8.5 (-22.1 to 6.5)	1.7 (1.5 to 1.9)	1.7 (1.6 to 1.9)	1.8 (1.6 to 2.2)	1.6 (-13.3 to 16.4)	5.7 (-12.7 to 25.8)
Occupational exposure to arsenic	0.4 (0.0 to 0.9)	0.4 (0.0 to 0.9)	0.4 (0.0 to 0.9)	-	-	0.3 (0.0 to 0.8)	0.3 (0.0 to 0.8)	0.3 (0.0 to 0.8)	-	-
Occupational exposure to benzene	0.6 (0.0 to 1.6)	0.6 (0.0 to 1.7)	0.6 (0.0 to 1.6)	1.1 (-7.3 to 13.4)	-2.7 (-9.0 to 6.5)	0.8 (0.0 to 2.2)	0.9 (0.0 to 2.7)	0.9 (0.0 to 2.7)	14.4 (3.0 to 34.4)	-1.1 (-8.4 to 11.0)
Occupational exposure to beryllium	0.0 (0.0 to 0.0)	0.0 (0.0 to 0.0)	0.0 (0.0 to 0.0)	-0.1 (-5.7 to 5.8)	0.4 (-4.4 to 5.1)	0.0 (0.0 to 0.0)	0.0 (0.0 to 0.0)	0.0 (0.0 to 0.0)	6.1 (-2.2 to 15.5)	-1.2 (-7.3 to 4.8)
Occupational exposure to cadmium	0.0 (0.0 to 0.0)	0.0 (0.0 to 0.0)	0.0 (0.0 to 0.0)	-1.2 (-12.3 to 10.8)	0.3 (-9.3 to 9.7)	0.0 (0.0 to 0.0)	0.0 (0.0 to 0.0)	0.0 (0.0 to 0.0)	-1.2 (-14.5 to 14.8)	-1.3 (-11.3 to 10.7)
Occupational exposure to chromium	0.1 (0.1 to 0.1)	0.1 (0.1 to 0.1)	0.1 (0.1 to 0.1)	1.8 (-8.0 to 12.6)	0.4 (-7.8 to 8.6)	0.1 (0.1 to 0.1)	0.1 (0.1 to 0.1)	0.1 (0.1 to 0.1)	2.6 (-9.2 to 16.8)	-1.8 (-10.4 to 8.1)
Occupational exposure to diesel engine exhaust	0.5 (0.4 to 0.5)	0.5 (0.5 to 0.6)	0.6 (0.5 to 0.6)	14.6 (4.8 to 24.9)	2.5 (-4.1 to 9.9)	0.4 (0.4 to 0.5)	0.5 (0.4 to 0.5)	0.5 (0.4 to 0.5)	14.5 (0.8 to 30.8)	-5.3 (-13.8 to 5.4)
Occupational exposure to formaldehyde	0.2 (0.2 to 0.2)	0.2 (0.2 to 0.2)	0.2 (0.2 to 0.2)	-3.1 (-14.2 to 9.7)	-1.2 (-11.1 to 8.6)	0.2 (0.2 to 0.2)	0.2 (0.2 to 0.2)	0.2 (0.2 to 0.2)	-0.3 (-13.2 to 14.6)	-1.2 (-10.7 to 9.9)
Occupational exposure to nickel	0.3 (0.0 to 1.4)	0.3 (0.0 to 1.3)	0.3 (0.0 to 1.3)	-	-	0.3 (0.0 to 1.1)	0.2 (0.0 to 1.1)	0.2 (0.0 to 1.1)	-	-
Occupational exposure to polycyclic aromatic hydrocarbons	0.2 (0.2 to 0.2)	0.2 (0.2 to 0.2)	0.2 (0.2 to 0.2)	1.9 (-7.6 to 12.3)	-0.3 (-8.1 to 7.8)	0.2 (0.2 to 0.2)	0.2 (0.2 to 0.2)	0.2 (0.2 to 0.2)	6.5 (-4.6 to 19.7)	-1.7 (-9.4 to 6.7)
Occupational exposure to silica	2.7 (0.4 to 8.4)	2.5 (0.4 to 7.8)	2.5 (0.3 to 8.2)	-7.7 (-19.3 to 6.3)	0.8 (-9.5 to 12.5)	2.2 (0.3 to 7.1)	1.9 (0.3 to 6.3)	1.9 (0.3 to 6.3)	-9.9 (-24.9 to 10.4)	-1.3 (-14.9 to 16.0)
Occupational exposure to sulphuric acid	0.6 (0.1 to 1.8)	0.6 (0.1 to 2.0)	0.6 (0.1 to 1.9)	10.5 (-1.6 to 24.5)	-1.9 (-10.2 to 7.7)	0.5 (0.1 to 1.6)	0.6 (0.1 to 2.0)	0.6 (0.1 to 1.9)	23.3 (4.3 to 45.5)	-5.6 (-17.8 to 6.8)
Occupational exposure to trichloroethylene	0.1 (0.1 to 0.1)	0.1 (0.1 to 0.1)	0.1 (0.1 to 0.1)	5.9 (-1.6 to 14.7)	1.2 (-4.9 to 7.2)	0.1 (0.0 to 0.1)	0.1 (0.1 to 0.1)	0.1 (0.0 to 0.1)	6.2 (-4.1 to 19.1)	-2.7 (-10.4 to 5.5)
Occupational asthmagens	15.9 (13.3 to 19.4)	14.7 (12.4 to 17.7)	14.4 (11.8 to 17.5)	-7.5 (-18.0 to 5.0)	-2.2 (-10.4 to 6.7)	7.4 (6.1 to 9.1)	7.5 (6.1 to 9.0)	7.0 (5.7 to 8.6)	0.4 (-12.3 to 15.2)	-5.7 (-15.8 to 5.0)
Occupational particulate matter, gases, and fumes	6.9 (5.0 to 9.9)	6.5 (4.7 to 9.4)	6.2 (4.5 to 9.0)	-5.3 (-10.3 to -0.8)	-4.7 (-8.4 to -1.2)	3.6 (2.5 to 5.5)	3.5 (2.5 to 5.2)	3.3 (2.3 to 4.9)	-5.2 (-12.1 to 2.5)	-5.3 (-11.0 to 0.1)
Occupational noise	6.0 (5.5 to 6.6)	5.8 (5.3 to 6.4)	5.6 (5.1 to 6.2)	-2.5 (-5.9 to 0.9)	-3.6 (-6.0 to -1.2)	3.9 (3.4 to 4.6)	4.2 (3.7 to 4.7)	4.2 (3.7 to 4.7)	5.7 (0.2 to 12.0)	-0.2 (-3.4 to 3.6)
Occupational ergonomic factors	9.4 (7.7 to 11.3)	8.1 (6.8 to 9.7)	7.8 (6.5 to 9.5)	-13.1 (-24.0 to -0.9)	-3.9 (-11.9 to 5.3)	5.3 (4.1 to 6.6)	5.3 (4.1 to 6.7)	5.1 (4.1 to 6.5)	0.3 (-14.8 to 19.0)	-3.2 (-14.3 to 9.9)
Non-exclusive breastfeeding	0.5 (0.5 to 0.6)	0.5 (0.5 to 0.6)	0.5 (0.4 to 0.6)	0.2 (-10.8 to 13.4)	-7.1 (-16.0 to 2.0)	0.5 (0.5 to 0.6)	0.5 (0.5 to 0.6)	0.5 (0.4 to 0.6)	0.0 (-10.9 to 13.2)	-7.4 (-16.4 to 1.6)
Discontinued breastfeeding	2.2 (2.1 to 2.3)	2.1 (2.0 to 2.3)	2.2 (2.1 to 2.3)	-3.2 (-10.0 to 3.8)	2.8 (-3.2 to 9.2)	2.2 (2.0 to 2.3)	2.1 (2.0 to 2.3)	2.2 (2.0 to 2.3)	-1.8 (-8.7 to 5.3)	1.5 (-4.5 to 7.9)
Child underweight	0.1 (0.1 to 0.1)	0.1 (0.1 to 0.1)	0.1 (0.0 to 0.1)	-5.2 (-19.2 to 10.4)	-12.7 (-22.1 to -3.1)	0.1 (0.0 to 0.1)	0.1 (0.0 to 0.1)	0.1 (0.0 to 0.1)	-3.1 (-20.2 to 14.0)	-3.9 (-14.5 to 8.1)
Child wasting	0.1 (0.0 to 0.1)	0.1 (0.0 to 0.1)	0.1 (0.0 to 0.1)	-9.6 (-29.2 to 11.2)	-3.7 (-15.7 to 11.4)	0.1 (0.0 to 0.1)	0.1 (0.0 to 0.1)	0.1 (0.0 to 0.1)	6.2 (-16.3 to 37.1)	-5.6 (-21.2 to 10.0)
Child stunting	0.3 (0.1 to 0.5)	0.2 (0.1 to 0.4)	0.2 (0.1 to 0.4)	-8.5 (-48.3 to 60.8)	-10.6 (-39.7 to 28.8)	0.2 (0.1 to 0.4)	0.2 (0.1 to 0.3)	0.2 (0.1 to 0.3)	-10.5 (-47.5 to 46.7)	-2.6 (-28.4 to 32.2)
Short gestation for birth weight	0.0 (0.0 to 0.0)	0.0 (0.0 to 0.0)	0.0 (0.0 to 0.0)	5.0 (-15.8 to 39.9)	4.4 (-13.8 to 31.1)	0.0 (0.0 to 0.0)	0.0 (0.0 to 0.0)	0.0 (0.0 to 0.0)	3.7 (-21.5 to 38.1)	2.2 (-18.2 to 30.0)
Low birth weight for gestation	0.0 (0.0 to 0.0)	0.0 (0.0 to 0.0)	0.0 (0.0 to 0.0)	1.5 (-19.9 to 27.1)	-2.9 (-19.6 to 14.3)	0.0 (0.0 to 0.0)	0.0 (0.0 to 0.0)	0.0 (0.0 to 0.0)	3.1 (-18.3 to 35.5)	-2.8 (-21.4 to 16.3)
Iron deficiency	-	-	-	-	-	4.1 (2.6 to 6.1)	3.9 (2.6 to 5.8)	4.0 (2.5 to 6.0)	-6.8 (-31.5 to 28.2)	3.0 (-19.9 to 34.1)
Vitamin A deficiency	0.5 (0.3 to 0.7)	0.5 (0.3 to 0.7)	0.5 (0.3 to 0.7)	12.8 (-7.6 to 42.2)	-5.3 (-24.5 to 13.7)	0.6 (0.4 to 0.8)	0.6 (0.4 to 0.7)	0.7 (0.4 to 1.1)	-2.9 (-25.7 to 27.2)	27.2 (-1.5 to 75.8)
Zinc deficiency	0.5 (0.1 to 1.2)	0.2 (0.0 to 1.0)	0.2 (0.0 to 1.0)	-56.5 (-88.3 to 251.9)	0.5 (-89.0 to 753.5)	0.5 (0.1 to 1.2)	0.2 (0.0 to 1.0)	0.2 (0.0 to 1.0)	-53.3 (-88.0 to 218.8)	-1.3 (-88.2 to 707.8)
Smoking	14.6 (11.9 to 17.4)	8.2 (6.5 to 9.9)	6.4 (5.0 to 8.1)	-43.9 (-51.9 to -34.5)	-21.2 (-30.0 to -10.5)	10.8 (8.2 to 13.7)	6.1 (4.8 to 7.7)	4.8 (3.7 to 6.1)	-43.1 (-53.3 to -29.6)	-22.3 (-31.0 to -12.2)
Chewing tobacco	1.0 (0.8 to 1.3)	1.1 (0.7 to 1.5)	1.1 (0.7 to 1.6)	0.8 (-31.5 to 46.0)	1.4 (-24.7 to 42.9)	0.1 (0.1 to 0.1)	0.1 (0.0 to 0.1)	0.1 (0.0 to 0.1)	-4.2 (-41.4 to 60.9)	3.6 (-29.0 to 54.3)
Second-hand smoke	46.0 (42.4 to 50.3)	28.0 (26.3 to 29.7)	28.8 (25.8 to 31.8)	-39.0 (-46.1 to -31.5)	2.5 (-7.7 to 13.1)	44.1 (41.0 to 47.6)	27.0 (25.7 to 28.2)	27.4 (25.5 to 29.4)	-38.8 (-45.5 to -32.0)	1.4 (-6.3 to 9.3)
Alcohol use	19.3 (10.3 to 30.9)	22.0 (11.8 to 34.3)	24.2 (13.2 to 38.5)	14.1 (-26.9 to 78.9)	10.1 (-25.0 to 63.7)	14.0 (7.1 to 24.6)	15.5 (8.0 to 25.6)	17.0 (8.7 to 29.1)	10.3 (-27.0 to 69.7)	9.9 (-24.9 to 61.3)
Drug use	1.6 (1.2 to 2.4)	2.1 (1.7 to 2.8)	3.2 (2.5 to 4.2)	32.6 (13.4 to 57.5)	50.7 (35.3 to 69.2)	1.4 (1.1 to 2.0)	1.9 (1.5 to 2.5)	2.4 (1.9 to 3.1)	35.2 (20.2 to 54.7)	25.8 (14.4 to 39.5)
Diet low in fruits	26.6 (20.1 to 32.1)	25.4 (19.0 to 30.7)	27.4 (20.7 to 33.3)	-4.4 (-16.7 to 8.1)	8.0 (-3.3 to 21.8)	22.9 (15.9 to 28.8)	23.4 (17.2 to 28.8)	25.3 (18.7 to 31.1)	2.1 (-11.3 to 21.0)	8.4 (-4.1 to 23.0)
Diet low in vegetables	18.6 (10.6 to 25.7)	24.2 (18.0 to 29.5)	26.2 (20.4 to 31.1)	30.0 (4.2 to 90.5)	8.1 (-4.3 to 24.9)	22.4 (14.4 to 29.3)	25.7 (18.4 to 31.7)	28.2 (20.9 to 33.8)	14.6 (-4.8 to 49.6)	10.0 (-1.9 to 28.2)
Diet low in legumes	24.0 (17.0 to 30.2)	24.2 (17.3 to 30.4)	25.6 (18.8 to 31.4)	0.9 (-15.4 to 21.1)	5.8 (-7.1 to 21.1)	28.6 (22.7 to 33.8)	29.3 (23.7 to 34.3)	30.7 (25.0 to 35.6)	2.6 (-9.6 to 17.4)	4.6 (-4.5 to 14.5)
Diet low in whole grains	38.4 (34.9 to 41.4)	39.6 (36.2 to 42.3)	39.1 (35.5 to 42.0)	3.0 (-1.8 to 8.6)	-1.2 (-5.0 to 2.6)	39.3 (36.2 to 42.2)	40.4 (37.3 to 43.1)	40.1 (37.0 to 42.9)	2.8 (-1.7 to 7.7)	-0.9 (-4.7 to 2.5)
Diet low in nuts and seeds	46.4 (43.5 to 48.8)	37.4 (32.8 to 41.4)	38.1 (32.8 to 42.1)	-19.4 (-27.3 to -13.3)	1.8 (-6.1 to 9.1)	47.2 (44.7 to 49.2)	38.6 (33.9 to 42.4)	39.1 (34.1 to 43.1)	-18.2 (-25.8 to -12.2)	1.3 (-5.3 to 8.3)
Diet low in milk	28.1 (21.7 to 33.2)	26.6 (20.2 to 32.0)	27.2 (20.8 to 32.7)	-5.3 (-14.9 to 4.4)	2.2 (-5.9 to 12.2)	29.8 (24.0 to 34.7)	27.7 (21.4 to 32.7)	28.1 (22.1 to 33.1)	-7.0 (-16.4 to 1.4)	1.4 (-6.0 to 9.9)
Diet high in red meat	40.6 (29.4 to 48.9)	35.0 (24.8 to 43.9)	33.3 (21.4 to 42.5)	-13.7 (-34.0 to 9.8)	-5.0 (-24.6 to 18.2)	22.2 (12.5 to 32.0)	16.4 (8.5 to 25.3)	15.3 (7.5 to 24.4)	-25.8 (-56.2 to 12.6)	-6.9 (-37.9 to 38.8)
Diet high in processed meat	38.1 (30.2 to 44.9)	34.9 (27.4 to 41.2)	27.7 (20.6 to 34.2)	-8.4 (-25.1 to 10.6)	-20.8 (-32.5 to -7.5)	20.5 (14.7 to 26.2)	19.9 (14.3 to 24.9)	17.2 (12.3 to 21.8)	-3.3 (-25.0 to 21.1)	-13.4 (-28.0 to 1.6)
Diet high in sugar-sweetened beverages	30.4 (14.8 to 40.5)	28.6 (14.5 to 39.5)	23.7 (11.9 to 34.6)	-5.9 (-37.2 to 37.1)	-17.0 (-43.0 to 10.6)	25.0 (11.8 to 35.8)	22.0 (10.6 to 32.7)	18.0 (8.3 to 28.1)	-11.7 (-45.2 to 46.0)	-18.3 (-45.5 to 15.4)
Diet low in fibre	33.5 (27.8 to 38.2)	25.6 (18.8 to 31.3)	27.2 (20.5 to 33.3)	-23.8 (-37.0 to -13.1)	6.4 (-4.7 to 19.6)	38.2 (33.3 to 42.1)	30.3 (24.2 to 35.4)	31.6 (25.3 to 36.9)	-20.8 (-31.3 to -12.5)	4.4 (-4.2 to 14.0)
Diet low in calcium	17.5 (10.3 to 23.9)	15.7 (8.8 to 22.1)	16.6 (9.4 to 23.1)	-10.2 (-24.3 to 2.6)	5.6 (-5.6 to 21.0)	21.5 (14.6 to 27.3)	19.7 (12.6 to 25.7)	20.5 (13.5 to 26.6)	-8.6 (-21.5 to 2.4)	4.5 (-3.4 to 15.3)
Diet low in seafood omega-3 fatty acids	29.8 (24.0 to 35.0)	25.3 (19.2 to 30.7)	27.0 (20.6 to 32.3)	-14.9 (-26.0 to -4.6)	6.5 (-3.0 to 19.1)	33.1 (27.9 to 37.6)	29.5 (23.9 to 34.6)	30.7 (25.3 to 35.4)	-10.8 (-19.3 to -2.3)	3.9 (-3.4 to 12.8)
Diet low in polyunsaturated fatty acids	8.9 (3.1 to 15.7)	5.7 (1.1 to 11.8)	4.4 (0.6 to 10.0)	-36.1 (-75.8 to 1.7)	-22.8 (-67.8 to 49.2)	9.7 (3.9 to 16.5)	6.2 (1.3 to 12.8)	4.7 (0.9 to 10.6)	-35.8 (-76.6 to 3.3)	-24.1 (-64.5 to 36.5)
Diet high in trans fatty acids	16.8 (12.2 to 22.2)	9.8 (5.8 to 14.0)	7.1 (3.4 to 11.1)	-41.4 (-56.9 to -28.5)	-27.7 (-43.7 to -16.5)	23.8 (18.3 to 32.5)	13.3 (9.1 to 17.9)	9.7 (5.8 to 13.7)	-44.1 (-56.0 to -31.9)	-26.8 (-39.8 to -16.7)
Diet high in sodium	7.1 (0.6 to 14.4)	12.4 (3.9 to 19.8)	12.9 (4.2 to 20.8)	75.7 (21.8 to 718.3)	4.0 (-23.7 to 47.4)	6.0 (0.3 to 13.3)	10.9 (2.5 to 18.3)	11.4 (3.0 to 19.1)	82.0 (27.1 to 992.9)	4.1 (-22.9 to 56.0)
Intimate partner violence	-	-	-	-	-	0.5 (0.3 to 0.7)	0.5 (0.4 to 0.6)	0.5 (0.4 to 0.7)	-8.9 (-20.6 to 8.6)	5.0 (-8.2 to 17.2)
Childhood sexual abuse	5.6 (4.6 to 6.9)	5.1 (4.1 to 6.2)	5.1 (4.1 to 6.2)	-9.8 (-15.1 to -4.3)	0.4 (-4.7 to 6.0)	13.8 (11.0 to 16.9)	12.1 (9.8 to 14.7)	12.2 (9.9 to 14.8)	-11.9 (-17.2 to -6.0)	0.4 (-4.8 to 6.5)

Supplementary Results Table 1. Maine age-standardised Summary Exposure Values (SEVs) for Level 4 risk factors and percentage change, by sex, 1990, 2007, and 2017.Data in parentheses are 95% uncertainty intervals.

	Male					Female				
	**1990**	**2007**	**2017**	**Percentage change 1990-2007**	**Percentage change 2007-2017**	**1990**	**2007**	**2017**	**Percentage change 1990-2007**	**Percentage change 2007-2017**
Bullying victimization	6.1 (2.5 to 13.1)	9.1 (3.9 to 18.6)	8.7 (3.7 to 18.6)	47.8 (32.7 to 67.2)	-3.8 (-11.7 to 3.8)	4.6 (1.9 to 9.6)	7.7 (3.3 to 16.2)	7.6 (3.2 to 16.2)	65.6 (46.1 to 92.7)	-1.8 (-9.8 to 7.9)
Low physical activity	0.4 (0.2 to 0.7)	0.4 (0.2 to 0.7)	0.4 (0.2 to 0.7)	0.1 (-2.3 to 2.6)	-0.1 (-2.0 to 2.0)	0.5 (0.2 to 0.9)	0.5 (0.2 to 0.9)	0.5 (0.2 to 0.9)	0.5 (-2.1 to 3.5)	-1.1 (-2.9 to 0.6)
High fasting plasma glucose	1.3 (0.8 to 2.2)	2.8 (1.7 to 4.2)	3.2 (2.0 to 5.0)	113.4 (27.1 to 282.6)	16.3 (-28.1 to 81.8)	1.5 (0.9 to 2.5)	2.7 (1.5 to 4.5)	2.5 (1.3 to 4.0)	86.6 (8.3 to 221.4)	-7.1 (-46.7 to 65.7)
High LDL cholesterol	21.5 (19.4 to 23.6)	11.6 (10.0 to 13.4)	10.2 (8.6 to 11.9)	-45.9 (-50.8 to -41.1)	-12.4 (-16.9 to -8.3)	19.9 (18.0 to 21.9)	11.5 (10.0 to 13.3)	10.6 (9.1 to 12.3)	-41.9 (-46.4 to -37.3)	-8.1 (-12.4 to -4.1)
High systolic blood pressure	6.1 (5.3 to 7.0)	4.7 (4.0 to 5.4)	4.8 (4.1 to 5.6)	-23.7 (-31.6 to -15.3)	2.4 (-6.4 to 11.7)	5.8 (5.1 to 6.7)	4.3 (3.7 to 4.8)	4.2 (3.6 to 4.8)	-26.9 (-34.8 to -17.6)	-1.7 (-12.3 to 9.3)
High body-mass index	13.1 (9.3 to 18.2)	19.2 (14.7 to 25.2)	19.3 (14.7 to 25.4)	46.5 (28.3 to 69.6)	0.4 (-8.2 to 9.4)	15.8 (11.8 to 21.1)	21.8 (17.3 to 28.0)	22.9 (17.8 to 29.2)	38.2 (21.1 to 60.0)	5.1 (-3.7 to 16.4)
Low bone mineral density	5.2 (4.3 to 6.3)	4.5 (3.7 to 5.3)	4.7 (3.9 to 5.7)	-14.5 (-26.4 to -0.0)	6.1 (-10.3 to 24.8)	5.8 (4.8 to 6.9)	5.7 (4.8 to 6.7)	6.3 (5.3 to 7.5)	-1.9 (-14.6 to 12.9)	9.8 (-3.4 to 25.4)
Impaired kidney function	2.3 (1.1 to 5.7)	2.3 (1.1 to 5.8)	2.3 (1.1 to 5.8)	-0.2 (-5.5 to 5.3)	1.9 (-4.0 to 8.3)	2.9 (1.4 to 6.9)	2.9 (1.4 to 6.8)	3.0 (1.4 to 7.0)	-1.7 (-6.8 to 3.7)	3.8 (-1.0 to 10.0)

Supplementary Results Table 1. Maryland age-standardised Summary Exposure Values (SEVs) for Level 4 risk factors and percentage change, by sex, 1990, 2007, and 2017.Data in parentheses are 95% uncertainty intervals.

	Male					Female				
	**1990**	**2007**	**2017**	**Percentage change 1990-2007**	**Percentage change 2007-2017**	**1990**	**2007**	**2017**	**Percentage change 1990-2007**	**Percentage change 2007-2017**
Unsafe water source	1.2 (0.7 to 1.9)	0.4 (0.3 to 0.7)	0.3 (0.2 to 0.4)	-64.7 (-81.3 to -32.0)	-33.5 (-57.0 to 2.1)	1.2 (0.7 to 1.9)	0.4 (0.3 to 0.7)	0.3 (0.2 to 0.4)	-64.7 (-81.3 to -32.0)	-33.5 (-57.0 to 2.1)
Unsafe sanitation	5.0 (2.4 to 9.0)	3.0 (1.4 to 5.7)	2.2 (0.9 to 4.3)	-40.2 (-74.5 to 37.6)	-26.6 (-62.7 to 43.9)	5.0 (2.4 to 9.0)	3.0 (1.4 to 5.7)	2.2 (0.9 to 4.3)	-40.2 (-74.5 to 37.6)	-26.6 (-62.7 to 43.9)
No access to handwashing facility	3.4 (2.4 to 4.6)	3.1 (2.2 to 4.3)	3.0 (2.1 to 4.0)	-8.3 (-34.9 to 30.9)	-3.2 (-25.2 to 24.3)	3.4 (2.4 to 4.6)	3.1 (2.2 to 4.3)	3.0 (2.1 to 4.0)	-8.3 (-34.9 to 30.9)	-3.2 (-25.2 to 24.3)
Ambient particulate matter pollution	26.9 (18.2 to 39.4)	27.0 (18.6 to 39.3)	20.7 (12.7 to 32.1)	0.2 (-19.9 to 28.6)	-23.4 (-45.8 to -3.3)	26.9 (18.2 to 39.3)	27.0 (18.6 to 39.3)	20.7 (12.7 to 32.1)	0.3 (-19.8 to 28.7)	-23.4 (-45.8 to -3.1)
Household air pollution from solid fuels	0.2 (0.0 to 0.5)	0.1 (0.0 to 0.3)	0.1 (0.0 to 0.3)	-35.6 (-78.0 to 51.3)	-3.5 (-62.8 to 113.7)	0.3 (0.1 to 1.6)	0.2 (0.0 to 1.0)	0.2 (0.0 to 1.3)	-30.3 (-78.1 to 50.9)	0.5 (-62.7 to 114.0)
Ambient ozone pollution	49.5 (20.8 to 54.8)	49.9 (21.4 to 54.8)	50.5 (22.5 to 54.8)	0.8 (0.0 to 3.0)	1.3 (0.0 to 5.2)	49.5 (20.8 to 54.8)	49.9 (21.4 to 54.8)	50.5 (22.5 to 54.8)	0.8 (0.0 to 3.0)	1.3 (0.0 to 5.2)
Residential radon	13.9 (0.8 to 42.0)	13.9 (0.8 to 42.0)	13.9 (0.8 to 42.0)	0.0 (0.0 to 0.0)	0.0 (0.0 to 0.0)	13.9 (0.8 to 42.0)	13.9 (0.8 to 42.0)	13.9 (0.8 to 42.0)	0.0 (0.0 to 0.0)	0.0 (0.0 to 0.0)
Lead exposure	8.7 (4.2 to 13.1)	5.4 (2.2 to 9.0)	3.6 (1.2 to 6.4)	-37.9 (-50.1 to -28.2)	-34.3 (-44.0 to -28.1)	5.6 (1.8 to 9.8)	3.6 (0.9 to 7.2)	2.4 (0.5 to 5.2)	-36.3 (-52.4 to -25.3)	-33.5 (-45.9 to -27.6)
Occupational exposure to asbestos	6.8 (5.7 to 7.7)	5.2 (4.8 to 5.6)	4.4 (3.7 to 5.2)	-24.5 (-34.2 to -9.7)	-14.2 (-28.7 to 2.4)	1.0 (0.9 to 1.1)	0.9 (0.8 to 0.9)	0.8 (0.7 to 1.0)	-12.1 (-24.9 to 1.9)	-7.4 (-24.6 to 12.6)
Occupational exposure to arsenic	0.3 (0.0 to 0.7)	0.3 (0.0 to 0.7)	0.3 (0.0 to 0.7)	-	-	0.3 (0.0 to 0.7)	0.3 (0.0 to 0.7)	0.3 (0.0 to 0.7)	-	-
Occupational exposure to benzene	0.6 (0.0 to 1.7)	0.6 (0.0 to 1.8)	0.6 (0.0 to 1.8)	3.4 (-5.3 to 16.2)	-3.8 (-10.6 to 4.4)	0.9 (0.0 to 2.5)	1.0 (0.1 to 2.8)	1.0 (0.1 to 2.9)	15.1 (4.5 to 34.9)	-0.9 (-8.9 to 8.7)
Occupational exposure to beryllium	0.0 (0.0 to 0.0)	0.0 (0.0 to 0.0)	0.0 (0.0 to 0.0)	0.5 (-5.1 to 6.2)	0.9 (-3.4 to 5.3)	0.0 (0.0 to 0.0)	0.0 (0.0 to 0.0)	0.0 (0.0 to 0.0)	6.1 (-1.2 to 14.0)	-0.6 (-6.1 to 5.2)
Occupational exposure to cadmium	0.0 (0.0 to 0.0)	0.0 (0.0 to 0.0)	0.0 (0.0 to 0.0)	1.4 (-10.4 to 13.6)	2.9 (-6.6 to 12.2)	0.0 (0.0 to 0.0)	0.0 (0.0 to 0.0)	0.0 (0.0 to 0.0)	5.4 (-6.7 to 20.1)	-0.6 (-10.3 to 10.3)
Occupational exposure to chromium	0.1 (0.1 to 0.1)	0.1 (0.1 to 0.1)	0.1 (0.1 to 0.1)	4.6 (-6.0 to 15.5)	2.3 (-6.0 to 10.2)	0.1 (0.1 to 0.1)	0.1 (0.1 to 0.1)	0.1 (0.1 to 0.1)	8.9 (-1.7 to 22.0)	-1.1 (-9.3 to 8.1)
Occupational exposure to diesel engine exhaust	0.5 (0.4 to 0.5)	0.5 (0.5 to 0.6)	0.5 (0.5 to 0.6)	12.0 (2.4 to 21.5)	1.9 (-4.7 to 9.4)	0.4 (0.4 to 0.5)	0.5 (0.4 to 0.5)	0.5 (0.4 to 0.5)	11.4 (-0.5 to 25.7)	-3.5 (-11.8 to 6.4)
Occupational exposure to formaldehyde	0.2 (0.2 to 0.2)	0.2 (0.2 to 0.2)	0.2 (0.2 to 0.2)	0.4 (-11.9 to 13.4)	1.1 (-8.8 to 11.1)	0.2 (0.2 to 0.2)	0.2 (0.2 to 0.2)	0.2 (0.2 to 0.2)	7.9 (-4.2 to 22.3)	-1.0 (-10.0 to 9.0)
Occupational exposure to nickel	0.2 (0.0 to 1.1)	0.2 (0.0 to 1.1)	0.2 (0.0 to 1.1)	-	-	0.2 (0.0 to 0.9)	0.2 (0.0 to 1.0)	0.2 (0.0 to 1.0)	-	-
Occupational exposure to polycyclic aromatic hydrocarbons	0.2 (0.2 to 0.2)	0.2 (0.2 to 0.2)	0.2 (0.2 to 0.2)	5.2 (-5.1 to 15.5)	1.3 (-6.4 to 9.0)	0.2 (0.2 to 0.2)	0.2 (0.2 to 0.2)	0.2 (0.2 to 0.2)	12.5 (2.2 to 24.9)	-1.0 (-8.1 to 6.9)
Occupational exposure to silica	2.2 (0.3 to 7.2)	2.1 (0.3 to 6.5)	2.2 (0.3 to 6.7)	-7.0 (-19.9 to 7.1)	4.1 (-7.0 to 17.5)	1.8 (0.2 to 5.7)	1.7 (0.2 to 5.6)	1.7 (0.2 to 5.6)	-2.8 (-17.3 to 17.4)	-0.6 (-13.5 to 16.4)
Occupational exposure to sulphuric acid	0.5 (0.1 to 1.5)	0.6 (0.1 to 1.7)	0.6 (0.1 to 1.8)	15.7 (1.6 to 31.7)	0.6 (-8.7 to 11.4)	0.4 (0.1 to 1.4)	0.6 (0.1 to 1.8)	0.5 (0.1 to 1.8)	28.0 (9.1 to 48.7)	-1.8 (-14.4 to 11.6)
Occupational exposure to trichloroethylene	0.1 (0.0 to 0.1)	0.1 (0.1 to 0.1)	0.1 (0.1 to 0.1)	7.2 (-1.0 to 16.0)	2.2 (-4.1 to 8.5)	0.1 (0.0 to 0.1)	0.1 (0.1 to 0.1)	0.1 (0.1 to 0.1)	9.1 (-0.2 to 21.1)	-1.7 (-9.3 to 6.3)
Occupational asthmagens	13.3 (11.2 to 16.0)	12.8 (10.7 to 15.1)	12.6 (10.5 to 15.1)	-3.9 (-14.2 to 6.4)	-1.3 (-9.8 to 7.0)	7.1 (5.8 to 8.7)	7.4 (6.0 to 9.1)	7.0 (5.7 to 8.8)	4.9 (-8.5 to 21.0)	-5.4 (-16.4 to 6.6)
Occupational particulate matter, gases, and fumes	5.5 (3.9 to 7.9)	5.2 (3.7 to 7.5)	5.0 (3.6 to 7.3)	-4.9 (-10.0 to -0.2)	-3.2 (-7.3 to 0.6)	3.0 (2.1 to 4.4)	2.9 (2.0 to 4.3)	2.8 (1.9 to 4.1)	-3.2 (-9.9 to 4.4)	-3.4 (-8.7 to 1.9)
Occupational noise	5.2 (4.8 to 5.7)	5.2 (4.8 to 5.7)	5.0 (4.6 to 5.6)	-0.9 (-4.4 to 2.5)	-2.4 (-5.0 to 0.1)	3.8 (3.4 to 4.3)	4.1 (3.7 to 4.5)	4.1 (3.7 to 4.5)	6.2 (1.3 to 12.0)	0.7 (-2.7 to 4.0)
Occupational ergonomic factors	8.4 (6.9 to 10.2)	7.7 (6.4 to 9.3)	7.4 (6.0 to 9.0)	-8.2 (-18.0 to 1.7)	-4.2 (-11.6 to 3.3)	4.9 (3.9 to 6.2)	5.0 (4.0 to 6.4)	5.0 (4.0 to 6.3)	3.5 (-12.2 to 21.9)	-1.6 (-13.0 to 10.8)
Non-exclusive breastfeeding	0.5 (0.5 to 0.6)	0.5 (0.5 to 0.6)	0.5 (0.4 to 0.6)	-0.5 (-11.3 to 13.0)	-7.1 (-15.8 to 2.9)	0.5 (0.5 to 0.6)	0.5 (0.5 to 0.6)	0.5 (0.4 to 0.6)	-0.1 (-10.9 to 13.5)	-7.3 (-16.0 to 2.6)
Discontinued breastfeeding	2.3 (2.1 to 2.4)	2.2 (2.1 to 2.3)	2.2 (2.1 to 2.3)	-3.5 (-10.4 to 4.1)	1.1 (-4.5 to 7.2)	2.3 (2.1 to 2.4)	2.2 (2.1 to 2.3)	2.2 (2.1 to 2.4)	-3.4 (-10.3 to 4.1)	2.1 (-3.6 to 8.2)
Child underweight	0.1 (0.1 to 0.1)	0.1 (0.0 to 0.1)	0.1 (0.0 to 0.1)	-4.9 (-18.6 to 10.7)	-12.4 (-21.2 to -3.5)	0.1 (0.0 to 0.1)	0.1 (0.0 to 0.1)	0.1 (0.0 to 0.1)	-2.6 (-19.4 to 14.6)	-4.3 (-16.9 to 7.5)
Child wasting	0.1 (0.0 to 0.1)	0.1 (0.0 to 0.1)	0.1 (0.0 to 0.1)	-9.8 (-30.5 to 9.0)	-3.1 (-15.8 to 11.7)	0.1 (0.0 to 0.1)	0.1 (0.0 to 0.1)	0.1 (0.0 to 0.1)	6.2 (-14.7 to 31.2)	-5.0 (-20.4 to 10.0)
Child stunting	0.3 (0.1 to 0.5)	0.2 (0.1 to 0.4)	0.2 (0.1 to 0.4)	-7.7 (-46.4 to 57.8)	-11.0 (-39.5 to 24.7)	0.2 (0.1 to 0.3)	0.2 (0.1 to 0.3)	0.2 (0.1 to 0.3)	-10.9 (-45.2 to 37.9)	-4.3 (-30.1 to 28.1)
Short gestation for birth weight	0.0 (0.0 to 0.0)	0.0 (0.0 to 0.0)	0.0 (0.0 to 0.0)	9.6 (-12.8 to 49.8)	-0.1 (-22.6 to 24.8)	0.0 (0.0 to 0.0)	0.0 (0.0 to 0.0)	0.0 (0.0 to 0.0)	9.3 (-22.0 to 41.2)	4.2 (-26.4 to 54.9)
Low birth weight for gestation	0.0 (0.0 to 0.0)	0.0 (0.0 to 0.0)	0.0 (0.0 to 0.0)	-0.7 (-25.3 to 25.2)	1.8 (-18.6 to 26.2)	0.0 (0.0 to 0.0)	0.0 (0.0 to 0.0)	0.0 (0.0 to 0.0)	-1.3 (-22.0 to 37.9)	-2.6 (-31.2 to 30.0)
Iron deficiency	-	-	-	-	-	3.8 (2.4 to 5.6)	3.5 (2.2 to 5.1)	3.5 (2.2 to 5.3)	-8.4 (-32.6 to 28.2)	0.6 (-22.8 to 29.6)
Vitamin A deficiency	0.4 (0.3 to 0.6)	0.4 (0.3 to 0.5)	0.5 (0.3 to 0.7)	-10.1 (-27.0 to 12.5)	32.0 (0.9 to 80.4)	0.6 (0.4 to 0.8)	0.5 (0.3 to 0.7)	0.6 (0.4 to 0.7)	-12.5 (-29.9 to 6.8)	7.5 (-7.4 to 26.5)
Zinc deficiency	0.5 (0.1 to 1.1)	0.2 (0.0 to 1.0)	0.2 (0.0 to 1.0)	-55.7 (-89.0 to 315.0)	-0.4 (-88.5 to 844.4)	0.5 (0.1 to 1.1)	0.2 (0.0 to 1.0)	0.2 (0.0 to 1.1)	-56.5 (-89.2 to 247.8)	5.4 (-88.4 to 980.1)
Smoking	12.9 (10.4 to 15.3)	6.9 (5.4 to 8.4)	5.5 (4.2 to 6.8)	-46.0 (-54.6 to -36.0)	-20.7 (-29.7 to -11.0)	9.6 (7.4 to 12.0)	5.3 (4.1 to 6.5)	3.9 (3.0 to 4.9)	-45.2 (-55.6 to -30.5)	-26.3 (-35.0 to -16.3)
Chewing tobacco	0.7 (0.5 to 0.9)	0.9 (0.6 to 1.3)	1.0 (0.6 to 1.5)	29.7 (-12.6 to 94.3)	7.7 (-22.8 to 48.8)	0.1 (0.1 to 0.1)	0.1 (0.0 to 0.1)	0.1 (0.1 to 0.1)	-6.5 (-43.4 to 53.3)	3.2 (-26.7 to 50.1)
Second-hand smoke	44.3 (40.7 to 48.1)	24.8 (23.3 to 26.3)	26.1 (23.5 to 29.2)	-43.9 (-50.6 to -37.0)	5.2 (-5.6 to 17.1)	41.5 (38.3 to 44.8)	23.3 (22.1 to 24.3)	24.1 (22.2 to 25.9)	-43.9 (-50.1 to -37.4)	3.6 (-4.9 to 12.6)
Alcohol use	20.8 (11.0 to 32.8)	23.2 (12.8 to 36.3)	25.3 (13.0 to 41.0)	11.5 (-27.6 to 76.3)	8.8 (-24.9 to 57.8)	14.5 (7.7 to 23.8)	15.2 (7.8 to 25.4)	15.3 (7.5 to 26.7)	4.9 (-31.3 to 56.7)	0.8 (-31.2 to 41.9)
Drug use	1.8 (1.2 to 2.7)	2.1 (1.6 to 2.9)	2.4 (1.7 to 3.3)	18.3 (3.8 to 36.5)	14.1 (3.8 to 24.9)	1.4 (1.0 to 2.0)	1.6 (1.2 to 2.2)	1.7 (1.2 to 2.5)	14.8 (5.7 to 27.0)	6.8 (-3.5 to 16.8)
Diet low in fruits	24.8 (18.3 to 30.1)	25.8 (19.6 to 31.2)	28.9 (22.0 to 34.2)	4.0 (-6.1 to 17.1)	11.9 (1.1 to 26.2)	23.2 (16.7 to 28.8)	22.6 (16.3 to 28.2)	23.5 (16.6 to 29.9)	-2.6 (-14.3 to 9.4)	3.9 (-9.8 to 19.9)
Diet low in vegetables	17.0 (8.8 to 24.3)	7.4 (4.5 to 10.0)	7.4 (4.9 to 9.4)	-56.6 (-69.5 to -31.2)	0.1 (-20.7 to 29.1)	19.4 (11.4 to 26.3)	9.8 (4.8 to 15.1)	9.8 (5.1 to 14.6)	-49.4 (-67.5 to -31.8)	0.3 (-27.2 to 43.5)
Diet low in legumes	23.8 (16.9 to 29.7)	24.0 (17.0 to 30.1)	25.4 (18.6 to 31.3)	0.8 (-16.2 to 19.8)	5.7 (-7.0 to 22.7)	28.6 (22.6 to 33.6)	29.3 (23.5 to 34.2)	30.6 (24.6 to 35.4)	2.3 (-9.9 to 16.5)	4.6 (-5.0 to 15.2)
Diet low in whole grains	37.5 (34.0 to 40.8)	38.8 (35.6 to 41.7)	38.3 (34.8 to 41.3)	3.4 (-1.9 to 9.4)	-1.2 (-5.3 to 2.4)	38.4 (34.9 to 41.5)	39.7 (36.5 to 42.4)	39.3 (36.1 to 42.2)	3.3 (-1.8 to 8.8)	-1.0 (-4.9 to 2.5)
Diet low in nuts and seeds	43.9 (40.5 to 46.9)	33.8 (28.6 to 38.3)	34.0 (27.9 to 38.9)	-22.9 (-31.7 to -15.7)	0.6 (-8.9 to 10.1)	44.9 (41.5 to 47.5)	34.9 (29.4 to 39.3)	35.0 (28.8 to 40.0)	-22.3 (-31.7 to -15.2)	0.5 (-8.5 to 9.3)
Diet low in milk	26.1 (19.5 to 31.7)	24.6 (17.4 to 30.6)	25.1 (18.0 to 31.2)	-5.8 (-18.4 to 5.0)	2.1 (-6.9 to 13.2)	27.9 (21.8 to 33.1)	25.6 (18.8 to 31.1)	25.9 (19.3 to 31.5)	-8.2 (-19.6 to 1.4)	1.1 (-8.1 to 11.6)
Diet high in red meat	38.4 (32.3 to 43.7)	37.5 (27.6 to 45.2)	36.9 (25.7 to 45.3)	-2.5 (-23.3 to 19.4)	-1.5 (-19.7 to 17.9)	14.8 (9.0 to 19.6)	17.1 (9.0 to 24.7)	17.8 (9.6 to 26.9)	16.0 (-29.5 to 79.9)	4.2 (-27.0 to 48.7)
Diet high in processed meat	38.3 (32.5 to 42.8)	47.7 (41.3 to 51.4)	43.0 (34.4 to 49.0)	24.6 (9.2 to 43.4)	-9.8 (-20.5 to -0.1)	18.4 (14.2 to 22.1)	29.6 (23.4 to 35.8)	28.0 (20.8 to 34.7)	60.7 (28.7 to 104.7)	-5.3 (-19.7 to 9.4)
Diet high in sugar-sweetened beverages	34.0 (16.7 to 44.1)	32.9 (17.1 to 43.3)	27.9 (14.0 to 39.1)	-3.0 (-31.1 to 36.1)	-15.4 (-38.7 to 10.1)	28.7 (14.2 to 39.1)	25.9 (13.3 to 36.3)	21.3 (10.4 to 32.0)	-9.8 (-42.2 to 41.9)	-17.5 (-43.5 to 13.6)
Diet low in fibre	32.4 (26.4 to 37.3)	24.3 (17.3 to 30.1)	25.9 (18.6 to 31.7)	-25.0 (-39.0 to -14.2)	6.6 (-5.7 to 20.8)	37.1 (31.9 to 41.1)	28.9 (22.4 to 34.5)	30.2 (24.0 to 35.5)	-22.0 (-33.7 to -13.1)	4.5 (-4.7 to 15.5)
Diet low in calcium	14.6 (7.7 to 21.0)	13.3 (6.9 to 19.2)	14.0 (7.6 to 20.1)	-9.0 (-23.6 to 8.4)	5.7 (-6.5 to 21.7)	18.6 (11.3 to 24.7)	16.8 (9.8 to 23.3)	17.6 (10.5 to 24.0)	-9.3 (-23.2 to 2.8)	4.6 (-5.0 to 16.4)
Diet low in seafood omega-3 fatty acids	25.8 (19.7 to 31.6)	21.6 (15.3 to 27.4)	23.1 (17.1 to 29.2)	-16.6 (-29.9 to -3.8)	7.2 (-3.9 to 21.8)	29.5 (23.7 to 34.5)	25.8 (19.7 to 31.0)	26.9 (21.3 to 32.1)	-12.8 (-24.6 to -2.5)	4.5 (-3.7 to 15.2)
Diet low in polyunsaturated fatty acids	6.8 (1.7 to 13.2)	4.0 (0.4 to 9.4)	3.0 (0.3 to 7.3)	-40.5 (-84.8 to 7.0)	-26.7 (-74.2 to 71.0)	7.5 (2.1 to 14.1)	4.6 (0.7 to 10.2)	3.4 (0.4 to 8.3)	-38.8 (-82.2 to 8.6)	-26.5 (-71.2 to 60.6)
Diet high in trans fatty acids	16.8 (11.9 to 22.5)	9.9 (5.9 to 14.1)	7.1 (3.5 to 11.3)	-41.2 (-55.2 to -28.6)	-27.4 (-43.9 to -16.2)	23.6 (18.0 to 32.5)	13.3 (9.1 to 18.2)	9.8 (5.7 to 13.9)	-43.7 (-54.9 to -32.7)	-26.5 (-40.3 to -16.3)
Diet high in sodium	7.6 (0.8 to 15.4)	13.2 (4.5 to 20.4)	13.7 (4.9 to 21.4)	72.9 (19.9 to 673.4)	3.9 (-23.1 to 44.3)	6.1 (0.4 to 13.4)	11.1 (2.9 to 18.5)	11.6 (3.1 to 19.2)	80.4 (28.2 to 1006.4)	4.7 (-20.6 to 58.2)
Intimate partner violence	-	-	-	-	-	1.4 (1.1 to 1.6)	1.0 (0.8 to 1.1)	1.2 (1.0 to 1.4)	-29.4 (-33.6 to -25.5)	24.6 (18.6 to 31.1)
Childhood sexual abuse	5.6 (4.6 to 6.9)	5.1 (4.1 to 6.2)	5.1 (4.1 to 6.2)	-9.8 (-15.1 to -4.3)	0.4 (-4.7 to 6.0)	13.8 (11.0 to 16.9)	12.1 (9.8 to 14.7)	12.2 (9.9 to 14.8)	-11.9 (-17.2 to -6.0)	0.4 (-4.8 to 6.5)

Supplementary Results Table 1. Maryland age-standardised Summary Exposure Values (SEVs) for Level 4 risk factors and percentage change, by sex, 1990, 2007, and 2017.Data in parentheses are 95% uncertainty intervals.

	Male					Female				
	1990	2007	2017	Percentage change 1990-2007	Percentage change 2007-2017	1990	2007	2017	Percentage change 1990-2007	Percentage change 2007-2017
Bullying victimization	14.7 (6.1 to 27.9)	19.5 (9.7 to 33.1)	19.1 (9.1 to 33.0)	32.6 (7.4 to 85.6)	-2.2 (-10.0 to 6.1)	13.1 (5.5 to 26.8)	16.4 (8.0 to 30.4)	15.7 (7.5 to 29.2)	25.5 (0.5 to 70.1)	-4.0 (-11.7 to 4.0)
Low physical activity	0.4 (0.2 to 0.8)	0.4 (0.2 to 0.8)	0.4 (0.2 to 0.8)	-0.2 (-2.4 to 1.9)	0.4 (-0.9 to 1.8)	0.4 (0.2 to 0.9)	0.4 (0.2 to 0.9)	0.4 (0.2 to 0.9)	-0.2 (-2.4 to 2.1)	-0.1 (-1.5 to 1.4)
High fasting plasma glucose	1.5 (0.9 to 2.6)	2.5 (1.5 to 3.9)	3.3 (2.0 to 4.9)	67.1 (-5.5 to 211.1)	29.6 (-17.5 to 107.9)	1.7 (1.0 to 2.7)	3.0 (1.7 to 4.8)	2.9 (1.5 to 4.7)	82.0 (5.3 to 233.5)	-4.6 (-46.6 to 67.4)
High LDL cholesterol	21.0 (19.1 to 23.1)	11.5 (9.9 to 13.2)	10.1 (8.7 to 11.9)	-45.1 (-49.8 to -40.4)	-12.1 (-16.6 to -7.6)	19.9 (18.0 to 21.9)	11.4 (9.9 to 13.0)	10.6 (9.1 to 12.2)	-42.8 (-47.7 to -38.4)	-7.3 (-11.4 to -3.0)
High systolic blood pressure	6.5 (5.7 to 7.4)	4.8 (4.2 to 5.6)	5.0 (4.3 to 5.8)	-25.3 (-33.1 to -16.7)	4.0 (-5.3 to 14.3)	6.0 (5.2 to 6.8)	4.6 (4.0 to 5.2)	4.6 (3.9 to 5.3)	-24.2 (-32.9 to -13.8)	-0.2 (-10.4 to 10.7)
High body-mass index	15.5 (11.6 to 21.1)	22.0 (17.2 to 27.9)	23.3 (18.5 to 29.0)	41.6 (25.5 to 62.5)	6.1 (-2.6 to 16.0)	16.9 (13.0 to 22.0)	23.1 (18.6 to 29.1)	24.4 (19.4 to 30.2)	36.8 (21.6 to 57.5)	5.6 (-4.1 to 14.6)
Low bone mineral density	5.1 (4.2 to 6.1)	4.3 (3.6 to 5.1)	4.6 (3.7 to 5.6)	-15.1 (-25.7 to -2.0)	5.7 (-9.0 to 21.5)	5.7 (4.8 to 6.9)	5.6 (4.7 to 6.6)	6.1 (5.1 to 7.2)	-3.0 (-15.3 to 12.3)	8.6 (-6.0 to 24.1)
Impaired kidney function	2.4 (1.2 to 6.0)	2.4 (1.2 to 6.1)	2.4 (1.2 to 5.9)	-1.3 (-7.2 to 3.7)	1.9 (-4.1 to 8.7)	3.1 (1.5 to 7.1)	3.0 (1.4 to 7.0)	3.1 (1.5 to 7.3)	-3.0 (-9.0 to 1.5)	4.0 (-1.2 to 10.0)

Supplementary Results Table 1. Massachusetts age-standardised Summary Exposure Values (SEVs) for Level 4 risk factors and percentage change, by sex, 1990, 2007, and 2017.Data in parentheses are 95% uncertainty intervals.										
	Male					Female				
	1990	2007	2017	Percentage change 1990-2007	Percentage change 2007-2017	1990	2007	2017	Percentage change 1990-2007	Percentage change 2007-2017
Unsafe water source	1.0 (0.6 to 1.7)	0.4 (0.2 to 0.6)	0.3 (0.2 to 0.4)	-63.5 (-80.6 to -28.7)	-27.7 (-52.9 to 8.6)	1.0 (0.6 to 1.7)	0.4 (0.2 to 0.6)	0.3 (0.2 to 0.4)	-63.5 (-80.6 to -28.7)	-27.7 (-52.9 to 8.6)
Unsafe sanitation	3.7 (1.9 to 6.6)	2.3 (1.0 to 4.5)	1.9 (0.8 to 3.6)	-39.1 (-72.7 to 29.6)	-18.1 (-58.2 to 66.0)	3.7 (1.9 to 6.6)	2.3 (1.0 to 4.5)	1.9 (0.8 to 3.6)	-39.1 (-72.7 to 29.6)	-18.1 (-58.2 to 66.0)
No access to handwashing facility	3.3 (2.4 to 4.5)	3.1 (2.2 to 4.2)	3.0 (2.1 to 4.1)	-8.0 (-34.7 to 29.8)	-2.5 (-24.5 to 26.2)	3.3 (2.4 to 4.5)	3.1 (2.2 to 4.2)	3.0 (2.1 to 4.1)	-8.0 (-34.7 to 29.8)	-2.5 (-24.5 to 26.2)
Ambient particulate matter pollution	19.5 (12.1 to 31.6)	19.6 (12.6 to 30.9)	12.9 (5.2 to 22.6)	0.4 (-28.1 to 45.7)	-34.3 (-71.3 to -7.8)	19.5 (12.0 to 31.6)	19.6 (12.6 to 30.9)	12.9 (5.2 to 22.6)	0.5 (-28.0 to 45.9)	-34.3 (-71.4 to -7.8)
Household air pollution from solid fuels	0.2 (0.0 to 0.6)	0.1 (0.0 to 0.3)	0.1 (0.0 to 0.3)	-34.7 (-81.6 to 55.2)	-8.7 (-73.5 to 94.9)	0.3 (0.0 to 1.7)	0.2 (0.0 to 1.1)	0.2 (0.0 to 0.8)	-29.4 (-81.6 to 54.1)	-11.6 (-74.6 to 93.8)
Ambient ozone pollution	46.2 (17.6 to 54.8)	45.8 (17.3 to 54.8)	46.0 (17.4 to 54.8)	-0.9 (-2.2 to 0.0)	0.4 (0.0 to 1.2)	46.2 (17.6 to 54.8)	45.8 (17.3 to 54.8)	46.0 (17.4 to 54.8)	-0.9 (-2.2 to 0.0)	0.4 (0.0 to 1.2)
Residential radon	15.9 (1.3 to 49.4)	15.9 (1.3 to 49.4)	15.9 (1.3 to 49.4)	0.0 (0.0 to 0.0)	0.0 (0.0 to 0.0)	15.9 (1.3 to 49.4)	15.9 (1.3 to 49.4)	15.9 (1.3 to 49.4)	0.0 (0.0 to 0.0)	0.0 (0.0 to 0.0)
Lead exposure	8.2 (3.8 to 12.6)	5.0 (2.0 to 8.6)	3.3 (1.1 to 6.1)	-39.0 (-51.6 to -28.6)	-35.0 (-45.0 to -28.7)	5.4 (1.6 to 9.5)	3.4 (0.8 to 6.9)	2.2 (0.4 to 4.9)	-37.2 (-53.5 to -25.4)	-34.2 (-45.9 to -27.8)
Occupational exposure to asbestos	8.0 (6.9 to 8.9)	6.1 (5.7 to 6.5)	5.4 (4.7 to 6.1)	-24.0 (-32.8 to -10.8)	-11.2 (-24.2 to 2.1)	1.1 (1.0 to 1.3)	1.1 (1.0 to 1.2)	1.1 (0.9 to 1.3)	-1.8 (-15.8 to 14.2)	-1.7 (-19.3 to 19.2)
Occupational exposure to arsenic	0.4 (0.0 to 0.9)	0.4 (0.0 to 0.9)	0.4 (0.0 to 0.9)	-	-	0.4 (0.0 to 0.8)	0.3 (0.0 to 0.8)	0.3 (0.0 to 0.8)	-	-
Occupational exposure to benzene	0.7 (0.0 to 1.9)	0.7 (0.0 to 1.9)	0.6 (0.0 to 1.8)	1.5 (-6.4 to 13.7)	-4.1 (-9.6 to 3.4)	0.9 (0.1 to 2.7)	1.1 (0.1 to 3.2)	1.1 (0.1 to 3.0)	13.2 (3.1 to 28.6)	-2.1 (-9.1 to 8.8)
Occupational exposure to beryllium	0.0 (0.0 to 0.0)	0.0 (0.0 to 0.0)	0.0 (0.0 to 0.0)	-0.5 (-6.4 to 5.1)	0.8 (-3.6 to 5.4)	0.0 (0.0 to 0.0)	0.0 (0.0 to 0.0)	0.0 (0.0 to 0.0)	4.9 (-2.4 to 13.4)	-1.6 (-6.6 to 3.8)
Occupational exposure to cadmium	0.0 (0.0 to 0.0)	0.0 (0.0 to 0.0)	0.0 (0.0 to 0.0)	-4.5 (-15.2 to 7.5)	2.0 (-7.3 to 12.0)	0.0 (0.0 to 0.0)	0.0 (0.0 to 0.0)	0.0 (0.0 to 0.0)	-1.3 (-14.0 to 14.1)	-2.0 (-12.3 to 10.2)
Occupational exposure to chromium	0.1 (0.1 to 0.1)	0.1 (0.1 to 0.1)	0.1 (0.1 to 0.1)	-1.4 (-11.5 to 8.7)	1.5 (-6.6 to 9.5)	0.1 (0.1 to 0.1)	0.1 (0.1 to 0.1)	0.1 (0.1 to 0.1)	2.2 (-8.6 to 15.3)	-2.4 (-10.7 to 7.4)
Occupational exposure to diesel engine exhaust	0.5 (0.4 to 0.5)	0.5 (0.5 to 0.6)	0.5 (0.5 to 0.6)	12.7 (2.9 to 22.5)	1.4 (-5.1 to 8.5)	0.5 (0.4 to 0.5)	0.5 (0.5 to 0.6)	0.5 (0.4 to 0.6)	10.4 (-2.1 to 24.8)	-4.1 (-12.4 to 4.3)
Occupational exposure to formaldehyde	0.2 (0.2 to 0.3)	0.2 (0.2 to 0.2)	0.2 (0.2 to 0.2)	-6.5 (-17.3 to 5.4)	0.2 (-9.2 to 10.1)	0.2 (0.2 to 0.2)	0.2 (0.2 to 0.2)	0.2 (0.2 to 0.2)	0.1 (-11.4 to 13.9)	-2.4 (-11.8 to 8.3)
Occupational exposure to nickel	0.3 (0.0 to 1.4)	0.3 (0.0 to 1.3)	0.3 (0.0 to 1.3)	-	-	0.3 (0.0 to 1.2)	0.3 (0.0 to 1.2)	0.3 (0.0 to 1.2)	-	-
Occupational exposure to polycyclic aromatic hydrocarbons	0.2 (0.2 to 0.2)	0.2 (0.2 to 0.2)	0.2 (0.2 to 0.2)	-1.0 (-10.3 to 8.6)	0.6 (-6.7 to 8.1)	0.2 (0.2 to 0.3)	0.2 (0.2 to 0.3)	0.2 (0.2 to 0.3)	5.8 (-4.0 to 17.4)	-2.3 (-9.6 to 5.6)
Occupational exposure to silica	2.8 (0.4 to 8.5)	2.4 (0.3 to 7.6)	2.5 (0.4 to 7.8)	-11.4 (-22.2 to 2.0)	2.9 (-7.7 to 15.7)	2.3 (0.3 to 7.3)	2.1 (0.3 to 6.5)	2.0 (0.3 to 6.7)	-9.9 (-24.6 to 11.0)	-2.3 (-16.3 to 17.5)
Occupational exposure to sulphuric acid	0.6 (0.1 to 1.9)	0.6 (0.1 to 2.0)	0.6 (0.1 to 2.0)	7.0 (-5.7 to 20.5)	-0.4 (-9.9 to 9.7)	0.5 (0.1 to 1.7)	0.6 (0.1 to 2.0)	0.6 (0.1 to 2.0)	18.7 (2.2 to 39.1)	-3.5 (-15.4 to 9.5)
Occupational exposure to trichloroethylene	0.1 (0.1 to 0.1)	0.1 (0.1 to 0.1)	0.1 (0.1 to 0.1)	3.2 (-4.6 to 11.2)	1.5 (-4.3 to 7.4)	0.1 (0.1 to 0.1)	0.1 (0.1 to 0.1)	0.1 (0.1 to 0.1)	4.6 (-4.8 to 16.1)	-2.7 (-9.8 to 5.4)
Occupational asthmagens	16.0 (13.3 to 19.4)	14.5 (12.2 to 17.4)	14.3 (11.7 to 17.3)	-9.5 (-20.2 to 2.8)	-1.4 (-9.6 to 8.1)	8.3 (6.9 to 10.1)	8.5 (6.9 to 10.6)	8.0 (6.5 to 10.0)	2.3 (-11.7 to 18.6)	-6.3 (-17.2 to 5.1)
Occupational particulate matter, gases, and fumes	6.6 (4.7 to 9.4)	6.1 (4.3 to 8.6)	5.7 (4.2 to 8.2)	-8.1 (-12.6 to -3.5)	-5.1 (-9.0 to -1.7)	3.9 (2.7 to 5.7)	3.6 (2.5 to 5.2)	3.4 (2.4 to 4.9)	-6.5 (-12.4 to 0.5)	-5.5 (-11.0 to 0.1)
Occupational noise	6.0 (5.5 to 6.5)	5.8 (5.3 to 6.3)	5.6 (5.1 to 6.1)	-3.2 (-6.2 to 0.2)	-3.7 (-5.9 to -1.5)	4.4 (3.9 to 5.0)	4.6 (4.2 to 5.1)	4.6 (4.2 to 5.1)	4.2 (-0.5 to 10.0)	-0.5 (-3.5 to 2.8)
Occupational ergonomic factors	8.2 (6.8 to 9.9)	7.6 (6.2 to 9.2)	7.3 (6.0 to 8.9)	-7.5 (-16.9 to 2.0)	-3.8 (-10.5 to 3.4)	4.9 (3.8 to 6.2)	5.1 (4.0 to 6.4)	5.0 (4.0 to 6.4)	4.4 (-11.3 to 23.9)	-1.9 (-13.6 to 11.7)
Non-exclusive breastfeeding	0.5 (0.5 to 0.6)	0.5 (0.5 to 0.6)	0.5 (0.4 to 0.6)	0.1 (-10.4 to 13.6)	-7.3 (-16.4 to 1.7)	0.5 (0.5 to 0.6)	0.5 (0.5 to 0.6)	0.5 (0.4 to 0.6)	-0.2 (-10.6 to 13.2)	-7.0 (-16.1 to 2.0)
Discontinued breastfeeding	2.3 (2.2 to 2.4)	2.2 (2.1 to 2.3)	2.2 (2.1 to 2.4)	-4.4 (-10.7 to 2.4)	2.6 (-2.6 to 8.6)	2.3 (2.2 to 2.4)	2.2 (2.1 to 2.3)	2.2 (2.1 to 2.4)	-4.2 (-10.5 to 2.6)	1.9 (-3.3 to 7.9)
Child underweight	0.1 (0.0 to 0.1)	0.1 (0.0 to 0.1)	0.1 (0.0 to 0.1)	-4.3 (-18.0 to 11.5)	-11.1 (-20.0 to -2.6)	0.1 (0.0 to 0.1)	0.1 (0.0 to 0.1)	0.1 (0.0 to 0.1)	-1.9 (-23.7 to 51.8)	-2.6 (-12.9 to 13.5)
Child wasting	0.1 (0.0 to 0.1)	0.1 (0.0 to 0.1)	0.1 (0.0 to 0.1)	-9.5 (-30.4 to 9.7)	-3.1 (-18.0 to 12.7)	0.1 (0.0 to 0.1)	0.1 (0.0 to 0.1)	0.1 (0.0 to 0.1)	5.6 (-15.1 to 31.3)	-4.4 (-17.2 to 11.2)
Child stunting	0.2 (0.1 to 0.5)	0.2 (0.1 to 0.4)	0.2 (0.1 to 0.4)	-7.5 (-47.2 to 58.8)	-9.5 (-39.1 to 30.2)	0.2 (0.1 to 0.3)	0.2 (0.1 to 0.3)	0.2 (0.1 to 0.3)	-8.7 (-41.8 to 38.0)	-2.1 (-27.8 to 30.8)
Short gestation for birth weight	0.0 (0.0 to 0.0)	0.0 (0.0 to 0.0)	0.0 (0.0 to 0.0)	10.6 (-15.4 to 43.2)	0.8 (-17.4 to 30.5)	0.0 (0.0 to 0.0)	0.0 (0.0 to 0.0)	0.0 (0.0 to 0.0)	5.6 (-20.4 to 34.2)	4.8 (-17.3 to 43.8)
Low birth weight for gestation	0.0 (0.0 to 0.0)	0.0 (0.0 to 0.0)	0.0 (0.0 to 0.0)	1.6 (-19.1 to 33.8)	-0.5 (-19.2 to 18.6)	0.0 (0.0 to 0.0)	0.0 (0.0 to 0.0)	0.0 (0.0 to 0.0)	4.6 (-15.4 to 36.4)	-3.2 (-31.5 to 21.1)
Iron deficiency	-	-	-	-	-	3.4 (2.1 to 5.2)	3.3 (2.1 to 4.9)	3.4 (2.1 to 5.3)	-4.1 (-31.3 to 35.6)	4.8 (-21.2 to 40.1)
Vitamin A deficiency	0.4 (0.3 to 0.5)	0.4 (0.3 to 0.6)	0.5 (0.3 to 0.6)	18.6 (-0.5 to 42.3)	10.1 (-7.2 to 31.0)	0.5 (0.3 to 0.6)	0.5 (0.3 to 0.6)	0.6 (0.3 to 0.8)	6.5 (-13.1 to 29.5)	11.9 (-6.2 to 36.4)
Zinc deficiency	0.5 (0.1 to 1.1)	0.2 (0.0 to 1.1)	0.2 (0.0 to 1.0)	-54.1 (-88.9 to 387.2)	-2.7 (-89.0 to 817.1)	0.5 (0.1 to 1.2)	0.2 (0.0 to 1.0)	0.2 (0.0 to 1.0)	-55.2 (-88.5 to 335.4)	2.7 (-88.5 to 881.9)
Smoking	13.2 (10.9 to 15.8)	7.1 (5.7 to 8.8)	5.4 (4.2 to 6.8)	-46.2 (-54.2 to -37.5)	-23.9 (-32.7 to -14.5)	10.9 (8.5 to 13.3)	6.2 (4.9 to 7.6)	4.4 (3.4 to 5.5)	-43.2 (-52.8 to -30.2)	-28.9 (-37.3 to -20.0)
Chewing tobacco	0.6 (0.5 to 0.8)	0.9 (0.7 to 1.3)	1.0 (0.7 to 1.6)	46.6 (-0.6 to 114.0)	11.5 (-20.8 to 55.7)	0.1 (0.1 to 0.1)	0.1 (0.1 to 0.1)	0.1 (0.1 to 0.1)	-4.3 (-42.6 to 60.1)	3.2 (-29.1 to 54.7)
Second-hand smoke	46.8 (42.9 to 51.3)	25.1 (23.7 to 26.7)	25.4 (22.9 to 28.3)	-46.3 (-52.7 to -39.4)	1.2 (-8.7 to 12.4)	42.3 (39.1 to 46.0)	23.2 (22.2 to 24.3)	23.3 (21.6 to 25.1)	-45.1 (-51.3 to -38.6)	0.5 (-7.1 to 8.8)
Alcohol use	22.2 (12.1 to 35.4)	24.6 (13.3 to 38.6)	26.1 (13.5 to 41.7)	11.1 (-27.9 to 68.1)	5.7 (-26.8 to 50.8)	14.7 (7.6 to 24.7)	15.9 (8.1 to 26.6)	16.6 (8.2 to 29.1)	8.3 (-26.8 to 66.1)	4.1 (-28.9 to 49.3)
Drug use	1.7 (1.2 to 2.4)	2.6 (2.1 to 3.3)	3.4 (2.8 to 4.4)	56.7 (33.0 to 89.2)	30.1 (18.4 to 44.4)	1.3 (1.0 to 1.9)	1.8 (1.4 to 2.3)	2.1 (1.7 to 2.7)	31.5 (17.4 to 50.9)	18.5 (8.8 to 29.4)
Diet low in fruits	21.4 (14.6 to 27.5)	23.9 (17.5 to 29.4)	25.5 (18.4 to 31.6)	12.1 (-1.7 to 33.7)	6.3 (-7.0 to 20.6)	18.2 (11.3 to 24.4)	20.8 (13.9 to 26.9)	21.9 (14.3 to 28.8)	14.5 (-2.6 to 42.0)	5.0 (-10.3 to 24.8)
Diet low in vegetables	20.6 (12.0 to 27.6)	26.1 (18.6 to 32.2)	28.2 (20.7 to 34.3)	26.7 (4.6 to 73.3)	8.1 (-4.2 to 23.3)	24.1 (16.4 to 31.0)	28.2 (20.9 to 33.8)	30.6 (23.8 to 36.2)	16.7 (0.0 to 45.2)	8.6 (-1.7 to 24.0)
Diet low in legumes	23.8 (16.9 to 29.7)	23.9 (17.0 to 29.8)	25.4 (18.4 to 31.2)	0.4 (-15.7 to 20.0)	6.1 (-6.3 to 21.4)	28.7 (22.7 to 33.6)	29.4 (23.8 to 34.0)	30.7 (25.0 to 35.7)	2.2 (-10.3 to 18.8)	4.6 (-4.3 to 15.2)
Diet low in whole grains	37.5 (33.7 to 40.6)	38.6 (35.0 to 41.6)	38.0 (34.2 to 41.0)	3.0 (-2.3 to 9.2)	-1.5 (-5.9 to 2.3)	38.5 (34.8 to 41.4)	39.6 (36.3 to 42.4)	39.0 (35.6 to 42.0)	2.8 (-1.9 to 8.0)	-1.4 (-5.3 to 2.5)
Diet low in nuts and seeds	44.0 (40.3 to 46.8)	33.2 (27.6 to 37.7)	32.7 (26.4 to 37.9)	-24.6 (-33.9 to -17.3)	-1.4 (-10.9 to 8.5)	44.9 (41.4 to 47.4)	34.2 (29.1 to 38.6)	33.7 (27.8 to 38.9)	-23.8 (-32.6 to -16.6)	-1.4 (-11.3 to 7.4)
Diet low in milk	26.2 (19.6 to 31.5)	24.2 (17.2 to 30.2)	24.5 (17.7 to 30.5)	-7.4 (-19.8 to 3.5)	0.9 (-8.7 to 11.9)	27.9 (21.3 to 33.1)	25.2 (18.3 to 30.9)	25.2 (18.4 to 31.0)	-9.4 (-22.0 to 0.9)	-0.1 (-9.7 to 10.9)
Diet high in red meat	37.1 (29.4 to 43.1)	37.3 (26.9 to 45.4)	37.5 (26.4 to 46.2)	0.6 (-20.8 to 25.2)	0.4 (-16.6 to 21.7)	13.7 (7.5 to 19.3)	16.9 (8.9 to 24.6)	17.9 (8.8 to 26.7)	23.2 (-27.6 to 100.2)	6.3 (-27.3 to 53.1)
Diet high in processed meat	34.8 (28.5 to 39.8)	44.0 (37.5 to 49.8)	39.3 (30.6 to 46.1)	26.7 (7.3 to 50.5)	-10.7 (-21.7 to 0.1)	16.1 (11.8 to 19.7)	25.6 (19.6 to 31.0)	24.7 (18.3 to 30.9)	59.2 (28.0 to 101.2)	-3.4 (-18.2 to 13.5)
Diet high in sugar-sweetened beverages	33.9 (17.0 to 43.9)	33.6 (17.5 to 43.3)	29.2 (15.1 to 40.2)	-1.0 (-29.0 to 36.1)	-13.0 (-35.0 to 12.2)	28.7 (15.0 to 39.4)	26.4 (12.8 to 36.6)	22.5 (10.6 to 32.3)	-8.0 (-39.4 to 40.7)	-14.8 (-40.6 to 15.5)
Diet low in fibre	32.4 (26.4 to 37.3)	24.8 (17.7 to 30.7)	26.0 (18.8 to 32.0)	-23.5 (-37.4 to -13.4)	5.1 (-5.9 to 17.8)	36.8 (31.9 to 40.8)	28.2 (21.8 to 33.7)	29.4 (23.0 to 34.8)	-23.3 (-34.7 to -14.4)	4.1 (-5.2 to 15.0)
Diet low in calcium	14.6 (6.7 to 21.4)	11.8 (4.8 to 18.7)	12.2 (5.0 to 19.1)	-19.0 (-41.4 to -4.2)	3.1 (-13.2 to 24.1)	18.9 (11.1 to 25.1)	16.0 (7.9 to 22.5)	16.4 (8.4 to 23.0)	-15.3 (-34.0 to -2.3)	2.2 (-9.3 to 17.8)
Diet low in seafood omega-3 fatty acids	24.1 (19.1 to 28.8)	17.7 (13.6 to 21.7)	18.5 (14.4 to 22.3)	-26.7 (-36.6 to -17.8)	4.5 (-4.6 to 16.2)	26.7 (22.3 to 30.9)	19.8 (15.8 to 23.9)	20.1 (16.2 to 24.0)	-25.7 (-33.3 to -18.7)	1.5 (-6.2 to 10.4)
Diet low in polyunsaturated fatty acids	7.5 (2.1 to 13.6)	5.5 (1.2 to 11.3)	4.0 (0.6 to 9.1)	-26.8 (-72.8 to 23.9)	-27.9 (-68.4 to 22.5)	8.2 (2.6 to 14.9)	5.9 (1.5 to 12.0)	4.3 (0.8 to 9.3)	-27.8 (-70.1 to 27.3)	-28.2 (-65.1 to 26.9)
Diet high in trans fatty acids	16.8 (12.1 to 22.4)	9.8 (5.8 to 14.2)	7.1 (3.4 to 11.0)	-41.3 (-55.9 to -29.0)	-27.7 (-44.4 to -16.1)	23.7 (18.0 to 32.0)	13.3 (9.0 to 18.2)	9.8 (5.8 to 13.8)	-43.7 (-55.4 to -31.2)	-26.9 (-40.1 to -17.2)
Diet high in sodium	7.5 (0.9 to 15.3)	13.2 (4.5 to 20.7)	13.9 (5.0 to 21.6)	75.9 (20.5 to 589.6)	5.5 (-21.4 to 49.5)	6.2 (0.3 to 13.5)	11.2 (2.9 to 19.0)	11.8 (3.4 to 19.7)	81.6 (29.3 to 1055.5)	5.1 (-17.2 to 49.3)
Intimate partner violence	-	-	-	-	-	1.2 (1.0 to 1.4)	0.8 (0.7 to 1.0)	1.0 (0.9 to 1.2)	-30.4 (-34.1 to -26.5)	25.2 (18.6 to 31.8)
Childhood sexual abuse	5.6 (4.6 to 6.9)	5.1 (4.1 to 6.2)	5.1 (4.1 to 6.2)	-9.8 (-15.1 to -4.3)	0.4 (-4.7 to 6.0)	13.8 (11.0 to 16.9)	12.1 (9.8 to 14.7)	12.2 (9.9 to 14.8)	-11.9 (-17.2 to -6.0)	0.4 (-4.8 to 6.5)

Supplementary Results Table 1. Massachusetts age-standardised Summary Exposure Values (SEVs) for Level 4 risk factors and percentage change, by sex, 1990, 2007, and 2017.Data in parentheses are 95% uncertainty intervals.

	Male					Female				
	**1990**	**2007**	**2017**	**Percentage change 1990-2007**	**Percentage change 2007-2017**	**1990**	**2007**	**2017**	**Percentage change 1990-2007**	**Percentage change 2007-2017**
Bullying victimization	6.1 (2.6 to 13.0)	9.0 (3.9 to 18.3)	8.7 (3.7 to 17.9)	48.0 (31.4 to 68.8)	-3.6 (-10.9 to 4.6)	4.6 (1.9 to 9.6)	7.6 (3.3 to 16.0)	7.5 (3.2 to 15.9)	65.2 (45.2 to 92.1)	-1.4 (-9.0 to 8.2)
Low physical activity	0.4 (0.2 to 0.8)	0.4 (0.2 to 0.8)	0.4 (0.2 to 0.8)	0.1 (-2.1 to 2.1)	0.1 (-1.2 to 1.5)	0.4 (0.2 to 0.8)	0.4 (0.2 to 0.8)	0.4 (0.2 to 0.8)	-0.8 (-3.0 to 1.5)	-0.1 (-1.5 to 1.3)
High fasting plasma glucose	1.3 (0.8 to 2.1)	2.4 (1.4 to 3.6)	3.1 (1.9 to 4.7)	90.8 (17.0 to 223.5)	29.3 (-19.4 to 114.9)	1.4 (0.8 to 2.3)	2.3 (1.2 to 3.7)	2.2 (1.1 to 3.7)	67.3 (-12.4 to 206.2)	-5.3 (-47.1 to 68.9)
High LDL cholesterol	21.4 (19.4 to 23.4)	11.6 (10.0 to 13.3)	10.1 (8.6 to 11.8)	-45.9 (-50.9 to -40.8)	-12.7 (-17.0 to -8.6)	20.1 (18.1 to 22.1)	11.4 (10.0 to 13.1)	10.5 (9.0 to 12.2)	-43.0 (-47.8 to -38.6)	-8.0 (-11.9 to -3.9)
High systolic blood pressure	6.2 (5.3 to 7.0)	4.5 (3.9 to 5.3)	4.7 (4.0 to 5.4)	-26.5 (-33.8 to -17.8)	3.3 (-6.0 to 13.1)	5.7 (5.0 to 6.5)	4.2 (3.6 to 4.7)	4.1 (3.5 to 4.8)	-26.8 (-35.5 to -17.1)	-0.5 (-10.8 to 10.7)
High body-mass index	16.0 (11.9 to 21.5)	21.8 (17.2 to 27.4)	23.3 (18.4 to 29.1)	36.0 (19.5 to 55.8)	6.7 (-1.6 to 16.2)	15.0 (11.3 to 19.4)	21.4 (17.3 to 27.0)	23.1 (18.7 to 28.6)	43.0 (25.3 to 67.3)	8.0 (-2.1 to 19.5)
Low bone mineral density	3.6 (2.8 to 4.4)	3.5 (2.8 to 4.2)	3.4 (2.7 to 4.2)	-2.7 (-17.6 to 15.6)	-1.9 (-16.5 to 14.8)	6.2 (5.1 to 7.3)	6.1 (5.1 to 7.1)	6.5 (5.4 to 7.6)	-1.4 (-14.2 to 12.9)	6.7 (-7.2 to 21.8)
Impaired kidney function	2.9 (1.3 to 6.8)	2.7 (1.3 to 6.6)	2.9 (1.4 to 6.8)	-5.4 (-11.8 to 0.5)	6.8 (1.1 to 14.9)	3.5 (1.6 to 7.8)	3.4 (1.6 to 7.8)	3.4 (1.6 to 7.8)	-2.5 (-7.9 to 2.6)	1.0 (-4.1 to 6.4)

Supplementary Results Table 1. Michigan age-standardised Summary Exposure Values (SEVs) for Level 4 risk factors and percentage change, by sex, 1990, 2007, and 2017.Data in parentheses are 95% uncertainty intervals.

	Male					Female				
	1990	2007	2017	Percentage change 1990-2007	Percentage change 2007-2017	1990	2007	2017	Percentage change 1990-2007	Percentage change 2007-2017
Unsafe water source	1.4 (0.8 to 2.3)	0.5 (0.3 to 0.7)	0.3 (0.2 to 0.5)	-66.5 (-82.6 to -35.1)	-33.7 (-58.6 to 5.5)	1.4 (0.8 to 2.3)	0.5 (0.3 to 0.7)	0.3 (0.2 to 0.5)	-66.5 (-82.6 to -35.1)	-33.7 (-58.6 to 5.5)
Unsafe sanitation	6.3 (3.4 to 10.7)	3.8 (1.8 to 7.4)	2.9 (1.3 to 5.7)	-39.7 (-72.3 to 24.1)	-24.4 (-59.9 to 46.9)	6.3 (3.4 to 10.7)	3.8 (1.8 to 7.4)	2.9 (1.3 to 5.7)	-39.7 (-72.3 to 24.1)	-24.4 (-59.9 to 46.9)
No access to handwashing facility	3.4 (2.5 to 4.7)	3.2 (2.3 to 4.4)	3.0 (2.2 to 4.1)	-8.5 (-34.5 to 29.5)	-3.9 (-25.8 to 25.5)	3.4 (2.5 to 4.7)	3.2 (2.3 to 4.4)	3.0 (2.2 to 4.1)	-8.5 (-34.5 to 29.5)	-3.9 (-25.8 to 25.5)
Ambient particulate matter pollution	24.9 (11.0 to 37.6)	24.9 (15.1 to 36.9)	18.6 (6.2 to 30.2)	-0.1 (-29.9 to 80.1)	-25.1 (-74.1 to 9.1)	24.8 (10.9 to 37.4)	24.8 (15.0 to 36.9)	18.6 (6.2 to 30.1)	-0.0 (-29.7 to 80.4)	-25.1 (-74.1 to 9.1)
Household air pollution from solid fuels	0.3 (0.1 to 0.7)	0.2 (0.0 to 0.4)	0.2 (0.0 to 0.4)	-34.4 (-78.3 to 61.7)	-10.1 (-72.2 to 100.4)	0.4 (0.1 to 3.0)	0.3 (0.0 to 1.2)	0.3 (0.0 to 1.1)	-31.2 (-78.2 to 61.7)	-10.1 (-70.8 to 101.2)
Ambient ozone pollution	46.8 (18.0 to 54.8)	47.2 (18.3 to 54.8)	48.3 (19.4 to 54.8)	0.9 (0.0 to 2.4)	2.3 (0.0 to 6.6)	46.8 (18.0 to 54.8)	47.2 (18.3 to 54.8)	48.3 (19.4 to 54.8)	0.9 (0.0 to 2.4)	2.3 (0.0 to 6.6)
Residential radon	18.0 (1.5 to 56.1)	18.0 (1.5 to 56.1)	18.0 (1.5 to 56.1)	0.0 (0.0 to 0.0)	0.0 (0.0 to 0.0)	18.0 (1.5 to 56.1)	18.0 (1.5 to 56.1)	18.0 (1.5 to 56.1)	0.0 (0.0 to 0.0)	0.0 (0.0 to 0.0)
Lead exposure	9.3 (4.6 to 13.8)	5.9 (2.5 to 9.7)	3.9 (1.5 to 7.0)	-36.9 (-48.1 to -27.6)	-33.4 (-42.1 to -27.2)	5.8 (1.9 to 9.9)	3.7 (1.0 to 7.4)	2.5 (0.5 to 5.4)	-35.2 (-50.6 to -24.1)	-32.5 (-44.3 to -26.3)
Occupational exposure to asbestos	5.9 (5.1 to 6.8)	5.2 (4.9 to 5.6)	4.7 (4.0 to 5.5)	-11.9 (-23.5 to 4.6)	-10.8 (-25.8 to 5.9)	0.9 (0.8 to 1.0)	1.0 (0.9 to 1.0)	1.0 (0.8 to 1.2)	6.6 (-8.0 to 22.7)	1.0 (-17.9 to 20.8)
Occupational exposure to arsenic	0.5 (0.0 to 1.1)	0.5 (0.0 to 1.1)	0.4 (0.0 to 1.1)	-	-	0.4 (0.0 to 0.9)	0.4 (0.0 to 0.9)	0.4 (0.0 to 0.8)	-	-
Occupational exposure to benzene	0.6 (0.0 to 1.8)	0.6 (0.0 to 1.8)	0.6 (0.0 to 1.7)	-0.0 (-7.0 to 10.9)	-2.3 (-8.1 to 5.7)	0.9 (0.0 to 2.6)	1.0 (0.1 to 2.7)	0.9 (0.1 to 2.7)	10.6 (0.7 to 27.3)	-0.6 (-8.4 to 10.3)
Occupational exposure to beryllium	0.0 (0.0 to 0.0)	0.0 (0.0 to 0.0)	0.0 (0.0 to 0.0)	-0.9 (-6.3 to 5.1)	-0.0 (-4.1 to 4.5)	0.0 (0.0 to 0.0)	0.0 (0.0 to 0.0)	0.0 (0.0 to 0.0)	5.4 (-2.5 to 14.6)	-1.6 (-7.5 to 4.6)
Occupational exposure to cadmium	0.1 (0.1 to 0.1)	0.1 (0.0 to 0.1)	0.1 (0.0 to 0.1)	-4.7 (-14.4 to 5.4)	-1.6 (-9.7 to 7.2)	0.0 (0.0 to 0.0)	0.0 (0.0 to 0.1)	0.0 (0.0 to 0.0)	4.0 (-11.1 to 20.6)	-3.8 (-14.5 to 7.9)
Occupational exposure to chromium	0.1 (0.1 to 0.1)	0.1 (0.1 to 0.1)	0.1 (0.1 to 0.1)	-2.4 (-11.0 to 6.8)	-1.2 (-8.4 to 6.5)	0.1 (0.1 to 0.1)	0.1 (0.1 to 0.1)	0.1 (0.1 to 0.1)	5.9 (-7.4 to 20.4)	-3.7 (-12.7 to 5.9)
Occupational exposure to diesel engine exhaust	0.5 (0.4 to 0.5)	0.5 (0.5 to 0.6)	0.5 (0.5 to 0.6)	12.8 (3.7 to 23.4)	2.5 (-4.4 to 9.5)	0.5 (0.4 to 0.5)	0.5 (0.5 to 0.6)	0.5 (0.4 to 0.5)	8.7 (-2.8 to 22.8)	-4.5 (-12.9 to 5.2)
Occupational exposure to formaldehyde	0.3 (0.3 to 0.3)	0.3 (0.2 to 0.3)	0.3 (0.2 to 0.3)	-7.0 (-16.6 to 3.1)	-3.0 (-11.4 to 6.0)	0.2 (0.2 to 0.2)	0.2 (0.2 to 0.2)	0.2 (0.2 to 0.2)	4.3 (-10.0 to 19.6)	-3.7 (-14.0 to 7.1)
Occupational exposure to nickel	0.4 (0.0 to 1.8)	0.4 (0.0 to 1.7)	0.4 (0.0 to 1.6)	-	-	0.3 (0.0 to 1.3)	0.3 (0.0 to 1.3)	0.3 (0.0 to 1.3)	-	-
Occupational exposure to polycyclic aromatic hydrocarbons	0.3 (0.2 to 0.3)	0.2 (0.2 to 0.3)	0.2 (0.2 to 0.3)	-2.3 (-10.7 to 6.6)	-1.6 (-8.4 to 5.7)	0.2 (0.2 to 0.2)	0.2 (0.2 to 0.3)	0.2 (0.2 to 0.3)	8.5 (-3.2 to 21.7)	-3.2 (-11.3 to 5.3)
Occupational exposure to silica	3.6 (0.5 to 11.3)	3.2 (0.4 to 10.2)	3.2 (0.4 to 10.3)	-10.5 (-20.1 to 0.6)	-1.2 (-10.0 to 9.3)	2.4 (0.3 to 7.8)	2.3 (0.3 to 7.4)	2.2 (0.3 to 7.3)	-3.1 (-20.0 to 19.6)	-4.7 (-18.1 to 11.5)
Occupational exposure to sulphuric acid	0.7 (0.1 to 2.3)	0.7 (0.2 to 2.4)	0.7 (0.2 to 2.3)	4.8 (-4.8 to 16.6)	-2.6 (-9.6 to 6.4)	0.6 (0.1 to 1.9)	0.7 (0.2 to 2.3)	0.7 (0.1 to 2.2)	22.6 (4.1 to 44.4)	-5.4 (-17.5 to 8.1)
Occupational exposure to trichloroethylene	0.1 (0.1 to 0.1)	0.1 (0.1 to 0.1)	0.1 (0.1 to 0.1)	2.2 (-4.7 to 10.2)	-0.0 (-5.4 to 6.0)	0.1 (0.1 to 0.1)	0.1 (0.1 to 0.1)	0.1 (0.1 to 0.1)	6.8 (-4.0 to 19.2)	-3.7 (-11.3 to 4.8)
Occupational asthmagens	19.9 (16.3 to 24.4)	17.5 (14.6 to 21.0)	16.8 (14.0 to 20.4)	-12.1 (-22.8 to 0.6)	-4.0 (-12.9 to 6.3)	8.1 (6.7 to 9.8)	8.3 (6.8 to 10.2)	7.8 (6.3 to 9.6)	2.9 (-9.6 to 17.8)	-6.7 (-16.9 to 5.2)
Occupational particulate matter, gases, and fumes	7.9 (5.7 to 11.1)	7.5 (5.3 to 10.6)	7.1 (5.0 to 10.1)	-5.6 (-10.0 to -1.3)	-5.4 (-9.0 to -2.0)	3.9 (2.7 to 5.7)	3.9 (2.7 to 5.7)	3.7 (2.6 to 5.5)	-0.1 (-6.8 to 7.6)	-4.0 (-9.3 to 1.9)
Occupational noise	6.6 (6.1 to 7.2)	6.4 (5.9 to 6.9)	6.1 (5.6 to 6.7)	-3.0 (-6.0 to 0.0)	-4.0 (-6.0 to -1.8)	4.3 (3.8 to 4.9)	4.6 (4.1 to 5.1)	4.5 (4.1 to 5.1)	5.3 (0.5 to 11.0)	-0.5 (-3.6 to 2.8)
Occupational ergonomic factors	8.6 (7.1 to 10.5)	7.8 (6.5 to 9.5)	7.5 (6.2 to 9.1)	-9.1 (-18.3 to 0.8)	-4.1 (-10.4 to 3.8)	4.9 (3.9 to 6.1)	5.0 (4.0 to 6.3)	4.9 (3.9 to 6.3)	2.8 (-13.0 to 20.7)	-1.8 (-13.3 to 11.0)
Non-exclusive breastfeeding	0.5 (0.5 to 0.6)	0.5 (0.5 to 0.6)	0.5 (0.4 to 0.6)	-0.5 (-11.1 to 12.3)	-7.1 (-15.9 to 2.2)	0.5 (0.5 to 0.6)	0.5 (0.5 to 0.6)	0.5 (0.4 to 0.6)	-1.0 (-11.5 to 11.7)	-6.8 (-15.6 to 2.5)
Discontinued breastfeeding	2.2 (2.1 to 2.3)	2.1 (2.0 to 2.2)	2.2 (2.0 to 2.3)	-4.9 (-11.6 to 2.6)	3.3 (-2.6 to 9.9)	2.2 (2.1 to 2.3)	2.1 (2.0 to 2.2)	2.2 (2.1 to 2.3)	-4.5 (-11.2 to 3.1)	3.9 (-1.9 to 10.6)
Child underweight	0.1 (0.1 to 0.1)	0.1 (0.1 to 0.1)	0.1 (0.0 to 0.1)	-4.9 (-18.6 to 10.2)	-12.8 (-21.7 to -3.7)	0.1 (0.0 to 0.1)	0.1 (0.0 to 0.1)	0.1 (0.0 to 0.1)	-3.2 (-19.0 to 14.3)	-3.9 (-13.9 to 7.1)
Child wasting	0.1 (0.0 to 0.1)	0.1 (0.0 to 0.1)	0.1 (0.0 to 0.1)	-10.2 (-31.3 to 9.1)	-3.0 (-16.6 to 10.8)	0.1 (0.0 to 0.1)	0.1 (0.0 to 0.1)	0.1 (0.0 to 0.1)	6.2 (-14.5 to 32.4)	-5.0 (-18.5 to 11.2)
Child stunting	0.3 (0.1 to 0.5)	0.2 (0.1 to 0.4)	0.2 (0.1 to 0.4)	-7.3 (-48.1 to 62.1)	-11.0 (-39.3 to 29.1)	0.2 (0.1 to 0.4)	0.2 (0.1 to 0.3)	0.2 (0.1 to 0.3)	-10.3 (-46.7 to 41.2)	-3.5 (-30.4 to 33.0)
Short gestation for birth weight	0.0 (0.0 to 0.0)	0.0 (0.0 to 0.0)	0.0 (0.0 to 0.0)	5.2 (-19.8 to 31.8)	3.2 (-16.3 to 30.1)	0.0 (0.0 to 0.0)	0.0 (0.0 to 0.0)	0.0 (0.0 to 0.0)	5.3 (-31.7 to 43.7)	4.1 (-21.6 to 57.0)
Low birth weight for gestation	0.0 (0.0 to 0.0)	0.0 (0.0 to 0.0)	0.0 (0.0 to 0.0)	2.0 (-15.1 to 30.9)	-1.3 (-20.8 to 17.1)	0.0 (0.0 to 0.0)	0.0 (0.0 to 0.0)	0.0 (0.0 to 0.0)	0.7 (-22.8 to 46.5)	-2.3 (-32.3 to 24.1)
Iron deficiency	-	-	-	-	-	4.1 (2.5 to 5.8)	3.8 (2.5 to 5.5)	3.9 (2.5 to 5.8)	-5.9 (-31.0 to 30.7)	3.1 (-19.8 to 30.6)
Vitamin A deficiency	0.4 (0.3 to 0.6)	0.5 (0.3 to 0.6)	0.5 (0.4 to 0.7)	13.4 (-3.7 to 36.4)	5.1 (-11.6 to 23.7)	0.6 (0.4 to 0.7)	0.5 (0.4 to 0.7)	0.7 (0.4 to 1.0)	-8.8 (-23.7 to 11.0)	26.2 (-9.4 to 60.1)
Zinc deficiency	0.5 (0.1 to 1.2)	0.2 (0.0 to 1.0)	0.2 (0.0 to 1.0)	-55.9 (-89.0 to 230.6)	2.2 (-88.0 to 862.4)	0.5 (0.1 to 1.1)	0.2 (0.0 to 1.0)	0.2 (0.0 to 1.0)	-52.8 (-87.7 to 405.1)	-2.4 (-89.9 to 841.1)
Smoking	14.5 (12.0 to 16.9)	7.8 (6.3 to 9.3)	6.1 (4.8 to 7.6)	-46.2 (-53.6 to -37.5)	-21.3 (-30.1 to -10.9)	10.5 (8.1 to 13.2)	6.1 (4.8 to 7.4)	4.5 (3.5 to 5.6)	-41.5 (-51.8 to -28.0)	-26.9 (-35.1 to -17.4)
Chewing tobacco	1.6 (1.2 to 2.0)	1.8 (1.2 to 2.4)	1.9 (1.3 to 2.8)	12.7 (-22.7 to 63.7)	7.7 (-21.3 to 44.1)	0.1 (0.1 to 0.1)	0.1 (0.1 to 0.1)	0.1 (0.1 to 0.1)	-7.3 (-43.7 to 54.4)	3.2 (-29.6 to 53.6)
Second-hand smoke	47.5 (43.7 to 51.4)	28.3 (26.8 to 30.0)	28.8 (26.1 to 31.7)	-40.3 (-46.7 to -33.1)	1.7 (-8.6 to 12.1)	45.0 (41.8 to 48.5)	27.5 (26.1 to 28.8)	27.7 (25.8 to 29.6)	-38.9 (-45.6 to -31.4)	0.7 (-6.6 to 8.7)
Alcohol use	20.4 (10.9 to 32.2)	22.3 (11.9 to 35.5)	24.1 (12.2 to 39.4)	9.6 (-29.1 to 69.6)	8.0 (-29.1 to 55.7)	12.9 (6.7 to 21.4)	13.9 (7.2 to 23.5)	14.7 (7.0 to 25.8)	7.6 (-29.1 to 63.2)	5.7 (-29.3 to 50.4)
Drug use	1.4 (1.0 to 2.2)	2.1 (1.6 to 2.8)	2.8 (2.2 to 3.7)	45.6 (27.4 to 68.9)	32.9 (20.0 to 48.5)	1.2 (0.9 to 1.6)	1.8 (1.5 to 2.3)	2.2 (1.7 to 2.8)	50.9 (34.2 to 72.8)	19.7 (8.3 to 31.3)
Diet low in fruits	23.1 (16.3 to 29.0)	26.5 (20.3 to 31.7)	28.4 (21.7 to 33.8)	15.0 (2.0 to 35.5)	7.1 (-3.0 to 20.6)	19.5 (12.6 to 25.7)	23.5 (16.9 to 29.1)	25.0 (18.2 to 31.3)	20.6 (3.2 to 54.0)	6.4 (-6.2 to 22.2)
Diet low in vegetables	19.4 (11.4 to 26.7)	28.1 (21.4 to 33.7)	30.8 (24.5 to 36.2)	44.9 (18.0 to 111.8)	9.5 (-0.6 to 23.2)	23.6 (14.9 to 30.3)	30.5 (23.9 to 35.7)	33.6 (27.3 to 38.4)	29.0 (9.0 to 66.5)	10.1 (1.3 to 23.3)
Diet low in legumes	24.0 (17.0 to 29.9)	24.1 (17.7 to 29.7)	25.5 (19.0 to 31.2)	0.5 (-15.5 to 21.4)	5.8 (-7.2 to 22.7)	28.7 (22.5 to 33.5)	29.3 (23.6 to 34.1)	30.7 (25.2 to 35.5)	2.3 (-9.5 to 17.4)	4.5 (-4.7 to 15.2)
Diet low in whole grains	38.1 (34.7 to 41.0)	39.6 (36.5 to 42.3)	39.2 (35.8 to 42.0)	3.8 (-1.4 to 9.3)	-0.9 (-4.6 to 2.7)	39.0 (35.8 to 41.9)	40.4 (37.2 to 43.0)	40.1 (37.1 to 42.8)	3.5 (-1.2 to 8.7)	-0.7 (-4.3 to 2.8)
Diet low in nuts and seeds	45.6 (42.7 to 48.1)	37.3 (32.8 to 41.1)	38.3 (33.3 to 42.3)	-18.3 (-25.8 to -12.1)	2.8 (-4.4 to 10.1)	46.5 (43.7 to 48.7)	38.4 (33.9 to 42.1)	39.3 (34.5 to 43.4)	-17.4 (-24.7 to -11.5)	2.4 (-4.1 to 9.6)
Diet low in milk	27.5 (21.2 to 32.8)	26.5 (20.3 to 32.0)	27.3 (20.7 to 32.7)	-3.6 (-14.0 to 6.7)	2.9 (-4.7 to 12.9)	29.3 (23.1 to 34.3)	27.6 (21.4 to 32.7)	28.1 (22.0 to 33.4)	-5.8 (-15.2 to 2.7)	1.8 (-5.6 to 9.7)
Diet high in red meat	41.8 (31.3 to 49.6)	35.3 (24.5 to 44.2)	33.3 (22.4 to 42.8)	-15.6 (-34.5 to 4.3)	-5.8 (-24.8 to 17.1)	23.2 (14.0 to 32.4)	16.2 (8.5 to 24.4)	14.8 (6.7 to 24.2)	-29.9 (-57.2 to 2.4)	-8.9 (-41.6 to 33.5)
Diet high in processed meat	39.6 (31.2 to 45.9)	35.3 (27.1 to 42.0)	27.6 (20.6 to 34.3)	-10.9 (-26.4 to 6.7)	-21.8 (-33.4 to -8.1)	21.6 (15.8 to 27.5)	19.9 (14.8 to 24.9)	16.9 (11.7 to 21.5)	-7.5 (-26.1 to 15.9)	-15.3 (-29.0 to -0.5)
Diet high in sugar-sweetened beverages	31.4 (15.8 to 42.0)	29.0 (14.1 to 39.7)	23.4 (11.3 to 34.1)	-7.8 (-37.8 to 34.1)	-19.2 (-43.3 to 9.6)	26.3 (12.1 to 38.1)	22.3 (10.3 to 33.4)	18.0 (8.0 to 28.9)	-15.3 (-45.5 to 31.9)	-19.3 (-46.1 to 16.1)
Diet low in fibre	33.1 (26.8 to 37.9)	25.4 (18.5 to 31.4)	27.1 (20.0 to 33.0)	-23.1 (-36.6 to -12.8)	6.8 (-2.9 to 20.2)	37.9 (33.0 to 41.8)	30.1 (23.8 to 35.4)	31.5 (25.0 to 36.6)	-20.5 (-31.3 to -11.4)	4.7 (-3.4 to 15.4)
Diet low in calcium	16.5 (9.4 to 22.8)	15.7 (9.0 to 22.1)	16.7 (9.7 to 23.3)	-4.8 (-18.5 to 9.7)	6.9 (-3.2 to 22.4)	20.6 (13.5 to 26.5)	19.6 (12.5 to 25.6)	20.7 (13.5 to 26.6)	-5.1 (-17.2 to 5.4)	5.7 (-2.2 to 17.5)
Diet low in seafood omega-3 fatty acids	28.5 (22.8 to 33.5)	25.2 (18.9 to 30.5)	27.2 (21.2 to 32.6)	-11.7 (-23.4 to -0.8)	8.0 (-1.0 to 19.8)	32.0 (26.9 to 36.7)	29.5 (23.7 to 34.5)	30.9 (25.5 to 35.7)	-7.9 (-17.9 to 1.0)	5.0 (-1.9 to 14.0)
Diet low in polyunsaturated fatty acids	8.1 (2.3 to 14.6)	5.6 (1.0 to 11.7)	4.5 (0.8 to 10.2)	-31.2 (-75.8 to 18.6)	-19.6 (-62.7 to 54.9)	8.8 (2.8 to 15.6)	6.2 (1.4 to 12.3)	4.9 (0.9 to 10.8)	-29.8 (-72.0 to 18.0)	-21.2 (-59.8 to 36.1)
Diet high in trans fatty acids	16.8 (12.3 to 22.3)	9.9 (5.8 to 14.2)	7.1 (3.5 to 11.2)	-41.1 (-55.7 to -28.6)	-27.5 (-44.0 to -15.7)	23.7 (18.0 to 32.4)	13.3 (9.0 to 17.9)	9.7 (5.8 to 13.9)	-43.8 (-54.6 to -31.5)	-27.0 (-40.3 to -17.2)
Diet high in sodium	7.2 (0.7 to 14.8)	12.5 (3.7 to 20.5)	12.9 (4.1 to 20.7)	72.8 (21.0 to 657.1)	3.3 (-21.9 to 47.0)	6.0 (0.3 to 13.2)	10.9 (2.5 to 18.5)	11.4 (2.9 to 19.1)	80.5 (26.8 to 931.9)	4.2 (-21.7 to 51.4)
Intimate partner violence	-	-	-	-	-	1.4 (1.2 to 1.6)	1.0 (0.8 to 1.1)	1.2 (1.0 to 1.4)	-30.8 (-34.6 to -26.5)	26.7 (19.3 to 33.9)
Childhood sexual abuse	5.6 (4.6 to 6.9)	5.1 (4.1 to 6.2)	5.1 (4.1 to 6.2)	-9.8 (-15.1 to -4.3)	0.4 (-4.7 to 6.0)	13.8 (11.0 to 16.9)	12.1 (9.8 to 14.7)	12.2 (9.9 to 14.8)	-11.9 (-17.2 to -6.0)	0.4 (-4.8 to 6.5)

Supplementary Results Table 1. Michigan age-standardised Summary Exposure Values (SEVs) for Level 4 risk factors and percentage change, by sex, 1990, 2007, and 2017.Data in parentheses are 95% uncertainty intervals.										
	**Male**					**Female**				
	**1990**	**2007**	**2017**	**Percentage change 1990-2007**	**Percentage change 2007-2017**	**1990**	**2007**	**2017**	**Percentage change 1990-2007**	**Percentage change 2007-2017**
Bullying victimization	6.1 (2.5 to 12.9)	9.0 (3.9 to 18.9)	8.7 (3.6 to 18.4)	47.3 (31.6 to 67.7)	-3.5 (-10.7 to 4.5)	4.6 (1.9 to 9.8)	7.6 (3.2 to 16.1)	7.5 (3.1 to 15.8)	65.0 (45.5 to 92.6)	-1.4 (-9.1 to 7.3)
Low physical activity	0.4 (0.2 to 0.8)	0.4 (0.2 to 0.8)	0.4 (0.2 to 0.7)	0.1 (-2.0 to 2.3)	-0.5 (-2.1 to 0.9)	0.4 (0.2 to 0.9)	0.4 (0.2 to 0.9)	0.4 (0.2 to 0.9)	-0.7 (-2.6 to 1.3)	-0.2 (-1.6 to 1.2)
High fasting plasma glucose	1.4 (0.9 to 2.4)	2.6 (1.5 to 4.0)	3.3 (2.1 to 4.9)	83.9 (6.1 to 226.7)	28.4 (-20.4 to 104.2)	1.6 (1.0 to 2.8)	3.4 (2.0 to 5.2)	3.5 (2.0 to 5.3)	110.1 (21.5 to 282.0)	0.3 (-40.1 to 60.0)
High LDL cholesterol	21.3 (19.2 to 23.4)	11.7 (10.0 to 13.5)	10.3 (8.7 to 12.1)	-45.1 (-49.9 to -39.9)	-11.8 (-15.9 to -7.8)	20.1 (18.2 to 22.2)	11.5 (10.0 to 13.3)	10.6 (9.1 to 12.3)	-42.6 (-47.5 to -38.0)	-7.8 (-11.9 to -4.0)
High systolic blood pressure	6.4 (5.6 to 7.3)	4.9 (4.2 to 5.6)	5.0 (4.2 to 5.8)	-23.5 (-31.6 to -15.3)	1.9 (-7.4 to 11.4)	5.9 (5.2 to 6.7)	4.7 (4.1 to 5.3)	4.6 (3.9 to 5.3)	-21.6 (-30.4 to -11.6)	-1.4 (-12.5 to 10.0)
High body-mass index	15.6 (11.7 to 21.1)	21.7 (17.0 to 27.0)	22.2 (17.4 to 27.7)	38.4 (22.3 to 58.7)	2.3 (-5.8 to 10.6)	17.1 (13.2 to 21.7)	23.0 (18.6 to 28.9)	23.9 (19.2 to 29.5)	34.7 (19.2 to 53.9)	3.8 (-5.4 to 13.6)
Low bone mineral density	5.1 (4.2 to 6.1)	4.4 (3.6 to 5.1)	4.6 (3.8 to 5.6)	-14.7 (-25.6 to -2.3)	6.3 (-8.4 to 22.5)	5.8 (4.8 to 6.9)	5.6 (4.8 to 6.6)	6.2 (5.2 to 7.3)	-2.2 (-13.6 to 12.0)	9.5 (-3.9 to 23.8)
Impaired kidney function	2.4 (1.2 to 6.0)	2.4 (1.1 to 6.1)	2.4 (1.2 to 6.1)	-0.3 (-6.1 to 5.3)	1.9 (-4.0 to 8.1)	3.1 (1.5 to 7.2)	3.1 (1.5 to 7.0)	3.2 (1.5 to 7.4)	-2.2 (-7.6 to 2.7)	4.2 (-0.9 to 10.2)

Supplementary Results Table 1. Minnesota age-standardised Summary Exposure Values (SEVs) for Level 4 risk factors and percentage change, by sex, 1990, 2007, and 2017.Data in parentheses are 95% uncertainty intervals.										
	Male					Female				
	1990	2007	2017	Percentage change 1990-2007	Percentage change 2007-2017	1990	2007	2017	Percentage change 1990-2007	Percentage change 2007-2017
Unsafe water source	1.1 (0.7 to 1.9)	0.4 (0.3 to 0.7)	0.3 (0.2 to 0.4)	-62.7 (-79.3 to -32.6)	-33.3 (-57.3 to 4.1)	1.1 (0.7 to 1.9)	0.4 (0.3 to 0.7)	0.3 (0.2 to 0.4)	-62.7 (-79.3 to -32.6)	-33.3 (-57.3 to 4.1)
Unsafe sanitation	4.7 (2.5 to 8.4)	3.1 (1.4 to 6.2)	2.2 (1.0 to 4.3)	-35.3 (-72.7 to 43.0)	-26.9 (-64.0 to 42.1)	4.7 (2.5 to 8.4)	3.1 (1.4 to 6.2)	2.2 (1.0 to 4.3)	-35.3 (-72.7 to 43.0)	-26.9 (-64.0 to 42.1)
No access to handwashing facility	3.4 (2.5 to 4.6)	3.1 (2.2 to 4.2)	3.0 (2.2 to 4.0)	-8.7 (-34.5 to 27.8)	-3.7 (-25.2 to 23.5)	3.4 (2.5 to 4.6)	3.1 (2.2 to 4.2)	3.0 (2.2 to 4.0)	-8.7 (-34.5 to 27.8)	-3.7 (-25.2 to 23.5)
Ambient particulate matter pollution	20.7 (9.9 to 33.0)	21.1 (11.4 to 32.6)	14.3 (3.4 to 25.7)	1.9 (-37.1 to 84.4)	-32.1 (-81.6 to 8.3)	20.6 (9.9 to 33.0)	21.1 (11.4 to 32.6)	14.3 (3.4 to 25.6)	2.0 (-37.4 to 86.3)	-32.1 (-81.6 to 8.5)
Household air pollution from solid fuels	0.2 (0.1 to 0.7)	0.2 (0.0 to 0.4)	0.1 (0.0 to 0.4)	-34.2 (-79.5 to 51.2)	-16.8 (-80.3 to 78.9)	0.4 (0.1 to 2.1)	0.3 (0.0 to 1.3)	0.2 (0.0 to 0.9)	-30.5 (-79.5 to 52.7)	-15.2 (-80.9 to 79.5)
Ambient ozone pollution	40.8 (14.3 to 54.8)	41.0 (14.4 to 54.8)	41.8 (14.8 to 54.8)	0.5 (0.0 to 1.2)	2.0 (0.0 to 3.7)	40.8 (14.3 to 54.8)	41.0 (14.4 to 54.8)	41.8 (14.8 to 54.8)	0.5 (0.0 to 1.2)	2.0 (0.0 to 3.7)
Residential radon	19.5 (1.6 to 60.9)	19.5 (1.6 to 60.9)	19.5 (1.6 to 60.9)	0.0 (0.0 to 0.0)	0.0 (0.0 to 0.0)	19.5 (1.6 to 60.9)	19.5 (1.6 to 60.9)	19.5 (1.6 to 60.9)	0.0 (0.0 to 0.0)	0.0 (0.0 to 0.0)
Lead exposure	6.9 (2.7 to 11.2)	4.4 (1.4 to 8.1)	2.9 (0.8 to 5.8)	-36.1 (-48.9 to -26.3)	-33.1 (-43.8 to -26.5)	4.1 (0.9 to 8.2)	2.7 (0.5 to 6.2)	1.9 (0.3 to 4.6)	-34.1 (-51.2 to -23.0)	-31.9 (-45.6 to -25.4)
Occupational exposure to asbestos	7.6 (6.5 to 8.5)	6.0 (5.6 to 6.4)	5.5 (4.8 to 6.3)	-21.3 (-31.2 to -6.2)	-7.8 (-21.8 to 7.7)	1.2 (1.1 to 1.4)	1.2 (1.1 to 1.3)	1.3 (1.0 to 1.5)	1.0 (-13.3 to 18.0)	4.9 (-14.9 to 27.7)
Occupational exposure to arsenic	0.4 (0.0 to 0.9)	0.4 (0.0 to 0.9)	0.4 (0.0 to 0.9)	-	-	0.3 (0.0 to 0.8)	0.4 (0.0 to 0.9)	0.3 (0.0 to 0.8)	-	-
Occupational exposure to benzene	0.6 (0.0 to 1.7)	0.6 (0.0 to 1.7)	0.6 (0.0 to 1.7)	2.1 (-5.7 to 12.8)	-3.2 (-9.0 to 5.0)	0.9 (0.1 to 2.5)	1.0 (0.1 to 2.9)	1.0 (0.1 to 2.8)	11.2 (1.4 to 25.8)	-0.3 (-8.5 to 10.7)
Occupational exposure to beryllium	0.0 (0.0 to 0.0)	0.0 (0.0 to 0.0)	0.0 (0.0 to 0.0)	0.7 (-4.9 to 6.8)	0.3 (-4.0 to 5.2)	0.0 (0.0 to 0.0)	0.0 (0.0 to 0.0)	0.0 (0.0 to 0.0)	5.1 (-2.6 to 13.0)	-1.0 (-6.7 to 5.1)
Occupational exposure to cadmium	0.0 (0.0 to 0.0)	0.0 (0.0 to 0.1)	0.0 (0.0 to 0.1)	2.7 (-8.5 to 15.2)	0.0 (-8.6 to 9.7)	0.0 (0.0 to 0.0)	0.0 (0.0 to 0.0)	0.0 (0.0 to 0.0)	3.8 (-10.1 to 20.7)	-2.6 (-13.0 to 10.6)
Occupational exposure to chromium	0.1 (0.1 to 0.1)	0.1 (0.1 to 0.1)	0.1 (0.1 to 0.1)	4.5 (-5.4 to 15.3)	0.0 (-7.0 to 8.4)	0.1 (0.1 to 0.1)	0.1 (0.1 to 0.1)	0.1 (0.1 to 0.1)	6.0 (-5.8 to 20.1)	-2.7 (-11.6 to 8.3)
Occupational exposure to diesel engine exhaust	0.5 (0.5 to 0.6)	0.6 (0.5 to 0.6)	0.6 (0.5 to 0.6)	7.8 (-1.1 to 17.0)	1.9 (-4.8 to 9.3)	0.5 (0.4 to 0.5)	0.5 (0.5 to 0.6)	0.5 (0.4 to 0.6)	7.5 (-4.8 to 22.1)	-4.4 (-13.1 to 5.5)
Occupational exposure to formaldehyde	0.2 (0.2 to 0.2)	0.2 (0.2 to 0.3)	0.2 (0.2 to 0.3)	1.1 (-10.6 to 13.2)	-1.6 (-10.2 to 8.0)	0.2 (0.2 to 0.2)	0.2 (0.2 to 0.2)	0.2 (0.2 to 0.2)	4.8 (-8.4 to 20.2)	-2.6 (-12.2 to 10.0)
Occupational exposure to nickel	0.3 (0.0 to 1.4)	0.3 (0.0 to 1.4)	0.3 (0.0 to 1.4)	-	-	0.3 (0.0 to 1.1)	0.3 (0.0 to 1.2)	0.3 (0.0 to 1.2)	-	-
Occupational exposure to polycyclic aromatic hydrocarbons	0.2 (0.2 to 0.2)	0.2 (0.2 to 0.2)	0.2 (0.2 to 0.2)	4.4 (-5.1 to 14.6)	-0.5 (-7.1 to 7.5)	0.2 (0.2 to 0.2)	0.2 (0.2 to 0.3)	0.2 (0.2 to 0.3)	8.4 (-2.3 to 21.2)	-2.1 (-9.9 to 7.2)
Occupational exposure to silica	2.9 (0.4 to 9.1)	2.7 (0.4 to 8.7)	2.8 (0.4 to 8.8)	-5.3 (-16.5 to 9.0)	0.2 (-9.1 to 12.3)	2.2 (0.3 to 6.9)	2.2 (0.3 to 6.7)	2.1 (0.3 to 6.5)	-3.4 (-19.5 to 18.9)	-3.2 (-17.1 to 16.3)
Occupational exposure to sulphuric acid	0.6 (0.1 to 1.8)	0.7 (0.1 to 2.1)	0.7 (0.1 to 2.0)	10.3 (-2.7 to 23.7)	-1.0 (-9.3 to 8.3)	0.6 (0.1 to 1.8)	0.7 (0.1 to 2.1)	0.6 (0.1 to 2.0)	18.5 (0.4 to 39.3)	-3.7 (-15.9 to 11.0)
Occupational exposure to trichloroethylene	0.1 (0.1 to 0.1)	0.1 (0.1 to 0.1)	0.1 (0.1 to 0.1)	6.1 (-1.4 to 14.1)	0.8 (-4.5 to 6.9)	0.1 (0.1 to 0.1)	0.1 (0.1 to 0.1)	0.1 (0.1 to 0.1)	6.5 (-3.6 to 17.9)	-3.0 (-10.6 to 6.4)
Occupational asthmagens	16.4 (13.8 to 19.9)	15.7 (13.1 to 18.7)	15.3 (12.6 to 18.5)	-4.2 (-16.0 to 8.3)	-2.9 (-11.8 to 7.4)	8.0 (6.6 to 9.6)	8.3 (6.8 to 10.1)	7.8 (6.2 to 9.5)	4.2 (-9.4 to 21.3)	-6.3 (-16.7 to 5.3)
Occupational particulate matter, gases, and fumes	6.9 (5.0 to 9.8)	6.6 (4.8 to 9.4)	6.3 (4.6 to 9.1)	-4.4 (-9.1 to 0.1)	-4.3 (-8.3 to -0.8)	3.7 (2.6 to 5.5)	3.6 (2.6 to 5.4)	3.5 (2.5 to 5.1)	-2.3 (-9.5 to 5.5)	-4.5 (-10.0 to 1.2)
Occupational noise	6.2 (5.7 to 6.8)	6.0 (5.5 to 6.6)	5.8 (5.3 to 6.4)	-2.7 (-5.7 to 0.4)	-3.6 (-6.0 to -1.4)	4.3 (3.8 to 4.8)	4.5 (4.1 to 5.0)	4.5 (4.1 to 5.0)	4.9 (-0.4 to 11.0)	-0.4 (-3.7 to 2.9)
Occupational ergonomic factors	8.5 (6.9 to 10.4)	7.7 (6.4 to 9.5)	7.4 (6.1 to 9.1)	-8.5 (-18.4 to 2.0)	-4.3 (-10.8 to 2.9)	5.0 (3.9 to 6.4)	5.1 (4.1 to 6.4)	5.0 (4.0 to 6.2)	2.2 (-12.7 to 21.8)	-2.1 (-13.5 to 11.1)
[truncated: 1,068,173 more chars]
